# Cellular Proteomic Profiling Using Proximity Labeling by TurboID-NES in Microglial and Neuronal Cell Lines

**DOI:** 10.1016/j.mcpro.2023.100546

**Published:** 2023-04-14

**Authors:** Sydney Sunna, Christine Bowen, Hollis Zeng, Sruti Rayaprolu, Prateek Kumar, Pritha Bagchi, Eric B. Dammer, Qi Guo, Duc M. Duong, Sara Bitarafan, Aditya Natu, Levi Wood, Nicholas T. Seyfried, Srikant Rangaraju

**Affiliations:** 1 Department of Neurology, Emory University, Atlanta Georgia, USA; 2 Center for Neurodegenerative Diseases, Emory University, Atlanta, Georgia, USA; 3 Department of Biochemistry, Emory University, Atlanta, Georgia, USA; 4 Emory Integrated Proteomics Core, Emory University, Atlanta, Georgia, USA; 5 George W. Woodruff School of Mechanical Engineering, Wallace H. Coulter Department of Biomedical Engineering, and Parker H. Petit Institute for Bioengineering and Bioscience, Georgia Institute of Technology, Atlanta, Georgia, USA

**Keywords:** Alzheimer’s disease, mass spectrometry, proximity labeling, TurboID

## Abstract

Different brain cell types play distinct roles in brain development and disease. Molecular characterization of cell-specific mechanisms using cell type–specific approaches at the protein (proteomic) level can provide biological and therapeutic insights. To overcome the barriers of conventional isolation-based methods for cell type–specific proteomics, *in vivo* proteomic labeling with proximity-dependent biotinylation of cytosolic proteins using biotin ligase TurboID, coupled with mass spectrometry (MS) of labeled proteins, emerged as a powerful strategy for cell type–specific proteomics in the native state of cells without the need for cellular isolation. To complement *in vivo* proximity labeling approaches, *in vitro* studies are needed to ensure that cellular proteomes using the TurboID approach are representative of the whole-cell proteome and capture cellular responses to stimuli without disruption of cellular processes. To address this, we generated murine neuroblastoma (N2A) and microglial (BV2) lines stably expressing cytosolic TurboID to biotinylate the cellular proteome for downstream purification and analysis using MS. TurboID-mediated biotinylation captured 59% of BV2 and 65% of N2A proteomes under homeostatic conditions. TurboID labeled endolysosome, translation, vesicle, and signaling proteins in BV2 microglia and synaptic, neuron projection, and microtubule proteins in N2A neurons. TurboID expression and biotinylation minimally impacted homeostatic cellular proteomes of BV2 and N2A cells and did not affect lipopolysaccharide-mediated cytokine production or resting cellular respiration in BV2 cells. MS analysis of the microglial biotin-labeled proteins captured the impact of lipopolysaccharide treatment (>500 differentially abundant proteins) including increased canonical proinflammatory proteins (Il1a, Irg1, and Oasl1) and decreased anti-inflammatory proteins (Arg1 and Mgl2).

## Highlights

• Cytosolic TurboID biotinylates >50% of the proteome in microglia and neuronal cells.
• TurboID-NES has minimal impacts on cellular proteomic composition and function.
• About 1340 proteins labeled by TurboID differentiate microglia from neurons *in vitro*.
• TurboID proteomic profiling captures microglial activation by lipopolysaccharide.

## Graphical Abstract

**Figure:**
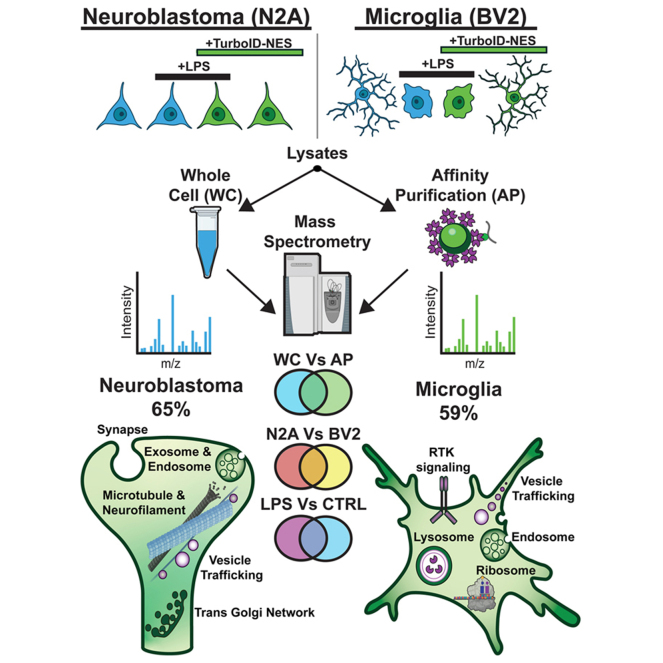

## In Brief

TurboID-based proximity labeling of cytosolic proteins coupled with mass spectrometry represents a promising approach for proteomic profiling of specific cell types *in vitro* and *in vivo*, although the breadth of proteomic coverage, the ability to distinguish cell types and to capture pathological responses, and potential impacts of TurboID-mediated proteomic labeling on cellular physiology, are unknown. To inform *in vivo* applications of TurboID-NES, we interrogated the proteomic coverage achieved by TurboID-NES in neuronal and microglial cell lines under homeostatic and inflammatory conditions.

The brain is a complex organ possessing heterogeneous populations of neurons, glia, and vascular cells. The orchestration of interactions within cell types (cell autonomous) and between cell types (noncell autonomous) support higher level processes critical to development, aging, and neurodegeneration. Protein-level analyses using mass spectrometry (MS) expand upon other systems-level analyses, including genomics and transcriptomics. Specifically, proteomics can profile total protein abundances, identify post-translational modifications, and resolve protein-level changes occurring in subcellular compartments. A central challenge to neuroproteomics is the difficulty in obtaining cell type–specific proteomes from brain tissue.

Traditional approaches to isolating cell type–specific proteomes for MS including fluorescence-activated cell sorting and magnetic-activated cell sorting require fresh brain tissue, and the harsh and laborious processing itself poses challenges (1, 2). A majority of adult neurons do not survive the isolation process, and sampling bias for healthier non-neuronal brain cells able to withstand the isolation process limits proteomic profiling in disease states. In addition, contamination from proteins derived from nontarget cell types persists. The challenges to maintaining cellular integrity with isolation methods motivated the field to innovate novel methods of applying cell type–specific labeling to *in vitro* and *in vivo* systems.

One approach to achieve cell type–specific proteomic labeling uses BioOrthogonal Non-Canonical Amino acid Tagging (BONCAT) in which a mutated methionyl-tRNA synthetase incorporates azidonorleucine, a methionine analog, into newly synthesized peptides (3, 4, 5). Subsequently, cell lysates or brain homogenates undergo click chemistry with biotin-alkyne to biotinylate the azidonorleucine-containing peptides. By driving methionyl-tRNA synthetase expression under a cell type–specific promoter and enriching biotinylated peptides by streptavidin affinity purification (AP), BONCAT can purify cell type–specific newly translated proteins (6, 7). One advantage of this strategy lies in its ability to label and purify low-abundant and newly synthesized proteins. A limitation may be low proteomic depth and biases toward proteins with high turnover. The BONCAT approach has thus far been applied to characterize excitatory and inhibitory neurons of mice and rats in both *in vivo*, *ex vivo*, and *in vitro* contexts (6, 7, 8, 9). To date, extension to other neuronal and glial cell types has not yet been published.

In contrast to the BONCAT approach that labels only newly synthesized proteins, proximity-labeling techniques rely on biotin ligases, which biotinylate nearby interactors. BioID is a promiscuous biotin ligase engineered from the site-specific biotin ligase, BirA, endogenously produced by *Escherichia coli* (10). Because BioID is nontoxic, this technology opened up opportunities for *in vivo* applications, though the reaction kinetics of biotin labeling takes place over 18 to 24 h (11). Alice Ting’s group used yeast surface display–mediated directed evolution to improve the reaction kinetics of BioID by introducing 15 mutations in the catalytic domain relative to wildtype BirA (12). This biotin ligase, termed TurboID, can robustly and promiscuously biotinylate proteomes in living cells and animal models without cellular toxicity, in as little as 10 min in cell culture systems (12, 13). Versatile in its applications, TurboID has been fused to proteins of interest to map protein interactomes, targeted to a subcellular compartment of interest, and has been exported out of the nucleus to label cytosolic proteins (14, 15, 16, 17, 18, 19). In addition, to biotin-label proteins proximal to intermembrane contact sites, split-TurboID was recently developed in which two inactive fragments of TurboID reconstitute in the presence of rapamycin (13, 20). When driven under a cell type–specific promoter, both TurboID and split-TurboID can label cellularly distinct proteomes in their native state for downstream affinity capture and MS. These advancements, which enable robust, targeted, and nontoxic biotinylation of cell type–specific proteomes, have yielded promising applications to neuroproteomics. Recently, split-TurboID has been applied to perisynaptic-cleft proteome discovery between astrocyte and neurons *in vivo* (18, 21). A novel transgenic mouse model for conditional expression of TurboID with a nuclear export sequence (TurboID-NES) was also recently developed to resolve region-specific proteomic signatures of CamKIIa neurons and Aldh1l1 astrocytes in adult mouse brain (21, 22). These recent advances position cell type–specific *in vivo* biotinylation of proteins (CIBOP) as a promising approach to resolve distinct cellular proteomes in different *in vivo* homeostatic and pathological contexts.

In anticipation of *in vivo* proximity labeling applications of TurboID-NES for cell type–specific proteomics, it is important to establish the effects of global cytosolic biotinylation on molecular and cellular processes in mammalian cells. It is also critical to characterize the breadth of cytosolic proteins labeled by TurboID-NES, determine how reflective these proteins are of untransduced whole-cell (WC) proteomes, and identify any inherent biases of the TurboID-NES approach. In order to support the use of TurboID-NES to label cytosolic proteins that are also relevant to cellular identity, it is important to test if TurboID-mediated proteomics can differentiate two distinct cell types, such as neurons and microglia. Finally, we need to ascertain whether proteomic changes induced by inflammatory stimuli can be efficiently and reliably captured by the TurboID-NES biotinylation of cytosolic proteins. These data are critical for interpreting proteomic results from TurboID-NES proximity labeling studies that aim to label the cellular proteome in mammalian systems *in vitro* and *in vivo*. To answer these questions under controlled experimental conditions, we generated neuroblastoma (N2A) and immortalized microglial (BV2) cell lines that stably express V5-TurboID-NES to label the cellular proteome excluding the nucleus. We examined the extent and coverage of cytosolic proteomic labeling by TurboID-NES in N2A and BV2 cells, under resting and lipopolysaccharide (LPS)-stimulated inflammatory conditions, using label-free quantitation (LFQ) MS. We found that TurboID-NES expression preserves the ability to resolve distinct cellular proteomes at the MS level under homeostatic states and inflammatory challenge.

## Experimental Procedures

### Antibodies, Buffers, and Reagents

A complete table of antibodies and reagents is provided (Tables 1 and 2).

**Table 1:** Antibodies used and their corresponding dilutions

Antibody	Manufacturer	Catalog number	Dilution
Rabbit anti-V5	Abcam	ab206566	1:500
Rat anti-α-tubulin	MilliporeSigma	MAB1864	1:1000
Goat anti-β-actin	Santa Cruz Biotechnology	sc-1615	1:1000
Rabbit anti-histone H3	Abcam	ab1791	1:5000
Donkey anti-rabbit 800	Invitrogen	A11374	1:10,000
Donkey anti-goat 680	LI-COR	A21084	1:10,000
Donkey anti-goat 800	LI-COR	A11370	1:10,000
Goat anti-rat 800	LI-COR	926-32219	1:10,000
Streptavidin DyLight 594	ThermoFisher	21842	1:1000
Streptavidin, Alexa-Fluor 680 conjugate	Invitrogen	S32358	1:10,000
DAPI	Roche	10236276001	1 μg/ml

**Table 2:** Reagents used and their manufacturer and catalog numbers

Reagent	Manufacturer	Catalog number
StartingBlockT20	ThermoFisher	37543
HALT protease & phosphatase inhibitor cocktail	ThermoFisher	78446
Dulbecco’s modified Eagle's medium	Gibco	11965-092
Penicillin–streptomycin	Gibco	15140-122
Fetal bovine serum (FBS)	Gibco	26140-7079
Biotin	Sigma–Aldrich	B4639-100mg
LPS	Sigma–Aldrich	L4391-1mg
Puromycin	Sigma–Aldrich	P9620
4% Paraformaldehyde in PBS	Thermo Scientific	J19943-K2
0.05% Trypsin–EDTA	Gibco	253000054
Reagent A	ThermoFisher	23222
Reagent B	ThermoFisher	23224
Bovine serum albumin standards	ThermoFisher	23208
LB medium	Sigma–Aldrich	28713-500G-F
Seahorse Flux Pack	Agilent	102601-100
Seahorse XF Media	Agilent	103575-100
Sodium pyruvate	Sigma–Aldrich	S8636-100mL
l-glutamine	Sigma–Aldrich	G7513-100mL
Glucose	Sigma–Aldrich	G8769-100mL
Oligomycin	Sigma–Aldrich	75351-5mg
Carbonyl cyanide-4 (trifluoromethoxy) phenylhydrazone	Sigma–Aldrich	C2920-10mg
Rotenone	Sigma–Aldrich	R8875-1G
Antimycin-A	Sigma–Aldrich	A8674-25mg

### Cell Culture

N2A and BV2 cells were cultured in filtered Dulbecco’s modified Eagle's medium supplemented with high glucose and l-glutamine containing 1% penicillin–streptomycin and 10% fetal bovine serum. All media were vacuum-filtered with 0.2 μm filters. The cells were incubated at 37 °C and 5% CO_2_ until reaching 80% confluency. The splitting regimen took place twice weekly, plating one million cells onto a 100 mm culture plate to a final volume of 10 ml culture media. In preparation for MS experiments, cells reached 95% confluency in 150 mm plates. Transduced cells expressing TurboID-NES were kept in 2 μg/ml puromycin media until being plated for MS, wherein they forwent puromycin treatment.

### Genetic Constructs and Gene Delivery

The V5-TurboID-NES_pCDNA3 and plasmid is a gift from Alice Ting (Addgene plasmid #107169). Plasmids were transformed using a competent *E. coli* strain DH5α according to the manufacturer protocols. Briefly, DH5 α cells were thawed on ice before aliquoting 50 μl into 1.5 ml tubes. Constructs were diluted 1:1000 into autoclaved Milli-Q water. LB medium was prepared and autoclaved by diluting 20 g of LB broth, Vegitone into 1000 ml of H_2_O. To the 50 μl aliquots of DH5α-competent cells, 5 μl of diluted constructs were mixed by turning the tubes upside down. The plasmids were incubated with the DH5α-competent cells for 30 min on ice. Following incubation, the samples underwent heat shock by for 42 °C for 20 s and were placed on ice for 2 min. About 500 μl of prewarmed LB medium were added to each sample before being placed on a rotor to shake at 225 rpm at 37 °C for 1 h. Plasmid DNAs were purified using QIAfilter Plasmid kits (Midi pre kit; Qiagen; catalog no.: 12243) following the manufacturer's protocol. Restriction sites (underlined) were introduced *via* the following PCR primers (V5.bstb.S; 5′-gcgcctactctagagctagcgaattcgaagccaccatgggcaagcccatccccaa-3′) (nes.Bam.A; 5′-agaaggcacagtcggcggccgcggatccttagtccagggtcaggcgctccagggg-3′). The V5-TurboID-NES sequence was subcloned into pCDH-EF1-MCS-BGH-PGK-GFP-T2A-Puro (CD550A-1) and sequenced. N2A and BV2 cells were transduced with a puromycin-resistant lentivirus construct of a V5-TurboID-NES containing an NES (V5-TurboID-NES) and a GFP connected *via* a T2A linker. Constructs were generated in Emory University’s Viral Vector Core. Given a titer of 1.5 × 10^9^ I.U./ml, we experimented with multiplicities of infection (MOIs) 5 and 10. In biological triplicates, three wells of each cell type received the lentivirus at either an MOI of 5 or 10 or received no virus (untransduced control). About 48 h following transduction, half of the media was replaced with fresh media, and the cells were split 1:3 on 72 h following transduction. Puromycin selection began 96 h after transduction, half of the media were replaced with 2 μg/ml of puromycin for a final concentration of 1 μg/ml. For the following week, half of the media were replaced every other day with fresh puromycin-containing media to remove nonadhering cells. After this, the cells were split twice weekly and maintained with media containing 1 μg/ml of puromycin. We validated the puromycin screening procedure by assessing the percentage of GFP-positive cells with a fluorescent microscope and flow cytometry. A majority of cells were GFP positive 3 days after addition of puromycin selection. After >90% of the cells were GFP positive, the cells were maintained in 10 cm dishes in media containing 1 μg/ml of puromycin. Cells receiving the lentivirus at an MOI of 5 reached confluency sooner, and we did not observe a difference in transduction efficacy between MOIs 5 and 10.

### Generation of WC Lysates and Supernatants and Confirmation of Labeling

BV2 and N2A cells transduced with V5-TurboID-NES or untransduced cells were seeded onto 10 cm plates. About 24 h after plating, media were replaced either with biotin-supplemented media (200 μM) or media containing both LPS (1 μg/ml) and biotin supplementation (n = 6/group). About 48 h after media replacement, the media were taken off, centrifuged for 2 min at 800 rpm at room temperature (RT) to remove cellular debris, and preserved in a 15 ml tube. Cells were dissociated using 2.5 ml of 0.05% trypsin–EDTA. After trypsin dissociation, cells were rinsed and collected *via* manually pipetting with 7.5 ml fresh media before being transferred to 15 ml tubes. Supernatants and cell pellets were flash-frozen on dry ice. Dissociated cells and supernatants were centrifuged at 800 rpm for 2 min at RT. Supernatants were transferred to a fresh 15 ml tube before being flash-frozen on dry ice. Cell pellets were washed twice with 10 ml of ice-cold 1× PBS. Finally, cell pellets were resuspended in 1 ml of ice-cold PBS and transferred to 2 ml LoBind Eppendorf tubes (Eppendorf; catalog no.: 022431102). Cells were centrifuged for 2 min at 800 rpm at RT before being flash-frozen on dry ice. Cell pellets were harvested in 500 μl urea lysis buffer (8 M urea, 10 mM Tris, 100 mM NaH_2_PO_4_, pH 8.5) with 1× HALT protease and phosphatase inhibitor cocktail without EDTA. Cell lysates were then sonicated at 30% amplitude thrice in 5 s on–off pulses to disrupt nucleic acids and cell membrane. All cell lysates were centrifuged at 4 °C for 2 min at 12,700 rpm. The supernatants were transferred to a fresh 1.5 ml LoBind Eppendorf tube. The protein concentrations of the cell lysates were determined by bicinchoninic acid assay reagents using bovine serum albumin standards. Of the six biological replicates prepared per experimental group, four samples per group were reserved for quality-control analyses performed prior and in addition to MS studies.

### Immunoblotting

In each well, 10 to 20 μg of protein from cell lysates resolved in a 4 to 12% polyacrylamide gel and transferred onto iBlot 2 Transfer Stack containing nitrocellulose membrane using the BOLT transfer system. The membranes incubated for 1 h at RT in StartingBlockT20 before receiving rabbit anti-V5 primary antibody overnight at 4 °C or 1 h at RT. After primary antibody incubation, the membranes underwent three rapid washes with 1× Tris-buffered saline with Tween-20 (TBST) followed by three 10-min washes with 1× TBST. Membranes then underwent three rapid washes with 1× TBS and three 10-min washes with 1× TBS. The membranes incubated for 1 h at RT in a secondary antibody cocktail of streptavidin 680 to visualize biotinylated proteins and donkey anti-rabbit 800 to visualize V5-tagged TurboID-NES. The membranes were then washed again as previously described before undergoing imaging *via* the Odyssey Infrared Imaging System (LI-COR Biosciences).

### Immunofluorescence

Immunofluorescent (IF) staining was performed as published previously with modifications for cultured cells (22). Briefly, BV2 and N2A cells were seeded at ∼50,000 cells onto autoclaved and HCl–ethanol-treated 25 mm coverslips in a 6-well dish. All cells received supplemental biotin treatment (200 μM) for the duration of maintenance. After reaching 50% confluency, cells were washed thrice with warm sterile PBS to remove media. Cells were fixed with 4% paraformaldehyde in PBS for 30 min and washed thrice in ice-cold PBS for 10 min, gently orbitally rotating at 50 rpm (IKA; catalog no.: KS 260). Fixed cells were permeabilized and blocked simultaneously in a solution of 5% normal horse serum in 0.25% TBST diluted in PBS for 1 h at RT on the orbital rotator. To each well, cells received 1 ml of rabbit anti-V5 (1:500 dilution) primary antibody solution in 1% normal horse serum diluted in PBS overnight at 4 °C. All cells received a 5 min incubation with 4′,6-diamidino-2-phenylindole (DAPI) for nuclear staining. All IF imagings were performed with a 60× oil-immersion objective taken using Keyence BZ-X810. Colocalization analysis was performed with slight modifications from previous methods (23, 24). Briefly, we subtracted the target area (μm^2^) of DAPI-signal overlap with V5 or biotinylation signal (nuclear-localizing TurboID-NES or biotinylated proteins, respectively) from the total area of V5 or streptavidin signal using the Keyence BZ-X810 Analyzer hybrid-cell-count colocalization software. Only cells positive for both DAPI and V5 or Streptavidin DyLight were analyzed for colocalization analysis. Significance *p* values were assessed using the two-tailed Mann–Whitney test. The total target area counts (number of cells counted because of the presence of a cell with overlapping of DAPI and V5 or Streptavidin DyLight signal) for transduced BV2 cells assessed for V5–DAPI colocalization were 1876 cells. The total target area counts for V5–DAPI colocalization for transduced BV2 cells receiving LPS challenge were 1945 cells. The total Streptavidin DyLight–DAPI colocalization target counts for transduced naïve BV2 cells were 2289 cells. The total target counts for Streptavidin DyLight–DAPI colocalization for transduced BV2 cells receiving LPS were 1638 cells.

### Subcellular Fractionation

Transduced and untransduced BV2 and N2A cells were plated in triplicates on 10 cm plates and grown to 70% confluency. Cellular monolayers were rinsed with 10 ml ice-cold PBS, and PBS was aspirated. Cell monolayers were scraped with a sterile cell scraper in 2.97 ml PBS with 30 μl of 100× HALT, added immediately prior to use. Cell slurries were transferred to 15 ml tubes and centrifuged at 1000*g* at 4 °C for 5 min. The supernatants were removed, and the pellet was washed once in 1 ml cold PBS. To obtain the WC fraction, 100 μl of the cell slurry was transferred to a fresh 0.5 ml Eppendorf tube. The remaining 900 μl of cell slurry was centrifuged at 1000*g* at 4 °C for 5 min. Supernatants were removed, and 150 μl of hypotonic lysis buffer (10 mM Hepes, pH 7.9, 20 mM KCl, 0.1 mM EDTA, 1 mM DTT, 5% glycerol, 0.5 mM PMSF, 10 μg/ml aprotinin, 10 μg/ml leupeptin, 0.1% NP-40, and 1× HALT) was added to cell pellets. Cell pellets incubated on ice for 5 min before being centrifuged at 15,600*g* at 4 °C for 10 min. The supernatants were transferred to a fresh 0.5 ml tube as cytoplasmic fractions. The cell pellets containing nuclei received 100 μl of high salt buffer (20 mM Hepes, pH 7.9, 0.4 M NaCl, 1 mM EDTA, 1 mM EGTA, 1 mM DTT, 0.5 mM PMSF, 10 μg/ml aprotinin, 10 μg/ml leupeptin, 1× HALT) and incubated for 30 min on ice. All fractions were sonicated for three 5 s on pulses followed by 5 s off pulses at 25% amplitude. Then, all samples were centrifuged for 10 min at 18,213*g* at 4 °C. All supernatants were transferred to fresh 0.5 ml Eppendorf tubes and stored at −80 °C until immunoblotting. To confirm the purity of subcellular fractionation, 20 μg of protein resolved onto 4 to 12% acrylamide PAGE gels. After transferring, nitrocellulose membranes were probed with goat anti-β-actin as a loading control, rat anti-α-tubulin as a cytoplasmic marker, rabbit anti-Histone H3, rabbit anti-V5 to visualize TurboID-NES, and streptavidin-680 to visualize biotinylation. Secondary antibodies included goat–anti-rat 800, donkey–anti-goat 680, and donkey–anti-rabbit 800. All primary and secondary antibodies incubated for 1 h at RT and were probed serially to ensure the specificity of the antibodies for their target.

### Sample Preparation for MS Studies

From each sample, 1 mg of lysate was set aside for streptavidin AP, 50 μg of protein were reserved as WC fractions, and the remaining protein was aliquoted and reserved for quality-control studies. Quality-control studies were conducted prior to submitting samples for MS analysis to confirm the presence of biotinylated proteins *via* Western blot (WB), equal loading *via* Coomassie, as well as ensure the specificity of streptavidin-purified preparations for biotinylated proteins *via* silver stain (Pierce, ThermoFisher; catalog no.: 24612) and WB.

Slightly modified from previous publications, the AP samples were processed as follows (13, 22): 1 mg of protein derived from transduced and untransduced BV2 and N2A lysates were affinity purified onto 83 μl of magnetic streptavidin beads (Thermo; catalog no.: 88817). Briefly, to each 1.5 ml Eppendorf LoBind tube, 1 ml of radioimmunoprecipitation assay (RIPA) buffer (150 mM NaCl, 50 mM Tris, 1% Triton X-100, 0.5% sodium deoxycholate, 0.1% SDS, pH 7.5) was added to the beads on rotation for 2 min at RT. Using a magnetic stand (PureProteome; Millipore; catalog no.: LSKMAGS08), the buffer was removed from the beads. To each tube-containing beads, 500 μl of fresh RIPA lysis buffer was added before adding 1 mg of protein. The samples incubated at 4 °C overnight on a rotator. Samples were then briefly centrifuged, placed on the magnetic stand, and the supernatants were preserved and frozen at −20 °C. After incubation, the beads containing the biotinylated proteins underwent series of washing procedures at RT. Beads were washed twice with 1 ml of RIPA lysis buffer for 8 min, 1 ml of 1 M KCL for 8 min, rapidly (∼10 s) in 1 ml of 0.1 M sodium carbonate (Na_2_CO_3_), rapidly in 1 ml of 2 M urea in 10 mM Tris–HCl (pH 8.0), and twice with 1 ml RIPA lysis buffer for 8 min. About 8 min washing steps took place on a rotator, whereas rapid washing procedures took place on the magnetic stand using pipette rinsing to briefly mix the samples. Each buffer removal step was performed *via* manual pipetting, as aspirating the buffer with vacuum systems may deplete bead volume. The beads were resuspended in 1 ml of 1× PBS before being transferred to a new Eppendorf Lo Bind tube where they were washed once more with 1 ml of 1× PBS. Finally, AP samples were resuspended into 83 μl of PBS, wherein 8 μl of beads-containing solution was transferred to a new tube and placed on a magnetic stand for >2 min. The remaining beads-containing solution was preserved. The PBS was removed and replaced with 30 μl of 4× Laemmli protein buffer (Bio-Rad; catalog no.: 1610747) supplemented with β-mercaptoethanol, 2 mM biotin, 20 mM DTT. Beads then incubated at 95 °C for 15 min and 20 μl were reserved for WB verification of biotinylated proteins *via* streptavidin 680 and 10 μl were reserved for separate silver stain to verify minimal nonspecific binding between untransduced AP samples and transduced AP samples.

To confirm the quality, equal loading, and biotinylation of proteins in the WC samples, 10 μg of protein from each sample resolved onto a 4 to 12% polyacrylamide gel for Coomassie blue staining, and 20 μg of protein was resolved on a separate gel to probe for actin and biotinylated proteins *via* immunoblotting. For Coomassie staining, gels were fixed in 50% methanol and 10% glacial acetic acid for 1 h at RT in a sealed container on a rocking incubator. The gels were stained for 20 min (0.1% Coomassie Brilliant Blue R-250, 50% methanol, 10% glacial acetic acid). Finally, a destaining solution (40% methanol and 10% glacial acetic acid) was applied at RT, whereas gels were incubated with gentle rocking. Coomassie gels were imaged in the 700 nm channel on the LiCor Odyssey imaging system. Confirmation of biotinylated proteins and equal loading of WC samples took place by immunoblotting with streptavidin-conjugated fluorophore 680 (Strep680) and goat antiactin, respectively.

### Peptide Digestion and Cleanup

Sample preparation for MS was performed according to our laboratory protocols modified from previous publications (25, 26, 27, 28, 29, 30, 31, 32). Briefly, 50 μg of protein from each cell lysate sample was digested. Samples were reduced with 5 mM DTT at RT for 30 min on a rotor, followed by alkylation with 10 mM iodoacetamide at RT for 30 min on a rotor in dark. Samples were diluted with 50 mM of ammonium bicarbonate to 4 M urea prior to undergoing overnight digestion with 2 μg of lysyl endopeptidase (Lys-C) (Wako; catalog no.: 127-06621) at RT. Samples were then diluted to reduce the concentration of urea to 1 M prior to trypsin digestion. Each sample received 2 μg of trypsin (Thermo; catalog no.: 90058) and was incubated overnight at RT. Acidifying buffer was added to the peptide solution for a final concentration of 1% formic acid and 0.1% TFA to stop the trypsin digestion. HLB columns were used to desalt samples (Waters; catalog no.: 186003908). The samples were dried overnight using a centrifugal vacuum concentrator (SpeedVac Vacuum Concentrator).

AP samples underwent on-bead digestion. Beads were resuspended in 150 μl ammonium bicarbonate. Application of DTT to a final concentration of 1 mM reduced the samples during a 30 min RT incubation on a rotator. A 5 mM application of iodoacetamide alkylated the samples during a 30 min incubation in the dark on a rotator. To each sample, 0.5 μg of LysC was added before incubating overnight at RT on a rotator. The digestion was completed overnight at RT on a rotator with the addition of 1 μg of trypsin to the samples. After overnight digestion, the samples were treated with acidifying buffer to stop the trypsin-mediated digestion. HLB columns were used to desalt samples. The samples were dried using the SpeedVac.

### Liquid Chromatography and MS

All samples were analyzed on the Evosep One system using an in-house packed 15 cm × 150 μm i.d. capillary column with 1.9 μm Reprosil-Pur C18 beads (Dr Maisch) using the preprogrammed 44 min gradient (30 samples per day). MS was performed with a Q-Exactive Plus (Thermo) in positive ion mode using data-dependent acquisition with a top 20 method. Each cycle consisted of one full MS scan followed up to 20 MS/MS events. MS scans were collected at a resolution of 70,000, 400 to 1600 *m/z* range, 3 × 10^6^ automatic gain control, and 100 ms maximum ion injection time. All higher energy collision-induced dissociation MS/MS spectra were acquired at a resolution of 17,500 (1.6 *m/z* isolation width, 28% collision energy, 1 × 10^5^ automatic gain control target, and 100 ms maximum ion time). Dynamic exclusion was set to exclude previously sequenced peaks for 30 s within a 10-ppm isolation window. MS raw files of WC and AP samples were searched together using the search engine Andromeda integrated into MaxQuant (version 1.6.17.0). Raw files were searched against the August 2020 UniProt *murine* database, wherein 91,442 entries were searched. Variable modifications include methionine oxidation, N-terminal acetylation, and deamidation of glutamine and asparagine residues. Fixed modifications include carbamidomethylation of cysteine residues. Only peptides with up to two missed cleavages were considered in the database search. Additional search parameters included a maximum peptide mass of 6000 Da and the minimum peptide length of six residues. The mass tolerance for precursor ions is 20 ppm, and the mass tolerance for fragment ions is 0.05 Da. Peptide spectral match false discovery rates (FDRs), protein FDR, and site FDR were all set at 1%.

Annotated spectra of proteins identified on the basis of a single unique peptide can be found in supplemental data S1 for the 550 proteins identified in the WC samples and supplemental Data S2 for the 561 proteins identified in the AP samples. supplemental Datasheet S3 contains specifications for all proteins identified on the basis of a single unique peptide. We devised an R script that automates the MS/MS annotation of single peptide identifications’ MaxQuant best-scored peptide spectral matches directly from Thermo RAW data files into pdf (supplemental datas S1 andS2) and a data table (supplemental Datasheet S3). This resource can be found at https://github.com/edammer/MQ1pepAnnotate and leverages the protViz and rawrr R packages (33).

### Data Normalization and Filtering, Principal Component Analyses, Differential Expression Analyses, Clustering, Gene Set Enrichment Analysis

To analyze large datasets generated by MS, we used principal component analysis (PCA) as a dimension reduction strategy, differential expression analysis (DEA) was used to identify significant differences in protein intensities between samples, clustering analyses (K-means) identified discrete proteins with related abundance values within and across samples, and gene over-representation analysis (ORA) functionally annotated enriched groups of proteins.

#### Filtering Missingness, Data Normalization, and Log Transformation

LFQ intensities and raw intensity values were uploaded onto Perseus (version: 1.6.15) for analyses. Categorical variables were removed, intensity values were log-2 transformed, transduced AP intensity values were normalized to TurboID intensity to adjust for variability in TurboID expression, and data were in general filtered based on 50% missingness across group of samples that were selected for each analysis. Missing values were imputed from normal distribution.

#### PCA

PCAs of LFQ MS data were performed and visualized using SPSS (IBM Statistics, version 28.0.1.0). The PCA on cytokine profiling with Luminex were performed using the PCA function from the Monte Carlo Reference-based Consensus Clustering (M3C) library (Bioconductor, version 3.15) (34).

#### DEA

DEAs were performed in Perseus using Student’s two-sample *t* test comparisons (unadjusted *p* value ≤0.05). For comparisons within AP samples, including untransduced AP *versus* transduced AP and transduced N2A AP *versus* transduced BV2 AP, and transduced BV2 + LPS AP *versus* transduced BV2 AP, TurboID-NES-normalized intensity values were used. For within WC comparisons, including transduced BV2 + LPS WC *versus* transduced BV2 WCs, LFQ intensity values were used. Differentially expressed proteins (DEPs) were visualized as volcano plots with Prism (GraphPad, version 9.3.1 for Windows, https://www.graphpad.com). The DEA of cluster-level significant changes in response to LPS was performed using the average intensity values of all proteins within a specific cluster for each biological replicate within a group. Then, the average cluster intensity across replicates within an experimental group was taken. The average cluster-level responses to LPS were compared between WC and transduced AP groups with Log2FC DEA using an unadjusted *p* value ≤0.05, visualized as *asterisks*.

#### Clustering

To identify discrete groups of differentially enriched proteins across samples or associated with LPS treatment, we performed K-means clustering. The elbow method was used to determine the optimal number of clusters for K-means clustering by using Integrated Differential Expression and Pathway analysis (iDEP, version 0.95 [http://bioinformatics.sdstate.edu/idep]). Clustering analyses were visualized as heatmaps generated using Morpheus (Broad Institute, Morpheus, https://software.broadinstitute.org/morpheus).

#### Gene Set Enrichment Analysis

Gene set enrichment analysis (GSEA) of DEPs was performed using ORA with the software AltAnalyze (version 2.0). Fisher exact significance threshold of *p* value ≤0.05 (Z-score greater than 1.96) was used to identify significant gene ontologies (GOs). Functional annotation of proteins biotinylated by TurboID-NES that significantly differ by cell type took place by using enriched N2A and BV2 proteins (404 and 936 proteins, respectively) as input lists and the list of proteins identified in the AP dataset as the background (2277 proteins). Over-represented terms and their corresponding Z-scores were visualized as bar graphs using Prism. SynGO was used to identify unique synaptic terms in N2A-enriched proteins (35). To functionally annotate K-means clusters, lists of gene symbols associated with each K-means cluster were input into AltAnalyze for ORA. The resulting Z-scores underwent gene GO and Kyoto Encyclopedia of Genes and Genomes (KEGG) hierarchical cosine–cosine clustering, and each cluster was functionally annotated and the associated Z-scores were represented in heatmaps. Mapping gene-associated risk loci with Alzheimer’s disease (AD), Parkinson’s disease (PD), and amyotrophic lateral sclerosis (ALS) relevant terms, MAGMA dataset sheets were directly obtained from a previous review publication from our laboratory (36).

### Mitochondrial Oxygen Consumption in Living BV2 Cells

To determine if TurboID-NES expression, LPS treatment, or biotin supplementation impacts mitochondrial function in BV2 cells, we directly measured oxygen consumption rates (OCRs) and extracellular acidification rate (ECAR) parameters using a mitochondrial stress test in a Seahorse XFe96 extracellular flux analyzer (Agilent). In a 96-well cell microplate, 5000 transduced or untransduced BV2 cells were seeded in 80 μl of complimented growth media and incubated at RT in sterile cell culture hood for 1 h. After cells adhere to the bottom of the well, 120 μl of complimented growth media was added and cells incubated overnight in the cell culture incubator at 37 °C and 5% CO_2_. After 24 h of incubation, cells underwent a complete media change and were exposed to 1000 ng/ml LPS or PBS as a vehicle control for 48 h. The sensor cartridge was hydrated overnight at 37 °C and 0% CO_2_ using 200 μl of sterile deionized water added to each well of the utility plate. After incubating overnight without CO_2_, water was removed from the utility plate and 200 μl of calibrant prewarmed to 37 °C was added to each well. The sensor plate incubated in calibrant at 37° in a CO_2_-free incubator for 1 h prior to loading the cartridge. Prior to assay, cells were washed thrice with 180 μl of Seahorse Media (phenol-free 5 mM Hepes Seahorse XF media, 10 mM glucose, 2 mM l-glutamine, and 1 mM sodium pyruvate, pH 7.4). Cells were incubated in a CO_2_-free incubator for 1 h at 37 °C. Sensor cartridges were loaded with 20 μl of oligomycin (1.5 μM/well), 22 μl of carbonyl cyanide-4 (trifluoromethoxy) phenylhydrazone (0.75 μM/well), and 25 μl of rotenone and antimycin (0.5 μM/well) and calibrated. After calibration, the seahorse-assay plate containing BV2 cells was run using the mitochondrial stress test in Wave (version 2.6). Cells were then stained with Hoechst 33342 dye for 20 min and imaged using ImageXpress Micro Confocal imaging, and cells were counted using the Find Blobs feature of MetaExpress Software’s Count Nuclei Application. OCRs and ECARs were normalized to cell counts.

### Cytokine Profiling of Supernatants

Cytokine profiling was performed as previously published with modifications (22). Luminex multiplexed immunoassays (catalog no.: MCYTMAG-70K-PX32) quantified cytokines from cultured supernatants of transduced and untransduced BV2 and N2A cells receiving LPS or PBS SHAM stimulus (n = 6/group). The cytokine panel detected eotaxin, GM-CSF, INF-γ, IL-1a, IL-1b, IL-2, IL-4, IL-3, IL-5, IL-6, IL-7, IL-9, IL-10, IL-12p40, IL12p70, LIF, IL-13, LIX, IL-15, IL-17, IP-10, KC, MCP-1, MIP-1a, MIP-1b, M-CSF, MIP-2, MIG, RANTES, VEGF, and TNF-α. The average background intensity reading from each cytokine panel was subtracted from the raw cytokine abundance values, and negative values were imputed to zero. To find appropriate loading volume for samples, linear ranging was performed as previously published (37) and 24% of total sample volume was loaded. Assays were read on a MAGPIX instrument (Luminex).

### Experimental Design and Statistical Rationale

The sample conditions were prepared as follows: cell type (microglia or neuroblastoma), inflammatory challenge (LPS or PBS), TurboID-NES expression status (transduced or untransduced), and biotin-enrichment status (AP or WC). For each individual sample condition, there were four biological replicates, except for the WC BV2 + LPS condition, in which there were three biological replicates. Overall, a total of 63 samples analyzed and described in the results. The maximum number of biological replicates was chosen within budgeted allowance; an *a priori* power analysis was not performed. Sample acquisition order was randomized by the random number generator function in Excel, maintaining the following conditions: (1) WC samples were randomized and acquired before AP samples and (2) randomized untransduced AP samples ran before randomized transduced AP samples, to prevent contamination of biotin-labeled proteins by TurboID in untransduced AP samples. Statistical rationale for each analysis is described in “Data normalization and filtering, principal component analyses, differential expression analyses, clustering, gene set enrichment analysis” section.

## Results

### Generation and Validation of Stably Transduced Microglial and Neuronal TurboID-NES Cell Lines

**Fig. 1:**
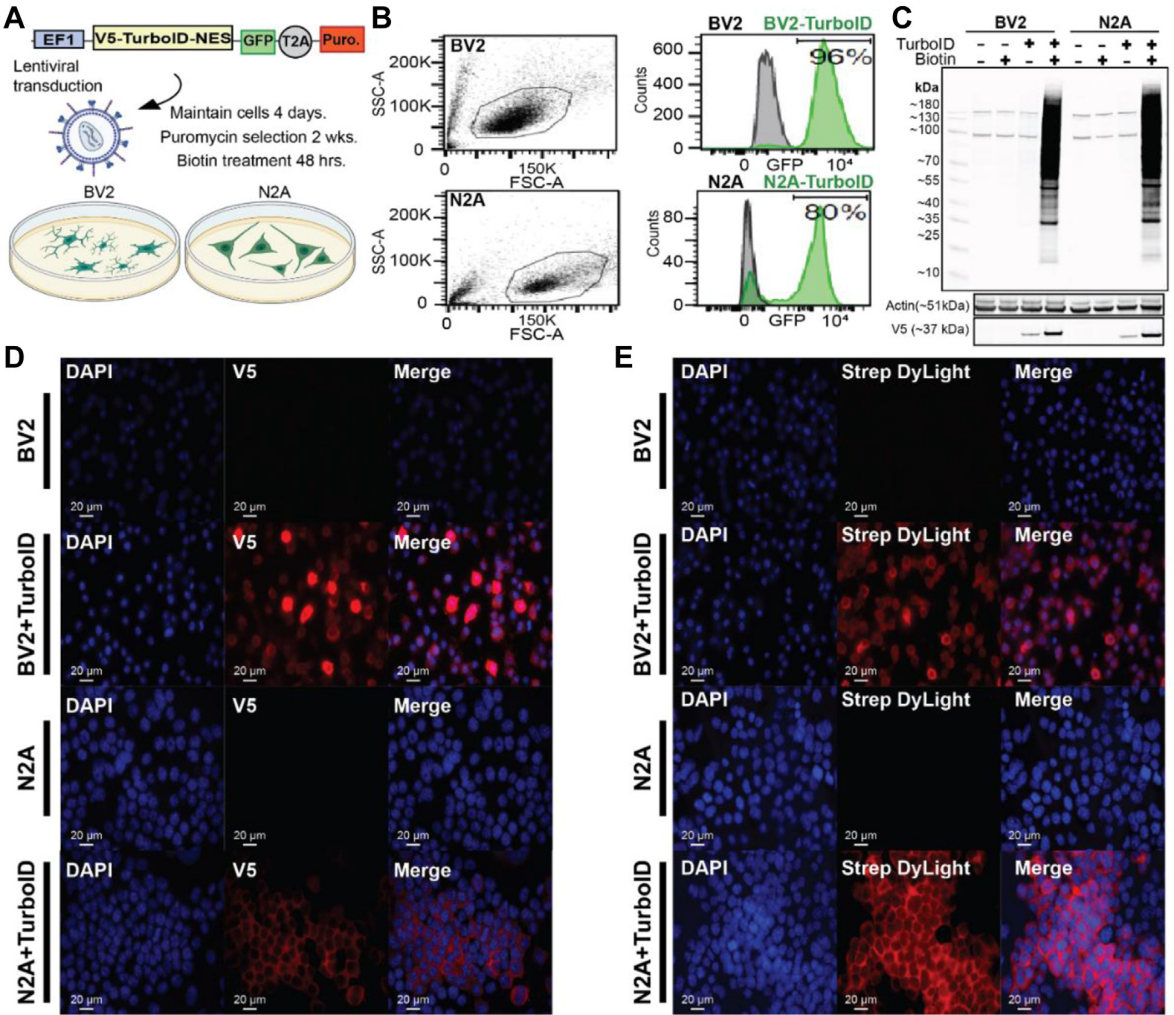
**Creation of stably transduced BV2 and N2A cells.***A*, schematic of transduction. The genetic construct packaged into a lentivirus contains V5-tagged TurboID-NES driven under an EF1 promoter and a GFP sequence separated by a T2A linker. N2A and BV2 cells were transduced and maintained for 4 days prior to 2 weeks of puromycin selection and biotin supplementation in media. *B*, following puromycin selection, flow cytometry confirms GFP positivity in a majority of BV2 (96%) and N2A (80%) cells. *C*, Western blot (WB) of transduced and untransduced cell lysates confirming the presence of TurboID (V5) and biotin-dependent robust biotinylation of proteins. Actin was used as a loading control. *D*, immunofluorescence (IF) confirming cytoplasmic localization of V5-TurboID-NES in transduced cells. *E*, IF confirming cytosolic biotinylation of proteins in transduced BV2 and N2A cells. NES, nuclear export sequence.

We created a lentiviral vector incorporating the V5-TurboID-NES sequence (Addgene plasmid #107169) including a GFP separated by a T2A linker and a puromycin resistance gene (Fig. 1*A*). The NES was incorporated to limit biotinylation to the extranuclear compartment. We then generated lentiviruses carrying this vector and transduced BV2 mouse microglia and N2A mouse neuroblastoma cells (MOI 5:1) followed by positive selection with puromycin for at least two passages and then fluorescent-activated cell sorting of GFP-positive cells. These sorted cells were then maintained in puromycin for passages to maintain stably transduced BV2-Turbo and N2A-Turbo lines (Fig. 1*B*) but cultured in puromycin-free medium prior to experimentation. After genetically screening successfully transduced cell lines with puromycin, we used WB and IF to confirm robust biotinylation of proteins in cell lysates (Fig. 1*C*) and confirm cytosolic localization of V5-TurboID-NES and biotinylated proteins (Fig. 1, *D* and *E*). WB probing for biotinylated proteins consistently identified bands in untransduced controls, which likely represent endogenously biotinylated carboxylases such as pyruvate carboxylase (∼130 kDa), 3-methylcrotonyl coA carboxylase (∼75 kDA), and propionyl coA carboxylase (72 kDa) (Fig. 1*C*) (38). The IF data in Figure 1, *D* and *E* confirmed the functionality of the NES, and the predominant biotinylation of cytosolic proteins in both BV2-TurboID-NES and N2A-TurboID-NES cells.

### TurboID-NES-Based MS Captures Representative Proteomes in Mammalian Microglial and Neuronal Cell Lines

**Fig. 2:**
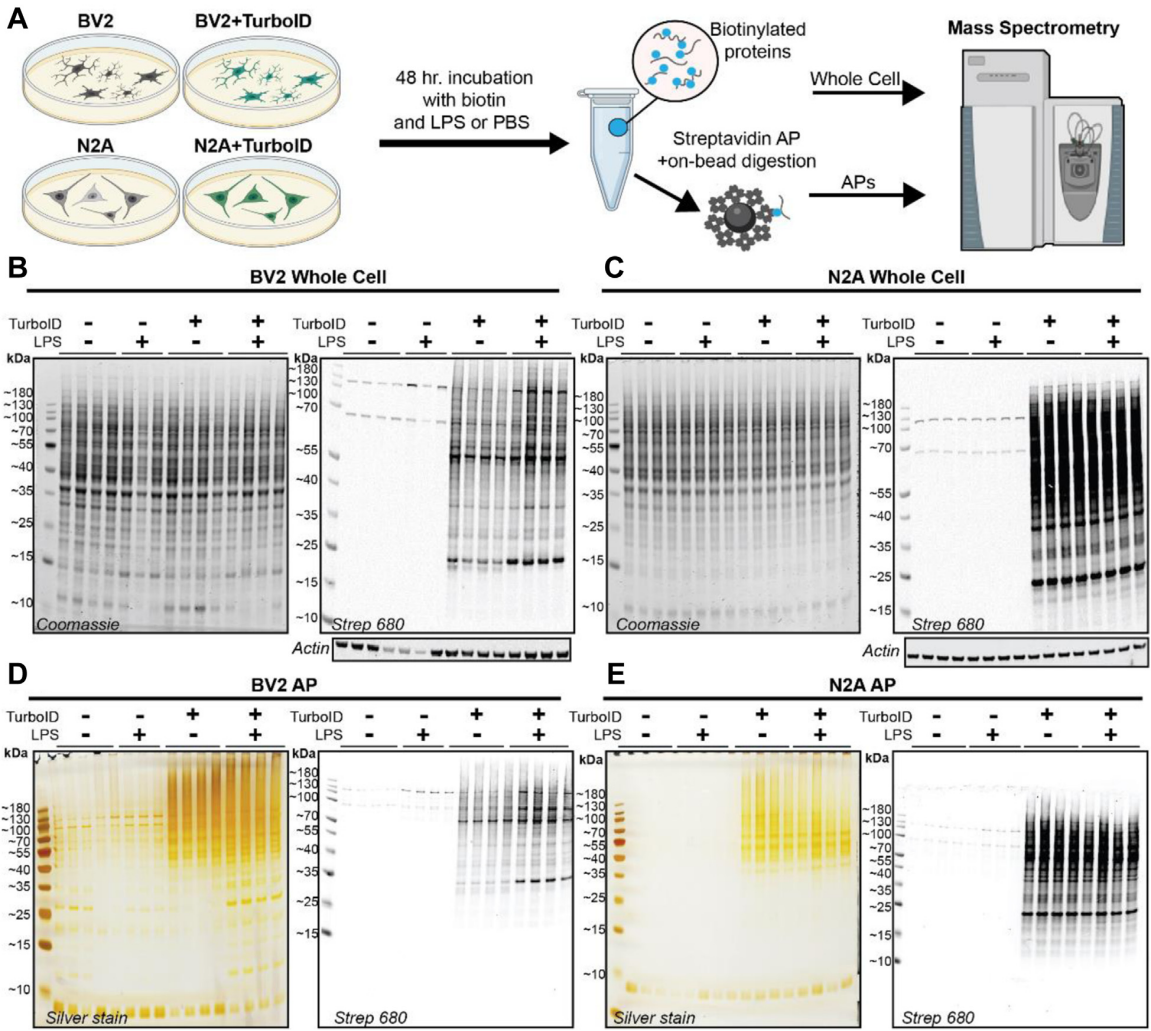
**Experimental design and quality control of whole-cell (WC) and affinity purification (AP) samples prior to mass spectrometry (MS).***A*, schematic of experimental design. Transduced and untransduced BV2 and N2A cells were treated with biotin and lipopolysaccharide (LPS) or PBS for 48 h. WC lysates and streptavidin-affinity purified (AP) samples were processed in parallel with MS. *B*, Western blot (WB) and Coomassie confirming biotinylation of proteins in transduced BV2 WC lysates. *C*, WB and Coomassie confirming biotinylation of proteins in transduced N2A WC lysates. *D*, WB and silver stain confirming biotinylation of proteins bound to streptavidin beads and specificity of streptavidin beads for biotinylated species in BV2 AP preparations. LPS impacts banding patterns (10–40 kDa) in transduced BV2 biotinylated proteins visualized with both silver stain and WB. *E*, WB and silver stain confirming biotinylation of proteins bound to streptavidin beads and specificity of streptavidin beads for biotinylated species in N2A AP preparations.

After confirming stable expression, cytosolic localization, and functionality of V5-TurboID-NES in BV2 and N2A cell lines, we prepared cell lysates for LFQ–MS studies. Transduced and untransduced BV2 and N2A cells received 1 μg/ml of LPS or equal volume of PBS in addition to 200 μM biotin supplementation for 48 h. LPS was used as a general immune stimulus to activate BV2 cells while anticipating marginal or no effects of LPS on N2A. WC lysates were prepared in parallel with lysates undergoing streptavidin AP to enrich for biotinylated proteins (n = 4/experimental condition) (Fig. 2*A*). WC lysates underwent Coomassie staining and were probed with Streptavidin-680 to confirm robust biotinylation of proteins in transduced cell lines (Fig. 2, *B* and *C*). Proteins bound to streptavidin beads were boiled and resolved with gel electrophoresis (Fig. 2, *D* and *E*). Proteins released from streptavidin beads were probed for biotinylation using streptavidin 680, and silver-stained gels were run to confirm minimal binding of nonbiotinylated proteins in untransduced lysates. In BV2 AP lysates, LPS treatment induced differential banding patterns visible in smaller molecular weight proteins (10–40 kDa) (Fig. 2*D*). The silver stain and WBs provided evidence for not only the capacity of TurboID-NES to biotinylate proteins altered by LPS treatment but also the ability to affinity-purify LPS-altered proteins biotinylated by TurboID-NES. Notably, LPS did not alter protein banding patterns in either the silver stain or the WBs of N2A samples. After confirming the enrichment of biotinylated proteins in TurboID-NES-transduced BV2 and N2A cell lines, we submitted these same samples for LFQ–MS. Raw LFQ and intensity values are found in supplemental Datasheet S1*A*.

First, we used PCA for dimension reduction of LFQ proteomic data obtained from all WC samples and transduced AP samples (supplemental Fig. S1, *A* and *B*). LFQ–MS data used for WC-level comparisons are available in supplemental Data S2*A*. and TurboID-normalized intensity values used for AP-level comparisons are available (supplemental Data S2*B*). WC samples included BV2 (n = 4), BV2 + LPS (n = 3), BV2 + TurboID-NES (n = 4), BV2 + TurboID-NES + LPS (n = 4) N2A (n = 4), N2A + LPS (n = 4), N2A + TurboID-NES (n = 4), and N2A + LPS + TurboID-NES (n = 4) (supplemental Fig. S1*A*). Because of the high number of missingness in the nontransduced + AP samples that only captures endogenously biotinylated proteins, we performed PCA on +TurboID-NES or + LPS + TurboID-NES BV2 and N2A AP samples (n = 4/group) (supplemental Fig. S1*B*). In the PCA of WC samples, PC1 described ∼32% of variance across WC proteomes and captured cell type differences between BV2 and N2A cells without contribution from TurboID-NES status or LPS treatment (supplemental Fig. S1*A*). The full WC principal component matrix can be found in supplemental Data S1*B*. In PCA of AP samples, PC1 accounted for 48% of the variance and also captured BV2 N2A cell type differences. PC2 accounted for 10% variance and captured the effect of LPS only in BV2 cells and not in N2A cells (supplemental Fig. S1*B*). The AP principal component matrix can be found in supplemental Data S1*C*. These PCA analyses of WC as well as AP proteomes confirmed that the cell type differences between BV2 and N2A proteomes, rather than TurboID-NES expression or proteomic biotinylation, explained the majority of variance in our data. Importantly, biotin-enriched proteomes from AP samples successfully resolved cell type differences as well as LPS-treatment effects within the BV2 cells.

**Fig. 3:**
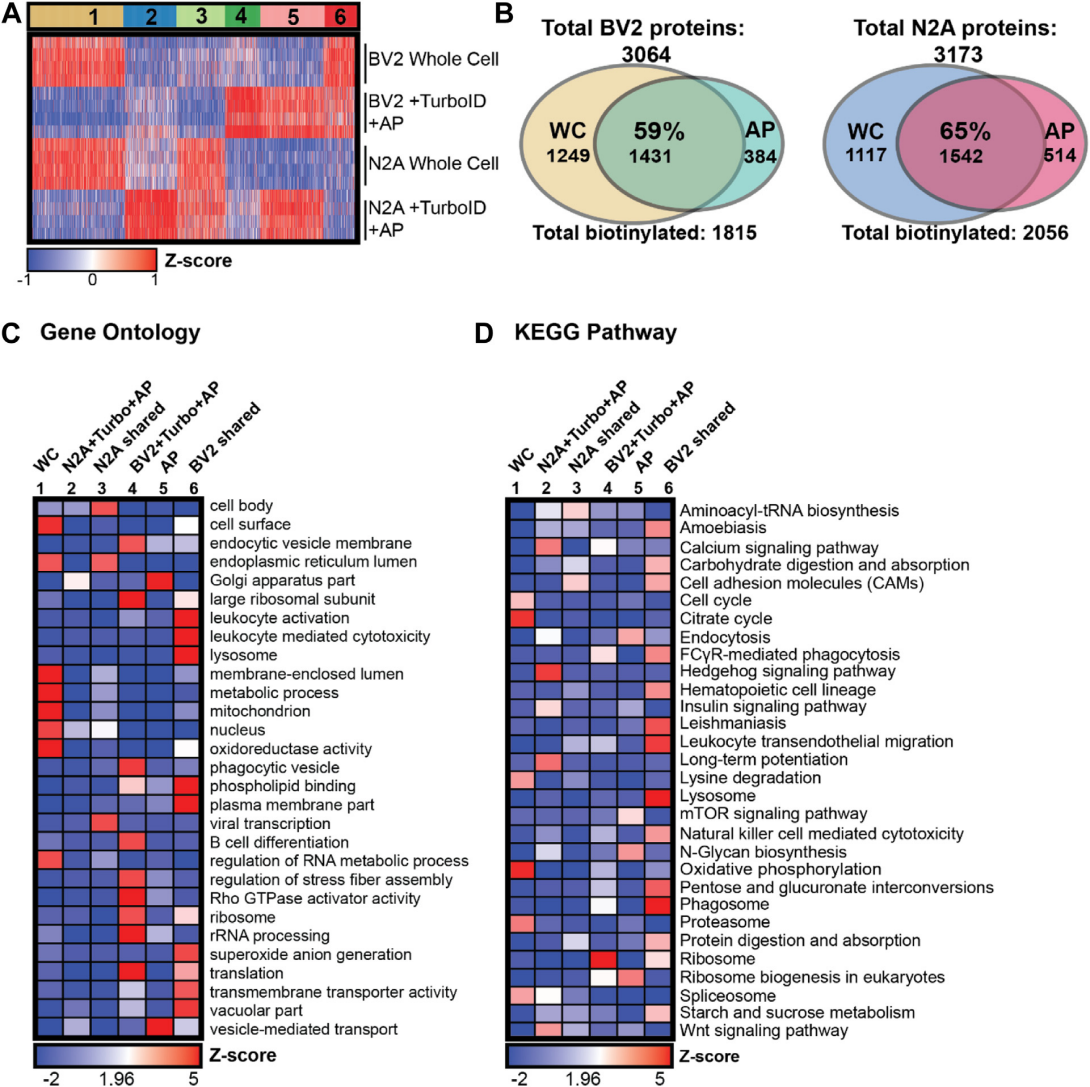
**Global profiling of TurboID labeling in microglial and neuronal cell lines**. *A,* K-means clustered heatmap representation of label-free quantitation (LFQ) intensity data of proteins identified by mass spectrometry (MS) in whole cell (WC) and biotinylated and affinity-purified (AP) proteomes (n = 4 per experimental group) in microglial and neuronal cell lines. Six distinct clusters represent labeling profile of TurboID, including proteomes enriched in WC preparations, AP preparations, and by cell type. *B*, Venn diagram of protein counts identified in BV2 WC samples and BV2 + TurboID + AP samples and N2A WC and N2A + TurboID + AP samples. In BV2 cells, TurboID-NES labels ∼59% of the total proteins captured by LFQ MS. In N2A cells, TurboID-NES captures ∼65% of the total proteins identified by LFQ MS. *C*, functional annotation of gene set enrichment based on Gene Ontology (GO) over-representation analysis (ORA). Heatmap color intensity is based on Z-score enrichment across the six clusters. *D*, heatmap representation and functional annotation of clusters derived from the KEGG pathways. KEGG, Kyoto Encyclopedia of Genes and Genomes; NES, nuclear export sequence.

To assess the global proteomic differences and similarities between untransduced WC proteomes (BV2 or N2A WC, n =4/group) and their TurboID-NES-transduced and biotin-enriched counterparts (BV2 + TurboID-NES_AP_ or N2A + TurboID-NES_AP_, n = 4/group), we performed K-means clustering analysis (Fig. 3*A*). We identified six distinct clusters using the elbow method for cluster number optimization (supplemental Fig. S2). We identified clusters of proteins preferentially abundant in WC proteomes (cluster 1) and clusters of proteins shared in abundance between TurboID-NES AP samples and their WC counterparts (clusters 3 and 6). Interestingly, we also identified clusters of proteins distinct to TurboID-NES AP samples, which TurboID-NES-mediated biotinylation showed preferential abundance for (clusters 2 and 4). Finally, we identified a cluster of proteins that are shared in enrichment between TurboID-NES AP cells (cluster 5). LFQ–MS identified 3064 proteins in BV2 proteomes, of which TurboID-NES biotinylated 1815 or ∼59% LFQ MS identified 3173 proteins in N2A proteomes, of which TurboID-NES biotinylated a total of 2056 proteins or ∼65% (Fig. 3*B*).

We performed GSEA using each of the six cluster sets compared with the background proteome of all proteins identified in the LFQ–MS dataset (∼2187 proteins; supplemental Data S2*A*), using KEGG and GO reference databases (39, 40, 41, 42, 43). GSEA of the WC cluster 1 (630 proteins) showed enrichment of nuclear, mitochondrial, cell surface, and RNA metabolic proteins (Fig. 3, *C* and *D*). Cluster 1 represented proteins derived from compartments less accessible to biotinylation by TurboID-NES in both cell types. In contrast, cluster 5 (425 proteins) contained proteins selectively abundant in both BV2- and N2A-transduced AP samples. Cluster 5 represented a group of proteins preferentially labeled by TurboID-NES in both cell types. GSEA of cluster 5 showed enrichment of Golgi apparatus, vesicle-mediated transport, and endocytosis-related proteins, indicating that TurboID-NES preferentially biotinylated Golgi, endocytic, and secretory compartments in both cell types. As TurboID-NES trafficked throughout the cytosol, it came in contact with vesicular compartments and labeled proteins involved in vesicular transport. Because vesicle-mediated transport is a biologically conserved function inherent to both microglial and neuronal cells, we expected that the abundance of vesicular-trafficking proteins labeled by TurboID-NES would not differ by cell type.

In contrast to clusters 1 and 5 that primarily differentiated WC from AP samples, we identified cell type–specific clusters (clusters 3 and 6) that were highly abundant in either N2A or BV2 cells, regardless of WC or AP status. Cluster 3 (323 proteins), the “N2A shared” cluster showed a high abundance of proteins shared between WC and transduced AP N2A cells. The “N2A shared” cluster contained proteins enriched in endoplasmic reticulum, cell body, and viral transcription proteins, potentially explained by the rapidly dividing stem cell–like origin of N2A cells. Cluster 6 (226 proteins), the “BV2 shared” cluster, was highly abundant in WC and AP samples from BV2 cells. The “BV2 shared cluster” was enriched in proteins associated with immune cell (leukocyte activation and trafficking), hematopoietic lineage, lysosomal, plasma membrane, translation, vacuole, metabolism, and phagosome functions as would be expected for BV2 microglial cells.

Clusters 2 and 4 showed cell type–specific proteomic differences apparent only in the AP samples but not in the WC lysates. These may be proteins that are expressed at low levels in the WC and are enriched by TurboID-NES labeling and AP. These clusters therefore represented proteomic features of N2A and BV2 cell types that were readily revealed by the TurboID-NES approach and are less apparent at the level of WC proteomes, potentially *via* preferential access of TurboID-NES to specific cellular compartments. Cluster 2 (350 proteins) was highly abundant in N2A AP samples compared with BV2 AP samples and were enriched in proteins involved in long-term potentiation, calcium signaling, Hedgehog signaling, consistent with ontologies expected in neuronal-origin N2A cells. Cluster 4 (233 proteins), on the other hand, was highly abundant in BV2 AP samples and were enriched in endocytosis, ribosomal subunits, phagocytosis, Rho GTPase activity, rRNA processing, and translation terms, consistent with a phenotype expected in immune and phagocytic cells such as BV2 microglia. The GSEA of clusters 2 and 4 shows that TurboID-NES-mediated biotinylation can enrich proteins in N2A and BV2 proteomes, respectively, which are not readily distinguished at the WC level.

Taken together, these proteomic analyses of N2A and BV2 TurboID-NES AP proteomes along with respective WC proteomes showed that TurboID-NES proteome was representative of the WC proteome. In addition, TurboID-NES preferentially biotinylated several classes of proteins shared across mammalian cell types as well as proteins unique to microglial or neuronal cell type origin. While many of these biotinylated proteins captured changes apparent at the WC level, several cell type–specific proteomic differences were more apparent in the TurboID-NES AP proteomes as compared with the WC proteomes.

### TurboID-NES Overexpression has Minimal Impact on Cellular Proteomic and Functional Profiles of BV2 and N2A Cells

**Fig. 4:**
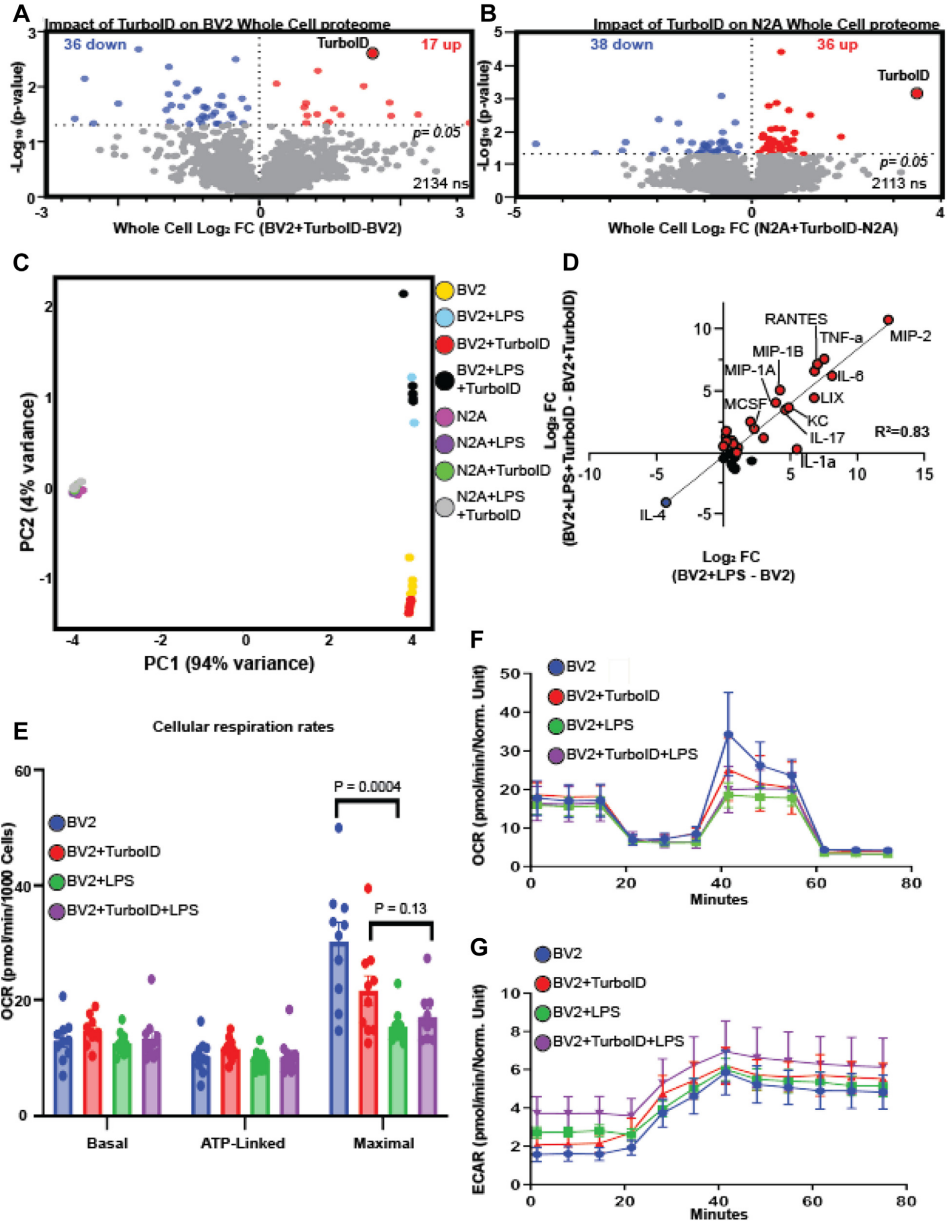
**TurboID does not impact cellular phenotypes.***A*, differential expression analysis (DEA) comparing whole cell (WC) transduced BV2 cell lysates with untransduced BV2 cell lysates identifies 53 differentially expressed proteins (DEPs) in BV2 with TurboID expression. *B*, DEA comparing WC transduced and untransduced N2A identifies 74 DEPs. *C*, principal component analysis (PCA) of cytokine profiles derived from cultured supernatants indicates PC1 captures 94% of the variance across samples, accounting for differences in cell type. PC2 captures 4% of the variance, accounting for lipopolysaccharide (LPS) impact on BV2 cytokines. *D*, linear regression of LPS cytokine fold-change induction of untransduced (*x*-axis) and transduced (*y*-axis) BV2 cells demonstrates high correlation (*R*^2^ = 0.83). *E*, bar graph depicting basal, ATP-linked, and maximal cellular respiration rates of transduced and untransduced BV2 cells. LPS significantly decreases the maximal respiration of untransduced BV2 cells (*p* = 0.0004) and decreases maximal respiration in transduced BV2 cells, though this finding is statistically insignificant (*p* = 0.13). *F*, oxygen consumption rate (OCR) traces for transduced and LPS exposed BV2 cells highlight LPS response in maximal respiration. *G*, extracellular acidification rate (ECAR) highlights the basal glycolytic rate as higher when cells are exposed to LPS but are not impacted by transduction status.

Lentiviral transduction and overexpression of TurboID-NES in mammalian cells could potentially impact basic cellular functions and phenotypes and therefore could have confounding implications for *in vivo* applications of TurboID-NES. To test whether TurboID-NES expression impacts cellular phenotypes under both homeostatic and inflammatory conditions, we compared WC transduced and untransduced proteomes, collected culture supernatants for cytokine profiling in response to LPS challenge, and we assessed the respiratory activity of living BV2 cells. To test the hypothesis that TurboID-NES expression itself impacts the WC proteome, we performed DEA comparing BV2 + TurboID-NES_WC_ – BV2_WC_ (Fig. 4*A*) and N2A + TurboID-NES_WC_ – N2A_WC_ (Fig. 4*B*). Only 53 BV2 proteins and 74 N2A proteins, including TurboID-NES, were significantly changed with TurboID-NES expression of 2187 total proteins. The DEA comparing transduced and untransduced BV2 or N2A proteomes can be found in supplemental Data S2, *C* and *D*. This proteomic result provided evidence that TurboID-NES expression minimally impacted WC proteomes of BV2 and N2A cells under resting conditions. The small sizes of the WC TurboID-DEP input lists did not yield GO terms in the ORA. However, the top five increased terms from the DEA comparing BV2 + TurboID-NES_WC_ – BV2_WC_ included histone H1.0; H1f0, phosphoserine phosphatase Psph, and kinases; Adrbk1, and Prkab1, and TurboID itself. The five most decreased proteins with TurboID transduction in BV2 include vesicle membrane protein, Vat1l, Rho-related GTP binding protein, Rhoc, mitochondrial tRNA ligase Tars2, U3 small nucleolar RNA-associated protein, Utp14a, and cell adhesion molecule 1, Cadm1. When assessing the impact of TurboID expression on N2A WC proteomes, N2A + TurboID-NES_WC_ – N2A_WC_, the DEA revealed the top five increased proteins included TurboID, dehydrogenase/reductase family member 7, Dhrs7, Niban, Fam129a, Nuclear valosin, Nvl, and disabled homolog 2, Dab2. The five most decreased proteins with TurboID transduction in N2A included putative methyltransferase Nsun7, COBW domain containing protein 1, Cbwd1, putative helicase MOV-10, Mov10, and AHNAK nucleoprotein 2, Ahnak2. Taken together, TurboID transduction in BV2 and N2A cells impacts the abundance of 2% and 3% of the proteins identified in the WC proteome, respectively. Although a minority of proteins are significantly impacted by TurboID, we did observe modest yet significant alterations in nuclear-associated proteins in both cell types, including histone H1.0 (−Log_10_ *p* value = 1.34, Log2FC = 2.98) in BV2 and nuclear valosin-containing protein, Nvl (−Log_10_ *p* value = 1.33, Log2FC = 1.10) in N2A.

In applying the CIBOP approach to immune cells and inflammatory disease models, it is important to confirm that TurboID expression has a minimal impact on both homeostatic and inflammatory cytokine release profiles. Then, we collected supernatants from TurboID-NES transduced and untransduced BV2 and N2A cells in response to 48 h of LPS challenge or PBS (n = 6/group) for profiling of 31 cytokines using a Luminex multiplexed immune assay. The raw intensity values, proceeding the subtraction of average background intensity, are found in supplemental Data S1*D*. PCA of secreted cytokines across all samples showed that the cellular identity of cytokines drives a majority of variance (captured by PC1; 94% variance) and that BV2-secreted cytokines robustly responded to LPS, whereas N2A cells did respond to LPS based on secreted cytokine profiles (captured by PC2; 4% variance). The complete principal component matrix can be found in supplemental Data S1*E*. Importantly, we also observed no separation of BV2 cells or N2A cells based on TurboID-NES status (Fig. 4*C*). To test if TurboID-NES expression impacts the magnitude of cytokine abundance in the presence of LPS, we compared the fold induction of LPS-driven cytokine abundance changes between transduced and untransduced BV2 cytokines (Fig. 4*D*). We observed strong concordance in LPS effects regardless of TurboID-NES status (*R*^2^ = 0.83). The secretion of proinflammatory cytokines (*e.g.*, macrophage inflammatory protein 2, interleukin 6, and tumor necrosis factor alpha) was increased to comparable extents in response to LPS while anti-inflammatory cytokine (IL-4) was suppressed by LPS to the same extent in BV2 control and BV2 + TurboID-NES cell lines (Fig. 4*D*). This result confirmed that TurboID-NES expression did not significantly influence the cytokine release profiles of BV2 cells, which received LPS.

To determine if either LPS or TurboID-NES expression impacted cellular respiration rates in transduced or untransduced BV2 cells, we used Seahorse assays of cellular respiration, OCR, and ECAR as a measure of glycolytic activity (Fig. 4, *E*–*G*). LPS significantly decreased the maximal respiration of untransduced BV2 cells (*p* = 0.0004), and we also observed a decrease in the maximal respiration in transduced BV2 cells, though the change was not significant (*p* = 0.13) (Fig. 4*E*). Neither LPS challenge nor transduction status significantly impacted OCR or ECAR in BV2 cells. Taken together, TurboID-NES expression in BV2 and N2A cells had a minimal impact on WC proteomes and does not impact LPS-driven cytokine release. TurboID-NES expression had significant impact neither on homeostatic cellular respiration nor on glycolytic activity. These *in vitro* findings are of critical importance in interpreting results derived from TurboID-NES-based proteomics by confirming the absence of undesired effects of TurboID-NES overexpression in mammalian cells.

### TurboID-NES Biotinylates a Variety of Subcellular Compartments Within BV2 and N2A Cells Including Several Neurodegenerative Disease–Relevant Proteins

**Fig. 5:**
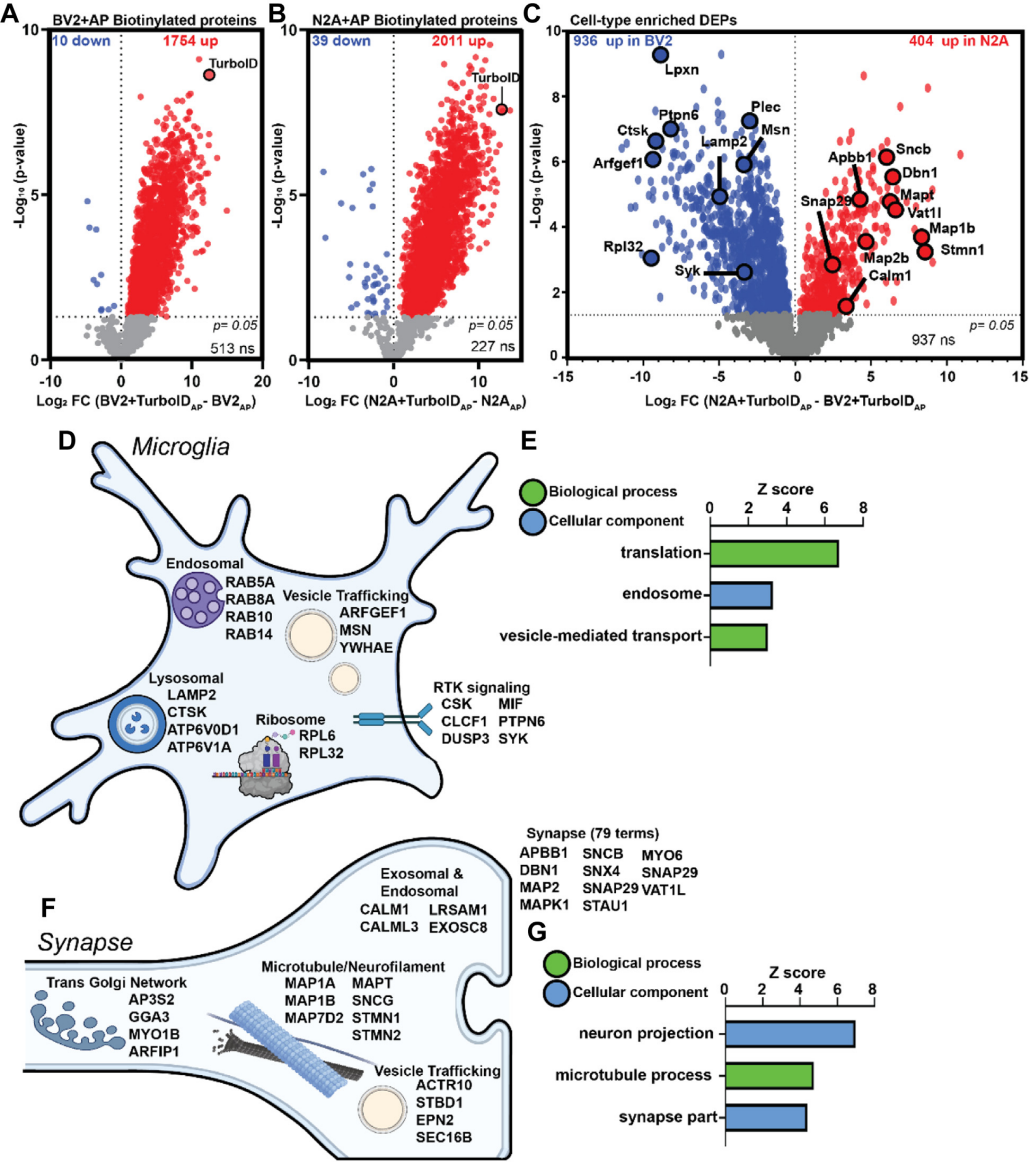
**TurboID labeling and streptavidin affinity-purified (AP) captures cellularly distinct proteomes**. *A*, differential expression analysis (DEA) of BV2 AP samples showing robust TurboID biotinylation of over >1700 proteins over endogenously biotinylated proteins derived from untransduced cell lines. *B*, DEA depicting biotin enrichment of N2A transduced AP proteome reveals >2000 proteins abelled by TurboID. *C*, DEA comparing transduced AP proteomes of BV2 (*left*) and N2A (*right*) TurboID-biotinylated proteomes. There are 936 proteins labeled by TurboID enriched in BV2 and 404 proteins enriched in N2A biotin-labeled proteomes. Proteins with disease relevance to neurodegenerative disease are highlighted. *D*, schematic of the variety of subcellular compartments abelled by TurboID in microglia (BV2). *E*, Gene Ontology (GO) of highly enriched cellular components and biological processes within the biotin-labeled BV2 proteome. TurboID biotinylates translational machinery, endosomal machinery, and vesicle-bound membranes in BV2 cells. *F*, schematic of diversity of subcellular compartments abelled by TurboID in synaptic compartment (N2A). *G*, GO of significantly enriched cellular components and biological processes within the biotin-labeled N2A proteome confirms that TurboID biotinylates neuronal processes including synaptic machinery and neuron projection.

The utility of expressing TurboID under cell type–specific promoters *in vivo* and the consequential purification of cellularly distinct proteins from total brain homogenate lies within the ability of TurboID-NES to label cellularly distinct proteins with disease relevance. Before comparing TurboID-NES-labeled proteomes of BV2 and N2A cells, we first assessed the enrichment of proteins biotinylated by TurboID-NES over endogenously biotinylated proteins. We used DEA to compare AP proteomes from untransduced BV2 lysates with TurboID-NES-transduced BV2 lysates (BV2 + TurboID-NES_AP_ – BV2_AP_), which showed that TurboID-NES biotinylated 1754 proteins, whereas 10 endogenously biotinylated proteins appear in the untransduced AP proteome (Fig. 5*A* and supplemental Data S2*E*). DEA comparing AP proteomes from untransduced N2A lysates with TurboID-NES-transduced N2A lysates (N2A + TurboID-NES_AP_ – N2A_AP_) showed that TurboID-NES biotinylated 2011 proteins, whereas 39 endogenously biotinylated proteins appeared in the untransduced AP proteome (Fig. 5*B* and supplemental Data S2*F*). TurboID-NES expression in BV2 and N2A cell lines and streptavidin-based AP yielded a robustly biotinylated proteome sufficient to overcome the background of endogenously-biotinylated proteins in untransduced AP samples.

To inform the application of CIBOP to future mouse models of disease, we tested the hypothesis that global cytosolic biotinylation of TurboID-NES can achieve a proteomic breadth sufficient for conclusive cellular distinction between glia and neurons. We performed DEA on AP proteomes from BV2 and N2A cell lines stably expressing TurboID-NES (N2A + TurboID-NES_AP_ – BV2 + TurboID-NES_AP_). We identified 936 proteins enriched in BV2 + TurboID-NES AP samples and 404 proteins enriched in N2A + TurboID-NES AP (Fig. 5*C* and supplemental Data S2*G*). Notably, TurboID-NES biotinylated proteins with relevance to AD in both microglial and neuronal cells. For example, moesin (Msn), protein tyrosine phosphatase nonreceptor 6 (Ptpn6), C-terminal Src Kinase (Csk), and plectin (Plec) were highly enriched in microglial proteomes labeled by TurboID-NES. Moesin has been identified as a hub protein for AD etiology in both human and 5xFAD mouse models (26, 28, 44, 45) and is necessary for P2X7R-dependent proteolytic processing of amyloid precursor protein (46). Both Csk and Ptpn6 have been identified as AD hub genes (47). Ptpn6 is associated *via* signaling pathways with CD33, a risk locus identified in human AD genome-wide association studies (47, 48, 49, 50). In N2A cells, TurboID-NES biotinylated AD-relevant proteins including microtubule-associated protein tau (Mapt), microtubule-associated protein 1A/B (Map1a; Map1b), amyloid beta precursor protein binding family B (Apbb1), and calmodulin 1 (Calm1).

Without being directed to a specific subcellular compartment, TurboID-NES biotinylated a variety of proteins in microglial and neuronal cell lines. In BV2 cells, TurboID-NES biotinylated lysosomal, ribosomal, endosomal, and vesicular proteins. In addition, TurboID-NES biotinylates proteins important to receptor tyrosine kinase signaling (Fig. 5*D*). GSEA of microglial-enriched proteins biotinylated by TurboID-NES highlighted significant enrichment for translational machinery, endosomal proteins, as well as intracellular trafficking of vesicles (Fig. 5*E*). In N2A cells, TurboID-NES labeled proteins involved with trans-Golgi network trafficking, microtubule and neurofilament elements, endosomal and exosomal machinery, vesicular trafficking, and over 75 synaptic terms out of 404 terms identified in the enriched N2A biotinylated proteome (Fig. 5*F*). GSEA of the N2A biotinylated proteome identified top terms associated with neuron projection, microtubule processes, and synaptic parts, supporting the ability of TurboID-NES to label synaptic proteins, which could contribute to cell-type distinction (Fig. 5*G*). Using the proteome of cellularly distinct proteins labeled by TurboID-NES identified in the DEA in Figure 4*A*, we mapped proteins to risk loci associated with neurodegenerative diseases (supplemental Fig. S3). Using risk loci published in the AD MAGMA (6), PD MAGMA (51), and ALS/frontotemporal dementia MAGMA (52) datasets, we identified 82 and 46 AD-relevant and cellularly distinct proteins labeled by TurboID-NES in BV2 and N2A proteomes, respectively (supplemental Fig. S3*A*). We identified 32 and 17 PD-relevant and 84 and 29 ALS/frontotemporal dementia–relevant proteins in BV2 and N2A proteomes labeled by TurboID-NES and enriched by streptavidin-based AP (supplemental Fig. S3, *B* and *C*). Taken together, TurboID-NES, when directed into the cytosol for global biotinylation of proteins, biotinylated a breadth of proteins sufficient to distinguish between microglial and neuroblastoma cell lines. In addition, TurboID-NES biotinylated proteins critical to neurodegenerative etiologies. These proof-of-principle analyses support the future direction of TurboID-NES into distinct brain cell types within living mouse models of neurodegeneration.

### BV2 Proteomes Biotinylated by TurboID-NES Capture LPS-Driven Changes, Partially Reflected in the WC BV2 Proteomes

**Fig. 6:**
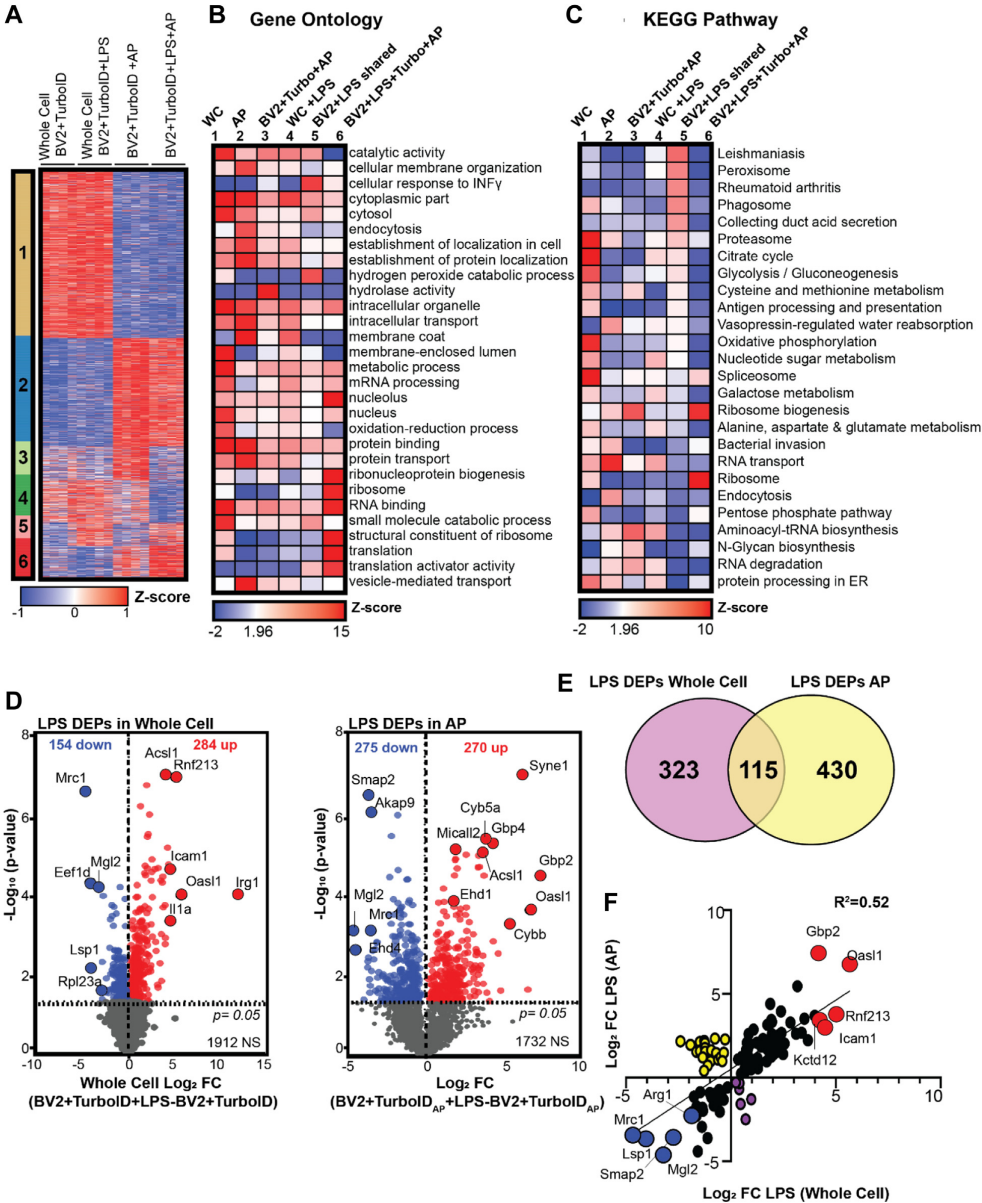
**TurboID-mediated biotinylation partially captures lipopolysaccharide (LPS)-driven proteomic changes**. *A*, K-means clustered heatmap representation of label-free quantitation (LFQ) intensity data of proteins differentially expressed by TurboID in whole-cell (WC) and transduced affinity-purified (AP) BV2 proteomes (n = 4 per experimental group). *B*, heatmap representation and functional annotation of Gene Ontology (GO) from *murine* GO database. Heatmap color intensity depicts Z-score values. *C*, heatmap representation of Z-scores associated with LPS differently expressed proteins (DEPs) derived from Kyoto Encyclopedia of Genes and Genomes (KEGG) pathways database. *D*, differential expression analysis (DEA) of LPS DEPs in WC (*left*) and affinity purification (AP) (*right*) identifies 438 and 545 LPS DEPs, respectively. *E*, Venn diagram depicting 323 LPS DEPs unique to WC BV2 samples, 115 shared LPS DEPs, and 430 unique LPS DEPs in BV2 AP samples. *F*, linear regression of Log2FC values of BV2 AP samples (*y*-axis) and BV2 WC samples (*x*-axis) reveals a modest correlation (*R*^2^ = 0.52) of log2FC of the shared 115 LPS DEPs. *Red points* depict proteins the top five proteins significantly upregulated with LPS treatment in AP and WC samples. *Yellow points* represent proteins significantly increased in AP samples and significantly decreased in WC samples with LPS treatment. *Blue points* illustrate the top five proteins significantly decreased with LPS in both AP and WC BV2 samples. *Purple points* depict proteins that are significantly increased in the WC proteome and significantly decreased in the AP proteome with LPS treatment.

After confirming that TurboID-NES robustly labeled distinct cellular proteomes with minimal impact on homeostatic phenotype, we hypothesized that TurboID-NES could label proteins impacted by LPS treatment. Our dimension reduction analyses confirmed our ability to resolve proteomic differences in transduced AP cells based on cell type and LPS challenge (supplemental Fig. S1*B*). To understand global differences between WC and AP proteomes differentially expressed by LPS treatment, we created a heatmap representation of LFQ intensity values (2350 proteins; supplemental Data S2*H*). We identified six distinct proteomic clusters (Fig. 6*A*), as determined by the elbow-method optimization of number of clusters (supplemental Fig. S4). Two large clusters (cluster 1 and cluster 2) showed specific abundance difference based on AP status. Cluster 1 (943 proteins) was highly abundant in WC lysates as compared with AP samples with minimal effect of LPS and was enriched in nuclear, mitochondrial, RNA binding, and metabolic proteins (Fig. 6, *B* and *C*); proteins not readily accessible by a cytosolic-directed TurboID-NES. Cluster 2 (608 proteins) was conversely more abundant in BV2 AP proteins compared with WC lysates with minimal effect of LPS and were enriched in cellular membrane organization, endocytic, cytoplasmic location, RNA transport, endoplasmic reticulum lumen, translation, and vesicle-mediated transport functions, consistent with groups of proteins preferentially biotinylated by TurboID-NES. More importantly, we identified clusters of proteins that captured LPS effects in WC lysates or AP samples. LPS significantly decreased protein abundances in BV2 AP in clusters 3 (191 proteins) and 4 (236 proteins) and significantly increased protein abundances 5 (131 proteins) and 6 (241 proteins) (supplemental Fig. S5). Cluster 3 was enriched in cytosolic, lysosomal (hydrolase activity), and ribosome proteins, suggesting that the TurboID-NES approach captured an effect of LPS on translation and lysosomal functions that cannot be resolved at the WC level. Cluster 5, the “BV2 + LPS shared cluster,” was increased by LPS treatment in both WC and AP samples and showed enrichment in terms such as response to IFN-γ, oxidative stress (hydrogen peroxide–related processes), and peroxisome and phagosome functions, indicative of expected LPS-driven proteomic changes in microglia. Several inflammatory terms in cluster 5 were previously reported in BV2 cells specifically in response to 1 μg/ml LPS for 48 h (53), which included antigen processing and presentation, and glycolysis/gluconeogenesis, reflecting a shift in bioenergetics induced by inflammatory stimuli (Fig. 6, *B* and *C*). Cluster 6 showed LPS-induced increased levels that were only apparent in AP samples and were enriched in RNA binding, nucleolus localization, ribosome, translational activity, and spliceosome functions. Cluster 6 may represent altered localization of RNA-interacting splicing proteins as well as potential nuclear speckle and nucleolar proteins from the nucleus to the cytoplasm because of LPS-induced stress. Such altered localization events are more likely to be captured using the TurboID-NES approach, rather than at the WC level. To rule out the possibility that LPS impacts TurboID-NES localization in BV2 cells, we performed immunocytochemistry and WB studies on cytoplasmic and nuclear fractions (supplemental Fig. S6, *A*, *B*, and *E*). We performed colocalization analyses comparing the DAPI signal as a nuclear marker with V5 for TurboID-NES or Streptavidin Dylight to determine the percent of cytosolic TurboID-NES signal or biotinylation signal, respectively. Interestingly, LPS significantly increased the percent of cytosolic TurboID-NES and cytosolic biotinylation (supplemental Fig. S6, *C* and *D*). We observed predominantly cytosolic localization of TurboID-NES (*via* V5 localization) and biotinylation in both immunocytochemistry studies as well as in WB analyses (supplemental Fig. S6). Taken together, it is likely that cytosolic direction of TurboID-NES can identify aberrantly trafficked proteins in response to LPS, though further studies are necessary.

We then compared the impact of LPS treatment on WC and AP proteomes using DEA. DEA of proteomic differences induced by LPS treatment in BV2 WC samples (BV2 + LPS + TurboID-NES_WC_ – BV2 + TurboID-NES_WC_) and AP samples (BV2 + LPS + TurboID-NES_AP_ – BV2 + TurboID-NES_AP_) identified 438 proteins impacted by LPS in the WC proteome and 535 proteins impacted by LPS in AP samples (Fig. 6*D* and supplemental Data S2, *J* and *I*). The top proteins increased with LPS treatment in the WC BV2 proteome included immune-responsive gene 1 (IRG1), oligoadenylate synthetase-like 1 (Oasl1), interleukin 1 a (Il1a), ring finger protein 213 (Rnf213), long-chain acyl-CoA synthetase family member 1 (Ascl1), and intracellular adhesion molecule 1 (Icam1). The proteins most downregulated by LPS treatment in BV2 WC samples included macrophage mannose receptor 1-like protein (Mrc1) macrophage galactose *N*-acetyl-galactosamine–specific lectin 2 (Mgl2), and eukaryotic translation elongation factor 1 delta (Eef1d) (Fig. 6*D*). The top terms increased with LPS in BV2 AP samples included proteins such as Oasl1, Gbp2, Syne1, Cyb5a, and Acsl1. Proteins in the BV2 AP proteome most downregulated by LPS treatment included Mgl2, Smap2, and Mrc2. LPS-increased proteins shared between the WC and AP samples, including Il1a, Irg1, Oasl1, corresponded with proinflammatory M1 phenotypic markers, as well as noncanonical inflammasome mediators such as Gbp2 (54, 55, 56, 57, 58, 59). Proteins similarly decreased with LPS treatment shared between WC and AP BV2 proteins including canonical M2 markers such as Arg1 and Mgl2 (60, 61, 62, 63). When comparing the overlap of proteins differentially expressed by LPS treatment in the WC and transduced AP BV2 proteomes, we identified 115 shared proteins that were differentially expressed in both AP and WC BV2. About 323 proteins were differentially expressed in response to LPS only in the WC proteome, whereas 430 proteins were differentially expressed in response to LPS only in the AP proteome (Fig. 6*E*). Of the 115 proteins with shared LPS-induced differential expression, we observed a moderate concordance based on magnitude (Log2FC LPS_WC_ *versus* Log2FC LPS_AP_) and direction of LPS effect (coefficient of determination, *R*^2^ = 0.52) (Fig. 6*F*). We identified 28 proteins that showed incongruent changes (*yellow points*, Fig. 6*F*) with LPS-induced increase in AP proteomes but LPS-induced decrease in WC proteomes.

## Discussion

Proteomics based on proximity-labeling strategies using biotin ligases (*e.g.*, BioID, TurboID-NES) are being increasingly used in *in vitro* and *in vivo* experimental contexts and in model systems ranging from plants (17, 64, 65, 66), yeast (67), zebrafish (68, 69), Drosophila (70, 71), *Caenorhabditis elegans* (16, 72), to mouse models (18, 21, 22, 73). TurboID is one of the most efficient biotin ligases that can effectively label several proteins within a 10 nm labeling radius in mammalian cells. The high catalytic activity, nontoxicity, and promiscuity of TurboID uniquely position TurboID as a powerful tool to obtain global proteomic snapshots of specific cells in homeostatic and disease states, particularly in multicellular models. Many recent studies have incorporated TurboID and split-TurboID (20) to characterize interactomes and secretomes *via* fusion with proteins, organelles, and intercellular contacts (14, 16, 21, 70, 73, 74, 75, 76, 77). While one application of TurboID-based proteomics is to identify protein–protein interactors of proteins of interest or within specific subcellular compartments, another application of TurboID is to broadly label the cellular proteome of a specific cell type, so that cell type–specific proteomics can be resolved from a complex mixture of proteins derived from multiple cell types. The viability of the latter application was recently tested *in vivo* using genetic Cre/lox strategies to resolve neuronal and astrocyte proteomes in the native state of these cells in mouse brain, a method referred to as CIBOP (22). Whether these neuronal or glial proteomes obtained using CIBOP reflect global cellular proteomes remains to be clarified. As interest in TurboID-based global cellular proteomics continues to grow, it is important to determine what fraction of the WC proteome in mammalian cells can be faithfully captured by the TurboID-NES approach, under both homeostatic (resting) conditions and following cellular perturbations (*e.g.*, immune stress by LPS to mimic neuroinflammatory disease conditions). It is also important to determine whether TurboID-NES overexpression or excessive biotinylation impacts molecular phenotypes and cellular functions, and whether TurboID-NES-biotinylated proteomes have inherent biases as compared with the WC proteome. These questions can be answered by performing well-controlled *in vitro* studies using distinct types of mammalian cells. The results from such studies can inform the interpretation of proteomic findings gained from *in vitro* and *in vivo* applications of TurboID-NES in mammalian model systems, while considering the relative biases of this strategy for cell type–specific proteomics. To address these questions, we directed TurboID-NES to the cytosol using an NES, rather than using protein-specific or cellular compartment–restricted localization of TurboID-NES. We hypothesized that TurboID-NES could globally biotinylate a breadth of the cellular proteome sufficient to distinguish two distinct brain cell types (neurons and microglia) and capture the effect of an inflammatory challenge, without significantly impacting functional or molecular phenotypes of cells that overexpress TurboID-NES.

We generated murine N2A neuroblastoma and BV2 microglial cell lines that stably express TurboID-NES and validated the expression and functionality of TurboID-NES using flow cytometry, biochemical, and immunocytochemical analyses. After validation of these stably transduced TurboID-NES cell lines, we analyzed the TurboID-NES-biotinylated as well as the WC reference proteomes of sham-treated or LPS-treated cells using MS-based quantitative proteomics. We confirmed that TurboID-NES biotinylates >50% of the WC proteome in both N2A and BV2 cells, including proteins in a variety of subcellular compartments in the cytosol (*e.g.*, endocytic machinery, ribosomal proteins, mRNA-binding proteins, membrane proteins, vesicle-related and transport proteins, and cytoskeletal proteins). While a large proportion of biotinylated proteins were common to both cell types, several neuron-enriched and microglia-enriched proteins were indeed identified in the respective biotinylated proteomes. For example, BV2 microglial biotinylated proteomes captured endolysosomal and phagocytic proteins, whereas neuron projection and axonal transport proteins were labeled in N2A neurons, further verifying the validity of this approach to study cell type–specific mechanisms of distinct cell types rather than just homeostatic cellular mechanisms that are shared across cell types. These neuron-enriched and microglia-enriched proteins captured by the TurboID-NES approach also included several neurodegenerative disease–related proteins with causal implications in AD, PD, and ALS. This suggests that the TurboID-NES approach can be used to investigate disease-relevant biology in neurons and glia in mammalian systems.

Using the TurboID-NES approach, we also captured immune effects of LPS on BV2 cells. Some of these were shared at the WC level (*e.g.*, increased expression of proinflammatory proteins Gbp2, Oasl1, Rnf213 and Icam1 and decreased expression of anti-inflammatory proteins such as Mrc1, Arg1, and Mgl2), indicating that TurboID-NES biotinylation captures core pathological transformations induced by long-term LPS stimulation in BV2 cells. KEGG terms in this cluster include antigen processing and presentation, glycolysis and gluconeogenesis, and alanine, aspartate, and glutamate metabolism (53). Previous studies assessing the longitudinal impact of LPS stimulation on BV2 proteomes similarly identified these major KEGG terms reflecting a strong metabolic shift in later LPS activation. Our results confirm that TurboID-NES expression does not impair metabolic functioning, and we confirm that biotinylation by TurboID-NES is able to biotinylate metabolic and immune responsive protein pathways impacted by long-term LPS stimulation. Importantly, LPS DEPs identified in transduced and affinity-purified BV2 cells correlate moderately with LPS DEPs at the WC level. Despite these consistent LPS-induced proteomic changes observed in WC and biotinylated BV2 proteomes, a relatively large group of protein changes because of LPS were identified only at the level of biotinylated proteins but not at the WC level. These LPS-induced proteomic changes preferentially captured by the TurboID-NES approach may be due to better access of TurboID-NES to specific cellular compartments, such as the cytosol, which allow capture of post-translational effects of LPS, such as altered protein trafficking, nucleocytoplasmic transport of proteins from the nucleus to the cytosol or vice versa, and altered localization of RNA binding, ribosomal, and ribonucleoprotein-related proteins. Consistent with this, proteins involved in RNA binding, nucleolar proteins, and ribosomal and translational machinery were selectively increased in the LPS-induced biotinylated proteome. We also observed a slight increase in trafficking of TurboID-NES to the cytosol with LPS stimulation, implying that the effects of LPS observed at the level of the biotinylated proteome are likely because of a combination of increased cytosolic localization of TurboID-NES and altered localization of biotinylated proteins. The LPS-induced changes in levels of ribonuclear proteins agree with reported ribosomal mechanisms involved in innate immune activation in microglia, which are responsible for translational repression and a divergence between mRNA and protein expression following LPS challenge (78). Furthermore, ribonuclear proteins within nuclear speckles may also be localized to the cytosol as a direct result of immune activation or a cell proliferative response to LPS stimulation, as has been reported previously (79, 80). Finally, several nucleus-resident RNA binding and ribonucleoproteins can translocate to the cytosol to regulate mRNA translation by the ribosome. The ability to use TurboID-NES-based proteomics to investigate protein trafficking and mislocalization is of particular relevance to neurodegenerative disorders. Mislocalization of nuclear proteins to the cytosol occur in several neurodegenerative diseases, where RNA-binding proteins such as TDP-43, Tau, and FUS can aberrantly localize to the cytosol where they become more prone to aggregation (36). Therefore, the TurboID-NES approach when employed *in vivo via* CIBOP, could be specifically used to interrogate mechanisms of neurodegeneration that involve dysfunction of nucleocytoplasmic transport, changes in protein trafficking, and cytosolic aggregation.

Another important result of our study is that TurboID-NES overexpression had a minimal impact on the proteomes of both N2A and BV2 cells at the WC level and did not significantly impact cellular respiration or secreted cytokine profiles in response to LPS in BV2 cells. These findings suggest that cellular molecular composition and function are not meaningfully compromised using the TurboID-NES approach. This finding is consistent with the lack of electrophysiological alterations observed in *in vivo* TurboID-NES studies in Camk2a excitatory neurons (22). These findings are indeed reassuring and support the use of TurboID-NES-based cellular proteomic profiling approaches to investigate mechanisms of disease with minimal effects on cellular functions because of TurboID-NES overexpression.

Despite the strengths of well-controlled *in vitro* studies, some limitations of our work need to be considered. Cell lines such as BV2 and N2A cells, despite their ability to recapitulate major cellular phenotypes of microglia and neuronal cells, display many well-known differences as compared with primary cells in the nervous system. Another limitation is that the LPS dose and duration used for 1 μg/ml for 48 h could have caused induced overstimulation, cell death, apoptosis, or stress-induced changes in BV2 cells, leading to biased proteomic findings. However, it is reassuring that our observed LPS effects on the BV2 WC proteome, generally agree with prior proteomics studies of BV2 cells using low dose (10–100 ng/ml) and high concentrations (1 μg/ml) (81, 82). Another limitation is related to the use of the NES in our TurboID studies. TurboID-NES was intentionally directed to the cytosol to increase the sampling of non-nuclear proteins as well as to minimize undesired effects of excessive biotinylation of nuclear or mitochondrial proteins that are involved in key cellular functions, such as chromosomal stability and gene regulation and mitochondrial metabolic processes. To minimize the chance of relative biotin deficiency because of TurboID-NES overexpression, all experimental conditions included biotin supplementation in the medium. While this controlled for biotin deficiency, this may have induced some metabolic alterations because of excessive biotin in experimental conditions. However, in doing so, we also biased the biotinylated proteome away from nuclear, cell-surface, mitochondrial, and intraluminal-directed proteins, which was evident when biotinylated AP proteomes were contrasted with WC lysate proteomes.

Beyond the scope of the methodologies published in this study, alternative applications of TurboID and other proximity labeling methods can be used to recover proteins enriched in the WC proteomes over the AP proteomes, including cell surface and nuclear proteins. TurboID can be directed to the nucleus *via* inclusion of a nuclear localization sequence (NLS) in lieu of an NES, wherein TurboID-NLS would remain localized within the nucleus to preferentially label nuclear proteins. Alternatively, TurboID without a localization sequence can be used. By agnostically expressing TurboID throughout the cell, TurboID could label both nuclear and cytosolic proteins. Subsequent biochemical fractionations upstream of MS can purify for nuclear, cytosolic, and synaptic proteins. Direction of TurboID to the nucleus using an NLS could pose a challenge to cellular toxicity. When directed to the nucleus, TurboID could biotinylate key proteins involved with gene regulation and potentially alter the structure and functionality of those proteins. Therefore, it is necessary that future studies rigorously investigate the possibility of toxicity with a nuclear direction of TurboID. Future studies may consider comparing proteomes using TurboID without NES to TurboID-NES to confirm whether cellular toxicity is indeed observed.

Furthermore, modifications to the proximity labeling approach may recover cell-surface proteins, otherwise not enriched in the AP samples within this study using the cytosolic TurboID-NES approach. Specifically, Split-TurboID and non–biotin-ligase proximity labeling methods can efficiently recover cell-surface proteins. A split-TurboID approach has been used to recover cell type–specific and cell-surface proteins interfacing at the tripartite synapse (18). The recent development of *in situ* cell-surface proteome extraction by extracellular labeling is an alternative proximity-labeling method to biotin-ligase approaches, which has been used to profile surface proteins of mature *murine* cerebellar Purkinje neurons with minute-temporal resolution (83). The Split-TurboID approach to enriching cell-surface proteins can be used *in vivo*, though its limitations include a low extracellular concentration of ATP needed for TurboID catalytic activity (84). Whereas, *in situ* cell-surface proteome extraction by extracellular labelin is currently limited in its applications in *ex vivo* environments. While both approaches have respective strengths and limitations, they can efficiently recover cellularly distinct cell-surface proteins.

Our results demonstrate the ability of TurboID-NES-based cellular proteomics to capture a representative portion of disease-relevant and immune-relevant proteins in two distinct brain cell types, namely microglia and neurons, using immortalized cell lines. These results directly impact future directions of TurboID-NES using the CIBOP approach in transgenic mouse models of inflammation and neurodegeneration.

In conclusion, we generated transduced neuroblastoma and microglial cell lines expressing cytosolic TurboID-NES, which yielded robustly labeled proteomes that covered a wide variety of subcellular compartments with no significant impact to cellular phenotypes. We identified a high representation of neurodegenerative disease–relevant protein pathways as well as a partial coverage of immune-relevant proteins in microglia. The breadth of the proteome labeled by TurboID-NES distinguished neuroblastoma cells from microglial cells, and TurboID-NES labeled over 50% of identified proteins within each cell type; supporting the *in vivo* application of TurboID-NES in its ability to purify cellularly distinct proteomes. Our results also highlight inherent biases of TurboID-NES-based proteomics approaches, which may be more suited to investigate post-translational mechanisms such as protein trafficking, which are not captured by WC proteomics.

## Data Availability

Data are publicly available *via* ProteomeXchange with the dataset accession number PXD036744, available at this link http://www.ebi.ac.uk/pride/archive/projects/PXD036744. Processed data are available as supplemental data files (85).

## Supplemental data

This article contains supplemental data.

## Conflict of interest

The authors declare no competing interests.

## Supplementary Data

Supplemental Data 2

Supplemental Datasheet 1

Supplemental Datasheet 2

Supplemental Datasheet 3
