# Supplementary material for: Cellular Proteomic Profiling Using Proximity Labeling by TurboID-NES in Microglial and Neuronal Cell Lines
Source: Mol Cell Proteomics. 2023 Apr 14;22(6):100546. doi: 10.1016/j.mcpro.2023.100546 (PMC10205547; doi:10.1016/j.mcpro.2023.100546)
Supplement: Supplemental Data 1 [file mmc1.pdf]

Annotated Spectra for protein identifications on the basis of a single unique peptide within the Whole Cell (WC) proteome.

Description:

b and y ion matches were annotated below and above each MS/MS spectrum and marked at the top of the centroided peak intensity by a small black or blue box, only for ions in the MS/MS spectrum within +/- 0.6 Da of the theoretical b or y ion mass.

(Ac) in the title of an annotated PSM PDF page, e.g.,"(Ac)PEPSEQ" indicates the primary amino acid sequence for the PSM, PEPSEQ, was matched by MaxQuant with a variablemodification monoisotopic mass shift for acetylation at the protein N-terminus (+42.010565 Da).

" in the annotated PSM title, e.g., "PEM*SEQ" indicates oxidation (+15.994915 Da) monoisotopic mass shift for the preceding methionine (M) residue.

^ in the title peptide sequence follows an asparagine (N) or glutamine (Q) residue and represents conversion to aspartate or glutamate, respectively, by deamidation, resulting in a +0.984016 Da monoisotopic mass increase for the preceding residue.

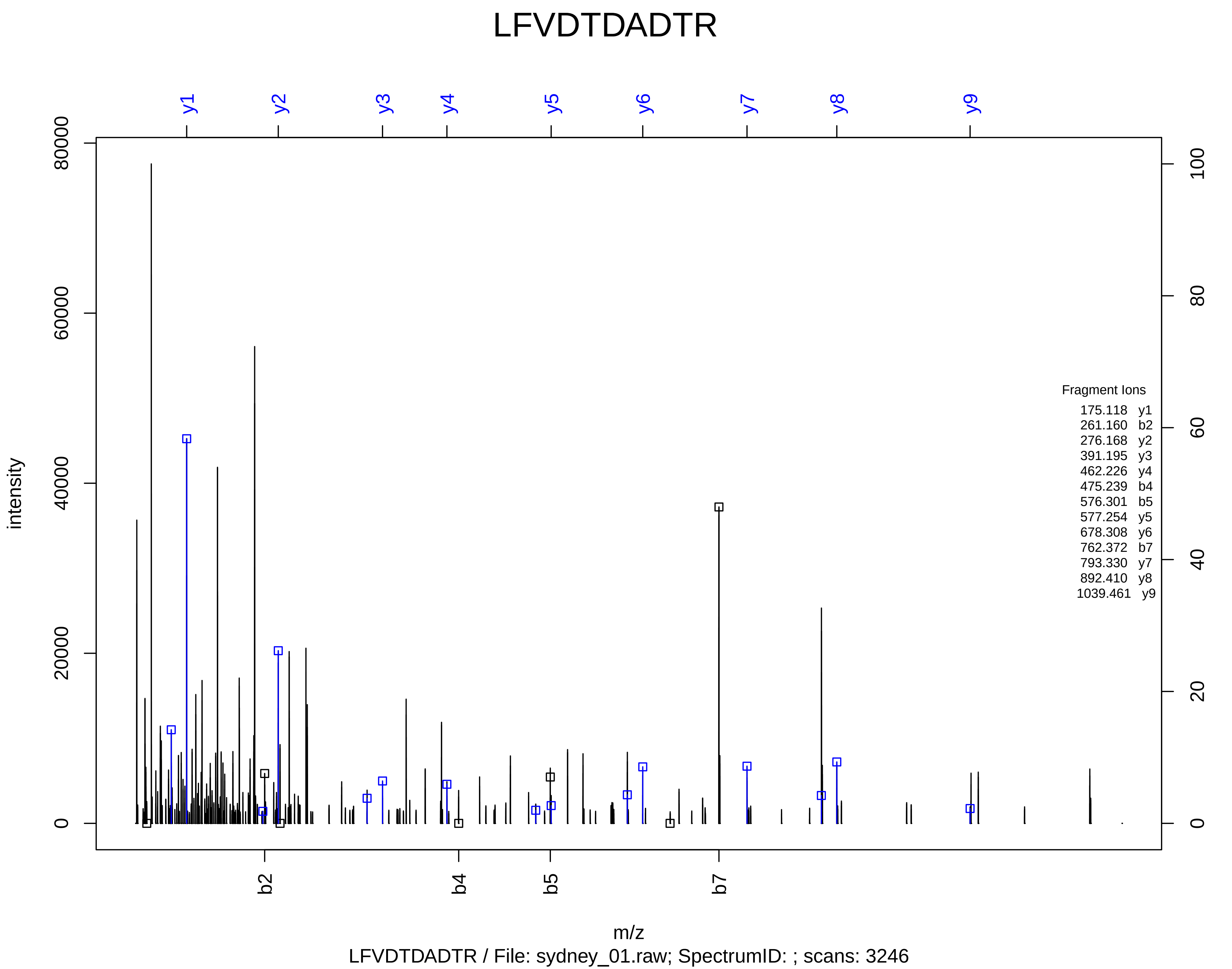
LFVDTDADTR
y1
y2
y3
y4
y5
y6
y7
y8
y9
80000
60000
40000
20000
0
intensity
100
80
60
40
20
0
Fragment Ions
175.118 y1
261.160 b2
276.168 y2
391.195 y3
462.226 y4
475.239 b4
576.301 b5
577.254 y5
678.308 y6
762.372 b7
793.330 y7
892.410 y8
1039.461 y9
b2
b4
b5
b7
m/z
LFVDTDADTR / File: sydney_01.raw; SpectrumID: ; scans: 3246

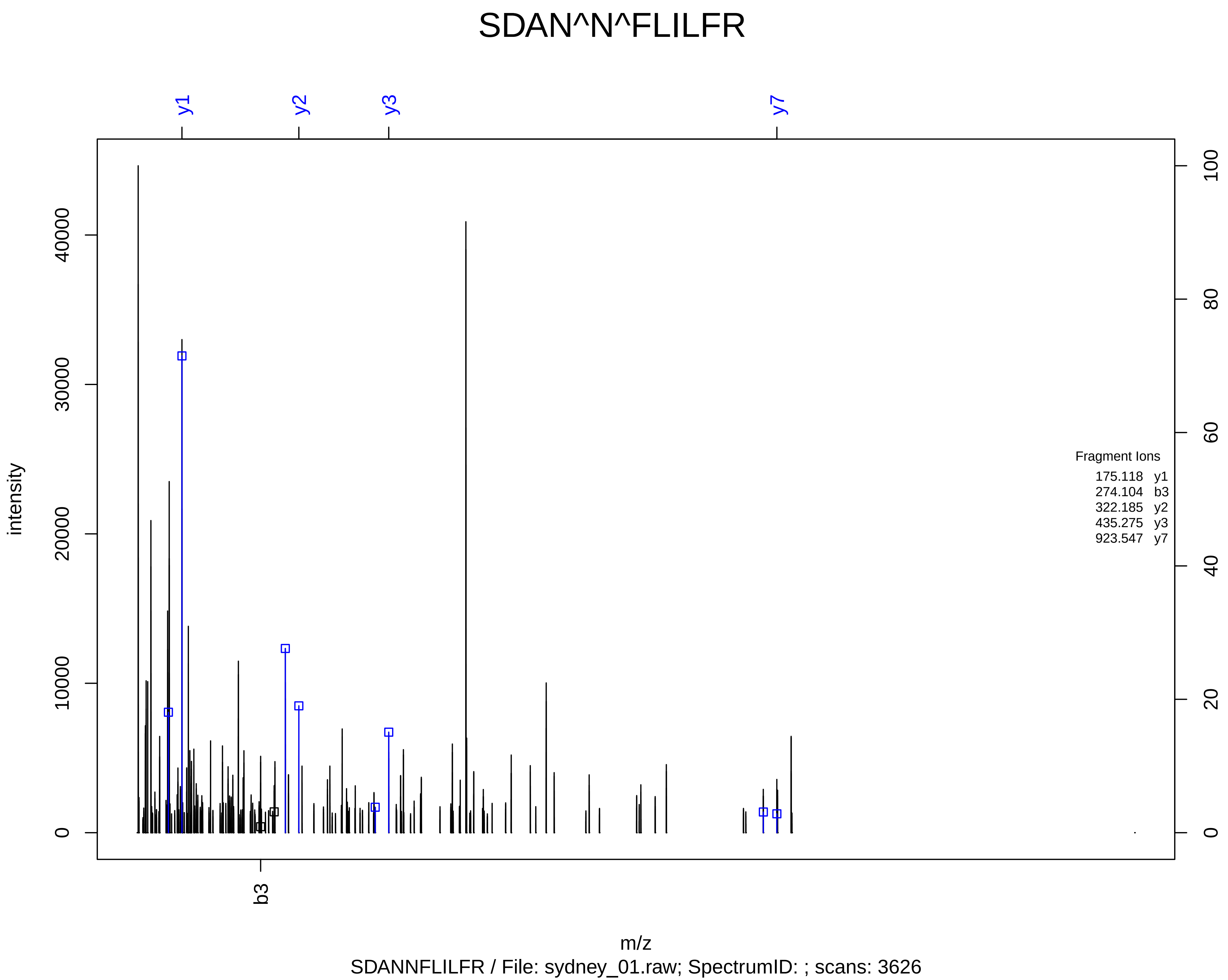
SDAN^N^FLILFR
y1
y2
y3
y7
b3
intensity
m/z
Fragment Ions
175.118 y1
274.104 b3
322.185 y2
435.275 y3
923.547 y7
SDANNFLILFR / File: sydney_01.raw; SpectrumID: ; scans: 3626

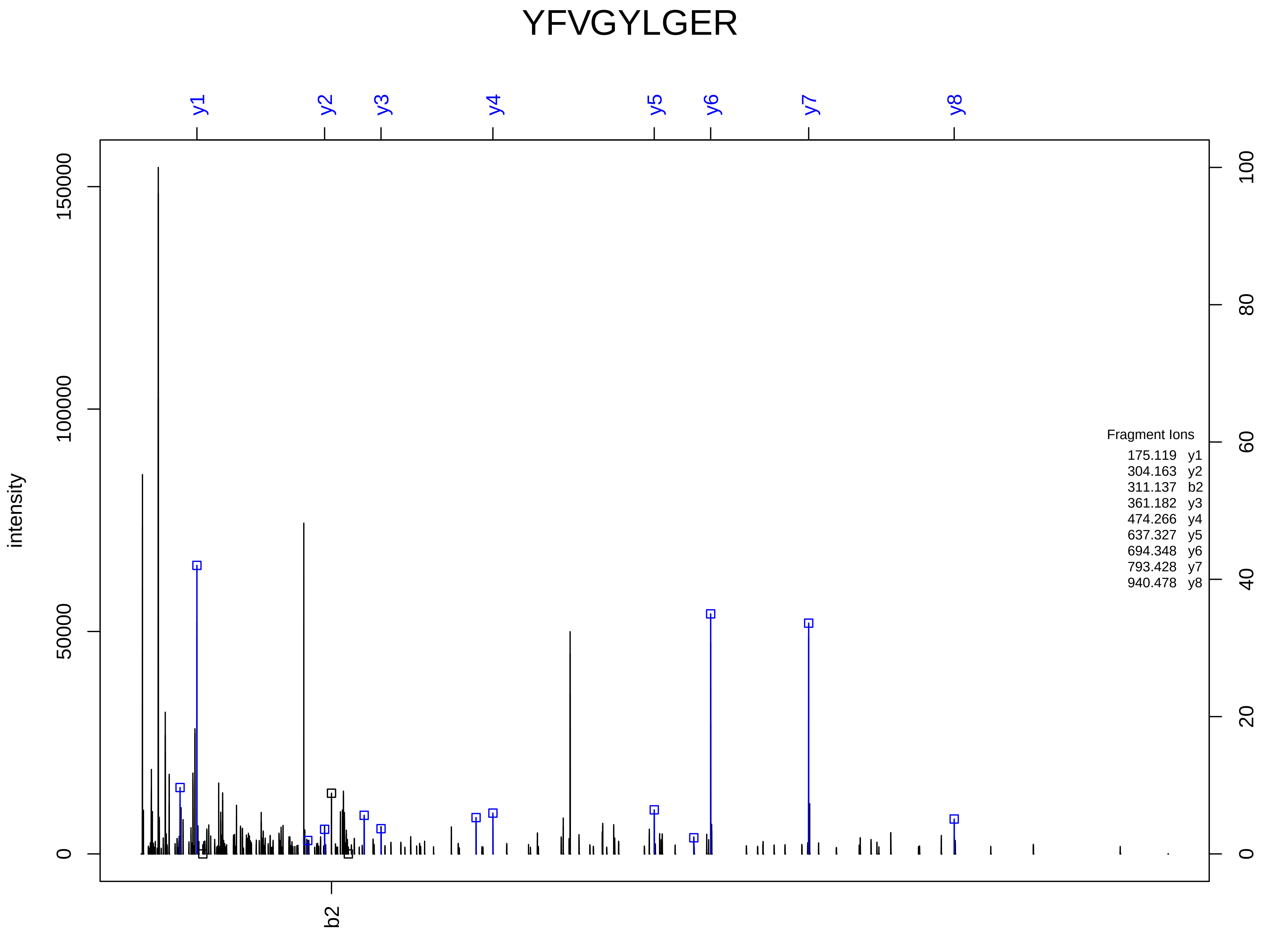

YFVGYLGER / File: sydney_01.raw; SpectrumID: ; scans: 5831

AANPGCFLEDFVR

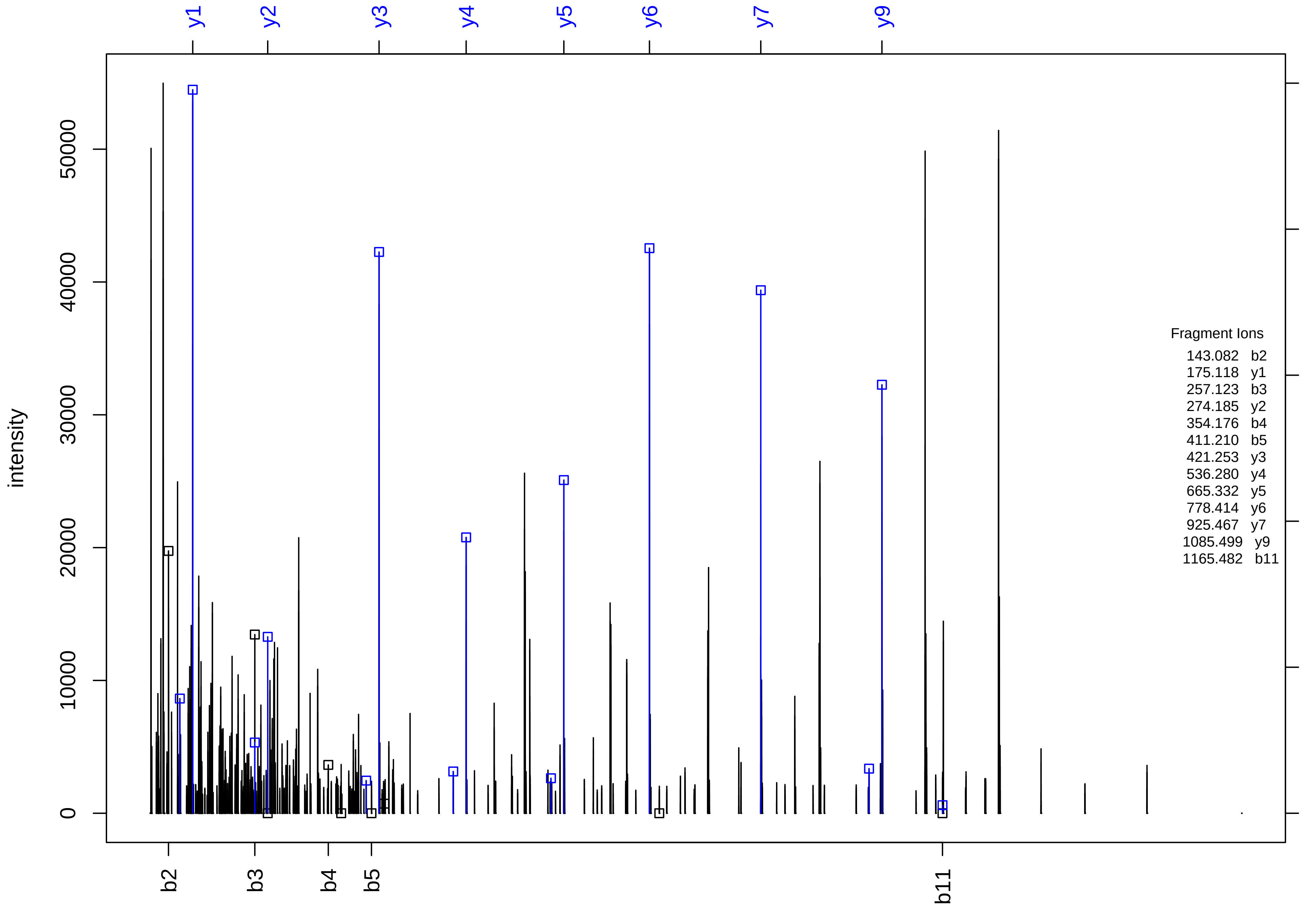

AANPGCFLEDFVR / File: sydney_01.raw; SpectrumID: ; scans: 11568

VVN^SN^KEKLK

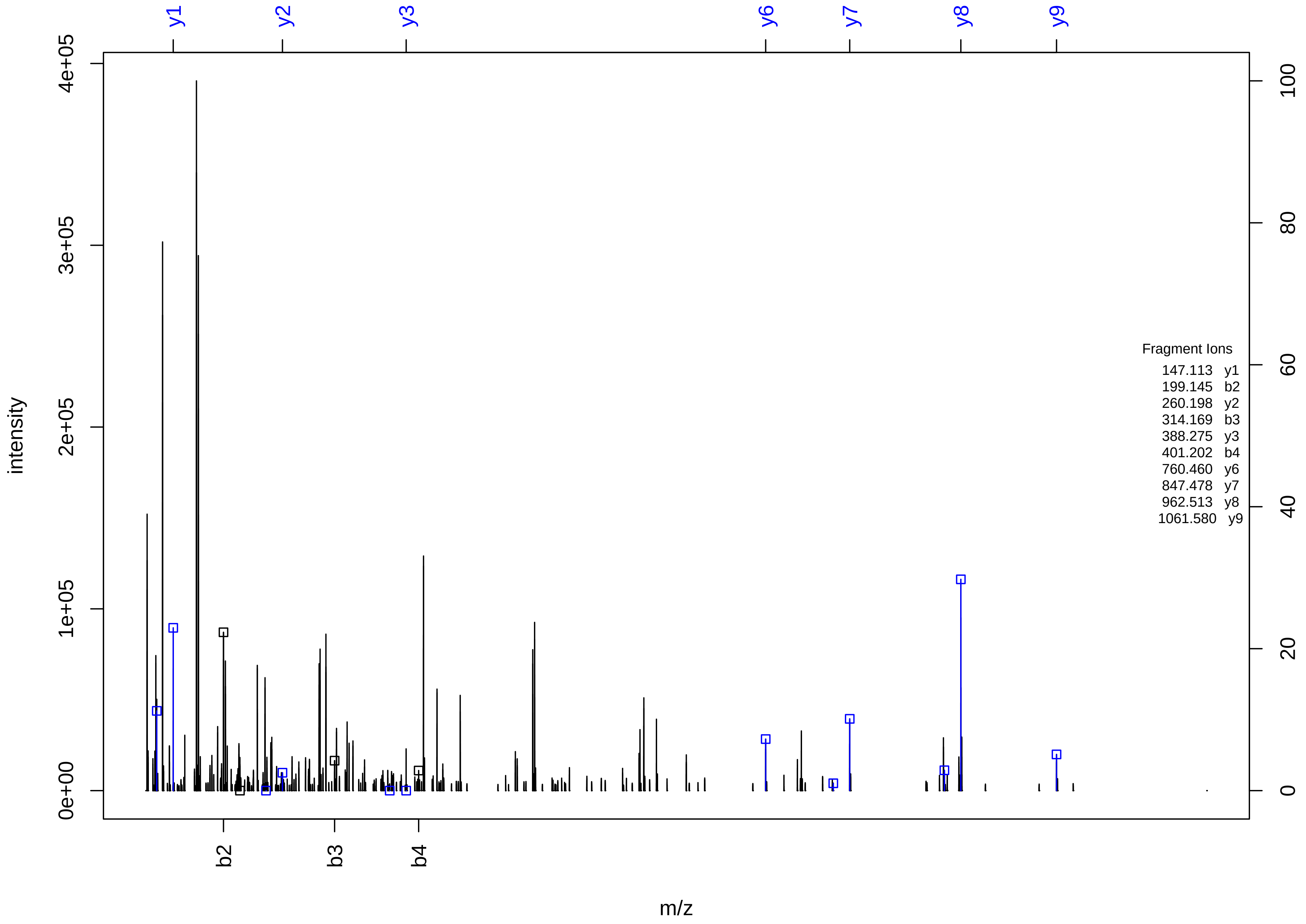

VVNSNKEKLK / File: sydney_01.raw; SpectrumID: ; scans: 3386

APETEAPASQAGTDEPEPEGTAVQVQENQDTRPK

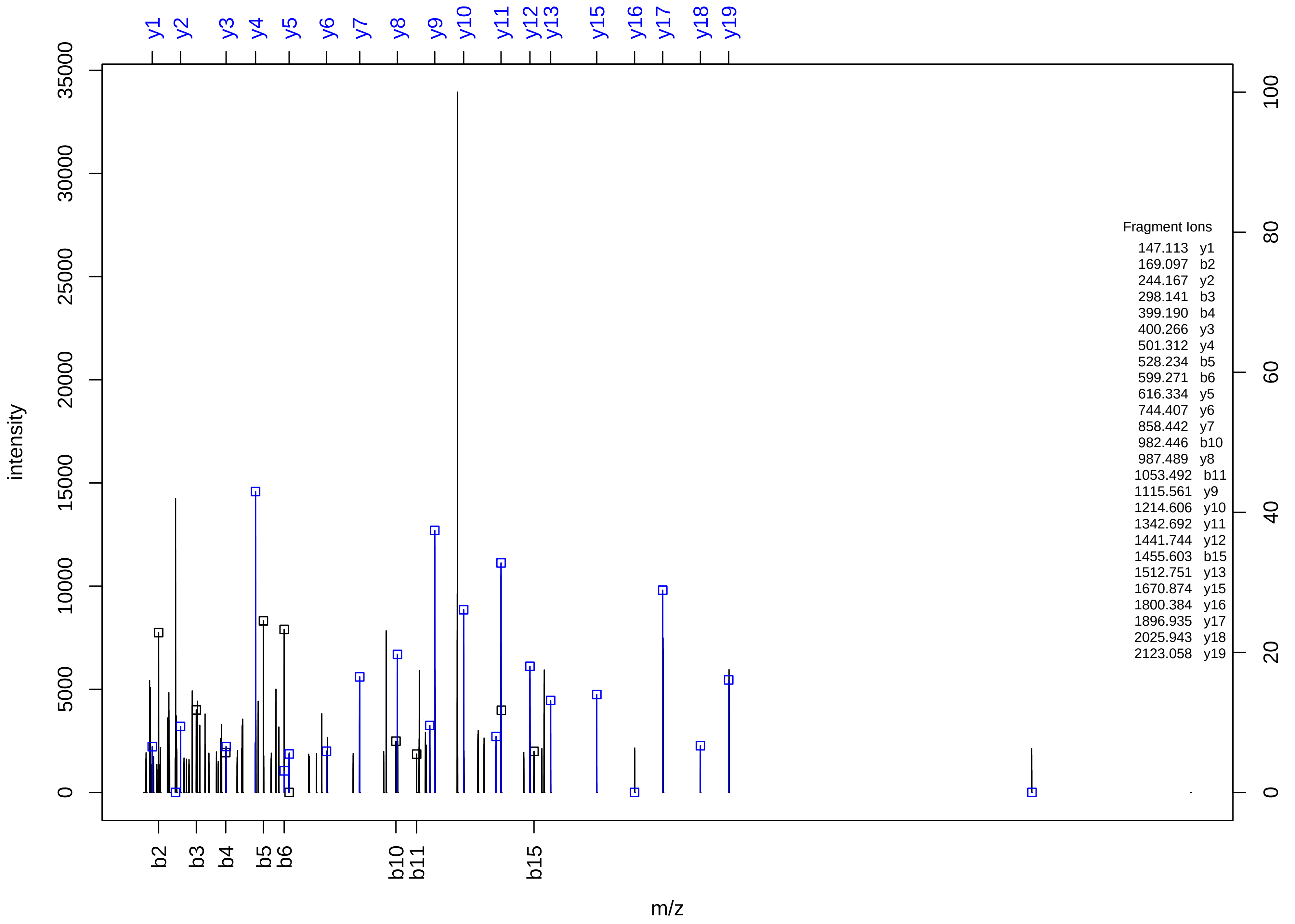

APETEAPASQAGTDEPEPEGTAVQVQENQDTRPK / File: sydney_01.raw; SpectrumID: ; scans: 2717

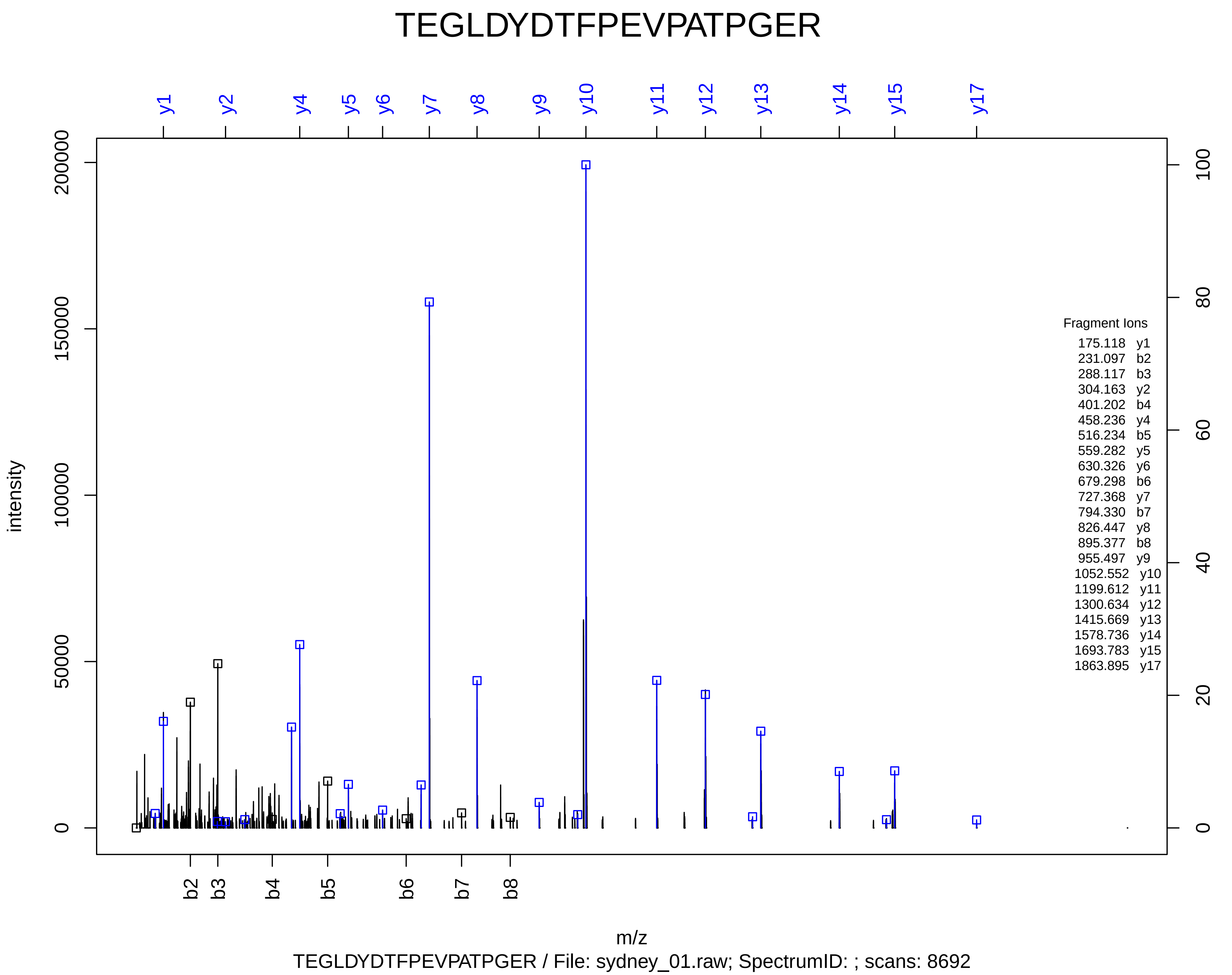

TEGLDYDTFPEVPATPGER
y1 y2 y4 y5 y6 y7 y8 y9 y10 y11 y12 y13 y14 y15 y17
b2 b3 b4 b5 b6 b7 b8
intensity
0 50000 100000 150000 200000
0 20 40 60 80 100
m/z
Fragment Ions
175.118 y1
231.097 b2
288.117 b3
304.163 y2
401.202 b4
458.236 y4
516.234 b5
559.282 y5
630.326 y6
679.298 b6
727.368 y7
794.330 b7
826.447 y8
895.377 b8
955.497 y9
1052.552 y10
1199.612 y11
1300.634 y12
1415.669 y13
1578.736 y14
1693.783 y15
1863.895 y17
TEGLDYDTFPEVPATPGER / File: sydney_01.raw; SpectrumID: ; scans: 8692

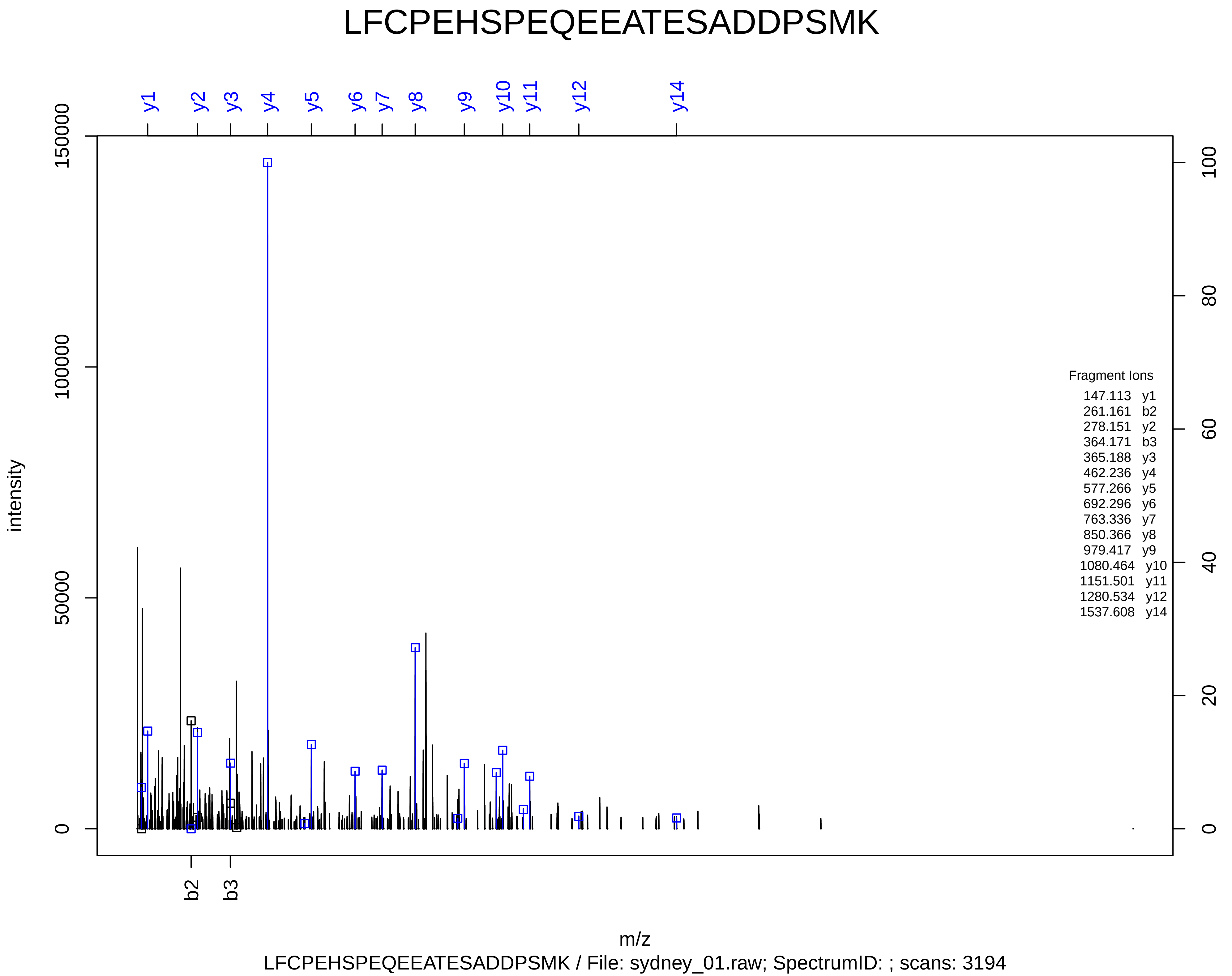
LFCPEHSPEQEEATESADDPSMK
y1
y2
y3
y4
y5
y6
y7
y8
y9
y10
y11
y12
y14
150000
100000
50000
0
intensity
100
80
60
40
20
0
b2
b3
m/z
Fragment Ions
147.113 y1
261.161 b2
278.151 y2
364.171 b3
365.188 y3
462.236 y4
577.266 y5
692.296 y6
763.336 y7
850.366 y8
979.417 y9
1080.464 y10
1151.501 y11
1280.534 y12
1537.608 y14
LFCPEHSPEQEEATESADDPSMK / File: sydney_01.raw; SpectrumID: ; scans: 3194

LDCPVVGWQQELR

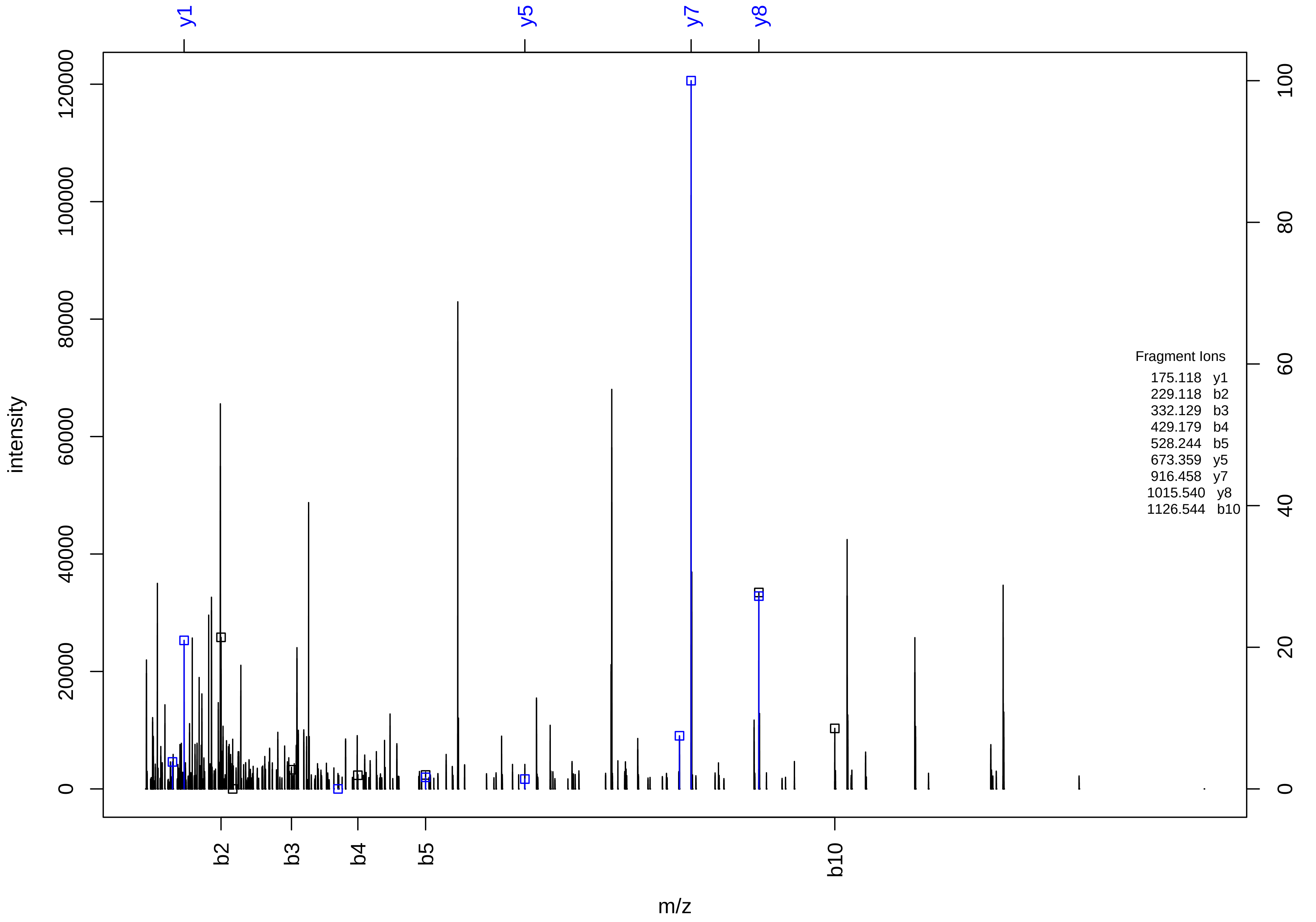

LDCPVVGWQQELR / File: sydney_01.raw; SpectrumID: ; scans: 7107

VQ^N^WFQRRR

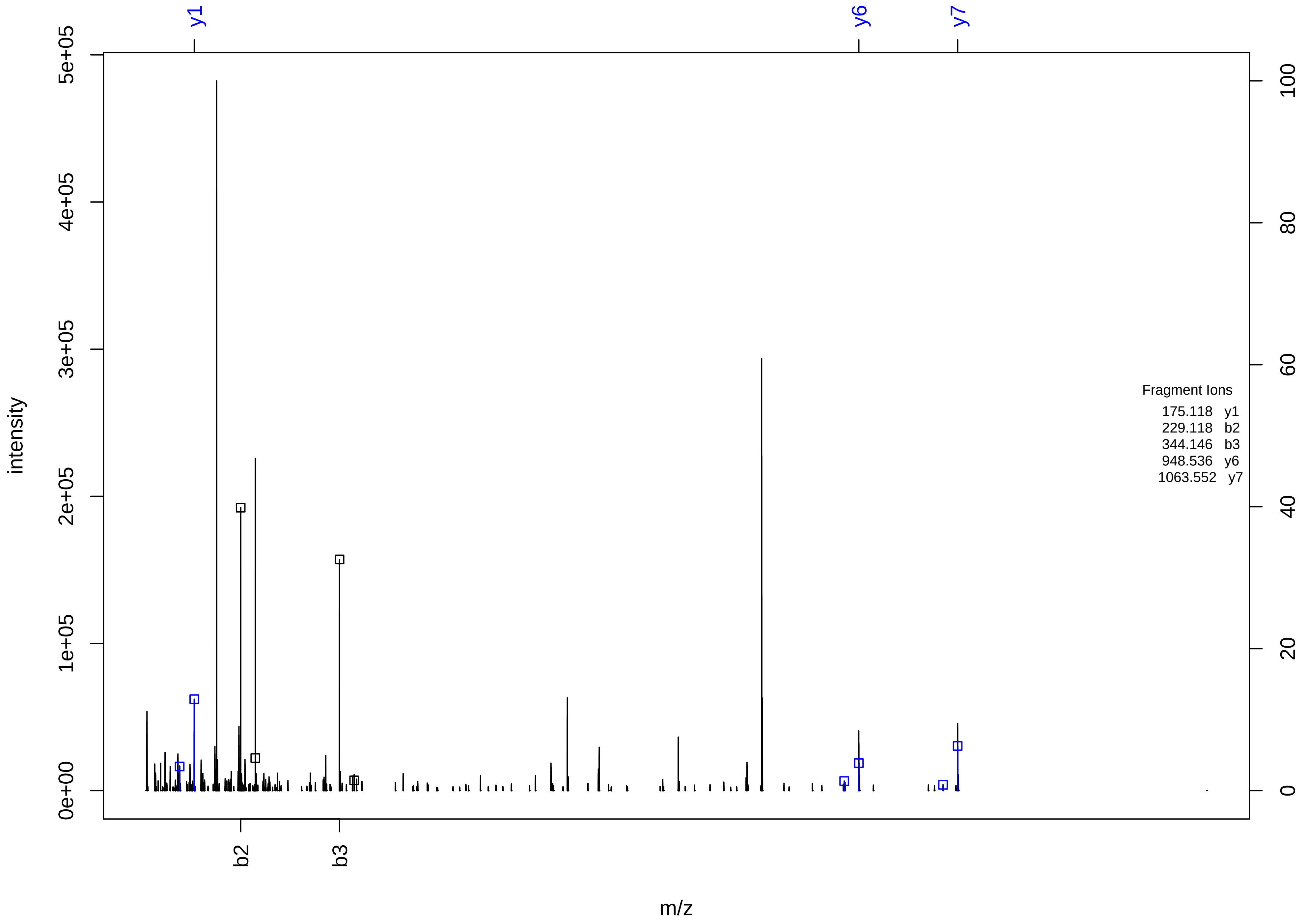

VQNWFQRRR / File: sydney_01.raw; SpectrumID: ; scans: 8301

DLPQ^PDSREN^R

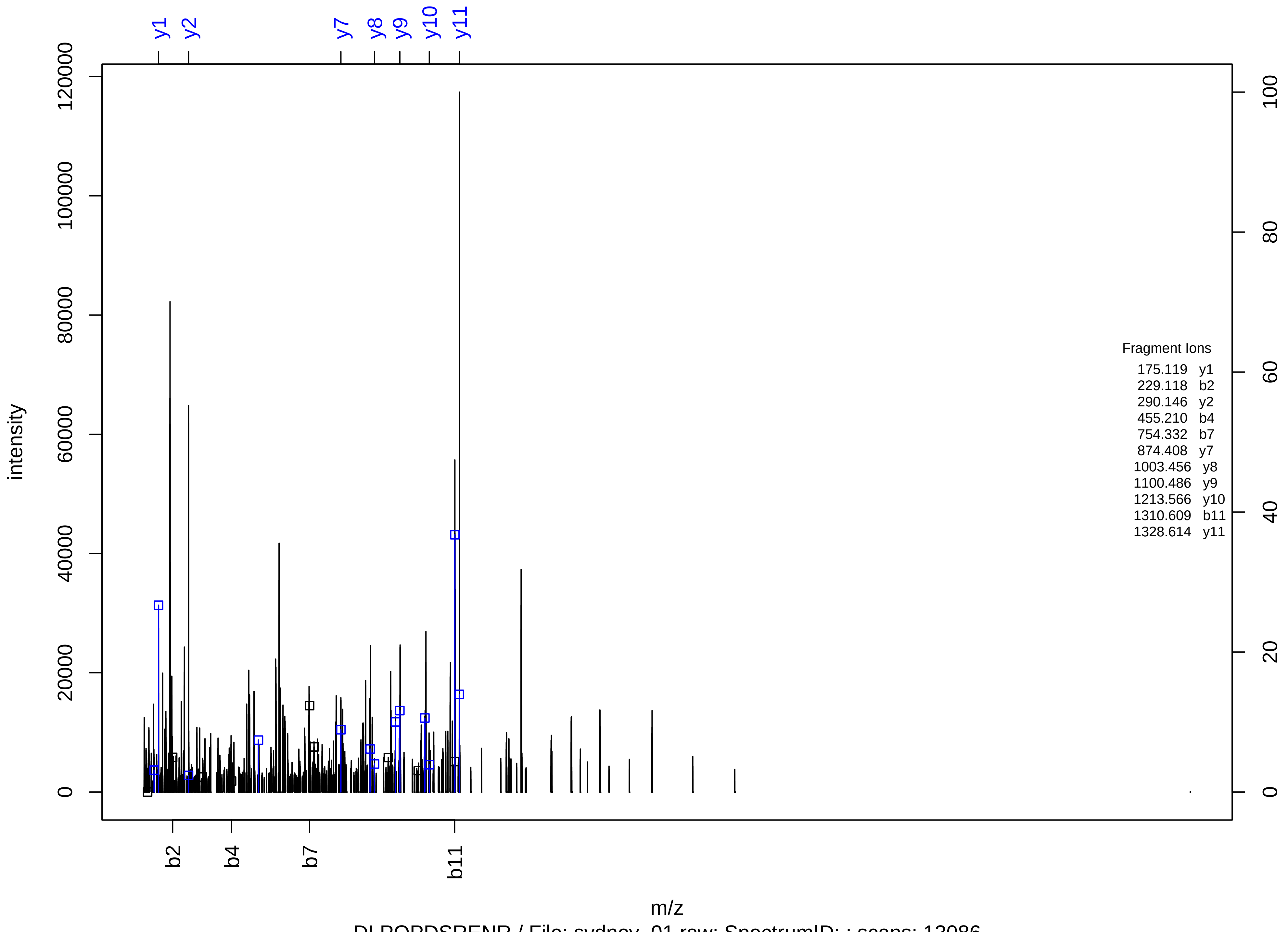

DLPQPDSRENR / File: sydney_01.raw; SpectrumID: ; scans: 13086

KWENEEEEEEPAPTSAEGEGNAEGPDAEAGSASTPR

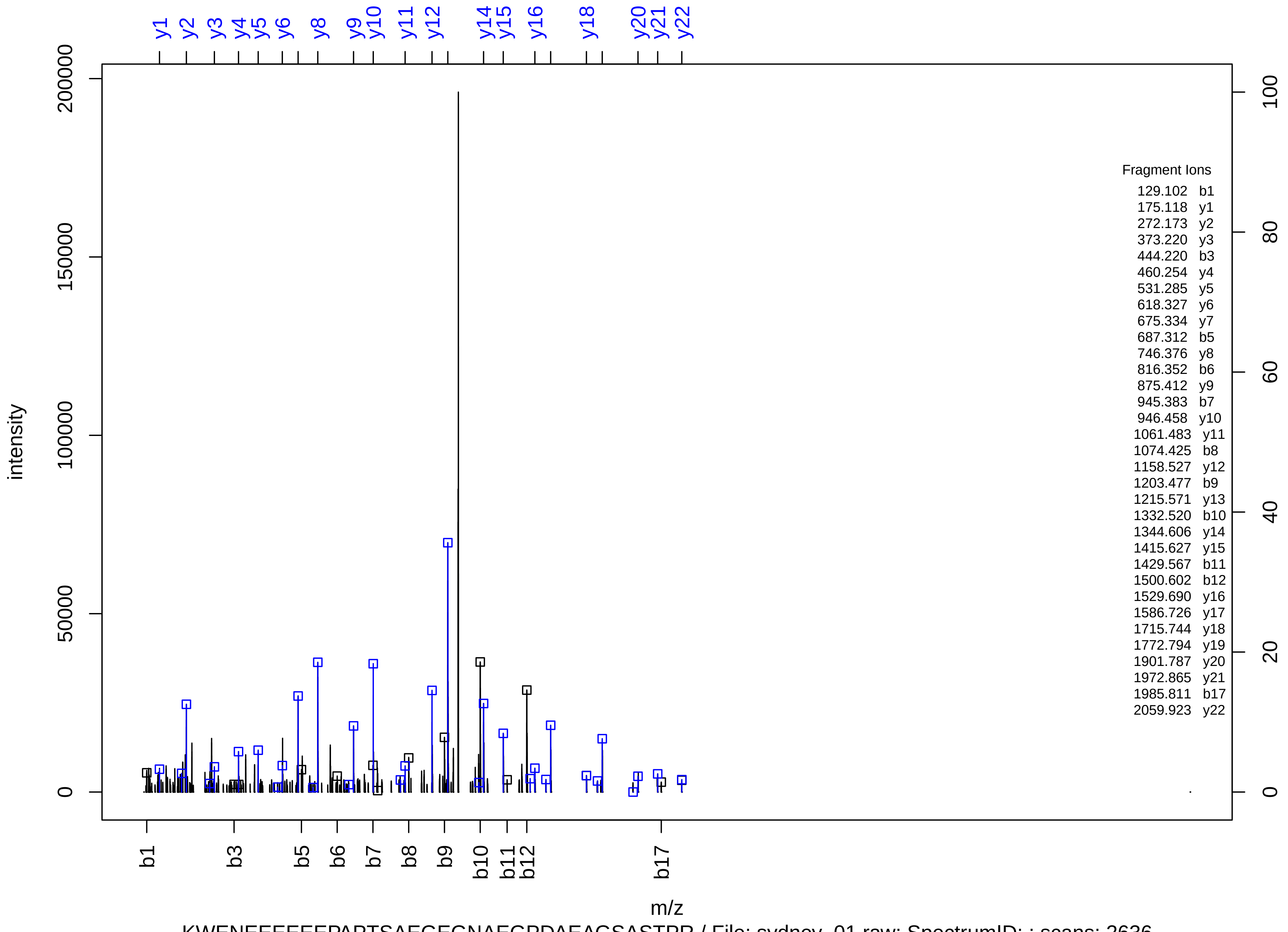

KWENEEEEEEPAPTSAEGEGNAEGPDAEAGSASTPR / File: sydney_01.raw; SpectrumID: ; scans: 2636

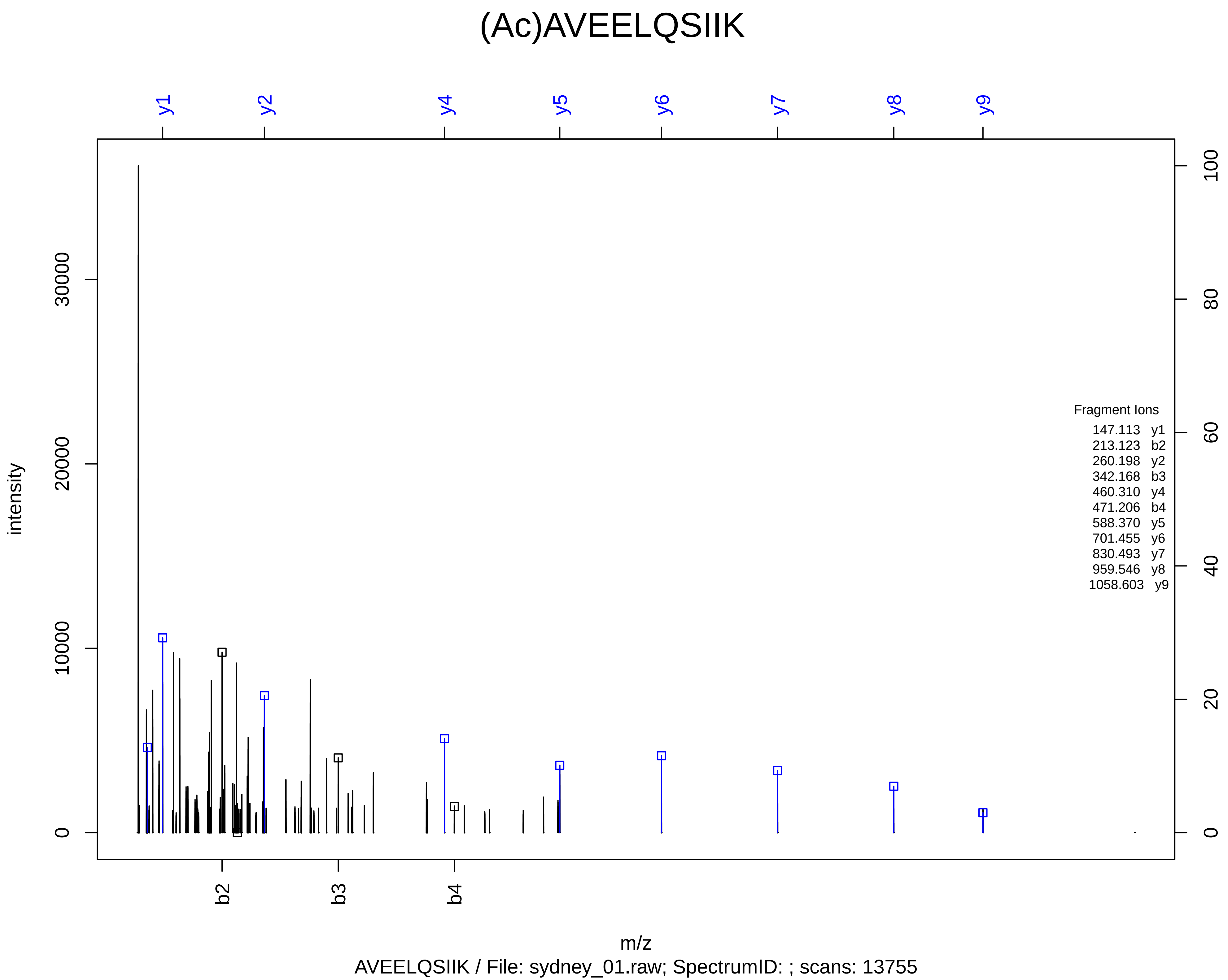

(Ac)AVEELQSIIK
y1
y2
y4
y5
y6
y7
y8
y9
intensity
0
10000
20000
30000
0
20
40
60
80
100
b2
b3
b4
m/z
Fragment Ions
147.113 y1
213.123 b2
260.198 y2
342.168 b3
460.310 y4
471.206 b4
588.370 y5
701.455 y6
830.493 y7
959.546 y8
1058.603 y9
AVEELQSIIK / File: sydney_01.raw; SpectrumID: ; scans: 13755

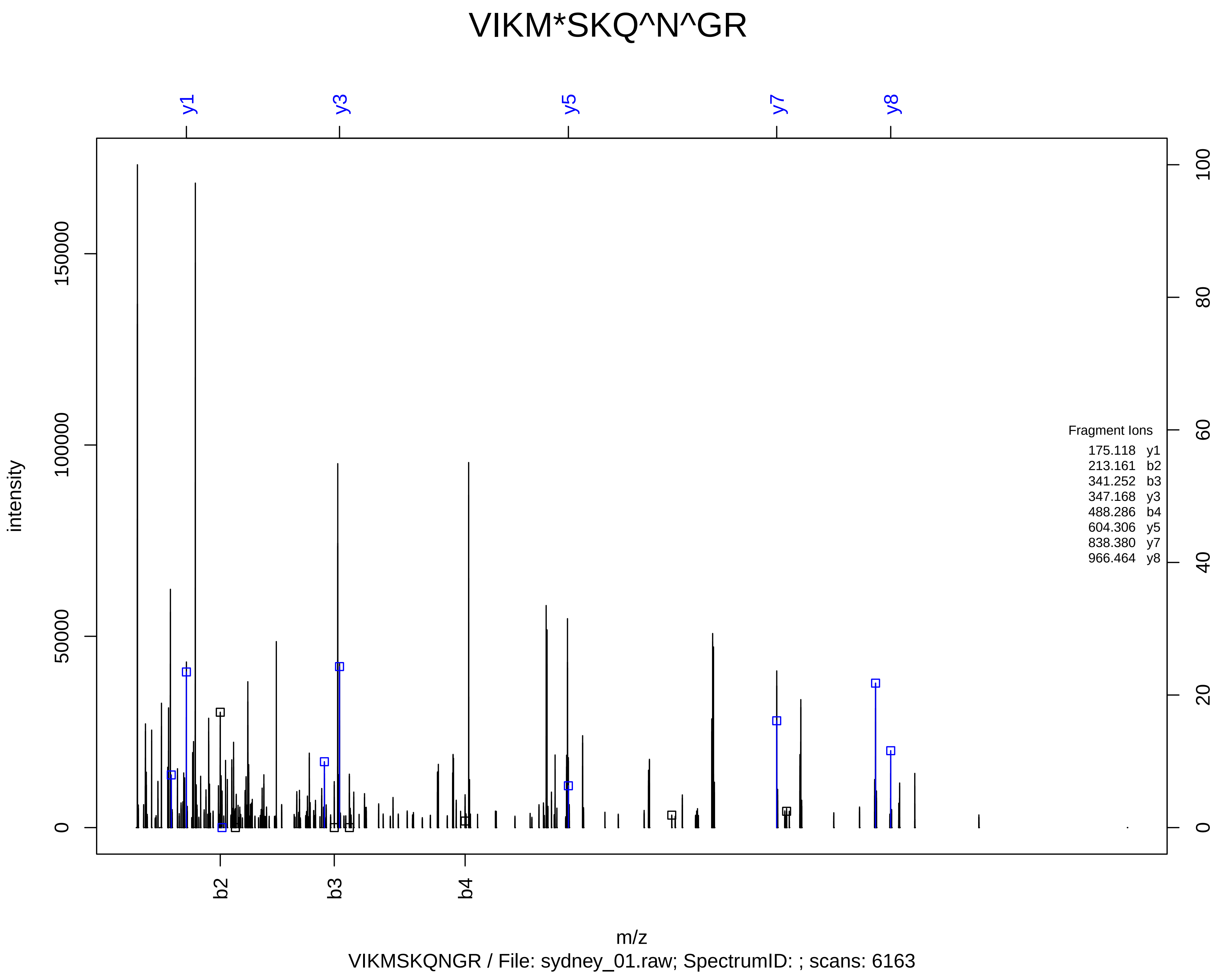
VIKM*SKQ^N^GR
y1
y3
y5
y7
y8
intensity
150000
100000
50000
0
100
80
60
40
20
0
Fragment Ions
175.118 y1
213.161 b2
341.252 b3
347.168 y3
488.286 b4
604.306 y5
838.380 y7
966.464 y8
b2
b3
b4
m/z
VIKMSKQNGR / File: sydney_01.raw; SpectrumID: ; scans: 6163

VSVVPDEVATIASEVNSFSR

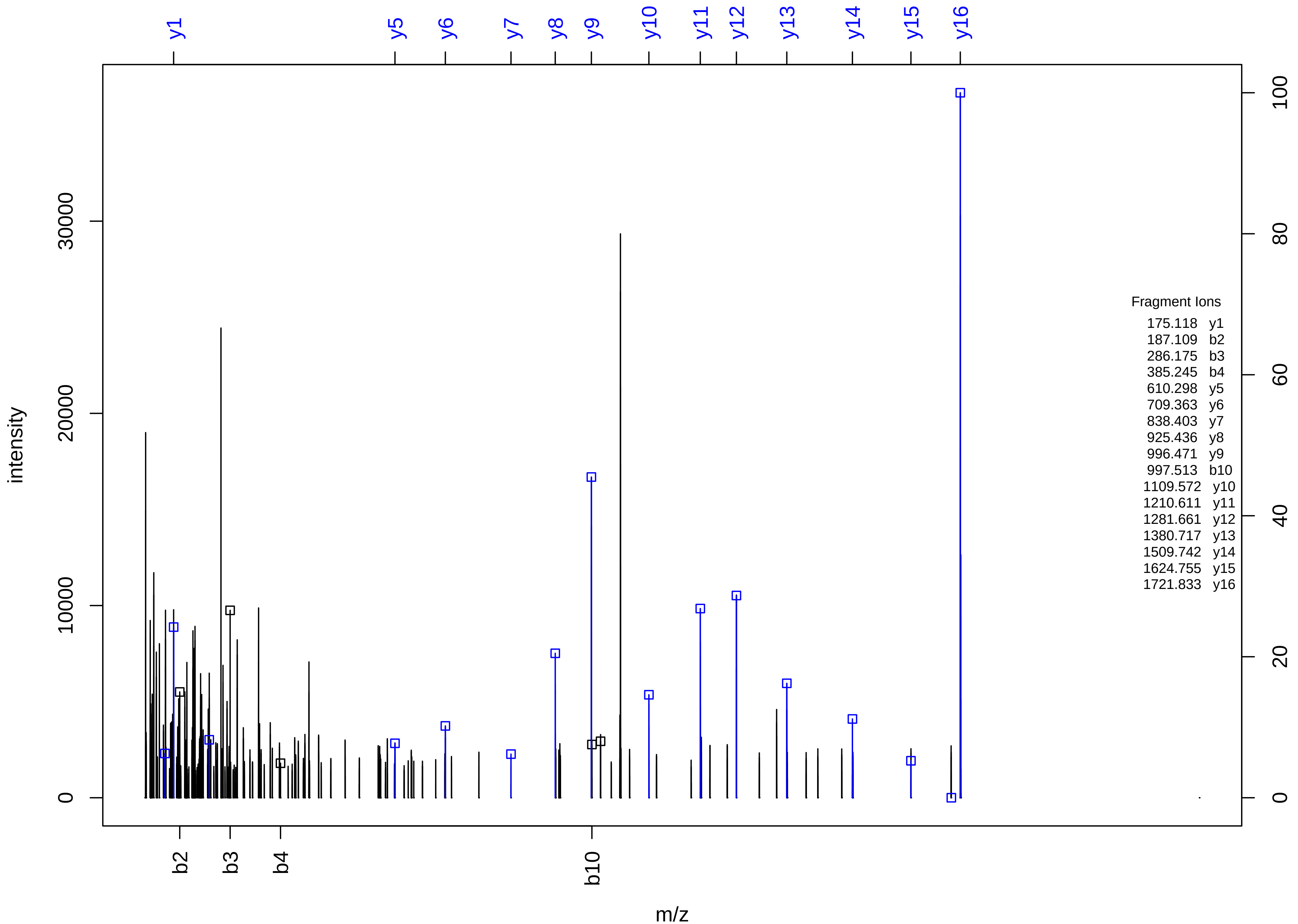

VSVVPDEVATIASEVNSFSR / File: sydney_01.raw; SpectrumID: ; scans: 14299

FYTEDSPGLK

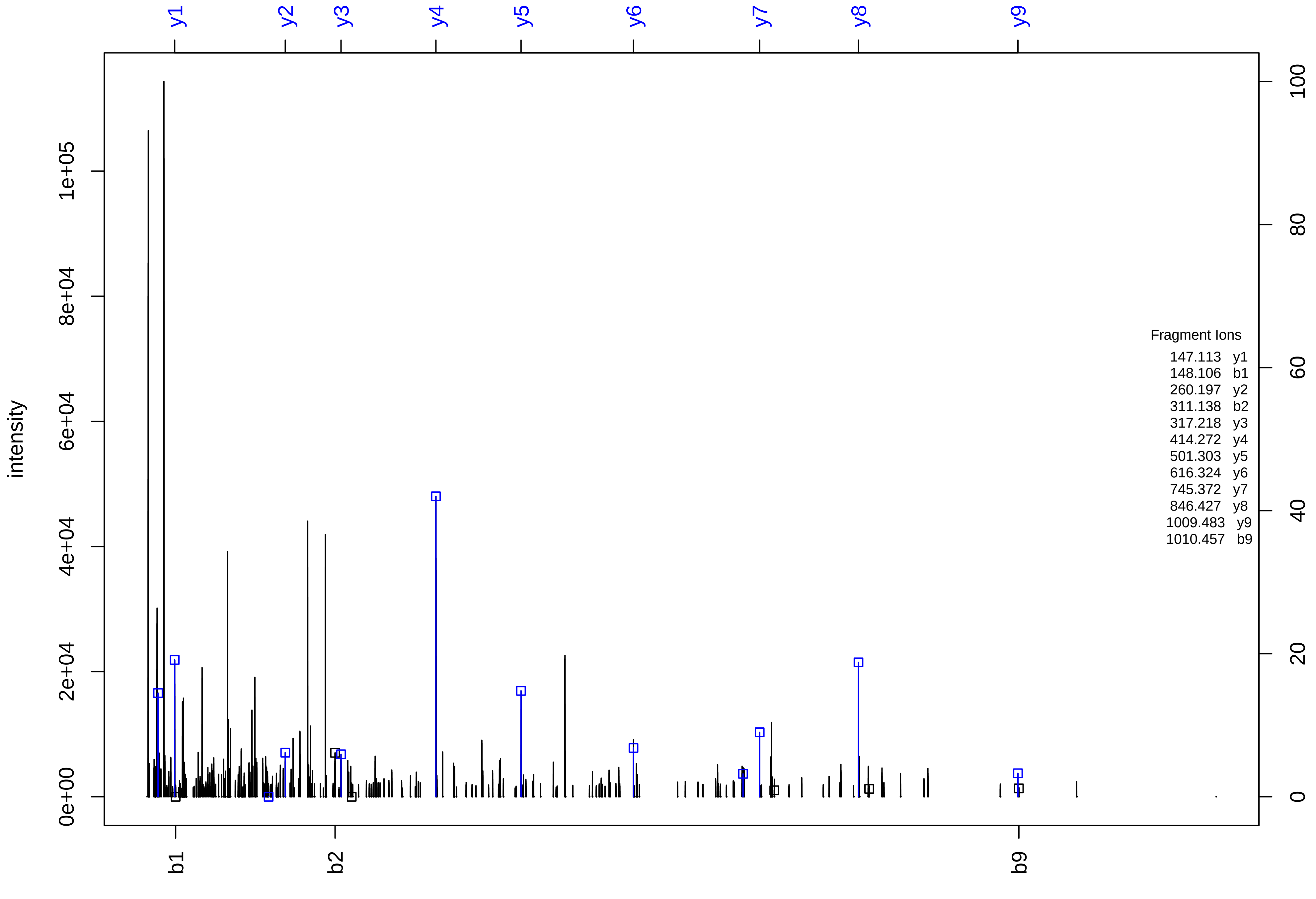

FYTEDSPGLK / File: sydney_01.raw; SpectrumID: ; scans: 2470

FLDPDMYSLIDDSTGDLR

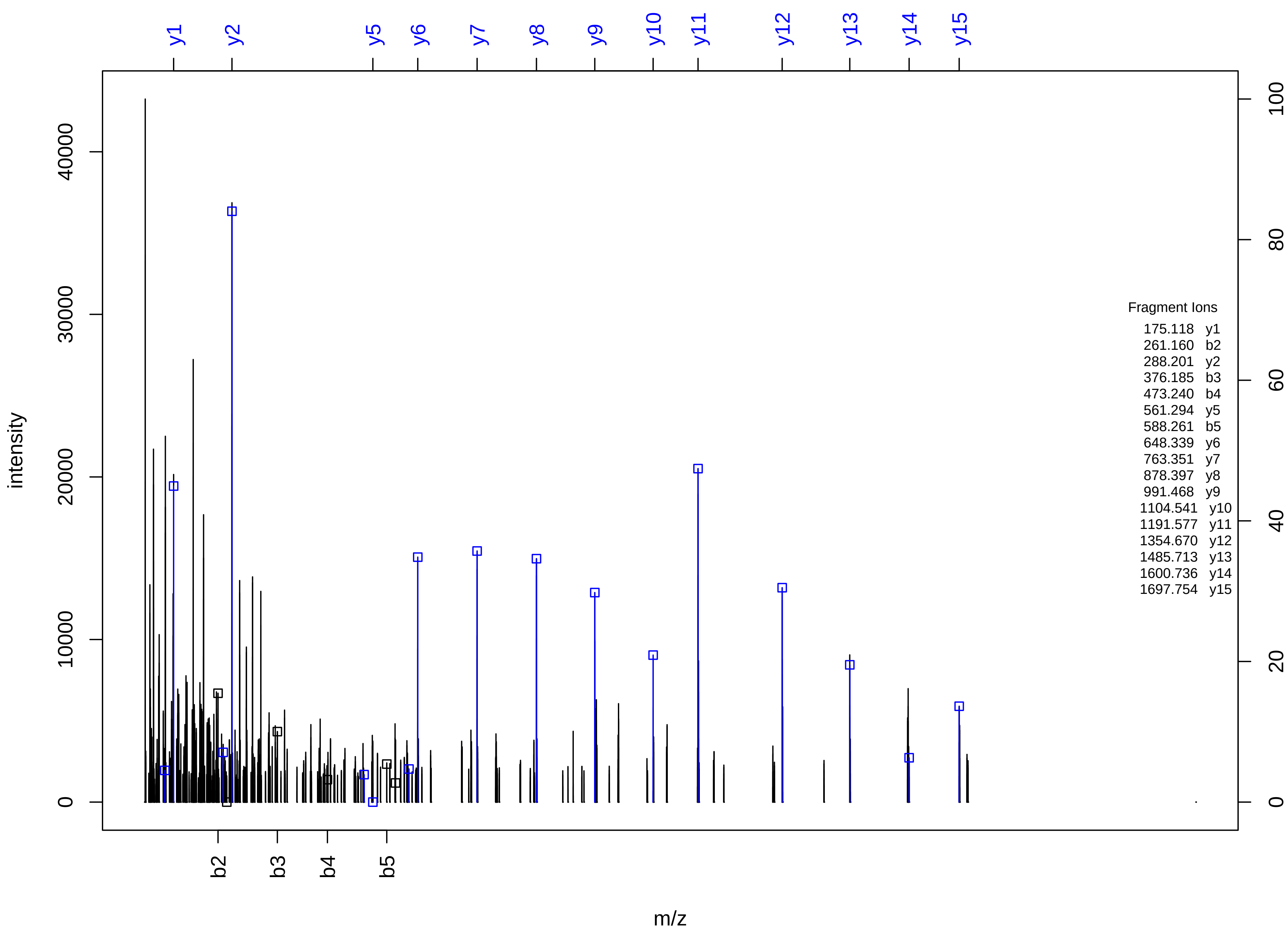

FLDPDMYSLIDDSTGDLR / File: sydney_01.raw; SpectrumID: ; scans: 13502

LLSVTPTSEGYSIK

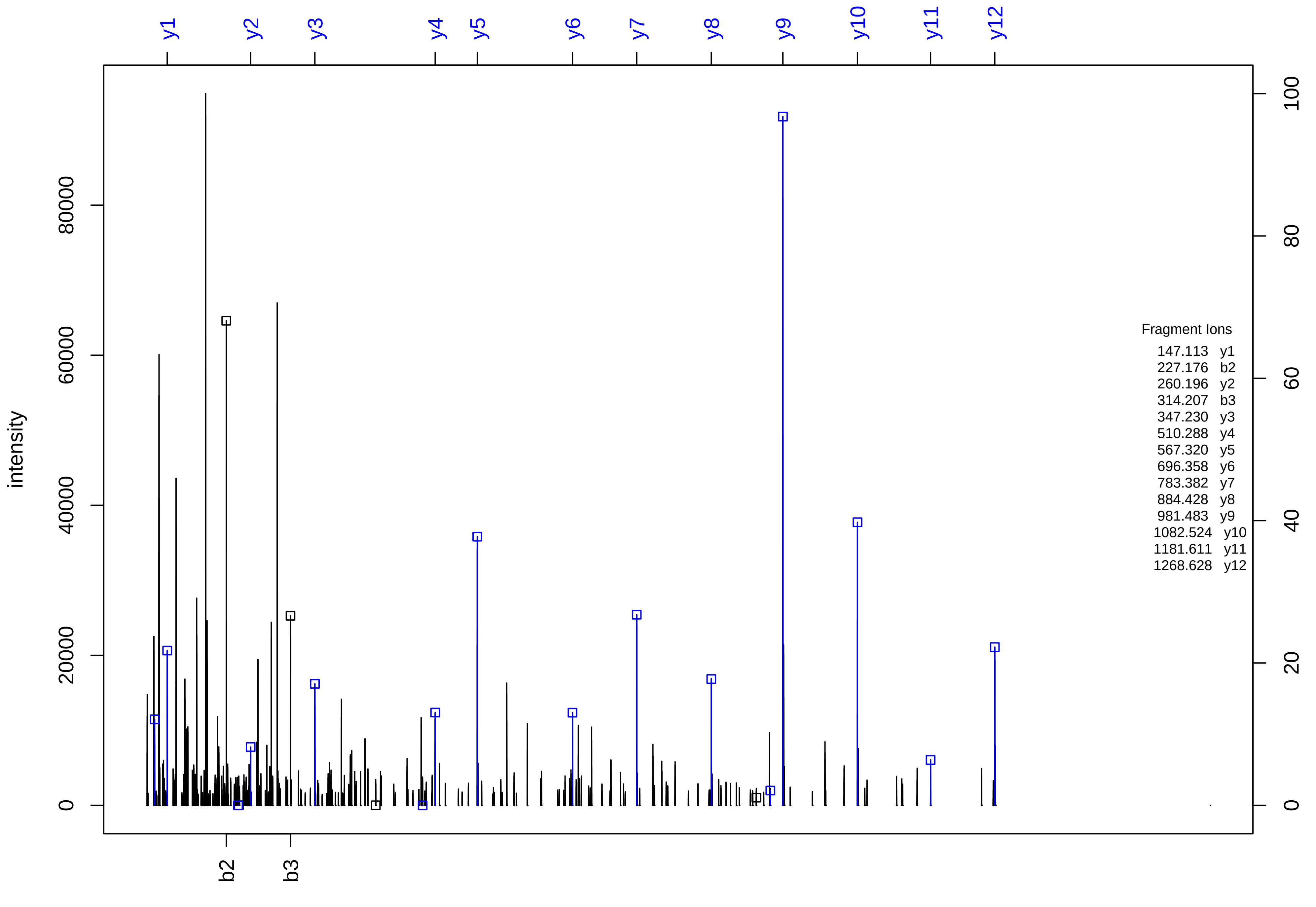

LLSVTPTSEGYSIK / File: sydney_01.raw; SpectrumID: ; scans: 6209

EFPGFLENQK

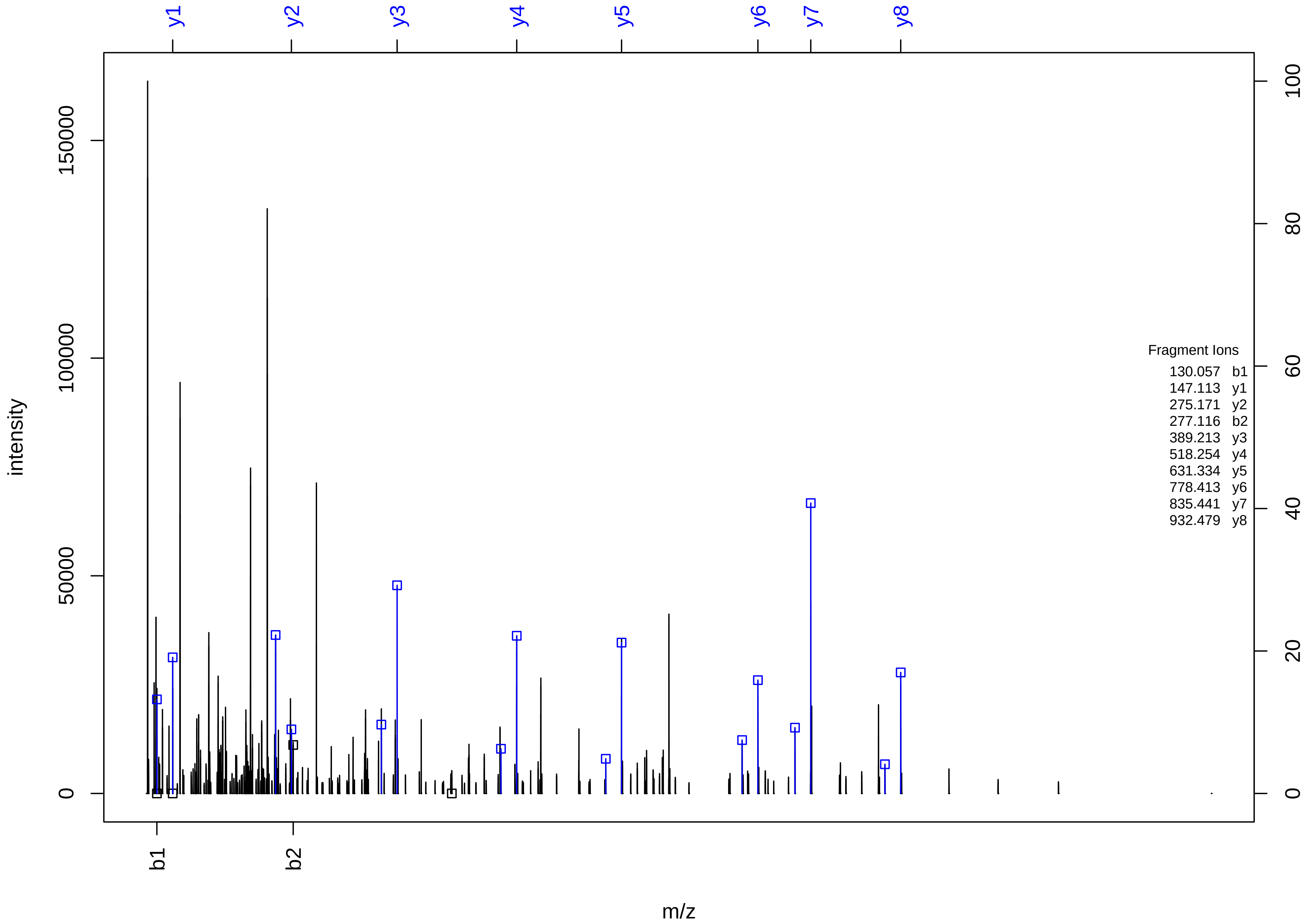

EFPGFLENQK / File: sydney_01.raw; SpectrumID: ; scans: 6618

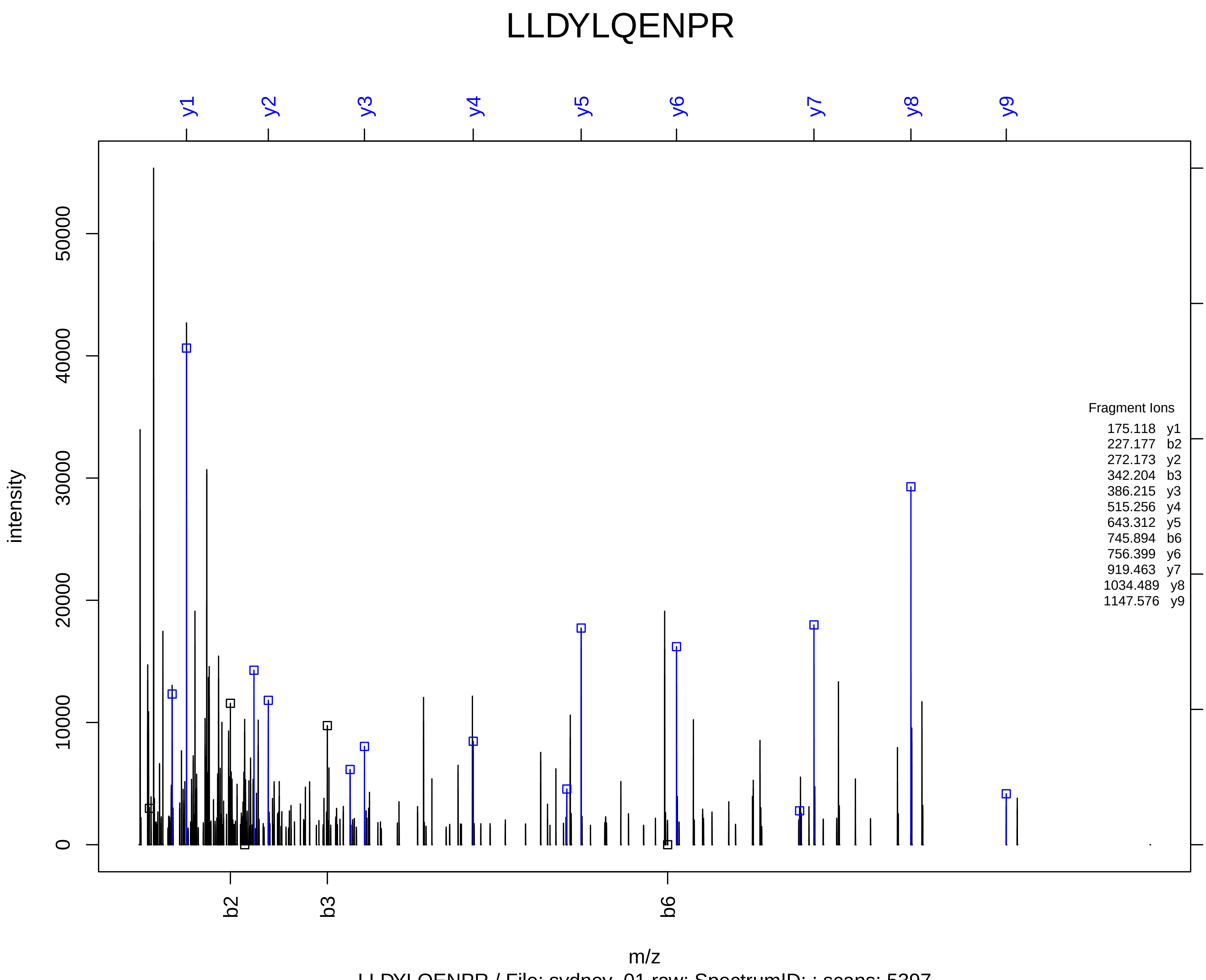

LLDYLQENPR / File: sydney_01.raw; SpectrumID: ; scans: 5397

LDIIQ^PLPETCEEISSDALTVR

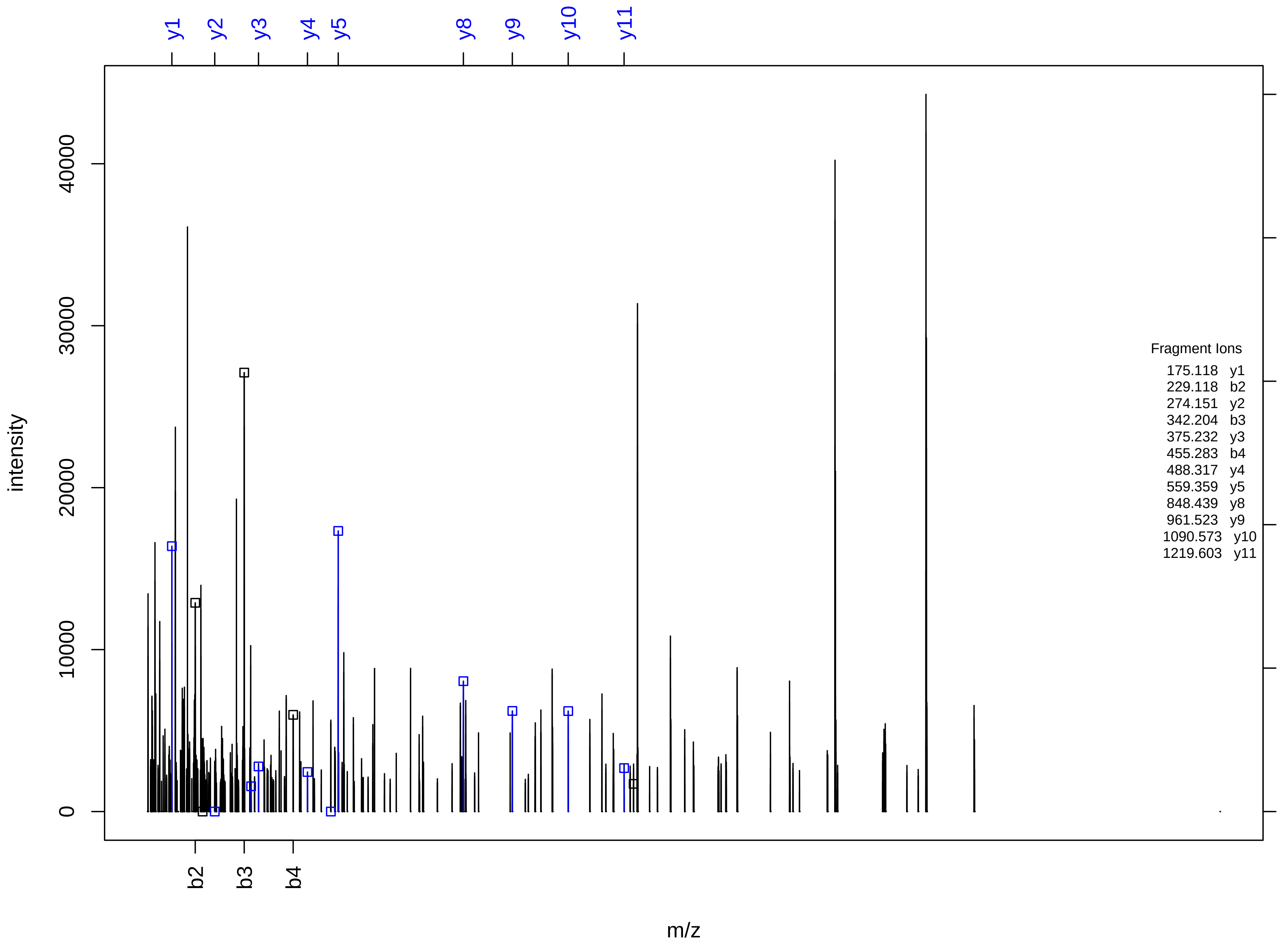

LDIIQPLPETCEEISSDALTVR / File: sydney_01.raw; SpectrumID: ; scans: 12241

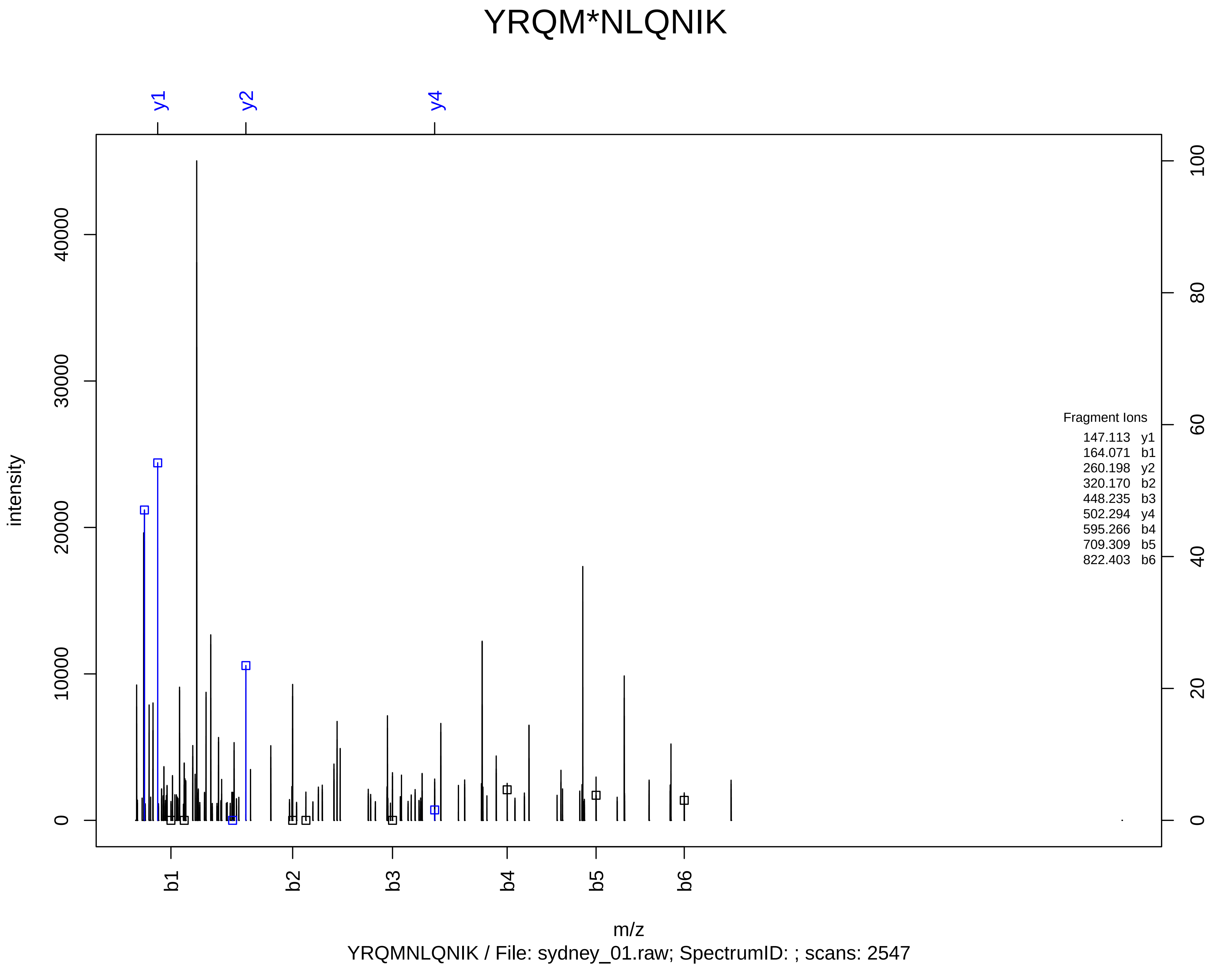

YRQM*NLQNIK
y1
y2
y4
intensity
0
10000
20000
30000
40000
0
20
40
60
80
100
Fragment Ions
147.113 y1
164.071 b1
260.198 y2
320.170 b2
448.235 b3
502.294 y4
595.266 b4
709.309 b5
822.403 b6
b1
b2
b3
b4
b5
b6
m/z
YRQMNLQNIK / File: sydney_01.raw; SpectrumID: ; scans: 2547

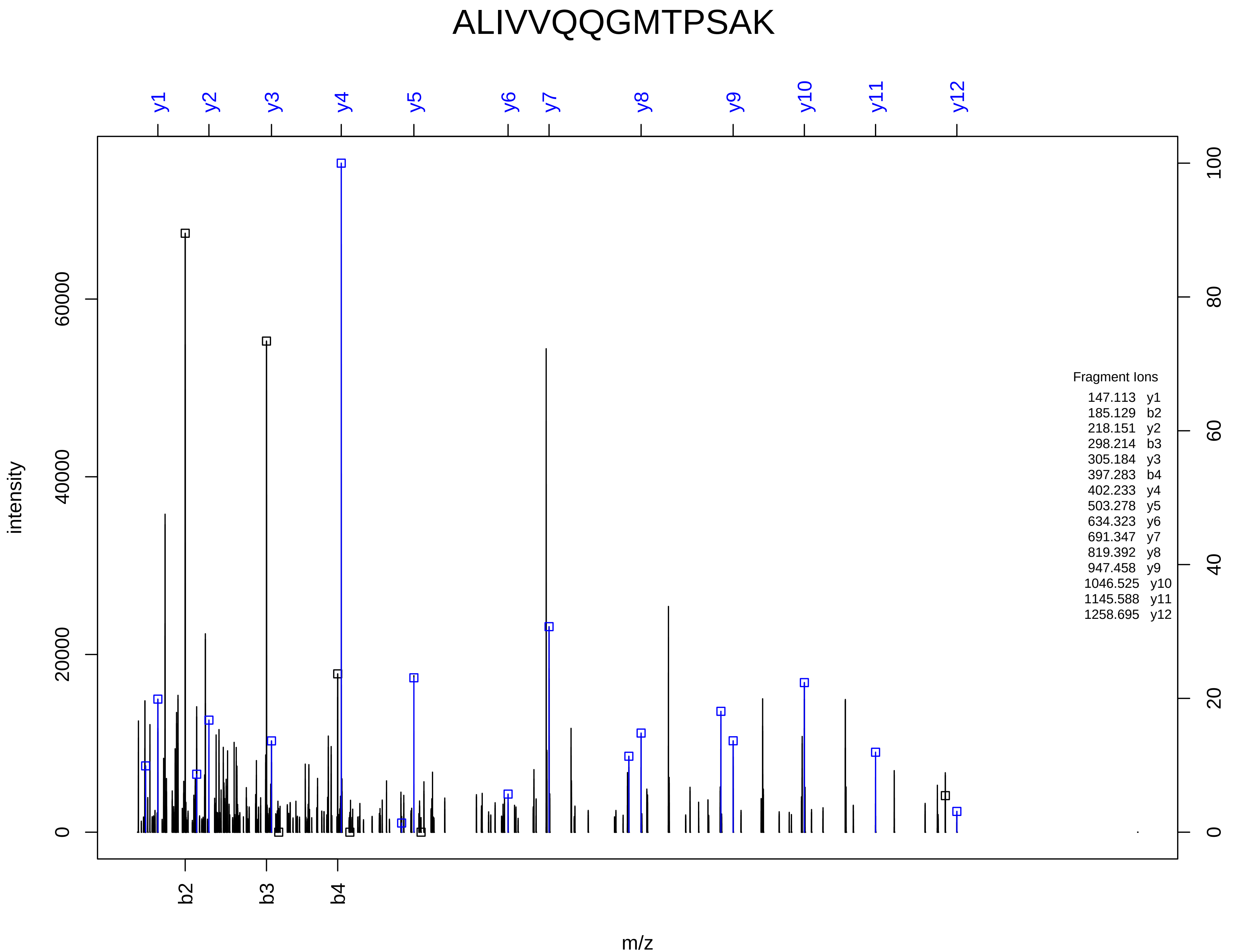

ALIVVQQGMTPSAK / File: sydney_01.raw; SpectrumID: ; scans: 5250

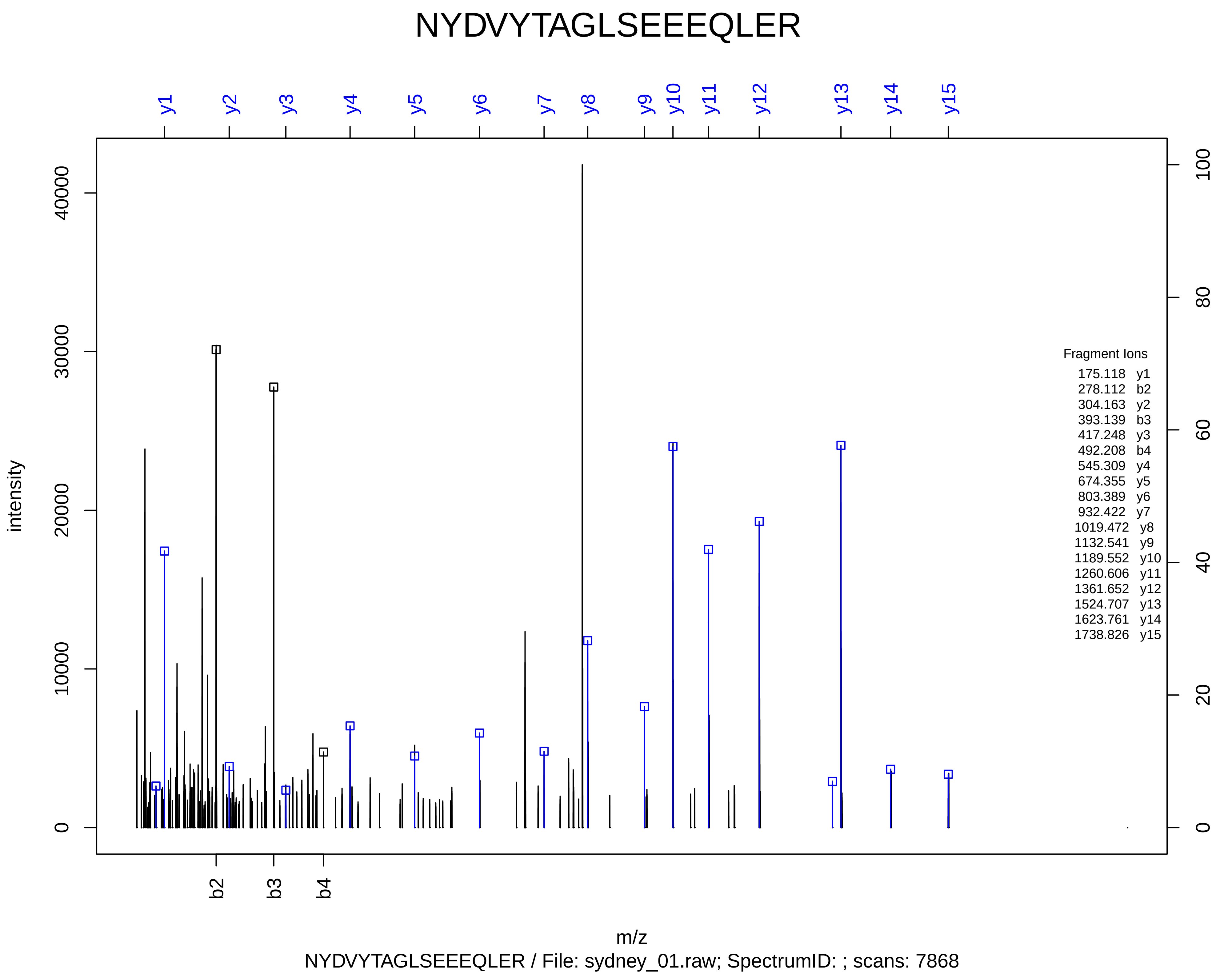
NYDVYTAGLSEEEQLER
intensity
m/z
y1 y2 y3 y4 y5 y6 y7 y8 y9 y10 y11 y12 y13 y14 y15
b2 b3 b4
0 10000 20000 30000 40000
0 20 40 60 80 100
Fragment Ions
175.118 y1
278.112 b2
304.163 y2
393.139 b3
417.248 y3
492.208 b4
545.309 y4
674.355 y5
803.389 y6
932.422 y7
1019.472 y8
1132.541 y9
1189.552 y10
1260.606 y11
1361.652 y12
1524.707 y13
1623.761 y14
1738.826 y15
NYDVYTAGLSEEEQLER / File: sydney_01.raw; SpectrumID: ; scans: 7868

TCHVGDENQEQQDVCSEPFEETSQK

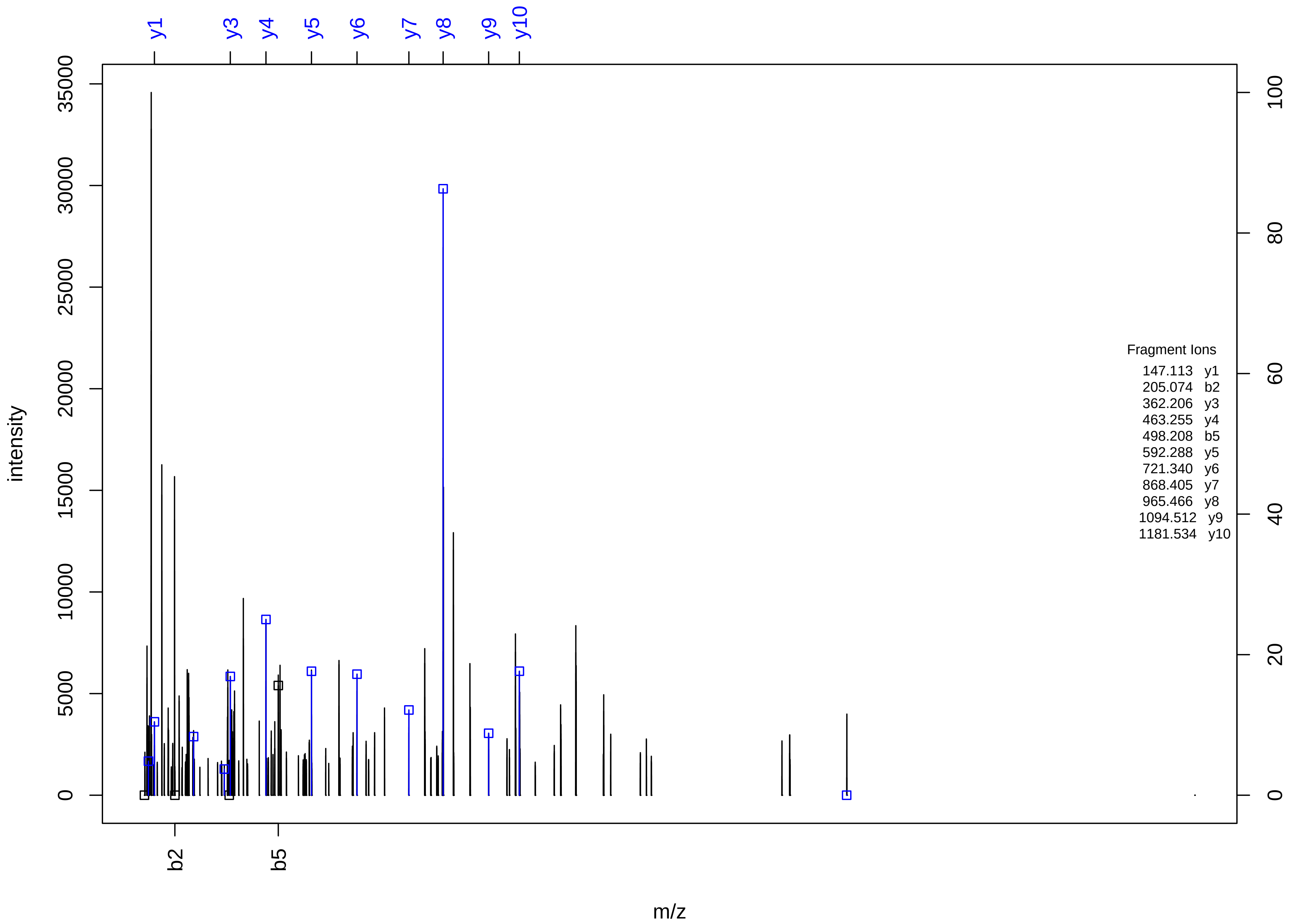

TCHVGDENQEQQDVCSEPFEETSQK / File: sydney_01.raw; SpectrumID: ; scans: 2474

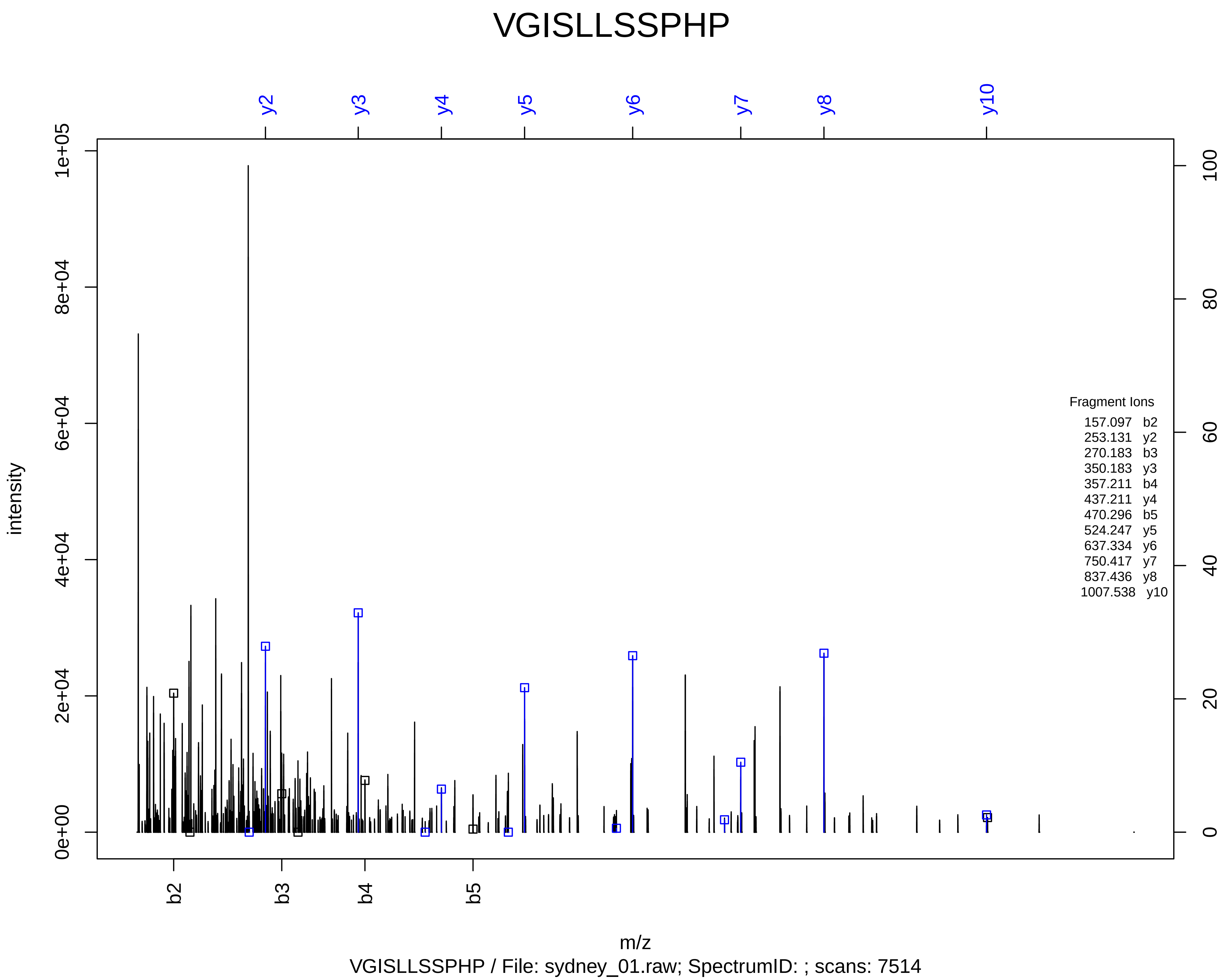
VGISLLSSPHP
y2
y3
y4
y5
y6
y7
y8
y10
intensity
0e+00
2e+04
4e+04
6e+04
8e+04
1e+05
0
20
40
60
80
100
b2
b3
b4
b5
m/z
Fragment Ions
157.097 b2
253.131 y2
270.183 b3
350.183 y3
357.211 b4
437.211 y4
470.296 b5
524.247 y5
637.334 y6
750.417 y7
837.436 y8
1007.538 y10
VGISLLSSPHP / File: sydney_01.raw; SpectrumID: ; scans: 7514

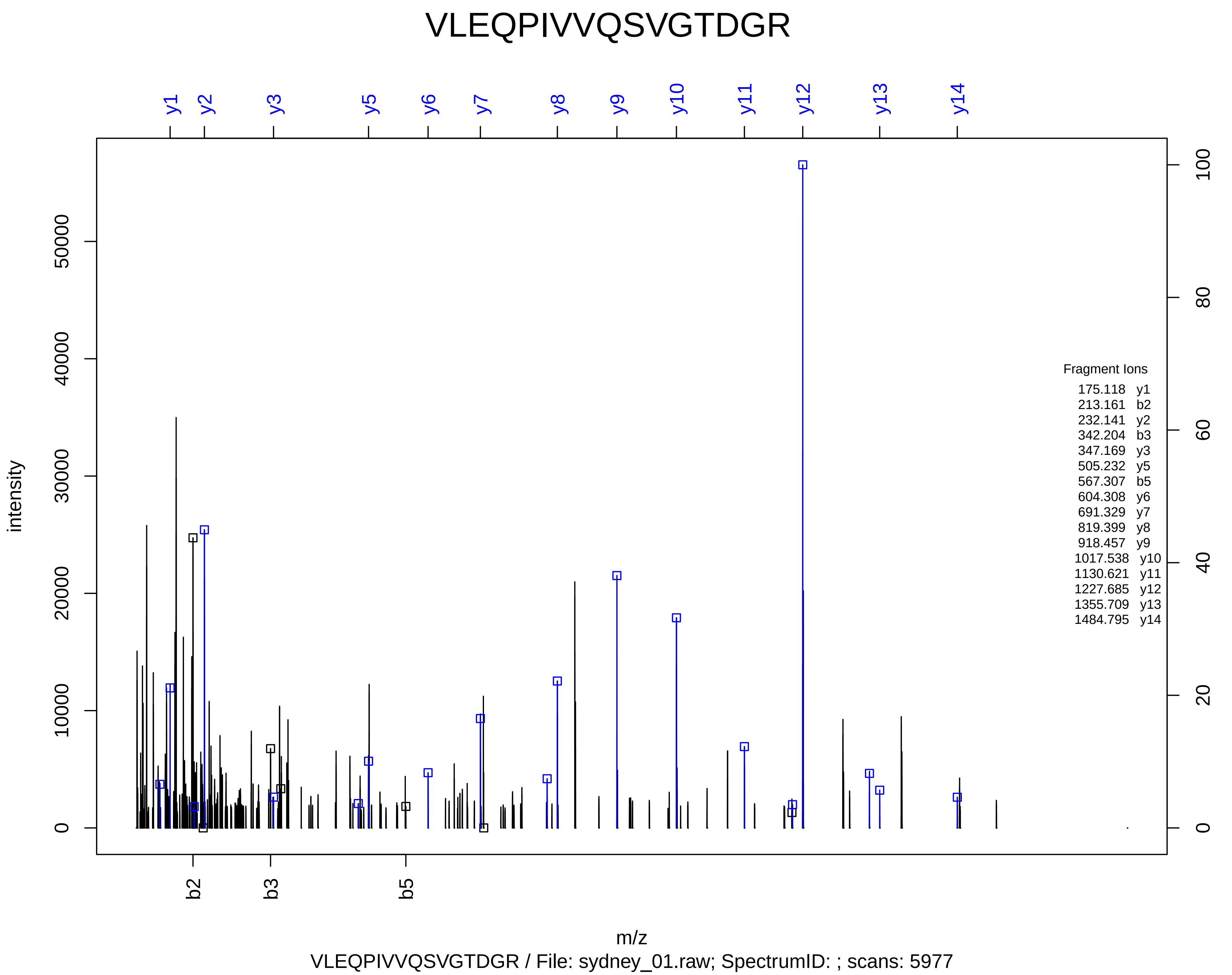
VLEQPIVVQSVGTDGR
y1 y2 y3 y5 y6 y7 y8 y9 y10 y11 y12 y13 y14
intensity
0 10000 20000 30000 40000 50000
0 20 40 60 80 100
b2 b3 b5
m/z
Fragment Ions
175.118 y1
213.161 b2
232.141 y2
342.204 b3
347.169 y3
505.232 y5
567.307 b5
604.308 y6
691.329 y7
819.399 y8
918.457 y9
1017.538 y10
1130.621 y11
1227.685 y12
1355.709 y13
1484.795 y14
VLEQPIVVQSVGTDGR / File: sydney_01.raw; SpectrumID: ; scans: 5977

GLITFESLEK

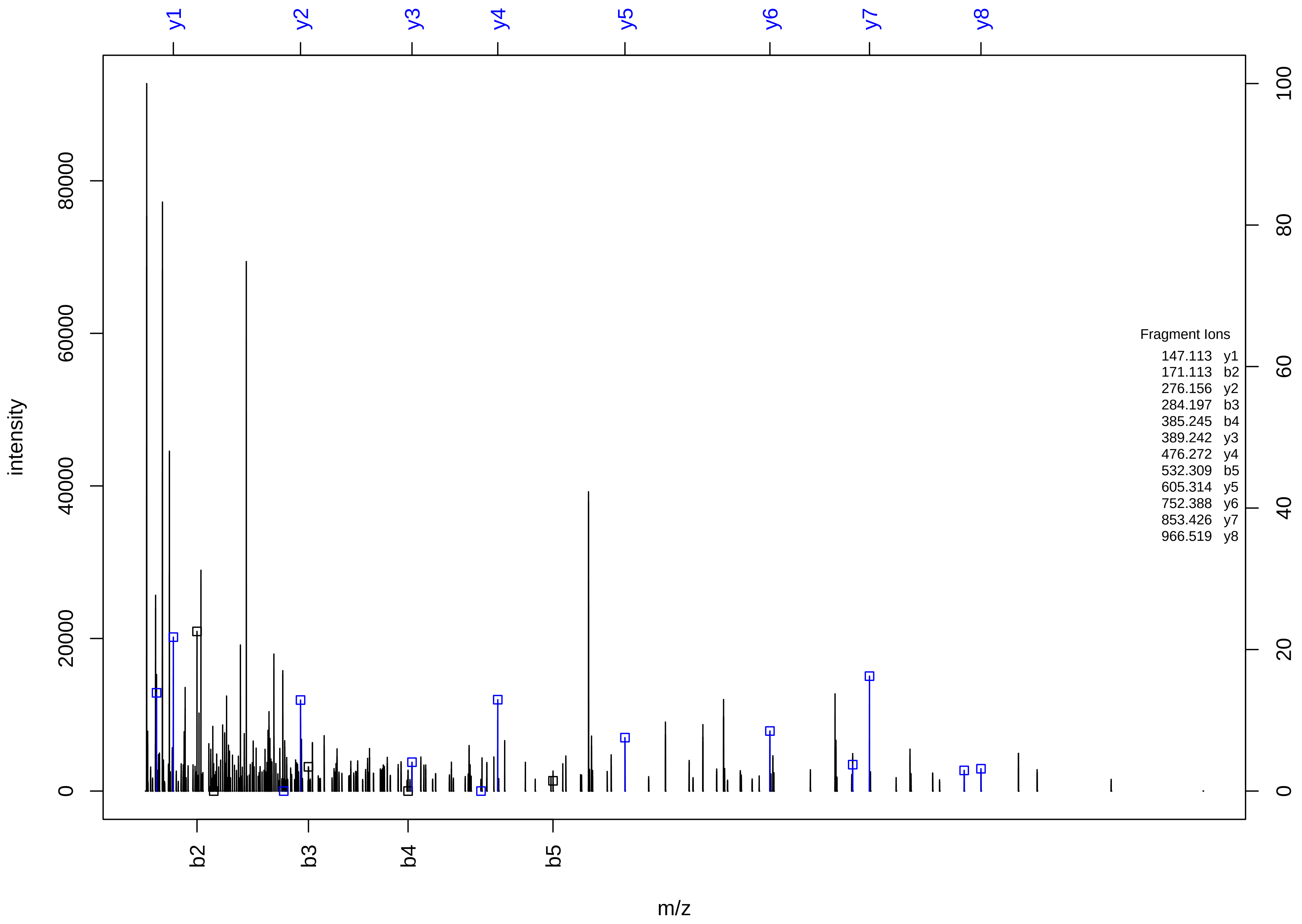

GLITFESLEK / File: sydney_01.raw; SpectrumID: ; scans: 8361

DAFLGQDLPYTEETLDEIAK

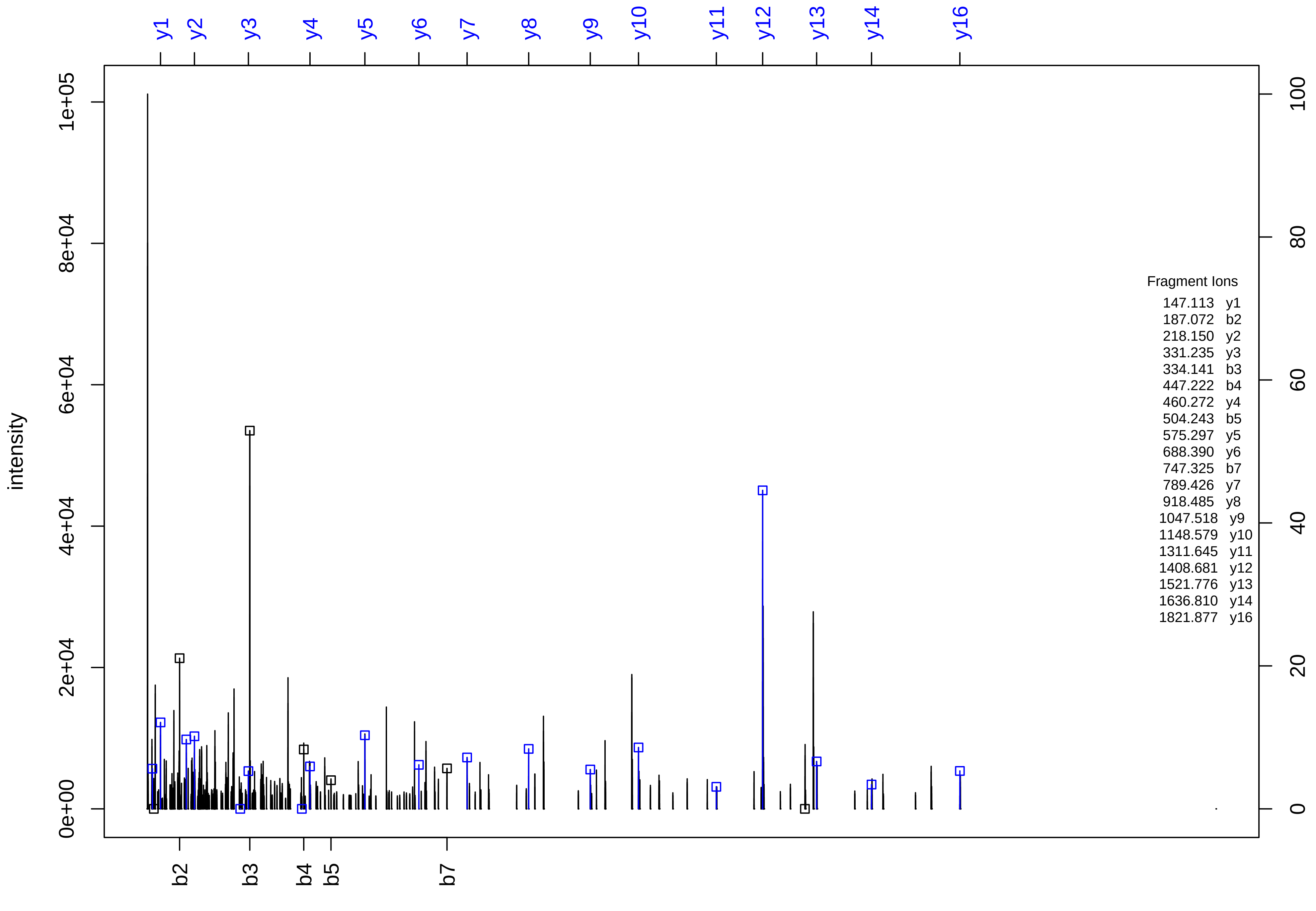

DAFLGQDLPYTEETLDEIAK / File: sydney_01.raw; SpectrumID: ; scans: 13660

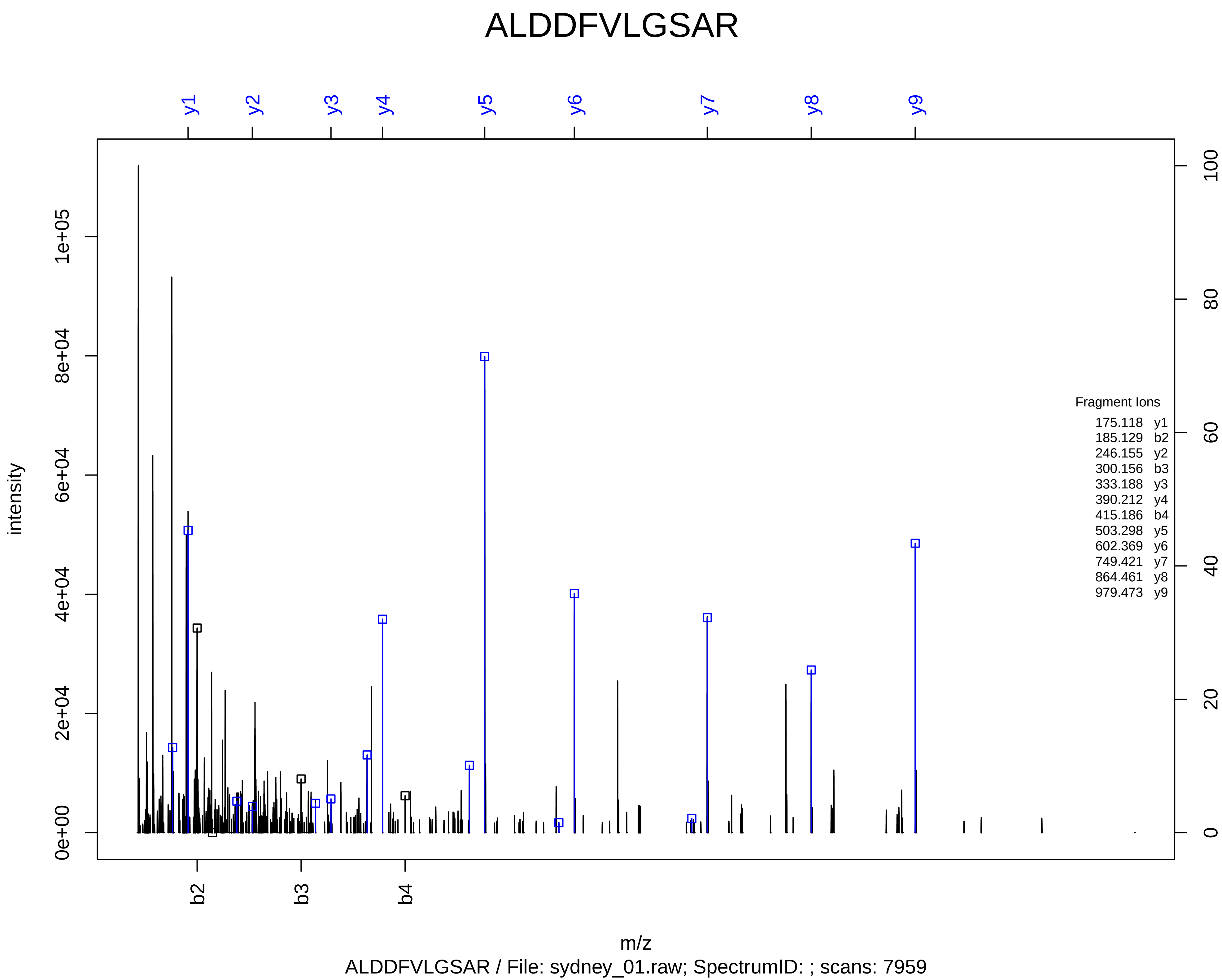
ALDDFVLGSAR
y1
y2
y3
y4
y5
y6
y7
y8
y9
b2
b3
b4
intensity
0e+00
2e+04
4e+04
6e+04
8e+04
1e+05
0
20
40
60
80
100
Fragment Ions
175.118 y1
185.129 b2
246.155 y2
300.156 b3
333.188 y3
390.212 y4
415.186 b4
503.298 y5
602.369 y6
749.421 y7
864.461 y8
979.473 y9
m/z
ALDDFVLGSAR / File: sydney_01.raw; SpectrumID: ; scans: 7959

ANPYECGFDPTSSAR

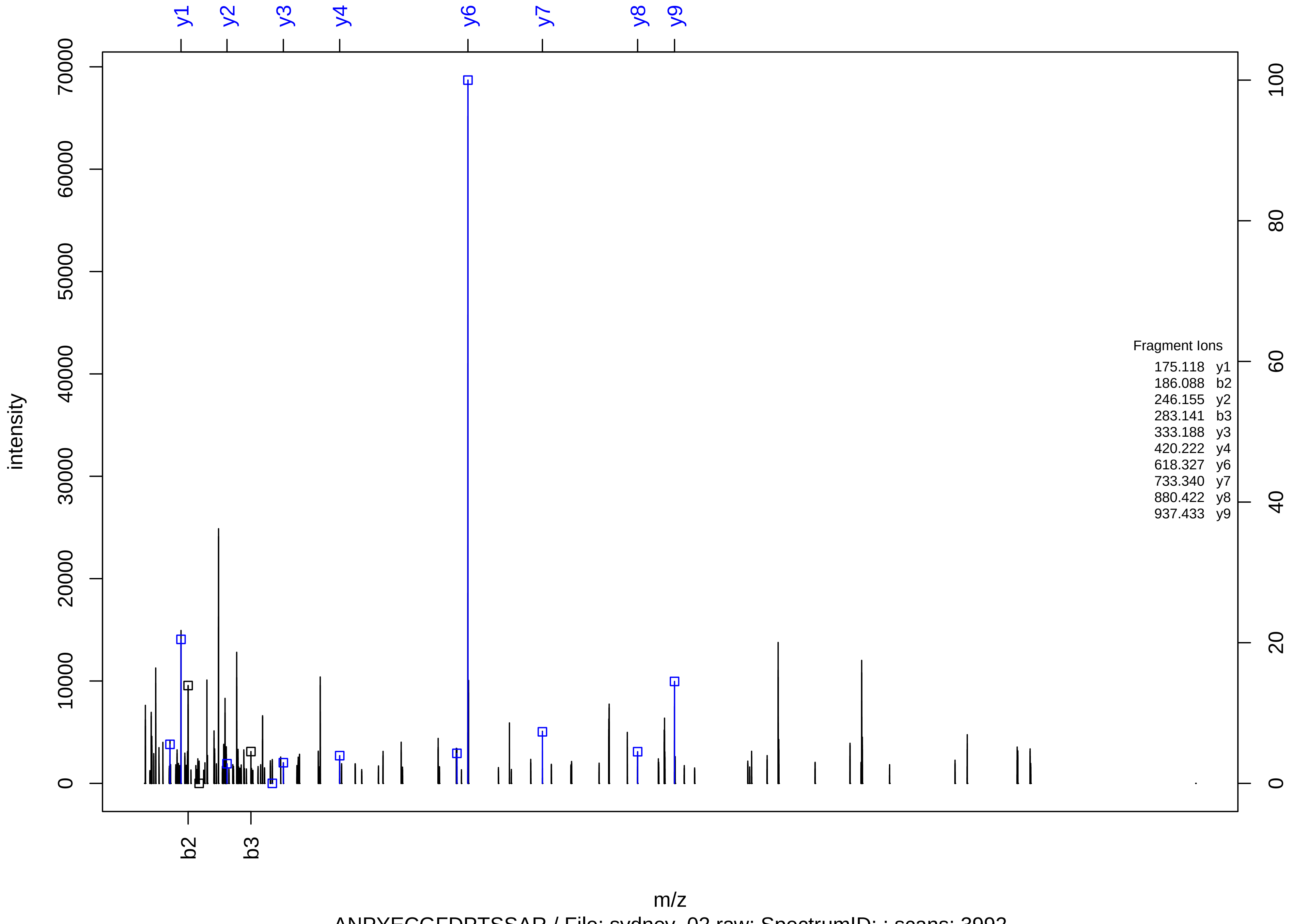

ANPYECGFDPTSSAR / File: sydney_02.raw; SpectrumID: ; scans: 3992

FILLACDGLFK

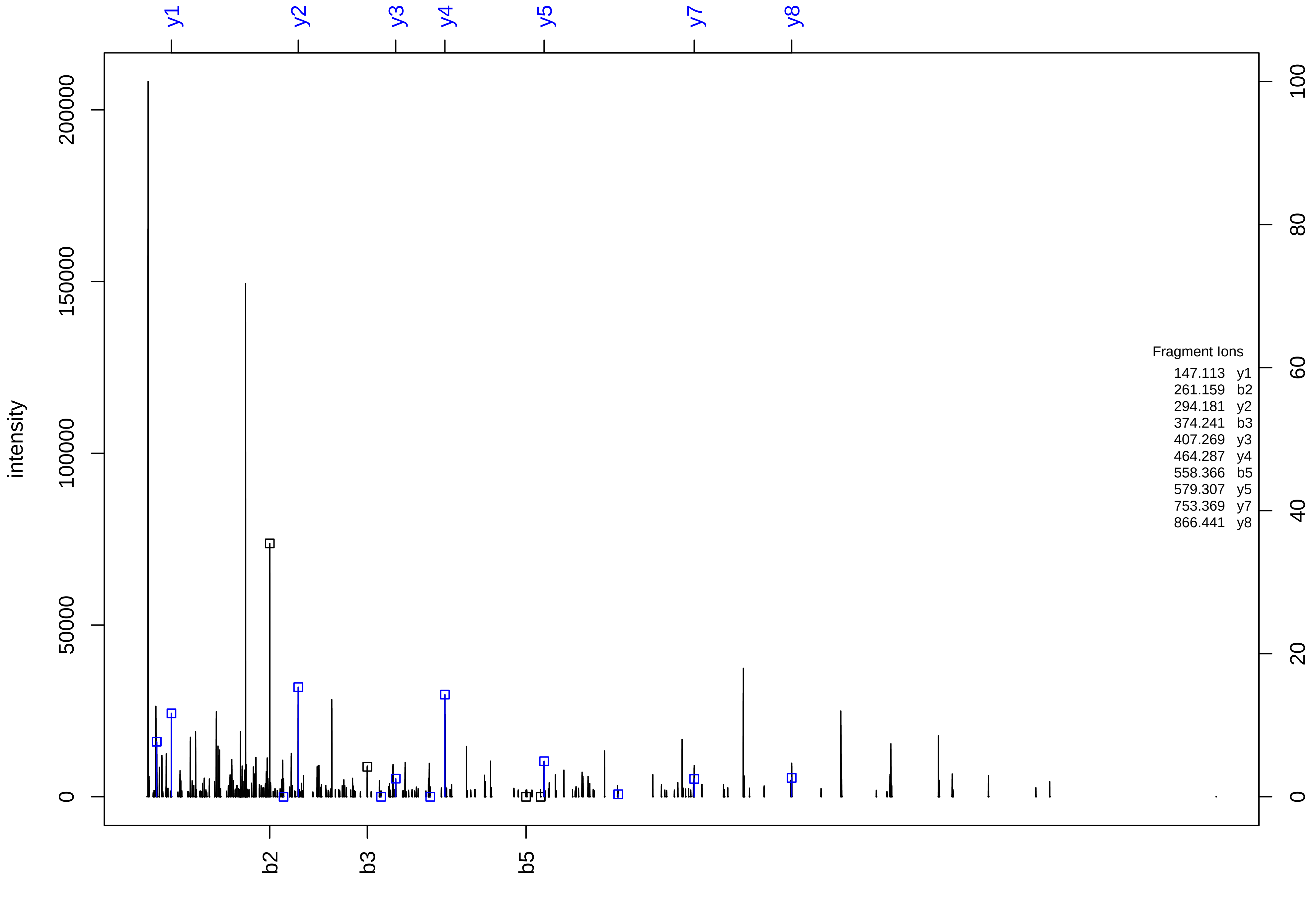

FILLACDGLFK / File: sydney_02.raw; SpectrumID: ; scans: 13080

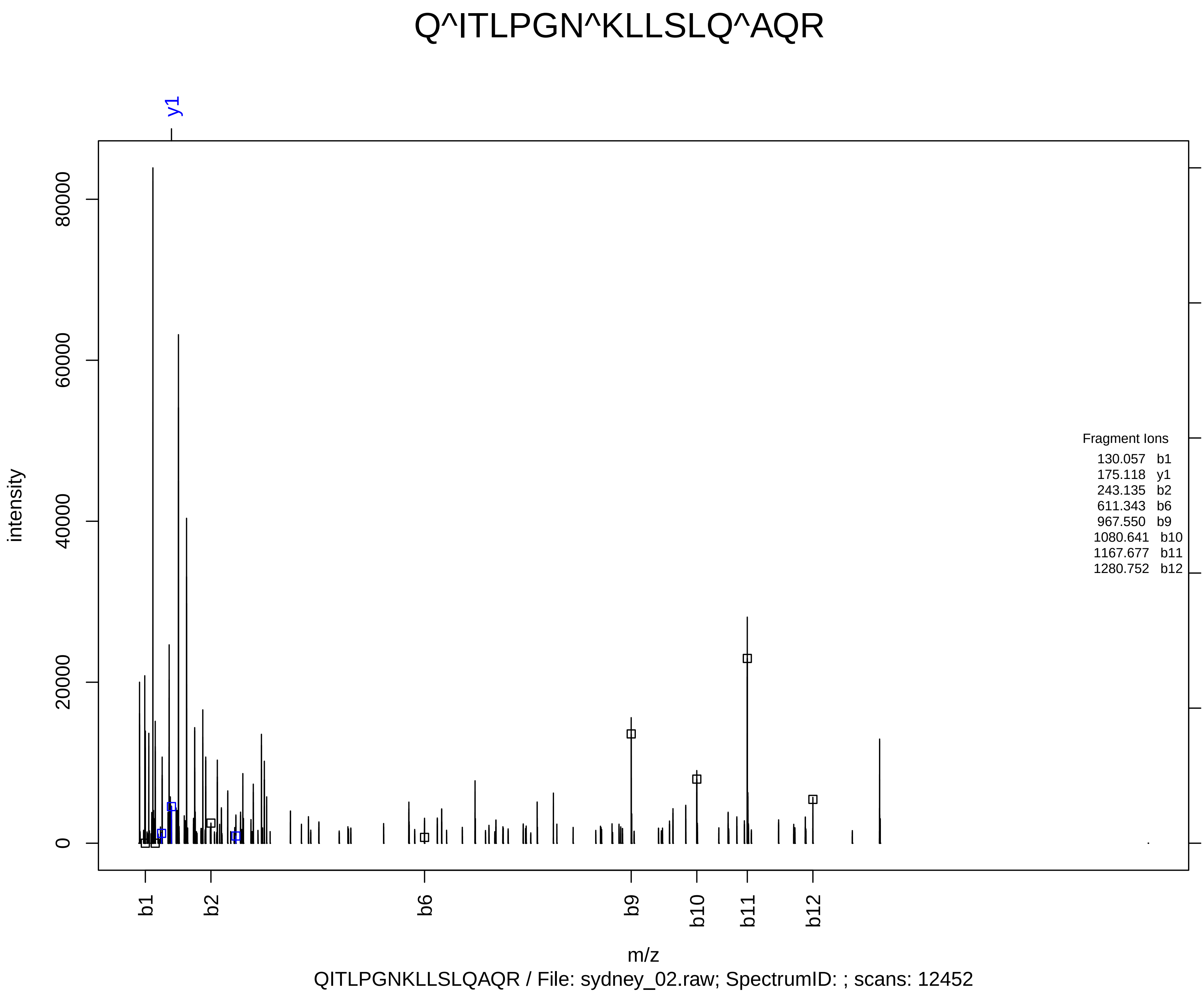
Q^ITLPGN^KLLSLQ^AQR
y1
intensity
0
20000
40000
60000
80000
0
20
40
60
80
100
Fragment Ions
130.057 b1
175.118 y1
243.135 b2
611.343 b6
967.550 b9
1080.641 b10
1167.677 b11
1280.752 b12
b1
b2
b6
b9
b10
b11
b12
m/z
QITLPGNKLLSLQAQR / File: sydney_02.raw; SpectrumID: ; scans: 12452

TVEPYVTWGFPNLK

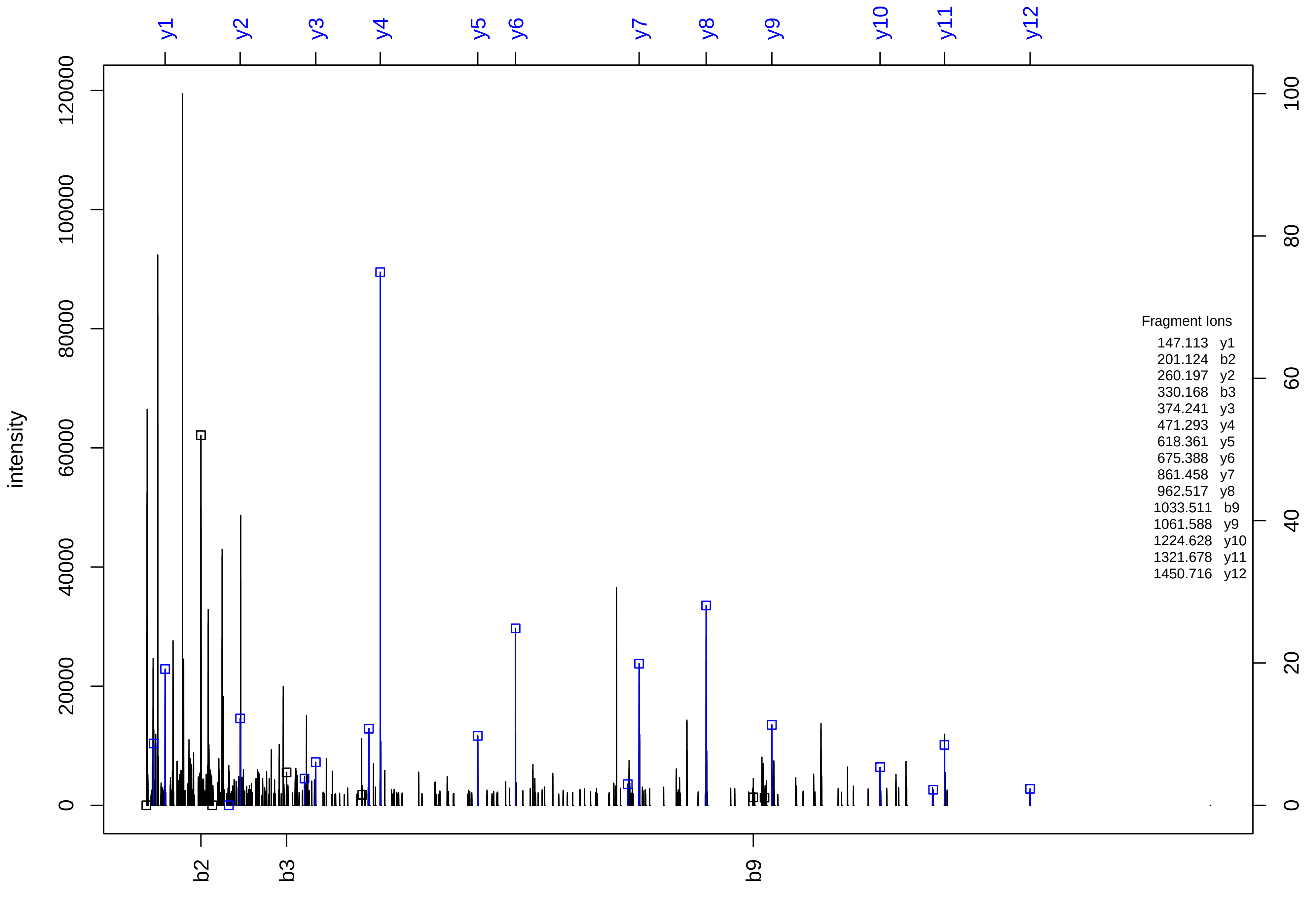

TVEPYVTWGFPNLK / File: sydney_02.raw; SpectrumID: ; scans: 12032

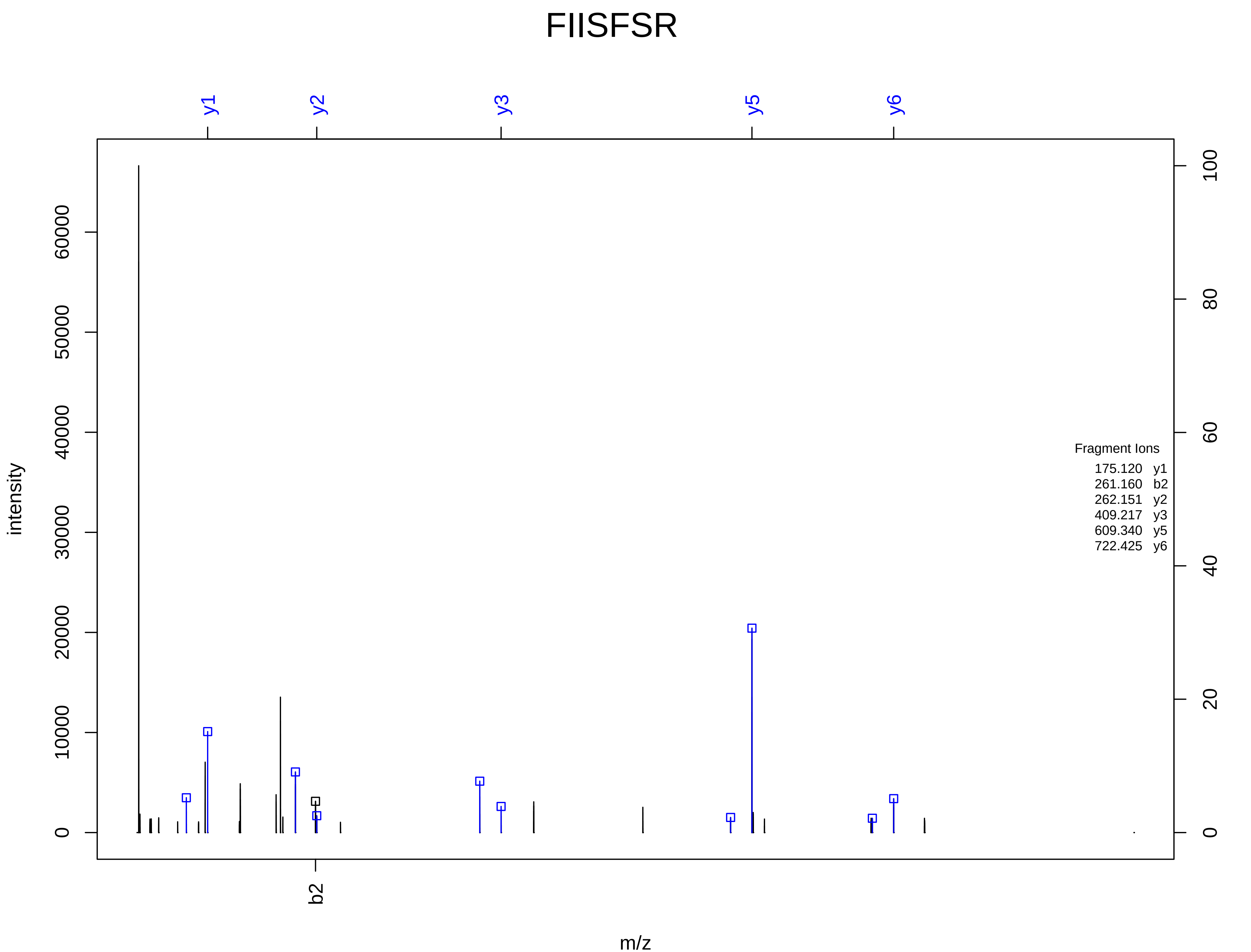

FIISFSR
y1
y2
y3
y5
y6
intensity
0
10000
20000
30000
40000
50000
60000
0
20
40
60
80
100
Fragment Ions
175.120 y1
261.160 b2
262.151 y2
409.217 y3
609.340 y5
722.425 y6
b2
m/z
FIISFSR / File: sydney_02.raw; SpectrumID: ; scans: 9198

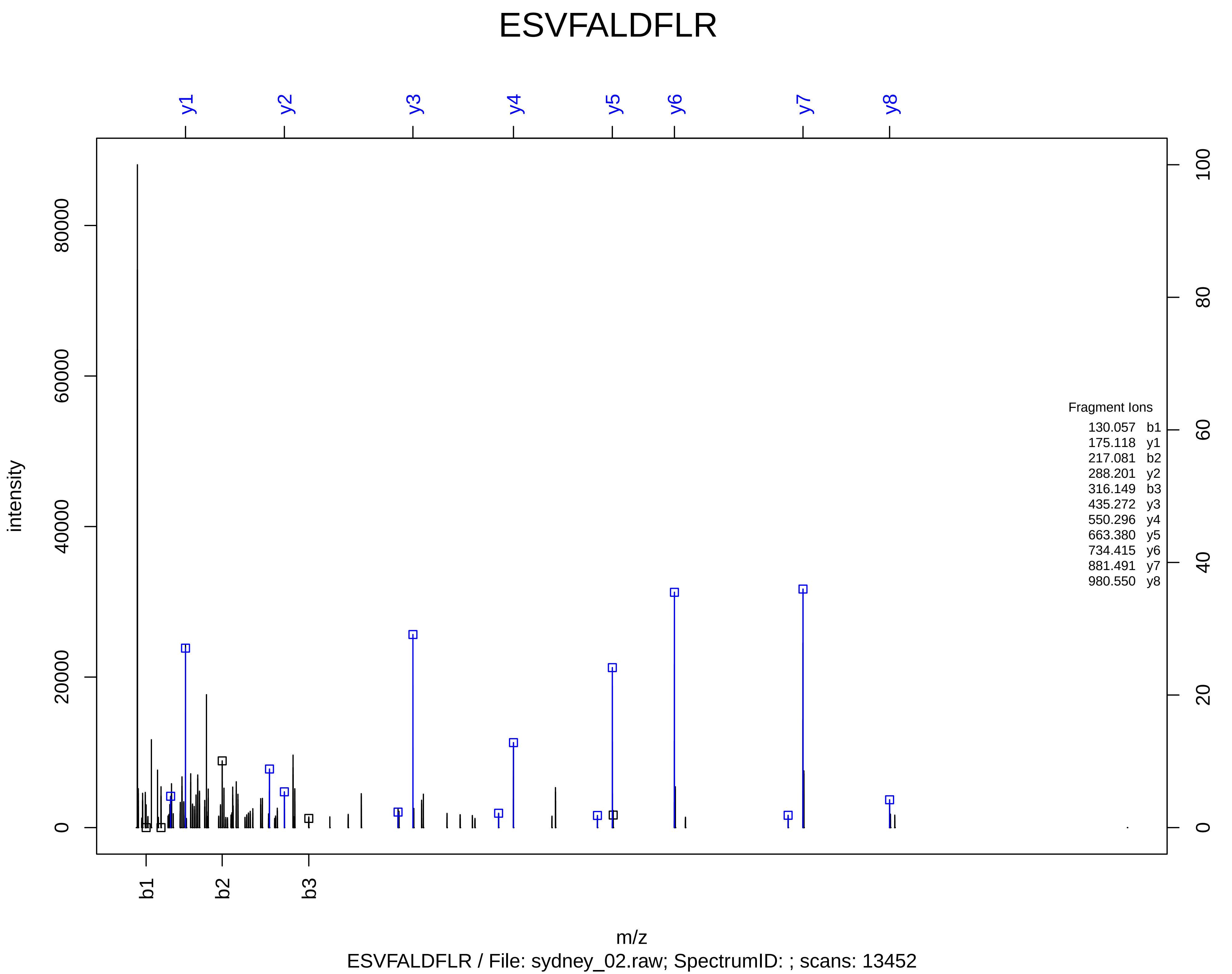
ESVFALDFLR
y1
y2
y3
y4
y5
y6
y7
y8
b1
b2
b3
intensity
0
20000
40000
60000
80000
0
20
40
60
80
100
Fragment Ions
130.057 b1
175.118 y1
217.081 b2
288.201 y2
316.149 b3
435.272 y3
550.296 y4
663.380 y5
734.415 y6
881.491 y7
980.550 y8
m/z
ESVFALDFLR / File: sydney_02.raw; SpectrumID: ; scans: 13452

(Ac)N^SVVSSNRSQVWN^EGNR

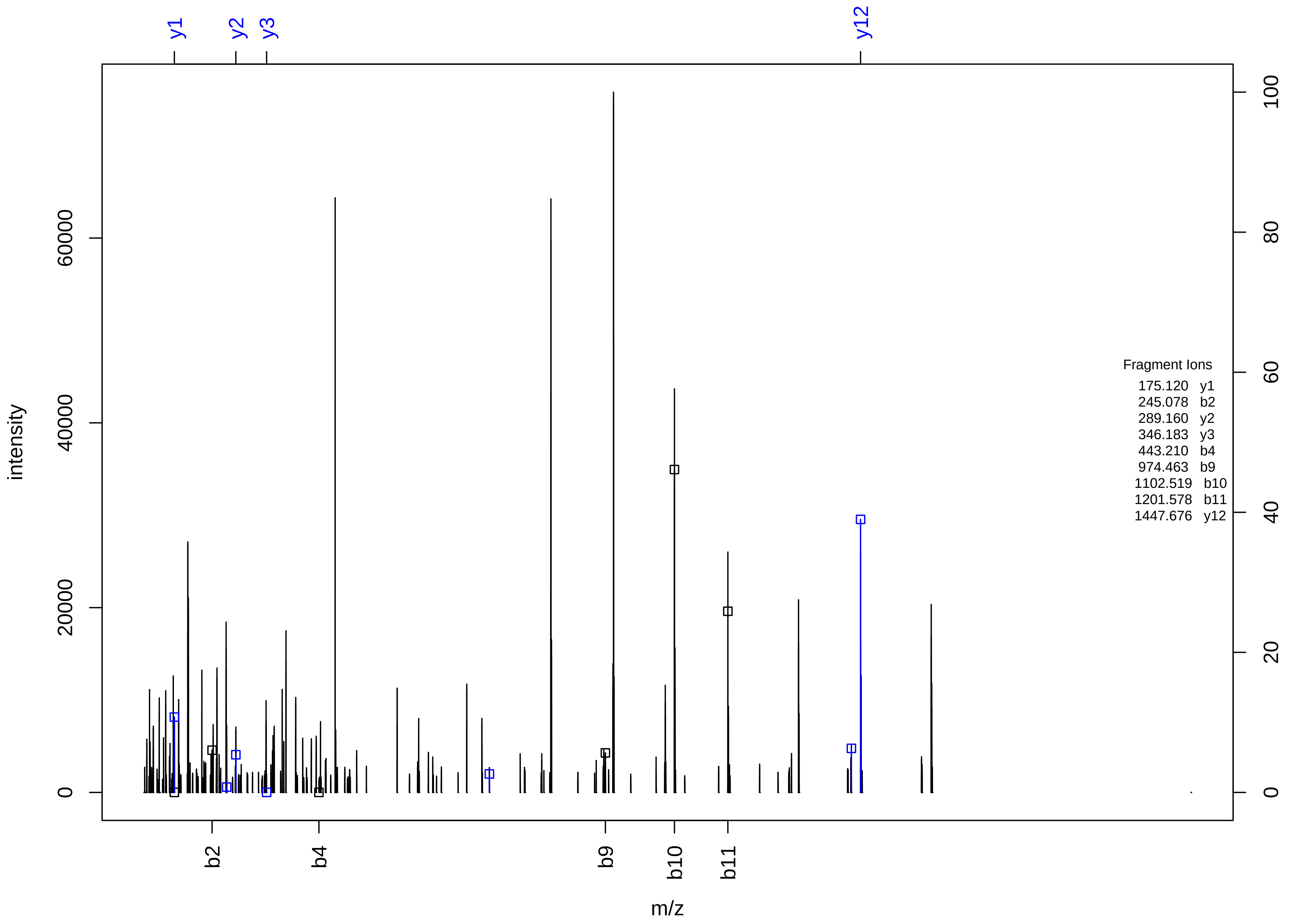

NSVVSSNRSQVWNEGNR / File: sydney_02.raw; SpectrumID: ; scans: 5987

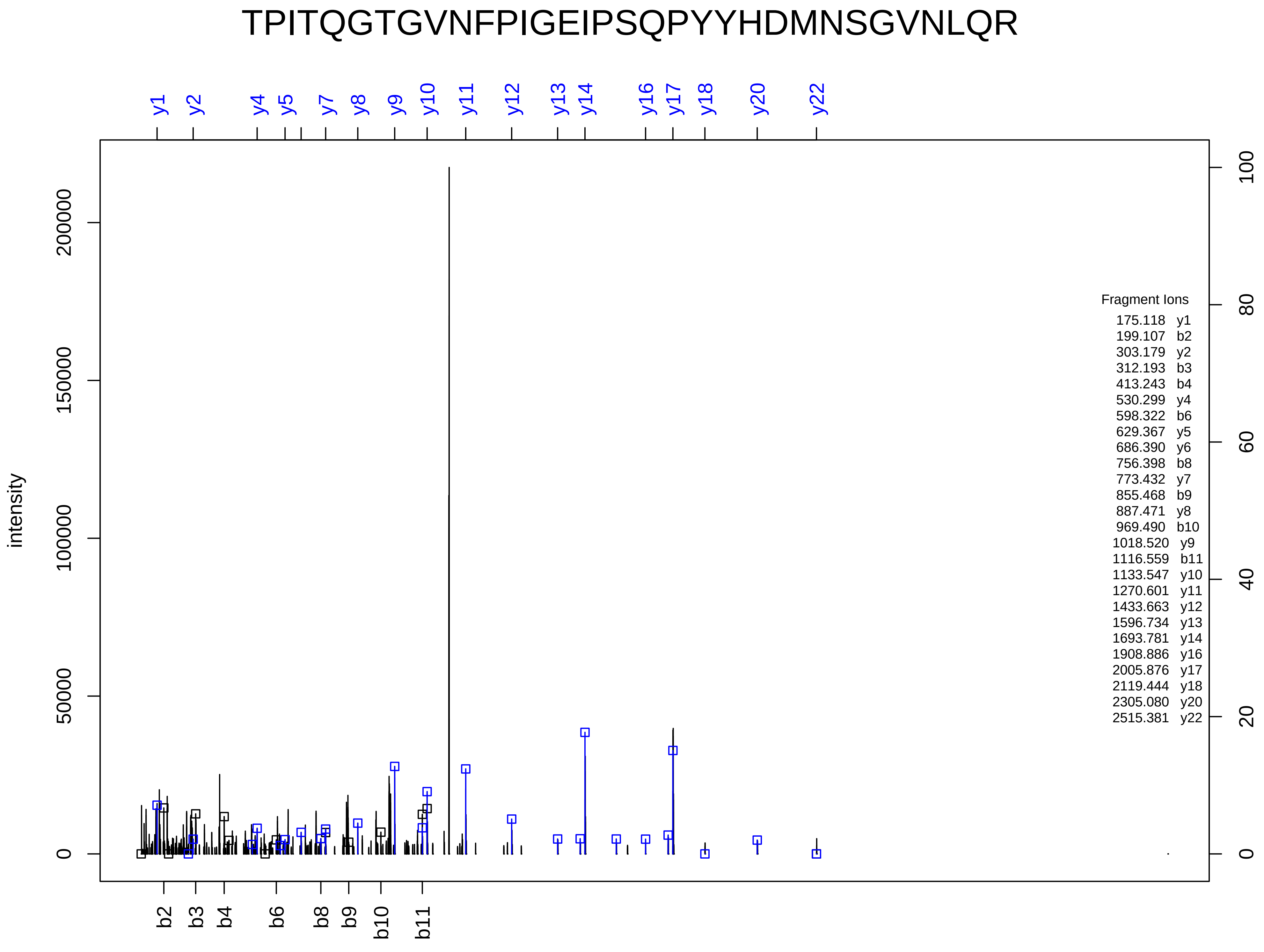

TPITQGTGVNFPIGEIPSQPYYHDMNSGVNLQR / File: sydney_02.raw; SpectrumID: ; scans: 10914

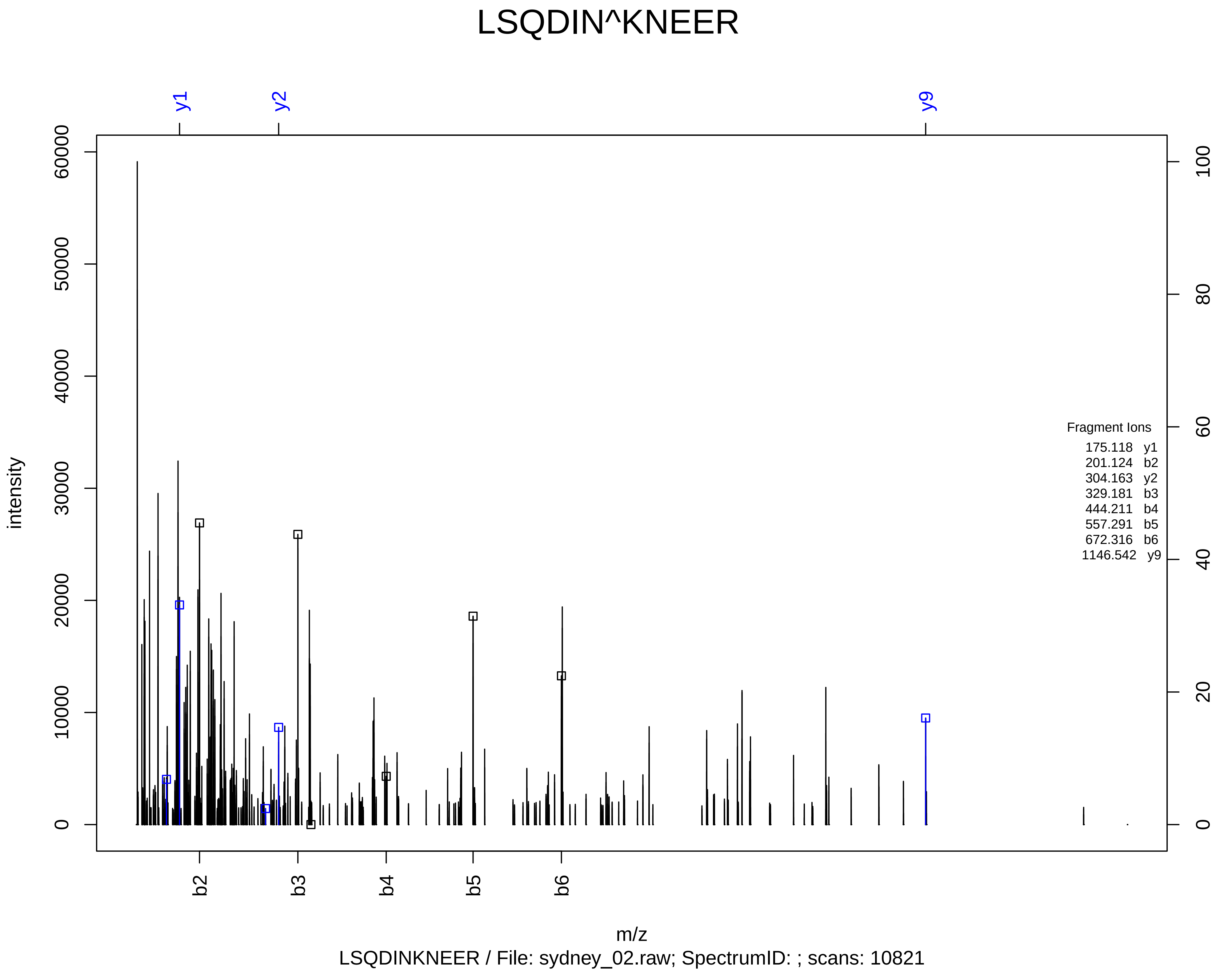
LSQDIN^KNEER
y1
y2
y9
intensity
60000
50000
40000
30000
20000
10000
0
100
80
60
40
20
0
Fragment Ions
175.118 y1
201.124 b2
304.163 y2
329.181 b3
444.211 b4
557.291 b5
672.316 b6
1146.542 y9
b2
b3
b4
b5
b6
m/z
LSQDINKNEER / File: sydney_02.raw; SpectrumID: ; scans: 10821

SNAVNQVVLWDK

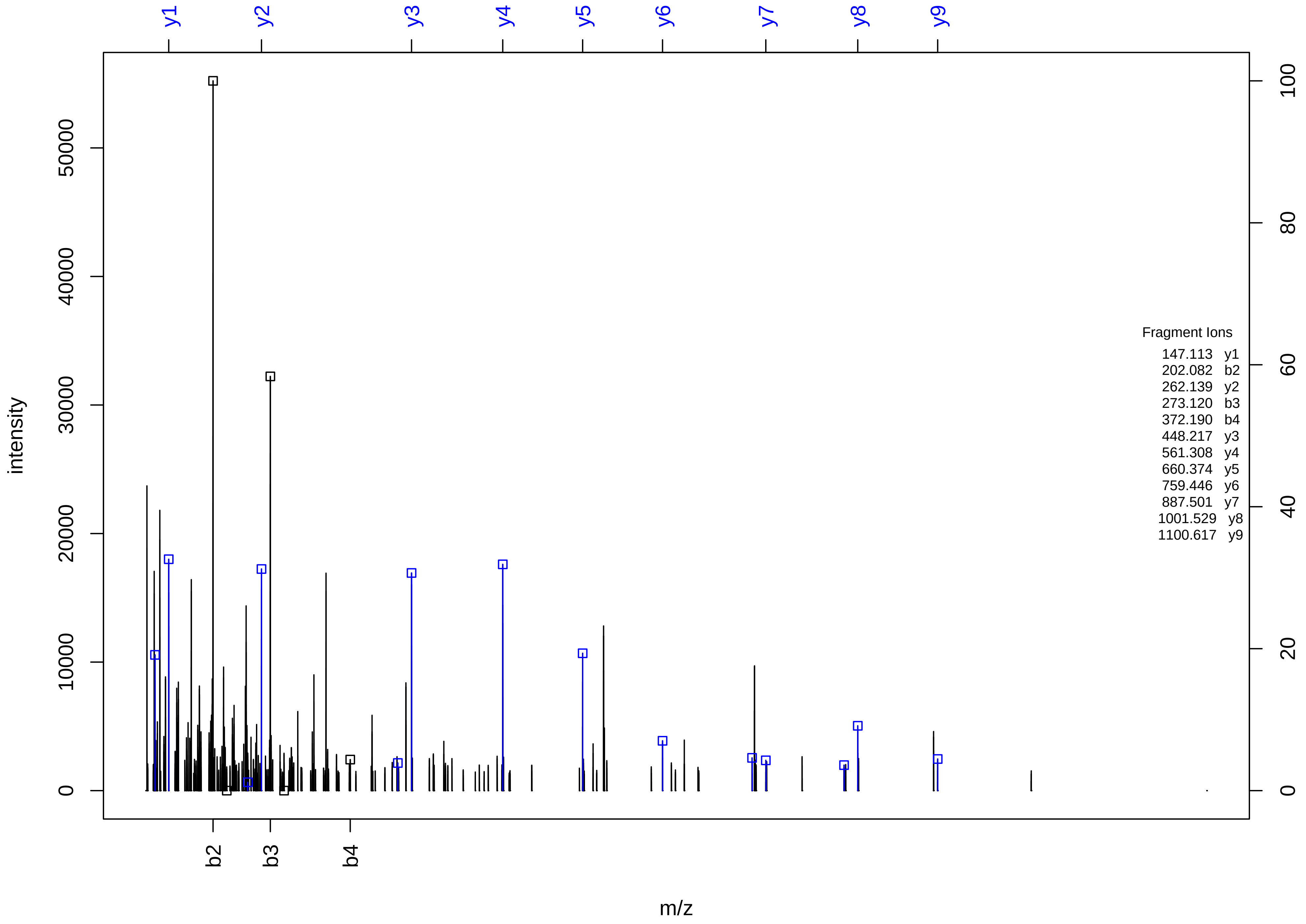

SNAVNQVVLWDK / File: sydney_02.raw; SpectrumID: ; scans: 6660

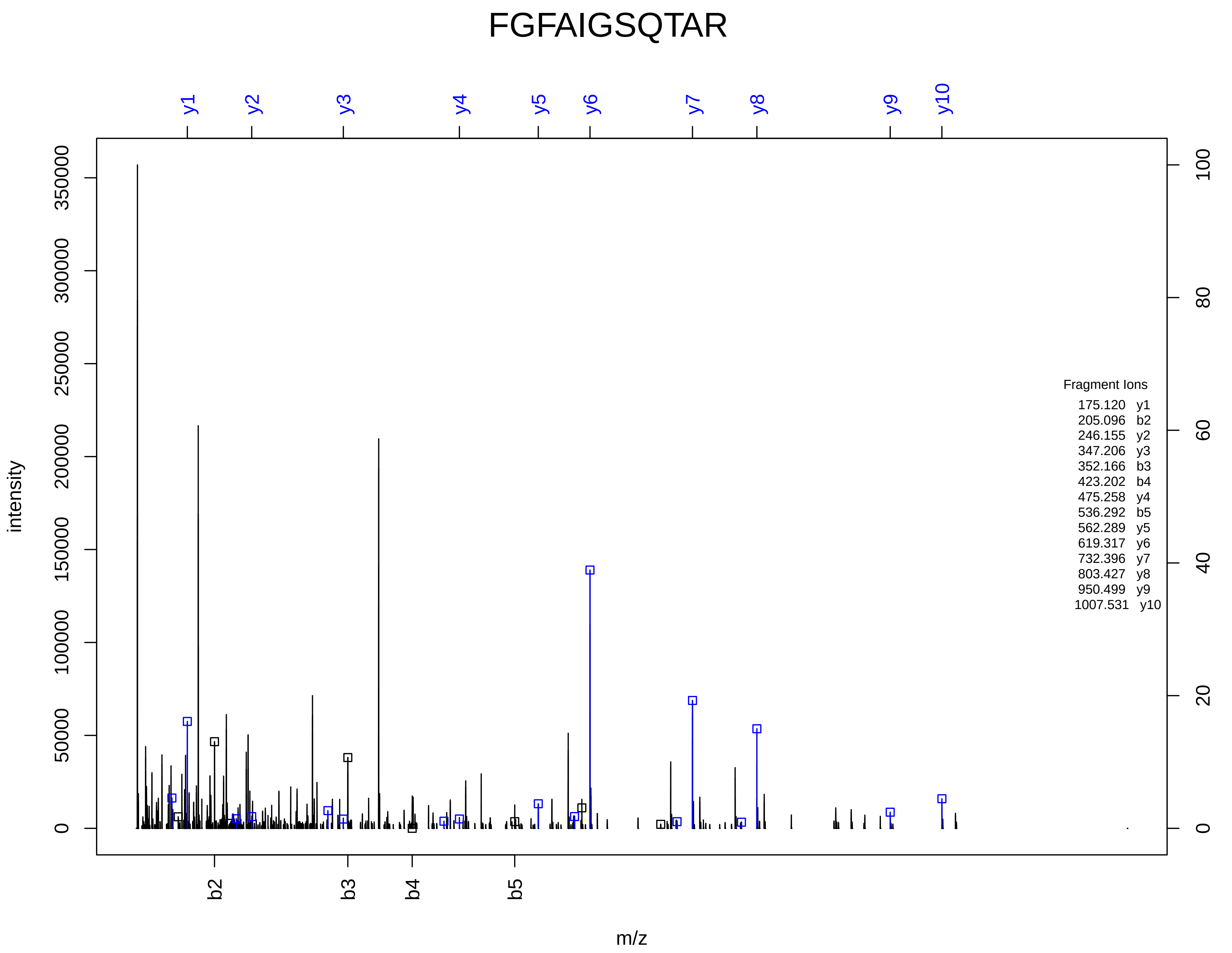

FGFAIGSQTAR
y1
y2
y3
y4
y5
y6
y7
y8
y9
y10
b2
b3
b4
b5
intensity
m/z
Fragment Ions
175.120 y1
205.096 b2
246.155 y2
347.206 y3
352.166 b3
423.202 b4
475.258 y4
536.292 b5
562.289 y5
619.317 y6
732.396 y7
803.427 y8
950.499 y9
1007.531 y10
FGFAIGSQTAR / File: sydney_02.raw; SpectrumID: ; scans: 6038

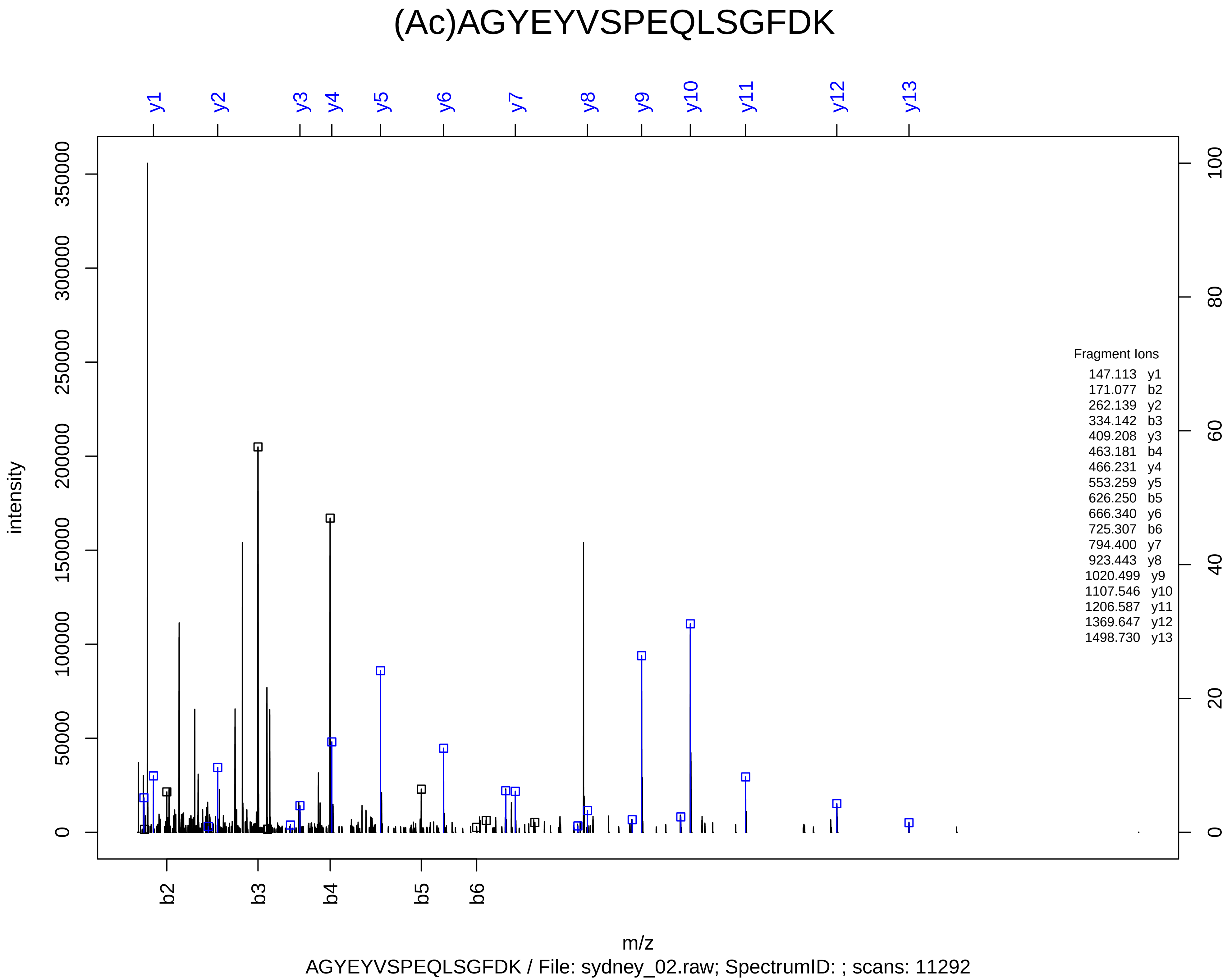

(Ac)AGYEYVSPEQLSGFDK
y1 y2 y3 y4 y5 y6 y7 y8 y9 y10 y11 y12 y13
b2 b3 b4 b5 b6
intensity
m/z
Fragment Ions
147.113 y1
171.077 b2
262.139 y2
334.142 b3
409.208 y3
463.181 b4
466.231 y4
553.259 y5
626.250 b5
666.340 y6
725.307 b6
794.400 y7
923.443 y8
1020.499 y9
1107.546 y10
1206.587 y11
1369.647 y12
1498.730 y13
AGYEYVSPEQLSGFDK / File: sydney_02.raw; SpectrumID: ; scans: 11292

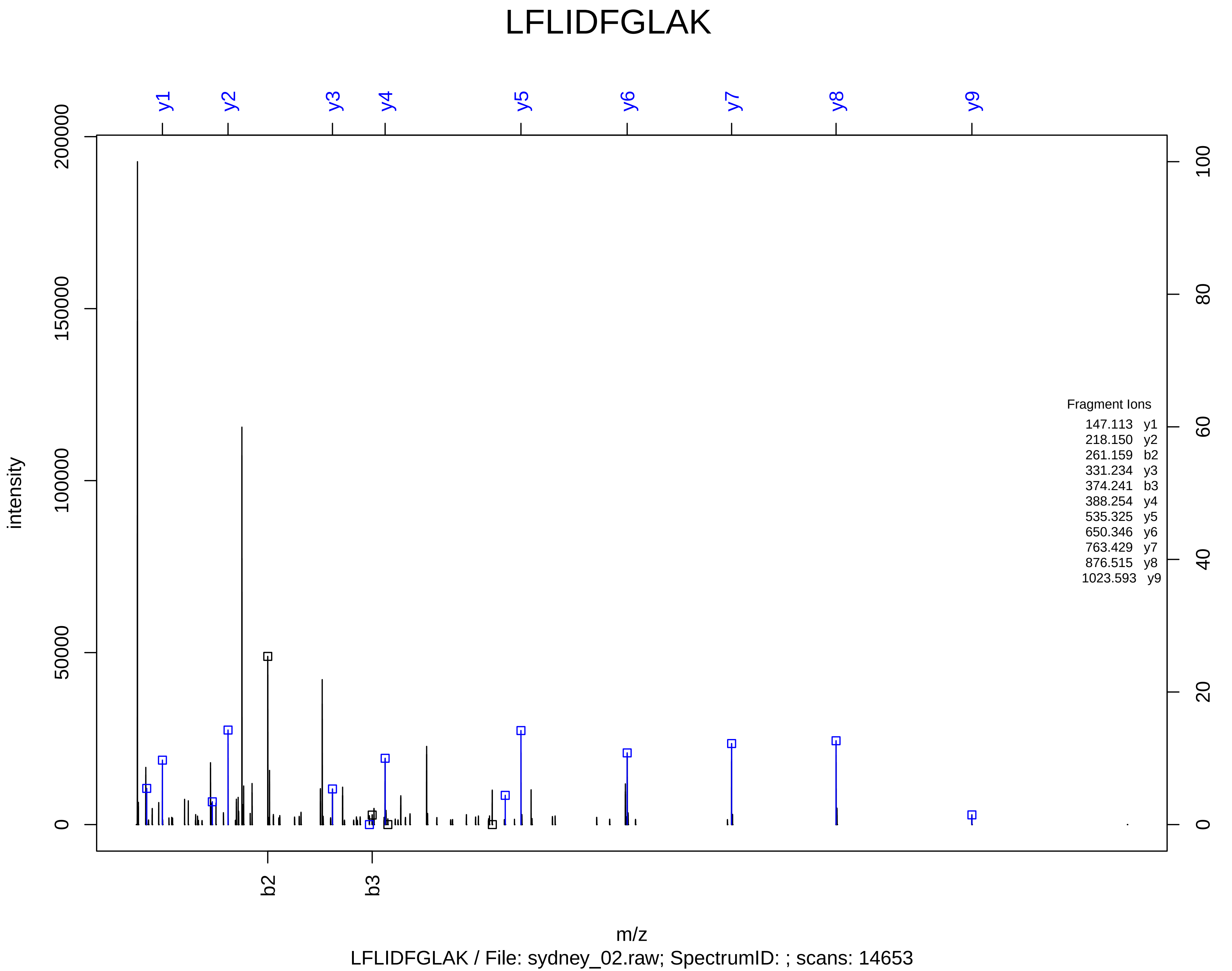
LFLIDFGLAK
y1
y2
y3
y4
y5
y6
y7
y8
y9
intensity
0
50000
100000
150000
200000
0
20
40
60
80
100
b2
b3
m/z
Fragment Ions
147.113 y1
218.150 y2
261.159 b2
331.234 y3
374.241 b3
388.254 y4
535.325 y5
650.346 y6
763.429 y7
876.515 y8
1023.593 y9
LFLIDFGLAK / File: sydney_02.raw; SpectrumID: ; scans: 14653

WWVWN^EVLKDIQ^QM*N^K

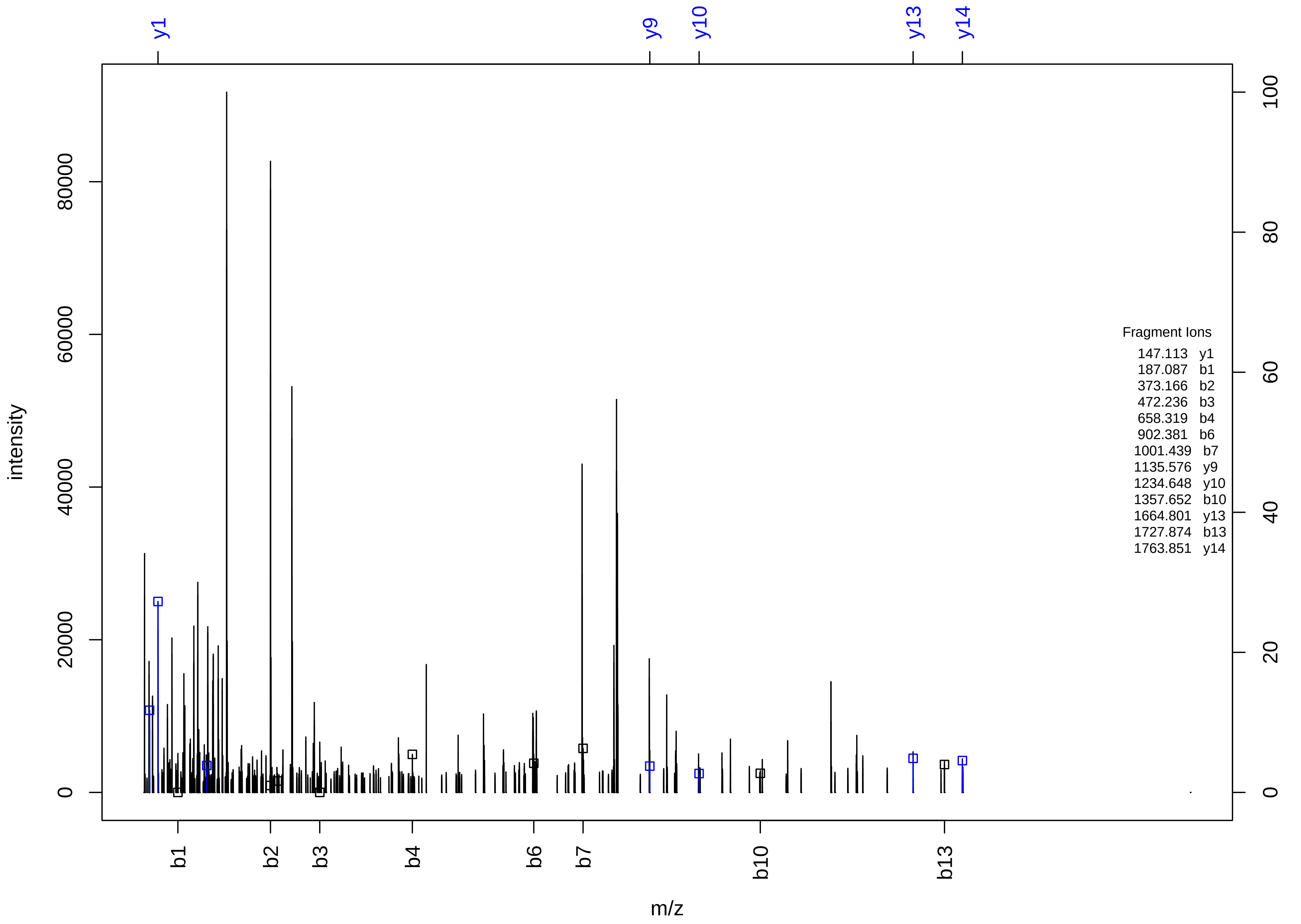

WWVWNEVLKDIQQMNK / File: sydney_02.raw; SpectrumID: ; scans: 9864

DIPDLYDDAAIFQAK

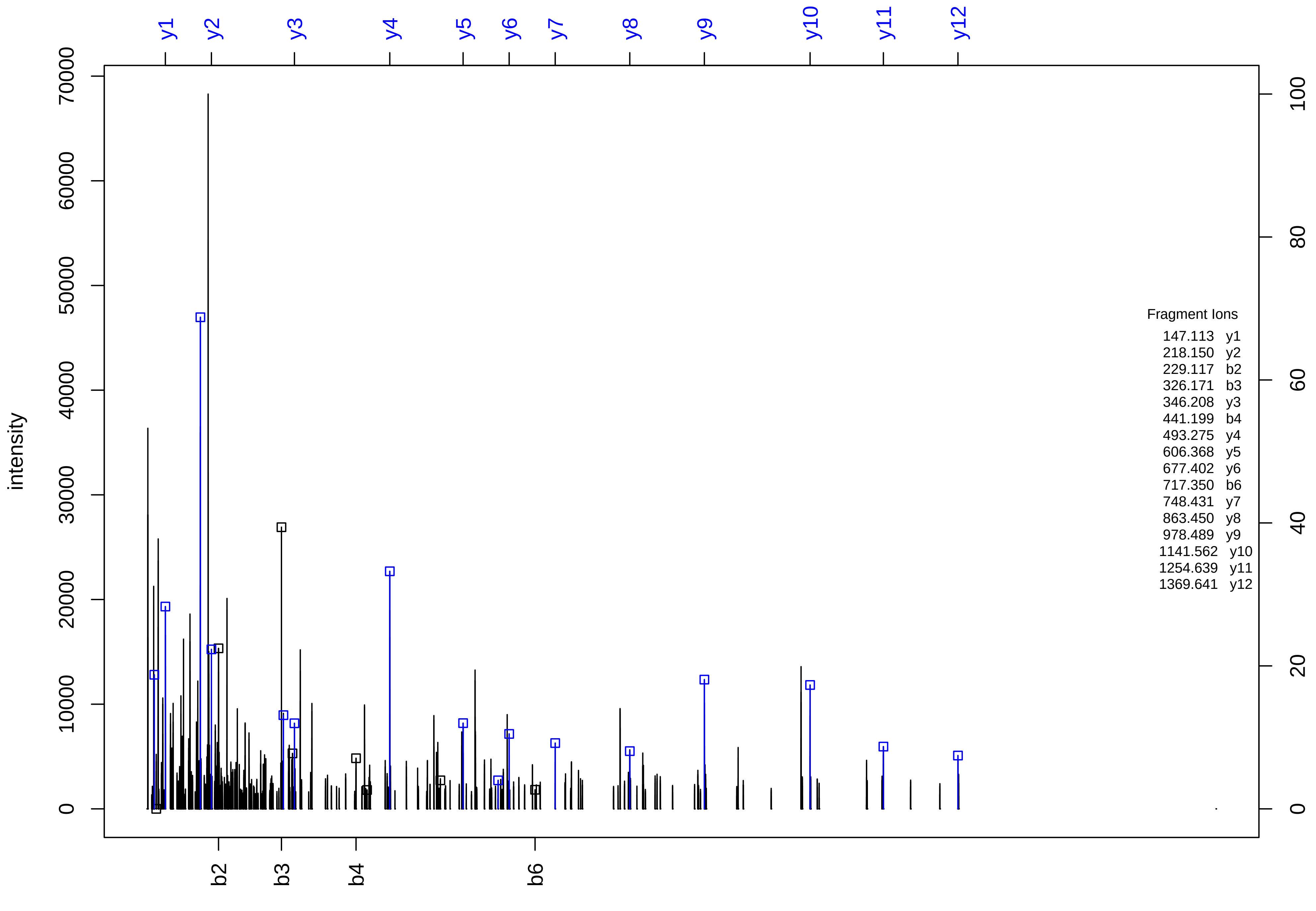

DIPDLYDDAAIFQAK / File: sydney_02.raw; SpectrumID: ; scans: 12538

VENGEGTIPVESSDIVPTWDGIR

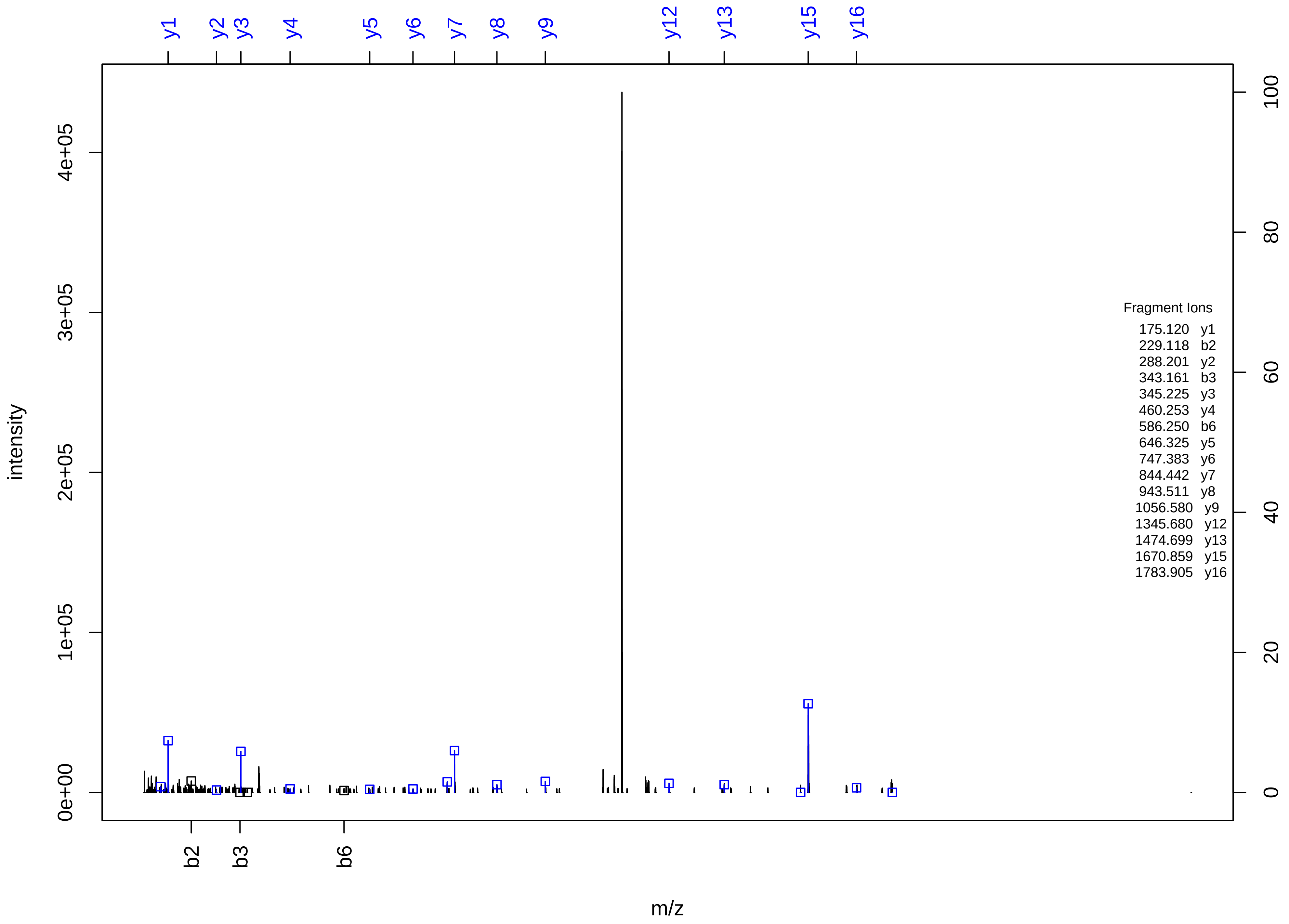

VENGEGTIPVESSDIVPTWDGIR / File: sydney_02.raw; SpectrumID: ; scans: 11299

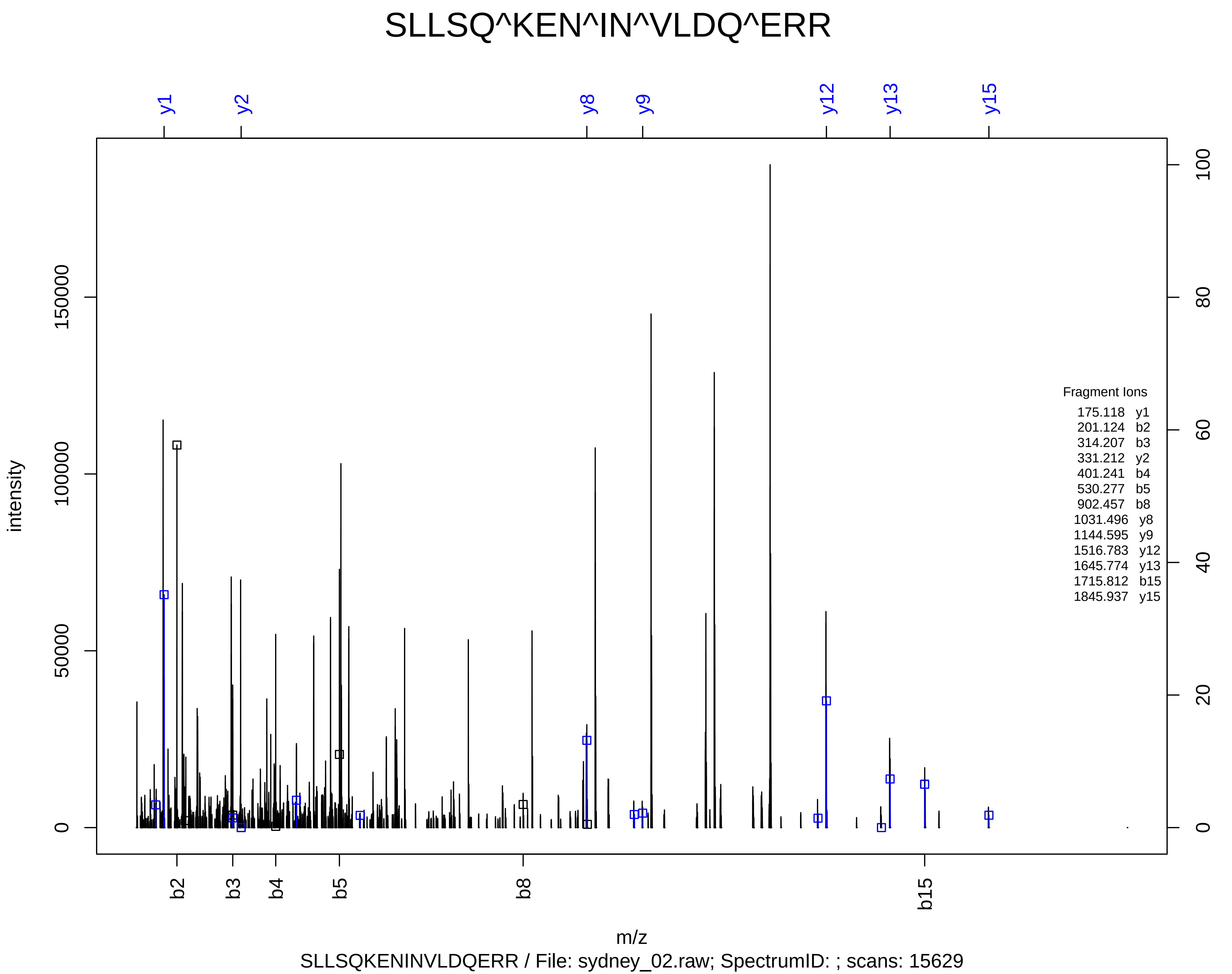
SLLSQ^KEN^IN^VLDQ^ERR
y1
y2
y8
y9
y12
y13
y15
intensity
0
50000
100000
150000
0
20
40
60
80
100
b2
b3
b4
b5
b8
b15
m/z
Fragment Ions
175.118 y1
201.124 b2
314.207 b3
331.212 y2
401.241 b4
530.277 b5
902.457 b8
1031.496 y8
1144.595 y9
1516.783 y12
1645.774 y13
1715.812 b15
1845.937 y15
SLLSQKENINVLDQERR / File: sydney_02.raw; SpectrumID: ; scans: 15629

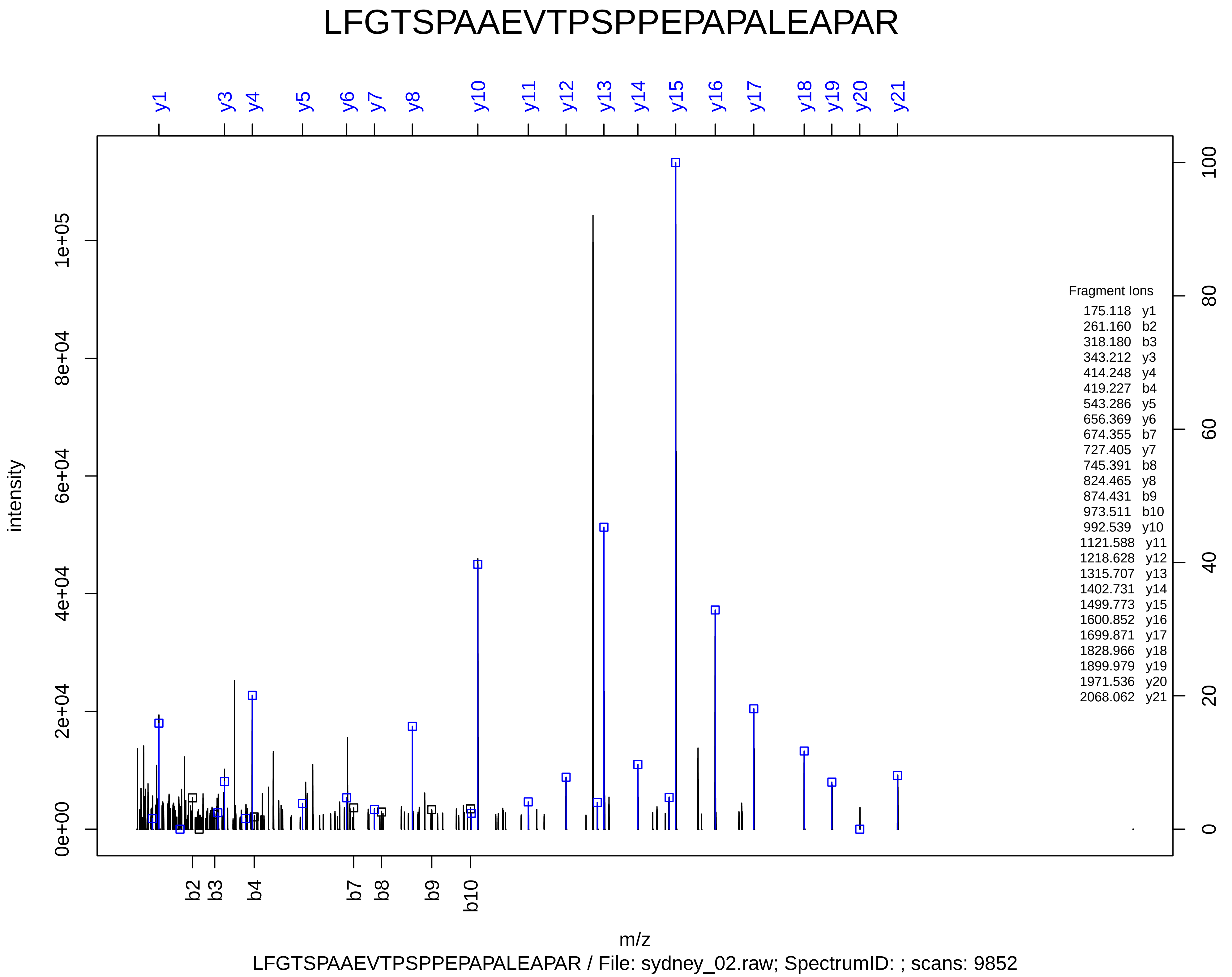
LFGTSPAAEVTPSPPEPAPALEAPAR
y1
y3
y4
y5
y6
y7
y8
y10
y11
y12
y13
y14
y15
y16
y17
y18
y19
y20
y21
intensity
0e+00
2e+04
4e+04
6e+04
8e+04
1e+05
0
20
40
60
80
100
b2
b3
b4
b7
b8
b9
b10
m/z
Fragment Ions
175.118 y1
261.160 b2
318.180 b3
343.212 y3
414.248 y4
419.227 b4
543.286 y5
656.369 y6
674.355 b7
727.405 y7
745.391 b8
824.465 y8
874.431 b9
973.511 b10
992.539 y10
1121.588 y11
1218.628 y12
1315.707 y13
1402.731 y14
1499.773 y15
1600.852 y16
1699.871 y17
1828.966 y18
1899.979 y19
1971.536 y20
2068.062 y21
LFGTSPAAEVTPSPPEPAPALEAPAR / File: sydney_02.raw; SpectrumID: ; scans: 9852

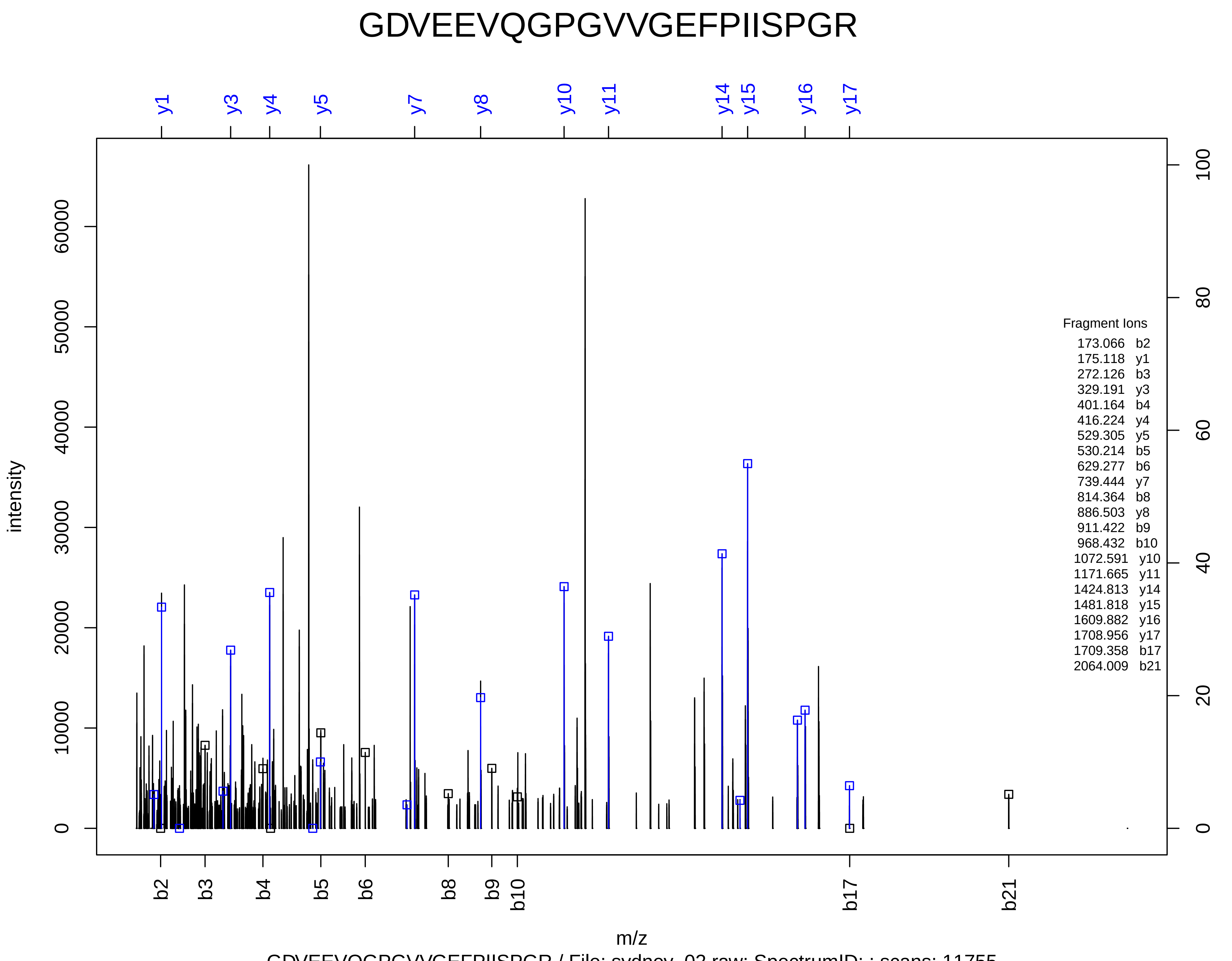
GDVEEVQGPGVVGEFPIISPGR
y1 y3 y4 y5 y7 y8 y10 y11 y14 y15 y16 y17
b2 b3 b4 b5 b6 b8 b9 b10 b17 b21
intensity
m/z
Fragment Ions
173.066 b2
175.118 y1
272.126 b3
329.191 y3
401.164 b4
416.224 y4
529.305 y5
530.214 b5
629.277 b6
739.444 y7
814.364 b8
886.503 y8
911.422 b9
968.432 b10
1072.591 y10
1171.665 y11
1424.813 y14
1481.818 y15
1609.882 y16
1708.956 y17
1709.358 b17
2064.009 b21
GDVEEVQGPGVVGEFPIISPGR / File: sydney_02.raw; SpectrumID: ; scans: 11755

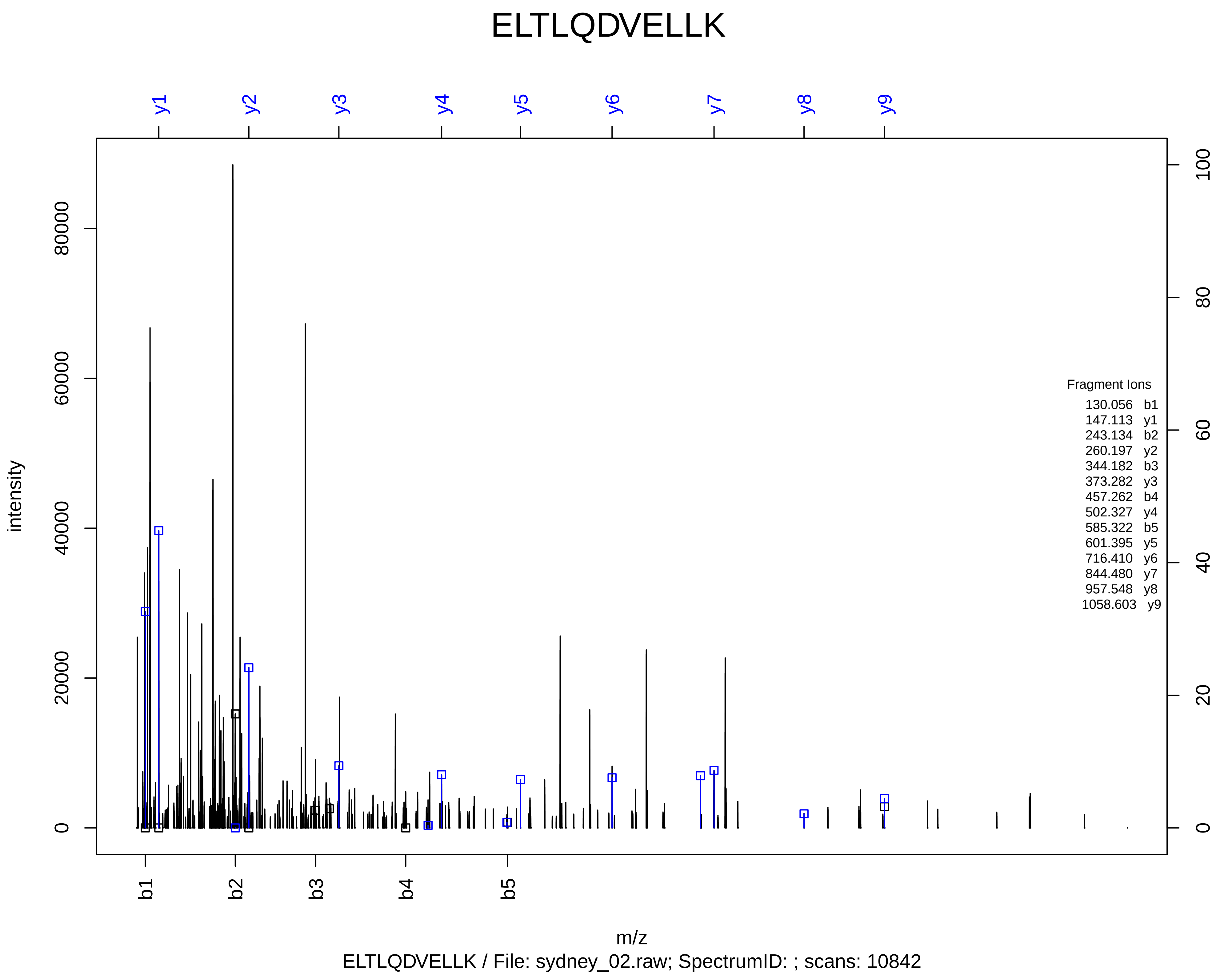
ELTLQDVELLK
y1 y2 y3 y4 y5 y6 y7 y8 y9
intensity
0 20000 40000 60000 80000
0 20 40 60 80 100
b1 b2 b3 b4 b5
m/z
Fragment Ions
130.056 b1
147.113 y1
243.134 b2
260.197 y2
344.182 b3
373.282 y3
457.262 b4
502.327 y4
585.322 b5
601.395 y5
716.410 y6
844.480 y7
957.548 y8
1058.603 y9
ELTLQDVELLK / File: sydney_02.raw; SpectrumID: ; scans: 10842

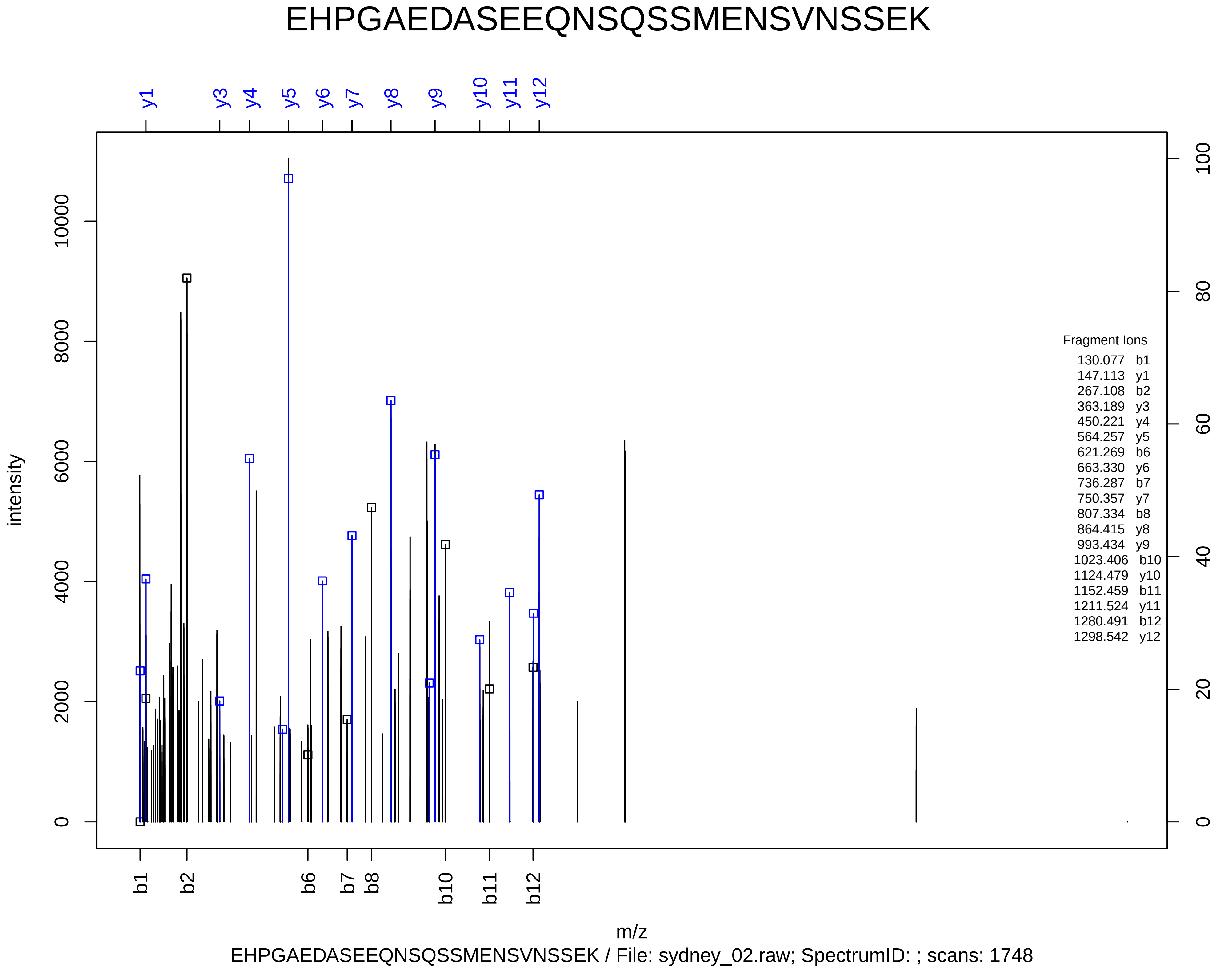
EHPGAEDASEEQNSQSSMENSVNSSEK
y1 y3 y4 y5 y6 y7 y8 y9 y10 y11 y12
b1 b2 b6 b7 b8 b10 b11 b12
intensity
m/z
Fragment Ions
130.077 b1
147.113 y1
267.108 b2
363.189 y3
450.221 y4
564.257 y5
621.269 b6
663.330 y6
736.287 b7
750.357 y7
807.334 b8
864.415 y8
993.434 y9
1023.406 b10
1124.479 y10
1152.459 b11
1211.524 y11
1280.491 b12
1298.542 y12
EHPGAEDASEEQNSQSSMENSVNSSEK / File: sydney_02.raw; SpectrumID: ; scans: 1748

DFISVLR

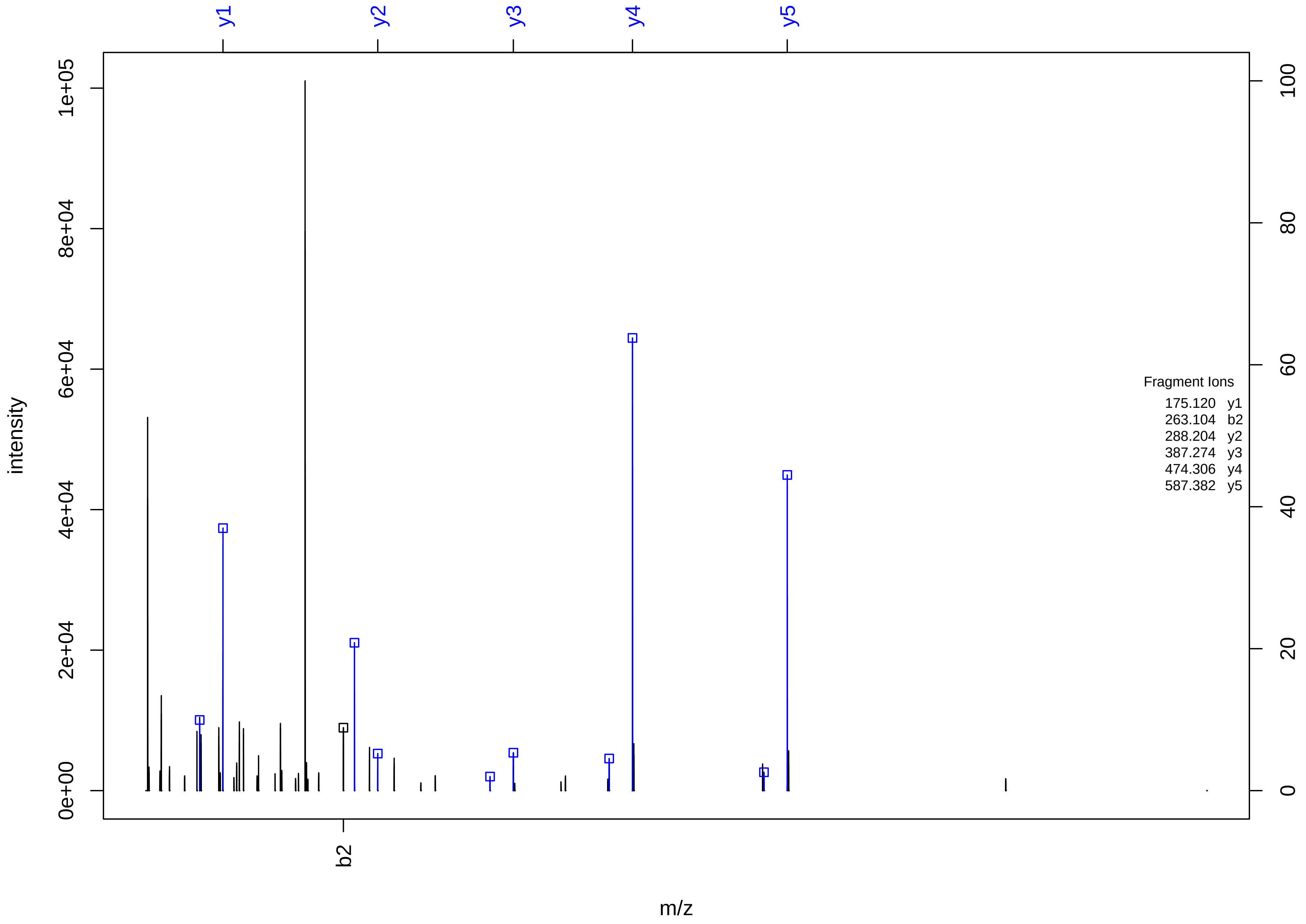

DFISVLR / File: sydney_02.raw; SpectrumID: ; scans: 8880

VSEGGPAEIAGLQIGDK

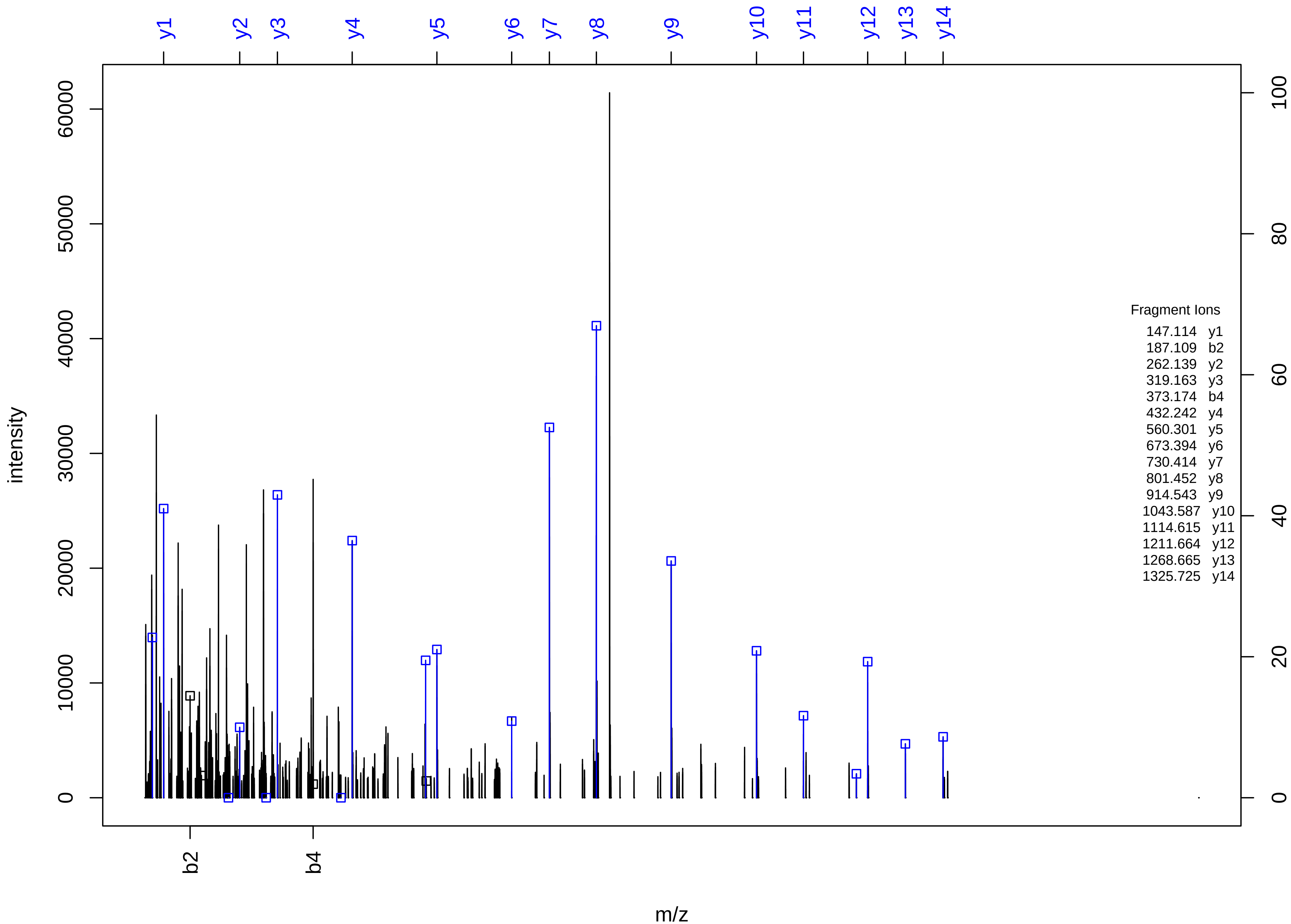

VSEGGPAEIAGLQIGDK / File: sydney_02.raw; SpectrumID: ; scans: 7345

(Ac)ADGQLPFPCSYPSR

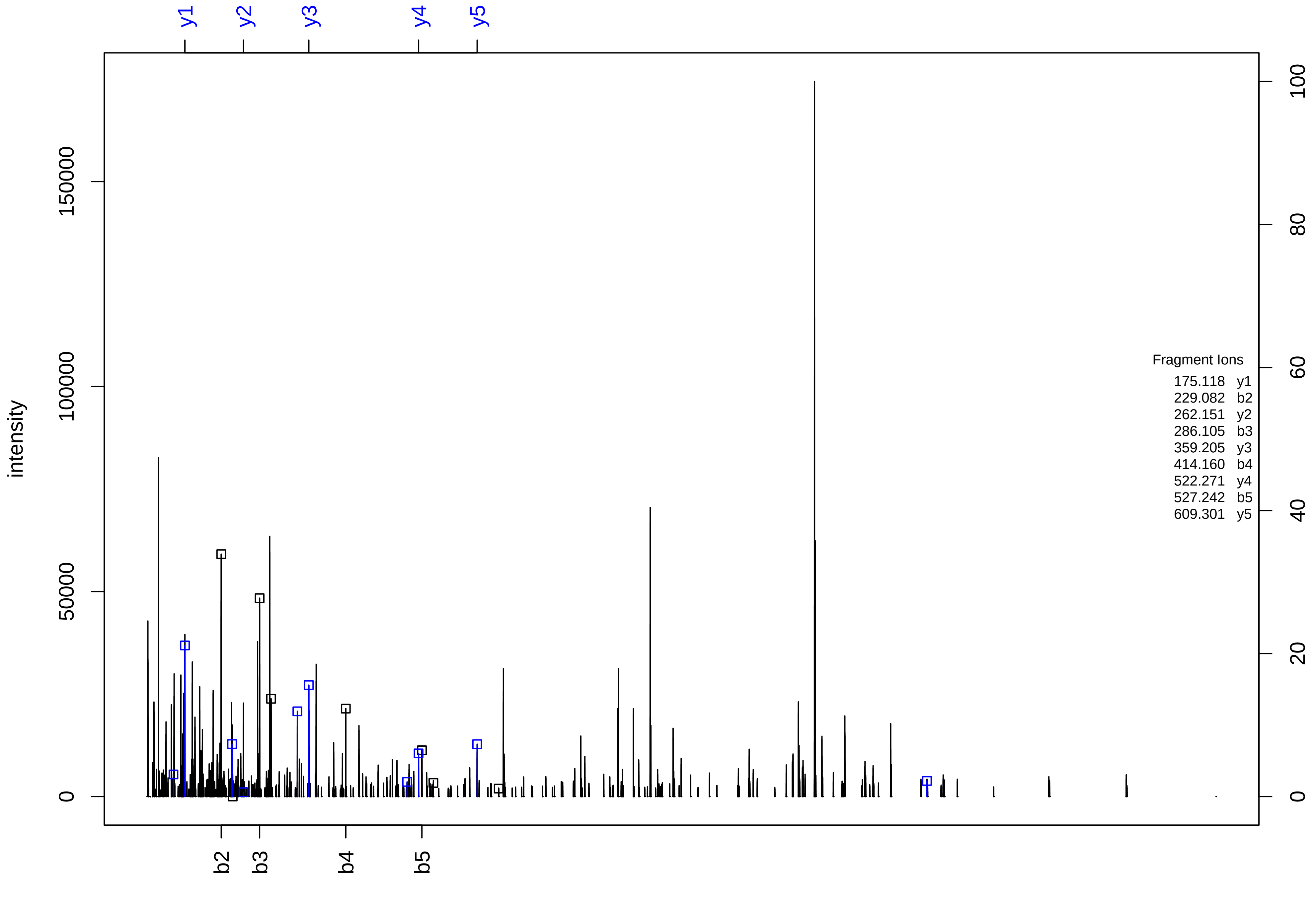

ADGQLPFPCSYPSR / File: sydney_02.raw; SpectrumID: ; scans: 10049

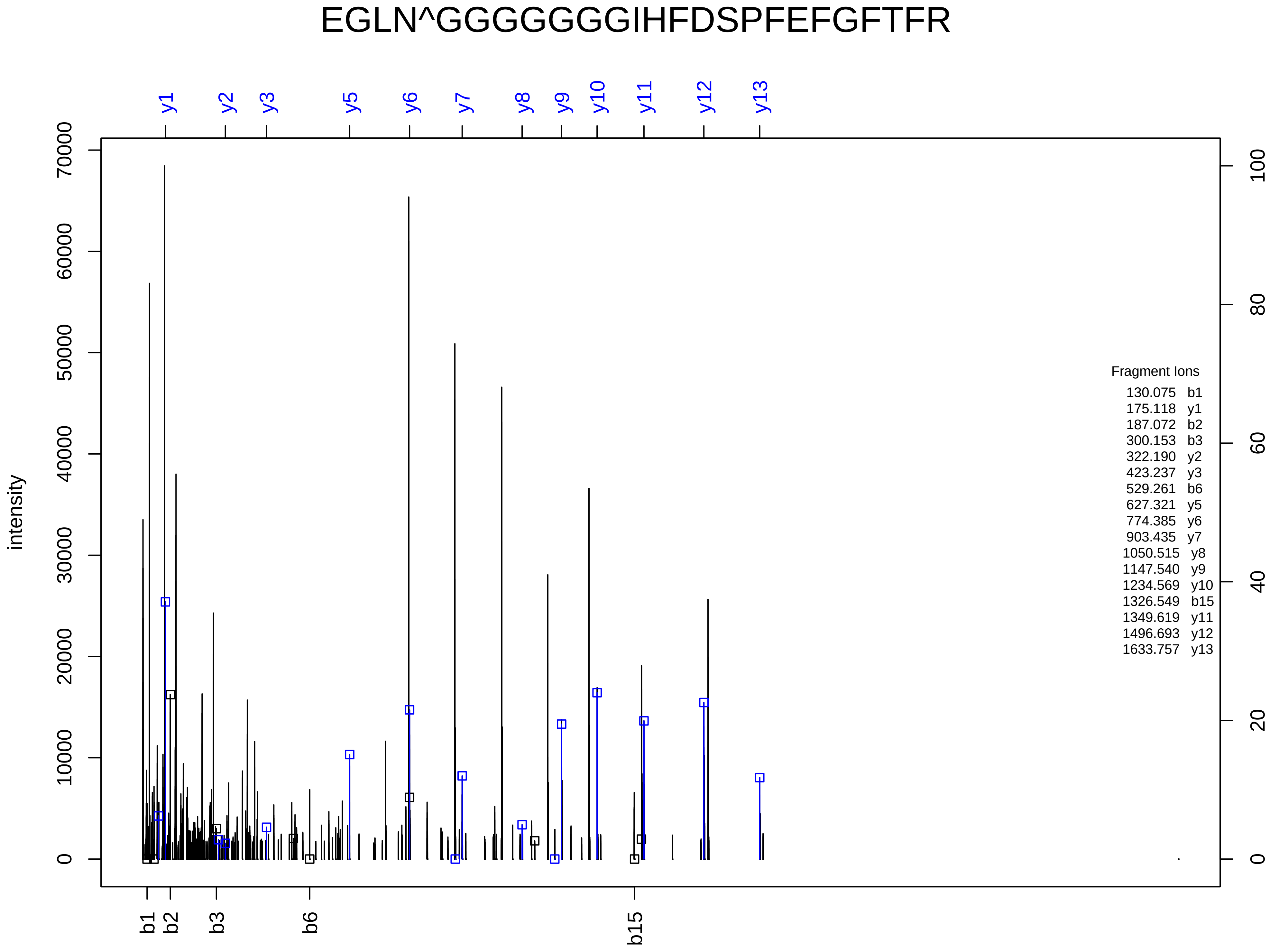

EGLNGGGGGGGIHFDSPFEFGFTFR / File: sydney_03.raw; SpectrumID: ; scans: 14972

LLGSTIPLCSAQWER

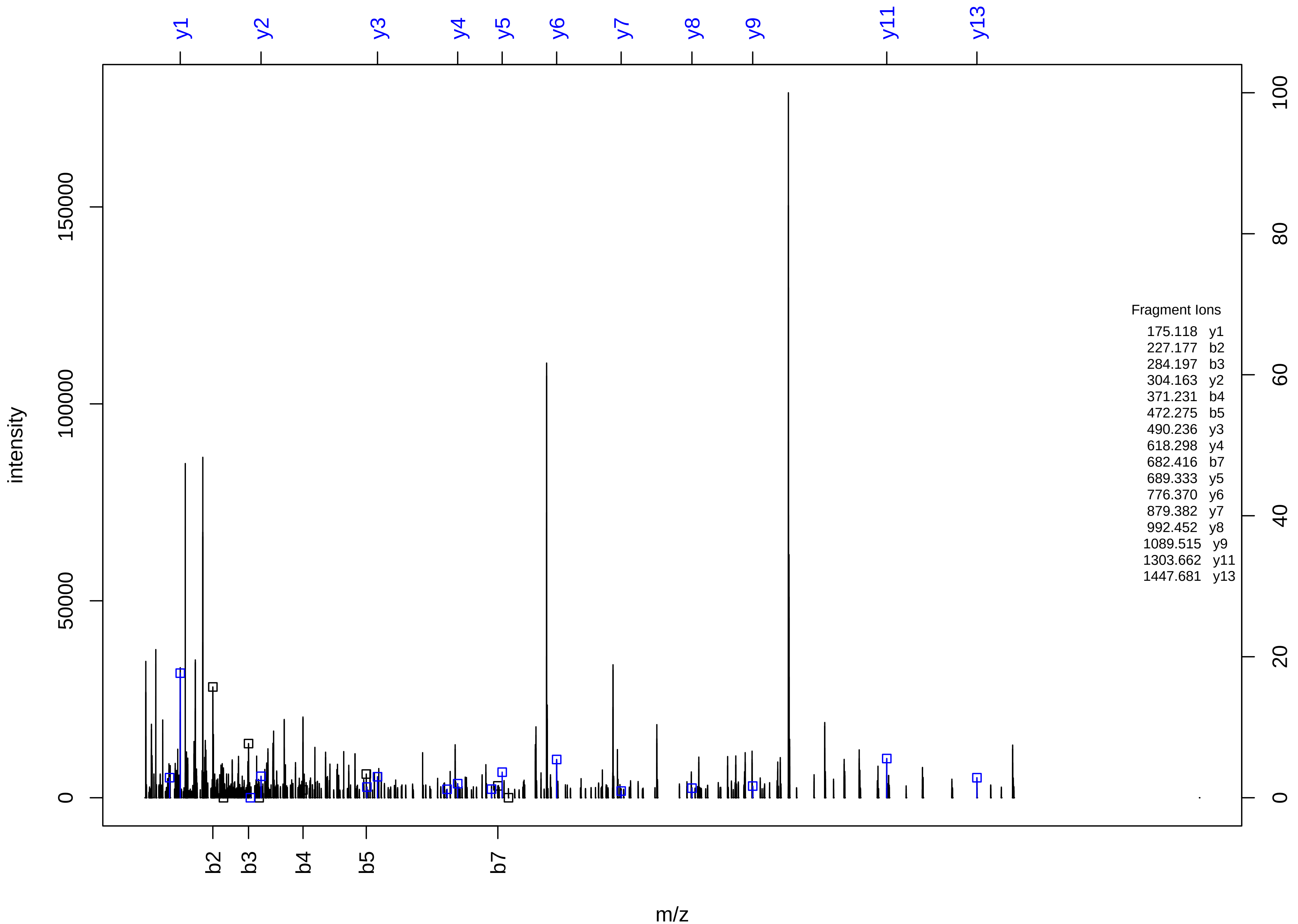

LLGSTIPLCSAQWER / File: sydney_03.raw; SpectrumID: ; scans: 9538

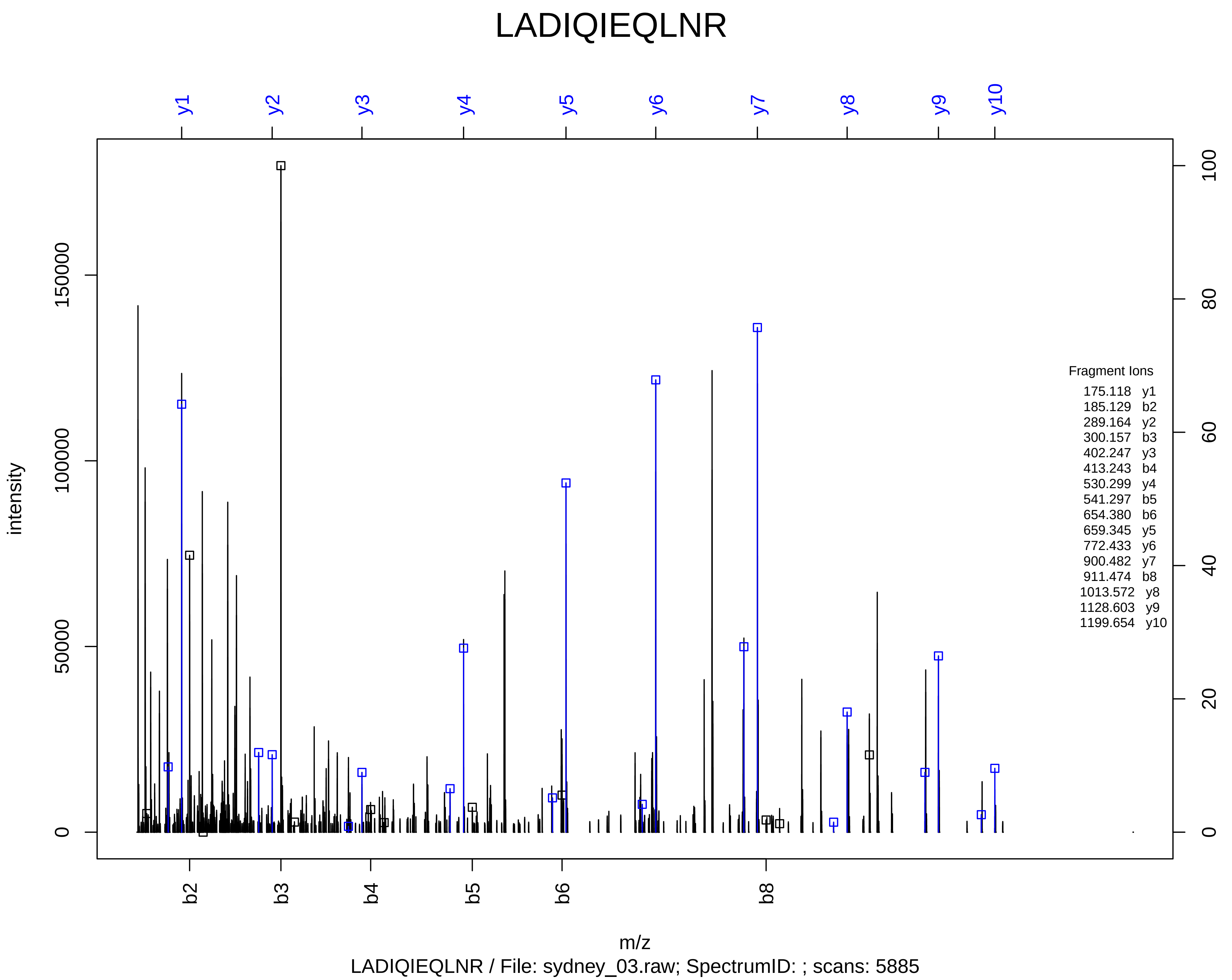
LADIQIEQLNR
y1
y2
y3
y4
y5
y6
y7
y8
y9
y10
intensity
0
50000
100000
150000
0
20
40
60
80
100
b2
b3
b4
b5
b6
b8
m/z
Fragment Ions
175.118 y1
185.129 b2
289.164 y2
300.157 b3
402.247 y3
413.243 b4
530.299 y4
541.297 b5
654.380 b6
659.345 y5
772.433 y6
900.482 y7
911.474 b8
1013.572 y8
1128.603 y9
1199.654 y10
LADIQIEQLNR / File: sydney_03.raw; SpectrumID: ; scans: 5885

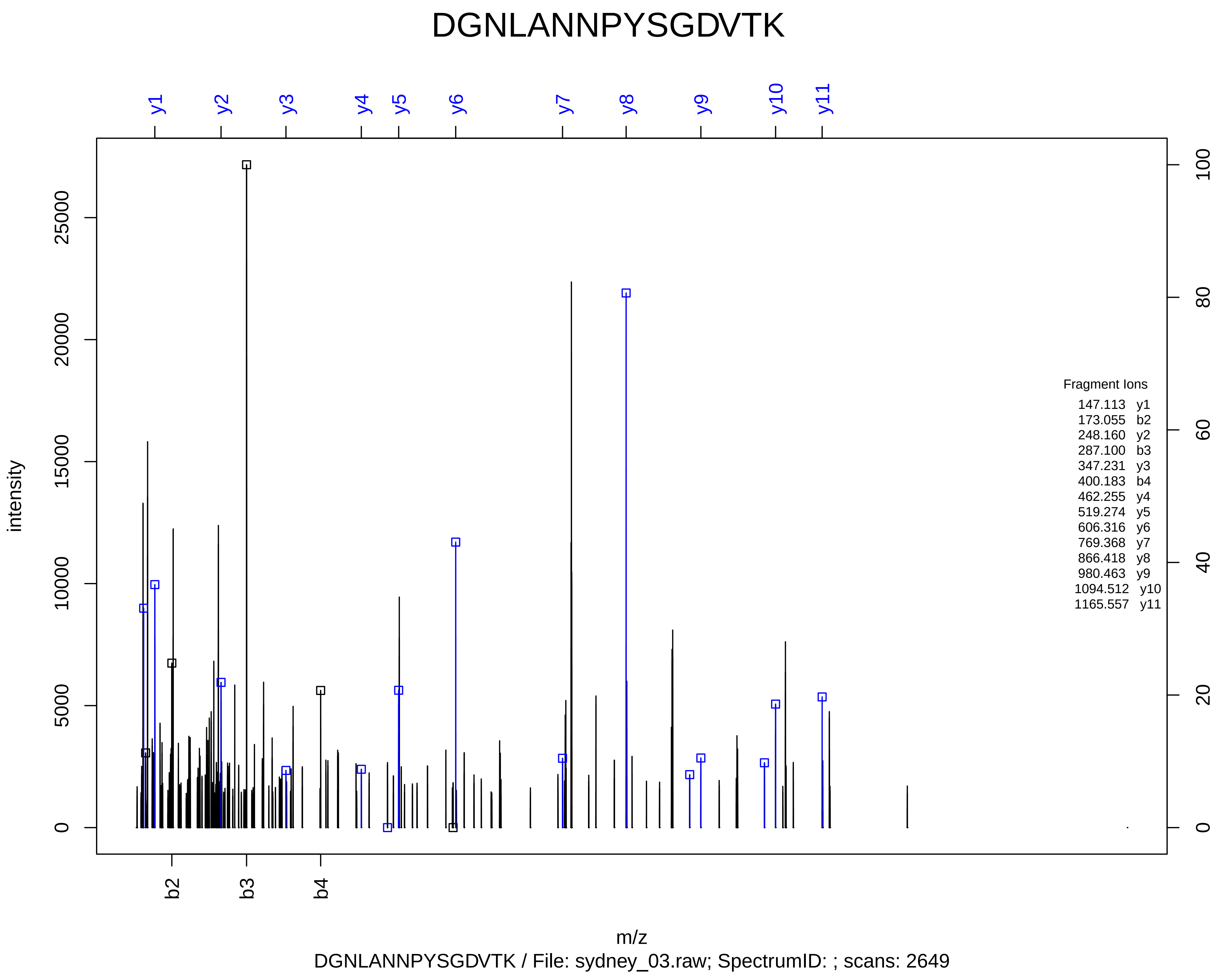

DGNLANNPYSGDVTK
y1
y2
y3
y4
y5
y6
y7
y8
y9
y10
y11
intensity
0
5000
10000
15000
20000
25000
0
20
40
60
80
100
b2
b3
b4
m/z
Fragment Ions
147.113 y1
173.055 b2
248.160 y2
287.100 b3
347.231 y3
400.183 b4
462.255 y4
519.274 y5
606.316 y6
769.368 y7
866.418 y8
980.463 y9
1094.512 y10
1165.557 y11
DGNLANNPYSGDVTK / File: sydney_03.raw; SpectrumID: ; scans: 2649

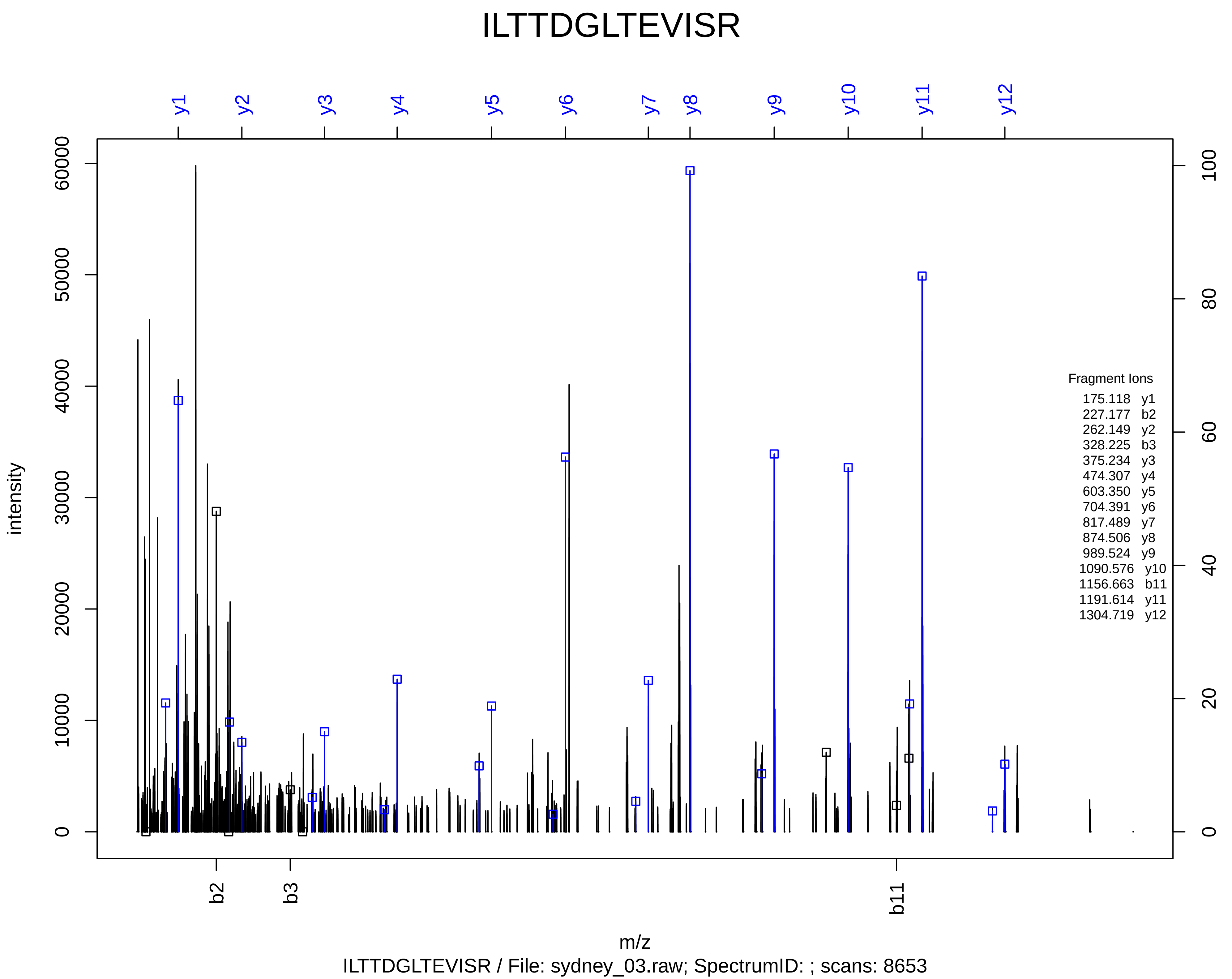
ILTTDGLTEVISR
y1
y2
y3
y4
y5
y6
y7
y8
y9
y10
y11
y12
intensity
0
10000
20000
30000
40000
50000
60000
0
20
40
60
80
100
b2
b3
b11
m/z
Fragment Ions
175.118 y1
227.177 b2
262.149 y2
328.225 b3
375.234 y3
474.307 y4
603.350 y5
704.391 y6
817.489 y7
874.506 y8
989.524 y9
1090.576 y10
1156.663 b11
1191.614 y11
1304.719 y12
ILTTDGLTEVISR / File: sydney_03.raw; SpectrumID: ; scans: 8653

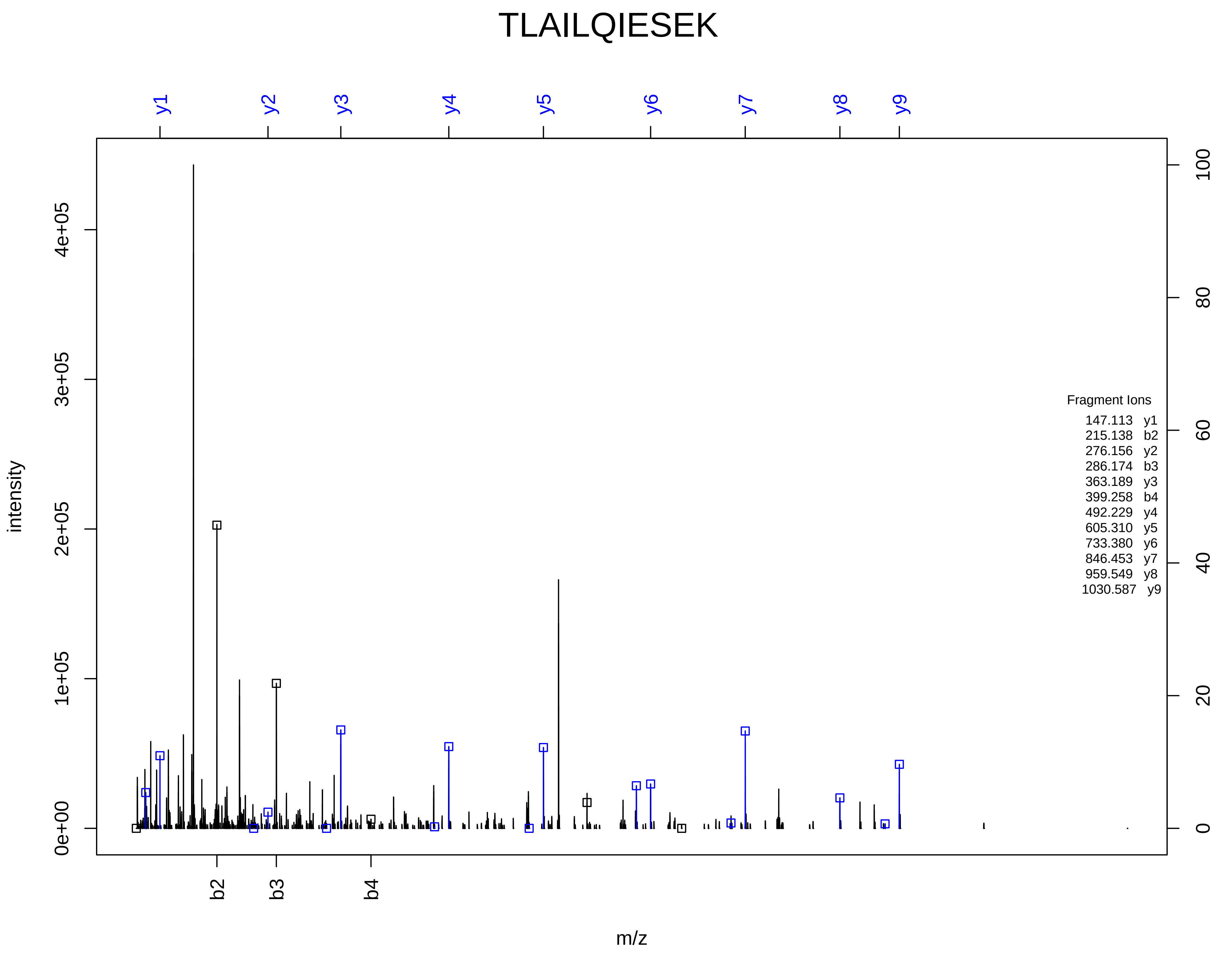
TLAILQIESEK
y1
y2
y3
y4
y5
y6
y7
y8
y9
b2
b3
b4
intensity
0e+00
1e+05
2e+05
3e+05
4e+05
0
20
40
60
80
100
m/z
Fragment Ions
147.113 y1
215.138 b2
276.156 y2
286.174 b3
363.189 y3
399.258 b4
492.229 y4
605.310 y5
733.380 y6
846.453 y7
959.549 y8
1030.587 y9
TLAILQIESEK / File: sydney_03.raw; SpectrumID: ; scans: 8263

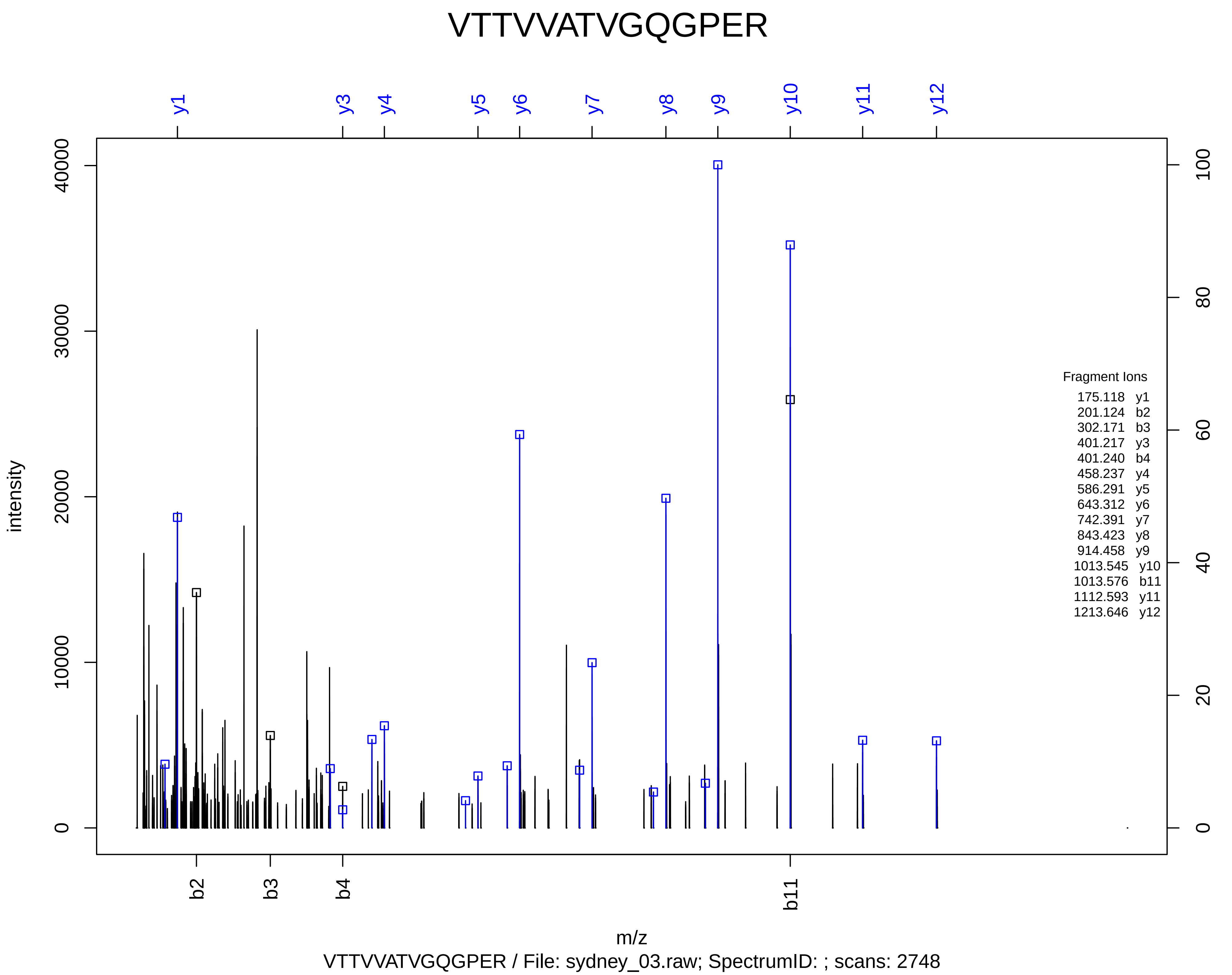

VTTVVATVGQGPER
y1
y3
y4
y5
y6
y7
y8
y9
y10
y11
y12
intensity
0
10000
20000
30000
40000
100
80
60
40
20
0
Fragment Ions
175.118 y1
201.124 b2
302.171 b3
401.217 y3
401.240 b4
458.237 y4
586.291 y5
643.312 y6
742.391 y7
843.423 y8
914.458 y9
1013.545 y10
1013.576 b11
1112.593 y11
1213.646 y12
b2
b3
b4
b11
m/z
VTTVVATVGQGPER / File: sydney_03.raw; SpectrumID: ; scans: 2748

NVLITDFFGSVR

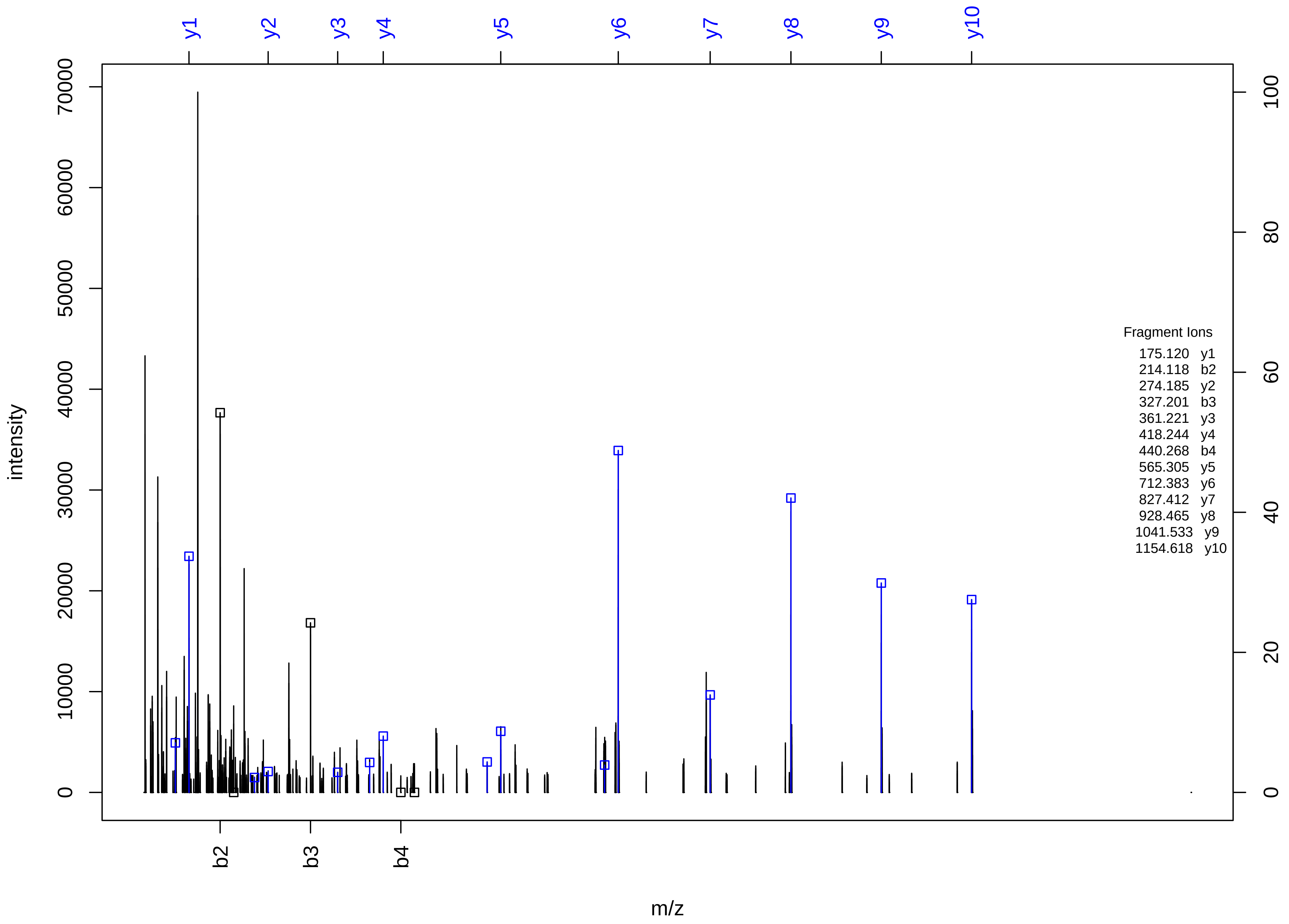

NVLITDFFGSVR / File: sydney_03.raw; SpectrumID: ; scans: 13131

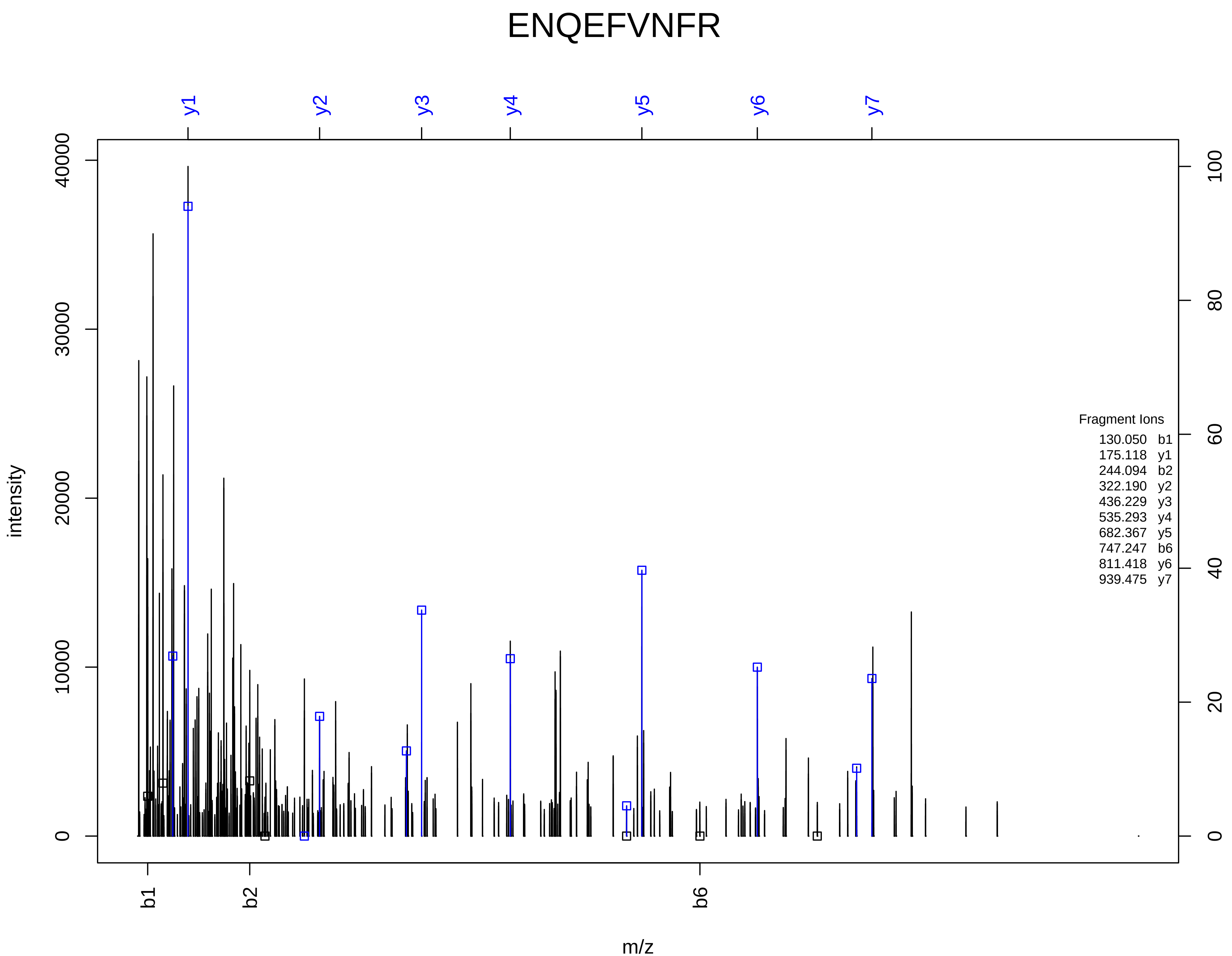
ENQEFVNFR
y1
y2
y3
y4
y5
y6
y7
intensity
0
10000
20000
30000
40000
0
20
40
60
80
100
b1
b2
b6
m/z
Fragment Ions
130.050 b1
175.118 y1
244.094 b2
322.190 y2
436.229 y3
535.293 y4
682.367 y5
747.247 b6
811.418 y6
939.475 y7
ENQEFVNFR / File: sydney_03.raw; SpectrumID: ; scans: 4072

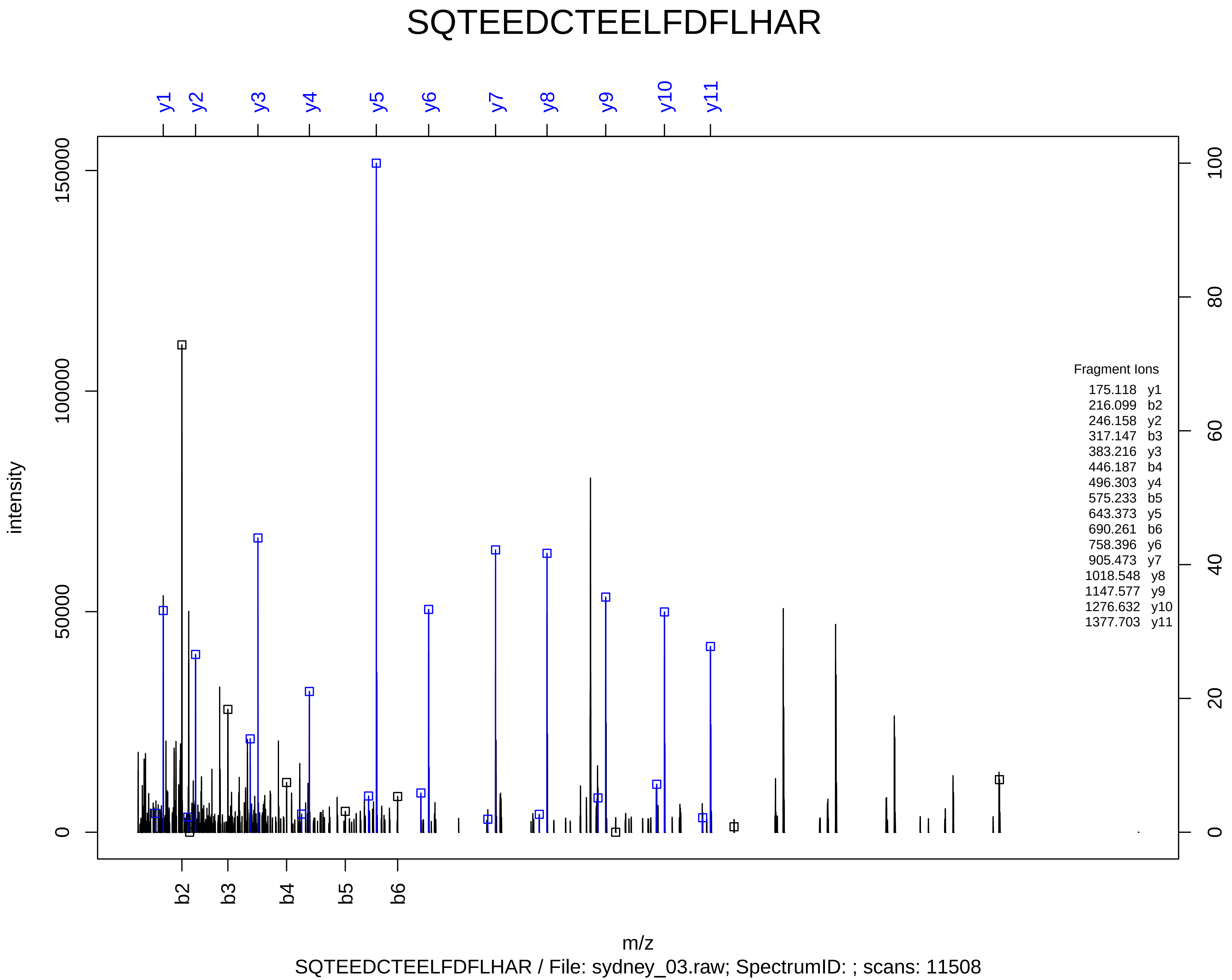
SQTEEDCTEELFDFLHAR
y1 y2 y3 y4 y5 y6 y7 y8 y9 y10 y11
b2 b3 b4 b5 b6
intensity
m/z
Fragment Ions
175.118 y1
216.099 b2
246.158 y2
317.147 b3
383.216 y3
446.187 b4
496.303 y4
575.233 b5
643.373 y5
690.261 b6
758.396 y6
905.473 y7
1018.548 y8
1147.577 y9
1276.632 y10
1377.703 y11
SQTEEDCTEELFDFLHAR / File: sydney_03.raw; SpectrumID: ; scans: 11508

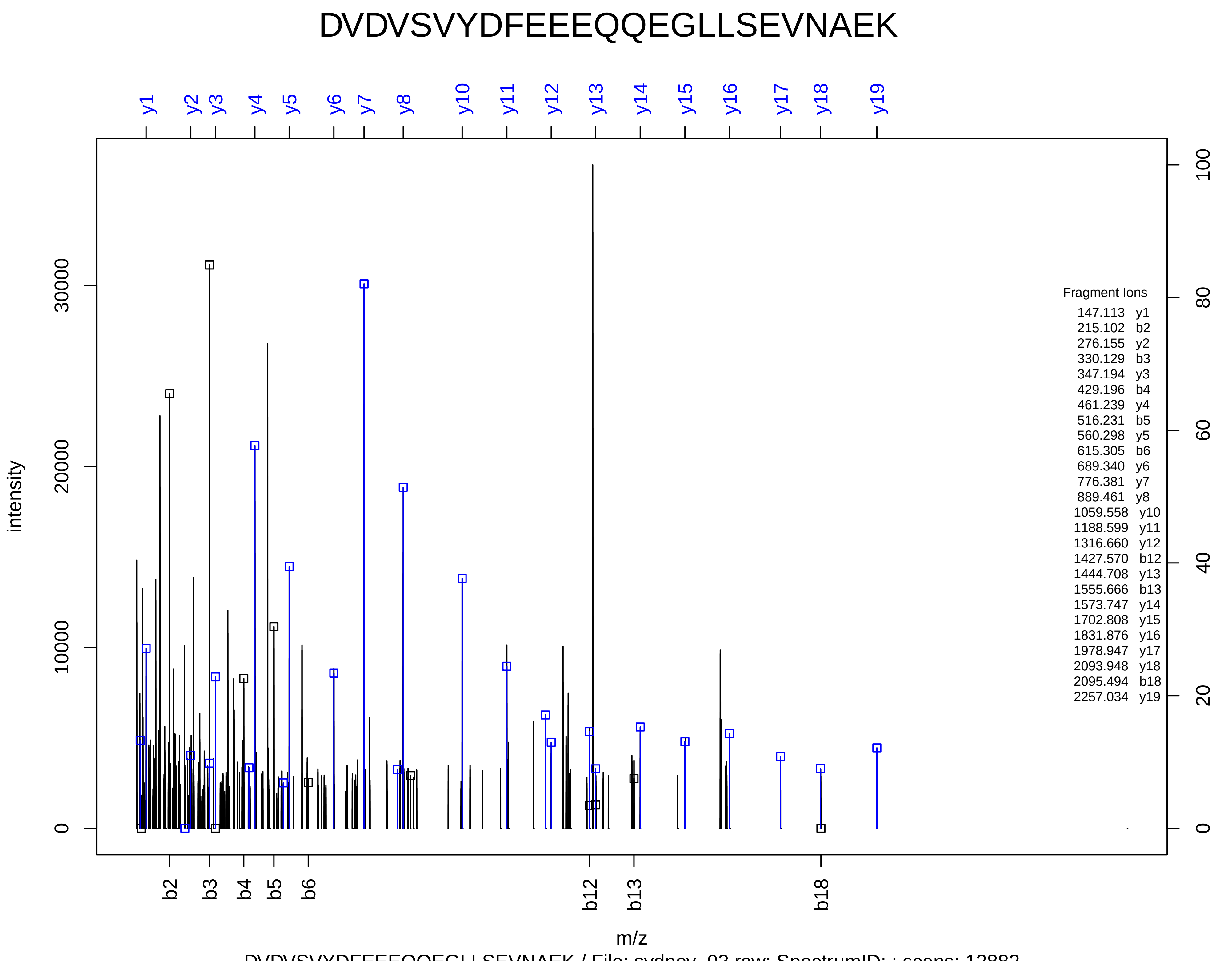
DVDVSVYDFEEEQQEGLLSEVNAEK
y1 y2 y3 y4 y5 y6 y7 y8 y10 y11 y12 y13 y14 y15 y16 y17 y18 y19
b2 b3 b4 b5 b6 b12 b13 b18
intensity
0
10000
20000
30000
0
20
40
60
80
100
m/z
Fragment Ions
147.113 y1
215.102 b2
276.155 y2
330.129 b3
347.194 y3
429.196 b4
461.239 y4
516.231 b5
560.298 y5
615.305 b6
689.340 y6
776.381 y7
889.461 y8
1059.558 y10
1188.599 y11
1316.660 y12
1427.570 b12
1444.708 y13
1555.666 b13
1573.747 y14
1702.808 y15
1831.876 y16
1978.947 y17
2093.948 y18
2095.494 b18
2257.034 y19
DVDVSVYDFEEEQQEGLLSEVNAEK / File: sydney_03.raw; SpectrumID: ; scans: 12882

EVWLLETPQADDFWCEGEPYPGPK

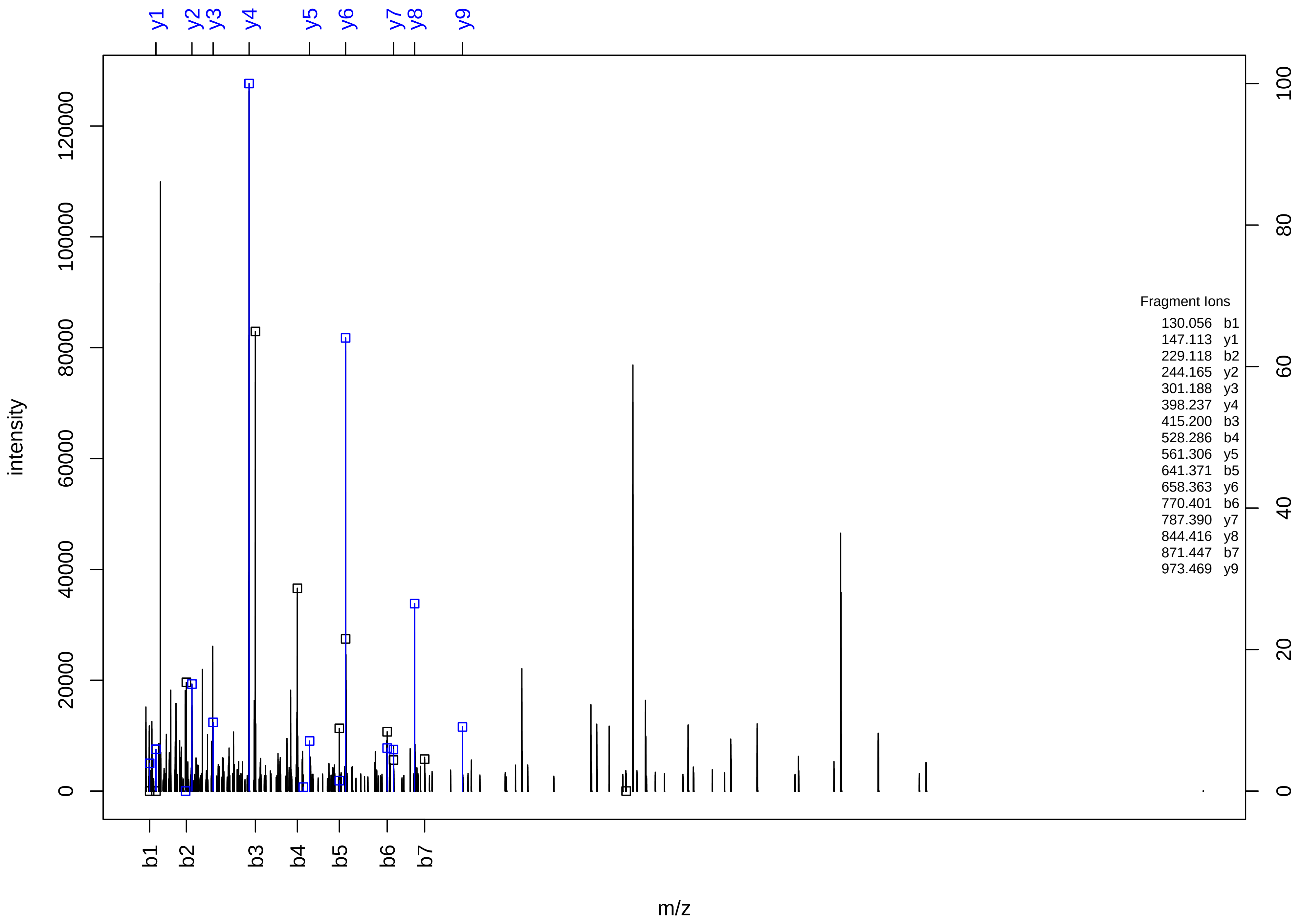

EVWLLETPQADDFWCEGEPYPGPK / File: sydney_03.raw; SpectrumID: ; scans: 14738

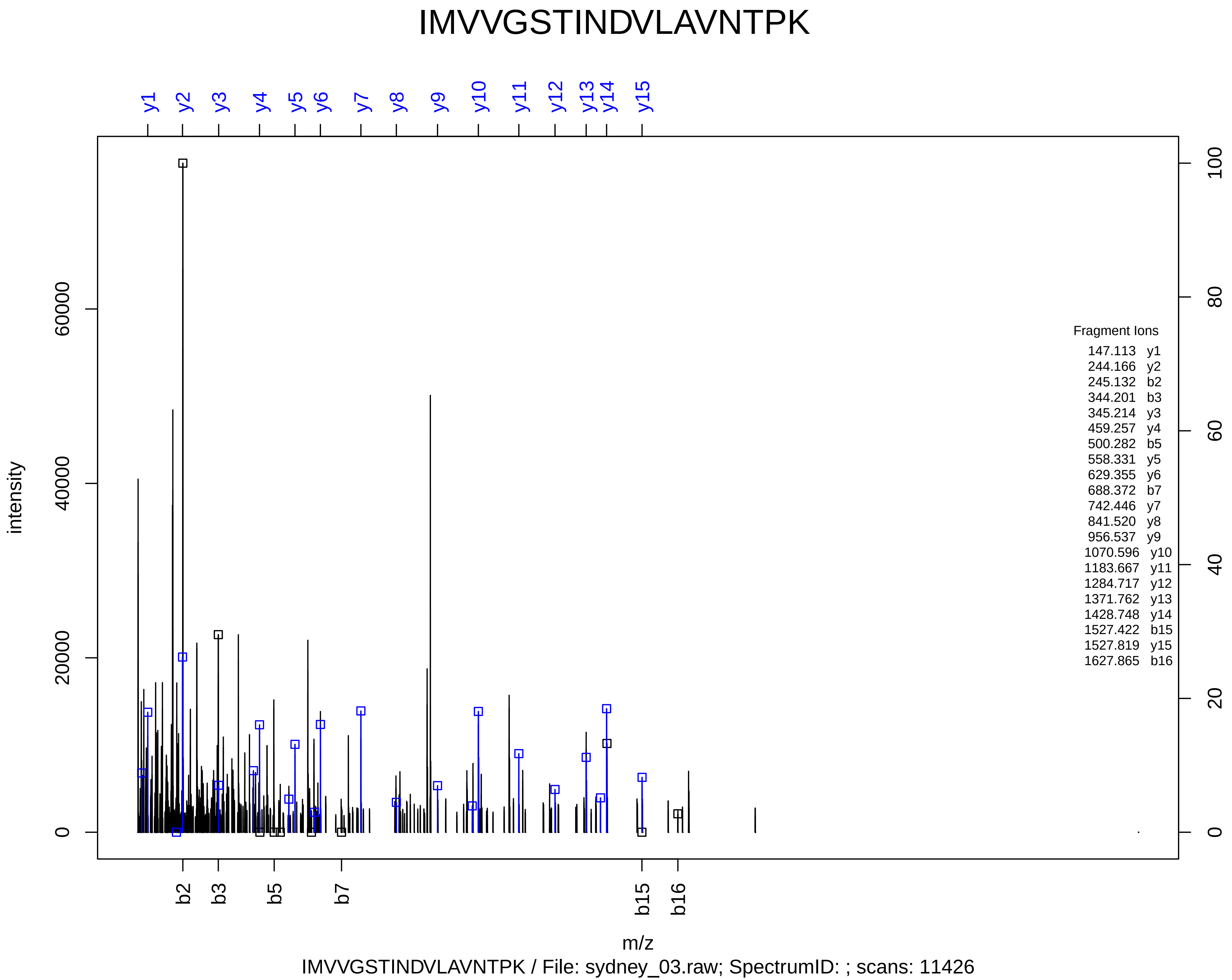
IMVVGSTINDVLAVNTPK
y1 y2 y3 y4 y5 y6 y7 y8 y9 y10 y11 y12 y13 y14 y15
intensity
0
20000
40000
60000
0
20
40
60
80
100
Fragment Ions
147.113 y1
244.166 y2
245.132 b2
344.201 b3
345.214 y3
459.257 y4
500.282 b5
558.331 y5
629.355 y6
688.372 b7
742.446 y7
841.520 y8
956.537 y9
1070.596 y10
1183.667 y11
1284.717 y12
1371.762 y13
1428.748 y14
1527.422 b15
1527.819 y15
1627.865 b16
b2 b3 b5 b7 b15 b16
m/z
IMVVGSTINDVLAVNTPK / File: sydney_03.raw; SpectrumID: ; scans: 11426

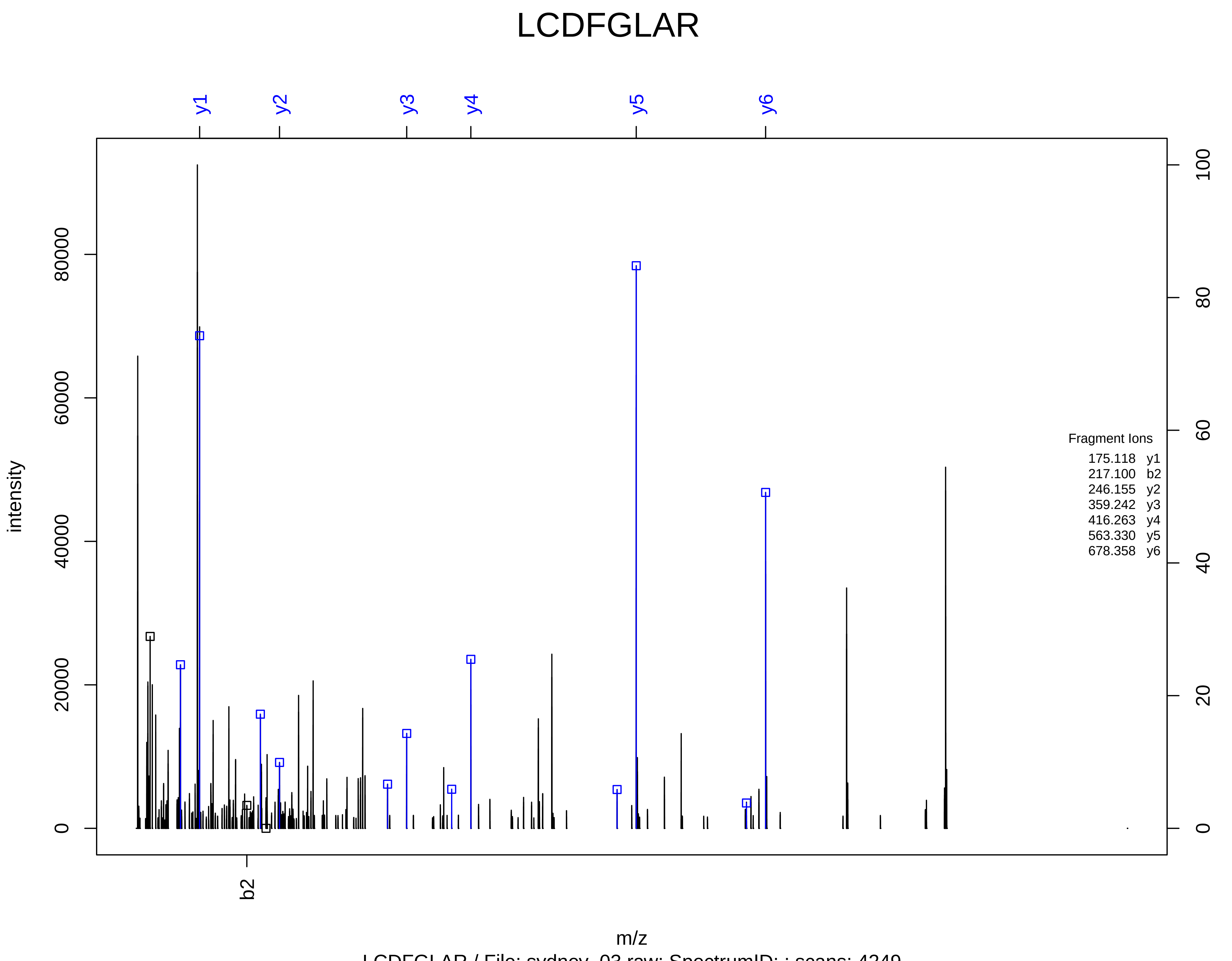
LCDFGLAR
y1
y2
y3
y4
y5
y6
intensity
0
20000
40000
60000
80000
0
20
40
60
80
100
Fragment Ions
175.118 y1
217.100 b2
246.155 y2
359.242 y3
416.263 y4
563.330 y5
678.358 y6
b2
m/z
LCDFGLAR / File: sydney_03.raw; SpectrumID: ; scans: 4249

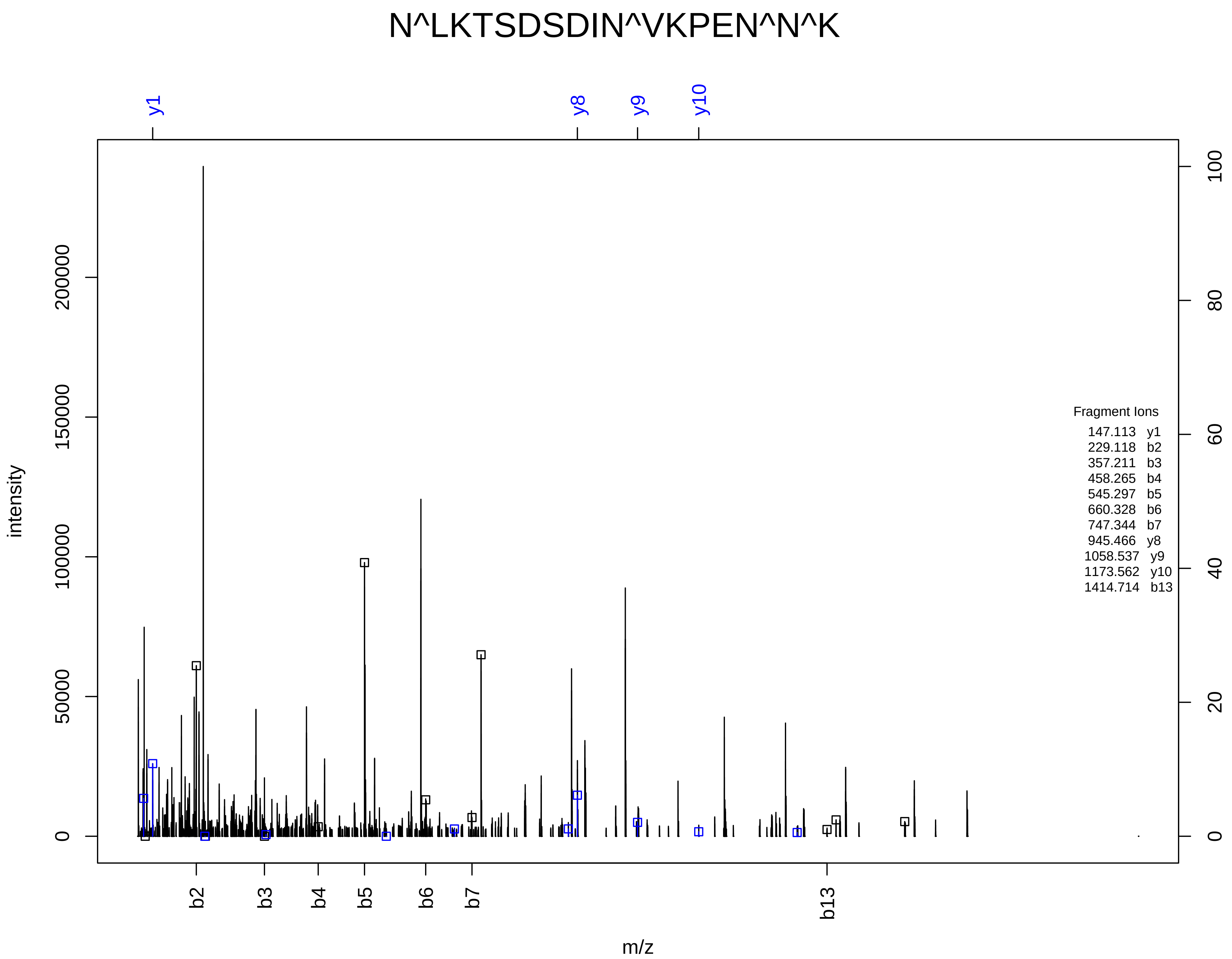

NLKTSDSDINVKPENNK / File: sydney_03.raw; SpectrumID: ; scans: 11717

FIDDVVSAVLR

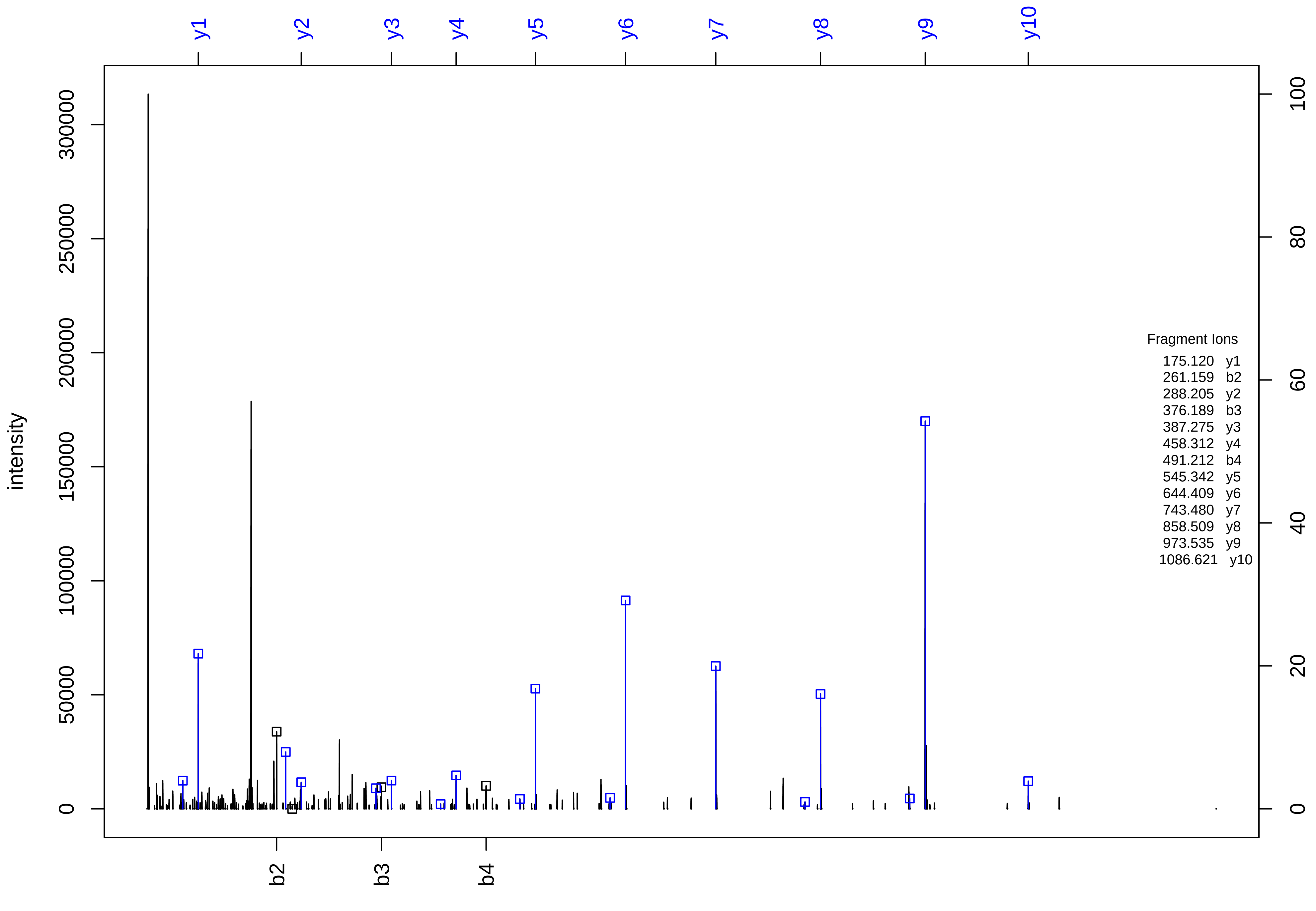

FIDDVVSAVLR / File: sydney_03.raw; SpectrumID: ; scans: 12352

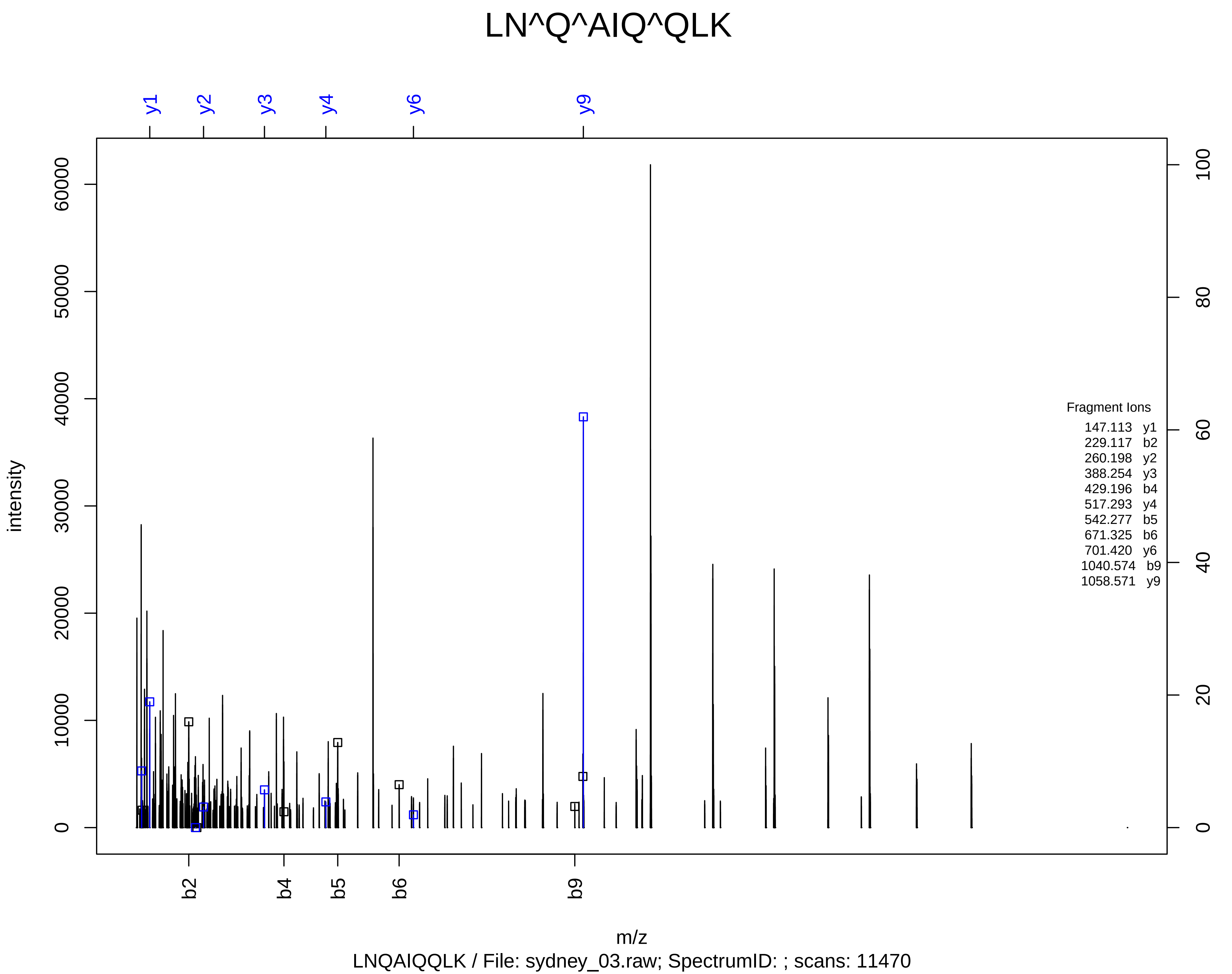
LN^Q^AIQ^QLK
y1
y2
y3
y4
y6
y9
intensity
60000
50000
40000
30000
20000
10000
0
100
80
60
40
20
0
Fragment Ions
147.113 y1
229.117 b2
260.198 y2
388.254 y3
429.196 b4
517.293 y4
542.277 b5
671.325 b6
701.420 y6
1040.574 b9
1058.571 y9
b2
b4
b5
b6
b9
m/z
LNQAIQQLK / File: sydney_03.raw; SpectrumID: ; scans: 11470

GISSYSVDIM*N^ILINDIFER

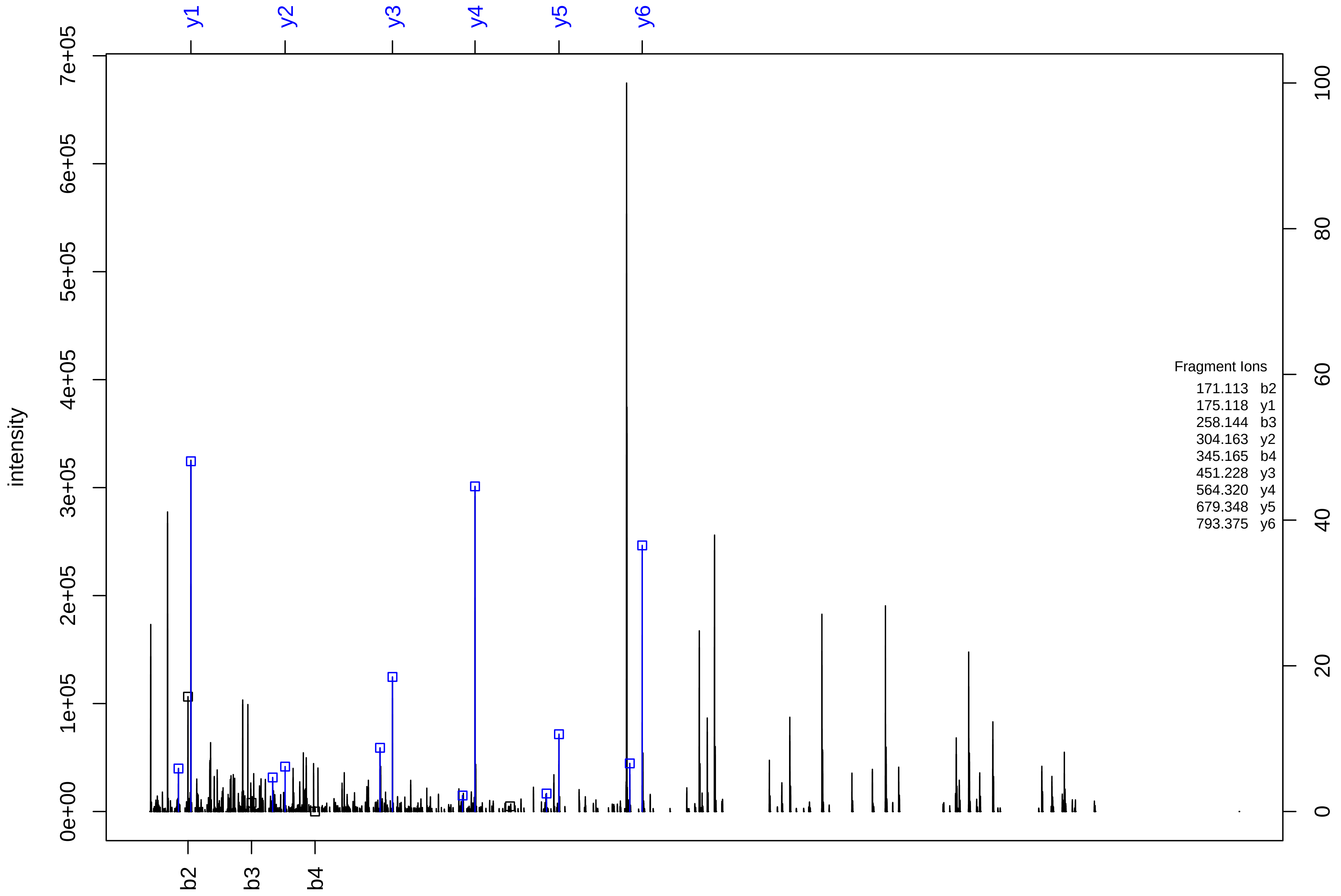

GISSYSVDIMNILINDIFER / File: sydney_03.raw; SpectrumID: ; scans: 13478

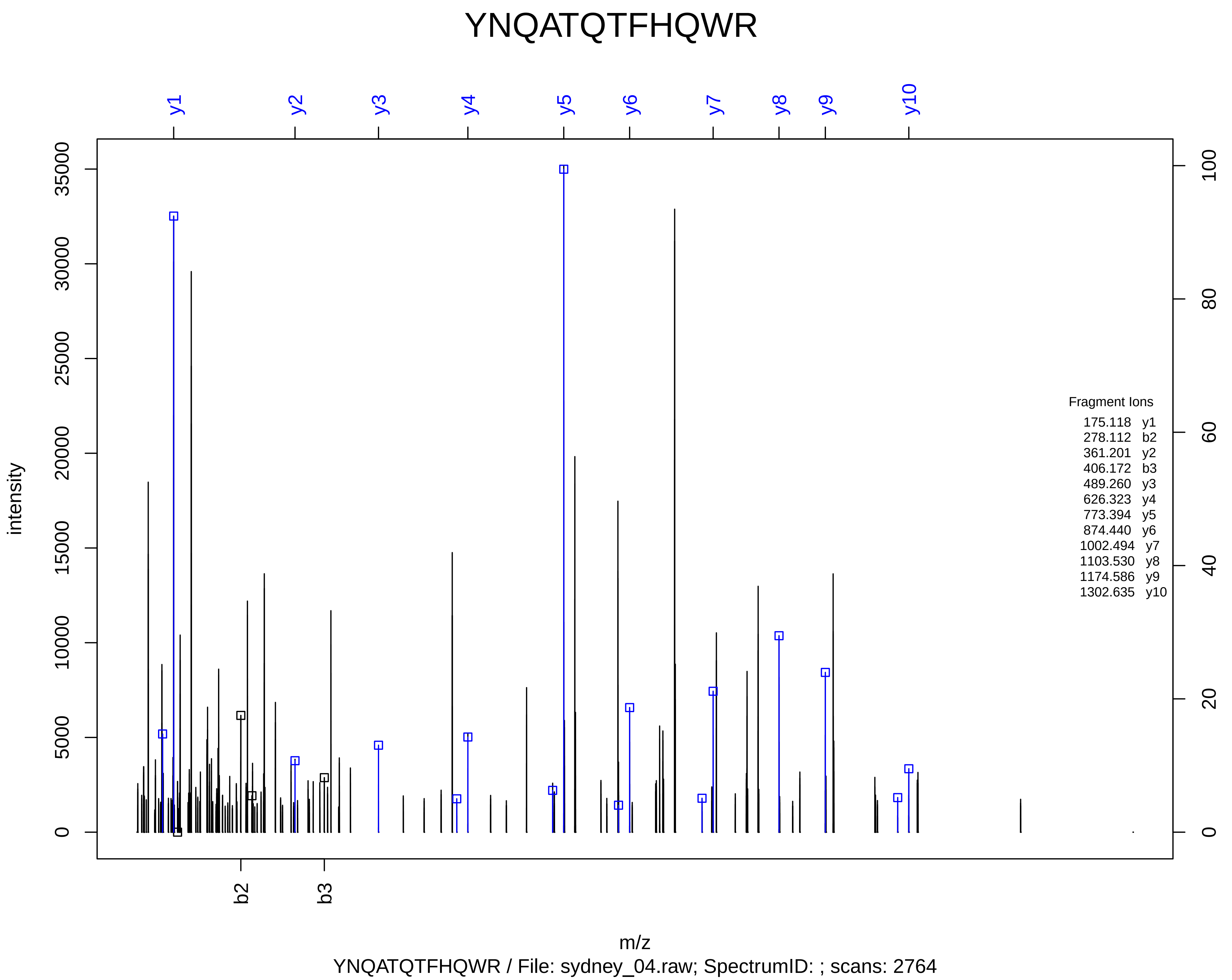
YNQATQTFHQWR
y1
y2
y3
y4
y5
y6
y7
y8
y9
y10
b2
b3
intensity
m/z
Fragment Ions
175.118 y1
278.112 b2
361.201 y2
406.172 b3
489.260 y3
626.323 y4
773.394 y5
874.440 y6
1002.494 y7
1103.530 y8
1174.586 y9
1302.635 y10
YNQATQTFHQWR / File: sydney_04.raw; SpectrumID: ; scans: 2764

(Ac)AANATTNPSQLLPLELVDK

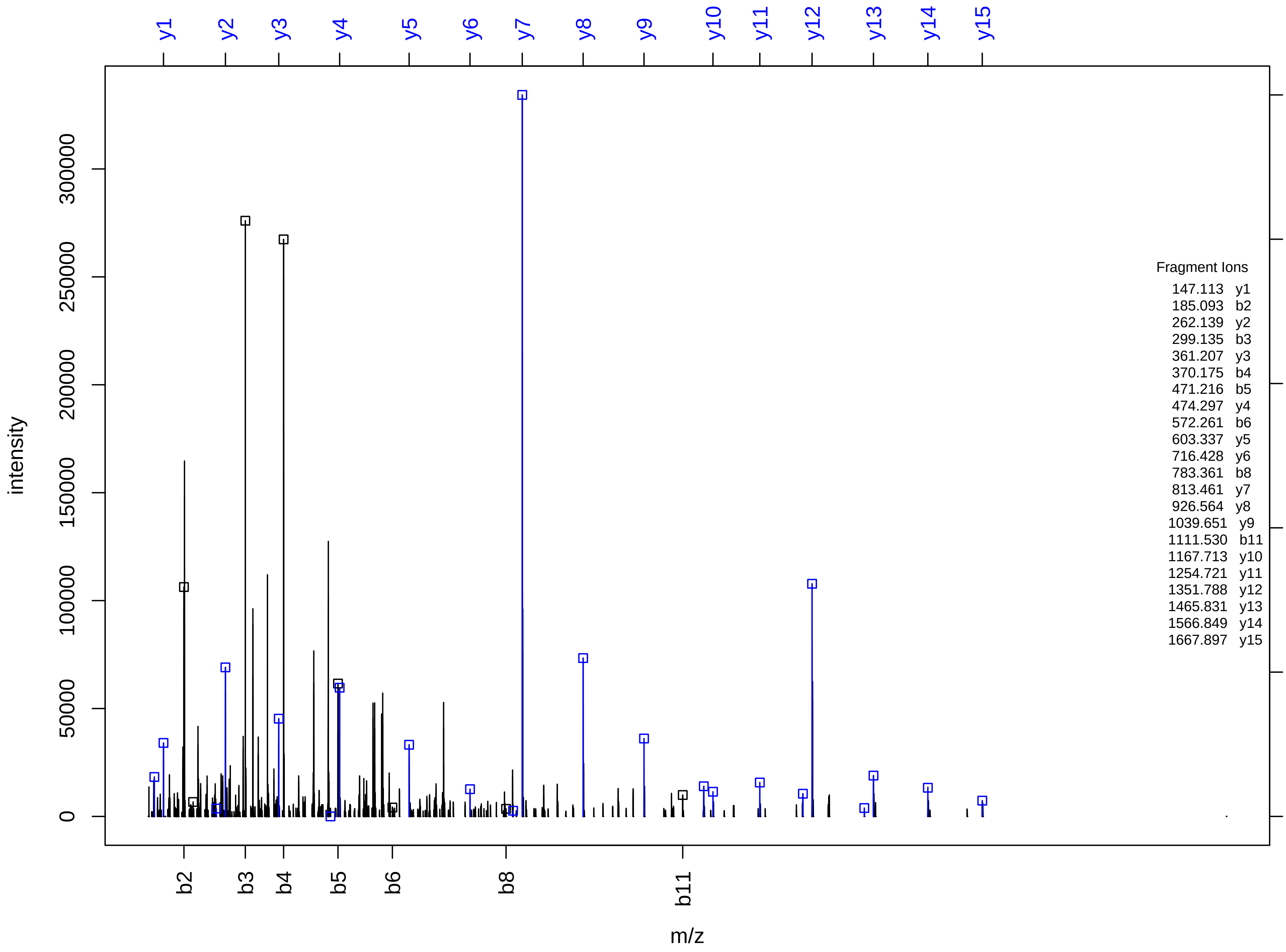

AANATTNPSQLLPLELVDK / File: sydney_04.raw; SpectrumID: ; scans: 16189

MATDFAENELDLFR

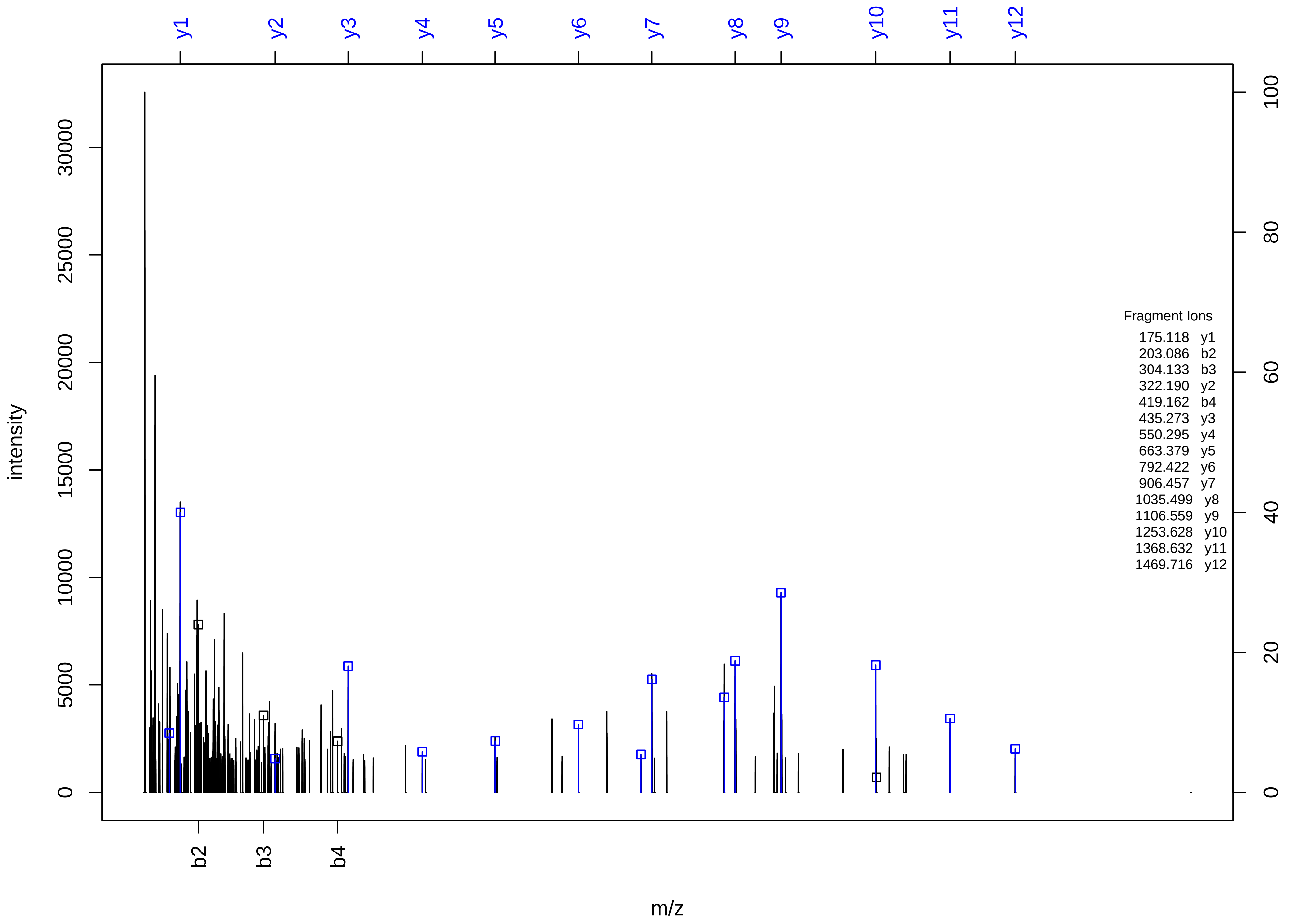

MATDFAENELDLFR / File: sydney_04.raw; SpectrumID: ; scans: 12819

AIECSYTSADGLK

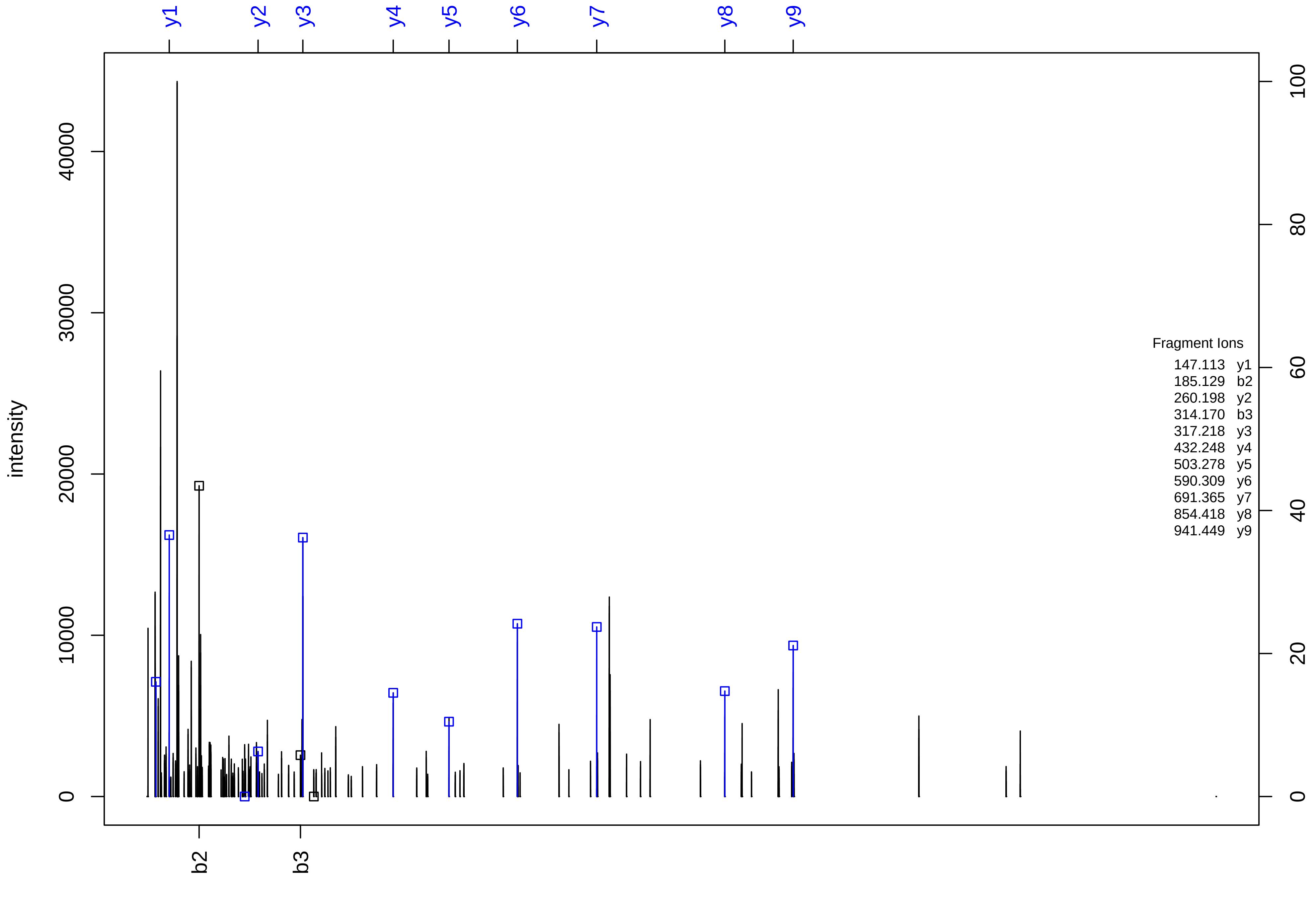

AIECSYTSADGLK / File: sydney_04.raw; SpectrumID: ; scans: 3103

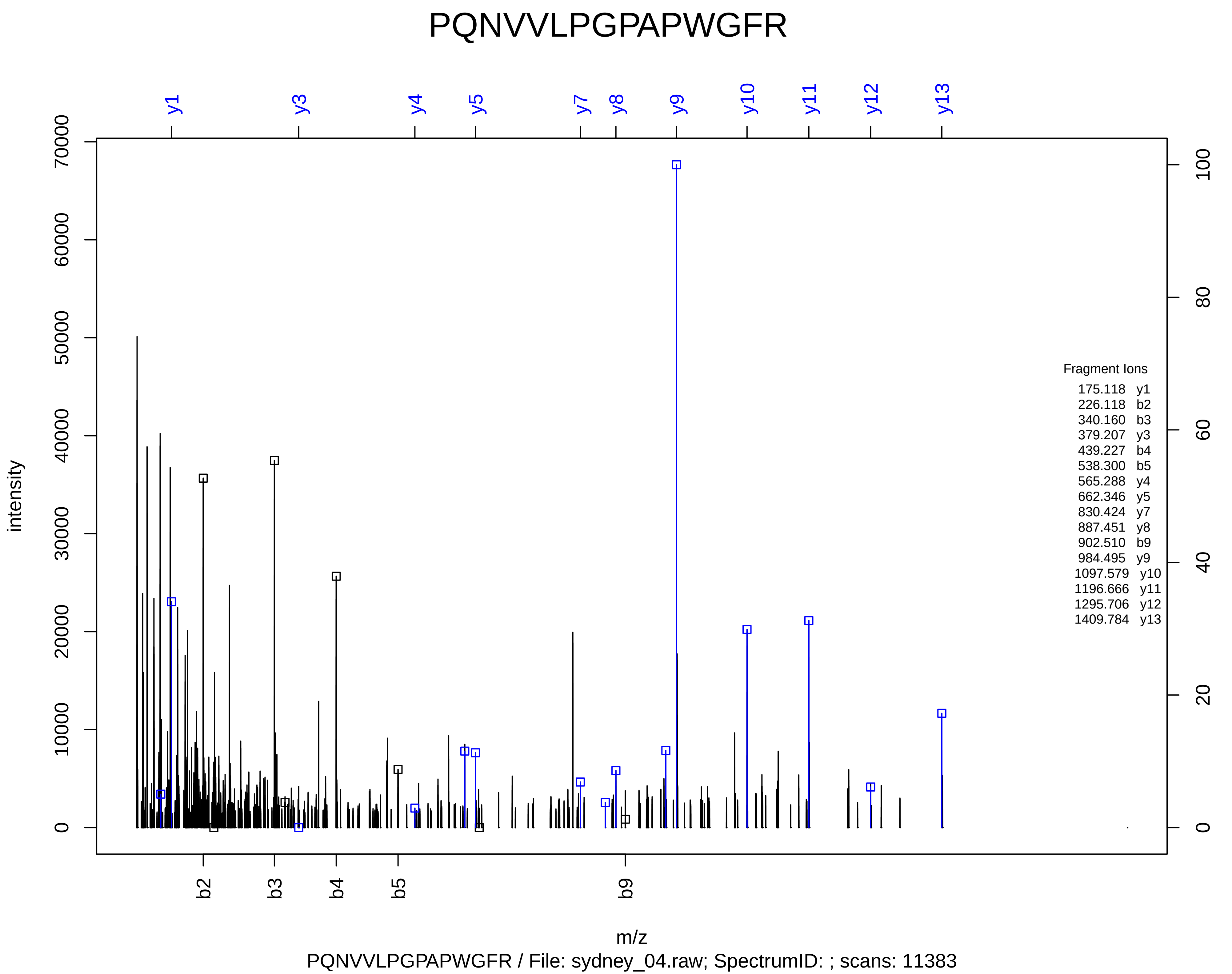
PQNVVLPGPAPWGFR
y1 y3 y4 y5 y7 y8 y9 y10 y11 y12 y13
b2 b3 b4 b5 b9
intensity
m/z
Fragment Ions
175.118 y1
226.118 b2
340.160 b3
379.207 y3
439.227 b4
538.300 b5
565.288 y4
662.346 y5
830.424 y7
887.451 y8
902.510 b9
984.495 y9
1097.579 y10
1196.666 y11
1295.706 y12
1409.784 y13
PQNVVLPGPAPWGFR / File: sydney_04.raw; SpectrumID: ; scans: 11383

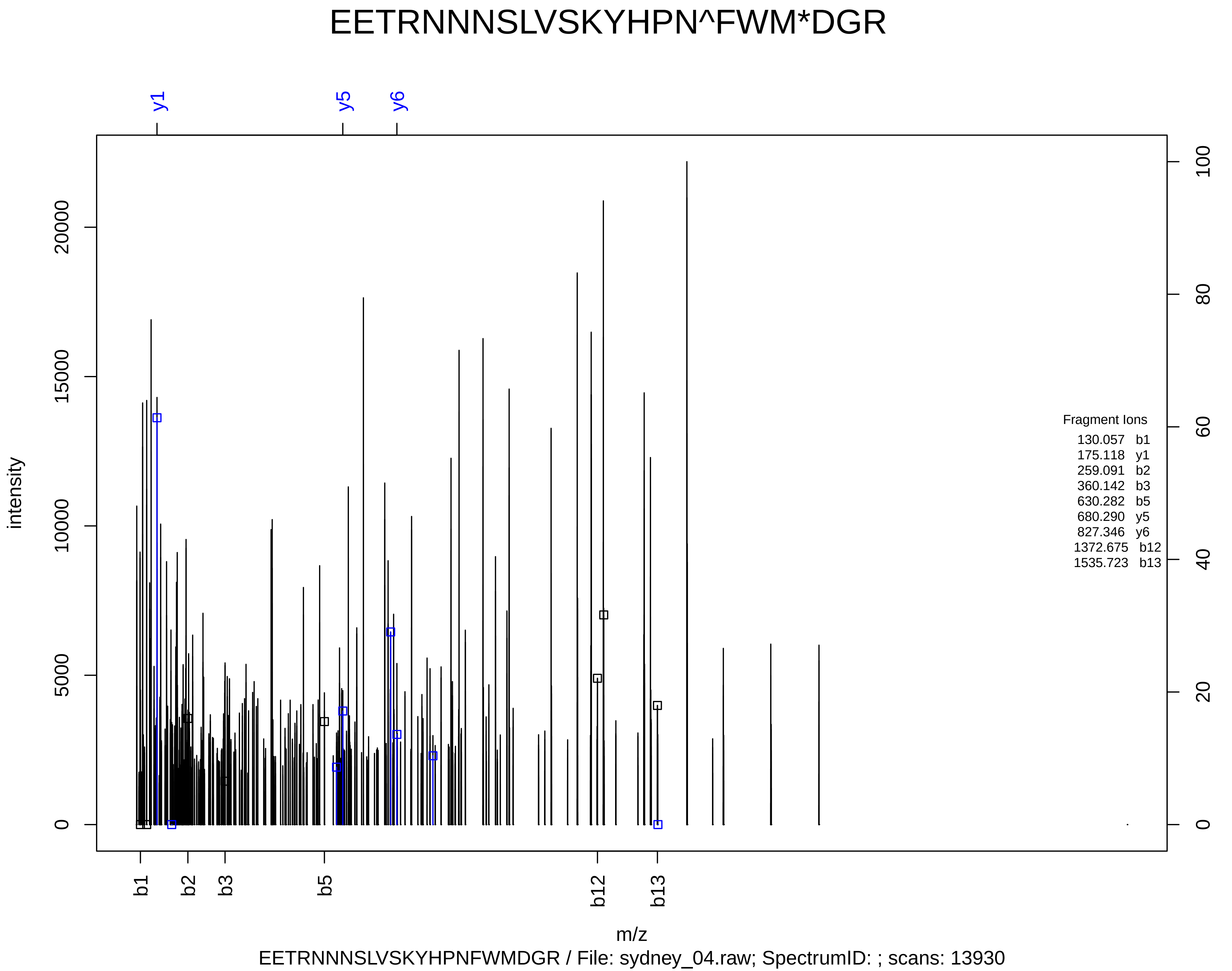
EETRNNNSLVSKYHPN^FWM*DGR
y1
y5
y6
intensity
0
5000
10000
15000
20000
0
20
40
60
80
100
Fragment Ions
130.057 b1
175.118 y1
259.091 b2
360.142 b3
630.282 b5
680.290 y5
827.346 y6
1372.675 b12
1535.723 b13
b1
b2
b3
b5
b12
b13
m/z
EETRNNNSLVSKYHPNFWMDGR / File: sydney_04.raw; SpectrumID: ; scans: 13930

SSIQVYADK

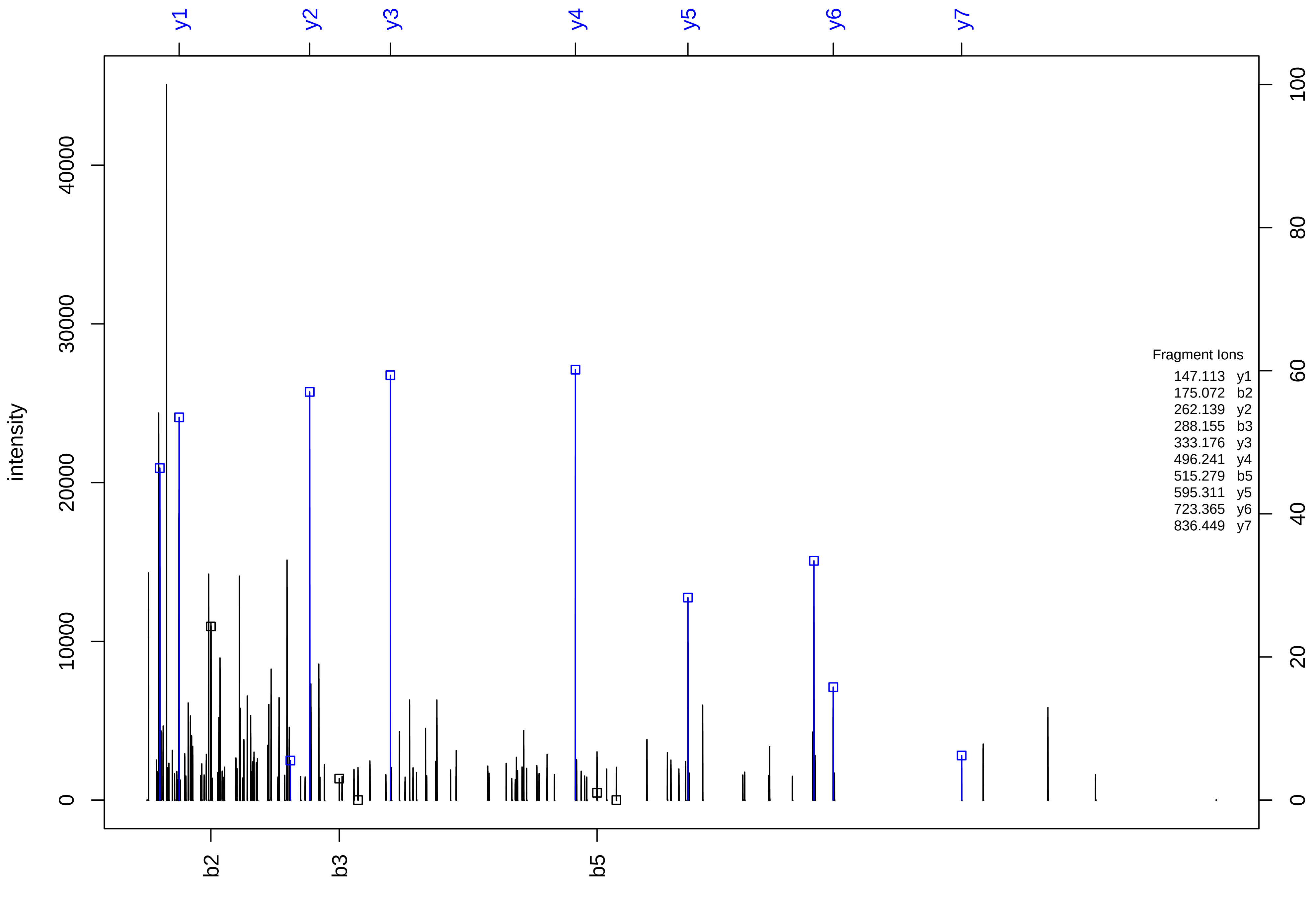

SSIQVYADK / File: sydney_04.raw; SpectrumID: ; scans: 1790

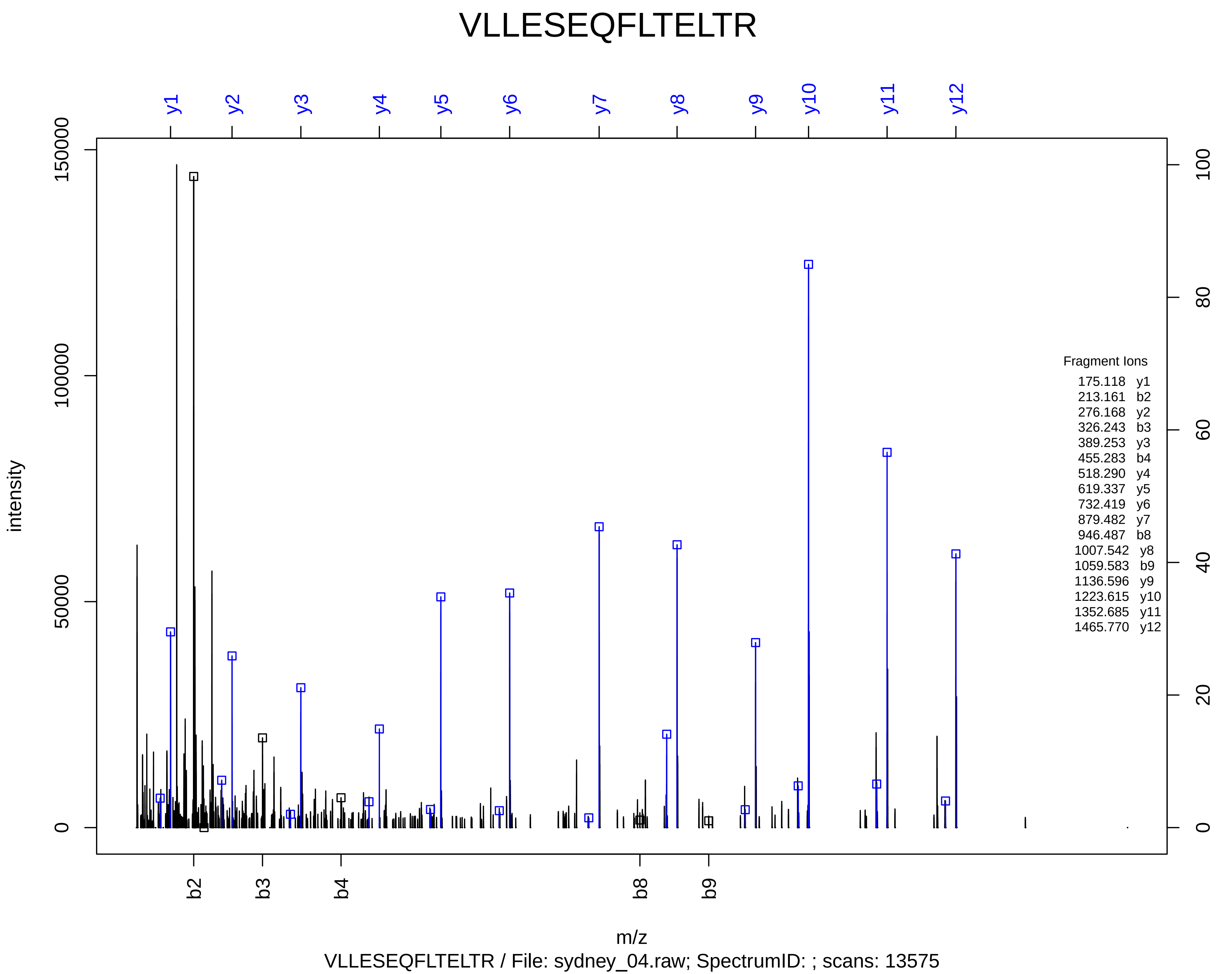
VLLESEQFLTELTR
y1
y2
y3
y4
y5
y6
y7
y8
y9
y10
y11
y12
intensity
150000
100000
50000
0
100
80
60
40
20
0
b2
b3
b4
b8
b9
m/z
Fragment Ions
175.118 y1
213.161 b2
276.168 y2
326.243 b3
389.253 y3
455.283 b4
518.290 y4
619.337 y5
732.419 y6
879.482 y7
946.487 b8
1007.542 y8
1059.583 b9
1136.596 y9
1223.615 y10
1352.685 y11
1465.770 y12
VLLESEQFLTELTR / File: sydney_04.raw; SpectrumID: ; scans: 13575

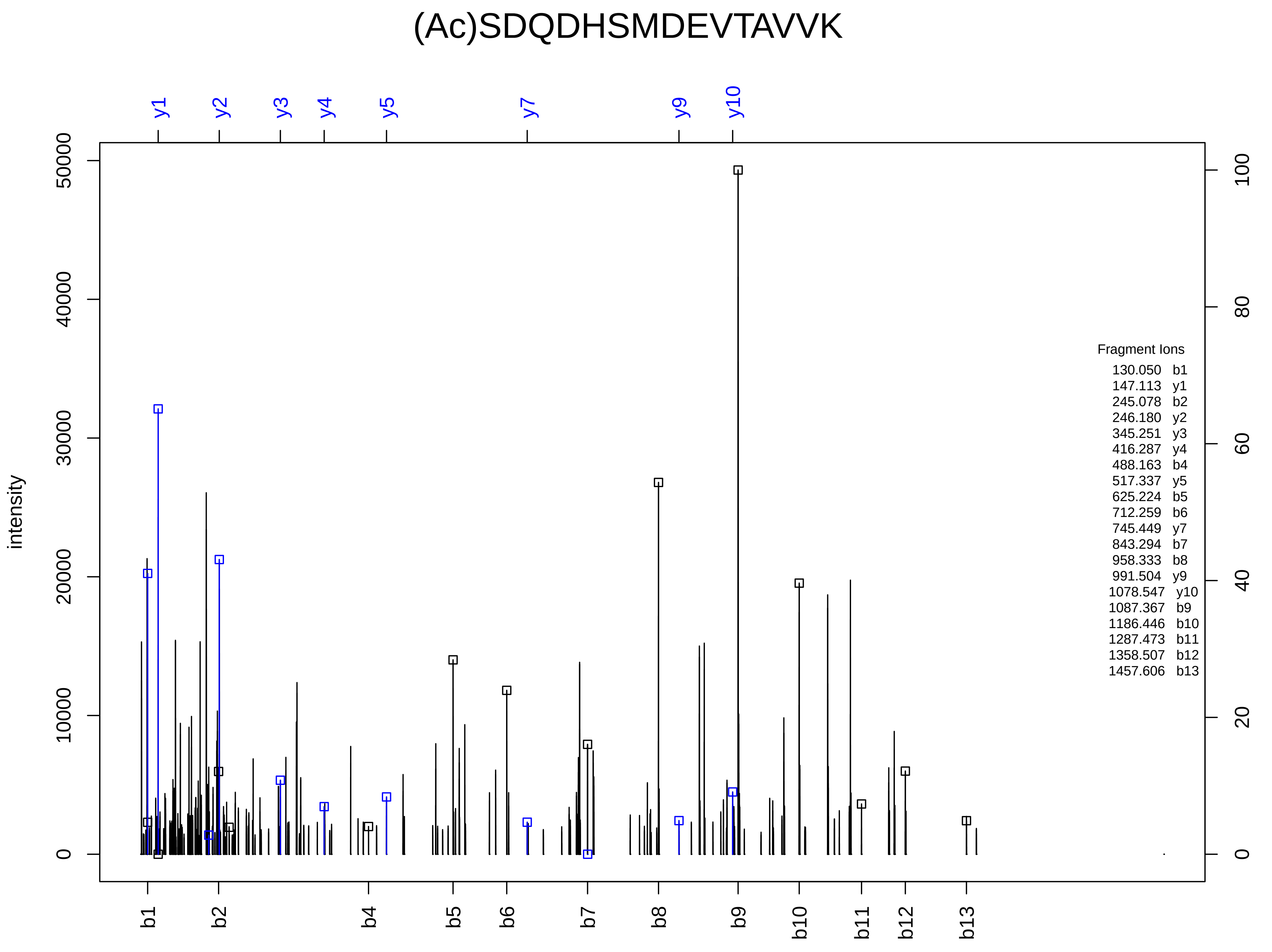

SDQDHSMDEVTAVVK / File: sydney_04.raw; SpectrumID: ; scans: 5924

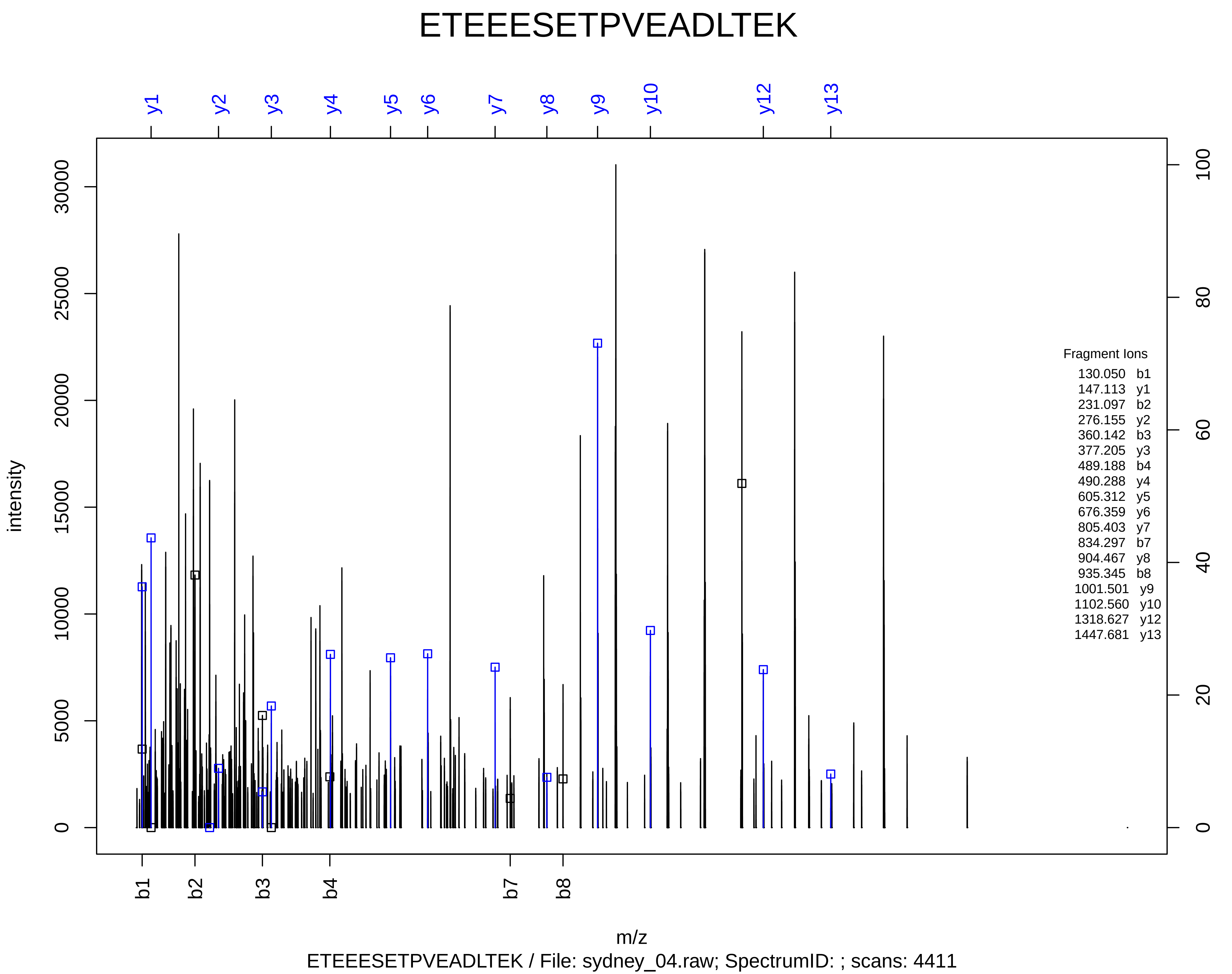
ETEEESETPVEADLTEK
y1
y2
y3
y4
y5
y6
y7
y8
y9
y10
y12
y13
intensity
0
5000
10000
15000
20000
25000
30000
0
20
40
60
80
100
b1
b2
b3
b4
b7
b8
m/z
Fragment Ions
130.050 b1
147.113 y1
231.097 b2
276.155 y2
360.142 b3
377.205 y3
489.188 b4
490.288 y4
605.312 y5
676.359 y6
805.403 y7
834.297 b7
904.467 y8
935.345 b8
1001.501 y9
1102.560 y10
1318.627 y12
1447.681 y13
ETEEESETPVEADLTEK / File: sydney_04.raw; SpectrumID: ; scans: 4411

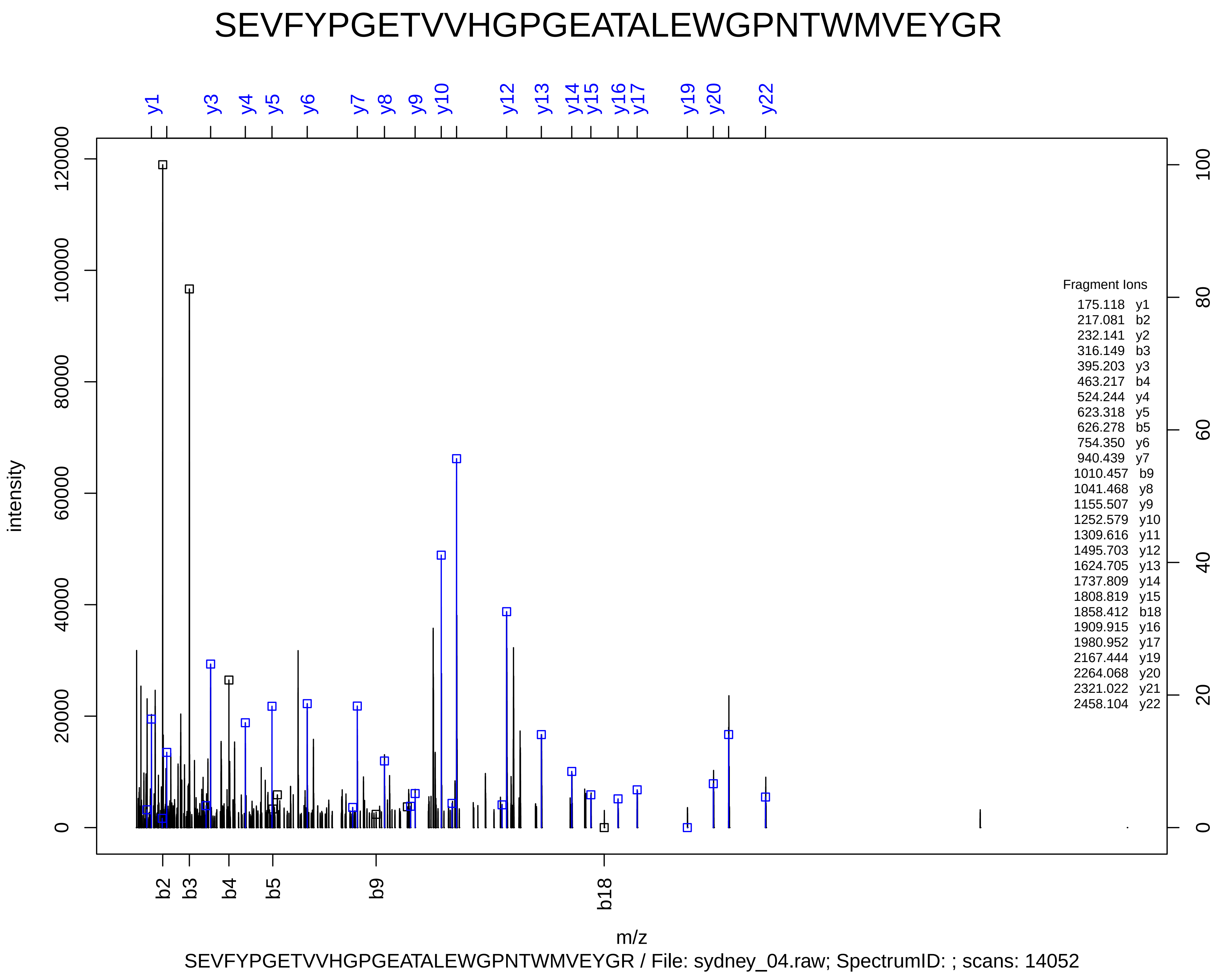

SEVFYPGETVVHGPGEATALEWGPNTWMVEYGR
y1 y3 y4 y5 y6 y7 y8 y9 y10 y12 y13 y14 y15 y16 y17 y19 y20 y22
intensity
120000 100000 80000 60000 40000 20000 0
100 80 60 40 20 0
Fragment Ions
175.118 y1
217.081 b2
232.141 y2
316.149 b3
395.203 y3
463.217 b4
524.244 y4
623.318 y5
626.278 b5
754.350 y6
940.439 y7
1010.457 b9
1041.468 y8
1155.507 y9
1252.579 y10
1309.616 y11
1495.703 y12
1624.705 y13
1737.809 y14
1808.819 y15
1858.412 b18
1909.915 y16
1980.952 y17
2167.444 y19
2264.068 y20
2321.022 y21
2458.104 y22
b2 b3 b4 b5 b9 b18
m/z
SEVFYPGETVVHGPGEATALEWGPNTWMVEYGR / File: sydney_04.raw; SpectrumID: ; scans: 14052

DMEANLLPGLLR

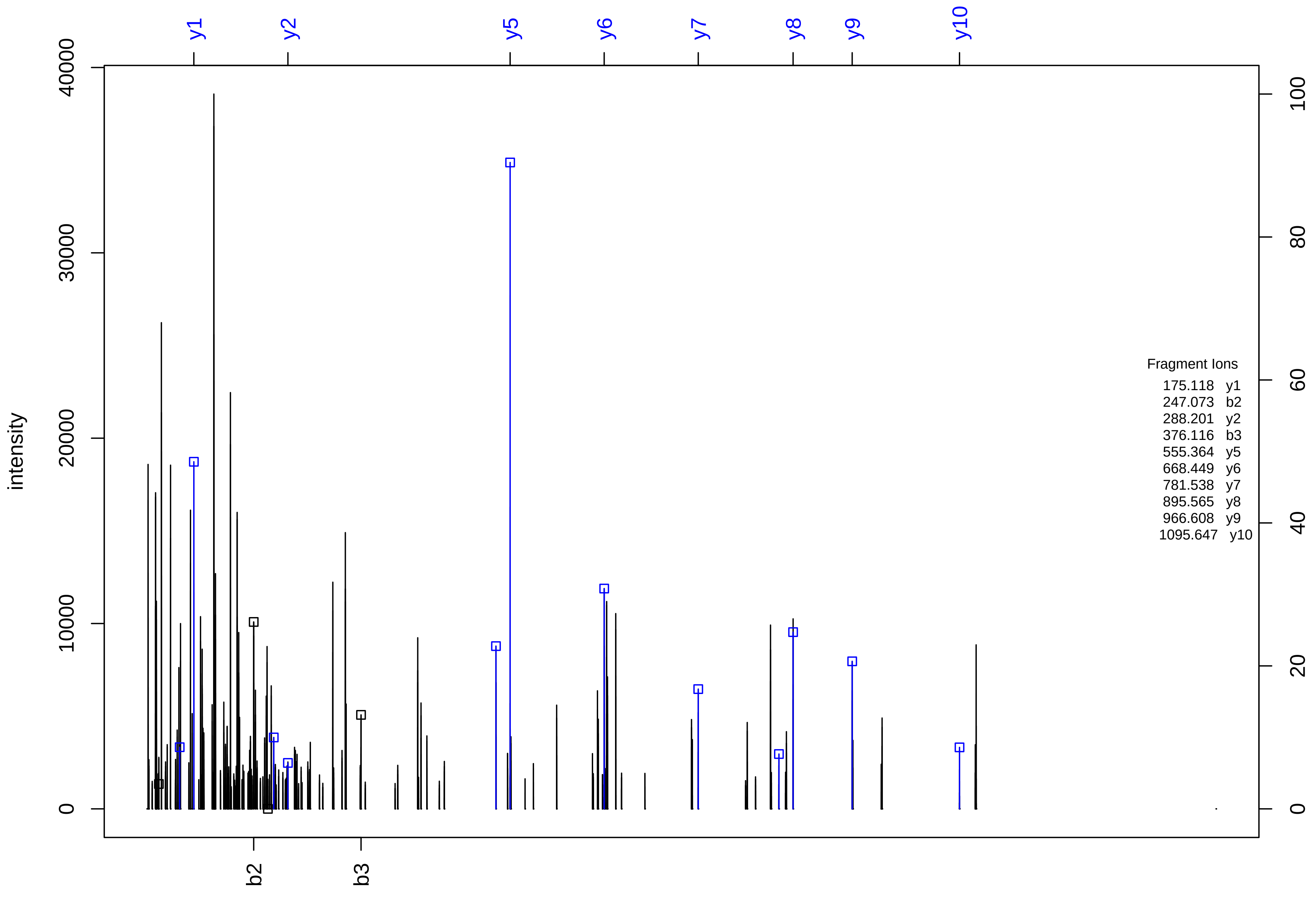

DMEANLLPGLLR / File: sydney_04.raw; SpectrumID: ; scans: 13672

TQSSLVPALTEFVR

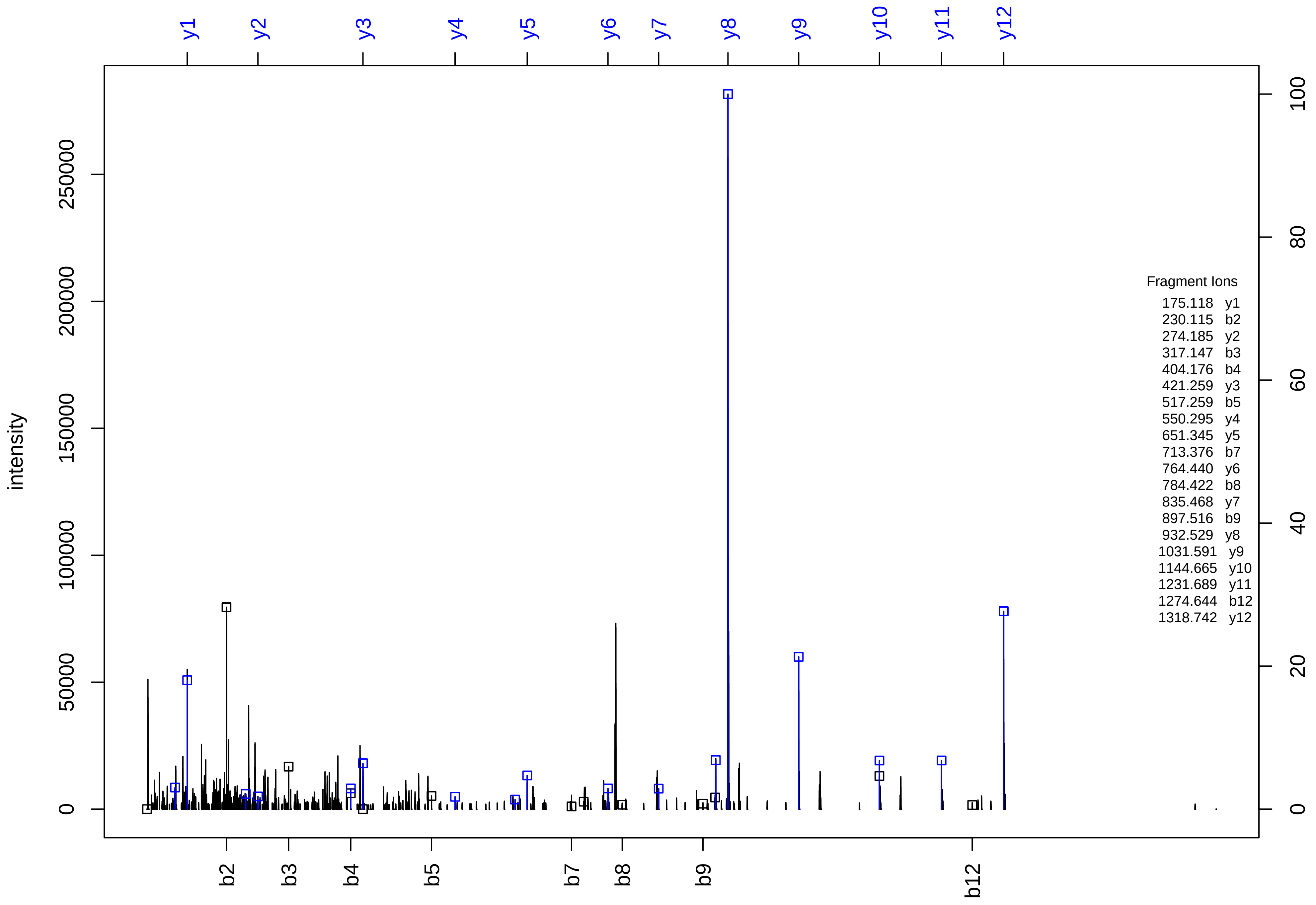

TQSSLVPALTEFVR / File: sydney_04.raw; SpectrumID: ; scans: 12702

(Ac)ALQALHSSGVGLR

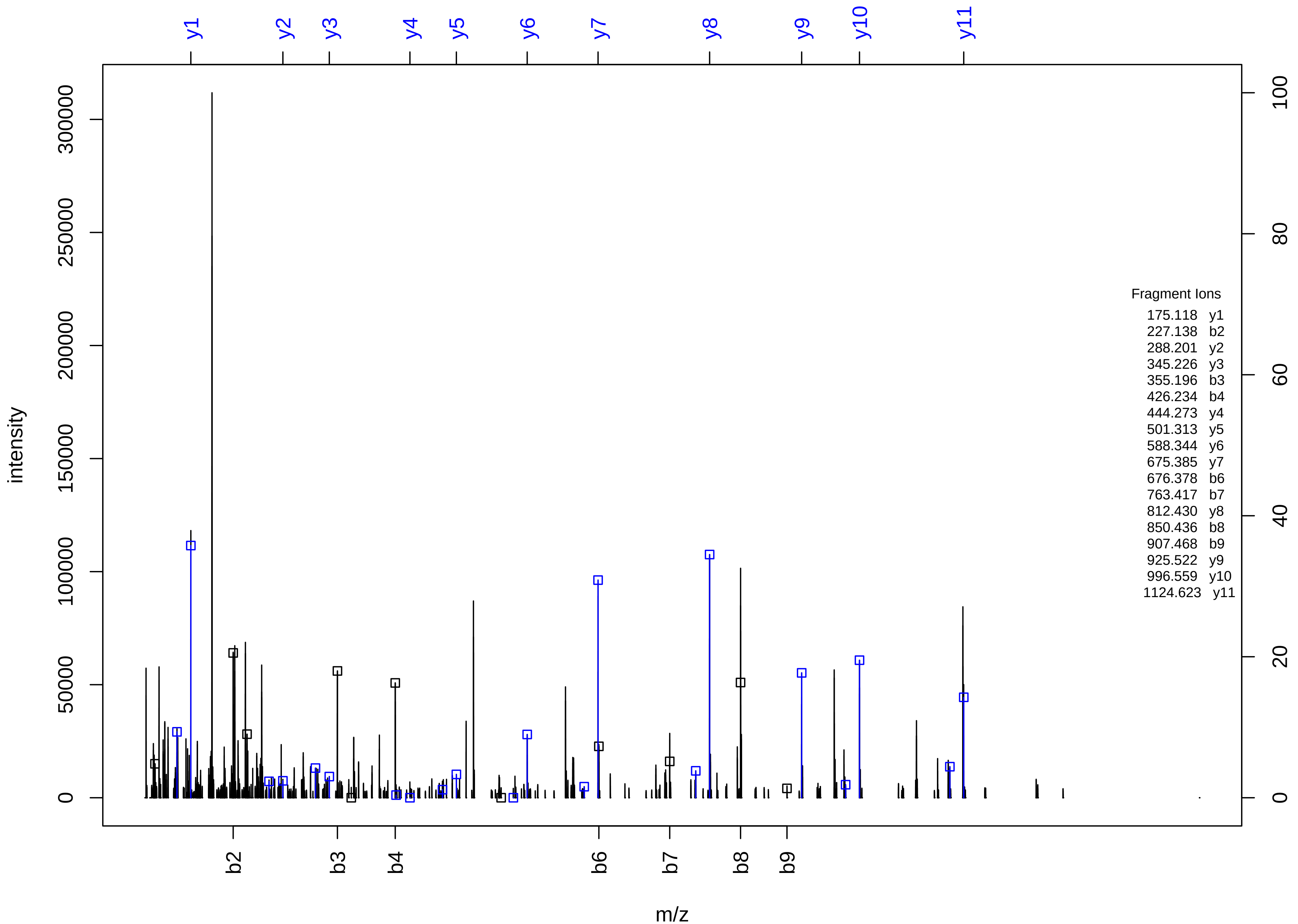

ALQALHSSGVGLR / File: sydney_04.raw; SpectrumID: ; scans: 8183

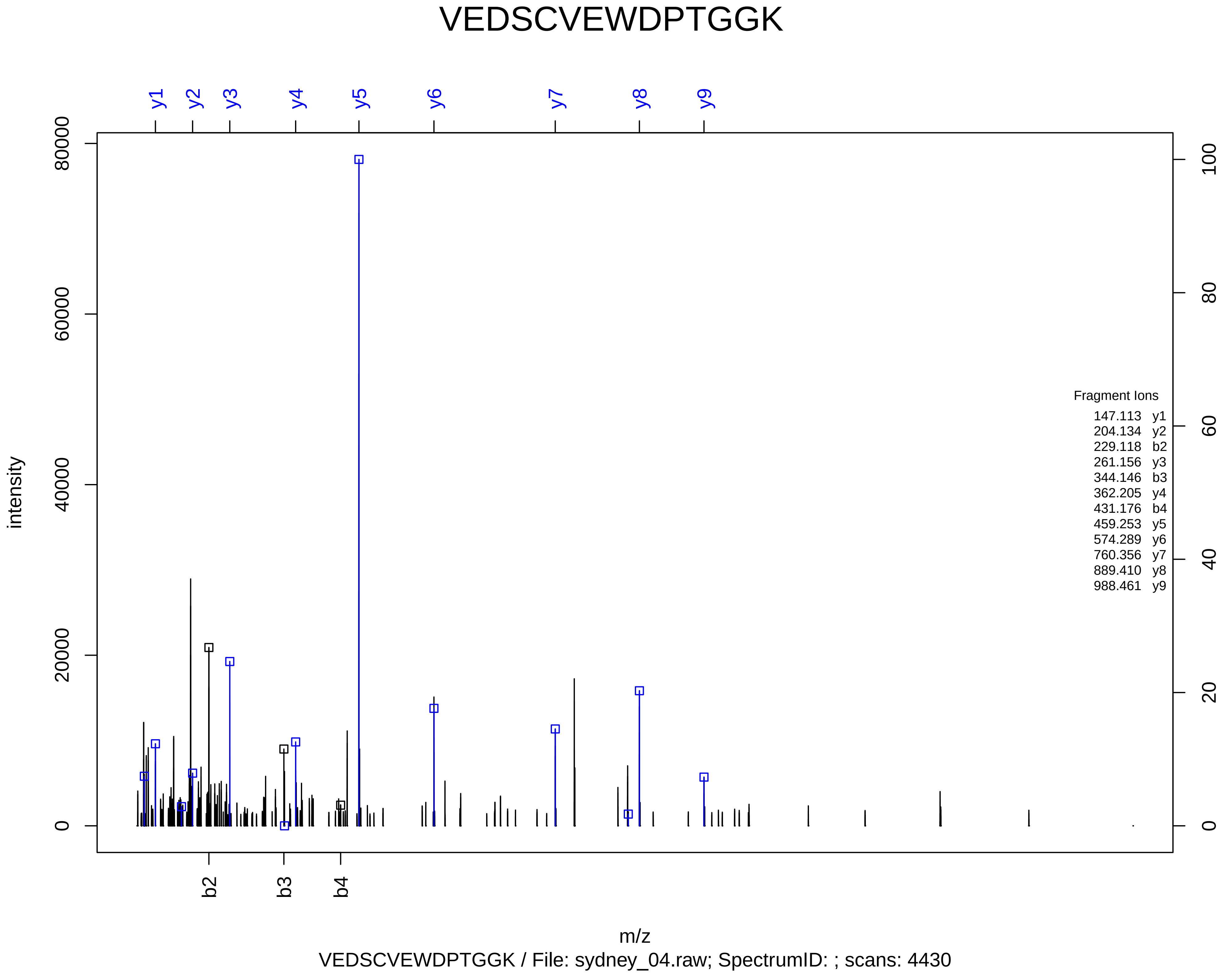
VEDSCVEWDPTGGK
y1
y2
y3
y4
y5
y6
y7
y8
y9
b2
b3
b4
intensity
0
20000
40000
60000
80000
0
20
40
60
80
100
Fragment Ions
147.113 y1
204.134 y2
229.118 b2
261.156 y3
344.146 b3
362.205 y4
431.176 b4
459.253 y5
574.289 y6
760.356 y7
889.410 y8
988.461 y9
m/z
VEDSCVEWDPTGGK / File: sydney_04.raw; SpectrumID: ; scans: 4430

N^LENEIYVNDQK

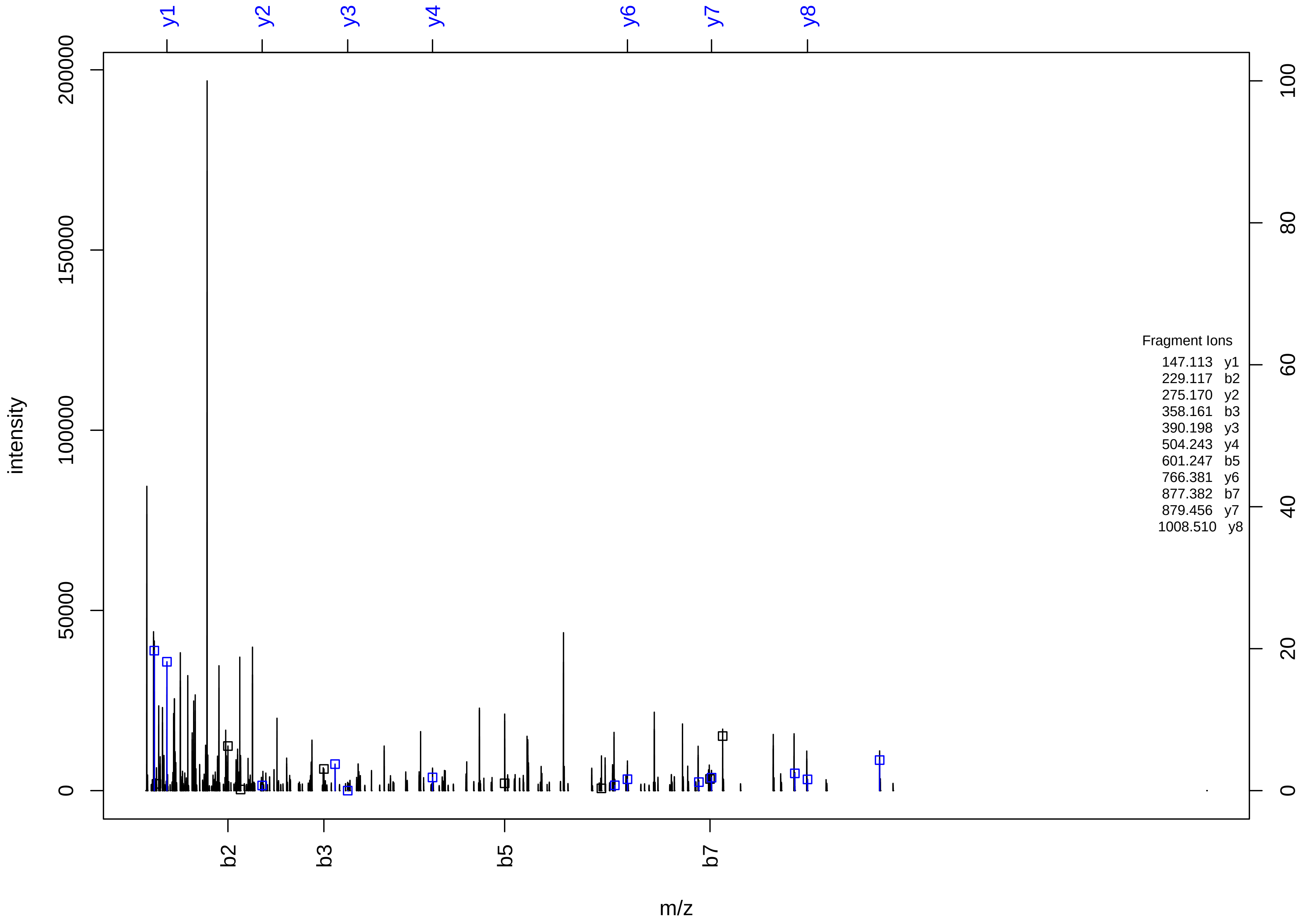

NLENEIYVNDQK / File: sydney_04.raw; SpectrumID: ; scans: 4052

ELTVDNSFEFEK

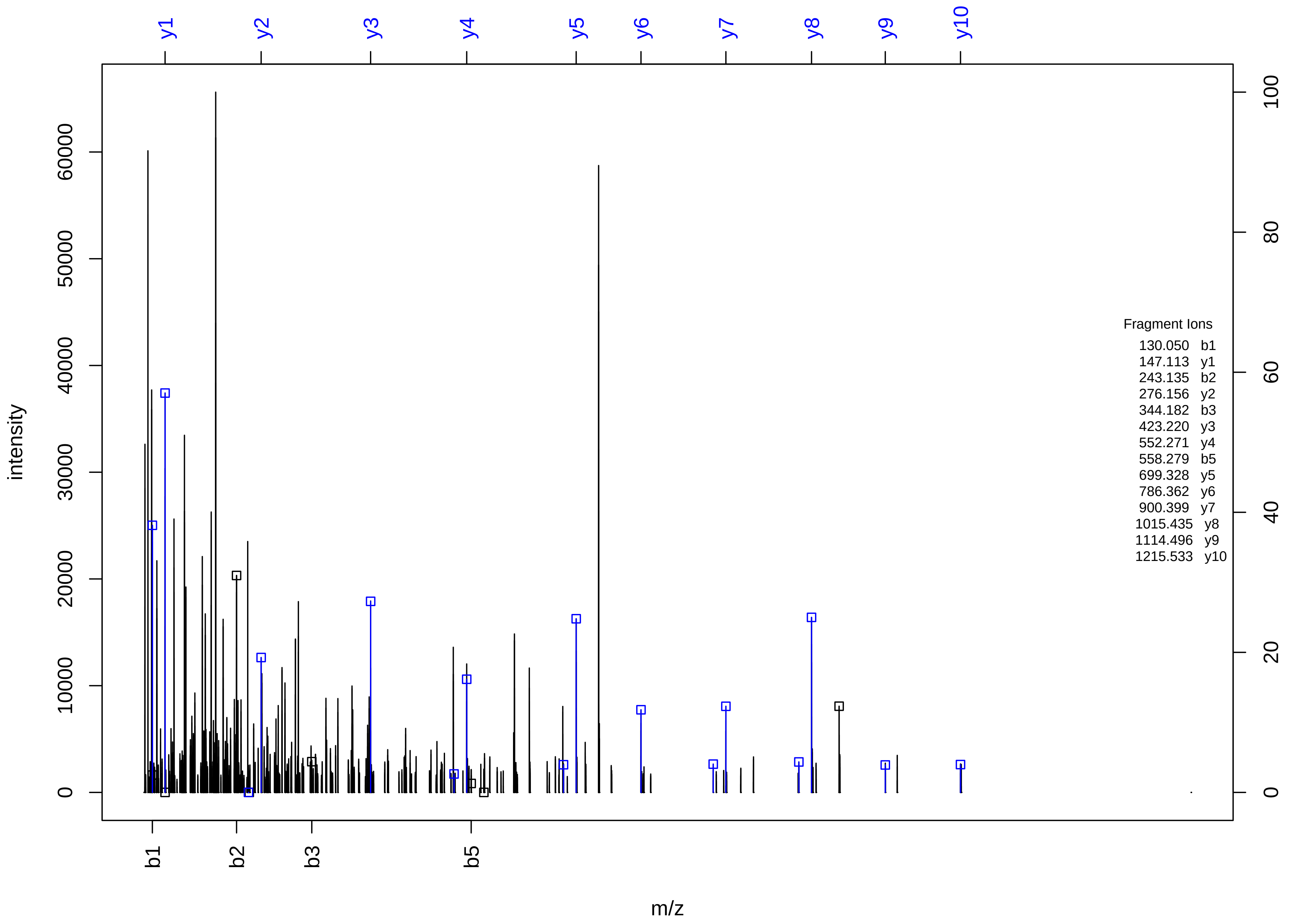

ELTVDNSFEFEK / File: sydney_04.raw; SpectrumID: ; scans: 7624

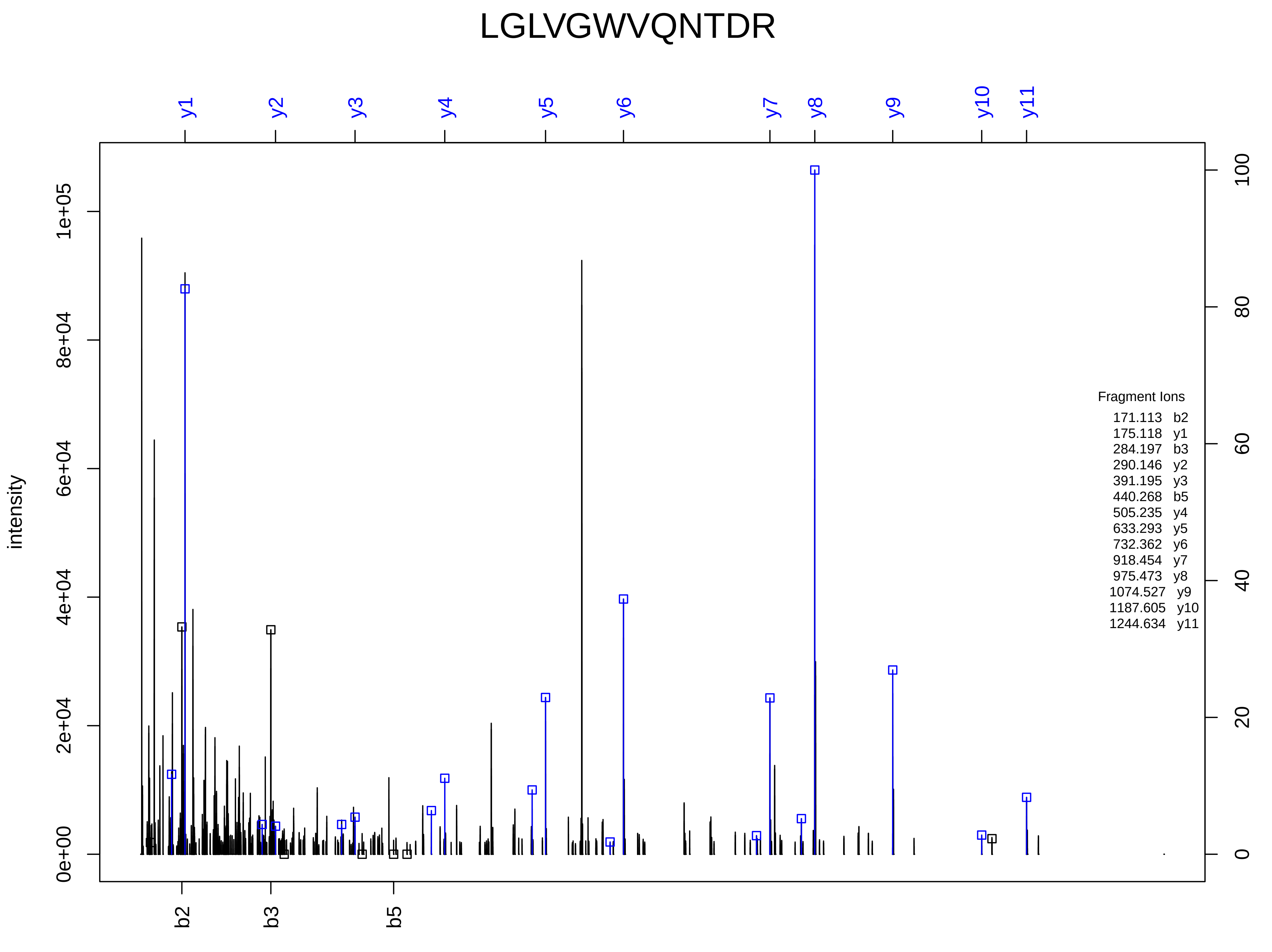

LGLVGWVQNTDR / File: sydney_04.raw; SpectrumID: ; scans: 9228

YTDQSGEEEEDYESEEQLQHR

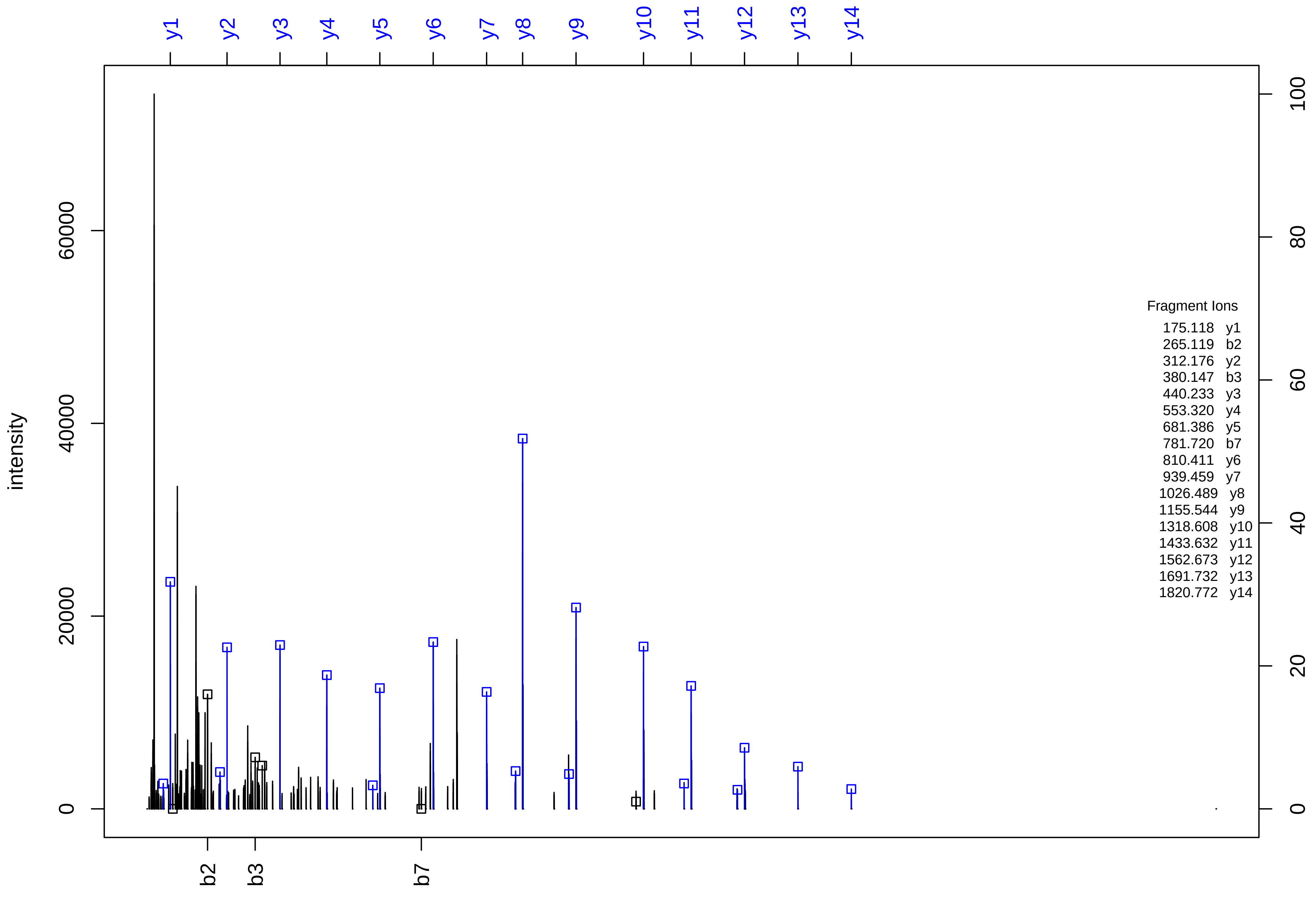

YTDQSGEEEEDYESEEQLQHR / File: sydney_04.raw; SpectrumID: ; scans: 2638

TQSEESRPQSLQQPATSTTETPASPAHTTPQTQNTSGR

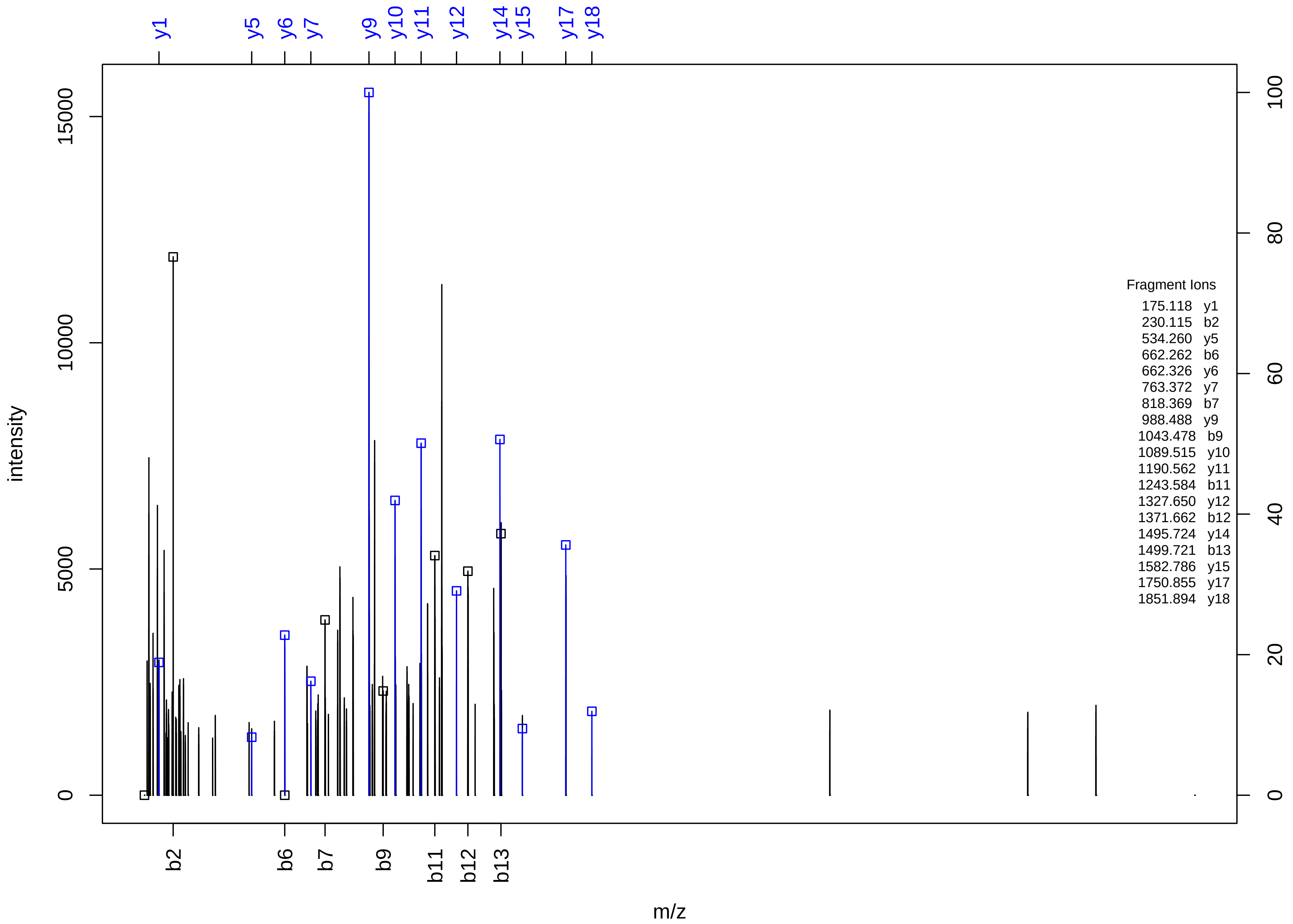

TQSEESRPQSLQQPATSTTETPASPAHTTPQTQNTSGR / File: sydney_04.raw; SpectrumID: ; scans: 2060

QTFLQDCEDDGETAAGGR

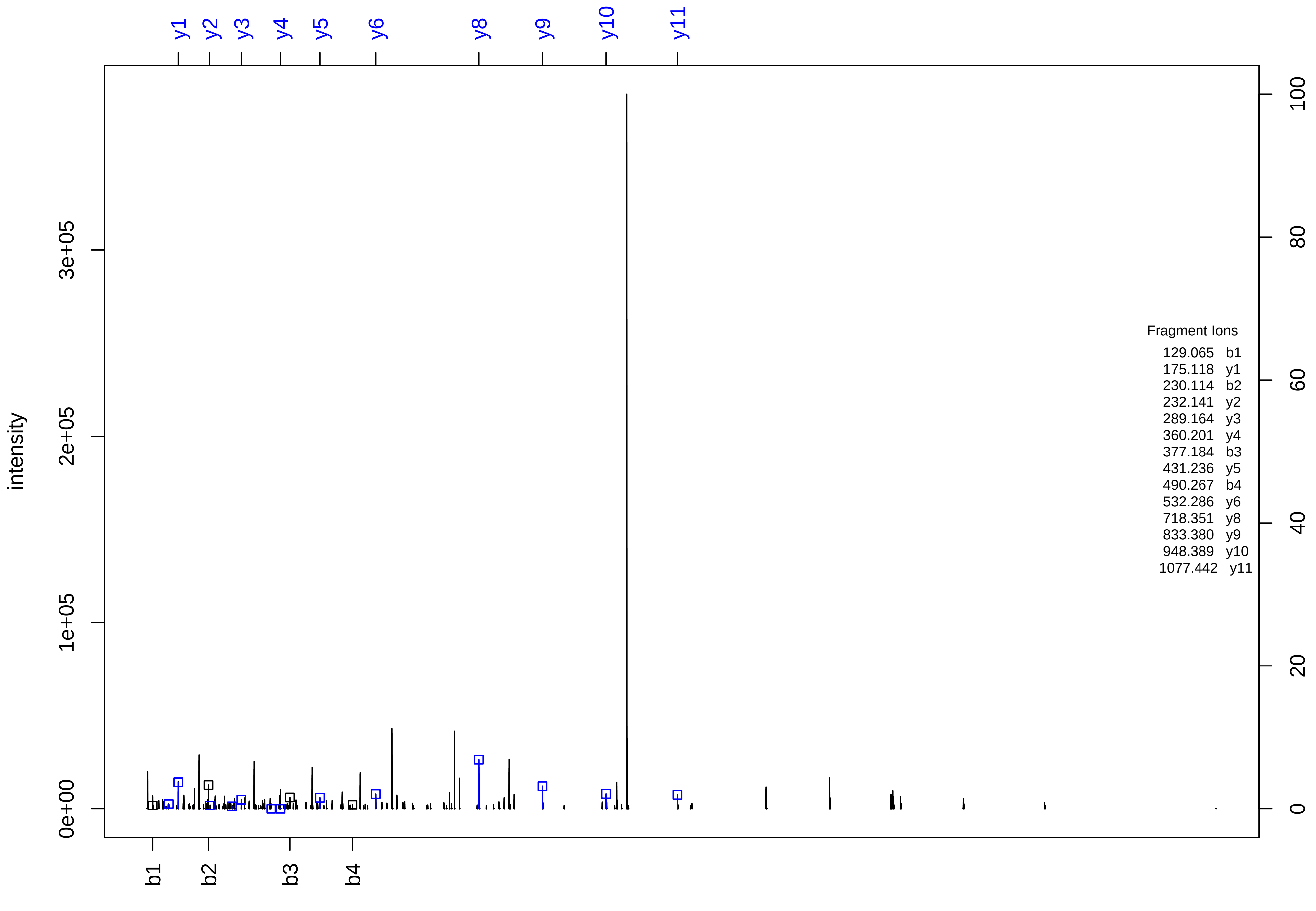

QTFLQDCEDDGETAAGGR / File: sydney_04.raw; SpectrumID: ; scans: 4310

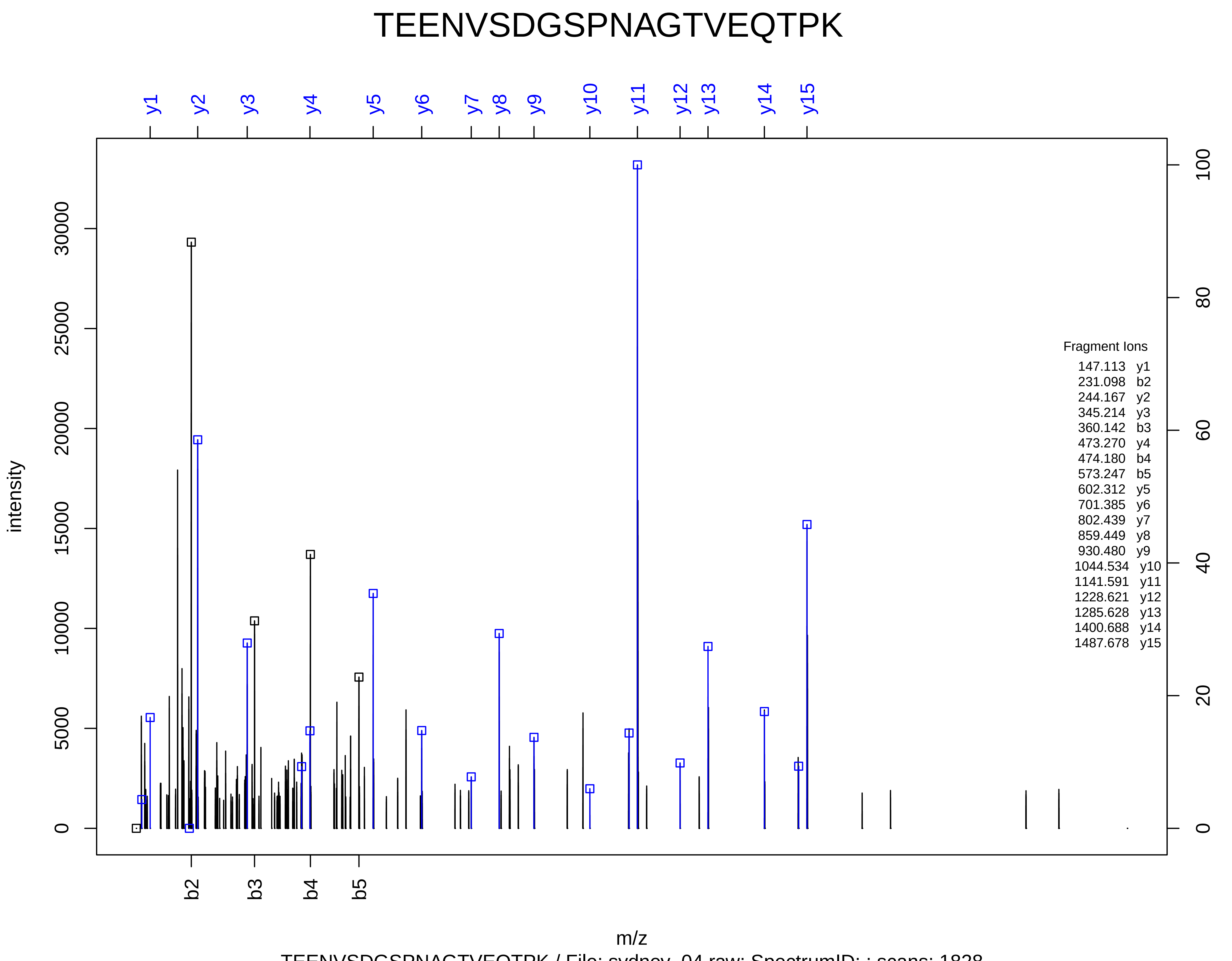

TEENVSDGSPNAGTVEQTPK
y1 y2 y3 y4 y5 y6 y7 y8 y9 y10 y11 y12 y13 y14 y15
b2 b3 b4 b5
intensity
0 5000 10000 15000 20000 25000 30000
0 20 40 60 80 100
m/z
Fragment Ions
147.113 y1
231.098 b2
244.167 y2
345.214 y3
360.142 b3
473.270 y4
474.180 b4
573.247 b5
602.312 y5
701.385 y6
802.439 y7
859.449 y8
930.480 y9
1044.534 y10
1141.591 y11
1228.621 y12
1285.628 y13
1400.688 y14
1487.678 y15
TEENVSDGSPNAGTVEQTPK / File: sydney_04.raw; SpectrumID: ; scans: 1828

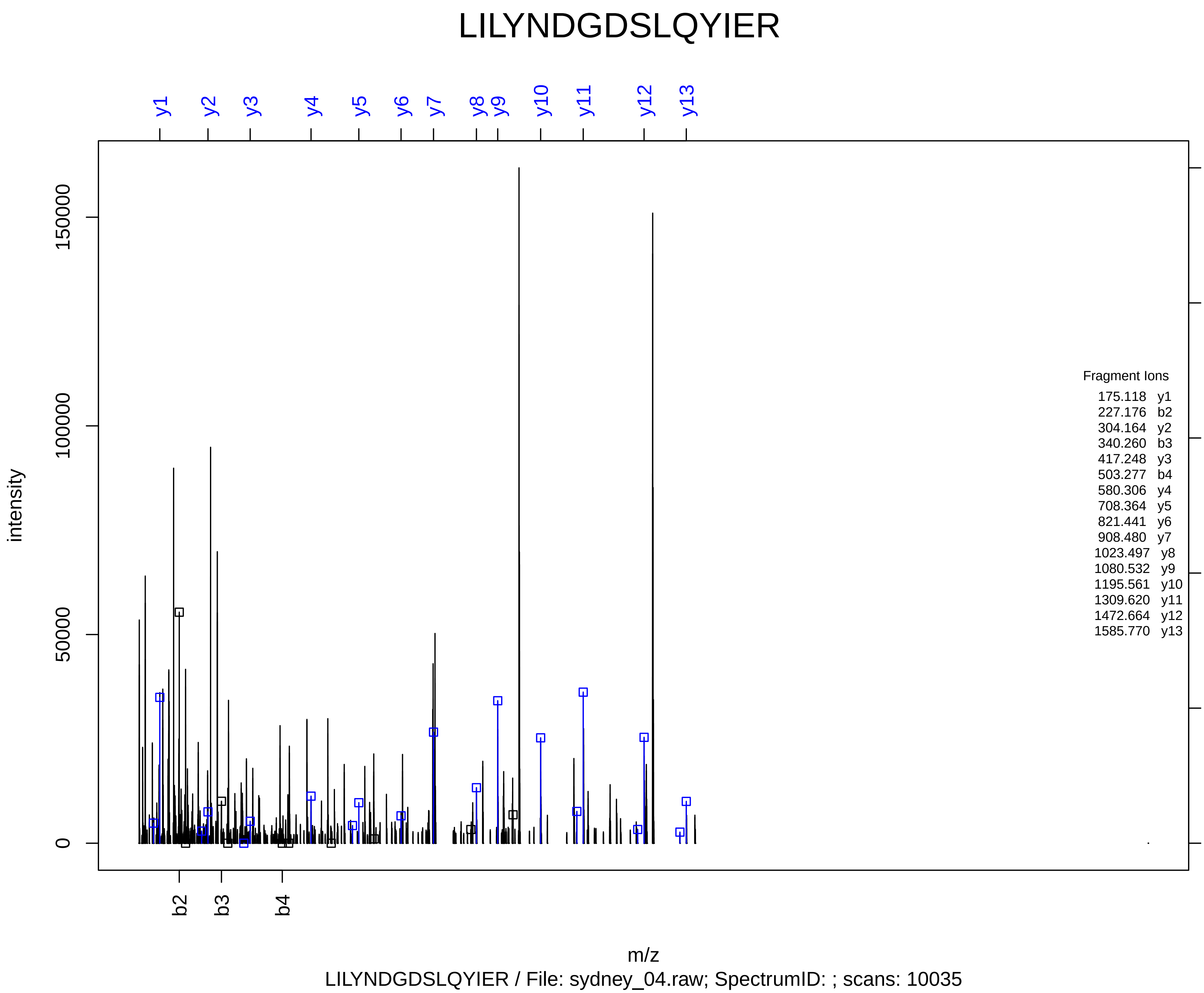

LILYNDGDSLQYIER
y1
y2
y3
y4
y5
y6
y7
y8
y9
y10
y11
y12
y13
intensity
0
50000
100000
150000
0
20
40
60
80
100
b2
b3
b4
m/z
Fragment Ions
175.118 y1
227.176 b2
304.164 y2
340.260 b3
417.248 y3
503.277 b4
580.306 y4
708.364 y5
821.441 y6
908.480 y7
1023.497 y8
1080.532 y9
1195.561 y10
1309.620 y11
1472.664 y12
1585.770 y13
LILYNDGDSLQYIER / File: sydney_04.raw; SpectrumID: ; scans: 10035

(Ac)MEVLATDTASQQER

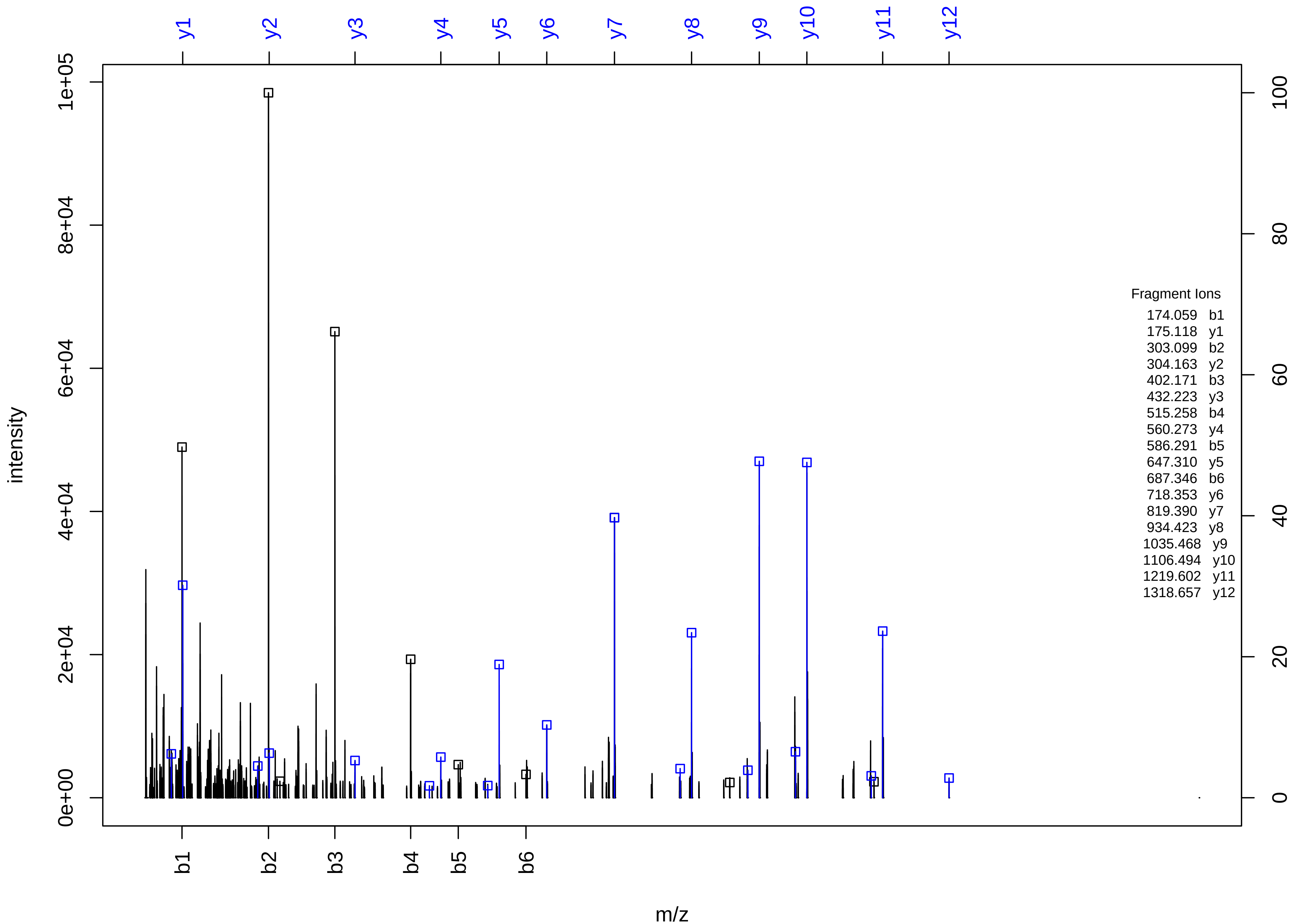

MEVLATDTASQQER / File: sydney_04.raw; SpectrumID: ; scans: 9532

VEEQLFPLIDDDTR

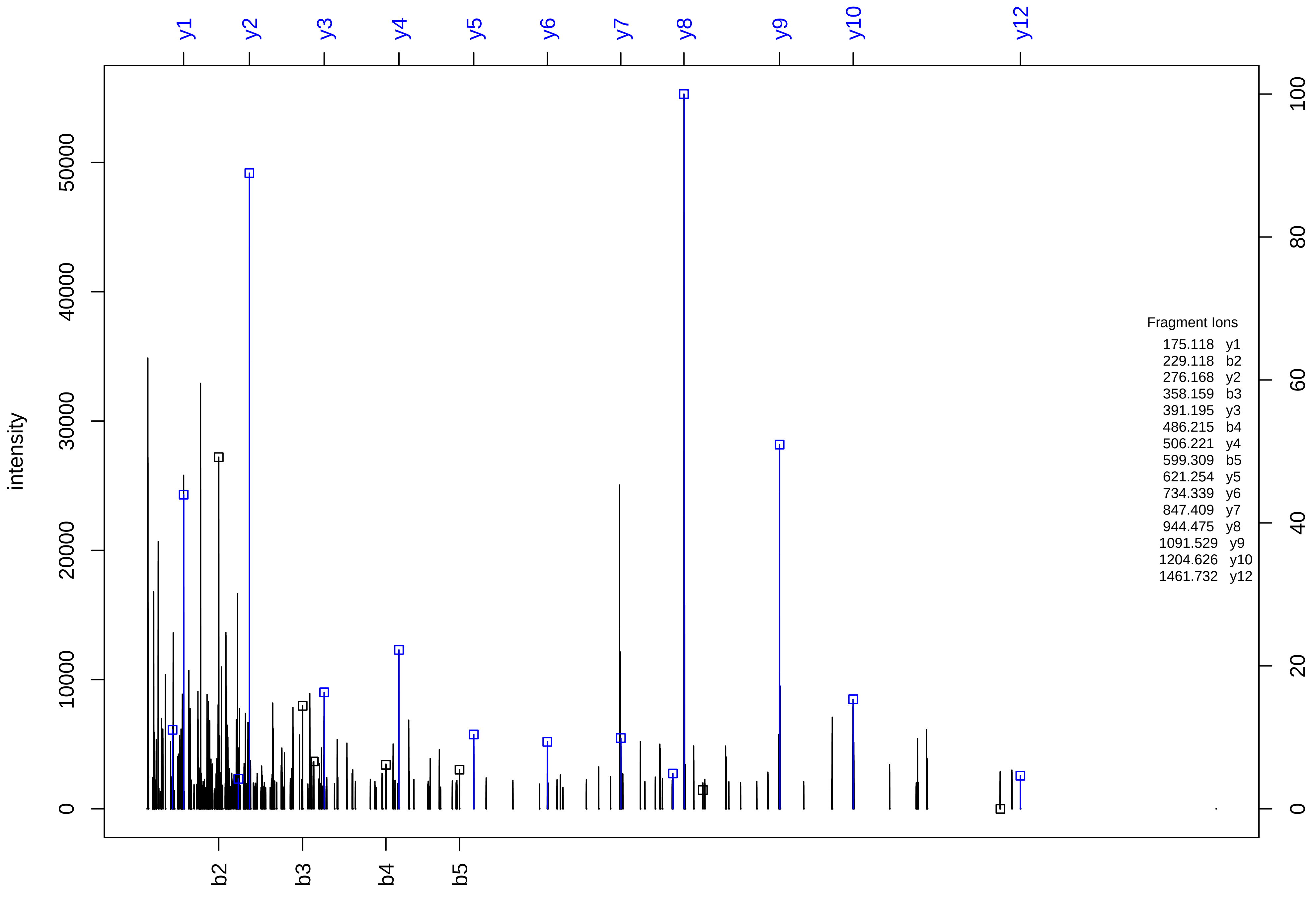

VEEQLFPLIDDDTR / File: sydney_04.raw; SpectrumID: ; scans: 11846

LAQELPDVTNEDSAFQK

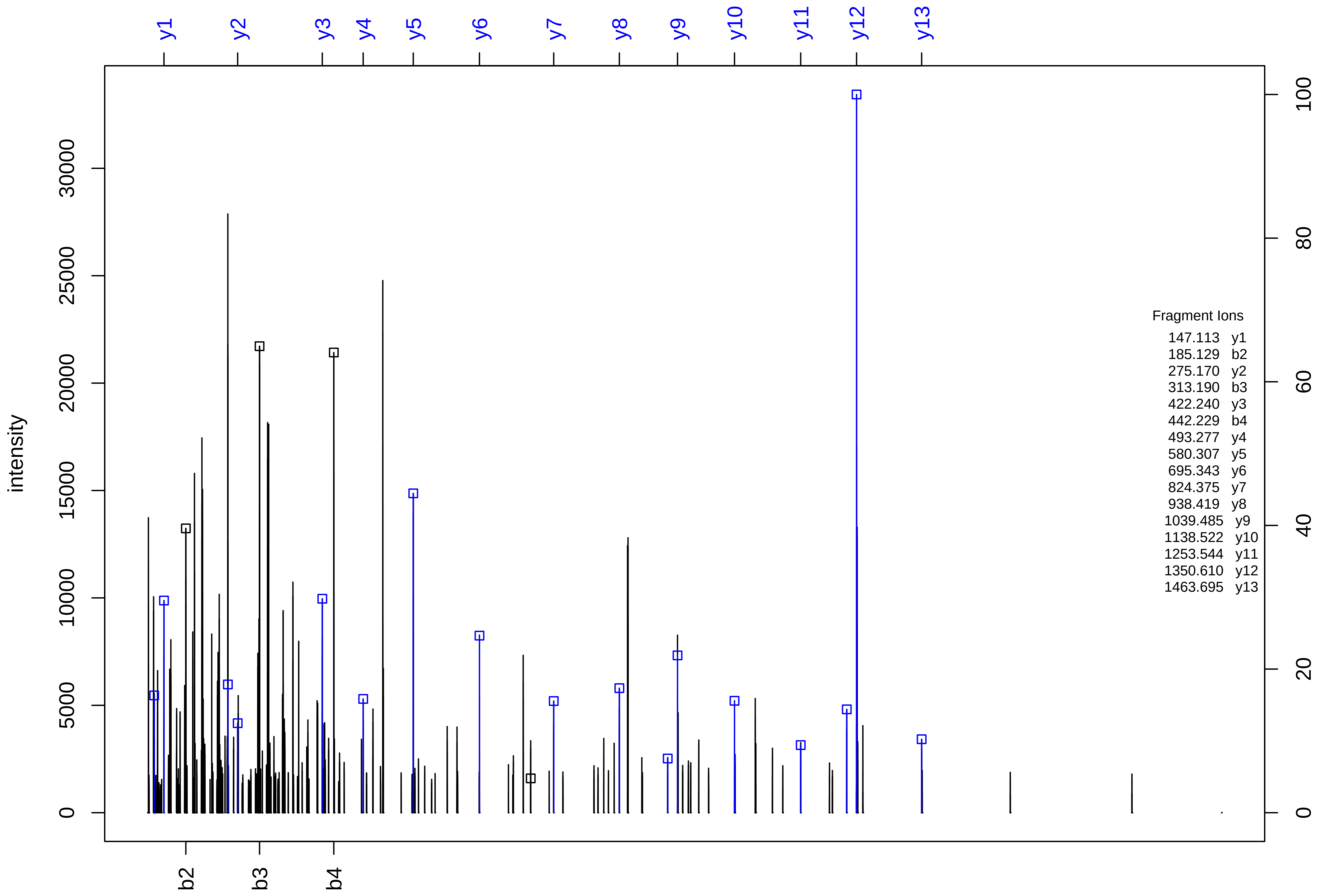

LAQELPDVTNEDSAFQK / File: sydney_04.raw; SpectrumID: ; scans: 6998

ITVVDDADTVELCGALK

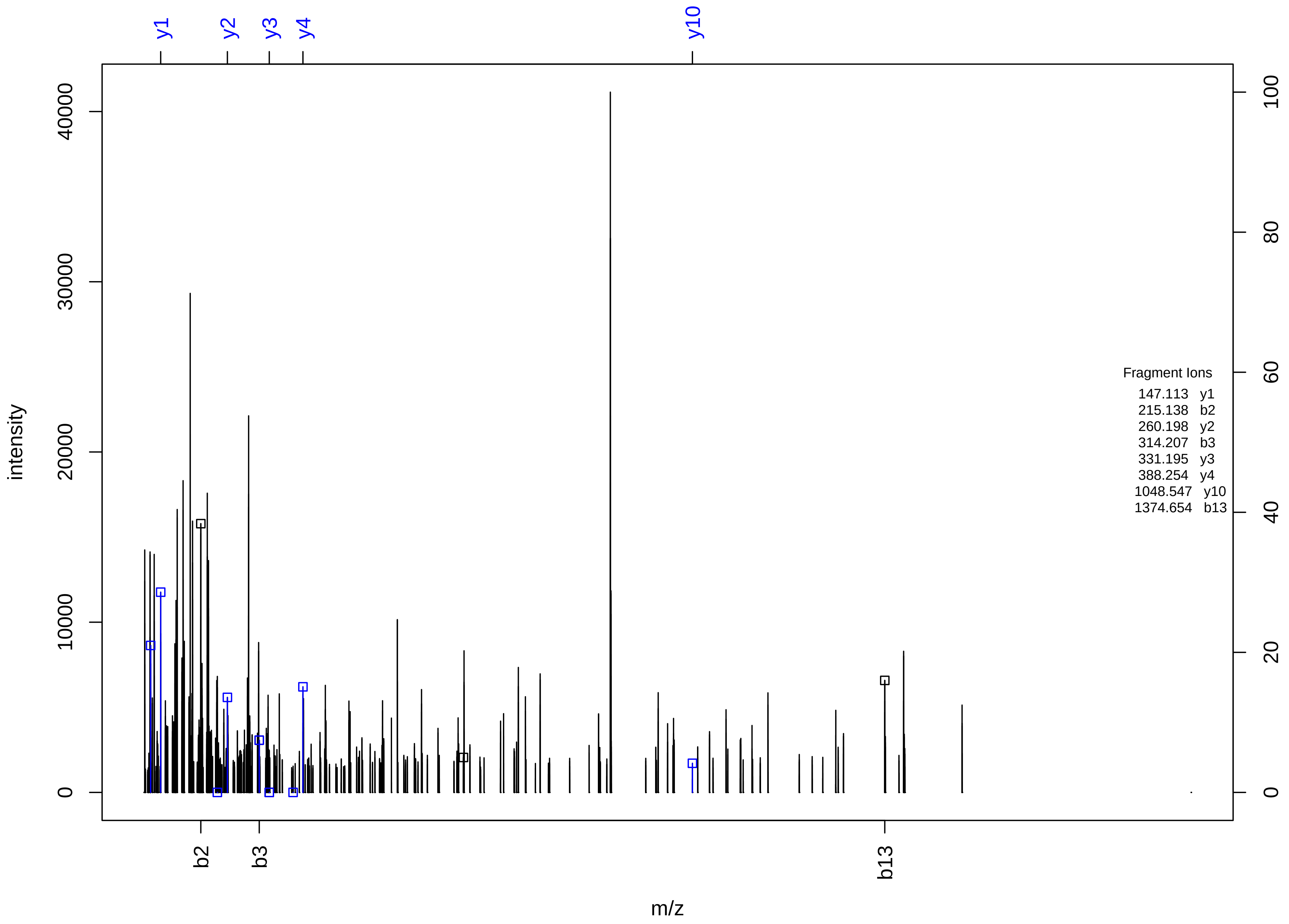

ITVVDDADTVELCGALK / File: sydney_04.raw; SpectrumID: ; scans: 9617

LAIQGPEDSPSR

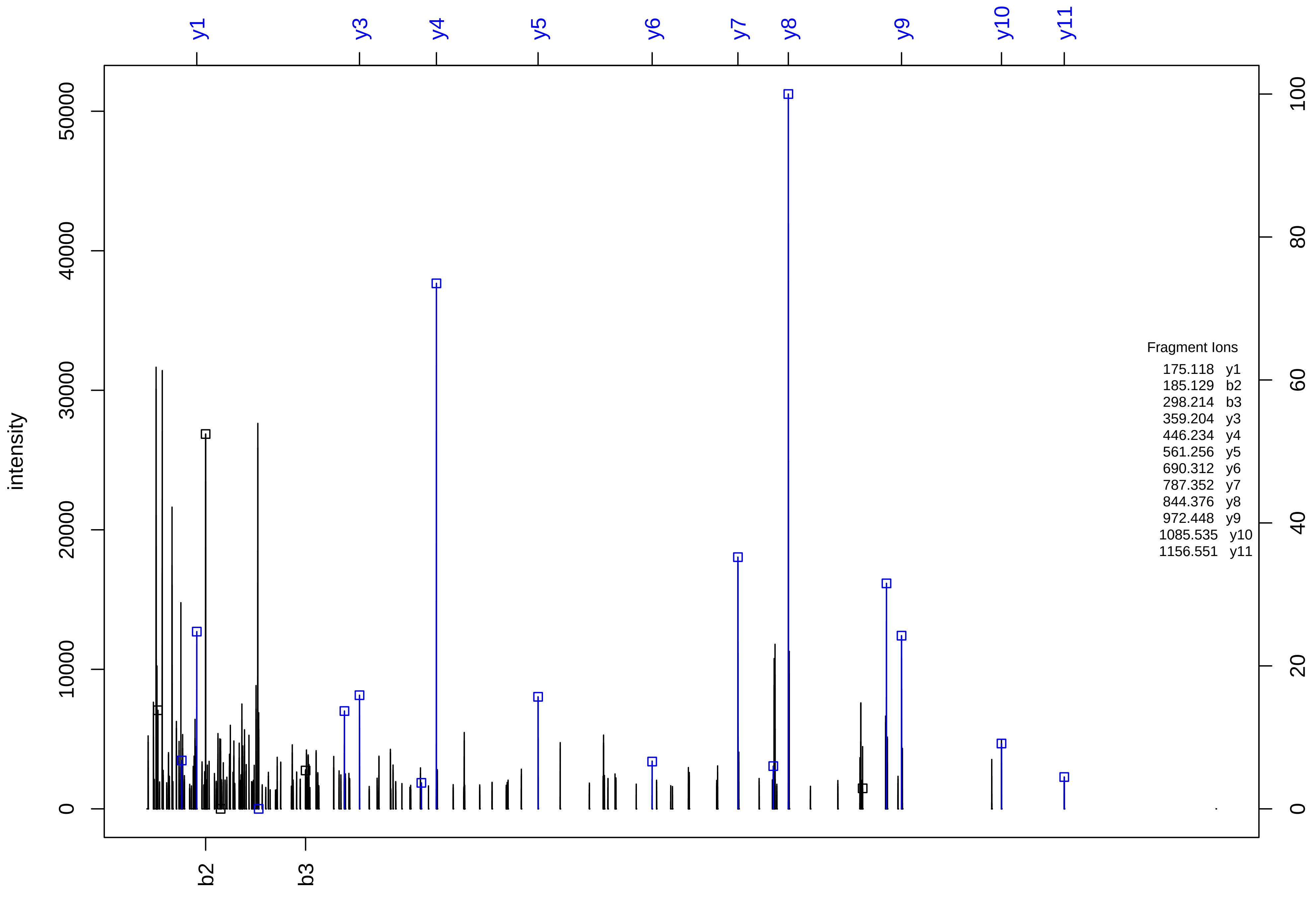

LAIQGPEDSPSR / File: sydney_04.raw; SpectrumID: ; scans: 2660

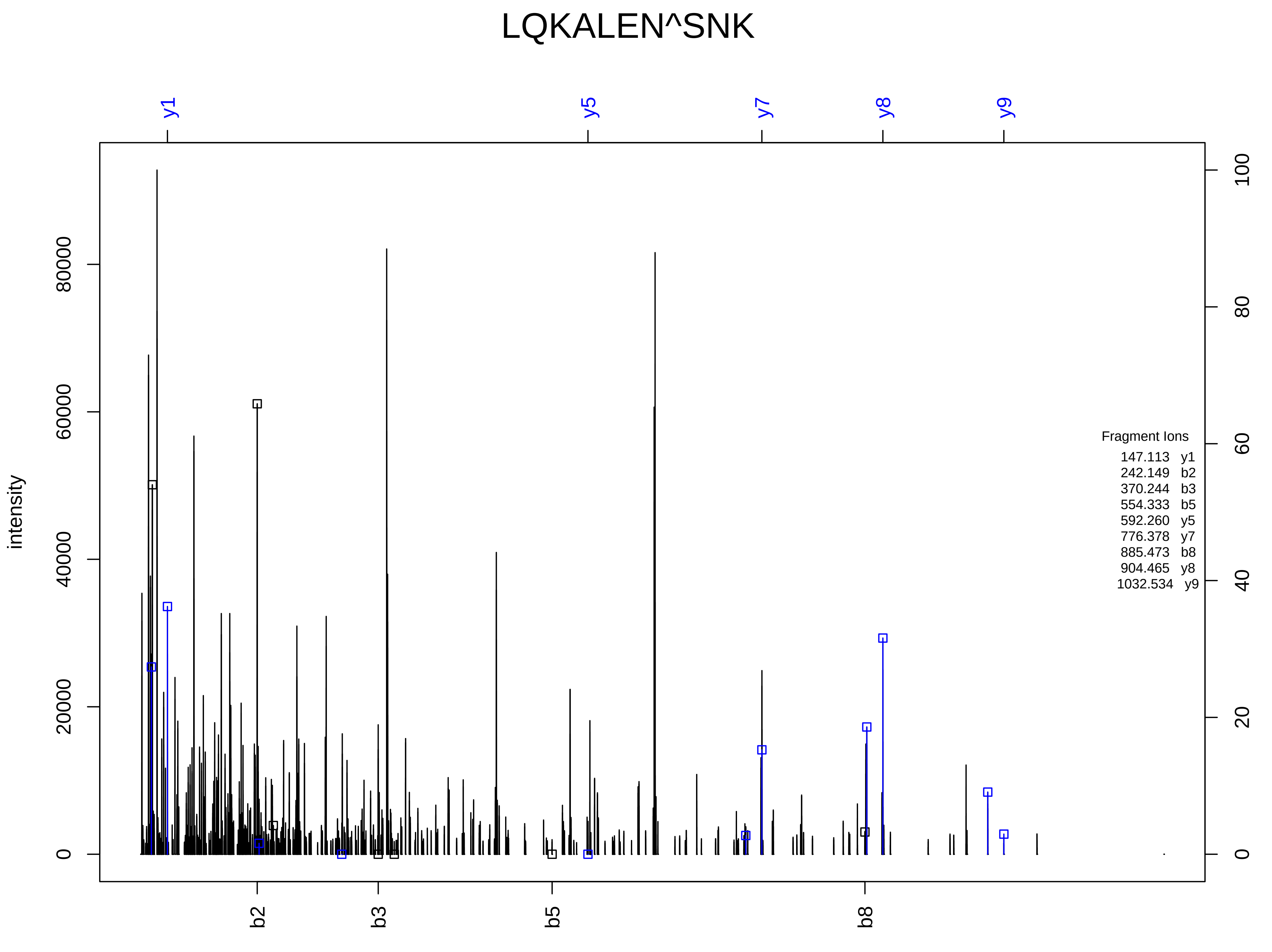

LQKALEN^SNK
y1
y5
y7
y8
y9
intensity
0
20000
40000
60000
80000
0
20
40
60
80
100
b2
b3
b5
b8
m/z
Fragment Ions
147.113 y1
242.149 b2
370.244 b3
554.333 b5
592.260 y5
776.378 y7
885.473 b8
904.465 y8
1032.534 y9
LQKALENSNK / File: sydney_04.raw; SpectrumID: ; scans: 5069

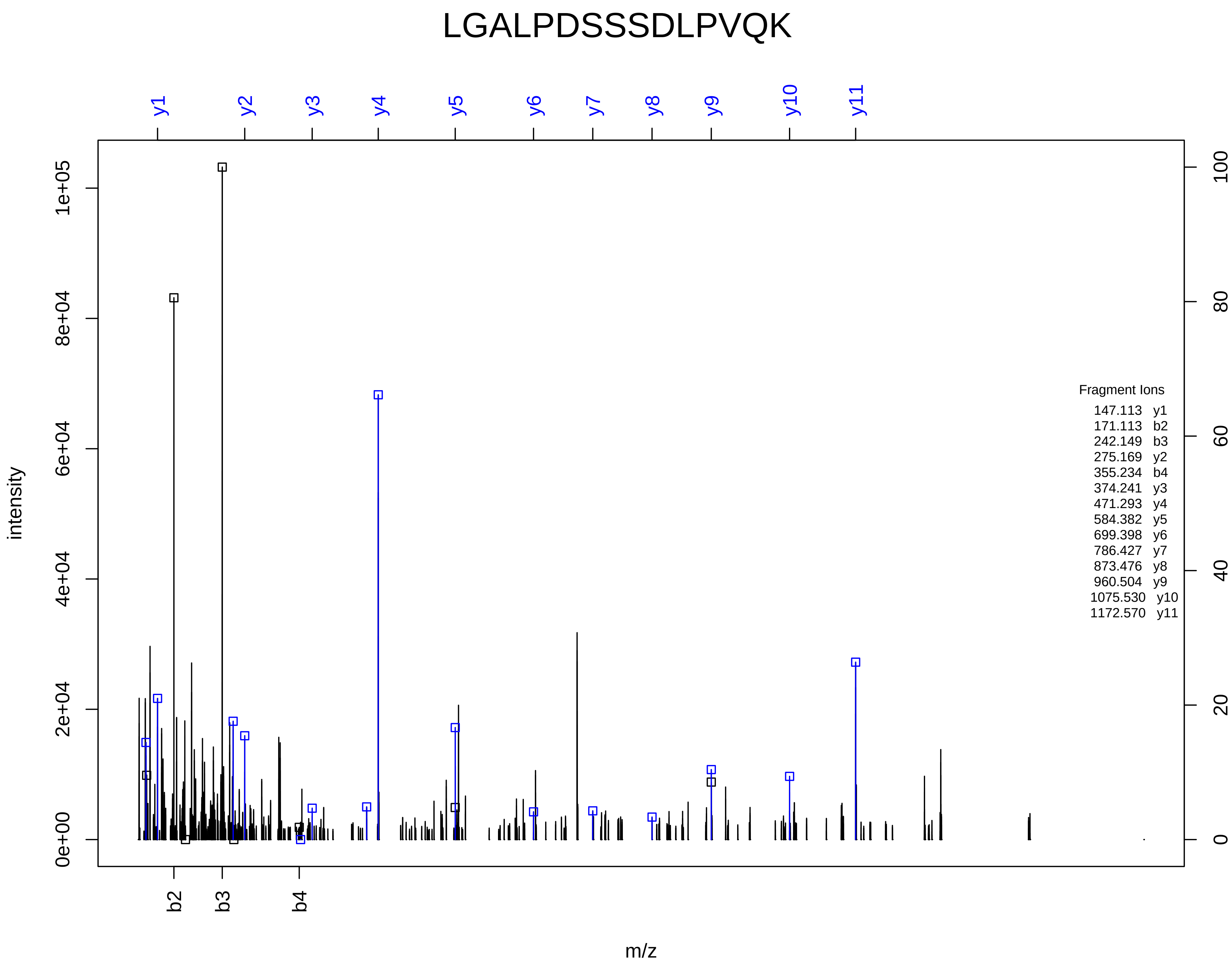
LGALPDSSSDLPVQK
y1
y2
y3
y4
y5
y6
y7
y8
y9
y10
y11
b2
b3
b4
intensity
0e+00
2e+04
4e+04
6e+04
8e+04
1e+05
0
20
40
60
80
100
Fragment Ions
147.113 y1
171.113 b2
242.149 b3
275.169 y2
355.234 b4
374.241 y3
471.293 y4
584.382 y5
699.398 y6
786.427 y7
873.476 y8
960.504 y9
1075.530 y10
1172.570 y11
m/z
LGALPDSSSDLPVQK / File: sydney_04.raw; SpectrumID: ; scans: 6128

IRGPEESQPPQLYAAEDDETPAAR

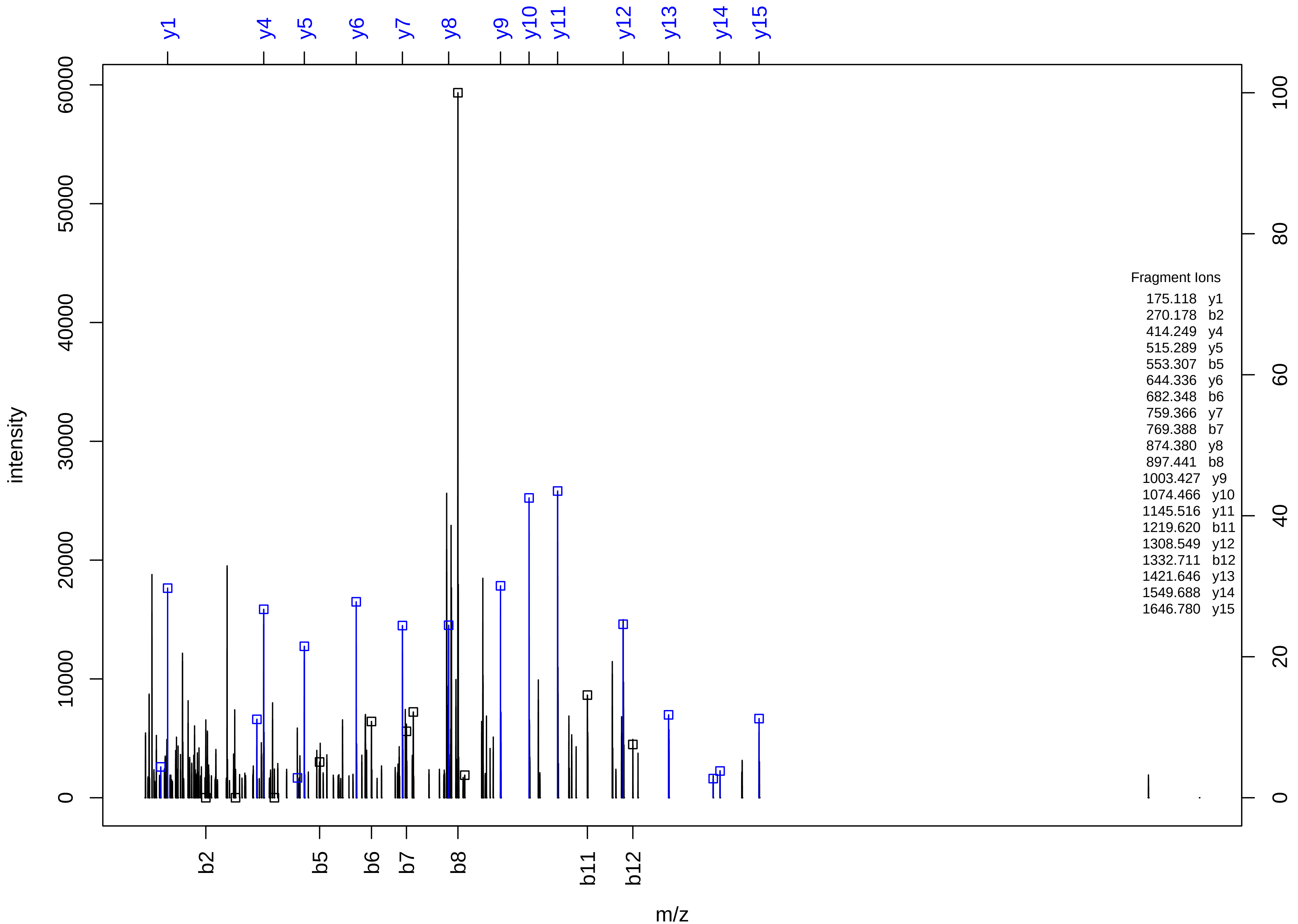

IRGPEESQPPQLYAAEDDETPAAR / File: sydney_04.raw; SpectrumID: ; scans: 4603

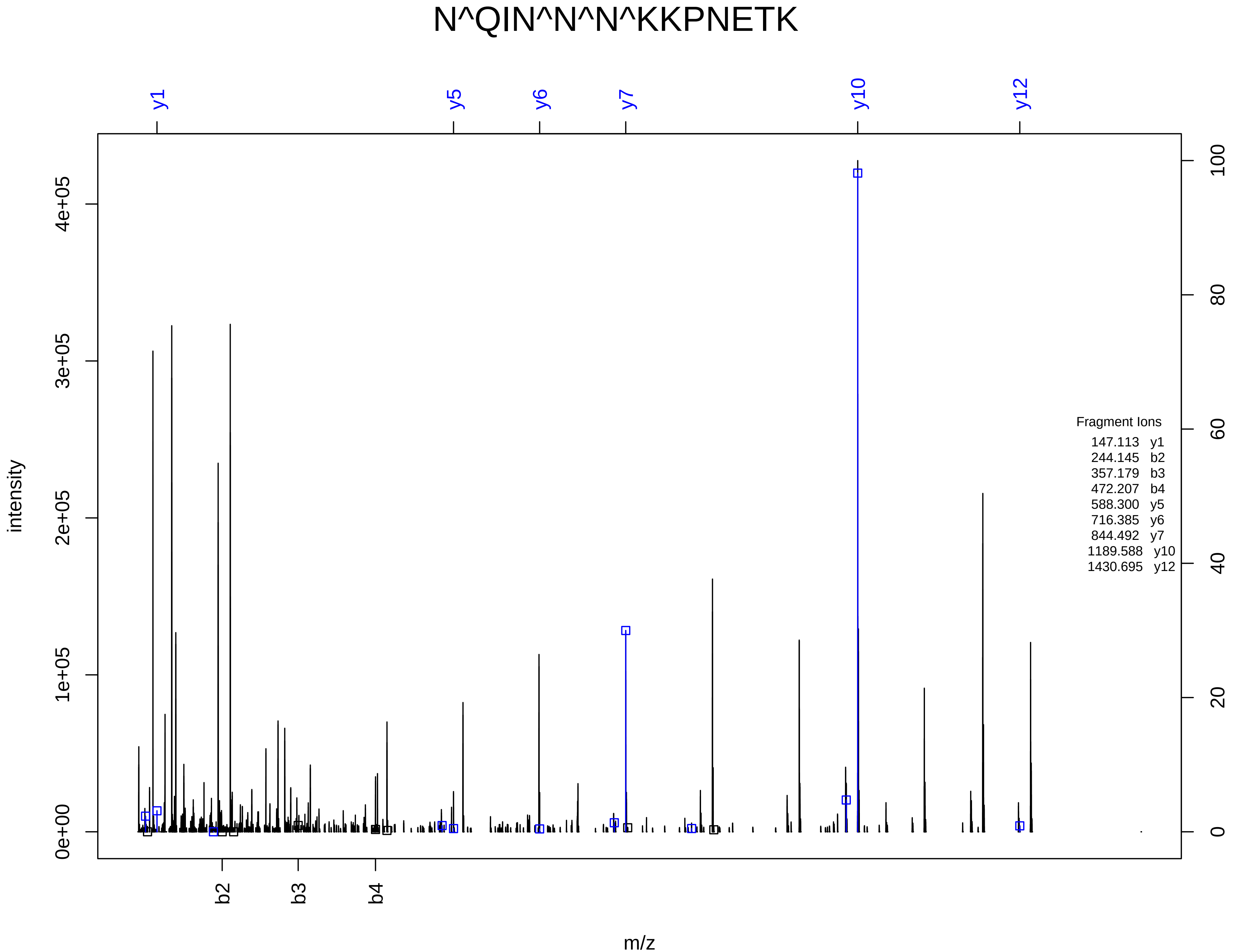

NQINNNKKPNETK / File: sydney_04.raw; SpectrumID: ; scans: 9145

SDSEPTPGCSGPGPAPVR

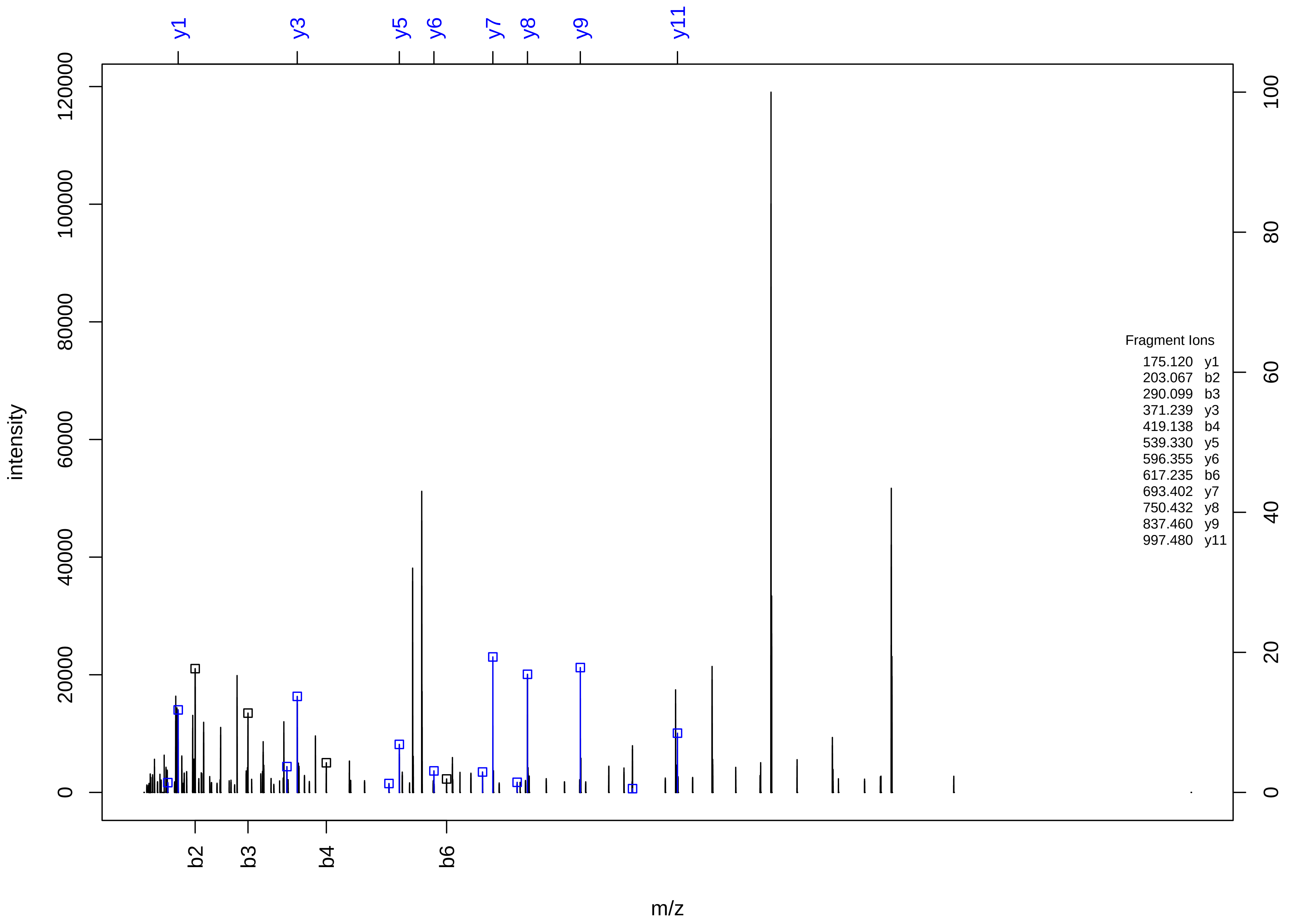

SDSEPTPGCSGPGPAPVR / File: sydney_04.raw; SpectrumID: ; scans: 2019

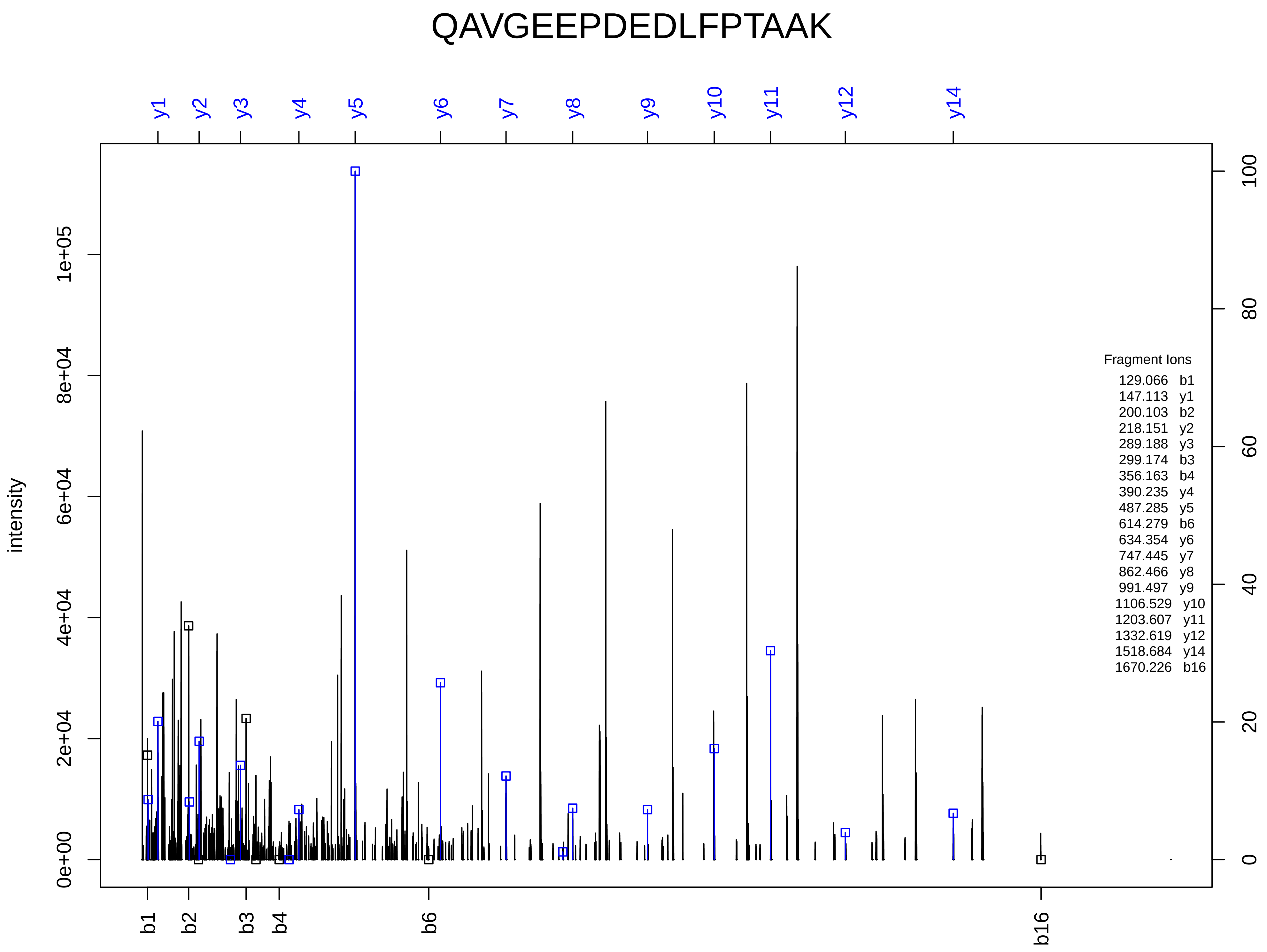

QAVGEEPDEDLFPTAAK / File: sydney_04.raw; SpectrumID: ; scans: 7124

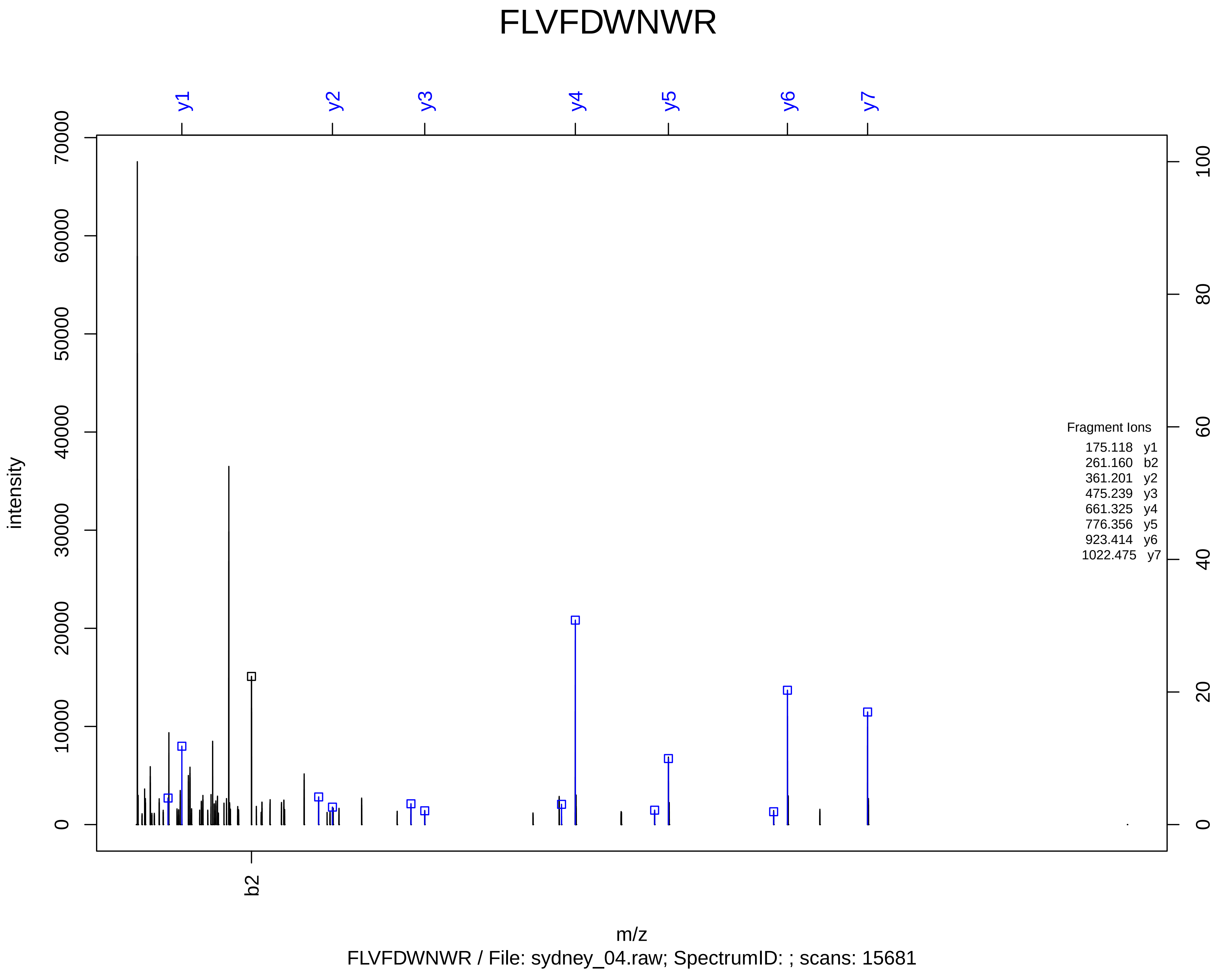
FLVFDWNWR
y1
y2
y3
y4
y5
y6
y7
b2
intensity
m/z
Fragment Ions
175.118 y1
261.160 b2
361.201 y2
475.239 y3
661.325 y4
776.356 y5
923.414 y6
1022.475 y7
FLVFDWNWR / File: sydney_04.raw; SpectrumID: ; scans: 15681

SCQEGRPGPEDGESTAQWR

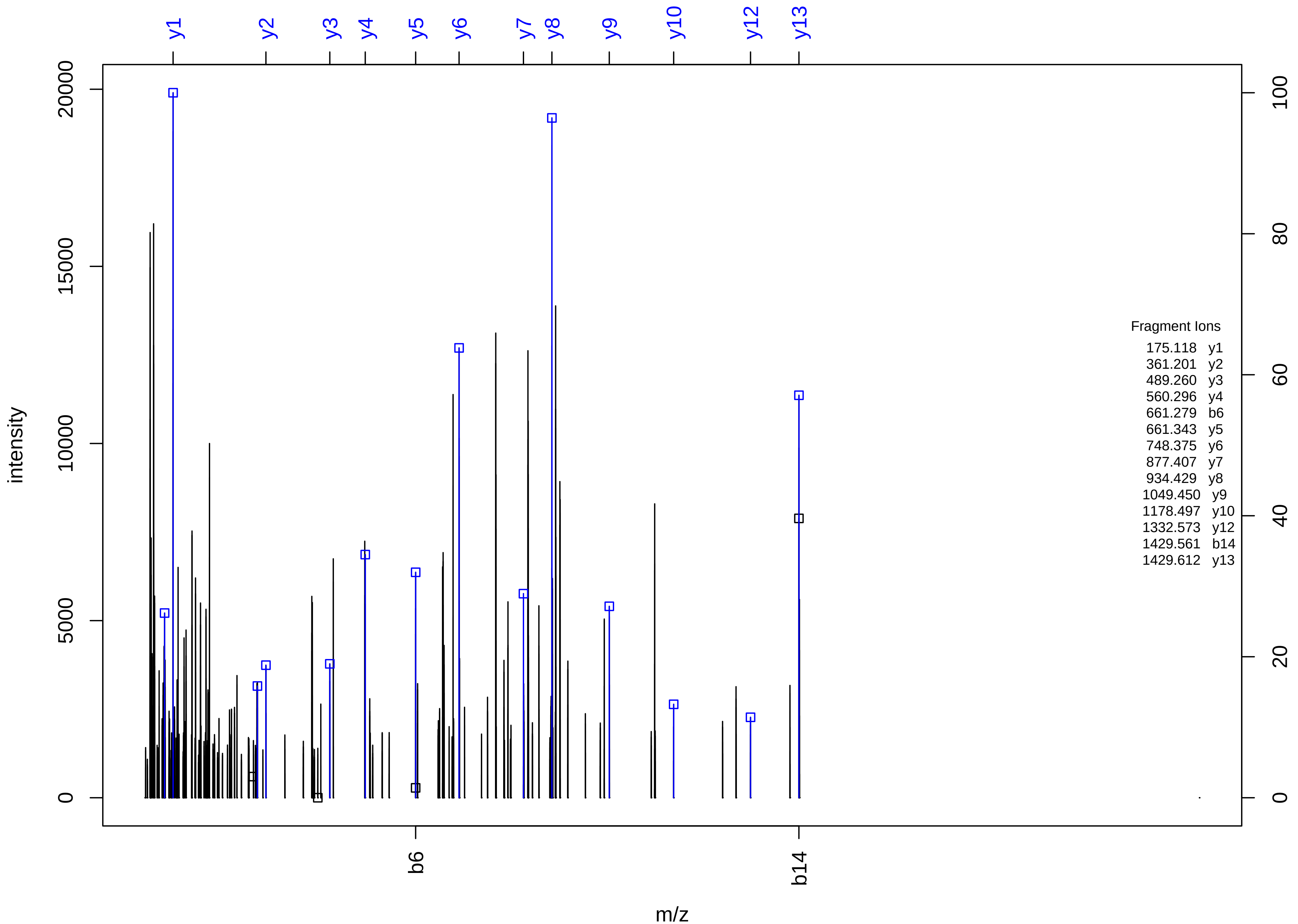

SCQEGRPGPEDGESTAQWR / File: sydney_05.raw; SpectrumID: ; scans: 2003

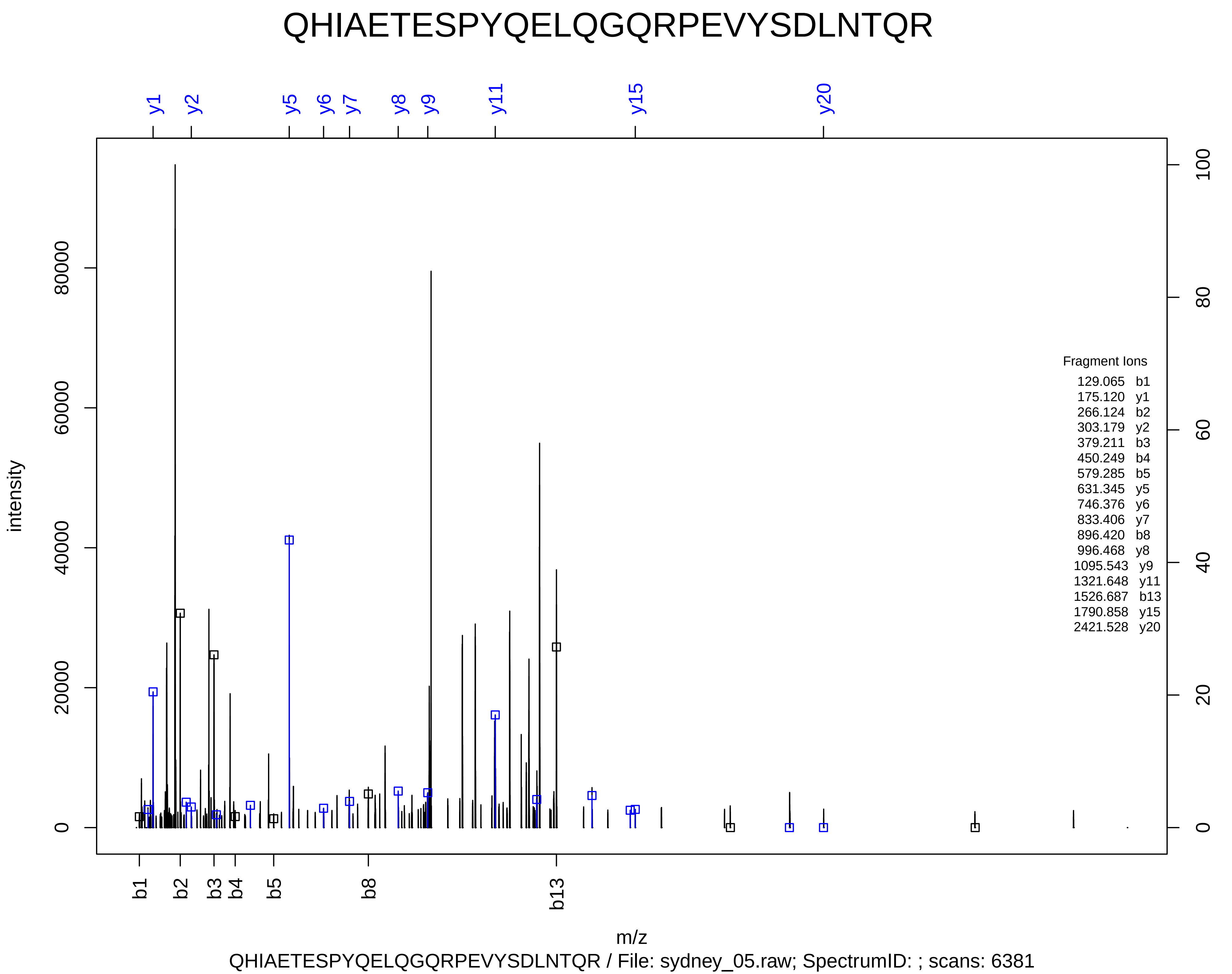
QHIAETESPYQELQGQRPEVYSDLNTQR
y1 y2 y5 y6 y7 y8 y9 y11 y15 y20
intensity
0
20000
40000
60000
80000
0
20
40
60
80
100
b1 b2 b3 b4 b5 b8 b13
m/z
Fragment Ions
129.065 b1
175.120 y1
266.124 b2
303.179 y2
379.211 b3
450.249 b4
579.285 b5
631.345 y5
746.376 y6
833.406 y7
896.420 b8
996.468 y8
1095.543 y9
1321.648 y11
1526.687 b13
1790.858 y15
2421.528 y20
QHIAETESPYQELQGQRPEVYSDLNTQR / File: sydney_05.raw; SpectrumID: ; scans: 6381

FNQDPEAEDEGR

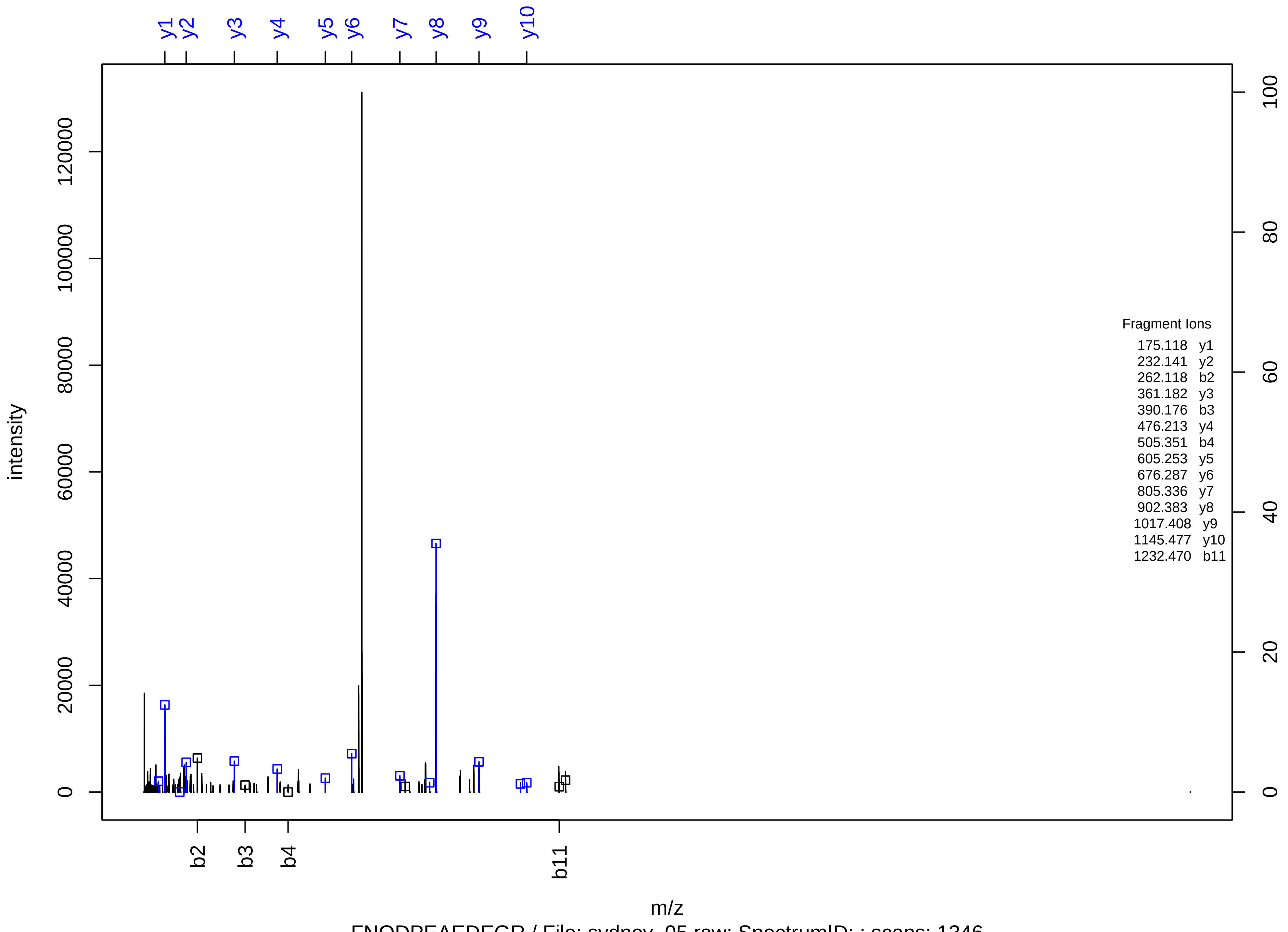

FNQDPEAEDEGR / File: sydney_05.raw; SpectrumID: ; scans: 1346

(Ac)MDPNCSCSTGGSCTCTSSCACK

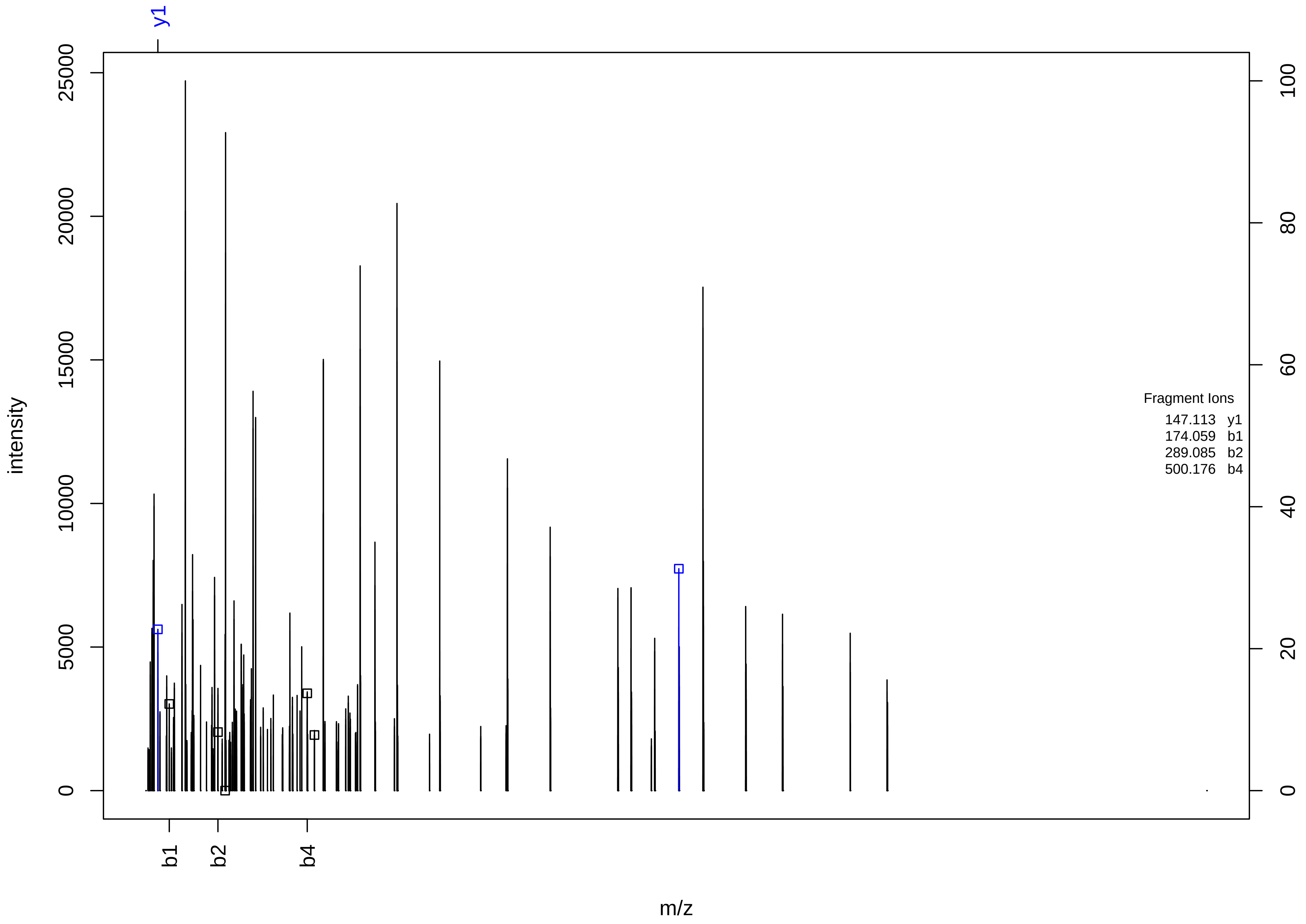

MDPNCSCSTGGSCTCTSSCACK / File: sydney_05.raw; SpectrumID: ; scans: 2348

ILGN^RVAELEK

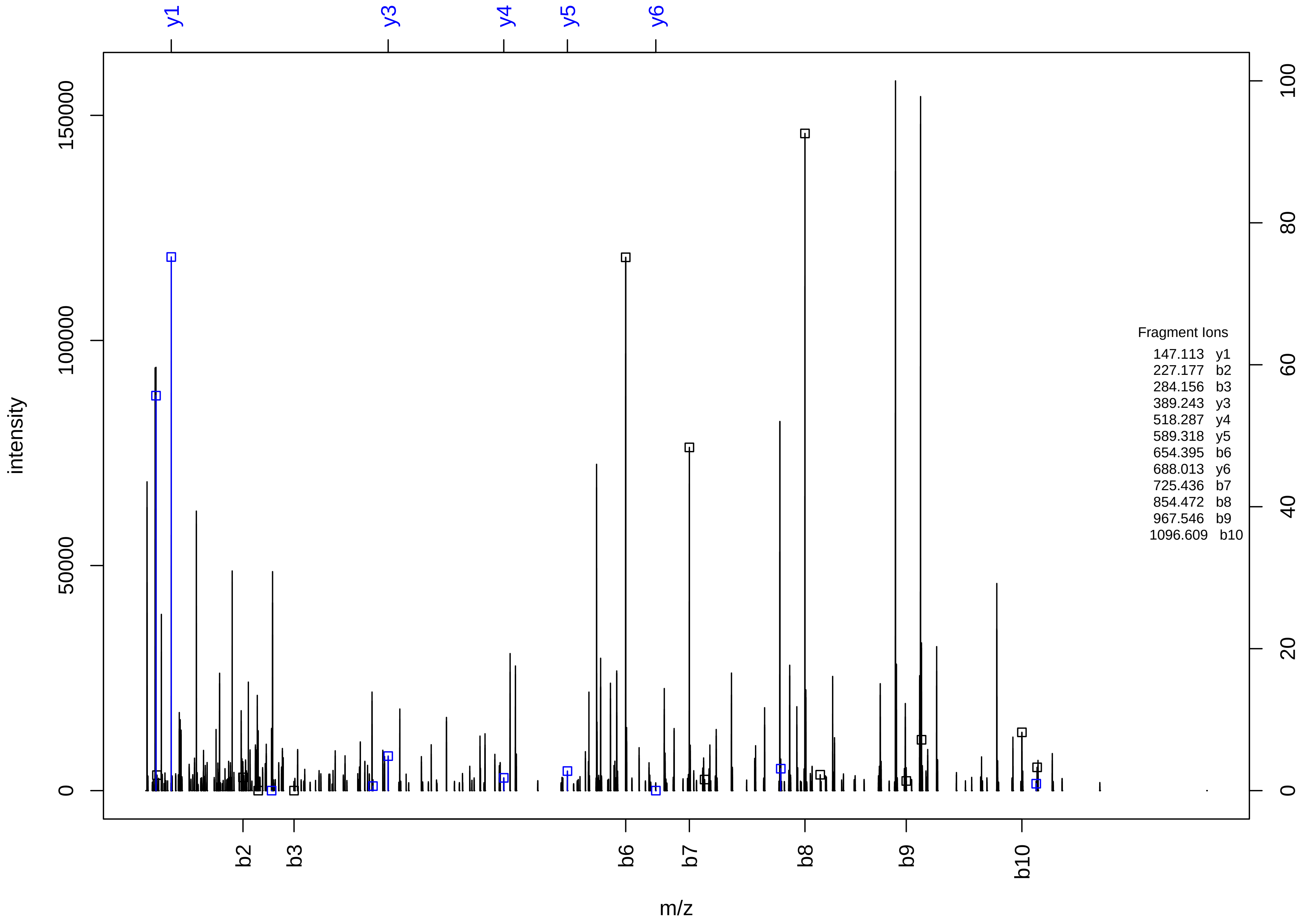

ILGNRVAELEK / File: sydney_05.raw; SpectrumID: ; scans: 3347

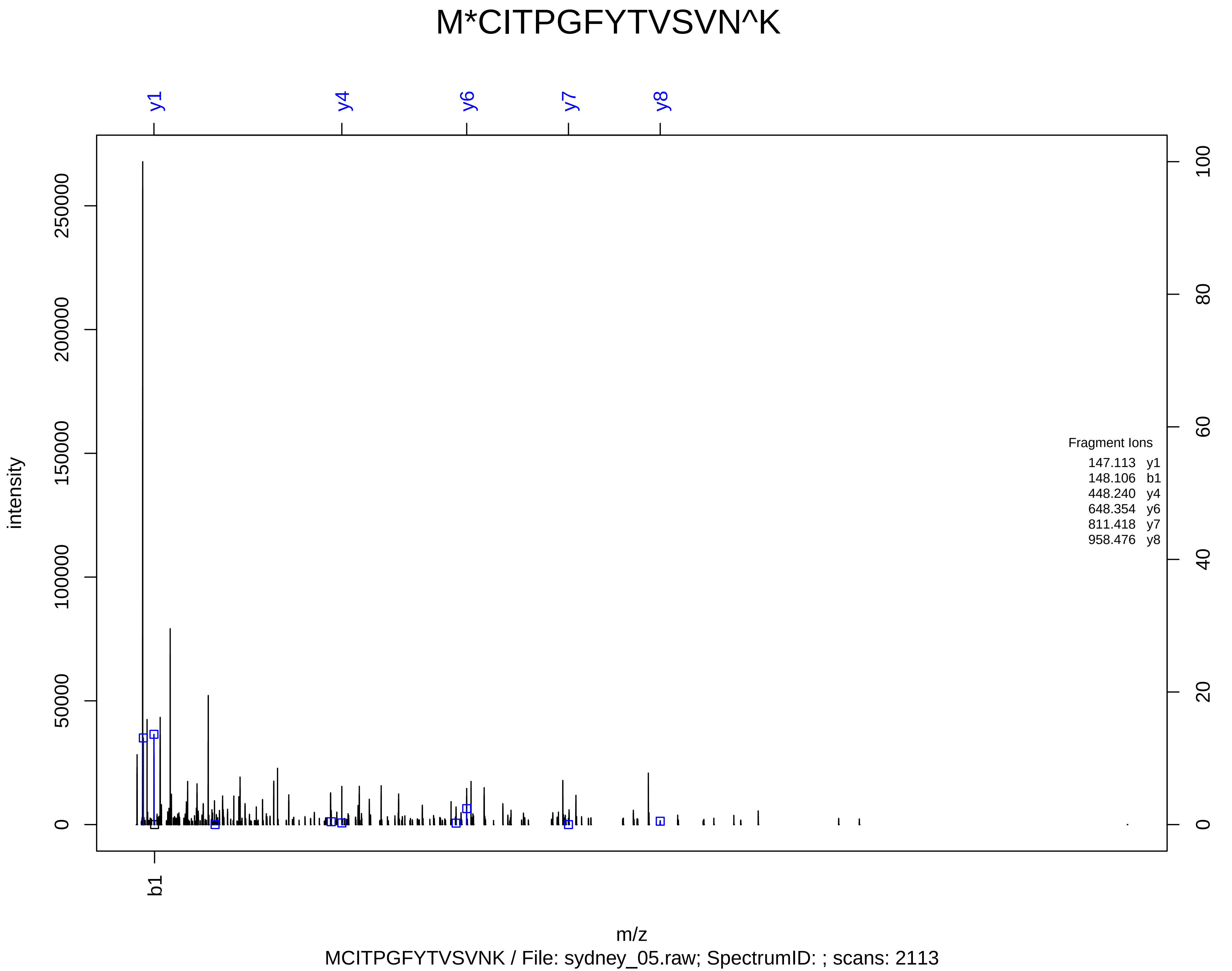
M*CITPGFYTVSVN^K
intensity
m/z
y1
y4
y6
y7
y8
b1
0
50000
100000
150000
200000
250000
20
40
60
80
100
Fragment Ions
147.113 y1
148.106 b1
448.240 y4
648.354 y6
811.418 y7
958.476 y8
MCITPGFYTVSVNK / File: sydney_05.raw; SpectrumID: ; scans: 2113

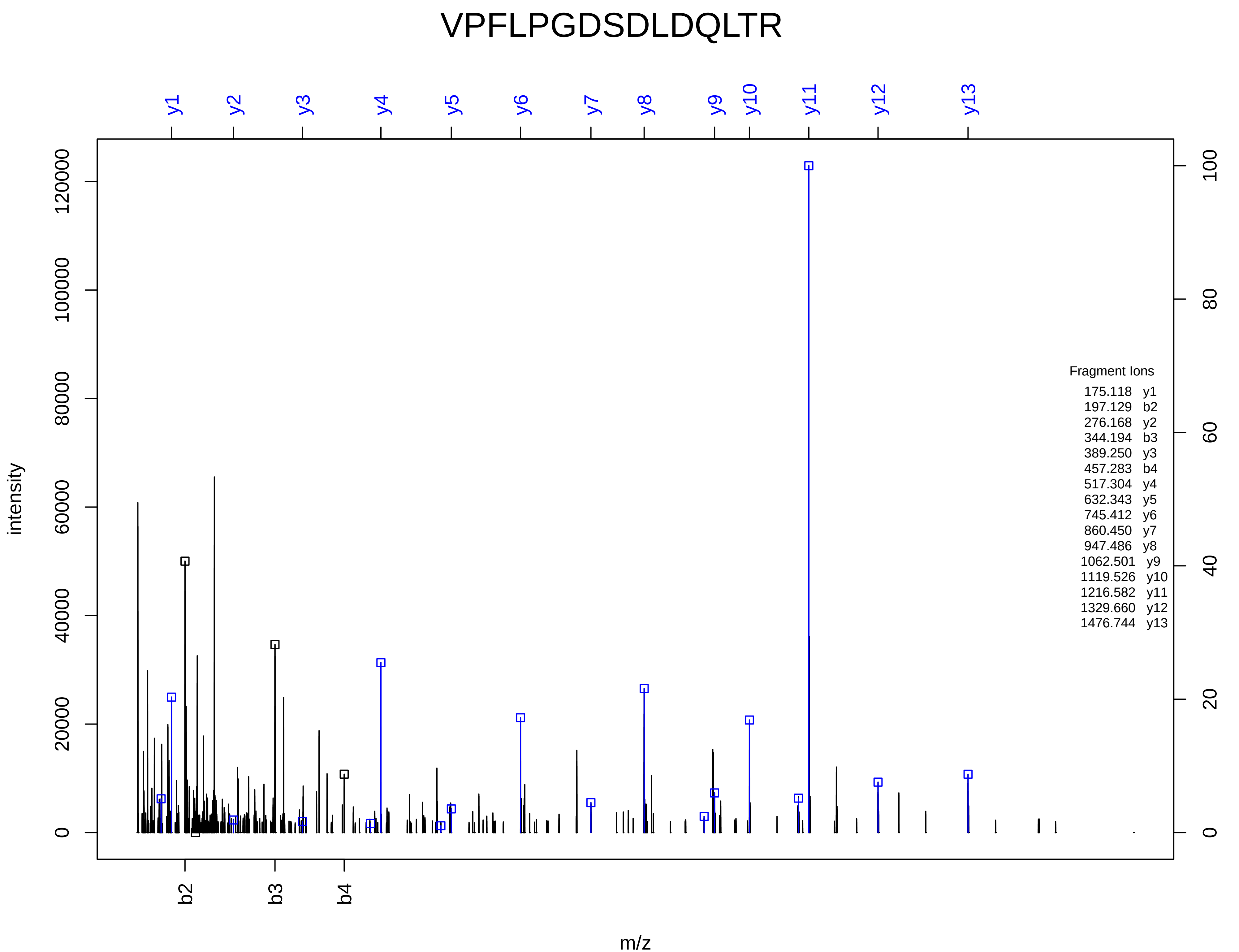
VPFLPGDSDLDQLTR
y1
y2
y3
y4
y5
y6
y7
y8
y9
y10
y11
y12
y13
intensity
0
20000
40000
60000
80000
100000
120000
0
20
40
60
80
100
b2
b3
b4
m/z
Fragment Ions
175.118 y1
197.129 b2
276.168 y2
344.194 b3
389.250 y3
457.283 b4
517.304 y4
632.343 y5
745.412 y6
860.450 y7
947.486 y8
1062.501 y9
1119.526 y10
1216.582 y11
1329.660 y12
1476.744 y13
VPFLPGDSDLDQLTR / File: sydney_05.raw; SpectrumID: ; scans: 11518

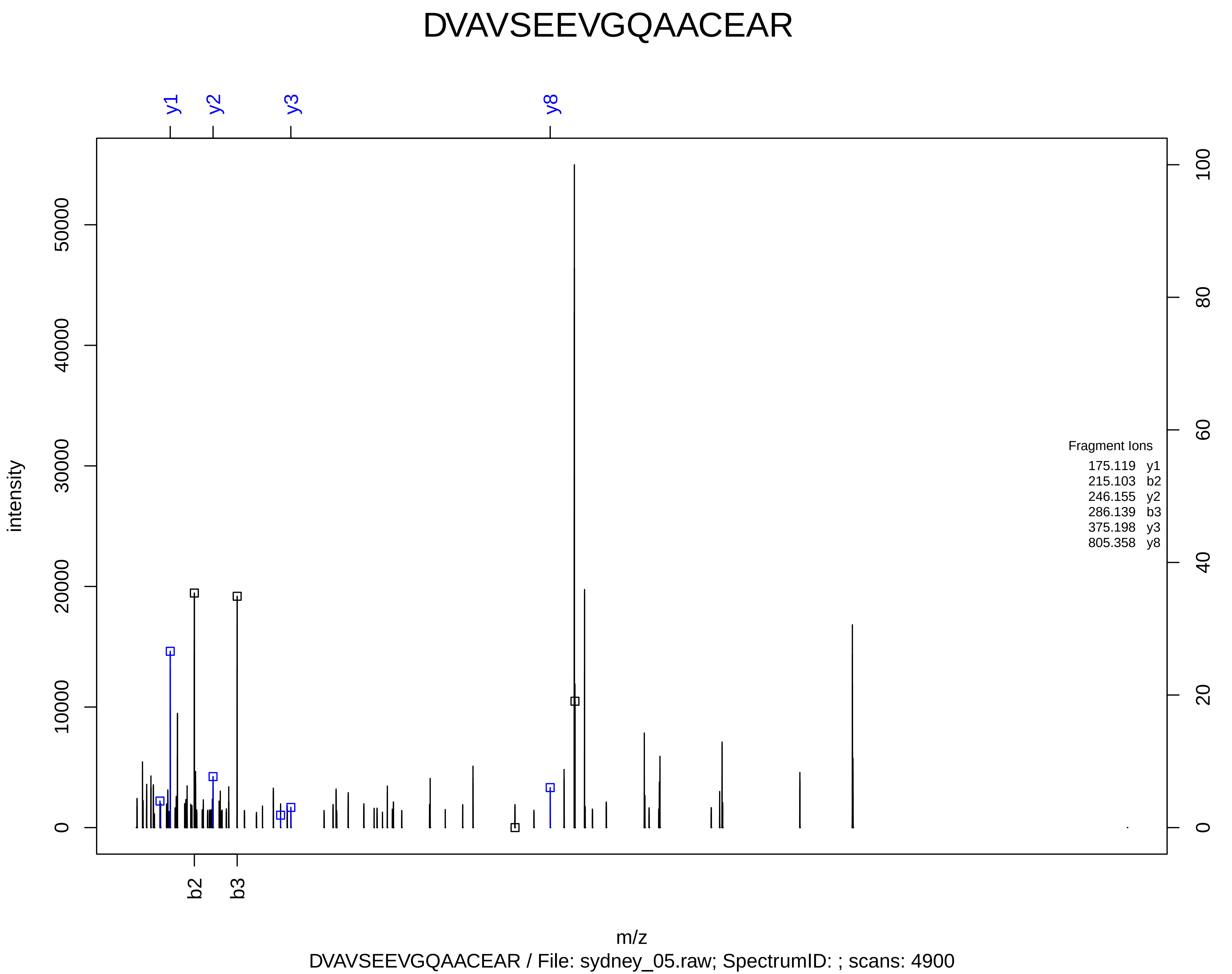

DVAVSEEVGQAACEAR
y1
y2
y3
y8
intensity
0
10000
20000
30000
40000
50000
0
20
40
60
80
100
b2
b3
m/z
Fragment Ions
175.119 y1
215.103 b2
246.155 y2
286.139 b3
375.198 y3
805.358 y8
DVAVSEEVGQAACEAR / File: sydney_05.raw; SpectrumID: ; scans: 4900

EVN^RLSDFEM*ESK

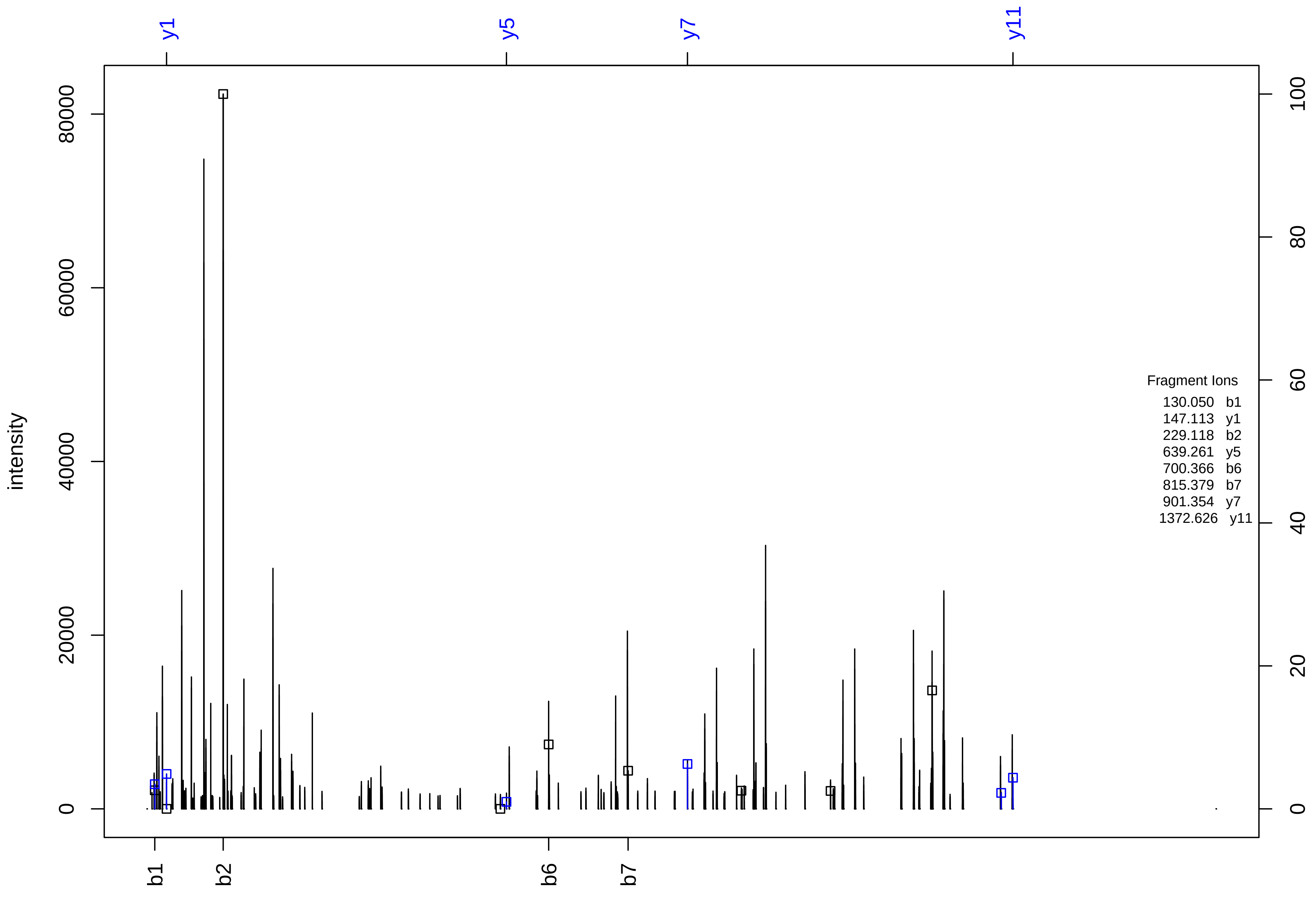

EVNRLSDFEMESK / File: sydney_05.raw; SpectrumID: ; scans: 1411

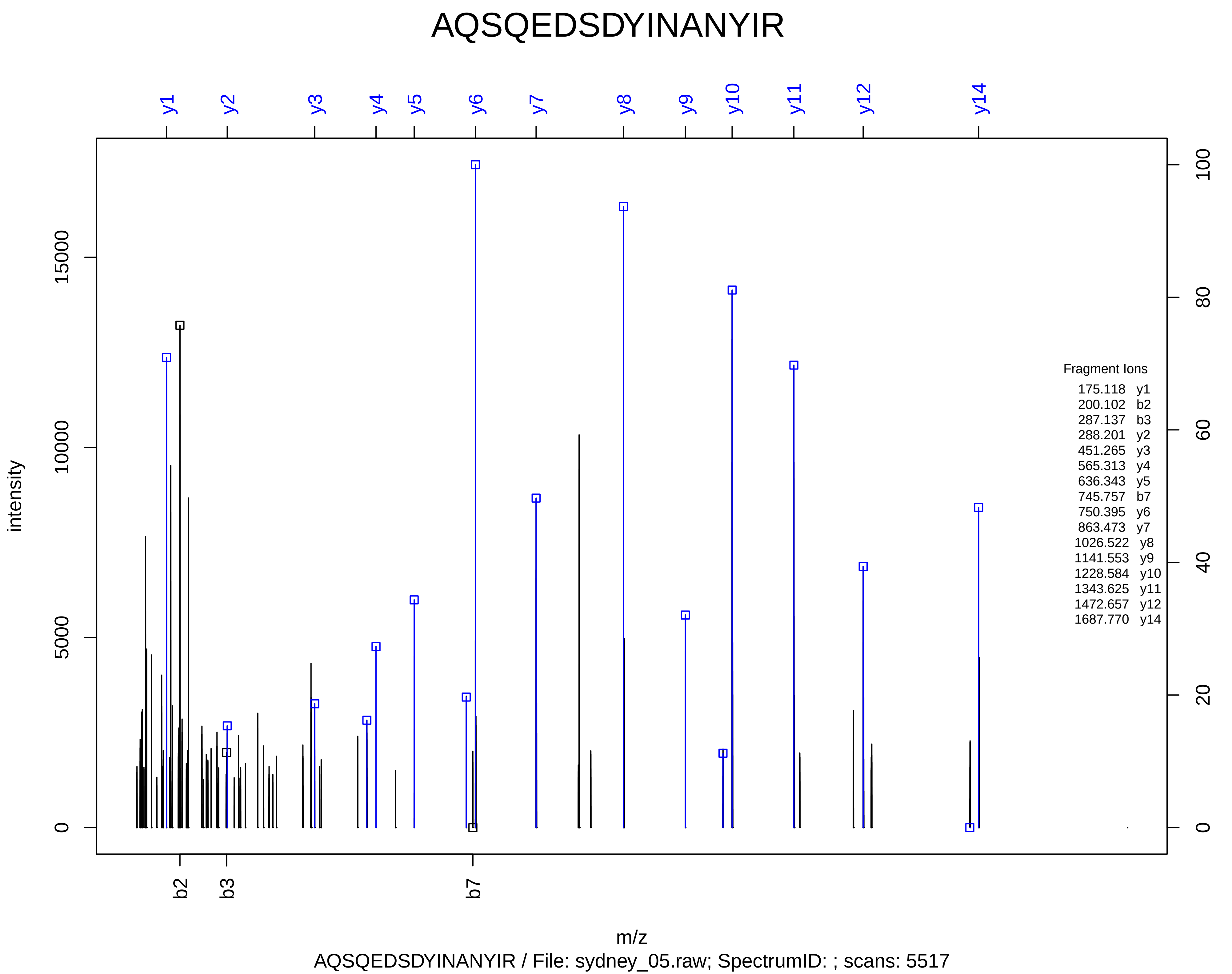

AQSQEDSDYINANYIR
y1
y2
y3
y4
y5
y6
y7
y8
y9
y10
y11
y12
y14
intensity
0
5000
10000
15000
0
20
40
60
80
100
b2
b3
b7
m/z
Fragment Ions
175.118 y1
200.102 b2
287.137 b3
288.201 y2
451.265 y3
565.313 y4
636.343 y5
745.757 b7
750.395 y6
863.473 y7
1026.522 y8
1141.553 y9
1228.584 y10
1343.625 y11
1472.657 y12
1687.770 y14
AQSQEDSDYINANYIR / File: sydney_05.raw; SpectrumID: ; scans: 5517

FVFEVIPASSDQNR

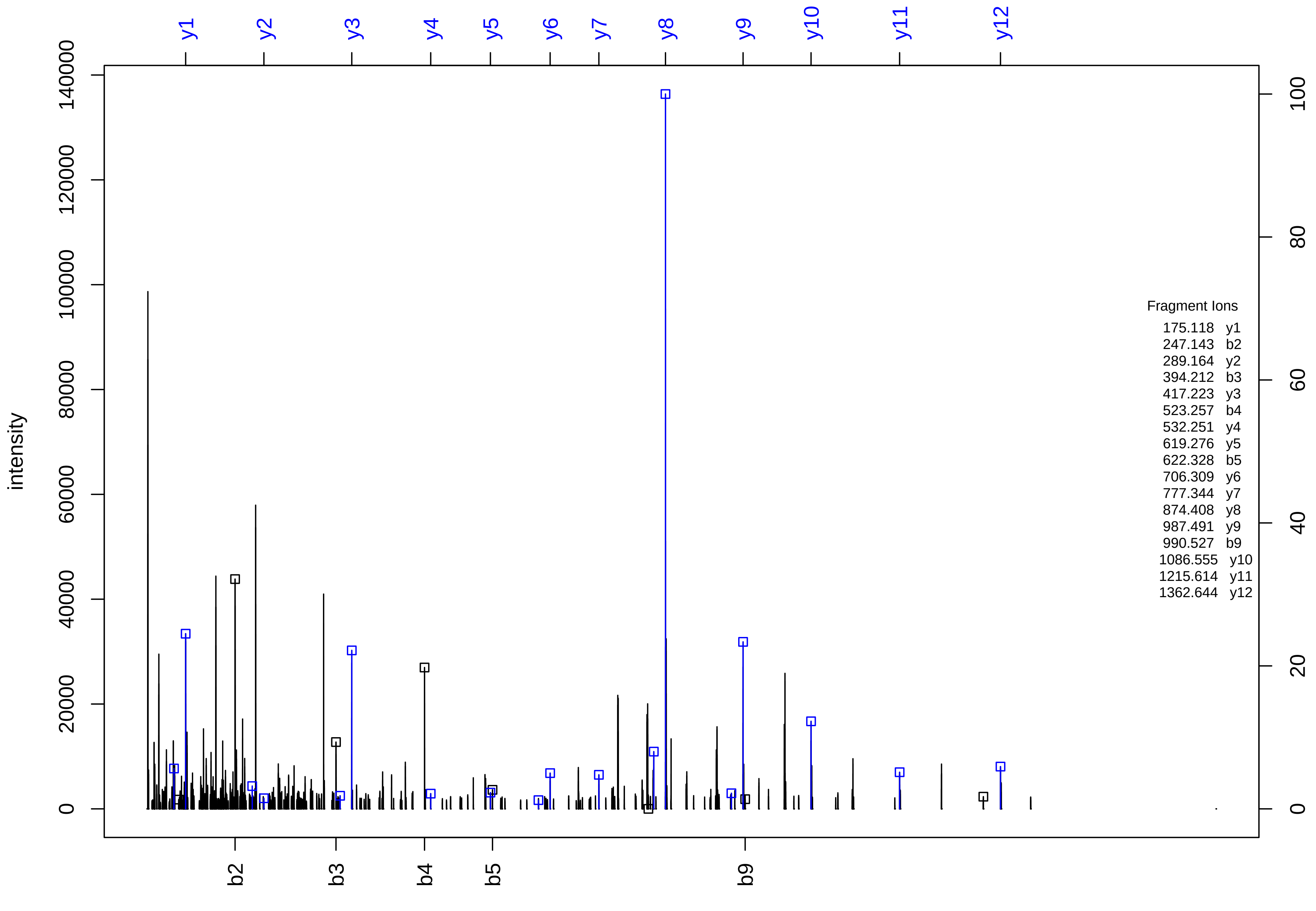

FVFEVIPASSDQNR / File: sydney_05.raw; SpectrumID: ; scans: 10140

(Ac)MMDQ^ARSAFSN^L

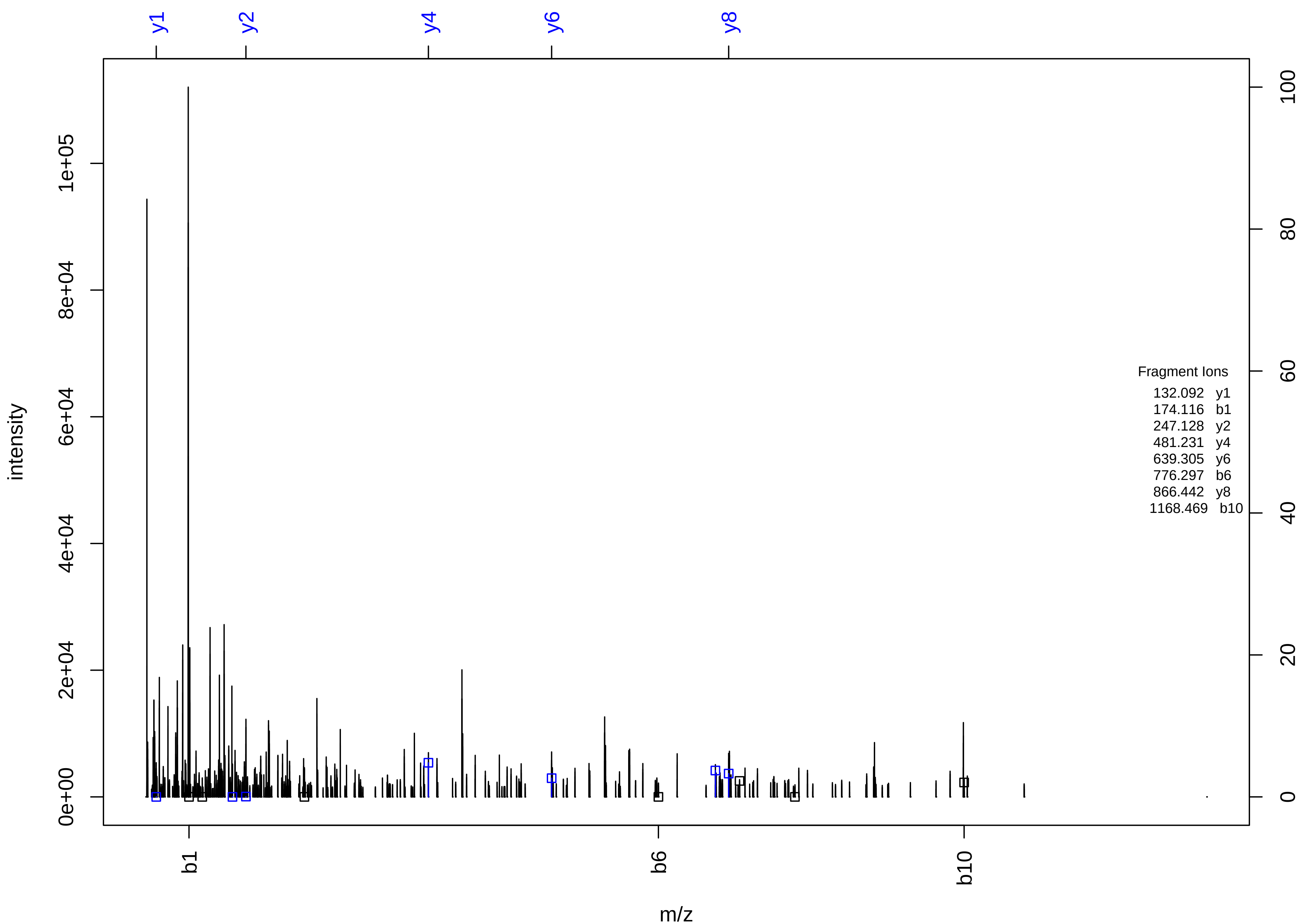

MMDQARSAFSNL / File: sydney_05.raw; SpectrumID: ; scans: 6853

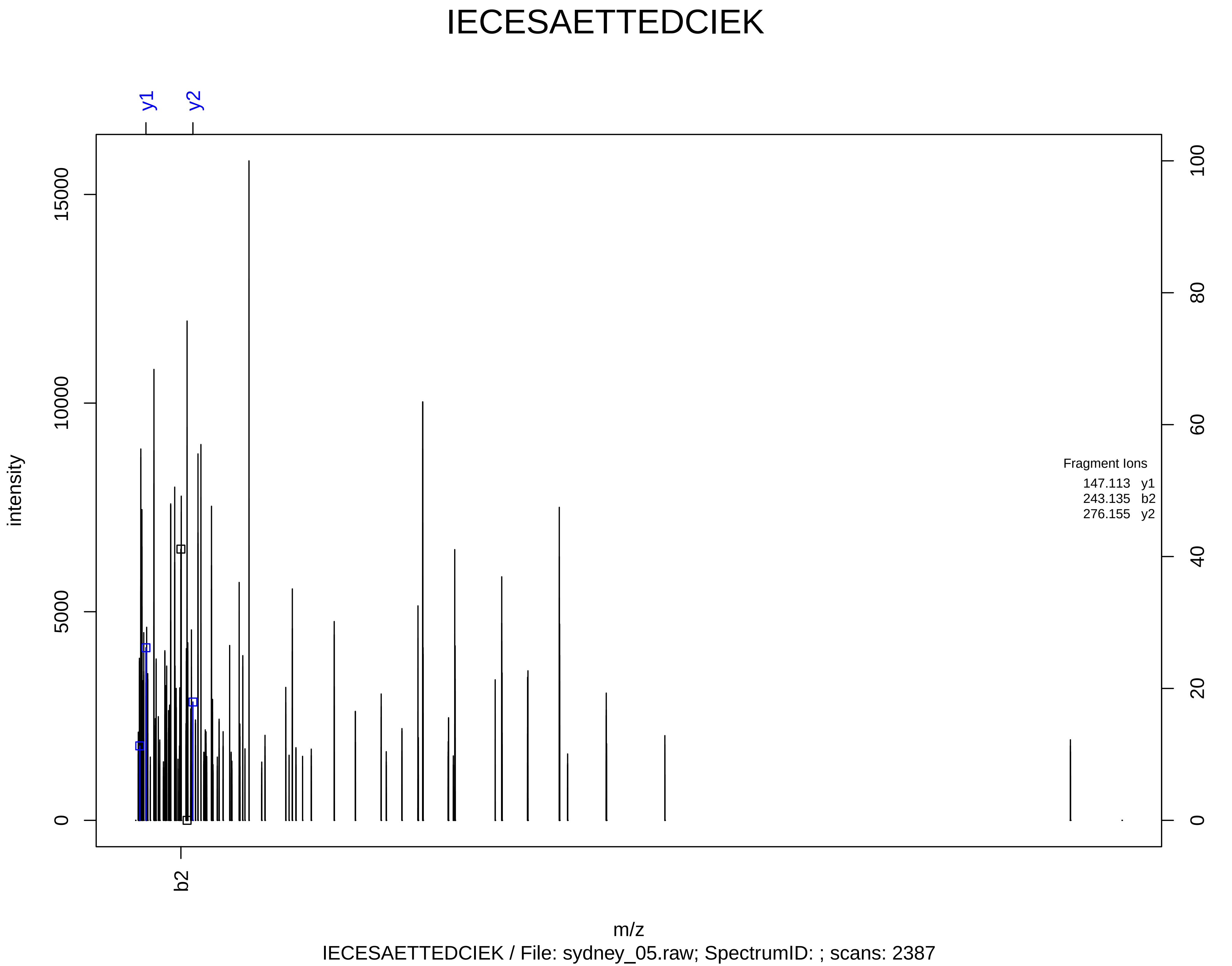

IECESAETTEDCIEK
y1
y2
intensity
15000
10000
5000
0
100
80
60
40
20
0
Fragment Ions
147.113 y1
243.135 b2
276.155 y2
b2
m/z
IECESAETTEDCIEK / File: sydney_05.raw; SpectrumID: ; scans: 2387

LEGWELFPTPK

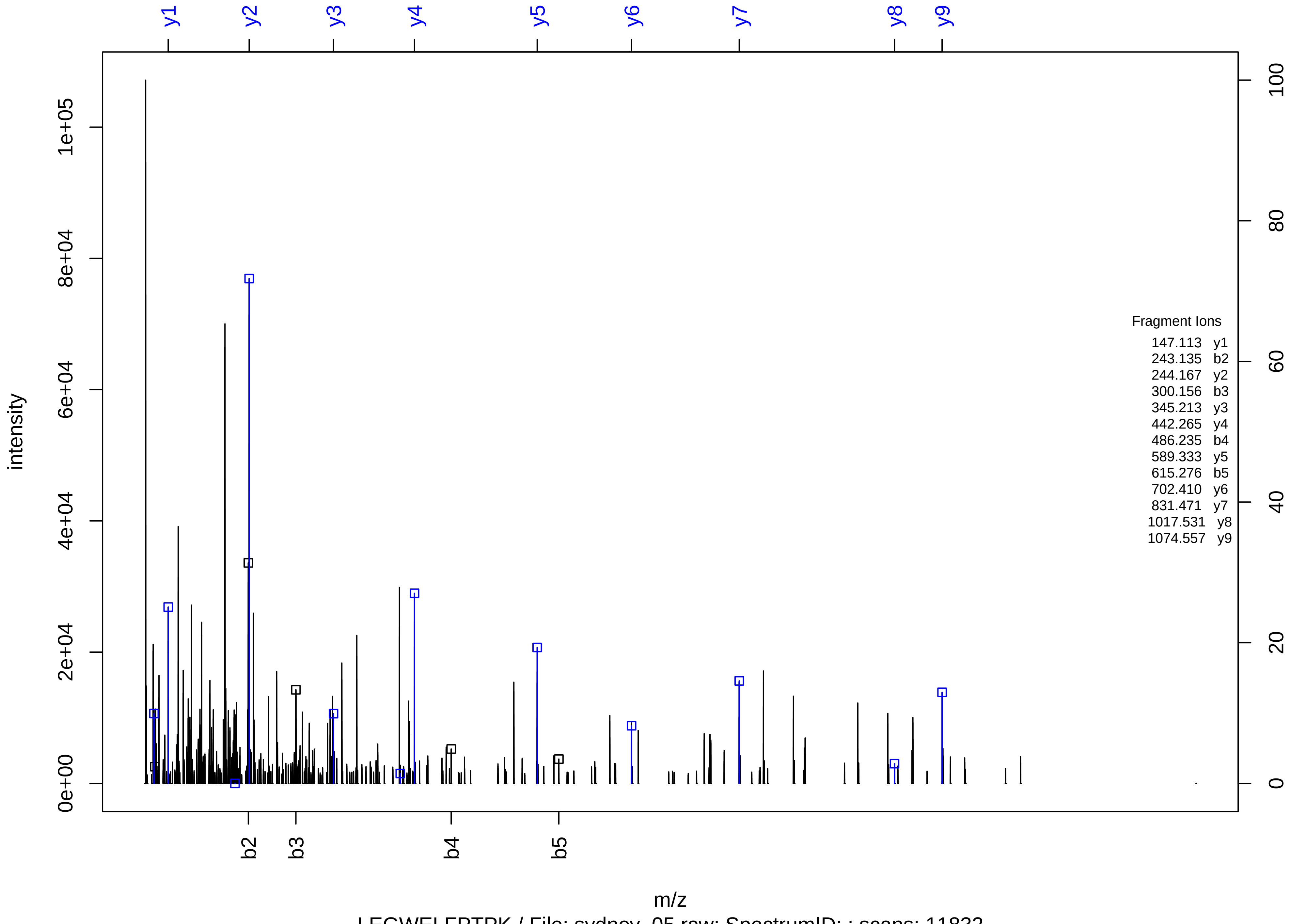

LEGWELFPTPK / File: sydney_05.raw; SpectrumID: ; scans: 11832

APVLLAGTADGNTWMWK

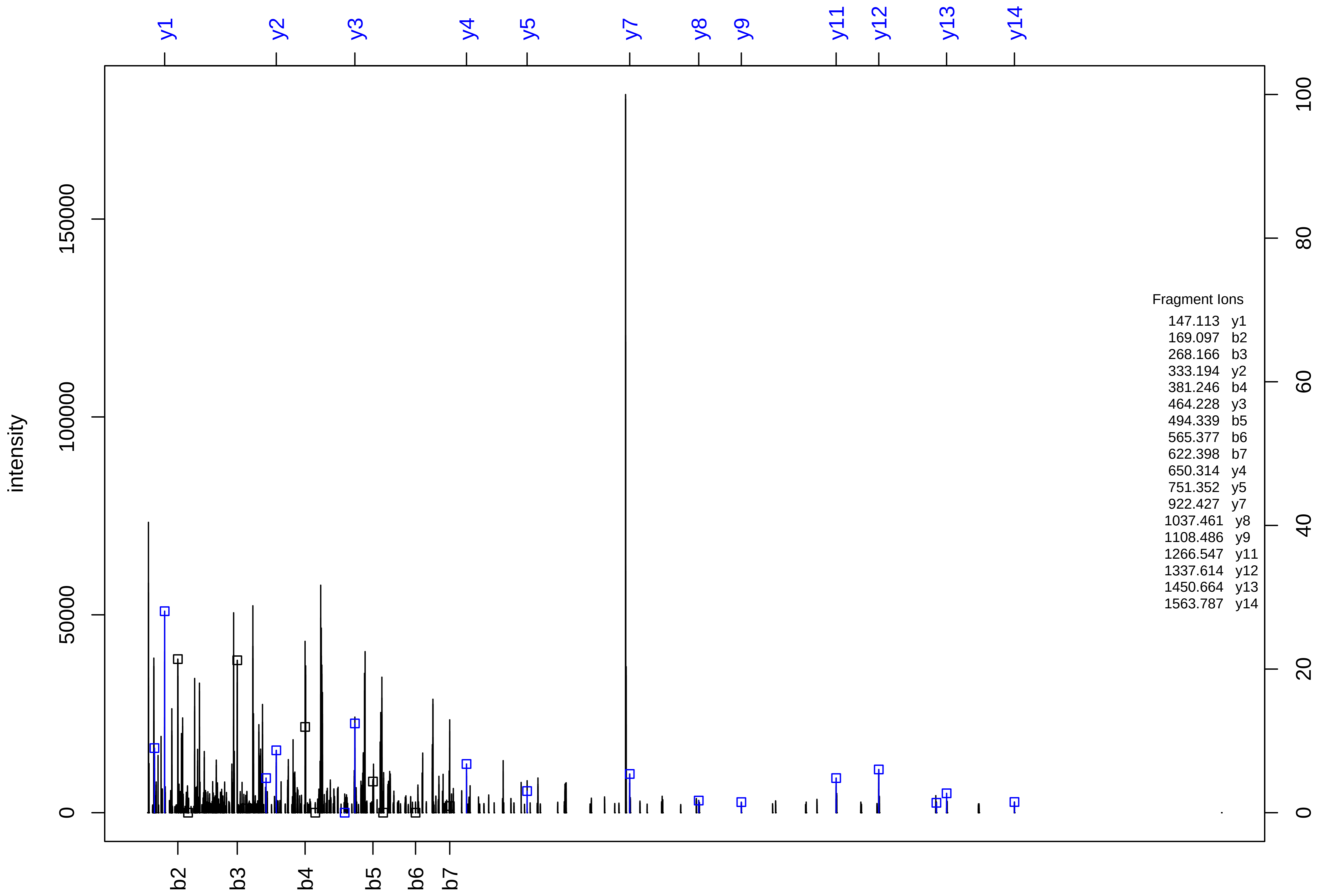

APVLLAGTADGNTWMWK / File: sydney_05.raw; SpectrumID: ; scans: 12168

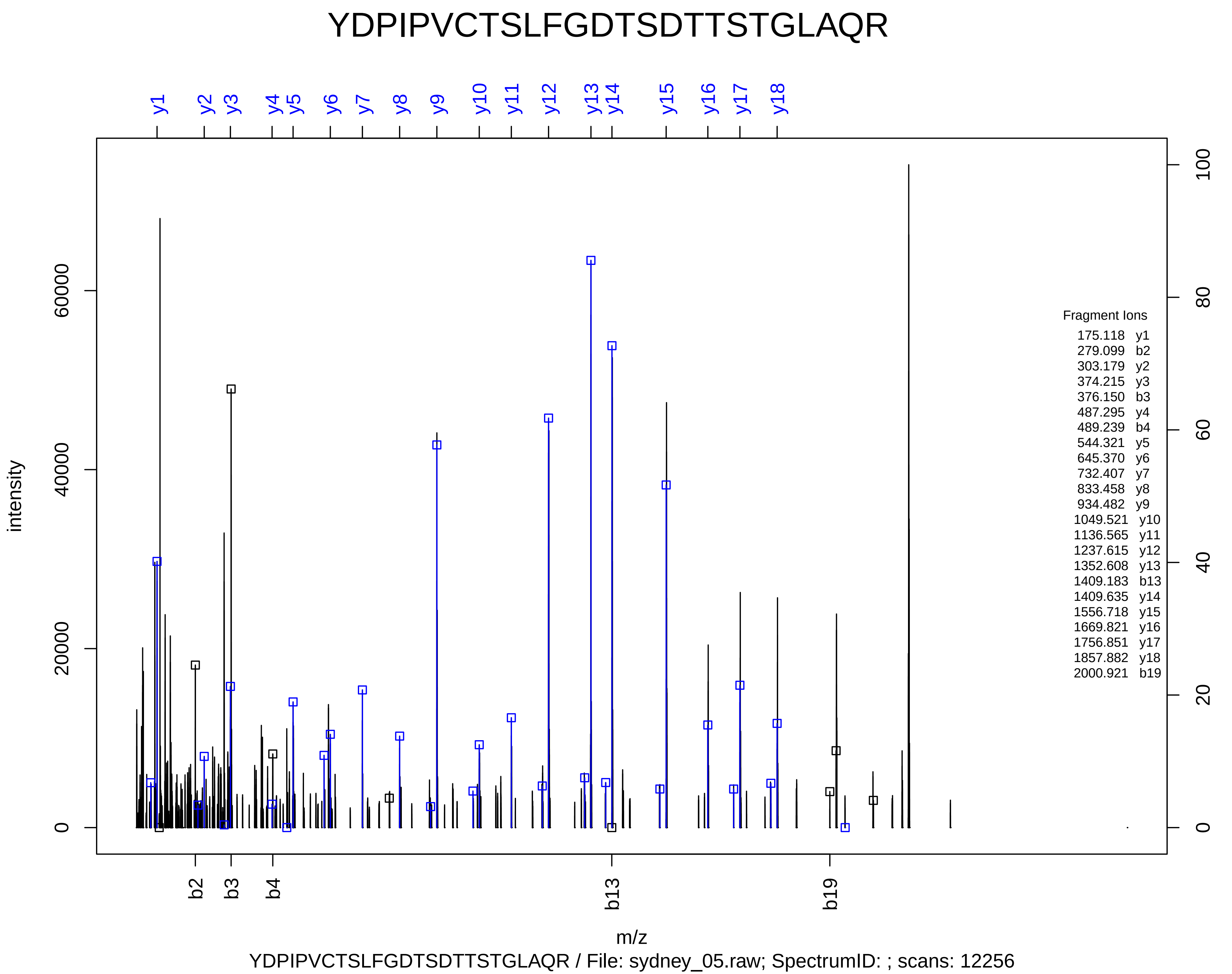
YDPIPVCTSLFGDTSDTTSTGLAQR
y1 y2 y3 y4 y5 y6 y7 y8 y9 y10 y11 y12 y13 y14 y15 y16 y17 y18
intensity
0
20000
40000
60000
0
20
40
60
80
100
b2 b3 b4 b13 b19
m/z
Fragment Ions
175.118 y1
279.099 b2
303.179 y2
374.215 y3
376.150 b3
487.295 y4
489.239 b4
544.321 y5
645.370 y6
732.407 y7
833.458 y8
934.482 y9
1049.521 y10
1136.565 y11
1237.615 y12
1352.608 y13
1409.183 b13
1409.635 y14
1556.718 y15
1669.821 y16
1756.851 y17
1857.882 y18
2000.921 b19
YDPIPVCTSLFGDTSDTTSTGLAQR / File: sydney_05.raw; SpectrumID: ; scans: 12256

TQQQVEAEVSNIK

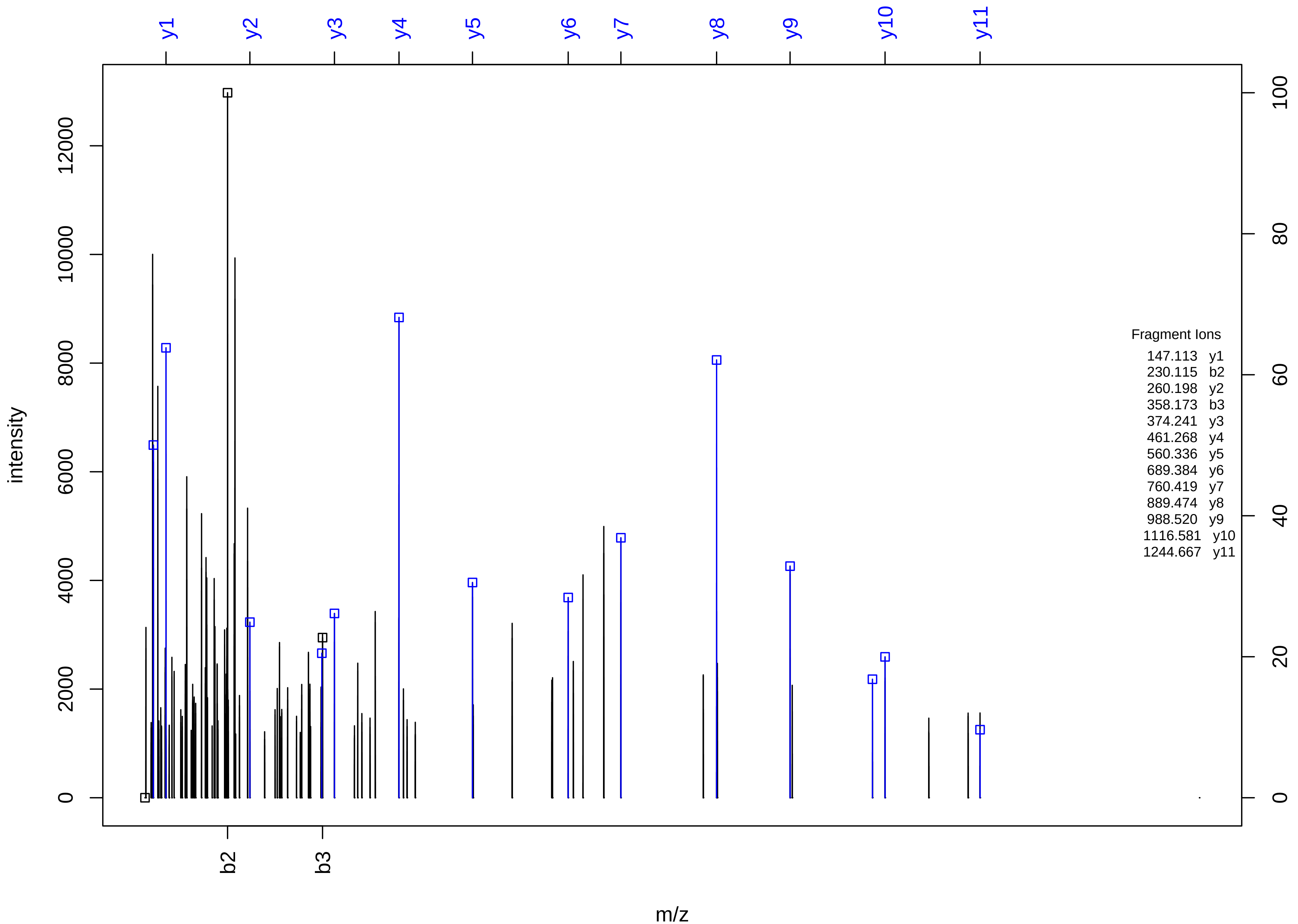

TQQQVEAEVSNIK / File: sydney_05.raw; SpectrumID: ; scans: 3414

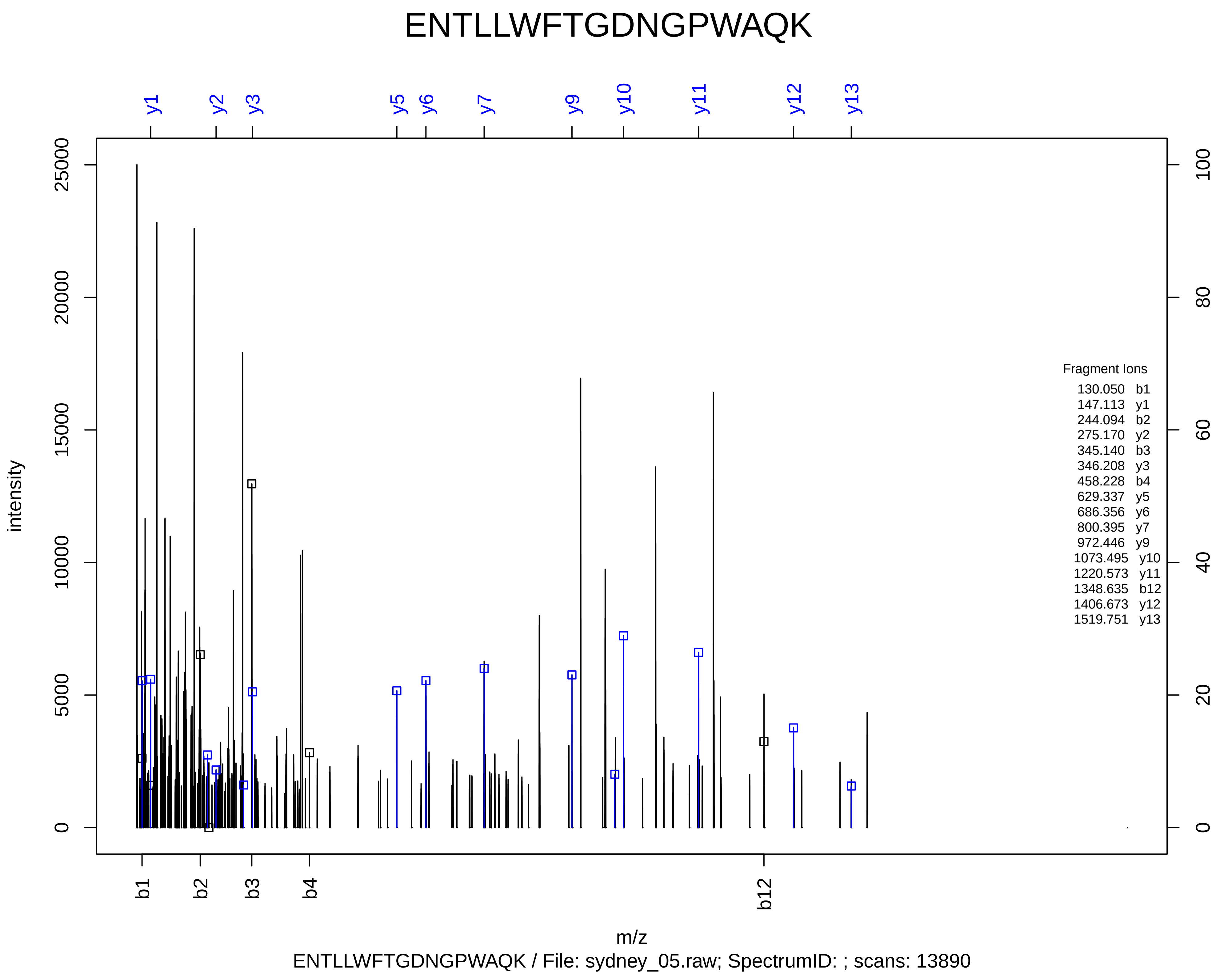
ENTLLWFTGDNGPWAQK
y1
y2
y3
y5
y6
y7
y9
y10
y11
y12
y13
intensity
0
5000
10000
15000
20000
25000
0
20
40
60
80
100
Fragment Ions
130.050 b1
147.113 y1
244.094 b2
275.170 y2
345.140 b3
346.208 y3
458.228 b4
629.337 y5
686.356 y6
800.395 y7
972.446 y9
1073.495 y10
1220.573 y11
1348.635 b12
1406.673 y12
1519.751 y13
b1
b2
b3
b4
b12
m/z
ENTLLWFTGDNGPWAQK / File: sydney_05.raw; SpectrumID: ; scans: 13890

TELPVTENVQTIPPPYVVR

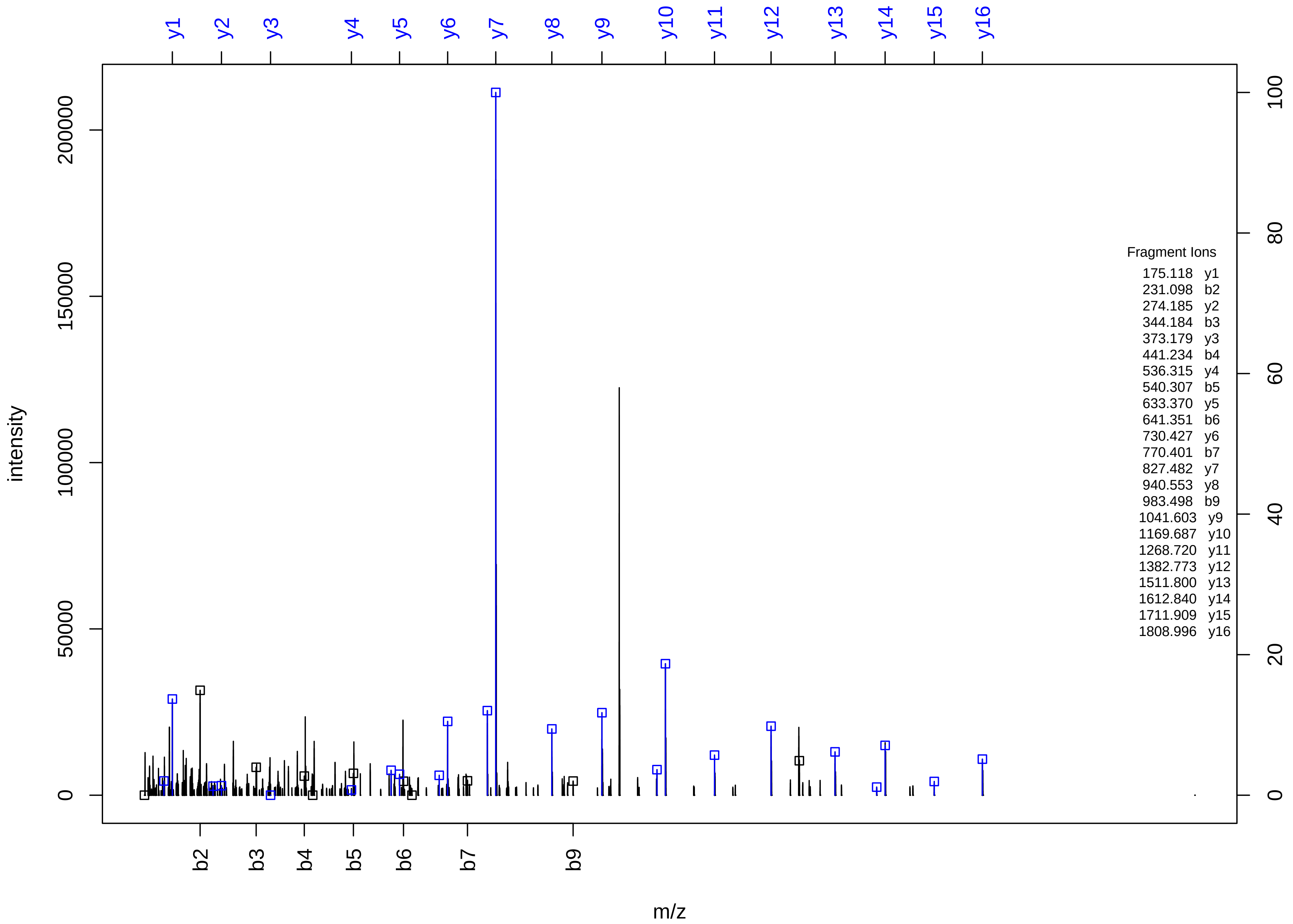

TELPVTENVQTIPPPYVVR / File: sydney_05.raw; SpectrumID: ; scans: 10593

QWLRPETHSK

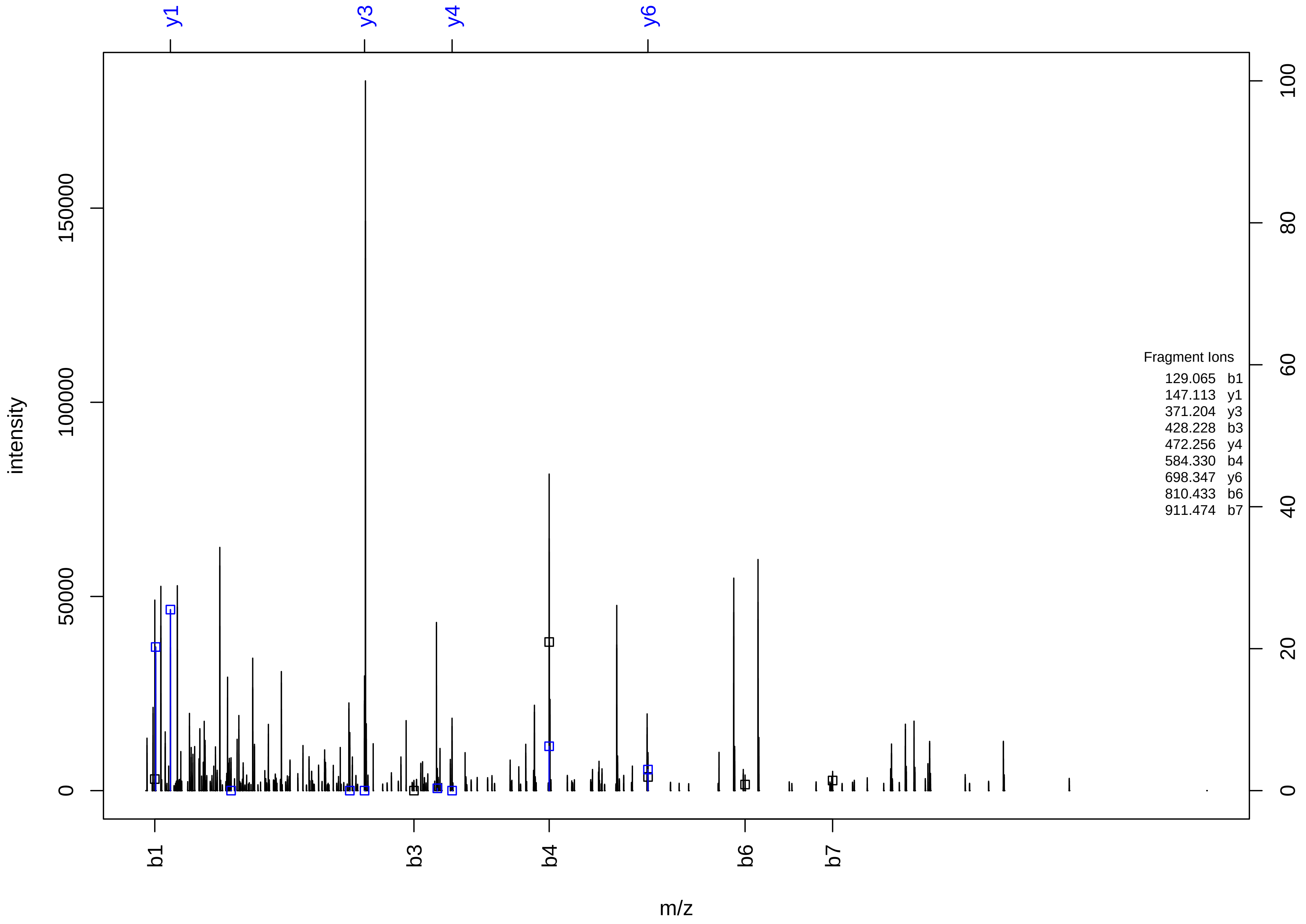

QWLRPETHSK / File: sydney_05.raw; SpectrumID: ; scans: 4893

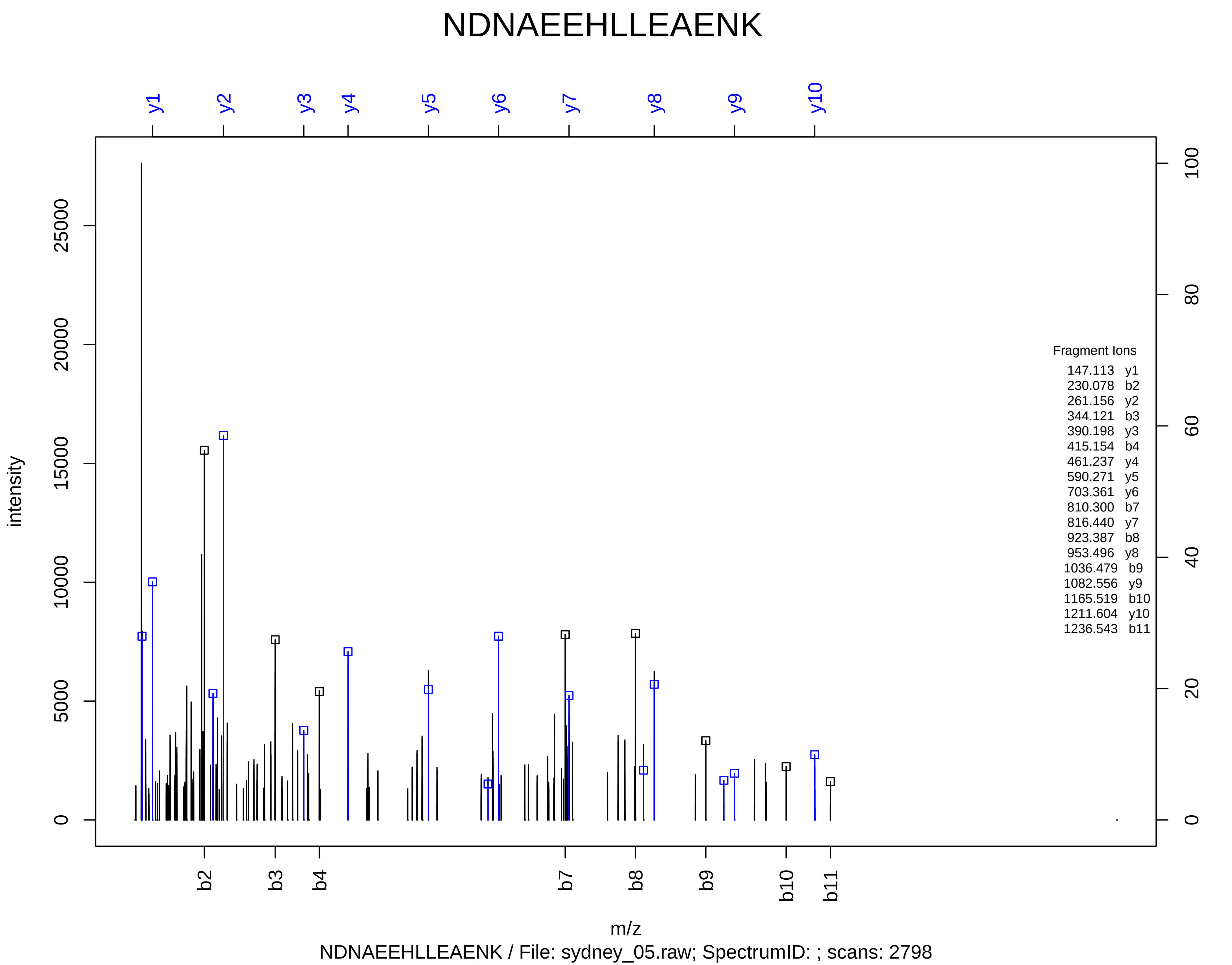

NDNAEEHLLEAENK
y1
y2
y3
y4
y5
y6
y7
y8
y9
y10
intensity
0
5000
10000
15000
20000
25000
0
20
40
60
80
100
b2
b3
b4
b7
b8
b9
b10
b11
m/z
Fragment Ions
147.113 y1
230.078 b2
261.156 y2
344.121 b3
390.198 y3
415.154 b4
461.237 y4
590.271 y5
703.361 y6
810.300 b7
816.440 y7
923.387 b8
953.496 y8
1036.479 b9
1082.556 y9
1165.519 b10
1211.604 y10
1236.543 b11
NDNAEEHLLEAENK / File: sydney_05.raw; SpectrumID: ; scans: 2798

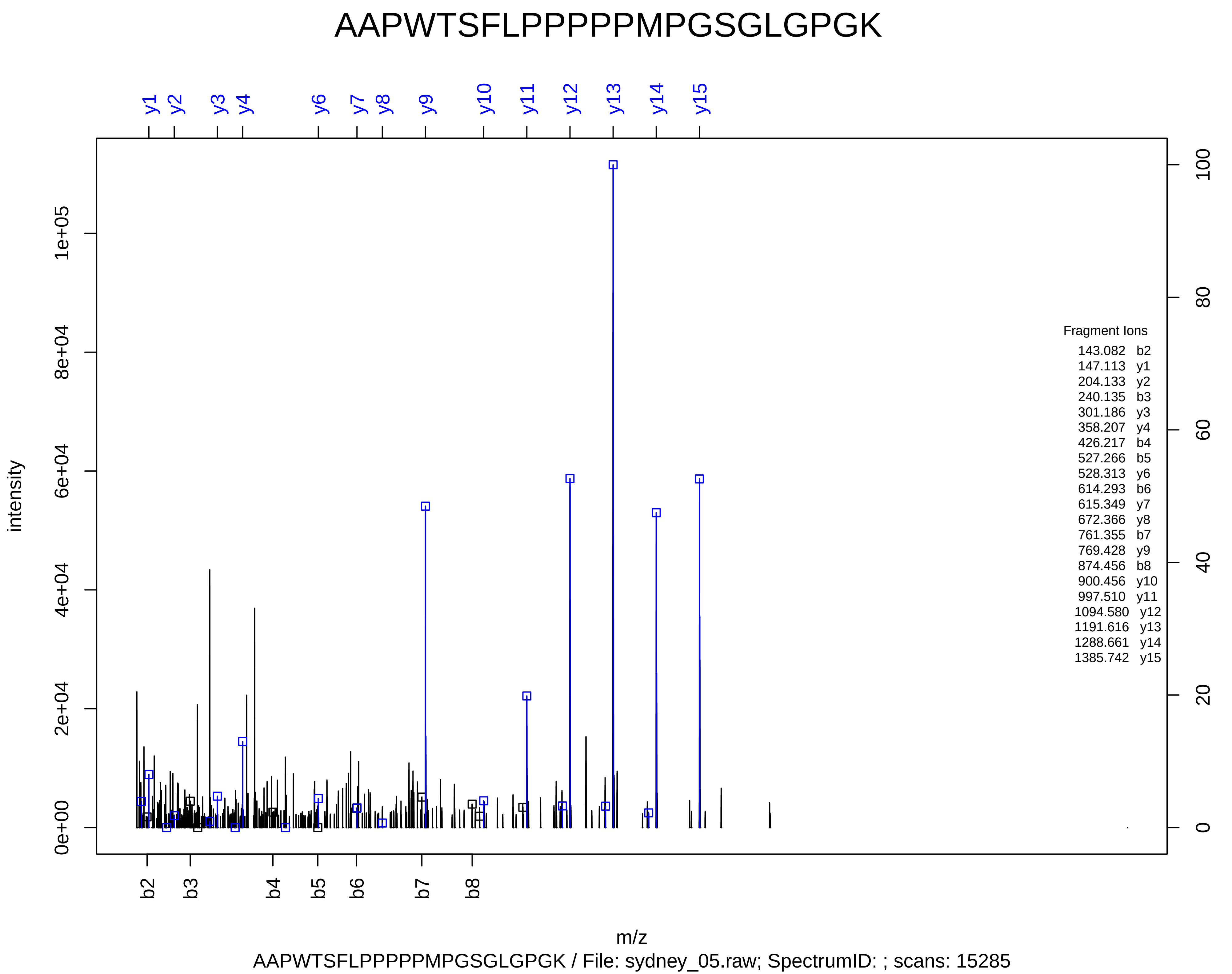

AAPWTSFLPPPPPMPGSGLGPGK
y1 y2 y3 y4 y6 y7 y8 y9 y10 y11 y12 y13 y14 y15
b2 b3 b4 b5 b6 b7 b8
intensity
0e+00
2e+04
4e+04
6e+04
8e+04
1e+05
0
20
40
60
80
100
m/z
Fragment Ions
143.082 b2
147.113 y1
204.133 y2
240.135 b3
301.186 y3
358.207 y4
426.217 b4
527.266 b5
528.313 y6
614.293 b6
615.349 y7
672.366 y8
761.355 b7
769.428 y9
874.456 b8
900.456 y10
997.510 y11
1094.580 y12
1191.616 y13
1288.661 y14
1385.742 y15
AAPWTSFLPPPPPMPGSGLGPGK / File: sydney_05.raw; SpectrumID: ; scans: 15285

AYAEFYR

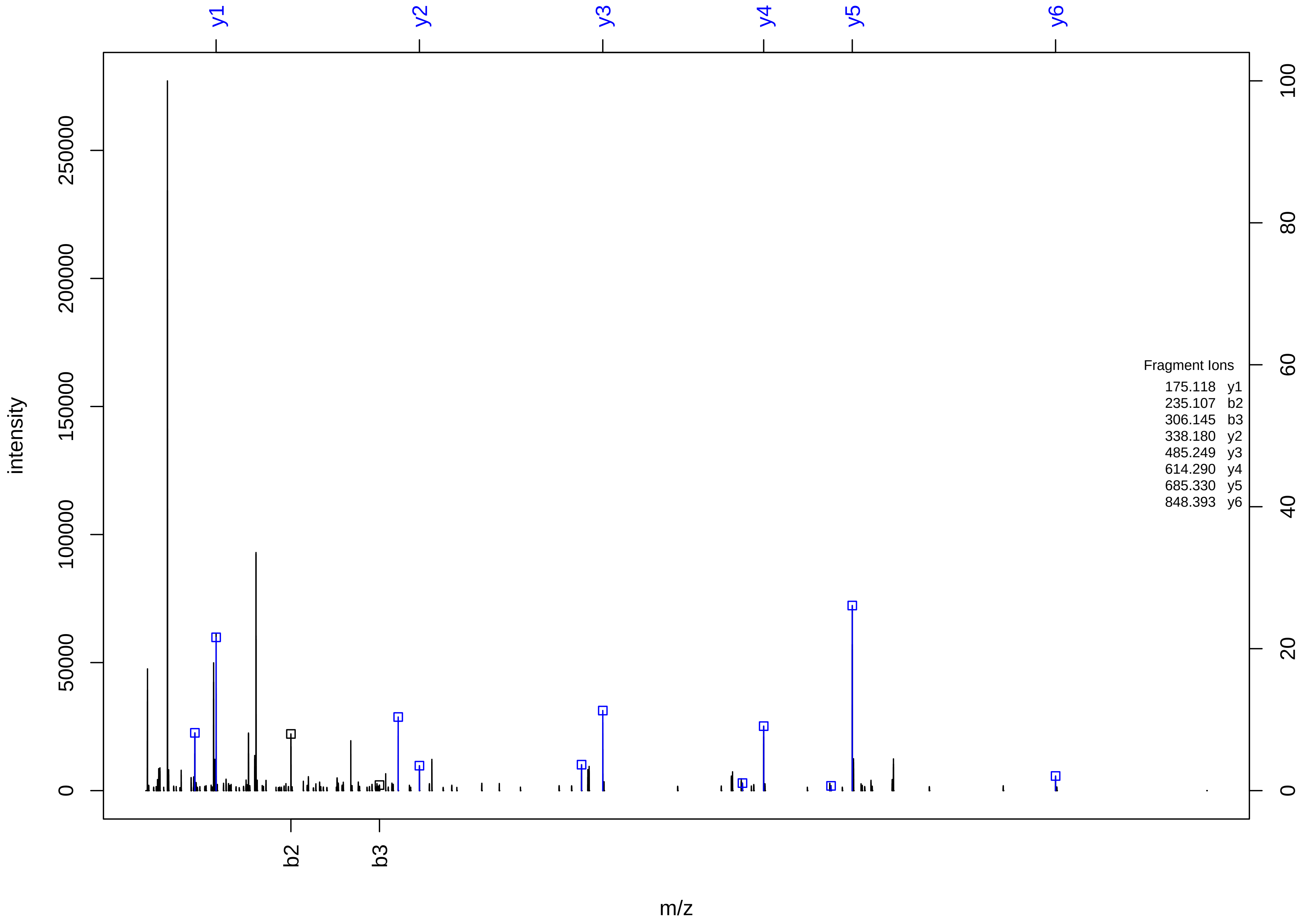

AYAEFYR / File: sydney_05.raw; SpectrumID: ; scans: 3117

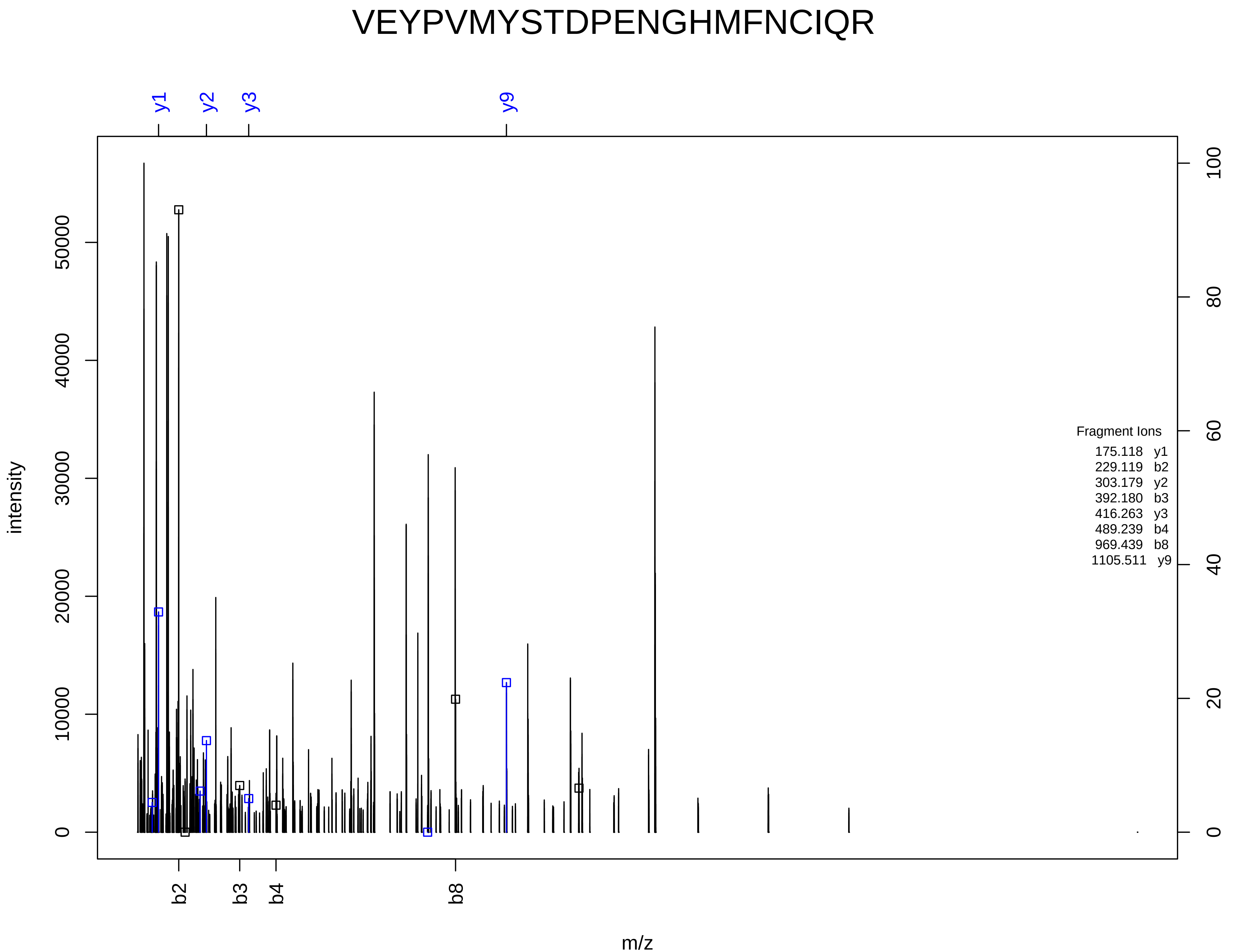

VEYPVMYSTDPENGHMFNCIQR / File: sydney_05.raw; SpectrumID: ; scans: 8517

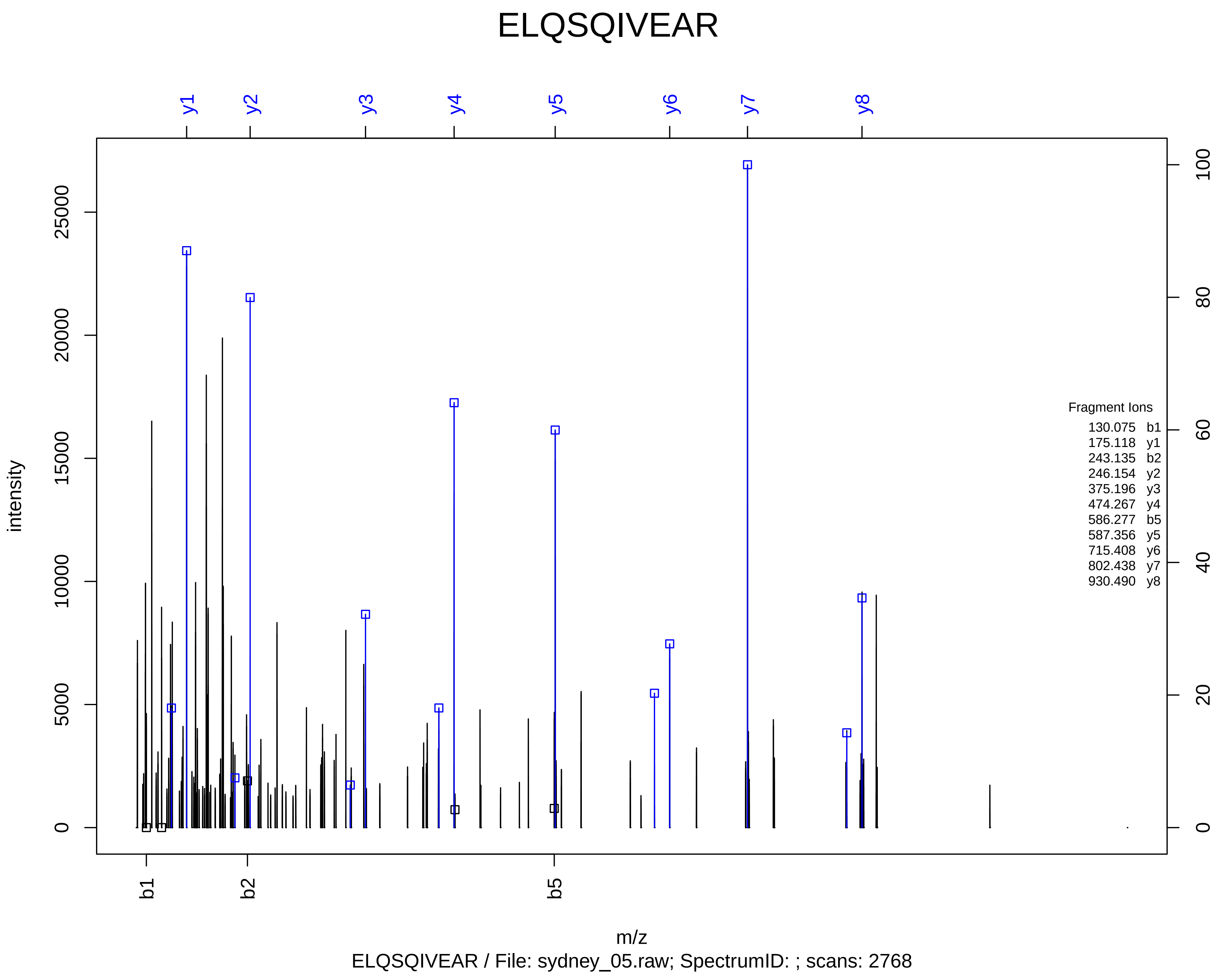
ELQSQIVEAR
y1
y2
y3
y4
y5
y6
y7
y8
intensity
0
5000
10000
15000
20000
25000
0
20
40
60
80
100
b1
b2
b5
m/z
Fragment Ions
130.075 b1
175.118 y1
243.135 b2
246.154 y2
375.196 y3
474.267 y4
586.277 b5
587.356 y5
715.408 y6
802.438 y7
930.490 y8
ELQSQIVEAR / File: sydney_05.raw; SpectrumID: ; scans: 2768

ELGENLTDEELQEMIDEADRDGDGEVNEQEFLR

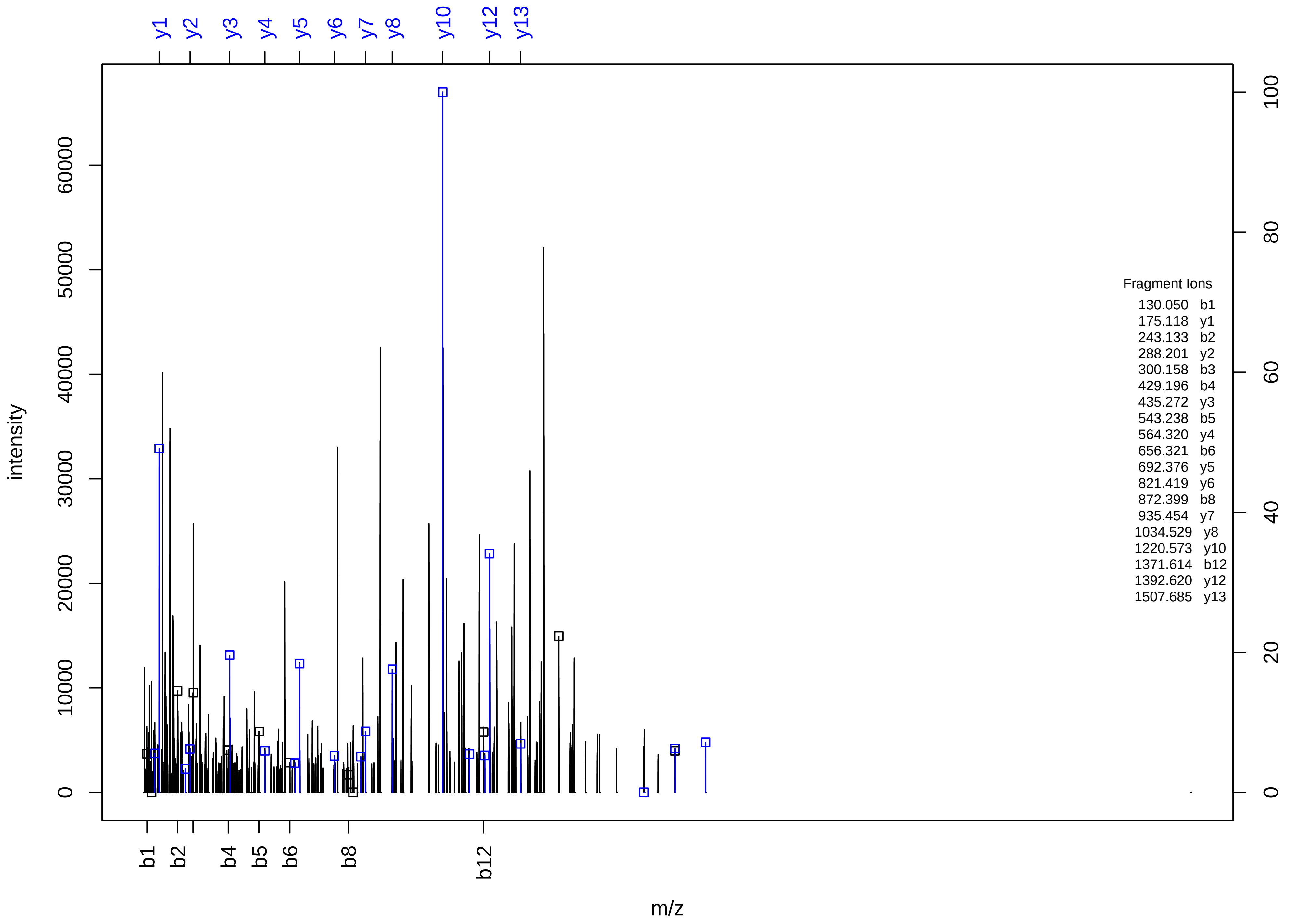

ELGENLTDEELQEMIDEADRDGDGEVNEQEFLR / File: sydney_05.raw; SpectrumID: ; scans: 14088

FASAQFPNGPQFTEDHSQLPSSQLPSSPFGDYR

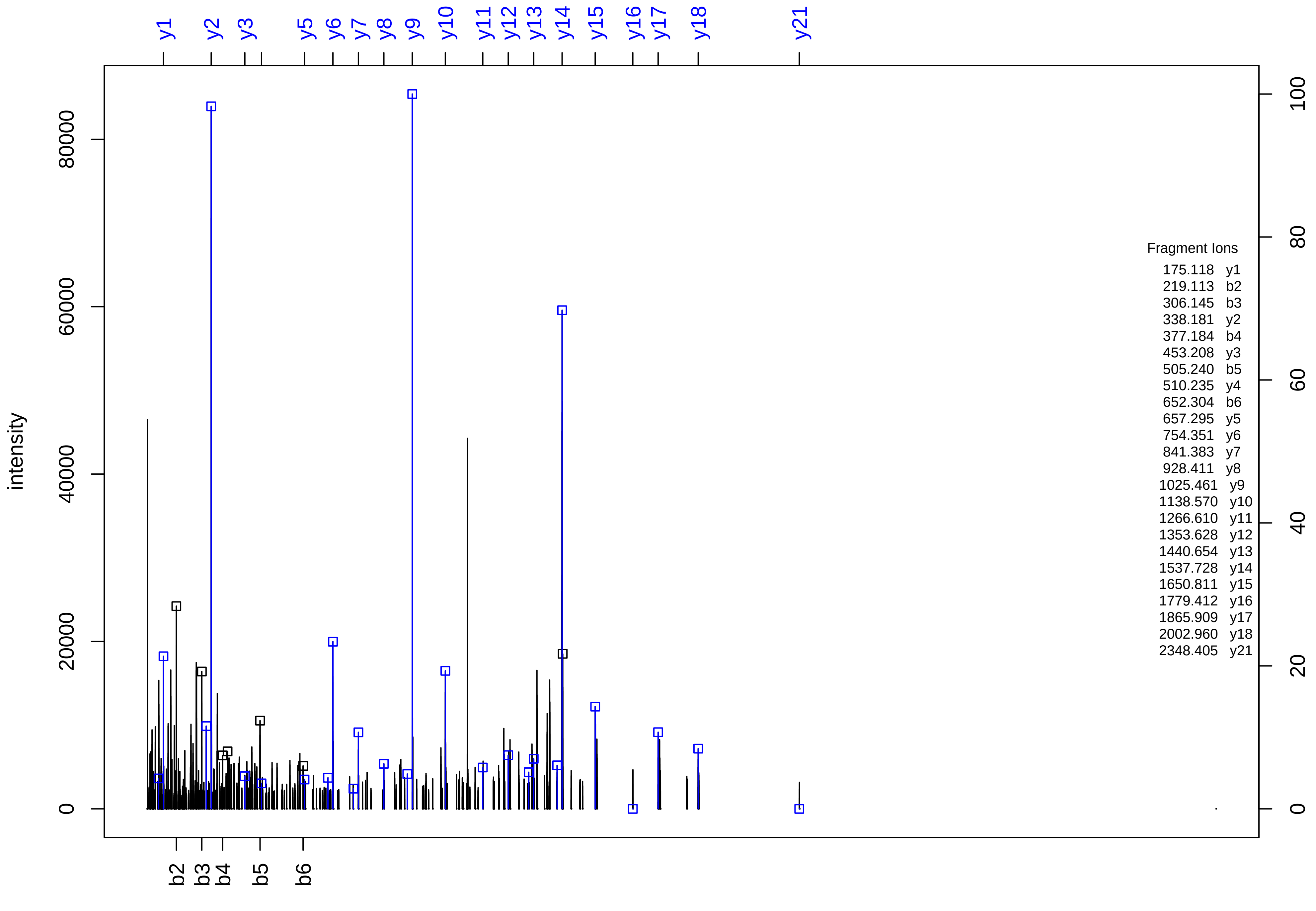

FASAQFPNGPQFTEDHSQLPSSQLPSSPFGDYR / File: sydney_05.raw; SpectrumID: ; scans: 10891

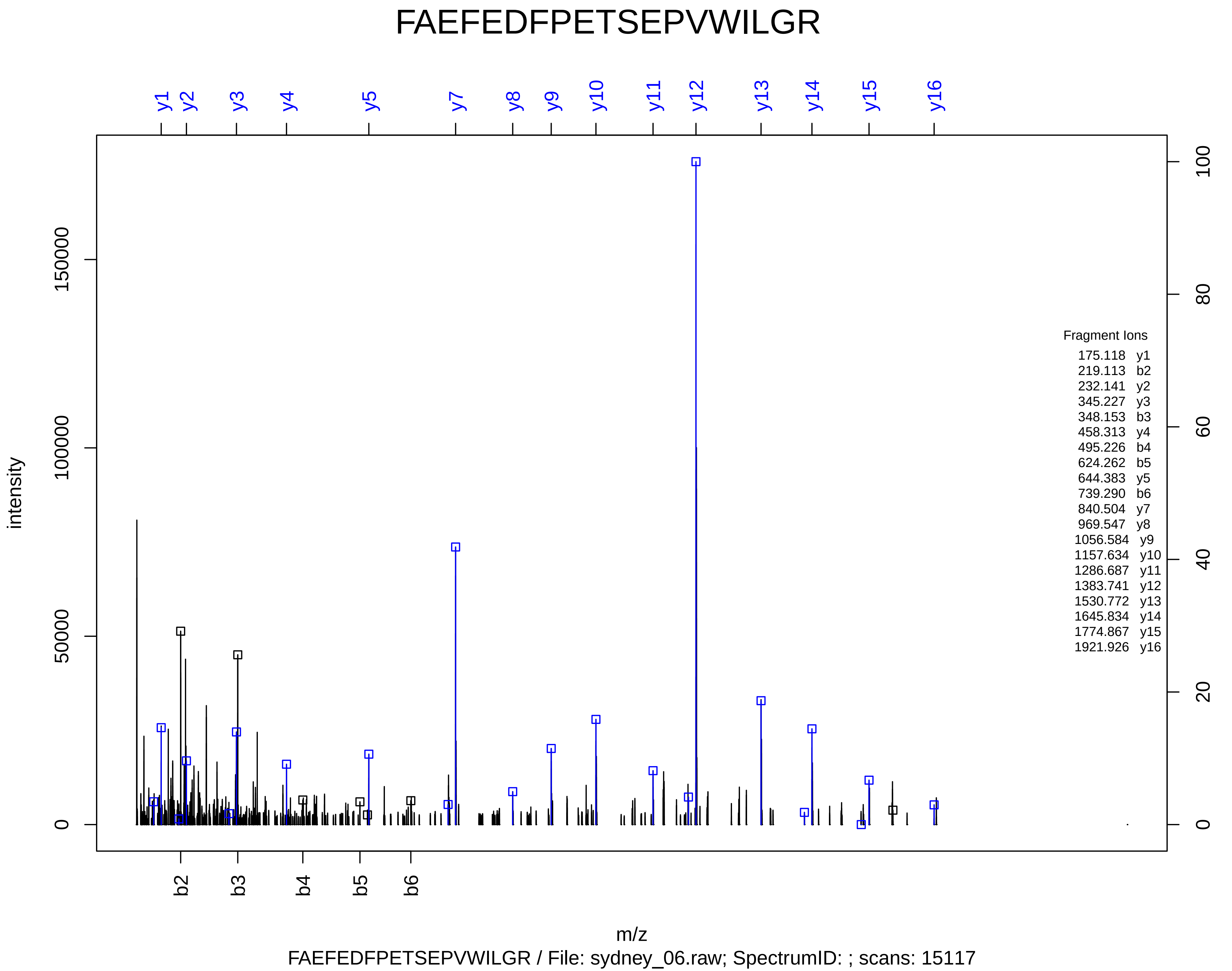

FAEFEDFPETSEPVWILGR
y1 y2 y3 y4 y5 y7 y8 y9 y10 y11 y12 y13 y14 y15 y16
intensity
0
50000
100000
150000
0
20
40
60
80
100
b2 b3 b4 b5 b6
m/z
Fragment Ions
175.118 y1
219.113 b2
232.141 y2
345.227 y3
348.153 b3
458.313 y4
495.226 b4
624.262 b5
644.383 y5
739.290 b6
840.504 y7
969.547 y8
1056.584 y9
1157.634 y10
1286.687 y11
1383.741 y12
1530.772 y13
1645.834 y14
1774.867 y15
1921.926 y16
FAEFEDFPETSEPVWILGR / File: sydney_06.raw; SpectrumID: ; scans: 15117

VMFSPFGQIEECR

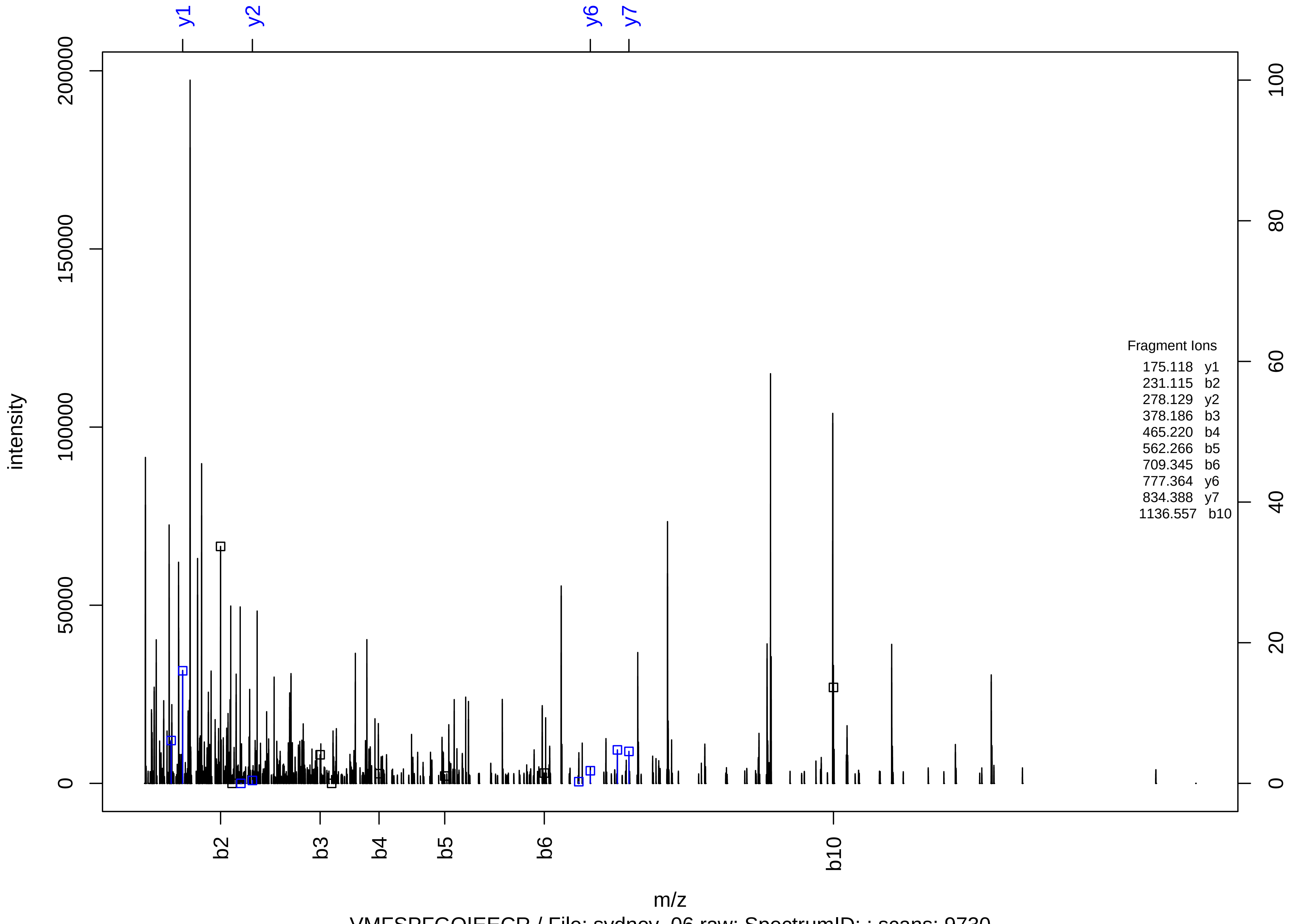

VMFSPFGQIEECR / File: sydney_06.raw; SpectrumID: ; scans: 9730

LLYPPETGLFLVR

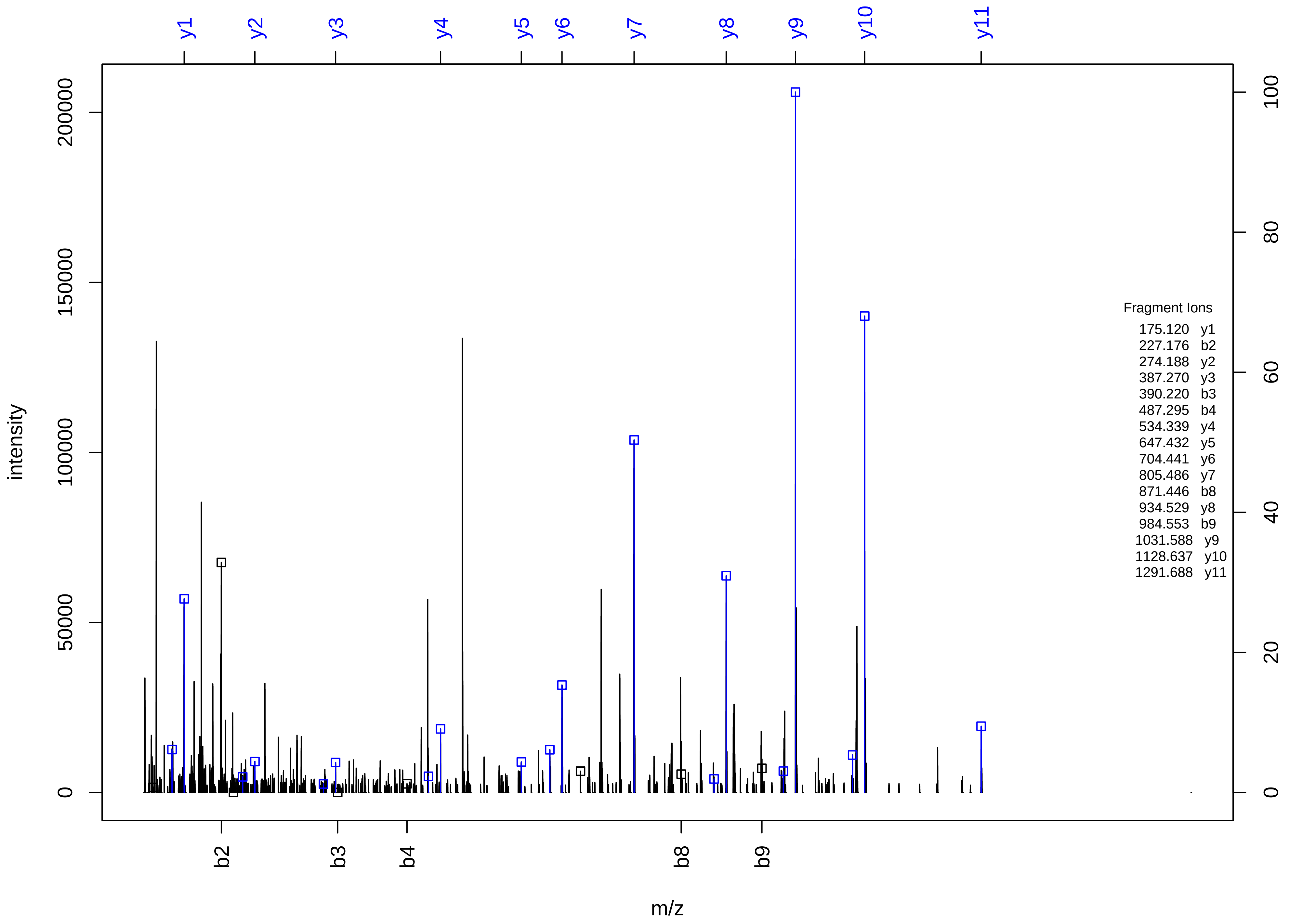

LLYPPETGLFLVR / File: sydney_06.raw; SpectrumID: ; scans: 13196

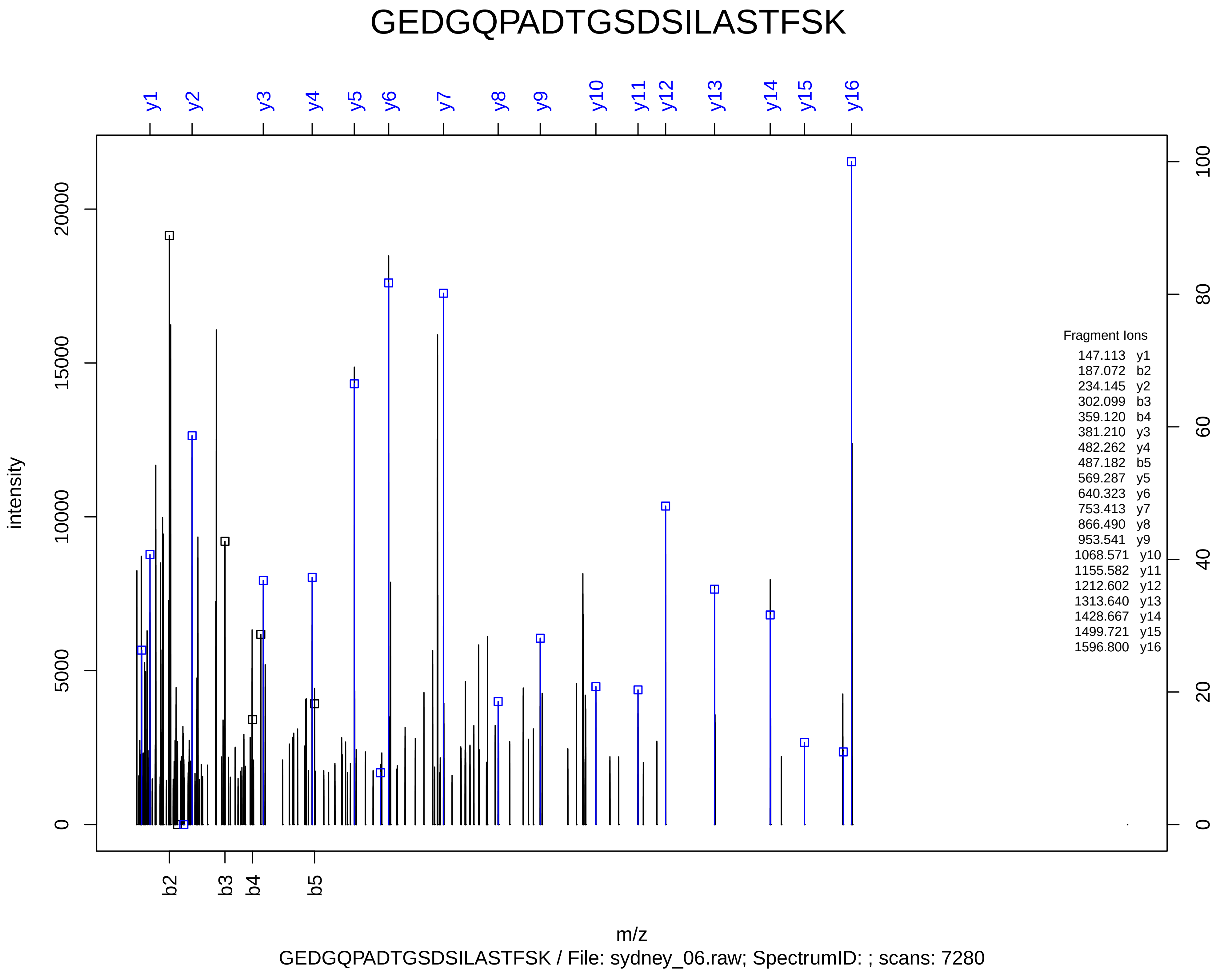
GEDGQPADTGSDSILASTFSK
y1
y2
y3
y4
y5
y6
y7
y8
y9
y10
y11
y12
y13
y14
y15
y16
intensity
0
5000
10000
15000
20000
0
20
40
60
80
100
b2
b3
b4
b5
Fragment Ions
147.113 y1
187.072 b2
234.145 y2
302.099 b3
359.120 b4
381.210 y3
482.262 y4
487.182 b5
569.287 y5
640.323 y6
753.413 y7
866.490 y8
953.541 y9
1068.571 y10
1155.582 y11
1212.602 y12
1313.640 y13
1428.667 y14
1499.721 y15
1596.800 y16
m/z
GEDGQPADTGSDSILASTFSK / File: sydney_06.raw; SpectrumID: ; scans: 7280

DLEEWNQR

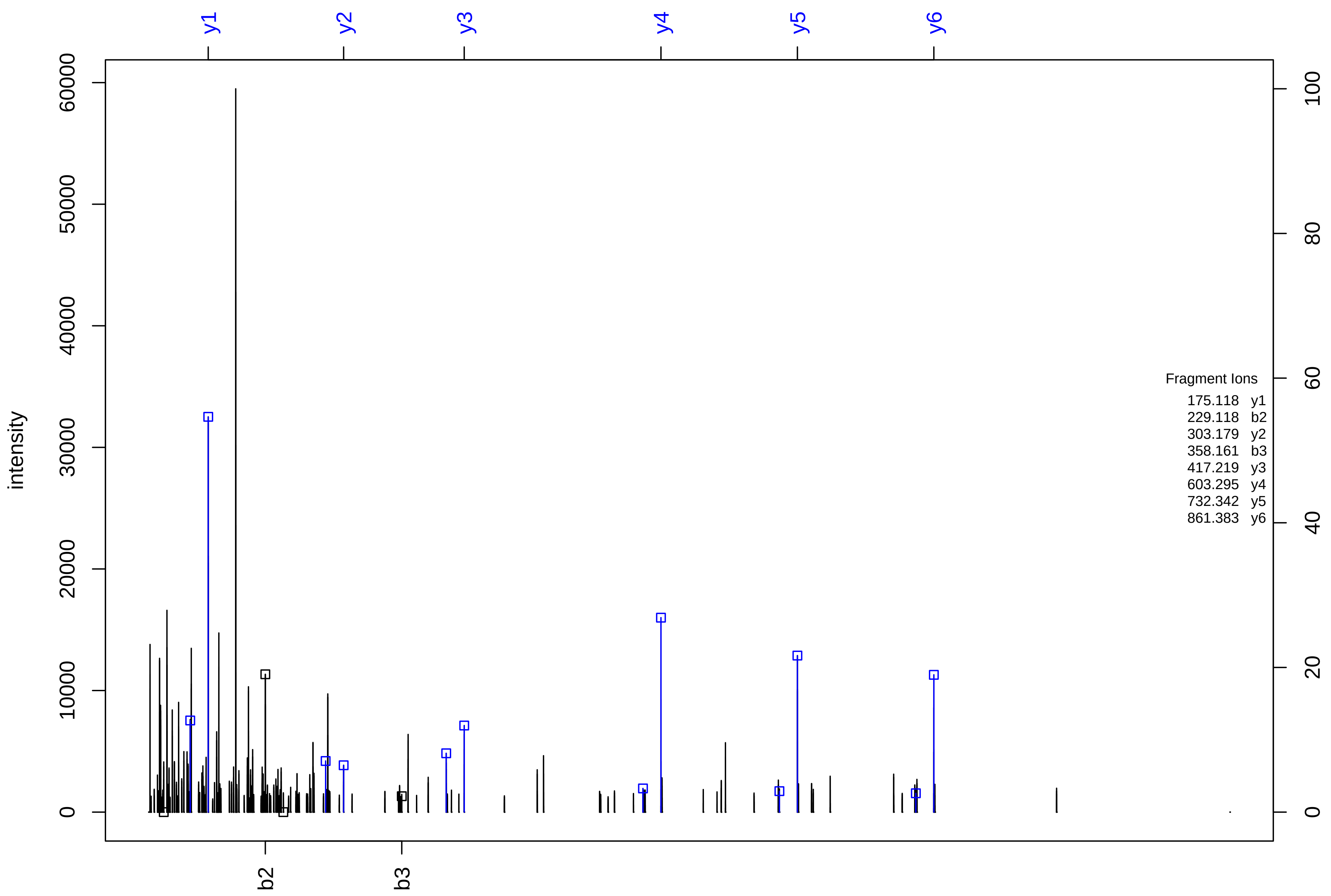

DLEEWNQR / File: sydney_06.raw; SpectrumID: ; scans: 2763

AGVDAFIQNYVDPTPDPNTAYASYPSASVENYQQPPFTQNVETTEGYQPPPVY

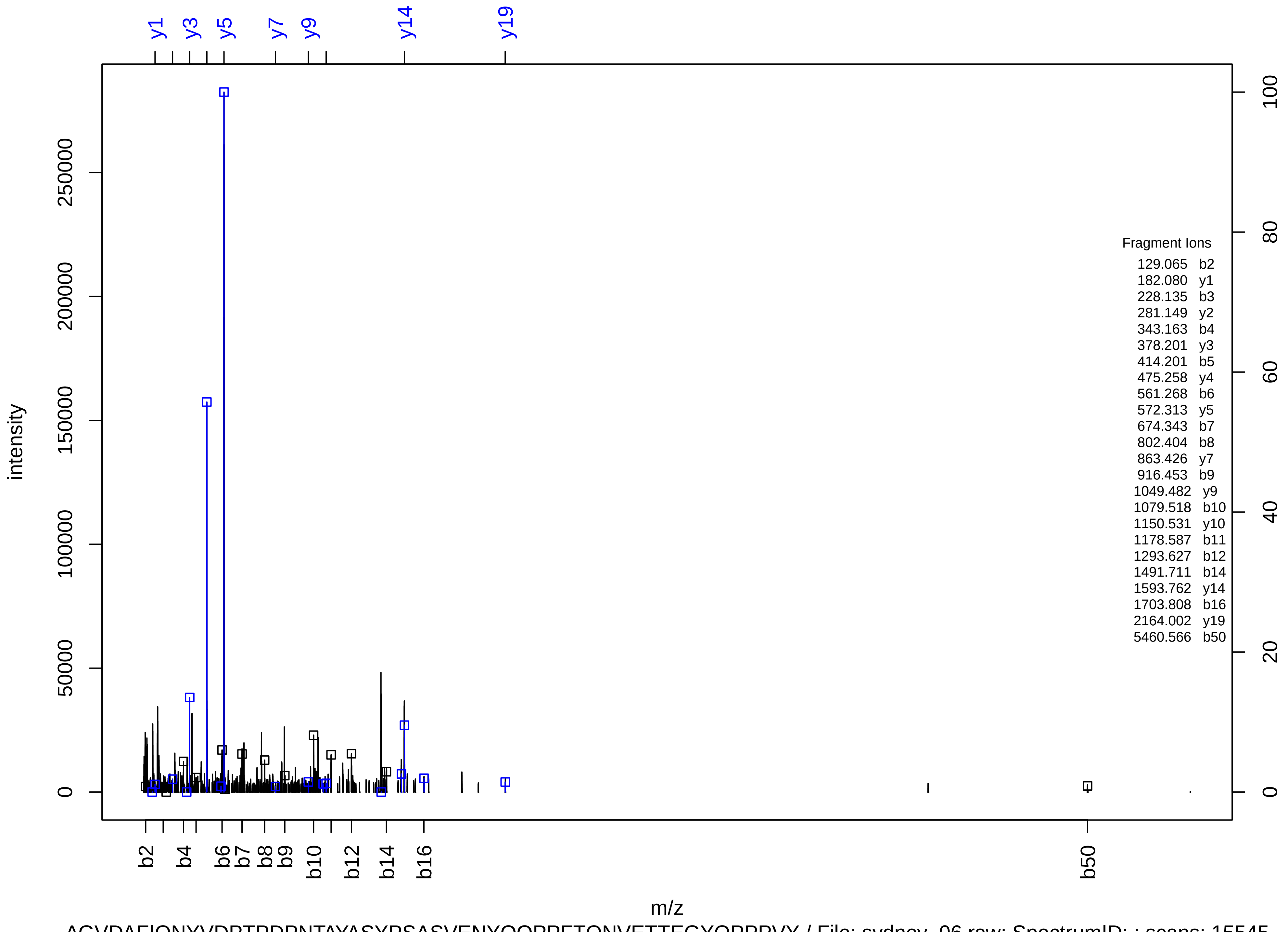

AGVDAFIQNYVDPTPDPNTAYASYPSASVENYQQPPFTQNVETTEGYQPPPVY / File: sydney_06.raw; SpectrumID: ; scans: 15545

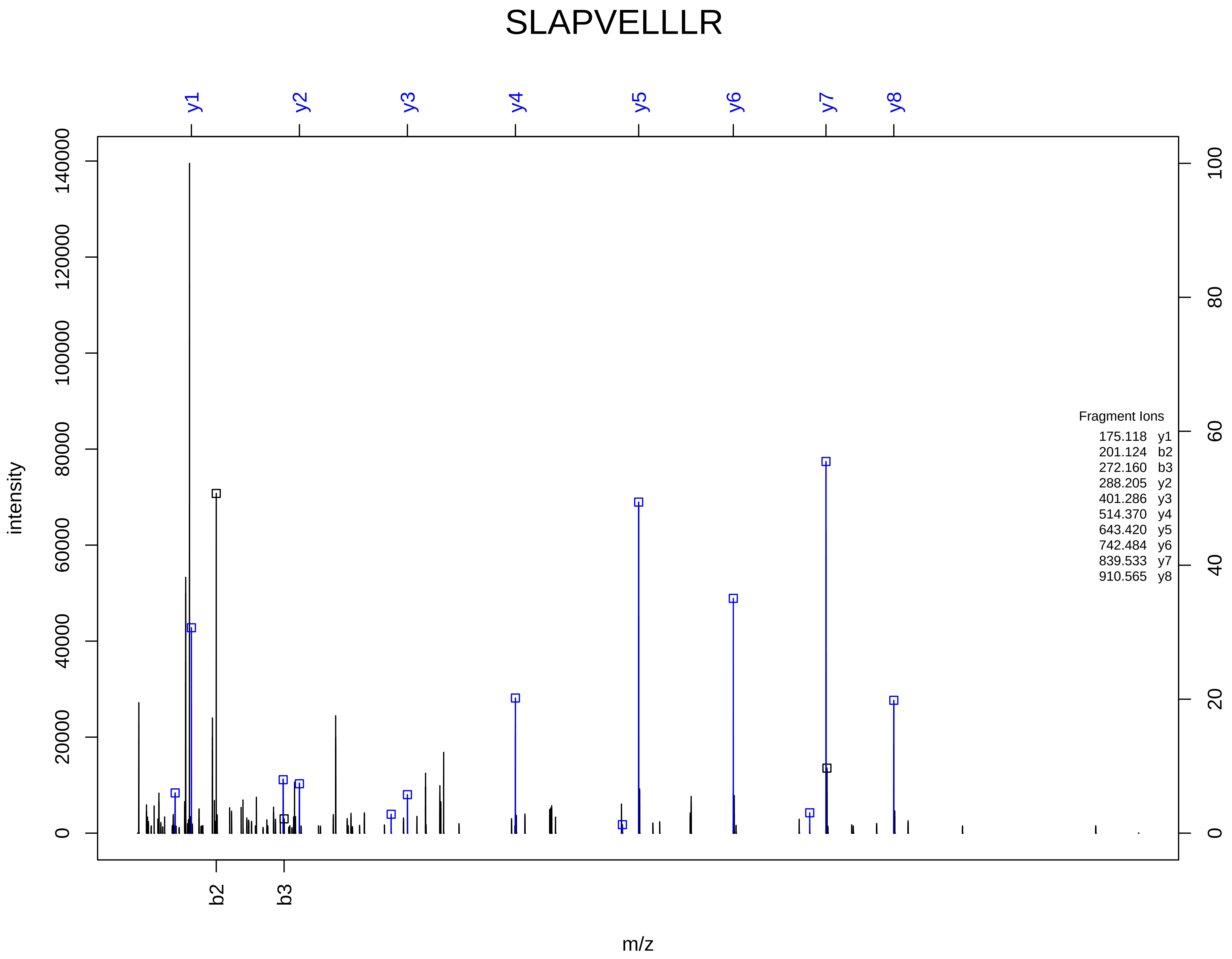
SLAPVELLLR
y1
y2
y3
y4
y5
y6
y7
y8
intensity
0
20000
40000
60000
80000
100000
120000
140000
0
20
40
60
80
100
Fragment Ions
175.118 y1
201.124 b2
272.160 b3
288.205 y2
401.286 y3
514.370 y4
643.420 y5
742.484 y6
839.533 y7
910.565 y8
b2
b3
m/z
SLAPVELLLR / File: sydney_06.raw; SpectrumID: ; scans: 11885

TLVTLGSQTPVQTQPTPK

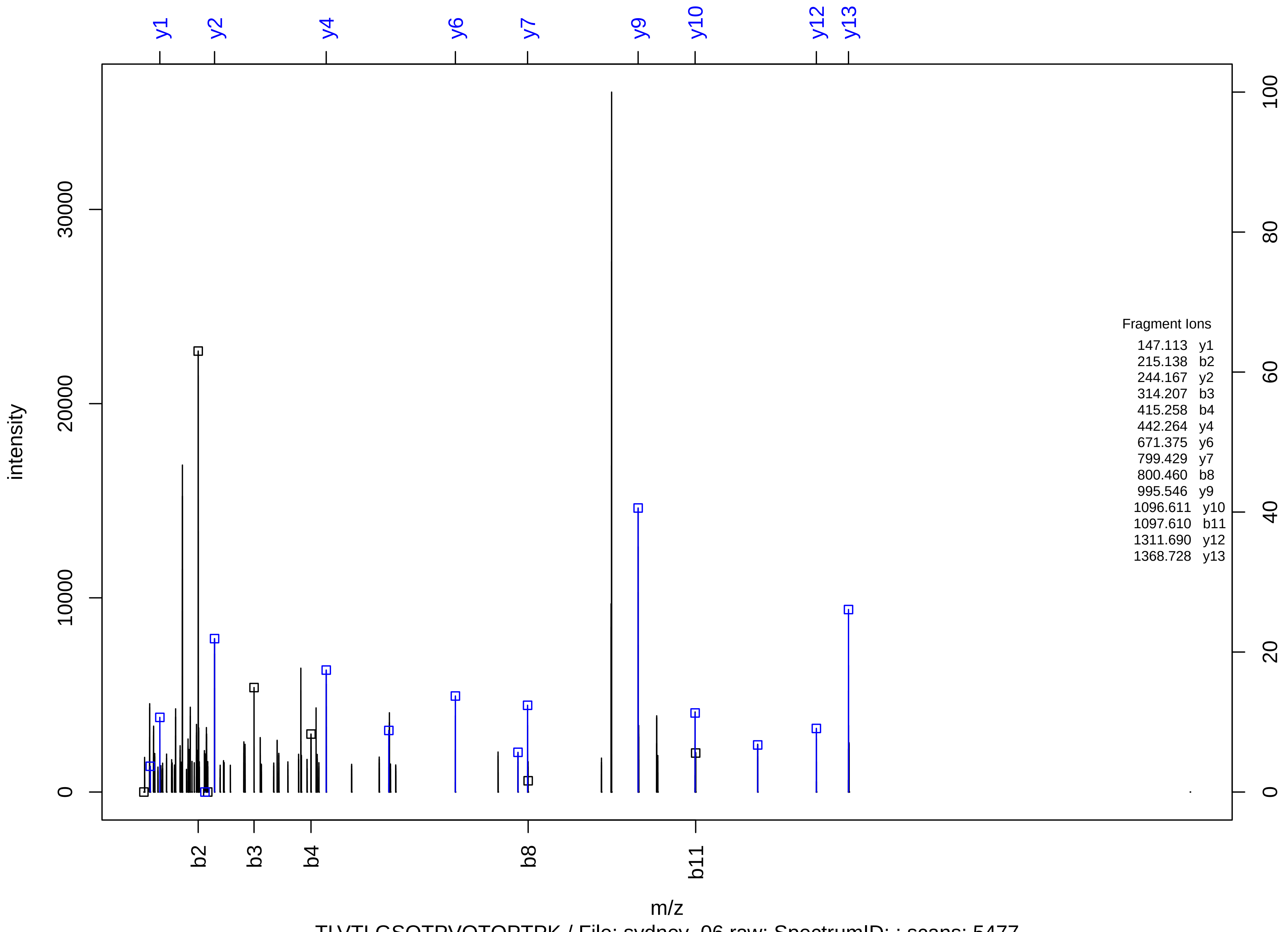

TLVTLGSQTPVQTQPTPK / File: sydney_06.raw; SpectrumID: ; scans: 5477

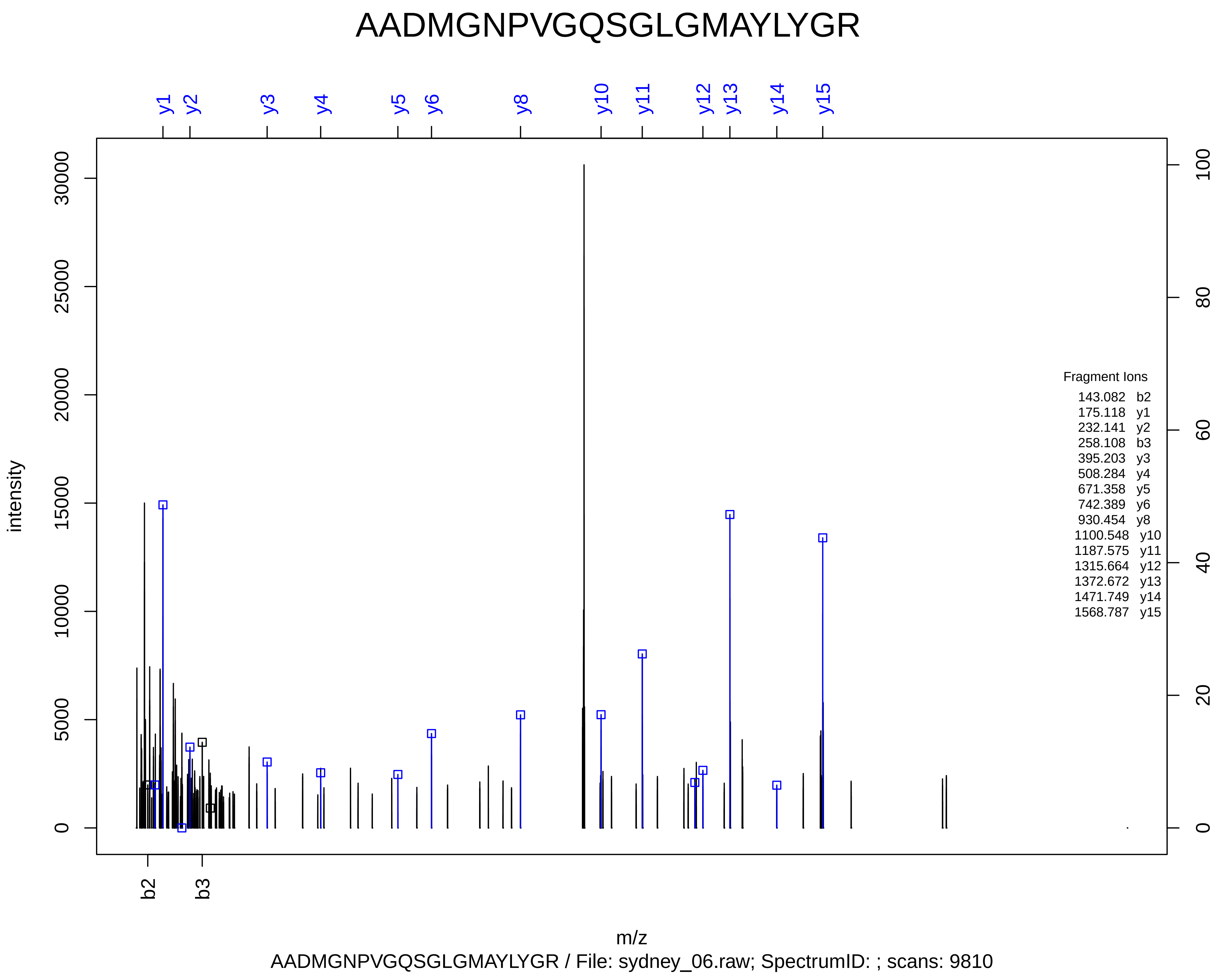

AADMGNPVGQSGLGMAYLYGR
y1
y2
y3
y4
y5
y6
y8
y10
y11
y12
y13
y14
y15
intensity
0
5000
10000
15000
20000
25000
30000
0
20
40
60
80
100
b2
b3
m/z
Fragment Ions
143.082 b2
175.118 y1
232.141 y2
258.108 b3
395.203 y3
508.284 y4
671.358 y5
742.389 y6
930.454 y8
1100.548 y10
1187.575 y11
1315.664 y12
1372.672 y13
1471.749 y14
1568.787 y15
AADMGNPVGQSGLGMAYLYGR / File: sydney_06.raw; SpectrumID: ; scans: 9810

(Ac)AGAACCFSDEQFR

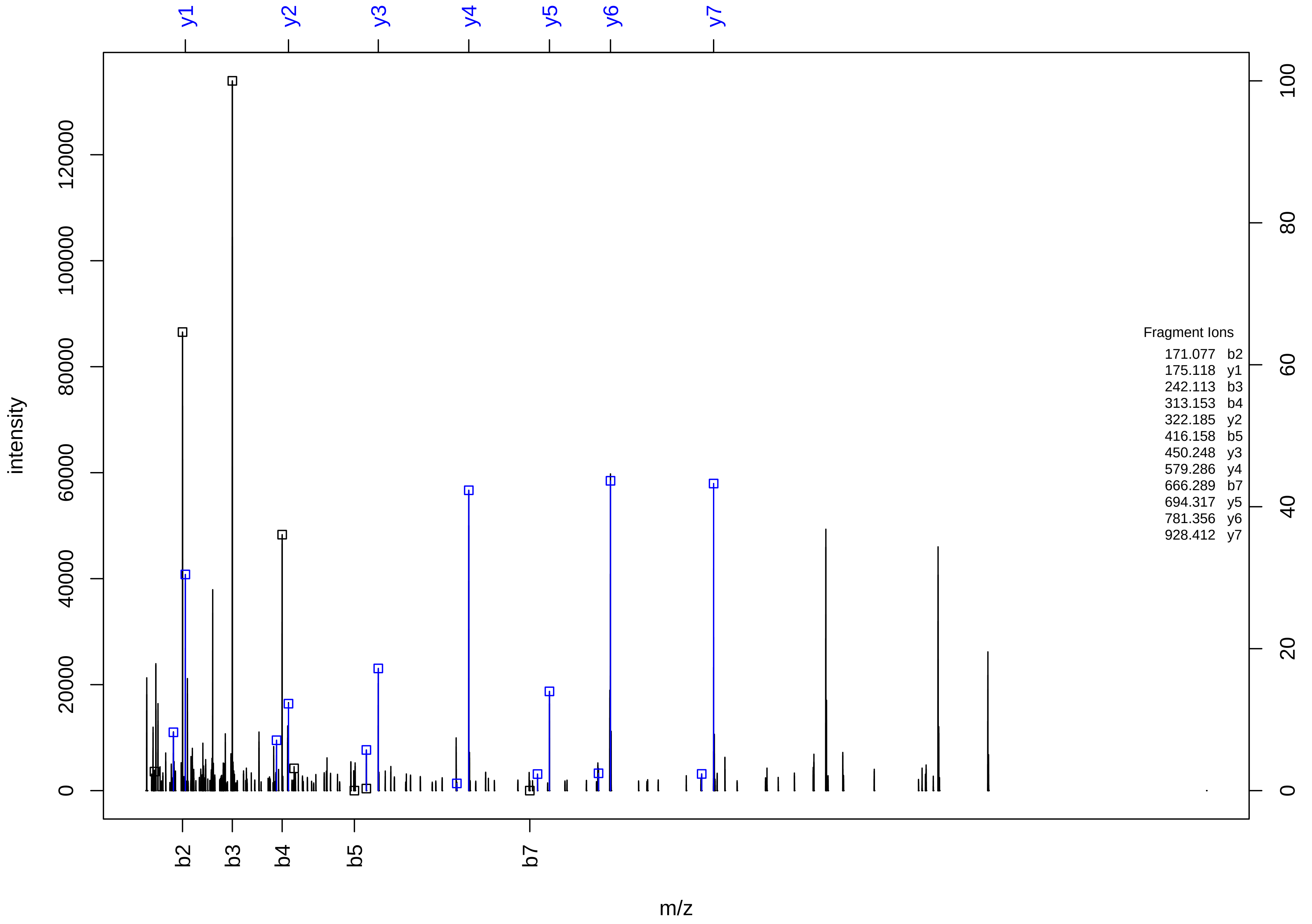

AGAACCFSDEQFR / File: sydney_06.raw; SpectrumID: ; scans: 6826

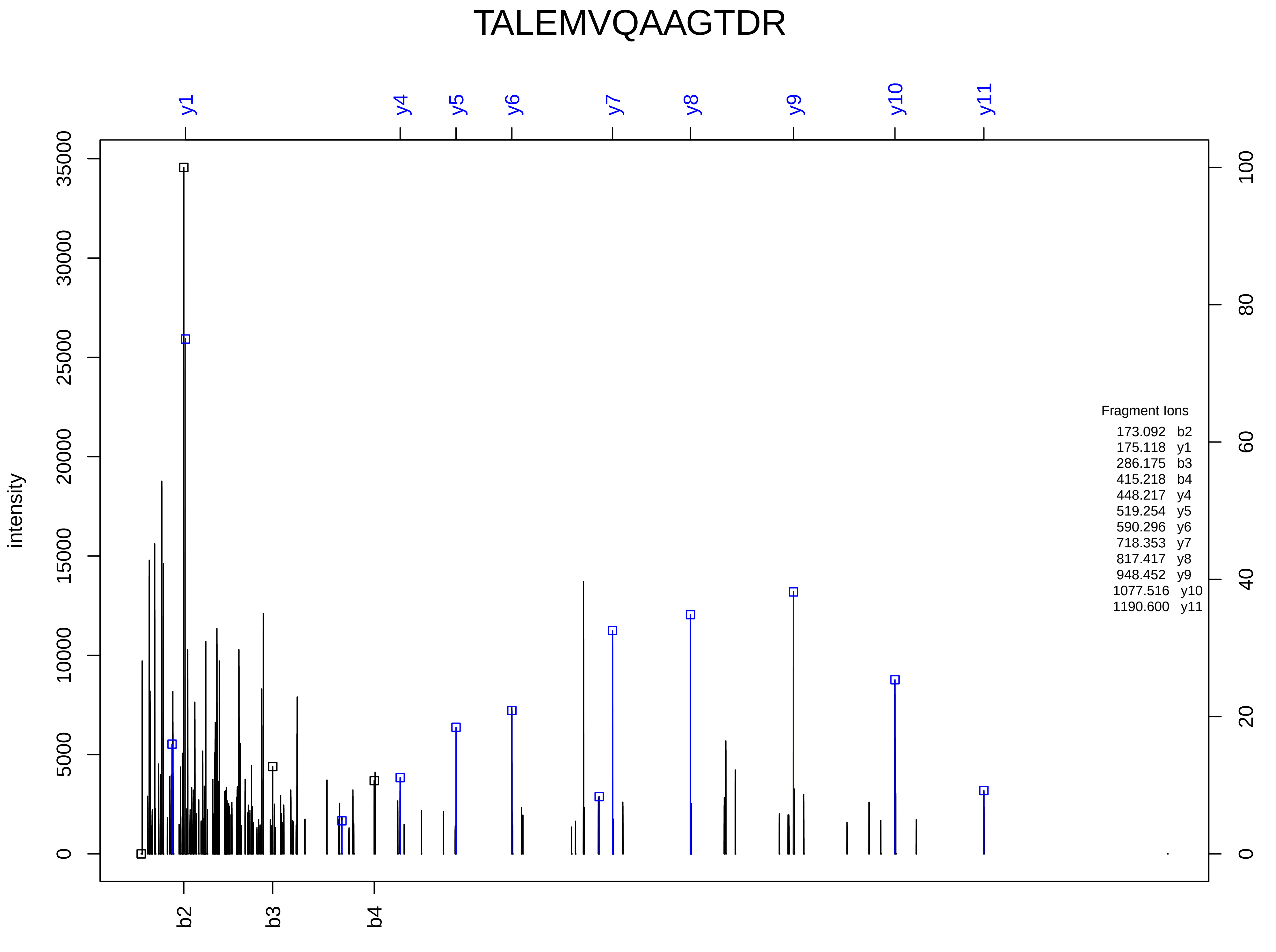

TALEMVQAAGTDR / File: sydney_06.raw; SpectrumID: ; scans: 4264

DQVFGCQLESLCQR

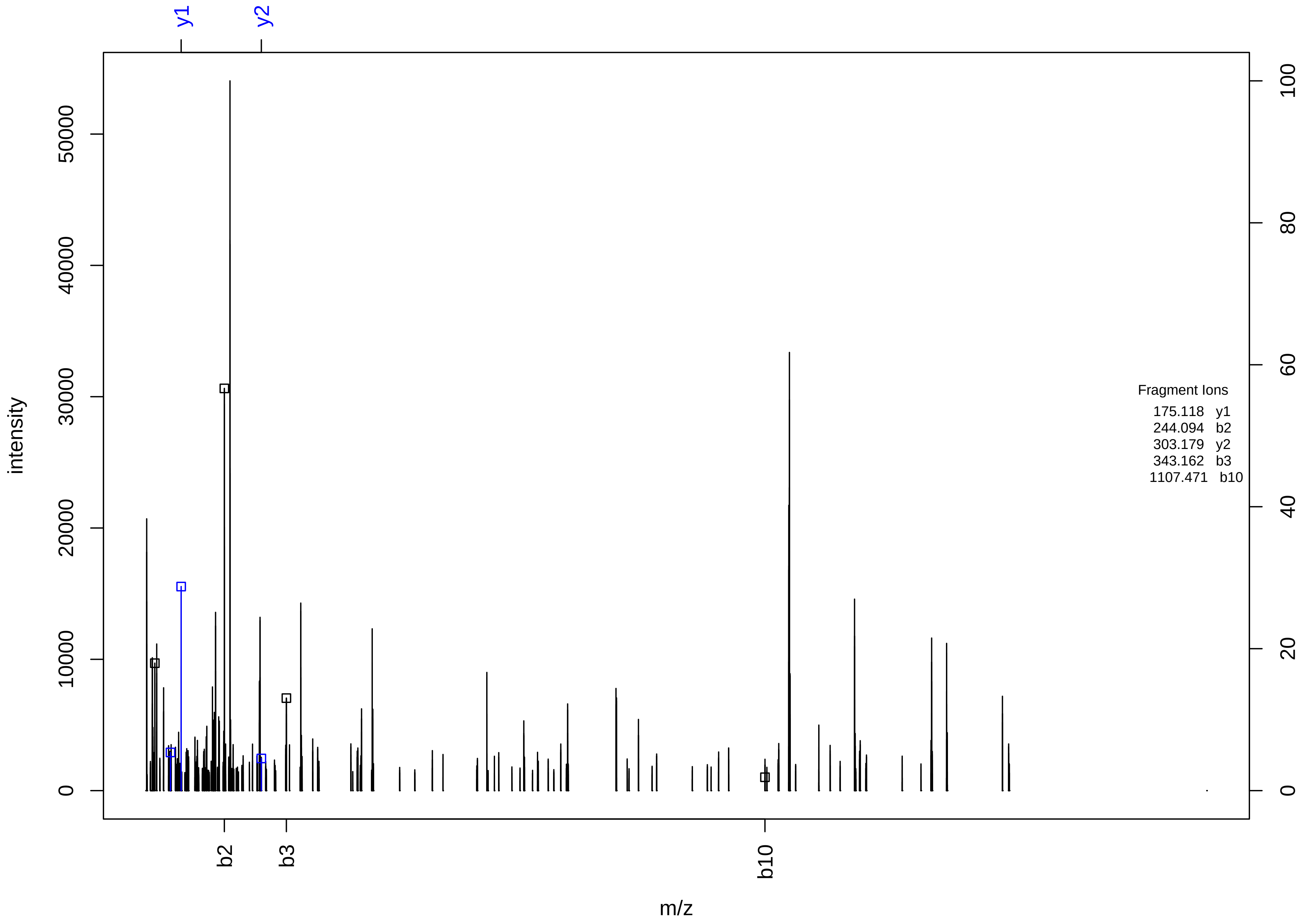

DQVFGCQLESLCQR / File: sydney_06.raw; SpectrumID: ; scans: 7832

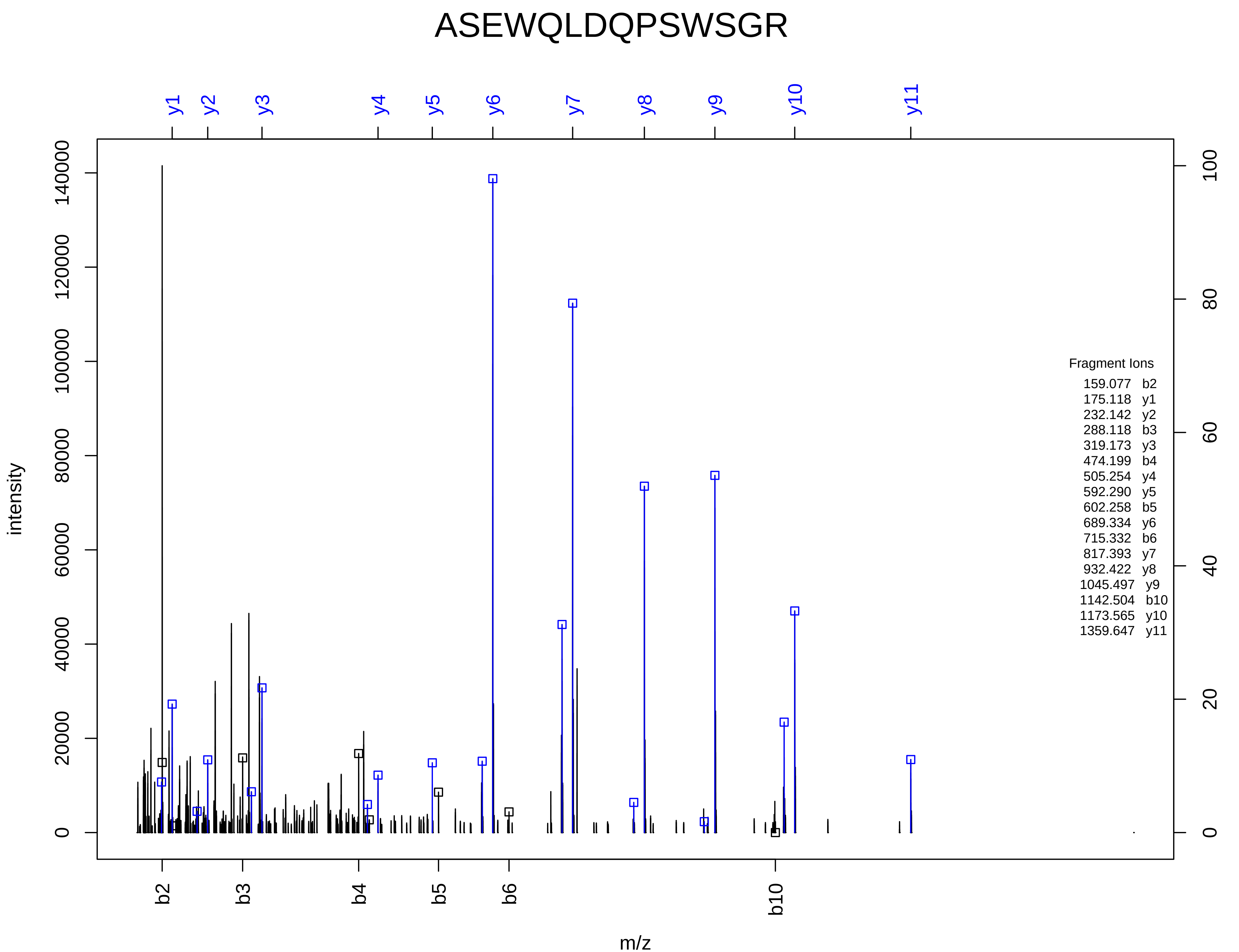

ASEWQLDQPSWSGR
y1
y2
y3
y4
y5
y6
y7
y8
y9
y10
y11
intensity
0
20000
40000
60000
80000
100000
120000
140000
0
20
40
60
80
100
b2
b3
b4
b5
b6
b10
m/z
Fragment Ions
159.077 b2
175.118 y1
232.142 y2
288.118 b3
319.173 y3
474.199 b4
505.254 y4
592.290 y5
602.258 b5
689.334 y6
715.332 b6
817.393 y7
932.422 y8
1045.497 y9
1142.504 b10
1173.565 y10
1359.647 y11
ASEWQLDQPSWSGR / File: sydney_06.raw; SpectrumID: ; scans: 6737

(Ac)M*PKPTHYYIK

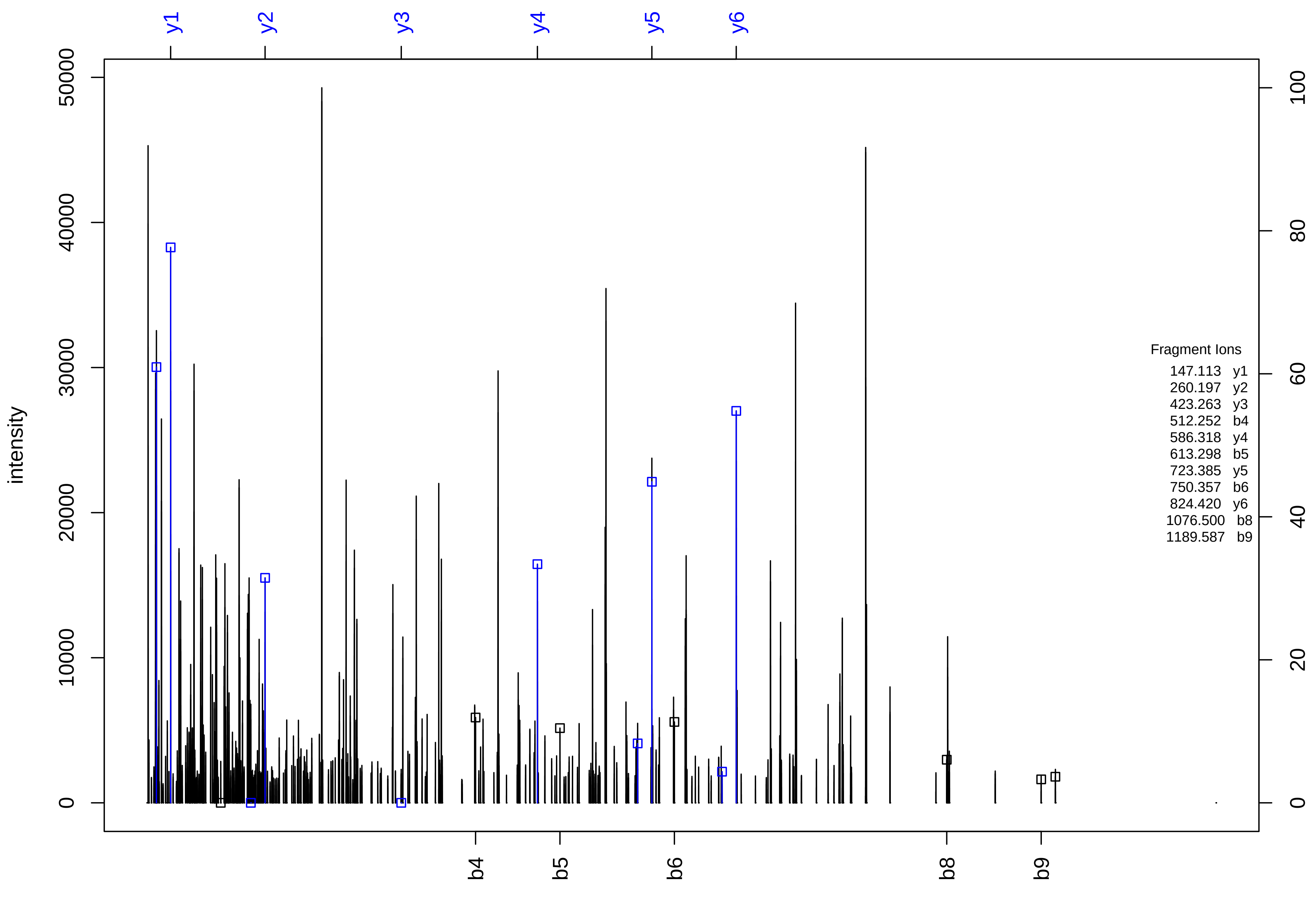

MPKPTHYYIK / File: sydney_06.raw; SpectrumID: ; scans: 8603

LPSSVFASEFEEDVGLLNK

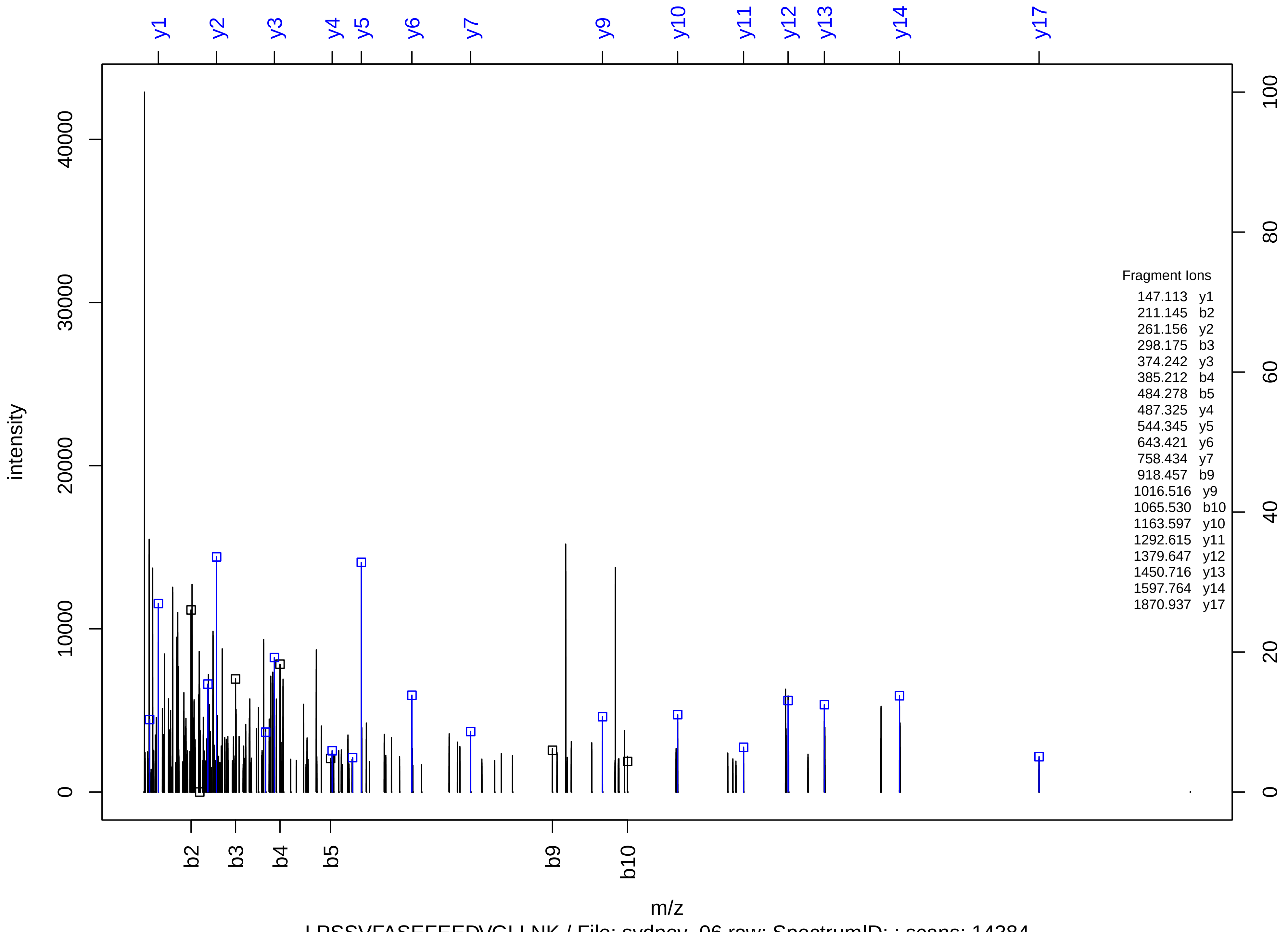

LPSSVFASEFEEDVGLLNK / File: sydney_06.raw; SpectrumID: ; scans: 14384

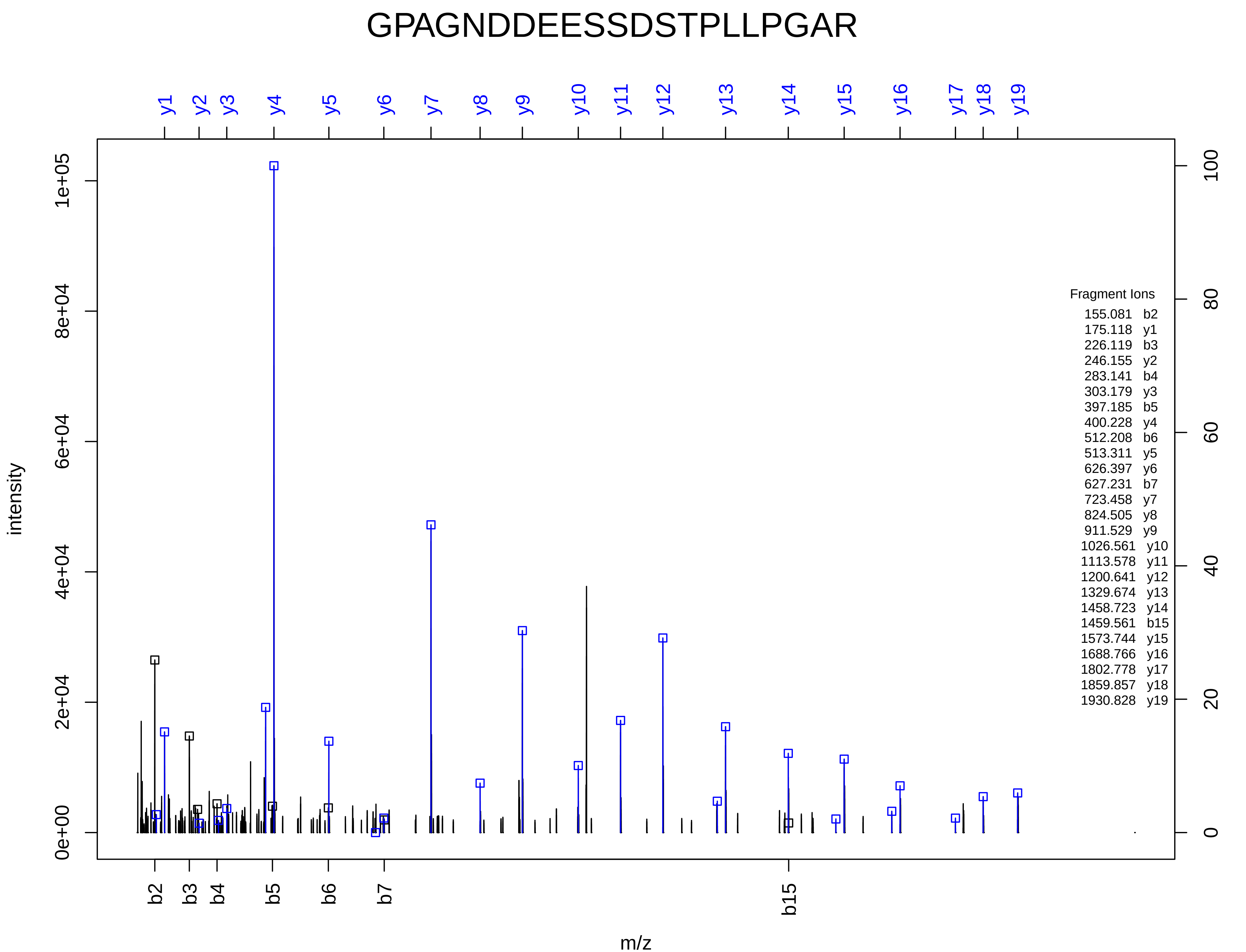
GPAGNDDEESSDSTPLLPGAR
y1 y2 y3 y4 y5 y6 y7 y8 y9 y10 y11 y12 y13 y14 y15 y16 y17 y18 y19
b2 b3 b4 b5 b6 b7 b15
intensity
m/z
0e+00
2e+04
4e+04
6e+04
8e+04
1e+05
0
20
40
60
80
100
Fragment Ions
155.081 b2
175.118 y1
226.119 b3
246.155 y2
283.141 b4
303.179 y3
397.185 b5
400.228 y4
512.208 b6
513.311 y5
626.397 y6
627.231 b7
723.458 y7
824.505 y8
911.529 y9
1026.561 y10
1113.578 y11
1200.641 y12
1329.674 y13
1458.723 y14
1459.561 b15
1573.744 y15
1688.766 y16
1802.778 y17
1859.857 y18
1930.828 y19
GPAGNDDEESSDSTPLLPGAR / File: sydney_06.raw; SpectrumID: ; scans: 4612

(Ac)M*DDSEKDFN^LK

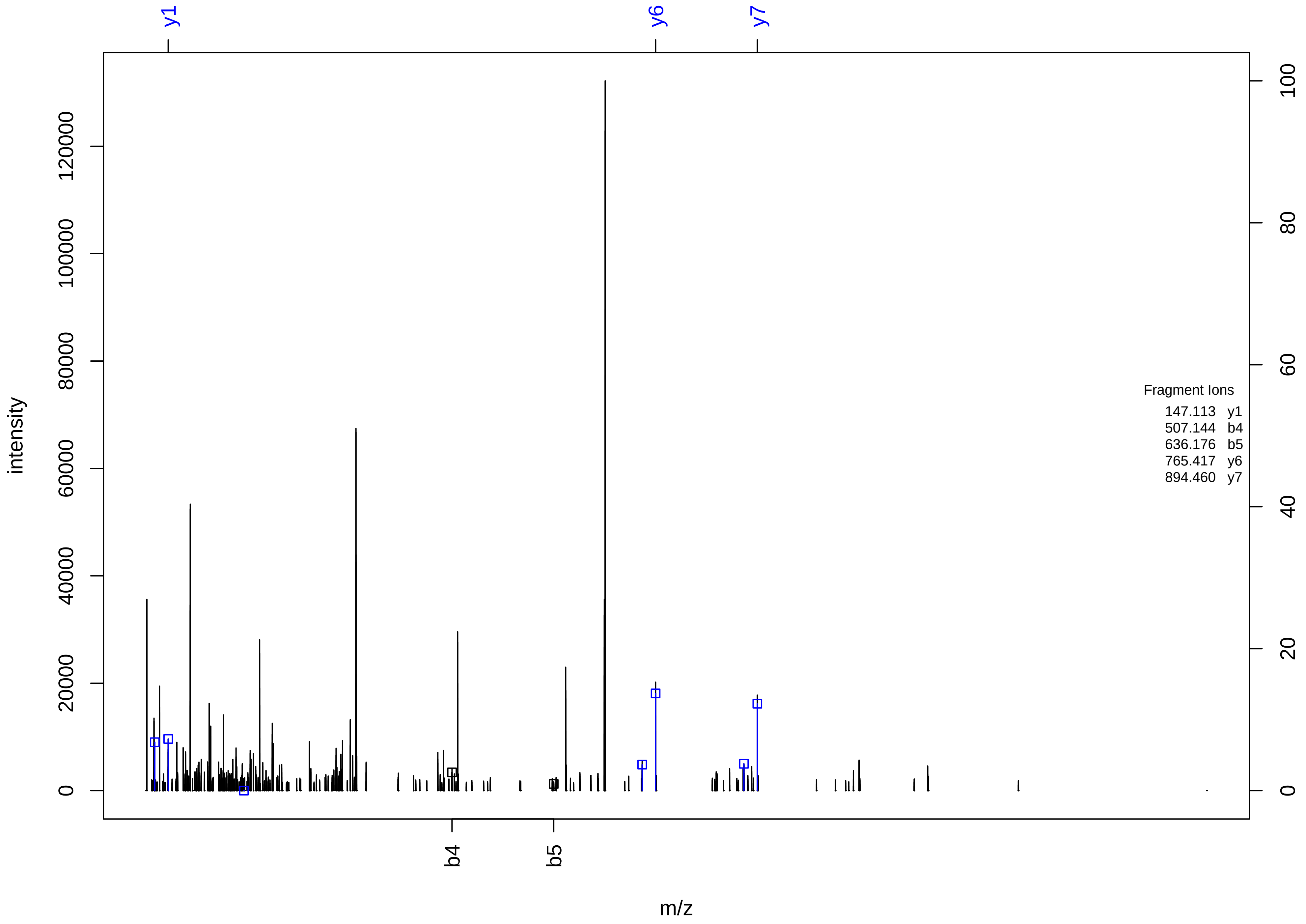

MDDSEKDFNLK / File: sydney_06.raw; SpectrumID: ; scans: 4142

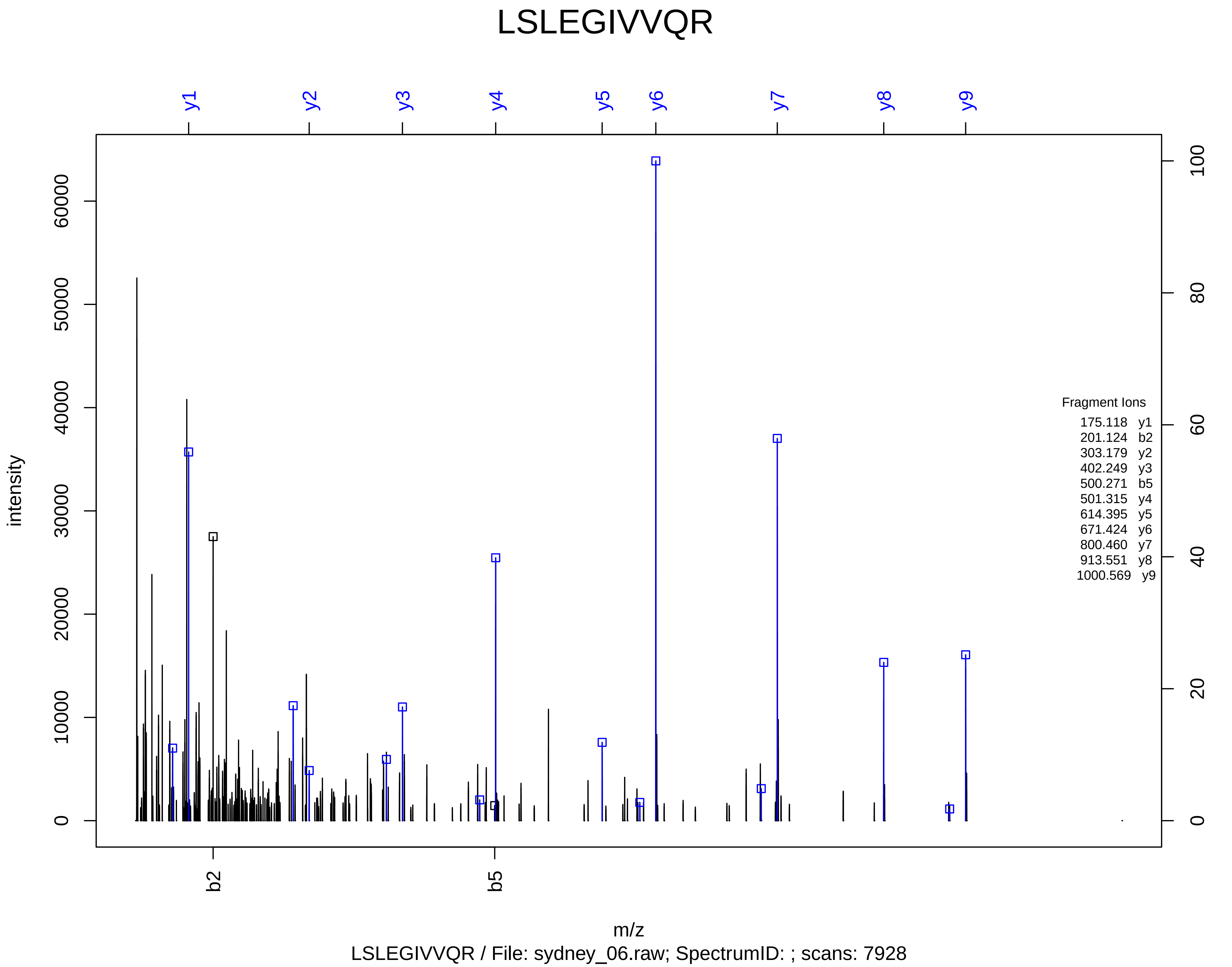
LSLEGIVVQR
y1
y2
y3
y4
y5
y6
y7
y8
y9
intensity
0
10000
20000
30000
40000
50000
60000
0
20
40
60
80
100
b2
b5
m/z
Fragment Ions
175.118 y1
201.124 b2
303.179 y2
402.249 y3
500.271 b5
501.315 y4
614.395 y5
671.424 y6
800.460 y7
913.551 y8
1000.569 y9
LSLEGIVVQR / File: sydney_06.raw; SpectrumID: ; scans: 7928

SSVDSQGPTPVCTPTFLER

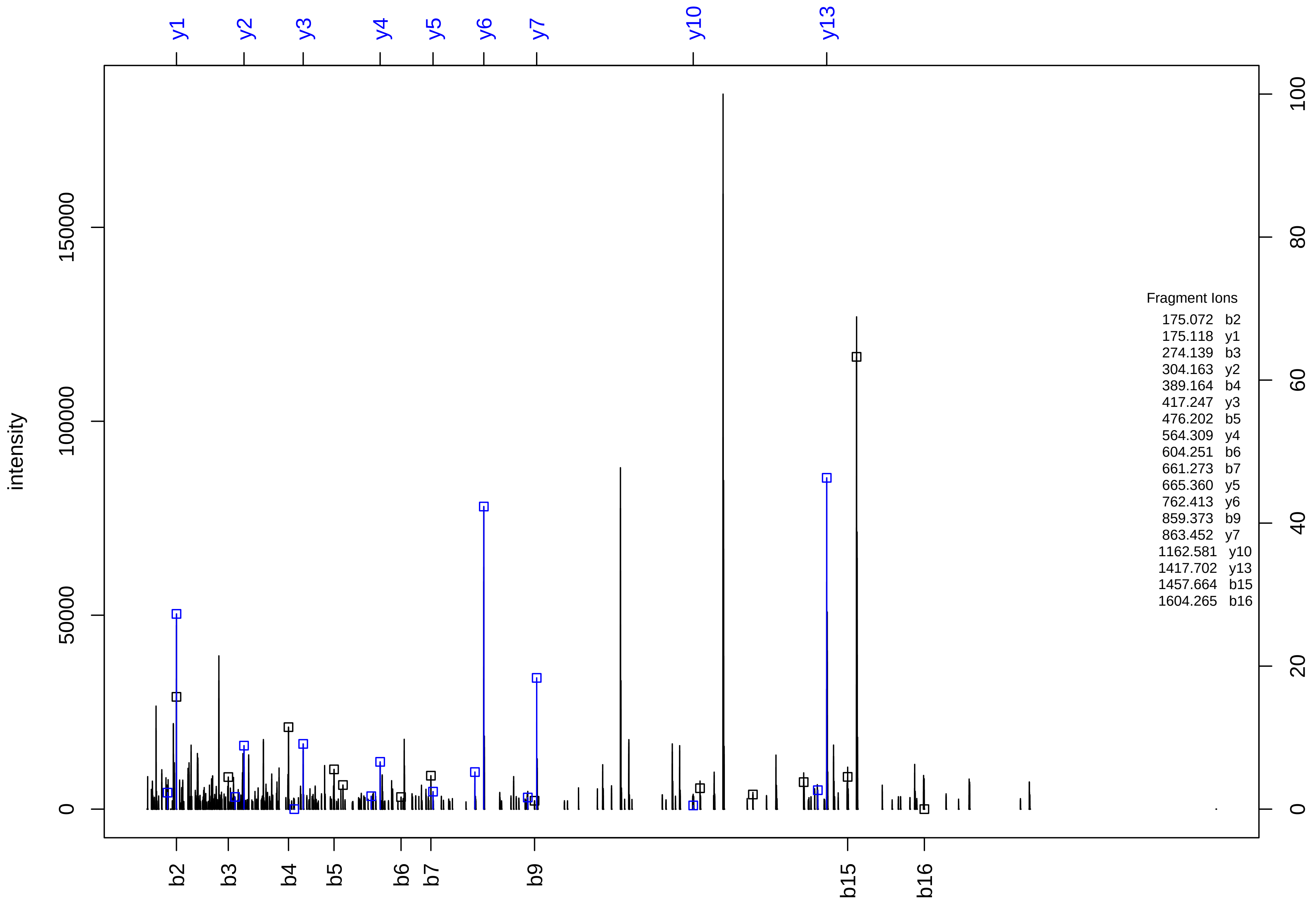

SSVDSQGPTPVCTPTFLER / File: sydney_06.raw; SpectrumID: ; scans: 7511

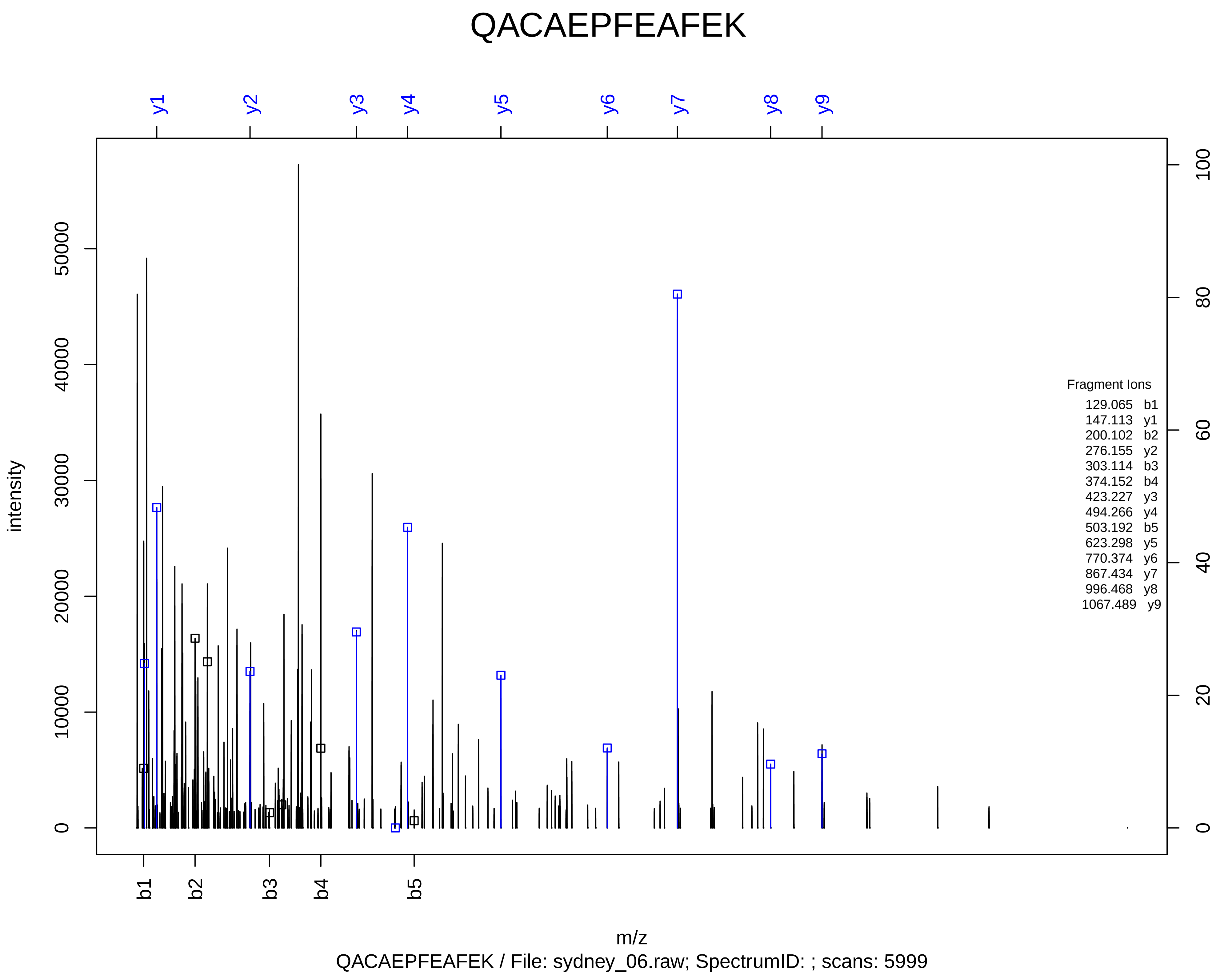
QACAEPFEAFEK
y1
y2
y3
y4
y5
y6
y7
y8
y9
b1
b2
b3
b4
b5
intensity
m/z
Fragment Ions
129.065 b1
147.113 y1
200.102 b2
276.155 y2
303.114 b3
374.152 b4
423.227 y3
494.266 y4
503.192 b5
623.298 y5
770.374 y6
867.434 y7
996.468 y8
1067.489 y9
QACAEPFEAFEK / File: sydney_06.raw; SpectrumID: ; scans: 5999

LQQGLAANPSGYGPLTELPDWSFADGRPAPPMK

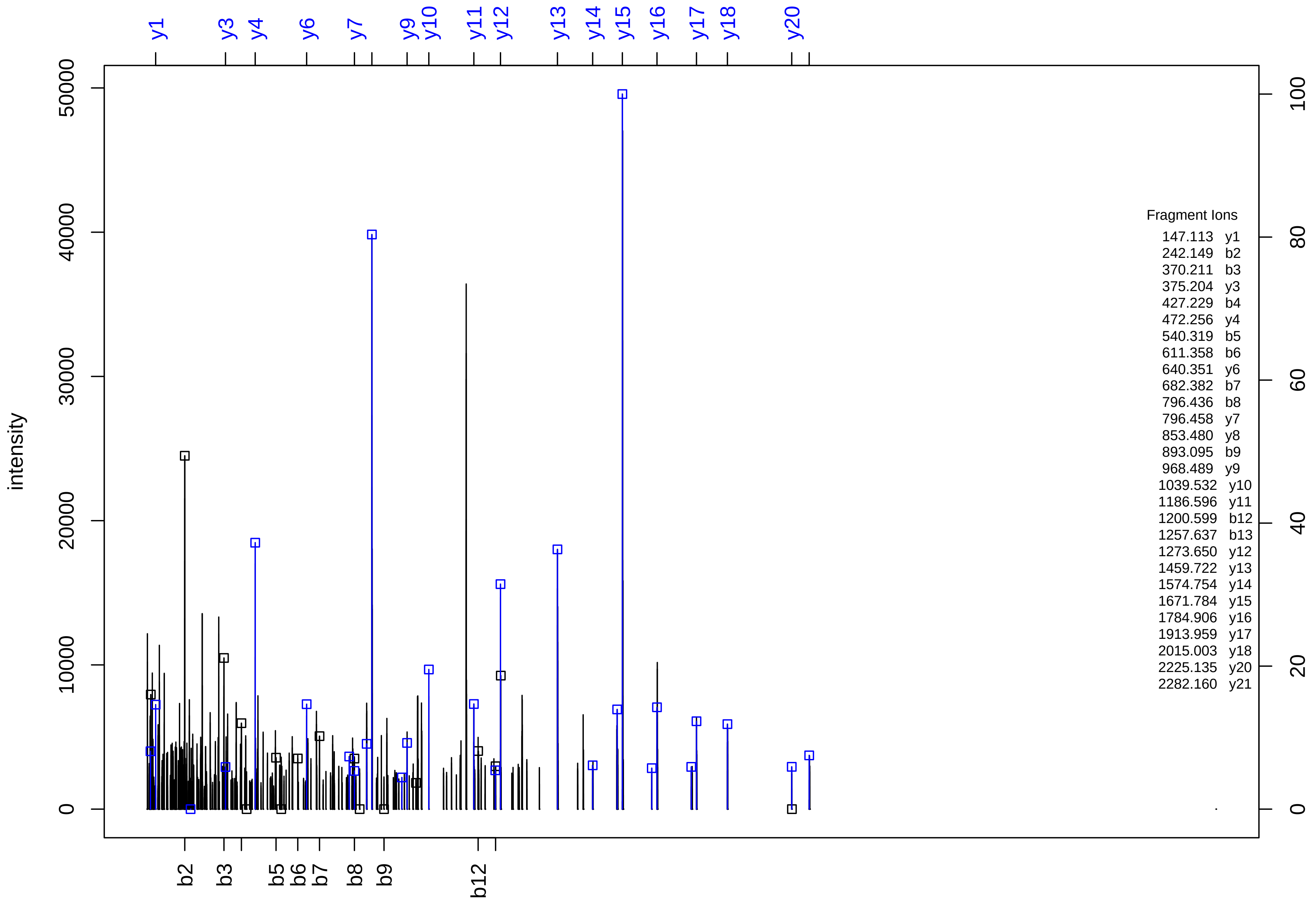

LQQGLAANPSGYGPLTELPDWSFADGRPAPPMK / File: sydney_06.raw; SpectrumID: ; scans: 12357

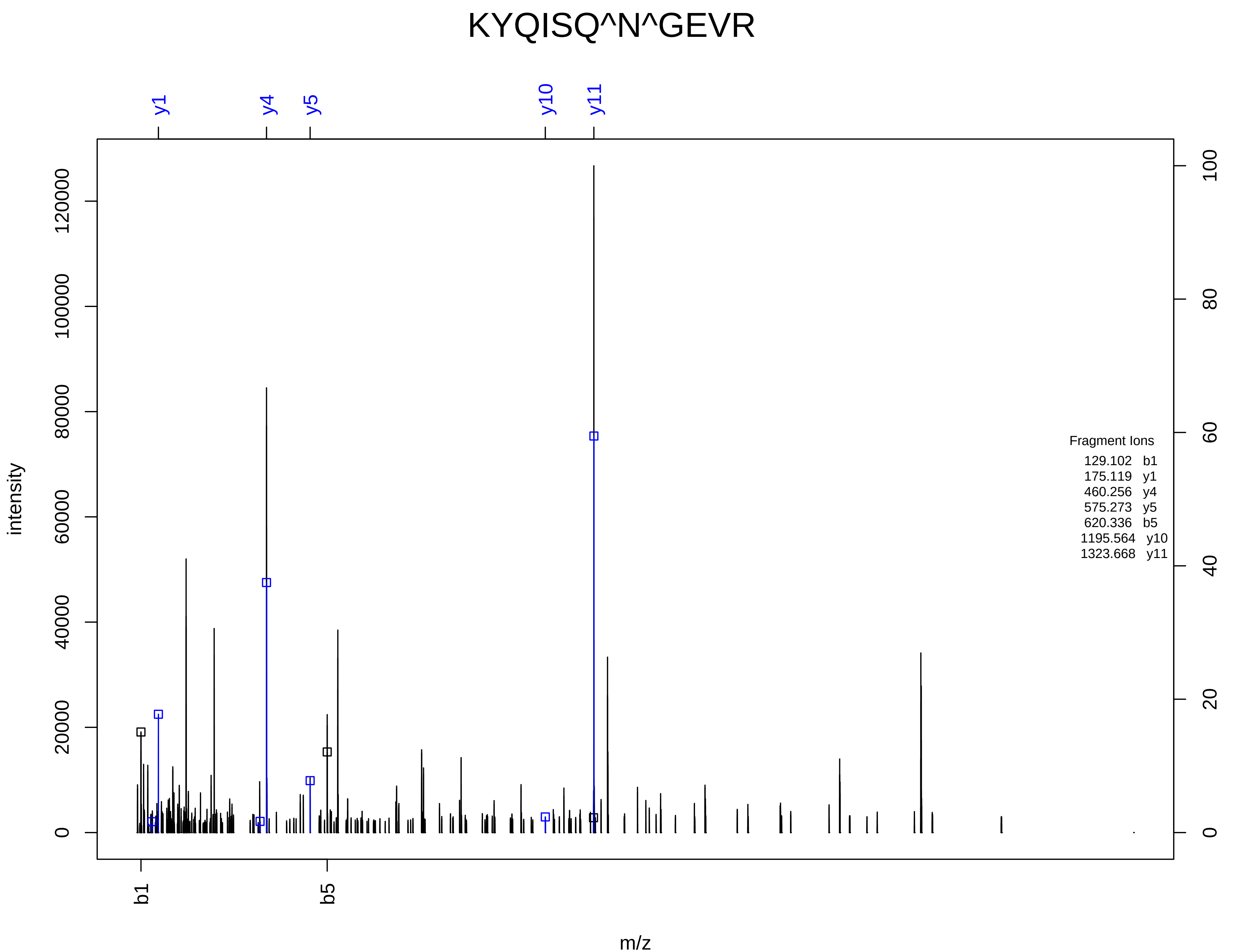

KYQISQ^N^GEVR
y1
y4
y5
y10
y11
intensity
0
20000
40000
60000
80000
100000
120000
0
20
40
60
80
100
b1
b5
m/z
Fragment Ions
129.102 b1
175.119 y1
460.256 y4
575.273 y5
620.336 b5
1195.564 y10
1323.668 y11
KYQISQNGEVR / File: sydney_07.raw; SpectrumID: ; scans: 10587

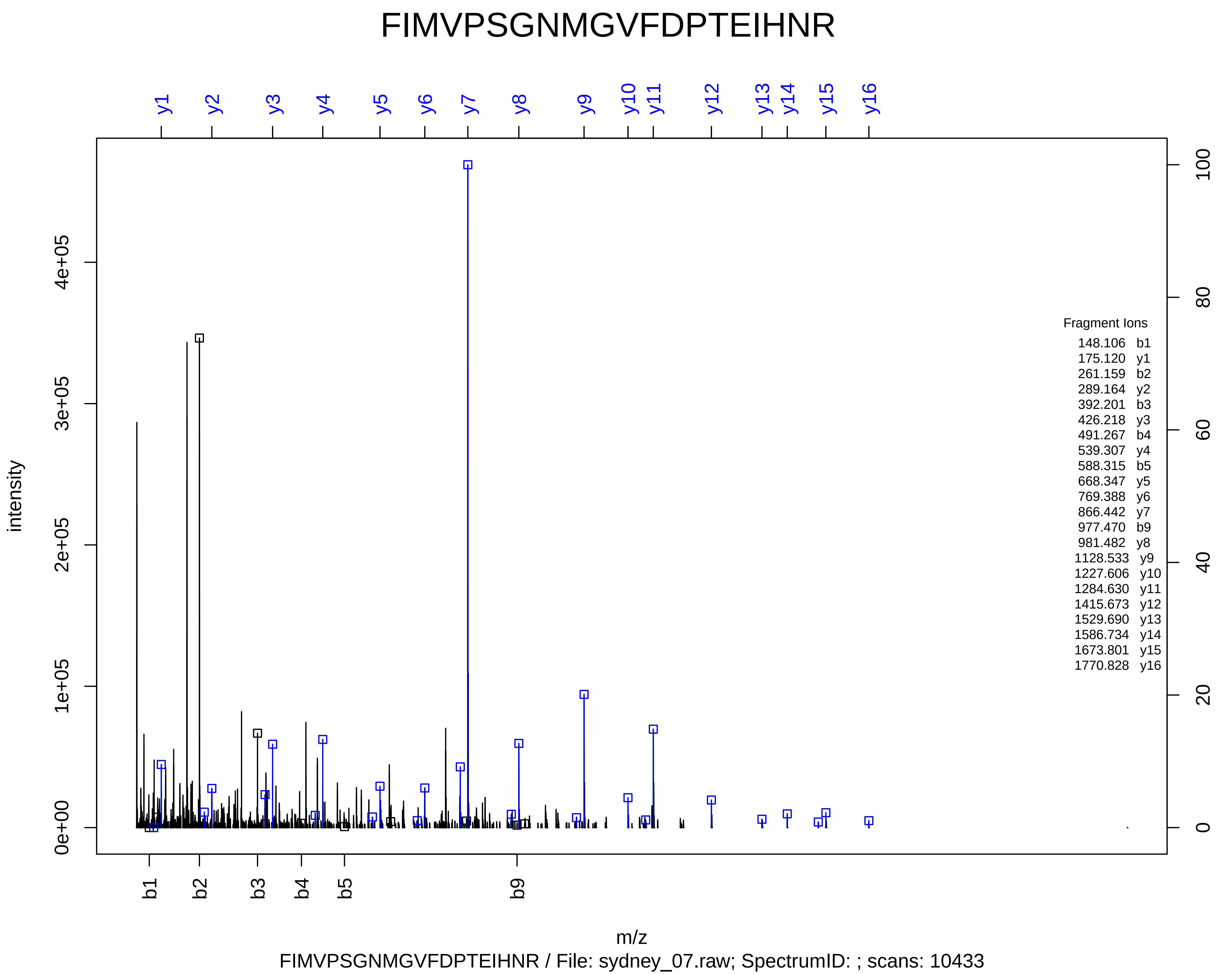

FIMVPSGNMGVFDPTEIHNR
y1 y2 y3 y4 y5 y6 y7 y8 y9 y10 y11 y12 y13 y14 y15 y16
b1 b2 b3 b4 b5 b9
intensity
m/z
Fragment Ions
148.106 b1
175.120 y1
261.159 b2
289.164 y2
392.201 b3
426.218 y3
491.267 b4
539.307 y4
588.315 b5
668.347 y5
769.388 y6
866.442 y7
977.470 b9
981.482 y8
1128.533 y9
1227.606 y10
1284.630 y11
1415.673 y12
1529.690 y13
1586.734 y14
1673.801 y15
1770.828 y16
FIMVPSGNMGVFDPTEIHNR / File: sydney_07.raw; SpectrumID: ; scans: 10433

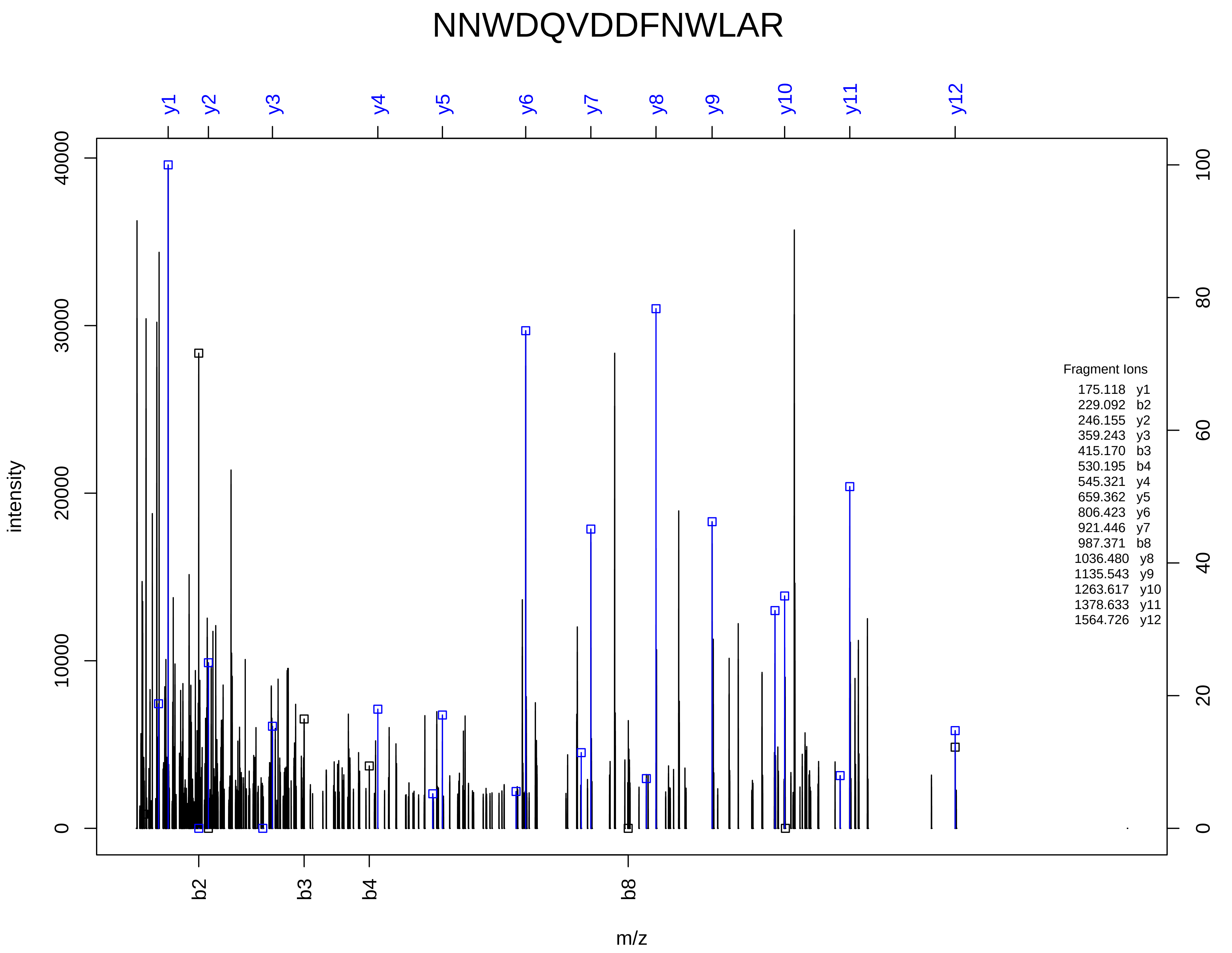
NNWDQVDDFNWLAR
y1
y2
y3
y4
y5
y6
y7
y8
y9
y10
y11
y12
b2
b3
b4
b8
intensity
m/z
Fragment Ions
175.118 y1
229.092 b2
246.155 y2
359.243 y3
415.170 b3
530.195 b4
545.321 y4
659.362 y5
806.423 y6
921.446 y7
987.371 b8
1036.480 y8
1135.543 y9
1263.617 y10
1378.633 y11
1564.726 y12
NNWDQVDDFNWLAR / File: sydney_07.raw; SpectrumID: ; scans: 11654

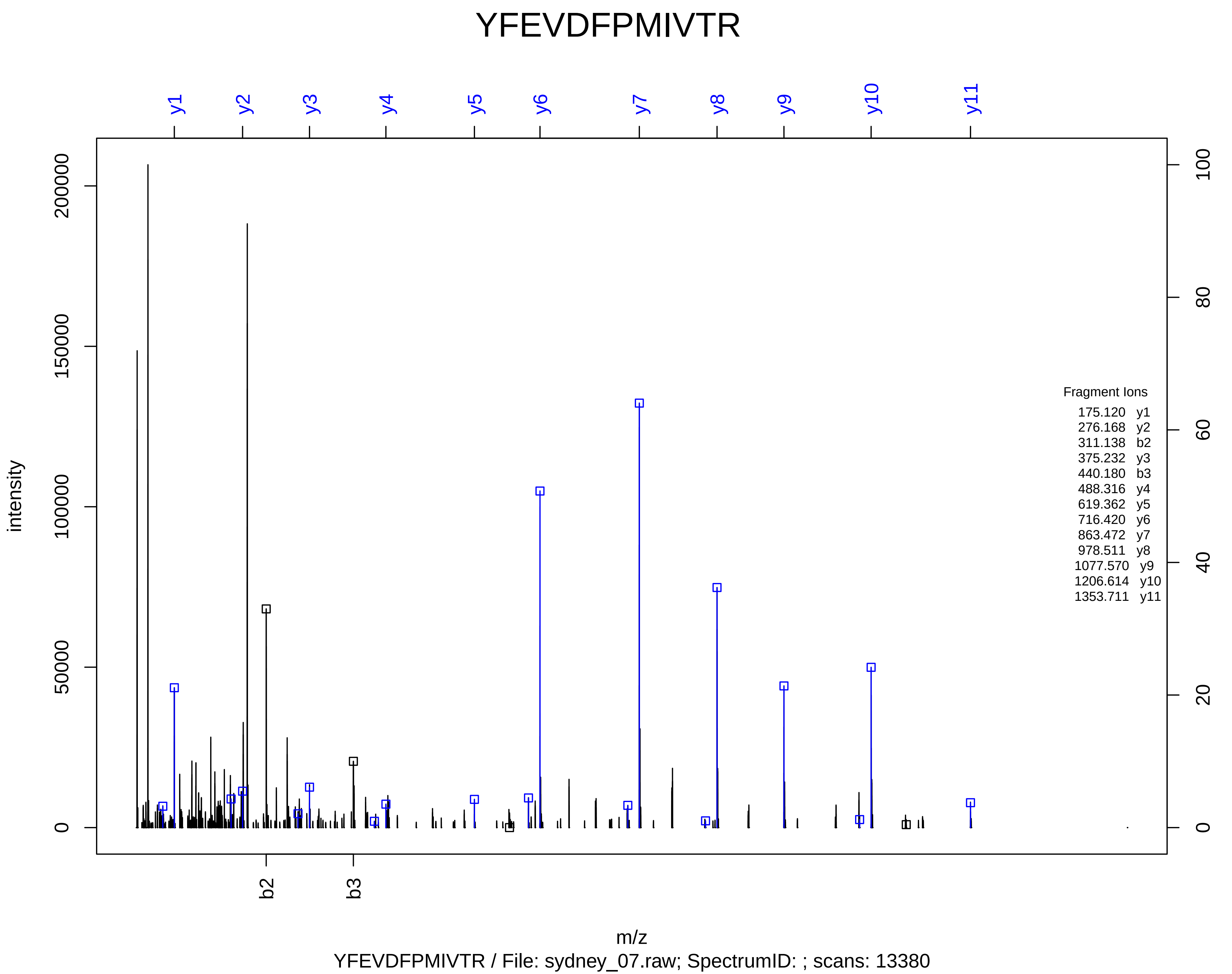
YFEVDFPMIVTR
y1
y2
y3
y4
y5
y6
y7
y8
y9
y10
y11
intensity
0
50000
100000
150000
200000
0
20
40
60
80
100
b2
b3
m/z
Fragment Ions
175.120 y1
276.168 y2
311.138 b2
375.232 y3
440.180 b3
488.316 y4
619.362 y5
716.420 y6
863.472 y7
978.511 y8
1077.570 y9
1206.614 y10
1353.711 y11
YFEVDFPMIVTR / File: sydney_07.raw; SpectrumID: ; scans: 13380

(Ac)M*KDRLAELQ^ELSR

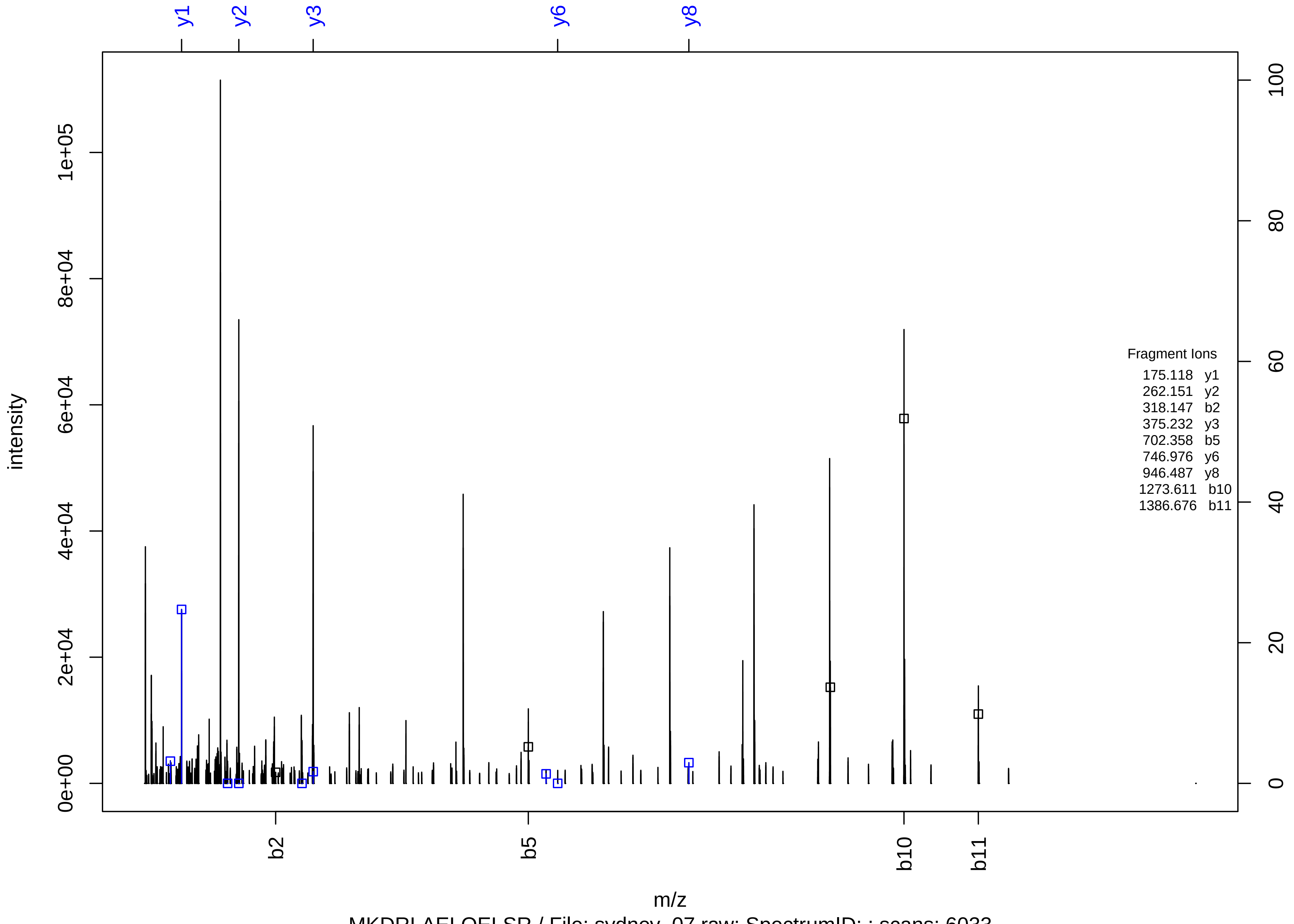

MKDRLAELQELSR / File: sydney_07.raw; SpectrumID: ; scans: 6033

GFFNALHSELGQYPGITFCNVYPGPVQSDIVK

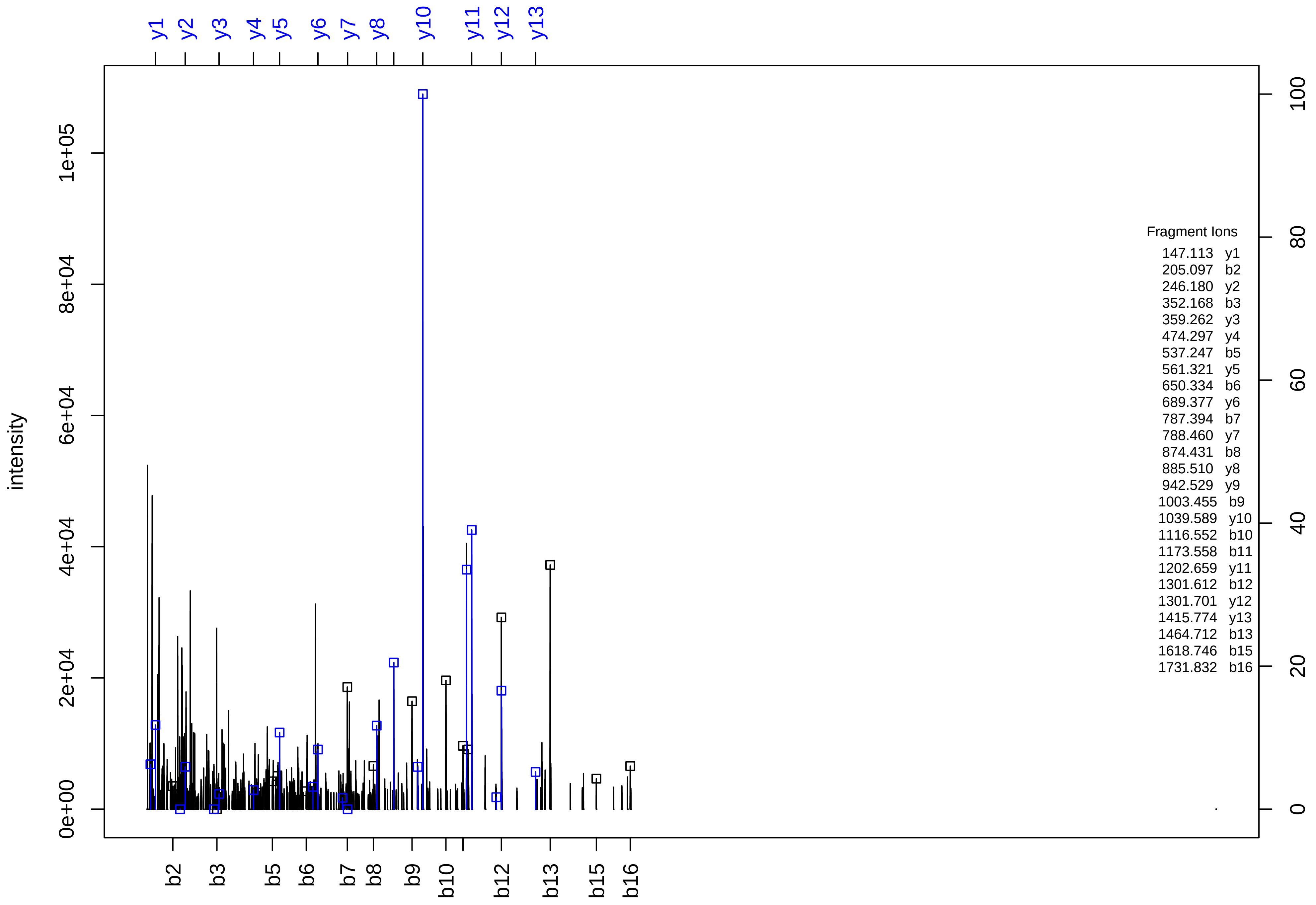

GFFNALHSELGQYPGITFCNVYPGPVQSDIVK / File: sydney_07.raw; SpectrumID: ; scans: 13874

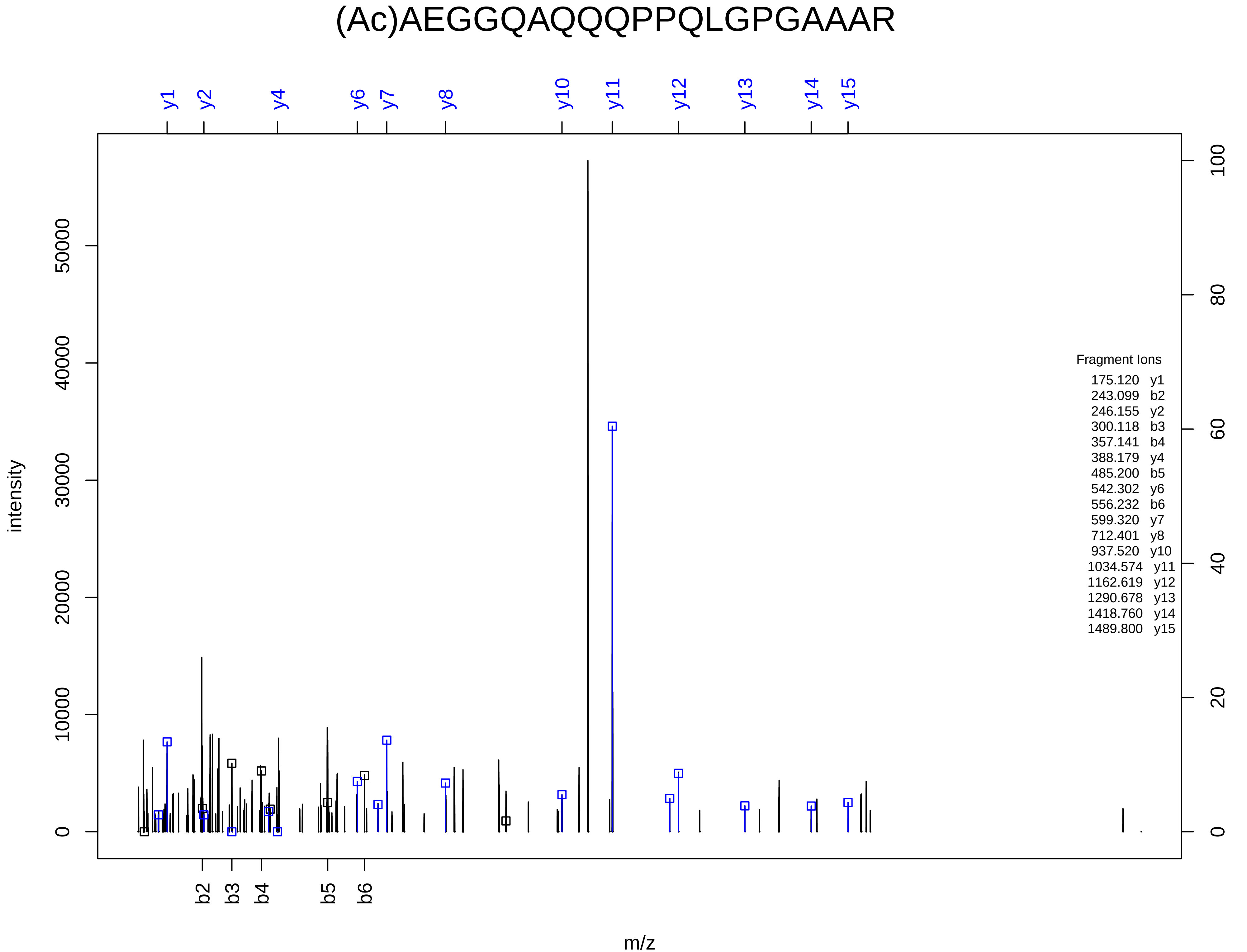
(Ac)AEGGQAQQQPPQLGPGAAAR
y1
y2
y4
y6
y7
y8
y10
y11
y12
y13
y14
y15
intensity
0
10000
20000
30000
40000
50000
0
20
40
60
80
100
b2
b3
b4
b5
b6
Fragment Ions
175.120 y1
243.099 b2
246.155 y2
300.118 b3
357.141 b4
388.179 y4
485.200 b5
542.302 y6
556.232 b6
599.320 y7
712.401 y8
937.520 y10
1034.574 y11
1162.619 y12
1290.678 y13
1418.760 y14
1489.800 y15
m/z
AEGGQAQQQPPQLGPGAAAR / File: sydney_07.raw; SpectrumID: ; scans: 3446

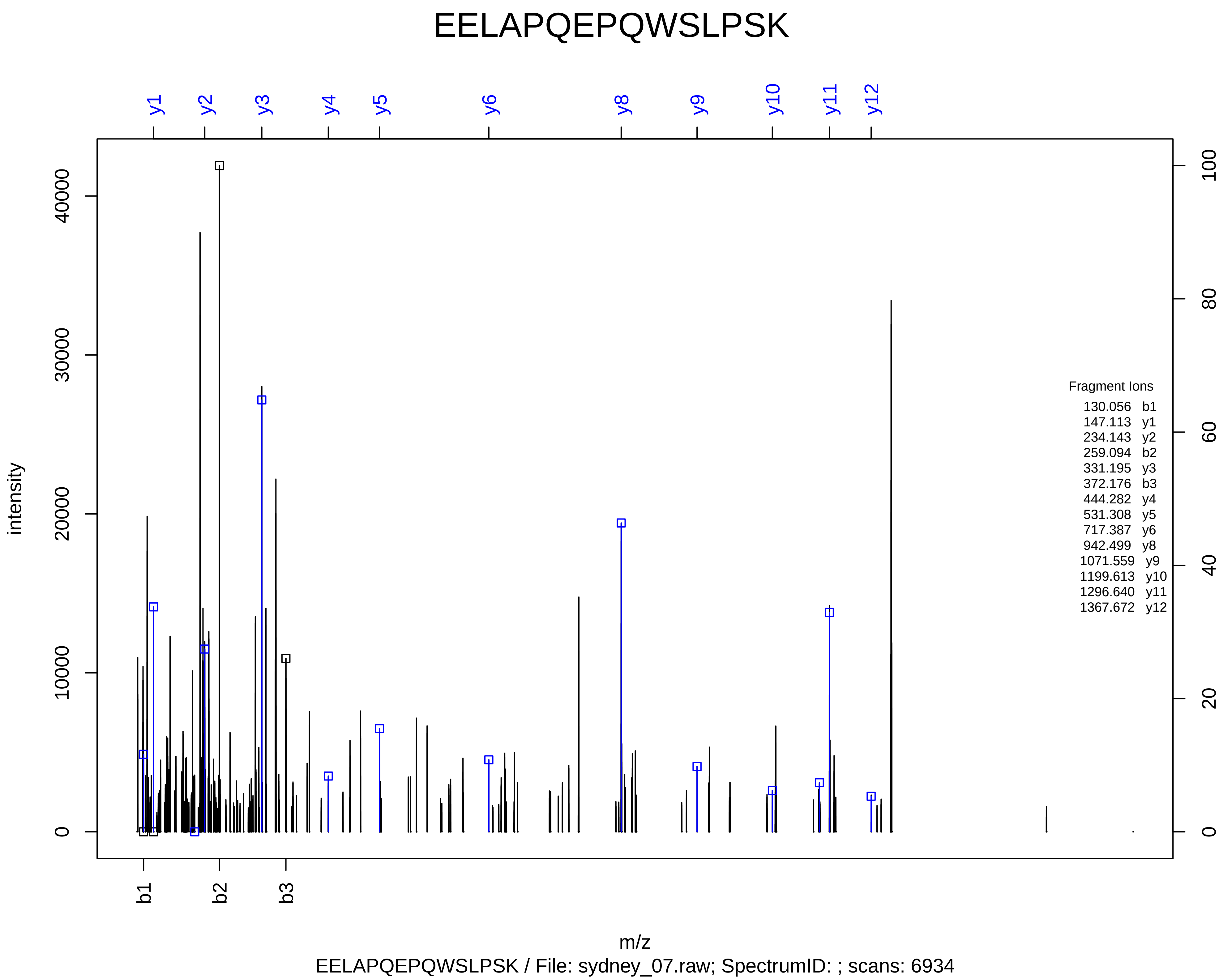
EELAPQEPQWSLPSK
y1 y2 y3 y4 y5 y6 y8 y9 y10 y11 y12
b1 b2 b3
intensity
m/z
0 10000 20000 30000 40000
0 20 40 60 80 100
Fragment Ions
130.056 b1
147.113 y1
234.143 y2
259.094 b2
331.195 y3
372.176 b3
444.282 y4
531.308 y5
717.387 y6
942.499 y8
1071.559 y9
1199.613 y10
1296.640 y11
1367.672 y12
EELAPQEPQWSLPSK / File: sydney_07.raw; SpectrumID: ; scans: 6934

ENDPSNVLLFLVGSK

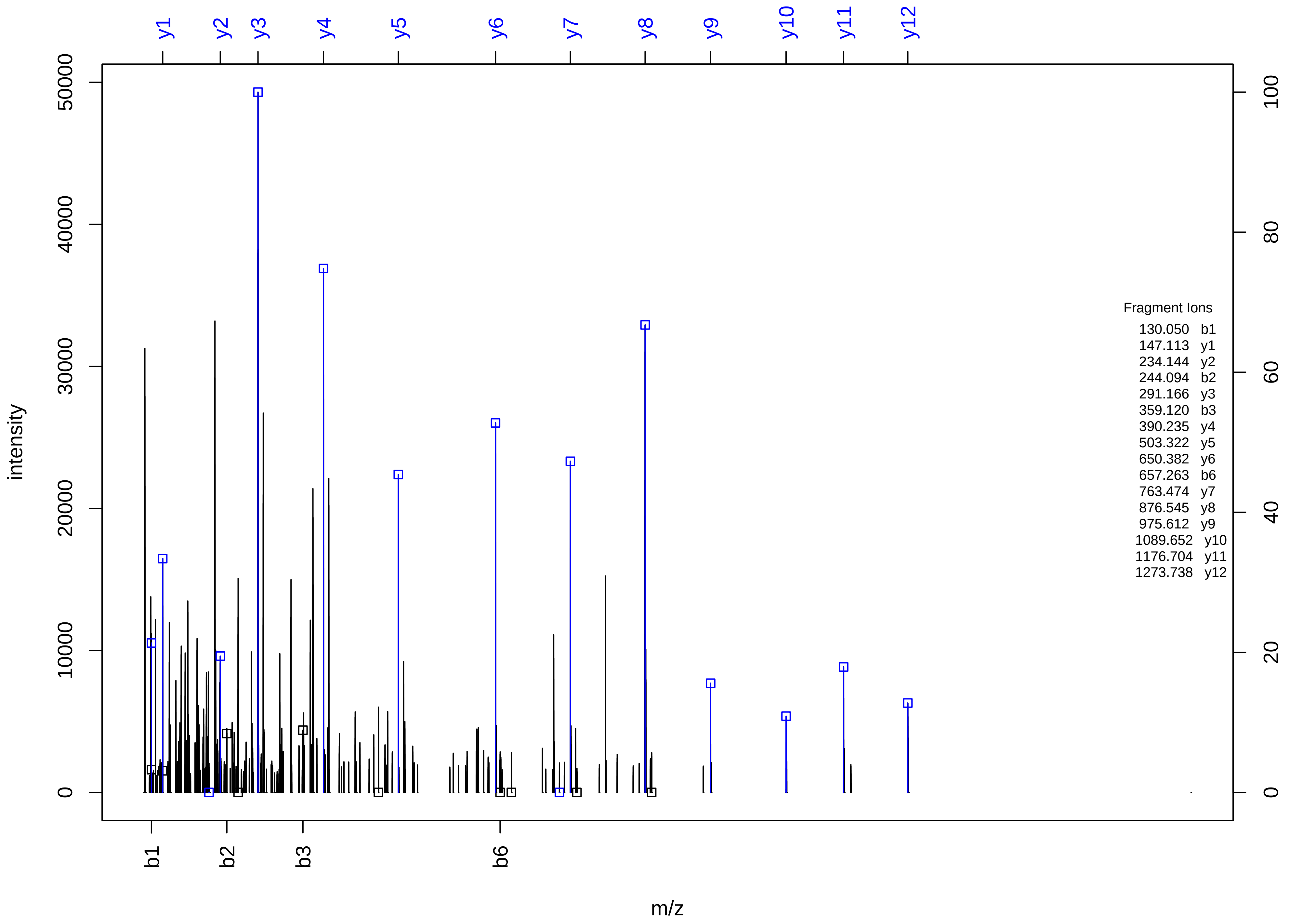

ENDPSNVLLFLVGSK / File: sydney_07.raw; SpectrumID: ; scans: 14581

SFKELHQ^IQ^ETERN^K

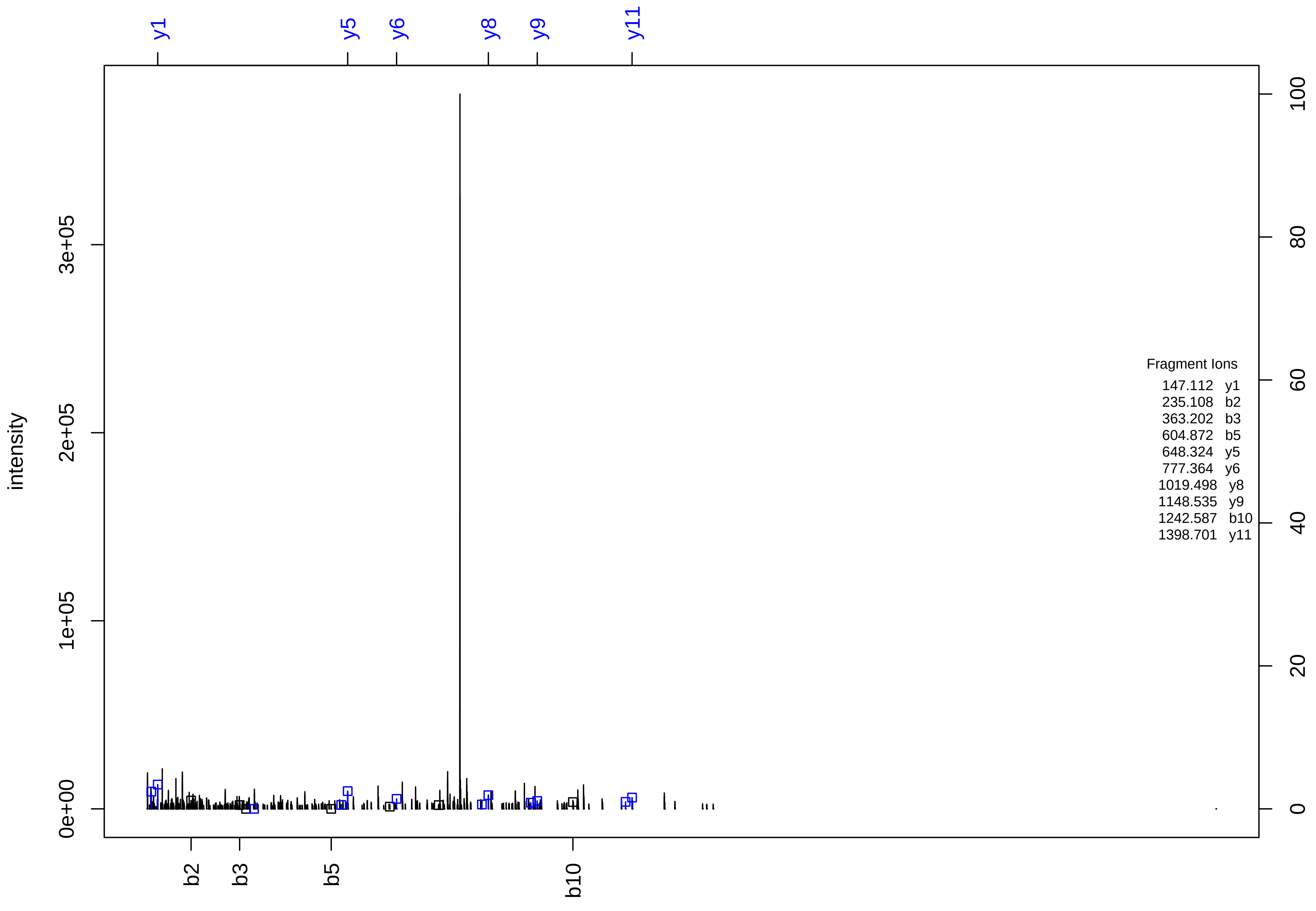

SFKELHQIQETERNK / File: sydney_07.raw; SpectrumID: ; scans: 5653

RPAGPEPQPPAAGSDGAAEDADEELADGEDRDPEAEEPLWASR

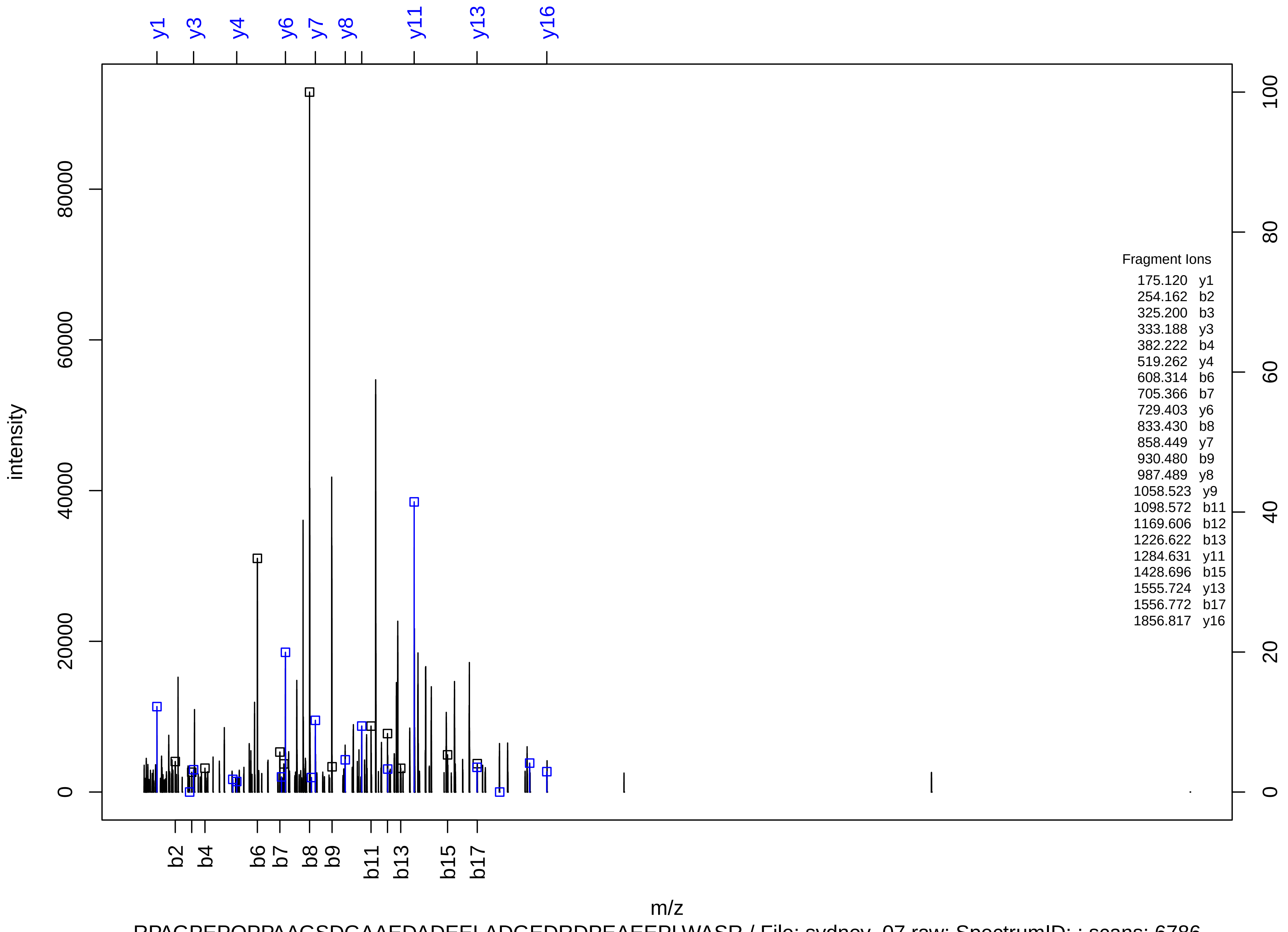

RPAGPEPQPPAAGSDGAAEDADEELADGEDRDPEAEEPLWASR / File: sydney_07.raw; SpectrumID: ; scans: 6786

DVTRPTITTSDEPDTLYK

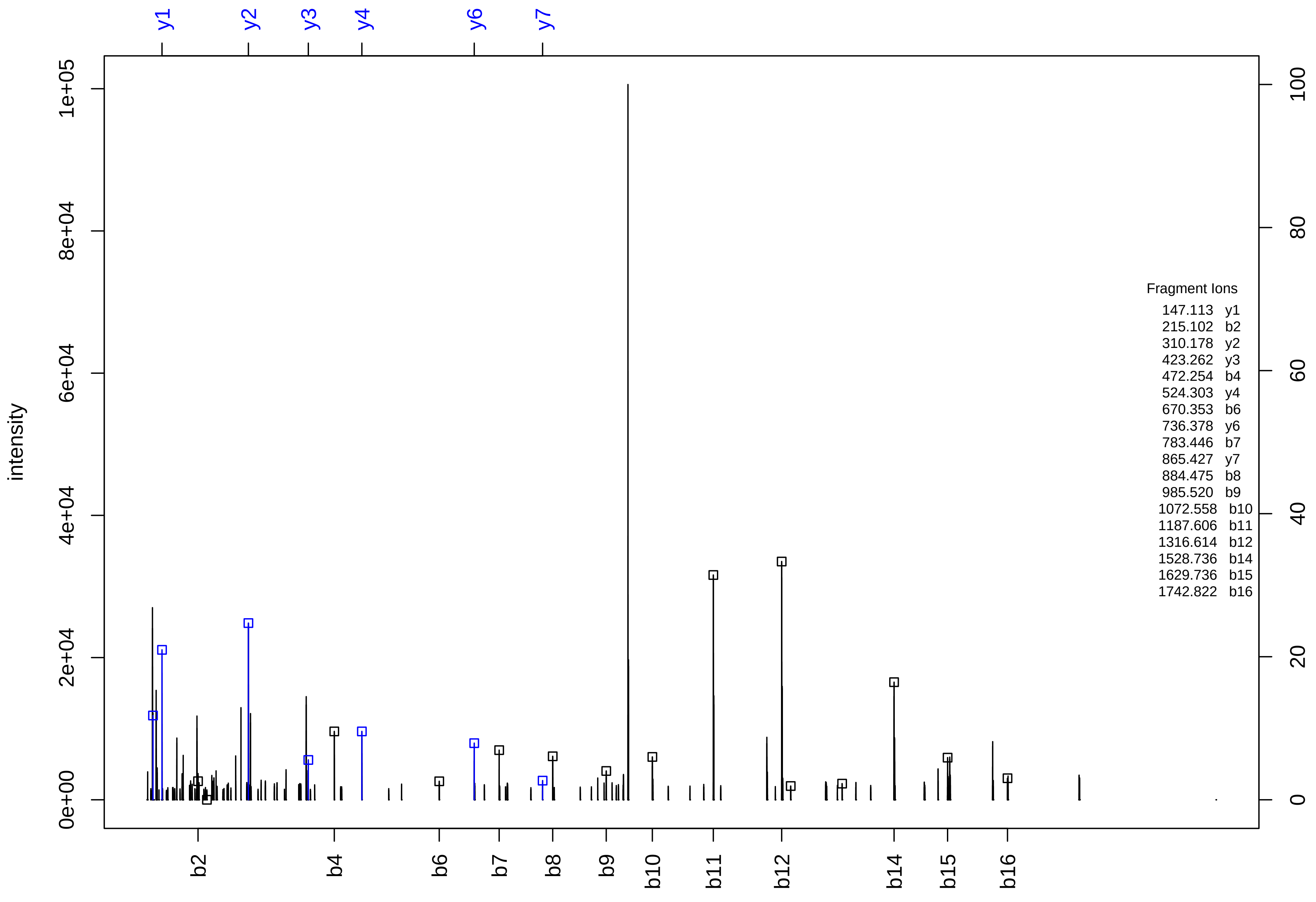

DVTRPTITTSDEPDTLYK / File: sydney_07.raw; SpectrumID: ; scans: 4334

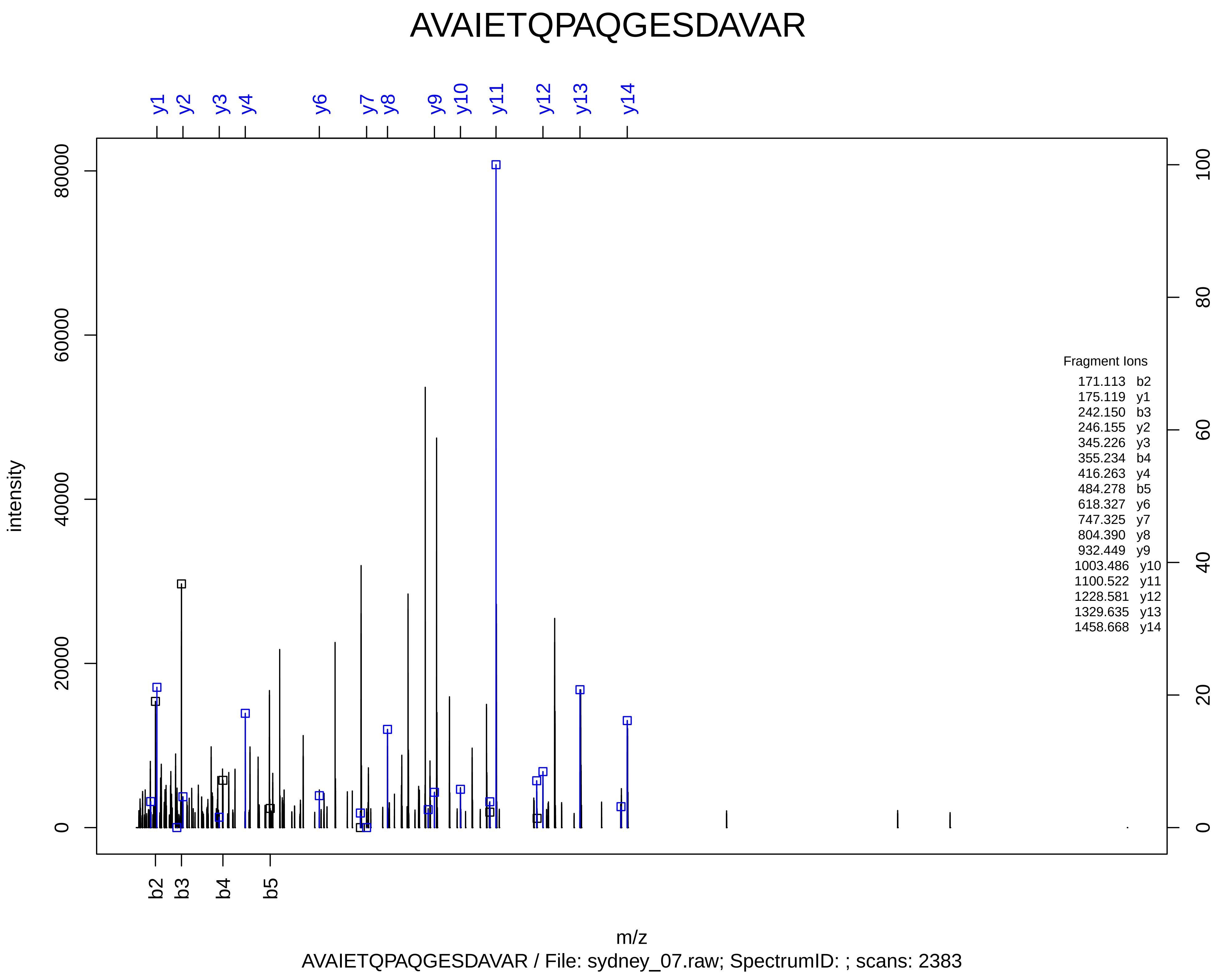
AVAIETQPAQGESDAVAR
y1 y2 y3 y4 y6 y7 y8 y9 y10 y11 y12 y13 y14
b2 b3 b4 b5
intensity
m/z
Fragment Ions
171.113 b2
175.119 y1
242.150 b3
246.155 y2
345.226 y3
355.234 b4
416.263 y4
484.278 b5
618.327 y6
747.325 y7
804.390 y8
932.449 y9
1003.486 y10
1100.522 y11
1228.581 y12
1329.635 y13
1458.668 y14
AVAIETQPAQGESDAVAR / File: sydney_07.raw; SpectrumID: ; scans: 2383

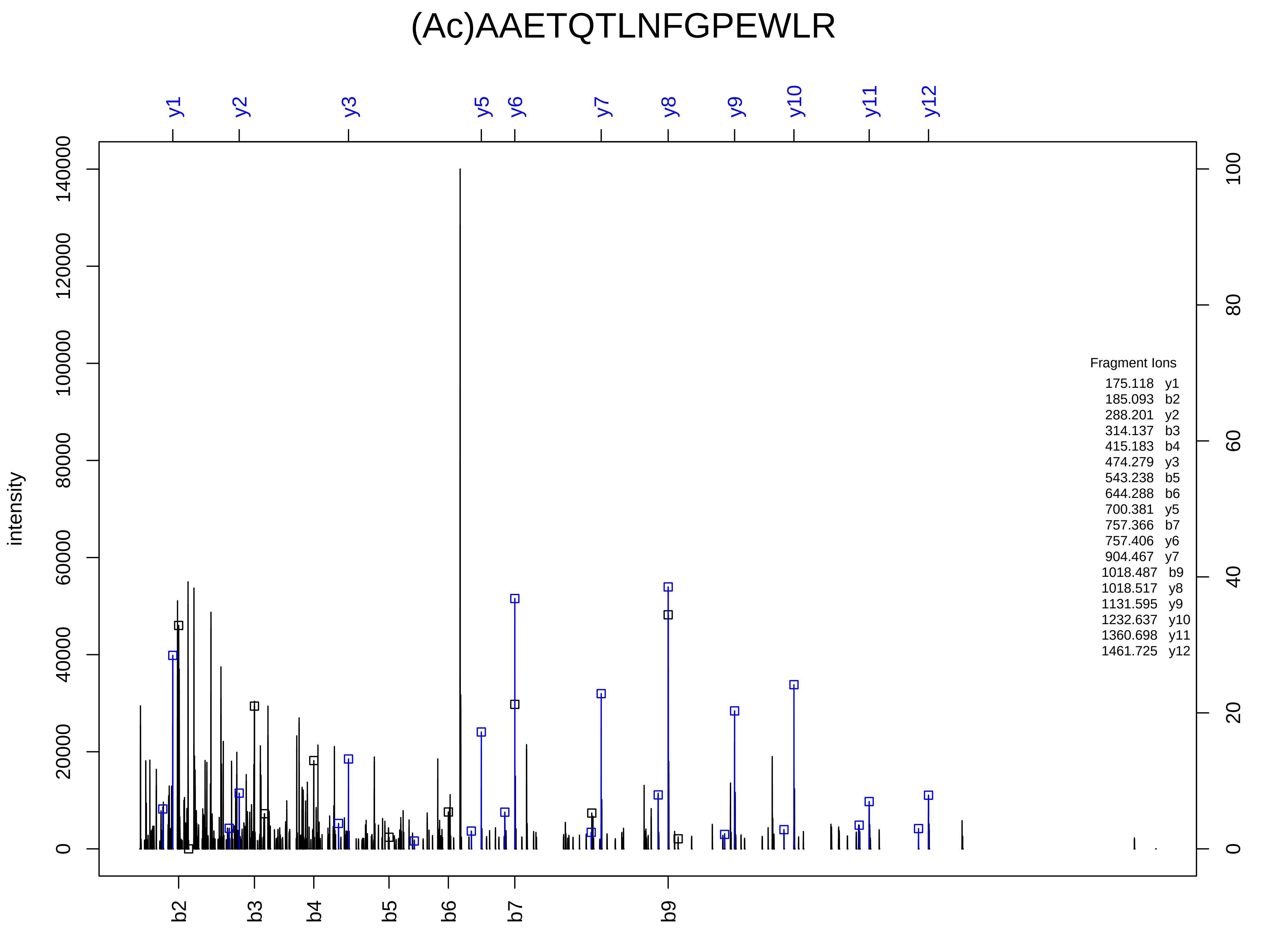
(Ac)AAETQTLNFGPEWLR
y1 y2 y3 y5 y6 y7 y8 y9 y10 y11 y12
b2 b3 b4 b5 b6 b7 b9
intensity
0 20000 40000 60000 80000 100000 120000 140000
0 20 40 60 80 100
m/z
Fragment Ions
175.118 y1
185.093 b2
288.201 y2
314.137 b3
415.183 b4
474.279 y3
543.238 b5
644.288 b6
700.381 y5
757.366 b7
757.406 y6
904.467 y7
1018.487 b9
1018.517 y8
1131.595 y9
1232.637 y10
1360.698 y11
1461.725 y12
AAETQTLNFGPEWLR / File: sydney_07.raw; SpectrumID: ; scans: 13921

M*LN^KM*TLHPQ^Q^

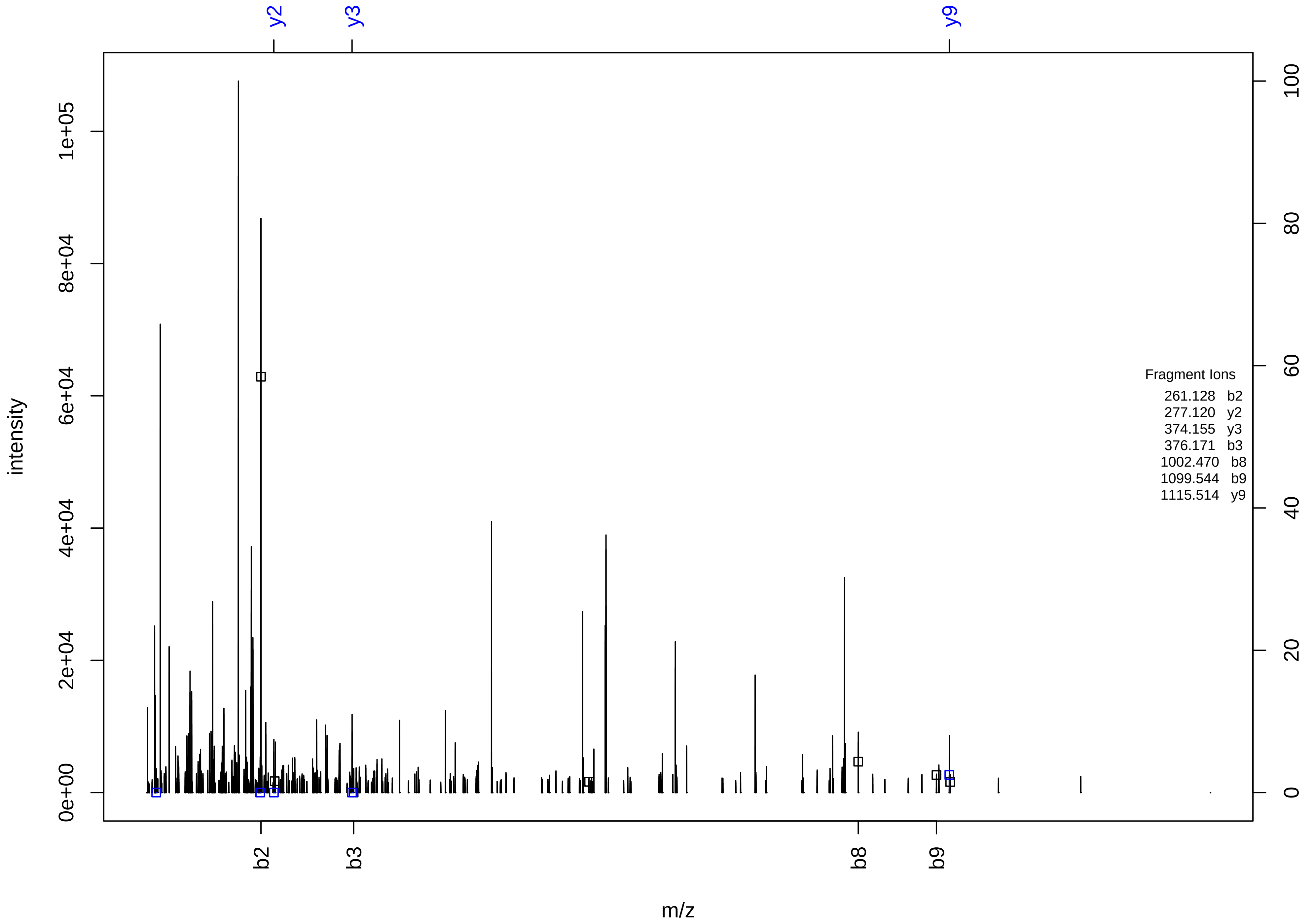

MLNKMTLHPQQ / File: sydney_07.raw; SpectrumID: ; scans: 5572

TLDPSIDGMESLEDLDK

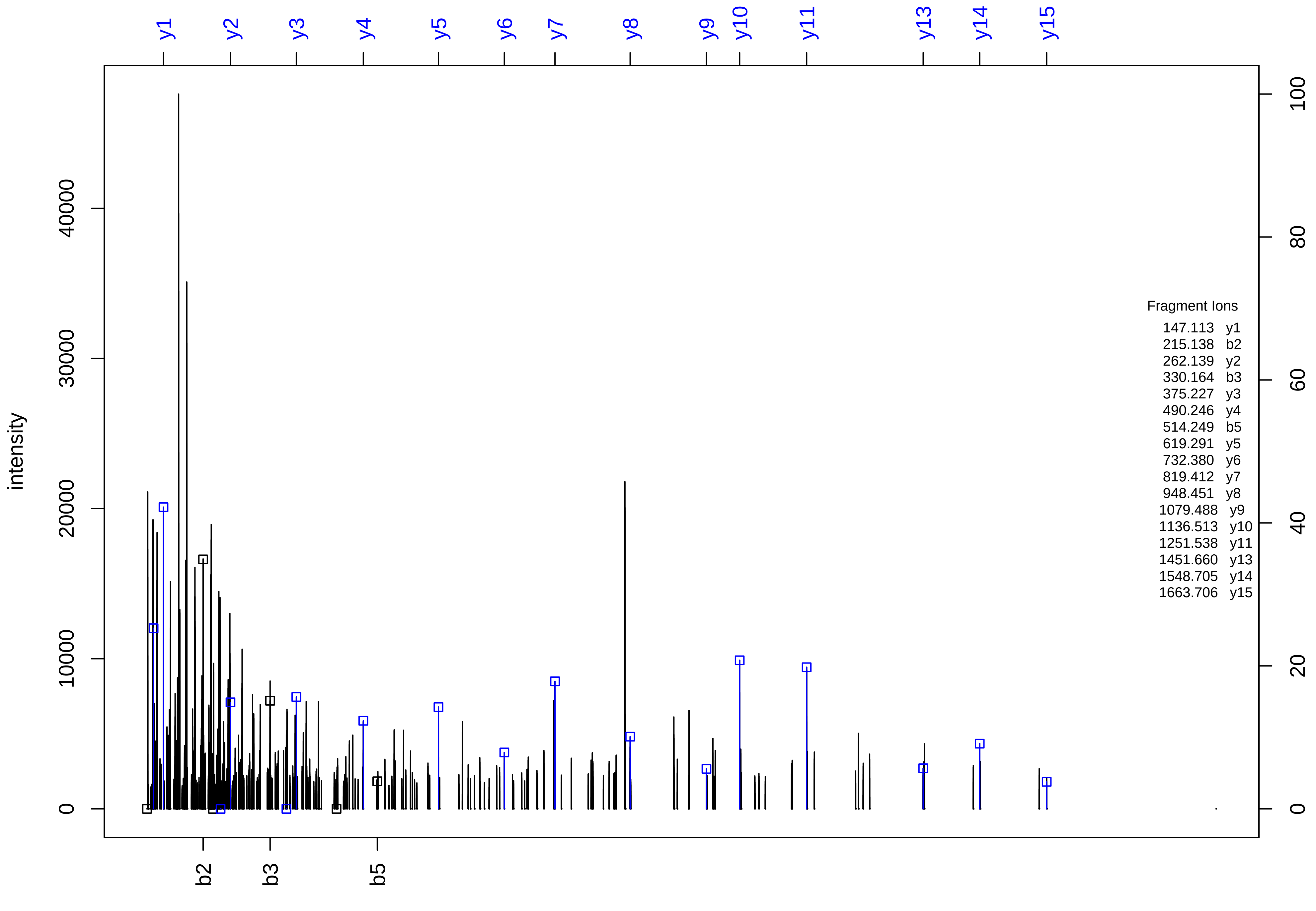

TLDPSIDGMESLEDLDK / File: sydney_07.raw; SpectrumID: ; scans: 10400

DDFSEFDNLR

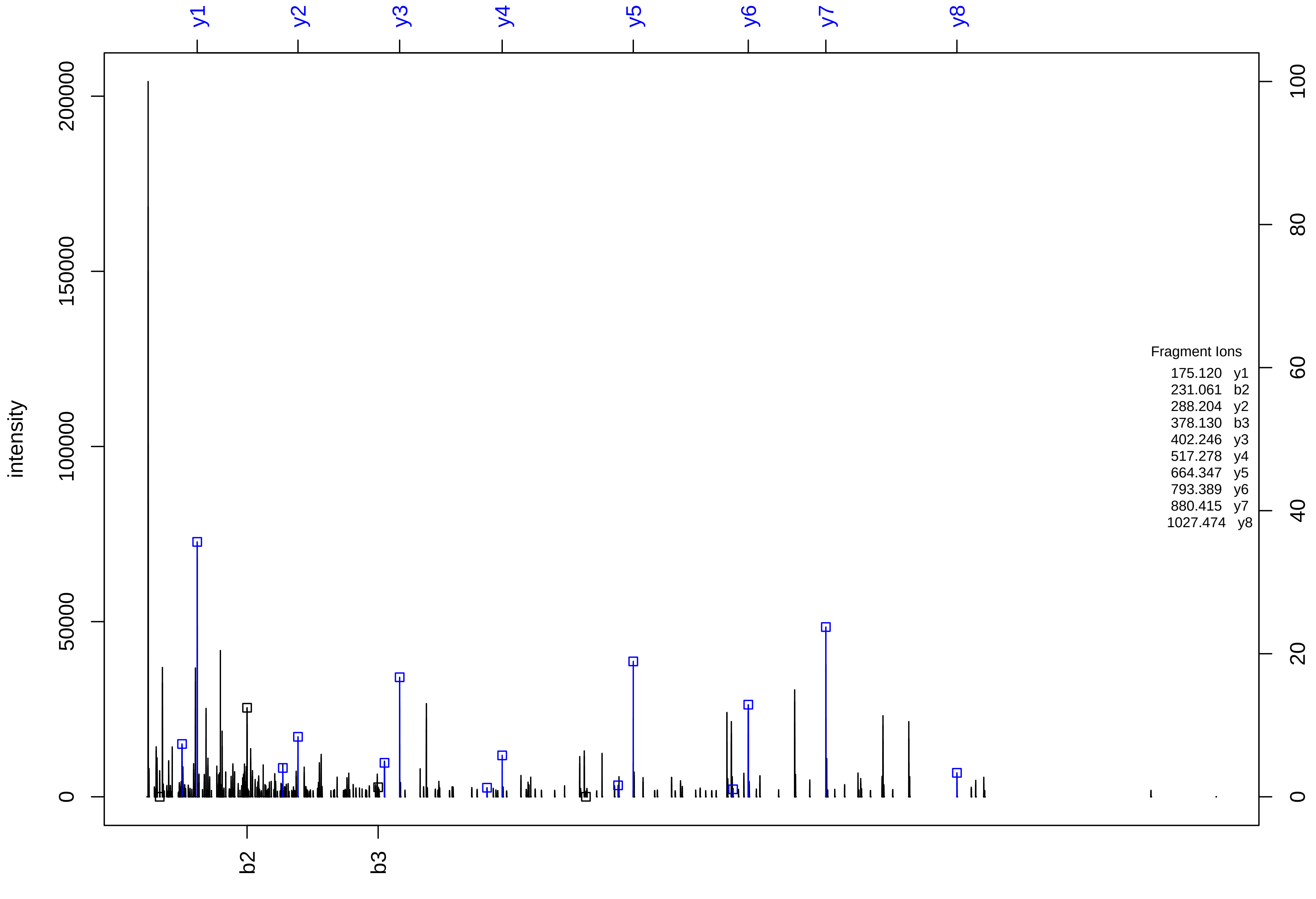

DDFSEFDNLR / File: sydney_07.raw; SpectrumID: ; scans: 7652

EN^EMDEN^LEQVSGIIGNLR

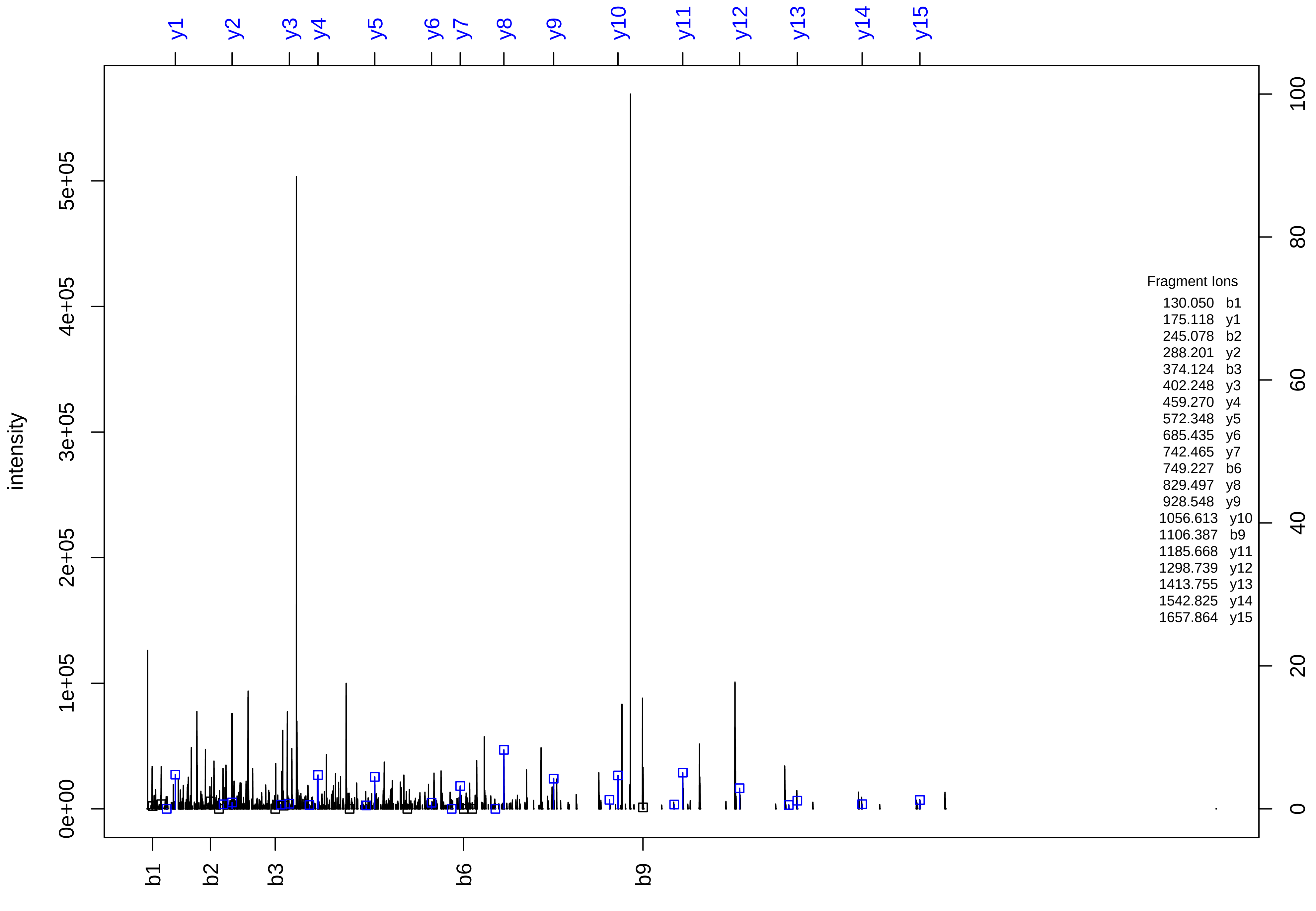

ENEMDENLEQVSGIIGNLR / File: sydney_07.raw; SpectrumID: ; scans: 12803

GVEEEEEDGEMRE

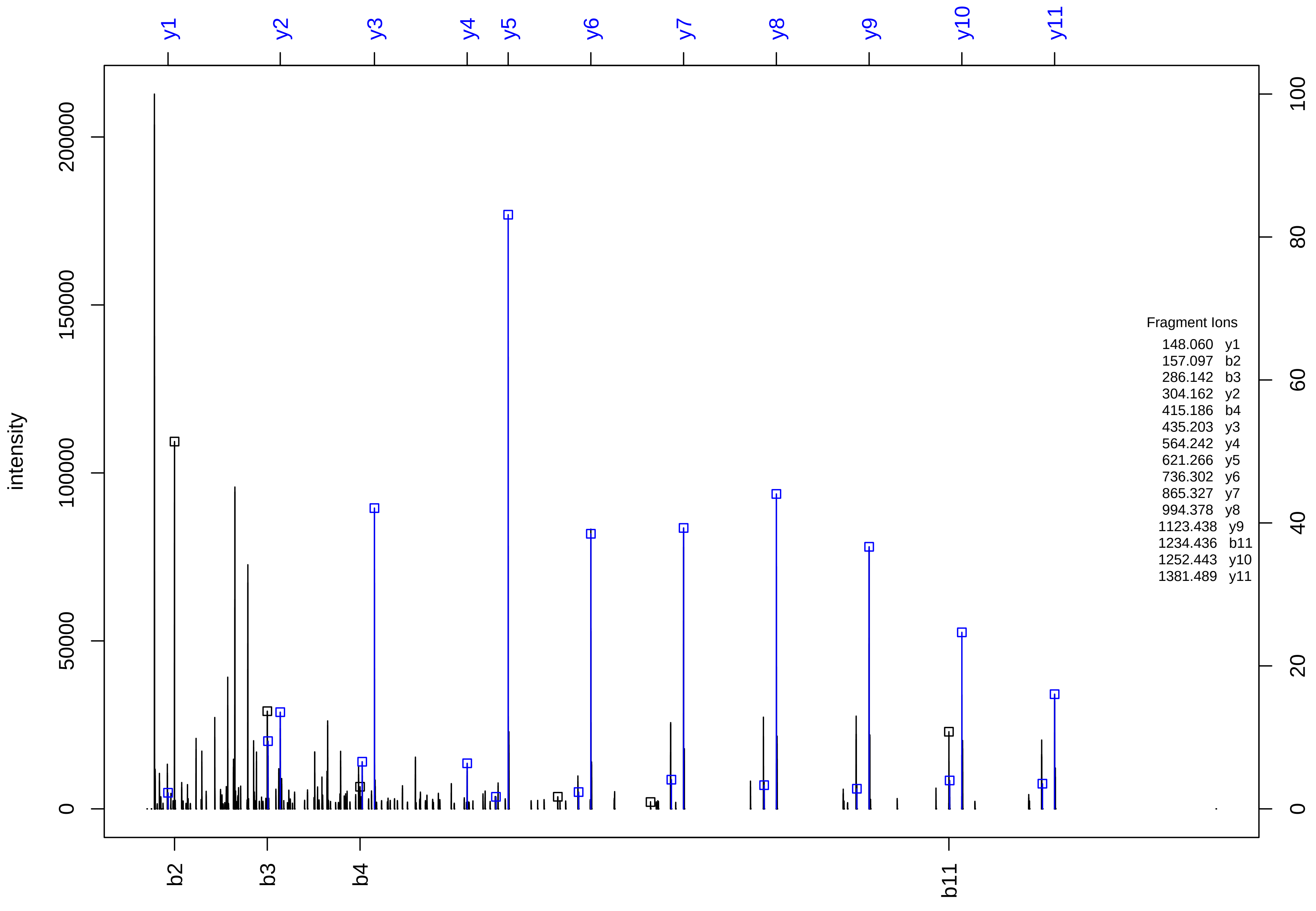

GVEEEEEDGEMRE / File: sydney_07.raw; SpectrumID: ; scans: 1510

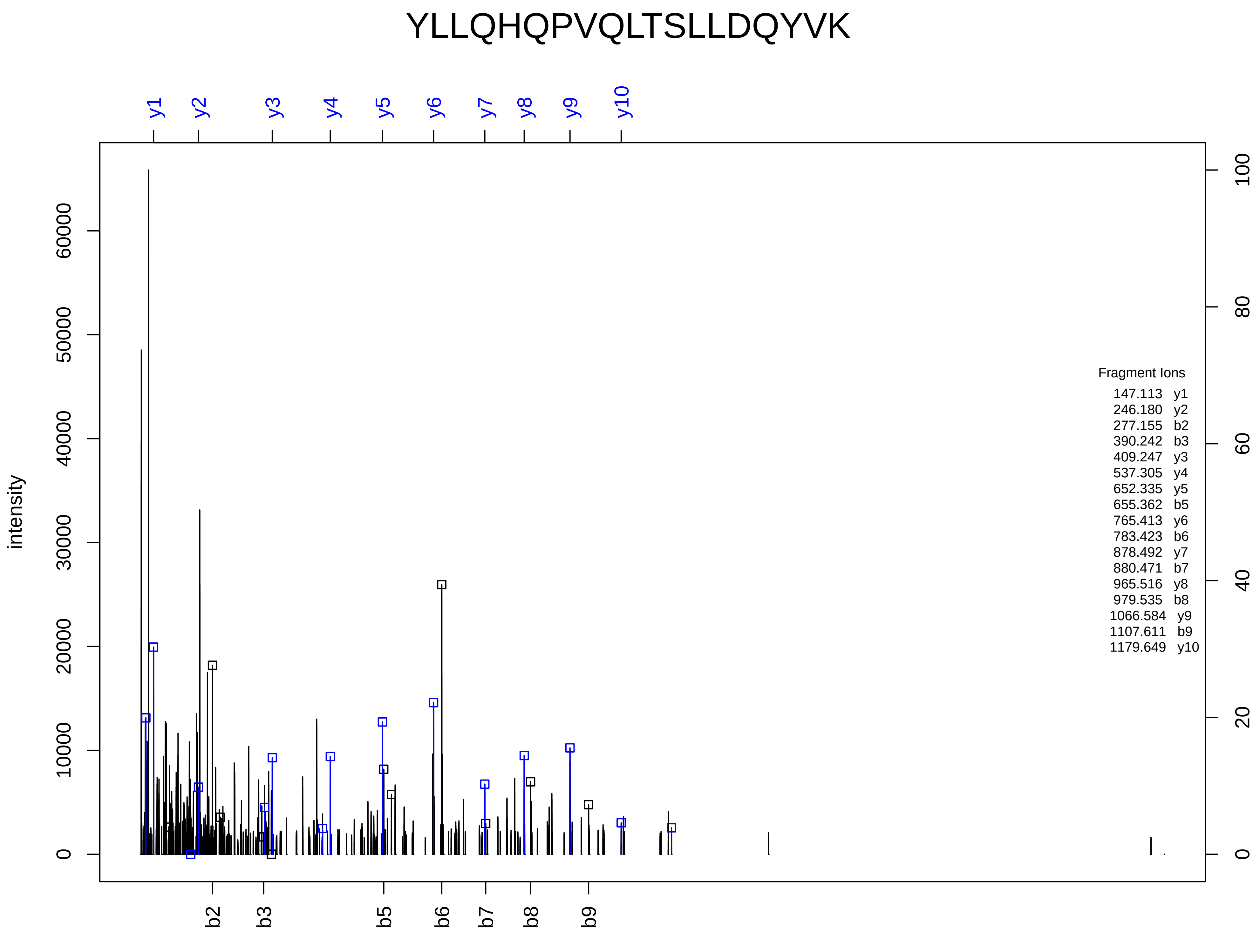

YLLQHQPVQLTSLLDQYVK / File: sydney_07.raw; SpectrumID: ; scans: 12185

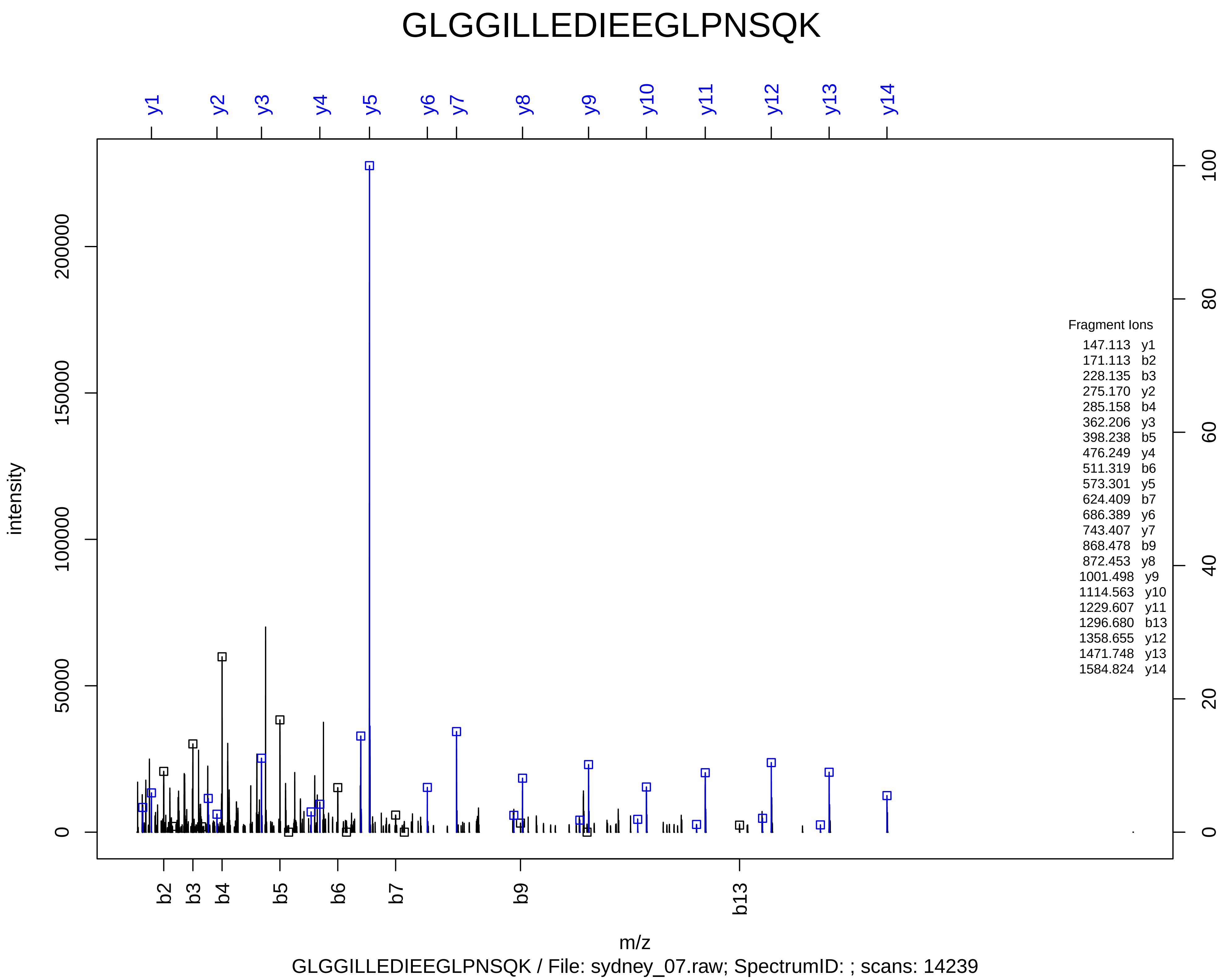
GLGGILLEDIEEGLPNSQK
y1
y2
y3
y4
y5
y6
y7
y8
y9
y10
y11
y12
y13
y14
intensity
0
50000
100000
150000
200000
0
20
40
60
80
100
Fragment Ions
147.113 y1
171.113 b2
228.135 b3
275.170 y2
285.158 b4
362.206 y3
398.238 b5
476.249 y4
511.319 b6
573.301 y5
624.409 b7
686.389 y6
743.407 y7
868.478 b9
872.453 y8
1001.498 y9
1114.563 y10
1229.607 y11
1296.680 b13
1358.655 y12
1471.748 y13
1584.824 y14
b2
b3
b4
b5
b6
b7
b9
b13
m/z
GLGGILLEDIEEGLPNSQK / File: sydney_07.raw; SpectrumID: ; scans: 14239

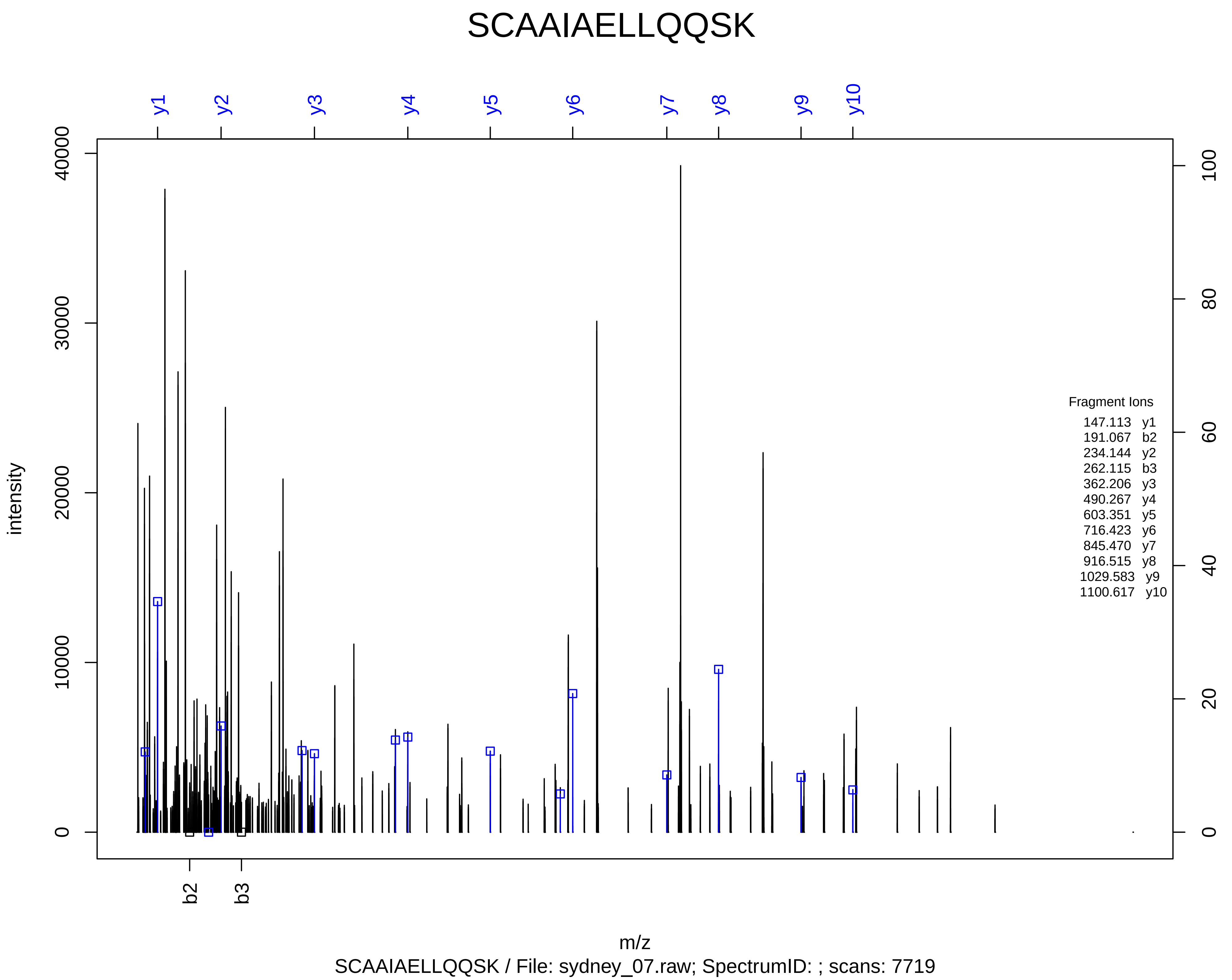
SCAAIAELLQQSK
y1 y2 y3 y4 y5 y6 y7 y8 y9 y10
b2 b3
intensity
m/z
Fragment Ions
147.113 y1
191.067 b2
234.144 y2
262.115 b3
362.206 y3
490.267 y4
603.351 y5
716.423 y6
845.470 y7
916.515 y8
1029.583 y9
1100.617 y10
SCAAIAELLQQSK / File: sydney_07.raw; SpectrumID: ; scans: 7719

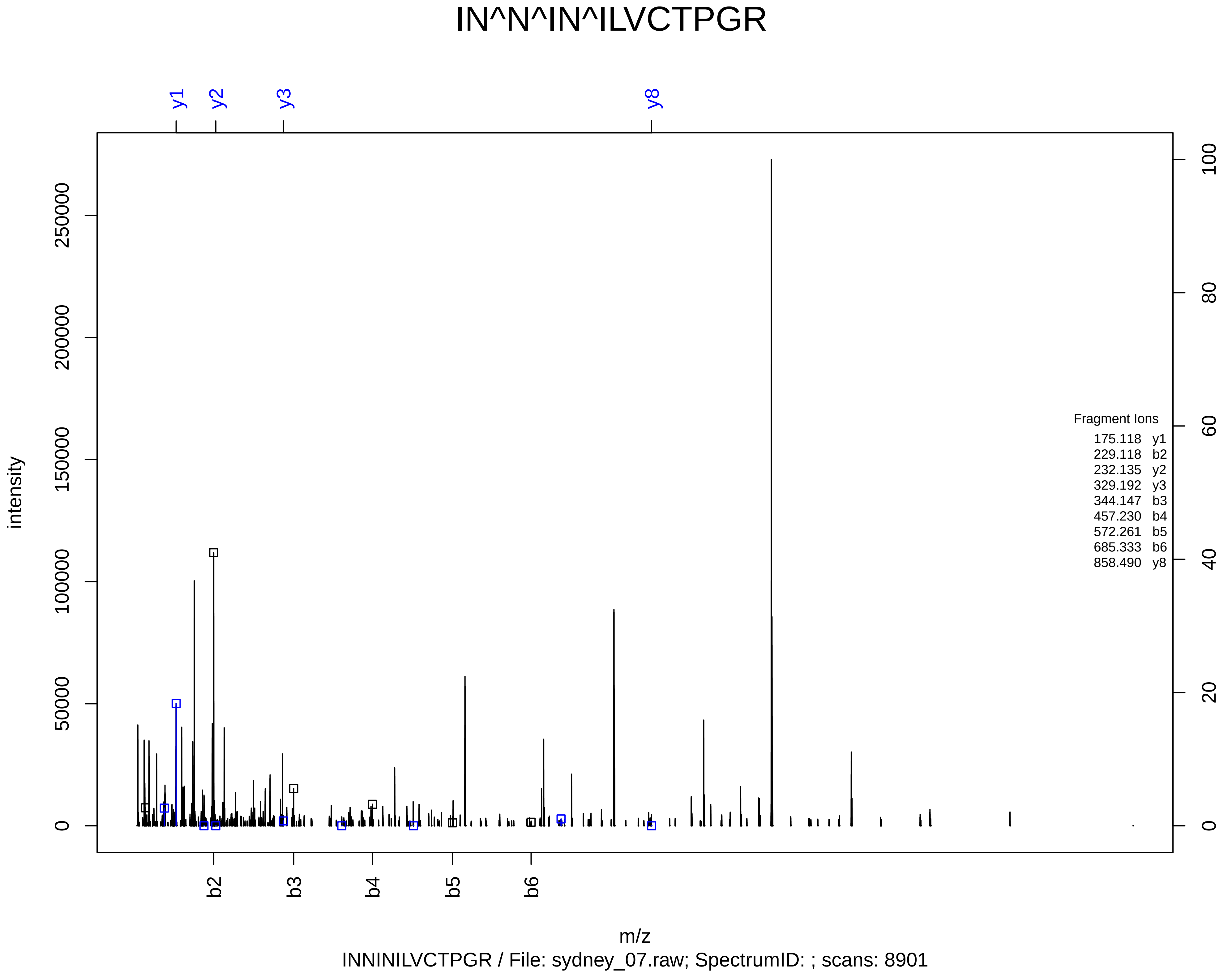
IN^N^IN^ILVCTPGR
y1
y2
y3
y8
intensity
0
50000
100000
150000
200000
250000
0
20
40
60
80
100
b2
b3
b4
b5
b6
m/z
Fragment Ions
175.118 y1
229.118 b2
232.135 y2
329.192 y3
344.147 b3
457.230 b4
572.261 b5
685.333 b6
858.490 y8
INNINILVCTPGR / File: sydney_07.raw; SpectrumID: ; scans: 8901

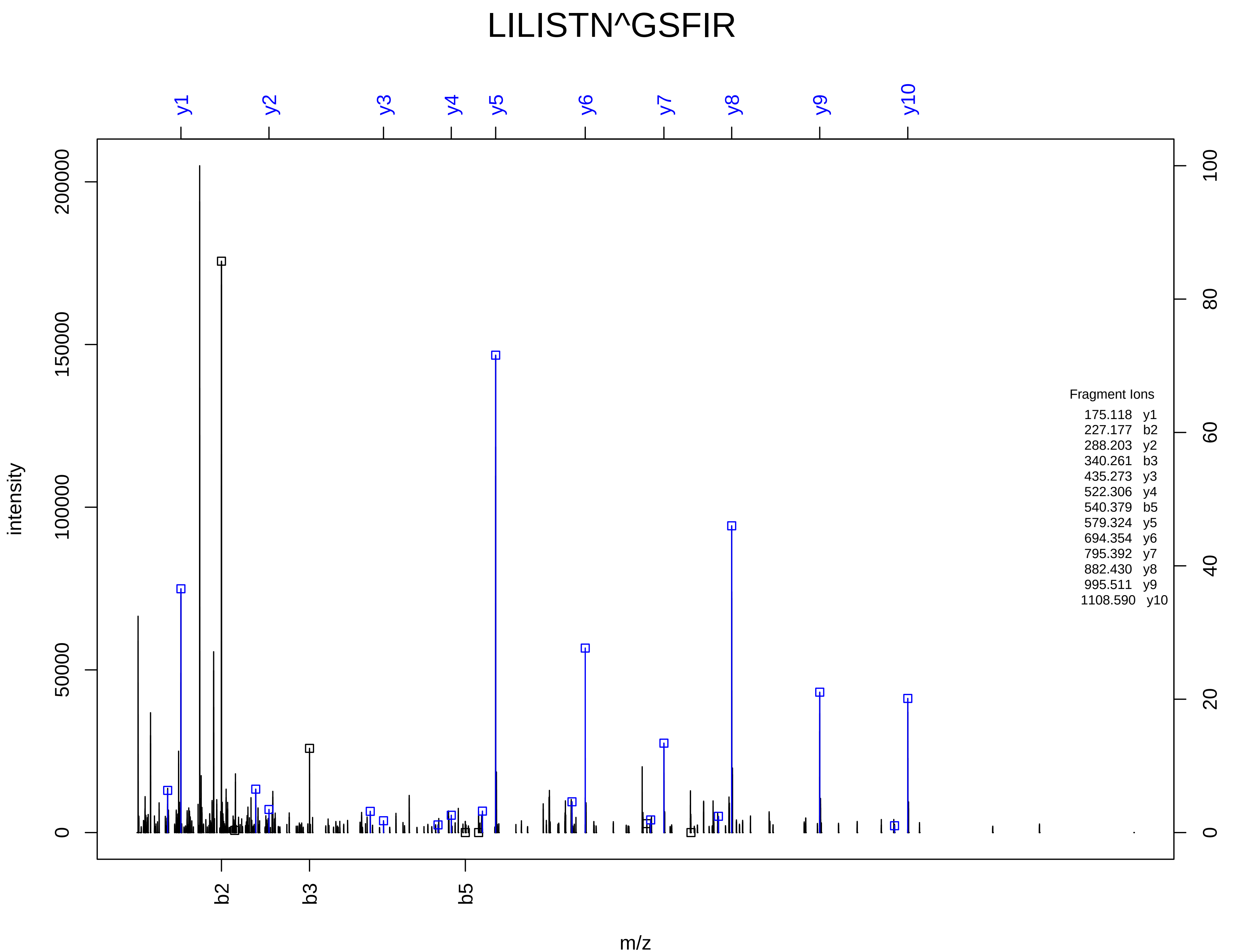
LILISTN^GSFIR
y1
y2
y3
y4
y5
y6
y7
y8
y9
y10
intensity
0
50000
100000
150000
200000
0
20
40
60
80
100
b2
b3
b5
m/z
Fragment Ions
175.118 y1
227.177 b2
288.203 y2
340.261 b3
435.273 y3
522.306 y4
540.379 b5
579.324 y5
694.354 y6
795.392 y7
882.430 y8
995.511 y9
1108.590 y10
LILISTNGSFIR / File: sydney_07.raw; SpectrumID: ; scans: 10690

DPSYSYNPSGQGLSR

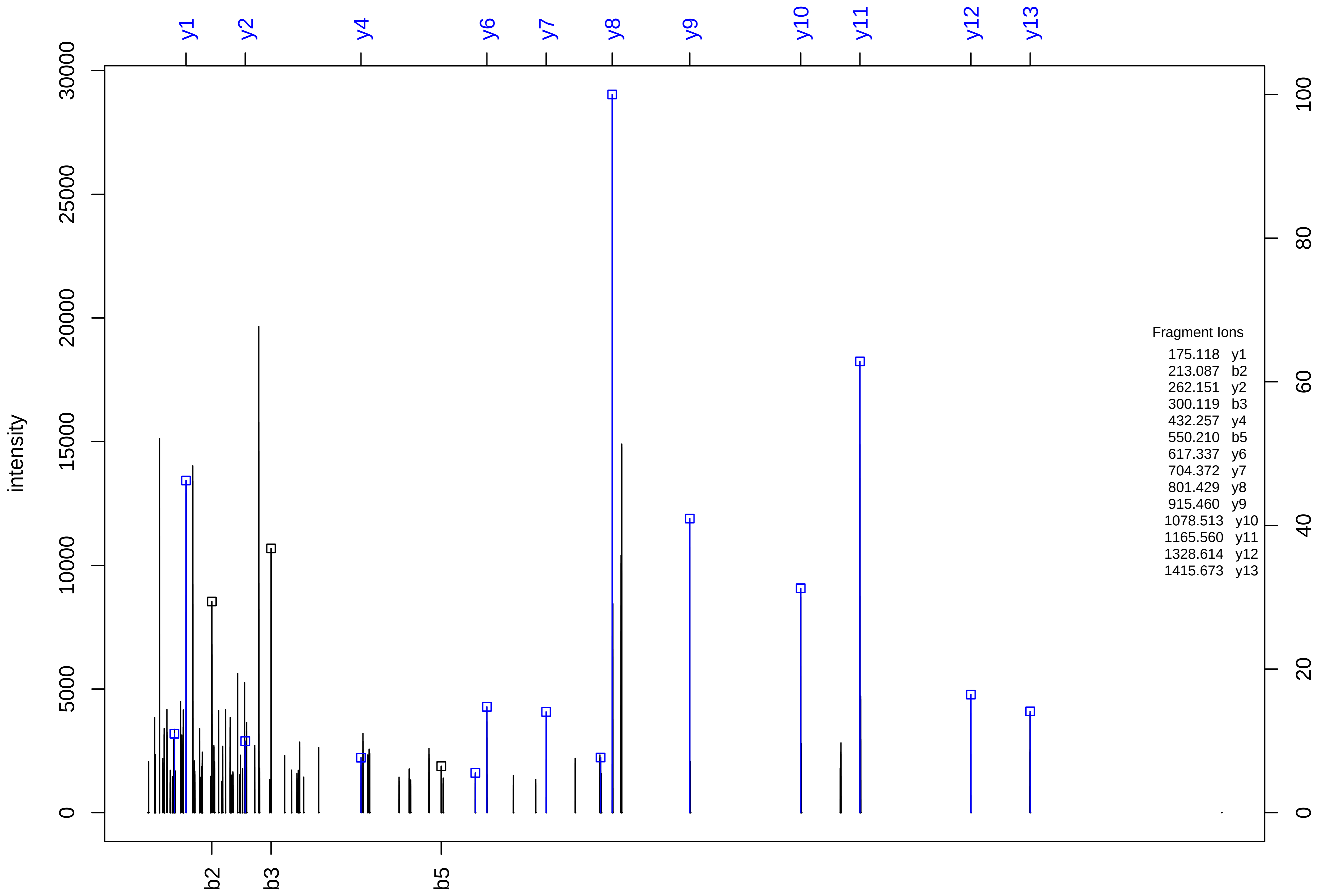

DPSYSYNPSGQGLSR / File: sydney_07.raw; SpectrumID: ; scans: 2708

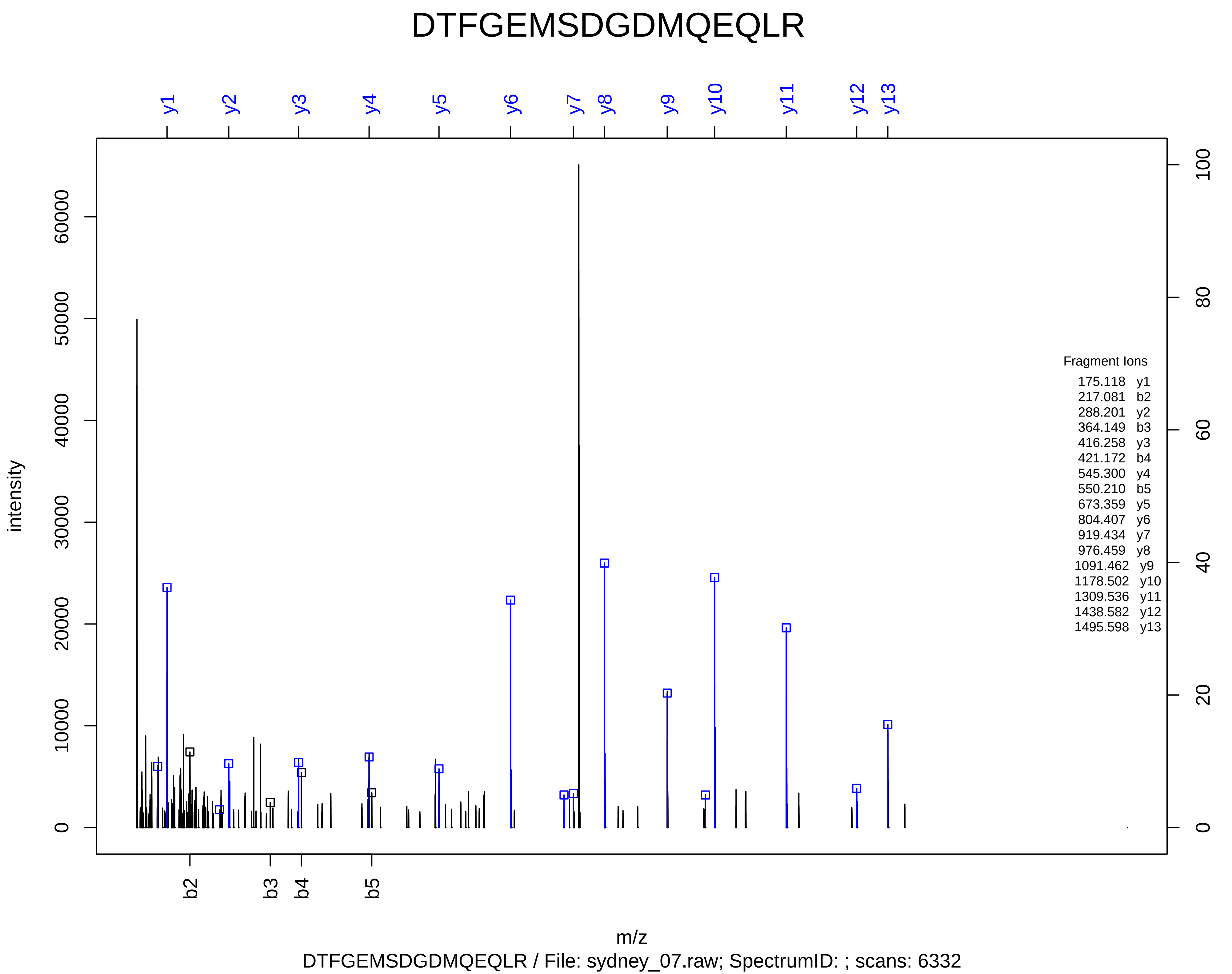

DTFGEMSDGDMQEQLR
y1 y2 y3 y4 y5 y6 y7 y8 y9 y10 y11 y12 y13
b2 b3 b4 b5
intensity
m/z
Fragment Ions
175.118 y1
217.081 b2
288.201 y2
364.149 b3
416.258 y3
421.172 b4
545.300 y4
550.210 b5
673.359 y5
804.407 y6
919.434 y7
976.459 y8
1091.462 y9
1178.502 y10
1309.536 y11
1438.582 y12
1495.598 y13
DTFGEMSDGDMQEQLR / File: sydney_07.raw; SpectrumID: ; scans: 6332

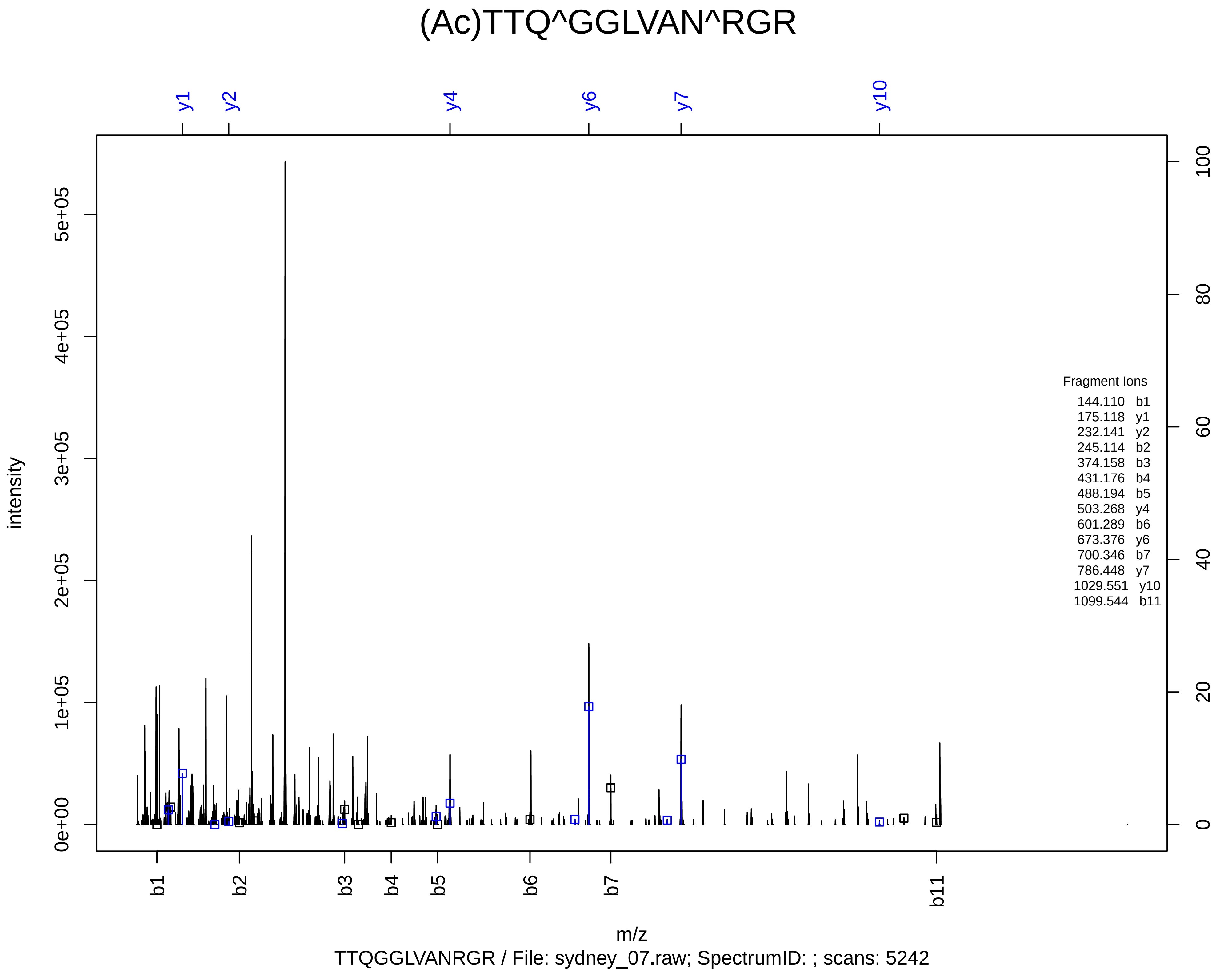
(Ac)TTQ^GGLVAN^RGR
y1
y2
y4
y6
y7
y10
intensity
m/z
b1
b2
b3
b4
b5
b6
b7
b11
Fragment Ions
144.110 b1
175.118 y1
232.141 y2
245.114 b2
374.158 b3
431.176 b4
488.194 b5
503.268 y4
601.289 b6
673.376 y6
700.346 b7
786.448 y7
1029.551 y10
1099.544 b11
TTQGGLVANRGR / File: sydney_07.raw; SpectrumID: ; scans: 5242

SDPLLMGIPTSENPFK

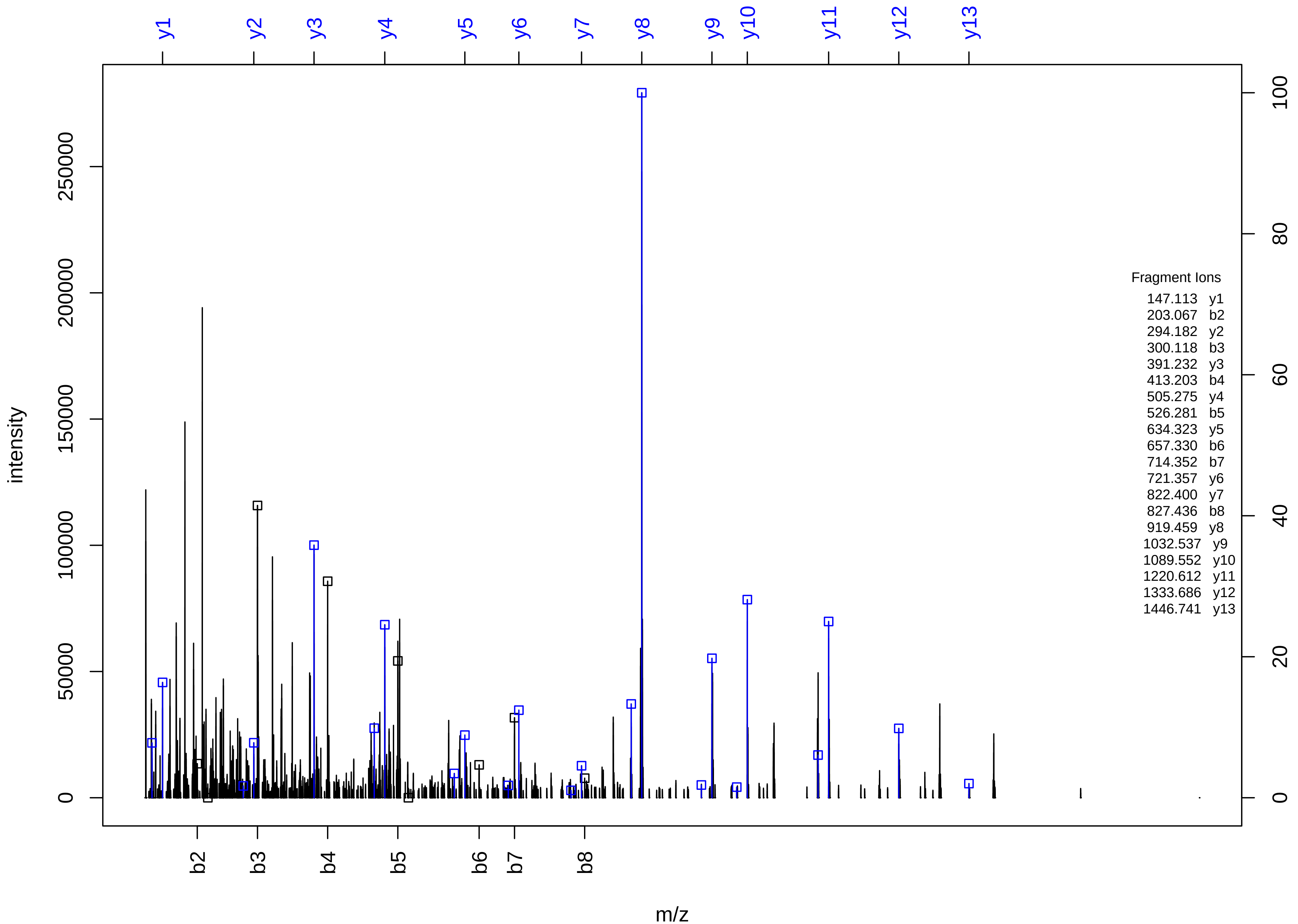

SDPLLMGIPTSENPFK / File: sydney_08.raw; SpectrumID: ; scans: 11308

LPDGSSFTNQFPSDAPLEEAR

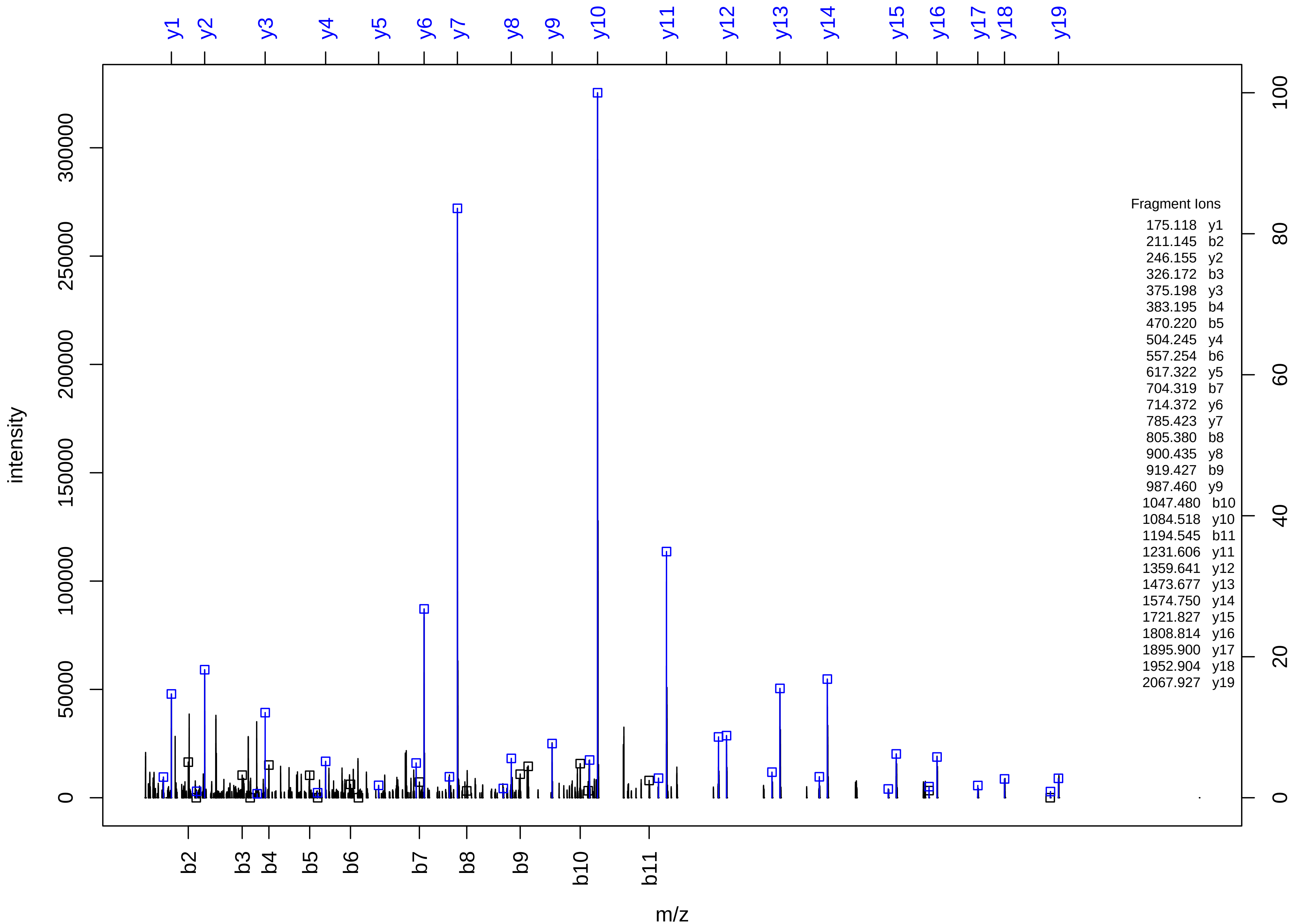

LPDGSSFTNQFPSDAPLEEAR / File: sydney_08.raw; SpectrumID: ; scans: 8228

SGERPVTAGEEDEEVPDSIDAR

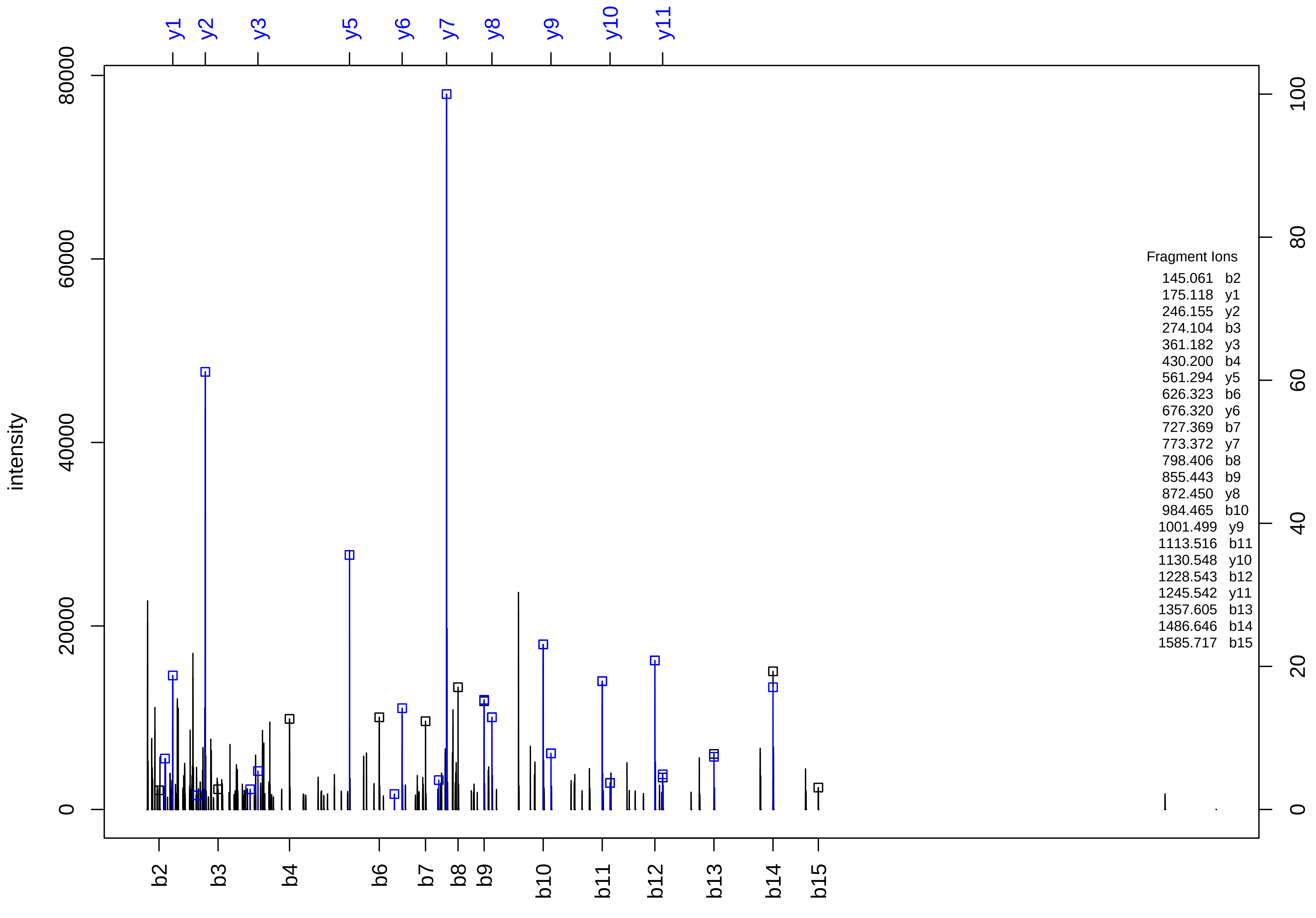

SGERPVTAGEEDEEVPDSIDAR / File: sydney_08.raw; SpectrumID: ; scans: 2654

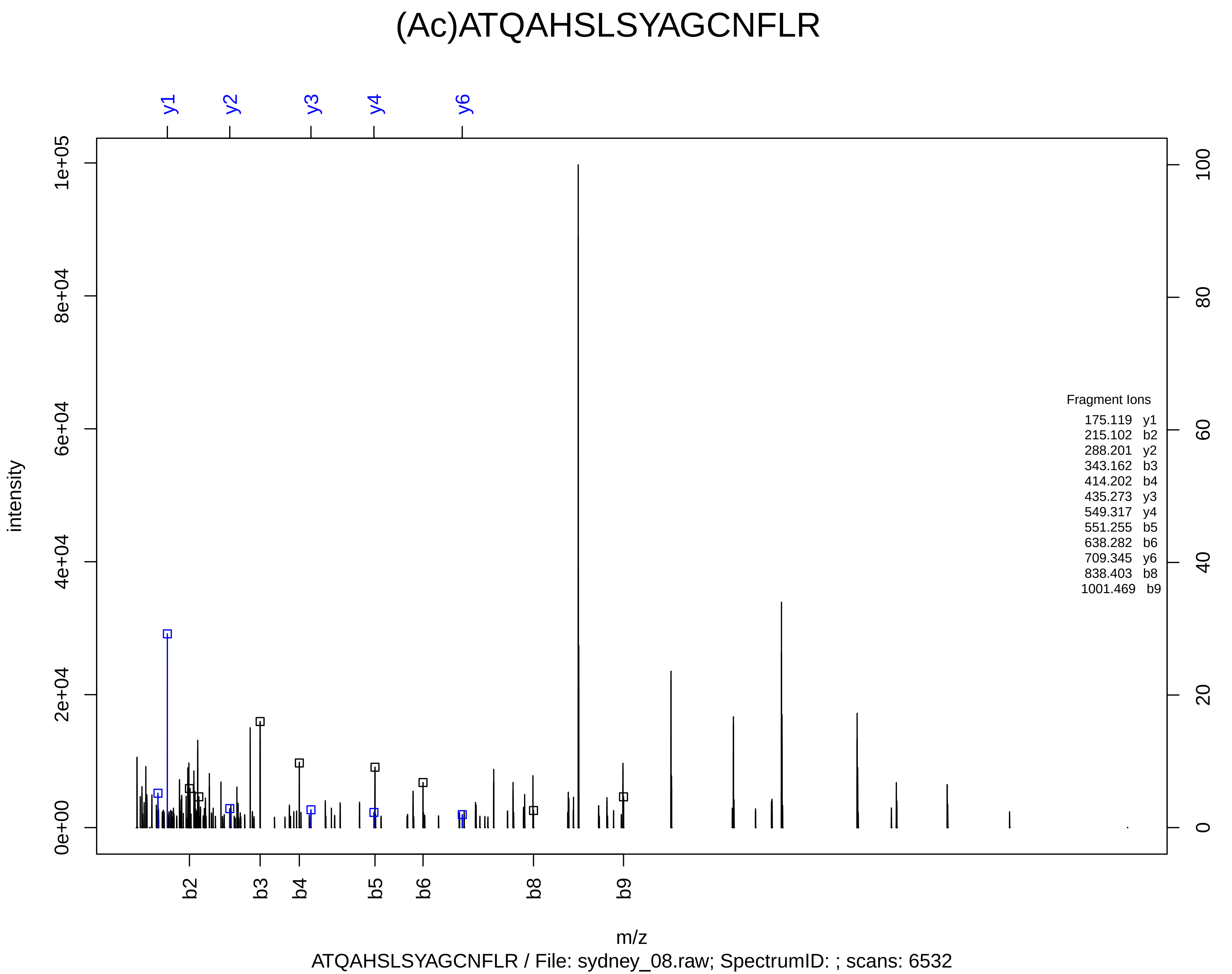

(Ac)ATQAHSLSYAGCNFLR
y1
y2
y3
y4
y6
intensity
0e+00
2e+04
4e+04
6e+04
8e+04
1e+05
0
20
40
60
80
100
b2
b3
b4
b5
b6
b8
b9
Fragment Ions
175.119 y1
215.102 b2
288.201 y2
343.162 b3
414.202 b4
435.273 y3
549.317 y4
551.255 b5
638.282 b6
709.345 y6
838.403 b8
1001.469 b9
m/z
ATQAHSLSYAGCNFLR / File: sydney_08.raw; SpectrumID: ; scans: 6532

AVEYFQDNSPDSPELNK

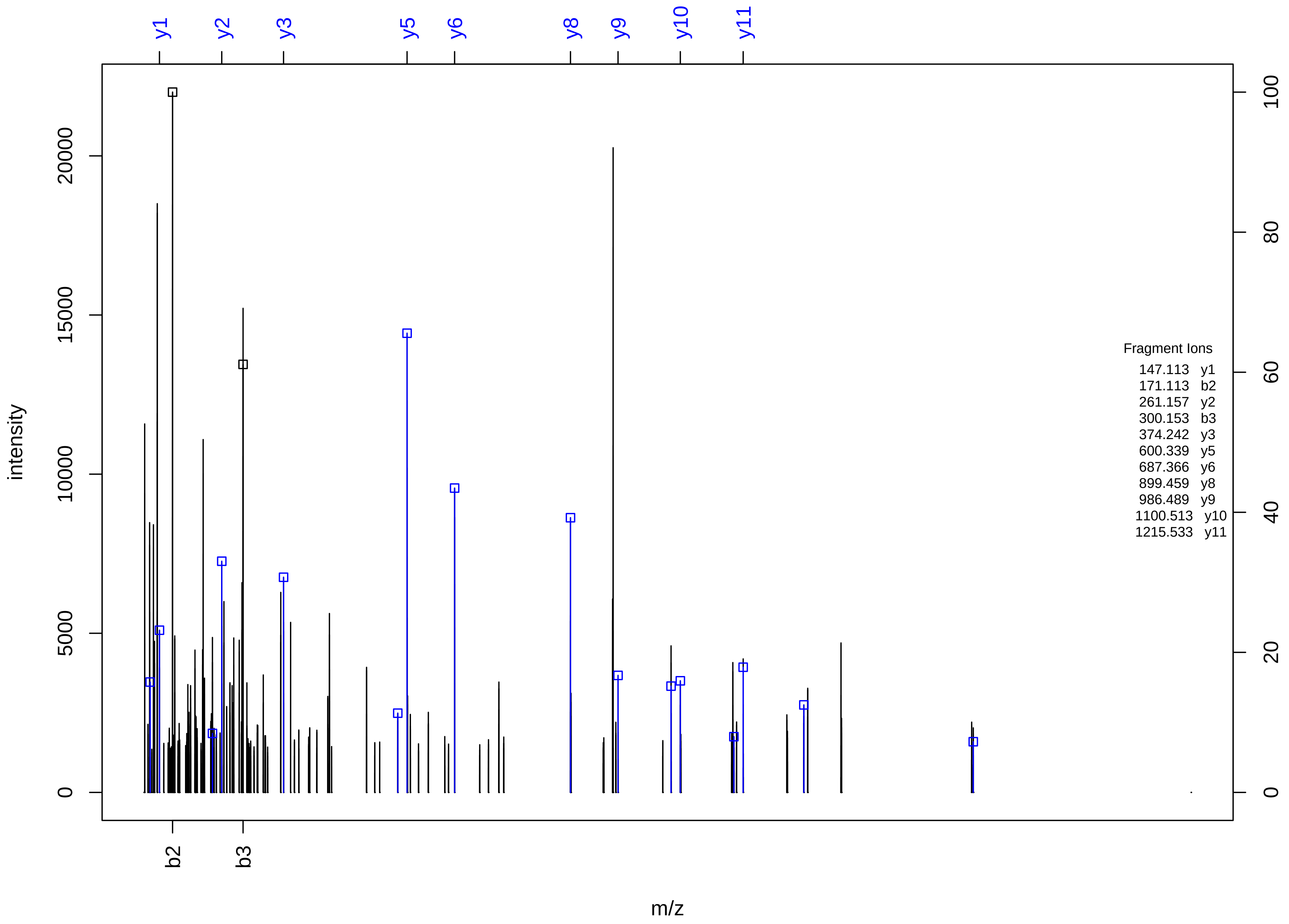

AVEYFQDNSPDSPELNK / File: sydney_08.raw; SpectrumID: ; scans: 4039

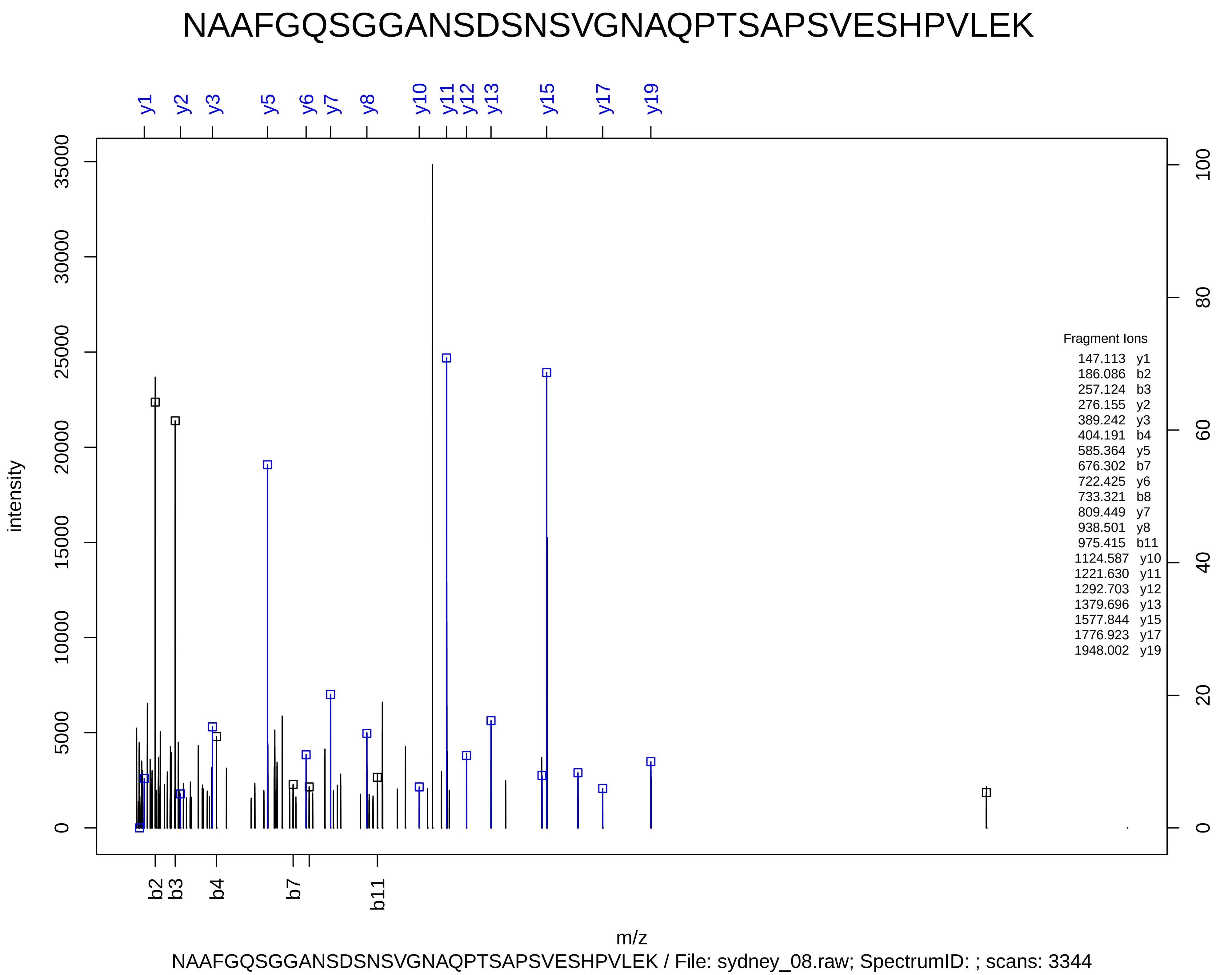
NAAFGQSGGANSDSNSVGNAQPTSAPSVESHPVLEK
y1 y2 y3 y5 y6 y7 y8 y10 y11 y12 y13 y15 y17 y19
b2 b3 b4 b7 b11
intensity
m/z
Fragment Ions
147.113 y1
186.086 b2
257.124 b3
276.155 y2
389.242 y3
404.191 b4
585.364 y5
676.302 b7
722.425 y6
733.321 b8
809.449 y7
938.501 y8
975.415 b11
1124.587 y10
1221.630 y11
1292.703 y12
1379.696 y13
1577.844 y15
1776.923 y17
1948.002 y19
NAAFGQSGGANSDSNSVGNAQPTSAPSVESHPVLEK / File: sydney_08.raw; SpectrumID: ; scans: 3344

(Ac)PLRN^VMAEN^NFKMLK

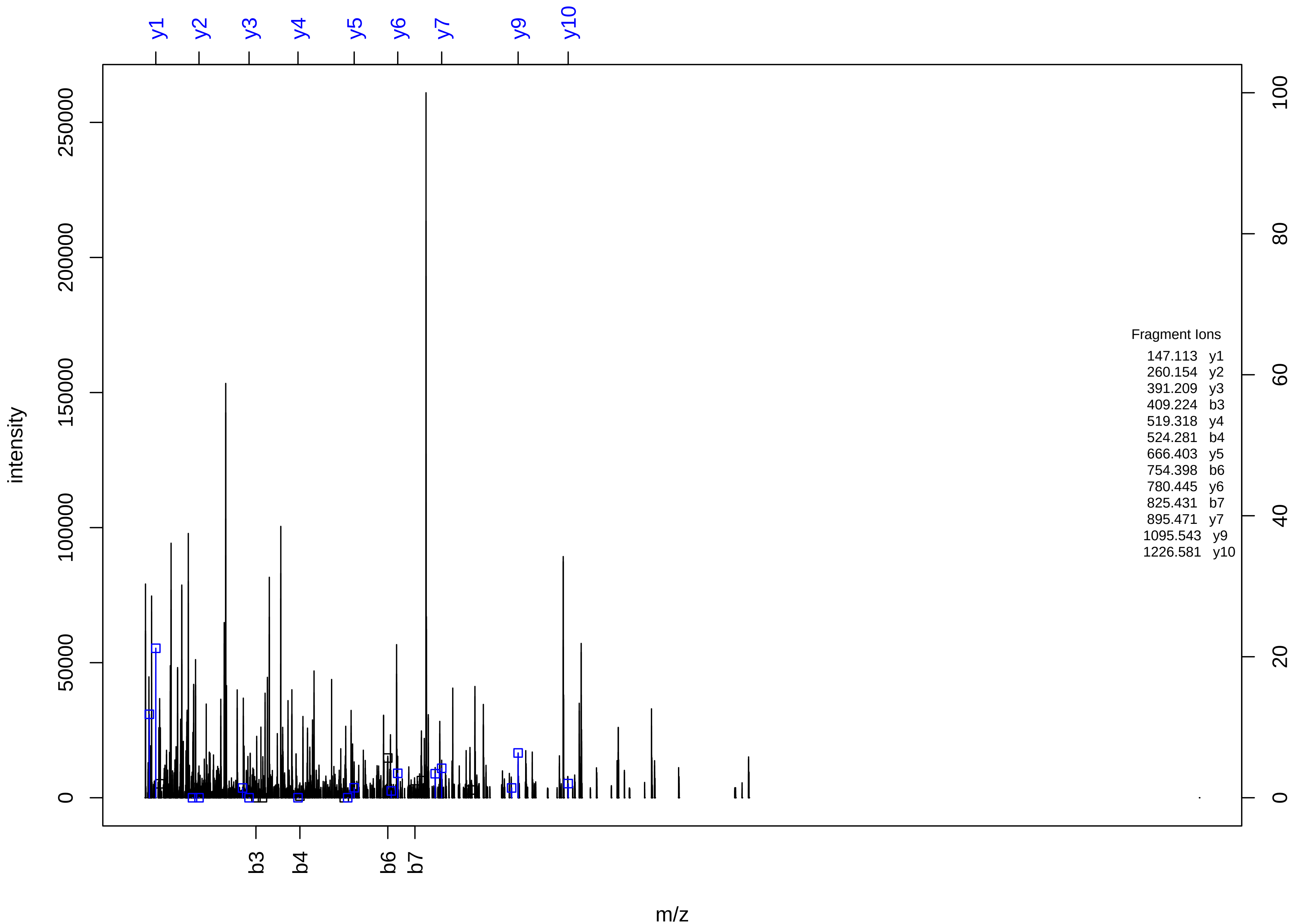

PLRNVMAENNFKMLK / File: sydney_08.raw; SpectrumID: ; scans: 10646

LYNVQDPETQQNLCR

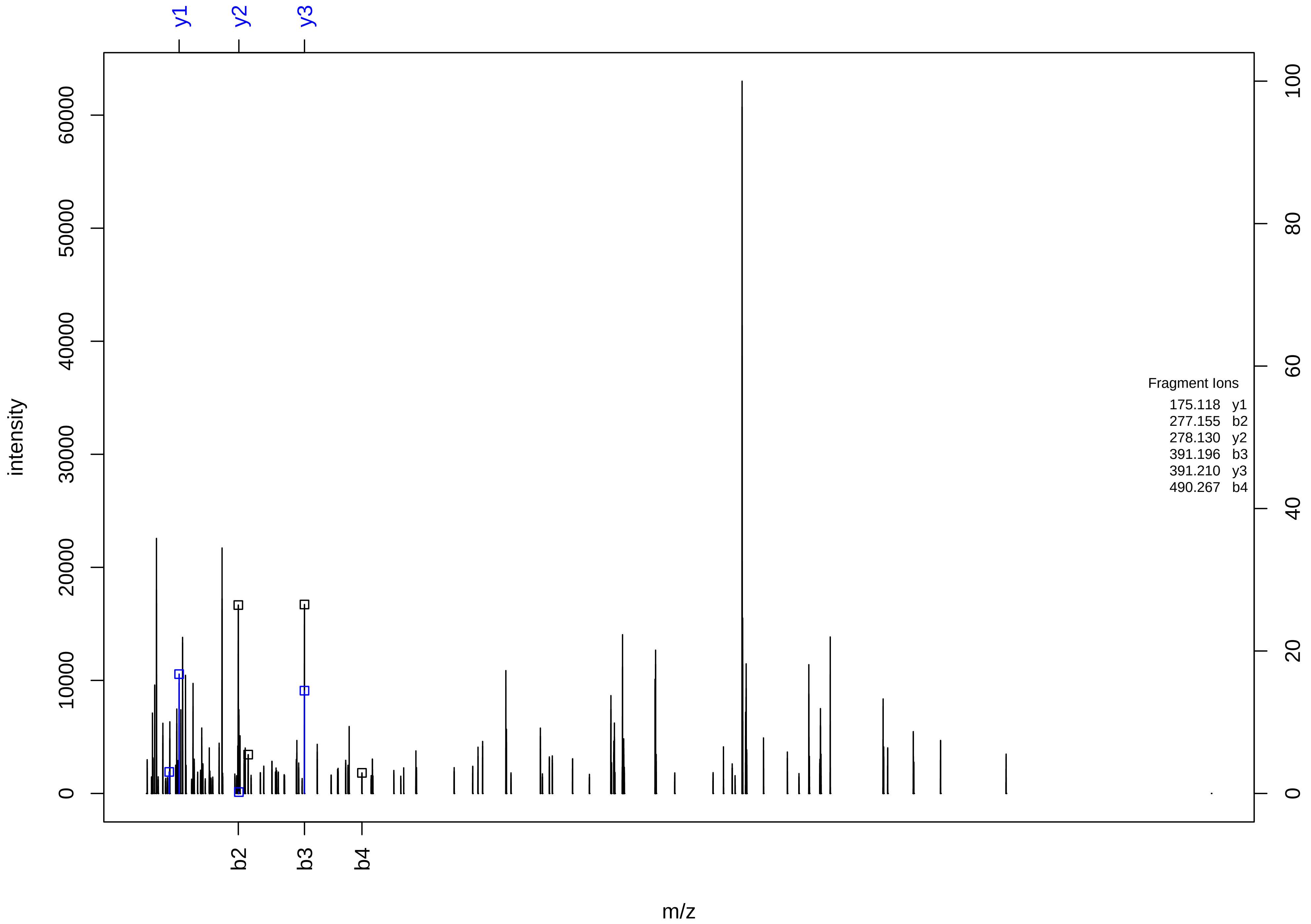

LYNVQDPETQQNLCR / File: sydney_08.raw; SpectrumID: ; scans: 2944

SLPLQPGMVITVEPGIYIPEDDRDAPEK

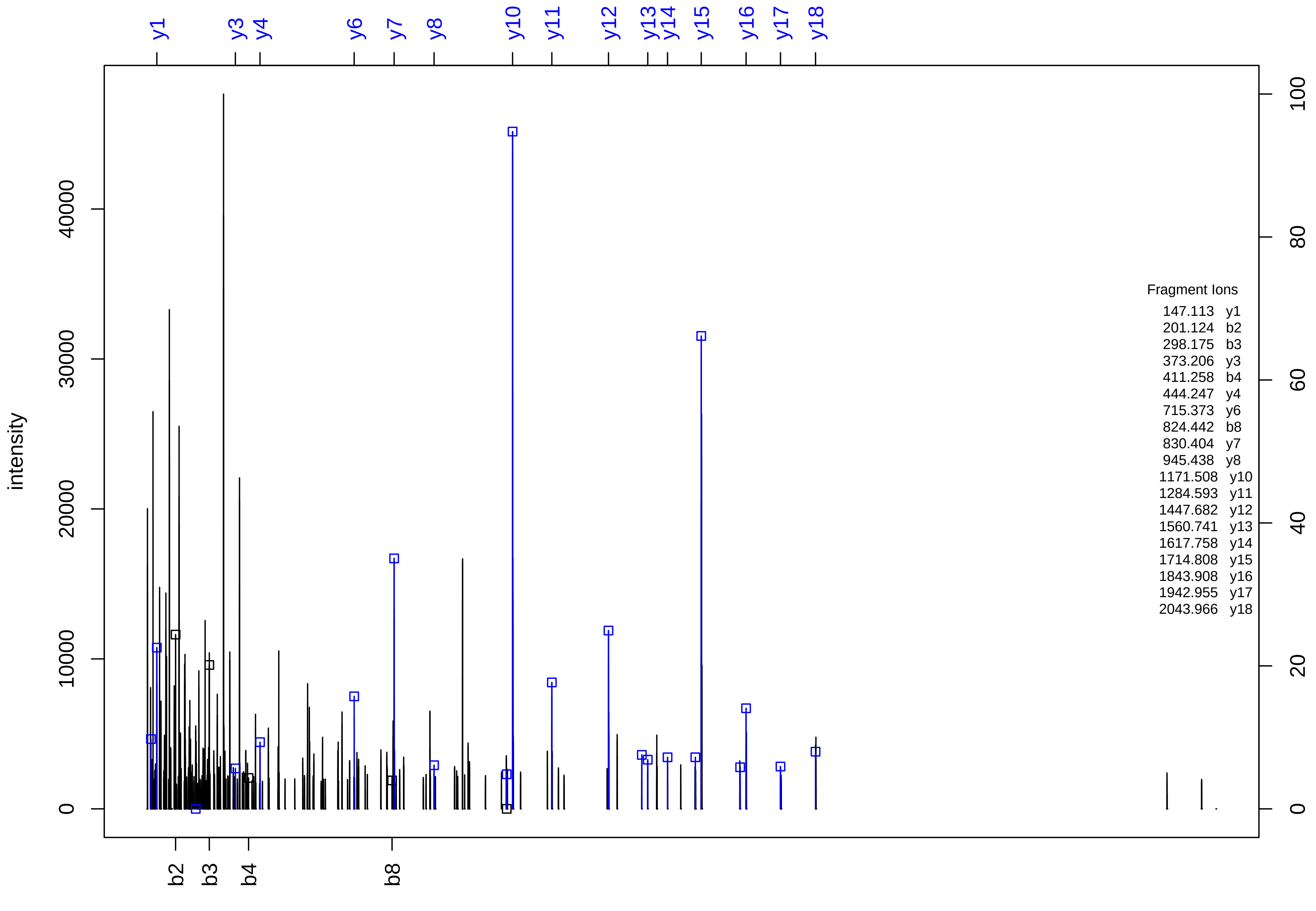

SLPLQPGMVITVEPGIYIPEDDRDAPEK / File: sydney_08.raw; SpectrumID: ; scans: 12071

DQYVSPETVTVR

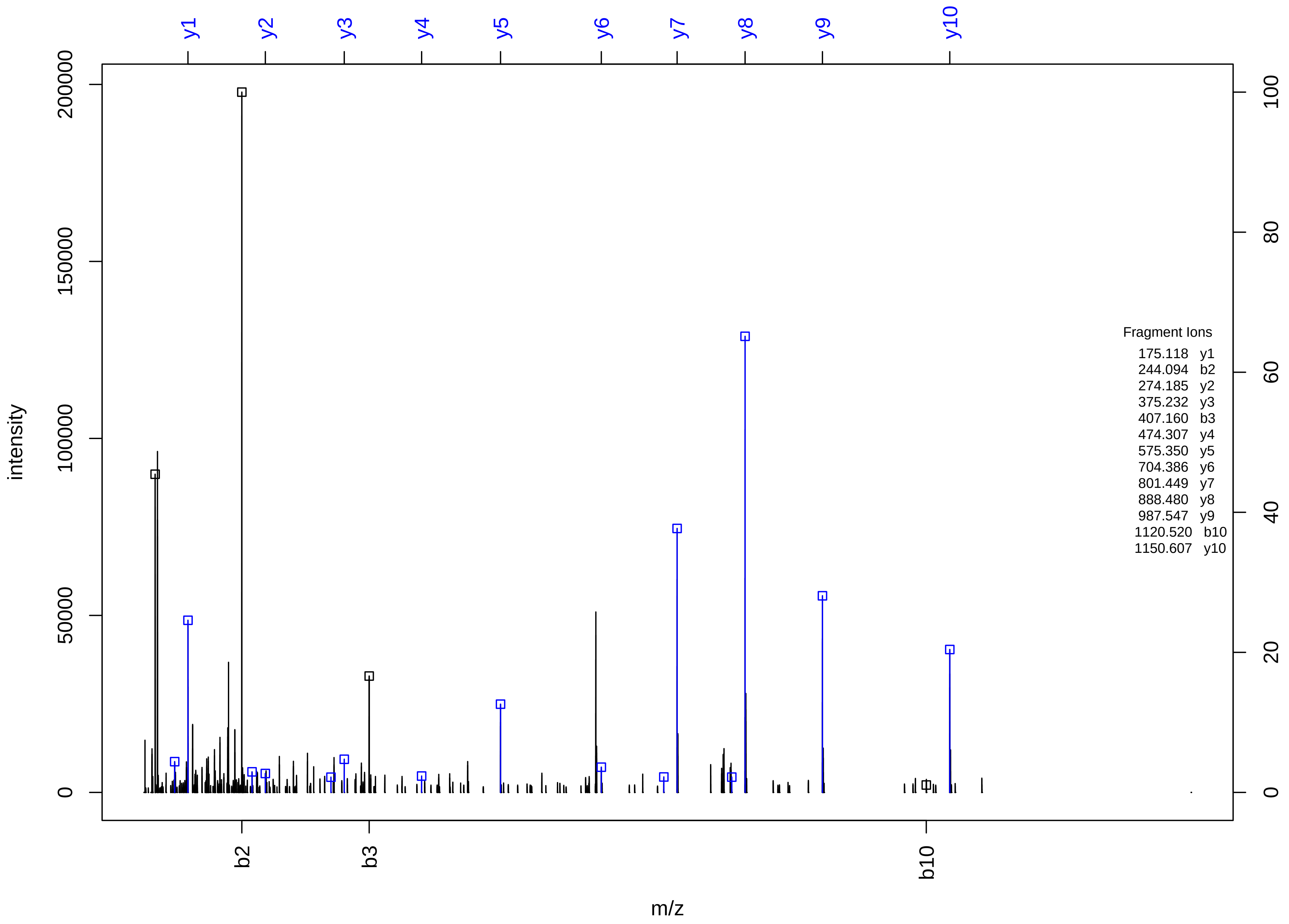

DQYVSPETVTVR / File: sydney_08.raw; SpectrumID: ; scans: 3255

ILEPPEGQDEGVWK

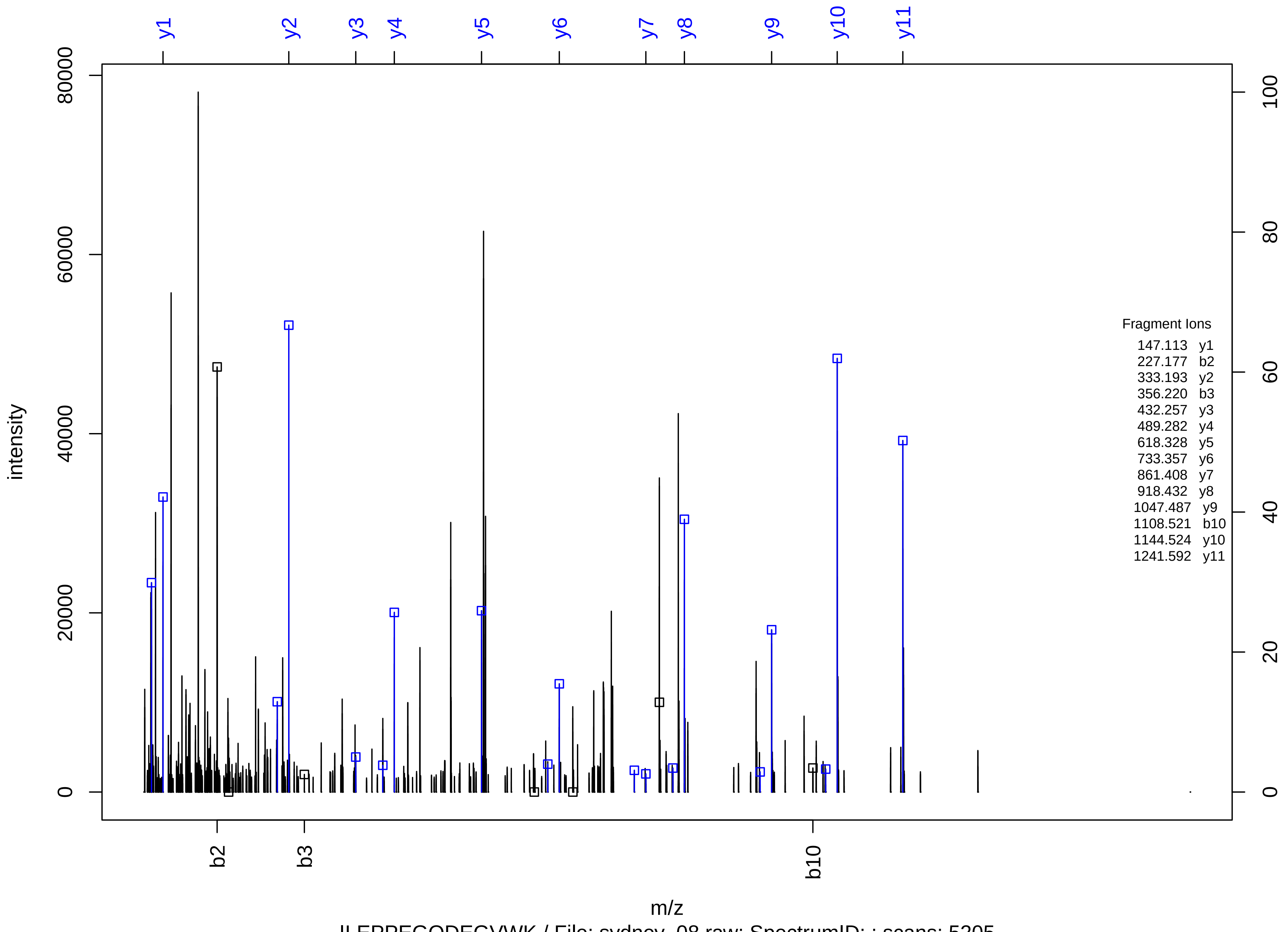

ILEPPEGQDEGVWK / File: sydney_08.raw; SpectrumID: ; scans: 5205

(Ac)MDPNCSCASDGSCSCAGACK

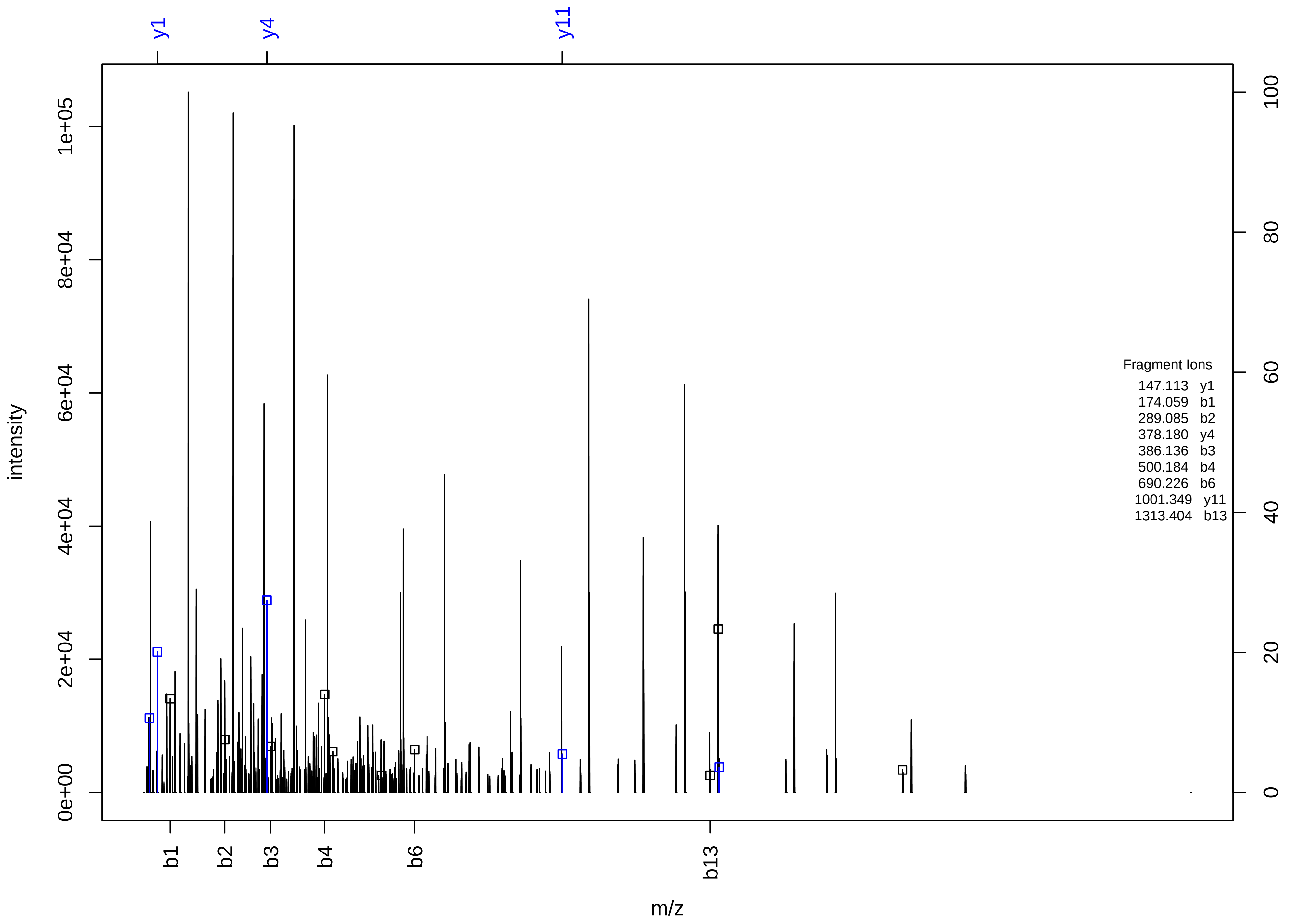

MDPNCSCASDGSCSCAGACK / File: sydney_08.raw; SpectrumID: ; scans: 1796

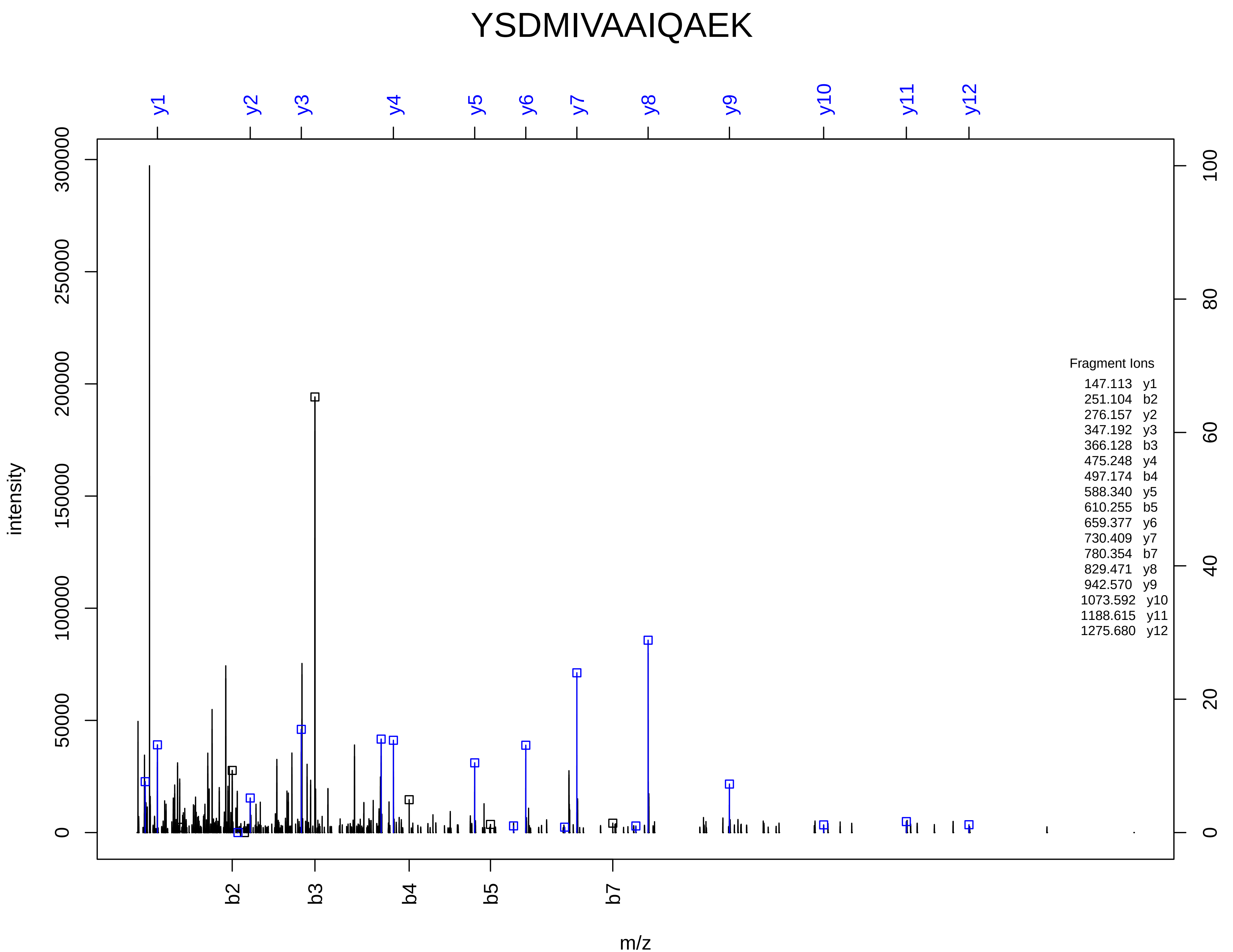

YSDMIVAAIQAEK / File: sydney_08.raw; SpectrumID: ; scans: 7653

FGESIANLGDIDNDGFEDIAIGAPQEDDLR

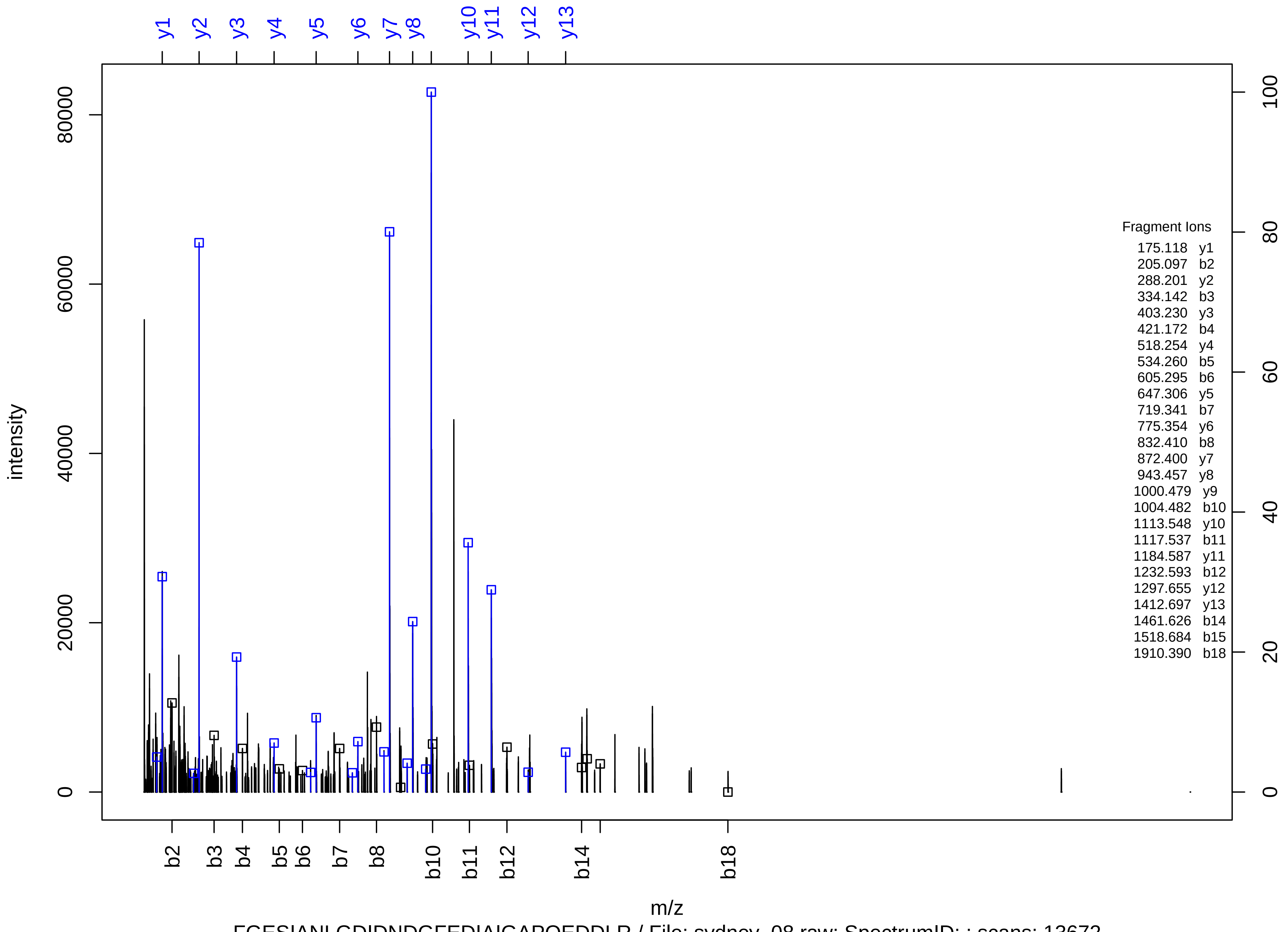

FGESIANLGDIDNDGFEDIAIGAPQEDDLR / File: sydney_08.raw; SpectrumID: ; scans: 13672

CDPIDFSNSPEALR

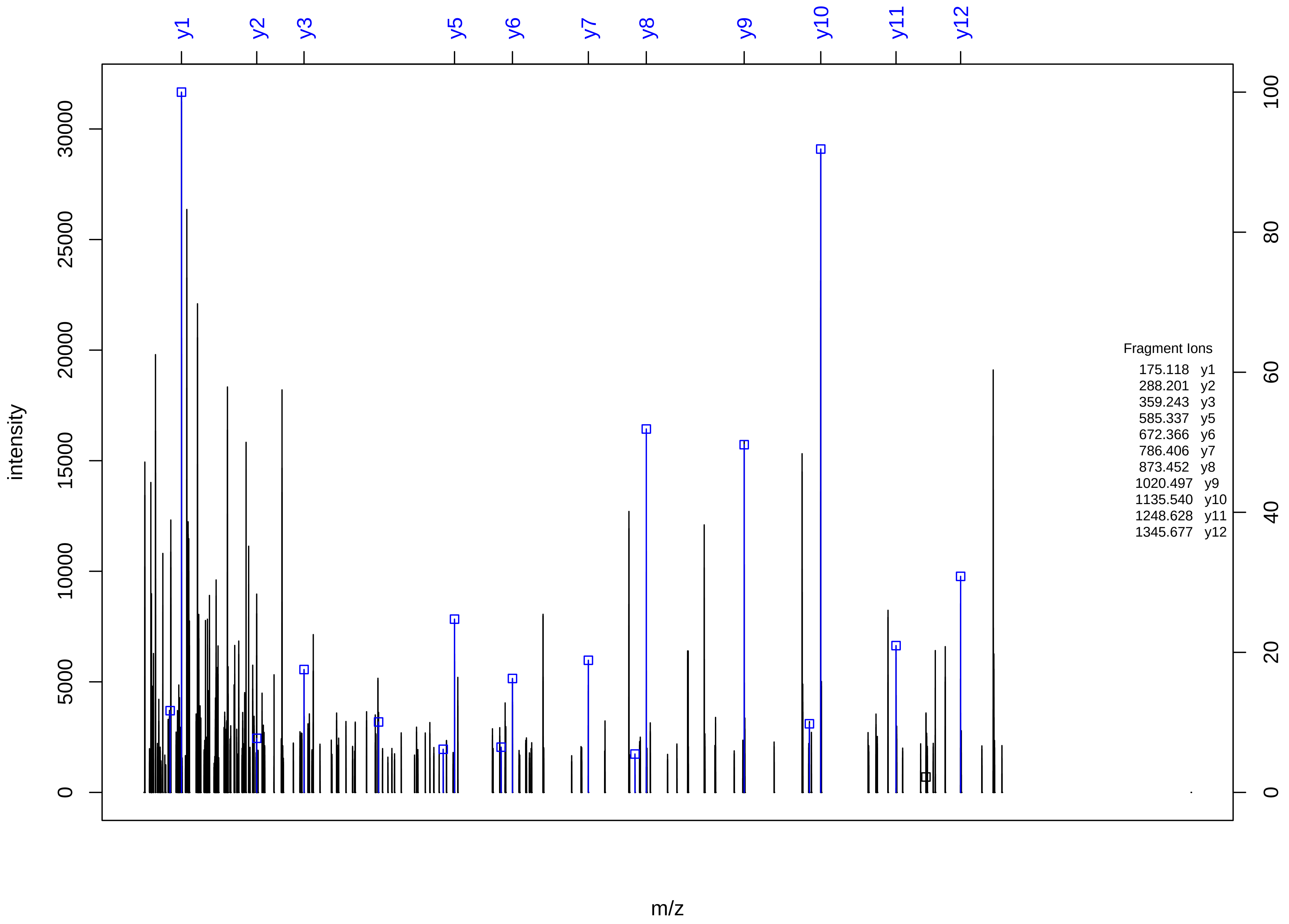

CDPIDFSNSPEALR / File: sydney_08.raw; SpectrumID: ; scans: 6129

YQPLDQVVVDDTFPDCTLLLR

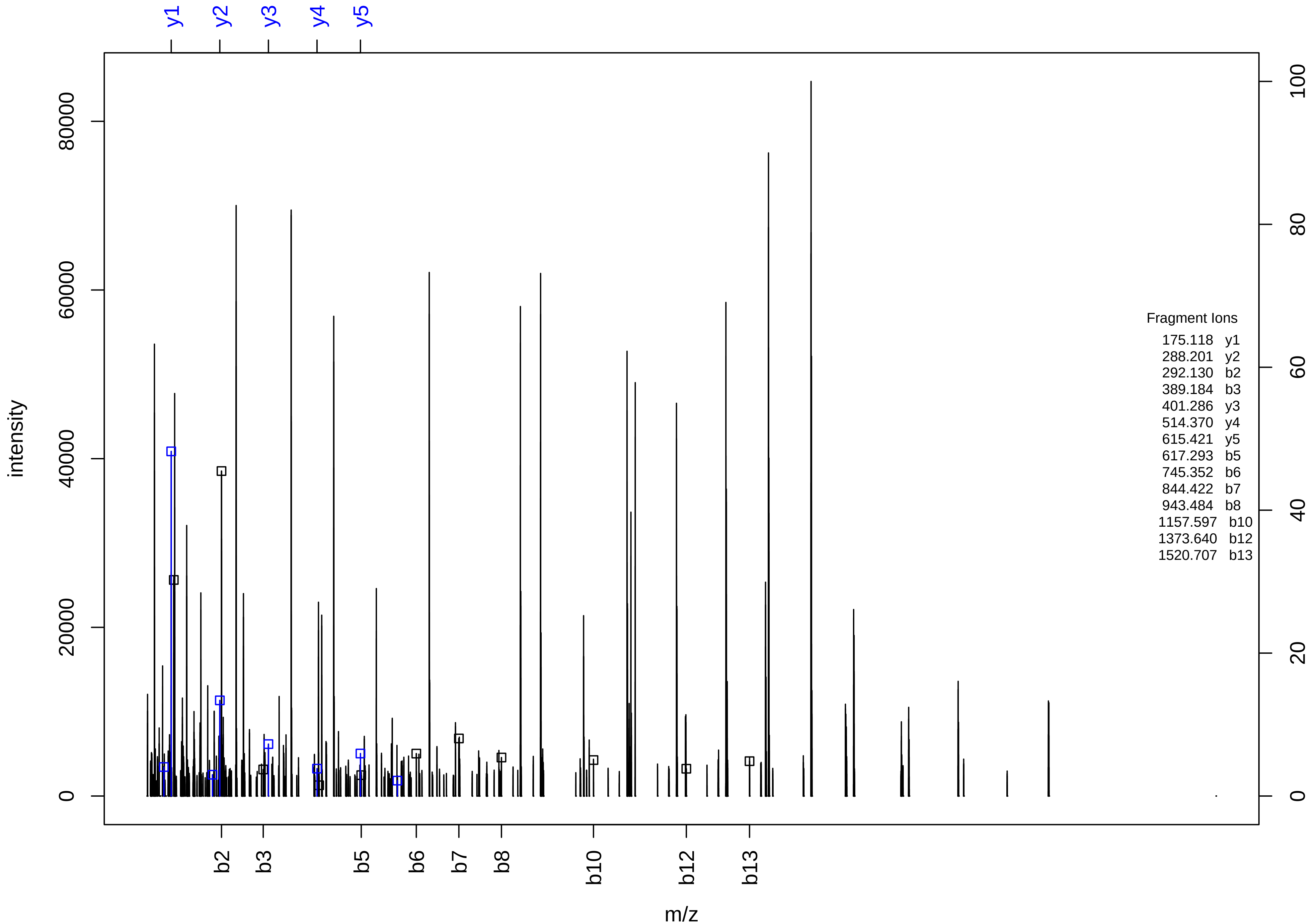

YQPLDQVVVDDTFPDCTLLLR / File: sydney_08.raw; SpectrumID: ; scans: 14105

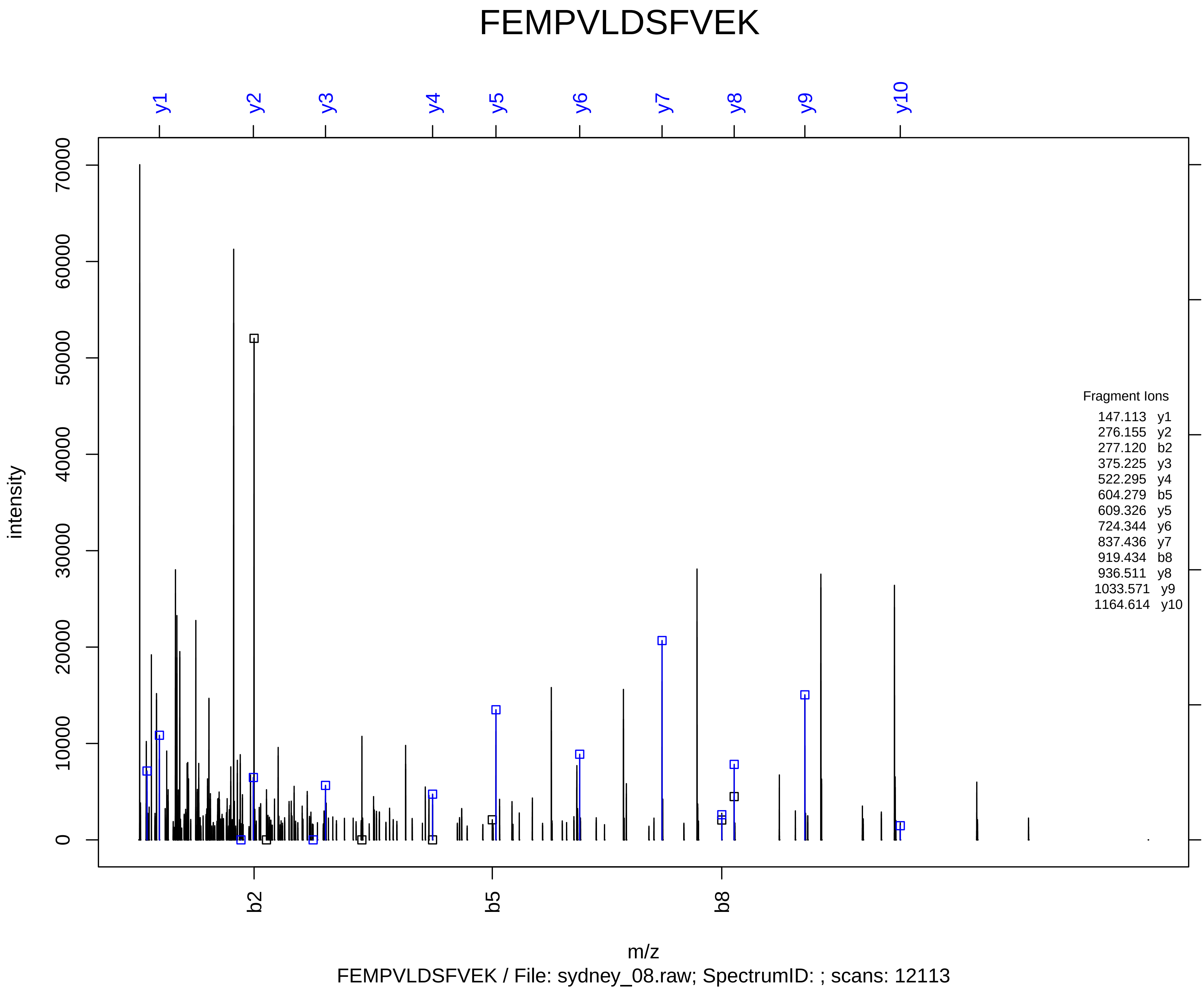
FEMPVLDSFVEK
y1
y2
y3
y4
y5
y6
y7
y8
y9
y10
intensity
0
10000
20000
30000
40000
50000
60000
70000
0
20
40
60
80
100
b2
b5
b8
m/z
Fragment Ions
147.113 y1
276.155 y2
277.120 b2
375.225 y3
522.295 y4
604.279 b5
609.326 y5
724.344 y6
837.436 y7
919.434 b8
936.511 y8
1033.571 y9
1164.614 y10
FEMPVLDSFVEK / File: sydney_08.raw; SpectrumID: ; scans: 12113

NNDGYIDYAEFAK

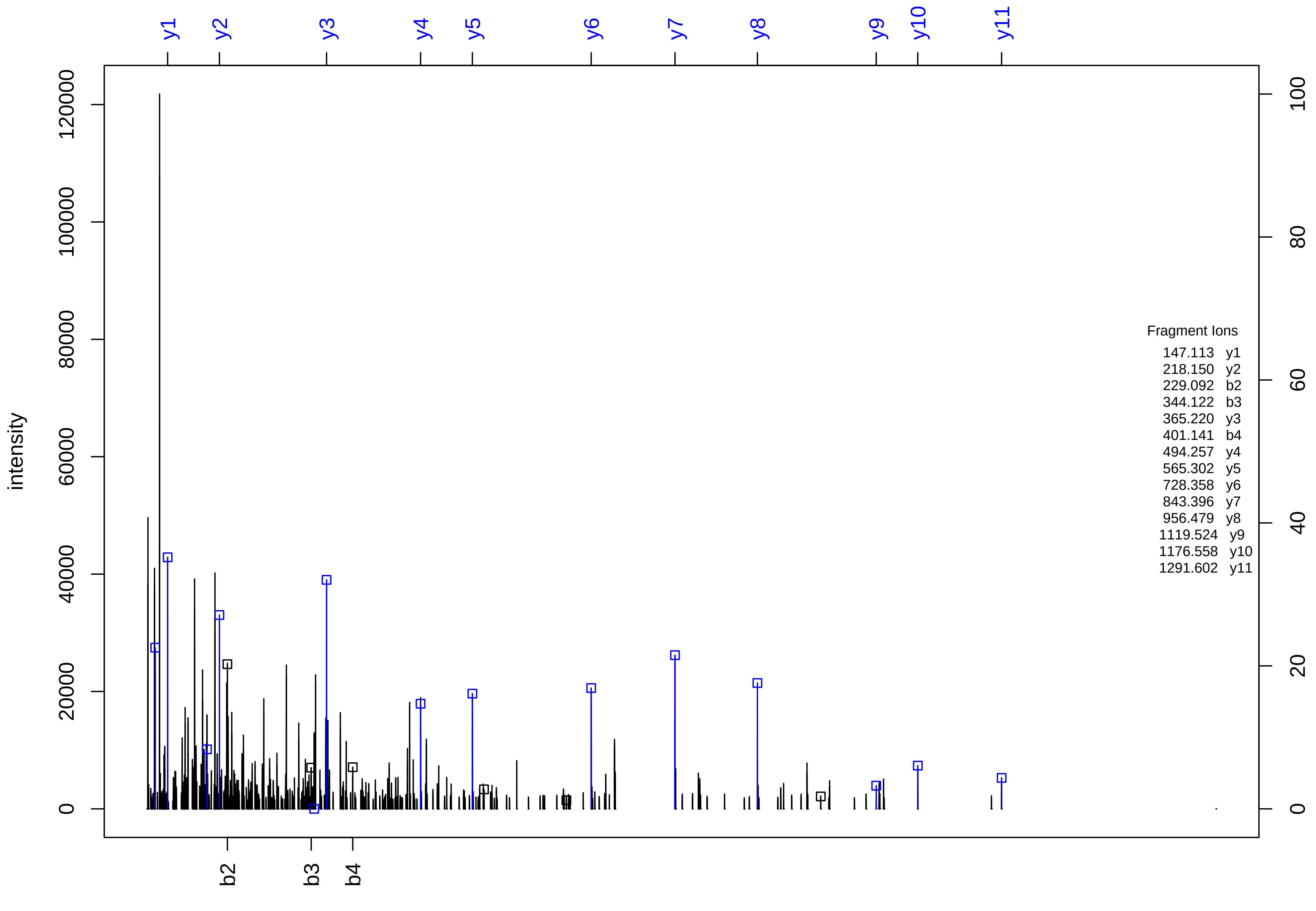

NNDGYIDYAEFAK / File: sydney_08.raw; SpectrumID: ; scans: 6095

LQDDILDSLGQGINELK

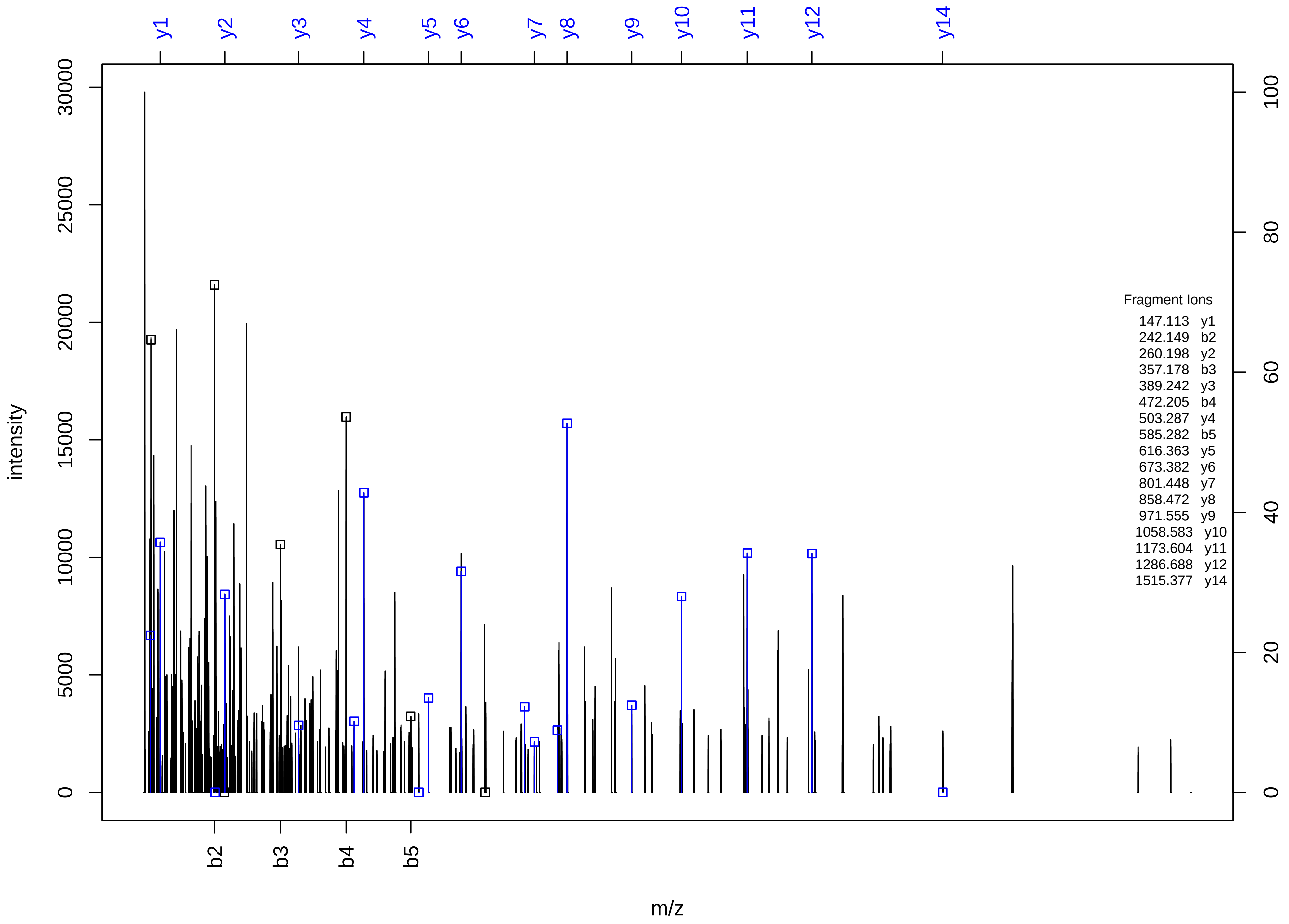

LQDDILDSLGQGINELK / File: sydney_08.raw; SpectrumID: ; scans: 12599

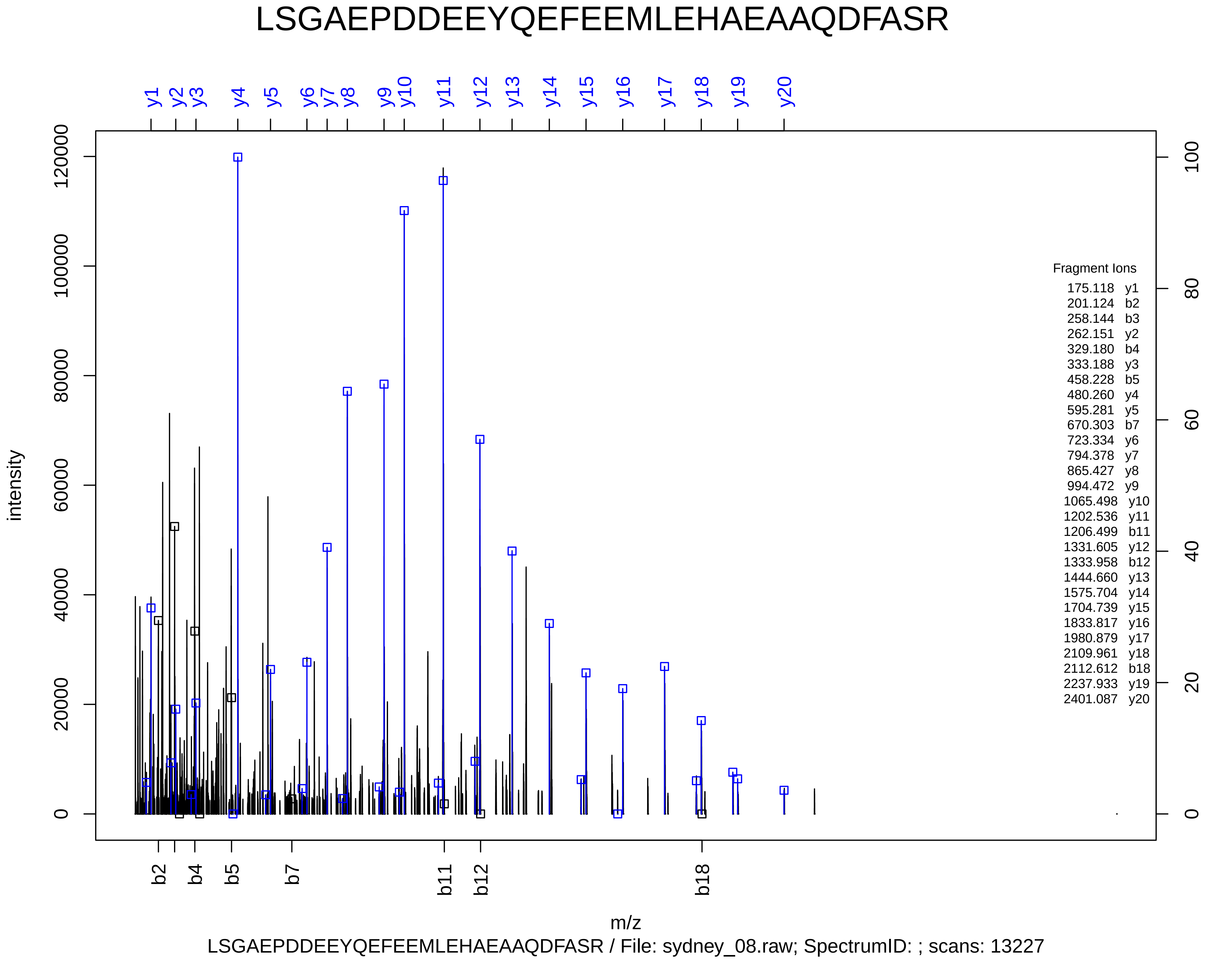

LSGAEPDDEEYQEFEEMLEHAEAAQDFASR
y1 y2 y3 y4 y5 y6 y7 y8 y9 y10 y11 y12 y13 y14 y15 y16 y17 y18 y19 y20
b2 b4 b5 b7 b11 b12 b18
intensity
m/z
Fragment Ions
175.118 y1
201.124 b2
258.144 b3
262.151 y2
329.180 b4
333.188 y3
458.228 b5
480.260 y4
595.281 y5
670.303 b7
723.334 y6
794.378 y7
865.427 y8
994.472 y9
1065.498 y10
1202.536 y11
1206.499 b11
1331.605 y12
1333.958 b12
1444.660 y13
1575.704 y14
1704.739 y15
1833.817 y16
1980.879 y17
2109.961 y18
2112.612 b18
2237.933 y19
2401.087 y20
LSGAEPDDEEYQEFEEMLEHAEAAQDFASR / File: sydney_08.raw; SpectrumID: ; scans: 13227

TIQGLIDFIK

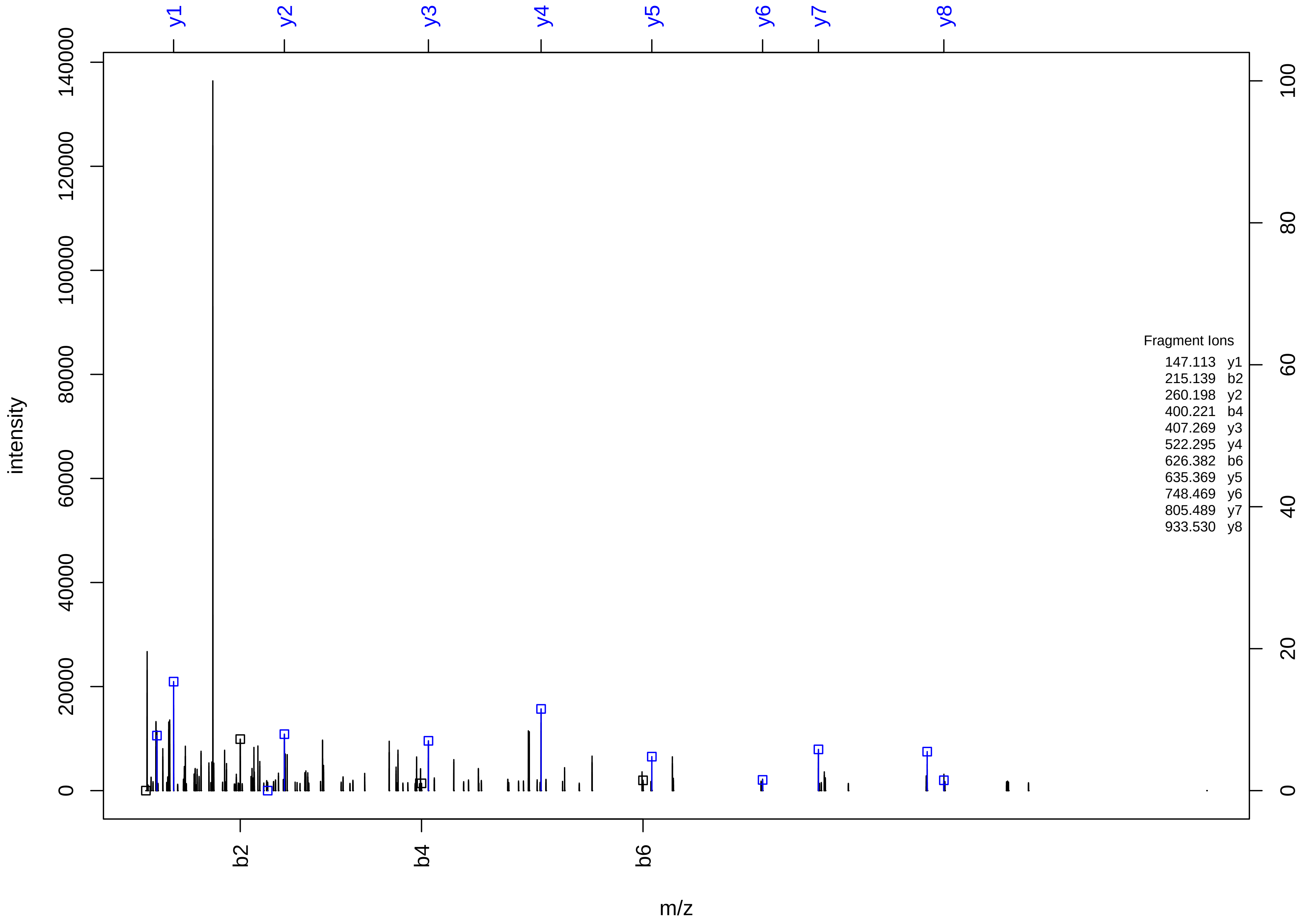

TIQGLIDFIK / File: sydney_08.raw; SpectrumID: ; scans: 11191

LPSMGDQEPPGQEK

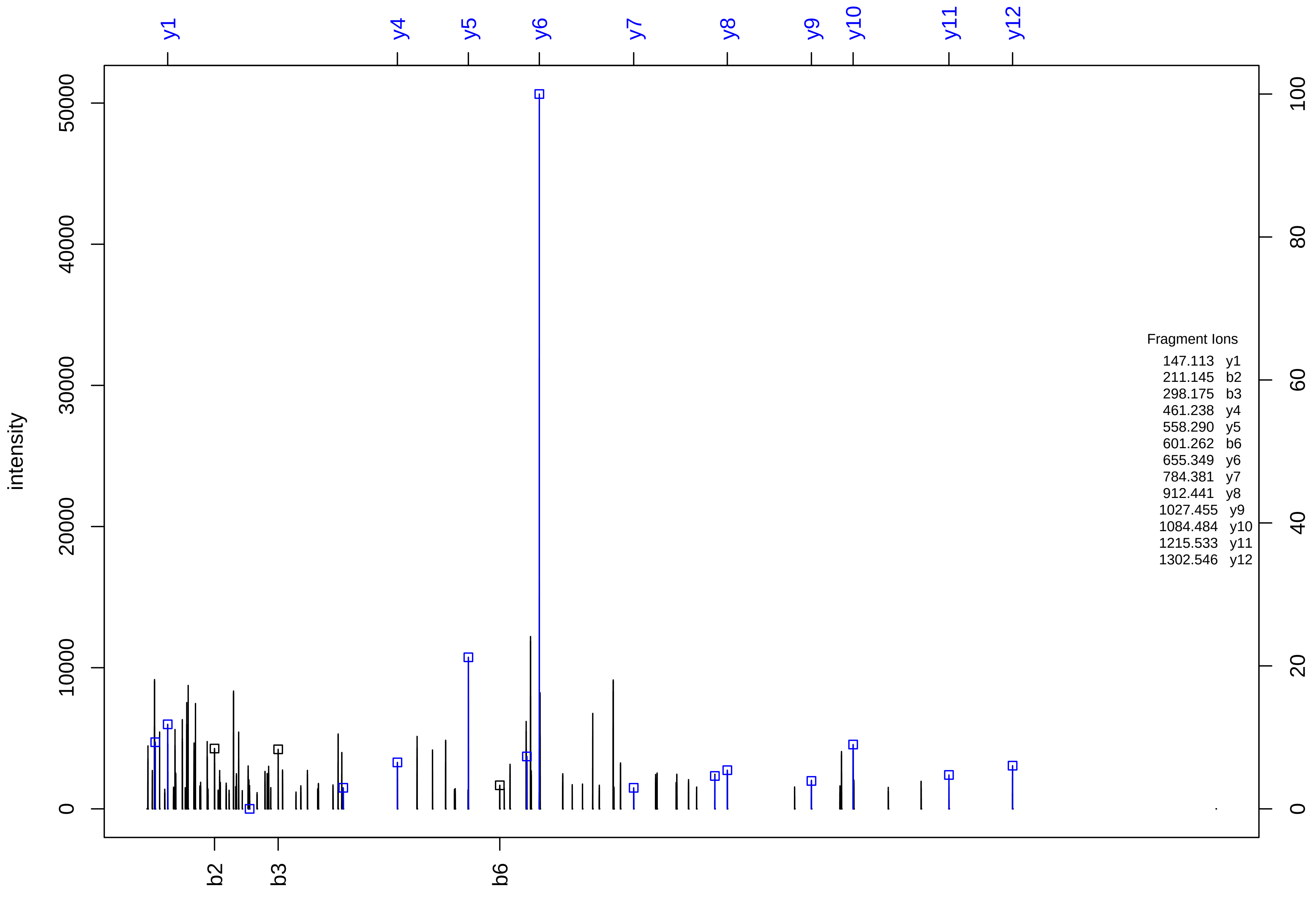

LPSMGDQEPPGQEK / File: sydney_09.raw; SpectrumID: ; scans: 2391

SILTVPWVELGGK

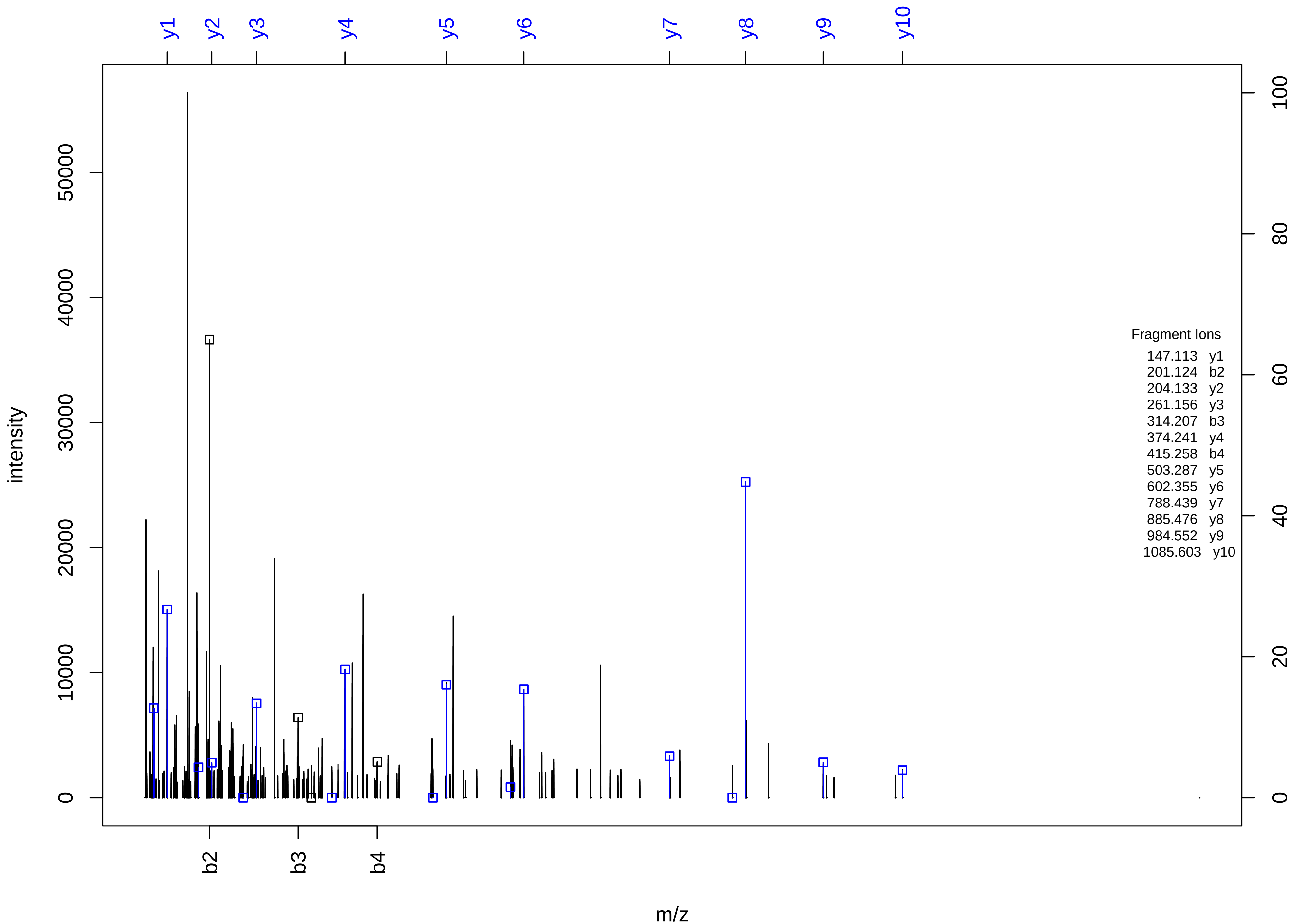

SILTVPWVELGGK / File: sydney_09.raw; SpectrumID: ; scans: 13530

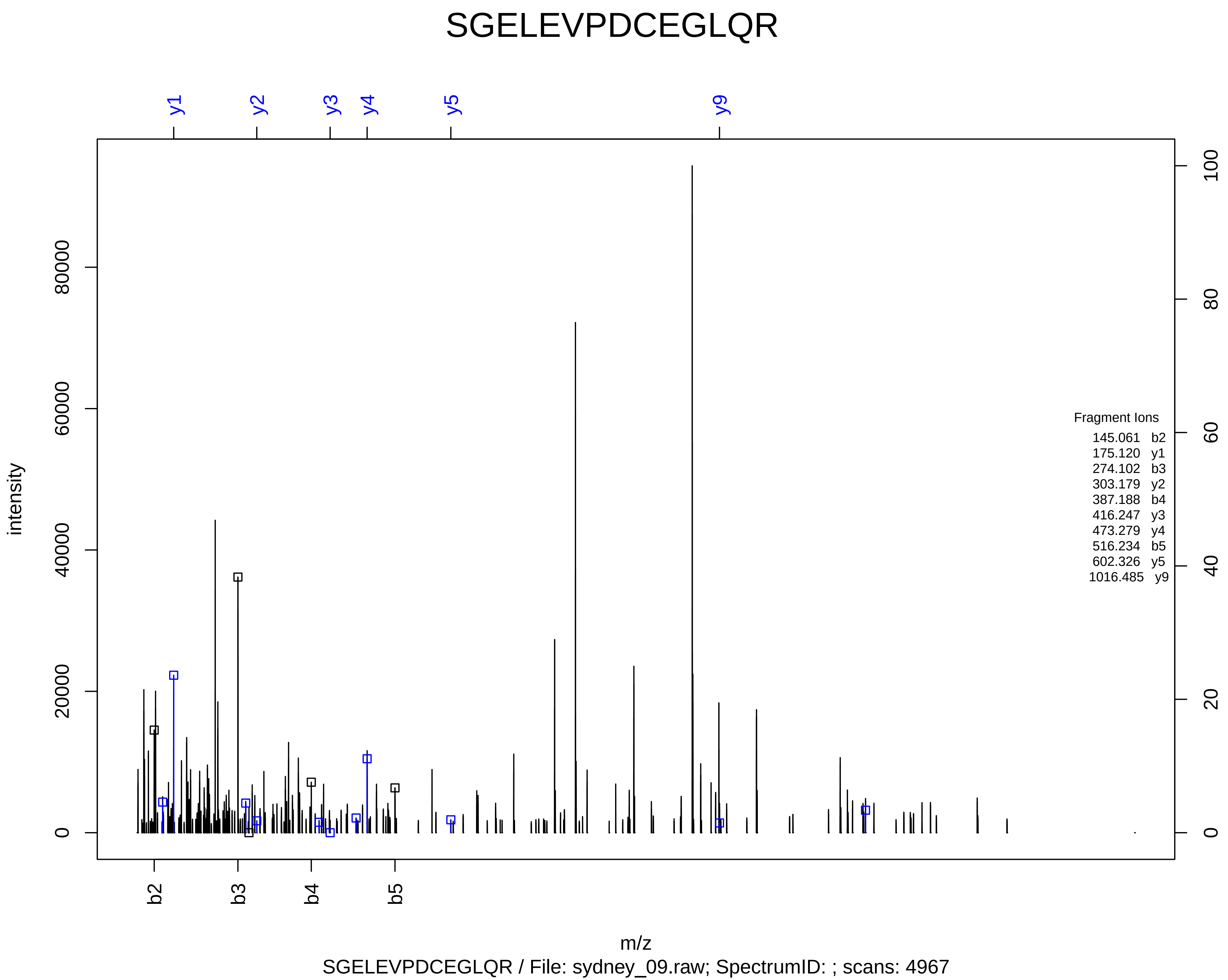

SGELEVPDCEGLQR
y1
y2
y3
y4
y5
y9
intensity
0
20000
40000
60000
80000
0
20
40
60
80
100
Fragment Ions
145.061 b2
175.120 y1
274.102 b3
303.179 y2
387.188 b4
416.247 y3
473.279 y4
516.234 b5
602.326 y5
1016.485 y9
b2
b3
b4
b5
m/z
SGELEVPDCEGLQR / File: sydney_09.raw; SpectrumID: ; scans: 4967

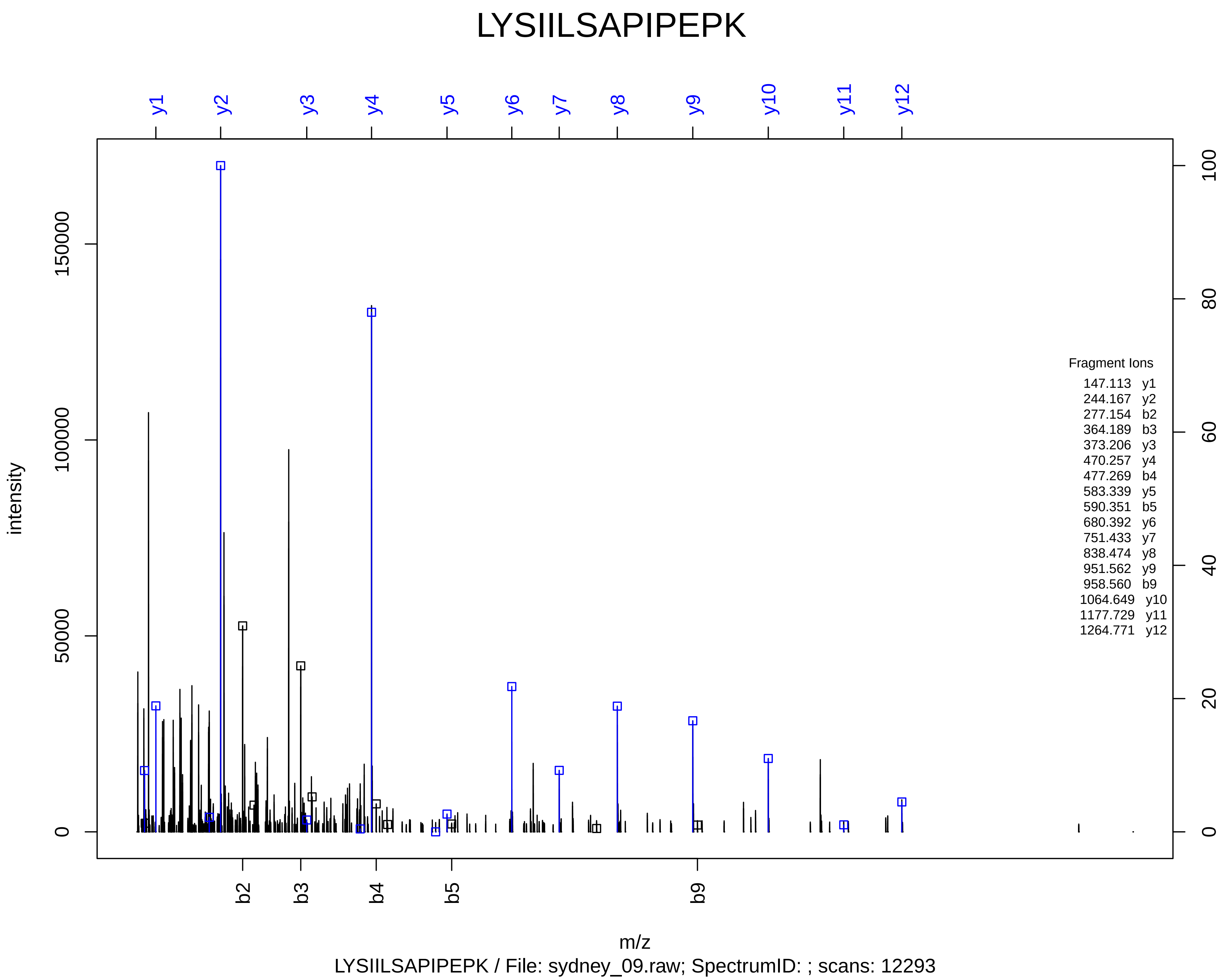
LYSIILSAPIPEPK
y1
y2
y3
y4
y5
y6
y7
y8
y9
y10
y11
y12
intensity
0
50000
100000
150000
0
20
40
60
80
100
b2
b3
b4
b5
b9
m/z
Fragment Ions
147.113 y1
244.167 y2
277.154 b2
364.189 b3
373.206 y3
470.257 y4
477.269 b4
583.339 y5
590.351 b5
680.392 y6
751.433 y7
838.474 y8
951.562 y9
958.560 b9
1064.649 y10
1177.729 y11
1264.771 y12
LYSIILSAPIPEPK / File: sydney_09.raw; SpectrumID: ; scans: 12293

(Ac)MWPPDAEPEPDPESAHGPR

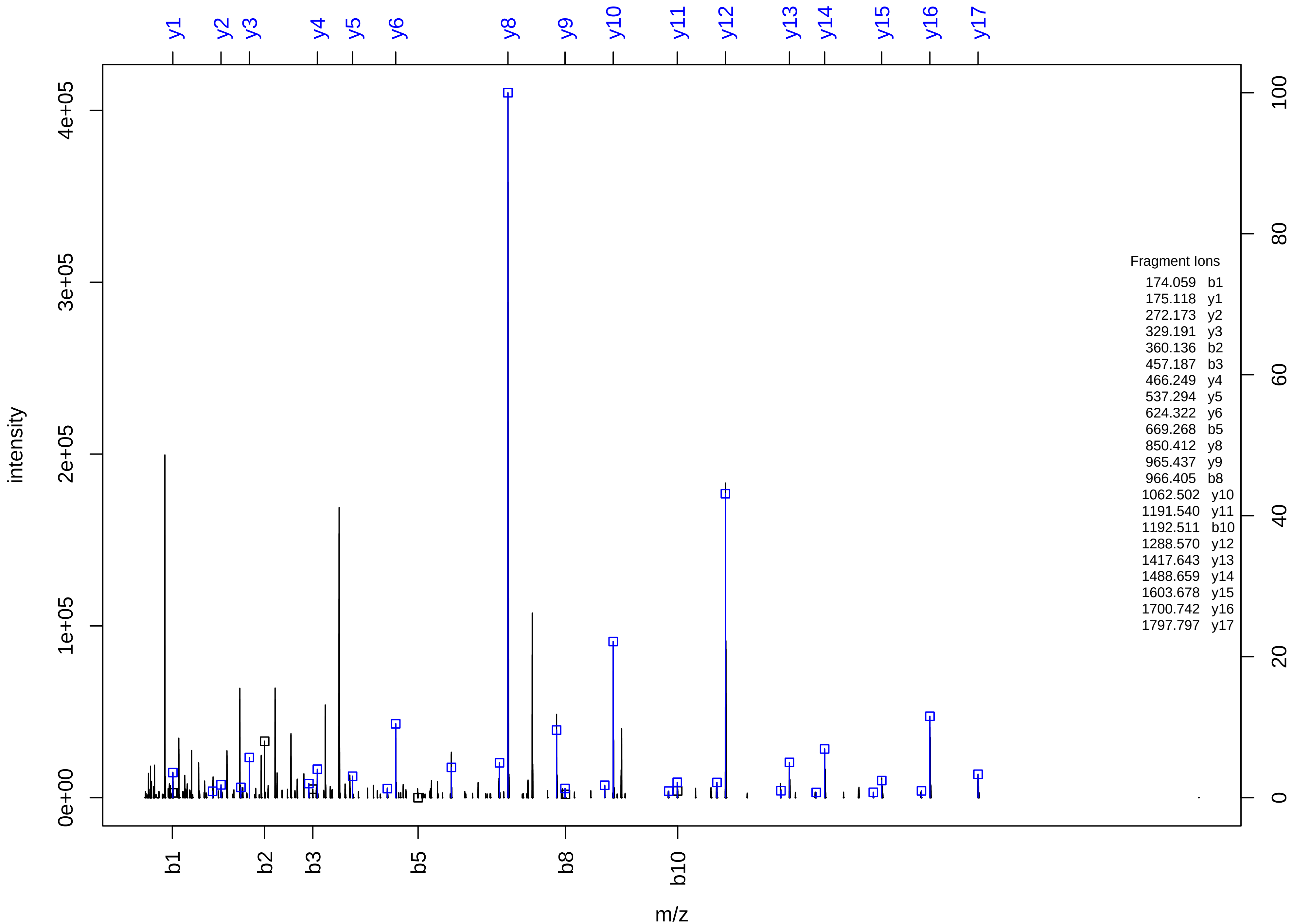

MWPPDAEPEPDPESAHGPR / File: sydney_09.raw; SpectrumID: ; scans: 8195

(Ac)AAAMDVDTPSGTNSGAGK

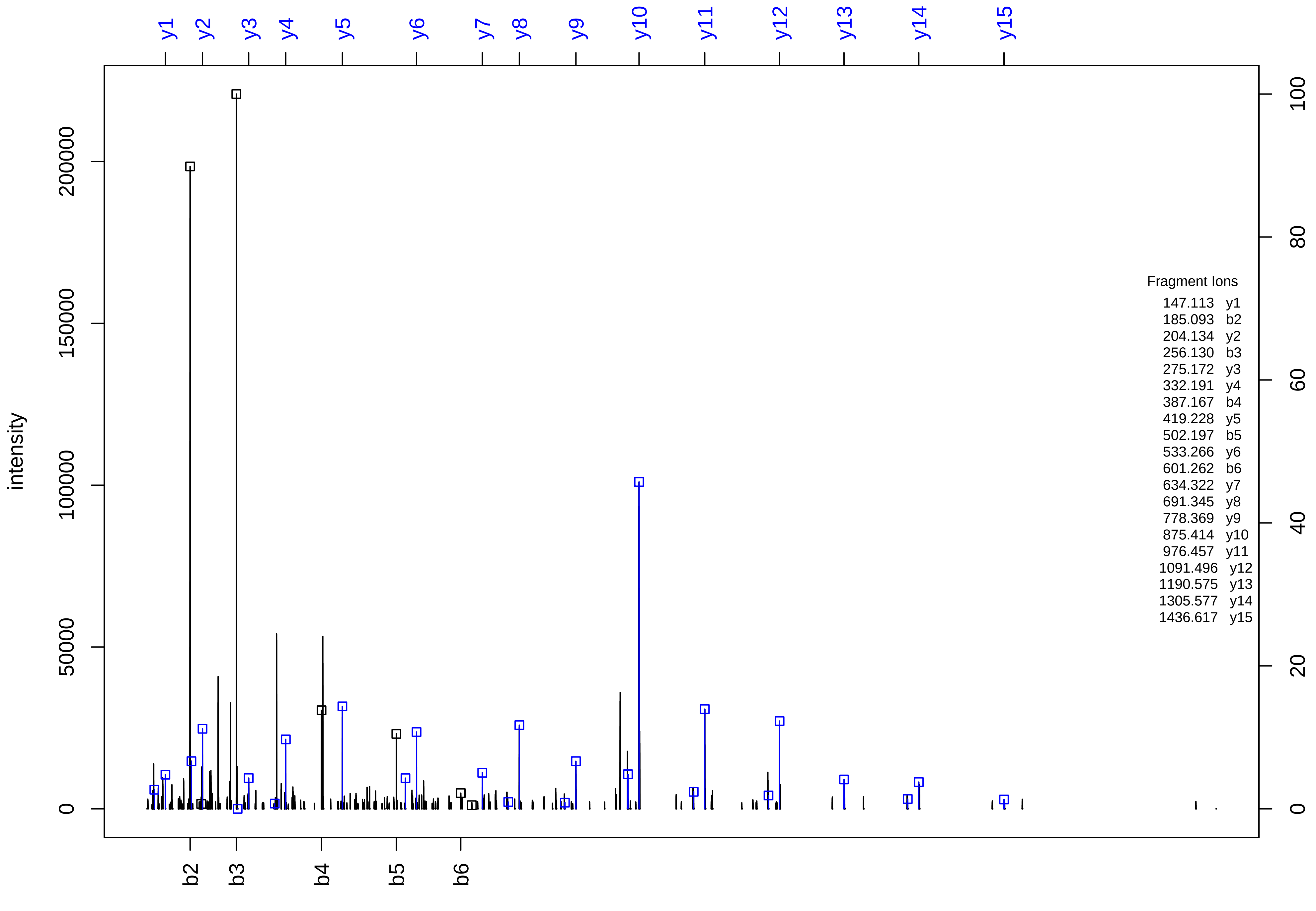

AAAMDVDTPSGTNSGAGK / File: sydney_09.raw; SpectrumID: ; scans: 4505

(Ac)AGILFEDIFDVK

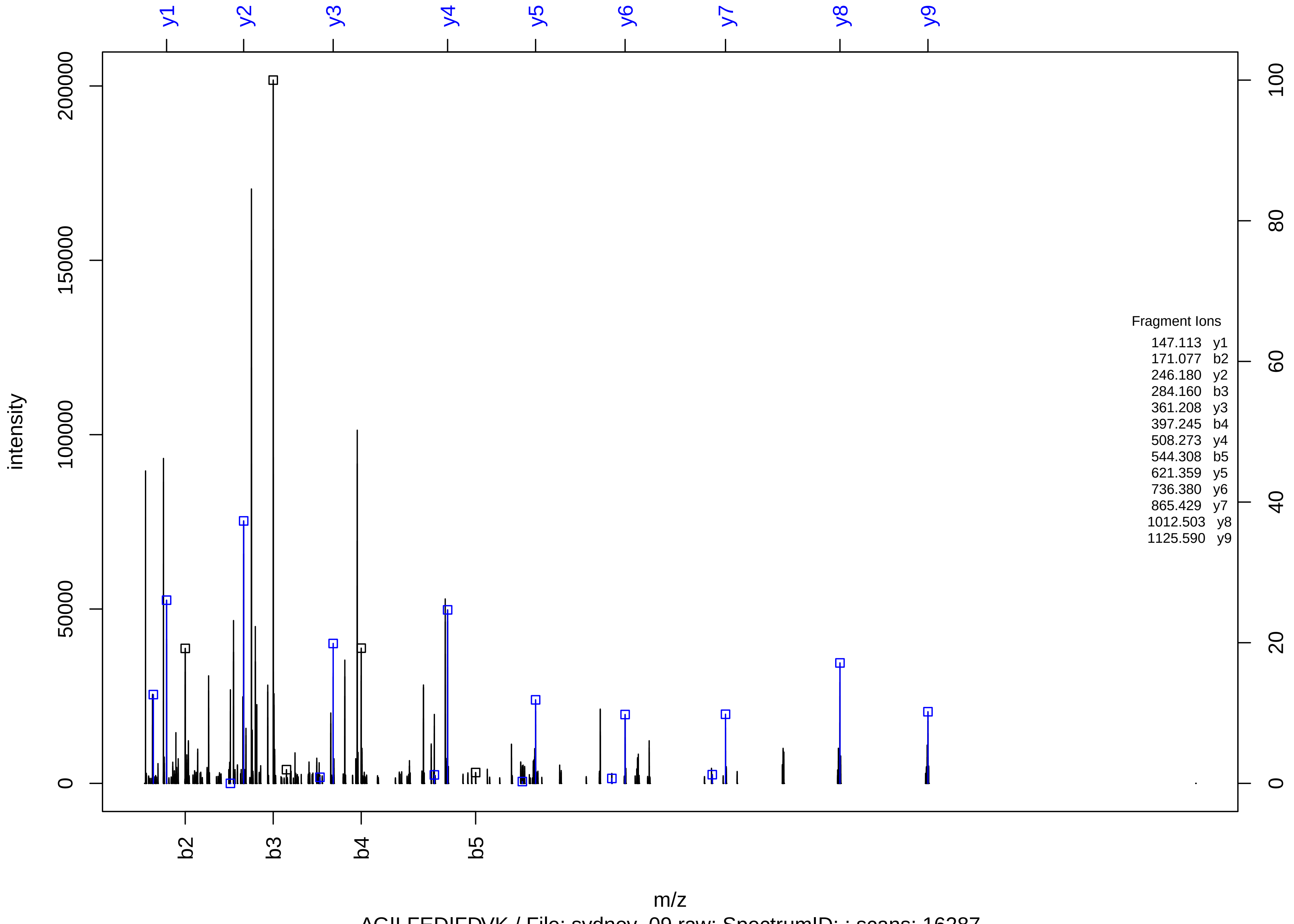

AGILFEDIFDVK / File: sydney_09.raw; SpectrumID: ; scans: 16287

SPAFQLVELGPGR

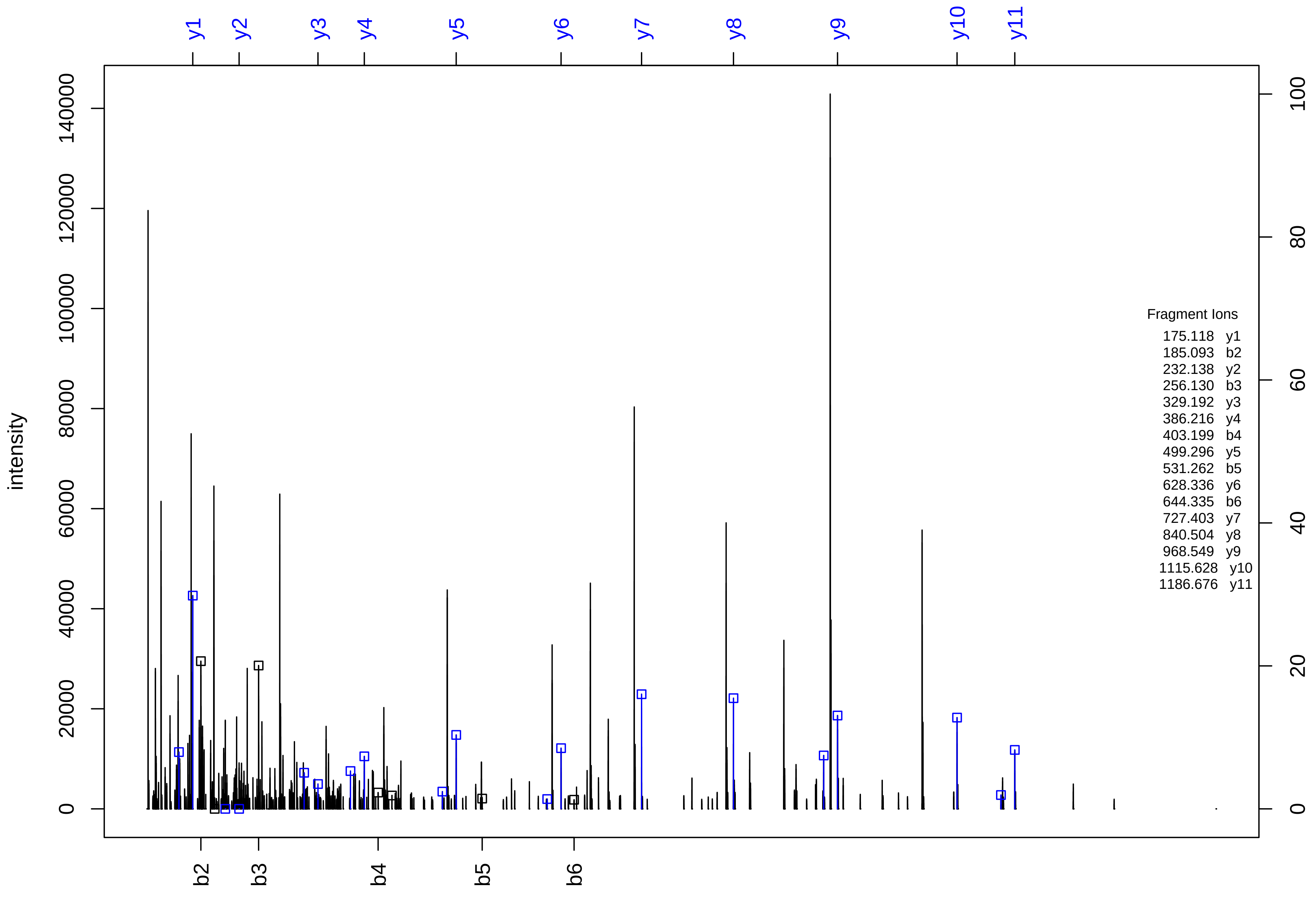

SPAFQLVELGPGR / File: sydney_09.raw; SpectrumID: ; scans: 9408

EIQ^Q^GVESWIALGN^R

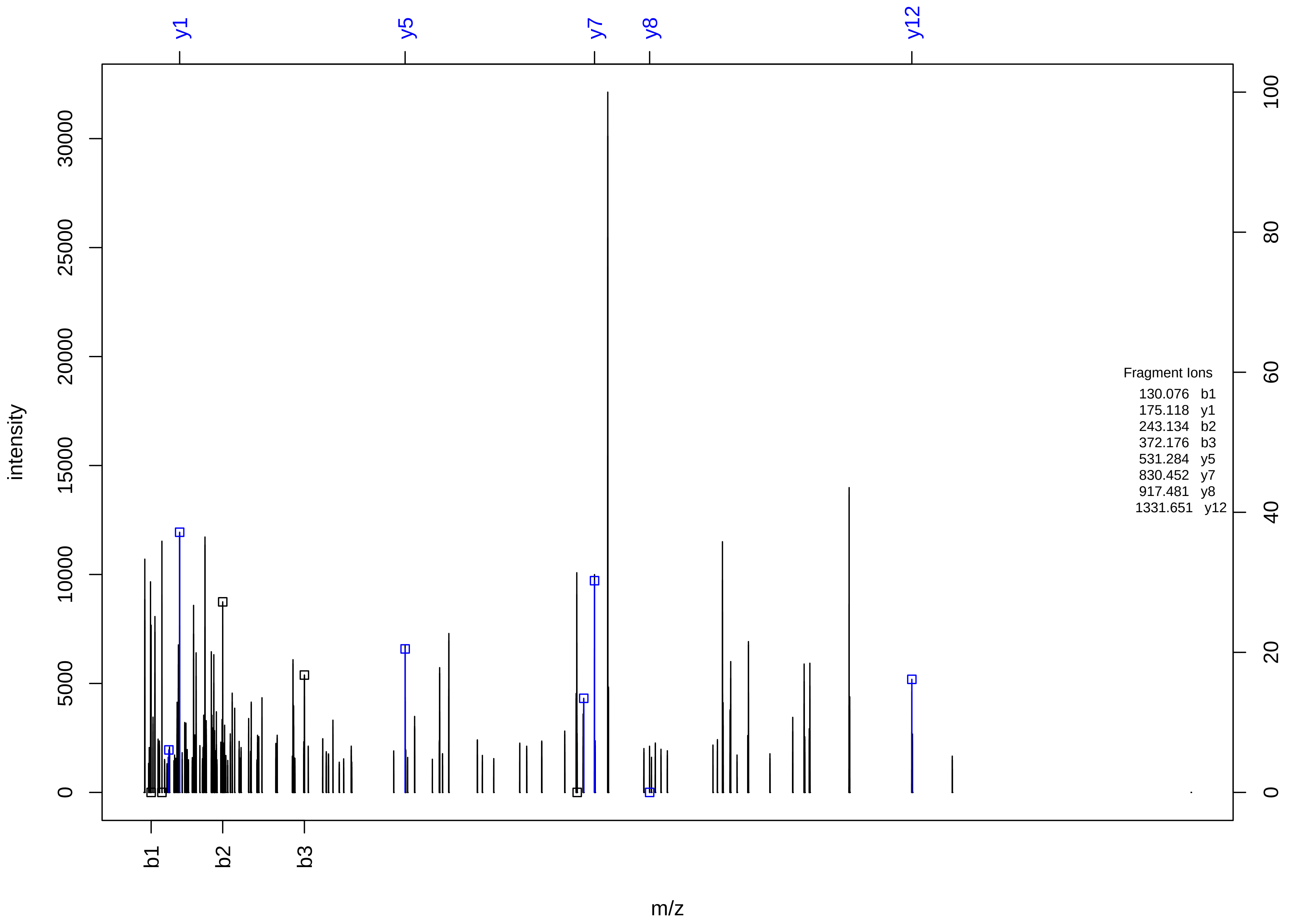

EIQQGVESWIALGNR / File: sydney_09.raw; SpectrumID: ; scans: 5875

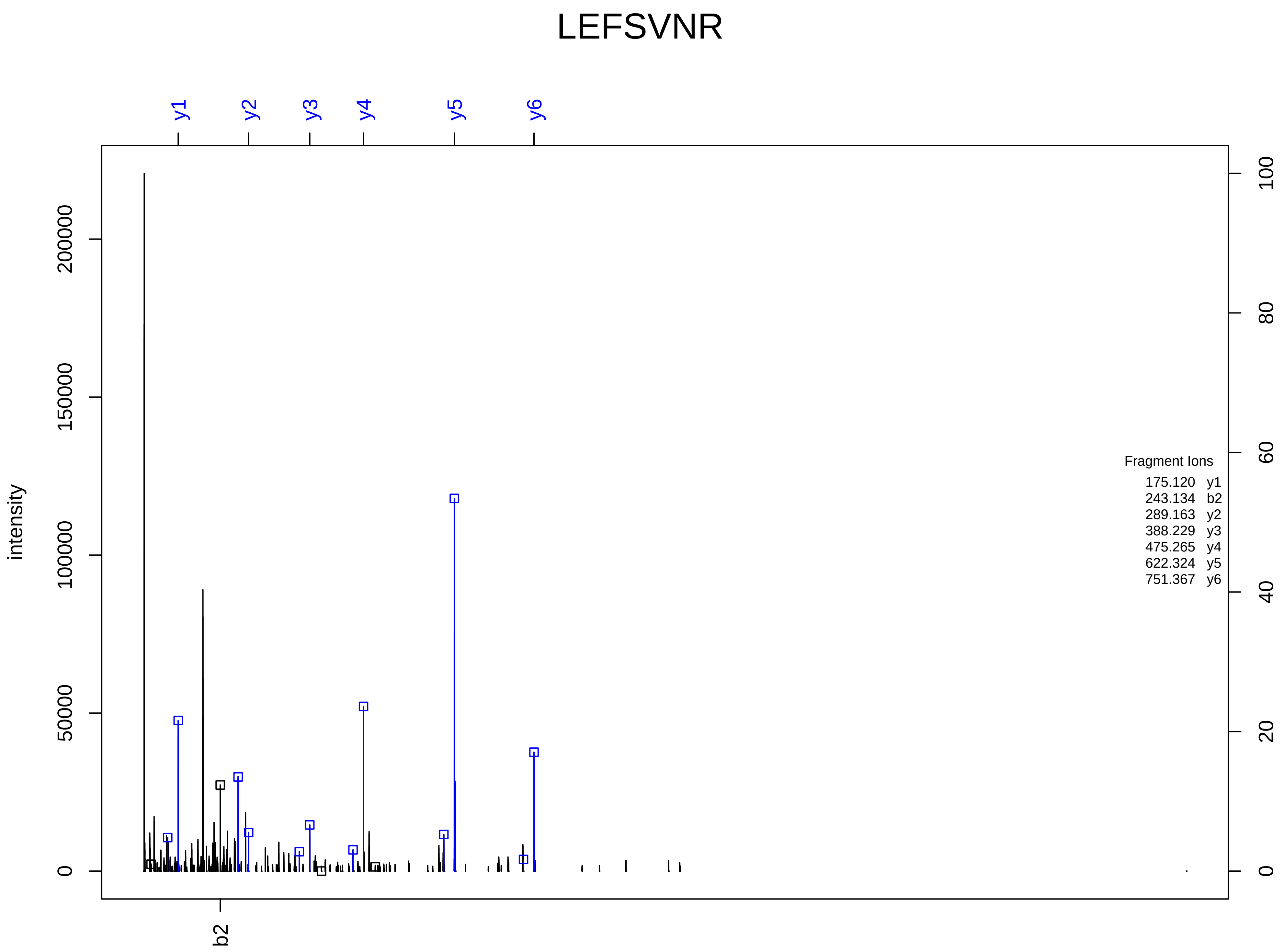

LEFSVNR / File: sydney_09.raw; SpectrumID: ; scans: 3457

N^AALNLVNSQNR

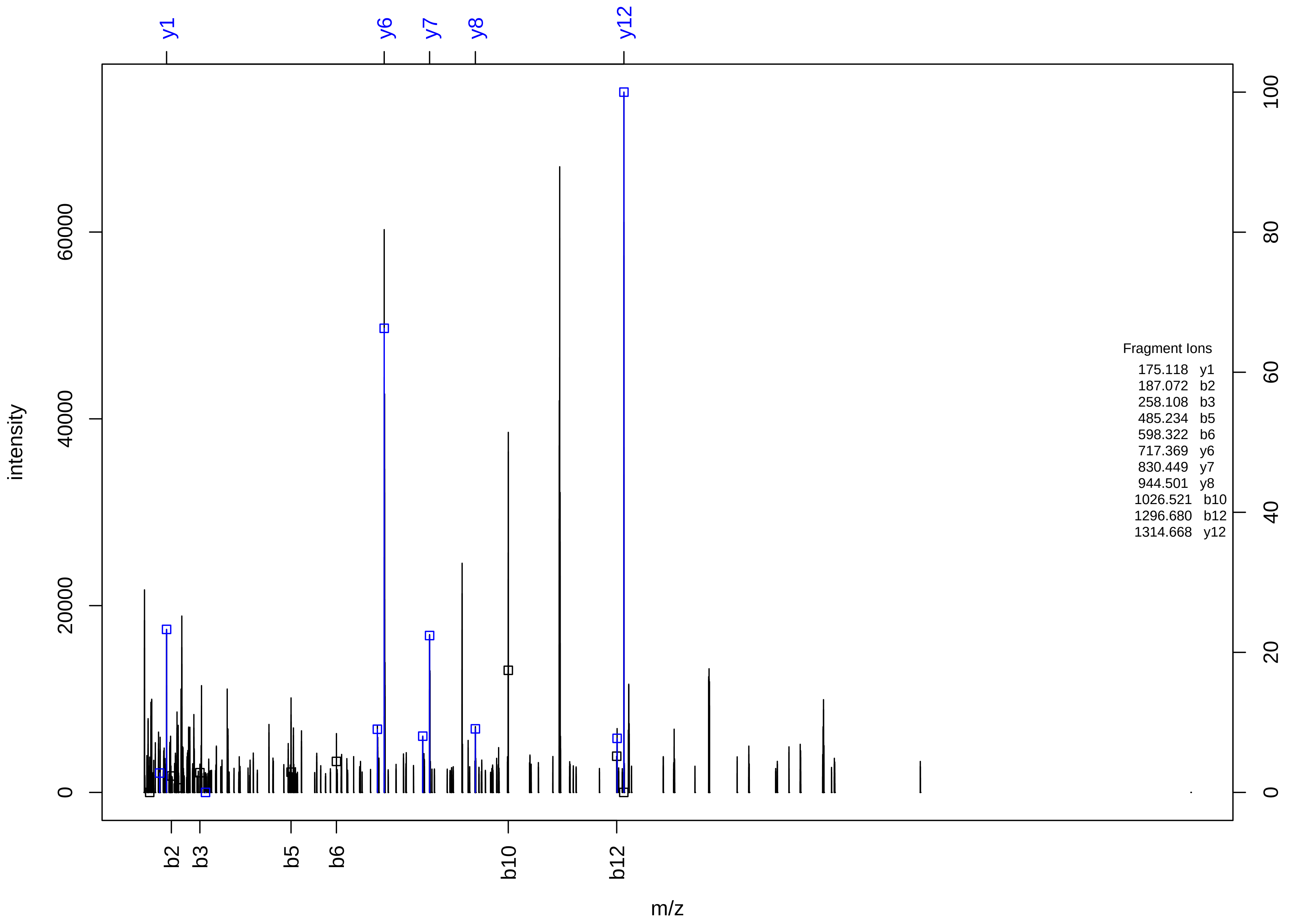

NAALNLVNSQNR / File: sydney_09.raw; SpectrumID: ; scans: 16066

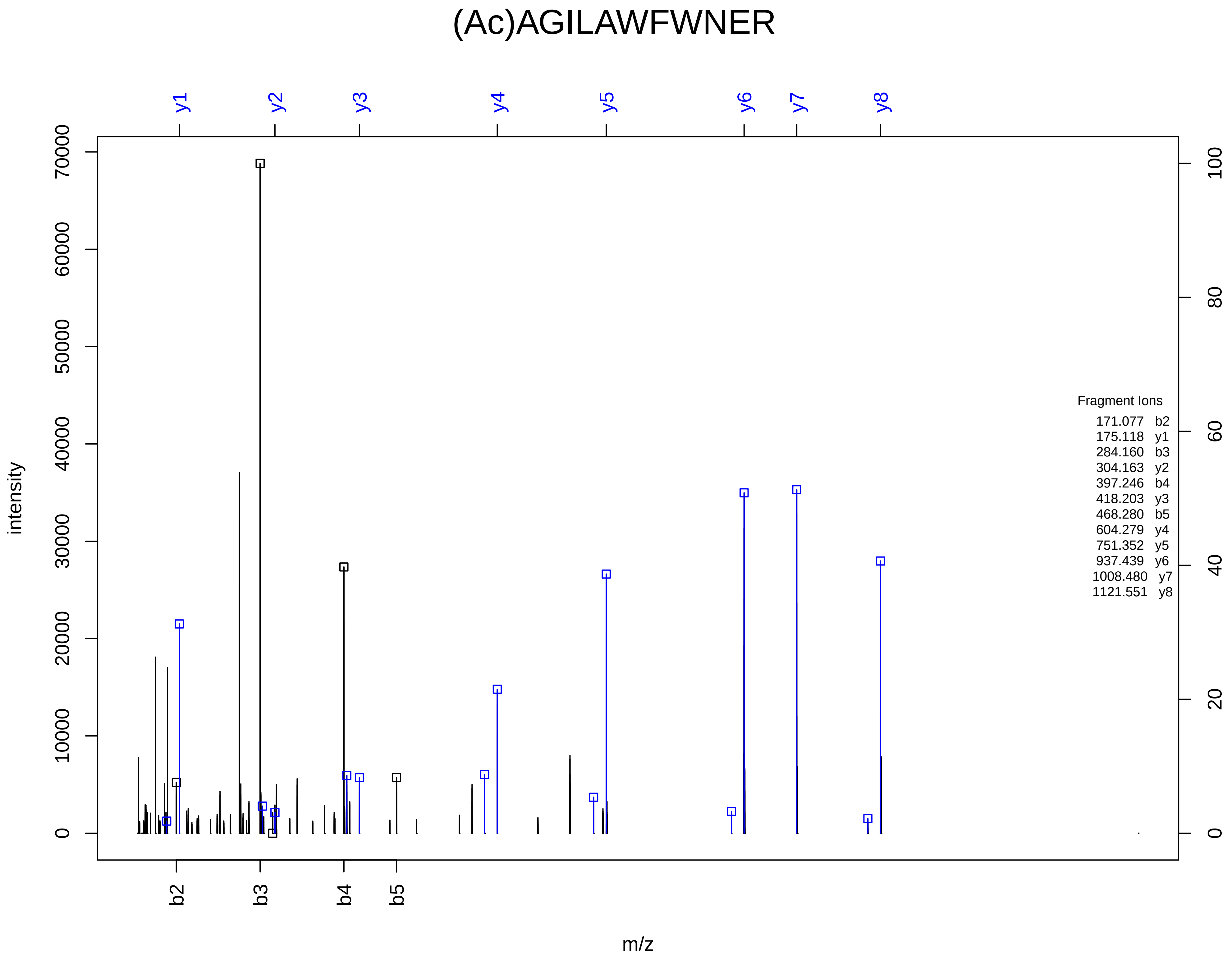

AGILAWFWNER / File: sydney_09.raw; SpectrumID: ; scans: 16290

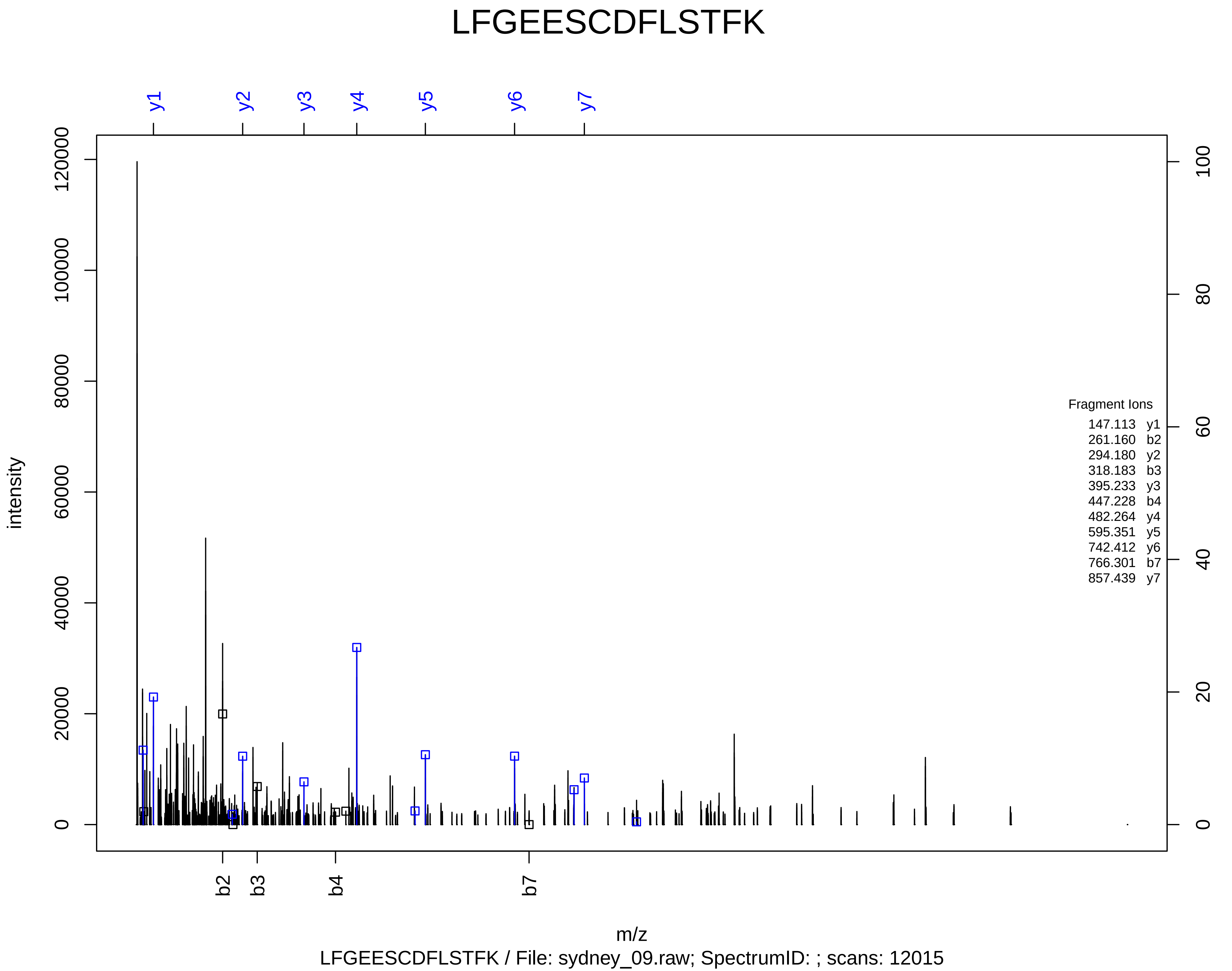
LFGEESCDFLSTFK
y1
y2
y3
y4
y5
y6
y7
intensity
0
20000
40000
60000
80000
100000
120000
0
20
40
60
80
100
b2
b3
b4
b7
m/z
Fragment Ions
147.113 y1
261.160 b2
294.180 y2
318.183 b3
395.233 y3
447.228 b4
482.264 y4
595.351 y5
742.412 y6
766.301 b7
857.439 y7
LFGEESCDFLSTFK / File: sydney_09.raw; SpectrumID: ; scans: 12015

ADQFANEWGR

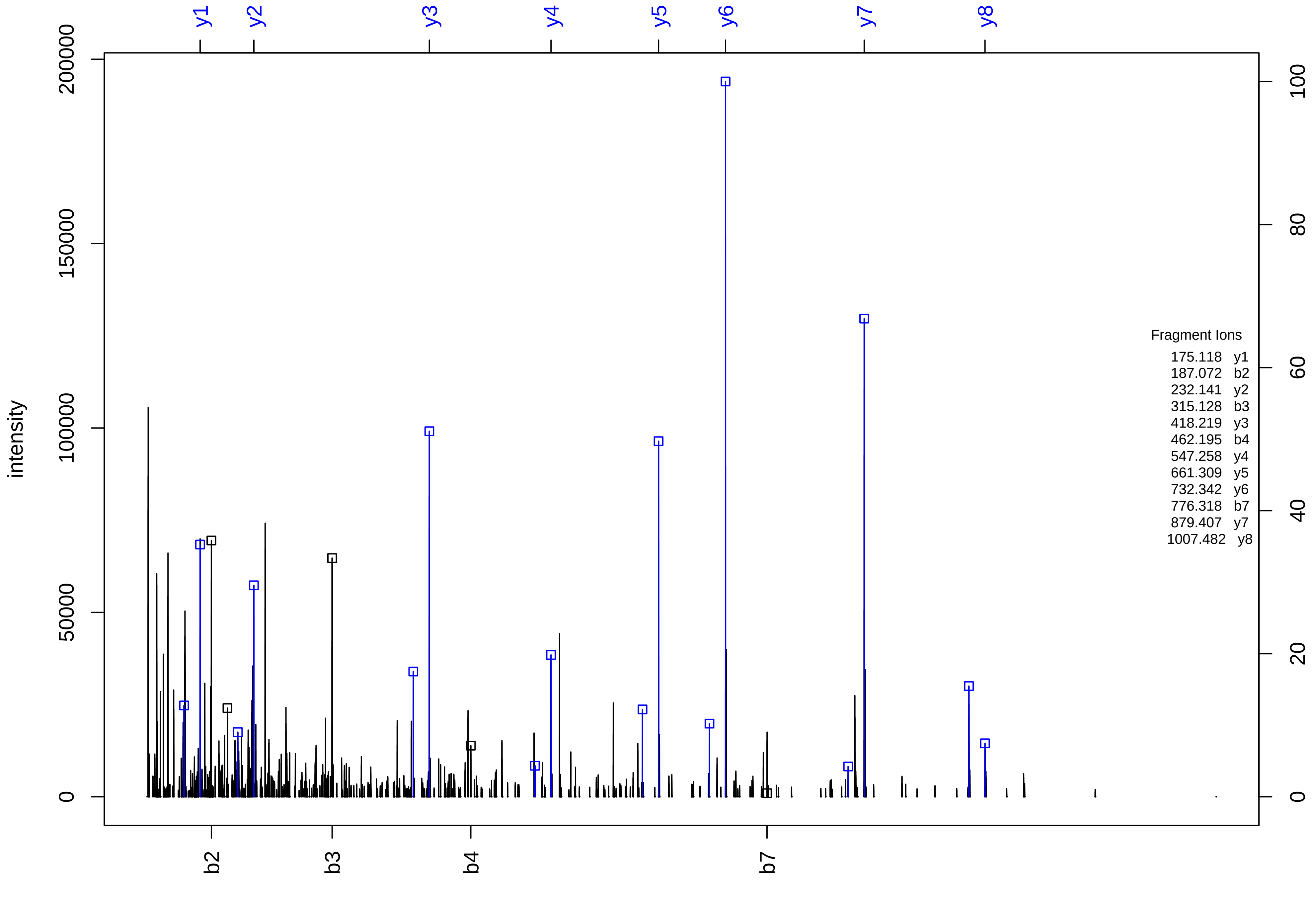

ADQFANEWGR / File: sydney_09.raw; SpectrumID: ; scans: 3713

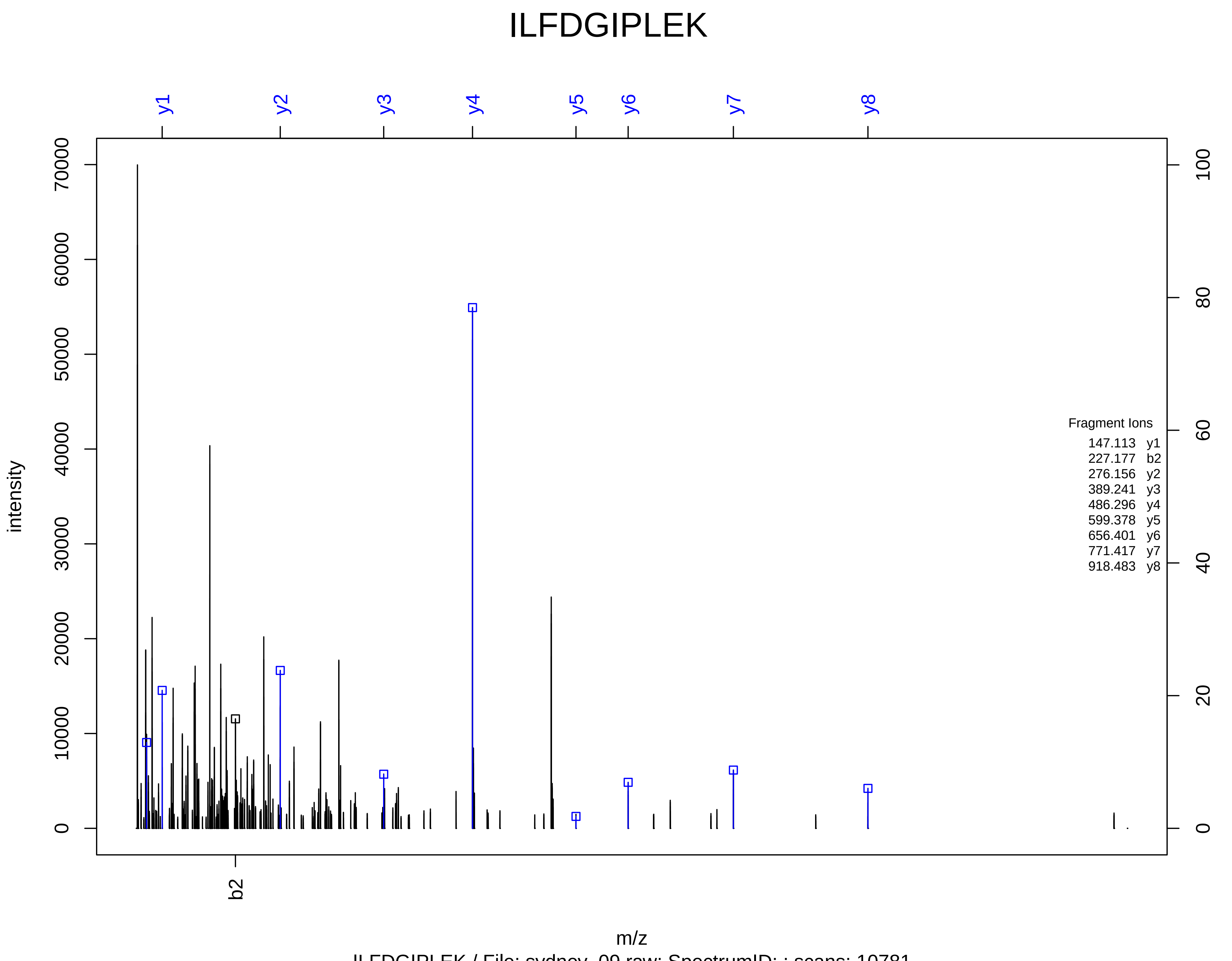
ILFDGIPLEK
y1
y2
y3
y4
y5
y6
y7
y8
b2
intensity
m/z
Fragment Ions
147.113 y1
227.177 b2
276.156 y2
389.241 y3
486.296 y4
599.378 y5
656.401 y6
771.417 y7
918.483 y8
ILFDGIPLEK / File: sydney_09.raw; SpectrumID: ; scans: 10781

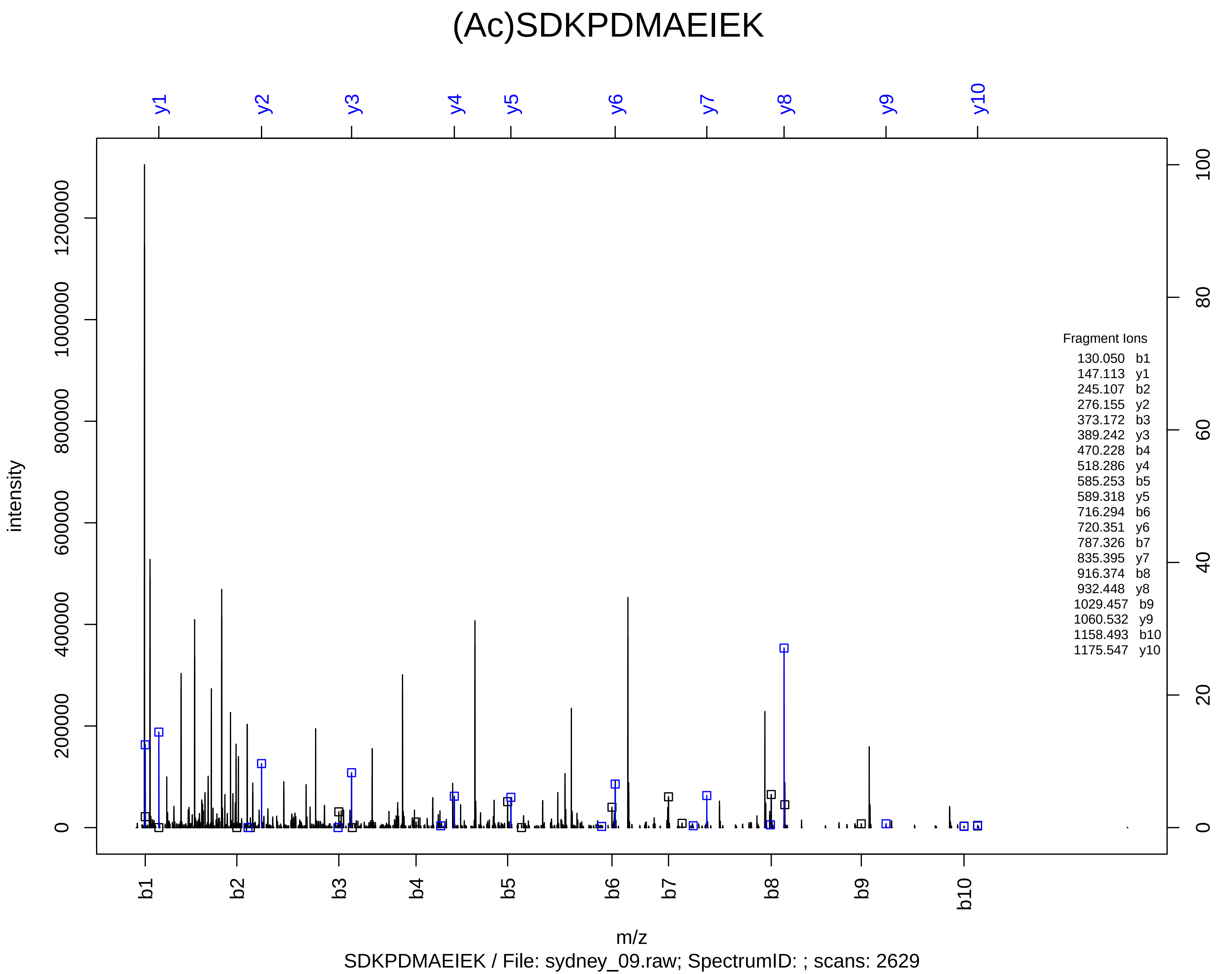

(Ac)SDKPDMAEIEK
y1 y2 y3 y4 y5 y6 y7 y8 y9 y10
b1 b2 b3 b4 b5 b6 b7 b8 b9 b10
intensity
0 200000 400000 600000 800000 1000000 1200000
0 20 40 60 80 100
m/z
Fragment Ions
130.050 b1
147.113 y1
245.107 b2
276.155 y2
373.172 b3
389.242 y3
470.228 b4
518.286 y4
585.253 b5
589.318 y5
716.294 b6
720.351 y6
787.326 b7
835.395 y7
916.374 b8
932.448 y8
1029.457 b9
1060.532 y9
1158.493 b10
1175.547 y10
SDKPDMAEIEK / File: sydney_09.raw; SpectrumID: ; scans: 2629

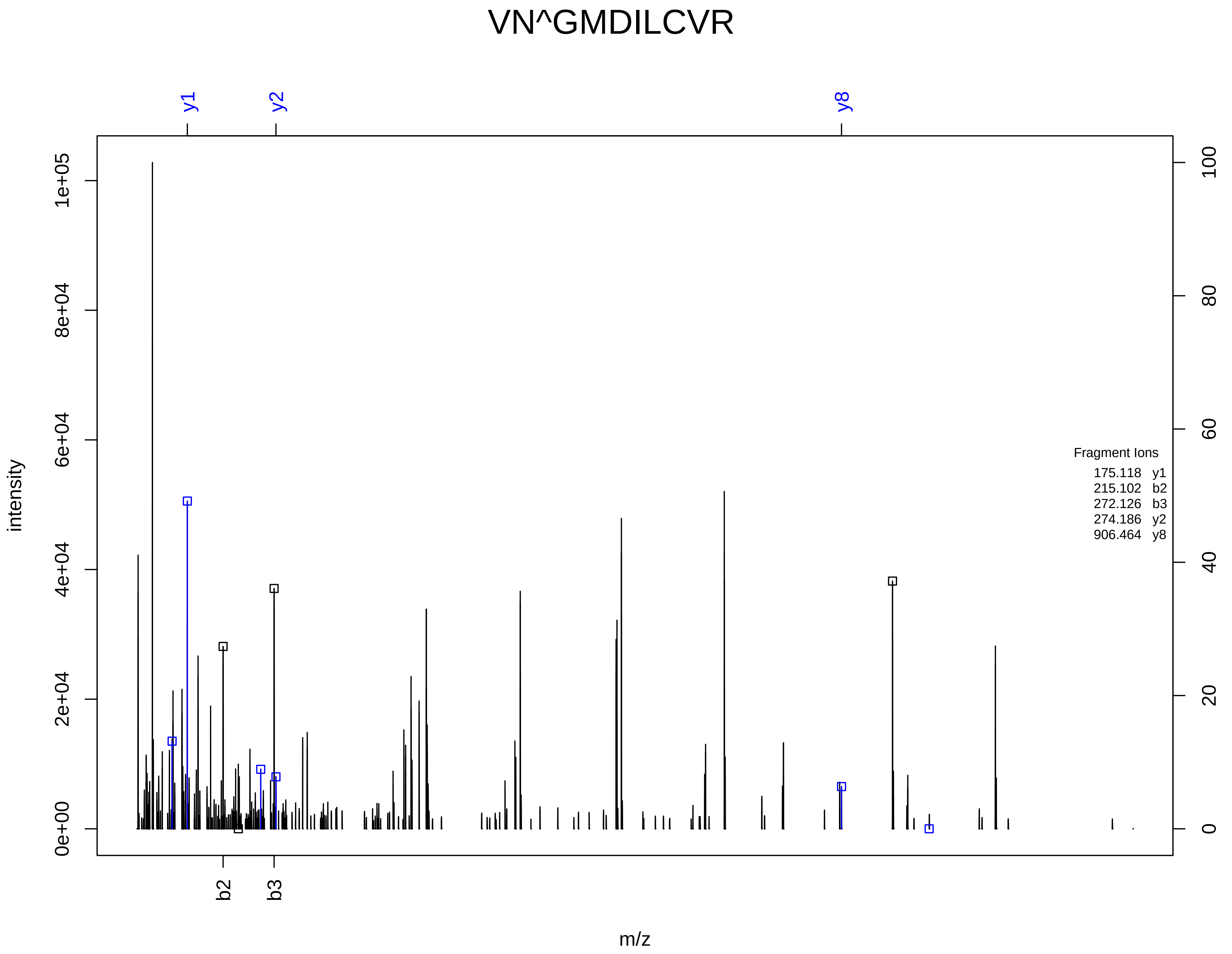

VNGMDILCVR / File: sydney_09.raw; SpectrumID: ; scans: 8657

GEGAGQPSTSAQGQPAAPVPQK

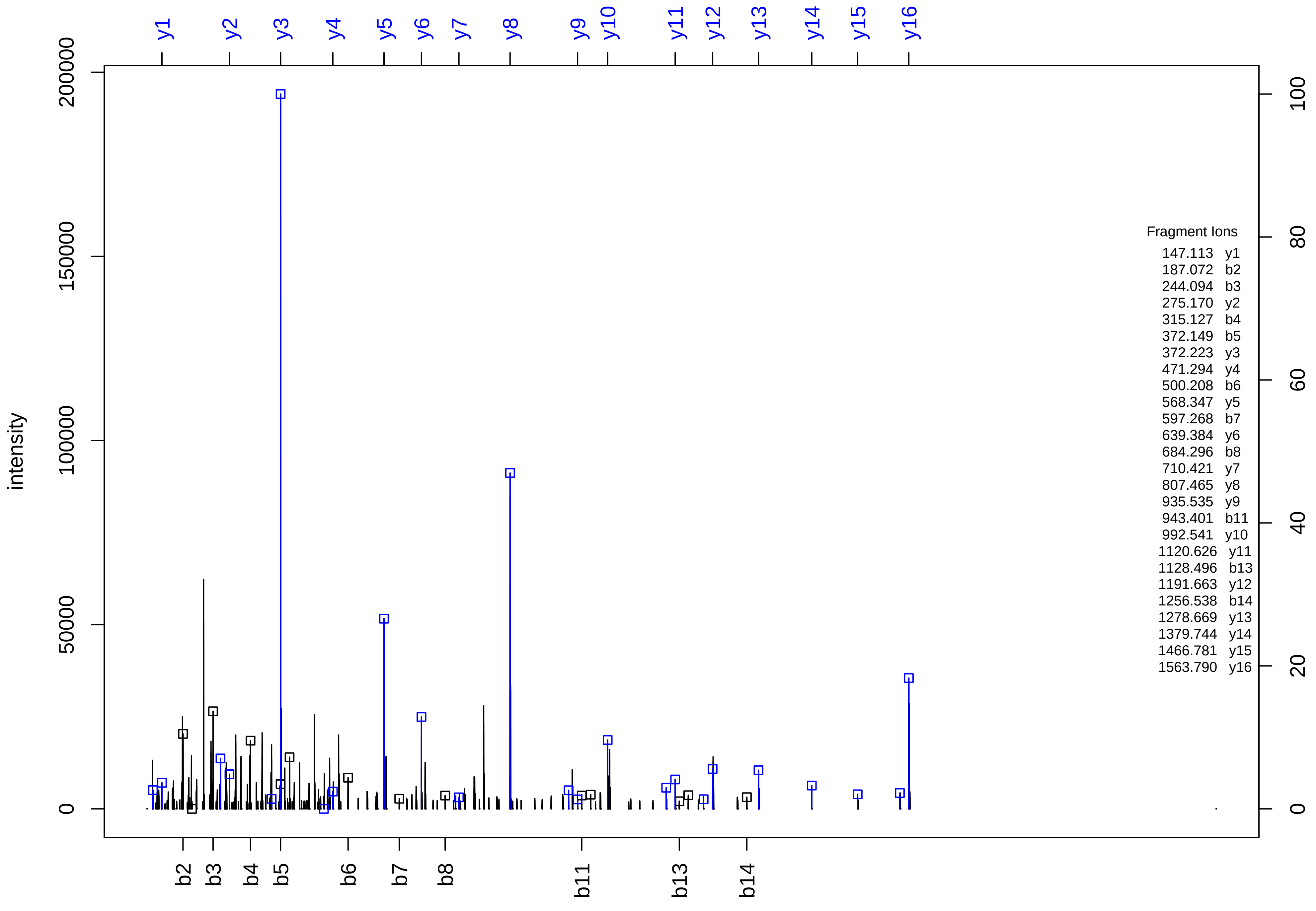

GEGAGQPSTSAQGQPAAPVPQK / File: sydney_09.raw; SpectrumID: ; scans: 1530

QAGGGAGPPNPSLN^GSAPR

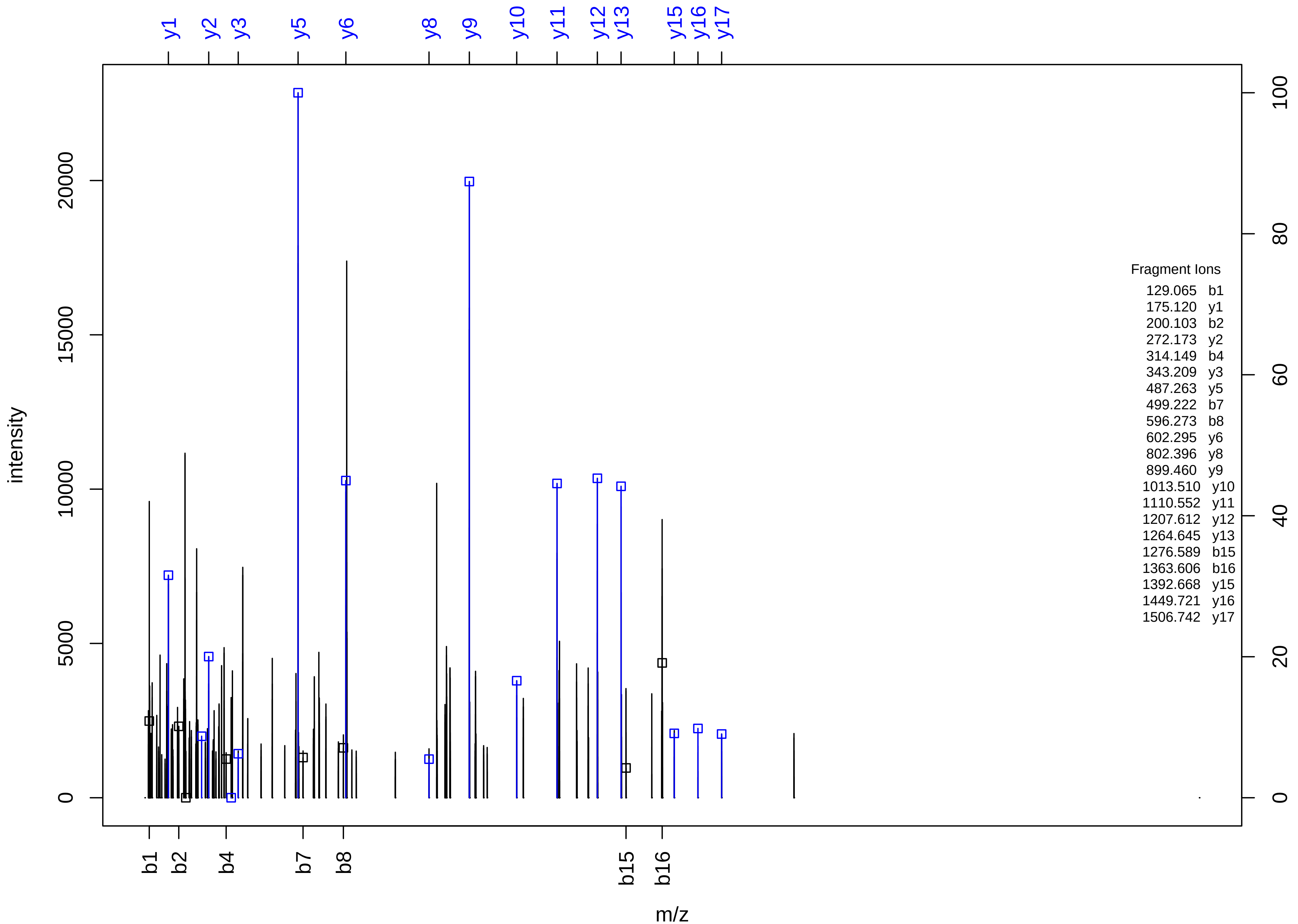

QAGGGAGPPNPSLNGSAPR / File: sydney_09.raw; SpectrumID: ; scans: 2212

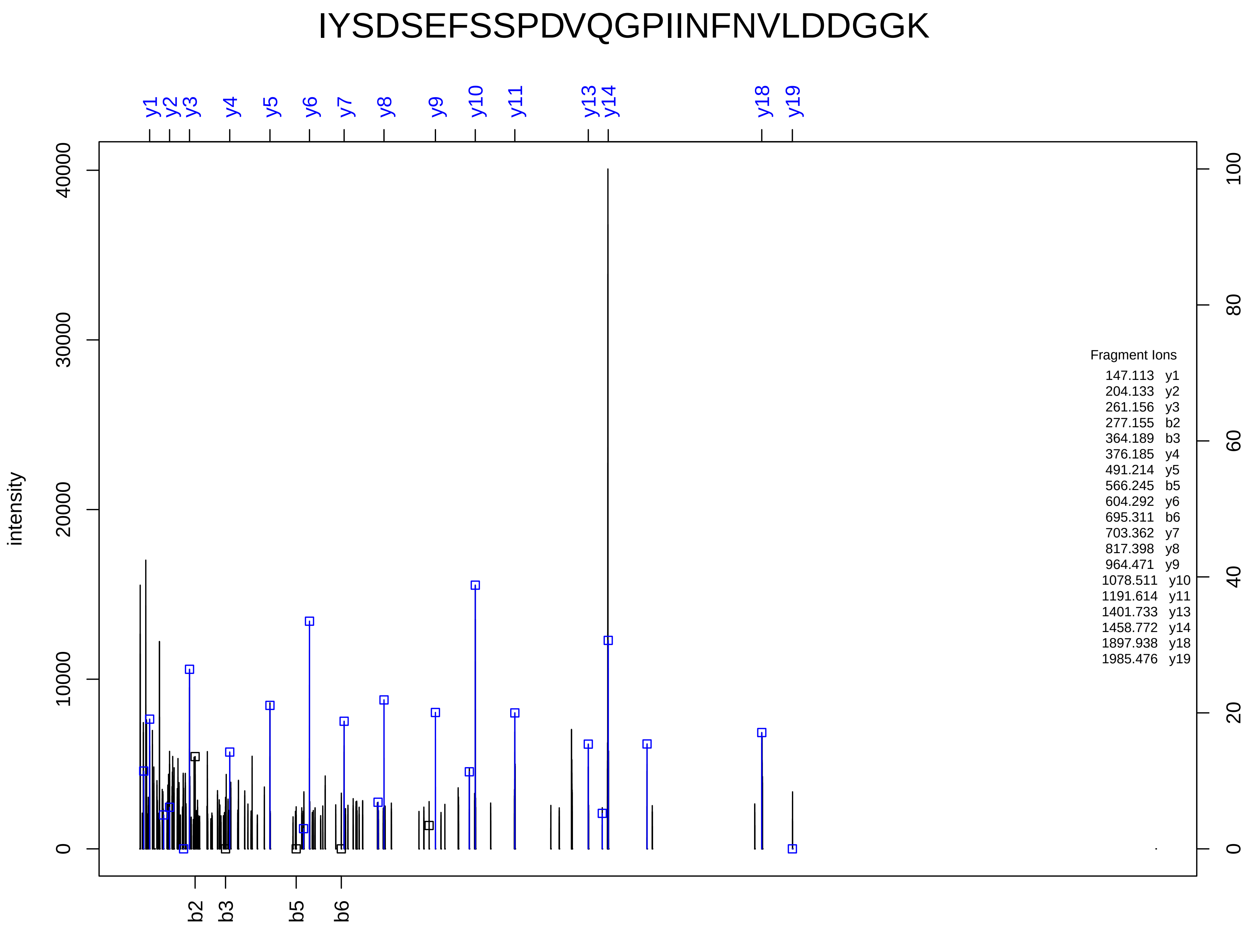

IYSDSEFSSPDVQGPIINFNVLDDGGK / File: sydney_09.raw; SpectrumID: ; scans: 14164

LYISQTIR

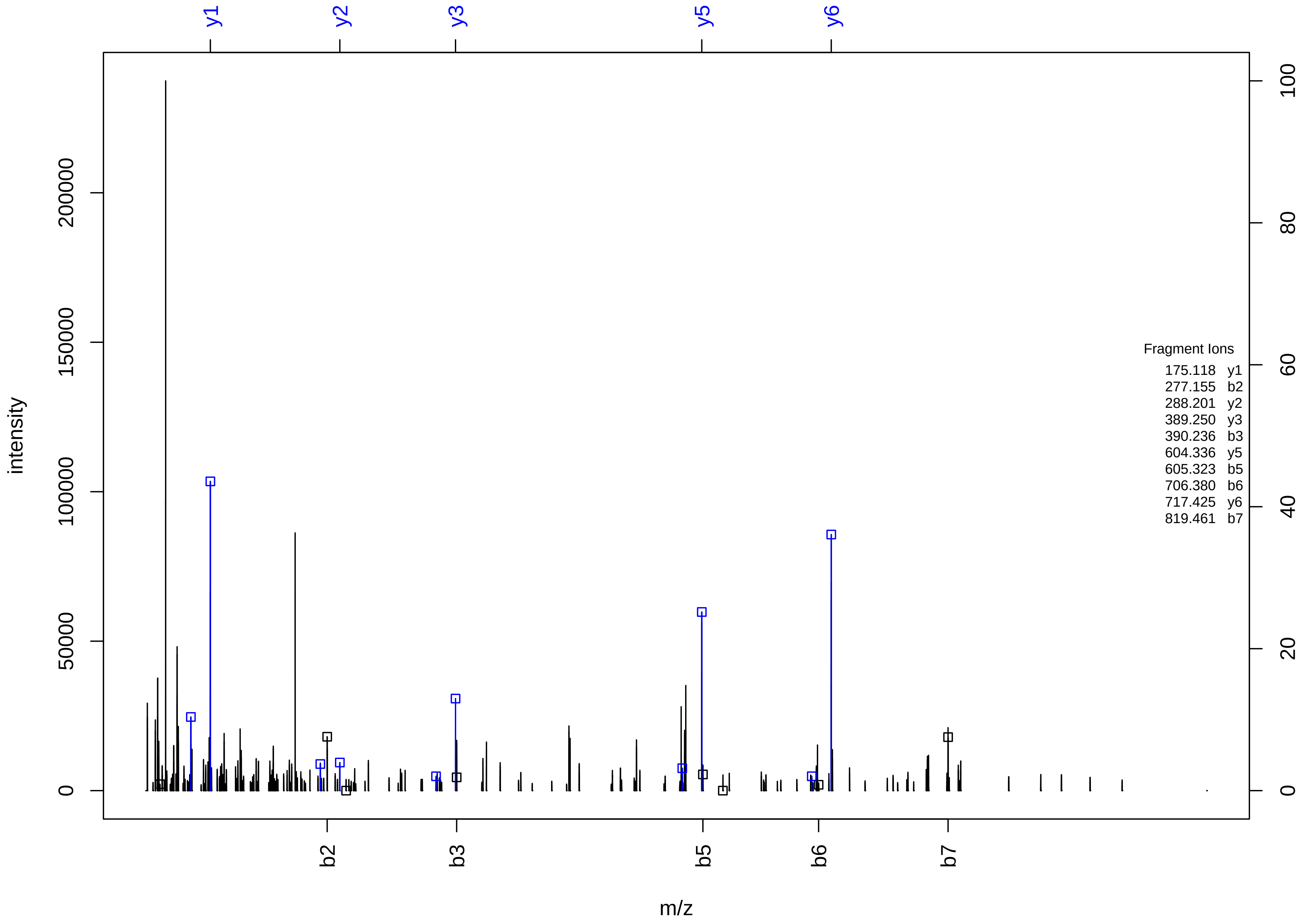

LYISQTIR / File: sydney_09.raw; SpectrumID: ; scans: 5814

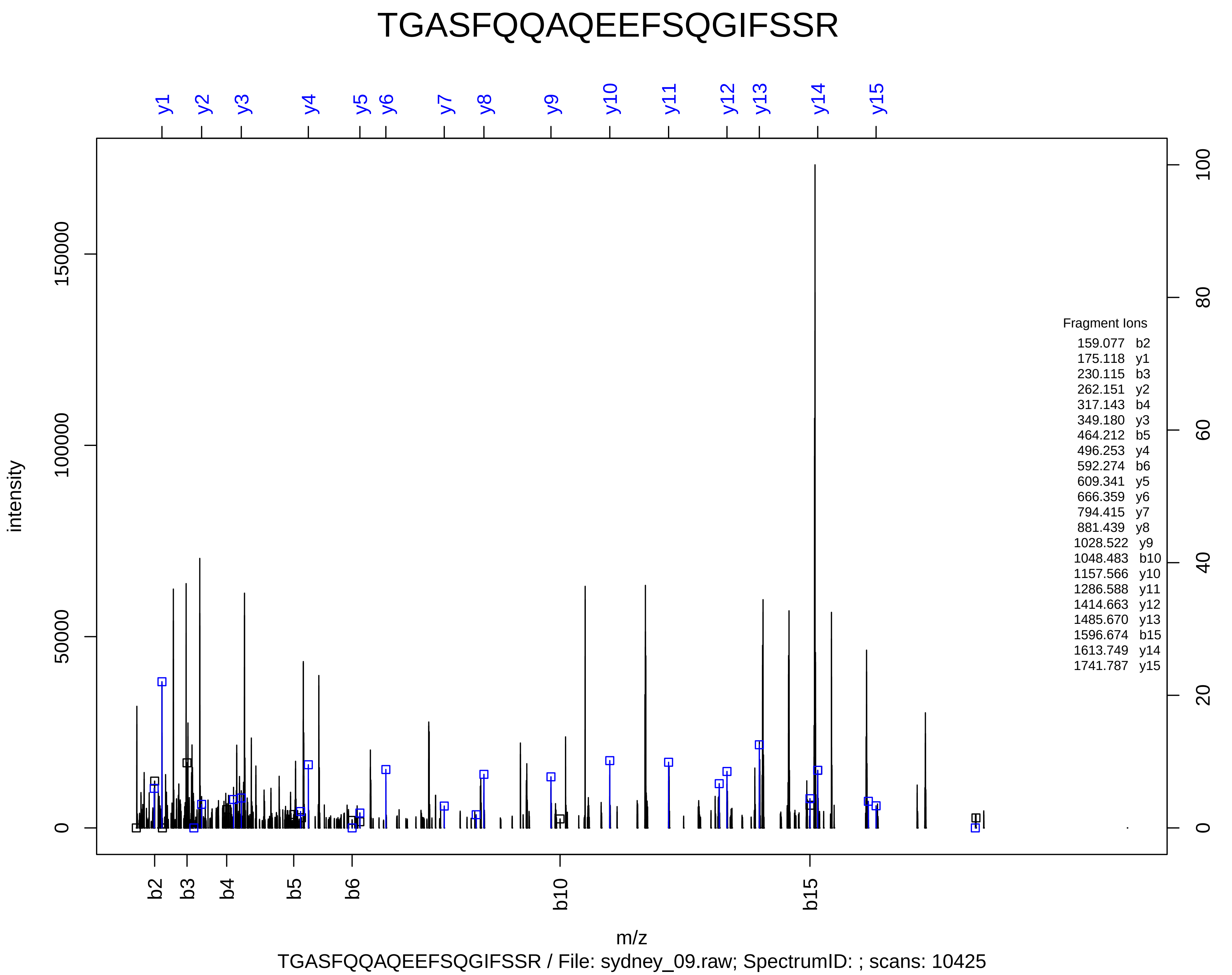
TGASFQQAQEEFSQGIFSSR
y1 y2 y3 y4 y5 y6 y7 y8 y9 y10 y11 y12 y13 y14 y15
intensity
0
50000
100000
150000
0 20 40 60 80 100
b2 b3 b4 b5 b6 b10 b15
m/z
Fragment Ions
159.077 b2
175.118 y1
230.115 b3
262.151 y2
317.143 b4
349.180 y3
464.212 b5
496.253 y4
592.274 b6
609.341 y5
666.359 y6
794.415 y7
881.439 y8
1028.522 y9
1048.483 b10
1157.566 y10
1286.588 y11
1414.663 y12
1485.670 y13
1596.674 b15
1613.749 y14
1741.787 y15
TGASFQQAQEEFSQGIFSSR / File: sydney_09.raw; SpectrumID: ; scans: 10425

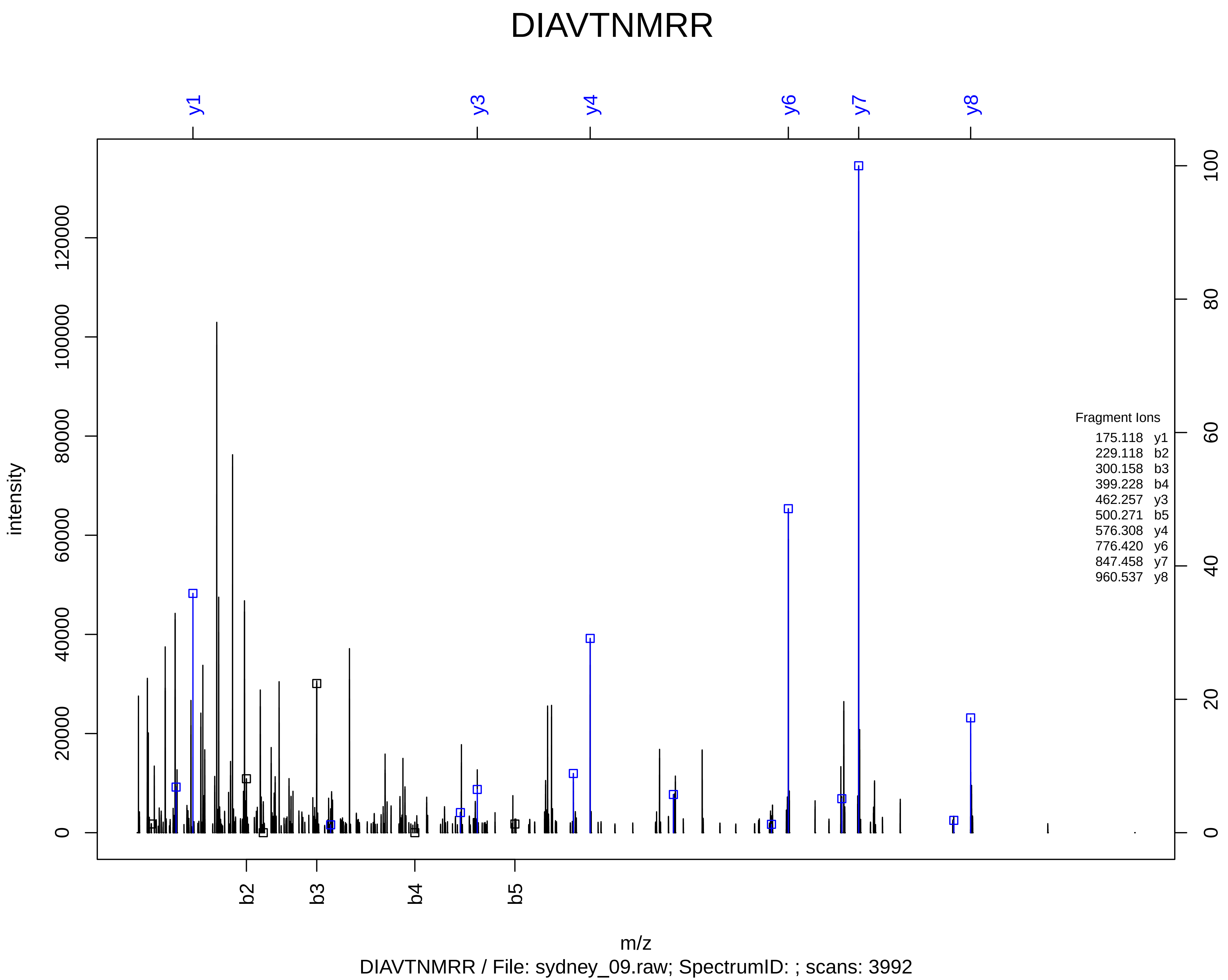
DIAVTNMRR
y1
y3
y4
y6
y7
y8
b2
b3
b4
b5
intensity
m/z
Fragment Ions
175.118 y1
229.118 b2
300.158 b3
399.228 b4
462.257 y3
500.271 b5
576.308 y4
776.420 y6
847.458 y7
960.537 y8
DIAVTNMRR / File: sydney_09.raw; SpectrumID: ; scans: 3992

APTSTDTPIYSQVAPR

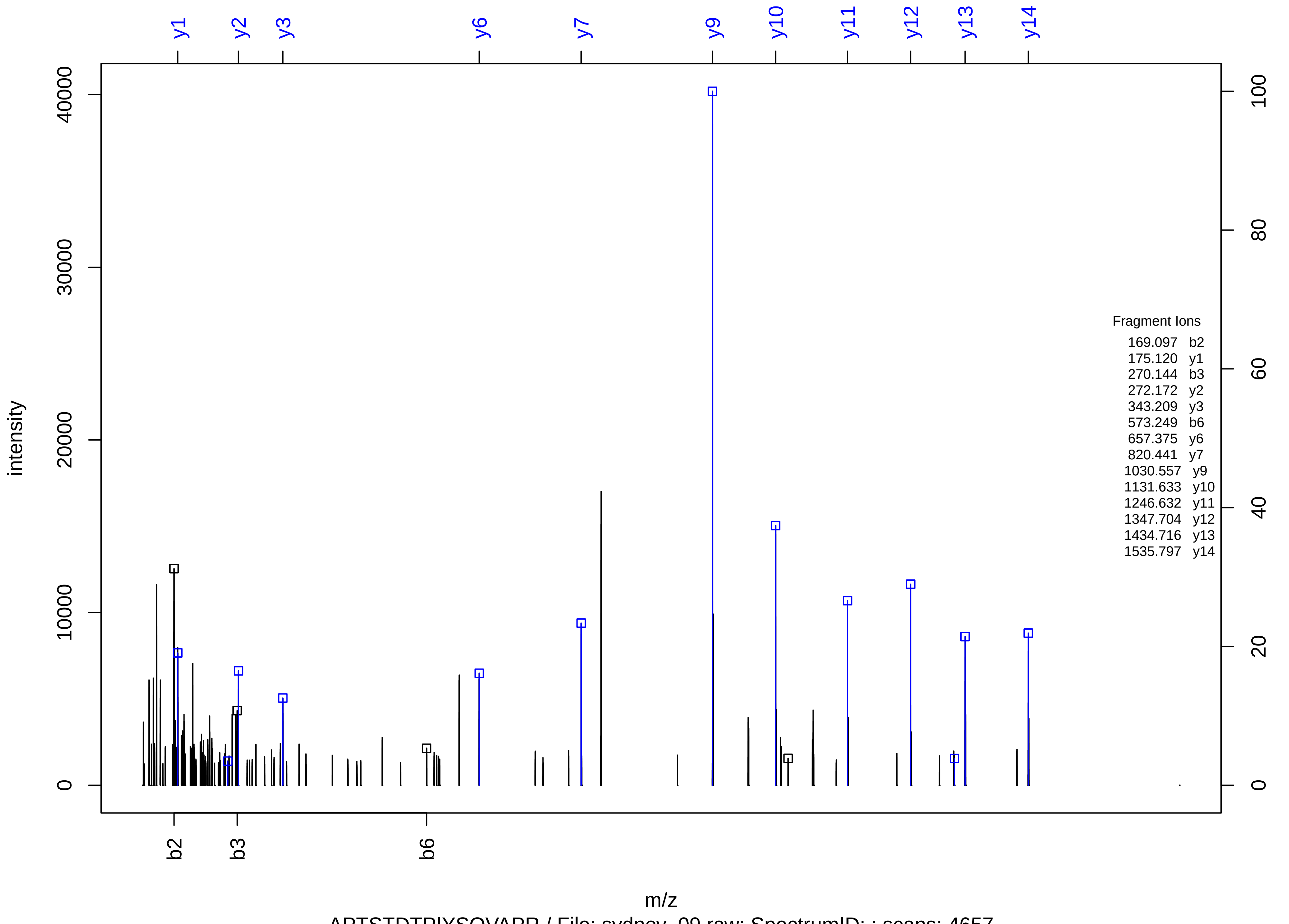

APTSTDTPIYSQVAPR / File: sydney_09.raw; SpectrumID: ; scans: 4657

LKLTN^NLMDDN^GHIILN^SMHRYQ^PR

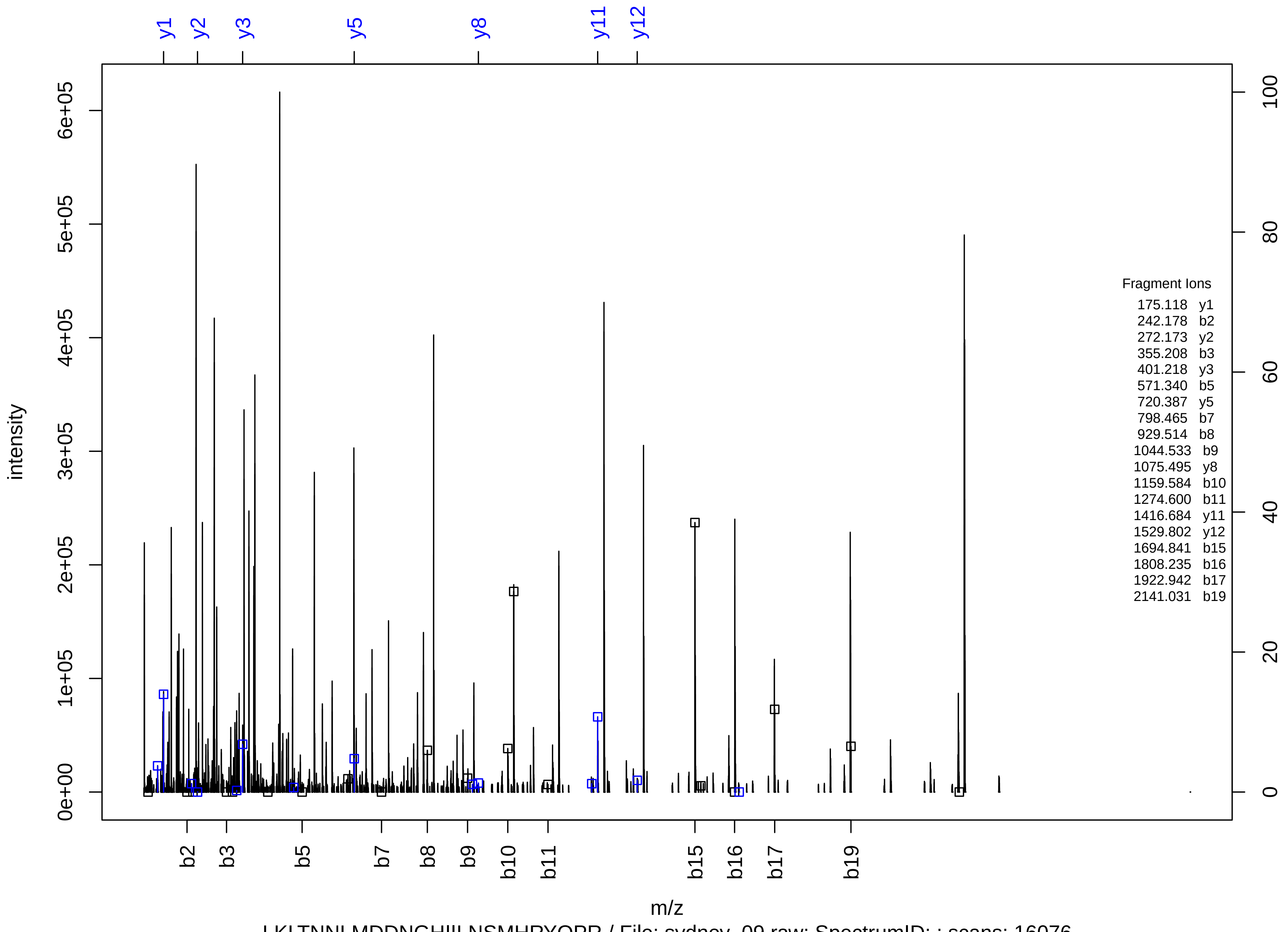

LKLTNNLMDDNGHIILNSMHRYQPR / File: sydney_09.raw; SpectrumID: ; scans: 16076

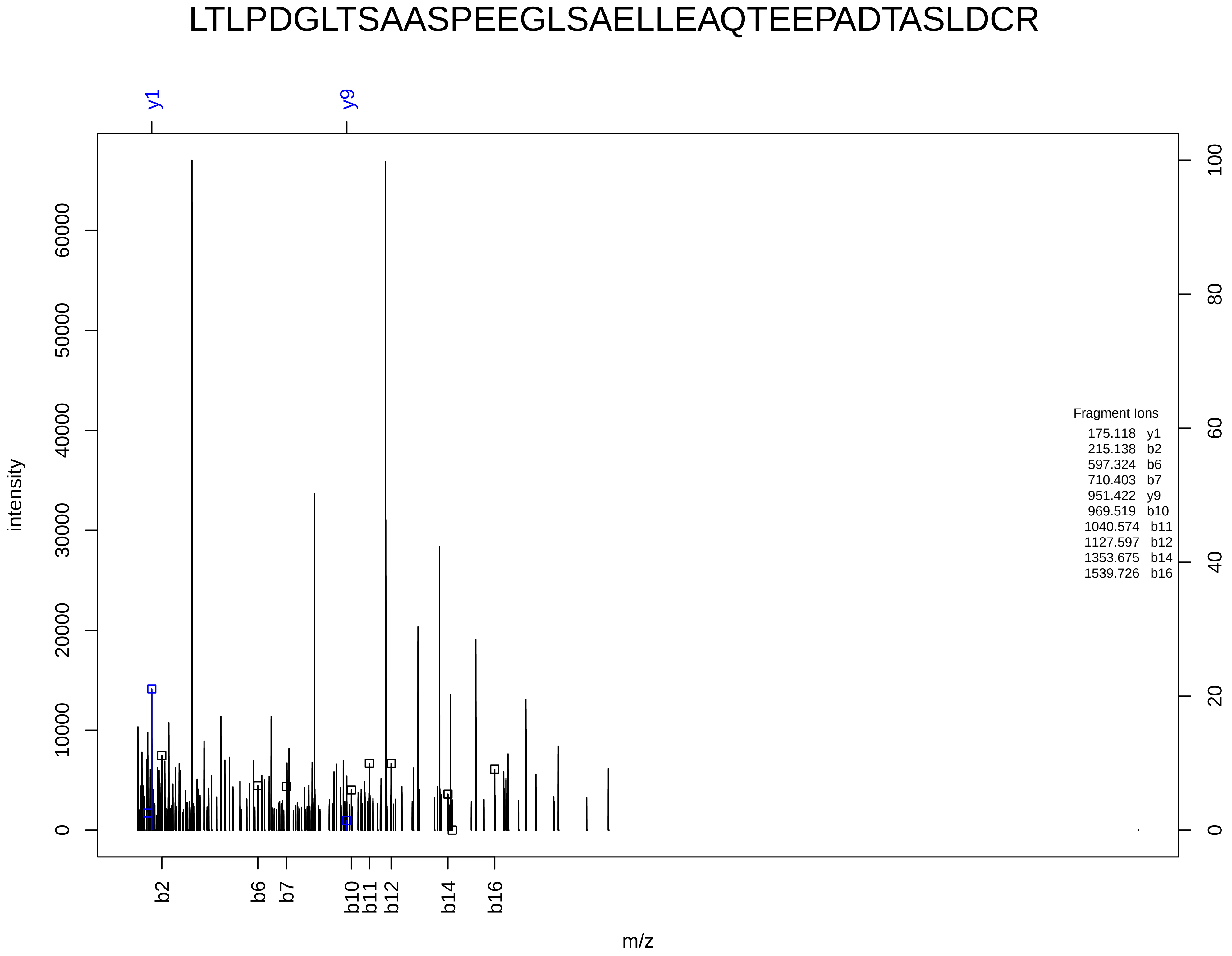

LTLPDGLTSAASPEEGLSAELLEAQTEEPADTASLDCR / File: sydney_09.raw; SpectrumID: ; scans: 14769

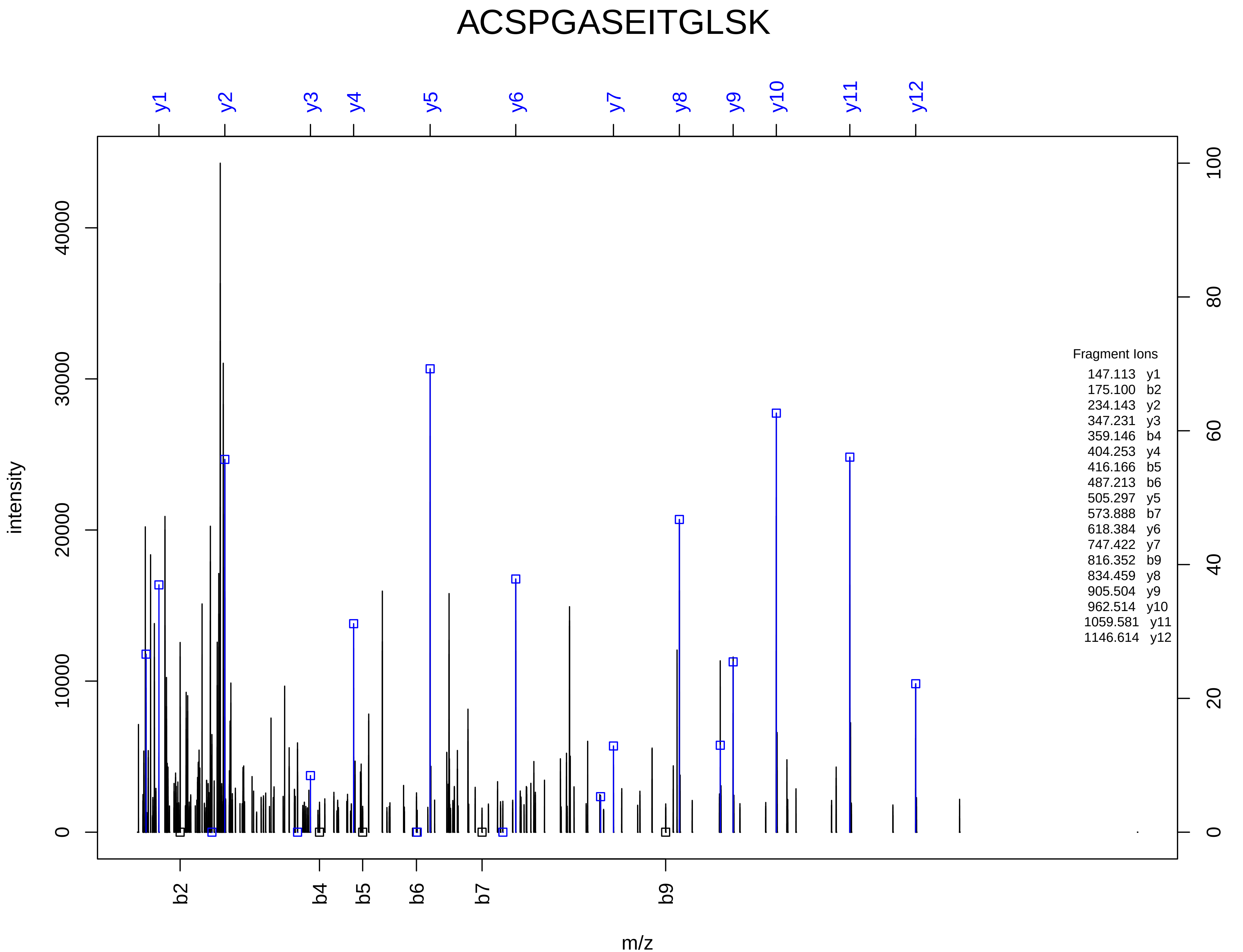
ACSPGASEITGLSK
intensity
m/z
y1
y2
y3
y4
y5
y6
y7
y8
y9
y10
y11
y12
b2
b4
b5
b6
b7
b9
Fragment Ions
147.113 y1
175.100 b2
234.143 y2
347.231 y3
359.146 b4
404.253 y4
416.166 b5
487.213 b6
505.297 y5
573.888 b7
618.384 y6
747.422 y7
816.352 b9
834.459 y8
905.504 y9
962.514 y10
1059.581 y11
1146.614 y12
ACSPGASEITGLSK / File: sydney_09.raw; SpectrumID: ; scans: 3246

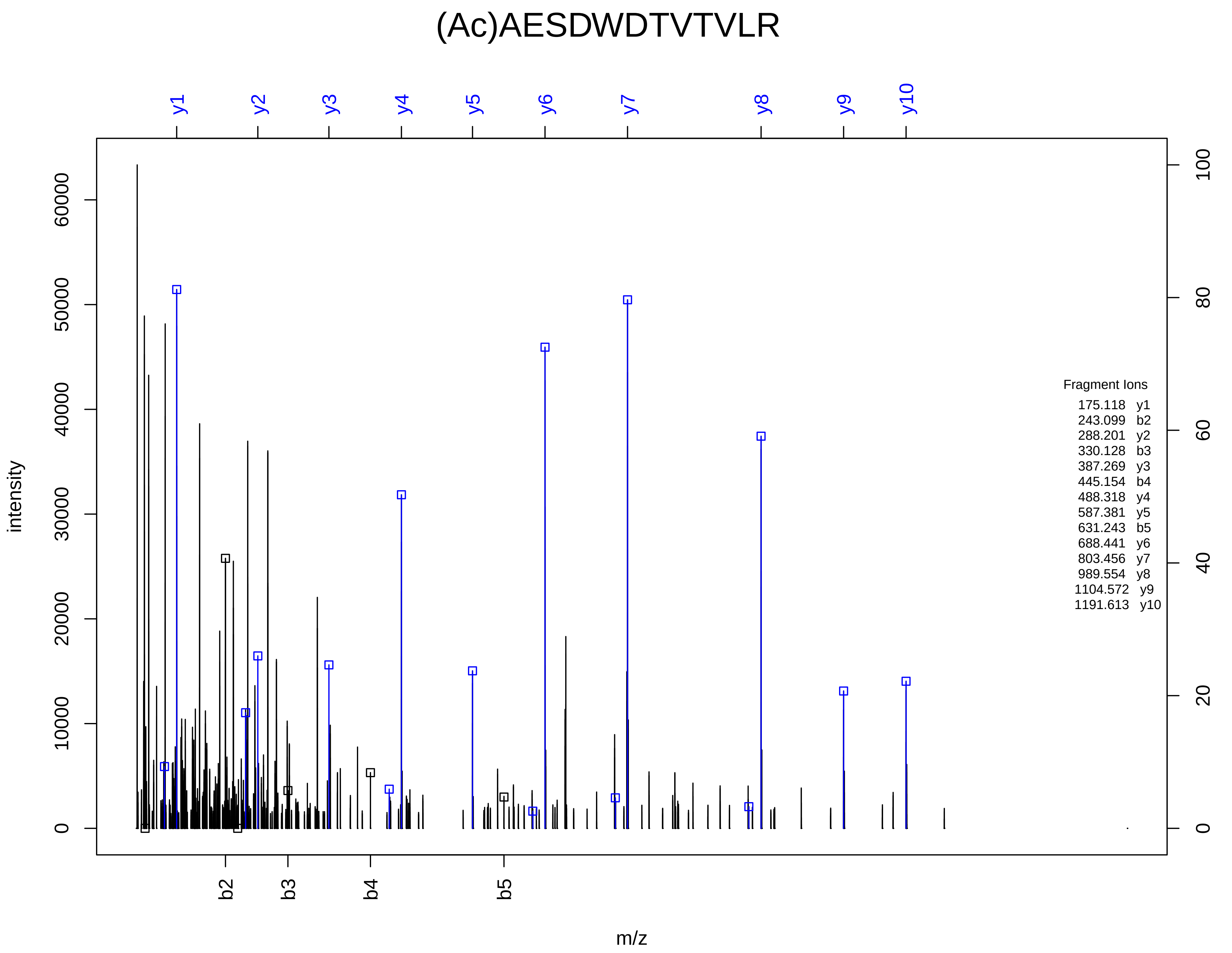

AESDWDTVTVLR / File: sydney_09.raw; SpectrumID: ; scans: 11559

ADGAGDEAAERTEEPESPESVDQTSPTPGDGNPR

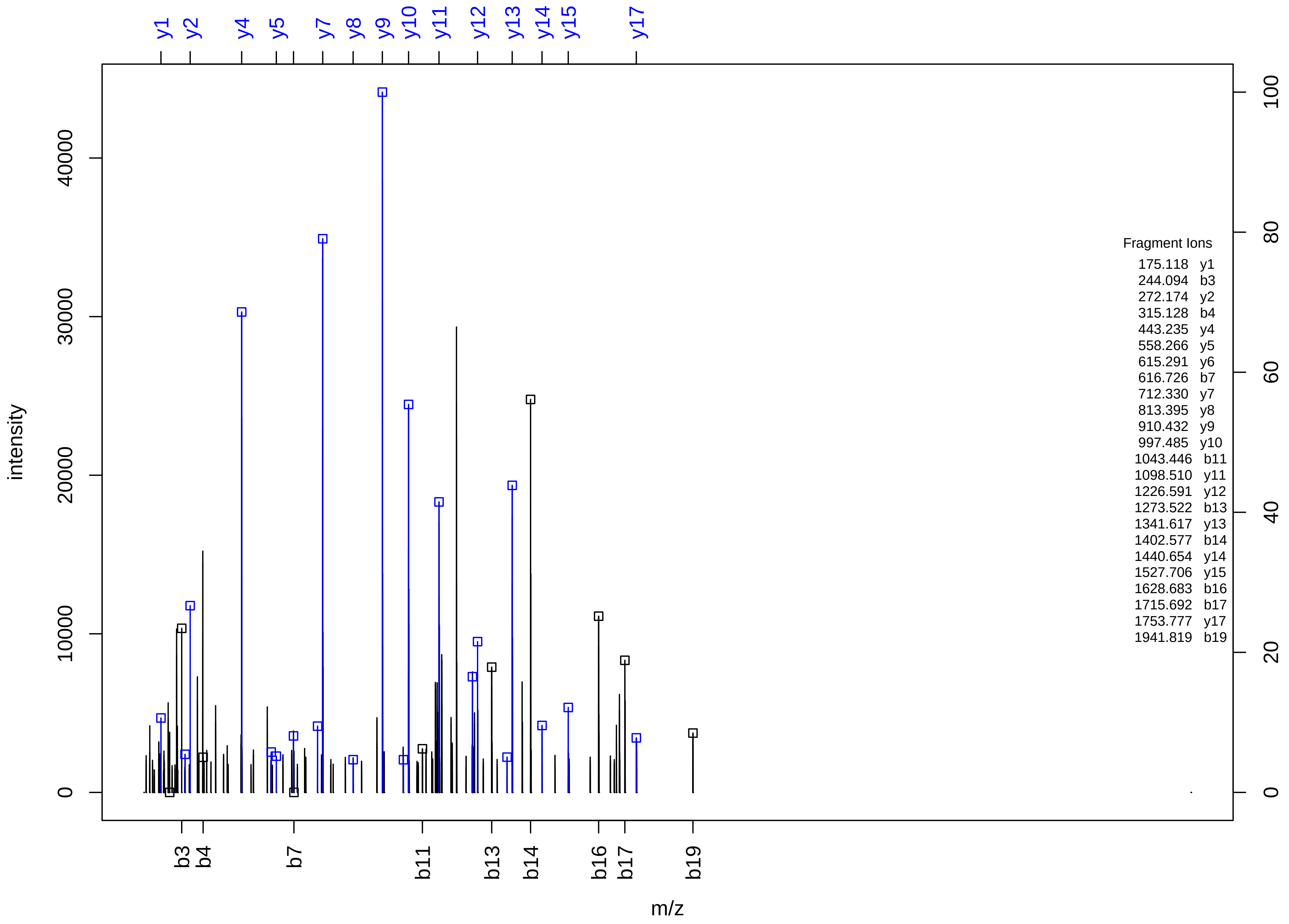

ADGAGDEAAERTEEPESPESVDQTSPTPGDGNPR / File: sydney_09.raw; SpectrumID: ; scans: 2642

VDDDFTAQDYR

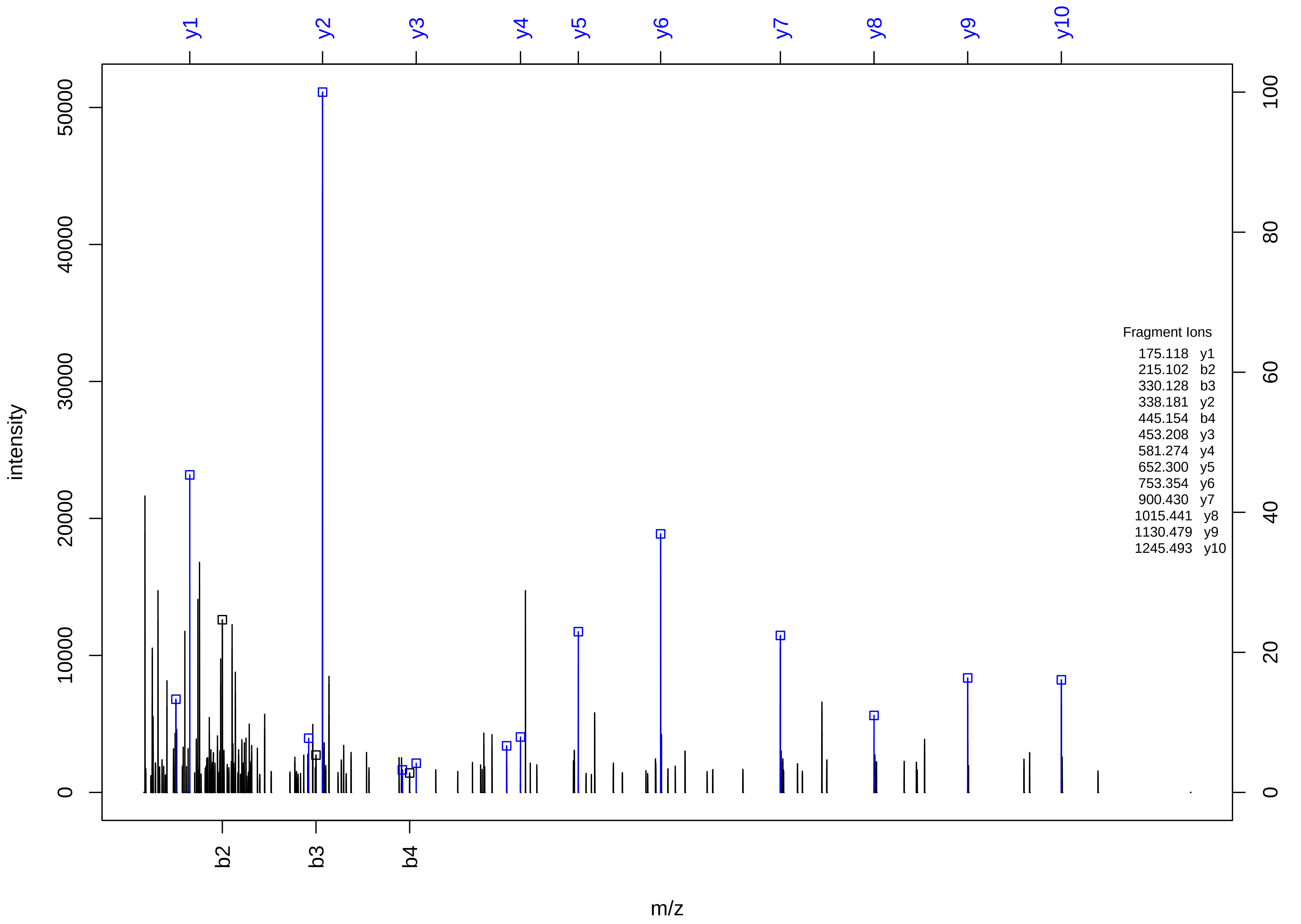

VDDDFTAQDYR / File: sydney_09.raw; SpectrumID: ; scans: 3259

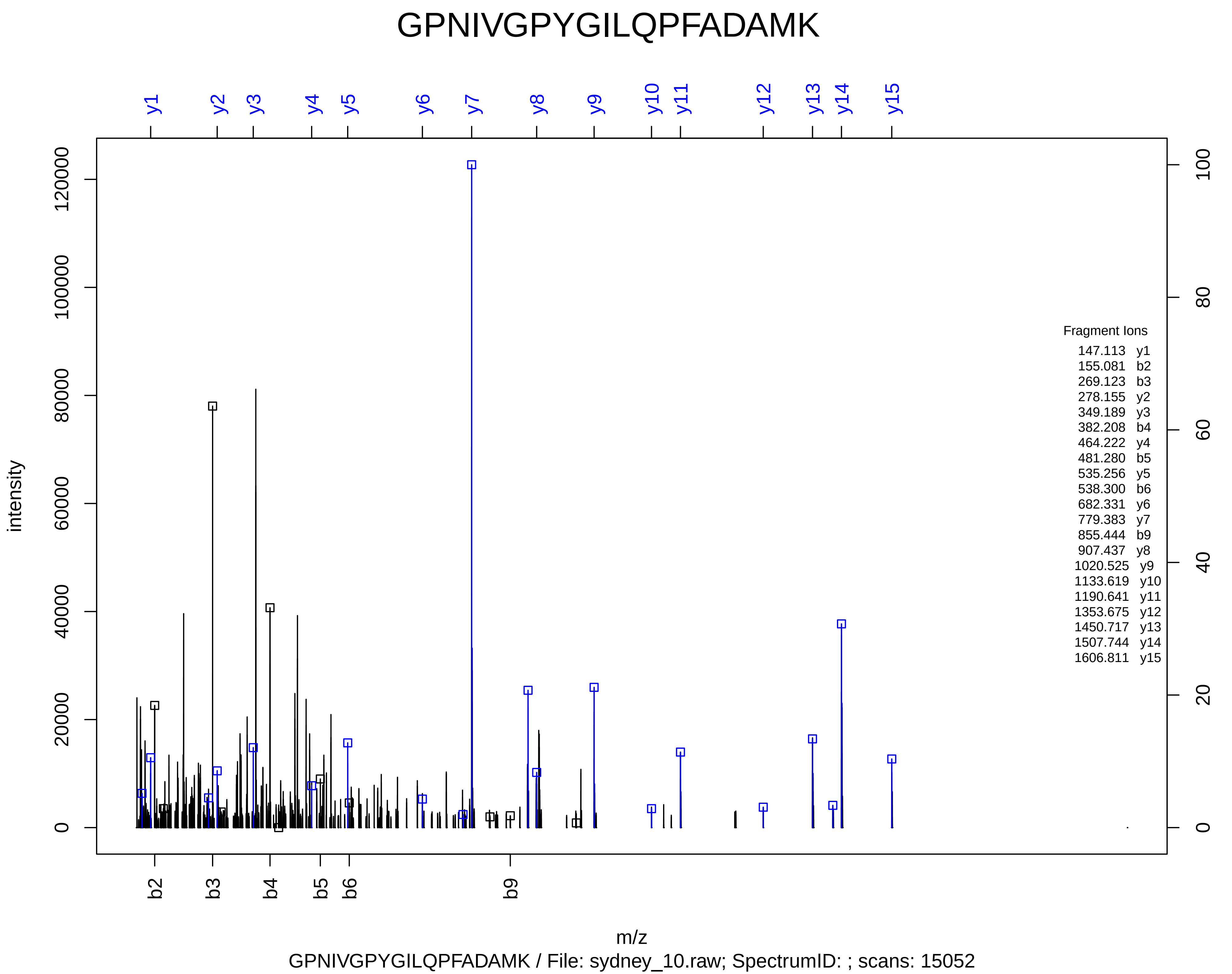

GPNIVGPYGILQPFADAMK
intensity
m/z
y1 y2 y3 y4 y5 y6 y7 y8 y9 y10 y11 y12 y13 y14 y15
b2 b3 b4 b5 b6 b9
Fragment Ions
147.113 y1
155.081 b2
269.123 b3
278.155 y2
349.189 y3
382.208 b4
464.222 y4
481.280 b5
535.256 y5
538.300 b6
682.331 y6
779.383 y7
855.444 b9
907.437 y8
1020.525 y9
1133.619 y10
1190.641 y11
1353.675 y12
1450.717 y13
1507.744 y14
1606.811 y15
GPNIVGPYGILQPFADAMK / File: sydney_10.raw; SpectrumID: ; scans: 15052

GTVIDVDHGIICENVPIITPAGEVVASR

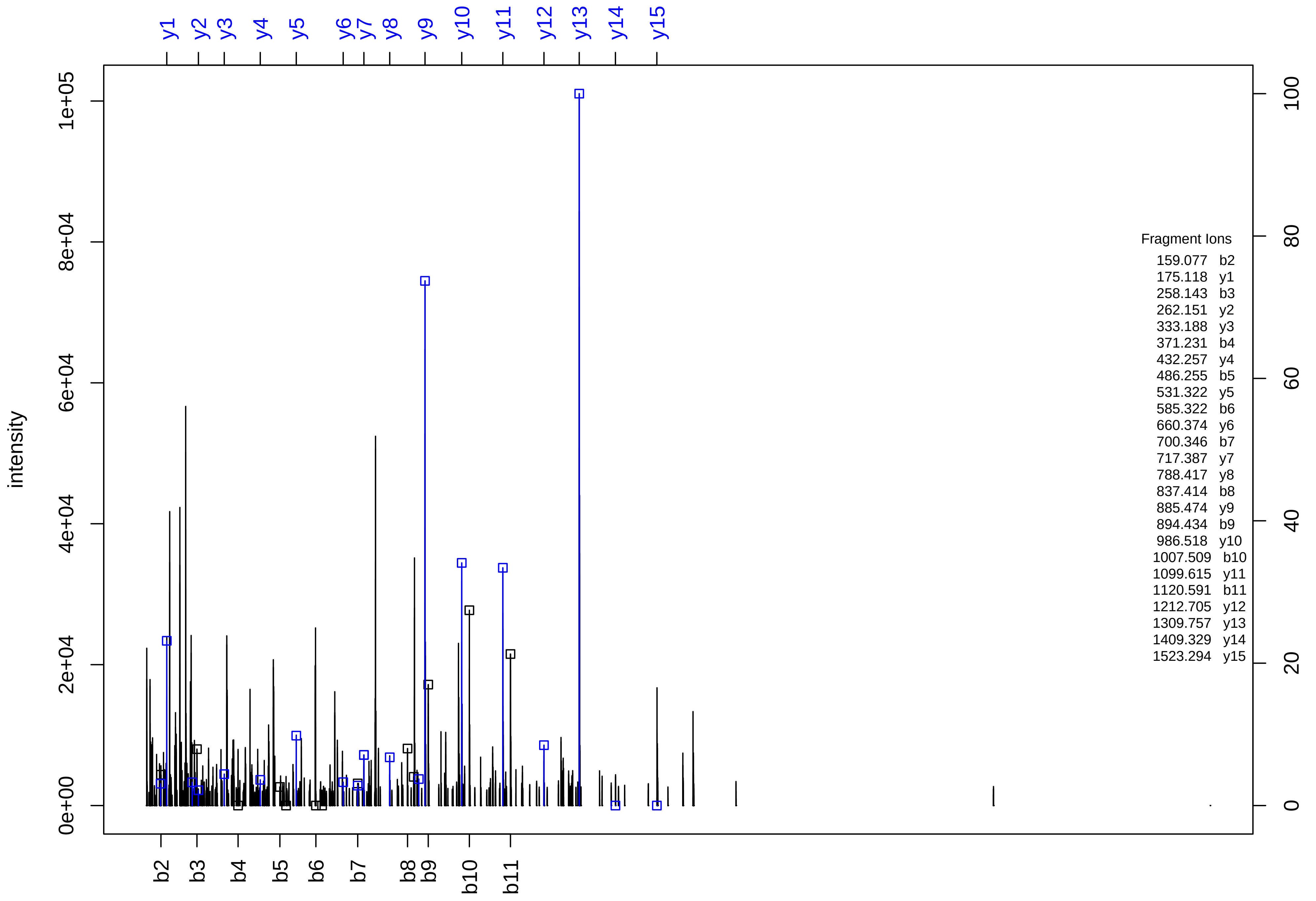

GTVIDVDHGIICENVPIITPAGEVVASR / File: sydney_10.raw; SpectrumID: ; scans: 11643

DQLAADMYNFMAK

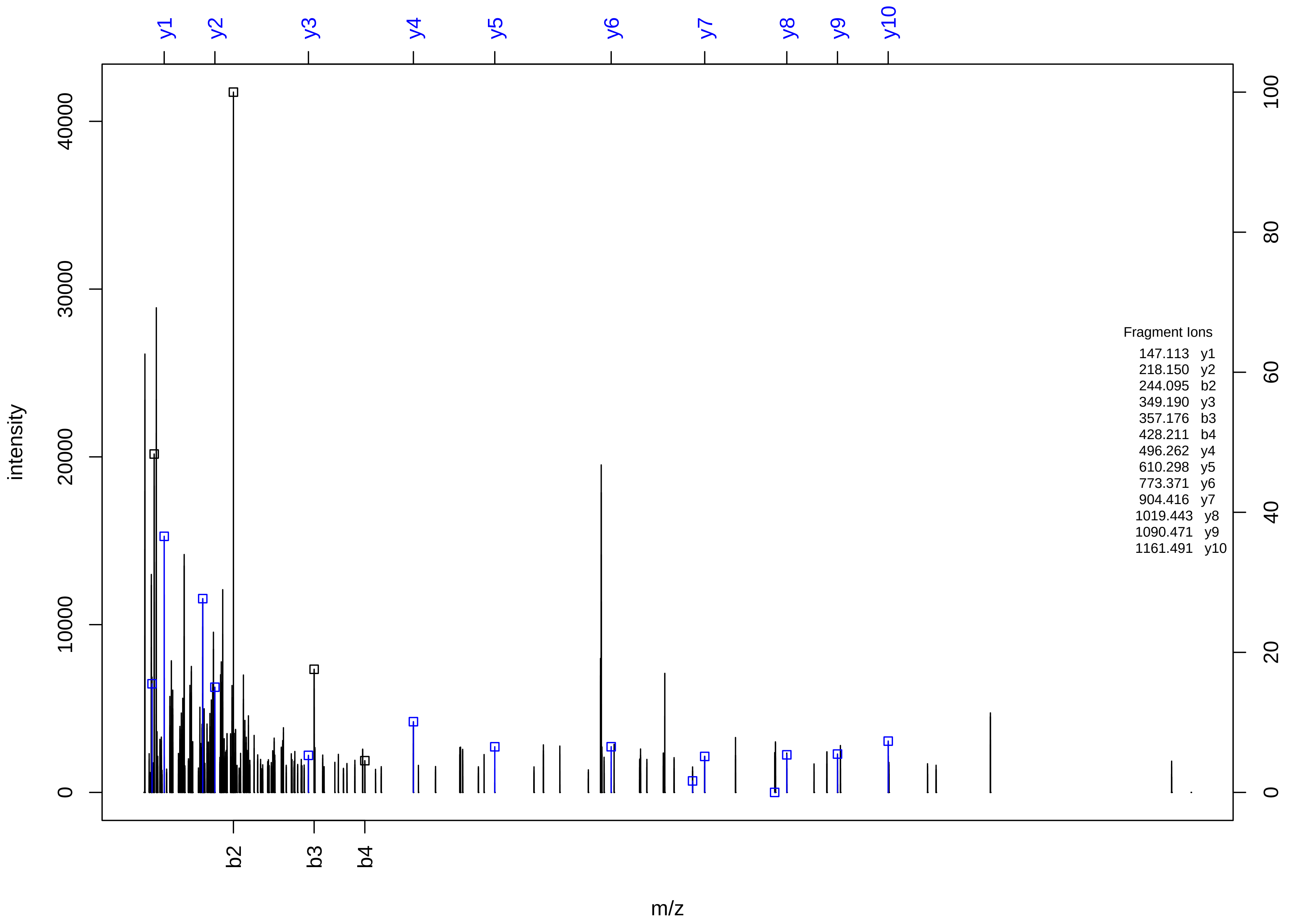

DQLAADMYNFMAK / File: sydney_10.raw; SpectrumID: ; scans: 10533

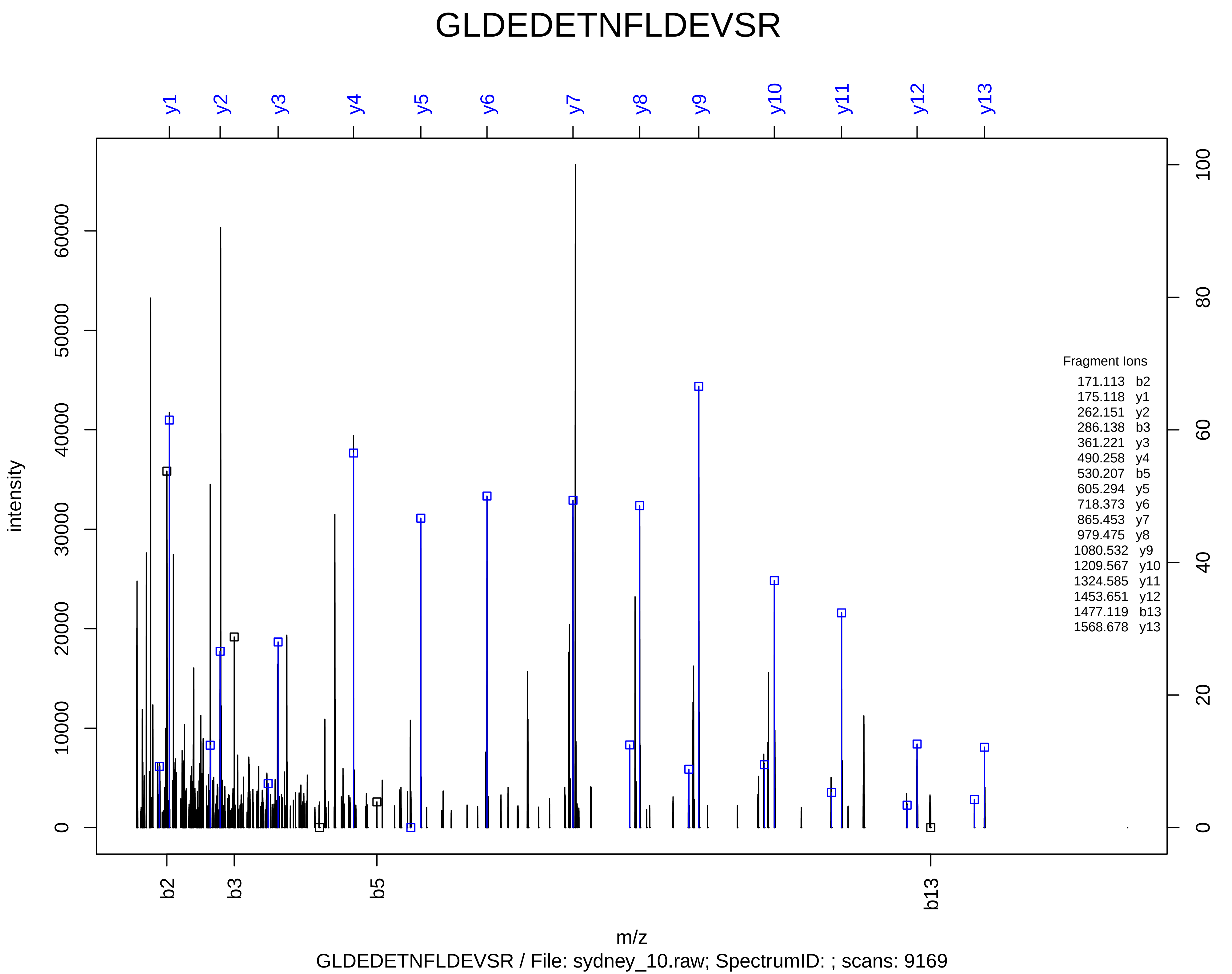
GLDEDETNFLDEVSR
y1
y2
y3
y4
y5
y6
y7
y8
y9
y10
y11
y12
y13
b2
b3
b5
b13
intensity
m/z
Fragment Ions
171.113 b2
175.118 y1
262.151 y2
286.138 b3
361.221 y3
490.258 y4
530.207 b5
605.294 y5
718.373 y6
865.453 y7
979.475 y8
1080.532 y9
1209.567 y10
1324.585 y11
1453.651 y12
1477.119 b13
1568.678 y13
GLDEDETNFLDEVSR / File: sydney_10.raw; SpectrumID: ; scans: 9169

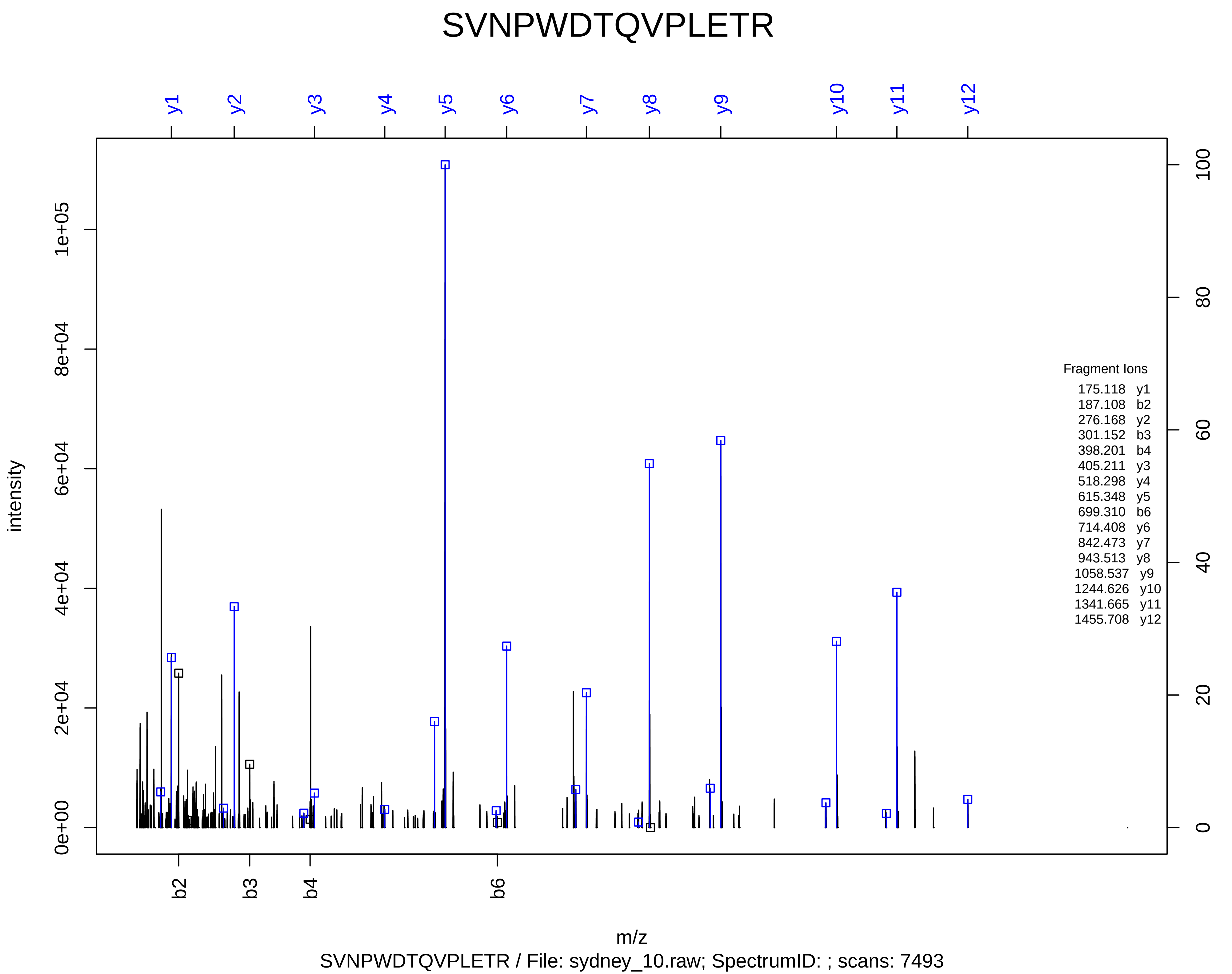
SVNPWDTQVPLETR
y1
y2
y3
y4
y5
y6
y7
y8
y9
y10
y11
y12
b2
b3
b4
b6
intensity
m/z
Fragment Ions
175.118 y1
187.108 b2
276.168 y2
301.152 b3
398.201 b4
405.211 y3
518.298 y4
615.348 y5
699.310 b6
714.408 y6
842.473 y7
943.513 y8
1058.537 y9
1244.626 y10
1341.665 y11
1455.708 y12
SVNPWDTQVPLETR / File: sydney_10.raw; SpectrumID: ; scans: 7493

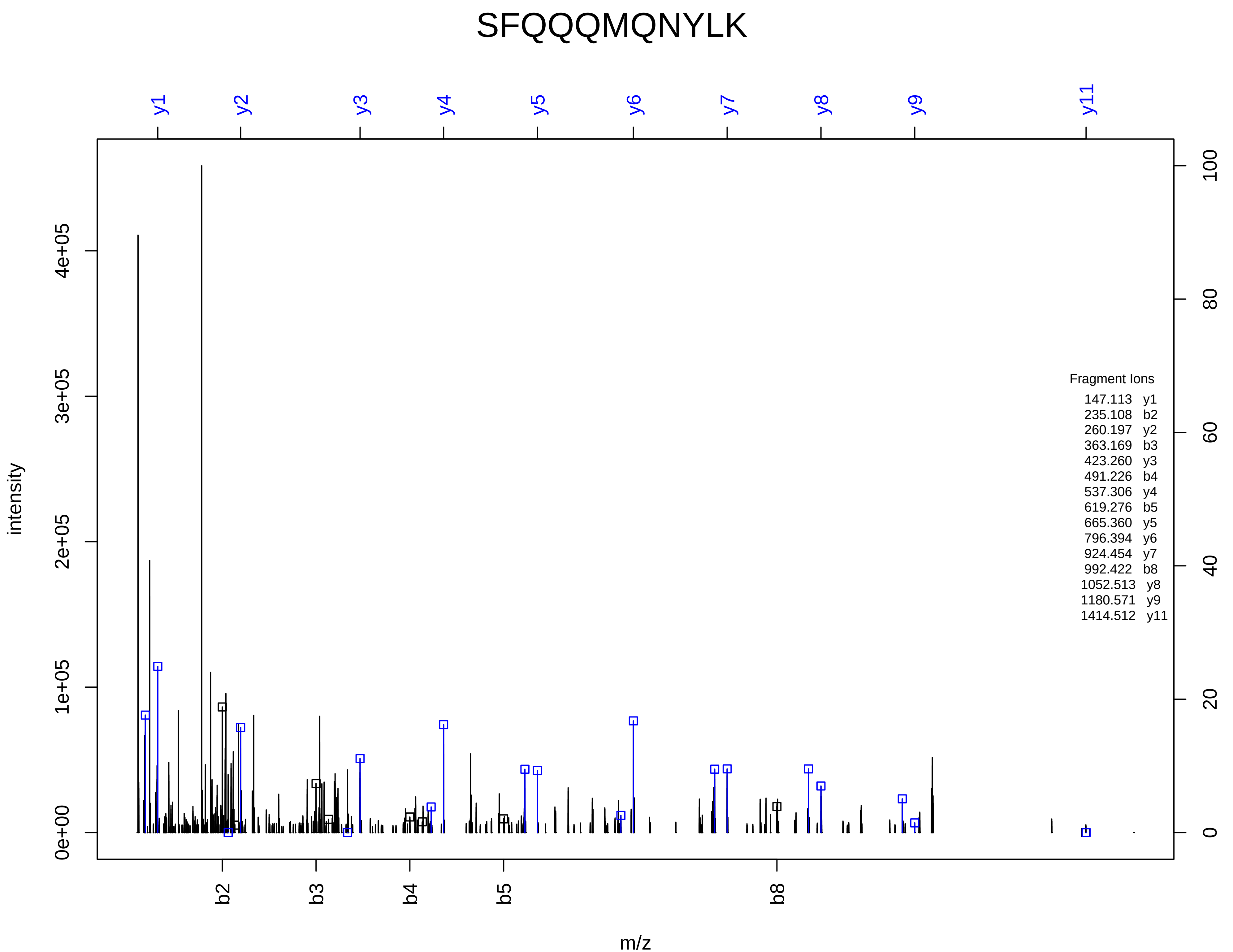

SFQQQMQNYLK
y1
y2
y3
y4
y5
y6
y7
y8
y9
y11
intensity
0e+00
1e+05
2e+05
3e+05
4e+05
0
20
40
60
80
100
b2
b3
b4
b5
b8
m/z
Fragment Ions
147.113 y1
235.108 b2
260.197 y2
363.169 b3
423.260 y3
491.226 b4
537.306 y4
619.276 b5
665.360 y5
796.394 y6
924.454 y7
992.422 b8
1052.513 y8
1180.571 y9
1414.512 y11
SFQQQMQNYLK / File: sydney_10.raw; SpectrumID: ; scans: 6053

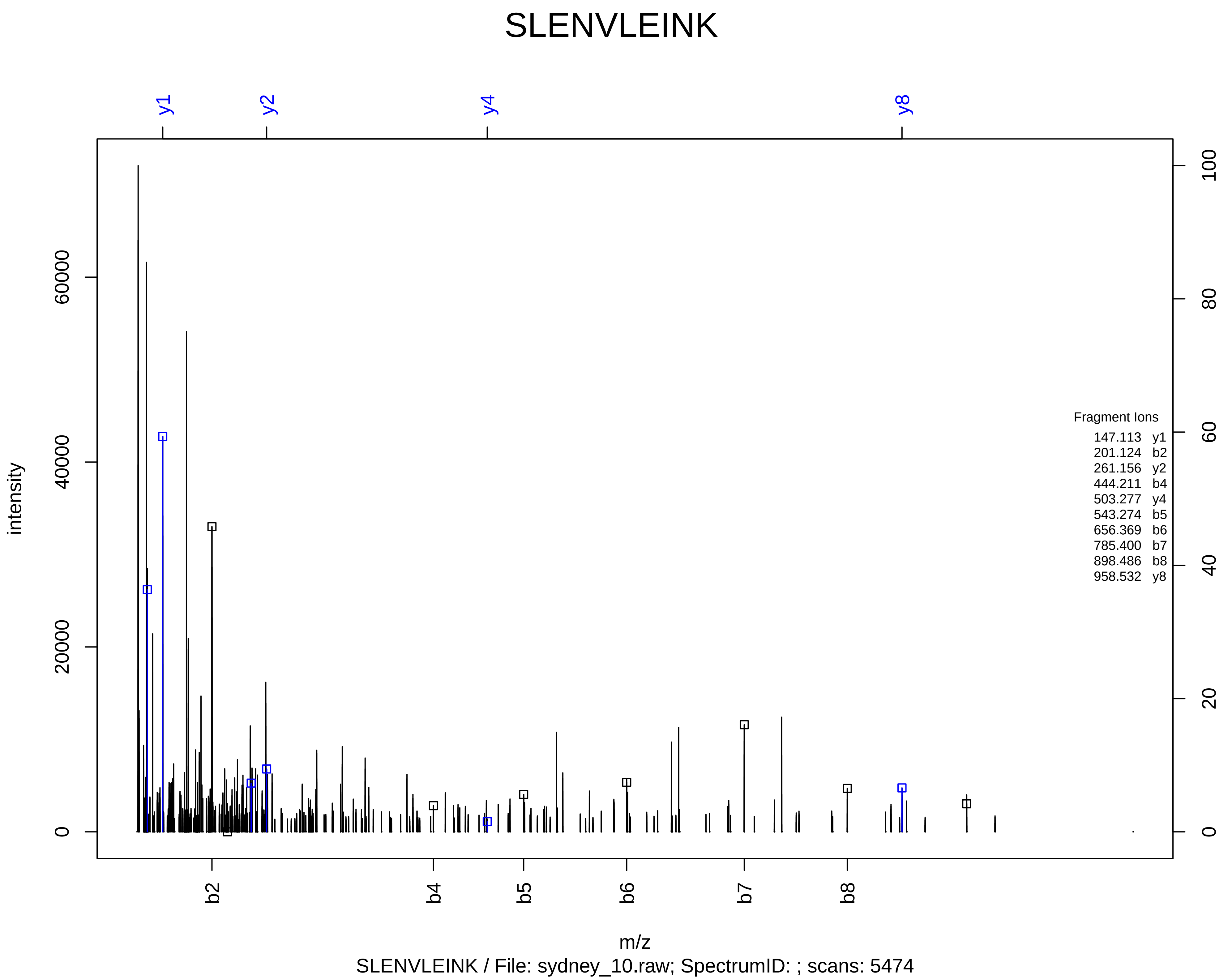
SLENVLEINK
y1
y2
y4
y8
intensity
0
20000
40000
60000
0
20
40
60
80
100
b2
b4
b5
b6
b7
b8
m/z
Fragment Ions
147.113 y1
201.124 b2
261.156 y2
444.211 b4
503.277 y4
543.274 b5
656.369 b6
785.400 b7
898.486 b8
958.532 y8
SLENVLEINK / File: sydney_10.raw; SpectrumID: ; scans: 5474

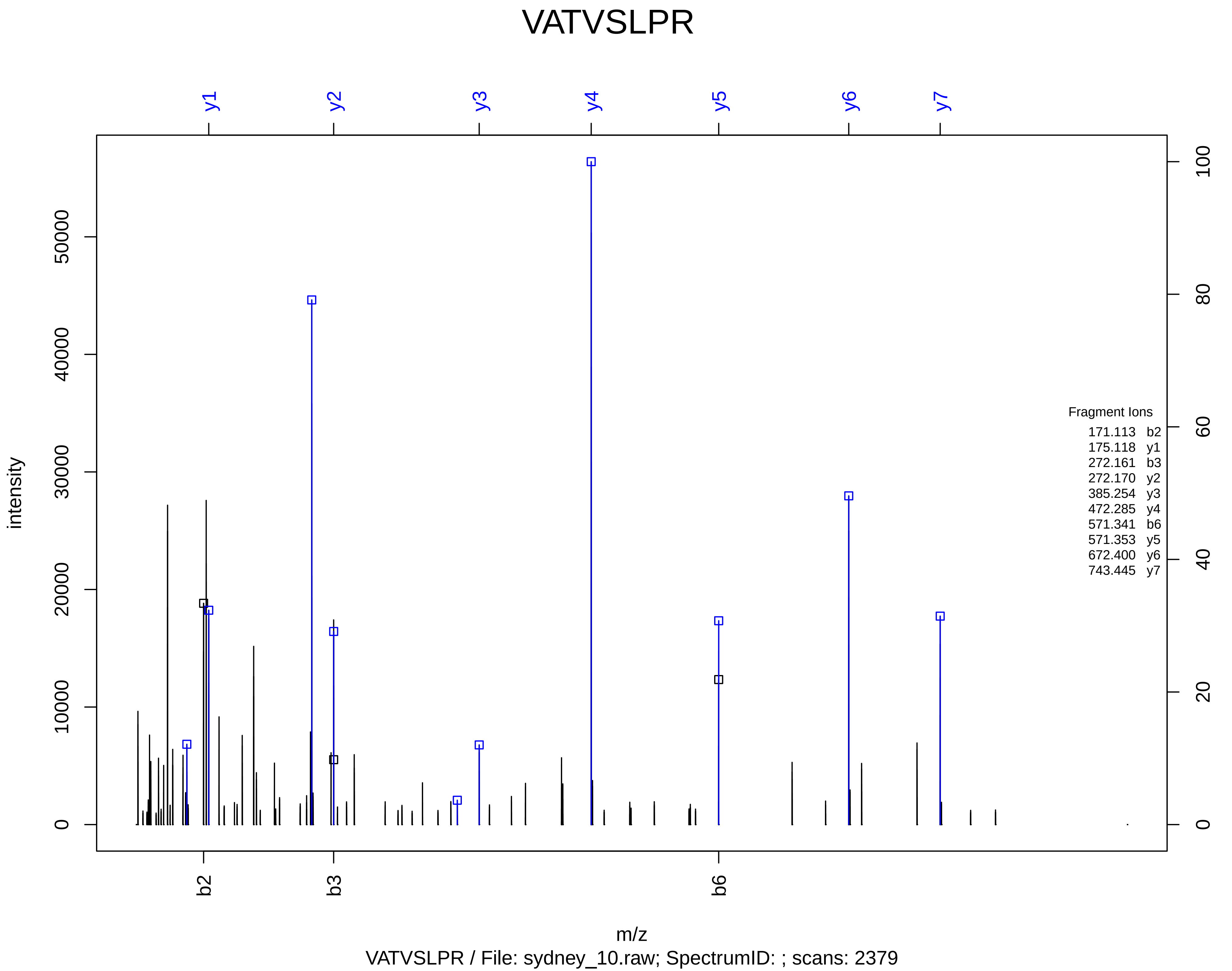
VATVSLPR
y1
y2
y3
y4
y5
y6
y7
intensity
0
10000
20000
30000
40000
50000
0
20
40
60
80
100
Fragment Ions
171.113 b2
175.118 y1
272.161 b3
272.170 y2
385.254 y3
472.285 y4
571.341 b6
571.353 y5
672.400 y6
743.445 y7
b2
b3
b6
m/z
VATVSLPR / File: sydney_10.raw; SpectrumID: ; scans: 2379

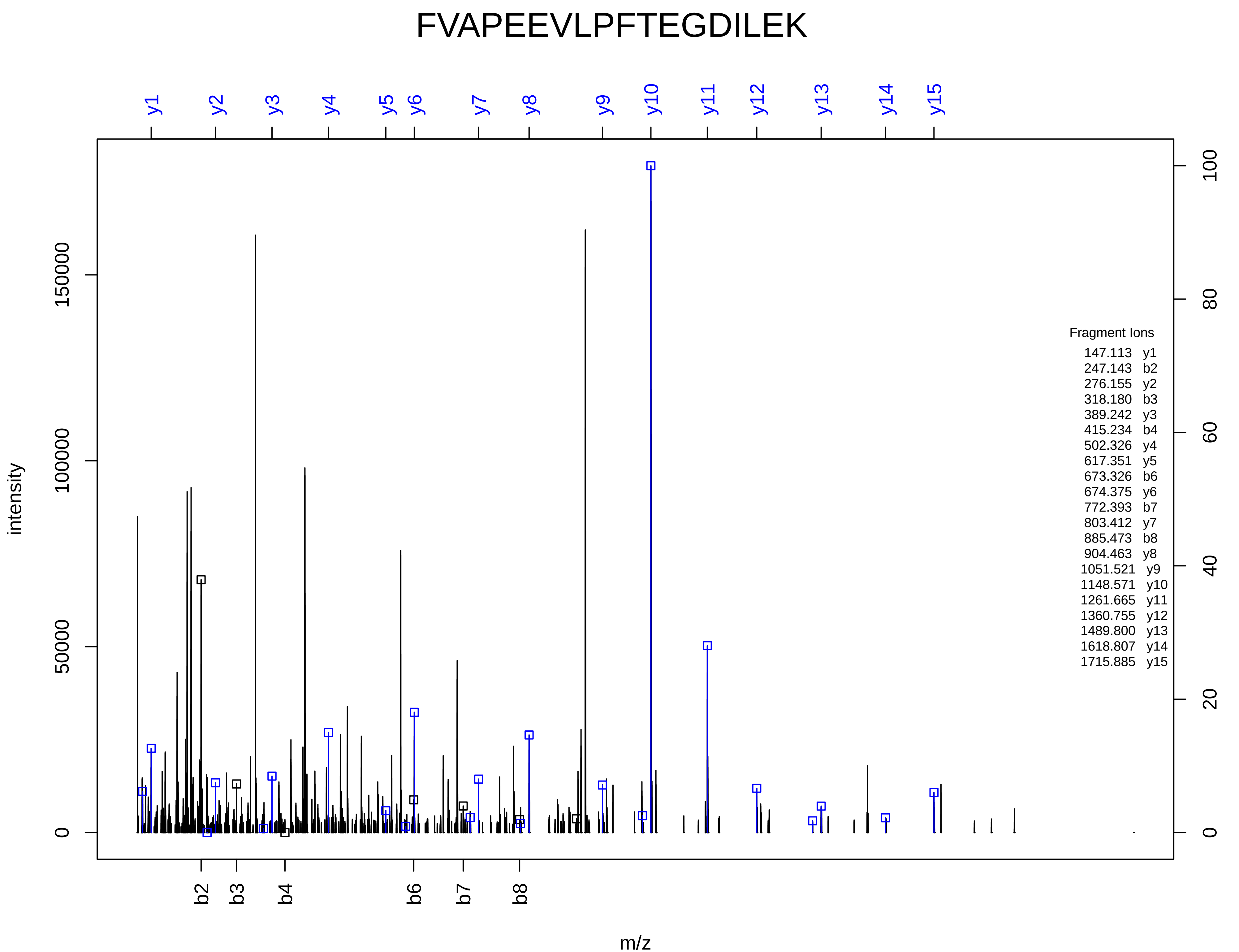

FVAPEEVLPFTEGDILEK / File: sydney_10.raw; SpectrumID: ; scans: 13692

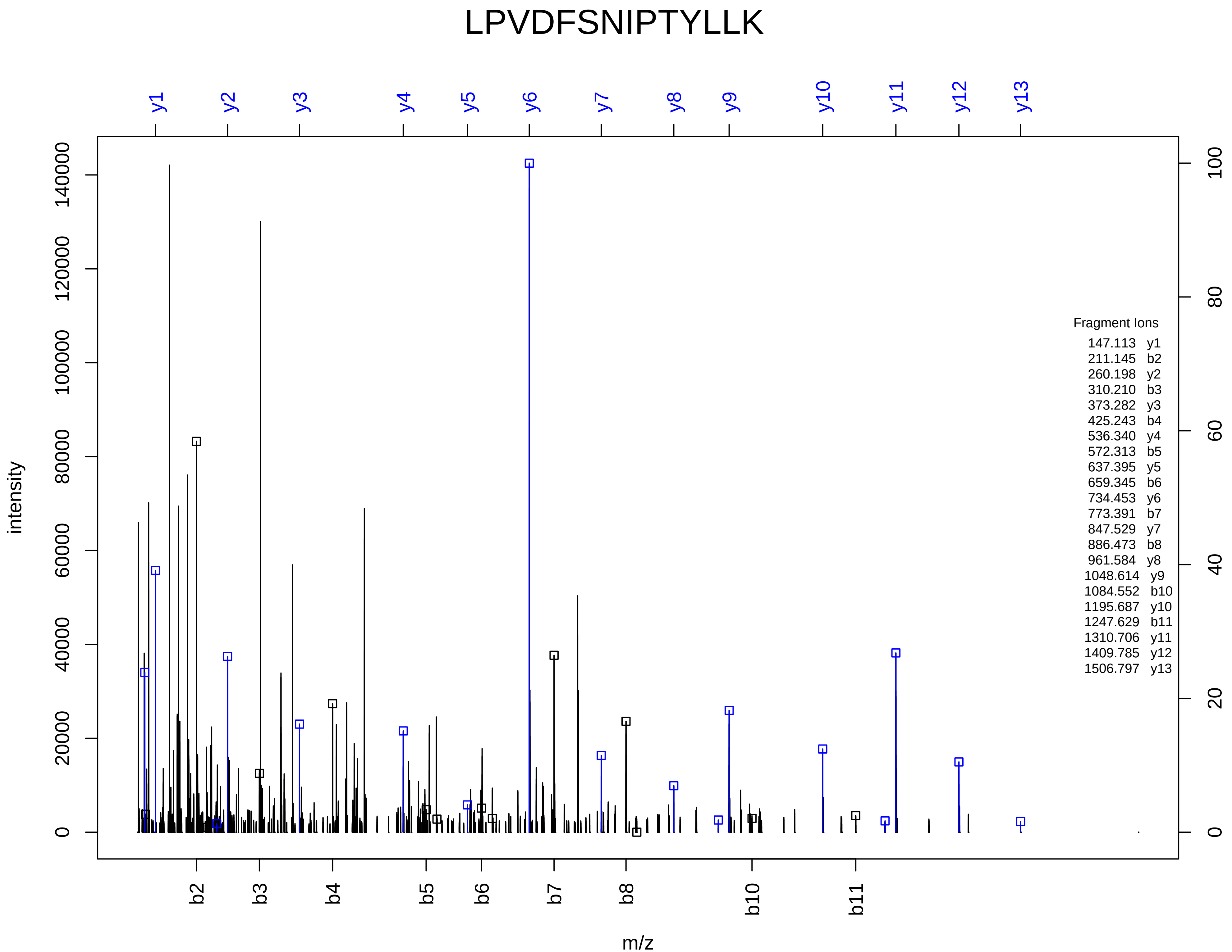

LPVDFSNIPTYLLK / File: sydney_10.raw; SpectrumID: ; scans: 14063

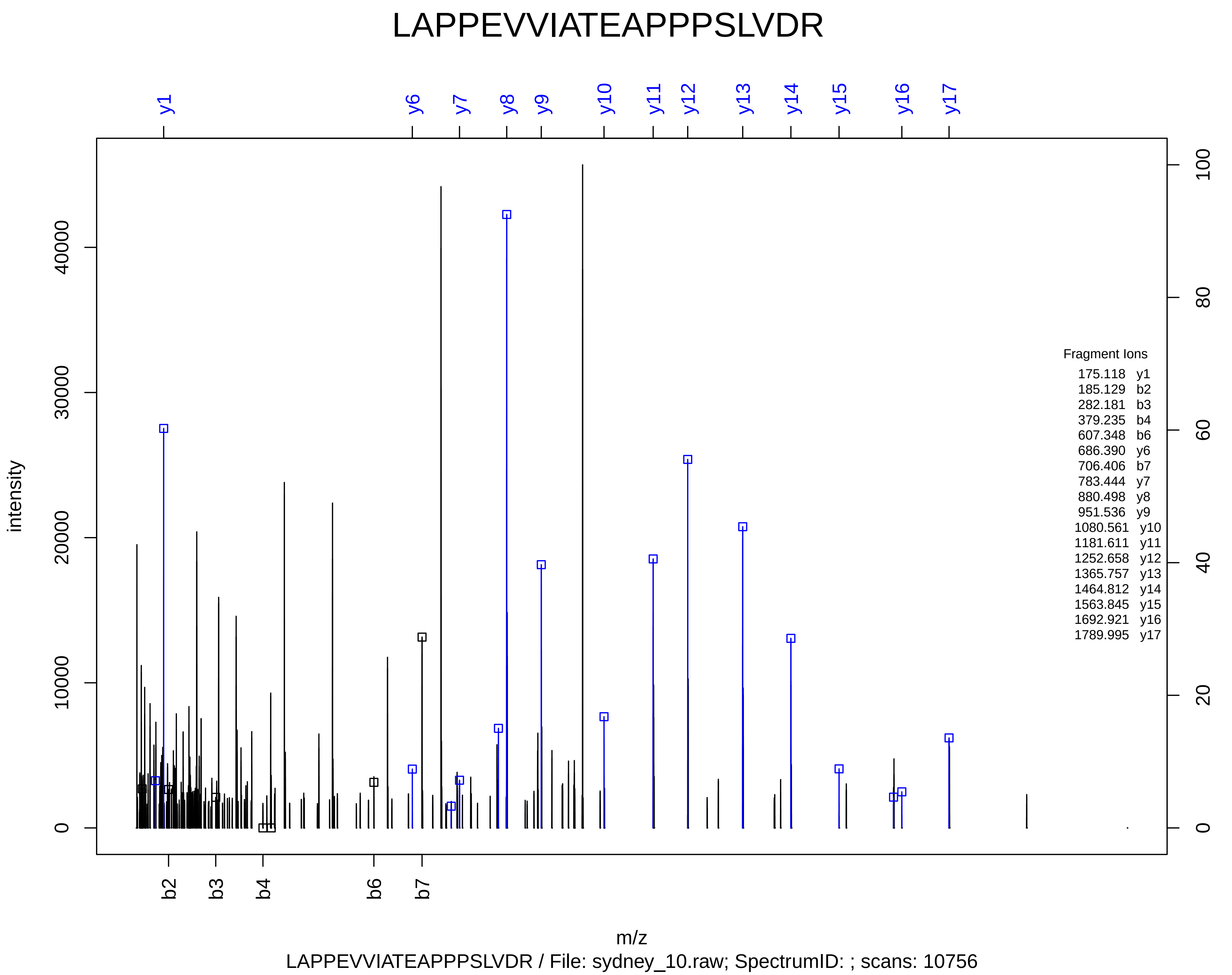

LAPPEVVIATEAPPPSLVDR
y1
y6
y7
y8
y9
y10
y11
y12
y13
y14
y15
y16
y17
intensity
0
10000
20000
30000
40000
0
20
40
60
80
100
b2
b3
b4
b6
b7
Fragment Ions
175.118 y1
185.129 b2
282.181 b3
379.235 b4
607.348 b6
686.390 y6
706.406 b7
783.444 y7
880.498 y8
951.536 y9
1080.561 y10
1181.611 y11
1252.658 y12
1365.757 y13
1464.812 y14
1563.845 y15
1692.921 y16
1789.995 y17
m/z
LAPPEVVIATEAPPPSLVDR / File: sydney_10.raw; SpectrumID: ; scans: 10756

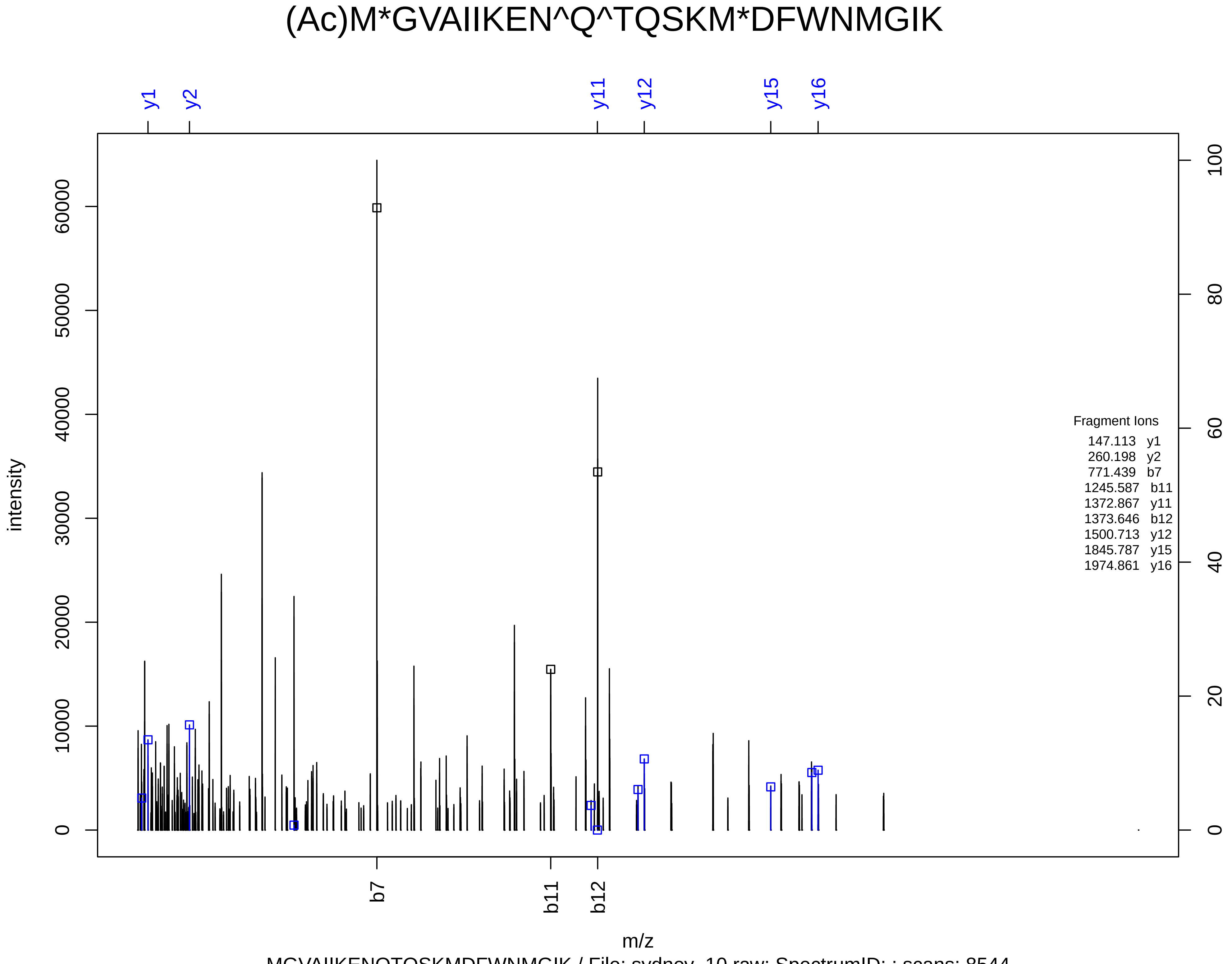

(Ac)M*GVAIIKEN^Q^TQSKM*DFWNMGIK
y1
y2
y11
y12
y15
y16
intensity
60000
50000
40000
30000
20000
10000
0
100
80
60
40
20
0
Fragment Ions
147.113 y1
260.198 y2
771.439 b7
1245.587 b11
1372.867 y11
1373.646 b12
1500.713 y12
1845.787 y15
1974.861 y16
b7
b11
b12
m/z
MGVAIIKENQTQSKMDFWNMGIK / File: sydney_10.raw; SpectrumID: ; scans: 8544

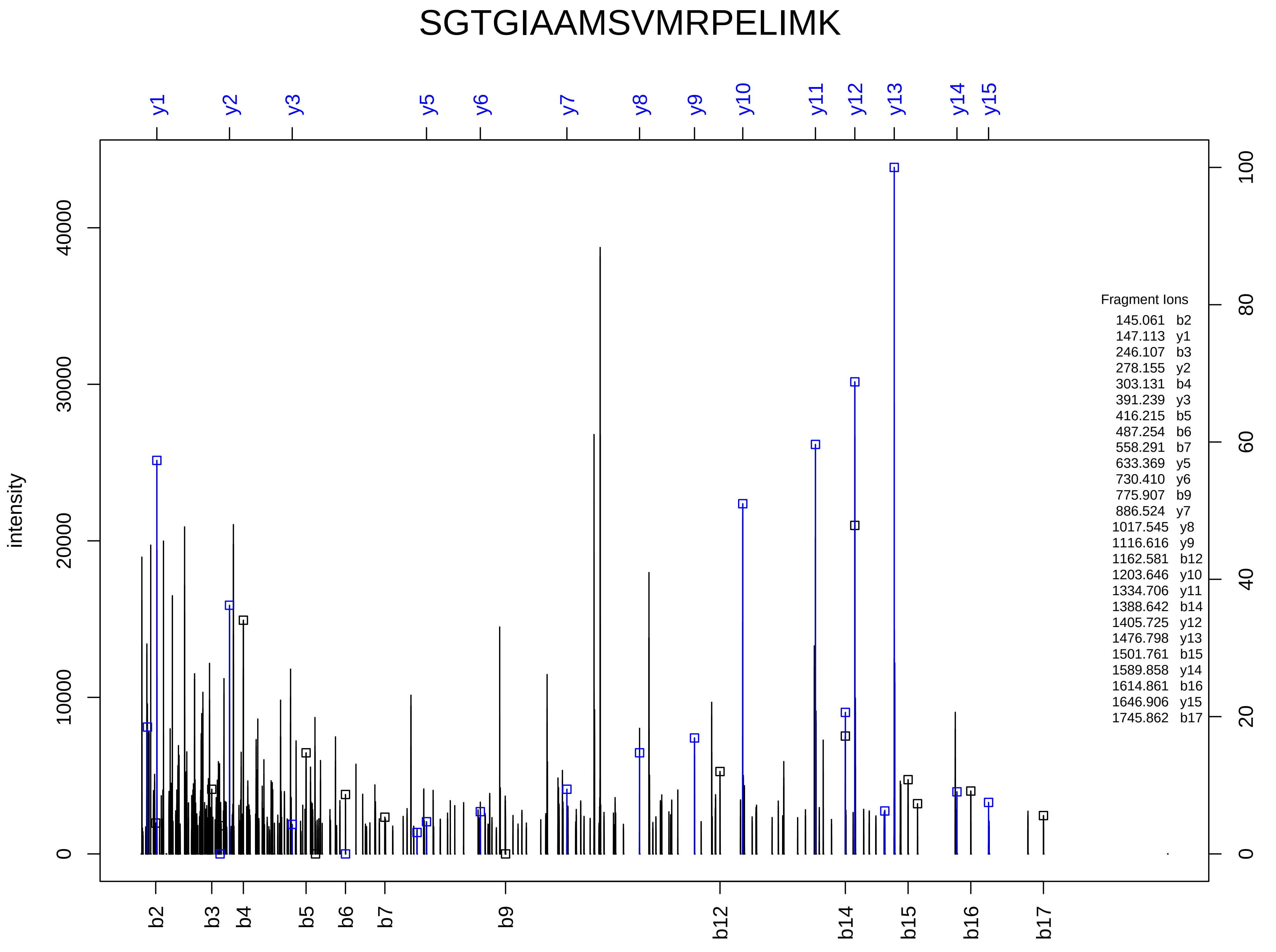
SGTGIAAMSVMRPELIMK
y1 y2 y3 y5 y6 y7 y8 y9 y10 y11 y12 y13 y14 y15
b2 b3 b4 b5 b6 b7 b9 b12 b14 b15 b16 b17
intensity
m/z
Fragment Ions
145.061 b2
147.113 y1
246.107 b3
278.155 y2
303.131 b4
391.239 y3
416.215 b5
487.254 b6
558.291 b7
633.369 y5
730.410 y6
775.907 b9
886.524 y7
1017.545 y8
1116.616 y9
1162.581 b12
1203.646 y10
1334.706 y11
1388.642 b14
1405.725 y12
1476.798 y13
1501.761 b15
1589.858 y14
1614.861 b16
1646.906 y15
1745.862 b17
SGTGIAAMSVMRPELIMK / File: sydney_10.raw; SpectrumID: ; scans: 9055

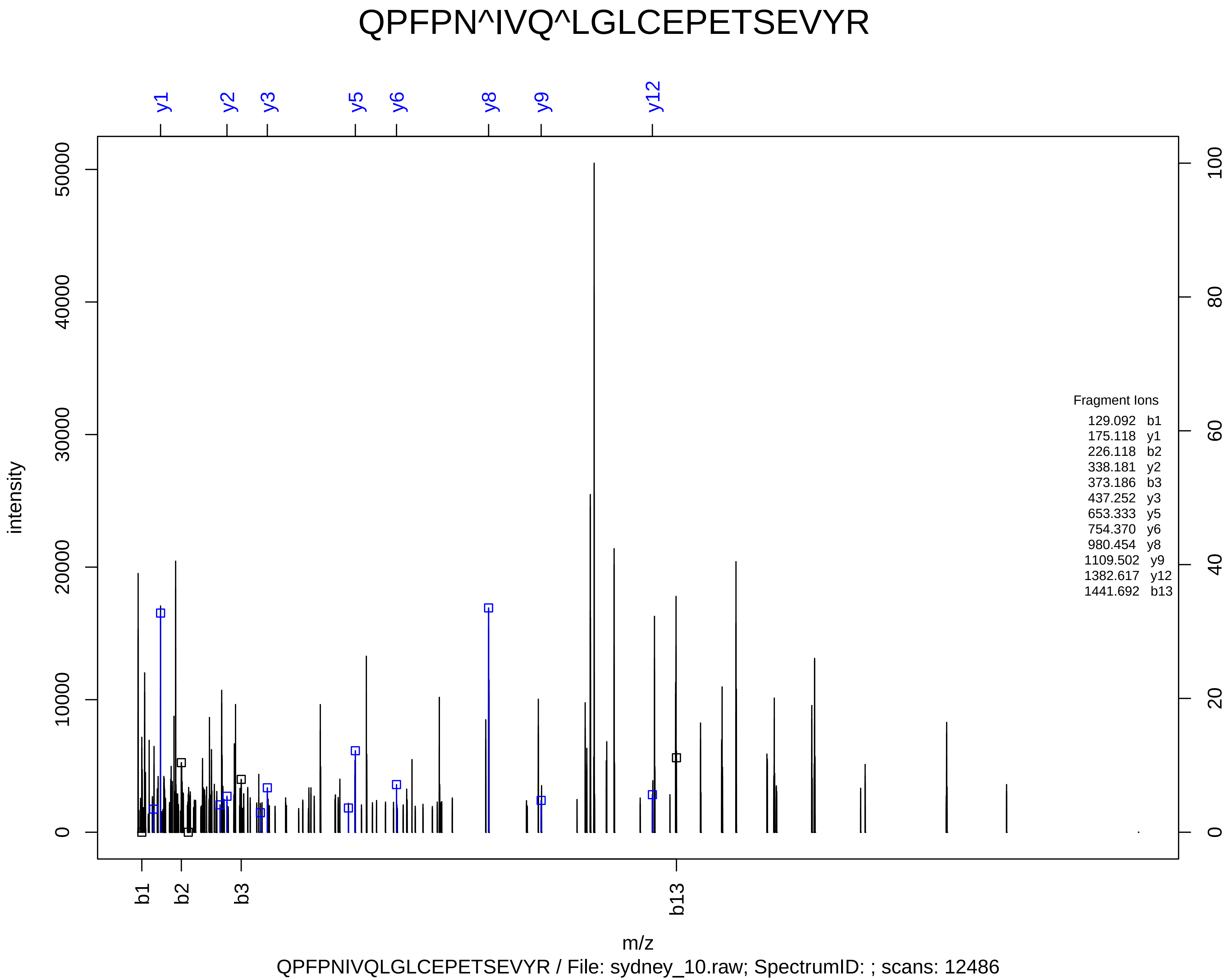
QPFPN^IVQ^LGLCEPETSEVYR
y1
y2
y3
y5
y6
y8
y9
y12
intensity
0
10000
20000
30000
40000
50000
0
20
40
60
80
100
Fragment Ions
129.092 b1
175.118 y1
226.118 b2
338.181 y2
373.186 b3
437.252 y3
653.333 y5
754.370 y6
980.454 y8
1109.502 y9
1382.617 y12
1441.692 b13
b1
b2
b3
b13
m/z
QPFPNIVQLGLCEPETSEVYR / File: sydney_10.raw; SpectrumID: ; scans: 12486

IEIQTLTGLFK

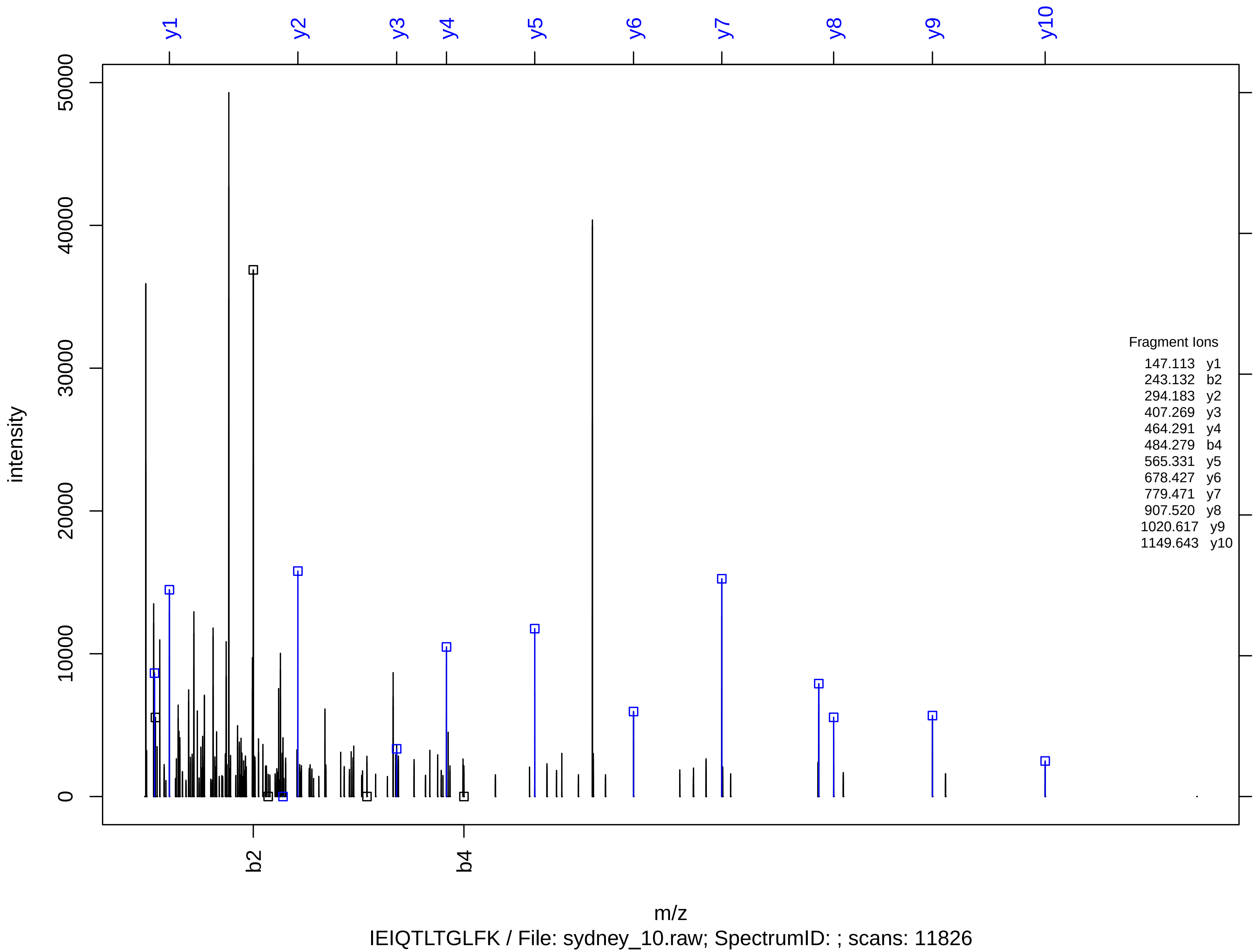

IEIQTLTGLFK / File: sydney_10.raw; SpectrumID: ; scans: 11826

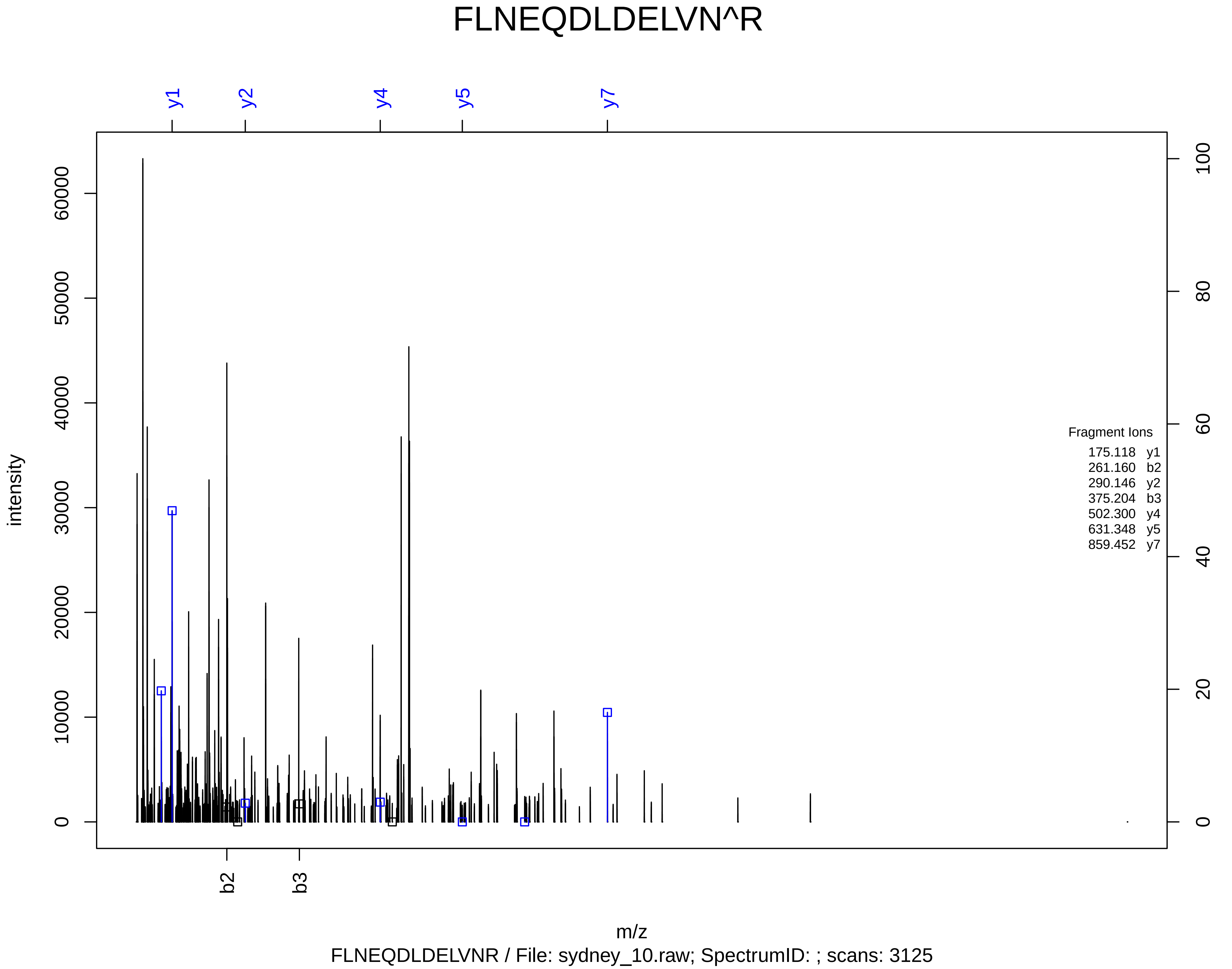

FLNEQDLDELVN^R
y1
y2
y4
y5
y7
intensity
0
10000
20000
30000
40000
50000
60000
0
20
40
60
80
100
Fragment Ions
175.118 y1
261.160 b2
290.146 y2
375.204 b3
502.300 y4
631.348 y5
859.452 y7
b2
b3
m/z
FLNEQDLDELVNR / File: sydney_10.raw; SpectrumID: ; scans: 3125

VAGFNPALQLILSR

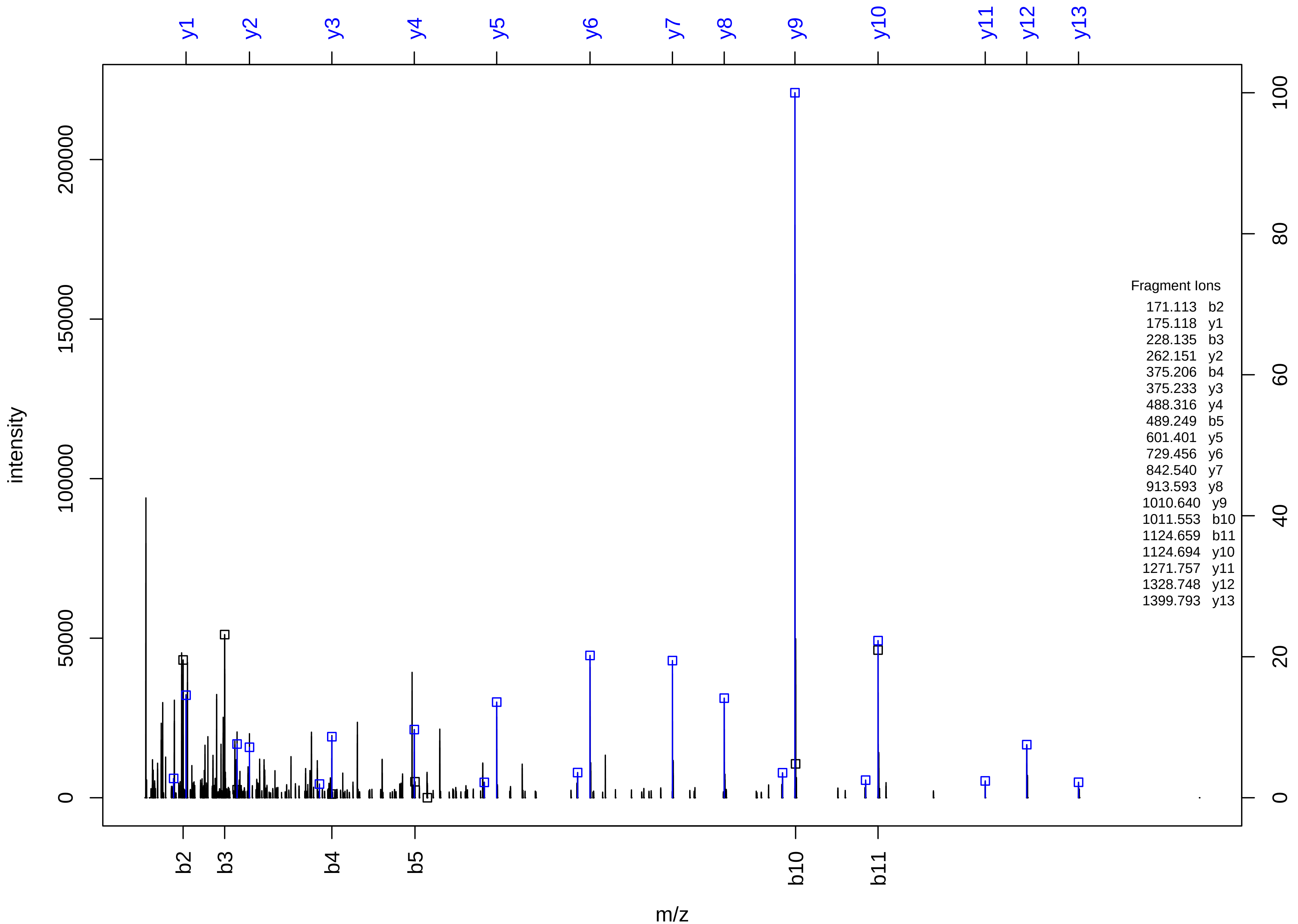

VAGFNPALQLILSR / File: sydney_10.raw; SpectrumID: ; scans: 12933

GANSSSGNPLVYLDVGADGQPLGR

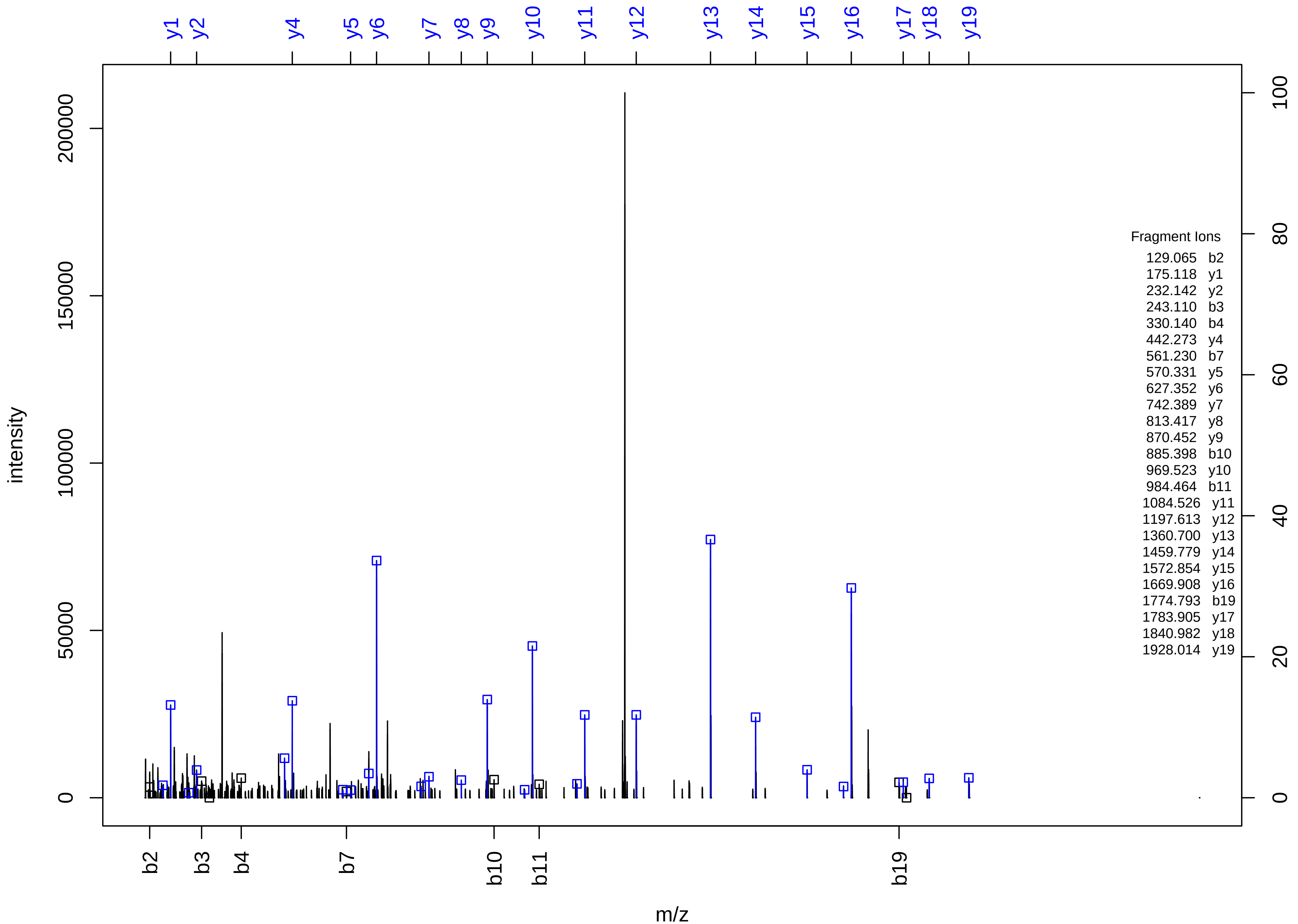

GANSSSGNPLVYLDVGADGQPLGR / File: sydney_10.raw; SpectrumID: ; scans: 10697

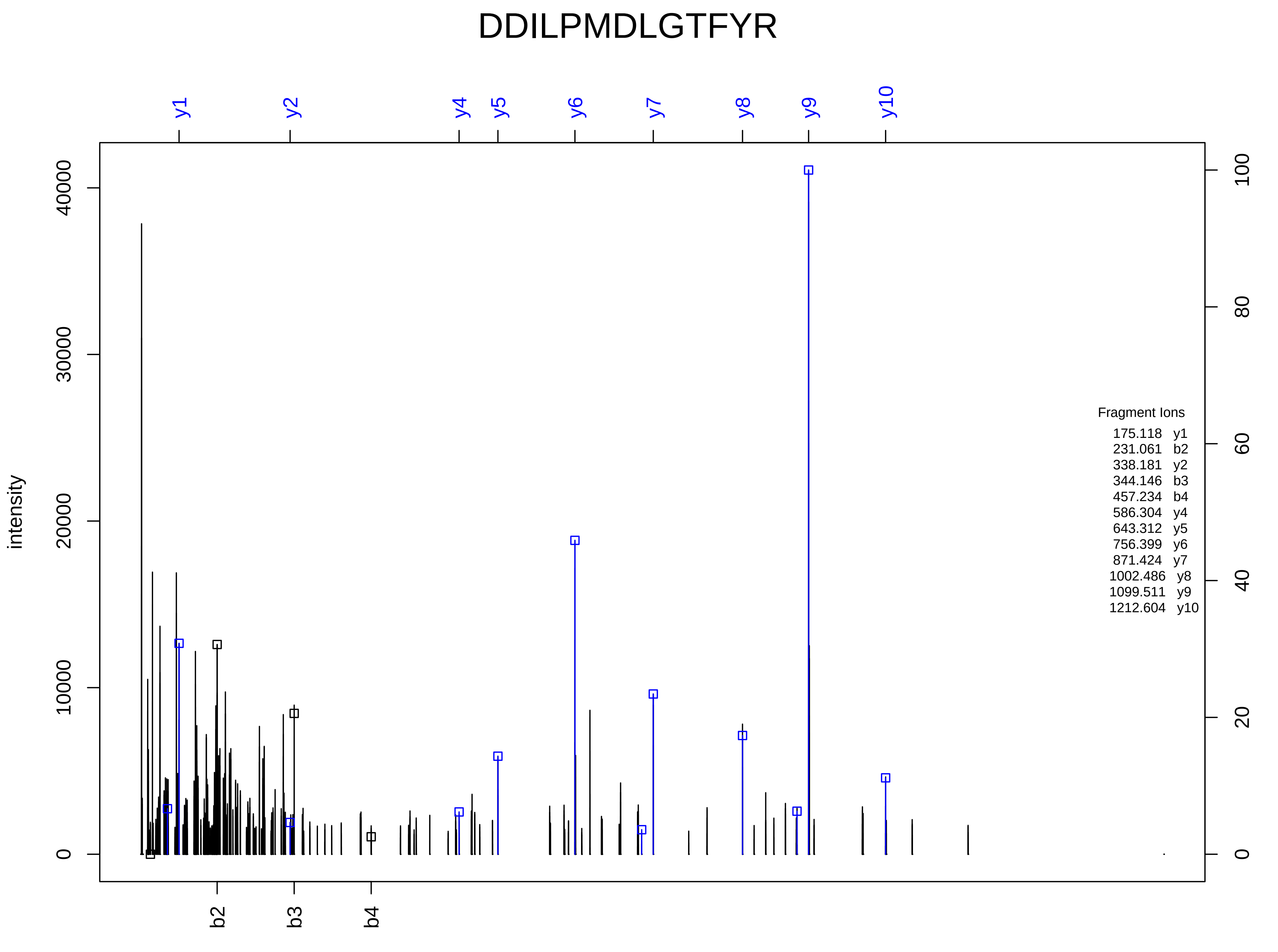

DDILPMDLGTFYR / File: sydney_10.raw; SpectrumID: ; scans: 13436

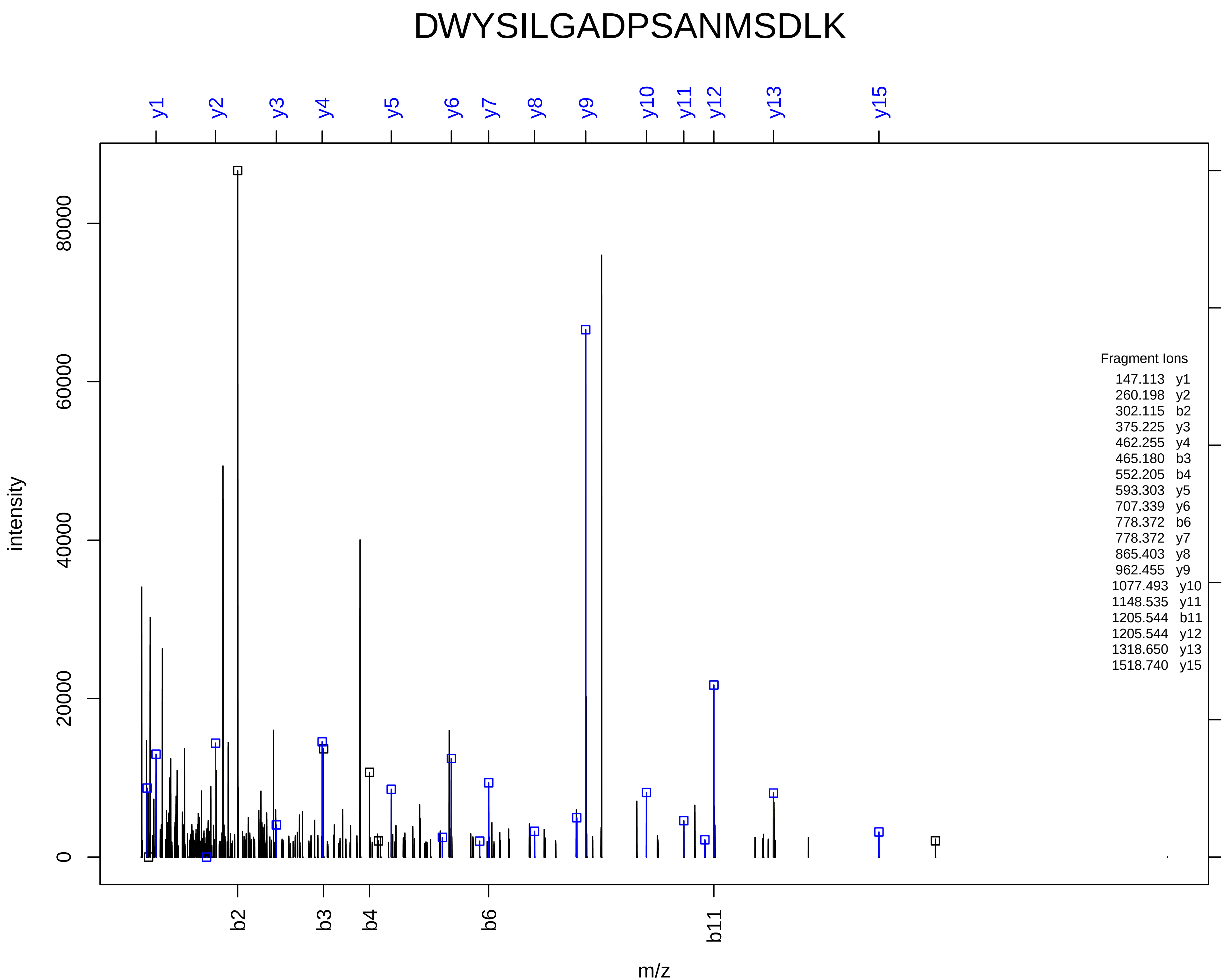
DWYSILGADPSANMSDLK
y1
y2
y3
y4
y5
y6
y7
y8
y9
y10
y11
y12
y13
y15
intensity
0
20000
40000
60000
80000
0
20
40
60
80
100
b2
b3
b4
b6
b11
m/z
Fragment Ions
147.113 y1
260.198 y2
302.115 b2
375.225 y3
462.255 y4
465.180 b3
552.205 b4
593.303 y5
707.339 y6
778.372 b6
778.372 y7
865.403 y8
962.455 y9
1077.493 y10
1148.535 y11
1205.544 b11
1205.544 y12
1318.650 y13
1518.740 y15
DWYSILGADPSANMSDLK / File: sydney_10.raw; SpectrumID: ; scans: 12859

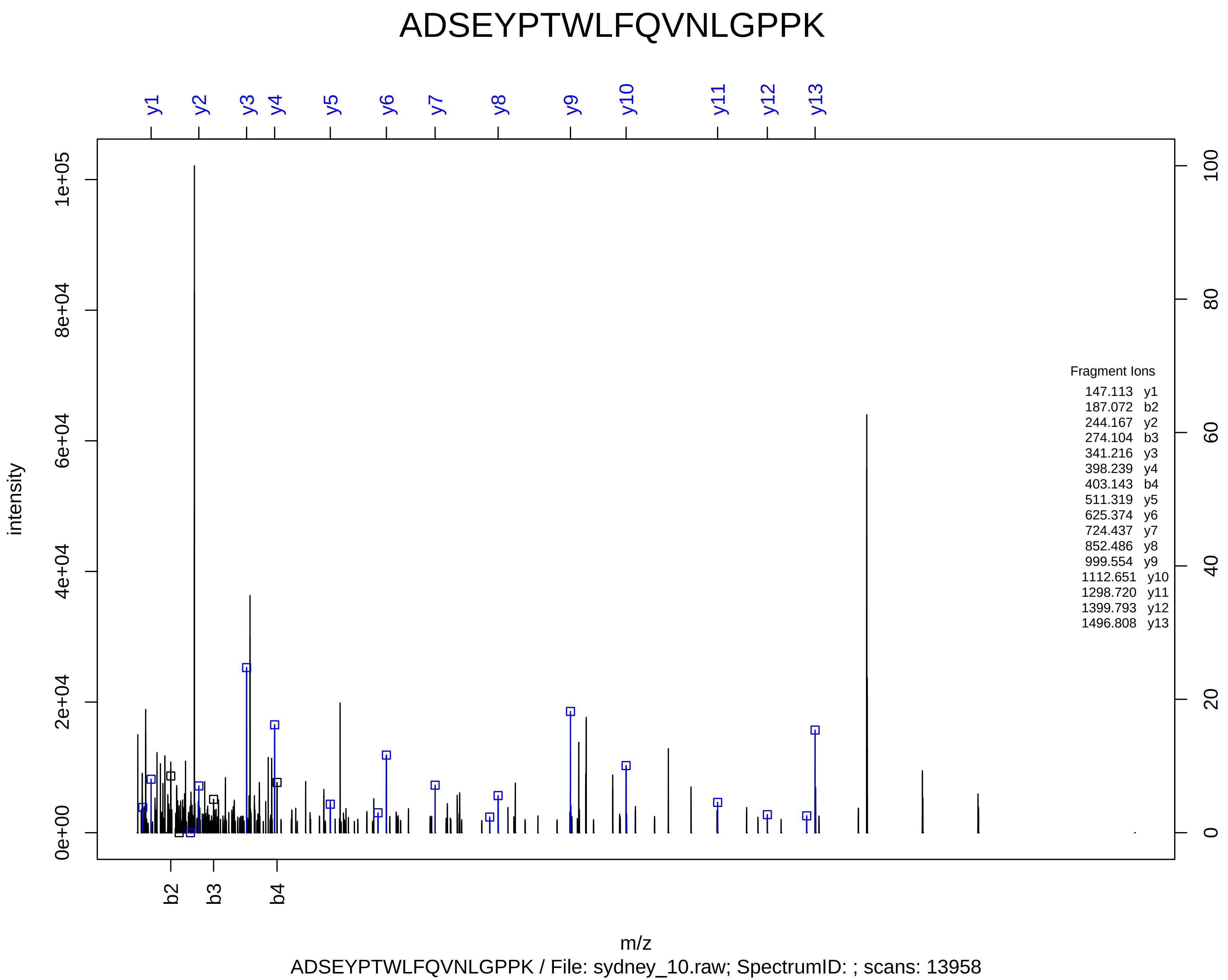
ADSEYPTWLFQVNLGPPK
y1 y2 y3 y4 y5 y6 y7 y8 y9 y10 y11 y12 y13
intensity
0e+00 2e+04 4e+04 6e+04 8e+04 1e+05
0 20 40 60 80 100
b2 b3 b4
Fragment Ions
147.113 y1
187.072 b2
244.167 y2
274.104 b3
341.216 y3
398.239 y4
403.143 b4
511.319 y5
625.374 y6
724.437 y7
852.486 y8
999.554 y9
1112.651 y10
1298.720 y11
1399.793 y12
1496.808 y13
m/z
ADSEYPTWLFQVNLGPPK / File: sydney_10.raw; SpectrumID: ; scans: 13958

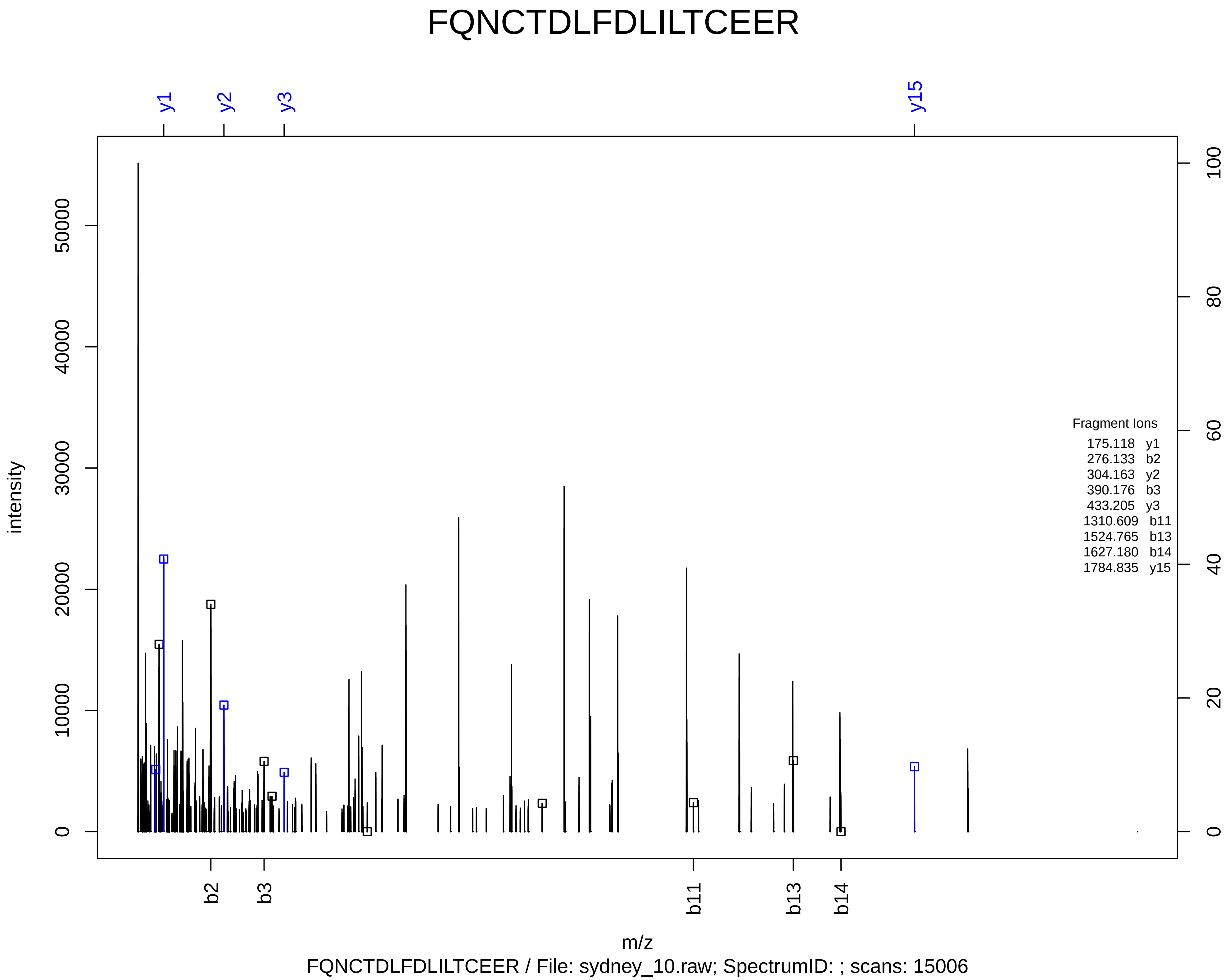
FQNCTDLFDLILTCEER
y1
y2
y3
y15
intensity
0
10000
20000
30000
40000
50000
0
20
40
60
80
100
b2
b3
b11
b13
b14
m/z
Fragment Ions
175.118 y1
276.133 b2
304.163 y2
390.176 b3
433.205 y3
1310.609 b11
1524.765 b13
1627.180 b14
1784.835 y15
FQNCTDLFDLILTCEER / File: sydney_10.raw; SpectrumID: ; scans: 15006

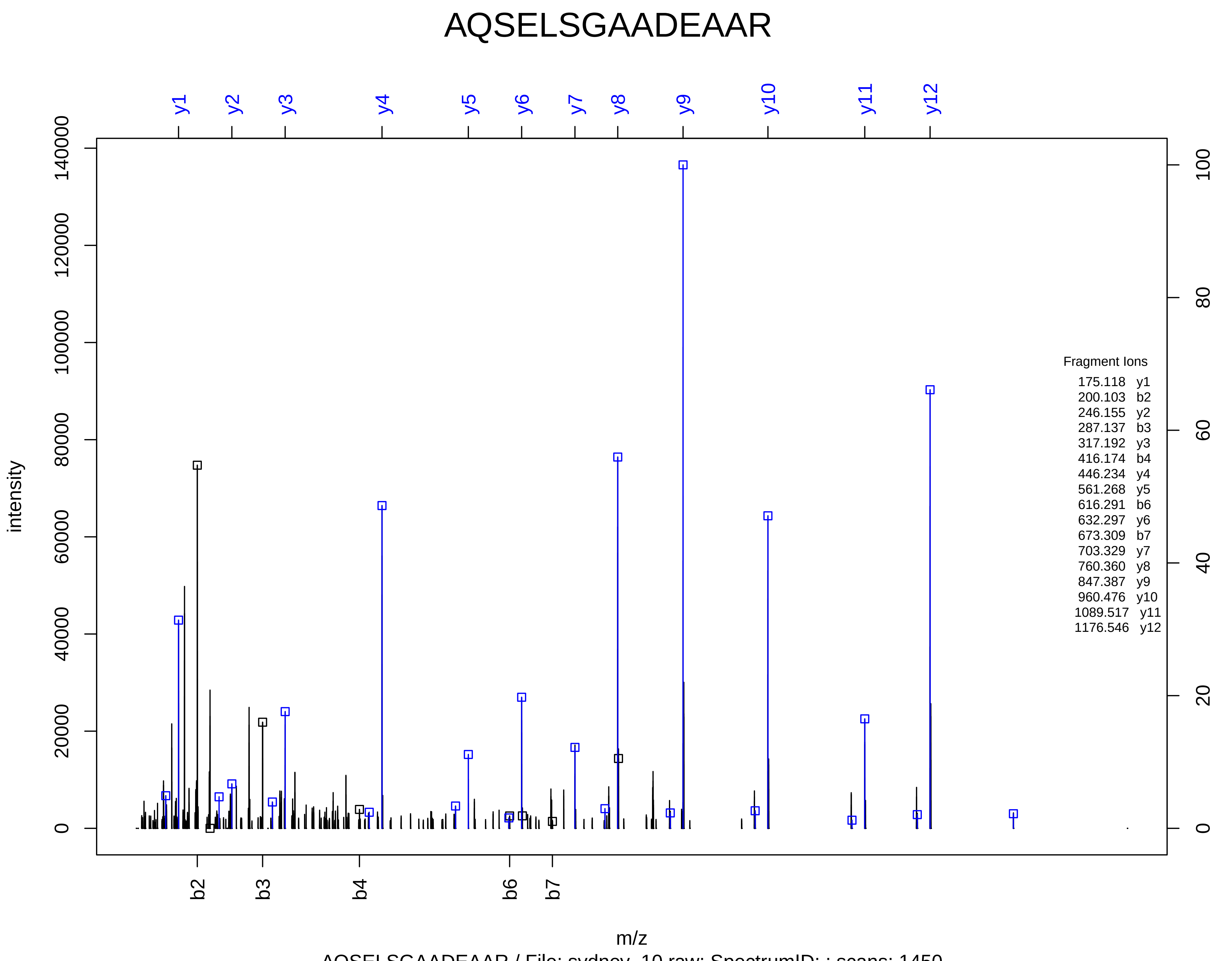

AQSELSGAADEAAR
y1
y2
y3
y4
y5
y6
y7
y8
y9
y10
y11
y12
intensity
0
20000
40000
60000
80000
100000
120000
140000
0
20
40
60
80
100
Fragment Ions
175.118 y1
200.103 b2
246.155 y2
287.137 b3
317.192 y3
416.174 b4
446.234 y4
561.268 y5
616.291 b6
632.297 y6
673.309 b7
703.329 y7
760.360 y8
847.387 y9
960.476 y10
1089.517 y11
1176.546 y12
b2
b3
b4
b6
b7
m/z
AQSELSGAADEAAR / File: sydney_10.raw; SpectrumID: ; scans: 1450

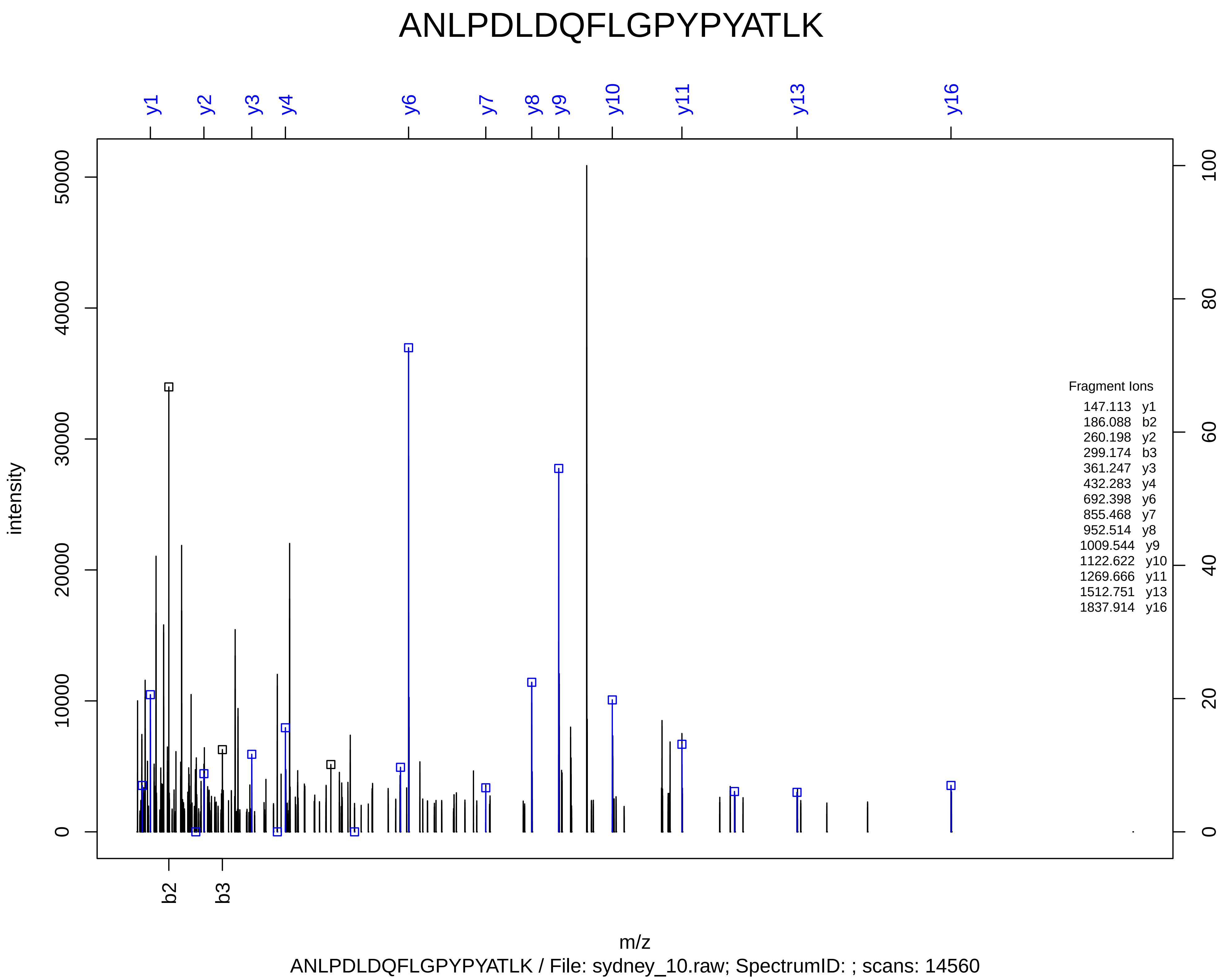
ANLPDLDQFLGPYPYATLK
y1
y2
y3
y4
y6
y7
y8
y9
y10
y11
y13
y16
intensity
0
10000
20000
30000
40000
50000
0
20
40
60
80
100
b2
b3
m/z
ANLPDLDQFLGPYPYATLK / File: sydney_10.raw; SpectrumID: ; scans: 14560
Fragment Ions
147.113 y1
186.088 b2
260.198 y2
299.174 b3
361.247 y3
432.283 y4
692.398 y6
855.468 y7
952.514 y8
1009.544 y9
1122.622 y10
1269.666 y11
1512.751 y13
1837.914 y16

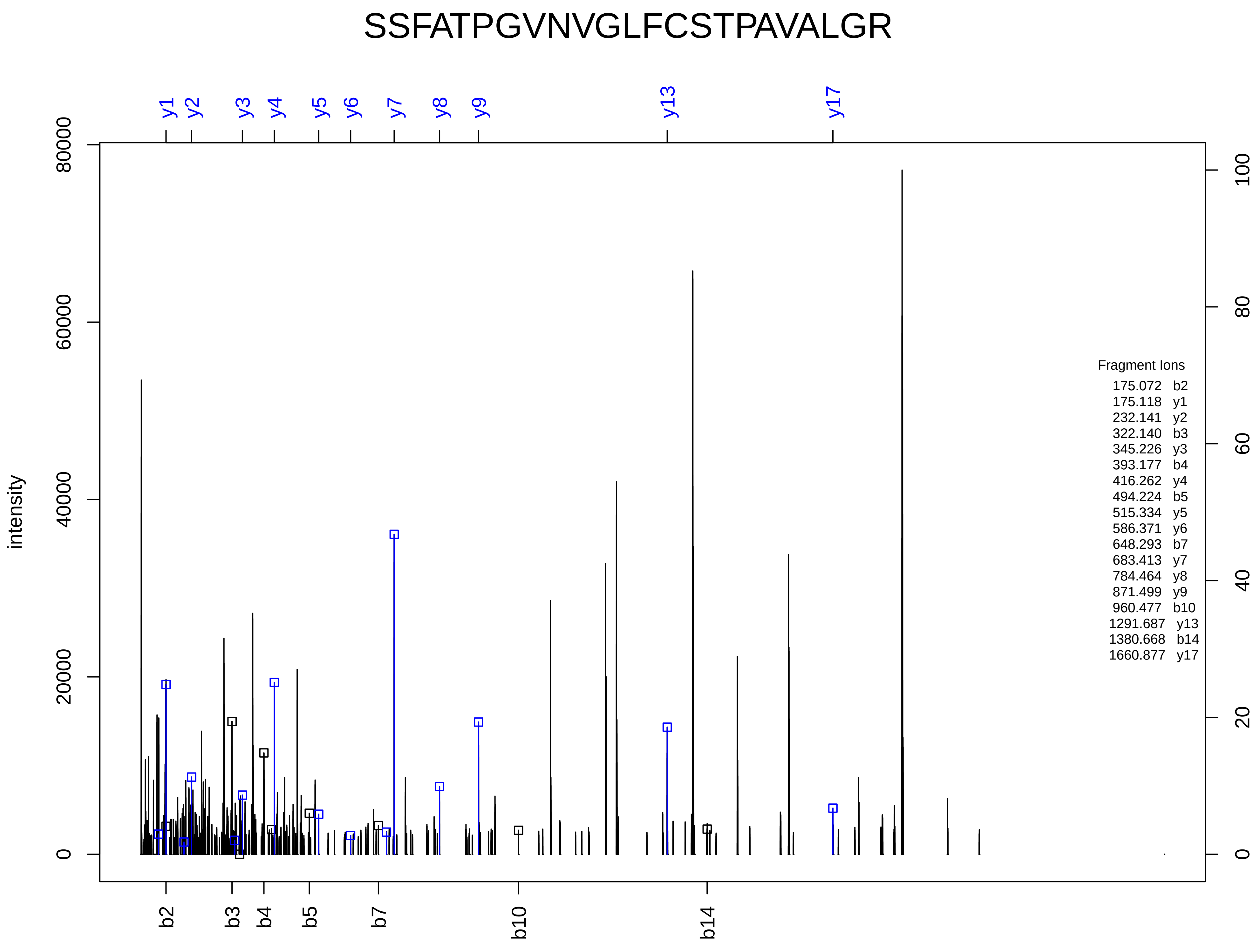

SSFATPGVNVGLFCSTPAVALGR / File: sydney_10.raw; SpectrumID: ; scans: 12450

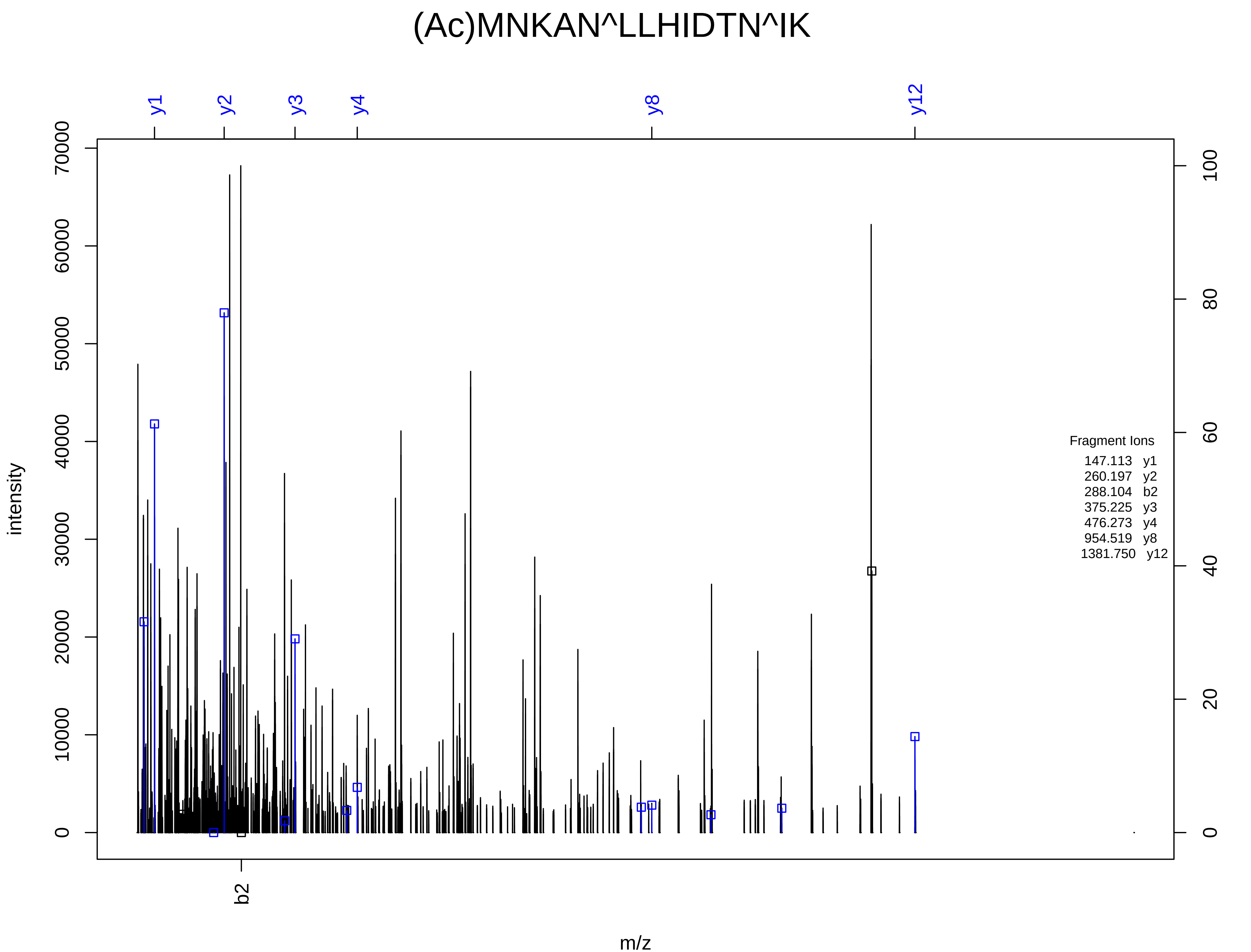
(Ac)MNKAN^LLHIDTN^IK
y1
y2
y3
y4
y8
y12
b2
intensity
m/z
Fragment Ions
147.113 y1
260.197 y2
288.104 b2
375.225 y3
476.273 y4
954.519 y8
1381.750 y12
MNKANLLHIDTNIK / File: sydney_10.raw; SpectrumID: ; scans: 10577

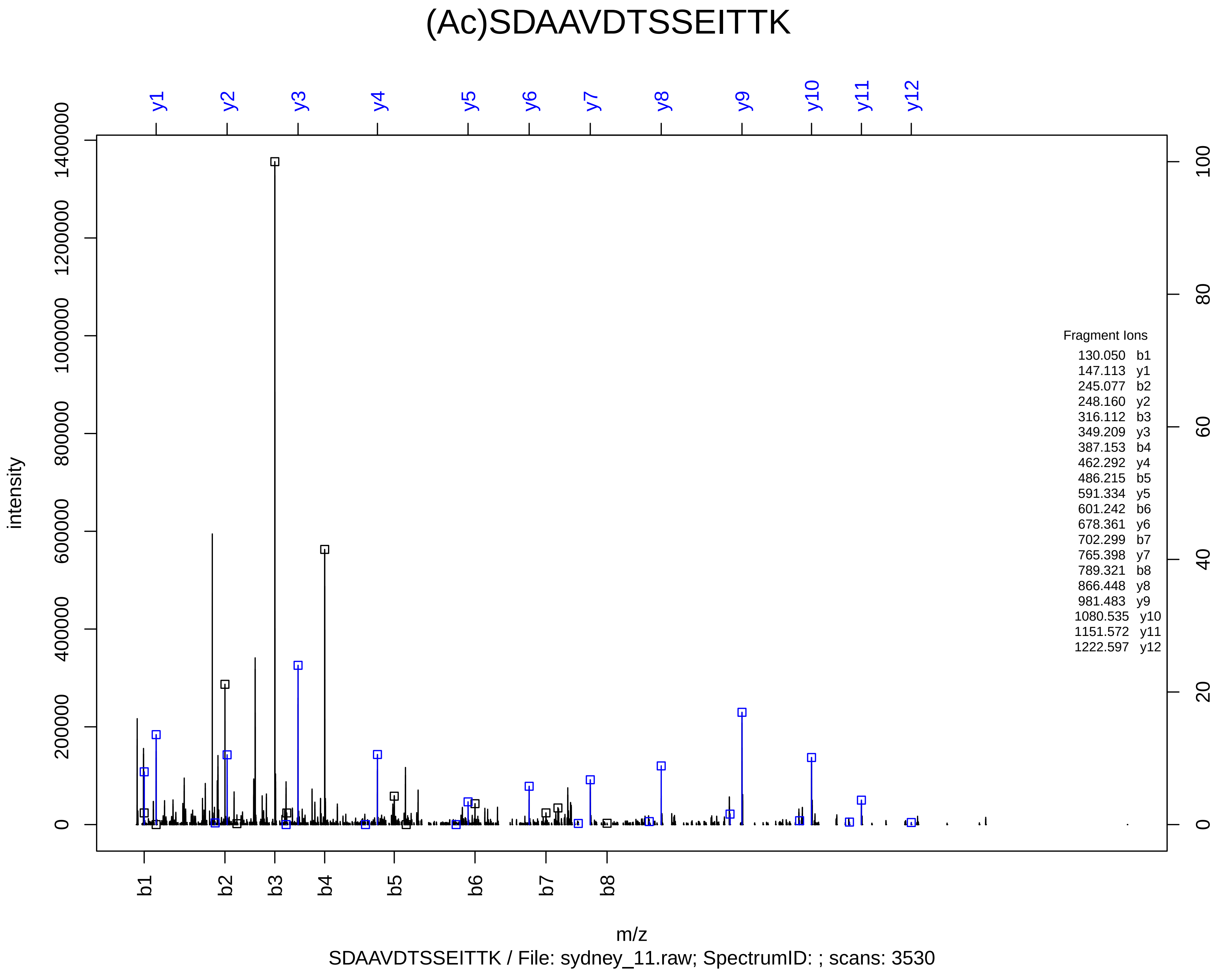
(Ac)SDAAVDTSSEITTK
y1
y2
y3
y4
y5
y6
y7
y8
y9
y10
y11
y12
intensity
0
200000
400000
600000
800000
1000000
1200000
1400000
0
20
40
60
80
100
Fragment Ions
130.050 b1
147.113 y1
245.077 b2
248.160 y2
316.112 b3
349.209 y3
387.153 b4
462.292 y4
486.215 b5
591.334 y5
601.242 b6
678.361 y6
702.299 b7
765.398 y7
789.321 b8
866.448 y8
981.483 y9
1080.535 y10
1151.572 y11
1222.597 y12
b1
b2
b3
b4
b5
b6
b7
b8
m/z
SDAAVDTSSEITTK / File: sydney_11.raw; SpectrumID: ; scans: 3530

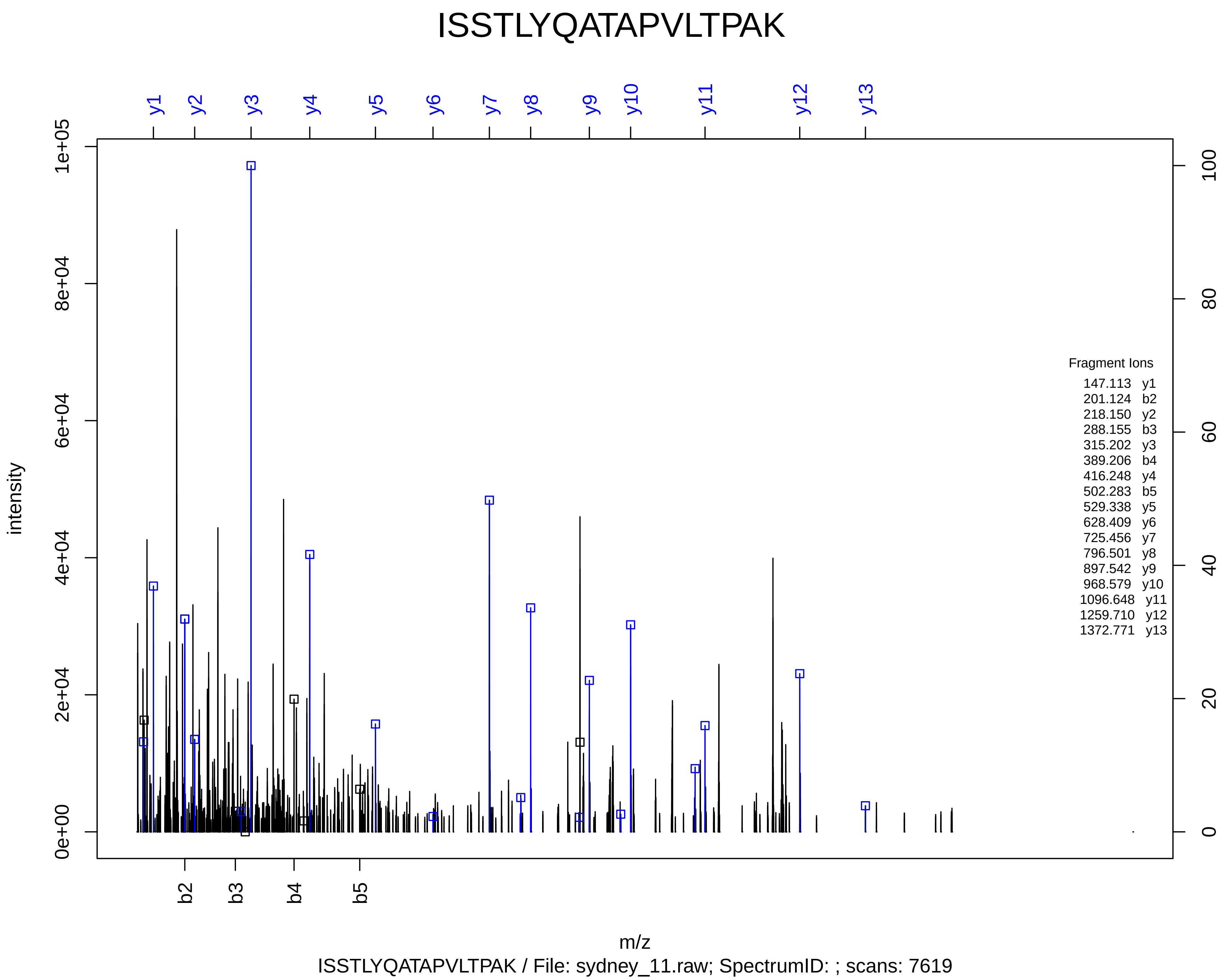
ISSTLYQATAPVLTPAK
y1
y2
y3
y4
y5
y6
y7
y8
y9
y10
y11
y12
y13
intensity
0e+00
2e+04
4e+04
6e+04
8e+04
1e+05
0
20
40
60
80
100
b2
b3
b4
b5
Fragment Ions
147.113 y1
201.124 b2
218.150 y2
288.155 b3
315.202 y3
389.206 b4
416.248 y4
502.283 b5
529.338 y5
628.409 y6
725.456 y7
796.501 y8
897.542 y9
968.579 y10
1096.648 y11
1259.710 y12
1372.771 y13
m/z
ISSTLYQATAPVLTPAK / File: sydney_11.raw; SpectrumID: ; scans: 7619

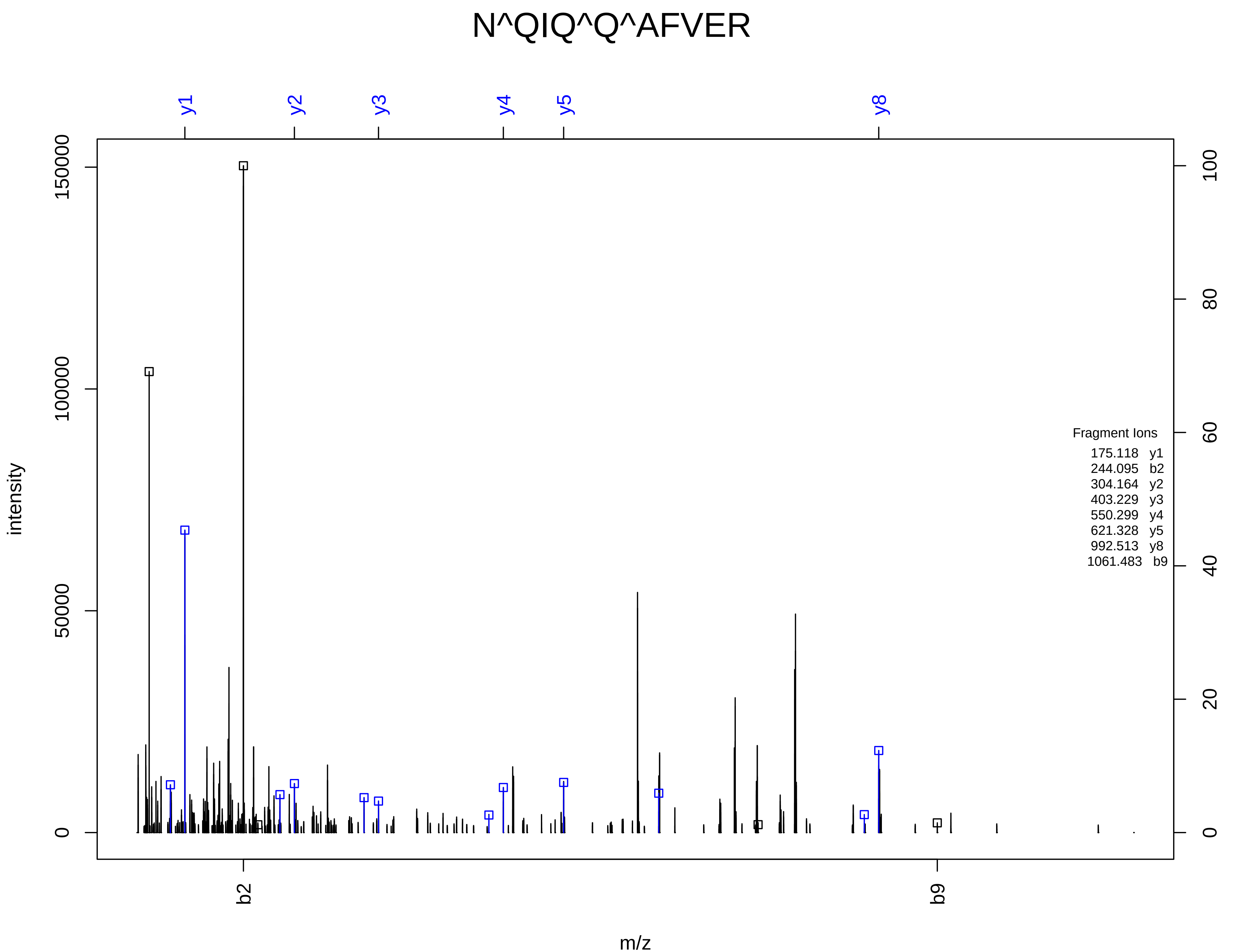
N^QIQ^Q^AFVER
y1
y2
y3
y4
y5
y8
intensity
150000
100000
50000
0
100
80
60
40
20
b2
b9
Fragment Ions
175.118 y1
244.095 b2
304.164 y2
403.229 y3
550.299 y4
621.328 y5
992.513 y8
1061.483 b9
m/z
NQIQQAFVER / File: sydney_11.raw; SpectrumID: ; scans: 2417

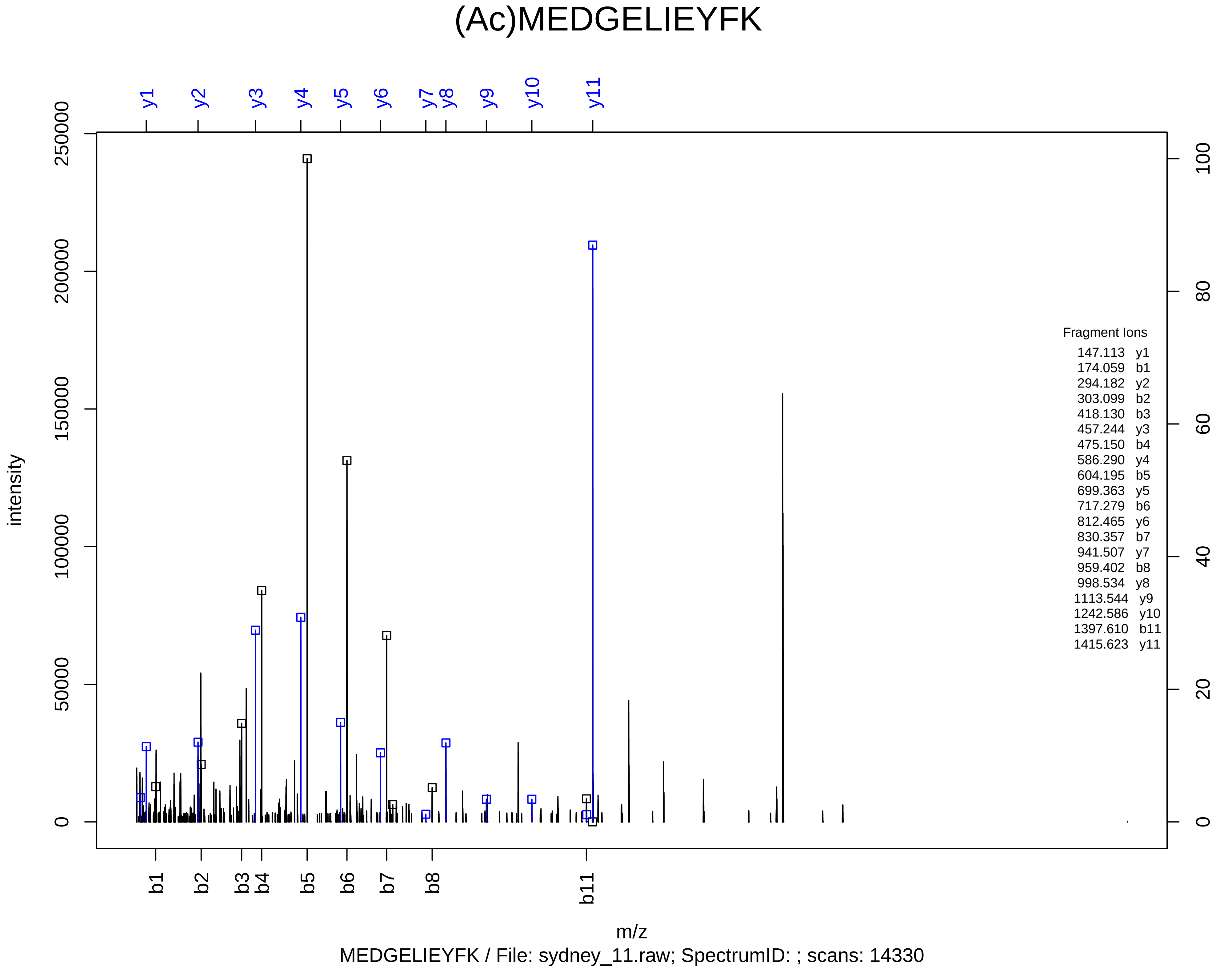

(Ac)MEDGELIEYFK
y1
y2
y3
y4
y5
y6
y7
y8
y9
y10
y11
intensity
250000
200000
150000
100000
50000
0
100
80
60
40
20
0
Fragment Ions
147.113 y1
174.059 b1
294.182 y2
303.099 b2
418.130 b3
457.244 y3
475.150 b4
586.290 y4
604.195 b5
699.363 y5
717.279 b6
812.465 y6
830.357 b7
941.507 y7
959.402 b8
998.534 y8
1113.544 y9
1242.586 y10
1397.610 b11
1415.623 y11
b1
b2
b3
b4
b5
b6
b7
b8
b11
m/z
MEDGELIEYFK / File: sydney_11.raw; SpectrumID: ; scans: 14330

TLGENEEEEDLADSGR

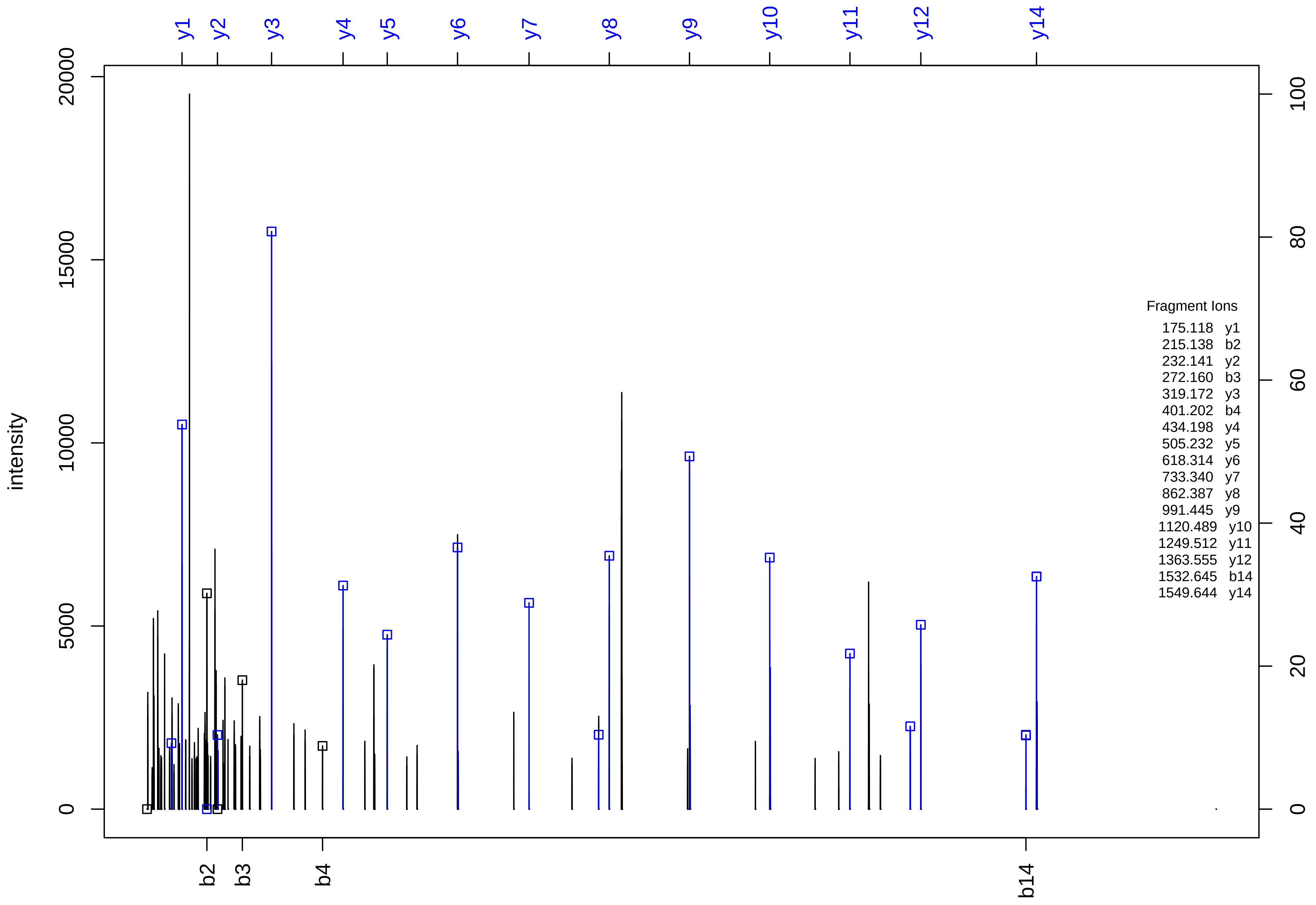

TLGENEEEEDLADSGR / File: sydney_11.raw; SpectrumID: ; scans: 2666

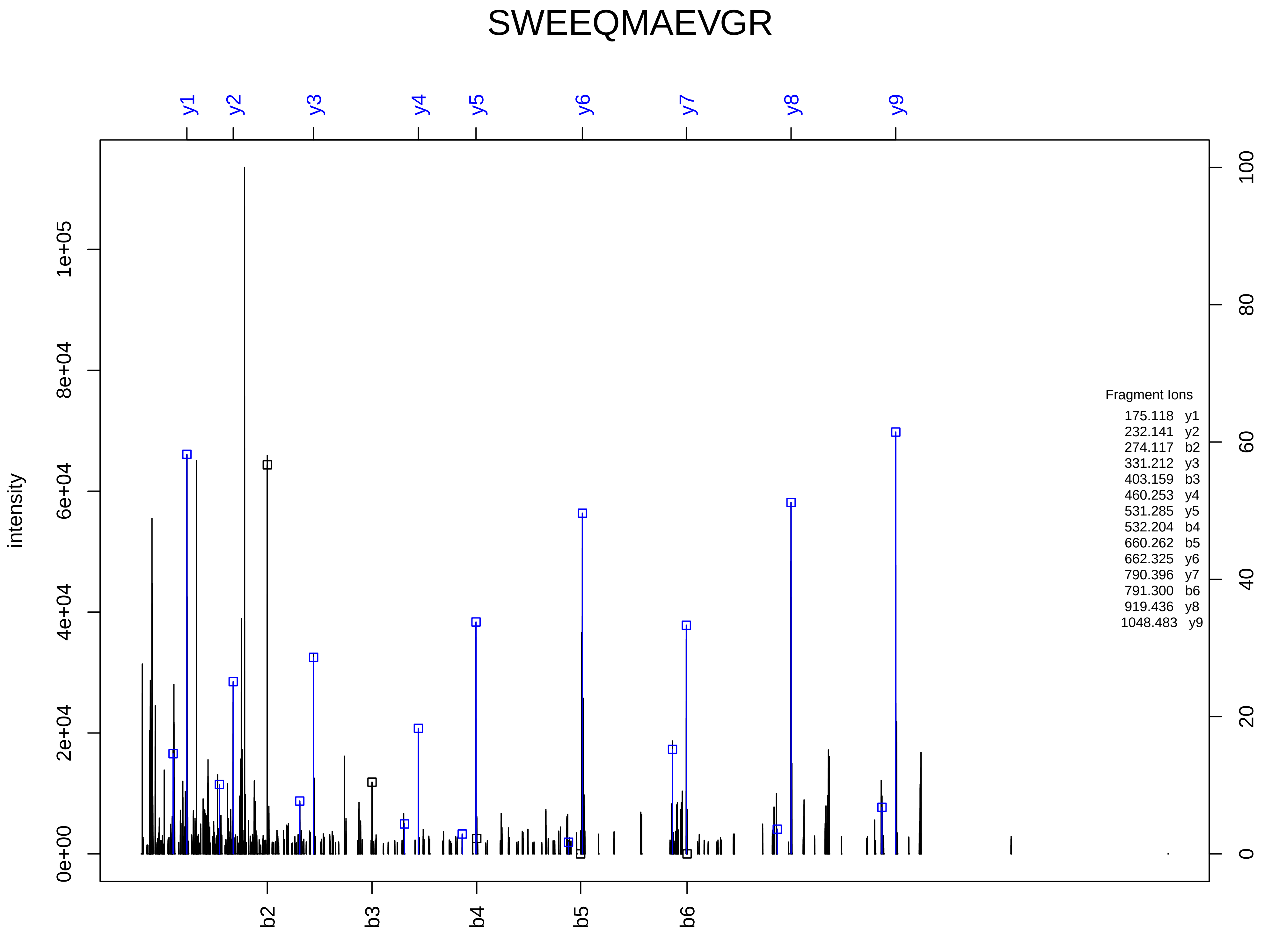

SWEEQMAEVGR / File: sydney_11.raw; SpectrumID: ; scans: 5119

EDLSLPELLDEEDVLQECK

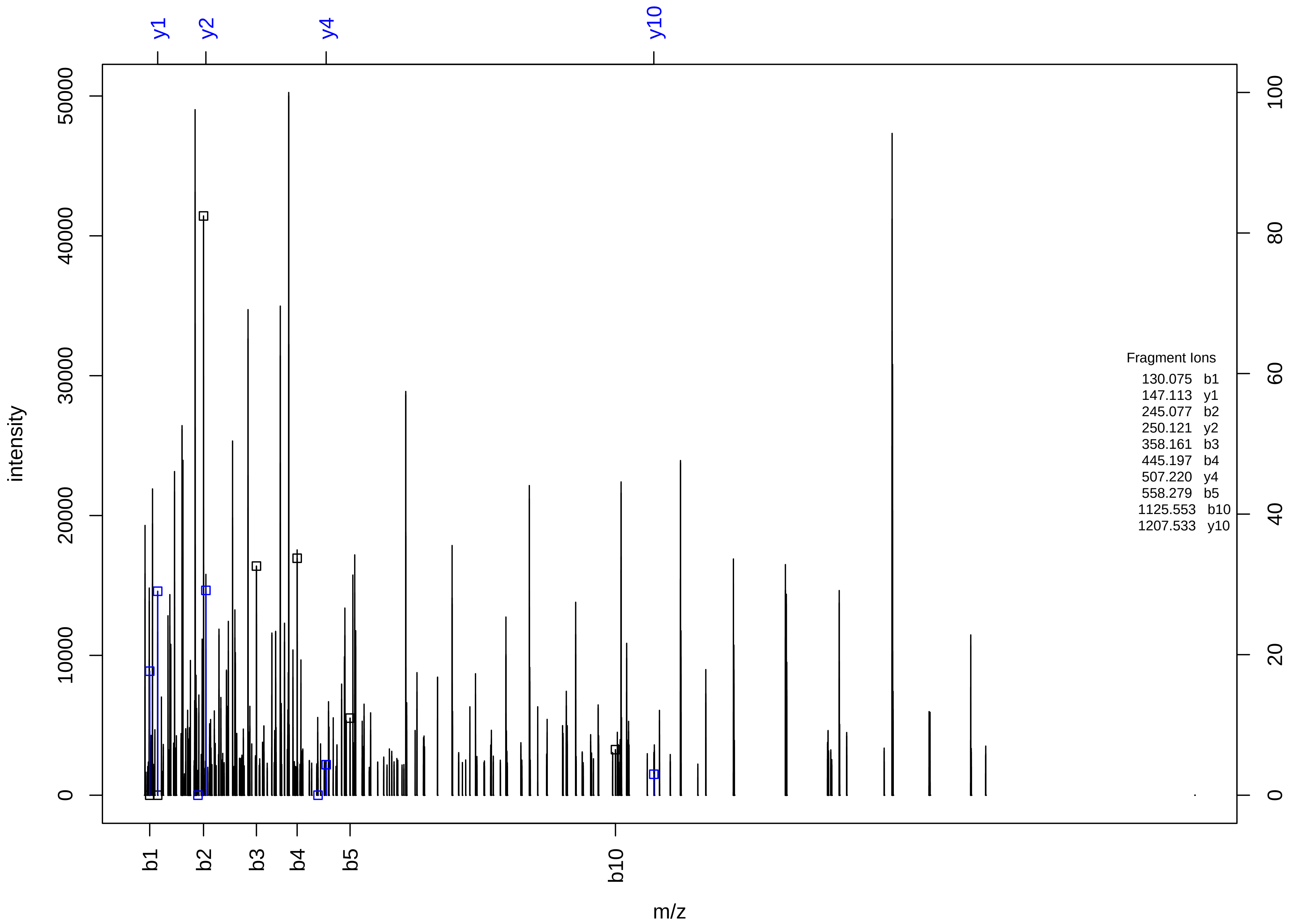

EDLSLPELLDEEDVLQECK / File: sydney_11.raw; SpectrumID: ; scans: 13951

N^DRN^NRWR

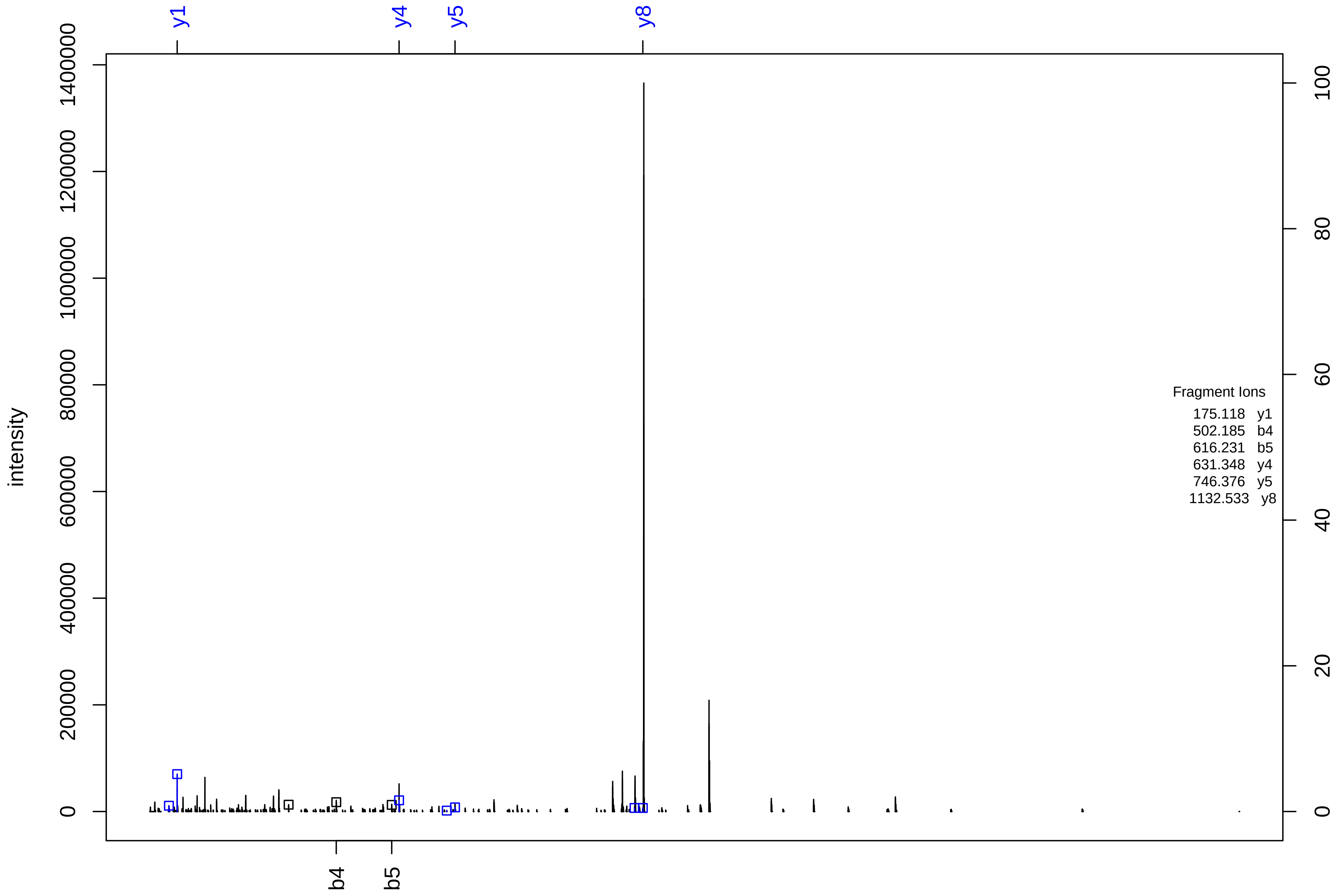

NDRNNRWR / File: sydney_11.raw; SpectrumID: ; scans: 2082

SMLALLLGR

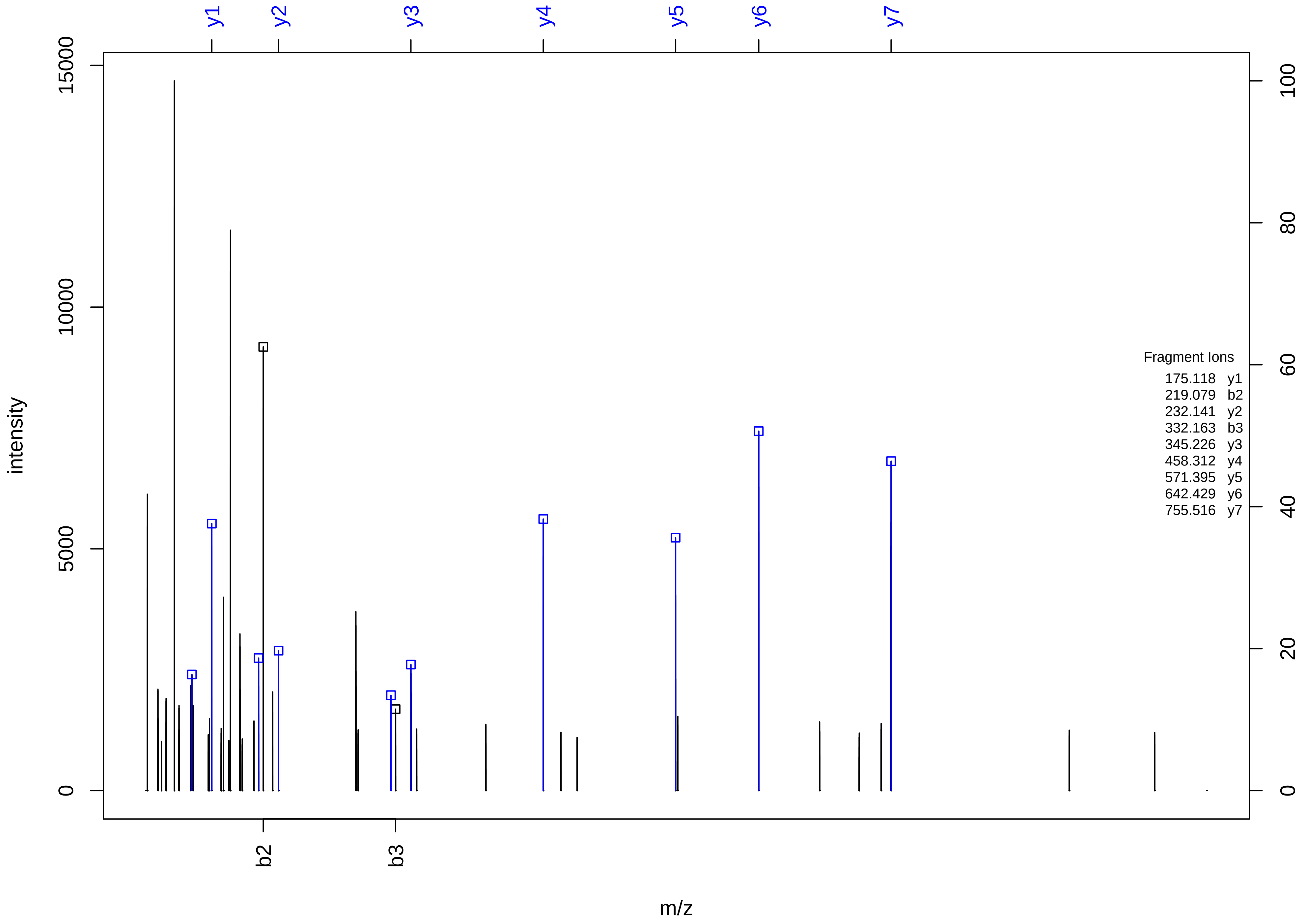

SMLALLLGR / File: sydney_11.raw; SpectrumID: ; scans: 12702

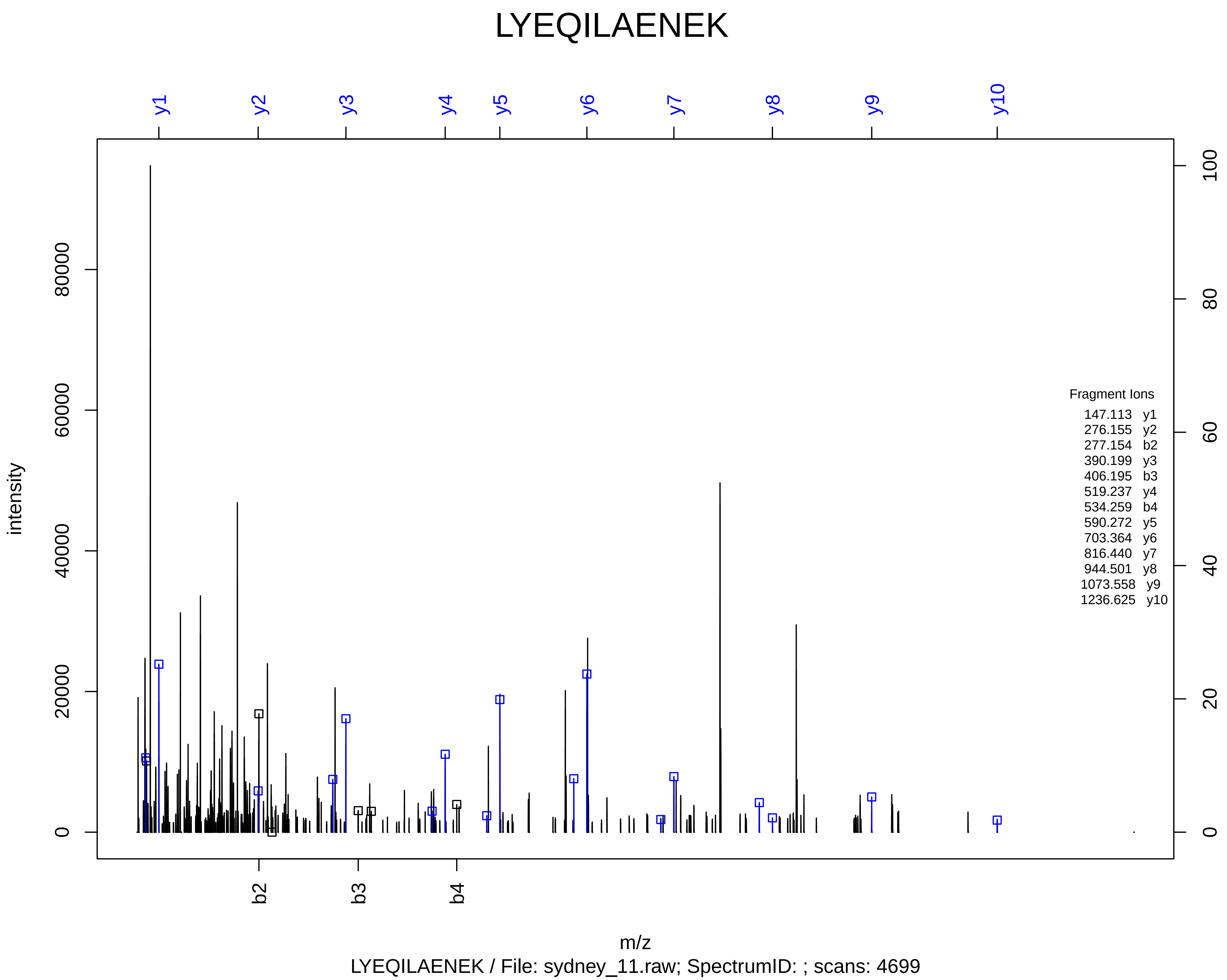

LYEQILAENEK
y1
y2
y3
y4
y5
y6
y7
y8
y9
y10
b2
b3
b4
intensity
0
20000
40000
60000
80000
100
80
60
40
20
0
m/z
Fragment Ions
147.113 y1
276.155 y2
277.154 b2
390.199 y3
406.195 b3
519.237 y4
534.259 b4
590.272 y5
703.364 y6
816.440 y7
944.501 y8
1073.558 y9
1236.625 y10
LYEQILAENEK / File: sydney_11.raw; SpectrumID: ; scans: 4699

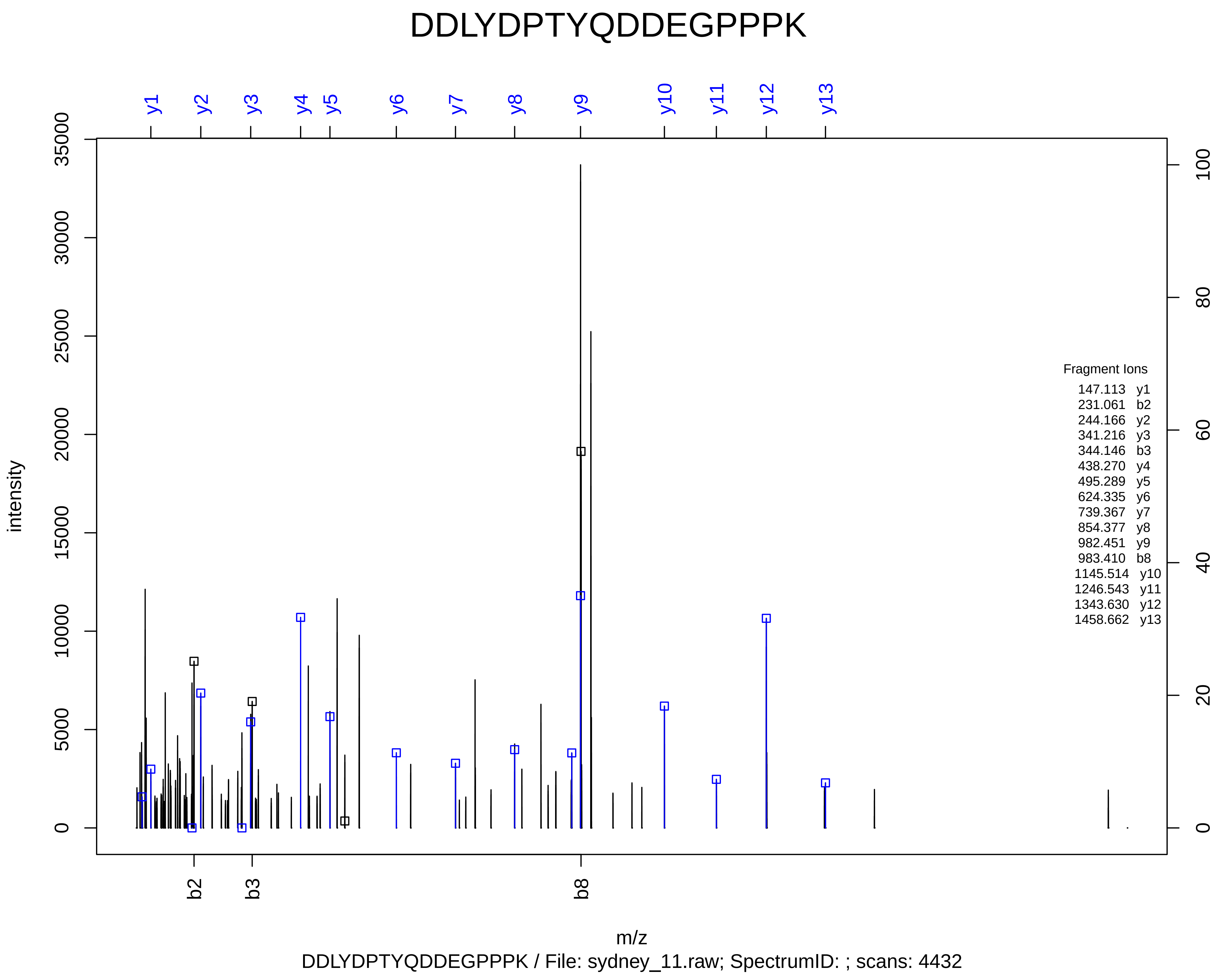
DDLYDPTYQDDEGPPPK
y1 y2 y3 y4 y5 y6 y7 y8 y9 y10 y11 y12 y13
b2 b3 b8
intensity
m/z
Fragment Ions
147.113 y1
231.061 b2
244.166 y2
341.216 y3
344.146 b3
438.270 y4
495.289 y5
624.335 y6
739.367 y7
854.377 y8
982.451 y9
983.410 b8
1145.514 y10
1246.543 y11
1343.630 y12
1458.662 y13
DDLYDPTYQDDEGPPPK / File: sydney_11.raw; SpectrumID: ; scans: 4432

ADEGHPSEDPPQQAGETLQASGENVR

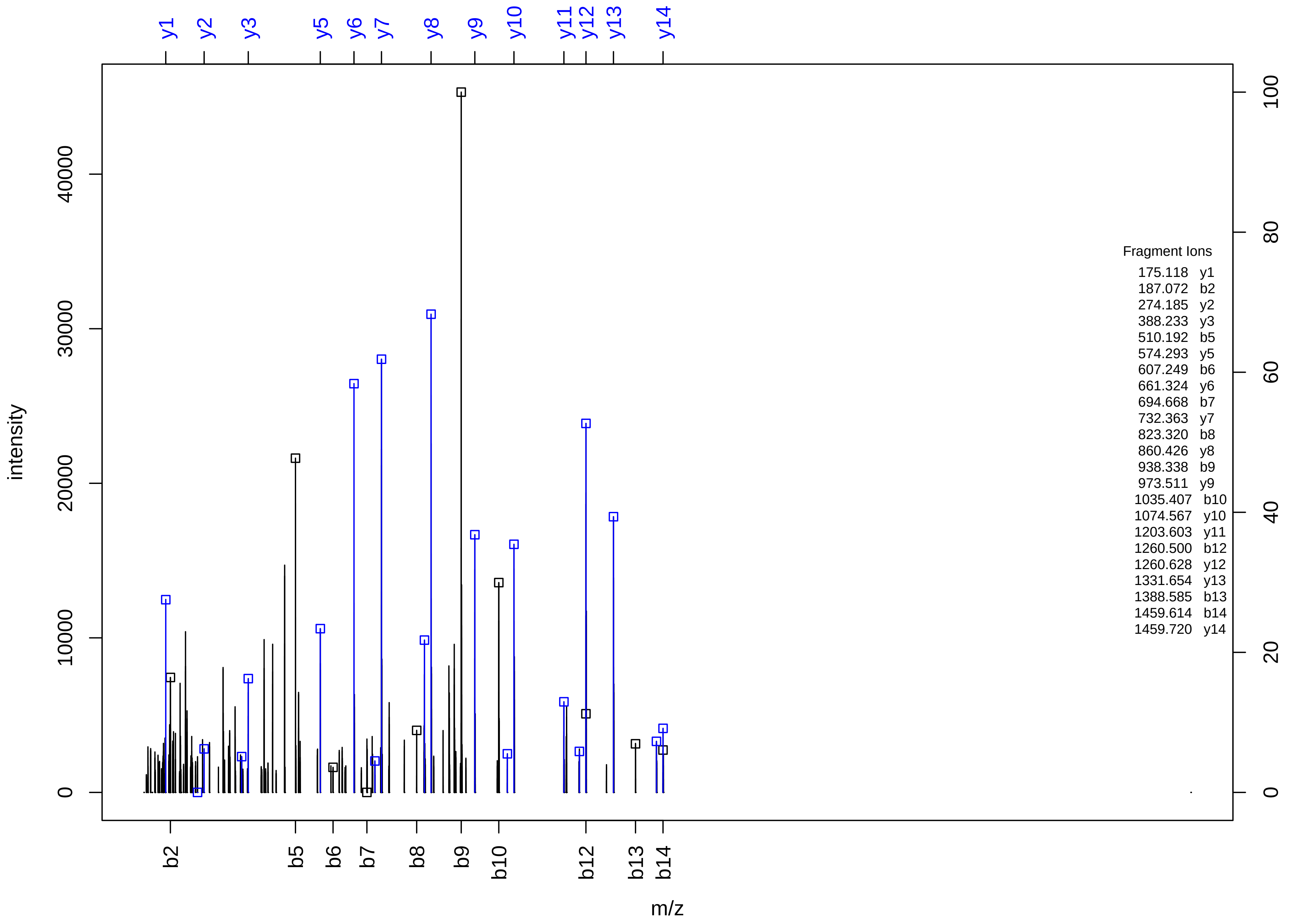

ADEGHPSEDPPQQAGETLQASGENVR / File: sydney_11.raw; SpectrumID: ; scans: 2430

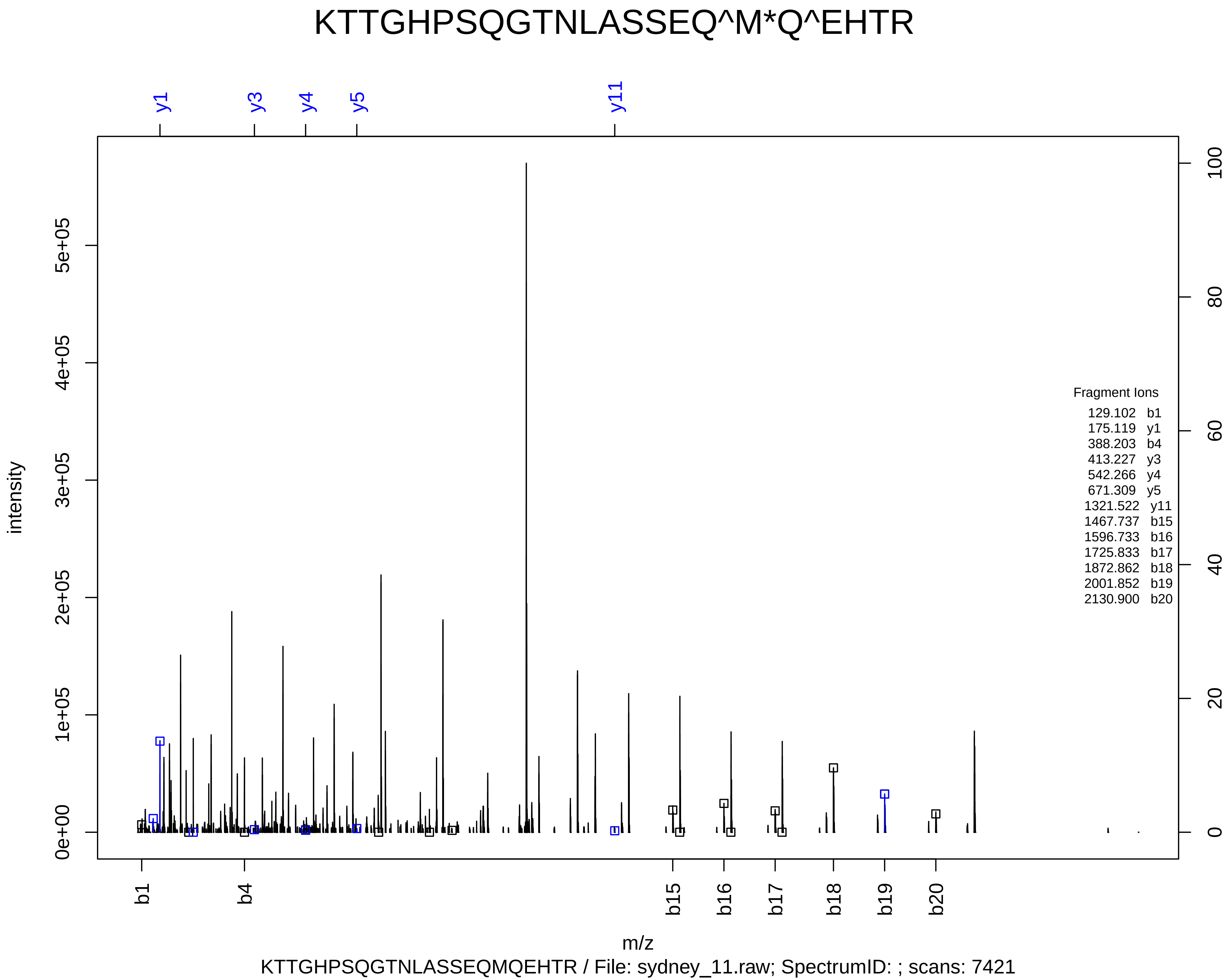

KTTGHPSQGTNLASSEQ^M*Q^EHTR
y1
y3
y4
y5
y11
intensity
0e+00
1e+05
2e+05
3e+05
4e+05
5e+05
0
20
40
60
80
100
b1
b4
b15
b16
b17
b18
b19
b20
m/z
Fragment Ions
129.102 b1
175.119 y1
388.203 b4
413.227 y3
542.266 y4
671.309 y5
1321.522 y11
1467.737 b15
1596.733 b16
1725.833 b17
1872.862 b18
2001.852 b19
2130.900 b20
KTTGHPSQGTNLASSEQMQEHTR / File: sydney_11.raw; SpectrumID: ; scans: 7421

MLPQ^ATQ^NRSQ^PPTRN^SSN^MEK

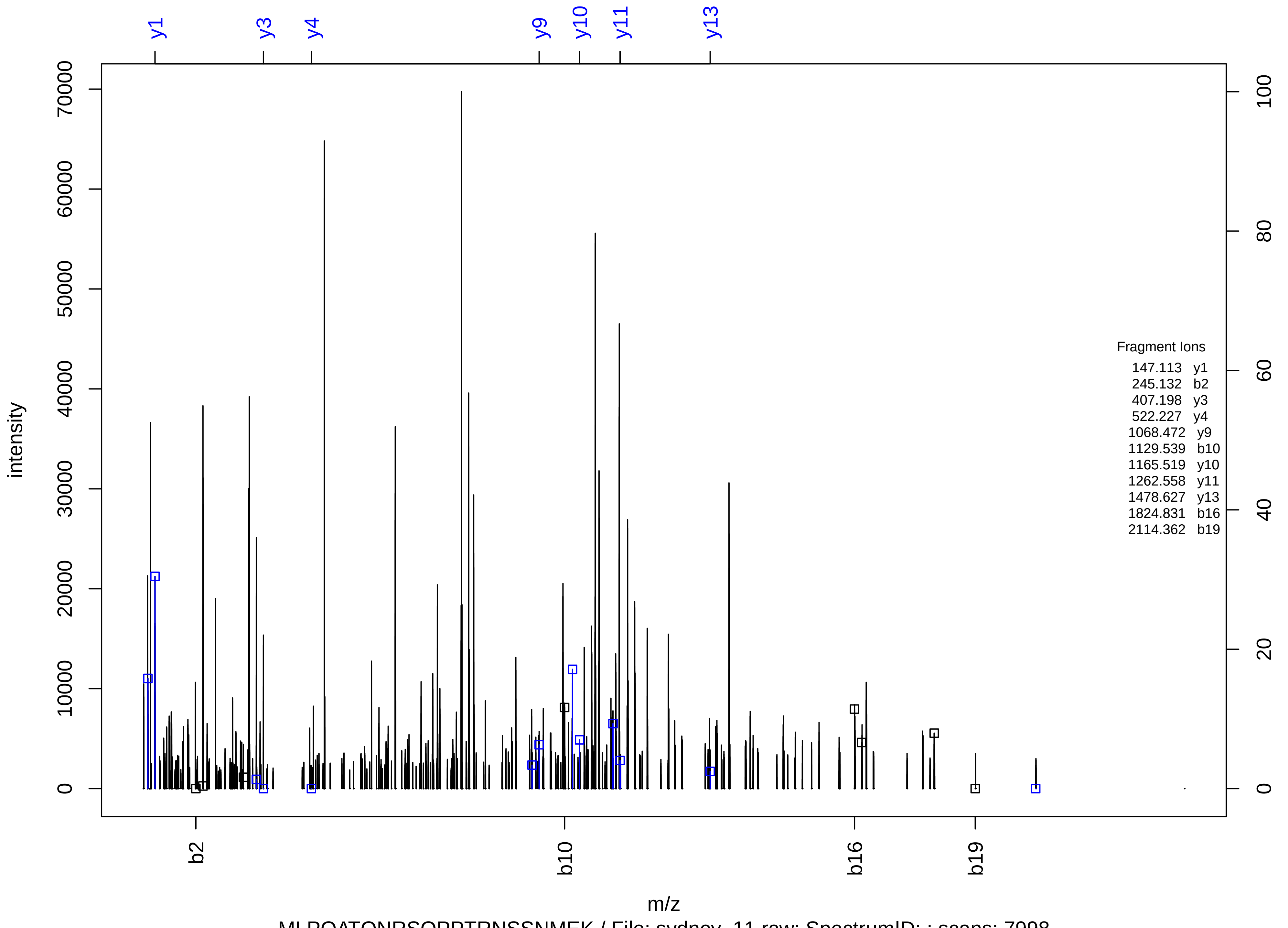

MLPQATQNRSQPPTRNSSNMEK / File: sydney_11.raw; SpectrumID: ; scans: 7998

DILYVFQGIDGK

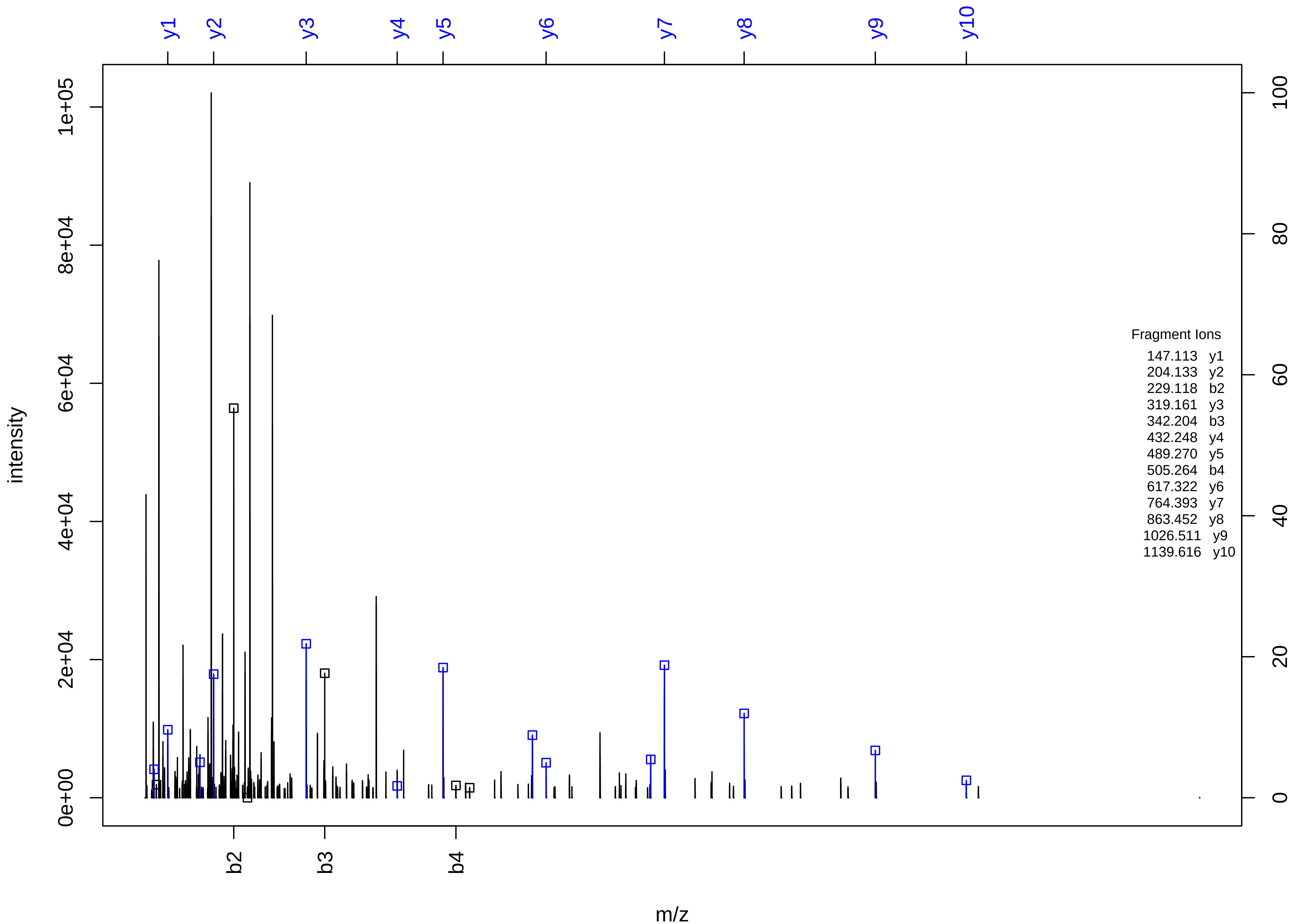

DILYVFQGIDGK / File: sydney_11.raw; SpectrumID: ; scans: 12429

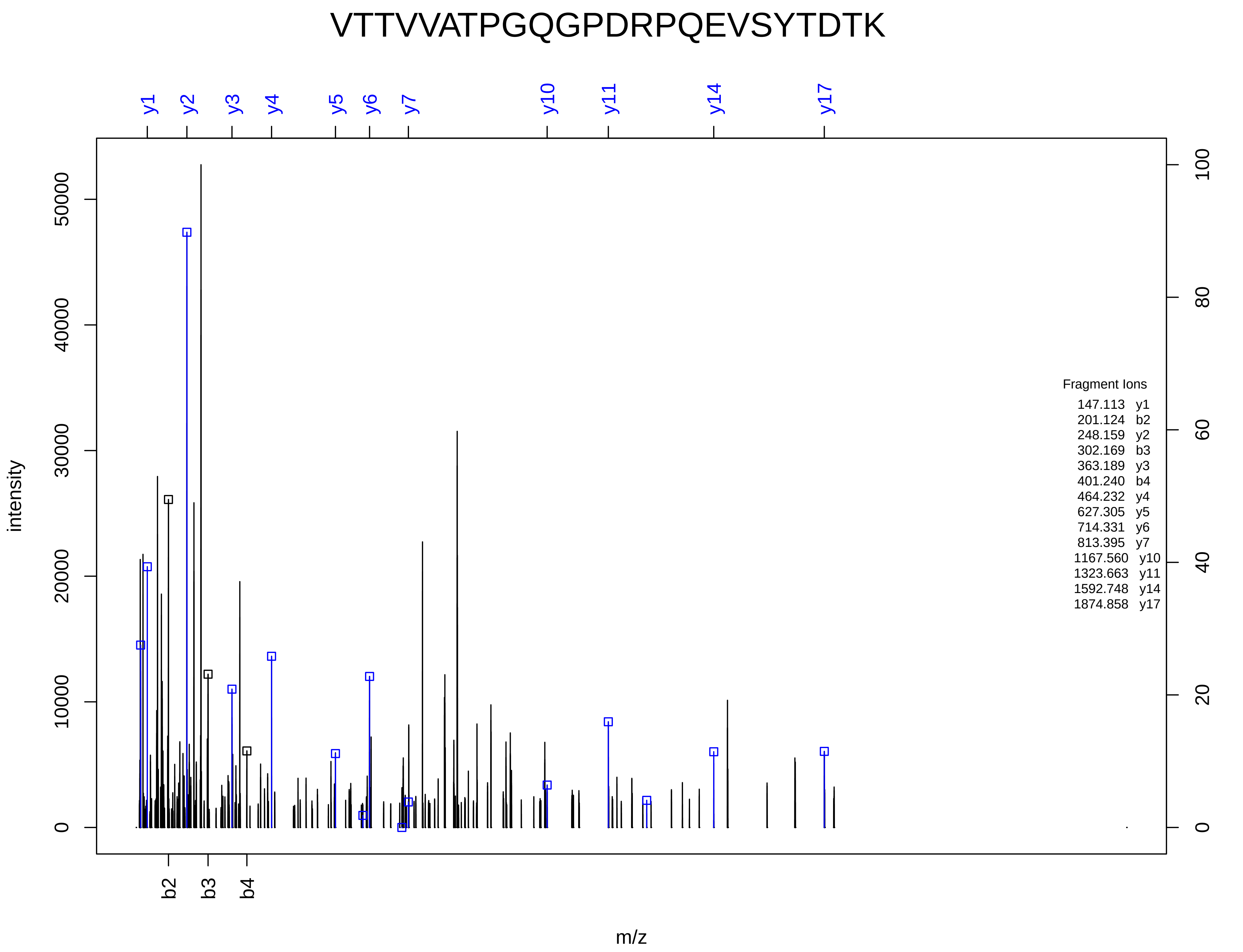

VTTVVATPGQGPDRPQEVSYTDTK / File: sydney_11.raw; SpectrumID: ; scans: 3088

LFVEQLEEFMTK

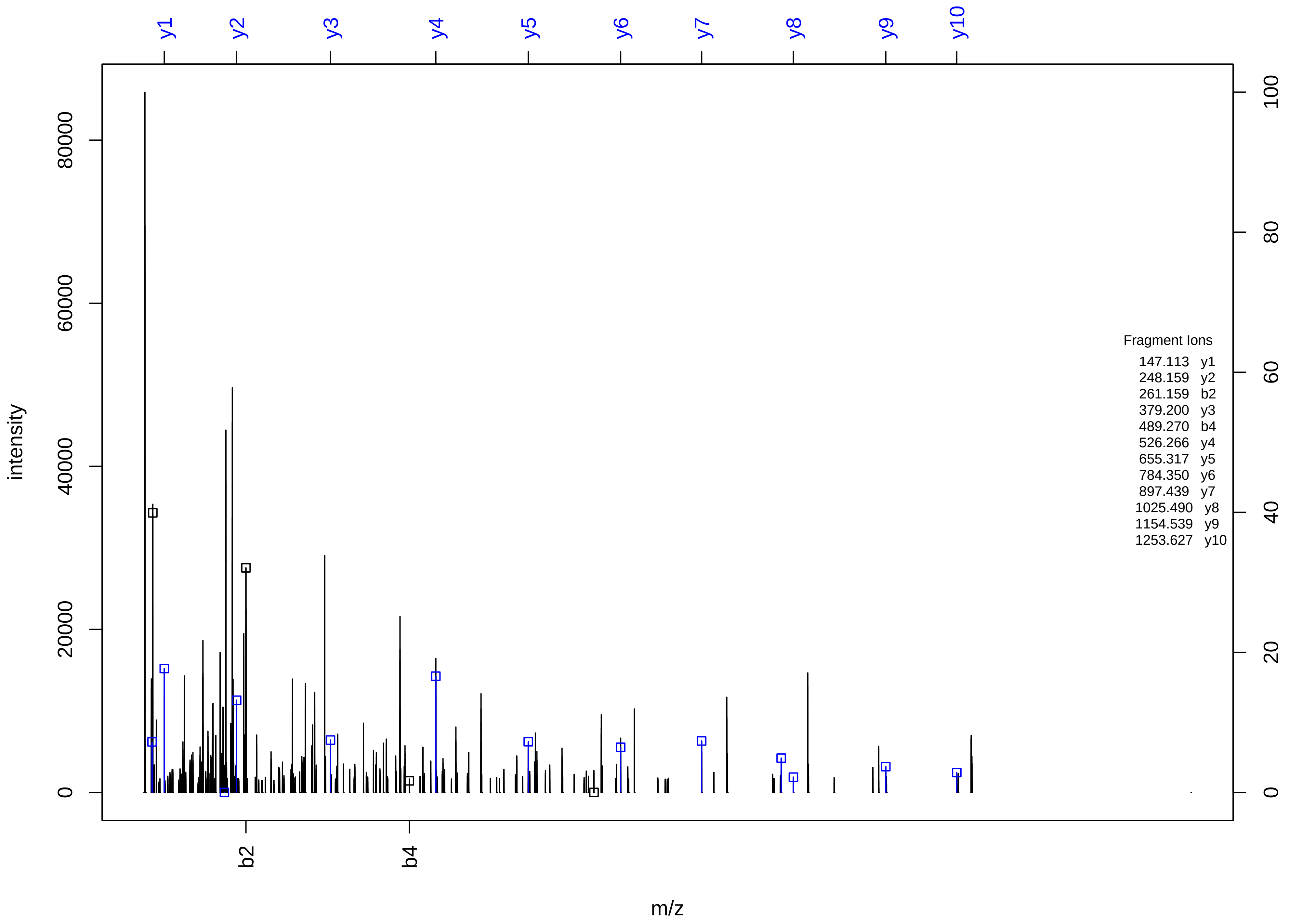

LFVEQLEEFMTK / File: sydney_11.raw; SpectrumID: ; scans: 14568

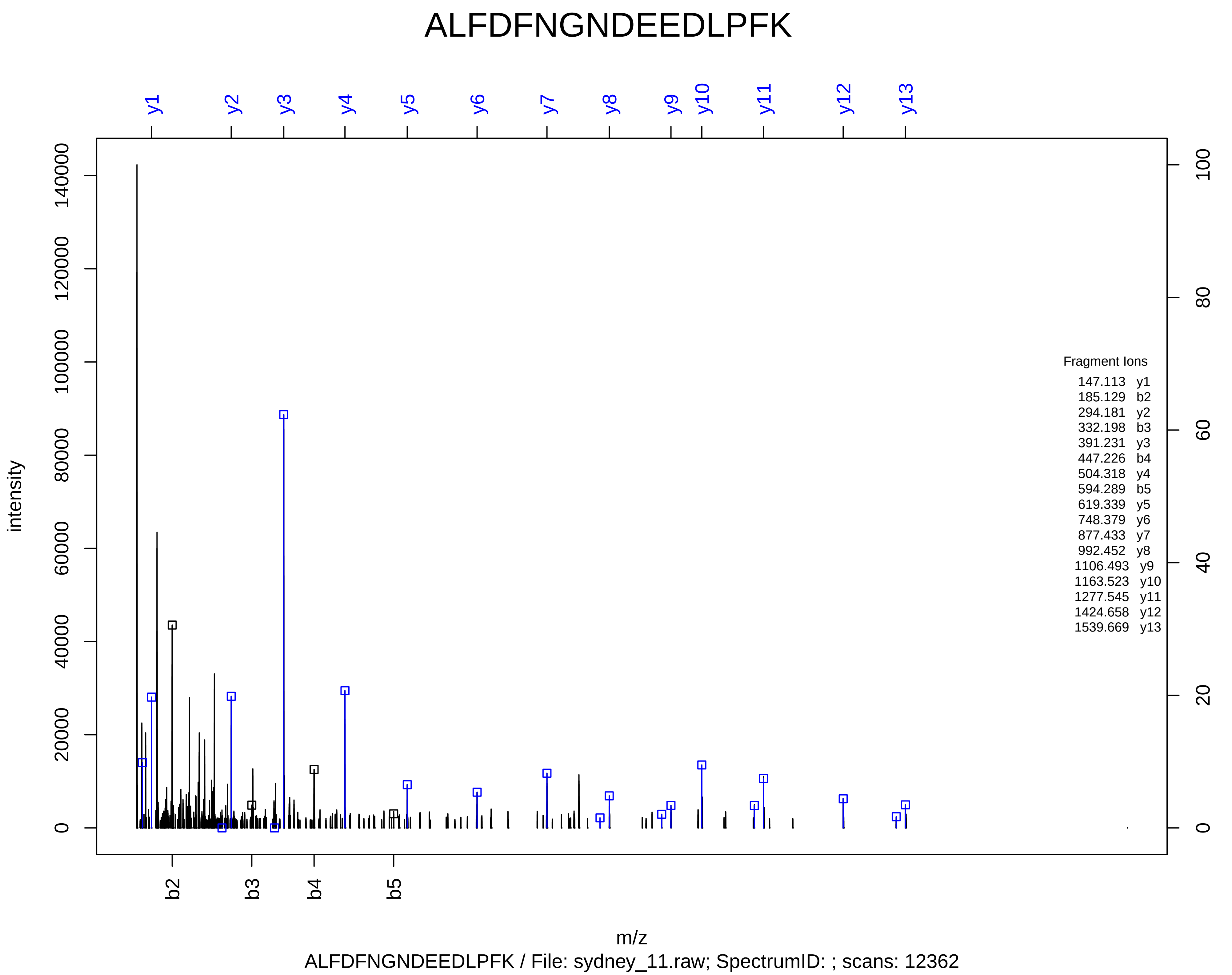
ALFDFNGNDEEDLPFK
y1
y2
y3
y4
y5
y6
y7
y8
y9
y10
y11
y12
y13
intensity
0
20000
40000
60000
80000
100000
120000
140000
0
20
40
60
80
100
b2
b3
b4
b5
m/z
Fragment Ions
147.113 y1
185.129 b2
294.181 y2
332.198 b3
391.231 y3
447.226 b4
504.318 y4
594.289 b5
619.339 y5
748.379 y6
877.433 y7
992.452 y8
1106.493 y9
1163.523 y10
1277.545 y11
1424.658 y12
1539.669 y13
ALFDFNGNDEEDLPFK / File: sydney_11.raw; SpectrumID: ; scans: 12362

FLVPDHVNMSELIK

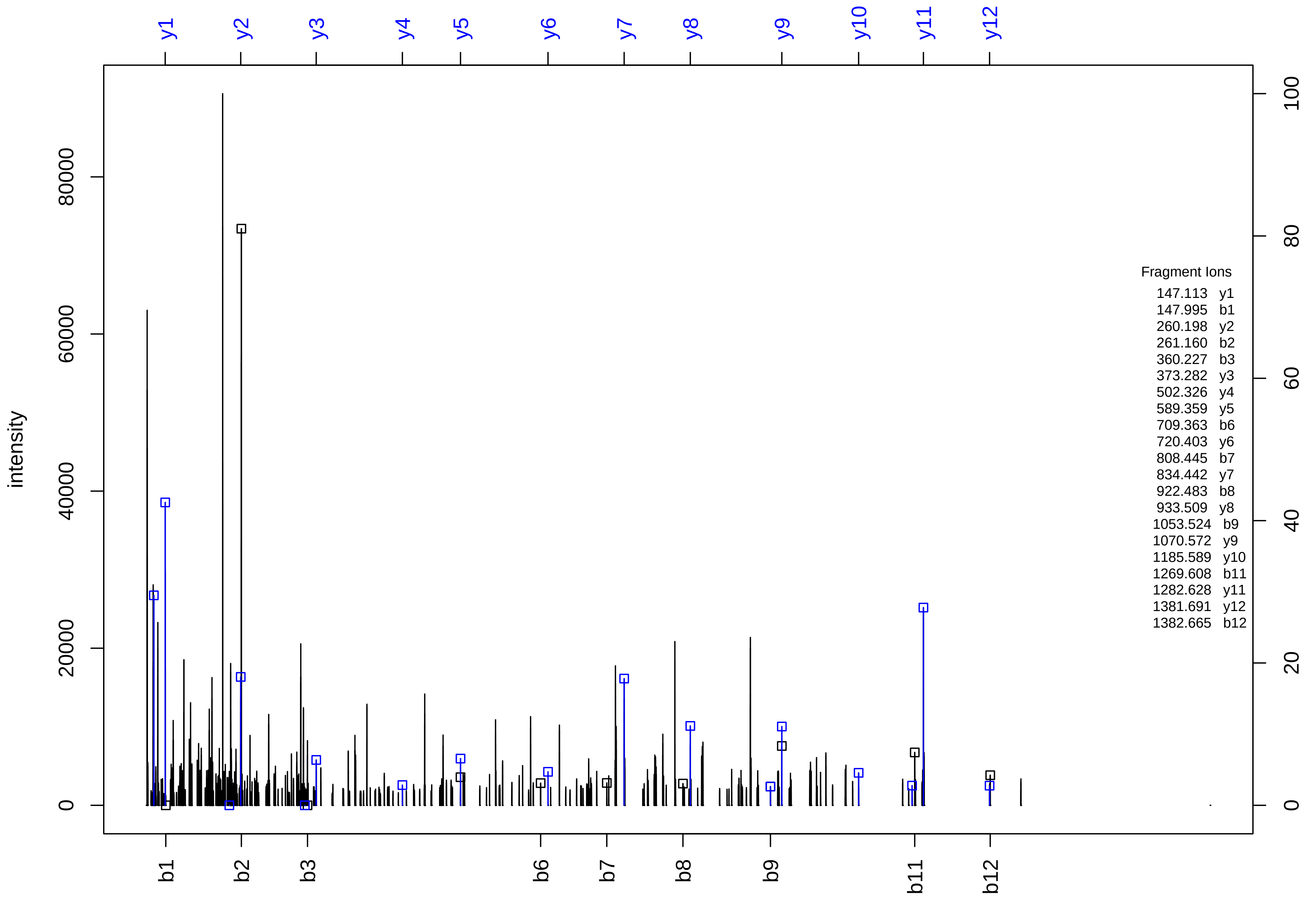

FLVPDHVNMSELIK / File: sydney_11.raw; SpectrumID: ; scans: 9862

IPLIVCGDFNAEPTEEVYK

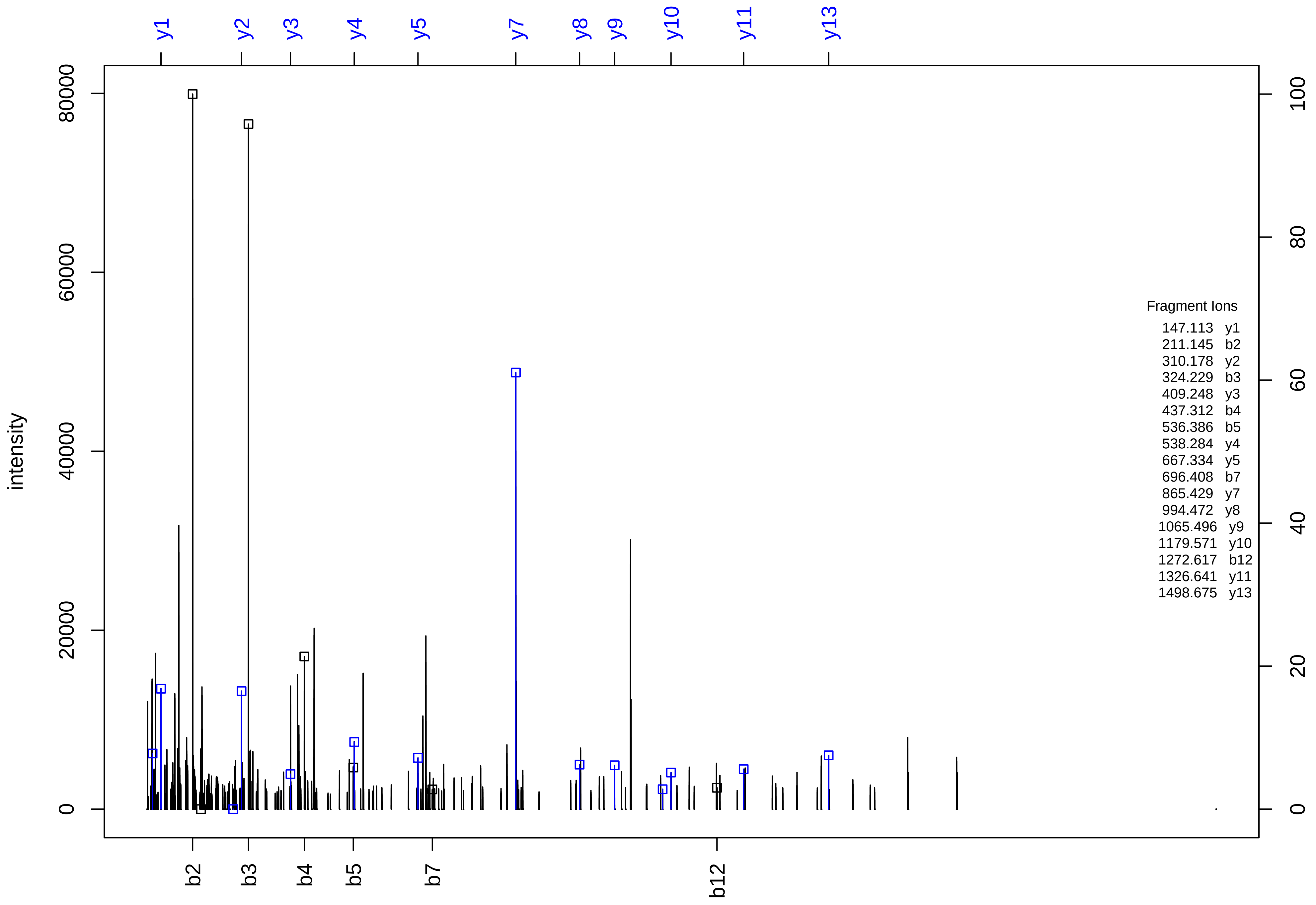

IPLIVCGDFNAEPTEEVYK / File: sydney_11.raw; SpectrumID: ; scans: 10607

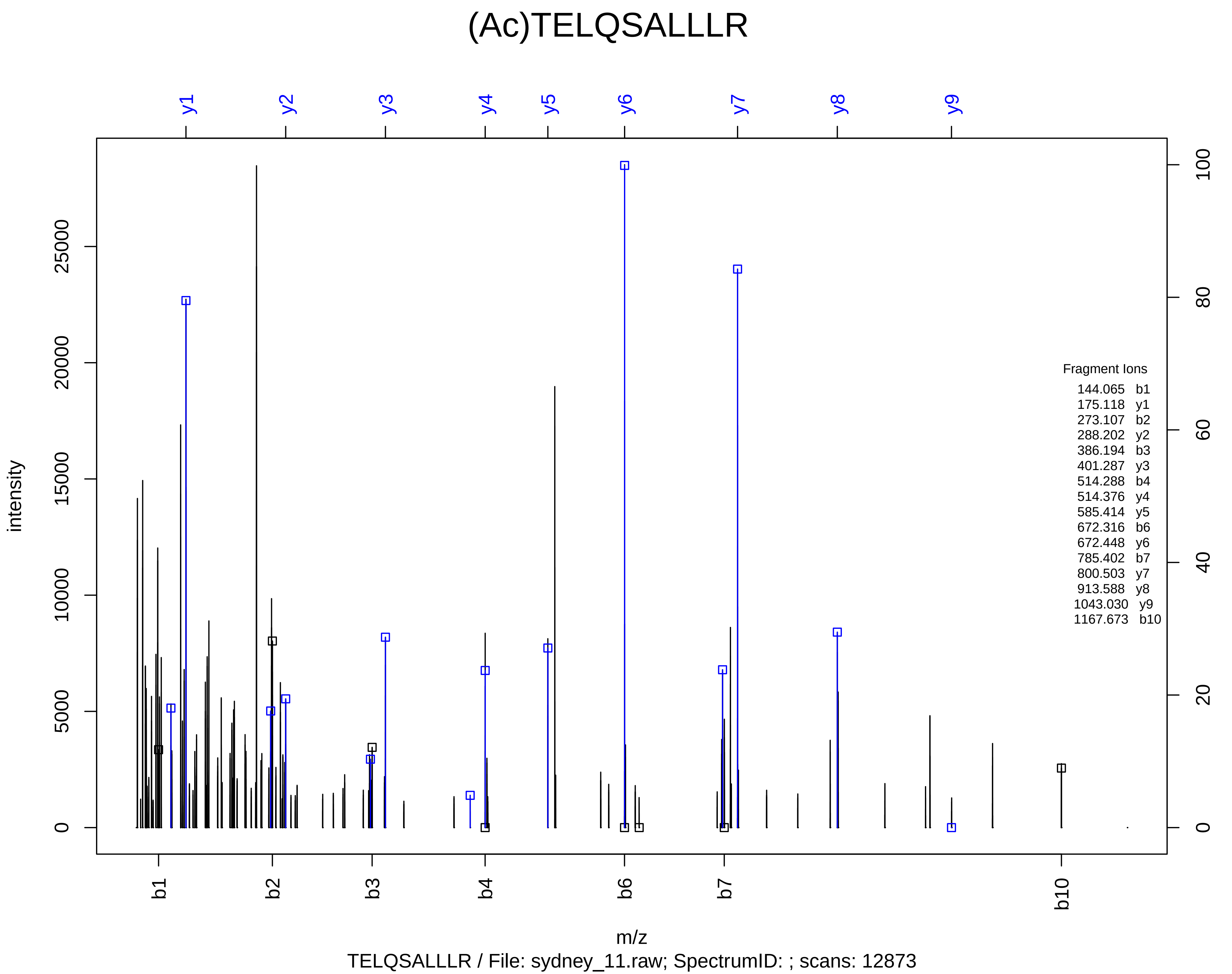
(Ac)TELQSALLLR
y1 y2 y3 y4 y5 y6 y7 y8 y9
intensity
0 5000 10000 15000 20000 25000
0 20 40 60 80 100
b1 b2 b3 b4 b6 b7 b10
m/z
Fragment Ions
144.065 b1
175.118 y1
273.107 b2
288.202 y2
386.194 b3
401.287 y3
514.288 b4
514.376 y4
585.414 y5
672.316 b6
672.448 y6
785.402 b7
800.503 y7
913.588 y8
1043.030 y9
1167.673 b10
TELQSALLLR / File: sydney_11.raw; SpectrumID: ; scans: 12873

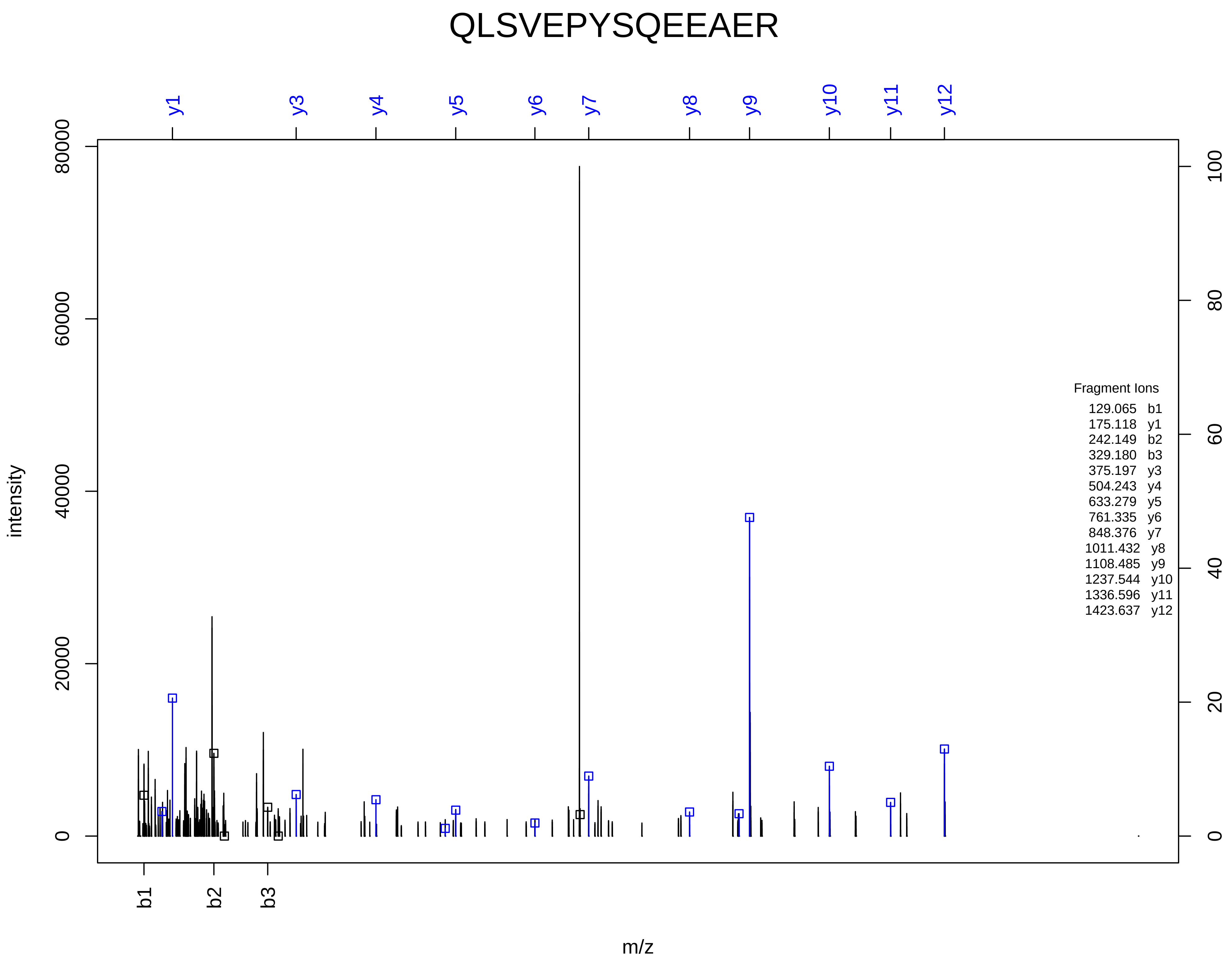

QLSVEPYSQEEAER / File: sydney_11.raw; SpectrumID: ; scans: 2979

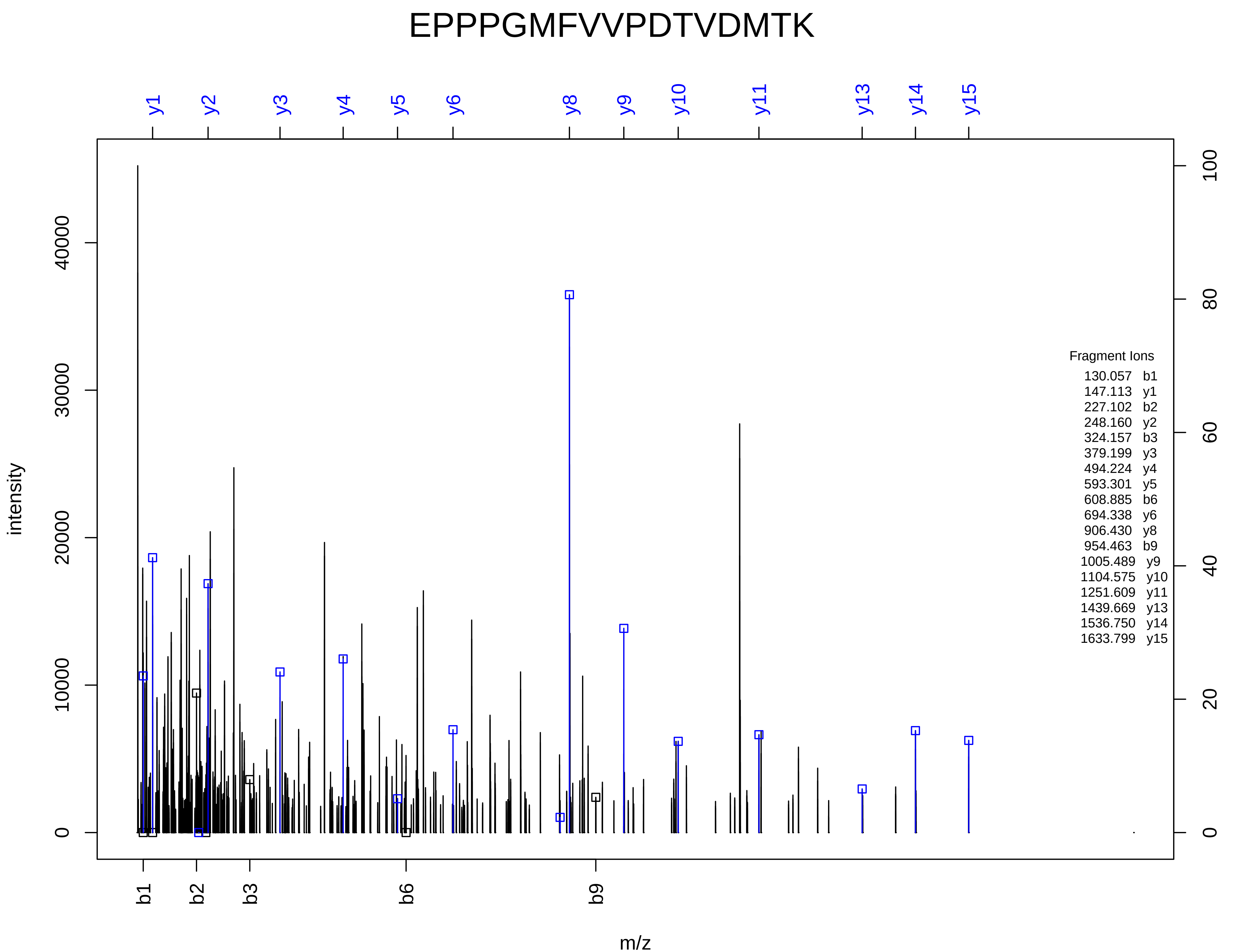
EPPPGMFVVPDTVDMTK
y1
y2
y3
y4
y5
y6
y8
y9
y10
y11
y13
y14
y15
intensity
0
10000
20000
30000
40000
0
20
40
60
80
100
Fragment Ions
130.057 b1
147.113 y1
227.102 b2
248.160 y2
324.157 b3
379.199 y3
494.224 y4
593.301 y5
608.885 b6
694.338 y6
906.430 y8
954.463 b9
1005.489 y9
1104.575 y10
1251.609 y11
1439.669 y13
1536.750 y14
1633.799 y15
b1
b2
b3
b6
b9
m/z
EPPPGMFVVPDTVDMTK / File: sydney_11.raw; SpectrumID: ; scans: 10755

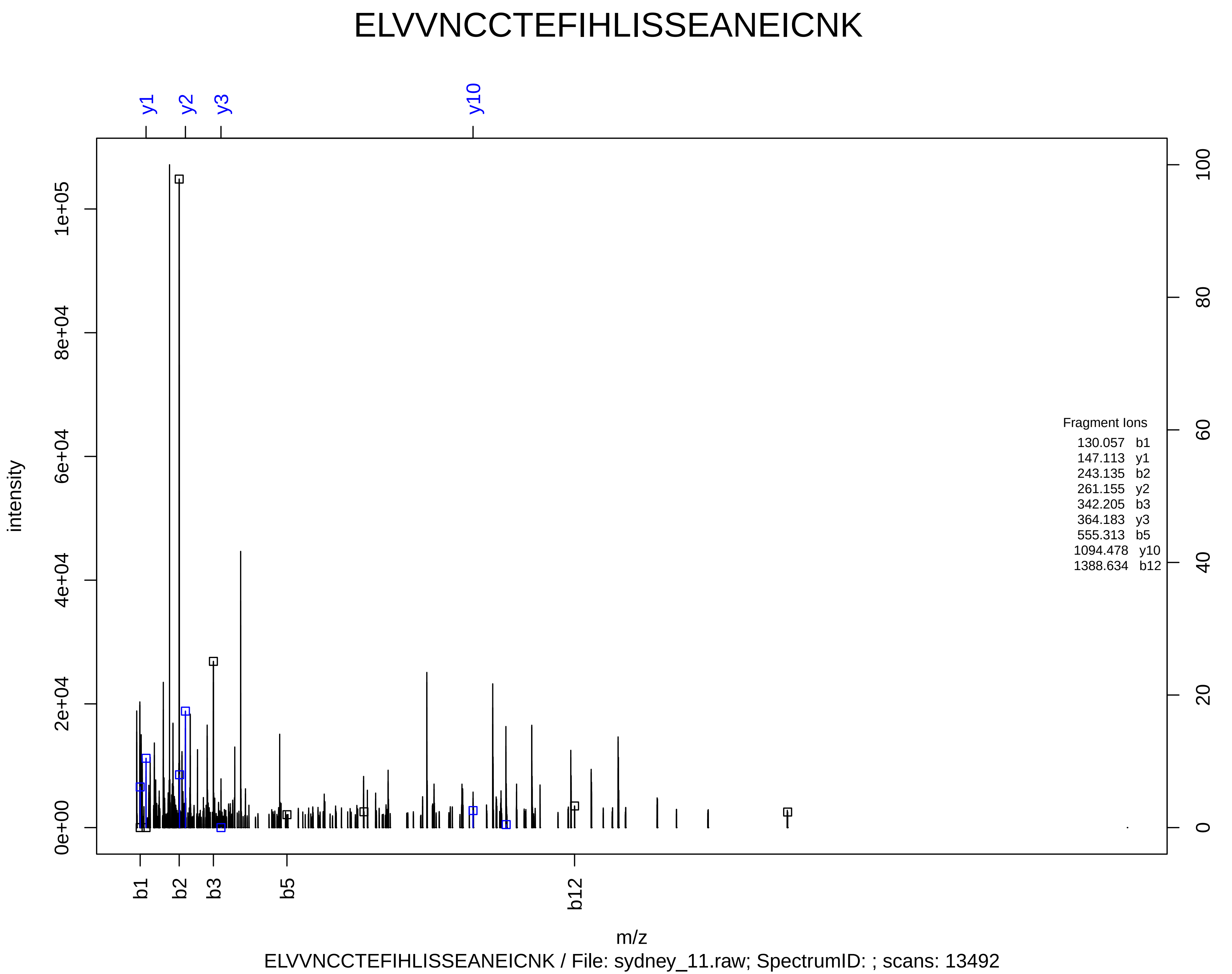
ELVVNCCTEFIHLISSEANEICNK
y1
y2
y3
y10
intensity
0e+00
2e+04
4e+04
6e+04
8e+04
1e+05
0
20
40
60
80
100
Fragment Ions
130.057 b1
147.113 y1
243.135 b2
261.155 y2
342.205 b3
364.183 y3
555.313 b5
1094.478 y10
1388.634 b12
b1
b2
b3
b5
b12
m/z
ELVVNCCTEFIHLISSEANEICNK / File: sydney_11.raw; SpectrumID: ; scans: 13492

LPGPAPLAHSR

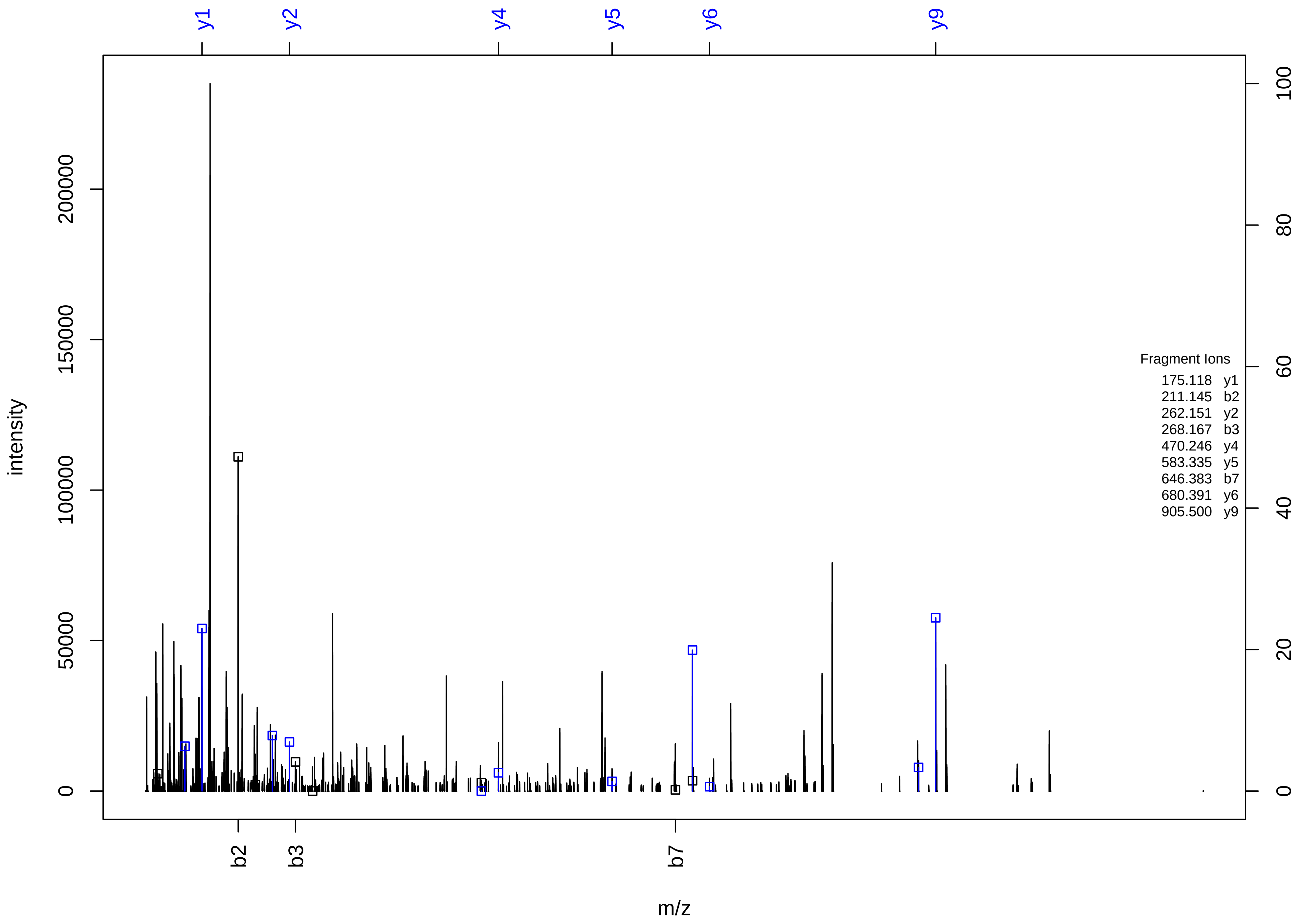

LPGPAPLAHSR / File: sydney_11.raw; SpectrumID: ; scans: 3713

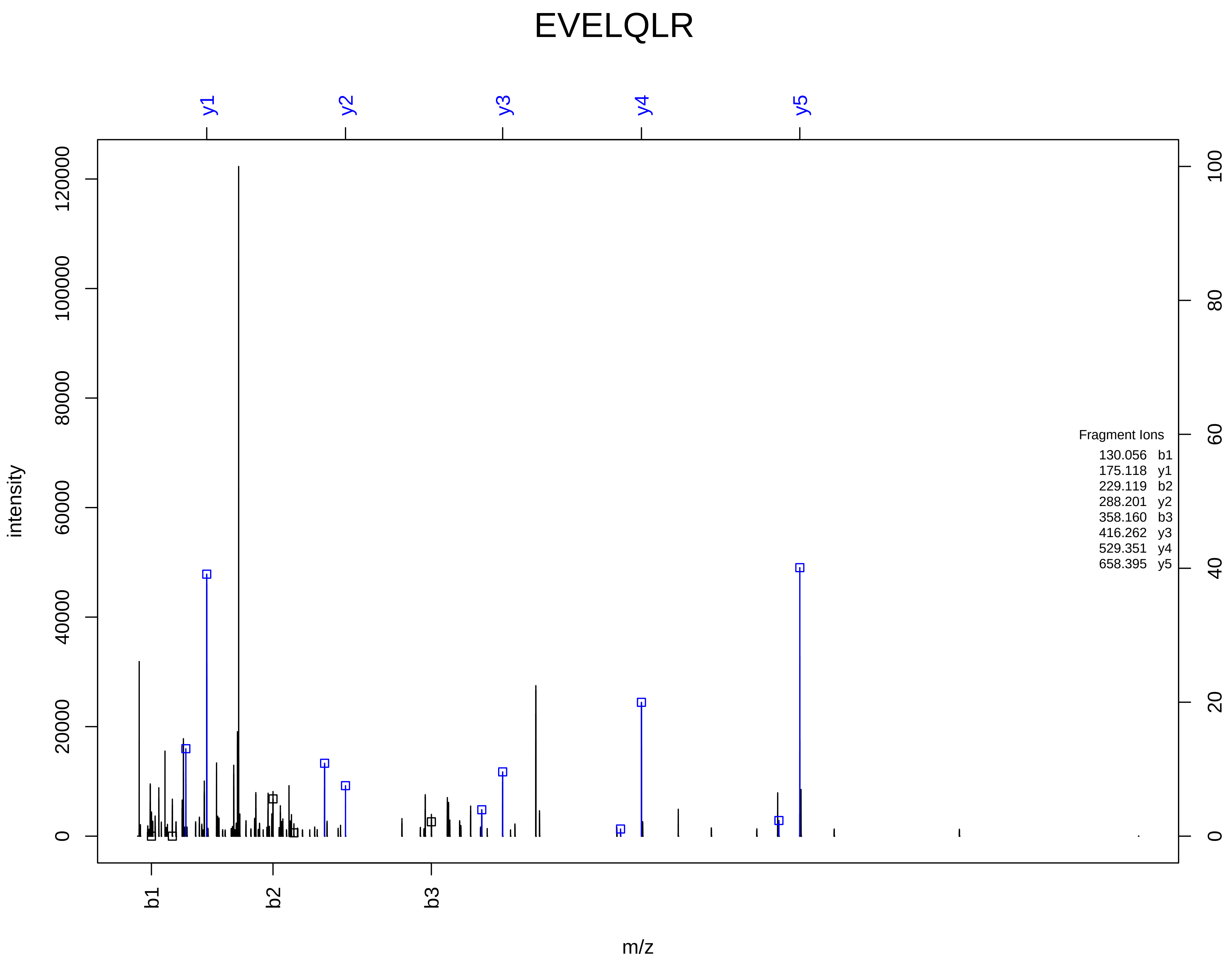

EVELQLR
y1
y2
y3
y4
y5
intensity
0
20000
40000
60000
80000
100000
120000
0
20
40
60
80
100
Fragment Ions
130.056 b1
175.118 y1
229.119 b2
288.201 y2
358.160 b3
416.262 y3
529.351 y4
658.395 y5
b1
b2
b3
m/z
EVELQLR / File: sydney_11.raw; SpectrumID: ; scans: 5564

FCPAGVYEFVPLEQGDGFR

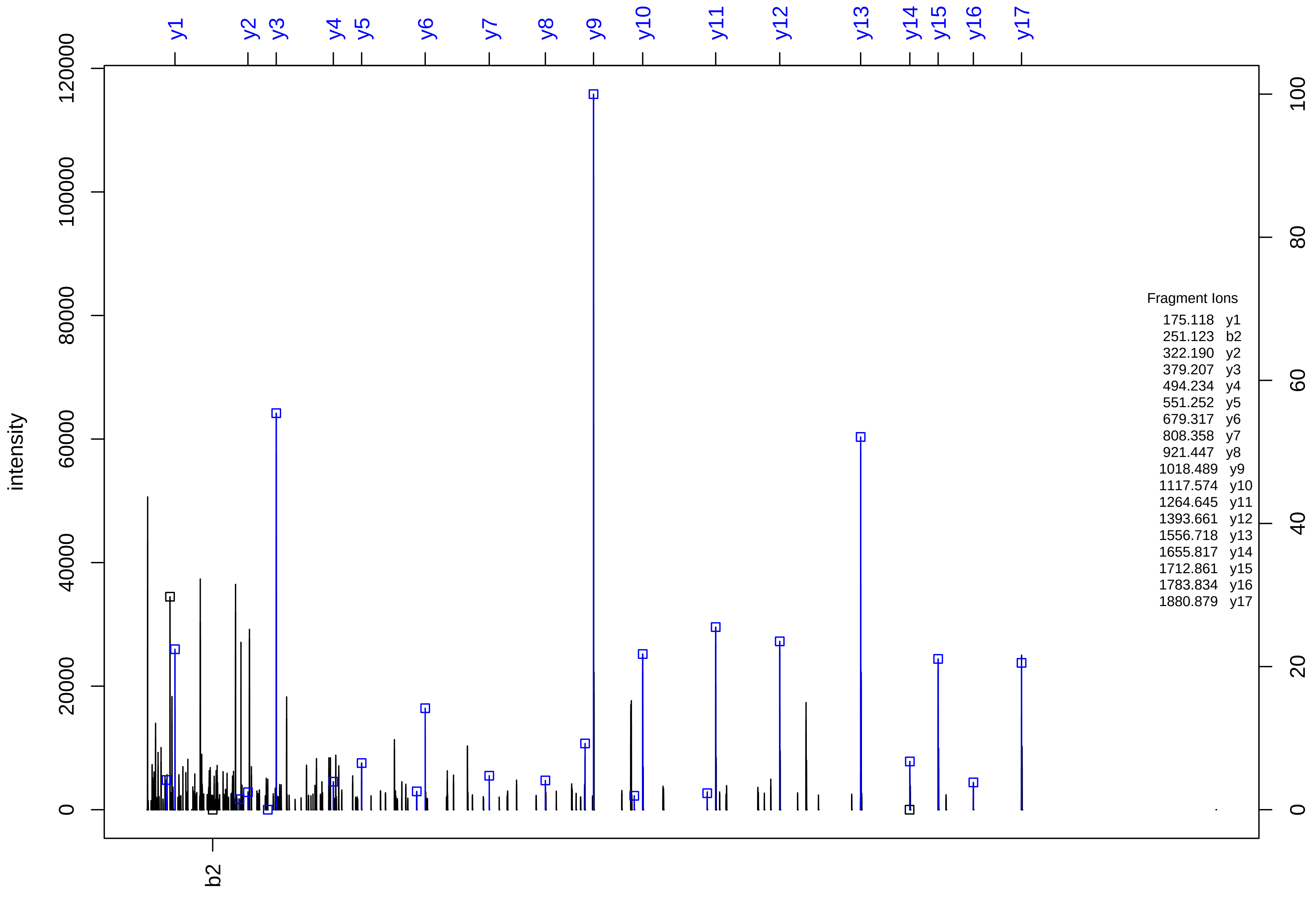

FCPAGVYEFVPLEQGDGFR / File: sydney_11.raw; SpectrumID: ; scans: 12795

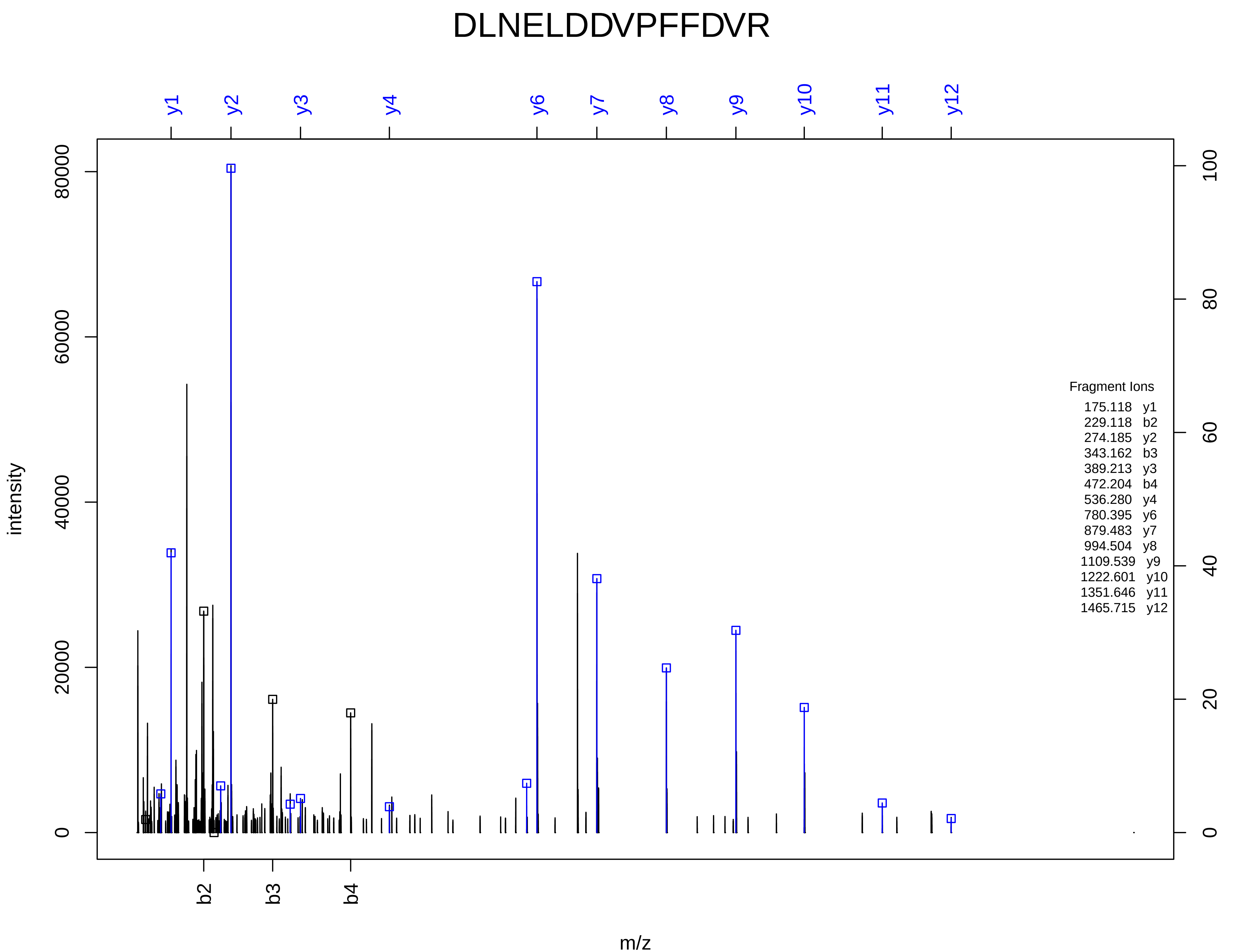
DLNELDDVPFFDVR
y1
y2
y3
y4
y6
y7
y8
y9
y10
y11
y12
b2
b3
b4
intensity
m/z
Fragment Ions
175.118 y1
229.118 b2
274.185 y2
343.162 b3
389.213 y3
472.204 b4
536.280 y4
780.395 y6
879.483 y7
994.504 y8
1109.539 y9
1222.601 y10
1351.646 y11
1465.715 y12
DLNELDDVPFFDVR / File: sydney_11.raw; SpectrumID: ; scans: 15054

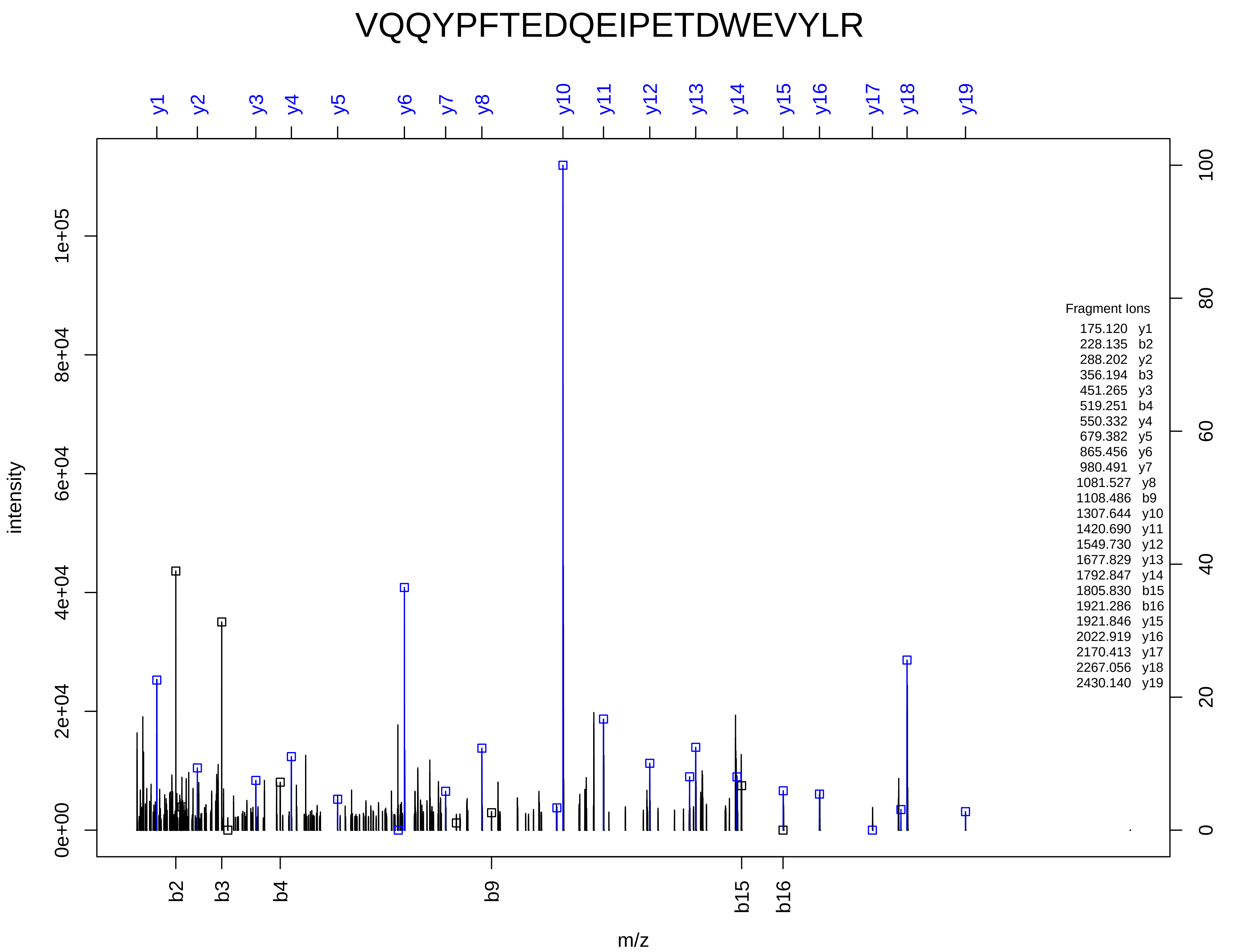

VQQYPFTEDQEIPETDWEVYLR / File: sydney_11.raw; SpectrumID: ; scans: 13317

MLQPSGPPADKPEEN

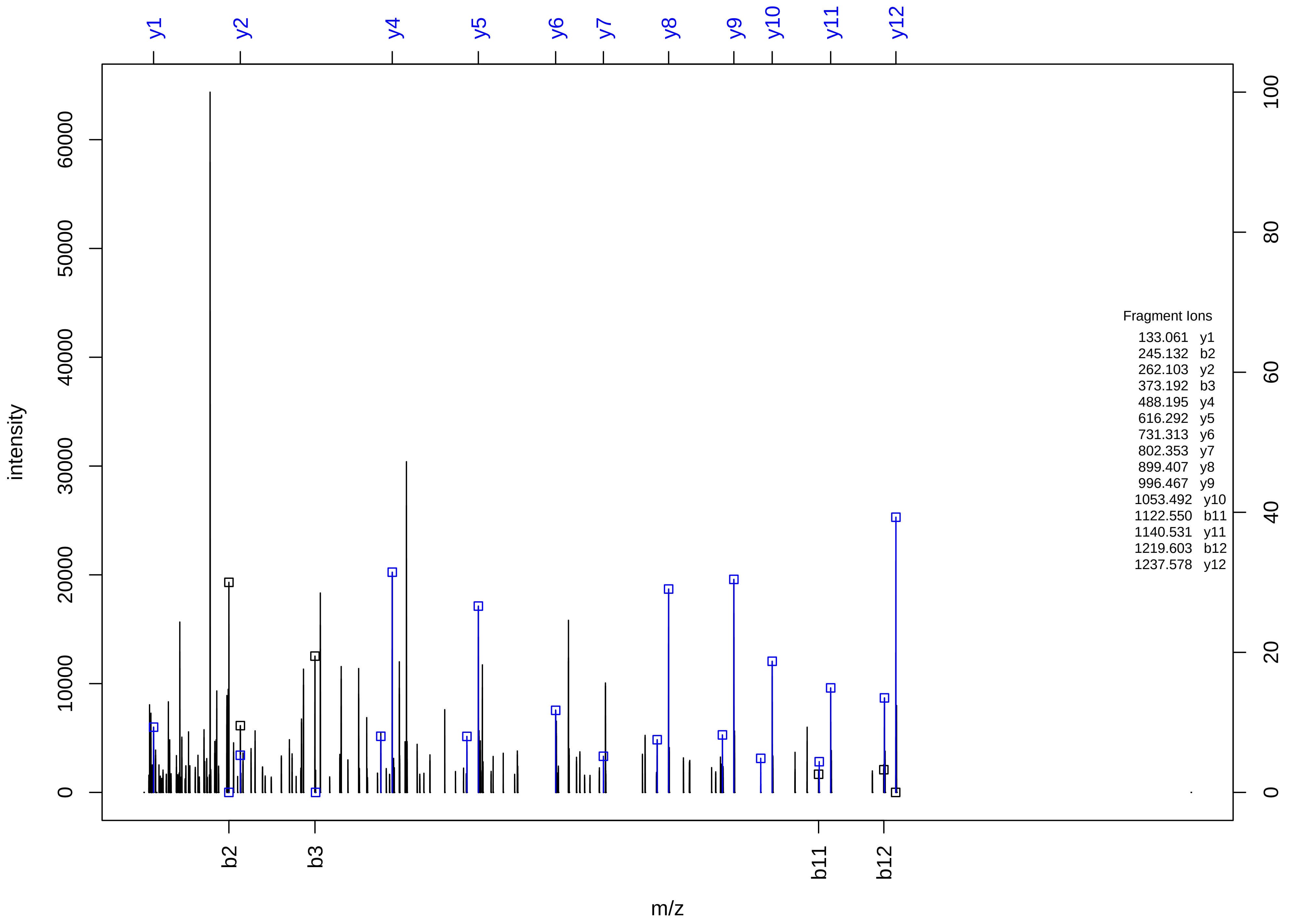

MLQPSGPPADKPEEN / File: sydney_11.raw; SpectrumID: ; scans: 1883

AVDDSYNVQPDTVAPIWNLR

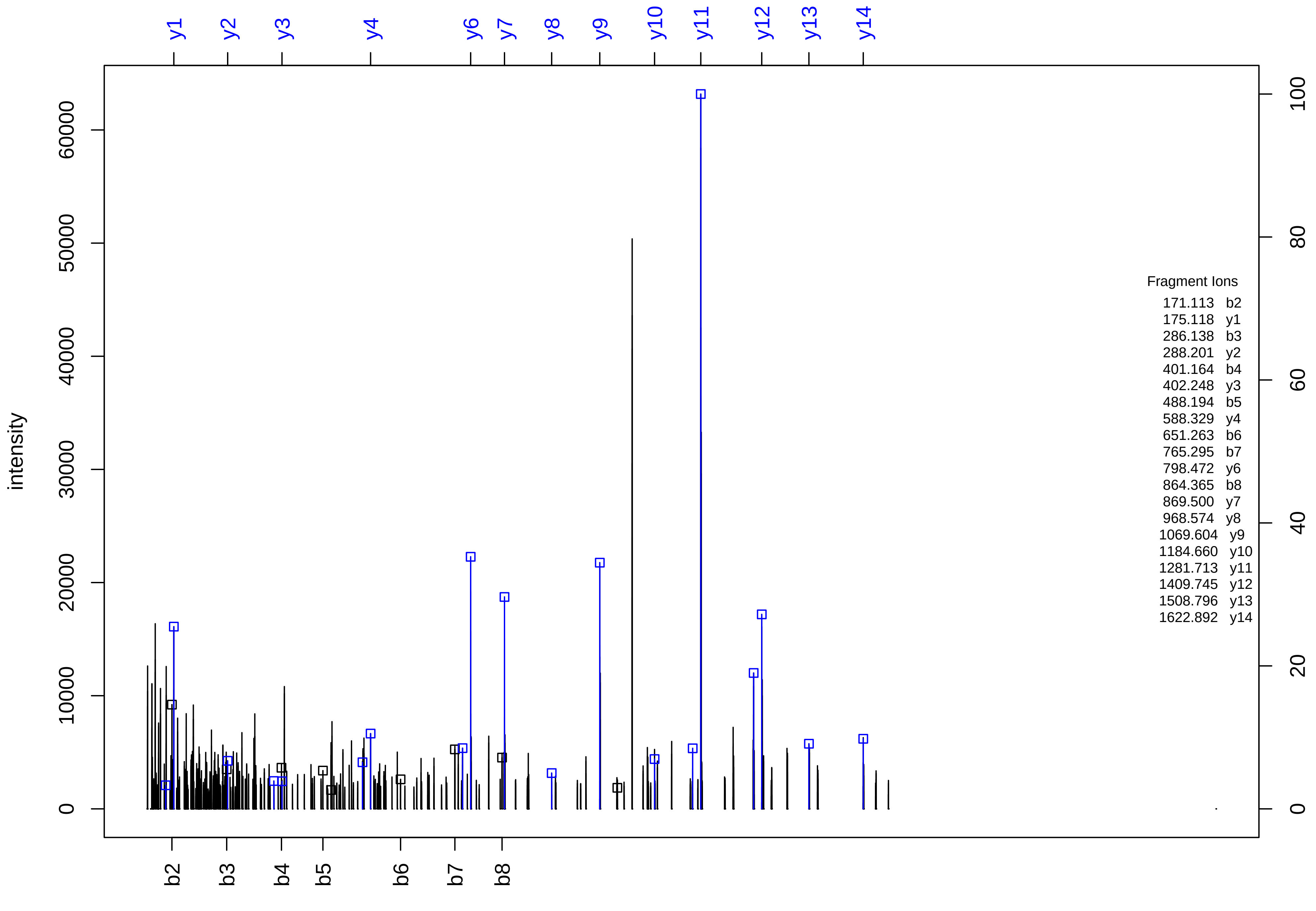

AVDDSYNVQPDTVAPIWNLR / File: sydney_11.raw; SpectrumID: ; scans: 11294

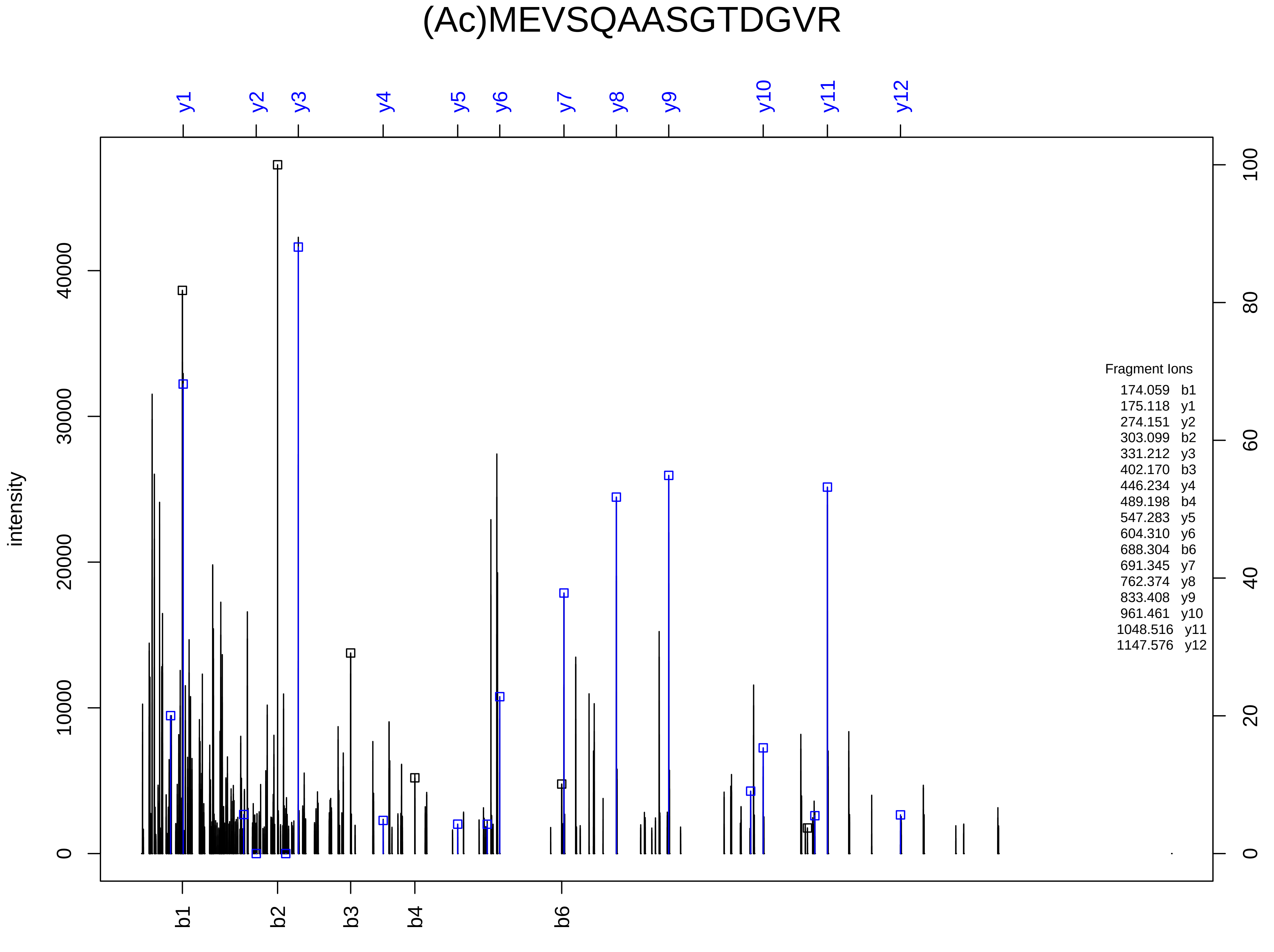

MEVSQAASGTDGVR / File: sydney_11.raw; SpectrumID: ; scans: 4812

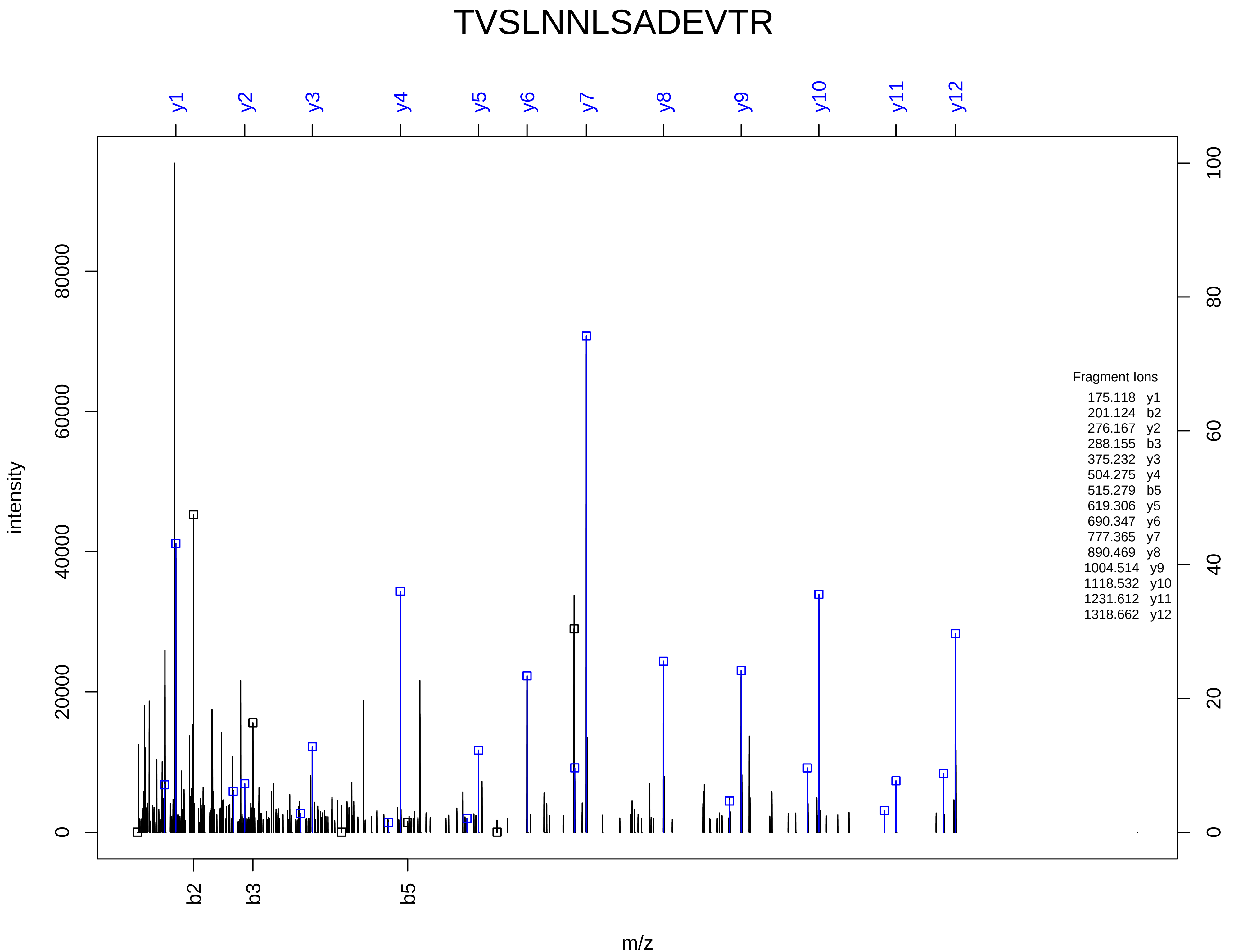
TVSLNNLSADEVTR
y1
y2
y3
y4
y5
y6
y7
y8
y9
y10
y11
y12
intensity
0
20000
40000
60000
80000
0
20
40
60
80
100
b2
b3
b5
m/z
Fragment Ions
175.118 y1
201.124 b2
276.167 y2
288.155 b3
375.232 y3
504.275 y4
515.279 b5
619.306 y5
690.347 y6
777.365 y7
890.469 y8
1004.514 y9
1118.532 y10
1231.612 y11
1318.662 y12
TVSLNNLSADEVTR / File: sydney_11.raw; SpectrumID: ; scans: 5272

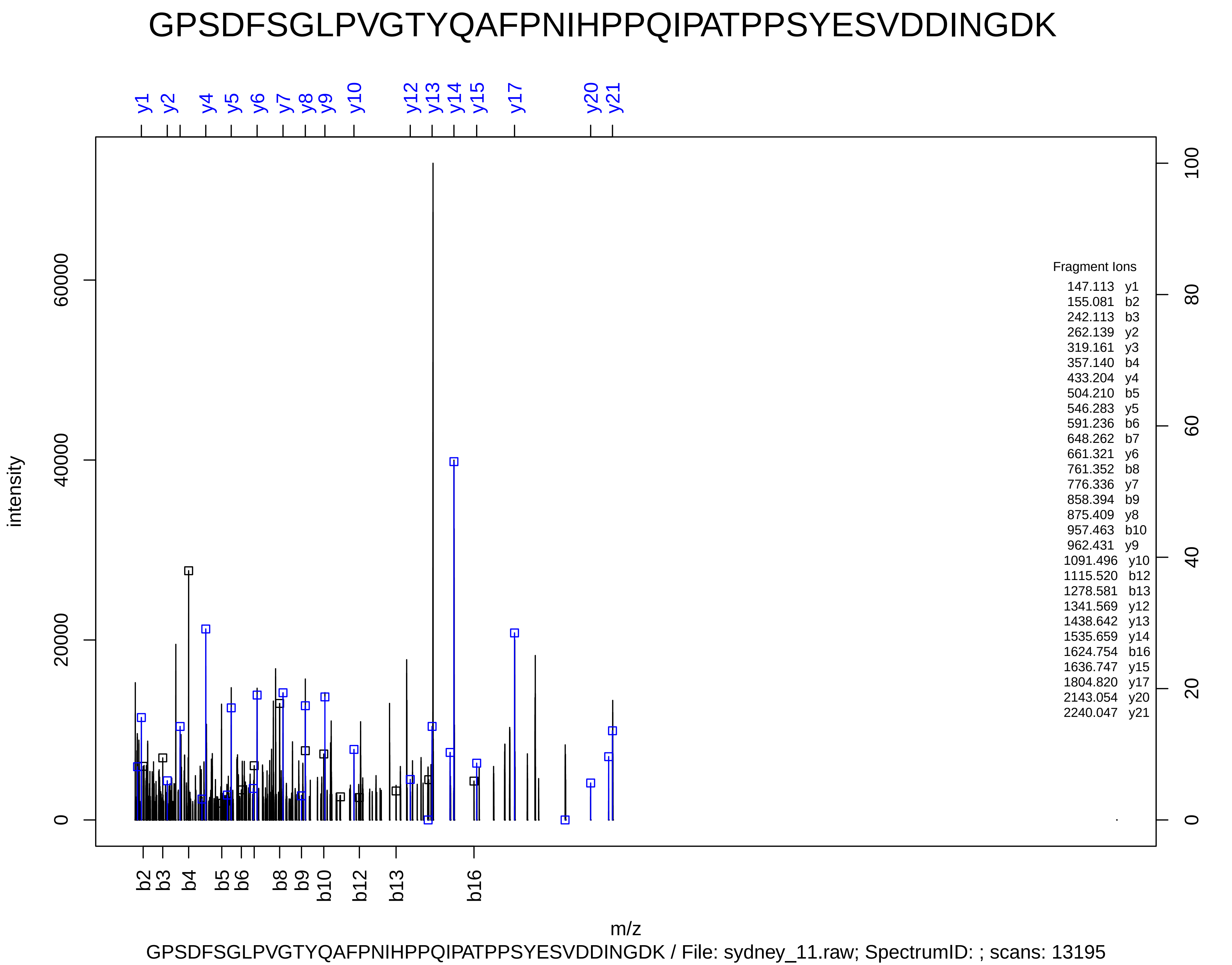

GPSDFSGLPVGTYQAFPNIHPPQIPATPPSYESVDDINGDK
y1 y2 y4 y5 y6 y7 y8 y9 y10 y12 y13 y14 y15 y17 y20 y21
intensity
0
20000
40000
60000
0
20
40
60
80
100
b2 b3 b4 b5 b6 b8 b9 b10 b12 b13 b16
m/z
Fragment Ions
147.113 y1
155.081 b2
242.113 b3
262.139 y2
319.161 y3
357.140 b4
433.204 y4
504.210 b5
546.283 y5
591.236 b6
648.262 b7
661.321 y6
761.352 b8
776.336 y7
858.394 b9
875.409 y8
957.463 b10
962.431 y9
1091.496 y10
1115.520 b12
1278.581 b13
1341.569 y12
1438.642 y13
1535.659 y14
1624.754 b16
1636.747 y15
1804.820 y17
2143.054 y20
2240.047 y21
GPSDFSGLPVGTYQAFPNIHPPQIPATPPSYESVDDINGDK / File: sydney_11.raw; SpectrumID: ; scans: 13195

ILESSQTLLSVLK

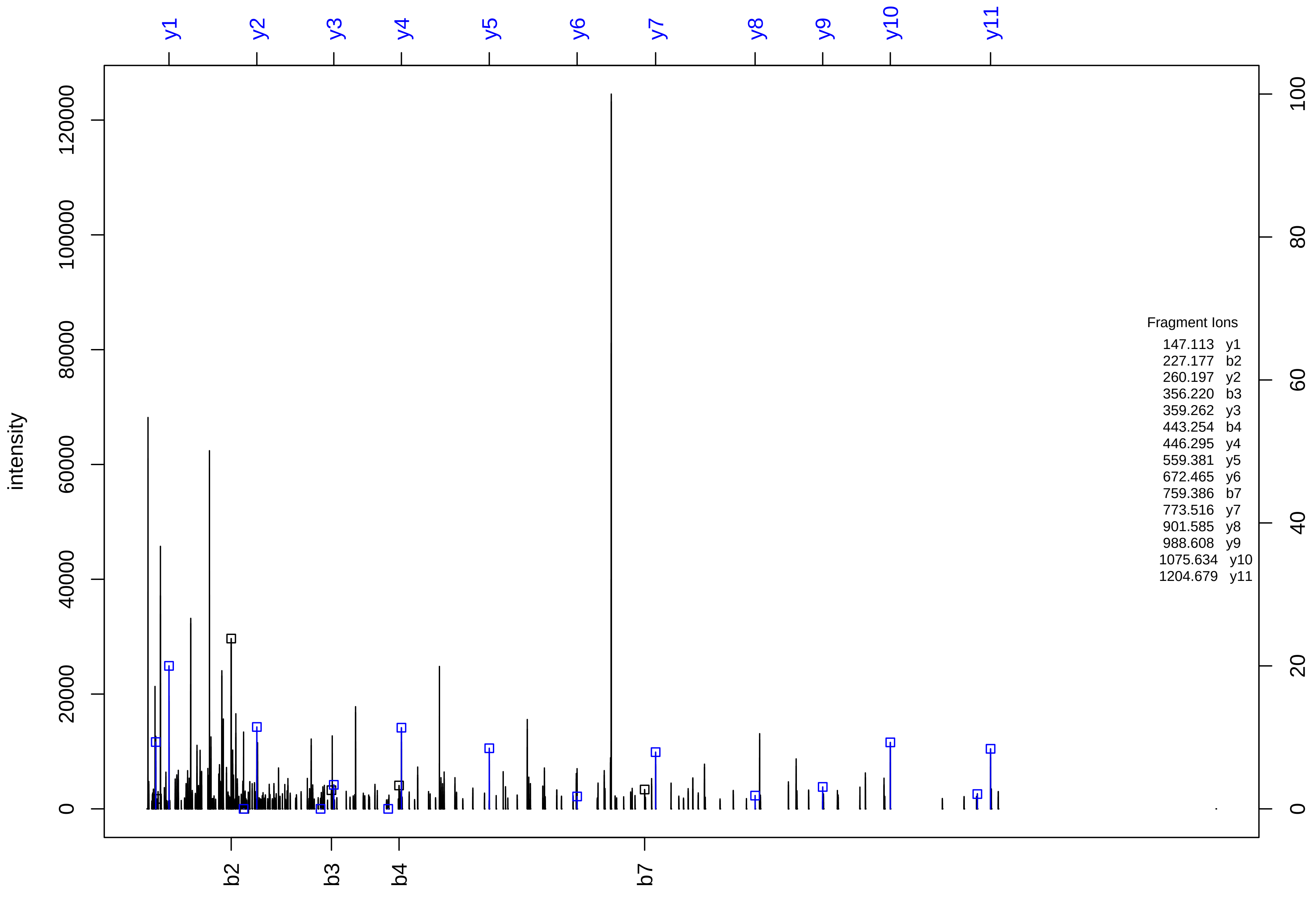

ILESSQTLLSVLK / File: sydney_11.raw; SpectrumID: ; scans: 11023

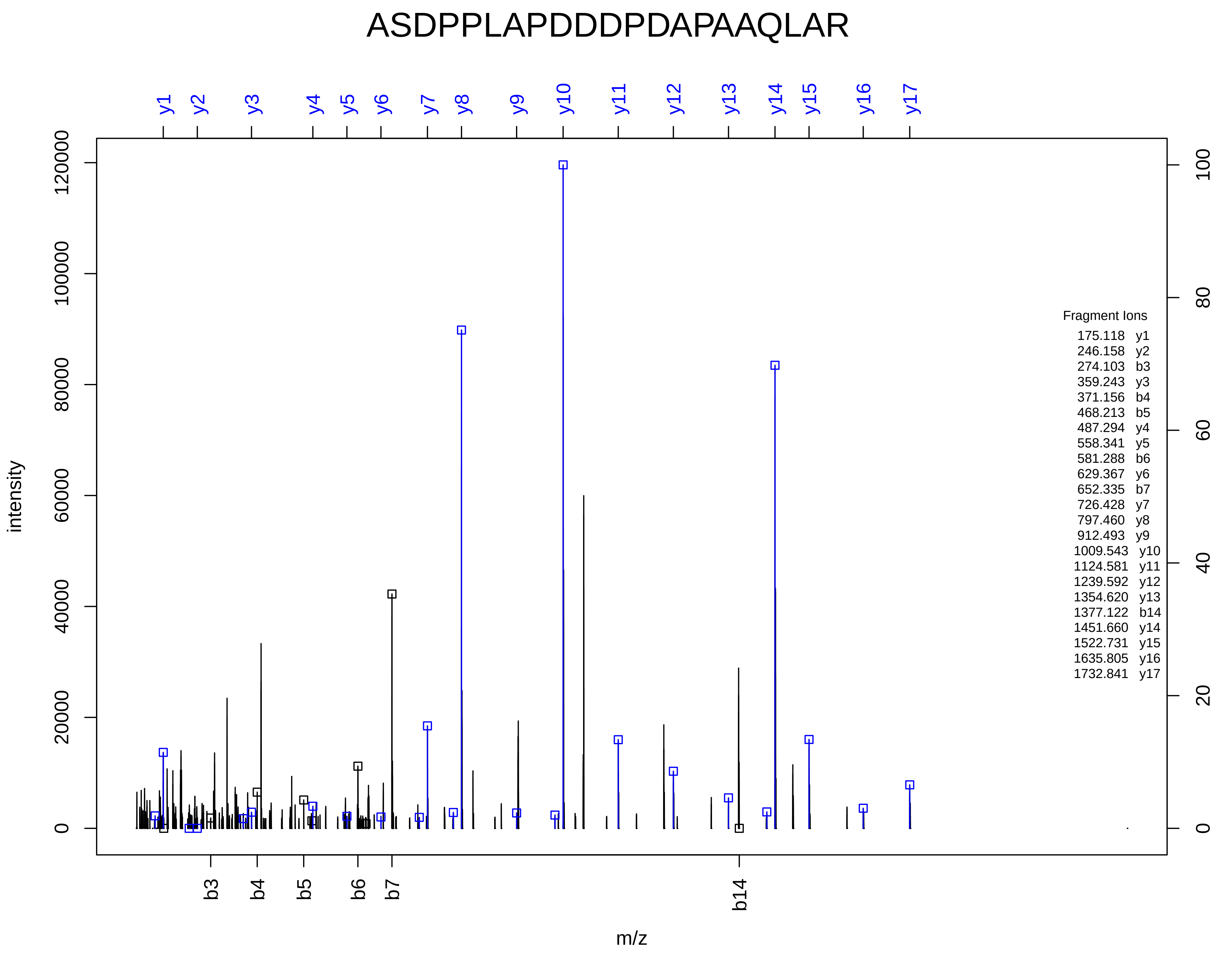
ASDPPLAPDDDPDAPAAQLAR
y1 y2 y3 y4 y5 y6 y7 y8 y9 y10 y11 y12 y13 y14 y15 y16 y17
b3 b4 b5 b6 b7 b14
intensity
m/z
Fragment Ions
175.118 y1
246.158 y2
274.103 b3
359.243 y3
371.156 b4
468.213 b5
487.294 y4
558.341 y5
581.288 b6
629.367 y6
652.335 b7
726.428 y7
797.460 y8
912.493 y9
1009.543 y10
1124.581 y11
1239.592 y12
1354.620 y13
1377.122 b14
1451.660 y14
1522.731 y15
1635.805 y16
1732.841 y17
ASDPPLAPDDDPDAPAAQLAR / File: sydney_11.raw; SpectrumID: ; scans: 6028

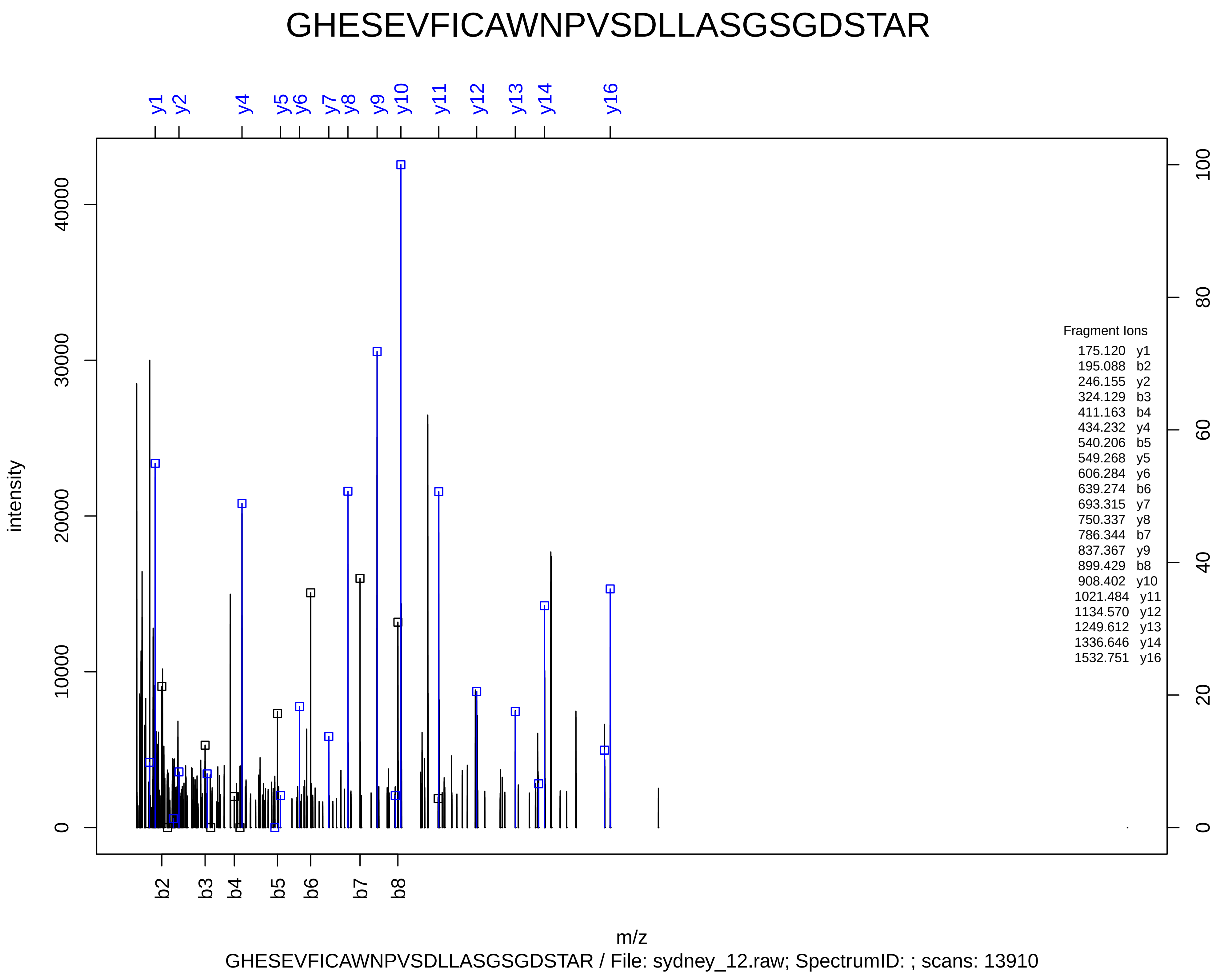
GHESEVFICAWNPVSDLLASGSGDSTAR
y1 y2 y4 y5 y6 y7 y8 y9 y10 y11 y12 y13 y14 y16
intensity
0
10000
20000
30000
40000
0
20
40
60
80
100
Fragment Ions
175.120 y1
195.088 b2
246.155 y2
324.129 b3
411.163 b4
434.232 y4
540.206 b5
549.268 y5
606.284 y6
639.274 b6
693.315 y7
750.337 y8
786.344 b7
837.367 y9
899.429 b8
908.402 y10
1021.484 y11
1134.570 y12
1249.612 y13
1336.646 y14
1532.751 y16
b2 b3 b4 b5 b6 b7 b8
m/z
GHESEVFICAWNPVSDLLASGSGDSTAR / File: sydney_12.raw; SpectrumID: ; scans: 13910

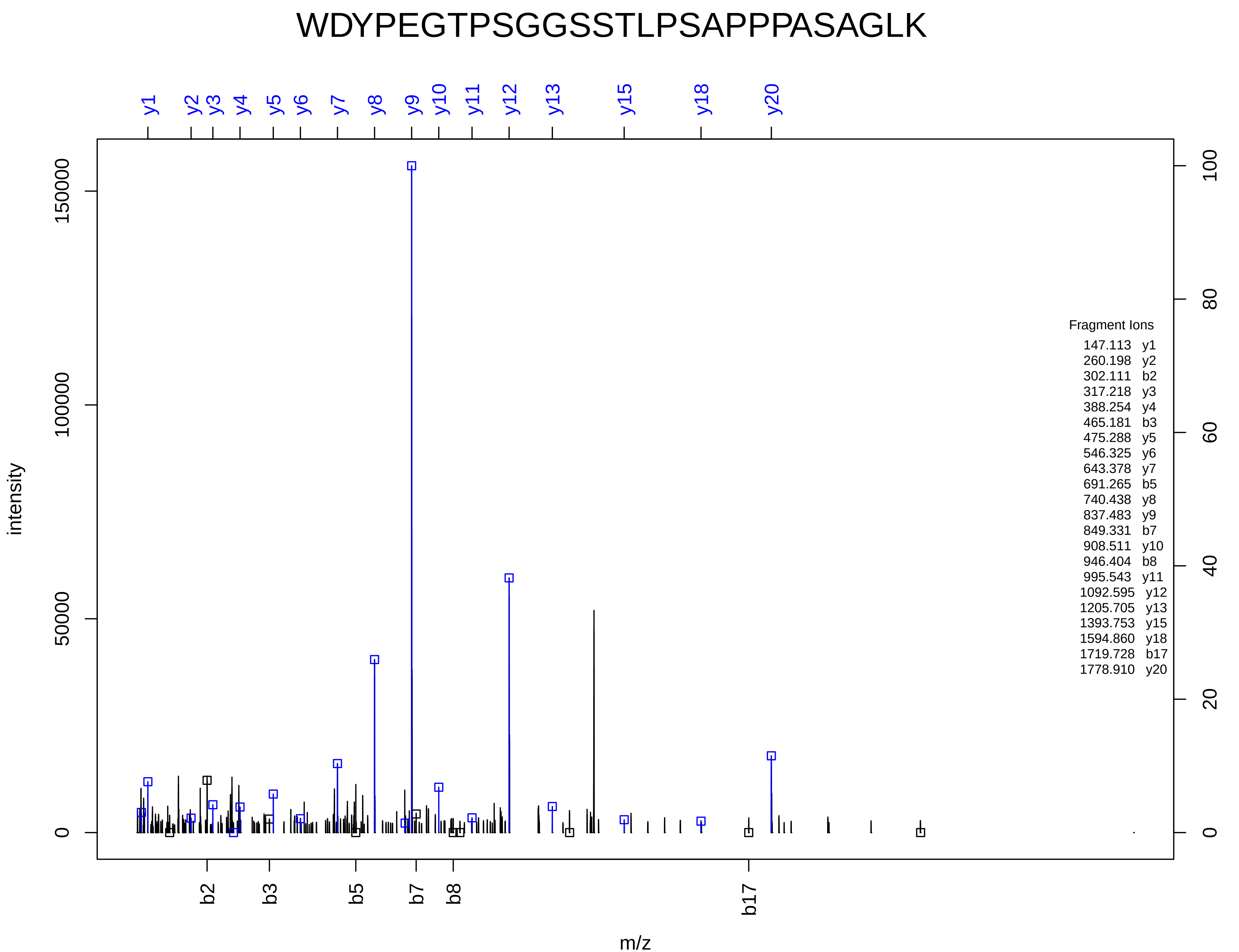

WDYPEGTPSGGSSTLPSAPPPASAGLK / File: sydney_12.raw; SpectrumID: ; scans: 7367

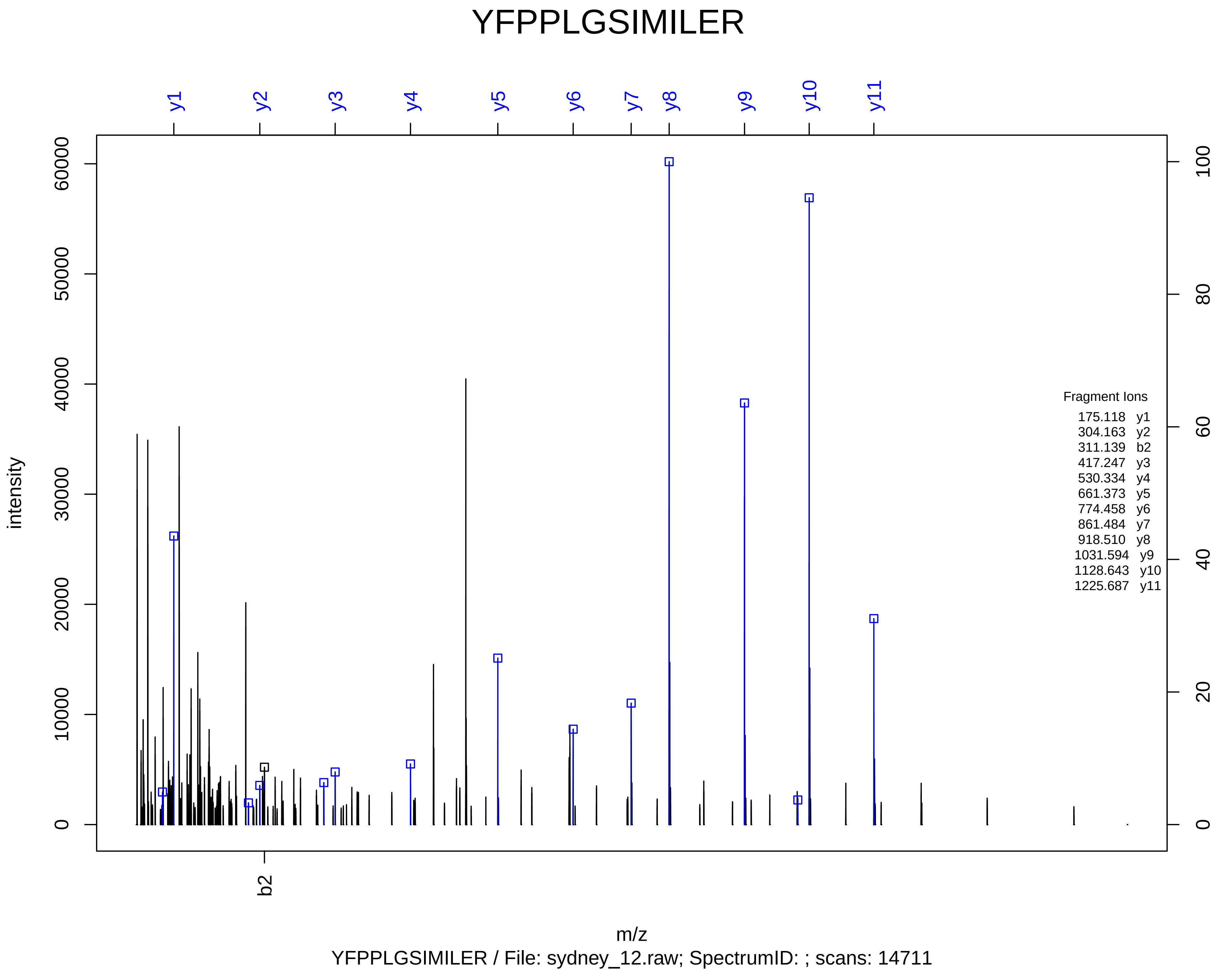

YFPPLGSIMILER
y1
y2
y3
y4
y5
y6
y7
y8
y9
y10
y11
intensity
0
10000
20000
30000
40000
50000
60000
0
20
40
60
80
100
b2
m/z
Fragment Ions
175.118 y1
304.163 y2
311.139 b2
417.247 y3
530.334 y4
661.373 y5
774.458 y6
861.484 y7
918.510 y8
1031.594 y9
1128.643 y10
1225.687 y11
YFPPLGSIMILER / File: sydney_12.raw; SpectrumID: ; scans: 14711

DEADVALLPTIVEK

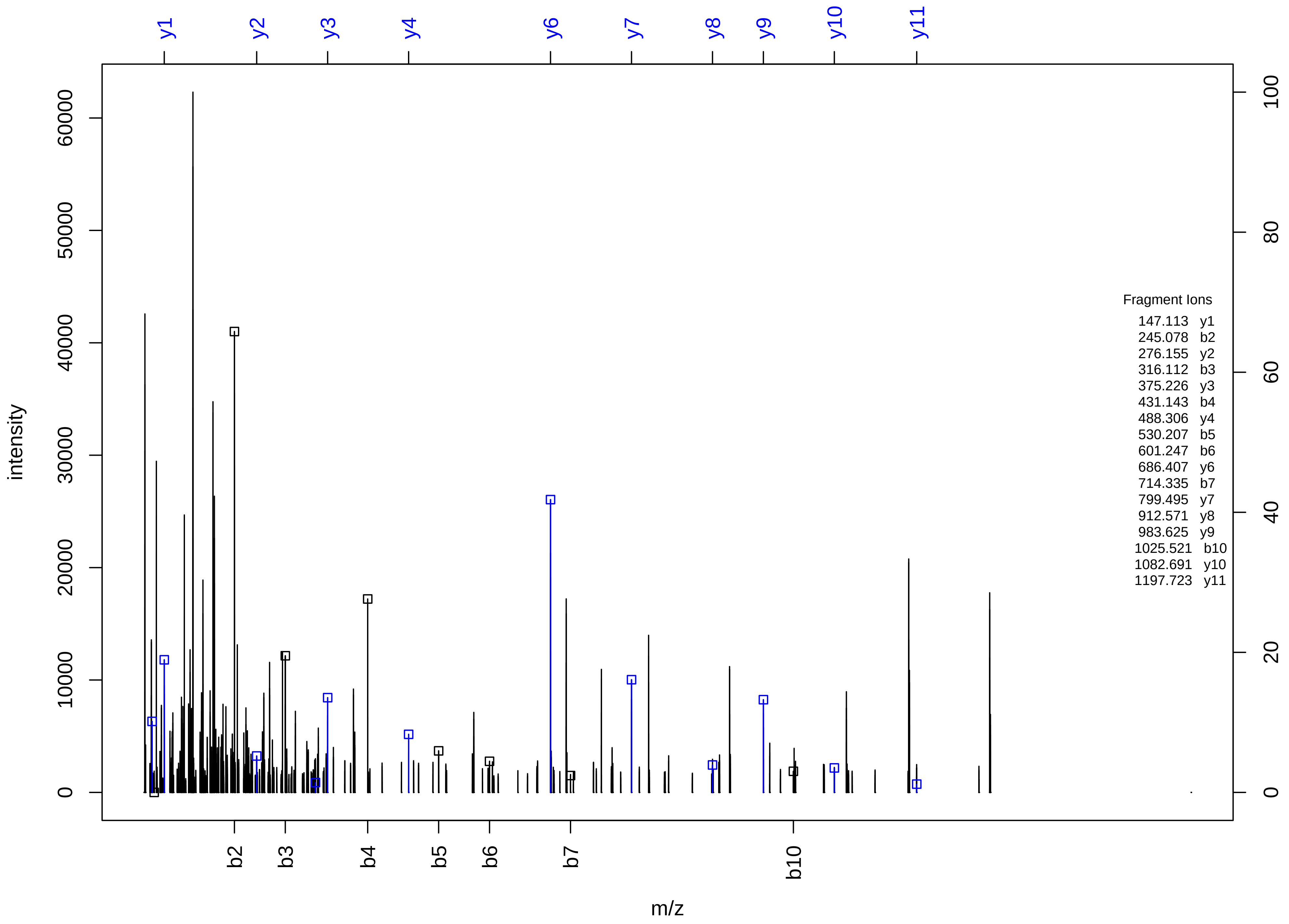

DEADVALLPTIVEK / File: sydney_12.raw; SpectrumID: ; scans: 10880

TLYDFPGNDAEDLPFK

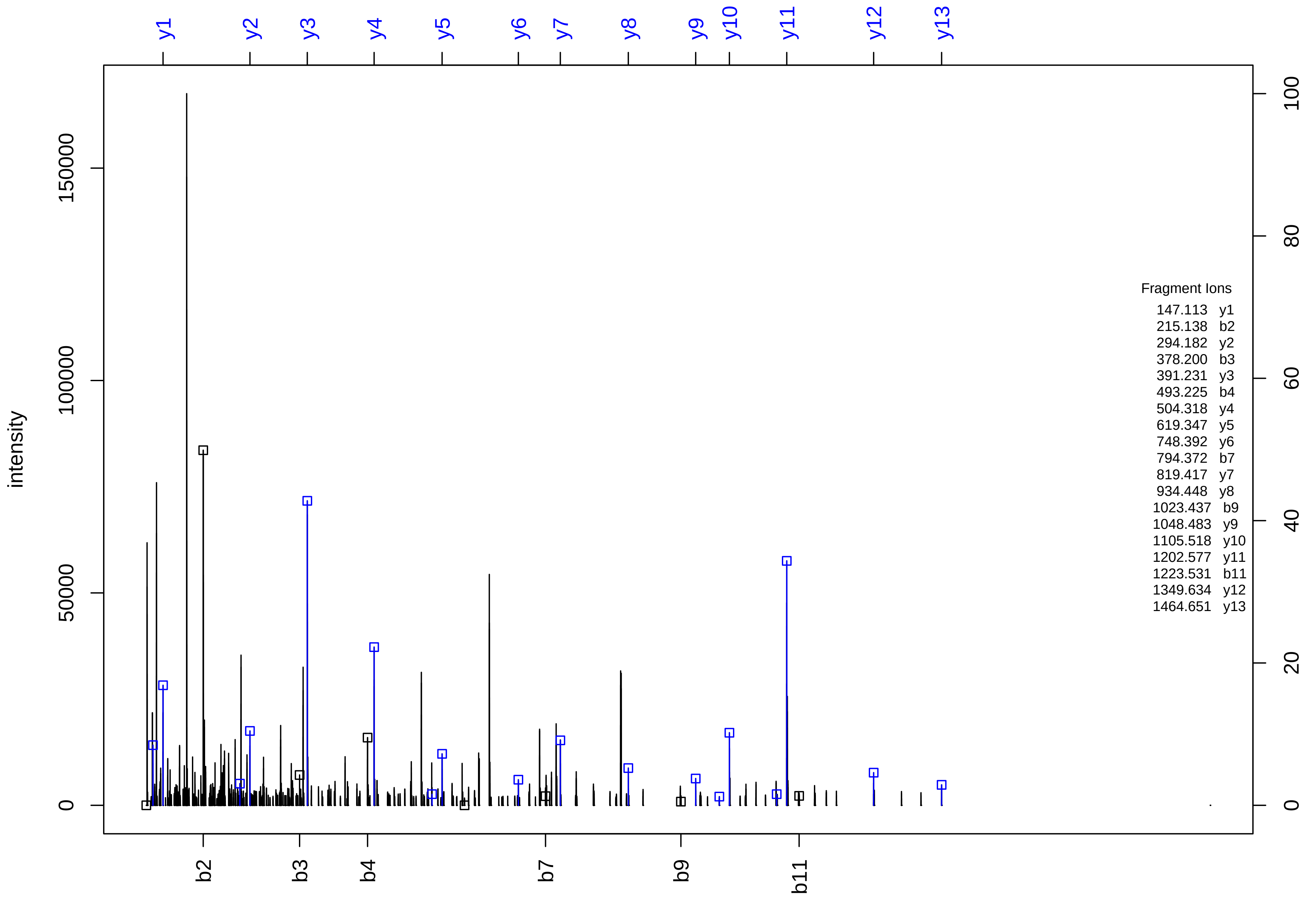

TLYDFPGNDAEDLPFK / File: sydney_12.raw; SpectrumID: ; scans: 10493

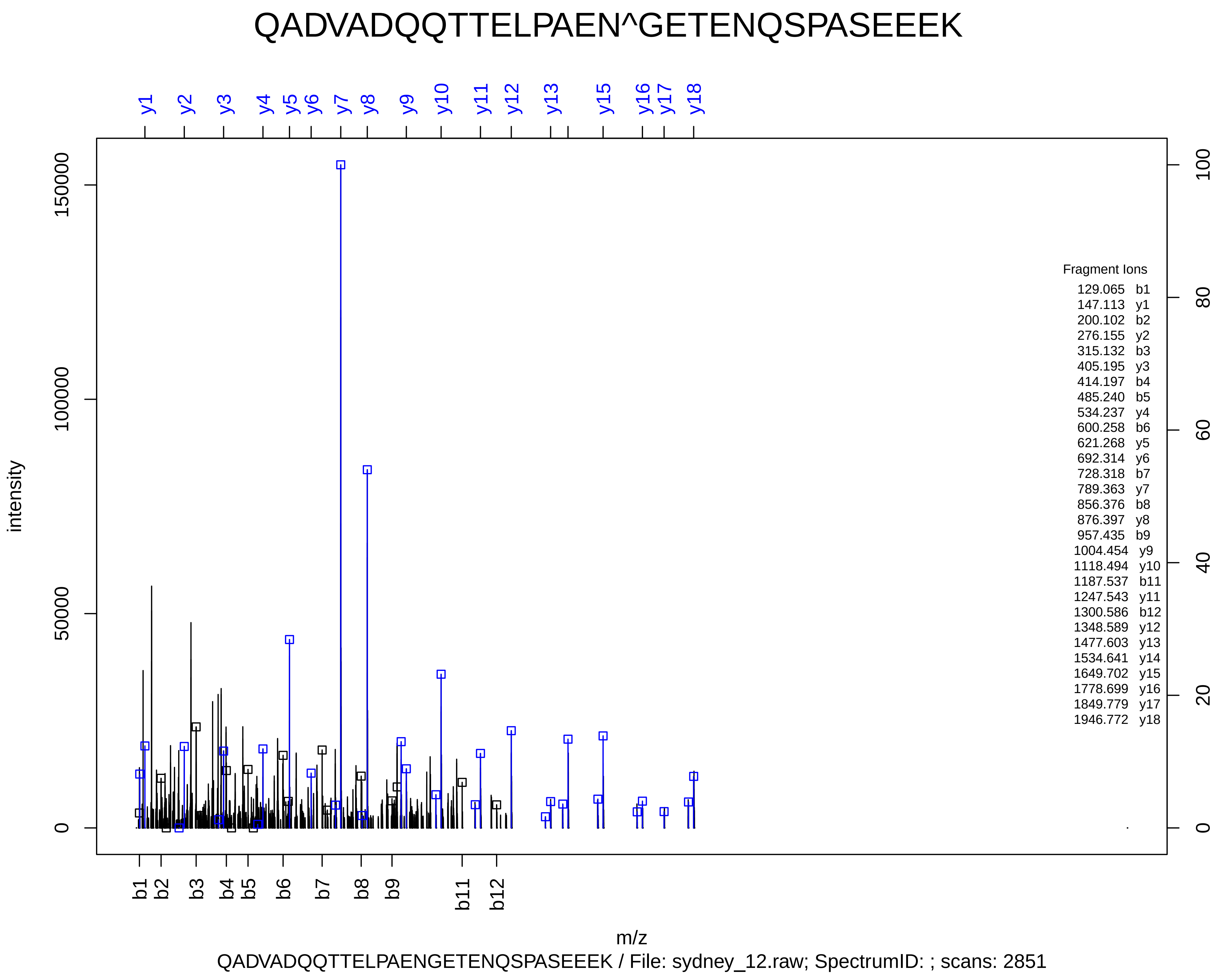

QADVADQQTTELPAEN^GETENQSPASEEEK
y1 y2 y3 y4 y5 y6 y7 y8 y9 y10 y11 y12 y13 y15 y16 y17 y18
b1 b2 b3 b4 b5 b6 b7 b8 b9 b11 b12
intensity
m/z
0
50000
100000
150000
0
20
40
60
80
100
Fragment Ions
129.065 b1
147.113 y1
200.102 b2
276.155 y2
315.132 b3
405.195 y3
414.197 b4
485.240 b5
534.237 y4
600.258 b6
621.268 y5
692.314 y6
728.318 b7
789.363 y7
856.376 b8
876.397 y8
957.435 b9
1004.454 y9
1118.494 y10
1187.537 b11
1247.543 y11
1300.586 b12
1348.589 y12
1477.603 y13
1534.641 y14
1649.702 y15
1778.699 y16
1849.779 y17
1946.772 y18
QADVADQQTTELPAENGETENQSPASEEEK / File: sydney_12.raw; SpectrumID: ; scans: 2851

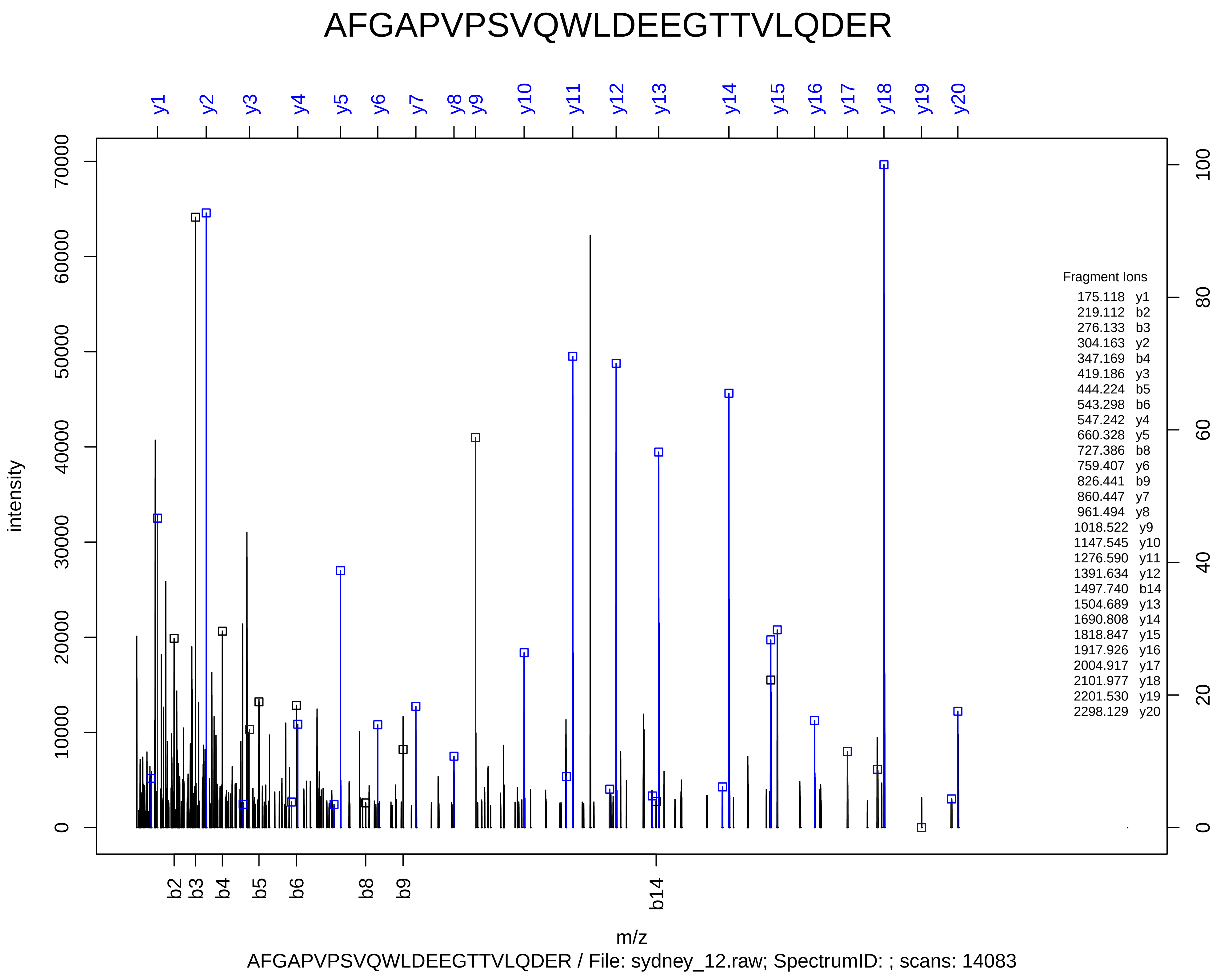
AFGAPVPSVQWLDEEGTTVLQDER
y1 y2 y3 y4 y5 y6 y7 y8 y9 y10 y11 y12 y13 y14 y15 y16 y17 y18 y19 y20
intensity
0 10000 20000 30000 40000 50000 60000 70000
0 20 40 60 80 100
b2 b3 b4 b5 b6 b8 b9 b14
Fragment Ions
175.118 y1
219.112 b2
276.133 b3
304.163 y2
347.169 b4
419.186 y3
444.224 b5
543.298 b6
547.242 y4
660.328 y5
727.386 b8
759.407 y6
826.441 b9
860.447 y7
961.494 y8
1018.522 y9
1147.545 y10
1276.590 y11
1391.634 y12
1497.740 b14
1504.689 y13
1690.808 y14
1818.847 y15
1917.926 y16
2004.917 y17
2101.977 y18
2201.530 y19
2298.129 y20
m/z
AFGAPVPSVQWLDEEGTTVLQDER / File: sydney_12.raw; SpectrumID: ; scans: 14083

STIWQFFSR

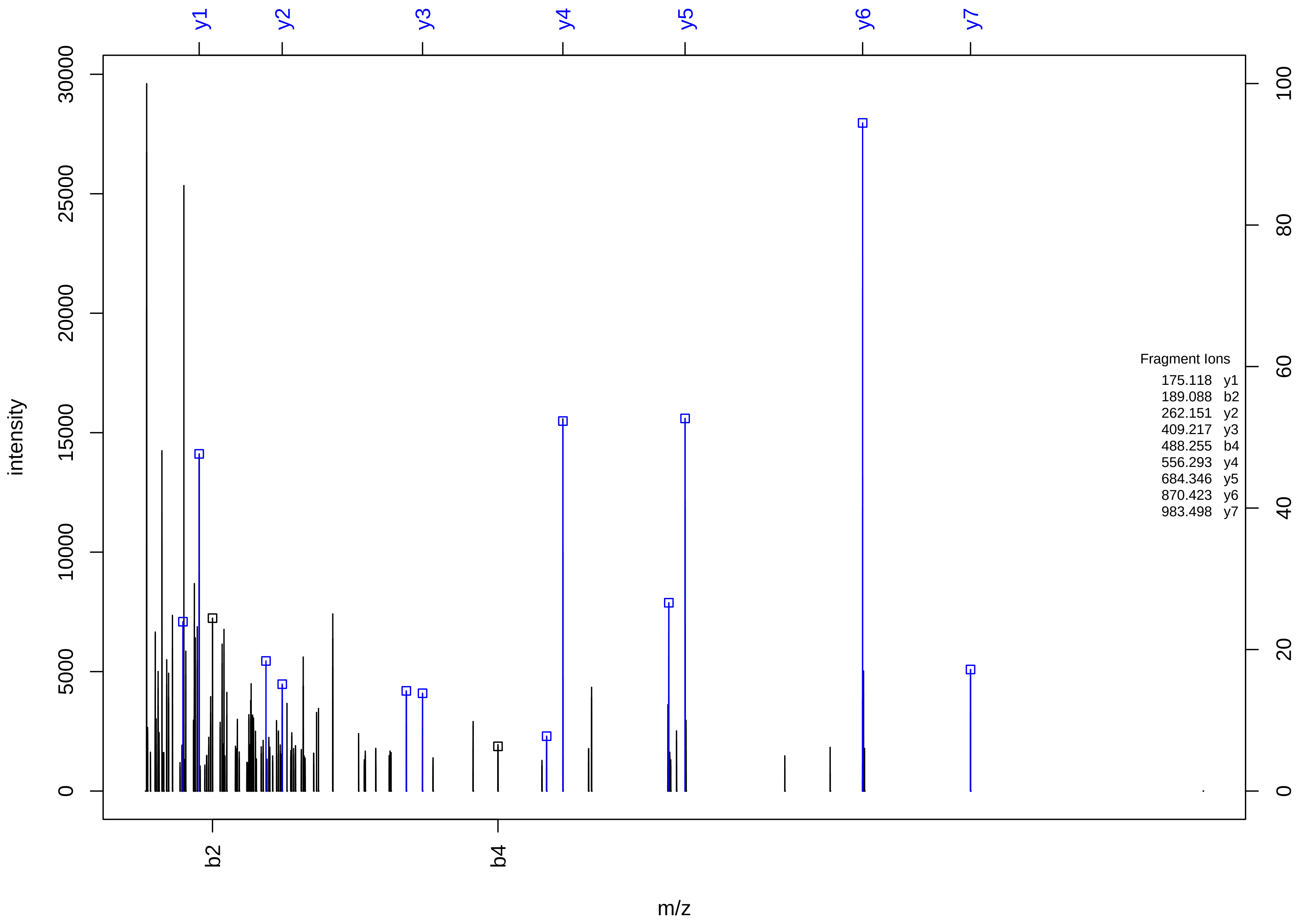

STIWQFFSR / File: sydney_12.raw; SpectrumID: ; scans: 11427

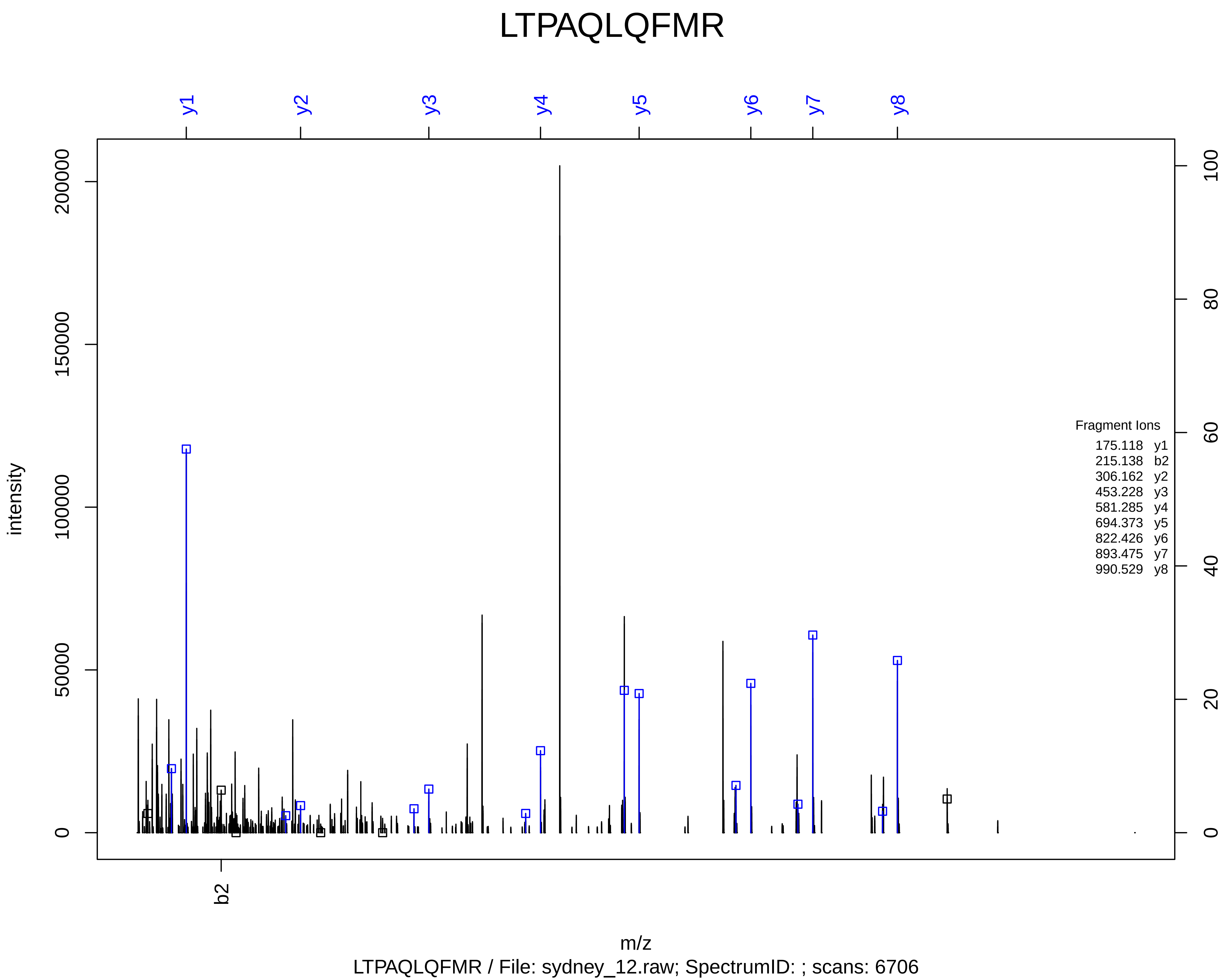

LTPAQLQFMR
y1
y2
y3
y4
y5
y6
y7
y8
b2
intensity
0
50000
100000
150000
200000
0
20
40
60
80
100
Fragment Ions
175.118 y1
215.138 b2
306.162 y2
453.228 y3
581.285 y4
694.373 y5
822.426 y6
893.475 y7
990.529 y8
m/z
LTPAQLQFMR / File: sydney_12.raw; SpectrumID: ; scans: 6706

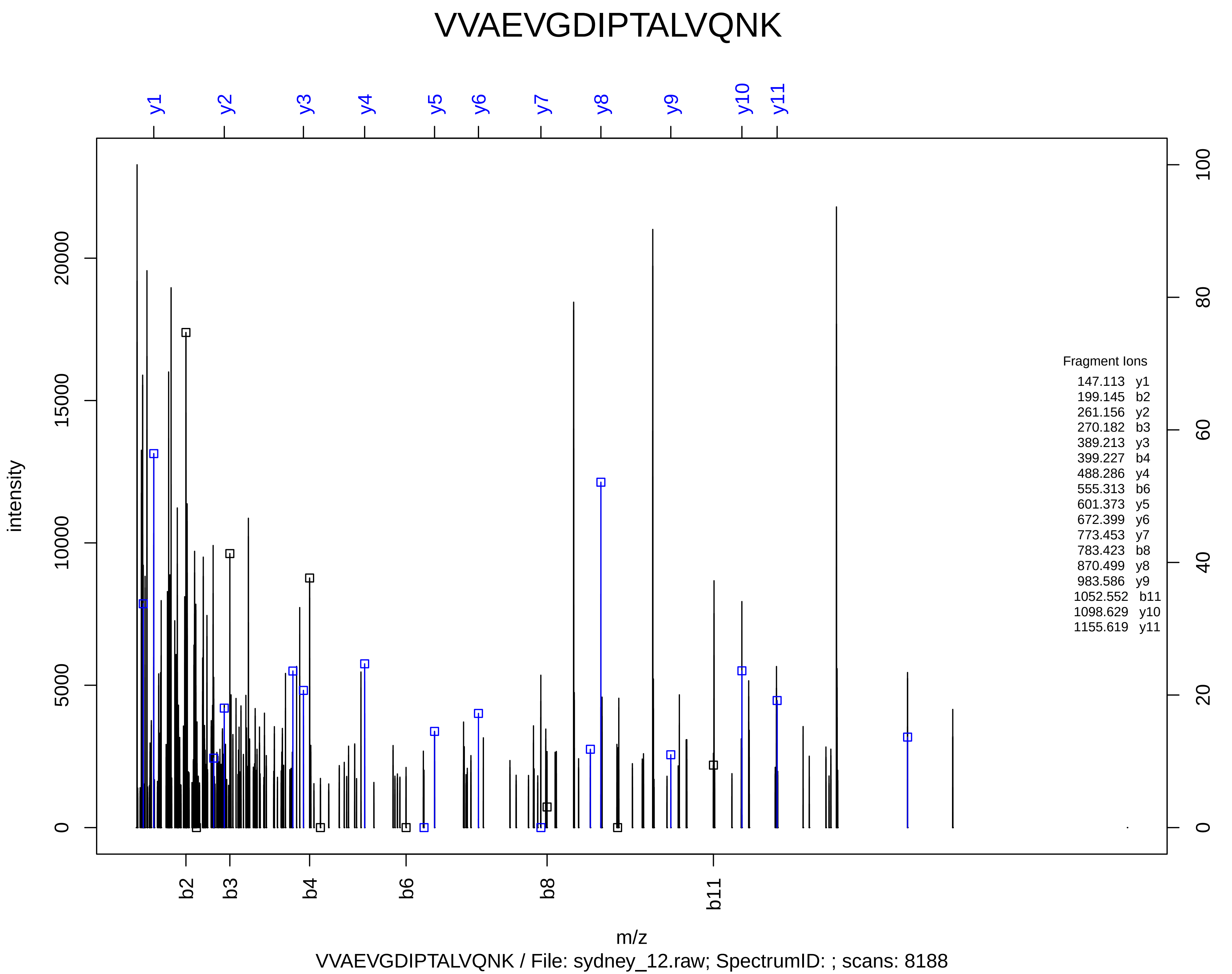
VVAEVGDIPTALVQNK
y1
y2
y3
y4
y5
y6
y7
y8
y9
y10
y11
intensity
0
5000
10000
15000
20000
0
20
40
60
80
100
b2
b3
b4
b6
b8
b11
m/z
Fragment Ions
147.113 y1
199.145 b2
261.156 y2
270.182 b3
389.213 y3
399.227 b4
488.286 y4
555.313 b6
601.373 y5
672.399 y6
773.453 y7
783.423 b8
870.499 y8
983.586 y9
1052.552 b11
1098.629 y10
1155.619 y11
VVAEVGDIPTALVQNK / File: sydney_12.raw; SpectrumID: ; scans: 8188

VVNVLAVCNMPFEIR

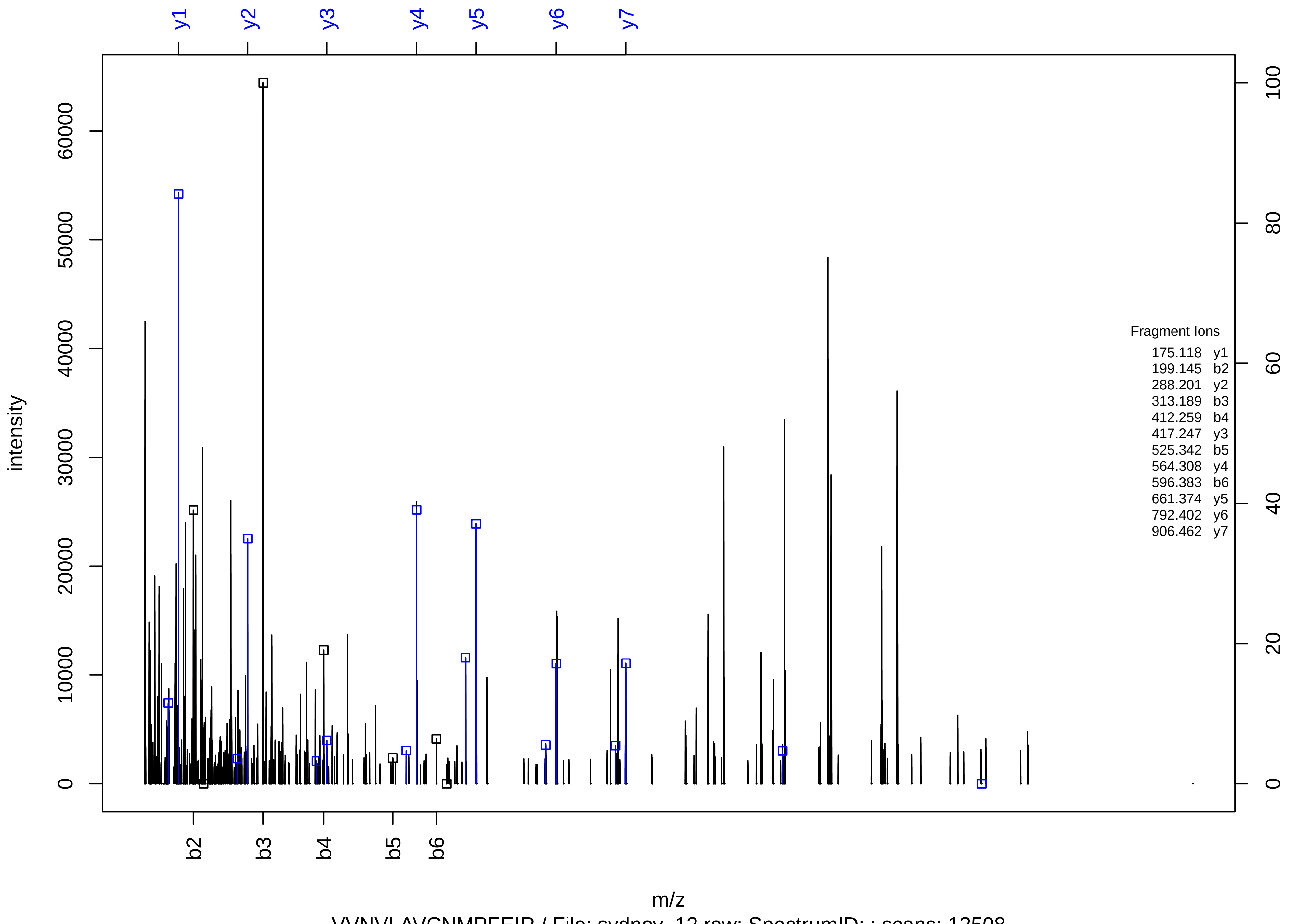

VVNVLAVCNMPFEIR / File: sydney_12.raw; SpectrumID: ; scans: 12508

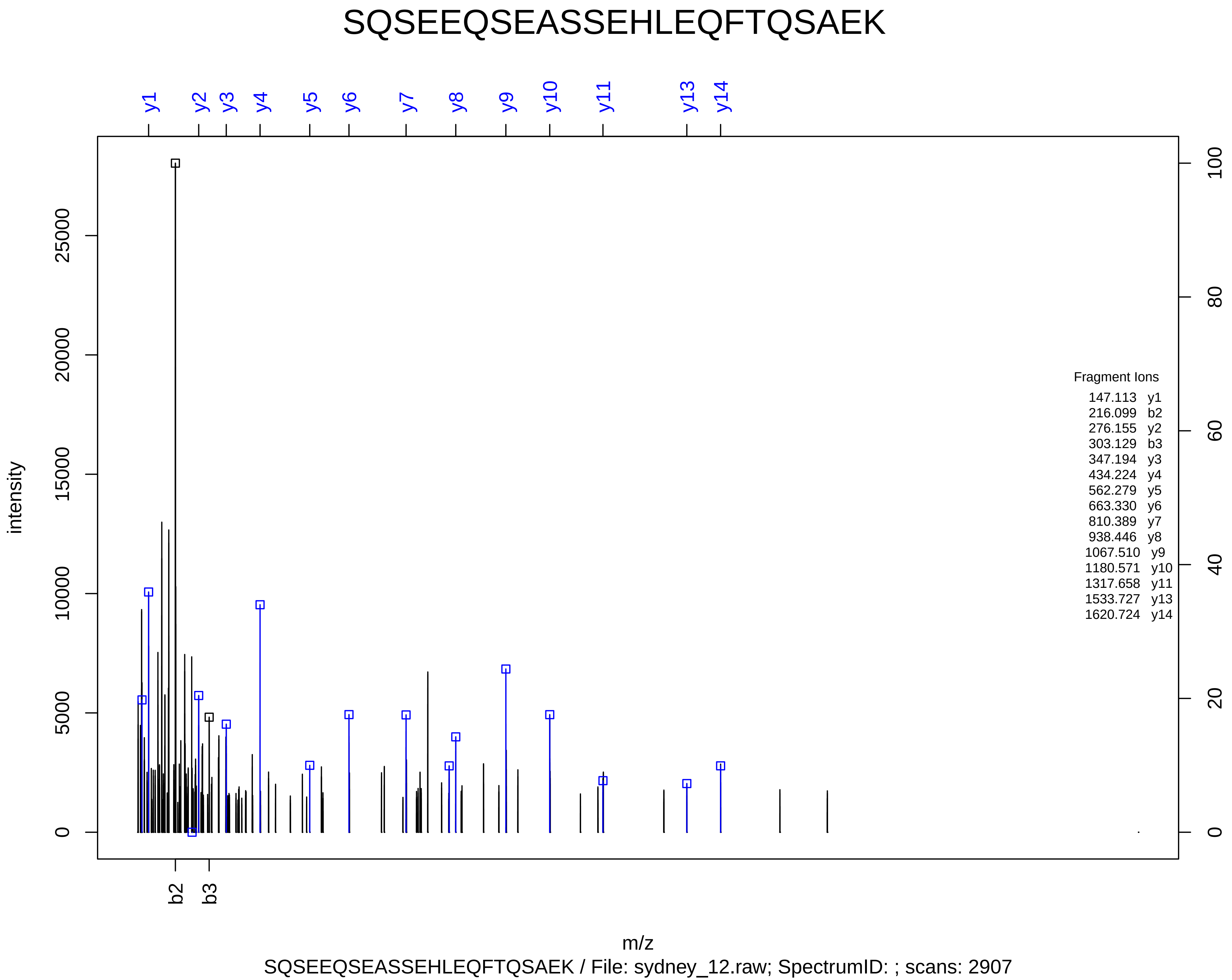
SQSEEQSEASSEHLEQFTQSAEK
y1
y2
y3
y4
y5
y6
y7
y8
y9
y10
y11
y13
y14
intensity
0
5000
10000
15000
20000
25000
0
20
40
60
80
100
b2
b3
m/z
Fragment Ions
147.113 y1
216.099 b2
276.155 y2
303.129 b3
347.194 y3
434.224 y4
562.279 y5
663.330 y6
810.389 y7
938.446 y8
1067.510 y9
1180.571 y10
1317.658 y11
1533.727 y13
1620.724 y14
SQSEEQSEASSEHLEQFTQSAEK / File: sydney_12.raw; SpectrumID: ; scans: 2907

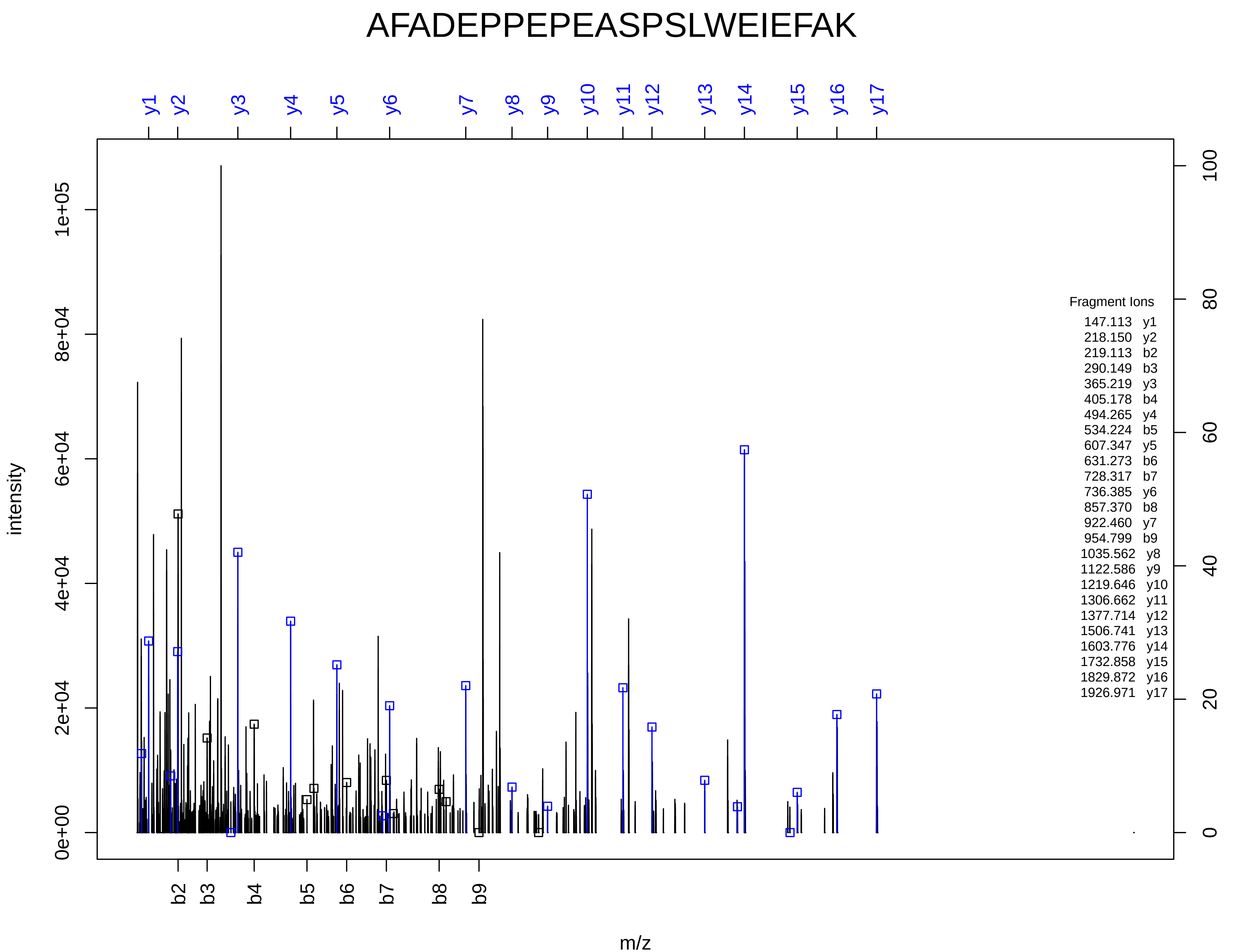
AFADEPPEPEASPSLWEIEFAK
y1 y2 y3 y4 y5 y6 y7 y8 y9 y10 y11 y12 y13 y14 y15 y16 y17
b2 b3 b4 b5 b6 b7 b8 b9
intensity
0e+00
2e+04
4e+04
6e+04
8e+04
1e+05
0
20
40
60
80
100
m/z
Fragment Ions
147.113 y1
218.150 y2
219.113 b2
290.149 b3
365.219 y3
405.178 b4
494.265 y4
534.224 b5
607.347 y5
631.273 b6
728.317 b7
736.385 y6
857.370 b8
922.460 y7
954.799 b9
1035.562 y8
1122.586 y9
1219.646 y10
1306.662 y11
1377.714 y12
1506.741 y13
1603.776 y14
1732.858 y15
1829.872 y16
1926.971 y17
AFADEPPEPEASPSLWEIEFAK / File: sydney_12.raw; SpectrumID: ; scans: 12354

YLQ^SQ^IDALIKEN^Q^EIR

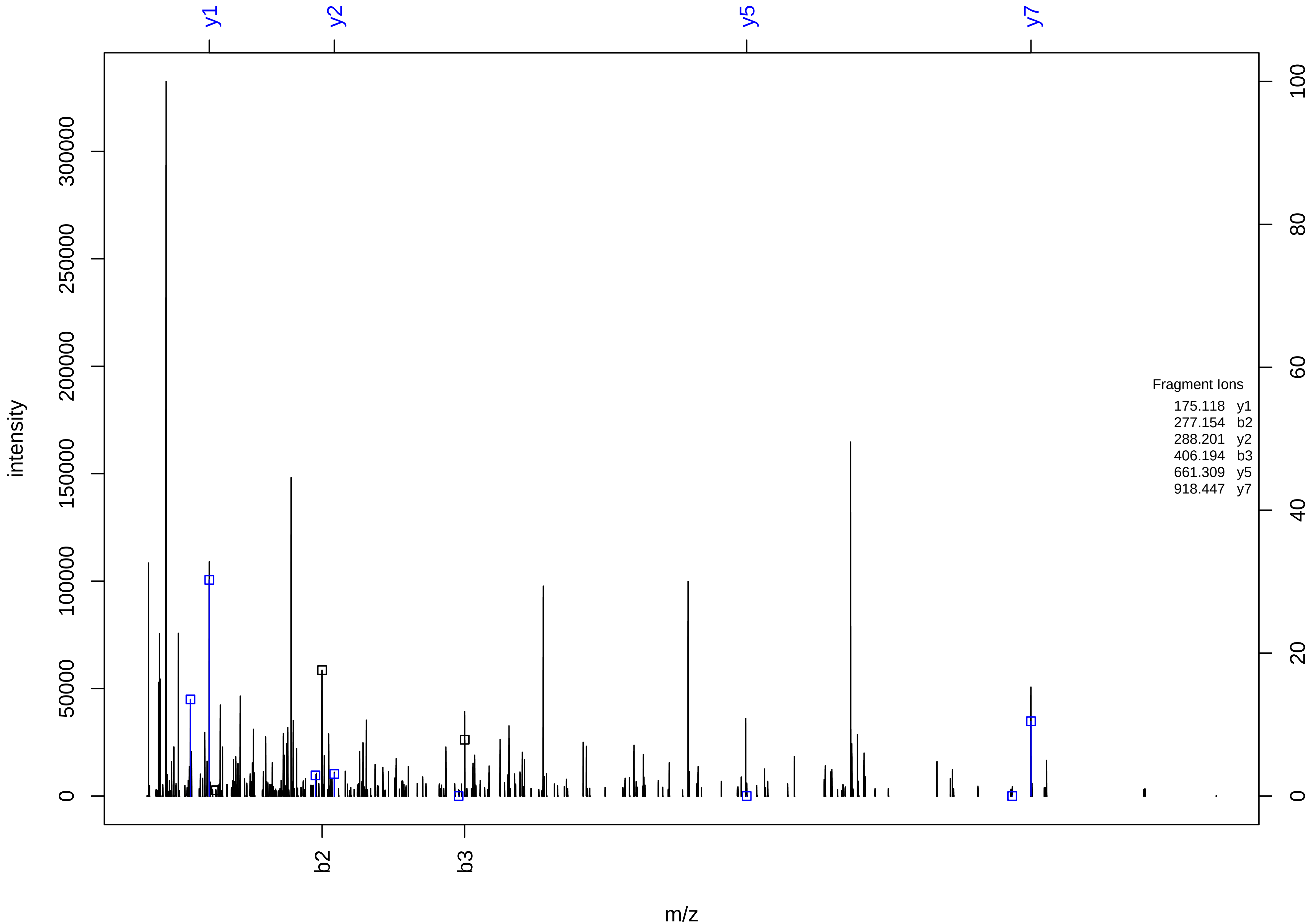

YLQSQIDALIKENQEIR / File: sydney_12.raw; SpectrumID: ; scans: 3683

RTLIYMVTEN^N^N^K

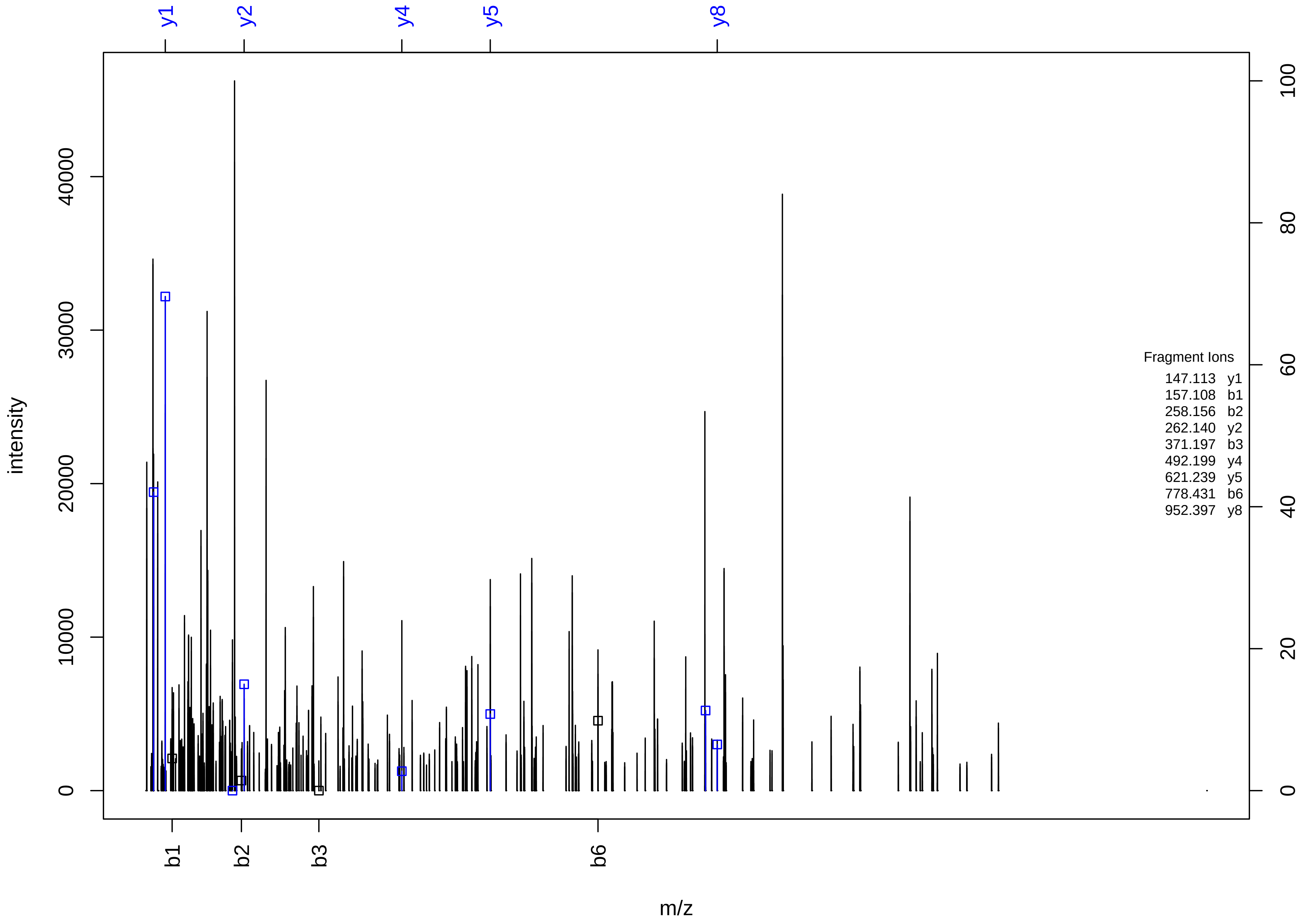

RTLIYMVTENNNK / File: sydney_12.raw; SpectrumID: ; scans: 5945

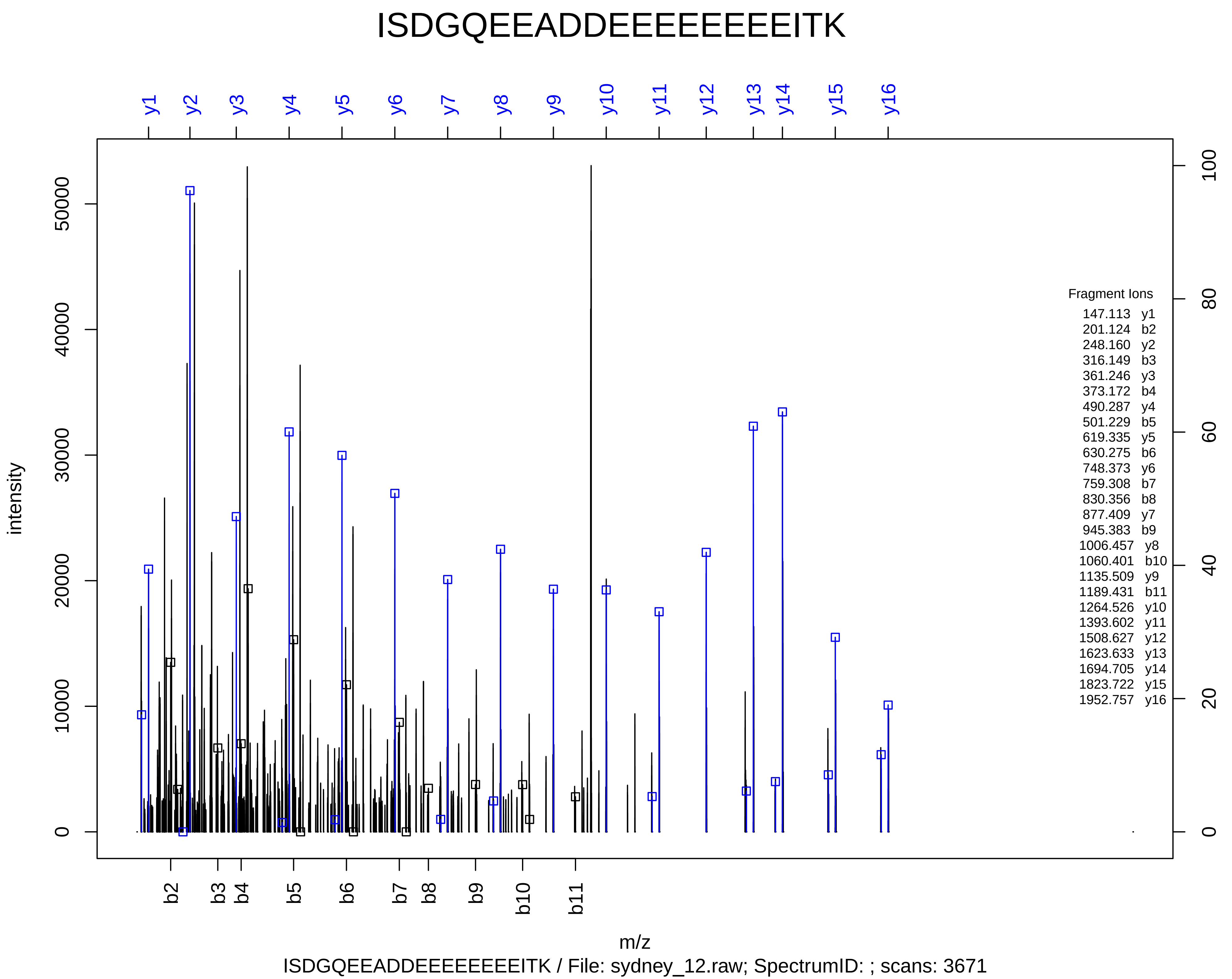

ISDGQEEADDEEEEEEEEITK
y1 y2 y3 y4 y5 y6 y7 y8 y9 y10 y11 y12 y13 y14 y15 y16
b2 b3 b4 b5 b6 b7 b8 b9 b10 b11
intensity
m/z
0
10000
20000
30000
40000
50000
0
20
40
60
80
100
Fragment Ions
147.113 y1
201.124 b2
248.160 y2
316.149 b3
361.246 y3
373.172 b4
490.287 y4
501.229 b5
619.335 y5
630.275 b6
748.373 y6
759.308 b7
830.356 b8
877.409 y7
945.383 b9
1006.457 y8
1060.401 b10
1135.509 y9
1189.431 b11
1264.526 y10
1393.602 y11
1508.627 y12
1623.633 y13
1694.705 y14
1823.722 y15
1952.757 y16
ISDGQEEADDEEEEEEEEITK / File: sydney_12.raw; SpectrumID: ; scans: 3671

DLPEPEFPFQYDPSYR

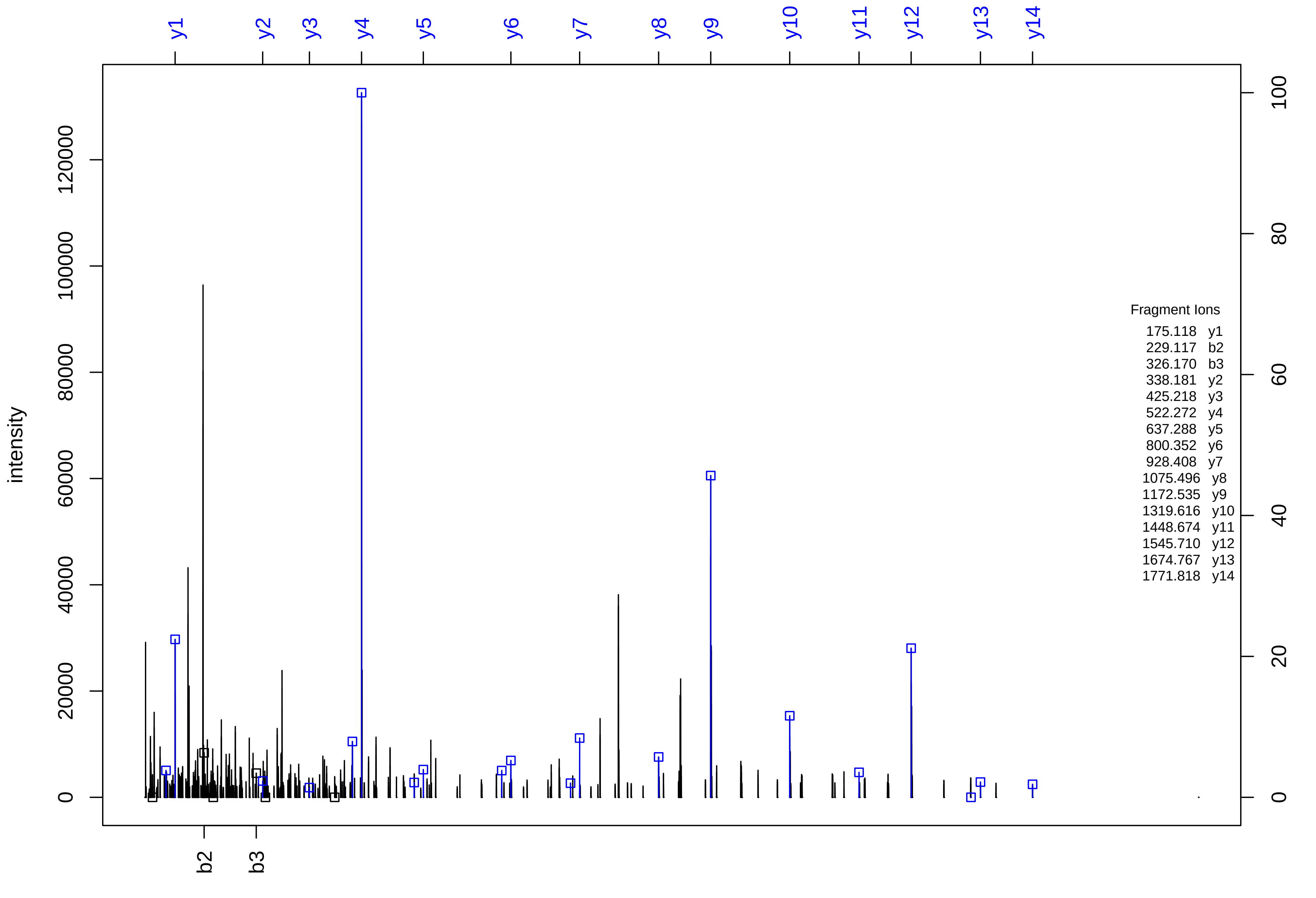

DLPEPEFPFQYDPSYR / File: sydney_12.raw; SpectrumID: ; scans: 11923

AIGTAYAVLSNPEK

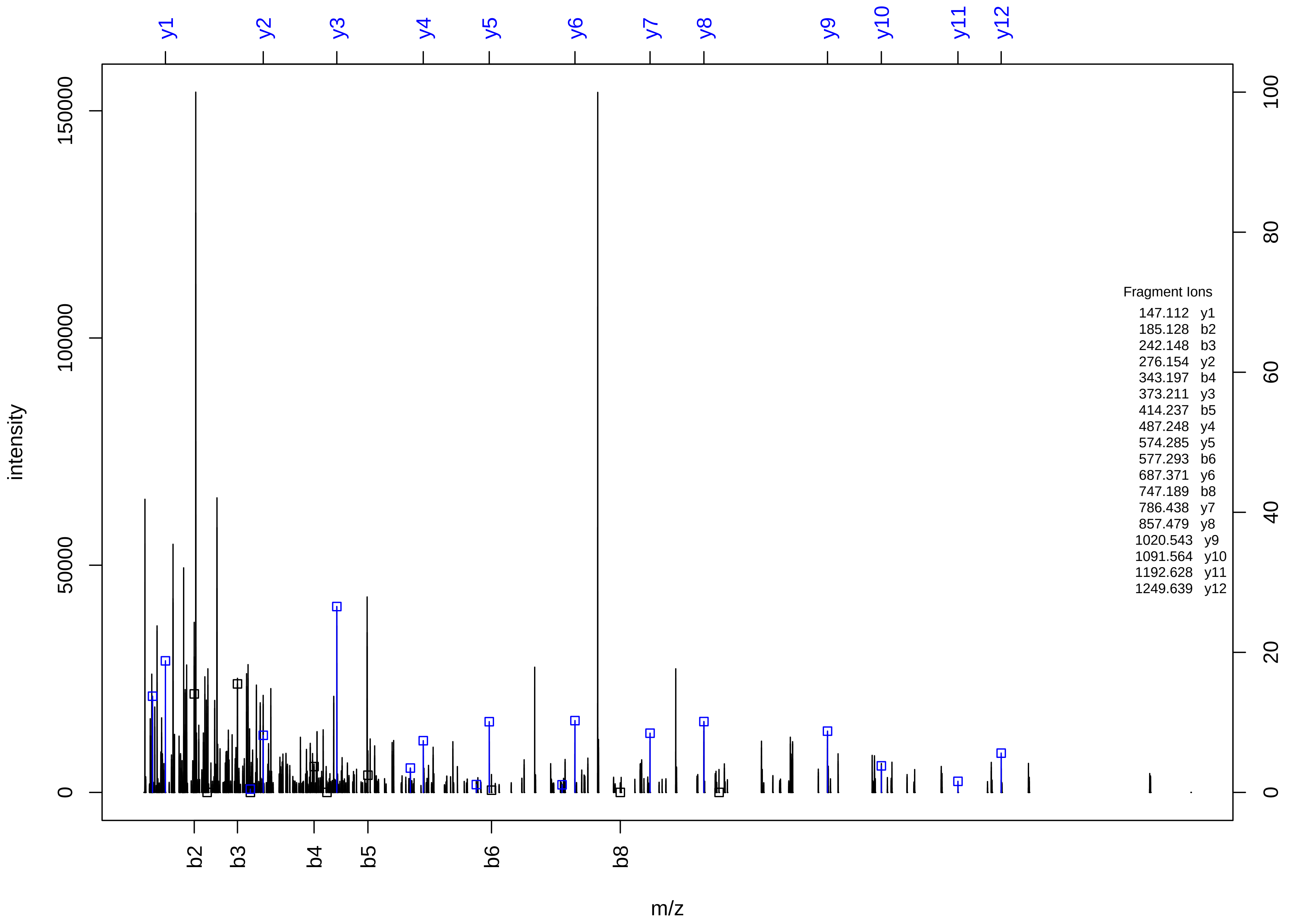

AIGTAYAVLSNPEK / File: sydney_12.raw; SpectrumID: ; scans: 5091

GEAYNLFEHNCNTFSNEVAQFLTGR

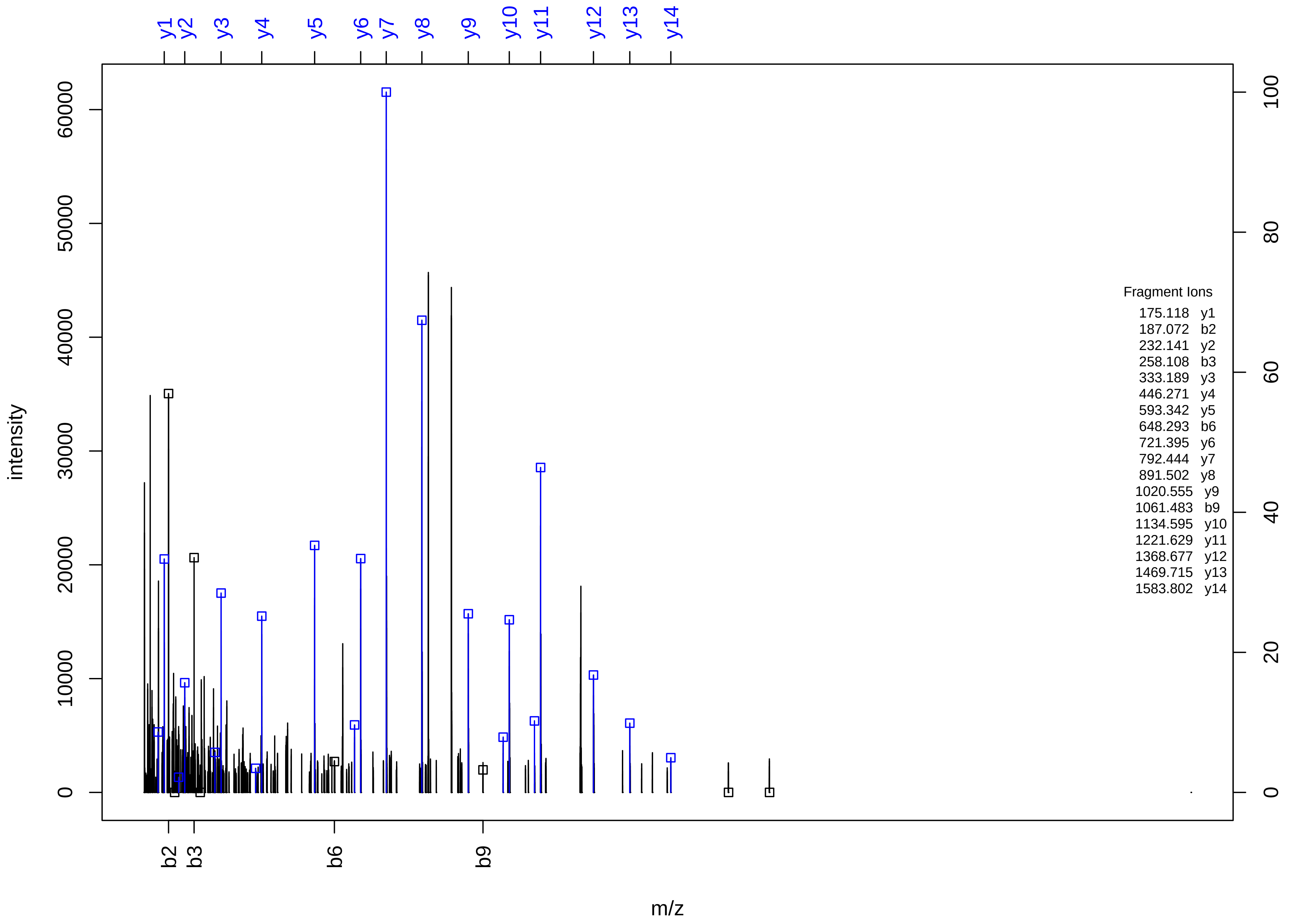

GEAYNLFEHNCNTFSNEVAQFLTGR / File: sydney_12.raw; SpectrumID: ; scans: 14548

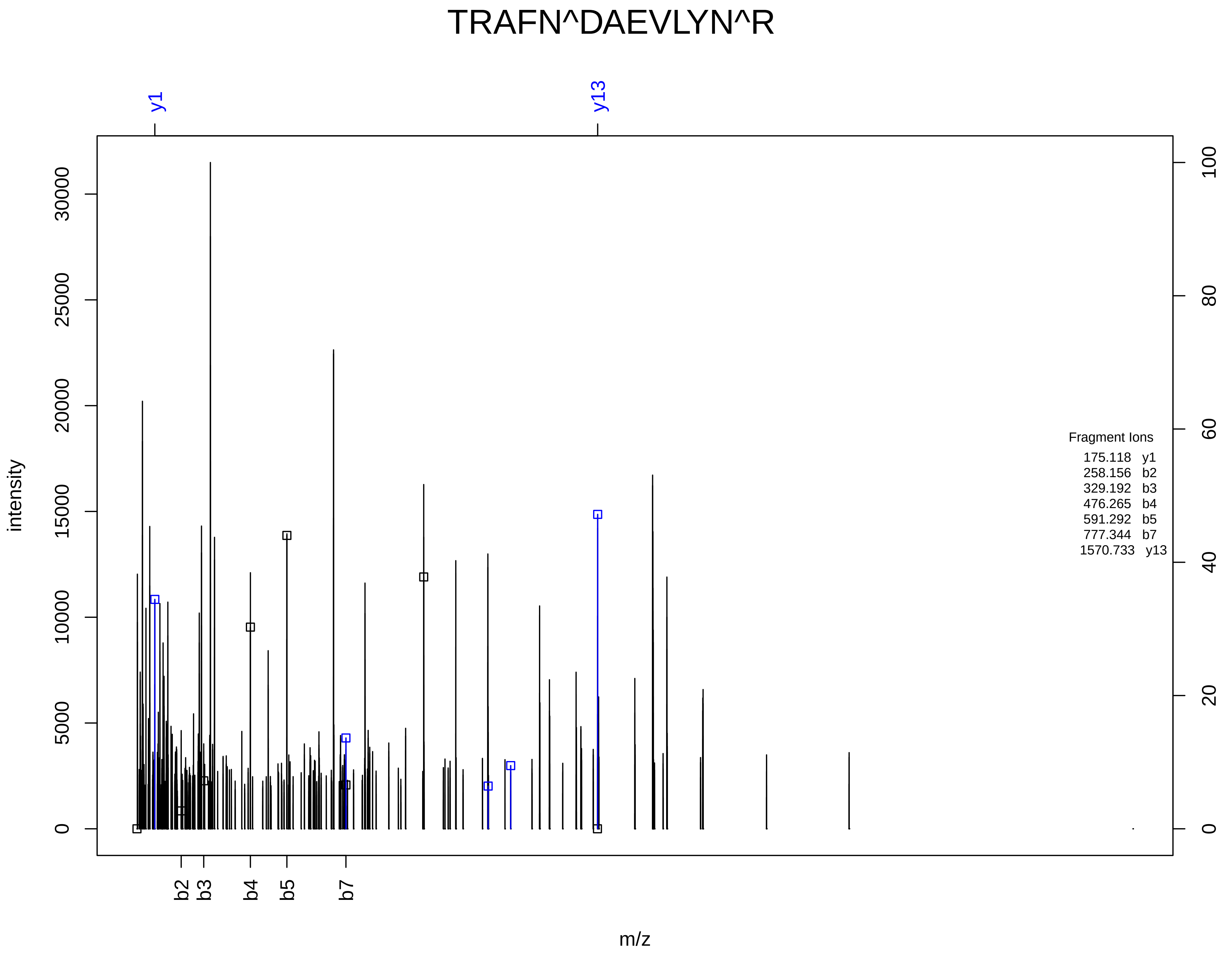

TRAFNDAEVLYNR / File: sydney_12.raw; SpectrumID: ; scans: 14675

TYAEILEPFHPVR

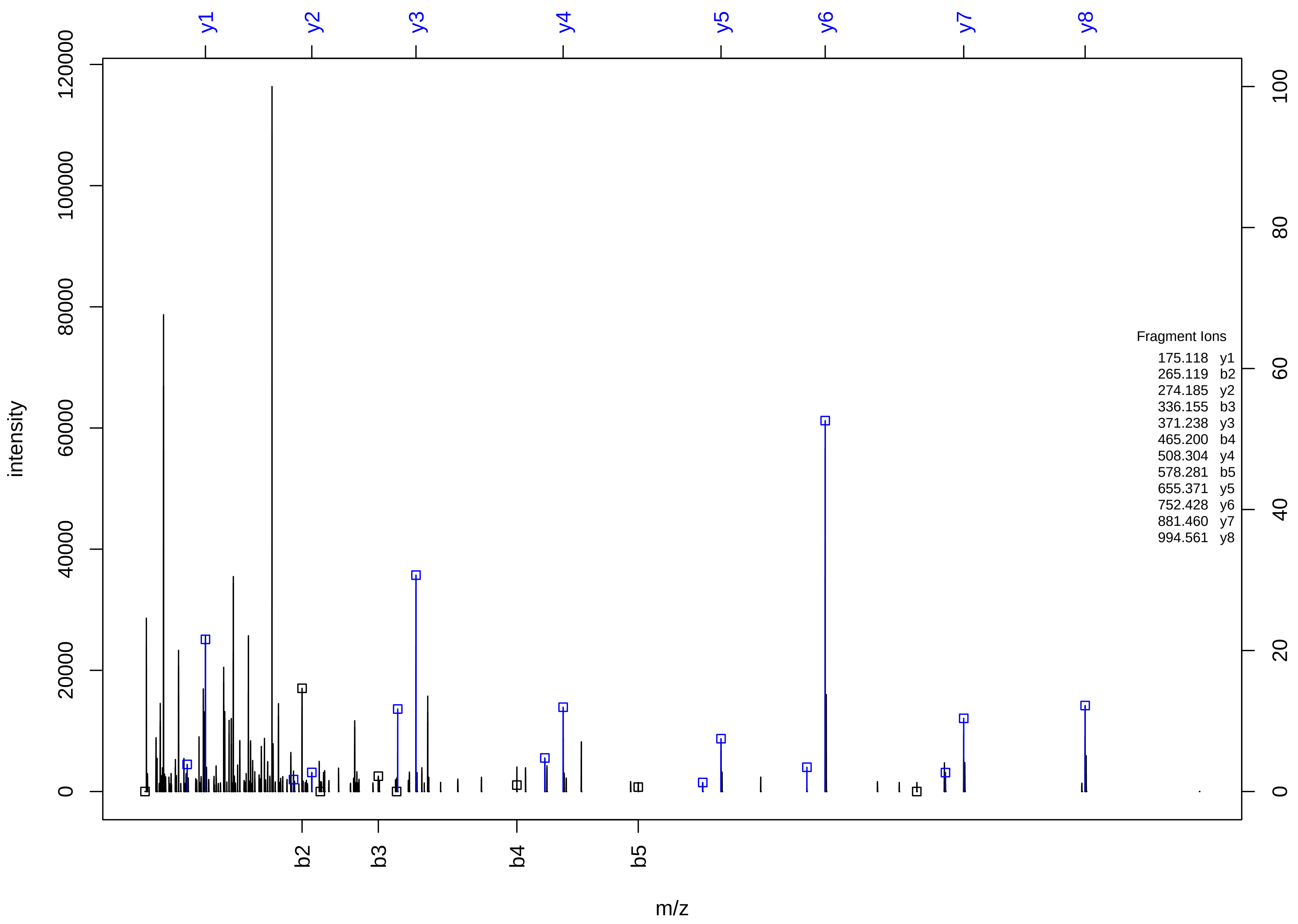

TYAEILEPFHPVR / File: sydney_12.raw; SpectrumID: ; scans: 7506

LNEIVGNEDTVSR

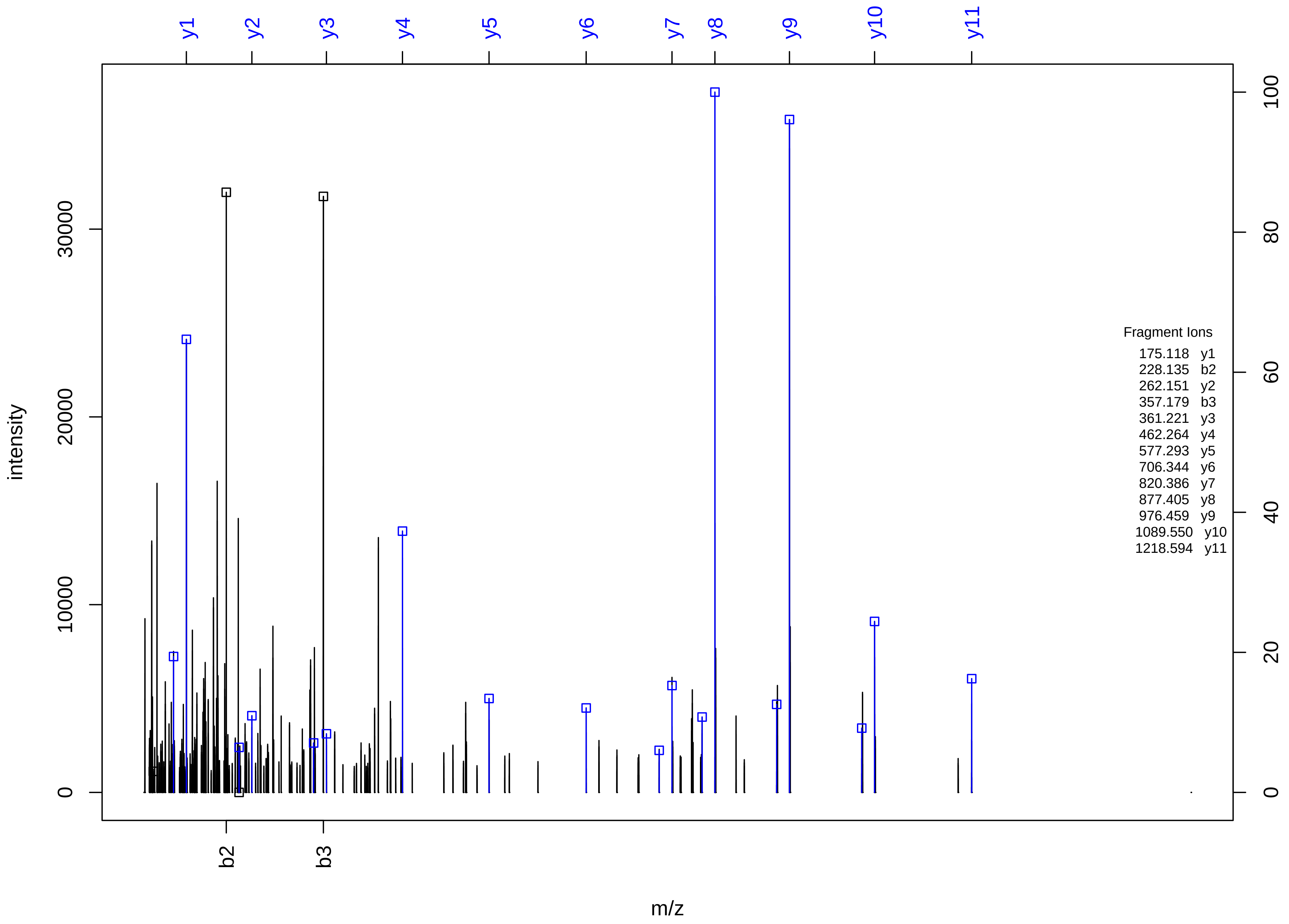

LNEIVGNEDTVSR / File: sydney_12.raw; SpectrumID: ; scans: 2300

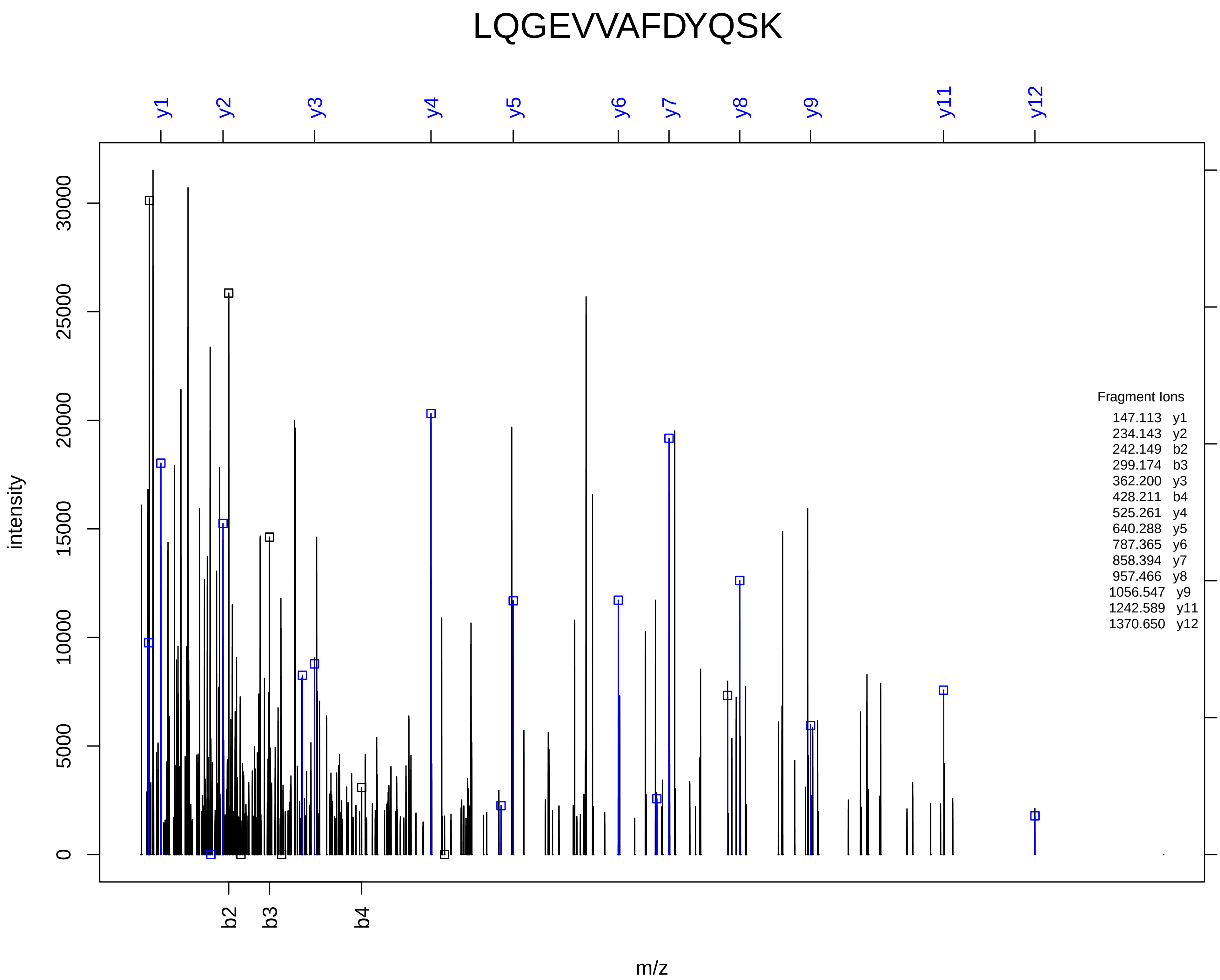

LQGEVVAFDYQSK / File: sydney_12.raw; SpectrumID: ; scans: 5265

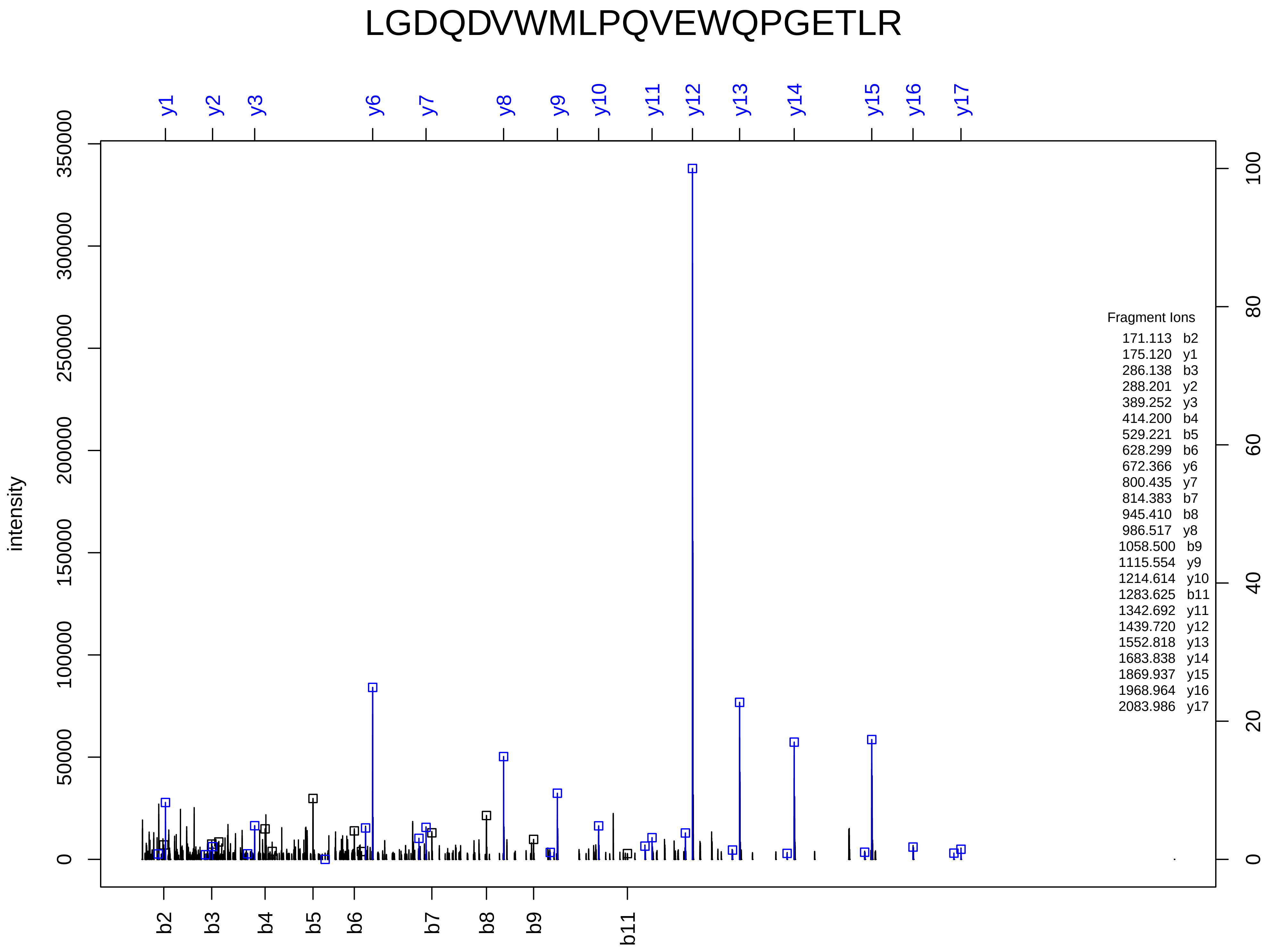

LGDQDVWMLPQVEWQPGETLR / File: sydney_12.raw; SpectrumID: ; scans: 14298

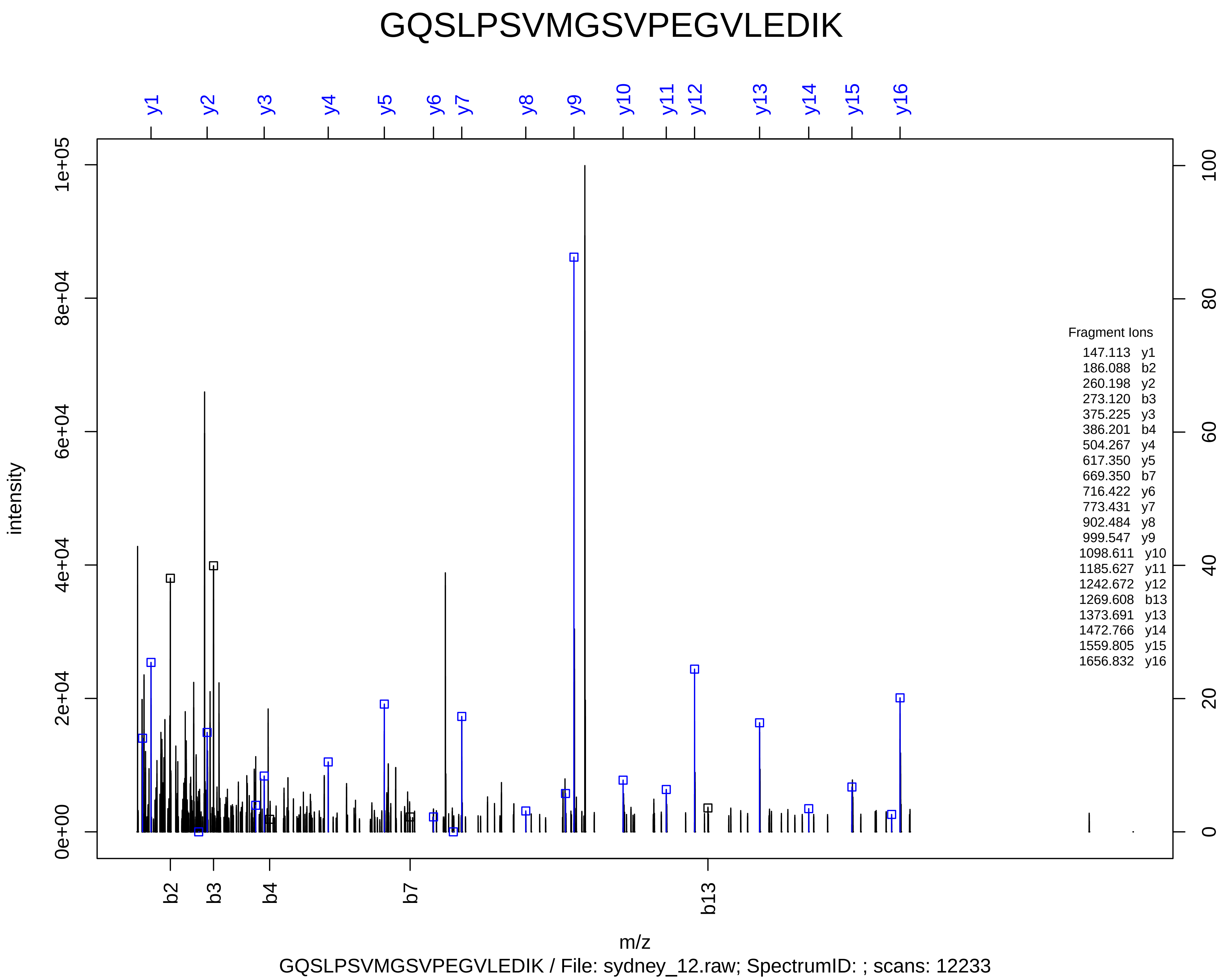
GQSLPSVMGSVPEGVLEDIK
y1
y2
y3
y4
y5
y6
y7
y8
y9
y10
y11
y12
y13
y14
y15
y16
intensity
0e+00
2e+04
4e+04
6e+04
8e+04
1e+05
0
20
40
60
80
100
b2
b3
b4
b7
b13
m/z
Fragment Ions
147.113 y1
186.088 b2
260.198 y2
273.120 b3
375.225 y3
386.201 b4
504.267 y4
617.350 y5
669.350 b7
716.422 y6
773.431 y7
902.484 y8
999.547 y9
1098.611 y10
1185.627 y11
1242.672 y12
1269.608 b13
1373.691 y13
1472.766 y14
1559.805 y15
1656.832 y16
GQSLPSVMGSVPEGVLEDIK / File: sydney_12.raw; SpectrumID: ; scans: 12233

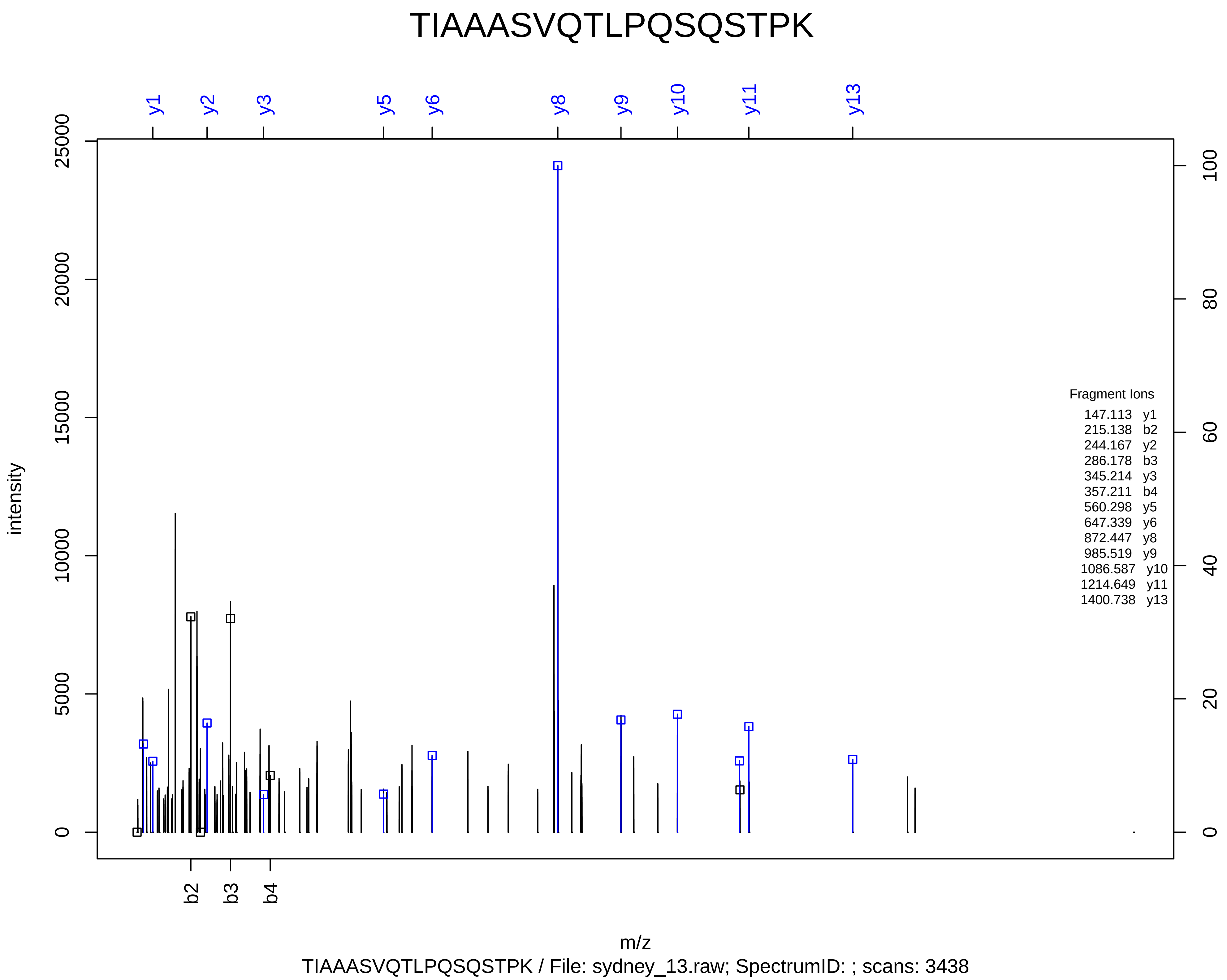
TIAAASVQTLPQSQSTPK
y1
y2
y3
y5
y6
y8
y9
y10
y11
y13
b2
b3
b4
intensity
m/z
Fragment Ions
147.113 y1
215.138 b2
244.167 y2
286.178 b3
345.214 y3
357.211 b4
560.298 y5
647.339 y6
872.447 y8
985.519 y9
1086.587 y10
1214.649 y11
1400.738 y13
TIAAASVQTLPQSQSTPK / File: sydney_13.raw; SpectrumID: ; scans: 3438

FAEDSAEAAVSVPESR

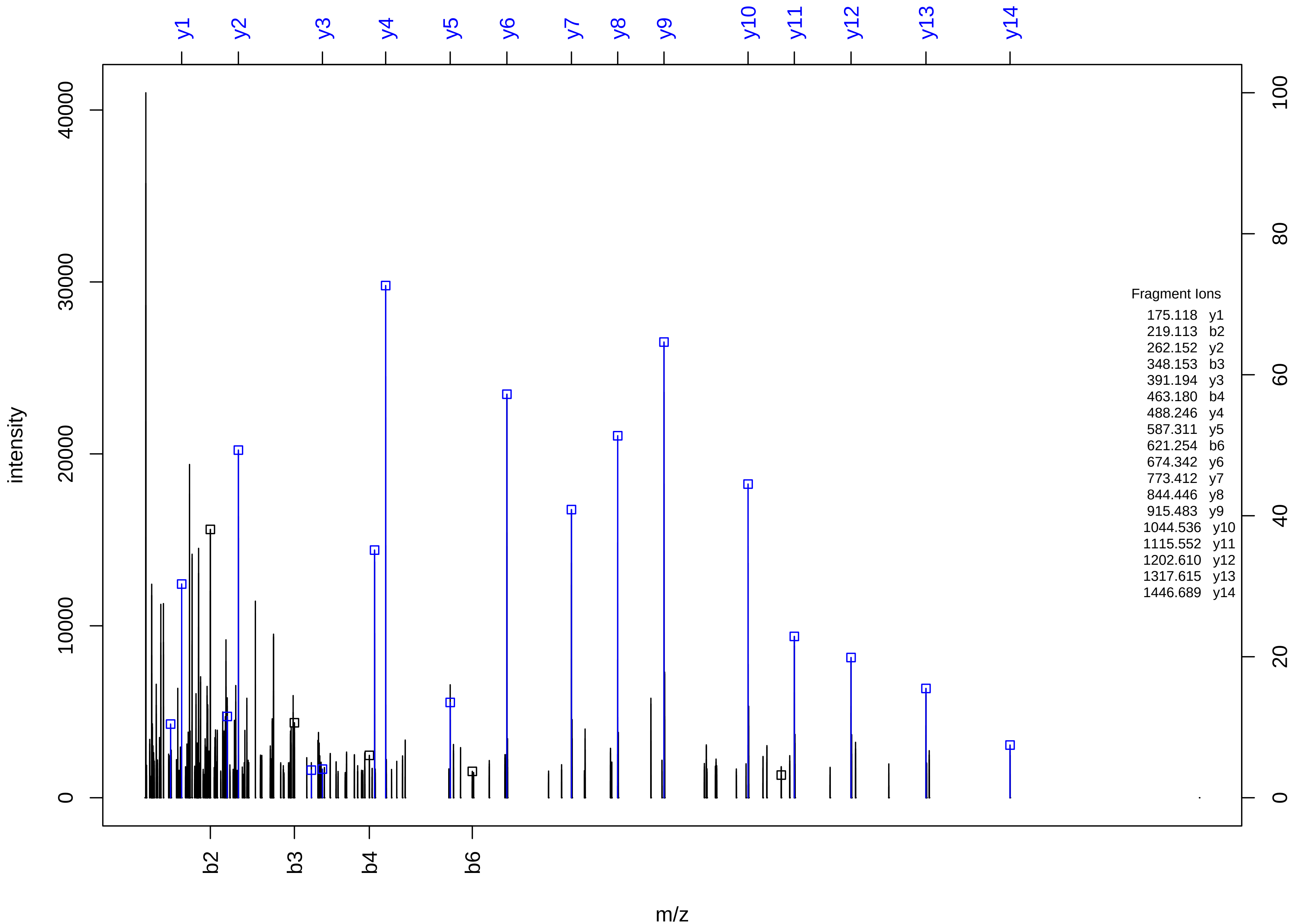

FAEDSAEAAVSVPESR / File: sydney_13.raw; SpectrumID: ; scans: 3959

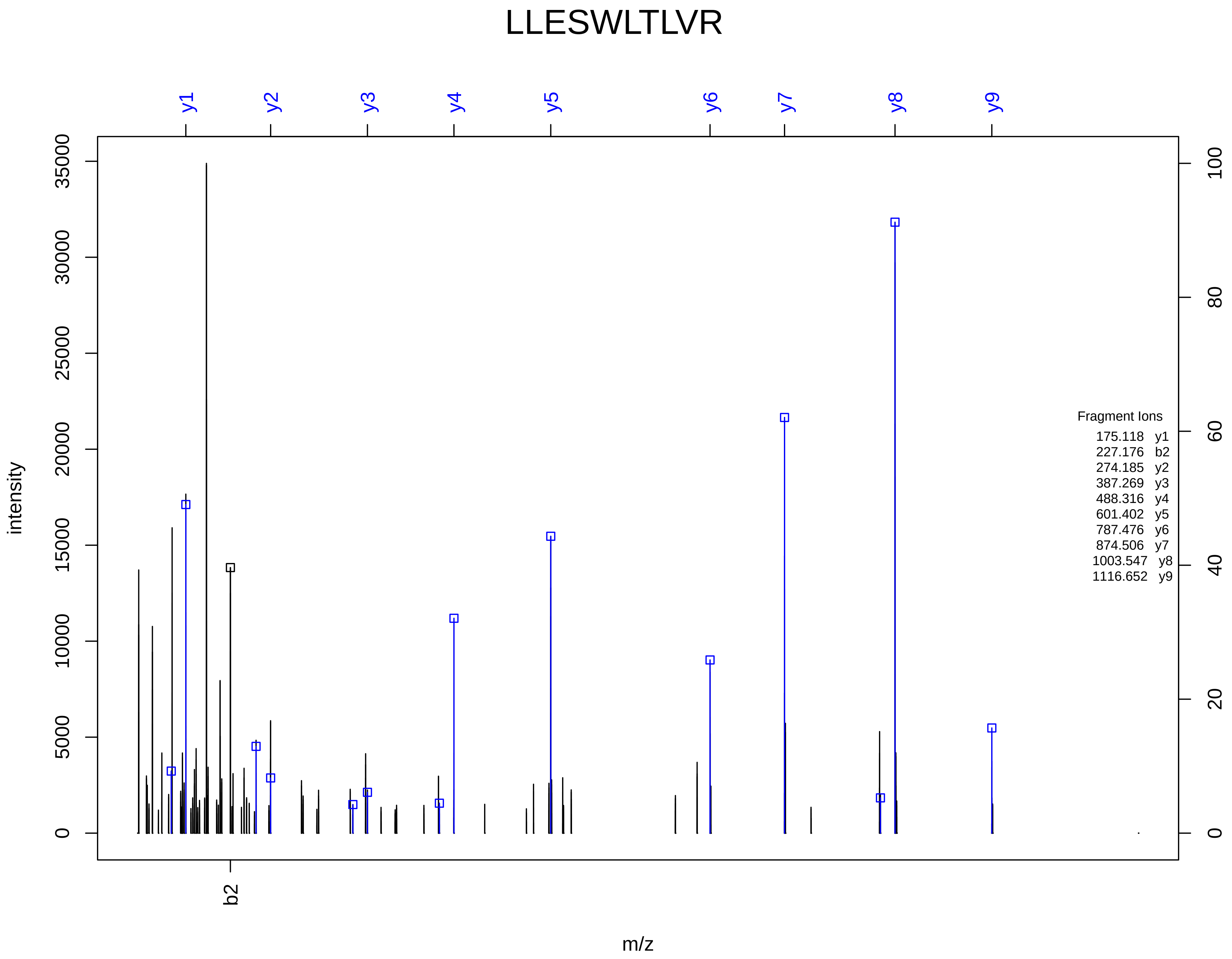

LLESWLTLVR / File: sydney_13.raw; SpectrumID: ; scans: 13238

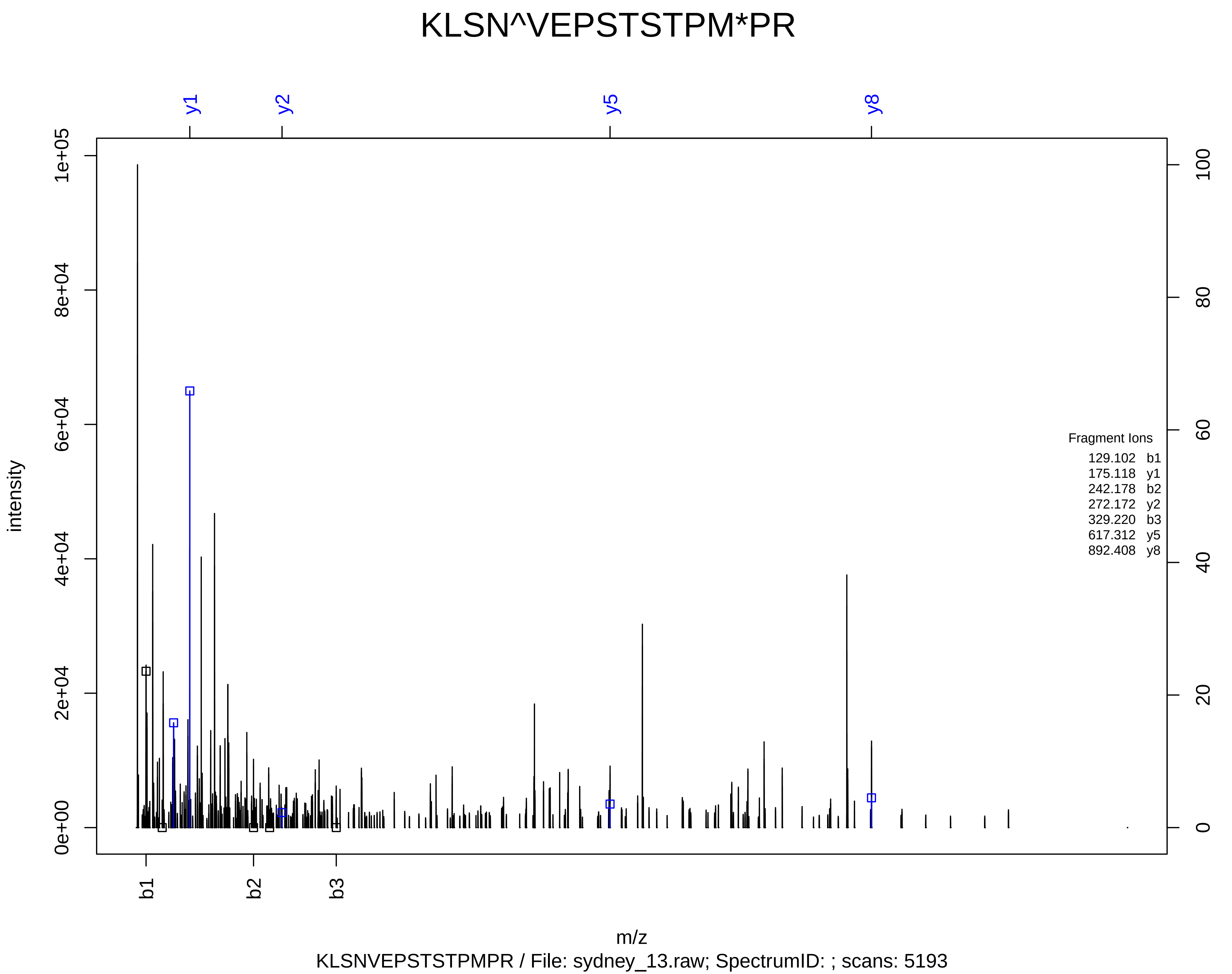

KLSN^VEPSTSTPM*PR
y1
y2
y5
y8
intensity
1e+05
8e+04
6e+04
4e+04
2e+04
0e+00
100
80
60
40
20
0
Fragment Ions
129.102 b1
175.118 y1
242.178 b2
272.172 y2
329.220 b3
617.312 y5
892.408 y8
b1
b2
b3
m/z
KLSNVEPSTSTPMPR / File: sydney_13.raw; SpectrumID: ; scans: 5193

(Ac)SDEFSLADALPEQSSAKPPAVTNTK

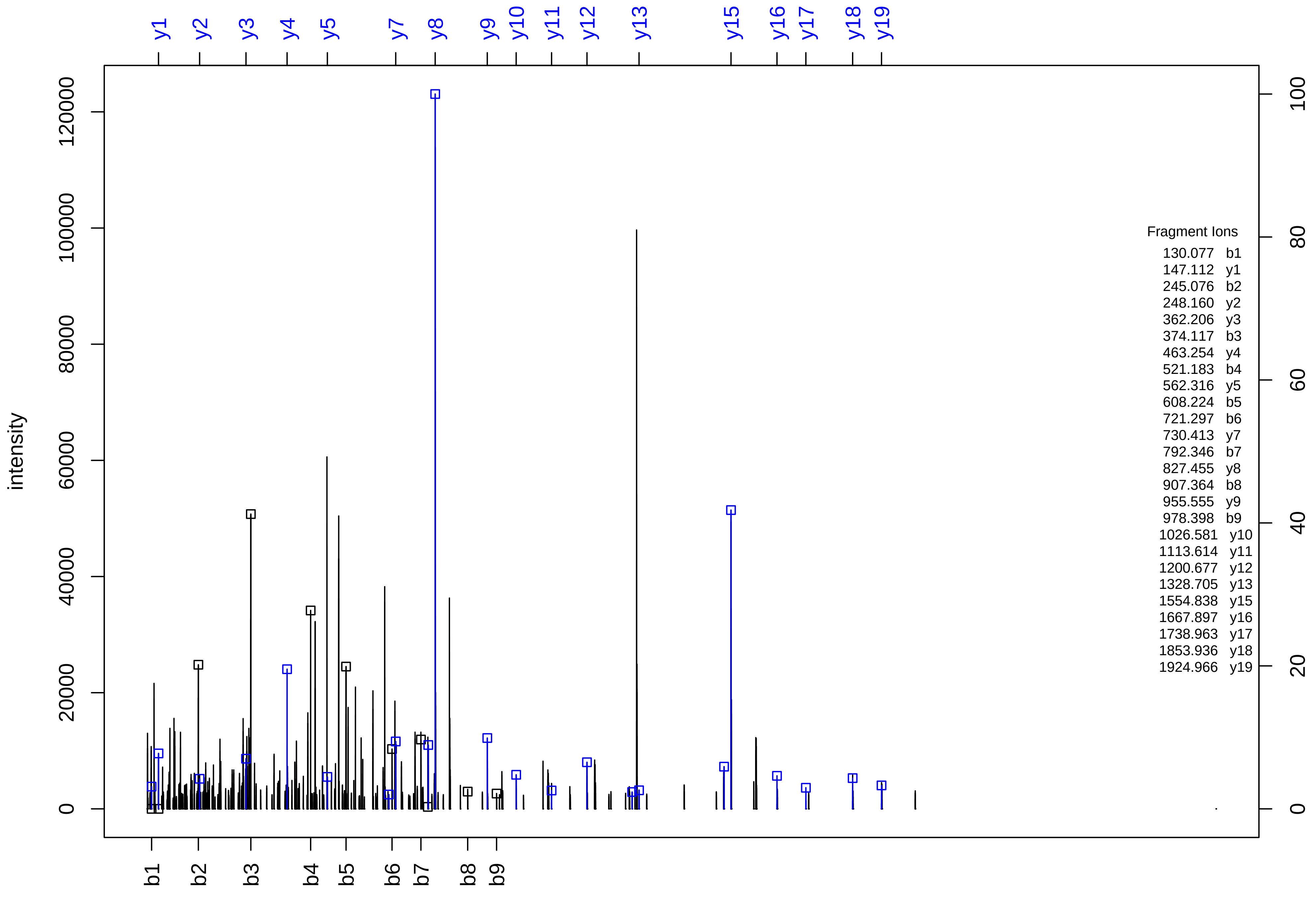

SDEFSLADALPEQSSAKPPAVTNTK / File: sydney_13.raw; SpectrumID: ; scans: 9634

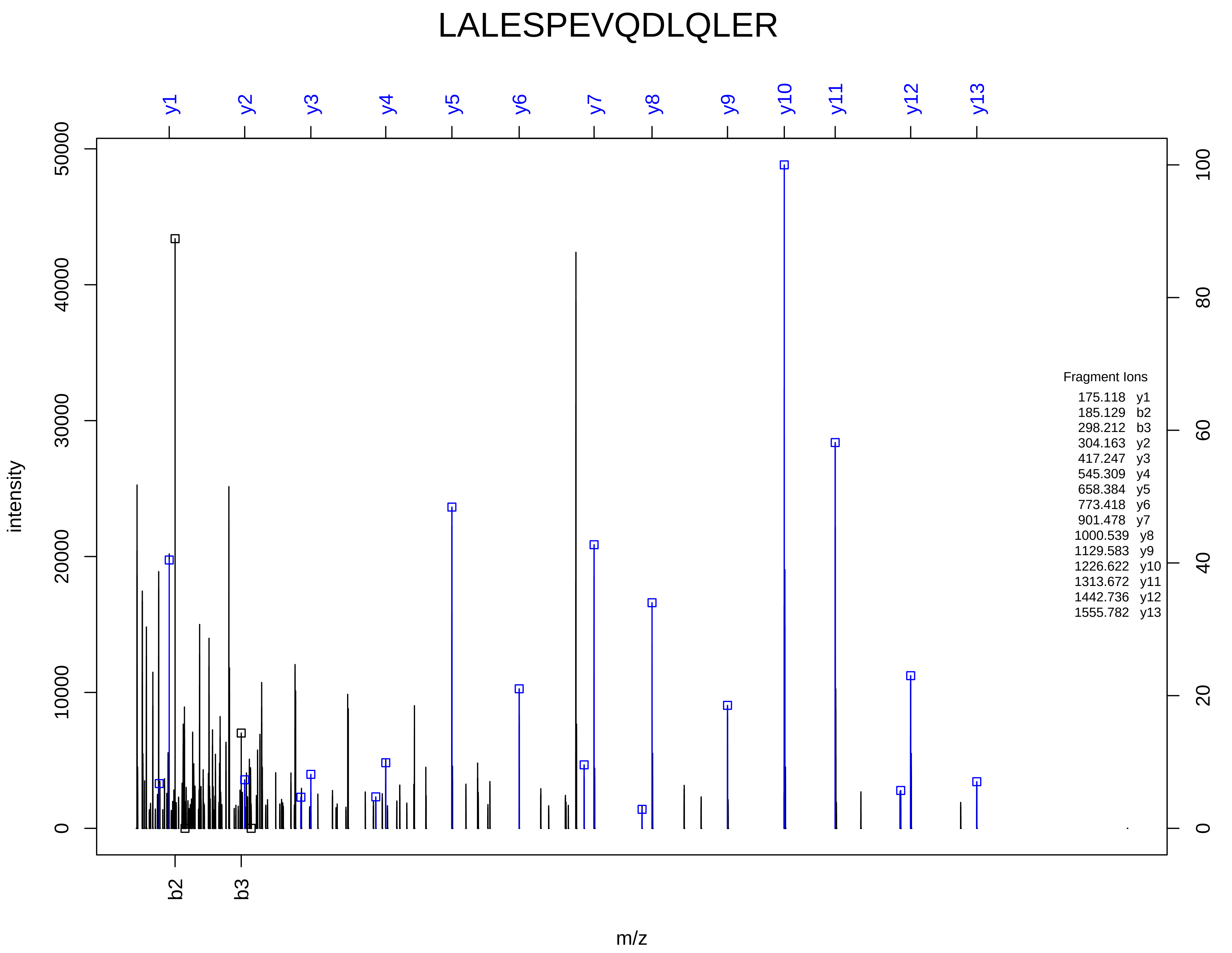
LALESPEVQDLQLER
y1
y2
y3
y4
y5
y6
y7
y8
y9
y10
y11
y12
y13
b2
b3
intensity
0
10000
20000
30000
40000
50000
0
20
40
60
80
100
Fragment Ions
175.118 y1
185.129 b2
298.212 b3
304.163 y2
417.247 y3
545.309 y4
658.384 y5
773.418 y6
901.478 y7
1000.539 y8
1129.583 y9
1226.622 y10
1313.672 y11
1442.736 y12
1555.782 y13
m/z
LALESPEVQDLQLER / File: sydney_13.raw; SpectrumID: ; scans: 8609

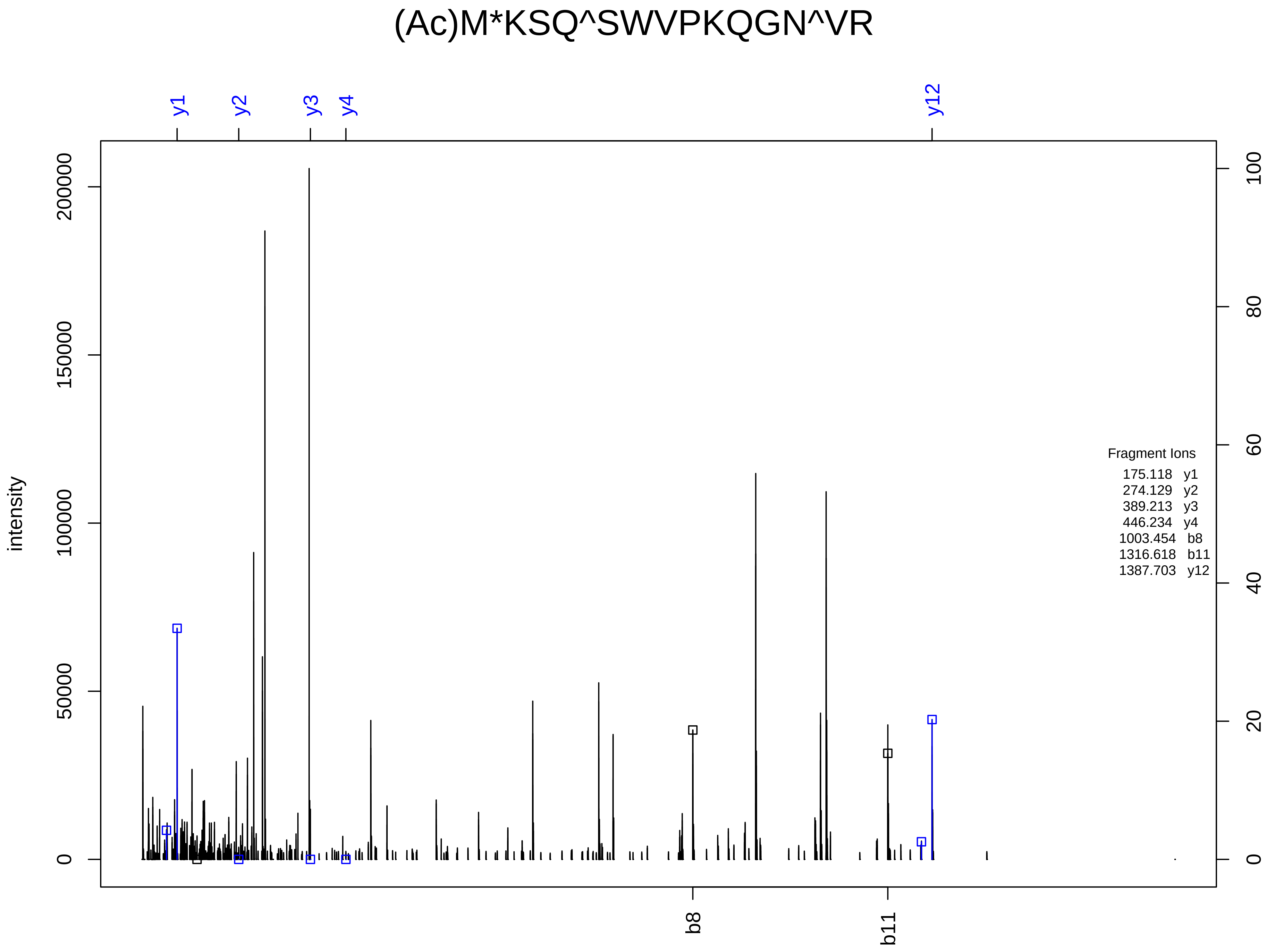

MKSQSWVPKQGNVR / File: sydney_13.raw; SpectrumID: ; scans: 8181

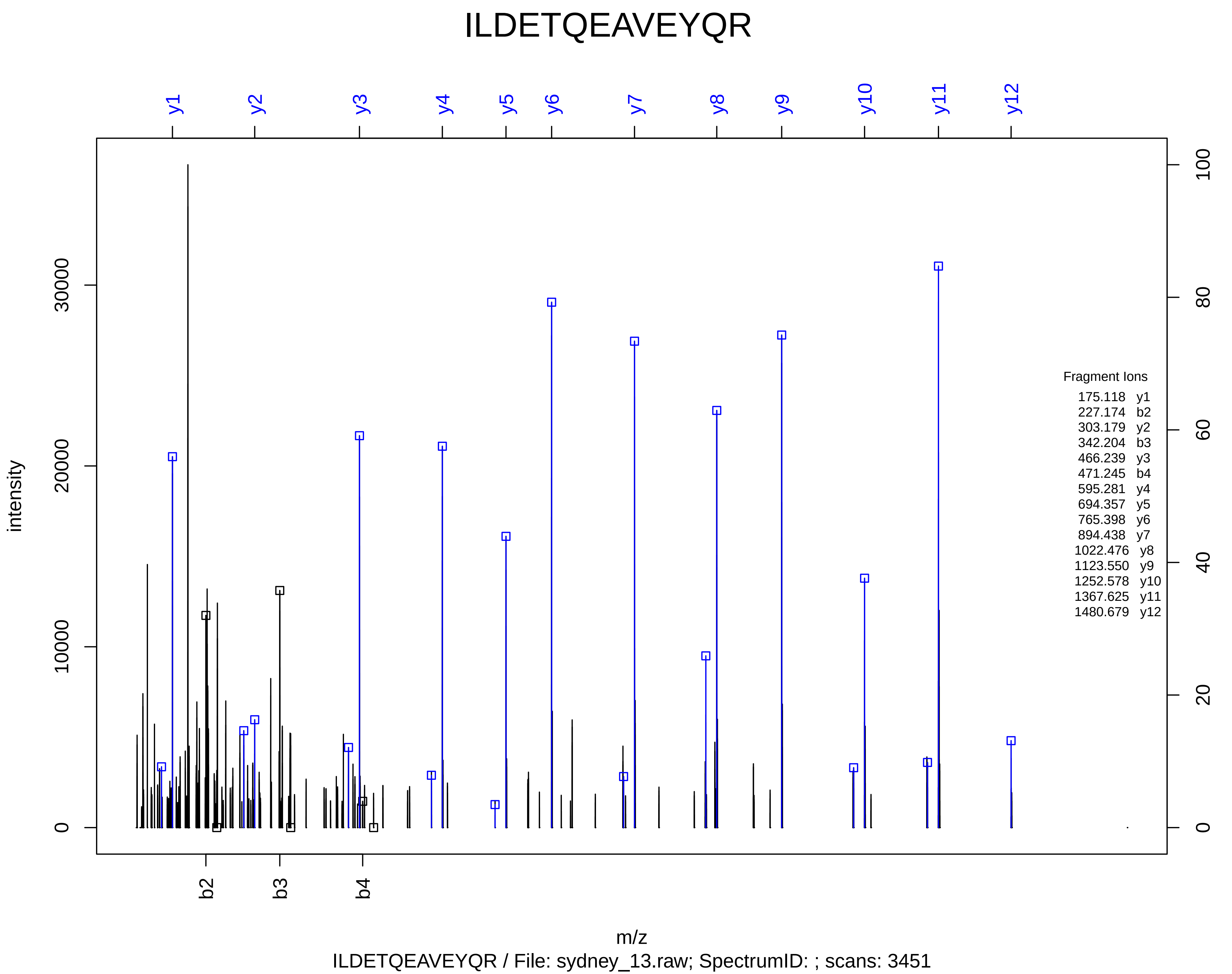
ILDETQEAVEYQR
y1 y2 y3 y4 y5 y6 y7 y8 y9 y10 y11 y12
b2 b3 b4
intensity
0
10000
20000
30000
0
20
40
60
80
100
Fragment Ions
175.118 y1
227.174 b2
303.179 y2
342.204 b3
466.239 y3
471.245 b4
595.281 y4
694.357 y5
765.398 y6
894.438 y7
1022.476 y8
1123.550 y9
1252.578 y10
1367.625 y11
1480.679 y12
m/z
ILDETQEAVEYQR / File: sydney_13.raw; SpectrumID: ; scans: 3451

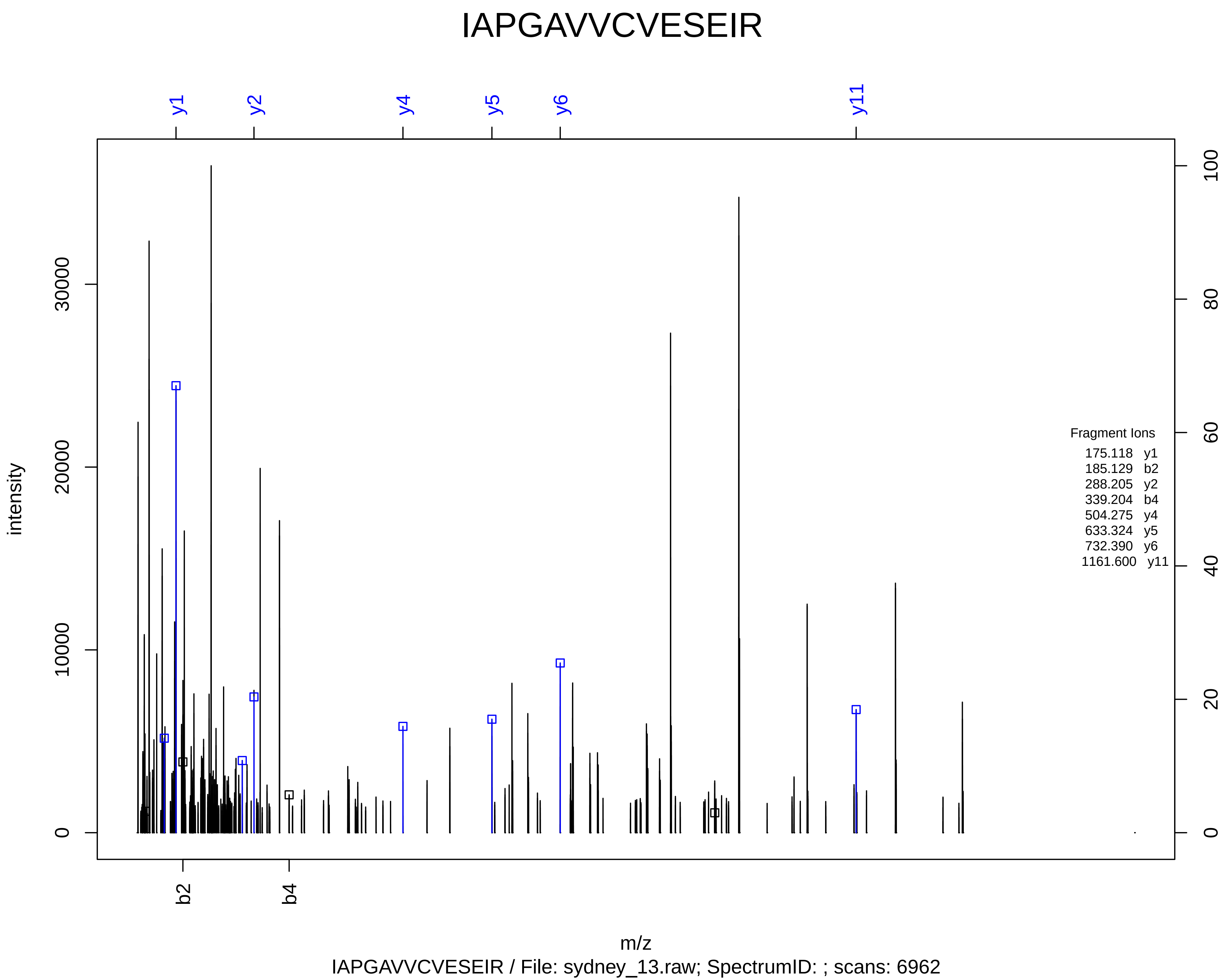
IAPGAVVCVESEIR
y1
y2
y4
y5
y6
y11
intensity
0
10000
20000
30000
0
20
40
60
80
100
b2
b4
m/z
Fragment Ions
175.118 y1
185.129 b2
288.205 y2
339.204 b4
504.275 y4
633.324 y5
732.390 y6
1161.600 y11
IAPGAVVCVESEIR / File: sydney_13.raw; SpectrumID: ; scans: 6962

LTEELTHTENEN^KK

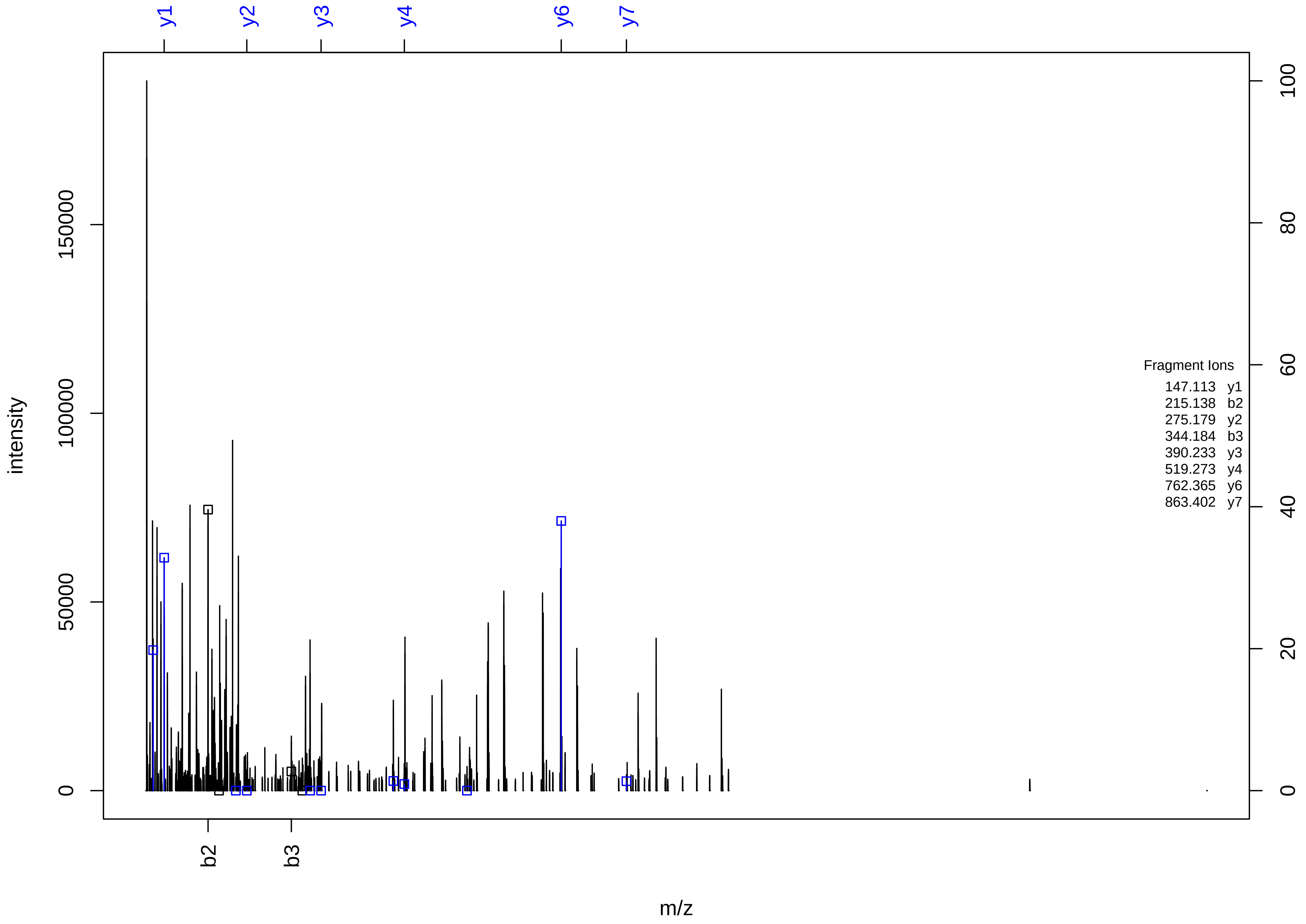

LTEELTHTENENKK / File: sydney_13.raw; SpectrumID: ; scans: 4816

EAEILEVLR

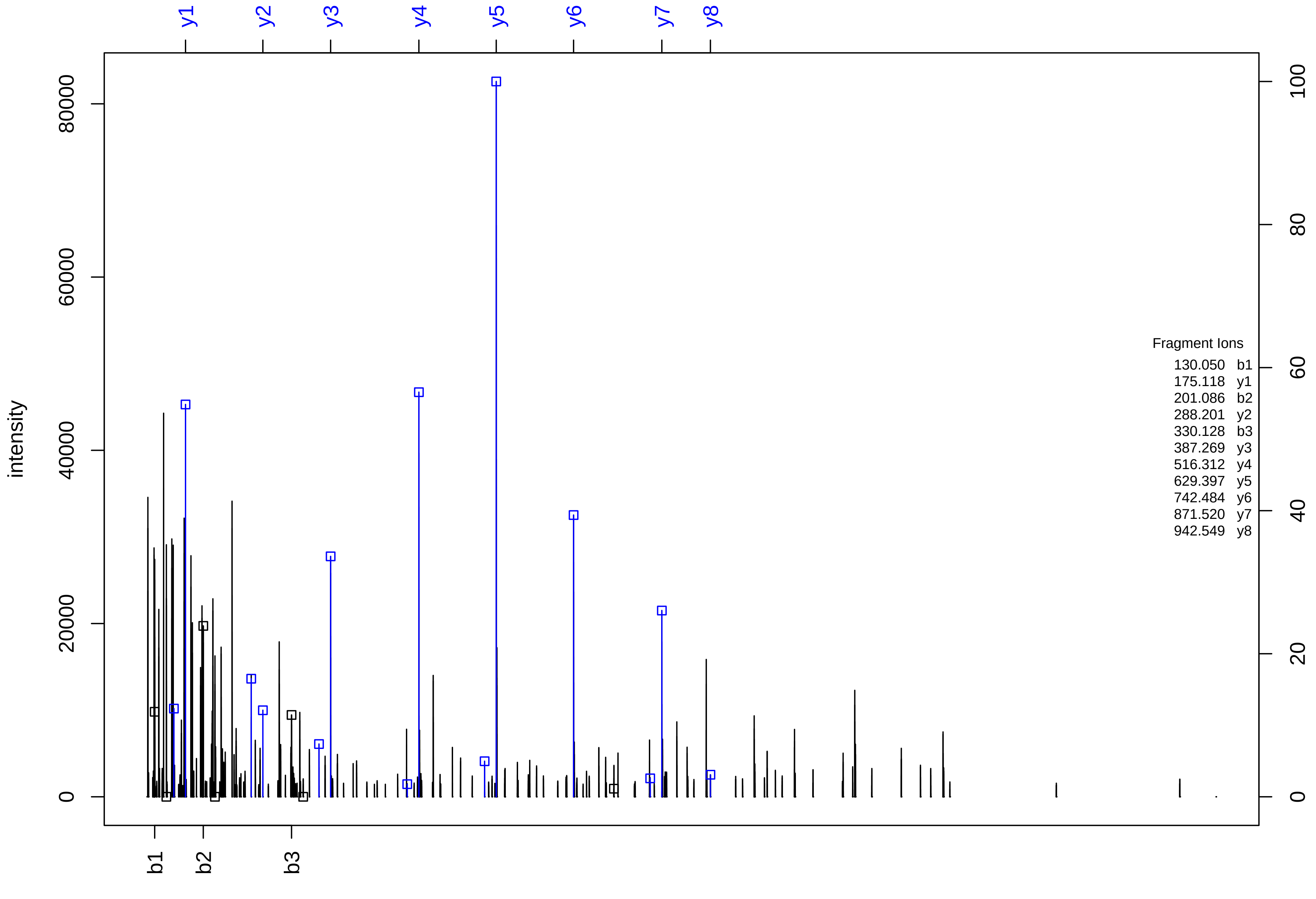

EAEILEVLR / File: sydney_13.raw; SpectrumID: ; scans: 9001

LQELGLLPLLTDEGSARPT

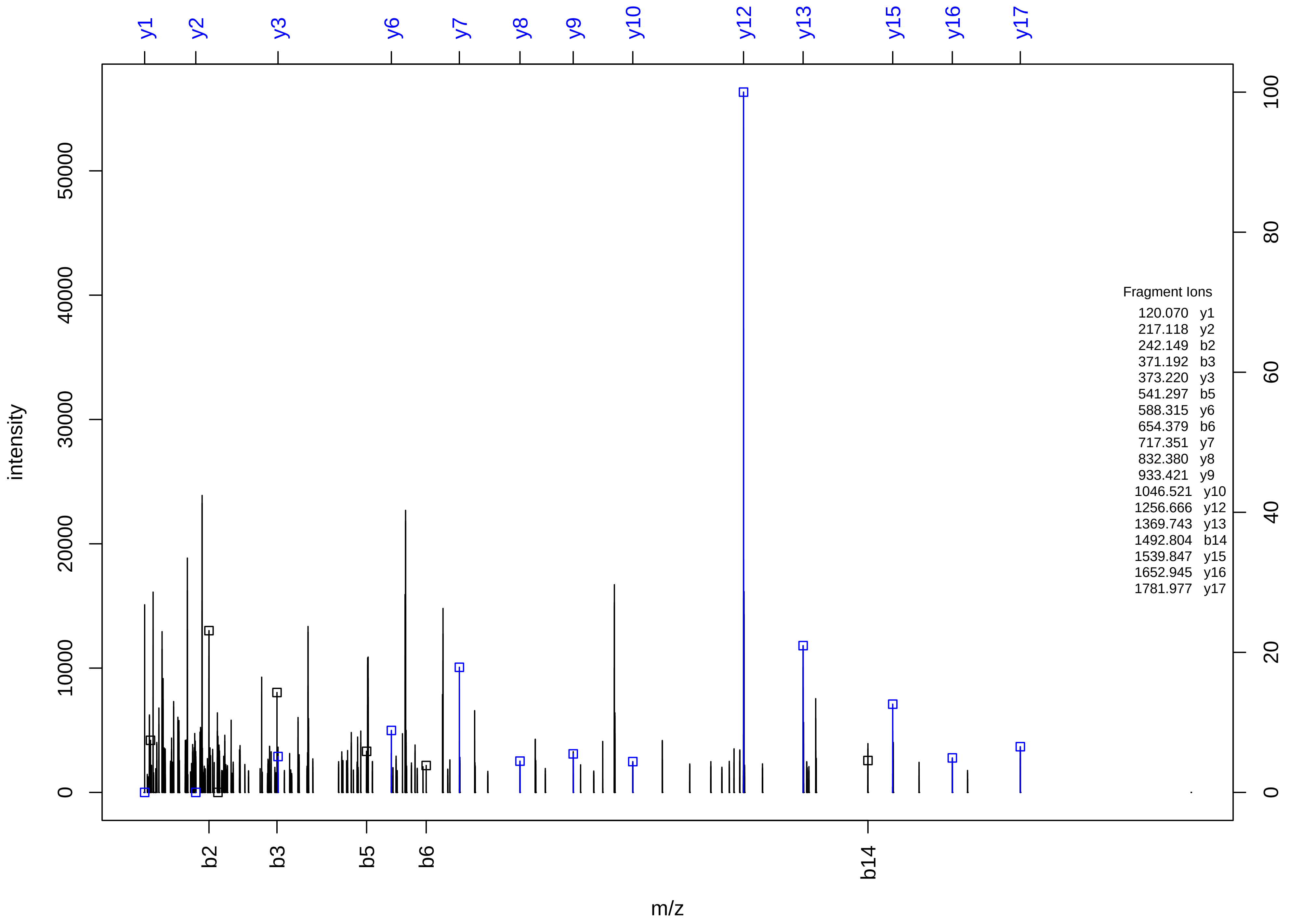

LQELGLLPLLTDEGSARPT / File: sydney_13.raw; SpectrumID: ; scans: 14328

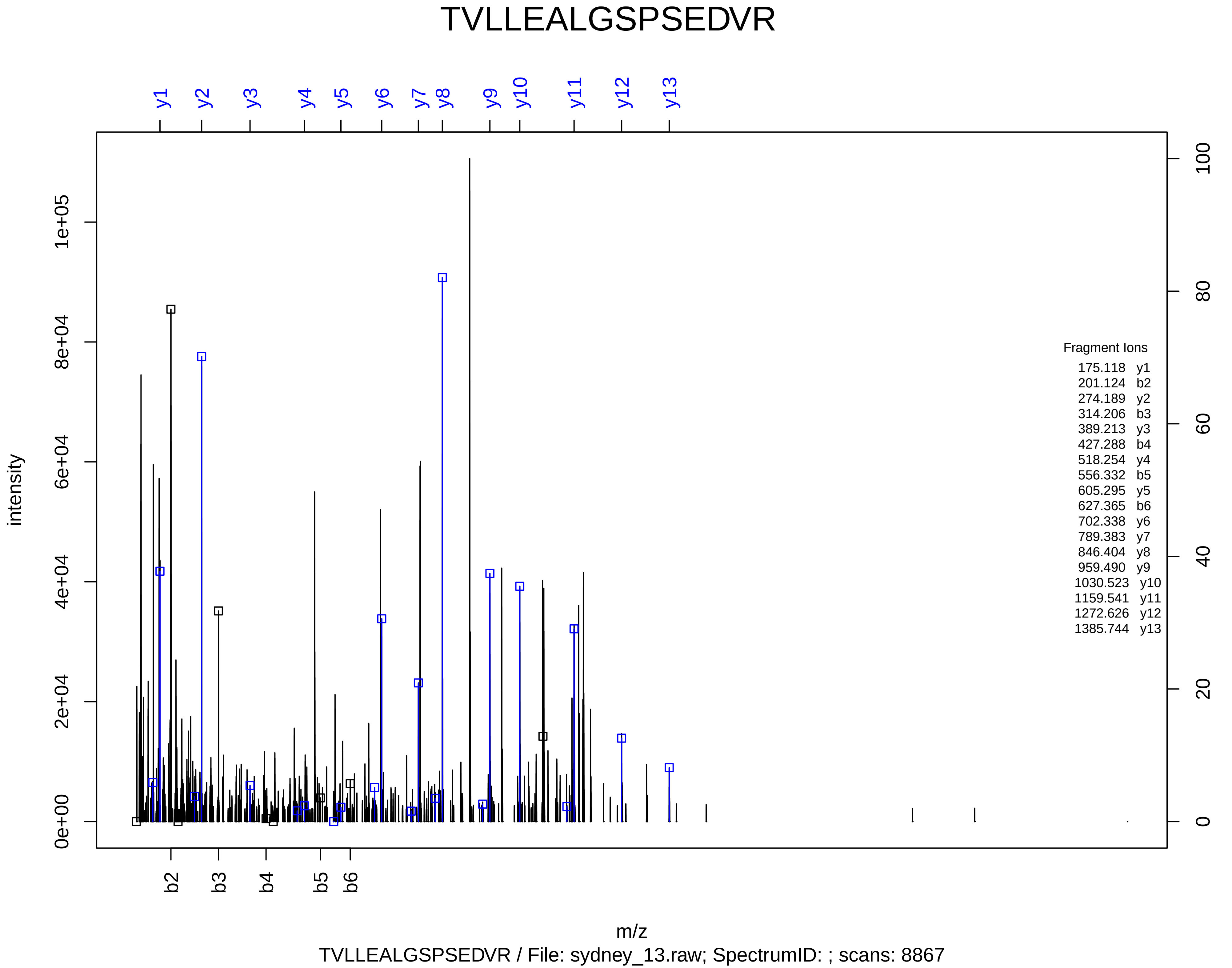
TVLLEALGSPSEDVR
y1 y2 y3 y4 y5 y6 y7 y8 y9 y10 y11 y12 y13
b2 b3 b4 b5 b6
intensity
m/z
Fragment Ions
175.118 y1
201.124 b2
274.189 y2
314.206 b3
389.213 y3
427.288 b4
518.254 y4
556.332 b5
605.295 y5
627.365 b6
702.338 y6
789.383 y7
846.404 y8
959.490 y9
1030.523 y10
1159.541 y11
1272.626 y12
1385.744 y13
0e+00
2e+04
4e+04
6e+04
8e+04
1e+05
0
20
40
60
80
100
TVLLEALGSPSEDVR / File: sydney_13.raw; SpectrumID: ; scans: 8867

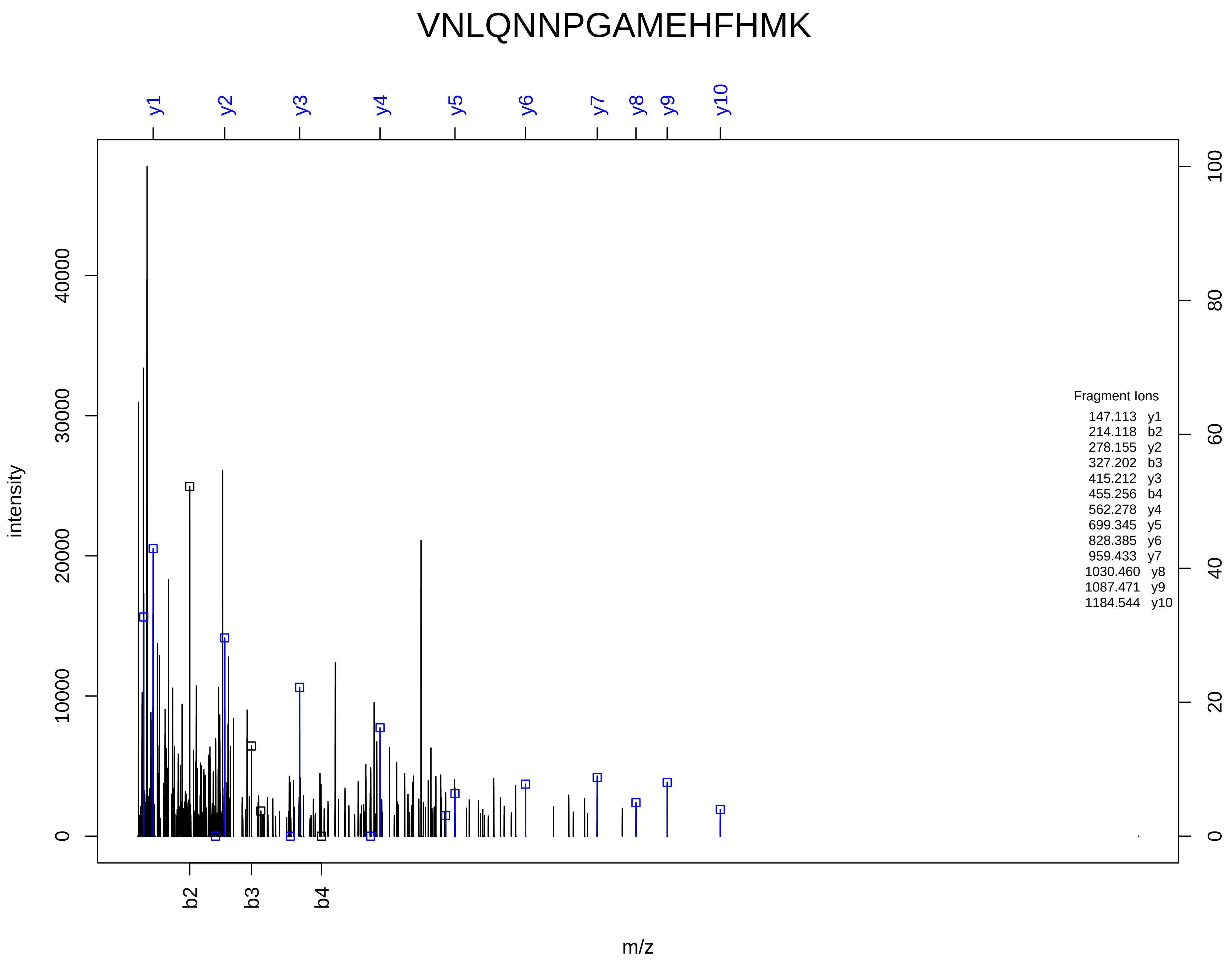

VNLQNNPGAMEHFHMK / File: sydney_13.raw; SpectrumID: ; scans: 3146

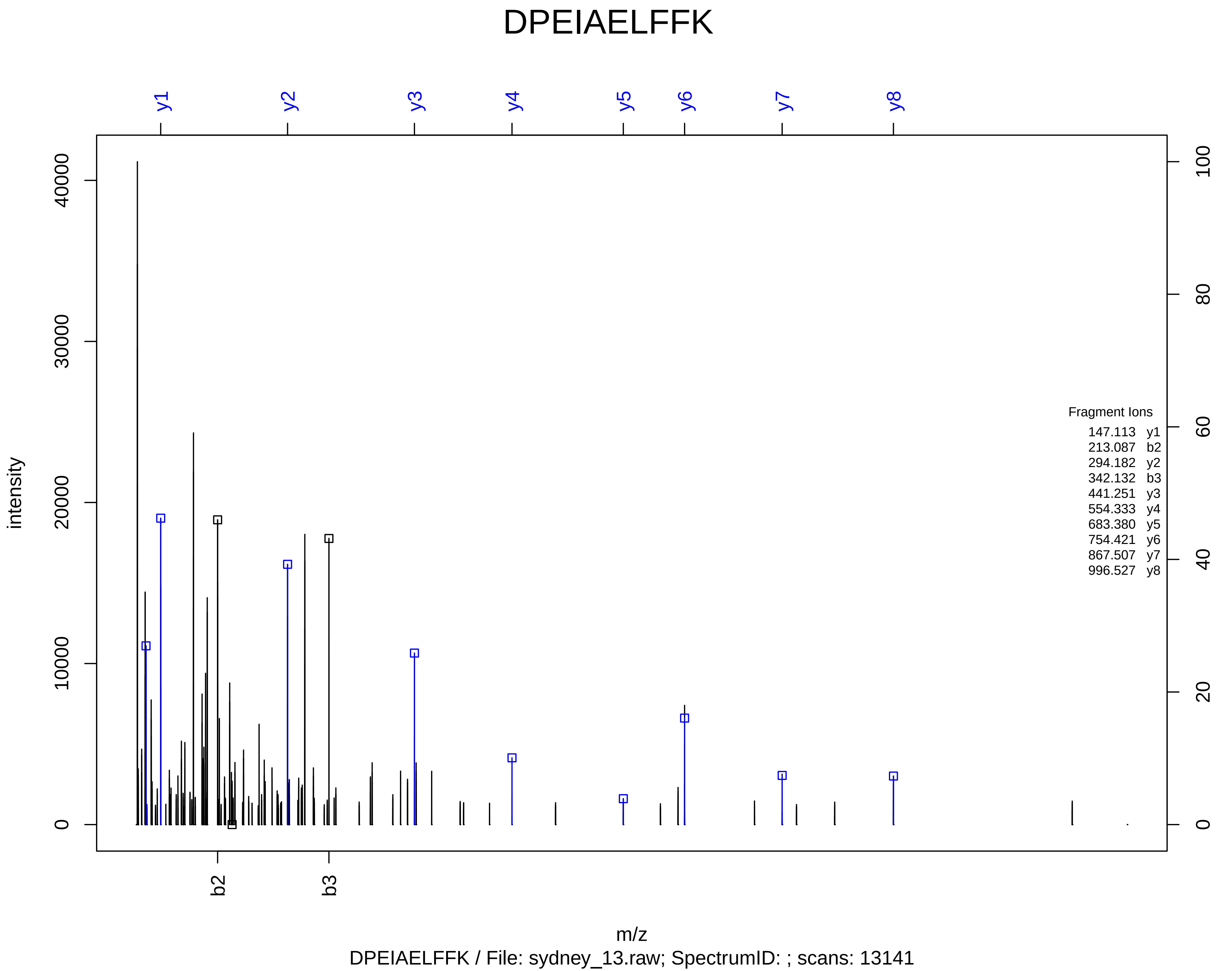
DPEIAELFFK
y1
y2
y3
y4
y5
y6
y7
y8
intensity
0
10000
20000
30000
40000
0
20
40
60
80
100
Fragment Ions
147.113 y1
213.087 b2
294.182 y2
342.132 b3
441.251 y3
554.333 y4
683.380 y5
754.421 y6
867.507 y7
996.527 y8
b2
b3
m/z
DPEIAELFFK / File: sydney_13.raw; SpectrumID: ; scans: 13141

VESTTTPDGPCVVSVQETEK

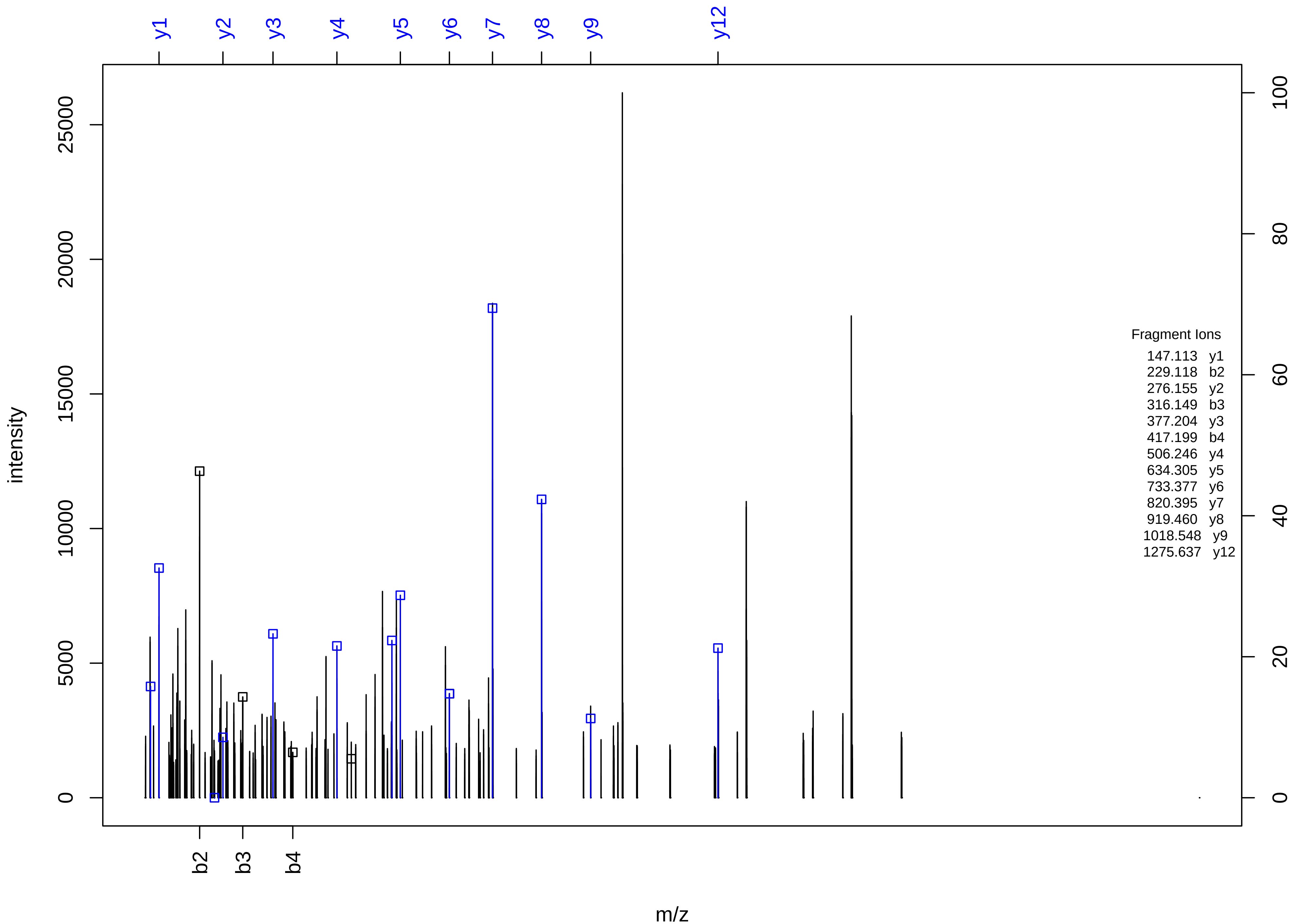

VESTTTPDGPCVVSVQETEK / File: sydney_13.raw; SpectrumID: ; scans: 3523

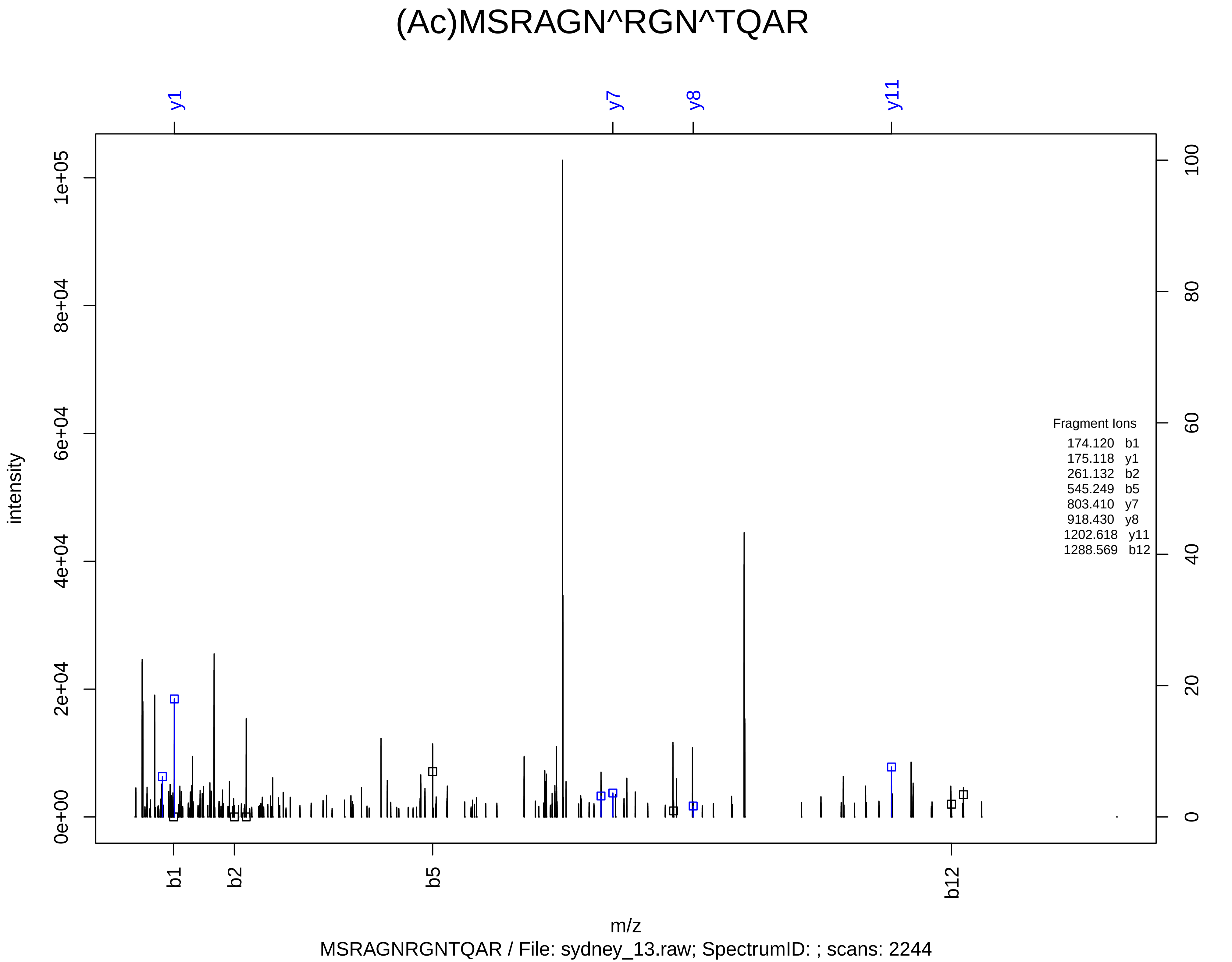
(Ac)MSRAGN^RGN^TQAR
y1
y7
y8
y11
intensity
0e+00
2e+04
4e+04
6e+04
8e+04
1e+05
0
20
40
60
80
100
Fragment Ions
174.120 b1
175.118 y1
261.132 b2
545.249 b5
803.410 y7
918.430 y8
1202.618 y11
1288.569 b12
b1
b2
b5
b12
m/z
MSRAGNRGNTQAR / File: sydney_13.raw; SpectrumID: ; scans: 2244

VQTPPAVDPAEFFVLTER

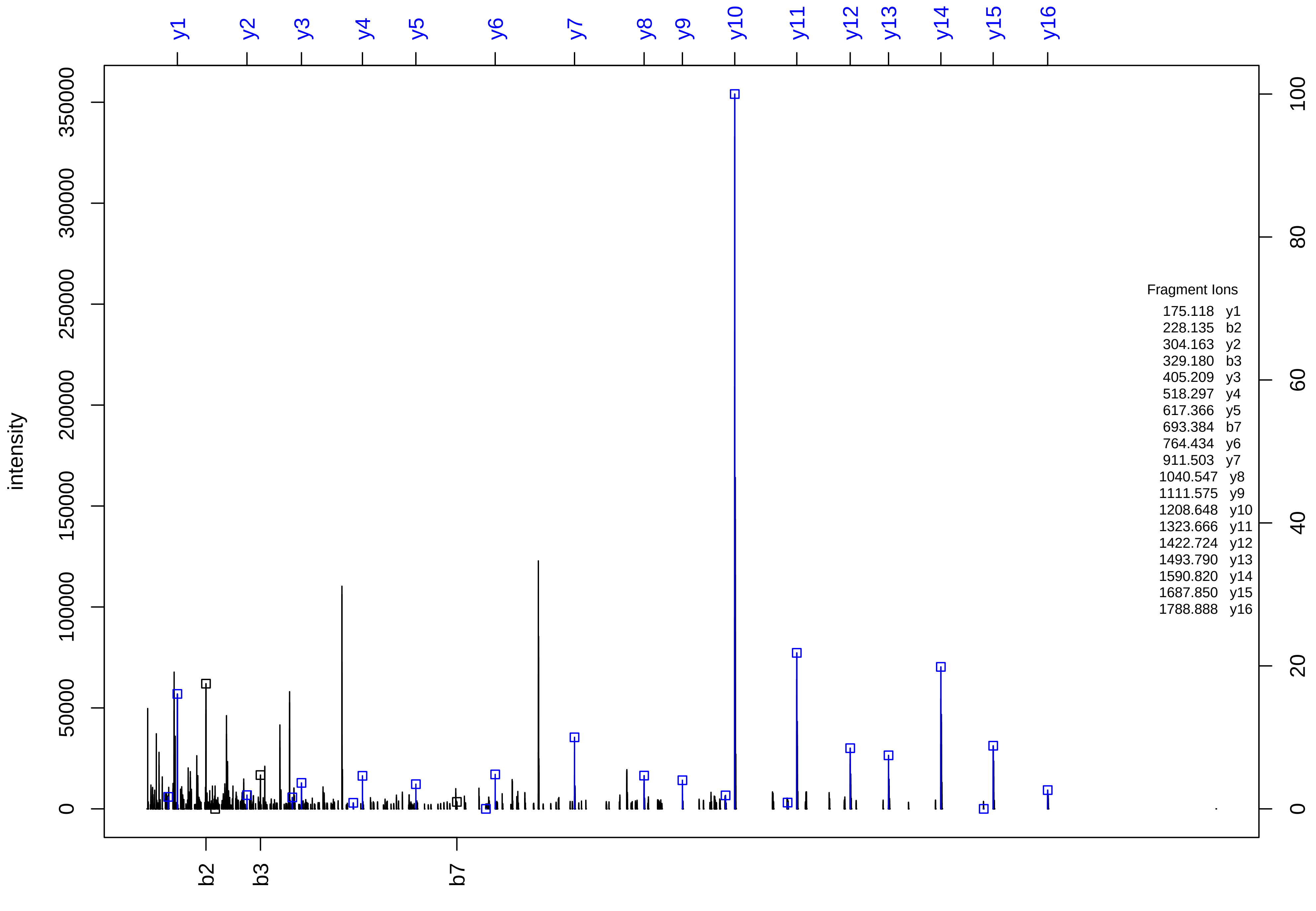

VQTPPAVDPAEFFVLTER / File: sydney_13.raw; SpectrumID: ; scans: 14284

FLCLEFGQVNDPLISR

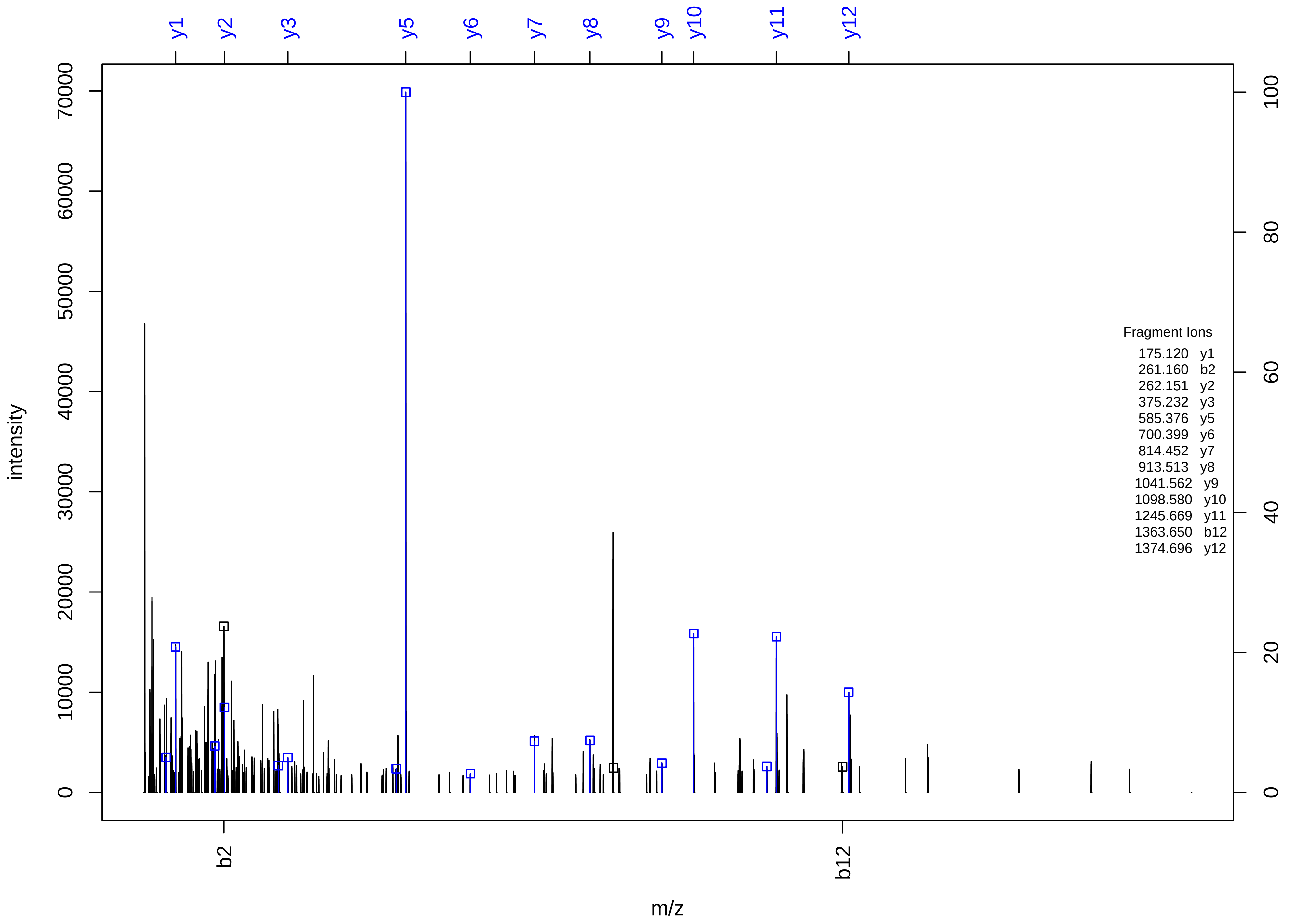

FLCLEFGQVNDPLISR / File: sydney_13.raw; SpectrumID: ; scans: 13281

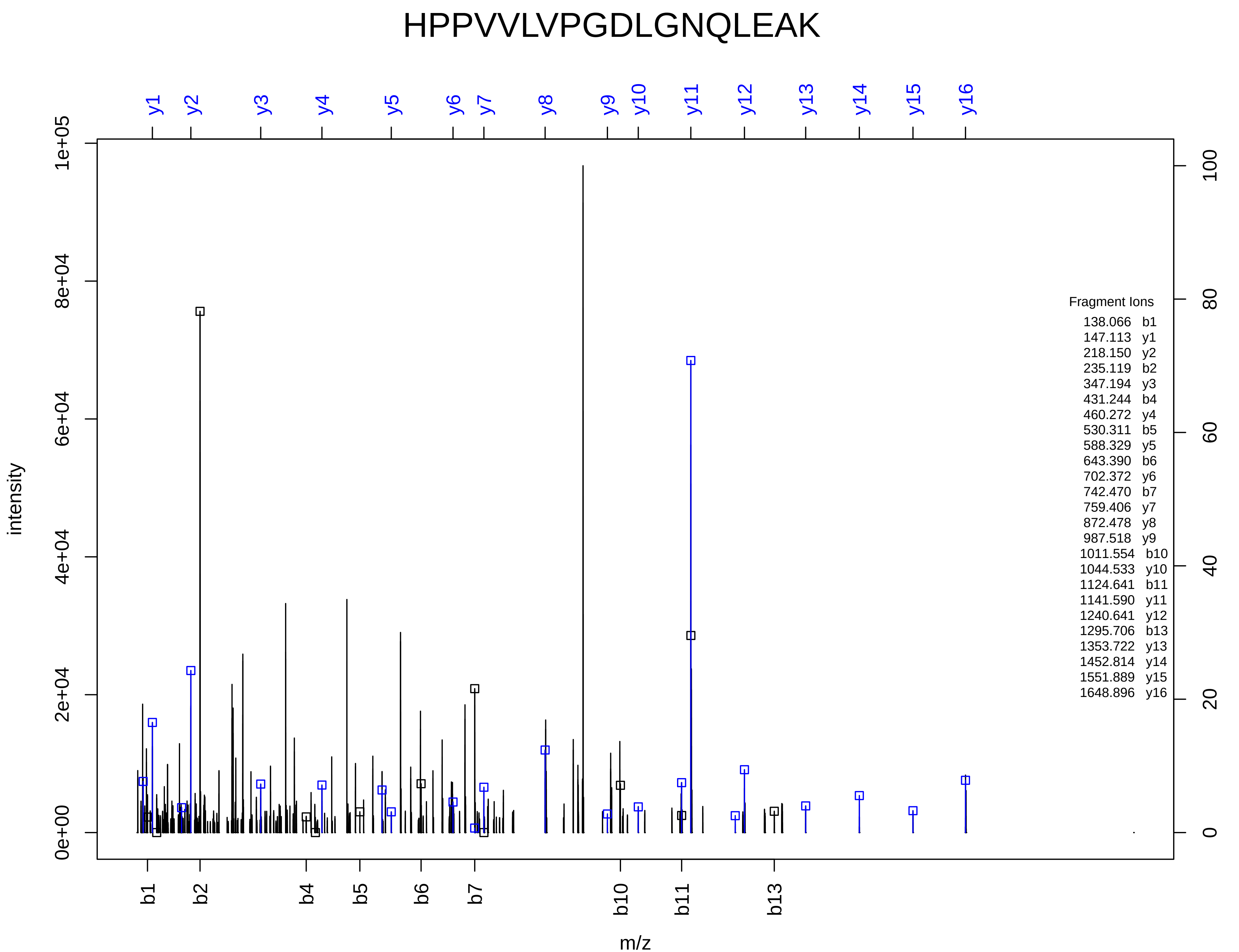

HPPVVLVPGDLGNQLEAK / File: sydney_14.raw; SpectrumID: ; scans: 8418

APVSALPLAPNNLGGGPIILGAESR

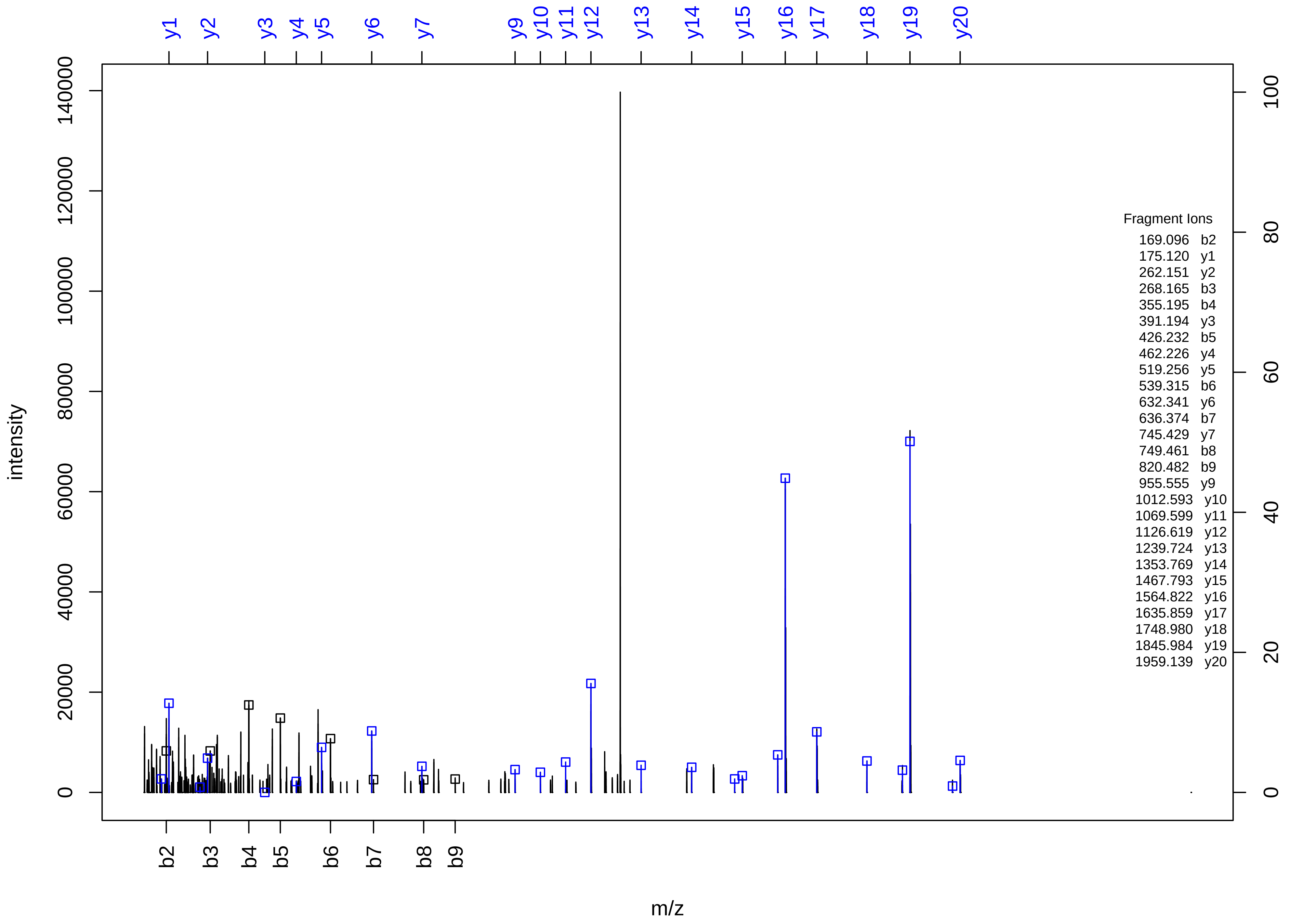

APVSALPLAPNNLGGGPIILGAESR / File: sydney_14.raw; SpectrumID: ; scans: 13576

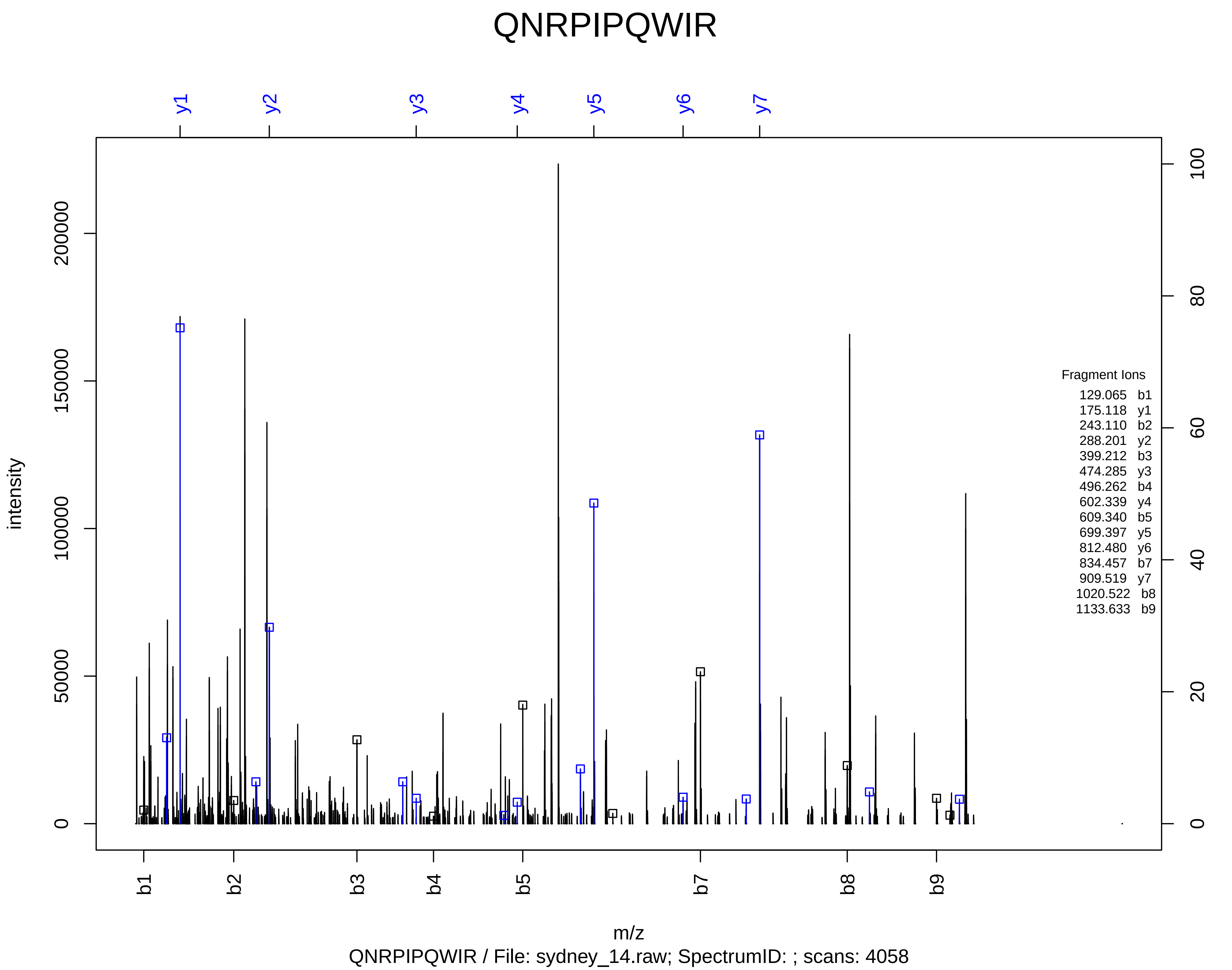
QNRPIPQWIR
y1
y2
y3
y4
y5
y6
y7
intensity
0
50000
100000
150000
200000
0
20
40
60
80
100
Fragment Ions
129.065 b1
175.118 y1
243.110 b2
288.201 y2
399.212 b3
474.285 y3
496.262 b4
602.339 y4
609.340 b5
699.397 y5
812.480 y6
834.457 b7
909.519 y7
1020.522 b8
1133.633 b9
b1
b2
b3
b4
b5
b7
b8
b9
m/z
QNRPIPQWIR / File: sydney_14.raw; SpectrumID: ; scans: 4058

DFMIQGGDPTGTGR

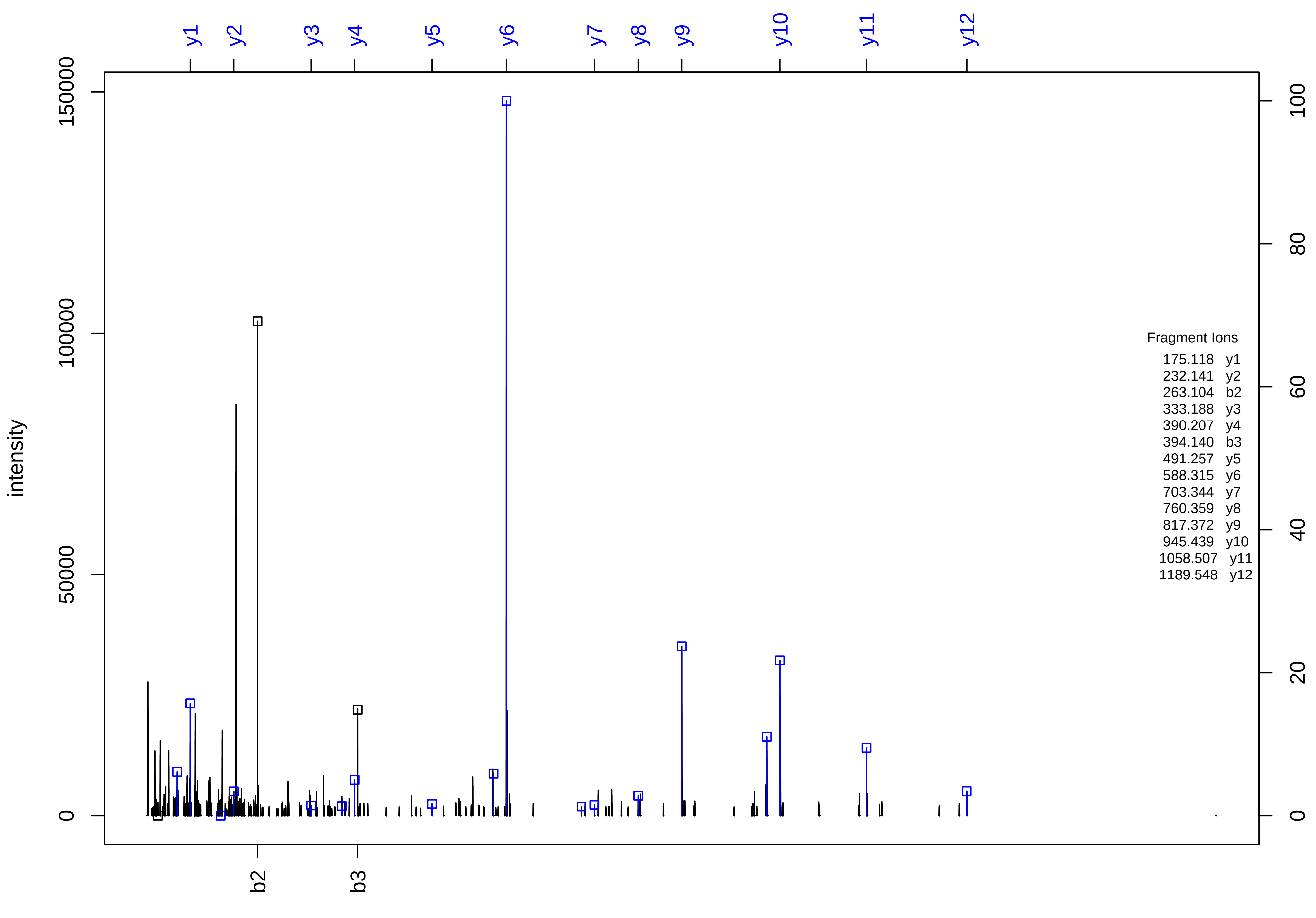

DFMIQGGDPTGTGR / File: sydney_14.raw; SpectrumID: ; scans: 5328

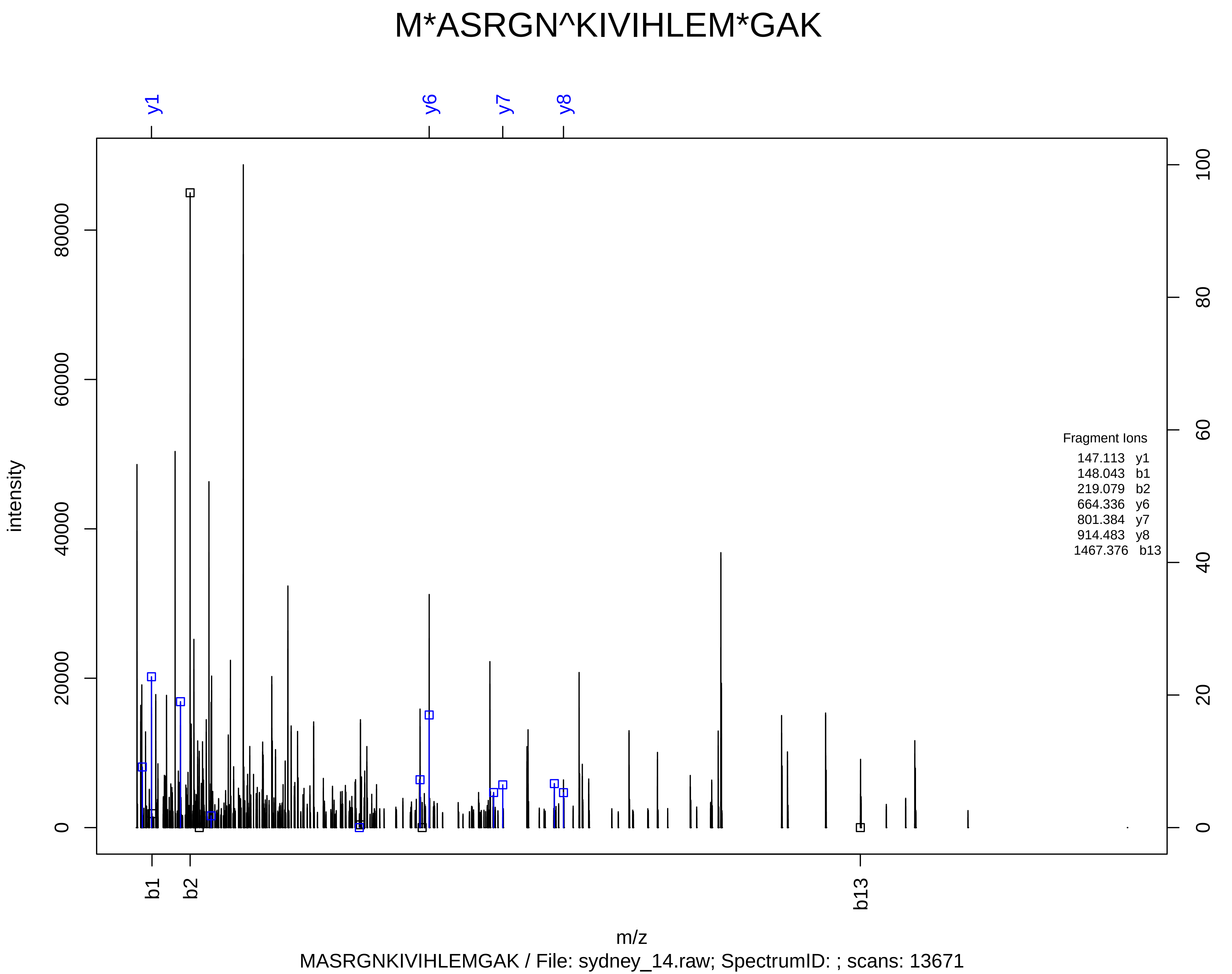
M*ASRGN^KIVIHLEM*GAK
y1
y6
y7
y8
intensity
0
20000
40000
60000
80000
0
20
40
60
80
100
Fragment Ions
147.113 y1
148.043 b1
219.079 b2
664.336 y6
801.384 y7
914.483 y8
1467.376 b13
b1
b2
b13
m/z
MASRGNKIVIHLEMGAK / File: sydney_14.raw; SpectrumID: ; scans: 13671

TIFDTPDEDPNYNPLPEERPGGFAWGEGQR

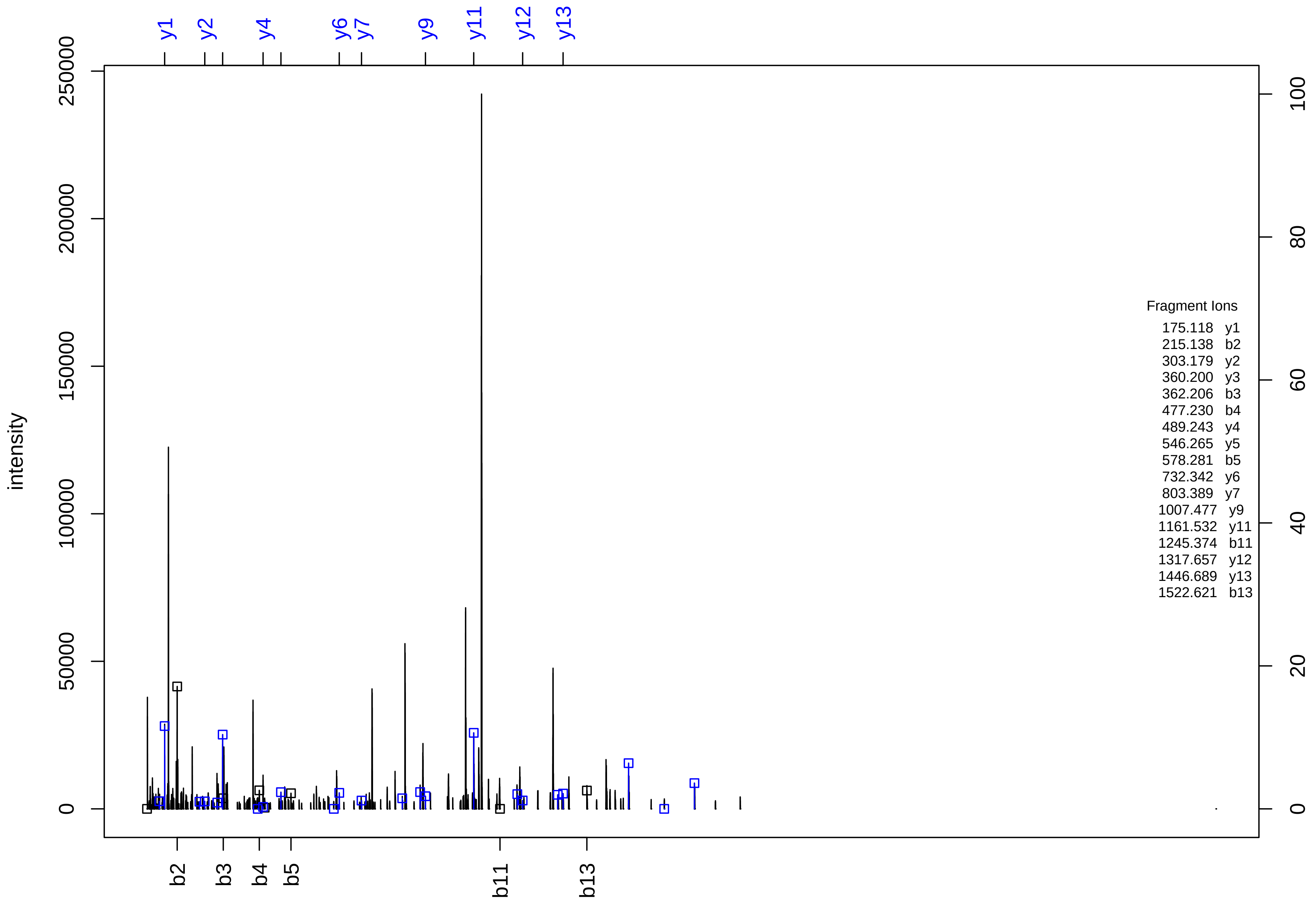

TIFDTPDEDPNYNPLPEERPGGFAWGEGQR / File: sydney_14.raw; SpectrumID: ; scans: 10619

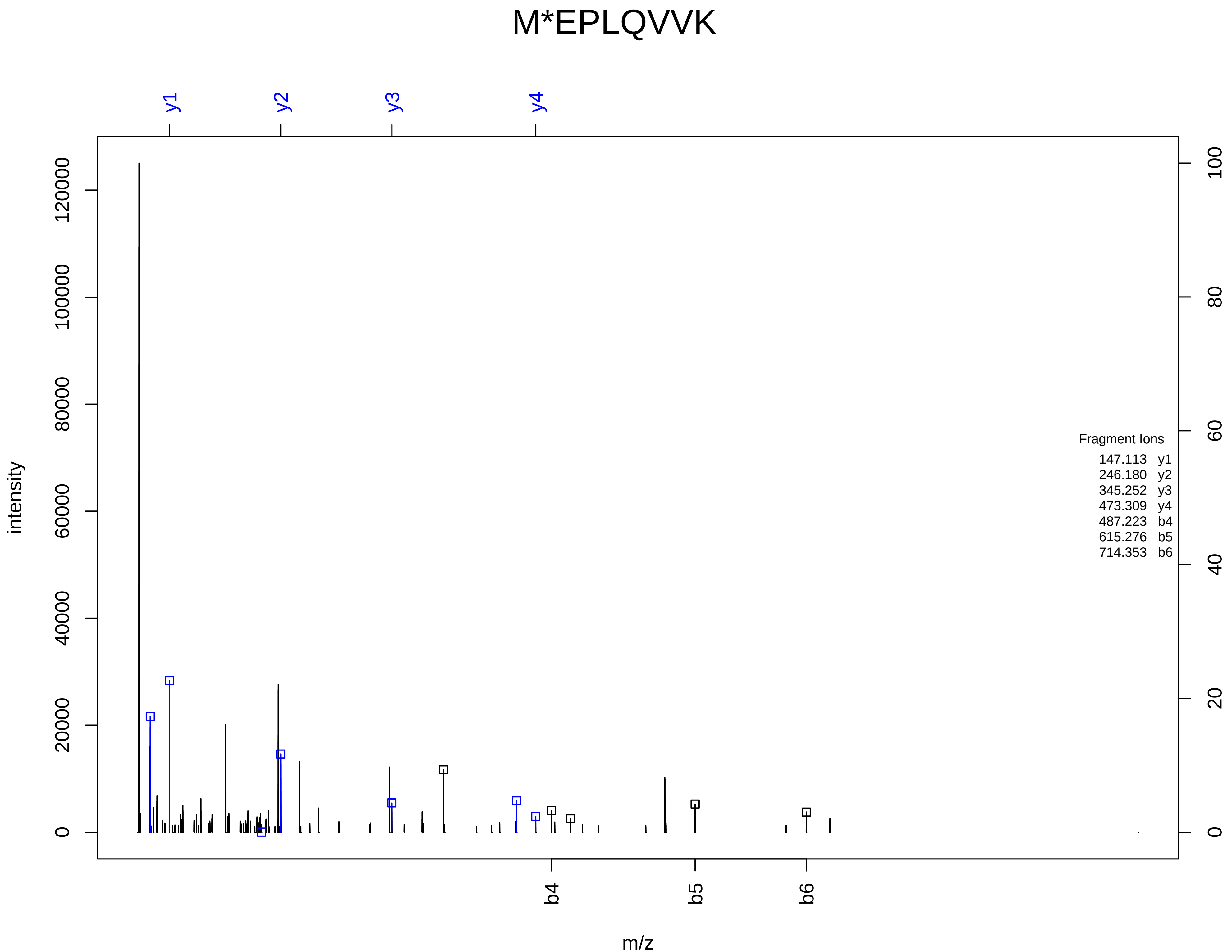

MEPLQVVK / File: sydney_14.raw; SpectrumID: ; scans: 10274

LFLSVGDDCLR

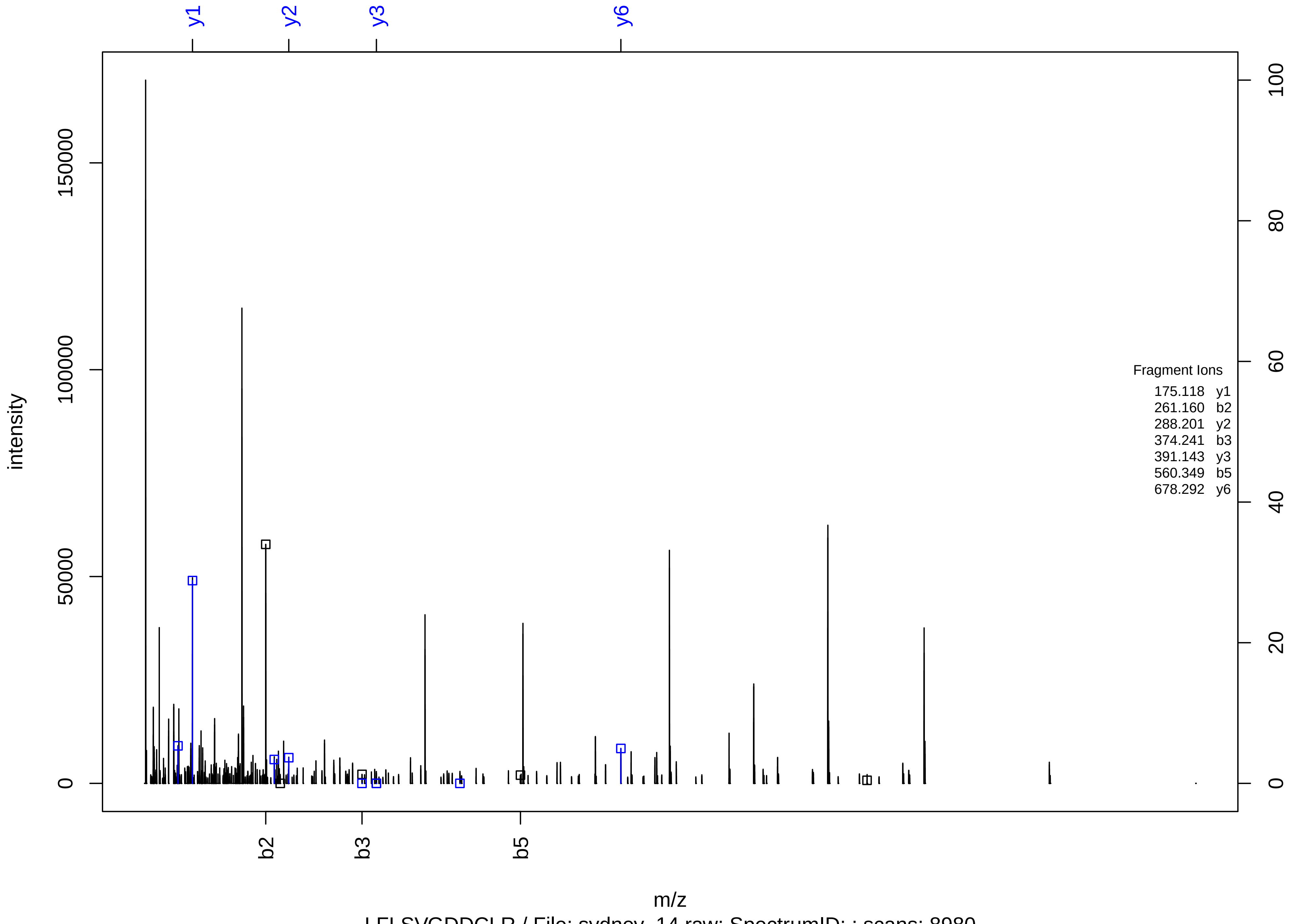

LFLSVGDDCLR / File: sydney_14.raw; SpectrumID: ; scans: 8980

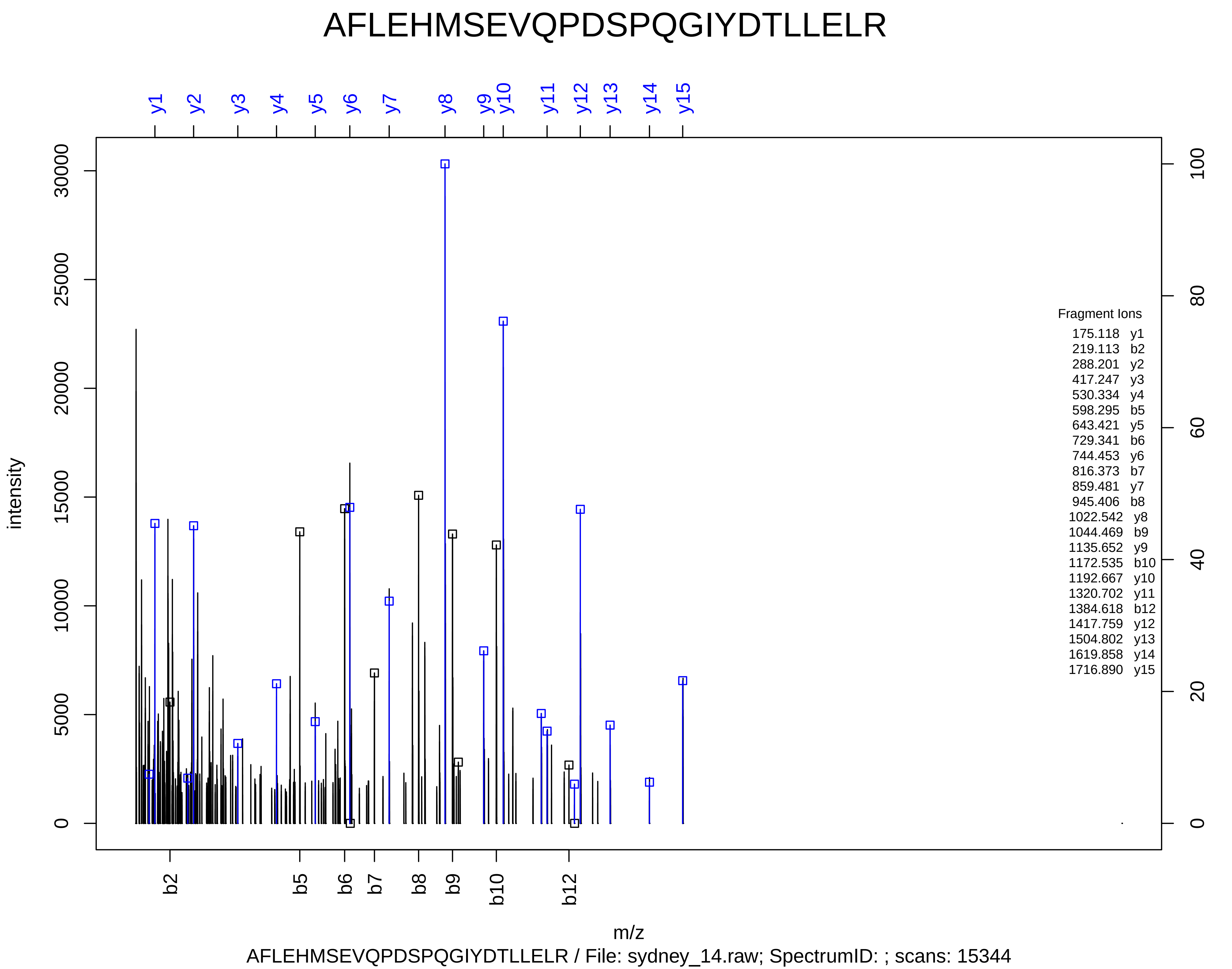
AFLEHMSEVQPDSPQGIYDTLLELR
y1
y2
y3
y4
y5
y6
y7
y8
y9
y10
y11
y12
y13
y14
y15
b2
b5
b6
b7
b8
b9
b10
b12
intensity
m/z
Fragment Ions
175.118 y1
219.113 b2
288.201 y2
417.247 y3
530.334 y4
598.295 b5
643.421 y5
729.341 b6
744.453 y6
816.373 b7
859.481 y7
945.406 b8
1022.542 y8
1044.469 b9
1135.652 y9
1172.535 b10
1192.667 y10
1320.702 y11
1384.618 b12
1417.759 y12
1504.802 y13
1619.858 y14
1716.890 y15
AFLEHMSEVQPDSPQGIYDTLLELR / File: sydney_14.raw; SpectrumID: ; scans: 15344

TELQGLIGQLDEVSLEK

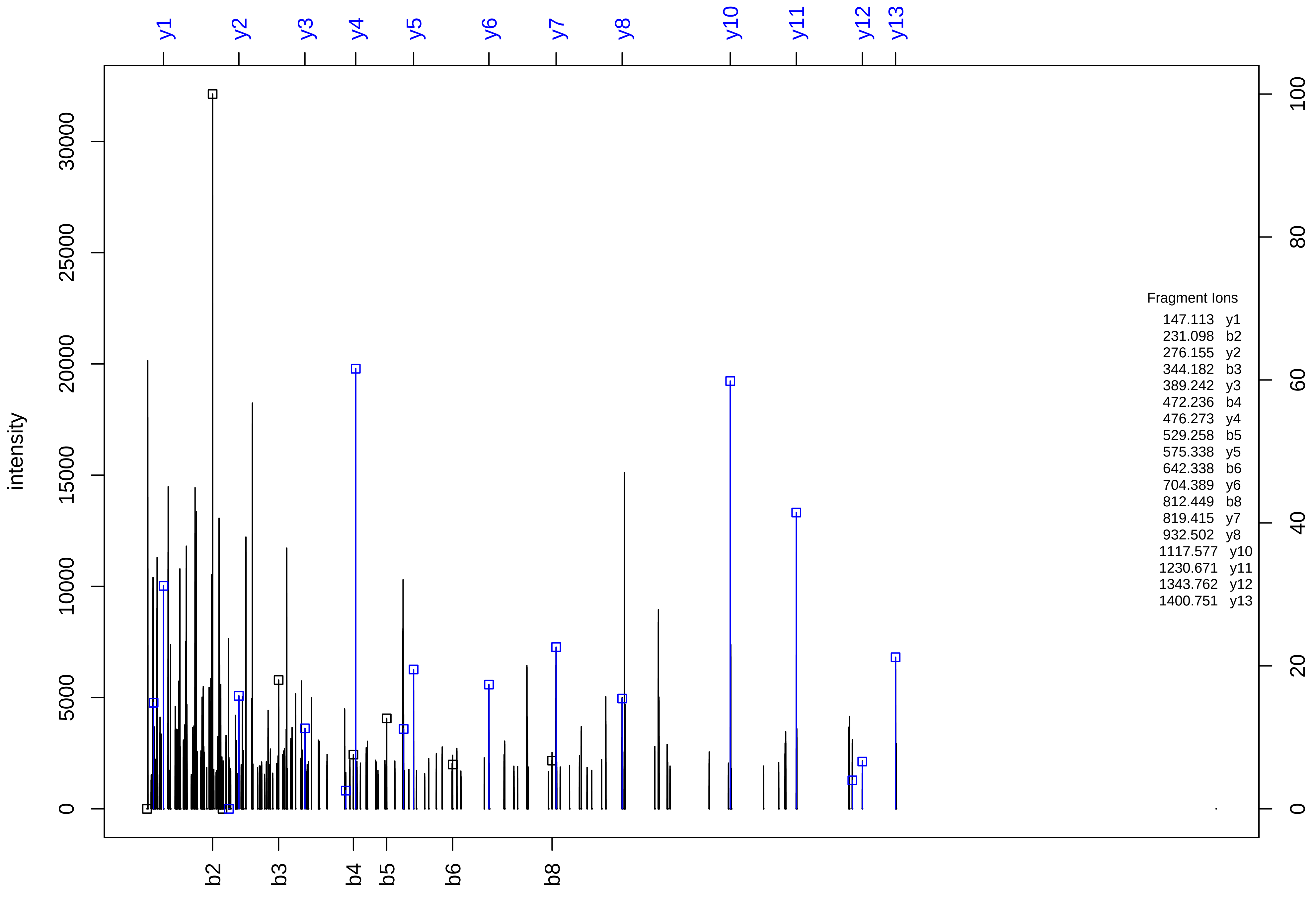

TELQGLIGQLDEVSLEK / File: sydney_14.raw; SpectrumID: ; scans: 13301

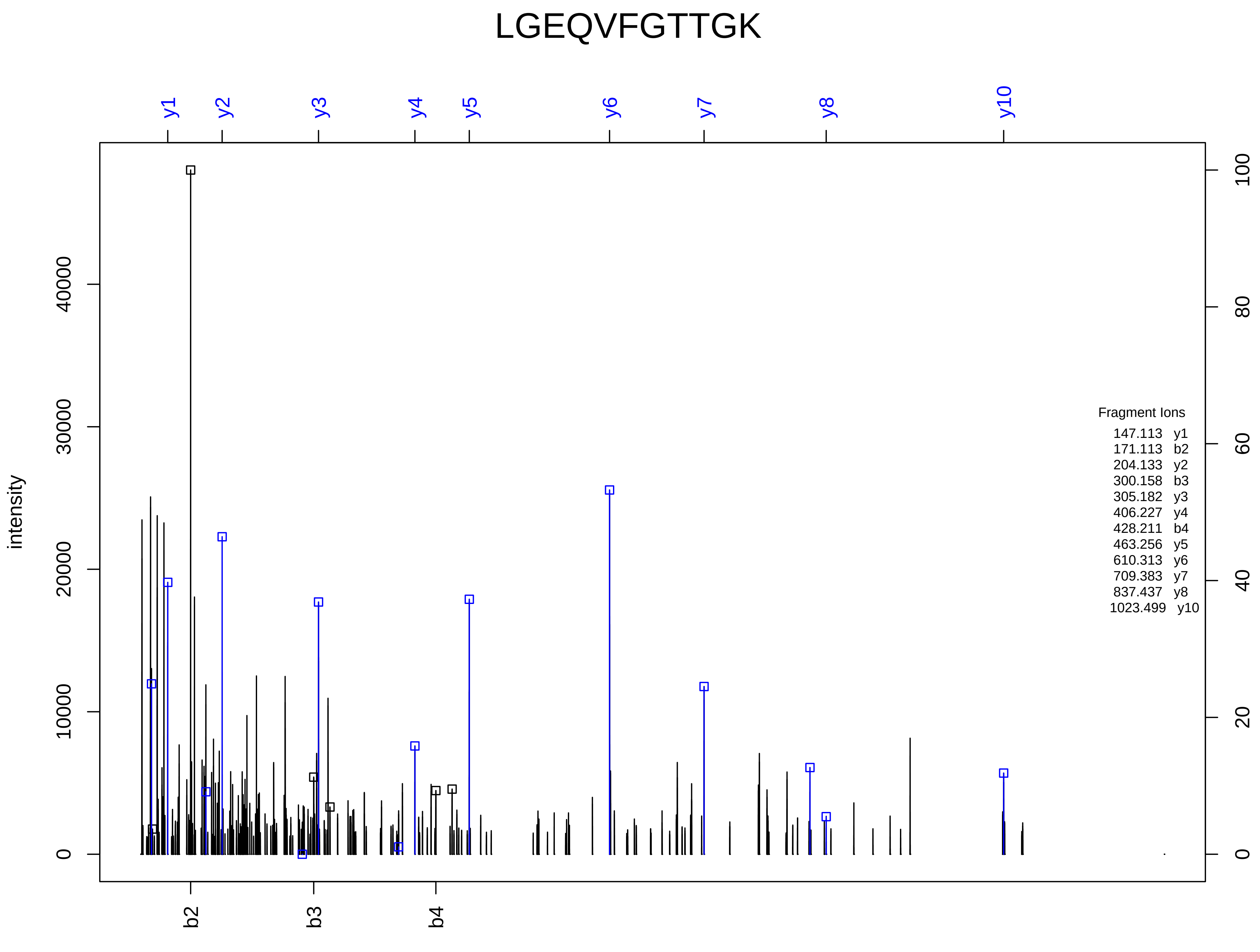

LGEQVFGTTGK / File: sydney_14.raw; SpectrumID: ; scans: 3545

FRENVQDVLPTLPNPDDYFLLR

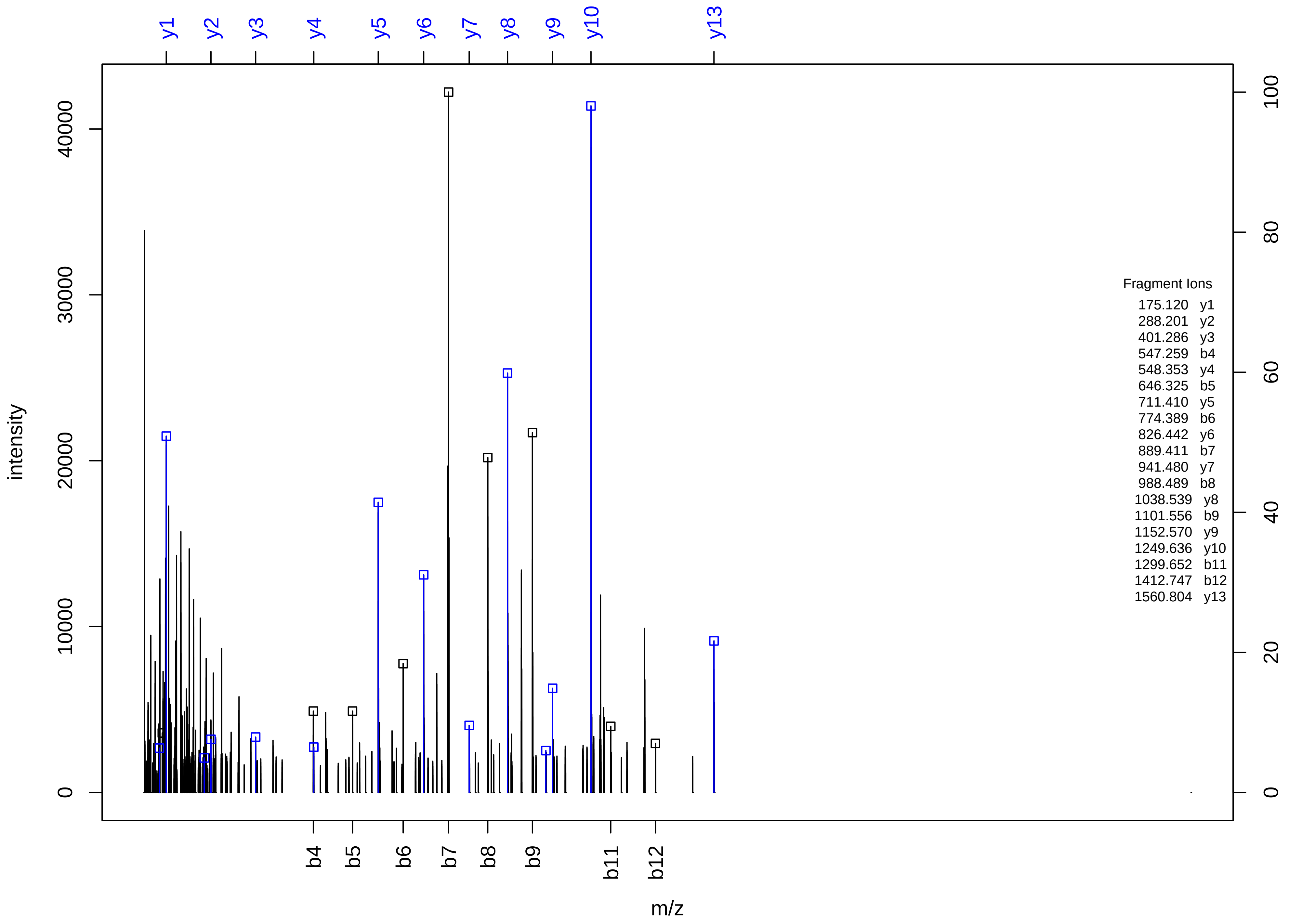

FRENVQDVLPTLPNPDDYFLLR / File: sydney_14.raw; SpectrumID: ; scans: 14618

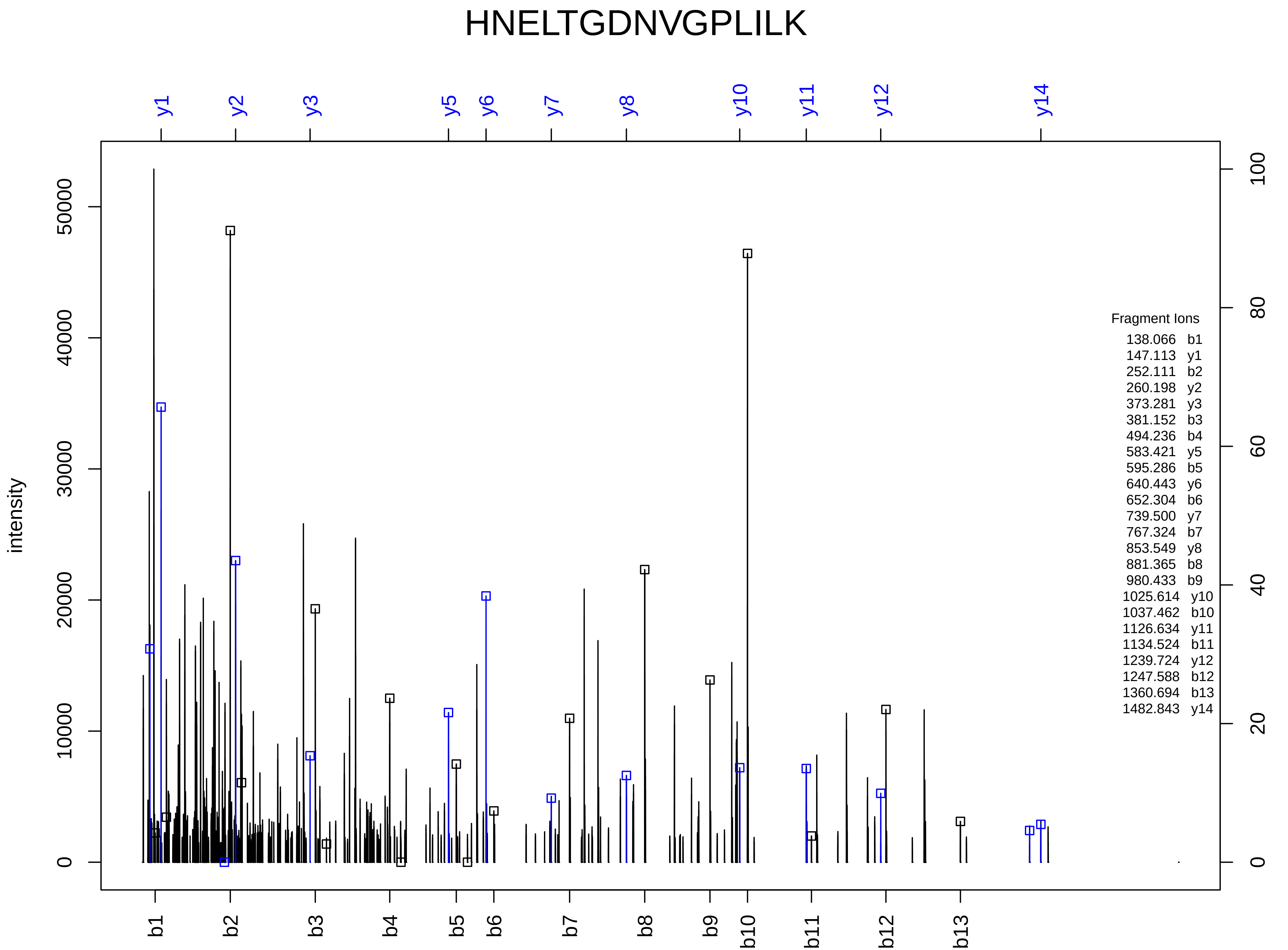

HNELTGDNVGPLILK / File: sydney_14.raw; SpectrumID: ; scans: 6970

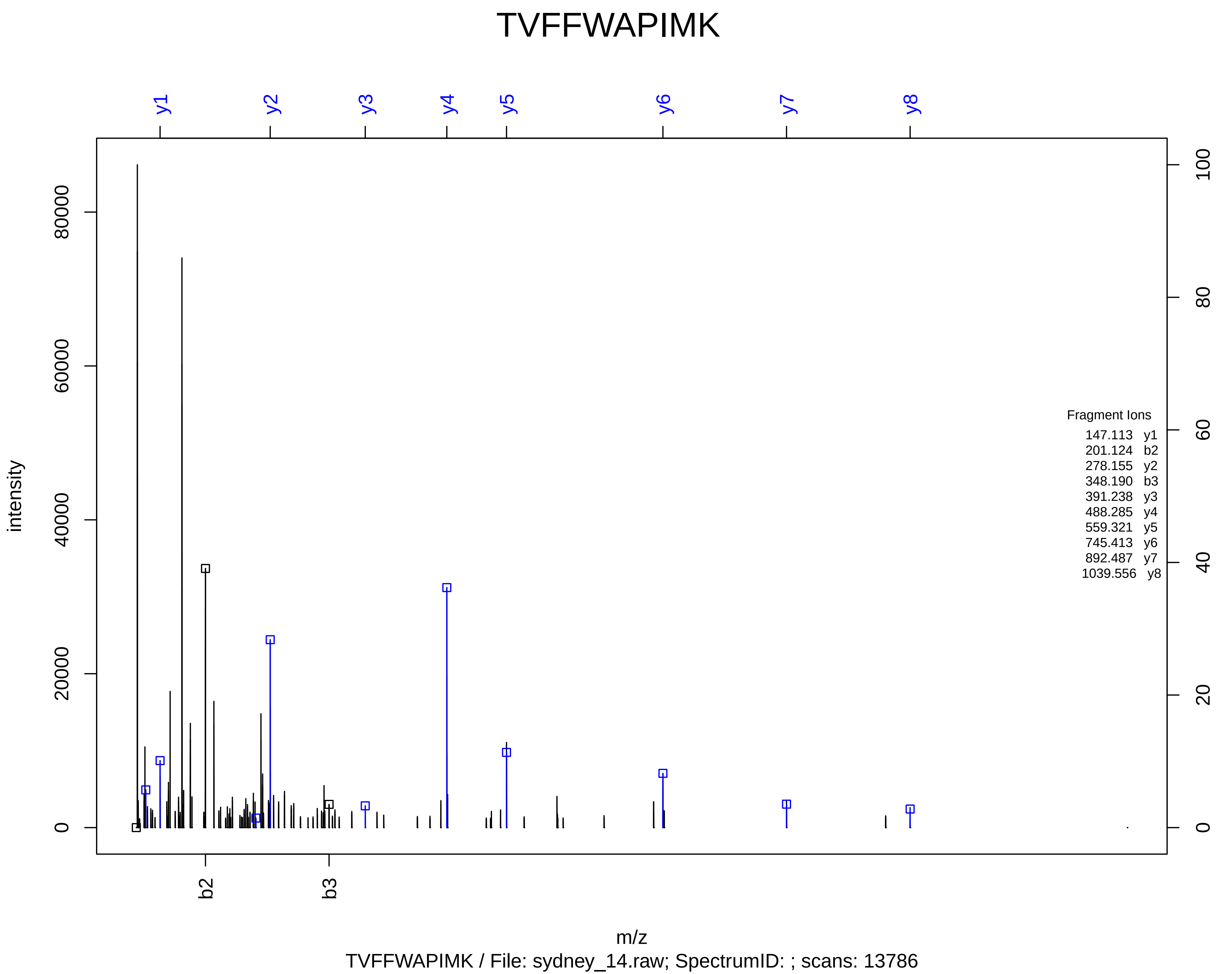

TVFFWAPIMK
y1
y2
y3
y4
y5
y6
y7
y8
intensity
0
20000
40000
60000
80000
0
20
40
60
80
100
b2
b3
m/z
Fragment Ions
147.113 y1
201.124 b2
278.155 y2
348.190 b3
391.238 y3
488.285 y4
559.321 y5
745.413 y6
892.487 y7
1039.556 y8
TVFFWAPIMK / File: sydney_14.raw; SpectrumID: ; scans: 13786

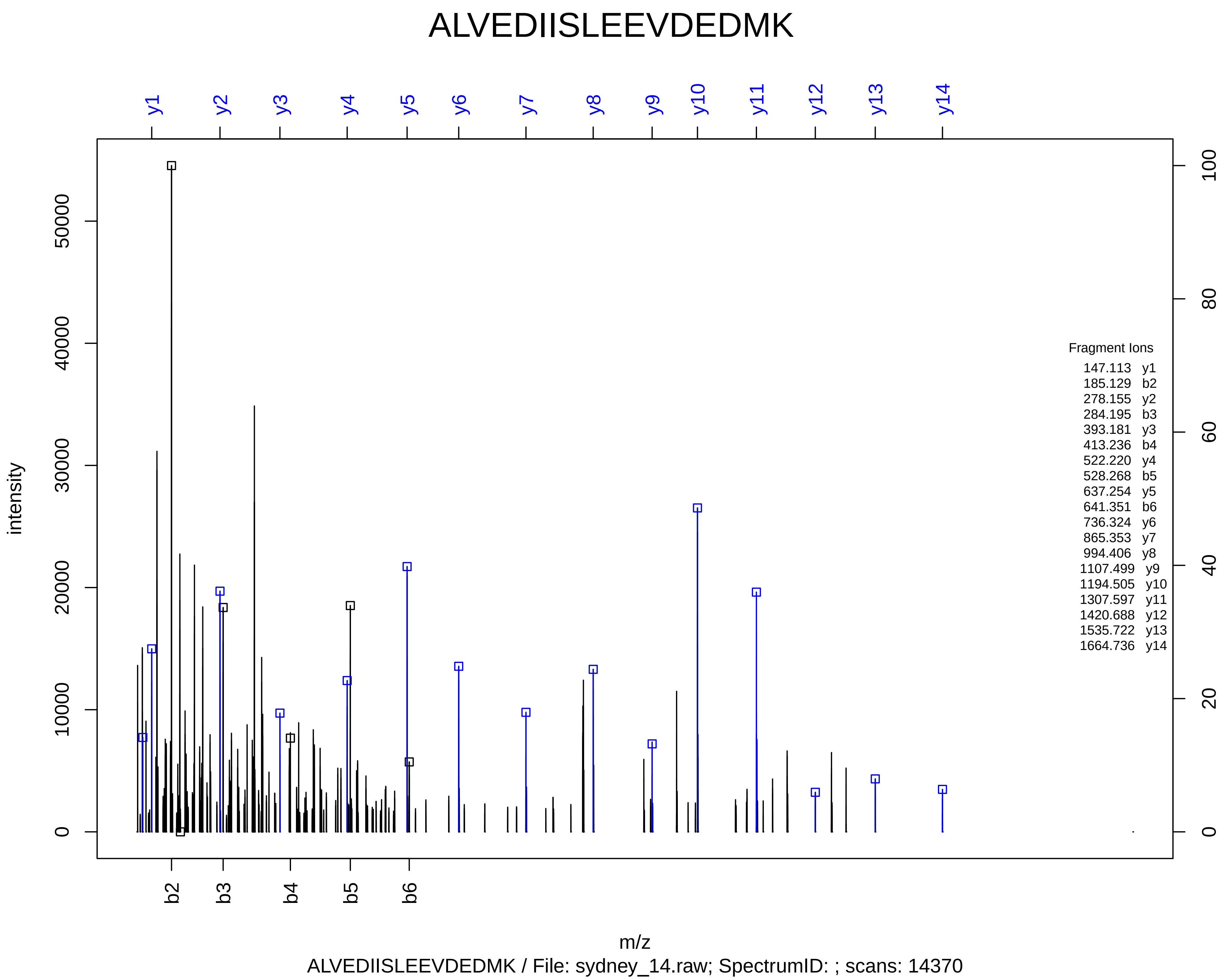
ALVEDIISLEEVDEDMK
y1
y2
y3
y4
y5
y6
y7
y8
y9
y10
y11
y12
y13
y14
b2
b3
b4
b5
b6
intensity
0
10000
20000
30000
40000
50000
0
20
40
60
80
100
m/z
Fragment Ions
147.113 y1
185.129 b2
278.155 y2
284.195 b3
393.181 y3
413.236 b4
522.220 y4
528.268 b5
637.254 y5
641.351 b6
736.324 y6
865.353 y7
994.406 y8
1107.499 y9
1194.505 y10
1307.597 y11
1420.688 y12
1535.722 y13
1664.736 y14
ALVEDIISLEEVDEDMK / File: sydney_14.raw; SpectrumID: ; scans: 14370

LPQGLPTEETMSSTCLK

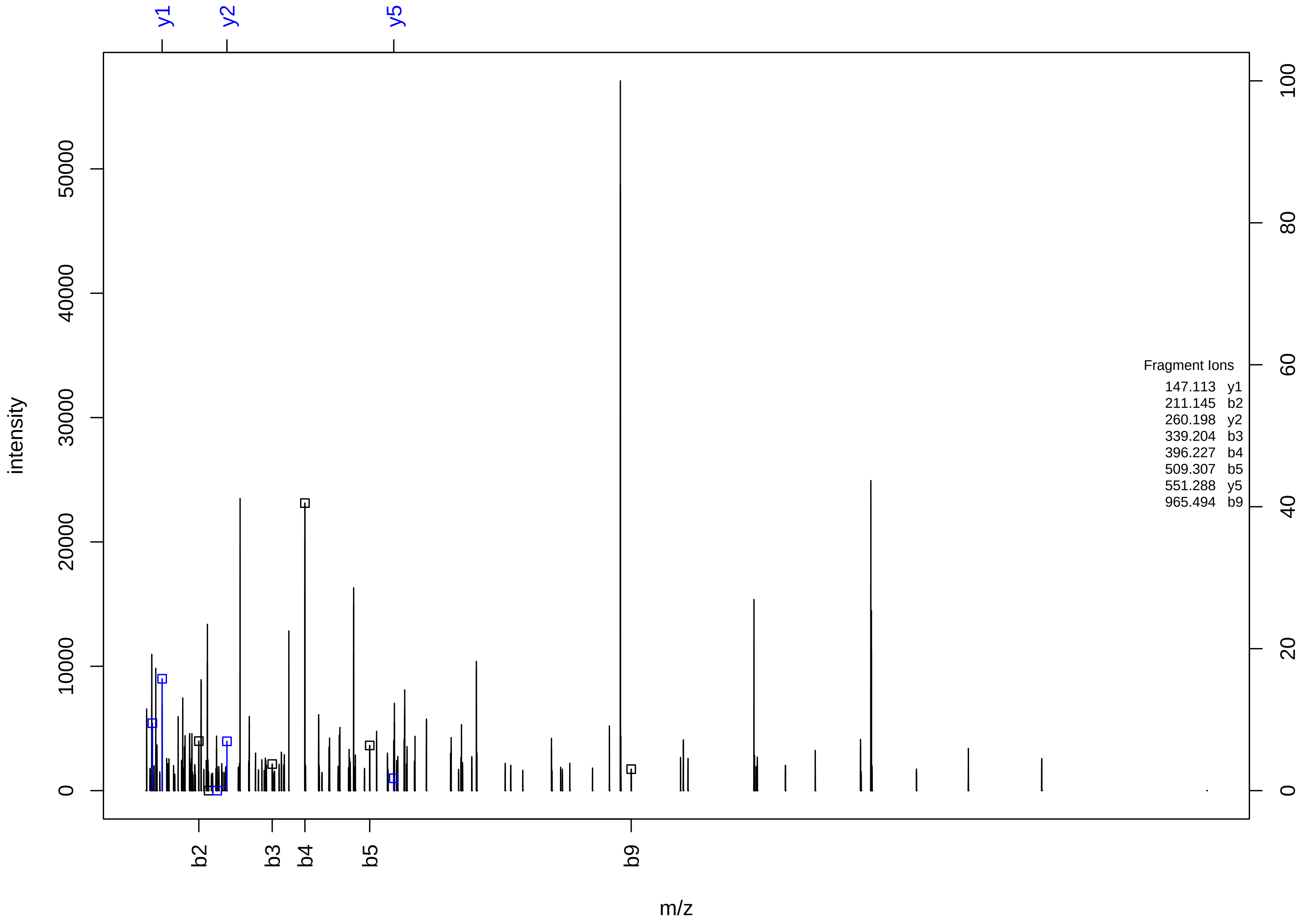

LPQGLPTEETMSSTCLK / File: sydney_14.raw; SpectrumID: ; scans: 7054

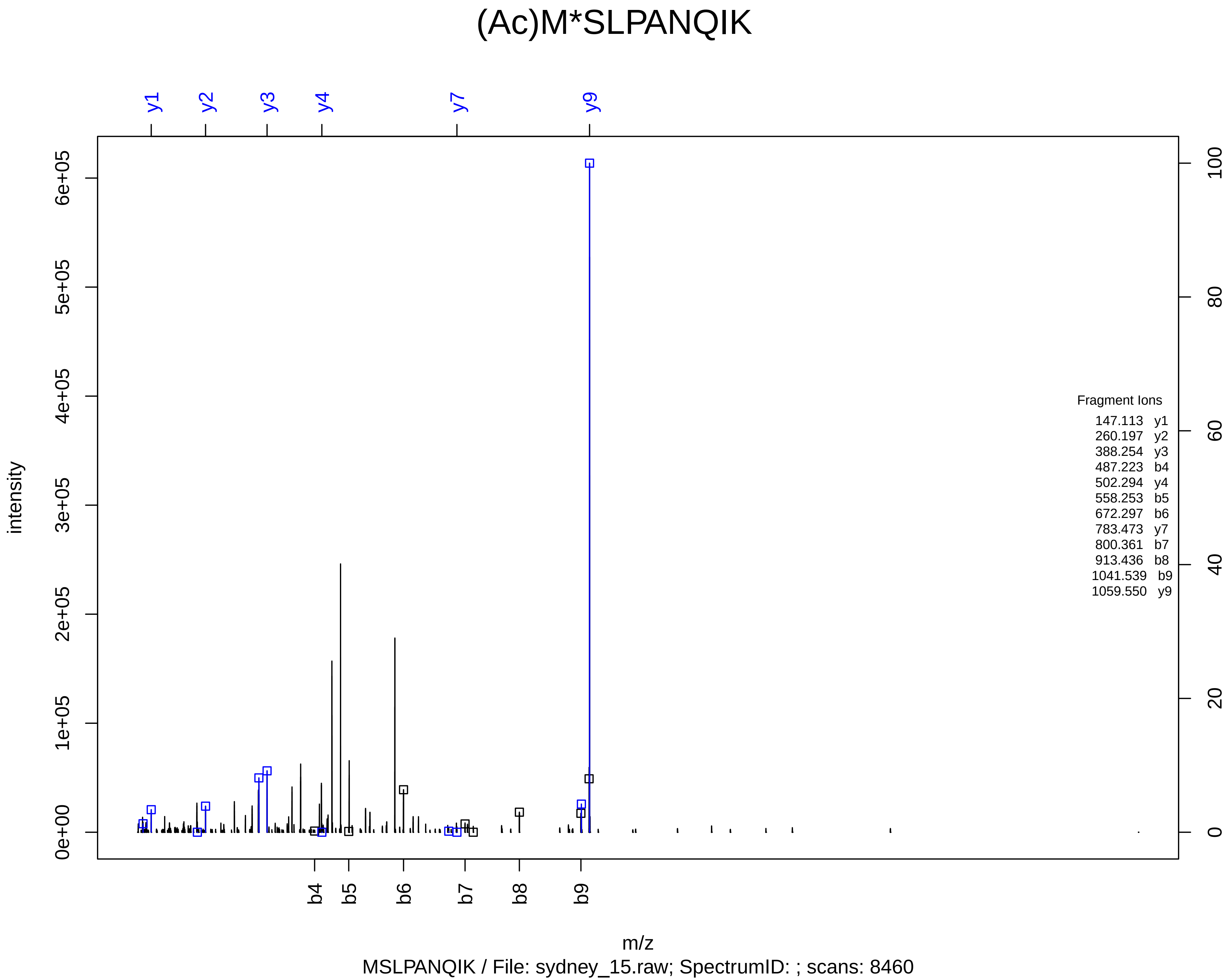
(Ac)M*SLPANQIK
y1
y2
y3
y4
y7
y9
intensity
0e+00
1e+05
2e+05
3e+05
4e+05
5e+05
6e+05
0
20
40
60
80
100
Fragment Ions
147.113 y1
260.197 y2
388.254 y3
487.223 b4
502.294 y4
558.253 b5
672.297 b6
783.473 y7
800.361 b7
913.436 b8
1041.539 b9
1059.550 y9
b4
b5
b6
b7
b8
b9
m/z
MSLPANQIK / File: sydney_15.raw; SpectrumID: ; scans: 8460

(Ac)STNTDLSLSSYDEGQGSK

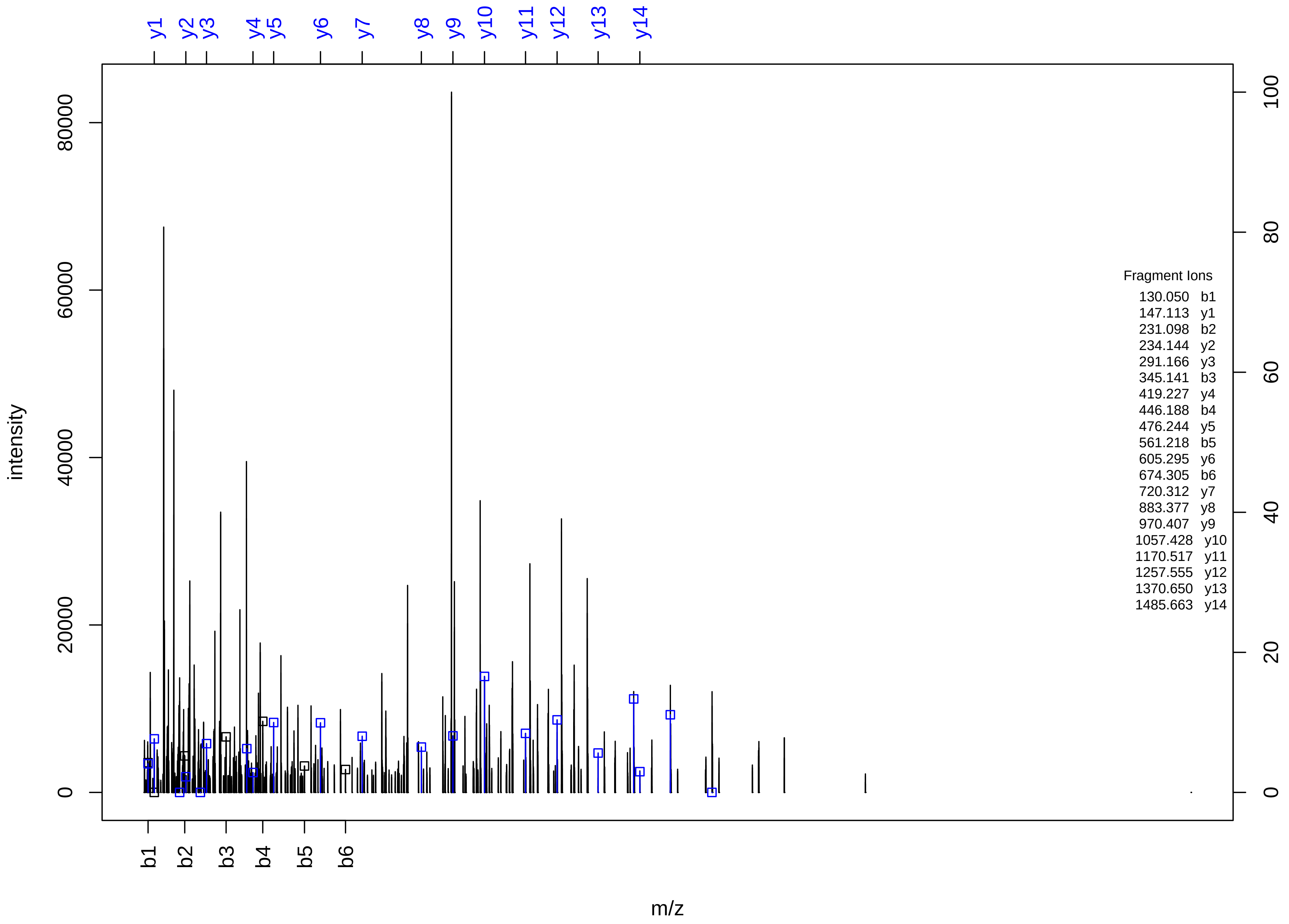

STNTDLSLSSYDEGQGSK / File: sydney_15.raw; SpectrumID: ; scans: 6734

(Ac)GAVTDDEVIRK

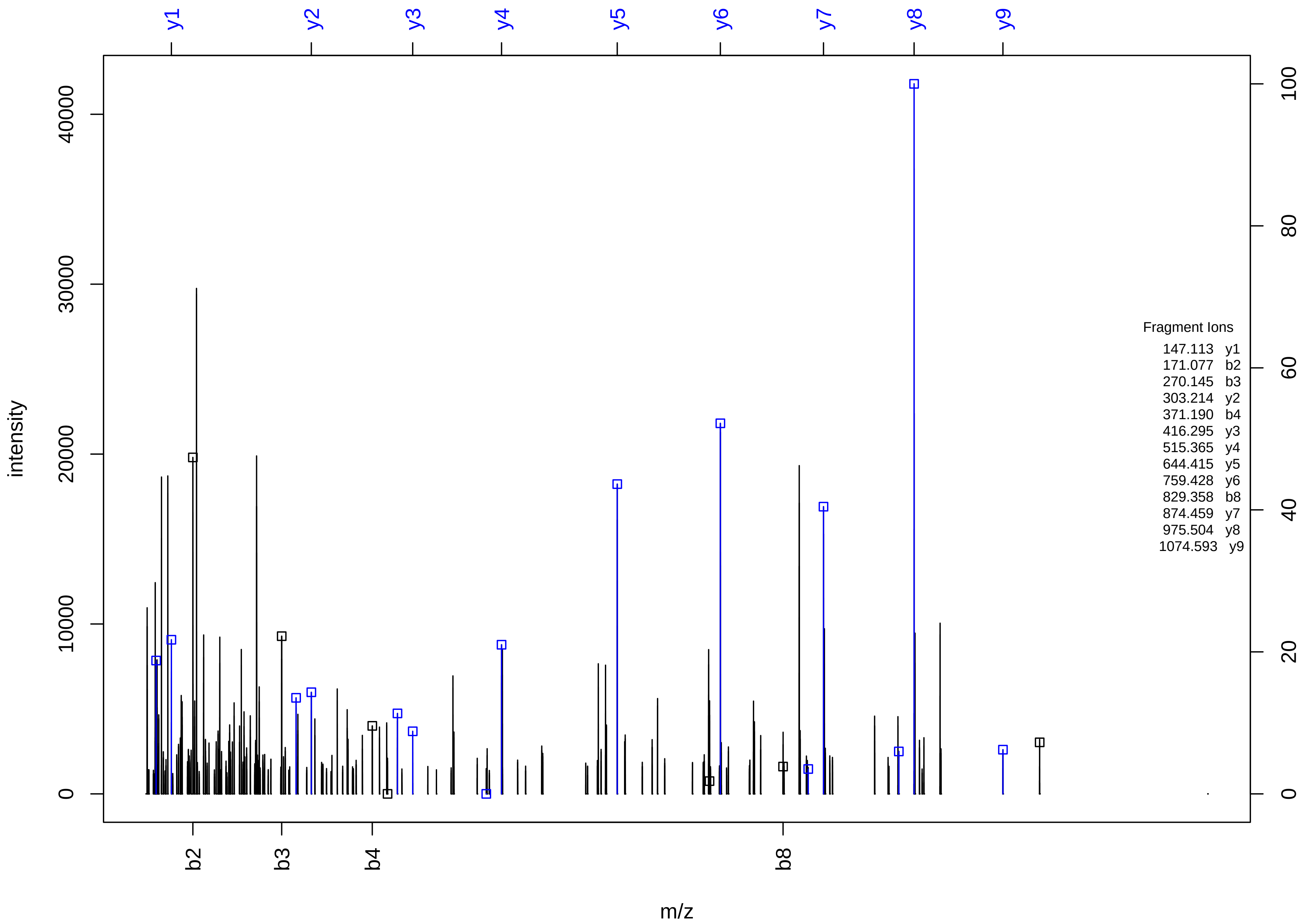

GAVTDDEVIRK / File: sydney_15.raw; SpectrumID: ; scans: 2986

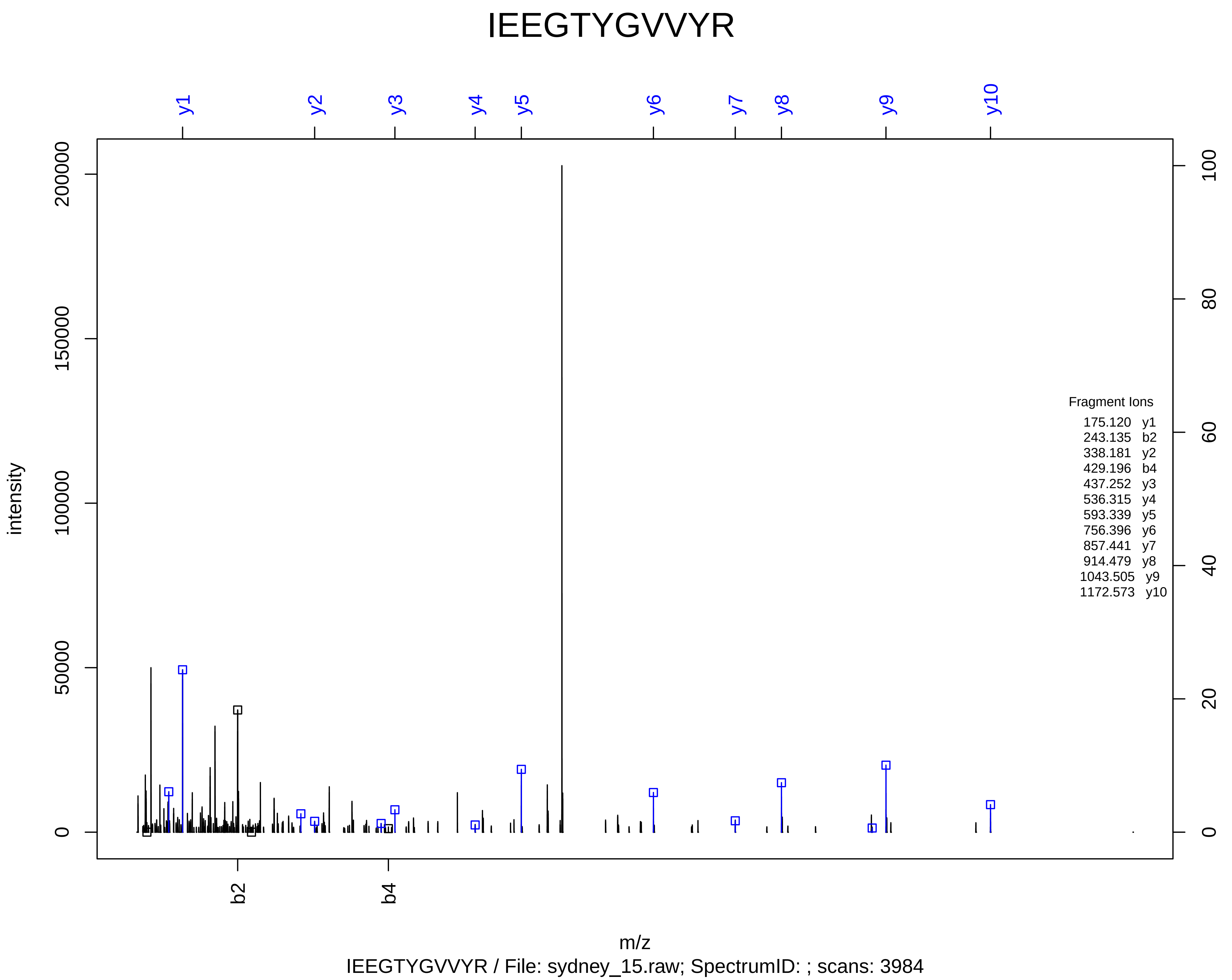
IEEGTYGVVYR
y1
y2
y3
y4
y5
y6
y7
y8
y9
y10
intensity
0
50000
100000
150000
200000
0
20
40
60
80
100
b2
b4
m/z
Fragment Ions
175.120 y1
243.135 b2
338.181 y2
429.196 b4
437.252 y3
536.315 y4
593.339 y5
756.396 y6
857.441 y7
914.479 y8
1043.505 y9
1172.573 y10
IEEGTYGVVYR / File: sydney_15.raw; SpectrumID: ; scans: 3984

LM*Q^GDQ^ILMVN^GEDVR

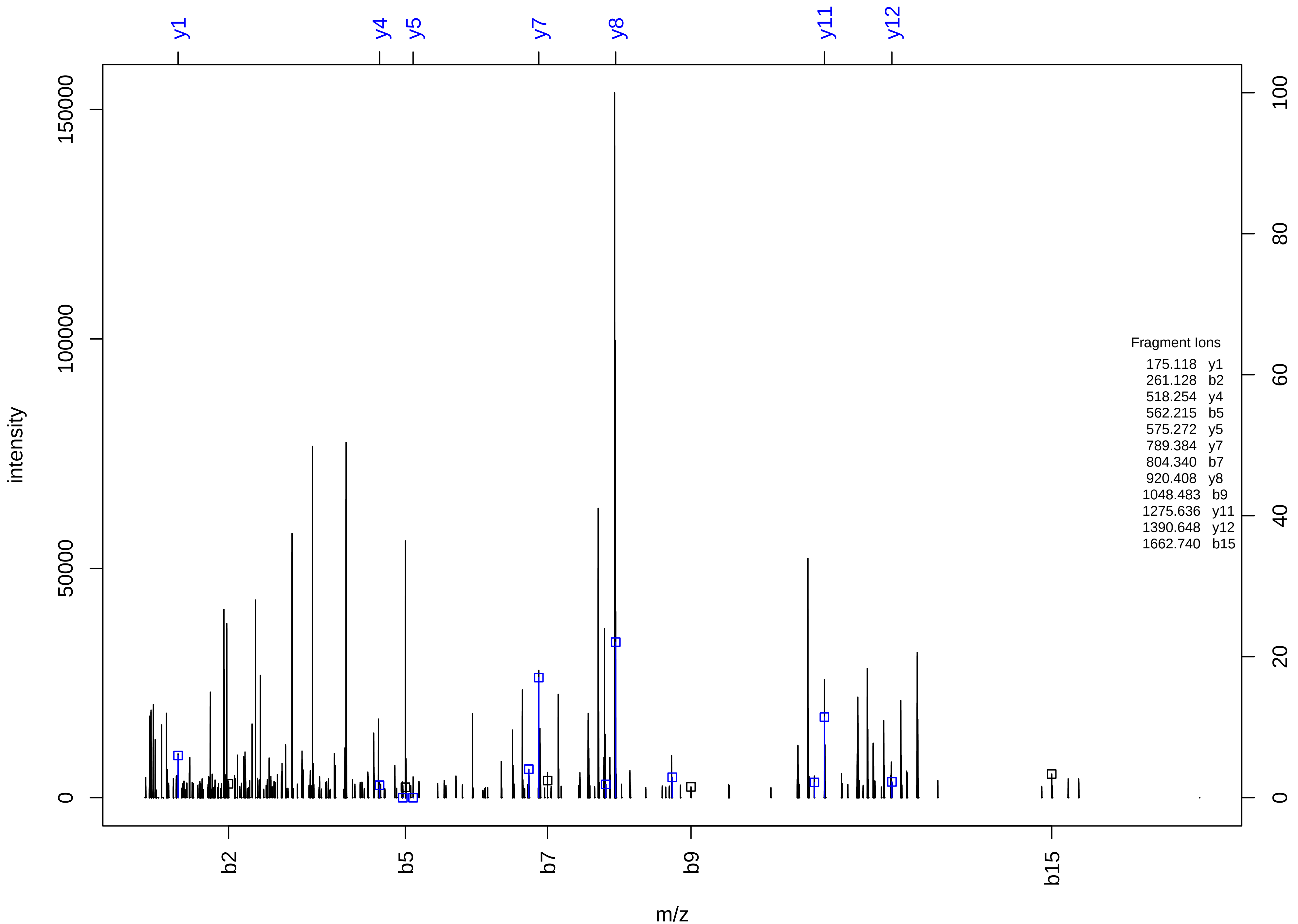

LMQGDQILMVNGEDVR / File: sydney_15.raw; SpectrumID: ; scans: 6021

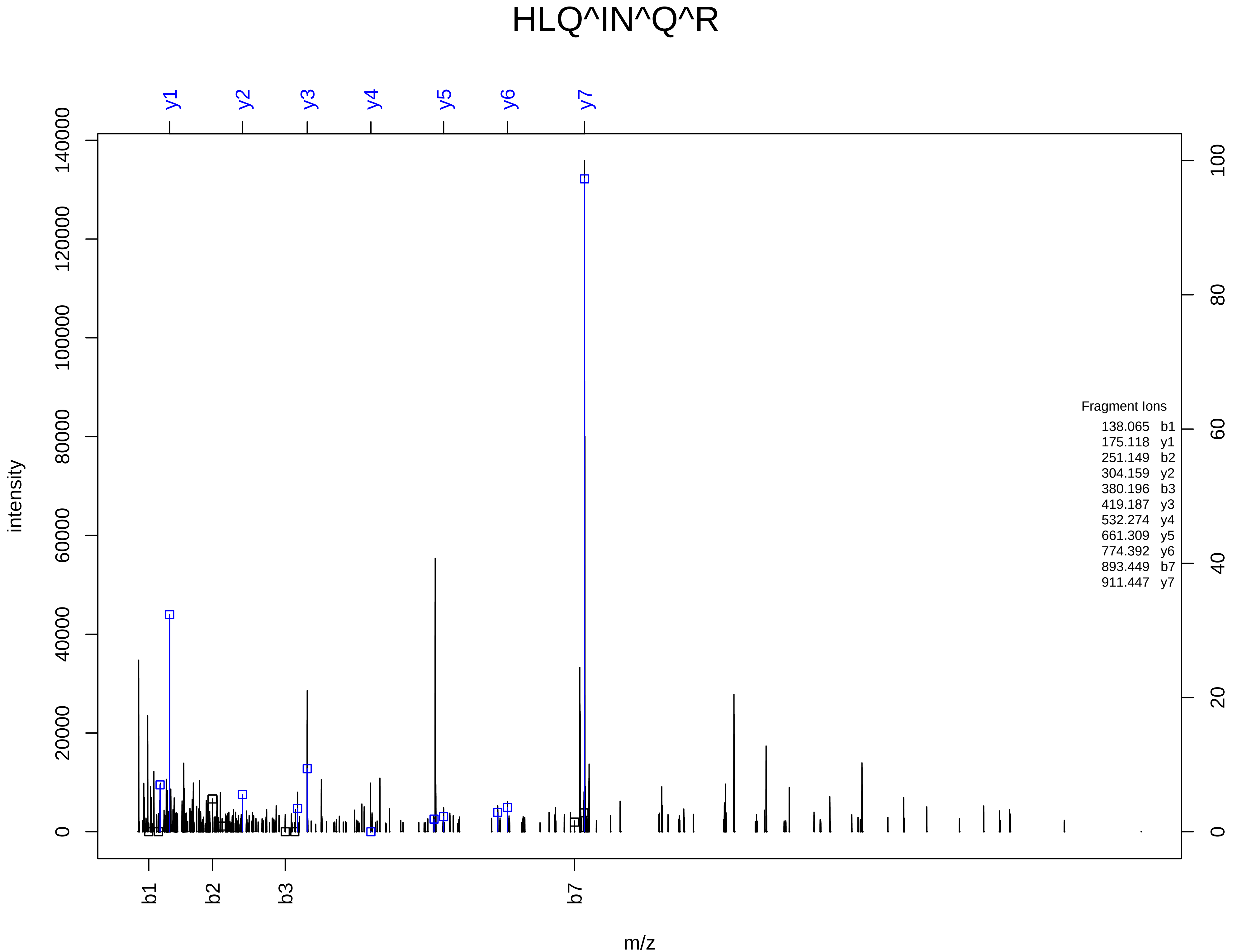

HLQ^IN^Q^R
y1
y2
y3
y4
y5
y6
y7
intensity
0
20000
40000
60000
80000
100000
120000
140000
0
20
40
60
80
100
Fragment Ions
138.065 b1
175.118 y1
251.149 b2
304.159 y2
380.196 b3
419.187 y3
532.274 y4
661.309 y5
774.392 y6
893.449 b7
911.447 y7
b1
b2
b3
b7
m/z
HLQINQR / File: sydney_15.raw; SpectrumID: ; scans: 10089

ISARN^TKQ^VQ^K

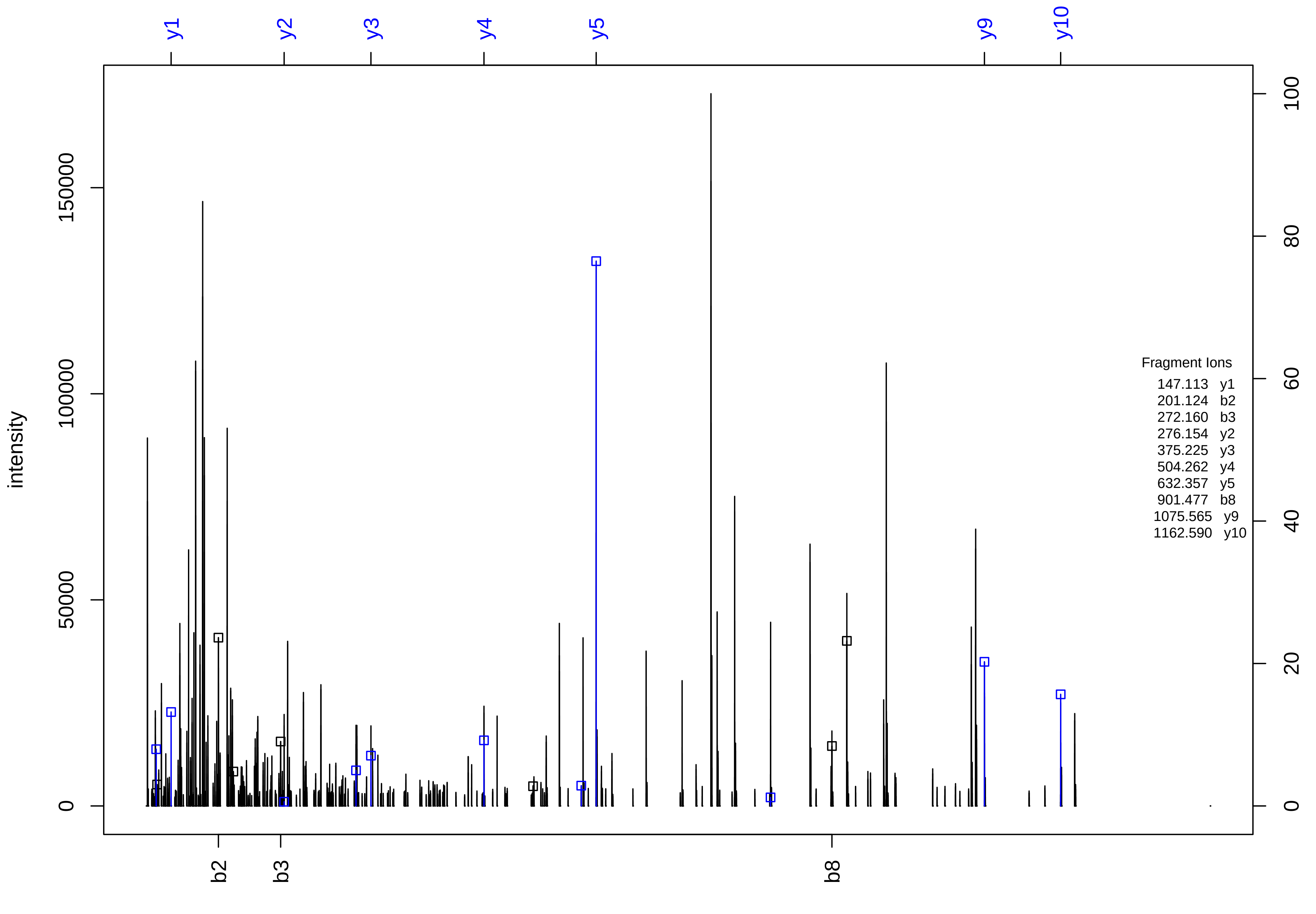

ISARNTKQVQK / File: sydney_15.raw; SpectrumID: ; scans: 8174

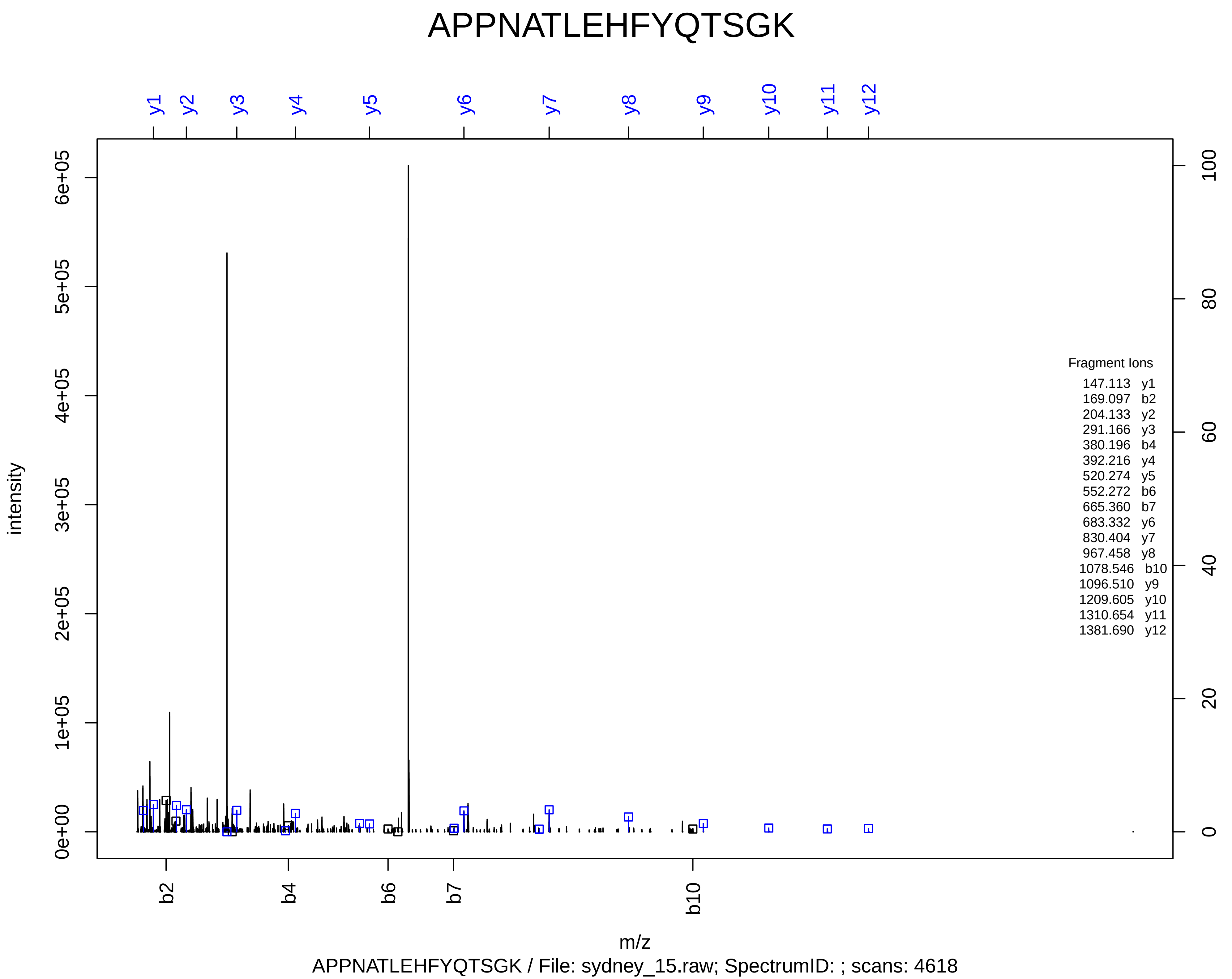

APPNATLEHFYQTSGK
y1
y2
y3
y4
y5
y6
y7
y8
y9
y10
y11
y12
intensity
0e+00
1e+05
2e+05
3e+05
4e+05
5e+05
6e+05
0
20
40
60
80
100
Fragment Ions
147.113 y1
169.097 b2
204.133 y2
291.166 y3
380.196 b4
392.216 y4
520.274 y5
552.272 b6
665.360 b7
683.332 y6
830.404 y7
967.458 y8
1078.546 b10
1096.510 y9
1209.605 y10
1310.654 y11
1381.690 y12
b2
b4
b6
b7
b10
m/z
APPNATLEHFYQTSGK / File: sydney_15.raw; SpectrumID: ; scans: 4618

TM*LDM*N^KMTQSIIGSM*QYSK

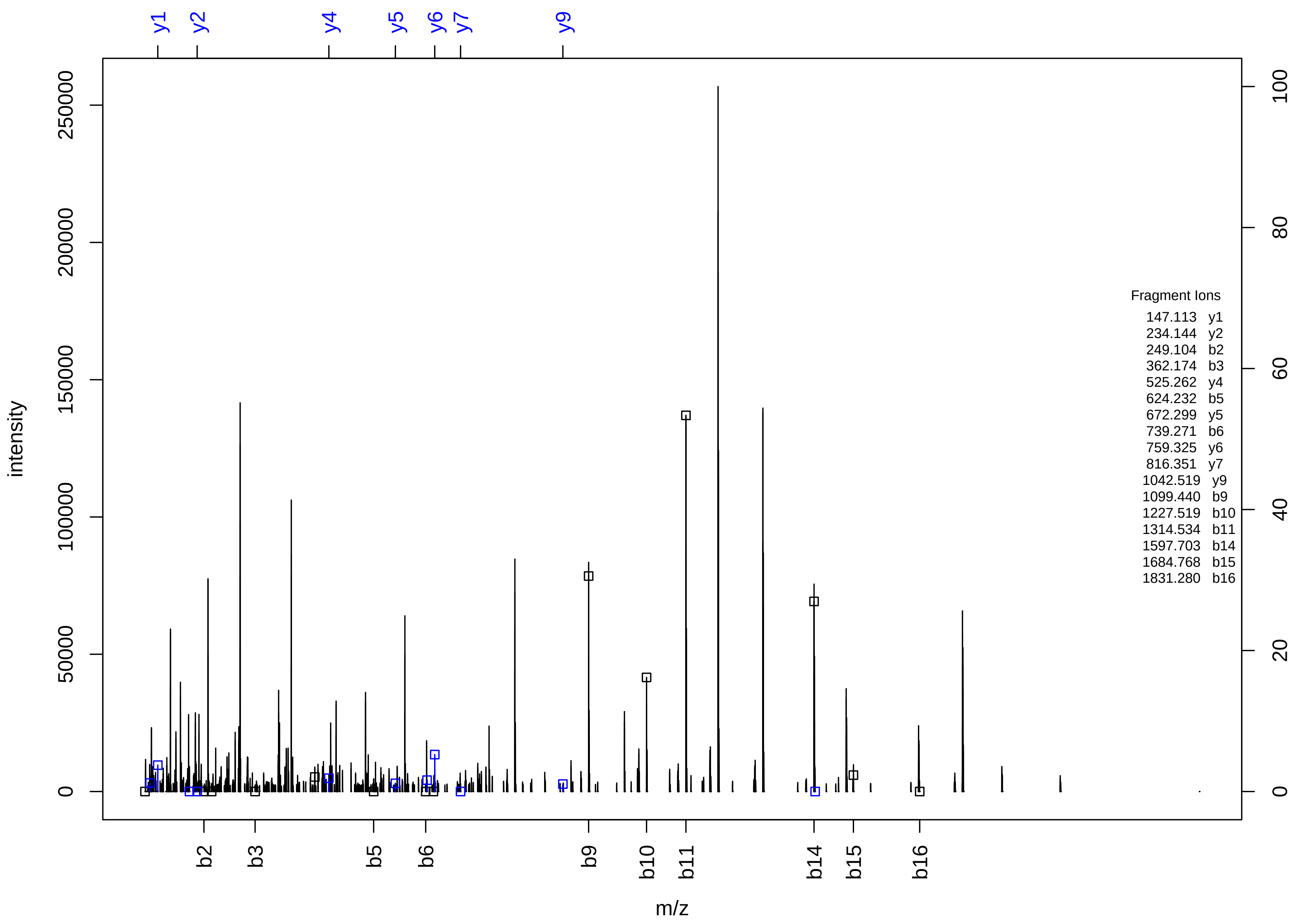

TMLDMNKMTQSIIGSMQYSK / File: sydney_15.raw; SpectrumID: ; scans: 9663

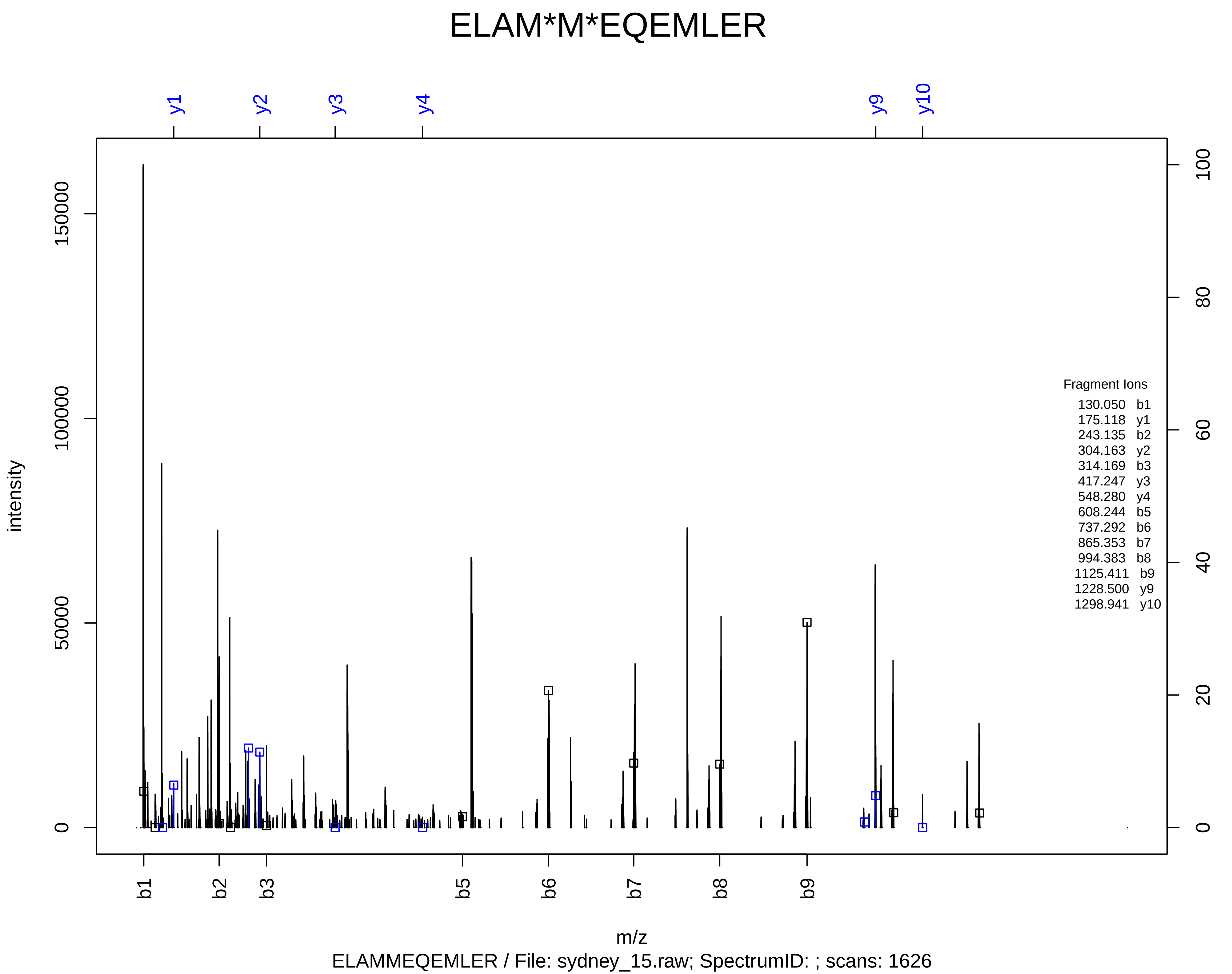
ELAM*M*EQEMLER
y1
y2
y3
y4
y9
y10
intensity
0
50000
100000
150000
0
20
40
60
80
100
b1
b2
b3
b5
b6
b7
b8
b9
m/z
Fragment Ions
130.050 b1
175.118 y1
243.135 b2
304.163 y2
314.169 b3
417.247 y3
548.280 y4
608.244 b5
737.292 b6
865.353 b7
994.383 b8
1125.411 b9
1228.500 y9
1298.941 y10
ELAMMEQEMLER / File: sydney_15.raw; SpectrumID: ; scans: 1626

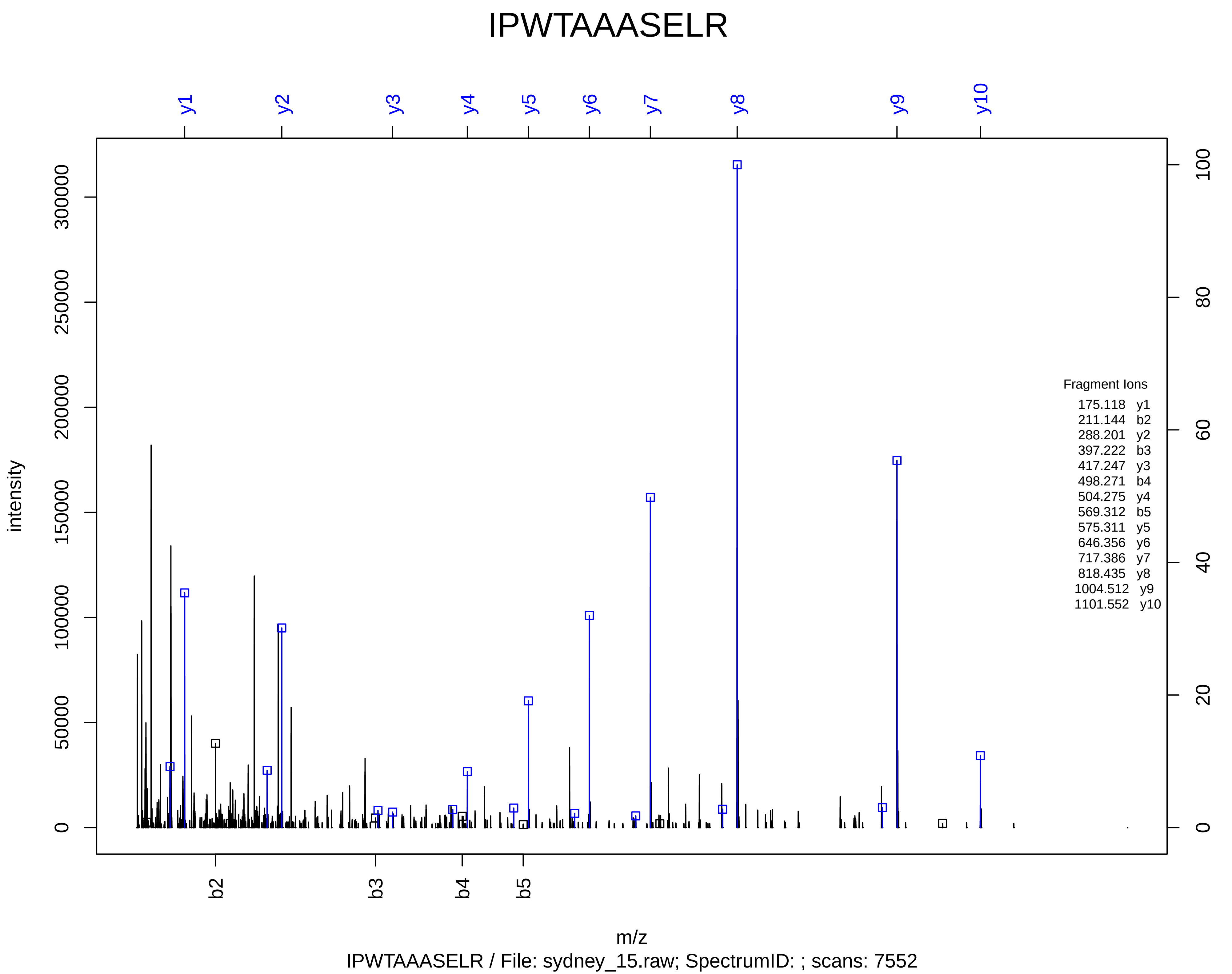
IPWTAAASELR
y1
y2
y3
y4
y5
y6
y7
y8
y9
y10
b2
b3
b4
b5
intensity
0
50000
100000
150000
200000
250000
300000
0
20
40
60
80
100
m/z
Fragment Ions
175.118 y1
211.144 b2
288.201 y2
397.222 b3
417.247 y3
498.271 b4
504.275 y4
569.312 b5
575.311 y5
646.356 y6
717.386 y7
818.435 y8
1004.512 y9
1101.552 y10
IPWTAAASELR / File: sydney_15.raw; SpectrumID: ; scans: 7552

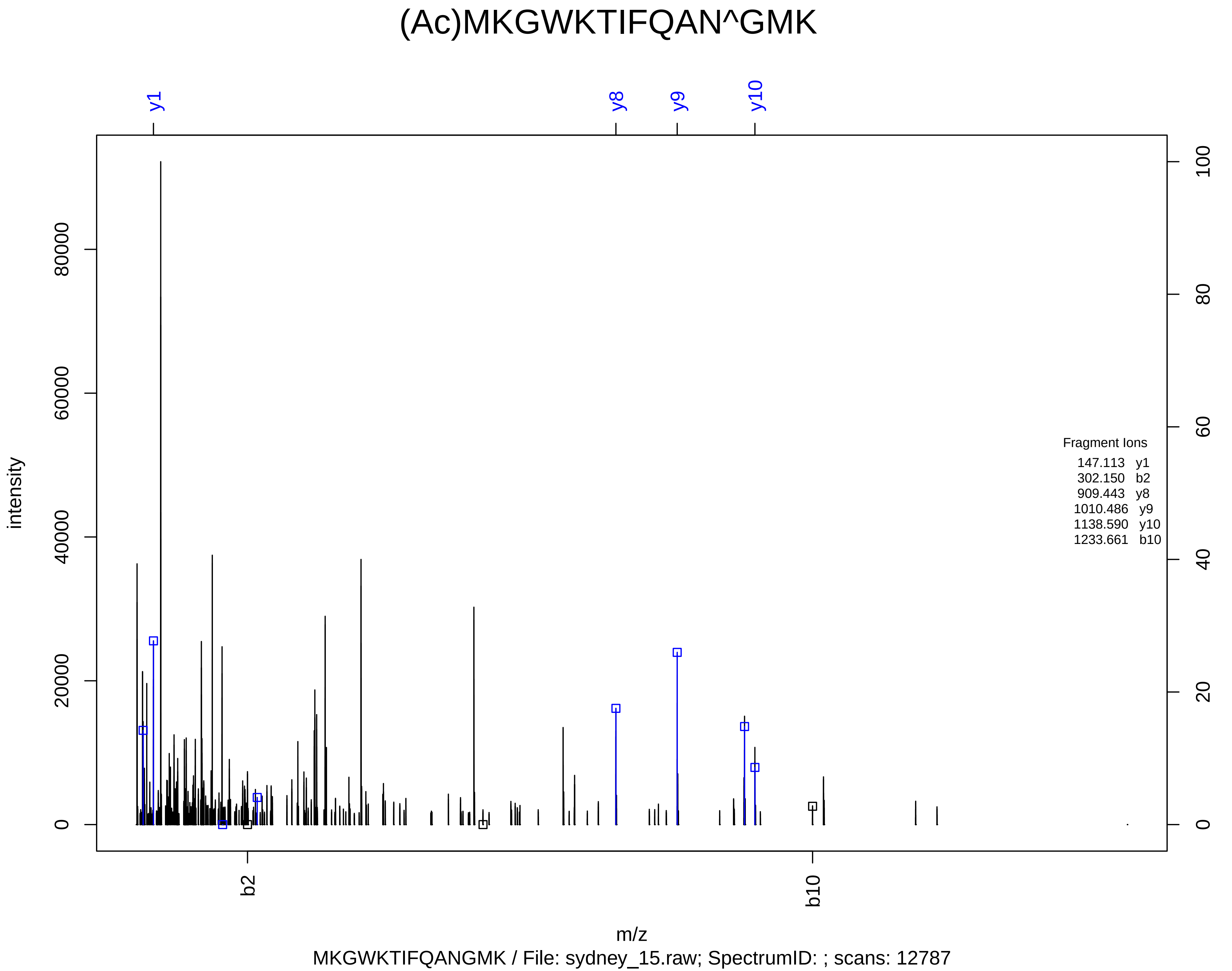
(Ac)MKGWKTIFQAN^GMK
y1
y8
y9
y10
intensity
0
20000
40000
60000
80000
0
20
40
60
80
100
Fragment Ions
147.113 y1
302.150 b2
909.443 y8
1010.486 y9
1138.590 y10
1233.661 b10
b2
b10
m/z
MKGWKTIFQANGMK / File: sydney_15.raw; SpectrumID: ; scans: 12787

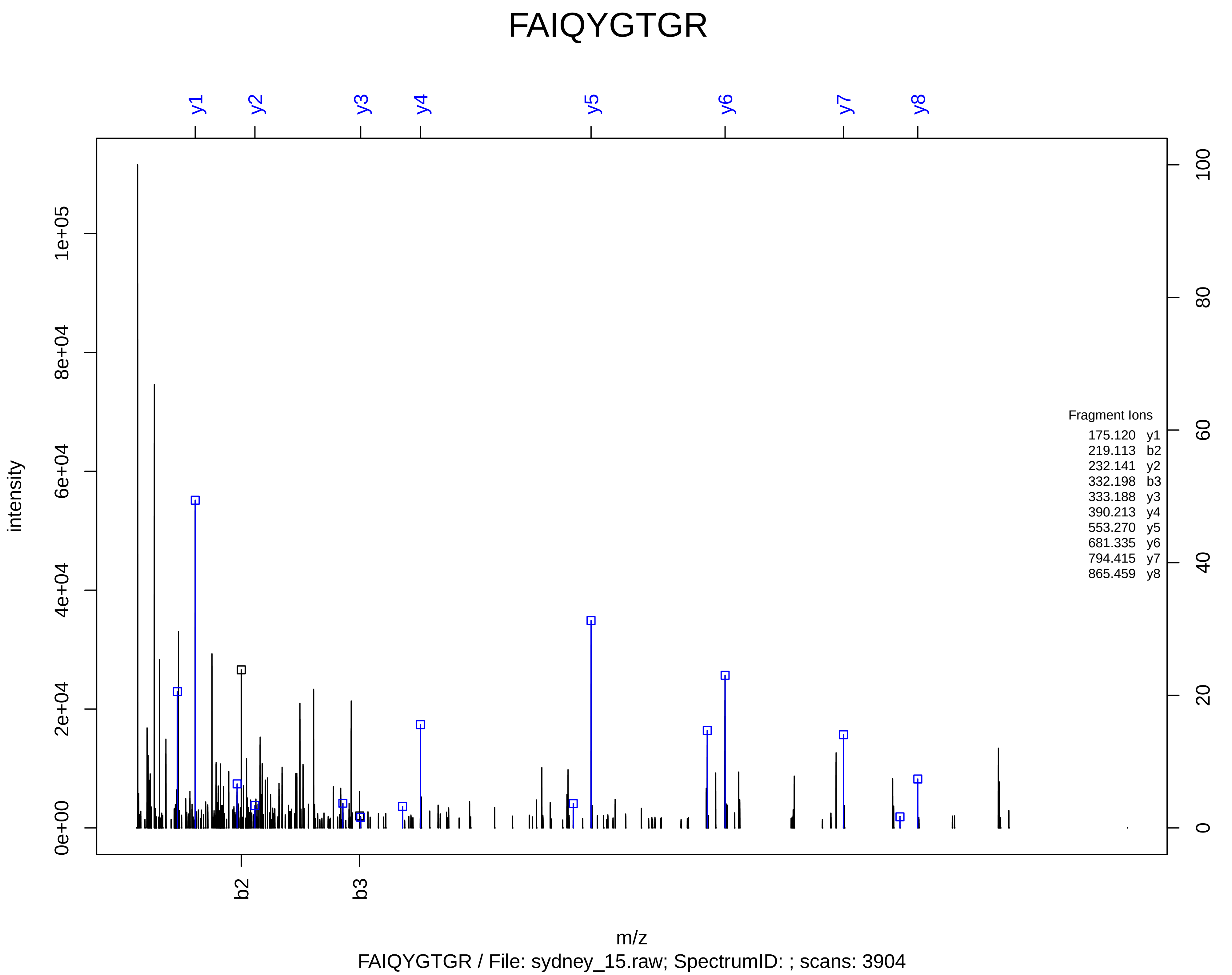
FAIQYGTGR
y1
y2
y3
y4
y5
y6
y7
y8
b2
b3
intensity
m/z
0e+00
2e+04
4e+04
6e+04
8e+04
1e+05
0
20
40
60
80
100
Fragment Ions
175.120 y1
219.113 b2
232.141 y2
332.198 b3
333.188 y3
390.213 y4
553.270 y5
681.335 y6
794.415 y7
865.459 y8
FAIQYGTGR / File: sydney_15.raw; SpectrumID: ; scans: 3904

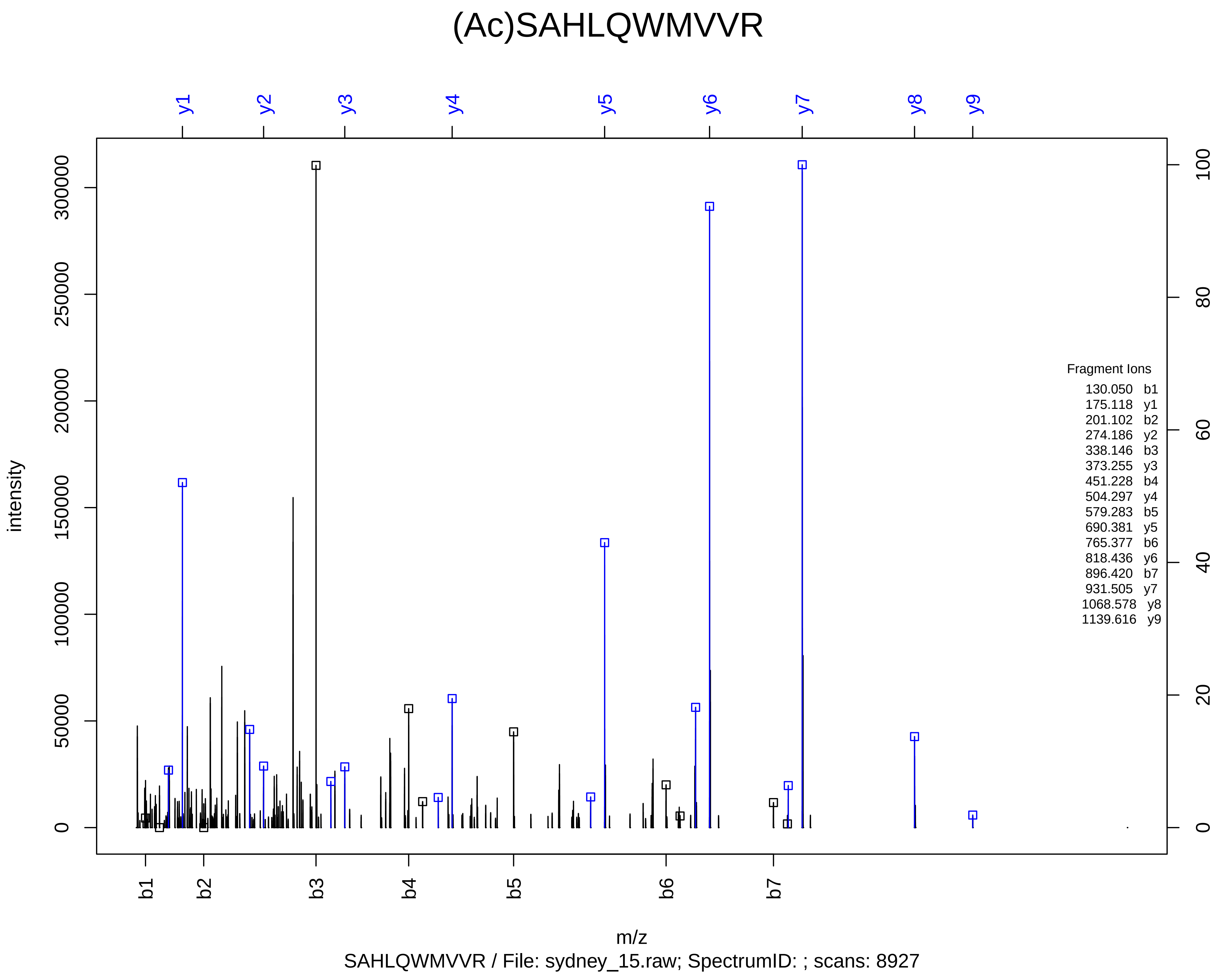

(Ac)SAHLQWMVVR
y1
y2
y3
y4
y5
y6
y7
y8
y9
intensity
300000
250000
200000
150000
100000
50000
0
100
80
60
40
20
0
Fragment Ions
130.050 b1
175.118 y1
201.102 b2
274.186 y2
338.146 b3
373.255 y3
451.228 b4
504.297 y4
579.283 b5
690.381 y5
765.377 b6
818.436 y6
896.420 b7
931.505 y7
1068.578 y8
1139.616 y9
b1
b2
b3
b4
b5
b6
b7
m/z
SAHLQWMVVR / File: sydney_15.raw; SpectrumID: ; scans: 8927

PVAVGPYGQSQPSCFDR

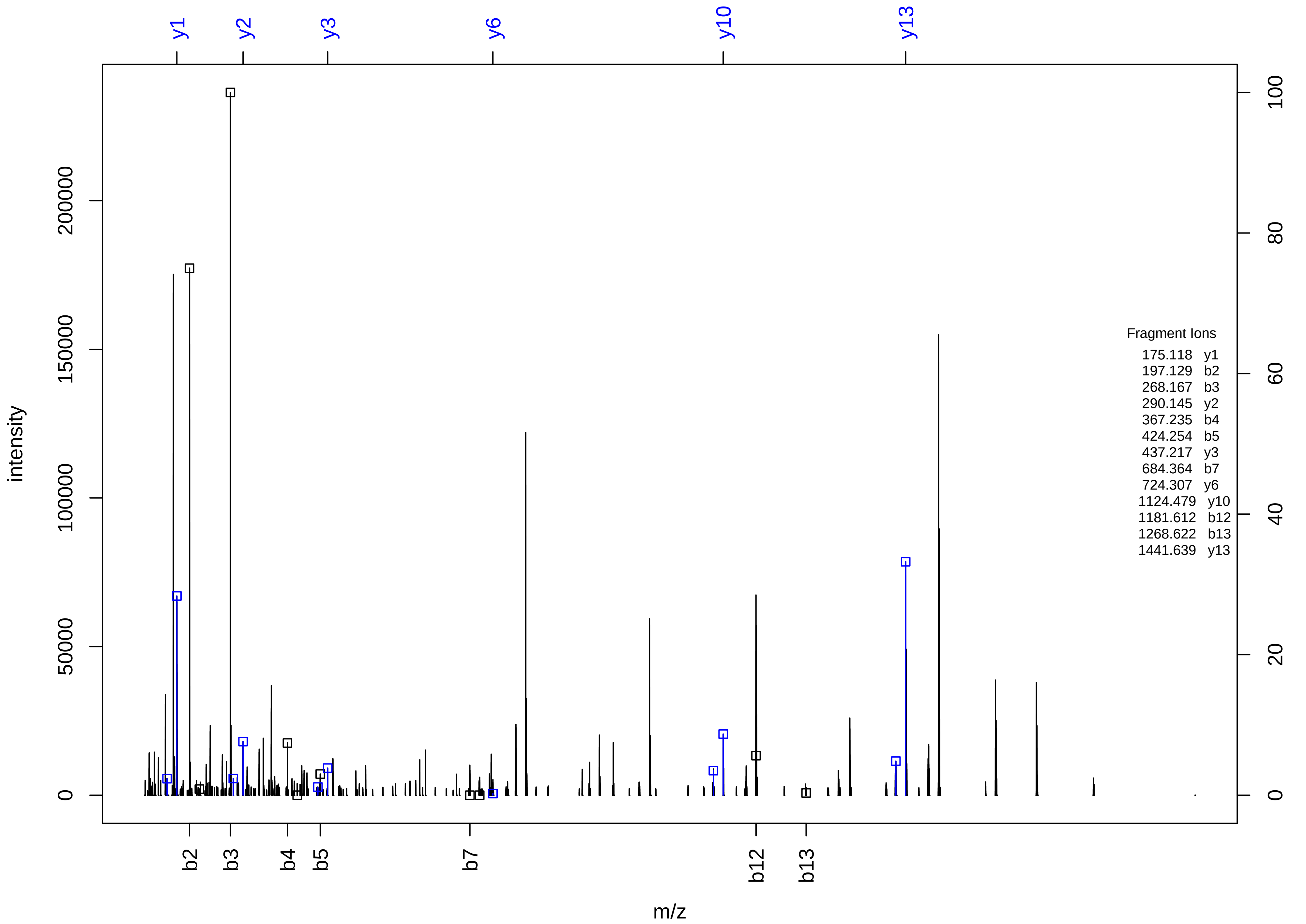

PVAVGPYGQSQPSCFDR / File: sydney_15.raw; SpectrumID: ; scans: 4911

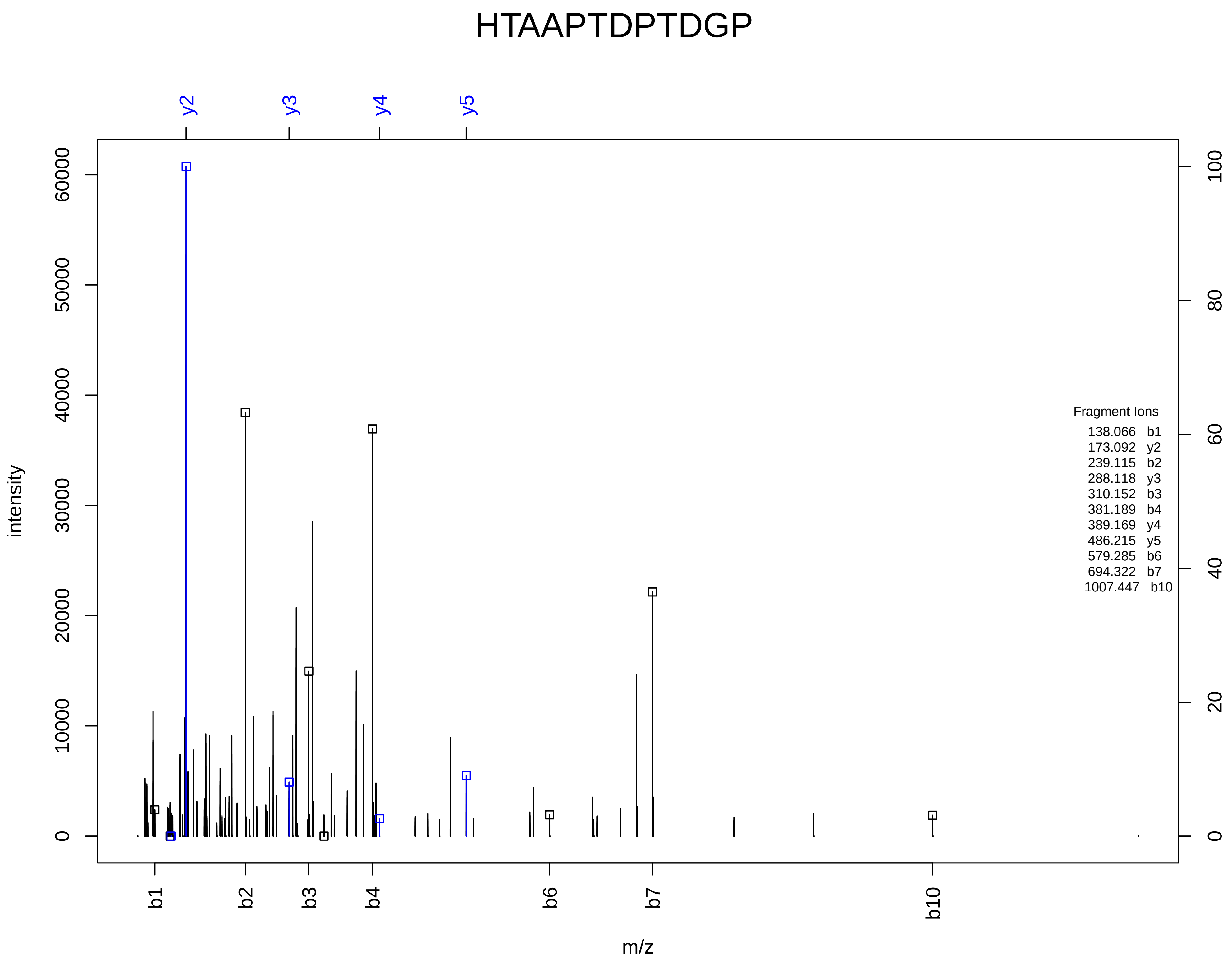

HTAAPTDPTDGP / File: sydney_15.raw; SpectrumID: ; scans: 1186

LLAEQEELER

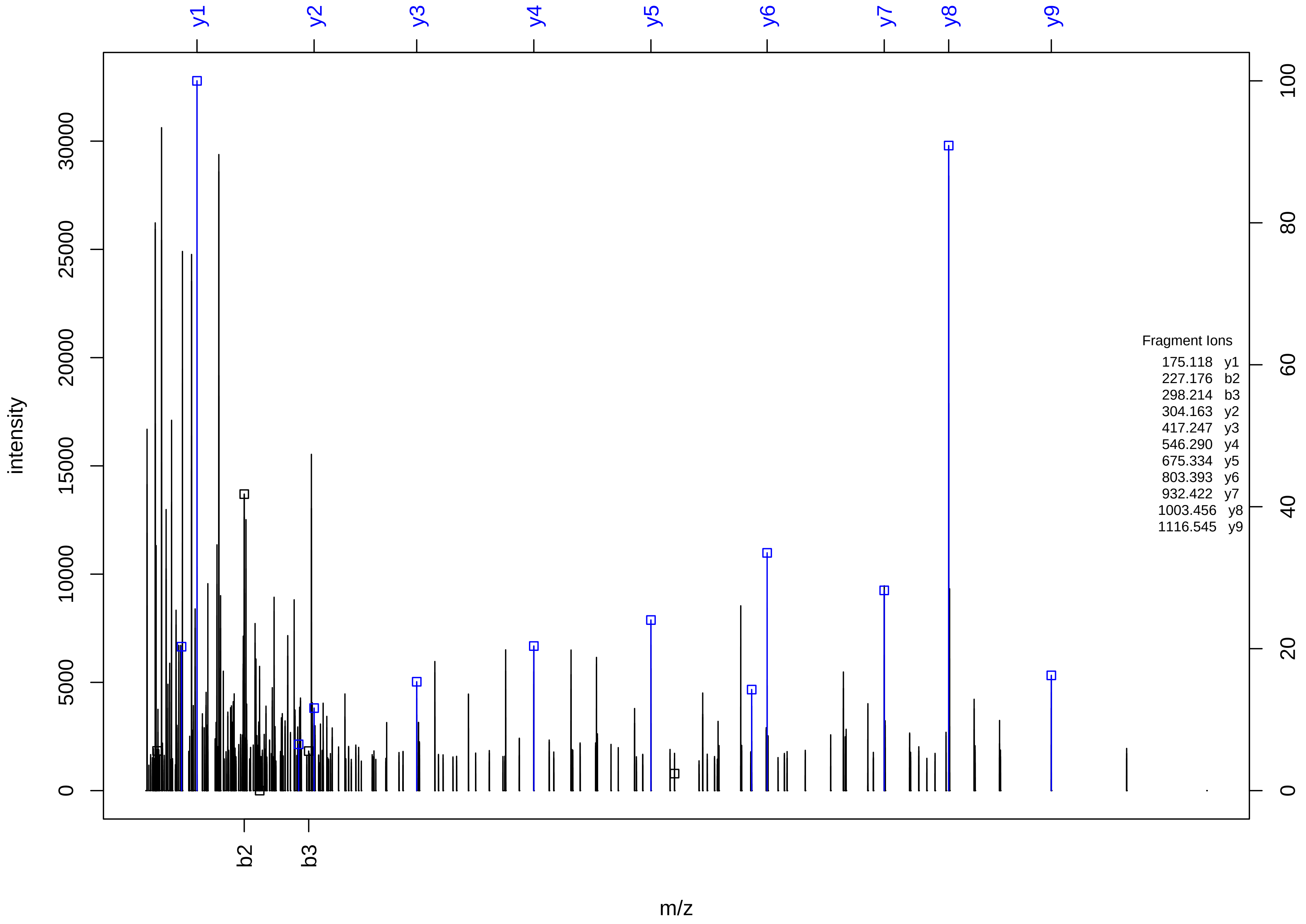

LLAEQEELER / File: sydney_15.raw; SpectrumID: ; scans: 3481

DALLLIFANK

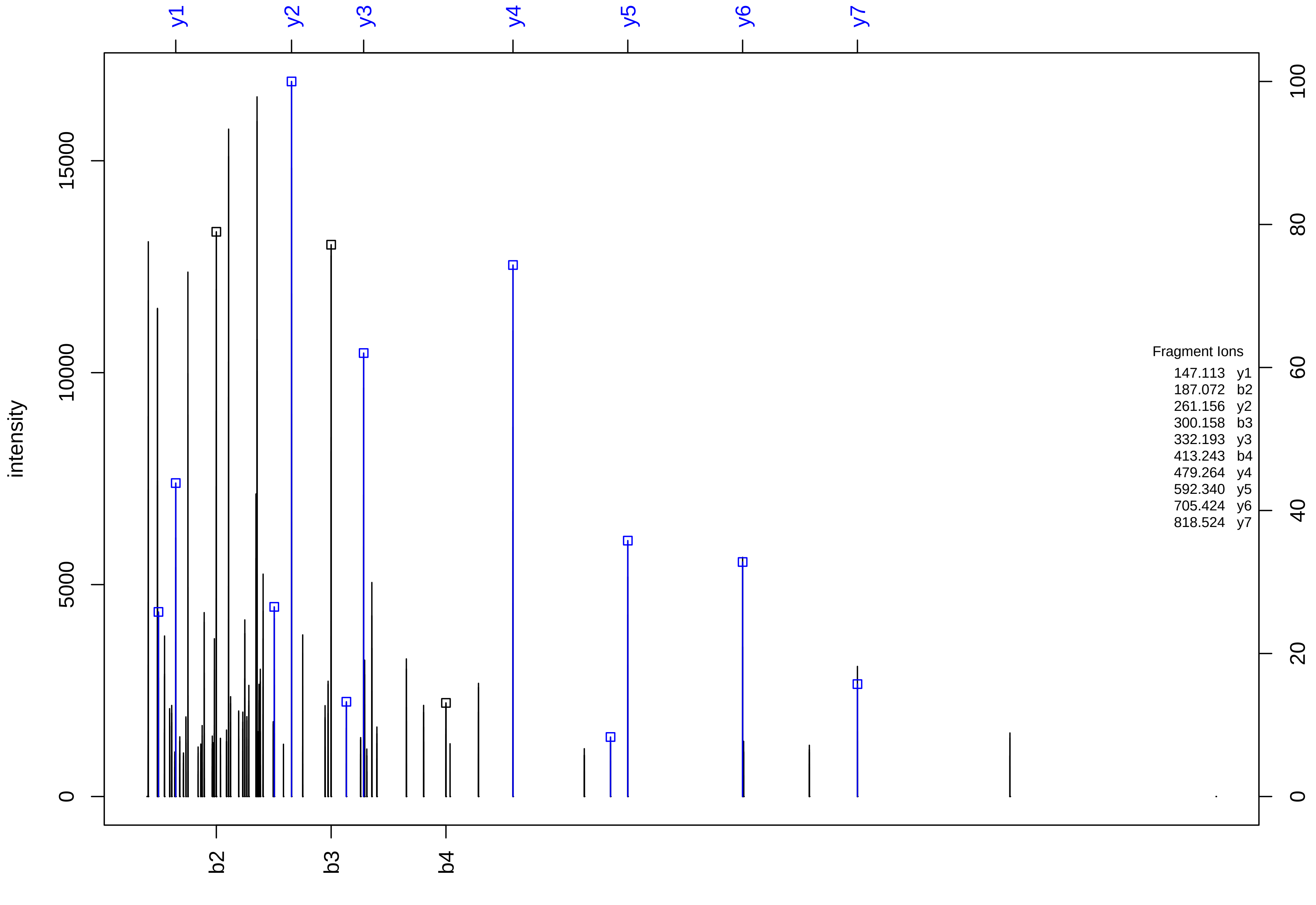

DALLLIFANK / File: sydney_15.raw; SpectrumID: ; scans: 12639

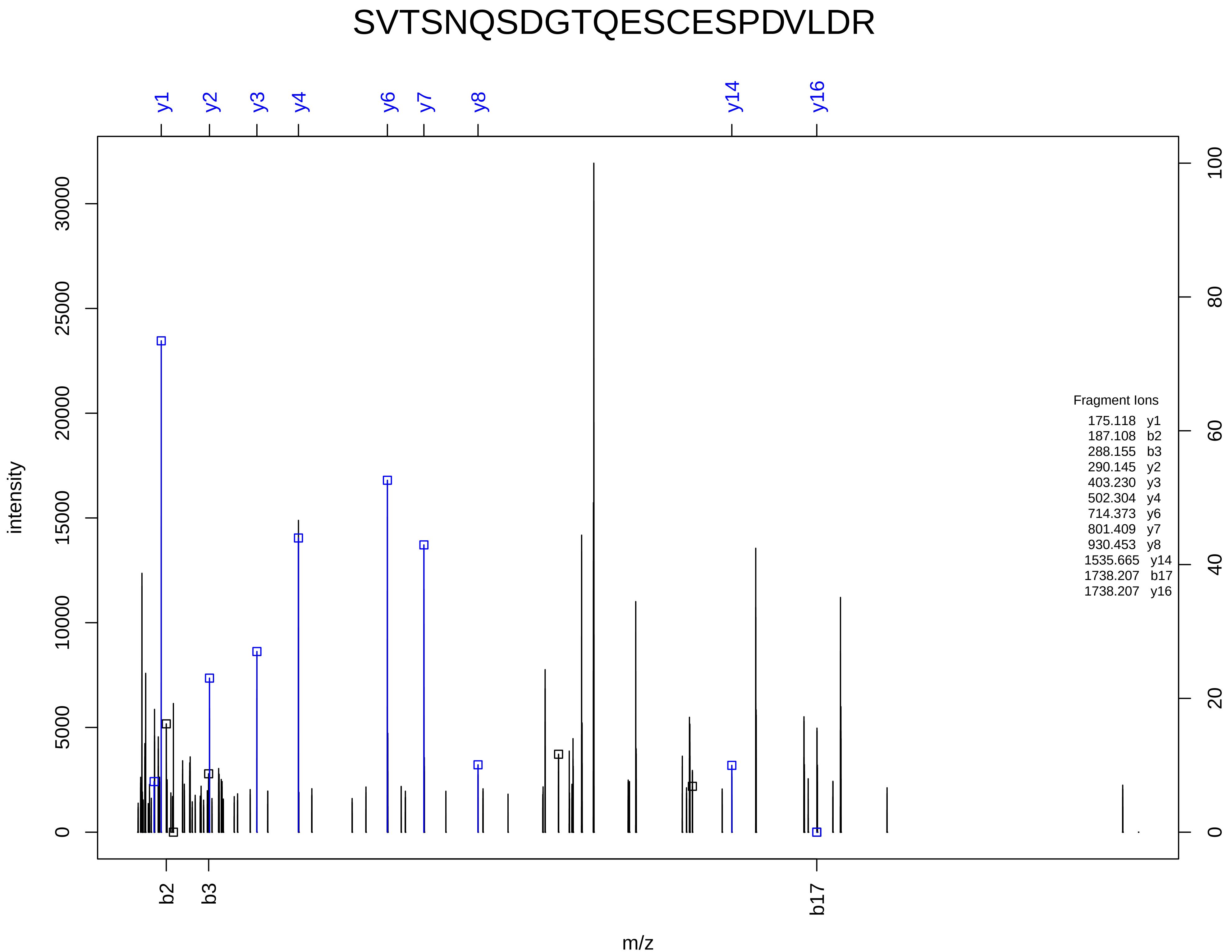
SVTSNQSDGTQESCESPDVLDR
y1
y2
y3
y4
y6
y7
y8
y14
y16
intensity
0
5000
10000
15000
20000
25000
30000
0
20
40
60
80
100
Fragment Ions
175.118 y1
187.108 b2
288.155 b3
290.145 y2
403.230 y3
502.304 y4
714.373 y6
801.409 y7
930.453 y8
1535.665 y14
1738.207 b17
1738.207 y16
b2
b3
b17
m/z
SVTSNQSDGTQESCESPDVLDR / File: sydney_15.raw; SpectrumID: ; scans: 3272

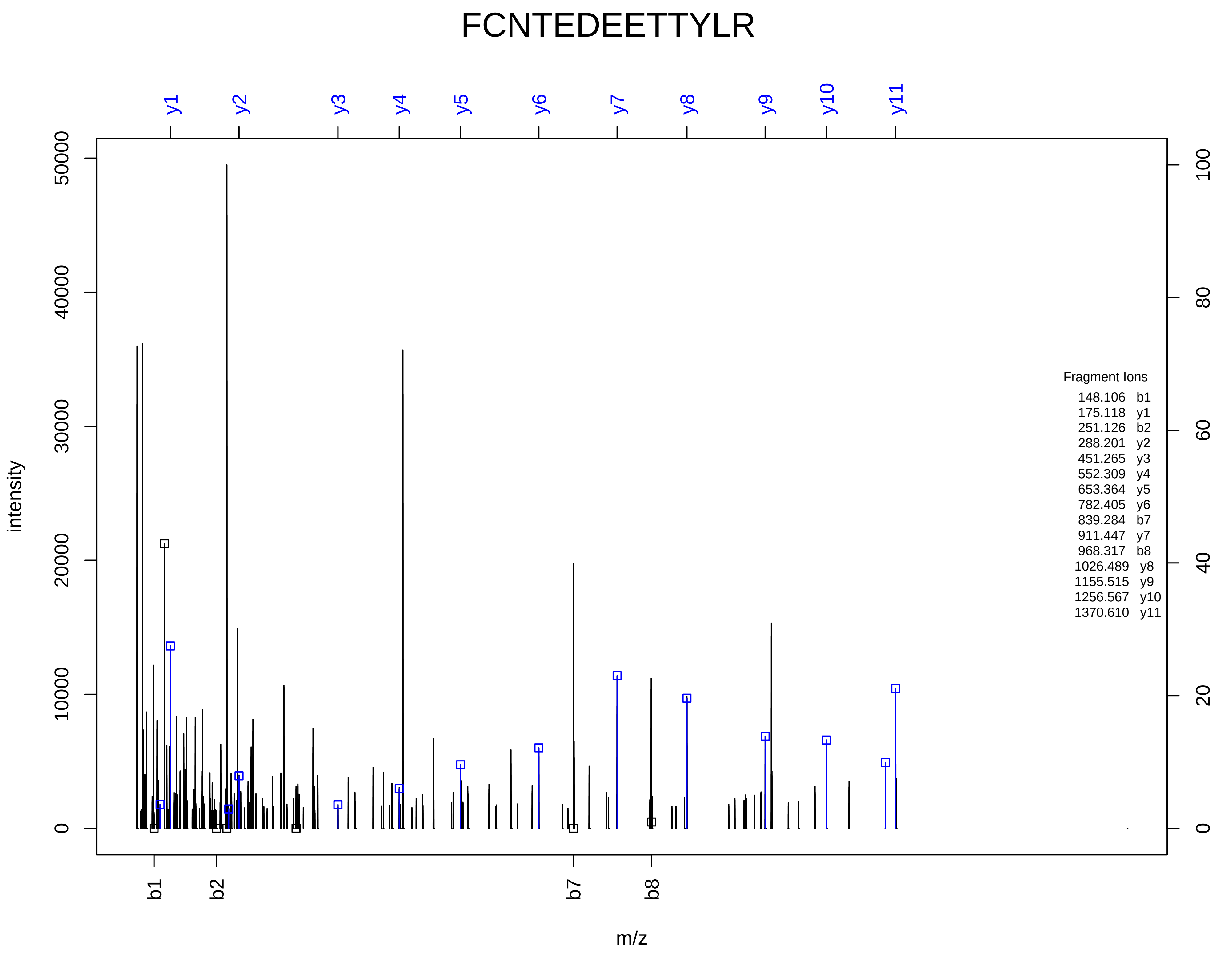

FCNTEDEETTYLR
y1
y2
y3
y4
y5
y6
y7
y8
y9
y10
y11
intensity
50000
40000
30000
20000
10000
0
100
80
60
40
20
0
Fragment Ions
148.106 b1
175.118 y1
251.126 b2
288.201 y2
451.265 y3
552.309 y4
653.364 y5
782.405 y6
839.284 b7
911.447 y7
968.317 b8
1026.489 y8
1155.515 y9
1256.567 y10
1370.610 y11
b1
b2
b7
b8
m/z
FCNTEDEETTYLR / File: sydney_15.raw; SpectrumID: ; scans: 4141

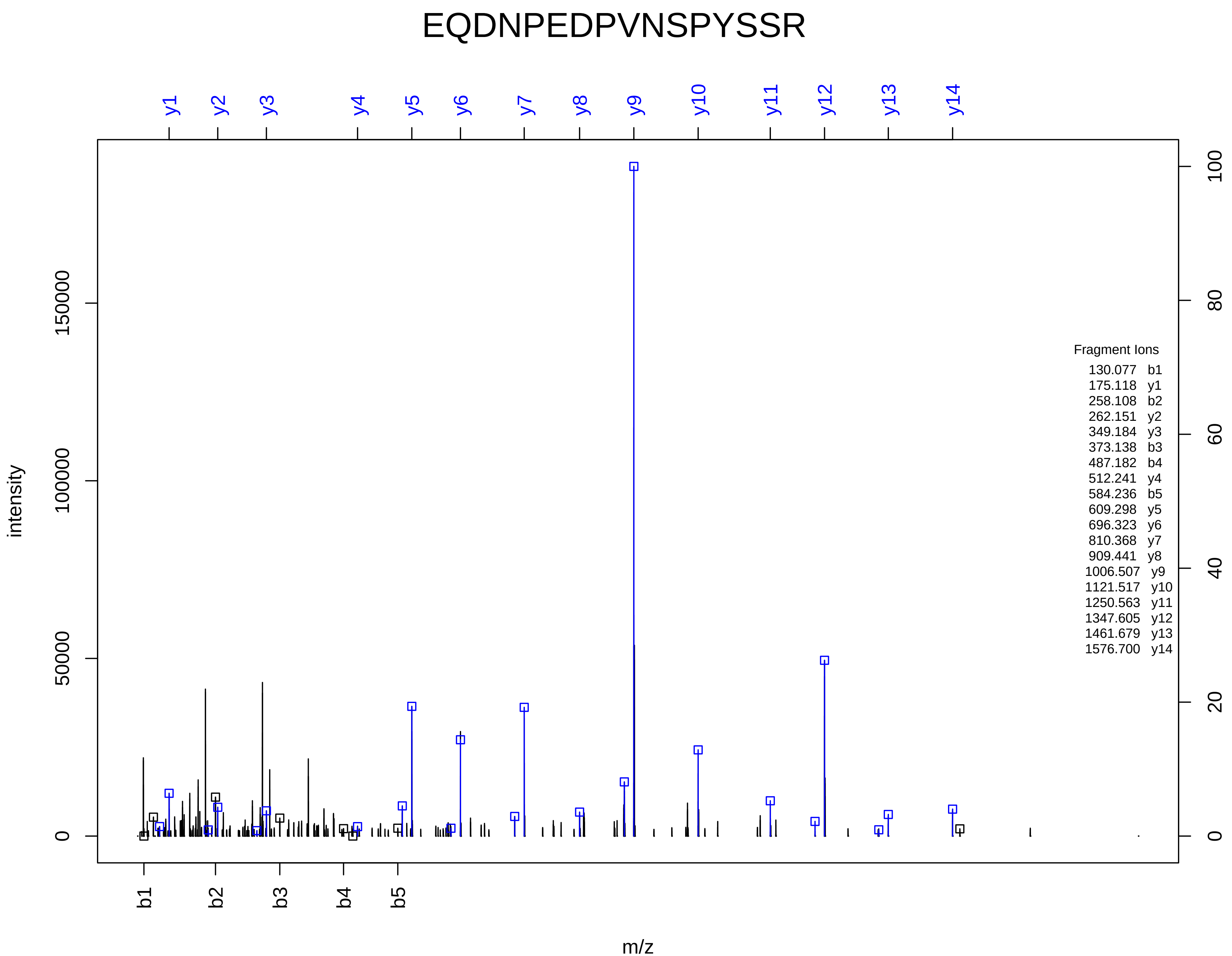

EQDNPEDPVNSPYSSR / File: sydney_15.raw; SpectrumID: ; scans: 2523

VLPPVSTEGLTPDDVPALADSVR

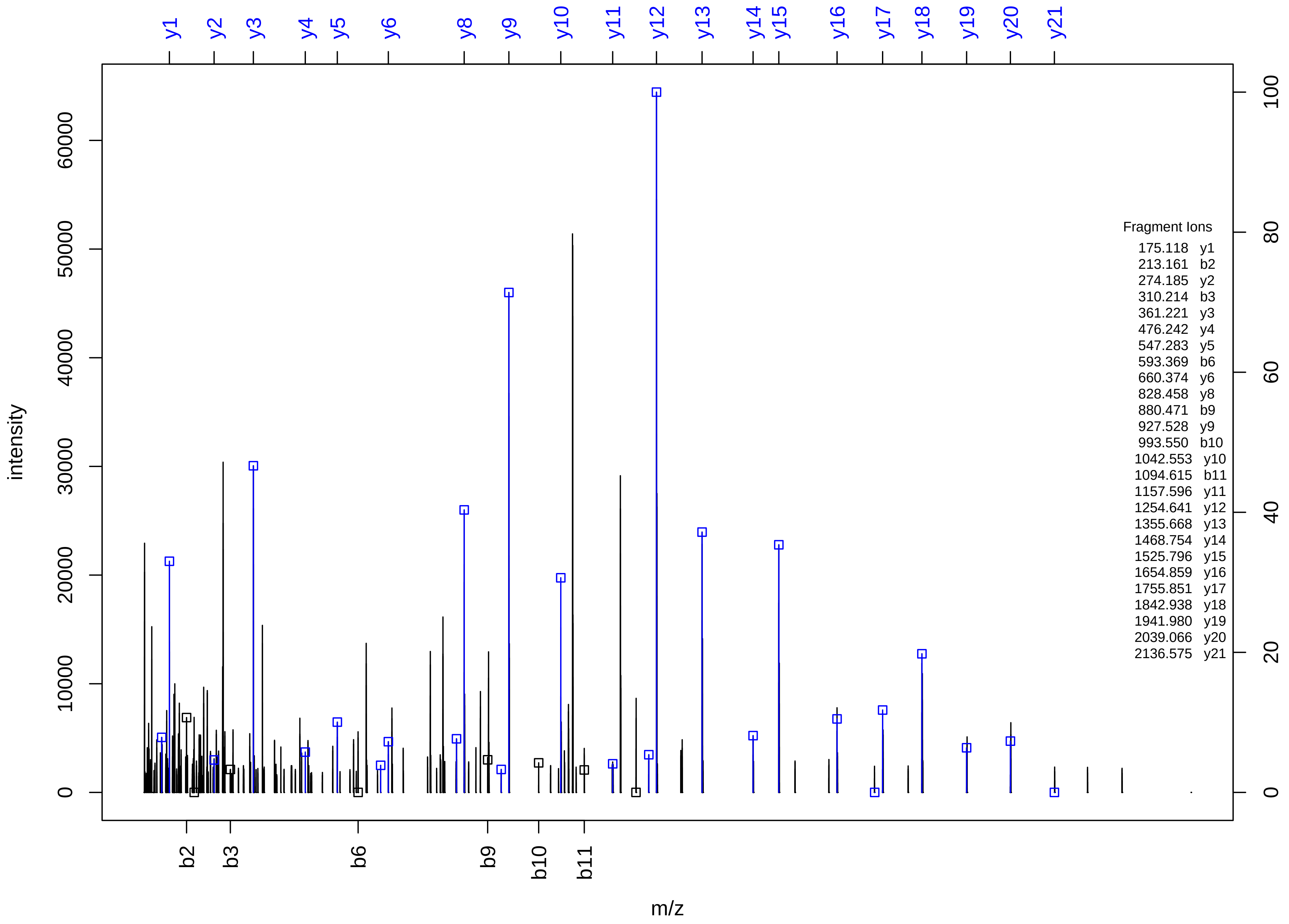

VLPPVSTEGLTPDDVPALADSVR / File: sydney_16.raw; SpectrumID: ; scans: 6430

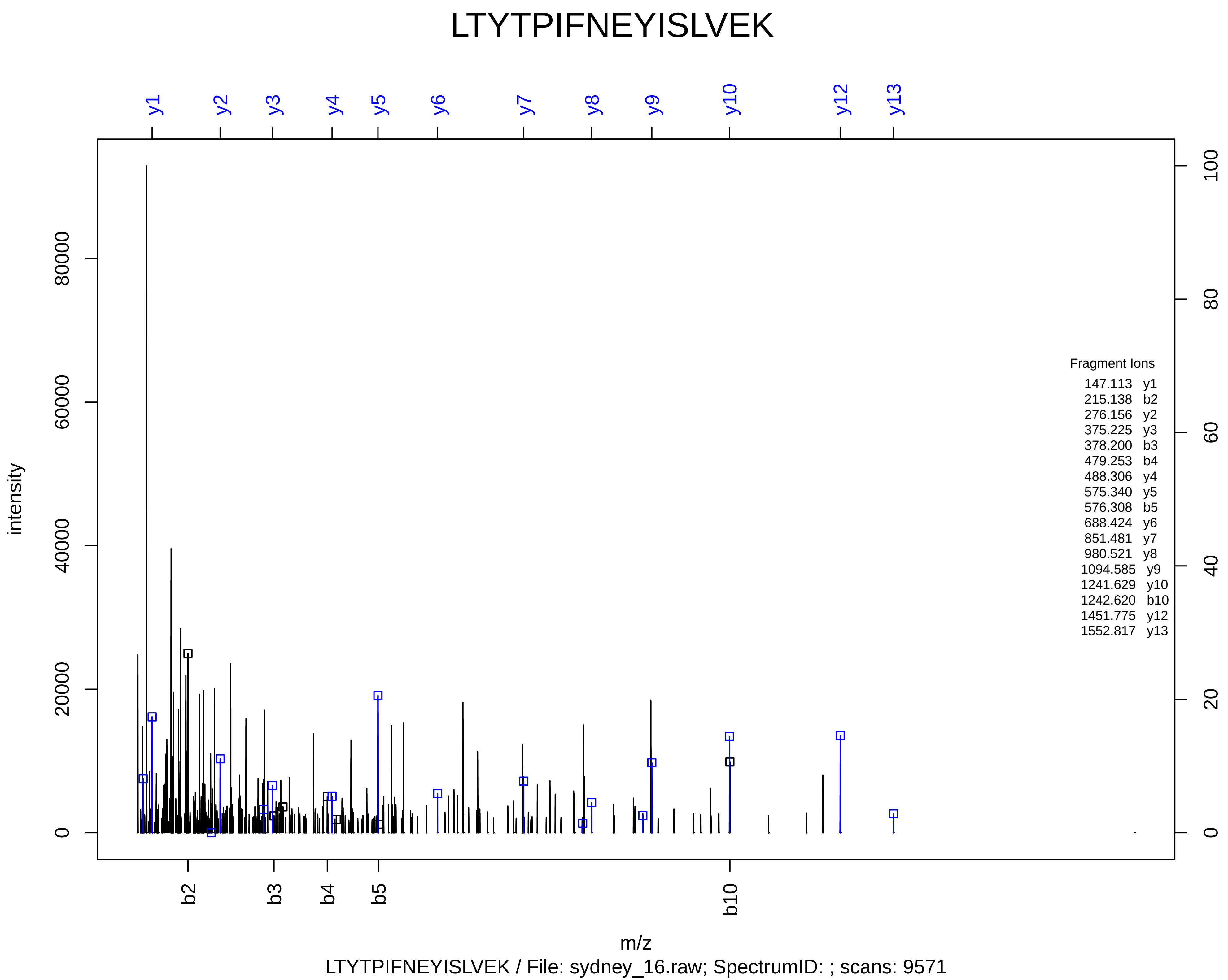
LTYTPIFNEYISLVEK
y1 y2 y3 y4 y5 y6 y7 y8 y9 y10 y12 y13
b2 b3 b4 b5 b10
intensity
m/z
Fragment Ions
147.113 y1
215.138 b2
276.156 y2
375.225 y3
378.200 b3
479.253 b4
488.306 y4
575.340 y5
576.308 b5
688.424 y6
851.481 y7
980.521 y8
1094.585 y9
1241.629 y10
1242.620 b10
1451.775 y12
1552.817 y13
LTYTPIFNEYISLVEK / File: sydney_16.raw; SpectrumID: ; scans: 9571

VPIVIQDDSLPTGPPPQIR

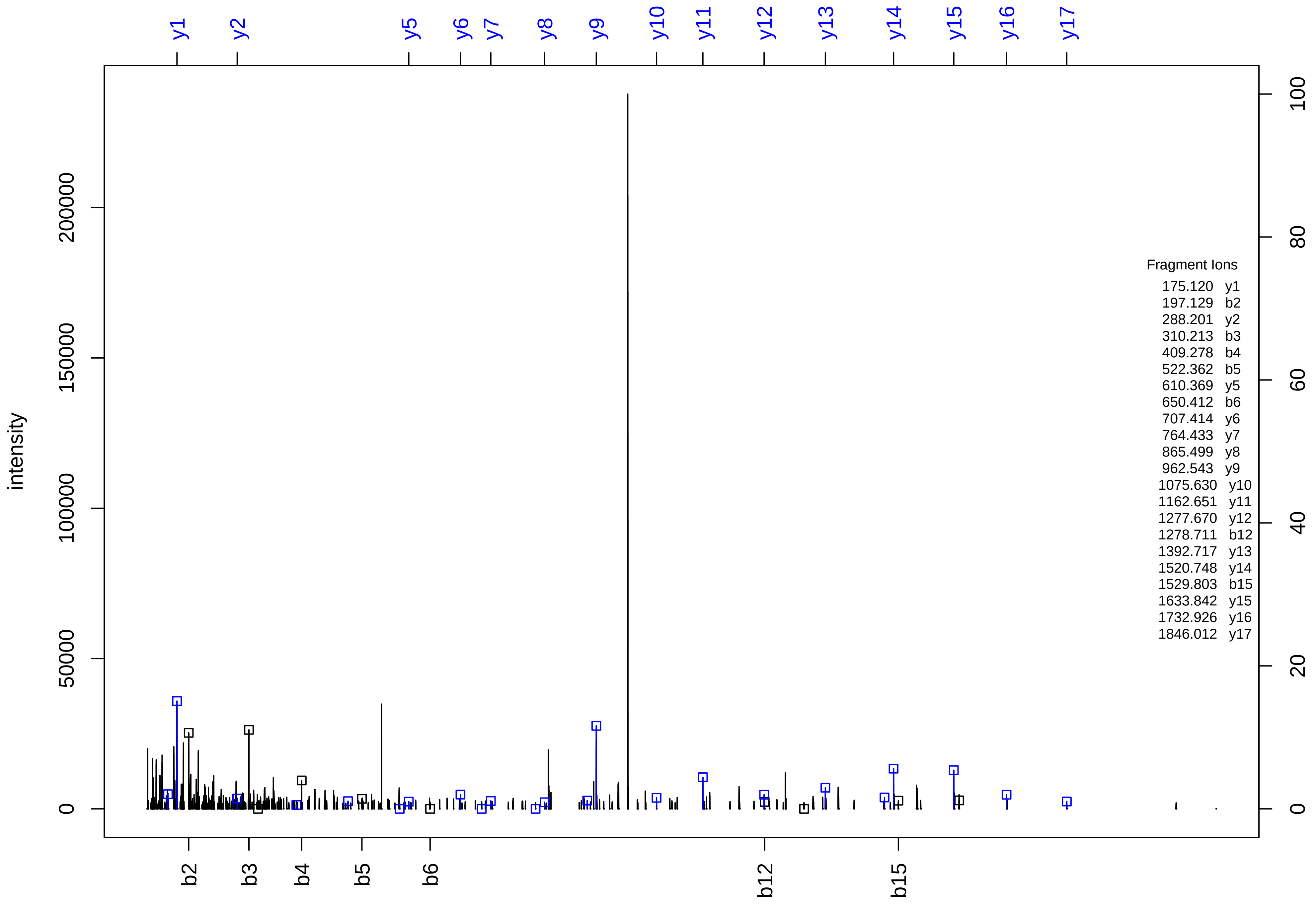

VPIVIQDDSLPTGPPPQIR / File: sydney_16.raw; SpectrumID: ; scans: 4789

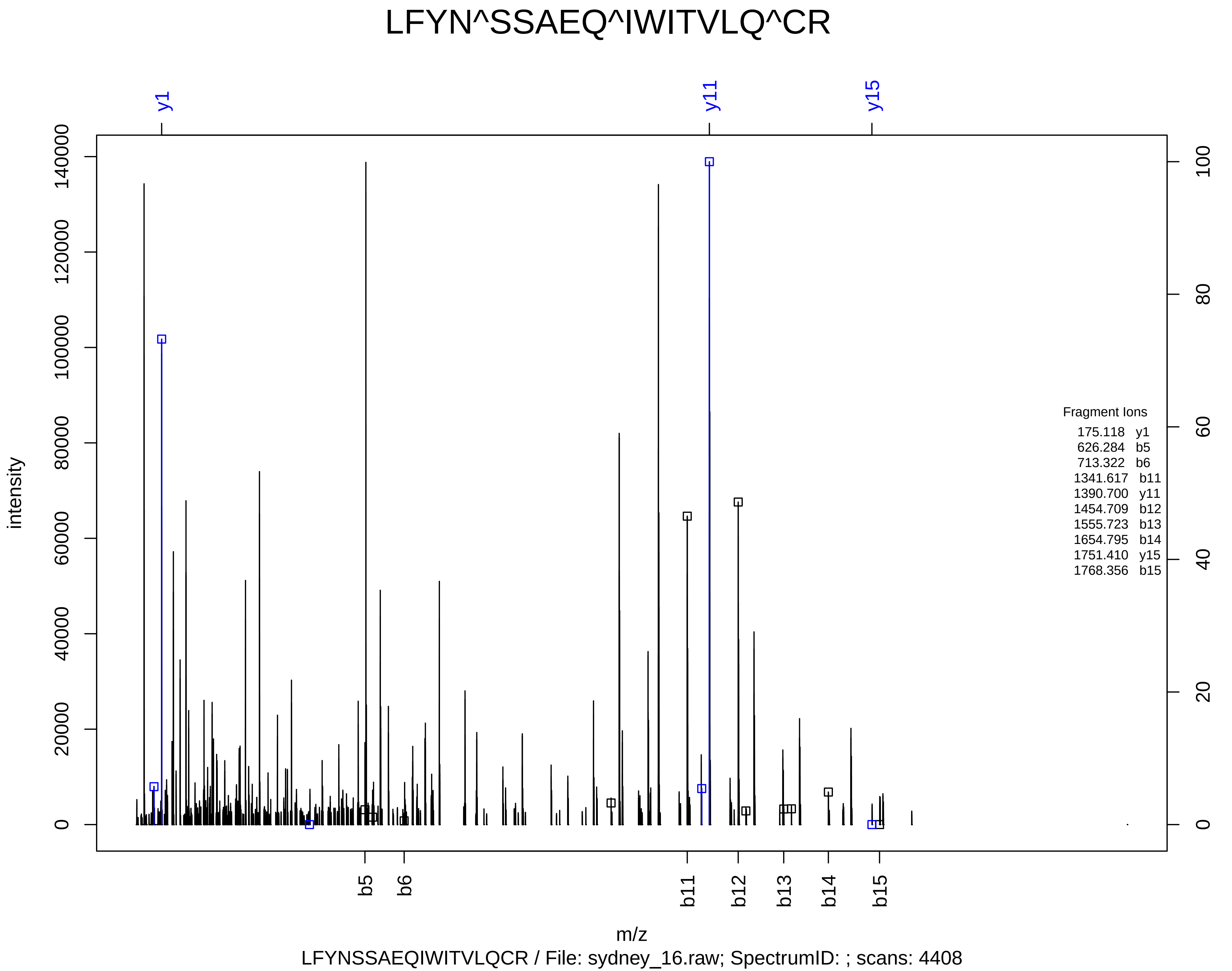

LFYN^SSAEQ^IWITVLQ^CR
y1
y11
y15
intensity
m/z
b5
b6
b11
b12
b13
b14
b15
Fragment Ions
175.118 y1
626.284 b5
713.322 b6
1341.617 b11
1390.700 y11
1454.709 b12
1555.723 b13
1654.795 b14
1751.410 y15
1768.356 b15
LFYNSSAEQIWITVLQCR / File: sydney_16.raw; SpectrumID: ; scans: 4408

AATITATSPGALWGLDR

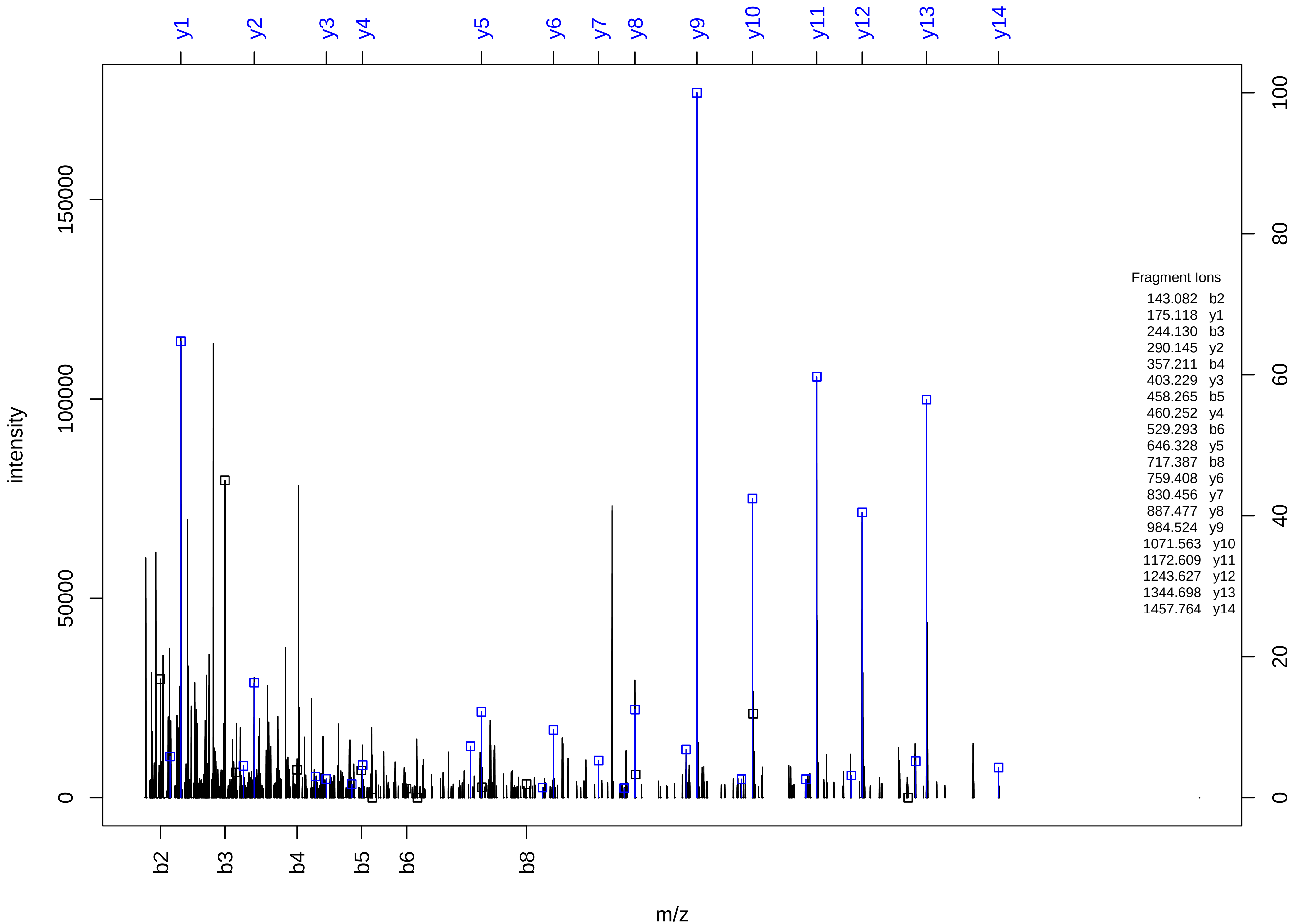

AATITATSPGALWGLDR / File: sydney_16.raw; SpectrumID: ; scans: 4721

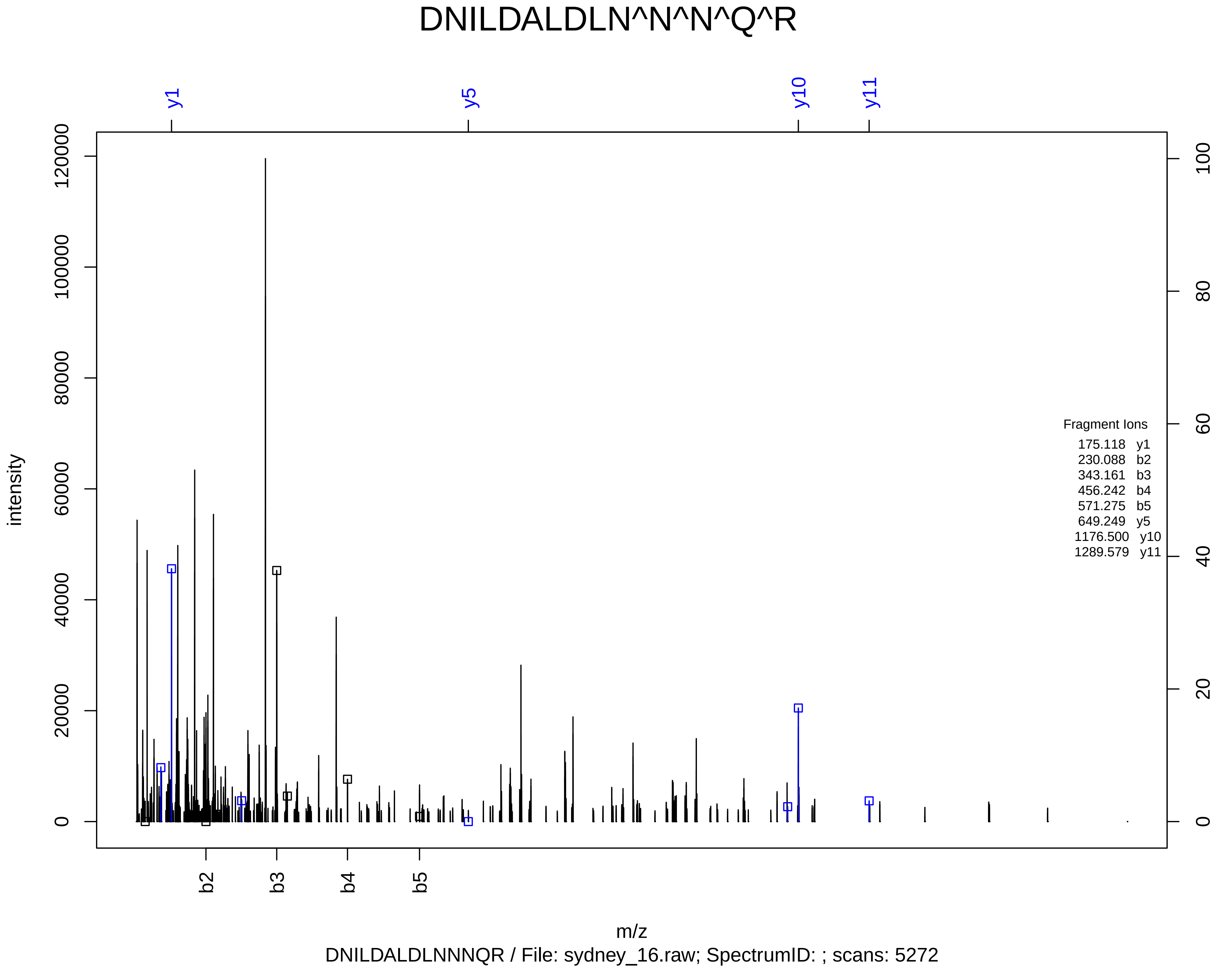
DNILDALDLN^N^N^Q^R
y1
y5
y10
y11
intensity
0
20000
40000
60000
80000
100000
120000
0
20
40
60
80
100
b2
b3
b4
b5
m/z
Fragment Ions
175.118 y1
230.088 b2
343.161 b3
456.242 b4
571.275 b5
649.249 y5
1176.500 y10
1289.579 y11
DNILDALDLNNNQR / File: sydney_16.raw; SpectrumID: ; scans: 5272

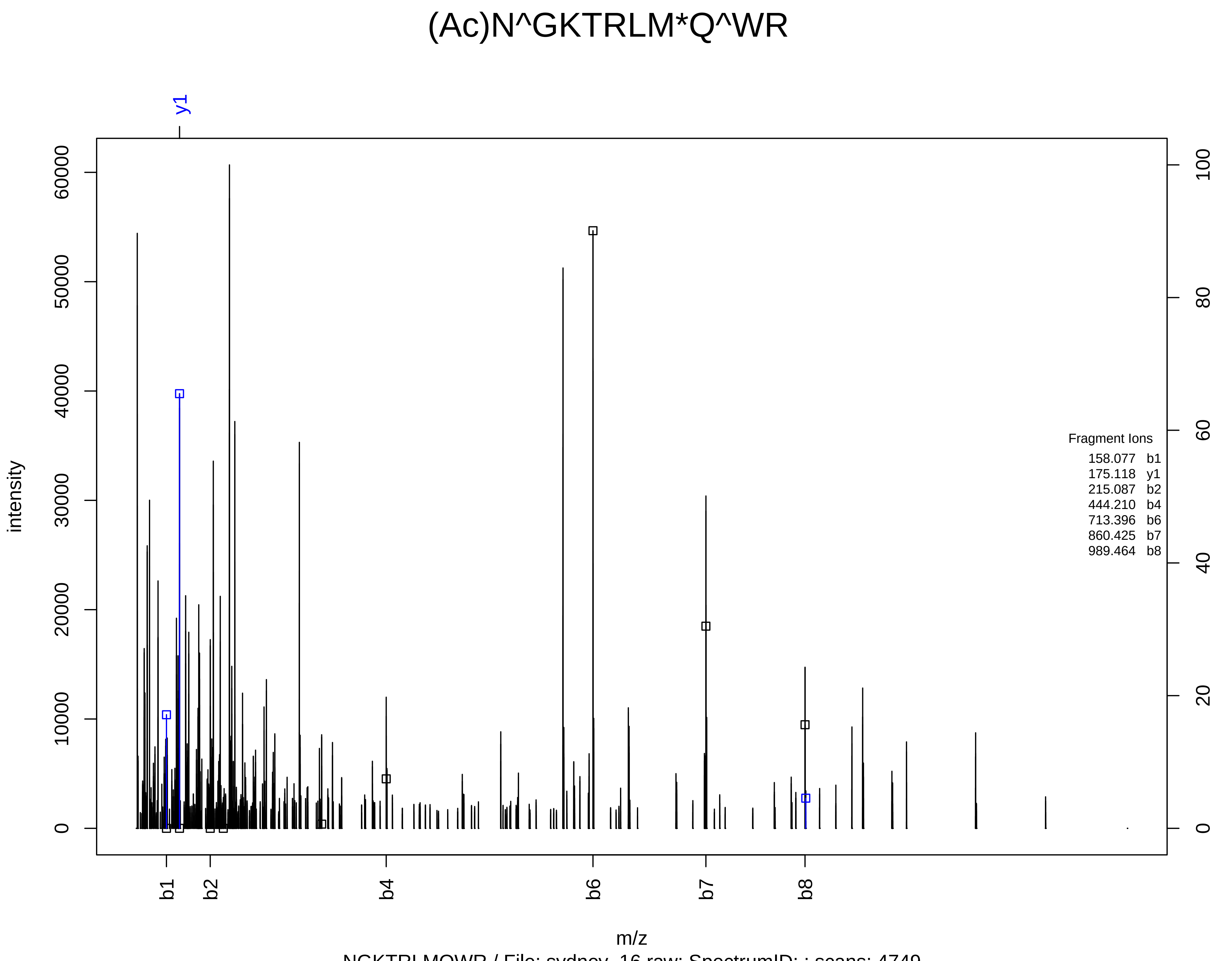
(Ac)N^GKTRLM*Q^WR
y1
intensity
0
10000
20000
30000
40000
50000
60000
0
20
40
60
80
100
Fragment Ions
158.077 b1
175.118 y1
215.087 b2
444.210 b4
713.396 b6
860.425 b7
989.464 b8
b1
b2
b4
b6
b7
b8
m/z
NGKTRLMQWR / File: sydney_16.raw; SpectrumID: ; scans: 4749

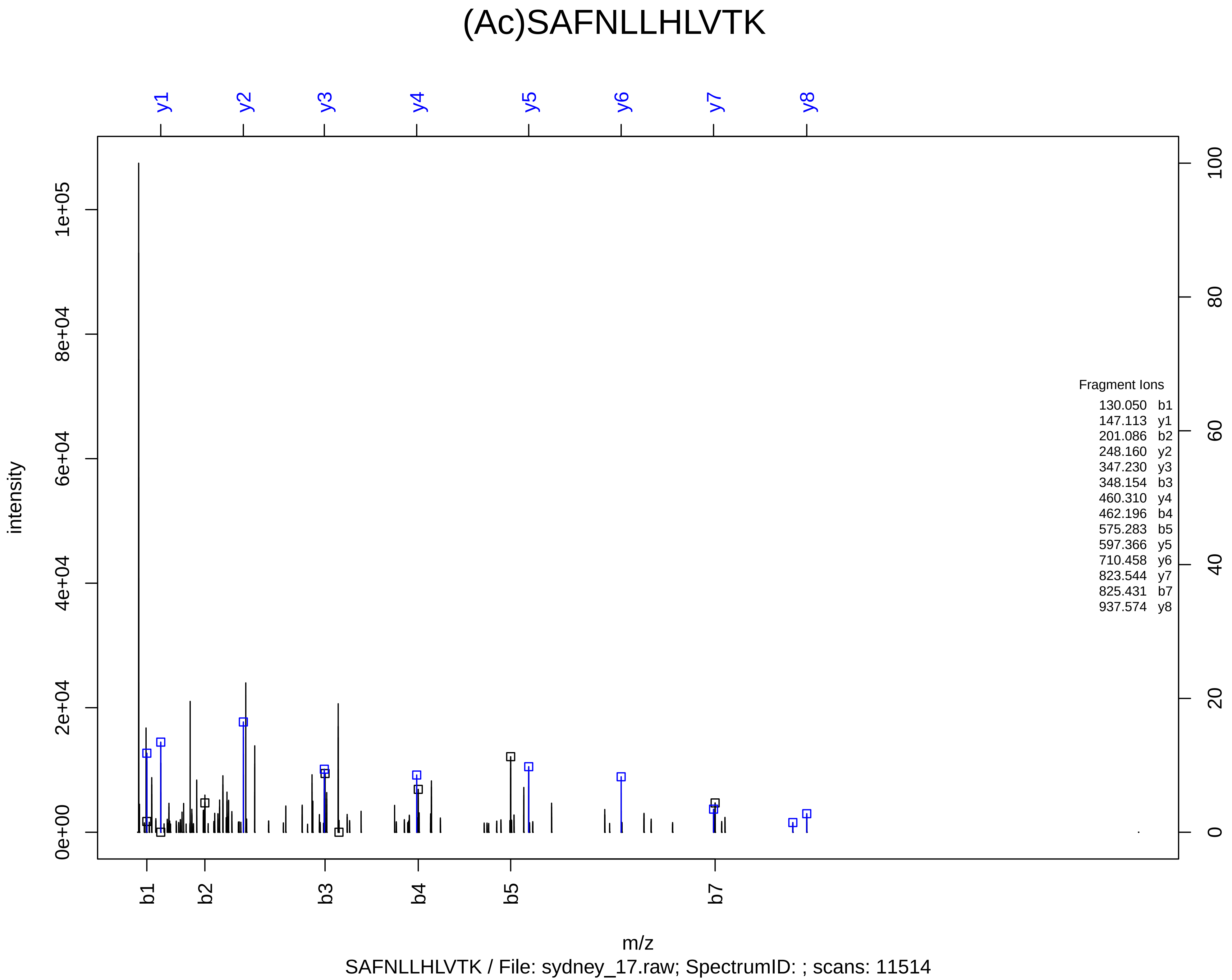

(Ac)SAFNLLHLVTK
y1
y2
y3
y4
y5
y6
y7
y8
intensity
0e+00
2e+04
4e+04
6e+04
8e+04
1e+05
0
20
40
60
80
100
Fragment Ions
130.050 b1
147.113 y1
201.086 b2
248.160 y2
347.230 y3
348.154 b3
460.310 y4
462.196 b4
575.283 b5
597.366 y5
710.458 y6
823.544 y7
825.431 b7
937.574 y8
b1
b2
b3
b4
b5
b7
m/z
SAFNLLHLVTK / File: sydney_17.raw; SpectrumID: ; scans: 11514

LLDLLEGLTGTSLPK

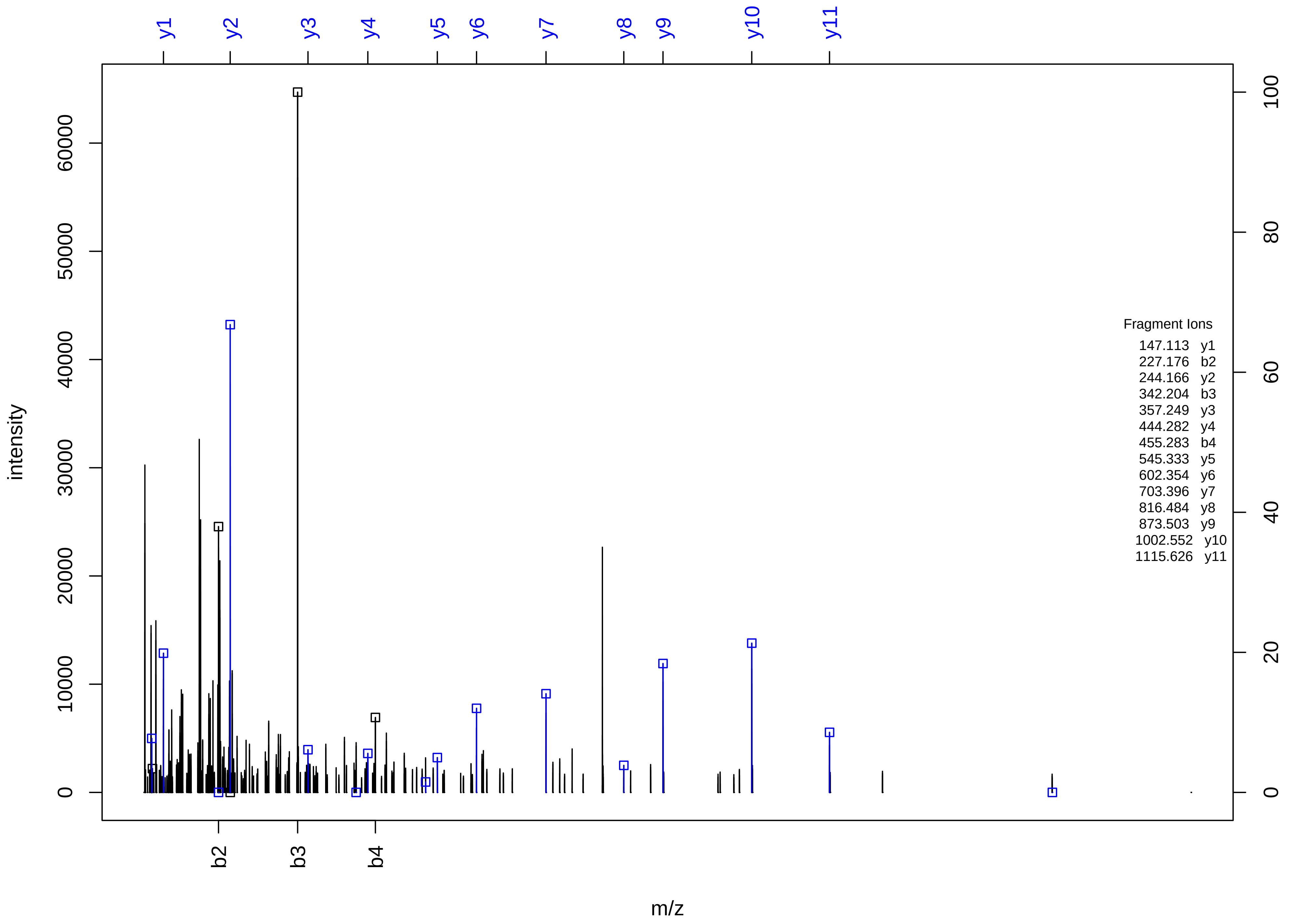

LLDLLEGLTGTSLPK / File: sydney_17.raw; SpectrumID: ; scans: 12706

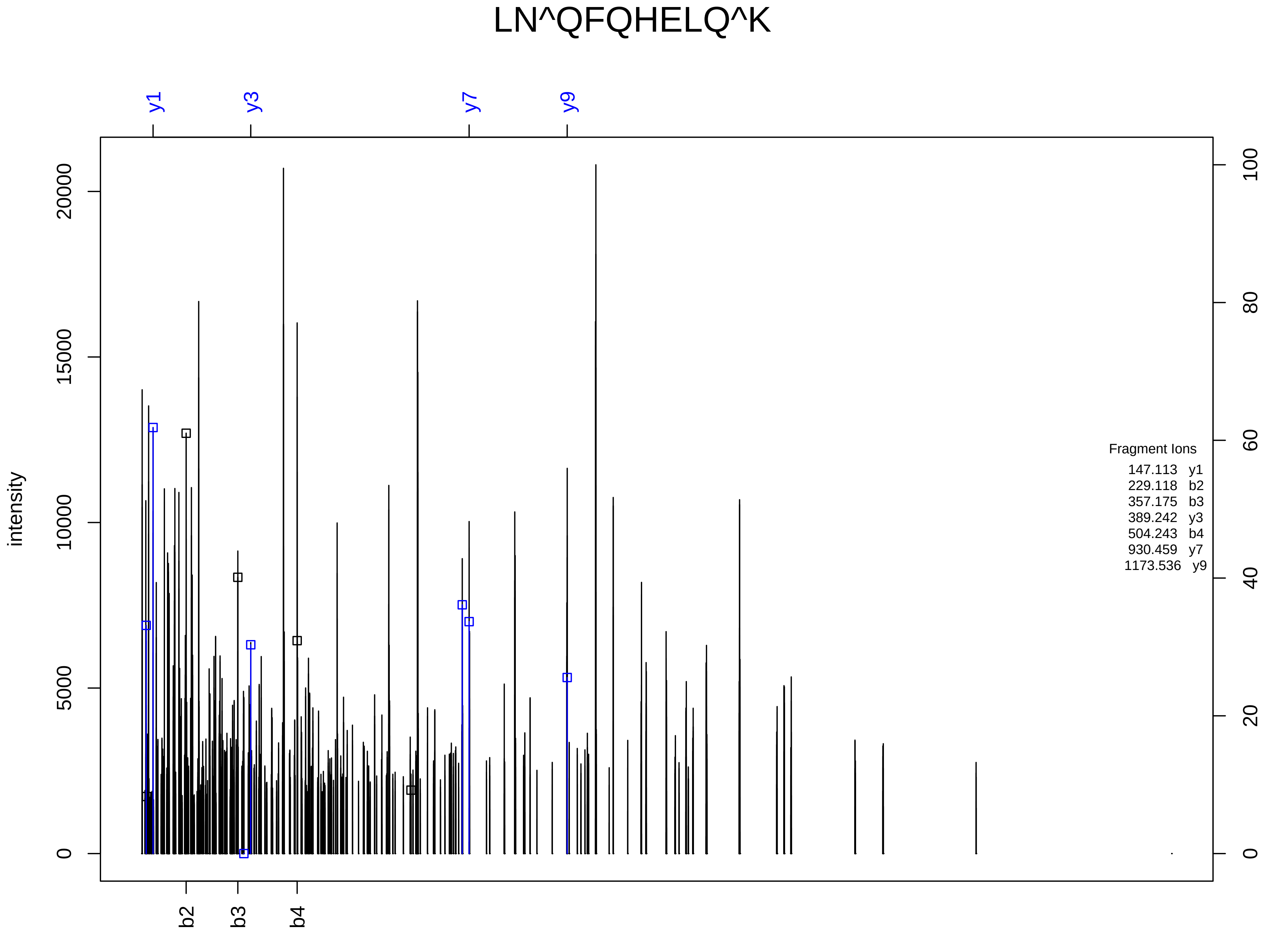

LNQFQHELQK / File: sydney_17.raw; SpectrumID: ; scans: 12494

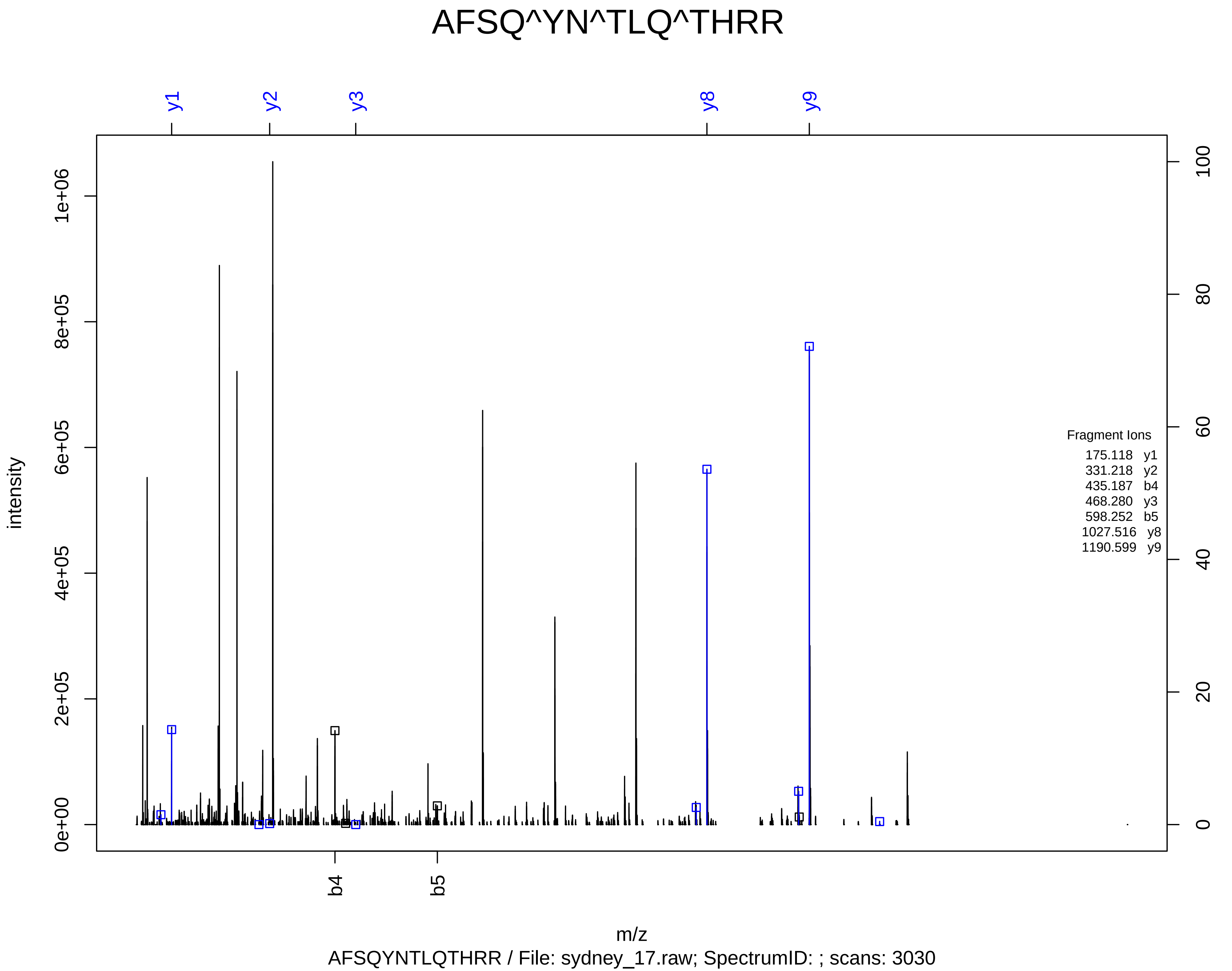
AFSQ^YN^TLQ^THRR
y1
y2
y3
y8
y9
b4
b5
intensity
m/z
0e+00
2e+05
4e+05
6e+05
8e+05
1e+06
0
20
40
60
80
100
Fragment Ions
175.118 y1
331.218 y2
435.187 b4
468.280 y3
598.252 b5
1027.516 y8
1190.599 y9
AFSQYNTLQTHRR / File: sydney_17.raw; SpectrumID: ; scans: 3030

DMLESPDFSTVLNTCLNR

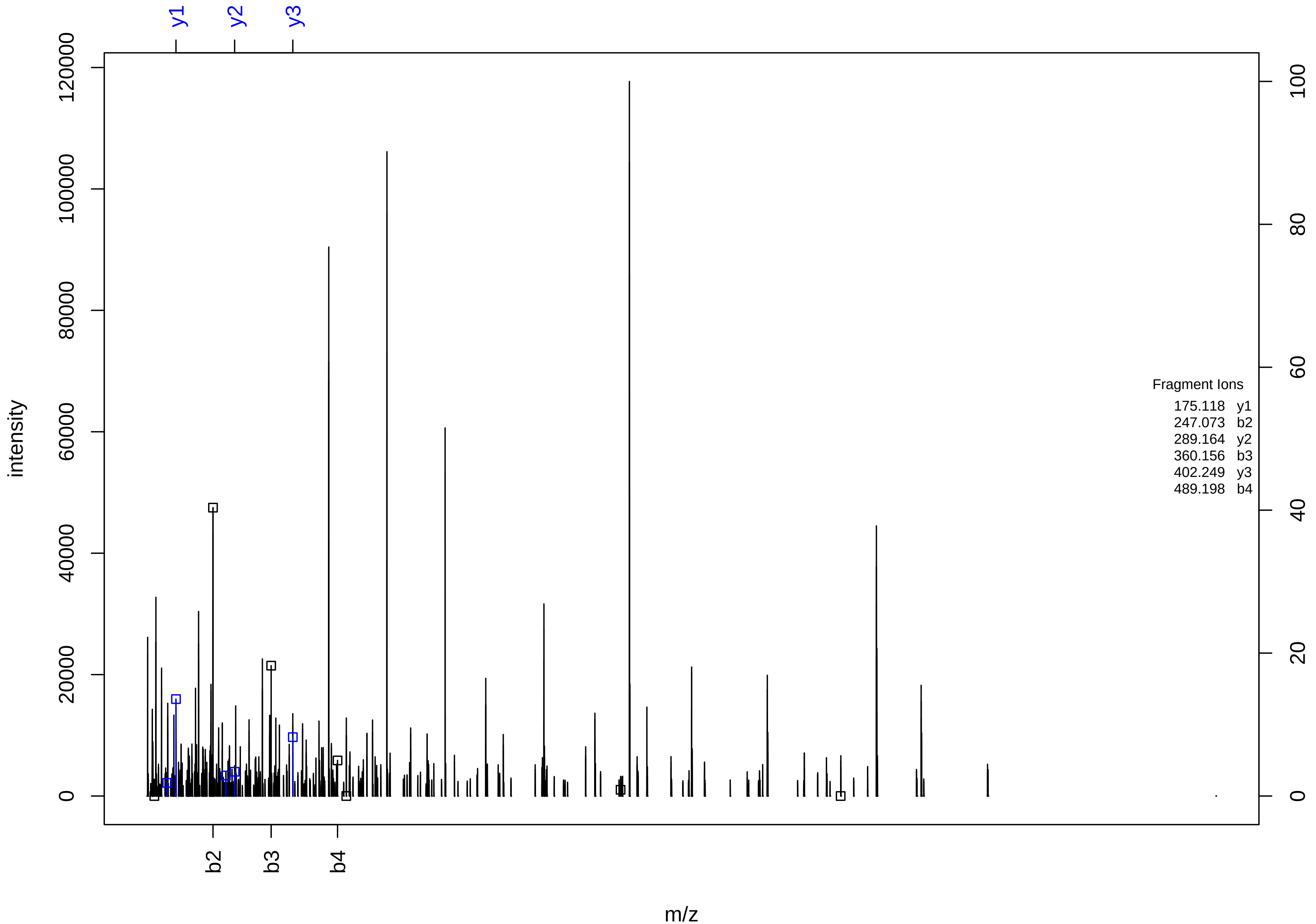

DMLESPDFSTVLNTCLNR / File: sydney_17.raw; SpectrumID: ; scans: 10000

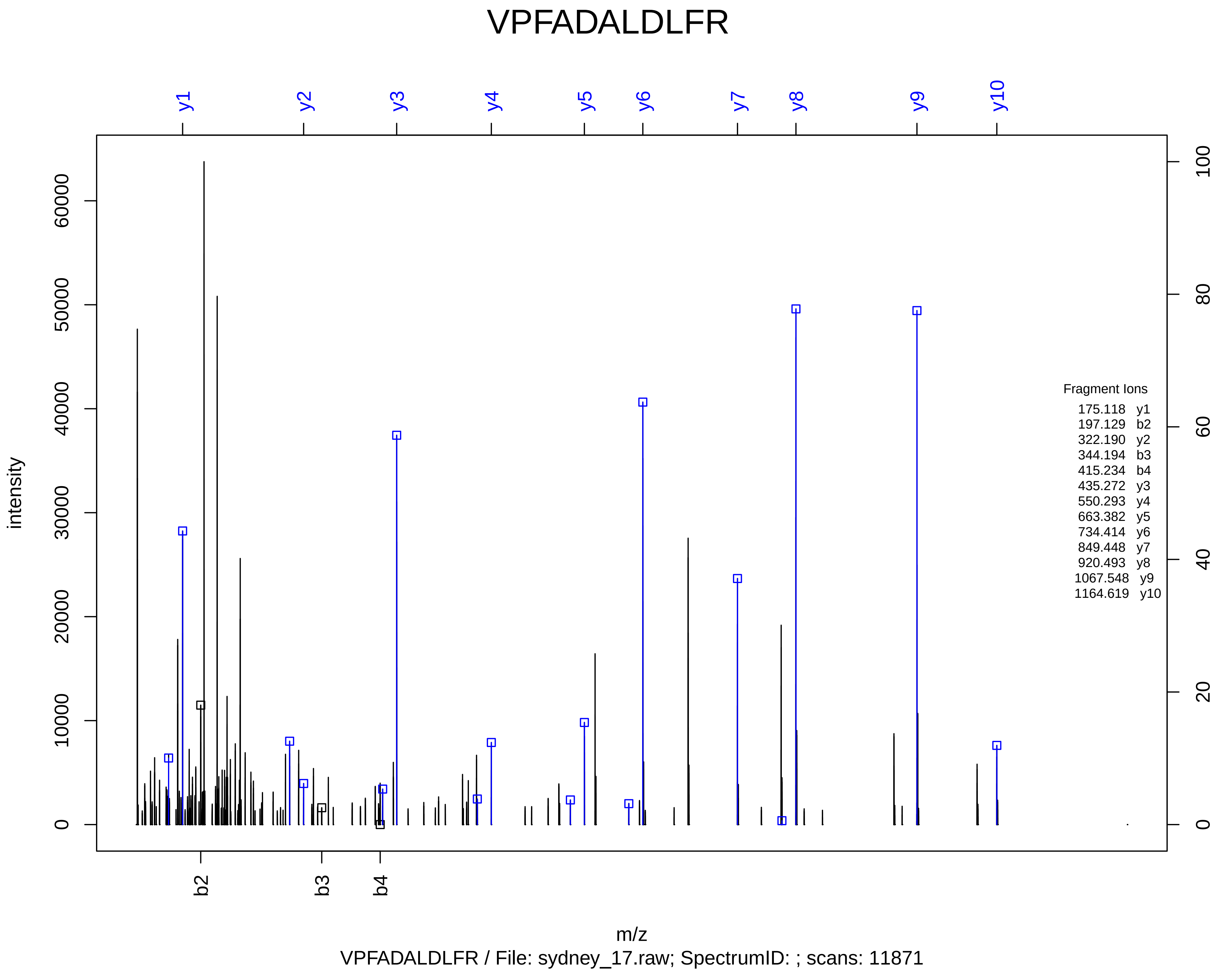

VPFADALDLFR
intensity
m/z
y1
y2
y3
y4
y5
y6
y7
y8
y9
y10
b2
b3
b4
Fragment Ions
175.118 y1
197.129 b2
322.190 y2
344.194 b3
415.234 b4
435.272 y3
550.293 y4
663.382 y5
734.414 y6
849.448 y7
920.493 y8
1067.548 y9
1164.619 y10
VPFADALDLFR / File: sydney_17.raw; SpectrumID: ; scans: 11871

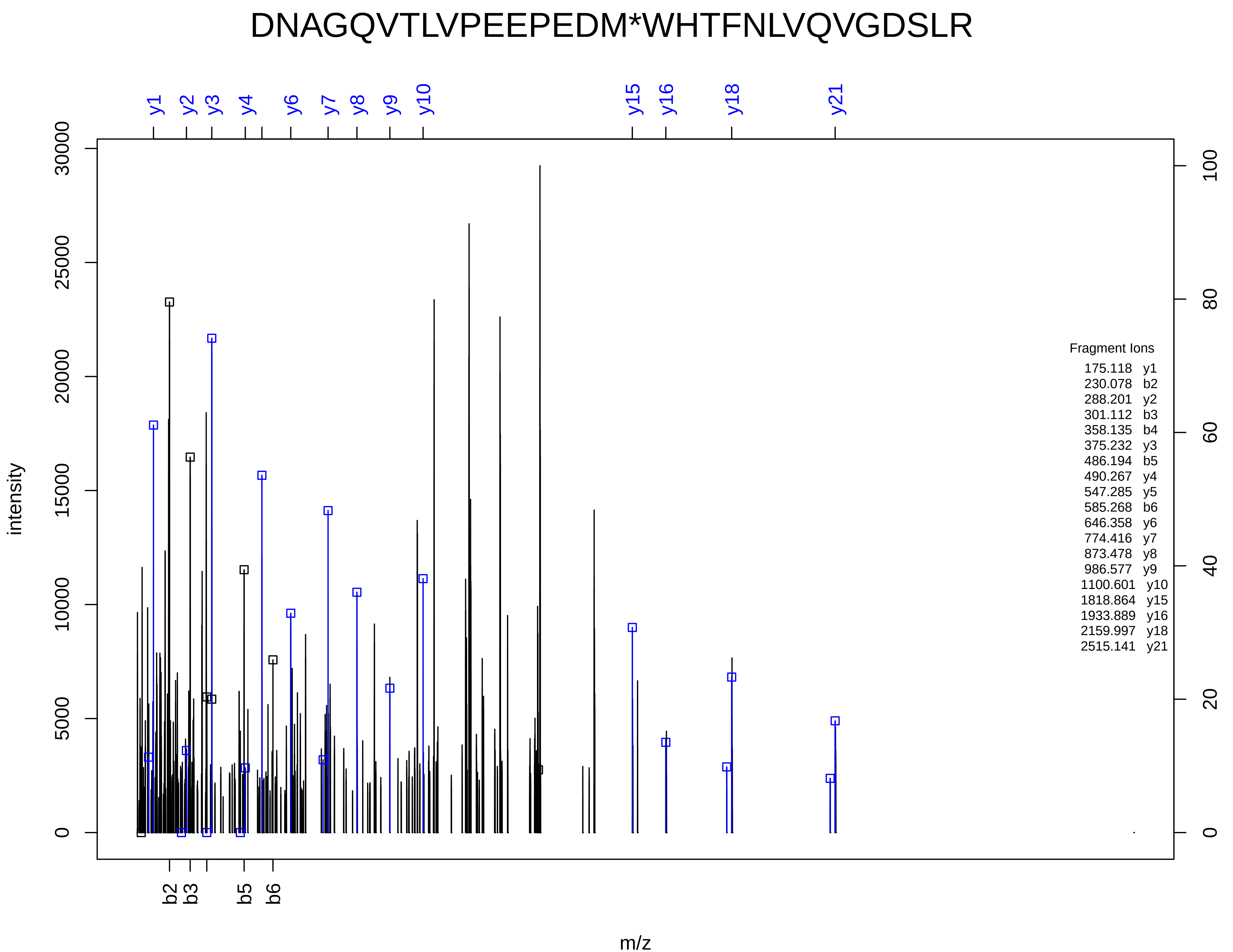

DNAGQVTLVPEEPEDMWHTFNLVQVGDSLR / File: sydney_17.raw; SpectrumID: ; scans: 11300

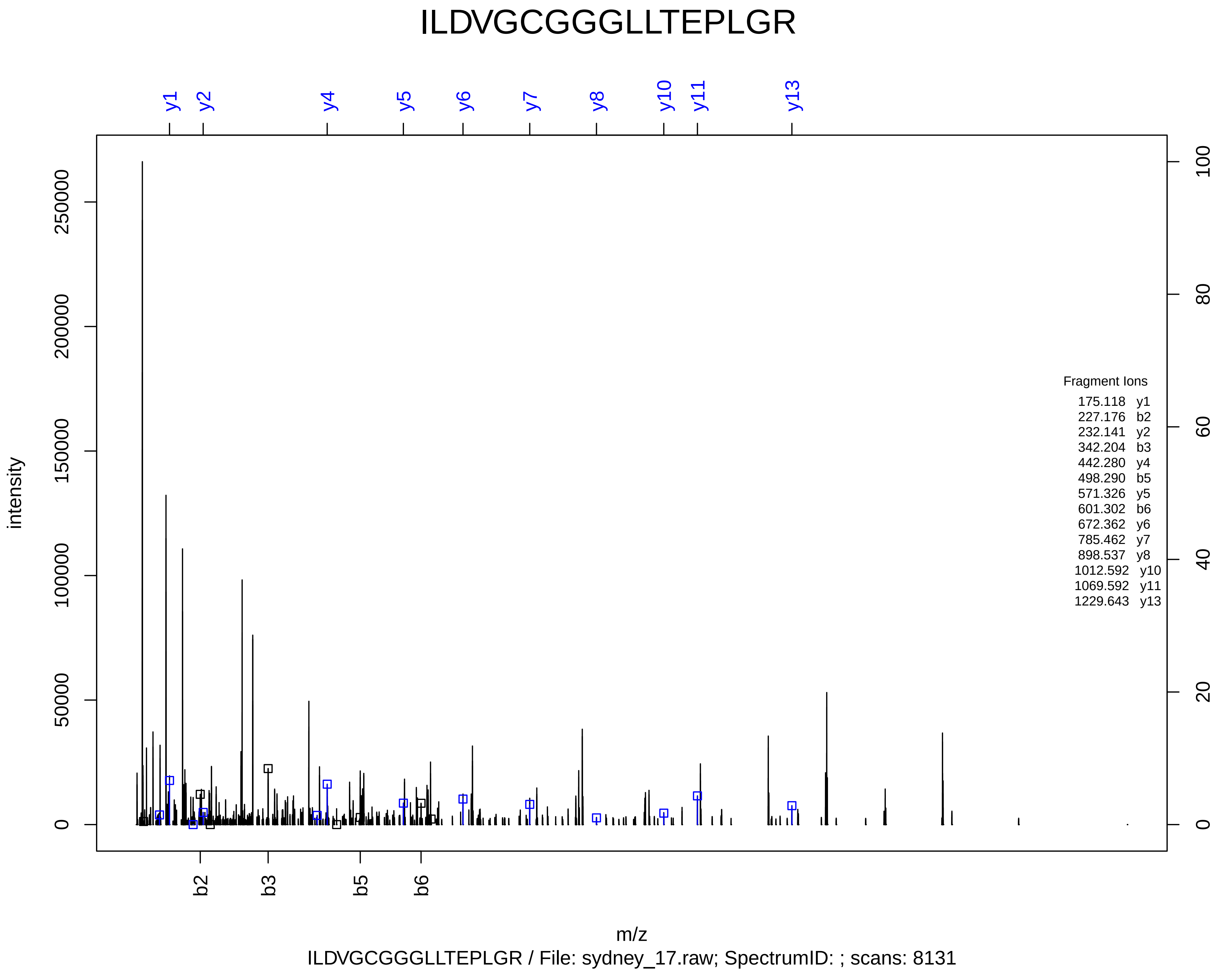
ILDVGCGGGLLTEPLGR
y1
y2
y4
y5
y6
y7
y8
y10
y11
y13
intensity
0
50000
100000
150000
200000
250000
0
20
40
60
80
100
b2
b3
b5
b6
m/z
Fragment Ions
175.118 y1
227.176 b2
232.141 y2
342.204 b3
442.280 y4
498.290 b5
571.326 y5
601.302 b6
672.362 y6
785.462 y7
898.537 y8
1012.592 y10
1069.592 y11
1229.643 y13
ILDVGCGGGLLTEPLGR / File: sydney_17.raw; SpectrumID: ; scans: 8131

LKFVVN^N^N^VAFSPGMKN^R

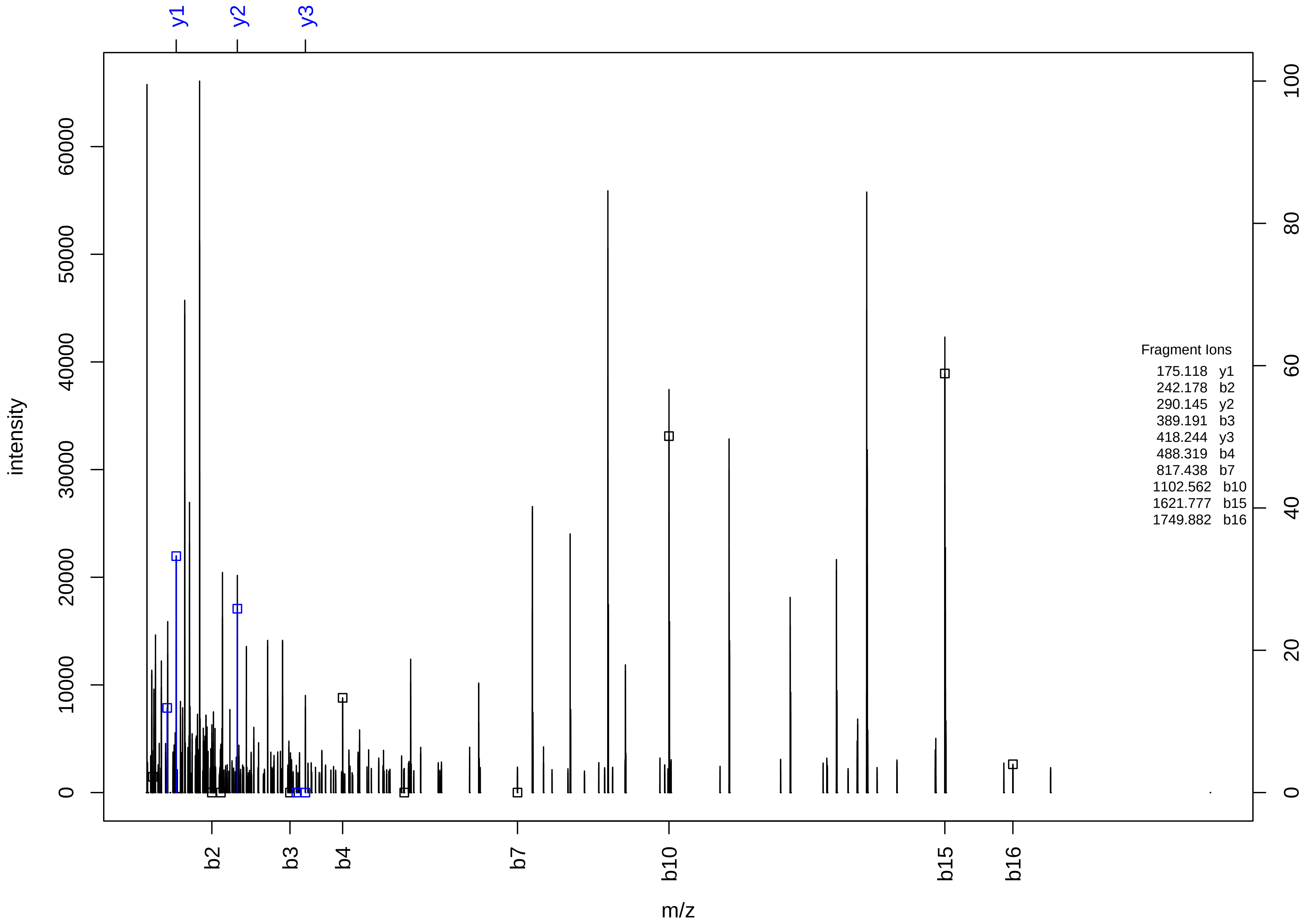

LKFVVNNNVAFSPGMKNR / File: sydney_17.raw; SpectrumID: ; scans: 10602

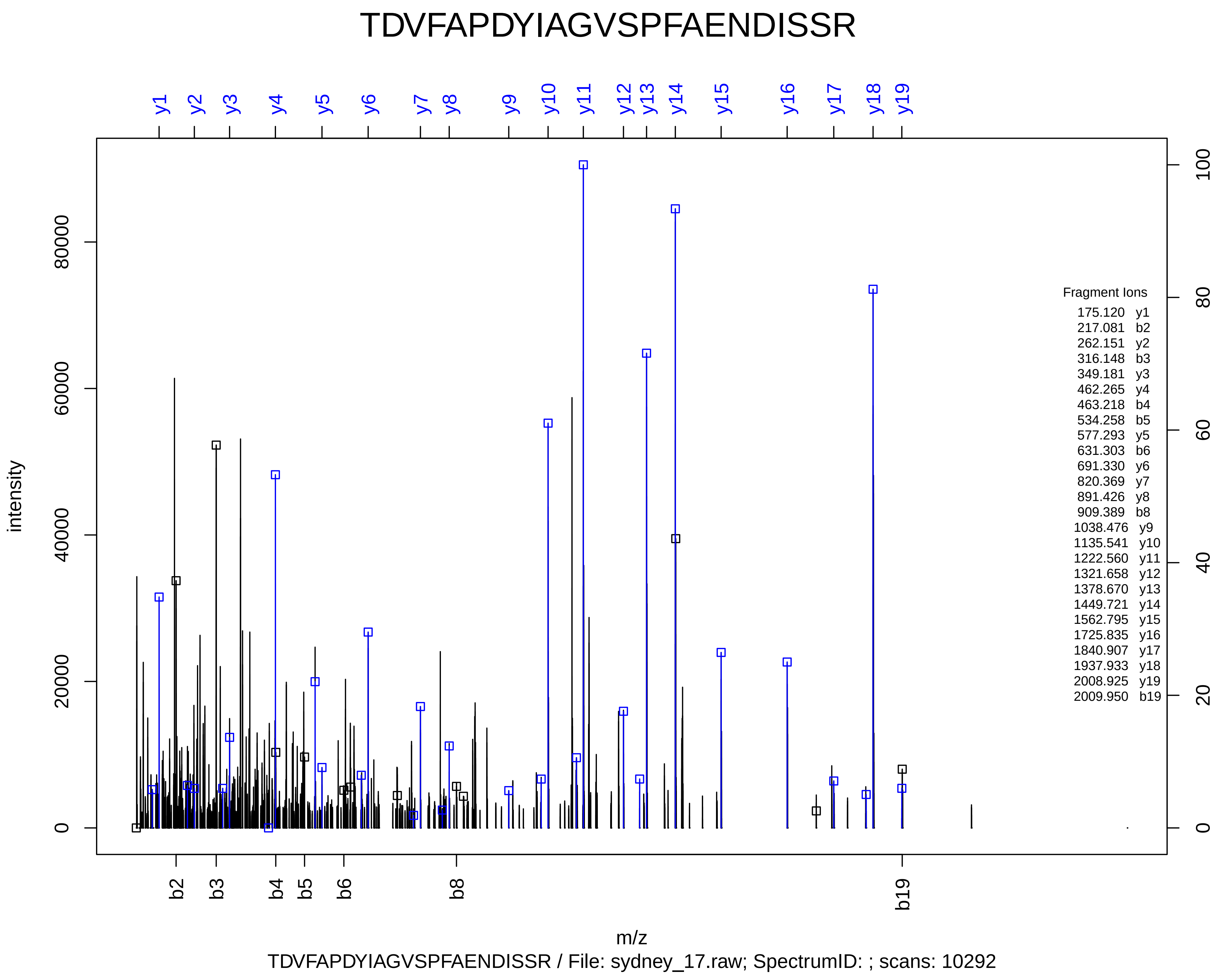

TDVFAPDYIAGVSPFAENDISSR
y1
y2
y3
y4
y5
y6
y7
y8
y9
y10
y11
y12
y13
y14
y15
y16
y17
y18
y19
b2
b3
b4
b5
b6
b8
b19
intensity
0
20000
40000
60000
80000
100
80
60
40
20
0
m/z
Fragment Ions
175.120 y1
217.081 b2
262.151 y2
316.148 b3
349.181 y3
462.265 y4
463.218 b4
534.258 b5
577.293 y5
631.303 b6
691.330 y6
820.369 y7
891.426 y8
909.389 b8
1038.476 y9
1135.541 y10
1222.560 y11
1321.658 y12
1378.670 y13
1449.721 y14
1562.795 y15
1725.835 y16
1840.907 y17
1937.933 y18
2008.925 y19
2009.950 b19
TDVFAPDYIAGVSPFAENDISSR / File: sydney_17.raw; SpectrumID: ; scans: 10292

AFLLESLLK

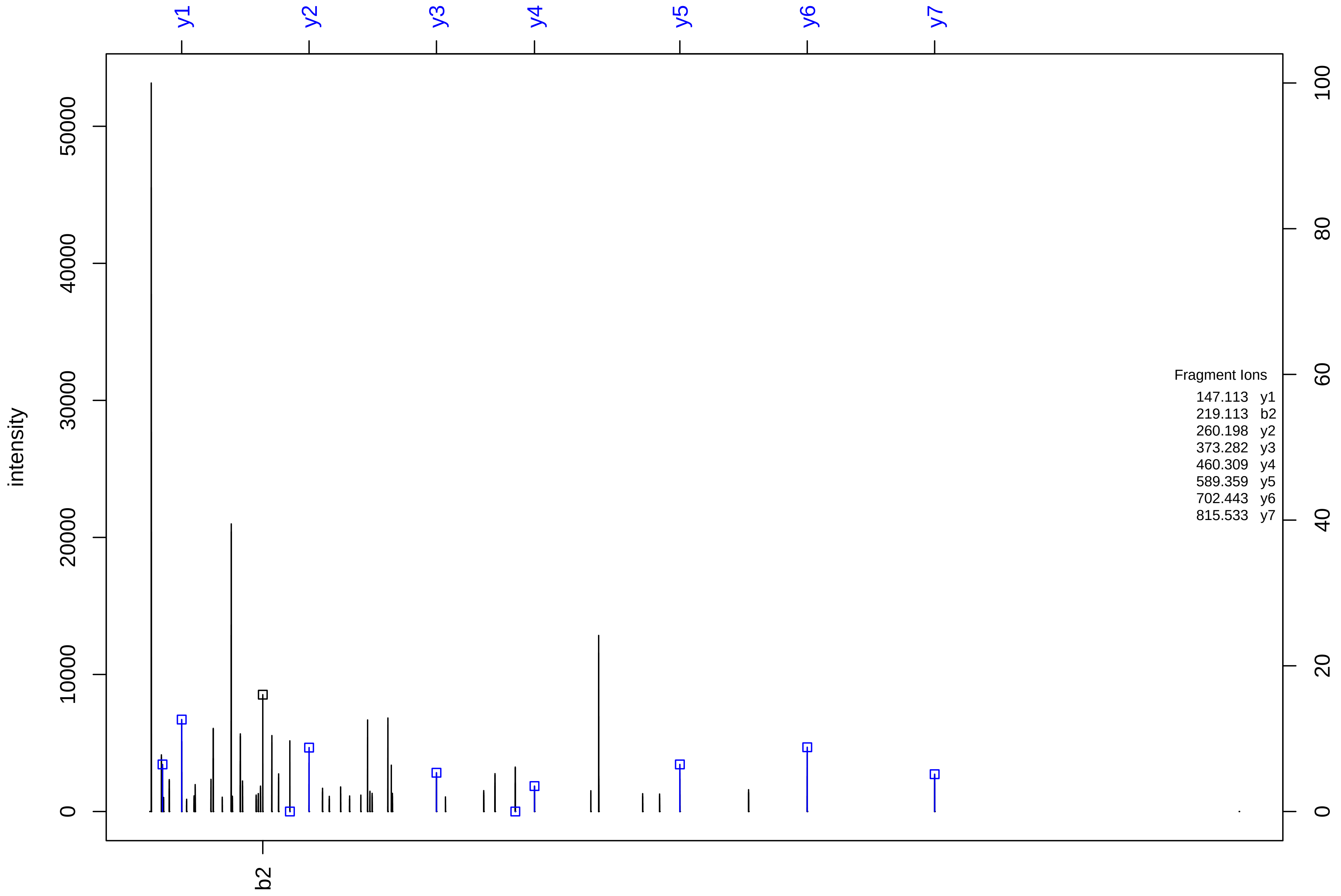

AFLLESLLK / File: sydney_17.raw; SpectrumID: ; scans: 10711

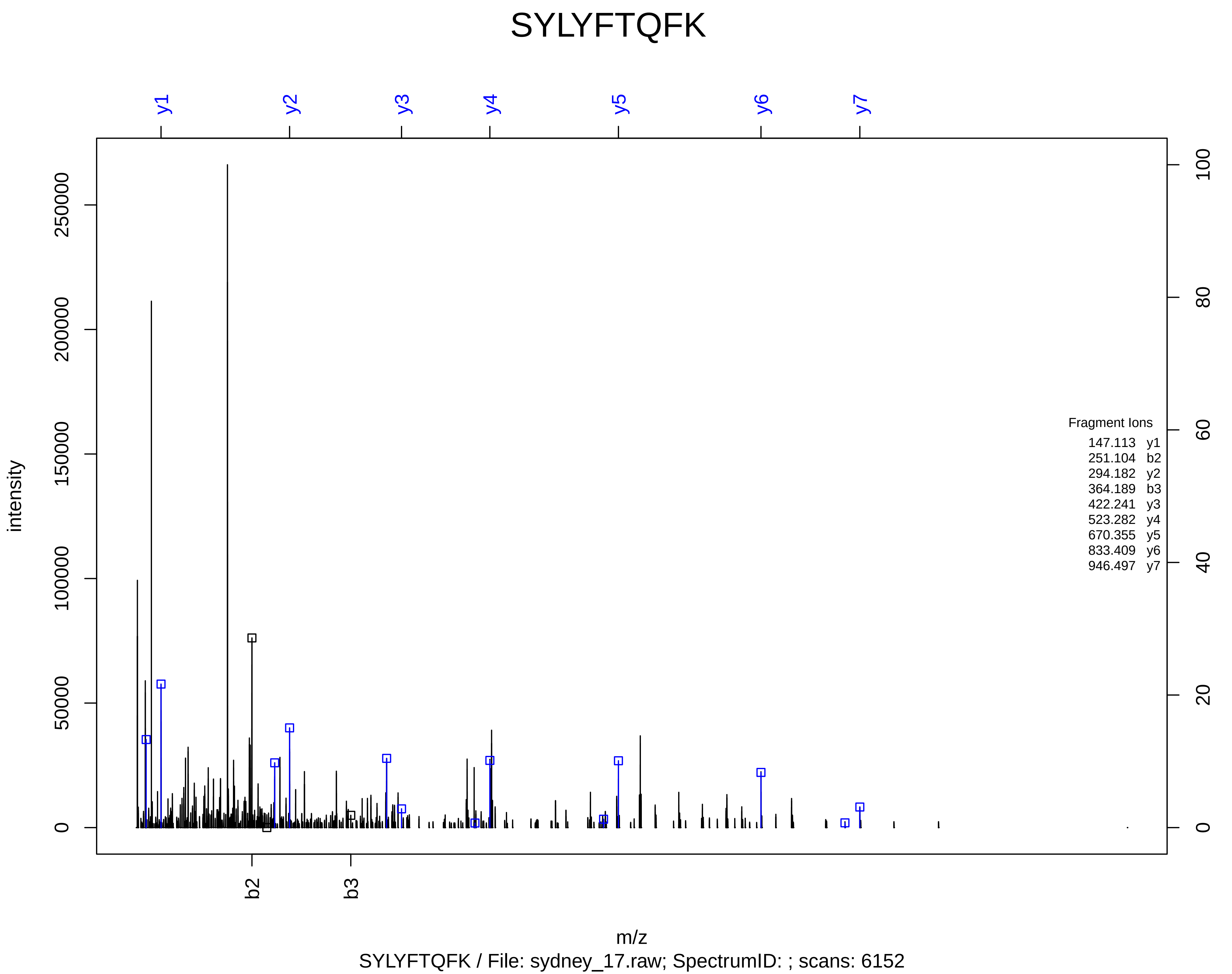
SYLYFTQFK
y1
y2
y3
y4
y5
y6
y7
b2
b3
intensity
m/z
Fragment Ions
147.113 y1
251.104 b2
294.182 y2
364.189 b3
422.241 y3
523.282 y4
670.355 y5
833.409 y6
946.497 y7
SYLYFTQFK / File: sydney_17.raw; SpectrumID: ; scans: 6152

NIADLMTQAGVEELDPENK

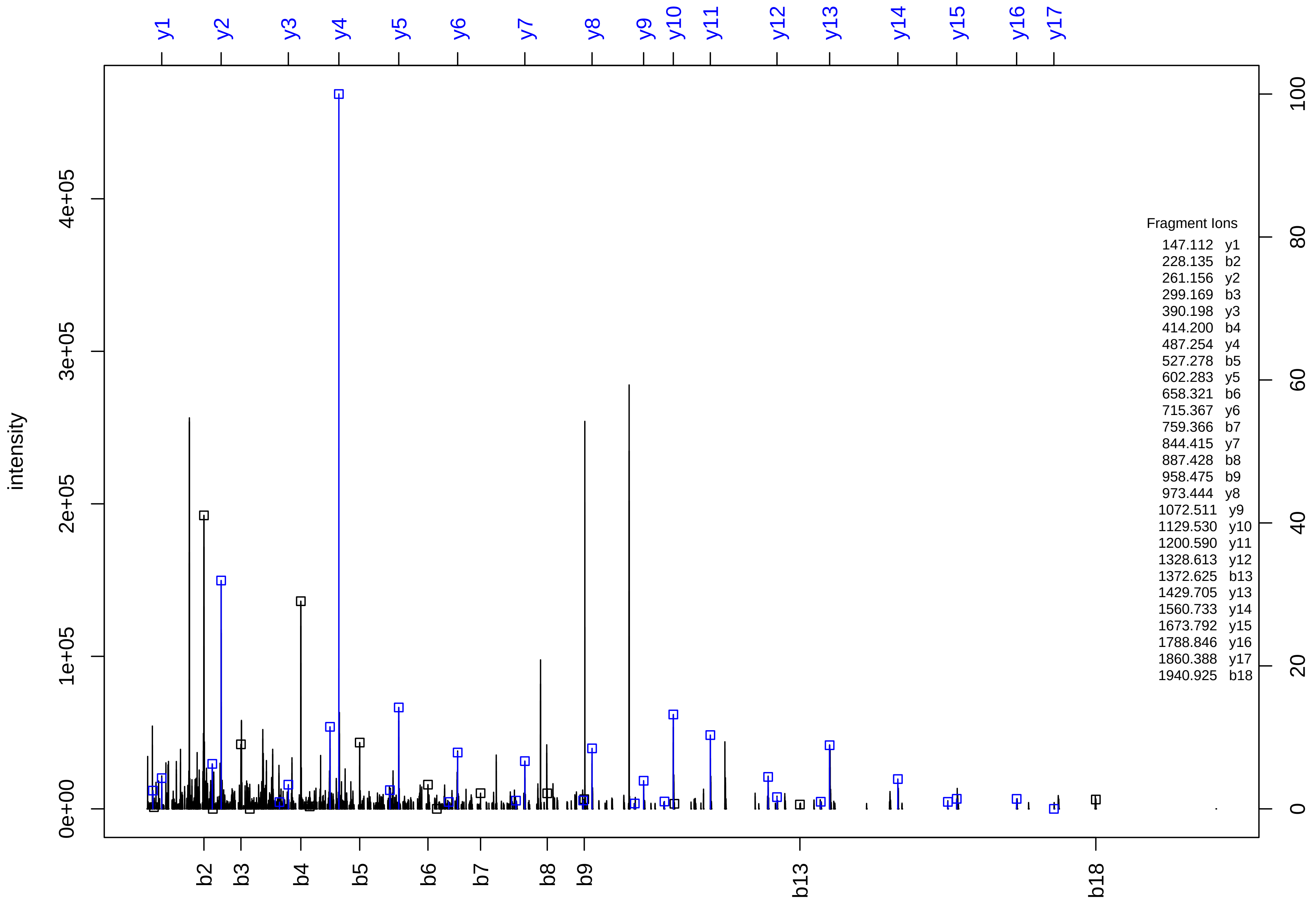

NIADLMTQAGVEELDPENK / File: sydney_17.raw; SpectrumID: ; scans: 9184

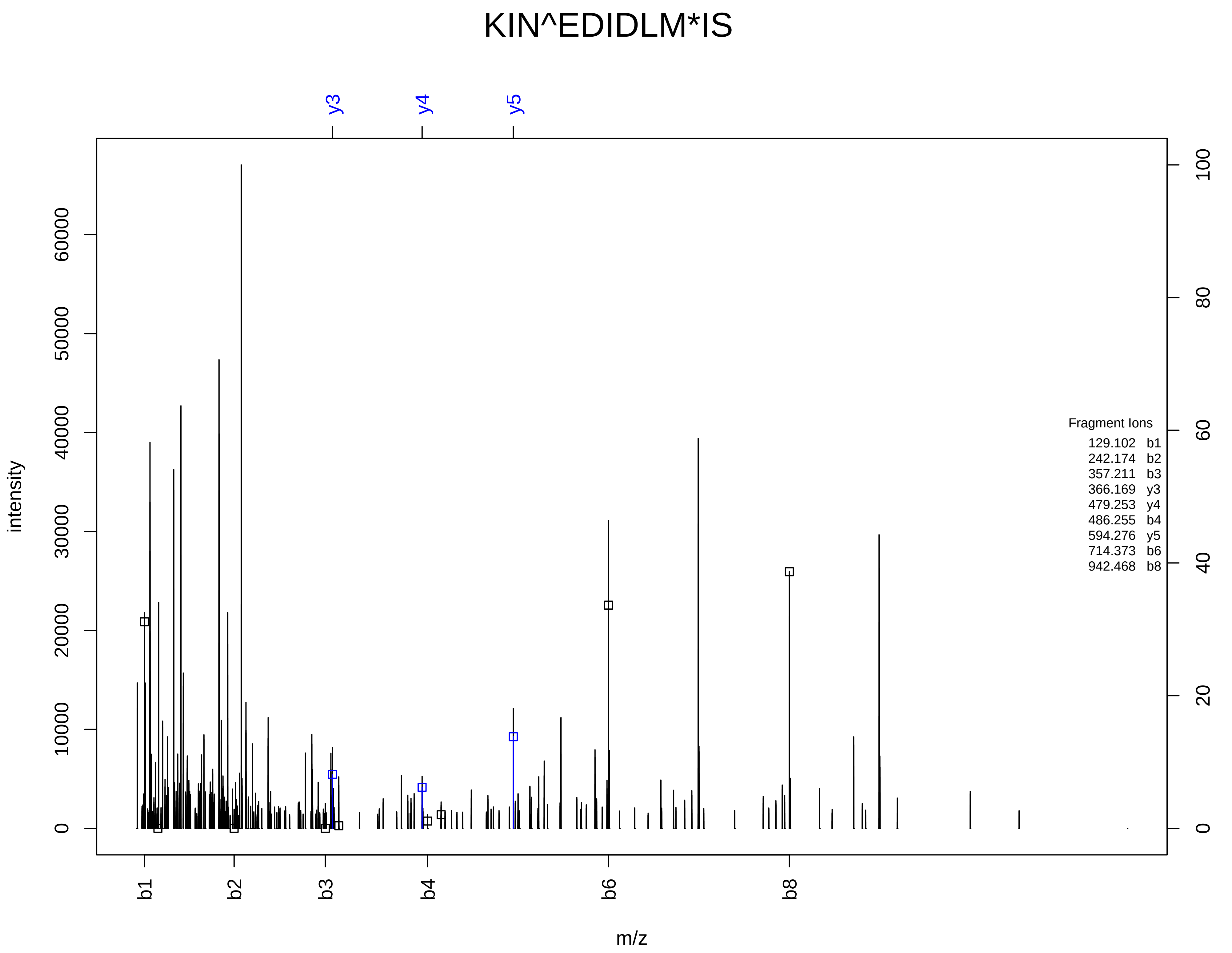
KIN^EDIDLM*IS
y3
y4
y5
intensity
m/z
Fragment Ions
129.102 b1
242.174 b2
357.211 b3
366.169 y3
479.253 y4
486.255 b4
594.276 y5
714.373 b6
942.468 b8
KINEDIDLMIS / File: sydney_18.raw; SpectrumID: ; scans: 3086

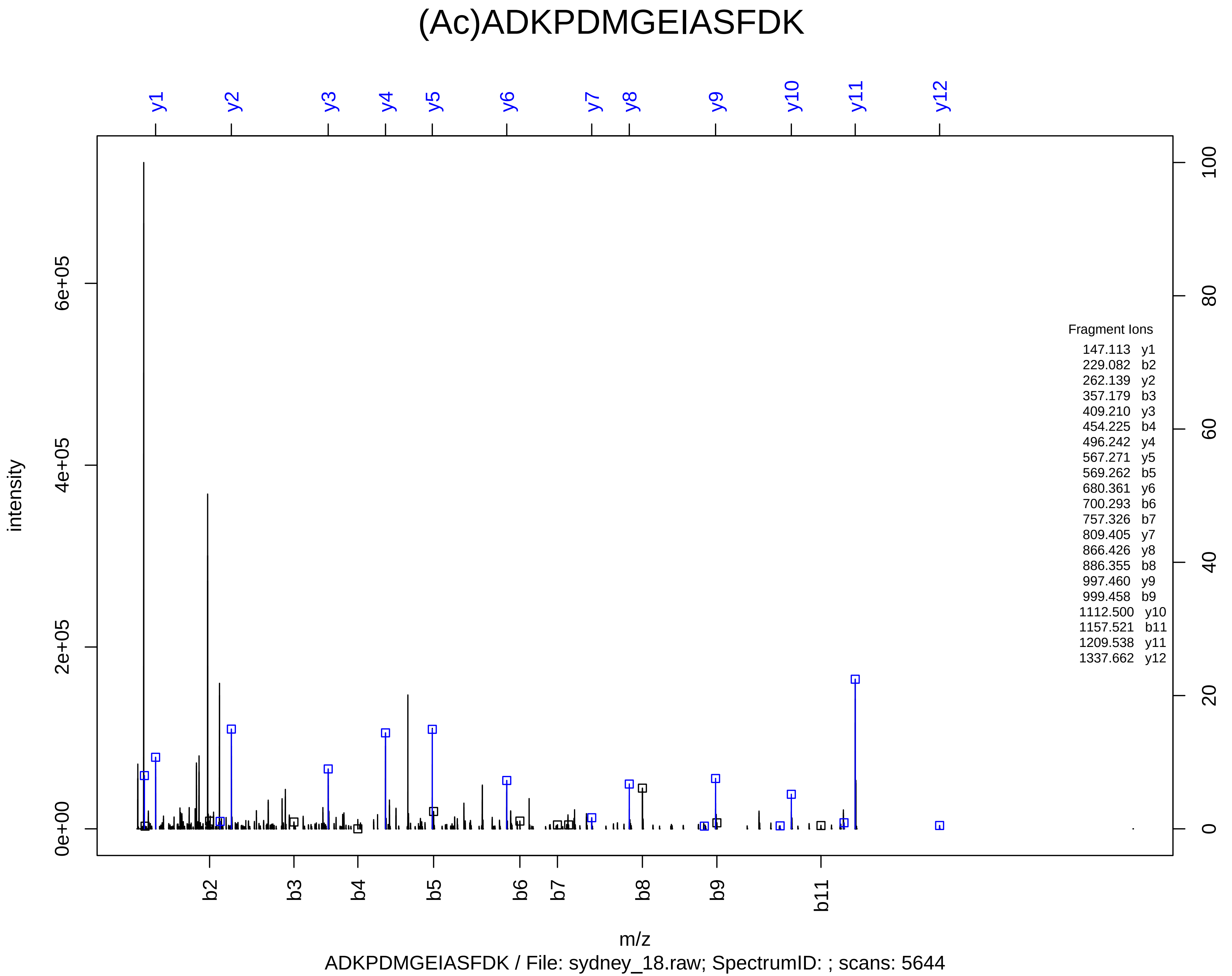

(Ac)ADKPDMGEIASFDK
y1
y2
y3
y4
y5
y6
y7
y8
y9
y10
y11
y12
intensity
0e+00
2e+05
4e+05
6e+05
0
20
40
60
80
100
Fragment Ions
147.113 y1
229.082 b2
262.139 y2
357.179 b3
409.210 y3
454.225 b4
496.242 y4
567.271 y5
569.262 b5
680.361 y6
700.293 b6
757.326 b7
809.405 y7
866.426 y8
886.355 b8
997.460 y9
999.458 b9
1112.500 y10
1157.521 b11
1209.538 y11
1337.662 y12
b2
b3
b4
b5
b6
b7
b8
b9
b11
m/z
ADKPDMGEIASFDK / File: sydney_18.raw; SpectrumID: ; scans: 5644

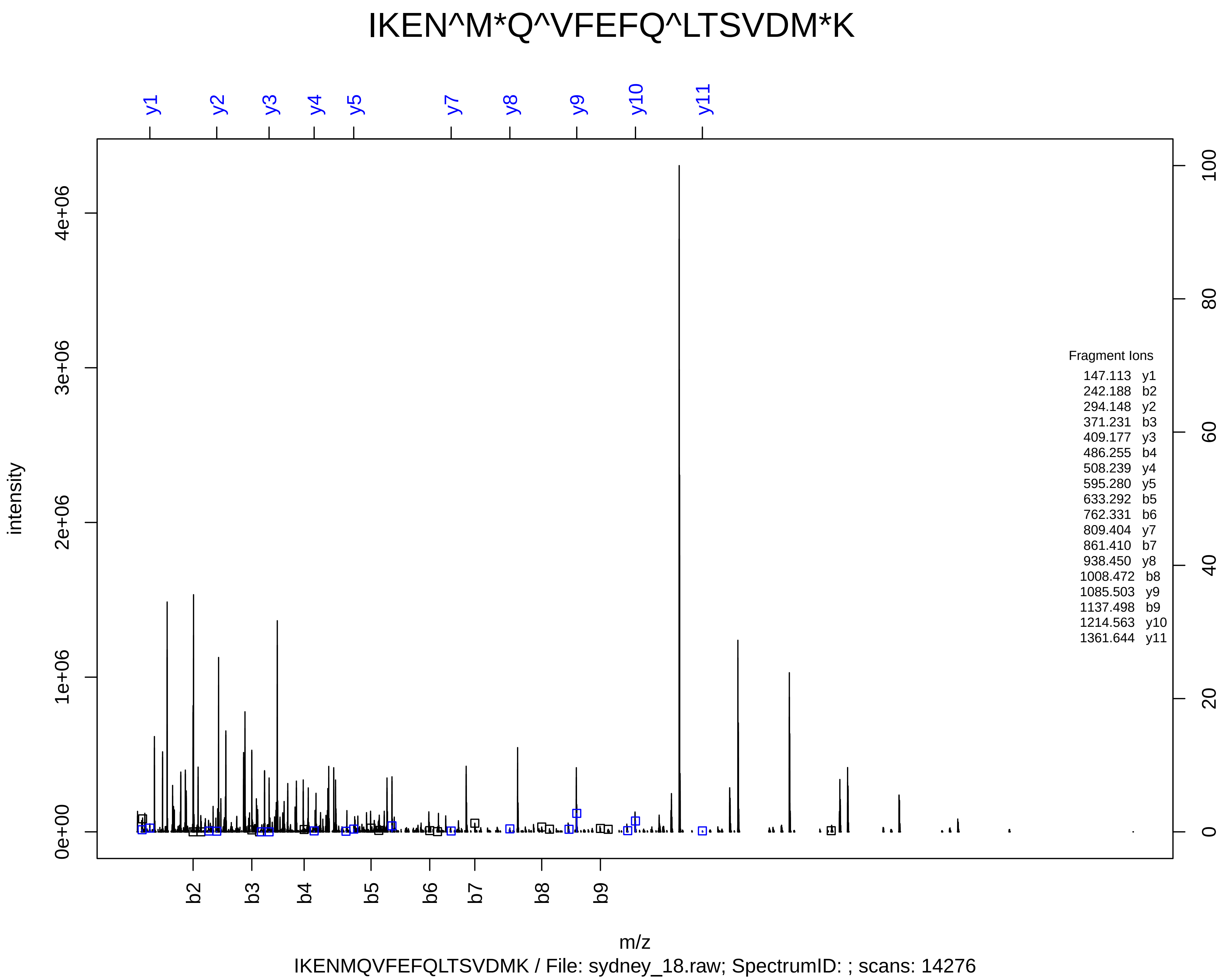

IKEN^M*Q^VFEFQ^LTSVDM*K
y1
y2
y3
y4
y5
y7
y8
y9
y10
y11
intensity
0e+00
1e+06
2e+06
3e+06
4e+06
0
20
40
60
80
100
b2
b3
b4
b5
b6
b7
b8
b9
m/z
Fragment Ions
147.113 y1
242.188 b2
294.148 y2
371.231 b3
409.177 y3
486.255 b4
508.239 y4
595.280 y5
633.292 b5
762.331 b6
809.404 y7
861.410 b7
938.450 y8
1008.472 b8
1085.503 y9
1137.498 b9
1214.563 y10
1361.644 y11
IKENMQVFEFQLTSVDMK / File: sydney_18.raw; SpectrumID: ; scans: 14276

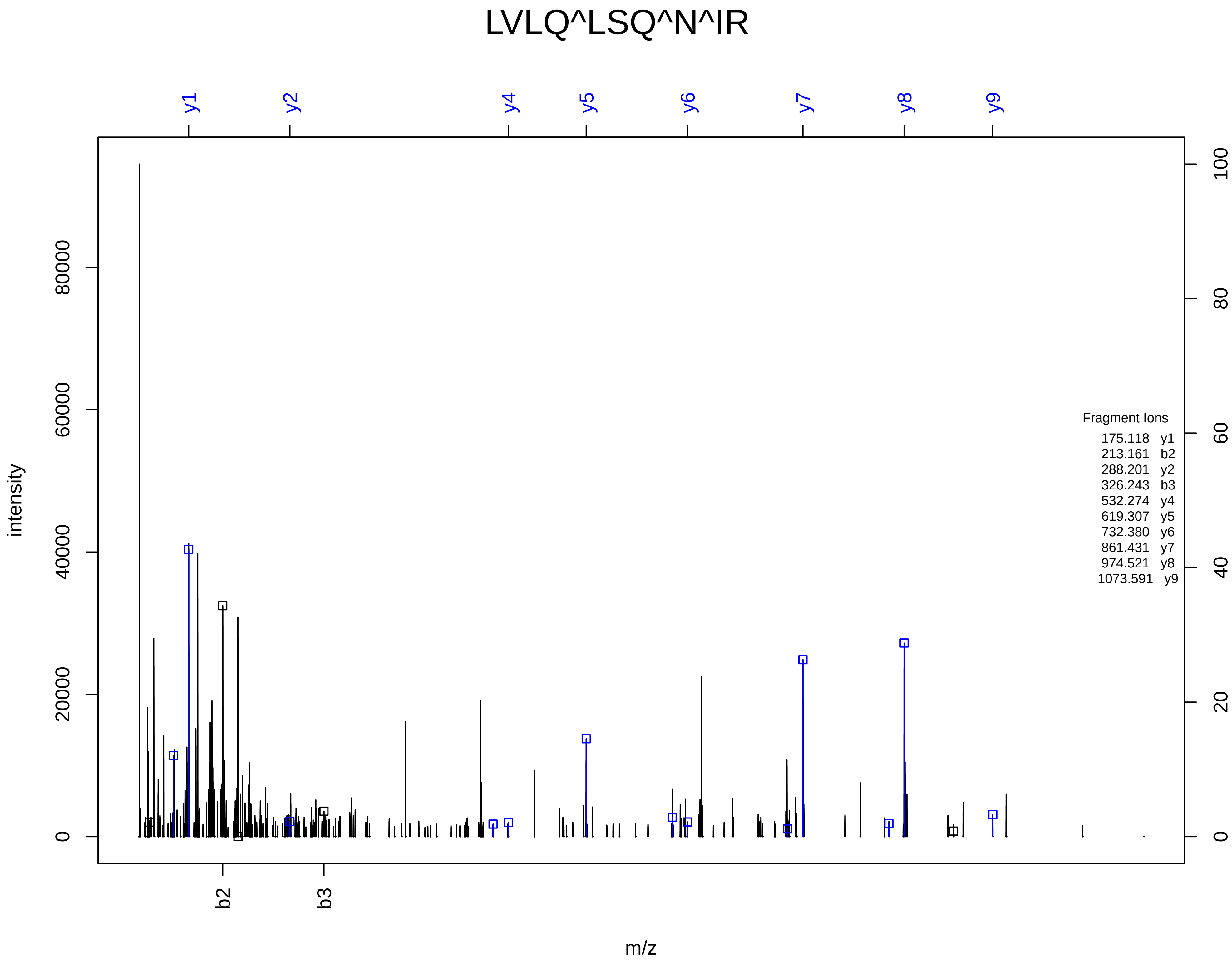
LVLQ^LSQ^N^IR
y1
y2
y4
y5
y6
y7
y8
y9
b2
b3
intensity
m/z
Fragment Ions
175.118 y1
213.161 b2
288.201 y2
326.243 b3
532.274 y4
619.307 y5
732.380 y6
861.431 y7
974.521 y8
1073.591 y9
LVLQLSQNIR / File: sydney_18.raw; SpectrumID: ; scans: 5865

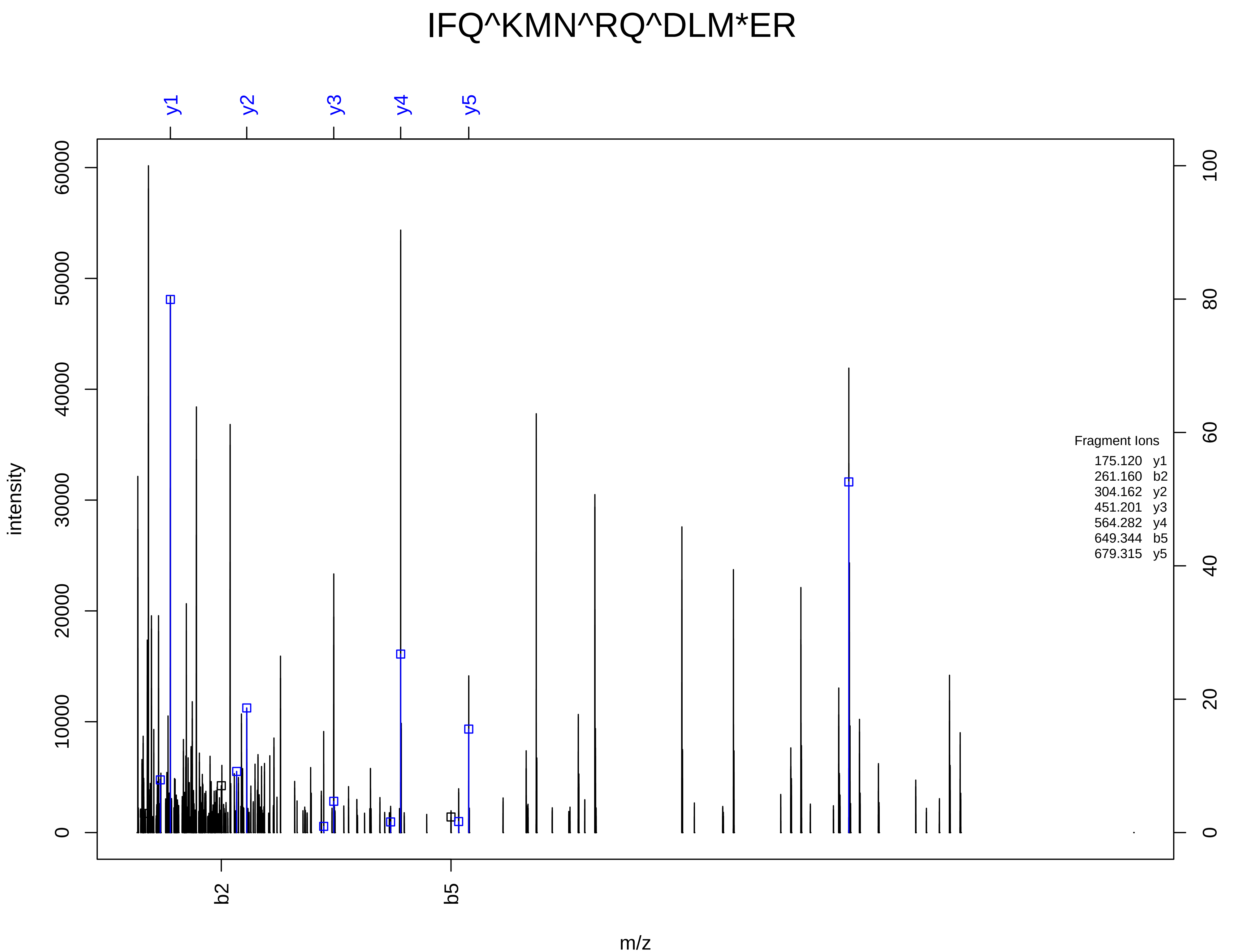
IFQ^KMN^RQ^DLM*ER
y1
y2
y3
y4
y5
intensity
0
10000
20000
30000
40000
50000
60000
0
20
40
60
80
100
b2
b5
m/z
Fragment Ions
175.120 y1
261.160 b2
304.162 y2
451.201 y3
564.282 y4
649.344 b5
679.315 y5
IFQKMNRQDLMER / File: sydney_18.raw; SpectrumID: ; scans: 12279

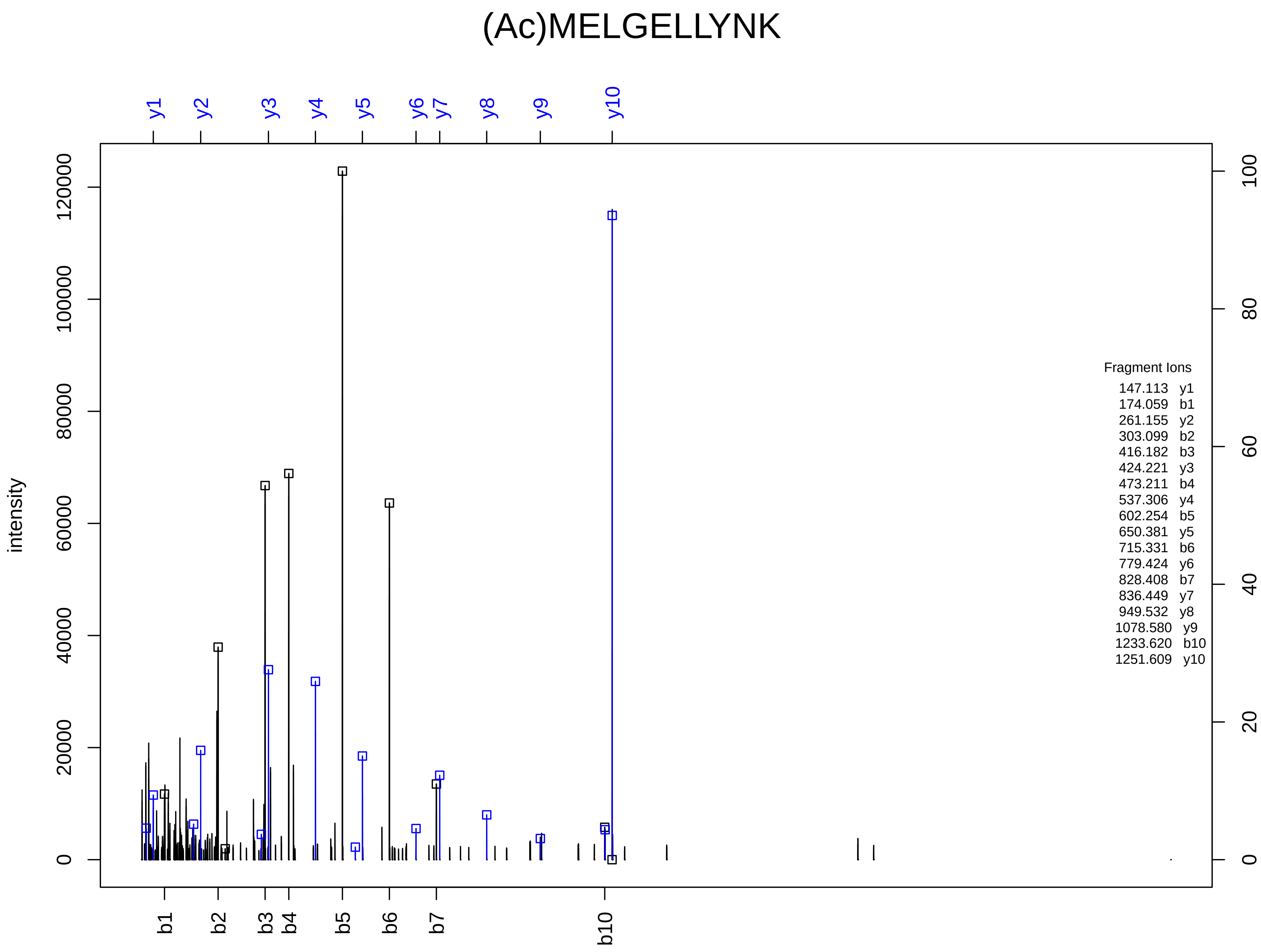

MELGELLYNK / File: sydney_18.raw; SpectrumID: ; scans: 14022

(Ac)ASFVTEVLAHSGSLEK

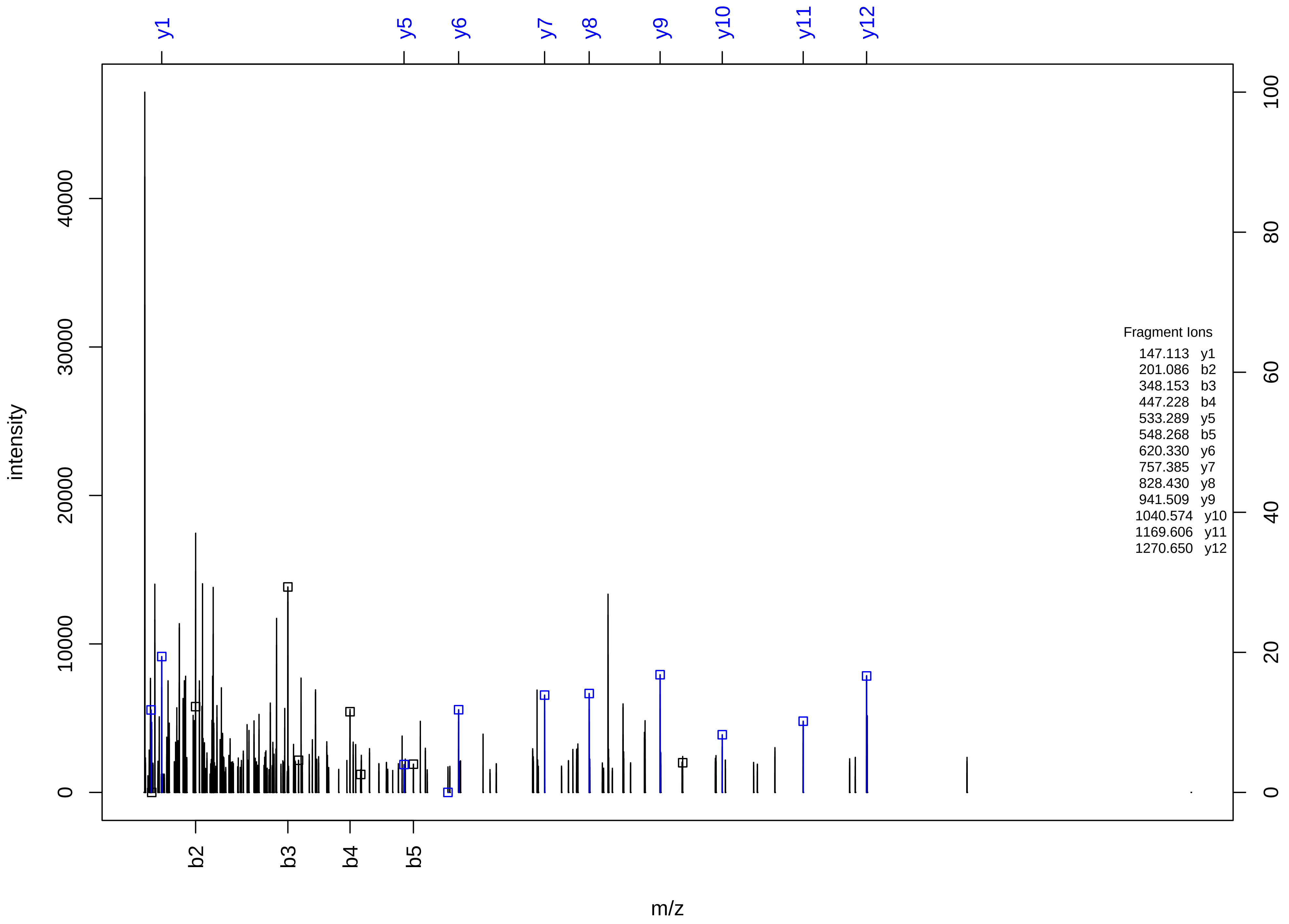

ASFVTEVLAHSGSLEK / File: sydney_18.raw; SpectrumID: ; scans: 13948

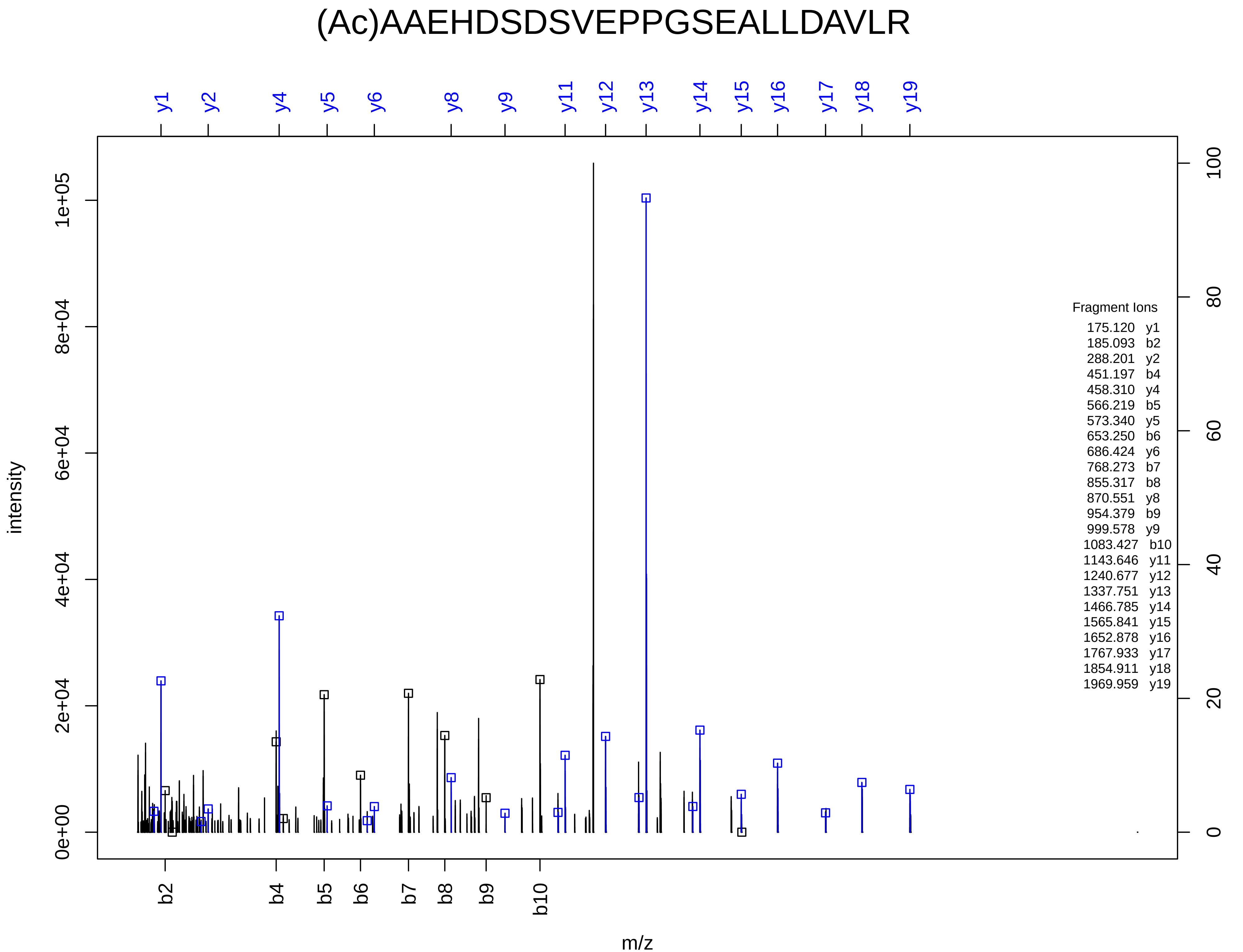

AAEHDSDSVEPPGSEALLDAVLR / File: sydney_18.raw; SpectrumID: ; scans: 12328

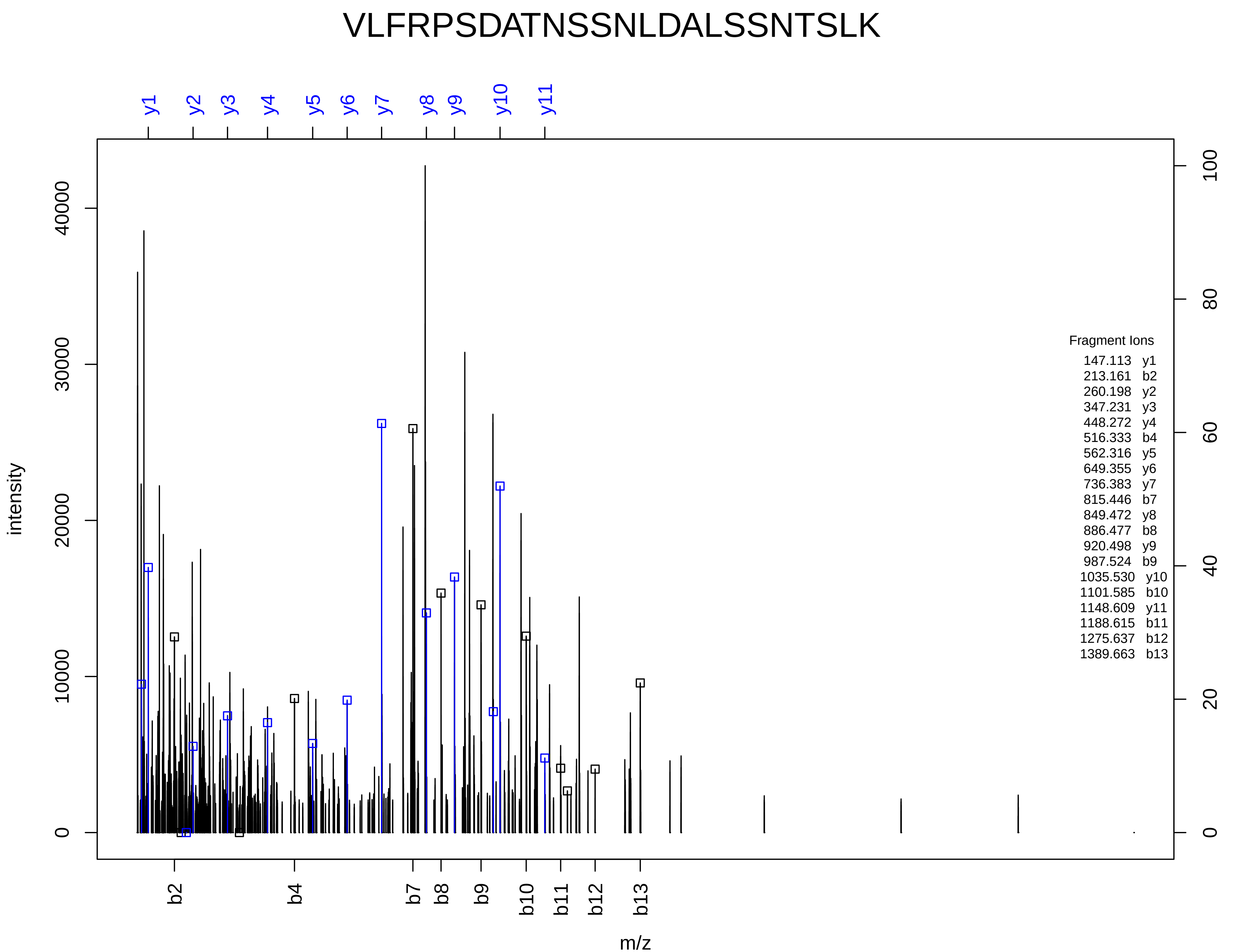

VLFRPSDATNSSNLDALSSNTSLK / File: sydney_18.raw; SpectrumID: ; scans: 6224

(Ac)MAVYQTYVNAM*N^DKIR

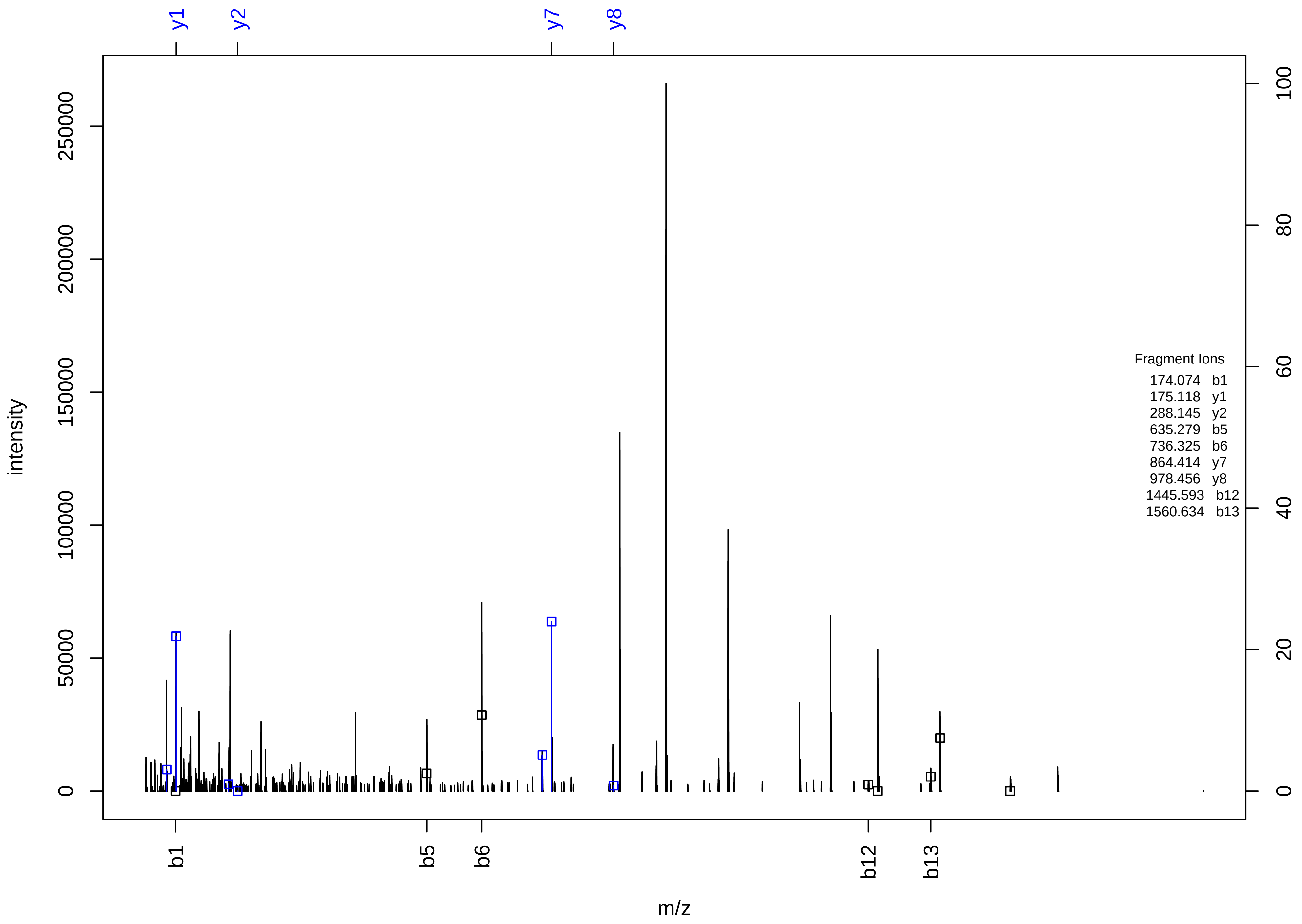

MAVYQTYVNAMNDKIR / File: sydney_18.raw; SpectrumID: ; scans: 7373

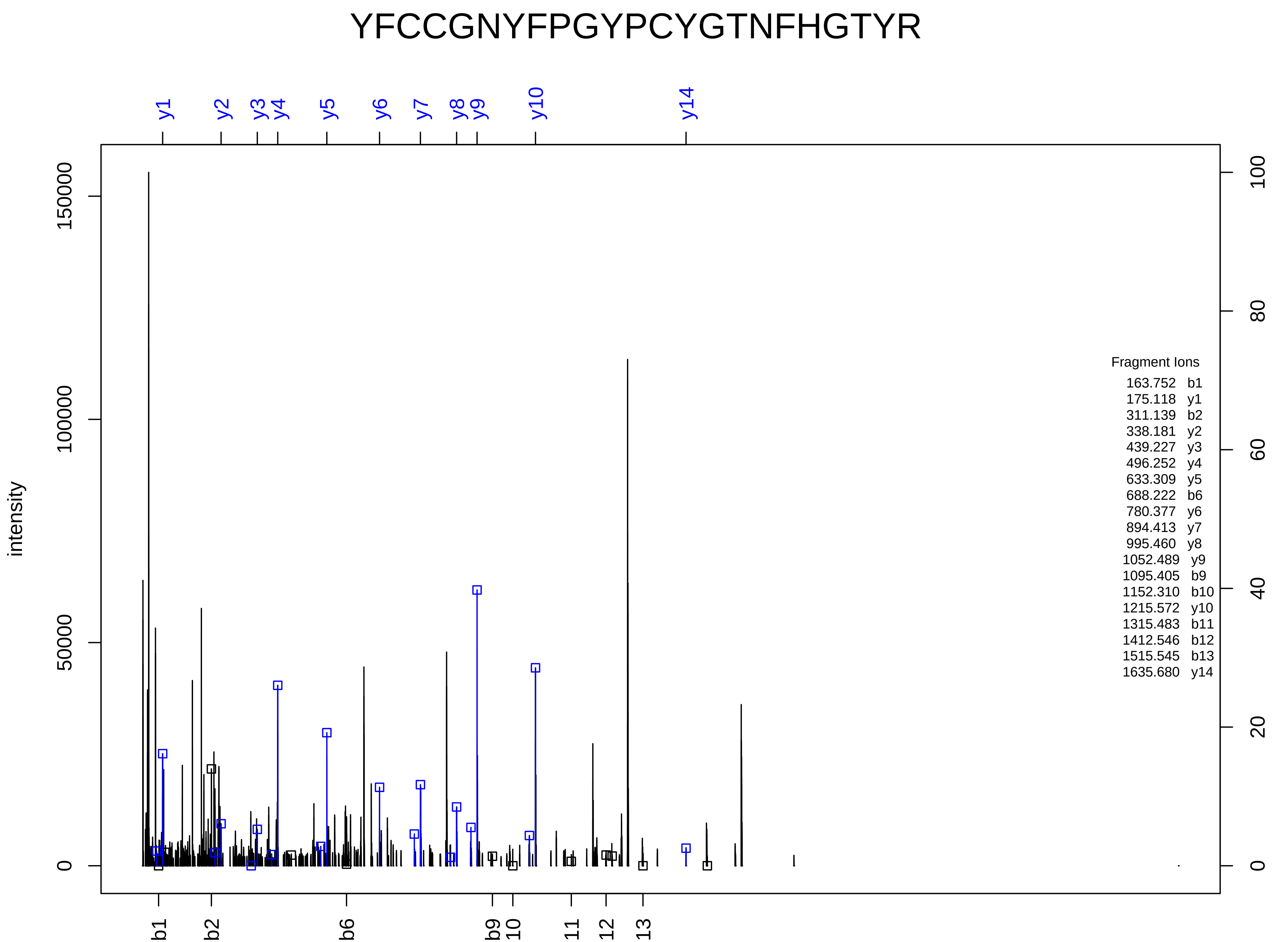

YFCCGNYFPGYPCYGTNFHGTYR / File: sydney_18.raw; SpectrumID: ; scans: 7228

THN^VEAPNLQ^NKM*CN^LR

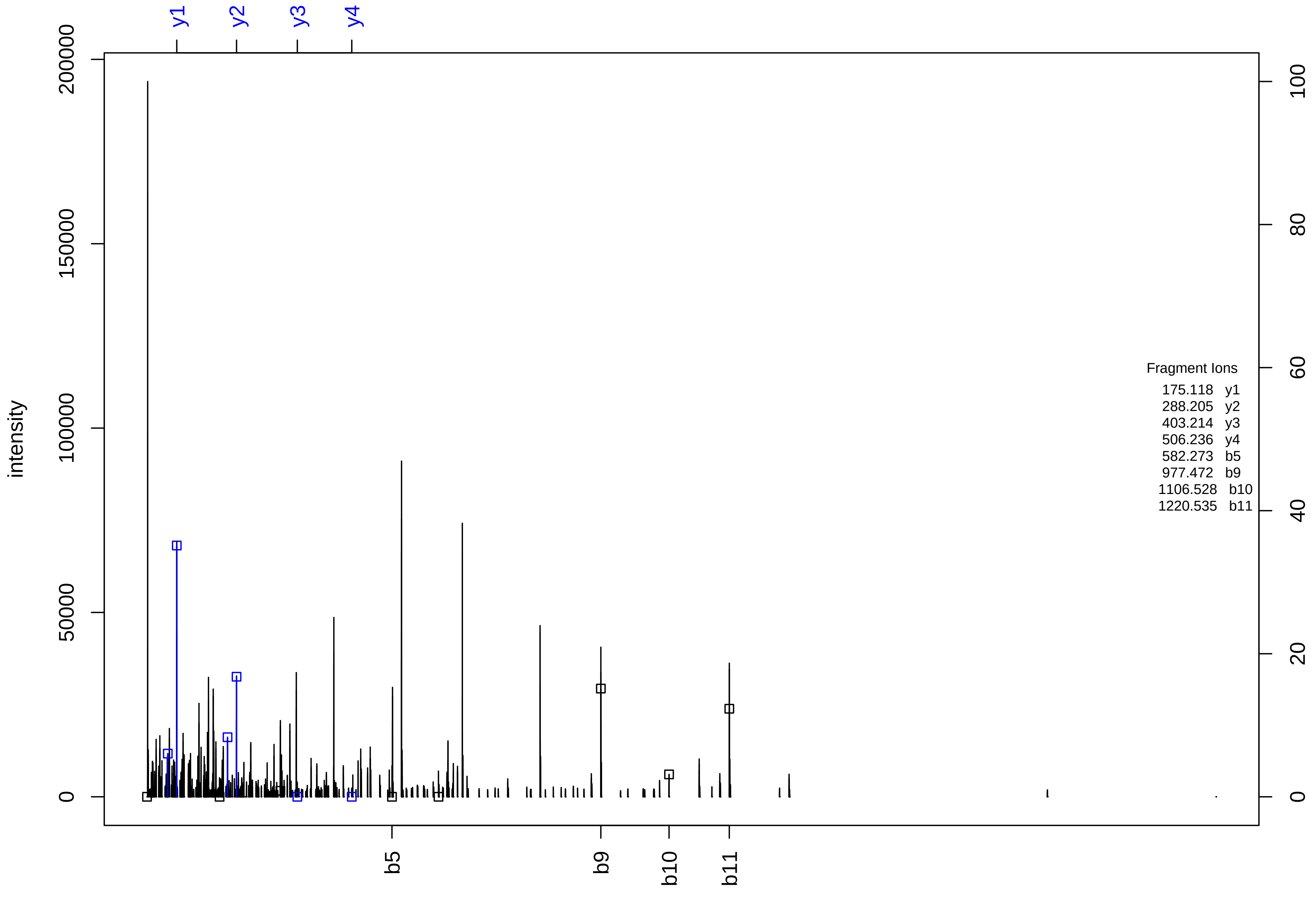

THNVEAPNLQNKMCNLR / File: sydney_19.raw; SpectrumID: ; scans: 10628

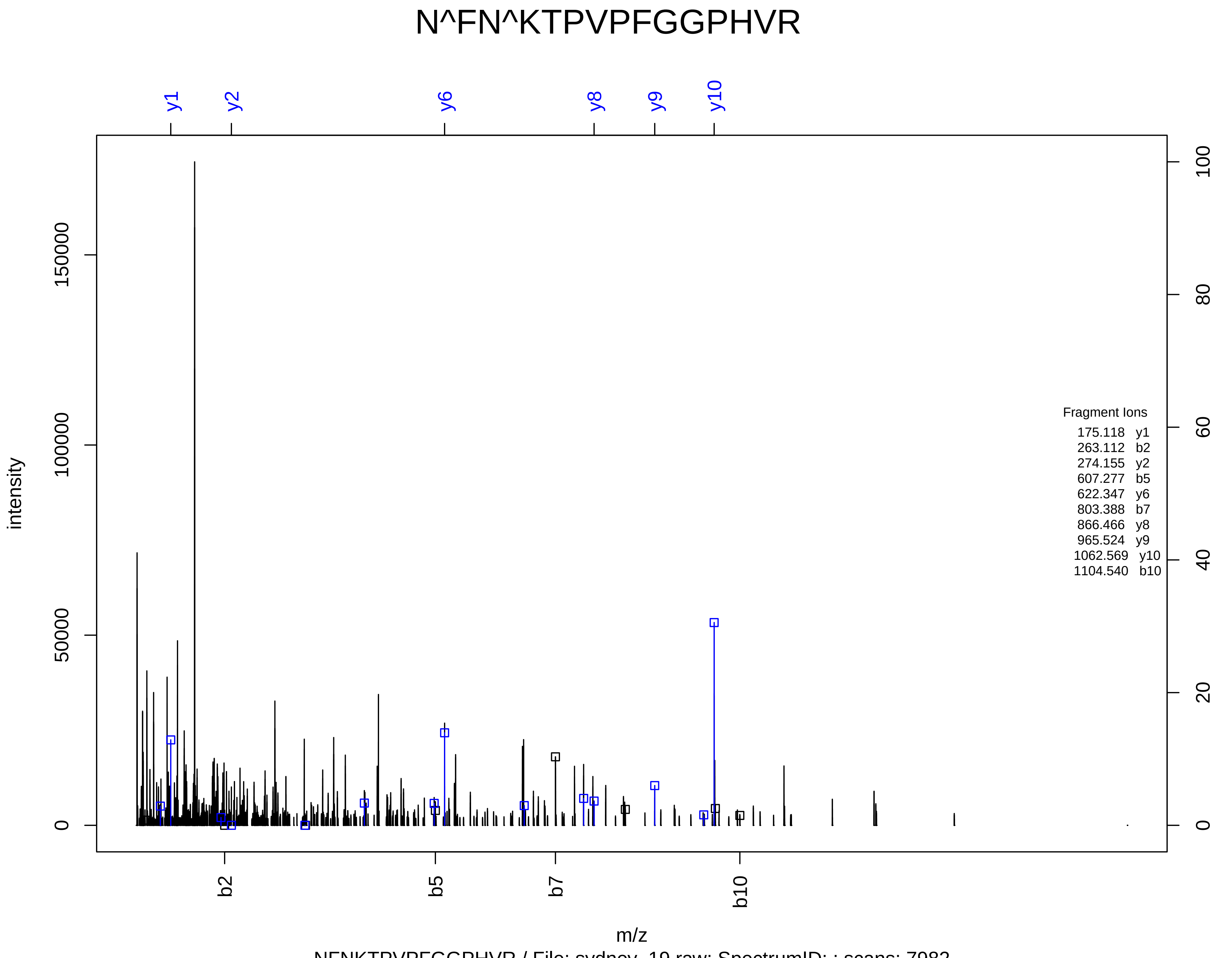
N^FN^KTPVPFGGPHVR
y1
y2
y6
y8
y9
y10
intensity
0
50000
100000
150000
0
20
40
60
80
100
Fragment Ions
175.118 y1
263.112 b2
274.155 y2
607.277 b5
622.347 y6
803.388 b7
866.466 y8
965.524 y9
1062.569 y10
1104.540 b10
b2
b5
b7
b10
m/z
NFNKTPVPFGGPHVR / File: sydney_19.raw; SpectrumID: ; scans: 7982

LPFTALGSGQGAAVALLEDR

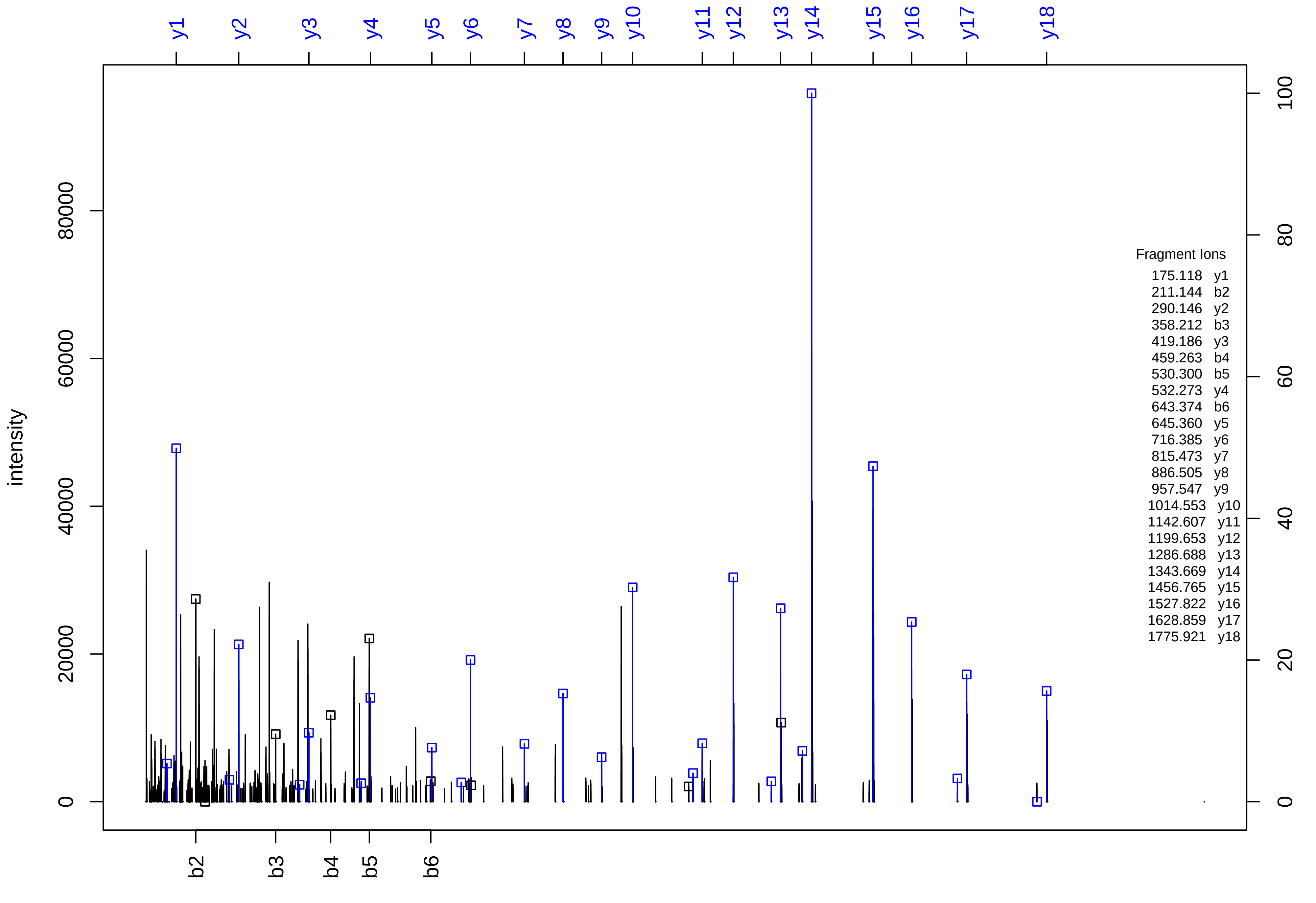

LPFTALGSGQGAAVALLEDR / File: sydney_19.raw; SpectrumID: ; scans: 11337

LFLVQTVQQASK

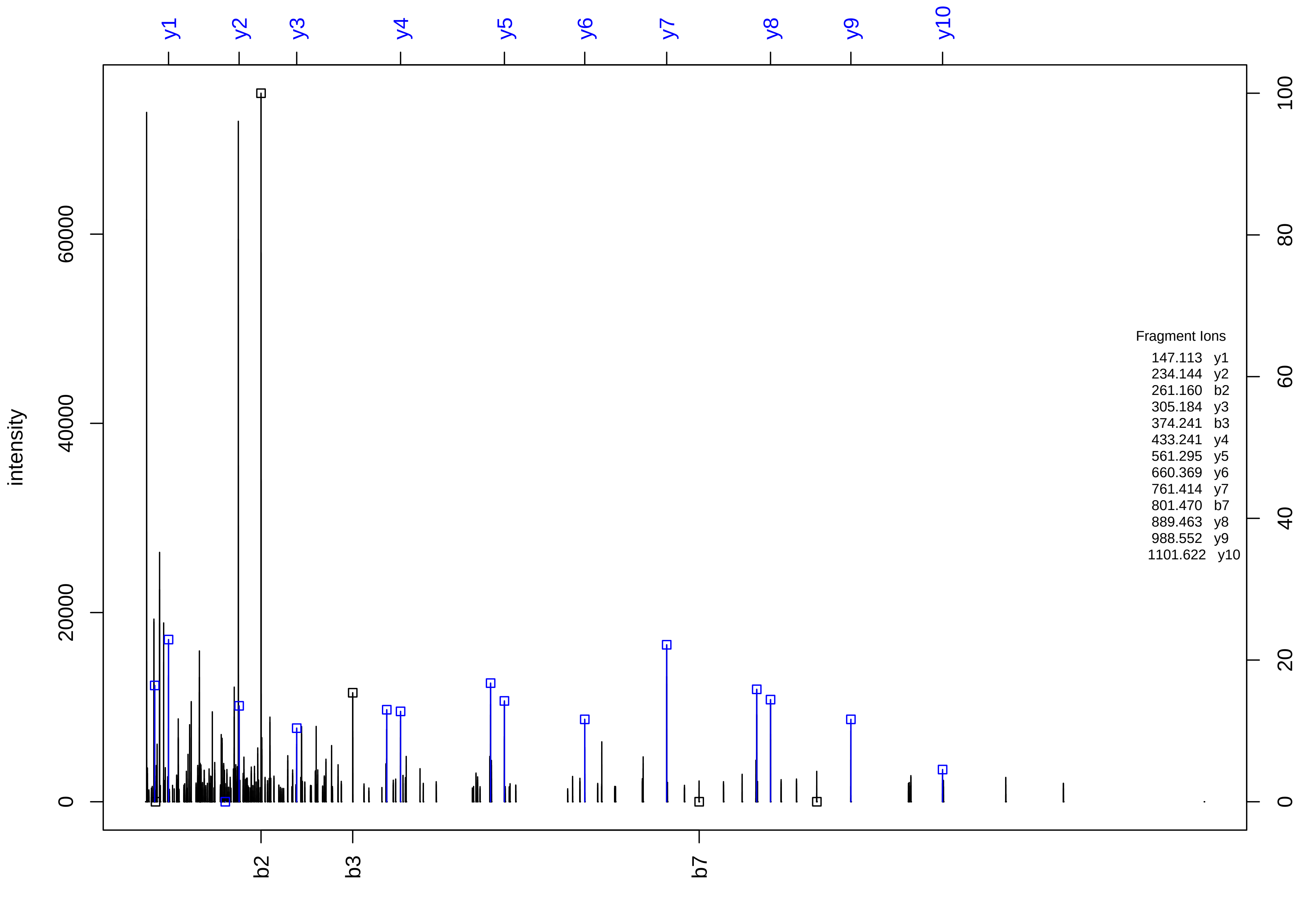

LFLVQTVQQASK / File: sydney_19.raw; SpectrumID: ; scans: 5953

HRATLVGLFN^N^LR

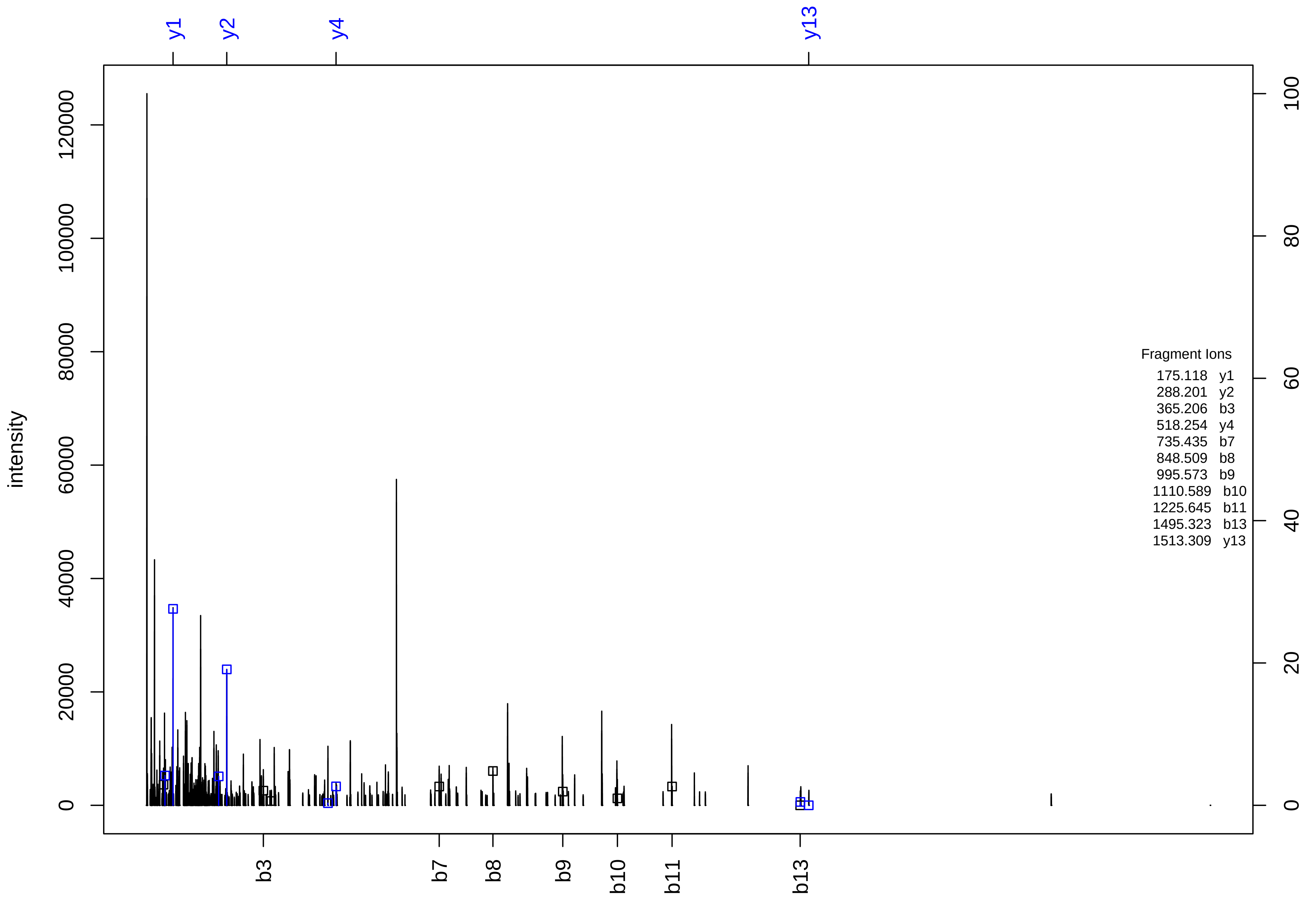

HRATLVGLFNNLR / File: sydney_19.raw; SpectrumID: ; scans: 9977

IDELSLYSVPEGQSK

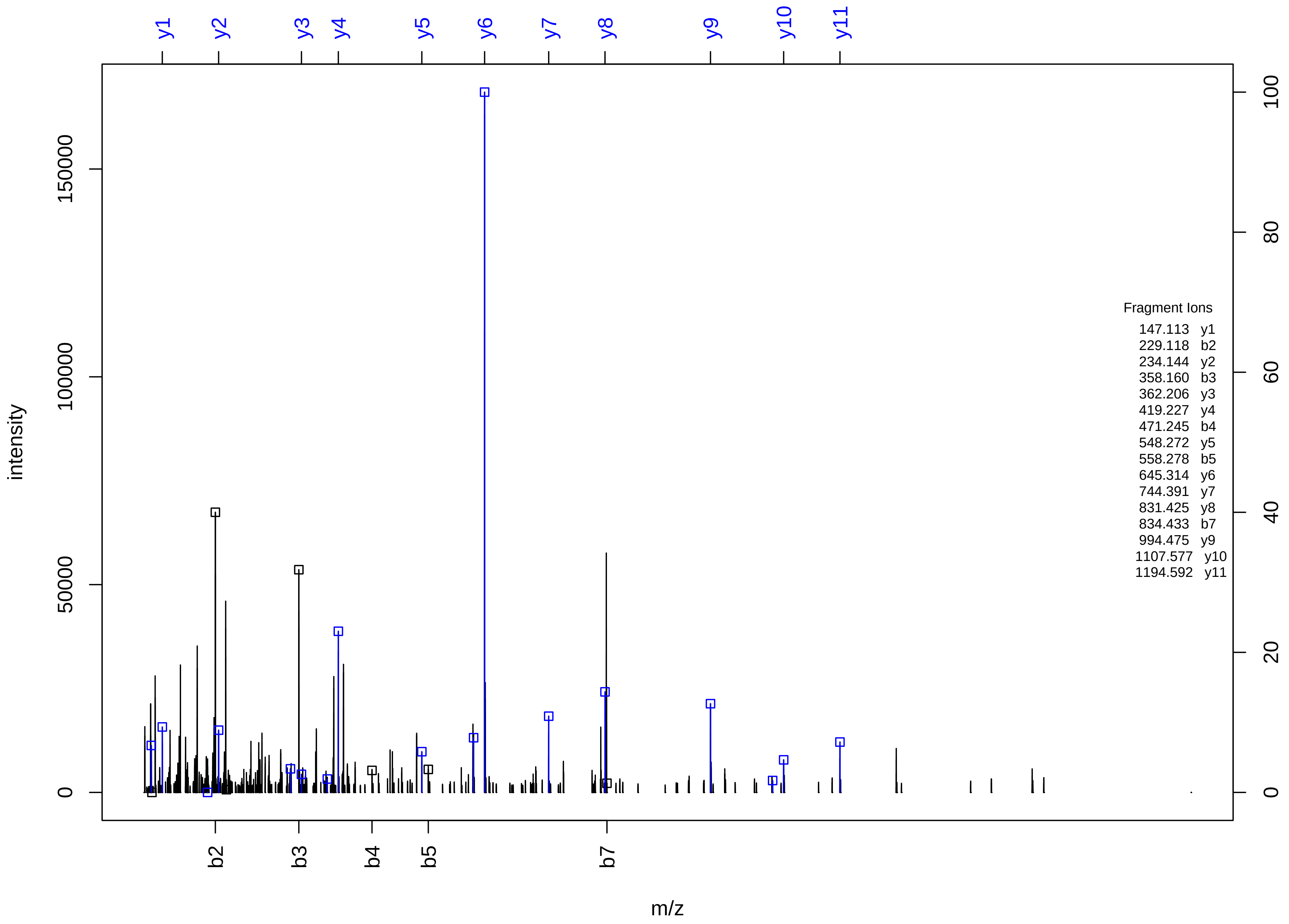

IDELSLYSVPEGQSK / File: sydney_19.raw; SpectrumID: ; scans: 5873

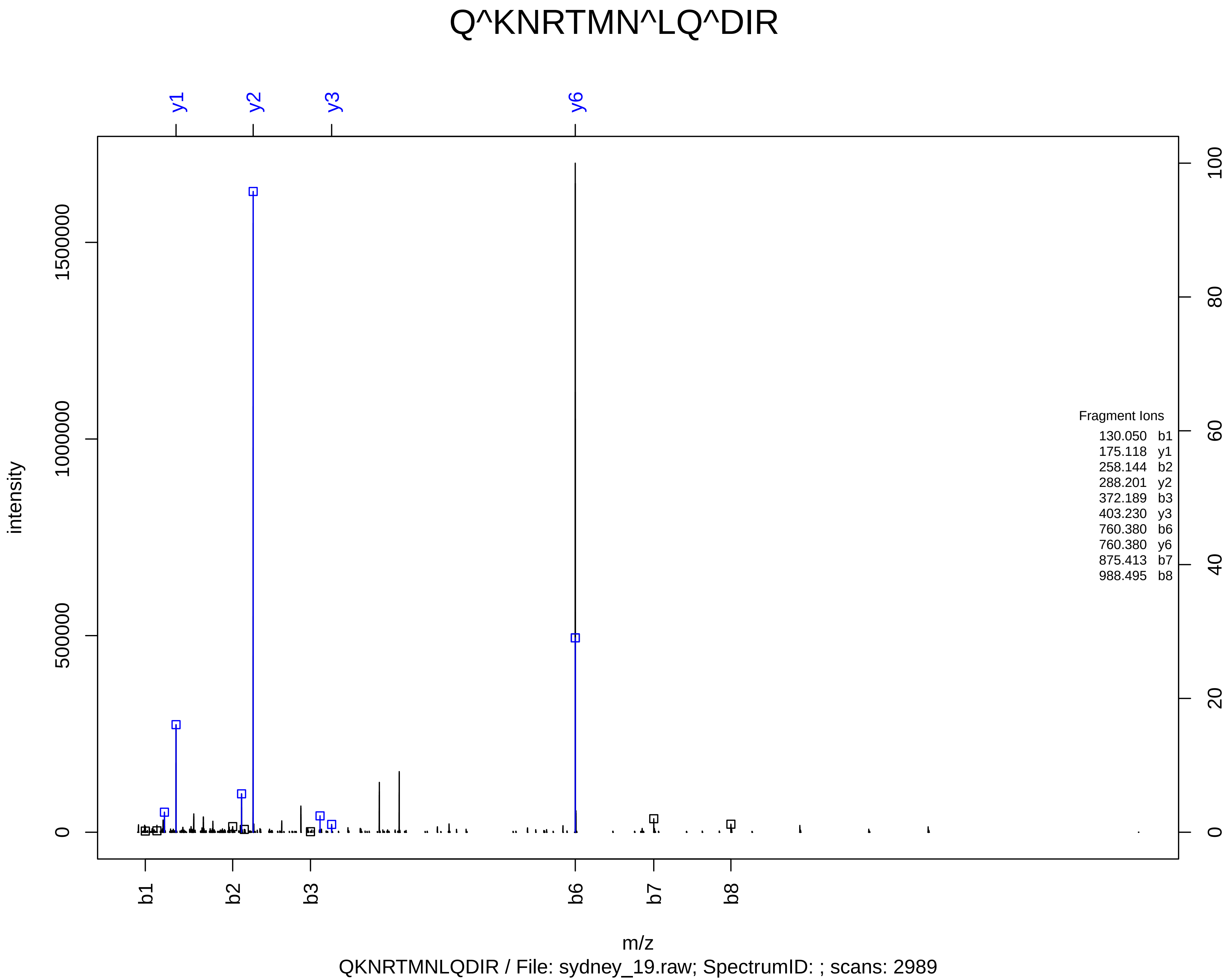

Q^KNRTMN^LQ^DIR
y1
y2
y3
y6
intensity
0
500000
1000000
1500000
0
20
40
60
80
100
Fragment Ions
130.050 b1
175.118 y1
258.144 b2
288.201 y2
372.189 b3
403.230 y3
760.380 b6
760.380 y6
875.413 b7
988.495 b8
b1
b2
b3
b6
b7
b8
m/z
QKNRTMNLQDIR / File: sydney_19.raw; SpectrumID: ; scans: 2989

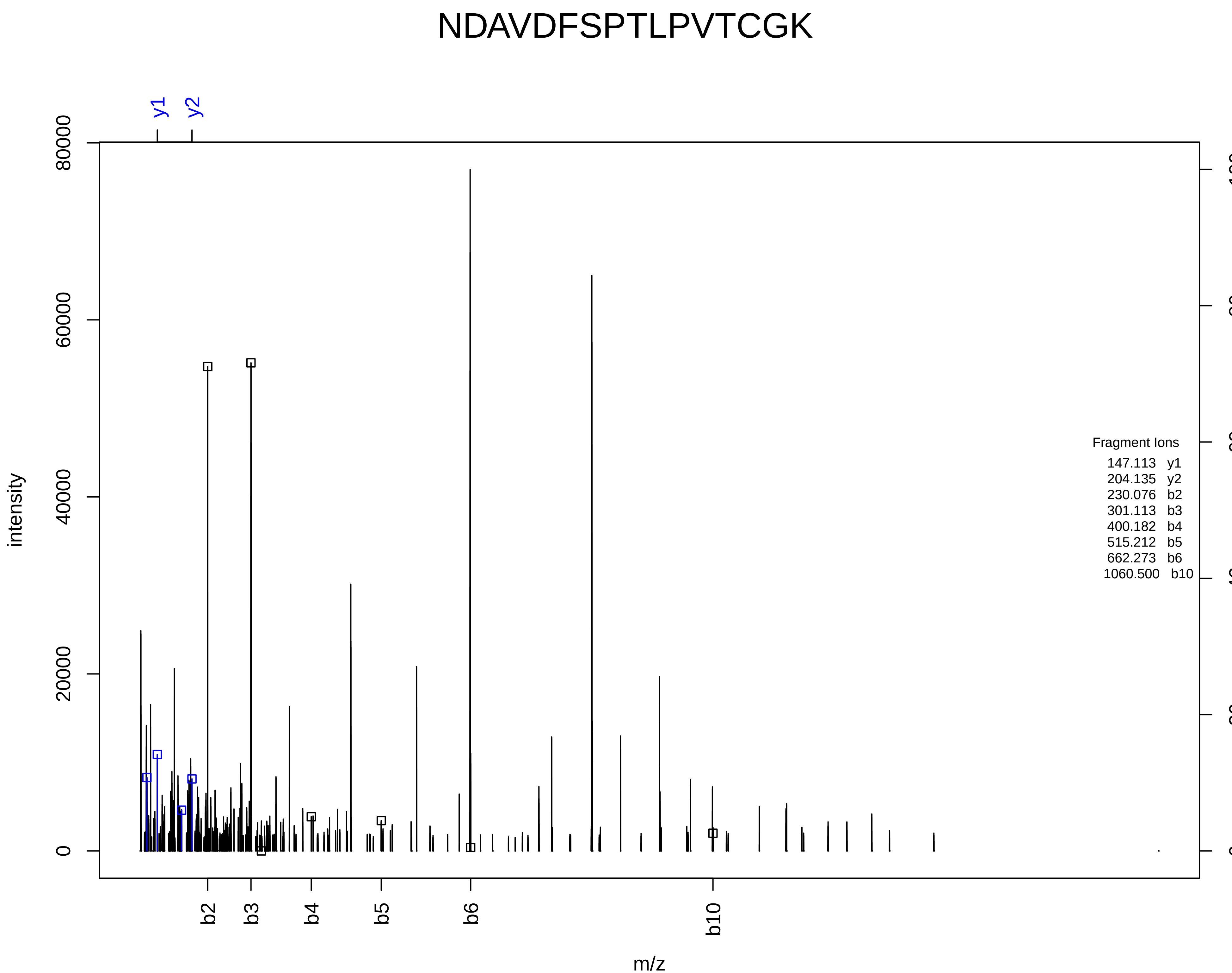
NDAVDFSPTLPVTCGK
y1
y2
80000
60000
40000
20000
0
intensity
100
80
60
40
20
0
Fragment Ions
147.113 y1
204.135 y2
230.076 b2
301.113 b3
400.182 b4
515.212 b5
662.273 b6
1060.500 b10
b2
b3
b4
b5
b6
b10
m/z
NDAVDFSPTLPVTCGK / File: sydney_19.raw; SpectrumID: ; scans: 6638

(Ac)MDDTLFQLK

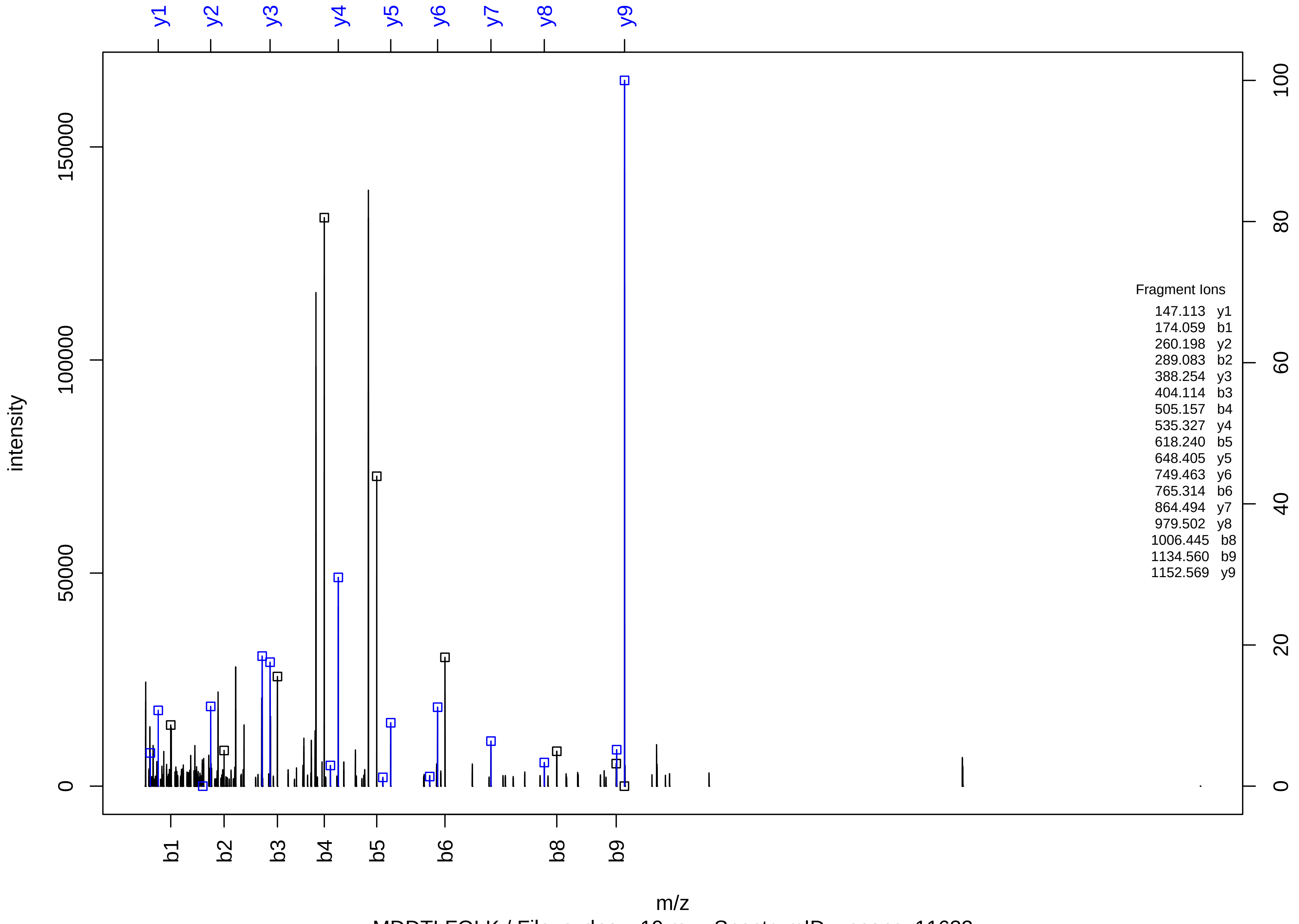

MDDTLFQLK / File: sydney_19.raw; SpectrumID: ; scans: 11633

NSEQIMEVGEELINDYASK

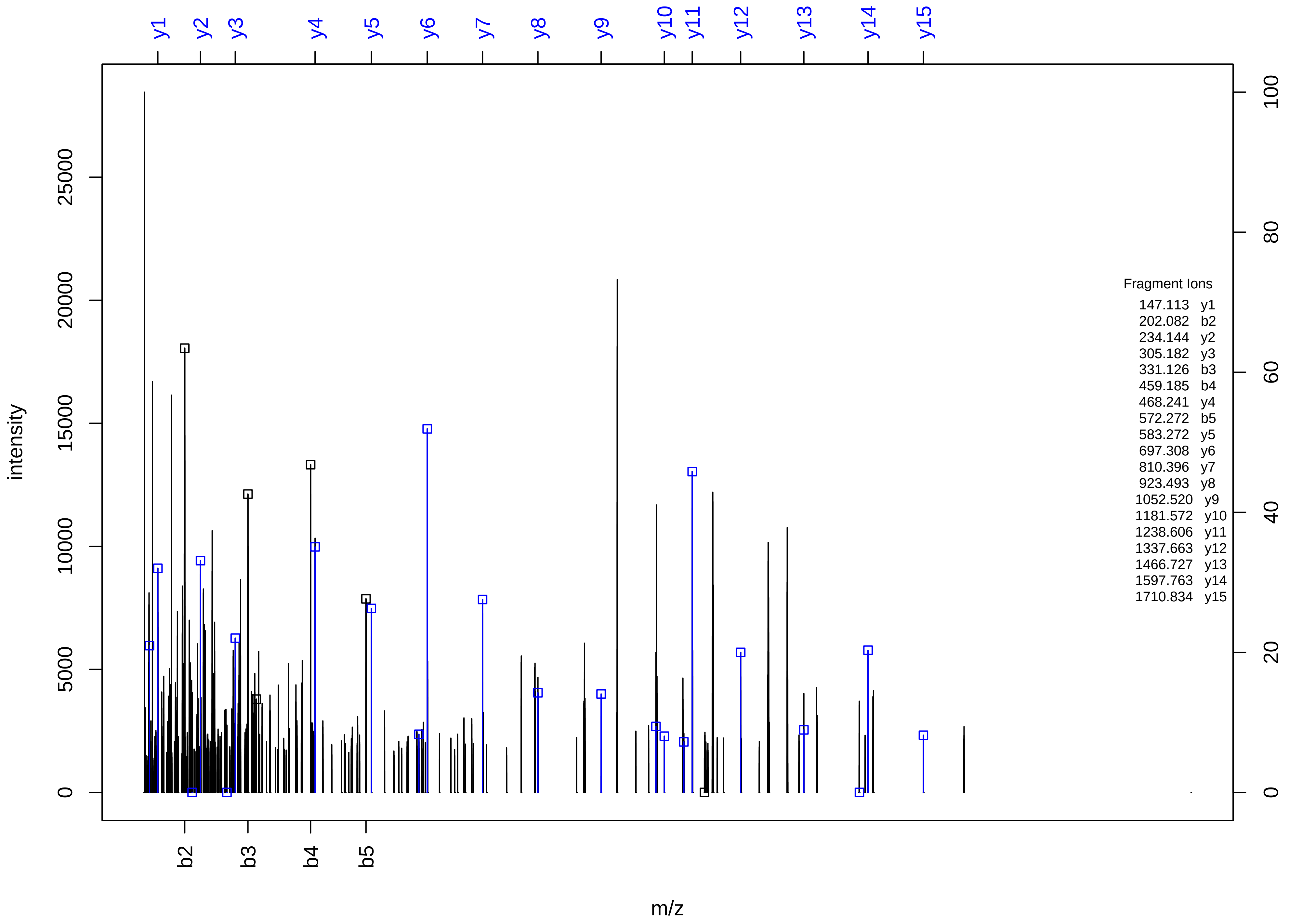

NSEQIMEVGEELINDYASK / File: sydney_19.raw; SpectrumID: ; scans: 12947

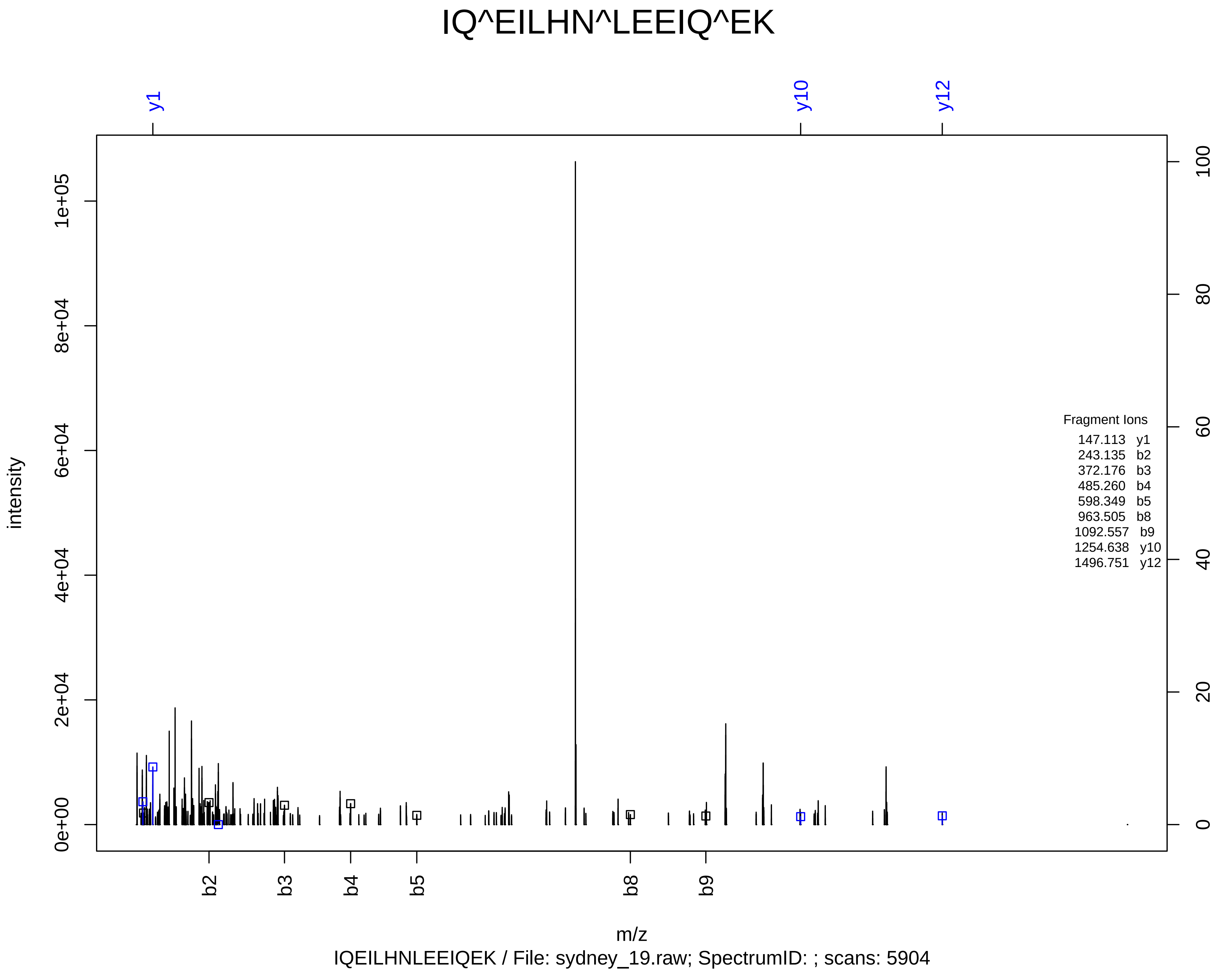
IQ^EILHN^LEEIQ^EK
y1
y10
y12
intensity
0e+00
2e+04
4e+04
6e+04
8e+04
1e+05
0
20
40
60
80
100
b2
b3
b4
b5
b8
b9
m/z
Fragment Ions
147.113 y1
243.135 b2
372.176 b3
485.260 b4
598.349 b5
963.505 b8
1092.557 b9
1254.638 y10
1496.751 y12
IQEILHNLEEIQEK / File: sydney_19.raw; SpectrumID: ; scans: 5904

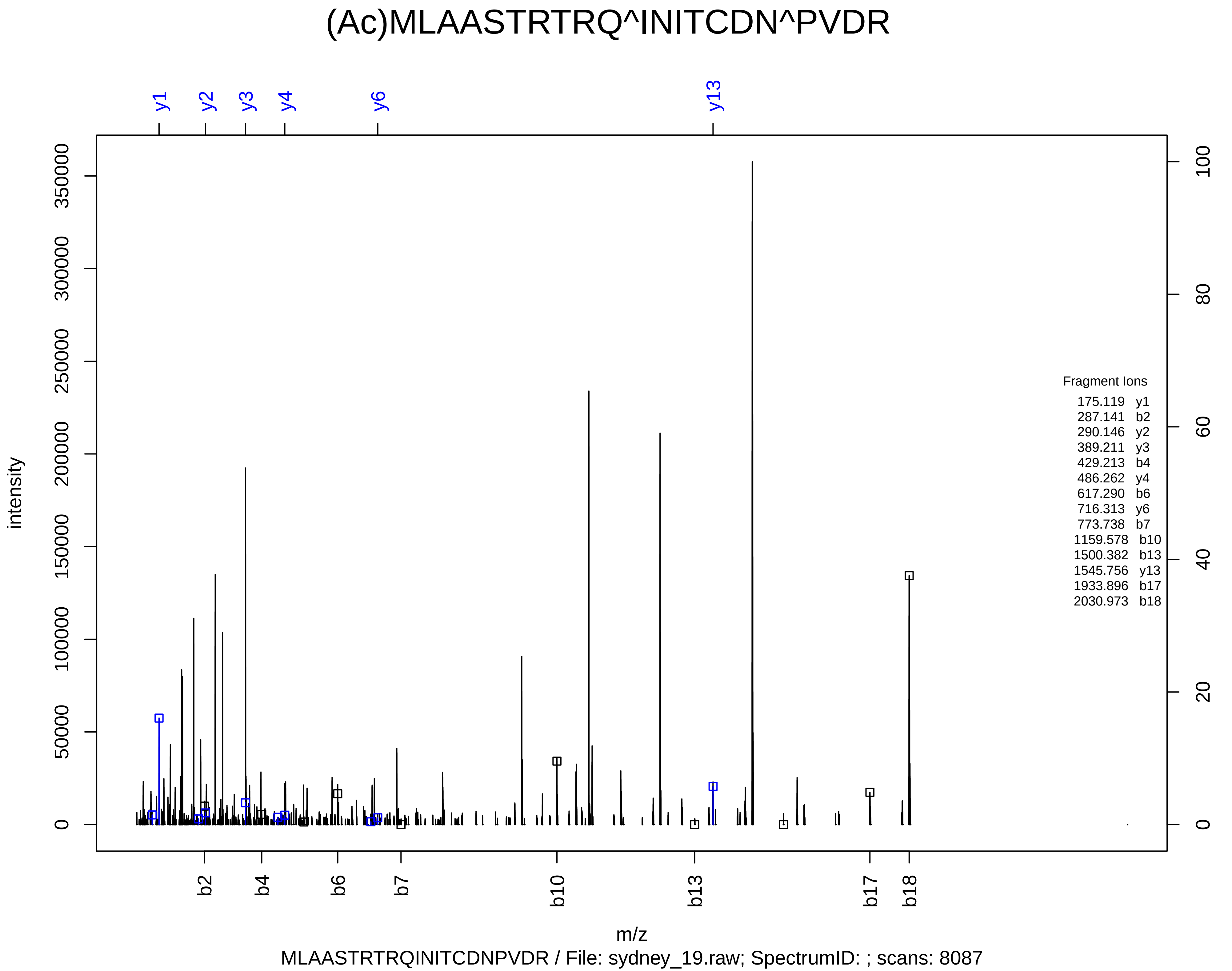
(Ac)MLAASTRTRQ^INITCDN^PVDR
y1
y2
y3
y4
y6
y13
intensity
0
50000
100000
150000
200000
250000
300000
350000
0
20
40
60
80
100
Fragment Ions
175.119 y1
287.141 b2
290.146 y2
389.211 y3
429.213 b4
486.262 y4
617.290 b6
716.313 y6
773.738 b7
1159.578 b10
1500.382 b13
1545.756 y13
1933.896 b17
2030.973 b18
b2
b4
b6
b7
b10
b13
b17
b18
m/z
MLAASTRTRQINITCDNPVDR / File: sydney_19.raw; SpectrumID: ; scans: 8087

AVAIDLPGLGR

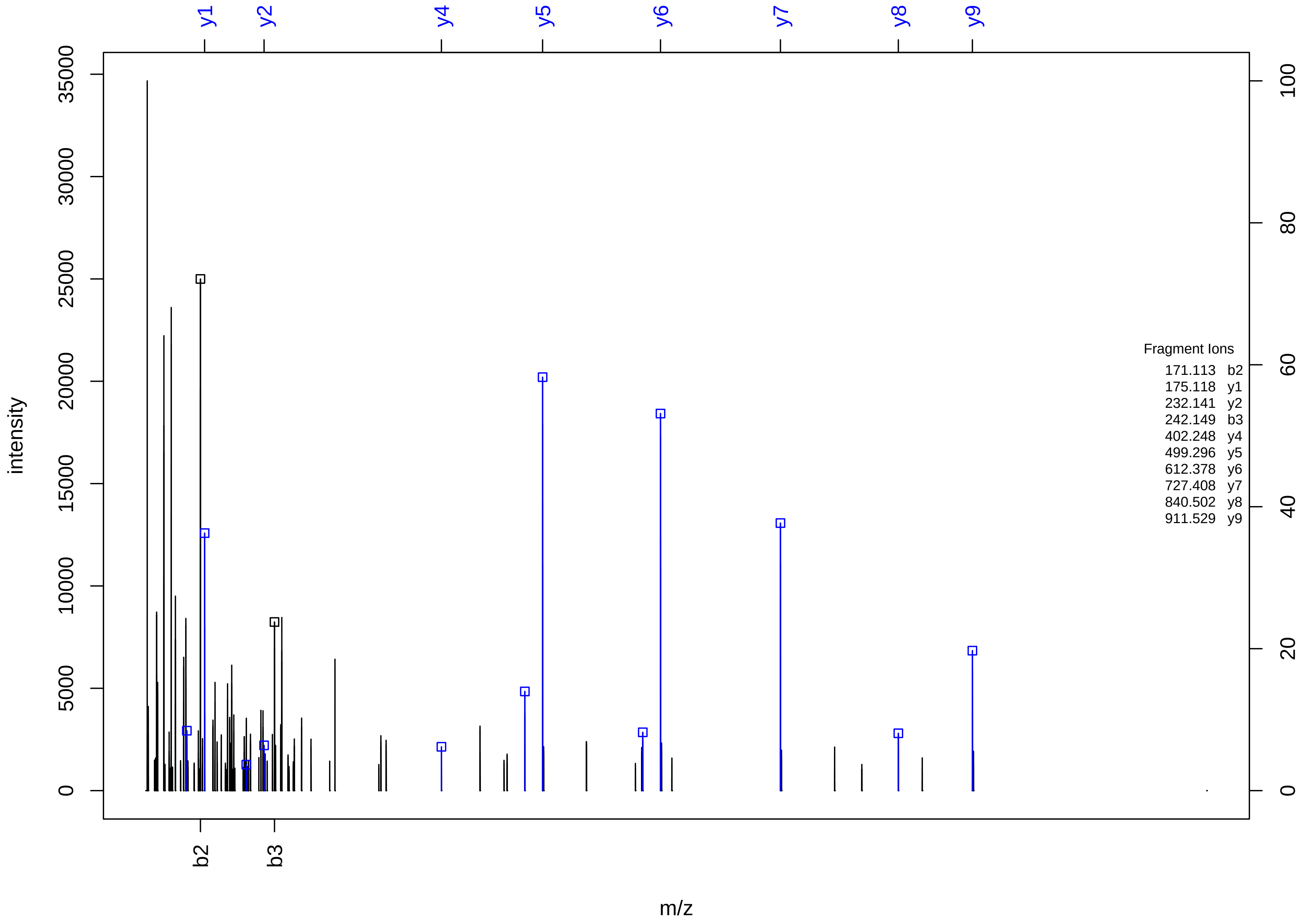

AVAIDLPGLGR / File: sydney_20.raw; SpectrumID: ; scans: 7584

GFLEKN^N^DLLYR

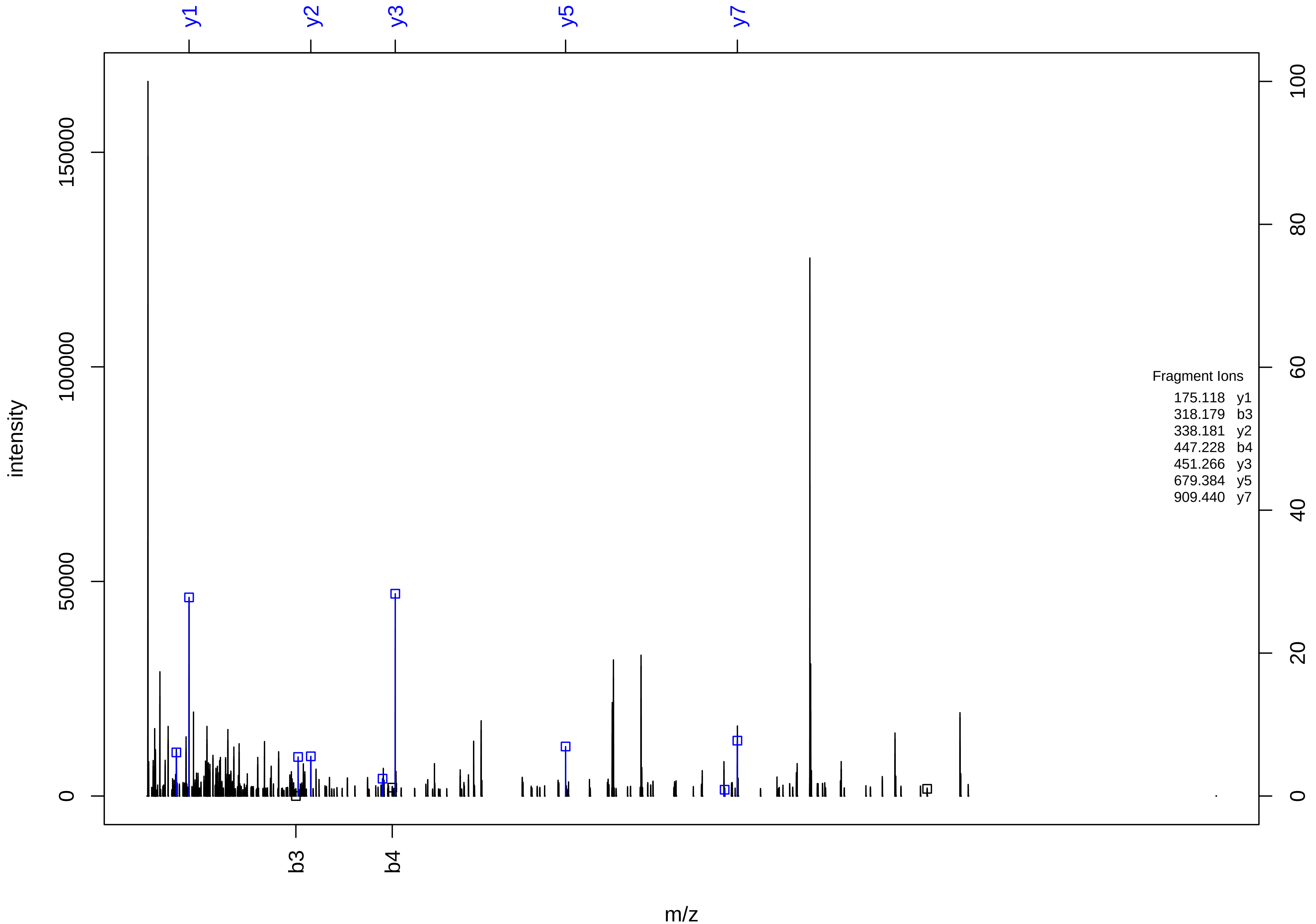

GFLEKNNDLLYR / File: sydney_20.raw; SpectrumID: ; scans: 6141

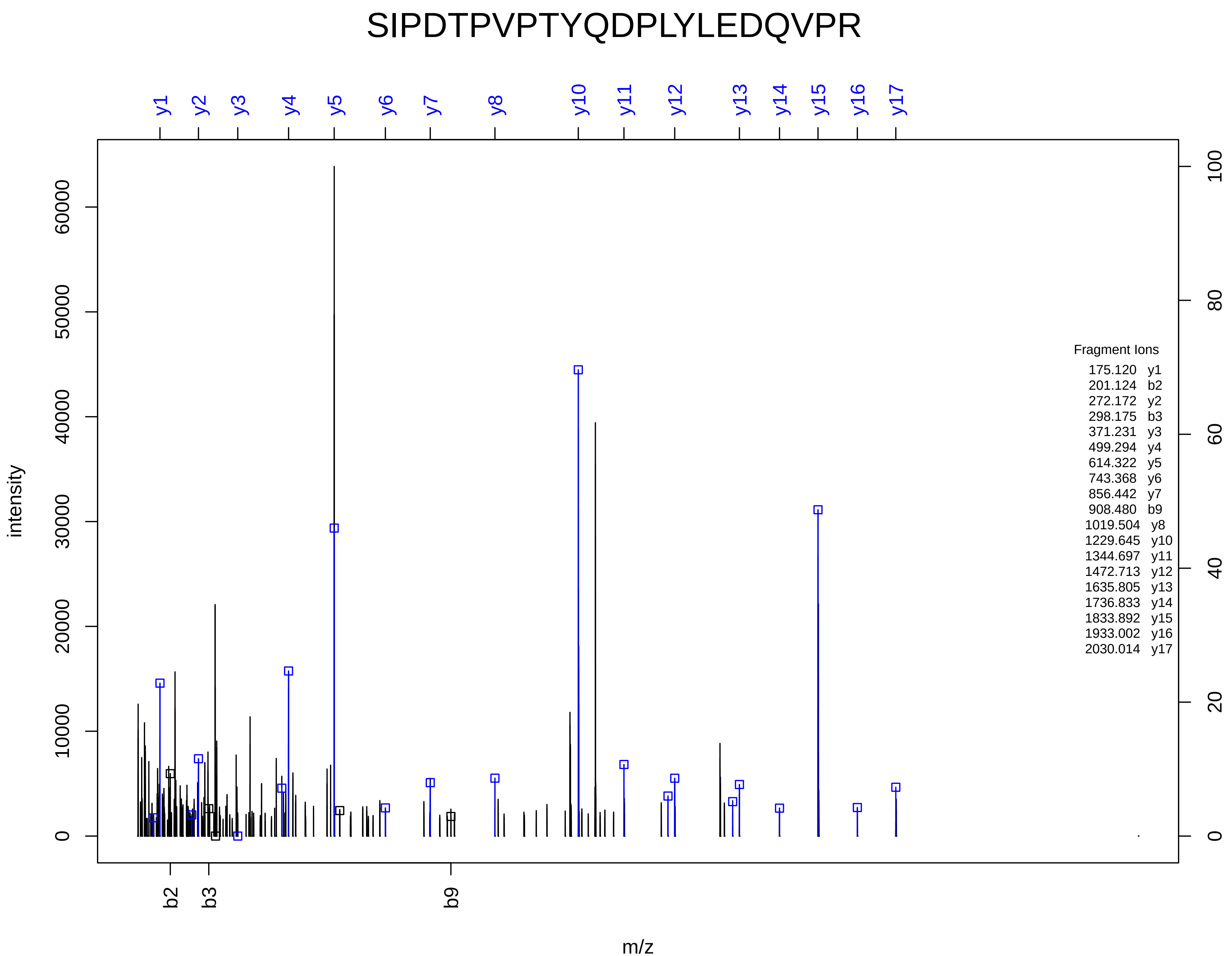

SIPDTPVPTYQDPLYLEDQVPR
y1
y2
y3
y4
y5
y6
y7
y8
y10
y11
y12
y13
y14
y15
y16
y17
b2
b3
b9
intensity
m/z
0
10000
20000
30000
40000
50000
60000
0
20
40
60
80
100
Fragment Ions
175.120 y1
201.124 b2
272.172 y2
298.175 b3
371.231 y3
499.294 y4
614.322 y5
743.368 y6
856.442 y7
908.480 b9
1019.504 y8
1229.645 y10
1344.697 y11
1472.713 y12
1635.805 y13
1736.833 y14
1833.892 y15
1933.002 y16
2030.014 y17
SIPDTPVPTYQDPLYLEDQVPR / File: sydney_20.raw; SpectrumID: ; scans: 9802

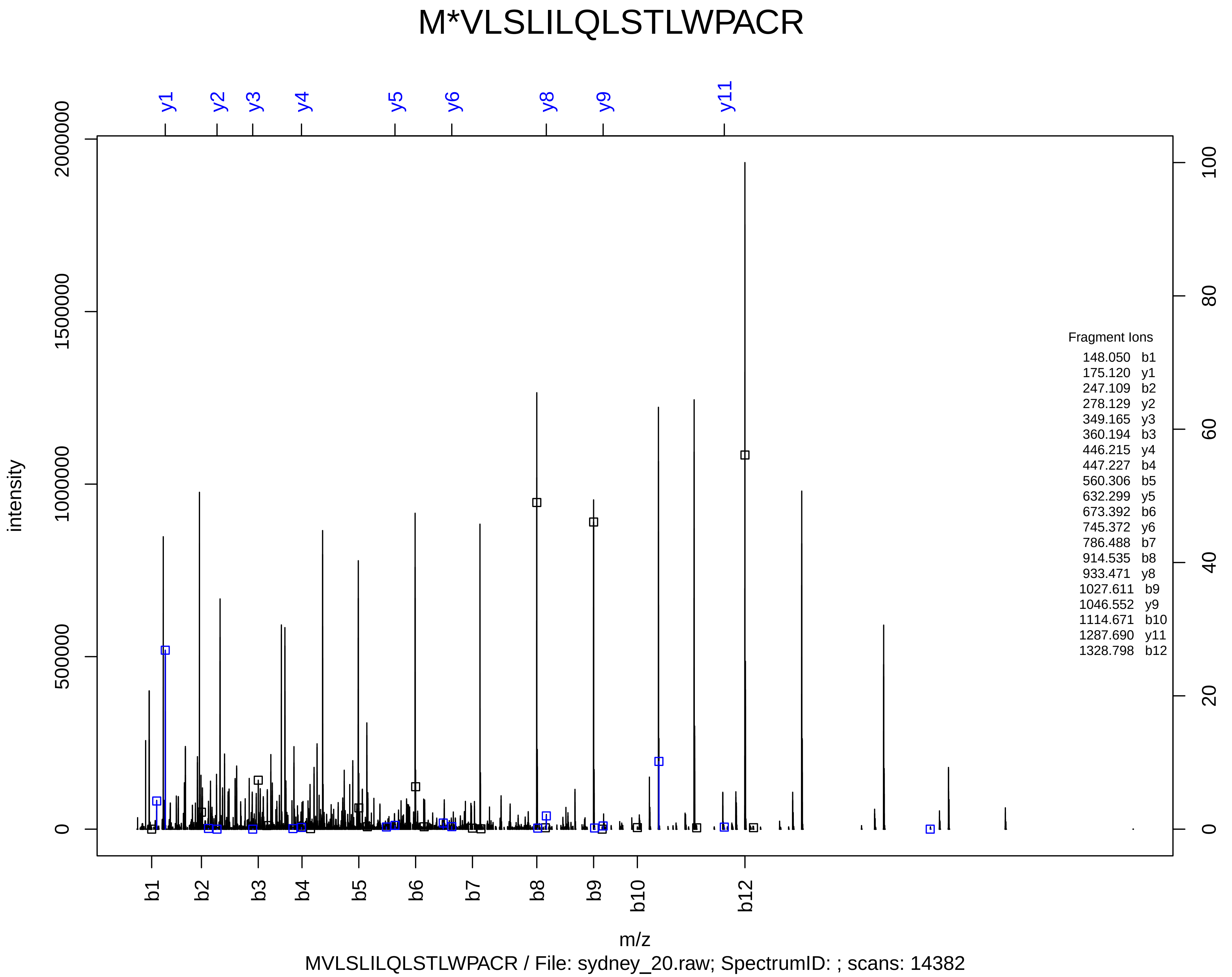
M*VLSLILQLSTLWPACR
y1 y2 y3 y4 y5 y6 y8 y9 y11
intensity
0
500000
1000000
1500000
2000000
0 20 40 60 80 100
Fragment Ions
148.050 b1
175.120 y1
247.109 b2
278.129 y2
349.165 y3
360.194 b3
446.215 y4
447.227 b4
560.306 b5
632.299 y5
673.392 b6
745.372 y6
786.488 b7
914.535 b8
933.471 y8
1027.611 b9
1046.552 y9
1114.671 b10
1287.690 y11
1328.798 b12
b1 b2 b3 b4 b5 b6 b7 b8 b9 b10 b12
m/z
MVLSLILQLSTLWPACR / File: sydney_20.raw; SpectrumID: ; scans: 14382

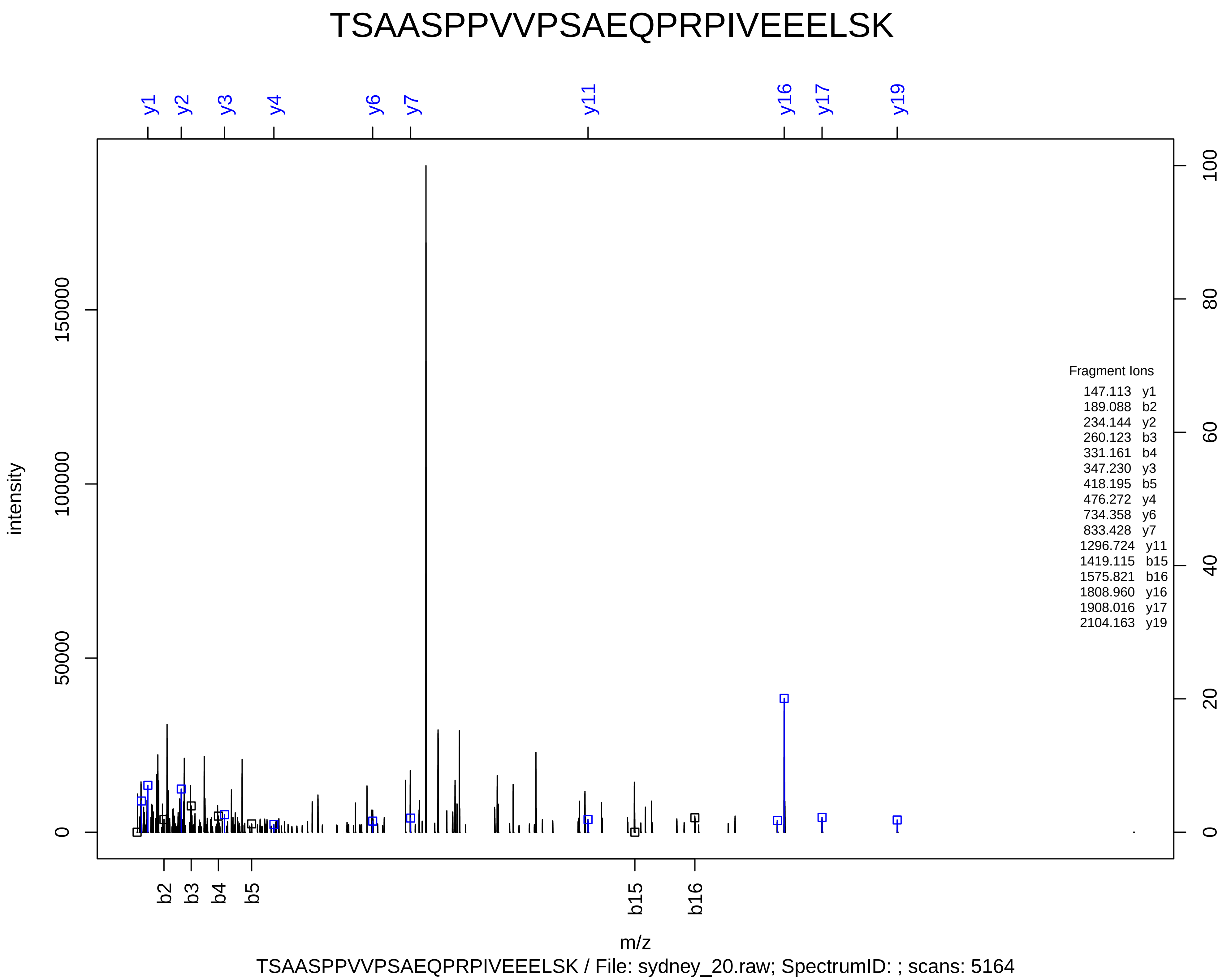
TSAASPPVVPSAEQPRPIVEEELSK
y1
y2
y3
y4
y6
y7
y11
y16
y17
y19
b2
b3
b4
b5
b15
b16
intensity
0
50000
100000
150000
0
20
40
60
80
100
m/z
Fragment Ions
147.113 y1
189.088 b2
234.144 y2
260.123 b3
331.161 b4
347.230 y3
418.195 b5
476.272 y4
734.358 y6
833.428 y7
1296.724 y11
1419.115 b15
1575.821 b16
1808.960 y16
1908.016 y17
2104.163 y19
TSAASPPVVPSAEQPRPIVEEELSK / File: sydney_20.raw; SpectrumID: ; scans: 5164

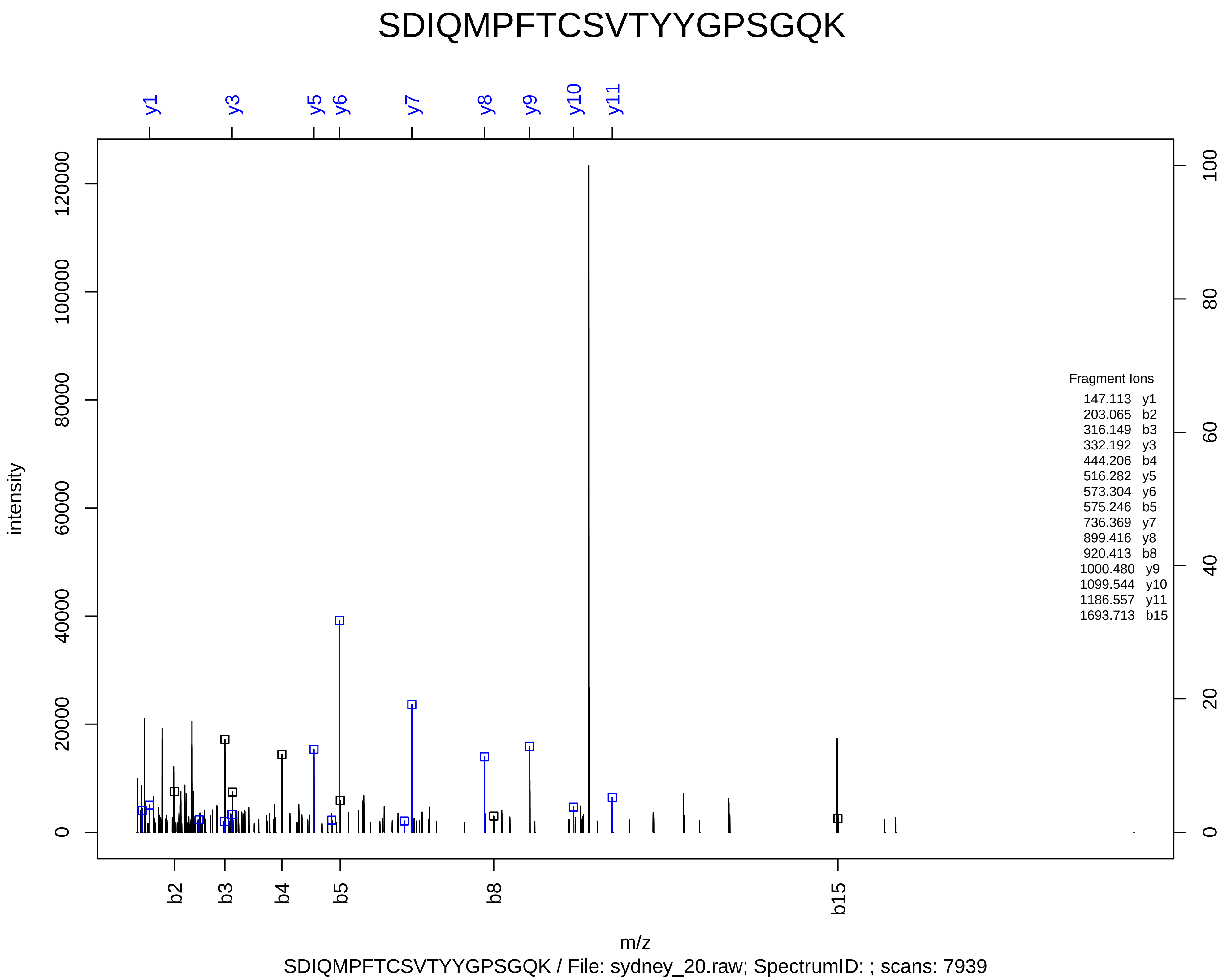
SDIQMPFTCSVTYYGPSGQK
y1
y3
y5
y6
y7
y8
y9
y10
y11
intensity
0
20000
40000
60000
80000
100000
120000
0
20
40
60
80
100
Fragment Ions
147.113 y1
203.065 b2
316.149 b3
332.192 y3
444.206 b4
516.282 y5
573.304 y6
575.246 b5
736.369 y7
899.416 y8
920.413 b8
1000.480 y9
1099.544 y10
1186.557 y11
1693.713 b15
b2
b3
b4
b5
b8
b15
m/z
SDIQMPFTCSVTYYGPSGQK / File: sydney_20.raw; SpectrumID: ; scans: 7939

VGQ^PMVSSSN^Q^R

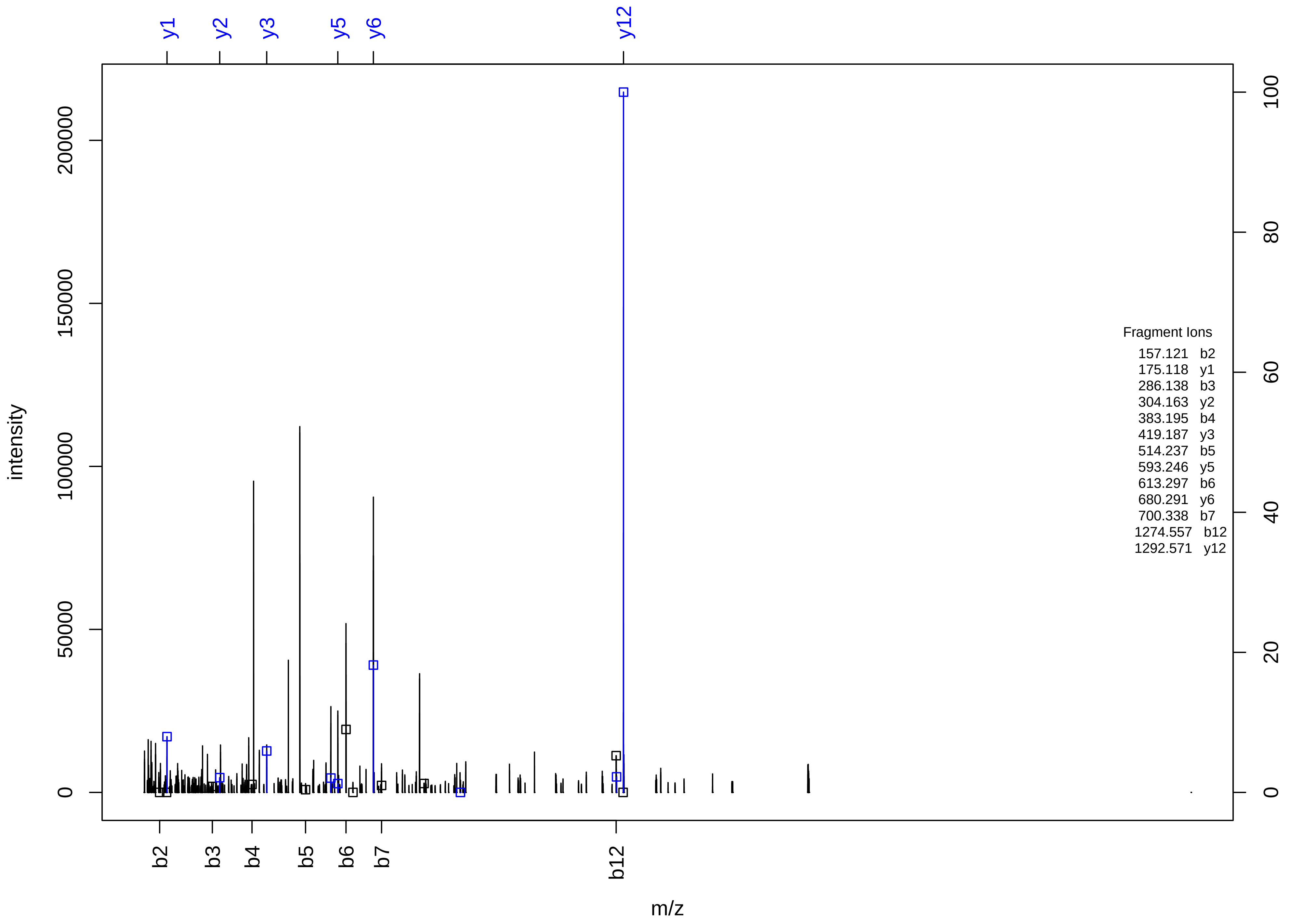

VGQPMVSSSNQR / File: sydney_20.raw; SpectrumID: ; scans: 13405

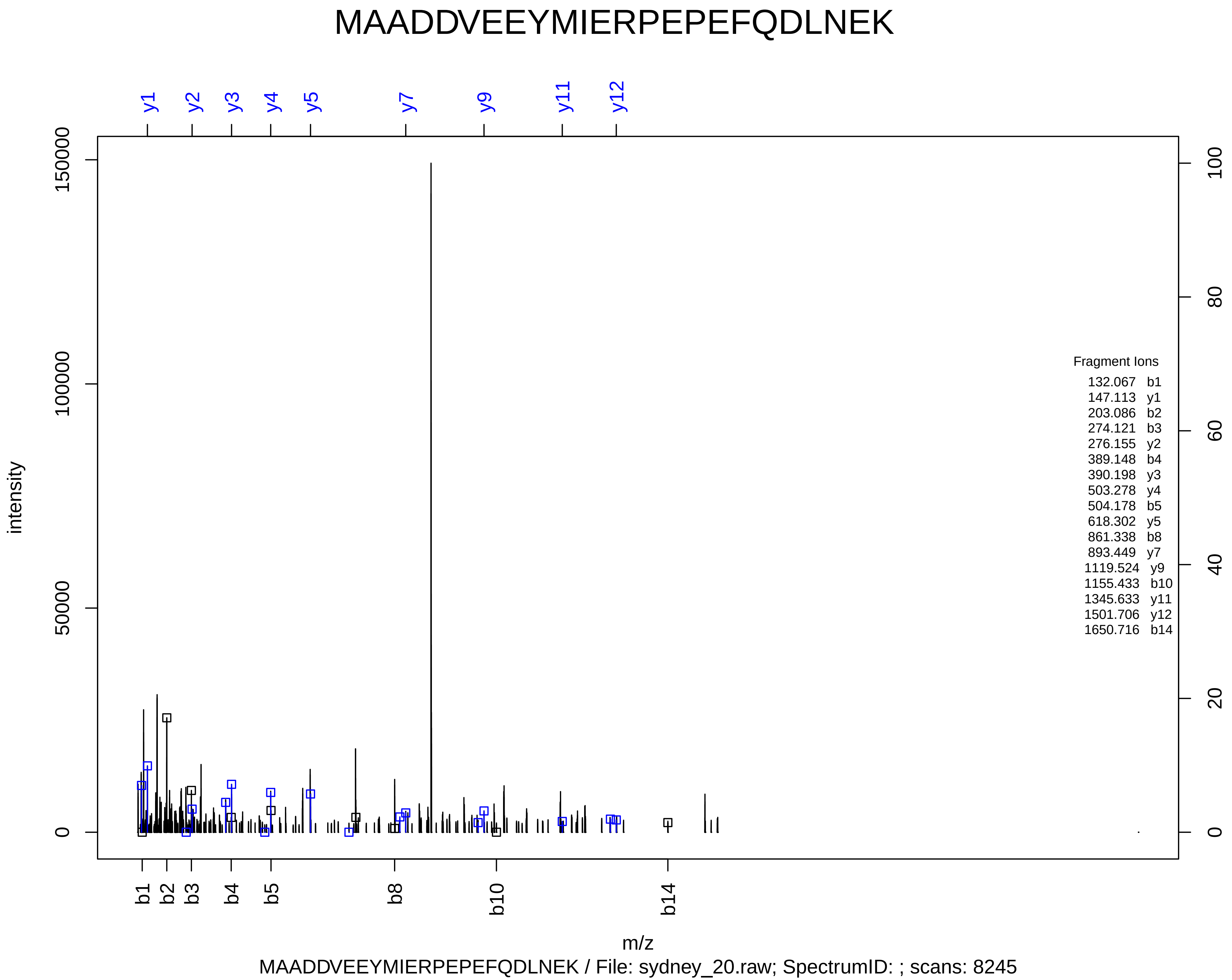

MAADDVEEYMIERPEPEFQDLNEK
y1 y2 y3 y4 y5 y7 y9 y11 y12
intensity
0
50000
100000
150000
0
20
40
60
80
100
Fragment Ions
132.067 b1
147.113 y1
203.086 b2
274.121 b3
276.155 y2
389.148 b4
390.198 y3
503.278 y4
504.178 b5
618.302 y5
861.338 b8
893.449 y7
1119.524 y9
1155.433 b10
1345.633 y11
1501.706 y12
1650.716 b14
b1 b2 b3 b4 b5 b8 b10 b14
m/z
MAADDVEEYMIERPEPEFQDLNEK / File: sydney_20.raw; SpectrumID: ; scans: 8245

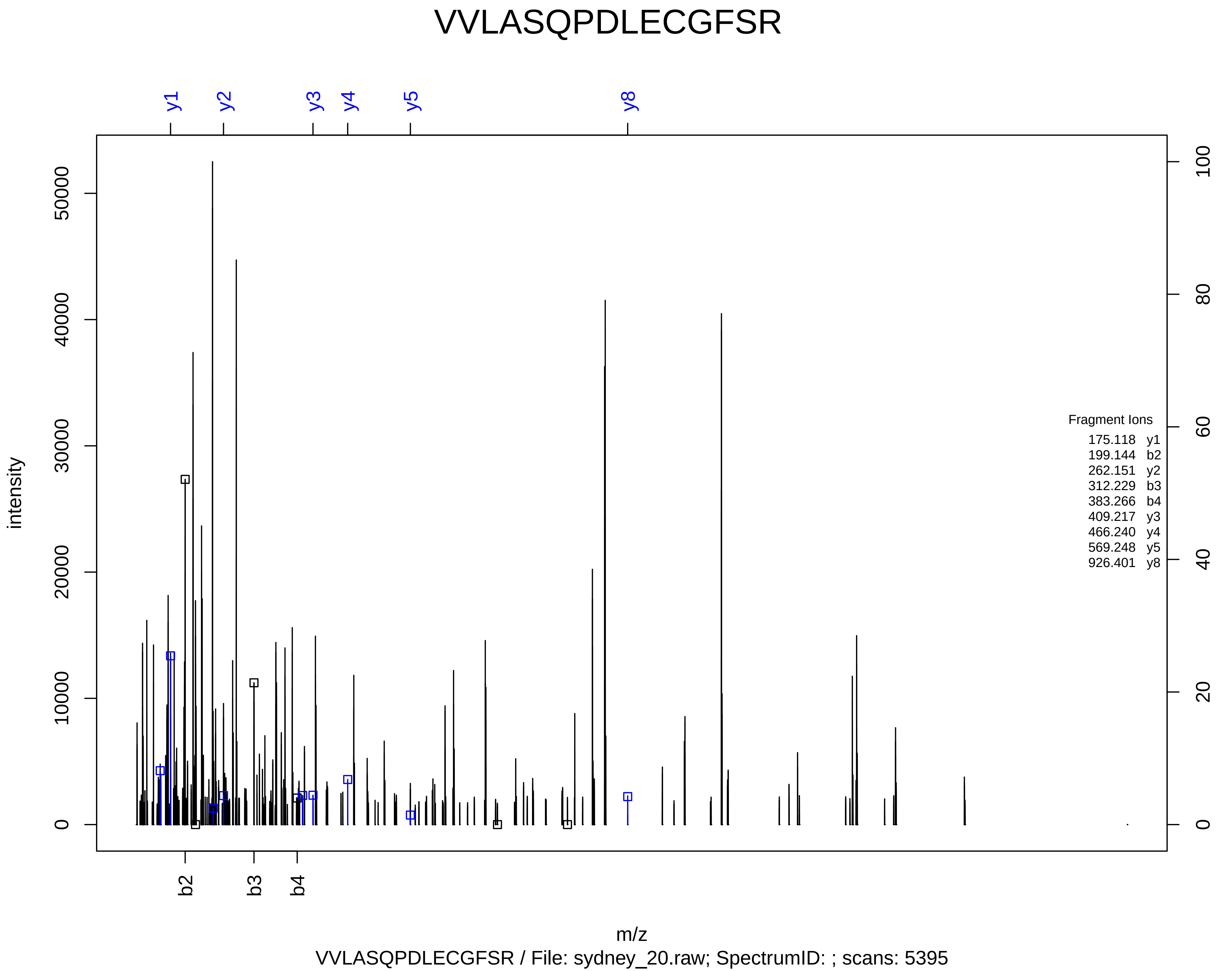
VVLASQPDLECGFSR
y1
y2
y3
y4
y5
y8
intensity
0
10000
20000
30000
40000
50000
0
20
40
60
80
100
b2
b3
b4
m/z
Fragment Ions
175.118 y1
199.144 b2
262.151 y2
312.229 b3
383.266 b4
409.217 y3
466.240 y4
569.248 y5
926.401 y8
VVLASQPDLECGFSR / File: sydney_20.raw; SpectrumID: ; scans: 5395

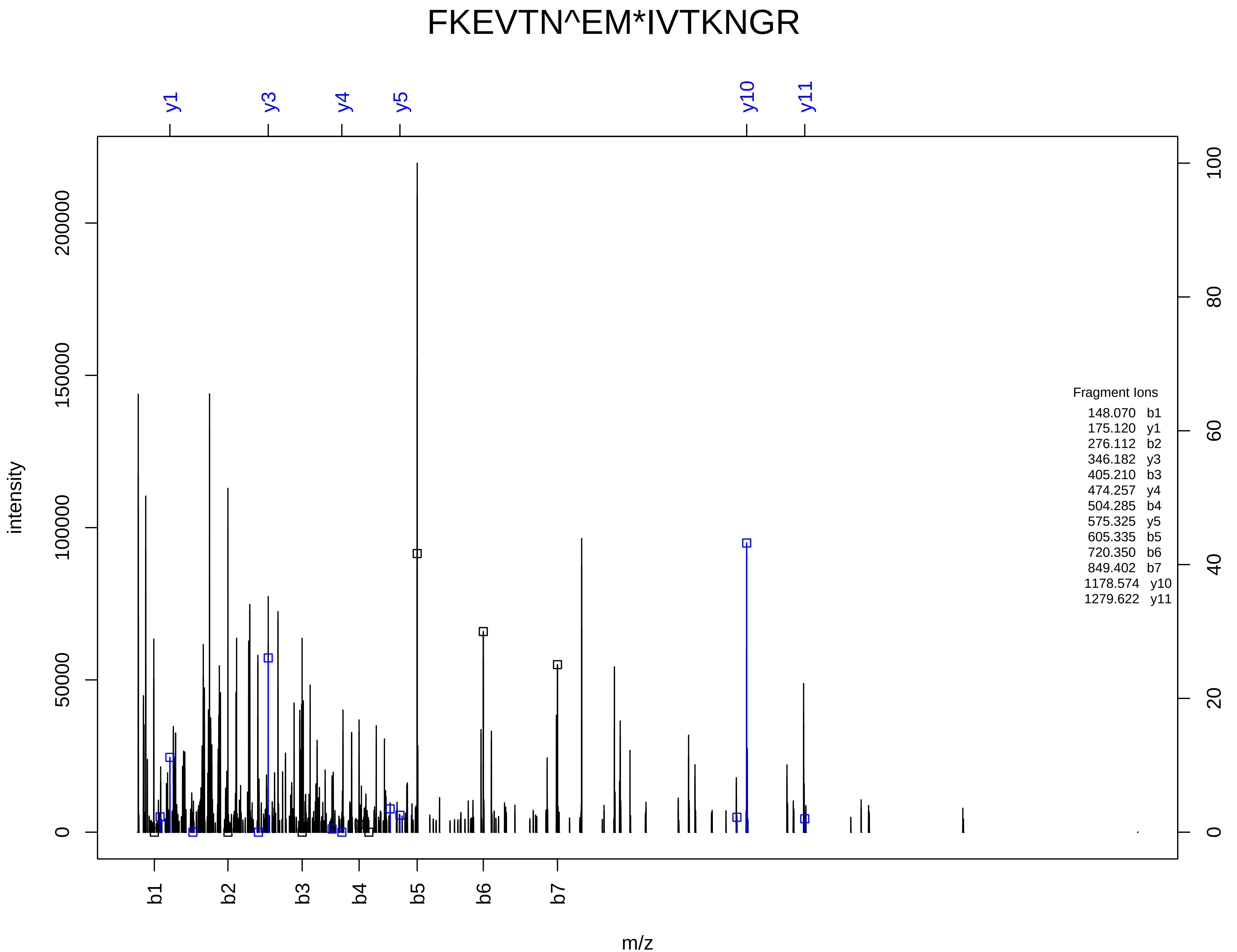

FKEVTNEMIVTKNGR / File: sydney_20.raw; SpectrumID: ; scans: 8278

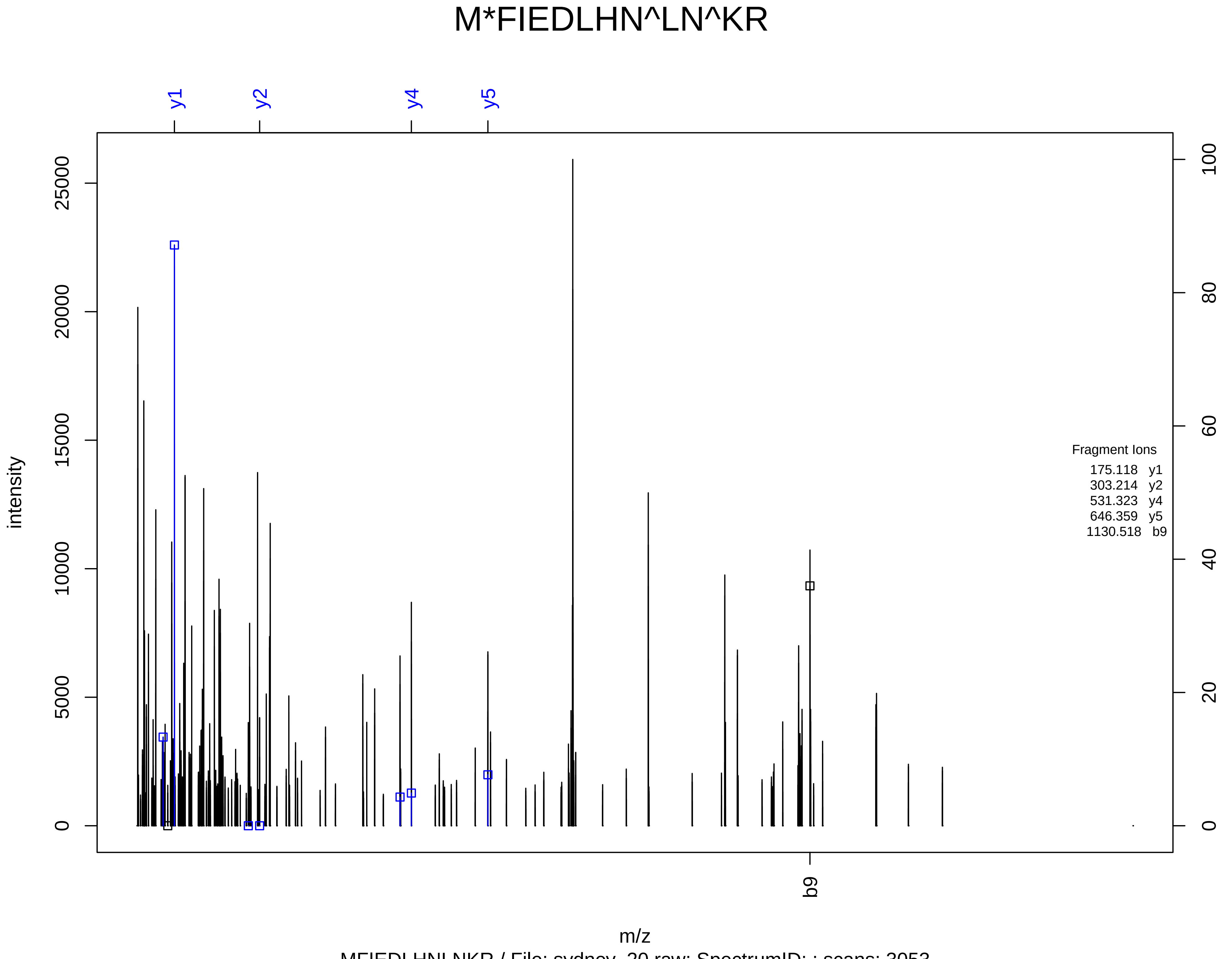
M*FIEDLHN^LN^KR
y1
y2
y4
y5
intensity
0
5000
10000
15000
20000
25000
0
20
40
60
80
100
Fragment Ions
175.118 y1
303.214 y2
531.323 y4
646.359 y5
1130.518 b9
b9
m/z
MFIEDLHNLNKR / File: sydney_20.raw; SpectrumID: ; scans: 3053

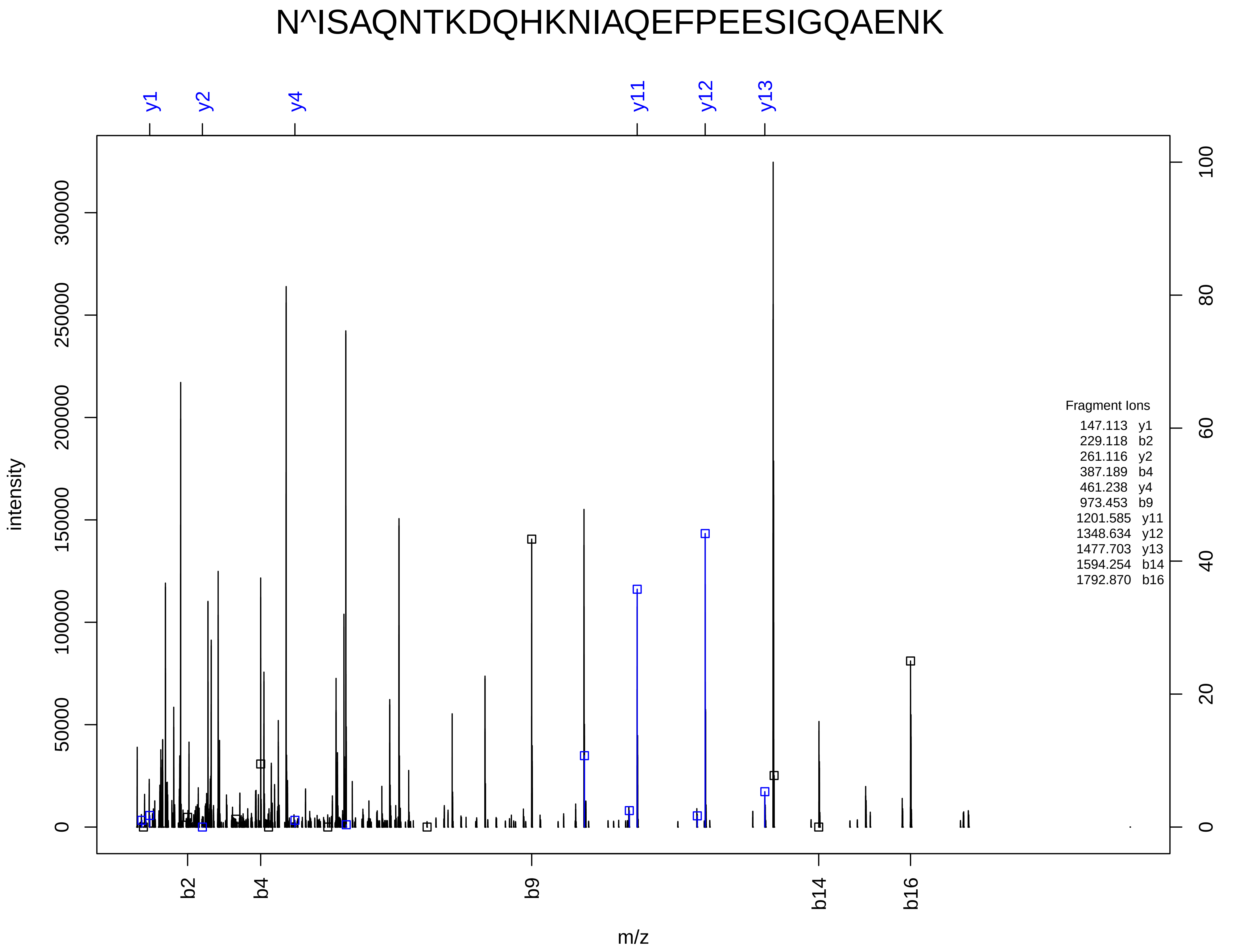
N^ISAQNTKDQHKNIAQEFPEESIGQAENK
y1
y2
y4
y11
y12
y13
intensity
0
50000
100000
150000
200000
250000
300000
0
20
40
60
80
100
Fragment Ions
147.113 y1
229.118 b2
261.116 y2
387.189 b4
461.238 y4
973.453 b9
1201.585 y11
1348.634 y12
1477.703 y13
1594.254 b14
1792.870 b16
b2
b4
b9
b14
b16
m/z
NISAQNTKDQHKNIAQEFPEESIGQAENK / File: sydney_21.raw; SpectrumID: ; scans: 14871

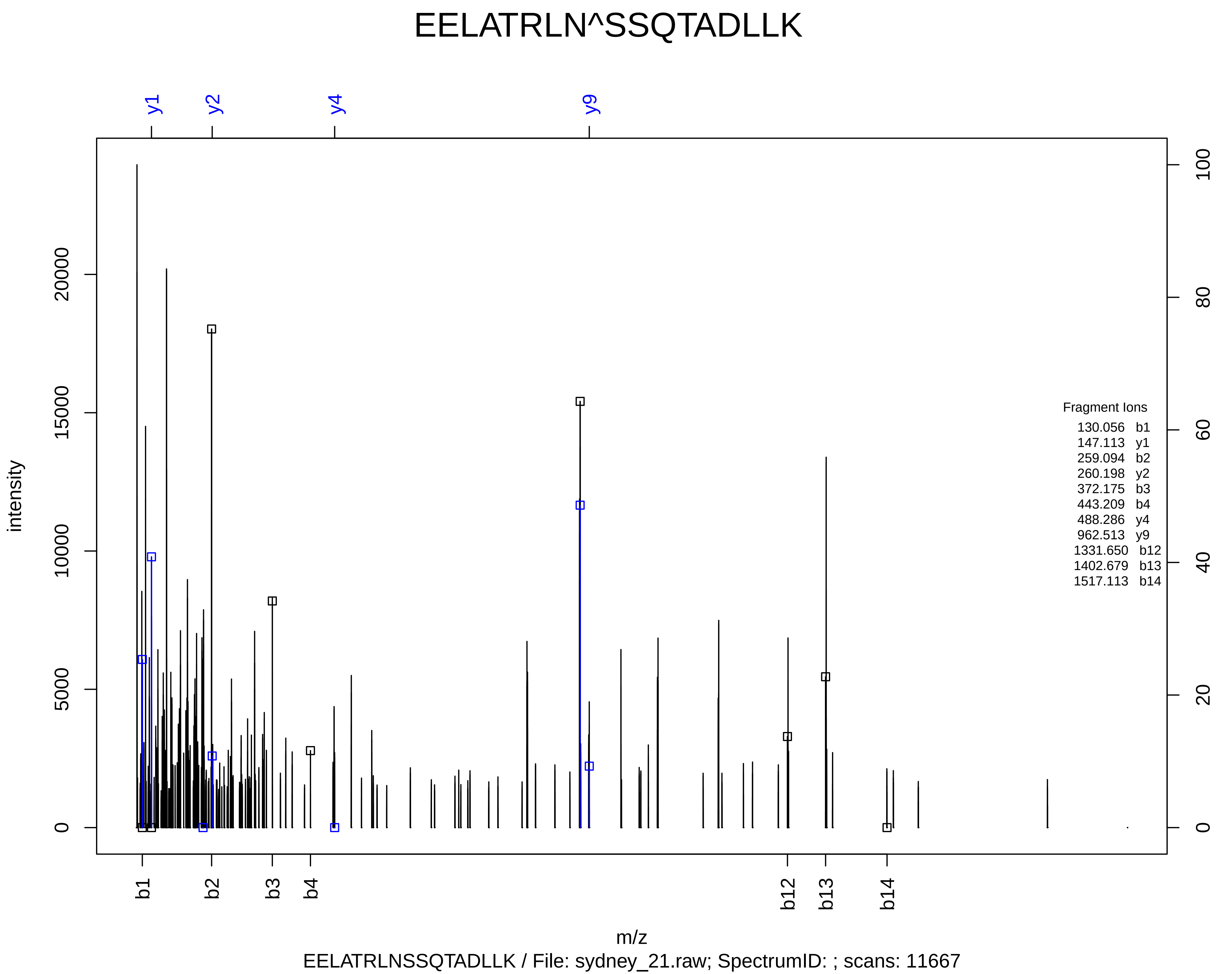

EELATRLN^SSQTADLLK
y1
y2
y4
y9
intensity
0
5000
10000
15000
20000
0
20
40
60
80
100
Fragment Ions
130.056 b1
147.113 y1
259.094 b2
260.198 y2
372.175 b3
443.209 b4
488.286 y4
962.513 y9
1331.650 b12
1402.679 b13
1517.113 b14
b1
b2
b3
b4
b12
b13
b14
m/z
EELATRLNSSQTADLLK / File: sydney_21.raw; SpectrumID: ; scans: 11667

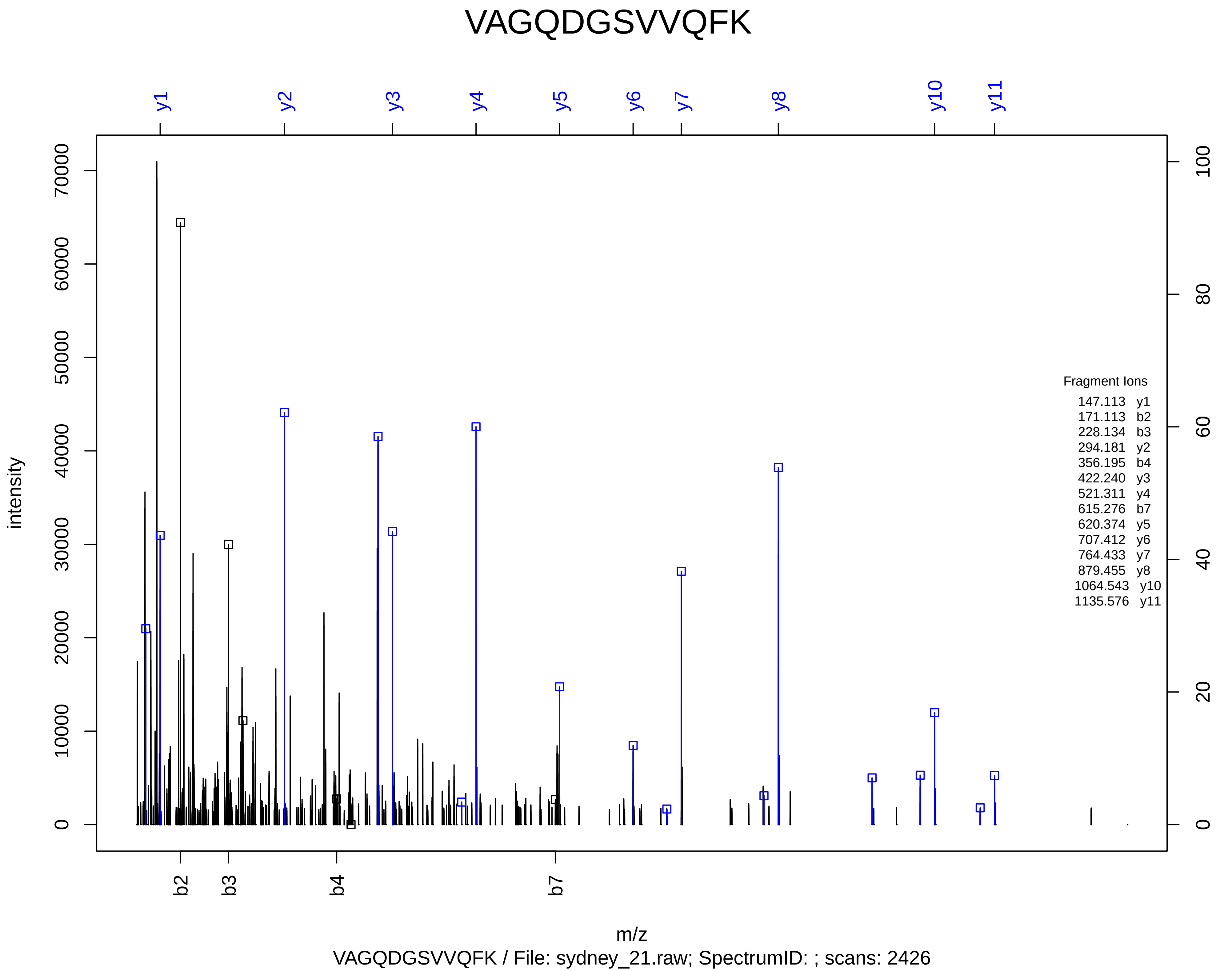
VAGQDGSVVQFK
y1
y2
y3
y4
y5
y6
y7
y8
y10
y11
intensity
0
10000
20000
30000
40000
50000
60000
70000
0
20
40
60
80
100
b2
b3
b4
b7
m/z
Fragment Ions
147.113 y1
171.113 b2
228.134 b3
294.181 y2
356.195 b4
422.240 y3
521.311 y4
615.276 b7
620.374 y5
707.412 y6
764.433 y7
879.455 y8
1064.543 y10
1135.576 y11
VAGQDGSVVQFK / File: sydney_21.raw; SpectrumID: ; scans: 2426

VVVTVEQTEEELQR

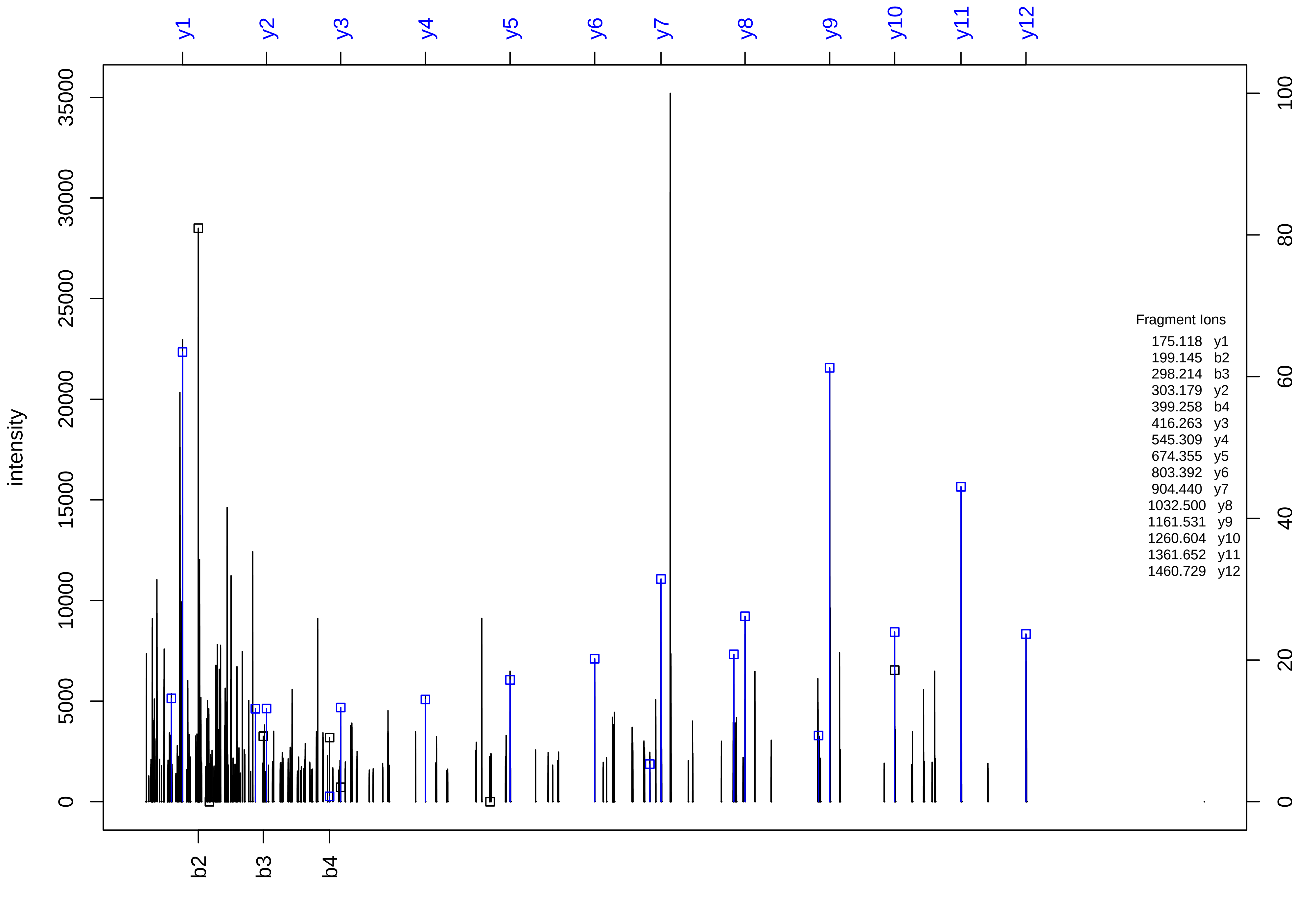

VVVTVEQTEEELQR / File: sydney_21.raw; SpectrumID: ; scans: 4932

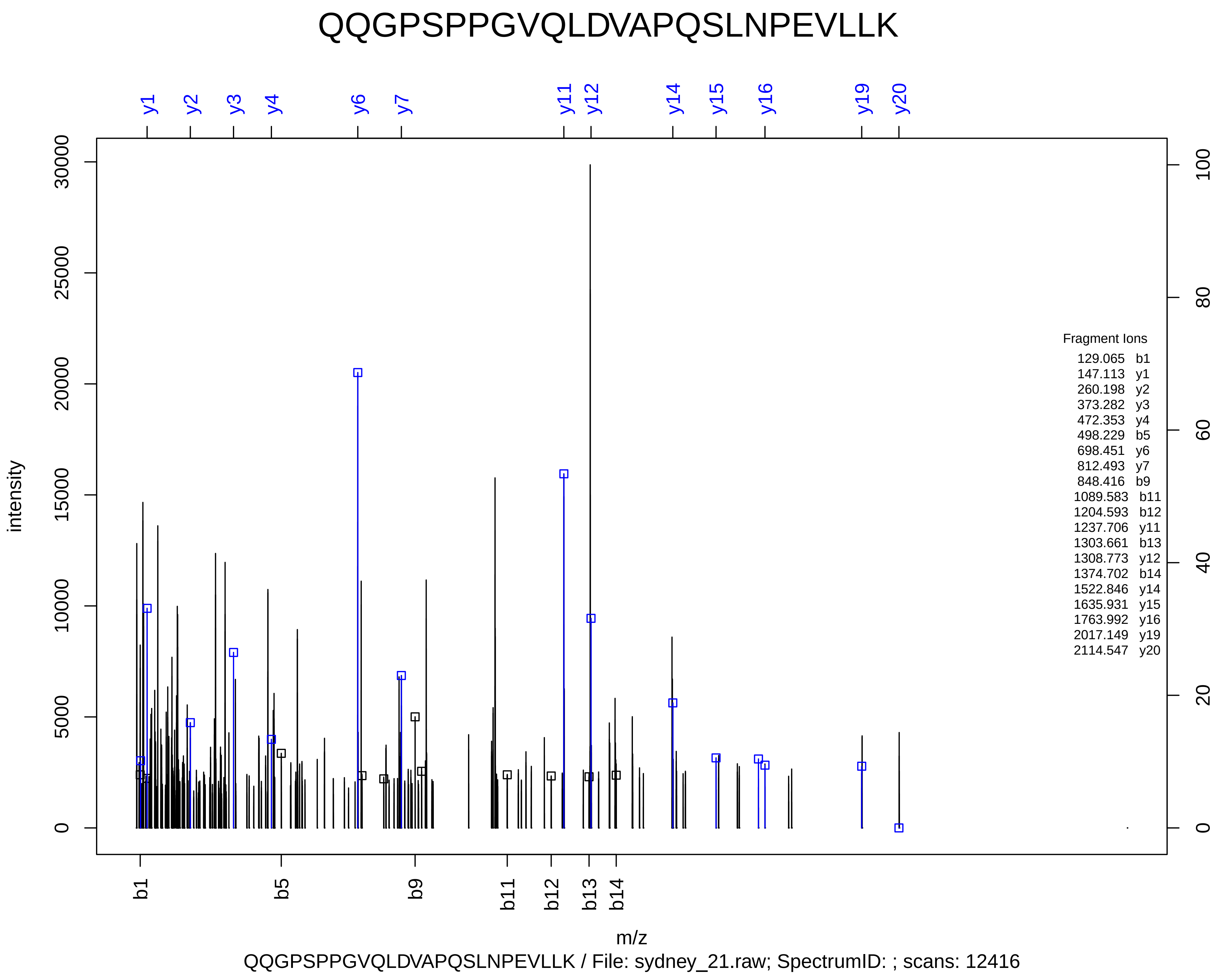
QQGPSPPGVQLDVAPQSLNPEVLLK
y1 y2 y3 y4 y6 y7 y11 y12 y14 y15 y16 y19 y20
b1 b5 b9 b11 b12 b13 b14
intensity
m/z
Fragment Ions
129.065 b1
147.113 y1
260.198 y2
373.282 y3
472.353 y4
498.229 b5
698.451 y6
812.493 y7
848.416 b9
1089.583 b11
1204.593 b12
1237.706 y11
1303.661 b13
1308.773 y12
1374.702 b14
1522.846 y14
1635.931 y15
1763.992 y16
2017.149 y19
2114.547 y20
QQGPSPPGVQLDVAPQSLNPEVLLK / File: sydney_21.raw; SpectrumID: ; scans: 12416

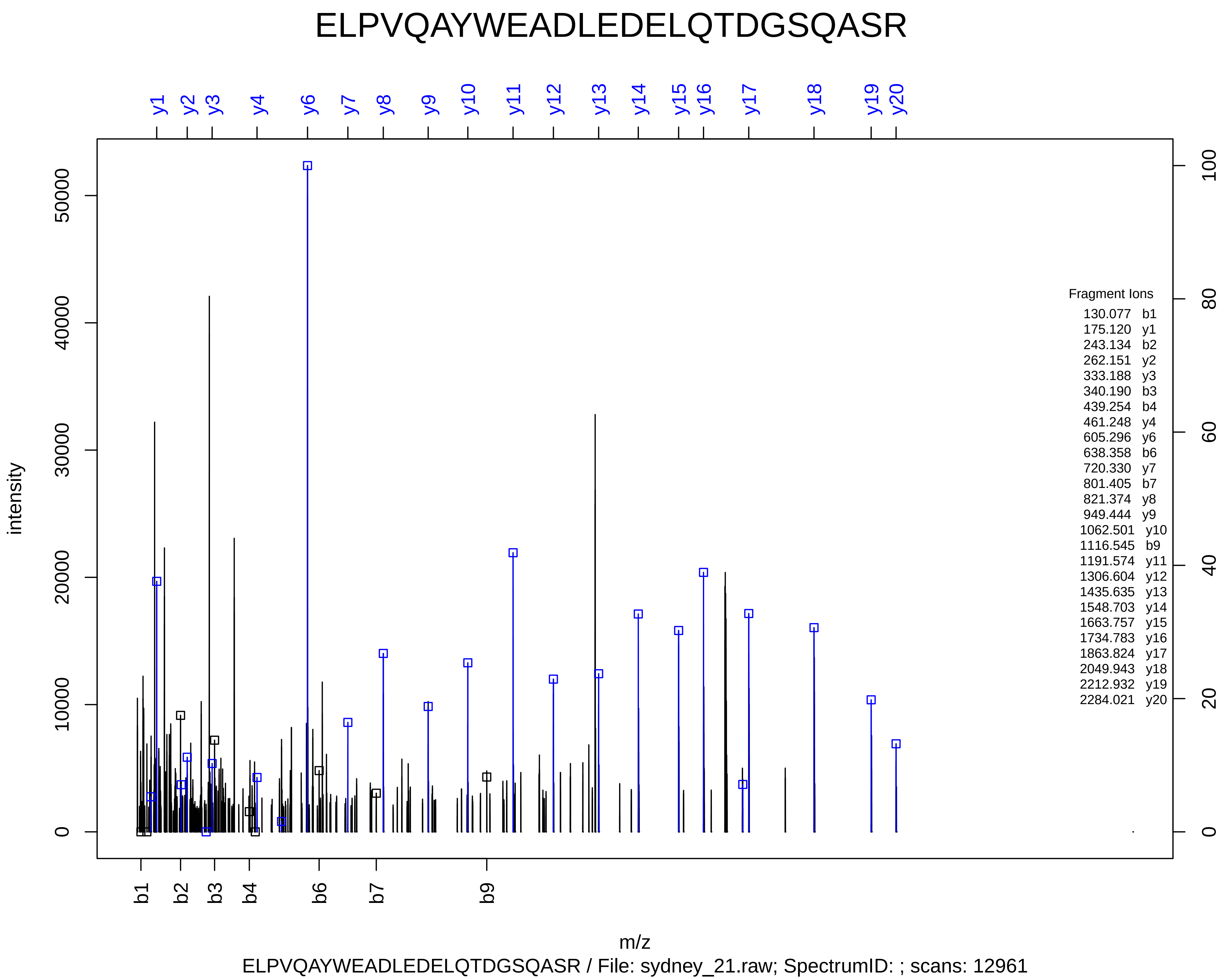

ELPVQAYWEADLEDELQTDGSQASR
y1 y2 y3 y4 y6 y7 y8 y9 y10 y11 y12 y13 y14 y15 y16 y17 y18 y19 y20
b1 b2 b3 b4 b6 b7 b9
intensity
m/z
0
10000
20000
30000
40000
50000
0
20
40
60
80
100
Fragment Ions
130.077 b1
175.120 y1
243.134 b2
262.151 y2
333.188 y3
340.190 b3
439.254 b4
461.248 y4
605.296 y6
638.358 b6
720.330 y7
801.405 b7
821.374 y8
949.444 y9
1062.501 y10
1116.545 b9
1191.574 y11
1306.604 y12
1435.635 y13
1548.703 y14
1663.757 y15
1734.783 y16
1863.824 y17
2049.943 y18
2212.932 y19
2284.021 y20
ELPVQAYWEADLEDELQTDGSQASR / File: sydney_21.raw; SpectrumID: ; scans: 12961

LLILVNPFGGR

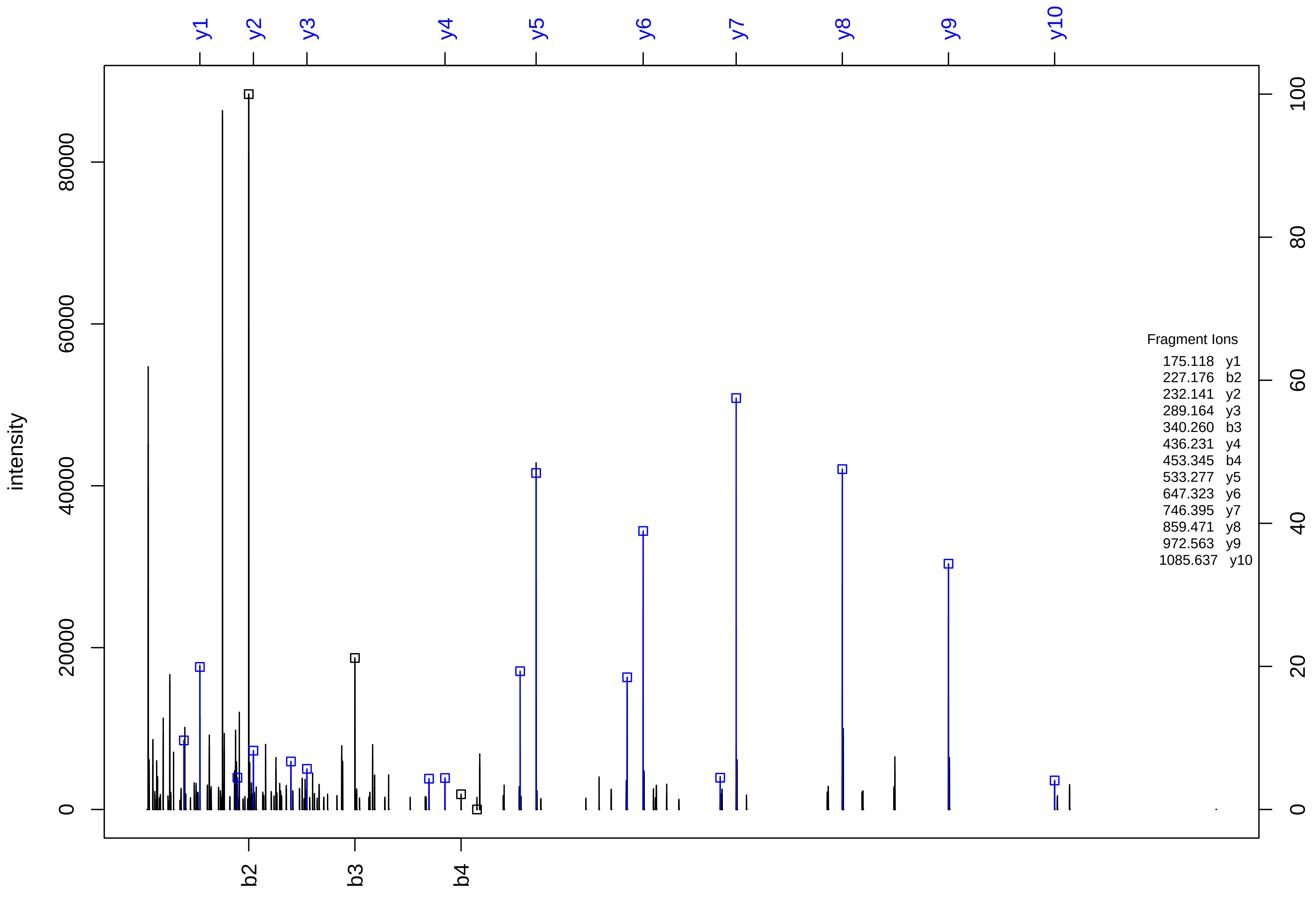

LLILVNPFGGR / File: sydney_21.raw; SpectrumID: ; scans: 12013

FLDEDFVFDIYR

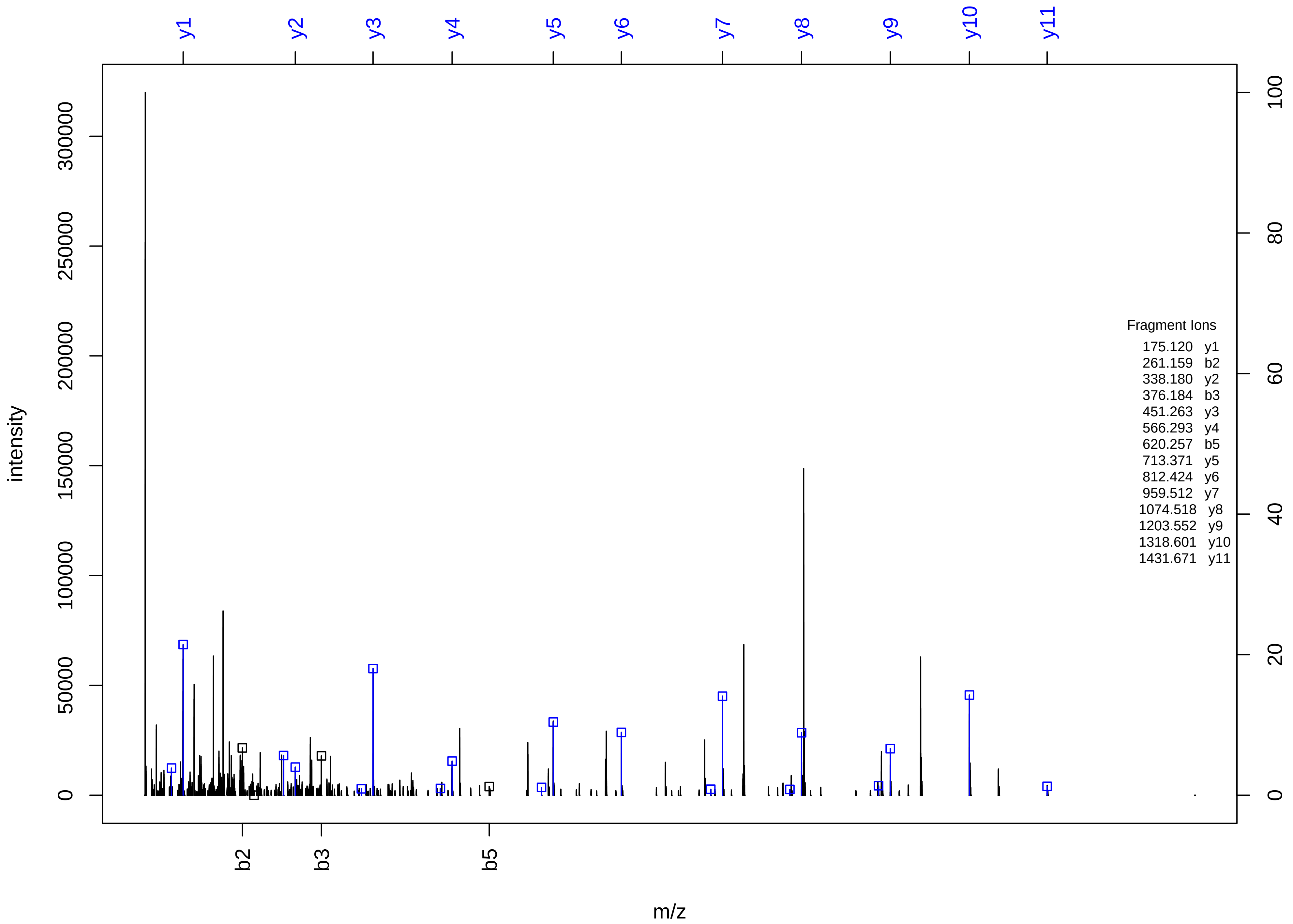

FLDEDFVFDIYR / File: sydney_21.raw; SpectrumID: ; scans: 13723

ELFDFVTDPSITADNMDAYLEK

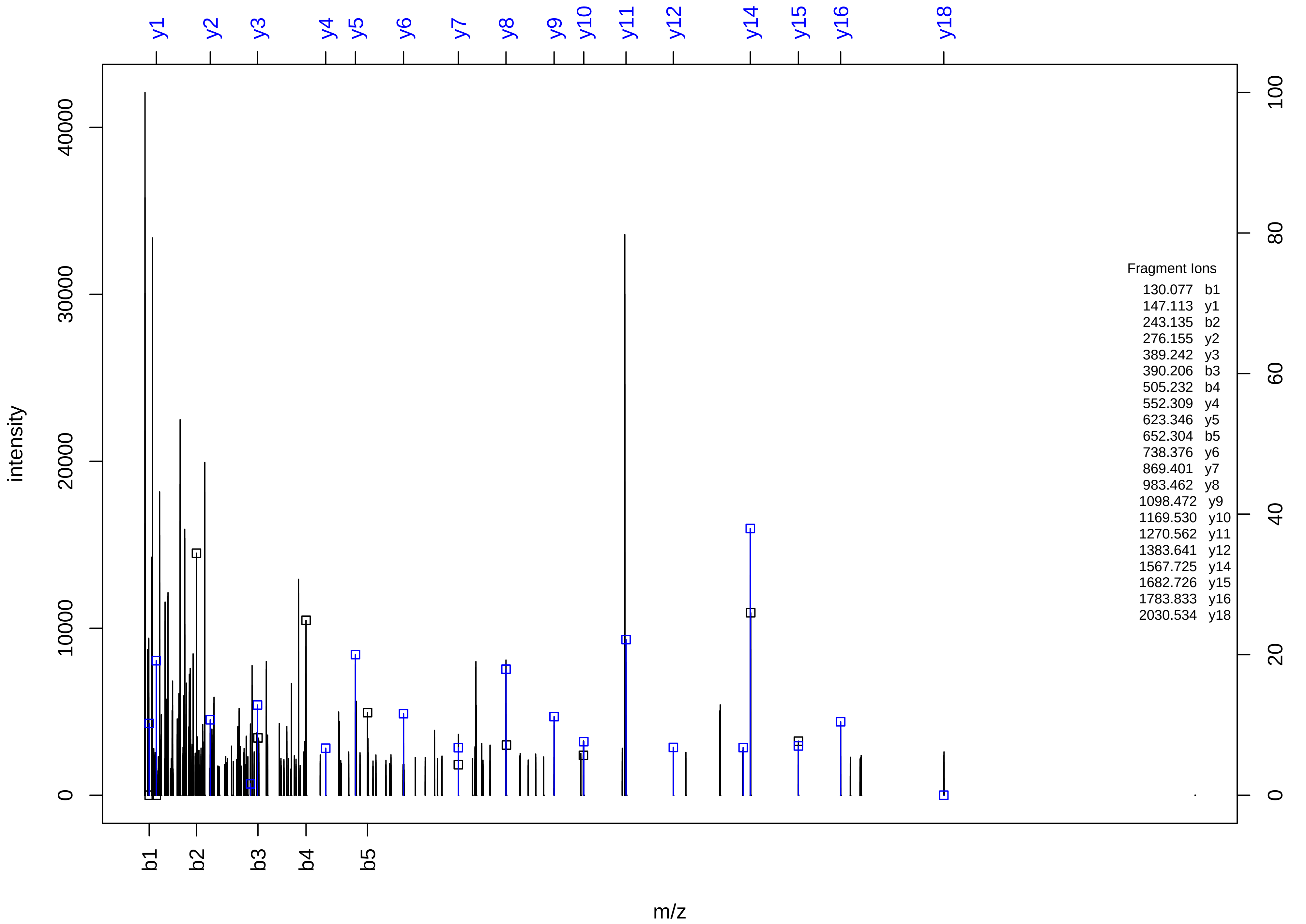

ELFDFVTDPSITADNMDAYLEK / File: sydney_21.raw; SpectrumID: ; scans: 14425

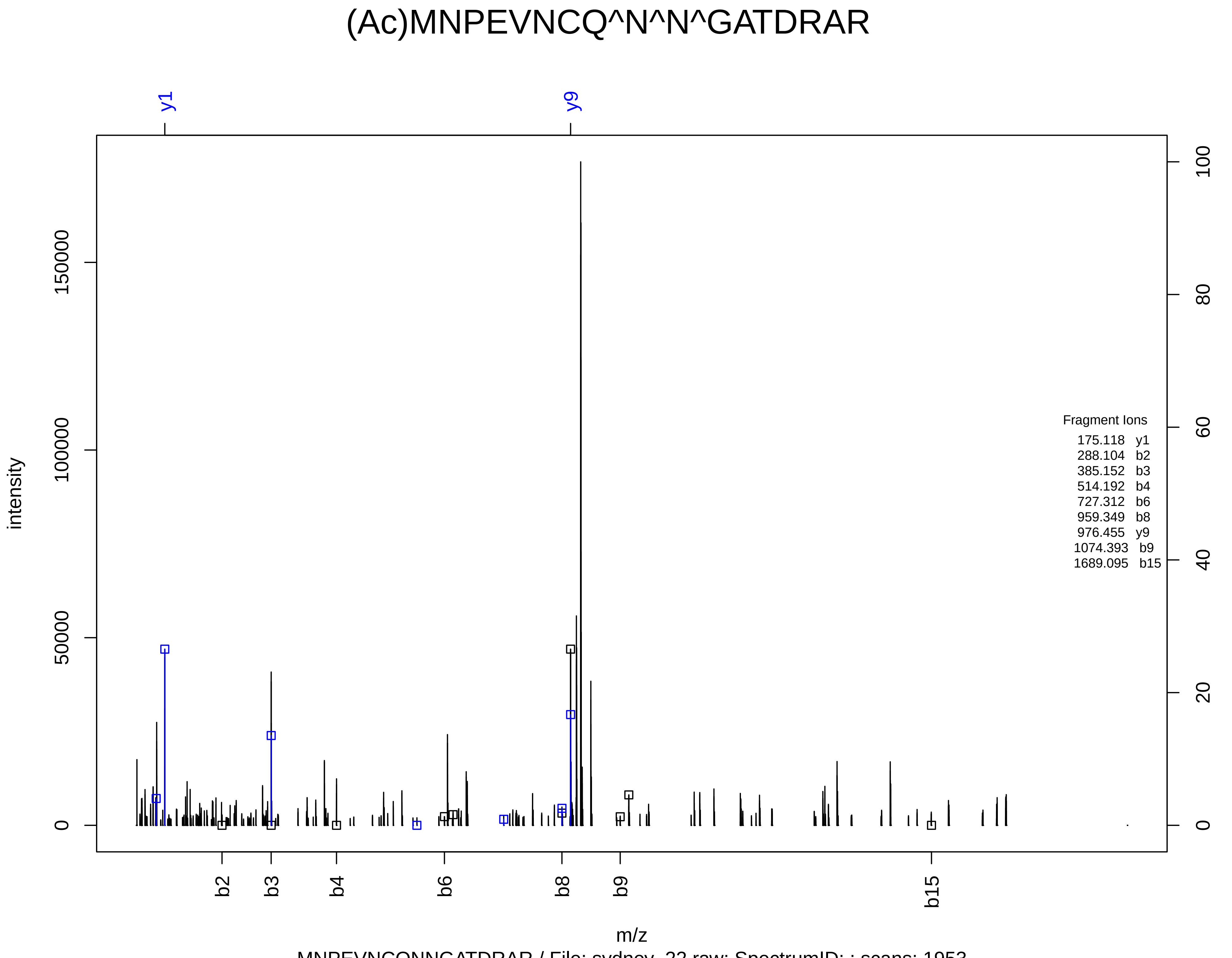
(Ac)MNPEVNCQ^N^N^GATDRAR
y1
y9
intensity
0
50000
100000
150000
0
20
40
60
80
100
Fragment Ions
175.118 y1
288.104 b2
385.152 b3
514.192 b4
727.312 b6
959.349 b8
976.455 y9
1074.393 b9
1689.095 b15
b2
b3
b4
b6
b8
b9
b15
m/z
MNPEVNCQNNGATDRAR / File: sydney_22.raw; SpectrumID: ; scans: 1953

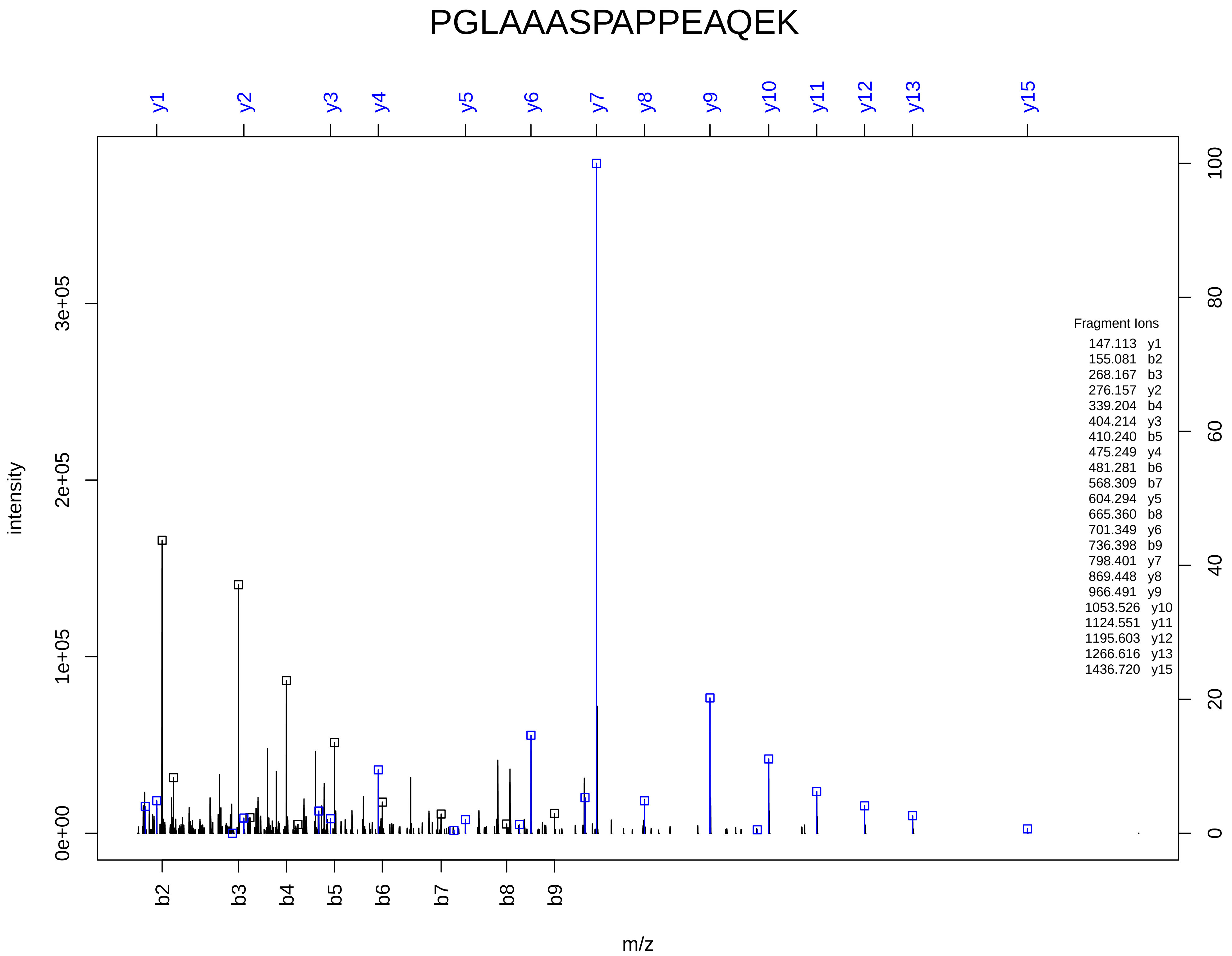

PGLAAASPAPPEAQEK / File: sydney_22.raw; SpectrumID: ; scans: 2096

(Ac)MSN^N^MAKIAEAR

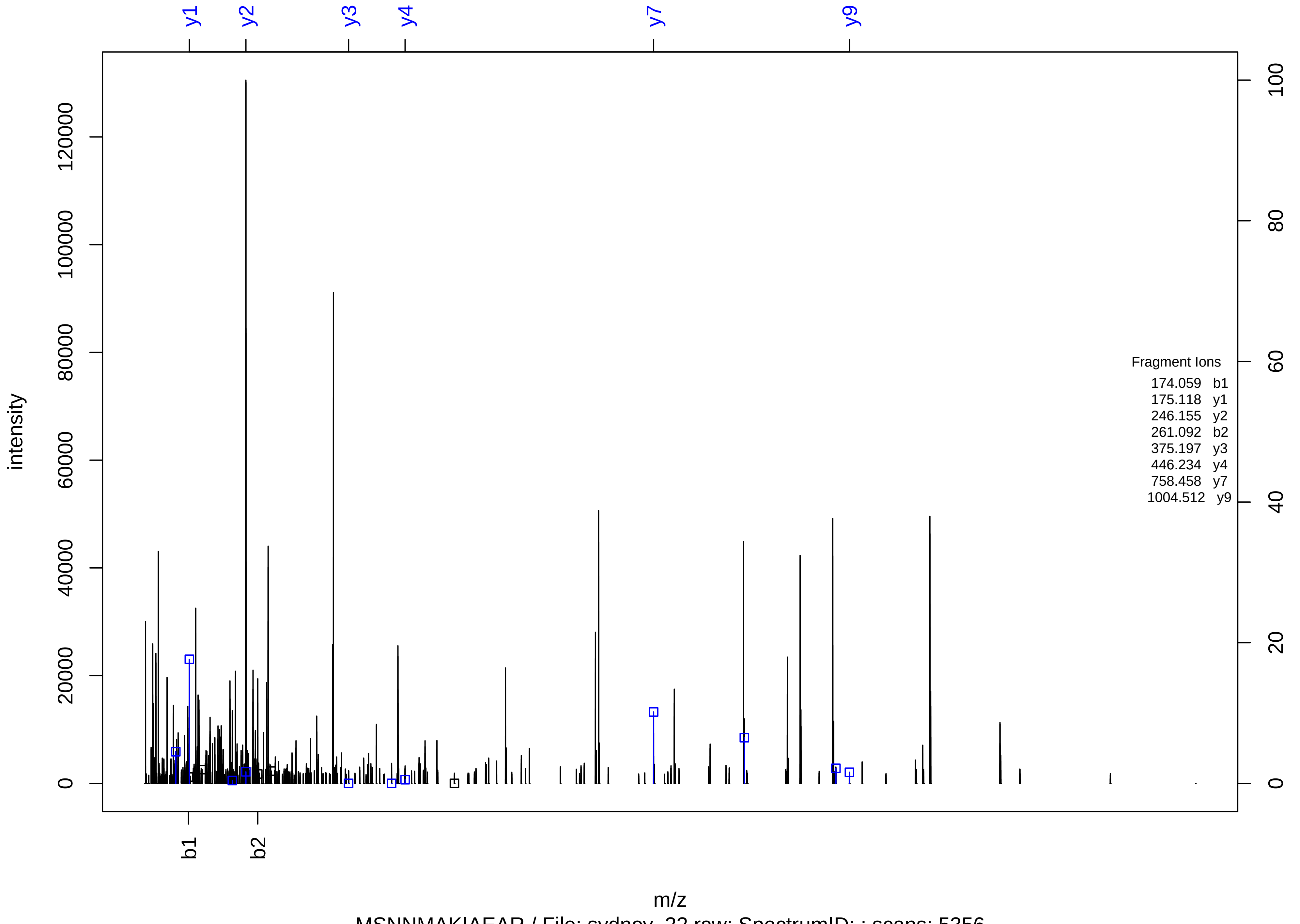

MSNNMAKIAEAR / File: sydney_22.raw; SpectrumID: ; scans: 5356

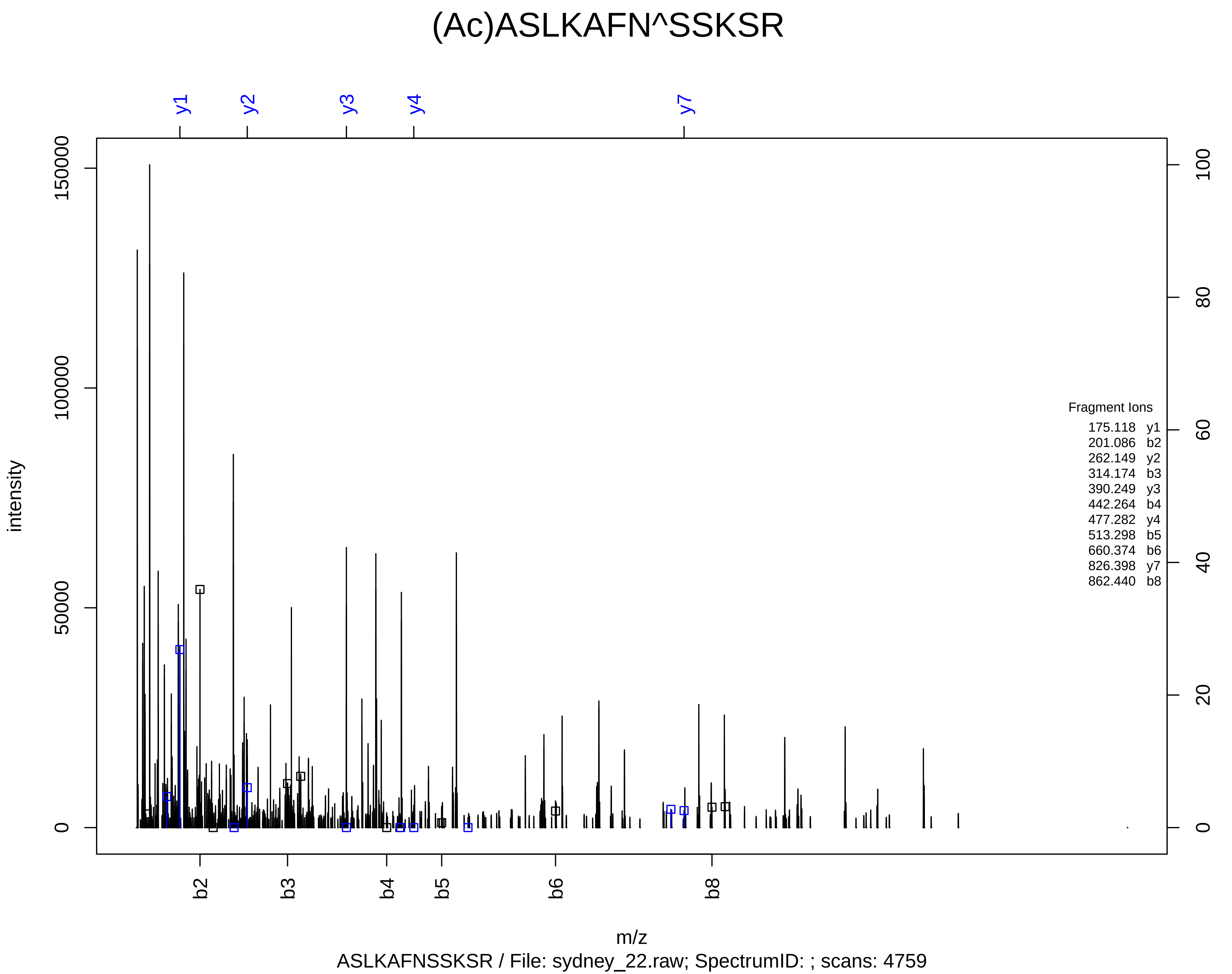

(Ac)ASLKAFN^SSKSR
y1
y2
y3
y4
y7
intensity
0
50000
100000
150000
0
20
40
60
80
100
b2
b3
b4
b5
b6
b8
m/z
Fragment Ions
175.118 y1
201.086 b2
262.149 y2
314.174 b3
390.249 y3
442.264 b4
477.282 y4
513.298 b5
660.374 b6
826.398 y7
862.440 b8
ASLKAFNSSKSR / File: sydney_22.raw; SpectrumID: ; scans: 4759

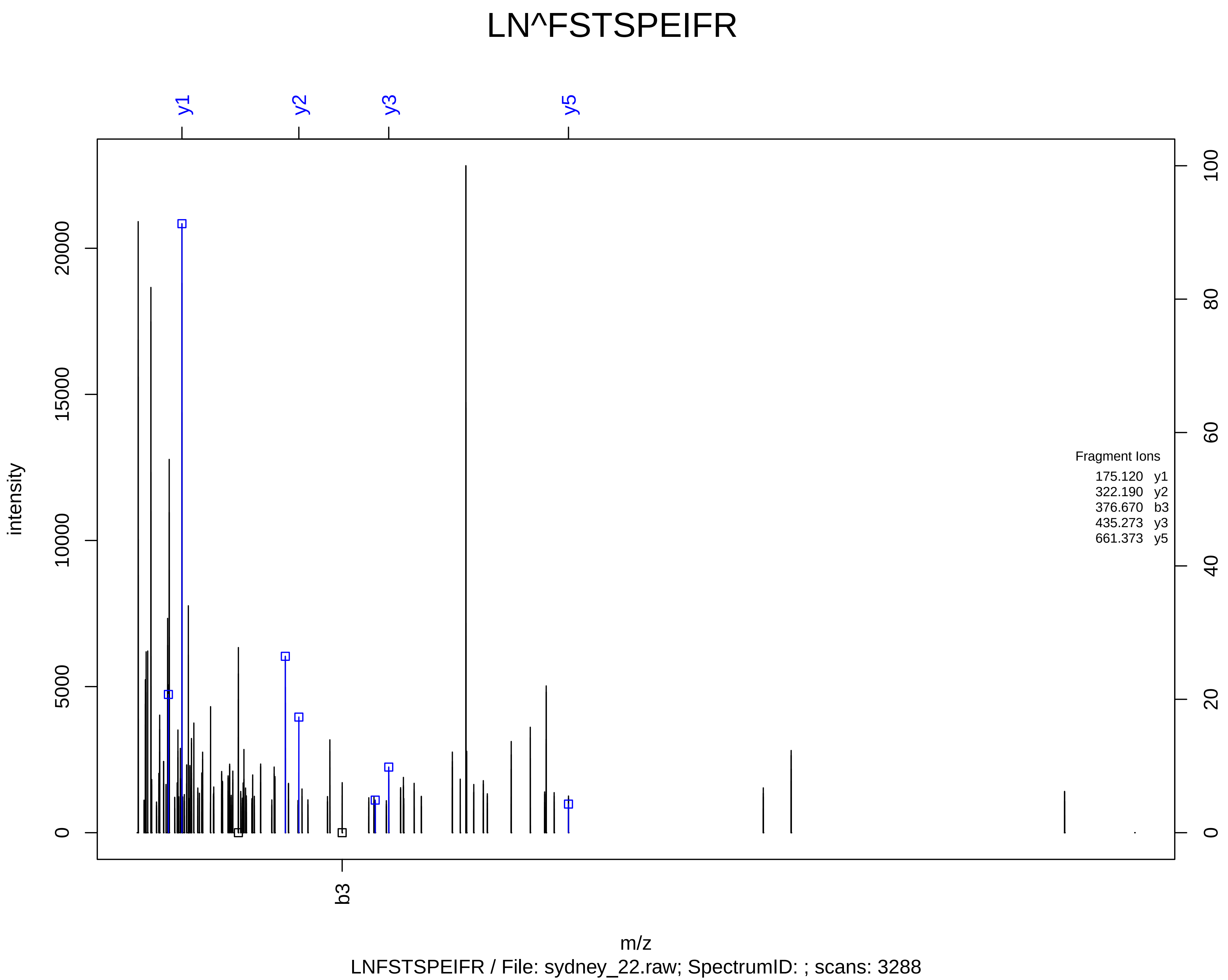

LN^FSTSPEIFR
y1
y2
y3
y5
b3
intensity
m/z
Fragment Ions
175.120 y1
322.190 y2
376.670 b3
435.273 y3
661.373 y5
LNFSTSPEIFR / File: sydney_22.raw; SpectrumID: ; scans: 3288

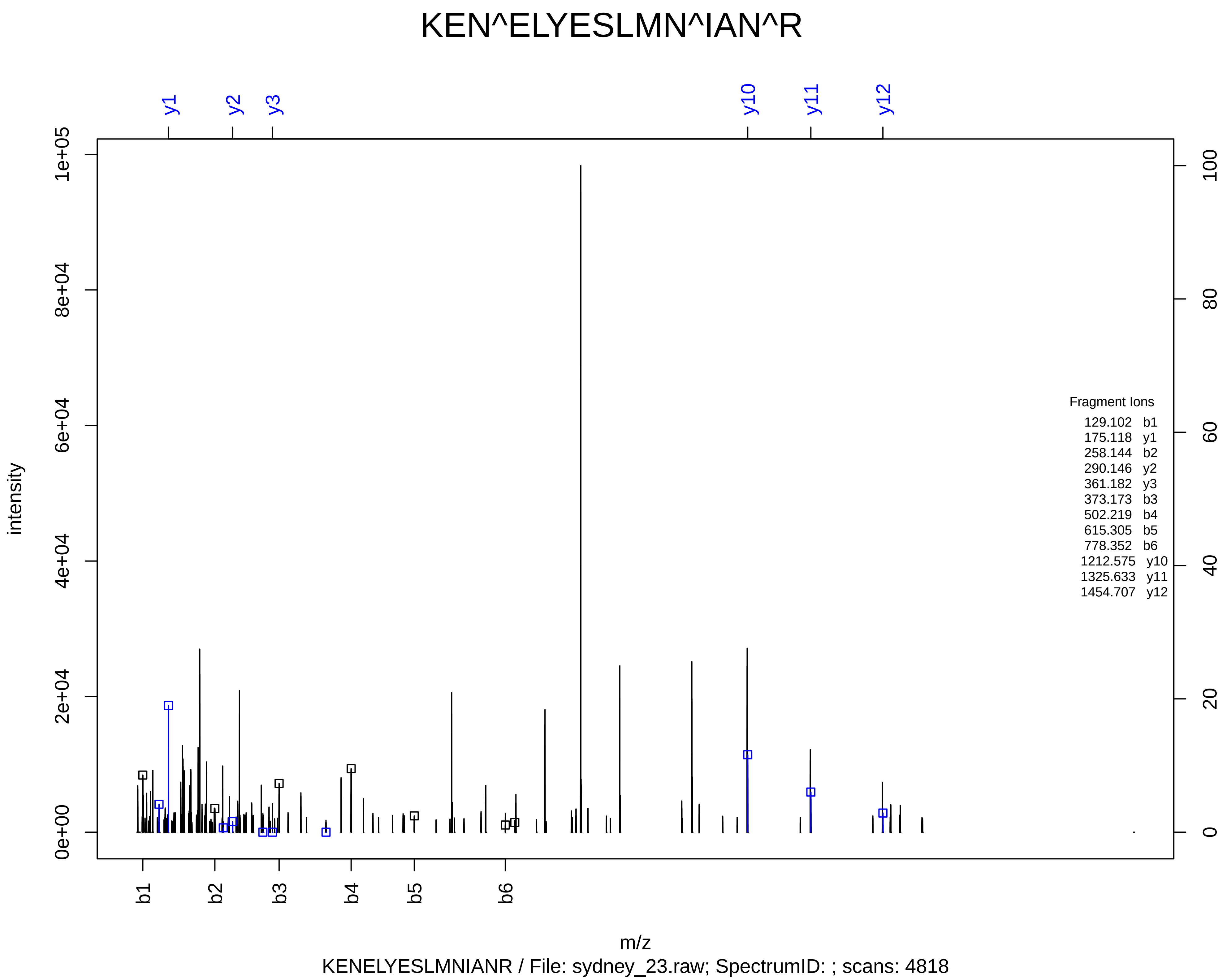
KEN^ELYESLMN^IAN^R
y1
y2
y3
y10
y11
y12
intensity
0e+00
2e+04
4e+04
6e+04
8e+04
1e+05
0
20
40
60
80
100
b1
b2
b3
b4
b5
b6
m/z
Fragment Ions
129.102 b1
175.118 y1
258.144 b2
290.146 y2
361.182 y3
373.173 b3
502.219 b4
615.305 b5
778.352 b6
1212.575 y10
1325.633 y11
1454.707 y12
KENELYESLMNIANR / File: sydney_23.raw; SpectrumID: ; scans: 4818

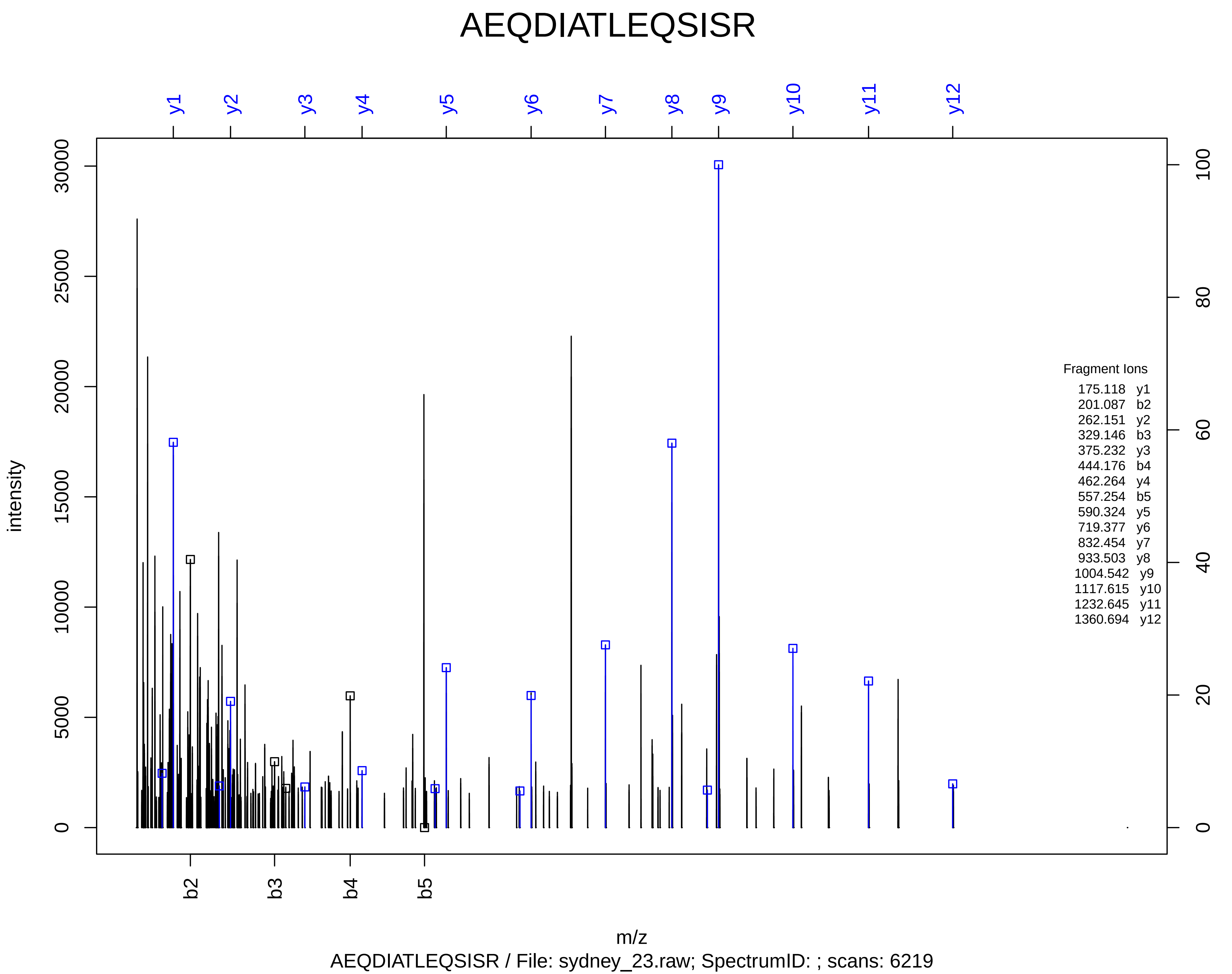

AEQDIATLEQSISR
y1 y2 y3 y4 y5 y6 y7 y8 y9 y10 y11 y12
b2 b3 b4 b5
intensity
m/z
0
5000
10000
15000
20000
25000
30000
0
20
40
60
80
100
Fragment Ions
175.118 y1
201.087 b2
262.151 y2
329.146 b3
375.232 y3
444.176 b4
462.264 y4
557.254 b5
590.324 y5
719.377 y6
832.454 y7
933.503 y8
1004.542 y9
1117.615 y10
1232.645 y11
1360.694 y12
AEQDIATLEQSISR / File: sydney_23.raw; SpectrumID: ; scans: 6219

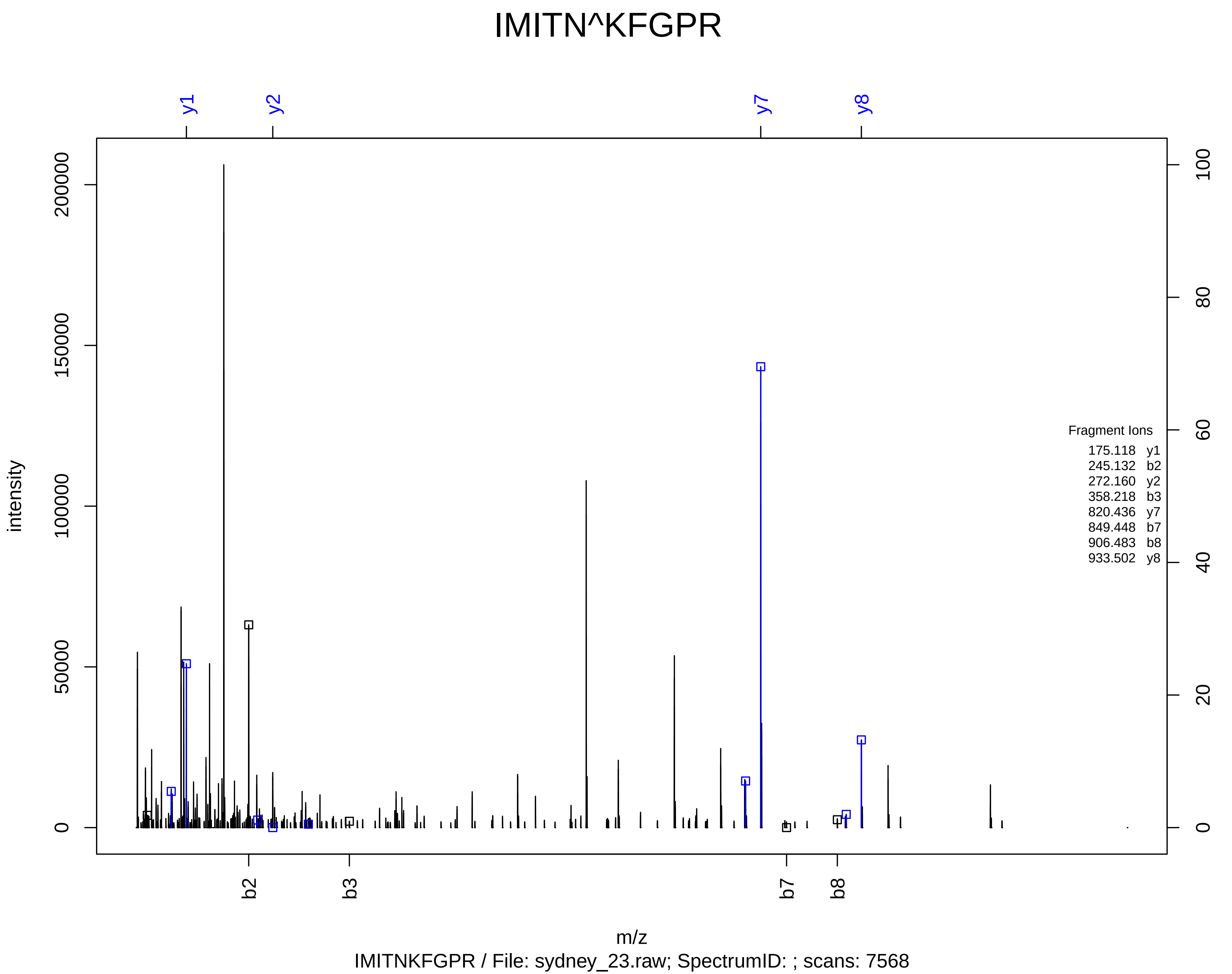
IMITN^KFGPR
y1
y2
y7
y8
intensity
0
50000
100000
150000
200000
0
20
40
60
80
100
Fragment Ions
175.118 y1
245.132 b2
272.160 y2
358.218 b3
820.436 y7
849.448 b7
906.483 b8
933.502 y8
b2
b3
b7
b8
m/z
IMITNKFGPR / File: sydney_23.raw; SpectrumID: ; scans: 7568

YEIQ^AQ^TLVDGRWQ^EFRTN^QIMQ^K

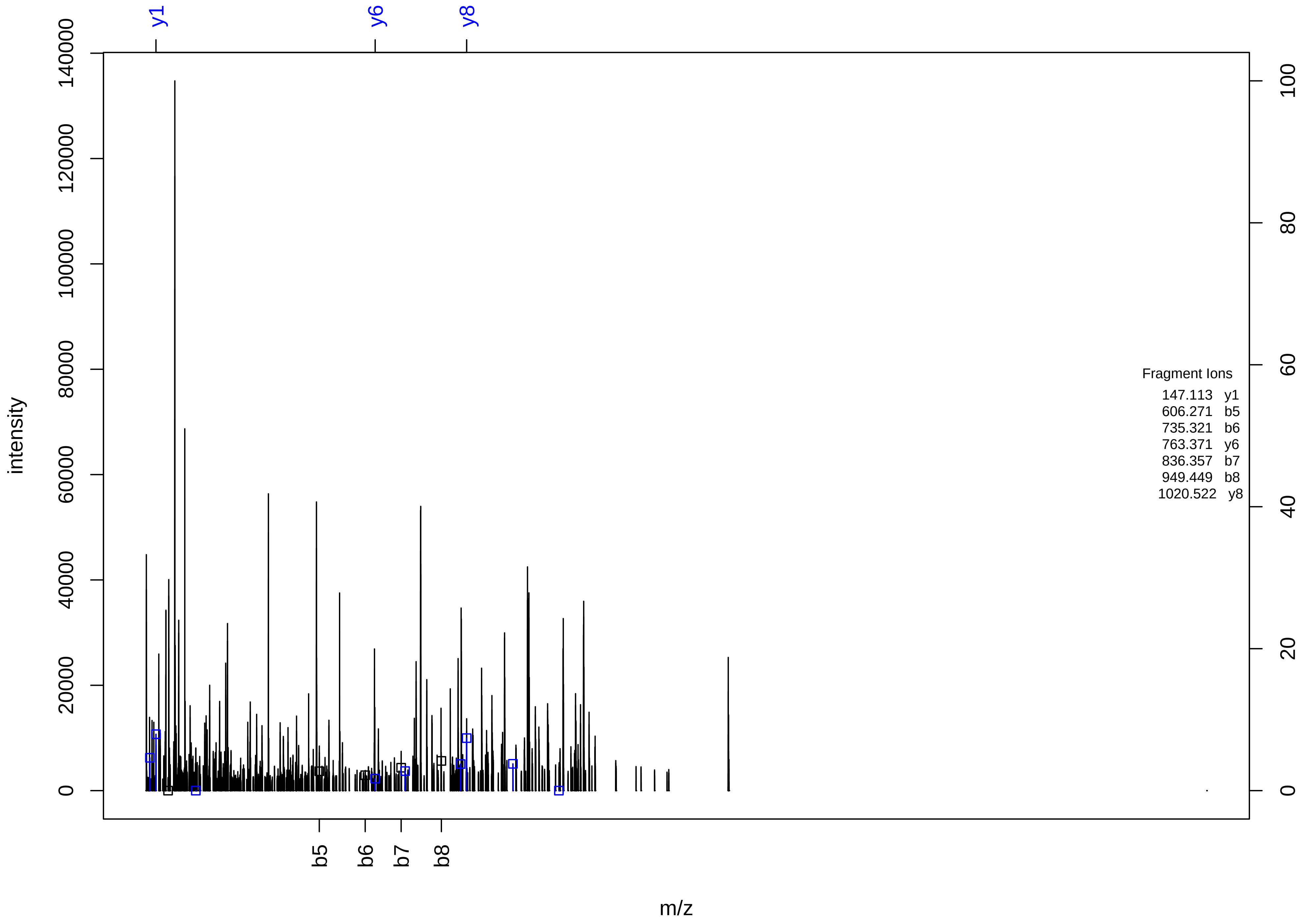

YEIQAQTLVDGRWQEFRTNQIMQK / File: sydney_23.raw; SpectrumID: ; scans: 14879

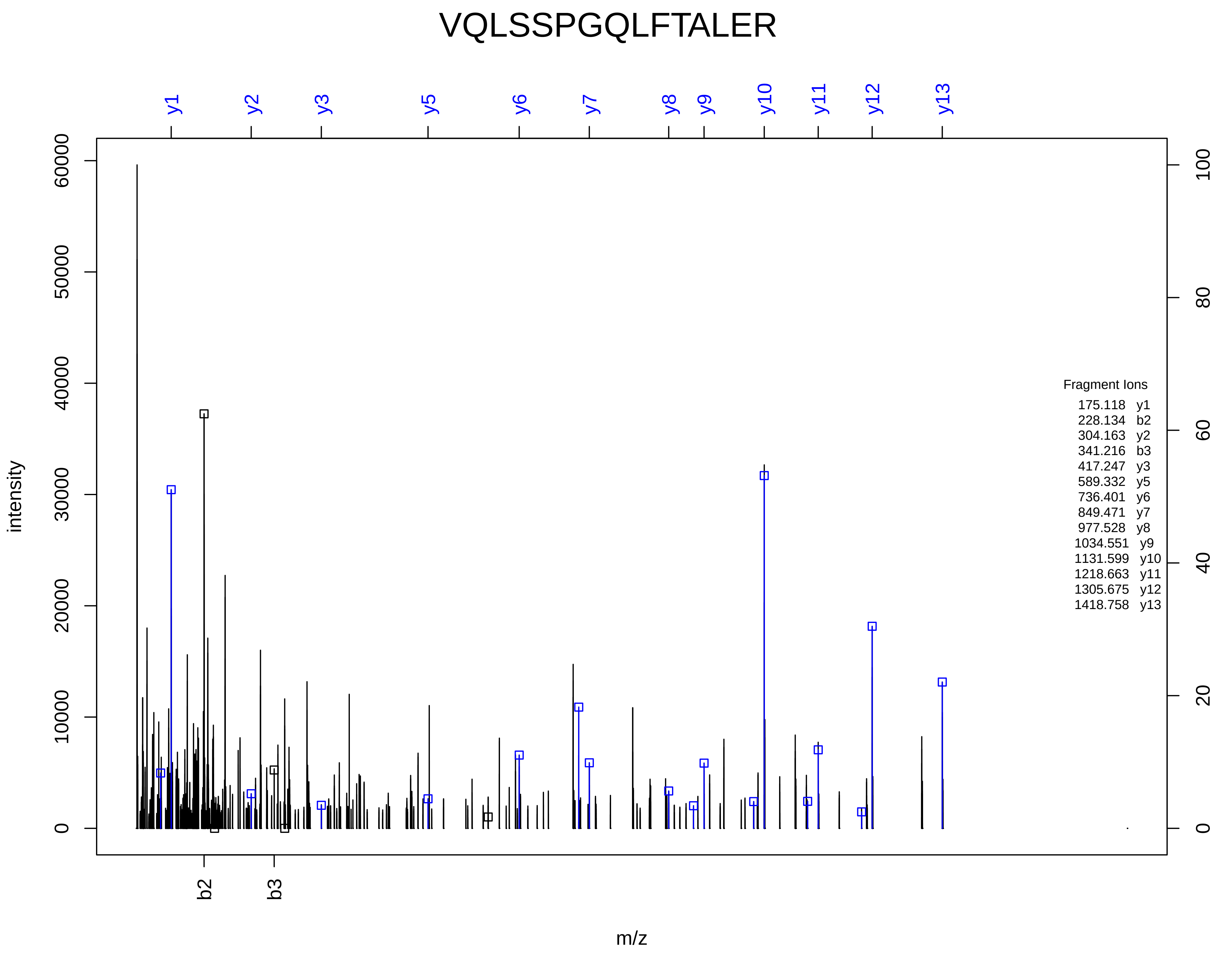

VQLSSPGQLFTALER / File: sydney_23.raw; SpectrumID: ; scans: 11037

YLVQDTDEFILPTGANK

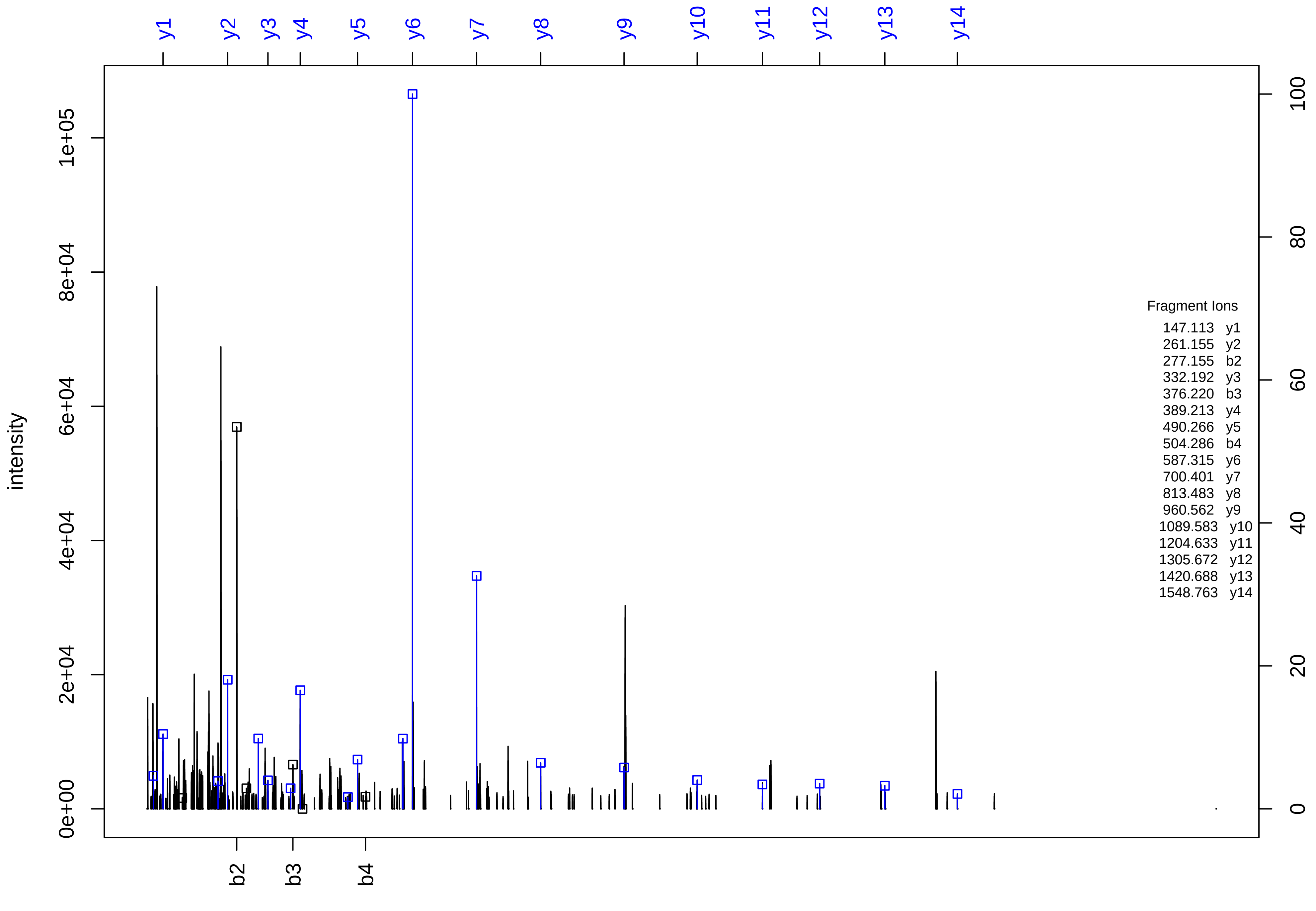

YLVQDTDEFILPTGANK / File: sydney_23.raw; SpectrumID: ; scans: 9132

DDAFAMQDVPLSSVVR

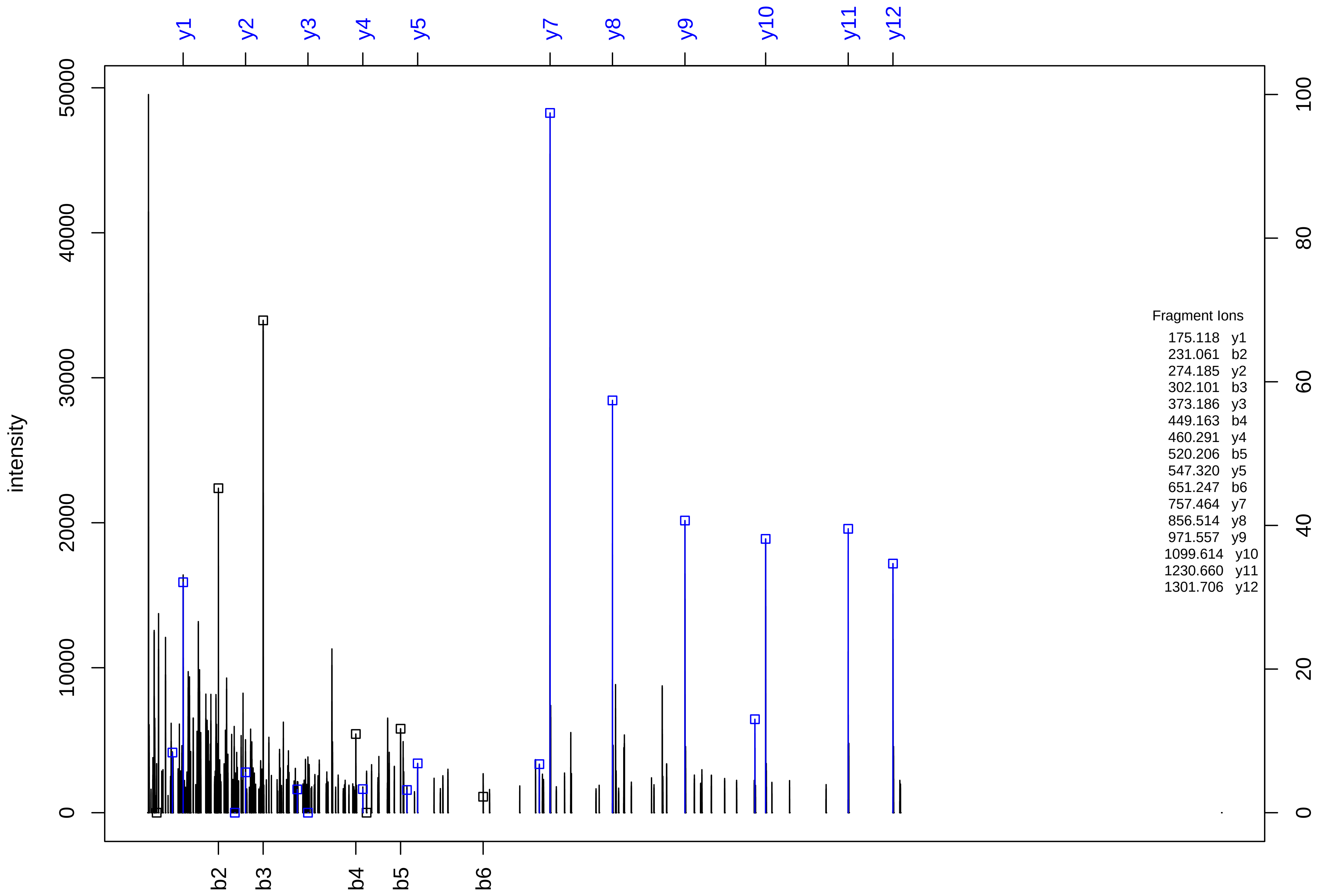

DDAFAMQDVPLSSVVR / File: sydney_23.raw; SpectrumID: ; scans: 10680

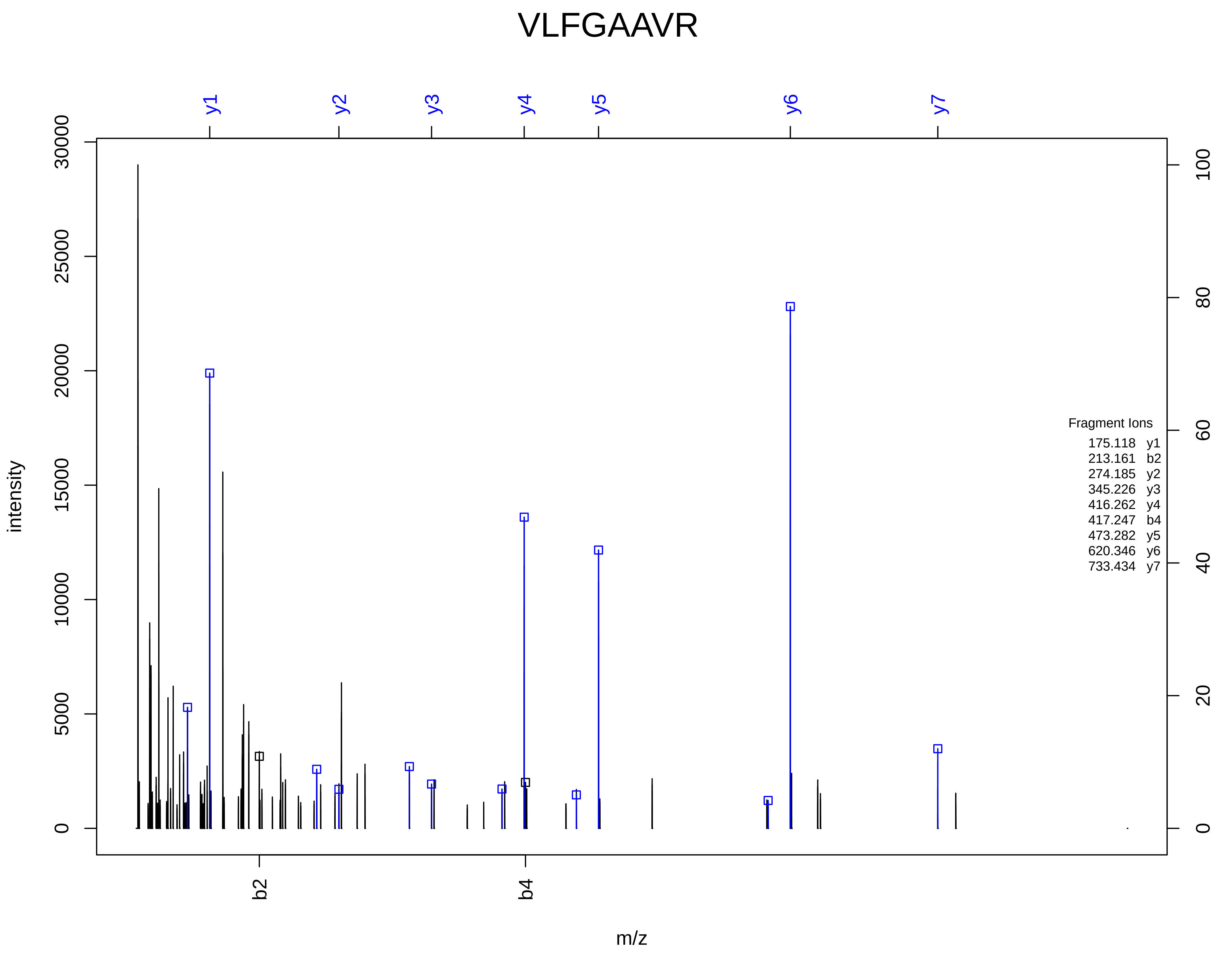

VLFGAAVR
y1
y2
y3
y4
y5
y6
y7
intensity
30000
25000
20000
15000
10000
5000
0
100
80
60
40
20
0
b2
b4
m/z
Fragment Ions
175.118 y1
213.161 b2
274.185 y2
345.226 y3
416.262 y4
417.247 b4
473.282 y5
620.346 y6
733.434 y7
VLFGAAVR / File: sydney_23.raw; SpectrumID: ; scans: 3722

EVKEN^SVVFN^NTPK

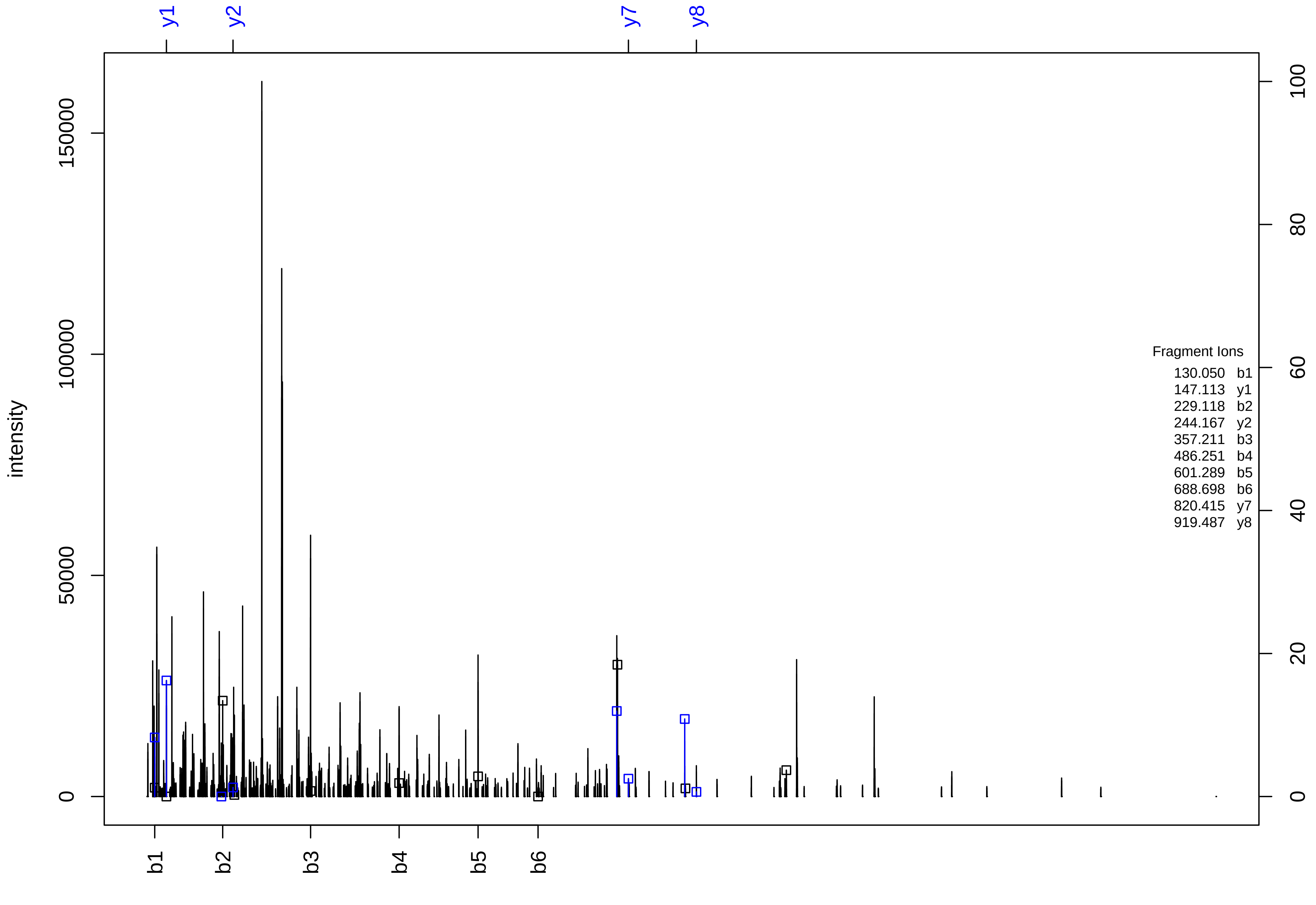

EVKENSVVFNNTPK / File: sydney_23.raw; SpectrumID: ; scans: 5832

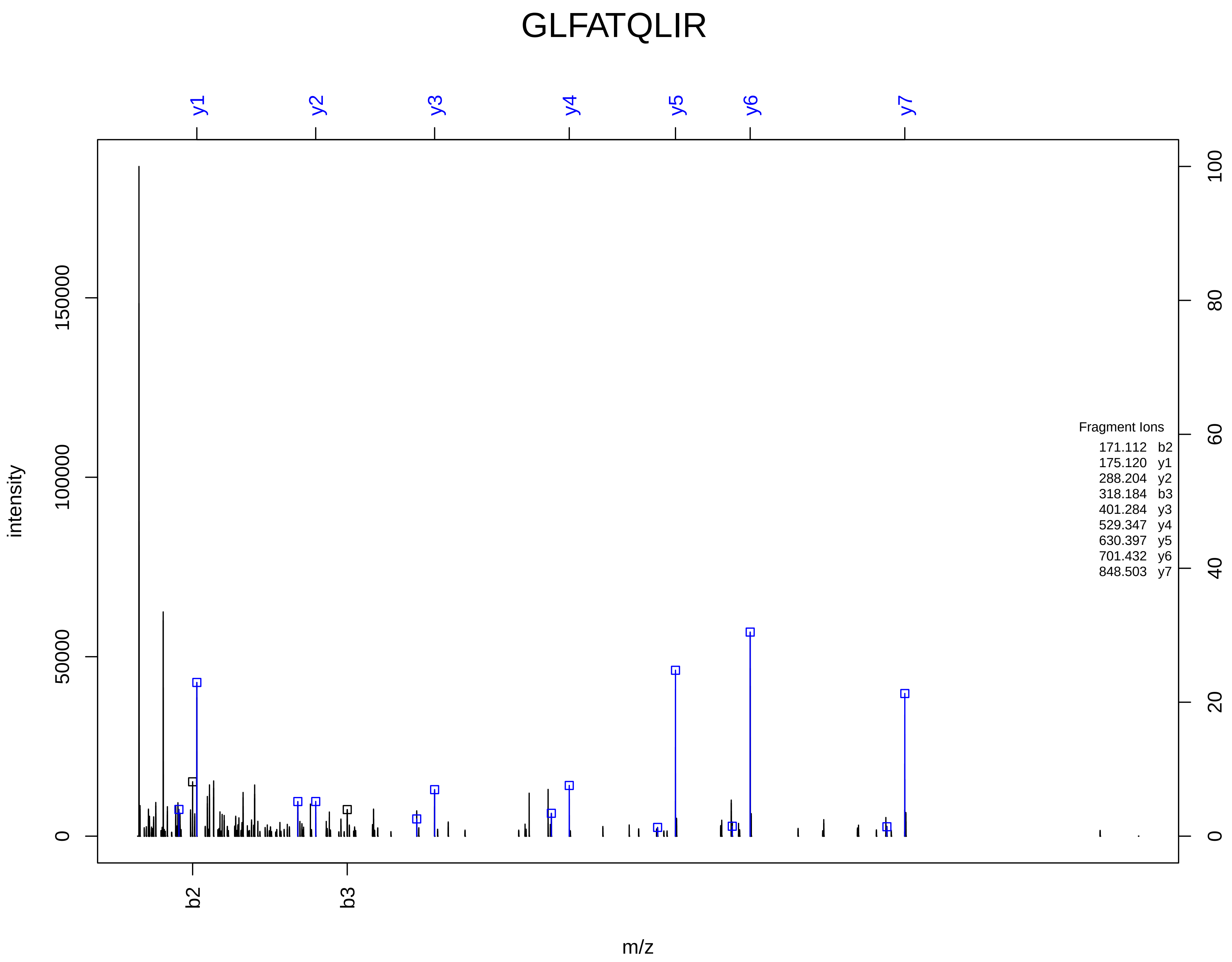
GLFATQLIR
y1
y2
y3
y4
y5
y6
y7
intensity
0
50000
100000
150000
0
20
40
60
80
100
b2
b3
m/z
Fragment Ions
171.112 b2
175.120 y1
288.204 y2
318.184 b3
401.284 y3
529.347 y4
630.397 y5
701.432 y6
848.503 y7
GLFATQLIR / File: sydney_23.raw; SpectrumID: ; scans: 7629

DRLSSSYN^N^PK

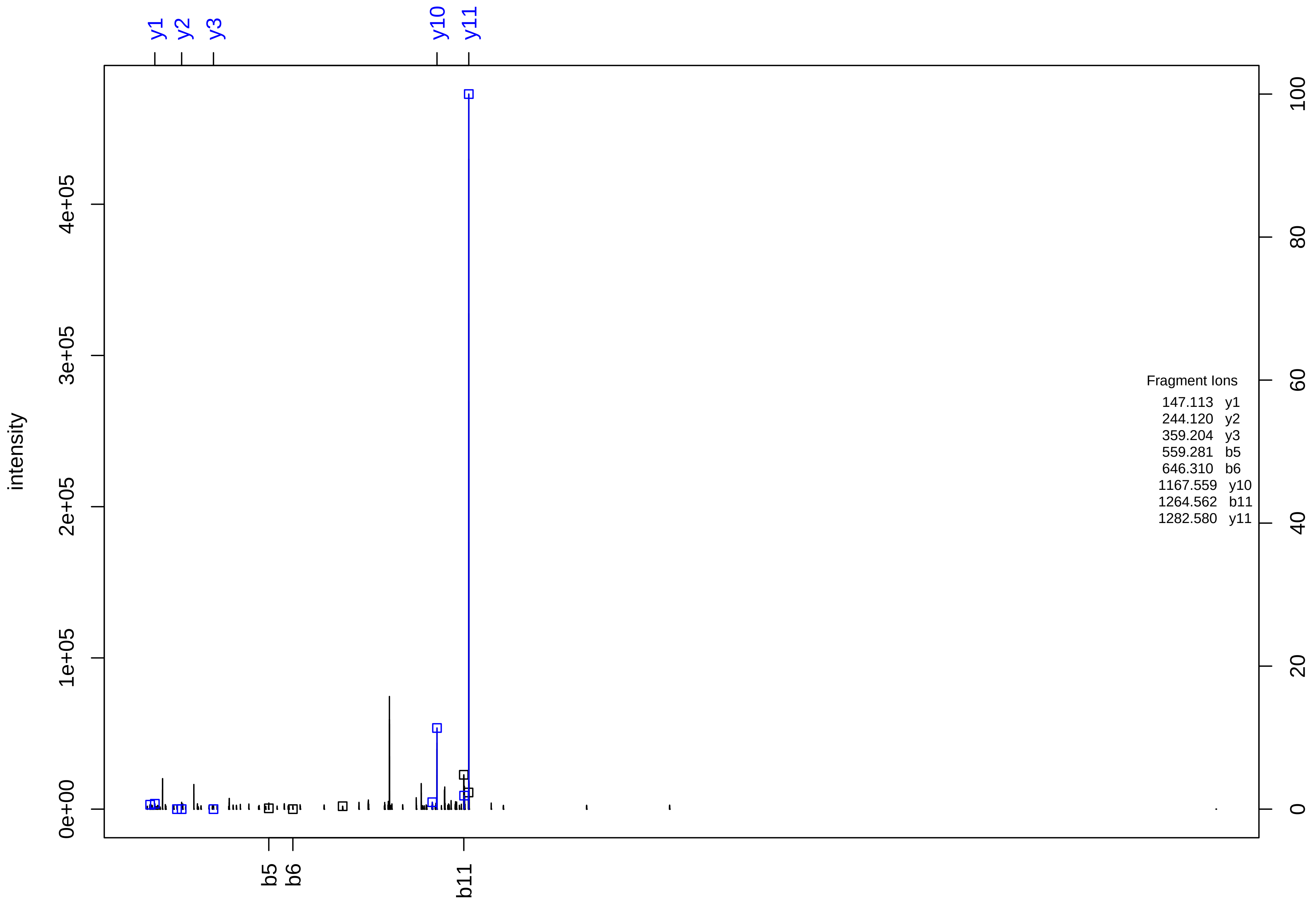

DRLSSSYNNPK / File: sydney_23.raw; SpectrumID: ; scans: 4880

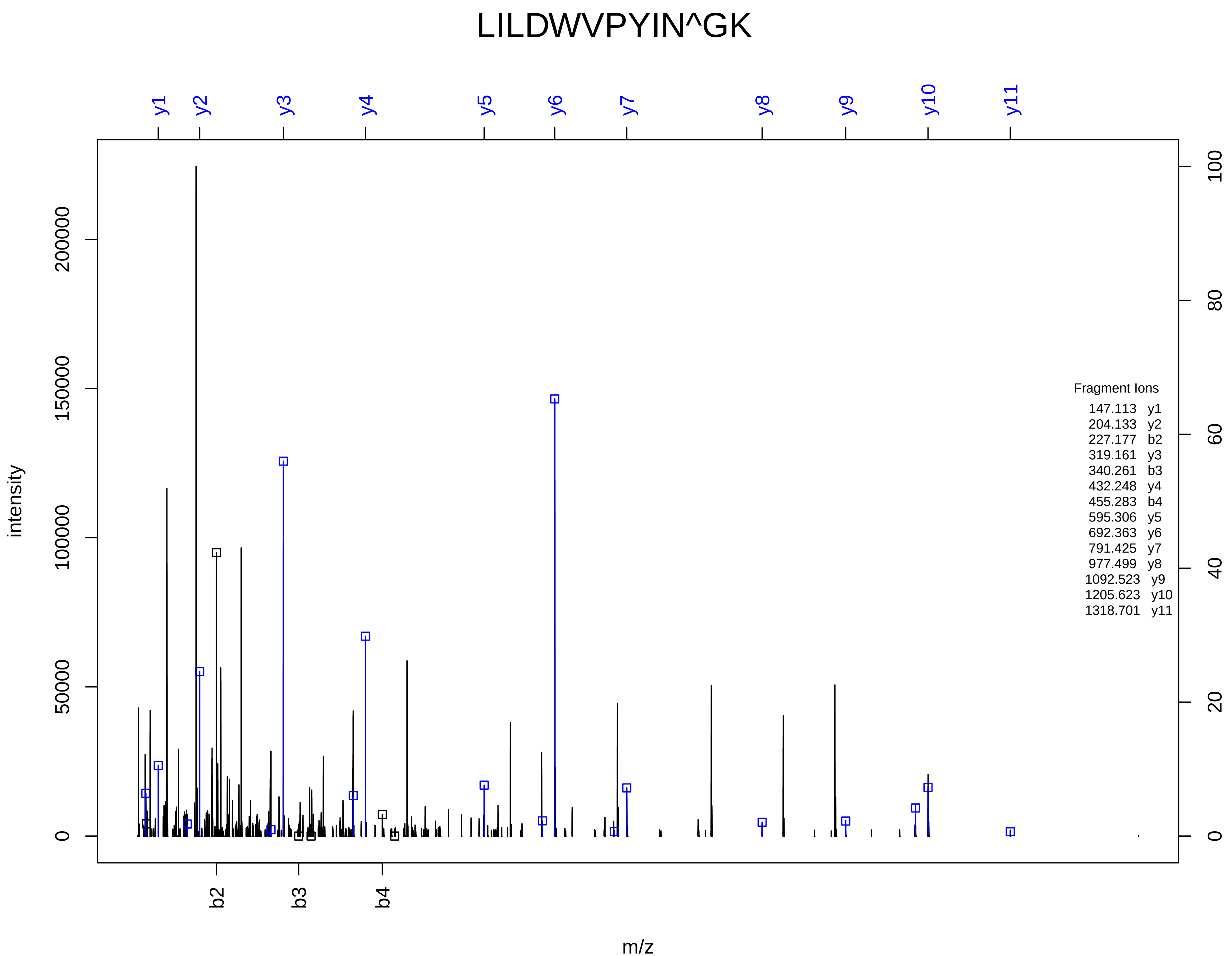

LILDWVPYINGK / File: sydney_23.raw; SpectrumID: ; scans: 13561

LPPTPLLLFPEEEATNGR

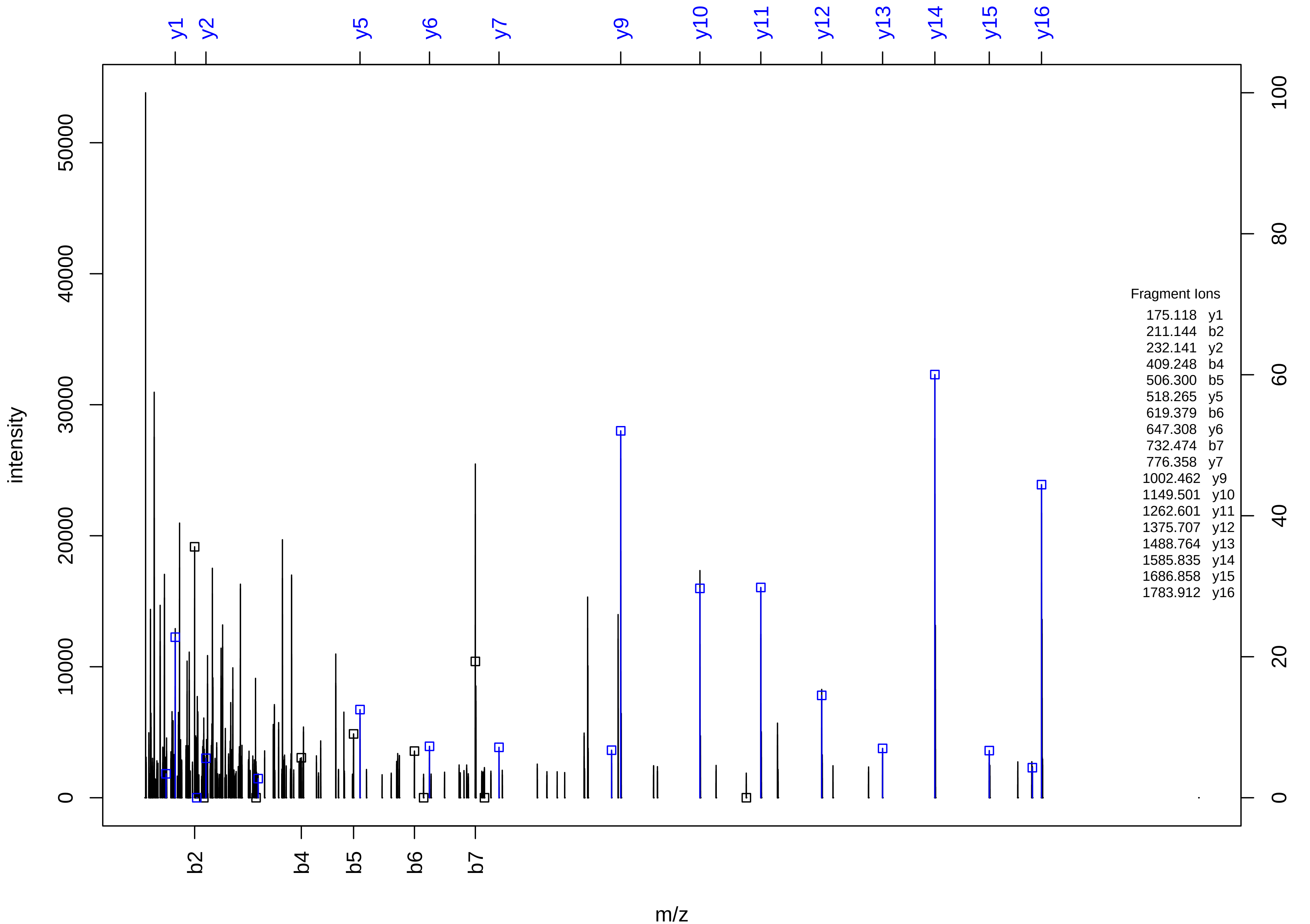

LPPTPLLLFPEEEATNGR / File: sydney_24.raw; SpectrumID: ; scans: 13167

DFLKEFNNK

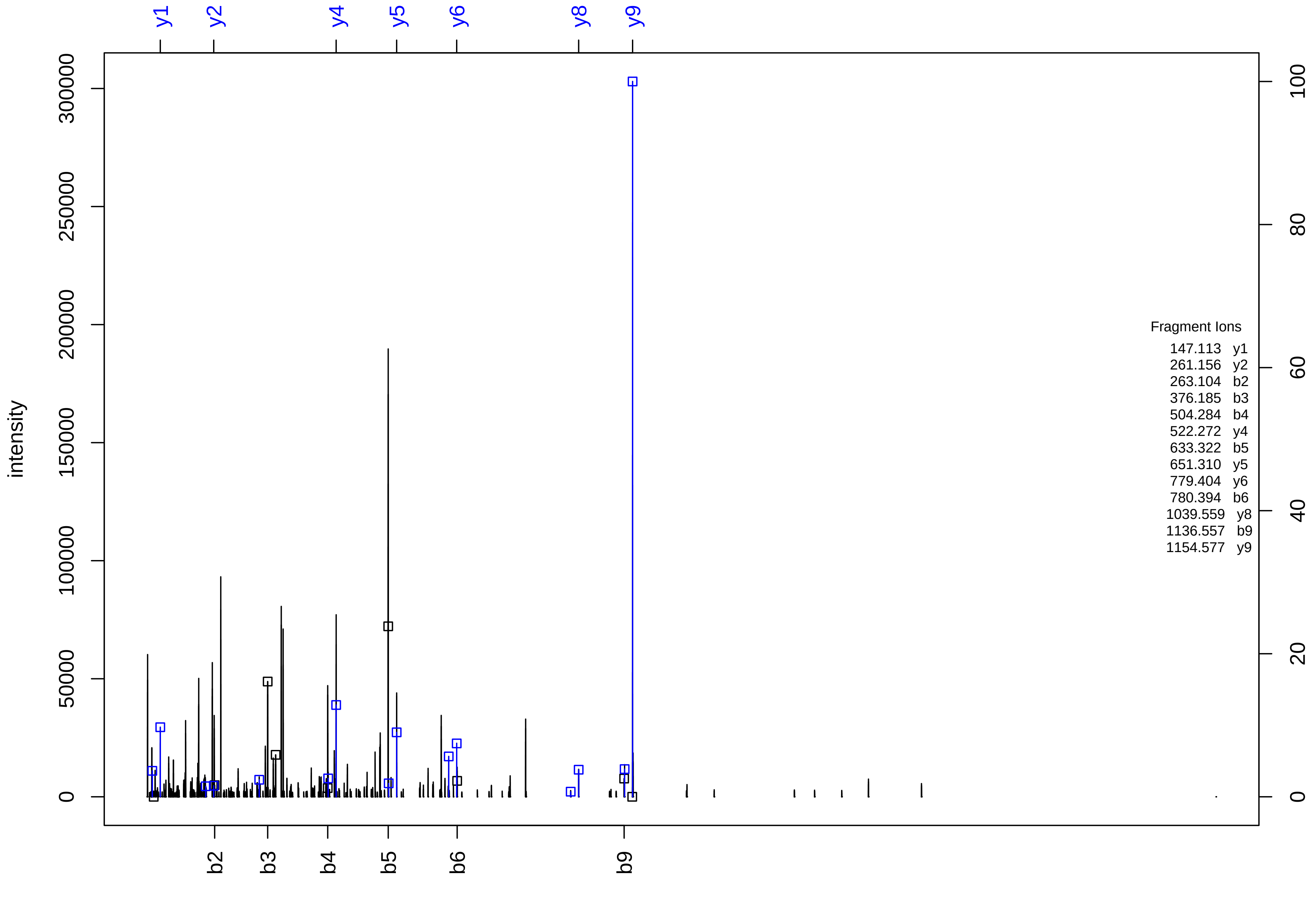

DFLKEFNNK / File: sydney_24.raw; SpectrumID: ; scans: 12558

DFVDLVLVK

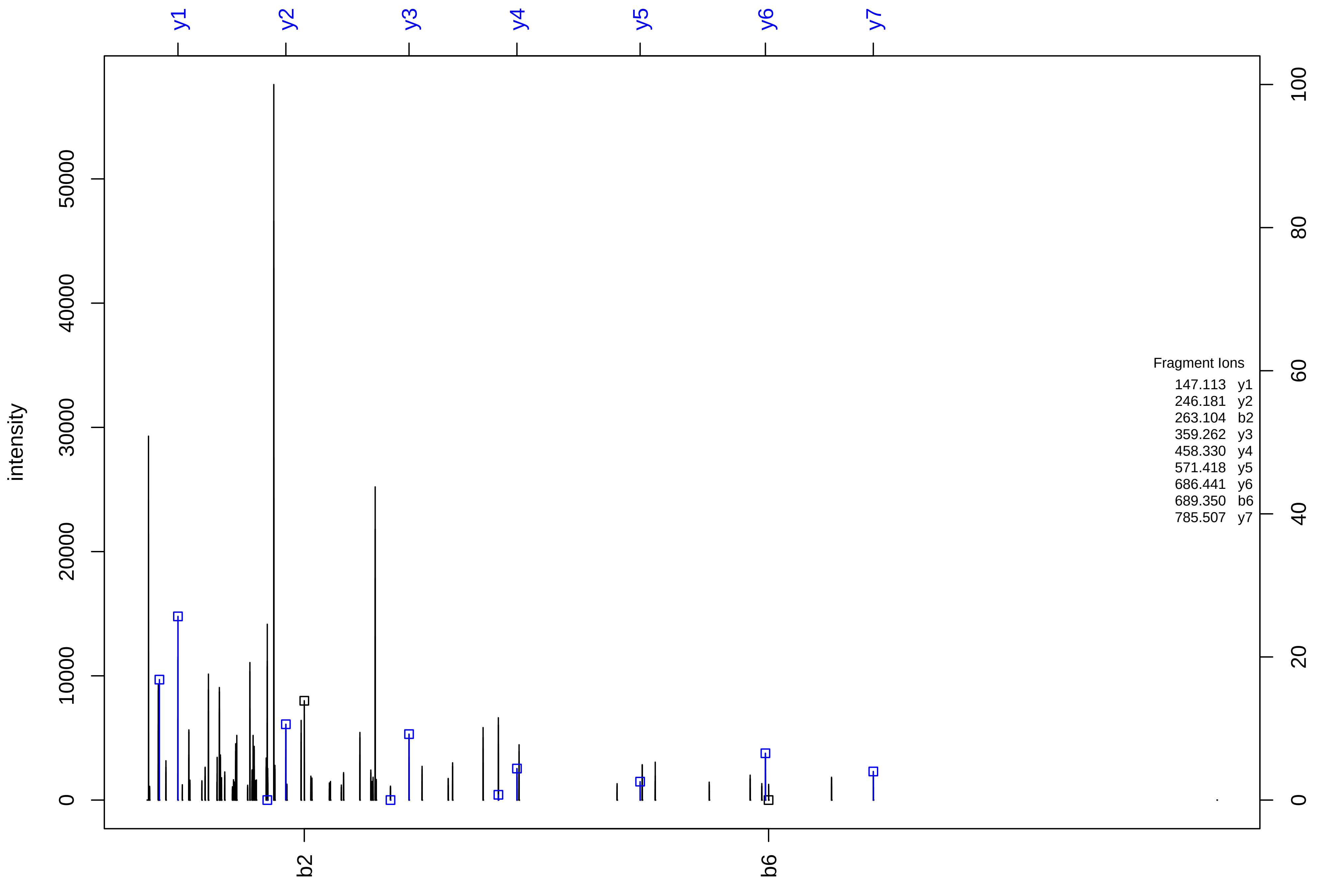

DFVDLVLVK / File: sydney_24.raw; SpectrumID: ; scans: 11648

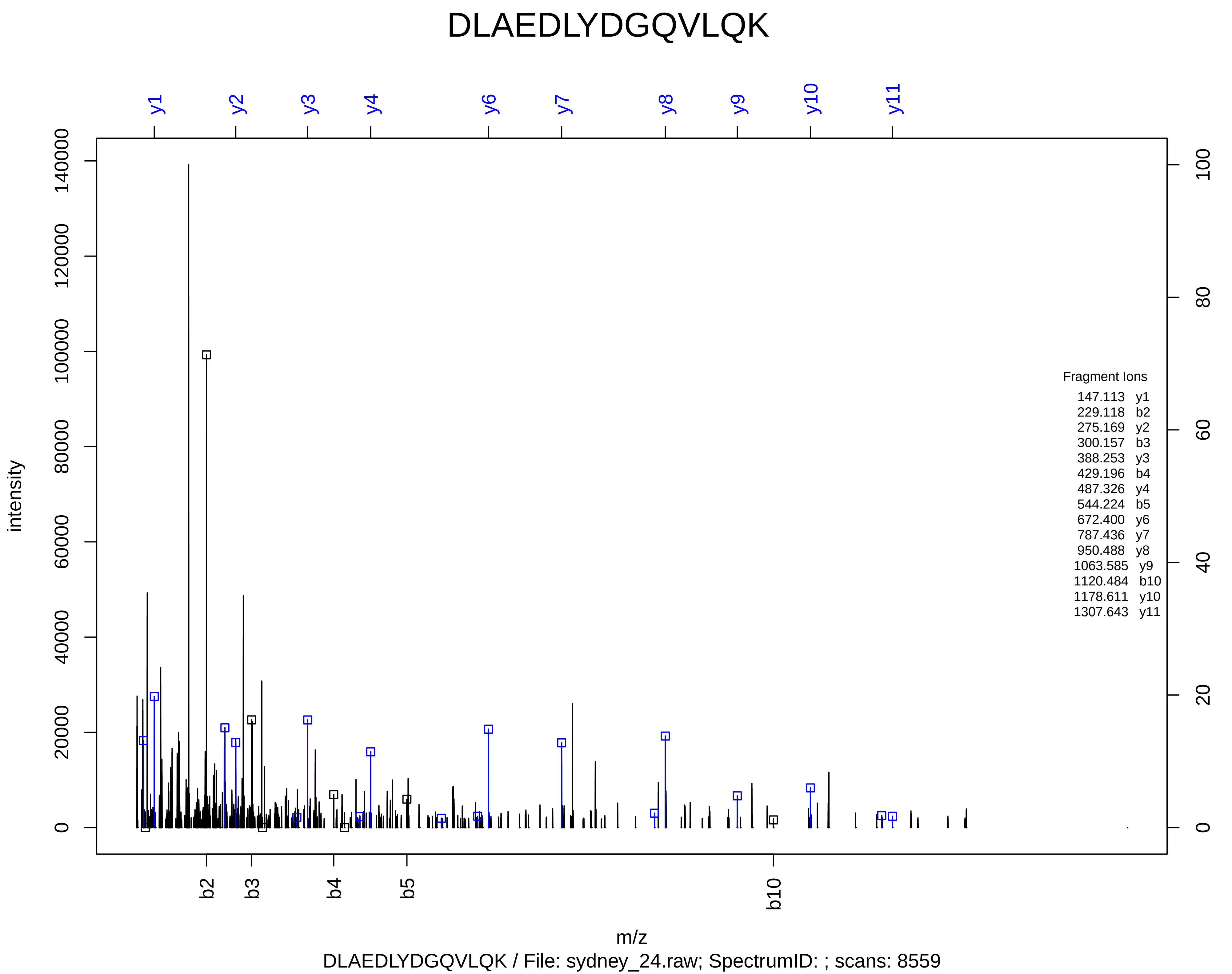

DLAEDLYDGQVLQK
y1
y2
y3
y4
y6
y7
y8
y9
y10
y11
intensity
0
20000
40000
60000
80000
100000
120000
140000
0
20
40
60
80
100
Fragment Ions
147.113 y1
229.118 b2
275.169 y2
300.157 b3
388.253 y3
429.196 b4
487.326 y4
544.224 b5
672.400 y6
787.436 y7
950.488 y8
1063.585 y9
1120.484 b10
1178.611 y10
1307.643 y11
b2
b3
b4
b5
b10
m/z
DLAEDLYDGQVLQK / File: sydney_24.raw; SpectrumID: ; scans: 8559

LQ^DKRMEIDK

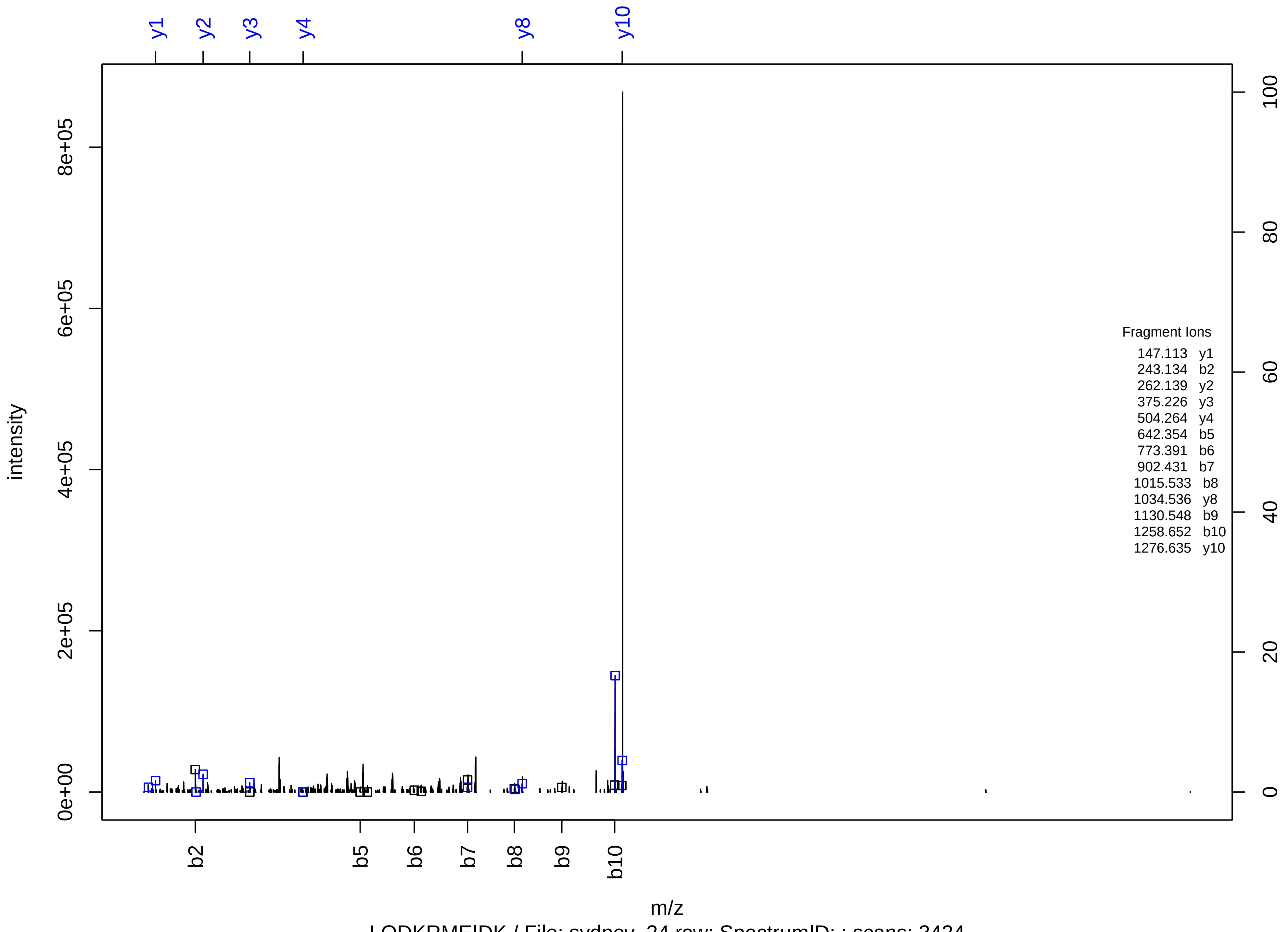

LQDKRMEIDK / File: sydney_24.raw; SpectrumID: ; scans: 3424

FIPSMALR

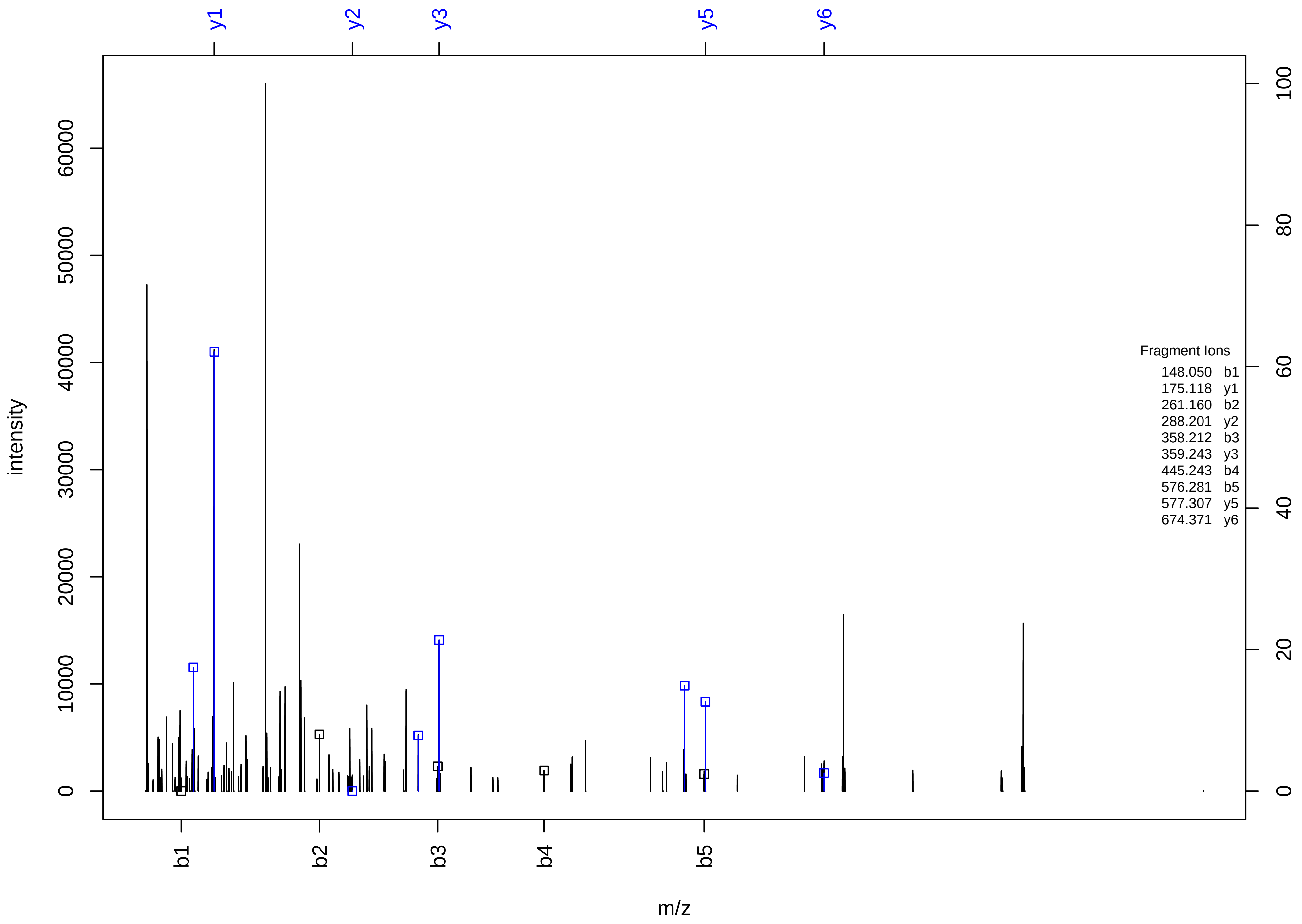

FIPSMALR / File: sydney_24.raw; SpectrumID: ; scans: 9201

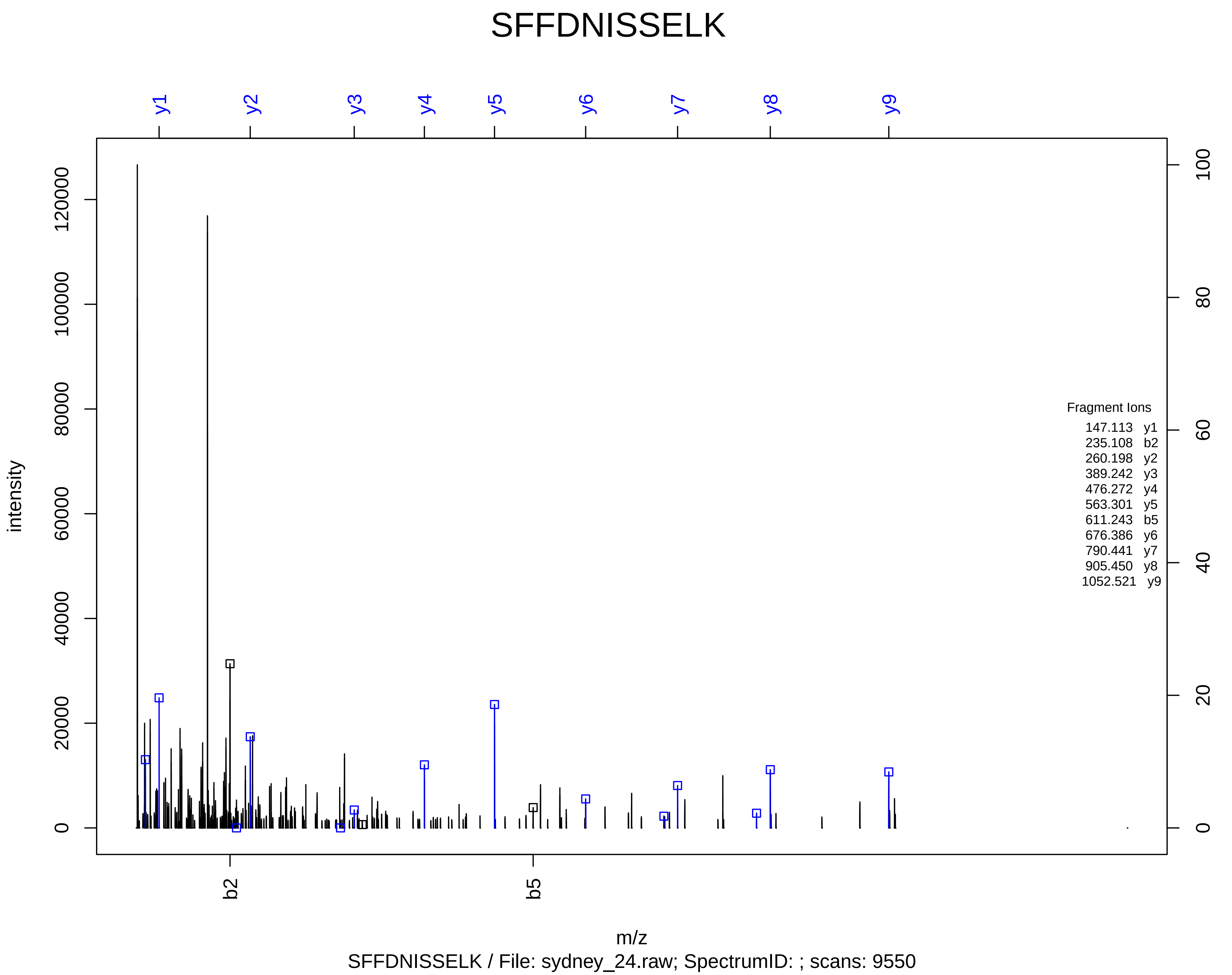
SFFDNISSELK
y1
y2
y3
y4
y5
y6
y7
y8
y9
intensity
0
20000
40000
60000
80000
100000
120000
0
20
40
60
80
100
b2
b5
m/z
Fragment Ions
147.113 y1
235.108 b2
260.198 y2
389.242 y3
476.272 y4
563.301 y5
611.243 b5
676.386 y6
790.441 y7
905.450 y8
1052.521 y9
SFFDNISSELK / File: sydney_24.raw; SpectrumID: ; scans: 9550

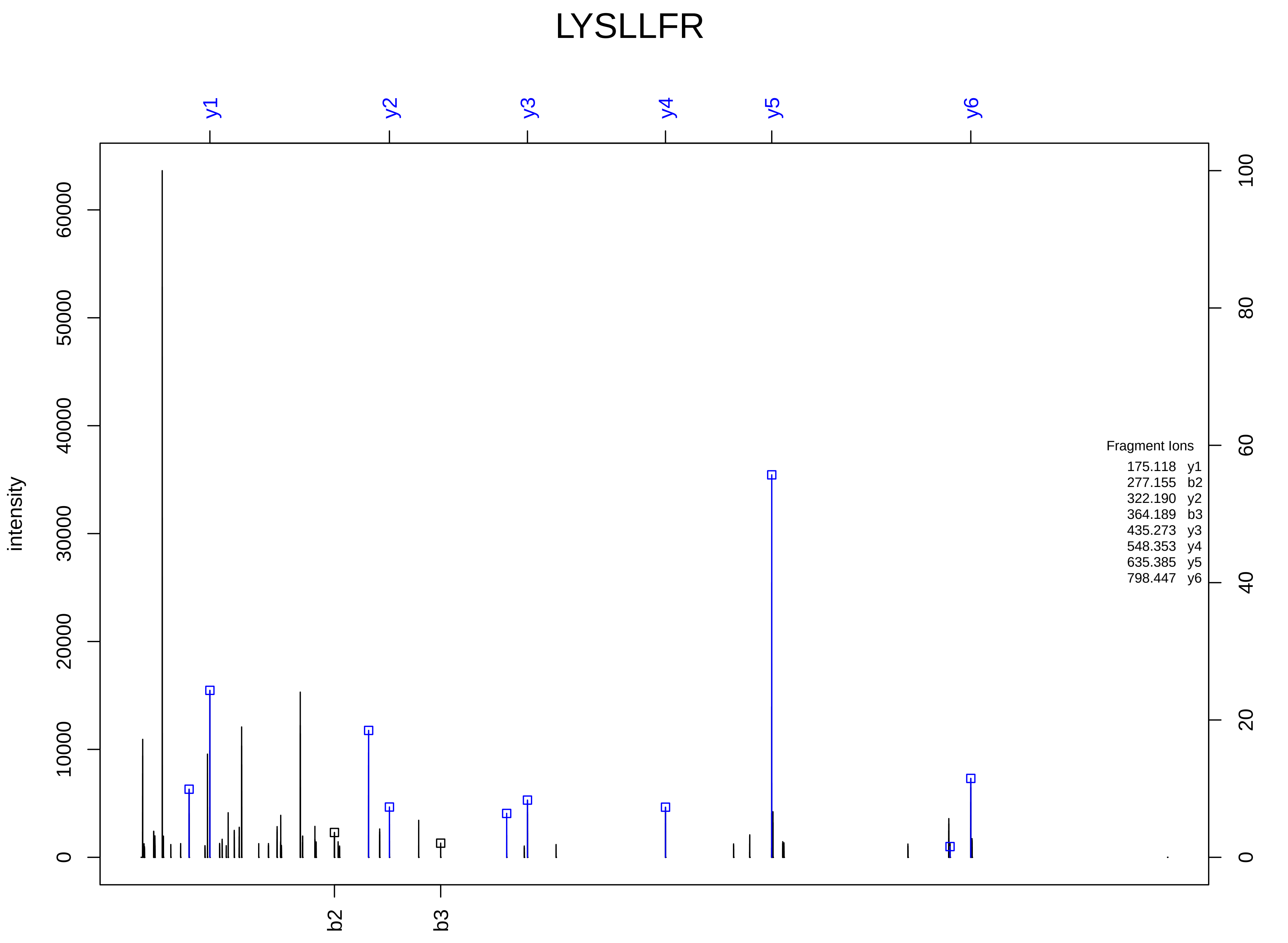

LYSLLFR / File: sydney_24.raw; SpectrumID: ; scans: 8137

ALCDVGTAISCSR

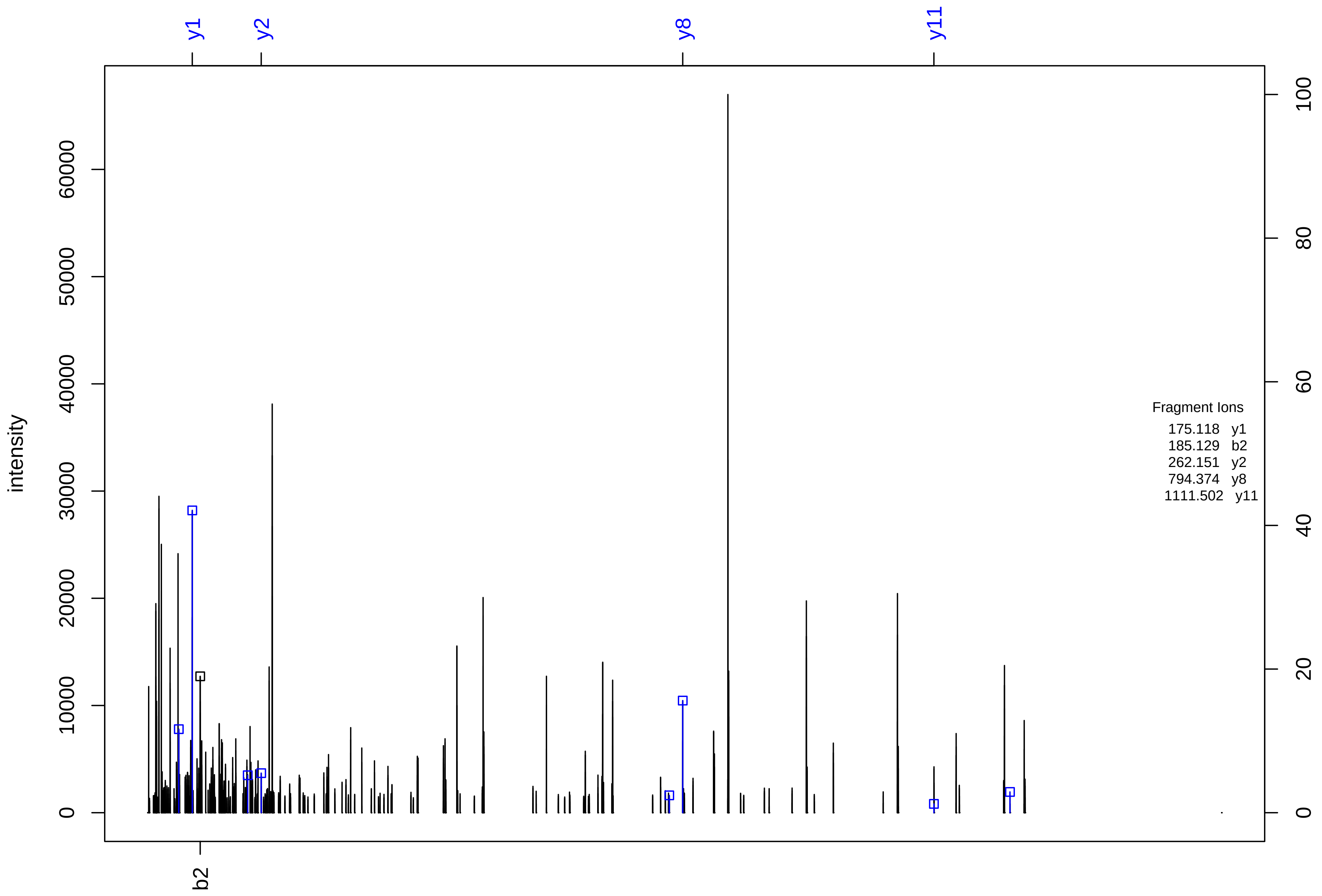

ALCDVGTAISCSR / File: sydney_24.raw; SpectrumID: ; scans: 3198

EEN^SAFIFKQ^EEK

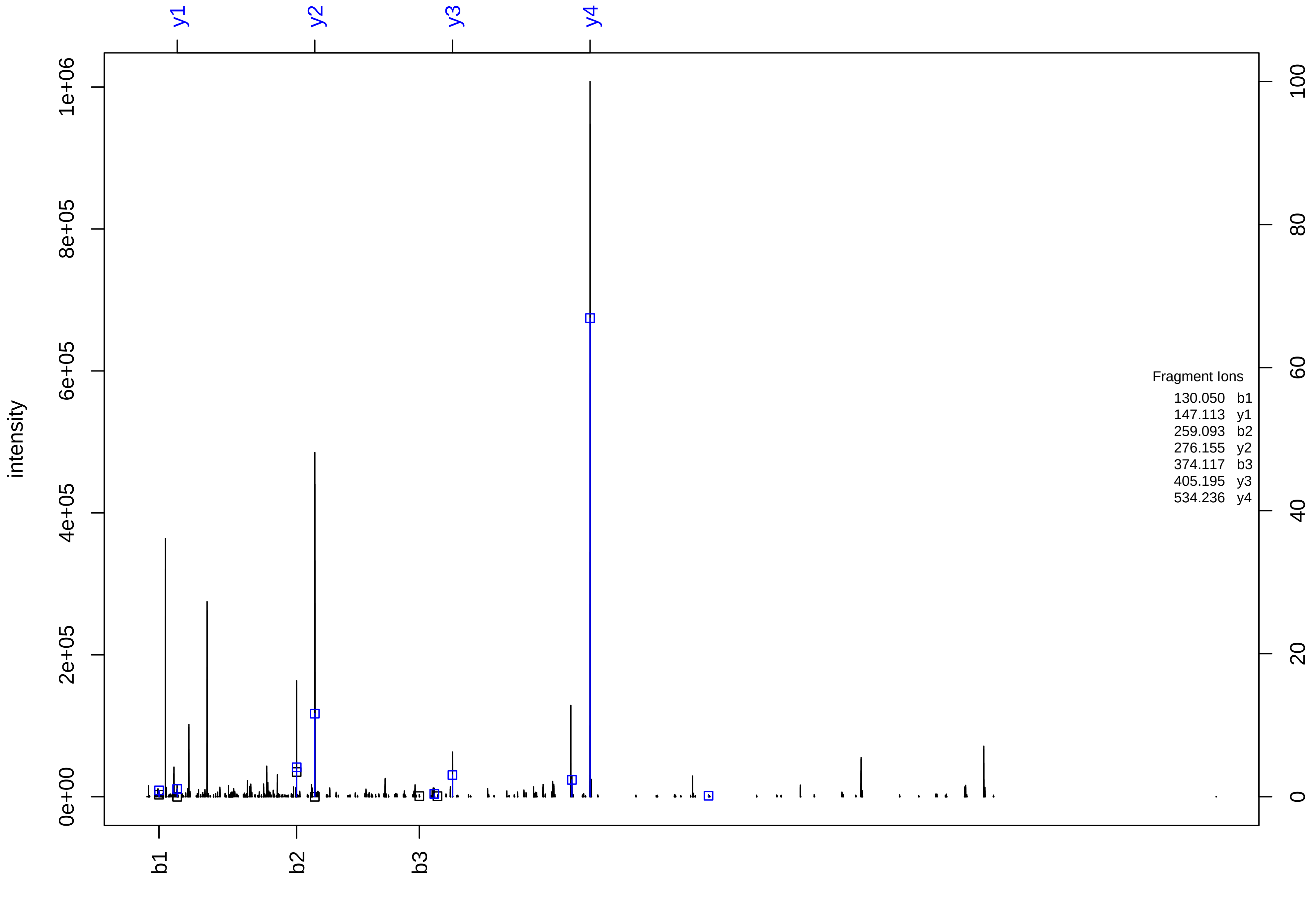

EENSAFIFKQEEK / File: sydney_25.raw; SpectrumID: ; scans: 2626

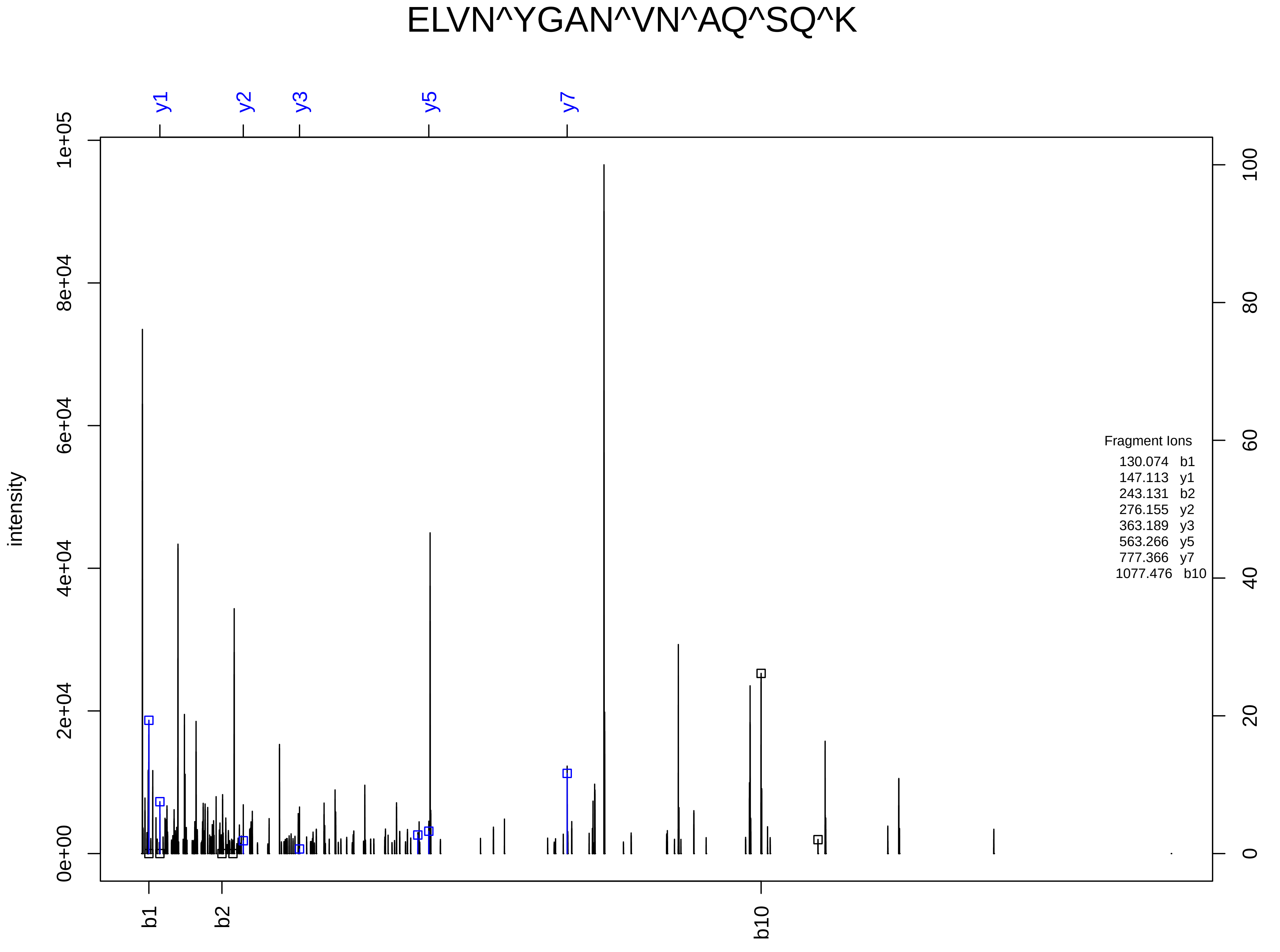
ELVN^YGAN^VN^AQ^SQ^K
y1
y2
y3
y5
y7
intensity
1e+05
8e+04
6e+04
4e+04
2e+04
0e+00
100
80
60
40
20
0
Fragment Ions
130.074 b1
147.113 y1
243.131 b2
276.155 y2
363.189 y3
563.266 y5
777.366 y7
1077.476 b10
b1
b2
b10
m/z
ELVNYGANVNAQSQK / File: sydney_25.raw; SpectrumID: ; scans: 4542

(Ac)MELEAMSR

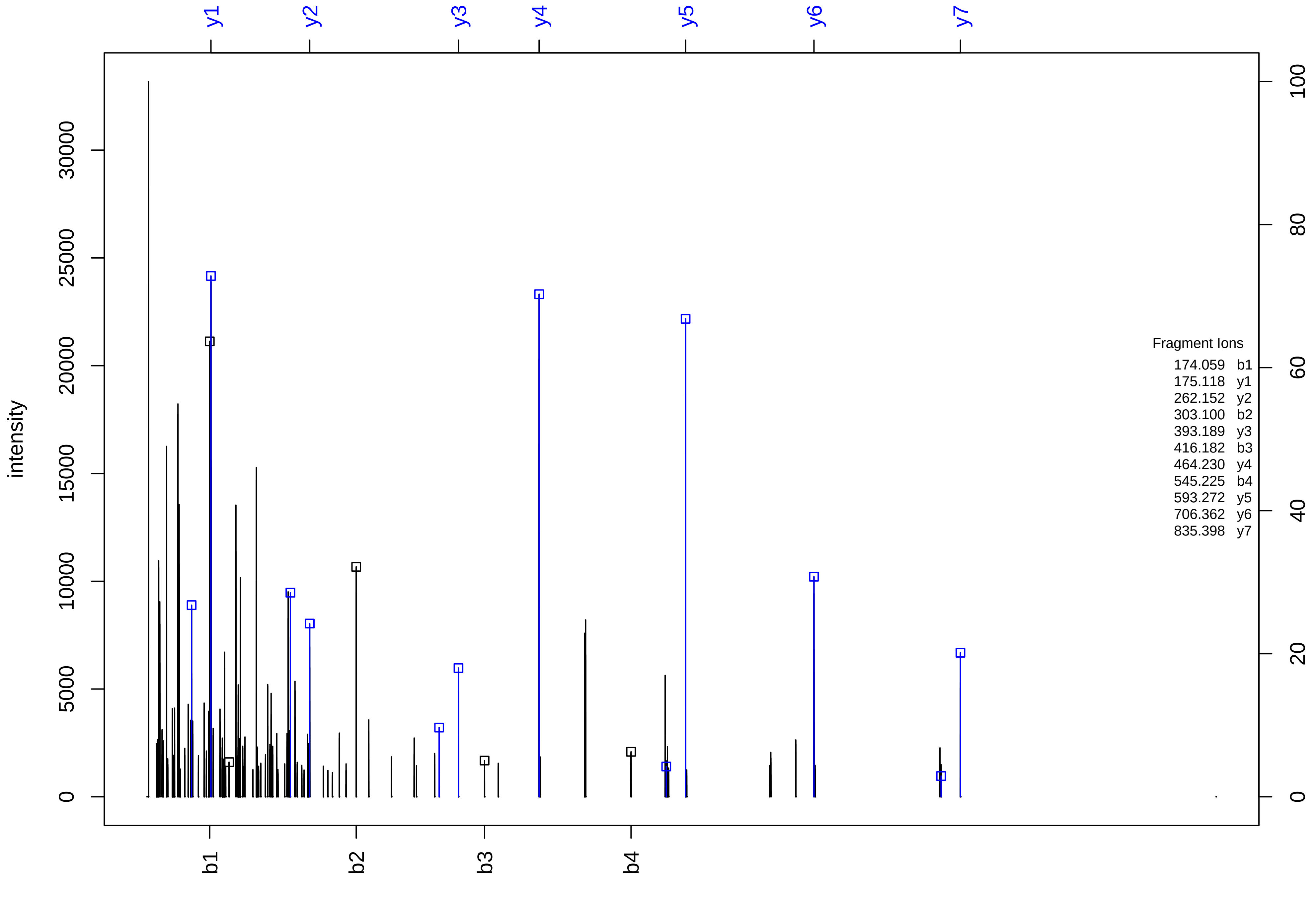

MELEAMSR / File: sydney_25.raw; SpectrumID: ; scans: 7761

WFNGQPIHAELSPVTDFR

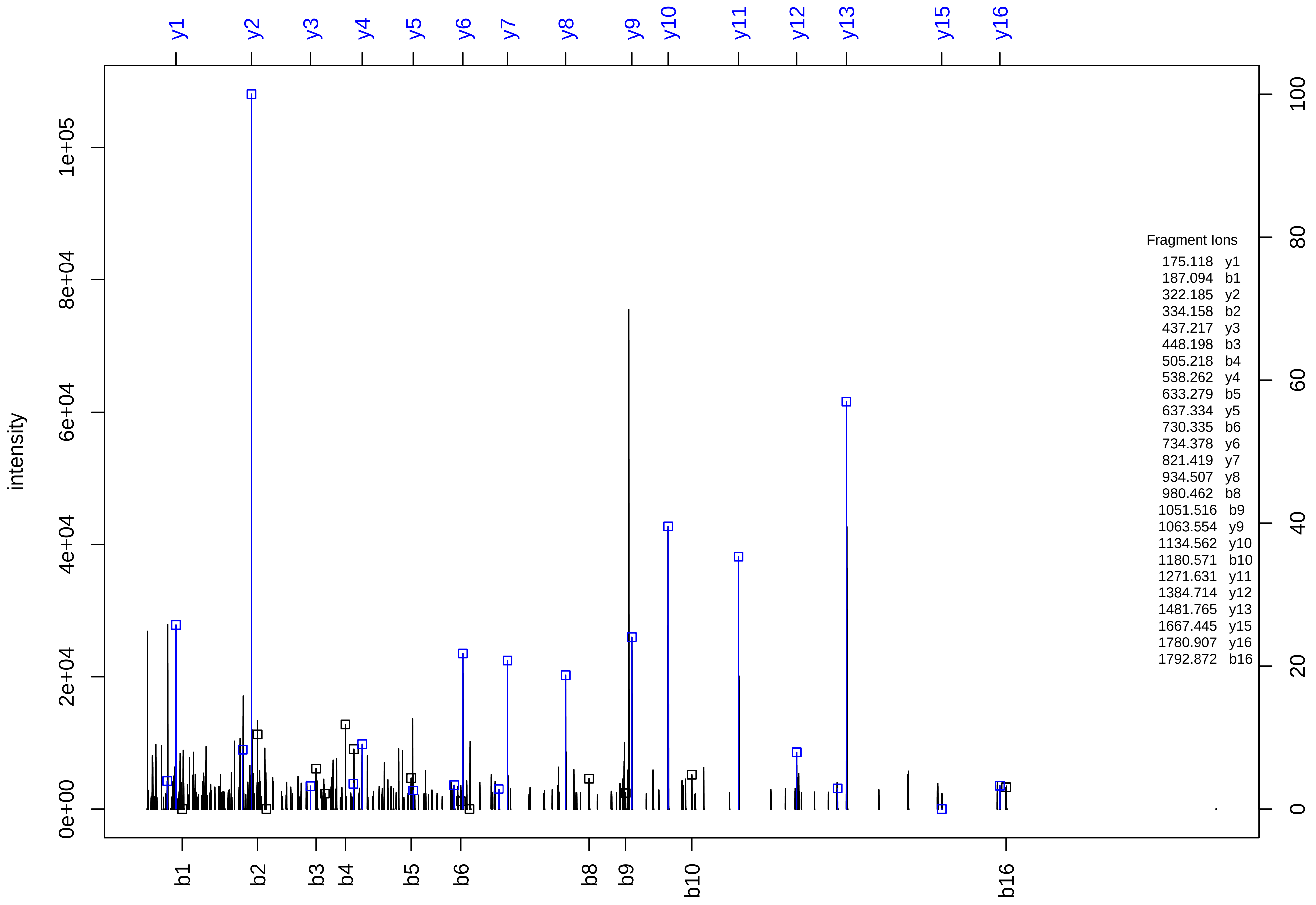

WFNGQPIHAELSPVTDFR / File: sydney_25.raw; SpectrumID: ; scans: 8680

HHTVLN^HSIKVM*SHVEN^LSK

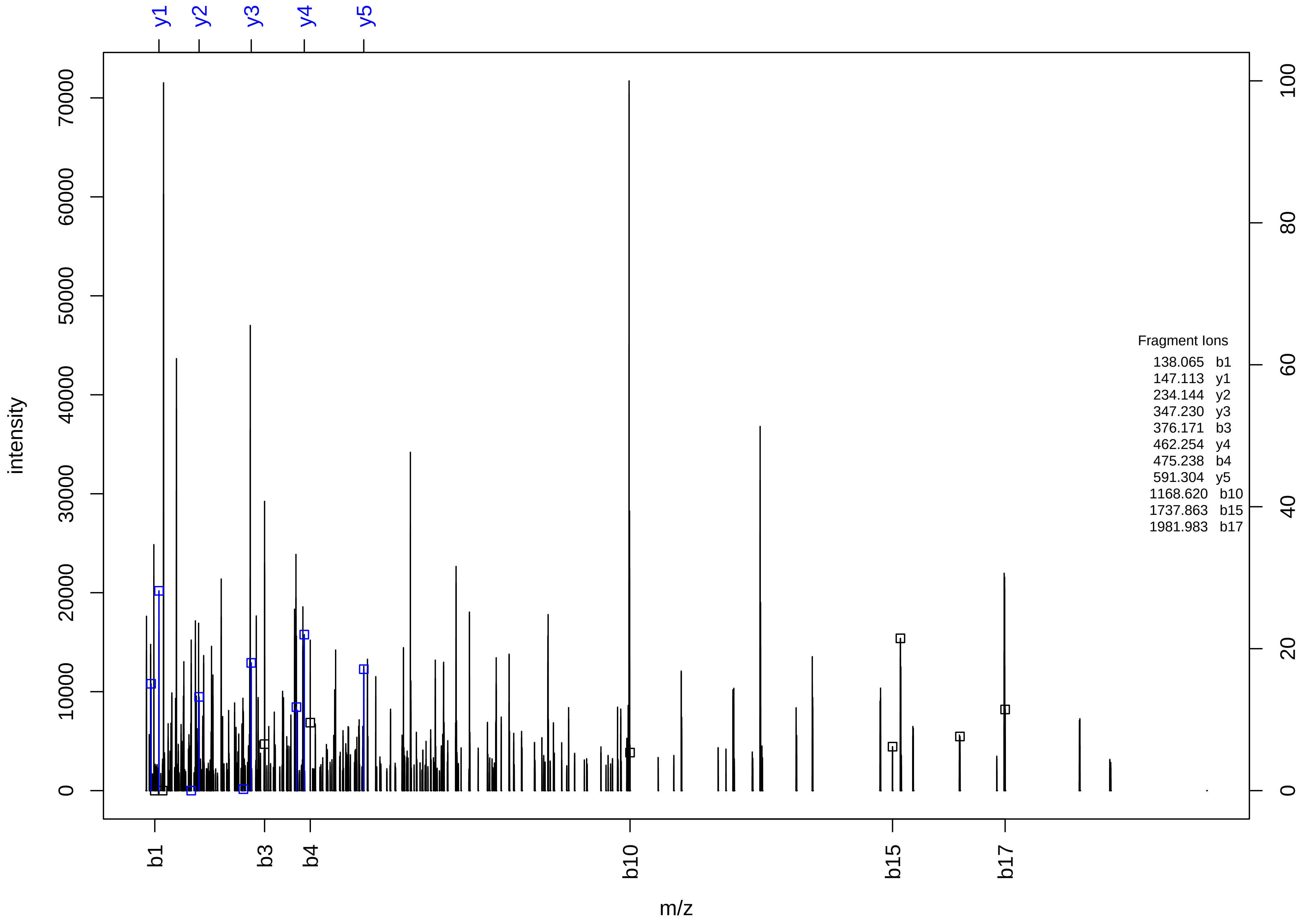

HHTVLNHSIKVMSHVENLSK / File: sydney_25.raw; SpectrumID: ; scans: 13322

LVILMEPFEDALR

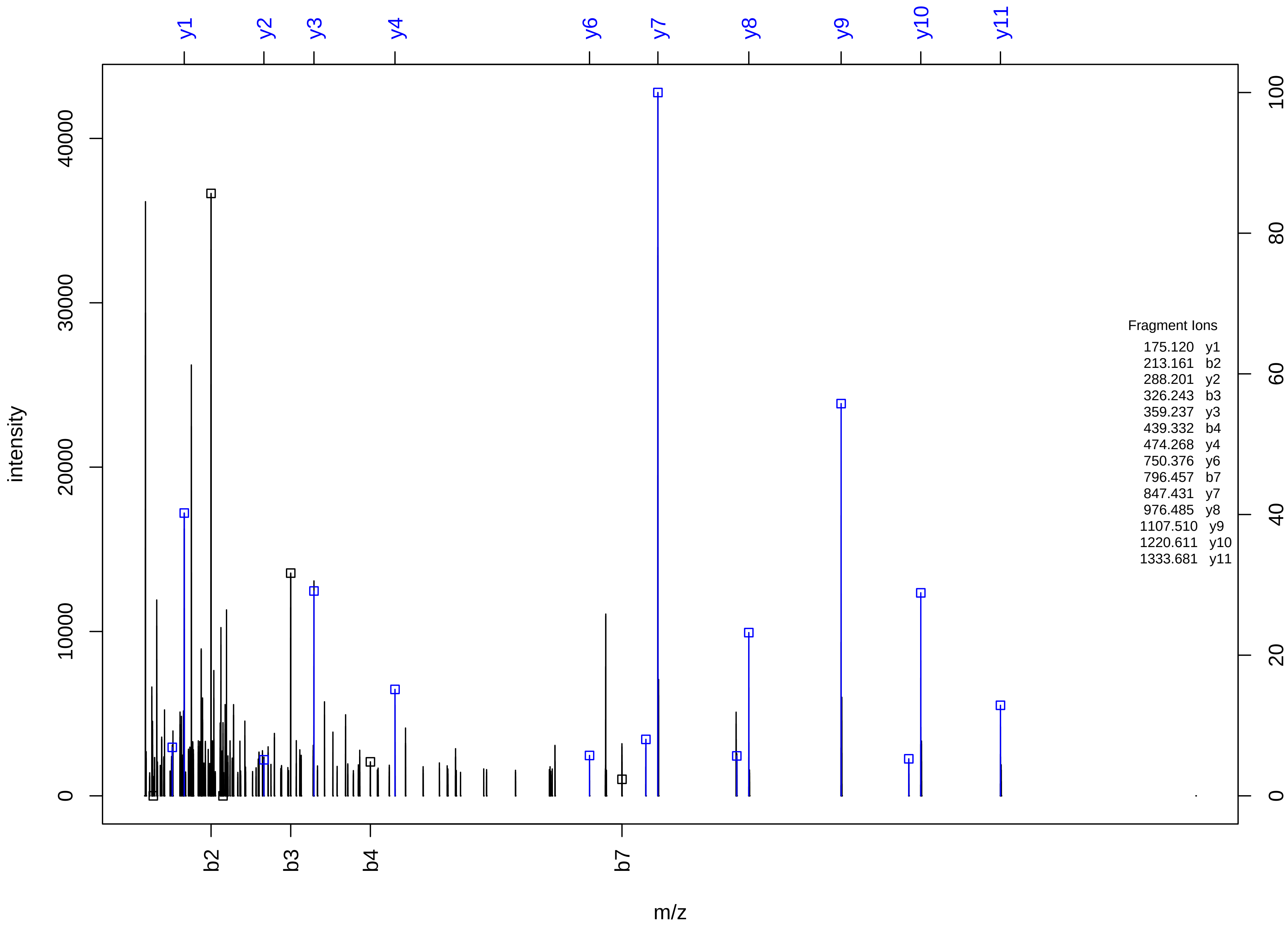

LVILMEPFEDALR / File: sydney_25.raw; SpectrumID: ; scans: 14317

LDFDLEPEPPPAPPR

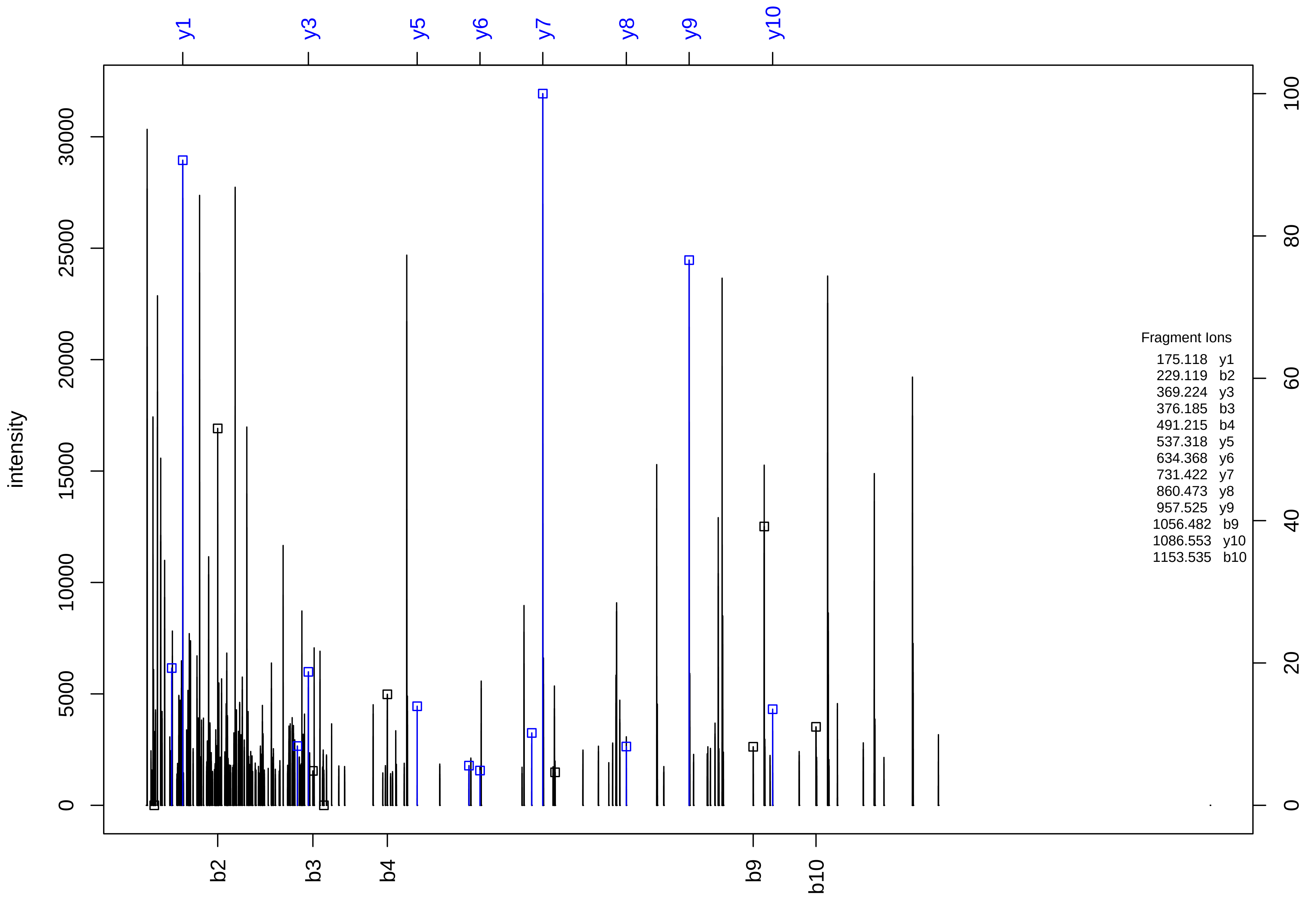

LDFDLEPEPPPAPPR / File: sydney_25.raw; SpectrumID: ; scans: 8544

ILLSSLLR

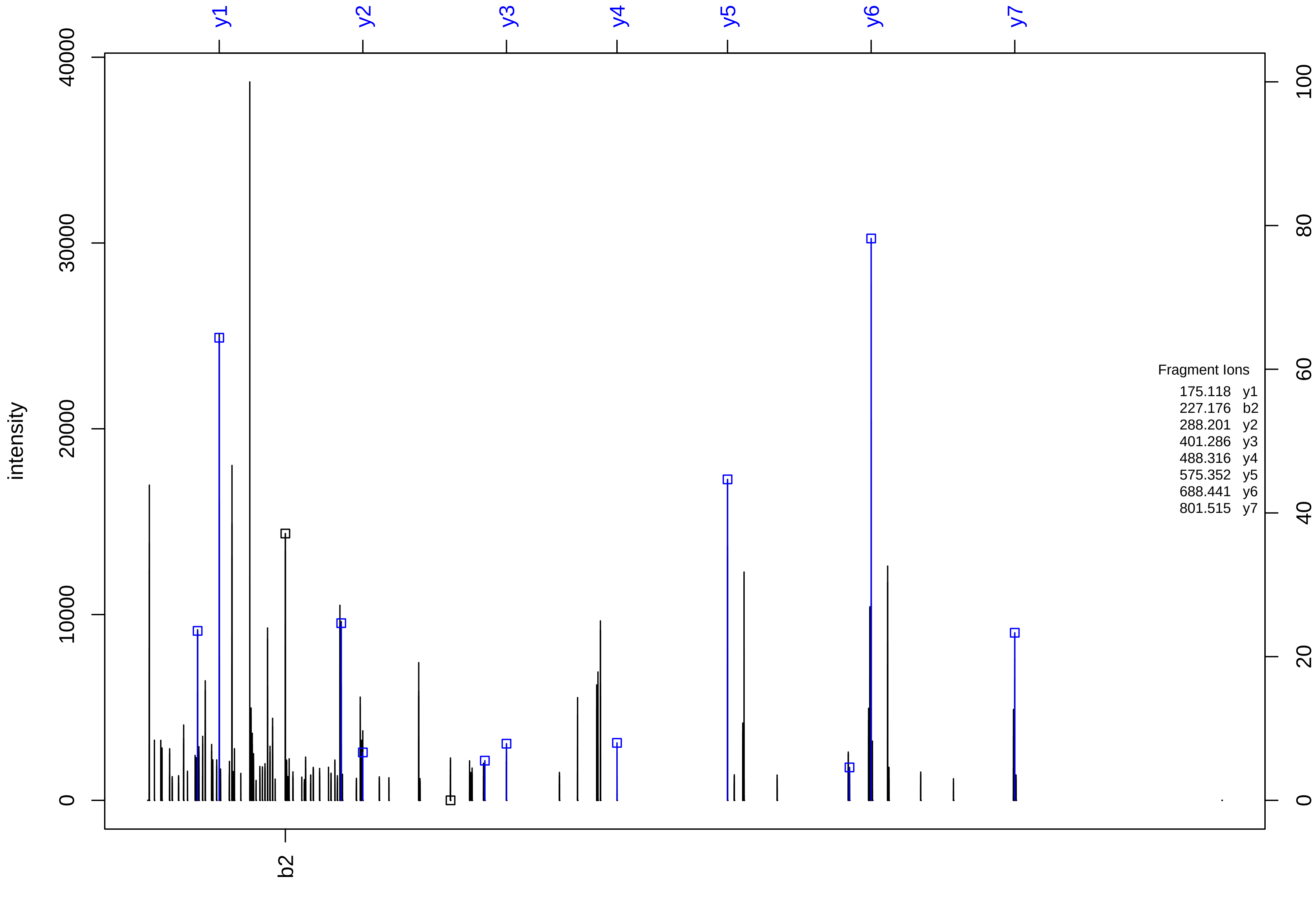

ILLSSLLR / File: sydney_25.raw; SpectrumID: ; scans: 9200

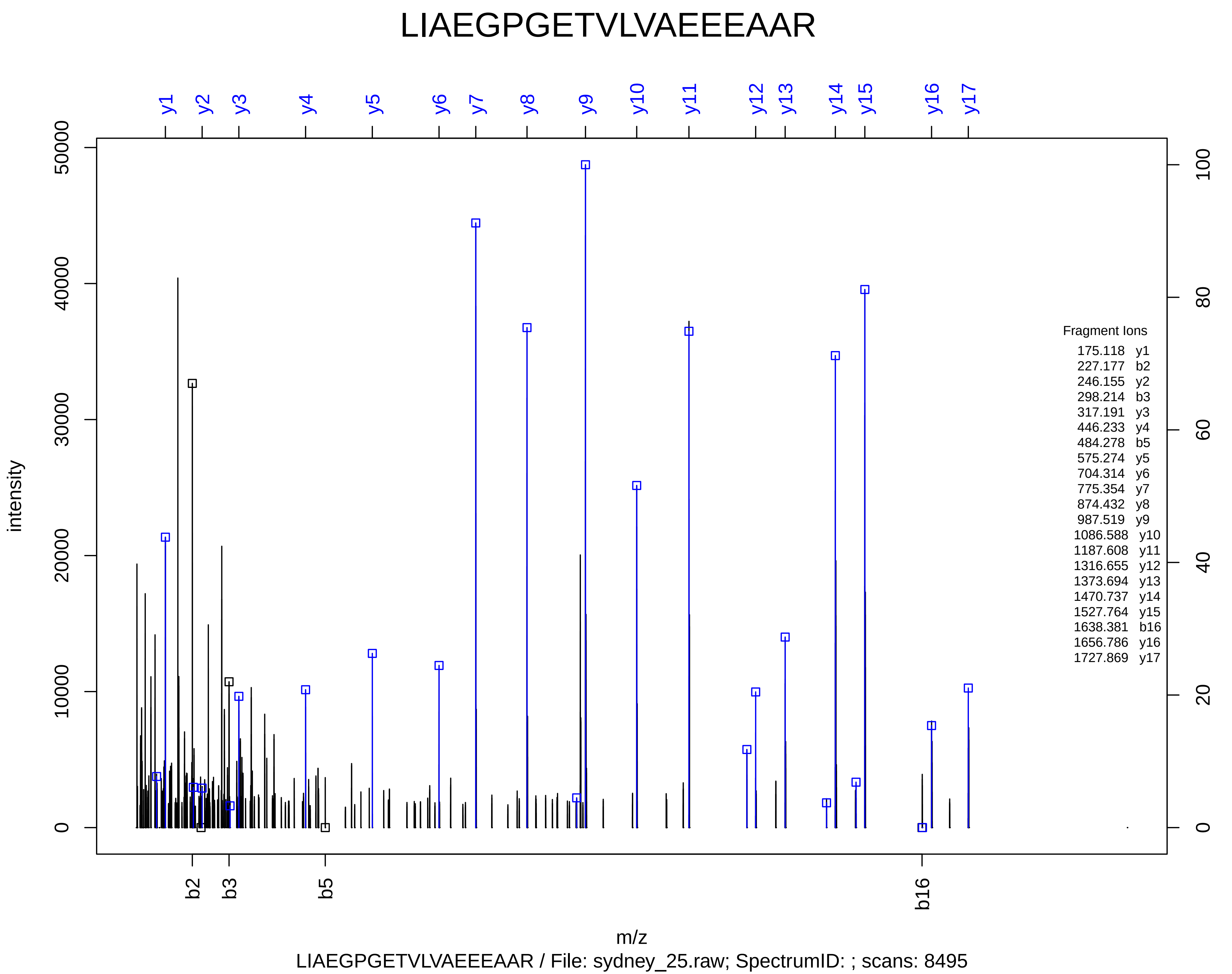
LIAEGPGETVLVAEEEAAR
y1 y2 y3 y4 y5 y6 y7 y8 y9 y10 y11 y12 y13 y14 y15 y16 y17
intensity
0 10000 20000 30000 40000 50000
0 20 40 60 80 100
b2 b3 b5 b16
m/z
Fragment Ions
175.118 y1
227.177 b2
246.155 y2
298.214 b3
317.191 y3
446.233 y4
484.278 b5
575.274 y5
704.314 y6
775.354 y7
874.432 y8
987.519 y9
1086.588 y10
1187.608 y11
1316.655 y12
1373.694 y13
1470.737 y14
1527.764 y15
1638.381 b16
1656.786 y16
1727.869 y17
LIAEGPGETVLVAEEEAAR / File: sydney_25.raw; SpectrumID: ; scans: 8495

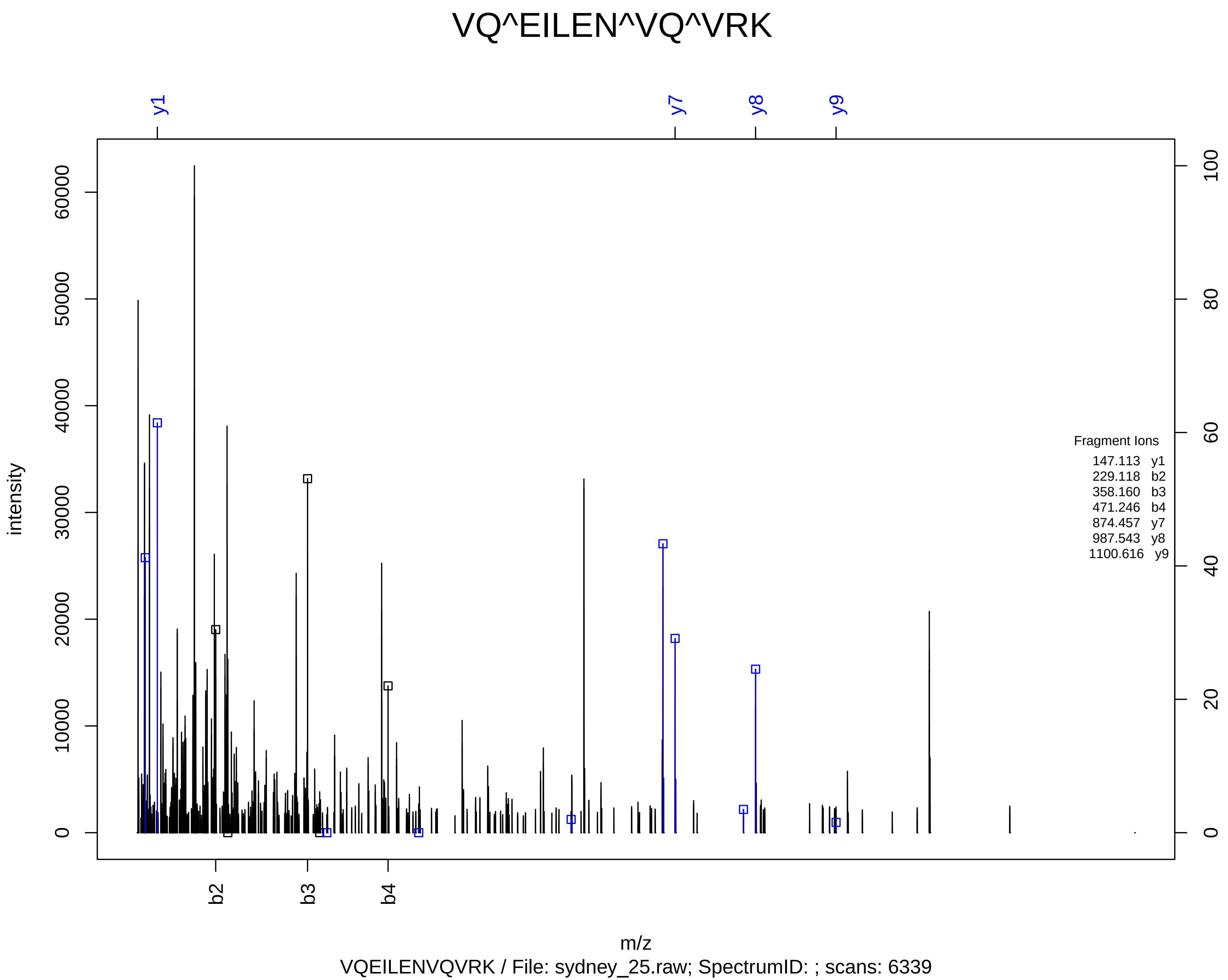
VQ^EILEN^VQ^VRK
y1
y7
y8
y9
intensity
0
10000
20000
30000
40000
50000
60000
0
20
40
60
80
100
Fragment Ions
147.113 y1
229.118 b2
358.160 b3
471.246 b4
874.457 y7
987.543 y8
1100.616 y9
b2
b3
b4
m/z
VQEILENVQVRK / File: sydney_25.raw; SpectrumID: ; scans: 6339

LDPDSIPSPIQVIENDR

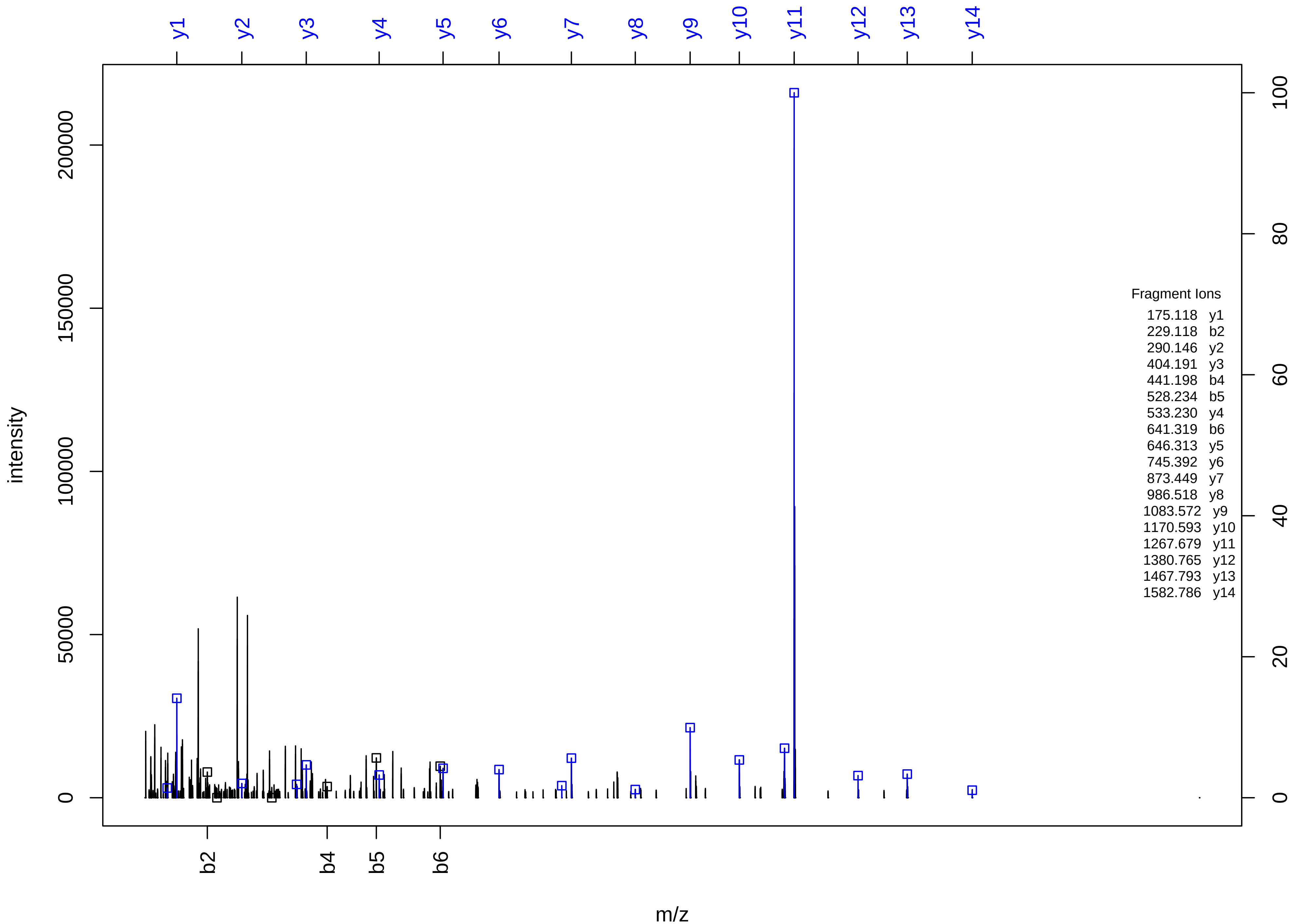

LDPDSIPSPIQVIENDR / File: sydney_25.raw; SpectrumID: ; scans: 11746

LGGLDVLEAEFSK

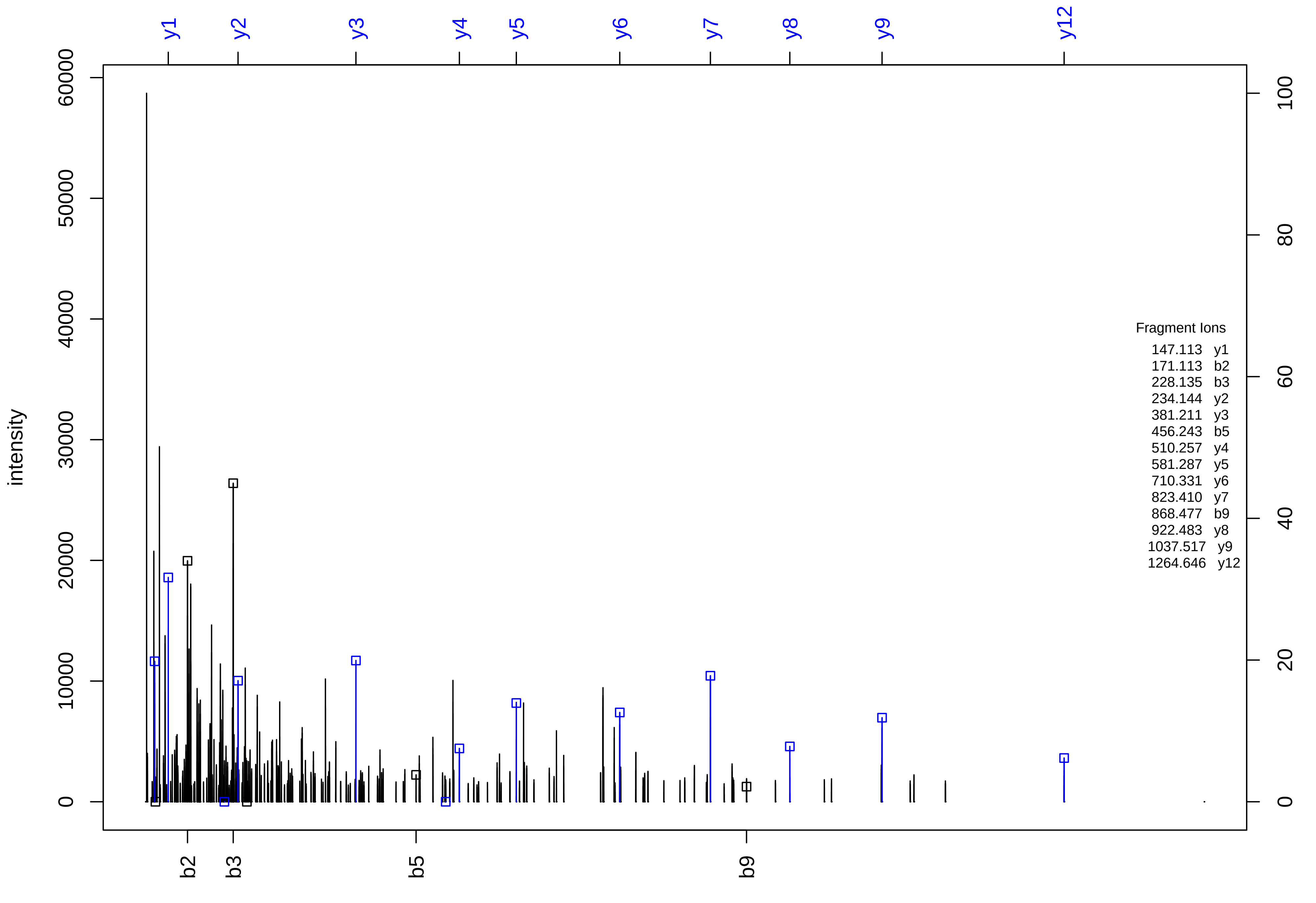

LGGLDVLEAEFSK / File: sydney_25.raw; SpectrumID: ; scans: 11159

ALGEPITLFGEGPAER

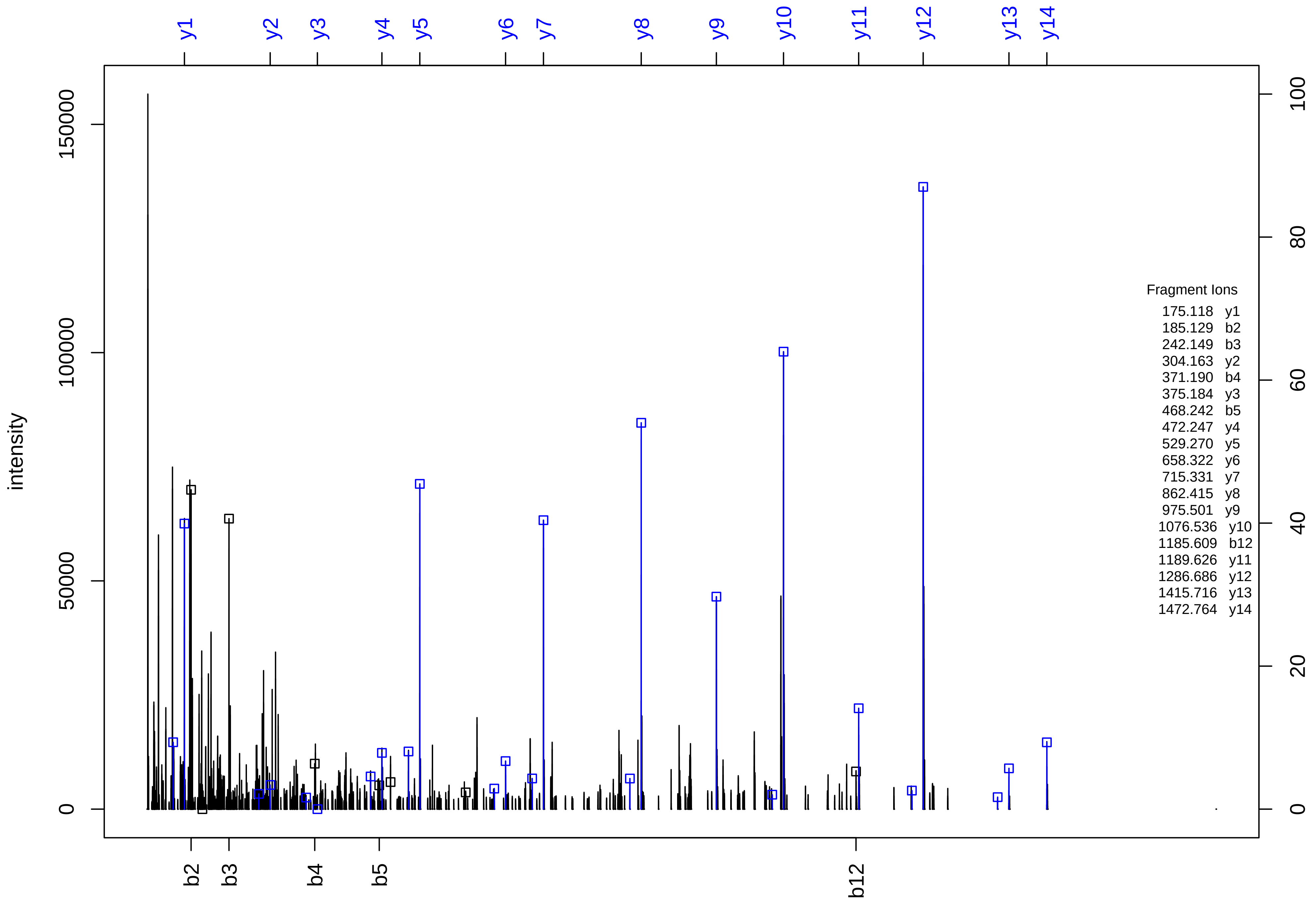

ALGEPITLFGEGPAER / File: sydney_25.raw; SpectrumID: ; scans: 9453

GVN^GKNM*Q^N^RK

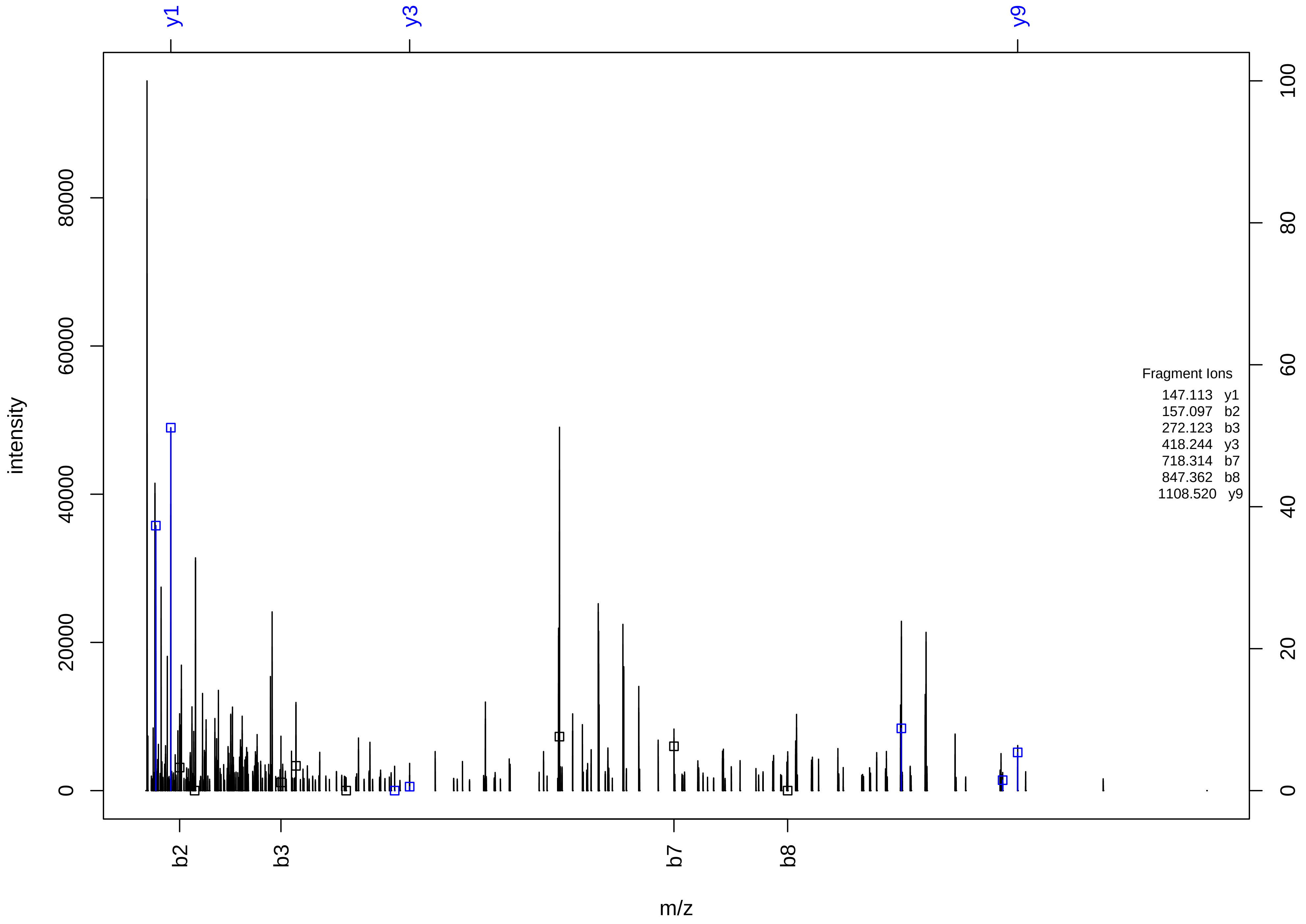

GVNGKNMQNRK / File: sydney_25.raw; SpectrumID: ; scans: 6448

GVTAEM*Q^NLVHRR

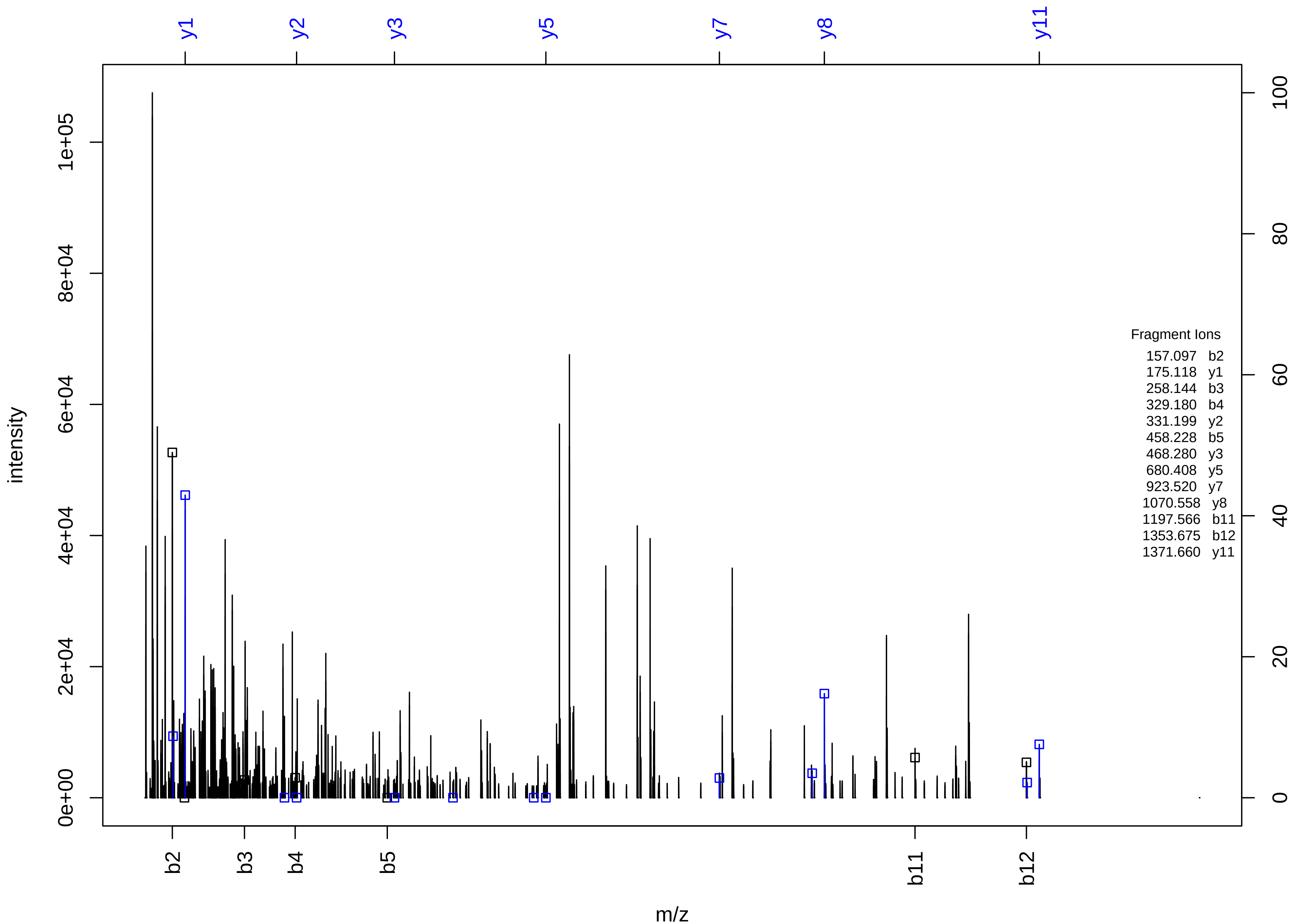

GVTAEMQNLVHRR / File: sydney_25.raw; SpectrumID: ; scans: 7531

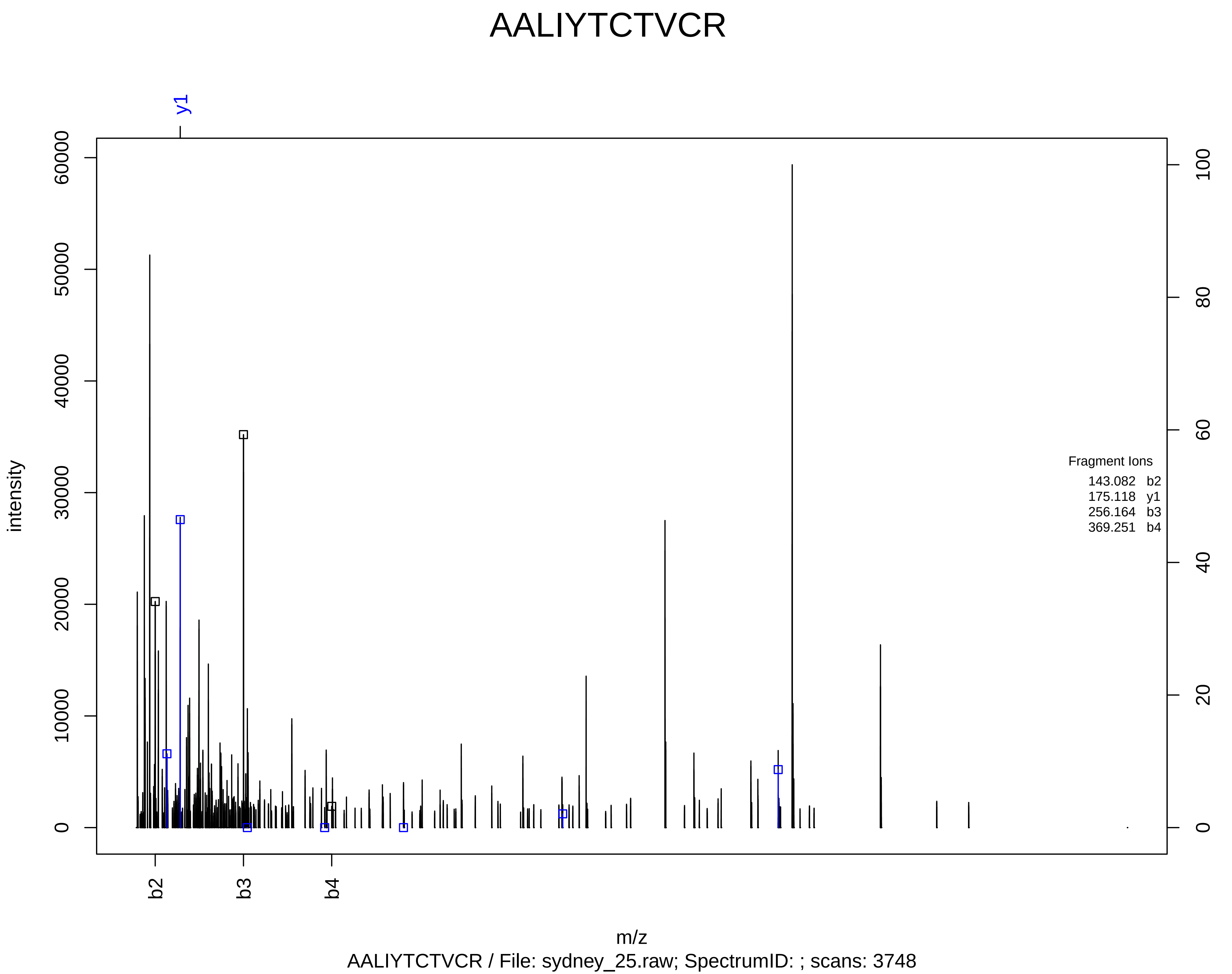
AALIYTCTVCR
intensity
m/z
y1
b2
b3
b4
Fragment Ions
143.082 b2
175.118 y1
256.164 b3
369.251 b4
AALIYTCTVCR / File: sydney_25.raw; SpectrumID: ; scans: 3748

N^LFQ^N^FTK

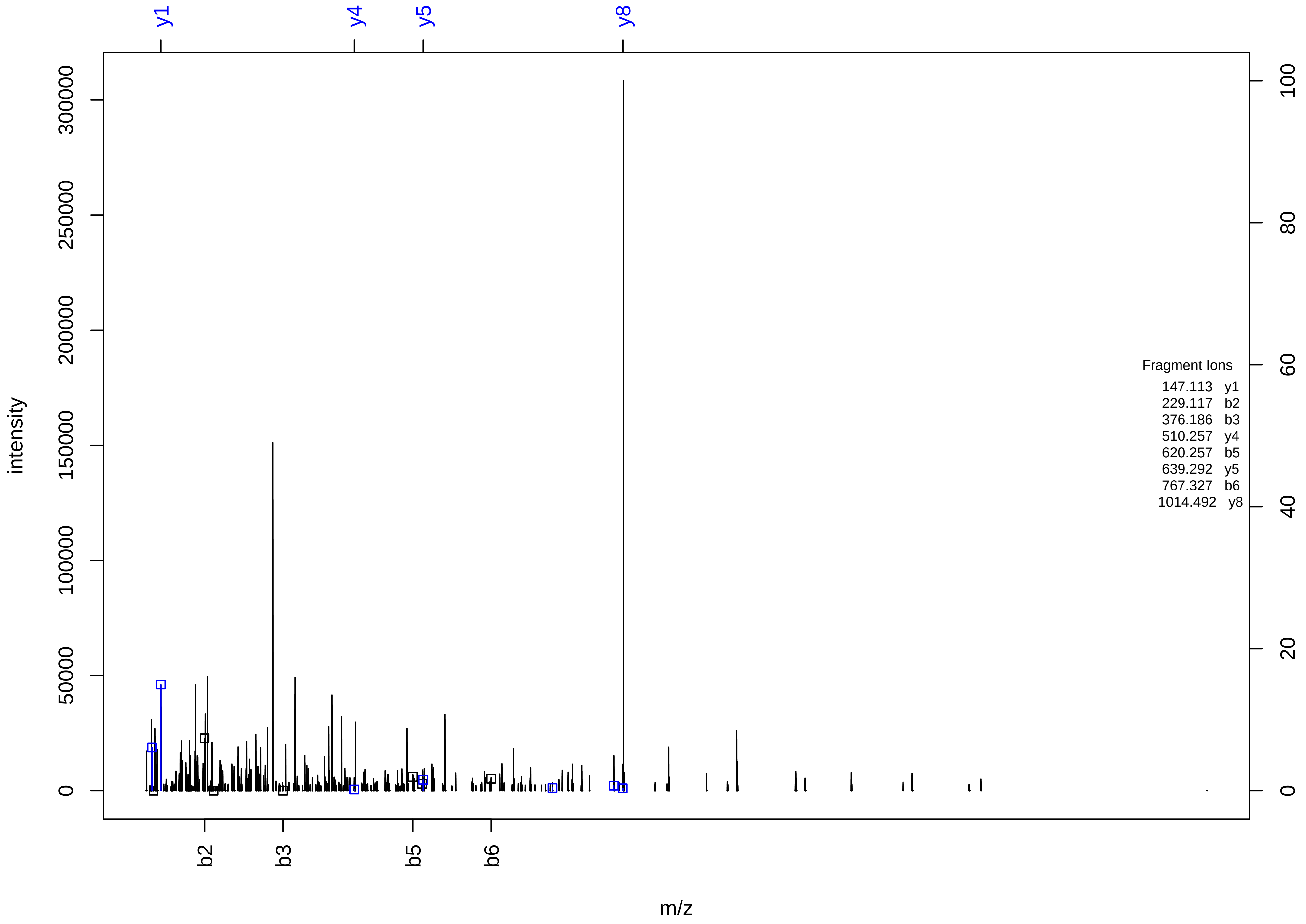

NLFQNFTK / File: sydney_26.raw; SpectrumID: ; scans: 5308

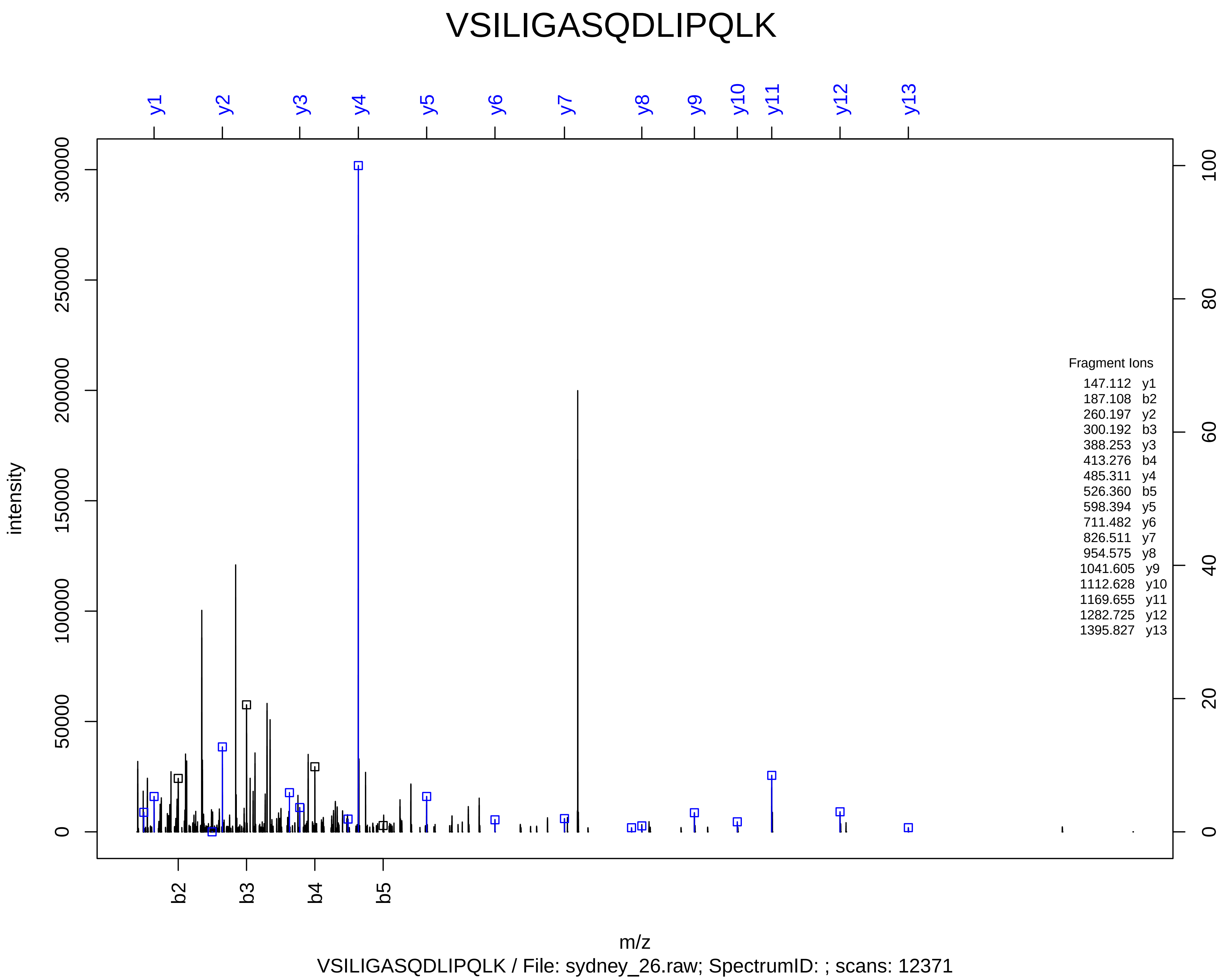
VSILIGASQDLIPQLK
y1 y2 y3 y4 y5 y6 y7 y8 y9 y10 y11 y12 y13
intensity
0 50000 100000 150000 200000 250000 300000
0 20 40 60 80 100
Fragment Ions
147.112 y1
187.108 b2
260.197 y2
300.192 b3
388.253 y3
413.276 b4
485.311 y4
526.360 b5
598.394 y5
711.482 y6
826.511 y7
954.575 y8
1041.605 y9
1112.628 y10
1169.655 y11
1282.725 y12
1395.827 y13
b2 b3 b4 b5
m/z
VSILIGASQDLIPQLK / File: sydney_26.raw; SpectrumID: ; scans: 12371

AALAQAADCEVEQWNSDDPIPR

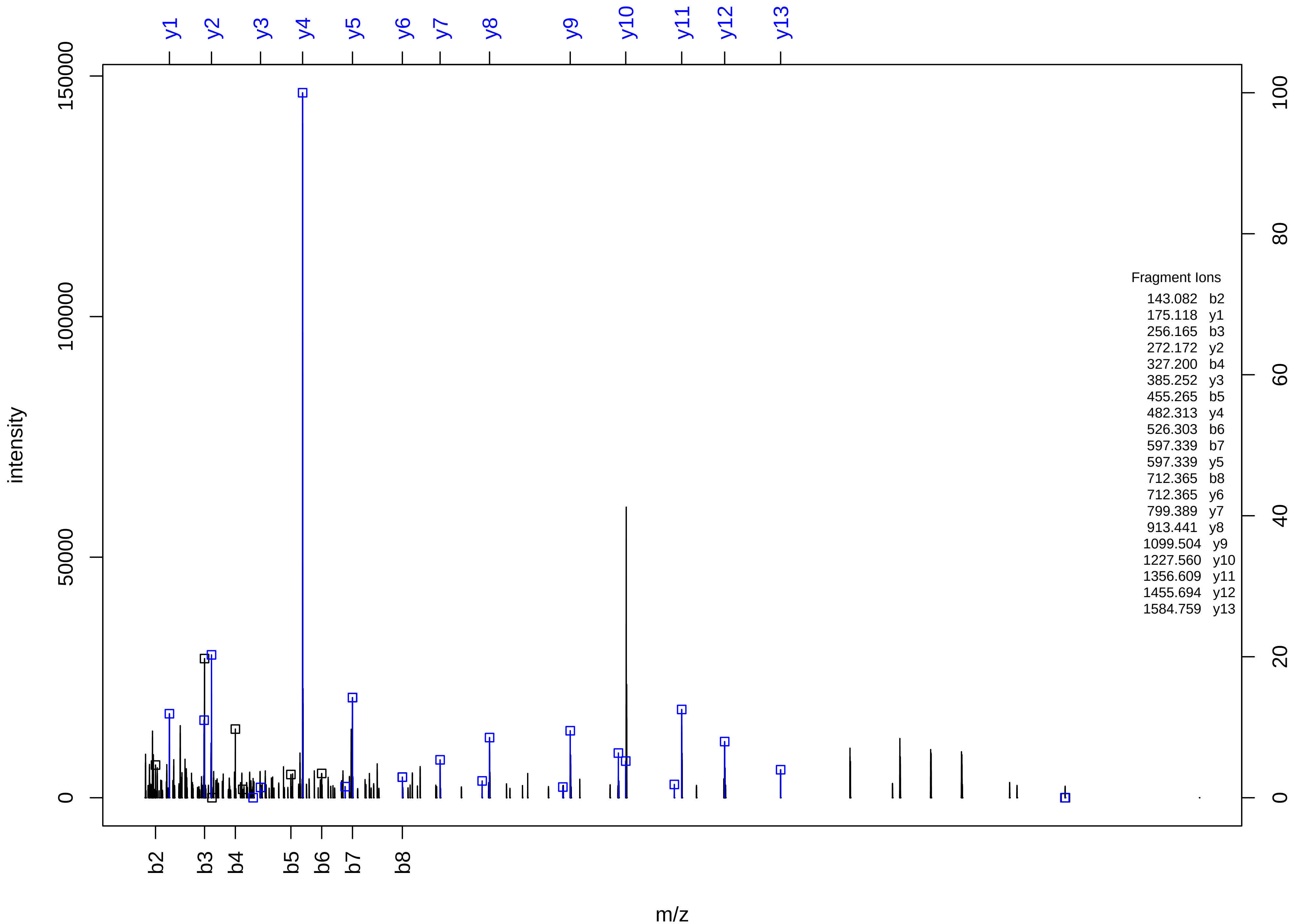

AALAQAADCEVEQWNSDDPIPR / File: sydney_26.raw; SpectrumID: ; scans: 8398

EDTSEEVVPVLVNNWK

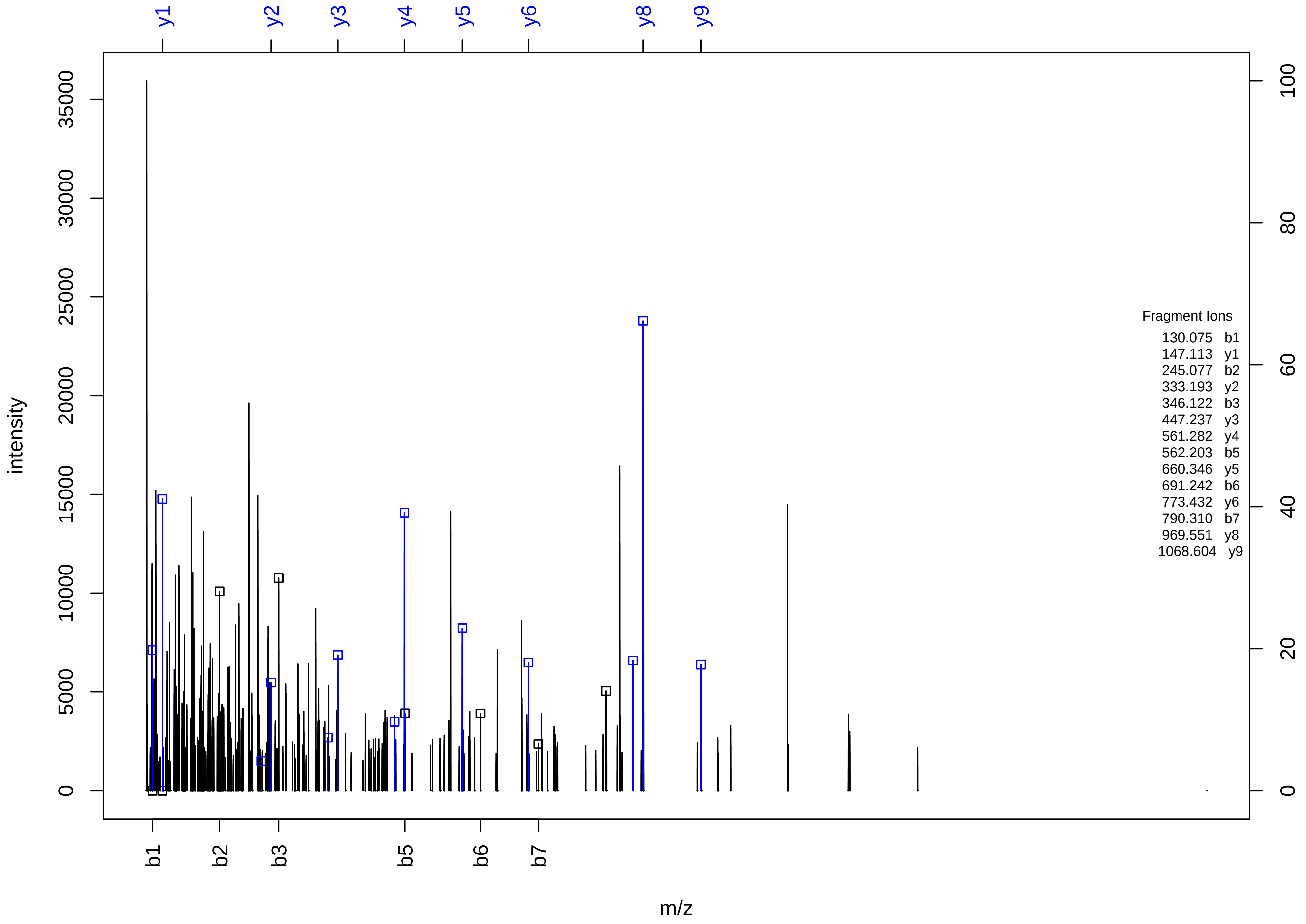

EDTSEEVVPVLVNNWK / File: sydney_26.raw; SpectrumID: ; scans: 9779

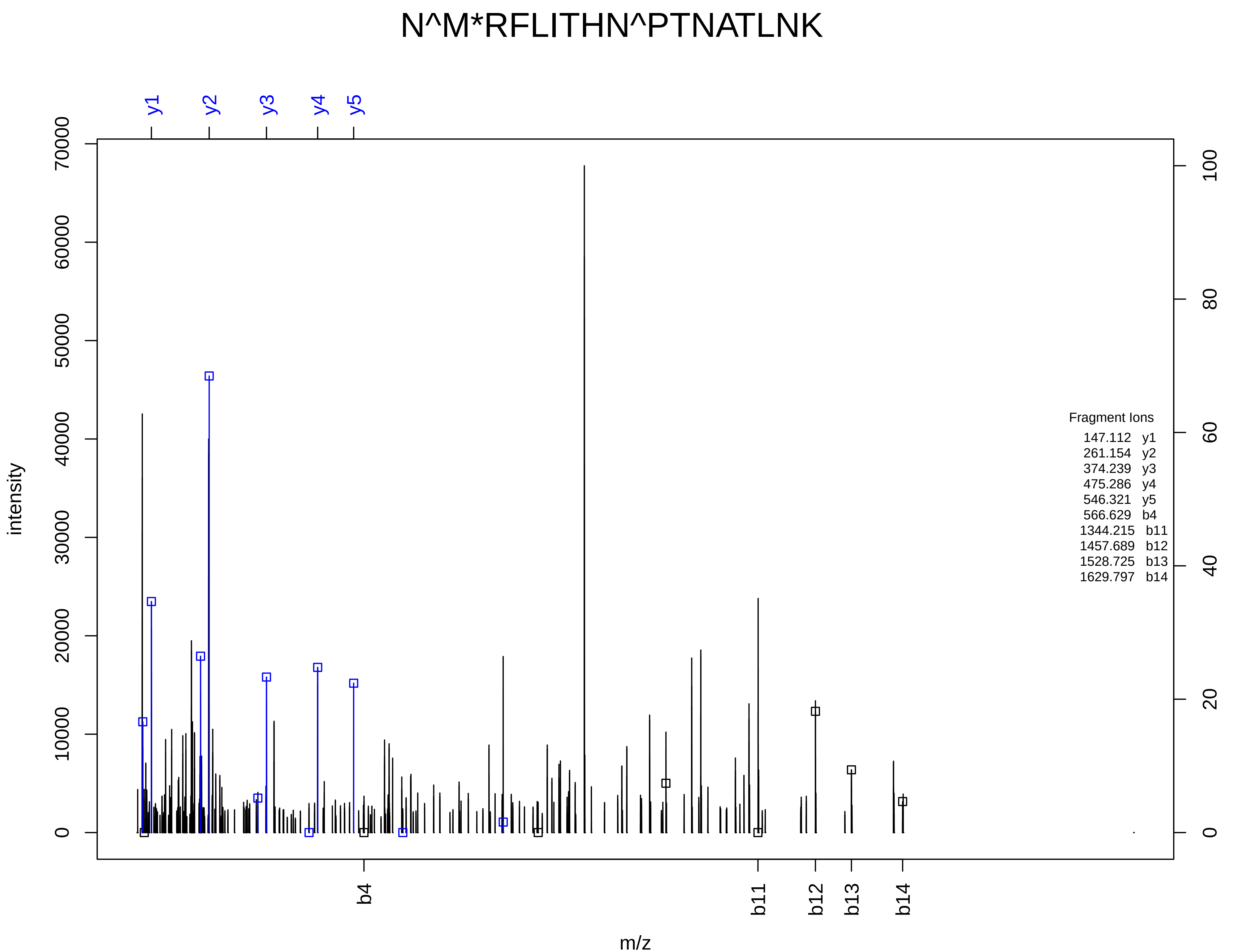

NMRFLITHNPTNATLNK / File: sydney_26.raw; SpectrumID: ; scans: 5760

VLLLEANR

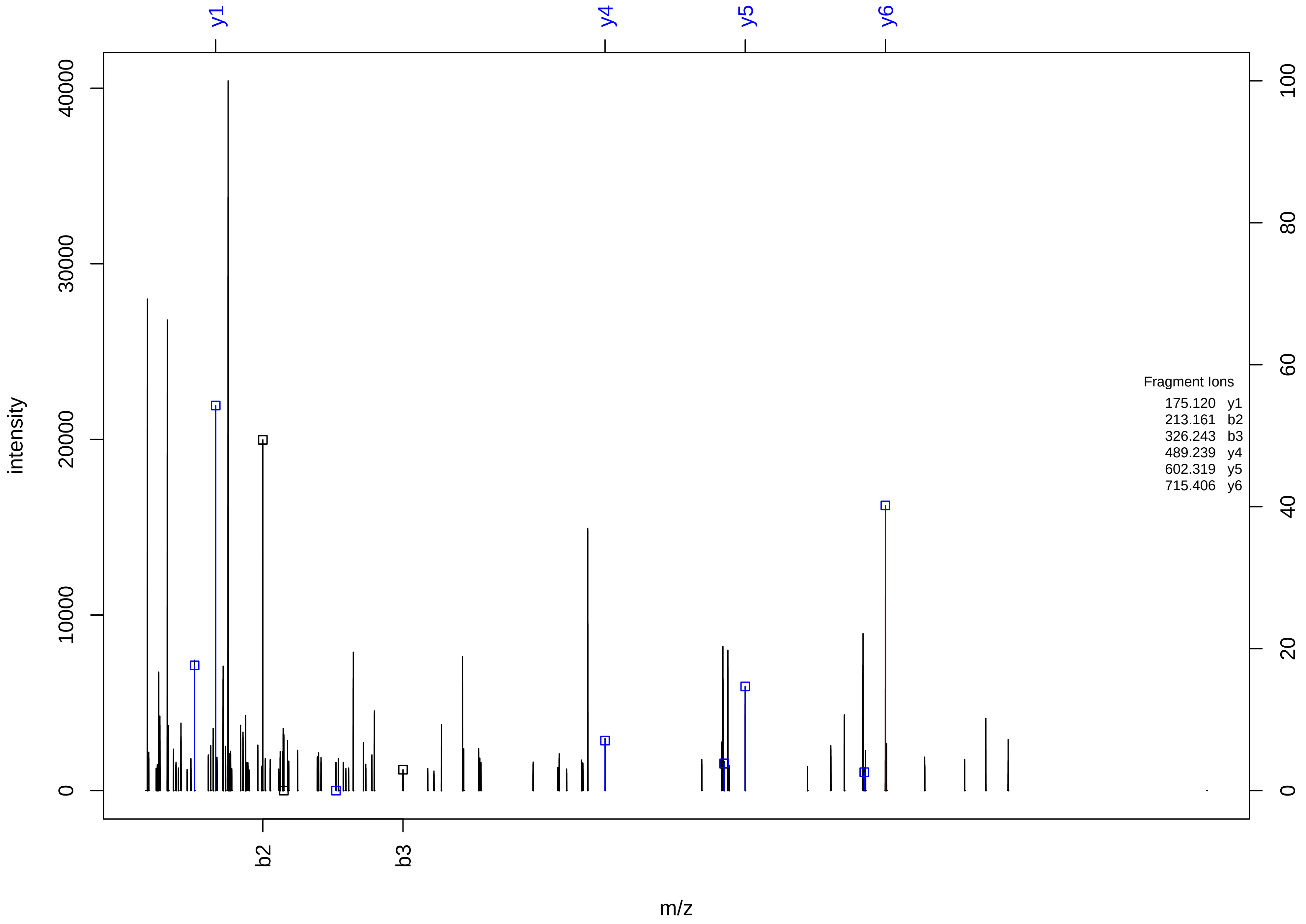

VLLLEANR / File: sydney_26.raw; SpectrumID: ; scans: 3495

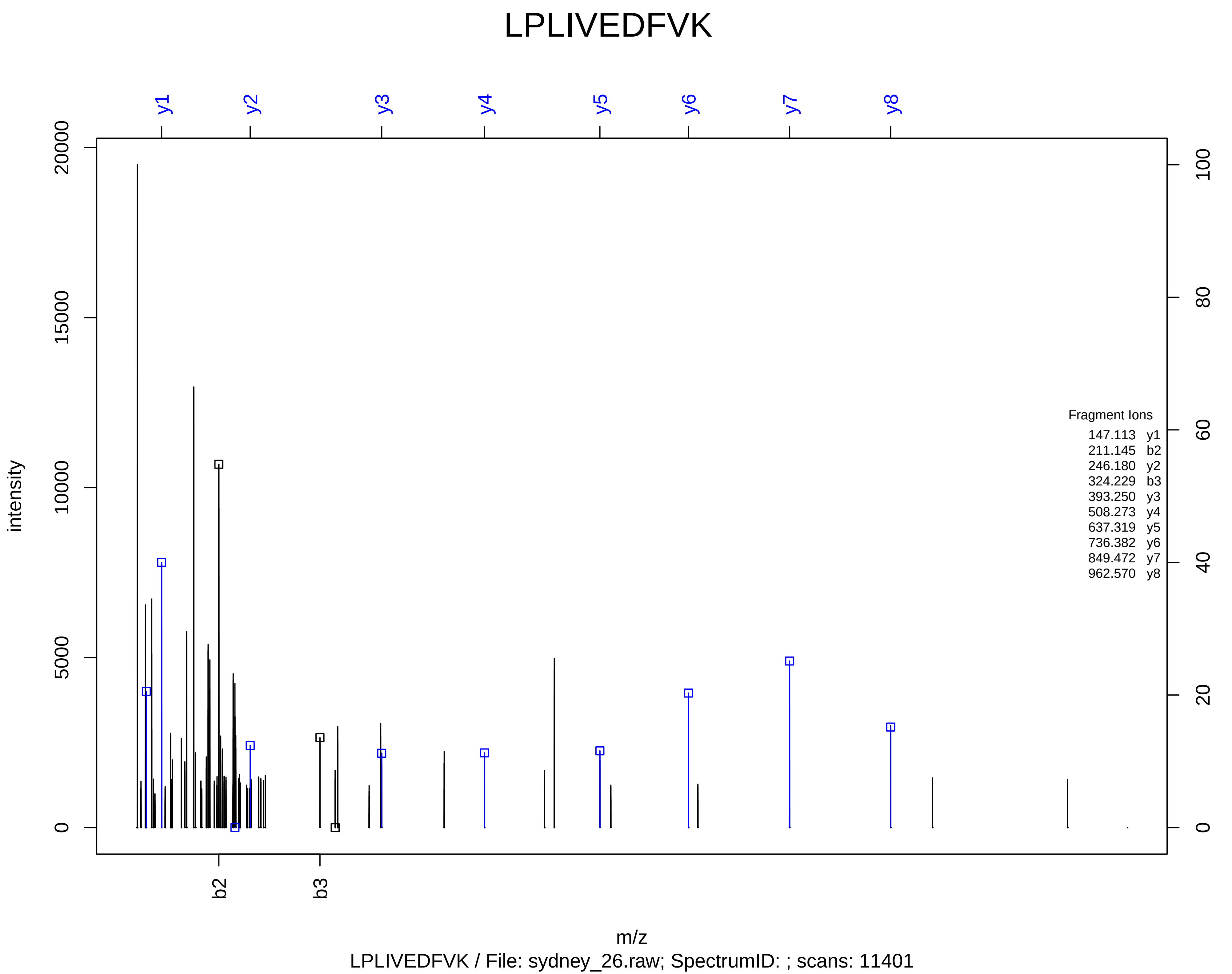
LPLIVEDFVK
y1
y2
y3
y4
y5
y6
y7
y8
b2
b3
intensity
0
5000
10000
15000
20000
0
20
40
60
80
100
Fragment Ions
147.113 y1
211.145 b2
246.180 y2
324.229 b3
393.250 y3
508.273 y4
637.319 y5
736.382 y6
849.472 y7
962.570 y8
m/z
LPLIVEDFVK / File: sydney_26.raw; SpectrumID: ; scans: 11401

DYDWTEPLSPTEDEFK

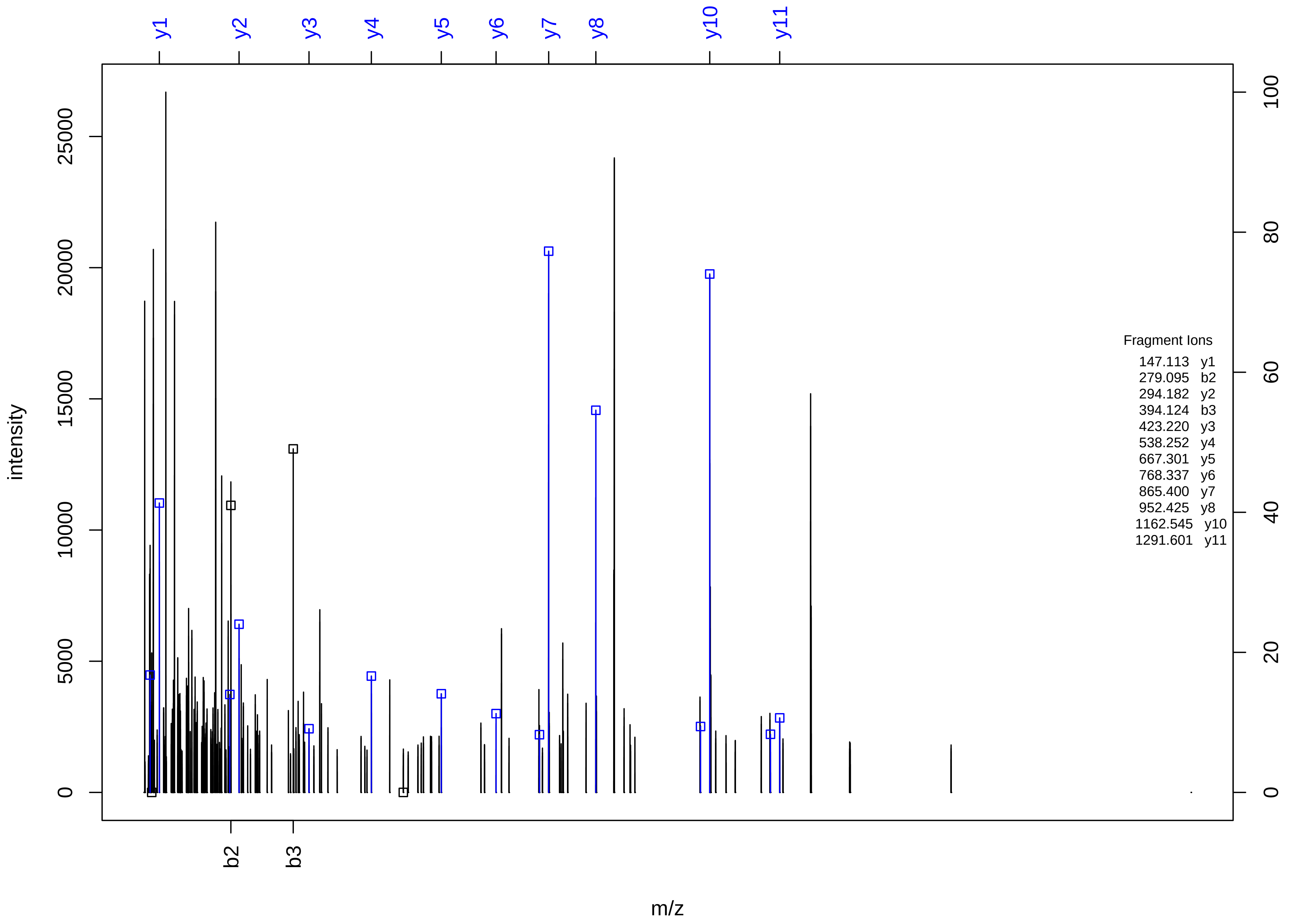

DYDWTEPLSPTEDEFK / File: sydney_26.raw; SpectrumID: ; scans: 9535

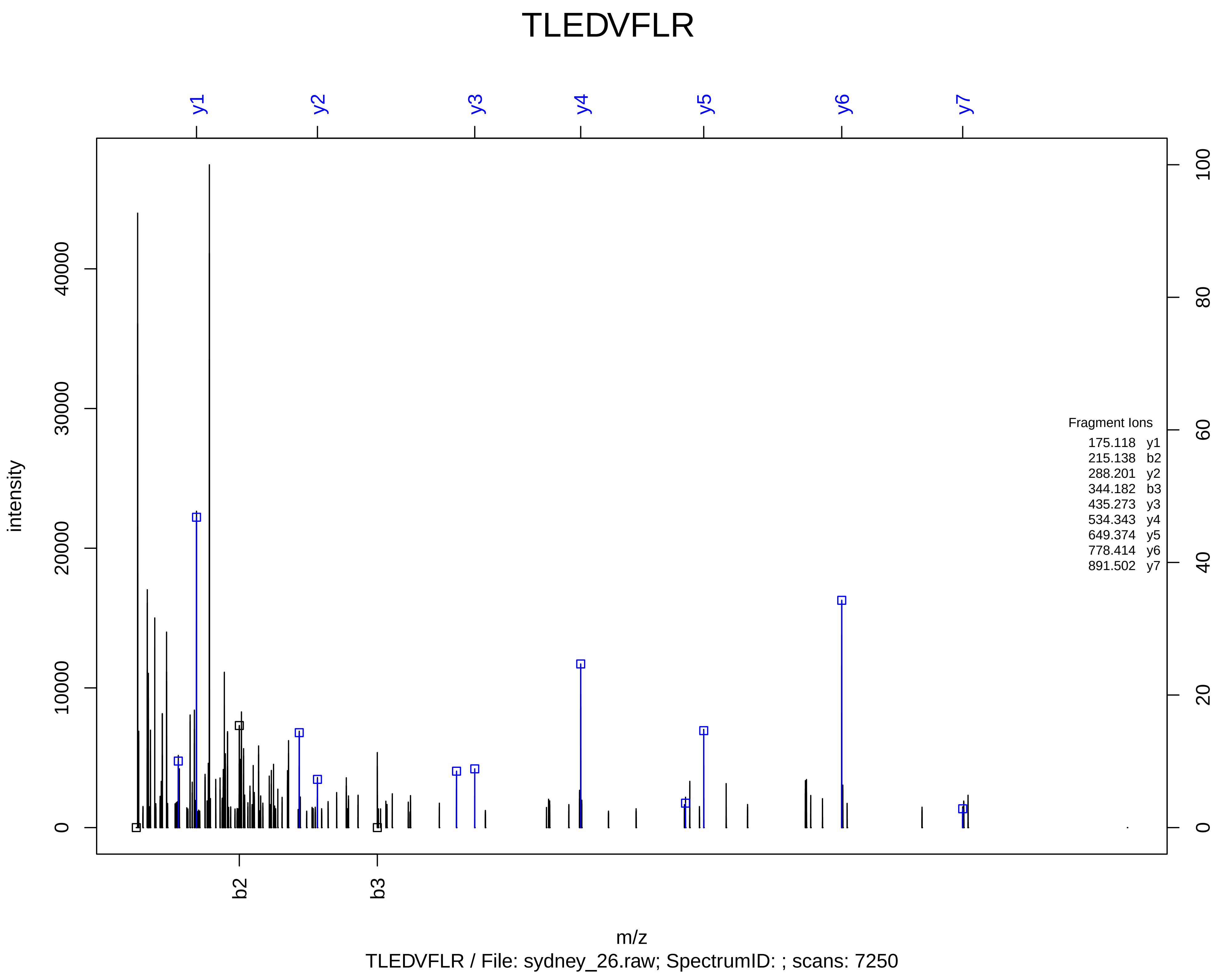
TLEDVFLR
y1
y2
y3
y4
y5
y6
y7
b2
b3
intensity
0
10000
20000
30000
40000
0
20
40
60
80
100
m/z
Fragment Ions
175.118 y1
215.138 b2
288.201 y2
344.182 b3
435.273 y3
534.343 y4
649.374 y5
778.414 y6
891.502 y7
TLEDVFLR / File: sydney_26.raw; SpectrumID: ; scans: 7250

Q^AQ^TLVFTDN^Q^M*DFR

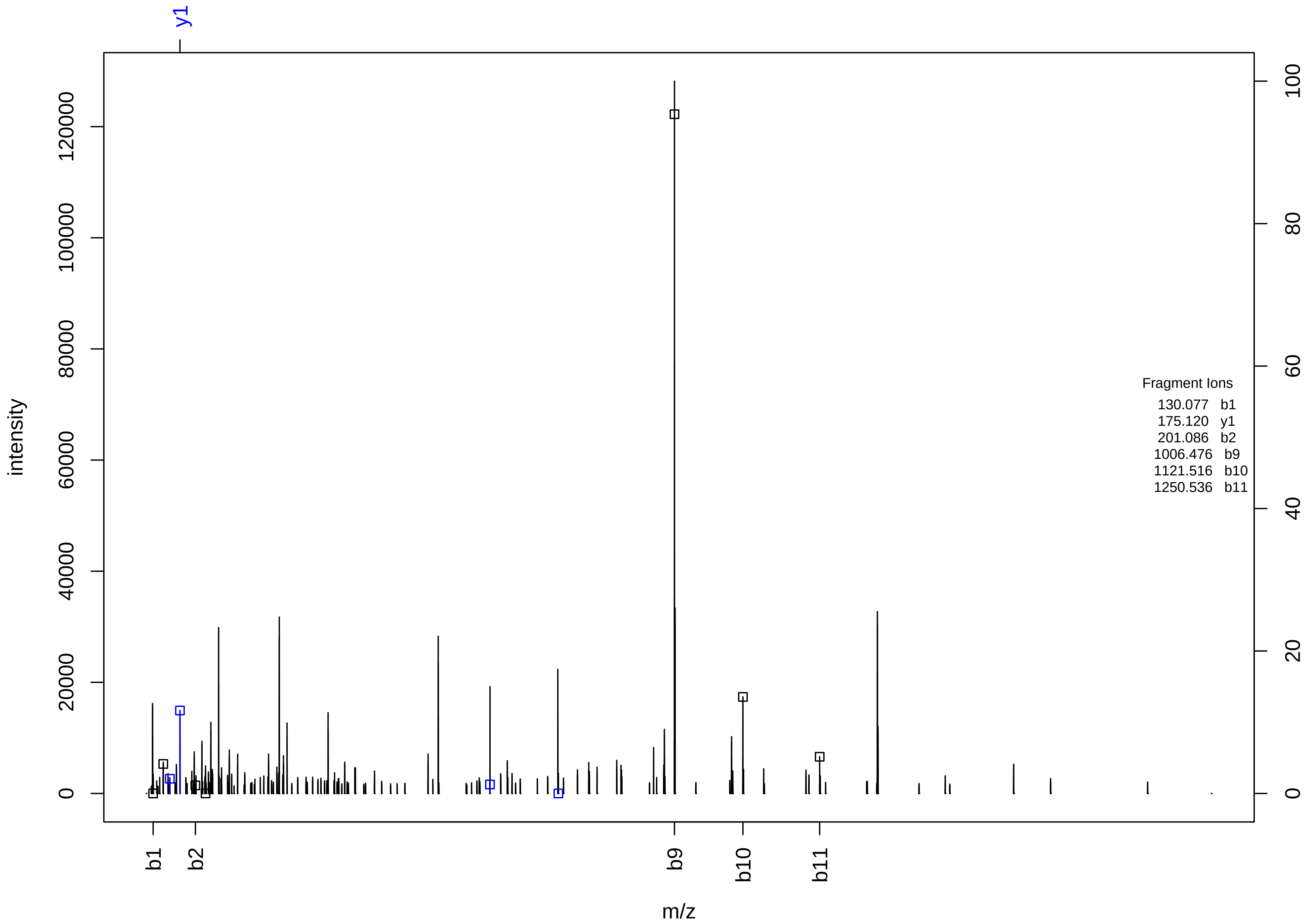

QAQTLVFTDNQMDFR / File: sydney_26.raw; SpectrumID: ; scans: 1943

VLAPDFDDEFDDEEPLPAIGTCK

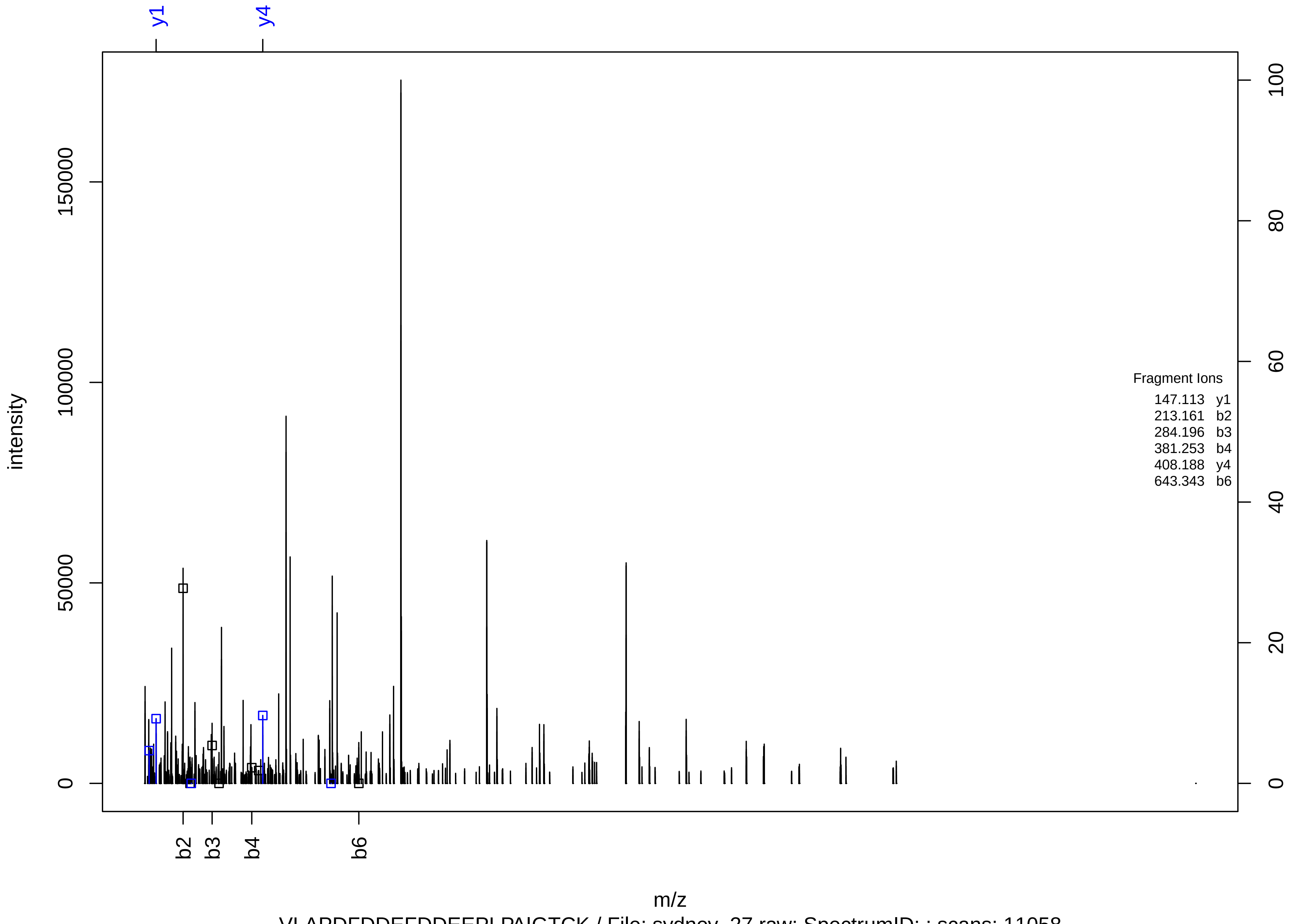

VLAPDFDDEFDDEEPLPAIGTCK / File: sydney_27.raw; SpectrumID: ; scans: 11058

LFQLPTPPLSR

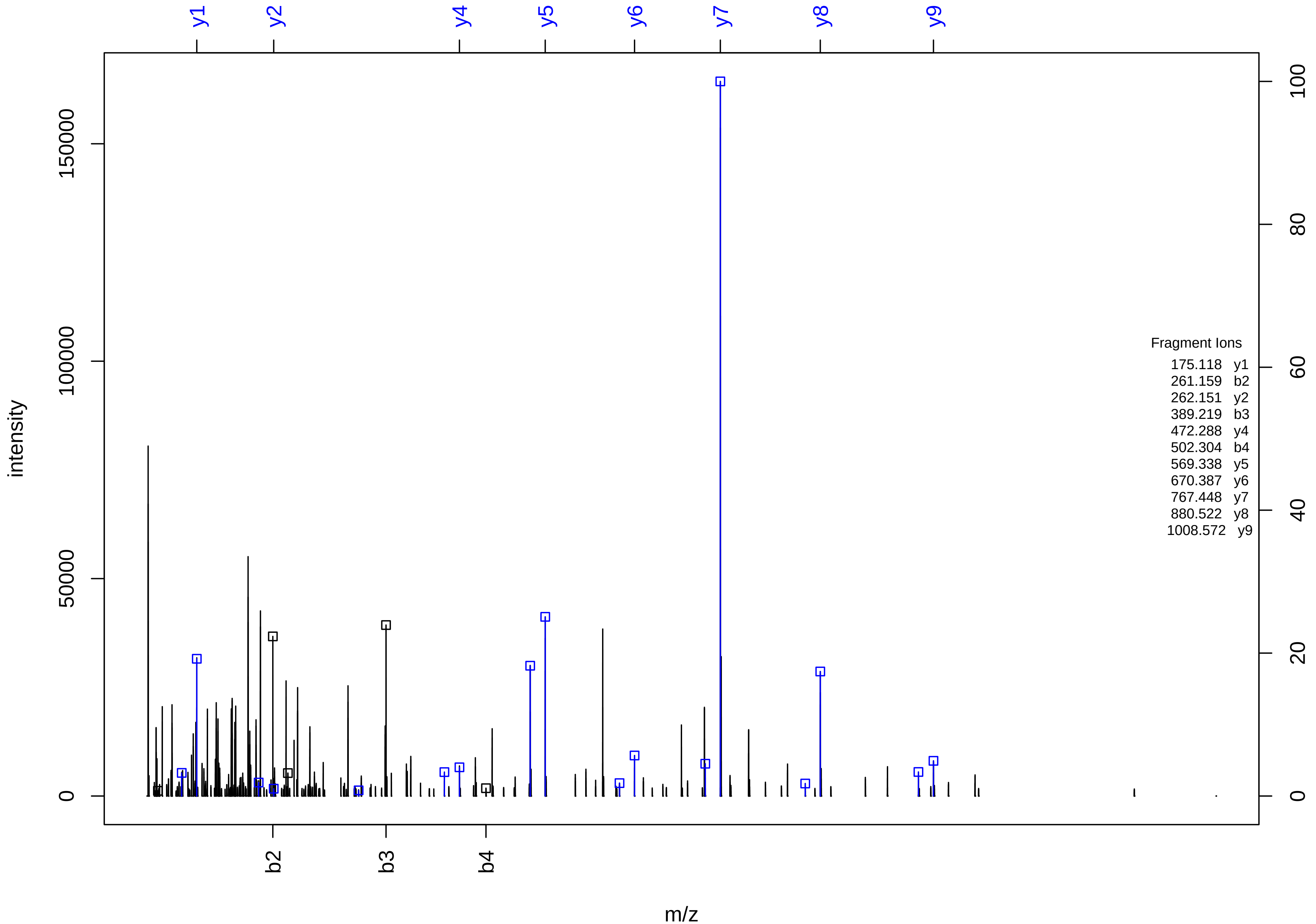

LFQLPTPPLSR / File: sydney_27.raw; SpectrumID: ; scans: 9379

TLWLFDINQDPEER

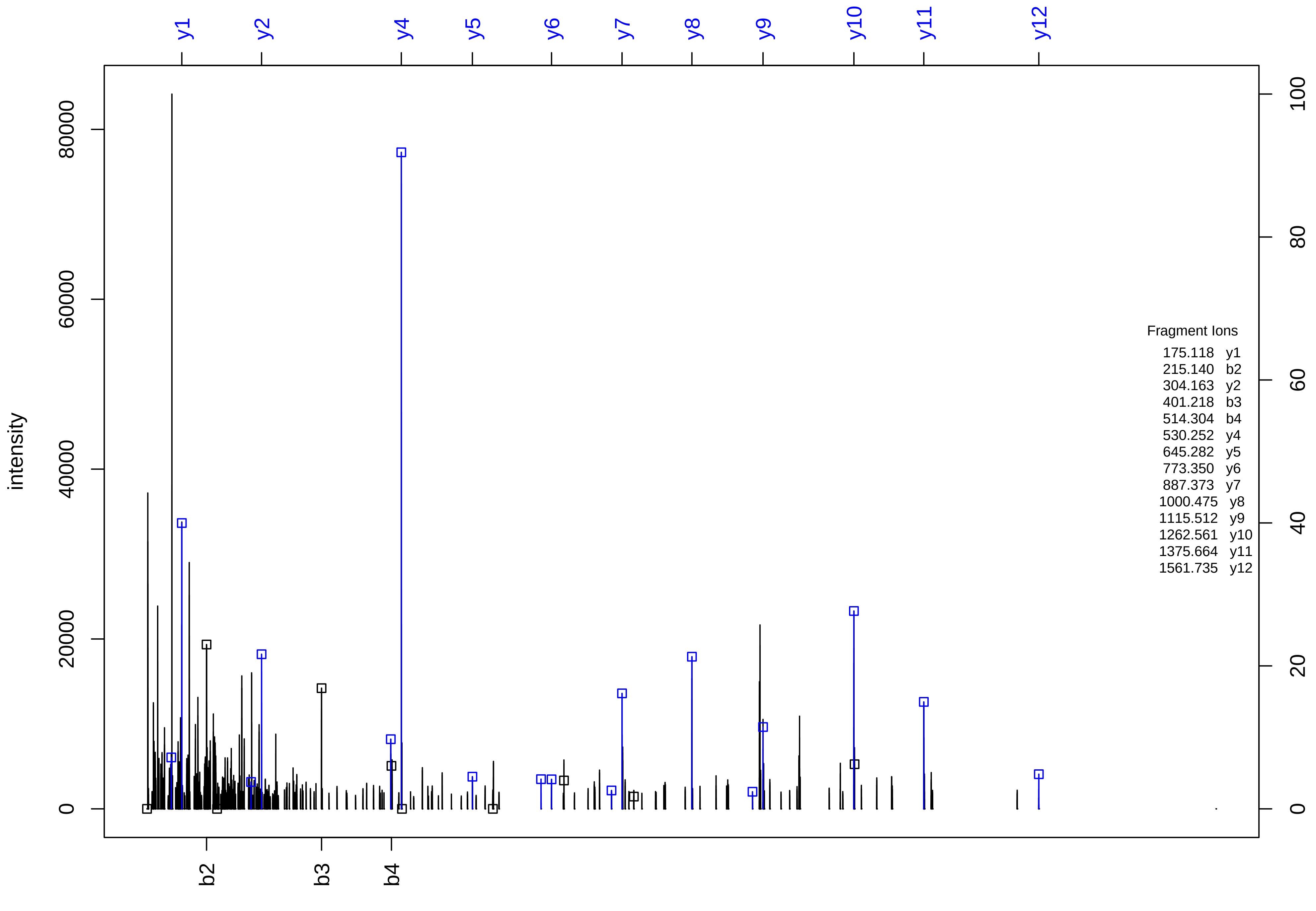

TLWLFDINQDPEER / File: sydney_27.raw; SpectrumID: ; scans: 12089

KELIEDLTSM*Q^M*QKR

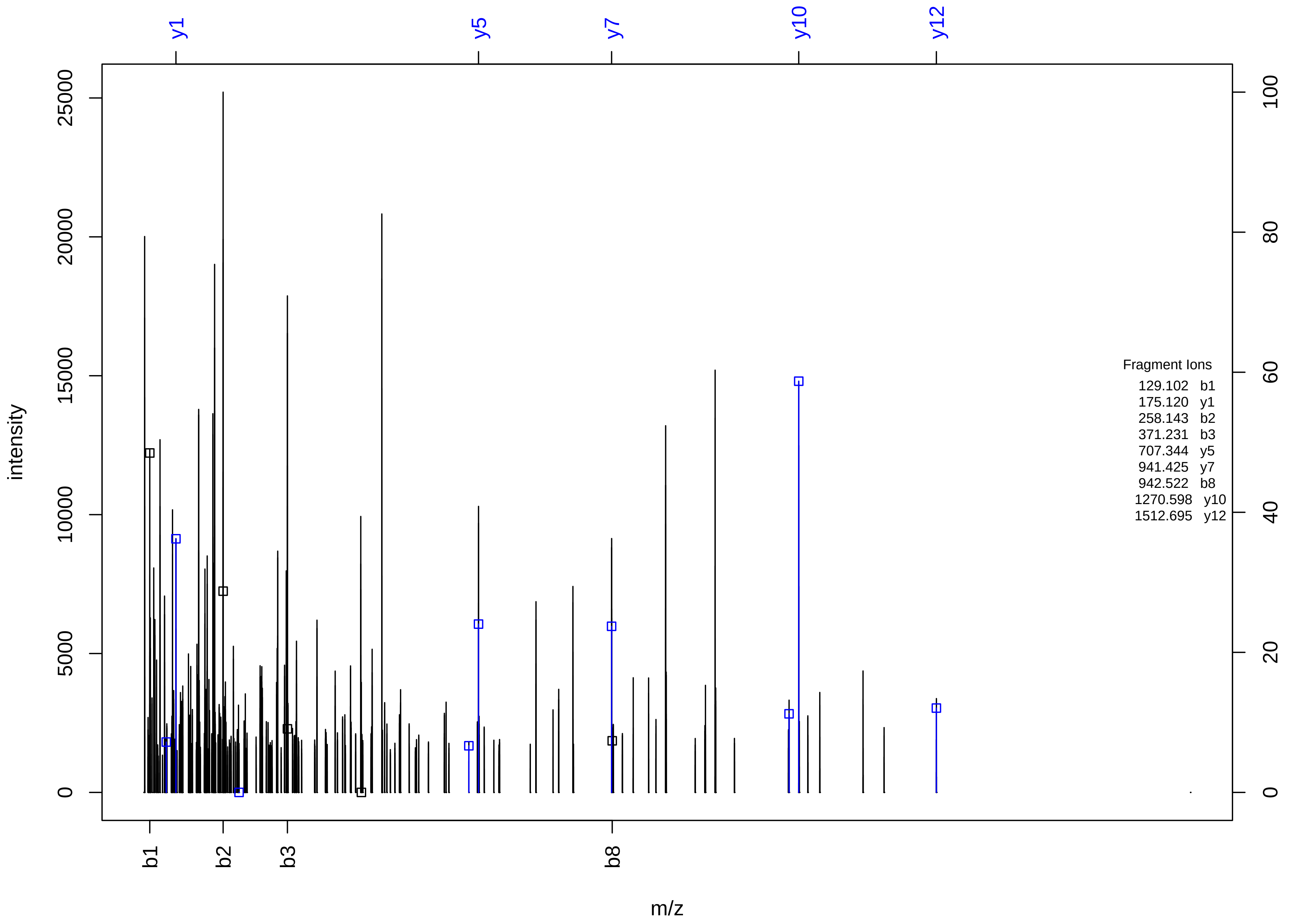

KELIEDLTSMQMQKR / File: sydney_27.raw; SpectrumID: ; scans: 7348

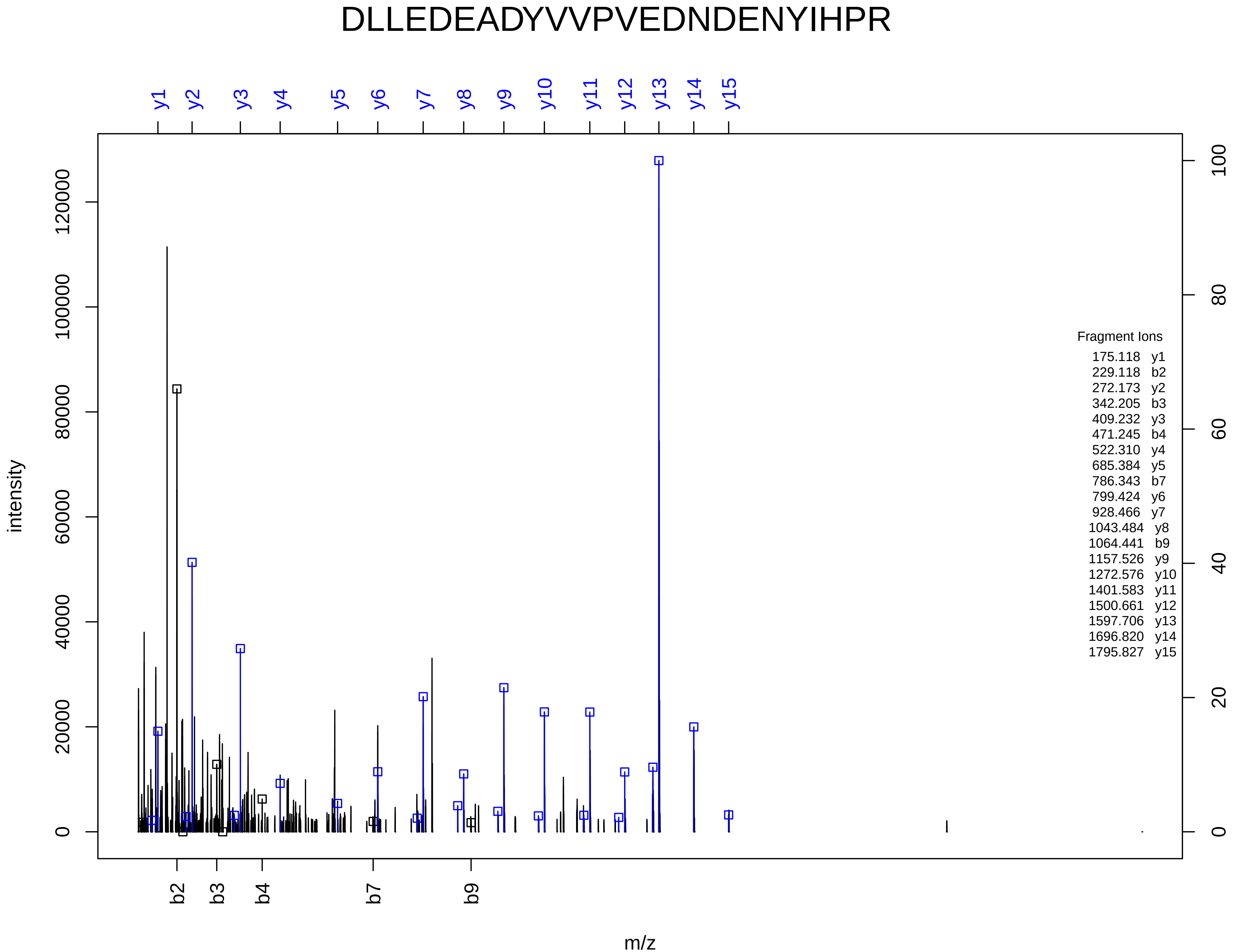

DLLEDEADYVVPVEDNDENYIHPR
y1 y2 y3 y4 y5 y6 y7 y8 y9 y10 y11 y12 y13 y14 y15
intensity
0 20000 40000 60000 80000 100000 120000
0 20 40 60 80 100
b2 b3 b4 b7 b9
m/z
Fragment Ions
175.118 y1
229.118 b2
272.173 y2
342.205 b3
409.232 y3
471.245 b4
522.310 y4
685.384 y5
786.343 b7
799.424 y6
928.466 y7
1043.484 y8
1064.441 b9
1157.526 y9
1272.576 y10
1401.583 y11
1500.661 y12
1597.706 y13
1696.820 y14
1795.827 y15
DLLEDEADYVVPVEDNDENYIHPR / File: sydney_27.raw; SpectrumID: ; scans: 9542

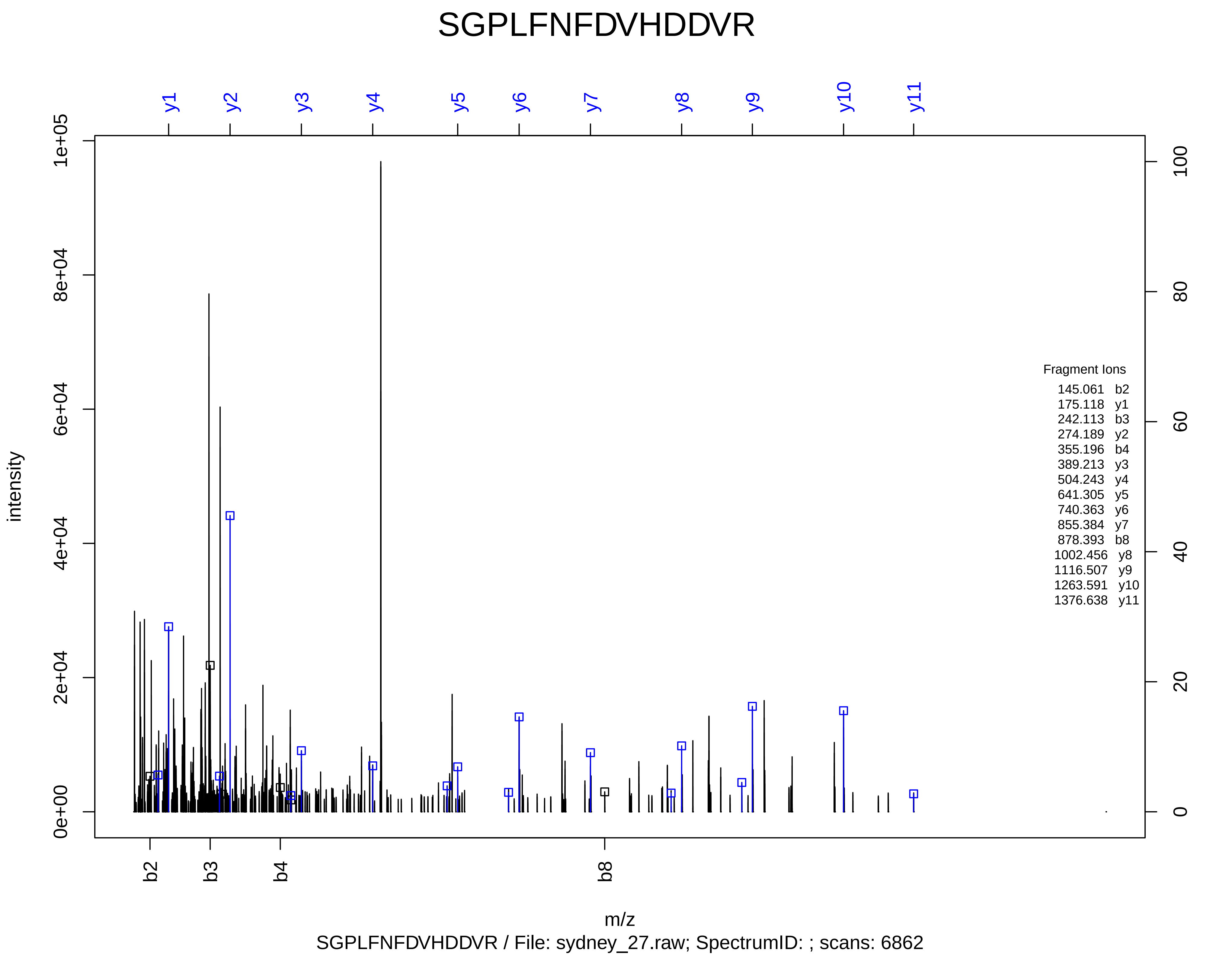
SGPLFNFDVHDDVR
y1
y2
y3
y4
y5
y6
y7
y8
y9
y10
y11
b2
b3
b4
b8
intensity
m/z
Fragment Ions
145.061 b2
175.118 y1
242.113 b3
274.189 y2
355.196 b4
389.213 y3
504.243 y4
641.305 y5
740.363 y6
855.384 y7
878.393 b8
1002.456 y8
1116.507 y9
1263.591 y10
1376.638 y11
SGPLFNFDVHDDVR / File: sydney_27.raw; SpectrumID: ; scans: 6862

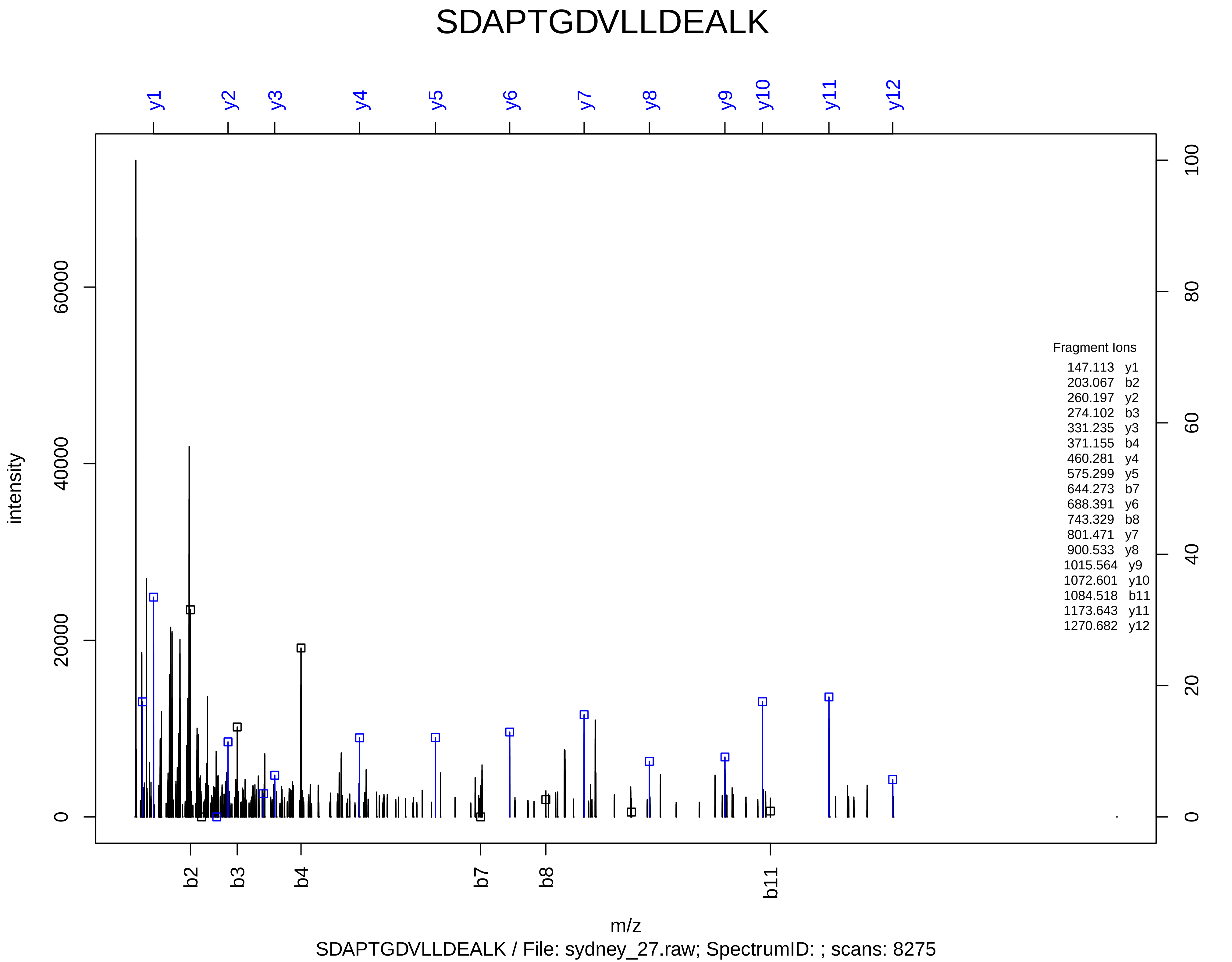
SDAPTGDVLLDEALK
y1
y2
y3
y4
y5
y6
y7
y8
y9
y10
y11
y12
intensity
0
20000
40000
60000
0
20
40
60
80
100
b2
b3
b4
b7
b8
b11
m/z
Fragment Ions
147.113 y1
203.067 b2
260.197 y2
274.102 b3
331.235 y3
371.155 b4
460.281 y4
575.299 y5
644.273 b7
688.391 y6
743.329 b8
801.471 y7
900.533 y8
1015.564 y9
1072.601 y10
1084.518 b11
1173.643 y11
1270.682 y12
SDAPTGDVLLDEALK / File: sydney_27.raw; SpectrumID: ; scans: 8275

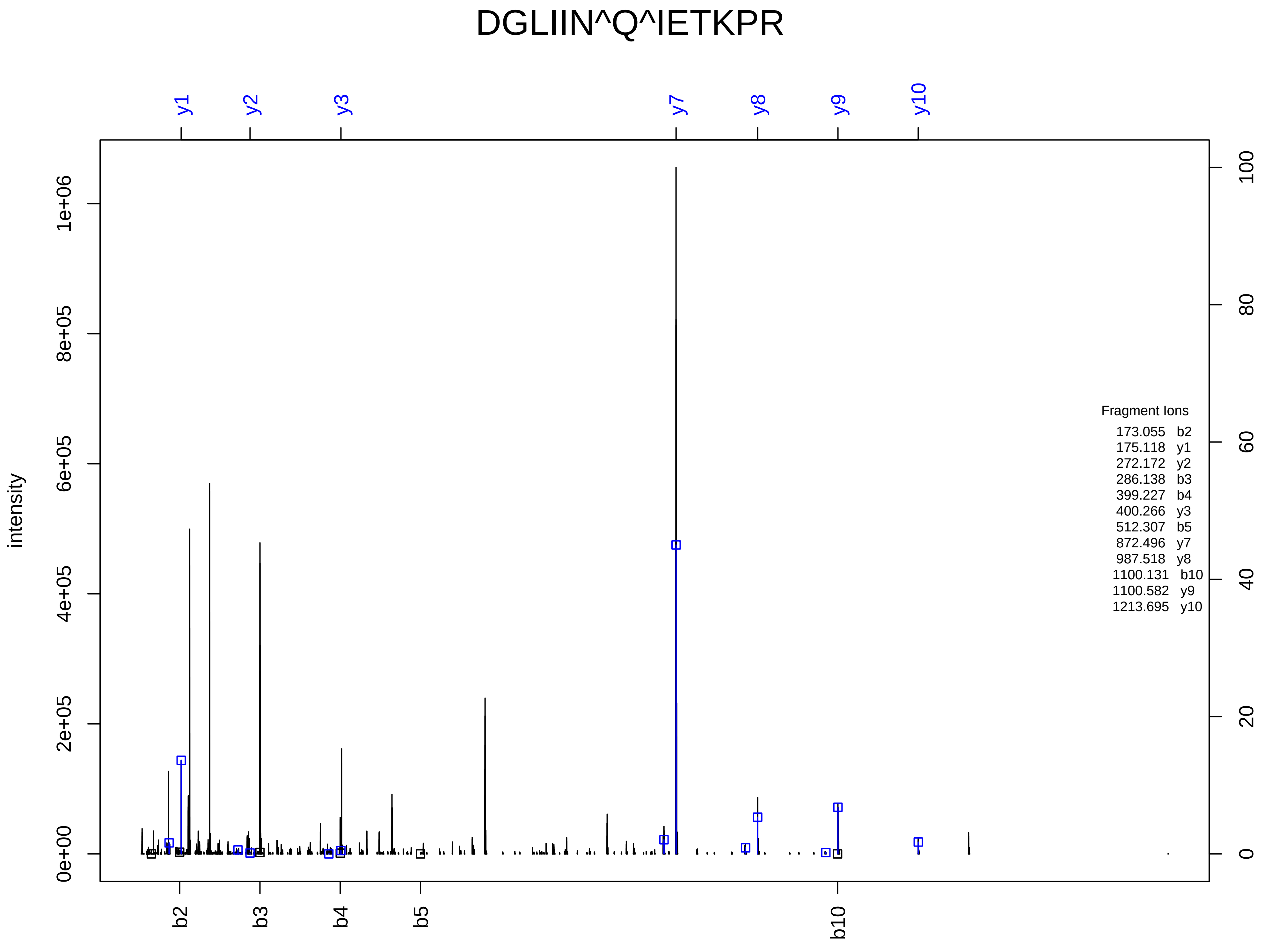

DGLIINQIETKPR / File: sydney_27.raw; SpectrumID: ; scans: 8795

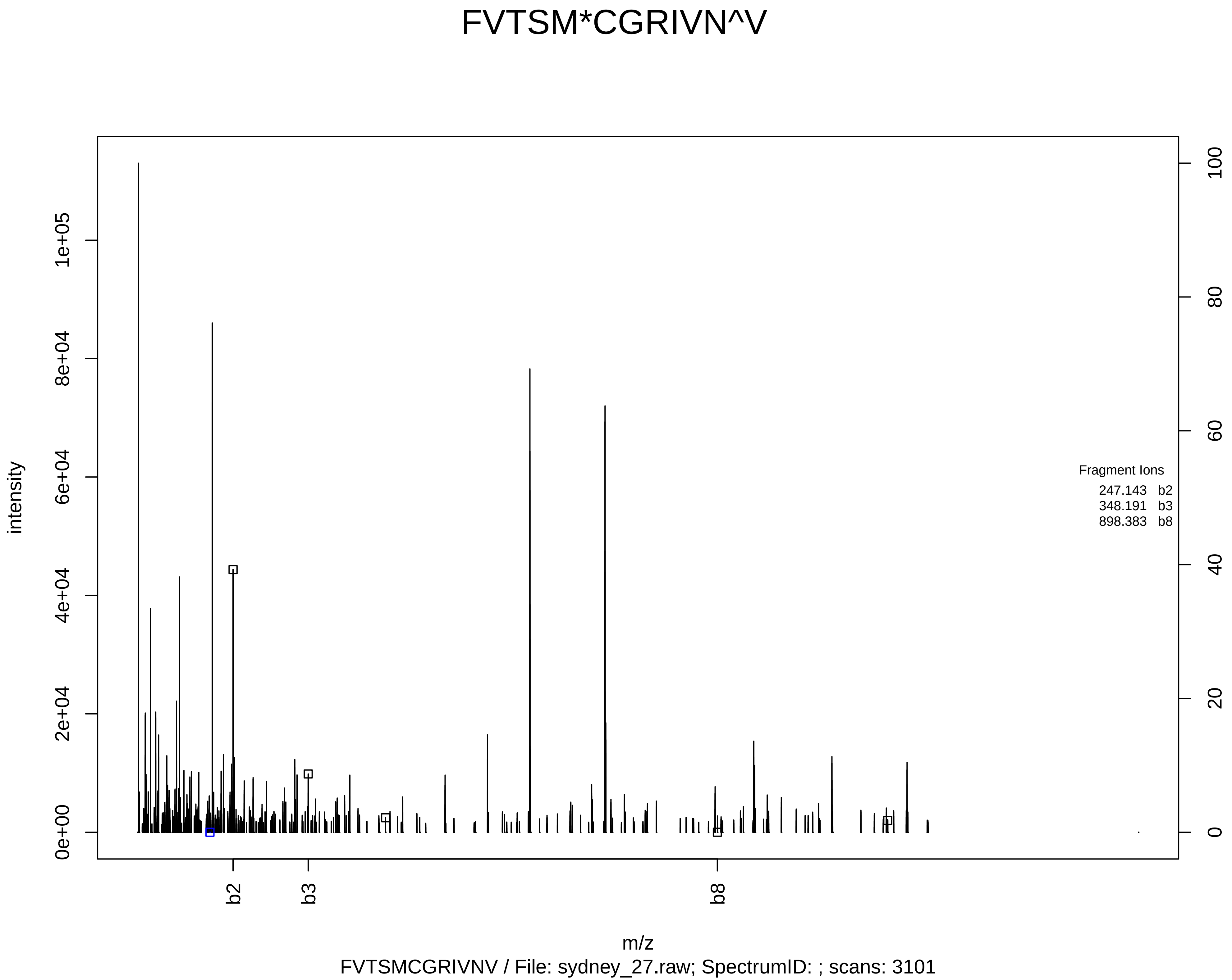

FVTSM*CGRIVN^V
intensity
0e+00
2e+04
4e+04
6e+04
8e+04
1e+05
0
20
40
60
80
100
Fragment Ions
247.143 b2
348.191 b3
898.383 b8
b2
b3
b8
m/z
FVTSMCGRIVNV / File: sydney_27.raw; SpectrumID: ; scans: 3101

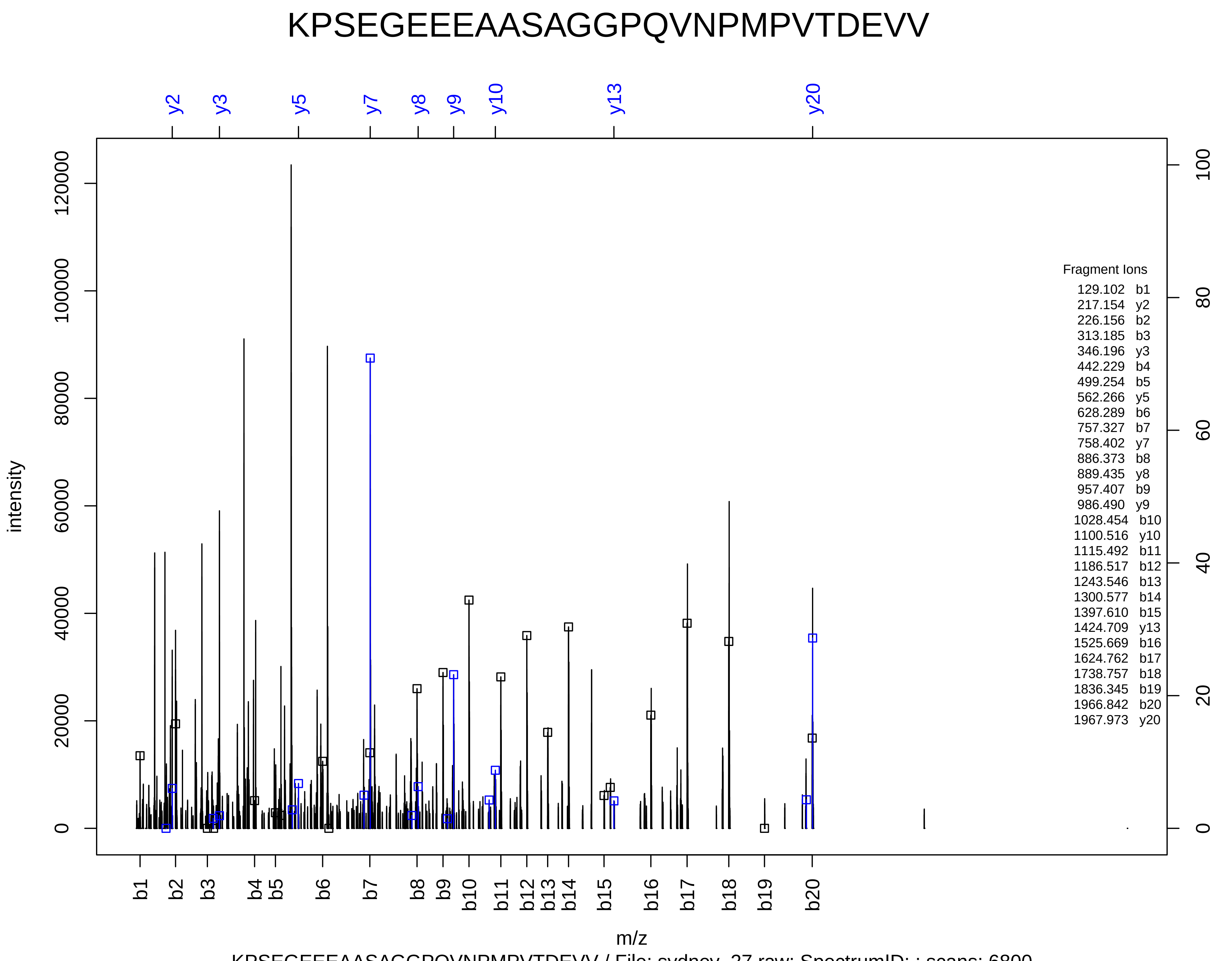
KPSEGEEEAASAGGPQVNPMPVTDEVV
y2 y3 y5 y7 y8 y9 y10 y13 y20
intensity
0 20000 40000 60000 80000 100000 120000
0 20 40 60 80 100
Fragment Ions
129.102 b1
217.154 y2
226.156 b2
313.185 b3
346.196 y3
442.229 b4
499.254 b5
562.266 y5
628.289 b6
757.327 b7
758.402 y7
886.373 b8
889.435 y8
957.407 b9
986.490 y9
1028.454 b10
1100.516 y10
1115.492 b11
1186.517 b12
1243.546 b13
1300.577 b14
1397.610 b15
1424.709 y13
1525.669 b16
1624.762 b17
1738.757 b18
1836.345 b19
1966.842 b20
1967.973 y20
b1 b2 b3 b4 b5 b6 b7 b8 b9 b10 b11 b12 b13 b14 b15 b16 b17 b18 b19 b20
m/z
KPSEGEEEAASAGGPQVNPMPVTDEVV / File: sydney_27.raw; SpectrumID: ; scans: 6800

DTLWGLFNNLQR

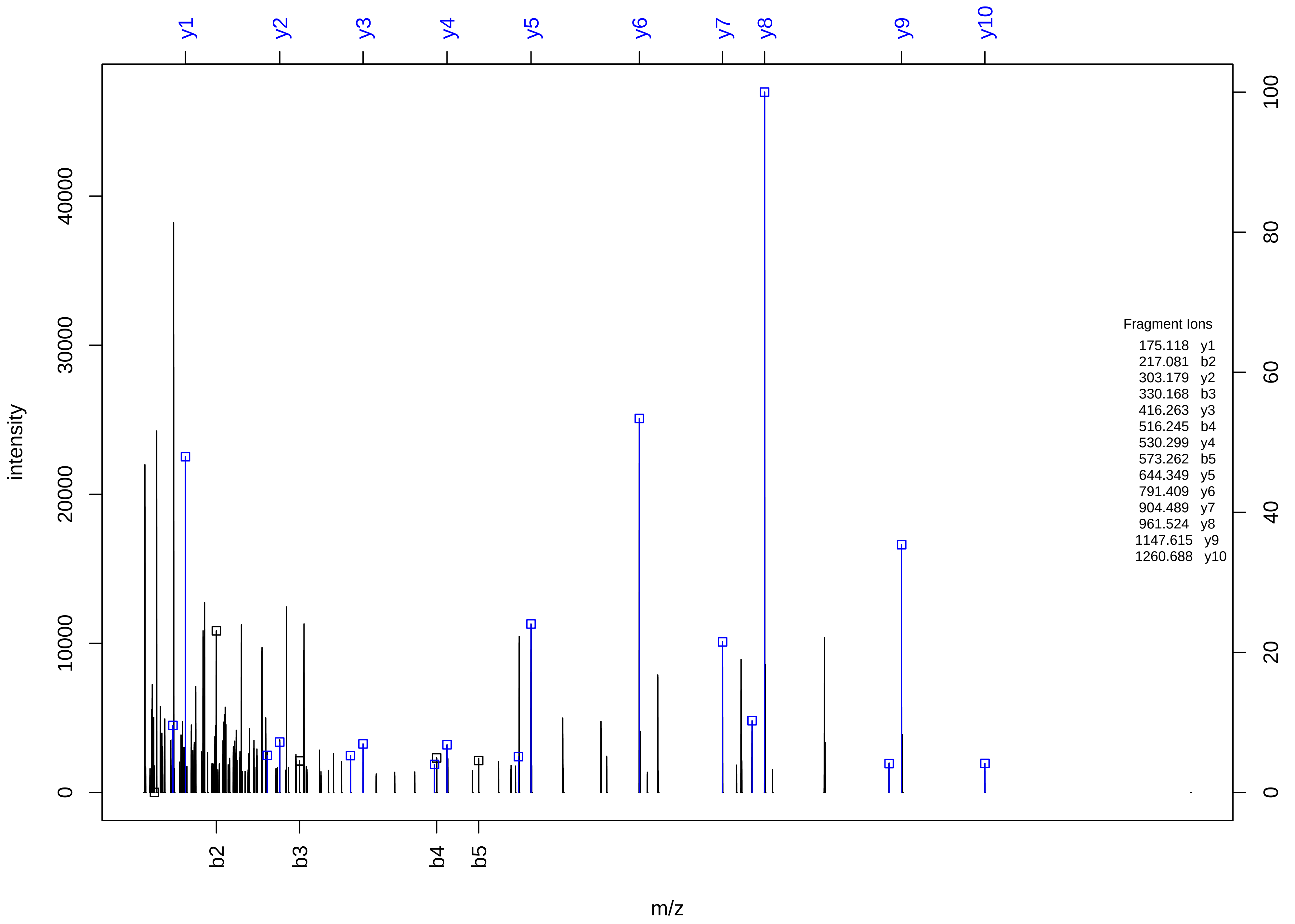

DTLWGLFNNLQR / File: sydney_27.raw; SpectrumID: ; scans: 13397

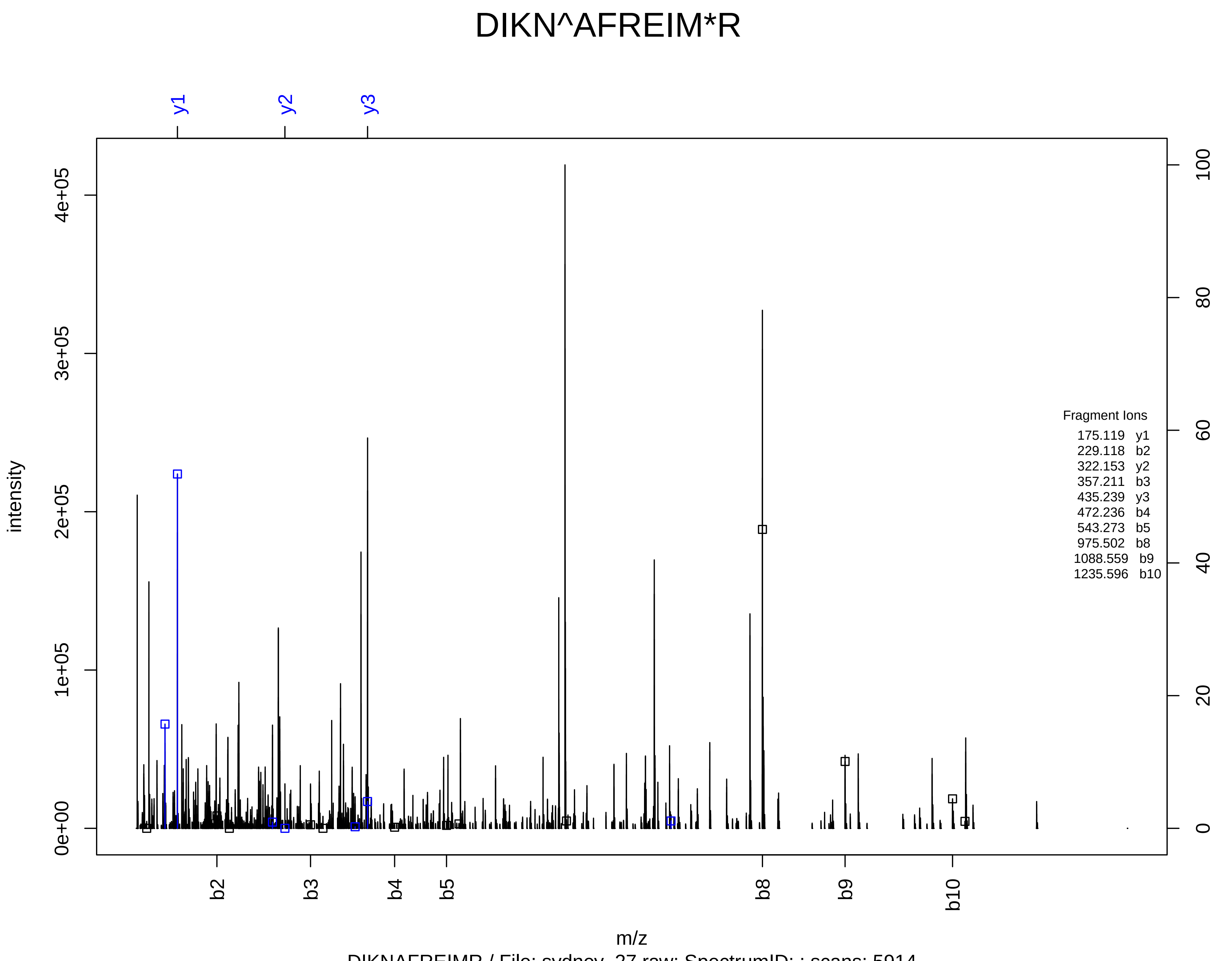

DIKN^AFREIM*R
y1
y2
y3
intensity
0e+00
1e+05
2e+05
3e+05
4e+05
0
20
40
60
80
100
Fragment Ions
175.119 y1
229.118 b2
322.153 y2
357.211 b3
435.239 y3
472.236 b4
543.273 b5
975.502 b8
1088.559 b9
1235.596 b10
b2
b3
b4
b5
b8
b9
b10
m/z
DIKNAFREIMR / File: sydney_27.raw; SpectrumID: ; scans: 5914

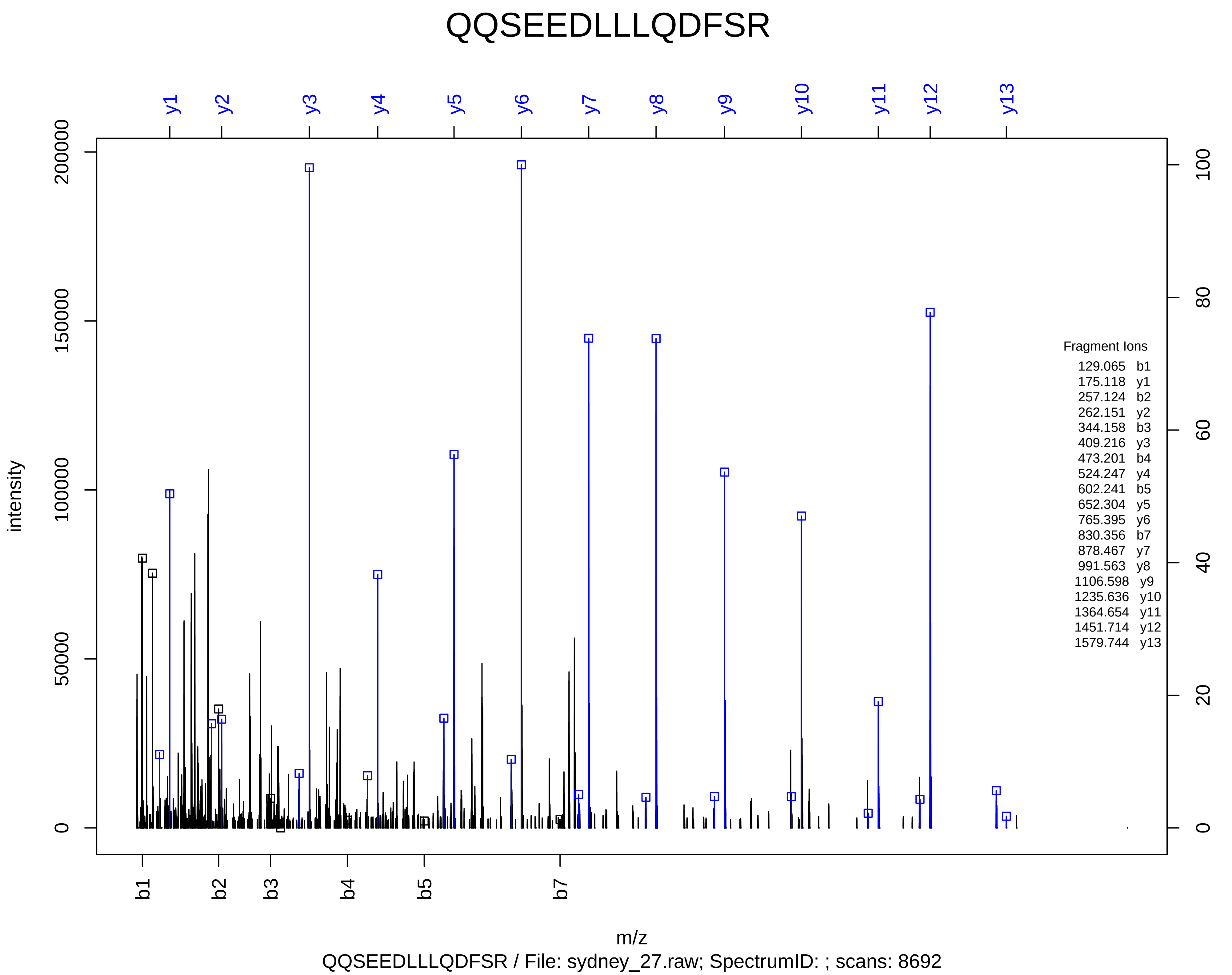
QQSEEDLLLQDFSR
y1 y2 y3 y4 y5 y6 y7 y8 y9 y10 y11 y12 y13
b1 b2 b3 b4 b5 b7
intensity
m/z
Fragment Ions
129.065 b1
175.118 y1
257.124 b2
262.151 y2
344.158 b3
409.216 y3
473.201 b4
524.247 y4
602.241 b5
652.304 y5
765.395 y6
830.356 b7
878.467 y7
991.563 y8
1106.598 y9
1235.636 y10
1364.654 y11
1451.714 y12
1579.744 y13
QQSEEDLLLQDFSR / File: sydney_27.raw; SpectrumID: ; scans: 8692

QLLLQ^LN^Q^Q^R

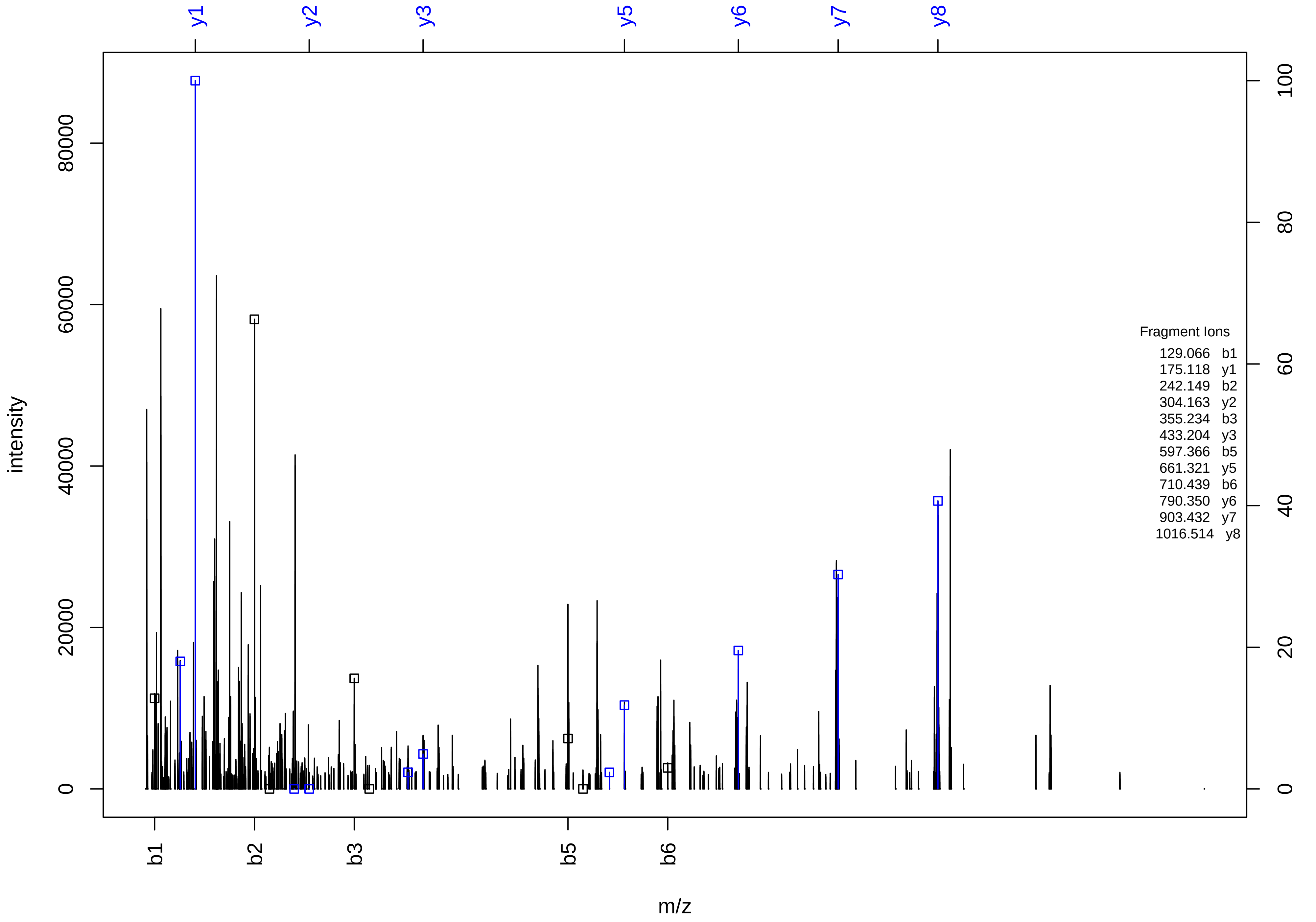

QLLLQLNQQR / File: sydney_27.raw; SpectrumID: ; scans: 6411

(Ac)AAETLNFGPEWLR

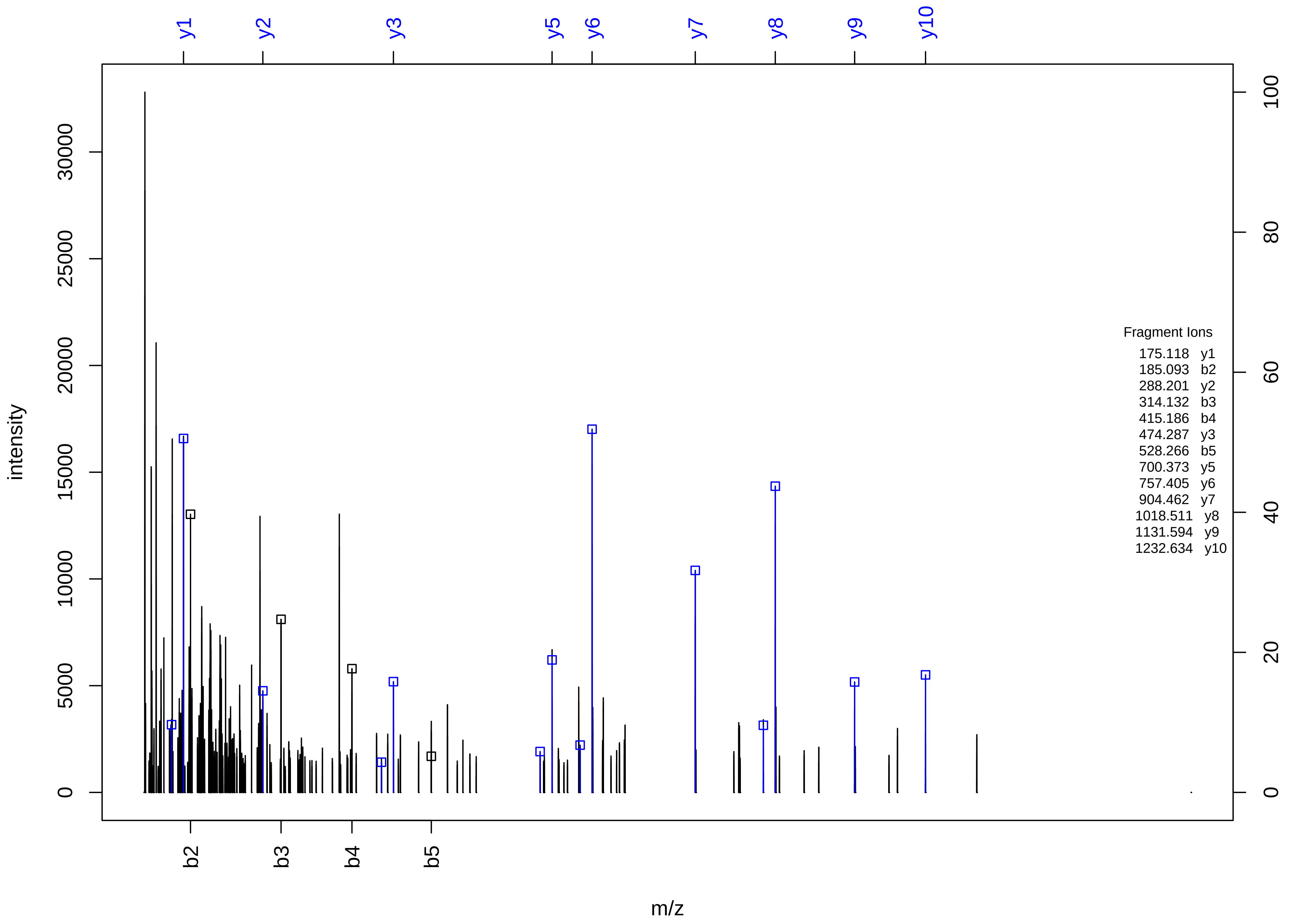

AAETLNFGPEWLR / File: sydney_28.raw; SpectrumID: ; scans: 14261

LAALFSTAEPSAPPVFVPVPQETSK

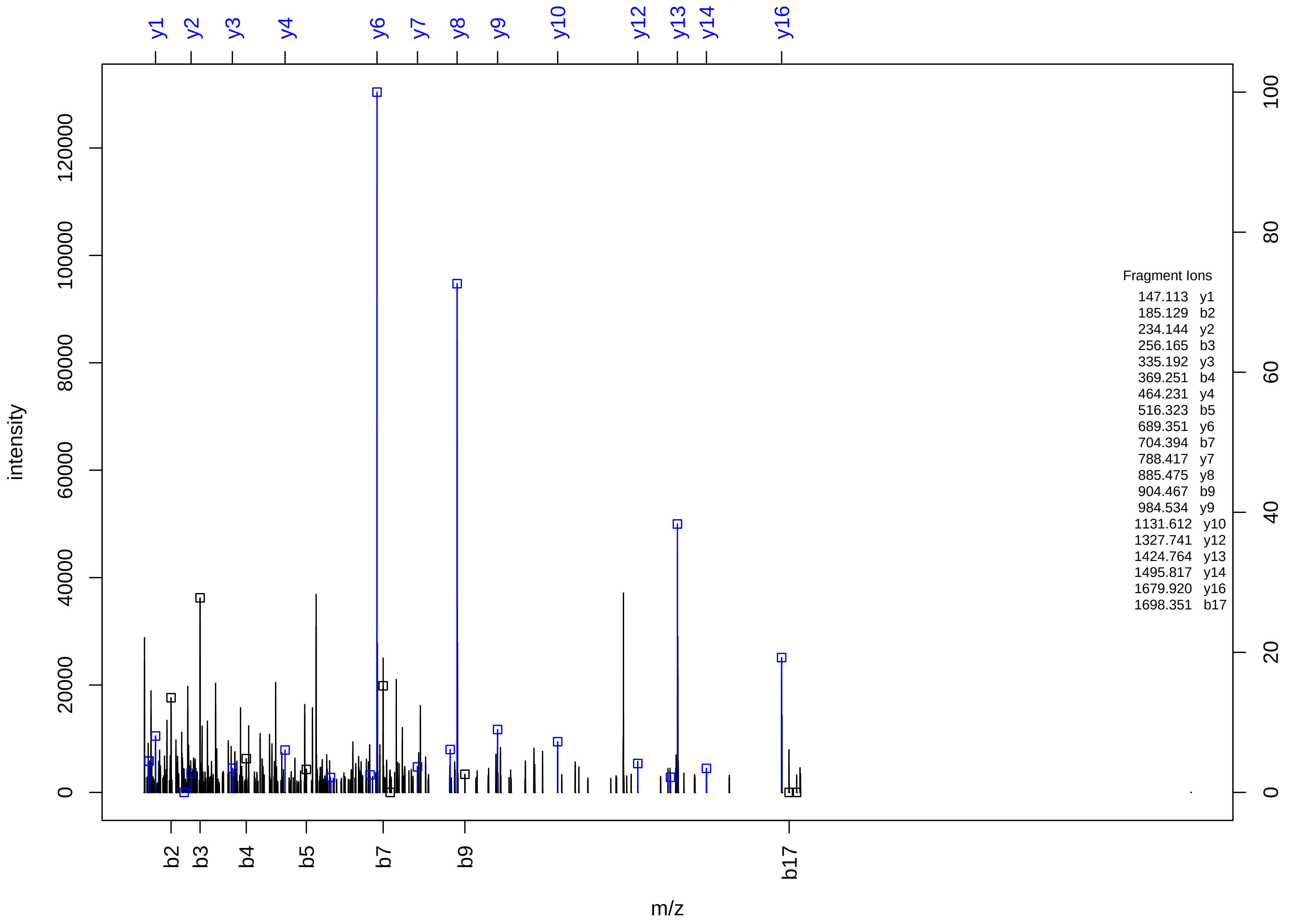

LAALFSTAEPSAPPVFVPVPQETSK / File: sydney_28.raw; SpectrumID: ; scans: 12959

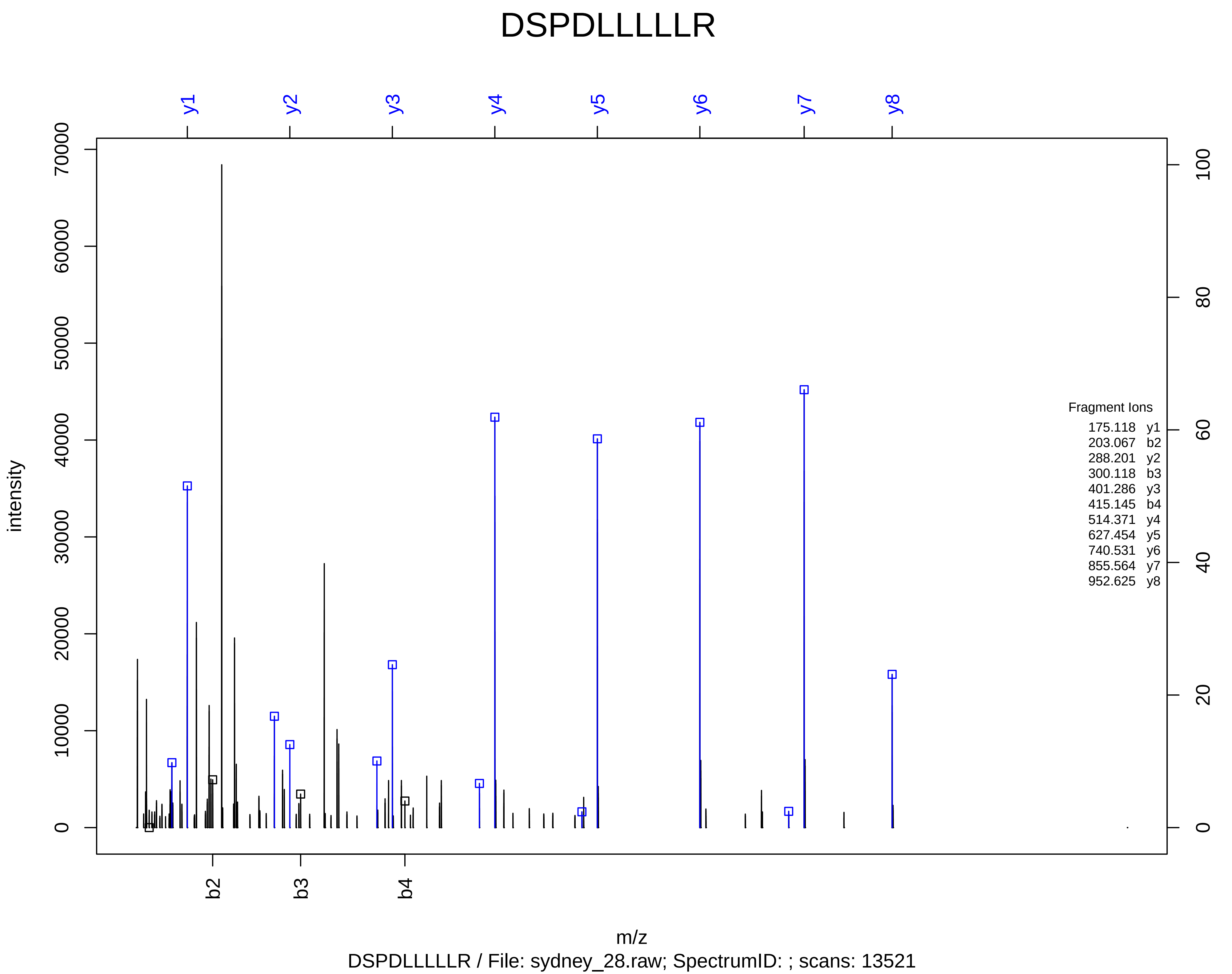
DSPDLLLLLR
y1
y2
y3
y4
y5
y6
y7
y8
b2
b3
b4
intensity
m/z
Fragment Ions
175.118 y1
203.067 b2
288.201 y2
300.118 b3
401.286 y3
415.145 b4
514.371 y4
627.454 y5
740.531 y6
855.564 y7
952.625 y8
0
10000
20000
30000
40000
50000
60000
70000
0
20
40
60
80
100
DSPDLLLLLR / File: sydney_28.raw; SpectrumID: ; scans: 13521

RQ^VSAESSSSM*N^SN^TPLVR

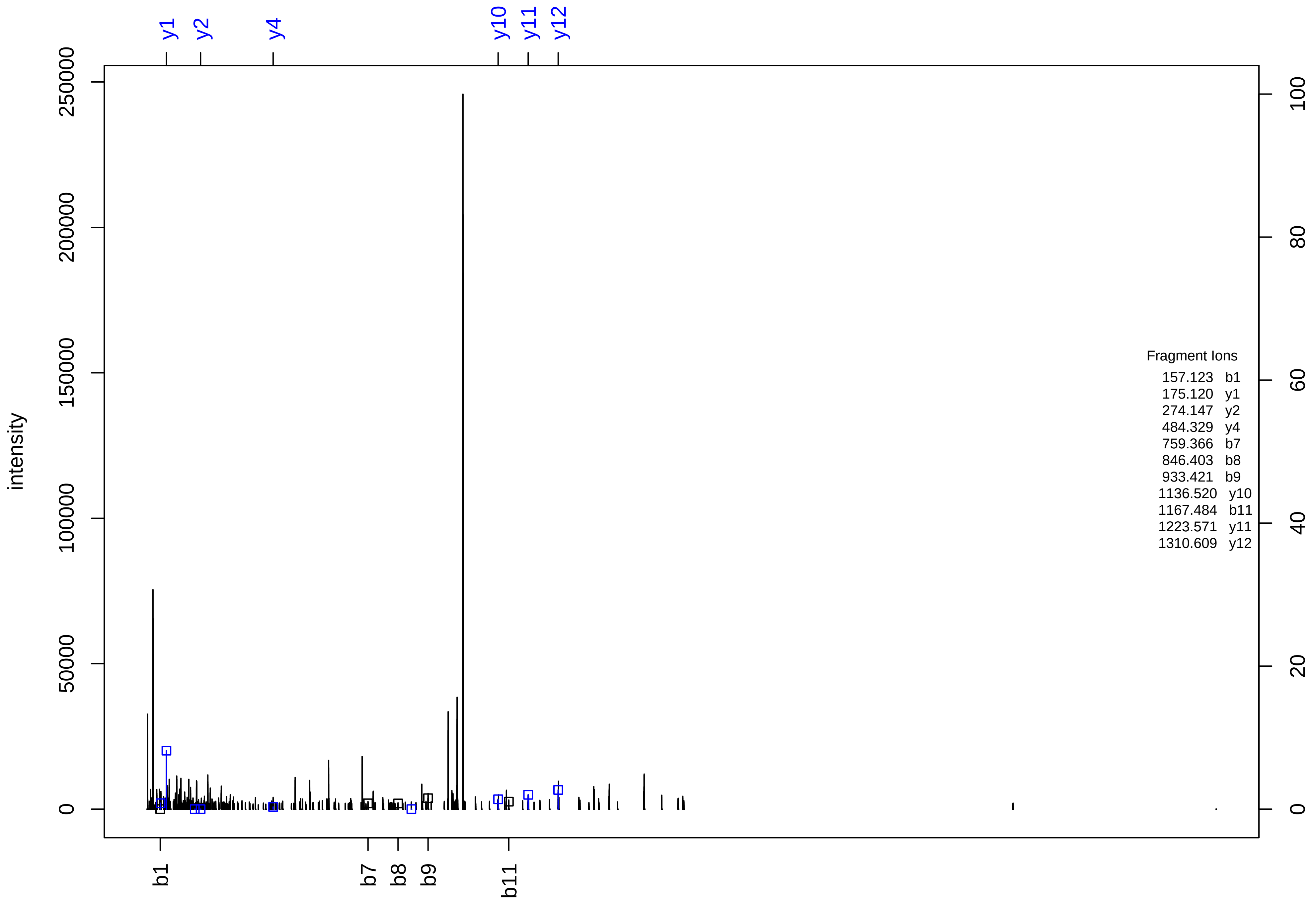

RQVSAESSSSMNSNTPLVR / File: sydney_28.raw; SpectrumID: ; scans: 13957

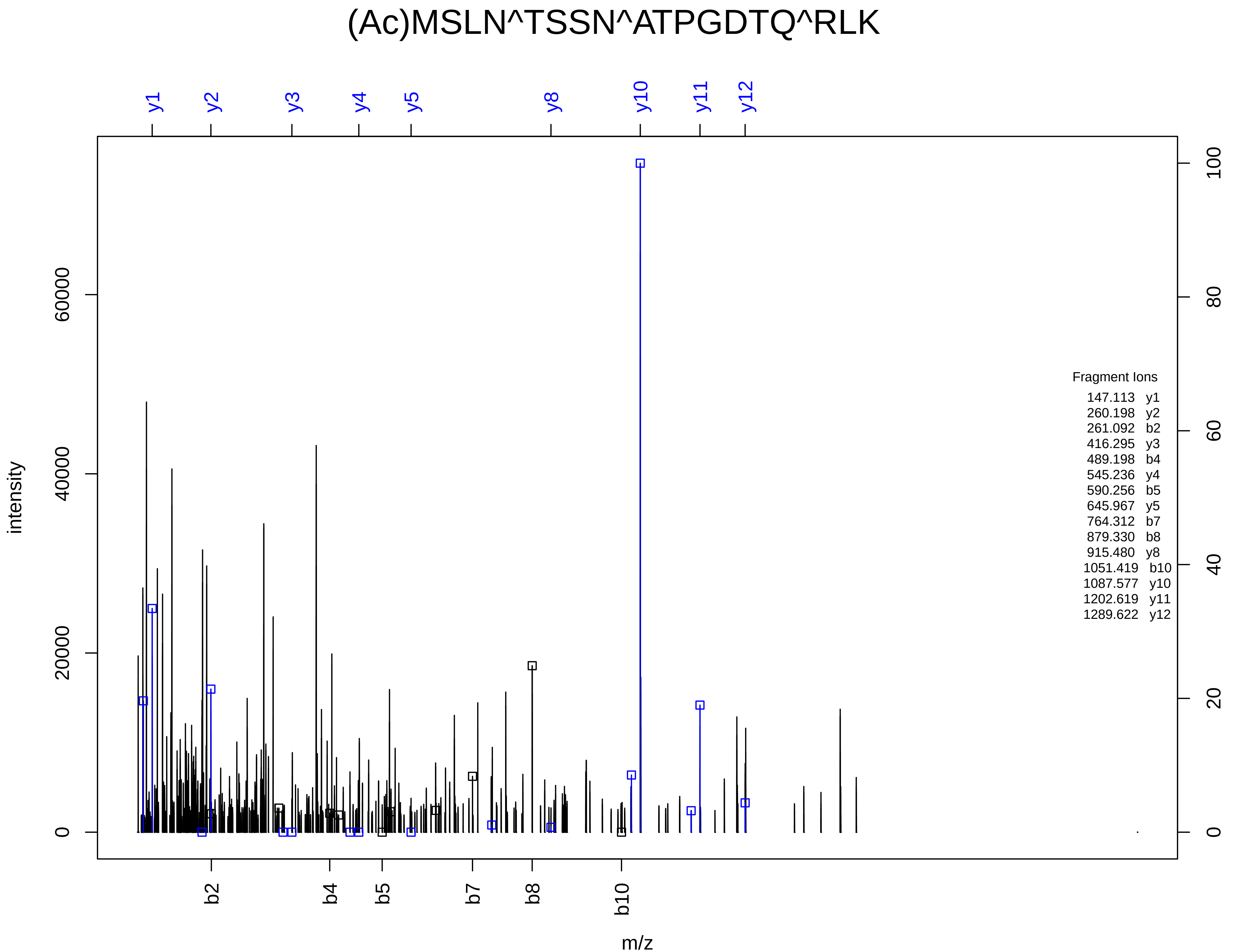

MSLNTSSNATPGDTQRLK / File: sydney_28.raw; SpectrumID: ; scans: 9167

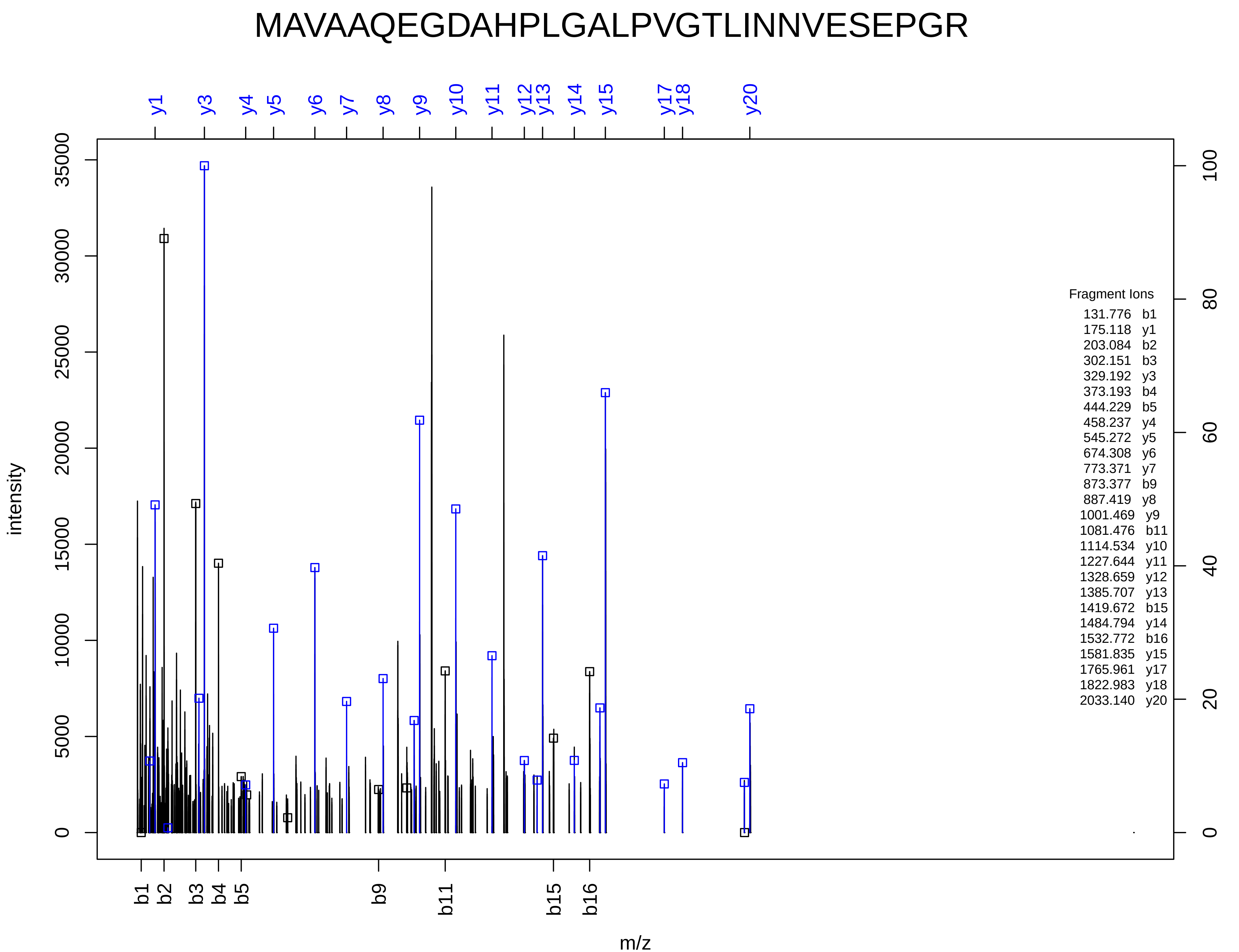

MAVAAQEGDAHPLGALPVGTLINNVESEPGR / File: sydney_28.raw; SpectrumID: ; scans: 10331

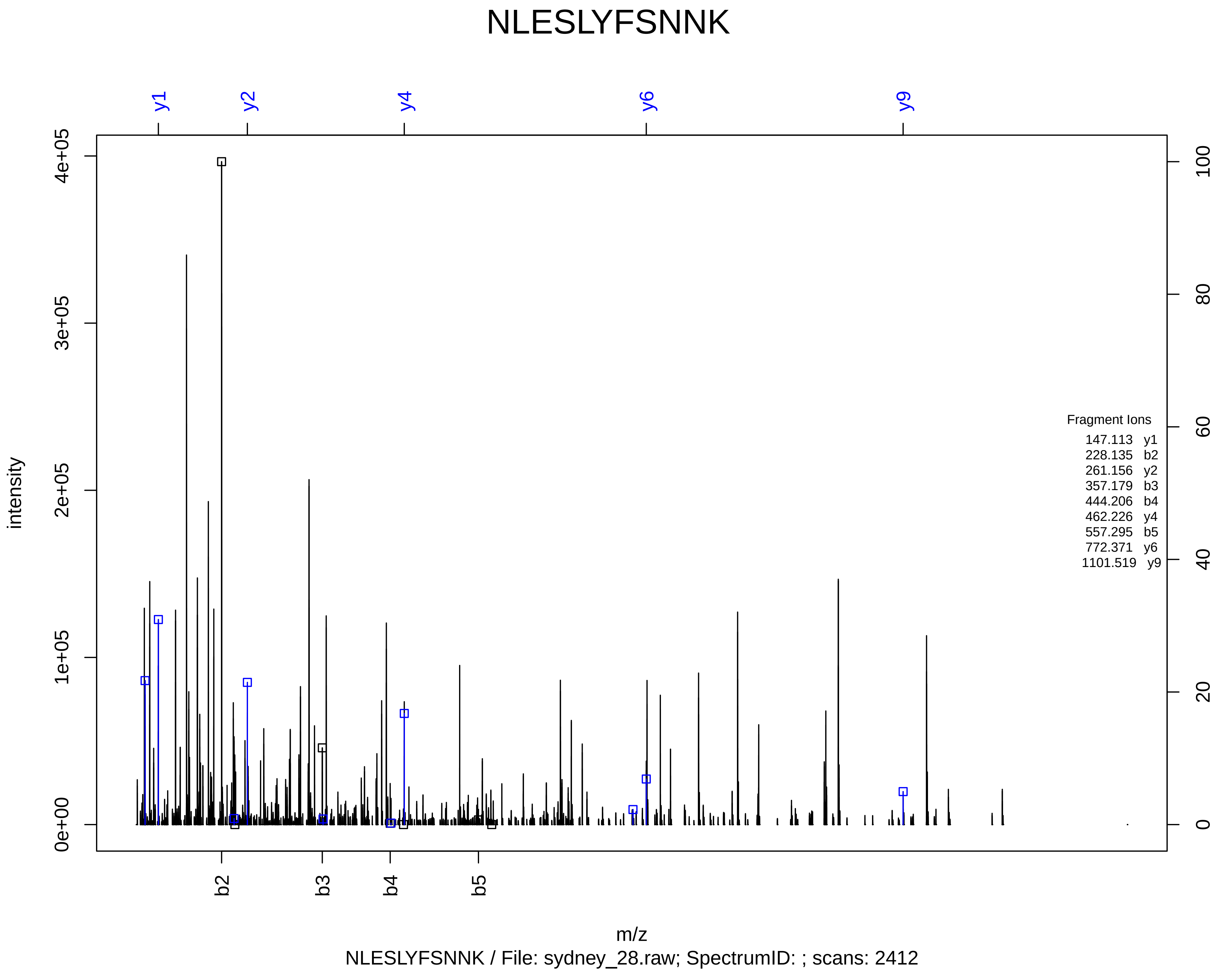
NLESLYFSNNK
y1
y2
y4
y6
y9
intensity
4e+05
3e+05
2e+05
1e+05
0e+00
100
80
60
40
20
0
Fragment Ions
147.113 y1
228.135 b2
261.156 y2
357.179 b3
444.206 b4
462.226 y4
557.295 b5
772.371 y6
1101.519 y9
b2
b3
b4
b5
m/z
NLESLYFSNNK / File: sydney_28.raw; SpectrumID: ; scans: 2412

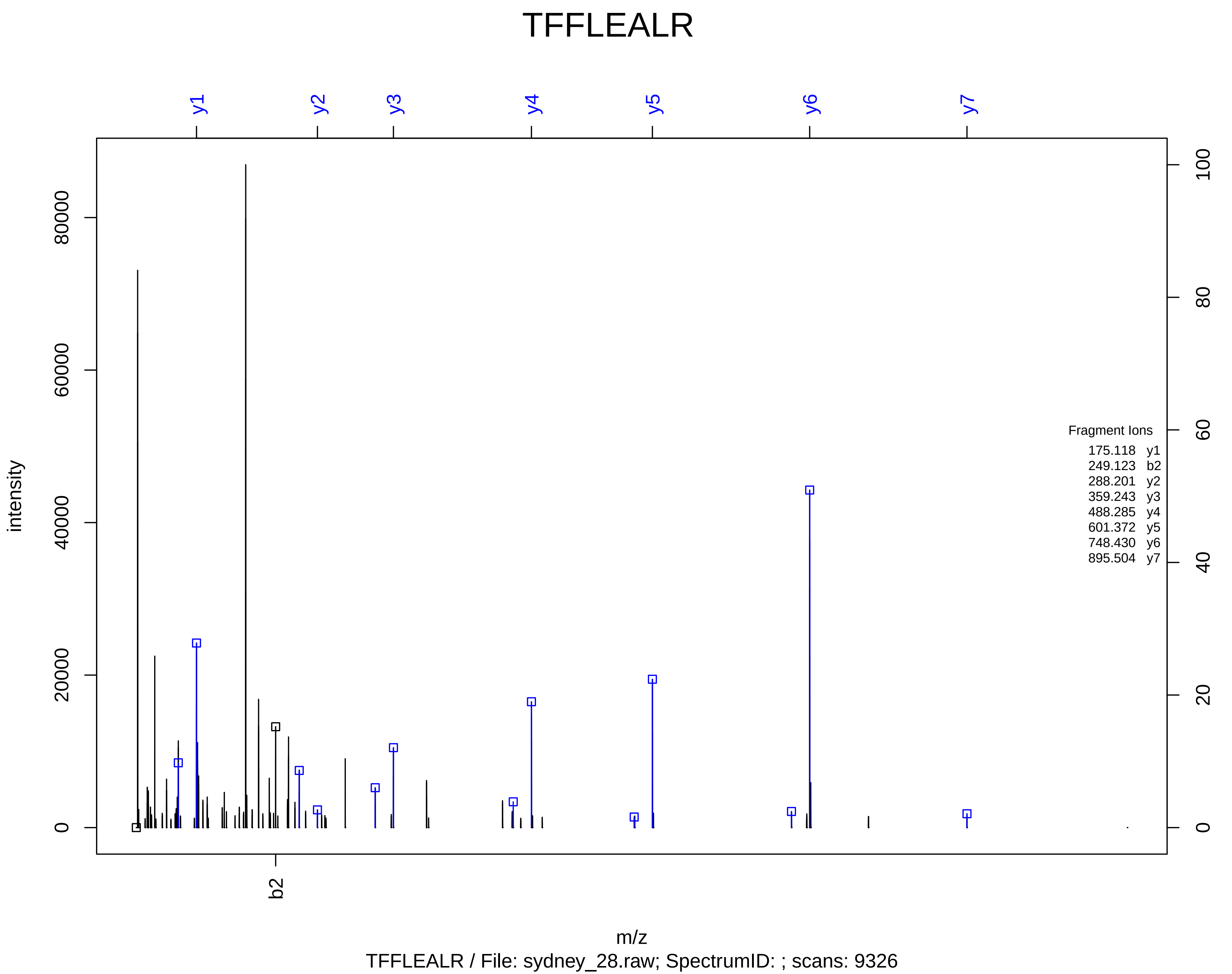
TFFLEALR
y1
y2
y3
y4
y5
y6
y7
b2
intensity
0
20000
40000
60000
80000
0
20
40
60
80
100
Fragment Ions
175.118 y1
249.123 b2
288.201 y2
359.243 y3
488.285 y4
601.372 y5
748.430 y6
895.504 y7
m/z
TFFLEALR / File: sydney_28.raw; SpectrumID: ; scans: 9326

WKIENDQLPR / File: sydney_28.raw; SpectrumID: ; scans: 9137

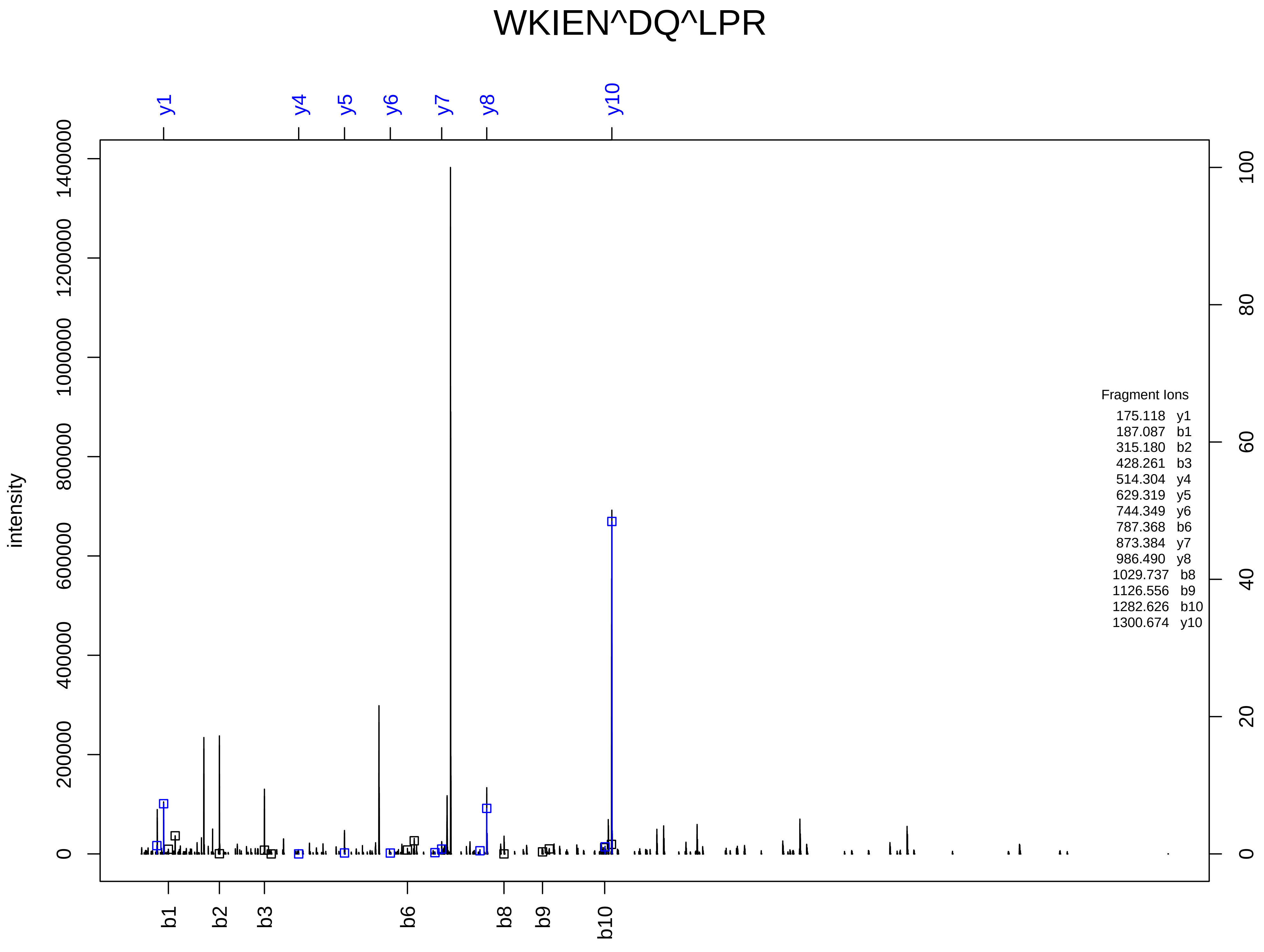

LCEPPVLSSVEDTYSPFFR

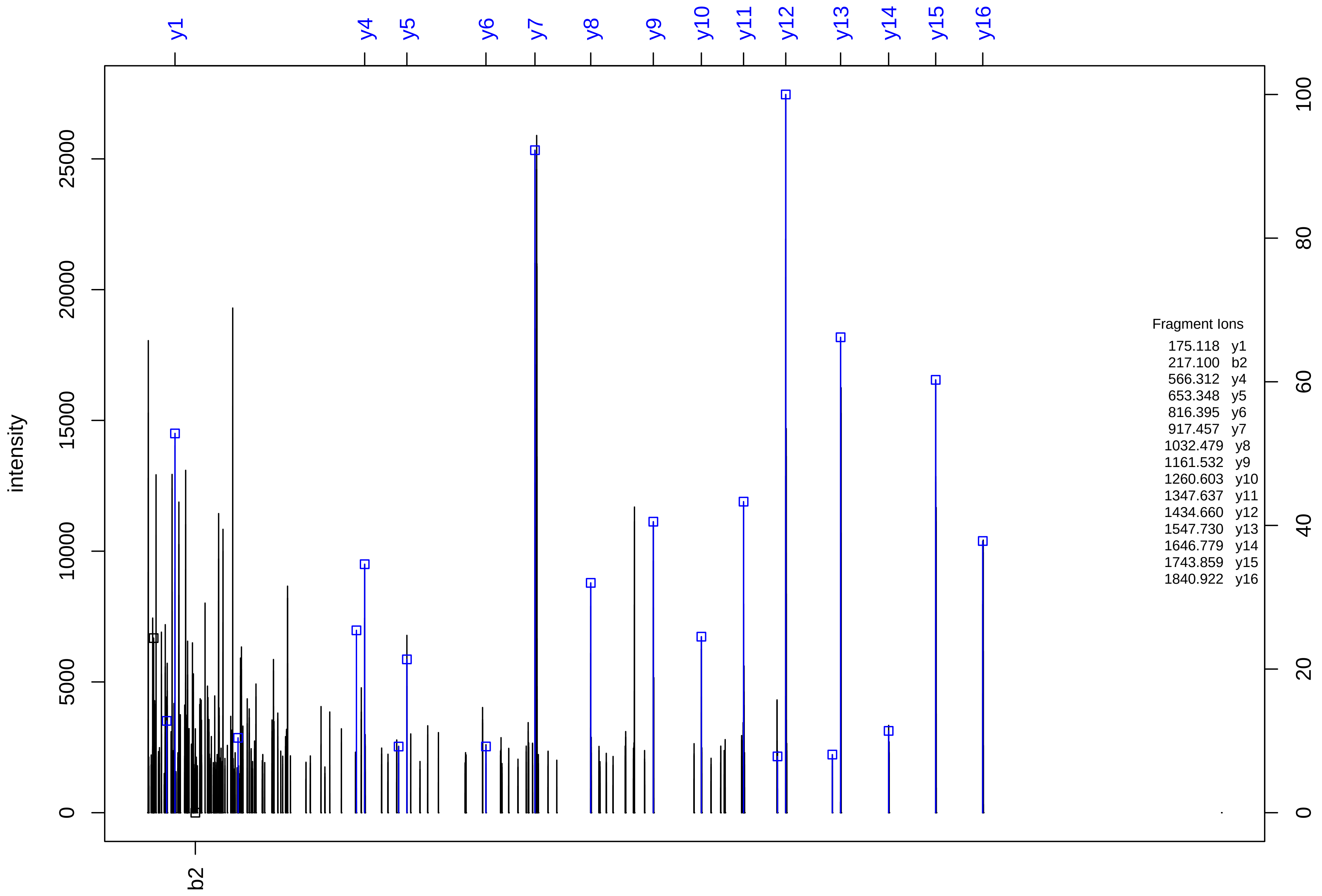

LCEPPVLSSVEDTYSPFFR / File: sydney_28.raw; SpectrumID: ; scans: 13281

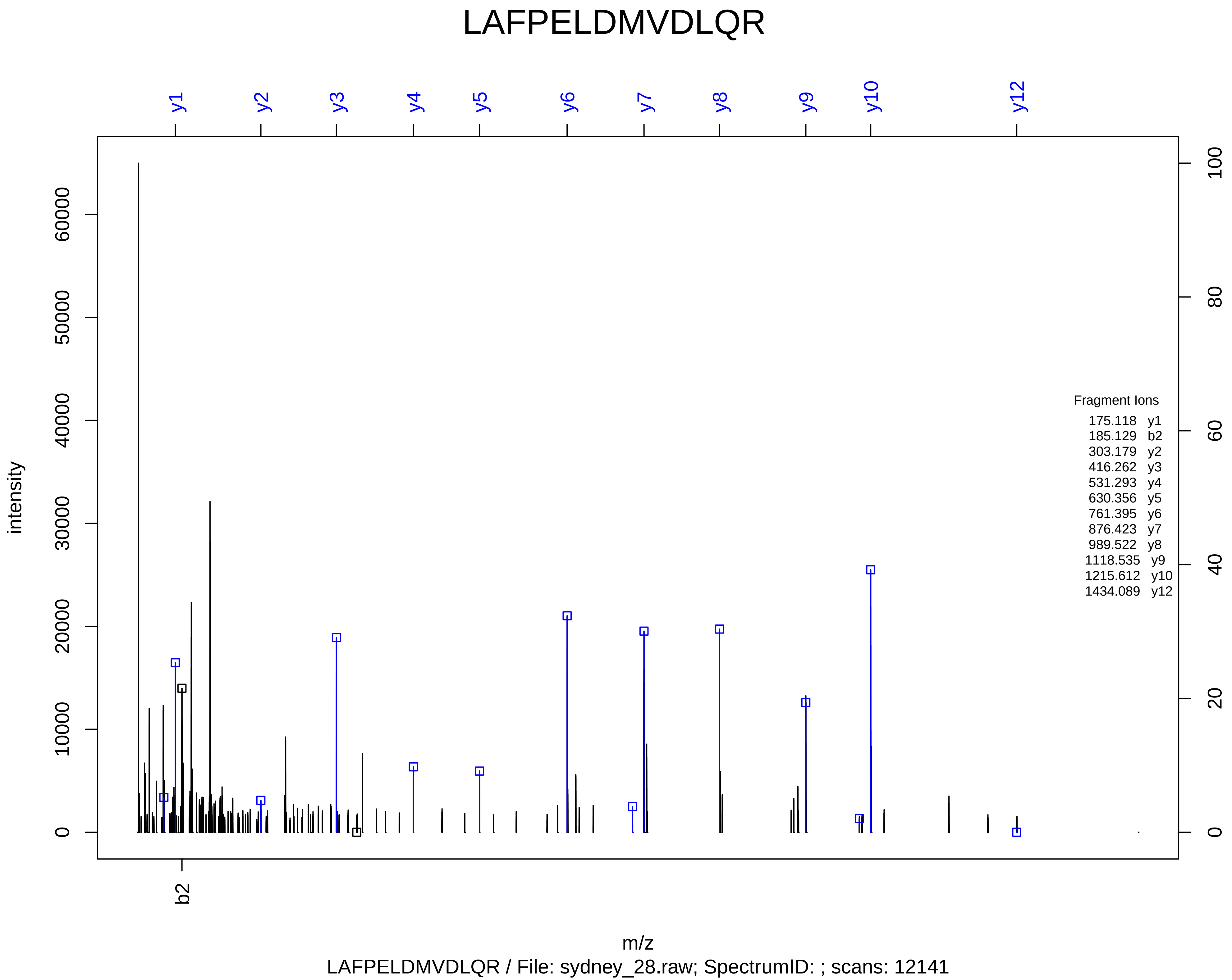
LAFPELDMVDLQR
y1
y2
y3
y4
y5
y6
y7
y8
y9
y10
y12
b2
intensity
0
10000
20000
30000
40000
50000
60000
0
20
40
60
80
100
m/z
Fragment Ions
175.118 y1
185.129 b2
303.179 y2
416.262 y3
531.293 y4
630.356 y5
761.395 y6
876.423 y7
989.522 y8
1118.535 y9
1215.612 y10
1434.089 y12
LAFPELDMVDLQR / File: sydney_28.raw; SpectrumID: ; scans: 12141

EGVLTSLR

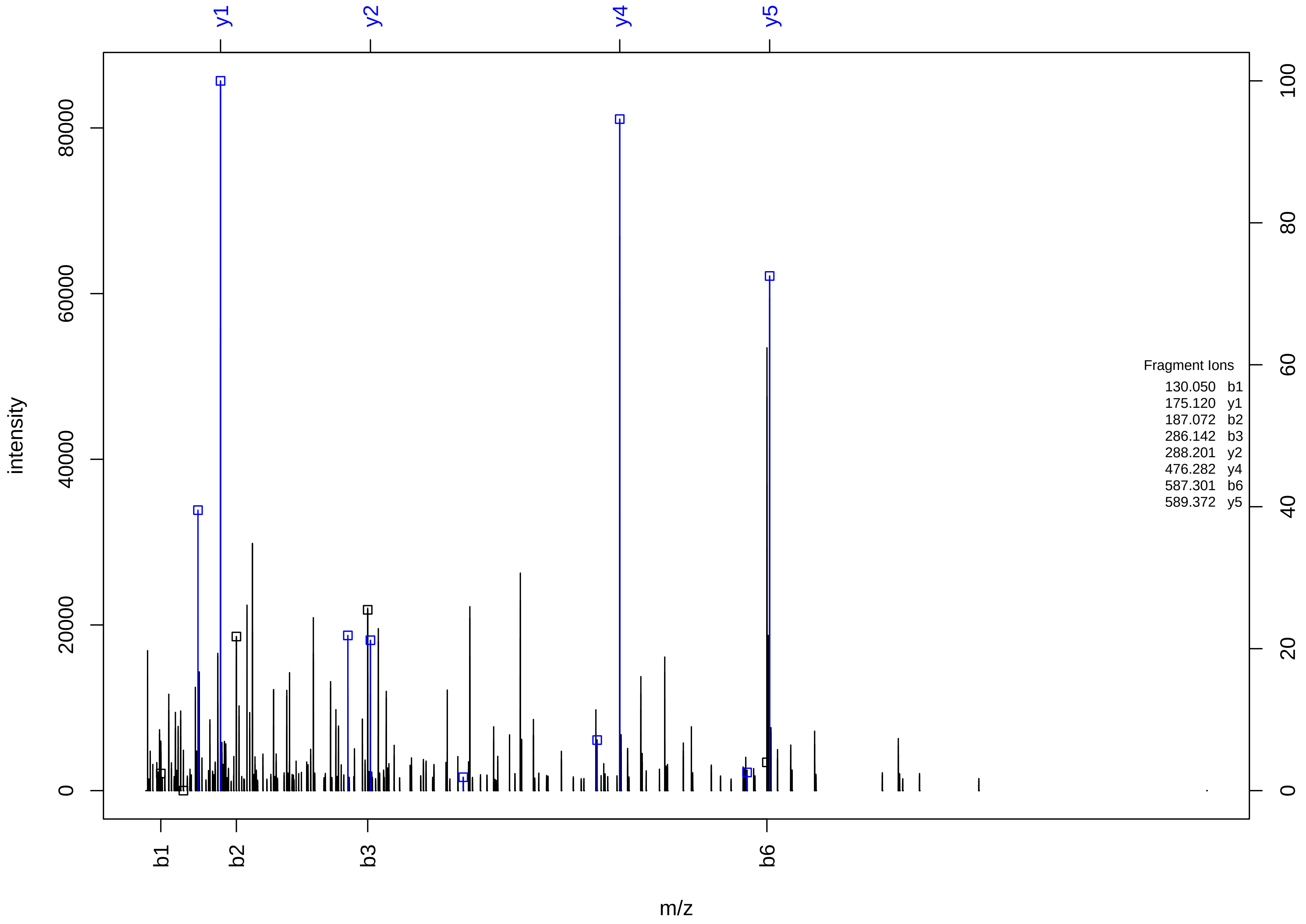

EGVLTSLR / File: sydney_28.raw; SpectrumID: ; scans: 3791

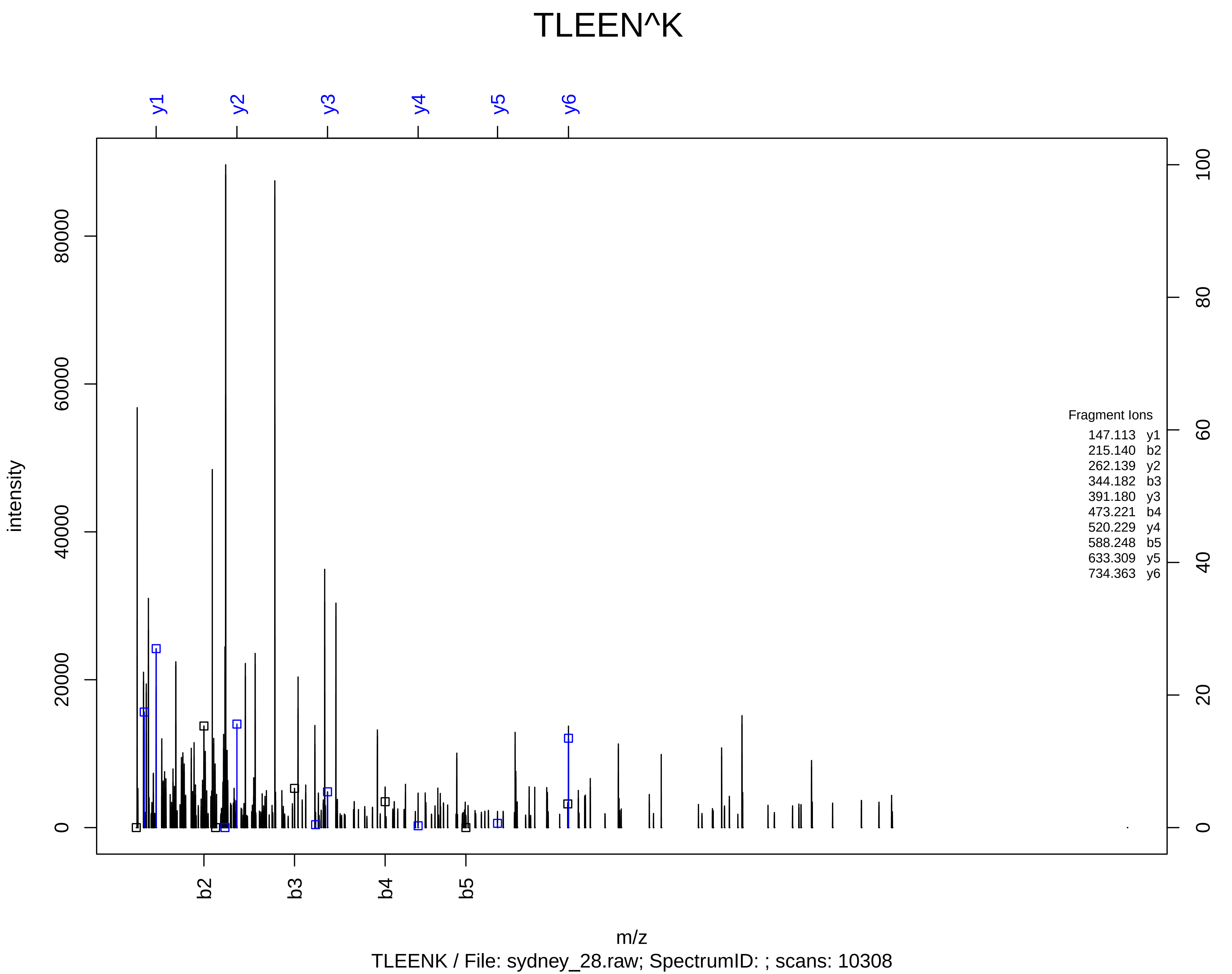
TLEEN^K
y1
y2
y3
y4
y5
y6
intensity
0
20000
40000
60000
80000
0
20
40
60
80
100
b2
b3
b4
b5
m/z
Fragment Ions
147.113 y1
215.140 b2
262.139 y2
344.182 b3
391.180 y3
473.221 b4
520.229 y4
588.248 b5
633.309 y5
734.363 y6
TLEENK / File: sydney_28.raw; SpectrumID: ; scans: 10308

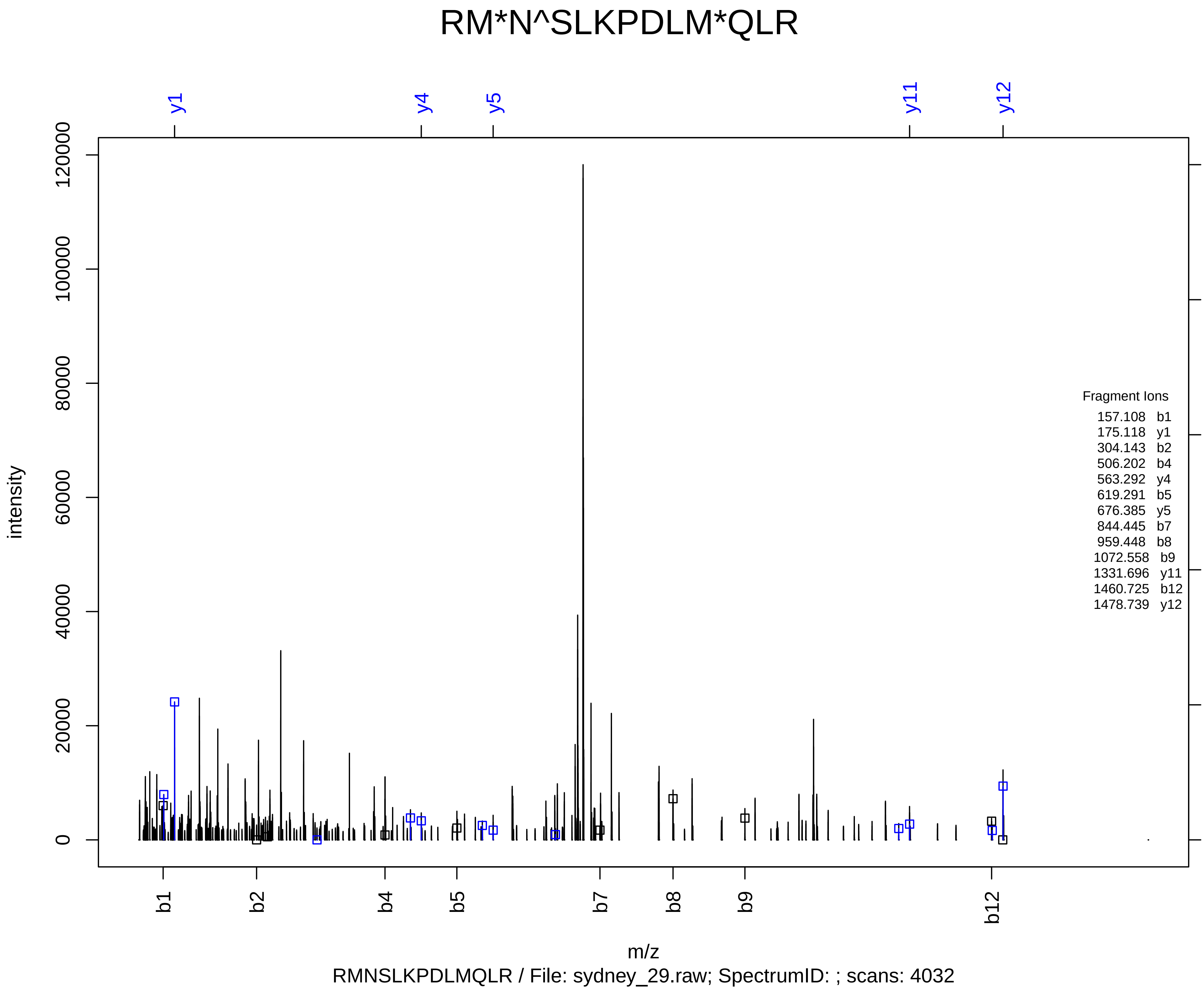
RM*N^SLKPDLM*QLR
y1
y4
y5
y11
y12
intensity
0
20000
40000
60000
80000
100000
120000
0
20
40
60
80
100
b1
b2
b4
b5
b7
b8
b9
b12
m/z
Fragment Ions
157.108 b1
175.118 y1
304.143 b2
506.202 b4
563.292 y4
619.291 b5
676.385 y5
844.445 b7
959.448 b8
1072.558 b9
1331.696 y11
1460.725 b12
1478.739 y12
RMNSLKPDLMQLR / File: sydney_29.raw; SpectrumID: ; scans: 4032

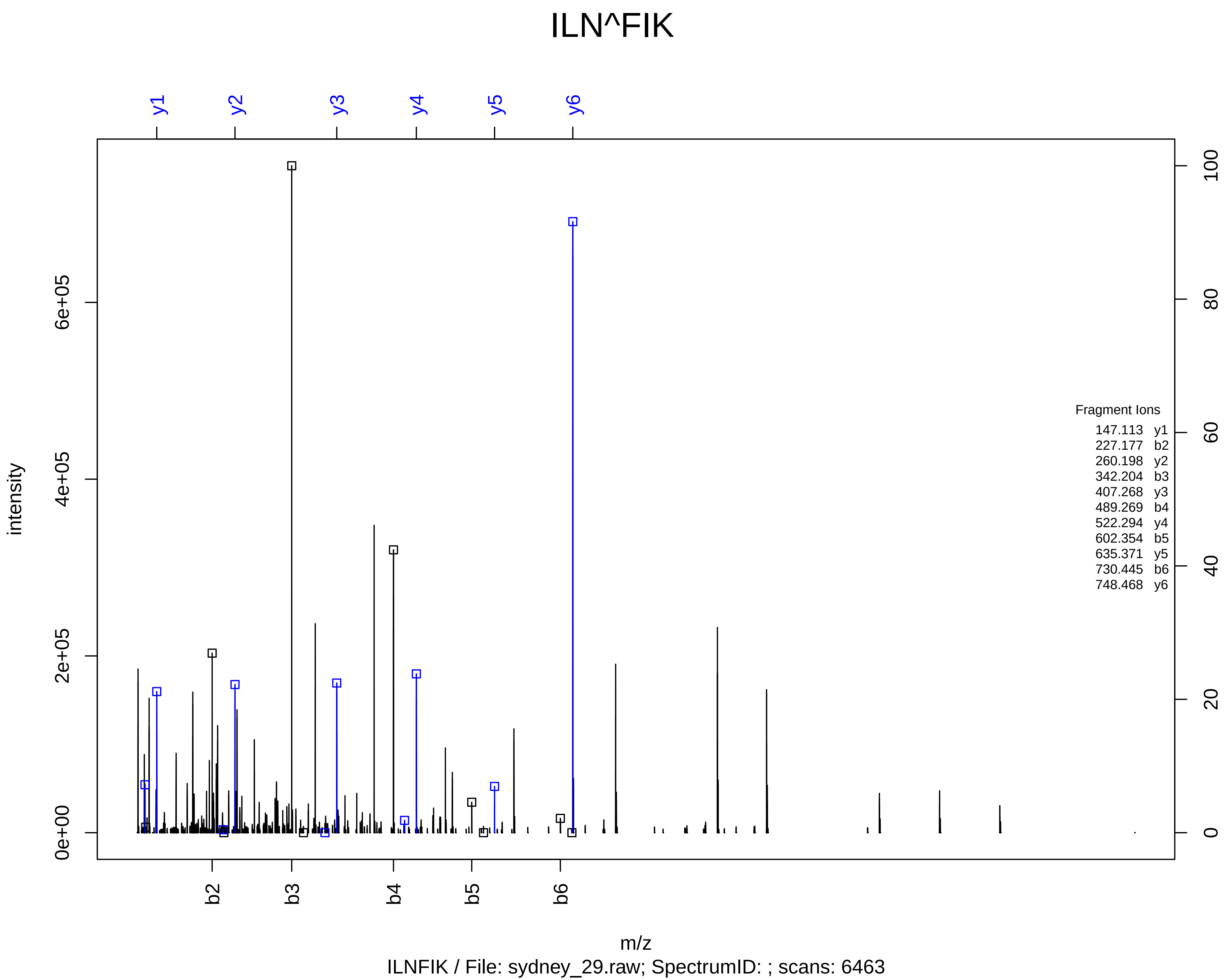
ILN^FIK
y1
y2
y3
y4
y5
y6
intensity
0e+00
2e+05
4e+05
6e+05
0
20
40
60
80
100
b2
b3
b4
b5
b6
Fragment Ions
147.113 y1
227.177 b2
260.198 y2
342.204 b3
407.268 y3
489.269 b4
522.294 y4
602.354 b5
635.371 y5
730.445 b6
748.468 y6
m/z
ILNFIK / File: sydney_29.raw; SpectrumID: ; scans: 6463

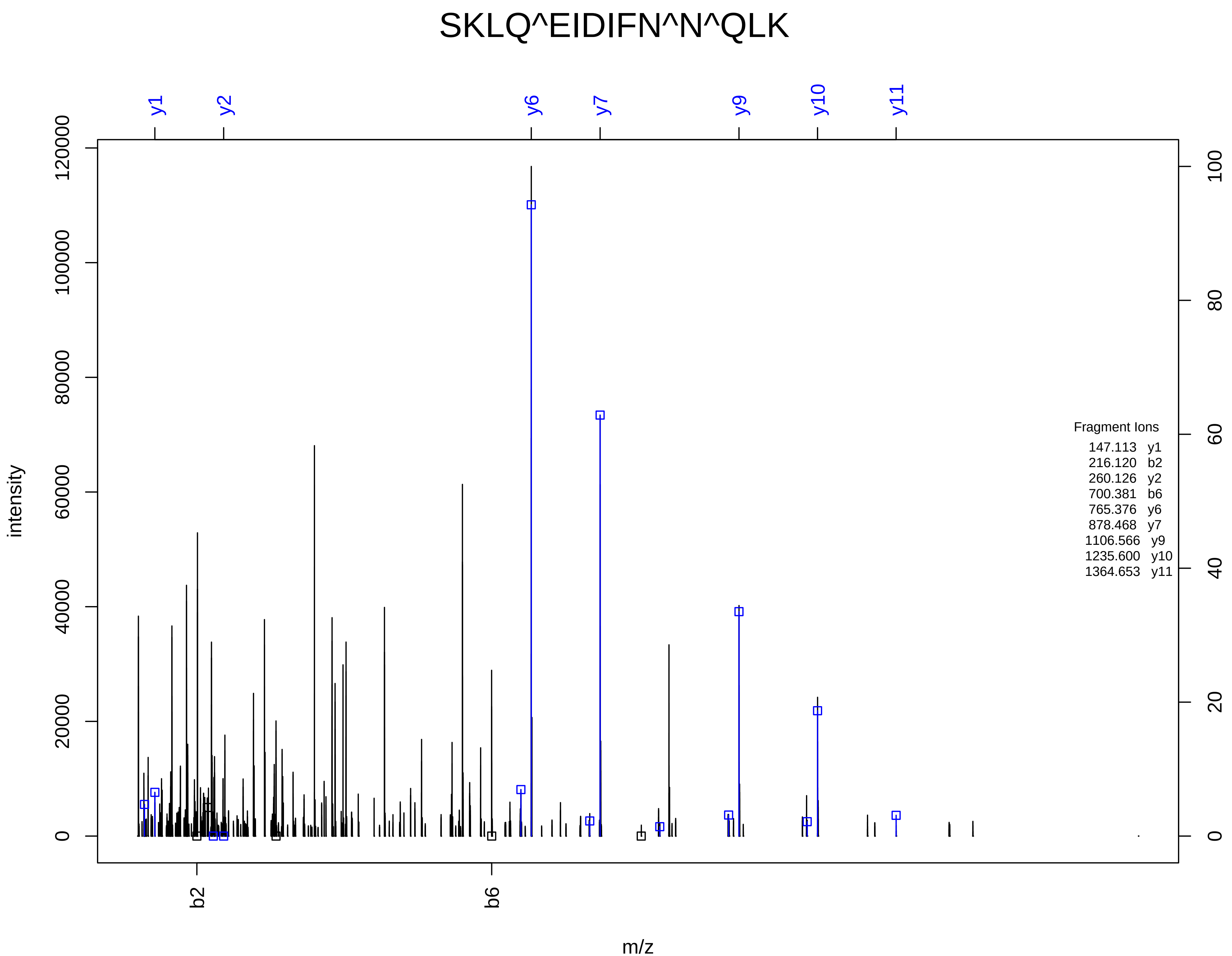

SKLQ^EIDIFN^N^QLK
y1
y2
y6
y7
y9
y10
y11
b2
b6
intensity
m/z
Fragment Ions
147.113 y1
216.120 b2
260.126 y2
700.381 b6
765.376 y6
878.468 y7
1106.566 y9
1235.600 y10
1364.653 y11
SKLQEIDIFNNQLK / File: sydney_29.raw; SpectrumID: ; scans: 11667

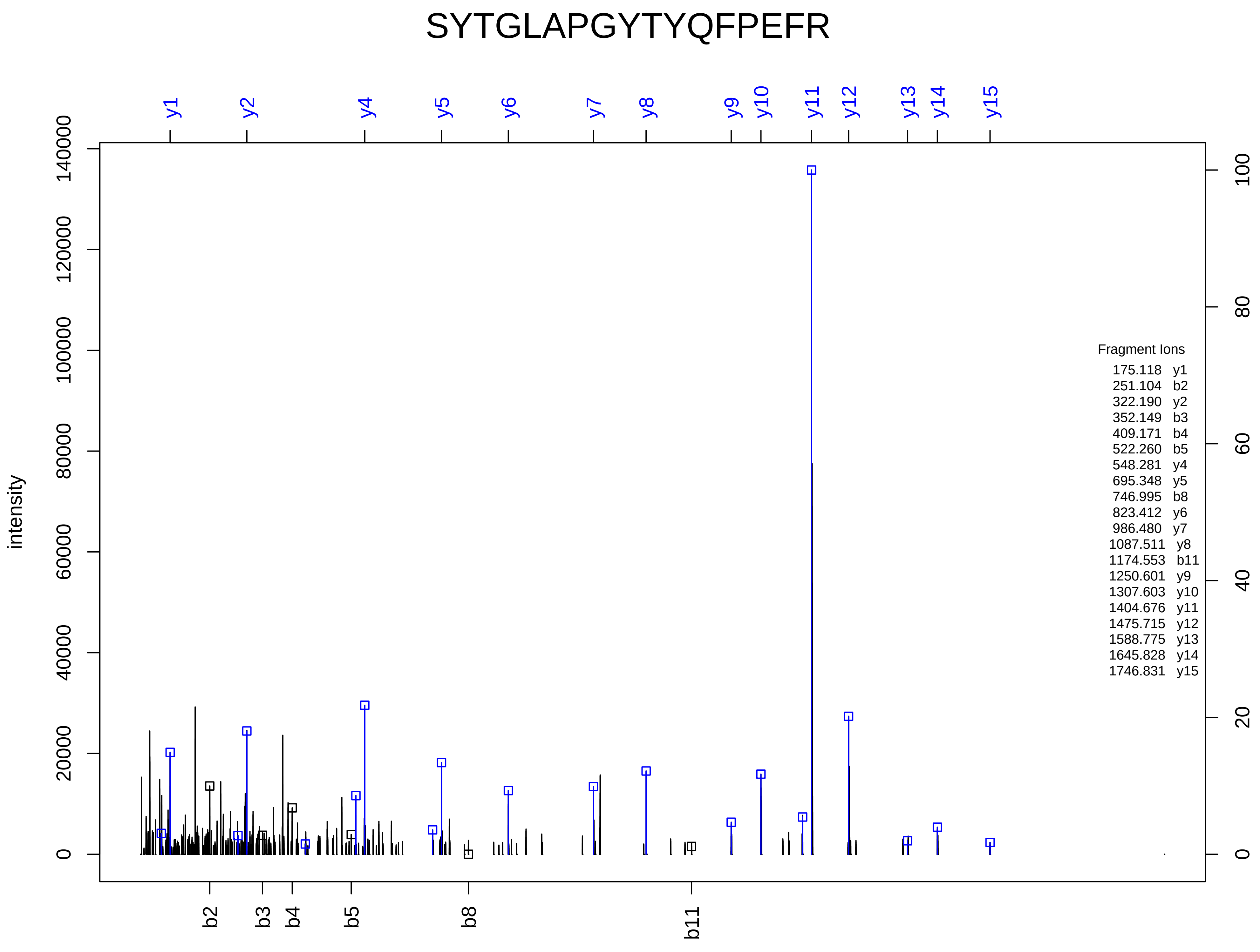

SYTGLAPGYTYQFPEFR / File: sydney_29.raw; SpectrumID: ; scans: 9344

AALSEELDAVDTGVGR

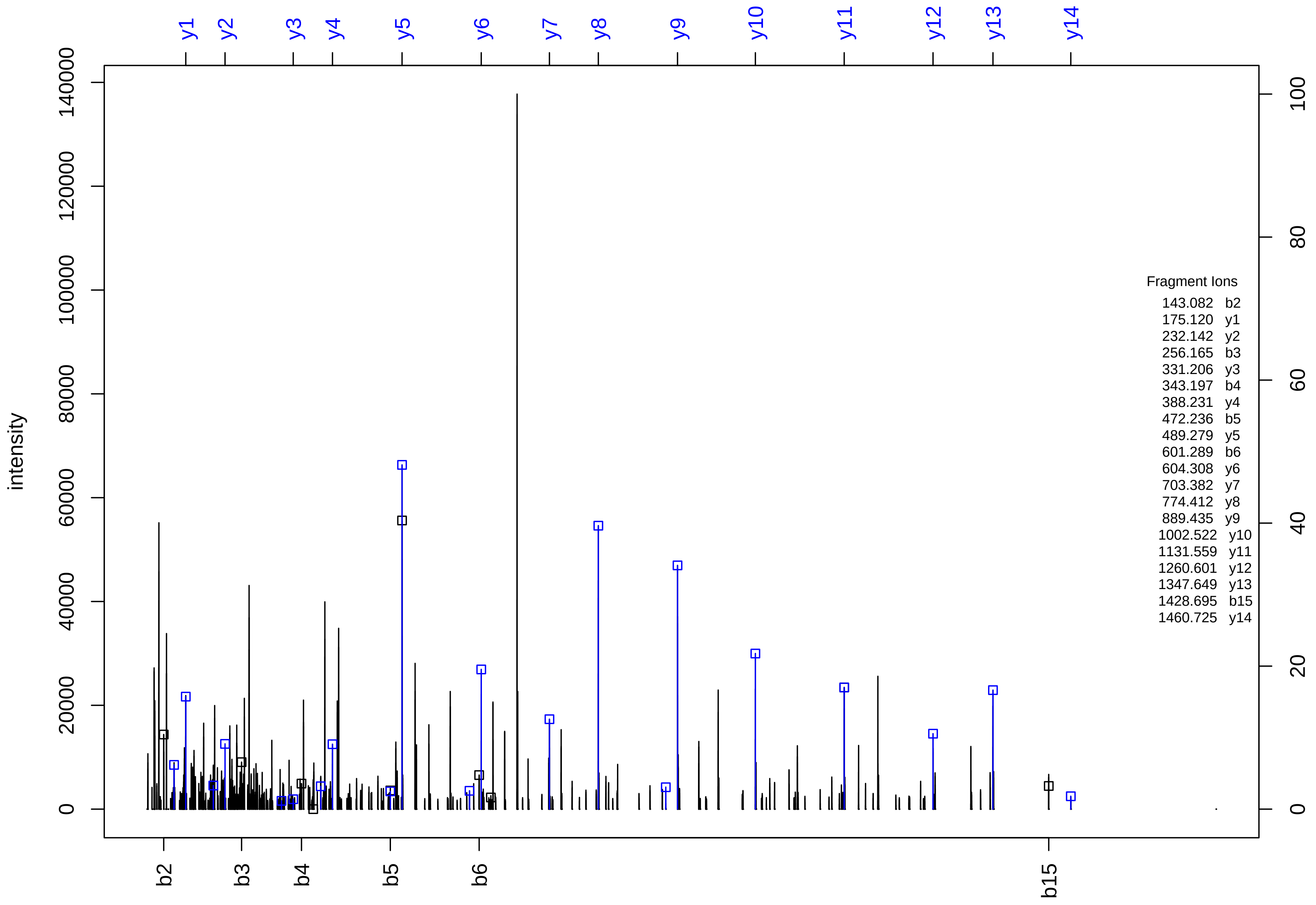

AALSEELDAVDTGVGR / File: sydney_29.raw; SpectrumID: ; scans: 6034

FIYEAWQGVER

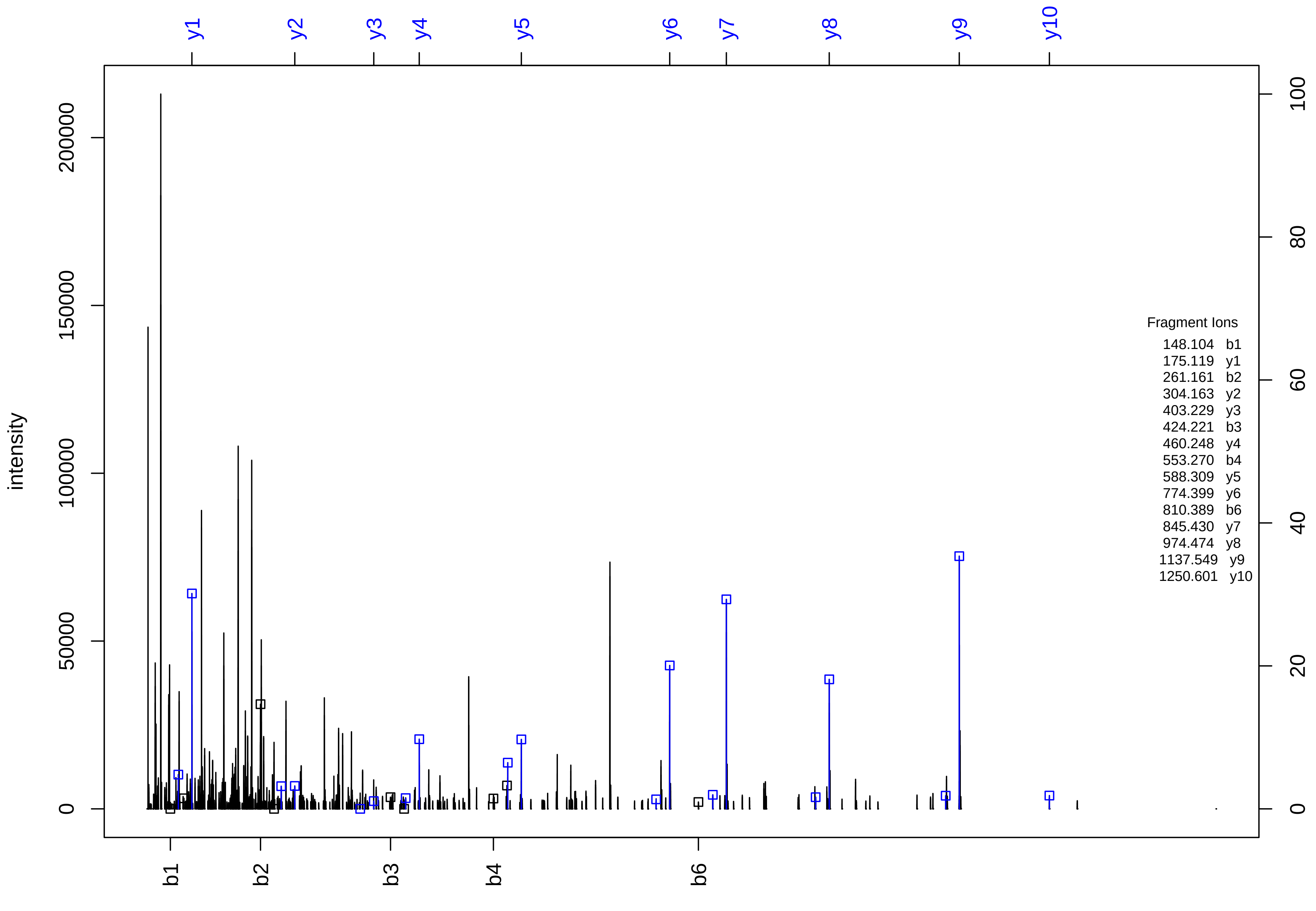

FIYEAWQGVER / File: sydney_29.raw; SpectrumID: ; scans: 6421

IYDPQEGAVVVLPAESQQR

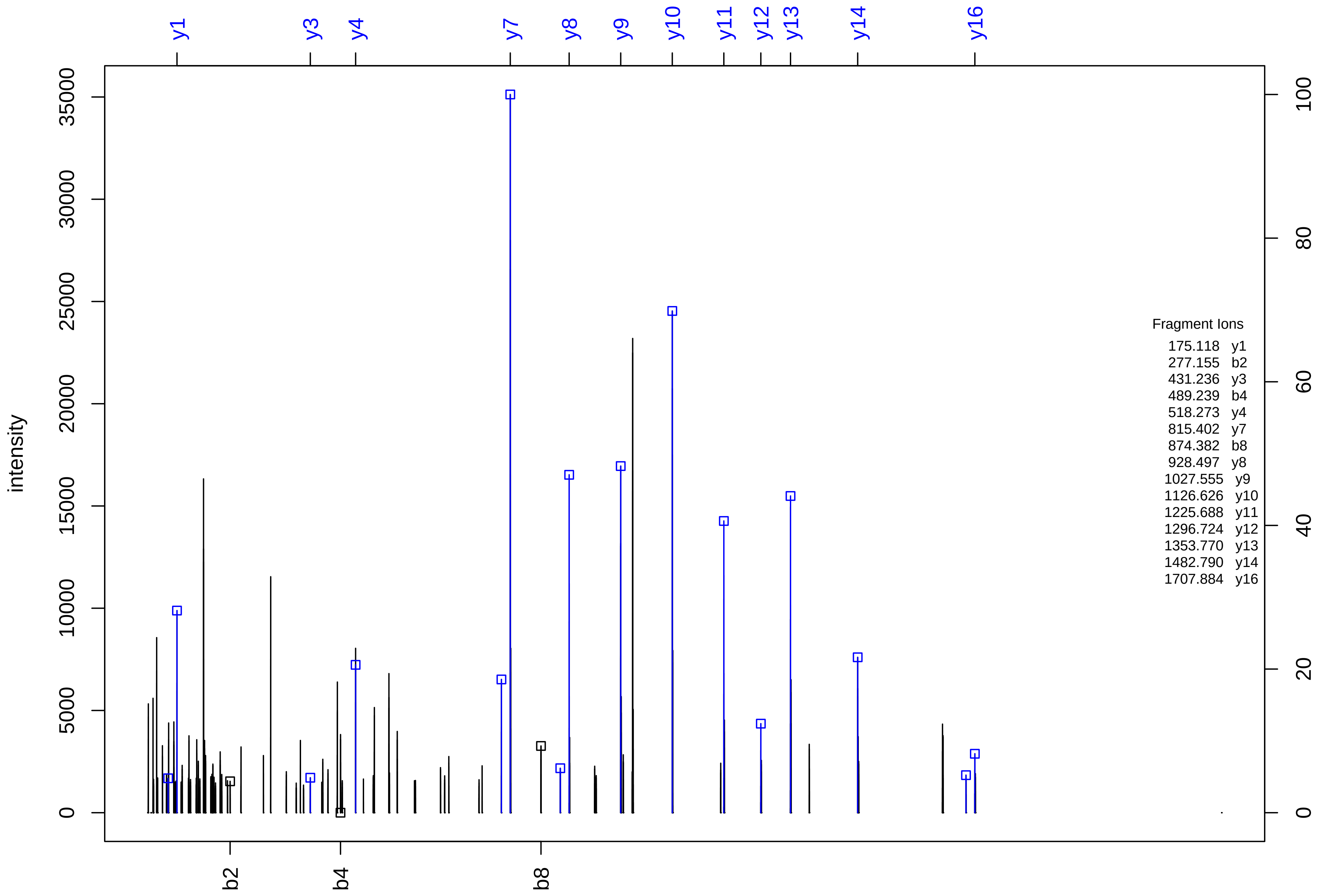

IYDPQEGAVVVLPAESQQR / File: sydney_29.raw; SpectrumID: ; scans: 6370

EIINTIDGVGGLGIGEGAPEIVTGSR

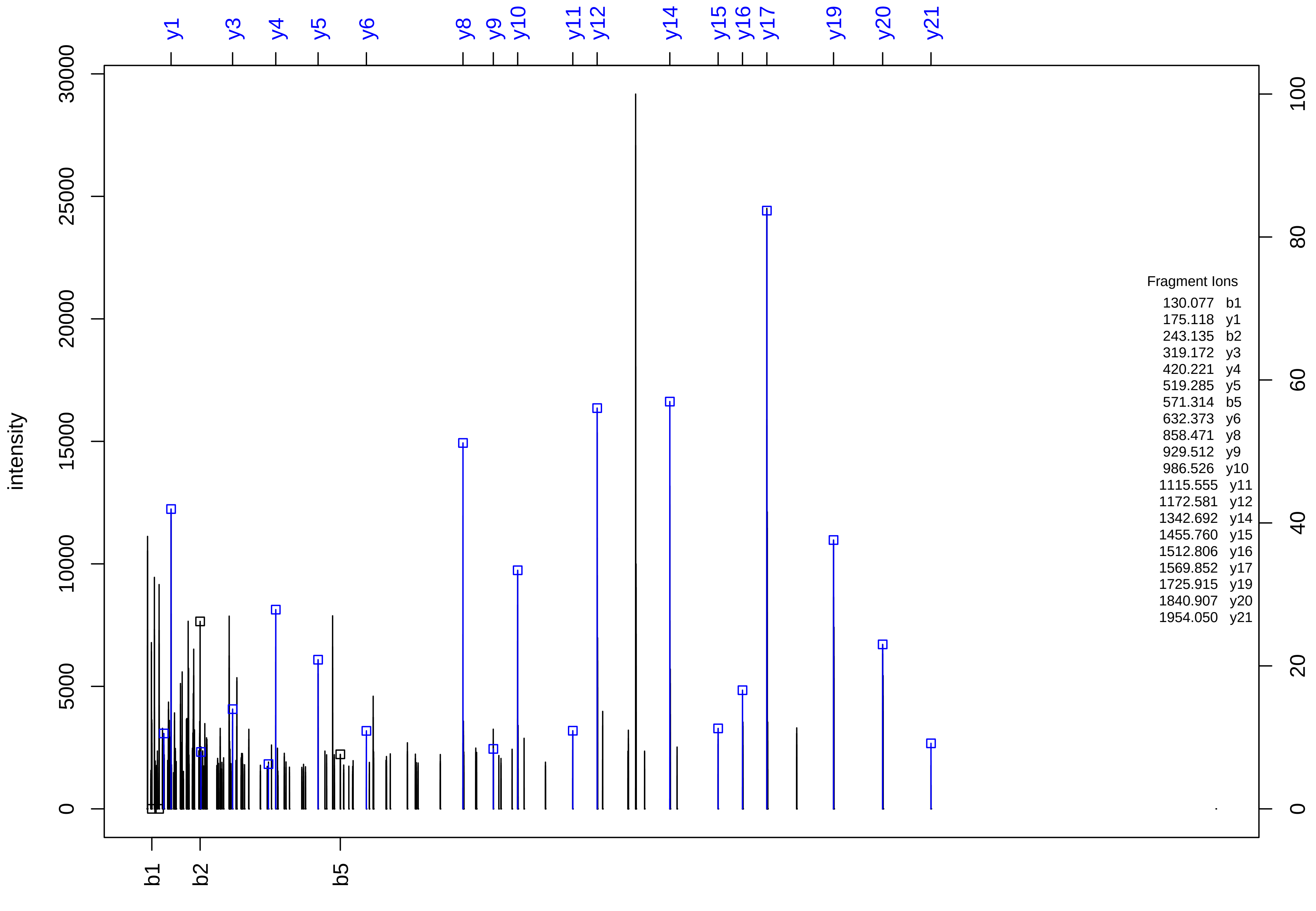

EIINTIDGVGGLGIGEGAPEIVTGSR / File: sydney_29.raw; SpectrumID: ; scans: 11120

LN^SLN^LTGN^MIGSLPKEVR

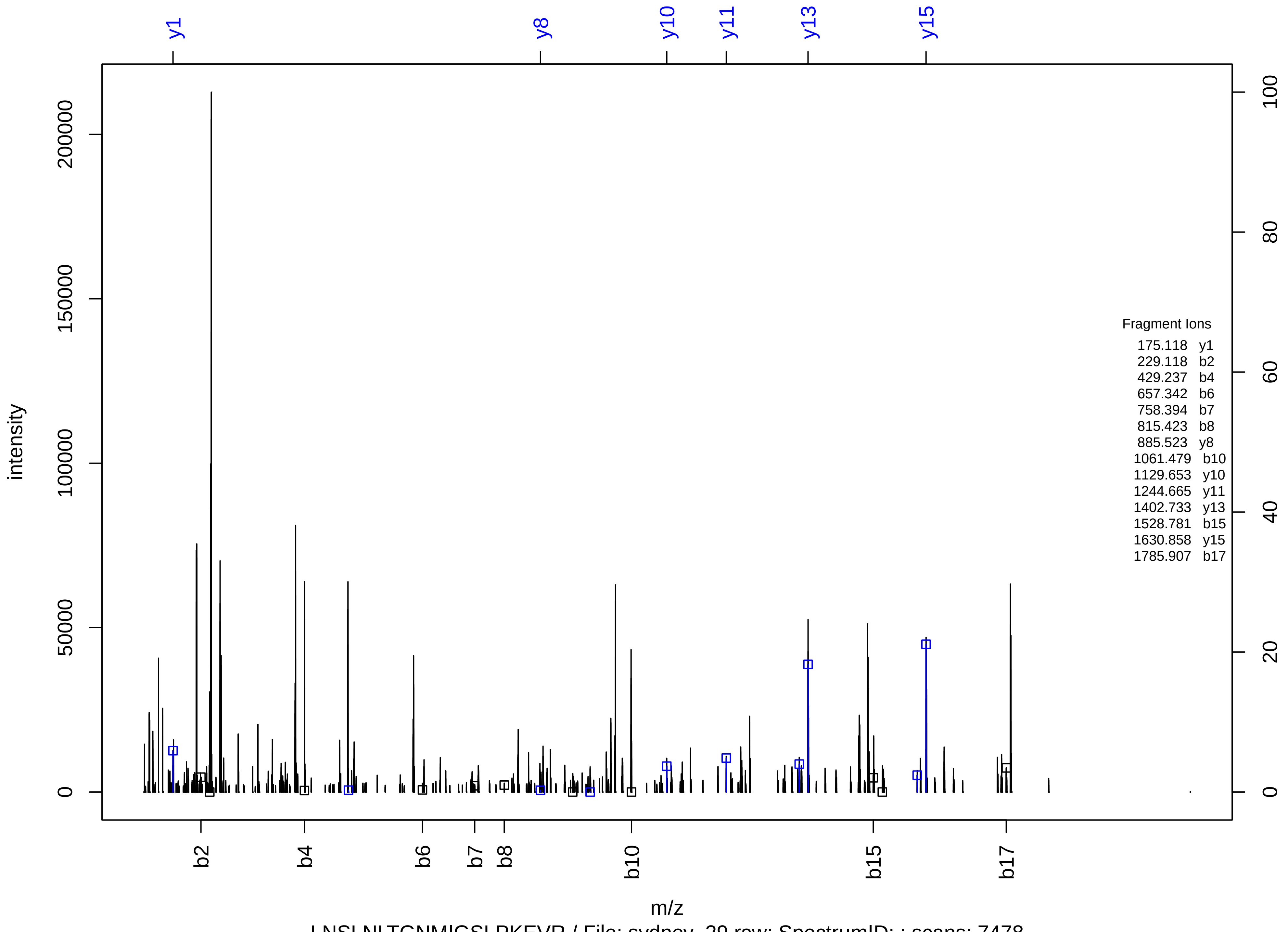

LNSLNLTGNMIGSLPKEVR / File: sydney_29.raw; SpectrumID: ; scans: 7478

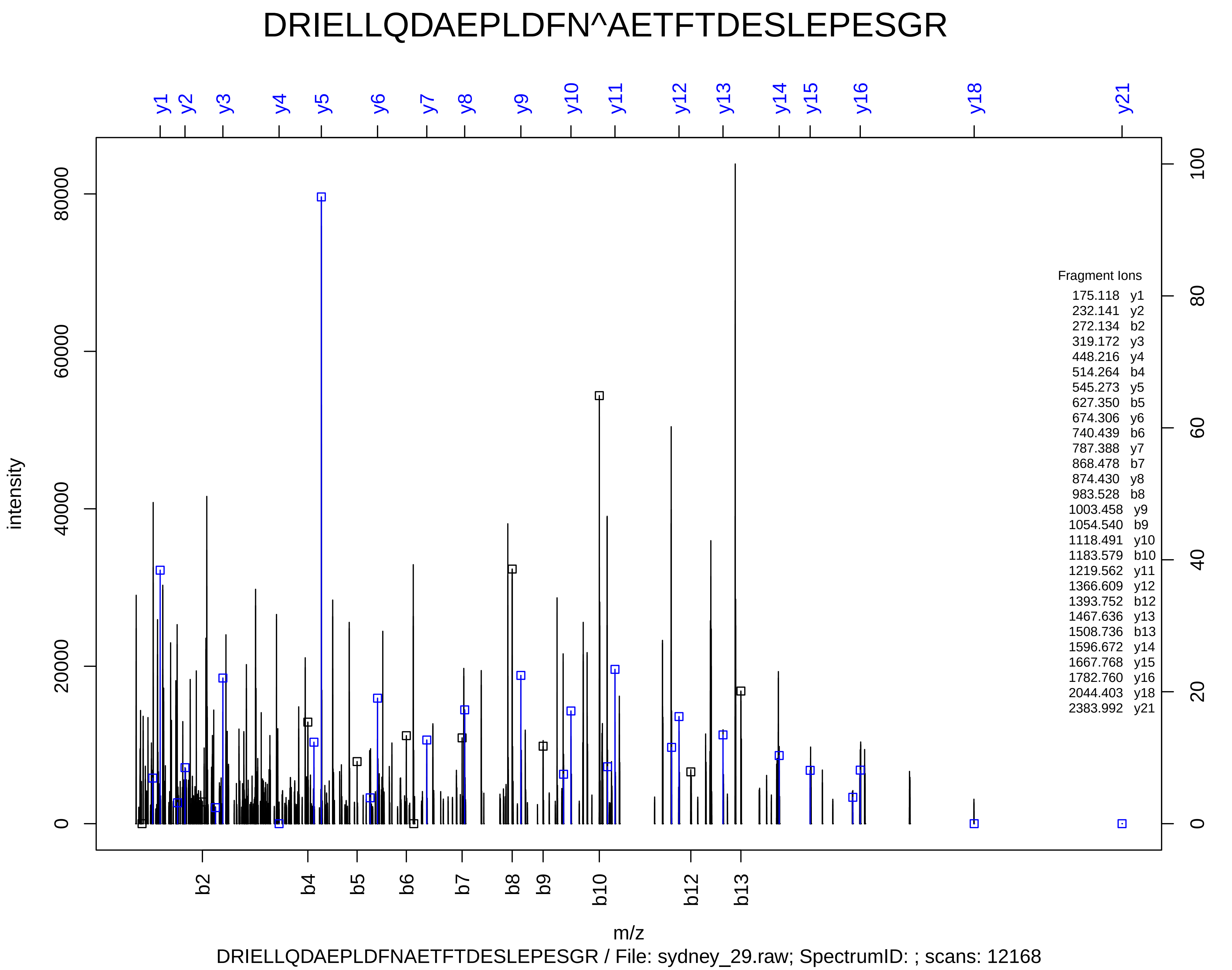
DRIELLQDAEPLDFN^AETFTDESLEPESGR
y1
y2
y3
y4
y5
y6
y7
y8
y9
y10
y11
y12
y13
y14
y15
y16
y18
y21
intensity
0
20000
40000
60000
80000
0
20
40
60
80
100
b2
b4
b5
b6
b7
b8
b9
b10
b12
b13
m/z
Fragment Ions
175.118 y1
232.141 y2
272.134 b2
319.172 y3
448.216 y4
514.264 b4
545.273 y5
627.350 b5
674.306 y6
740.439 b6
787.388 y7
868.478 b7
874.430 y8
983.528 b8
1003.458 y9
1054.540 b9
1118.491 y10
1183.579 b10
1219.562 y11
1366.609 y12
1393.752 b12
1467.636 y13
1508.736 b13
1596.672 y14
1667.768 y15
1782.760 y16
2044.403 y18
2383.992 y21
DRIELLQDAEPLDFNAETFTDESLEPESGR / File: sydney_29.raw; SpectrumID: ; scans: 12168

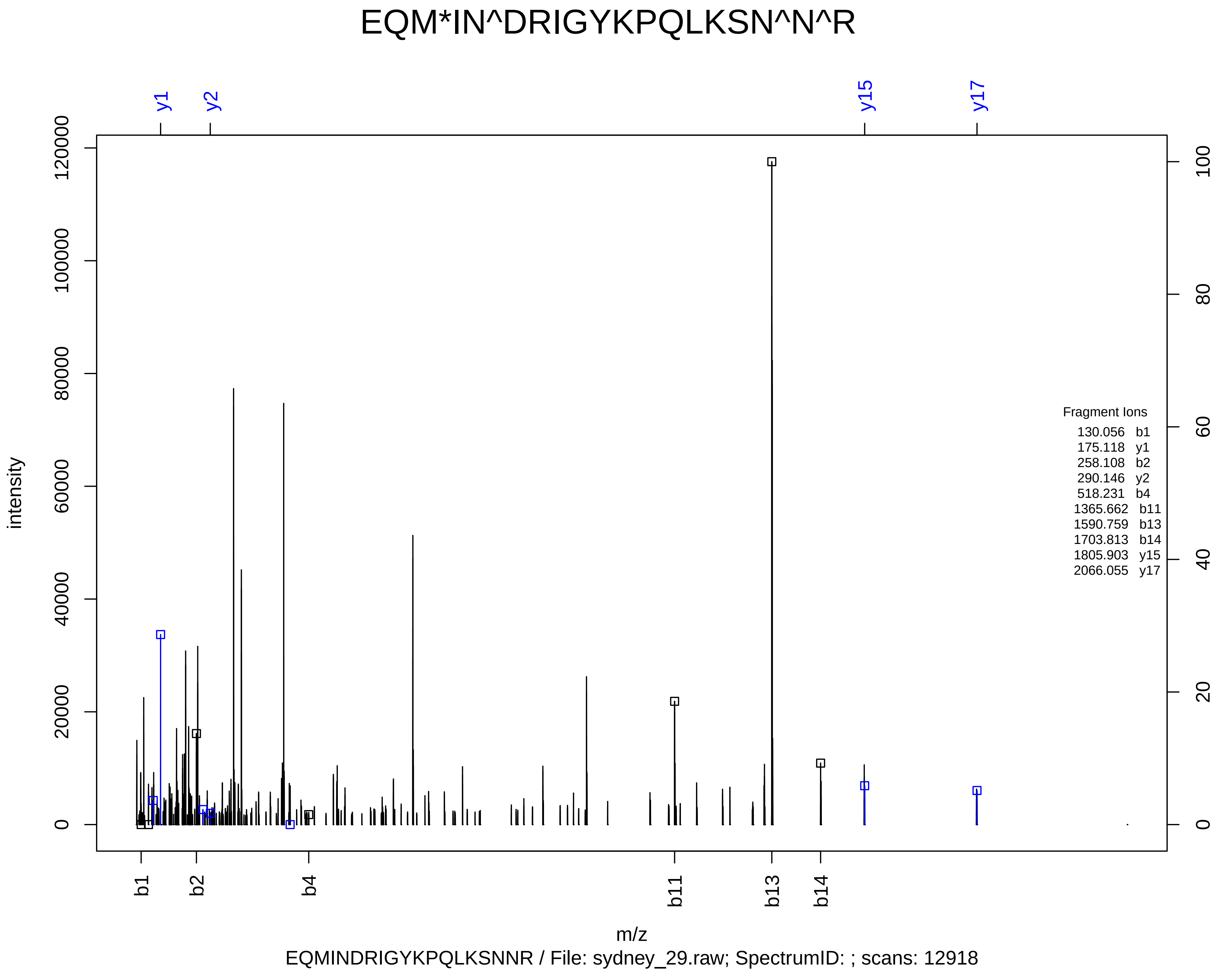
EQM*IN^DRIGYKPQLKSN^N^R
y1
y2
y15
y17
intensity
0
20000
40000
60000
80000
100000
120000
0
20
40
60
80
100
b1
b2
b4
b11
b13
b14
m/z
Fragment Ions
130.056 b1
175.118 y1
258.108 b2
290.146 y2
518.231 b4
1365.662 b11
1590.759 b13
1703.813 b14
1805.903 y15
2066.055 y17
EQMINDRIGYKPQLKSNNR / File: sydney_29.raw; SpectrumID: ; scans: 12918

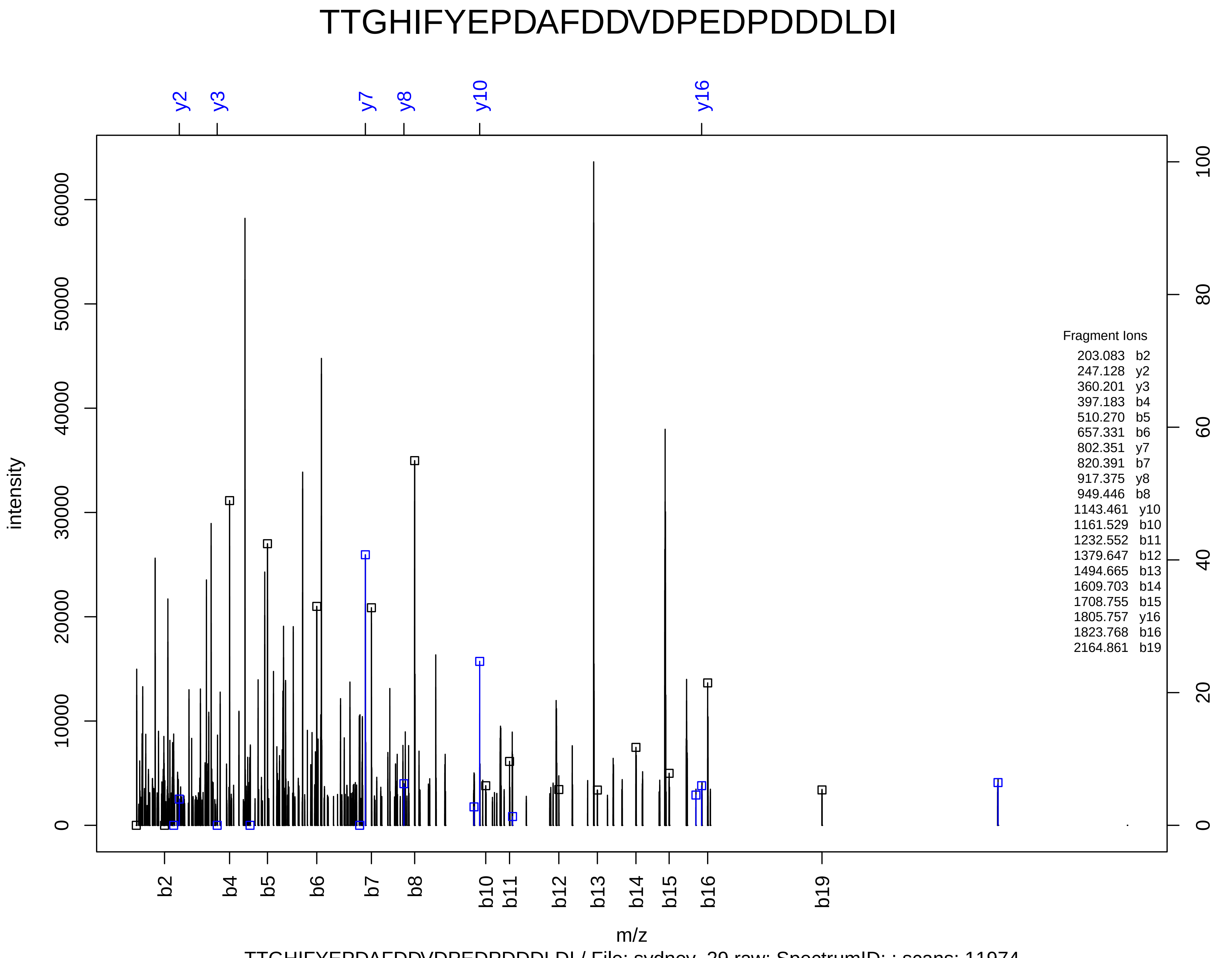

TTGHIFYEPDAFDDVDPEDPDDDLDI
y2
y3
y7
y8
y10
y16
intensity
0
10000
20000
30000
40000
50000
60000
0
20
40
60
80
100
Fragment Ions
203.083 b2
247.128 y2
360.201 y3
397.183 b4
510.270 b5
657.331 b6
802.351 y7
820.391 b7
917.375 y8
949.446 b8
1143.461 y10
1161.529 b10
1232.552 b11
1379.647 b12
1494.665 b13
1609.703 b14
1708.755 b15
1805.757 y16
1823.768 b16
2164.861 b19
b2
b4
b5
b6
b7
b8
b10
b11
b12
b13
b14
b15
b16
b19
m/z
TTGHIFYEPDAFDDVDPEDPDDDLDI / File: sydney_29.raw; SpectrumID: ; scans: 11974

EEYPGIEIESR

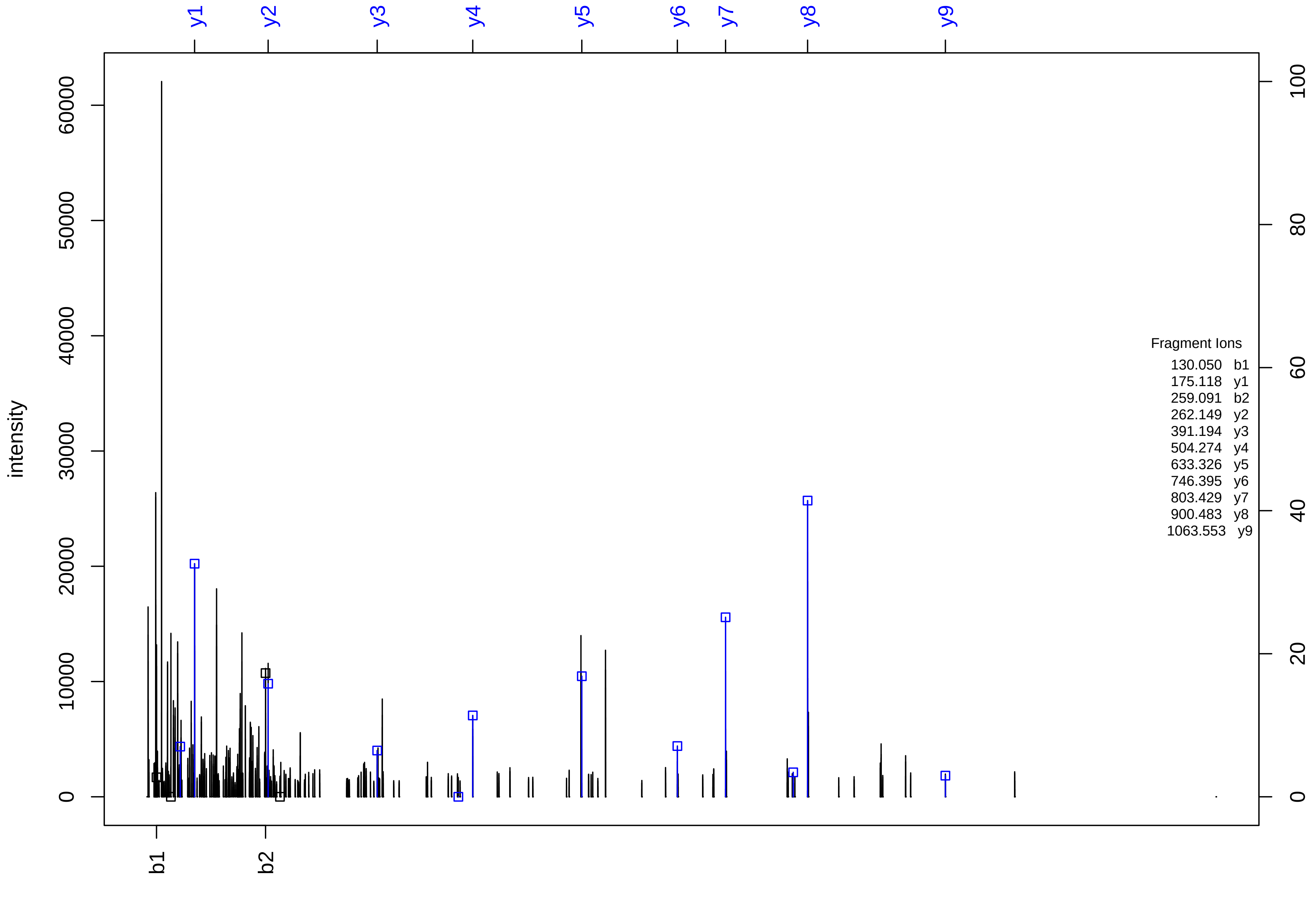

EEYPGIEIESR / File: sydney_29.raw; SpectrumID: ; scans: 3852

GTSVVLICPQDGMEAIPNPFVQQQDT

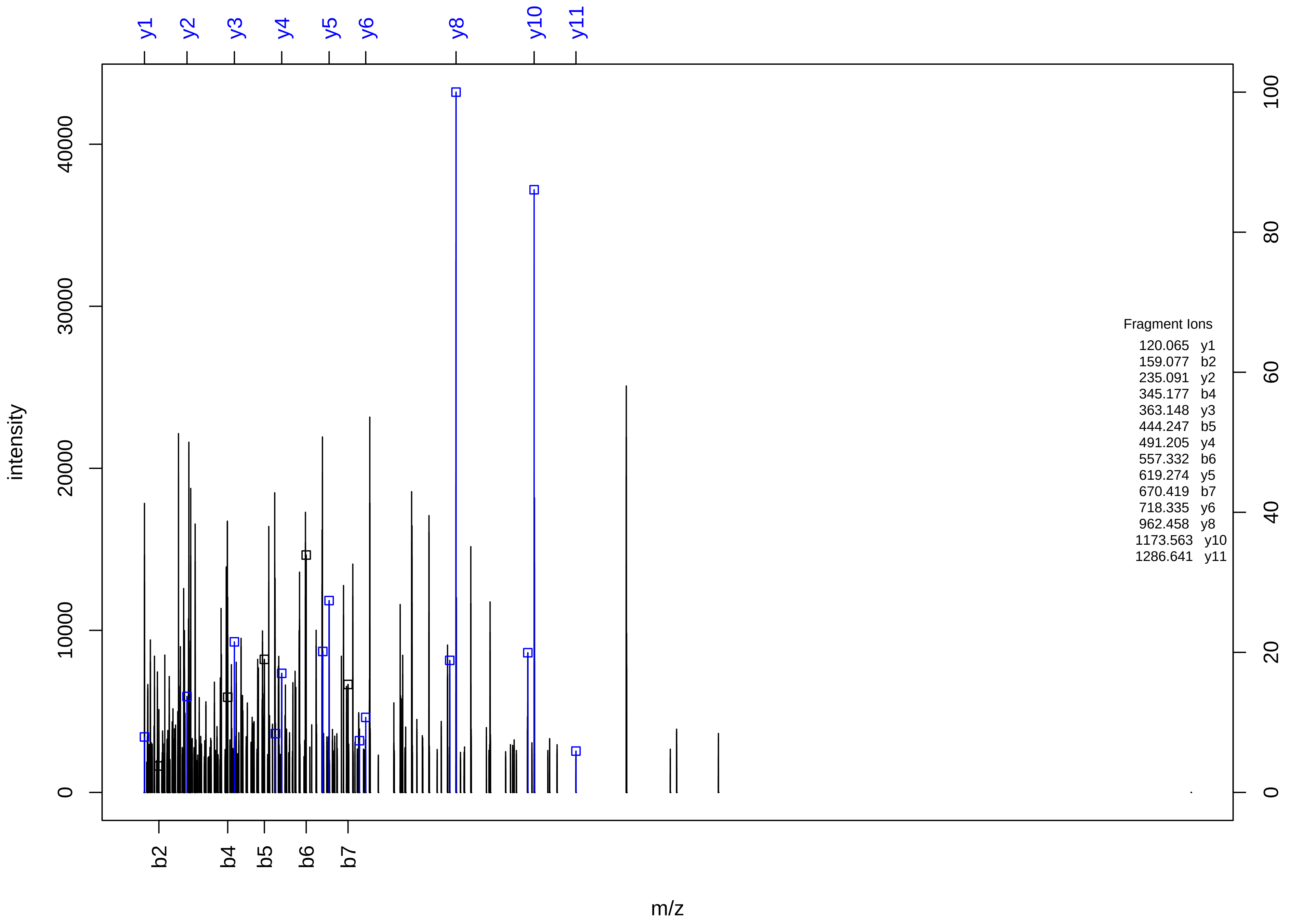

GTSVVLICPQDGMEAIPNPFVQQQDT / File: sydney_29.raw; SpectrumID: ; scans: 14265

IGPSILNSDLANLGAECLR

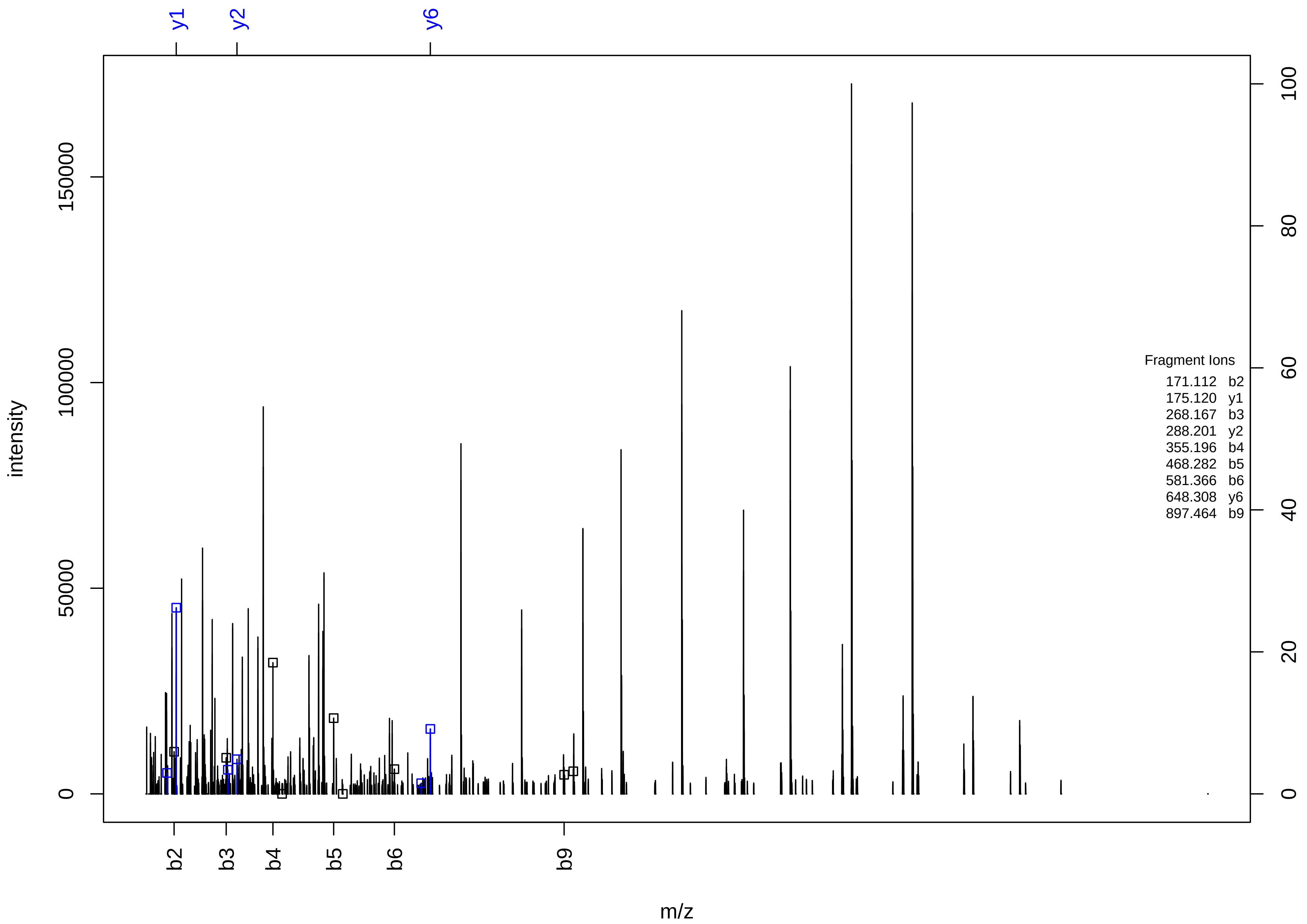

IGPSILNSDLANLGAECLR / File: sydney_30.raw; SpectrumID: ; scans: 11230

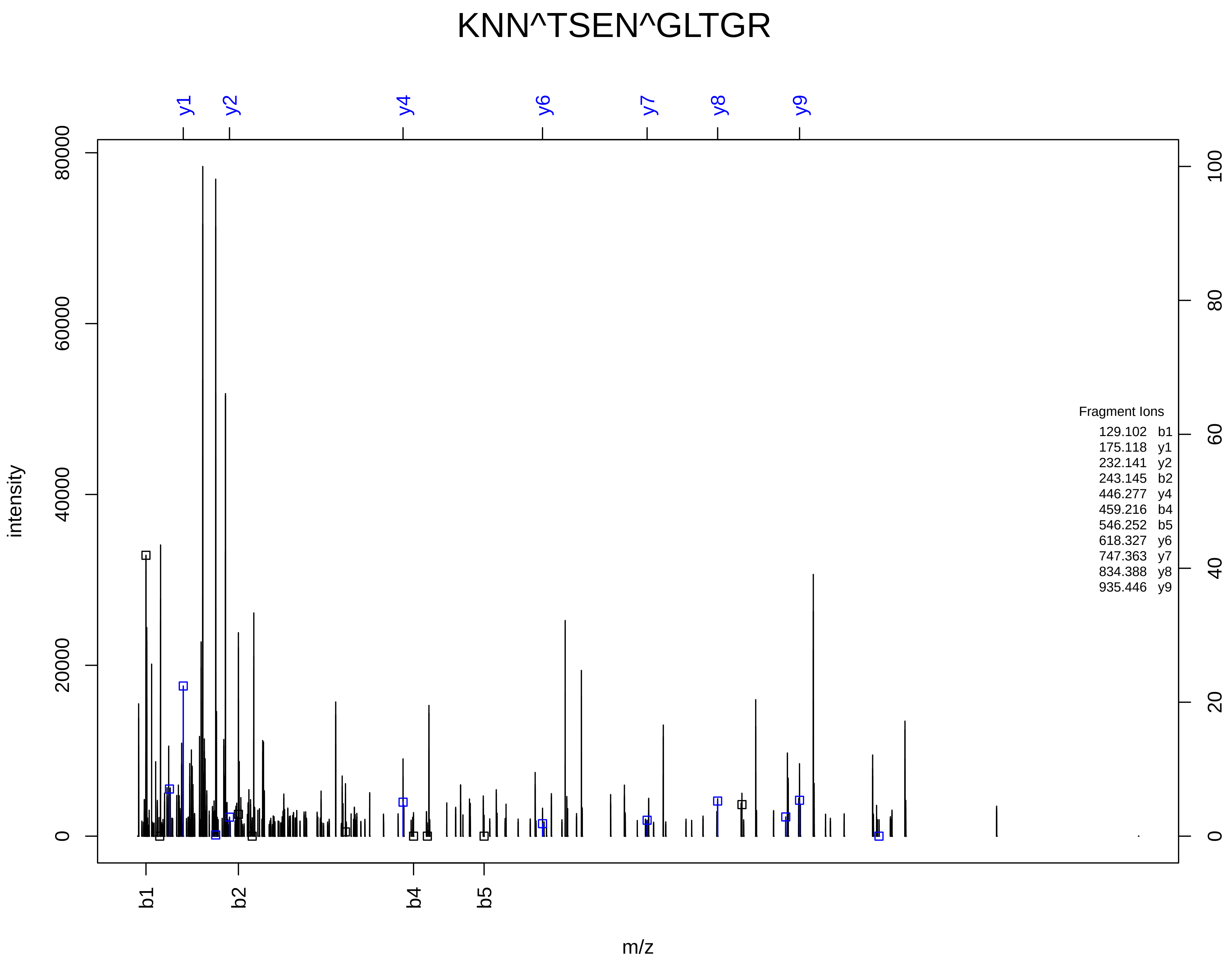

KNN^TSEN^GLTGR
y1
y2
y4
y6
y7
y8
y9
intensity
0
20000
40000
60000
80000
0
20
40
60
80
100
Fragment Ions
129.102 b1
175.118 y1
232.141 y2
243.145 b2
446.277 y4
459.216 b4
546.252 b5
618.327 y6
747.363 y7
834.388 y8
935.446 y9
b1
b2
b4
b5
m/z
KNNTSENGLTGR / File: sydney_30.raw; SpectrumID: ; scans: 3439

DLQEVIELTK

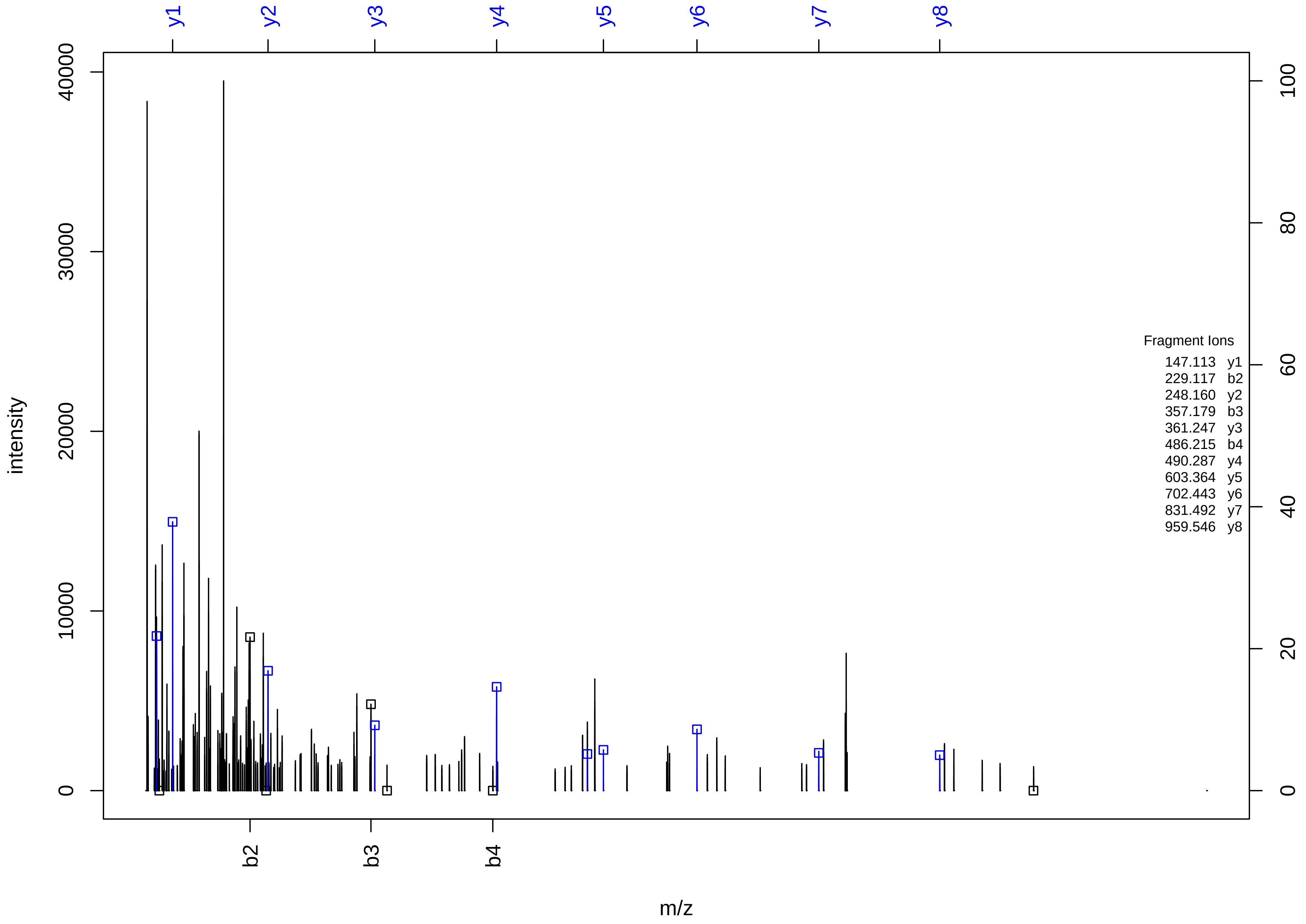

DLQEVIELTK / File: sydney_30.raw; SpectrumID: ; scans: 8535

CKM*DM*MSLN^SQLLDAIQ^QK

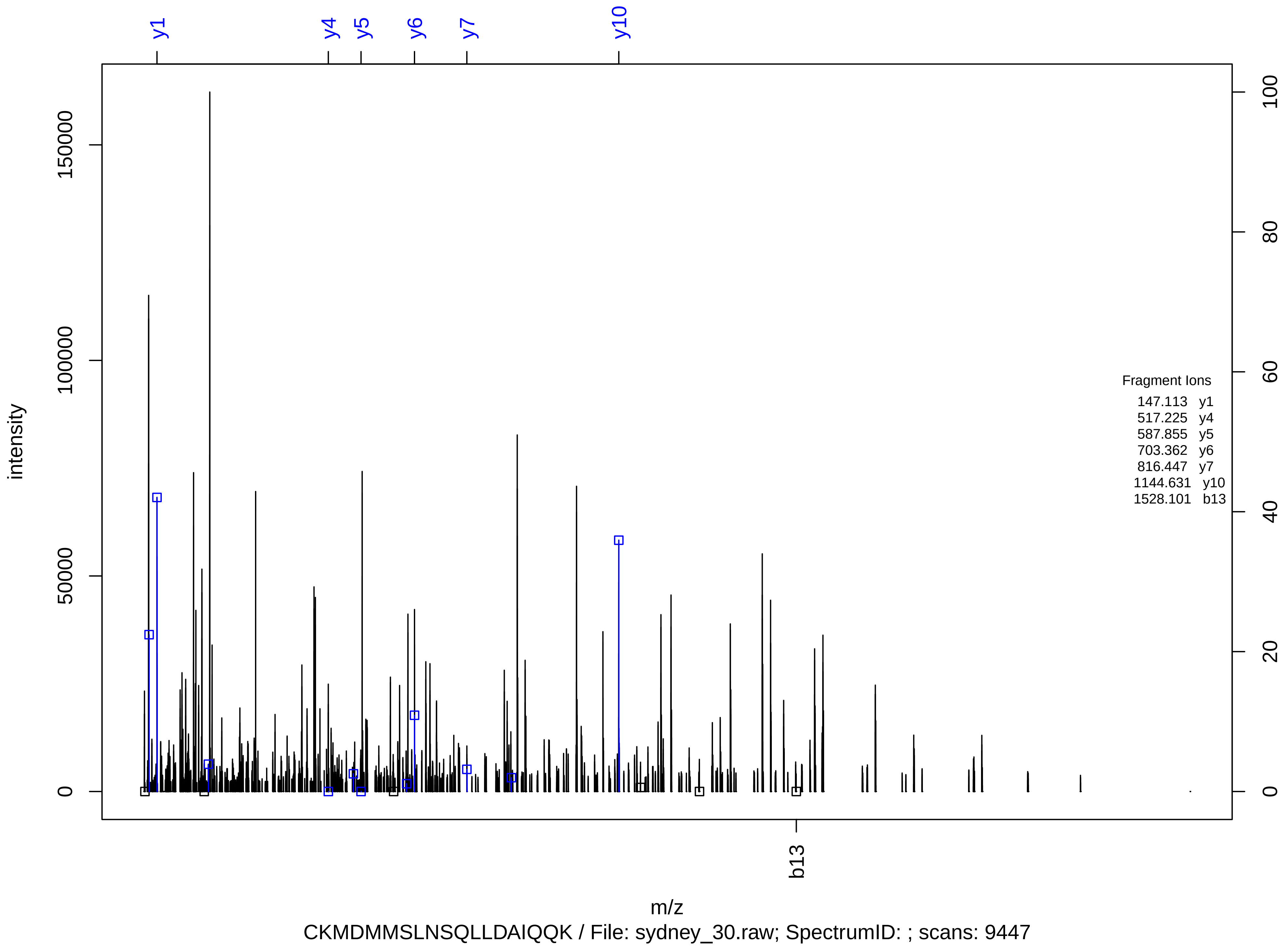

CKMDMMSLNSQLLDAIQQK / File: sydney_30.raw; SpectrumID: ; scans: 9447

ADEIESYLENLK

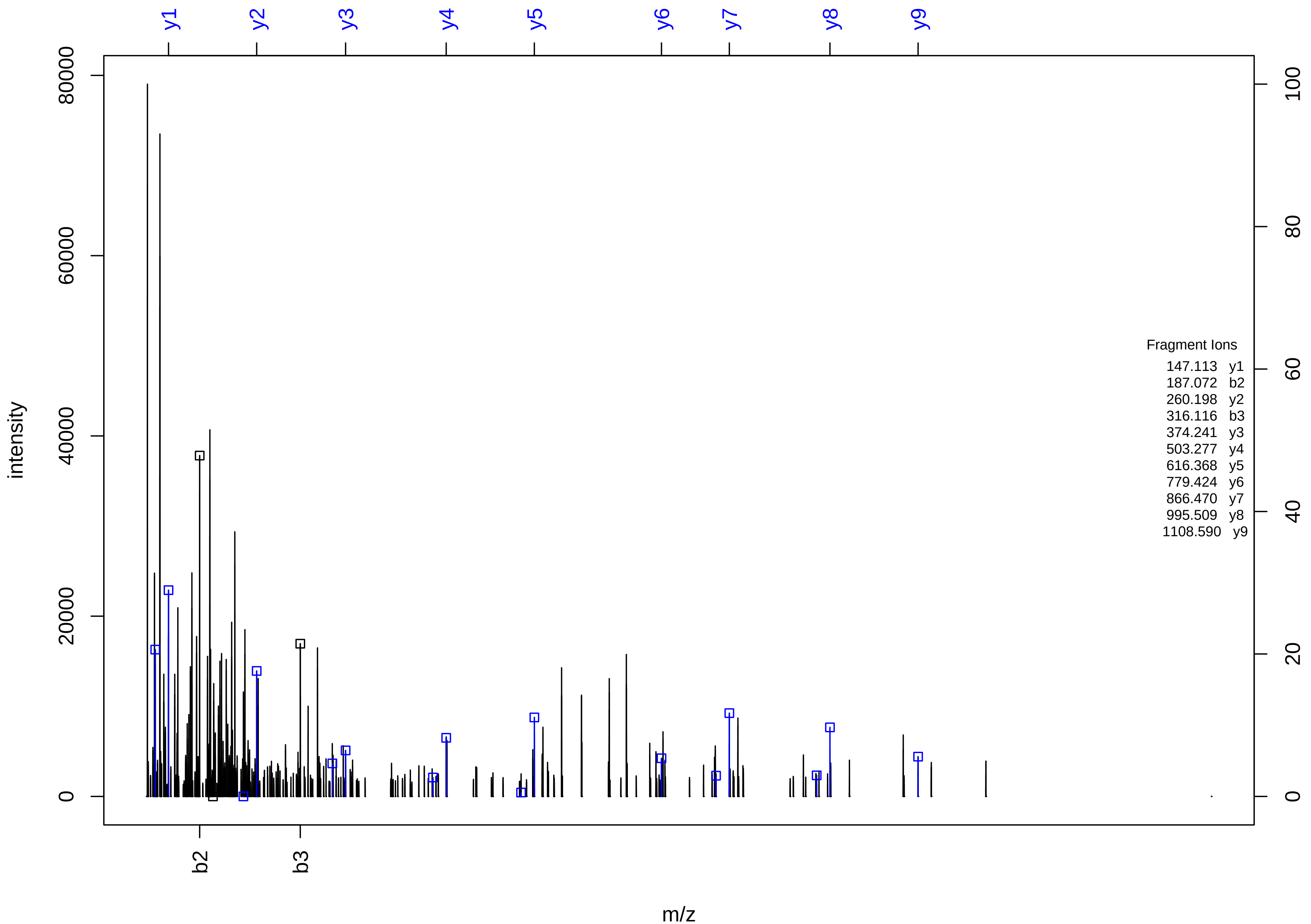

ADEIESYLENLK / File: sydney_30.raw; SpectrumID: ; scans: 9271

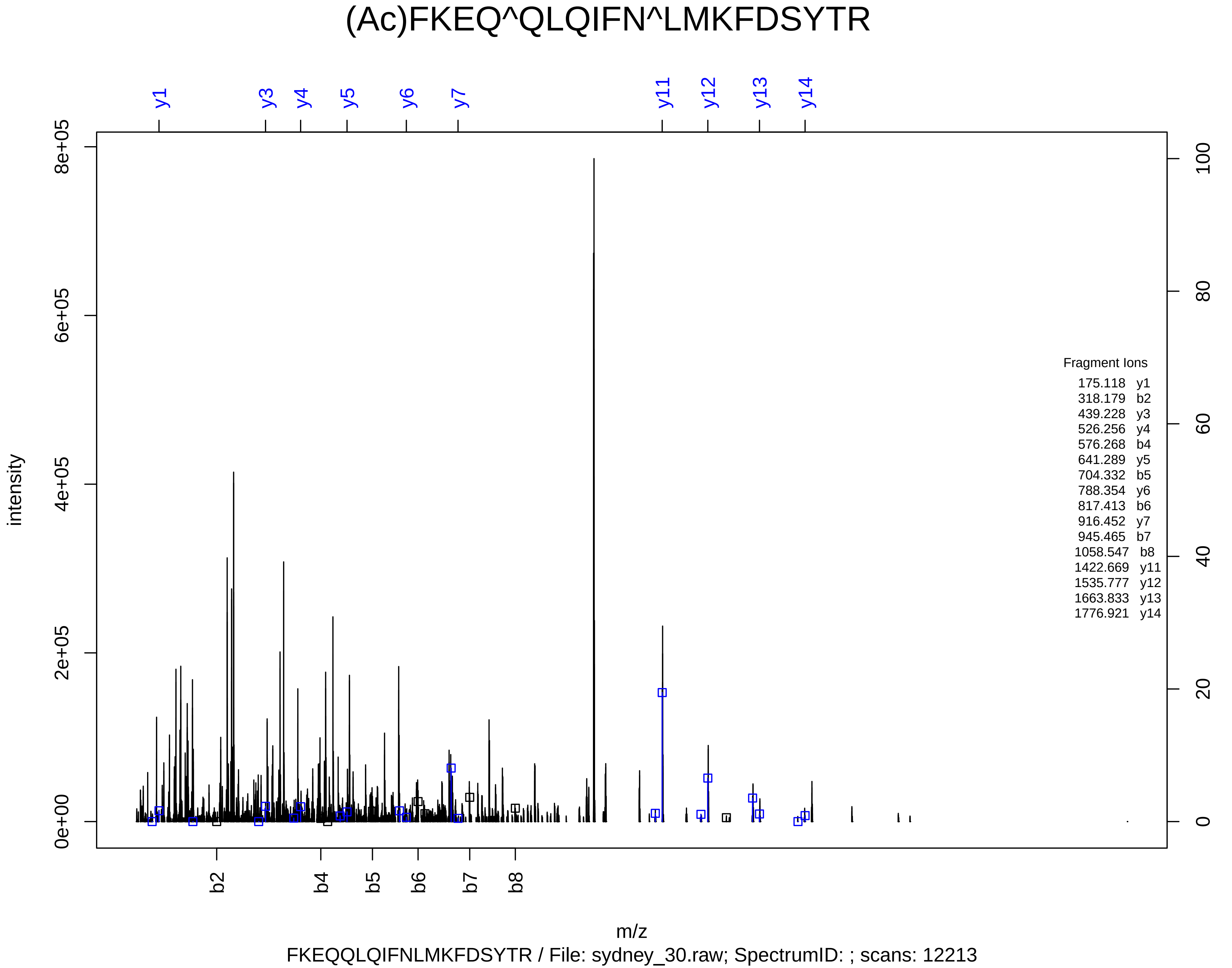
(Ac)FKEQ^QLQIFN^LMKFDSYTR
y1
y3
y4
y5
y6
y7
y11
y12
y13
y14
intensity
0e+00
2e+05
4e+05
6e+05
8e+05
0
20
40
60
80
100
b2
b4
b5
b6
b7
b8
m/z
Fragment Ions
175.118 y1
318.179 b2
439.228 y3
526.256 y4
576.268 b4
641.289 y5
704.332 b5
788.354 y6
817.413 b6
916.452 y7
945.465 b7
1058.547 b8
1422.669 y11
1535.777 y12
1663.833 y13
1776.921 y14
FKEQQLQIFNLMKFDSYTR / File: sydney_30.raw; SpectrumID: ; scans: 12213

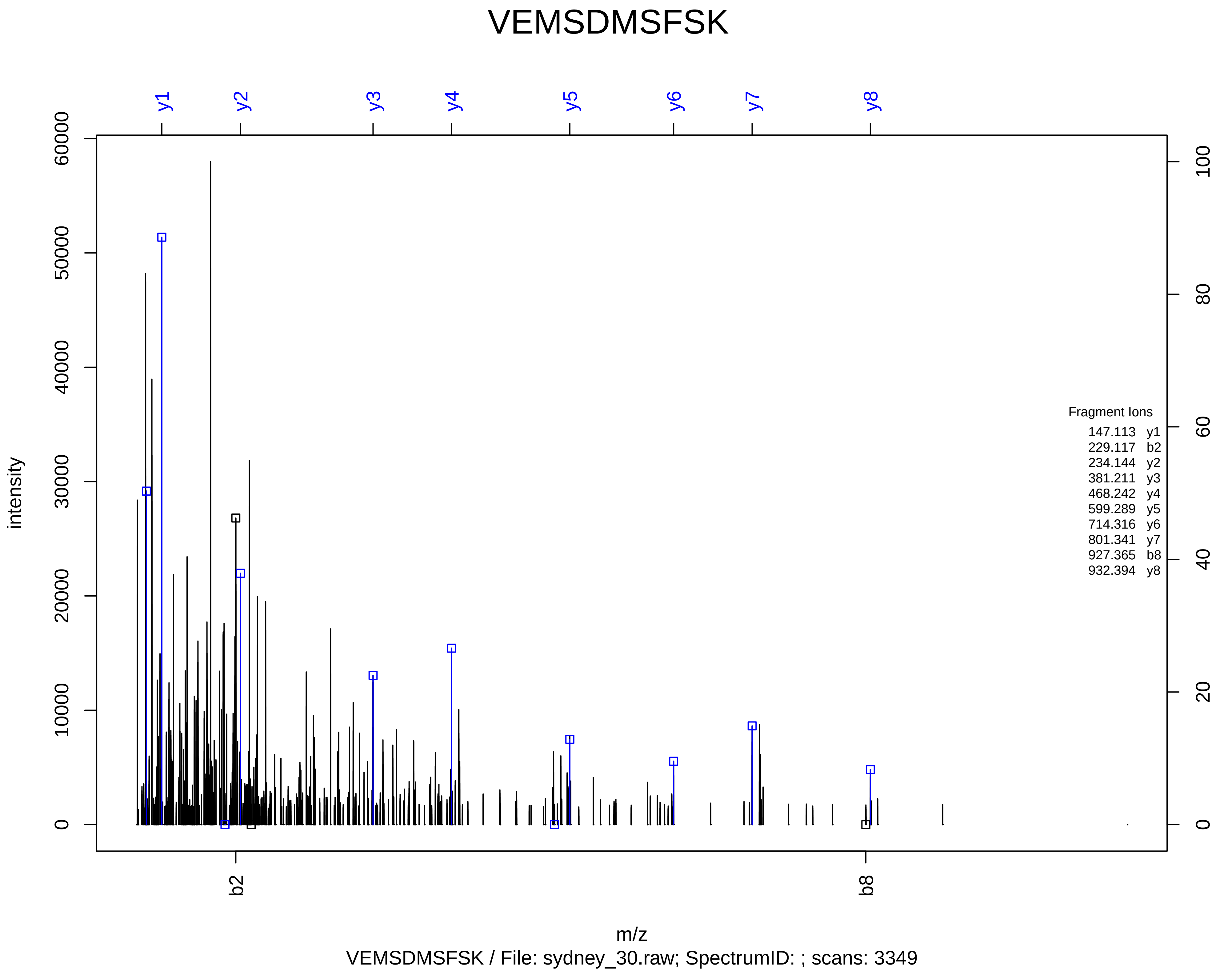

VEMSDMSFSK
y1
y2
y3
y4
y5
y6
y7
y8
b2
b8
intensity
m/z
Fragment Ions
147.113 y1
229.117 b2
234.144 y2
381.211 y3
468.242 y4
599.289 y5
714.316 y6
801.341 y7
927.365 b8
932.394 y8
VEMSDMSFSK / File: sydney_30.raw; SpectrumID: ; scans: 3349

MIITQYIPK

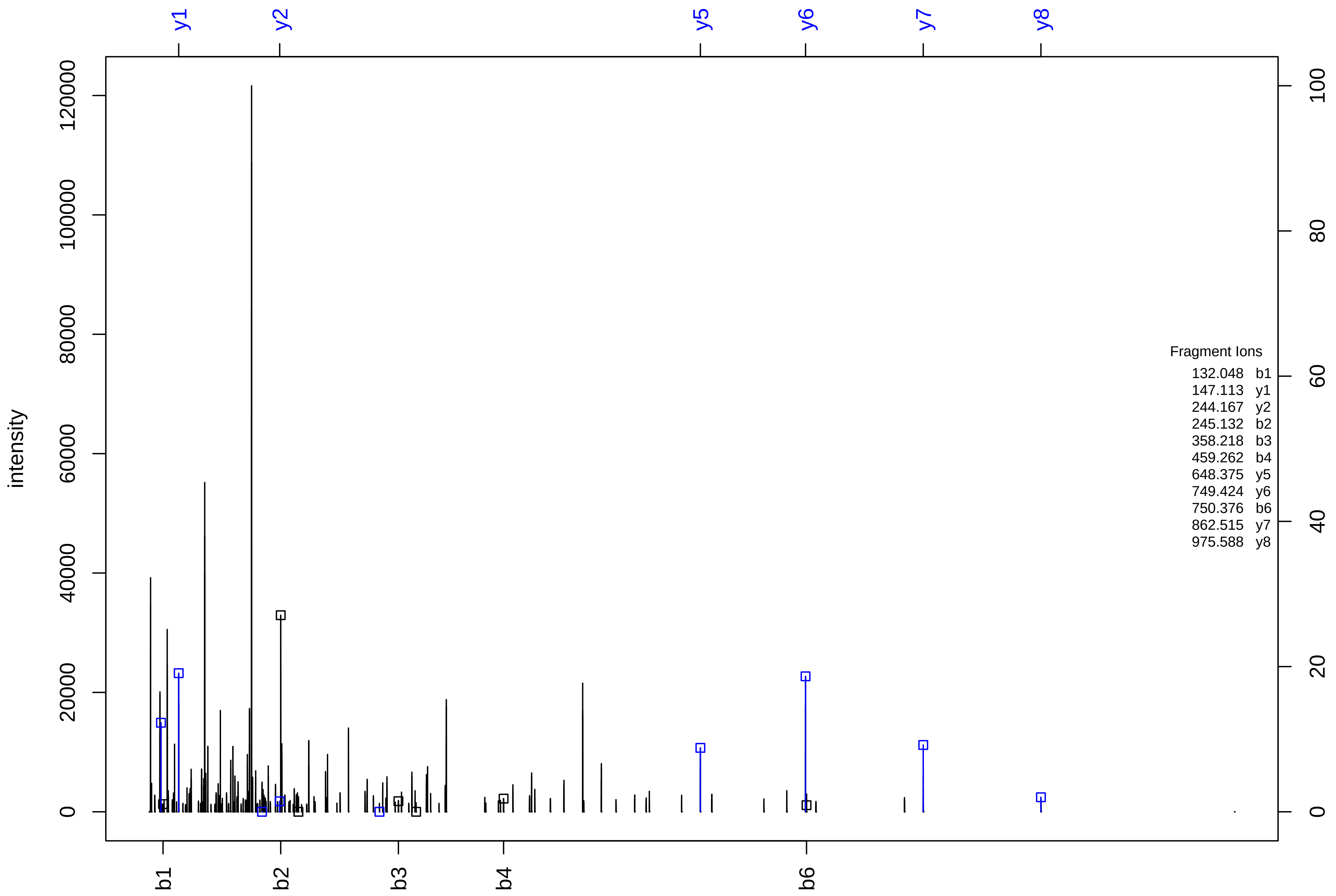

MIITQYIPK / File: sydney_30.raw; SpectrumID: ; scans: 8442

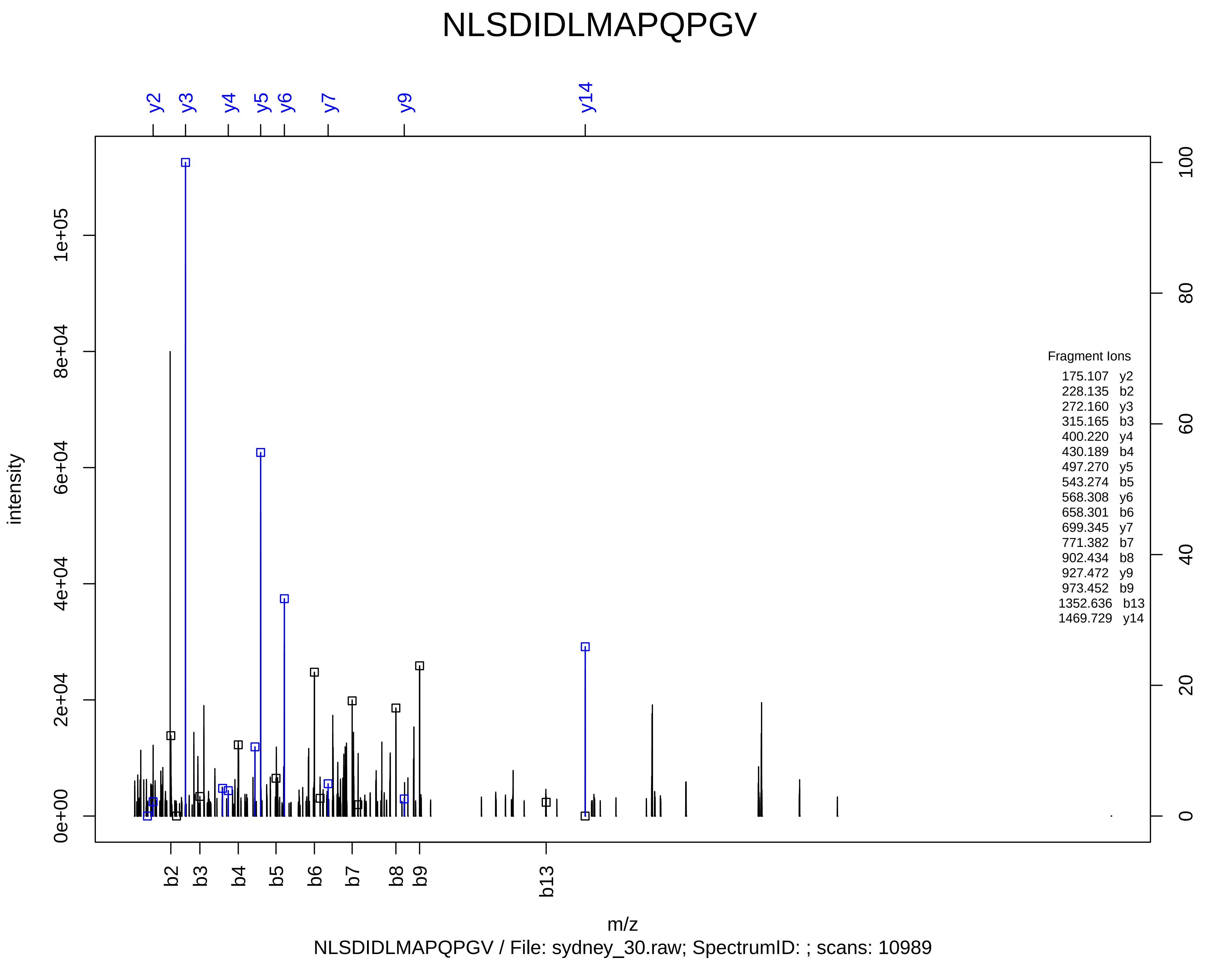
NLSDIDLMAPQPGV
y2
y3
y4
y5
y6
y7
y9
y14
intensity
0e+00
2e+04
4e+04
6e+04
8e+04
1e+05
0
20
40
60
80
100
b2
b3
b4
b5
b6
b7
b8
b9
b13
m/z
Fragment Ions
175.107 y2
228.135 b2
272.160 y3
315.165 b3
400.220 y4
430.189 b4
497.270 y5
543.274 b5
568.308 y6
658.301 b6
699.345 y7
771.382 b7
902.434 b8
927.472 y9
973.452 b9
1352.636 b13
1469.729 y14
NLSDIDLMAPQPGV / File: sydney_30.raw; SpectrumID: ; scans: 10989

LKHLPEKYM*MNSVLENFTILQVVTN^R

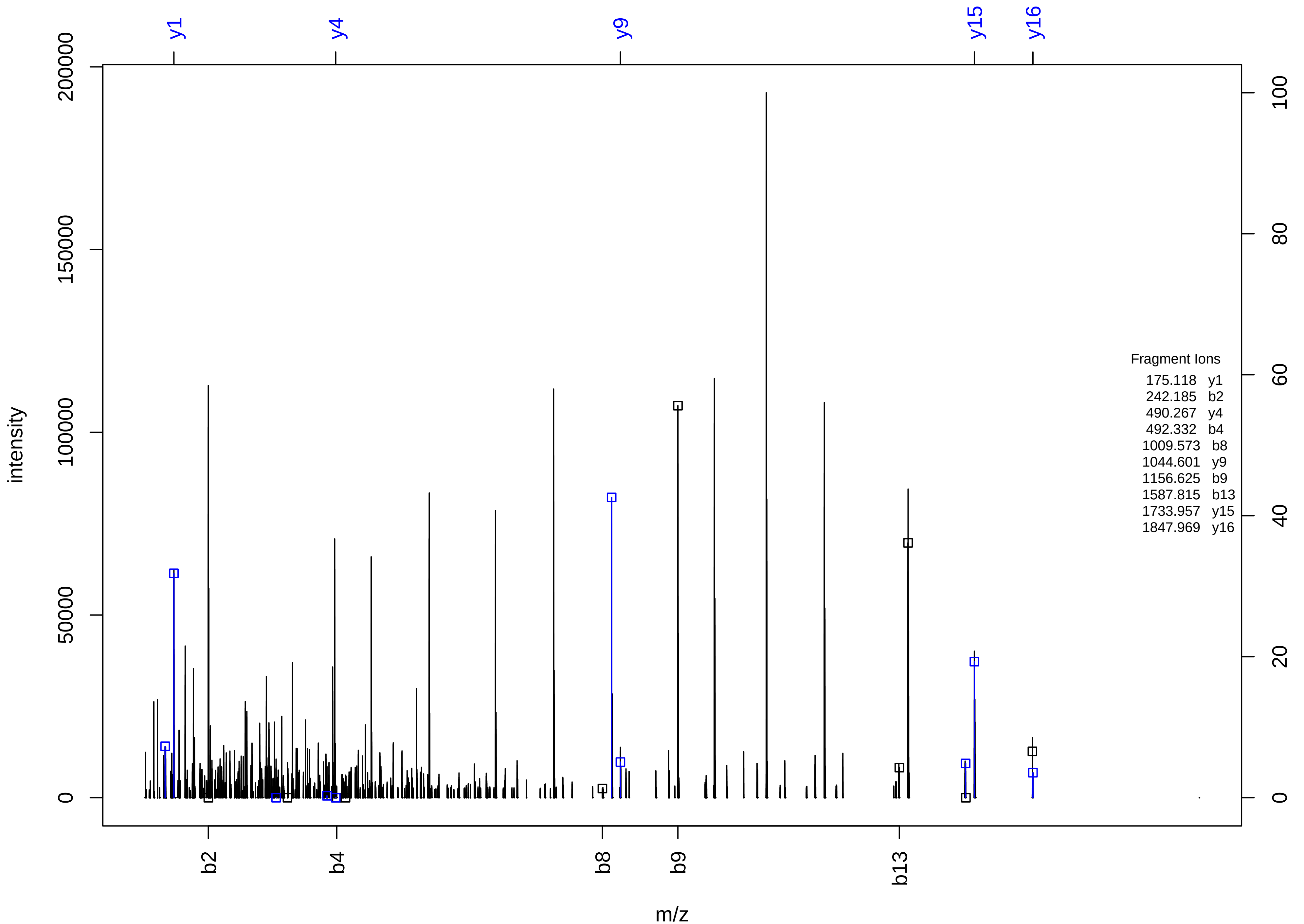

LKHLPEKYMMNSVLENFTILQVVTNR / File: sydney_31.raw; SpectrumID: ; scans: 14755

LN^ITEEQQ^AVLNRK

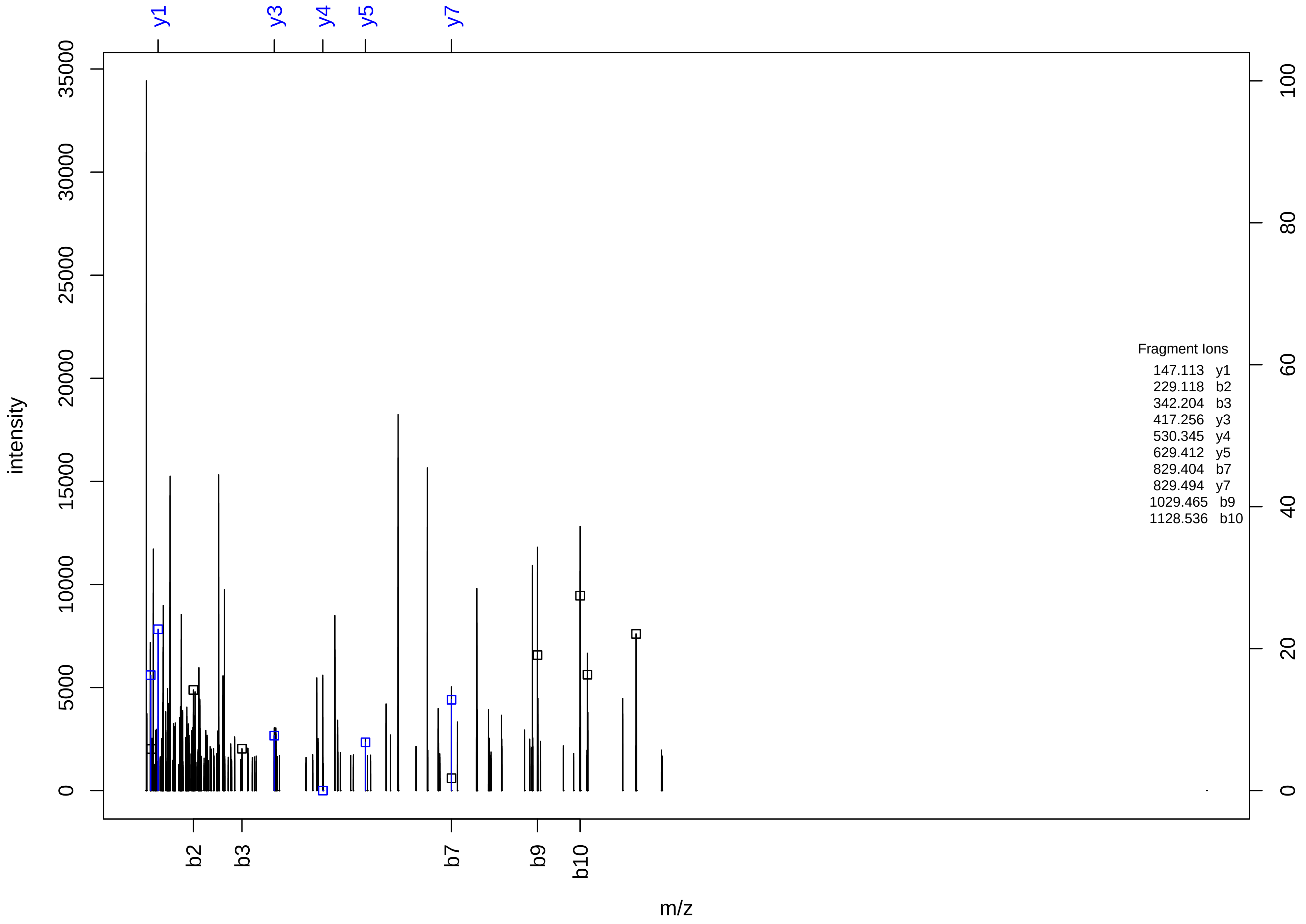

LNITEEQQAVLNRK / File: sydney_31.raw; SpectrumID: ; scans: 11889

DNˆQˆRVLVNTK

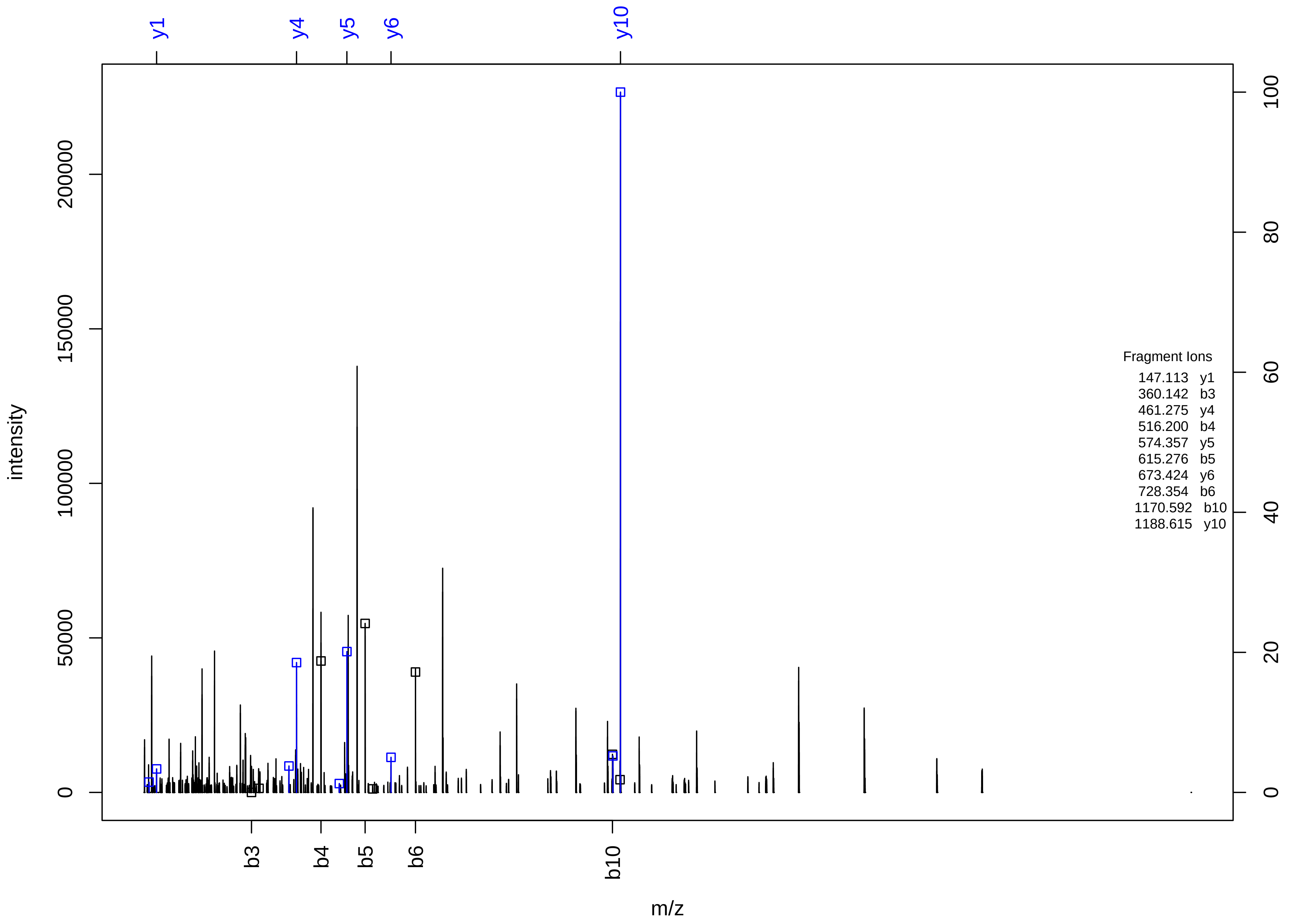

DNQRVLVNTK / File: sydney_31.raw; SpectrumID: ; scans: 10299

FQIGDYLDIAITPPNR

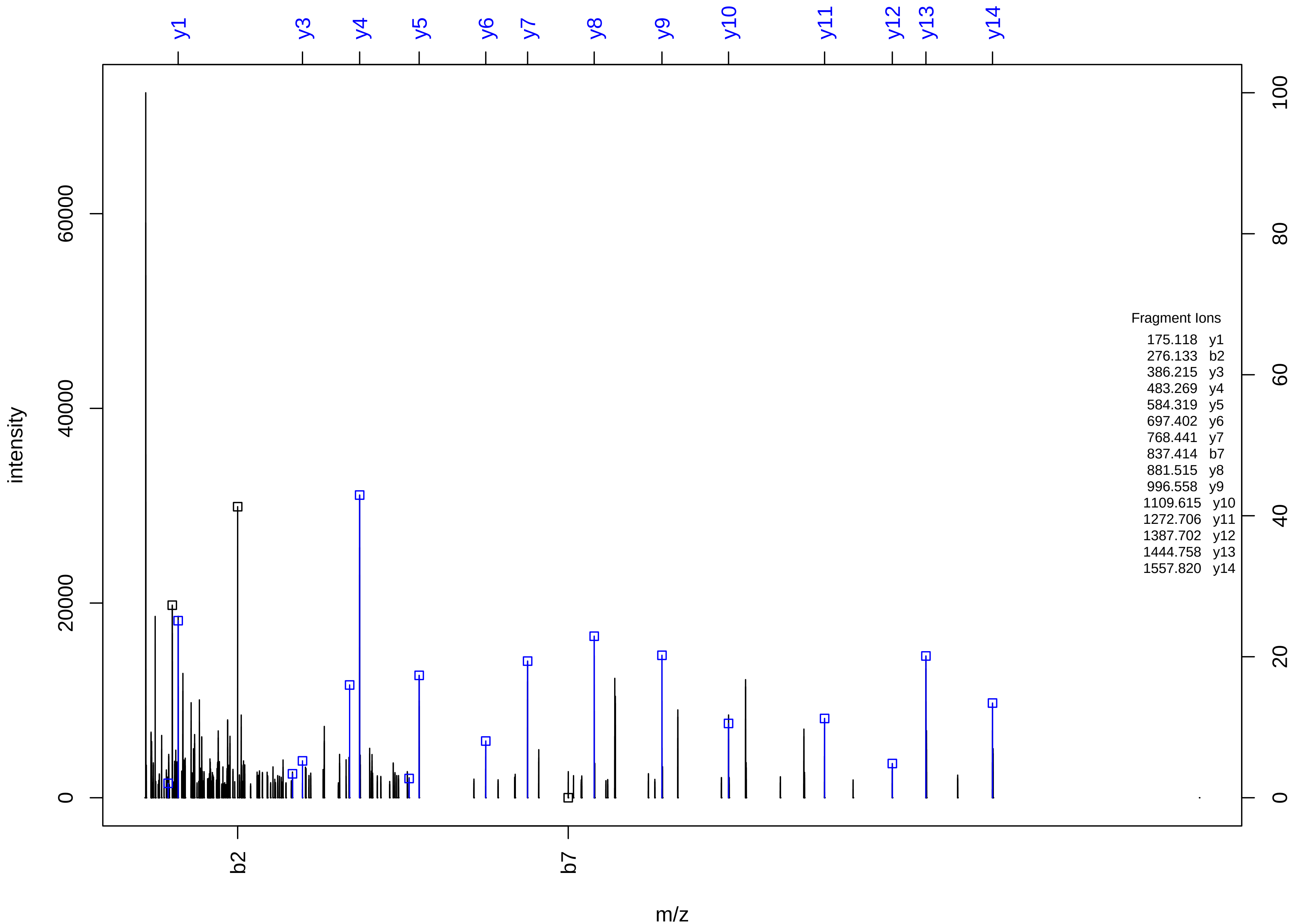

FQIGDYLDIAITPPNR / File: sydney_31.raw; SpectrumID: ; scans: 12771

VPAATSAIITNDGIGINPAQTAGNVFLK

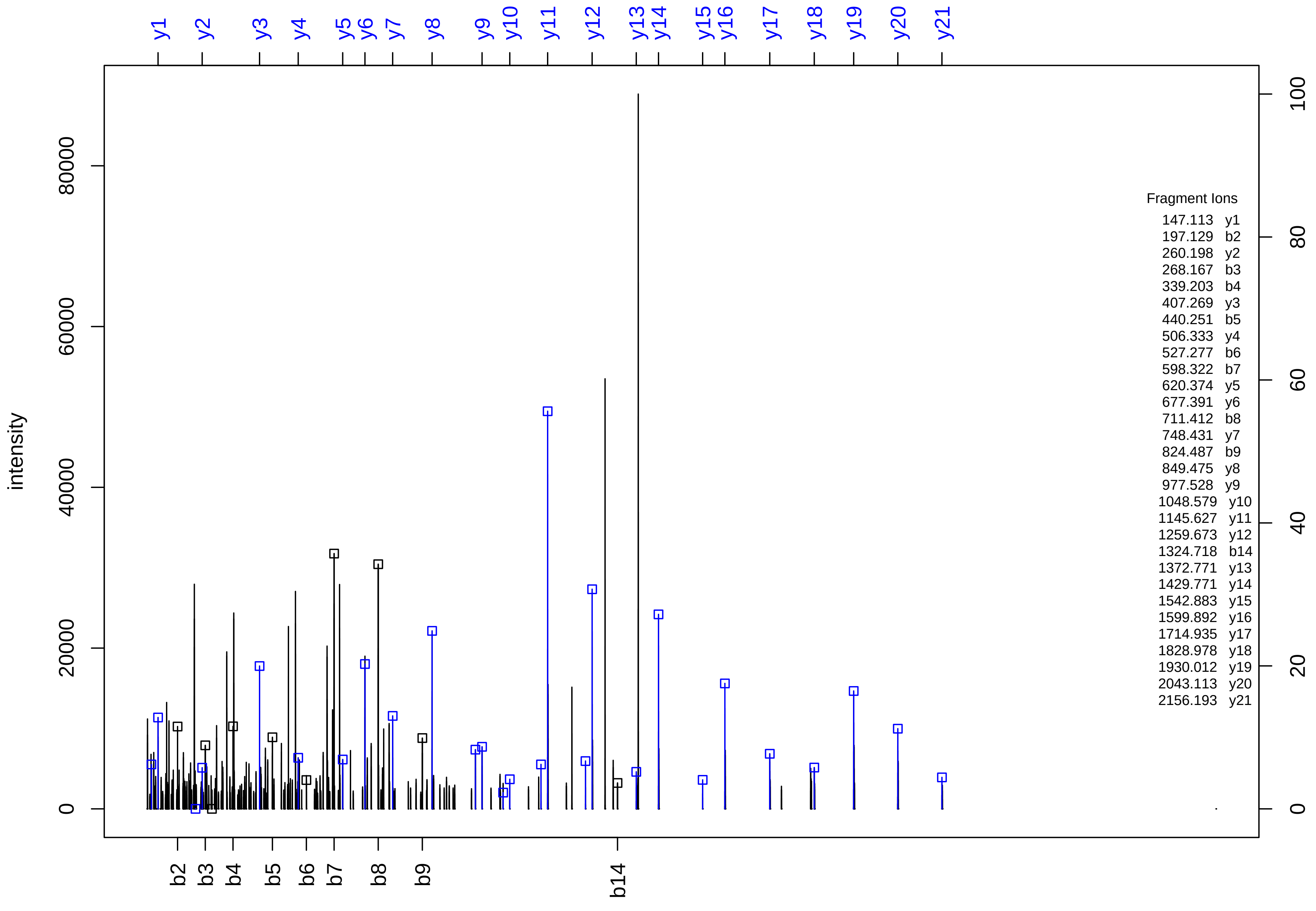

VPAATSAIITNDGIGINPAQTAGNVFLK / File: sydney_31.raw; SpectrumID: ; scans: 11625

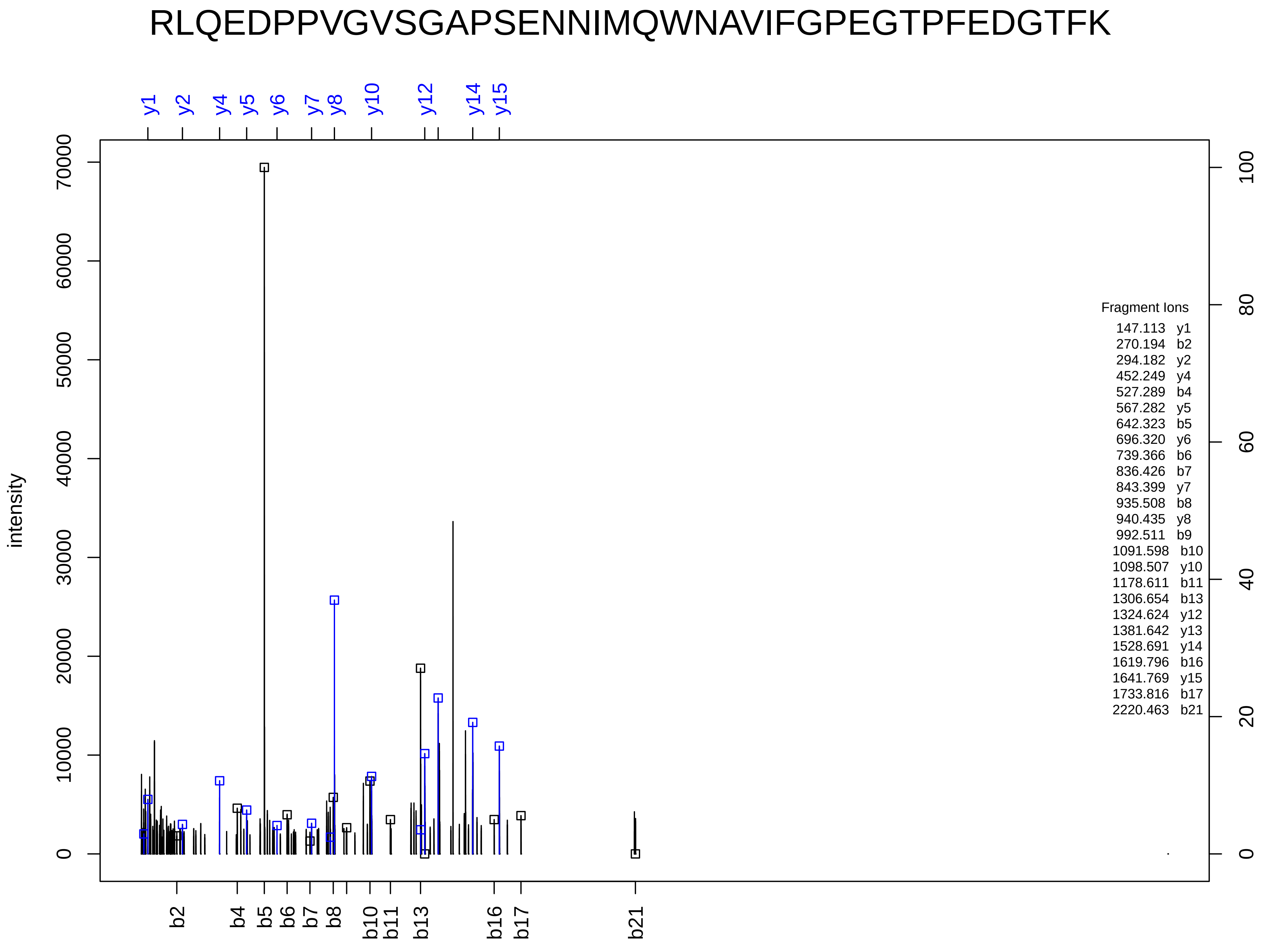

RLQEDPPVGVSGAPSENNIMQWNAVIFGPEGTPFEDGTFK / File: sydney_31.raw; SpectrumID: ; scans: 14137

ILGSLYAASEDQEK

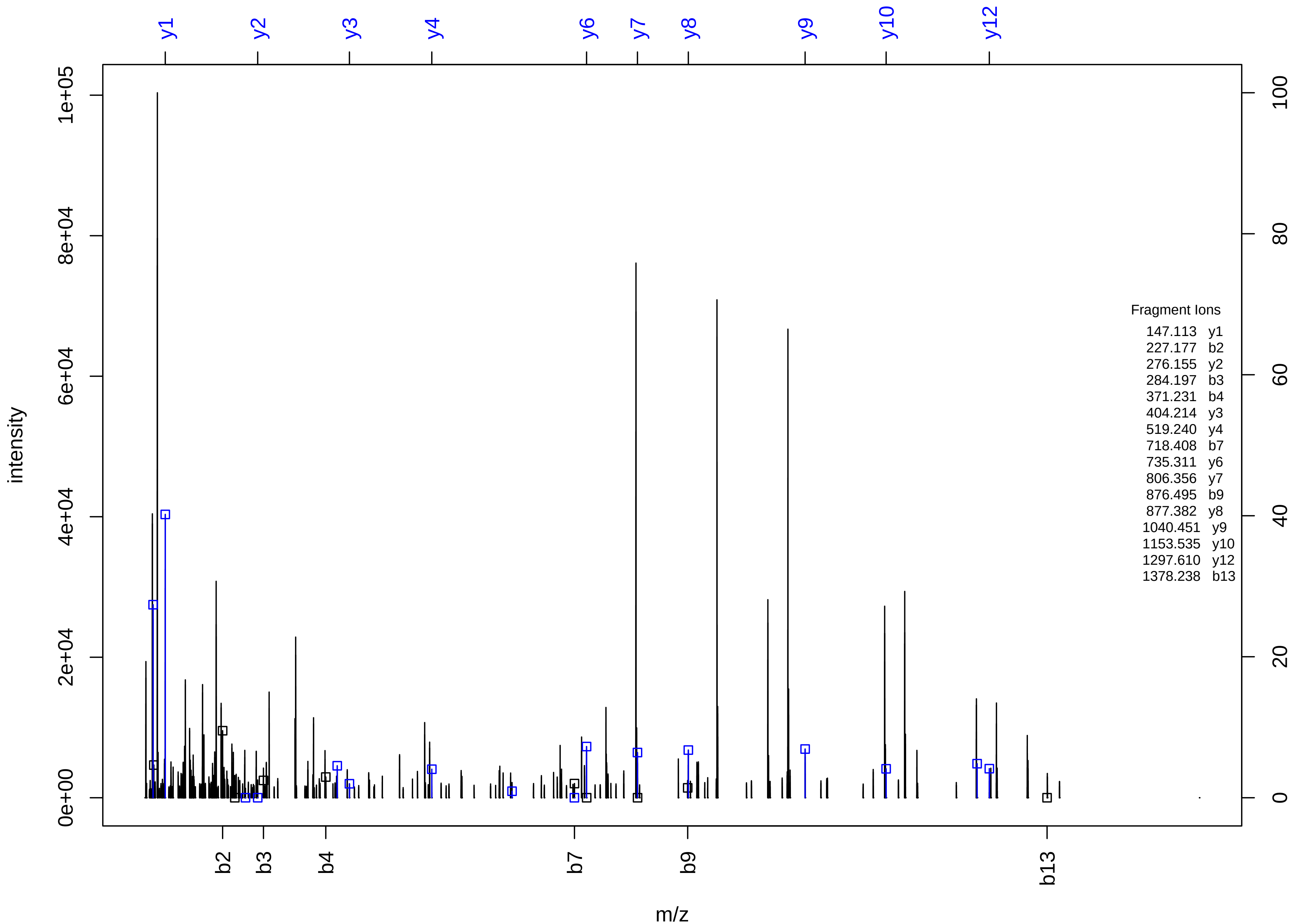

ILGSLYAASEDQEK / File: sydney_31.raw; SpectrumID: ; scans: 3942

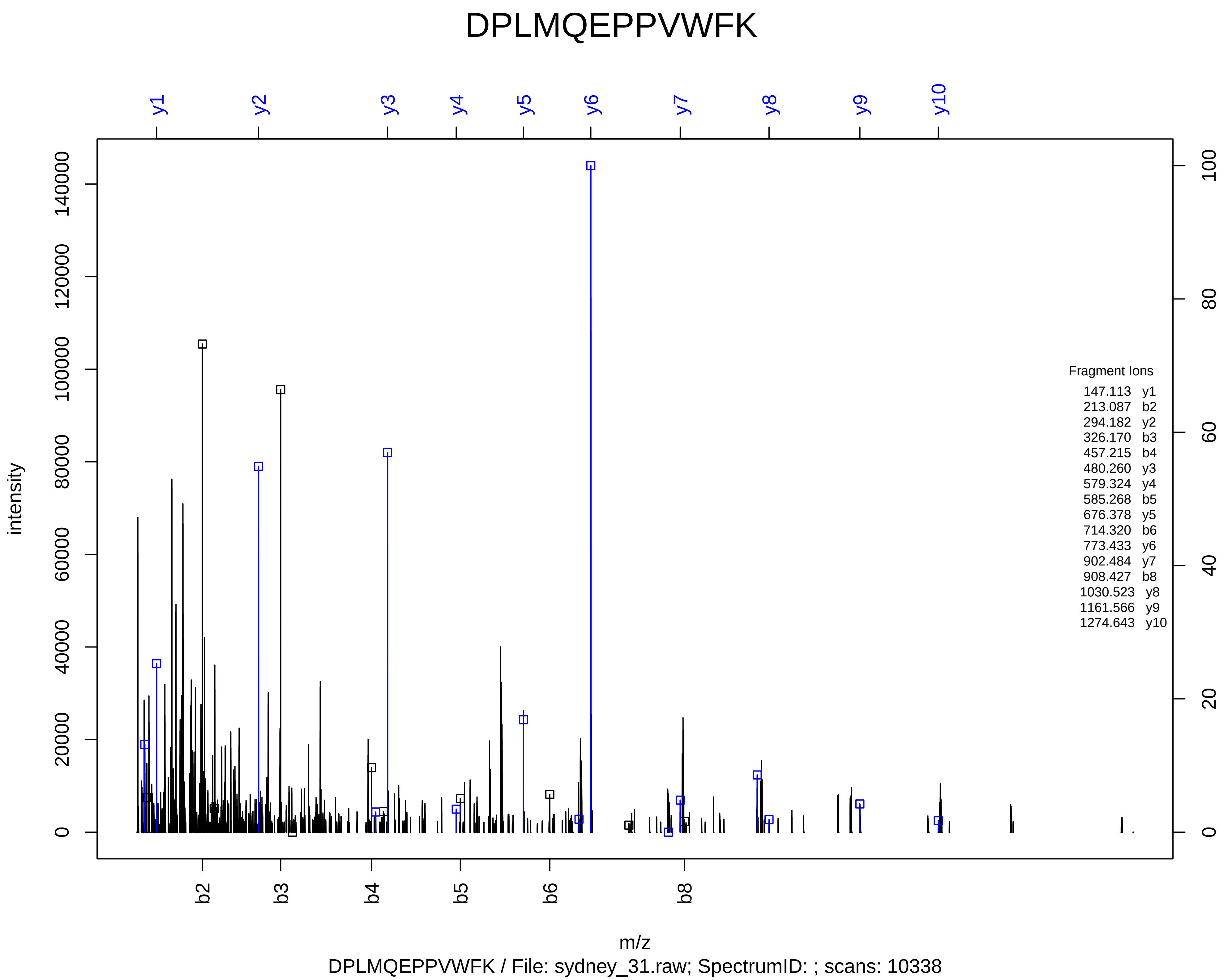
DPLMQEPPVWFK
y1
y2
y3
y4
y5
y6
y7
y8
y9
y10
intensity
0
20000
40000
60000
80000
100000
120000
140000
0
20
40
60
80
100
b2
b3
b4
b5
b6
b8
m/z
Fragment Ions
147.113 y1
213.087 b2
294.182 y2
326.170 b3
457.215 b4
480.260 y3
579.324 y4
585.268 b5
676.378 y5
714.320 b6
773.433 y6
902.484 y7
908.427 b8
1030.523 y8
1161.566 y9
1274.643 y10
DPLMQEPPVWFK / File: sydney_31.raw; SpectrumID: ; scans: 10338

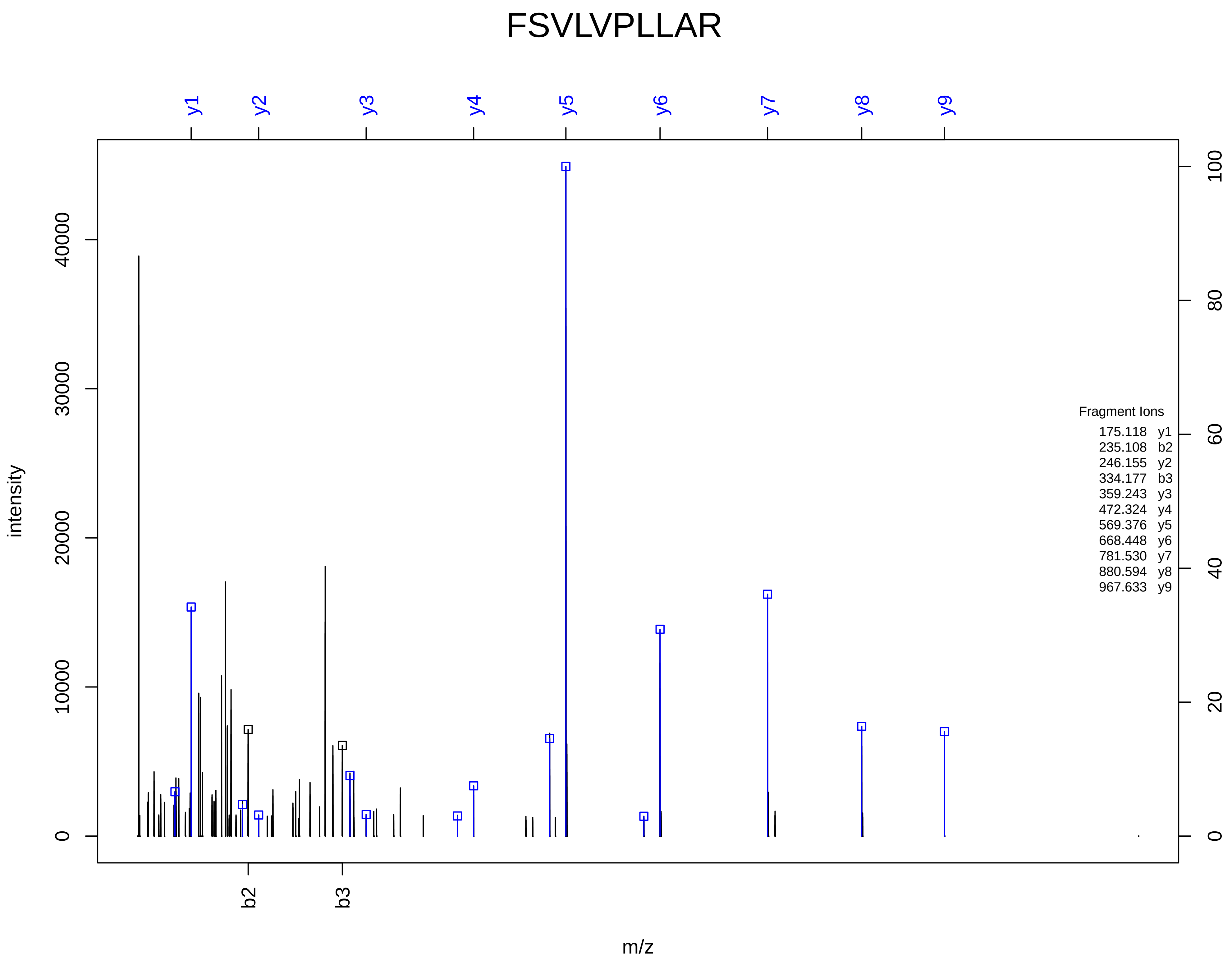
FSVLVPLLAR
y1
y2
y3
y4
y5
y6
y7
y8
y9
b2
b3
intensity
m/z
Fragment Ions
175.118 y1
235.108 b2
246.155 y2
334.177 b3
359.243 y3
472.324 y4
569.376 y5
668.448 y6
781.530 y7
880.594 y8
967.633 y9
FSVLVPLLAR / File: sydney_31.raw; SpectrumID: ; scans: 12100
