# Supplementary material for: Cellular Proteomic Profiling Using Proximity Labeling by TurboID-NES in Microglial and Neuronal Cell Lines
Source: Mol Cell Proteomics. 2023 Apr 14;22(6):100546. doi: 10.1016/j.mcpro.2023.100546 (PMC10205547; doi:10.1016/j.mcpro.2023.100546)
Supplement: Supplemental Data 2 [file mmc2.pdf]

Annotated Spectra for protein identifications on the basis of a single unique peptide within the Affinity Purified (AP) proteome.

Description:

b and y ion matches were annotated below and above each MS/MS spectrum and marked at the top of the centroided peak intensity by a small black or blue box, only for ions in the MS/MS spectrum within +/- 0.6 Da of the theoretical b or y ion mass.

(Ac) in the title of an annotated PSM PDF page, e.g.,“(Ac)PEPSEQ” indicates the primary amino acid sequence for the PSM, PEPSEQ, was matched by MaxQuant with a variablemodification monoisotopic mass shift for acetylation at the protein N-terminus (+42.010565 Da).

“ in the annotated PSM title, e.g., “PEM*SEQ” indicates oxidation (+15.994915 Da) monoisotopic mass shift for the preceding methionine (M) residue.

^ in the title peptide sequence follows an asparagine (N) or glutamine (Q) residue and represents conversion to aspartate or glutamate, respectively, by deamidation, resulting in a +0.984016 Da monoisotopic mass increase for the preceding residue.

QVVGAVIDEGLTTK

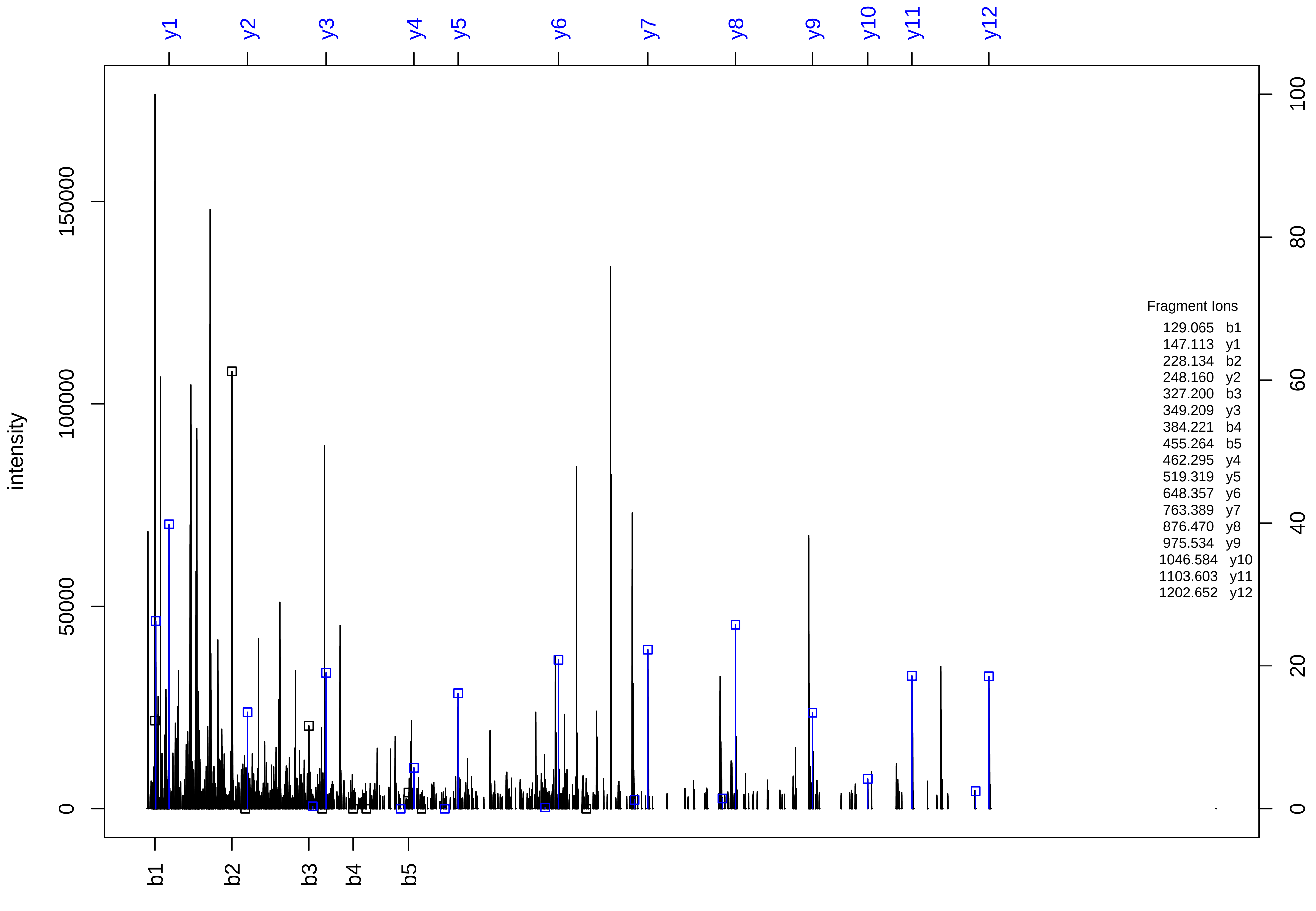

QVVGAVIDEGLTTK / File: sydney_ip_01.raw; SpectrumID: ; scans: 9859

IAIYELLFK

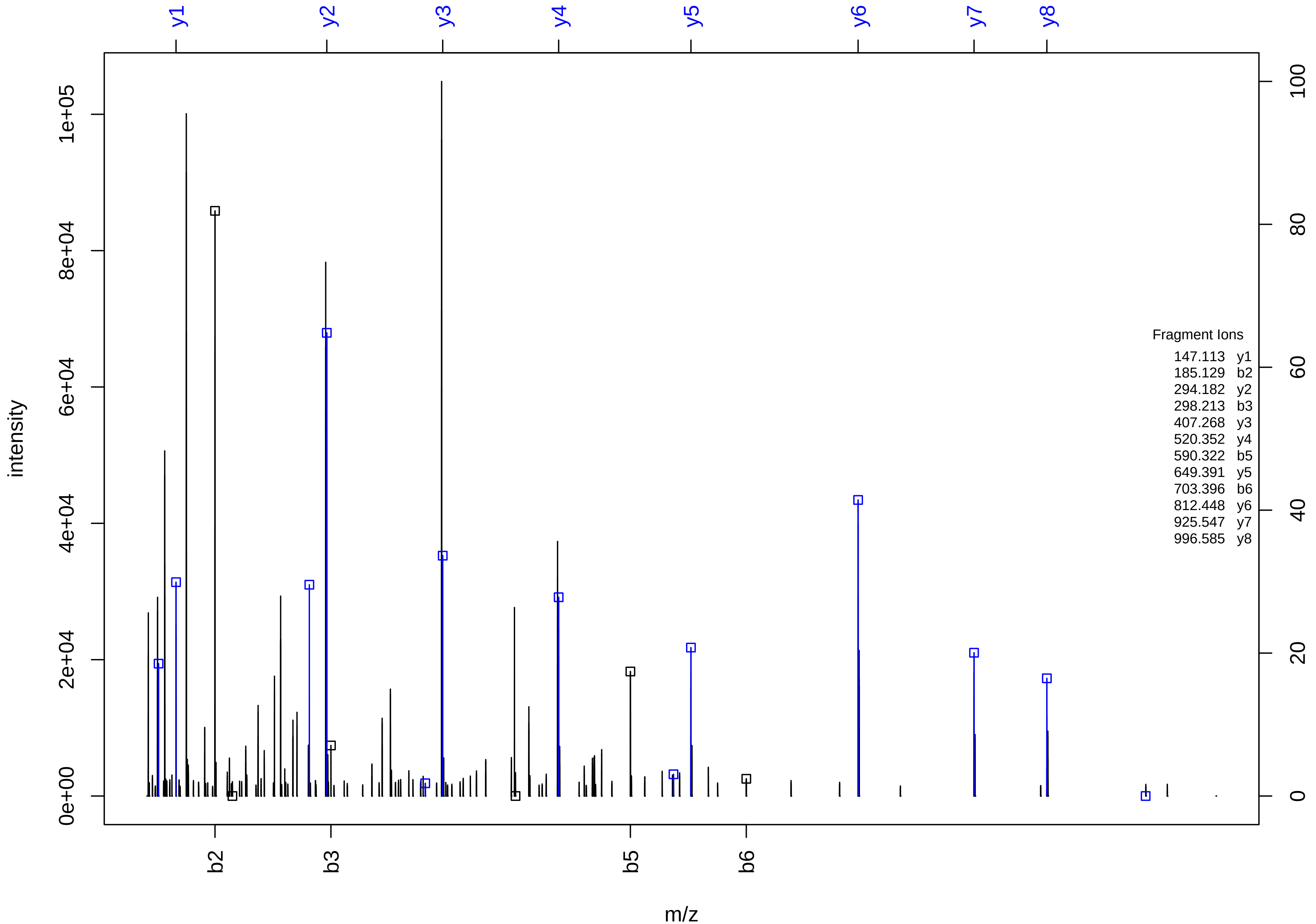

IAIYELLFK / File: sydney_ip_01.raw; SpectrumID: ; scans: 18771

ESEEKLKQ^Q^QQESAR

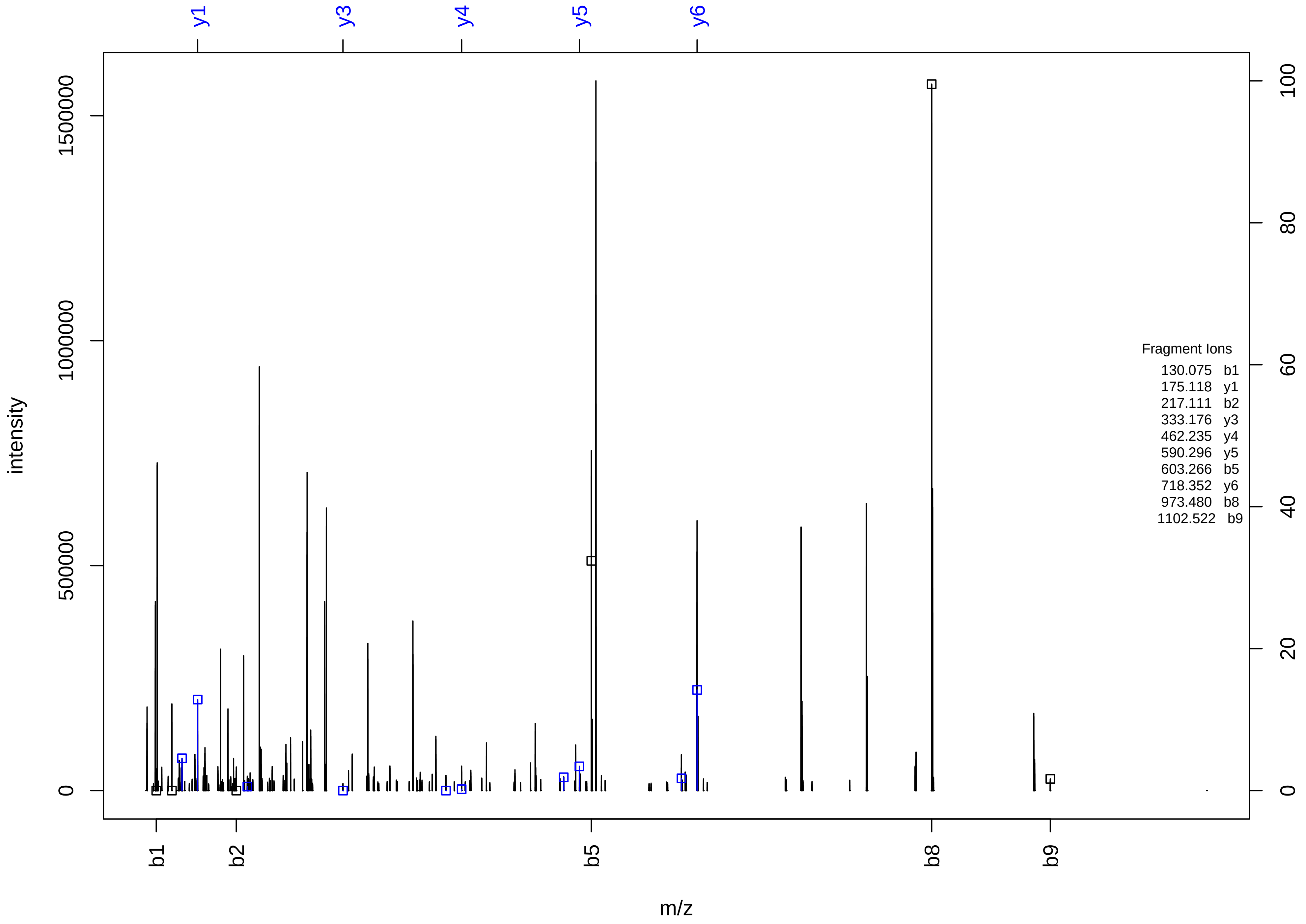

ESEEKLKQQQQESAR / File: sydney_ip_01.raw; SpectrumID: ; scans: 3199

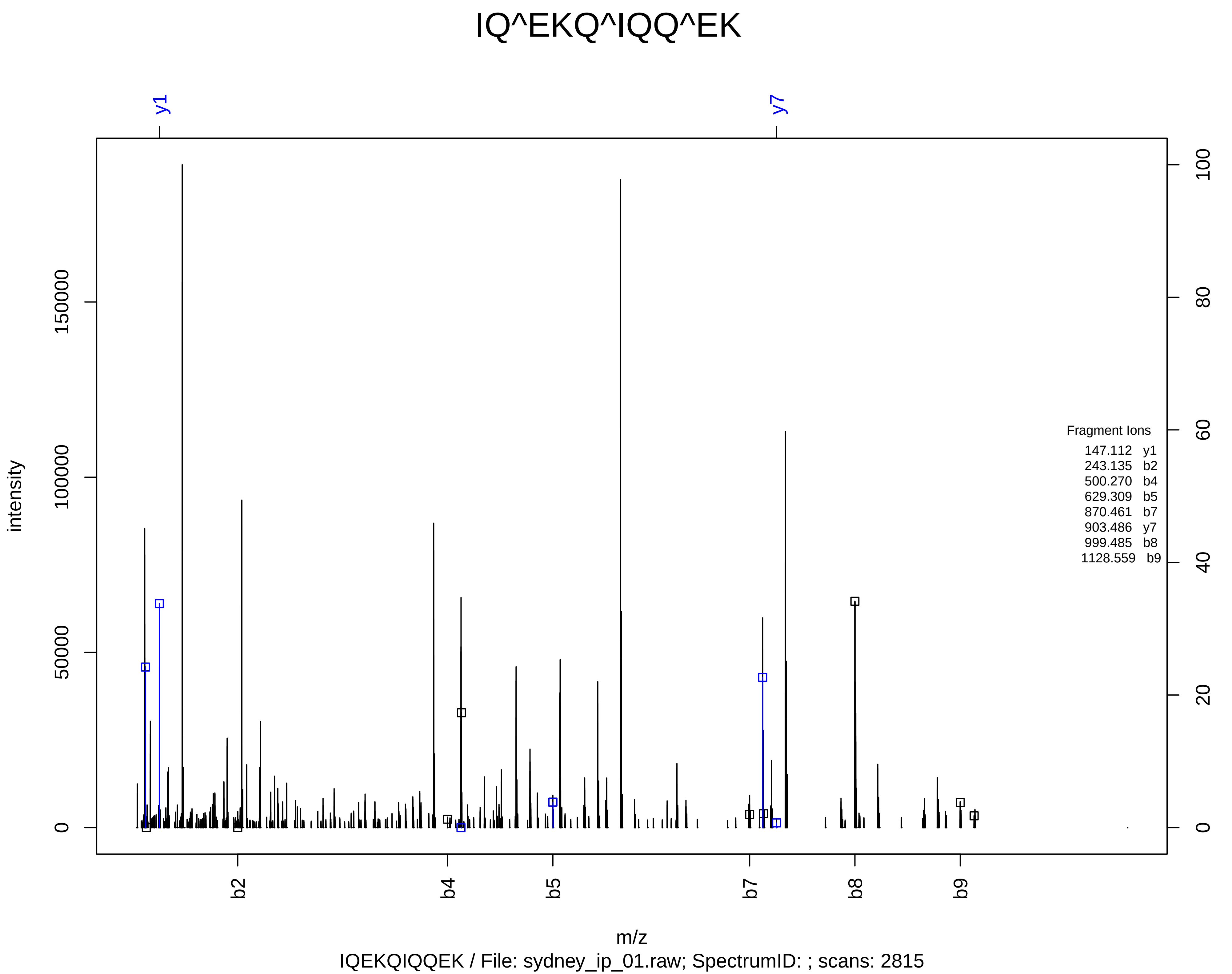
IQ^EKQ^IQQ^EK
y1
y7
intensity
0
50000
100000
150000
0
20
40
60
80
100
b2
b4
b5
b7
b8
b9
m/z
Fragment Ions
147.112 y1
243.135 b2
500.270 b4
629.309 b5
870.461 b7
903.486 y7
999.485 b8
1128.559 b9
IQEKQIQQEK / File: sydney_ip_01.raw; SpectrumID: ; scans: 2815

HPTGN^M*DVGFTR

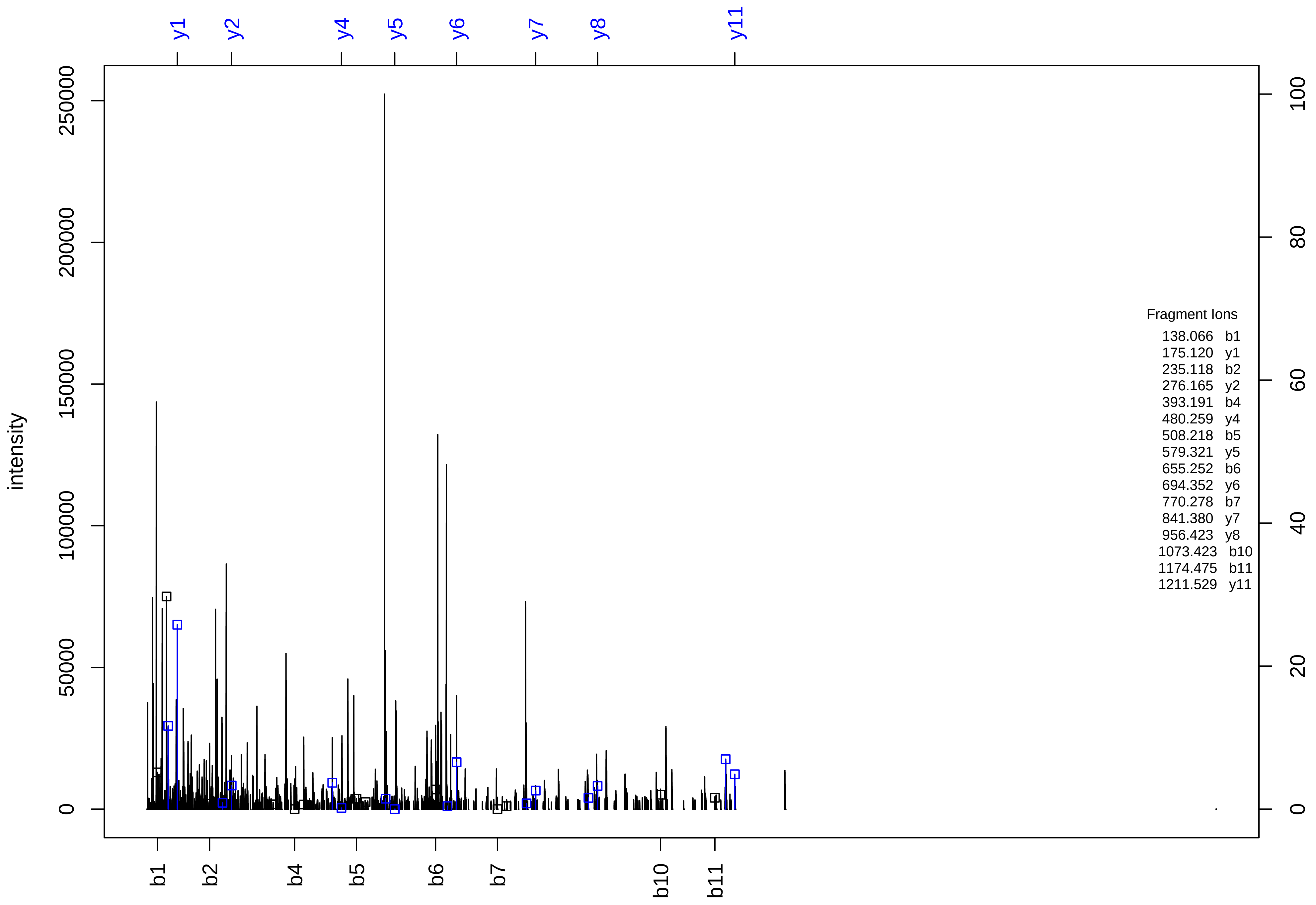

HPTGNMDVGFTR / File: sydney_ip_01.raw; SpectrumID: ; scans: 3213

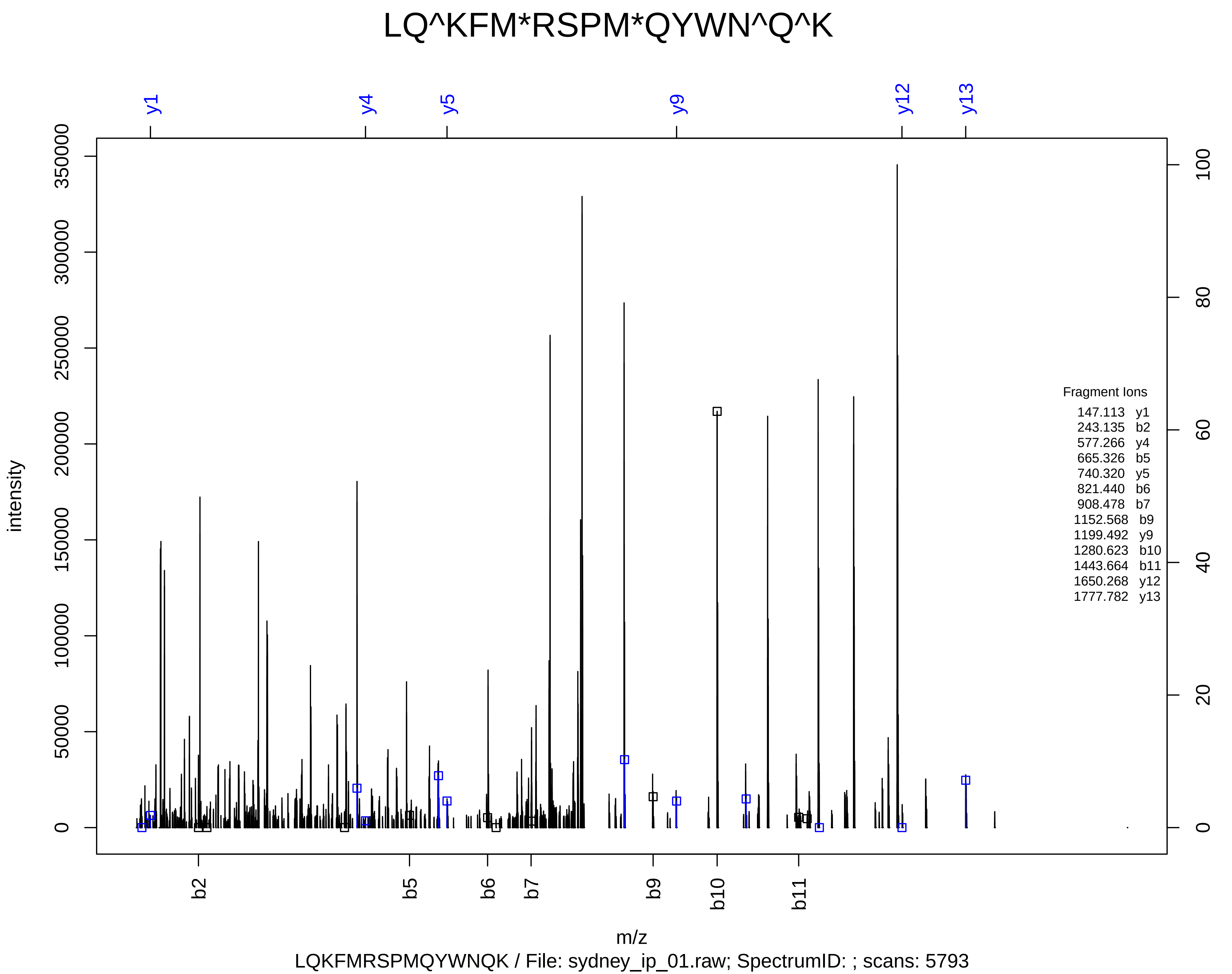
LQ^KFM*RSPM*QYWN^Q^K
y1
y4
y5
y9
y12
y13
intensity
350000
300000
250000
200000
150000
100000
50000
0
100
80
60
40
20
0
Fragment Ions
147.113 y1
243.135 b2
577.266 y4
665.326 b5
740.320 y5
821.440 b6
908.478 b7
1152.568 b9
1199.492 y9
1280.623 b10
1443.664 b11
1650.268 y12
1777.782 y13
b2
b5
b6
b7
b9
b10
b11
m/z
LQKFMRSPMQYWNQK / File: sydney_ip_01.raw; SpectrumID: ; scans: 5793

DGLGSDN^IGSRM*LQ^AM*GWKEGSGLGR

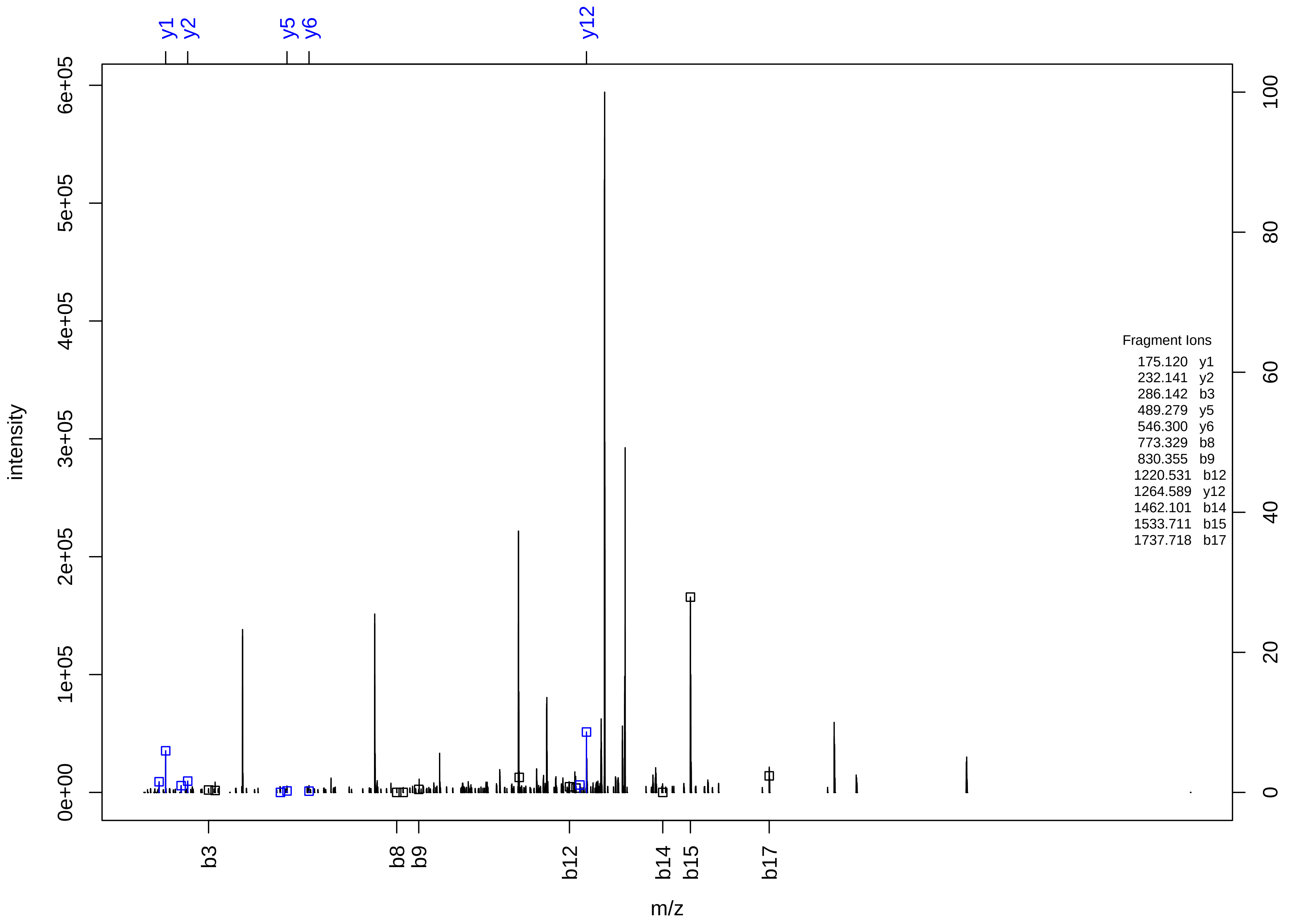

DGLGSDNIGSRMLQAMGWKEGSGLGR / File: sydney_ip_01.raw; SpectrumID: ; scans: 5301

ELTSQ^IR

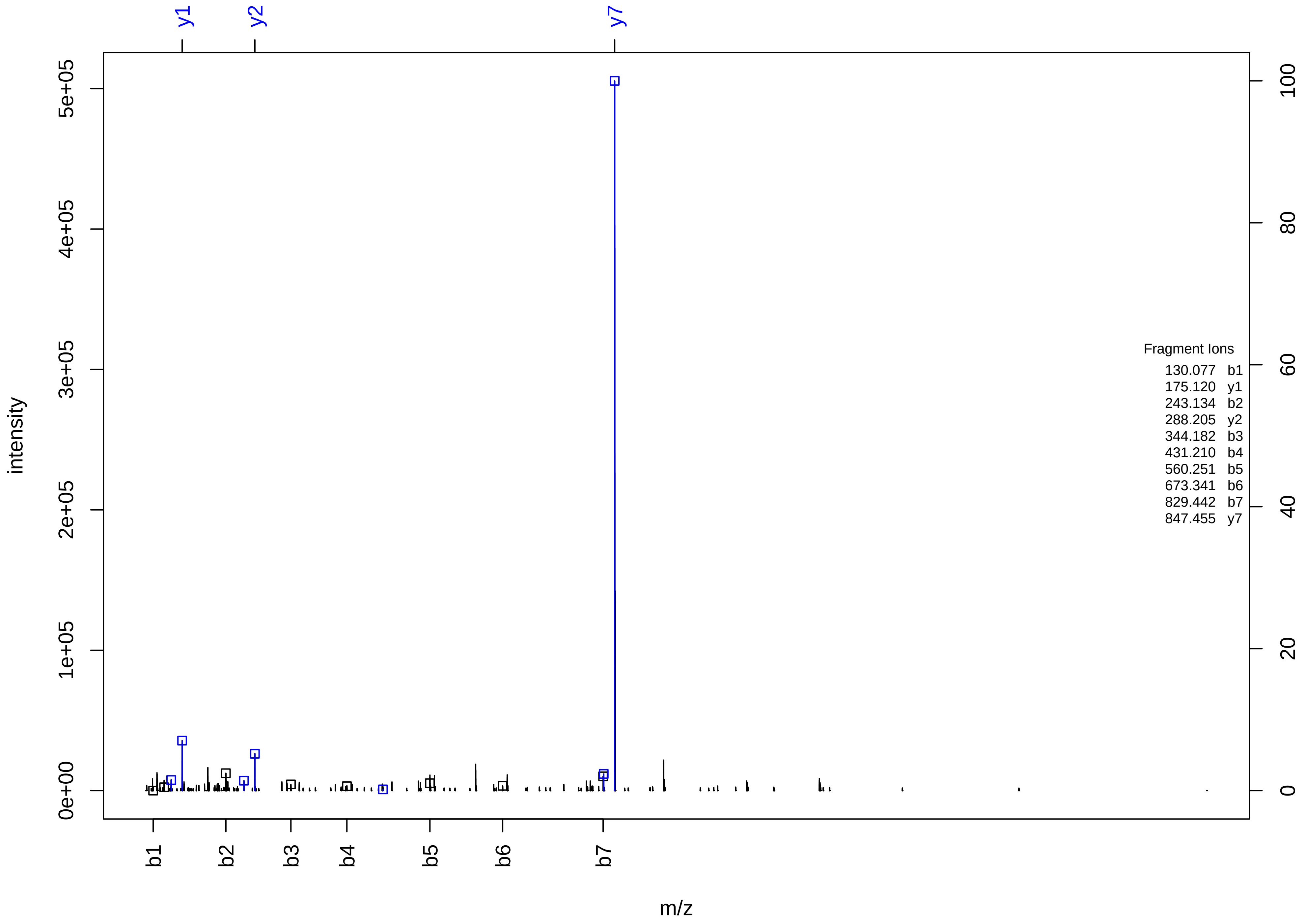

ELTSQIR / File: sydney_ip_02.raw; SpectrumID: ; scans: 3960

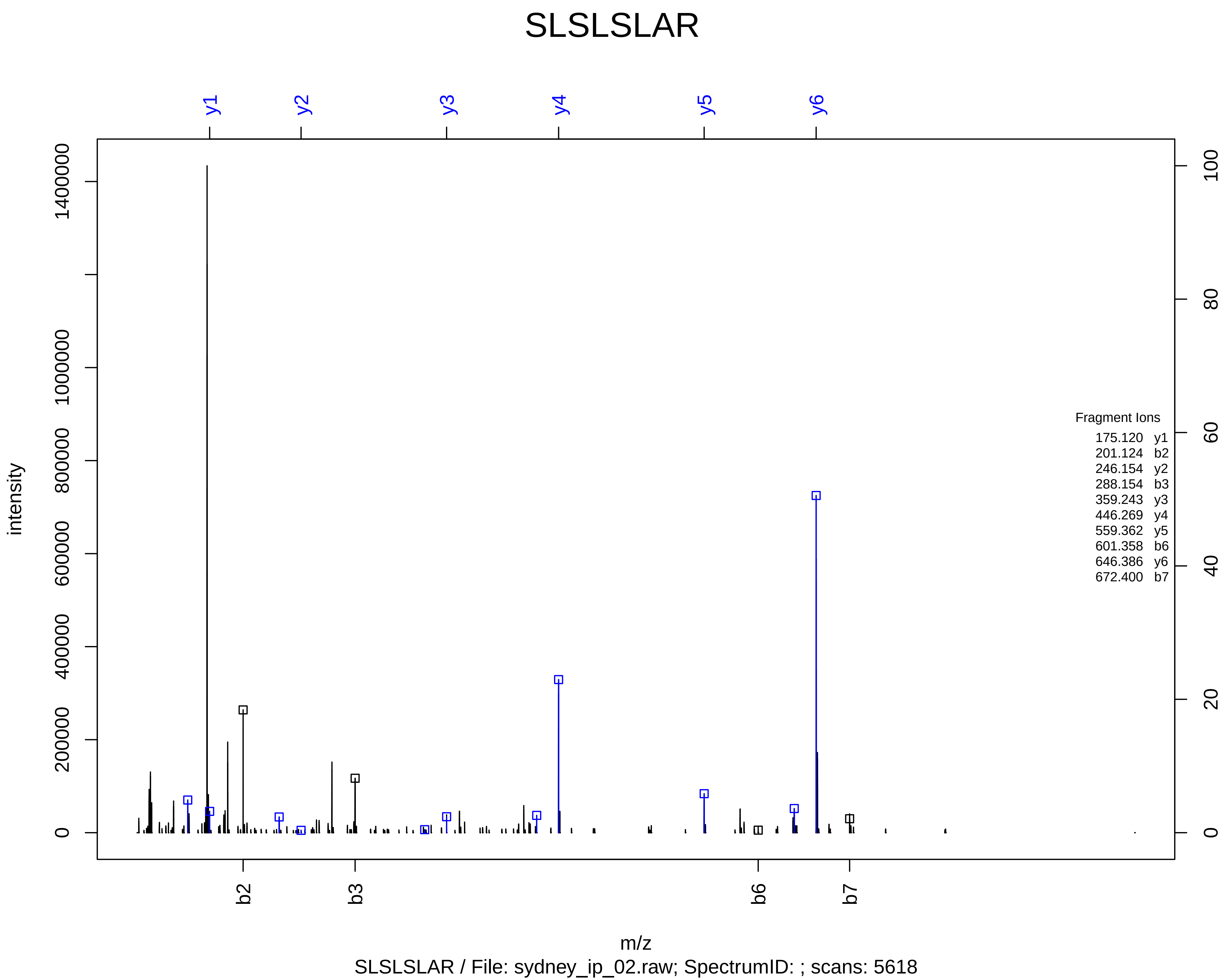
SLSLSLAR
y1
y2
y3
y4
y5
y6
b2
b3
b6
b7
intensity
m/z
0
200000
400000
600000
800000
1000000
1400000
0
20
40
60
80
100
Fragment Ions
175.120 y1
201.124 b2
246.154 y2
288.154 b3
359.243 y3
446.269 y4
559.362 y5
601.358 b6
646.386 y6
672.400 b7
SLSLSLAR / File: sydney_ip_02.raw; SpectrumID: ; scans: 5618

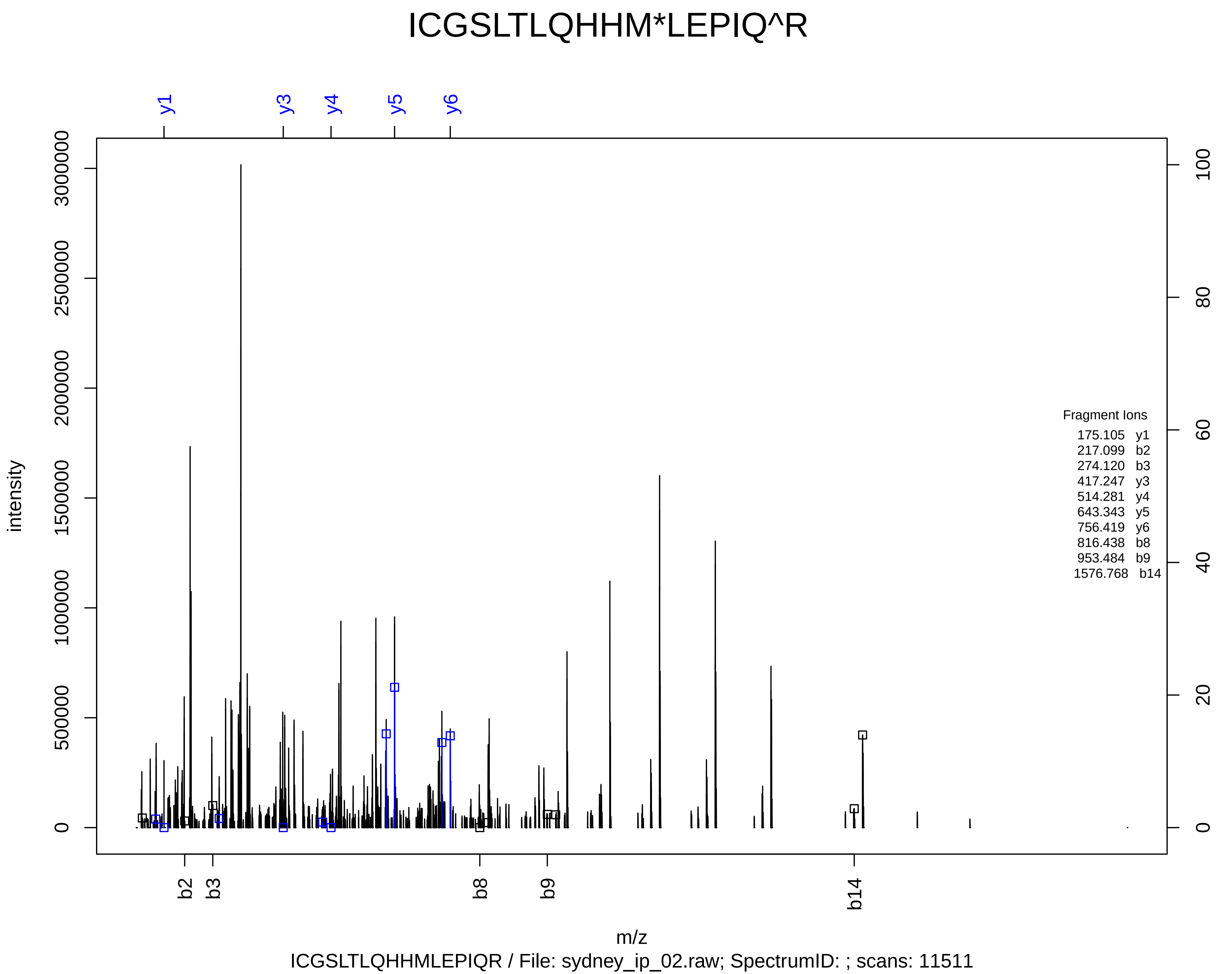
ICGSLTLQHHM*LEPIQ^R
y1
y3
y4
y5
y6
intensity
0
500000
1000000
1500000
2000000
2500000
3000000
0
20
40
60
80
100
Fragment Ions
175.105 y1
217.099 b2
274.120 b3
417.247 y3
514.281 y4
643.343 y5
756.419 y6
816.438 b8
953.484 b9
1576.768 b14
b2
b3
b8
b9
b14
m/z
ICGSLTLQHHMLEPIQR / File: sydney_ip_02.raw; SpectrumID: ; scans: 11511

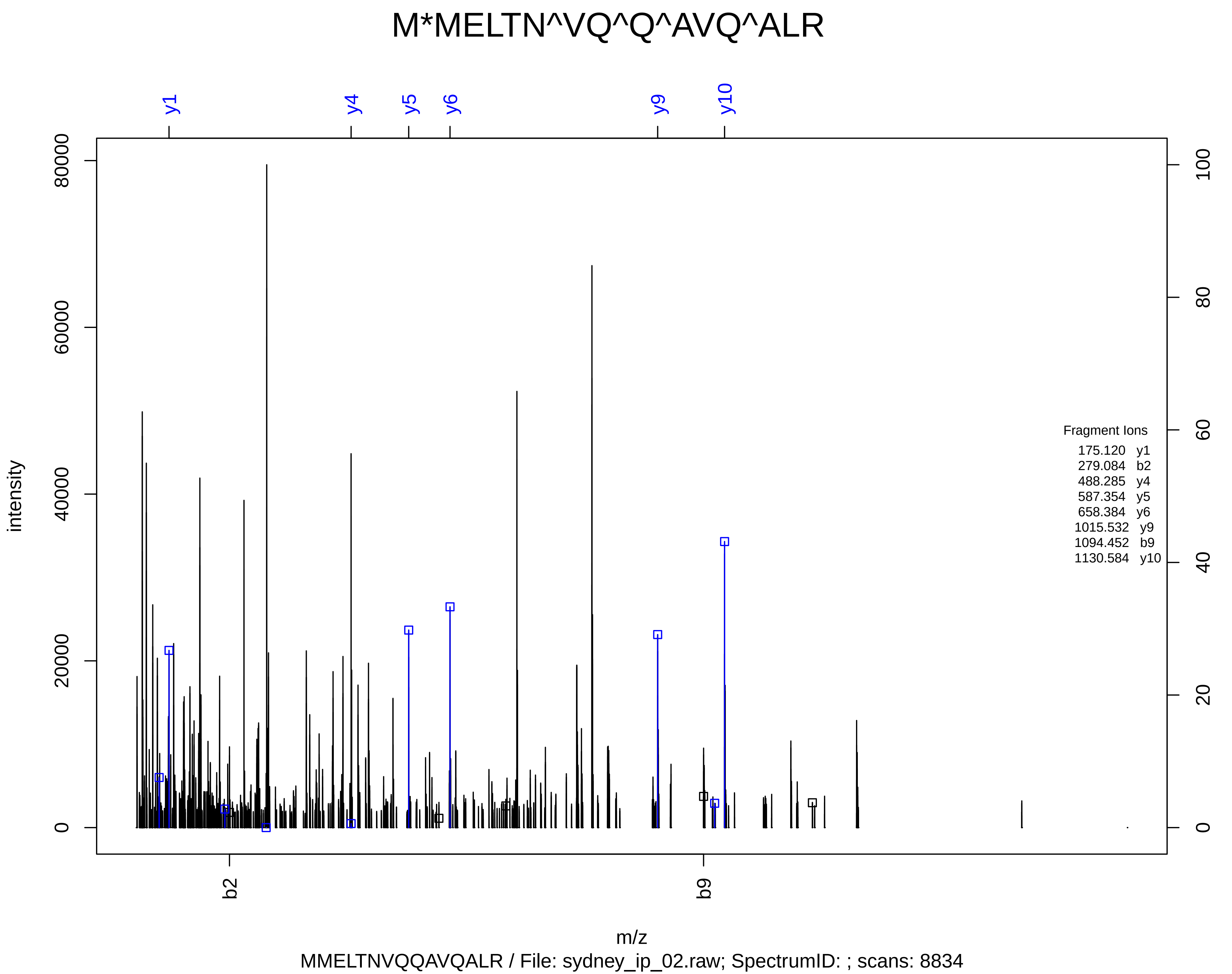
M*MELTN^VQ^Q^AVQ^ALR
y1
y4
y5
y6
y9
y10
intensity
80000
60000
40000
20000
0
100
80
60
40
20
0
Fragment Ions
175.120 y1
279.084 b2
488.285 y4
587.354 y5
658.384 y6
1015.532 y9
1094.452 b9
1130.584 y10
b2
b9
m/z
MMELTNVQQAVQALR / File: sydney_ip_02.raw; SpectrumID: ; scans: 8834

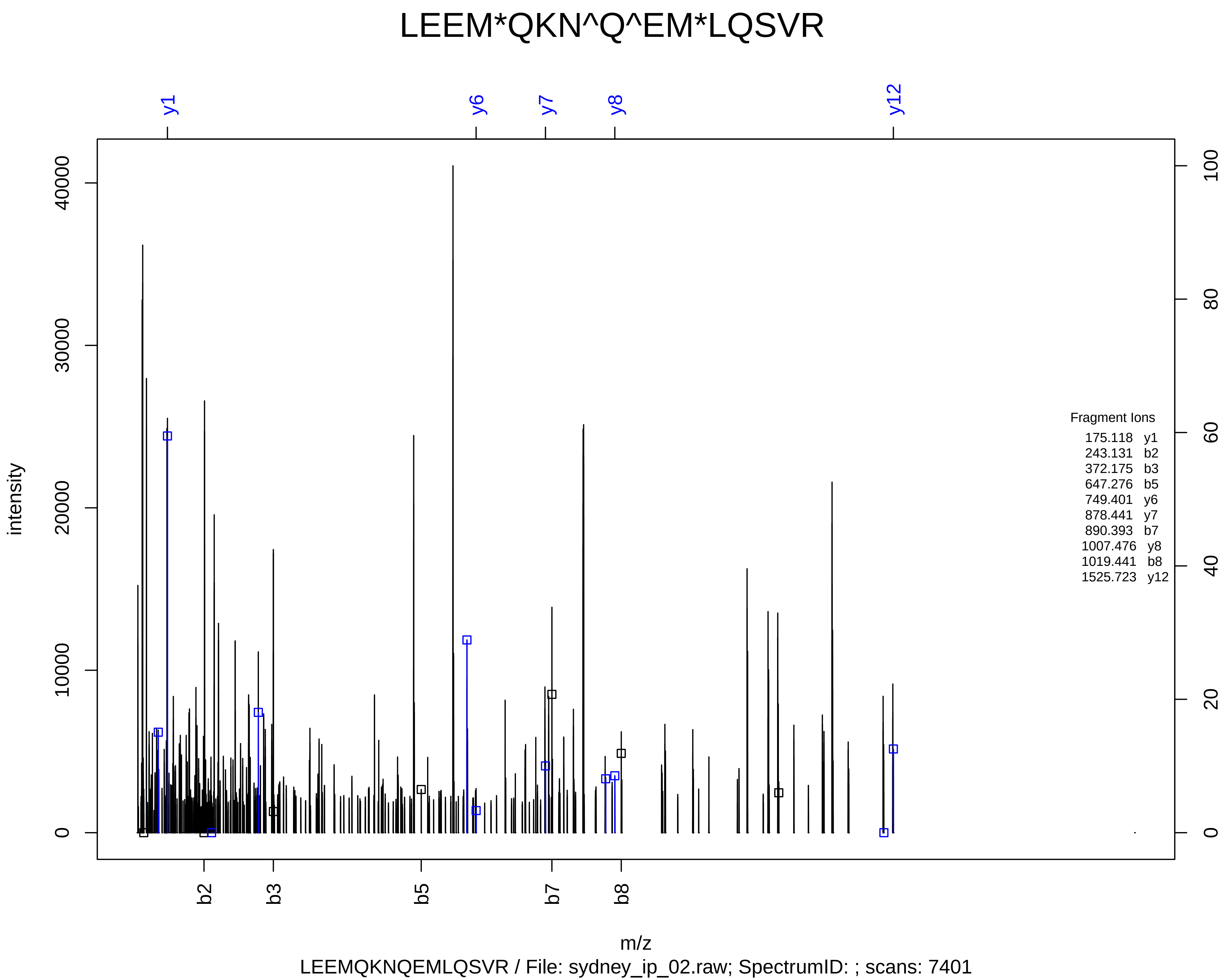
LEEM*QKN^Q^EM*LQSVR
y1
y6
y7
y8
y12
intensity
0
10000
20000
30000
40000
0
20
40
60
80
100
b2
b3
b5
b7
b8
m/z
Fragment Ions
175.118 y1
243.131 b2
372.175 b3
647.276 b5
749.401 y6
878.441 y7
890.393 b7
1007.476 y8
1019.441 b8
1525.723 y12
LEEMQKNQEMLQSVR / File: sydney_ip_02.raw; SpectrumID: ; scans: 7401

VITNFNSAHDTDR

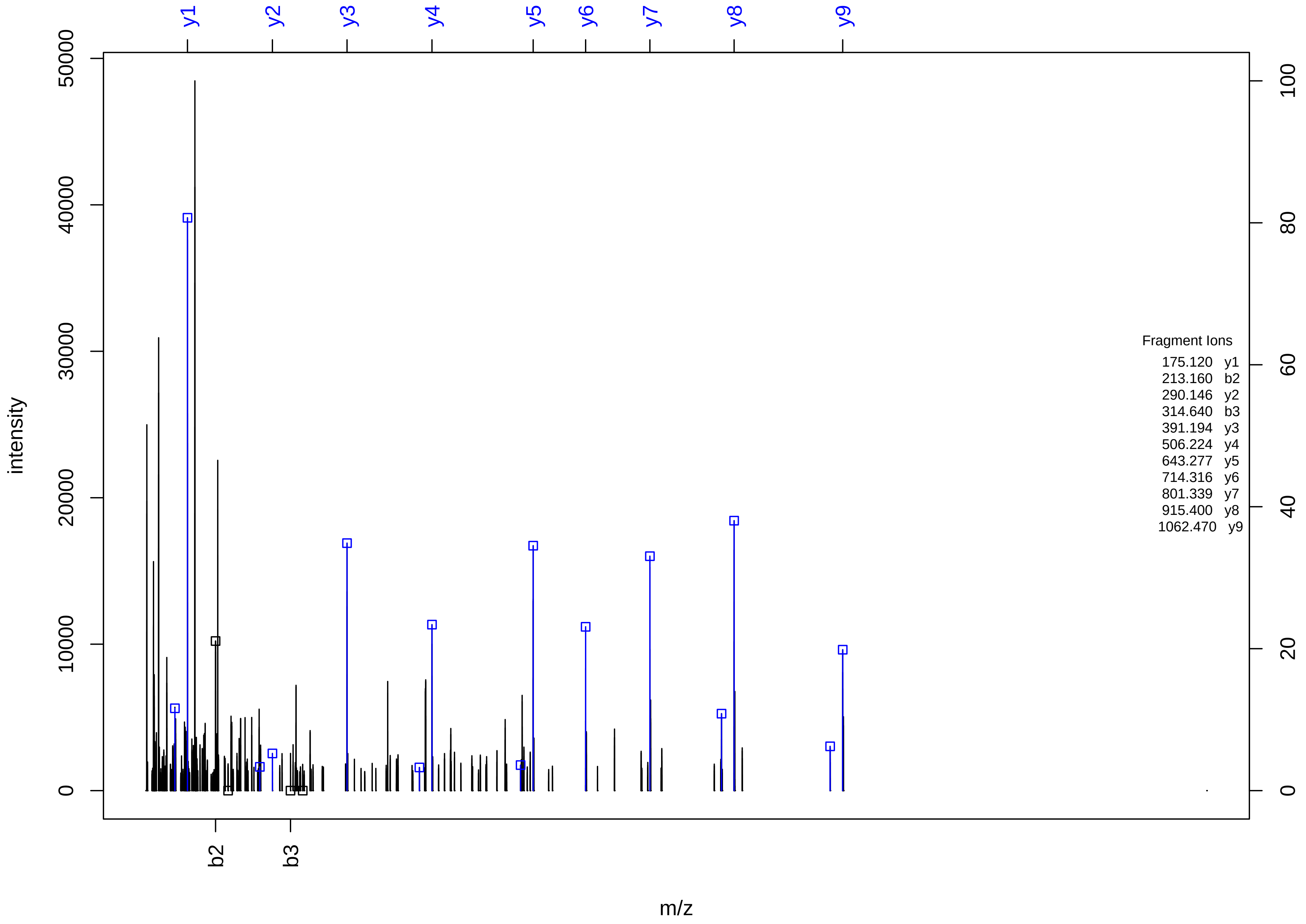

VITNFNSAHDTDR / File: sydney_ip_02.raw; SpectrumID: ; scans: 2726

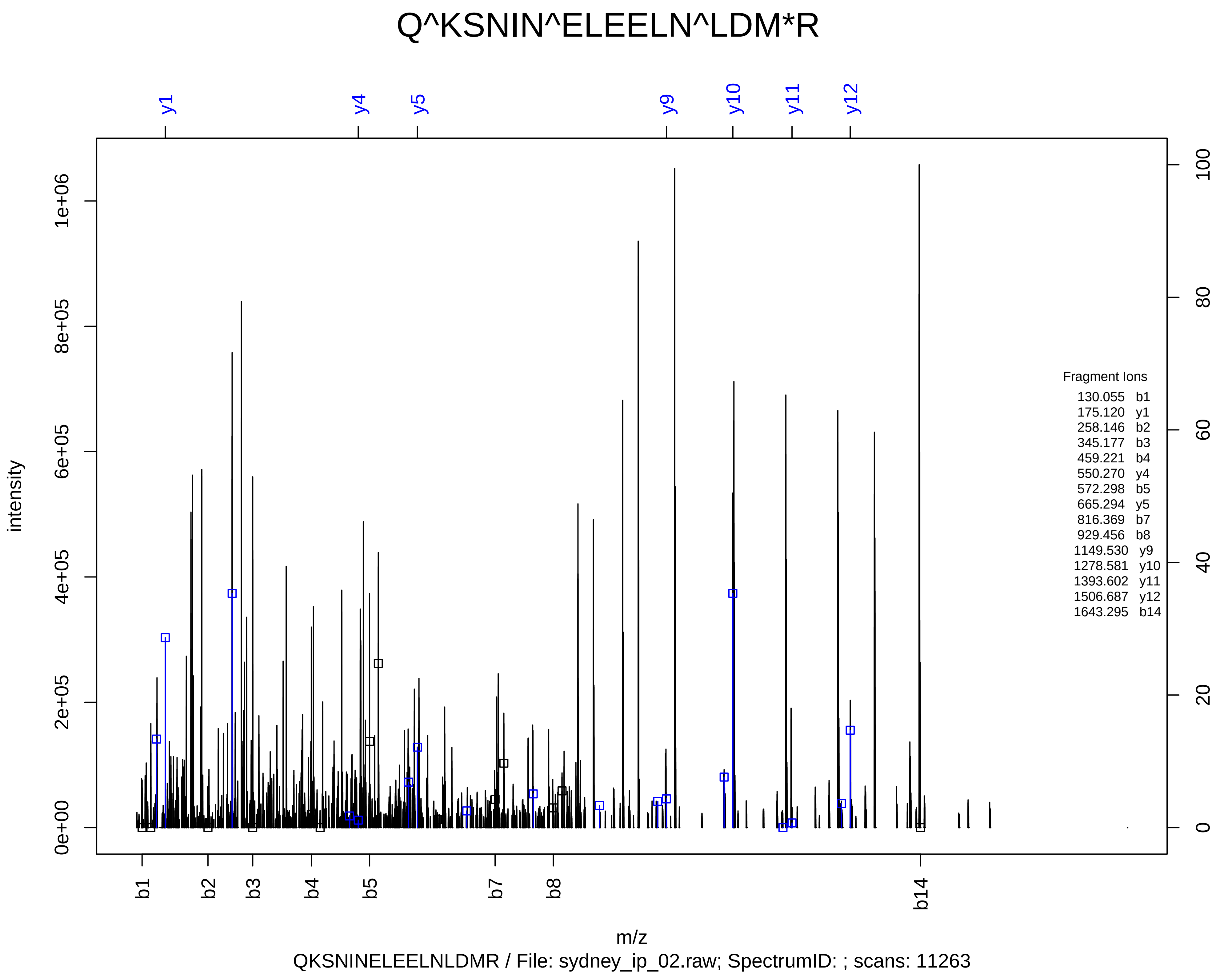

Q^KSNIN^ELEELN^LDM*R
y1
y4
y5
y9
y10
y11
y12
intensity
0e+00
2e+05
4e+05
6e+05
8e+05
1e+06
0
20
40
60
80
100
Fragment Ions
130.055 b1
175.120 y1
258.146 b2
345.177 b3
459.221 b4
550.270 y4
572.298 b5
665.294 y5
816.369 b7
929.456 b8
1149.530 y9
1278.581 y10
1393.602 y11
1506.687 y12
1643.295 b14
b1
b2
b3
b4
b5
b7
b8
b14
m/z
QKSNINELEELNLDMR / File: sydney_ip_02.raw; SpectrumID: ; scans: 11263

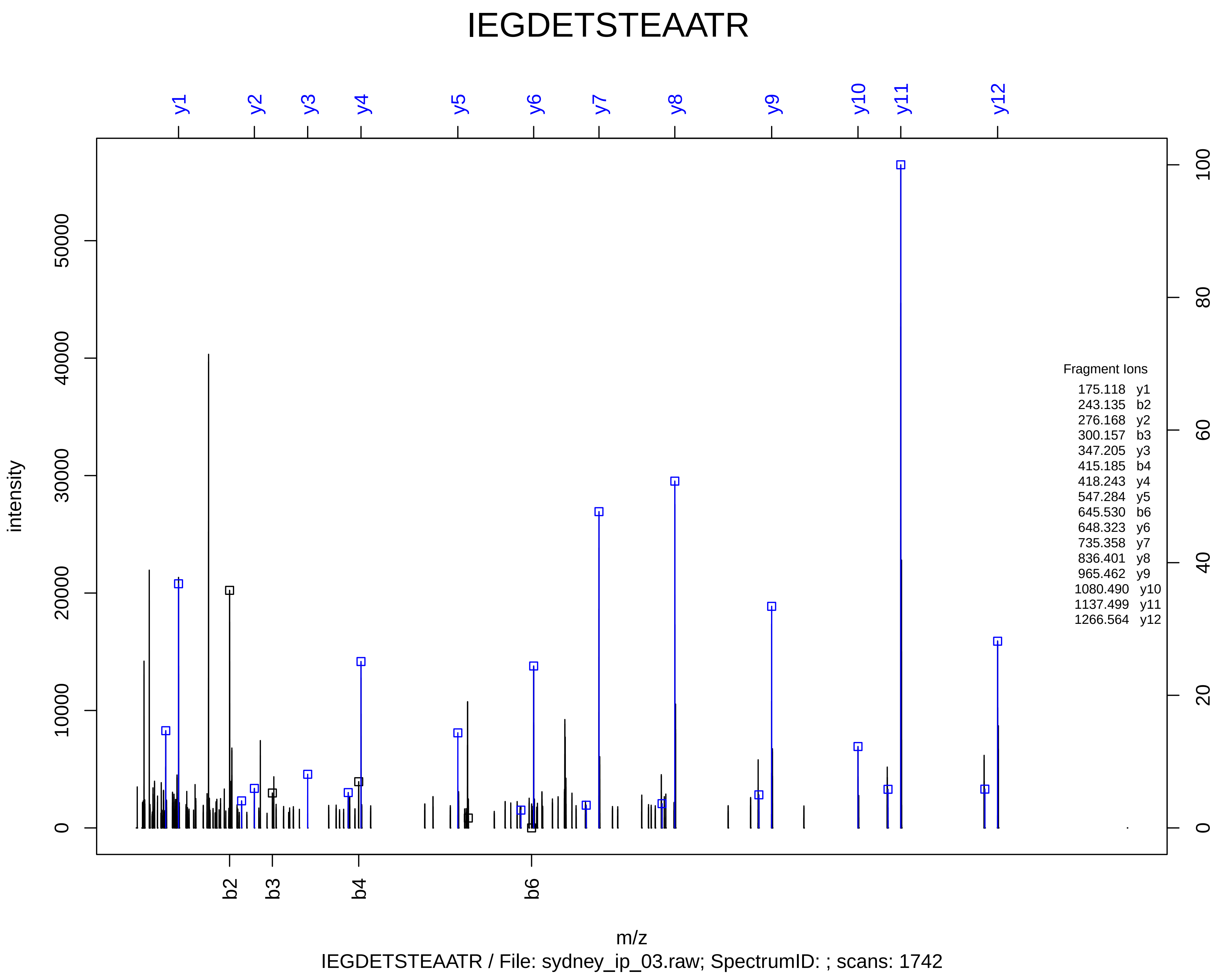
IEGDETSTEAATR
y1
y2
y3
y4
y5
y6
y7
y8
y9
y10
y11
y12
intensity
0
10000
20000
30000
40000
50000
0
20
40
60
80
100
b2
b3
b4
b6
Fragment Ions
175.118 y1
243.135 b2
276.168 y2
300.157 b3
347.205 y3
415.185 b4
418.243 y4
547.284 y5
645.530 b6
648.323 y6
735.358 y7
836.401 y8
965.462 y9
1080.490 y10
1137.499 y11
1266.564 y12
m/z
IEGDETSTEAATR / File: sydney_ip_03.raw; SpectrumID: ; scans: 1742

ILM*SLKQ^LN^LN^FHVS

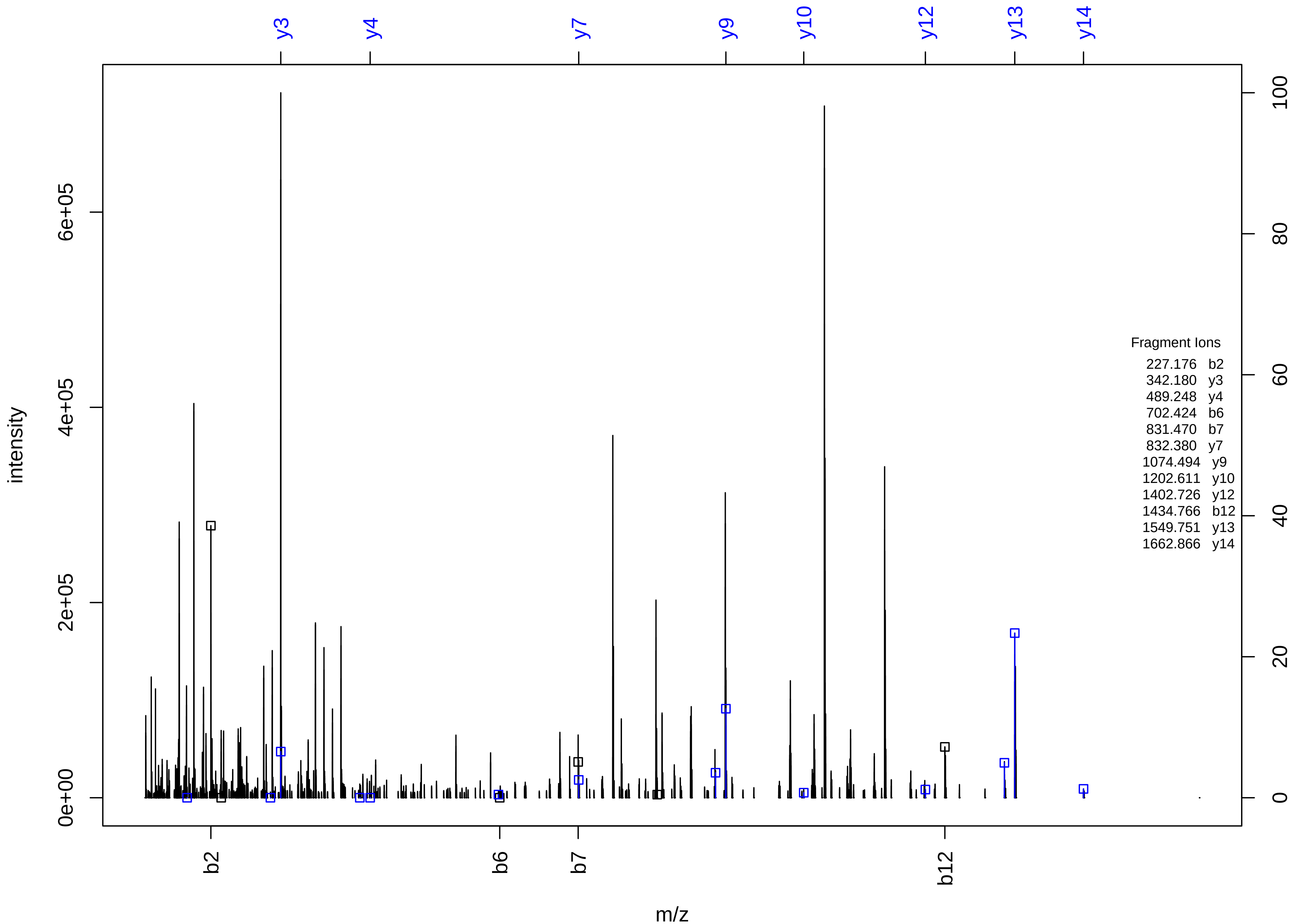

ILMSLKQLNLNFHVS / File: sydney_ip_03.raw; SpectrumID: ; scans: 11547

ELTVDNSFEFEK

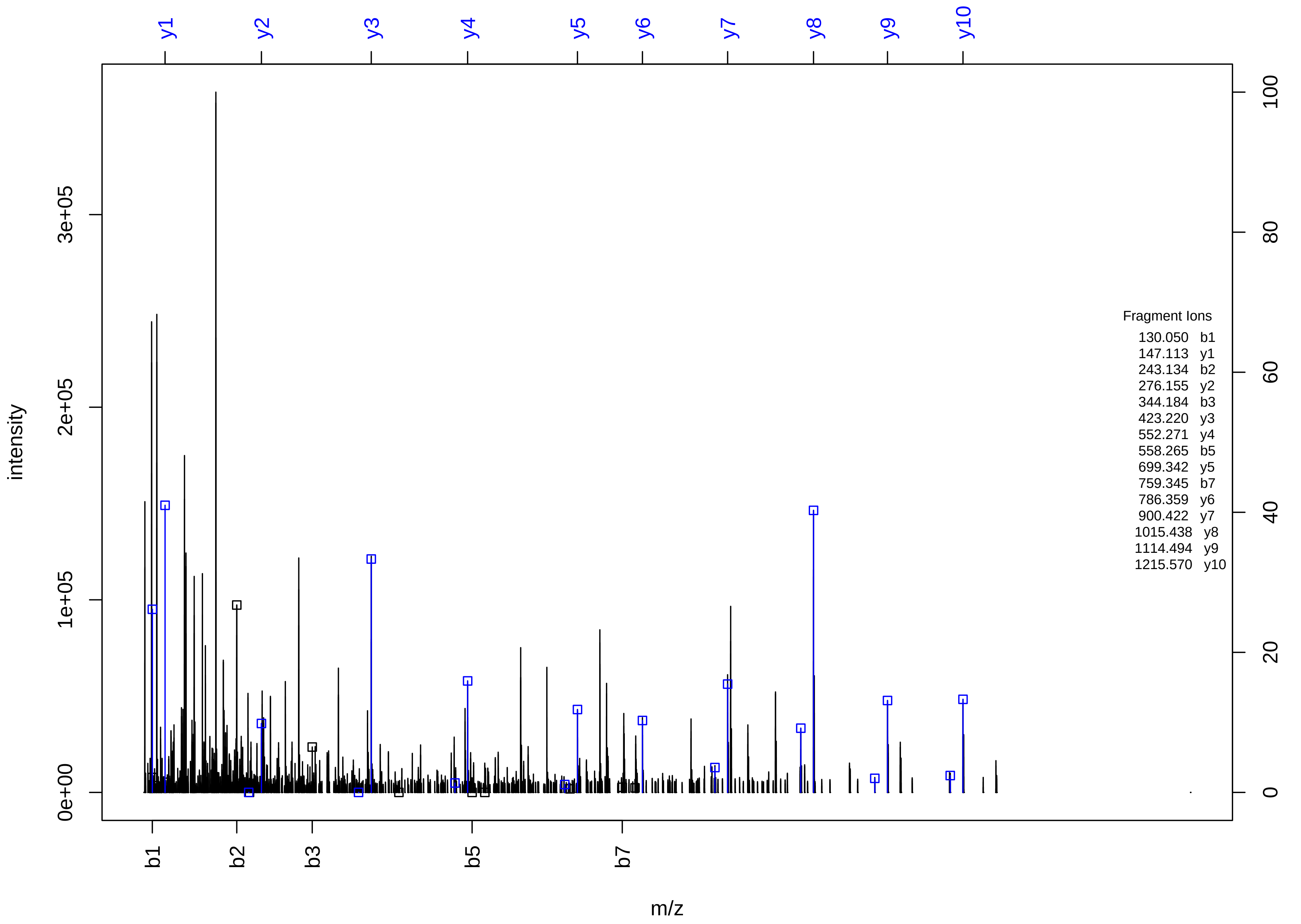

ELTVDNSFEFEK / File: sydney_ip_03.raw; SpectrumID: ; scans: 10998

RPLQSIR

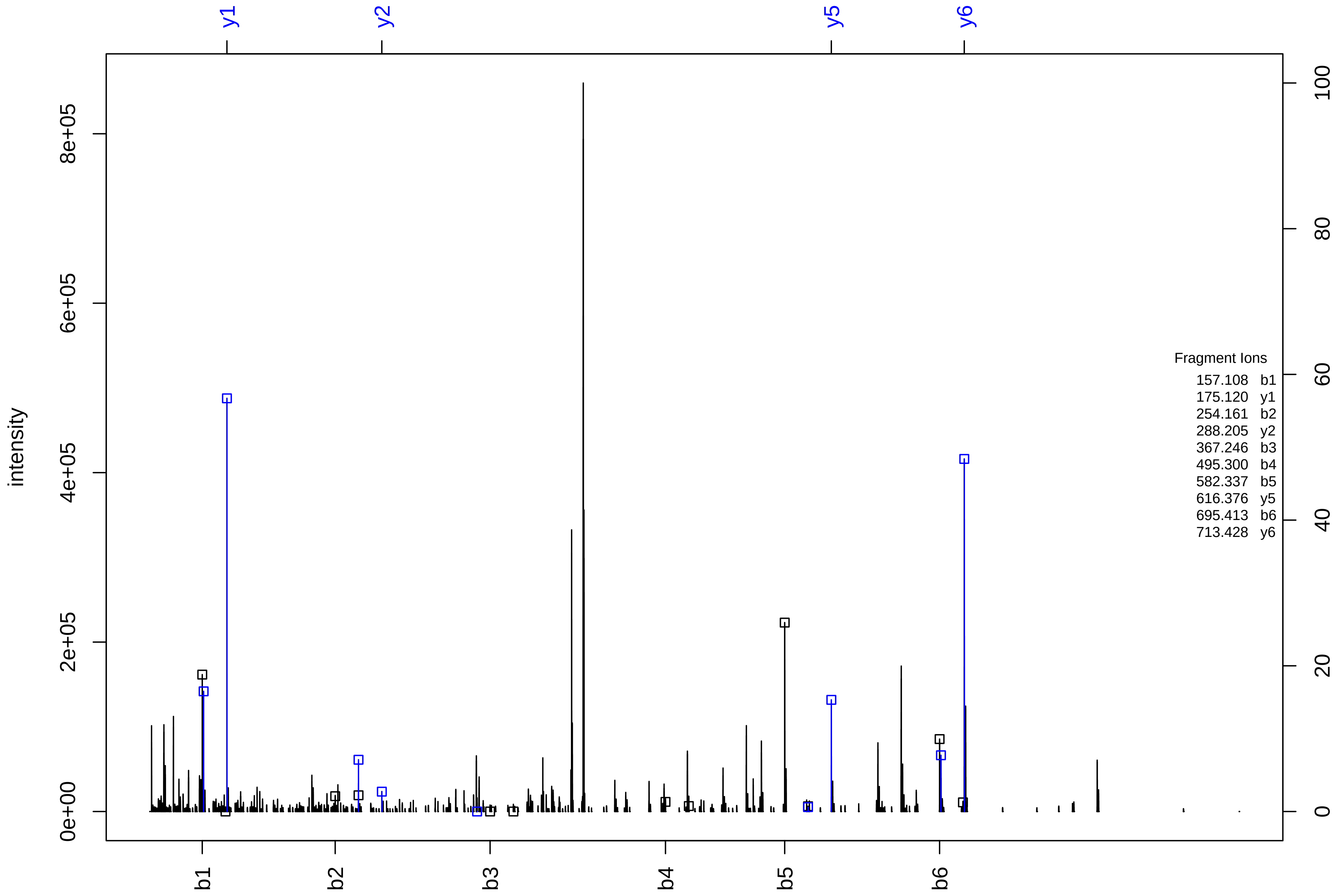

RPLQSIR / File: sydney_ip_03.raw; SpectrumID: ; scans: 2812

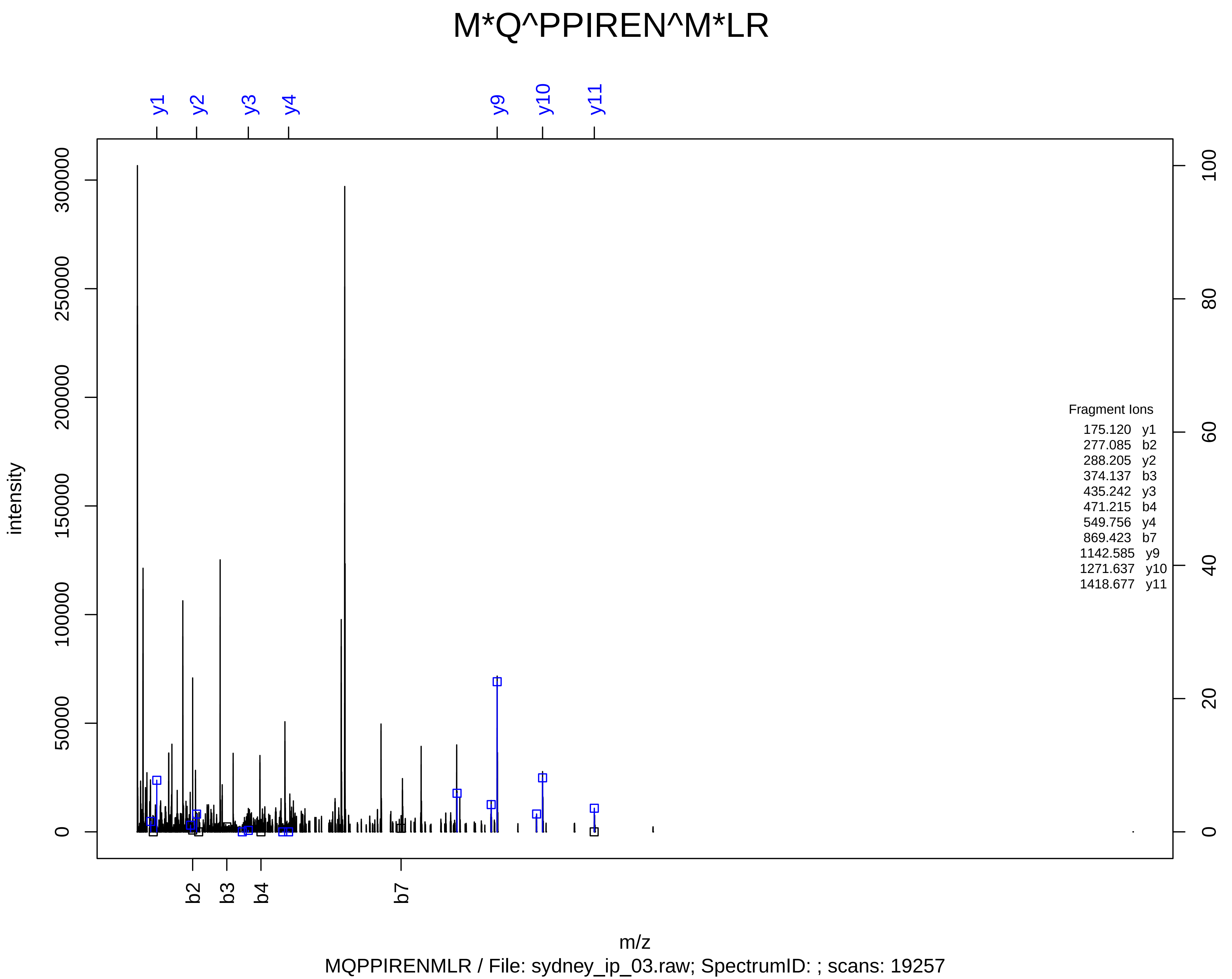
M*Q^PPIREN^M*LR
y1
y2
y3
y4
y9
y10
y11
intensity
b2
b3
b4
b7
m/z
Fragment Ions
175.120 y1
277.085 b2
288.205 y2
374.137 b3
435.242 y3
471.215 b4
549.756 y4
869.423 b7
1142.585 y9
1271.637 y10
1418.677 y11
MQPPIRENMLR / File: sydney_ip_03.raw; SpectrumID: ; scans: 19257

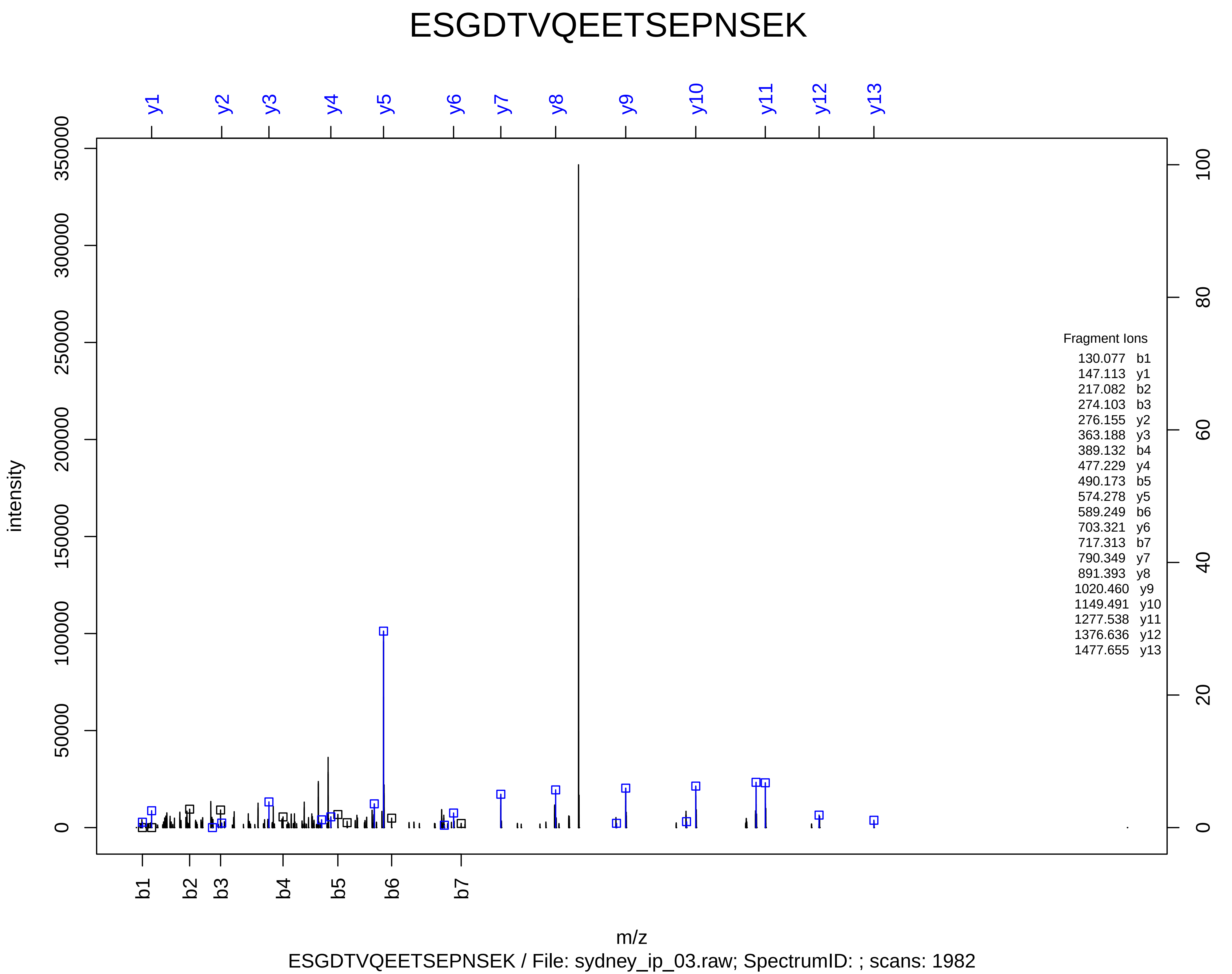
ESGDTVQEETSEPNSEK
y1
y2
y3
y4
y5
y6
y7
y8
y9
y10
y11
y12
y13
intensity
0
50000
100000
150000
200000
250000
300000
350000
0
20
40
60
80
100
b1
b2
b3
b4
b5
b6
b7
m/z
Fragment Ions
130.077 b1
147.113 y1
217.082 b2
274.103 b3
276.155 y2
363.188 y3
389.132 b4
477.229 y4
490.173 b5
574.278 y5
589.249 b6
703.321 y6
717.313 b7
790.349 y7
891.393 y8
1020.460 y9
1149.491 y10
1277.538 y11
1376.636 y12
1477.655 y13
ESGDTVQEETSEPNSEK / File: sydney_ip_03.raw; SpectrumID: ; scans: 1982

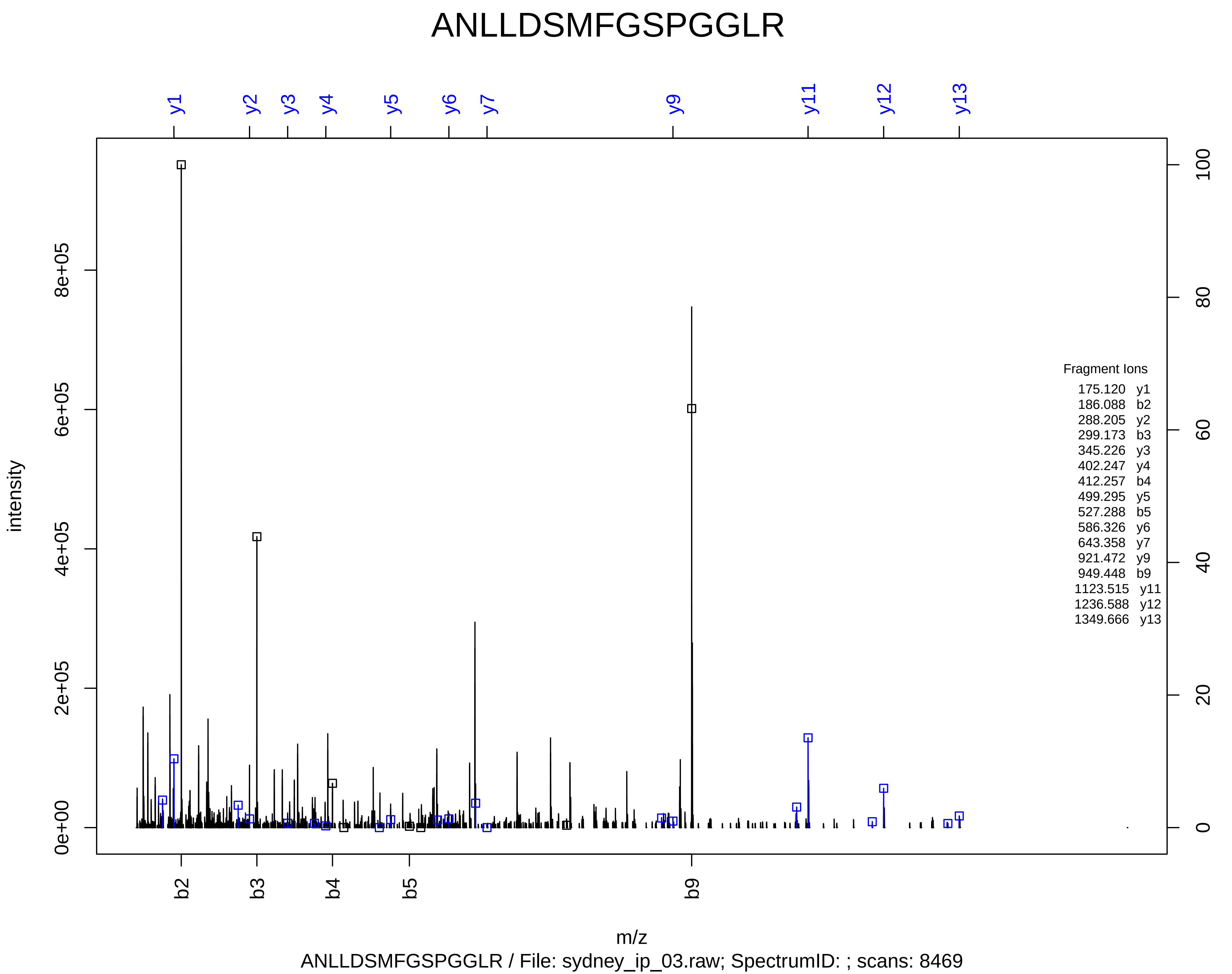
ANLLDSMFGSPGGLR
y1
y2
y3
y4
y5
y6
y7
y9
y11
y12
y13
intensity
0e+00
2e+05
4e+05
6e+05
8e+05
0
20
40
60
80
100
b2
b3
b4
b5
b9
Fragment Ions
175.120 y1
186.088 b2
288.205 y2
299.173 b3
345.226 y3
402.247 y4
412.257 b4
499.295 y5
527.288 b5
586.326 y6
643.358 y7
921.472 y9
949.448 b9
1123.515 y11
1236.588 y12
1349.666 y13
m/z
ANLLDSMFGSPGGLR / File: sydney_ip_03.raw; SpectrumID: ; scans: 8469

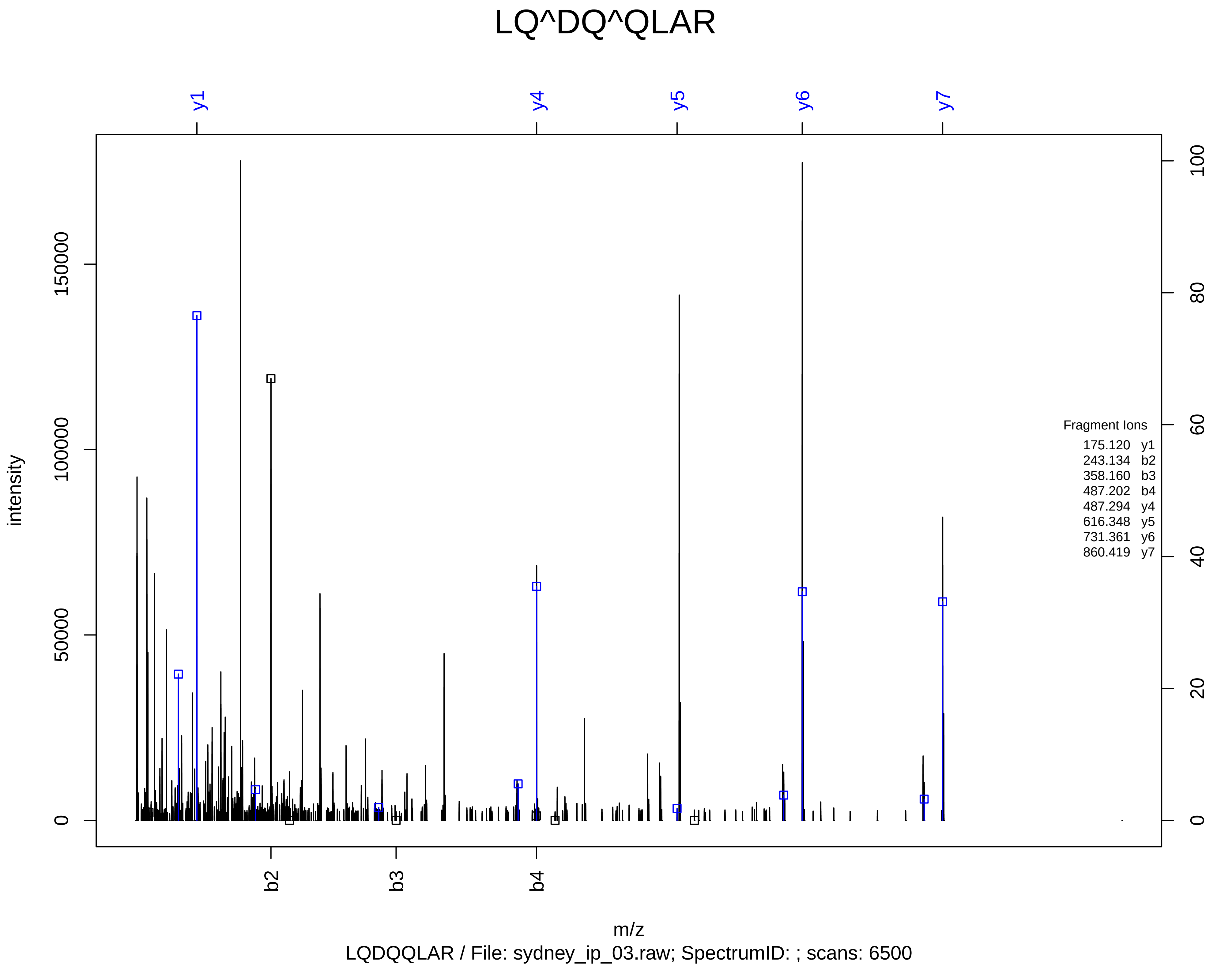
LQ^DQ^QLAR
y1
y4
y5
y6
y7
intensity
0
50000
100000
150000
0
20
40
60
80
100
b2
b3
b4
m/z
Fragment Ions
175.120 y1
243.134 b2
358.160 b3
487.202 b4
487.294 y4
616.348 y5
731.361 y6
860.419 y7
LQDQQLAR / File: sydney_ip_03.raw; SpectrumID: ; scans: 6500

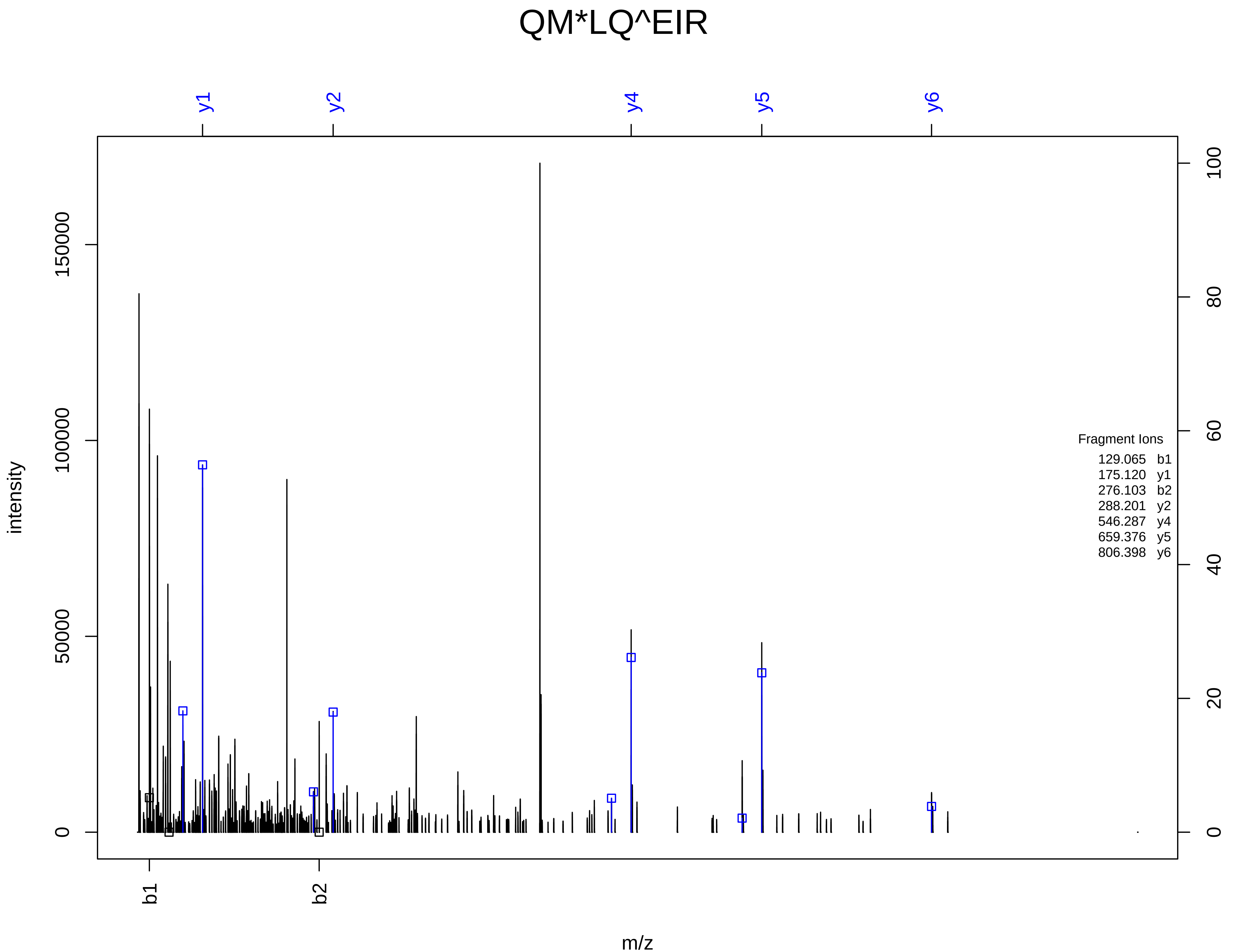

QMLQEIR / File: sydney_ip_03.raw; SpectrumID: ; scans: 10260

VVSLFSEQPLAK

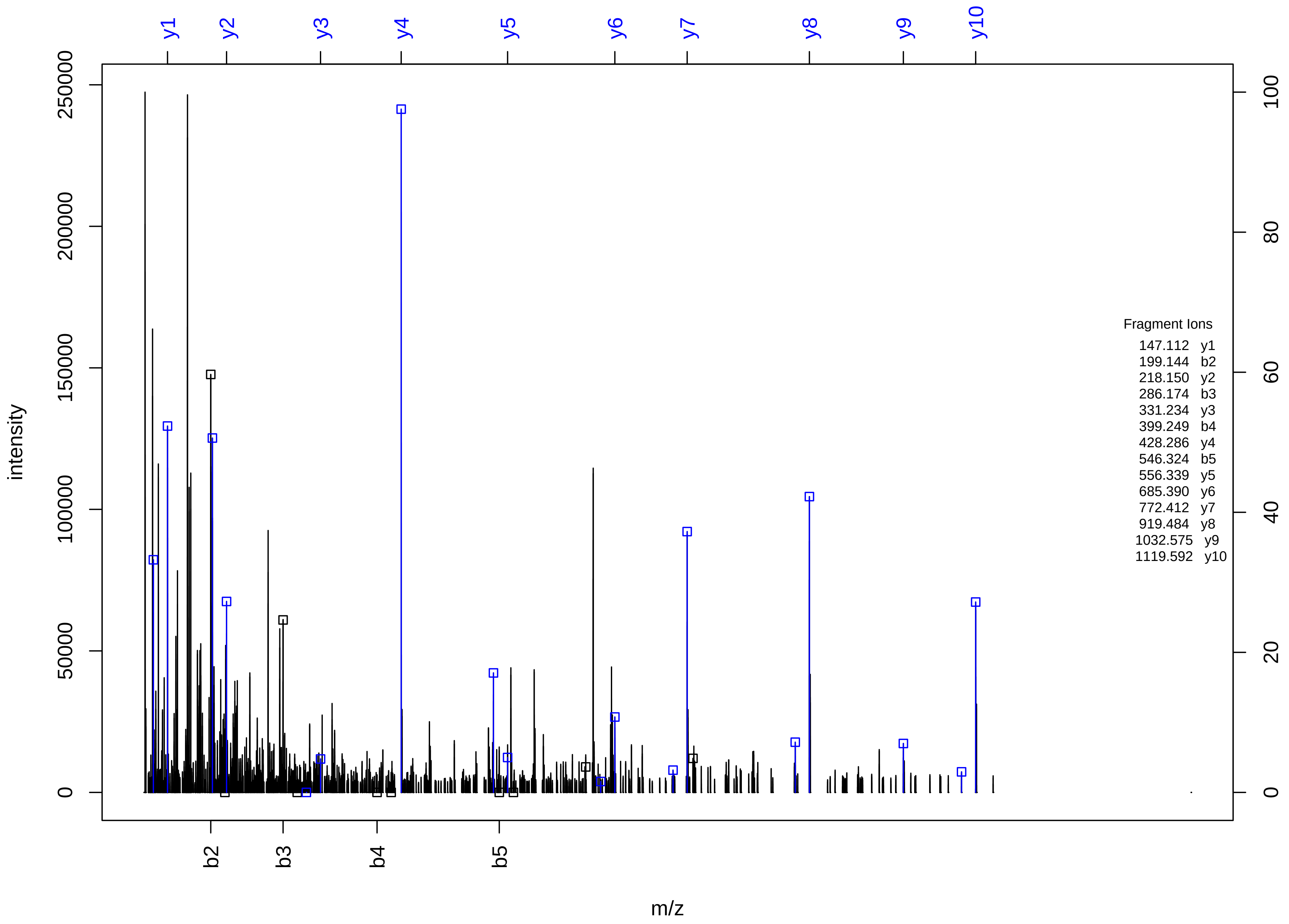

VVSLFSEQPLAK / File: sydney_ip_03.raw; SpectrumID: ; scans: 12478

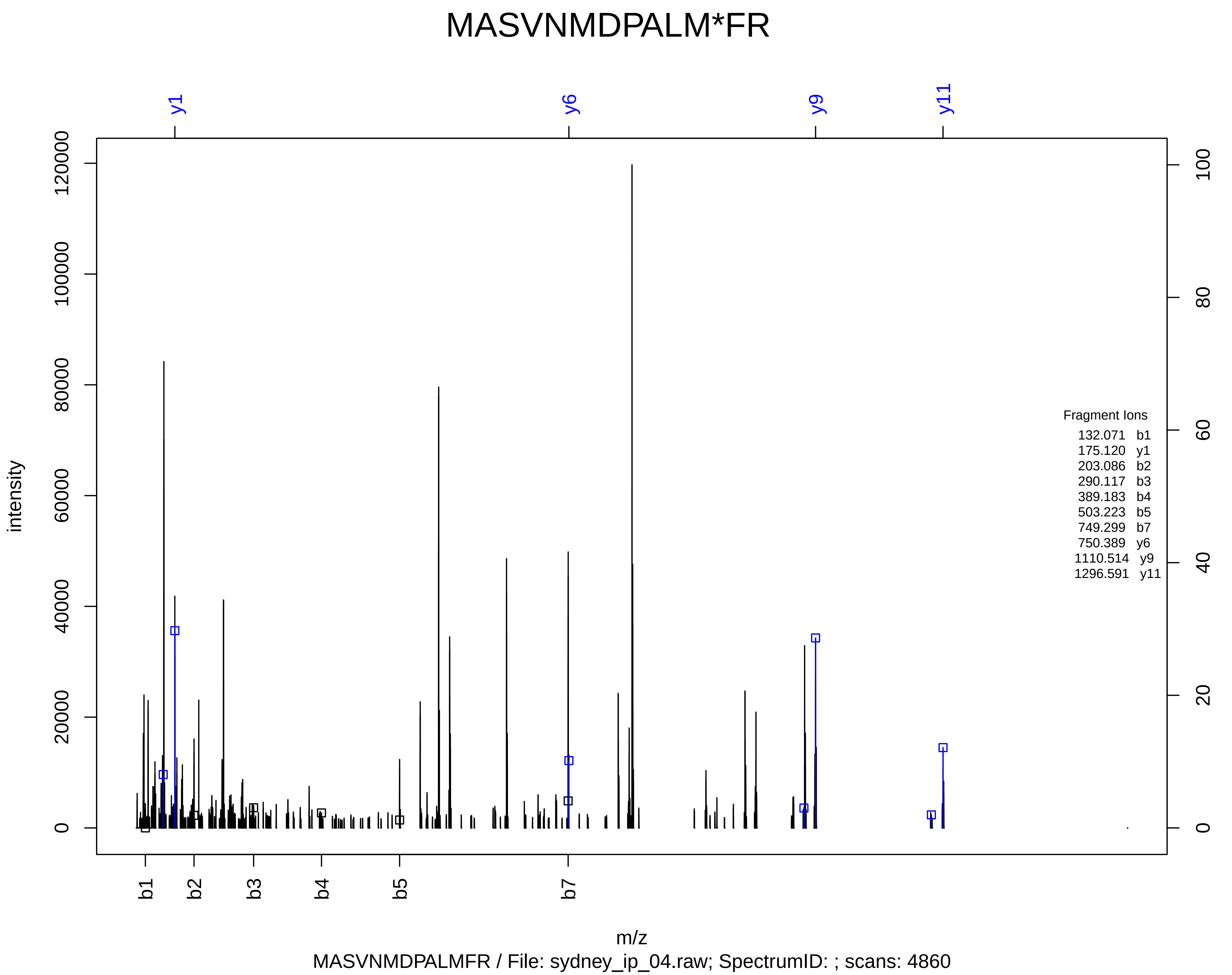
MASVNMDPALM*FR
y1
y6
y9
y11
intensity
120000
100000
80000
60000
40000
20000
0
100
80
60
40
20
0
Fragment Ions
132.071 b1
175.120 y1
203.086 b2
290.117 b3
389.183 b4
503.223 b5
749.299 b7
750.389 y6
1110.514 y9
1296.591 y11
b1
b2
b3
b4
b5
b7
m/z
MASVNMDPALMFR / File: sydney_ip_04.raw; SpectrumID: ; scans: 4860

EDVISTLQ^YLN^LINYYK

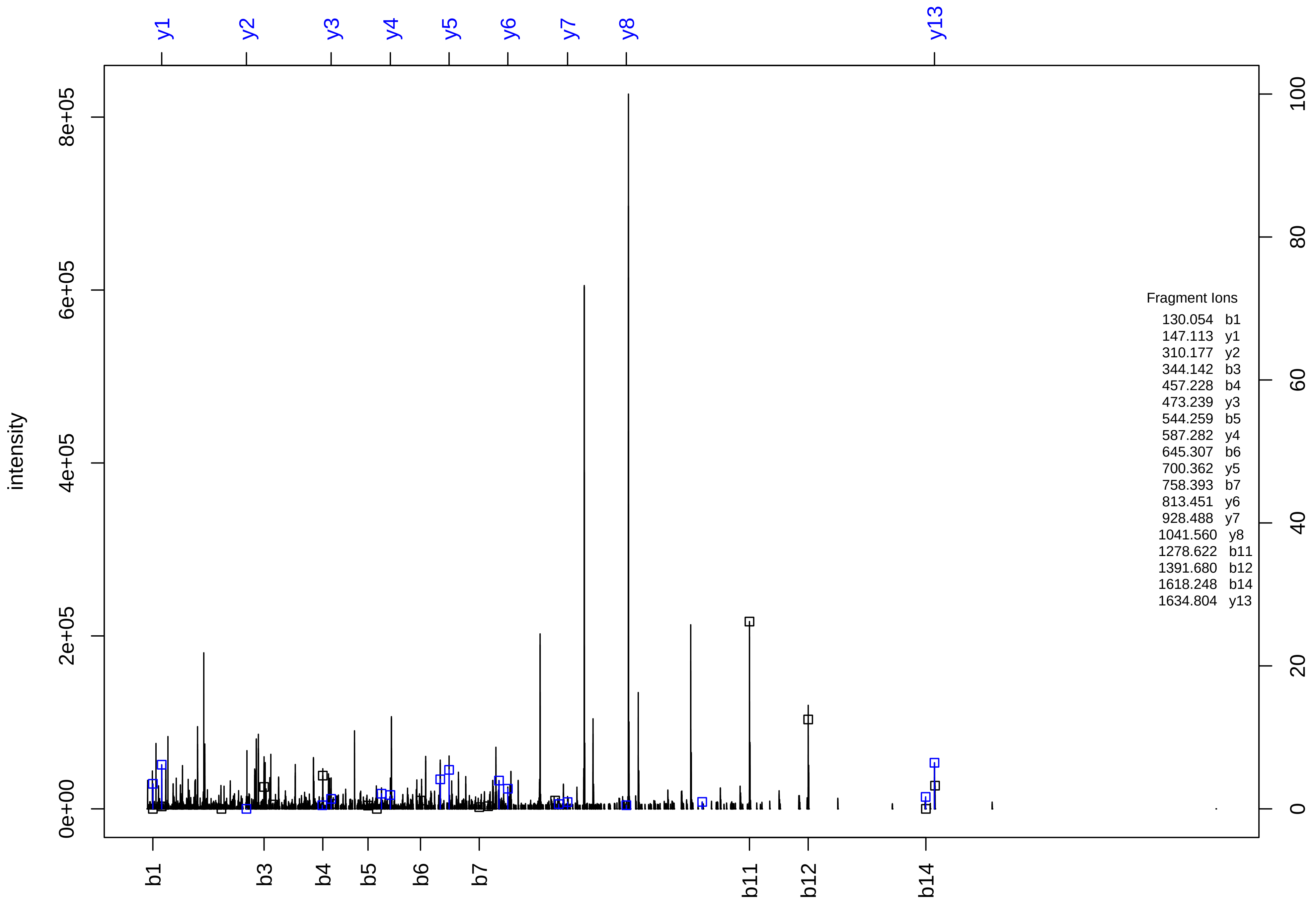

EDVISTLQYLNLINYYK / File: sydney_ip_04.raw; SpectrumID: ; scans: 12564

CPEPCPPPK

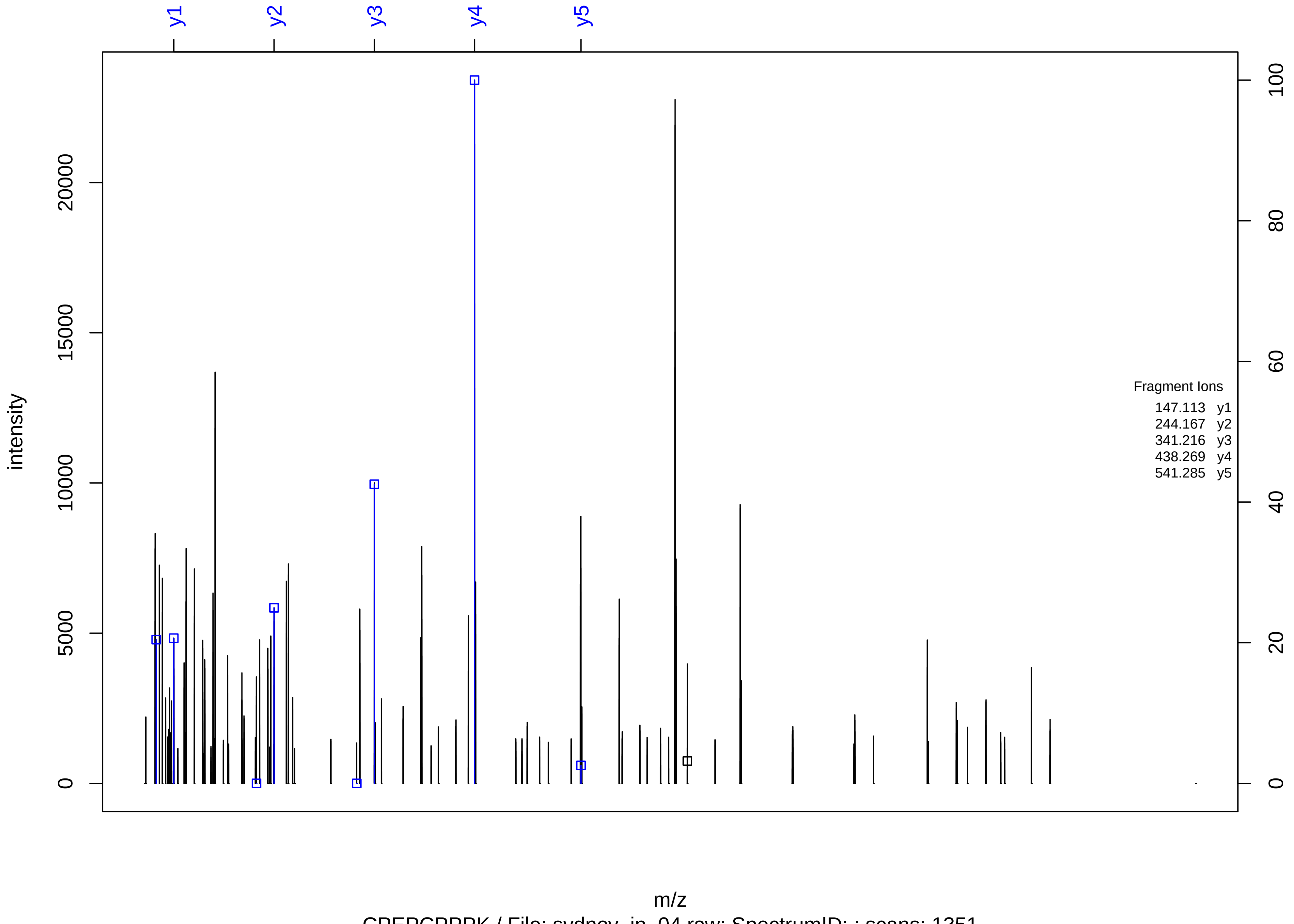

CPEPCPPPK / File: sydney_ip_04.raw; SpectrumID: ; scans: 1351

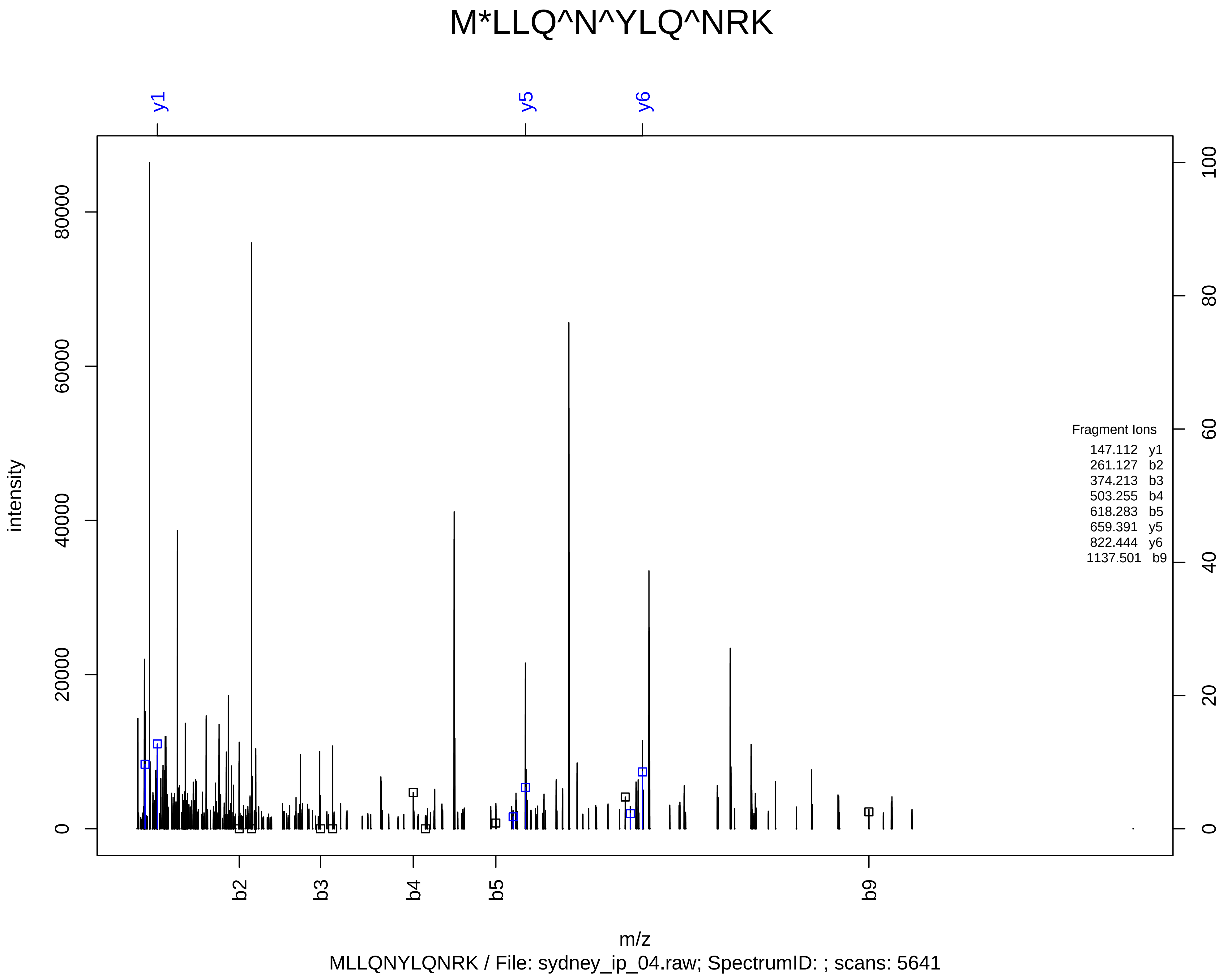
M*LLQ^N^YLQ^NRK
y1
y5
y6
intensity
80000
60000
40000
20000
0
100
80
60
40
20
0
Fragment Ions
147.112 y1
261.127 b2
374.213 b3
503.255 b4
618.283 b5
659.391 y5
822.444 y6
1137.501 b9
b2
b3
b4
b5
b9
m/z
MLLQNYLQNRK / File: sydney_ip_04.raw; SpectrumID: ; scans: 5641

SN^VLN^LN^SHISQ^YVK

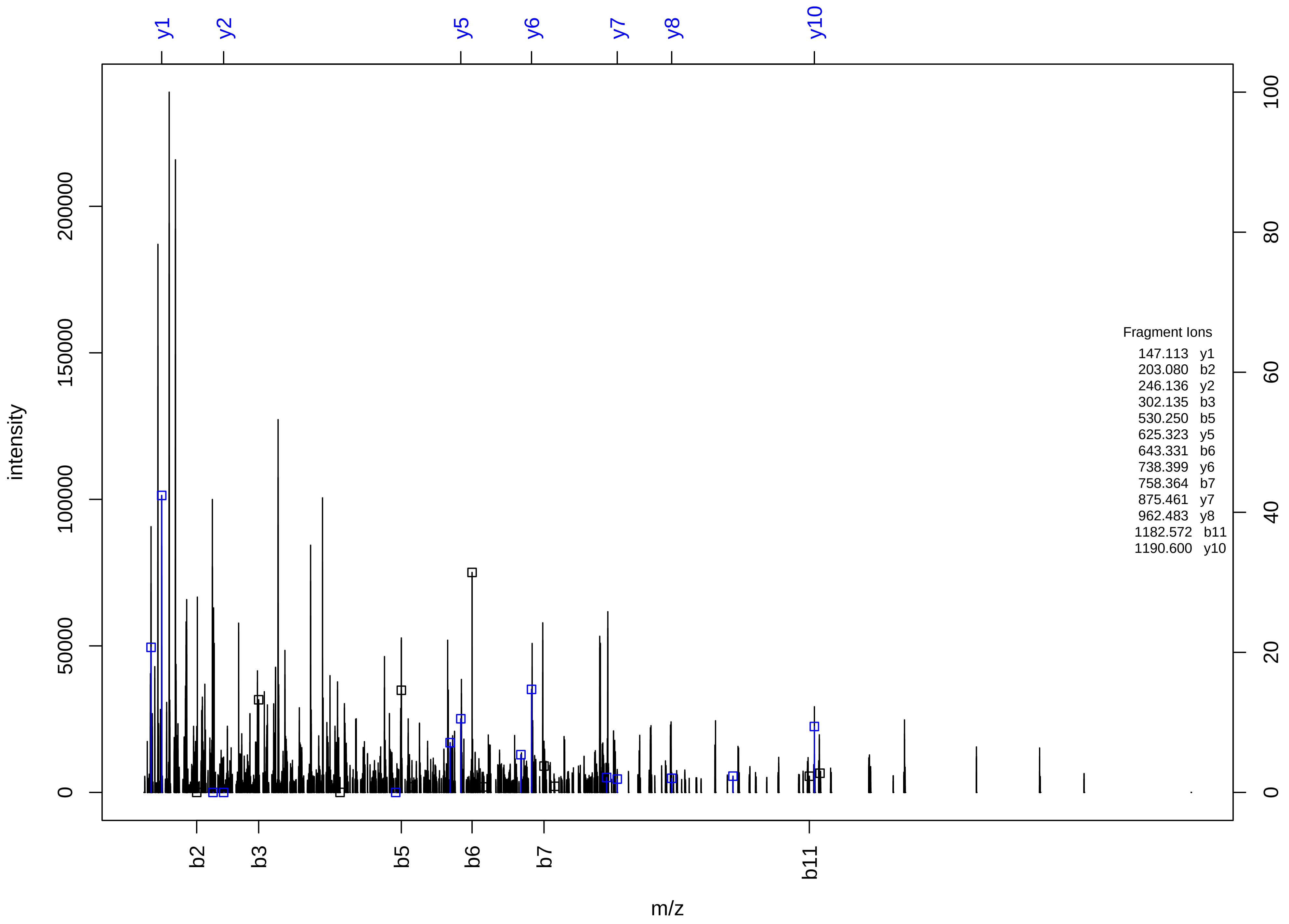

SNVLNLNSHISQYVK / File: sydney_ip_04.raw; SpectrumID: ; scans: 12800

VFGNLSQDHDVCNFLM*QK

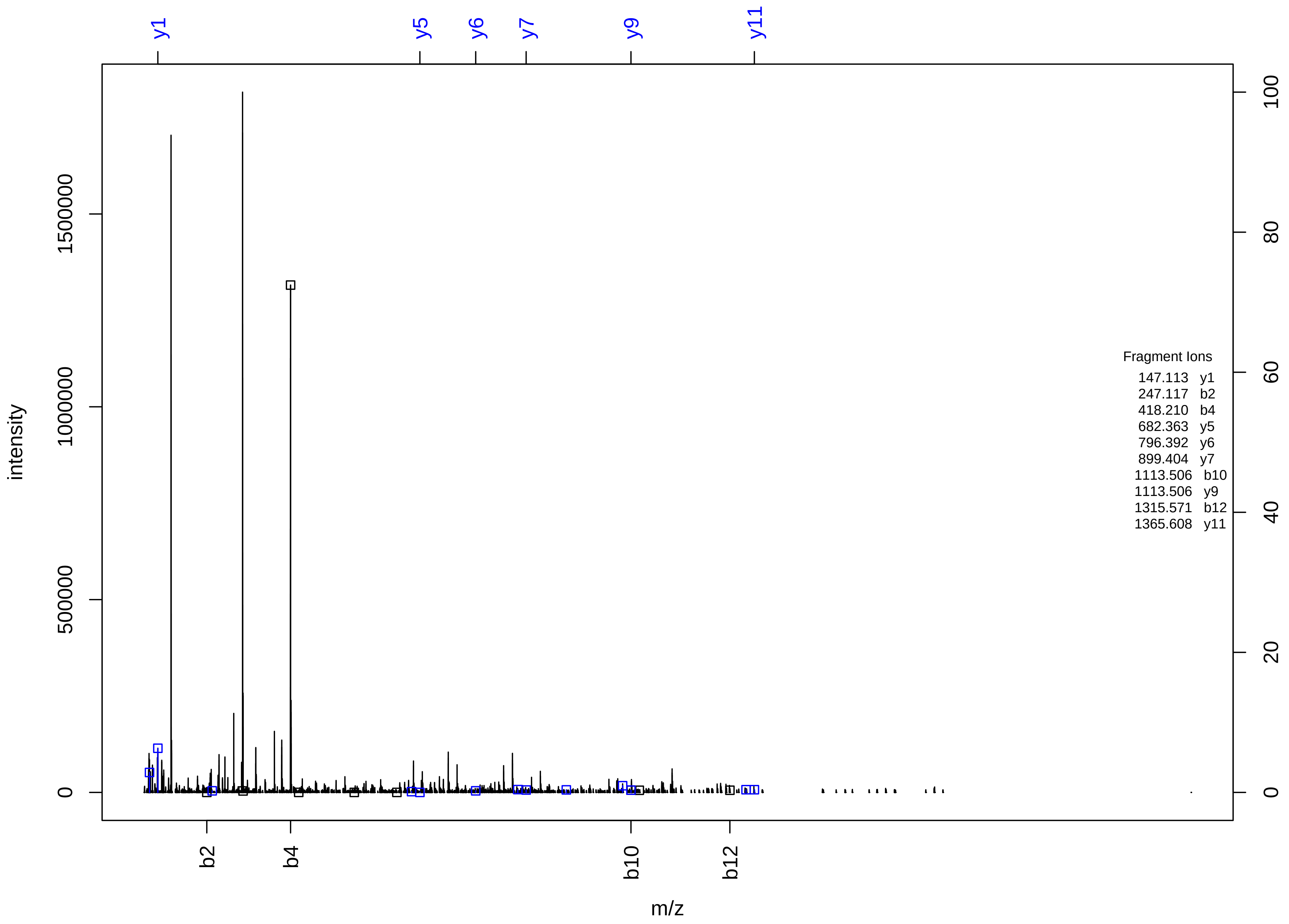

VFGNLSQDHDVCNFLMQK / File: sydney_ip_04.raw; SpectrumID: ; scans: 14871

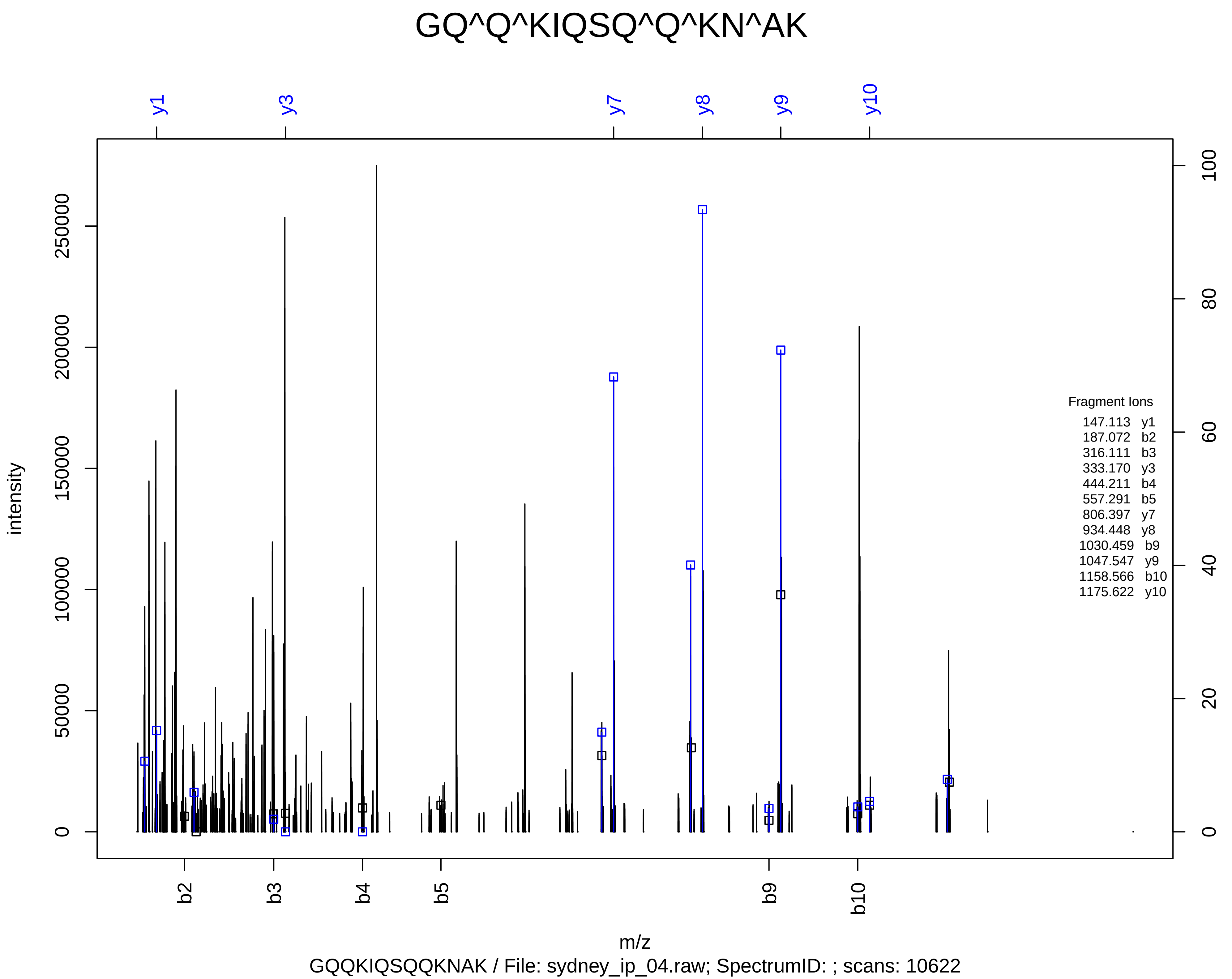
GQ^Q^KIQSQ^Q^KN^AK
y1
y3
y7
y8
y9
y10
intensity
m/z
b2
b3
b4
b5
b9
b10
Fragment Ions
147.113 y1
187.072 b2
316.111 b3
333.170 y3
444.211 b4
557.291 b5
806.397 y7
934.448 y8
1030.459 b9
1047.547 y9
1158.566 b10
1175.622 y10
GQQKIQSQQKNAK / File: sydney_ip_04.raw; SpectrumID: ; scans: 10622

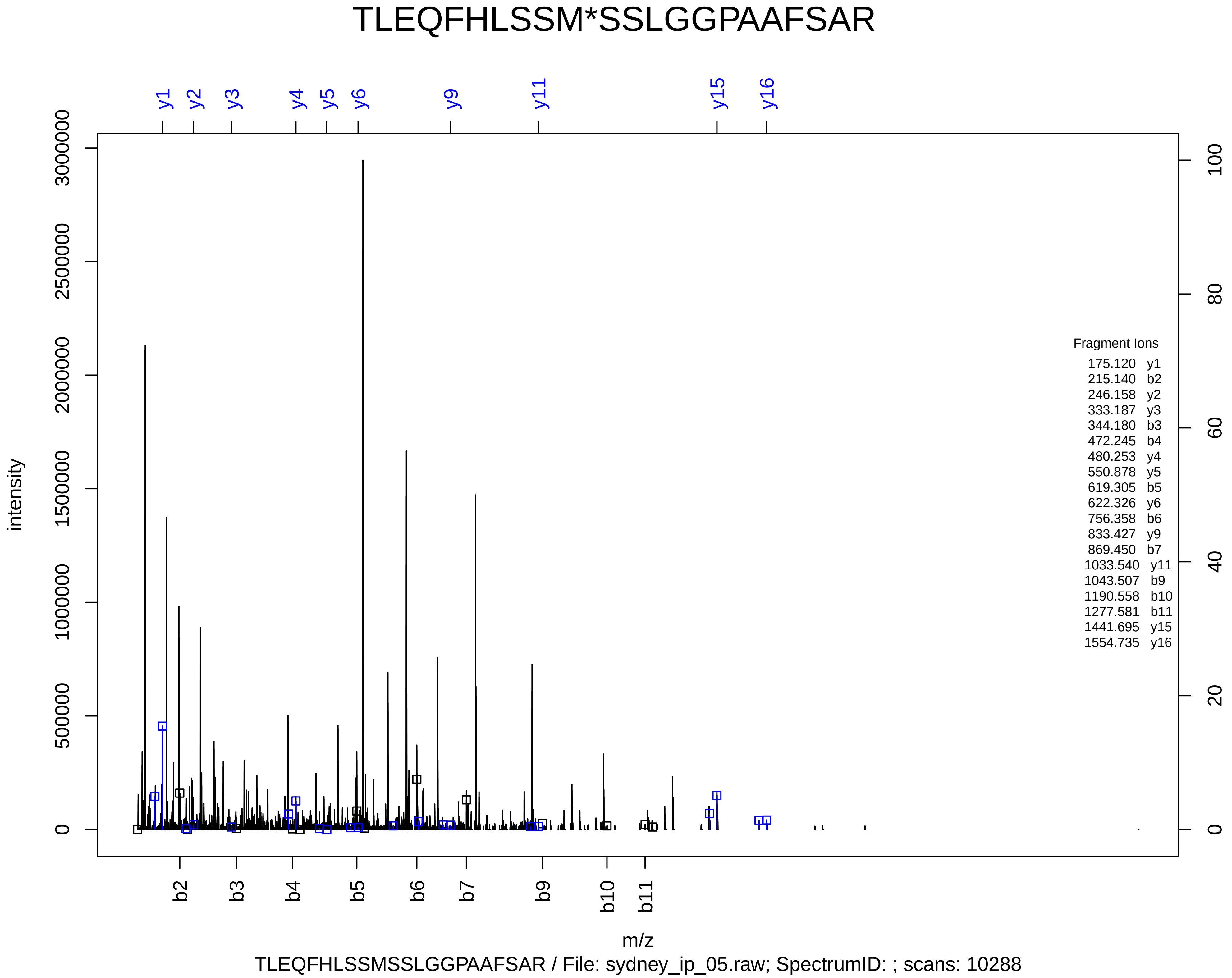
TLEQFHLSSM*SSLGGPAAFSAR
y1 y2 y3 y4 y5 y6 y9 y11 y15 y16
b2 b3 b4 b5 b6 b7 b9 b10 b11
intensity
m/z
Fragment Ions
175.120 y1
215.140 b2
246.158 y2
333.187 y3
344.180 b3
472.245 b4
480.253 y4
550.878 y5
619.305 b5
622.326 y6
756.358 b6
833.427 y9
869.450 b7
1033.540 y11
1043.507 b9
1190.558 b10
1277.581 b11
1441.695 y15
1554.735 y16
TLEQFHLSSMSSLGGPAAFSAR / File: sydney_ip_05.raw; SpectrumID: ; scans: 10288

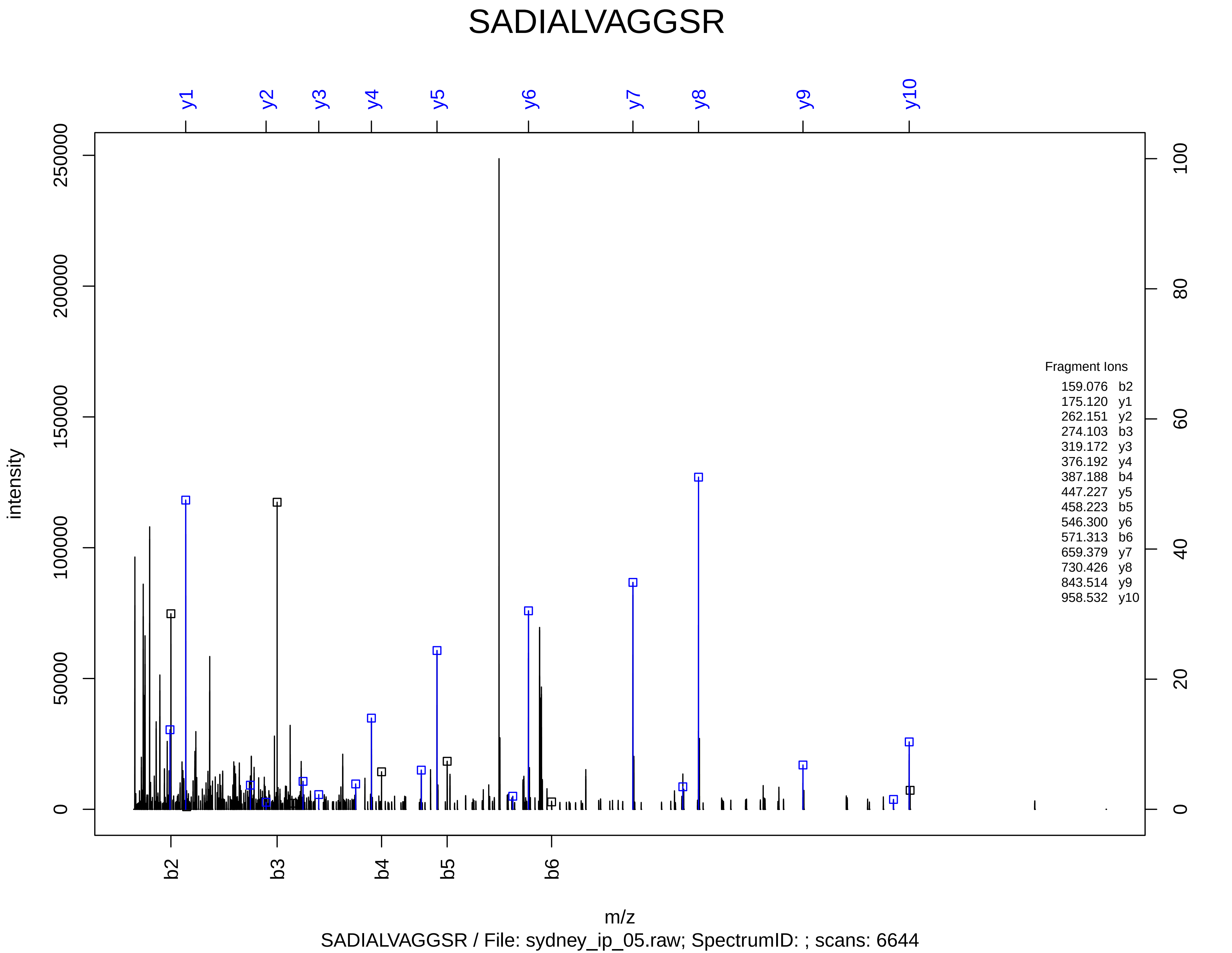
SADIALVAGGSR
y1 y2 y3 y4 y5 y6 y7 y8 y9 y10
b2 b3 b4 b5 b6
intensity
m/z
Fragment Ions
159.076 b2
175.120 y1
262.151 y2
274.103 b3
319.172 y3
376.192 y4
387.188 b4
447.227 y5
458.223 b5
546.300 y6
571.313 b6
659.379 y7
730.426 y8
843.514 y9
958.532 y10
SADIALVAGGSR / File: sydney_ip_05.raw; SpectrumID: ; scans: 6644

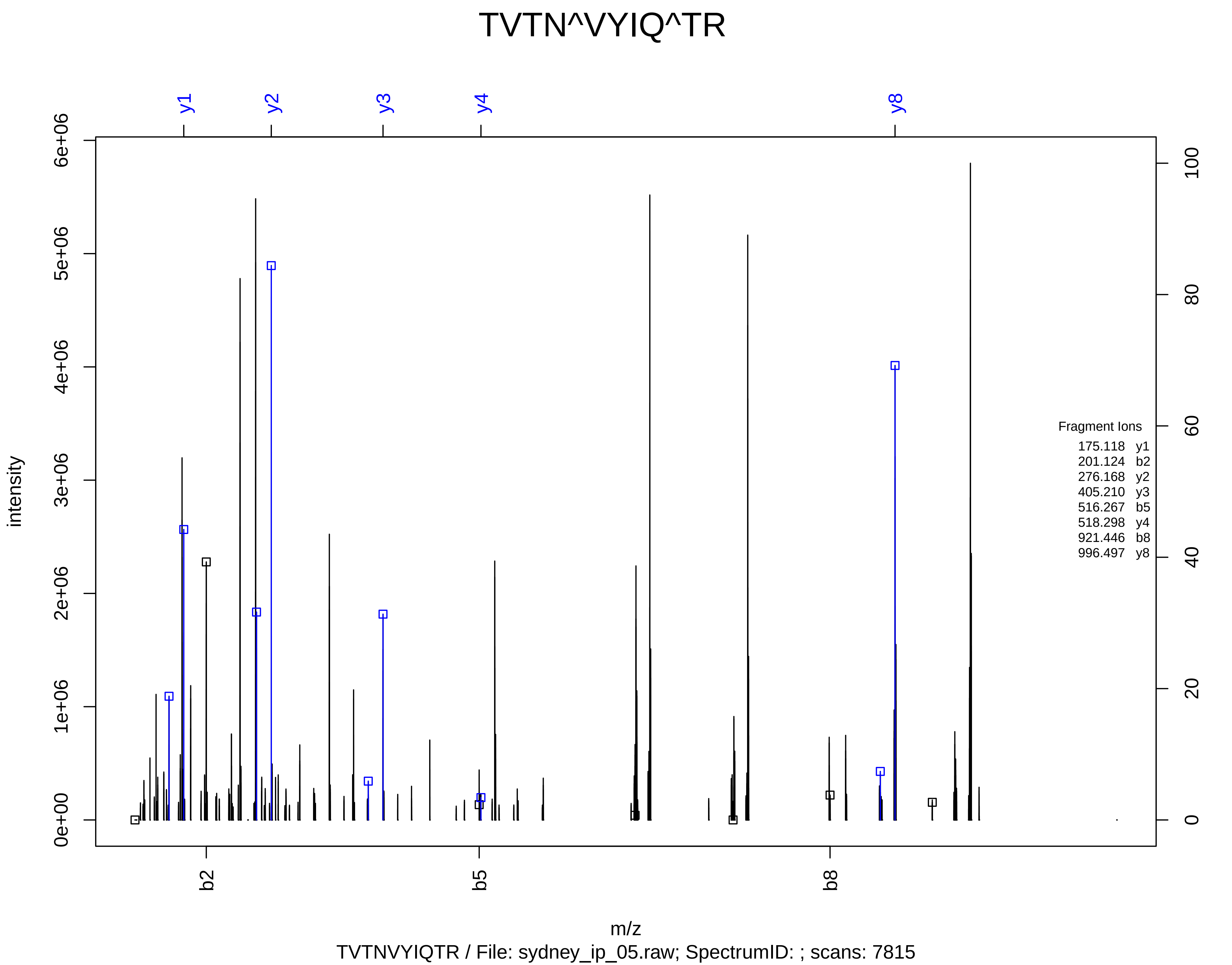
TVTN^VYIQ^TR
y1
y2
y3
y4
y8
intensity
0e+00
1e+06
2e+06
3e+06
4e+06
5e+06
6e+06
0
20
40
60
80
100
b2
b5
b8
m/z
Fragment Ions
175.118 y1
201.124 b2
276.168 y2
405.210 y3
516.267 b5
518.298 y4
921.446 b8
996.497 y8
TVTNVYIQTR / File: sydney_ip_05.raw; SpectrumID: ; scans: 7815

(Ac)MDSTLAVQQ^YIQ^QNIR

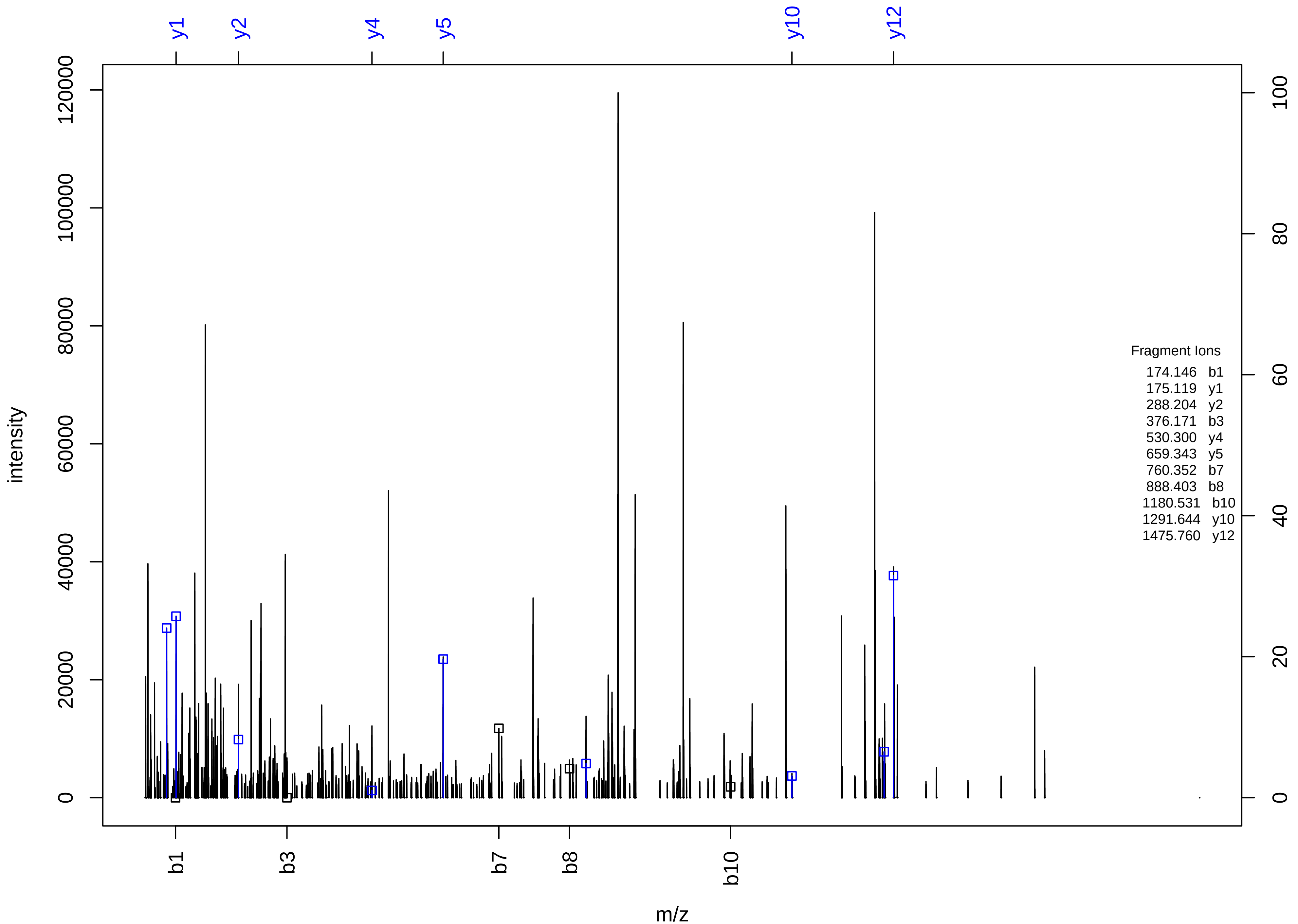

MDSTLAVQQYIQQNIR / File: sydney_ip_05.raw; SpectrumID: ; scans: 6587

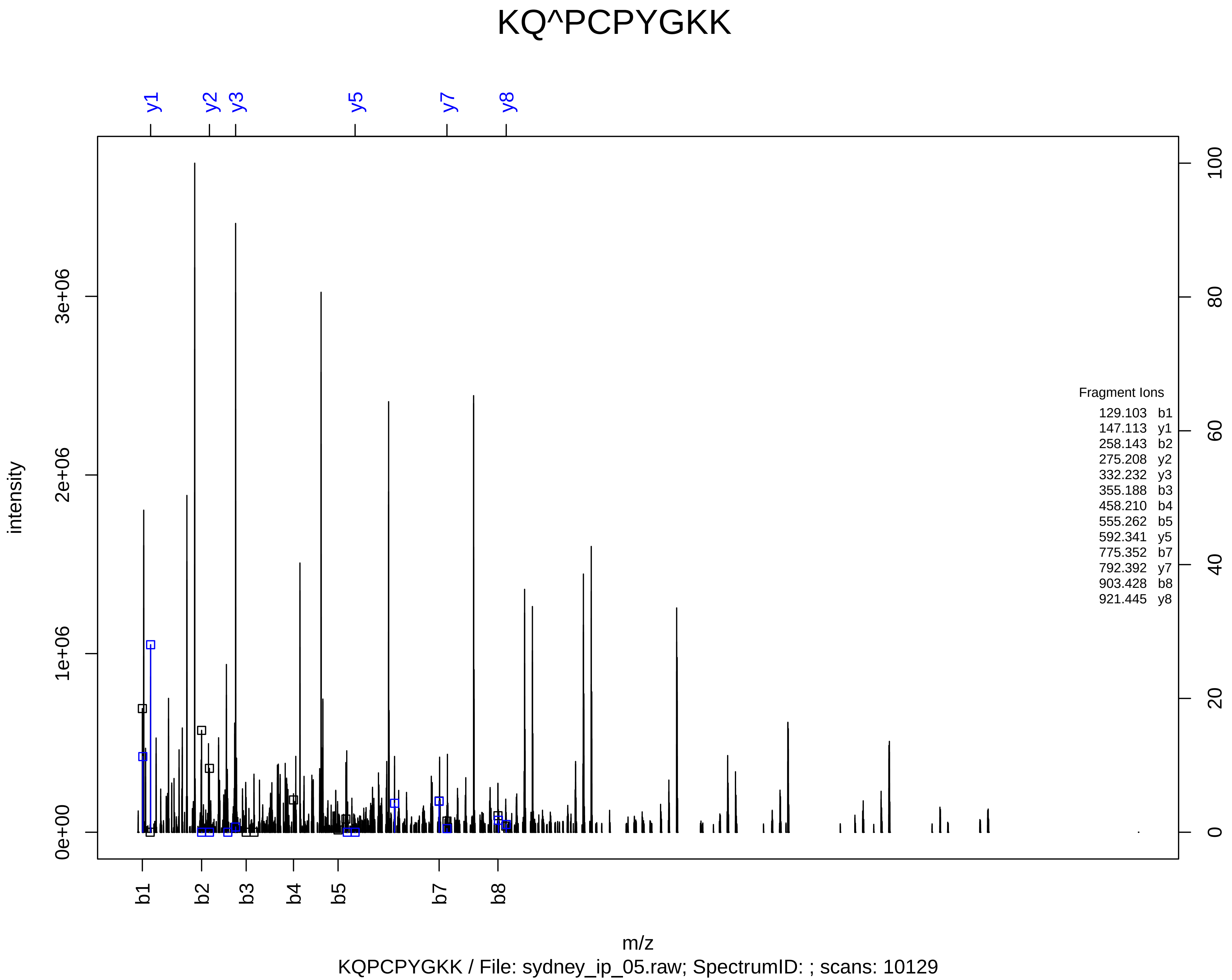
KQ^PCPYGKK
y1 y2 y3 y5 y7 y8
b1 b2 b3 b4 b5 b7 b8
intensity
0e+00
1e+06
2e+06
3e+06
0
20
40
60
80
100
m/z
Fragment Ions
129.103 b1
147.113 y1
258.143 b2
275.208 y2
332.232 y3
355.188 b3
458.210 b4
555.262 b5
592.341 y5
775.352 b7
792.392 y7
903.428 b8
921.445 y8
KQPCPYGKK / File: sydney_ip_05.raw; SpectrumID: ; scans: 10129

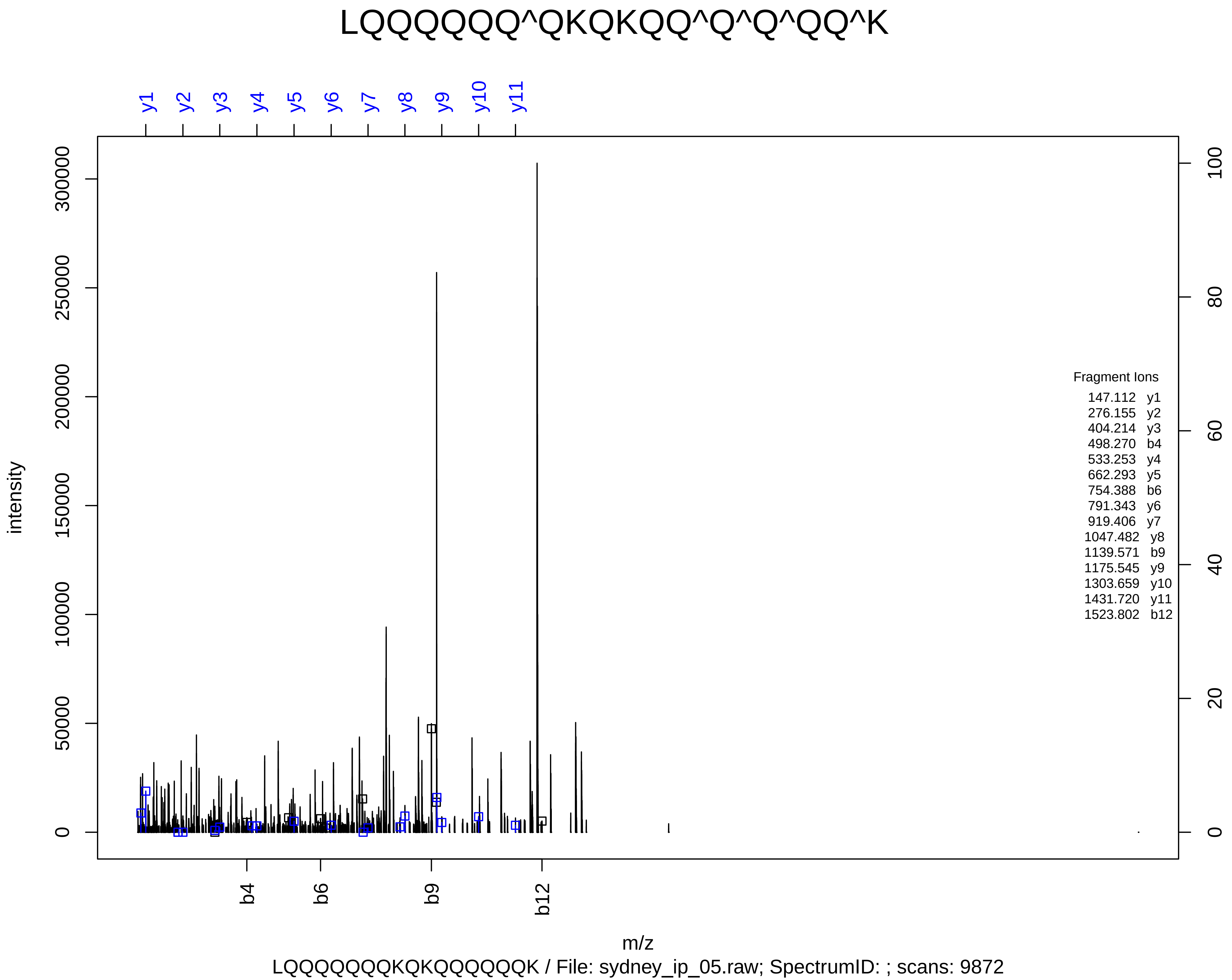

LQQQQQ^QKQKQQ^Q^Q^QQ^K
y1 y2 y3 y4 y5 y6 y7 y8 y9 y10 y11
intensity
0 50000 100000 150000 200000 250000 300000
0 20 40 60 80 100
b4 b6 b9 b12
m/z
Fragment Ions
147.112 y1
276.155 y2
404.214 y3
498.270 b4
533.253 y4
662.293 y5
754.388 b6
791.343 y6
919.406 y7
1047.482 y8
1139.571 b9
1175.545 y9
1303.659 y10
1431.720 y11
1523.802 b12
LQQQQQQKQKQQQQQK / File: sydney_ip_05.raw; SpectrumID: ; scans: 9872

M*IQEQLAEVAVSAK

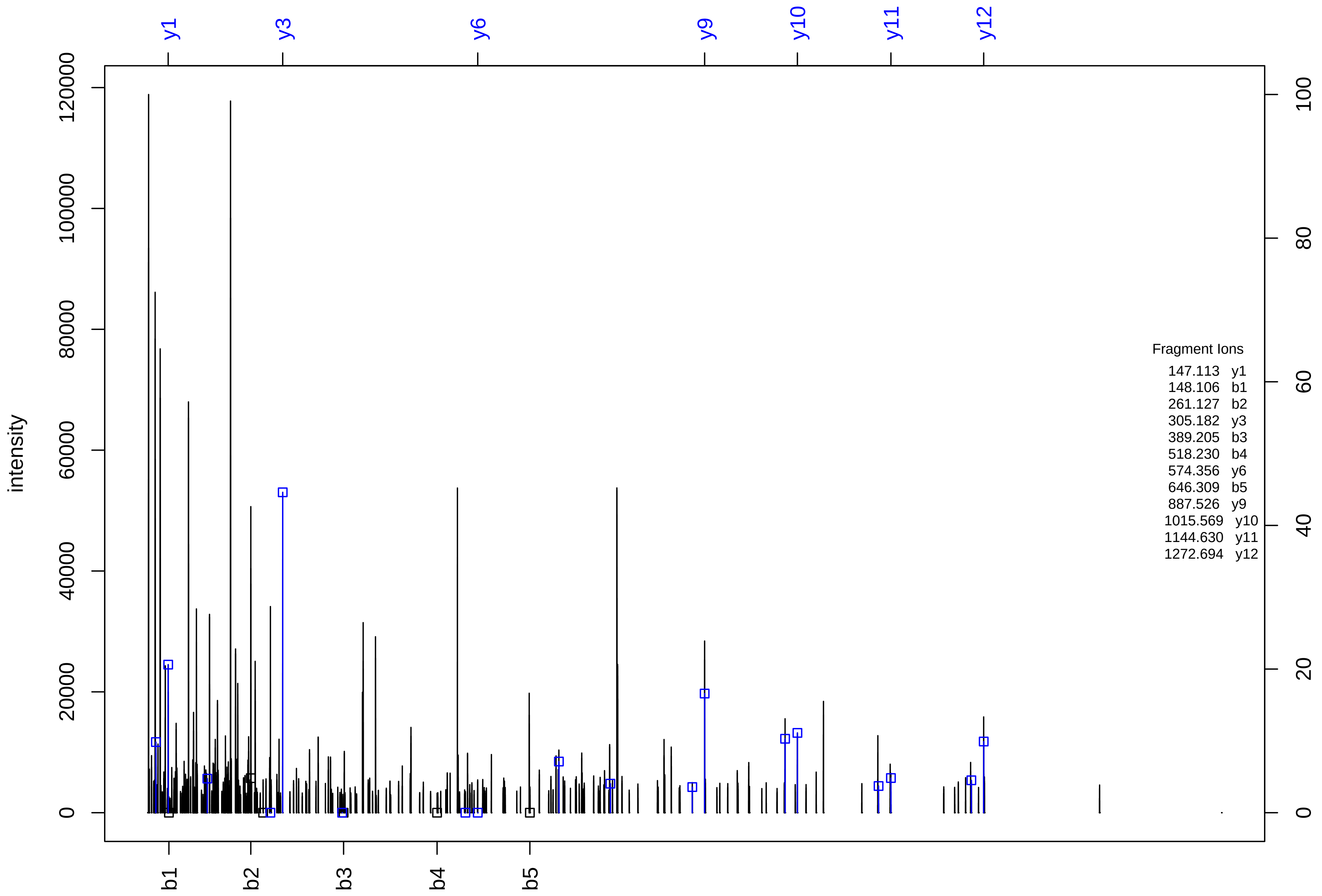

MIQEQLAEVAVSAK / File: sydney_ip_05.raw; SpectrumID: ; scans: 6233

ILLLFGESELSPTATPSTLK

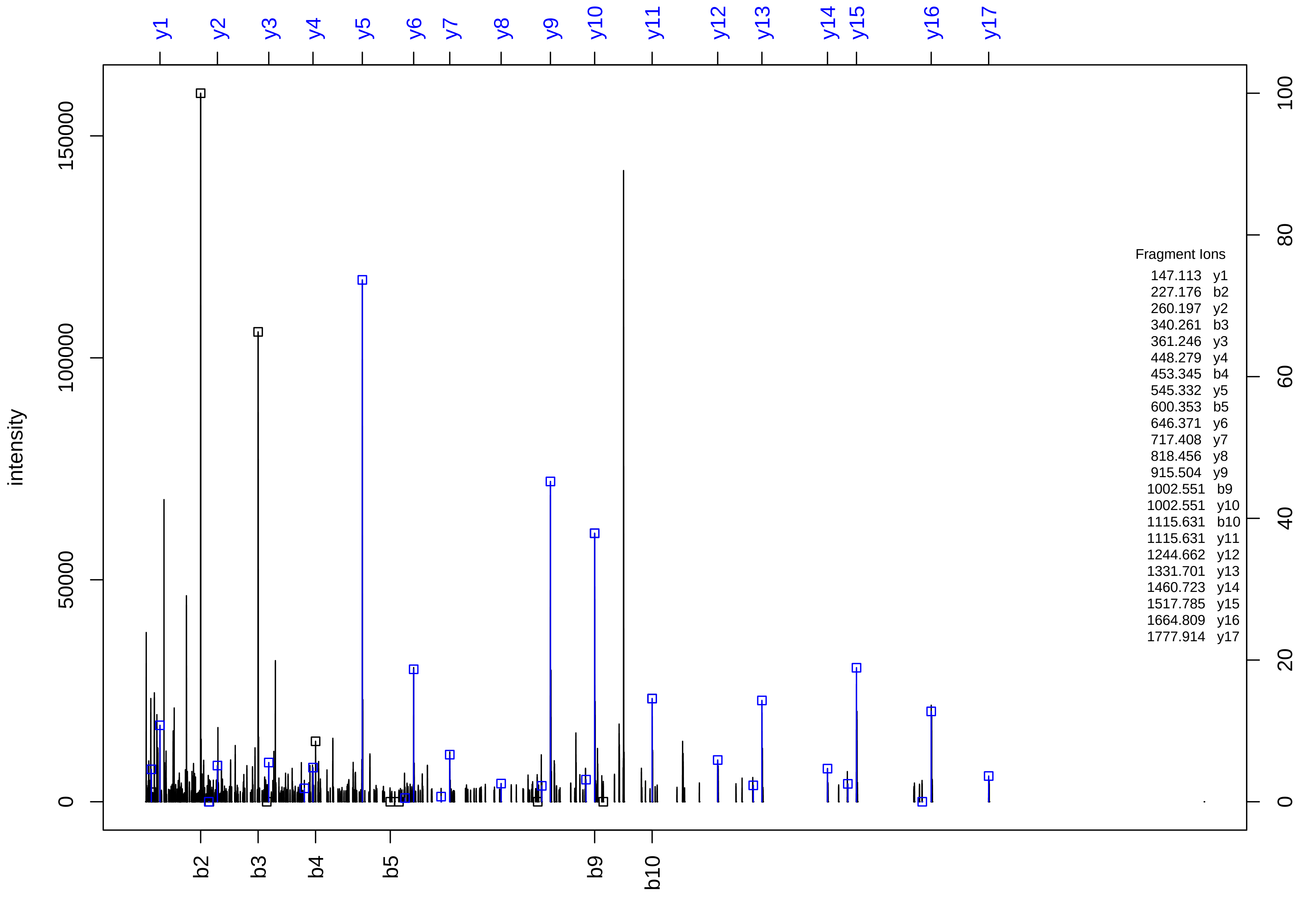

ILLLFGESELSPTATPSTLK / File: sydney_ip_05.raw; SpectrumID: ; scans: 18801

RQ^QQ^LRQ^PR

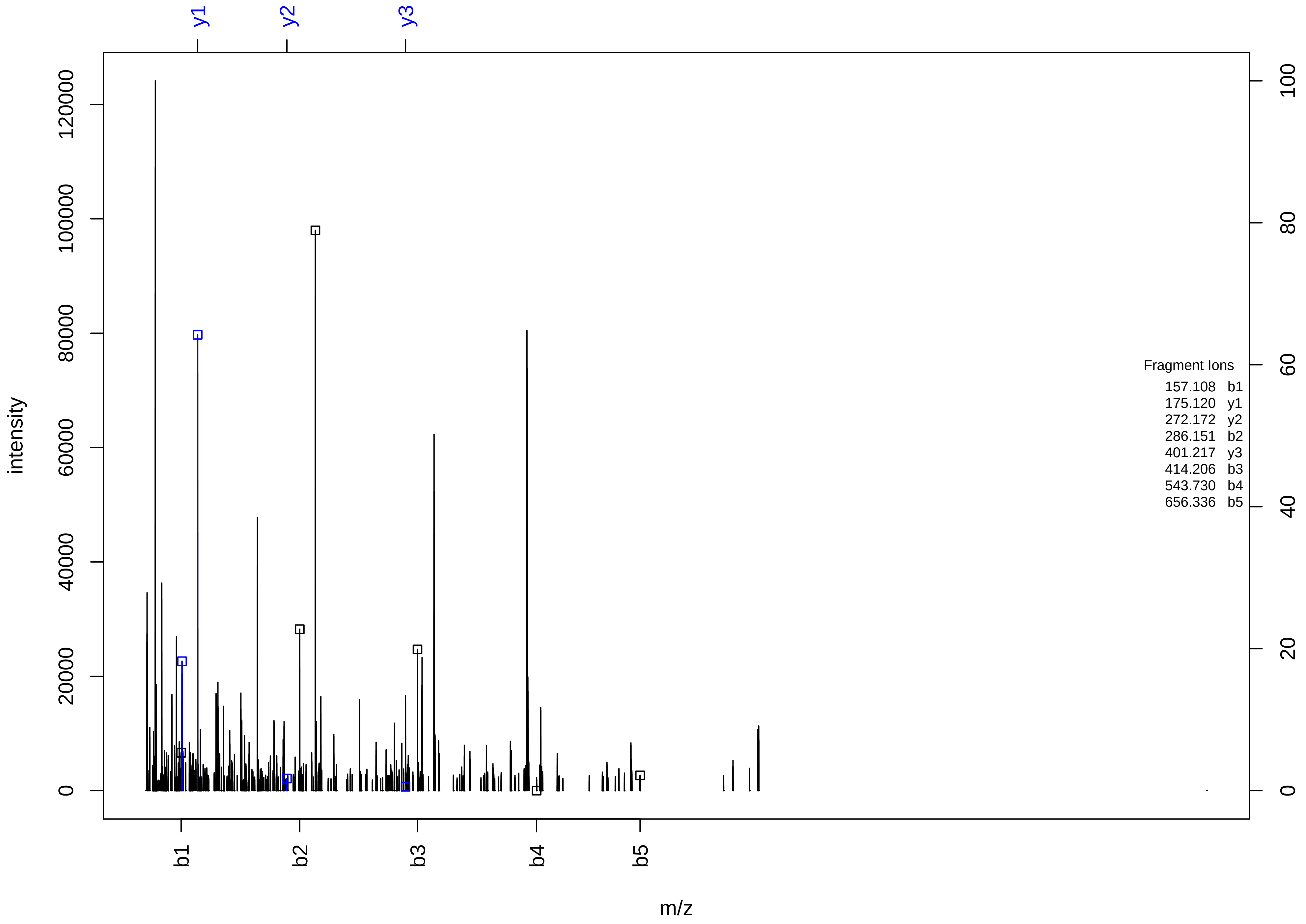

RQQQLRQPR / File: sydney_ip_05.raw; SpectrumID: ; scans: 2067

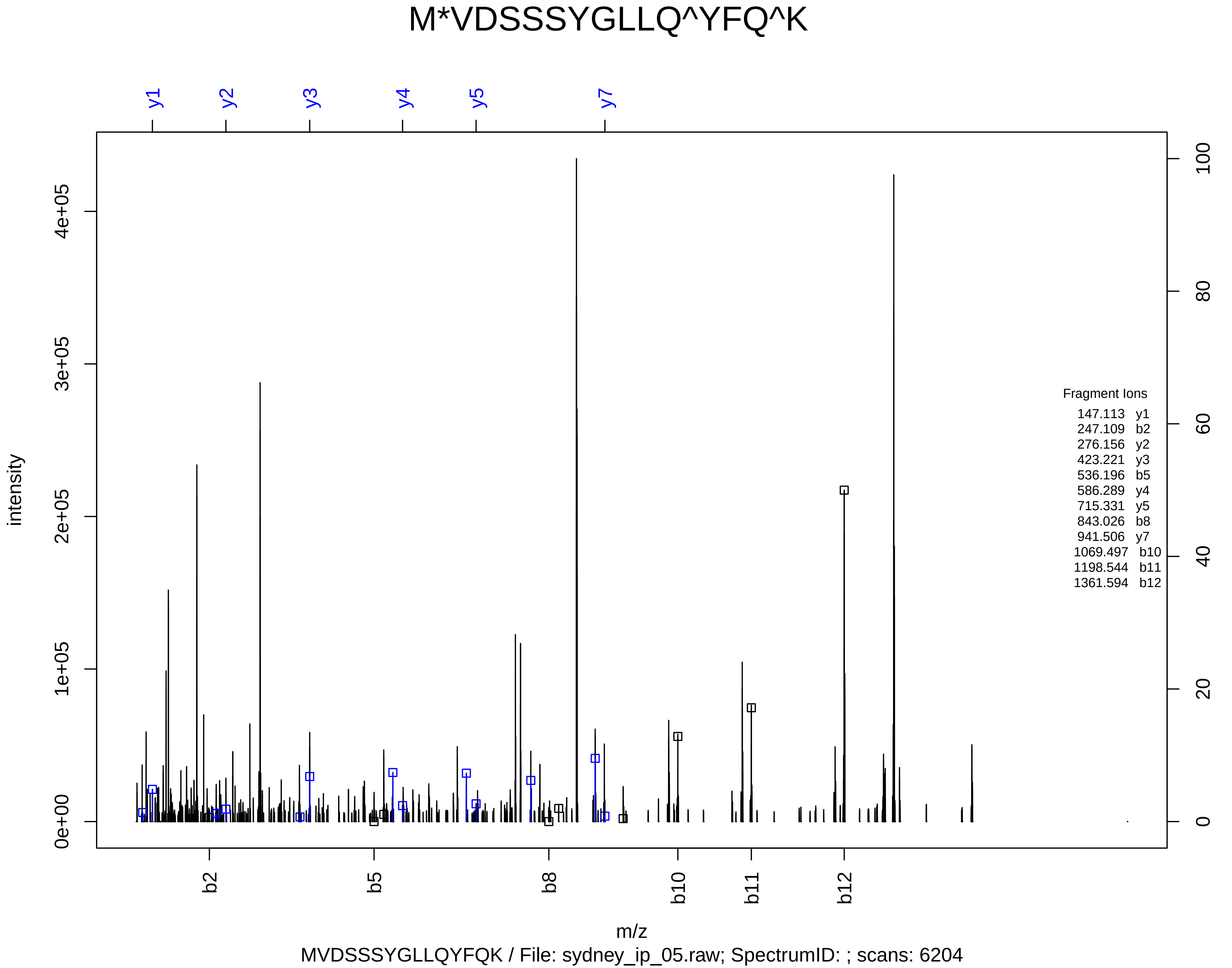
M*VDSSSYGLLQ^YFQ^K
y1
y2
y3
y4
y5
y7
intensity
0e+00
1e+05
2e+05
3e+05
4e+05
0
20
40
60
80
100
Fragment Ions
147.113 y1
247.109 b2
276.156 y2
423.221 y3
536.196 b5
586.289 y4
715.331 y5
843.026 b8
941.506 y7
1069.497 b10
1198.544 b11
1361.594 b12
b2
b5
b8
b10
b11
b12
m/z
MVDSSSYGLLQYFQK / File: sydney_ip_05.raw; SpectrumID: ; scans: 6204

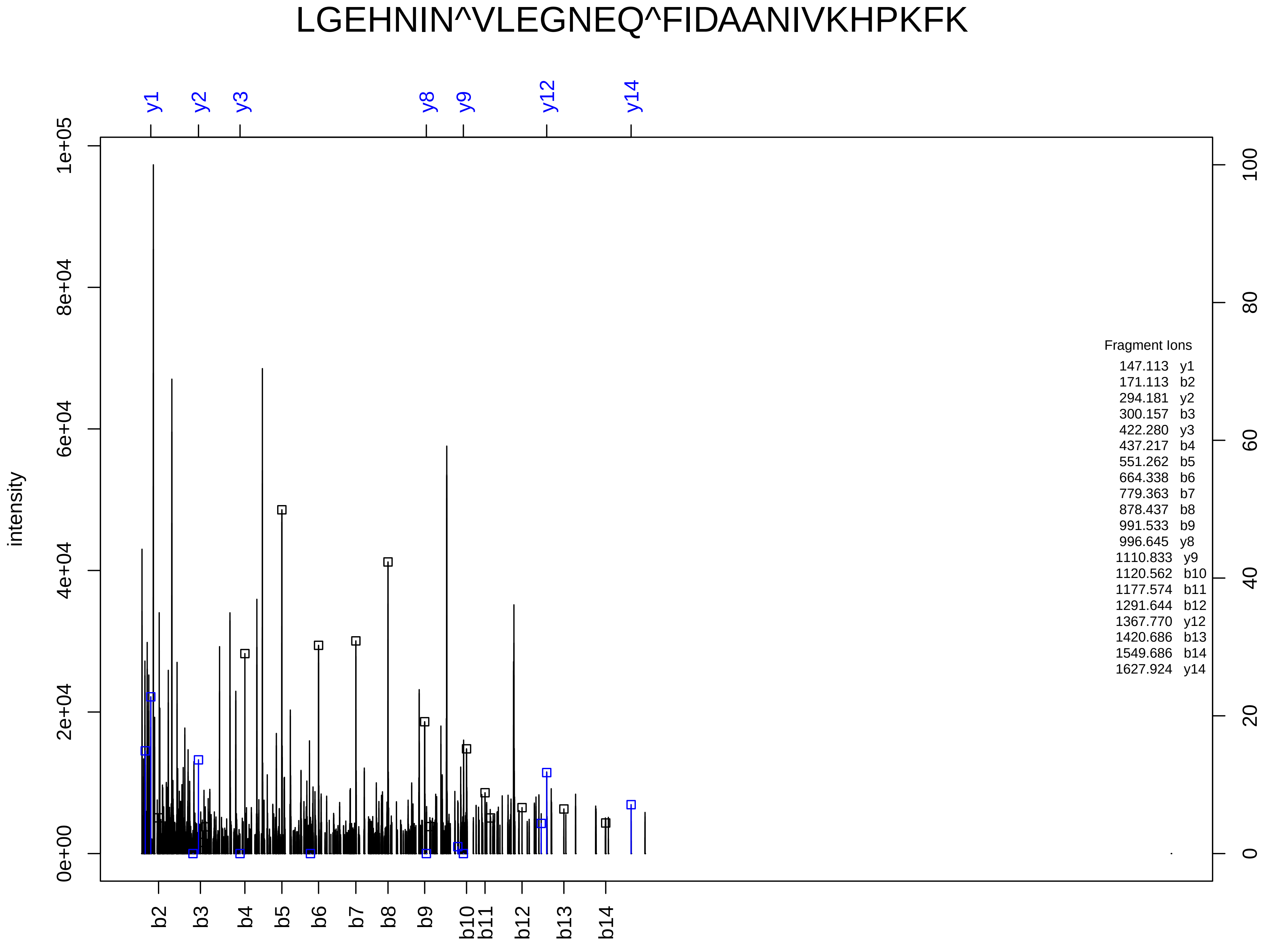

LGEHNINVLEGNEQFIDAANIVKHPKFK / File: sydney_ip_05.raw; SpectrumID: ; scans: 16731

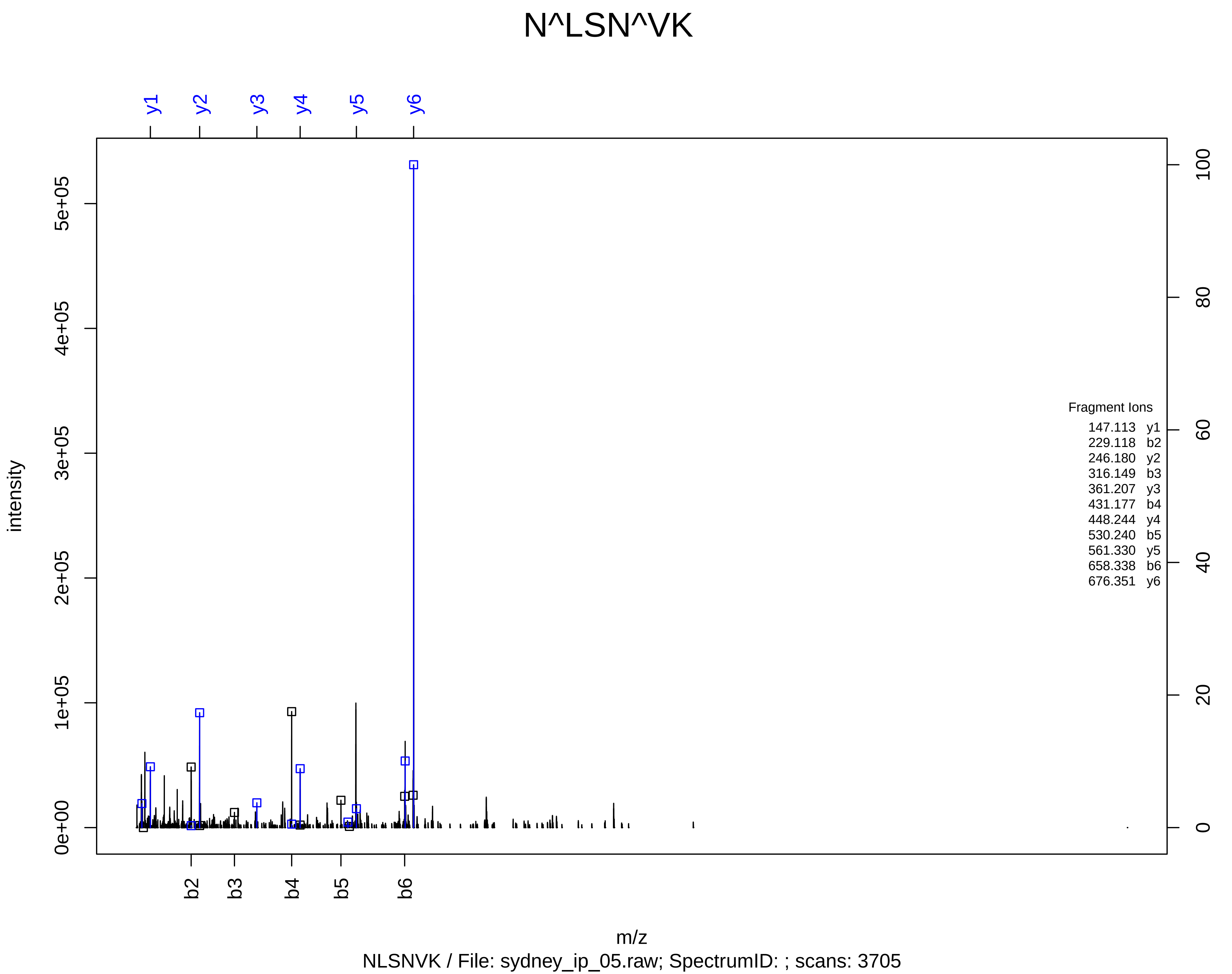
N^LSN^VK
y1
y2
y3
y4
y5
y6
intensity
0e+00
1e+05
2e+05
3e+05
4e+05
5e+05
0
20
40
60
80
100
b2
b3
b4
b5
b6
m/z
Fragment Ions
147.113 y1
229.118 b2
246.180 y2
316.149 b3
361.207 y3
431.177 b4
448.244 y4
530.240 b5
561.330 y5
658.338 b6
676.351 y6
NLSNVK / File: sydney_ip_05.raw; SpectrumID: ; scans: 3705

N^KEVNN^AFK

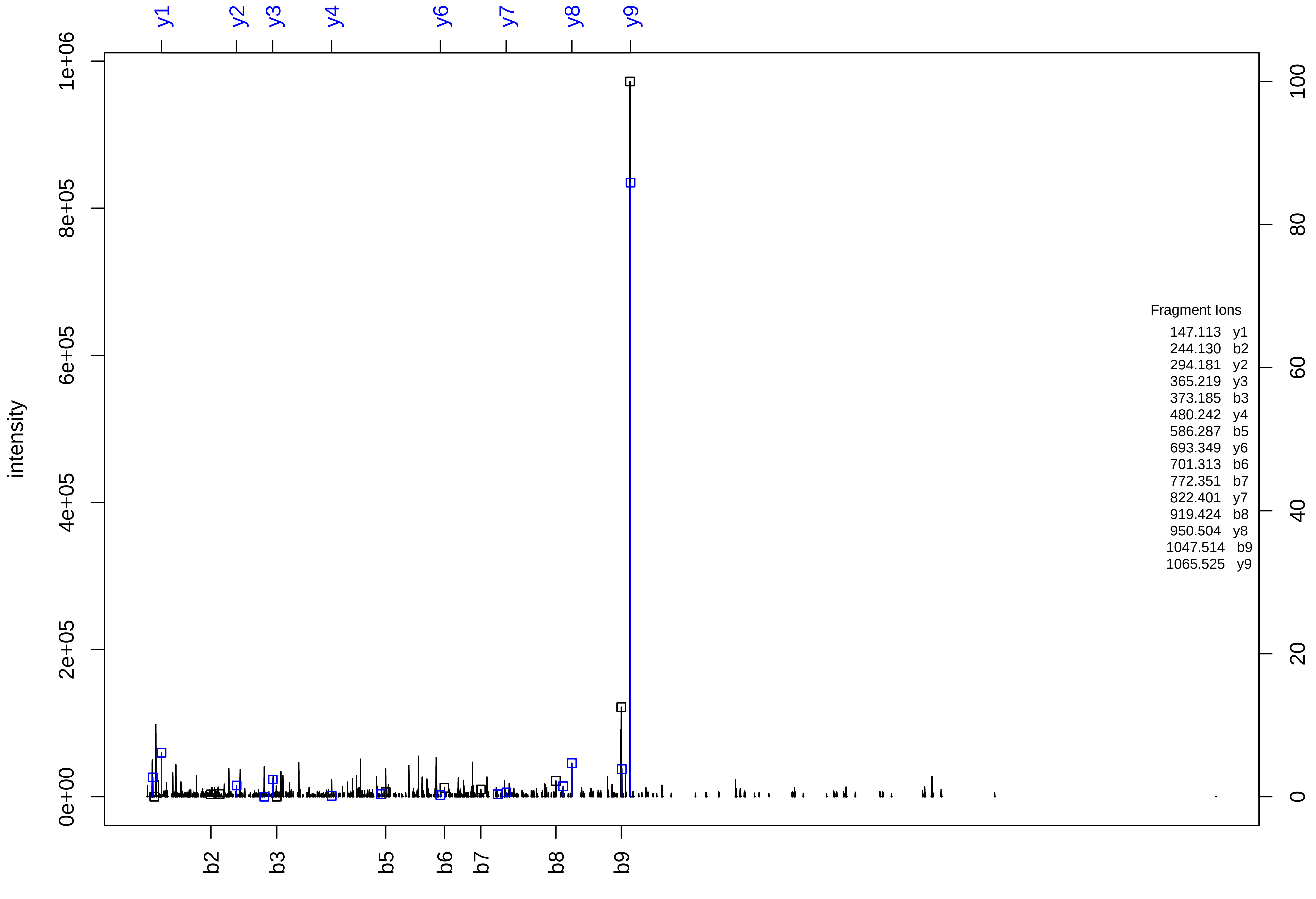

NKEVNNAFK / File: sydney_ip_05.raw; SpectrumID: ; scans: 9104

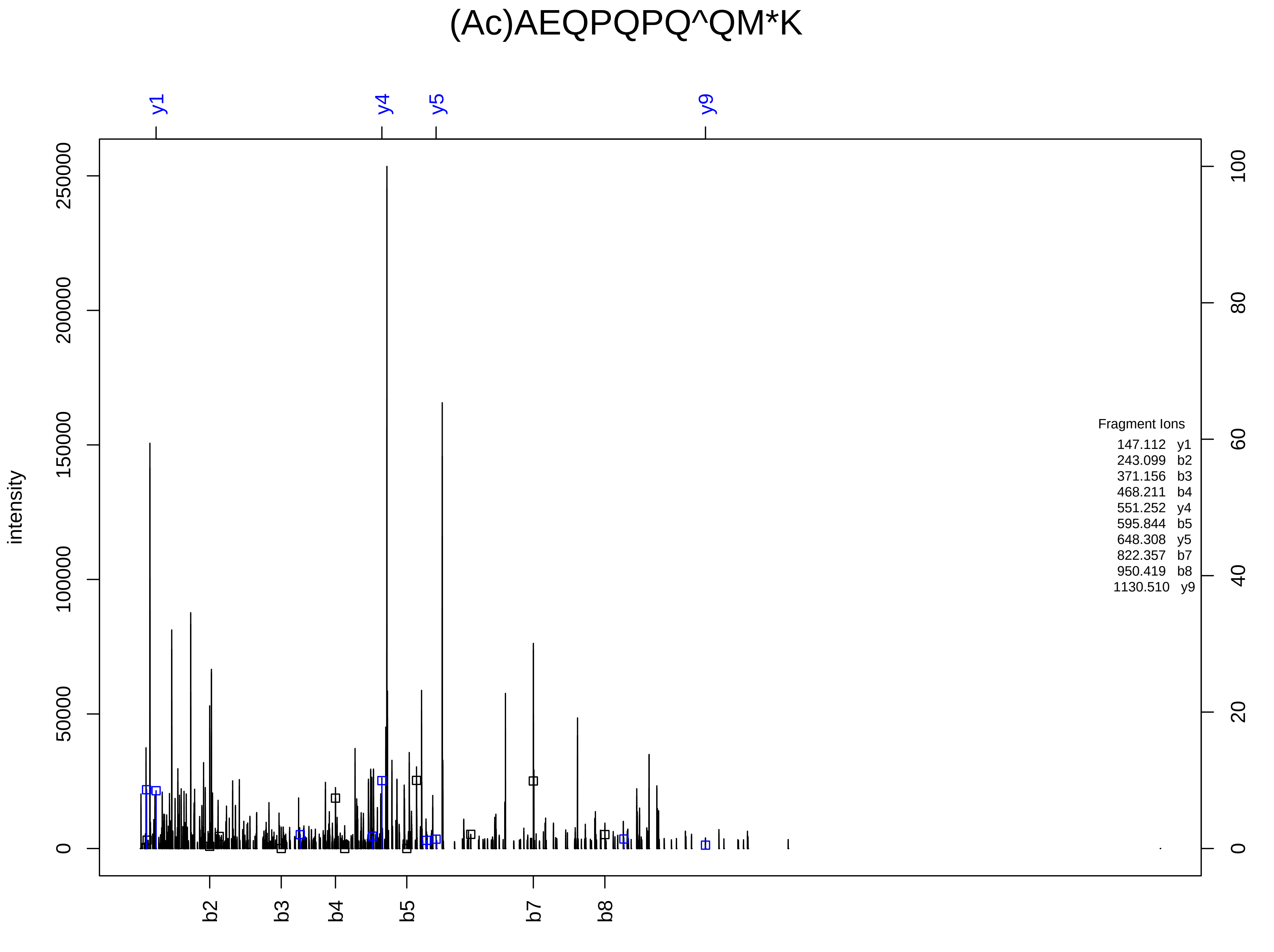

AEQPQPQQMK / File: sydney_ip_06.raw; SpectrumID: ; scans: 3071

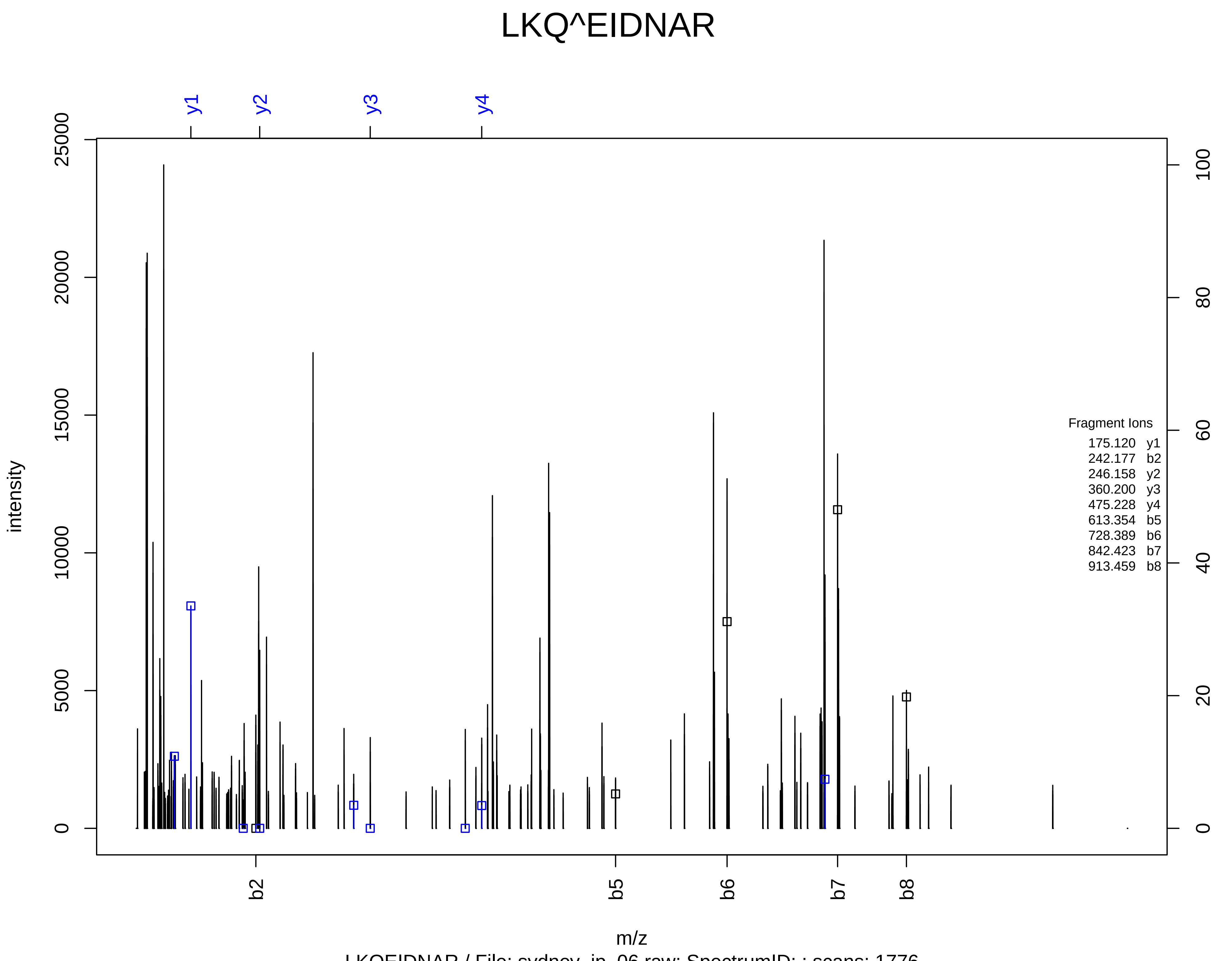
LKQ^EIDNAR
y1
y2
y3
y4
intensity
m/z
b2
b5
b6
b7
b8
Fragment Ions
175.120 y1
242.177 b2
246.158 y2
360.200 y3
475.228 y4
613.354 b5
728.389 b6
842.423 b7
913.459 b8
LKQEIDNAR / File: sydney_ip_06.raw; SpectrumID: ; scans: 1776

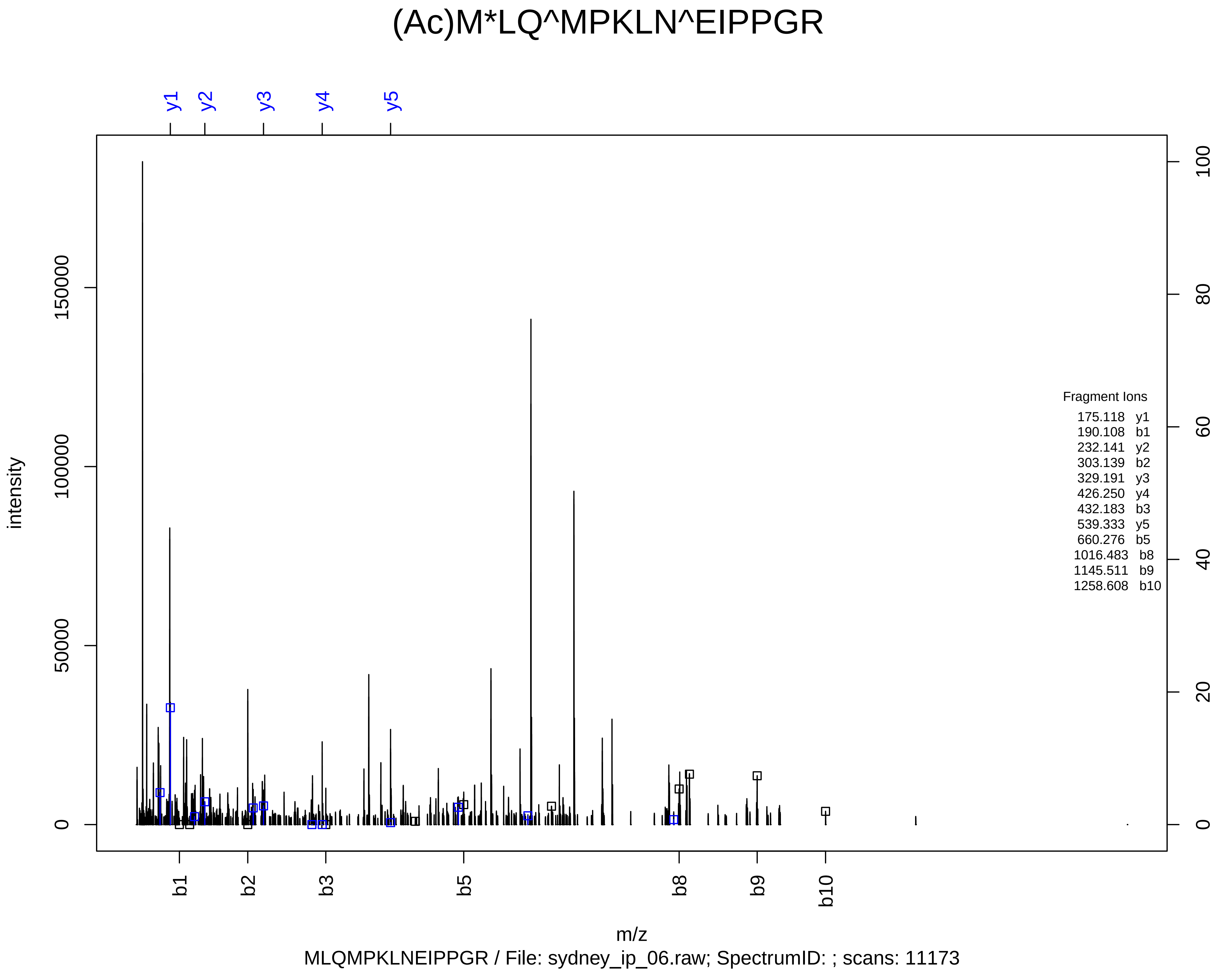

(Ac)M*LQ^MPKLN^EIPPGR
y1
y2
y3
y4
y5
intensity
0
50000
100000
150000
0
20
40
60
80
100
b1
b2
b3
b5
b8
b9
b10
m/z
Fragment Ions
175.118 y1
190.108 b1
232.141 y2
303.139 b2
329.191 y3
426.250 y4
432.183 b3
539.333 y5
660.276 b5
1016.483 b8
1145.511 b9
1258.608 b10
MLQMPKLNEIPPGR / File: sydney_ip_06.raw; SpectrumID: ; scans: 11173

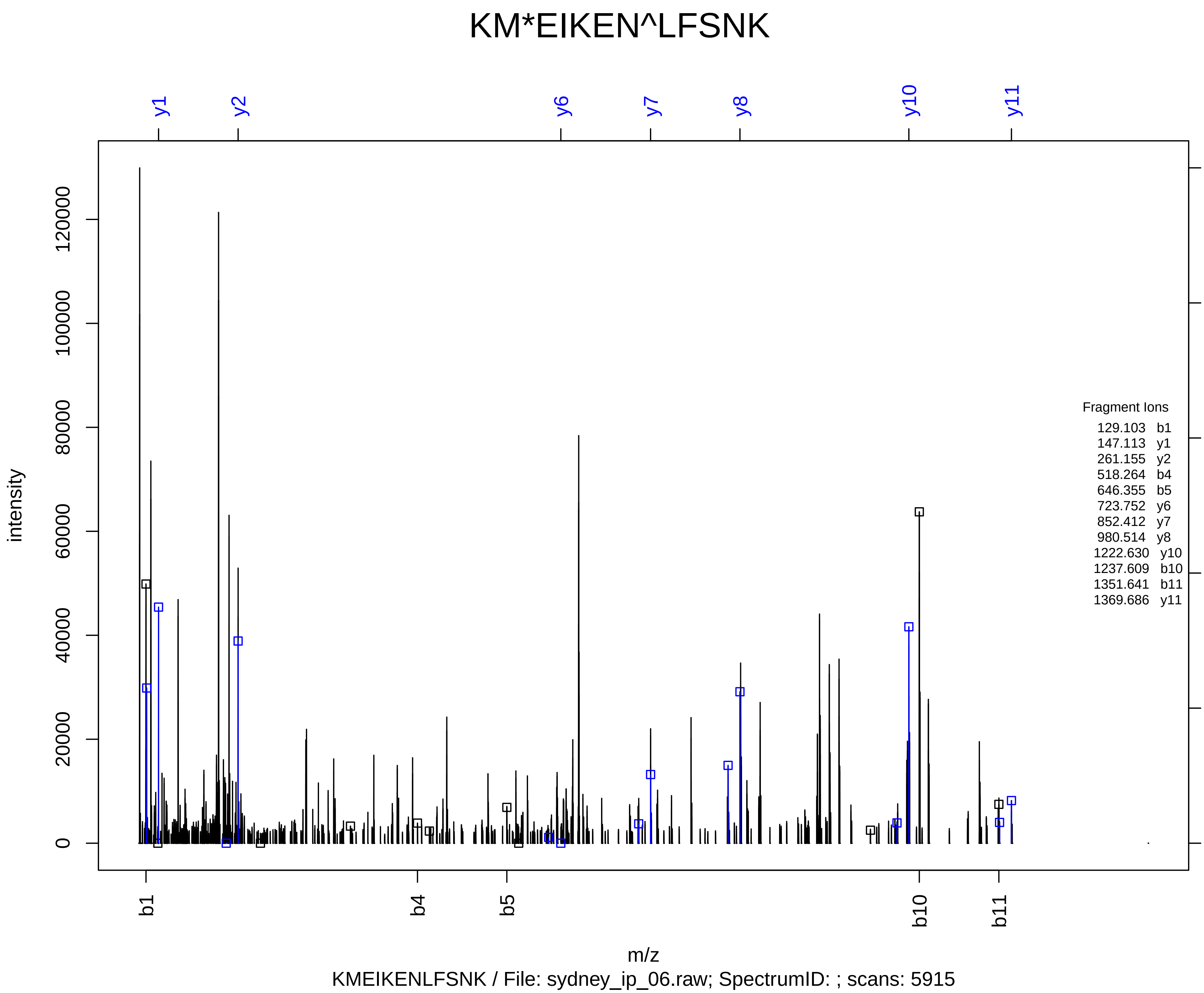

KM*EIKEN^LFSNK
y1
y2
y6
y7
y8
y10
y11
intensity
0
20000
40000
60000
80000
100000
120000
0
20
40
60
80
100
Fragment Ions
129.103 b1
147.113 y1
261.155 y2
518.264 b4
646.355 b5
723.752 y6
852.412 y7
980.514 y8
1222.630 y10
1237.609 b10
1351.641 b11
1369.686 y11
b1
b4
b5
b10
b11
m/z
KMEIKENLFSNK / File: sydney_ip_06.raw; SpectrumID: ; scans: 5915

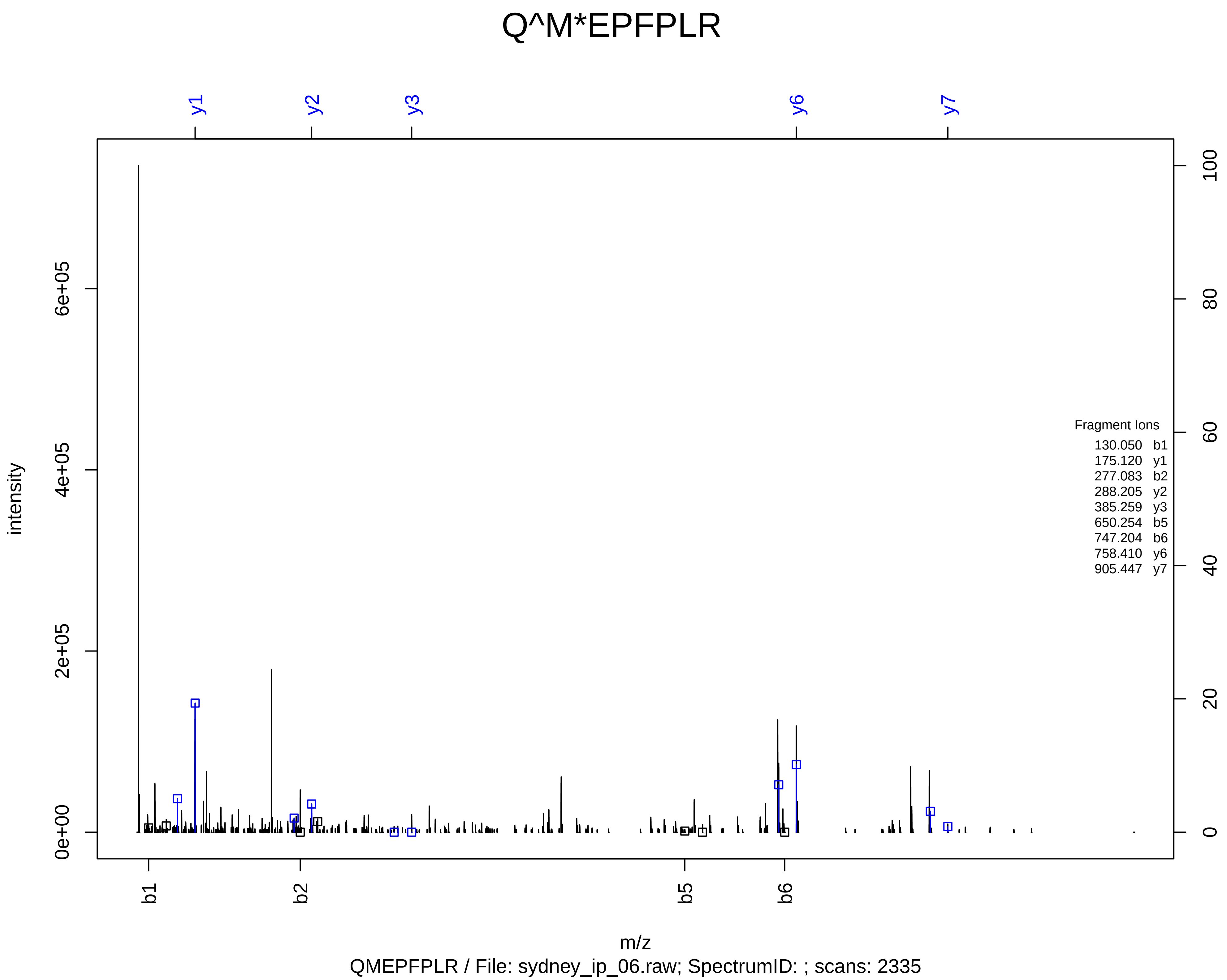
Q^M*EPFPLR
y1
y2
y3
y6
y7
intensity
0e+00
2e+05
4e+05
6e+05
0
20
40
60
80
100
Fragment Ions
130.050 b1
175.120 y1
277.083 b2
288.205 y2
385.259 y3
650.254 b5
747.204 b6
758.410 y6
905.447 y7
b1
b2
b5
b6
m/z
QMEPFPLR / File: sydney_ip_06.raw; SpectrumID: ; scans: 2335

FEIDQLSKN^LQ^N^CKQ^EN^ETLK

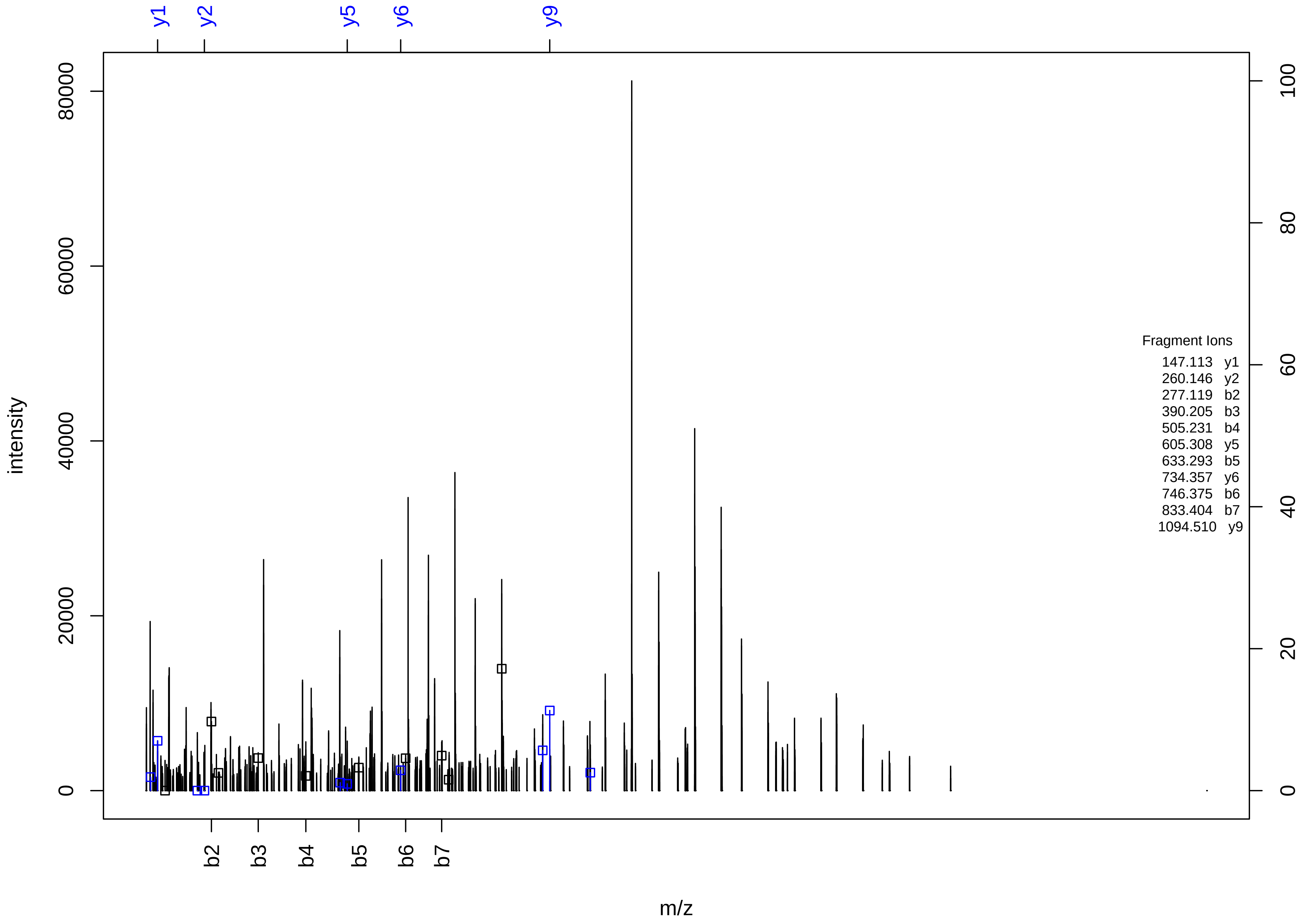

FEIDQLSKNLQNCKQENETLK / File: sydney_ip_07.raw; SpectrumID: ; scans: 8669

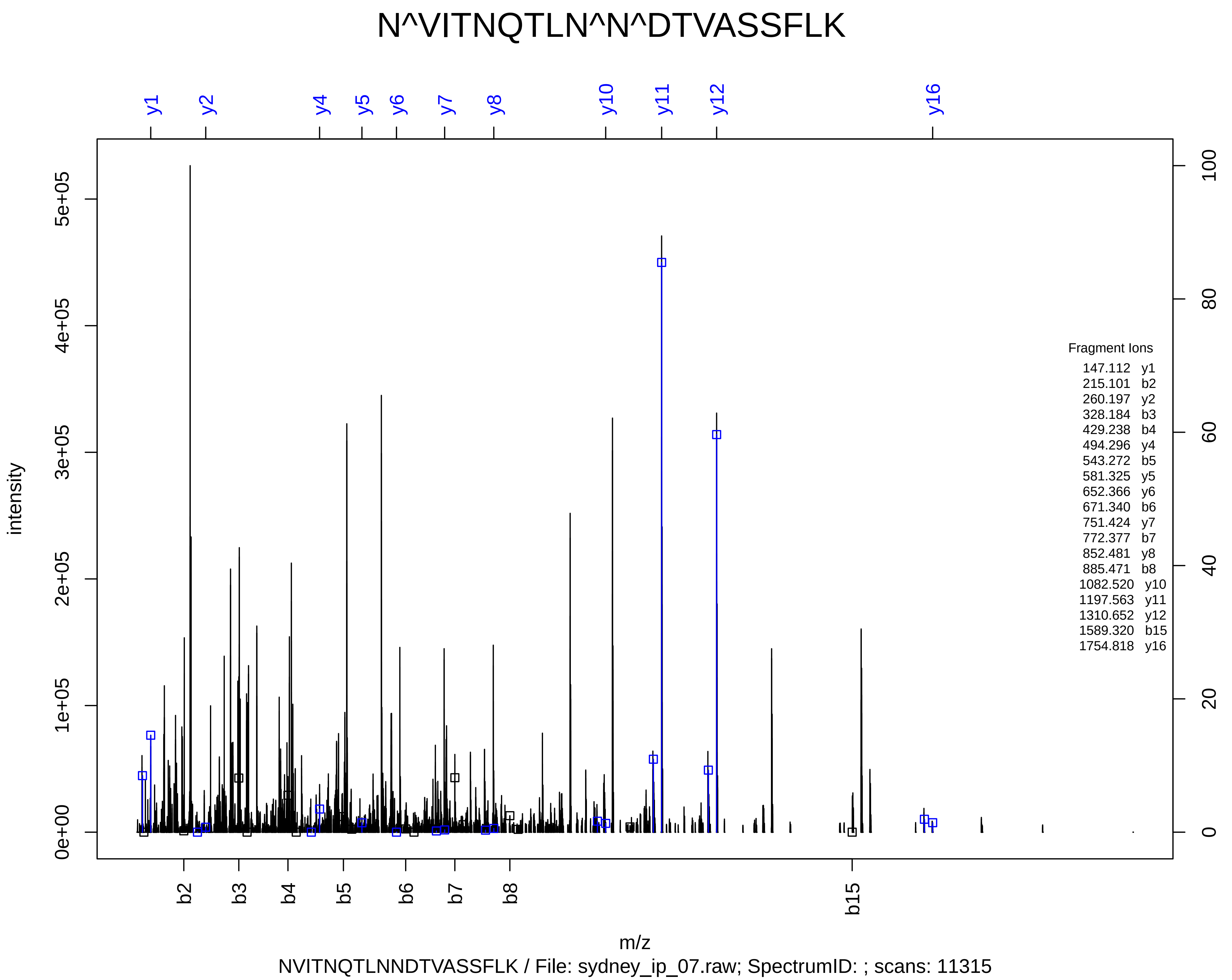

N^VITNQTLN^N^DTVASSFLK
y1
y2
y4
y5
y6
y7
y8
y10
y11
y12
y16
intensity
0e+00
1e+05
2e+05
3e+05
4e+05
5e+05
0
20
40
60
80
100
b2
b3
b4
b5
b6
b7
b8
b15
m/z
Fragment Ions
147.112 y1
215.101 b2
260.197 y2
328.184 b3
429.238 b4
494.296 y4
543.272 b5
581.325 y5
652.366 y6
671.340 b6
751.424 y7
772.377 b7
852.481 y8
885.471 b8
1082.520 y10
1197.563 y11
1310.652 y12
1589.320 b15
1754.818 y16
NVITNQTLNNDTVASSFLK / File: sydney_ip_07.raw; SpectrumID: ; scans: 11315

NRTSSLLPKM*SSLWEN^AR

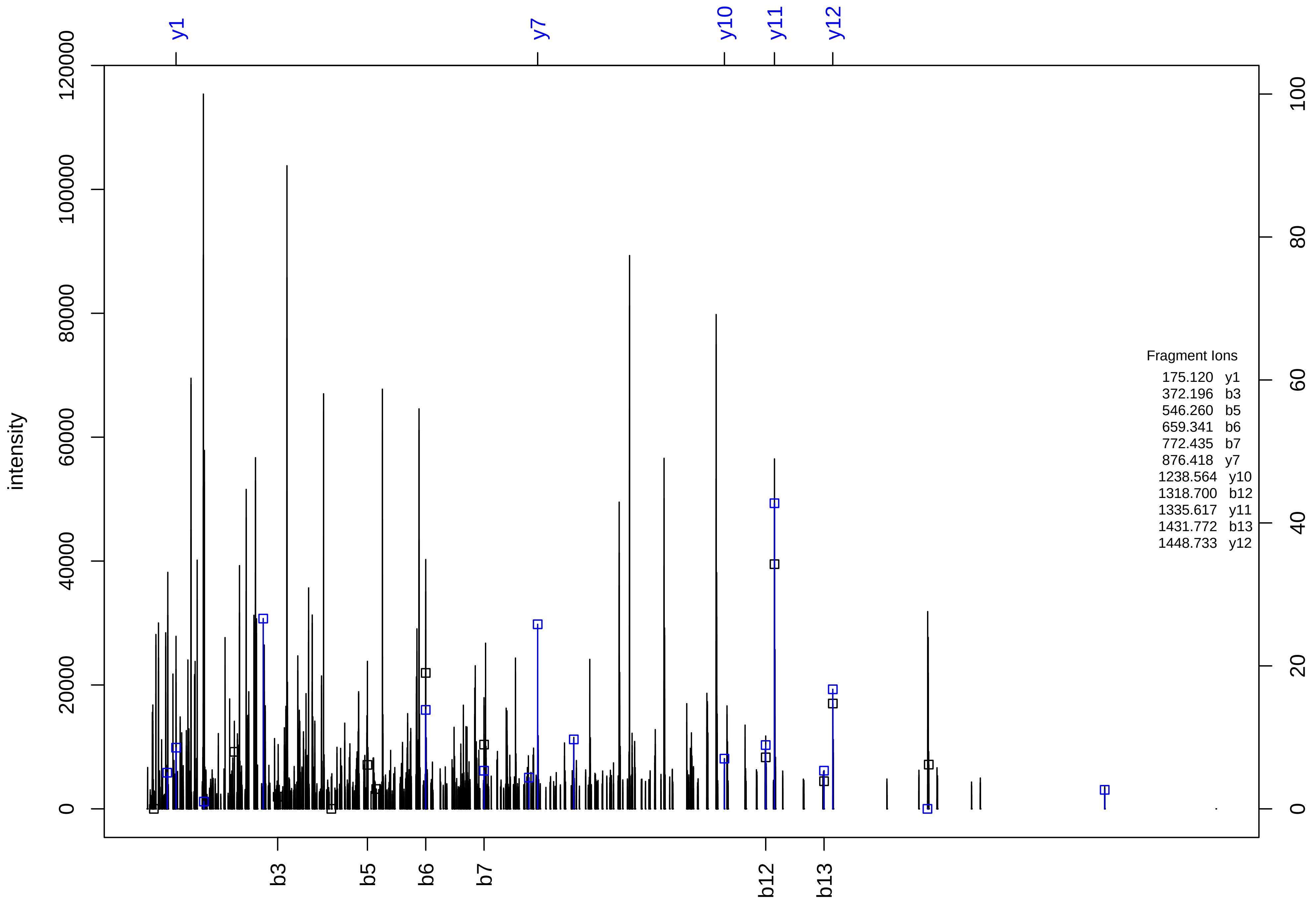

NRTSSLLPKMSSLWENAR / File: sydney_ip_07.raw; SpectrumID: ; scans: 11924

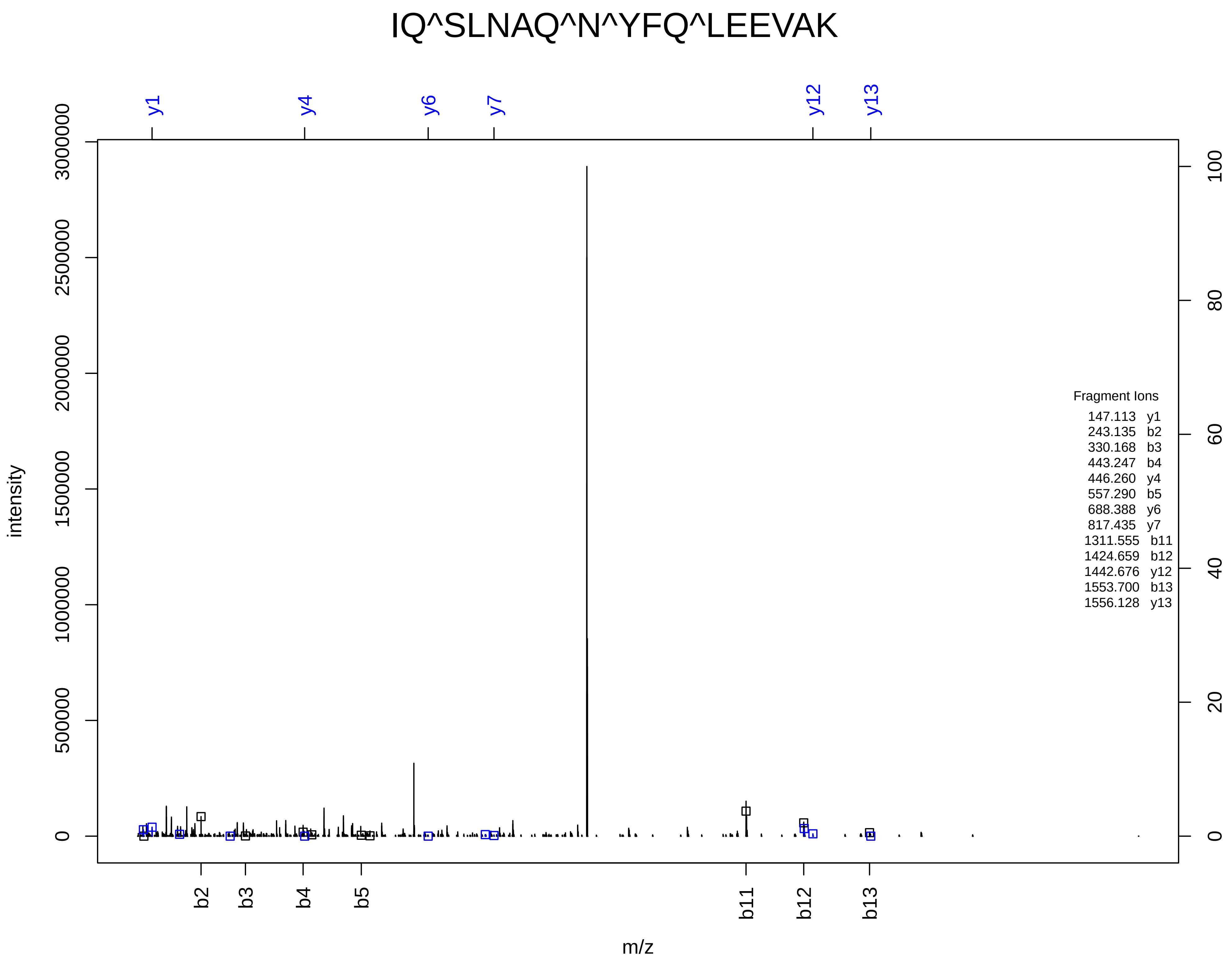

IQSLNAQNYFQLEEVAK / File: sydney_ip_07.raw; SpectrumID: ; scans: 10255

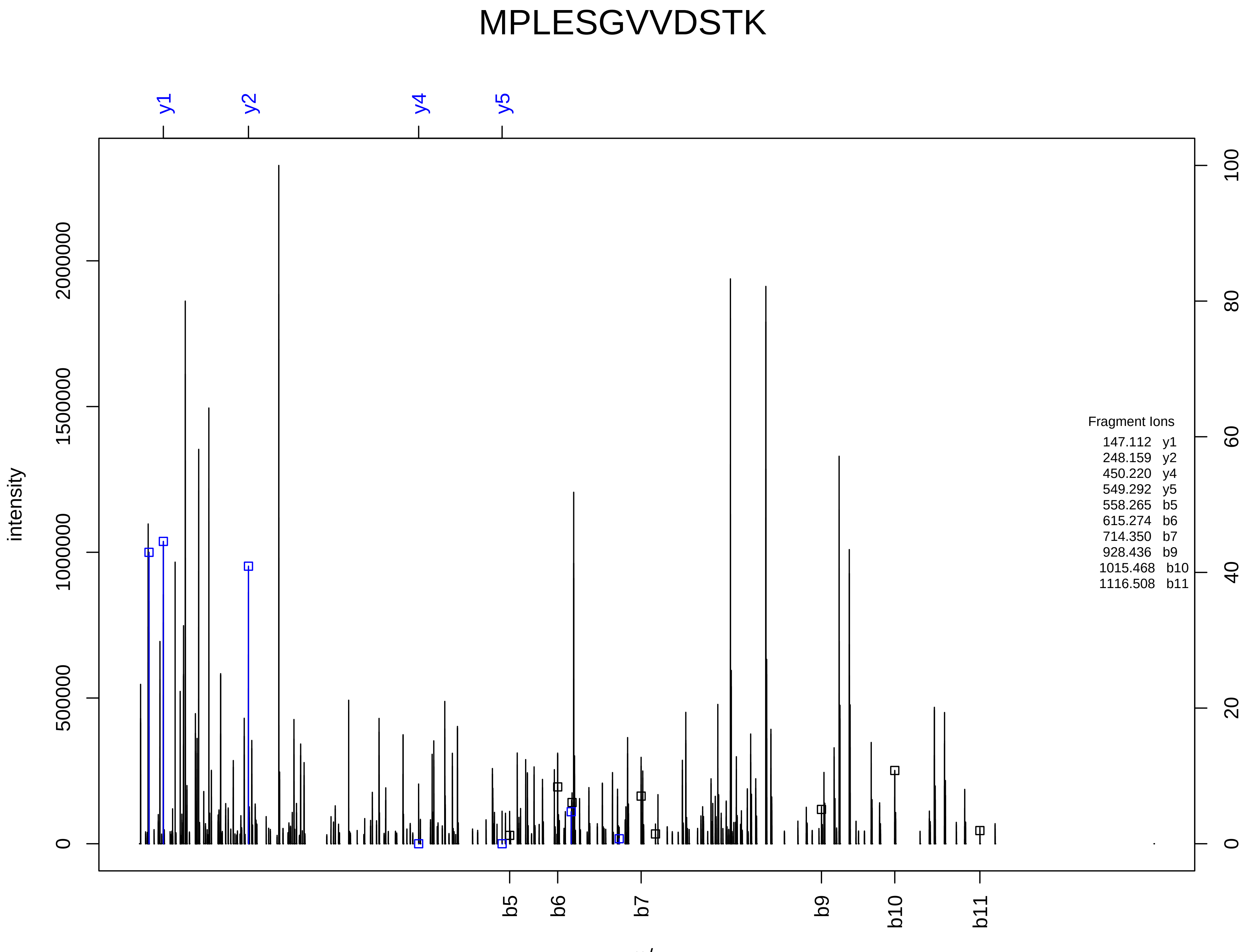

MPLESGVVDSTK / File: sydney_ip_07.raw; SpectrumID: ; scans: 3002

NDIQ^LEN^AKAAGK

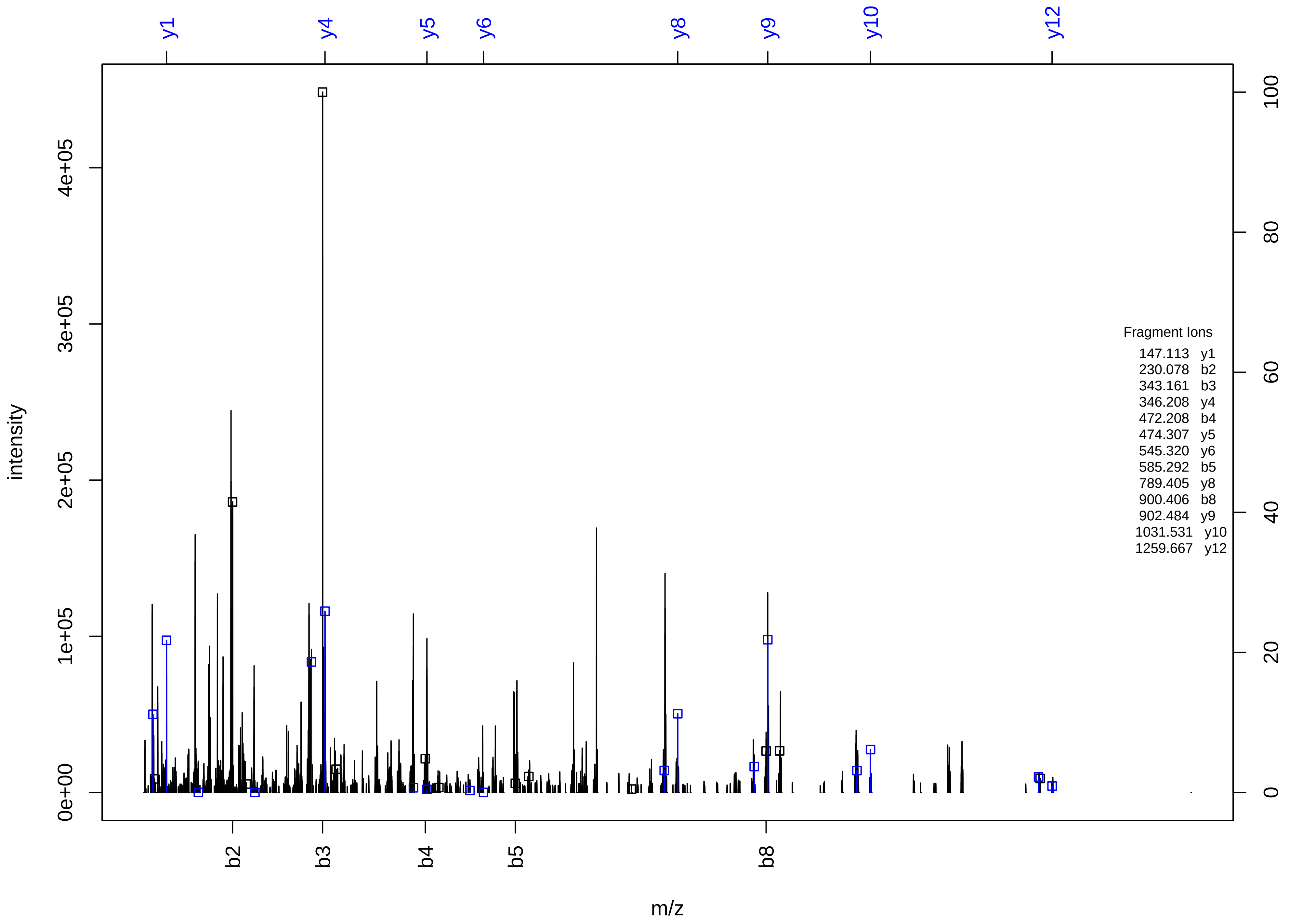

NDIQLENAKAAGK / File: sydney_ip_07.raw; SpectrumID: ; scans: 8620

GARIHHN^GN^K

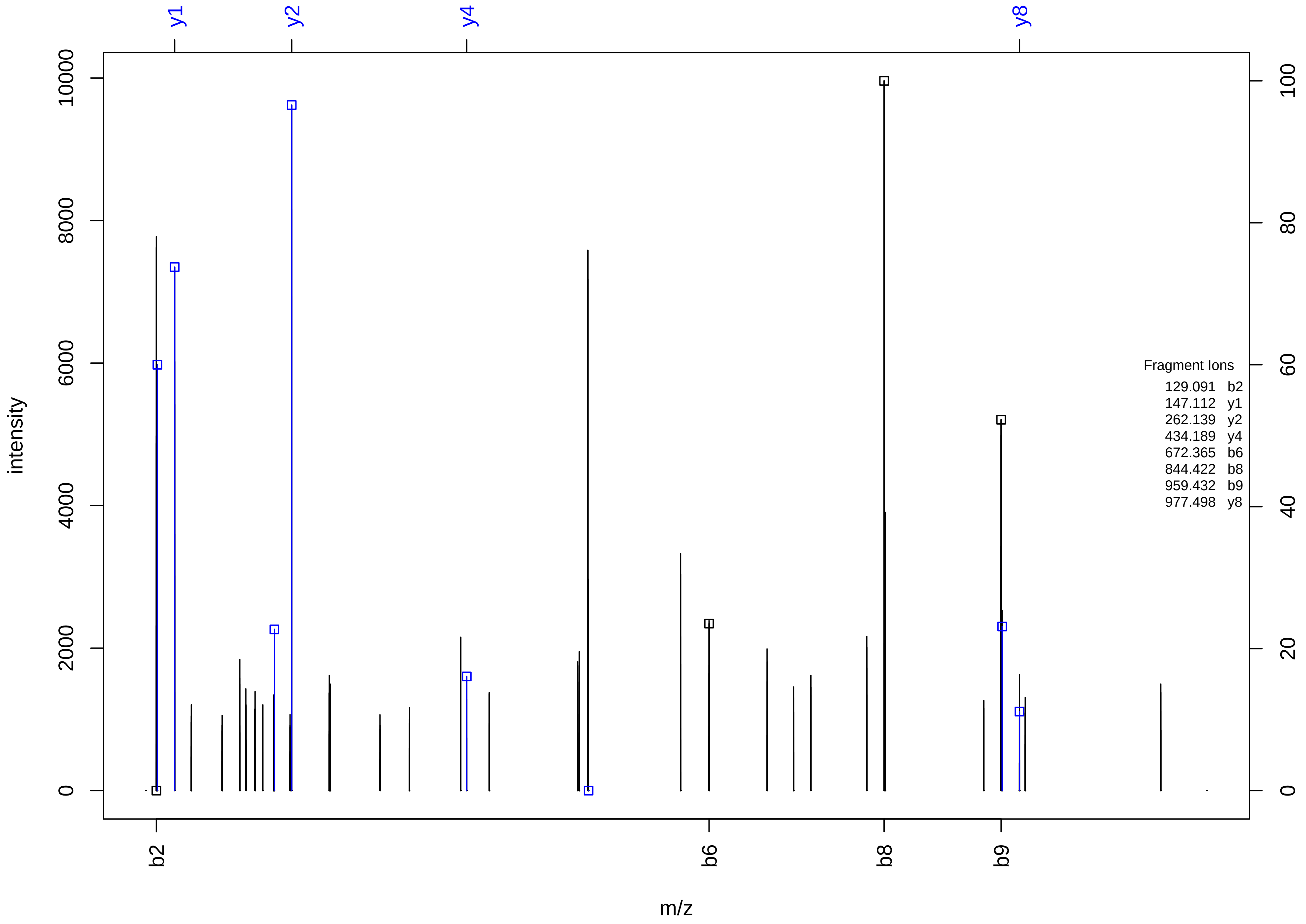

GARIHHNGNK / File: sydney_ip_08.raw; SpectrumID: ; scans: 878

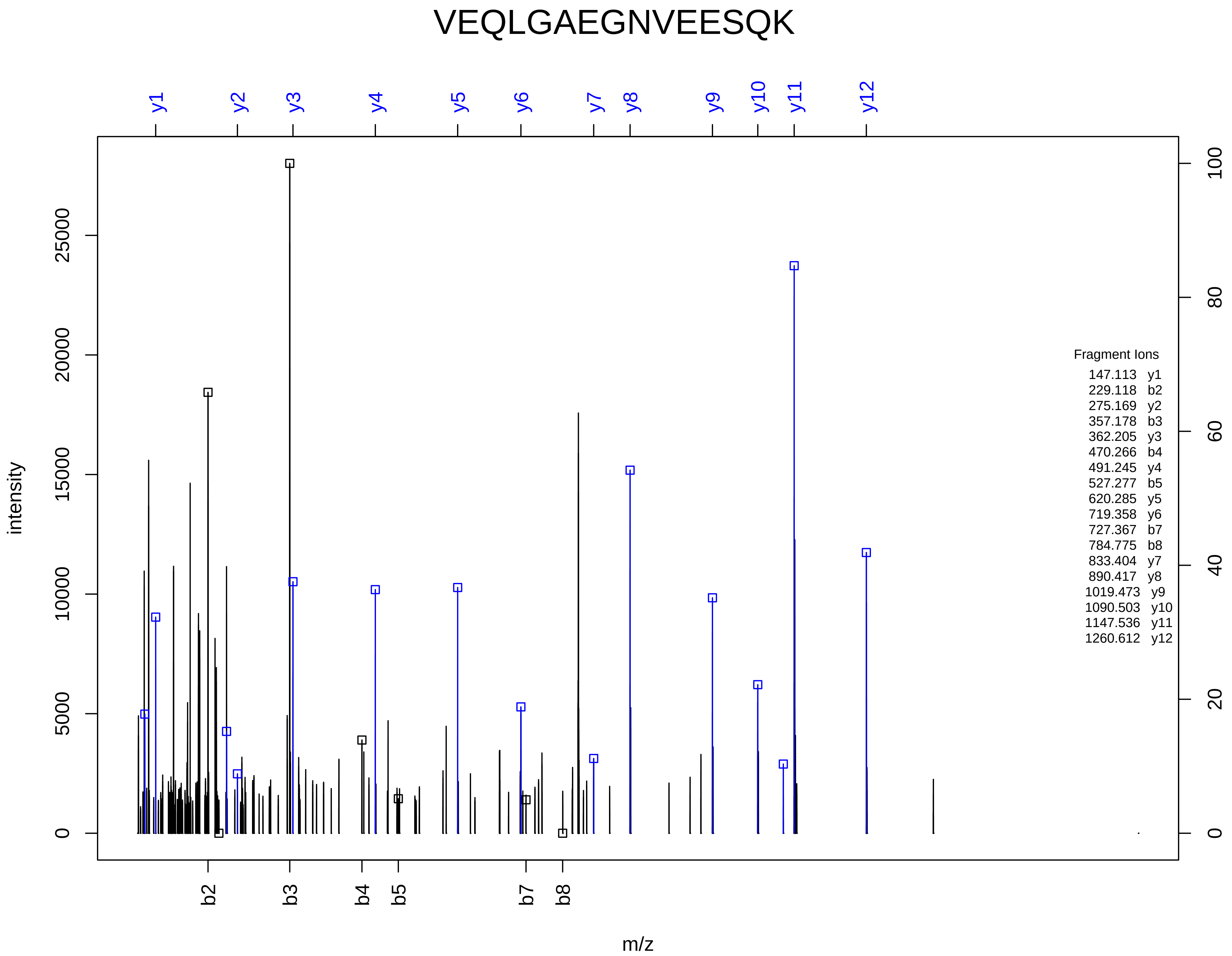

VEQLGAEGNVEESQK / File: sydney_ip_08.raw; SpectrumID: ; scans: 2930

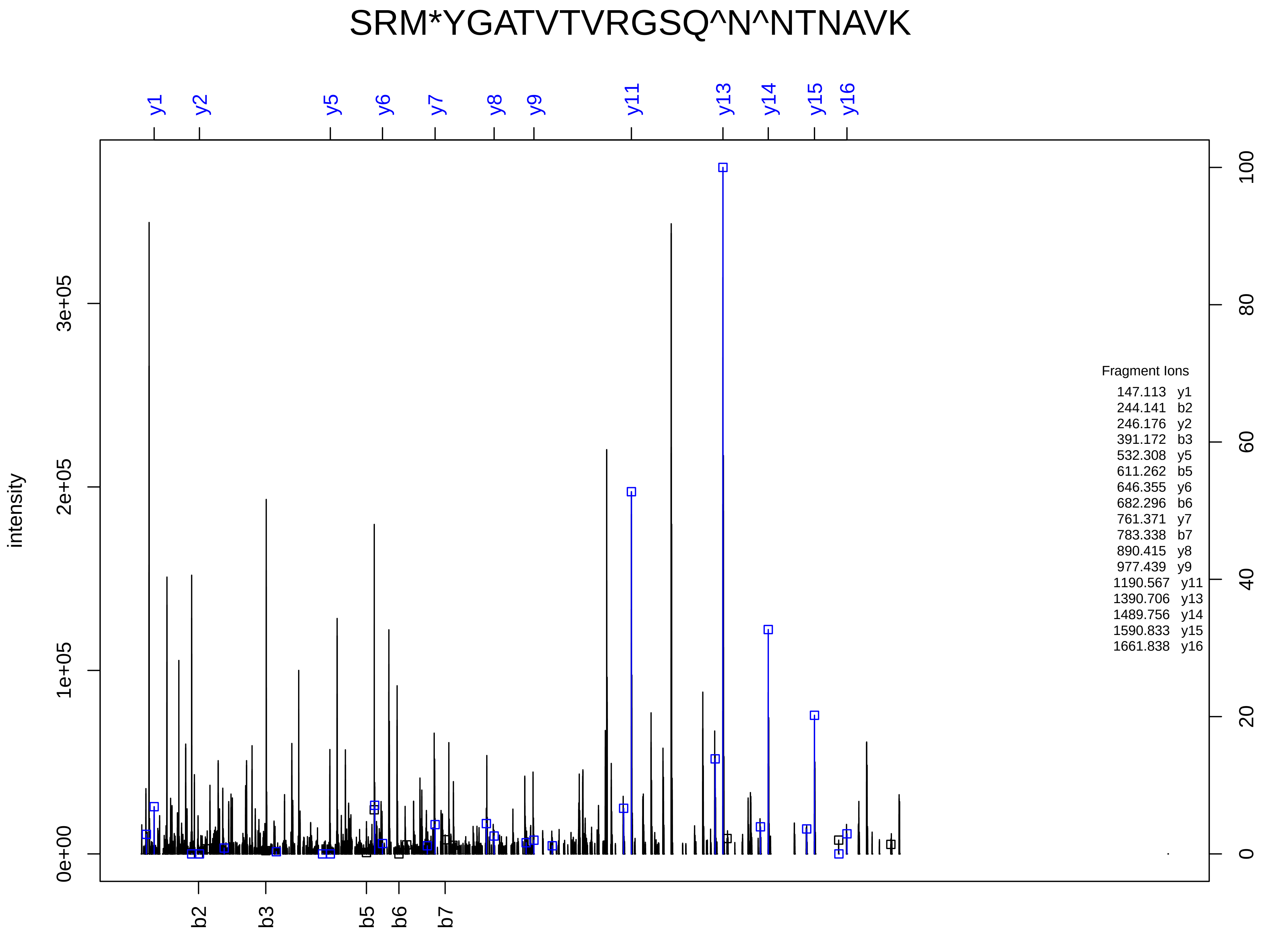

SRMYGATVTVRGSQNNTNAVK / File: sydney_ip_08.raw; SpectrumID: ; scans: 10552

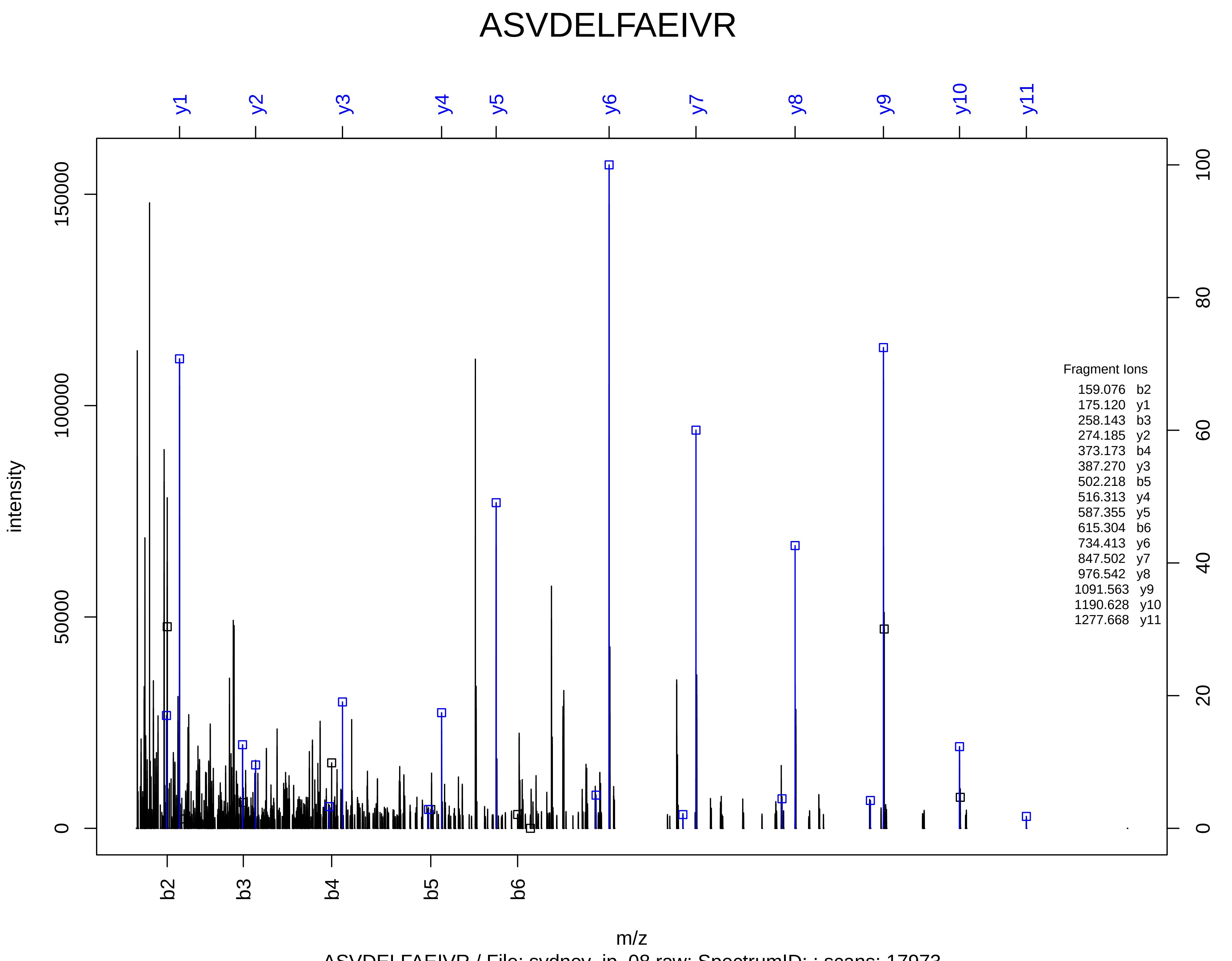
ASVDELFAEIVR
y1 y2 y3 y4 y5 y6 y7 y8 y9 y10 y11
b2 b3 b4 b5 b6
intensity
m/z
Fragment Ions
159.076 b2
175.120 y1
258.143 b3
274.185 y2
373.173 b4
387.270 y3
502.218 b5
516.313 y4
587.355 y5
615.304 b6
734.413 y6
847.502 y7
976.542 y8
1091.563 y9
1190.628 y10
1277.668 y11
ASVDELFAEIVR / File: sydney_ip_08.raw; SpectrumID: ; scans: 17973

LFQVEYAIEAIK

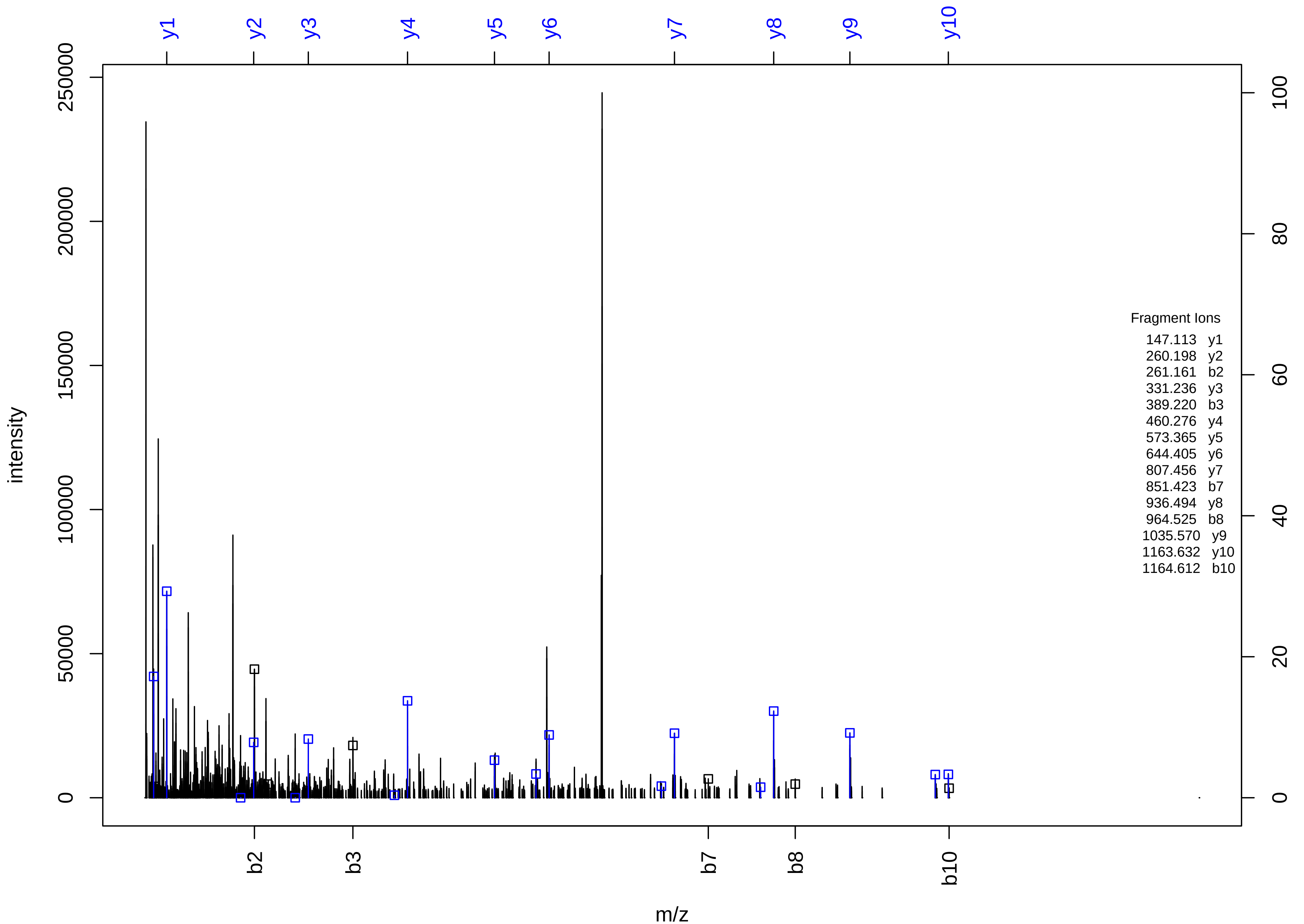

LFQVEYAIEAIK / File: sydney_ip_08.raw; SpectrumID: ; scans: 15500

YVNWIQQTIAAN

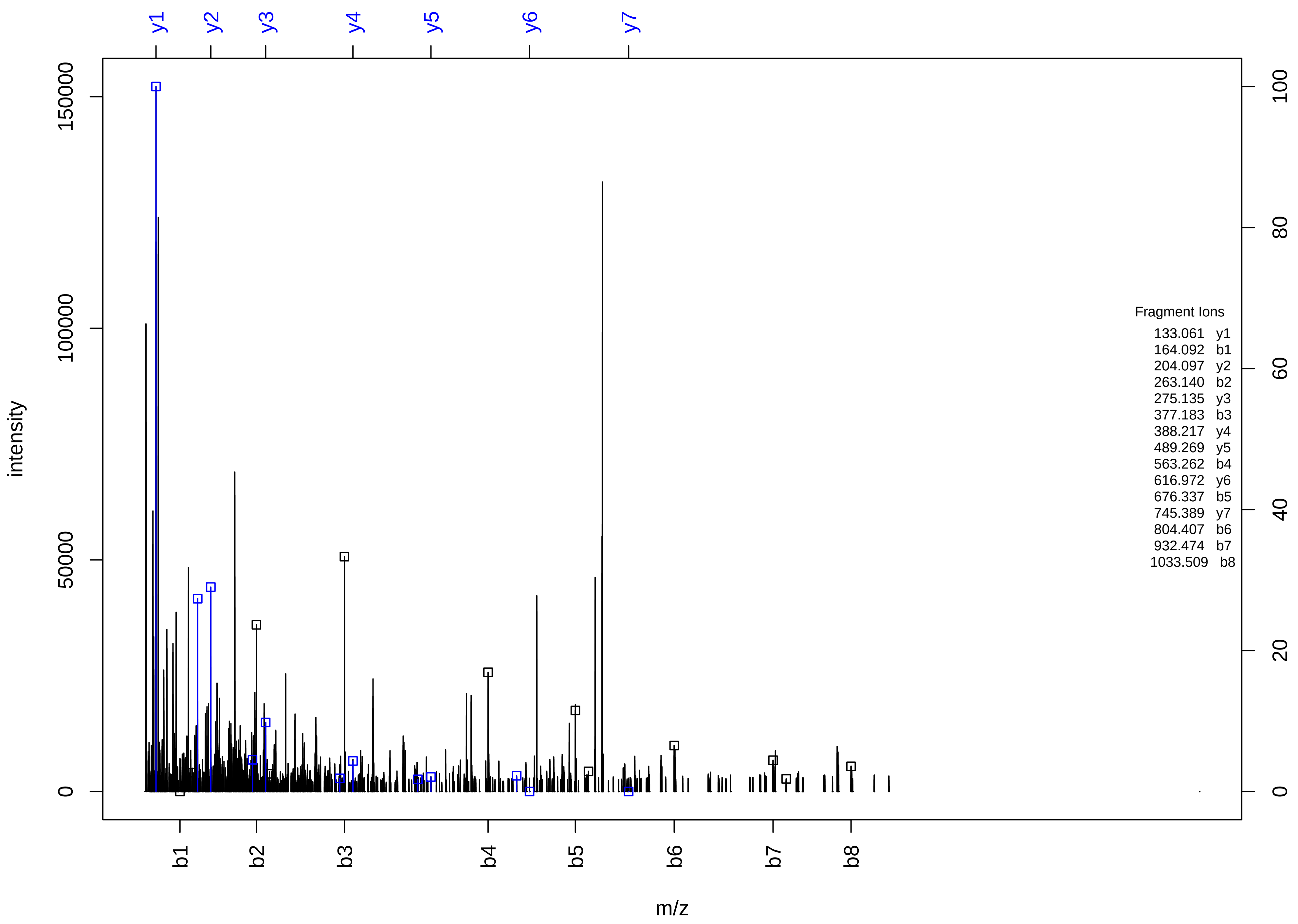

YVNWIQQTIAAN / File: sydney_ip_08.raw; SpectrumID: ; scans: 15104

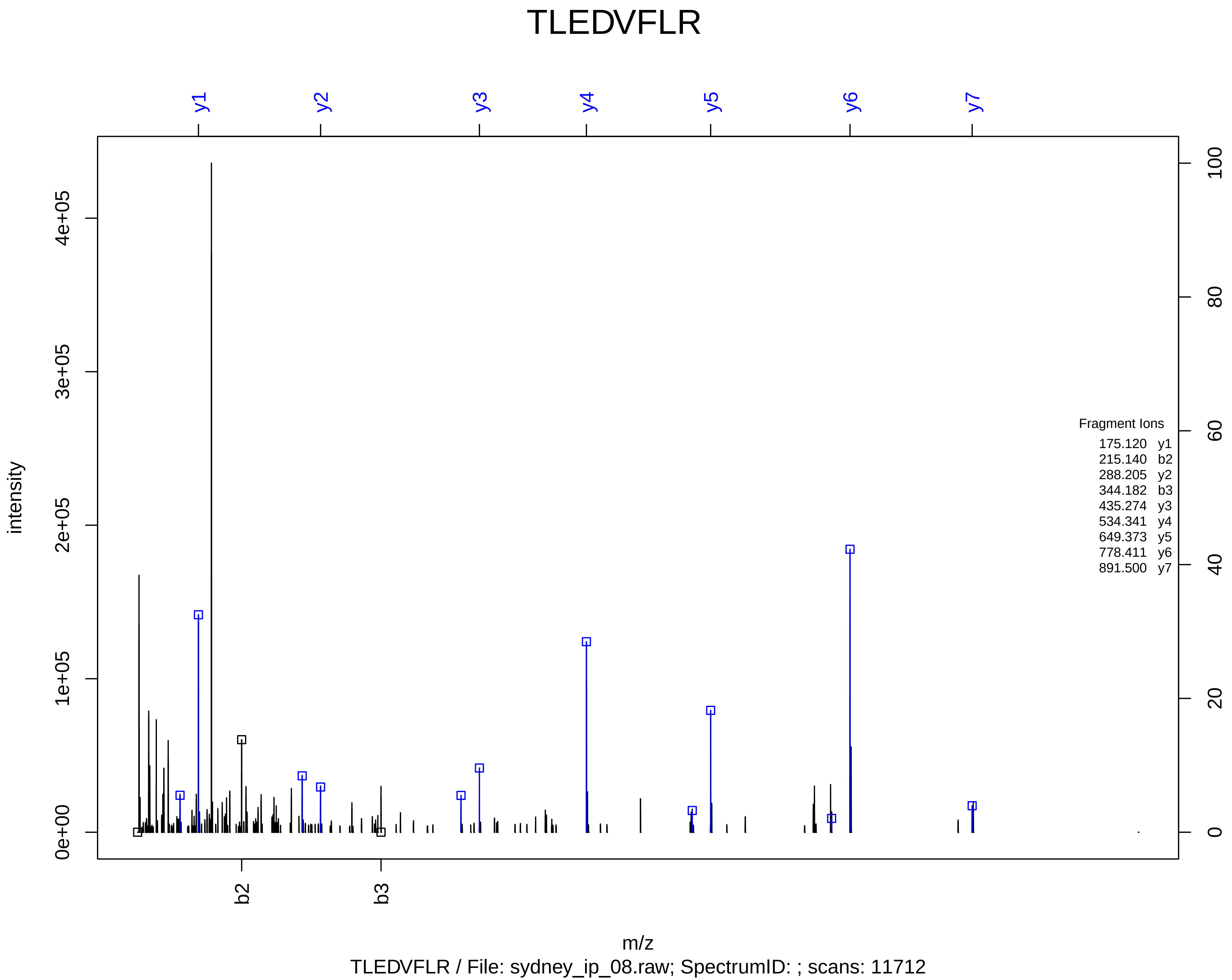

TLEDVFLR
y1
y2
y3
y4
y5
y6
y7
intensity
4e+05
3e+05
2e+05
1e+05
0e+00
100
80
60
40
20
0
Fragment Ions
175.120 y1
215.140 b2
288.205 y2
344.182 b3
435.274 y3
534.341 y4
649.373 y5
778.411 y6
891.500 y7
b2
b3
m/z
TLEDVFLR / File: sydney_ip_08.raw; SpectrumID: ; scans: 11712

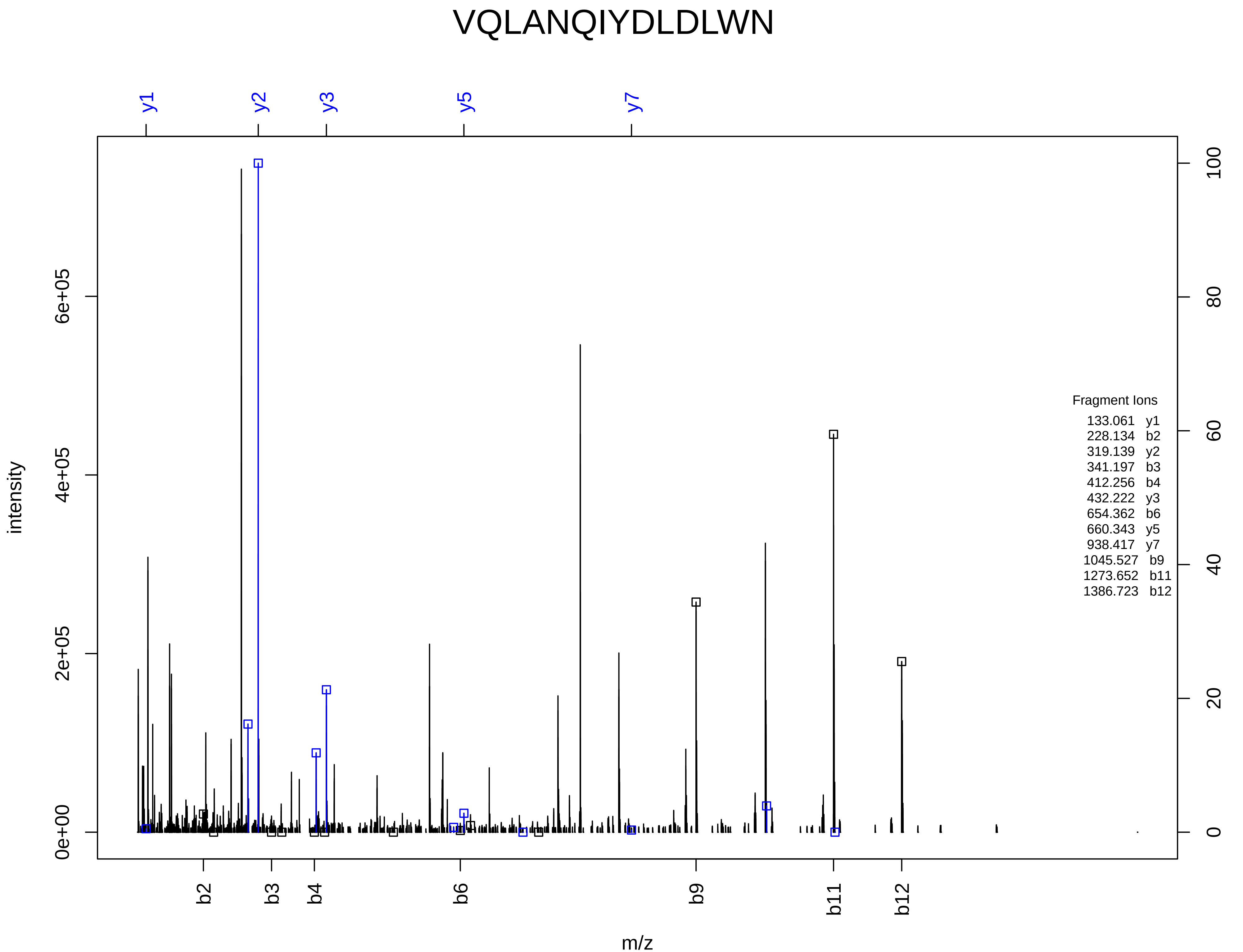

VQLANQIYDLDLWN / File: sydney_ip_08.raw; SpectrumID: ; scans: 10349

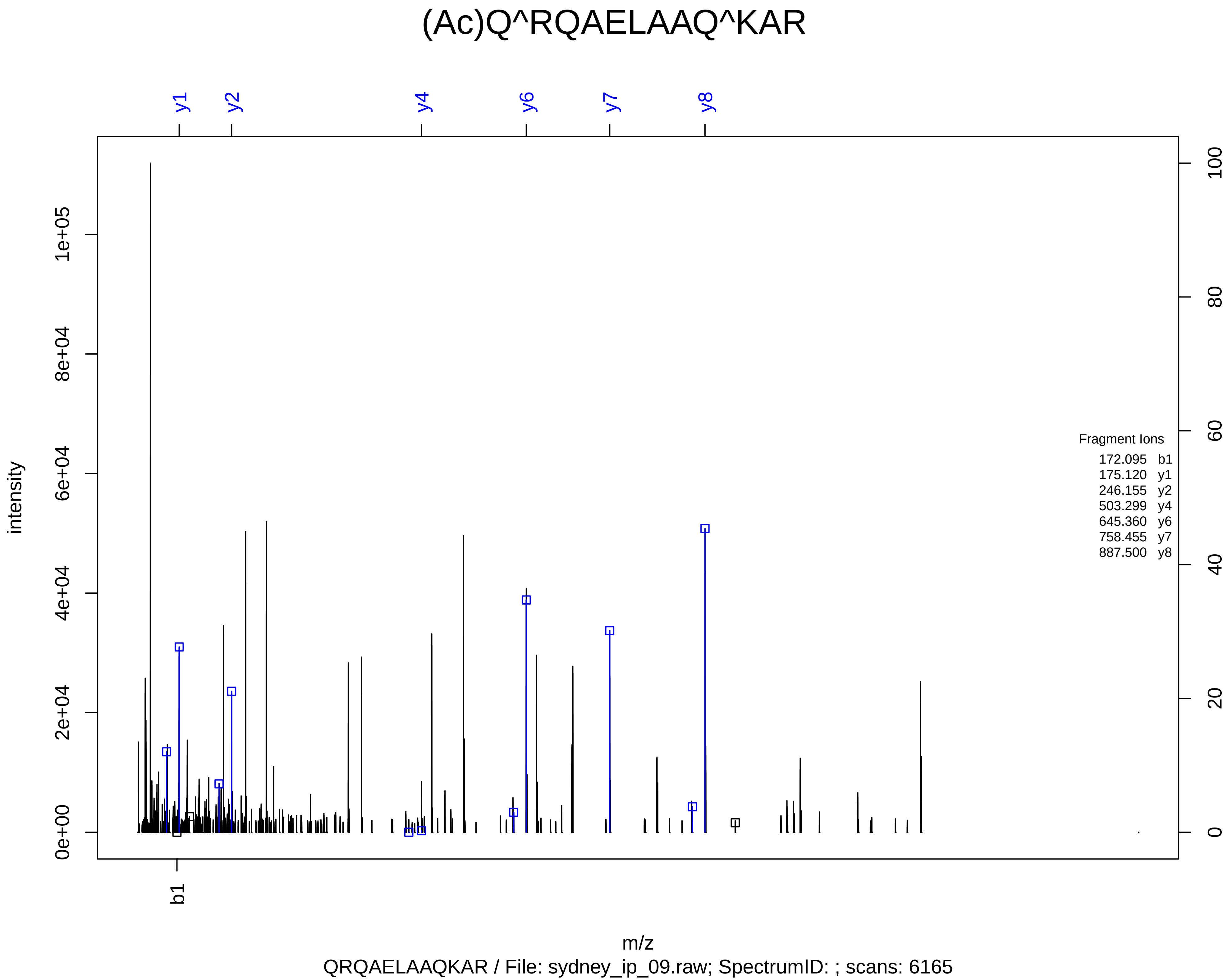

(Ac)Q^RQAELAAQ^KAR
y1
y2
y4
y6
y7
y8
intensity
0e+00
2e+04
4e+04
6e+04
8e+04
1e+05
0
20
40
60
80
100
b1
m/z
Fragment Ions
172.095 b1
175.120 y1
246.155 y2
503.299 y4
645.360 y6
758.455 y7
887.500 y8
QRQAELAAQKAR / File: sydney_ip_09.raw; SpectrumID: ; scans: 6165

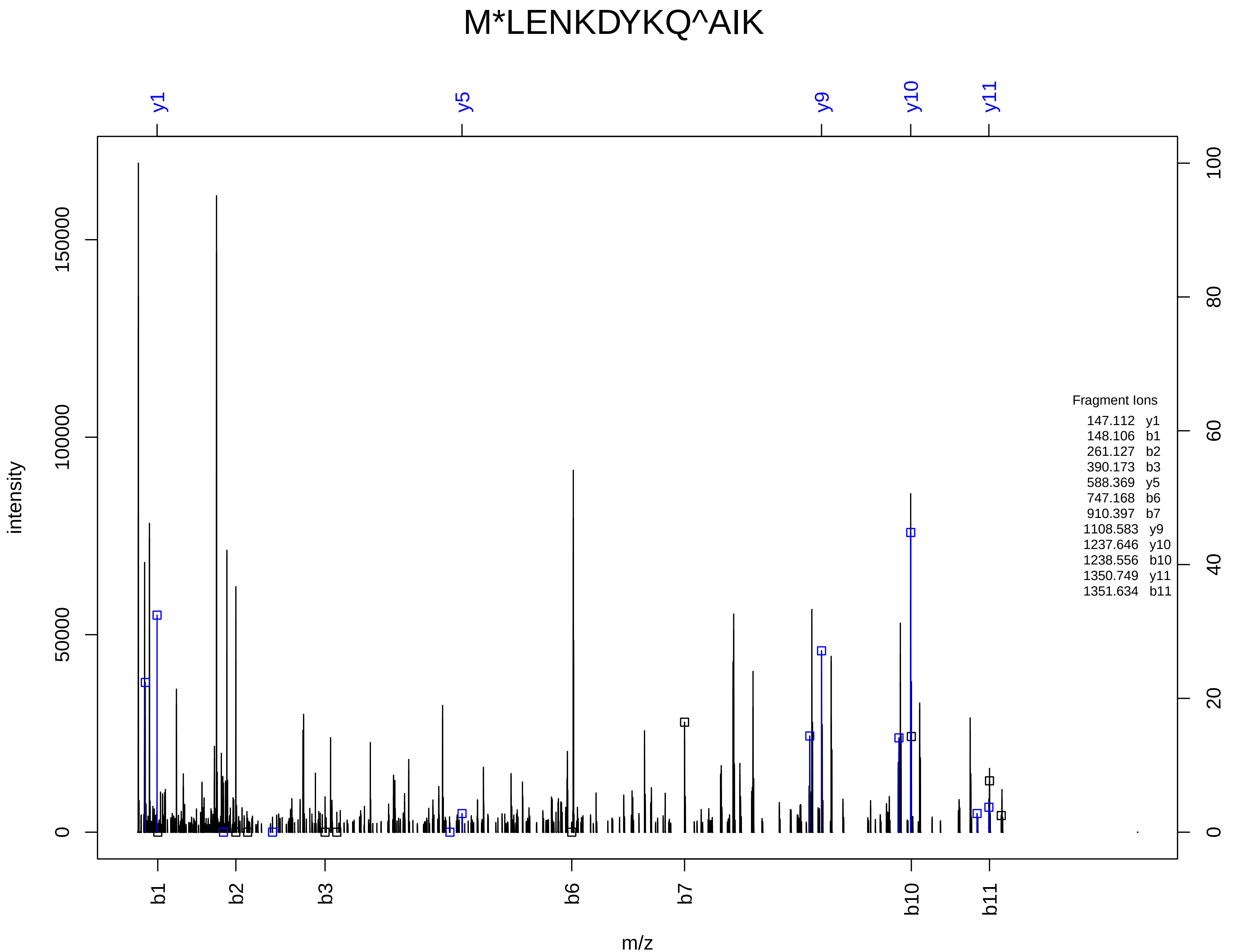

MLENKDYKQAIK / File: sydney_ip_09.raw; SpectrumID: ; scans: 5992

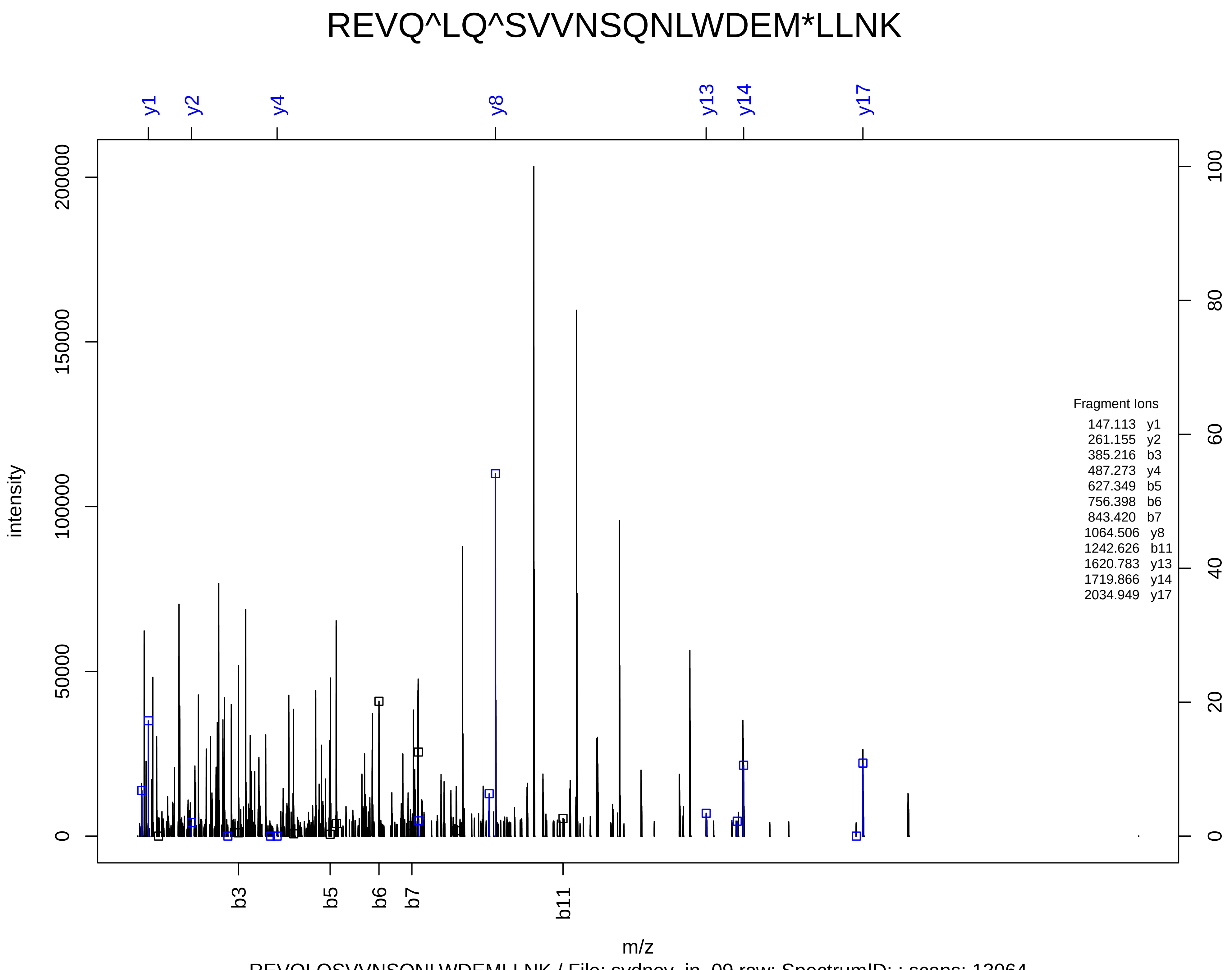

REVQ^LQ^SVVNSQNLWDEM*LLNK
y1
y2
y4
y8
y13
y14
y17
intensity
0
50000
100000
150000
200000
0
20
40
60
80
100
b3
b5
b6
b7
b11
m/z
Fragment Ions
147.113 y1
261.155 y2
385.216 b3
487.273 y4
627.349 b5
756.398 b6
843.420 b7
1064.506 y8
1242.626 b11
1620.783 y13
1719.866 y14
2034.949 y17
REVQLQSVVNSQNLWDEMLLNK / File: sydney_ip_09.raw; SpectrumID: ; scans: 13064

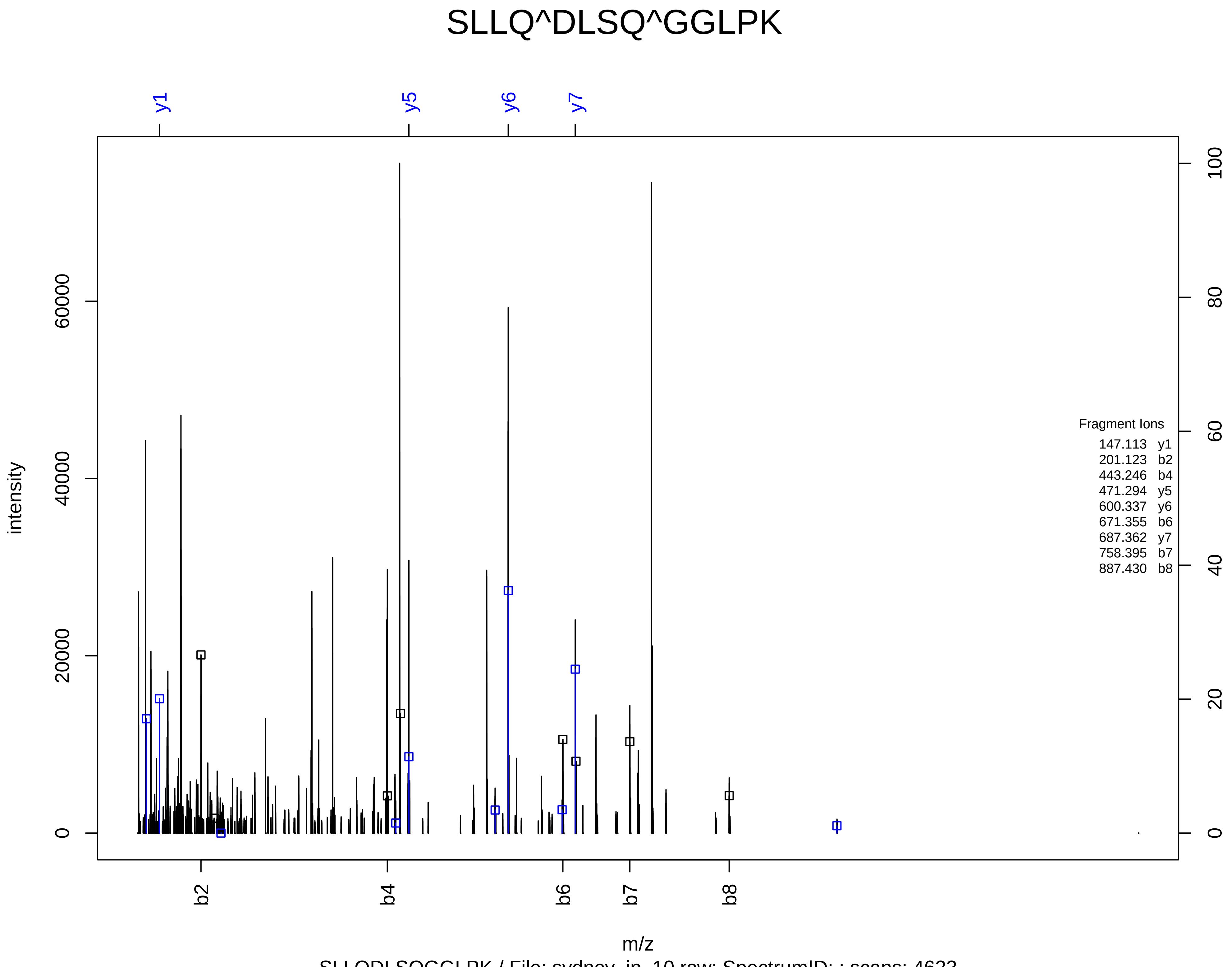

SLLQ^DLSQ^GGLPK
y1
y5
y6
y7
intensity
0
20000
40000
60000
0
20
40
60
80
100
Fragment Ions
147.113 y1
201.123 b2
443.246 b4
471.294 y5
600.337 y6
671.355 b6
687.362 y7
758.395 b7
887.430 b8
b2
b4
b6
b7
b8
m/z
SLLQDLSQGGLPK / File: sydney_ip_10.raw; SpectrumID: ; scans: 4623

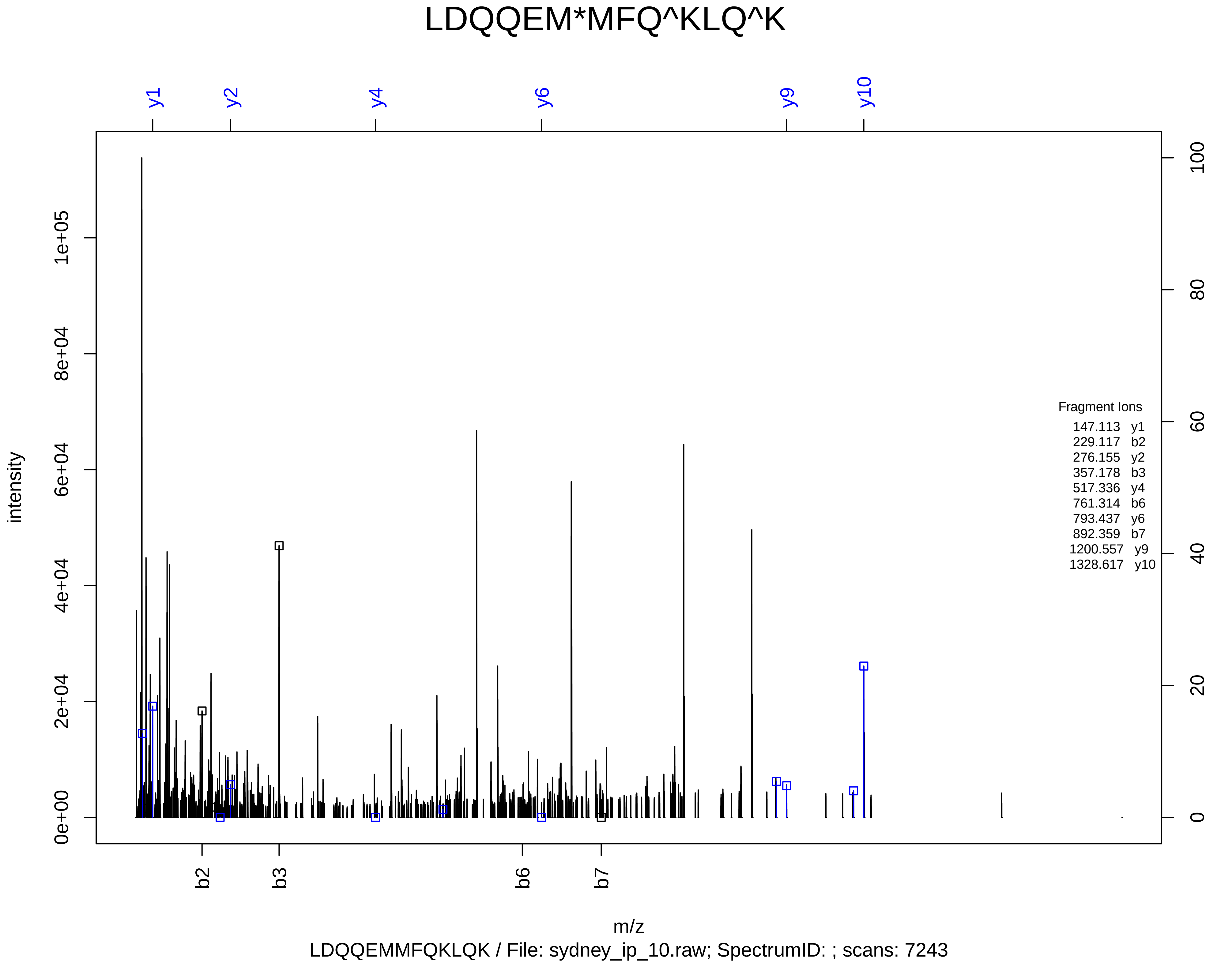
LDQQEM*MFQ^KLQ^K
y1
y2
y4
y6
y9
y10
intensity
0e+00
2e+04
4e+04
6e+04
8e+04
1e+05
0
20
40
60
80
100
b2
b3
b6
b7
m/z
Fragment Ions
147.113 y1
229.117 b2
276.155 y2
357.178 b3
517.336 y4
761.314 b6
793.437 y6
892.359 b7
1200.557 y9
1328.617 y10
LDQQEMMFQKLQK / File: sydney_ip_10.raw; SpectrumID: ; scans: 7243

LRYLQ^Q^Q^LQ^DETPRR

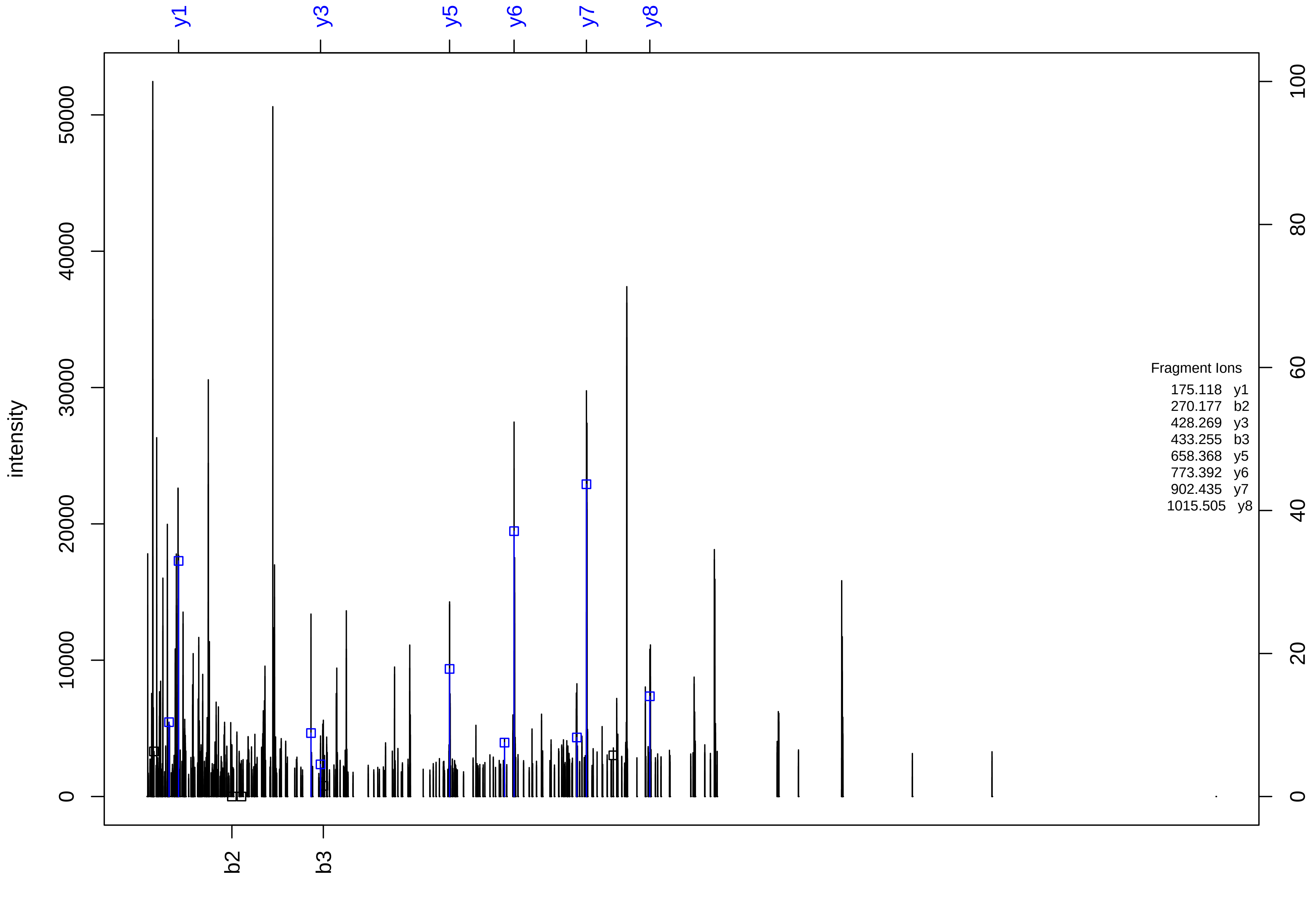

LRYLQQQLQDETPRR / File: sydney_ip_10.raw; SpectrumID: ; scans: 8540

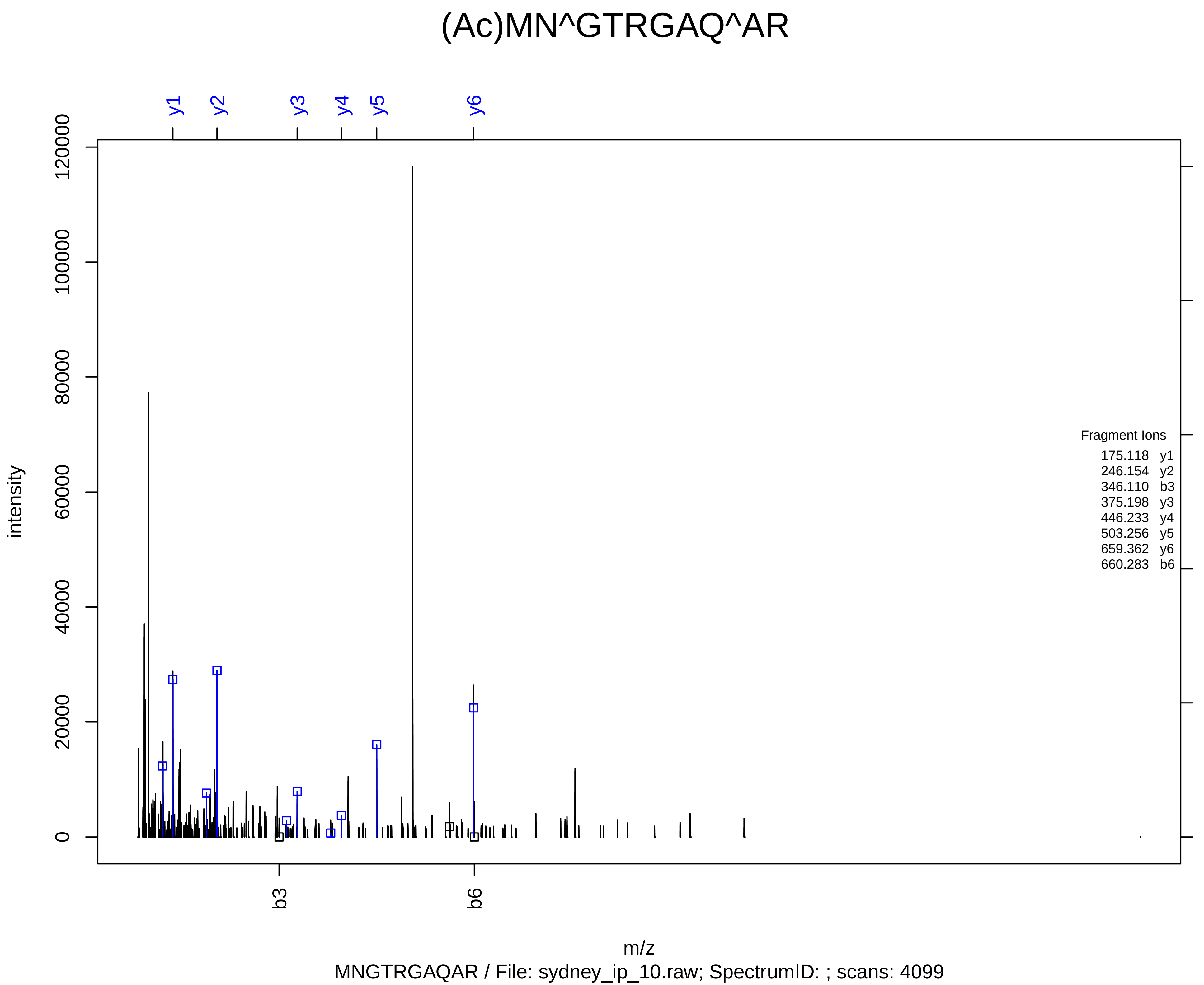

(Ac)MN^GTRGAQ^AR
y1
y2
y3
y4
y5
y6
intensity
0
20000
40000
60000
80000
100000
120000
0
20
40
60
80
100
Fragment Ions
175.118 y1
246.154 y2
346.110 b3
375.198 y3
446.233 y4
503.256 y5
659.362 y6
660.283 b6
b3
b6
m/z
MNGTRGAQAR / File: sydney_ip_10.raw; SpectrumID: ; scans: 4099

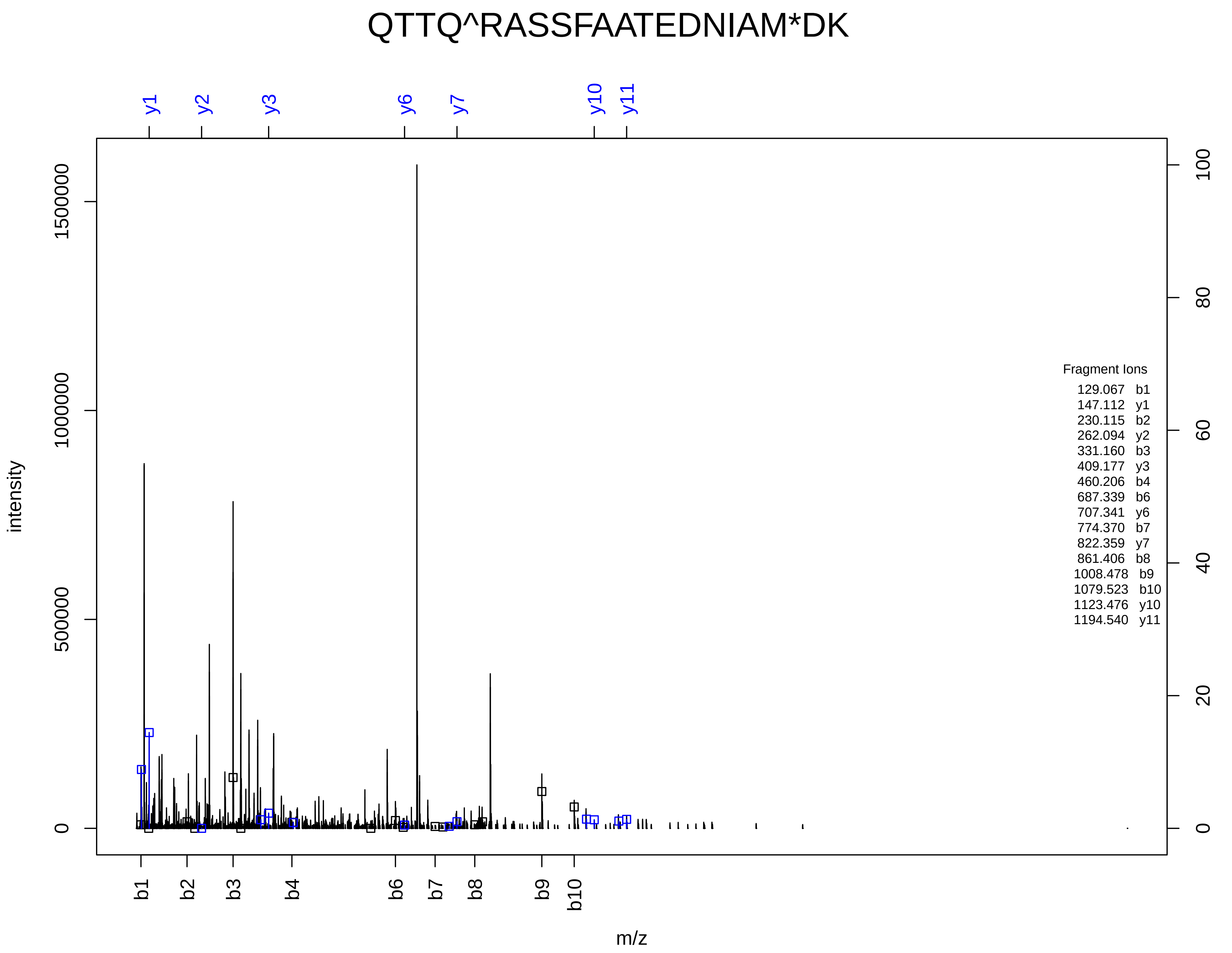

QTTQ^RASSFAATEDNIAM*DK
y1
y2
y3
y6
y7
y10
y11
intensity
1500000
1000000
500000
0
100
80
60
40
20
0
Fragment Ions
129.067 b1
147.112 y1
230.115 b2
262.094 y2
331.160 b3
409.177 y3
460.206 b4
687.339 b6
707.341 y6
774.370 b7
822.359 y7
861.406 b8
1008.478 b9
1079.523 b10
1123.476 y10
1194.540 y11
b1
b2
b3
b4
b6
b7
b8
b9
b10
m/z
QTTQRASSFAATEDNIAMDK / File: sydney_ip_10.raw; SpectrumID: ; scans: 10752

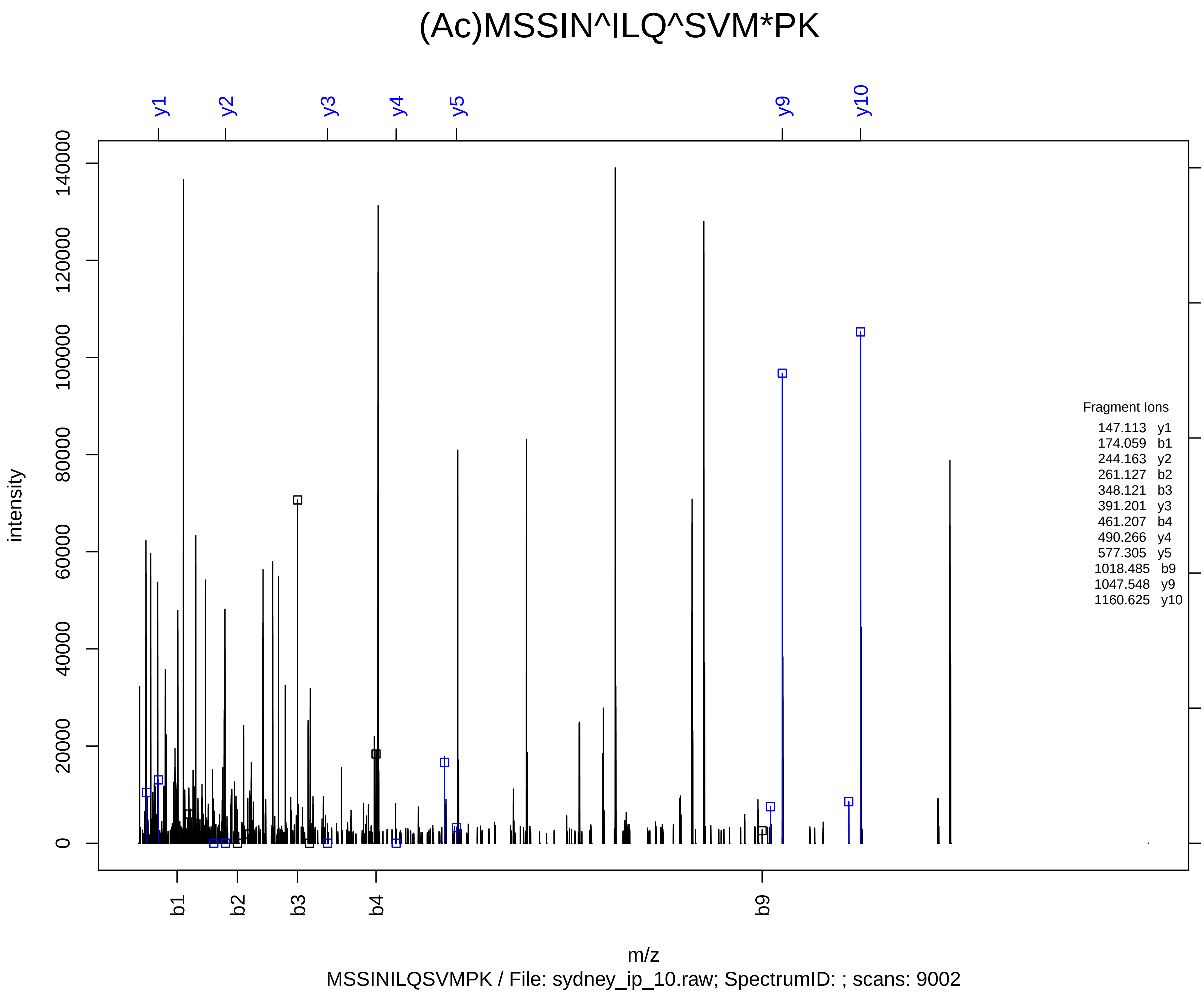

(Ac)MSSIN^ILQ^SVM*PK
y1
y2
y3
y4
y5
y9
y10
intensity
0
20000
40000
60000
80000
100000
120000
140000
0
20
40
60
80
100
b1
b2
b3
b4
b9
m/z
Fragment Ions
147.113 y1
174.059 b1
244.163 y2
261.127 b2
348.121 b3
391.201 y3
461.207 b4
490.266 y4
577.305 y5
1018.485 b9
1047.548 y9
1160.625 y10
MSSINILQSVMPK / File: sydney_ip_10.raw; SpectrumID: ; scans: 9002

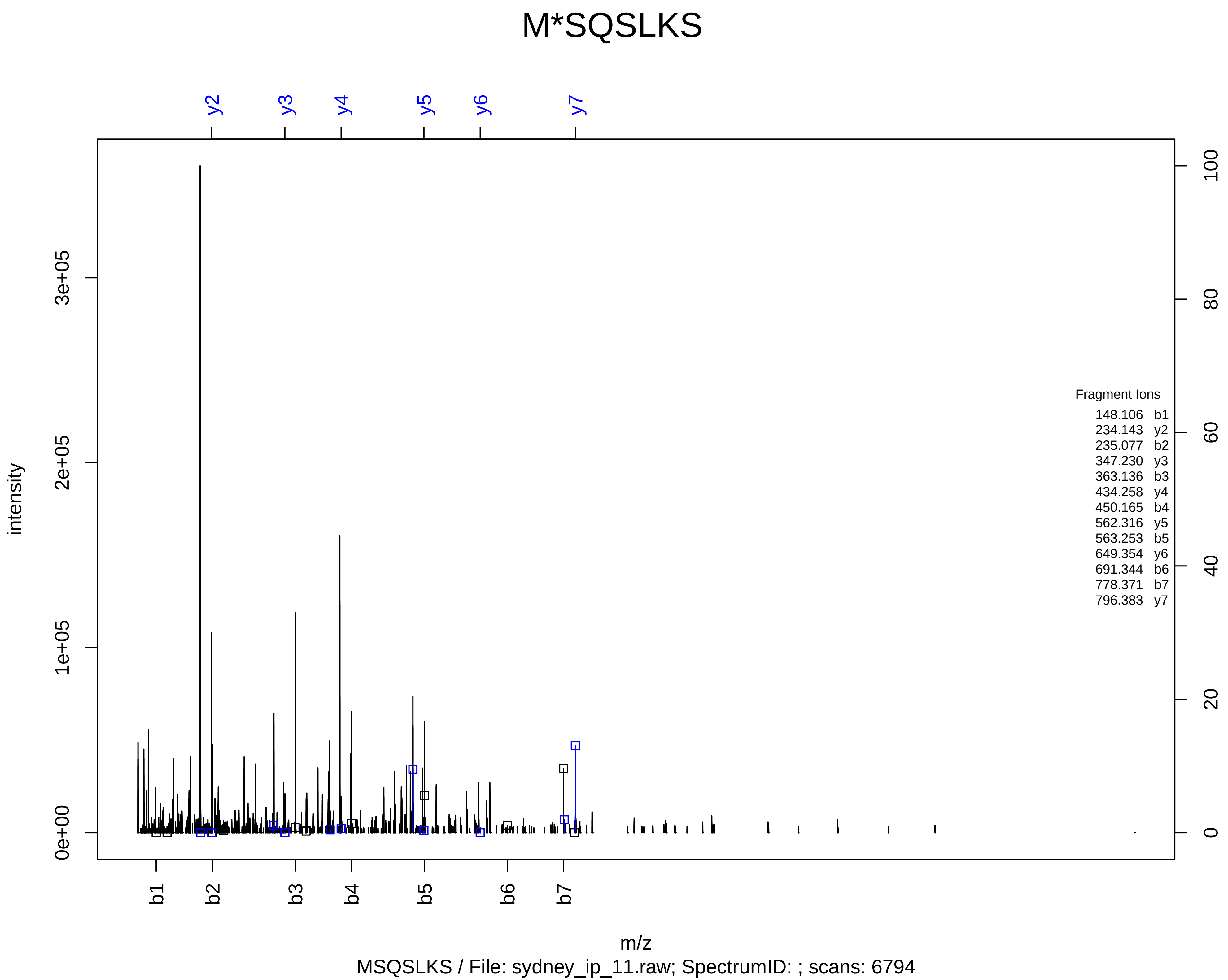
M*SQSLKS
y2
y3
y4
y5
y6
y7
intensity
0e+00
1e+05
2e+05
3e+05
0
20
40
60
80
100
Fragment Ions
148.106 b1
234.143 y2
235.077 b2
347.230 y3
363.136 b3
434.258 y4
450.165 b4
562.316 y5
563.253 b5
649.354 y6
691.344 b6
778.371 b7
796.383 y7
b1
b2
b3
b4
b5
b6
b7
m/z
MSQSLKS / File: sydney_ip_11.raw; SpectrumID: ; scans: 6794

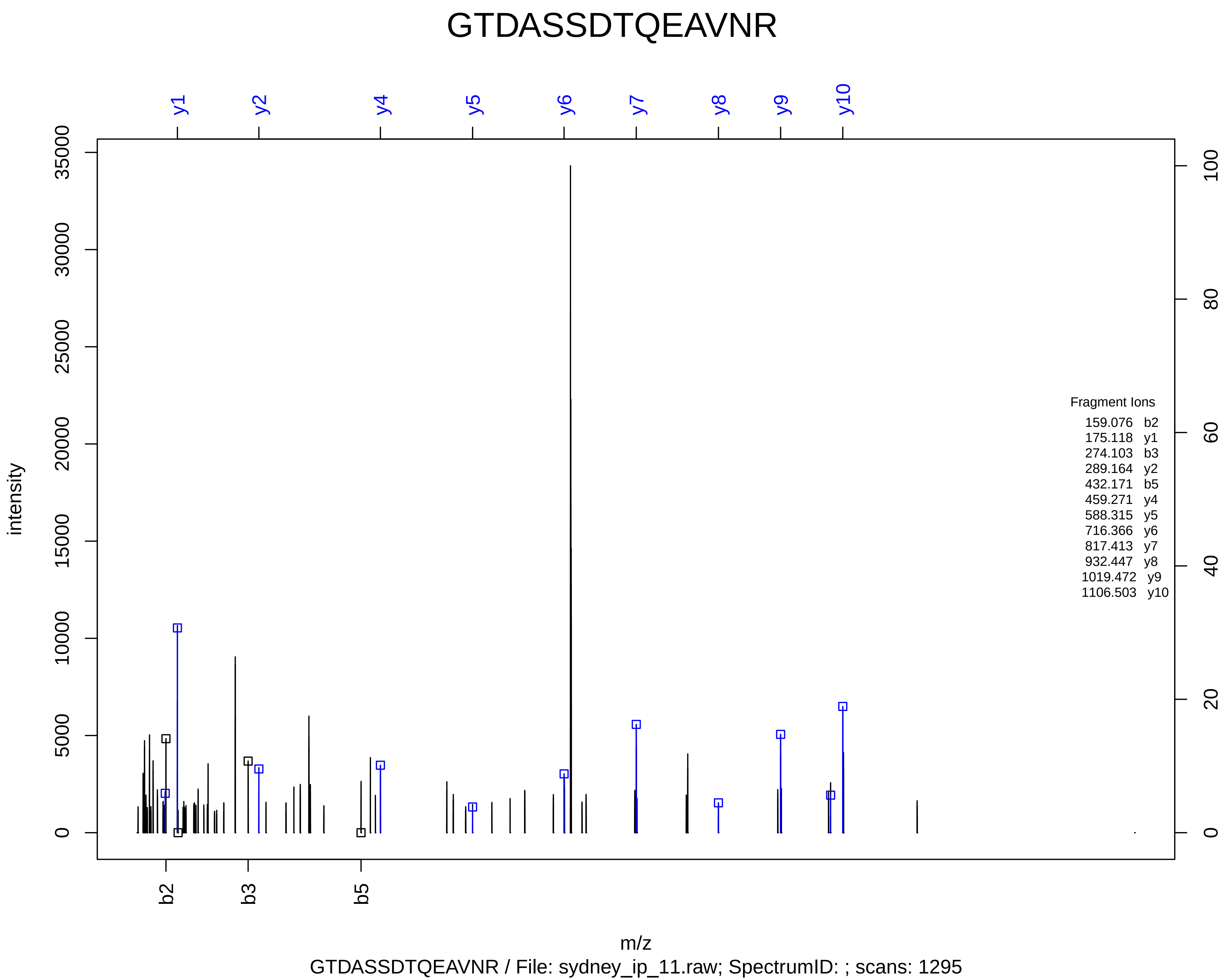
GTDASSDTQEAVNR
y1
y2
y4
y5
y6
y7
y8
y9
y10
intensity
0
5000
10000
15000
20000
25000
30000
35000
0
20
40
60
80
100
b2
b3
b5
Fragment Ions
159.076 b2
175.118 y1
274.103 b3
289.164 y2
432.171 b5
459.271 y4
588.315 y5
716.366 y6
817.413 y7
932.447 y8
1019.472 y9
1106.503 y10
m/z
GTDASSDTQEAVNR / File: sydney_ip_11.raw; SpectrumID: ; scans: 1295

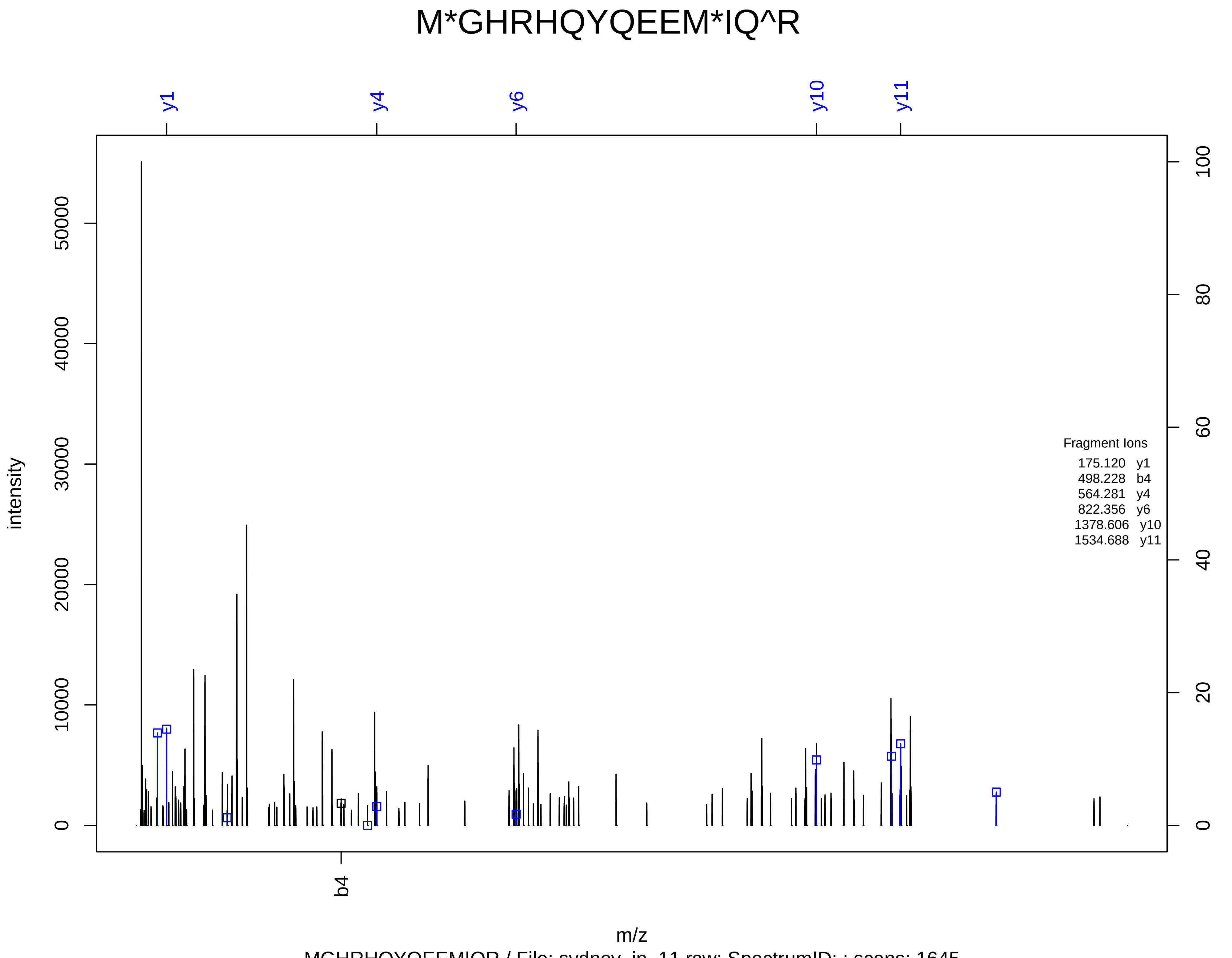
M*GHRHQYQEEM*IQ^R
y1
y4
y6
y10
y11
b4
intensity
m/z
0
10000
20000
30000
40000
50000
0
20
40
60
80
100
Fragment Ions
175.120 y1
498.228 b4
564.281 y4
822.356 y6
1378.606 y10
1534.688 y11
MGHRHQYQEEMIQR / File: sydney_ip_11.raw; SpectrumID: ; scans: 1645

(Ac)M*VNCTIKSEEK

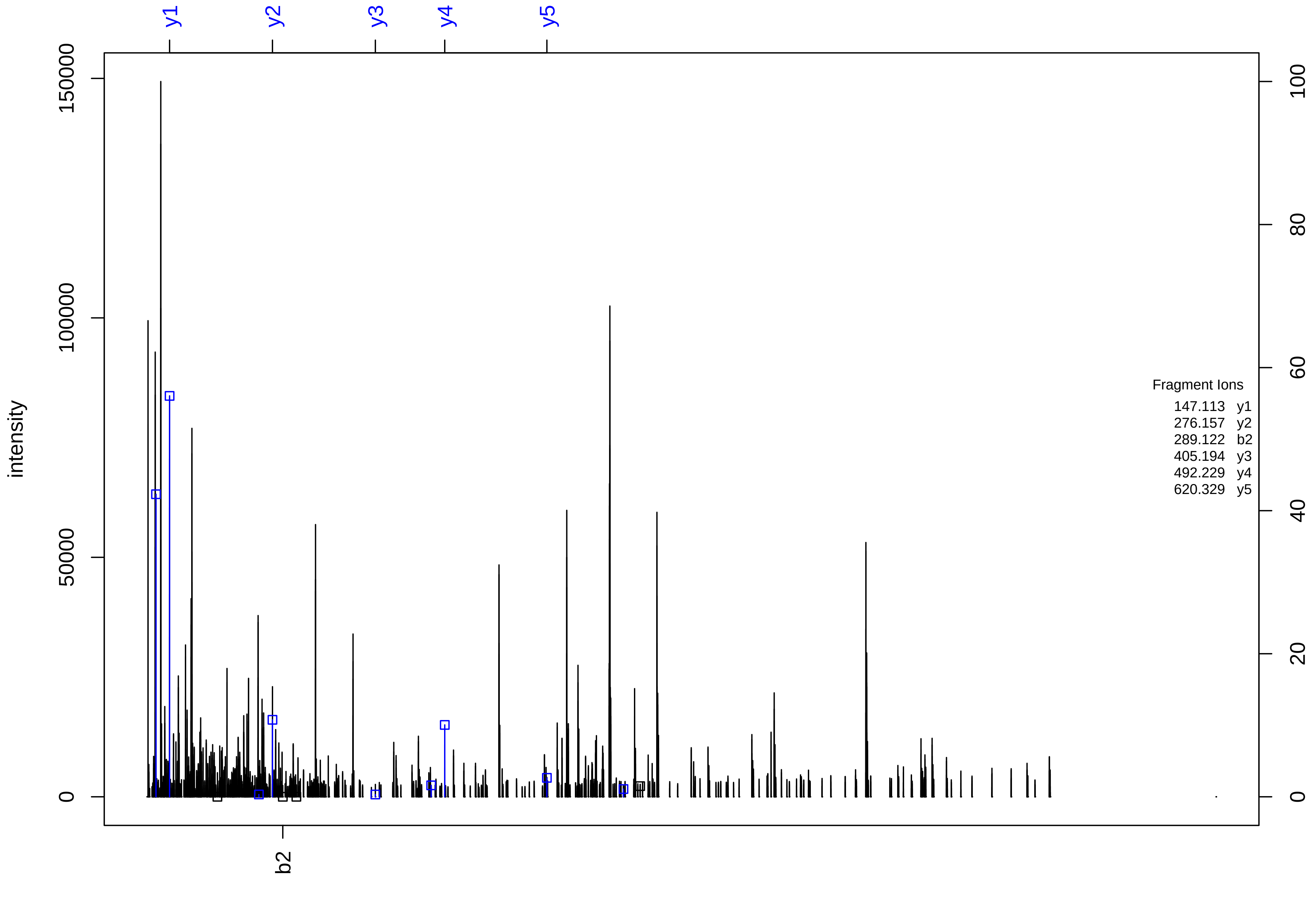

MVNCTIKSEEK / File: sydney_ip_11.raw; SpectrumID: ; scans: 5769

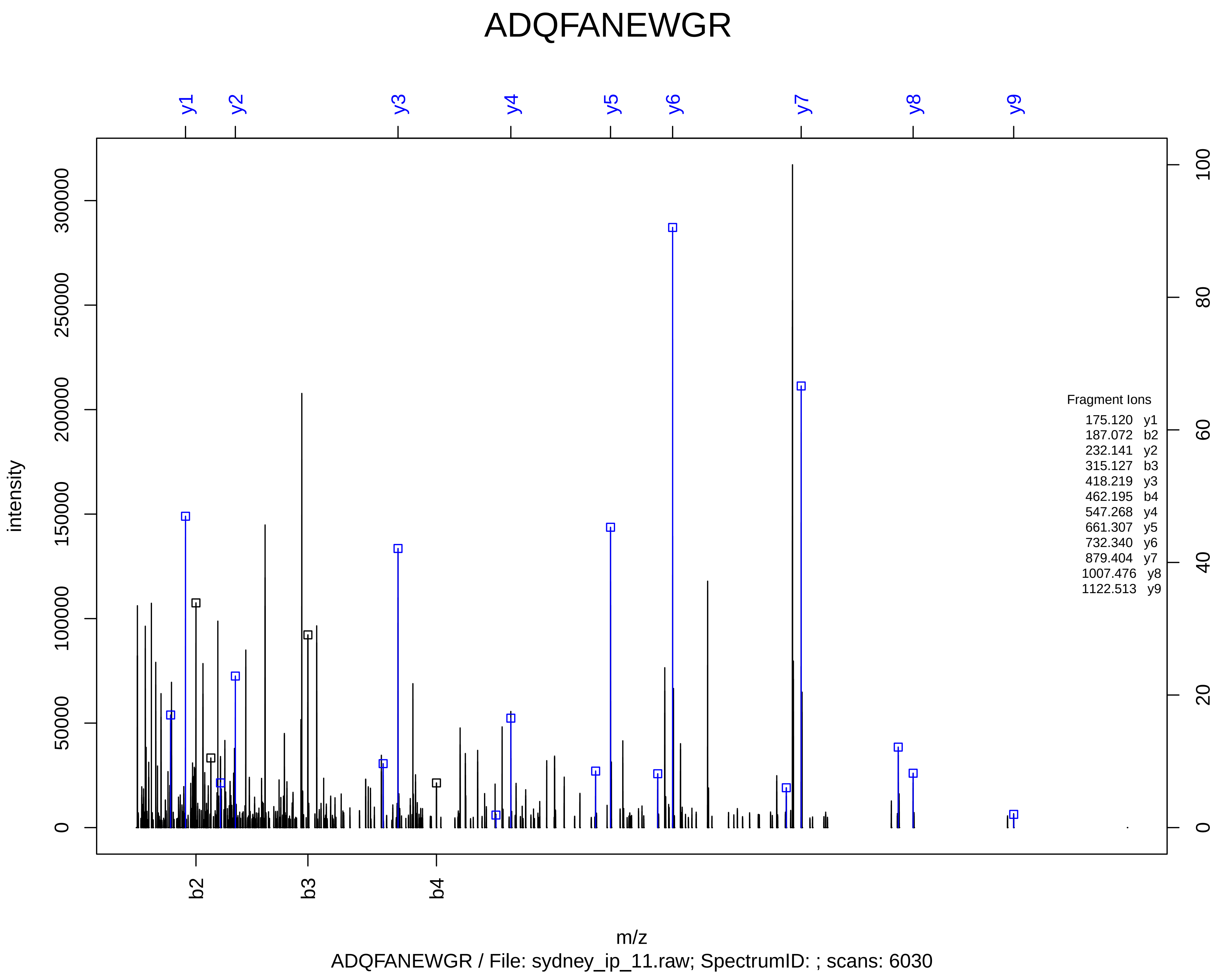

ADQFANEWGR
y1
y2
y3
y4
y5
y6
y7
y8
y9
intensity
0
50000
100000
150000
200000
250000
300000
0
20
40
60
80
100
Fragment Ions
175.120 y1
187.072 b2
232.141 y2
315.127 b3
418.219 y3
462.195 b4
547.268 y4
661.307 y5
732.340 y6
879.404 y7
1007.476 y8
1122.513 y9
b2
b3
b4
m/z
ADQFANEWGR / File: sydney_ip_11.raw; SpectrumID: ; scans: 6030

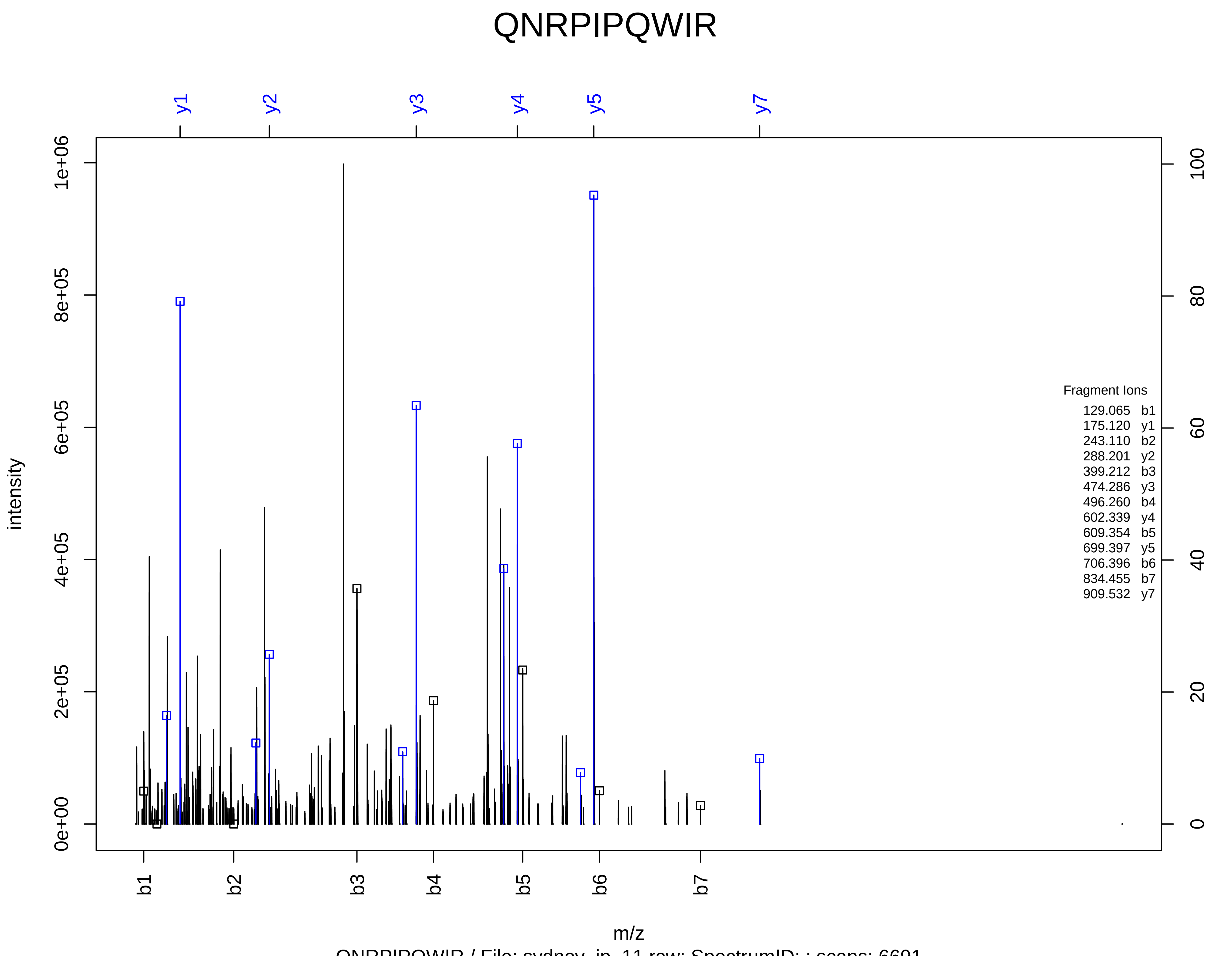
QNRPIPQWIR
y1
y2
y3
y4
y5
y7
intensity
0e+00
2e+05
4e+05
6e+05
8e+05
1e+06
0
20
40
60
80
100
b1
b2
b3
b4
b5
b6
b7
m/z
Fragment Ions
129.065 b1
175.120 y1
243.110 b2
288.201 y2
399.212 b3
474.286 y3
496.260 b4
602.339 y4
609.354 b5
699.397 y5
706.396 b6
834.455 b7
909.532 y7
QNRPIPQWIR / File: sydney_ip_11.raw; SpectrumID: ; scans: 6691

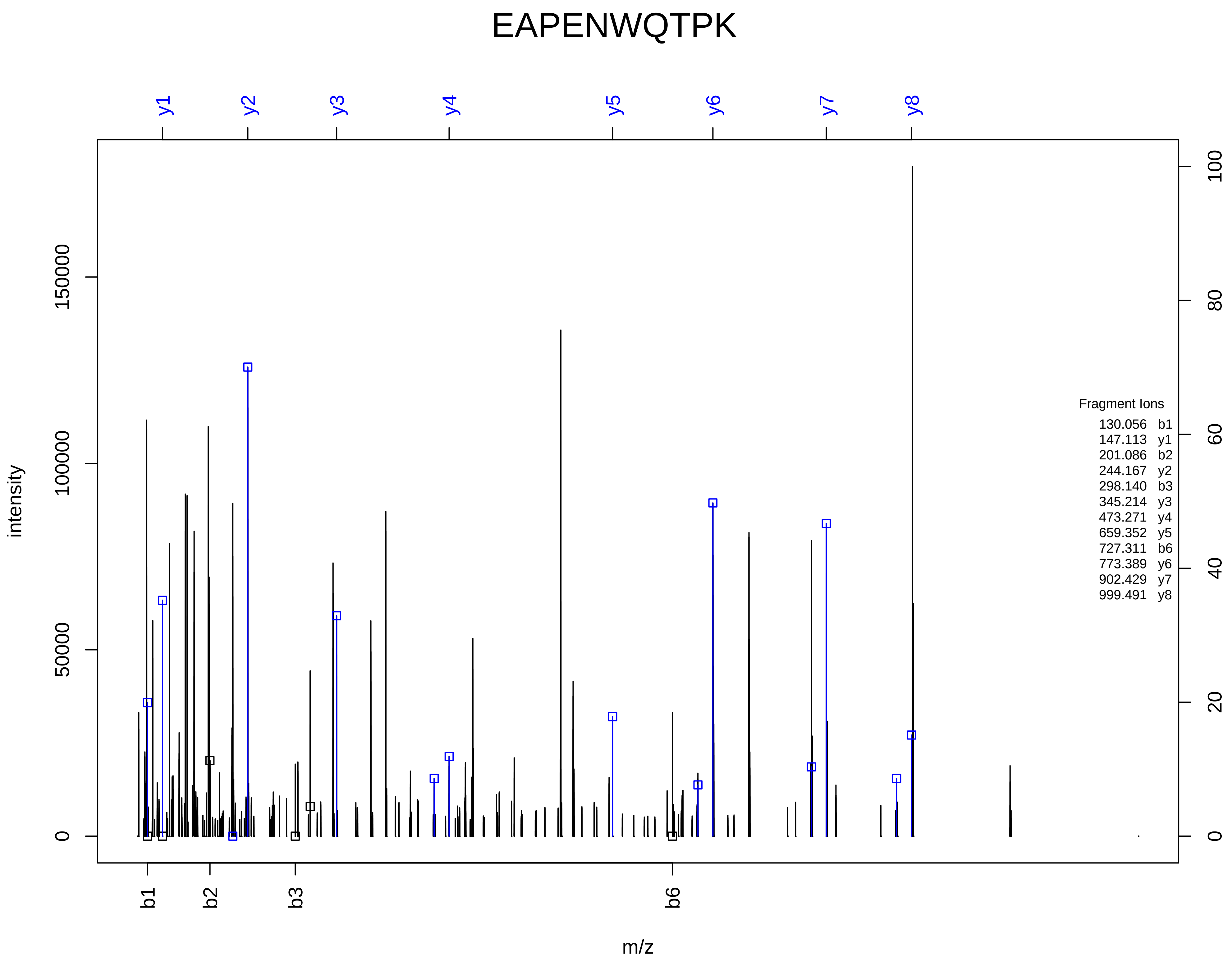

EAPENWQTPK / File: sydney_ip_11.raw; SpectrumID: ; scans: 3750

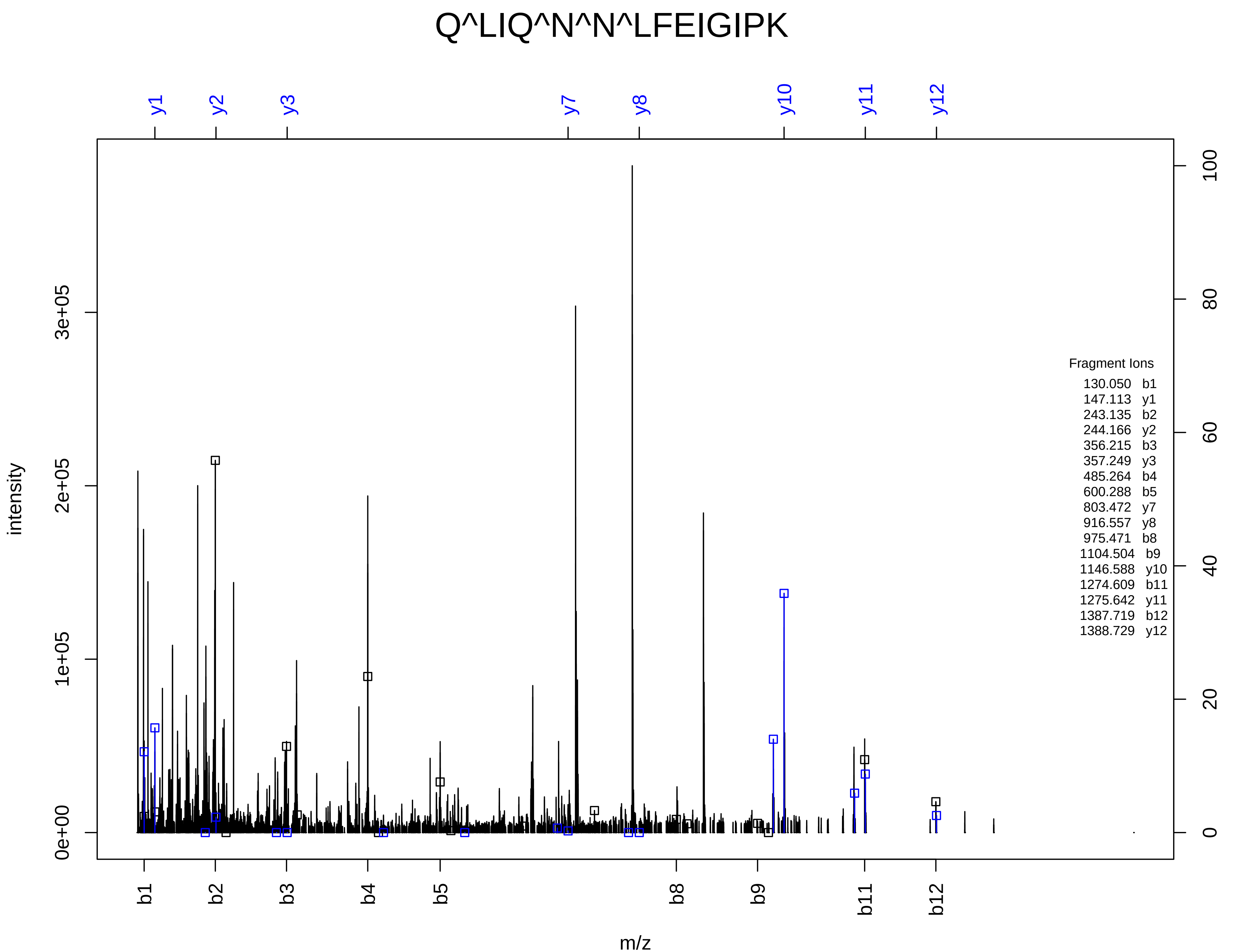

QLIQNNLFEIGIPK / File: sydney_ip_12.raw; SpectrumID: ; scans: 13289

IDLLGEFQSALPK

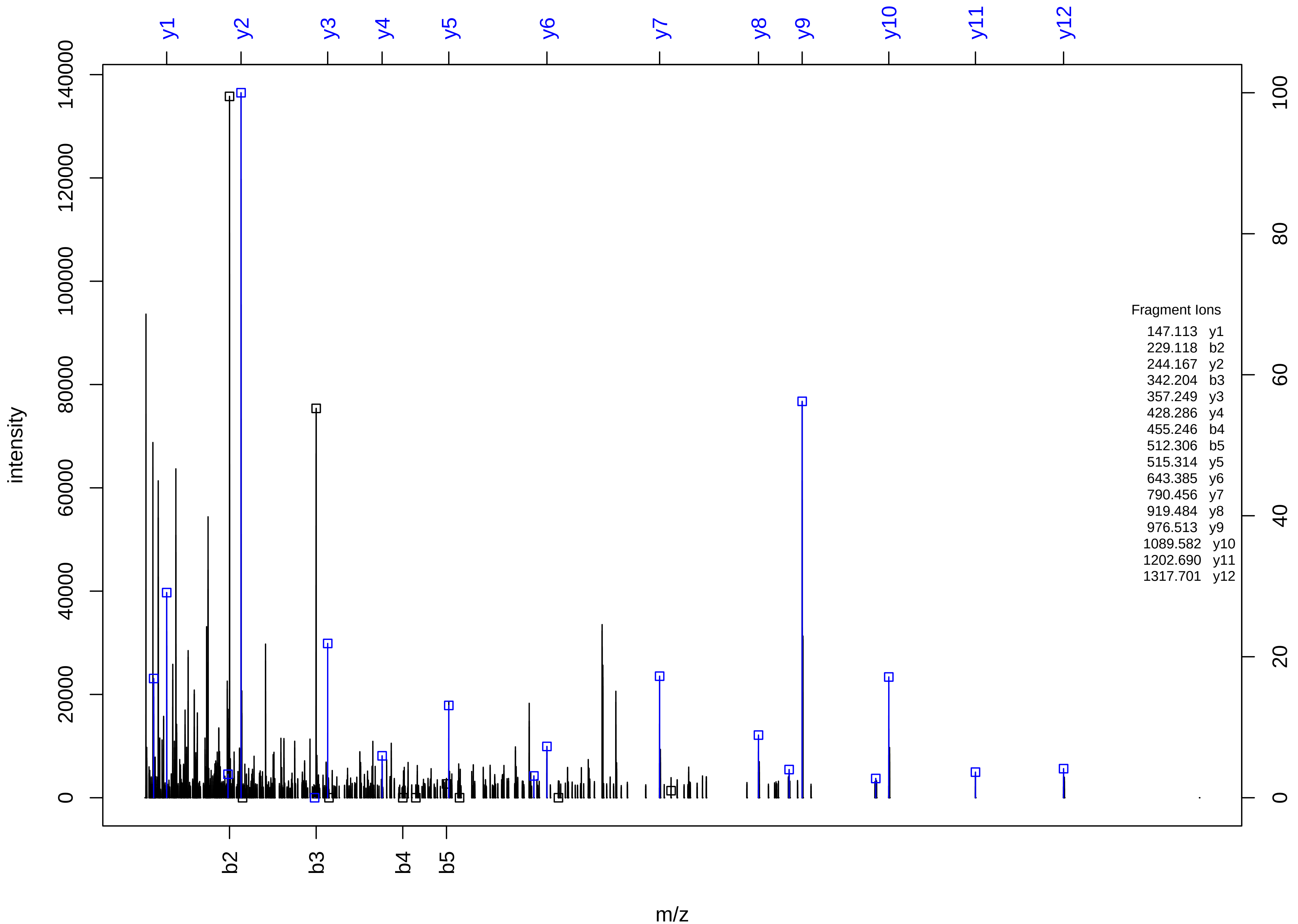

IDLLGEFQSALPK / File: sydney_ip_12.raw; SpectrumID: ; scans: 17402

NKLM*RYESELLIMAQ^ELELEDHQ^SR

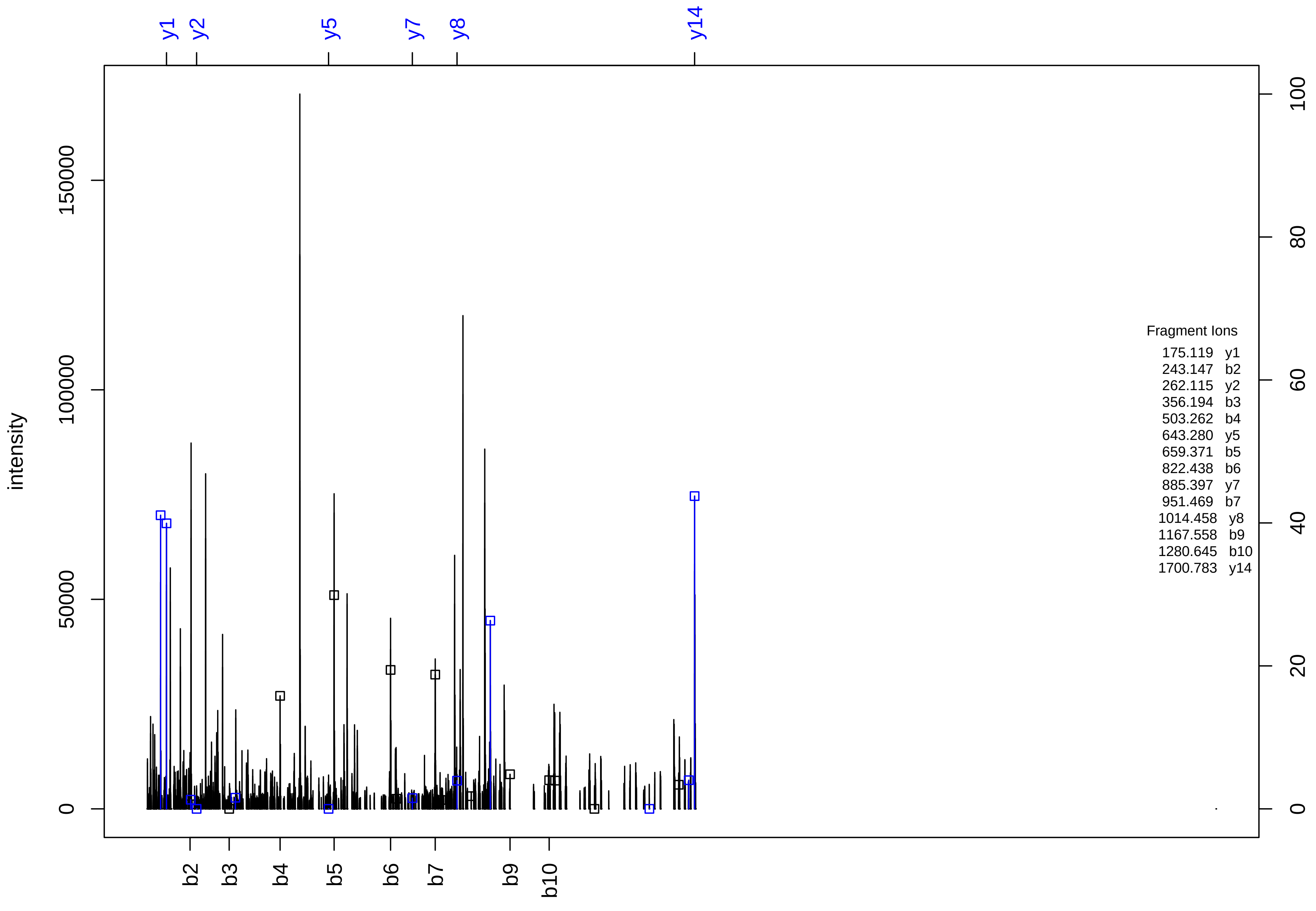

NKLMRYESELLIMAQELELEDHQSR / File: sydney_ip_12.raw; SpectrumID: ; scans: 5588

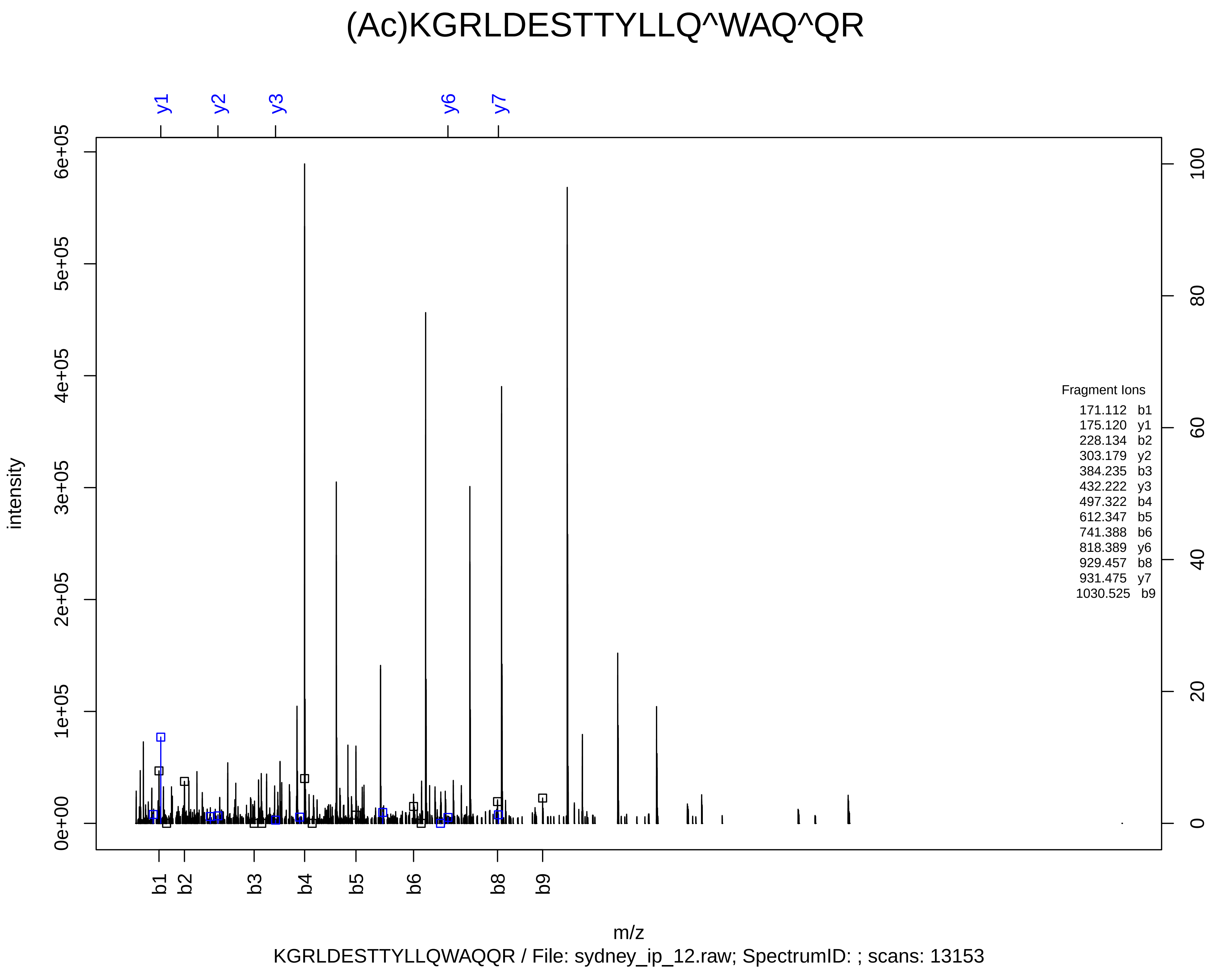

(Ac)KGRLDESTTYLLQ^WAQ^QR
y1
y2
y3
y6
y7
intensity
Fragment Ions
171.112 b1
175.120 y1
228.134 b2
303.179 y2
384.235 b3
432.222 y3
497.322 b4
612.347 b5
741.388 b6
818.389 y6
929.457 b8
931.475 y7
1030.525 b9
b1
b2
b3
b4
b5
b6
b8
b9
m/z
KGRLDESTTYLLQWAQQR / File: sydney_ip_12.raw; SpectrumID: ; scans: 13153

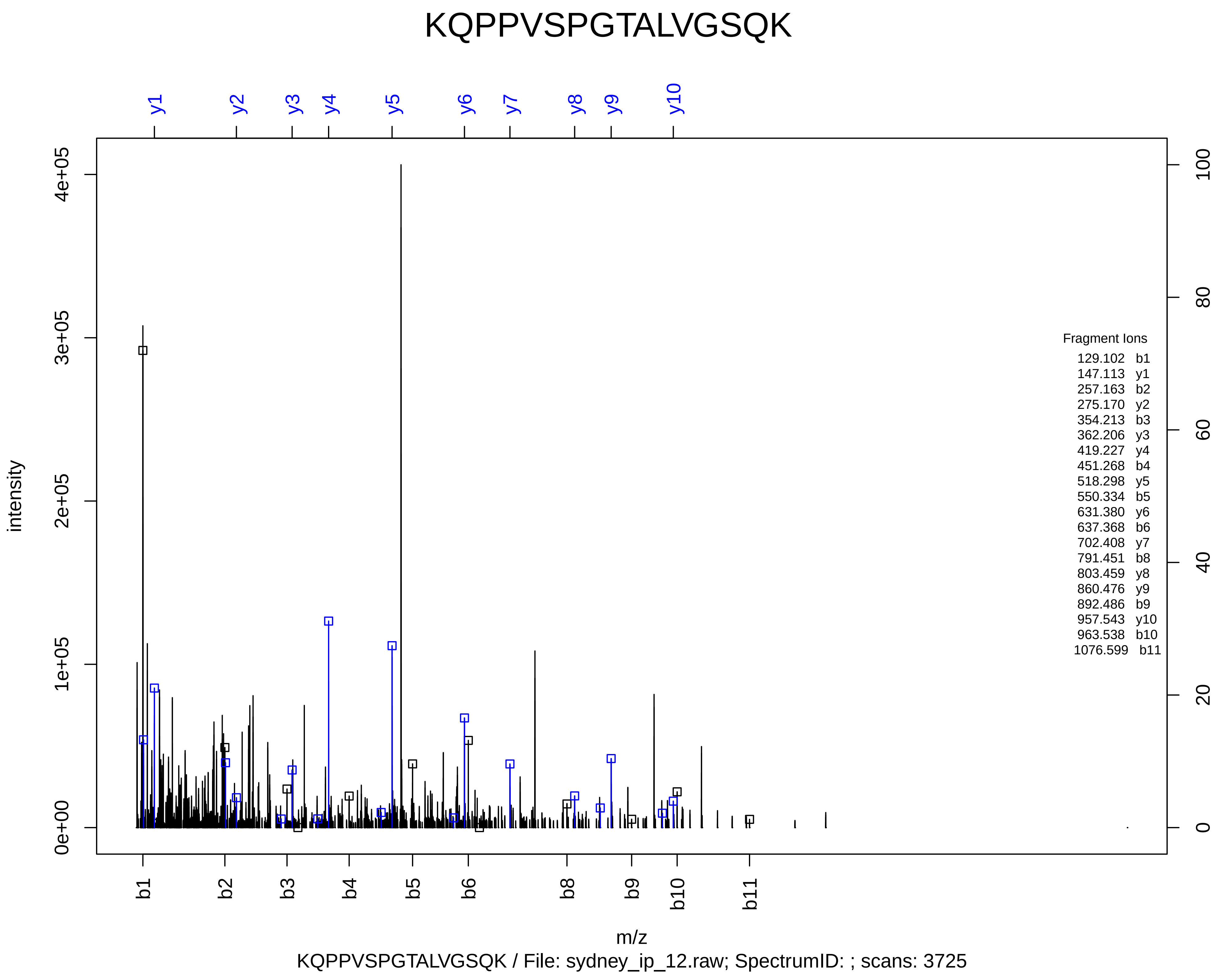
KQPPVSPGTALVGSQK
y1
y2
y3
y4
y5
y6
y7
y8
y9
y10
intensity
0e+00
1e+05
2e+05
3e+05
4e+05
0
20
40
60
80
100
Fragment Ions
129.102 b1
147.113 y1
257.163 b2
275.170 y2
354.213 b3
362.206 y3
419.227 y4
451.268 b4
518.298 y5
550.334 b5
631.380 y6
637.368 b6
702.408 y7
791.451 b8
803.459 y8
860.476 y9
892.486 b9
957.543 y10
963.538 b10
1076.599 b11
b1
b2
b3
b4
b5
b6
b8
b9
b10
b11
m/z
KQPPVSPGTALVGSQK / File: sydney_ip_12.raw; SpectrumID: ; scans: 3725

WQKEALQ^N^Q^K

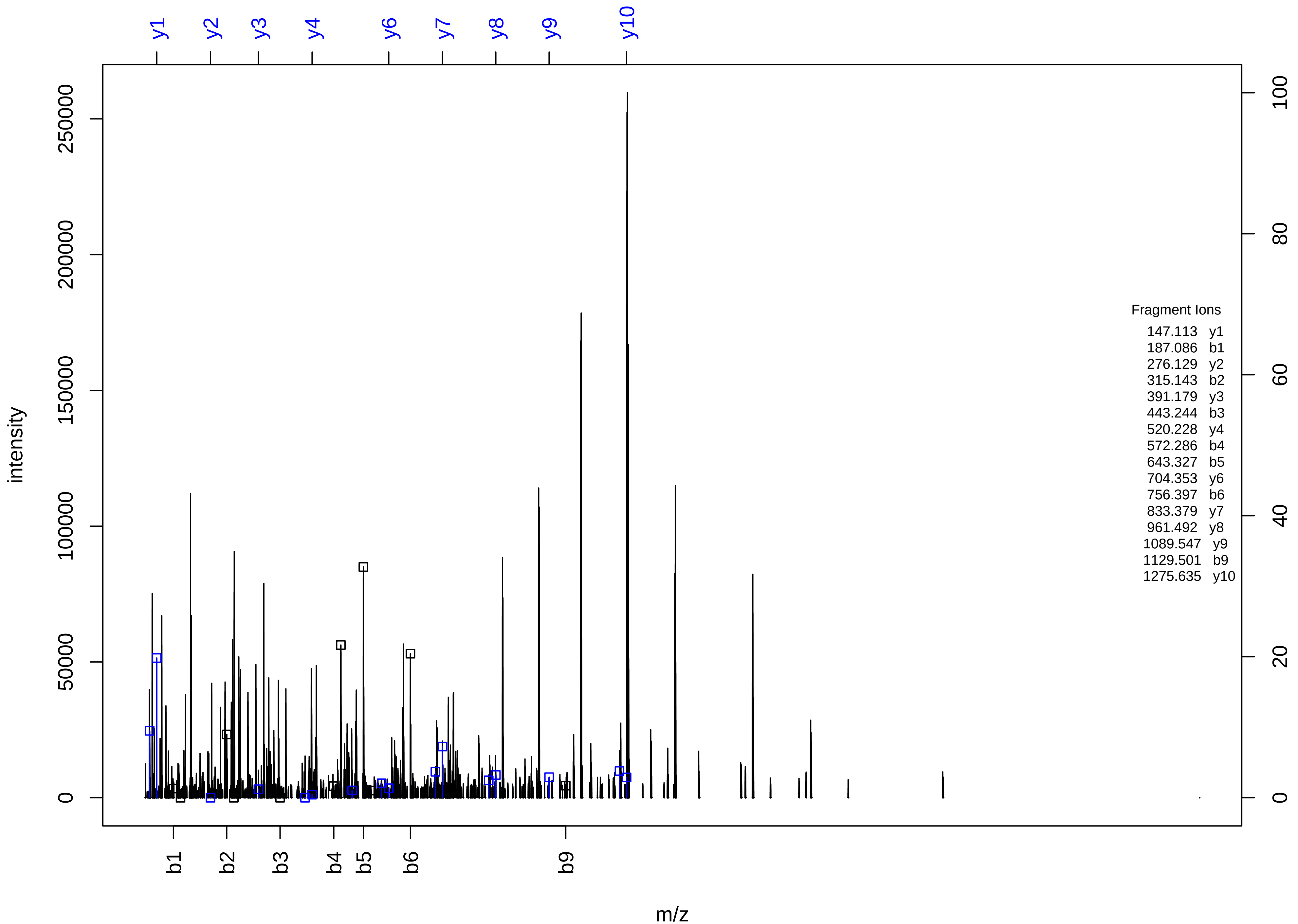

WQKEALQNQK / File: sydney_ip_12.raw; SpectrumID: ; scans: 13657

VLQ^DLLSEK

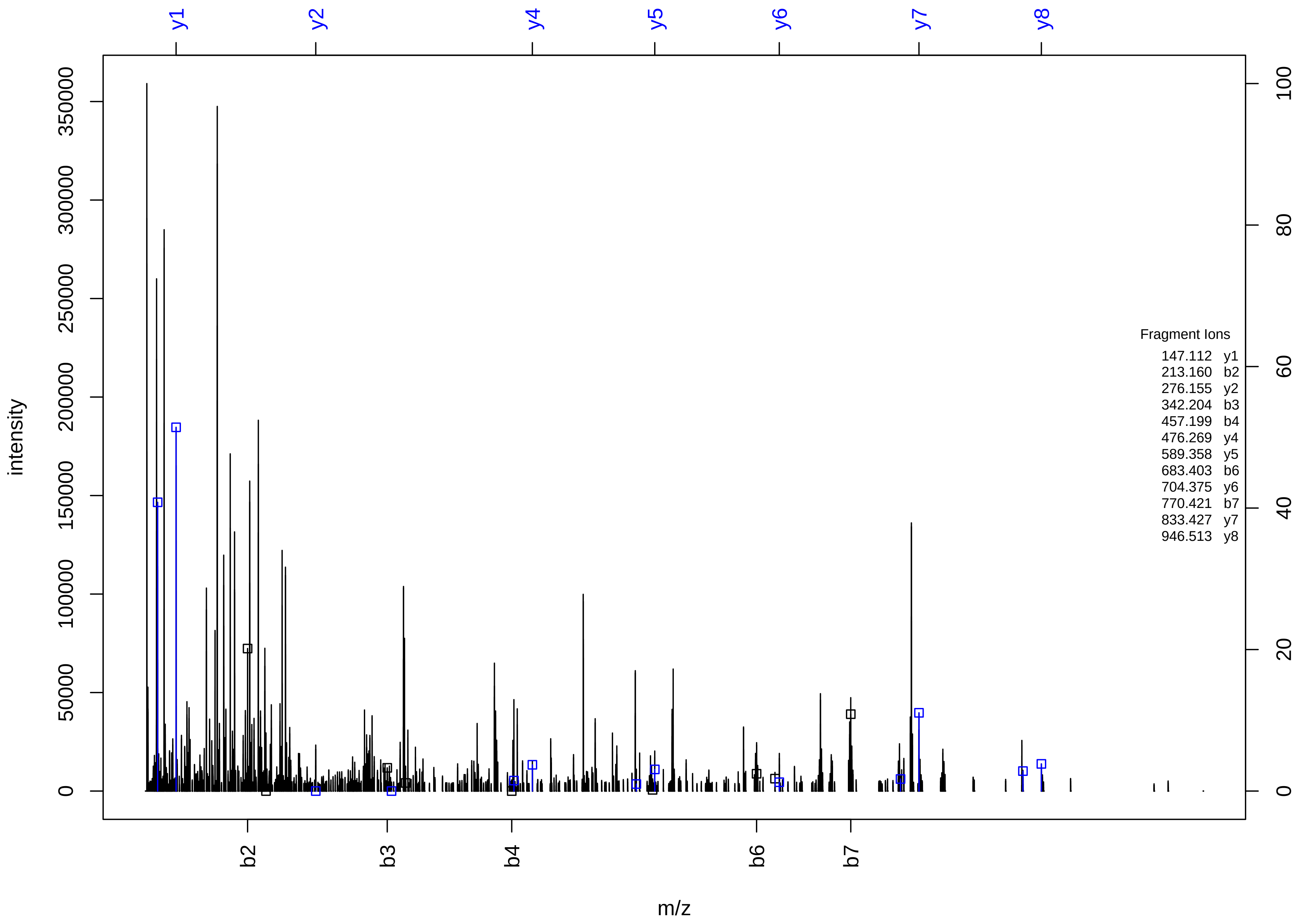

VLQDLLSEK / File: sydney_ip_12.raw; SpectrumID: ; scans: 8473

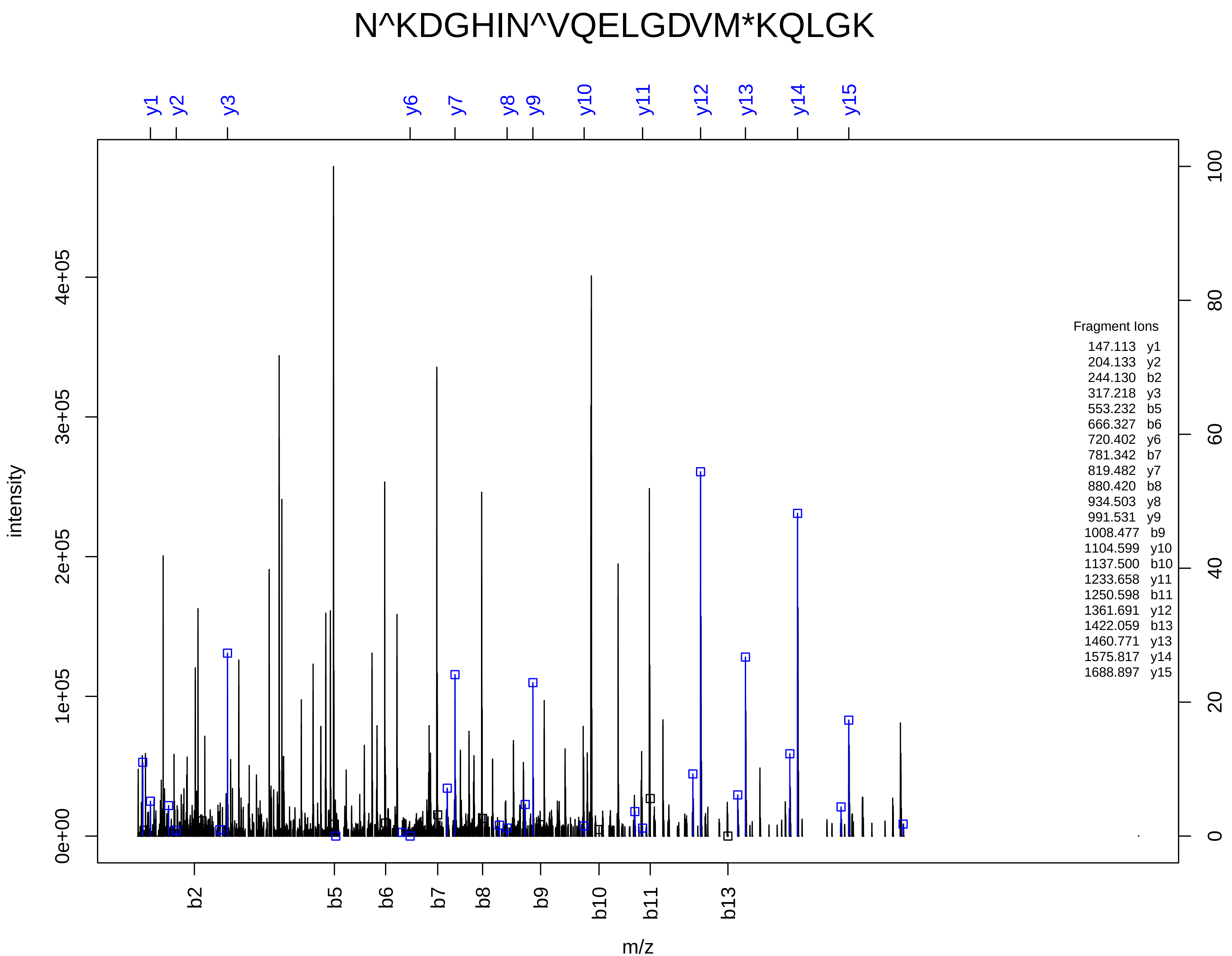

NKDGHINVQELGDVMKQLGK / File: sydney_ip_12.raw; SpectrumID: ; scans: 10752

(Ac)HQ^VVEEQMNTGSLM*R

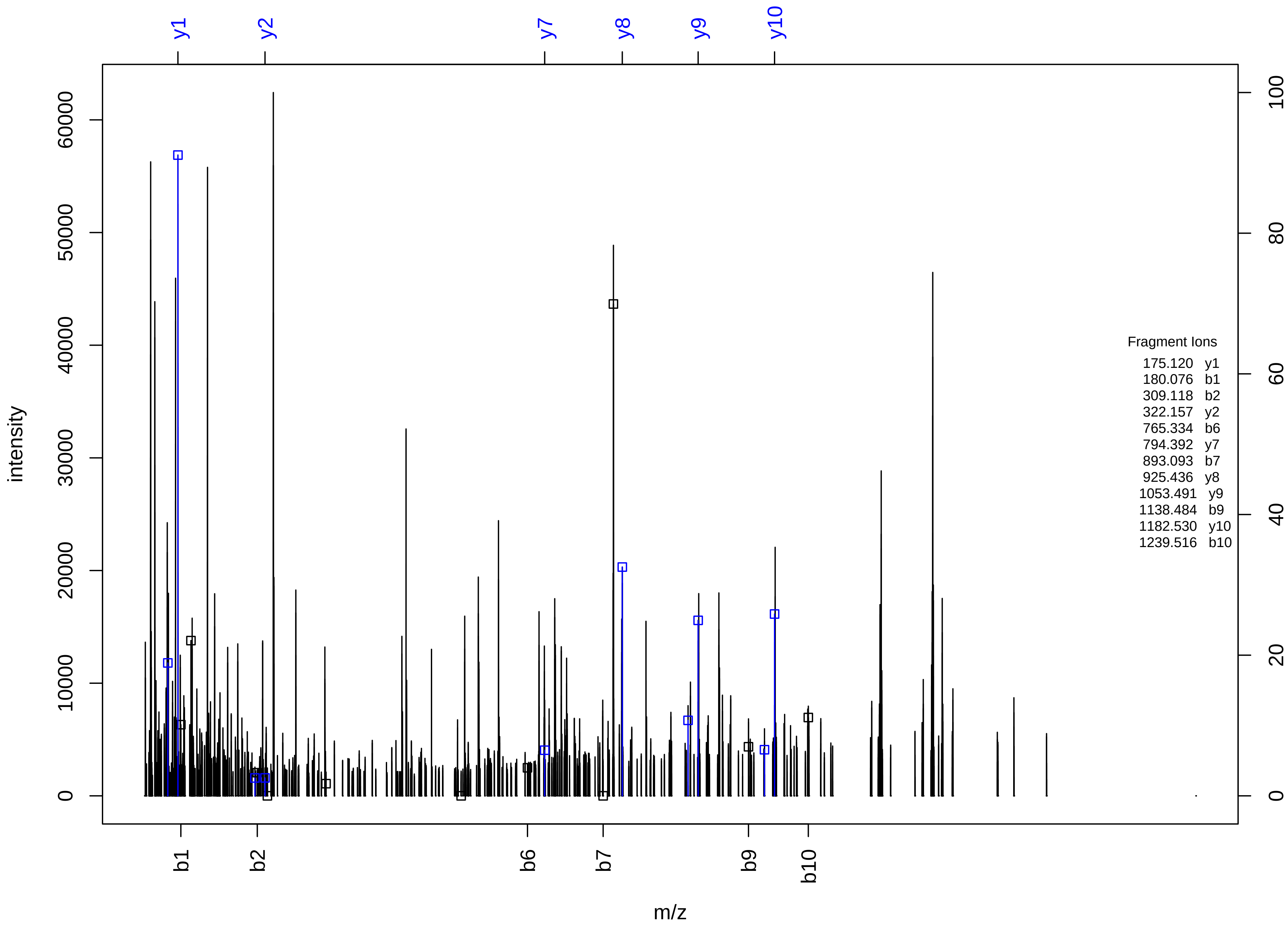

HQVVEEQMNTGSLMR / File: sydney_ip_12.raw; SpectrumID: ; scans: 5641

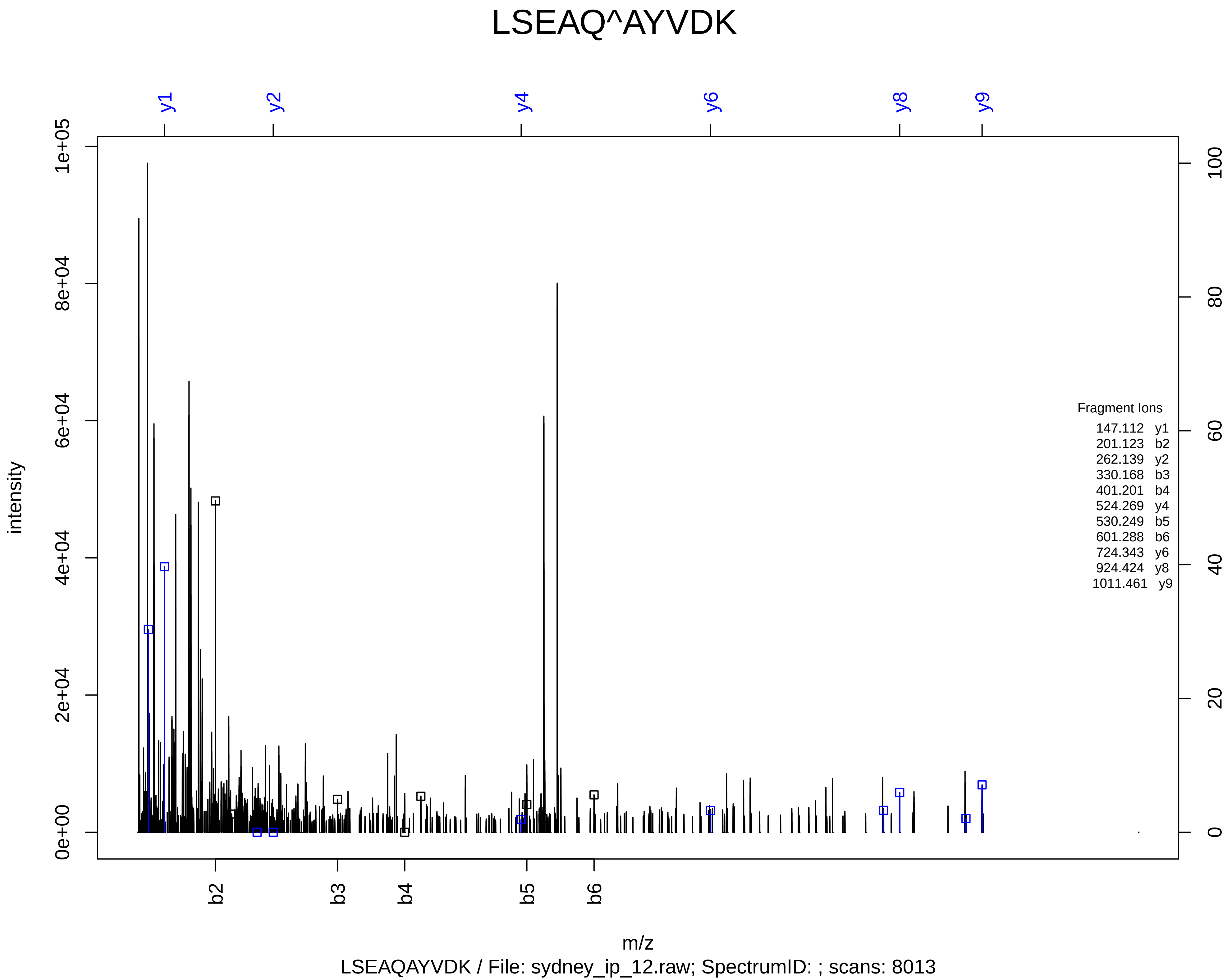

LSEAQ^AYVDK
y1
y2
y4
y6
y8
y9
intensity
0e+00
2e+04
4e+04
6e+04
8e+04
1e+05
0
20
40
60
80
100
b2
b3
b4
b5
b6
Fragment Ions
147.112 y1
201.123 b2
262.139 y2
330.168 b3
401.201 b4
524.269 y4
530.249 b5
601.288 b6
724.343 y6
924.424 y8
1011.461 y9
m/z
LSEAQAYVDK / File: sydney_ip_12.raw; SpectrumID: ; scans: 8013

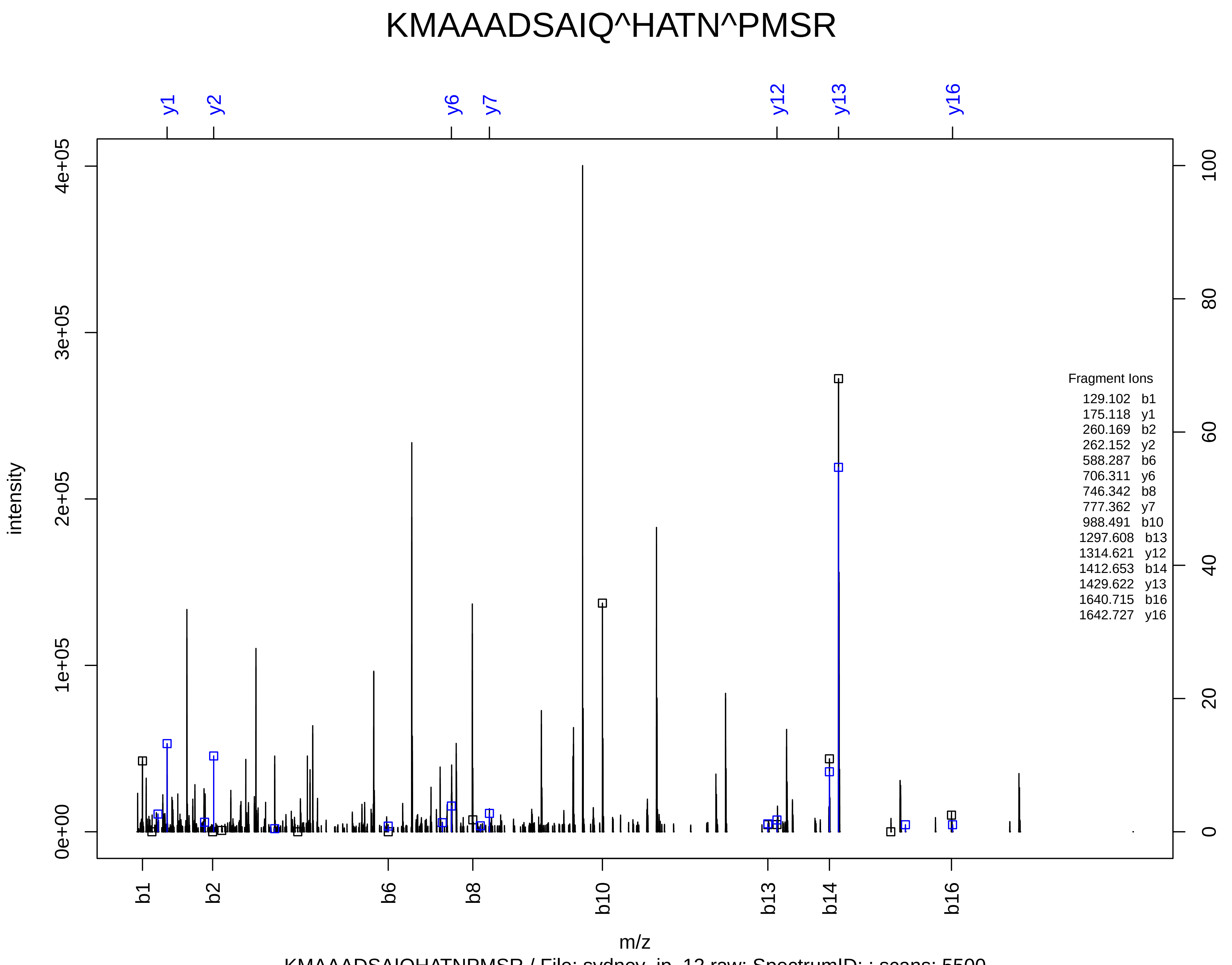
KMAAADSAIQ^HATN^PMSR
y1
y2
y6
y7
y12
y13
y16
intensity
0e+00
1e+05
2e+05
3e+05
4e+05
0
20
40
60
80
100
b1
b2
b6
b8
b10
b13
b14
b16
m/z
Fragment Ions
129.102 b1
175.118 y1
260.169 b2
262.152 y2
588.287 b6
706.311 y6
746.342 b8
777.362 y7
988.491 b10
1297.608 b13
1314.621 y12
1412.653 b14
1429.622 y13
1640.715 b16
1642.727 y16
KMAAADSAIQHATNPMSR / File: sydney_ip_12.raw; SpectrumID: ; scans: 5500

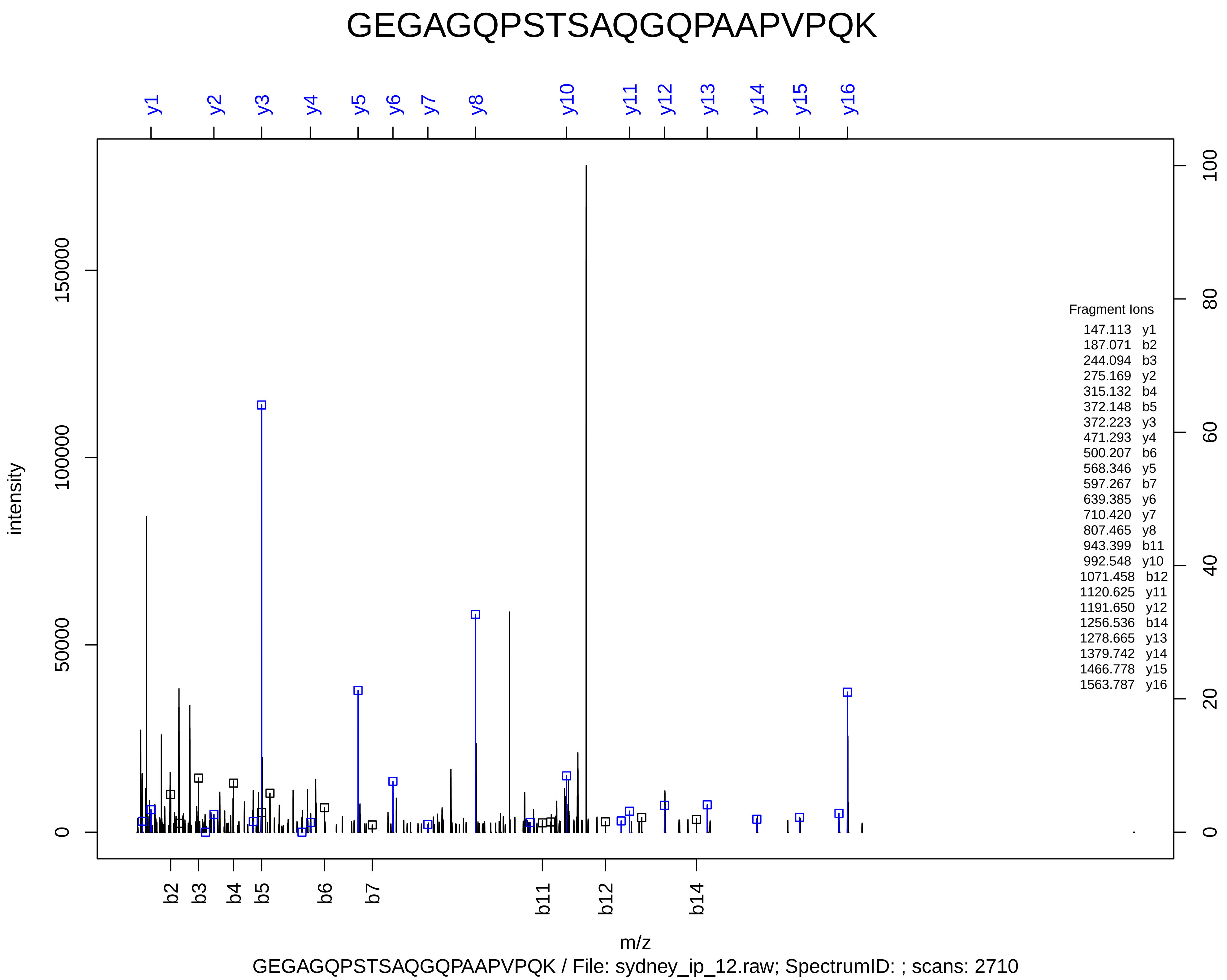
GEGAGQPSTSAQGQPAAPVPQK
y1 y2 y3 y4 y5 y6 y7 y8 y10 y11 y12 y13 y14 y15 y16
b2 b3 b4 b5 b6 b7 b11 b12 b14
intensity
m/z
Fragment Ions
147.113 y1
187.071 b2
244.094 b3
275.169 y2
315.132 b4
372.148 b5
372.223 y3
471.293 y4
500.207 b6
568.346 y5
597.267 b7
639.385 y6
710.420 y7
807.465 y8
943.399 b11
992.548 y10
1071.458 b12
1120.625 y11
1191.650 y12
1256.536 b14
1278.665 y13
1379.742 y14
1466.778 y15
1563.787 y16
GEGAGQPSTSAQGQPAAPVPQK / File: sydney_ip_12.raw; SpectrumID: ; scans: 2710

KILNSIQVM*RAQMNQIQ^SVEV

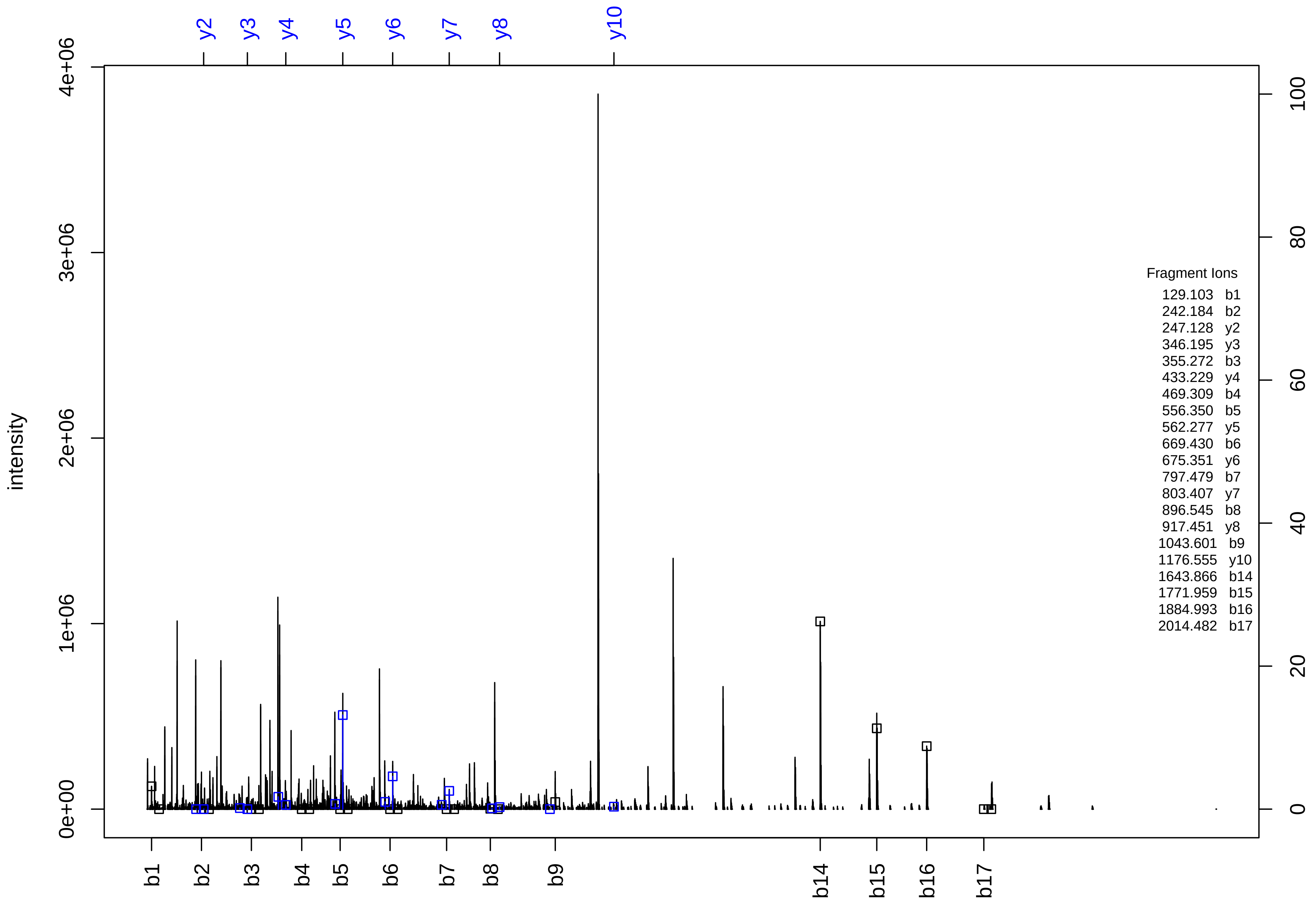

KILNSIQVMRAQMNQIQSVEV / File: sydney_ip_12.raw; SpectrumID: ; scans: 18040

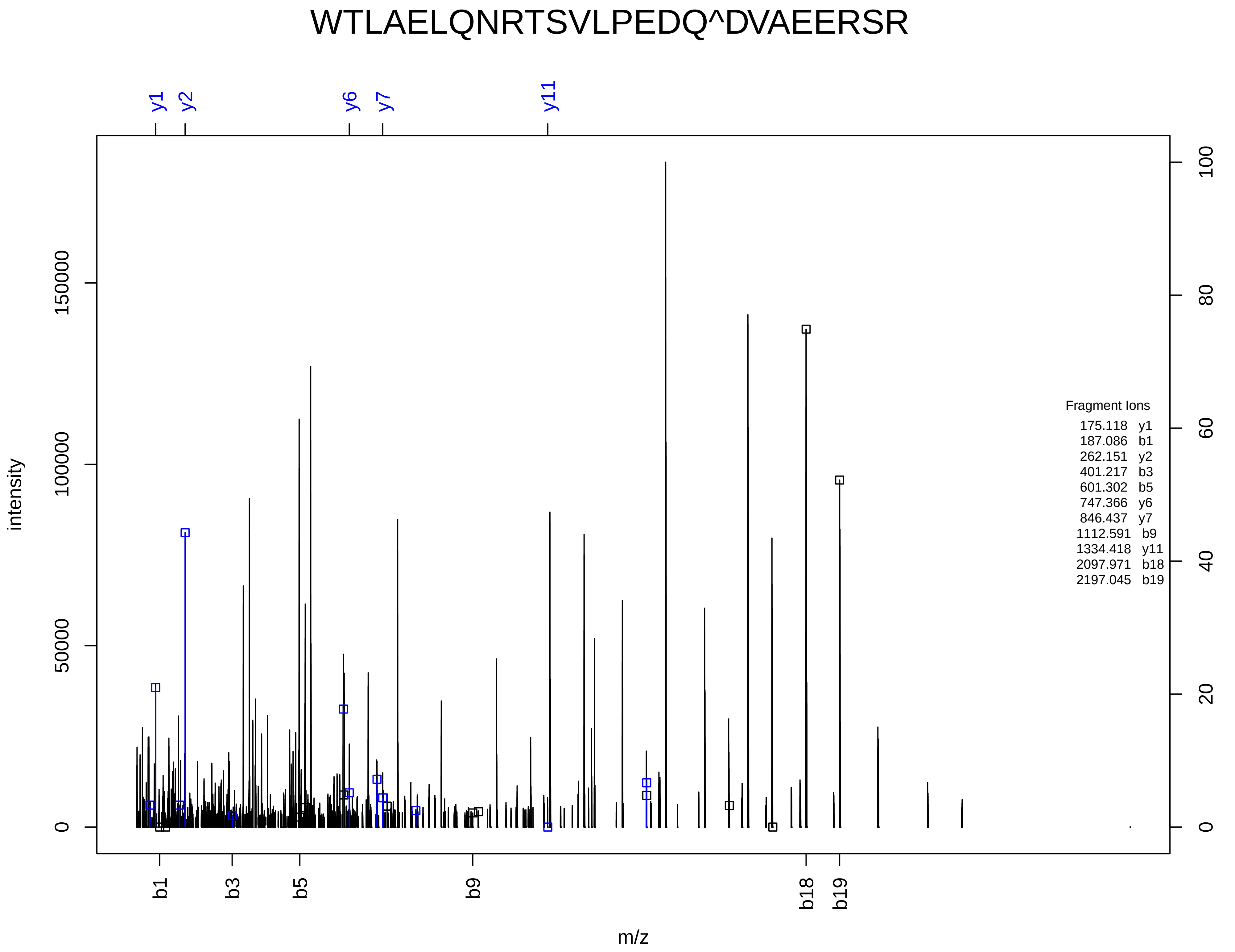

WTLAELQNRTSVLPEDQ^DVAEERSR
y1
y2
y6
y7
y11
intensity
0
50000
100000
150000
0
20
40
60
80
100
b1
b3
b5
b9
b18
b19
m/z
Fragment Ions
175.118 y1
187.086 b1
262.151 y2
401.217 b3
601.302 b5
747.366 y6
846.437 y7
1112.591 b9
1334.418 y11
2097.971 b18
2197.045 b19
WTLAELQNRTSVLPEDQDVAEERSR / File: sydney_ip_12.raw; SpectrumID: ; scans: 14499

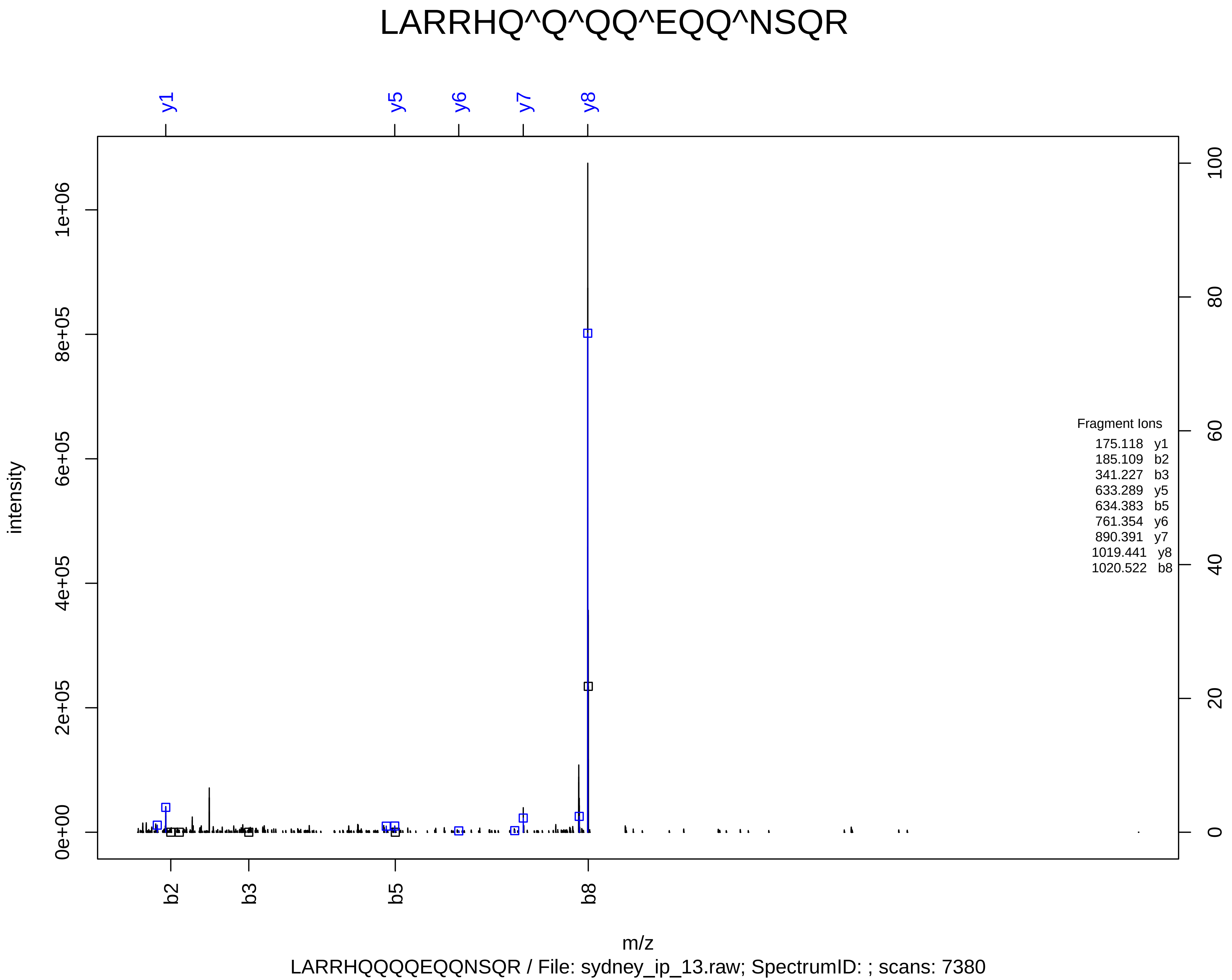

LARRHQ^Q^QQ^EQQ^NSQR
y1
y5
y6
y7
y8
intensity
1e+06
8e+05
6e+05
4e+05
2e+05
0e+00
100
80
60
40
20
0
Fragment Ions
175.118 y1
185.109 b2
341.227 b3
633.289 y5
634.383 b5
761.354 y6
890.391 y7
1019.441 y8
1020.522 b8
b2
b3
b5
b8
m/z
LARRHQQQQEQQNSQR / File: sydney_ip_13.raw; SpectrumID: ; scans: 7380

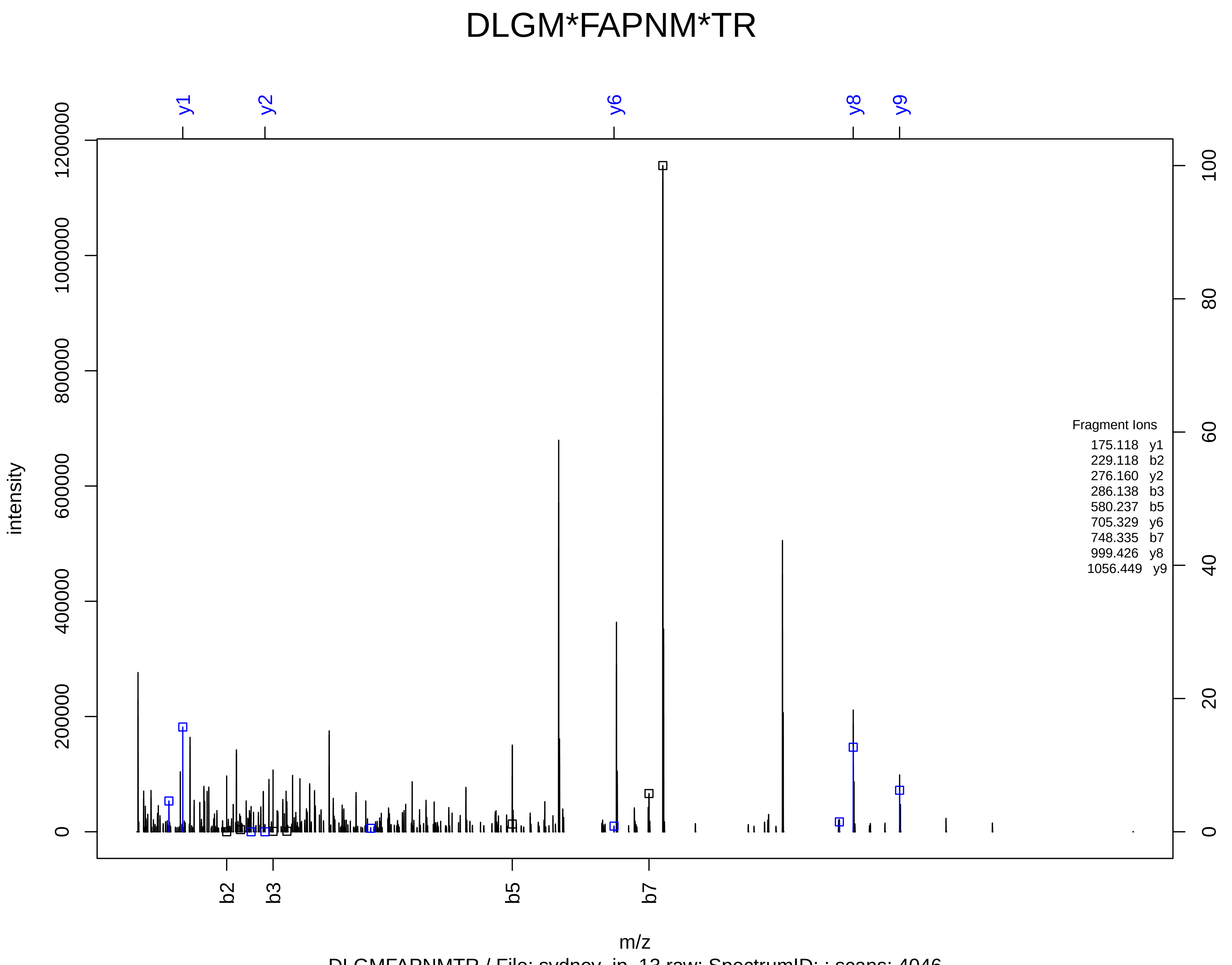

DLGM*FAPNM*TR
y1
y2
y6
y8
y9
intensity
1200000
1000000
800000
600000
400000
200000
0
100
80
60
40
20
0
Fragment Ions
175.118 y1
229.118 b2
276.160 y2
286.138 b3
580.237 b5
705.329 y6
748.335 b7
999.426 y8
1056.449 y9
b2
b3
b5
b7
m/z
DLGMFAPNMTR / File: sydney_ip_13.raw; SpectrumID: ; scans: 4046

LM*PAVLQ^SAN^HR

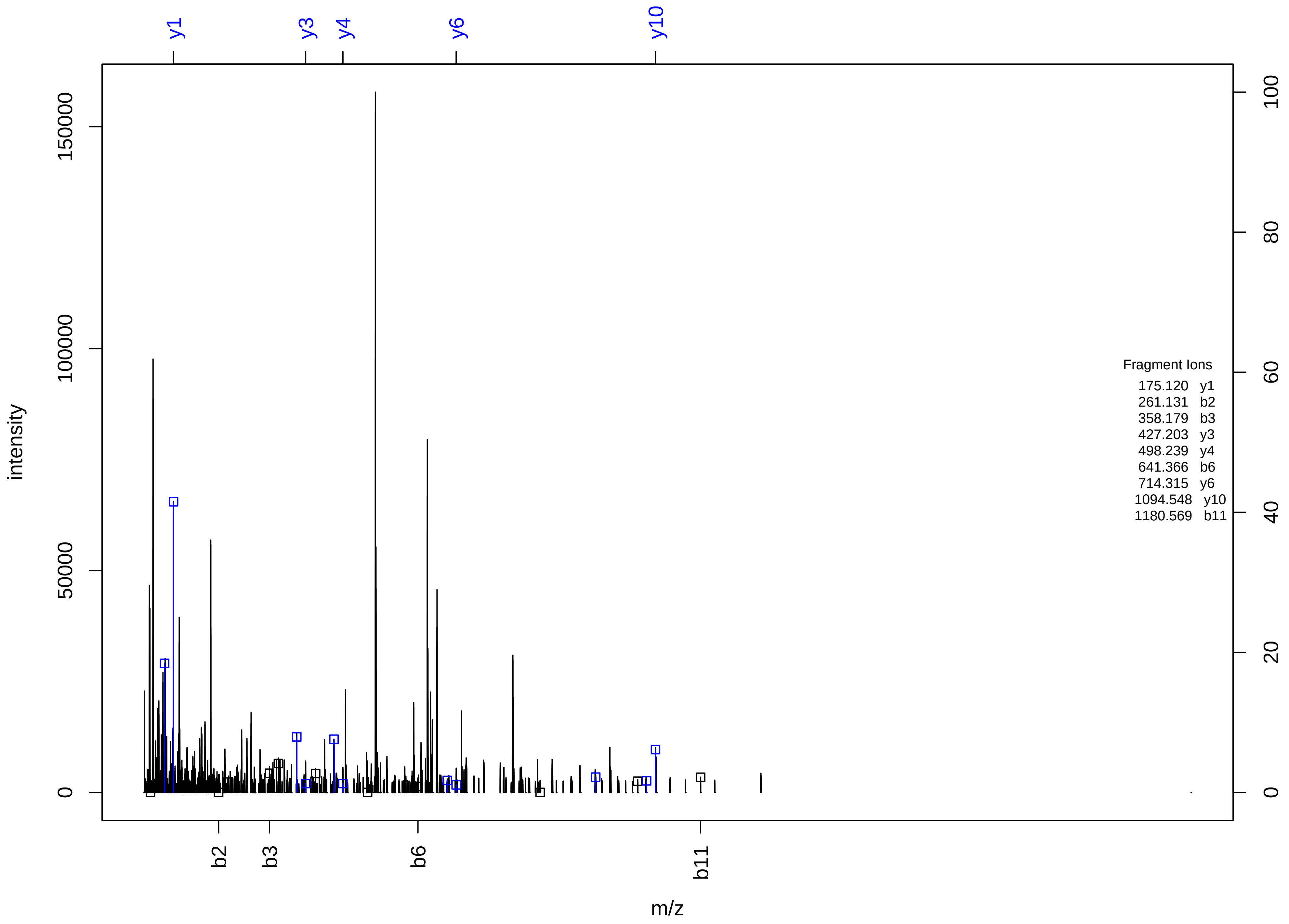

LMPAVLQSANHR / File: sydney_ip_13.raw; SpectrumID: ; scans: 7731

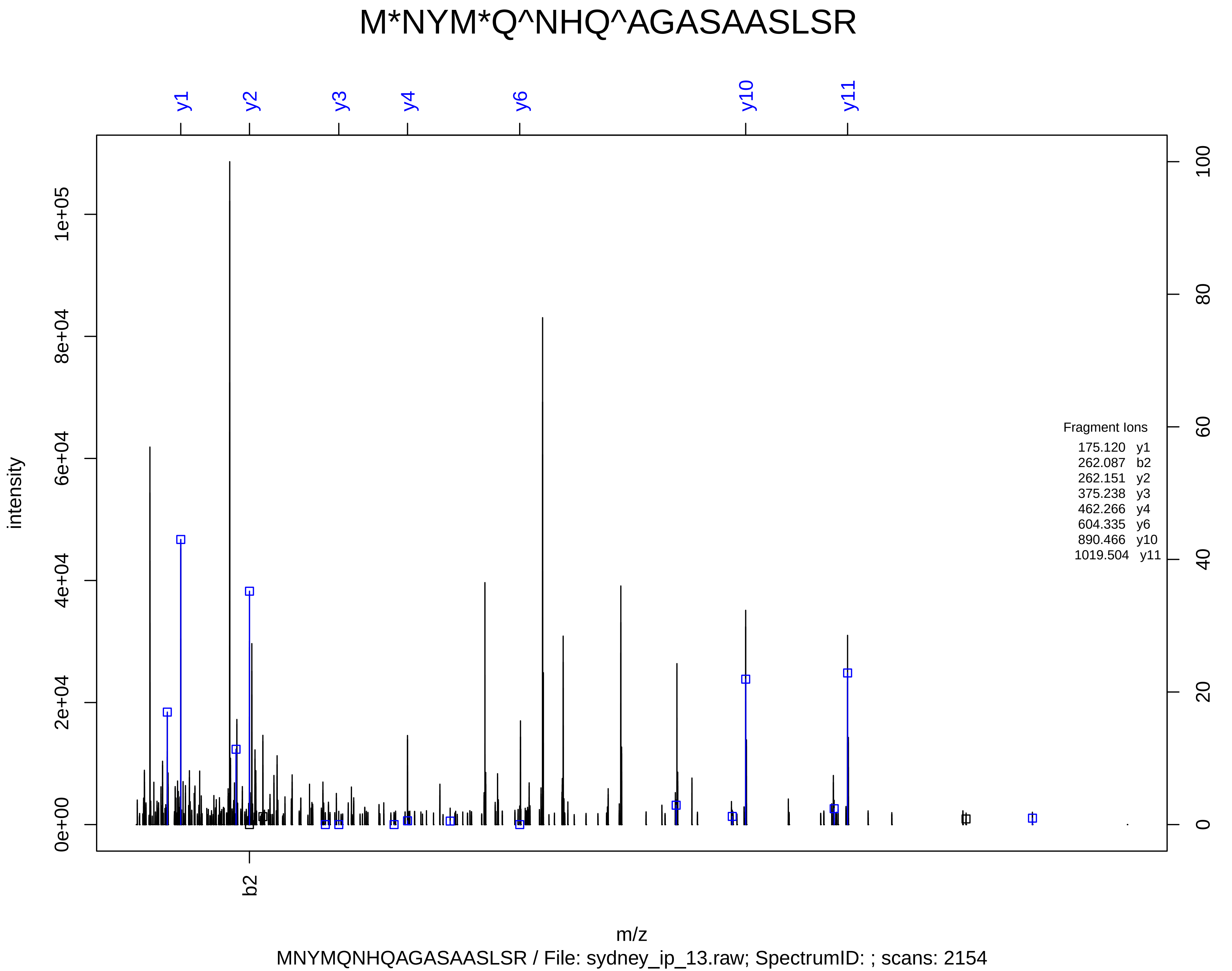
M*NYM*Q^NHQ^AGASAASLSR
y1
y2
y3
y4
y6
y10
y11
intensity
0e+00
2e+04
4e+04
6e+04
8e+04
1e+05
0
20
40
60
80
100
b2
Fragment Ions
175.120 y1
262.087 b2
262.151 y2
375.238 y3
462.266 y4
604.335 y6
890.466 y10
1019.504 y11
m/z
MNYMQNHQAGASAASLSR / File: sydney_ip_13.raw; SpectrumID: ; scans: 2154

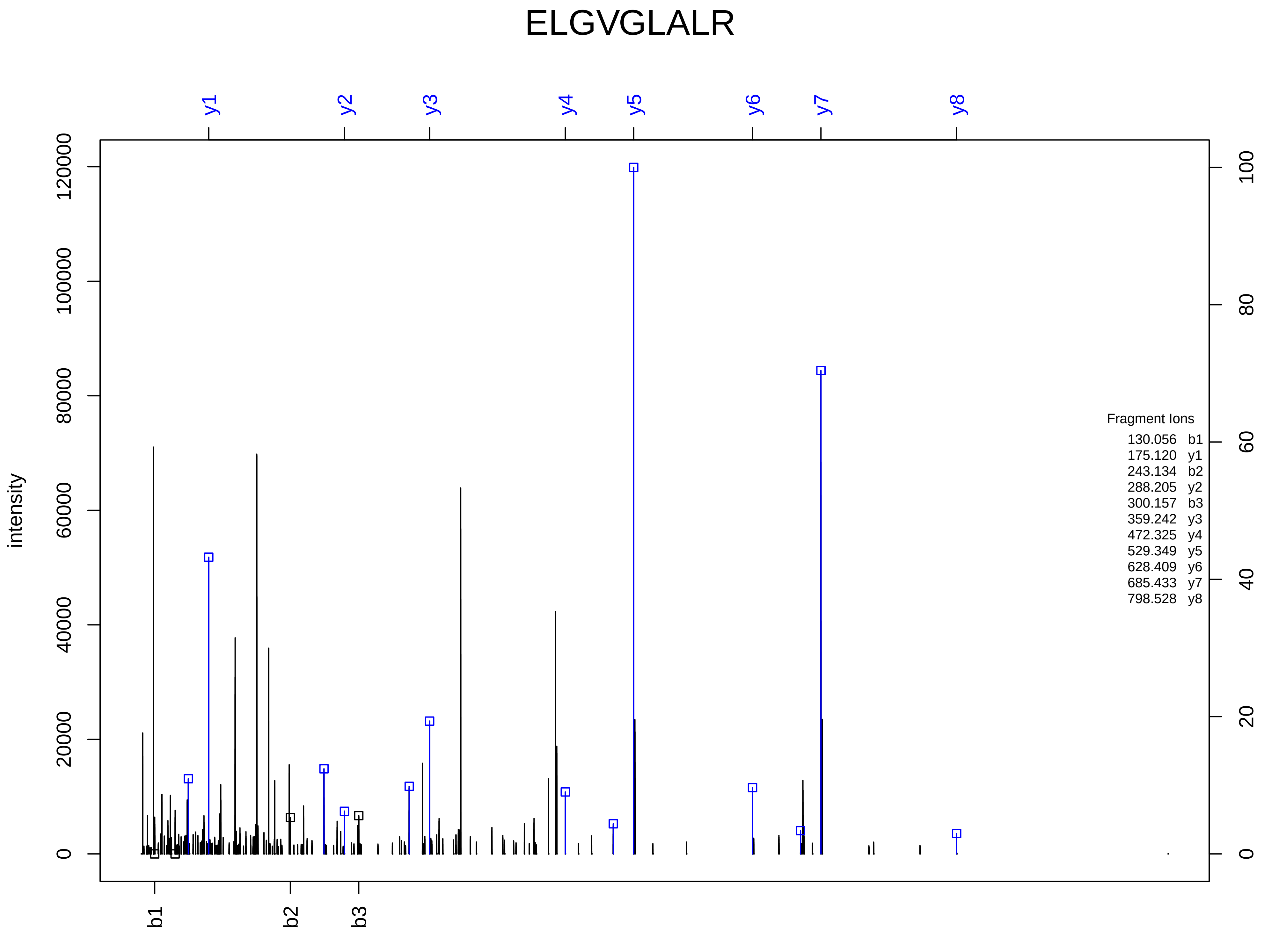

ELGVGLALR / File: sydney_ip_13.raw; SpectrumID: ; scans: 9389

LGAETLPR

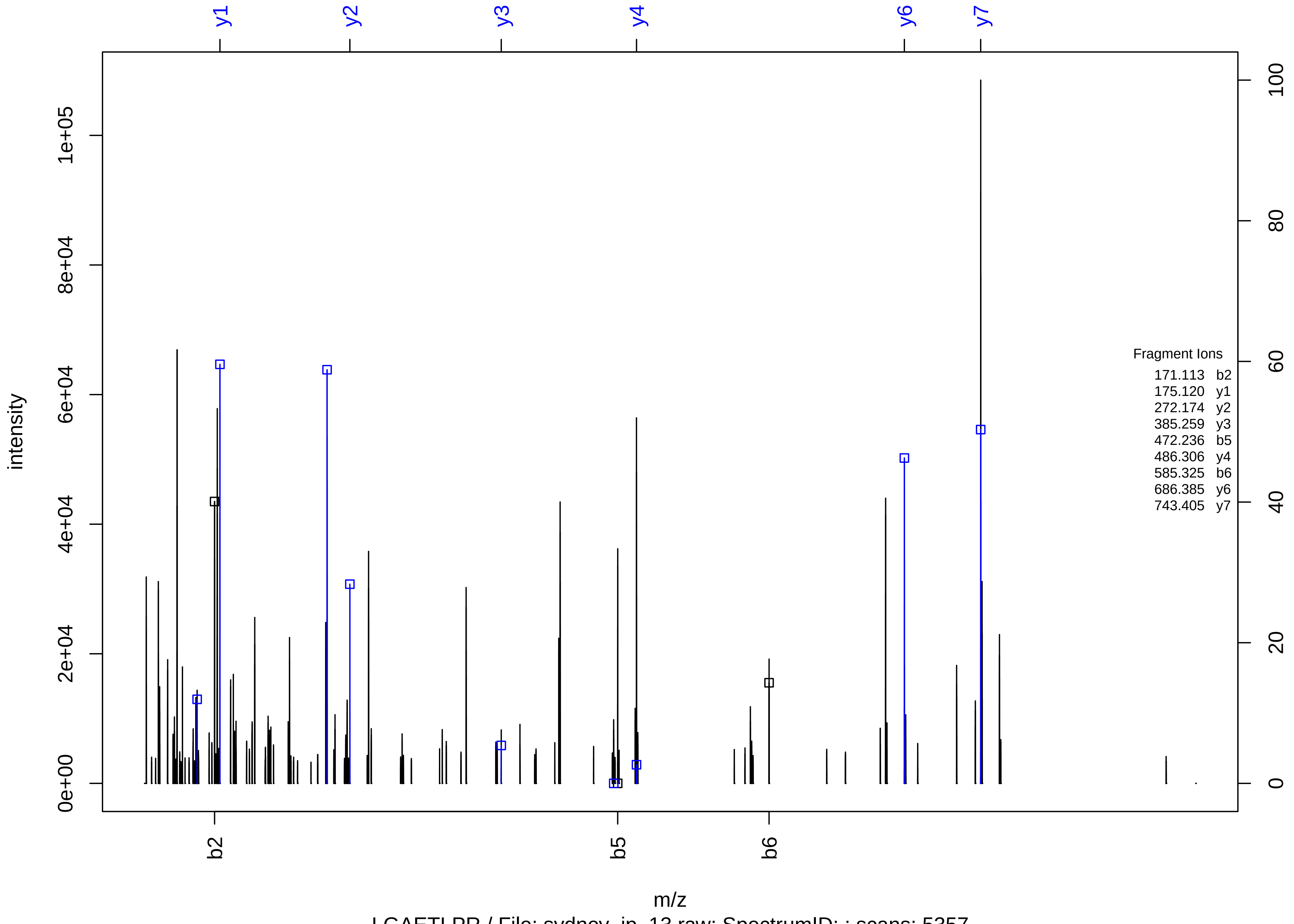

LGAETLPR / File: sydney_ip_13.raw; SpectrumID: ; scans: 5357

RASYIPASDGVQ^N^FFQIVSNLLDEEN^K

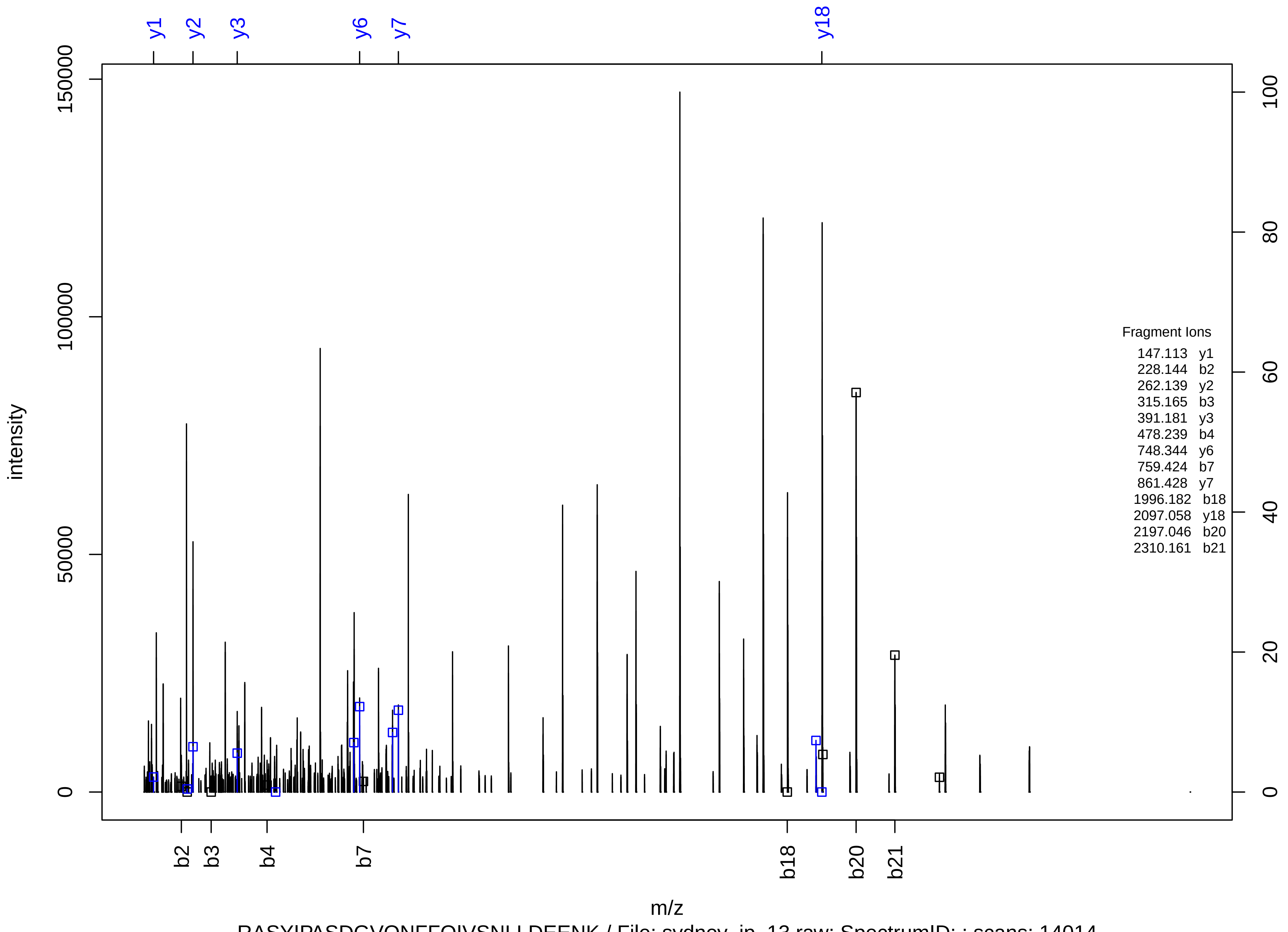

RASYIPASDGVQNFFQIVSNLLDEENK / File: sydney_ip_13.raw; SpectrumID: ; scans: 14014

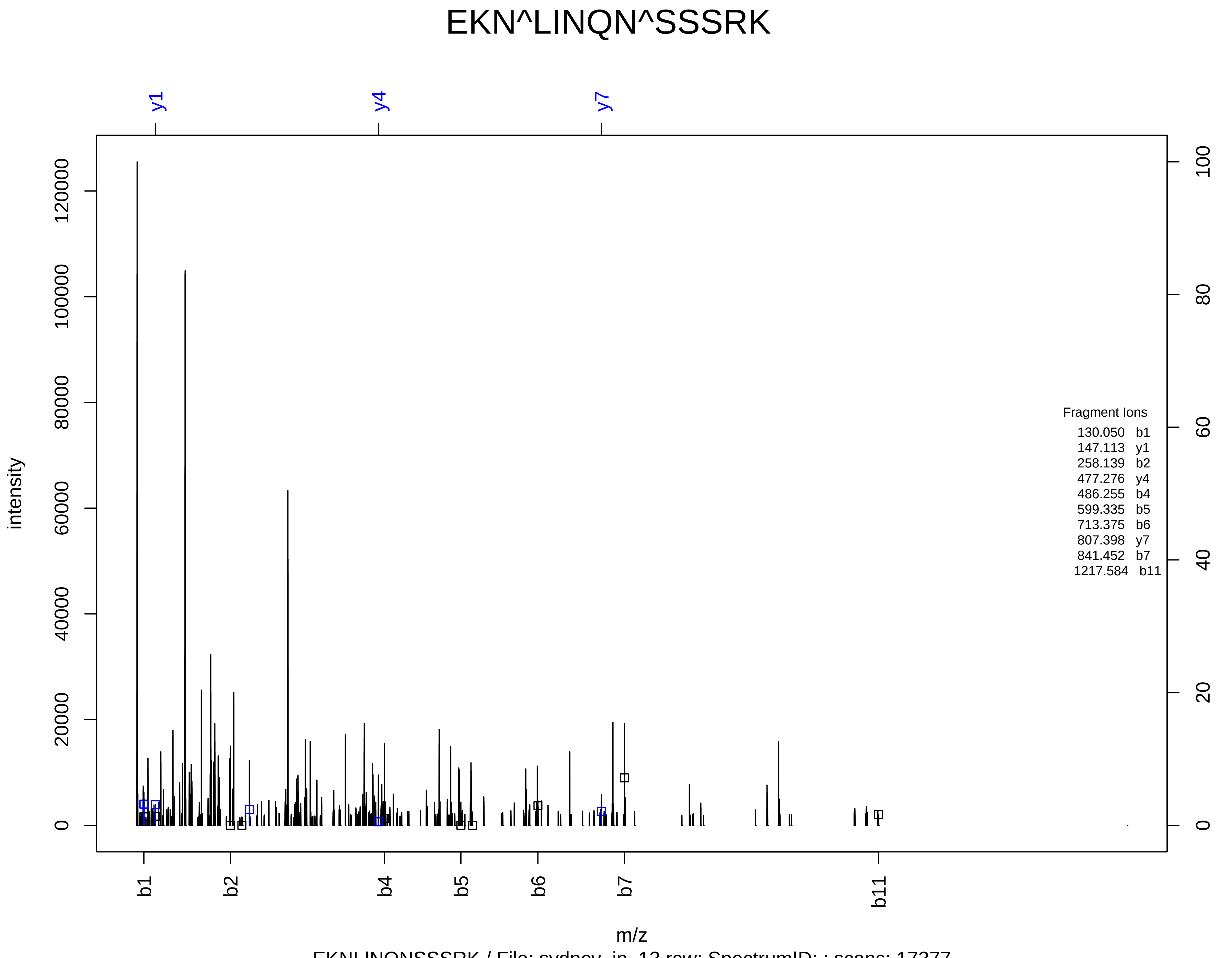
EKN^LINQN^SSSRK
y1
y4
y7
intensity
0
20000
40000
60000
80000
100000
120000
0
20
40
60
80
100
Fragment Ions
130.050 b1
147.113 y1
258.139 b2
477.276 y4
486.255 b4
599.335 b5
713.375 b6
807.398 y7
841.452 b7
1217.584 b11
b1
b2
b4
b5
b6
b7
b11
m/z
EKNLINQNSSSRK / File: sydney_ip_13.raw; SpectrumID: ; scans: 17377

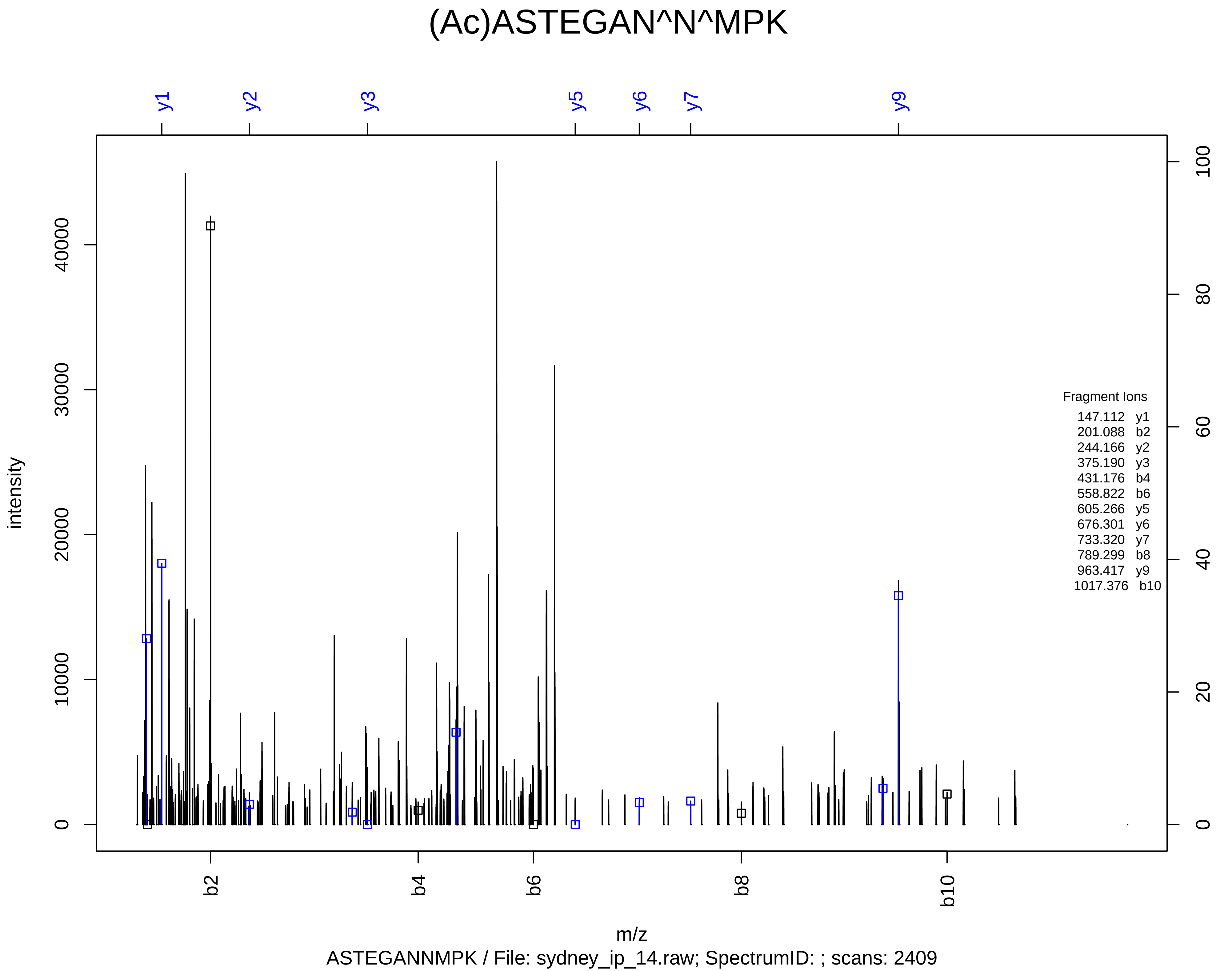
(Ac)ASTEGAN^N^MPK
y1
y2
y3
y5
y6
y7
y9
intensity
0
10000
20000
30000
40000
0
20
40
60
80
100
Fragment Ions
147.112 y1
201.088 b2
244.166 y2
375.190 y3
431.176 b4
558.822 b6
605.266 y5
676.301 y6
733.320 y7
789.299 b8
963.417 y9
1017.376 b10
b2
b4
b6
b8
b10
m/z
ASTEGANNMPK / File: sydney_ip_14.raw; SpectrumID: ; scans: 2409

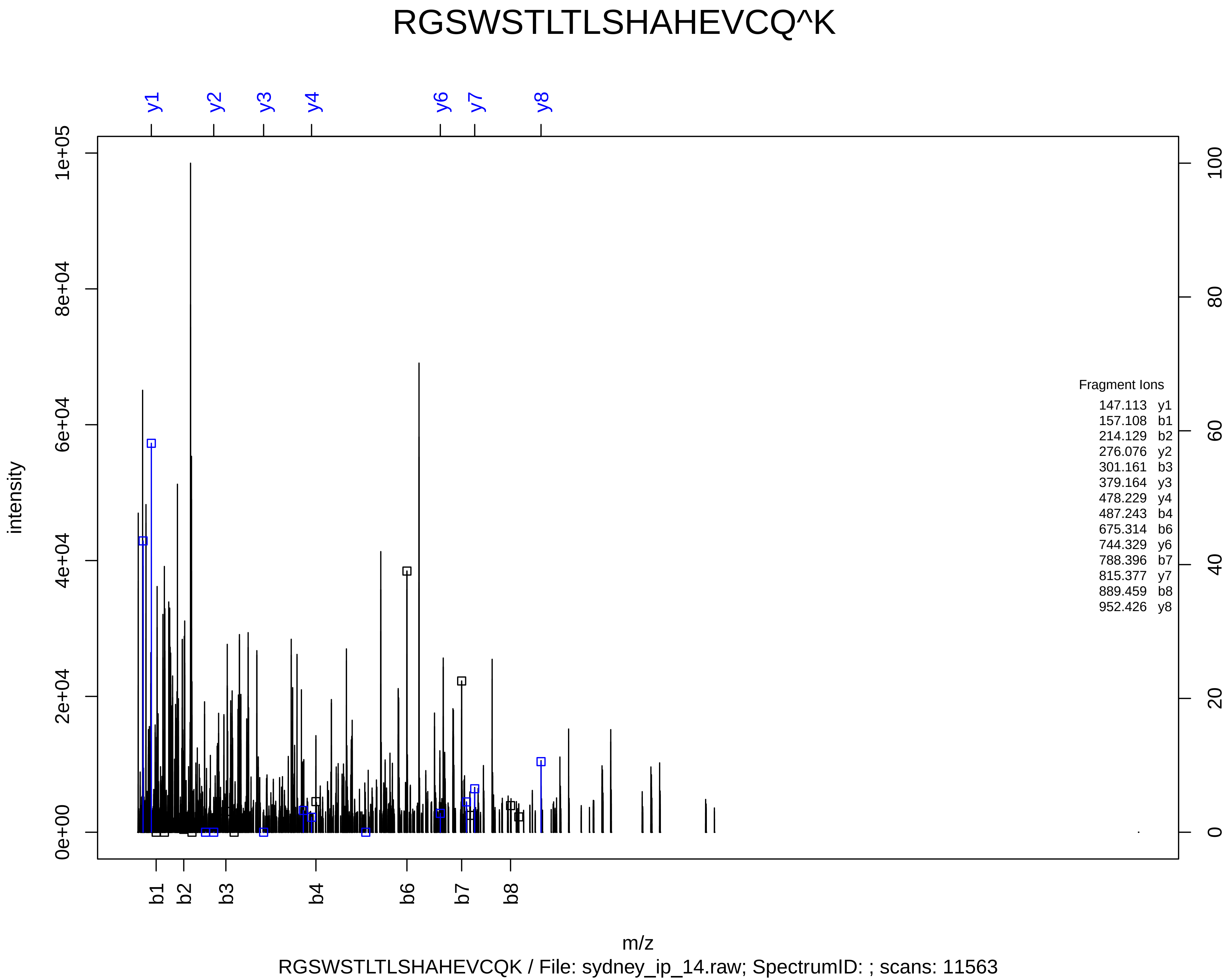
RGSWSTLTLSHAHEVCQ^K
y1
y2
y3
y4
y6
y7
y8
b1
b2
b3
b4
b6
b7
b8
intensity
m/z
Fragment Ions
147.113 y1
157.108 b1
214.129 b2
276.076 y2
301.161 b3
379.164 y3
478.229 y4
487.243 b4
675.314 b6
744.329 y6
788.396 b7
815.377 y7
889.459 b8
952.426 y8
RGSWSTLTLSHAHEVCQK / File: sydney_ip_14.raw; SpectrumID: ; scans: 11563

EGQ^DPNLSSAGRAPTLQ^RPPHQPPLRPSSGPR

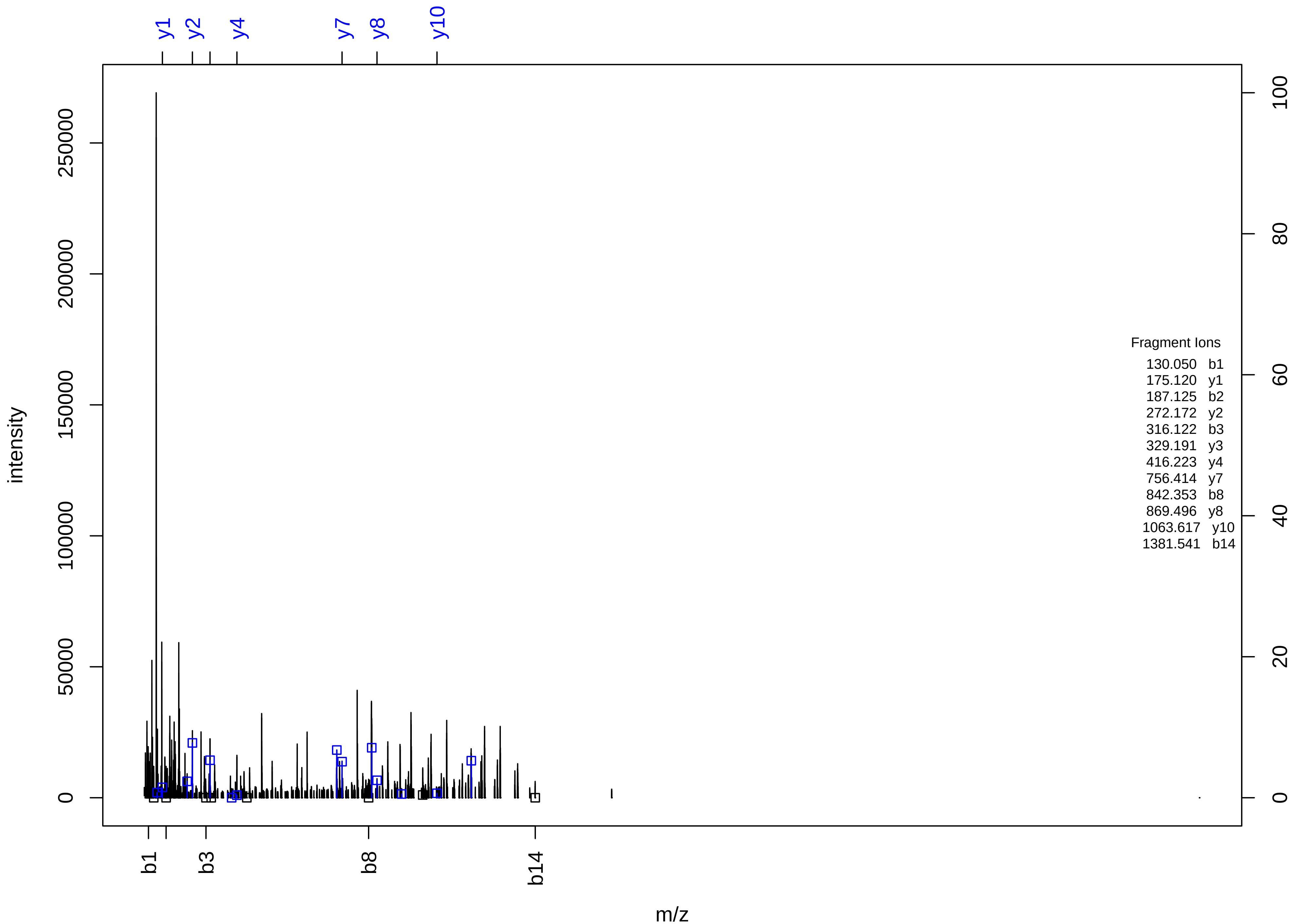

EGQDPNLSSAGRAPTLQRPPHQPPLRPSSGPR / File: sydney_ip_14.raw; SpectrumID: ; scans: 16213

LDIQ^M*IM*IM*NR

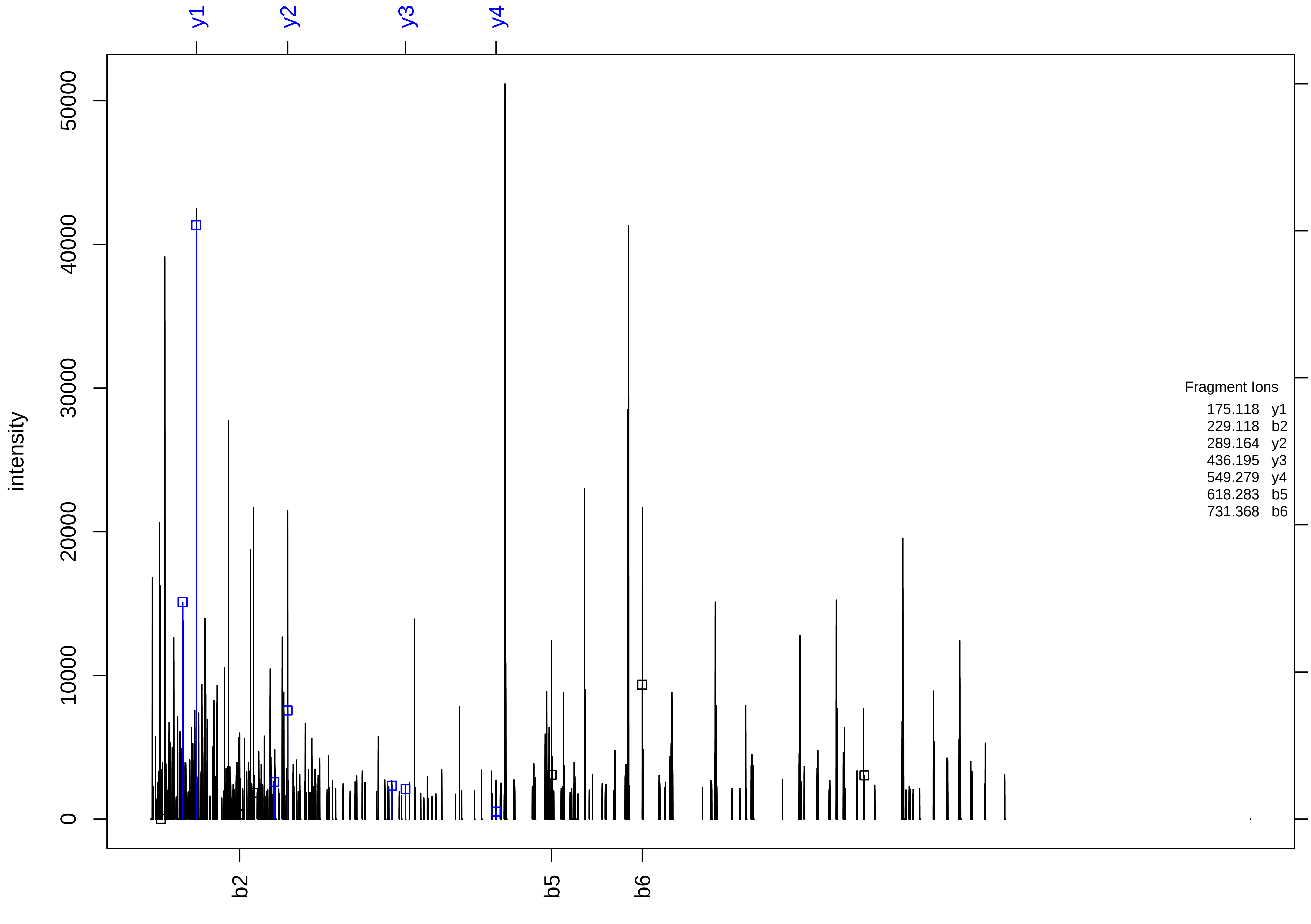

LDIQMIMIMNR / File: sydney_ip_14.raw; SpectrumID: ; scans: 5705

RAAELEVQRLQ^Q^R

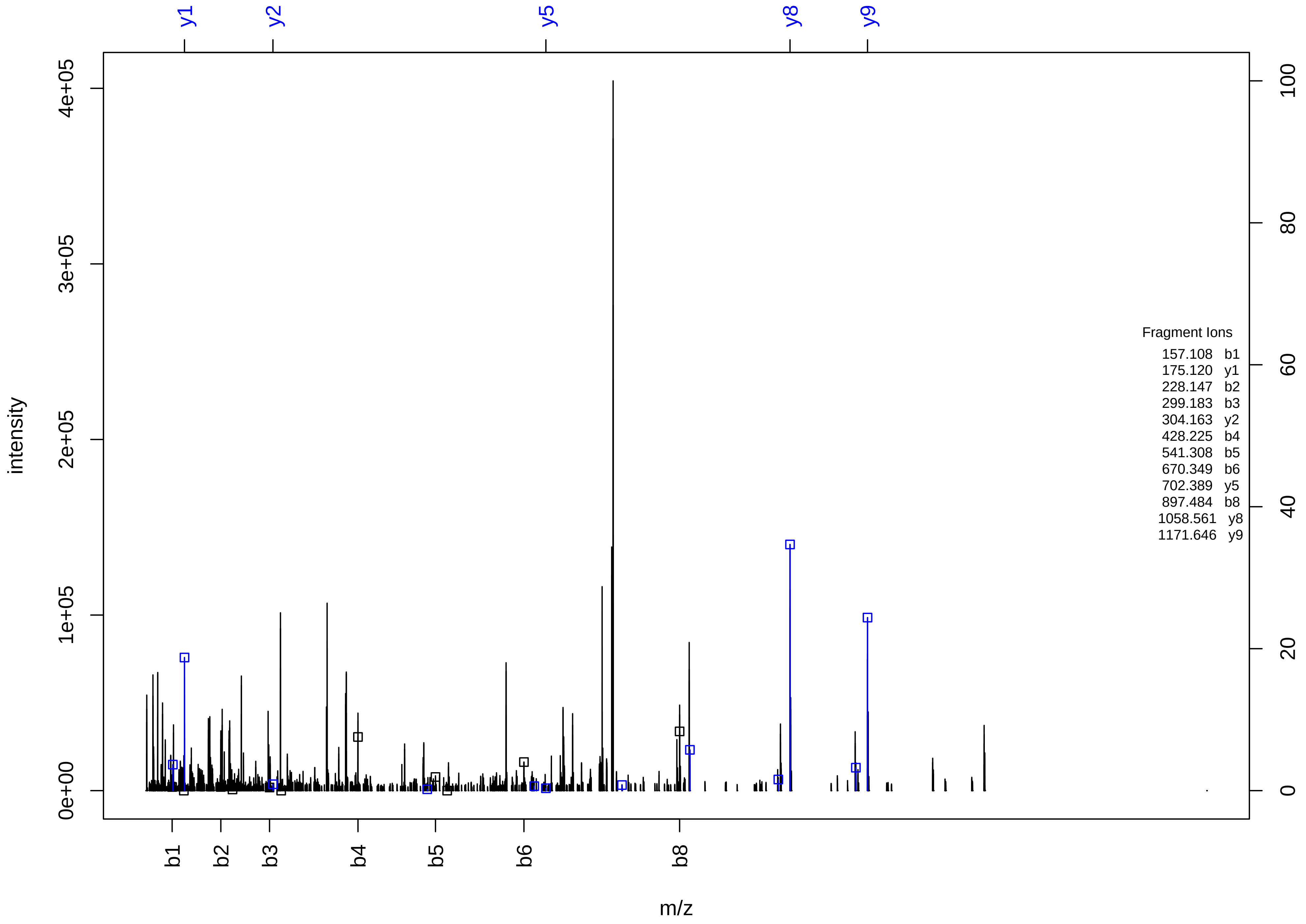

RAAELEVQRLQQR / File: sydney_ip_14.raw; SpectrumID: ; scans: 12665

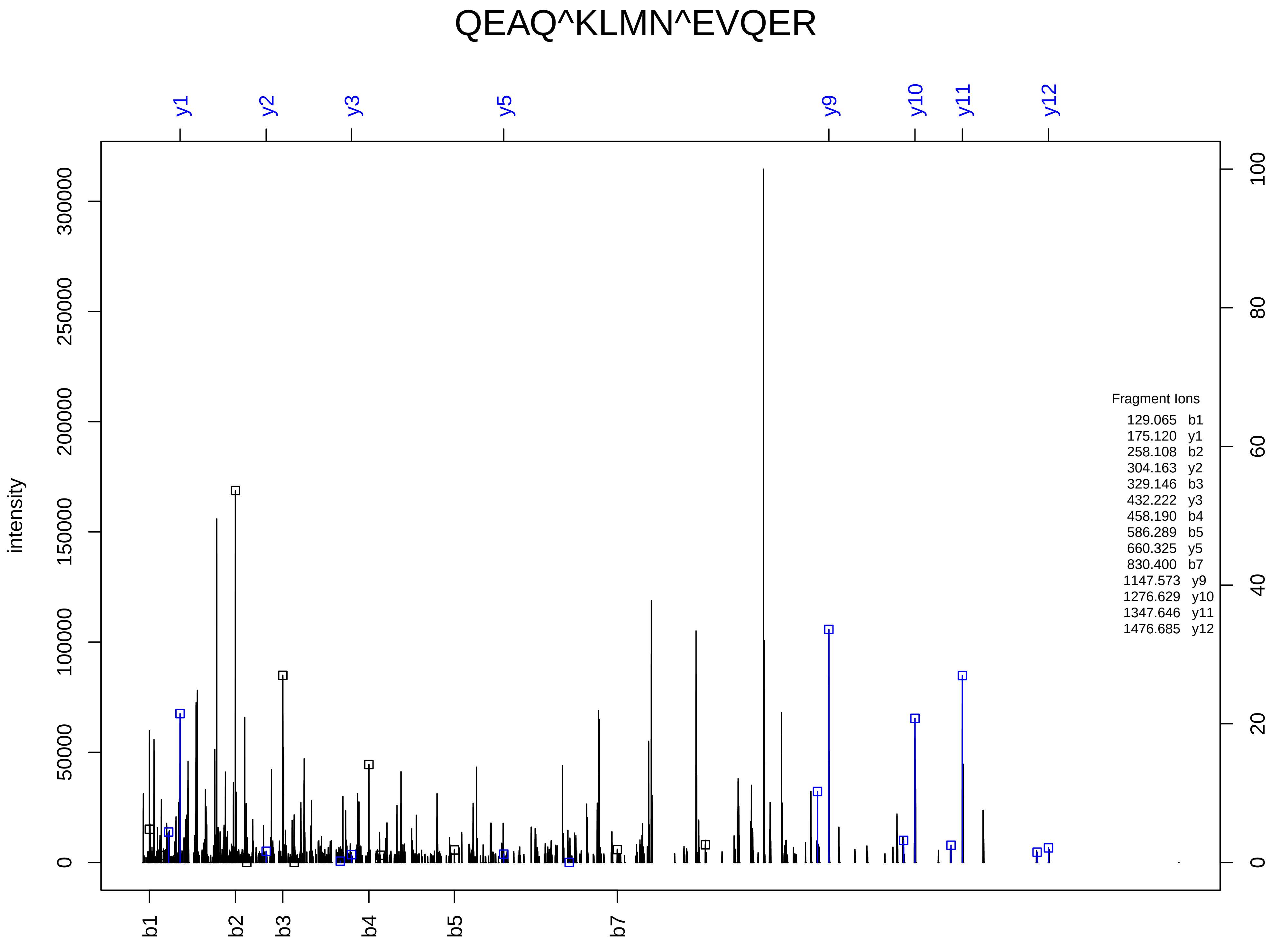

QEAQKLMNEVQER / File: sydney_ip_14.raw; SpectrumID: ; scans: 7860

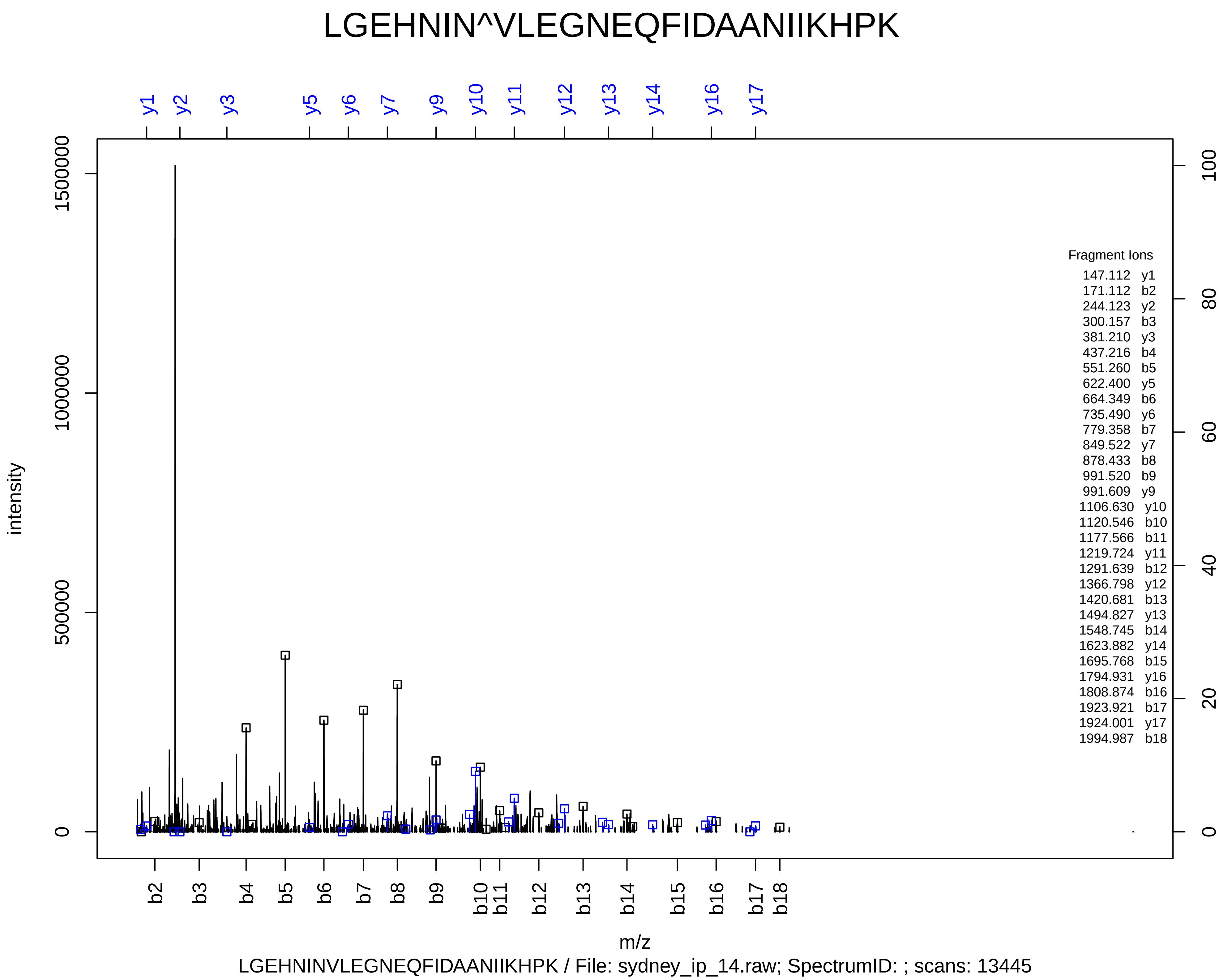
LGEHNIN^VLEGNEQFIDAANIIKHPK
y1 y2 y3 y5 y6 y7 y9 y10 y11 y12 y13 y14 y16 y17
intensity
0
500000
1000000
1500000
0
20
40
60
80
100
b2 b3 b4 b5 b6 b7 b8 b9 b10 b11 b12 b13 b14 b15 b16 b17 b18
m/z
Fragment Ions
147.112 y1
171.112 b2
244.123 y2
300.157 b3
381.210 y3
437.216 b4
551.260 b5
622.400 y5
664.349 b6
735.490 y6
779.358 b7
849.522 y7
878.433 b8
991.520 b9
991.609 y9
1106.630 y10
1120.546 b10
1177.566 b11
1219.724 y11
1291.639 b12
1366.798 y12
1420.681 b13
1494.827 y13
1548.745 b14
1623.882 y14
1695.768 b15
1794.931 y16
1808.874 b16
1923.921 b17
1924.001 y17
1994.987 b18
LGEHNINVLEGNEQFIDAANIIKHPK / File: sydney_ip_14.raw; SpectrumID: ; scans: 13445

M*KKGGGASGGVSVGGSGVGGQ^QPPPQ^Q^PR

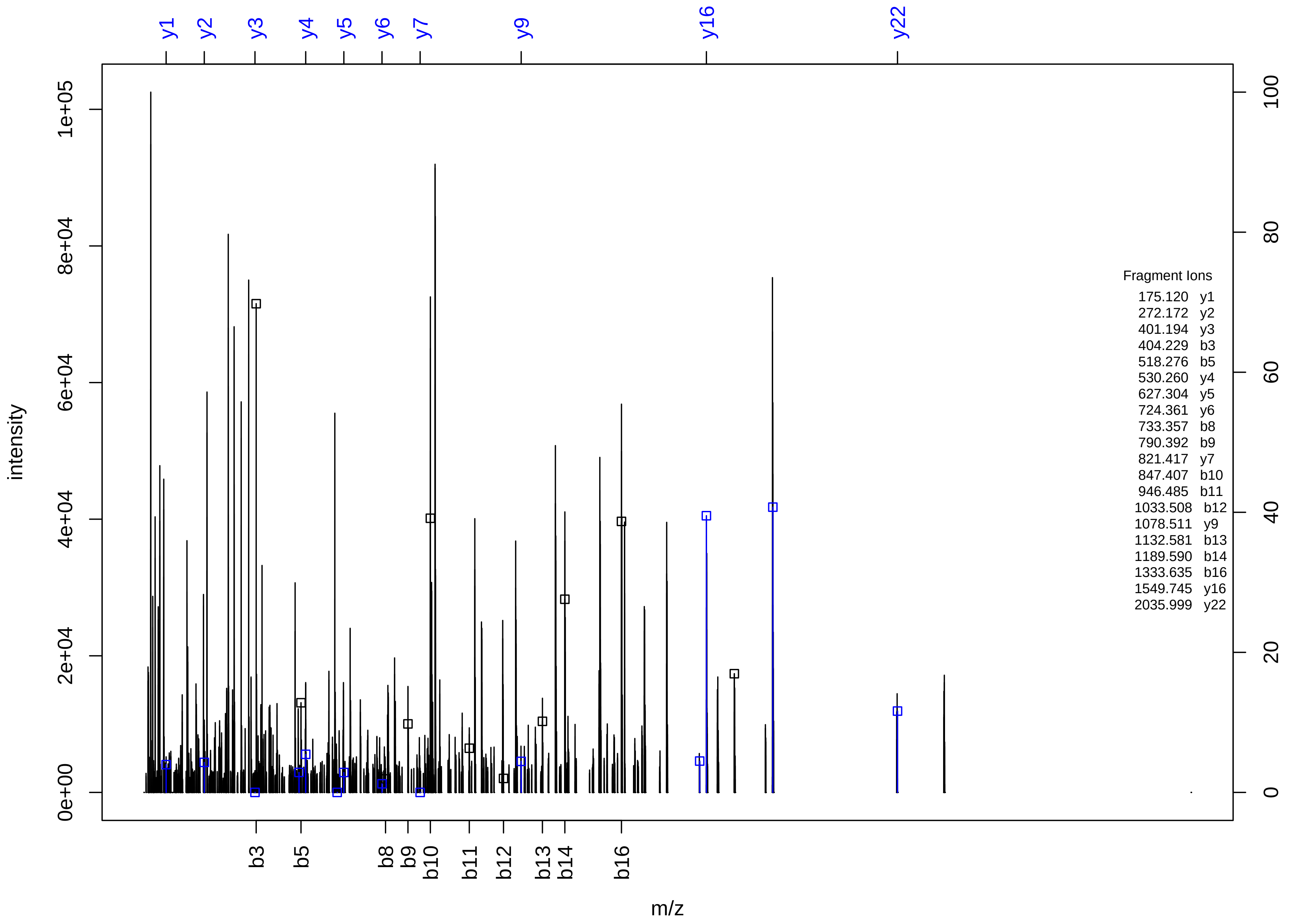

MKKGGGASGGVSVGGSGVGGQQPPPQQPR / File: sydney_ip_14.raw; SpectrumID: ; scans: 12995

M*RSQ^Q^STTQ^RR

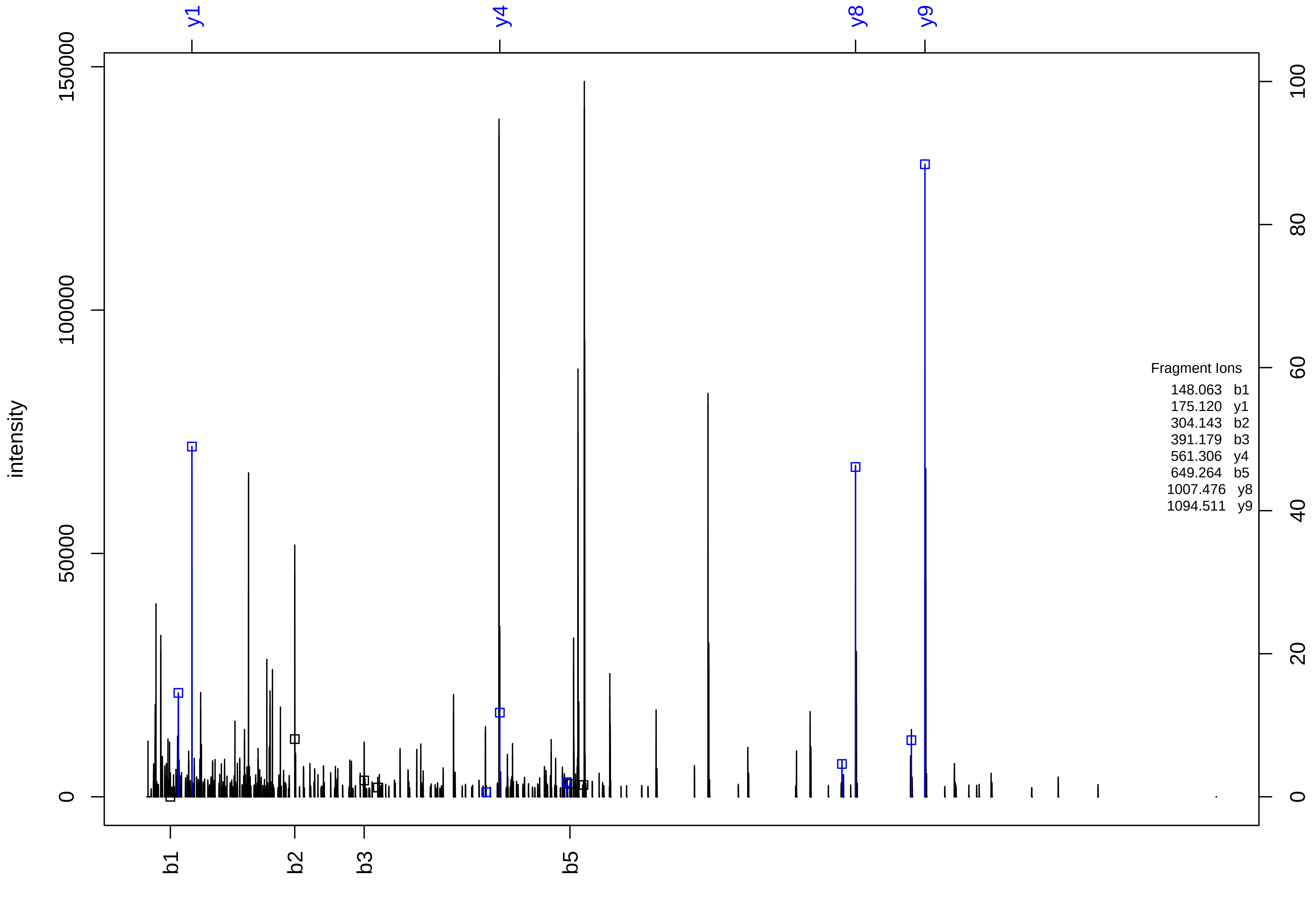

MRSQQSTTQRR / File: sydney_ip_15.raw; SpectrumID: ; scans: 3504

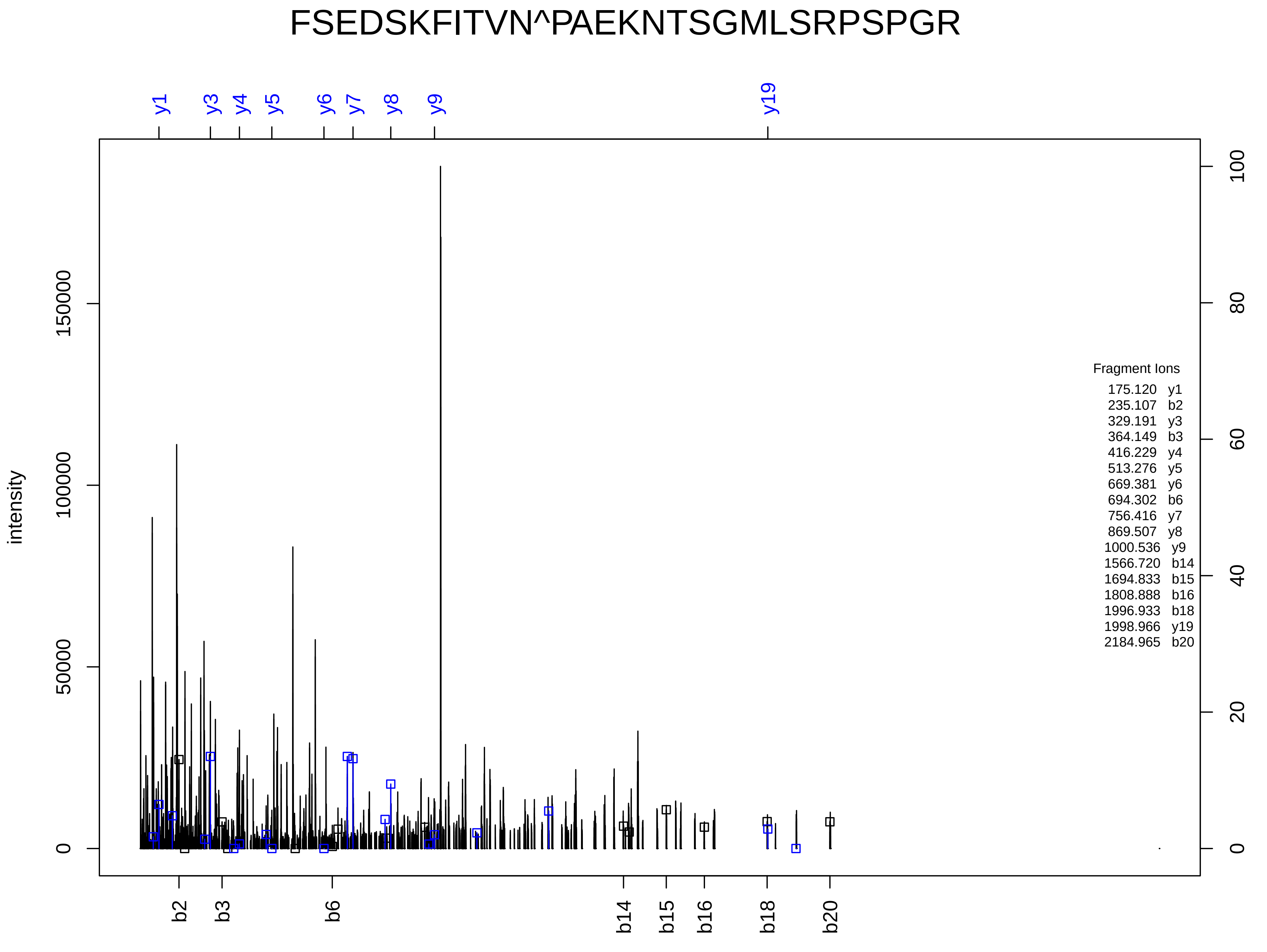

FSEDSKFITVNPAEKNTSGMLSRPSPGR / File: sydney_ip_15.raw; SpectrumID: ; scans: 17869

EALSAAMEEAQAWR

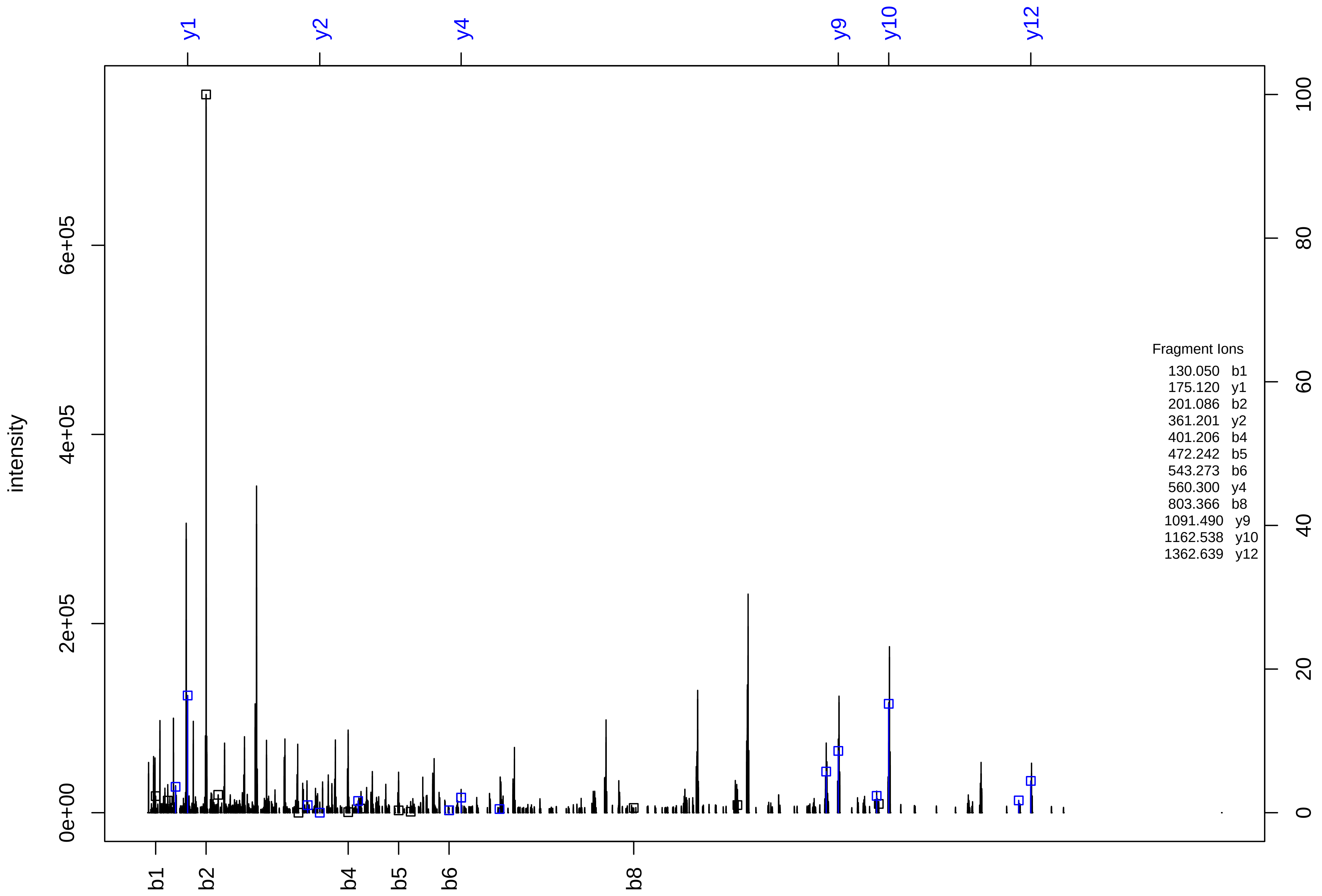

EALSAAMEEAQAWR / File: sydney_ip_15.raw; SpectrumID: ; scans: 8099

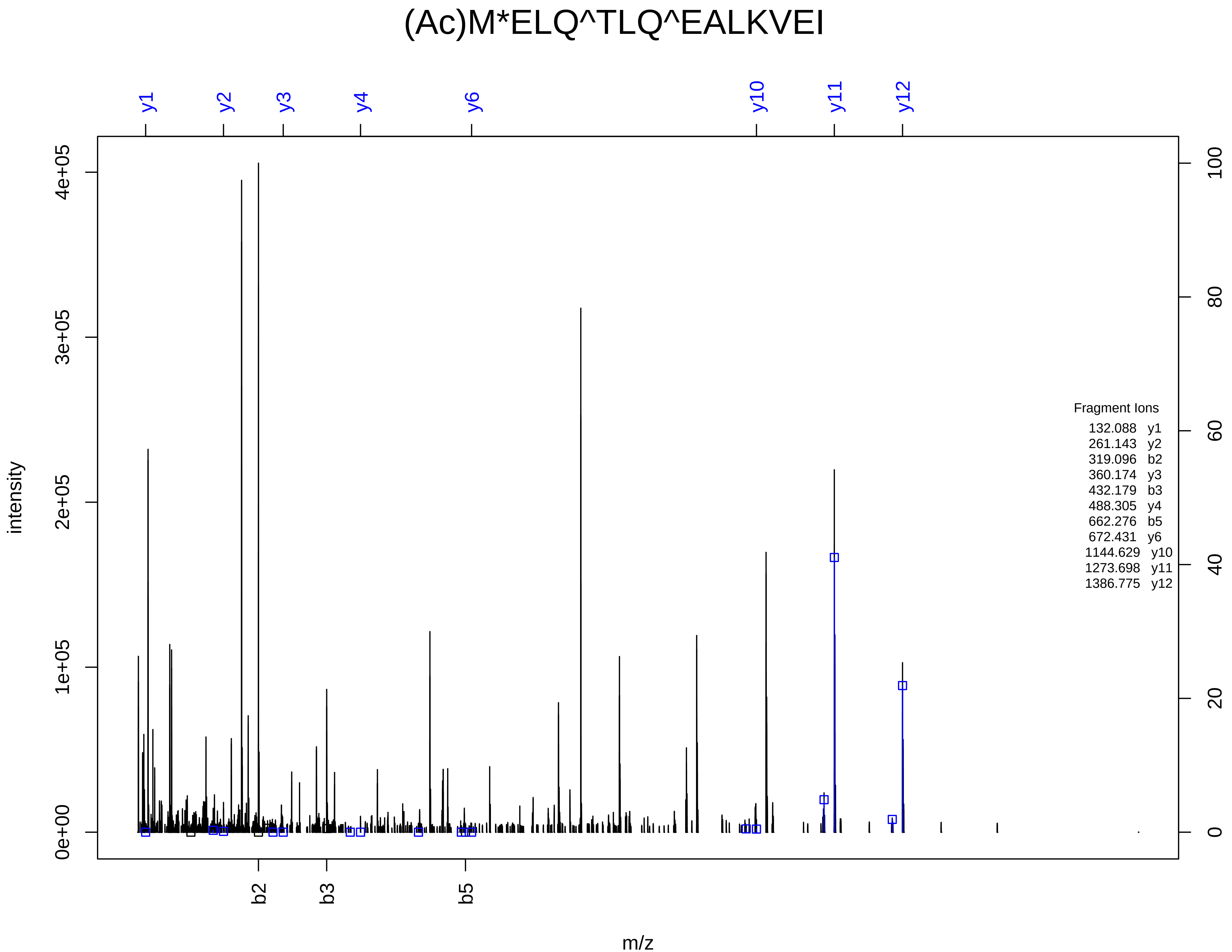

MELQTLQEALKVEI / File: sydney_ip_16.raw; SpectrumID: ; scans: 10652

N^QNLRN^LQ^VSN^N^K

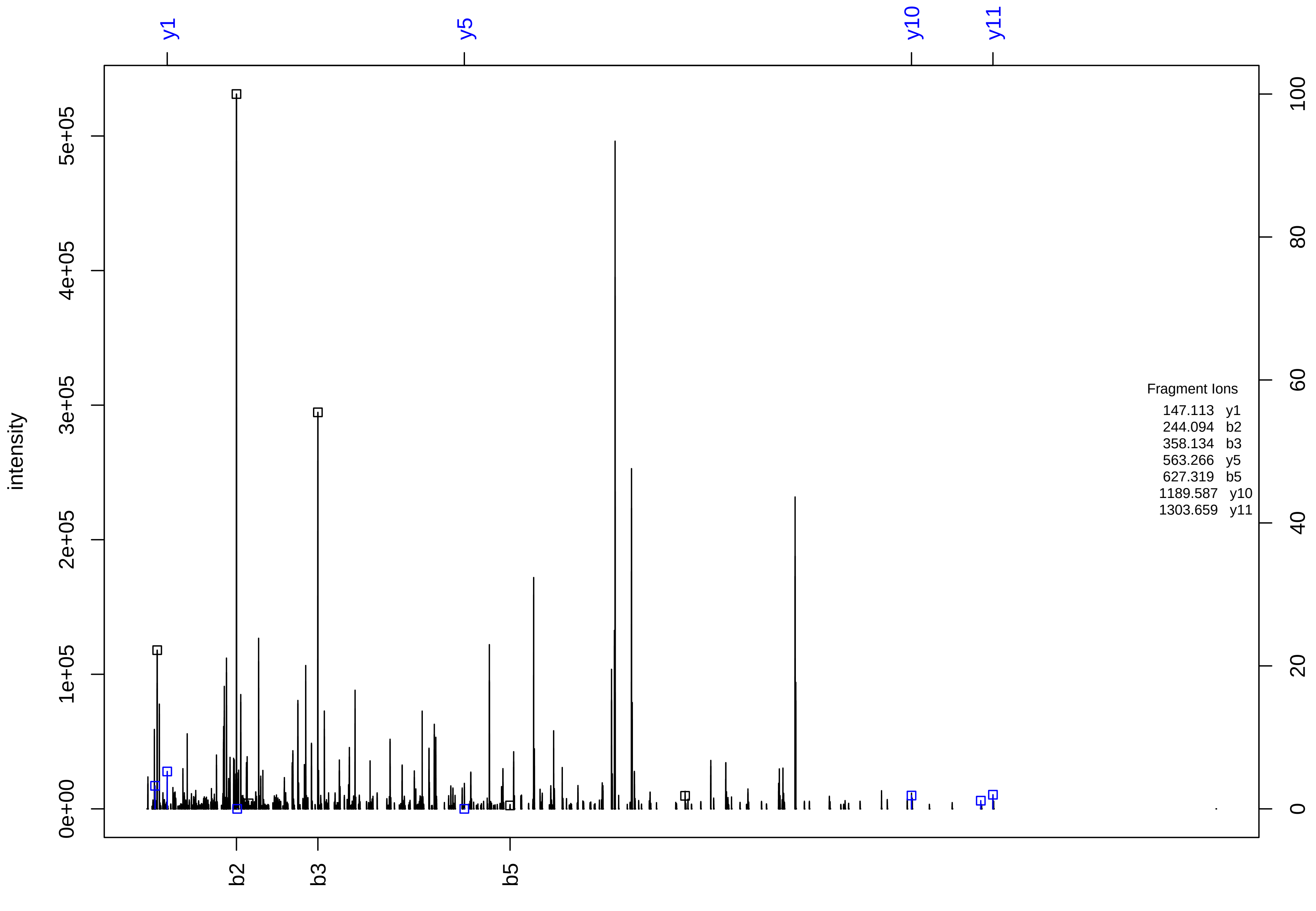

NQNLRNLQVSNNK / File: sydney_ip_16.raw; SpectrumID: ; scans: 5227

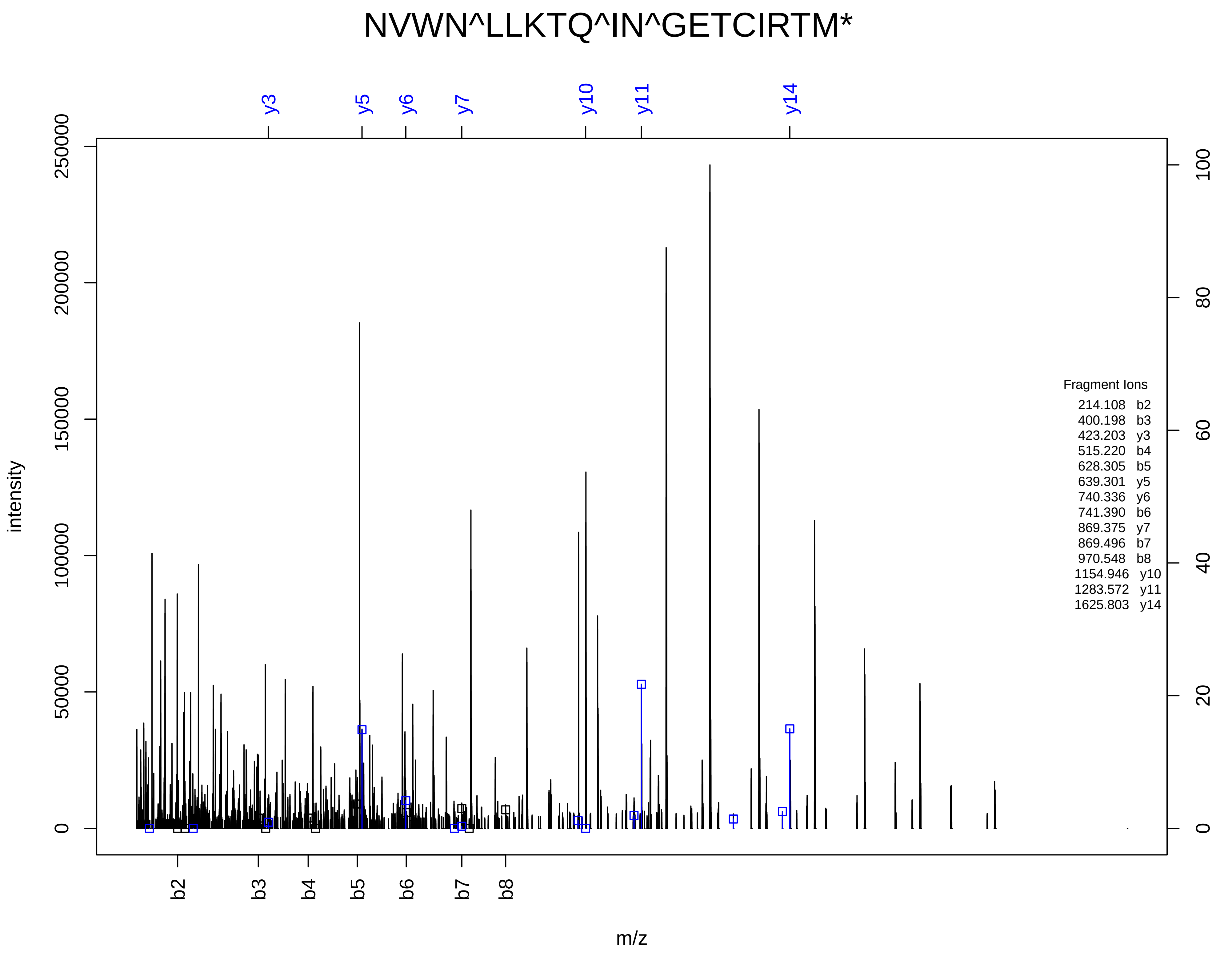
NVWN^LLKTQ^IN^GETCIRTM*
y3
y5
y6
y7
y10
y11
y14
intensity
0
50000
100000
150000
200000
250000
0
20
40
60
80
100
b2
b3
b4
b5
b6
b7
b8
Fragment Ions
214.108 b2
400.198 b3
423.203 y3
515.220 b4
628.305 b5
639.301 y5
740.336 y6
741.390 b6
869.375 y7
869.496 b7
970.548 b8
1154.946 y10
1283.572 y11
1625.803 y14
m/z
NVWNLLKTQINGETCIRTM / File: sydney_ip_16.raw; SpectrumID: ; scans: 16996

KVN^AIFQ^LDITK

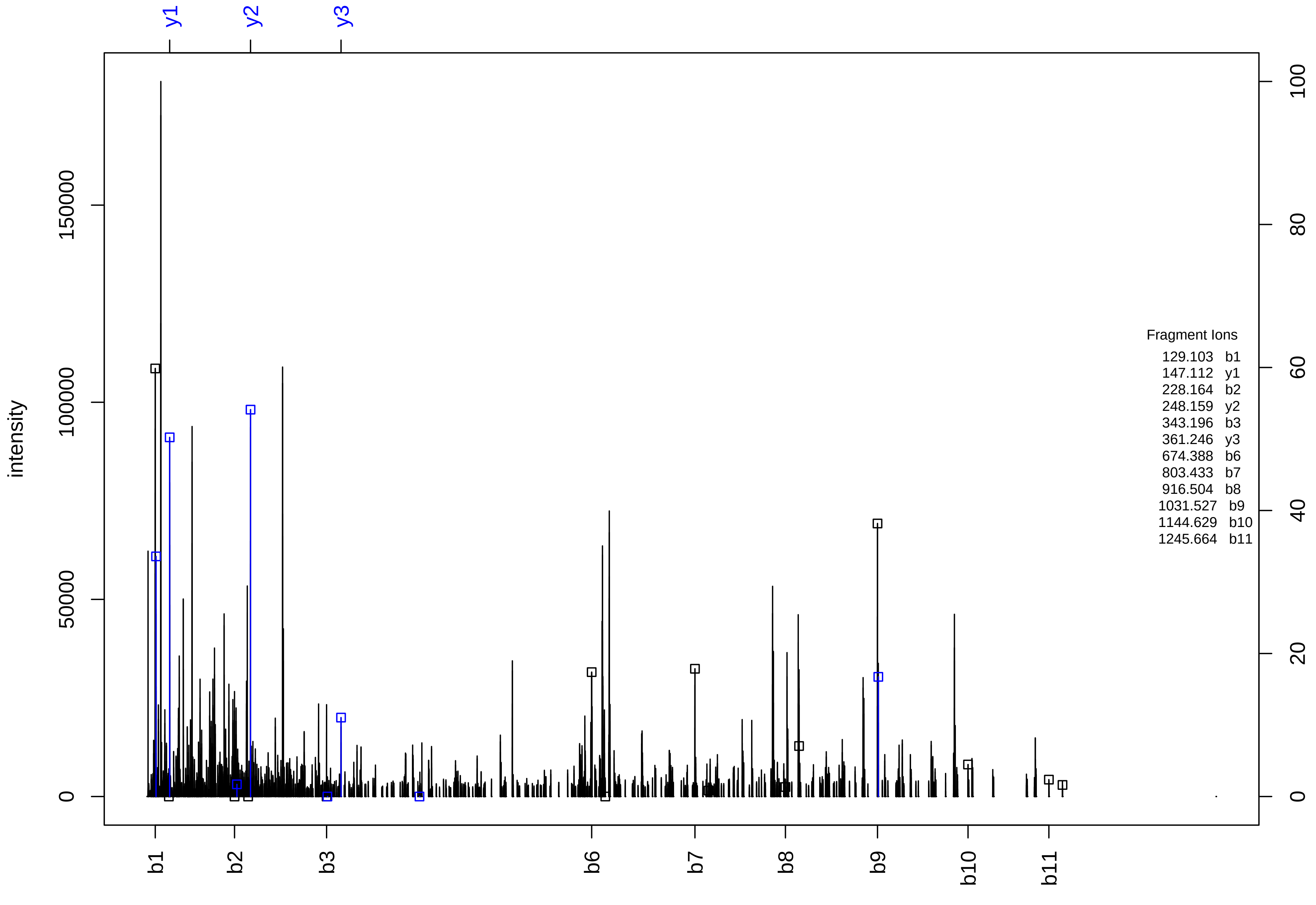

KVNAIFQLDITK / File: sydney_ip_16.raw; SpectrumID: ; scans: 9611

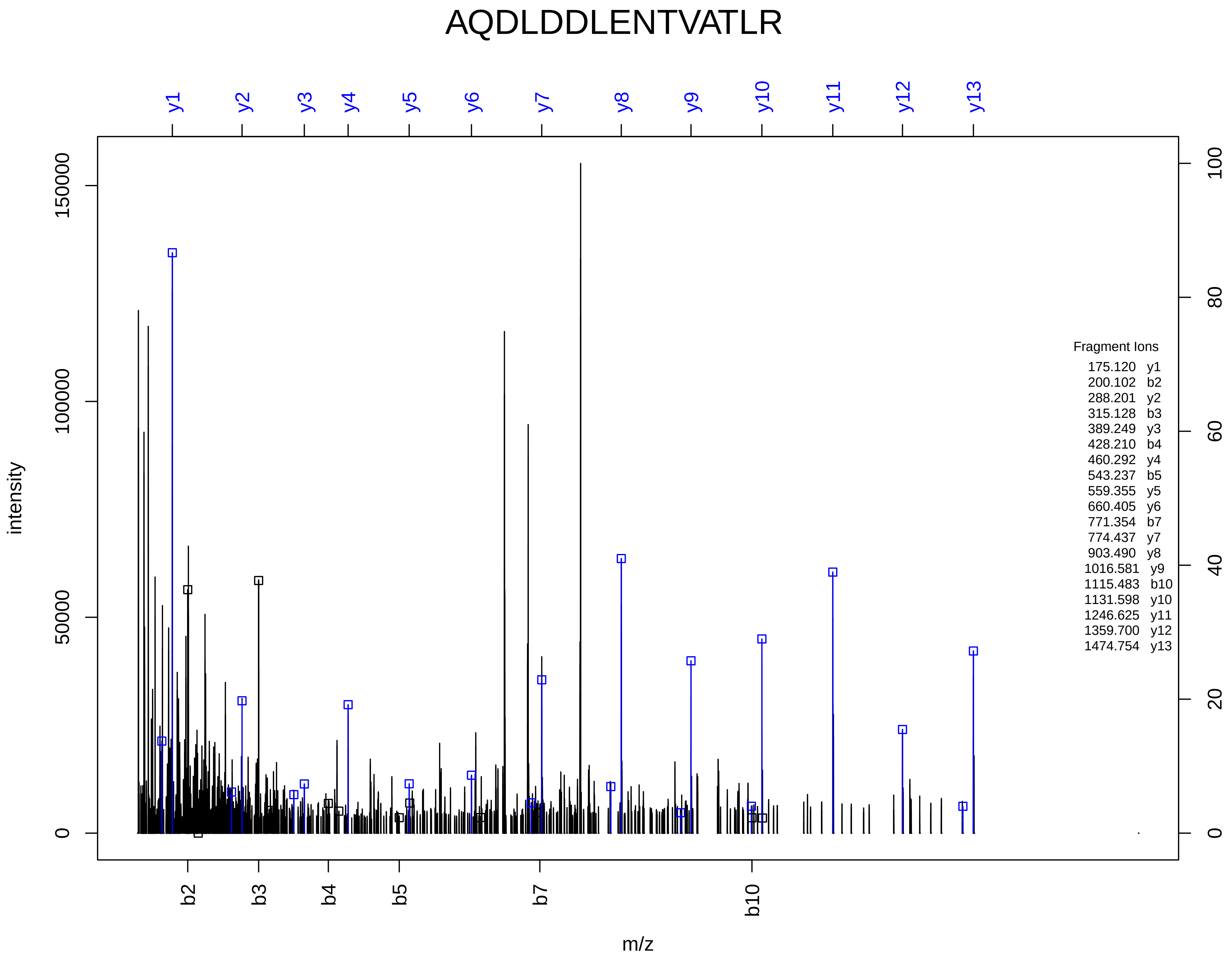

AQDLDDLENTVATLR / File: sydney_ip_17.raw; SpectrumID: ; scans: 14775

ASEPEAQGEVTACSDQK

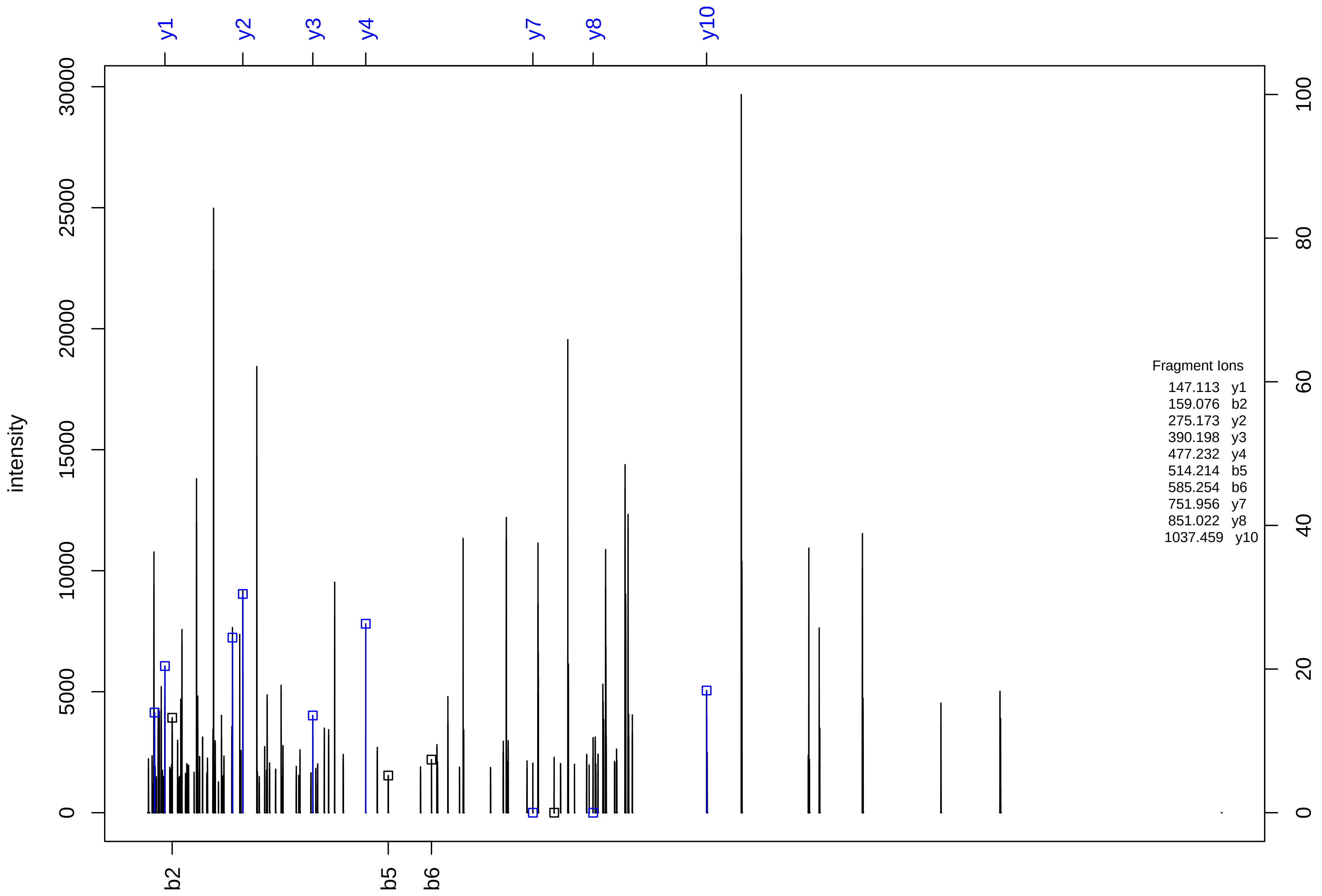

ASEPEAQGEVTACSDQK / File: sydney_ip_17.raw; SpectrumID: ; scans: 2181

VLEDPAIVSDENFGPVK

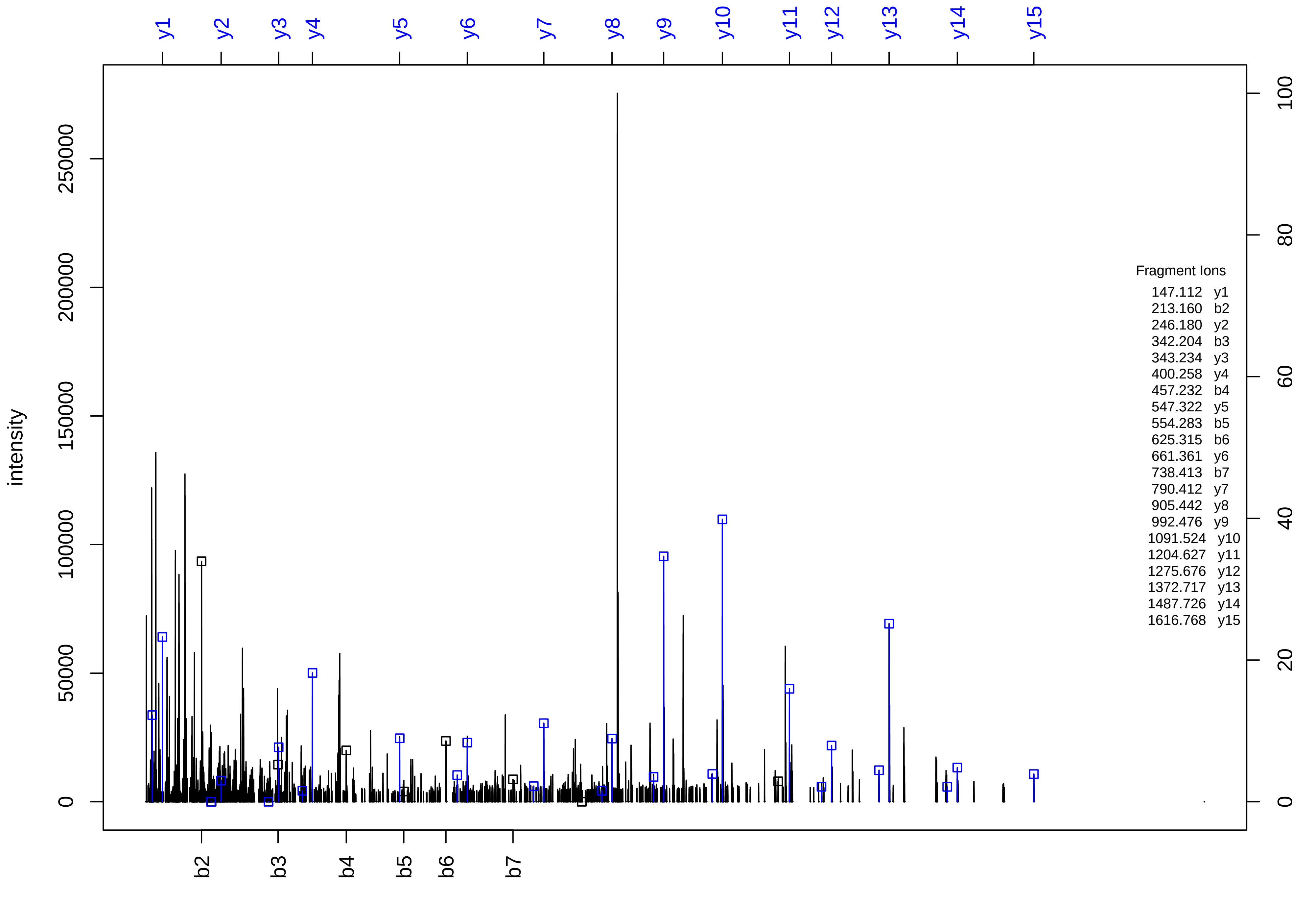

VLEDPAIVSDENFGPVK / File: sydney_ip_17.raw; SpectrumID: ; scans: 13459

HPDGEEDGNSDQSQASEATGGR

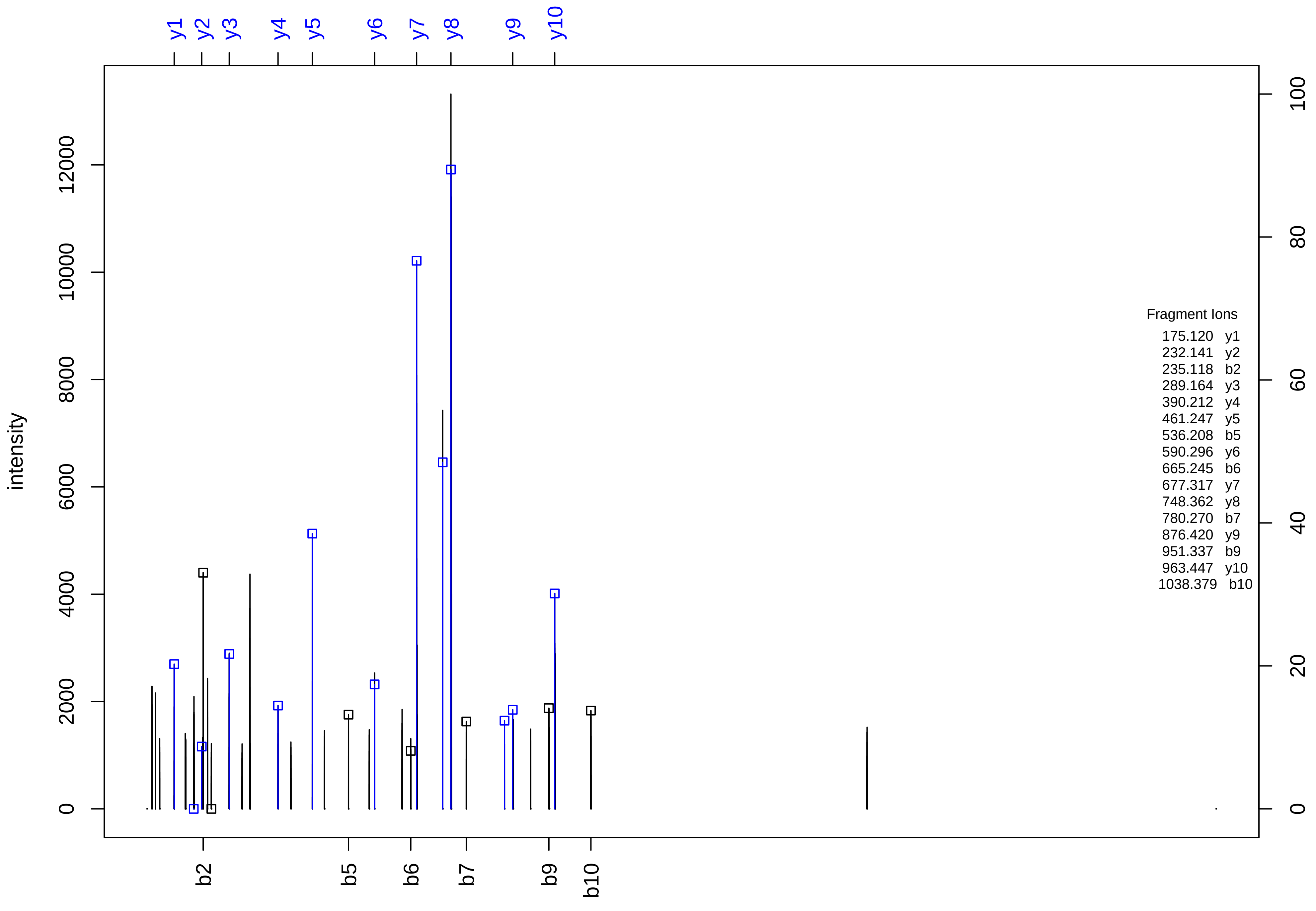

HPDGEEDGNSDQSQASEATGGR / File: sydney_ip_17.raw; SpectrumID: ; scans: 1032

EAPPM*EKPEVVK

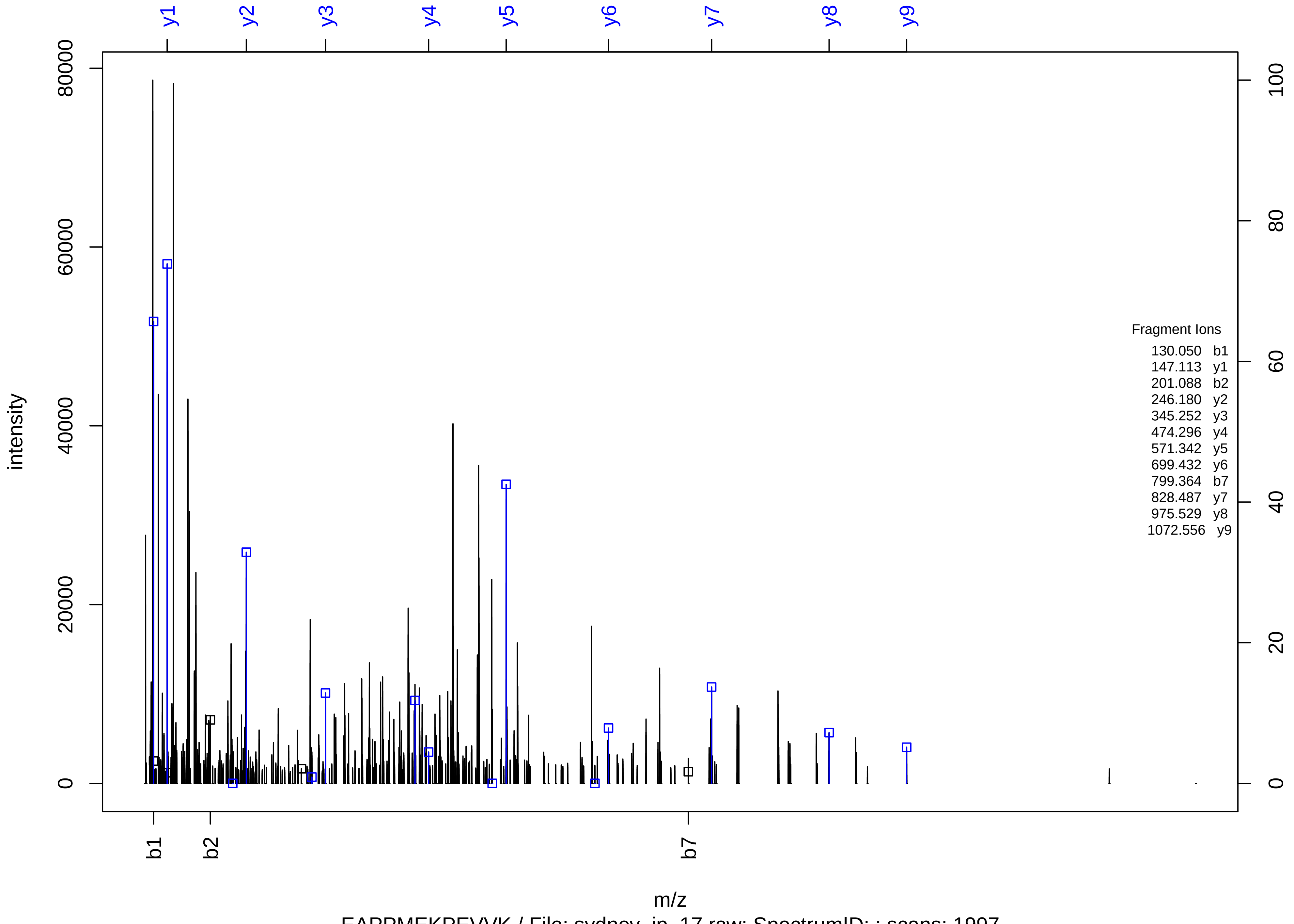

EAPPMEKPEVVK / File: sydney_ip_17.raw; SpectrumID: ; scans: 1997

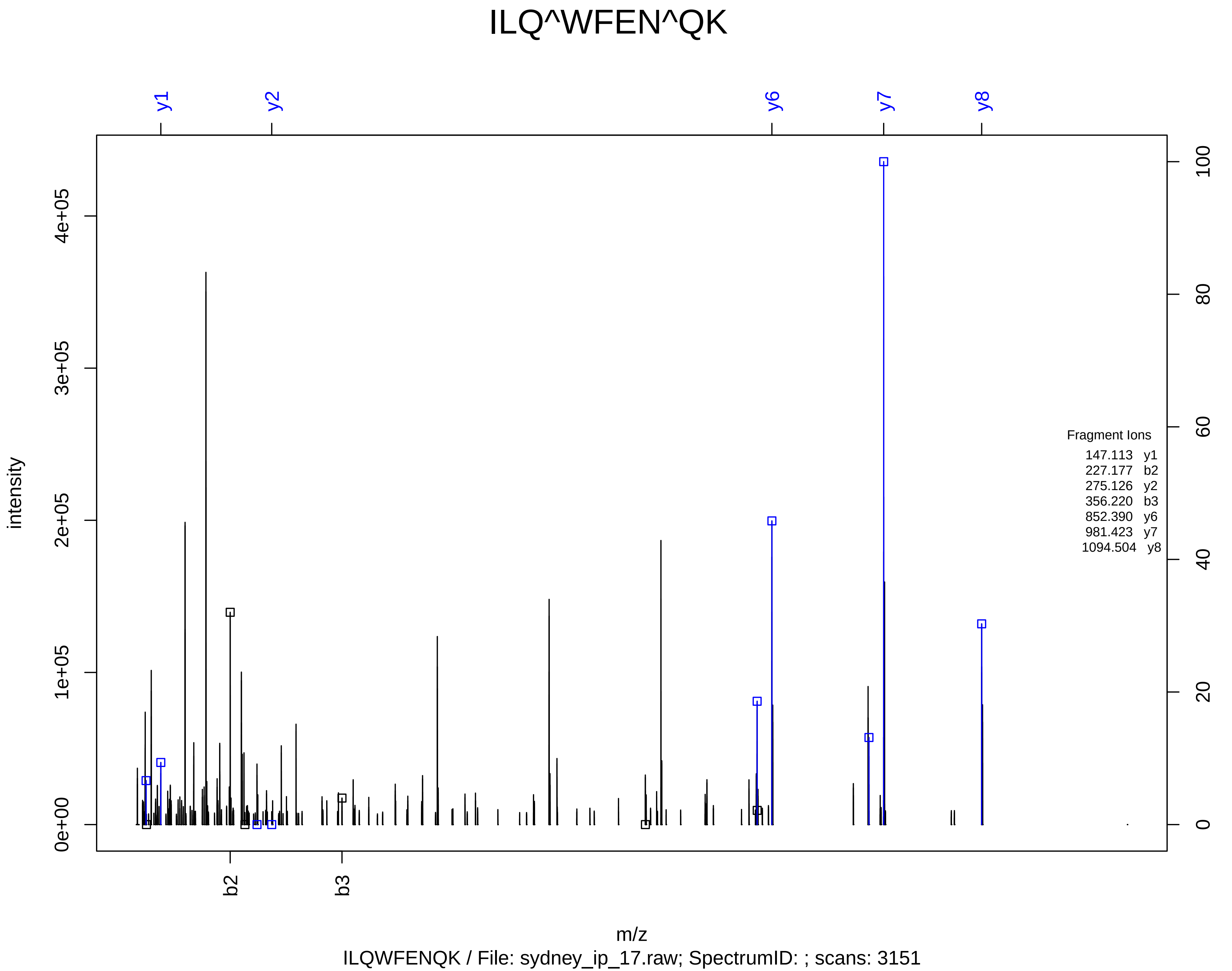
ILQ^WFEN^QK
y1
y2
y6
y7
y8
intensity
0e+00
1e+05
2e+05
3e+05
4e+05
0
20
40
60
80
100
Fragment Ions
147.113 y1
227.177 b2
275.126 y2
356.220 b3
852.390 y6
981.423 y7
1094.504 y8
b2
b3
m/z
ILQWFENQK / File: sydney_ip_17.raw; SpectrumID: ; scans: 3151

TAPAPTSSSTININTSTSK

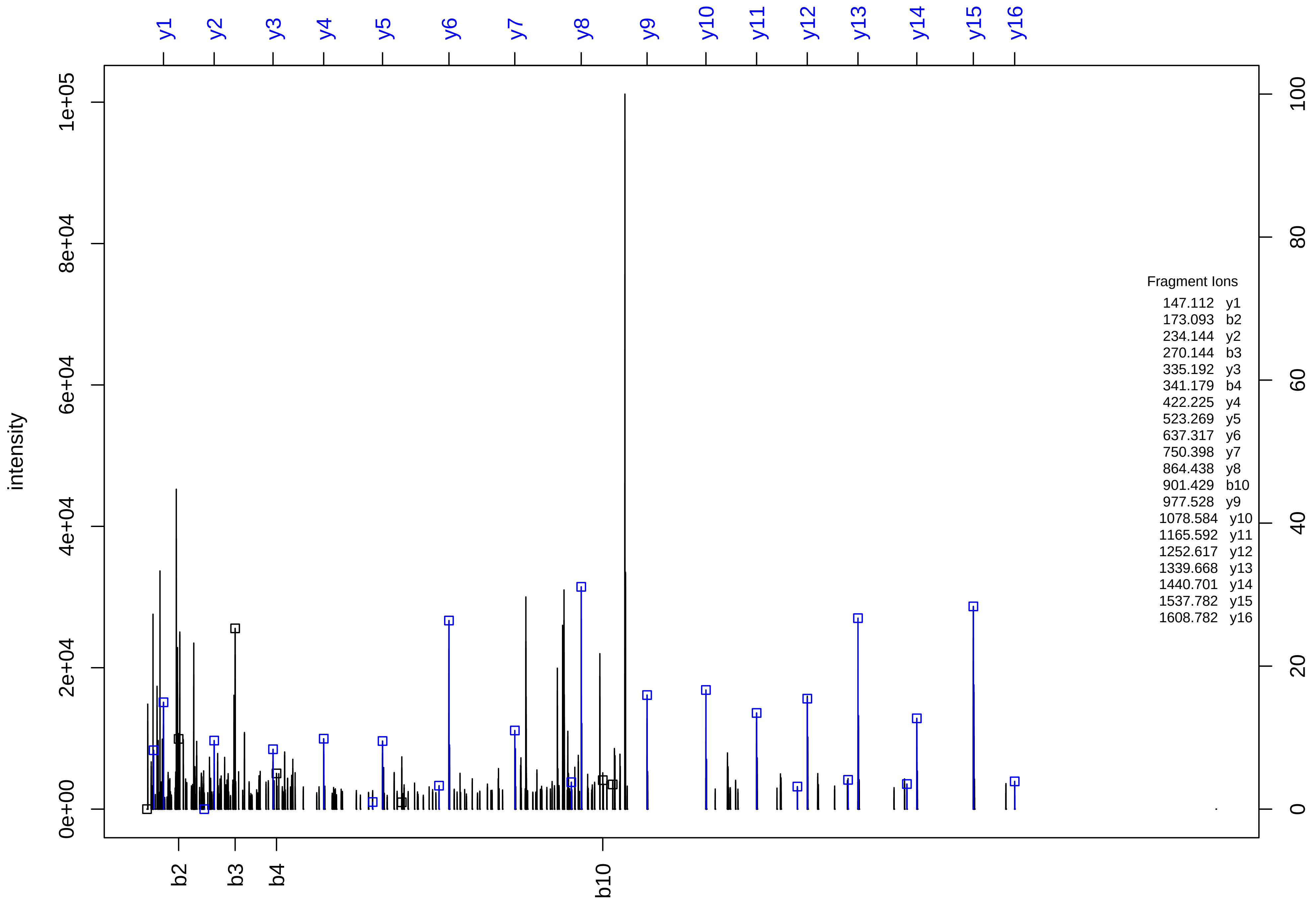

TAPAPTSSSTININTSTSK / File: sydney_ip_17.raw; SpectrumID: ; scans: 5260

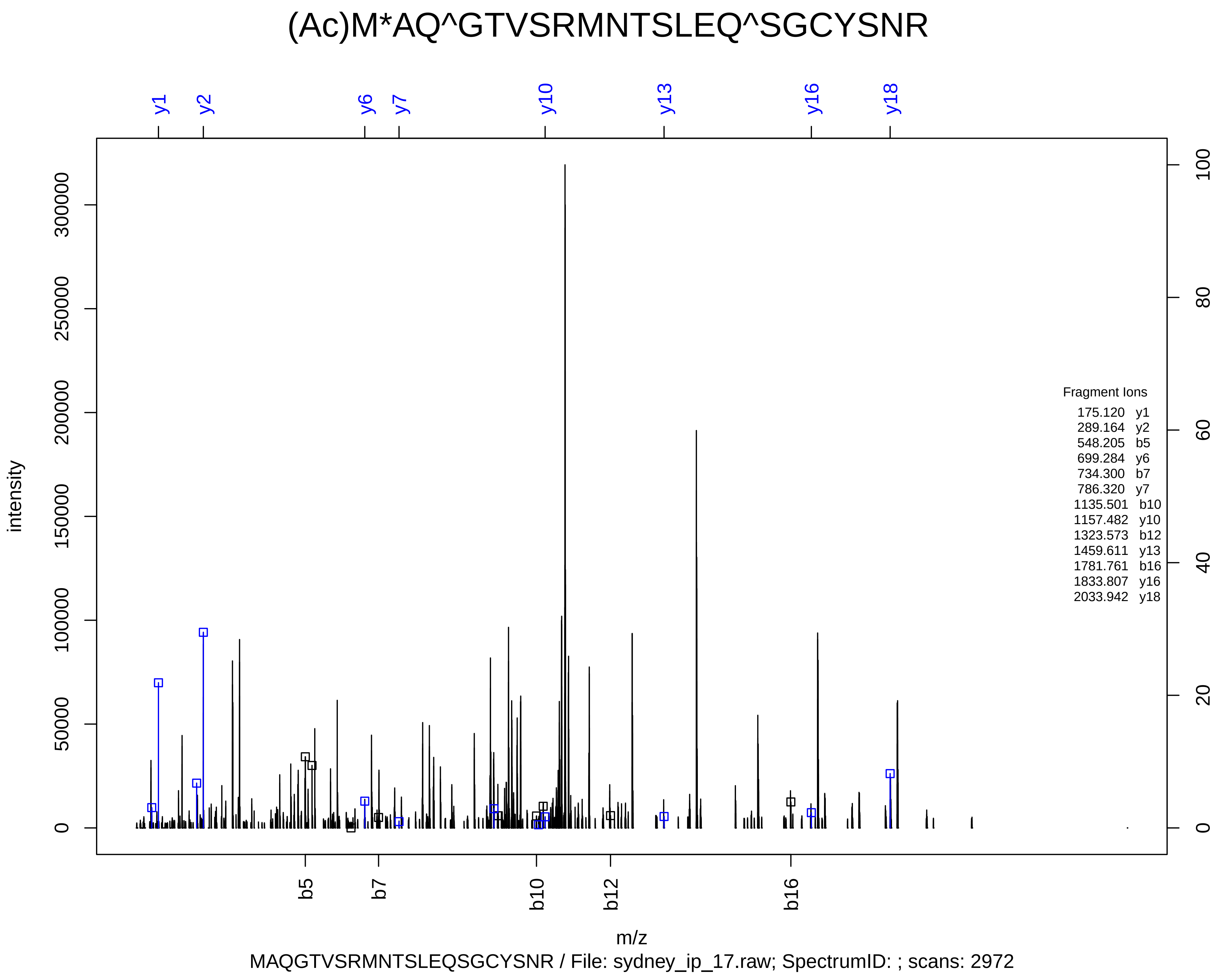
(Ac)M*AQ^GTVSRMNTSLEQ^SGCYSNR
y1
y2
y6
y7
y10
y13
y16
y18
intensity
0
50000
100000
150000
200000
250000
300000
0
20
40
60
80
100
b5
b7
b10
b12
b16
m/z
Fragment Ions
175.120 y1
289.164 y2
548.205 b5
699.284 y6
734.300 b7
786.320 y7
1135.501 b10
1157.482 y10
1323.573 b12
1459.611 y13
1781.761 b16
1833.807 y16
2033.942 y18
MAQGTVSRMNTSLEQSGCYSNR / File: sydney_ip_17.raw; SpectrumID: ; scans: 2972

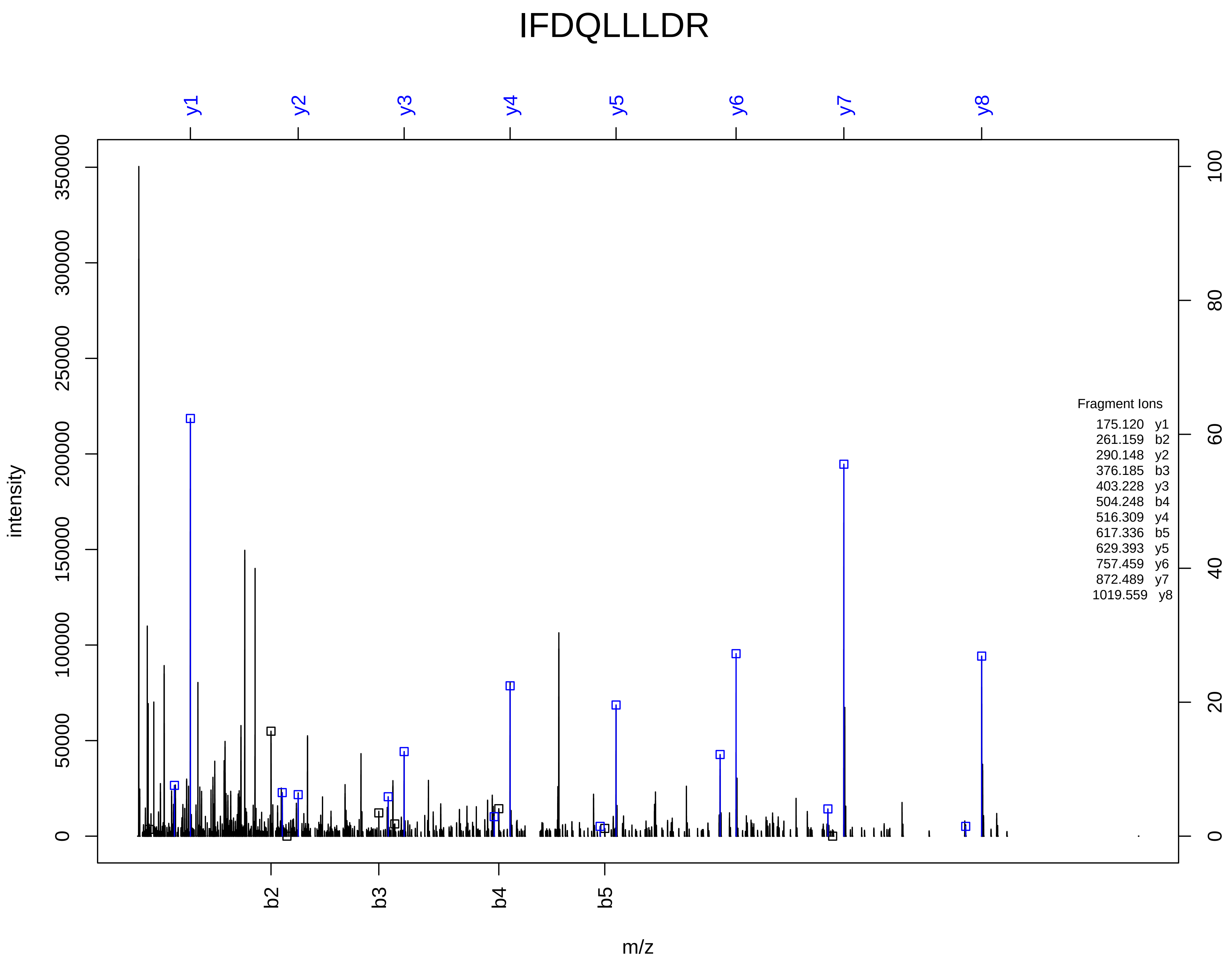

IFDQLLLDR / File: sydney_ip_17.raw; SpectrumID: ; scans: 15316

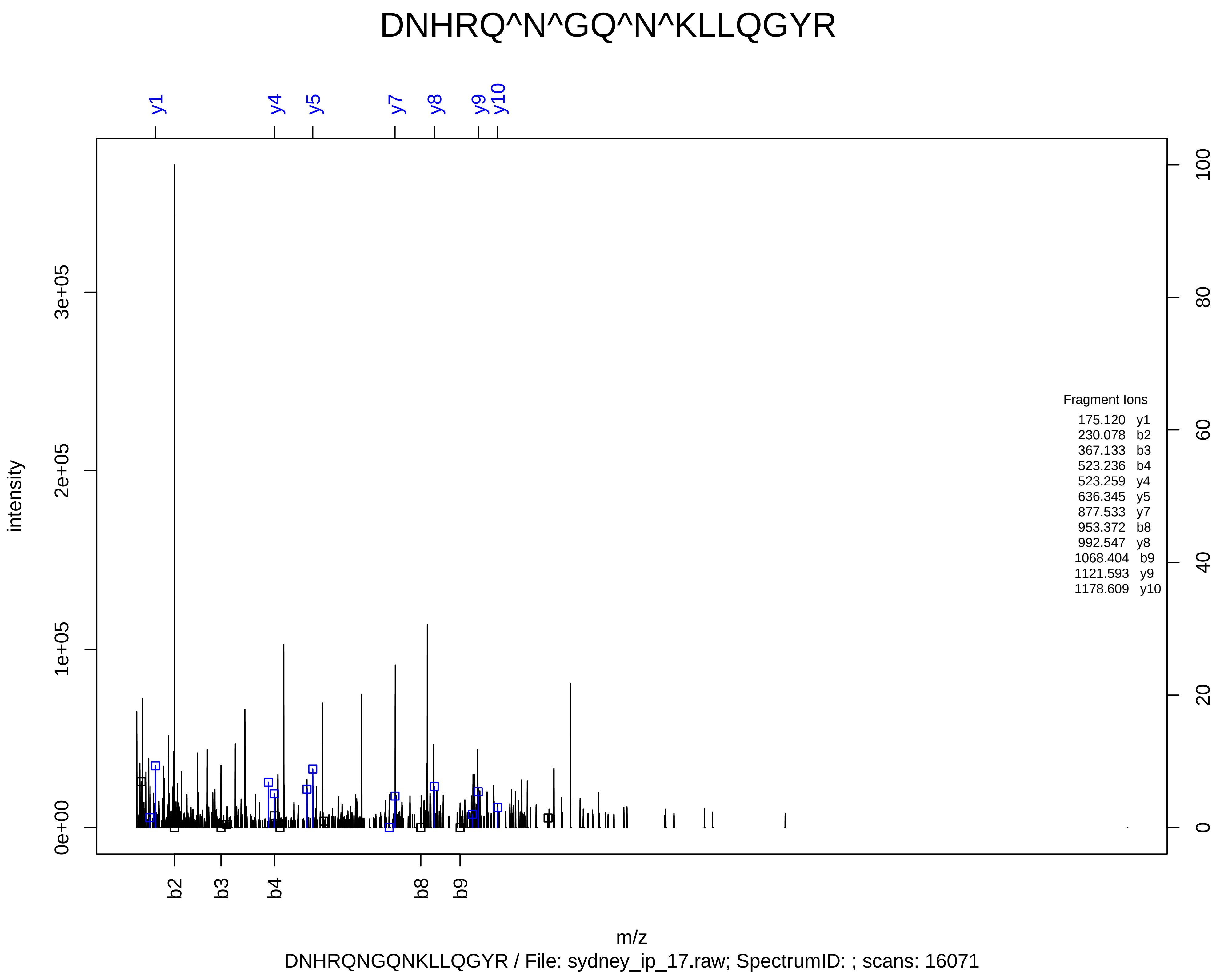
DNHRQ^N^GQ^N^KLLQGYR
y1
y4
y5
y7
y8
y9
y10
intensity
0e+00
1e+05
2e+05
3e+05
0
20
40
60
80
100
Fragment Ions
175.120 y1
230.078 b2
367.133 b3
523.236 b4
523.259 y4
636.345 y5
877.533 y7
953.372 b8
992.547 y8
1068.404 b9
1121.593 y9
1178.609 y10
b2
b3
b4
b8
b9
m/z
DNHRQNGQNKLLQGYR / File: sydney_ip_17.raw; SpectrumID: ; scans: 16071

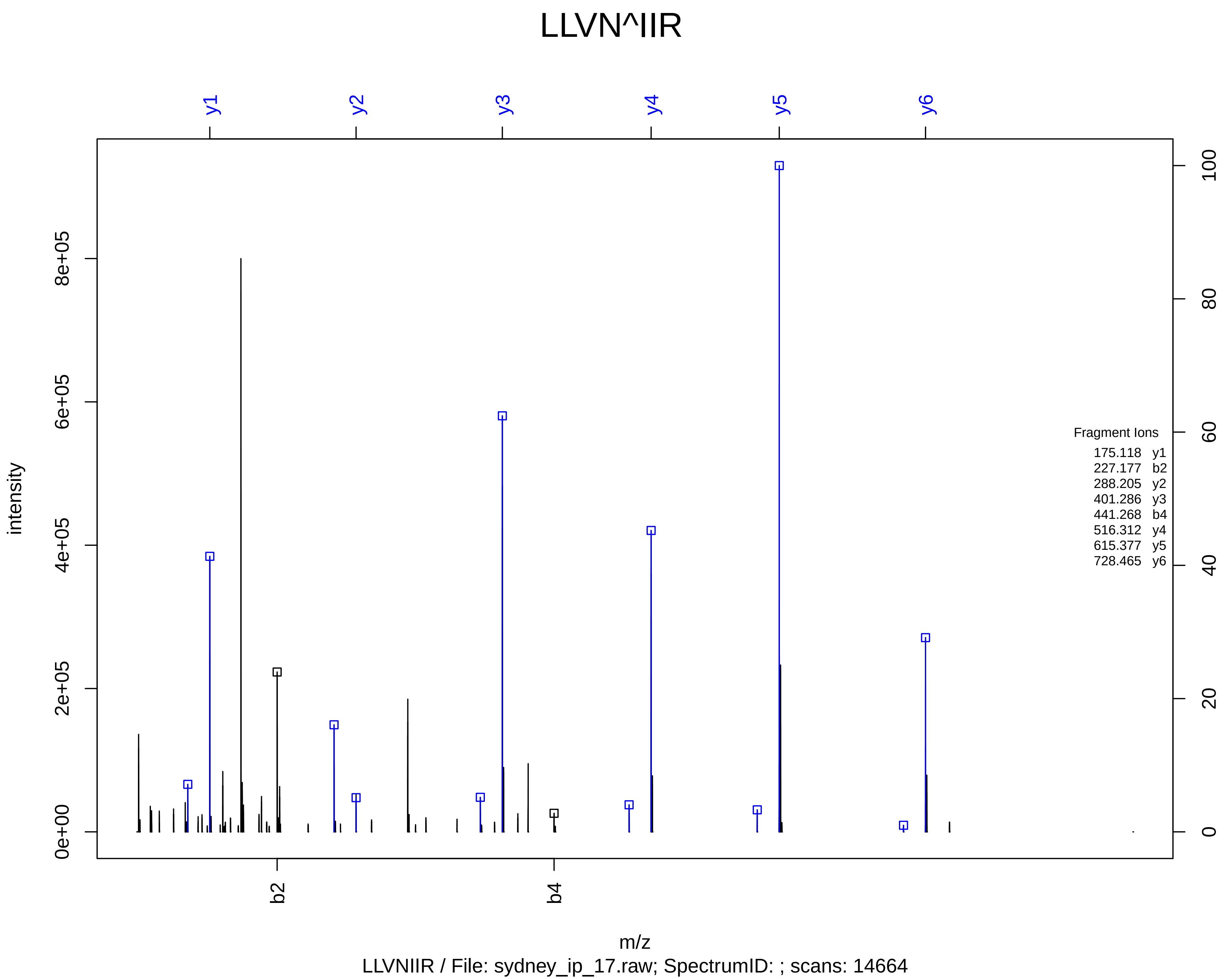

LLVN^IIR
y1
y2
y3
y4
y5
y6
intensity
0e+00
2e+05
4e+05
6e+05
8e+05
0
20
40
60
80
100
Fragment Ions
175.118 y1
227.177 b2
288.205 y2
401.286 y3
441.268 b4
516.312 y4
615.377 y5
728.465 y6
b2
b4
m/z
LLVNIIR / File: sydney_ip_17.raw; SpectrumID: ; scans: 14664

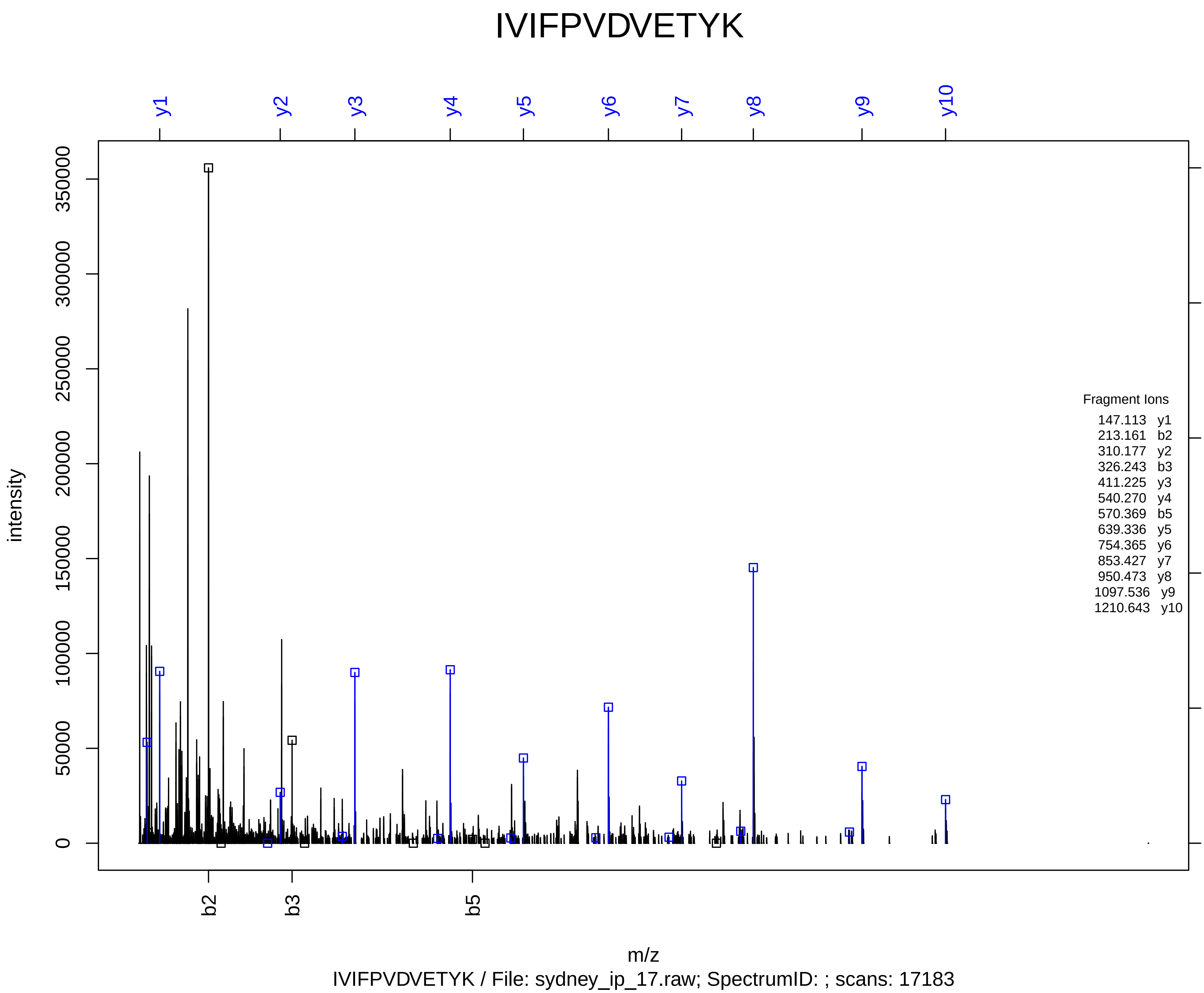
IVIFPVDVETYK
y1
y2
y3
y4
y5
y6
y7
y8
y9
y10
b2
b3
b5
intensity
m/z
Fragment Ions
147.113 y1
213.161 b2
310.177 y2
326.243 b3
411.225 y3
540.270 y4
570.369 b5
639.336 y5
754.365 y6
853.427 y7
950.473 y8
1097.536 y9
1210.643 y10
IVIFPVDVETYK / File: sydney_ip_17.raw; SpectrumID: ; scans: 17183

(Ac)M*TELQ^SALLLR

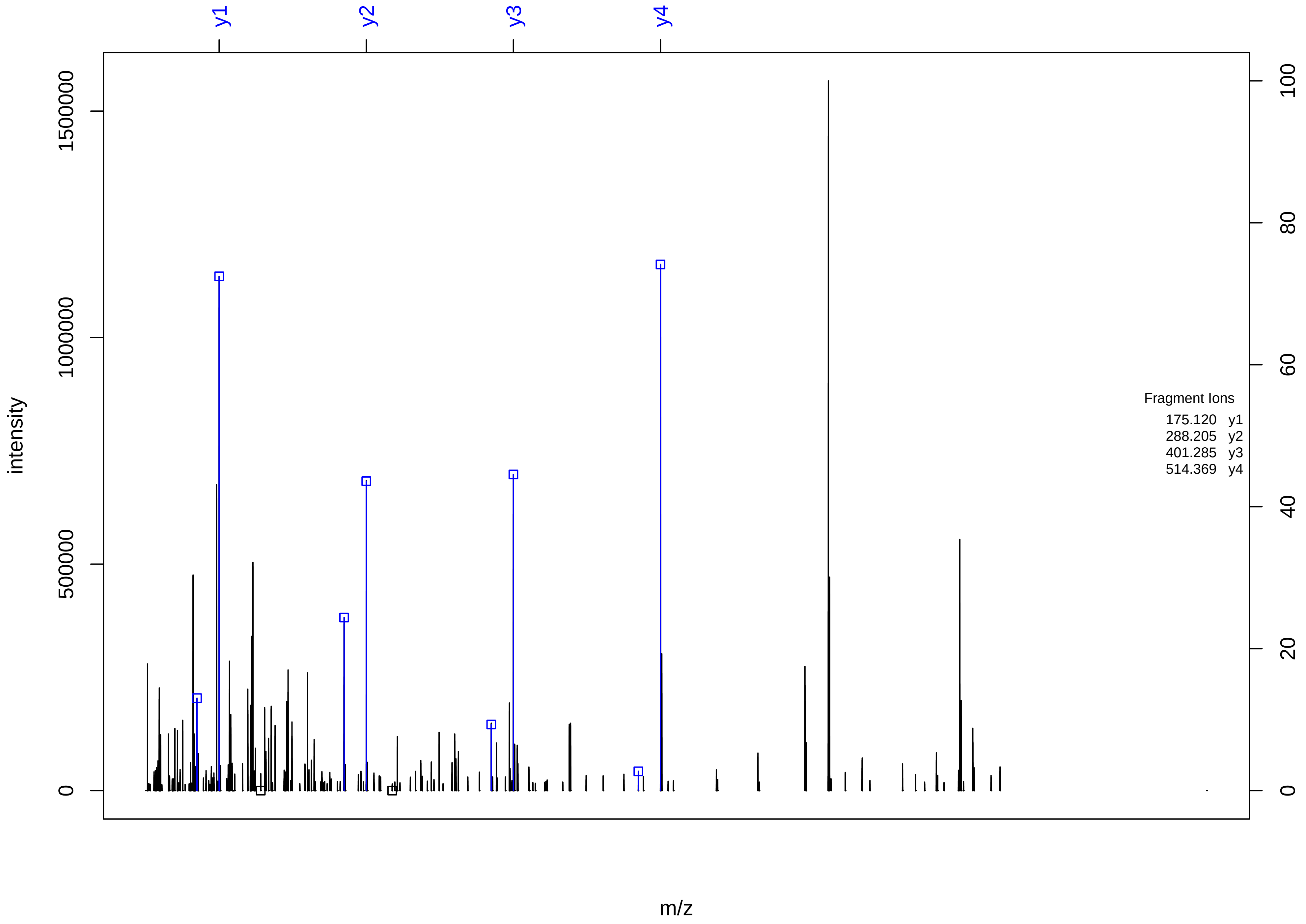

MTELQSALLLR / File: sydney_ip_17.raw; SpectrumID: ; scans: 7411

LITSFLELHR

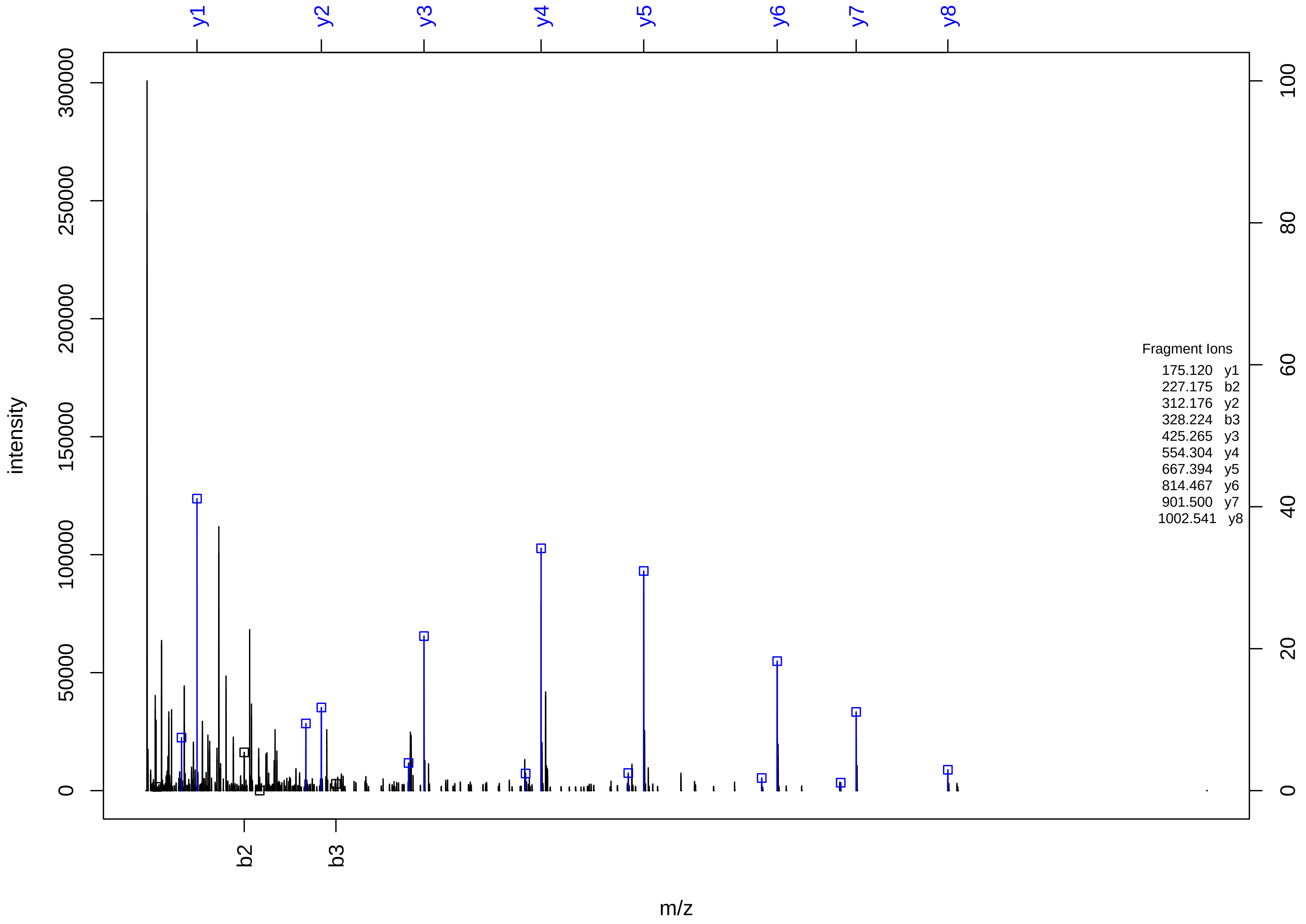

LITSFLELHR / File: sydney_ip_17.raw; SpectrumID: ; scans: 13011

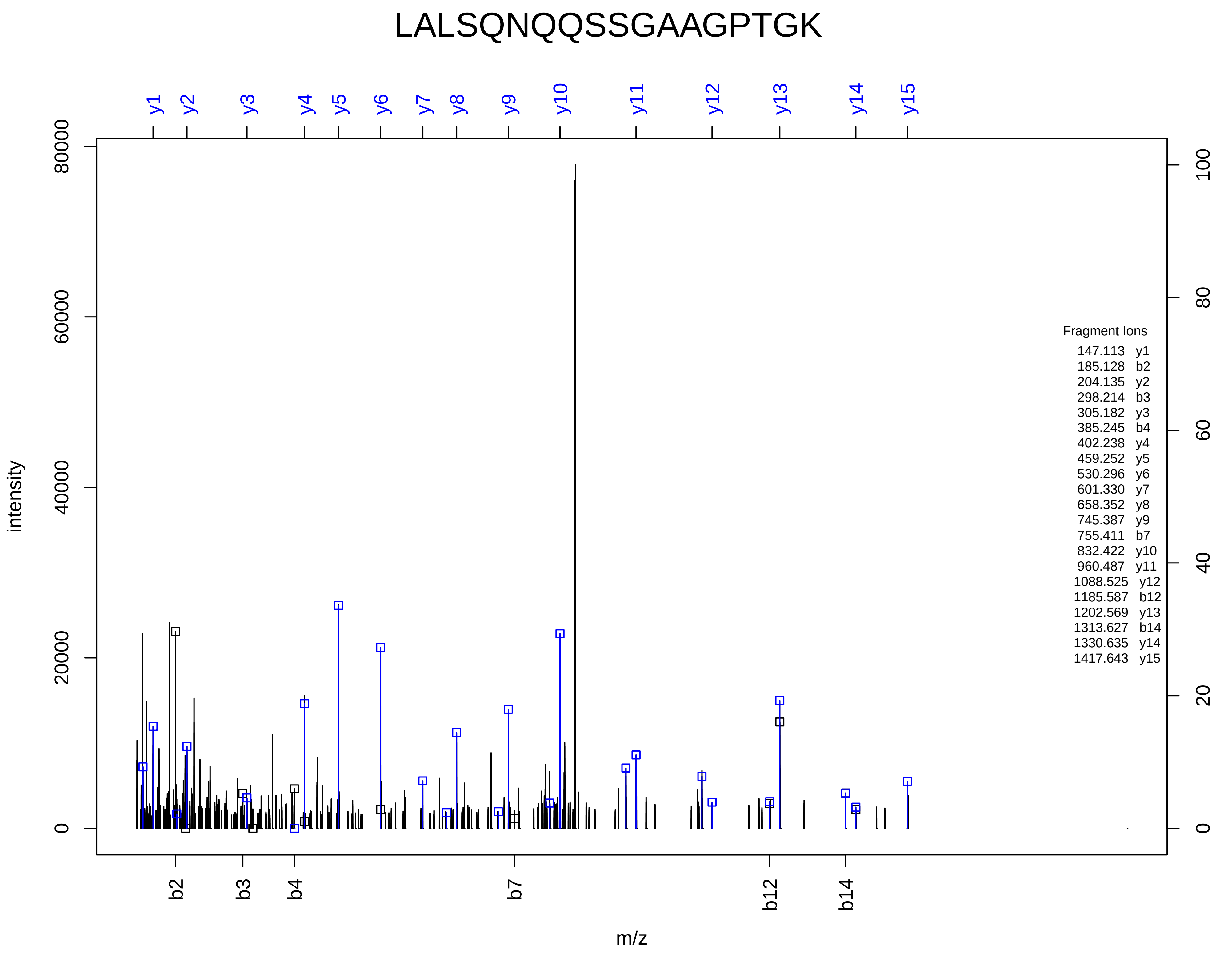
LALSQNQQSSGAAGPTGK
y1
y2
y3
y4
y5
y6
y7
y8
y9
y10
y11
y12
y13
y14
y15
intensity
0
20000
40000
60000
80000
0
20
40
60
80
100
b2
b3
b4
b7
b12
b14
m/z
Fragment Ions
147.113 y1
185.128 b2
204.135 y2
298.214 b3
305.182 y3
385.245 b4
402.238 y4
459.252 y5
530.296 y6
601.330 y7
658.352 y8
745.387 y9
755.411 b7
832.422 y10
960.487 y11
1088.525 y12
1185.587 b12
1202.569 y13
1313.627 b14
1330.635 y14
1417.643 y15
LALSQNQQSSGAAGPTGK / File: sydney_ip_17.raw; SpectrumID: ; scans: 3082

LKLTN^NLMDDN^GHIILN^SMHRYQ^PR

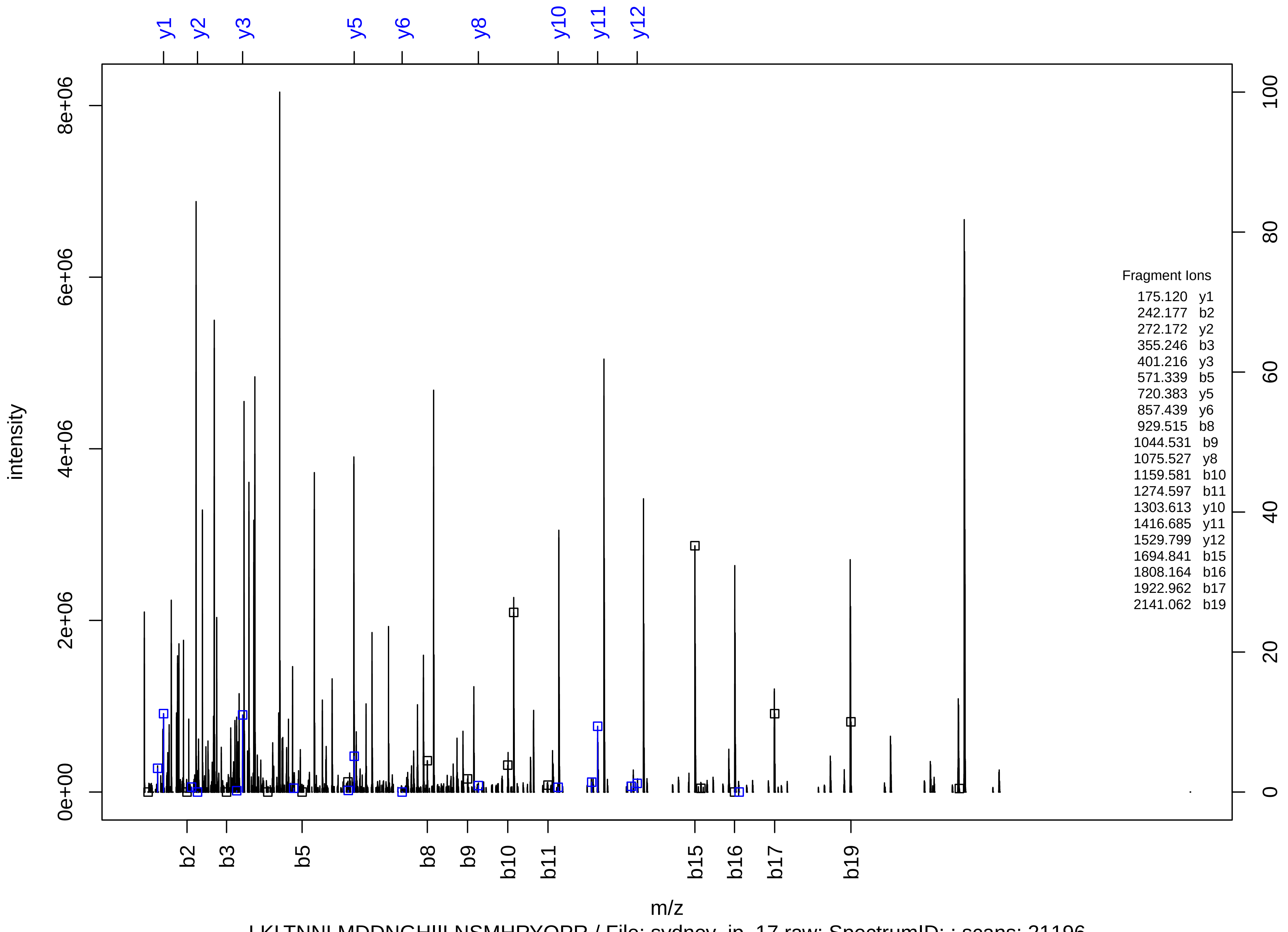

LKLTNNLMDDNGHIILNSMHRYQPR / File: sydney_ip_17.raw; SpectrumID: ; scans: 21196

DALLLIFANK

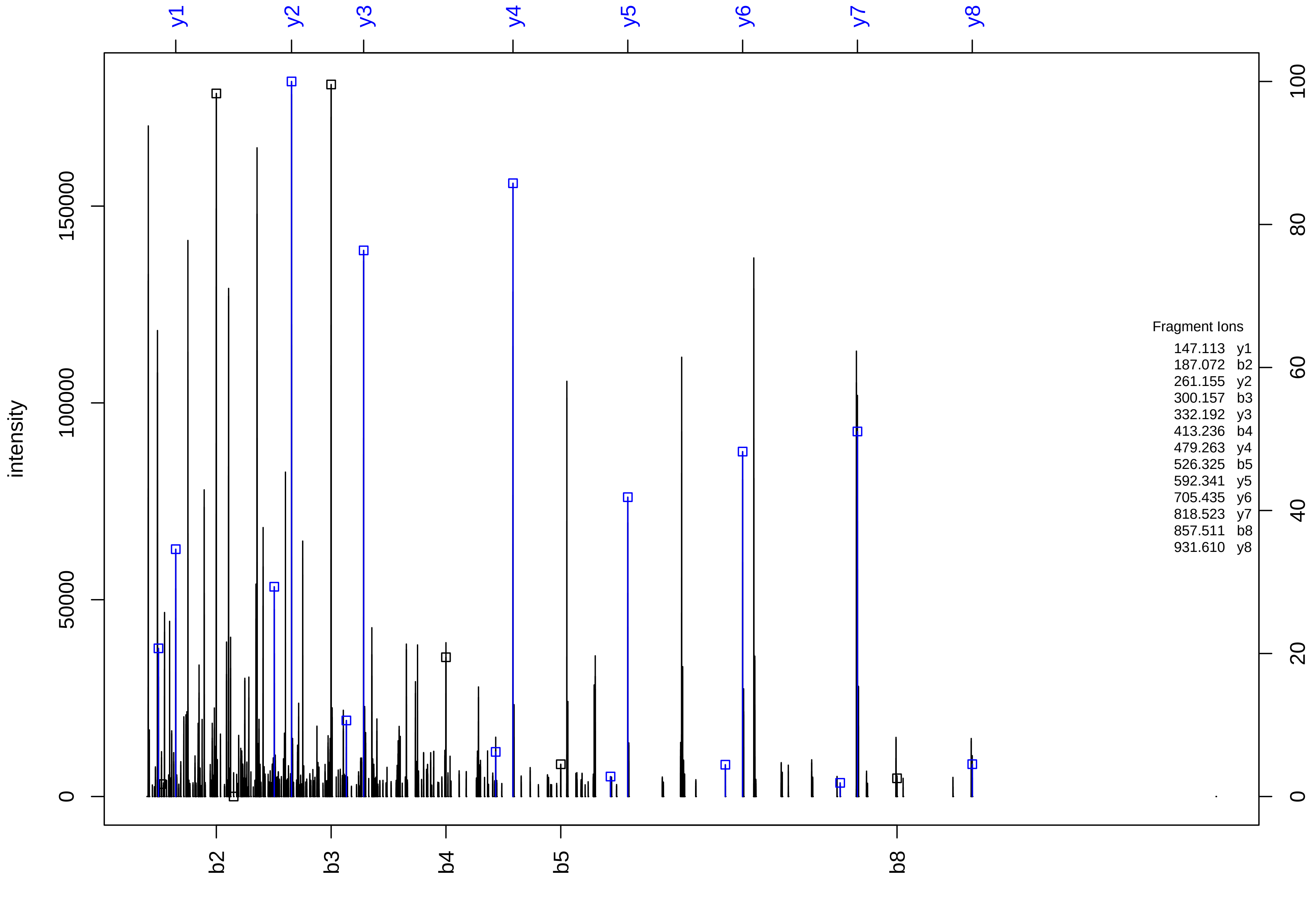

DALLLIFANK / File: sydney_ip_17.raw; SpectrumID: ; scans: 18175

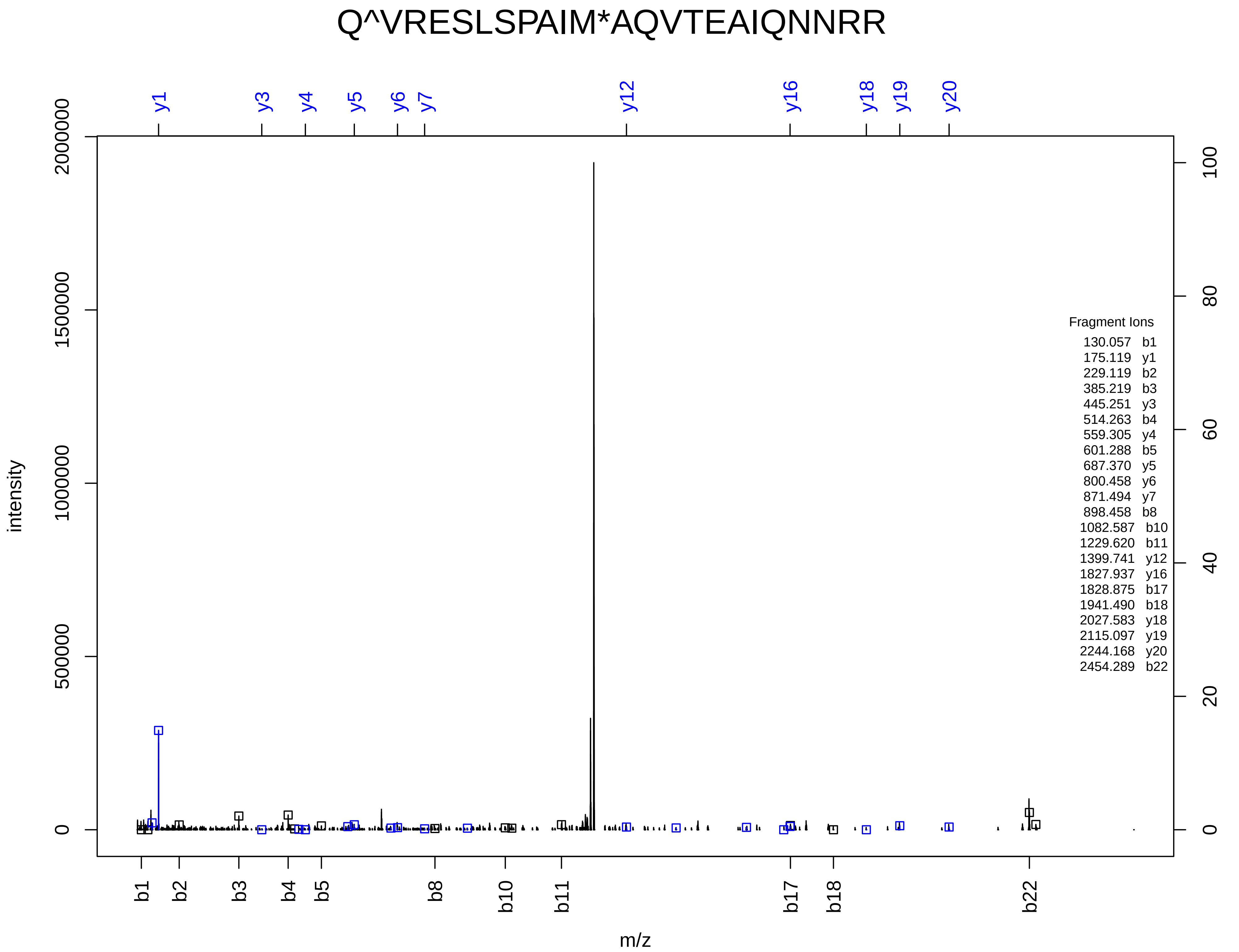

QVRESLSPAIMAQVTEAIQNNRR / File: sydney_ip_17.raw; SpectrumID: ; scans: 17673

Q^LHQ^RLLDLK

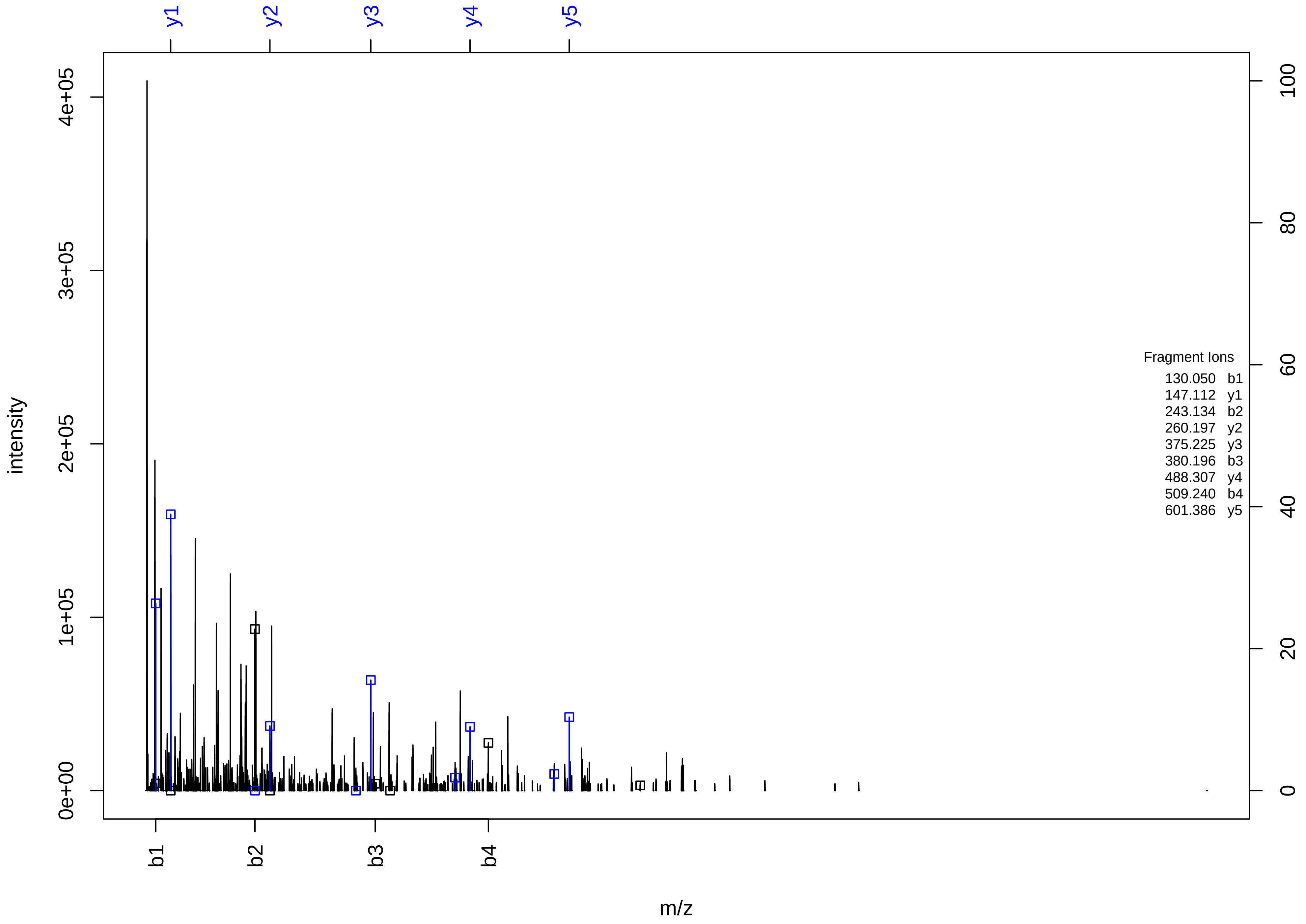

QLHQRLLDLK / File: sydney_ip_17.raw; SpectrumID: ; scans: 10830

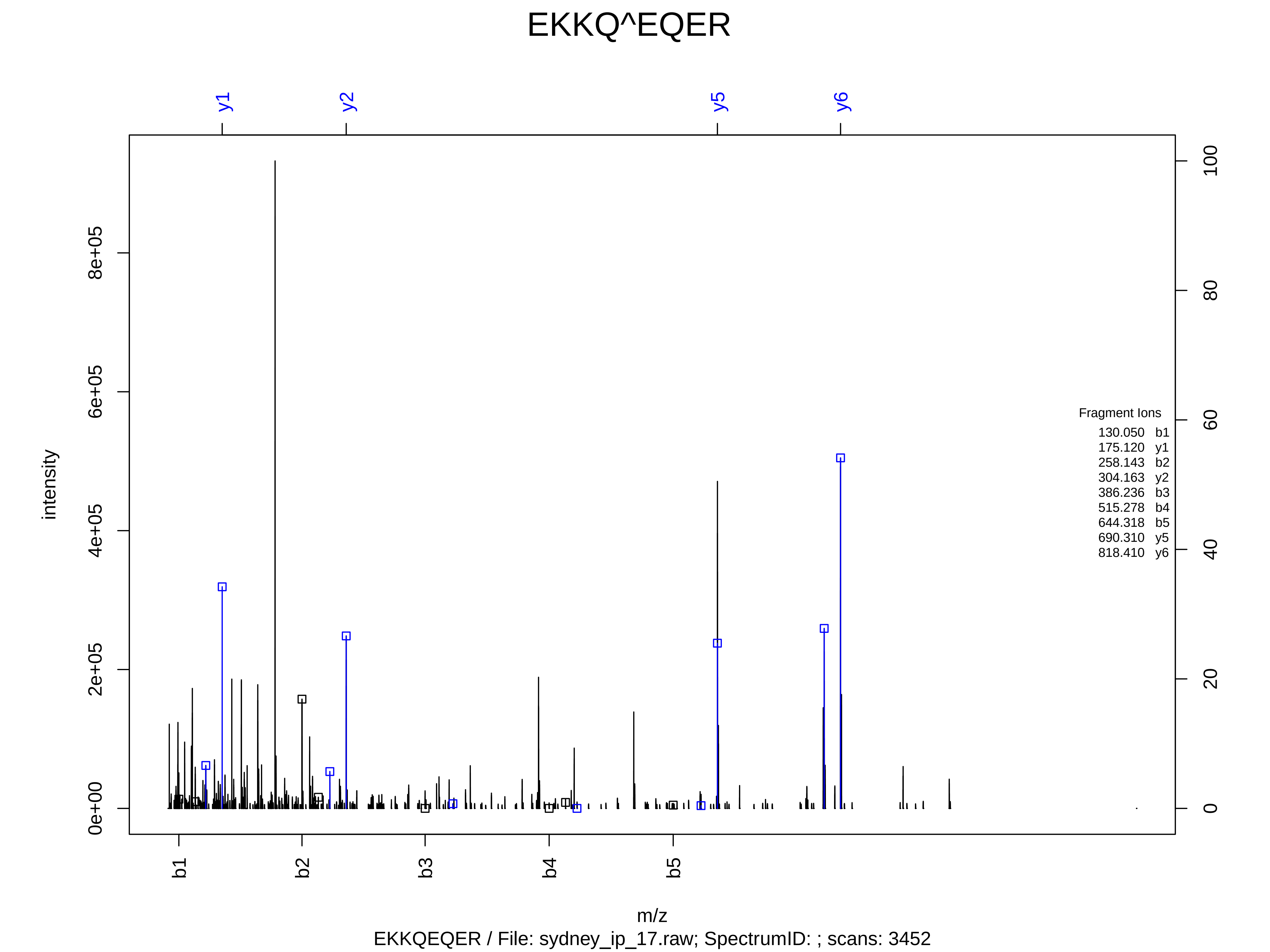
EKKQ^EQER
y1
y2
y5
y6
intensity
0e+00
2e+05
4e+05
6e+05
8e+05
0
20
40
60
80
100
Fragment Ions
130.050 b1
175.120 y1
258.143 b2
304.163 y2
386.236 b3
515.278 b4
644.318 b5
690.310 y5
818.410 y6
b1
b2
b3
b4
b5
m/z
EKKQEQER / File: sydney_ip_17.raw; SpectrumID: ; scans: 3452

QQTVM*AIATKIALQ^M*NCK

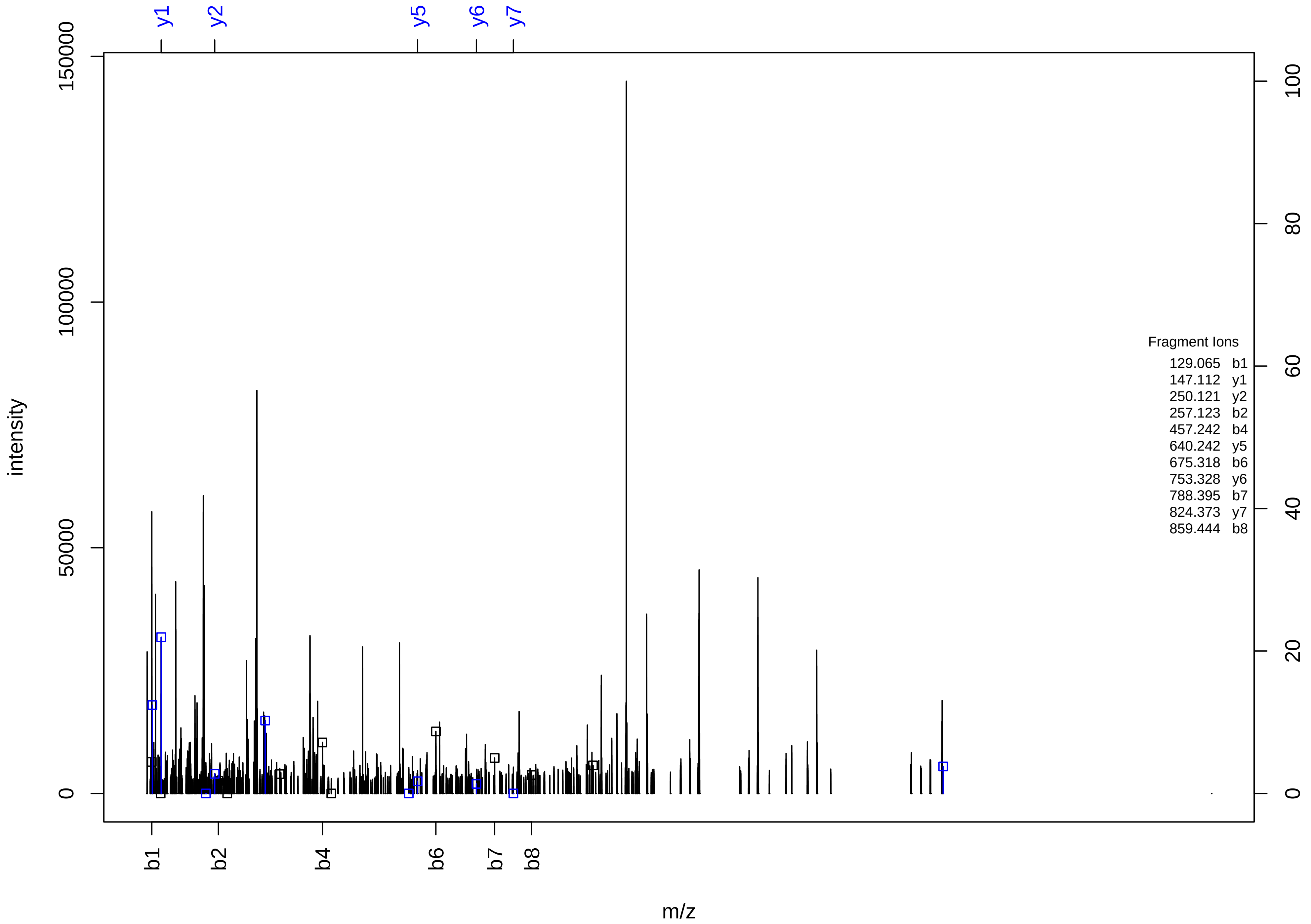

QQTVMAIATKIALQMNCK / File: sydney_ip_17.raw; SpectrumID: ; scans: 10779

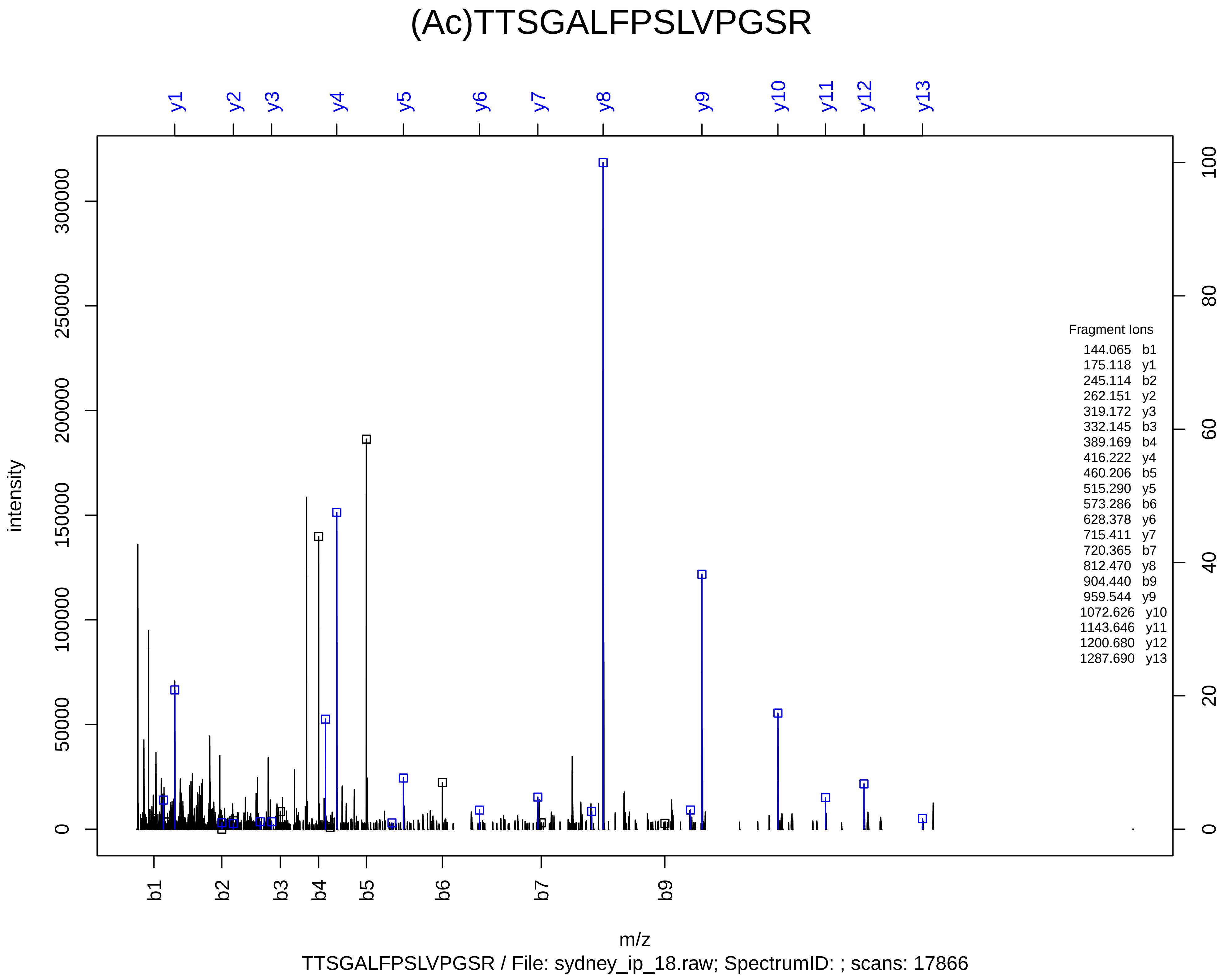

(Ac)TTSGALFPSLVPGSR
y1 y2 y3 y4 y5 y6 y7 y8 y9 y10 y11 y12 y13
intensity
0 50000 100000 150000 200000 250000 300000
0 20 40 60 80 100
b1 b2 b3 b4 b5 b6 b7 b9
m/z
Fragment Ions
144.065 b1
175.118 y1
245.114 b2
262.151 y2
319.172 y3
332.145 b3
389.169 b4
416.222 y4
460.206 b5
515.290 y5
573.286 b6
628.378 y6
715.411 y7
720.365 b7
812.470 y8
904.440 b9
959.544 y9
1072.626 y10
1143.646 y11
1200.680 y12
1287.690 y13
TTSGALFPSLVPGSR / File: sydney_ip_18.raw; SpectrumID: ; scans: 17866

ALGNILAYLSDAK

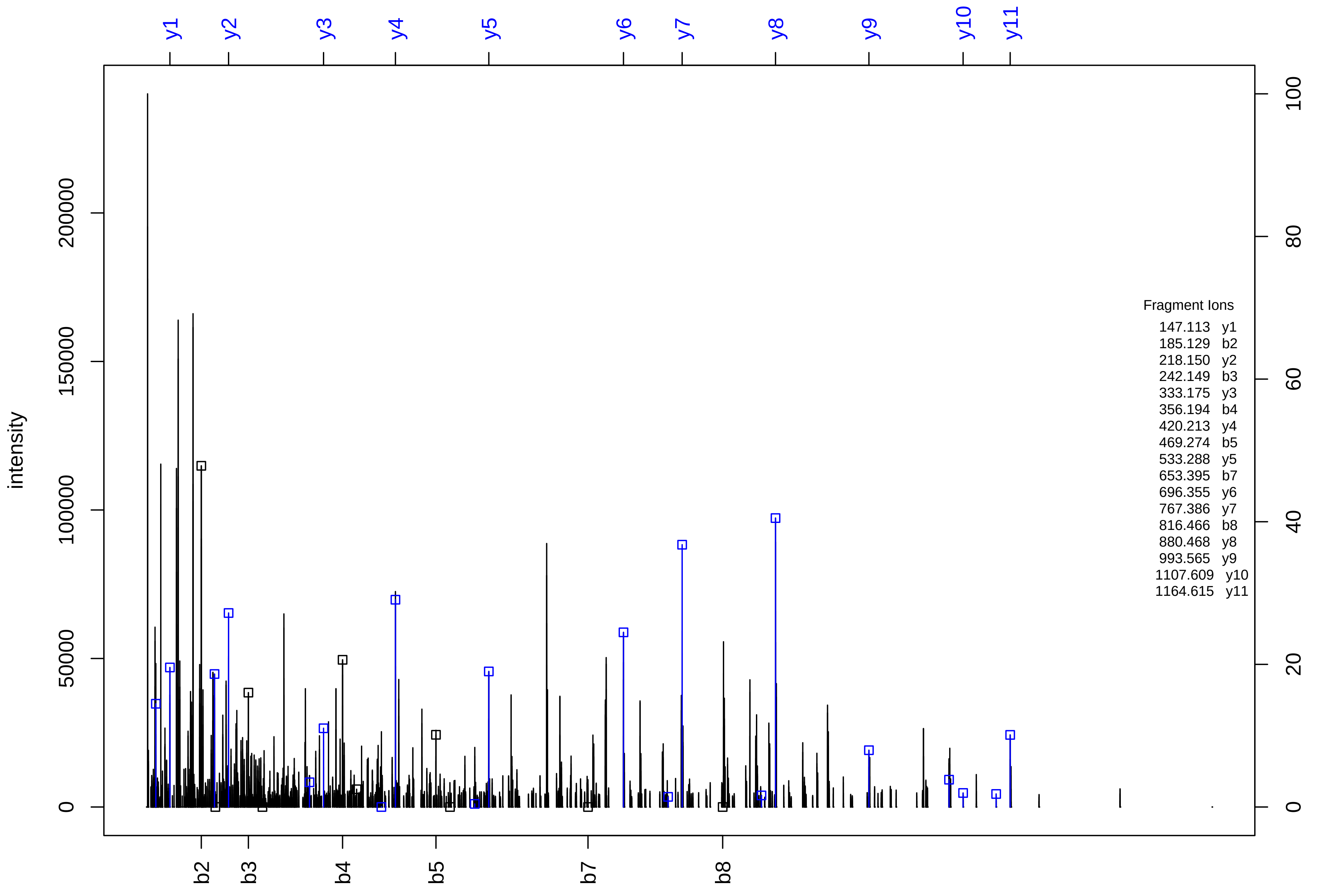

ALGNILAYLSDAK / File: sydney_ip_18.raw; SpectrumID: ; scans: 17661

VTSLVVDIVPR

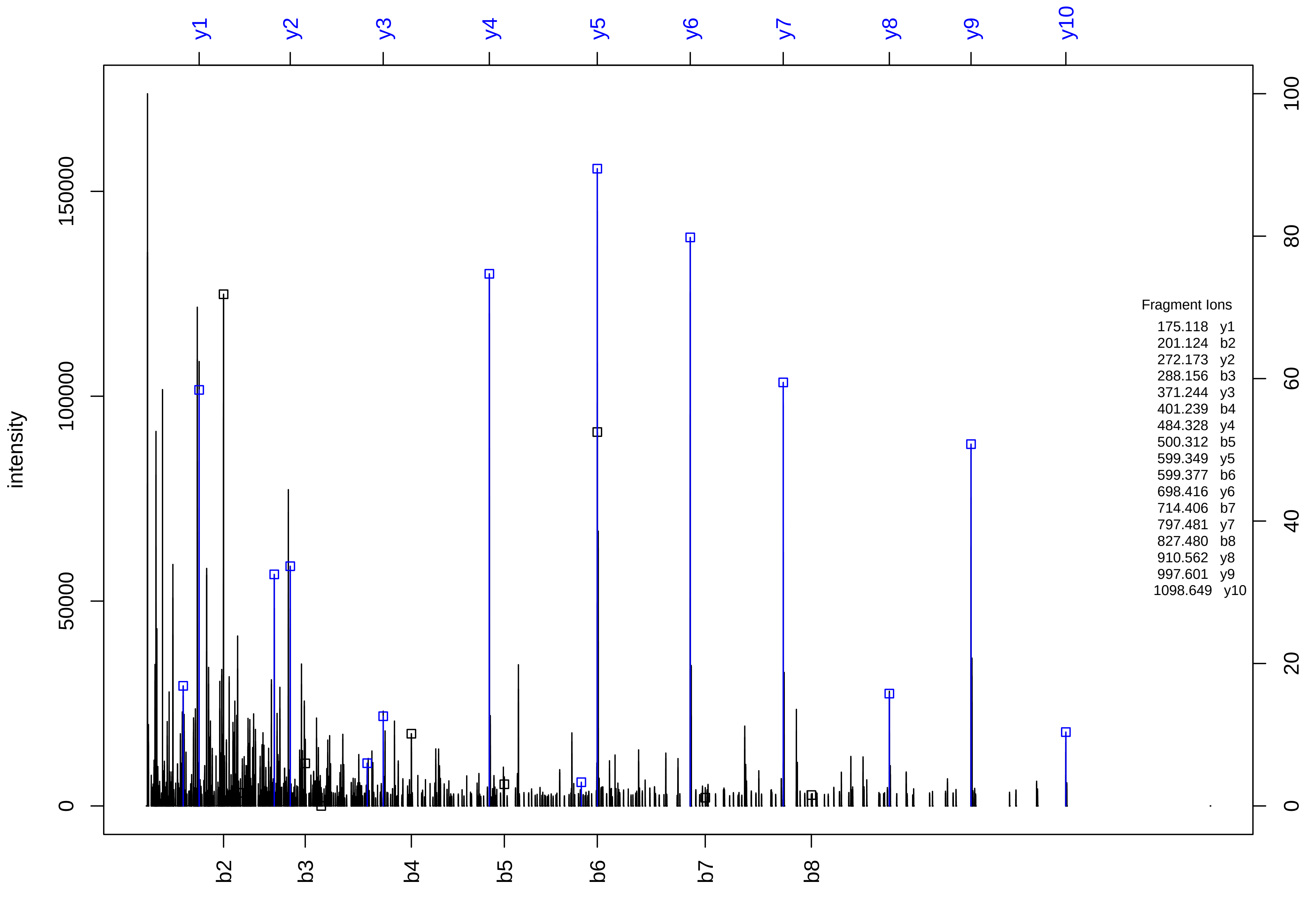

VTSLVVDIVPR / File: sydney_ip_18.raw; SpectrumID: ; scans: 13079

LLDAVDTYIPVPTR

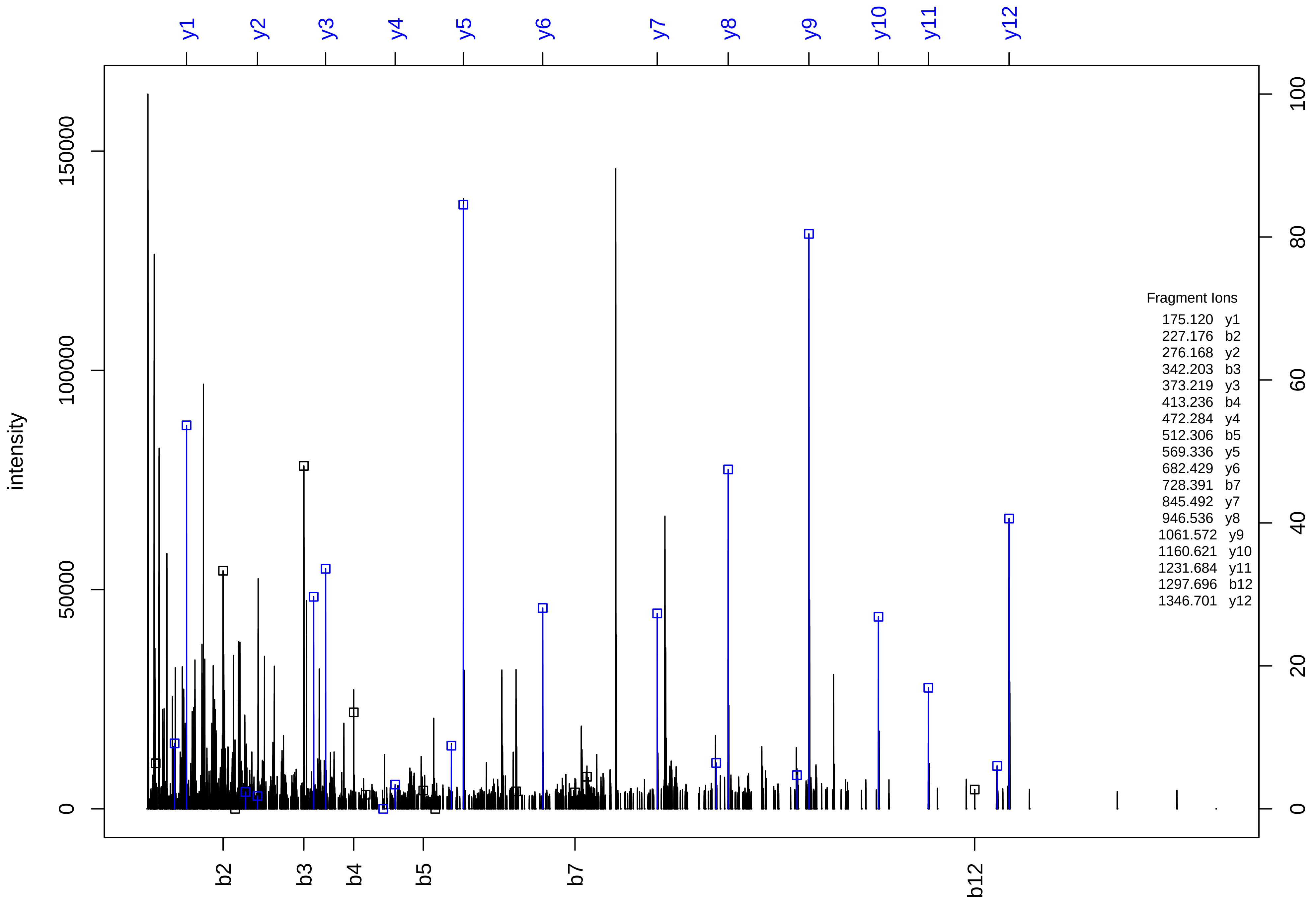

LLDAVDTYIPVPTR / File: sydney_ip_18.raw; SpectrumID: ; scans: 14825

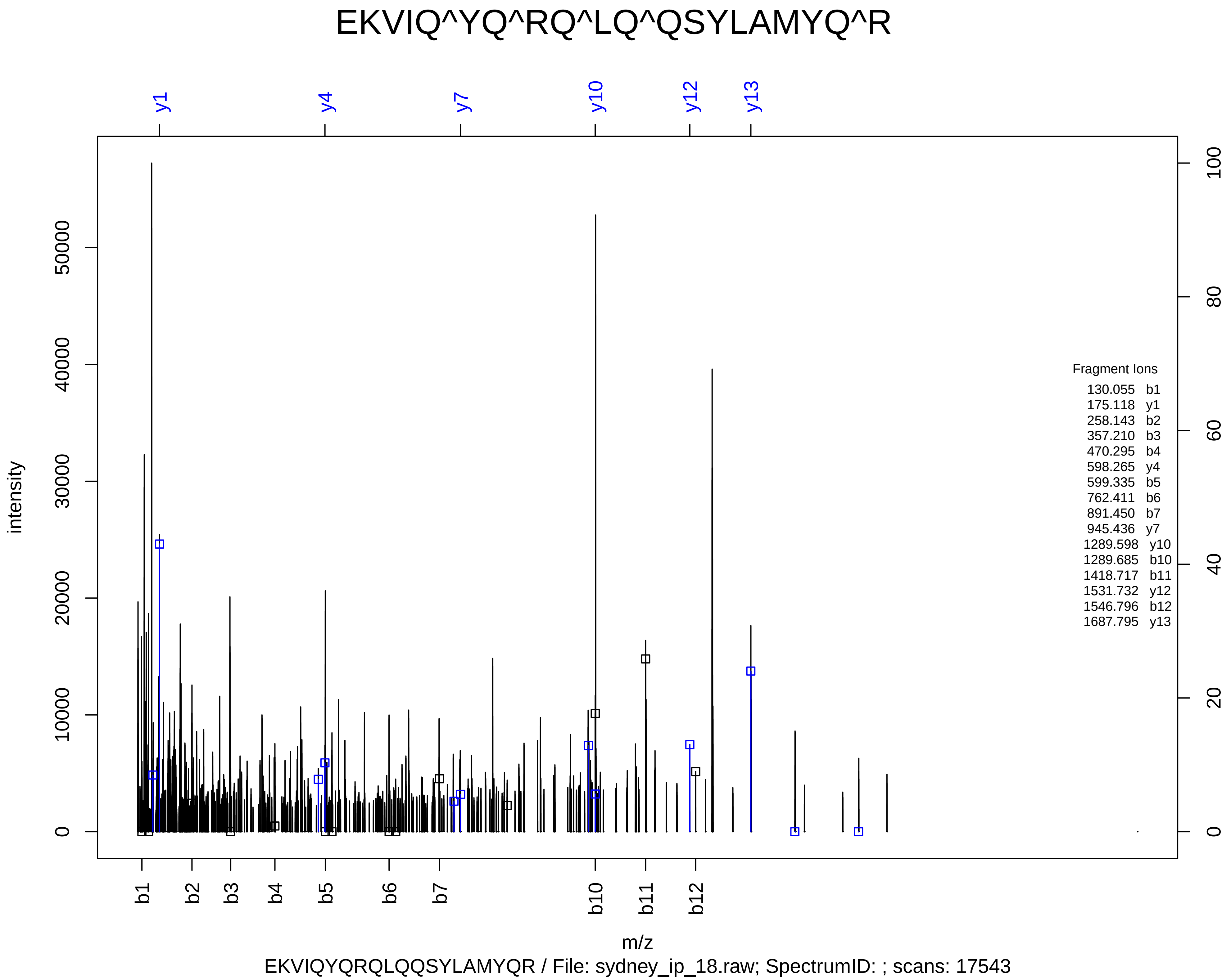

EKVIQ^YQ^RQ^LQ^QSYLAMYQ^R
y1
y4
y7
y10
y12
y13
intensity
0
10000
20000
30000
40000
50000
0
20
40
60
80
100
b1
b2
b3
b4
b5
b6
b7
b10
b11
b12
m/z
Fragment Ions
130.055 b1
175.118 y1
258.143 b2
357.210 b3
470.295 b4
598.265 y4
599.335 b5
762.411 b6
891.450 b7
945.436 y7
1289.598 y10
1289.685 b10
1418.717 b11
1531.732 y12
1546.796 b12
1687.795 y13
EKVIQYQRQLQQSYLAMYQR / File: sydney_ip_18.raw; SpectrumID: ; scans: 17543

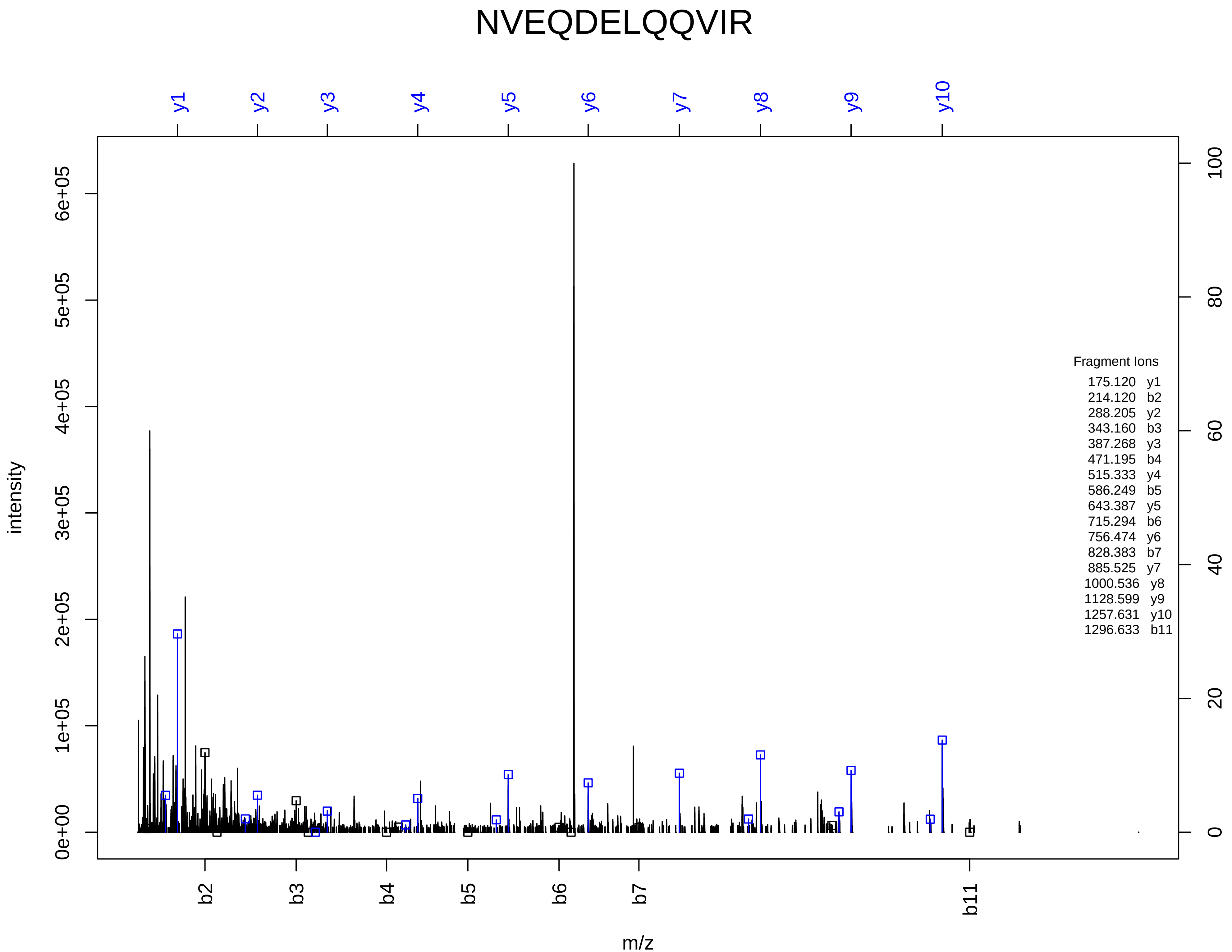

NVEQDELQQVIR / File: sydney_ip_18.raw; SpectrumID: ; scans: 8759

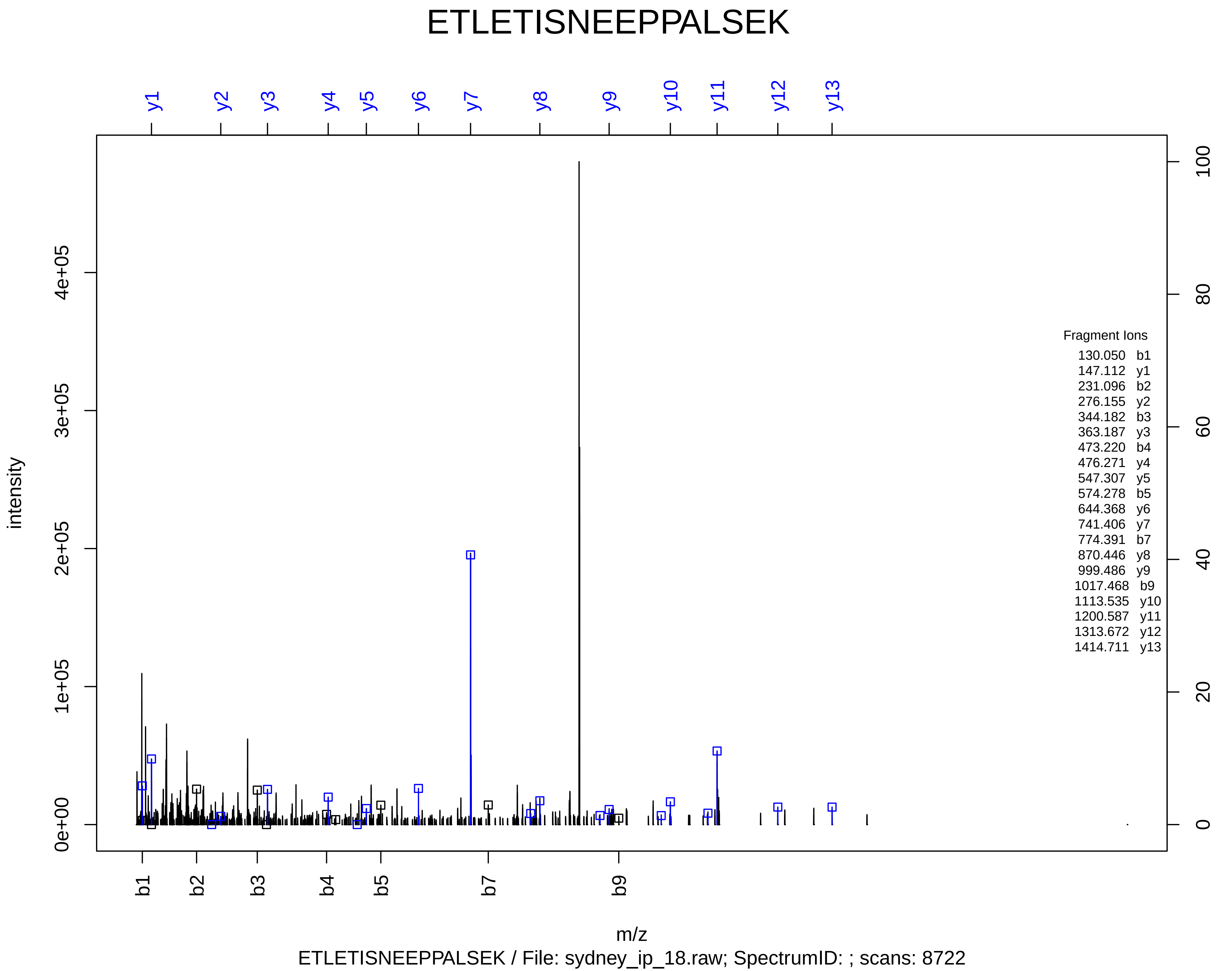
ETLETISNEEPPALSEK
y1 y2 y3 y4 y5 y6 y7 y8 y9 y10 y11 y12 y13
b1 b2 b3 b4 b5 b7 b9
intensity
0e+00
1e+05
2e+05
3e+05
4e+05
0
20
40
60
80
100
m/z
Fragment Ions
130.050 b1
147.112 y1
231.096 b2
276.155 y2
344.182 b3
363.187 y3
473.220 b4
476.271 y4
547.307 y5
574.278 b5
644.368 y6
741.406 y7
774.391 b7
870.446 y8
999.486 y9
1017.468 b9
1113.535 y10
1200.587 y11
1313.672 y12
1414.711 y13
ETLETISNEEPPALSEK / File: sydney_ip_18.raw; SpectrumID: ; scans: 8722

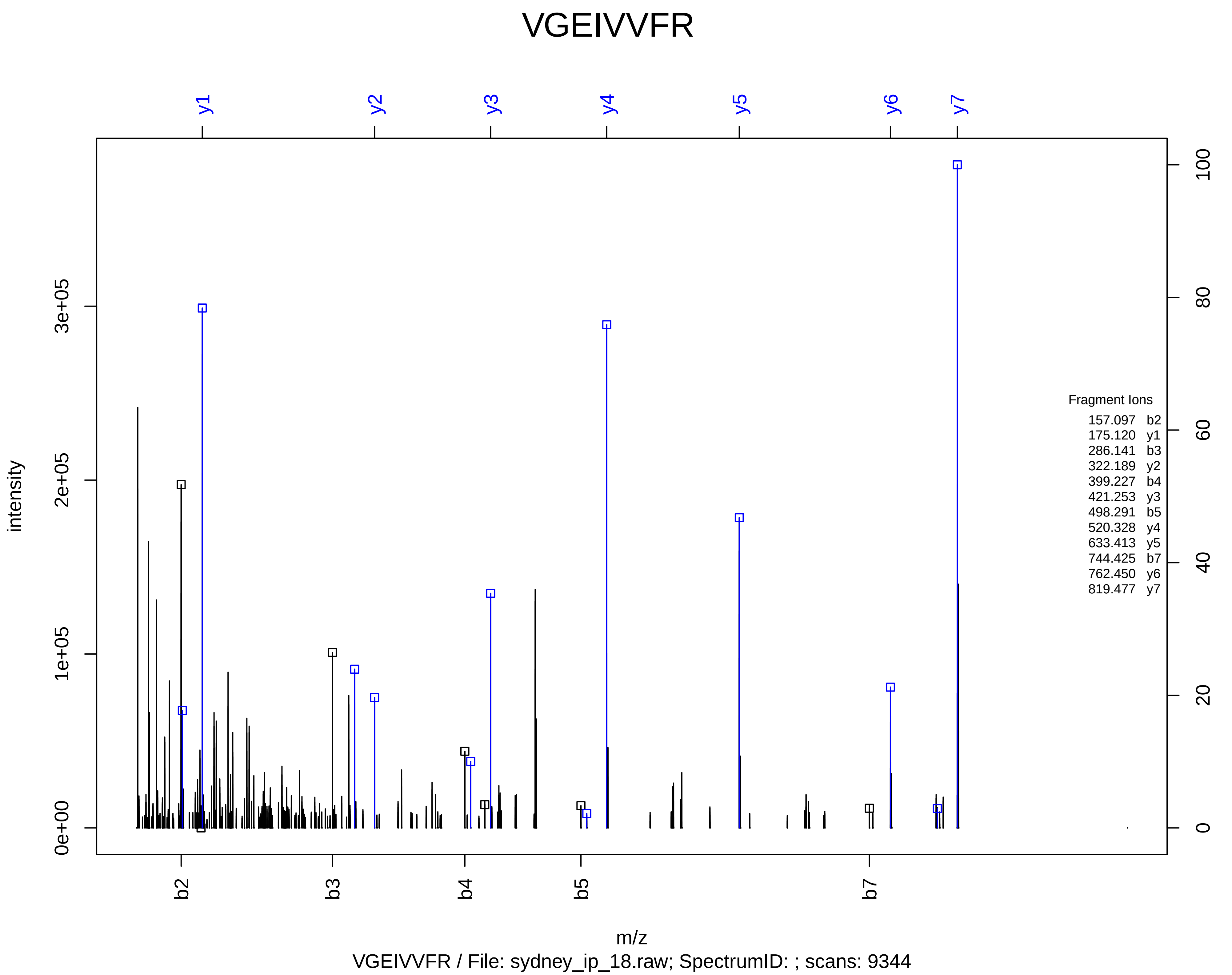

VGEIVVFR
y1
y2
y3
y4
y5
y6
y7
intensity
3e+05
2e+05
1e+05
0e+00
100
80
60
40
20
0
Fragment Ions
157.097 b2
175.120 y1
286.141 b3
322.189 y2
399.227 b4
421.253 y3
498.291 b5
520.328 y4
633.413 y5
744.425 b7
762.450 y6
819.477 y7
b2
b3
b4
b5
b7
m/z
VGEIVVFR / File: sydney_ip_18.raw; SpectrumID: ; scans: 9344

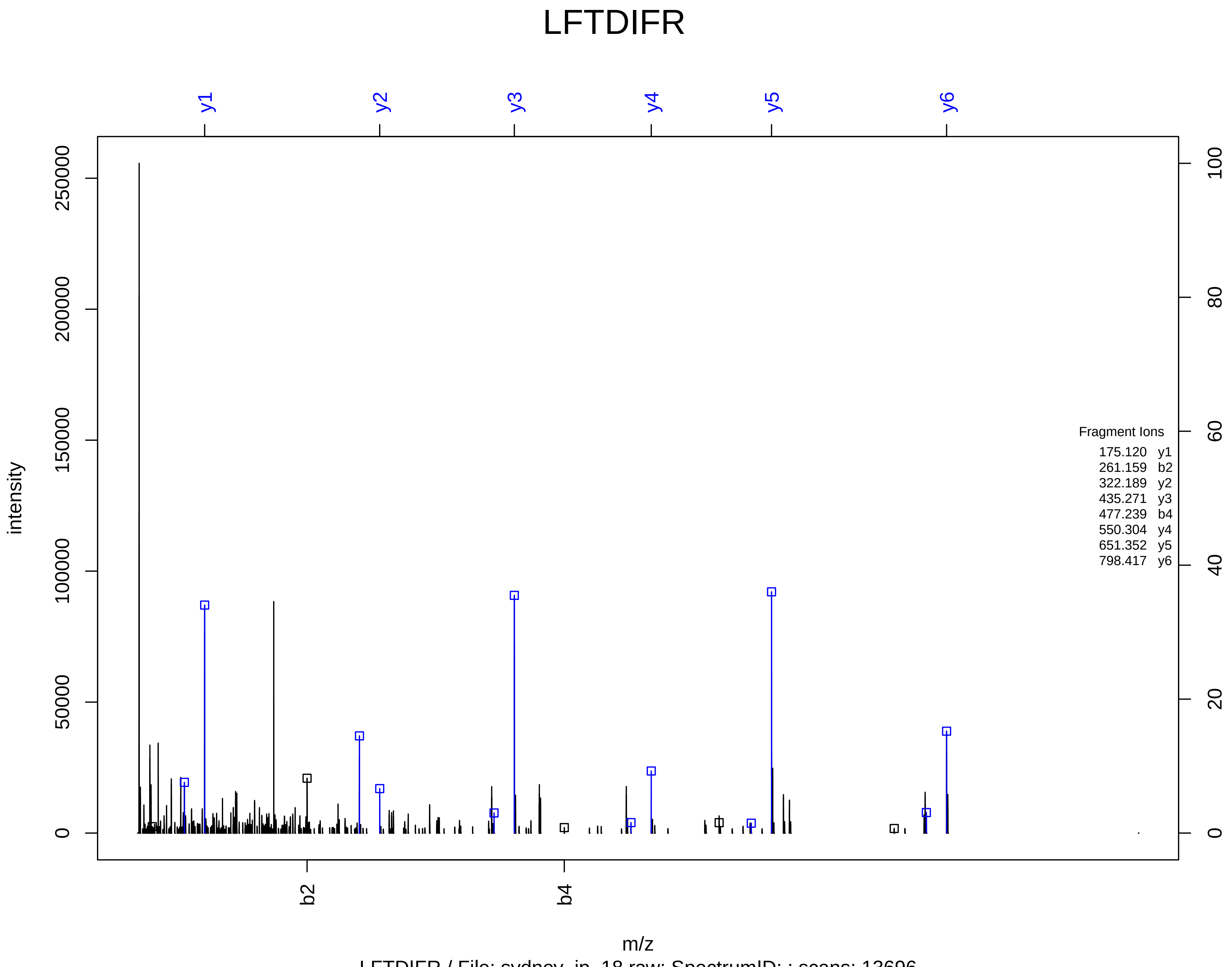
LFTDIFR
y1
y2
y3
y4
y5
y6
b2
b4
intensity
m/z
0
50000
100000
150000
200000
250000
0
20
40
60
80
100
Fragment Ions
175.120 y1
261.159 b2
322.189 y2
435.271 y3
477.239 b4
550.304 y4
651.352 y5
798.417 y6
LFTDIFR / File: sydney_ip_18.raw; SpectrumID: ; scans: 13696

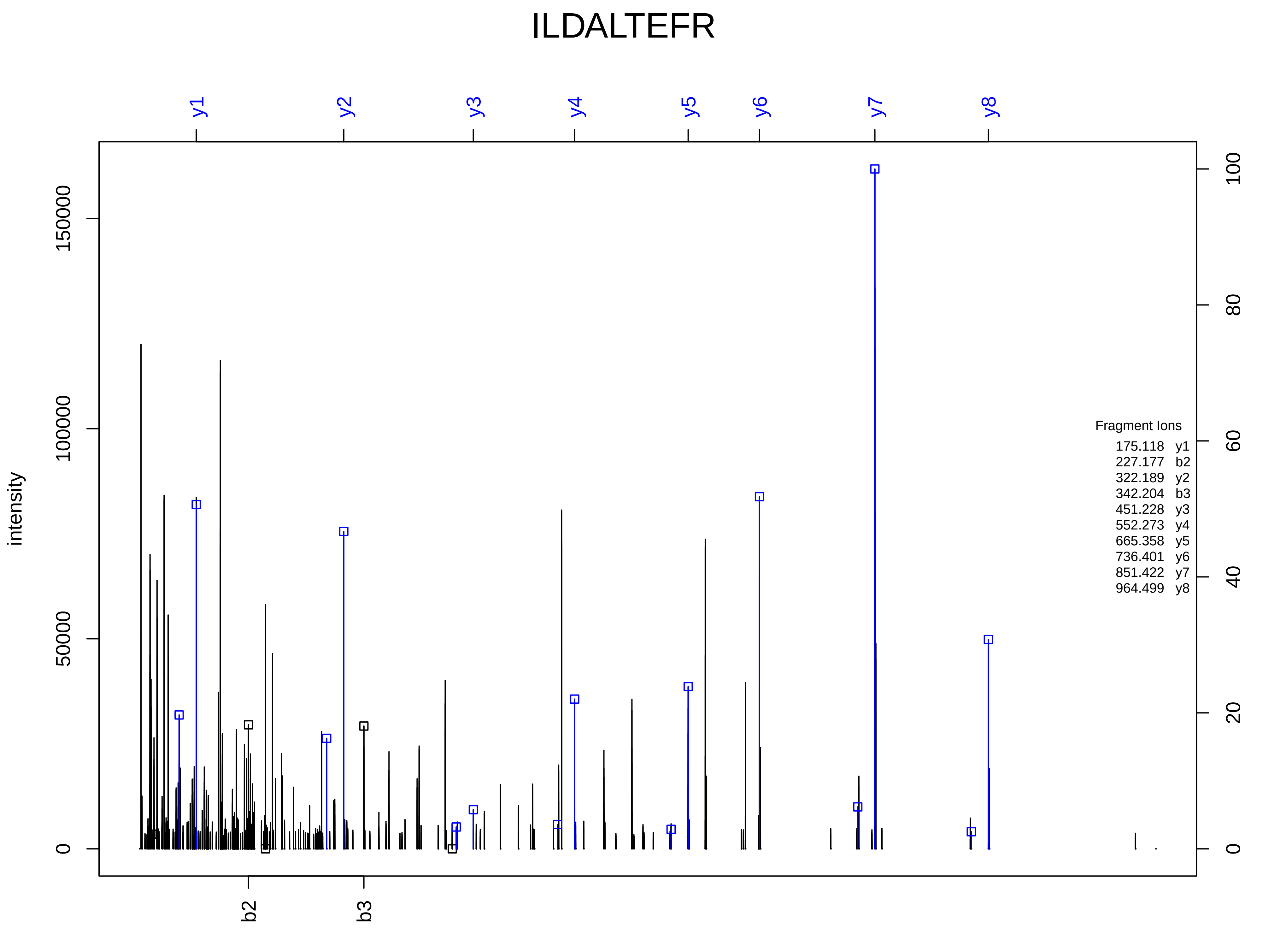

ILDALTEFR / File: sydney_ip_18.raw; SpectrumID: ; scans: 12826

ALLLVGDVAQAAEVAIEHR

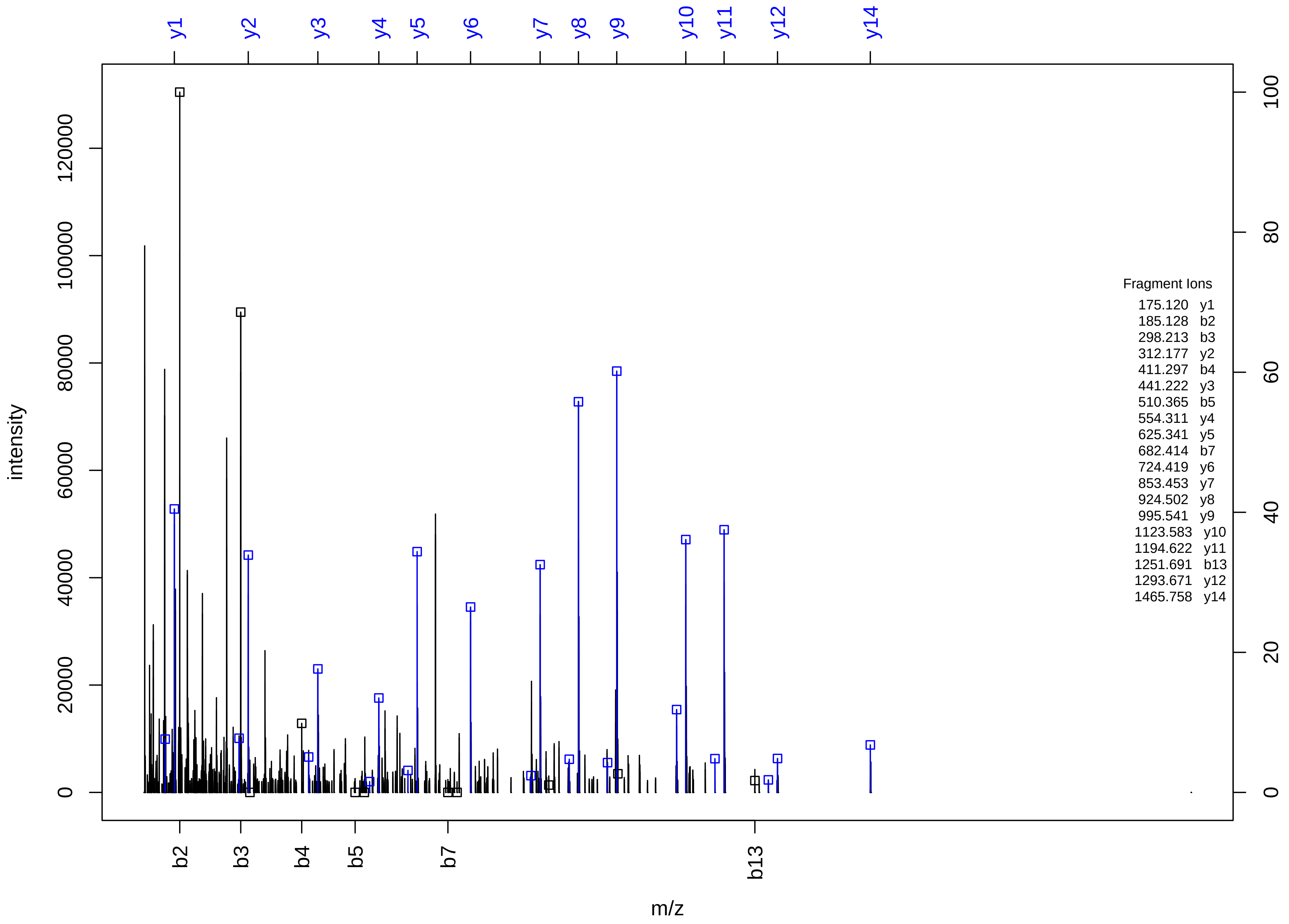

ALLLVGDVAQAAEVAIEHR / File: sydney_ip_18.raw; SpectrumID: ; scans: 18813

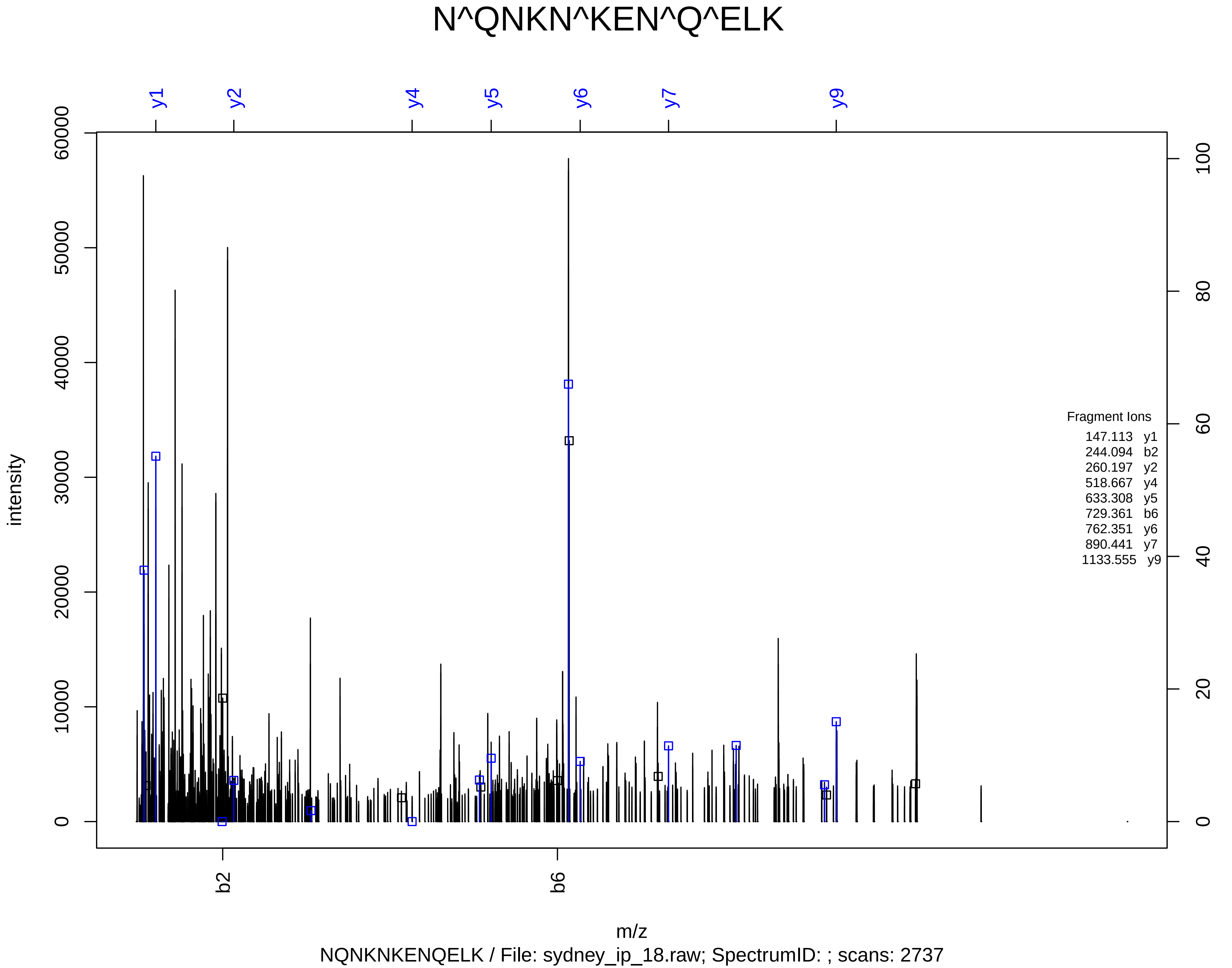
N^QNKN^KEN^Q^ELK
y1
y2
y4
y5
y6
y7
y9
intensity
60000
50000
40000
30000
20000
10000
0
100
80
60
40
20
0
Fragment Ions
147.113 y1
244.094 b2
260.197 y2
518.667 y4
633.308 y5
729.361 b6
762.351 y6
890.441 y7
1133.555 y9
b2
b6
m/z
NQNKNKENQELK / File: sydney_ip_18.raw; SpectrumID: ; scans: 2737

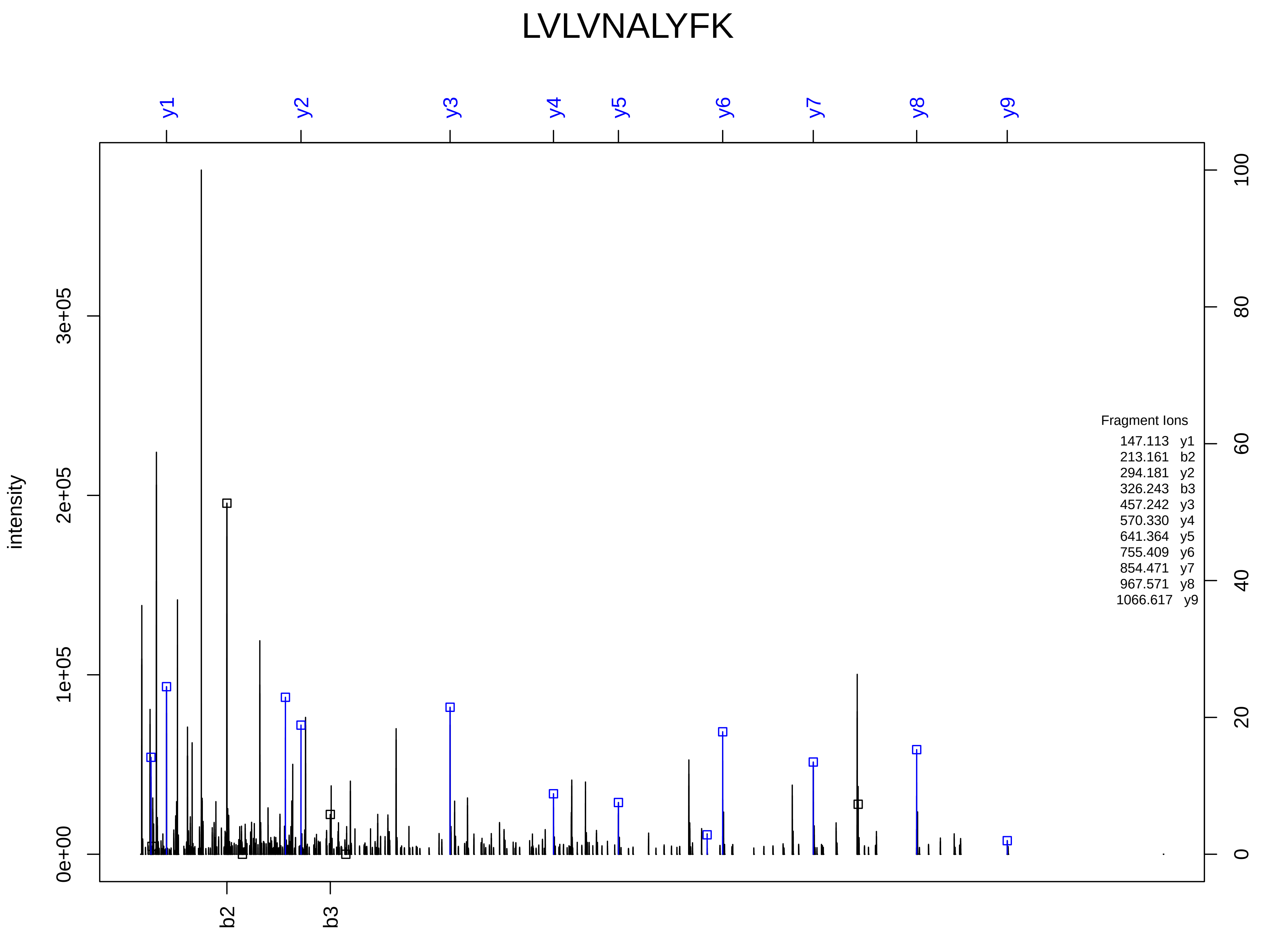

LVLVNALYFK / File: sydney_ip_18.raw; SpectrumID: ; scans: 15532

SNLQLPLQFLSR

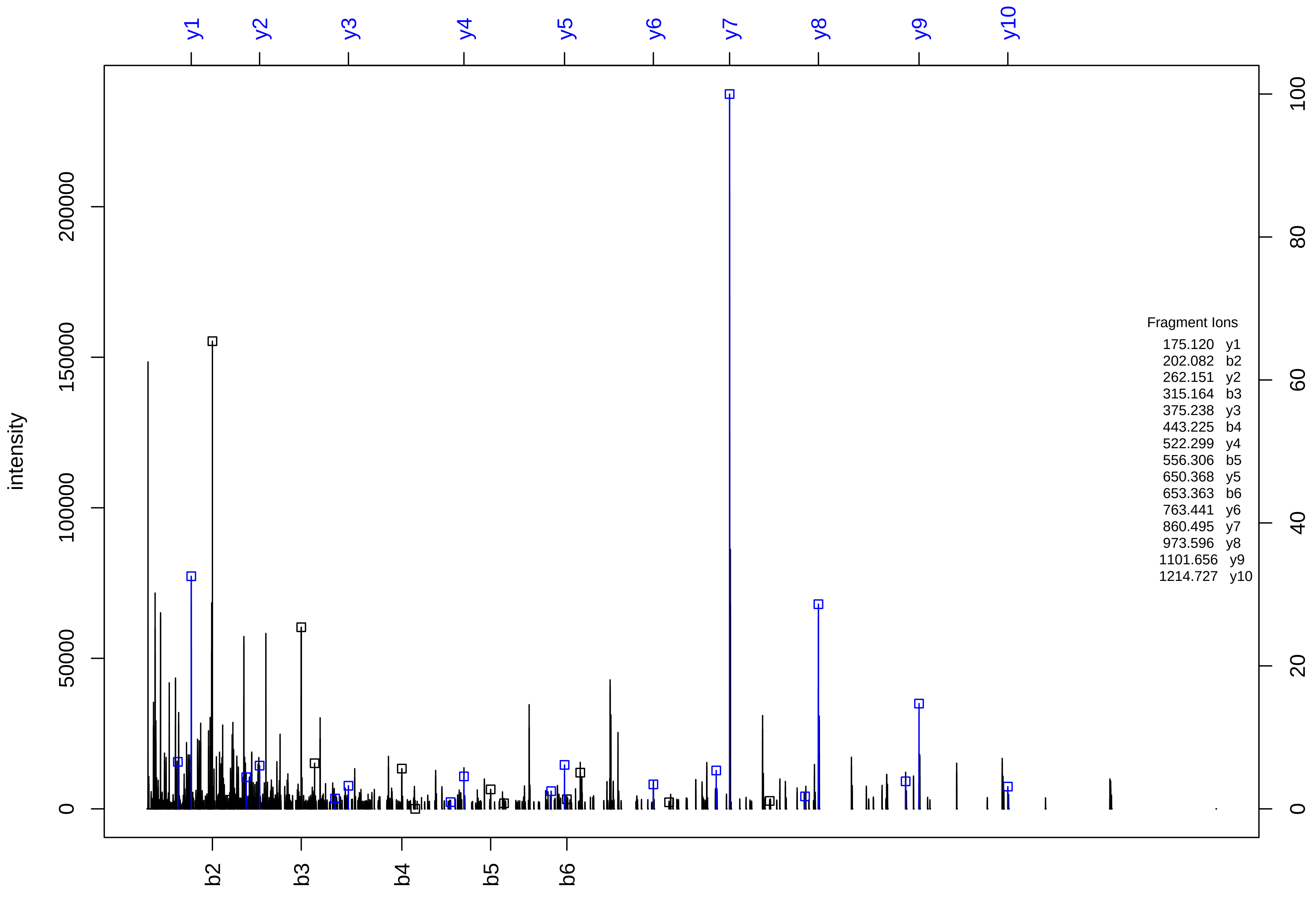

SNLQLPLQFLSR / File: sydney_ip_18.raw; SpectrumID: ; scans: 17159

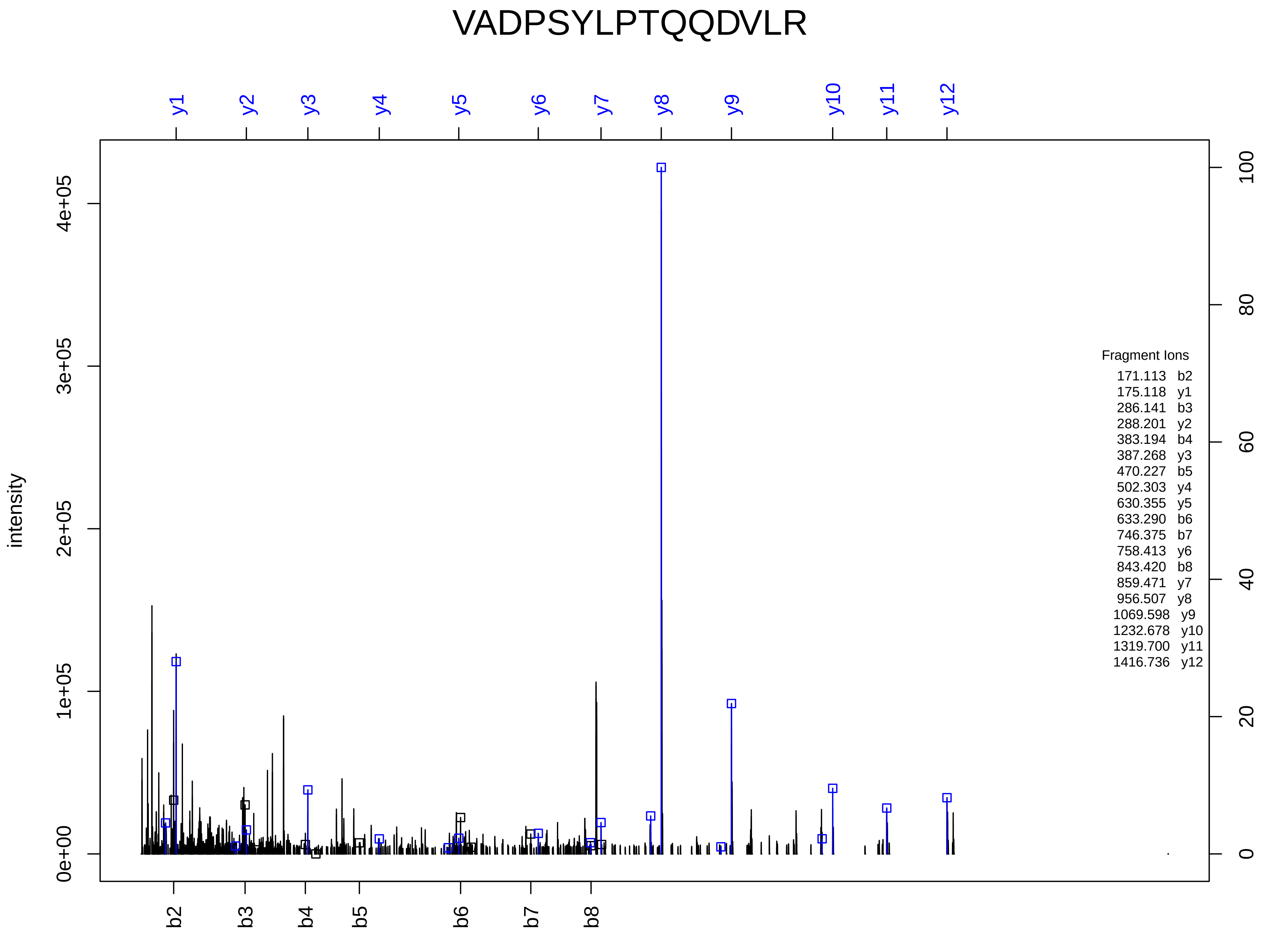

VADPSYLPTQQDVLR / File: sydney_ip_18.raw; SpectrumID: ; scans: 9892

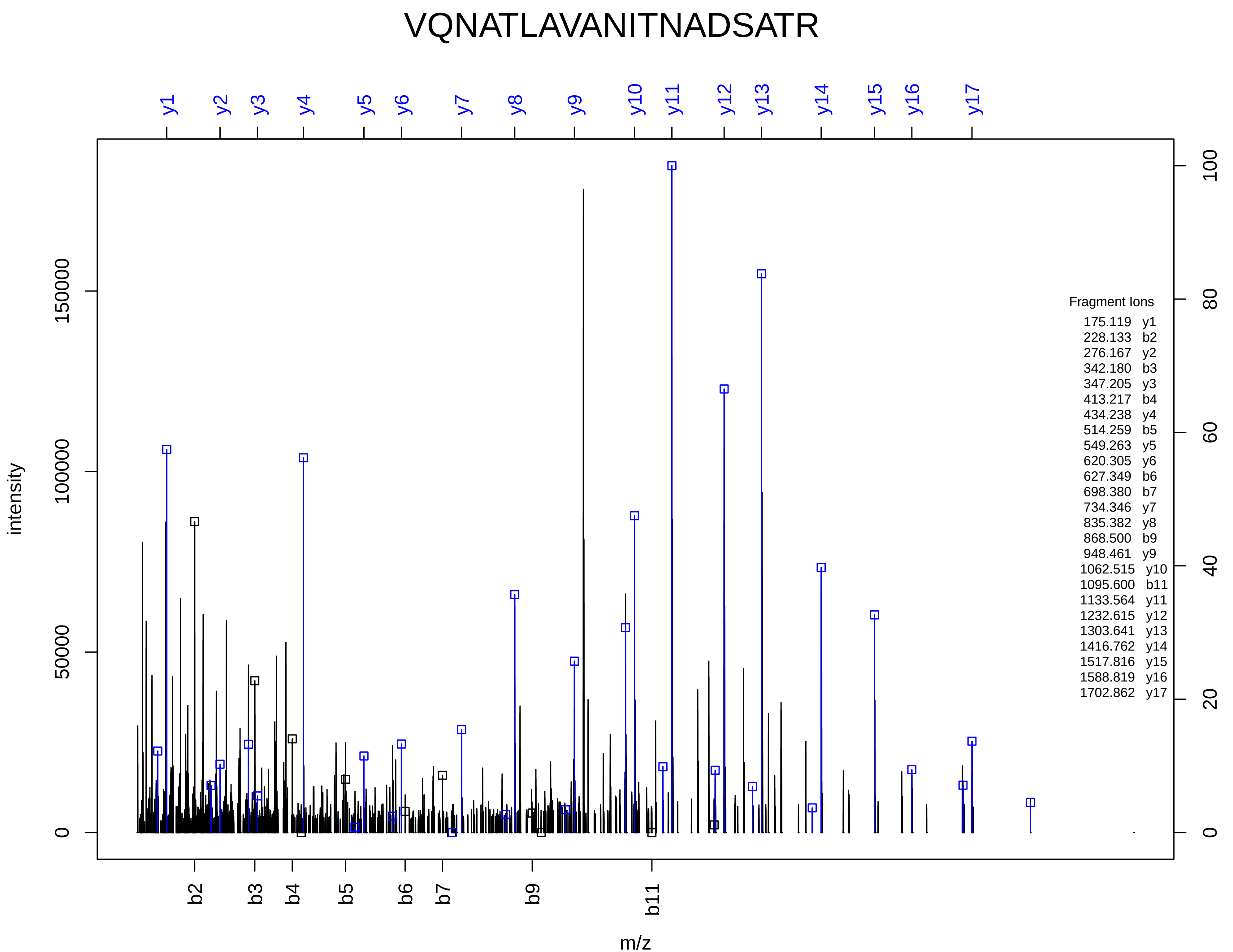
VQNATLAVANITNADSATR
y1 y2 y3 y4 y5 y6 y7 y8 y9 y10 y11 y12 y13 y14 y15 y16 y17
b2 b3 b4 b5 b6 b7 b9 b11
intensity
0
50000
100000
150000
0
20
40
60
80
100
m/z
Fragment Ions
175.119 y1
228.133 b2
276.167 y2
342.180 b3
347.205 y3
413.217 b4
434.238 y4
514.259 b5
549.263 y5
620.305 y6
627.349 b6
698.380 b7
734.346 y7
835.382 y8
868.500 b9
948.461 y9
1062.515 y10
1095.600 b11
1133.564 y11
1232.615 y12
1303.641 y13
1416.762 y14
1517.816 y15
1588.819 y16
1702.862 y17
VQNATLAVANITNADSATR / File: sydney_ip_18.raw; SpectrumID: ; scans: 8838

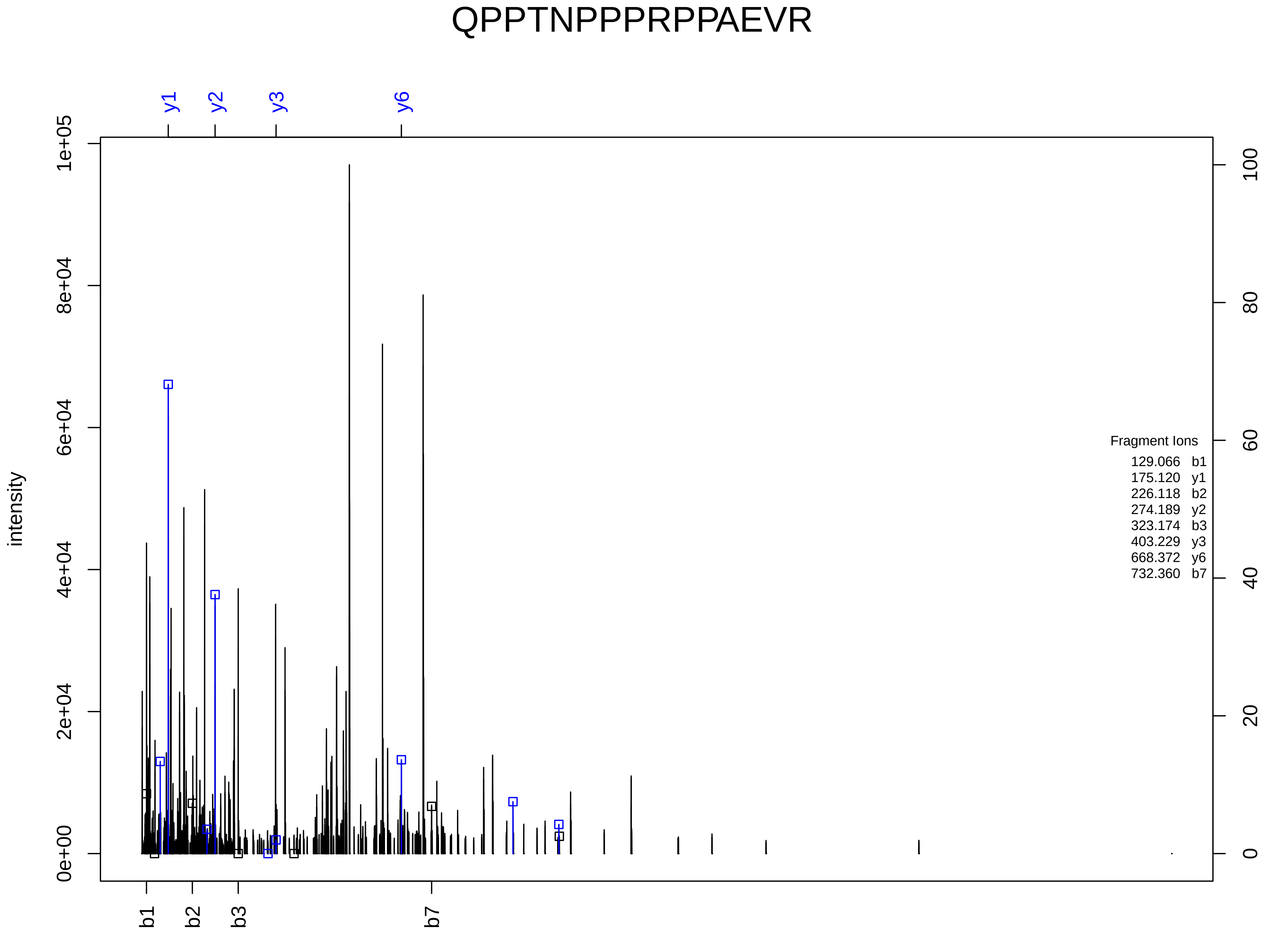

QPPTNPPPRPPAEVR / File: sydney_ip_18.raw; SpectrumID: ; scans: 2471

N^LDM*SDLKAEKLVM*FQ^R

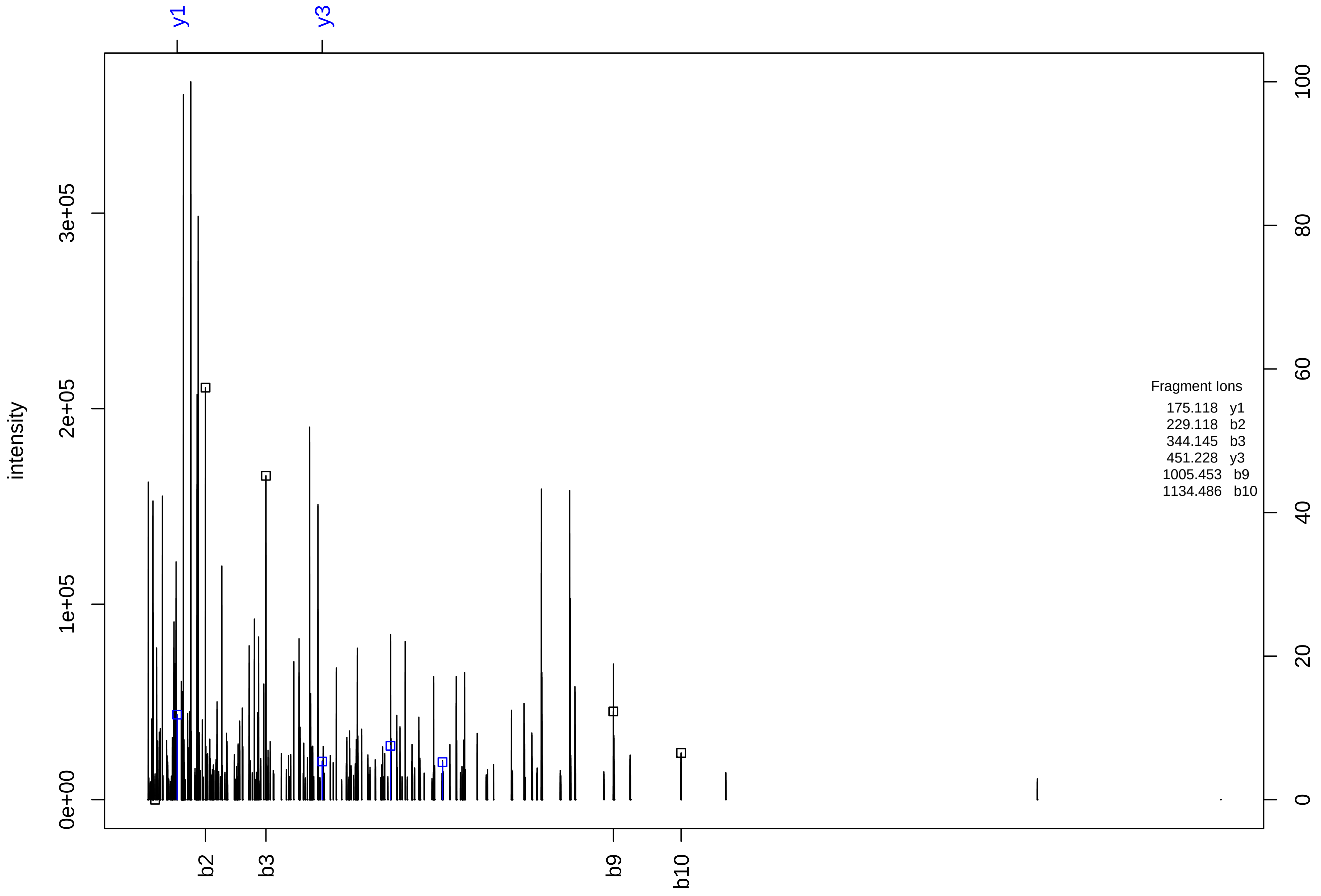

NLDMSDLKAEKLVMFQR / File: sydney_ip_18.raw; SpectrumID: ; scans: 12674

QQSLQQ^Q^LEQLQ^GLPGAR

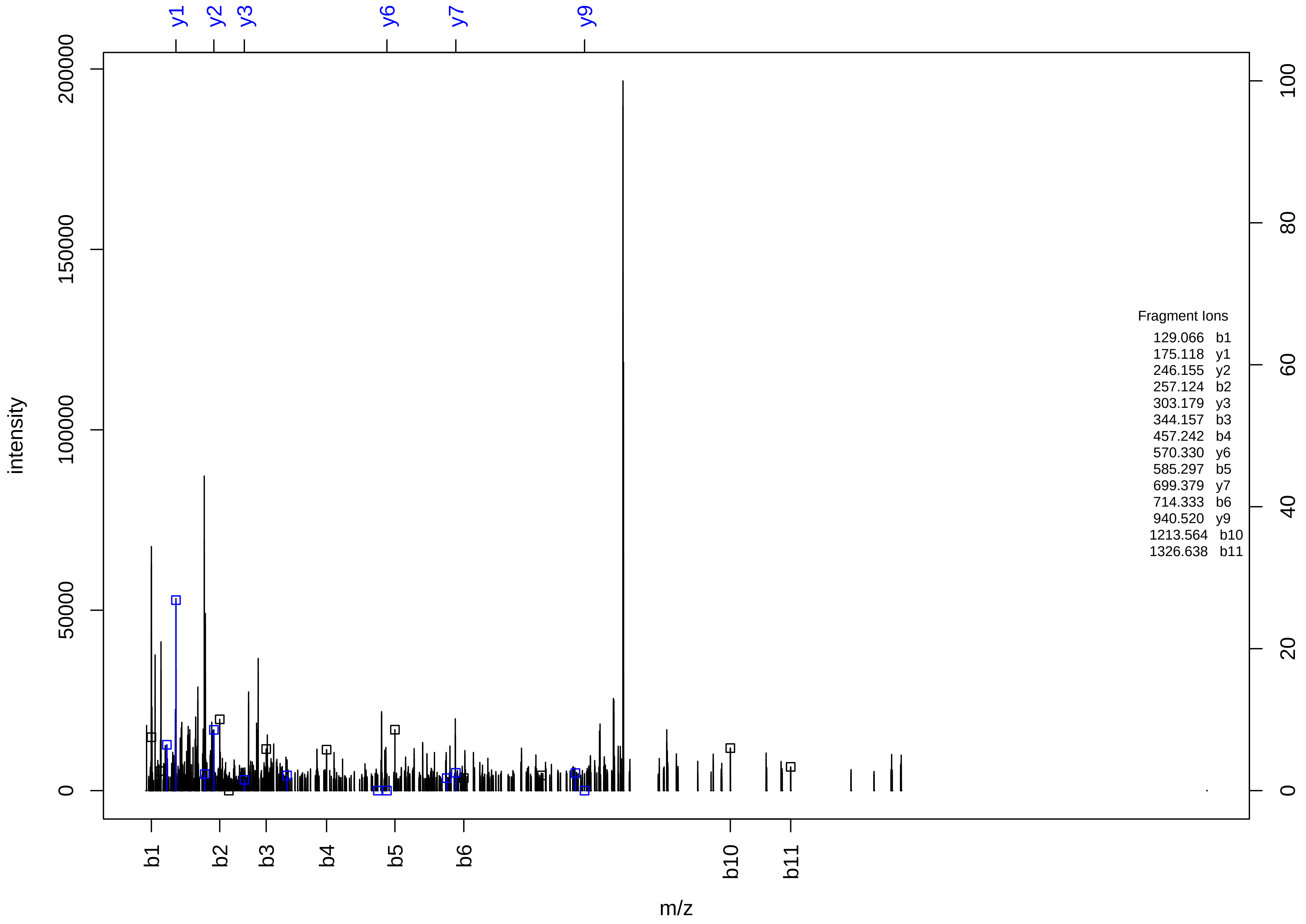

QQSLQQQLEQLQGLPGAR / File: sydney_ip_18.raw; SpectrumID: ; scans: 7663

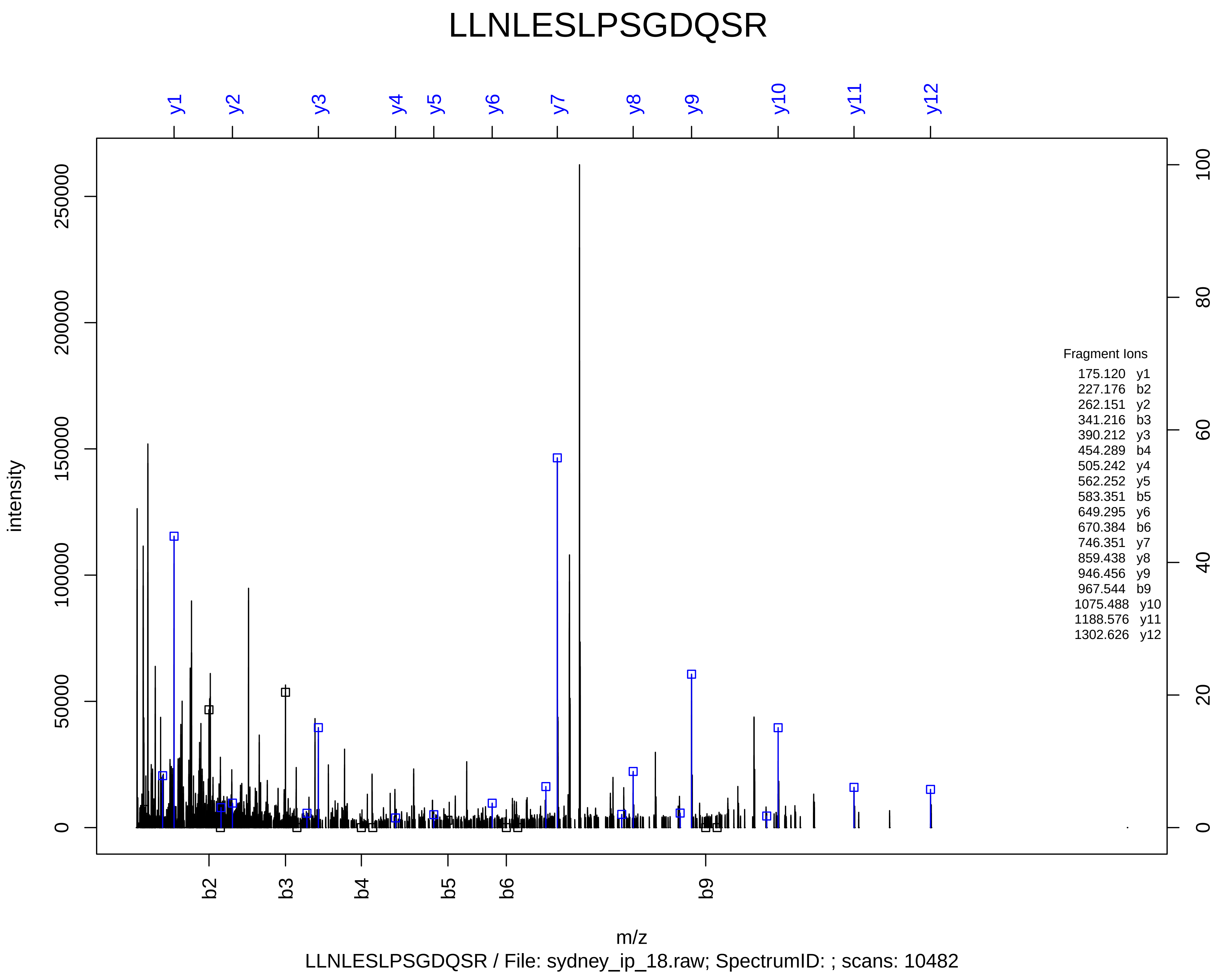
LLNLESLPSGDQSR
y1
y2
y3
y4
y5
y6
y7
y8
y9
y10
y11
y12
intensity
0
50000
100000
150000
200000
250000
0
20
40
60
80
100
Fragment Ions
175.120 y1
227.176 b2
262.151 y2
341.216 b3
390.212 y3
454.289 b4
505.242 y4
562.252 y5
583.351 b5
649.295 y6
670.384 b6
746.351 y7
859.438 y8
946.456 y9
967.544 b9
1075.488 y10
1188.576 y11
1302.626 y12
b2
b3
b4
b5
b6
b9
m/z
LLNLESLPSGDQSR / File: sydney_ip_18.raw; SpectrumID: ; scans: 10482

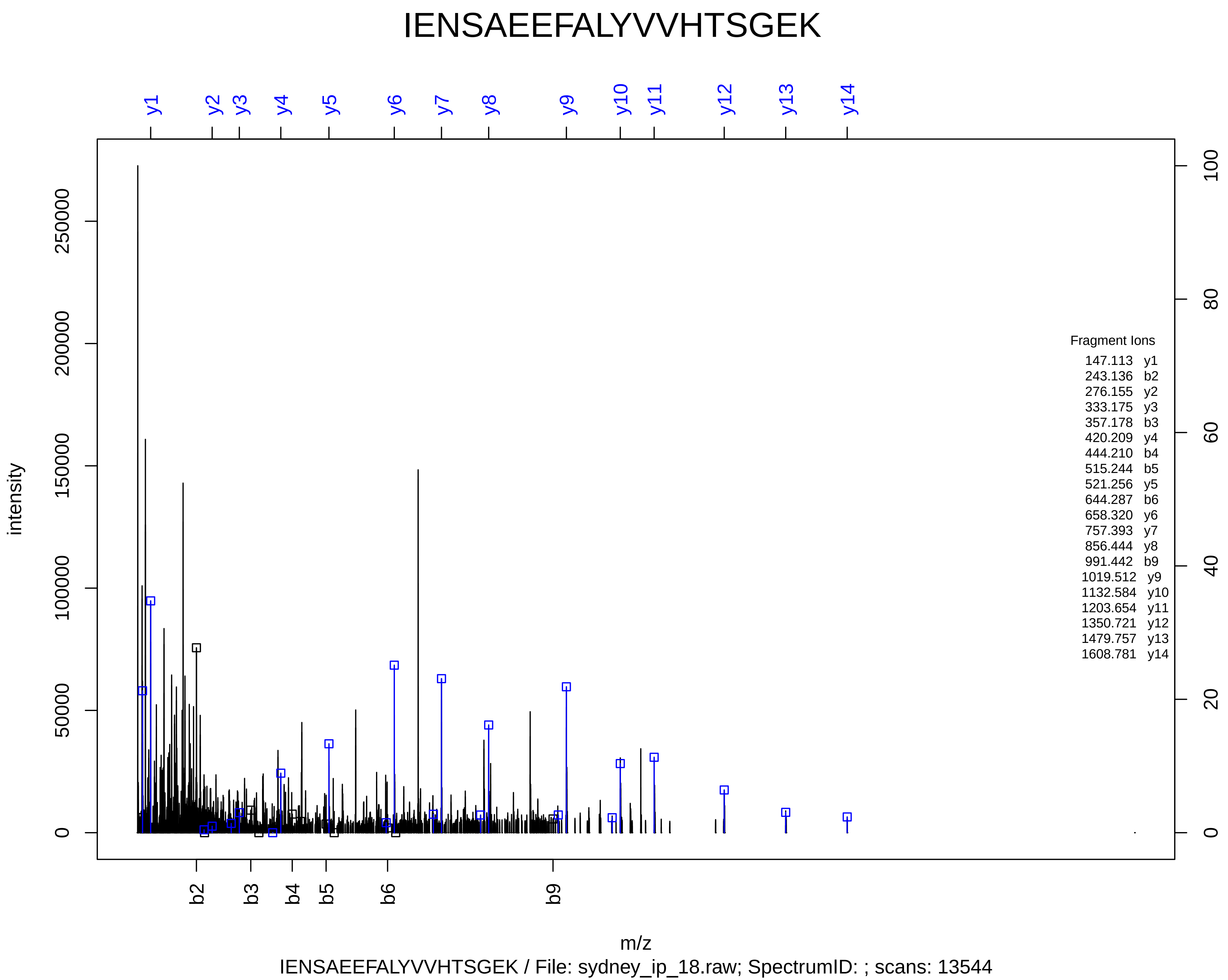
IENSAEEFALYVVHTSGEK
y1
y2
y3
y4
y5
y6
y7
y8
y9
y10
y11
y12
y13
y14
b2
b3
b4
b5
b6
b9
intensity
m/z
Fragment Ions
147.113 y1
243.136 b2
276.155 y2
333.175 y3
357.178 b3
420.209 y4
444.210 b4
515.244 b5
521.256 y5
644.287 b6
658.320 y6
757.393 y7
856.444 y8
991.442 b9
1019.512 y9
1132.584 y10
1203.654 y11
1350.721 y12
1479.757 y13
1608.781 y14
IENSAEEFALYVVHTSGEK / File: sydney_ip_18.raw; SpectrumID: ; scans: 13544

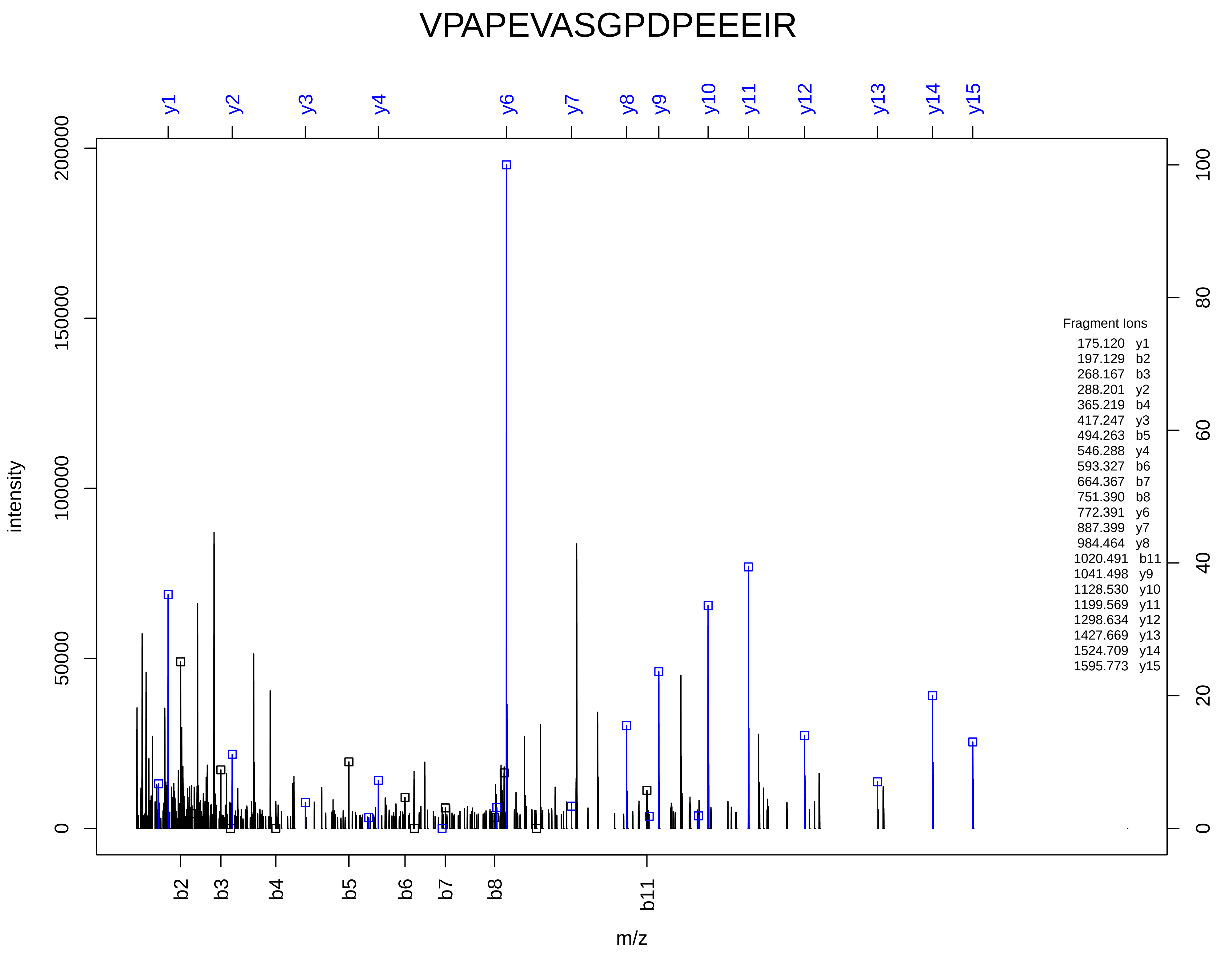
VPAPEVASGPDPEEEIR
y1
y2
y3
y4
y6
y7
y8
y9
y10
y11
y12
y13
y14
y15
intensity
0
50000
100000
150000
200000
0
20
40
60
80
100
b2
b3
b4
b5
b6
b7
b8
b11
m/z
Fragment Ions
175.120 y1
197.129 b2
268.167 b3
288.201 y2
365.219 b4
417.247 y3
494.263 b5
546.288 y4
593.327 b6
664.367 b7
751.390 b8
772.391 y6
887.399 y7
984.464 y8
1020.491 b11
1041.498 y9
1128.530 y10
1199.569 y11
1298.634 y12
1427.669 y13
1524.709 y14
1595.773 y15
VPAPEVASGPDPEEEIR / File: sydney_ip_18.raw; SpectrumID: ; scans: 7469

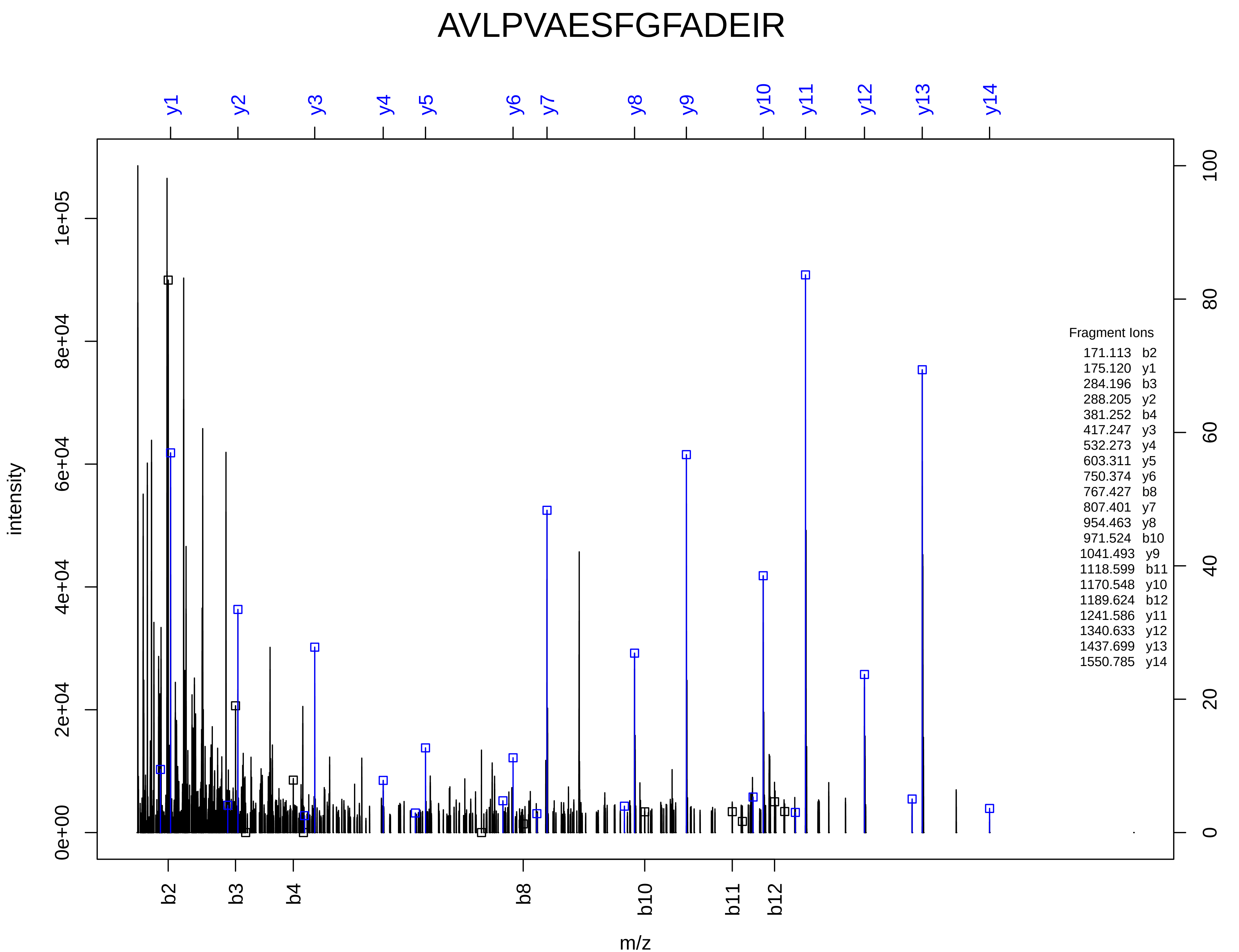

AVLPVAESFGFADEIR / File: sydney_ip_18.raw; SpectrumID: ; scans: 17143

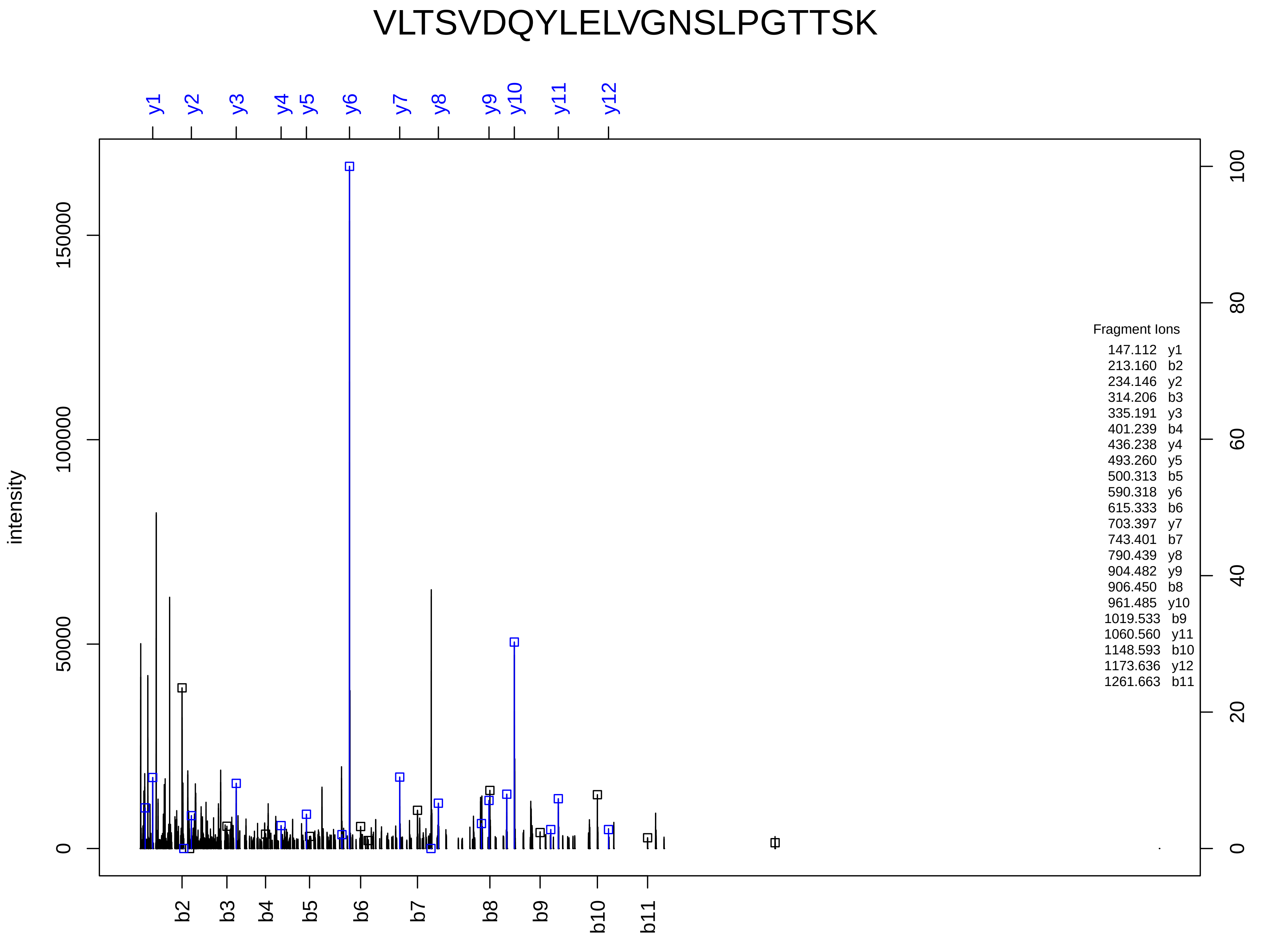

VLTSVDQYLELVGNSLPGTTSK / File: sydney_ip_18.raw; SpectrumID: ; scans: 18852

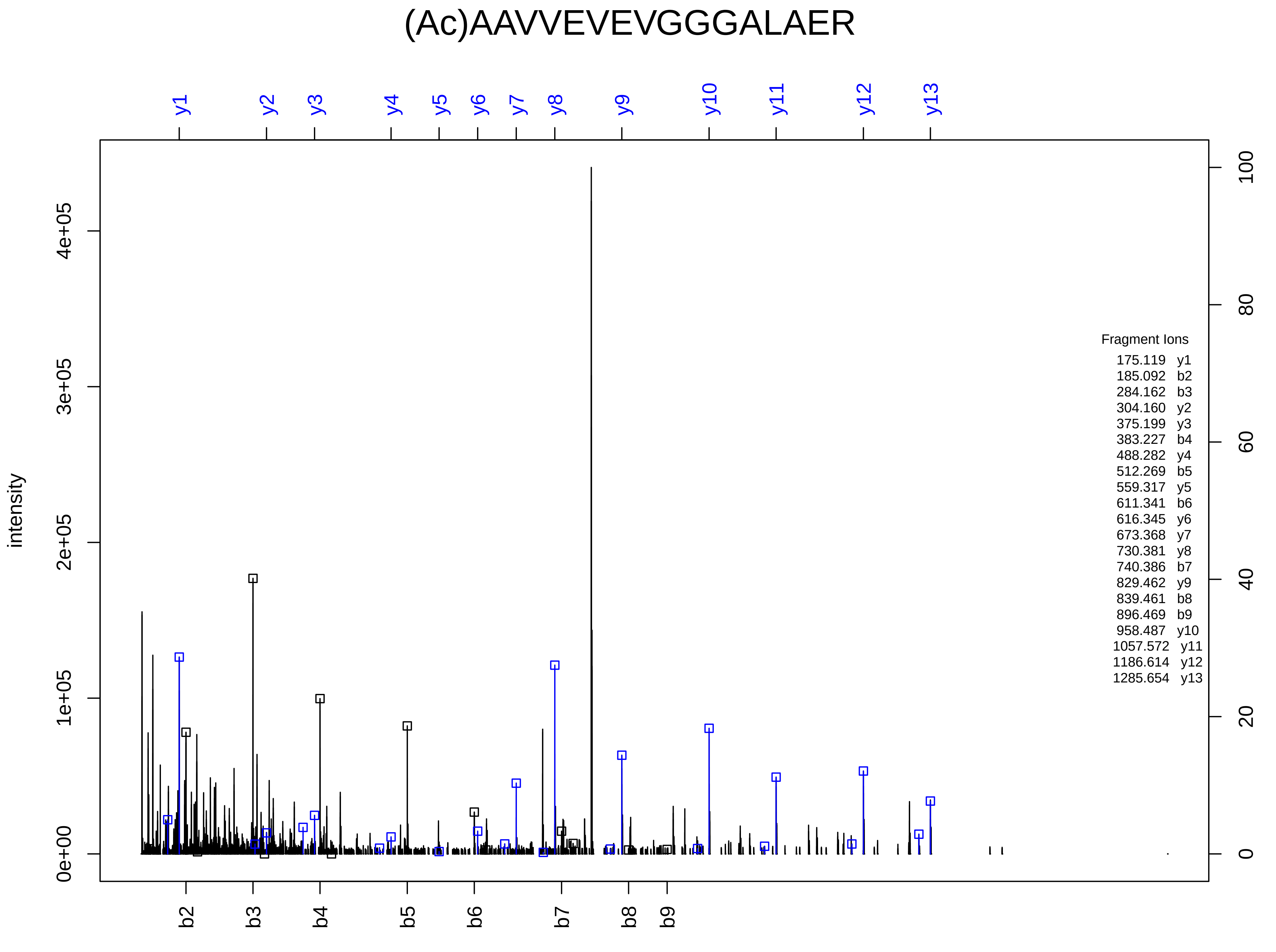

AAVVEVEVGGGALAER / File: sydney_ip_18.raw; SpectrumID: ; scans: 16135

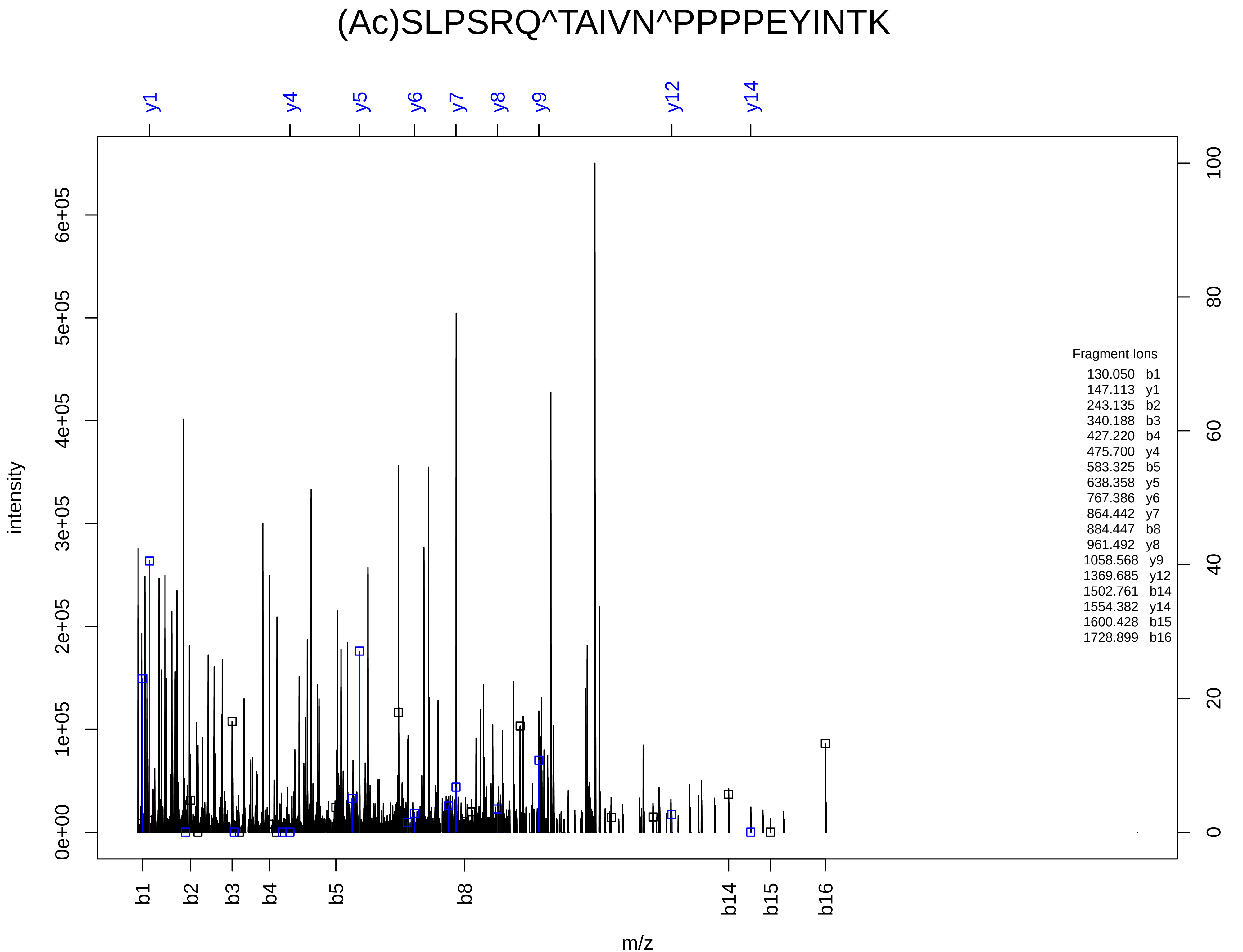

SLPSRQTAIVNPPPPEYINTK / File: sydney_ip_18.raw; SpectrumID: ; scans: 13956

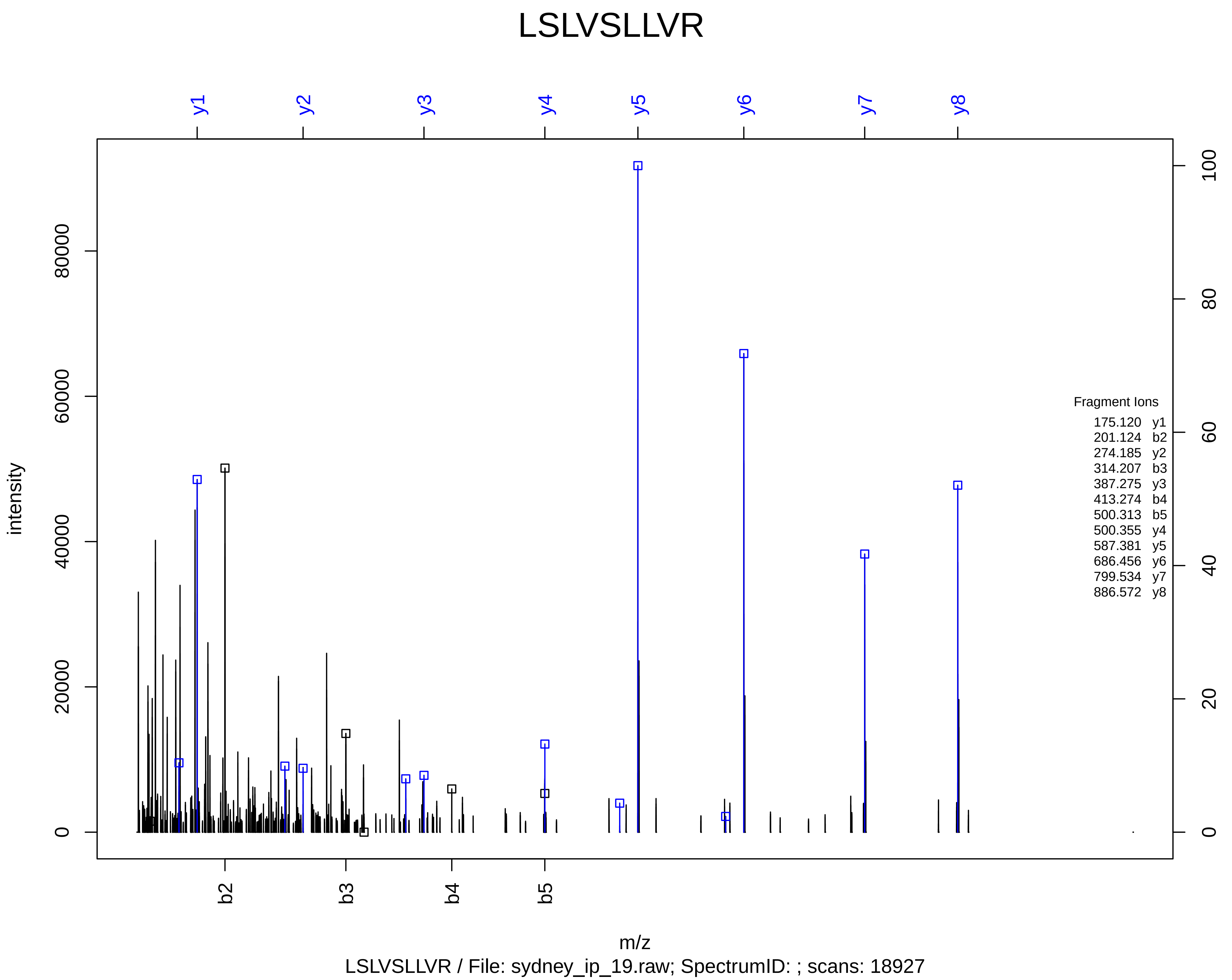

LSLVSLLVR
y1
y2
y3
y4
y5
y6
y7
y8
intensity
0
20000
40000
60000
80000
0
20
40
60
80
100
b2
b3
b4
b5
m/z
Fragment Ions
175.120 y1
201.124 b2
274.185 y2
314.207 b3
387.275 y3
413.274 b4
500.313 b5
500.355 y4
587.381 y5
686.456 y6
799.534 y7
886.572 y8
LSLVSLLVR / File: sydney_ip_19.raw; SpectrumID: ; scans: 18927

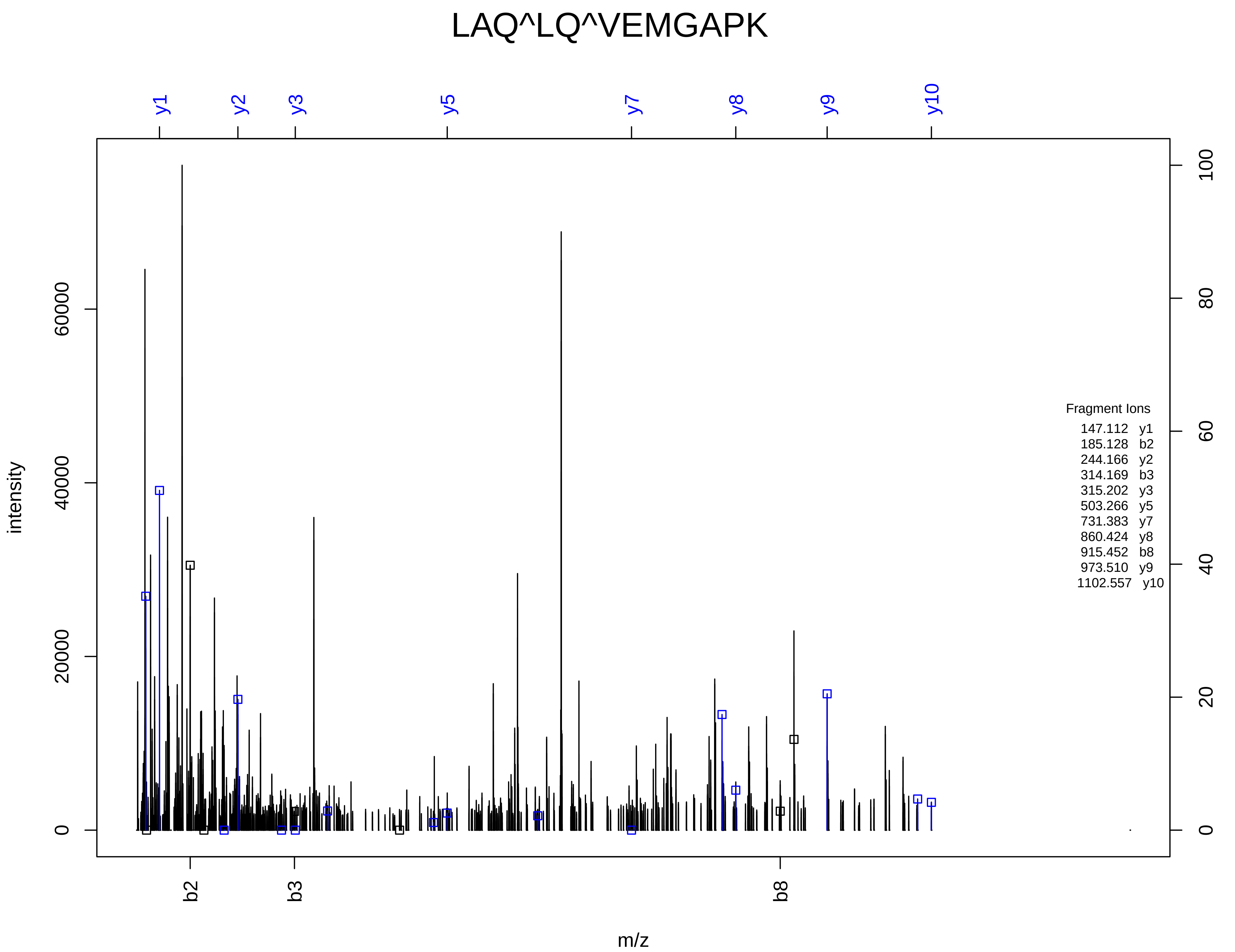

LAQLQVEMGAPK / File: sydney_ip_19.raw; SpectrumID: ; scans: 2772

SPLSSPQQTQLLGELEK

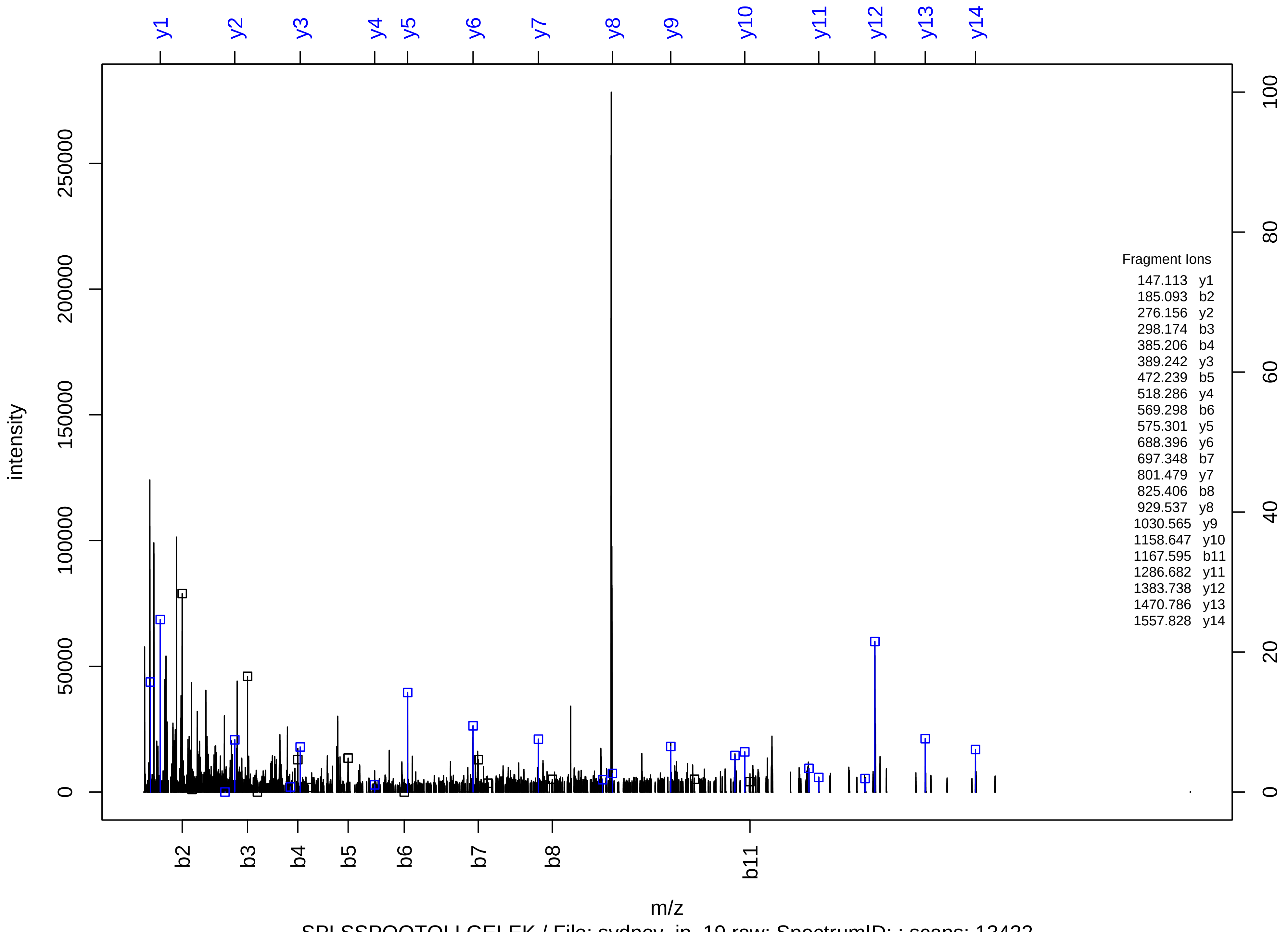

SPLSSPQQTQLLGELEK / File: sydney_ip_19.raw; SpectrumID: ; scans: 13422

LLLPLFR

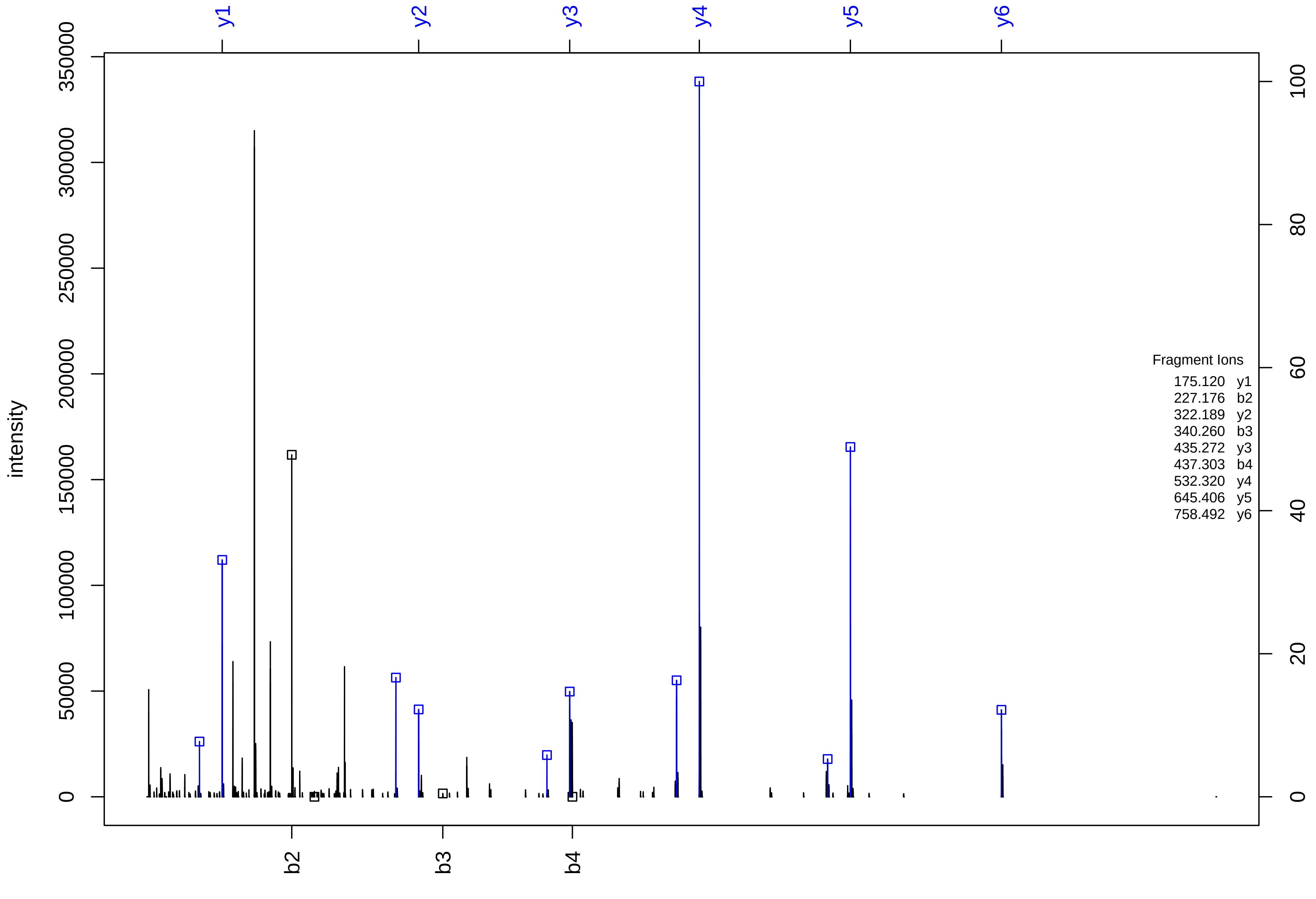

LLLPLFR / File: sydney_ip_19.raw; SpectrumID: ; scans: 18313

(Ac)AFVAGVIR

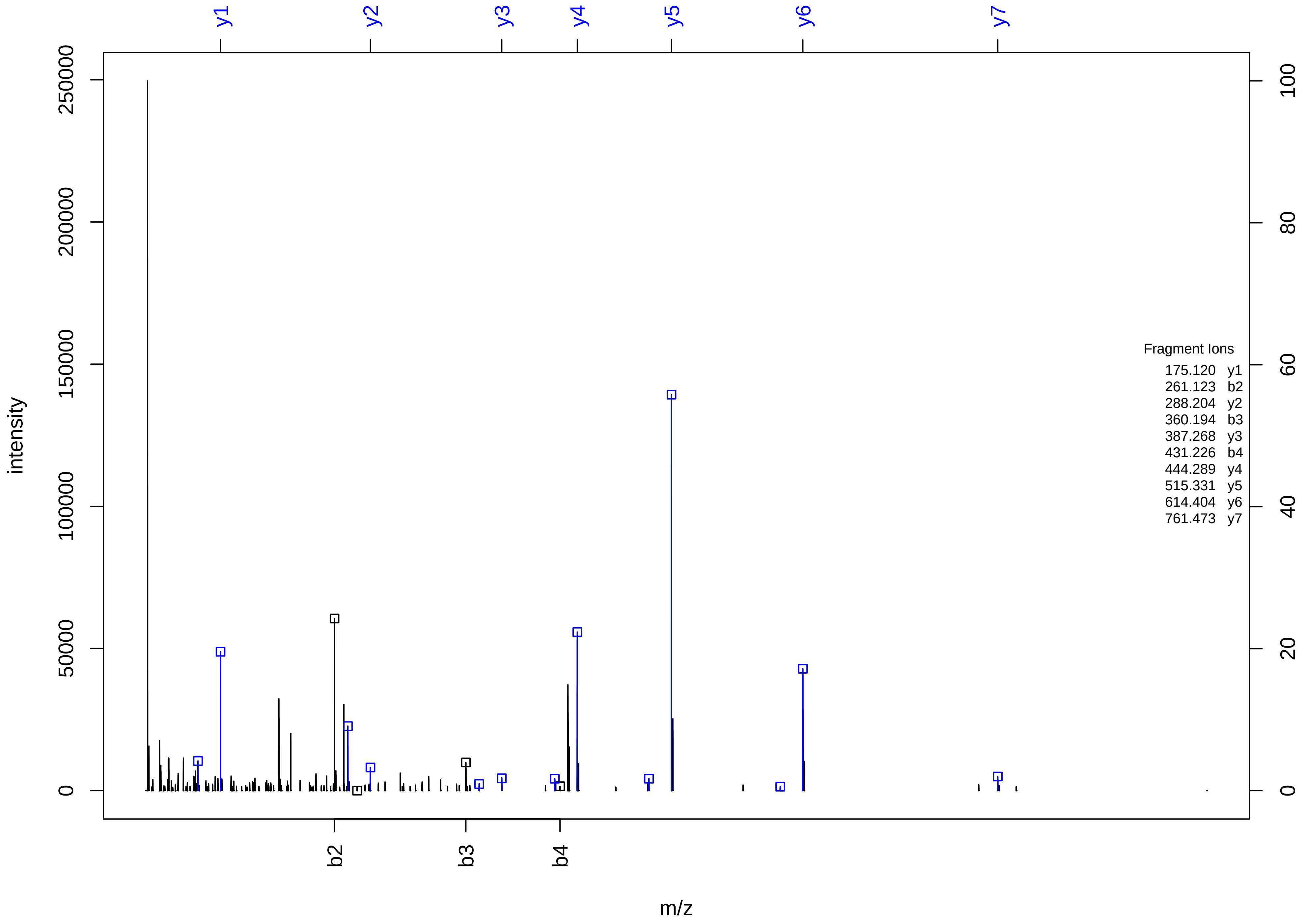

AFVAGVIR / File: sydney_ip_19.raw; SpectrumID: ; scans: 17543

TSQTVATFLDELAQK

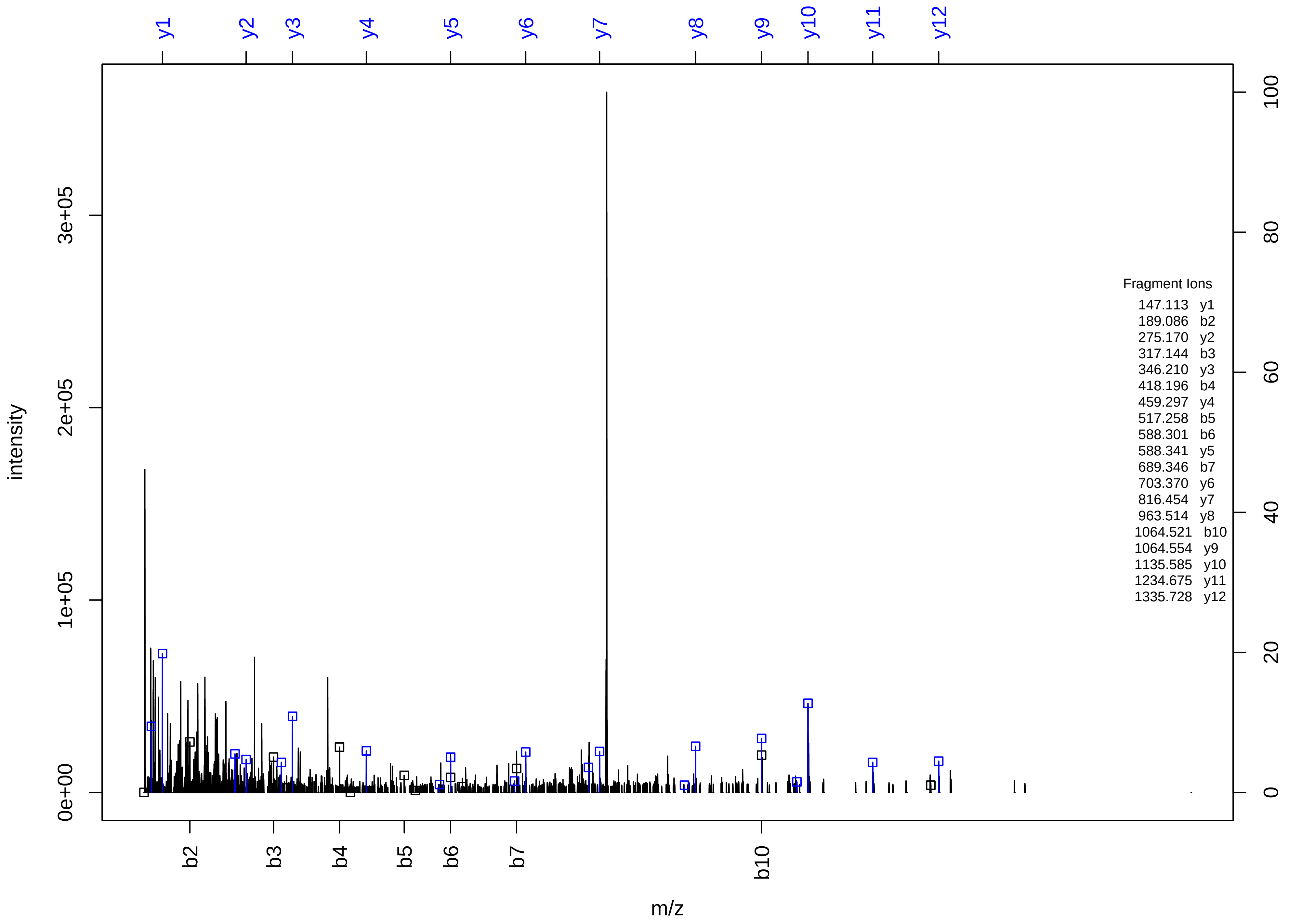

TSQTVATFLDELAQK / File: sydney_ip_19.raw; SpectrumID: ; scans: 18004

GLDEDETNFLDEVSR

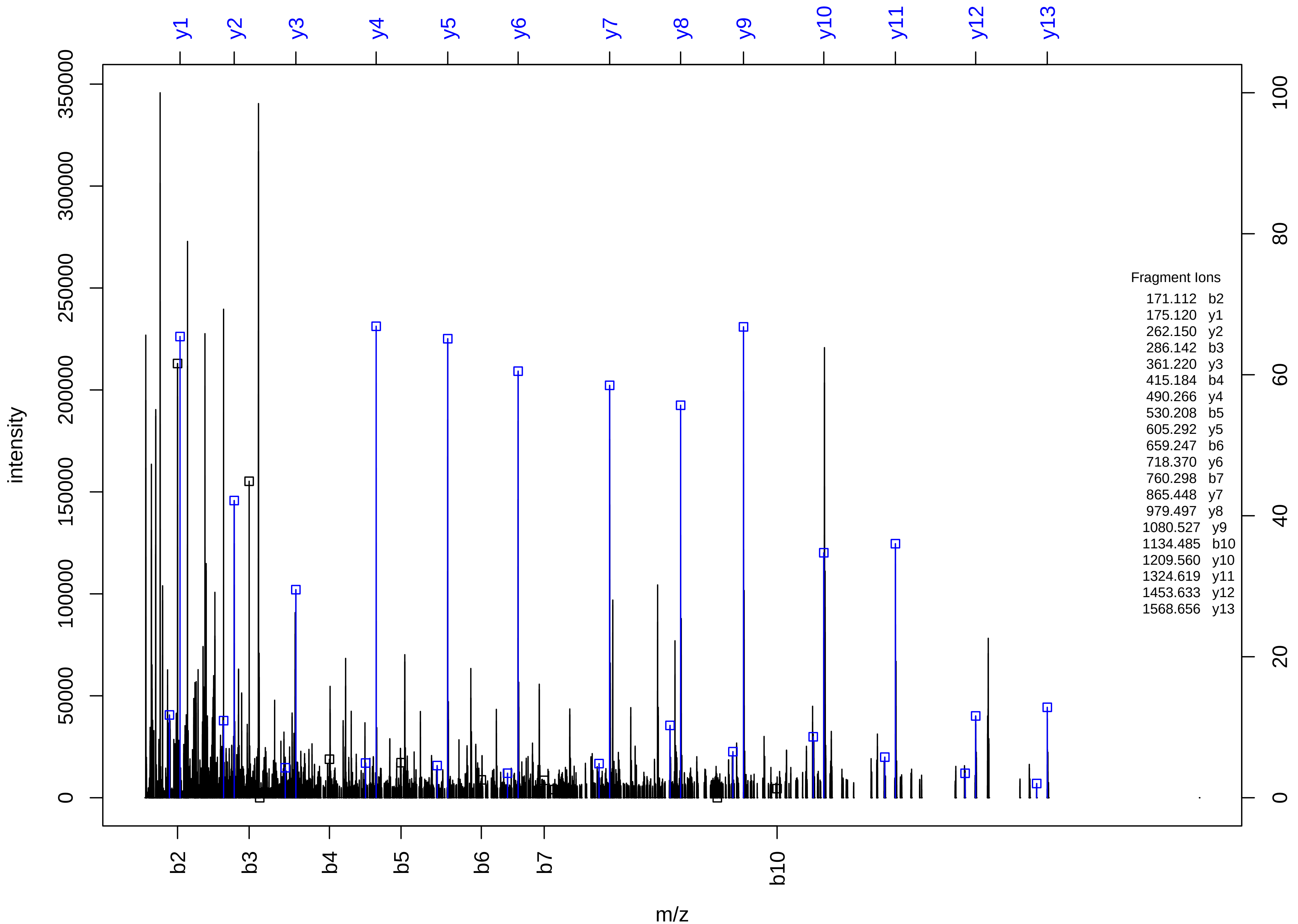

GLDEDETNFLDEVSR / File: sydney_ip_19.raw; SpectrumID: ; scans: 14427

AYAEELASHGLNVILISQEEEK

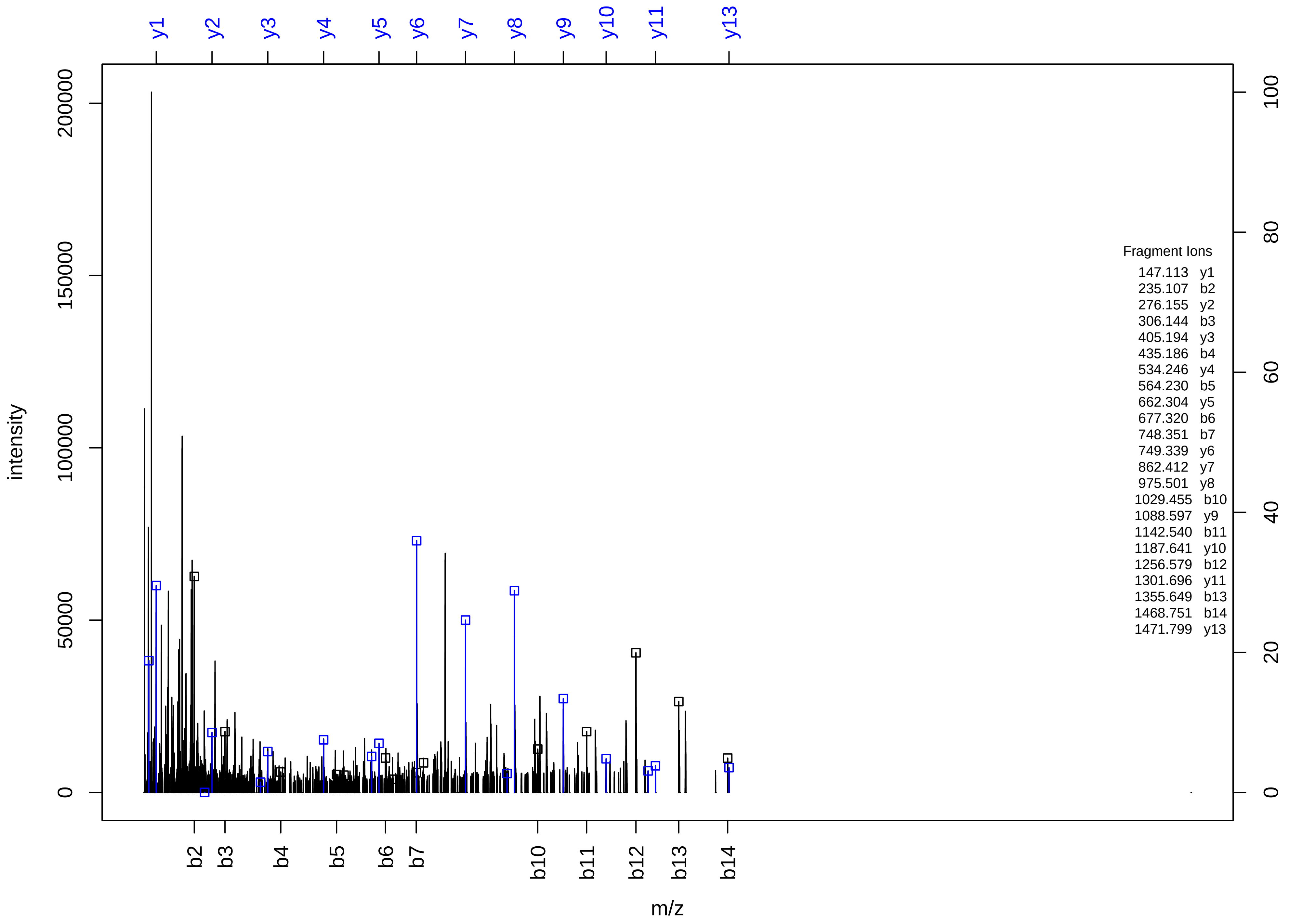

AYAEELASHGLNVILISQEEEK / File: sydney_ip_19.raw; SpectrumID: ; scans: 15702

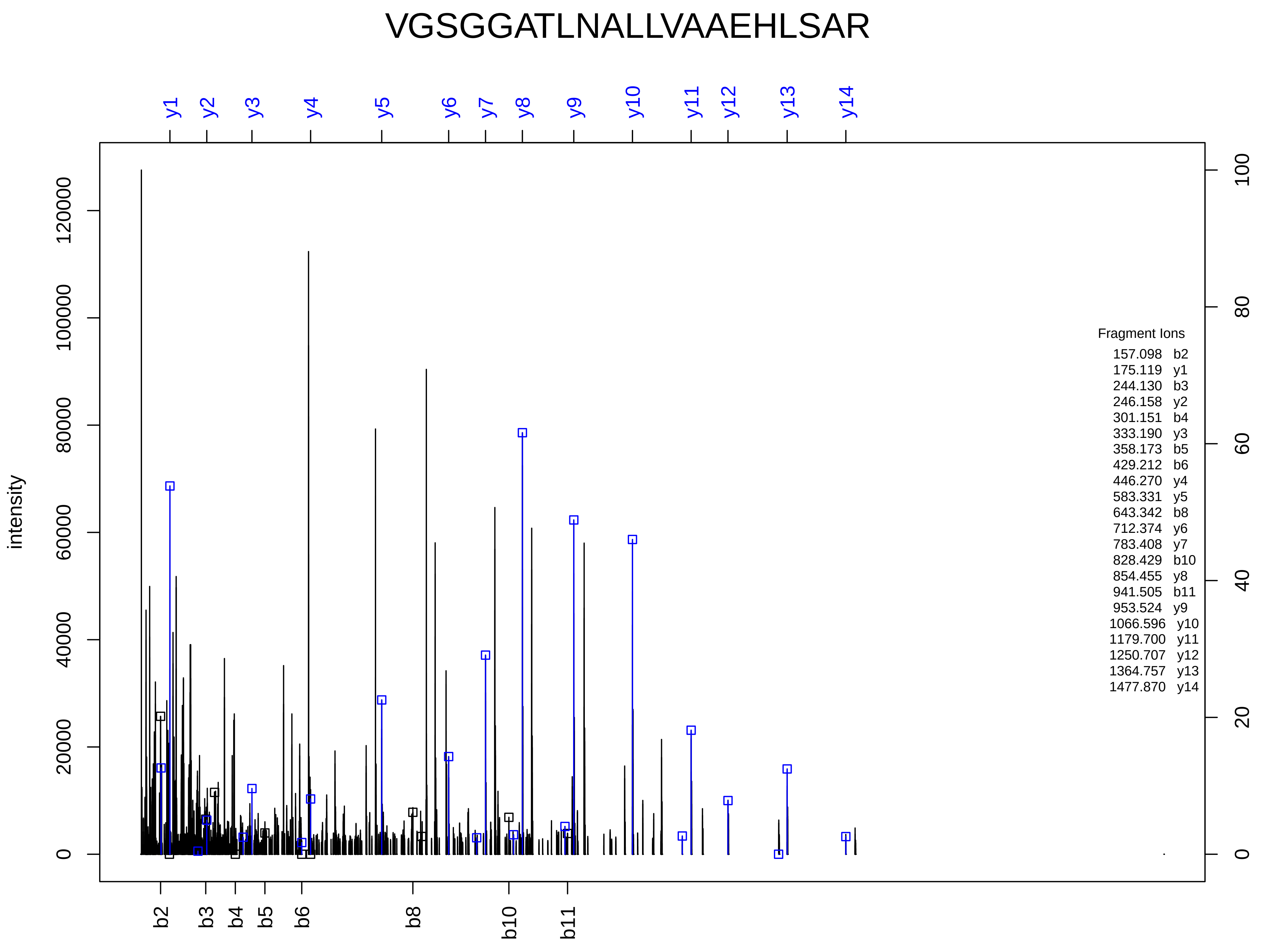

VGSGGATLNALLVAAEHLSAR / File: sydney_ip_19.raw; SpectrumID: ; scans: 19529

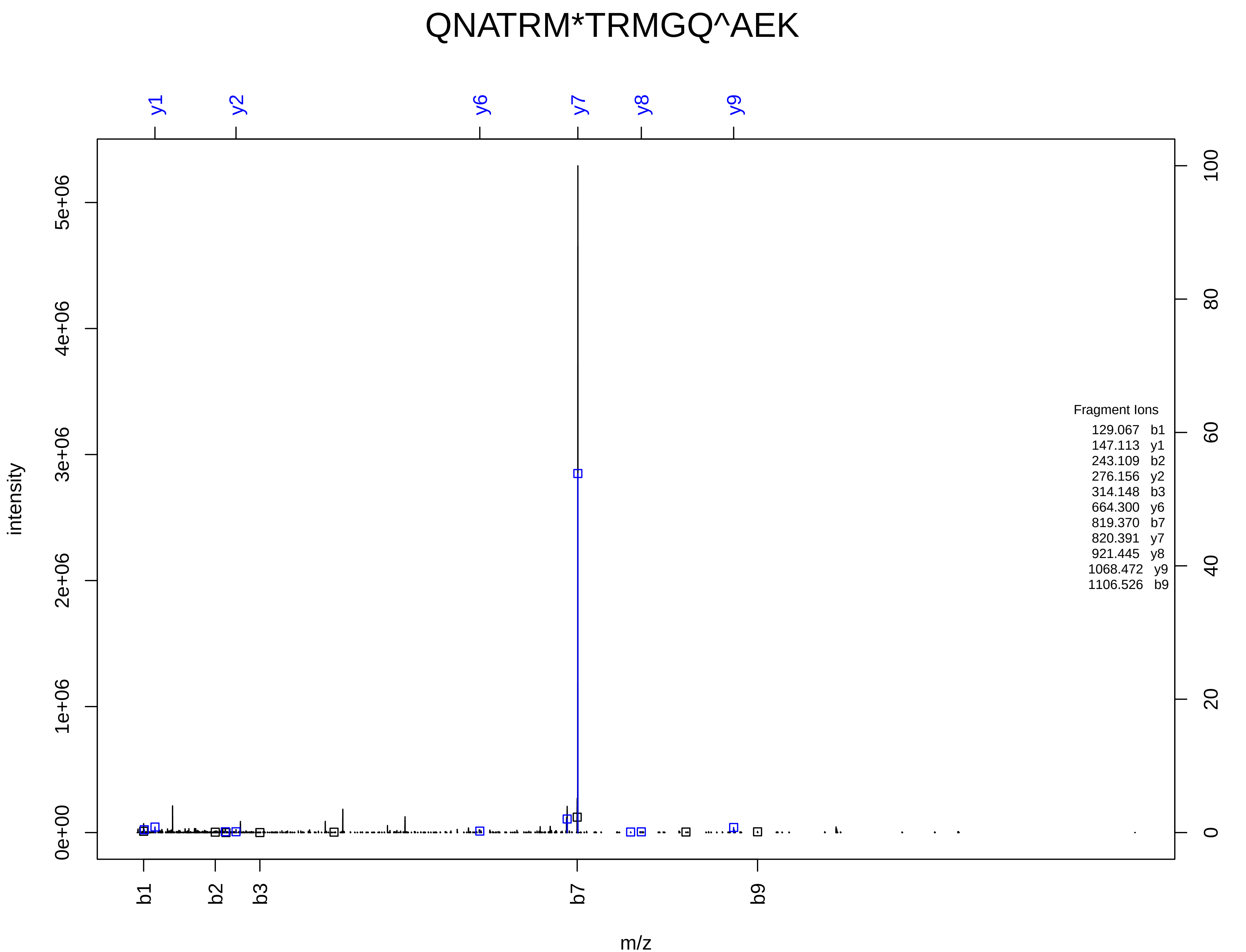
QNATRM*TRMGQ^AEK
y1
y2
y6
y7
y8
y9
intensity
5e+06
4e+06
3e+06
2e+06
1e+06
0e+00
100
80
60
40
20
0
Fragment Ions
129.067 b1
147.113 y1
243.109 b2
276.156 y2
314.148 b3
664.300 y6
819.370 b7
820.391 y7
921.445 y8
1068.472 y9
1106.526 b9
b1
b2
b3
b7
b9
m/z
QNATRMTRMGQAEK / File: sydney_ip_19.raw; SpectrumID: ; scans: 6073

ALFNAQFGSIFR

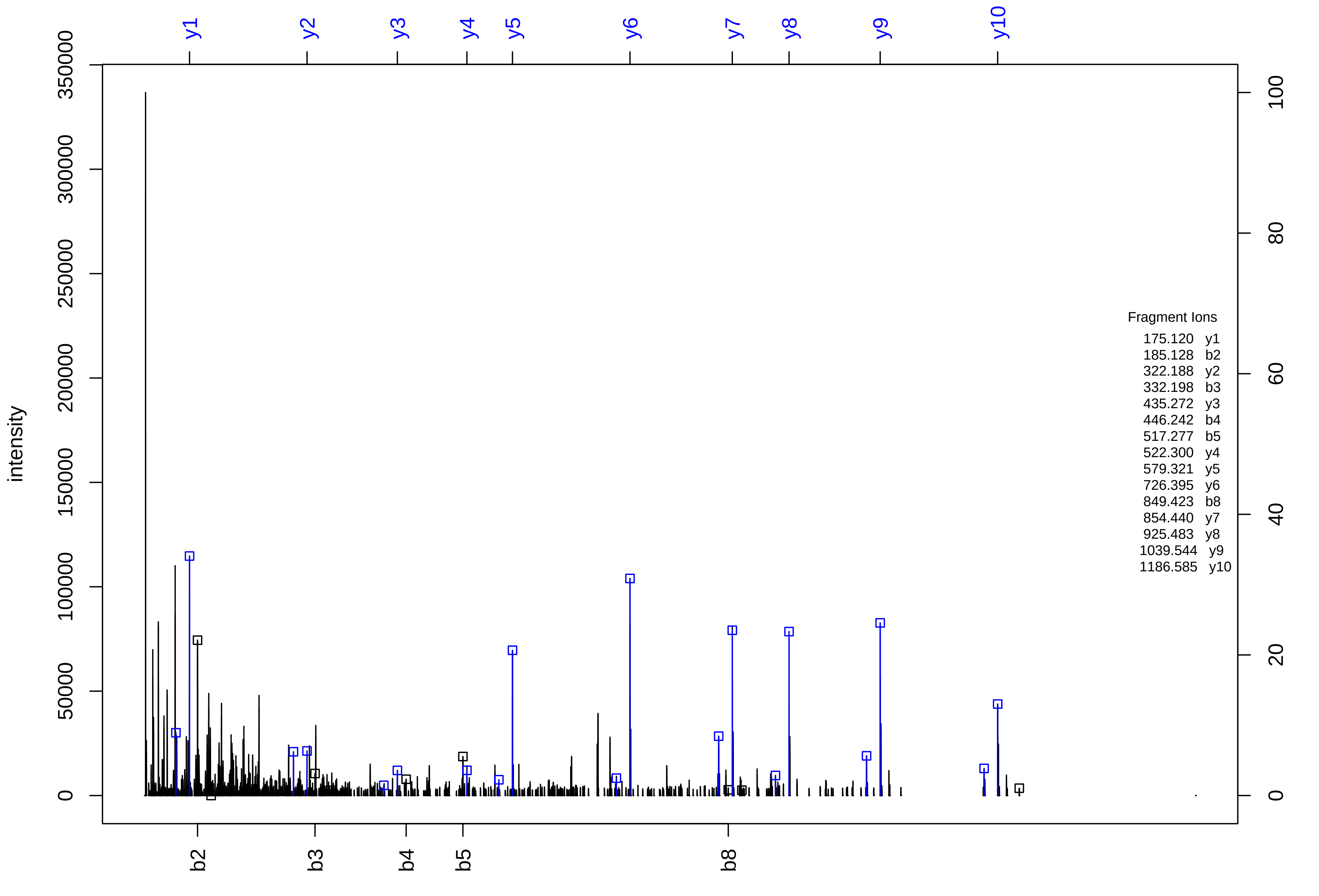

ALFNAQFGSIFR / File: sydney_ip_19.raw; SpectrumID: ; scans: 17976

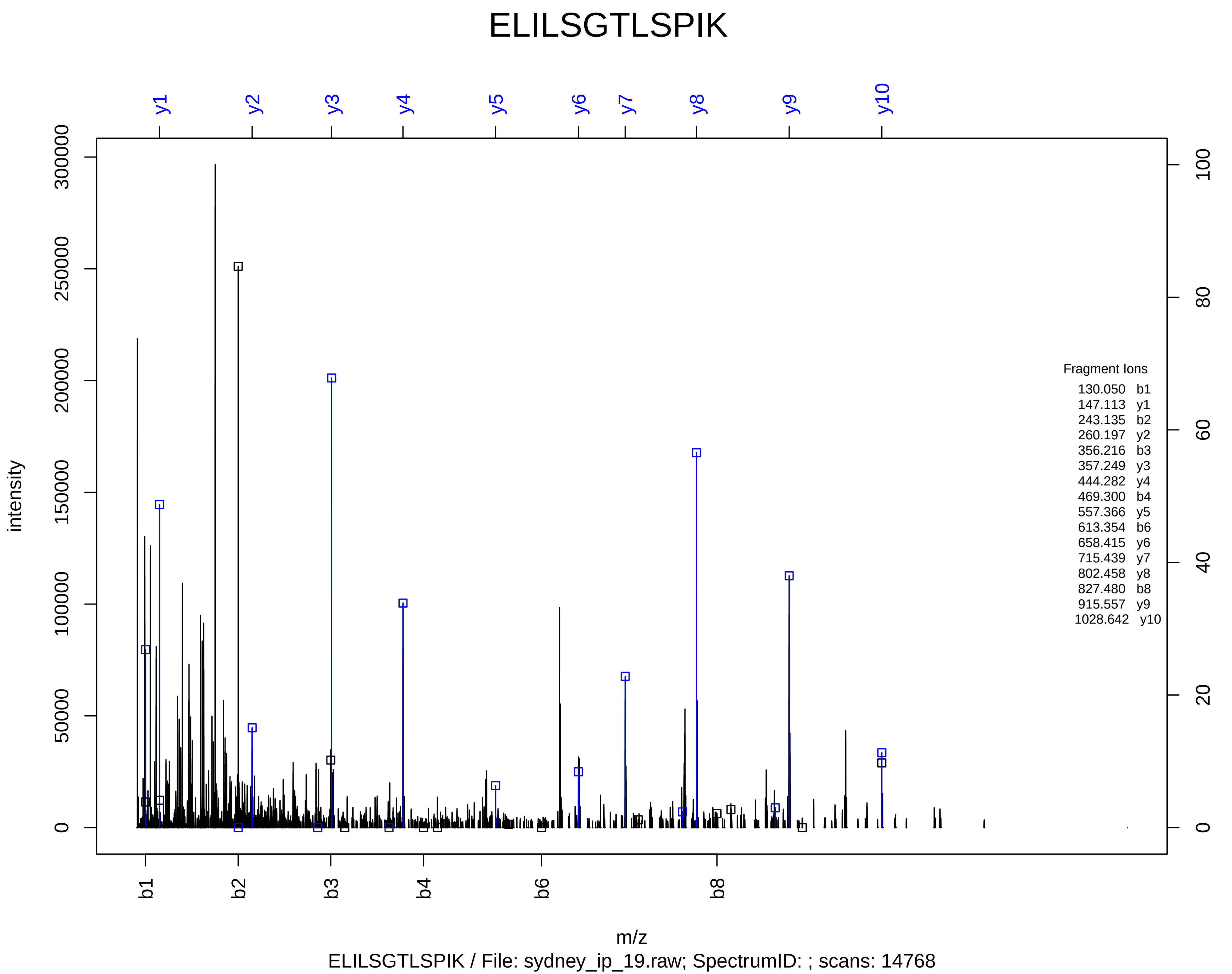
ELILSGTLSPIK
y1
y2
y3
y4
y5
y6
y7
y8
y9
y10
intensity
0
50000
100000
150000
200000
250000
300000
0
20
40
60
80
100
b1
b2
b3
b4
b6
b8
m/z
Fragment Ions
130.050 b1
147.113 y1
243.135 b2
260.197 y2
356.216 b3
357.249 y3
444.282 y4
469.300 b4
557.366 y5
613.354 b6
658.415 y6
715.439 y7
802.458 y8
827.480 b8
915.557 y9
1028.642 y10
ELILSGTLSPIK / File: sydney_ip_19.raw; SpectrumID: ; scans: 14768

MAGWIQ^AQ^Q^LQGDALR

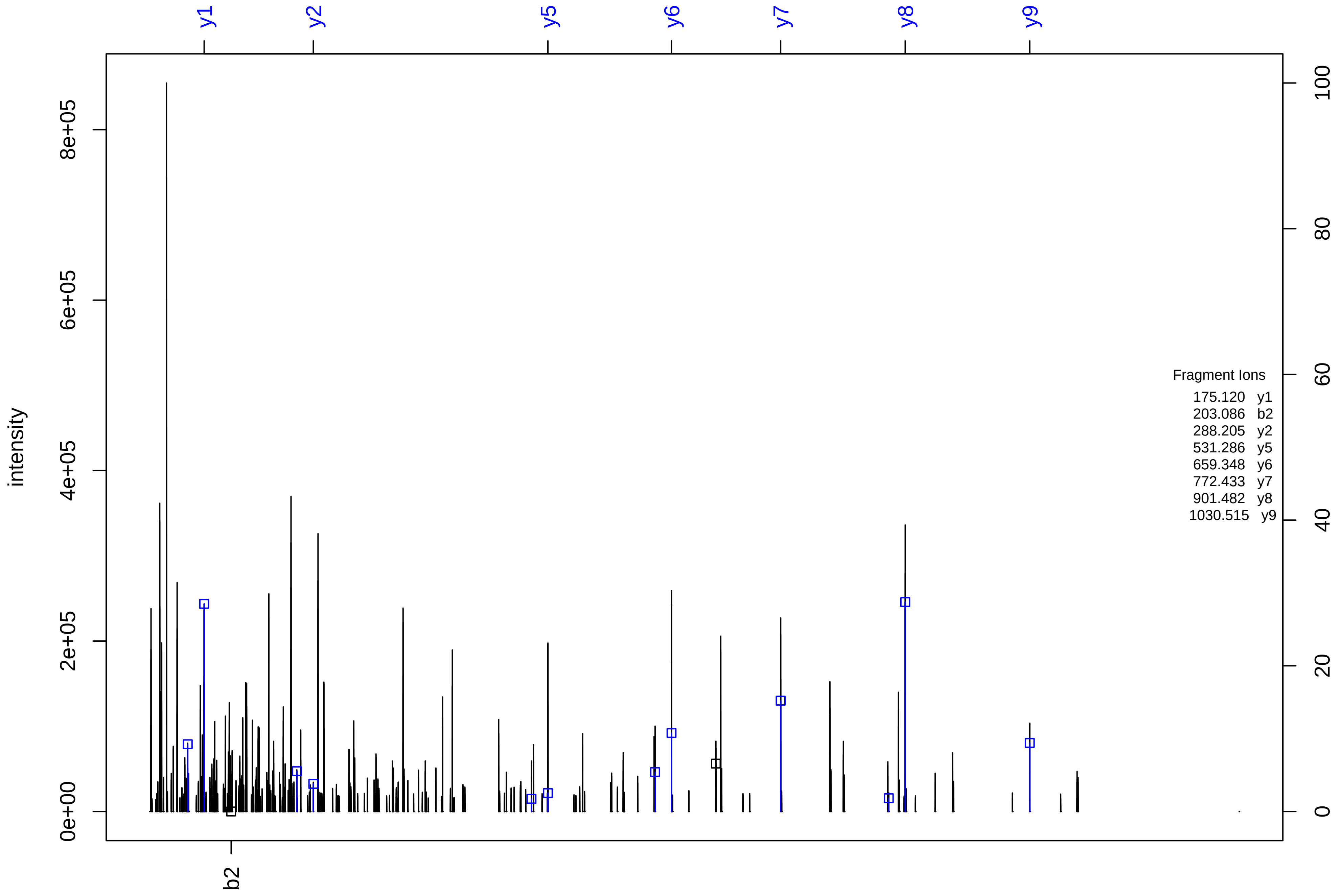

MAGWIQAQQLQGDALR / File: sydney_ip_19.raw; SpectrumID: ; scans: 7797

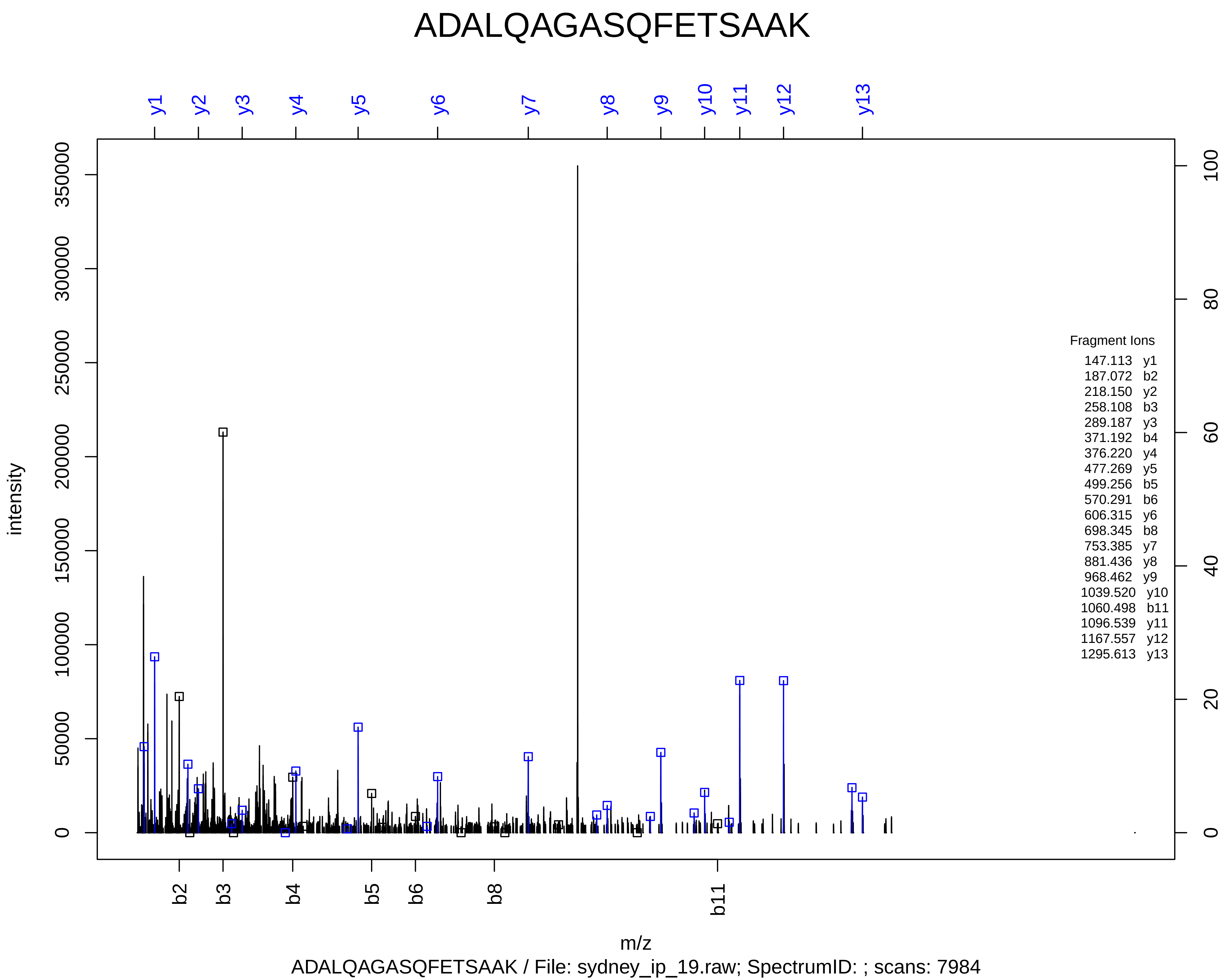
ADALQAGASQFETSAAK
y1
y2
y3
y4
y5
y6
y7
y8
y9
y10
y11
y12
y13
intensity
0
50000
100000
150000
200000
250000
300000
350000
0
20
40
60
80
100
b2
b3
b4
b5
b6
b8
b11
m/z
Fragment Ions
147.113 y1
187.072 b2
218.150 y2
258.108 b3
289.187 y3
371.192 b4
376.220 y4
477.269 y5
499.256 b5
570.291 b6
606.315 y6
698.345 b8
753.385 y7
881.436 y8
968.462 y9
1039.520 y10
1060.498 b11
1096.539 y11
1167.557 y12
1295.613 y13
ADALQAGASQFETSAAK / File: sydney_ip_19.raw; SpectrumID: ; scans: 7984

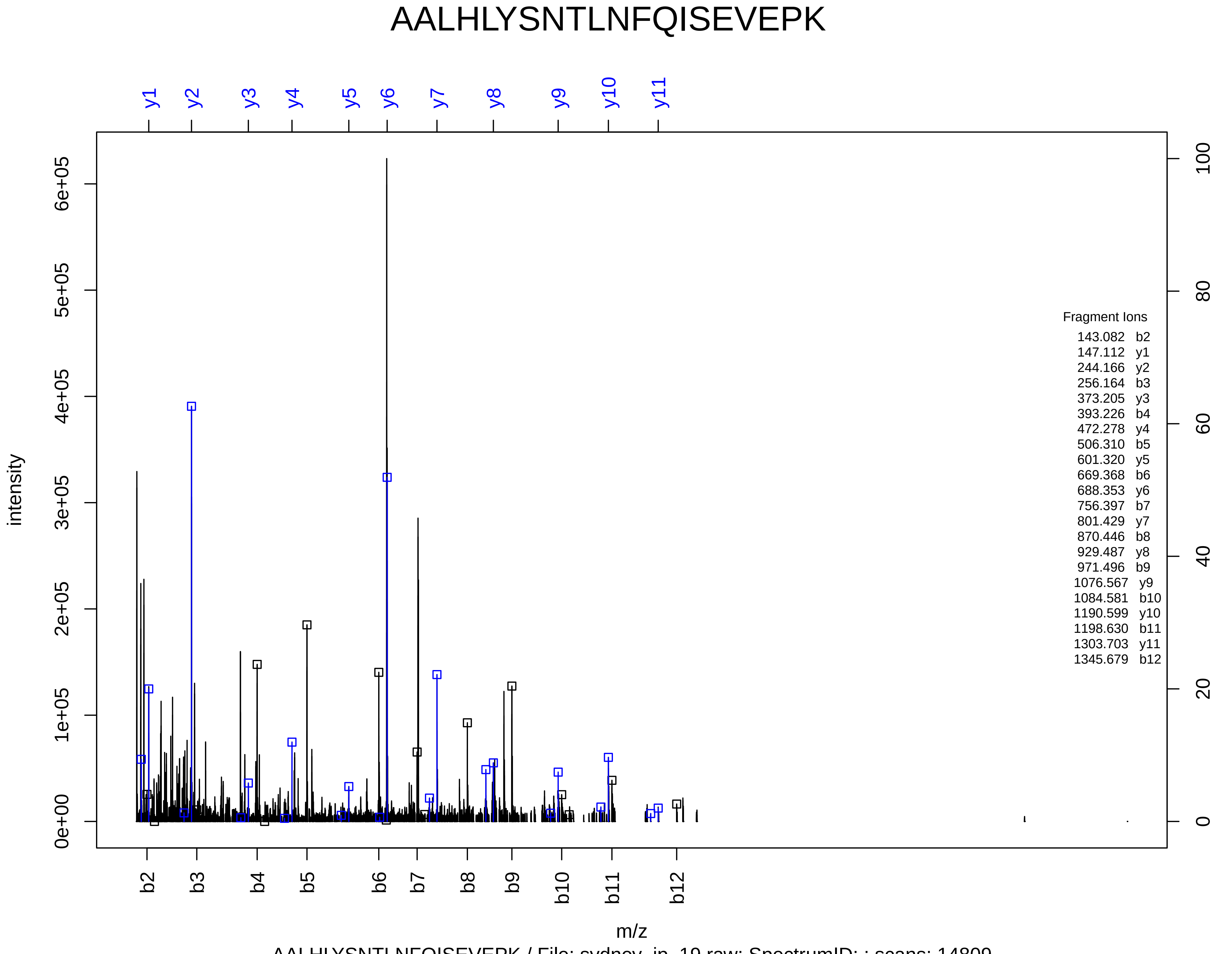
AALHLYSNTLNFQISEVEPK
y1
y2
y3
y4
y5
y6
y7
y8
y9
y10
y11
intensity
0e+00
1e+05
2e+05
3e+05
4e+05
5e+05
6e+05
0
20
40
60
80
100
Fragment Ions
143.082 b2
147.112 y1
244.166 y2
256.164 b3
373.205 y3
393.226 b4
472.278 y4
506.310 b5
601.320 y5
669.368 b6
688.353 y6
756.397 b7
801.429 y7
870.446 b8
929.487 y8
971.496 b9
1076.567 y9
1084.581 b10
1190.599 y10
1198.630 b11
1303.703 y11
1345.679 b12
b2
b3
b4
b5
b6
b7
b8
b9
b10
b11
b12
m/z
AALHLYSNTLNFQISEVEPK / File: sydney_ip_19.raw; SpectrumID: ; scans: 14809

NKTEQ^FQ^AR

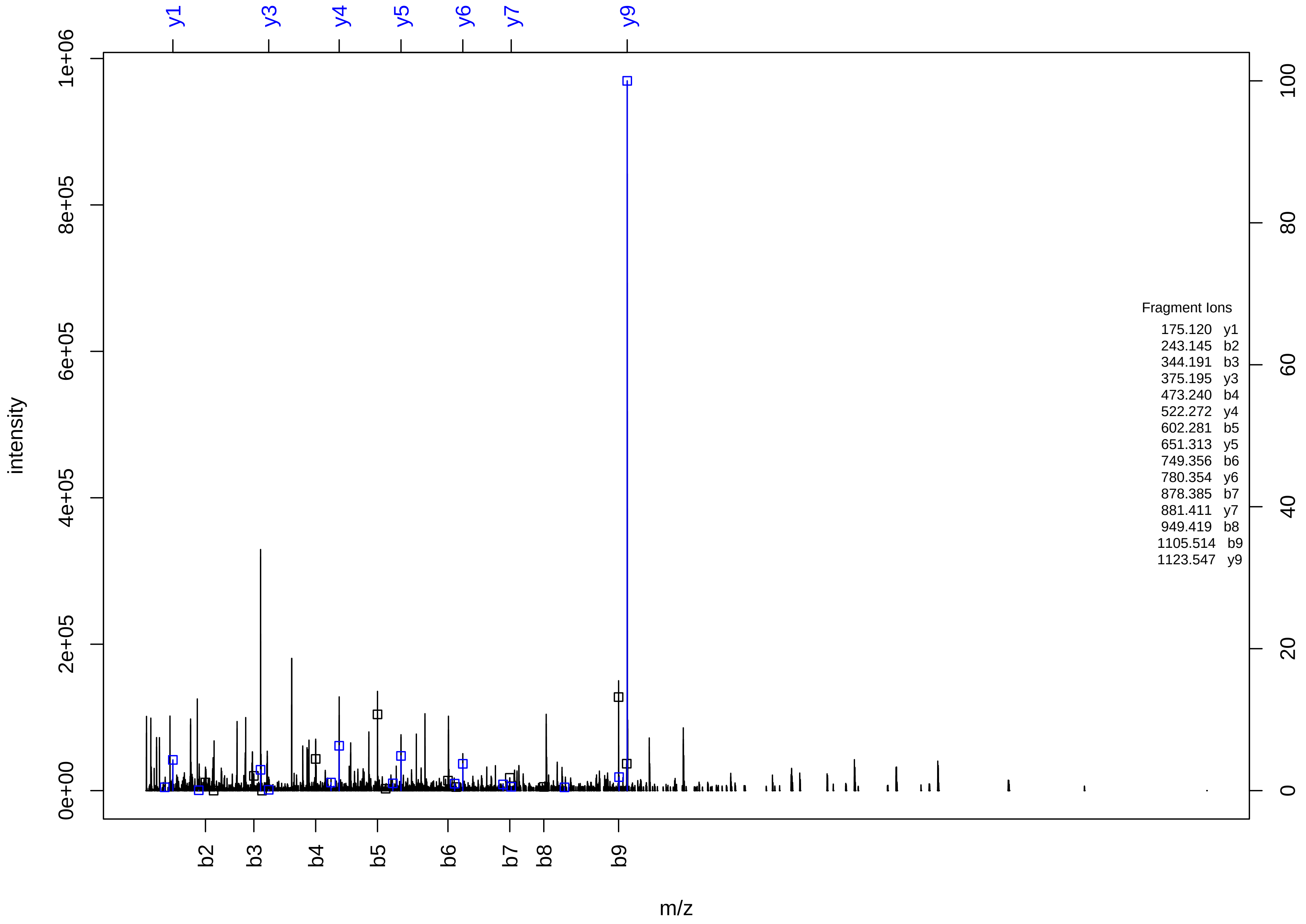

NKTEQFQAR / File: sydney_ip_19.raw; SpectrumID: ; scans: 10872

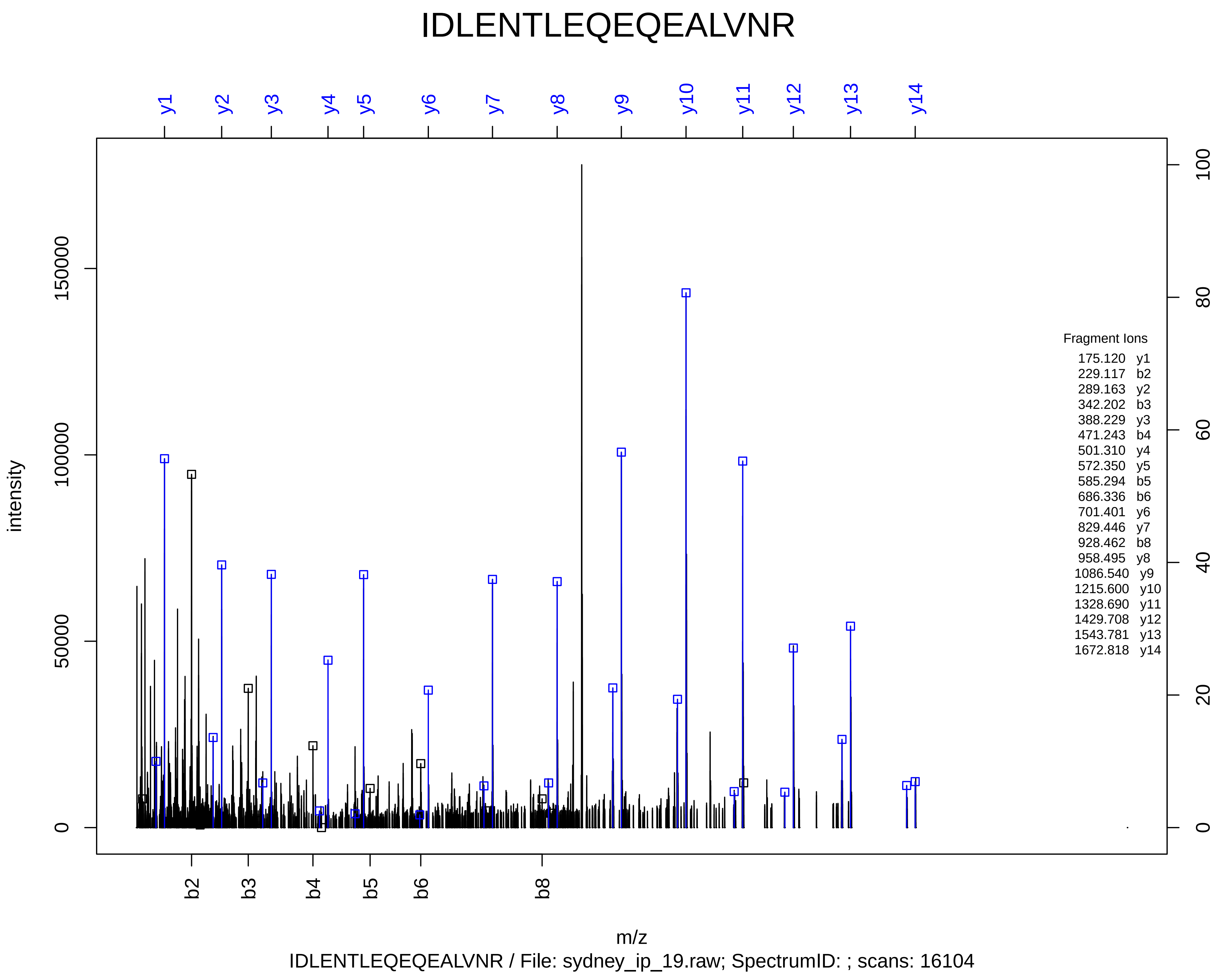
IDLENTLEQEQEALVNR
y1
y2
y3
y4
y5
y6
y7
y8
y9
y10
y11
y12
y13
y14
b2
b3
b4
b5
b6
b8
intensity
0
50000
100000
150000
0
20
40
60
80
100
m/z
Fragment Ions
175.120 y1
229.117 b2
289.163 y2
342.202 b3
388.229 y3
471.243 b4
501.310 y4
572.350 y5
585.294 b5
686.336 b6
701.401 y6
829.446 y7
928.462 b8
958.495 y8
1086.540 y9
1215.600 y10
1328.690 y11
1429.708 y12
1543.781 y13
1672.818 y14
IDLENTLEQEQEALVNR / File: sydney_ip_19.raw; SpectrumID: ; scans: 16104

INLLSHDYGDIVAQELLYR

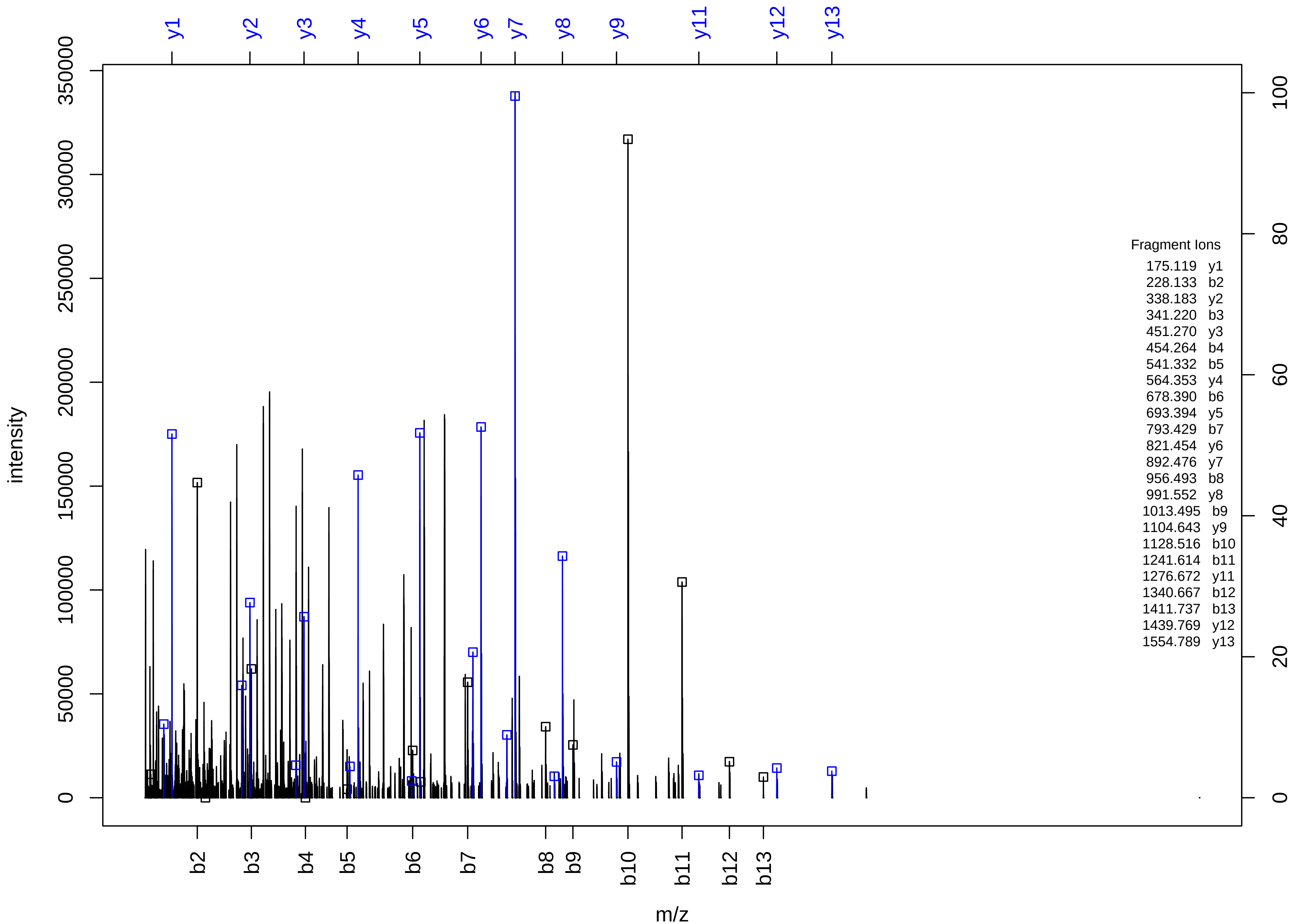

INLLSHDYGDIVAQELLYR / File: sydney_ip_19.raw; SpectrumID: ; scans: 18583

FVAPEEVLPFTEGDILEK

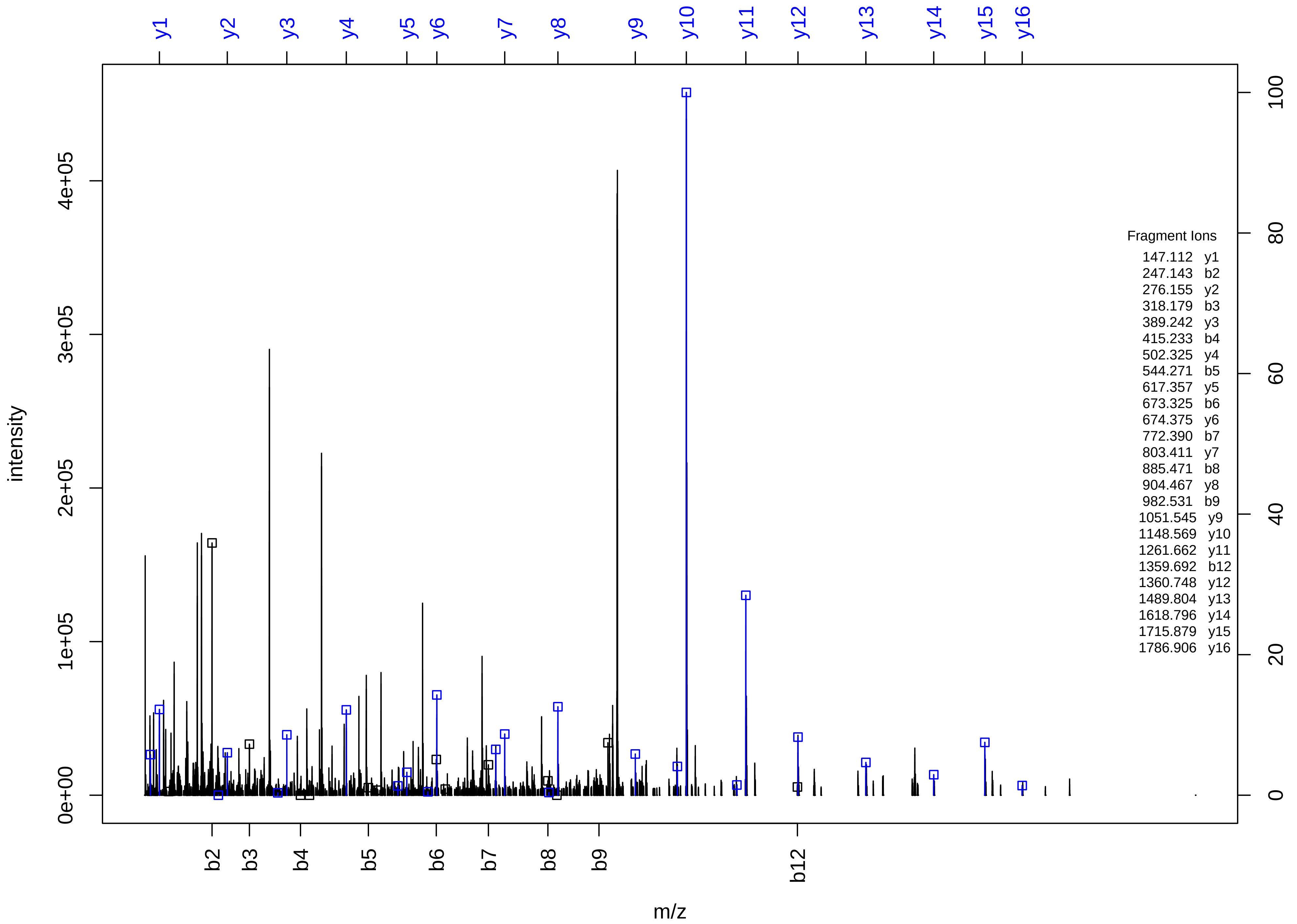

FVAPEEVLPFTEGDILEK / File: sydney_ip_19.raw; SpectrumID: ; scans: 20056

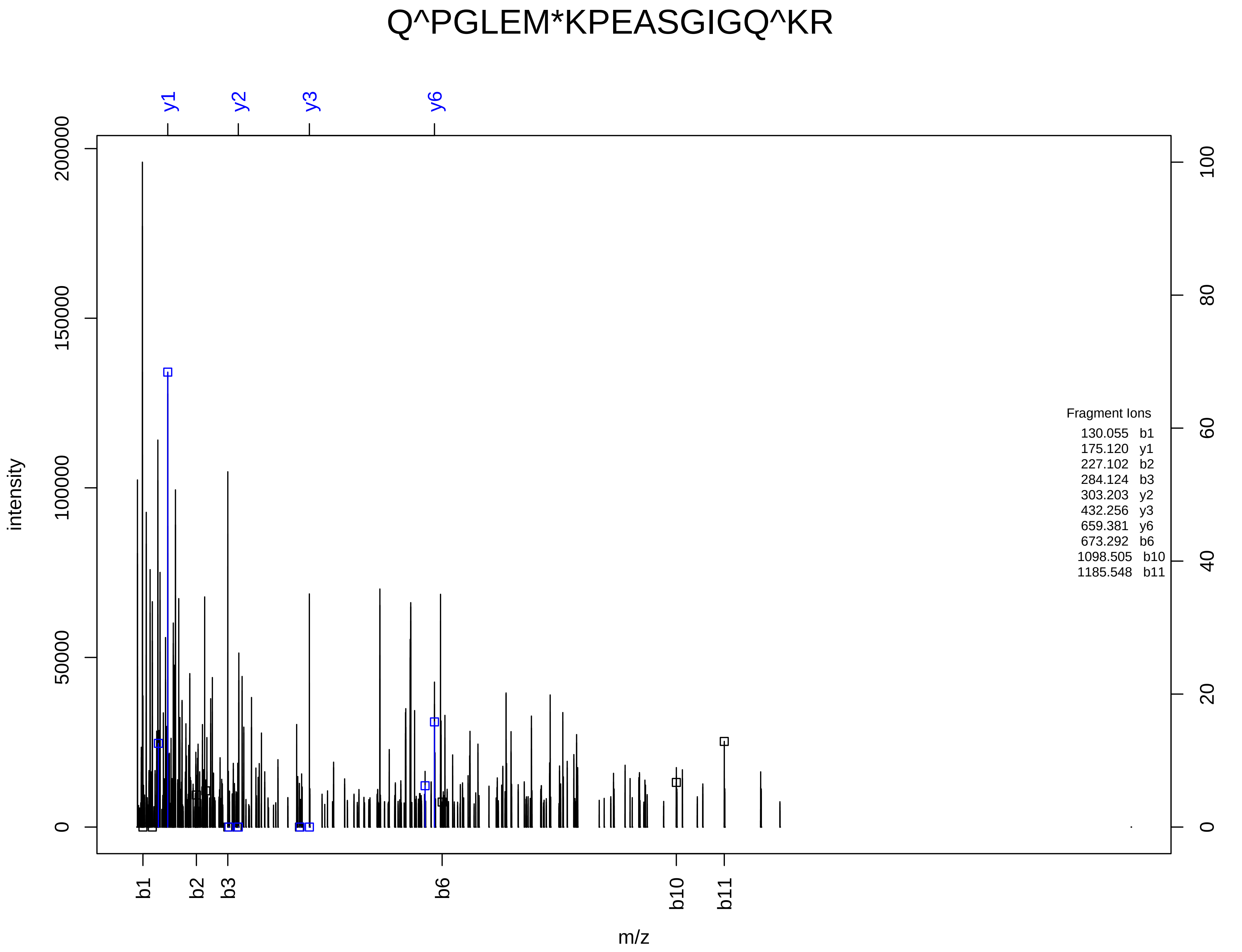

Q^PGLEM*KPEASGIGQ^KR
y1
y2
y3
y6
intensity
200000
150000
100000
50000
0
100
80
60
40
20
0
Fragment Ions
130.055 b1
175.120 y1
227.102 b2
284.124 b3
303.203 y2
432.256 y3
659.381 y6
673.292 b6
1098.505 b10
1185.548 b11
b1
b2
b3
b6
b10
b11
m/z
QPGLEMKPEASGIGQKR / File: sydney_ip_19.raw; SpectrumID: ; scans: 4752

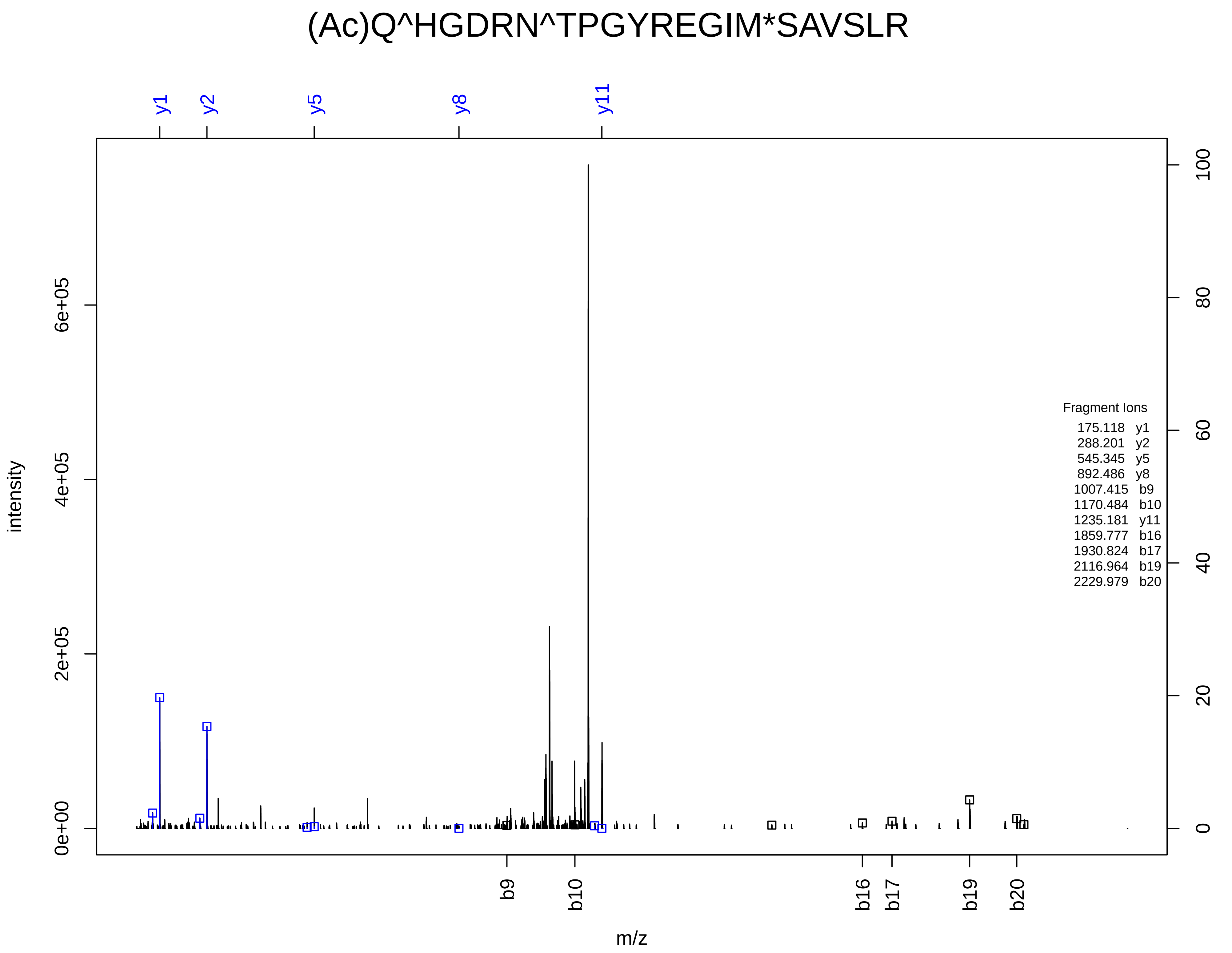
(Ac)Q^HGDRN^TPGYREGIM*SAVSLR
y1
y2
y5
y8
y11
intensity
0e+00
2e+05
4e+05
6e+05
0
20
40
60
80
100
Fragment Ions
175.118 y1
288.201 y2
545.345 y5
892.486 y8
1007.415 b9
1170.484 b10
1235.181 y11
1859.777 b16
1930.824 b17
2116.964 b19
2229.979 b20
b9
b10
b16
b17
b19
b20
m/z
QHGDRNTPGYREGIMSAVSLR / File: sydney_ip_19.raw; SpectrumID: ; scans: 5634

ESGEGEEEVADSAR

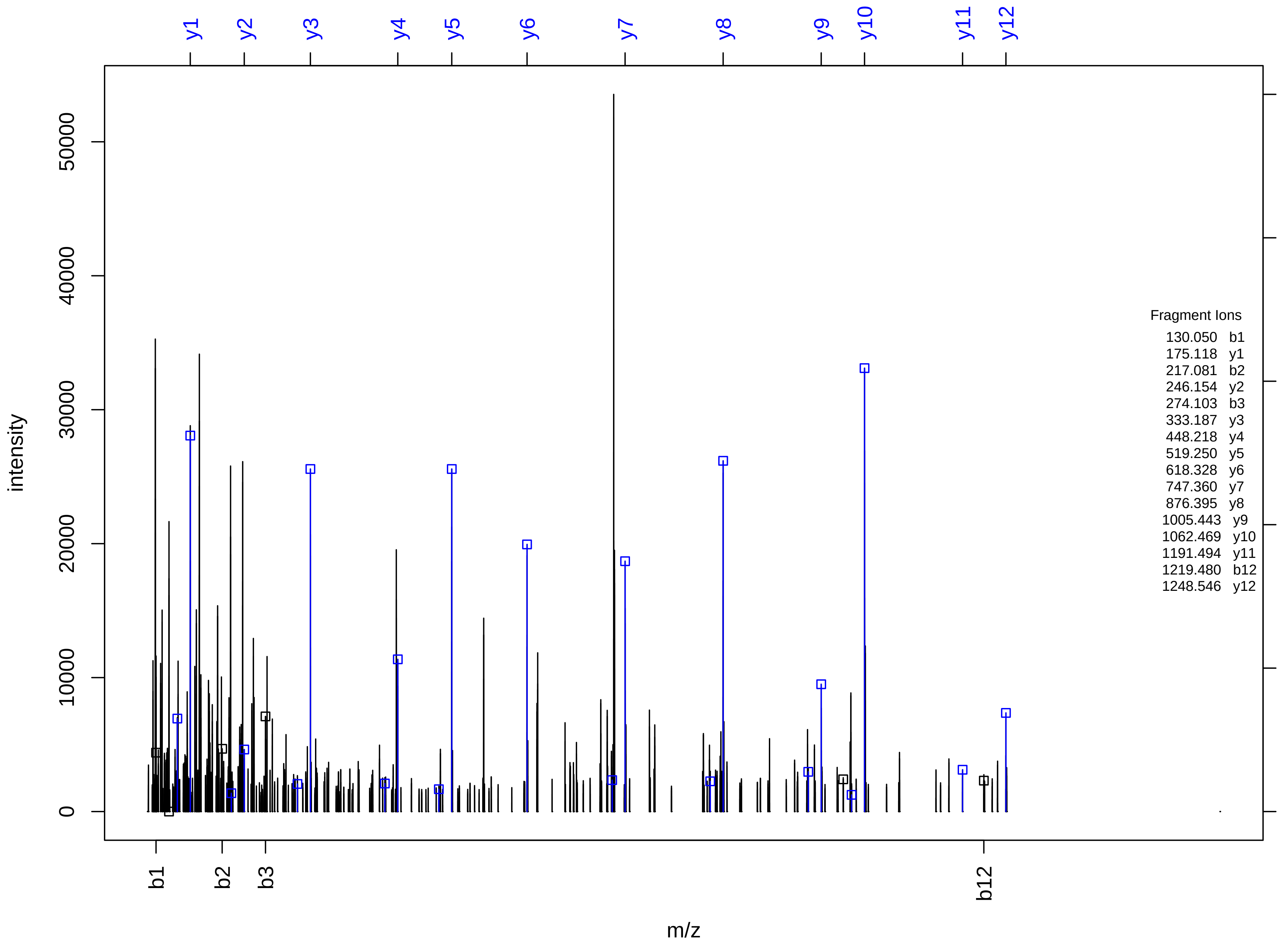

ESGEGEEEVADSAR / File: sydney_ip_19.raw; SpectrumID: ; scans: 2238

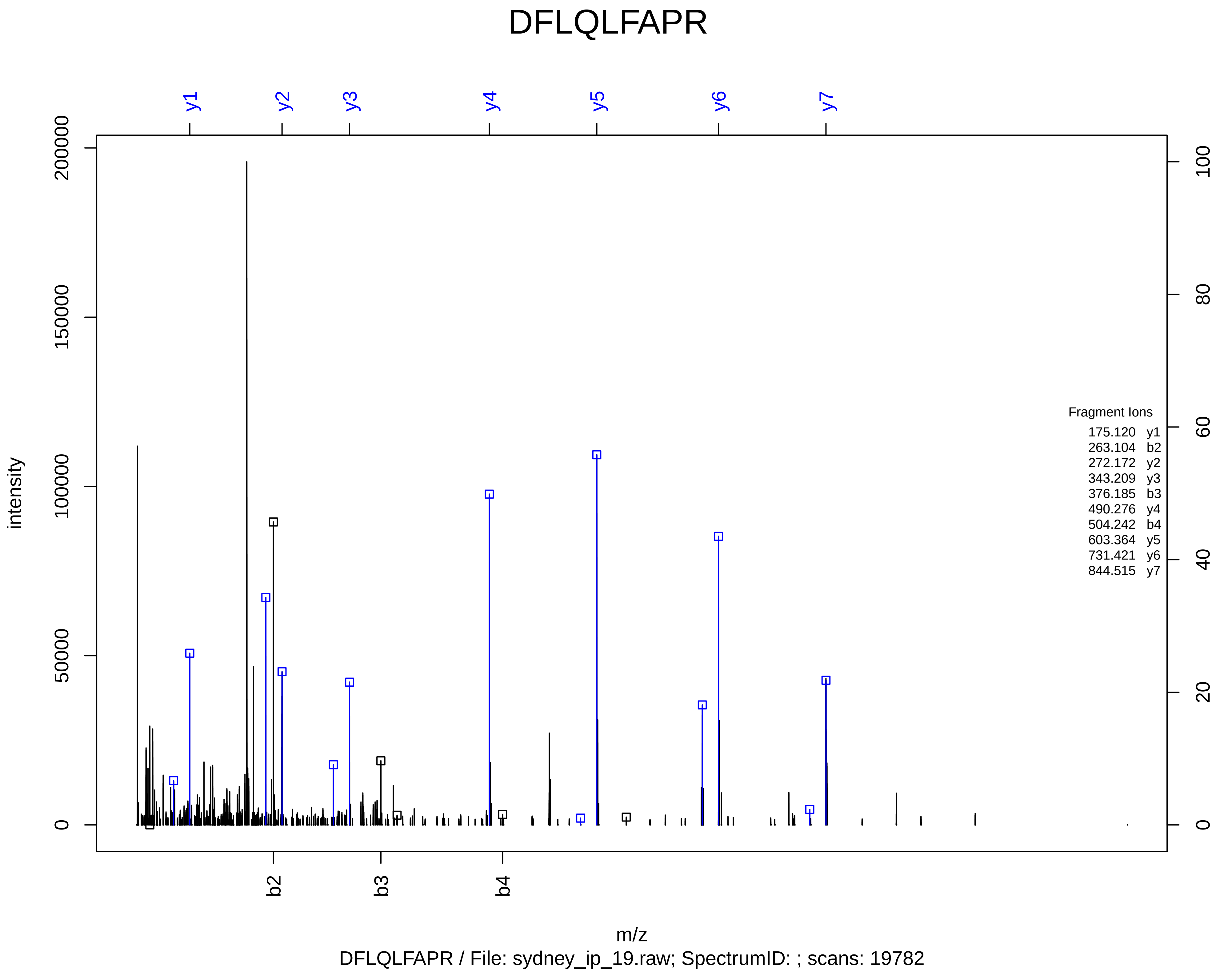
DFLQLFAPR
y1
y2
y3
y4
y5
y6
y7
intensity
0
50000
100000
150000
200000
0
20
40
60
80
100
b2
b3
b4
m/z
Fragment Ions
175.120 y1
263.104 b2
272.172 y2
343.209 y3
376.185 b3
490.276 y4
504.242 b4
603.364 y5
731.421 y6
844.515 y7
DFLQLFAPR / File: sydney_ip_19.raw; SpectrumID: ; scans: 19782

NLVPLLLAPENLVEK

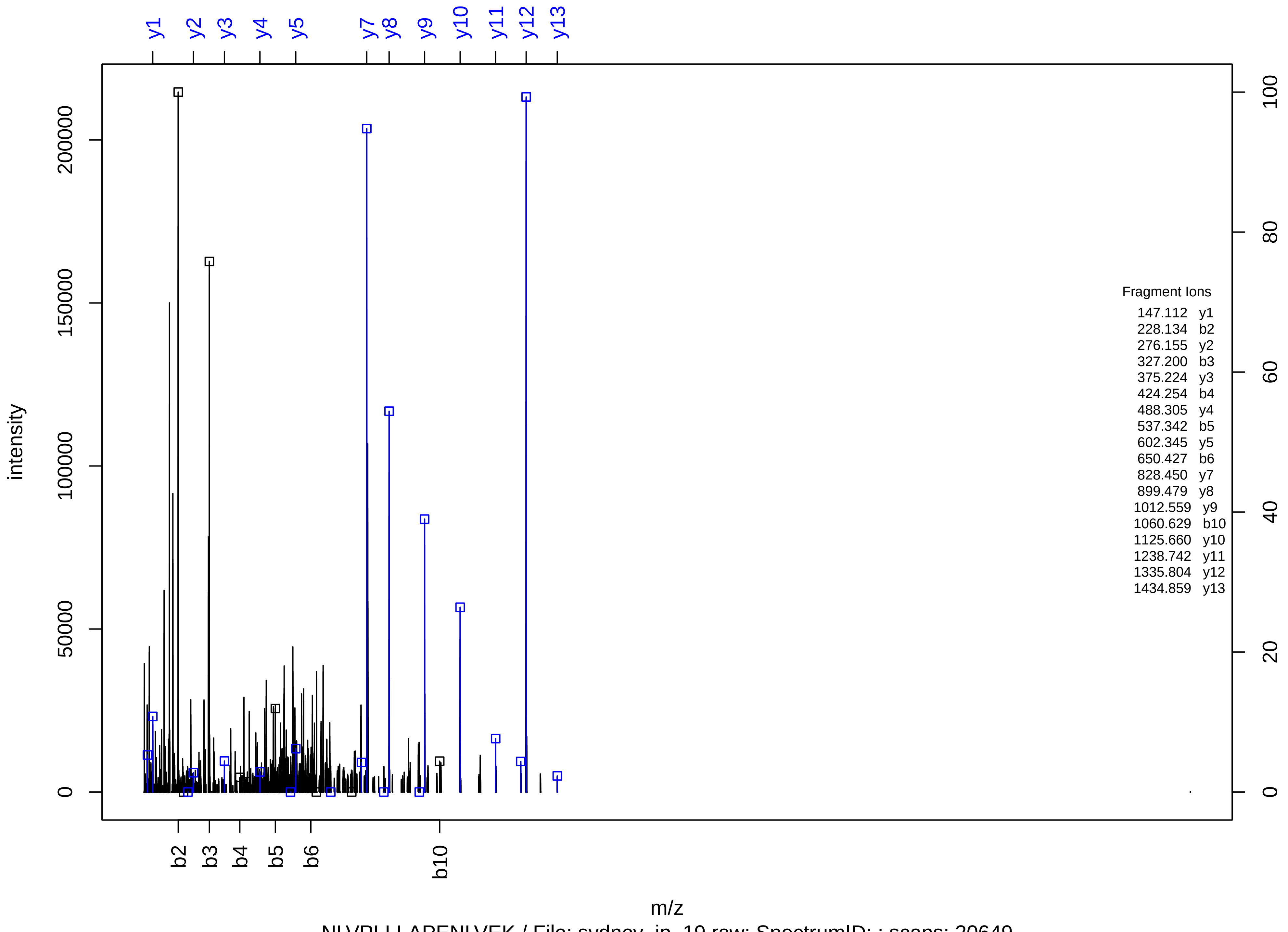

NLVPLLLAPENLVEK / File: sydney_ip_19.raw; SpectrumID: ; scans: 20649

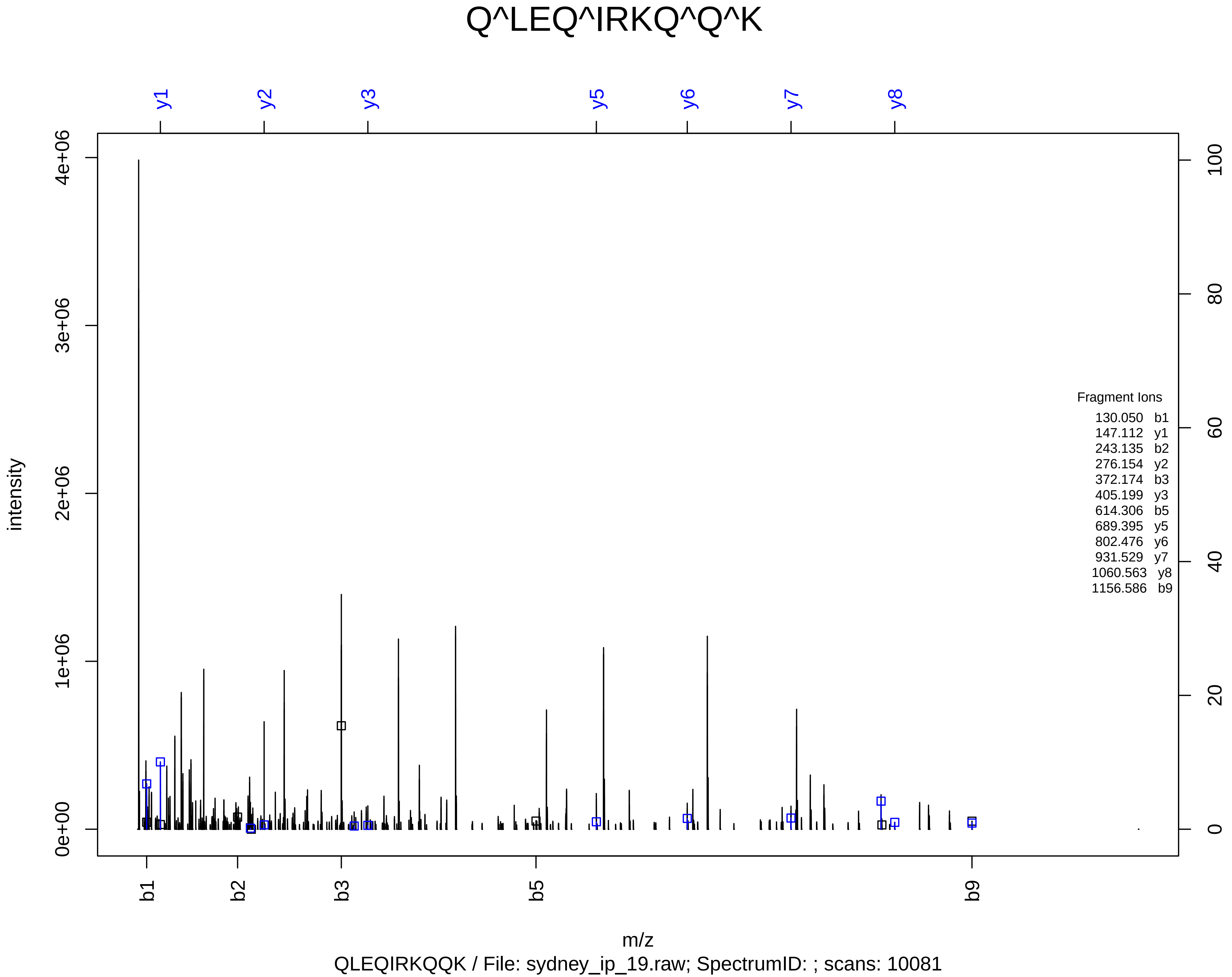
Q^LEQ^IRKQ^Q^K
y1
y2
y3
y5
y6
y7
y8
intensity
4e+06
3e+06
2e+06
1e+06
0e+00
100
80
60
40
20
0
Fragment Ions
130.050 b1
147.112 y1
243.135 b2
276.154 y2
372.174 b3
405.199 y3
614.306 b5
689.395 y5
802.476 y6
931.529 y7
1060.563 y8
1156.586 b9
b1
b2
b3
b5
b9
m/z
QLEQIRKQQK / File: sydney_ip_19.raw; SpectrumID: ; scans: 10081

(Ac)M*KTFPQ^SPTLR

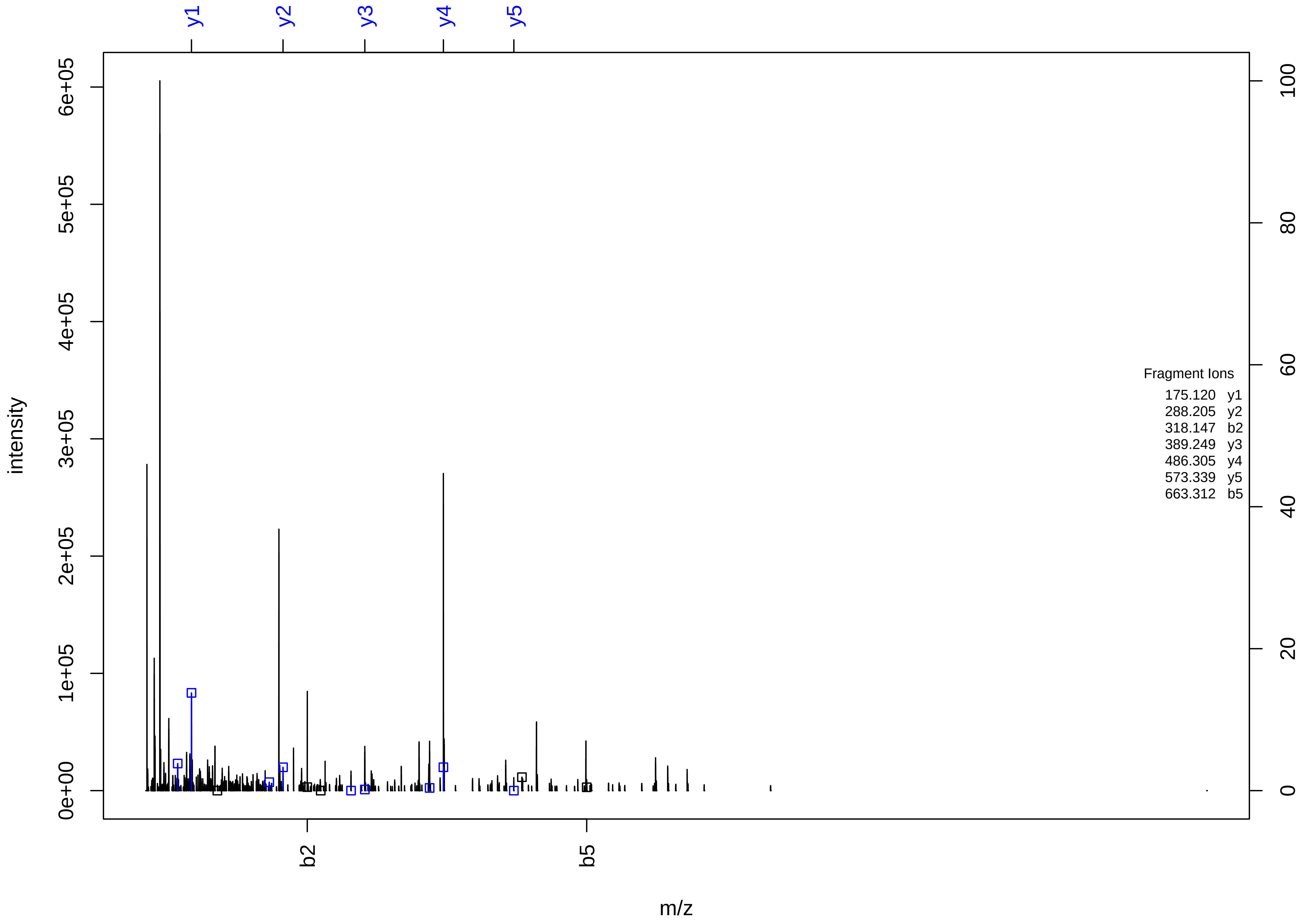

MKTFPQSPTLR / File: sydney_ip_19.raw; SpectrumID: ; scans: 3331

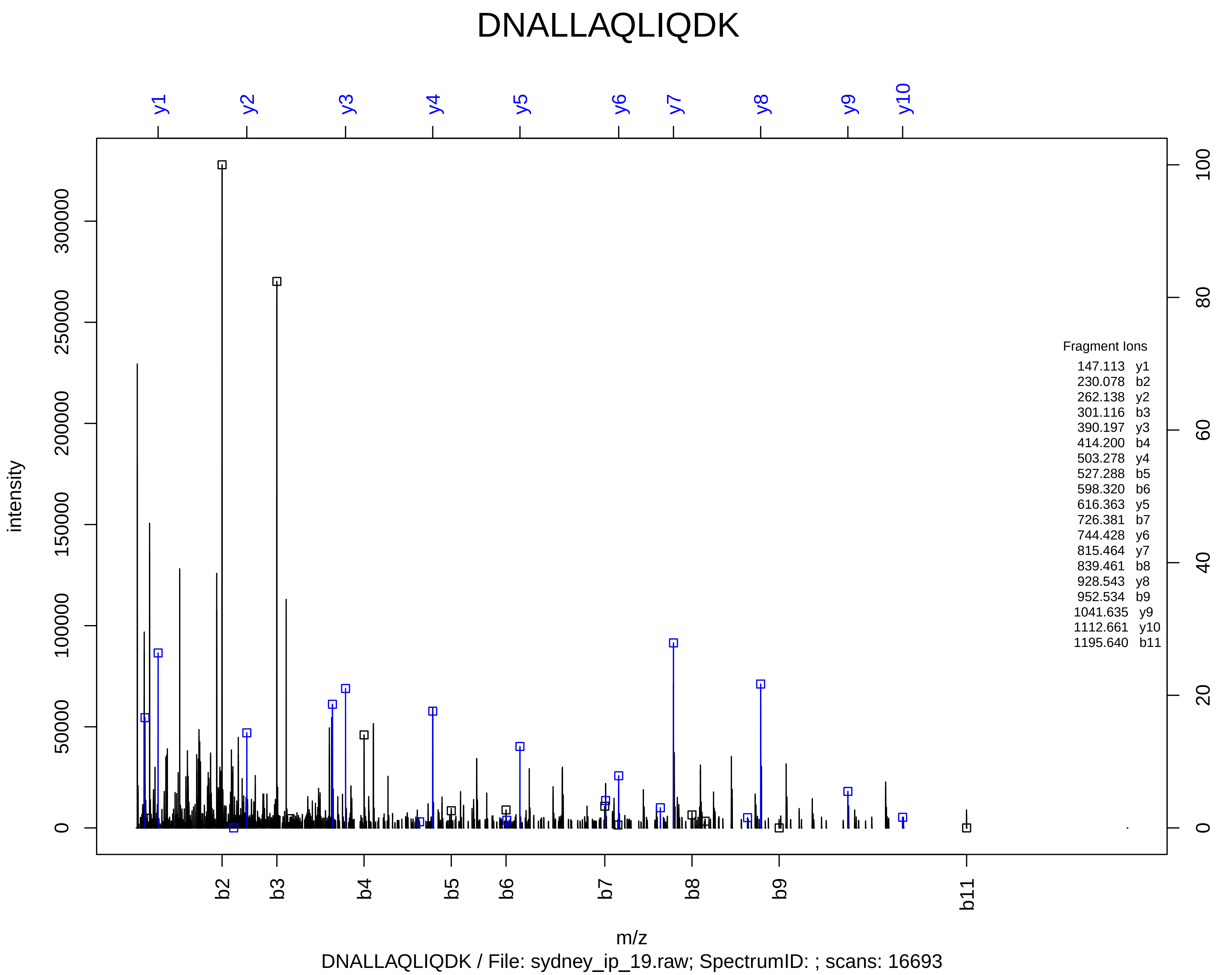

DNALLAQLIQDK
y1 y2 y3 y4 y5 y6 y7 y8 y9 y10
b2 b3 b4 b5 b6 b7 b8 b9 b11
intensity
m/z
0
50000
100000
150000
200000
250000
300000
0
20
40
60
80
100
Fragment Ions
147.113 y1
230.078 b2
262.138 y2
301.116 b3
390.197 y3
414.200 b4
503.278 y4
527.288 b5
598.320 b6
616.363 y5
726.381 b7
744.428 y6
815.464 y7
839.461 b8
928.543 y8
952.534 b9
1041.635 y9
1112.661 y10
1195.640 b11
DNALLAQLIQDK / File: sydney_ip_19.raw; SpectrumID: ; scans: 16693

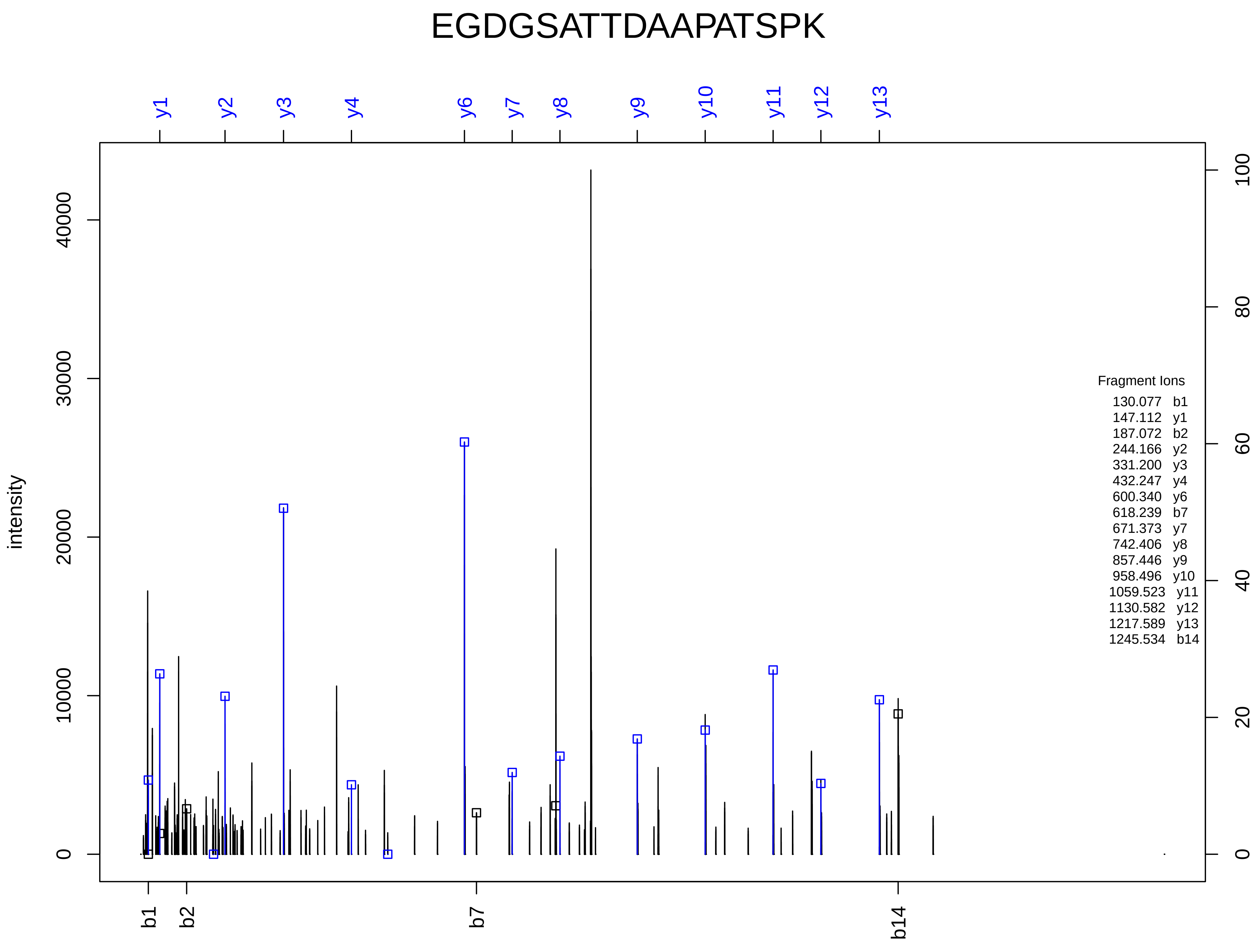

EGDGSATTDAAPATSPK / File: sydney_ip_19.raw; SpectrumID: ; scans: 1814

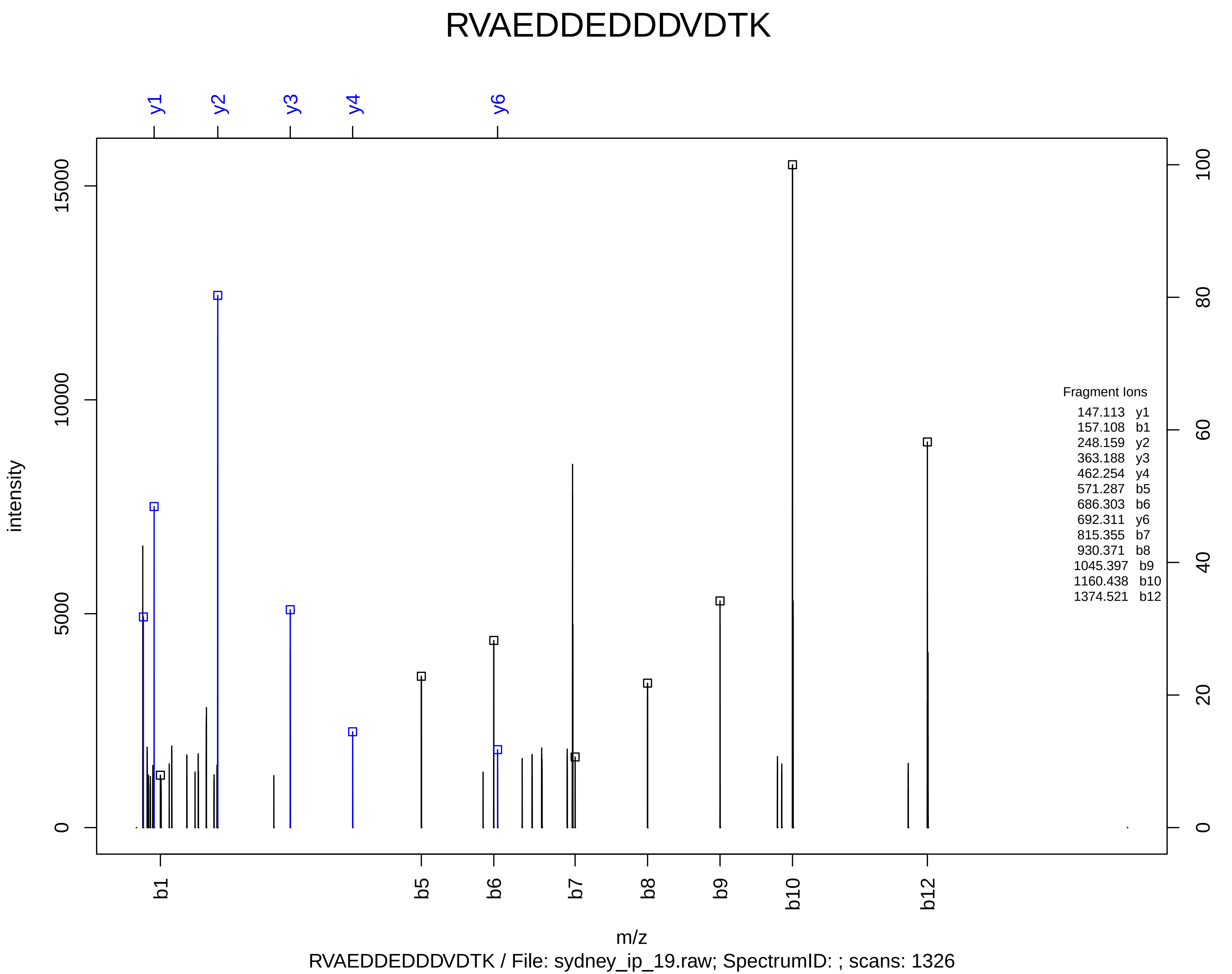
RVAEDDEDDDVDTK
y1
y2
y3
y4
y6
intensity
0
5000
10000
15000
0
20
40
60
80
100
b1
b5
b6
b7
b8
b9
b10
b12
m/z
Fragment Ions
147.113 y1
157.108 b1
248.159 y2
363.188 y3
462.254 y4
571.287 b5
686.303 b6
692.311 y6
815.355 b7
930.371 b8
1045.397 b9
1160.438 b10
1374.521 b12
RVAEDDEDDDVDTK / File: sydney_ip_19.raw; SpectrumID: ; scans: 1326

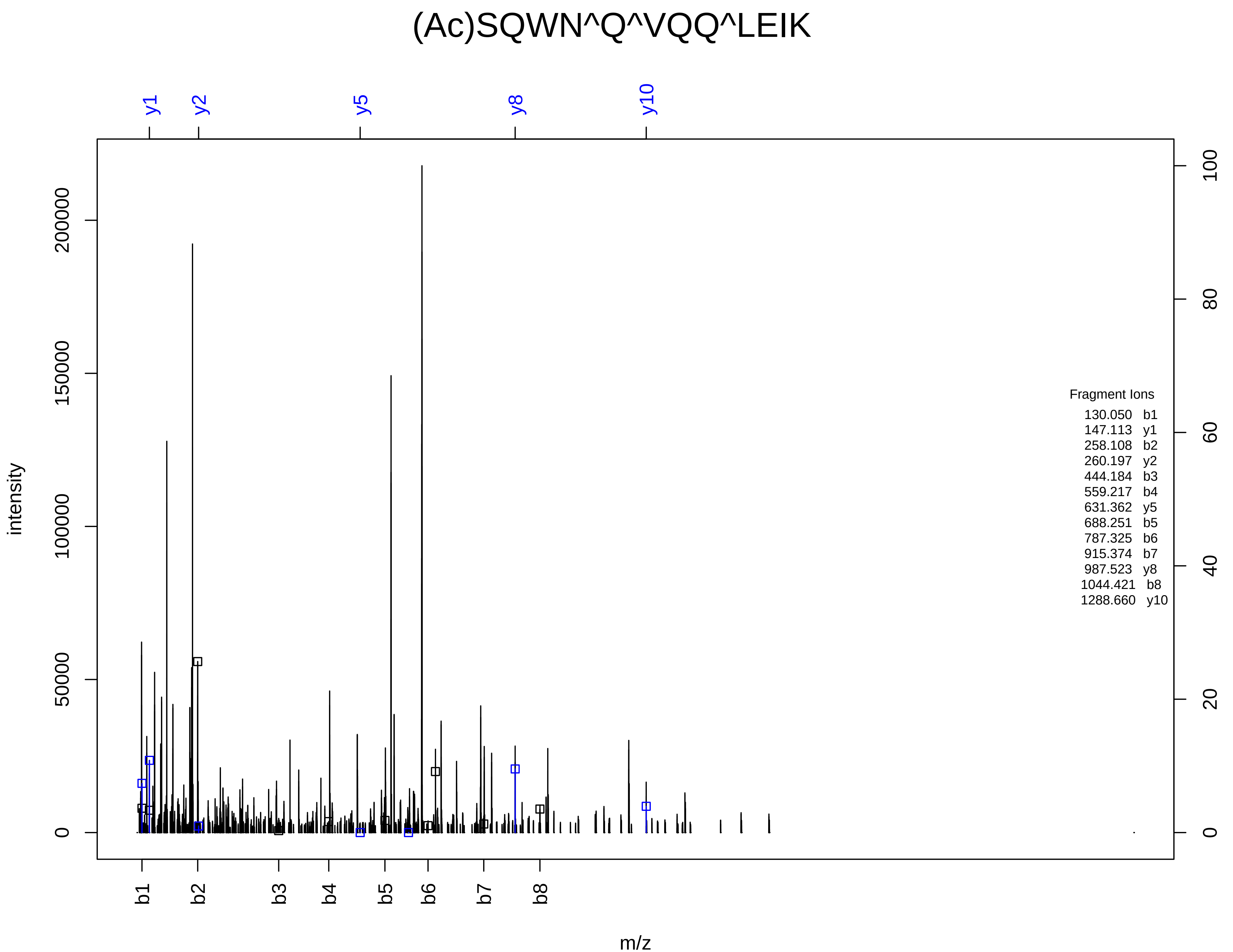

(Ac)SQWN^Q^VQQ^LEIK
y1
y2
y5
y8
y10
intensity
0
50000
100000
150000
200000
0
20
40
60
80
100
Fragment Ions
130.050 b1
147.113 y1
258.108 b2
260.197 y2
444.184 b3
559.217 b4
631.362 y5
688.251 b5
787.325 b6
915.374 b7
987.523 y8
1044.421 b8
1288.660 y10
b1
b2
b3
b4
b5
b6
b7
b8
m/z
SQWNQVQQLEIK / File: sydney_ip_19.raw; SpectrumID: ; scans: 2206

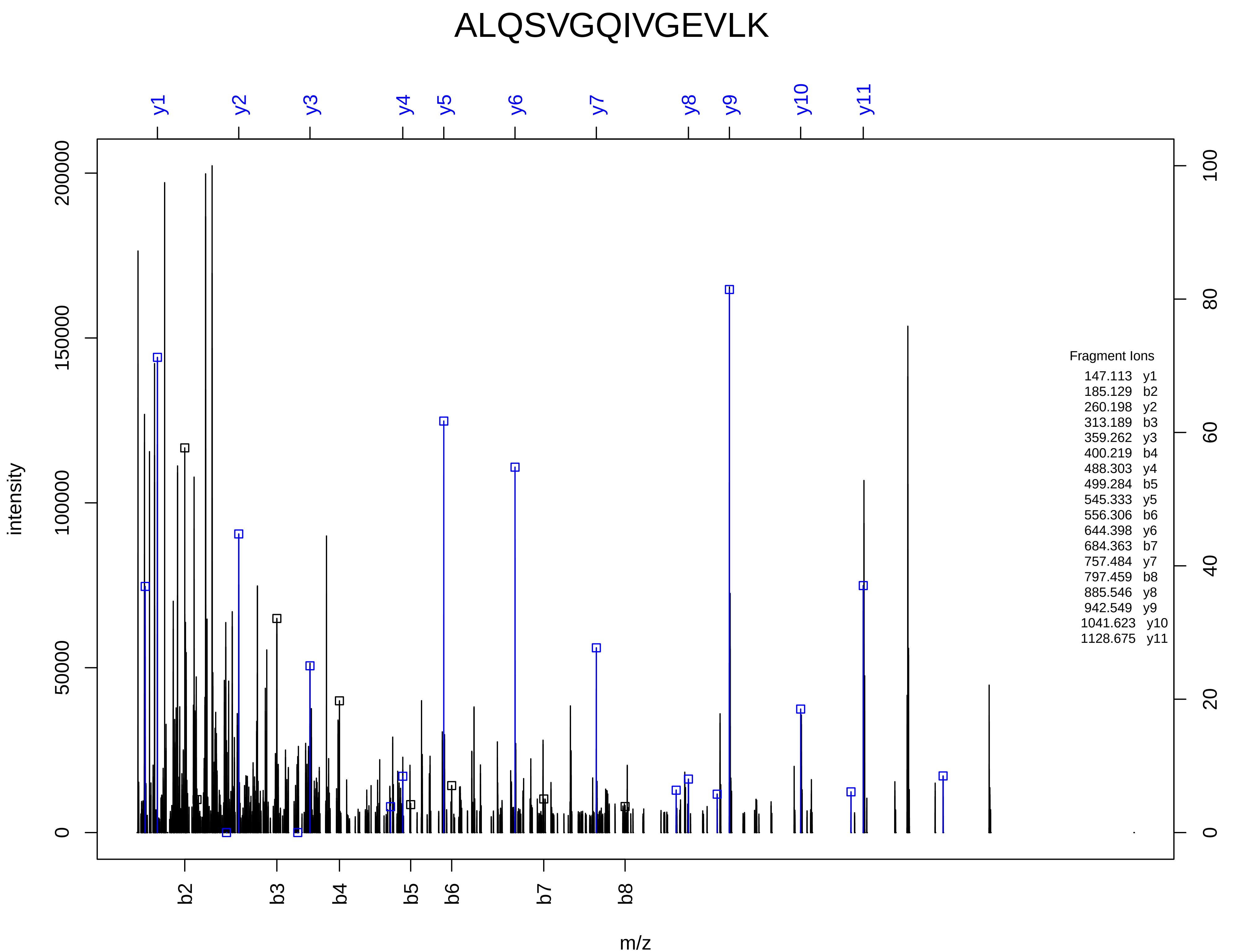

ALQSVGQIVGEVLK / File: sydney_ip_19.raw; SpectrumID: ; scans: 17926

GPDGLTALEATDNQAIK

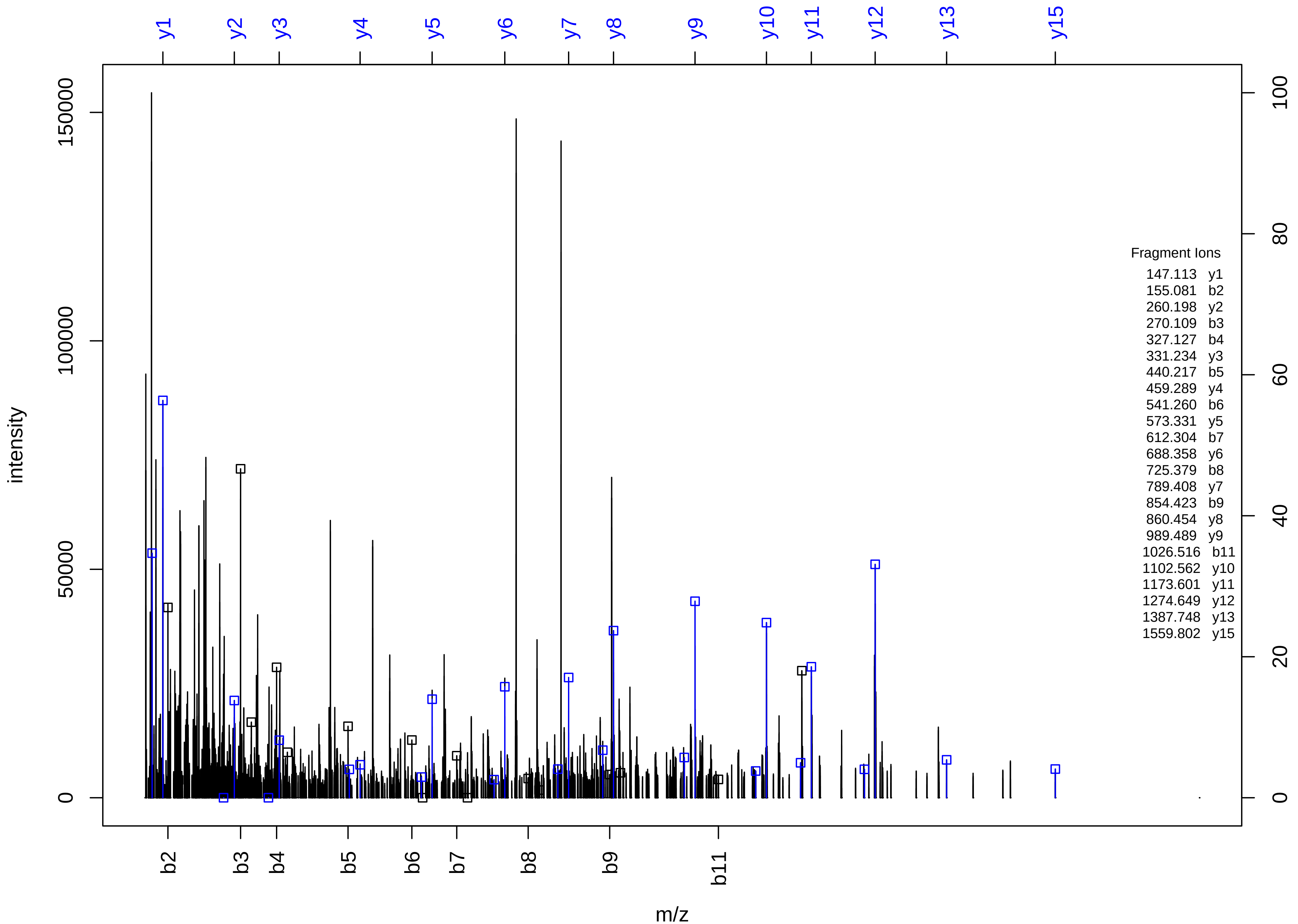

GPDGLTALEATDNQAIK / File: sydney_ip_19.raw; SpectrumID: ; scans: 10829

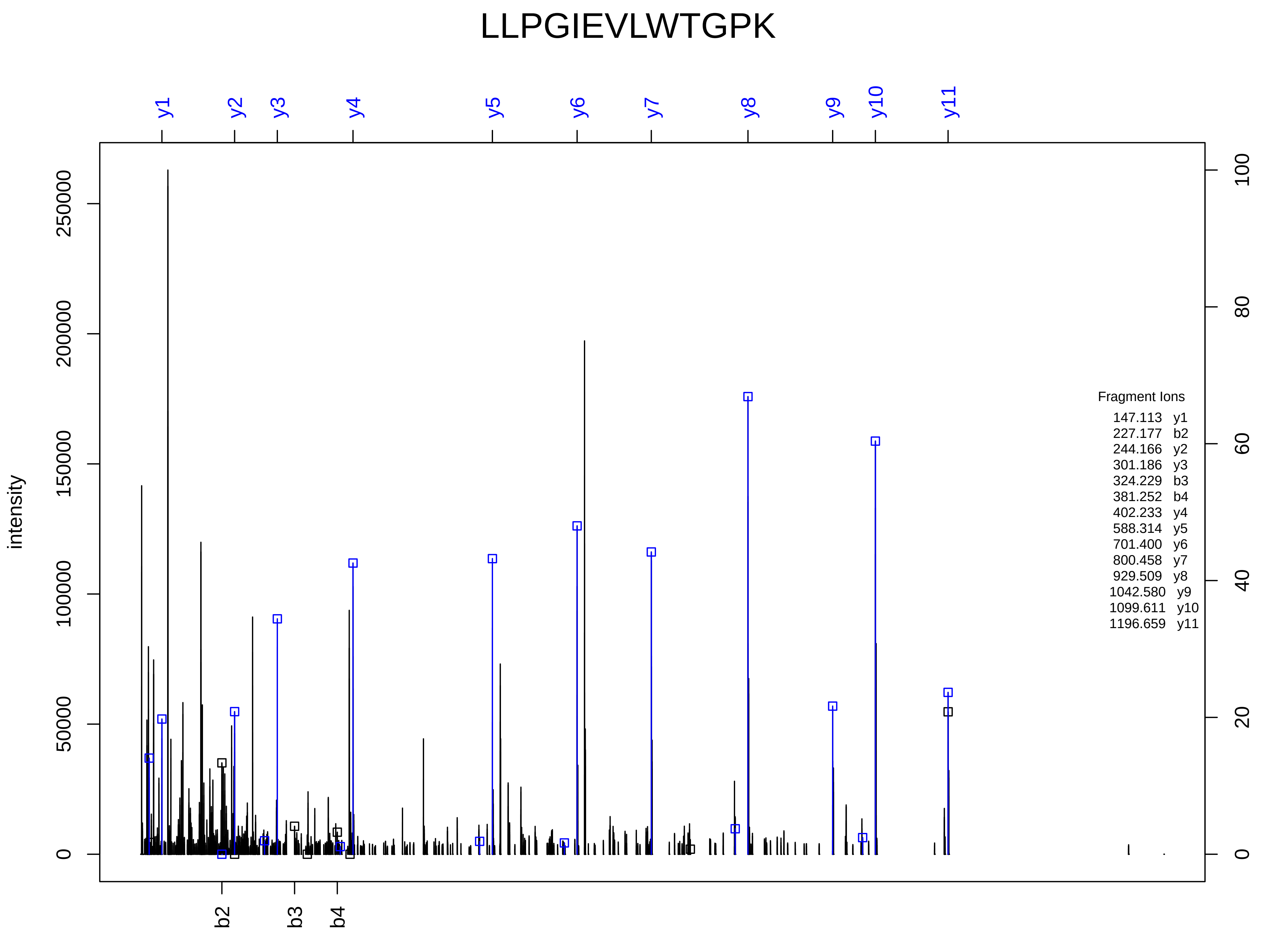

LLPGIEVLWTGPK / File: sydney_ip_19.raw; SpectrumID: ; scans: 19992

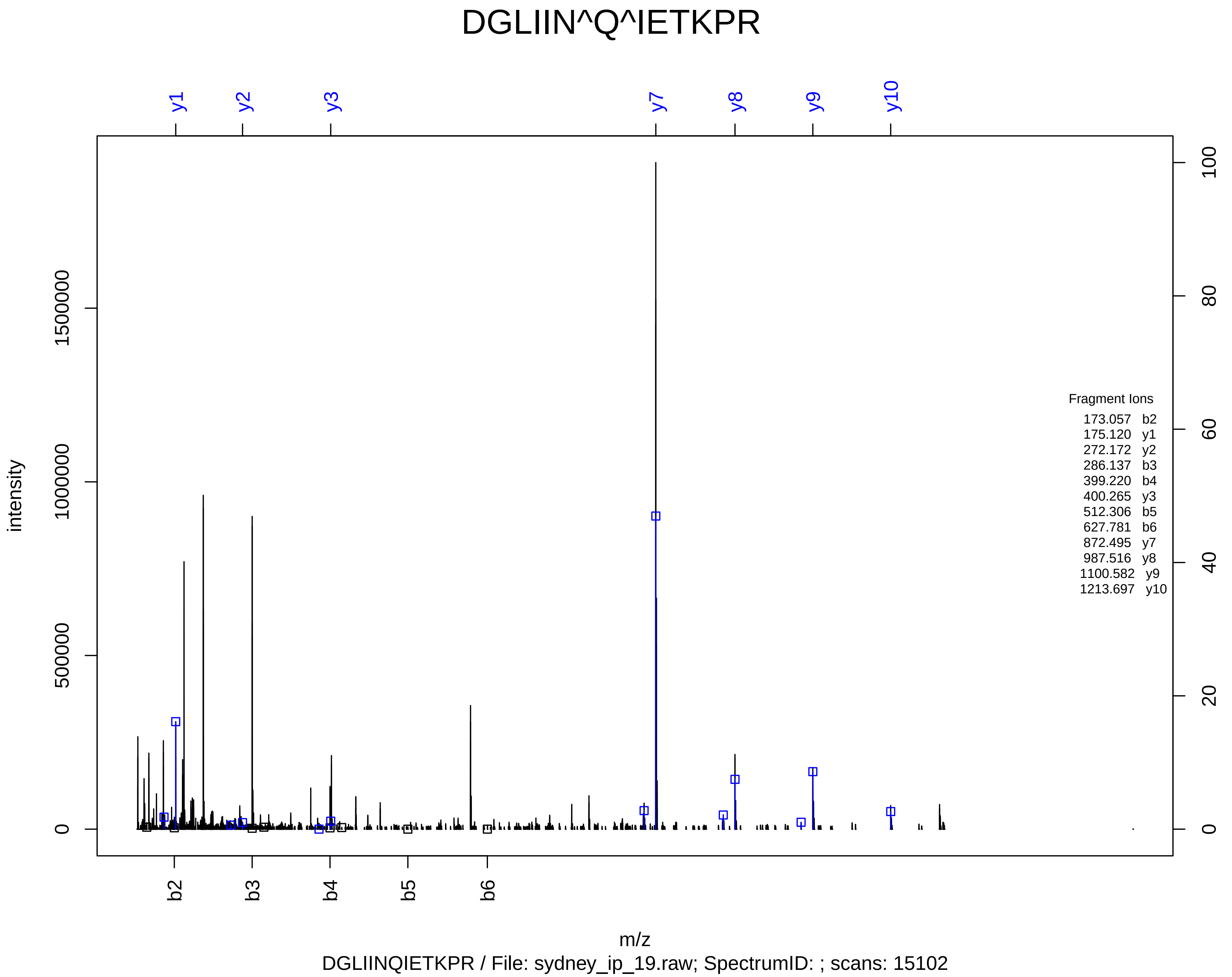
DGLIIN^Q^IETKPR
y1
y2
y3
y7
y8
y9
y10
intensity
0
500000
1000000
1500000
0
20
40
60
80
100
b2
b3
b4
b5
b6
m/z
Fragment Ions
173.057 b2
175.120 y1
272.172 y2
286.137 b3
399.220 b4
400.265 y3
512.306 b5
627.781 b6
872.495 y7
987.516 y8
1100.582 y9
1213.697 y10
DGLIINQIETKPR / File: sydney_ip_19.raw; SpectrumID: ; scans: 15102

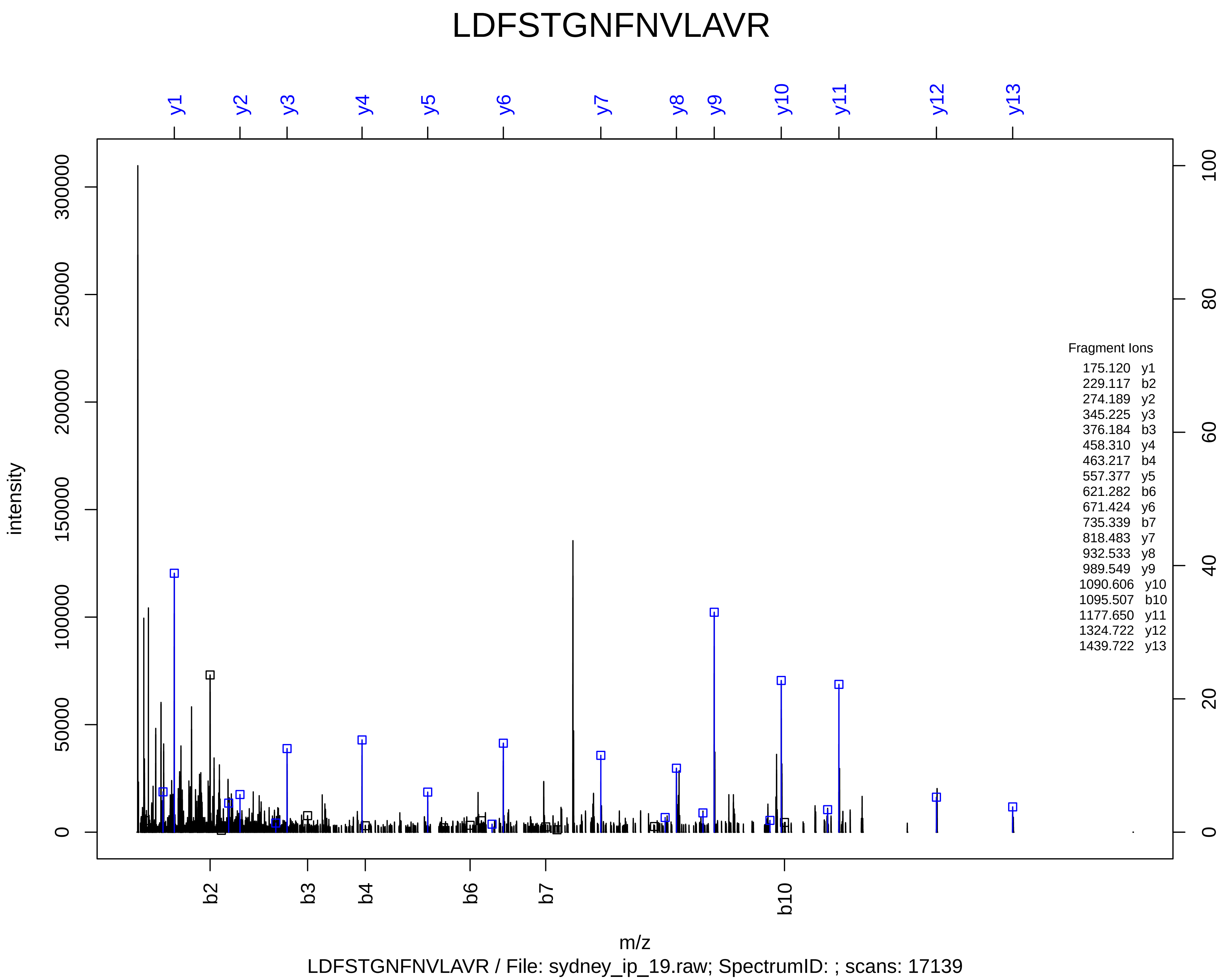
LDFSTGNFNVLAVR
y1 y2 y3 y4 y5 y6 y7 y8 y9 y10 y11 y12 y13
b2 b3 b4 b6 b7 b10
intensity
m/z
Fragment Ions
175.120 y1
229.117 b2
274.189 y2
345.225 y3
376.184 b3
458.310 y4
463.217 b4
557.377 y5
621.282 b6
671.424 y6
735.339 b7
818.483 y7
932.533 y8
989.549 y9
1090.606 y10
1095.507 b10
1177.650 y11
1324.722 y12
1439.722 y13
LDFSTGNFNVLAVR / File: sydney_ip_19.raw; SpectrumID: ; scans: 17139

VLGDIHTTLLSAVIPNAFR

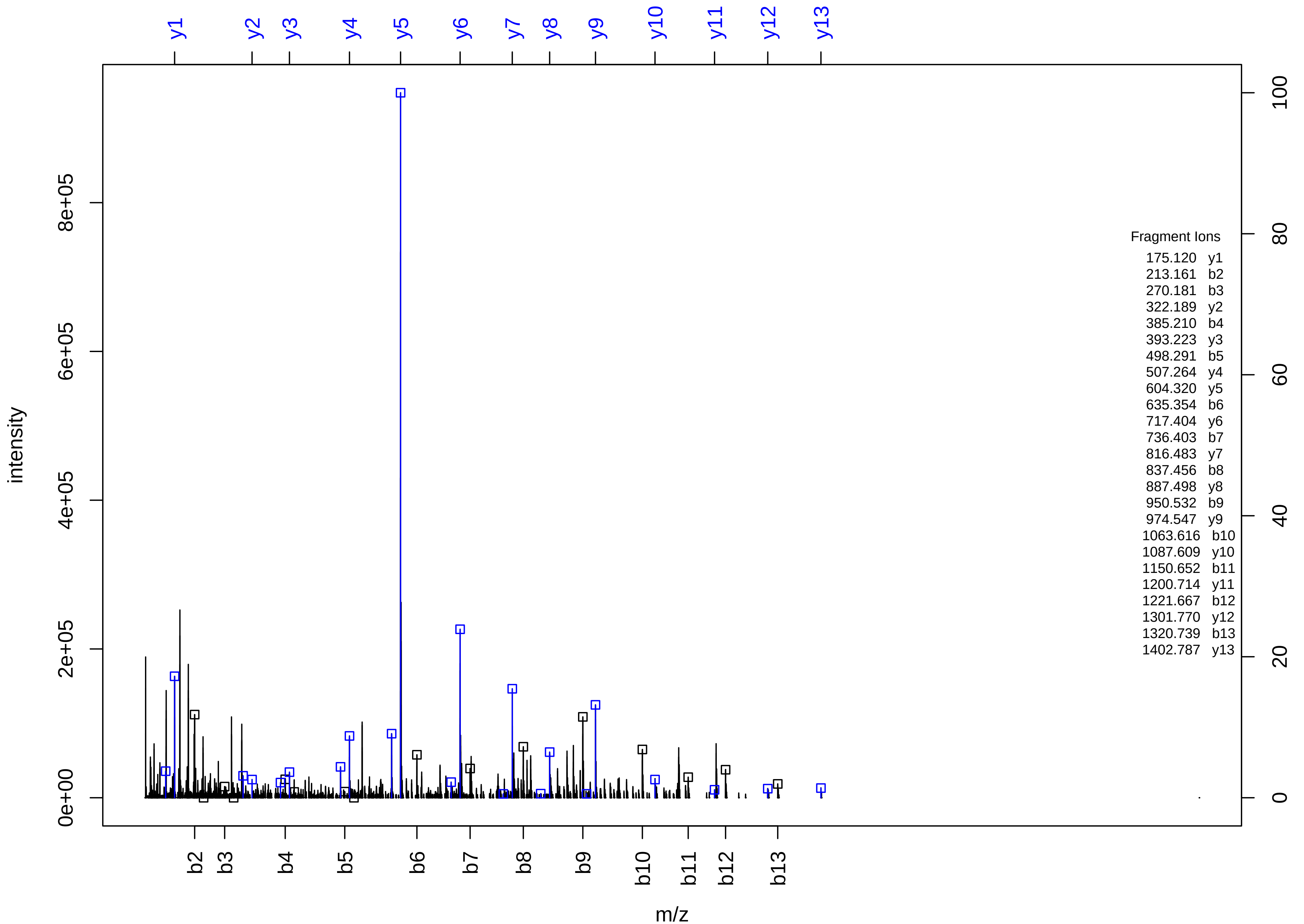

VLGDIHTTLLSAVIPNAFR / File: sydney_ip_19.raw; SpectrumID: ; scans: 20213

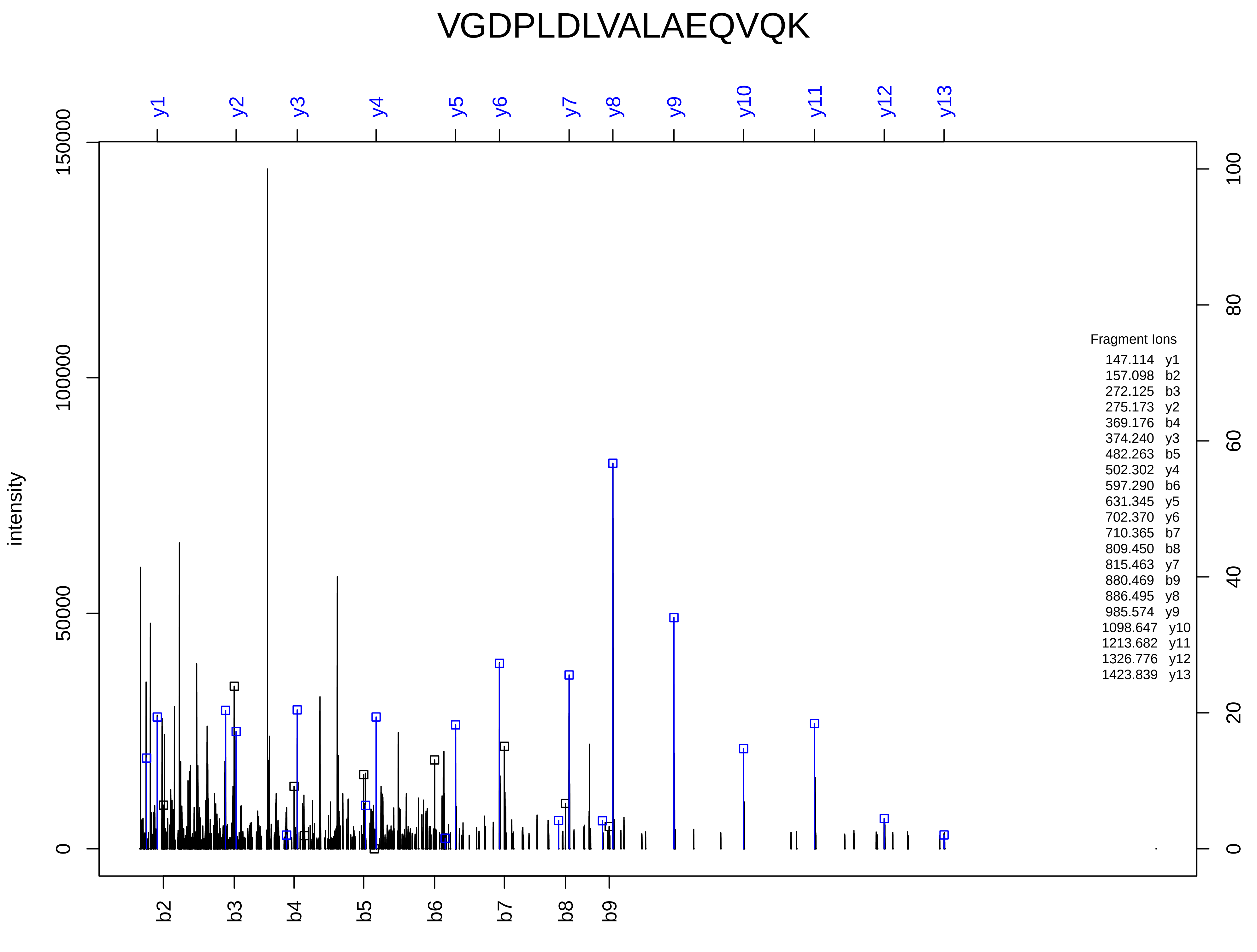

VGDPLDLVALAEQVQK / File: sydney_ip_19.raw; SpectrumID: ; scans: 20444

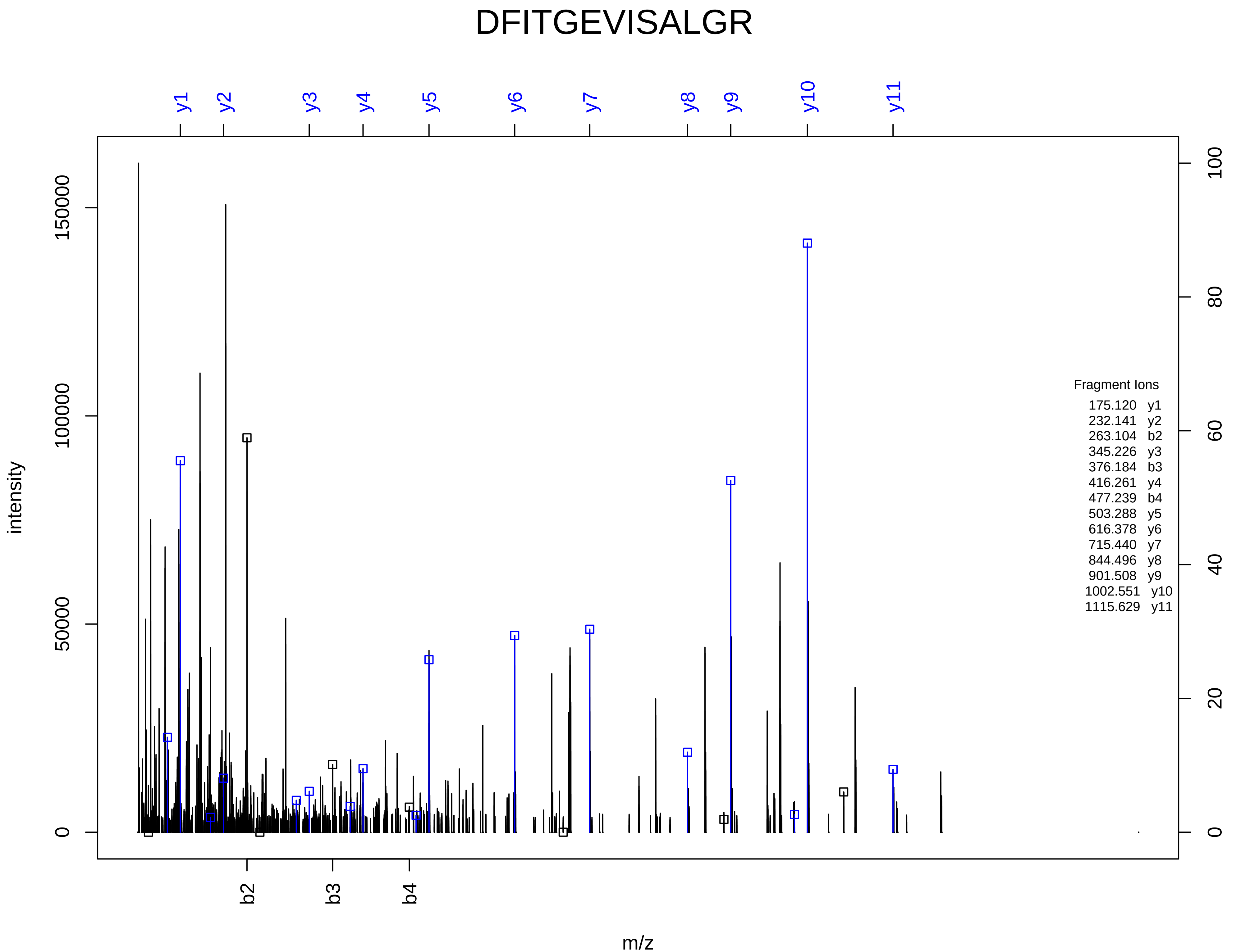

DFITGEVISALGR / File: sydney_ip_19.raw; SpectrumID: ; scans: 19094

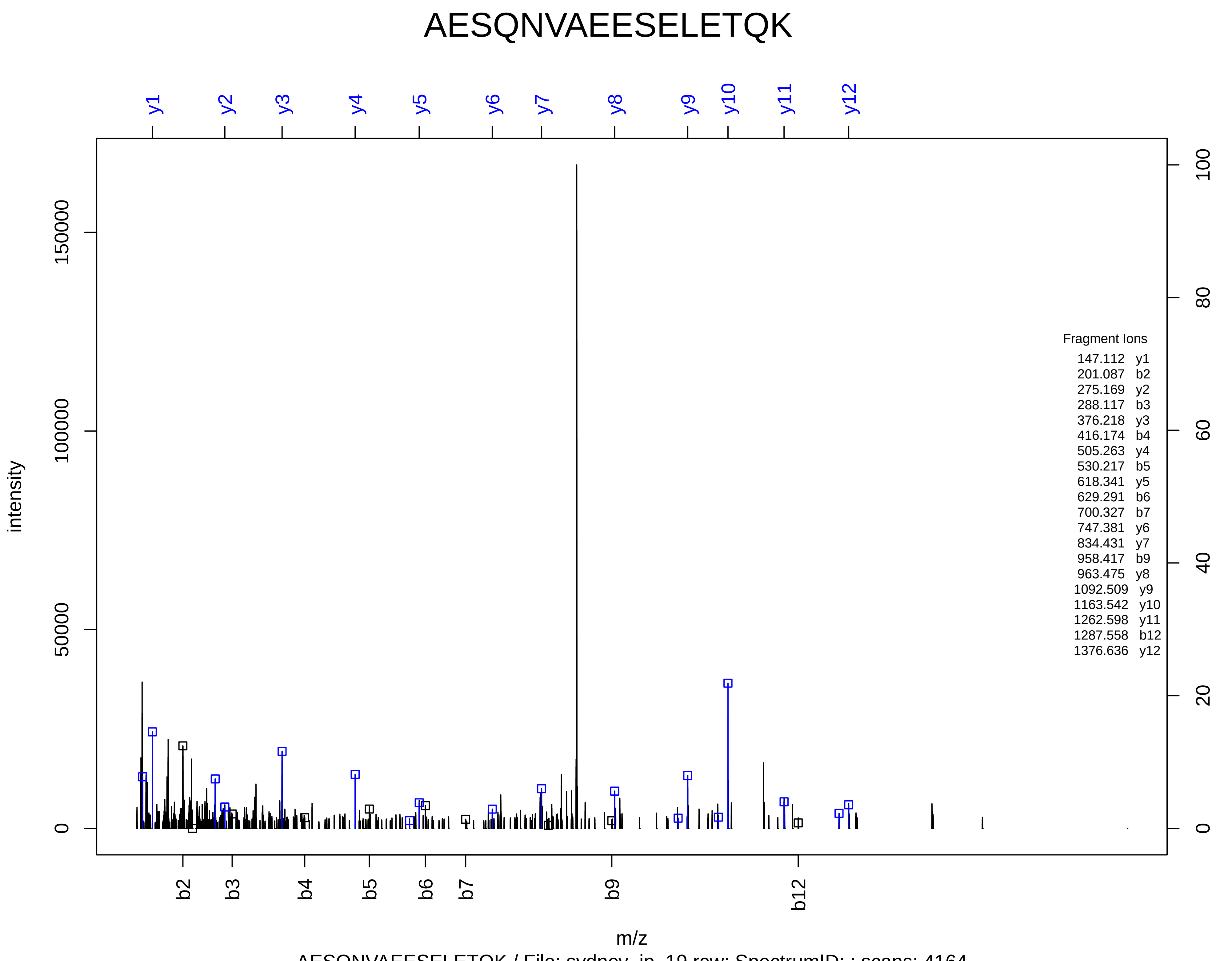

AESQNVAEESELETQK
y1
y2
y3
y4
y5
y6
y7
y8
y9
y10
y11
y12
intensity
0
50000
100000
150000
0
20
40
60
80
100
b2
b3
b4
b5
b6
b7
b9
b12
m/z
Fragment Ions
147.112 y1
201.087 b2
275.169 y2
288.117 b3
376.218 y3
416.174 b4
505.263 y4
530.217 b5
618.341 y5
629.291 b6
700.327 b7
747.381 y6
834.431 y7
958.417 b9
963.475 y8
1092.509 y9
1163.542 y10
1262.598 y11
1287.558 b12
1376.636 y12
AESQNVAEESELETQK / File: sydney_ip_19.raw; SpectrumID: ; scans: 4164

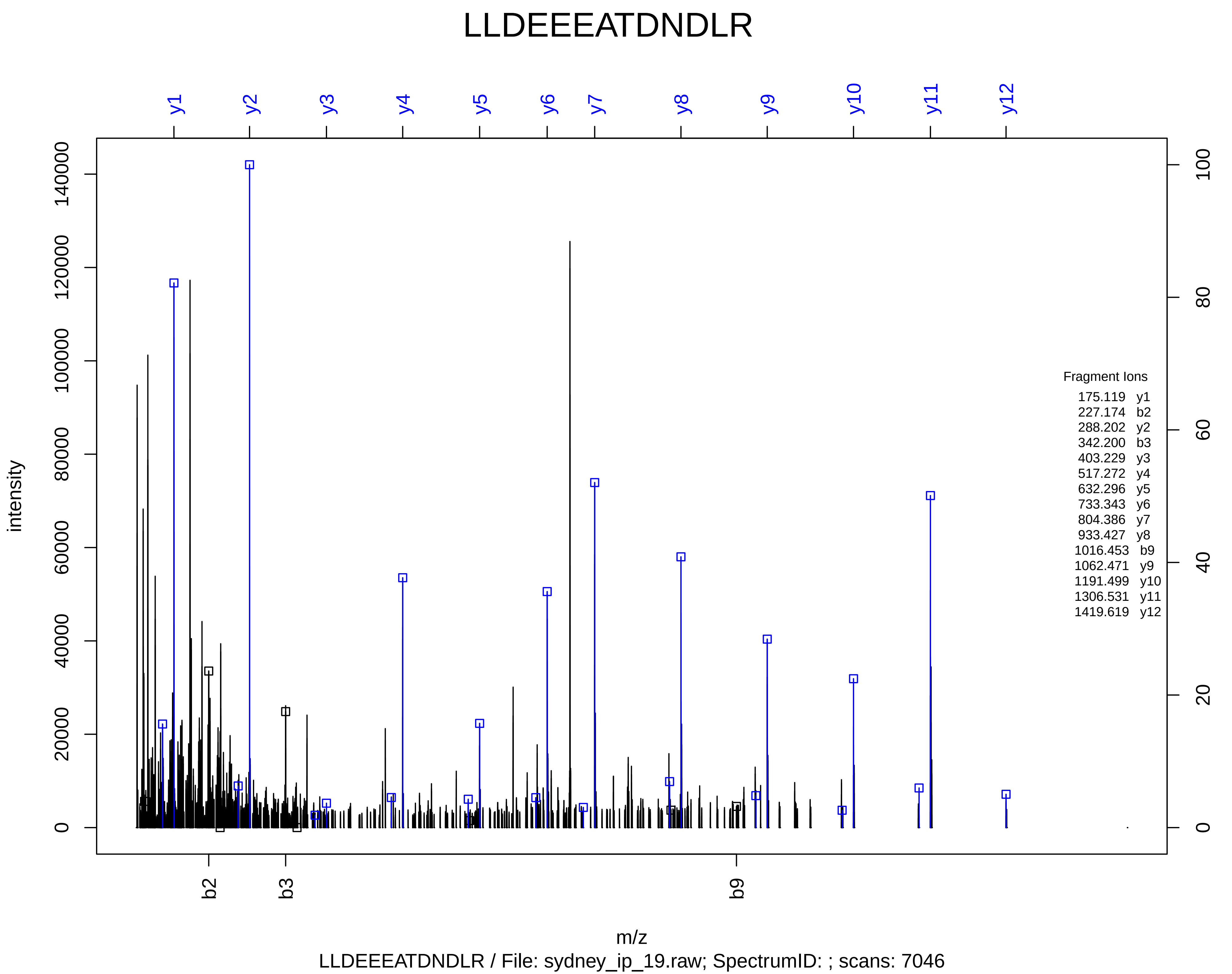
LLDEEEATDNDLR
y1
y2
y3
y4
y5
y6
y7
y8
y9
y10
y11
y12
intensity
b2
b3
b9
m/z
Fragment Ions
175.119 y1
227.174 b2
288.202 y2
342.200 b3
403.229 y3
517.272 y4
632.296 y5
733.343 y6
804.386 y7
933.427 y8
1016.453 b9
1062.471 y9
1191.499 y10
1306.531 y11
1419.619 y12
LLDEEEATDNDLR / File: sydney_ip_19.raw; SpectrumID: ; scans: 7046

VARQ^VPHPQYQPQAHDNDLM*LLK

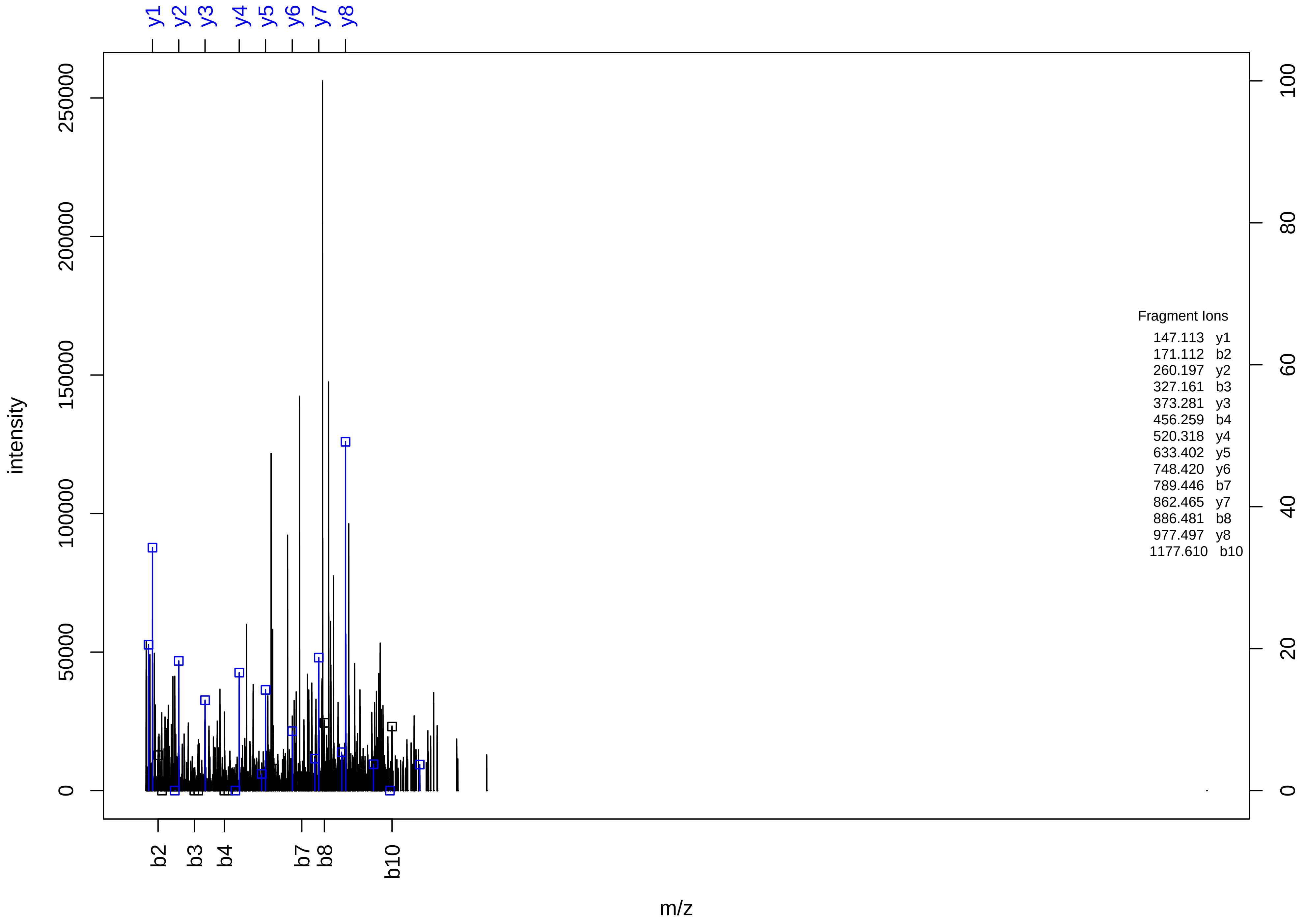

VARQVPHPQYQPQAHDNDLMLLK / File: sydney_ip_19.raw; SpectrumID: ; scans: 19716

APPPSLTDCIGTVDSR

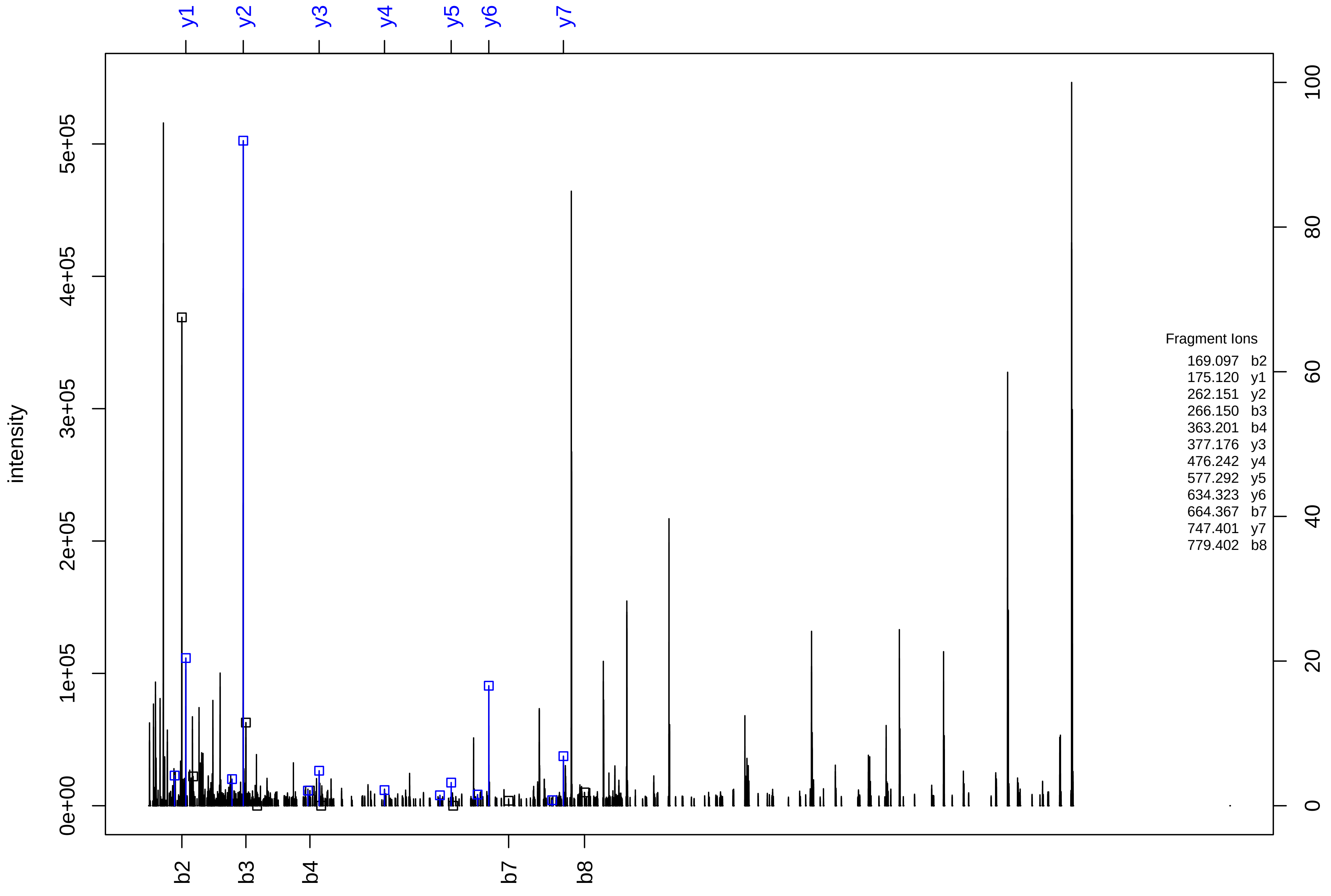

APPPSLTDCIGTVDSR / File: sydney_ip_19.raw; SpectrumID: ; scans: 9689

SDKGTSFMYVPEEQEATPPLSPEQ^EIK

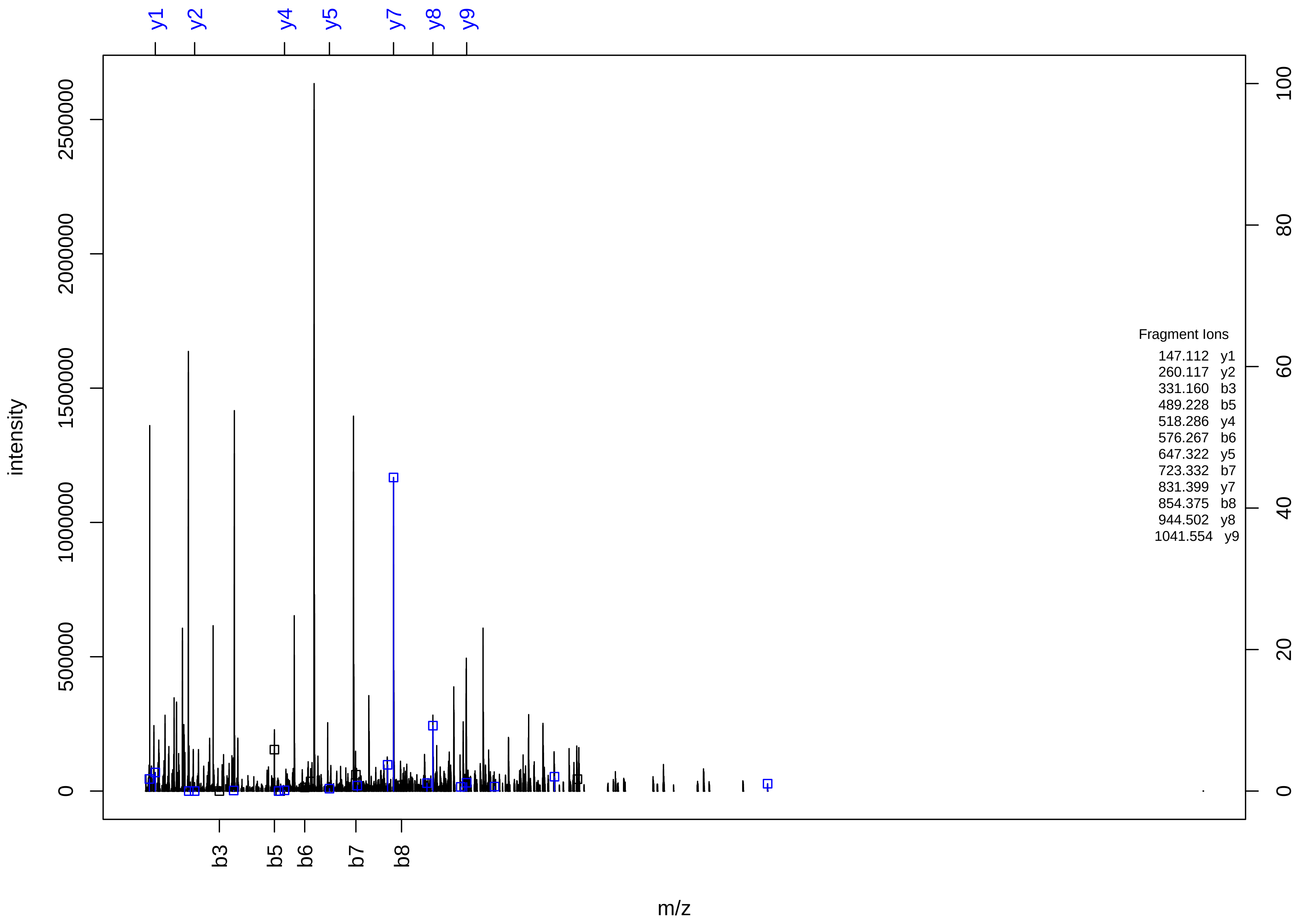

SDKGTSFMYVPEEQEATPPLSPEQEIK / File: sydney_ip_20.raw; SpectrumID: ; scans: 7060

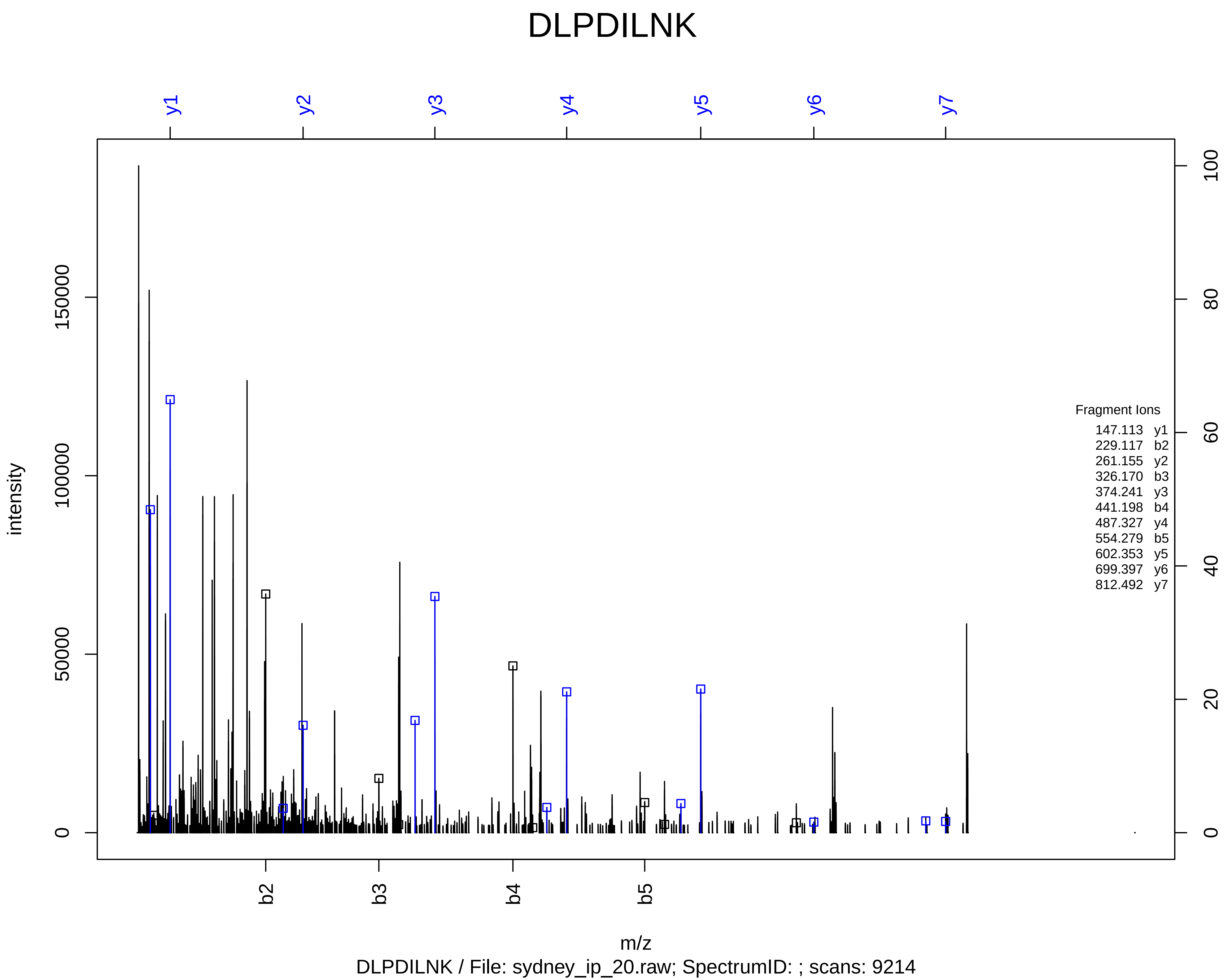
DLPDILNK
y1
y2
y3
y4
y5
y6
y7
b2
b3
b4
b5
intensity
0
50000
100000
150000
0
20
40
60
80
100
m/z
Fragment Ions
147.113 y1
229.117 b2
261.155 y2
326.170 b3
374.241 y3
441.198 b4
487.327 y4
554.279 b5
602.353 y5
699.397 y6
812.492 y7
DLPDILNK / File: sydney_ip_20.raw; SpectrumID: ; scans: 9214

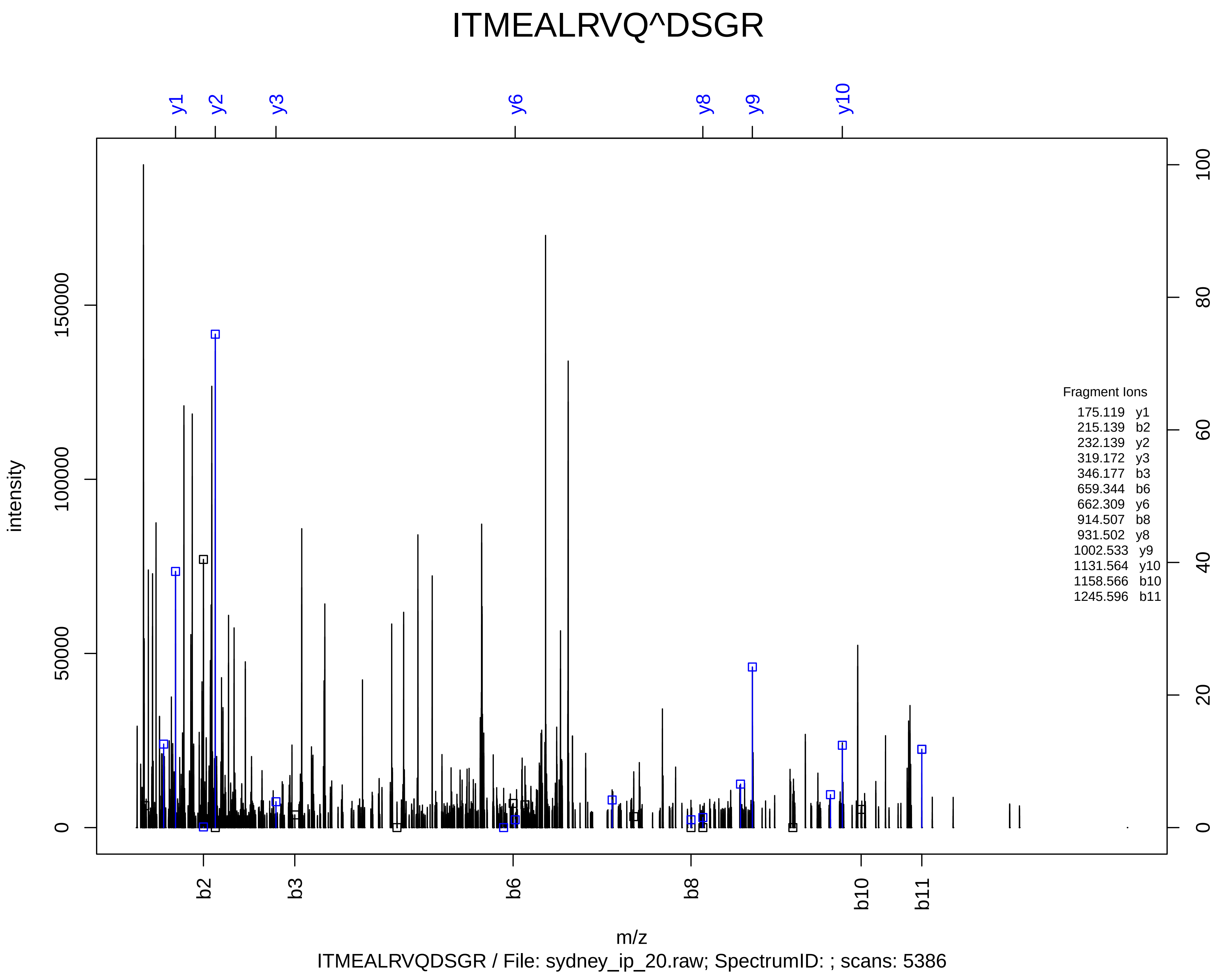
ITMEALRVQ^DSGR
y1
y2
y3
y6
y8
y9
y10
intensity
0
50000
100000
150000
0
20
40
60
80
100
b2
b3
b6
b8
b10
b11
m/z
Fragment Ions
175.119 y1
215.139 b2
232.139 y2
319.172 y3
346.177 b3
659.344 b6
662.309 y6
914.507 b8
931.502 y8
1002.533 y9
1131.564 y10
1158.566 b10
1245.596 b11
ITMEALRVQDSGR / File: sydney_ip_20.raw; SpectrumID: ; scans: 5386

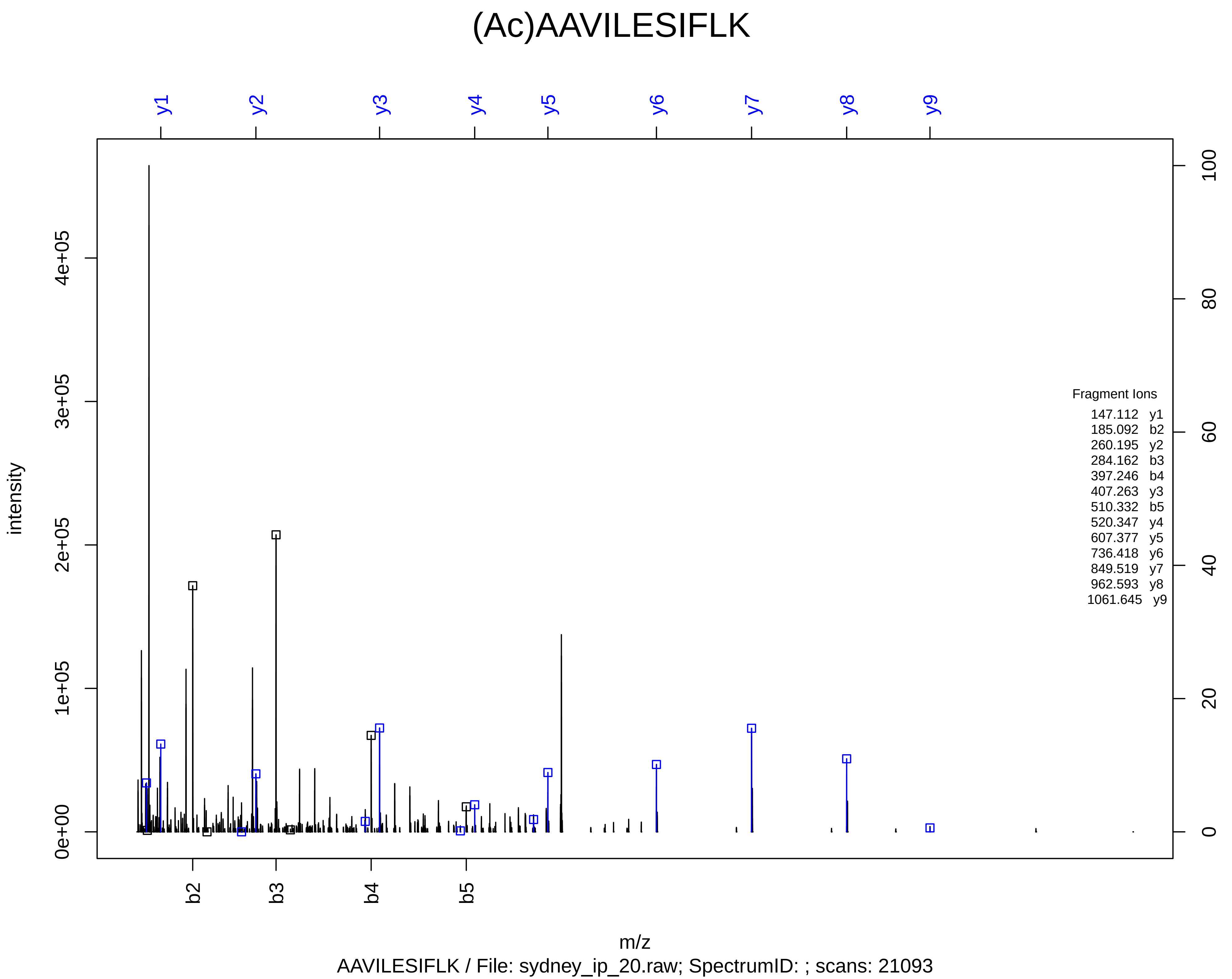
(Ac)AAVILESIFLK
y1
y2
y3
y4
y5
y6
y7
y8
y9
intensity
0e+00
1e+05
2e+05
3e+05
4e+05
0
20
40
60
80
100
b2
b3
b4
b5
m/z
Fragment Ions
147.112 y1
185.092 b2
260.195 y2
284.162 b3
397.246 b4
407.263 y3
510.332 b5
520.347 y4
607.377 y5
736.418 y6
849.519 y7
962.593 y8
1061.645 y9
AAVILESIFLK / File: sydney_ip_20.raw; SpectrumID: ; scans: 21093

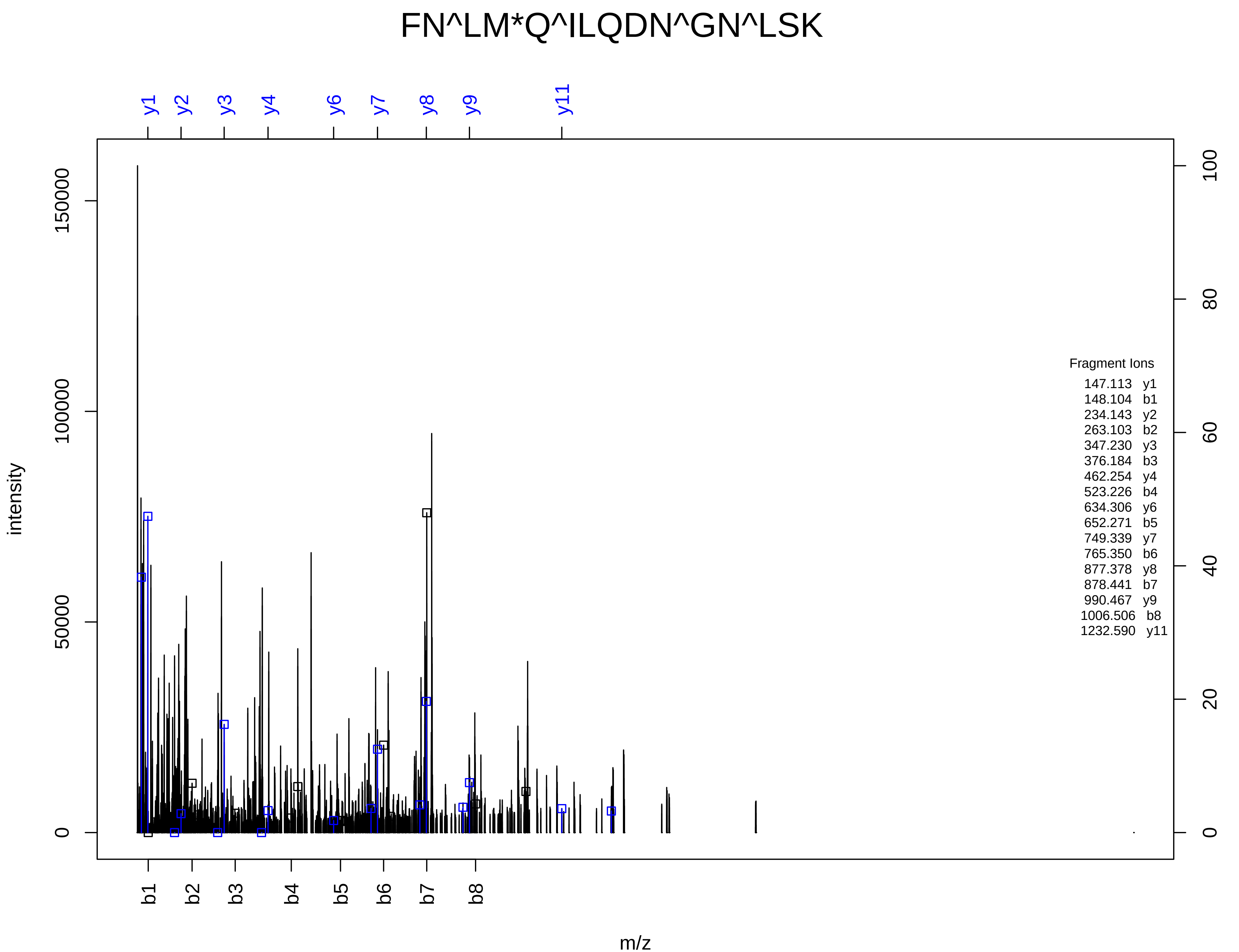

FNLMQILQDNGNLSK / File: sydney_ip_20.raw; SpectrumID: ; scans: 19456

M*ASSPSAAEADGESR

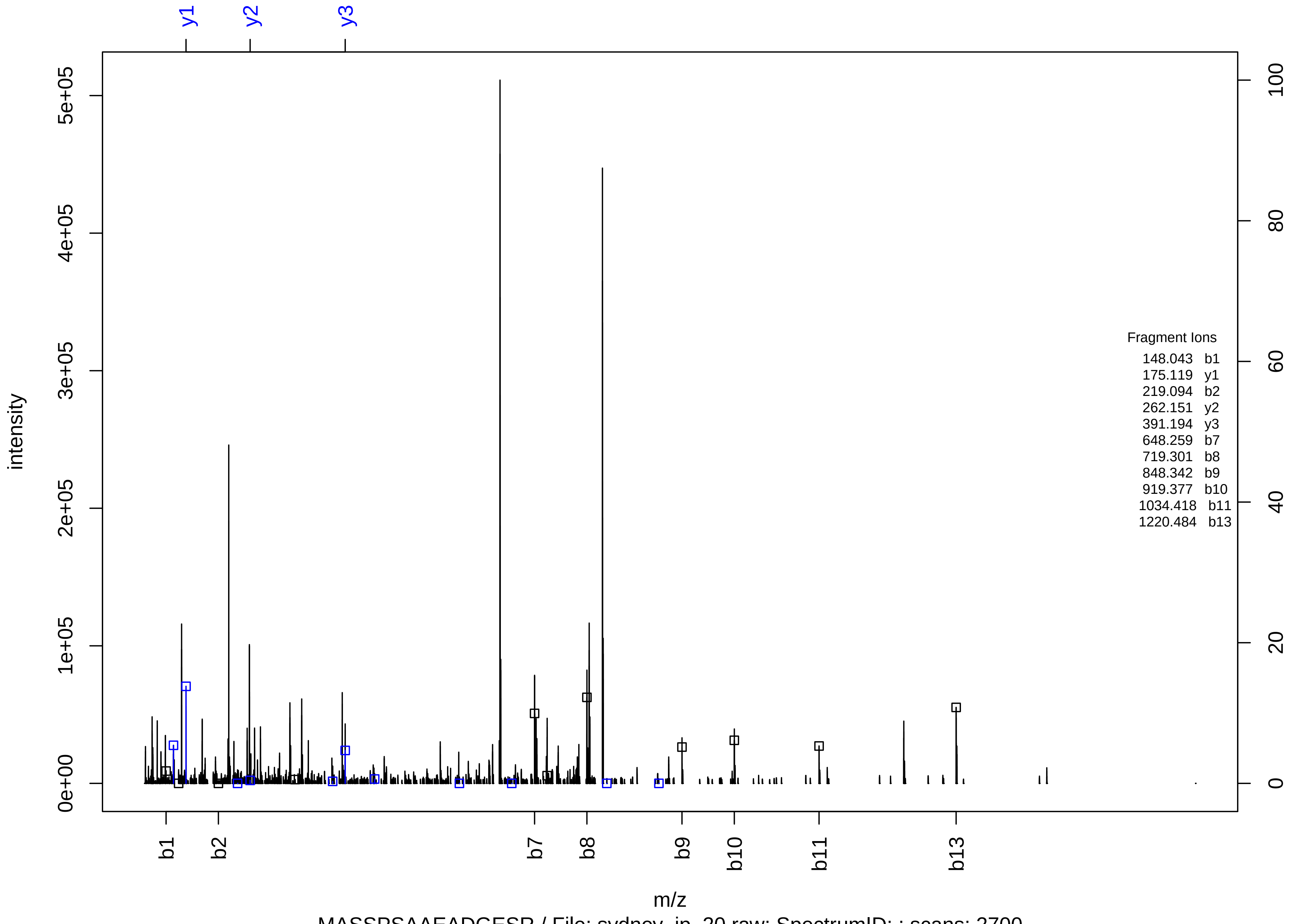

MASSPSAAEADGESR / File: sydney_ip_20.raw; SpectrumID: ; scans: 2700

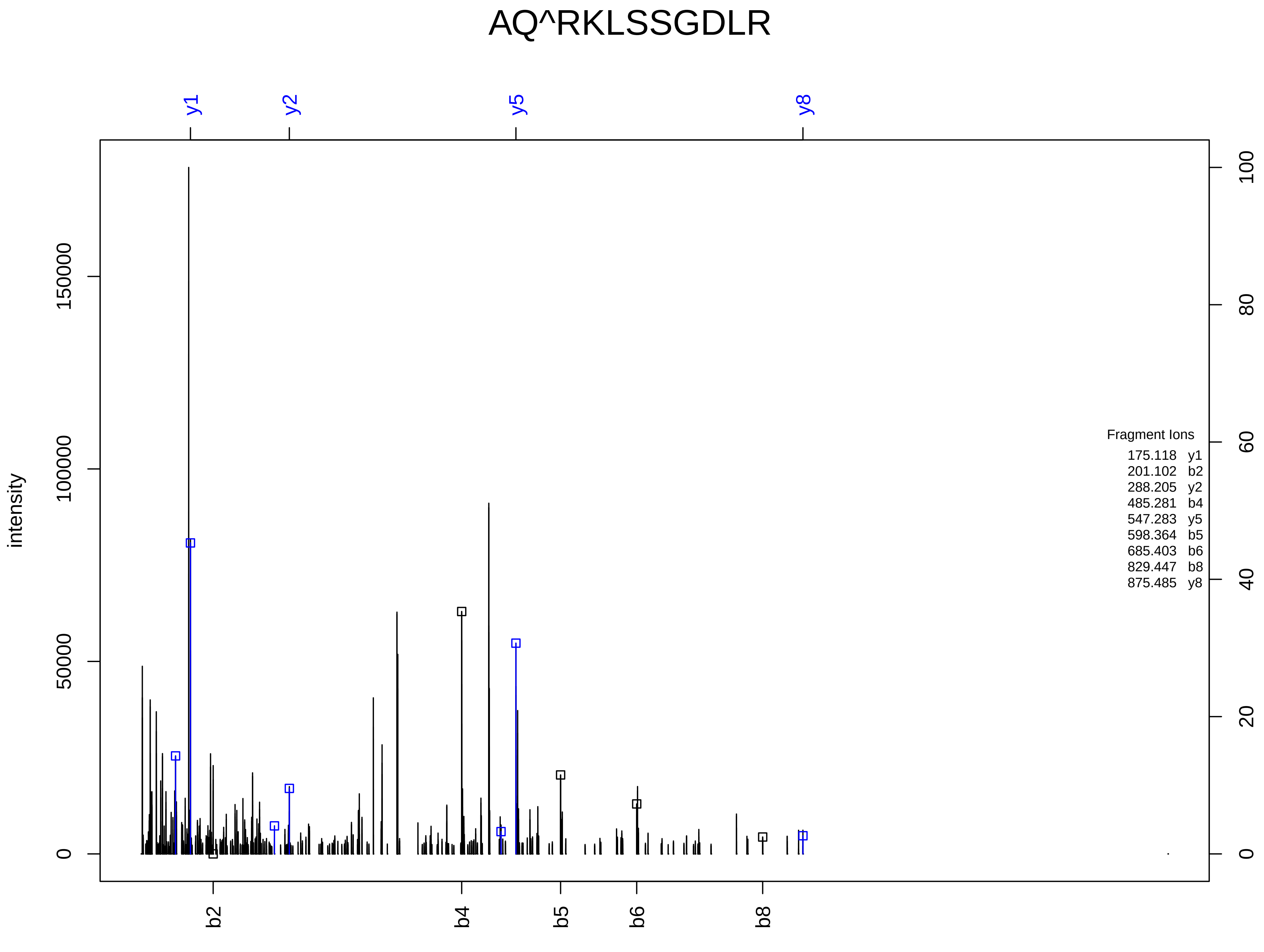

AQRKLSSGDLR / File: sydney_ip_20.raw; SpectrumID: ; scans: 2852

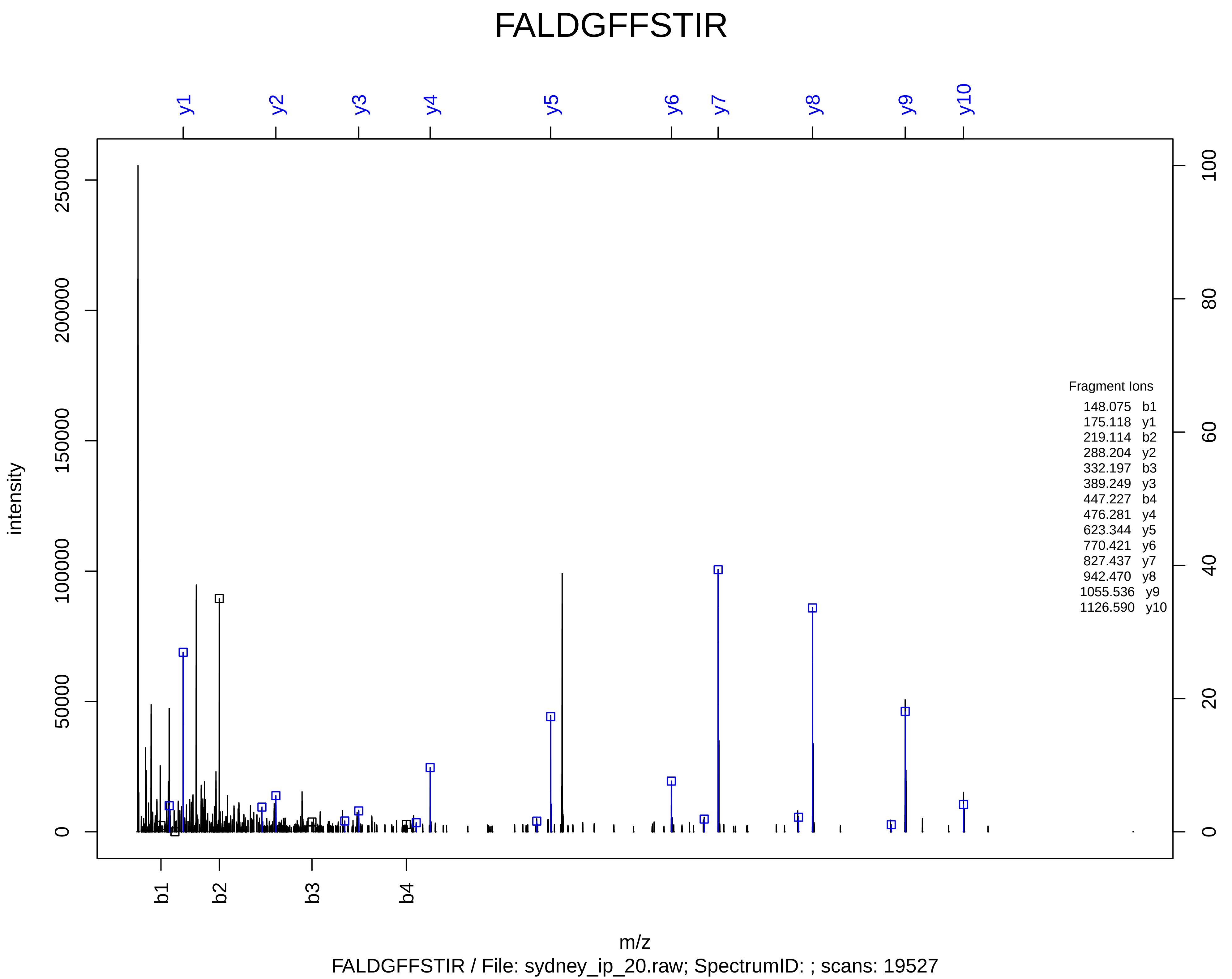

FALDGFFSTIR
y1
y2
y3
y4
y5
y6
y7
y8
y9
y10
intensity
0
50000
100000
150000
200000
250000
0
20
40
60
80
100
b1
b2
b3
b4
m/z
Fragment Ions
148.075 b1
175.118 y1
219.114 b2
288.204 y2
332.197 b3
389.249 y3
447.227 b4
476.281 y4
623.344 y5
770.421 y6
827.437 y7
942.470 y8
1055.536 y9
1126.590 y10
FALDGFFSTIR / File: sydney_ip_20.raw; SpectrumID: ; scans: 19527

SLFVPALDKPETDLLSHLDR

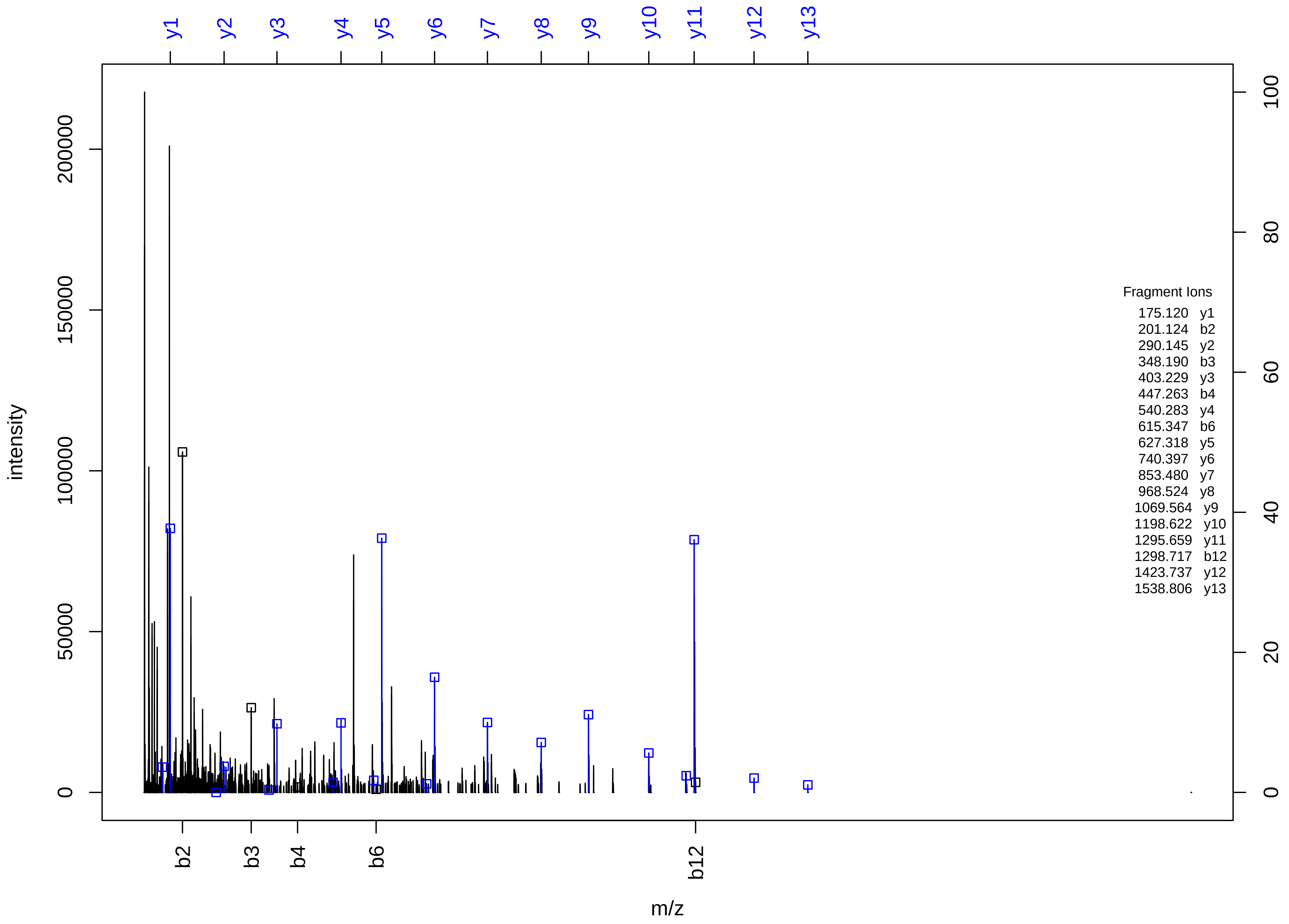

SLFVPALDKPETDLLSHLDR / File: sydney_ip_20.raw; SpectrumID: ; scans: 16995

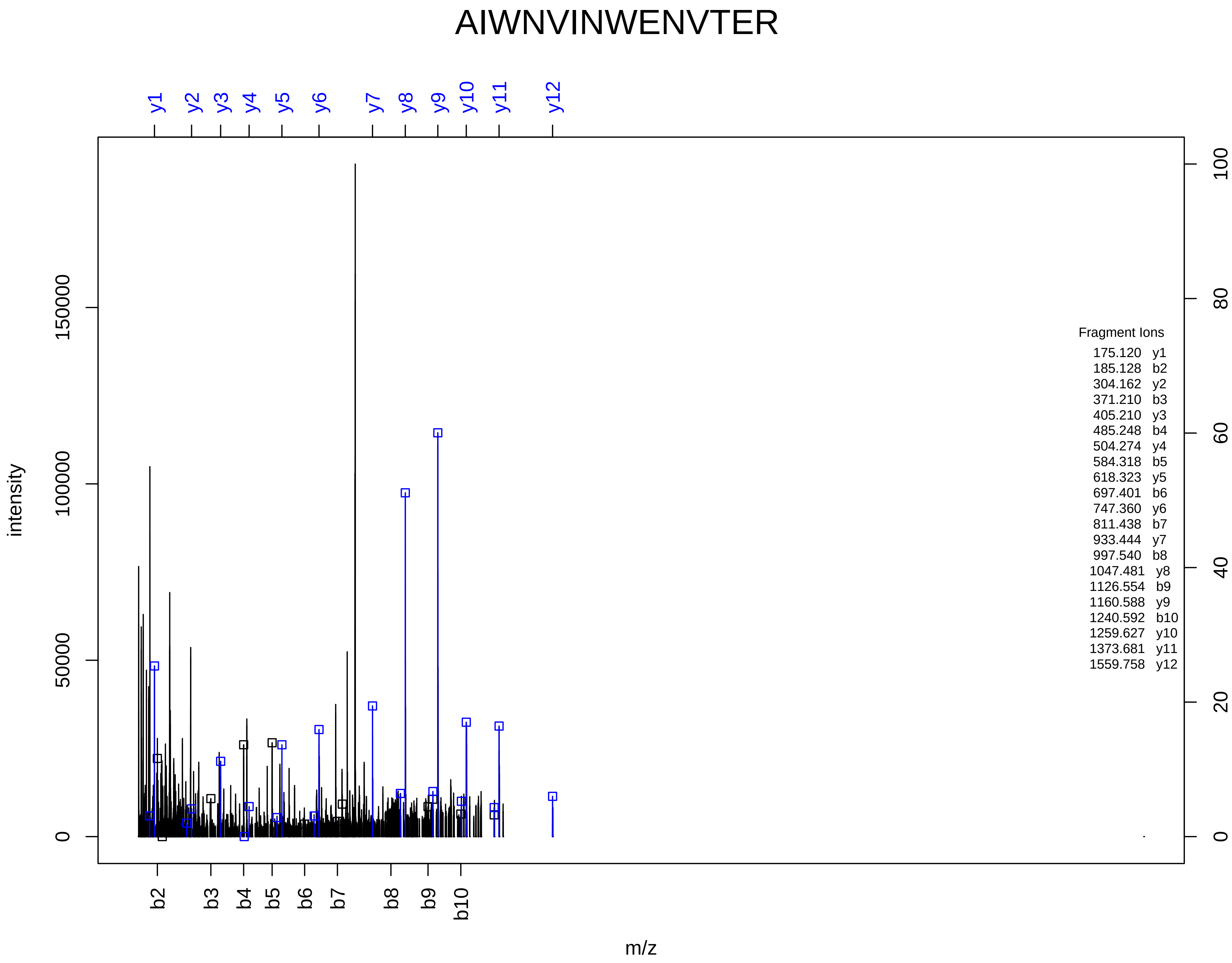

AIWNVINWENVTER
y1 y2 y3 y4 y5 y6 y7 y8 y9 y10 y11 y12
b2 b3 b4 b5 b6 b7 b8 b9 b10
intensity
0
50000
100000
150000
0
20
40
60
80
100
m/z
Fragment Ions
175.120 y1
185.128 b2
304.162 y2
371.210 b3
405.210 y3
485.248 b4
504.274 y4
584.318 b5
618.323 y5
697.401 b6
747.360 y6
811.438 b7
933.444 y7
997.540 b8
1047.481 y8
1126.554 b9
1160.588 y9
1240.592 b10
1259.627 y10
1373.681 y11
1559.758 y12
AIWNVINWENVTER / File: sydney_ip_20.raw; SpectrumID: ; scans: 18572

FGM*HLQ^AATPKGYEPDPNIVK

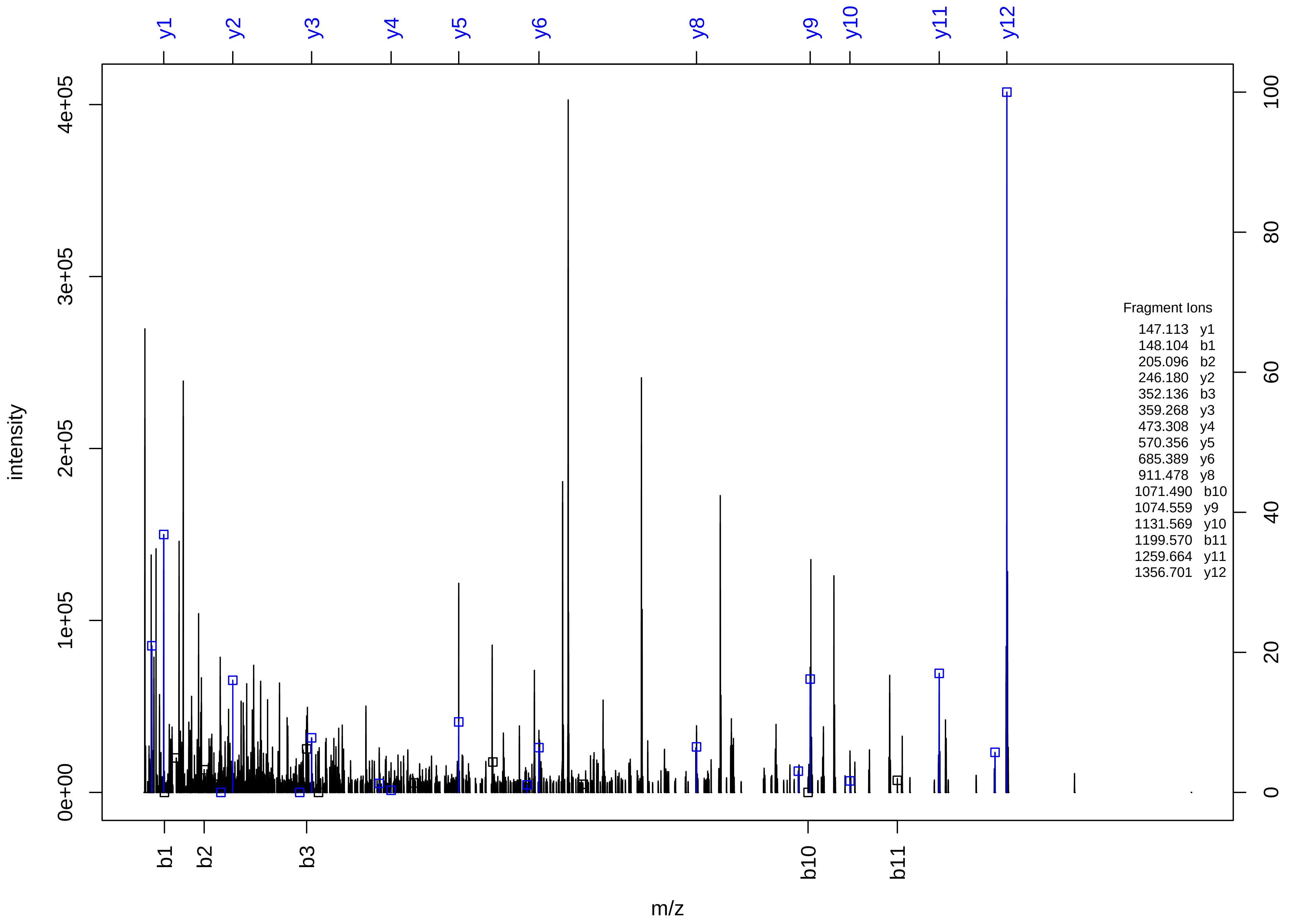

FGMHLQAATPKGYEPDPNIVK / File: sydney_ip_20.raw; SpectrumID: ; scans: 15175

(Ac)SDKPDM*AEIEK

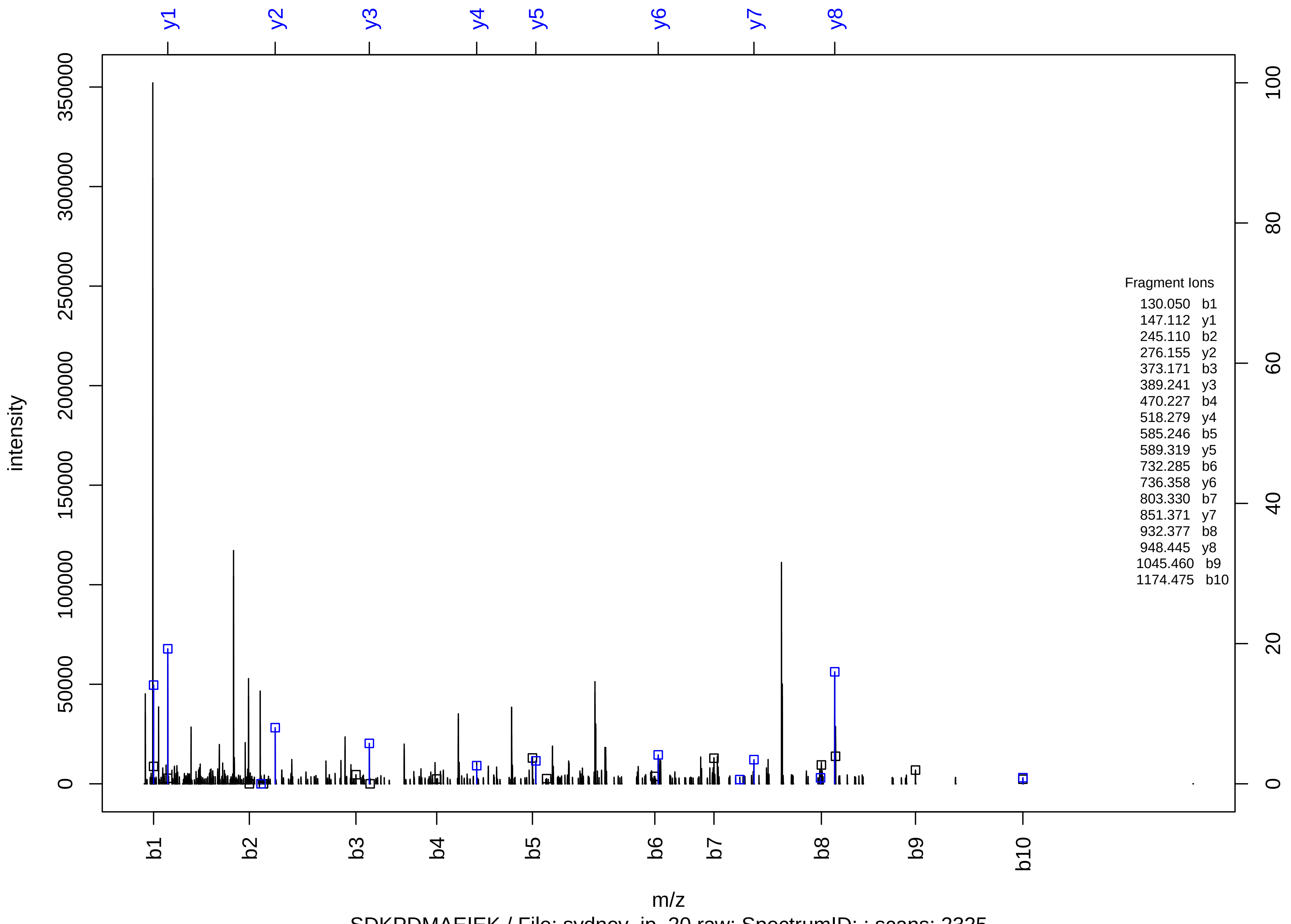

SDKPDMAEIEK / File: sydney_ip_20.raw; SpectrumID: ; scans: 2325

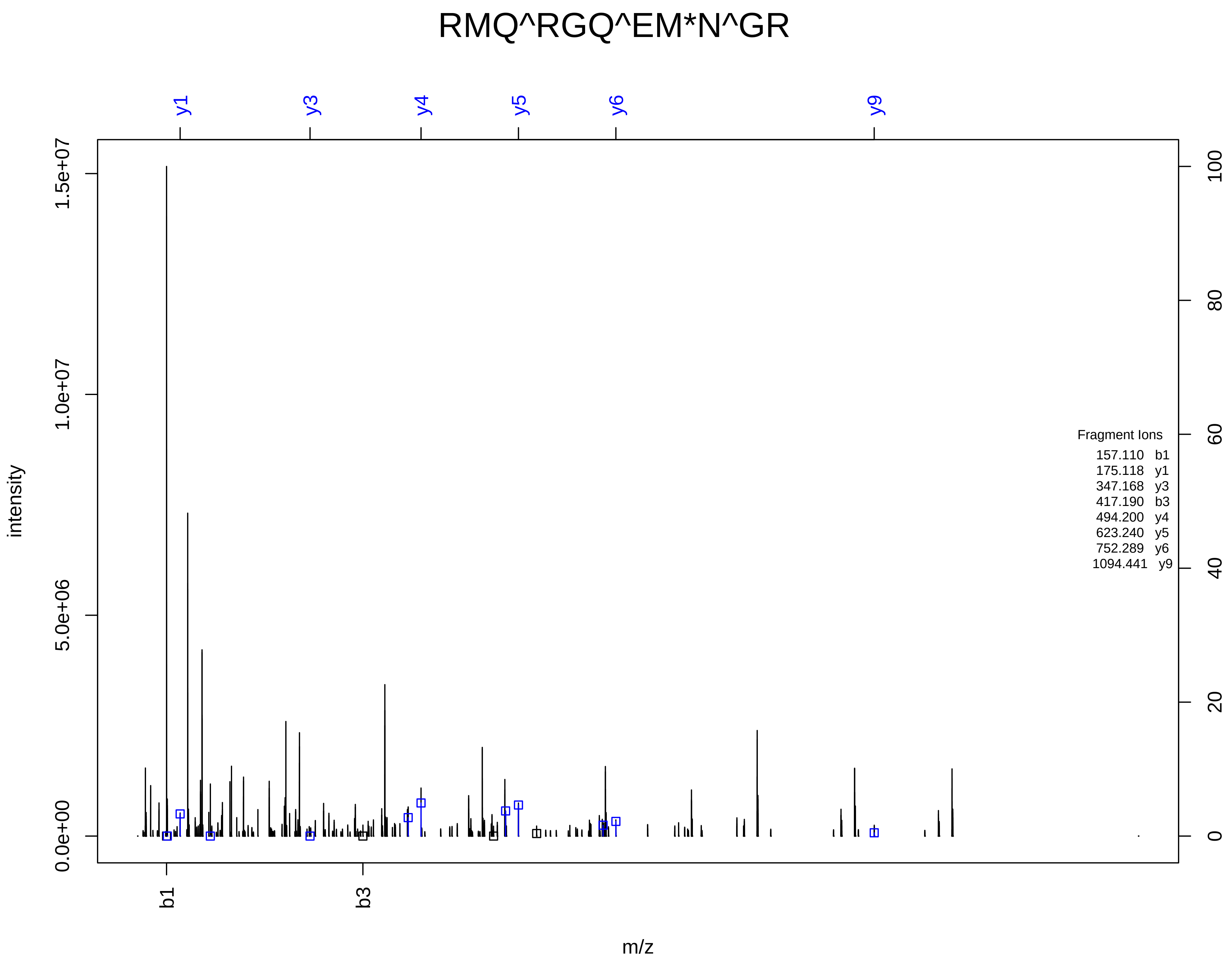

RMQRGQEMNGR / File: sydney_ip_20.raw; SpectrumID: ; scans: 5073

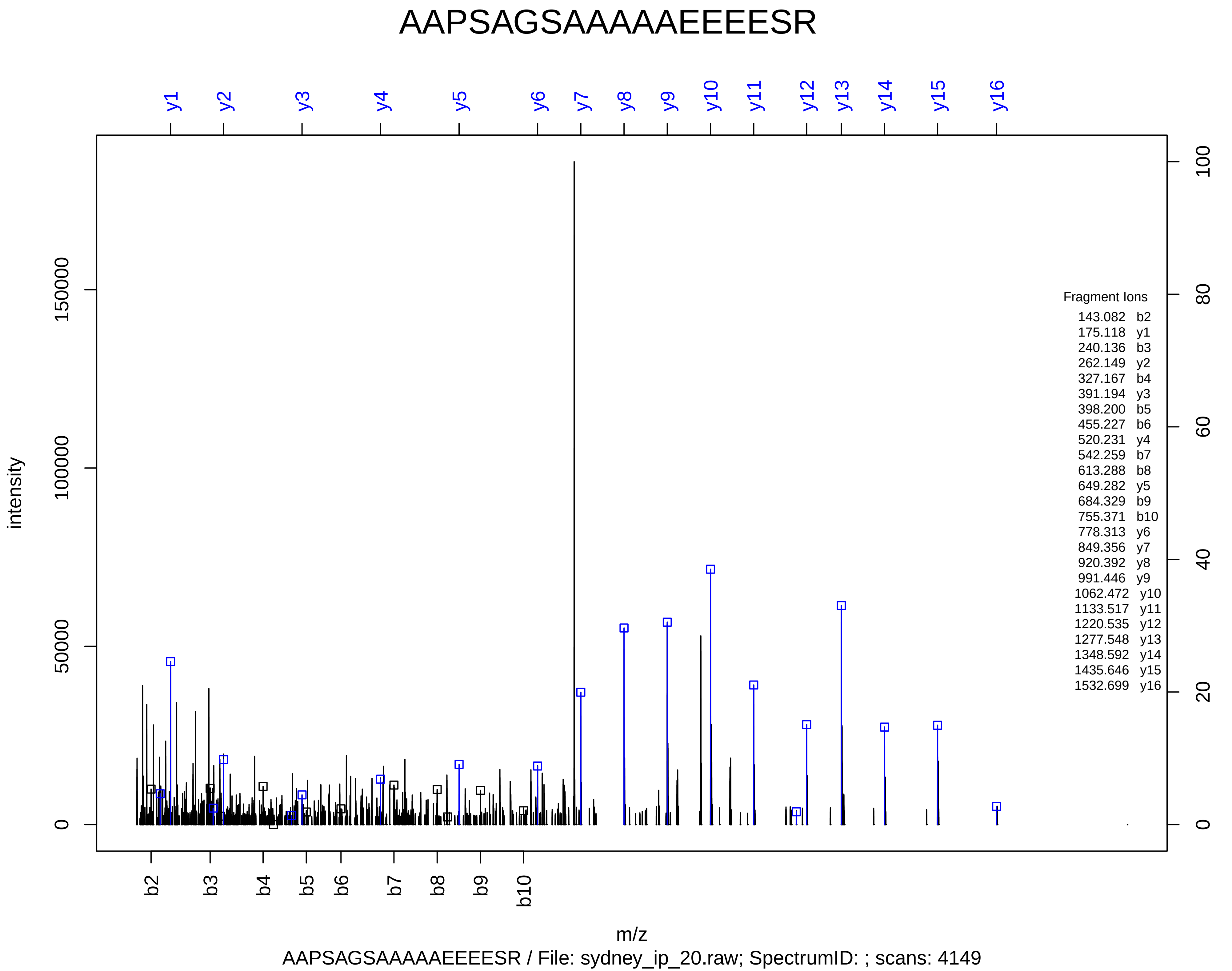
AAPSAGSAAAAAEEEESR
intensity
m/z
y1 y2 y3 y4 y5 y6 y7 y8 y9 y10 y11 y12 y13 y14 y15 y16
b2 b3 b4 b5 b6 b7 b8 b9 b10
0 50000 100000 150000
0 20 40 60 80 100
Fragment Ions
143.082 b2
175.118 y1
240.136 b3
262.149 y2
327.167 b4
391.194 y3
398.200 b5
455.227 b6
520.231 y4
542.259 b7
613.288 b8
649.282 y5
684.329 b9
755.371 b10
778.313 y6
849.356 y7
920.392 y8
991.446 y9
1062.472 y10
1133.517 y11
1220.535 y12
1277.548 y13
1348.592 y14
1435.646 y15
1532.699 y16
AAPSAGSAAAAAEEEESR / File: sydney_ip_20.raw; SpectrumID: ; scans: 4149

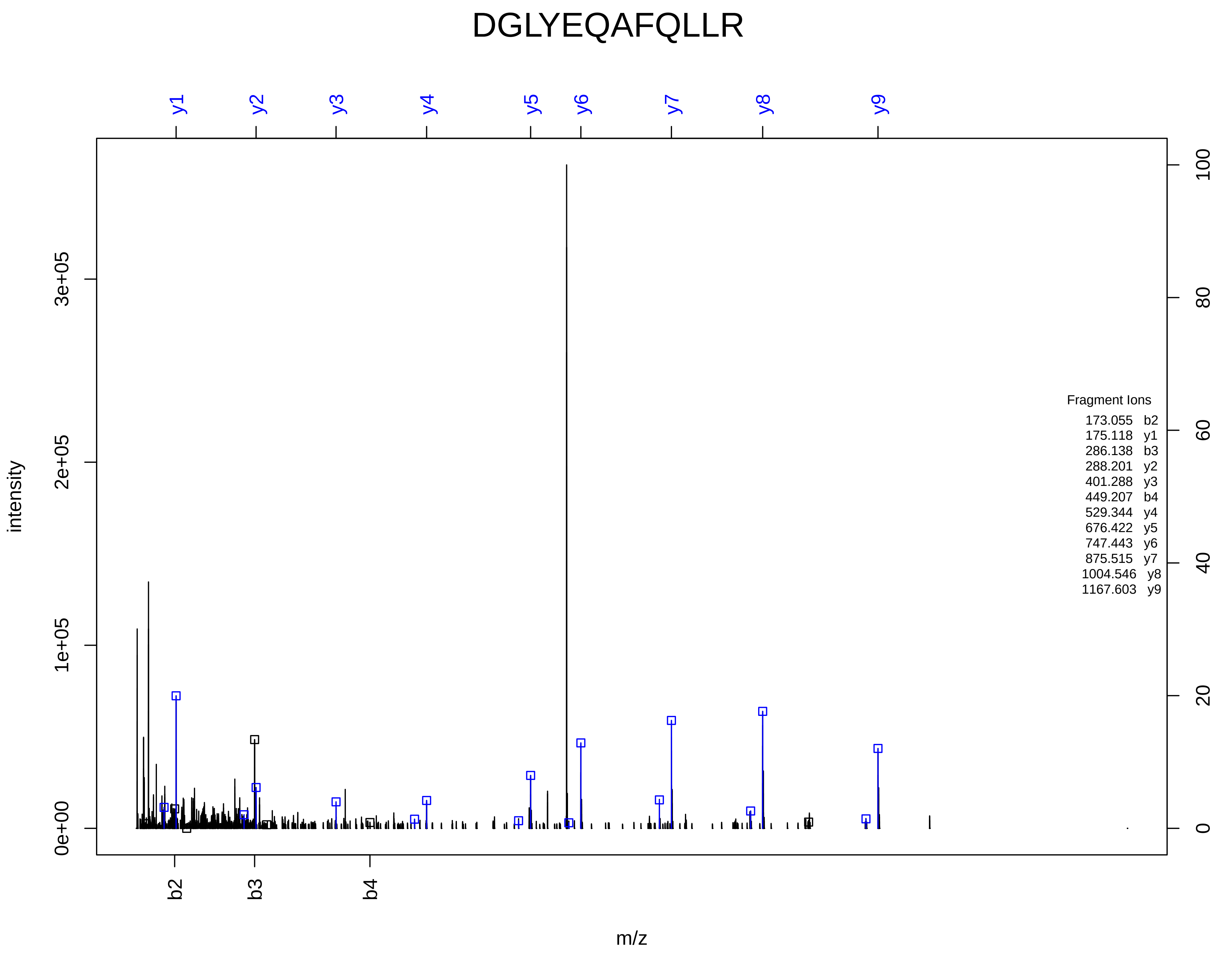
DGLYEQAFQLLR
y1
y2
y3
y4
y5
y6
y7
y8
y9
b2
b3
b4
intensity
0e+00
1e+05
2e+05
3e+05
0
20
40
60
80
100
m/z
Fragment Ions
173.055 b2
175.118 y1
286.138 b3
288.201 y2
401.288 y3
449.207 b4
529.344 y4
676.422 y5
747.443 y6
875.515 y7
1004.546 y8
1167.603 y9
DGLYEQAFQLLR / File: sydney_ip_20.raw; SpectrumID: ; scans: 19138

DVTSVLIDLER

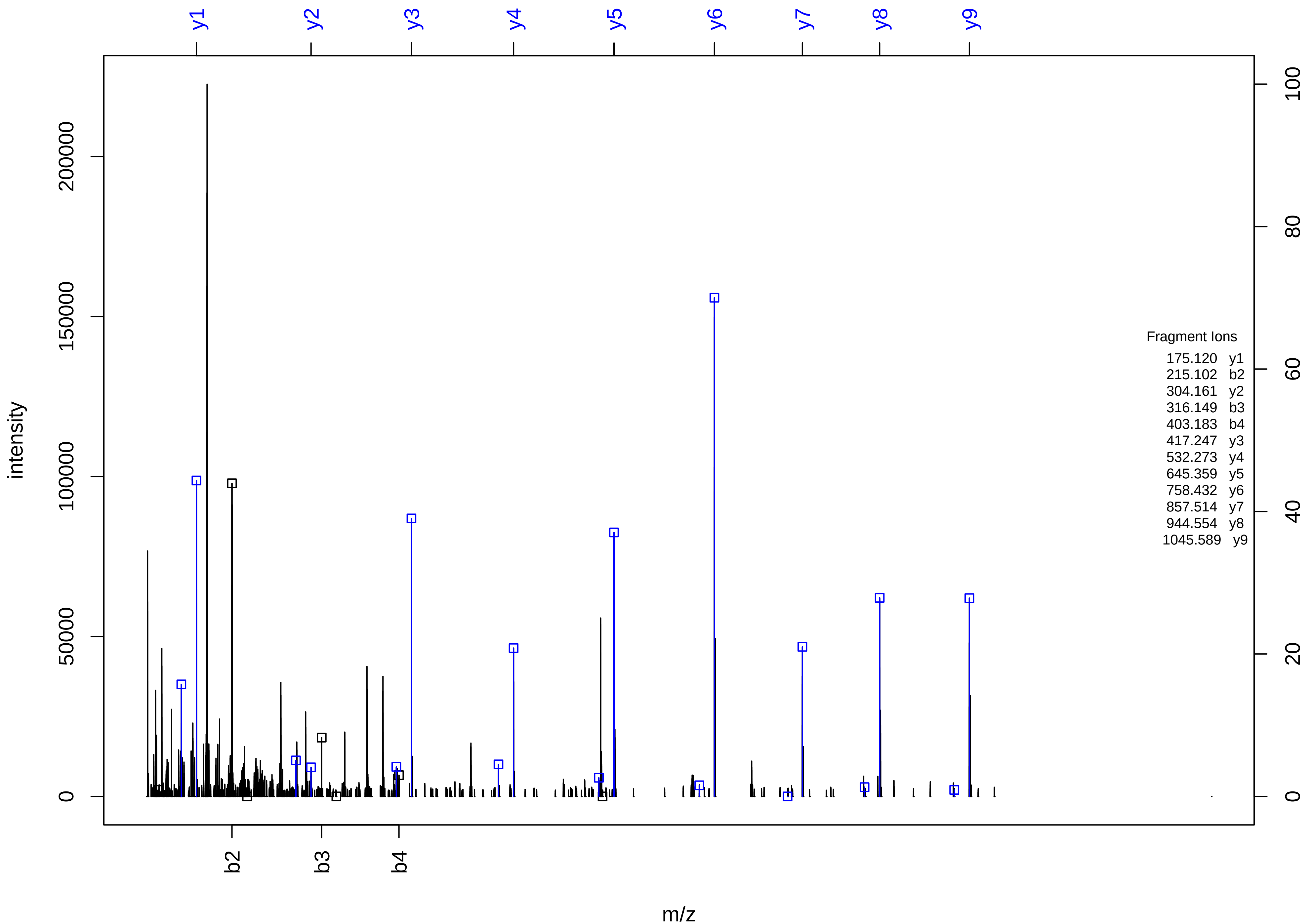

DVTSVLIDLER / File: sydney_ip_20.raw; SpectrumID: ; scans: 18008

AGAESDGQFQFTGIK

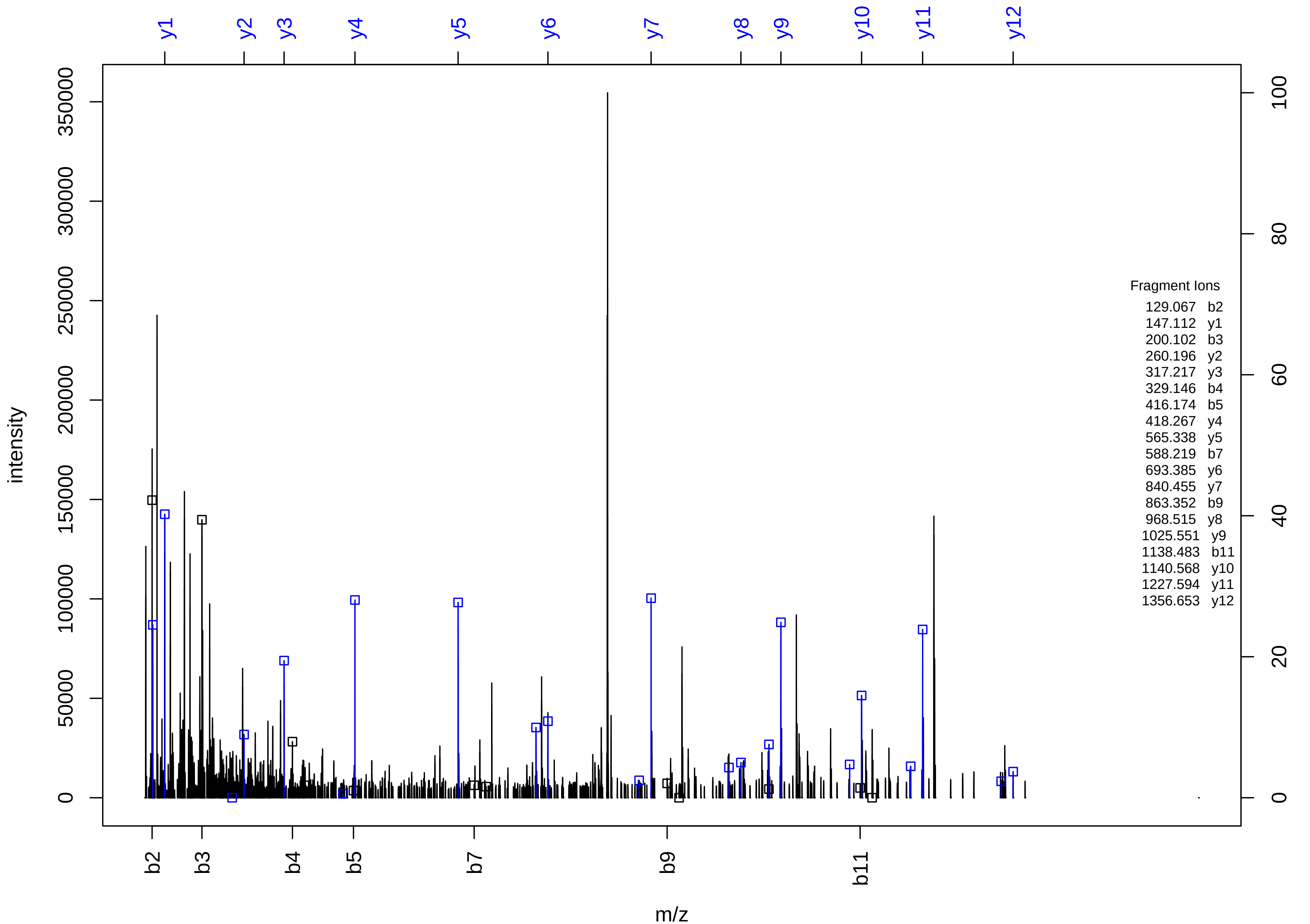

AGAESDGQFQFTGIK / File: sydney_ip_20.raw; SpectrumID: ; scans: 9761

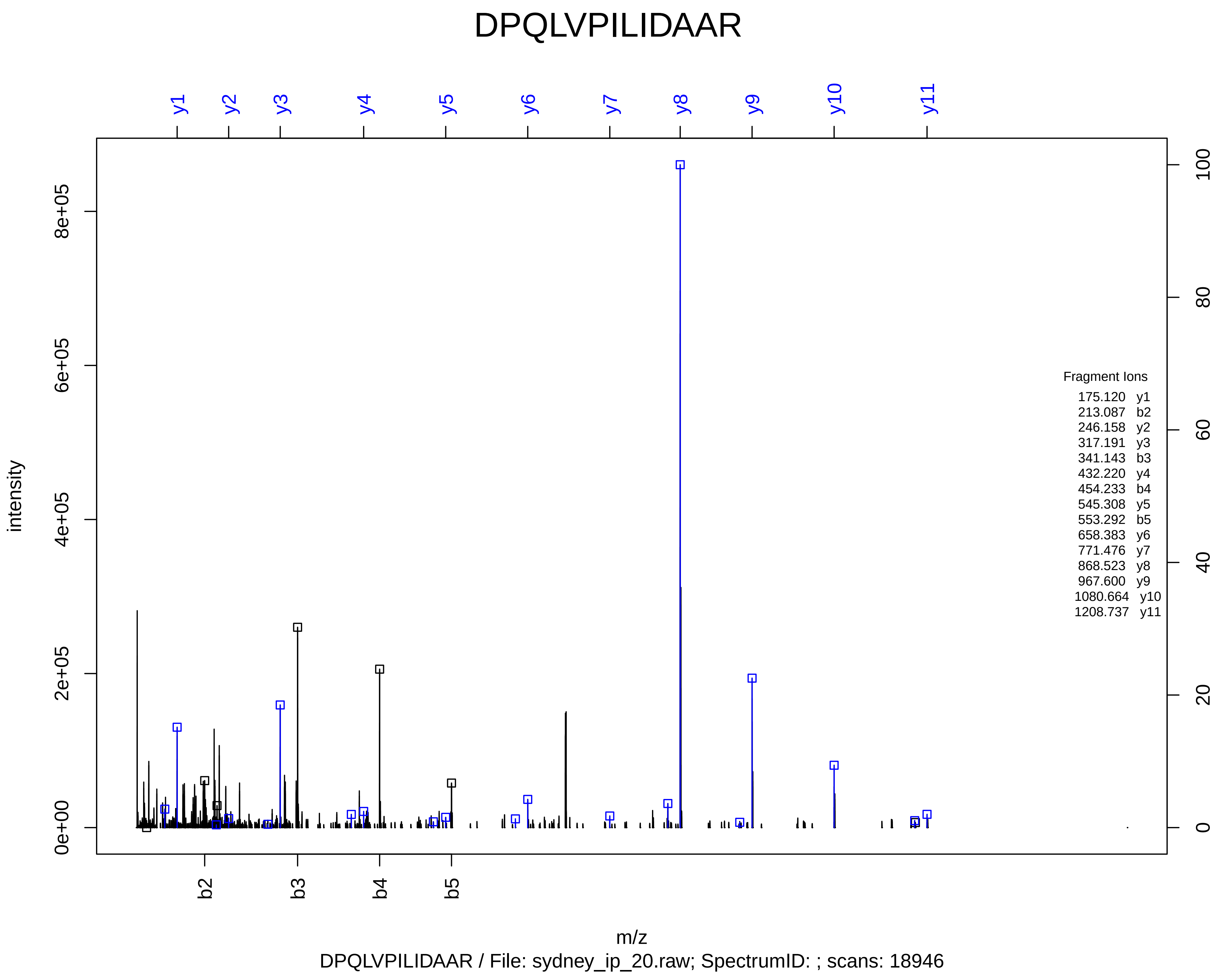

DPQLVPILIDAAR
y1
y2
y3
y4
y5
y6
y7
y8
y9
y10
y11
b2
b3
b4
b5
intensity
0e+00
2e+05
4e+05
6e+05
8e+05
0
20
40
60
80
100
m/z
Fragment Ions
175.120 y1
213.087 b2
246.158 y2
317.191 y3
341.143 b3
432.220 y4
454.233 b4
545.308 y5
553.292 b5
658.383 y6
771.476 y7
868.523 y8
967.600 y9
1080.664 y10
1208.737 y11
DPQLVPILIDAAR / File: sydney_ip_20.raw; SpectrumID: ; scans: 18946

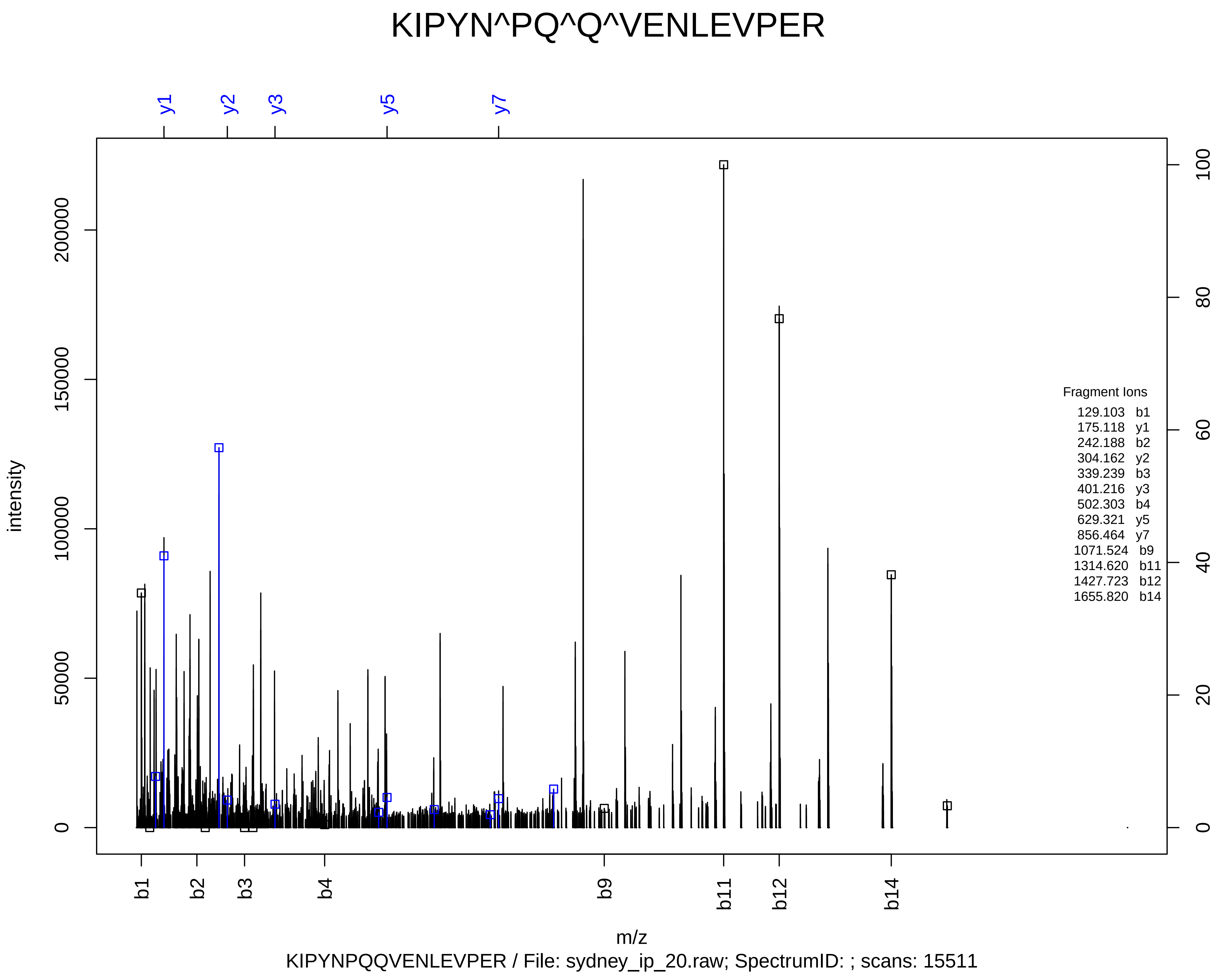

KIPYN^PQ^Q^VENLEVPER
y1
y2
y3
y5
y7
intensity
0
50000
100000
150000
200000
0
20
40
60
80
100
b1
b2
b3
b4
b9
b11
b12
b14
m/z
Fragment Ions
129.103 b1
175.118 y1
242.188 b2
304.162 y2
339.239 b3
401.216 y3
502.303 b4
629.321 y5
856.464 y7
1071.524 b9
1314.620 b11
1427.723 b12
1655.820 b14
KIPYNPQQVENLEVPER / File: sydney_ip_20.raw; SpectrumID: ; scans: 15511

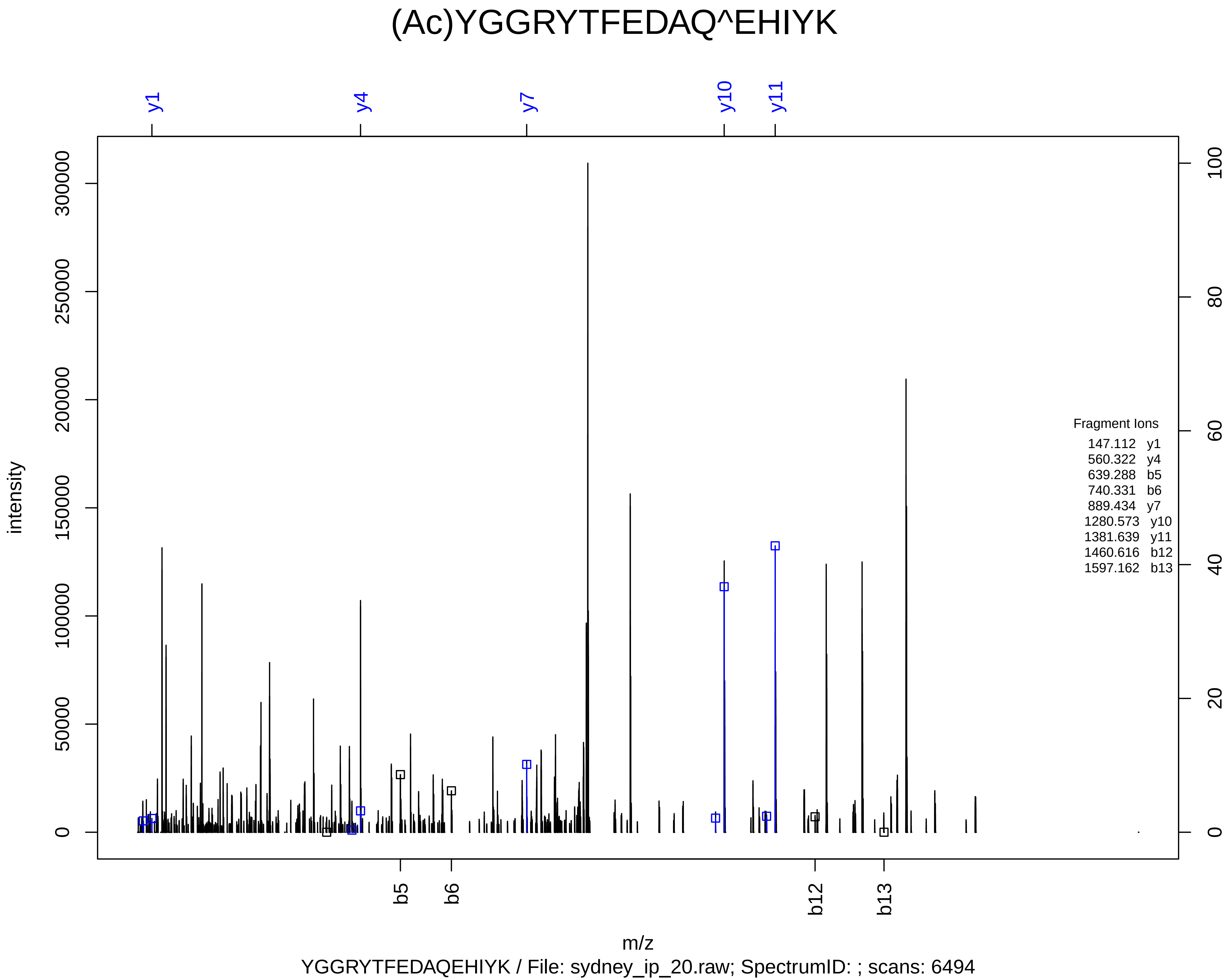

(Ac)YGGRYTFEDAQ^EHIYK
y1
y4
y7
y10
y11
b5
b6
b12
b13
intensity
m/z
Fragment Ions
147.112 y1
560.322 y4
639.288 b5
740.331 b6
889.434 y7
1280.573 y10
1381.639 y11
1460.616 b12
1597.162 b13
YGGRYTFEDAQEHIYK / File: sydney_ip_20.raw; SpectrumID: ; scans: 6494

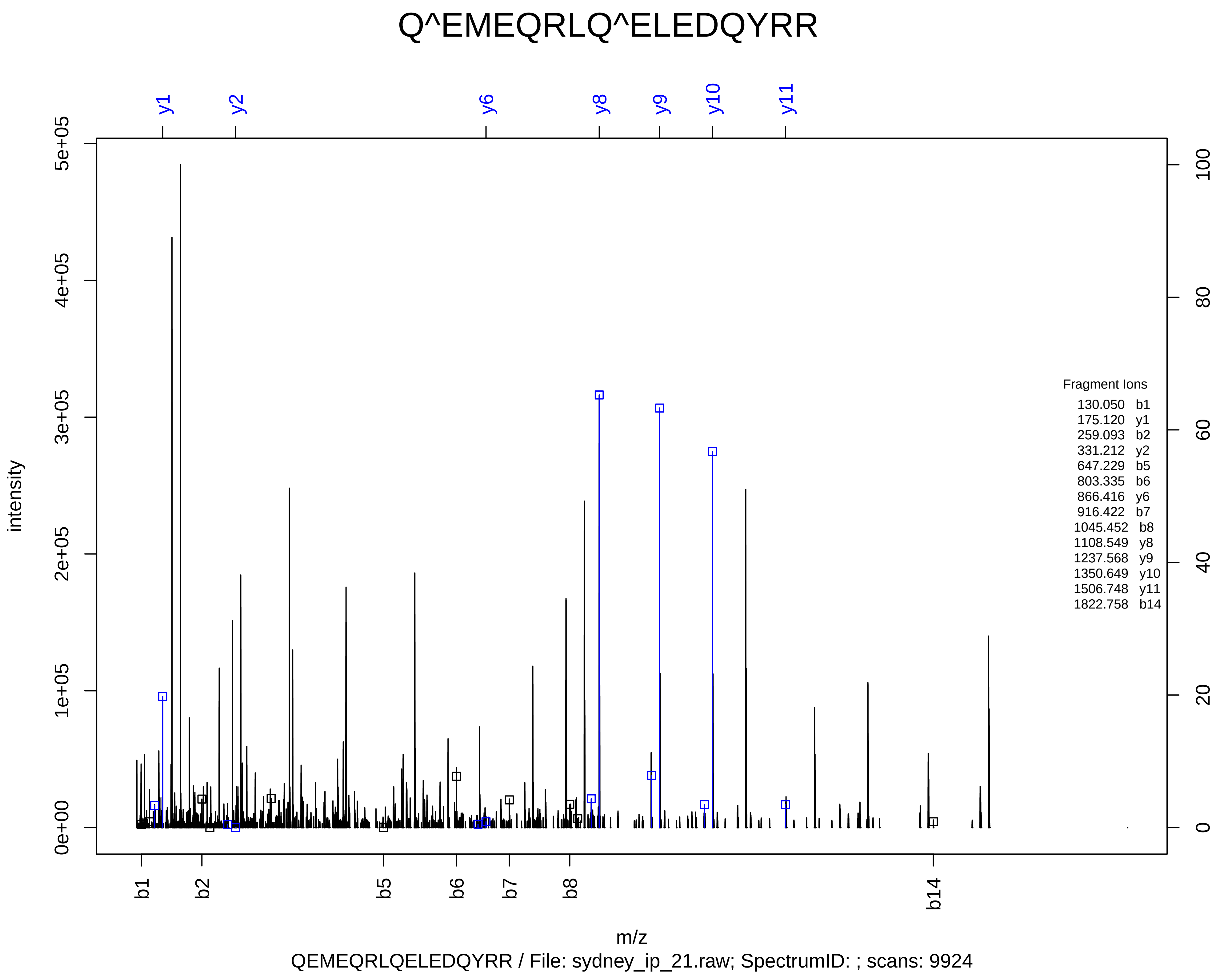
Q^EMEQRLQ^ELEDQYRR
y1
y2
y6
y8
y9
y10
y11
intensity
0e+00
1e+05
2e+05
3e+05
4e+05
5e+05
0
20
40
60
80
100
b1
b2
b5
b6
b7
b8
b14
m/z
Fragment Ions
130.050 b1
175.120 y1
259.093 b2
331.212 y2
647.229 b5
803.335 b6
866.416 y6
916.422 b7
1045.452 b8
1108.549 y8
1237.568 y9
1350.649 y10
1506.748 y11
1822.758 b14
QEMEQRLQELEDQYRR / File: sydney_ip_21.raw; SpectrumID: ; scans: 9924

DPILYLFGR

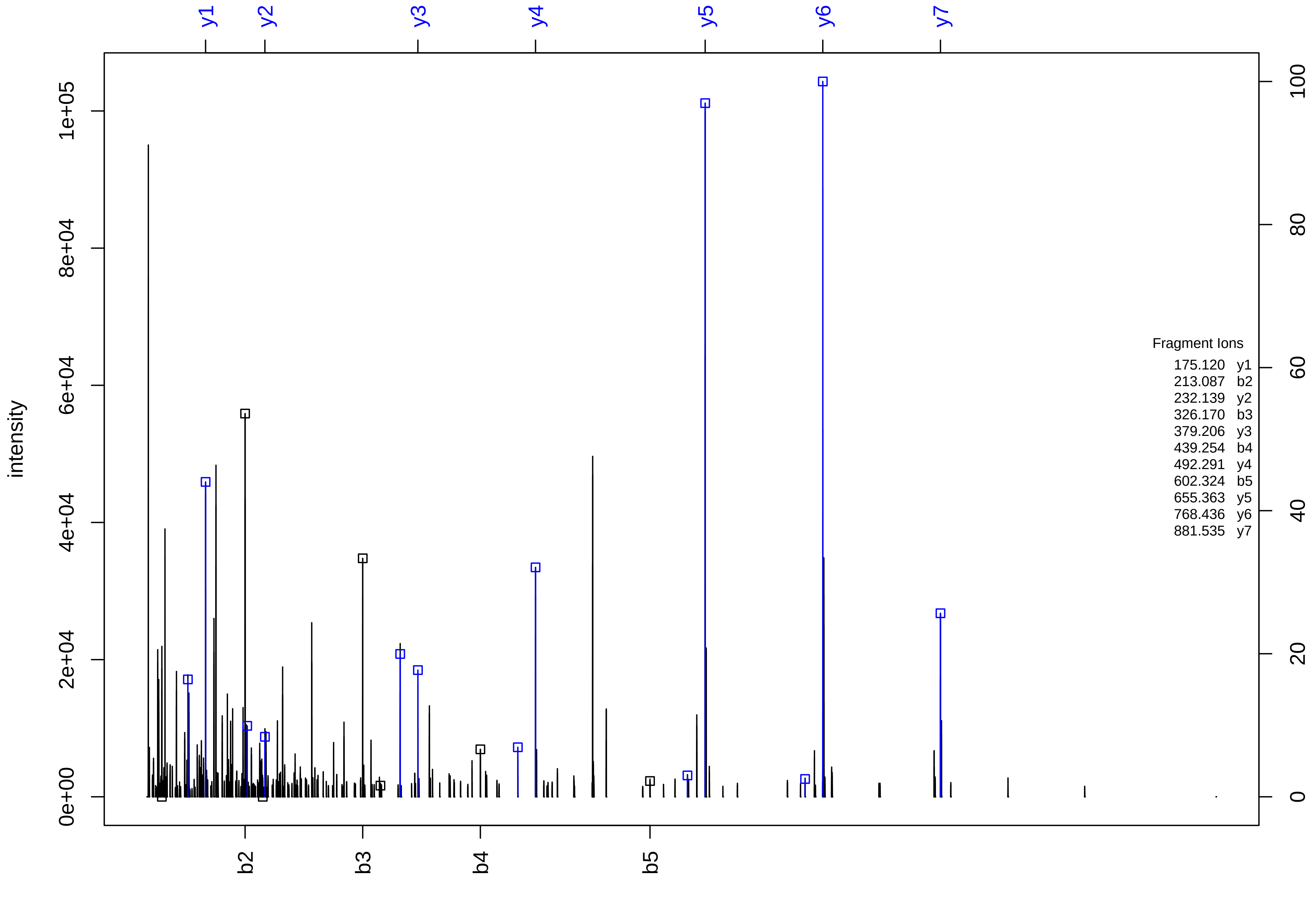

DPILYLFGR / File: sydney_ip_21.raw; SpectrumID: ; scans: 17906

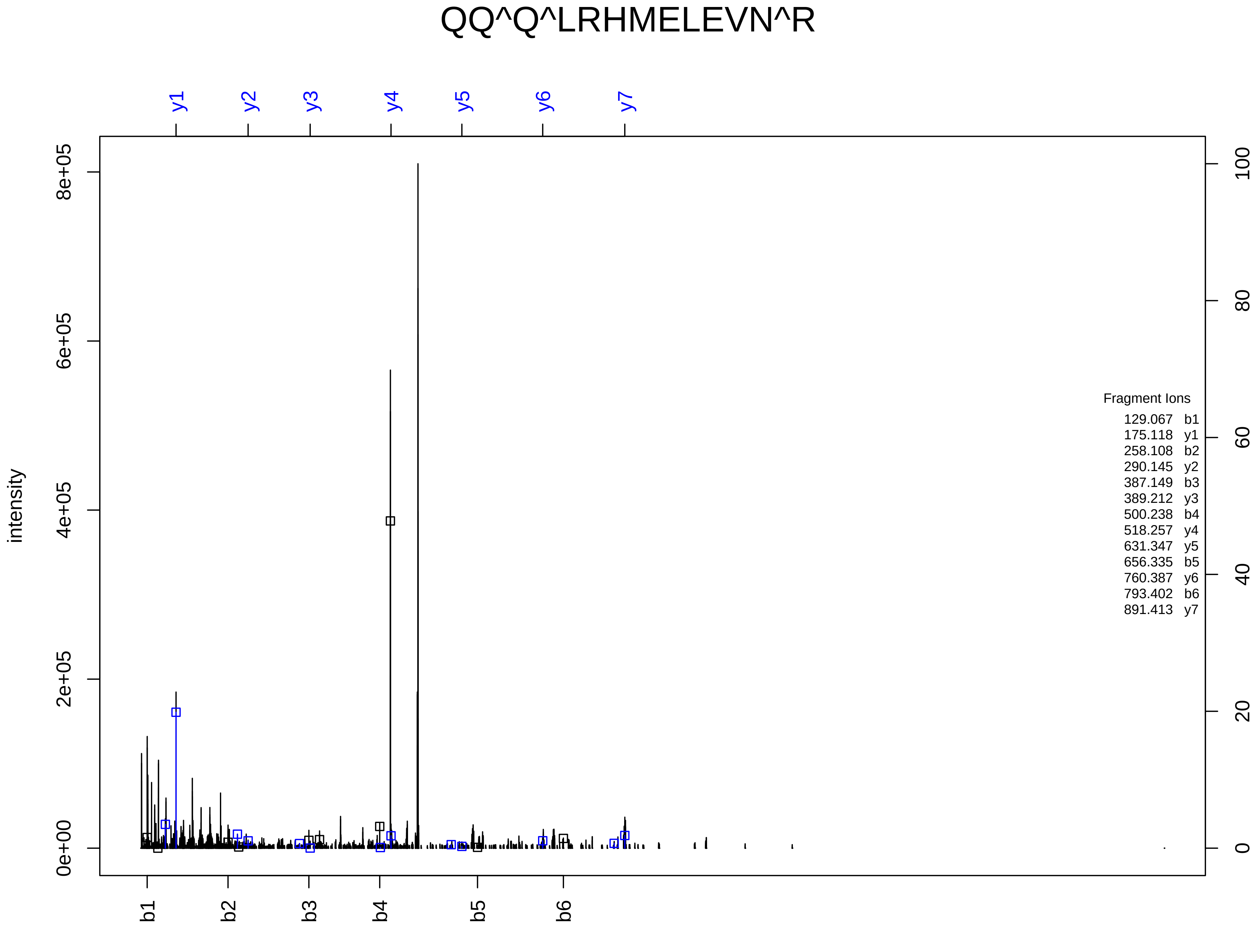

QQQLRHMELEVNR / File: sydney_ip_21.raw; SpectrumID: ; scans: 5364

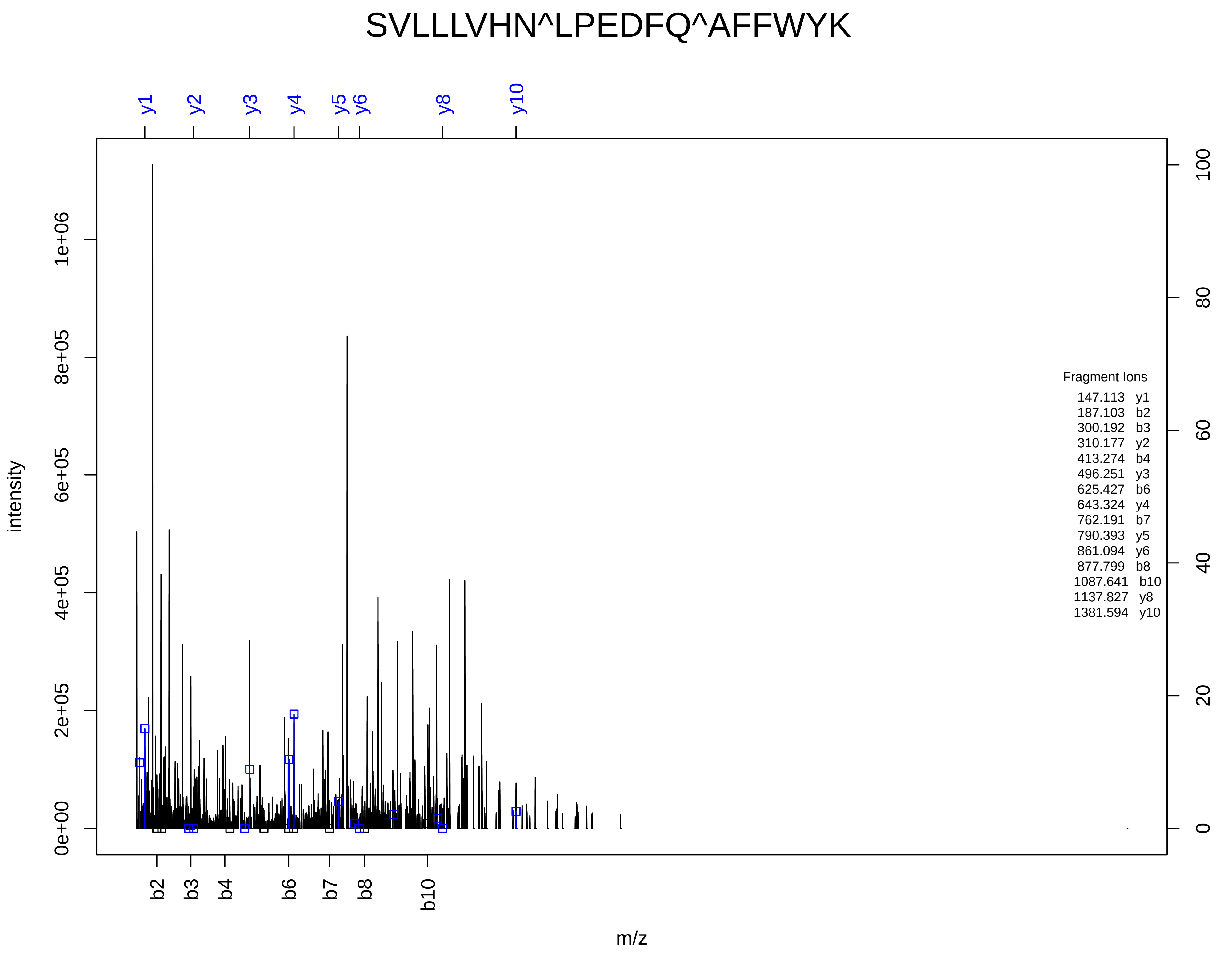
SVLLLVHN^LPEDFQ^AFFWYK
y1
y2
y3
y4
y5
y6
y8
y10
intensity
0e+00
2e+05
4e+05
6e+05
8e+05
1e+06
0
20
40
60
80
100
b2
b3
b4
b6
b7
b8
b10
m/z
Fragment Ions
147.113 y1
187.103 b2
300.192 b3
310.177 y2
413.274 b4
496.251 y3
625.427 b6
643.324 y4
762.191 b7
790.393 y5
861.094 y6
877.799 b8
1087.641 b10
1137.827 y8
1381.594 y10
SVLLLVHNLPEDFQAFFWYK / File: sydney_ip_21.raw; SpectrumID: ; scans: 18028

ELWTILLGR

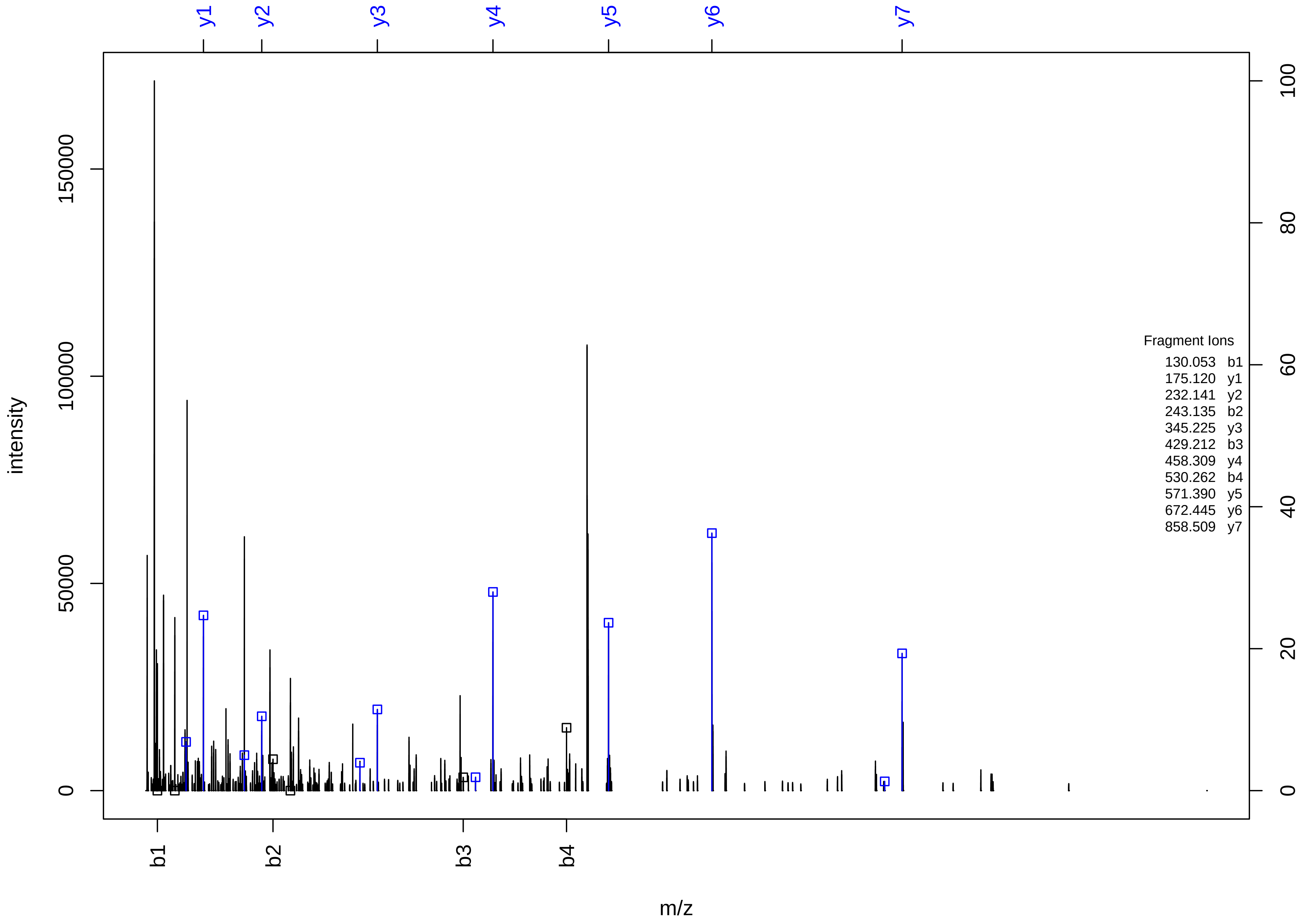

ELWTILLGR / File: sydney_ip_21.raw; SpectrumID: ; scans: 18829

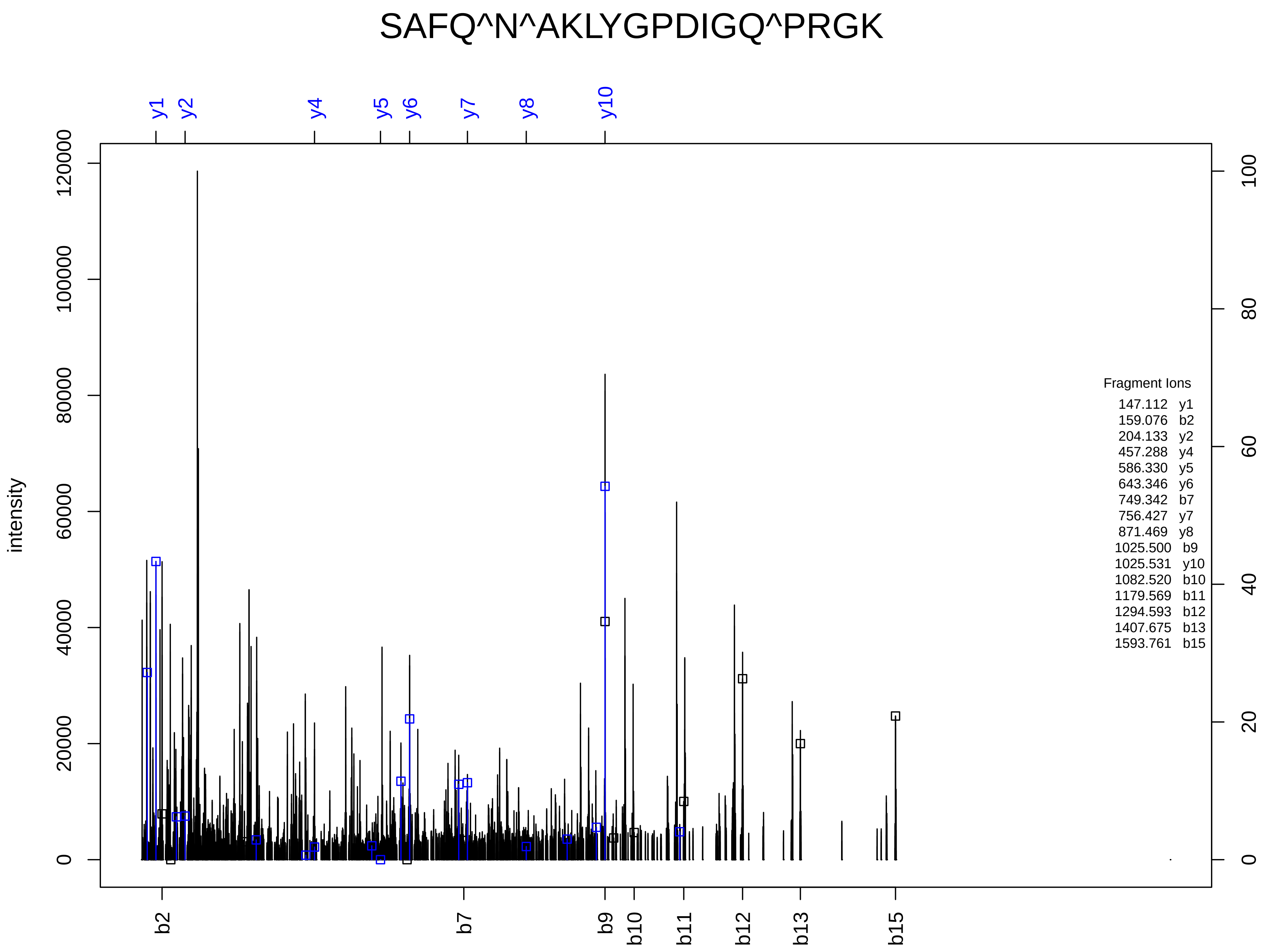

SAFQNAKLYGPDIGQPRGK / File: sydney_ip_21.raw; SpectrumID: ; scans: 15211

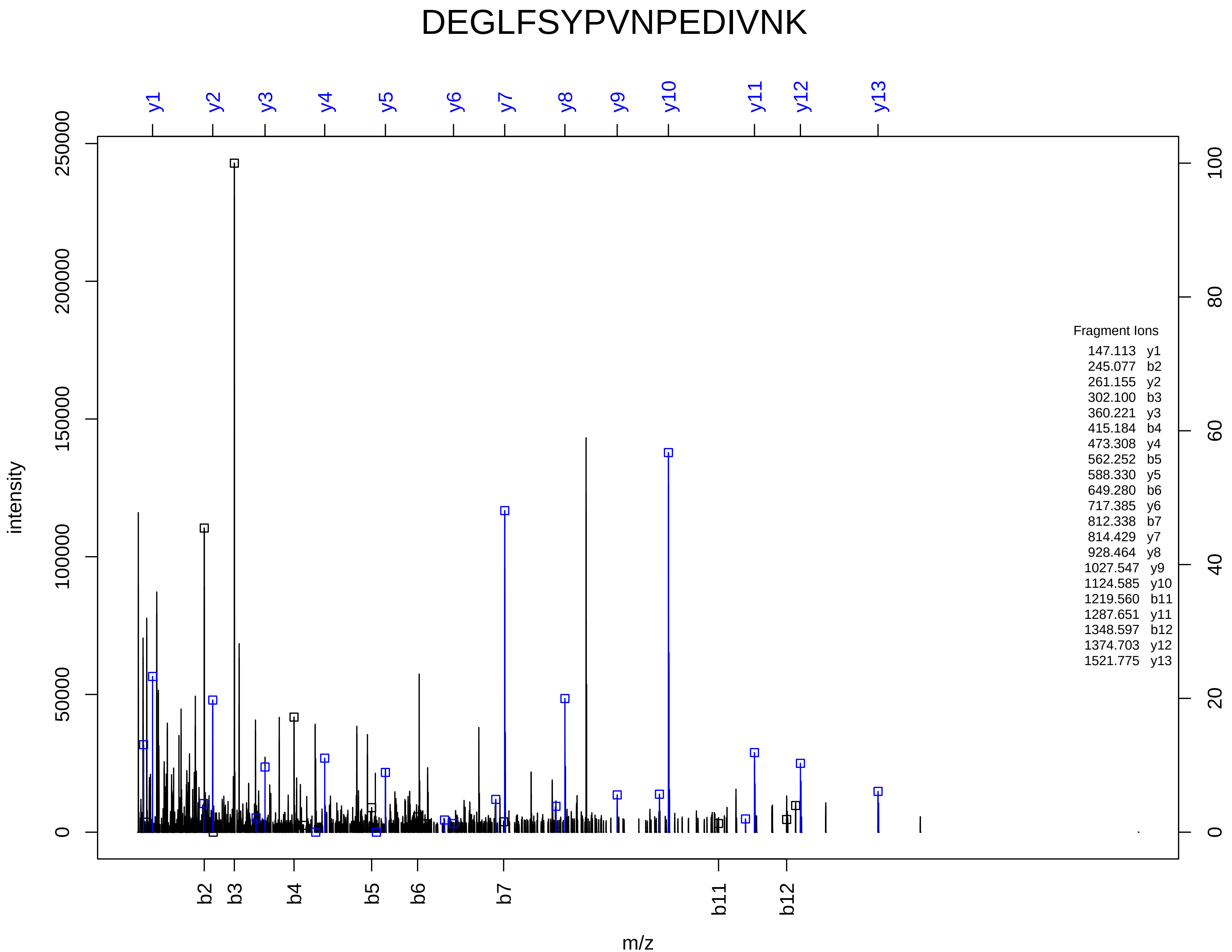

DEGLFSYPVNPEDIVNK / File: sydney_ip_21.raw; SpectrumID: ; scans: 16765

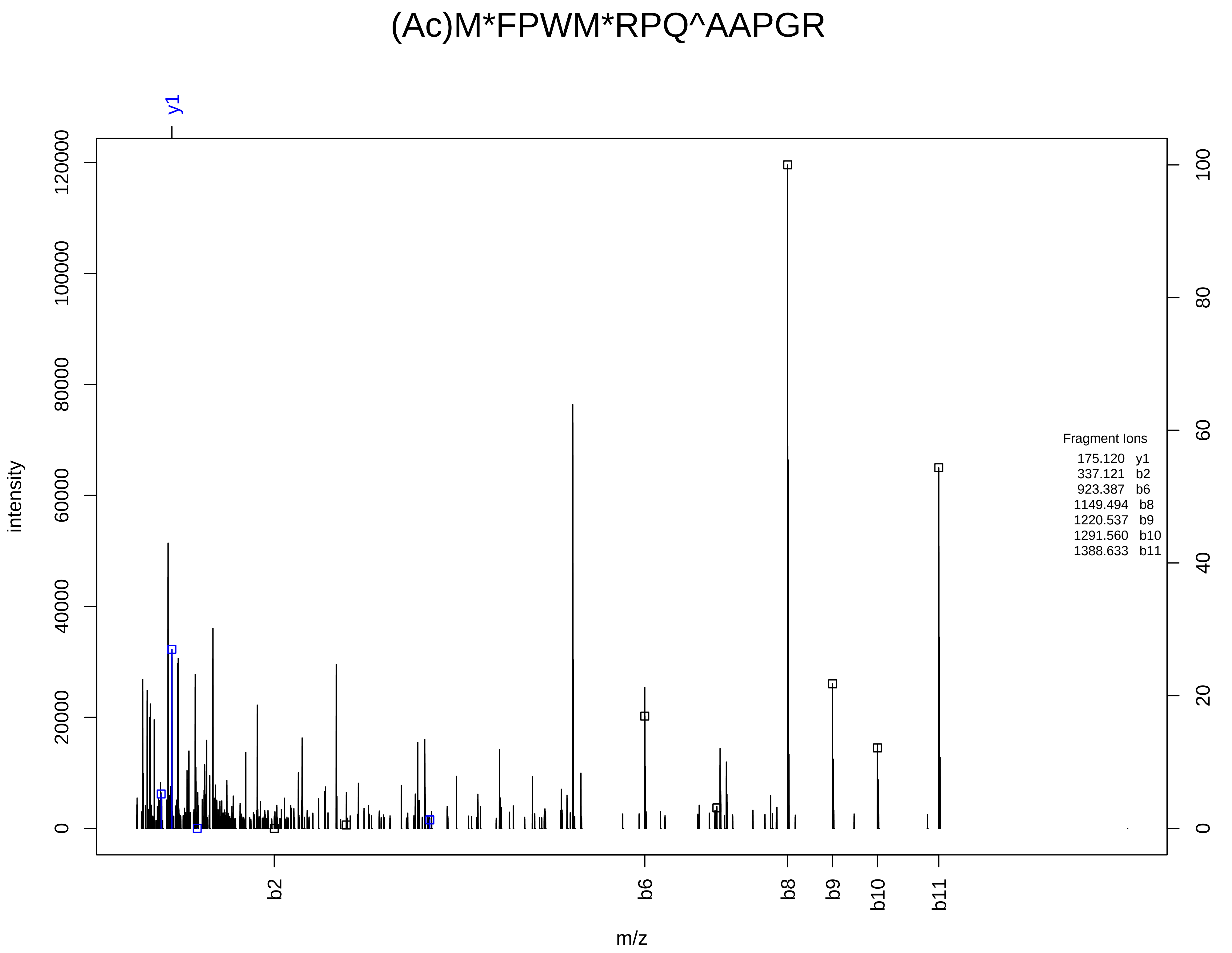

(Ac)M*FPWM*RPQ^AAPGR
y1
intensity
0
20000
40000
60000
80000
100000
120000
0
20
40
60
80
100
Fragment Ions
175.120 y1
337.121 b2
923.387 b6
1149.494 b8
1220.537 b9
1291.560 b10
1388.633 b11
b2
b6
b8
b9
b10
b11
m/z
MFPWMRPQAAPGR / File: sydney_ip_21.raw; SpectrumID: ; scans: 3826

IIEESTQCGPQAVK

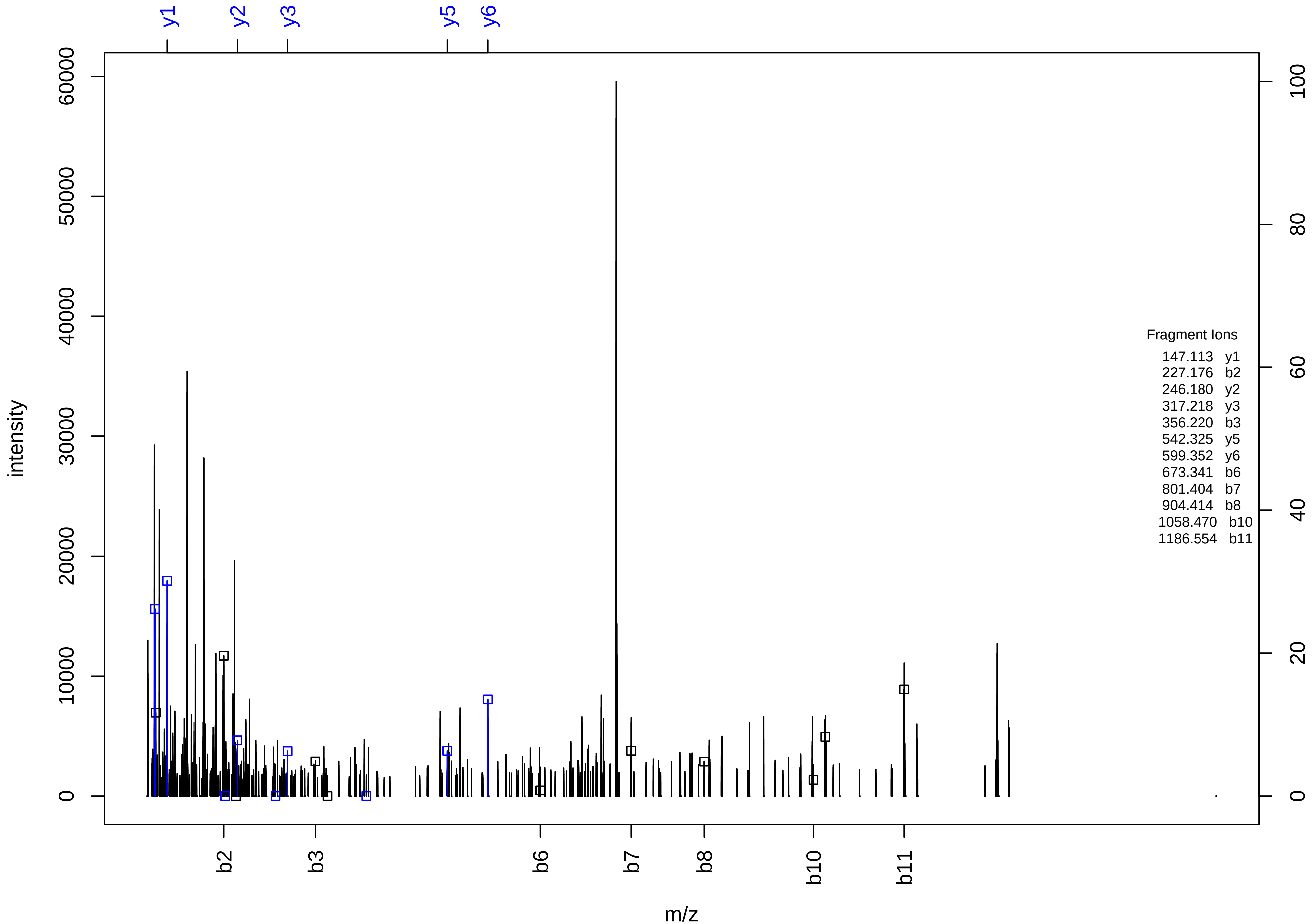

IIEESTQCGPQAVK / File: sydney_ip_21.raw; SpectrumID: ; scans: 3526

SQHLLHLQQ^Q^Q^K

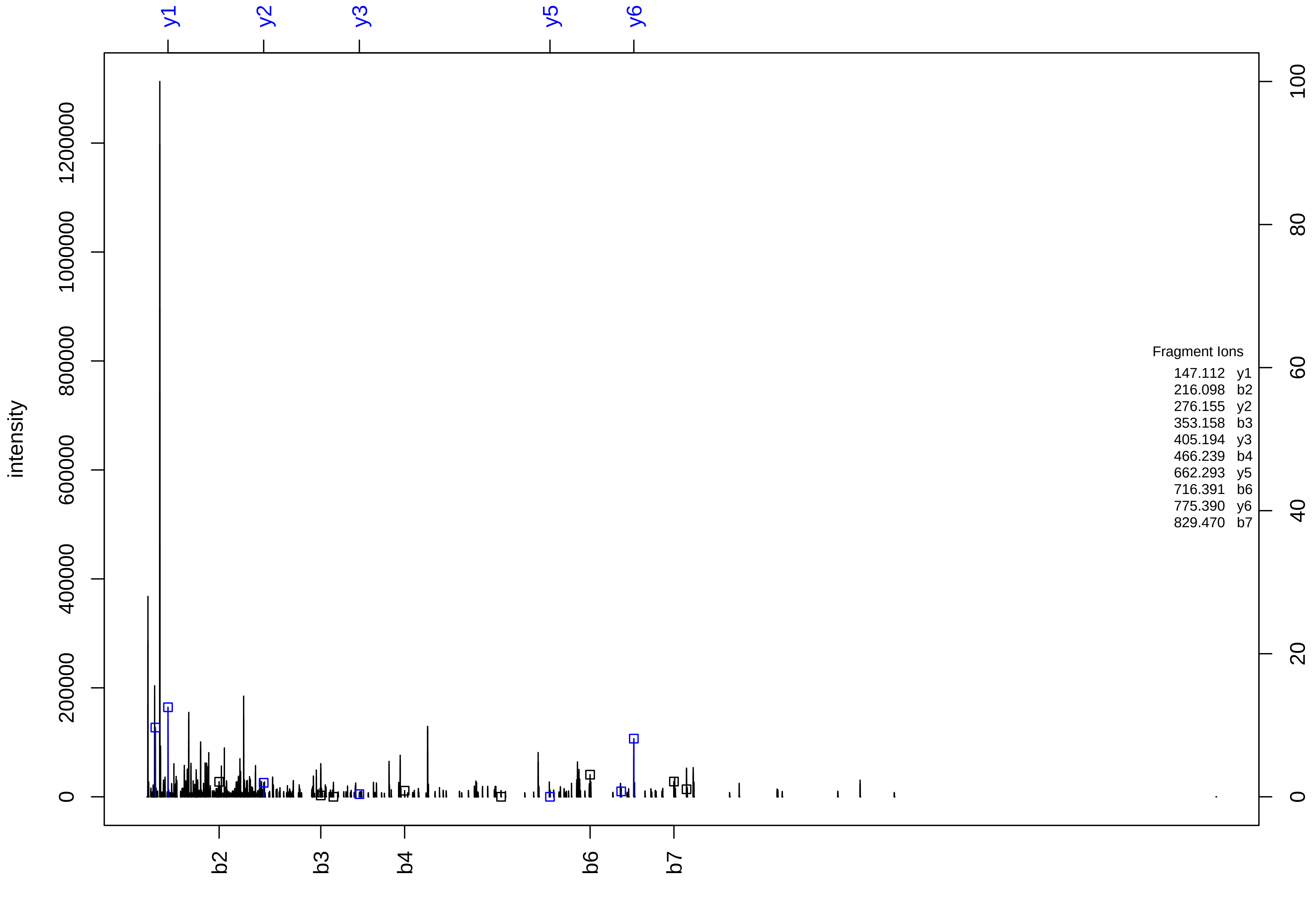

SQHLLHLQQQK / File: sydney_ip_21.raw; SpectrumID: ; scans: 3957

VVATTQM*QAADAR

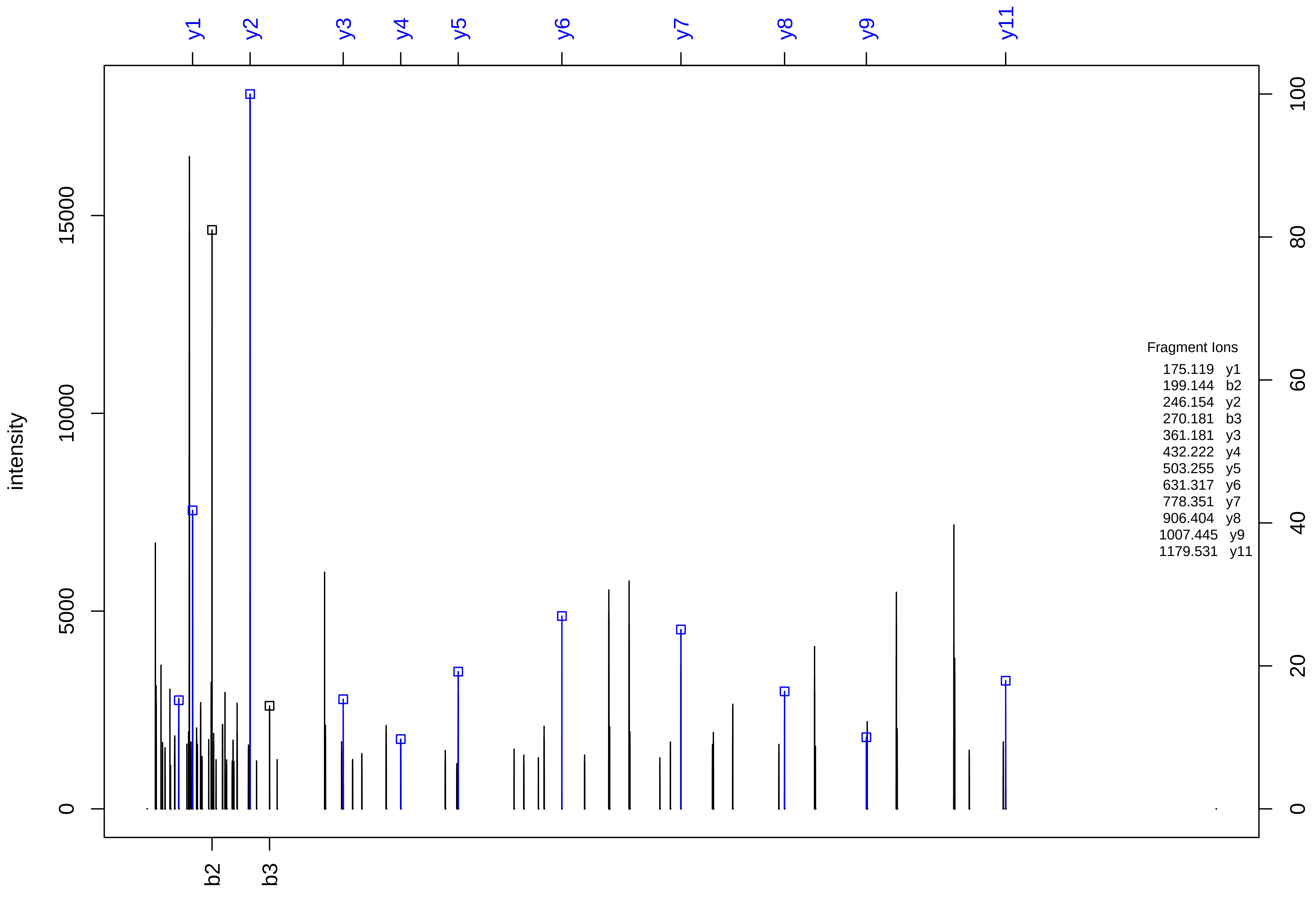

VVATTQMQAADAR / File: sydney_ip_21.raw; SpectrumID: ; scans: 1284

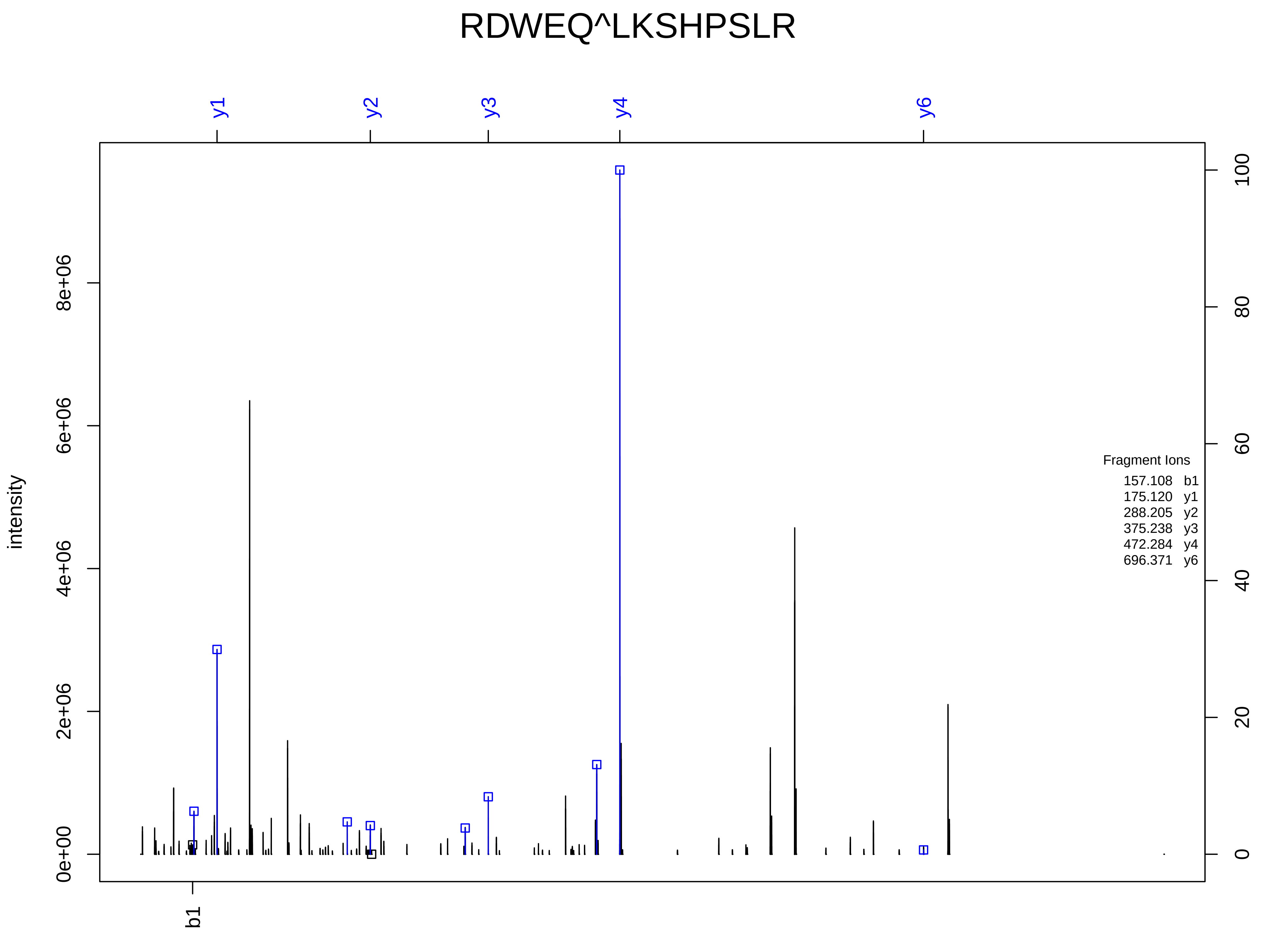
RDWEQ^LKSHPSLR
y1
y2
y3
y4
y6
intensity
0e+00
2e+06
4e+06
6e+06
8e+06
0
20
40
60
80
100
b1
m/z
Fragment Ions
157.108 b1
175.120 y1
288.205 y2
375.238 y3
472.284 y4
696.371 y6
RDWEQLKSHPSLR / File: sydney_ip_21.raw; SpectrumID: ; scans: 5654

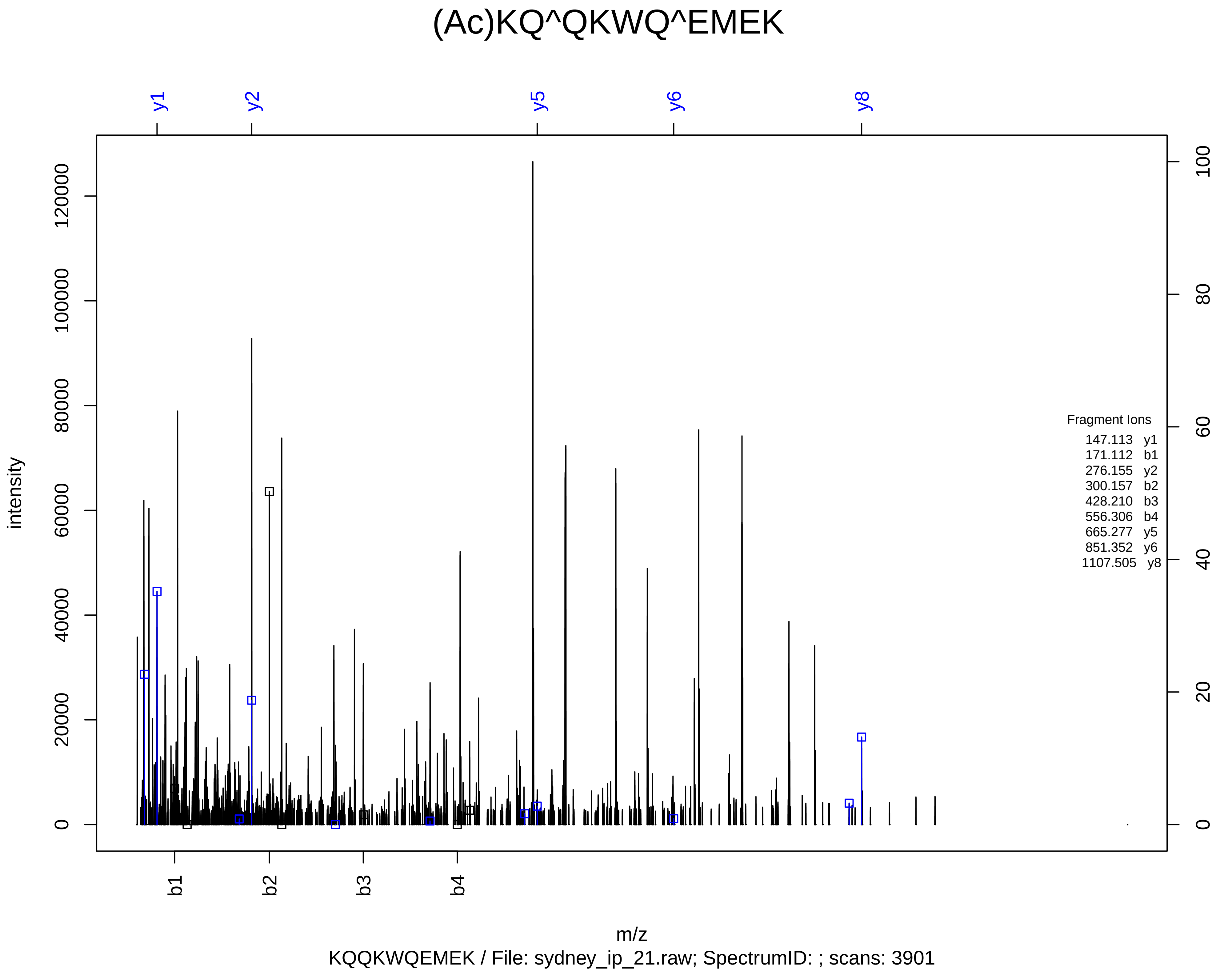

(Ac)KQ^QKWQ^EMEK
y1
y2
y5
y6
y8
intensity
0
20000
40000
60000
80000
100000
120000
0
20
40
60
80
100
b1
b2
b3
b4
m/z
Fragment Ions
147.113 y1
171.112 b1
276.155 y2
300.157 b2
428.210 b3
556.306 b4
665.277 y5
851.352 y6
1107.505 y8
KQQKWQEMEK / File: sydney_ip_21.raw; SpectrumID: ; scans: 3901

VVLVTGANSGIGFETAK

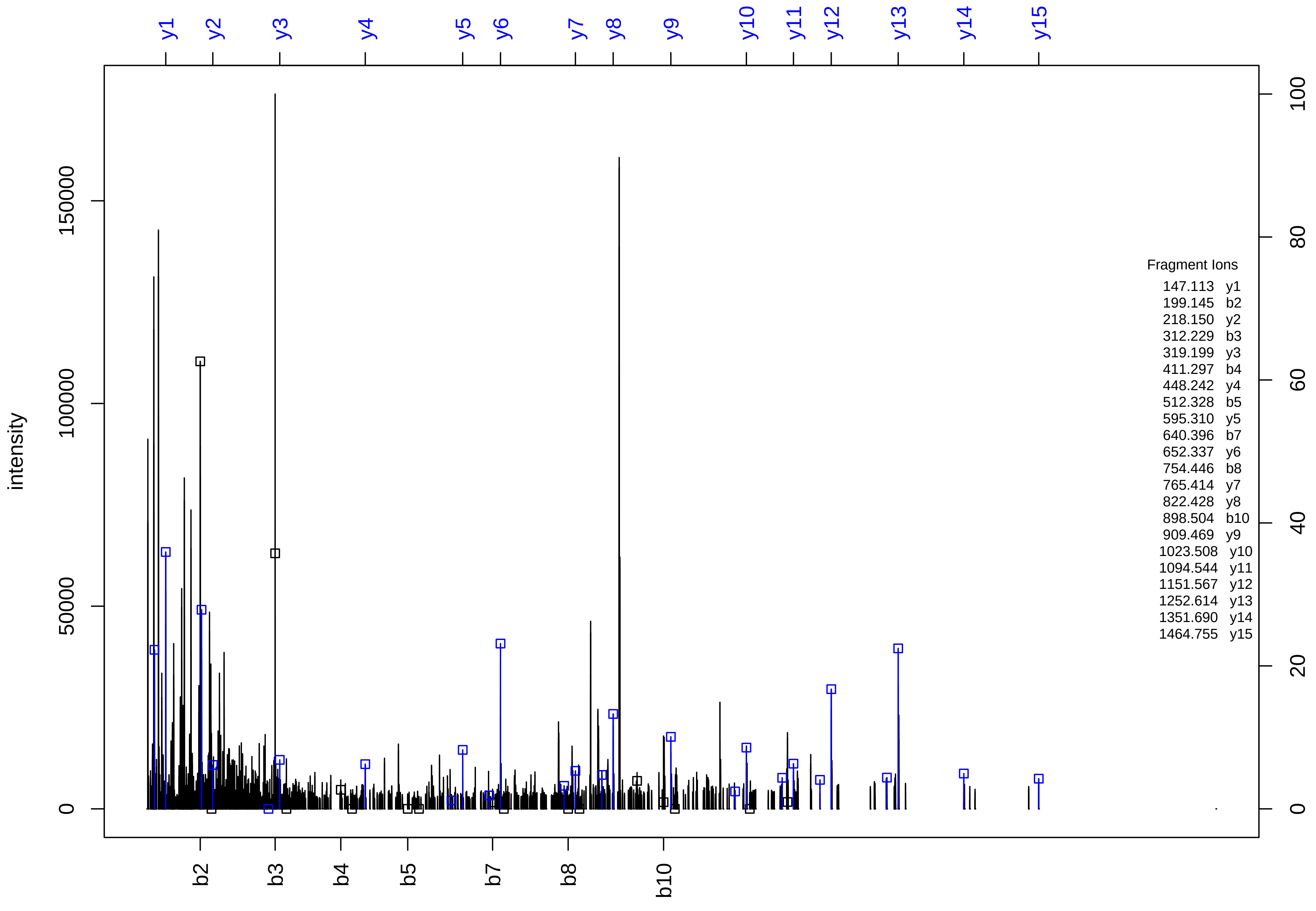

VVLVTGANSGIGFETAK / File: sydney_ip_21.raw; SpectrumID: ; scans: 11509

LQ^KALDSVM*SIR

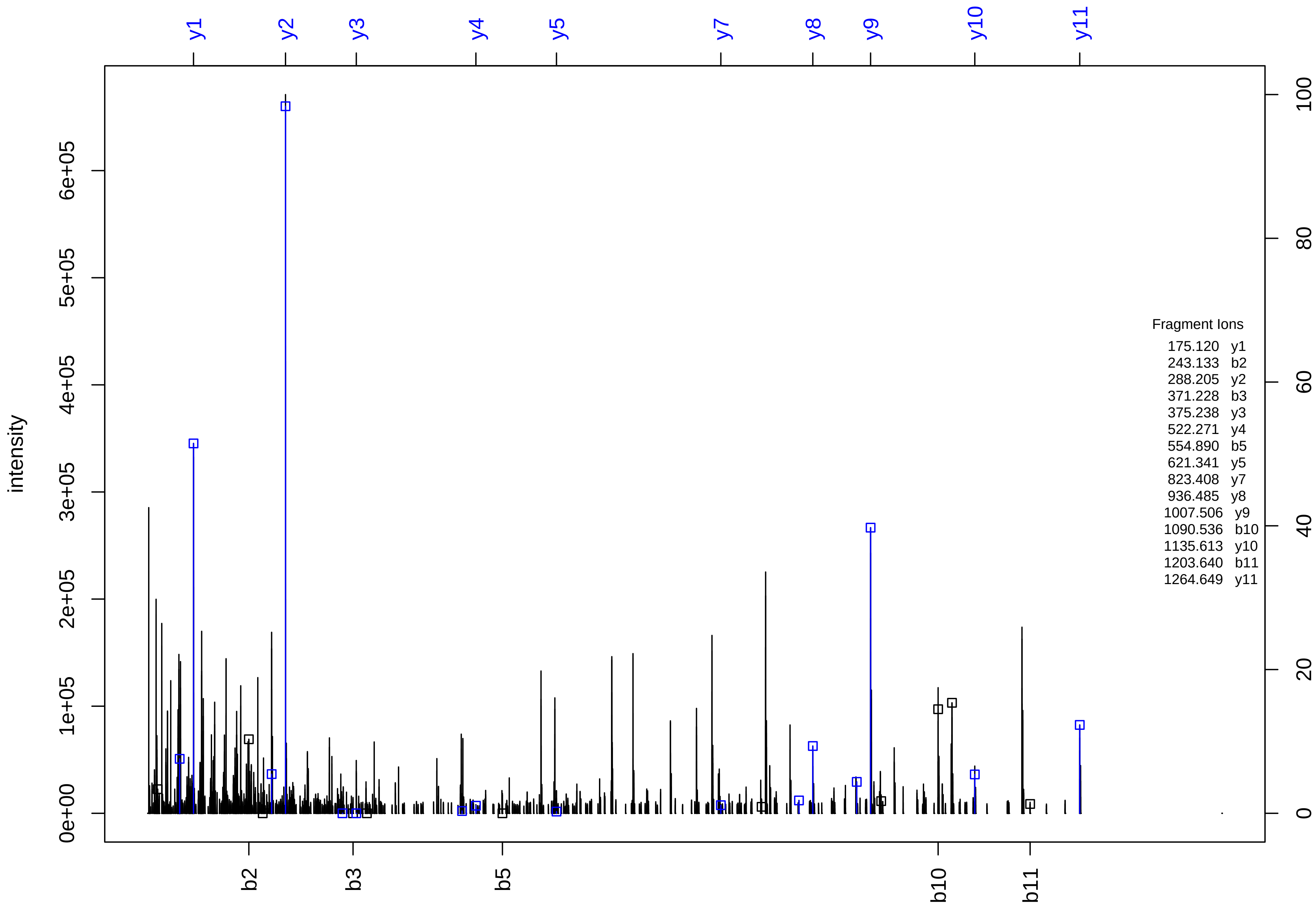

LQKALDSVMSIR / File: sydney_ip_22.raw; SpectrumID: ; scans: 11231

(Ac)MSLPAN^QIKEPGKQ^SQ^PAK

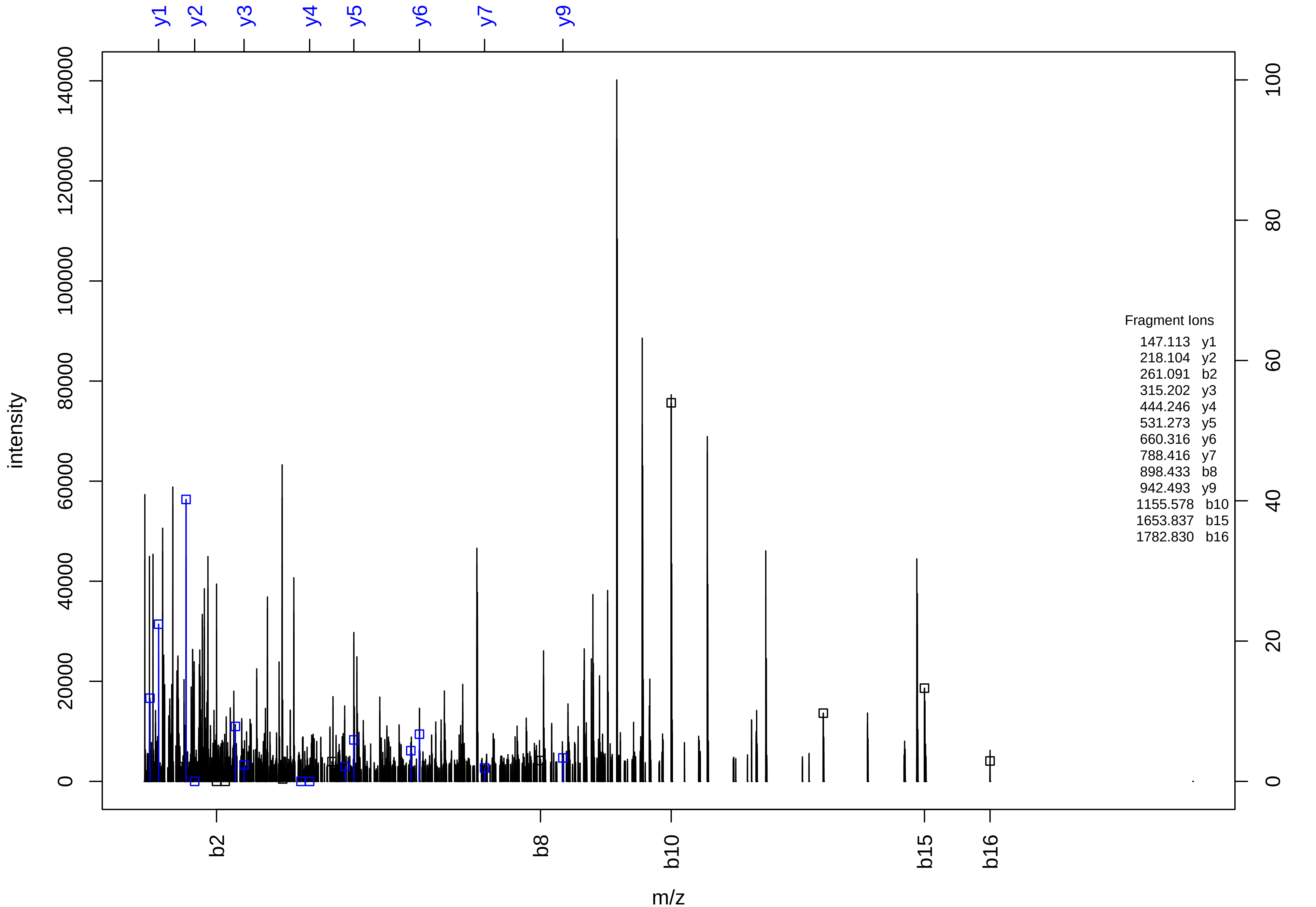

MSLPANQIKEPGKQSQPAK / File: sydney_ip_22.raw; SpectrumID: ; scans: 17734

DIQYFVN^Q^RK

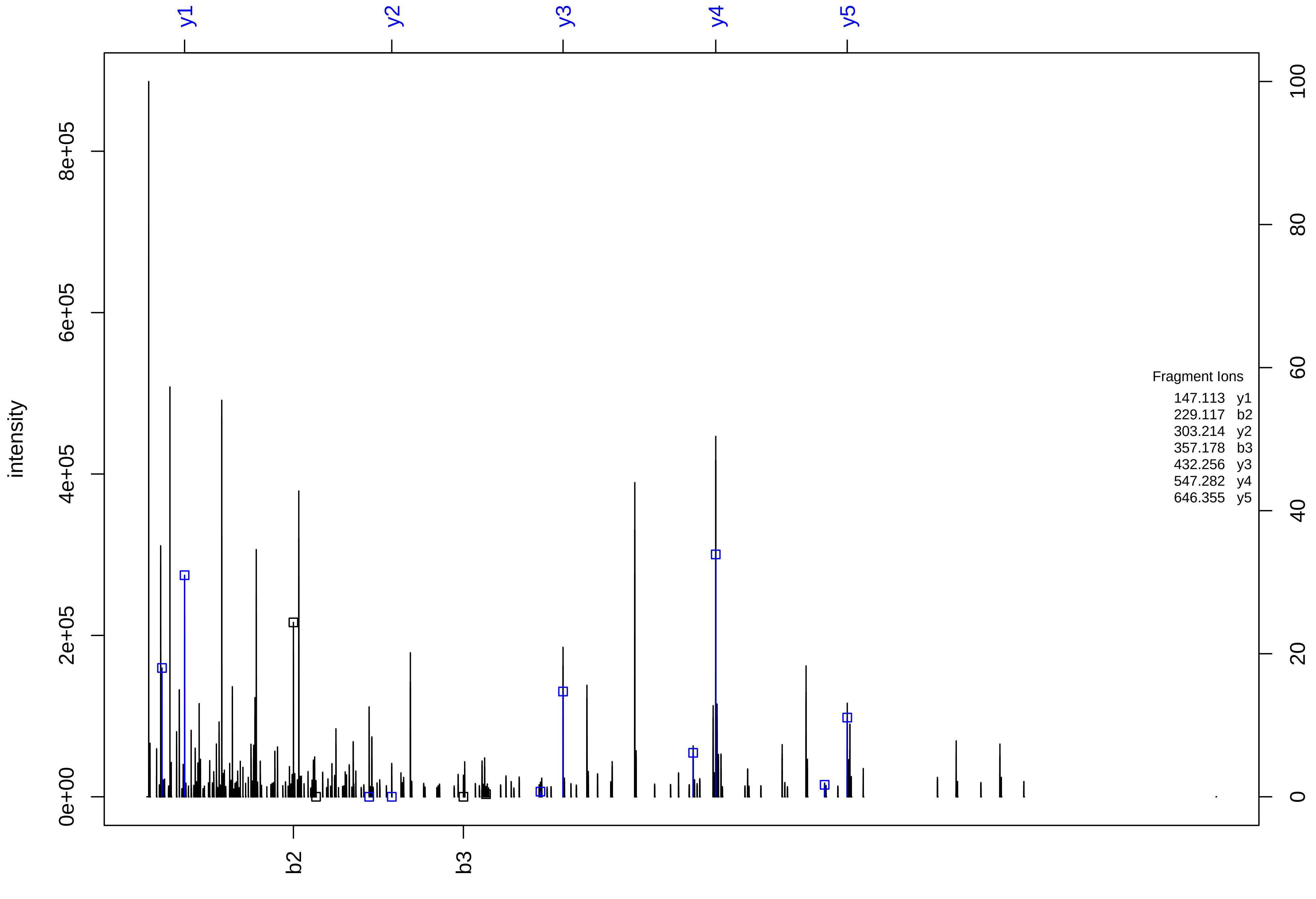

DIQYFVNQRK / File: sydney_ip_22.raw; SpectrumID: ; scans: 8261

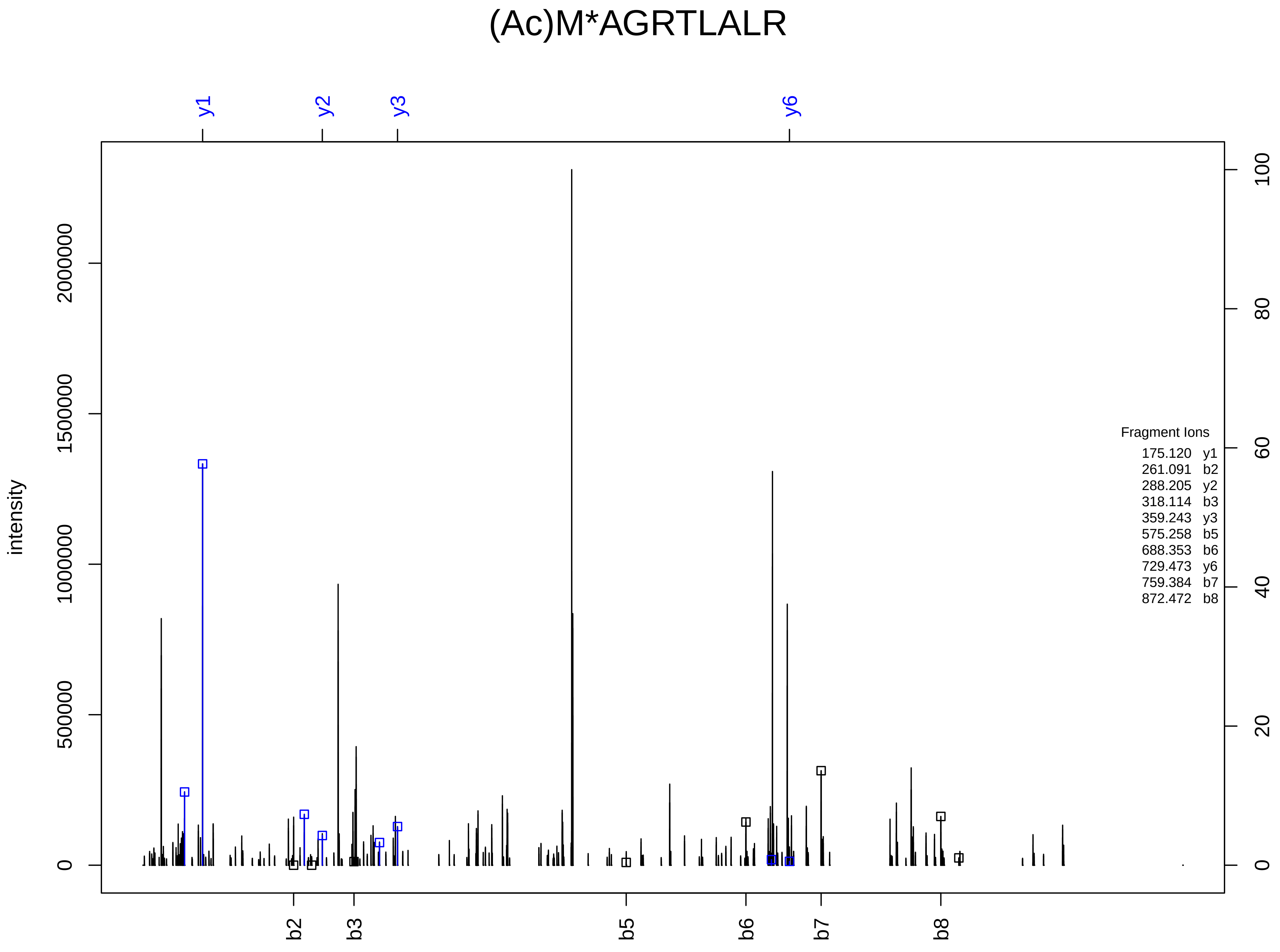

MAGRTLALR / File: sydney_ip_22.raw; SpectrumID: ; scans: 2935

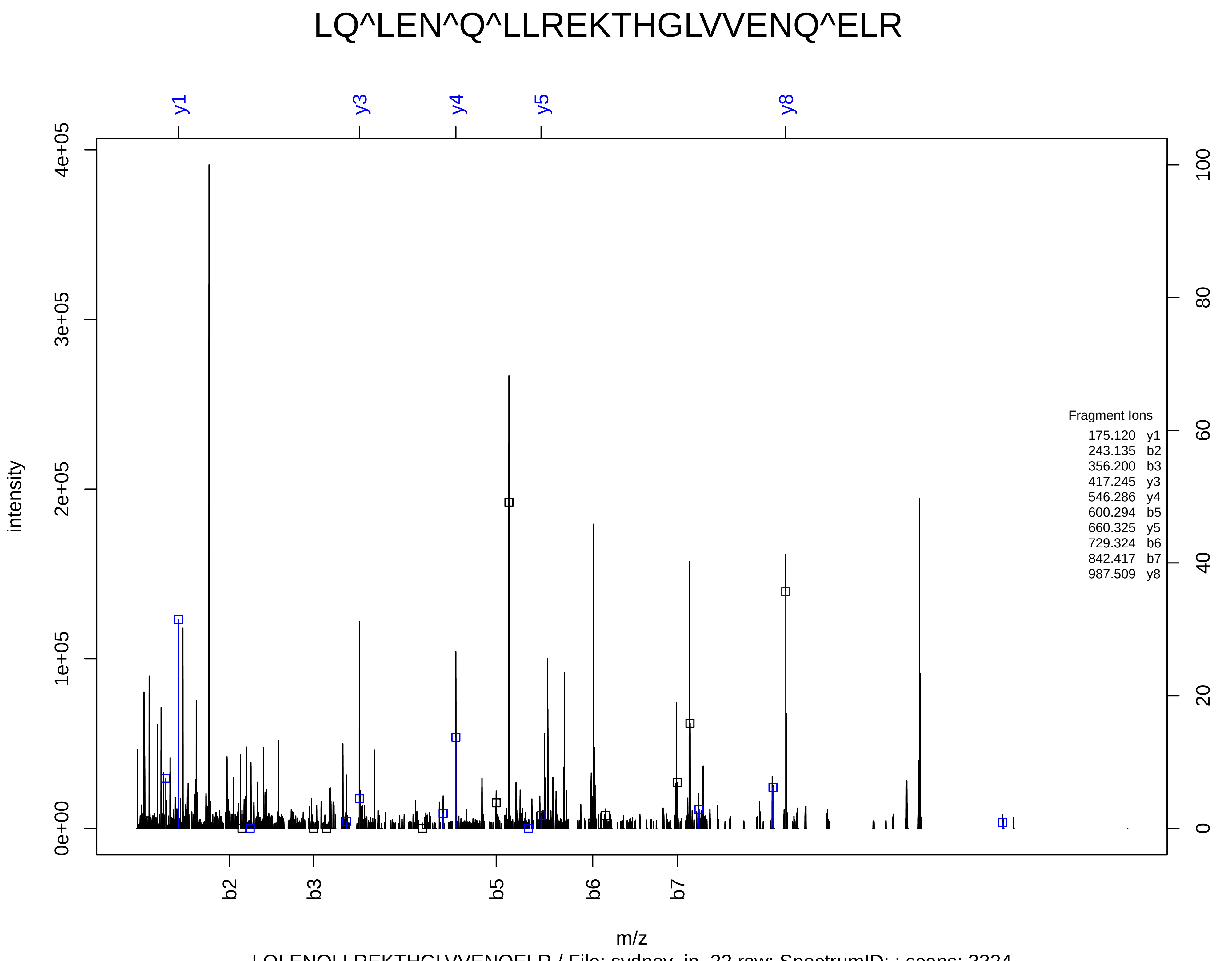
LQ^LEN^Q^LLREKTHGLVVENQ^ELR
y1
y3
y4
y5
y8
b2
b3
b5
b6
b7
intensity
m/z
Fragment Ions
175.120 y1
243.135 b2
356.200 b3
417.245 y3
546.286 y4
600.294 b5
660.325 y5
729.324 b6
842.417 b7
987.509 y8
0e+00
1e+05
2e+05
3e+05
4e+05
0
20
40
60
80
100
LQLENQLLREKTHGLVVENQELR / File: sydney_ip_22.raw; SpectrumID: ; scans: 3324

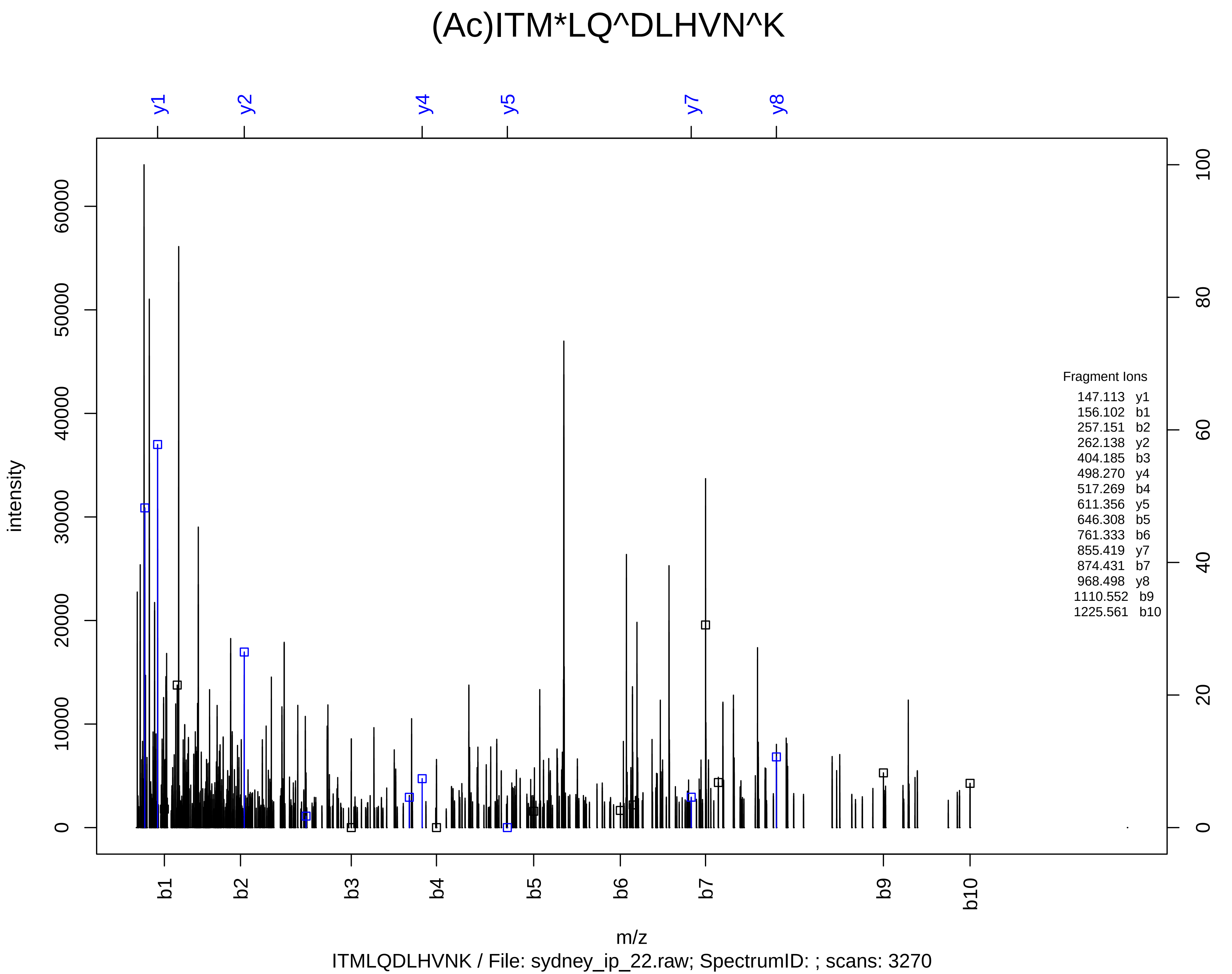

(Ac)ITM*LQ^DLHVN^K
y1
y2
y4
y5
y7
y8
intensity
Fragment Ions
147.113 y1
156.102 b1
257.151 b2
262.138 y2
404.185 b3
498.270 y4
517.269 b4
611.356 y5
646.308 b5
761.333 b6
855.419 y7
874.431 b7
968.498 y8
1110.552 b9
1225.561 b10
b1
b2
b3
b4
b5
b6
b7
b9
b10
m/z
ITMLQDLHVNK / File: sydney_ip_22.raw; SpectrumID: ; scans: 3270

DAYHHLQ^GQFHEQ^NKEKEN^IADQ^LR

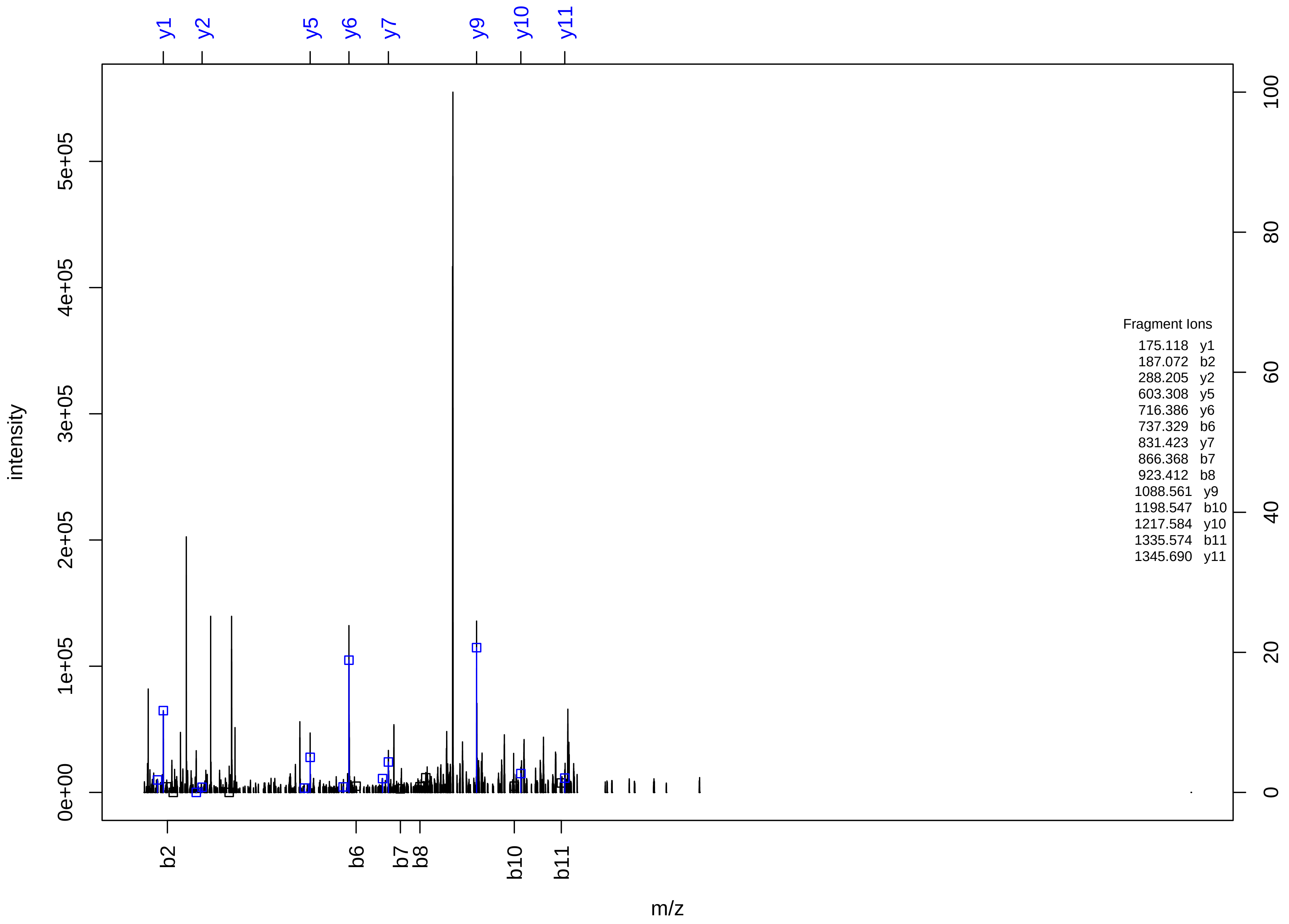

DAYHHLQGQFHEQNKEKENIADQLR / File: sydney_ip_22.raw; SpectrumID: ; scans: 7059

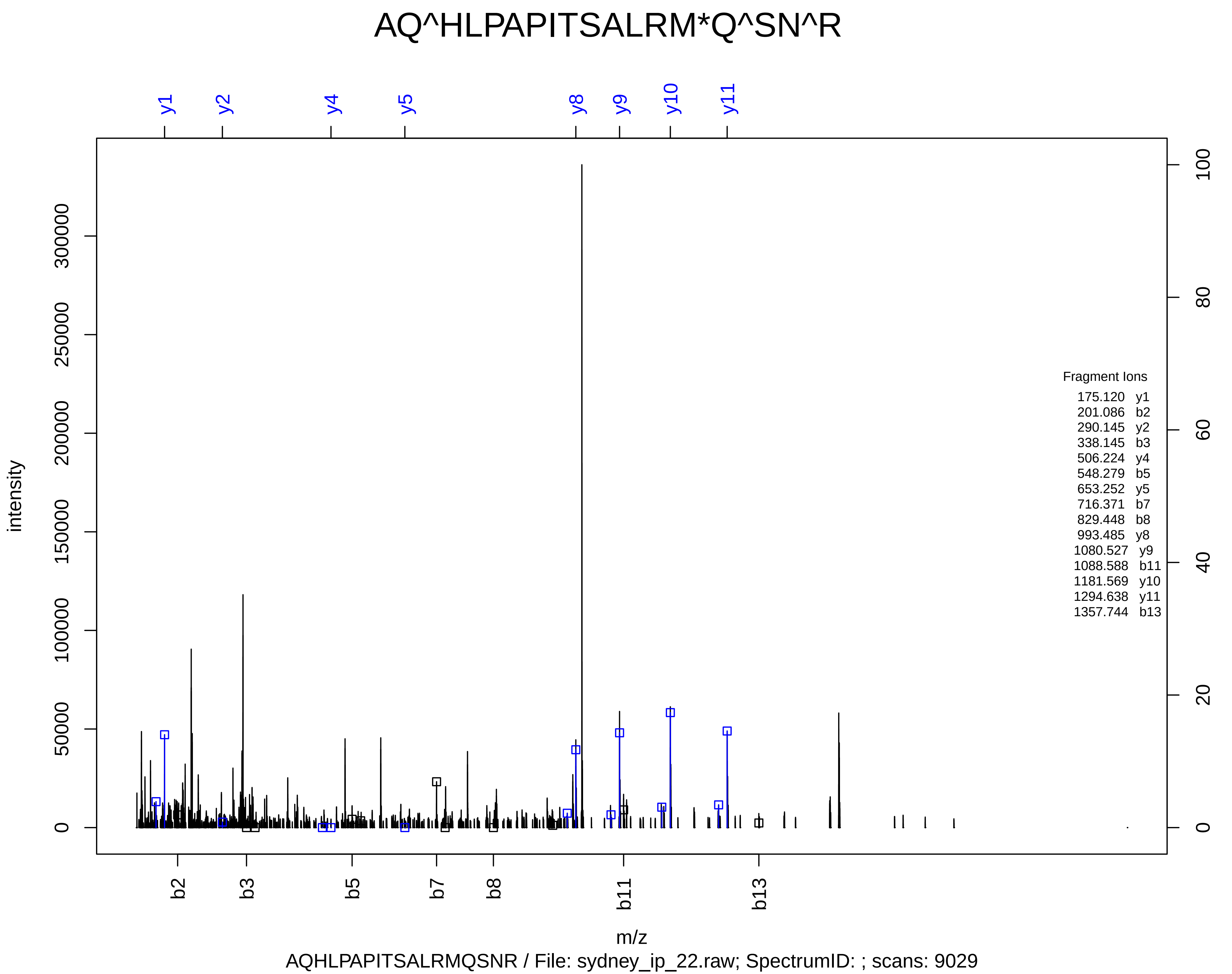
AQ^HLPAPITSALRM*Q^SN^R
y1
y2
y4
y5
y8
y9
y10
y11
intensity
0
50000
100000
150000
200000
250000
300000
0
20
40
60
80
100
Fragment Ions
175.120 y1
201.086 b2
290.145 y2
338.145 b3
506.224 y4
548.279 b5
653.252 y5
716.371 b7
829.448 b8
993.485 y8
1080.527 y9
1088.588 b11
1181.569 y10
1294.638 y11
1357.744 b13
b2
b3
b5
b7
b8
b11
b13
m/z
AQHLPAPITSALRMQSNR / File: sydney_ip_22.raw; SpectrumID: ; scans: 9029

LRPSSGLQ^ILNVHFR

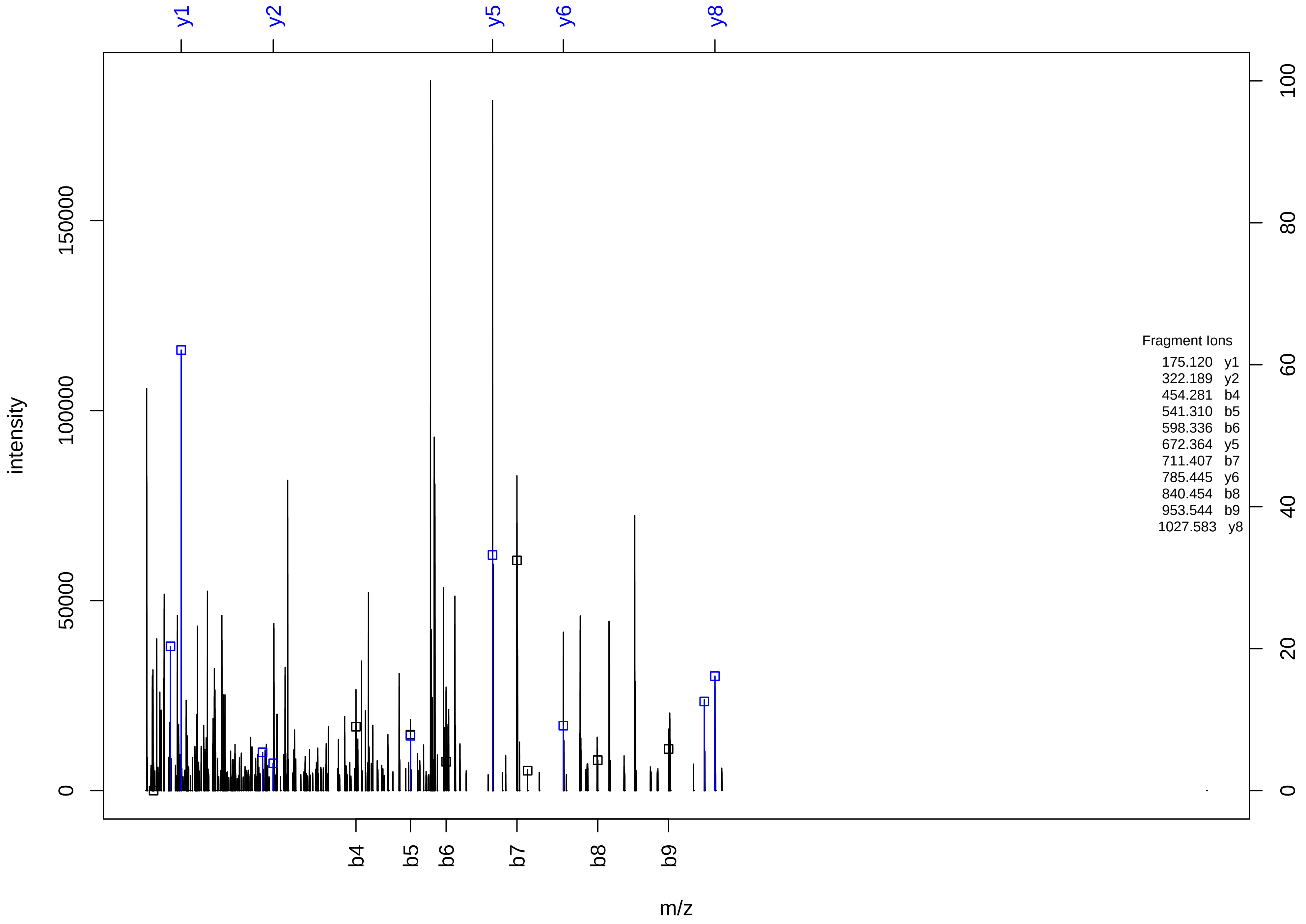

LRPSSGLQILNVHFR / File: sydney_ip_22.raw; SpectrumID: ; scans: 18521

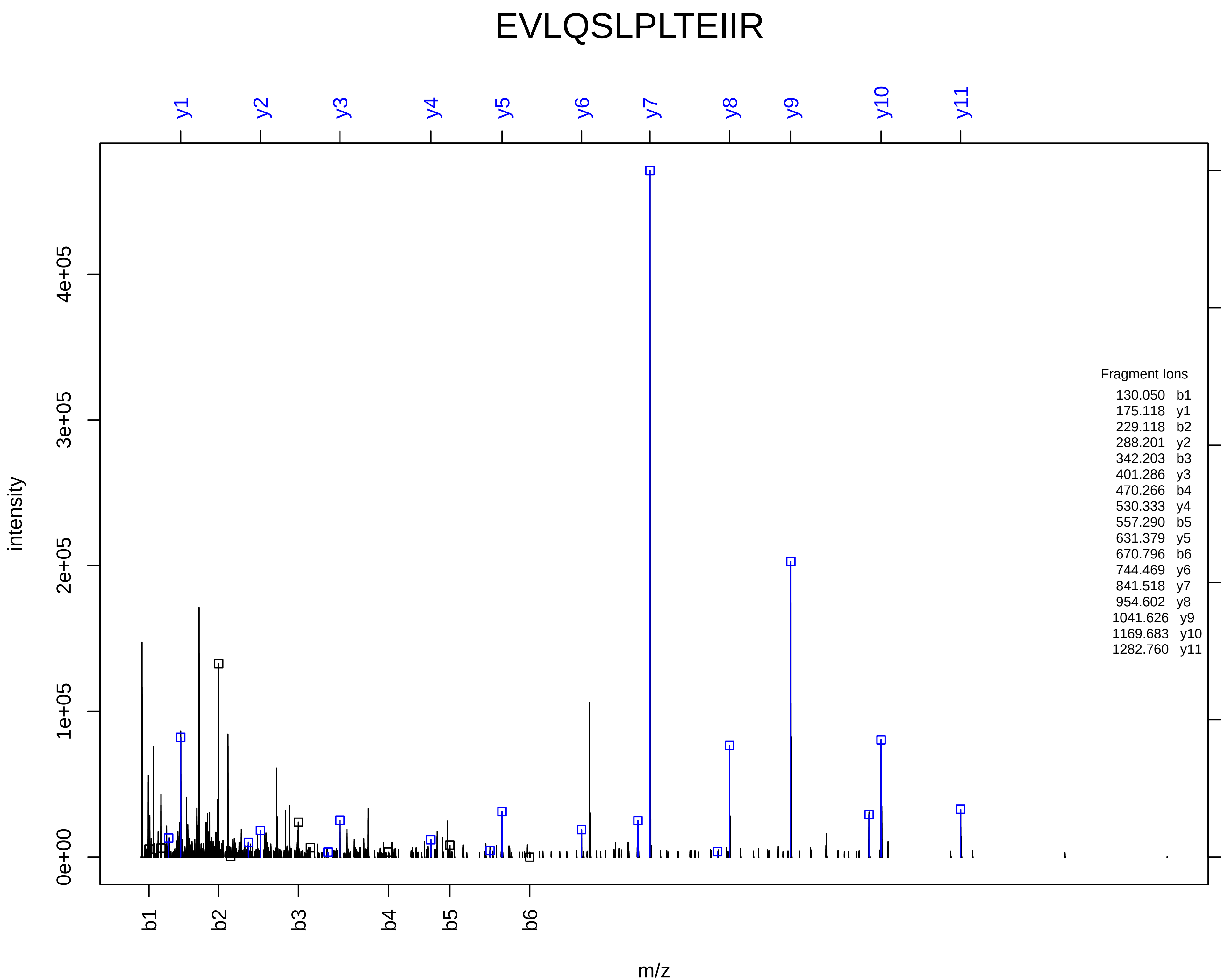
EVLQSLPLTEIIR
y1 y2 y3 y4 y5 y6 y7 y8 y9 y10 y11
b1 b2 b3 b4 b5 b6
intensity
m/z
Fragment Ions
130.050 b1
175.118 y1
229.118 b2
288.201 y2
342.203 b3
401.286 y3
470.266 b4
530.333 y4
557.290 b5
631.379 y5
670.796 b6
744.469 y6
841.518 y7
954.602 y8
1041.626 y9
1169.683 y10
1282.760 y11
EVLQSLPLTEIIR / File: sydney_ip_22.raw; SpectrumID: ; scans: 18461

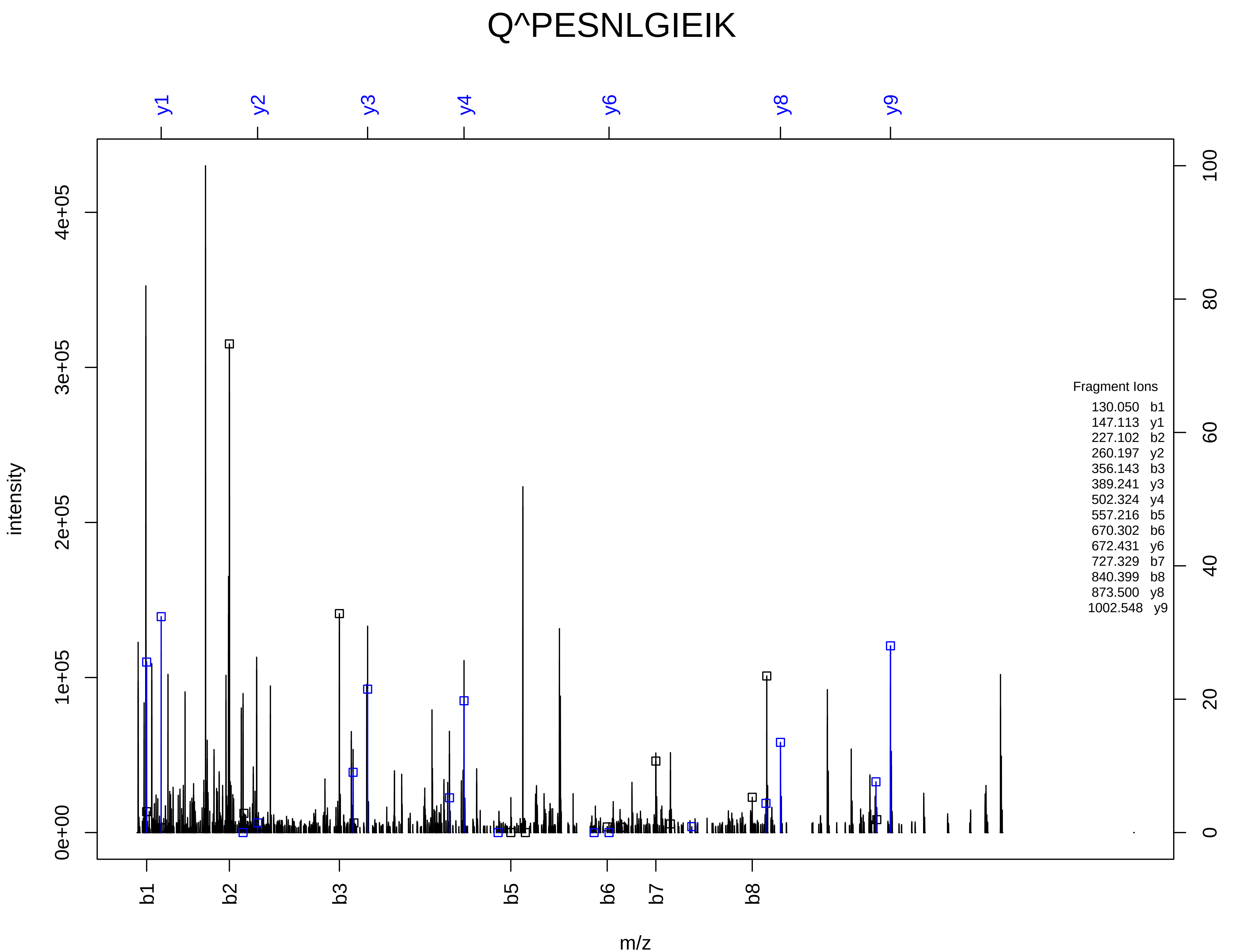

Q^PESNLGIEIK
y1
y2
y3
y4
y6
y8
y9
intensity
0e+00
1e+05
2e+05
3e+05
4e+05
0
20
40
60
80
100
b1
b2
b3
b5
b6
b7
b8
Fragment Ions
130.050 b1
147.113 y1
227.102 b2
260.197 y2
356.143 b3
389.241 y3
502.324 y4
557.216 b5
670.302 b6
672.431 y6
727.329 b7
840.399 b8
873.500 y8
1002.548 y9
m/z
QPESNLGIEIK / File: sydney_ip_22.raw; SpectrumID: ; scans: 5436

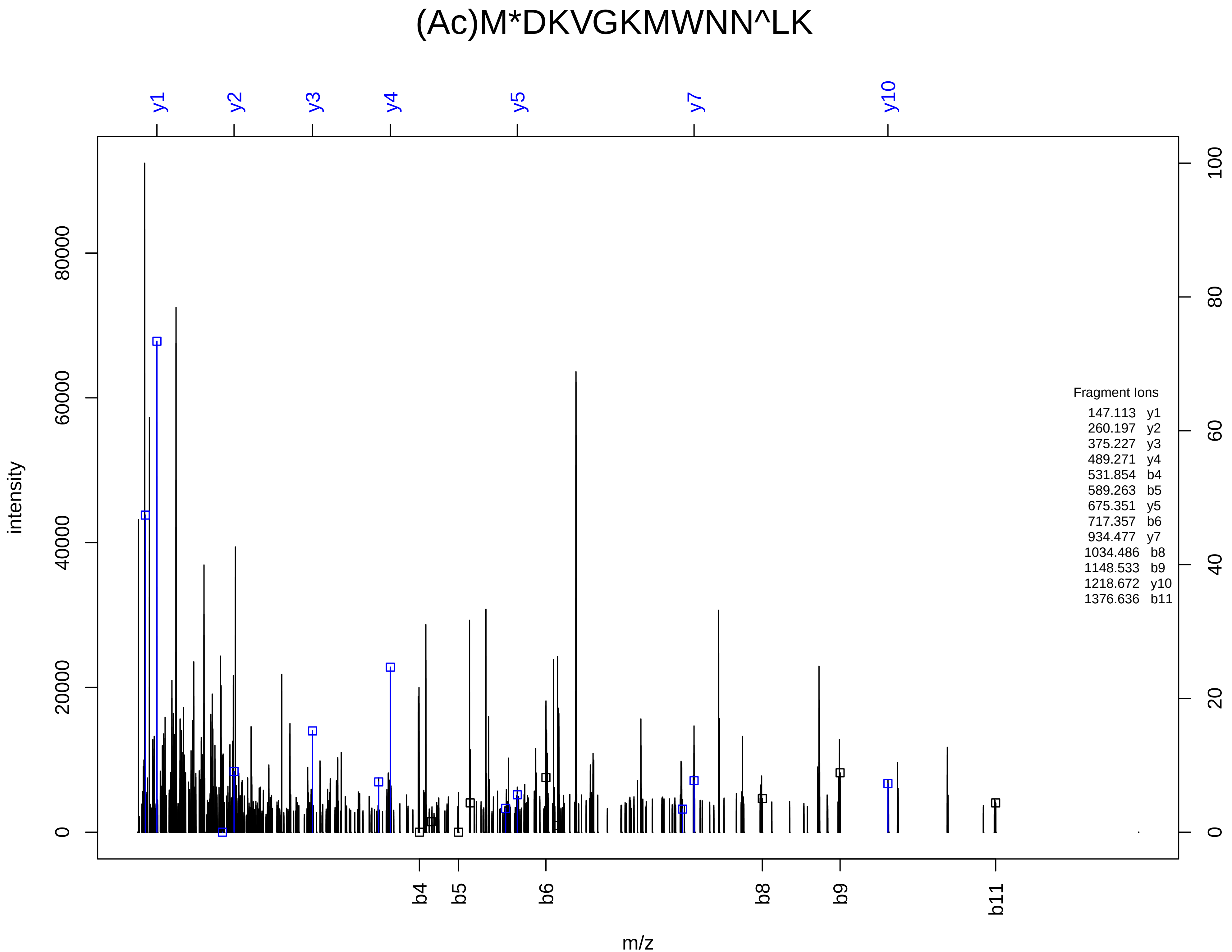

MDKVGKMWNNLK / File: sydney_ip_22.raw; SpectrumID: ; scans: 5407

ILGGSVLHLVLALR

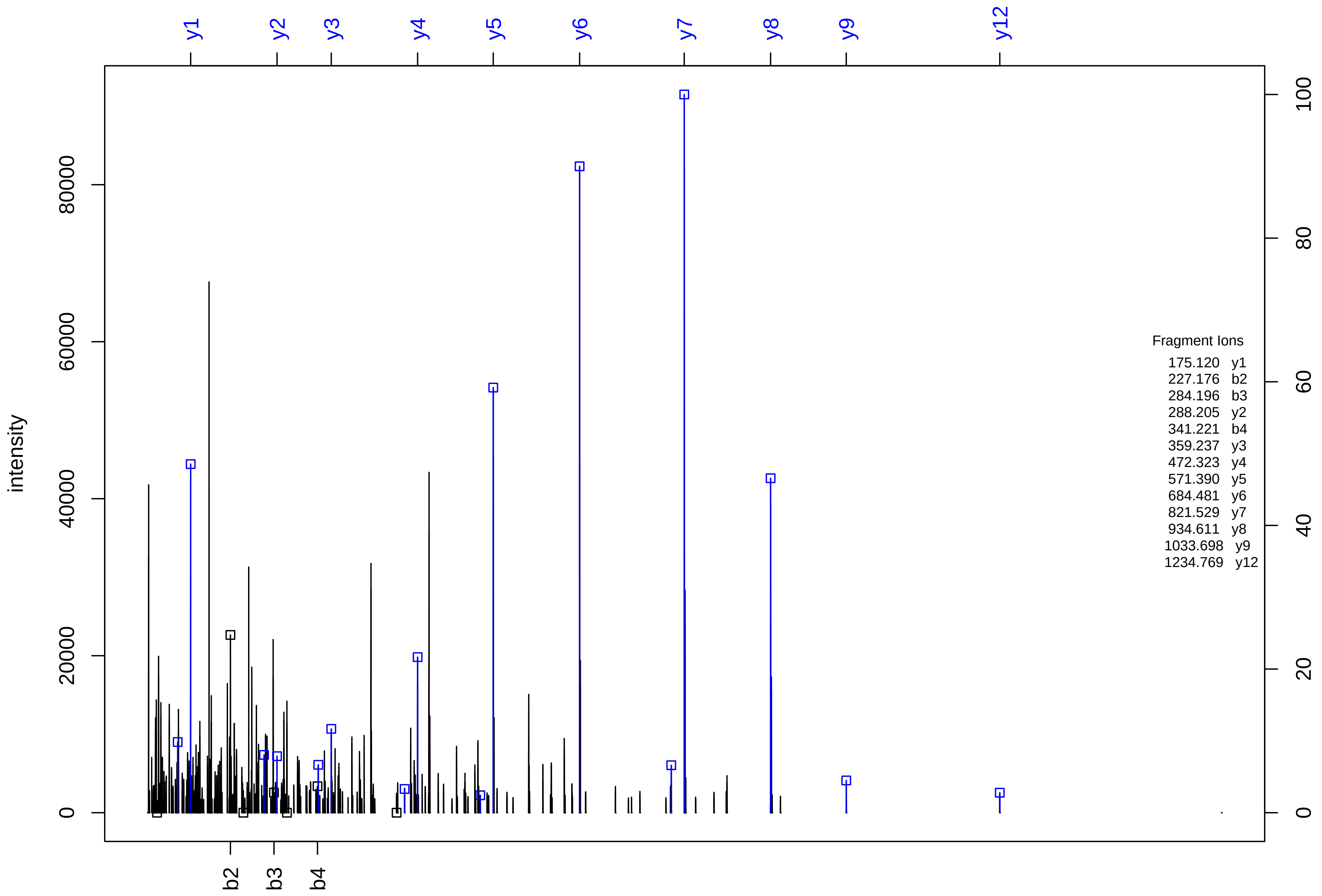

ILGGSVLHLVLALR / File: sydney_ip_22.raw; SpectrumID: ; scans: 19706

ITEEYYVHLIADNLPVATR

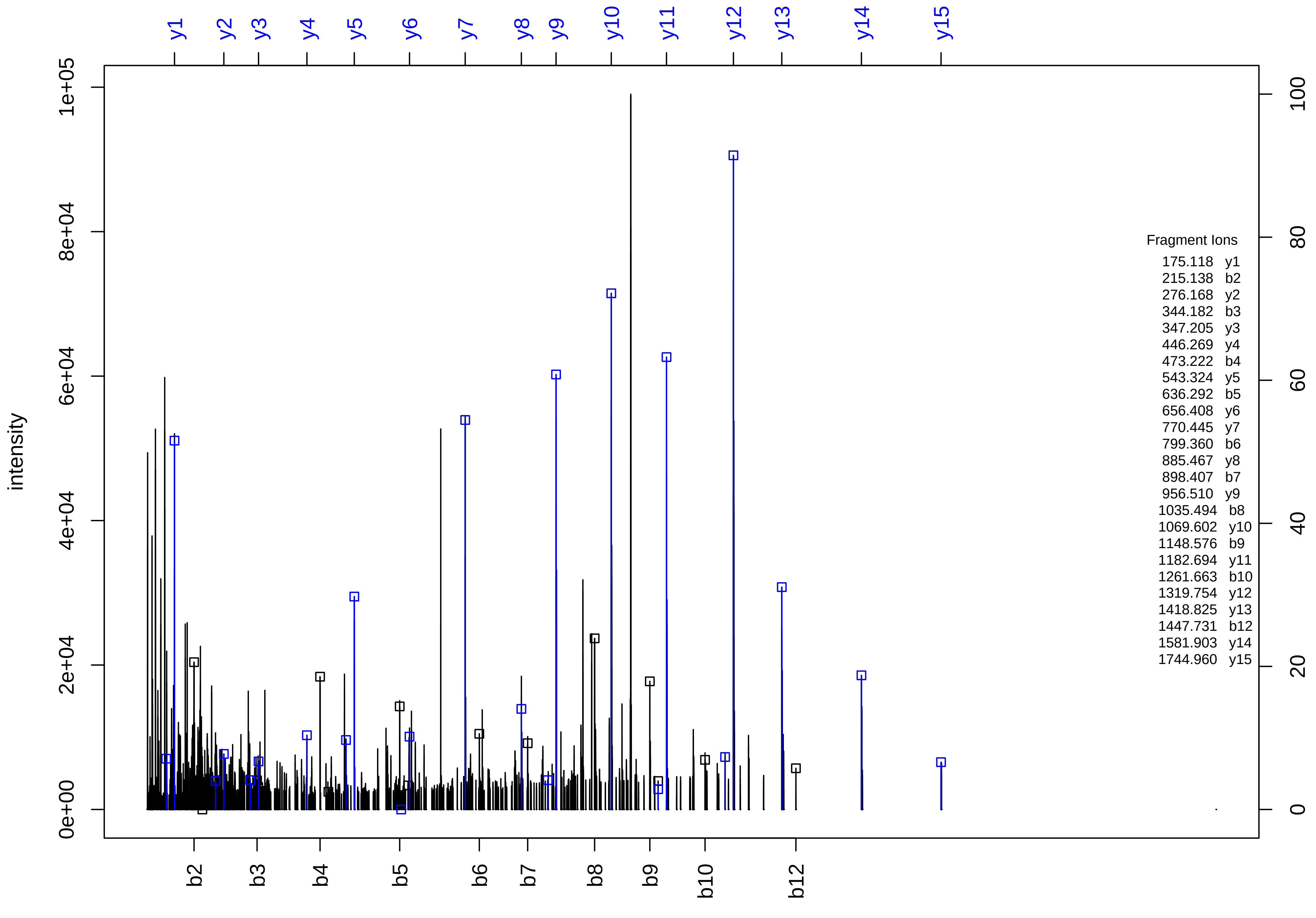

ITEEYYVHLIADNLPVATR / File: sydney_ip_22.raw; SpectrumID: ; scans: 17251

TLQIILEVVPEDSK

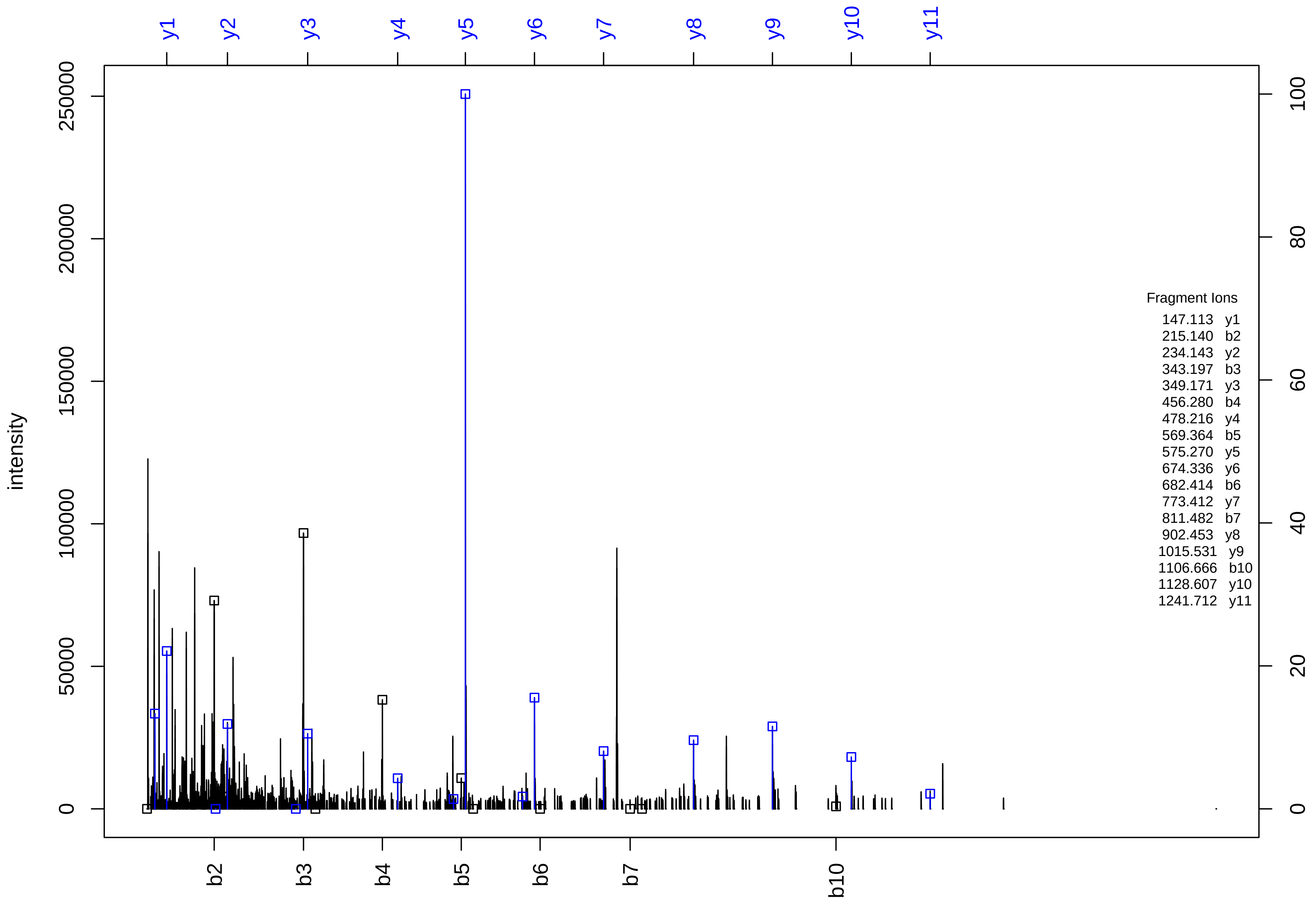

TLQIILEVVPEDSK / File: sydney_ip_22.raw; SpectrumID: ; scans: 17057

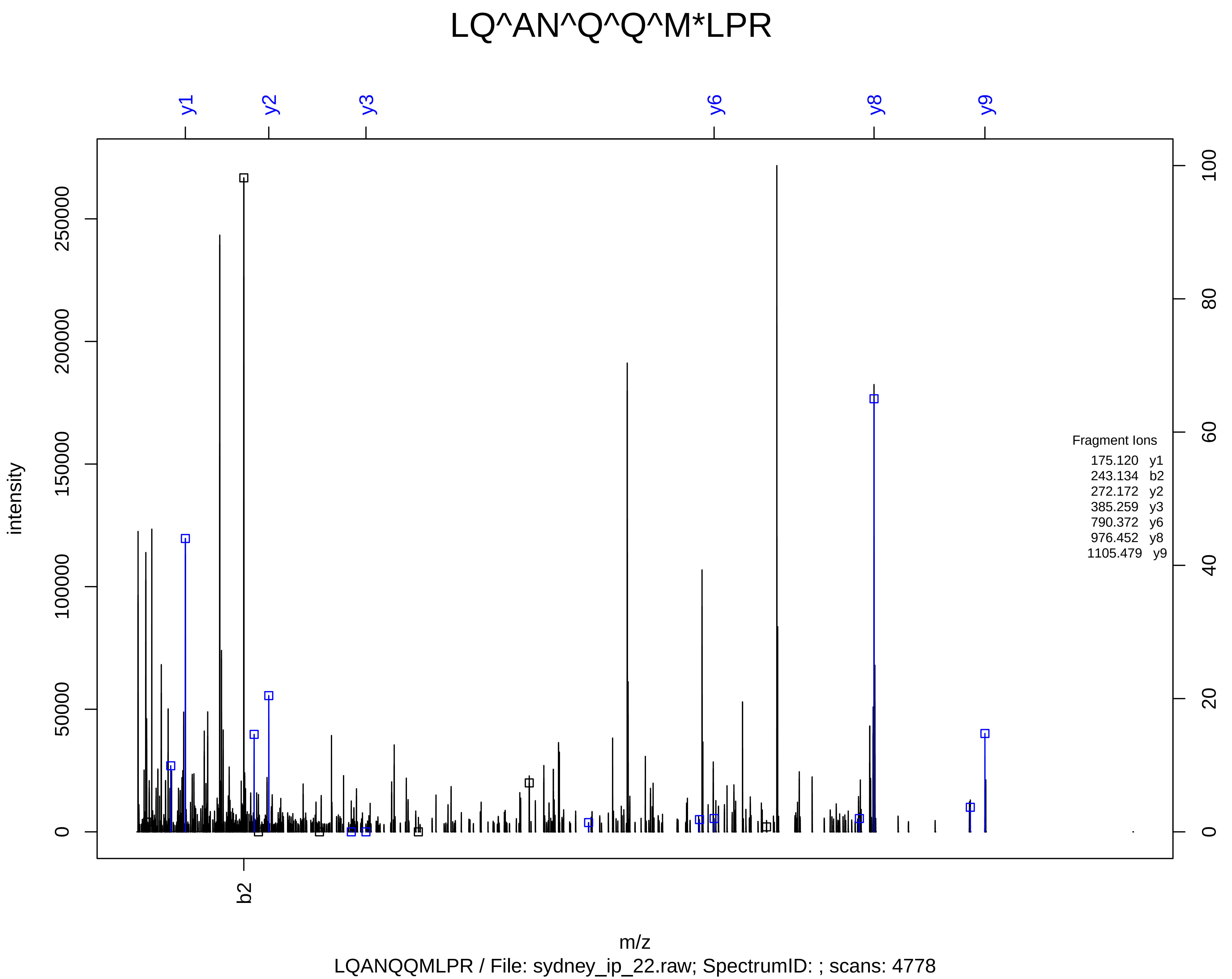
LQ^AN^Q^Q^M*LPR
y1
y2
y3
y6
y8
y9
b2
intensity
m/z
Fragment Ions
175.120 y1
243.134 b2
272.172 y2
385.259 y3
790.372 y6
976.452 y8
1105.479 y9
LQANQQMLPR / File: sydney_ip_22.raw; SpectrumID: ; scans: 4778

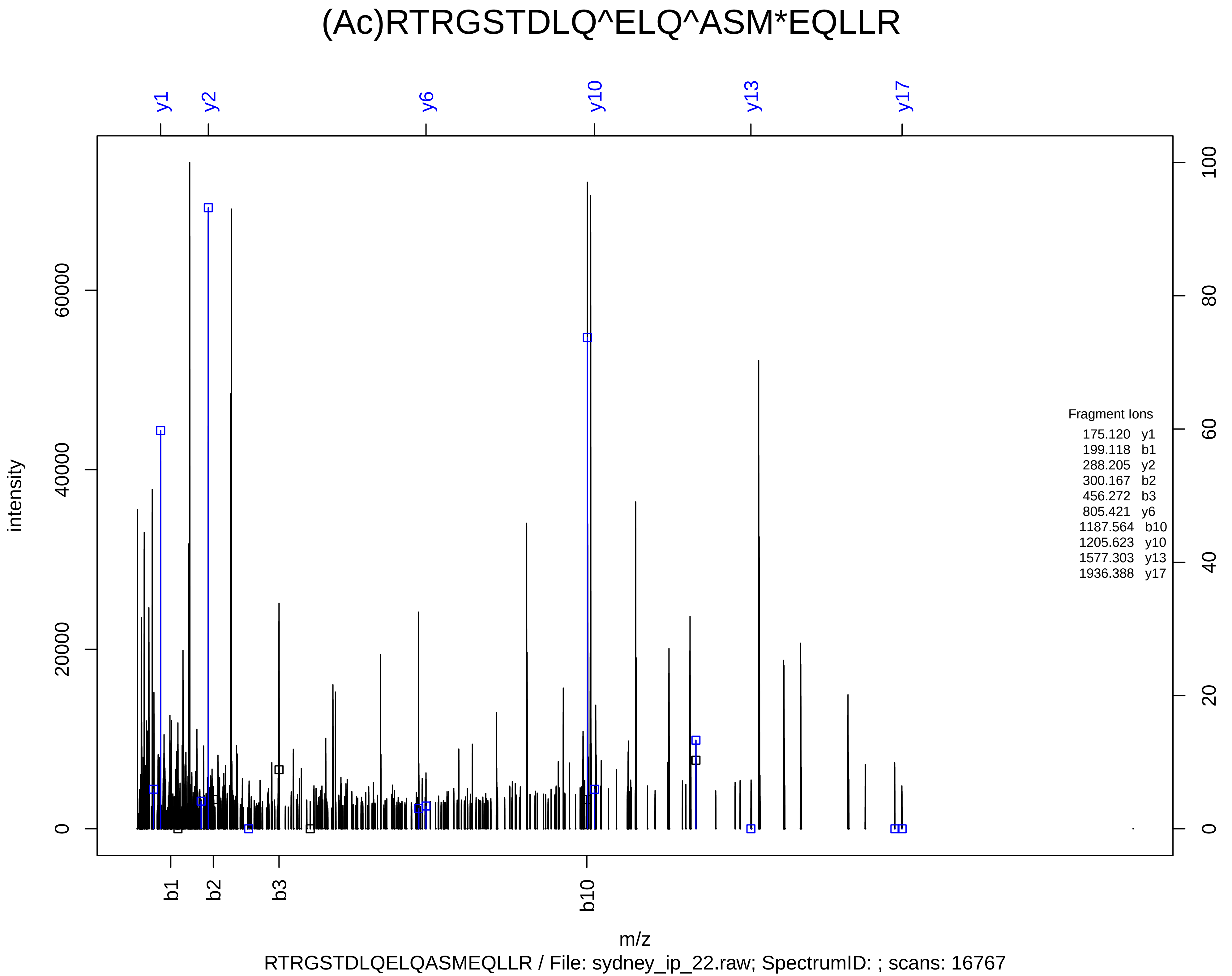

(Ac)RTRGSTDLQ^ELQ^ASM*EQLLR
y1
y2
y6
y10
y13
y17
intensity
0
20000
40000
60000
0
20
40
60
80
100
Fragment Ions
175.120 y1
199.118 b1
288.205 y2
300.167 b2
456.272 b3
805.421 y6
1187.564 b10
1205.623 y10
1577.303 y13
1936.388 y17
b1
b2
b3
b10
m/z
RTRGSTDLQELQASMEQLLR / File: sydney_ip_22.raw; SpectrumID: ; scans: 16767

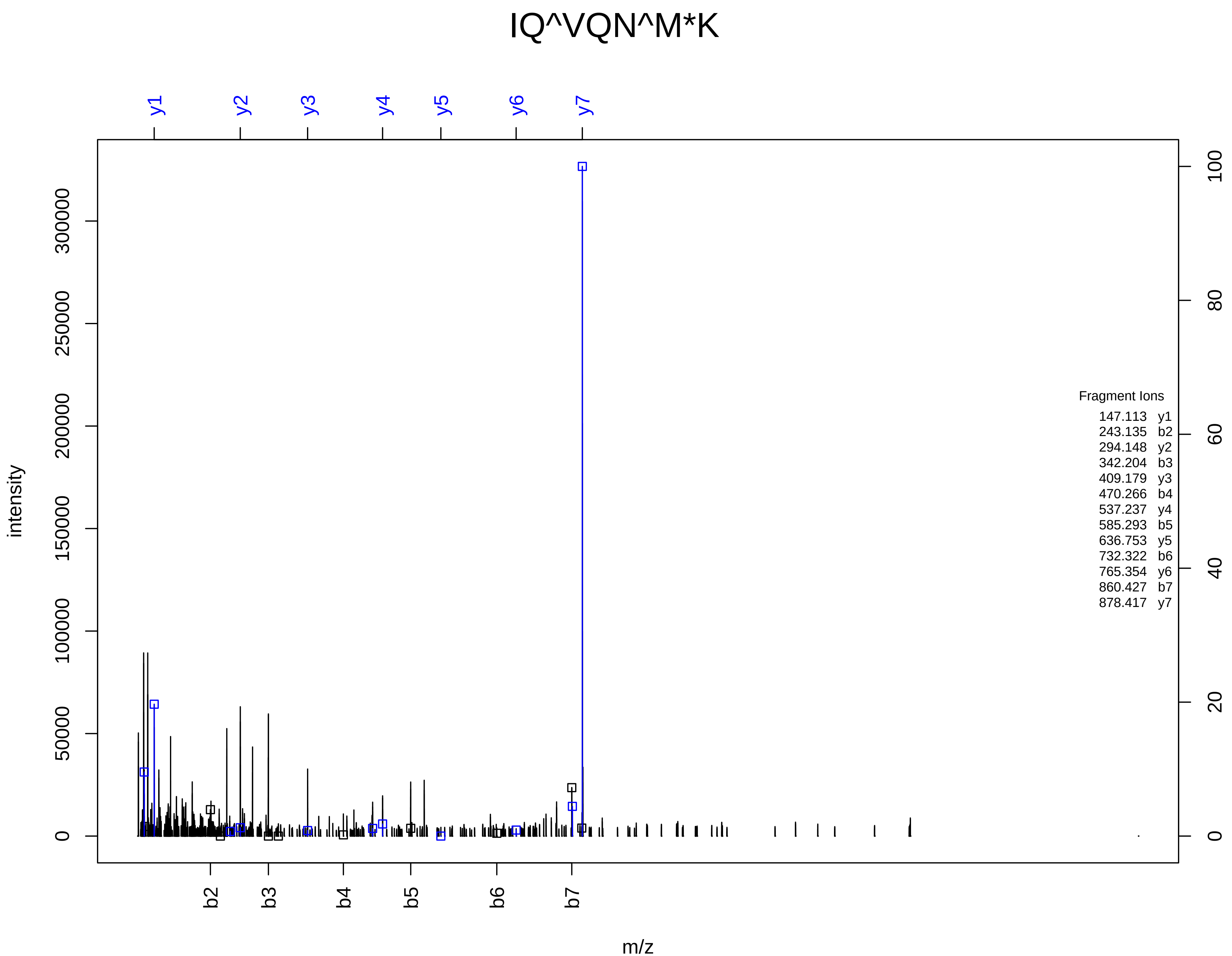

IQVQNMK / File: sydney_ip_22.raw; SpectrumID: ; scans: 9281

FSEVATQYSEDK

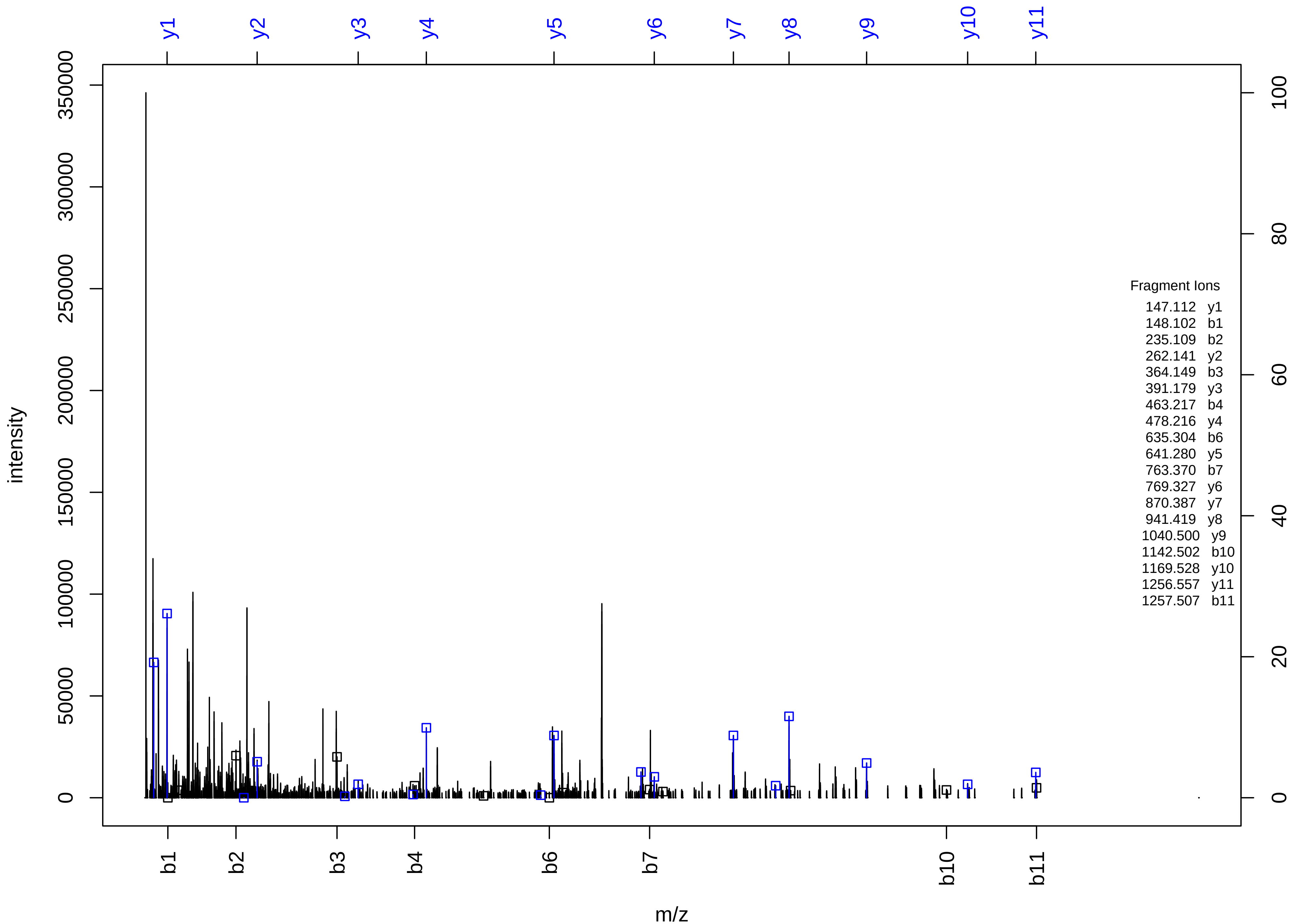

FSEVATQYSEDK / File: sydney_ip_22.raw; SpectrumID: ; scans: 4323

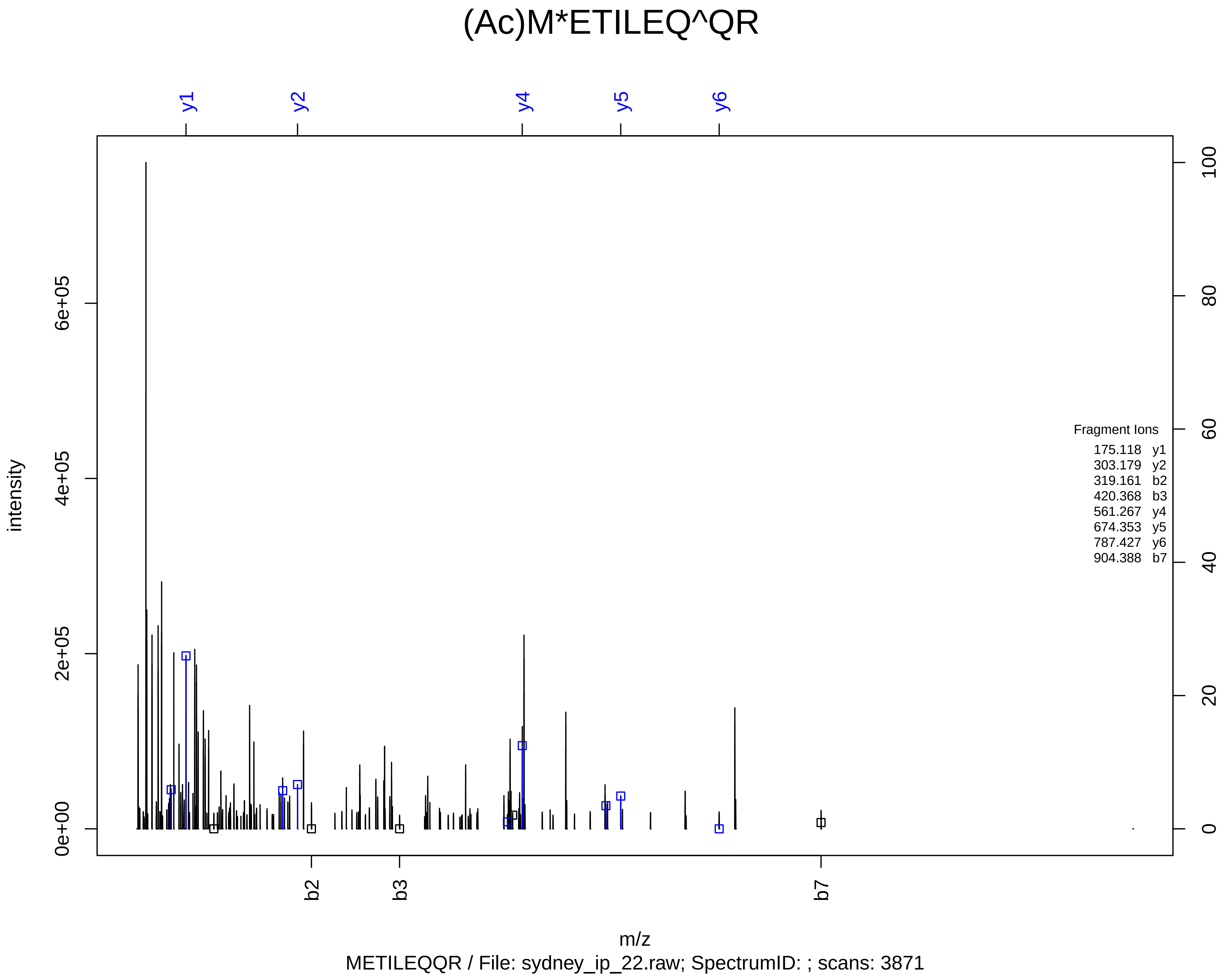
(Ac)M*ETILEQ^QR
y1
y2
y4
y5
y6
intensity
0e+00
2e+05
4e+05
6e+05
0
20
40
60
80
100
Fragment Ions
175.118 y1
303.179 y2
319.161 b2
420.368 b3
561.267 y4
674.353 y5
787.427 y6
904.388 b7
b2
b3
b7
m/z
METILEQQR / File: sydney_ip_22.raw; SpectrumID: ; scans: 3871

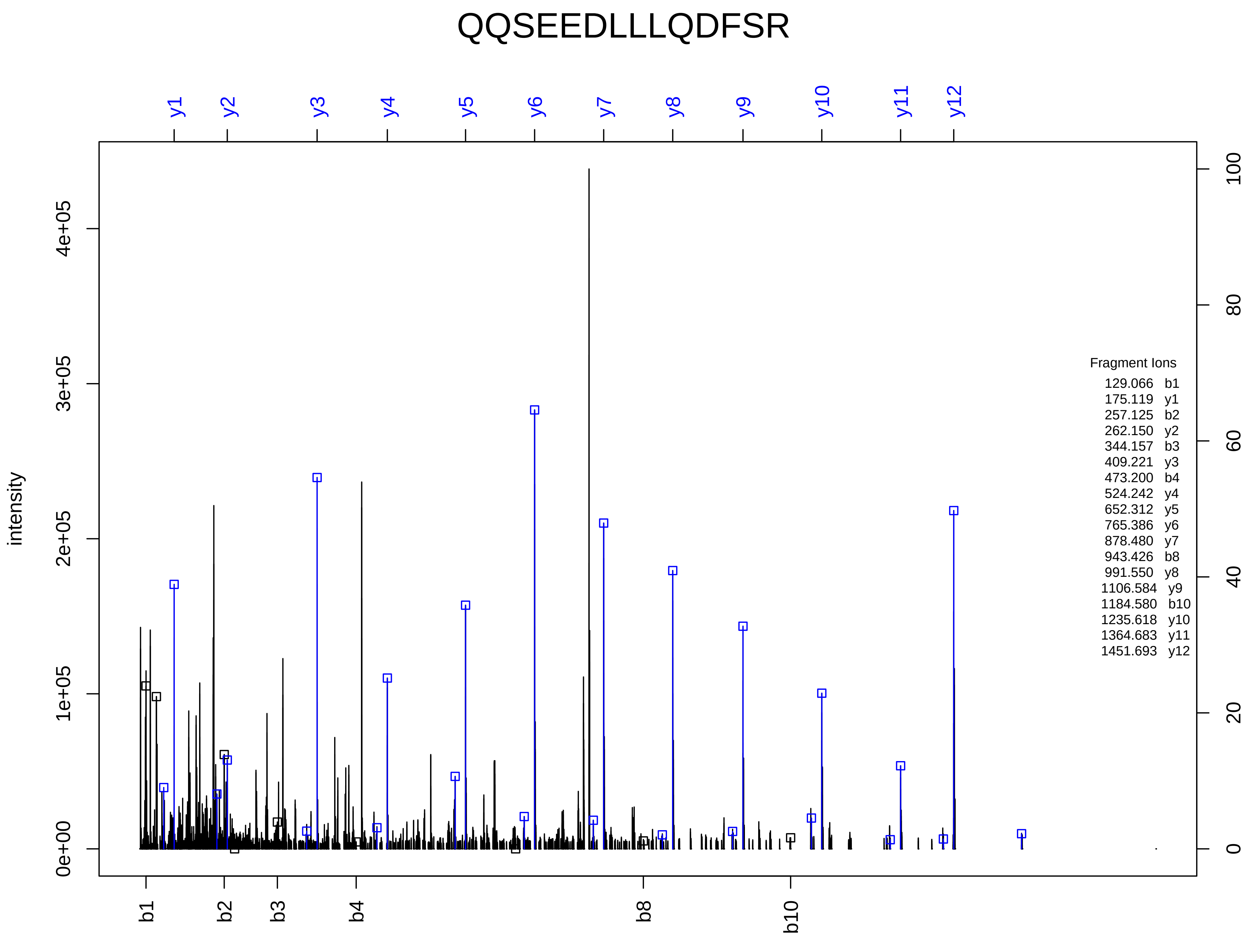

QQSEEDLLLQDFSR / File: sydney_ip_22.raw; SpectrumID: ; scans: 14489

GTSEGSNSSVSEFSPGIGIR

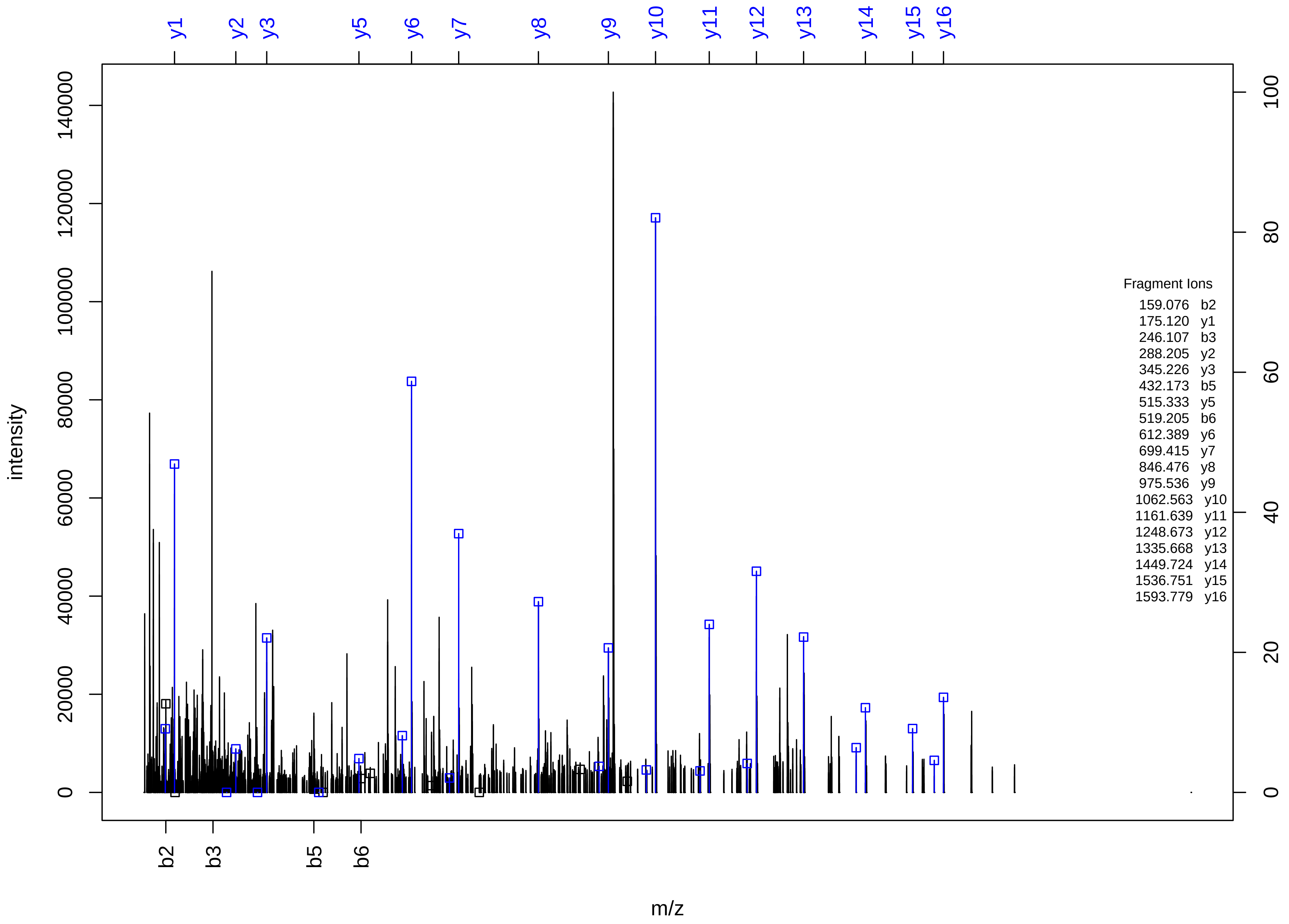

GTSEGSNSSVSEFSPGIGIR / File: sydney_ip_23.raw; SpectrumID: ; scans: 10162

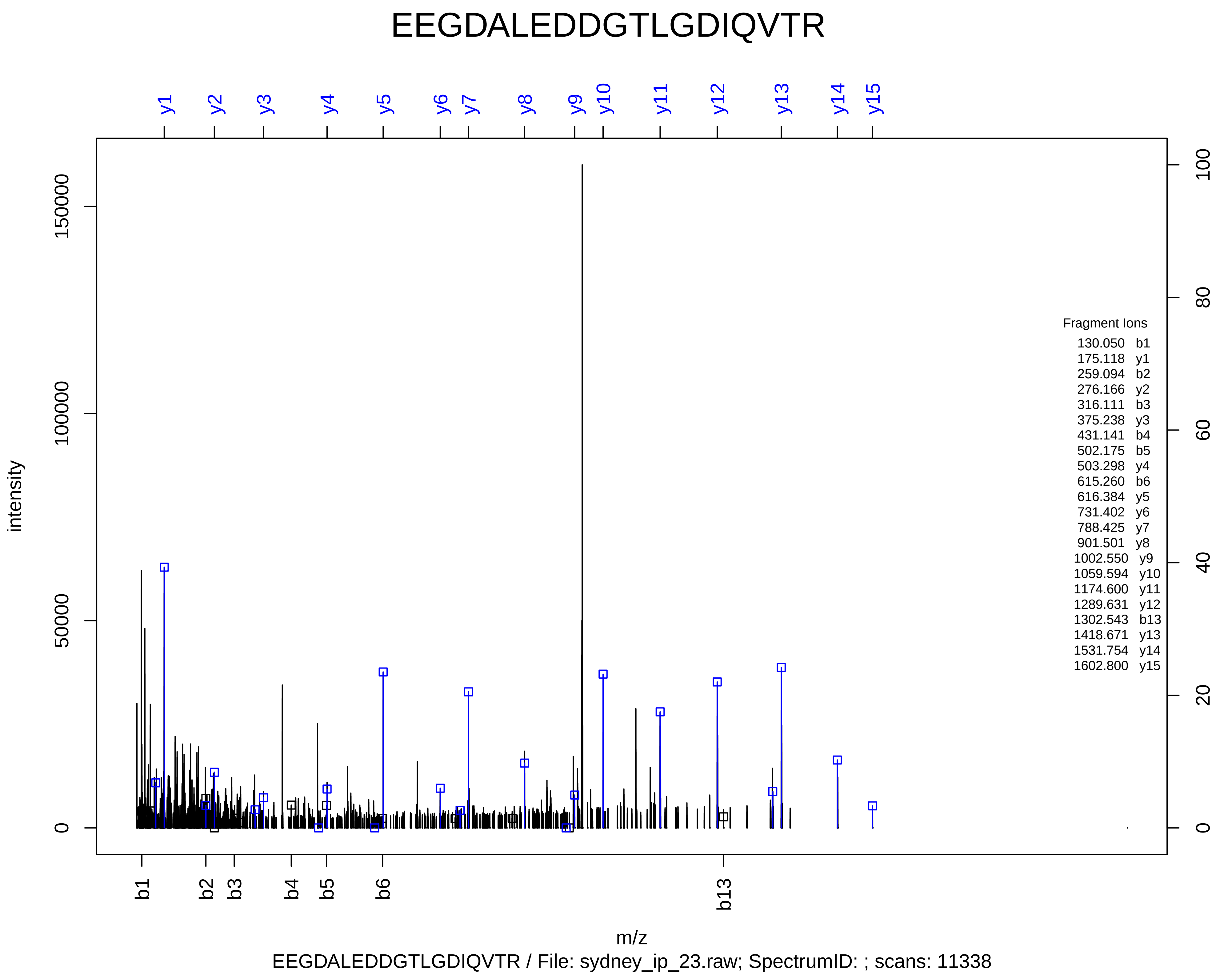

EEGDALEDDGTLGDIQVTR
y1 y2 y3 y4 y5 y6 y7 y8 y9 y10 y11 y12 y13 y14 y15
intensity
0
50000
100000
150000
0 20 40 60 80 100
b1 b2 b3 b4 b5 b6 b13
m/z
Fragment Ions
130.050 b1
175.118 y1
259.094 b2
276.166 y2
316.111 b3
375.238 y3
431.141 b4
502.175 b5
503.298 y4
615.260 b6
616.384 y5
731.402 y6
788.425 y7
901.501 y8
1002.550 y9
1059.594 y10
1174.600 y11
1289.631 y12
1302.543 b13
1418.671 y13
1531.754 y14
1602.800 y15
EEGDALEDDGTLGDIQVTR / File: sydney_ip_23.raw; SpectrumID: ; scans: 11338

YSPTSPTYSPTSPK

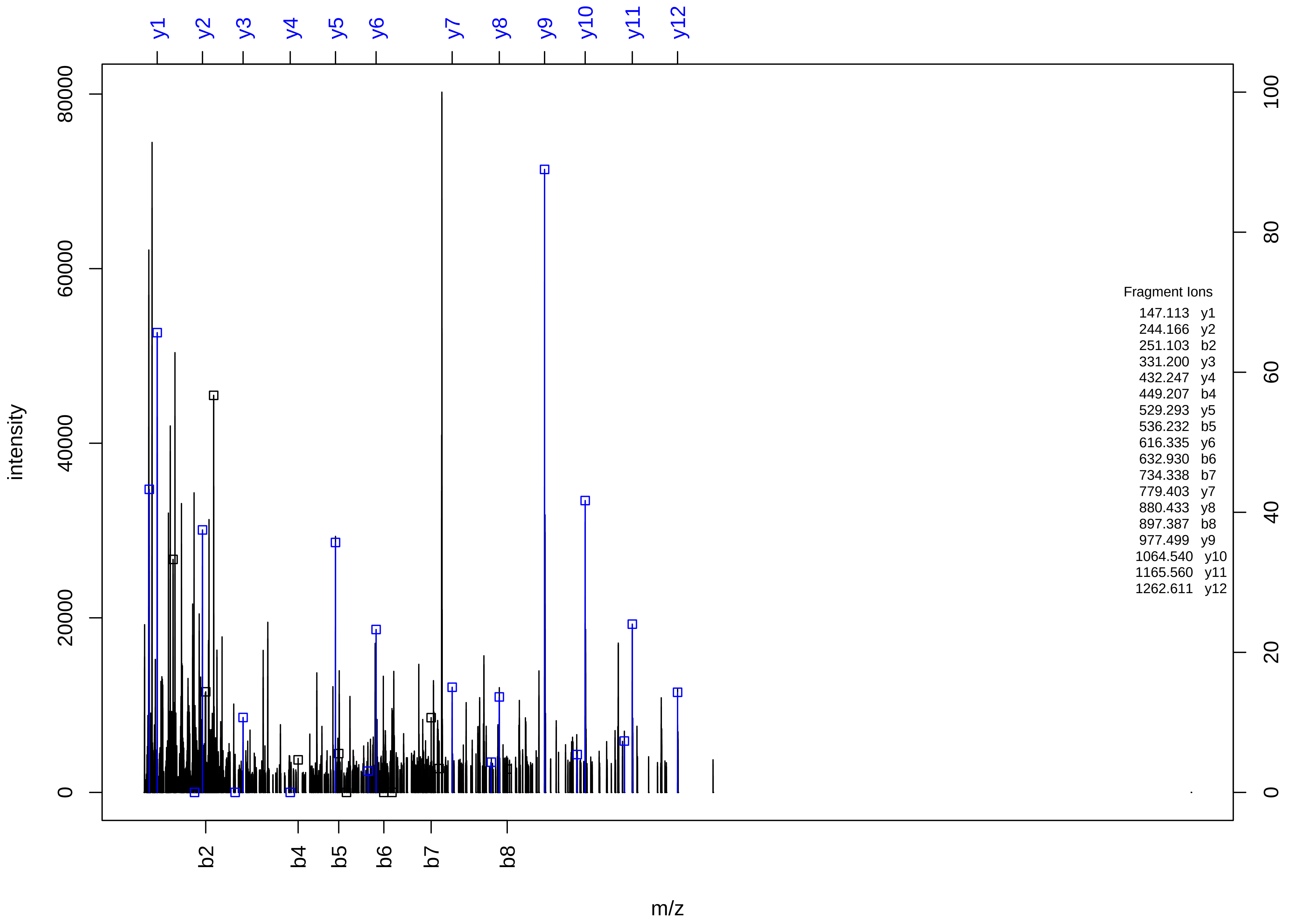

YSPTSPTYSPTSPK / File: sydney_ip_23.raw; SpectrumID: ; scans: 4036

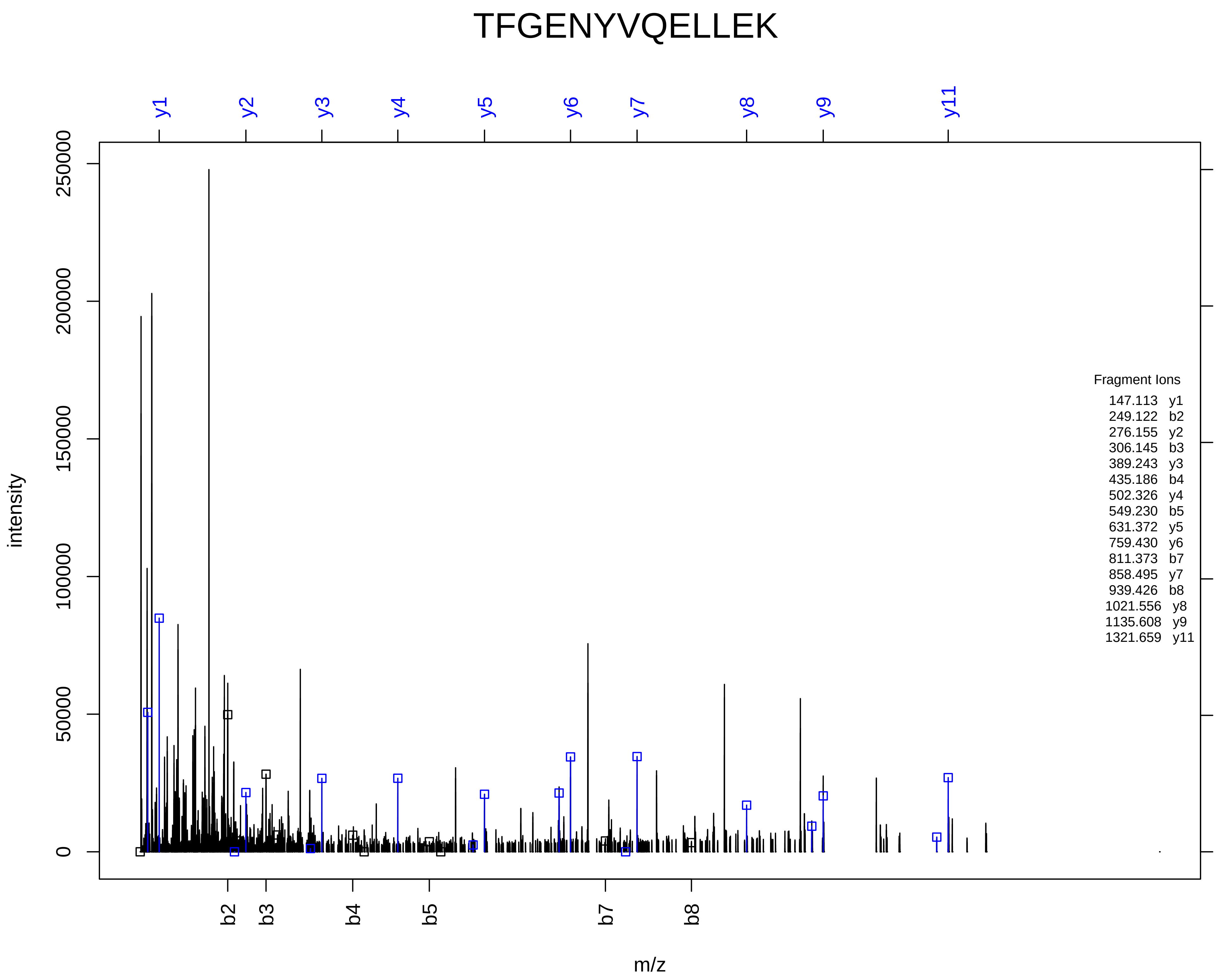

TFGENYVQELLEK / File: sydney_ip_23.raw; SpectrumID: ; scans: 16297

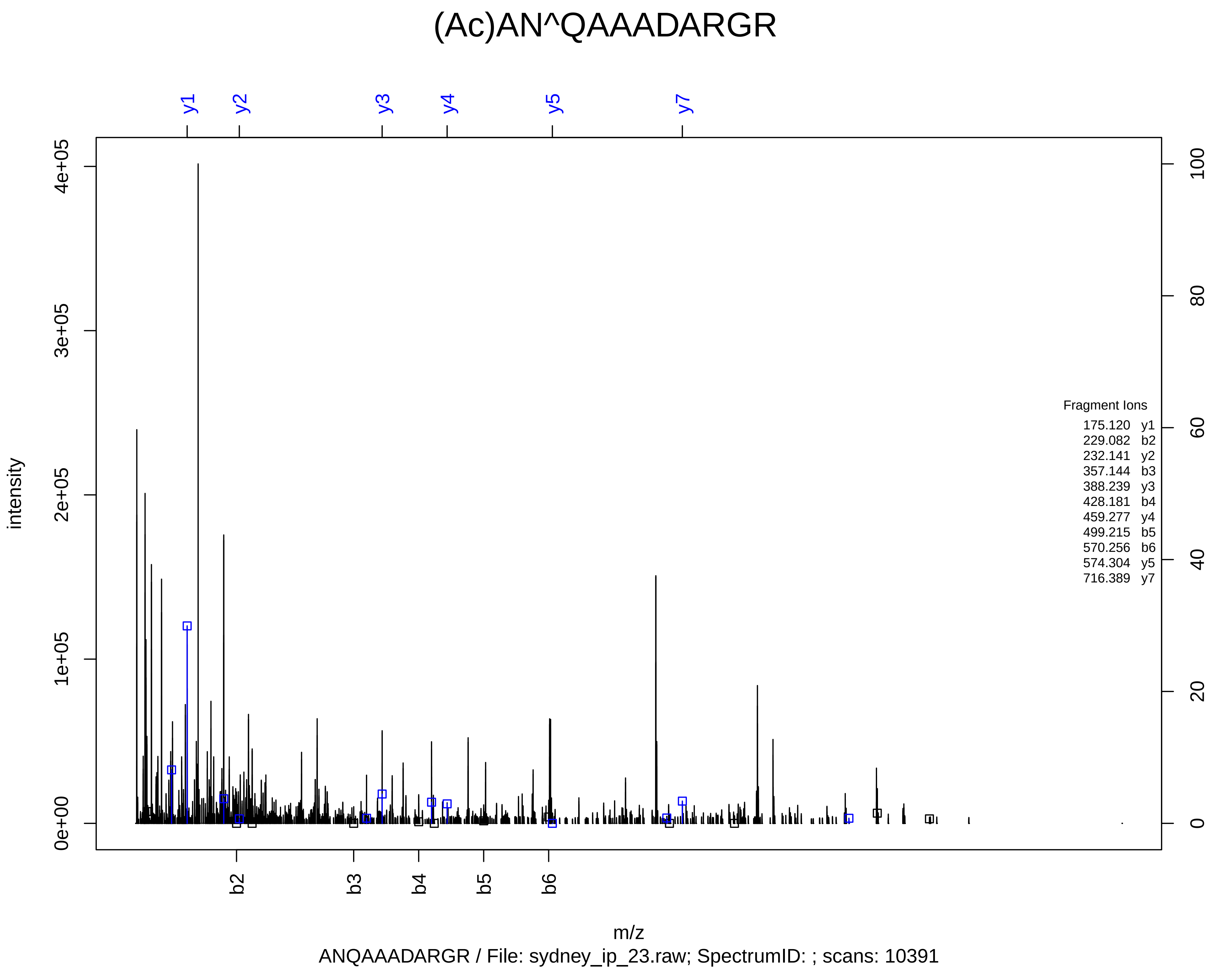
(Ac)AN^QAAADARGR
y1
y2
y3
y4
y5
y7
intensity
4e+05
3e+05
2e+05
1e+05
0e+00
100
80
60
40
20
0
Fragment Ions
175.120 y1
229.082 b2
232.141 y2
357.144 b3
388.239 y3
428.181 b4
459.277 y4
499.215 b5
570.256 b6
574.304 y5
716.389 y7
b2
b3
b4
b5
b6
m/z
ANQAAADARGR / File: sydney_ip_23.raw; SpectrumID: ; scans: 10391

IPQGFGNLLEGLTR

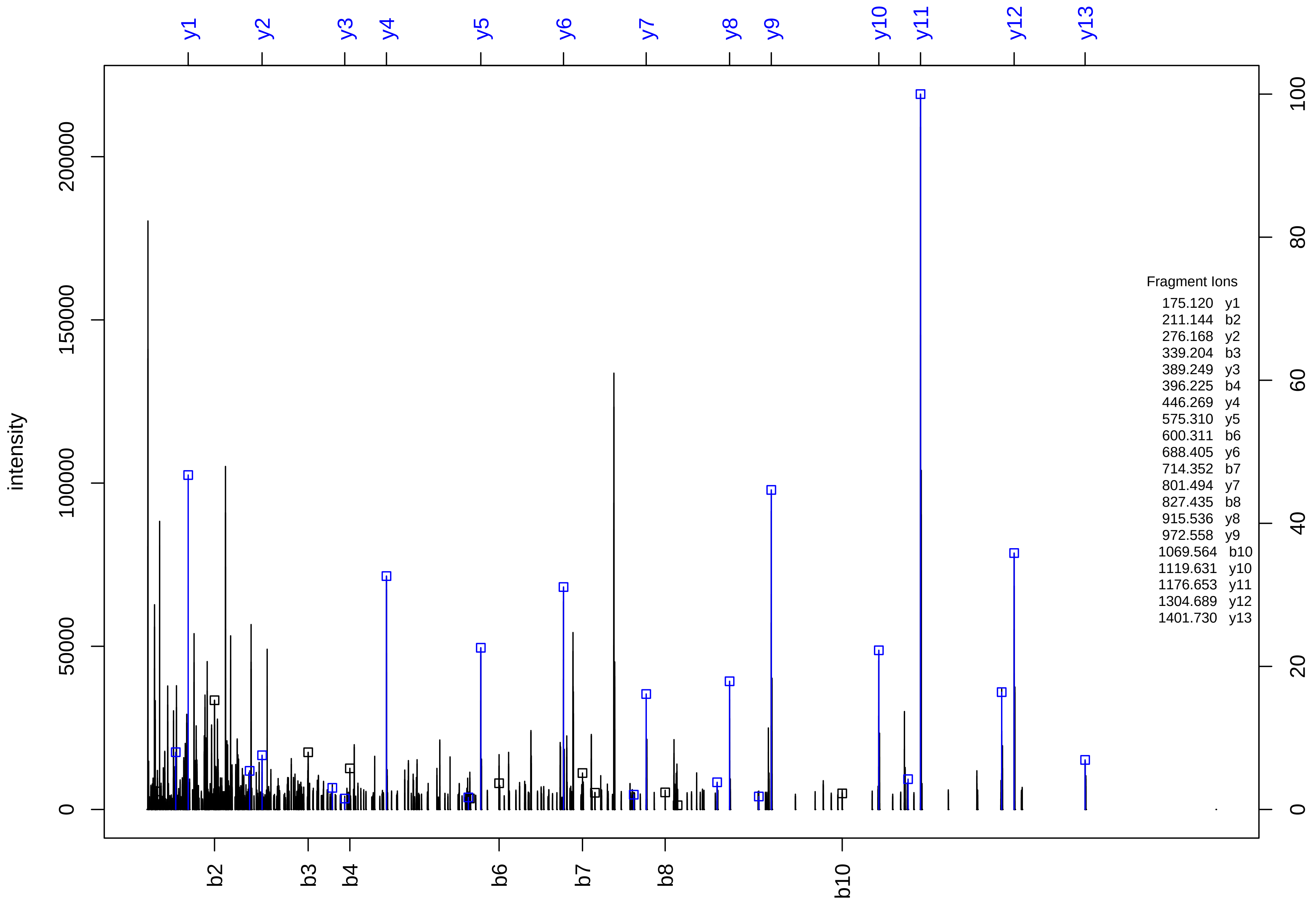

IPQGFGNLLEGLTR / File: sydney_ip_23.raw; SpectrumID: ; scans: 18345

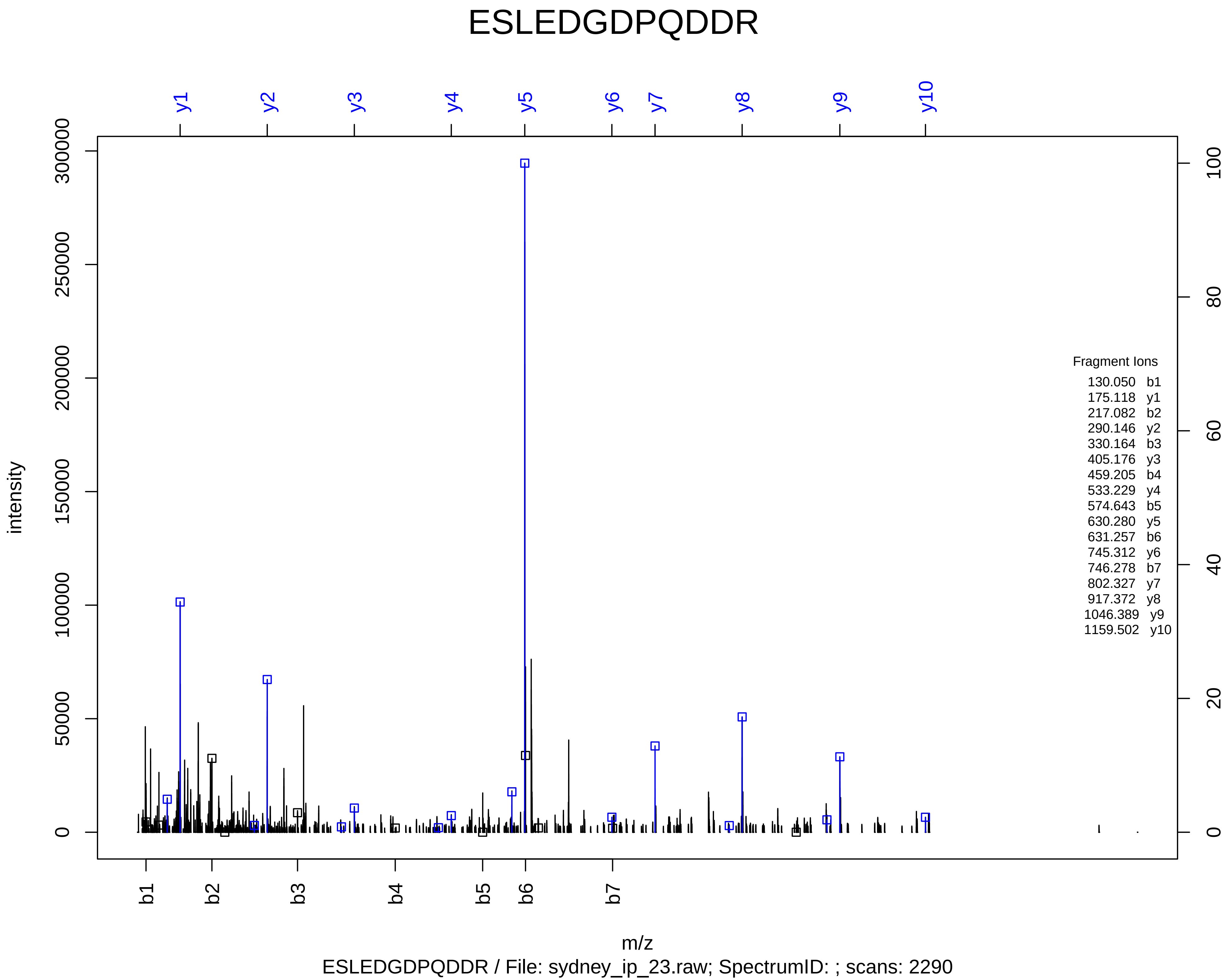

ESLEDGDPQDDR
y1
y2
y3
y4
y5
y6
y7
y8
y9
y10
intensity
0
50000
100000
150000
200000
250000
300000
0
20
40
60
80
100
Fragment Ions
130.050 b1
175.118 y1
217.082 b2
290.146 y2
330.164 b3
405.176 y3
459.205 b4
533.229 y4
574.643 b5
630.280 y5
631.257 b6
745.312 y6
746.278 b7
802.327 y7
917.372 y8
1046.389 y9
1159.502 y10
b1
b2
b3
b4
b5
b6
b7
m/z
ESLEDGDPQDDR / File: sydney_ip_23.raw; SpectrumID: ; scans: 2290

DILVLPLDLTDTSSHEAATK

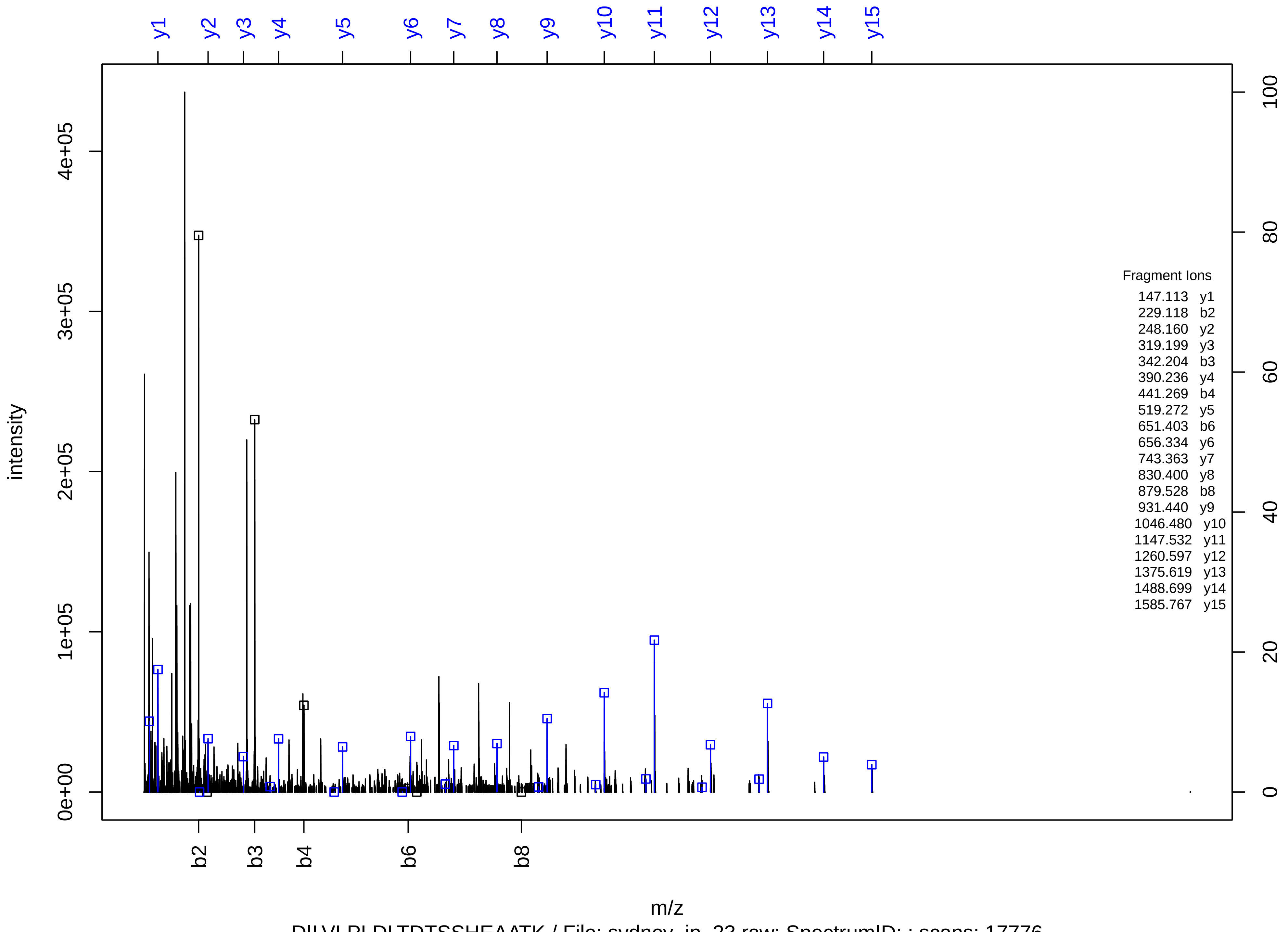

DILVLPLDLTDTSSHEAATK / File: sydney_ip_23.raw; SpectrumID: ; scans: 17776

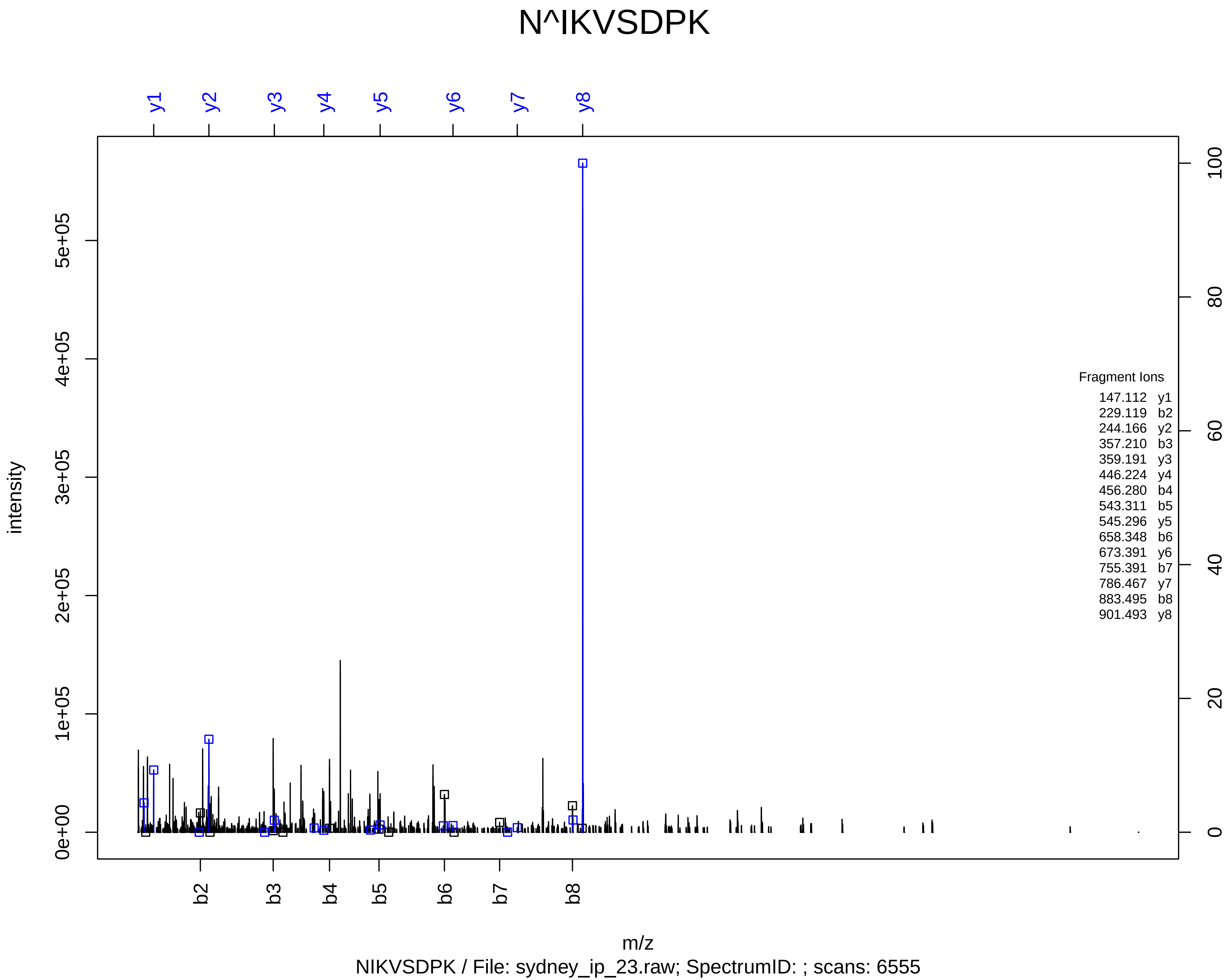
N^IKVSDPK
y1
y2
y3
y4
y5
y6
y7
y8
b2
b3
b4
b5
b6
b7
b8
intensity
0e+00
1e+05
2e+05
3e+05
4e+05
5e+05
0
20
40
60
80
100
m/z
Fragment Ions
147.112 y1
229.119 b2
244.166 y2
357.210 b3
359.191 y3
446.224 y4
456.280 b4
543.311 b5
545.296 y5
658.348 b6
673.391 y6
755.391 b7
786.467 y7
883.495 b8
901.493 y8
NIKVSDPK / File: sydney_ip_23.raw; SpectrumID: ; scans: 6555

TANLAEANASEEDK

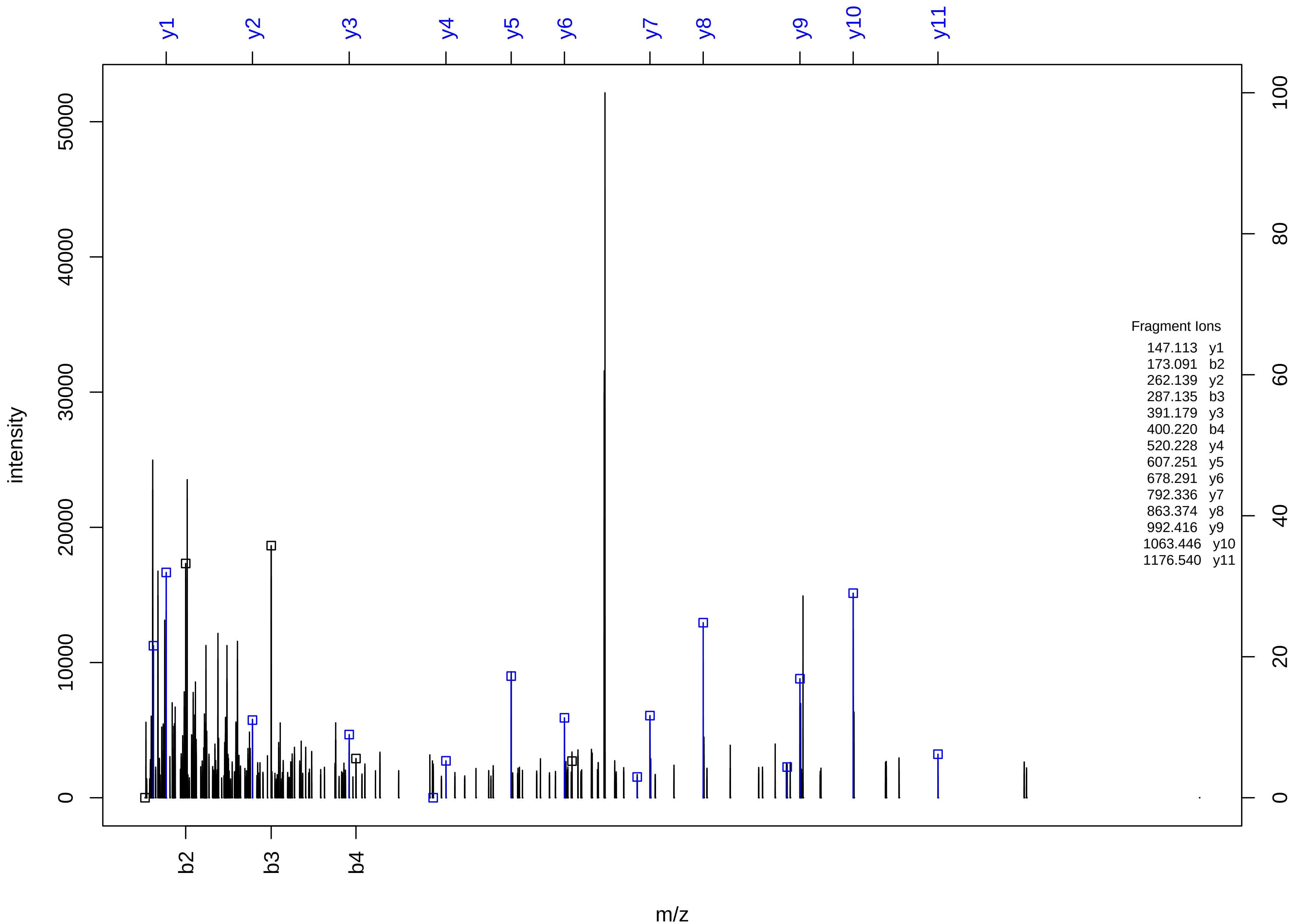

TANLAEANASEEDK / File: sydney_ip_23.raw; SpectrumID: ; scans: 2272

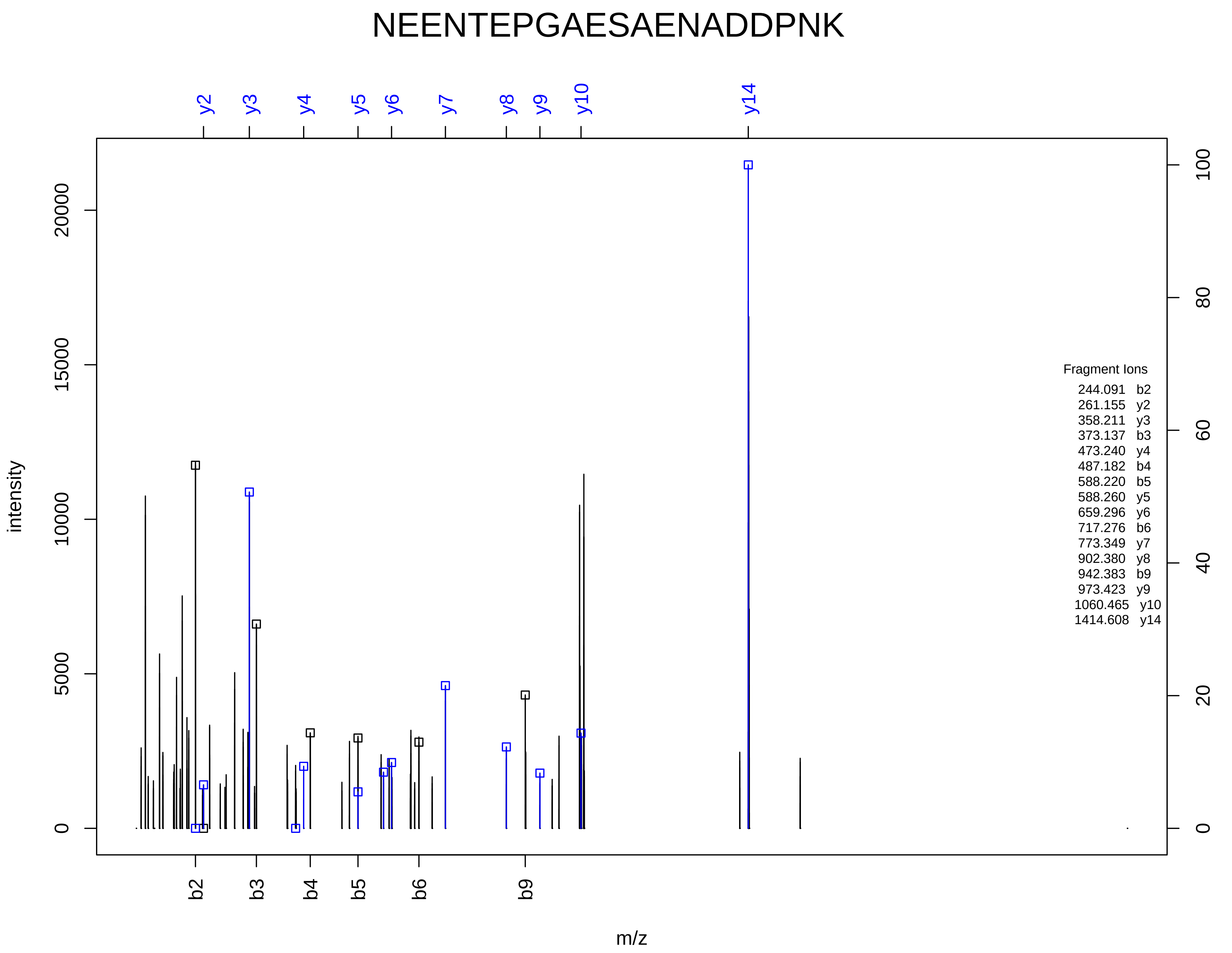
NEENTEPGAESAENADDPNK
y2
y3
y4
y5
y6
y7
y8
y9
y10
y14
intensity
0
5000
10000
15000
20000
0
20
40
60
80
100
Fragment Ions
244.091 b2
261.155 y2
358.211 y3
373.137 b3
473.240 y4
487.182 b4
588.220 b5
588.260 y5
659.296 y6
717.276 b6
773.349 y7
902.380 y8
942.383 b9
973.423 y9
1060.465 y10
1414.608 y14
b2
b3
b4
b5
b6
b9
m/z
NEENTEPGAESAENADDPNK / File: sydney_ip_23.raw; SpectrumID: ; scans: 2029

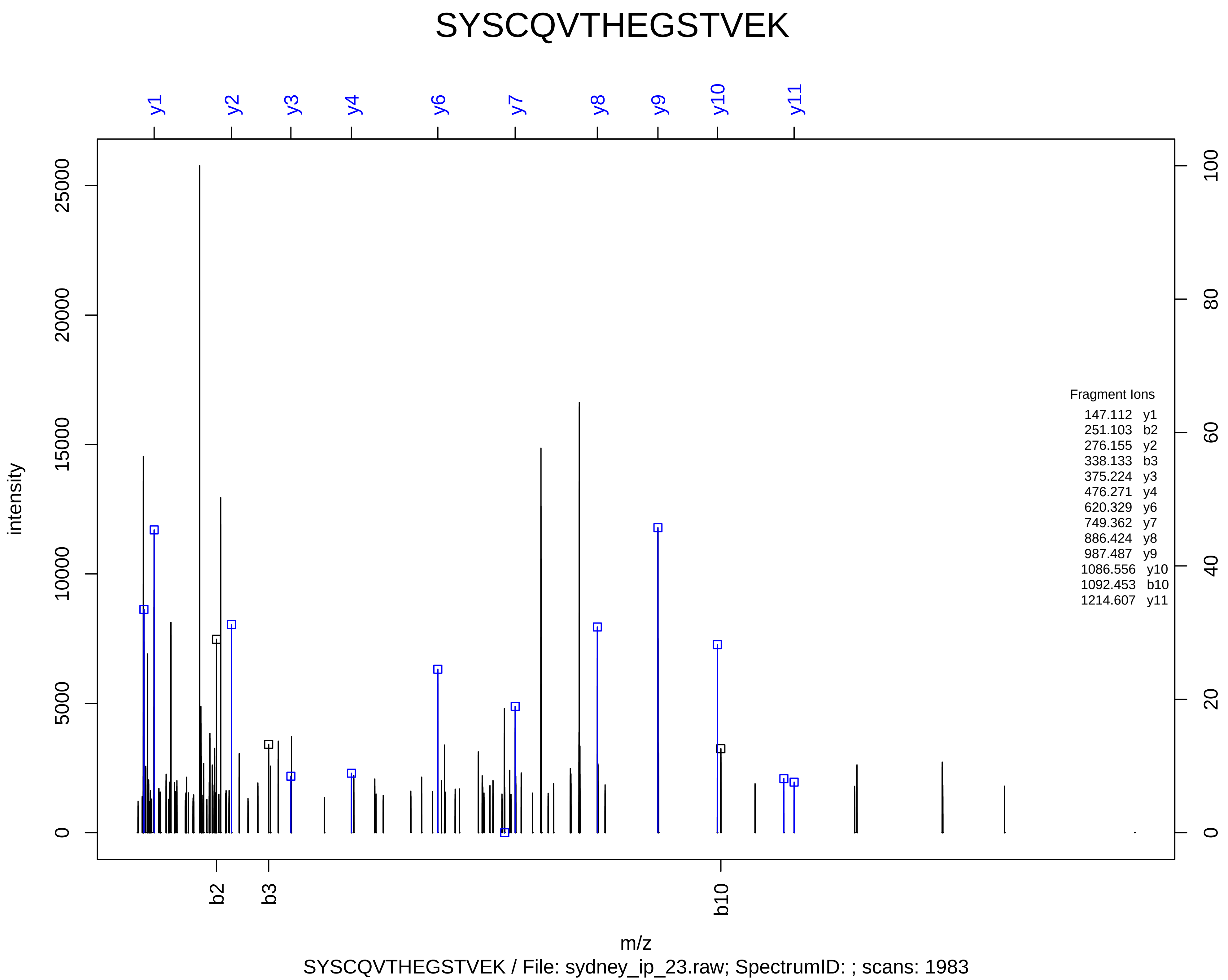

SYSCQVTHEGSTVEK
y1
y2
y3
y4
y6
y7
y8
y9
y10
y11
intensity
0
5000
10000
15000
20000
25000
0
20
40
60
80
100
b2
b3
b10
Fragment Ions
147.112 y1
251.103 b2
276.155 y2
338.133 b3
375.224 y3
476.271 y4
620.329 y6
749.362 y7
886.424 y8
987.487 y9
1086.556 y10
1092.453 b10
1214.607 y11
m/z
SYSCQVTHEGSTVEK / File: sydney_ip_23.raw; SpectrumID: ; scans: 1983

Q^VVQ^Q^AAWSPEK

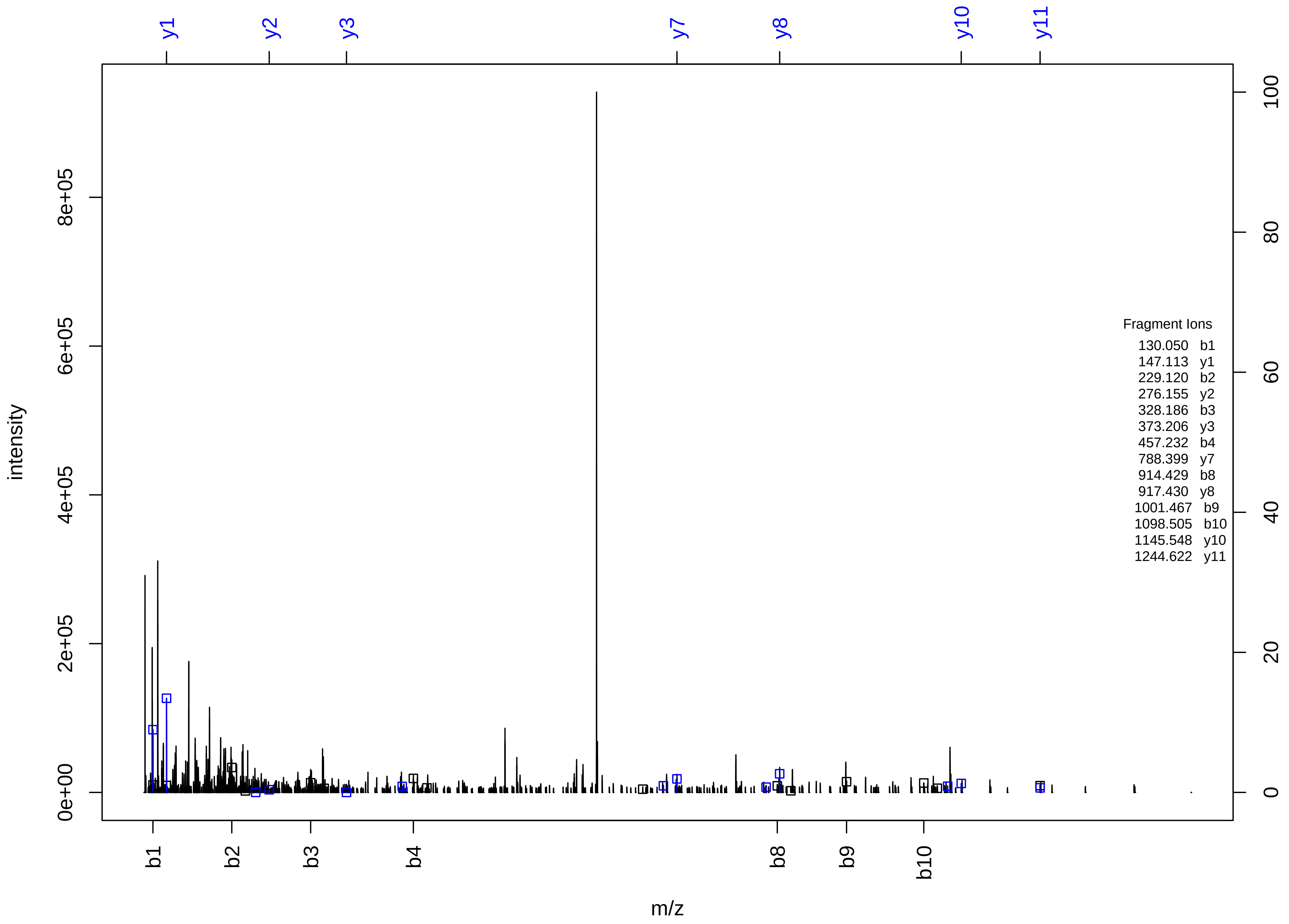

QVVQQAAWSPEK / File: sydney_ip_23.raw; SpectrumID: ; scans: 11318

THQLSLDAFLVALK

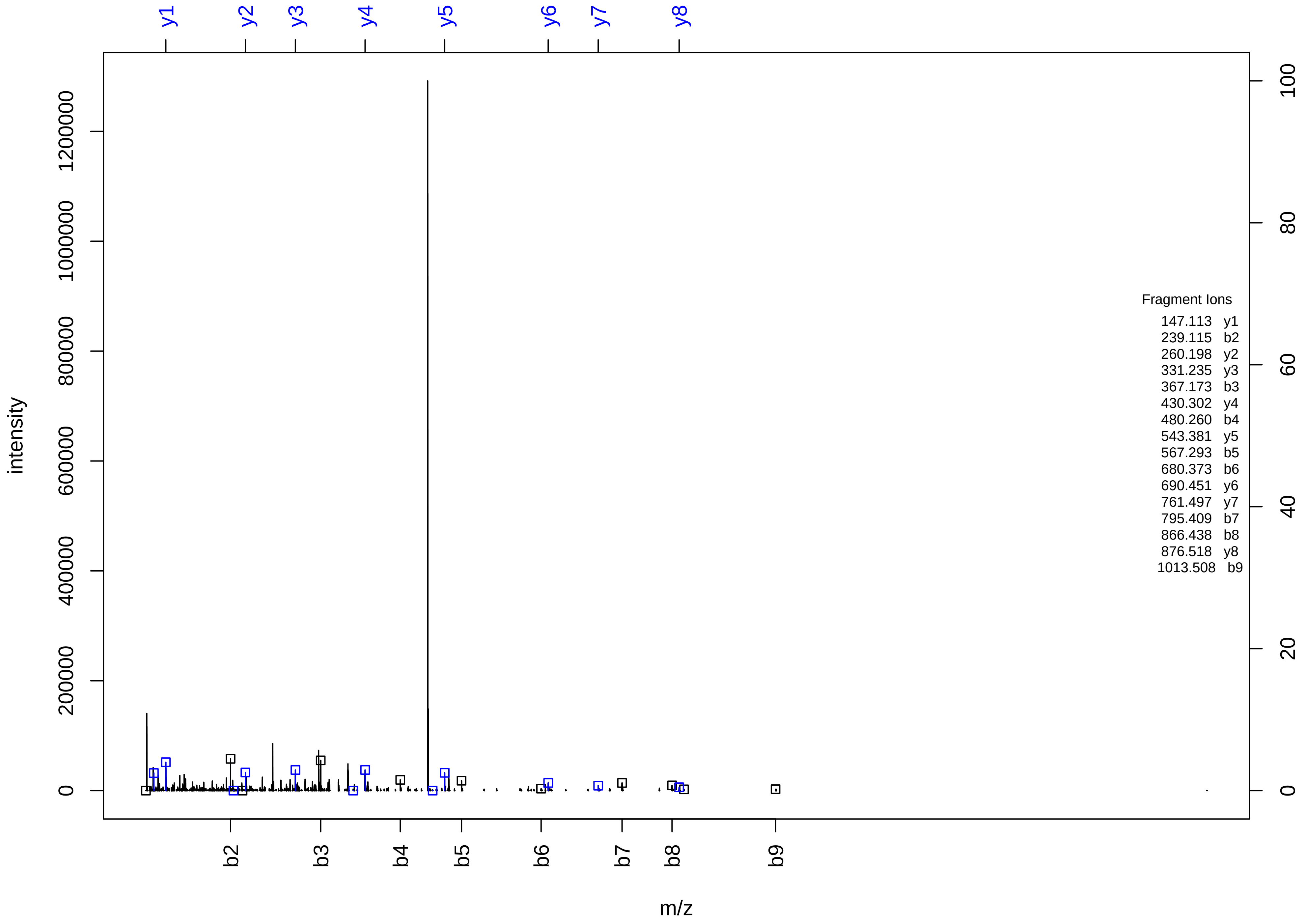

THQLSLDAFLVALK / File: sydney_ip_23.raw; SpectrumID: ; scans: 18139

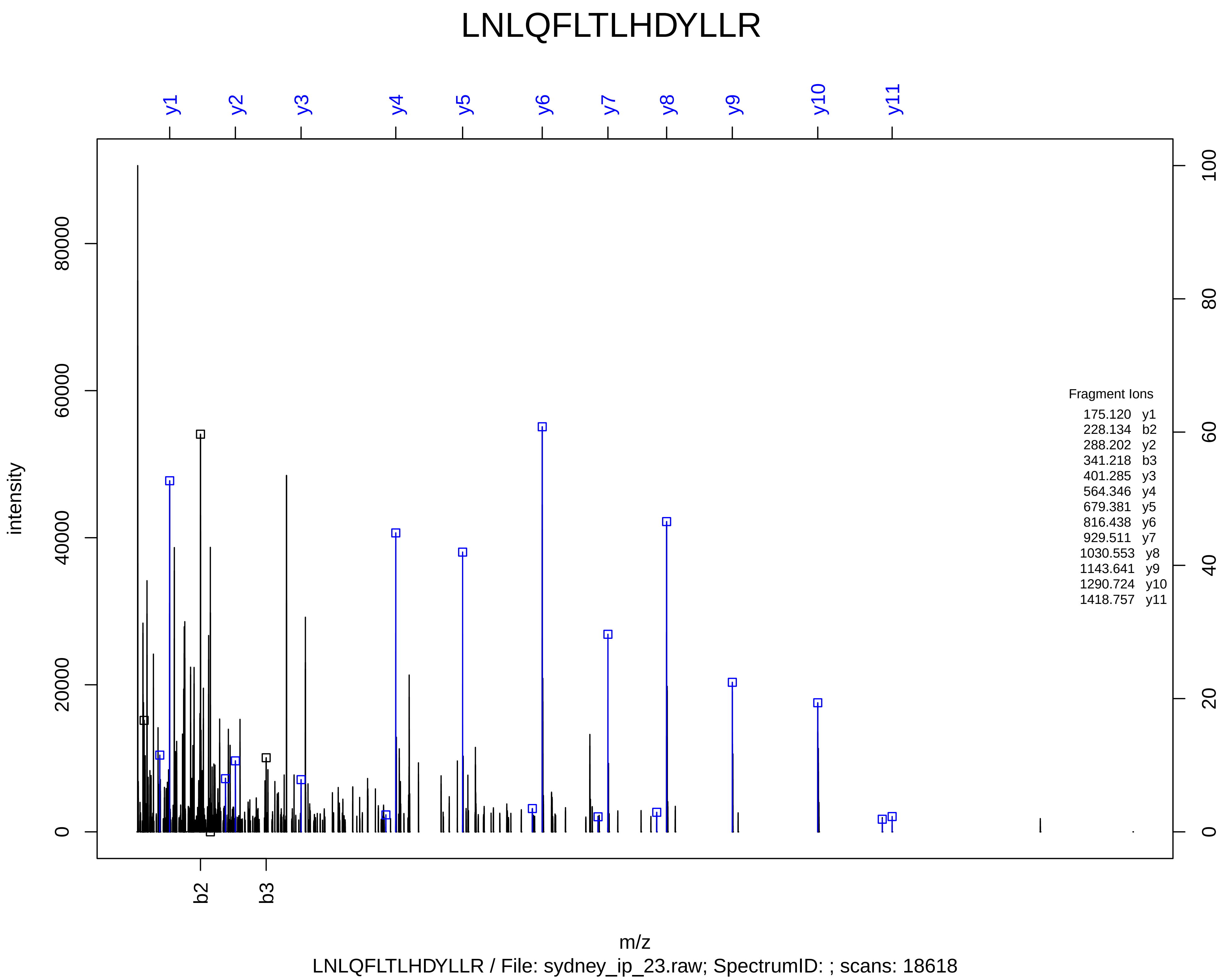
LNLQFLTLHDYLLR
y1
y2
y3
y4
y5
y6
y7
y8
y9
y10
y11
intensity
0
20000
40000
60000
80000
0
20
40
60
80
100
b2
b3
m/z
Fragment Ions
175.120 y1
228.134 b2
288.202 y2
341.218 b3
401.285 y3
564.346 y4
679.381 y5
816.438 y6
929.511 y7
1030.553 y8
1143.641 y9
1290.724 y10
1418.757 y11
LNLQFLTLHDYLLR / File: sydney_ip_23.raw; SpectrumID: ; scans: 18618

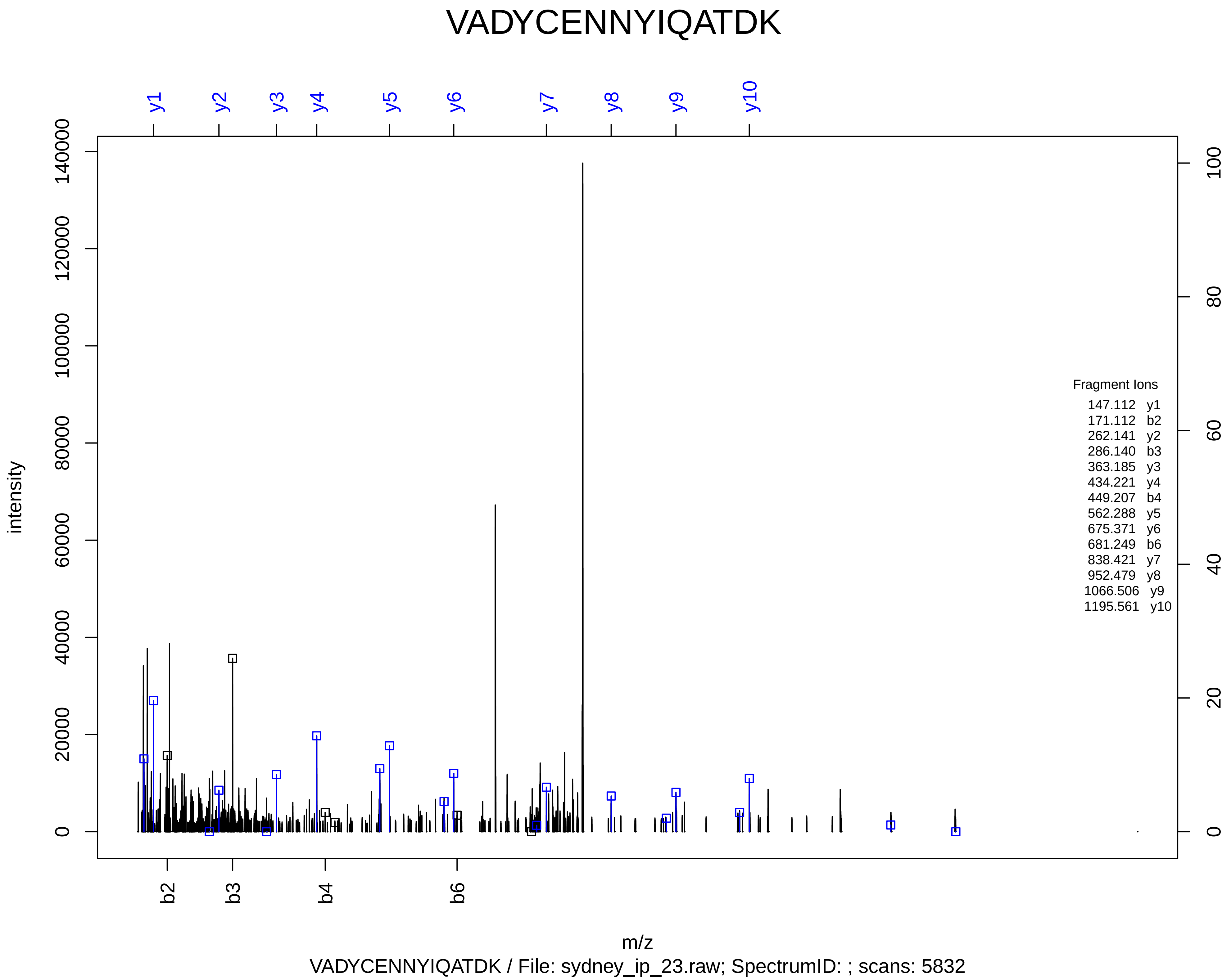
VADYCENNYIQATDK
y1
y2
y3
y4
y5
y6
y7
y8
y9
y10
b2
b3
b4
b6
intensity
m/z
Fragment Ions
147.112 y1
171.112 b2
262.141 y2
286.140 b3
363.185 y3
434.221 y4
449.207 b4
562.288 y5
675.371 y6
681.249 b6
838.421 y7
952.479 y8
1066.506 y9
1195.561 y10
VADYCENNYIQATDK / File: sydney_ip_23.raw; SpectrumID: ; scans: 5832

VELVPEEHPPFTAPVSFCDLGGR

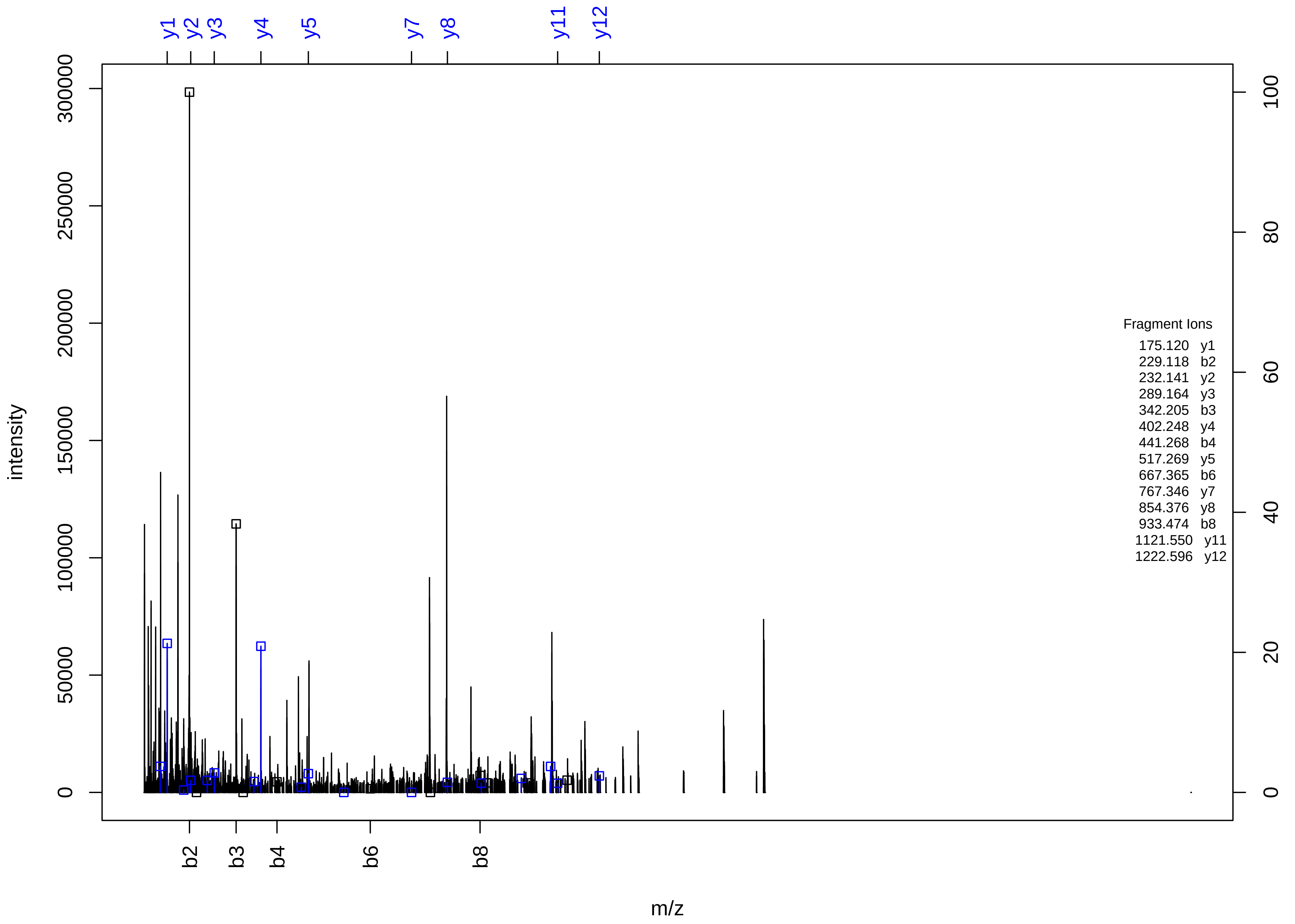

VELVPEEHPPFTAPVSFCDLGGR / File: sydney_ip_23.raw; SpectrumID: ; scans: 15894

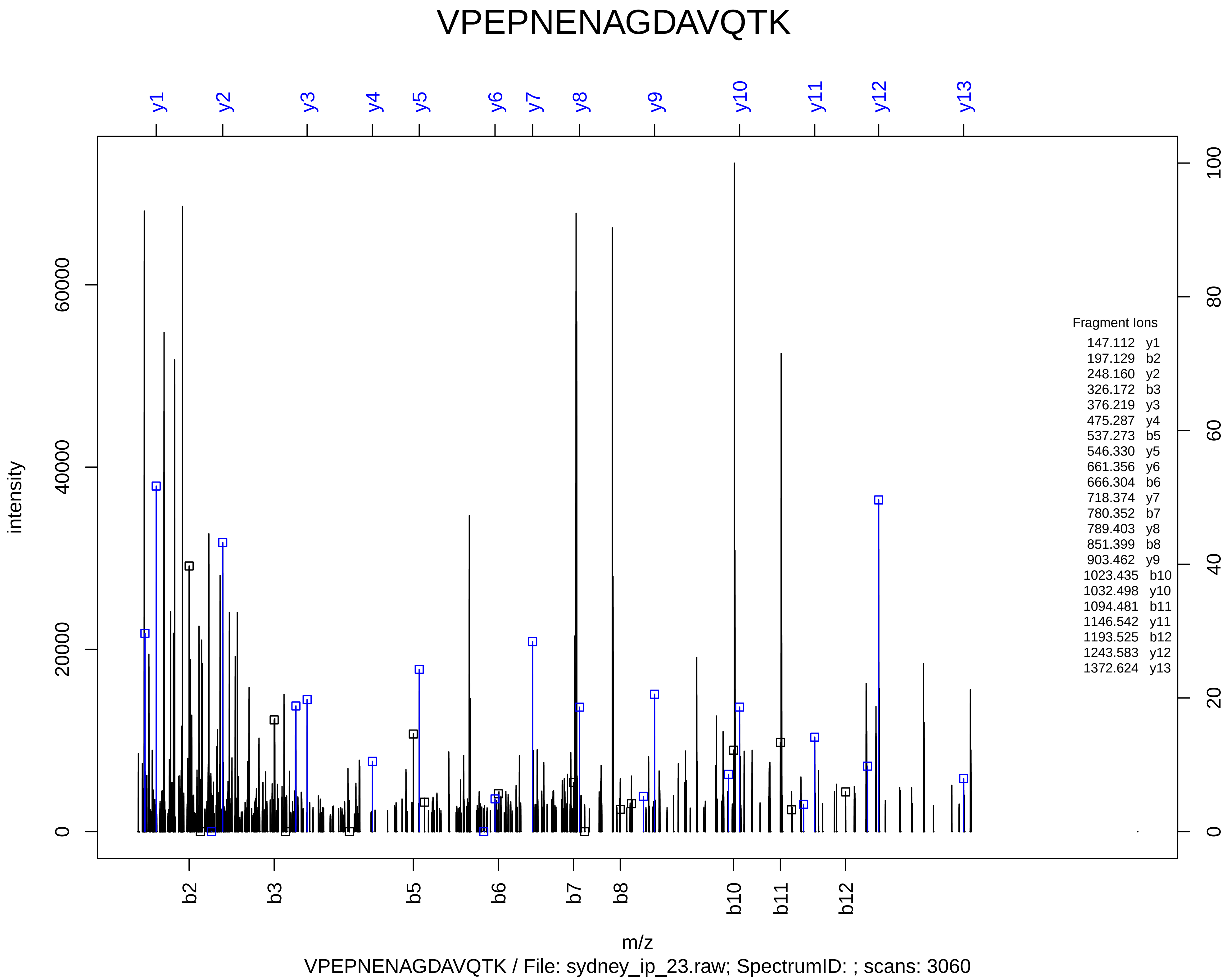
VPEPNENAGDAVQTK
y1
y2
y3
y4
y5
y6
y7
y8
y9
y10
y11
y12
y13
intensity
0
20000
40000
60000
0
20
40
60
80
100
b2
b3
b5
b6
b7
b8
b10
b11
b12
m/z
Fragment Ions
147.112 y1
197.129 b2
248.160 y2
326.172 b3
376.219 y3
475.287 y4
537.273 b5
546.330 y5
661.356 y6
666.304 b6
718.374 y7
780.352 b7
789.403 y8
851.399 b8
903.462 y9
1023.435 b10
1032.498 y10
1094.481 b11
1146.542 y11
1193.525 b12
1243.583 y12
1372.624 y13
VPEPNENAGDAVQTK / File: sydney_ip_23.raw; SpectrumID: ; scans: 3060

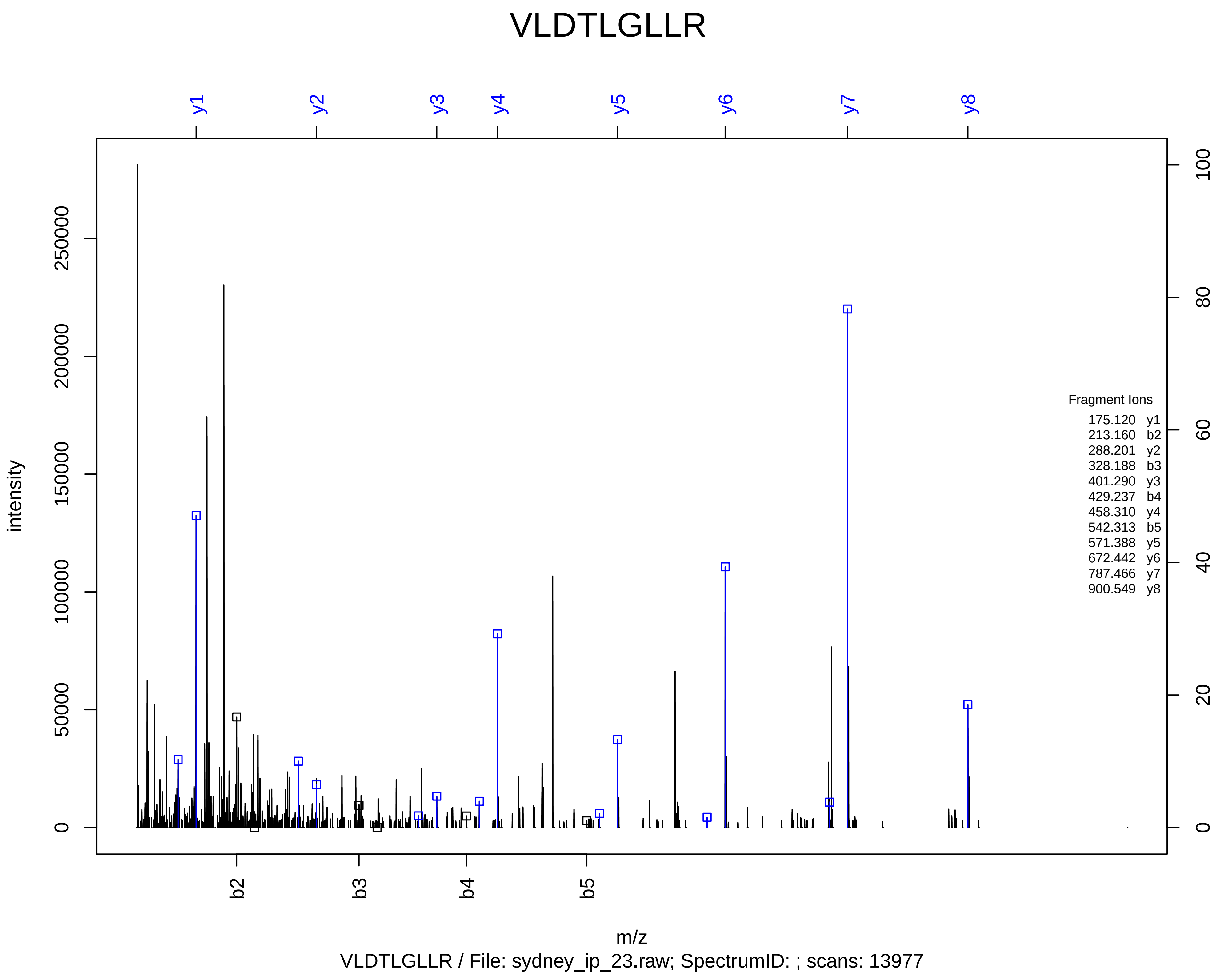
VLDTLGLLR
y1
y2
y3
y4
y5
y6
y7
y8
b2
b3
b4
b5
intensity
m/z
Fragment Ions
175.120 y1
213.160 b2
288.201 y2
328.188 b3
401.290 y3
429.237 b4
458.310 y4
542.313 b5
571.388 y5
672.442 y6
787.466 y7
900.549 y8
VLDTLGLLR / File: sydney_ip_23.raw; SpectrumID: ; scans: 13977

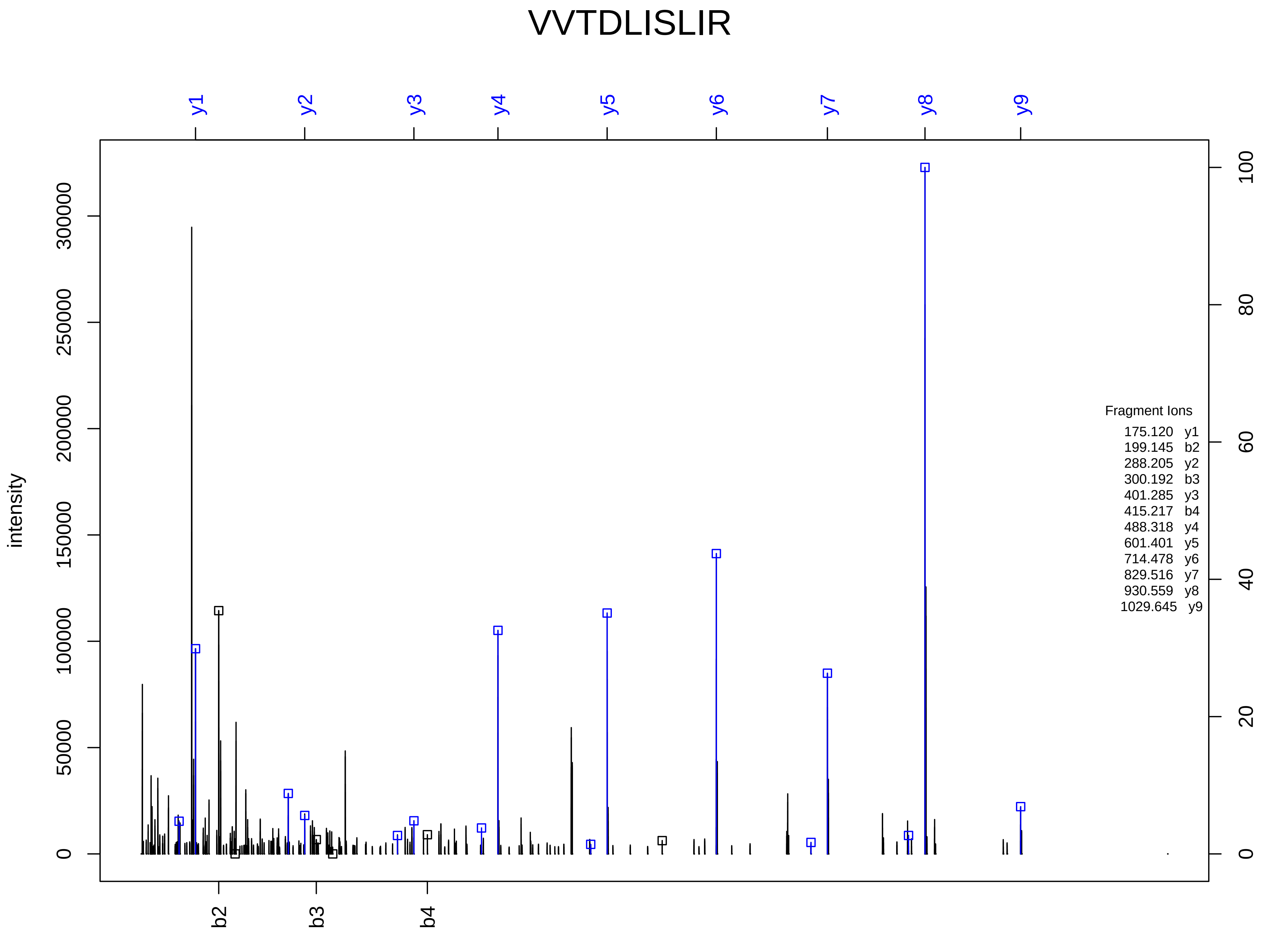

VVTDLISLIR / File: sydney_ip_23.raw; SpectrumID: ; scans: 17366

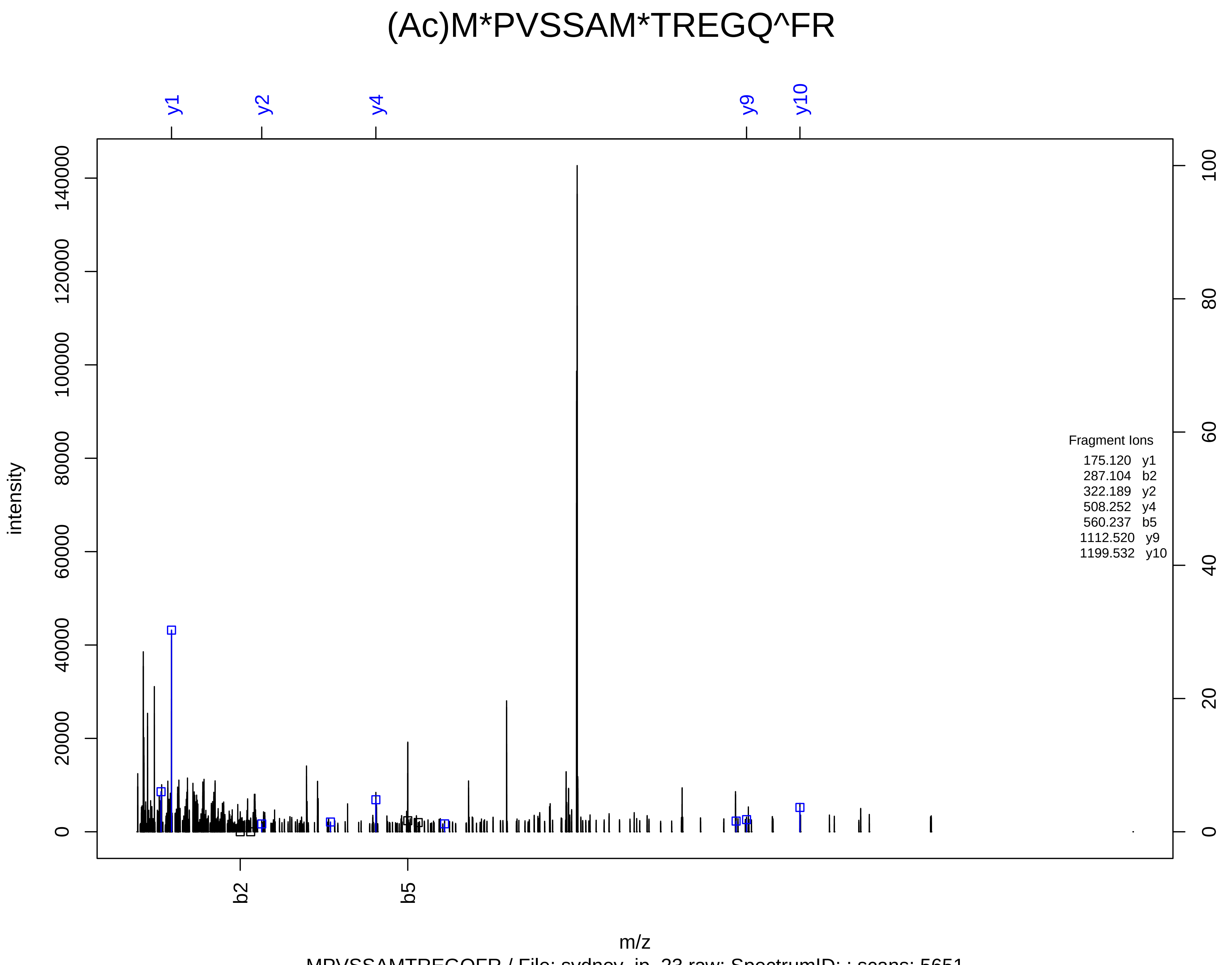
(Ac)M*PVSSAM*TREGQ^FR
y1
y2
y4
y9
y10
intensity
Fragment Ions
175.120 y1
287.104 b2
322.189 y2
508.252 y4
560.237 b5
1112.520 y9
1199.532 y10
b2
b5
m/z
MPVSSAMTREGQFR / File: sydney_ip_23.raw; SpectrumID: ; scans: 5651

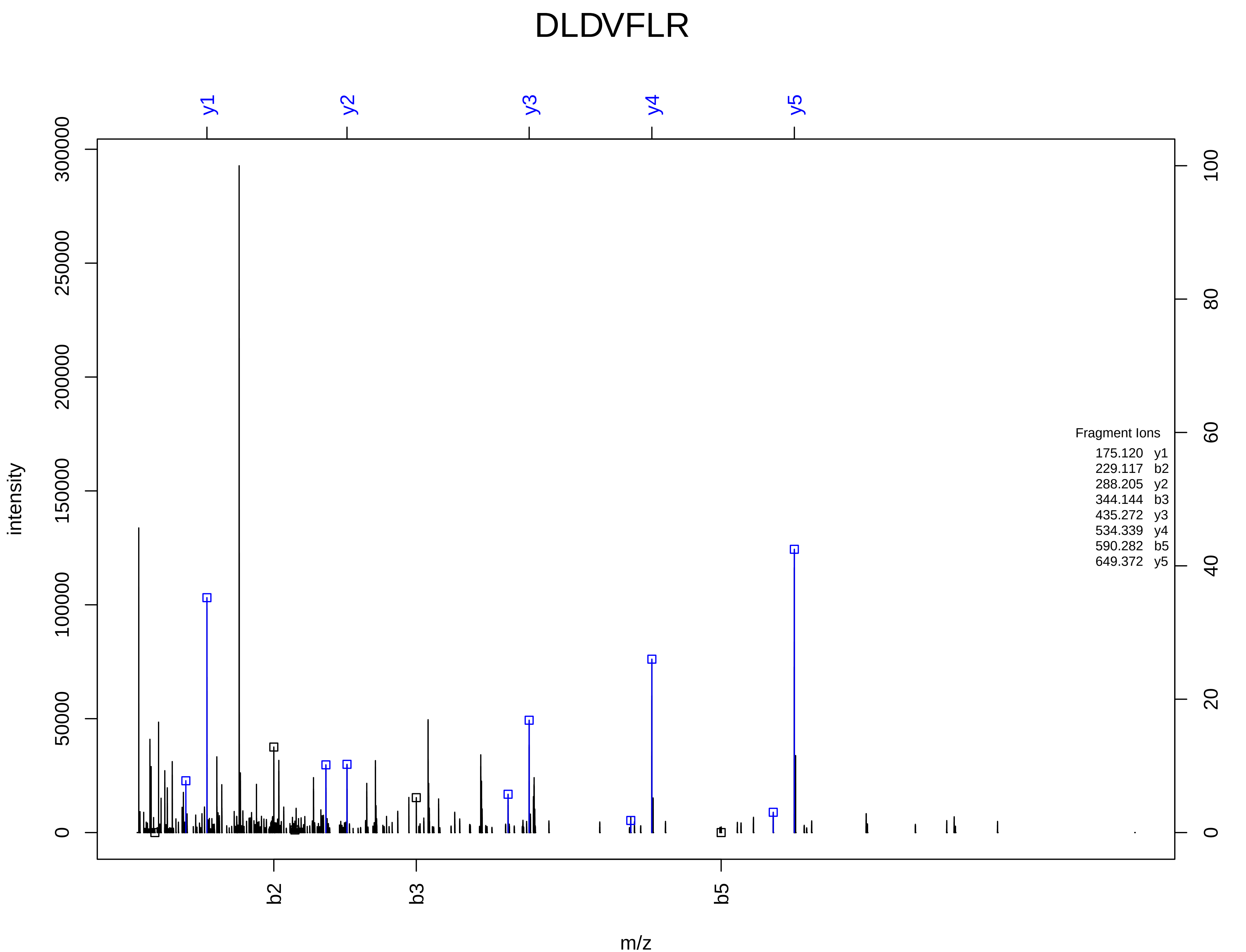
DLDVFLR
y1
y2
y3
y4
y5
intensity
300000
250000
200000
150000
100000
50000
0
100
80
60
40
20
0
Fragment Ions
175.120 y1
229.117 b2
288.205 y2
344.144 b3
435.272 y3
534.339 y4
590.282 b5
649.372 y5
b2
b3
b5
m/z
DLDVFLR / File: sydney_ip_23.raw; SpectrumID: ; scans: 13855

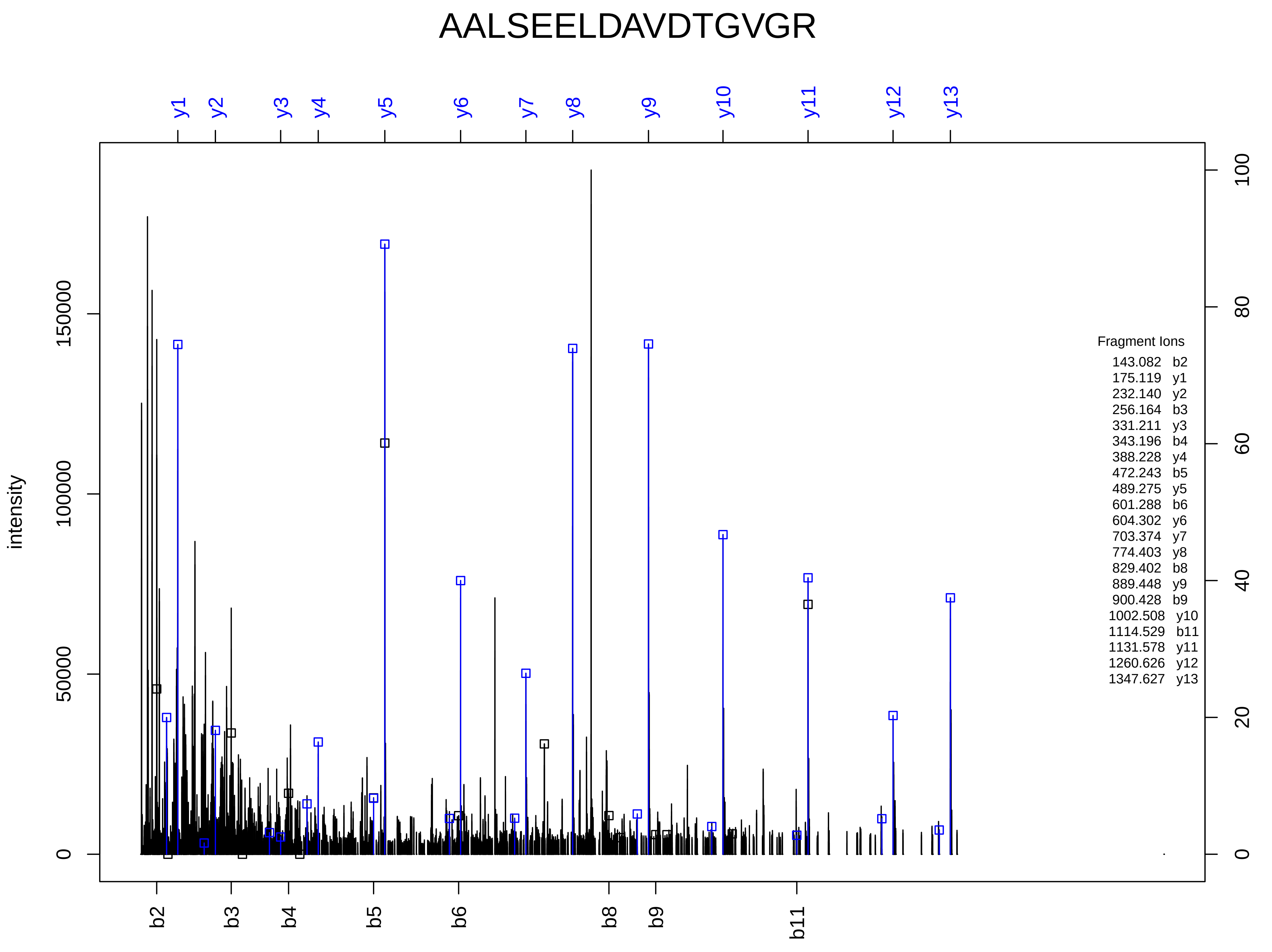

AALSEELDAVDTGVGR / File: sydney_ip_23.raw; SpectrumID: ; scans: 11189

ILEEIQDNLSEVK

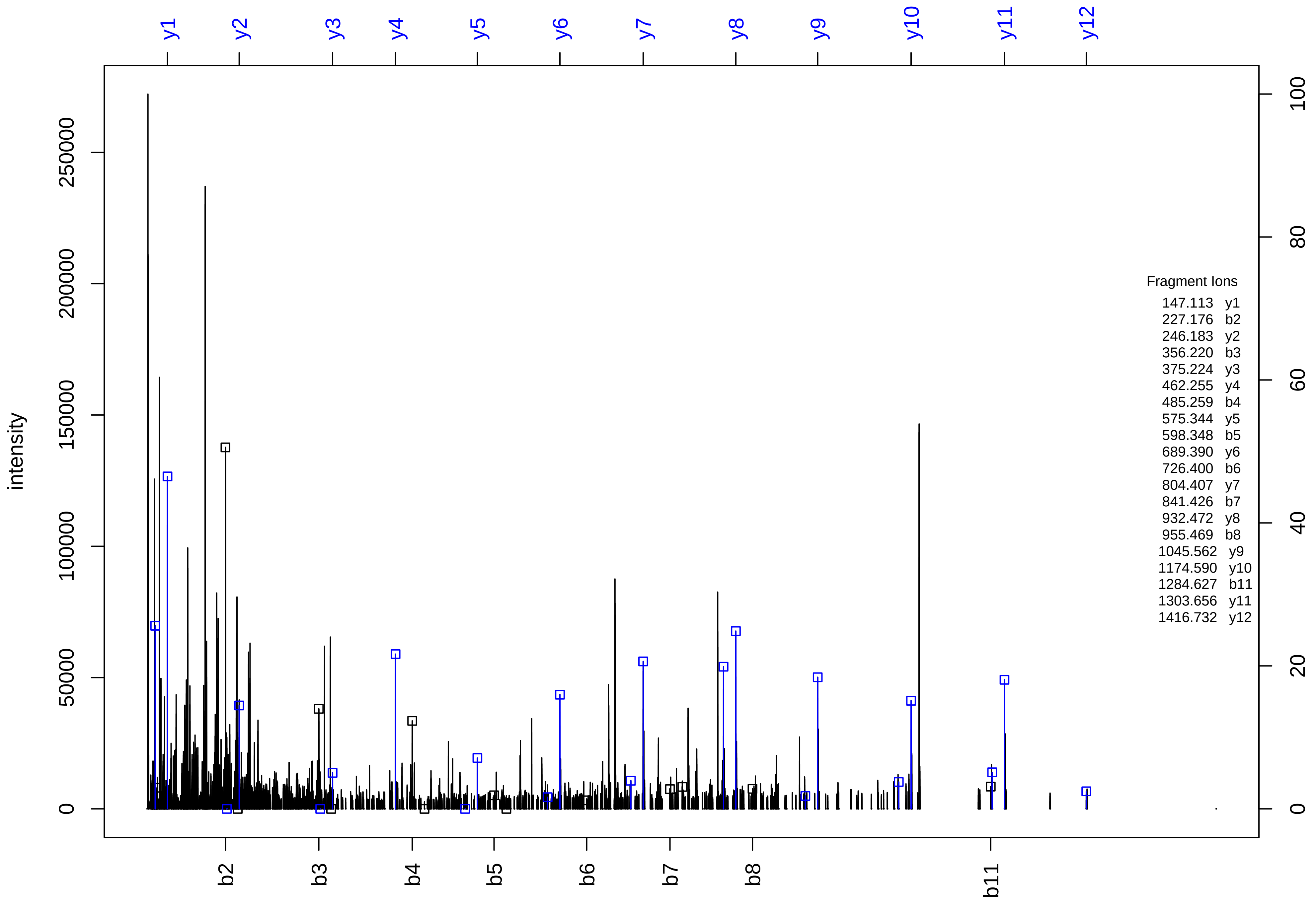

ILEEIQDNLSEVK / File: sydney_ip_23.raw; SpectrumID: ; scans: 11759

STDYGIFQINSR

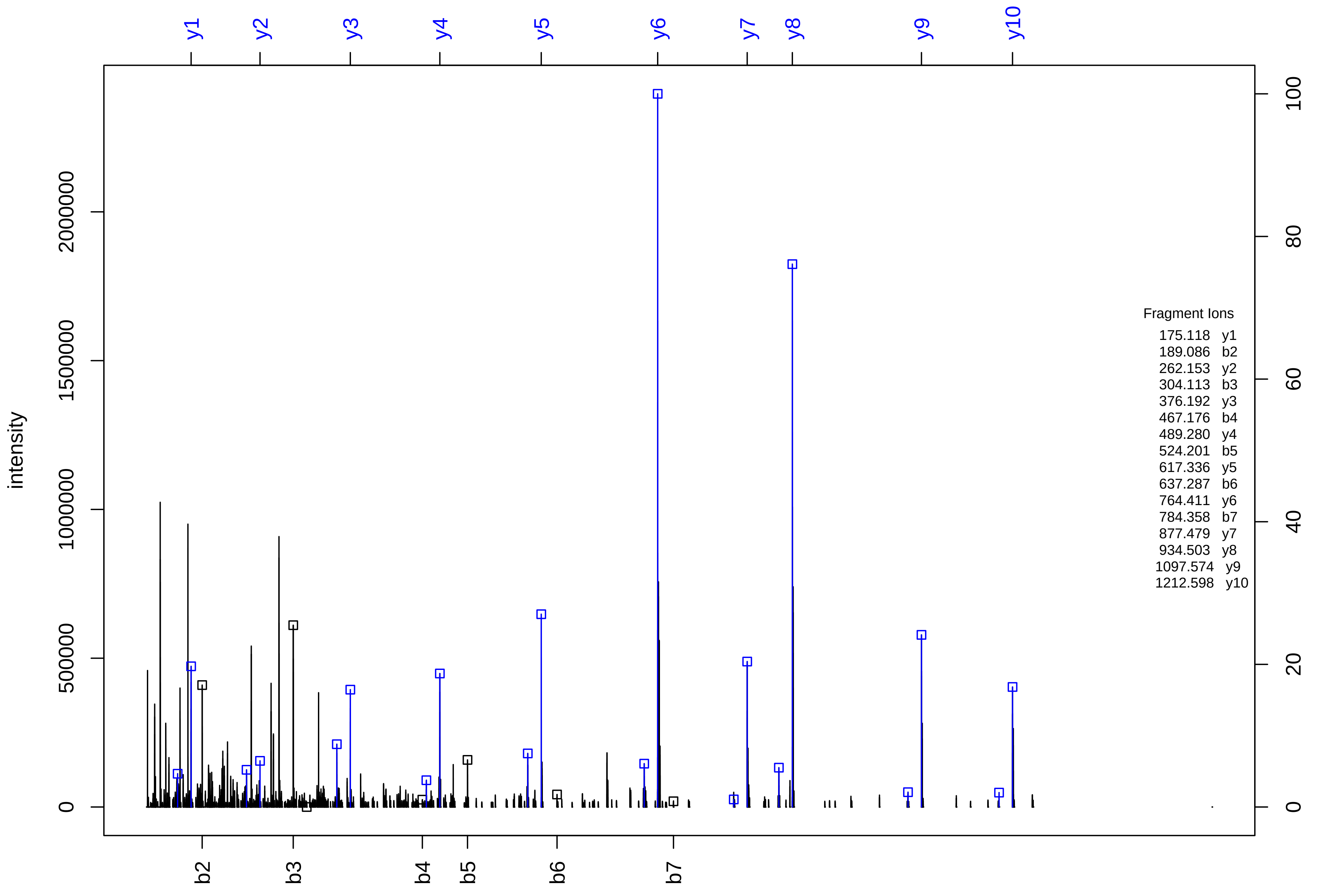

STDYGIFQINSR / File: sydney_ip_23.raw; SpectrumID: ; scans: 11368

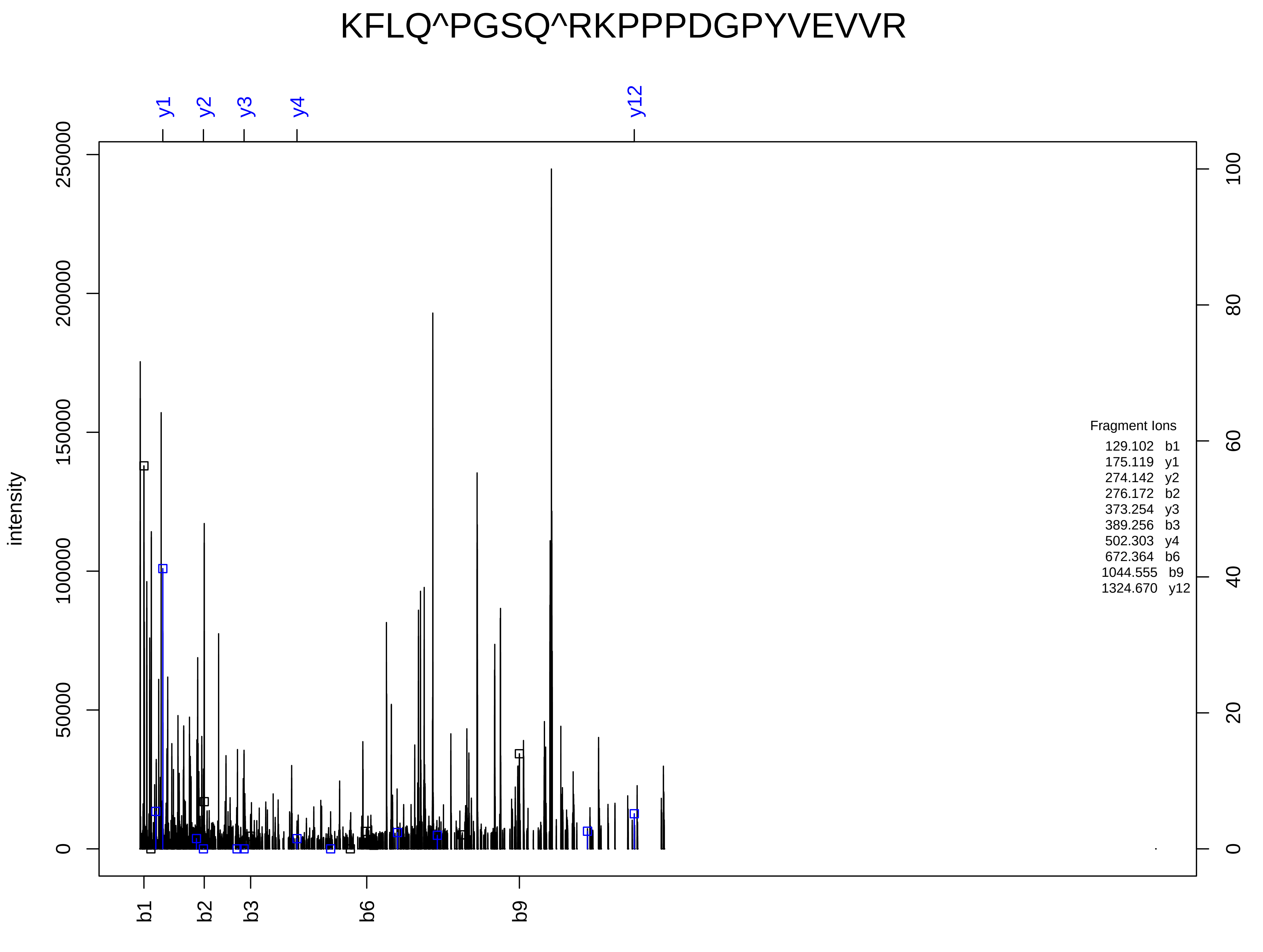

KFLQPGSQRKPPPDGPYVEVVR / File: sydney_ip_23.raw; SpectrumID: ; scans: 10771

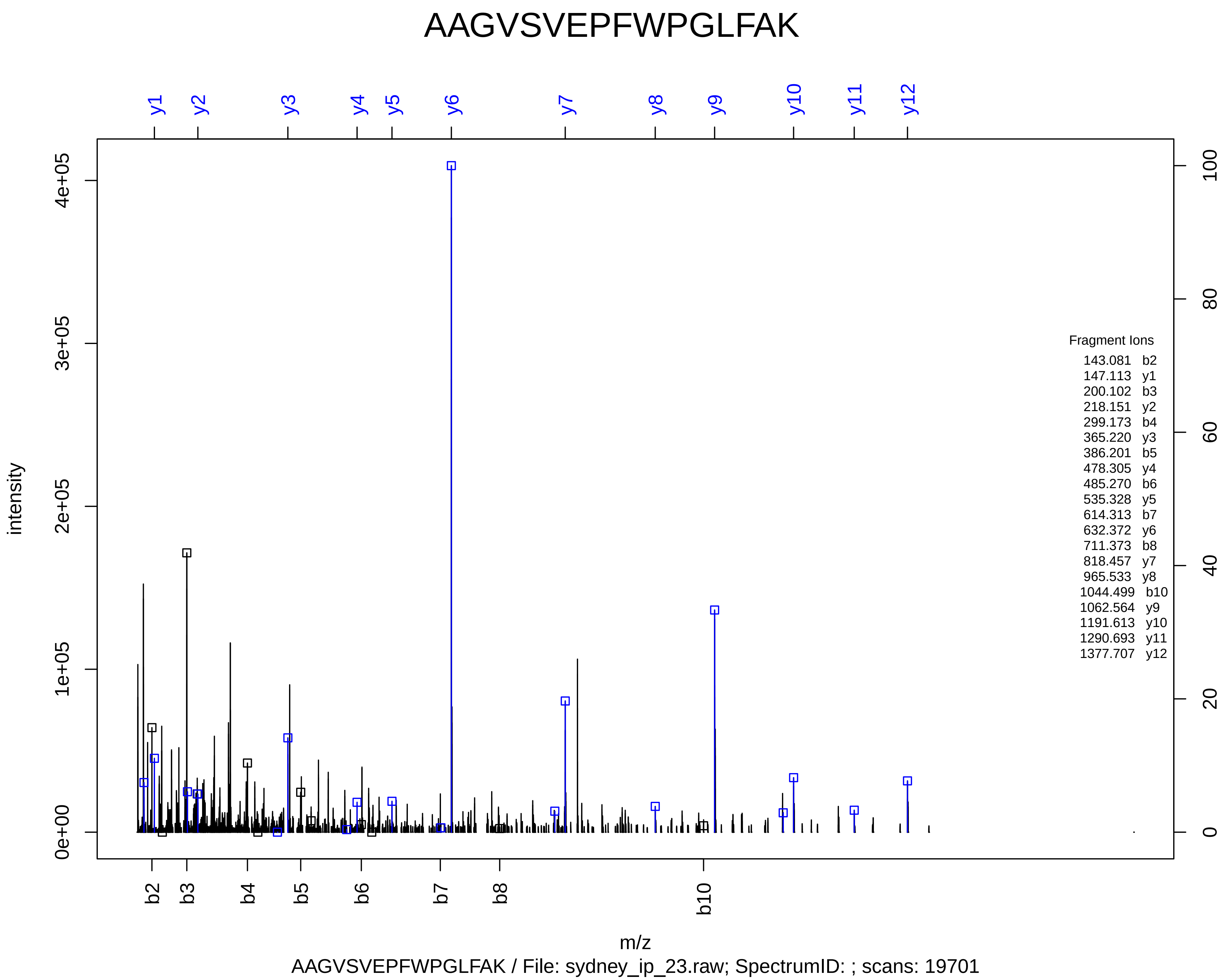
AAGVSVEPFWPGLFAK
y1 y2 y3 y4 y5 y6 y7 y8 y9 y10 y11 y12
intensity
0e+00
1e+05
2e+05
3e+05
4e+05
0
20
40
60
80
100
Fragment Ions
143.081 b2
147.113 y1
200.102 b3
218.151 y2
299.173 b4
365.220 y3
386.201 b5
478.305 y4
485.270 b6
535.328 y5
614.313 b7
632.372 y6
711.373 b8
818.457 y7
965.533 y8
1044.499 b10
1062.564 y9
1191.613 y10
1290.693 y11
1377.707 y12
b2 b3 b4 b5 b6 b7 b8 b10
m/z
AAGVSVEPFWPGLFAK / File: sydney_ip_23.raw; SpectrumID: ; scans: 19701

FDVQFPENNWINPDK

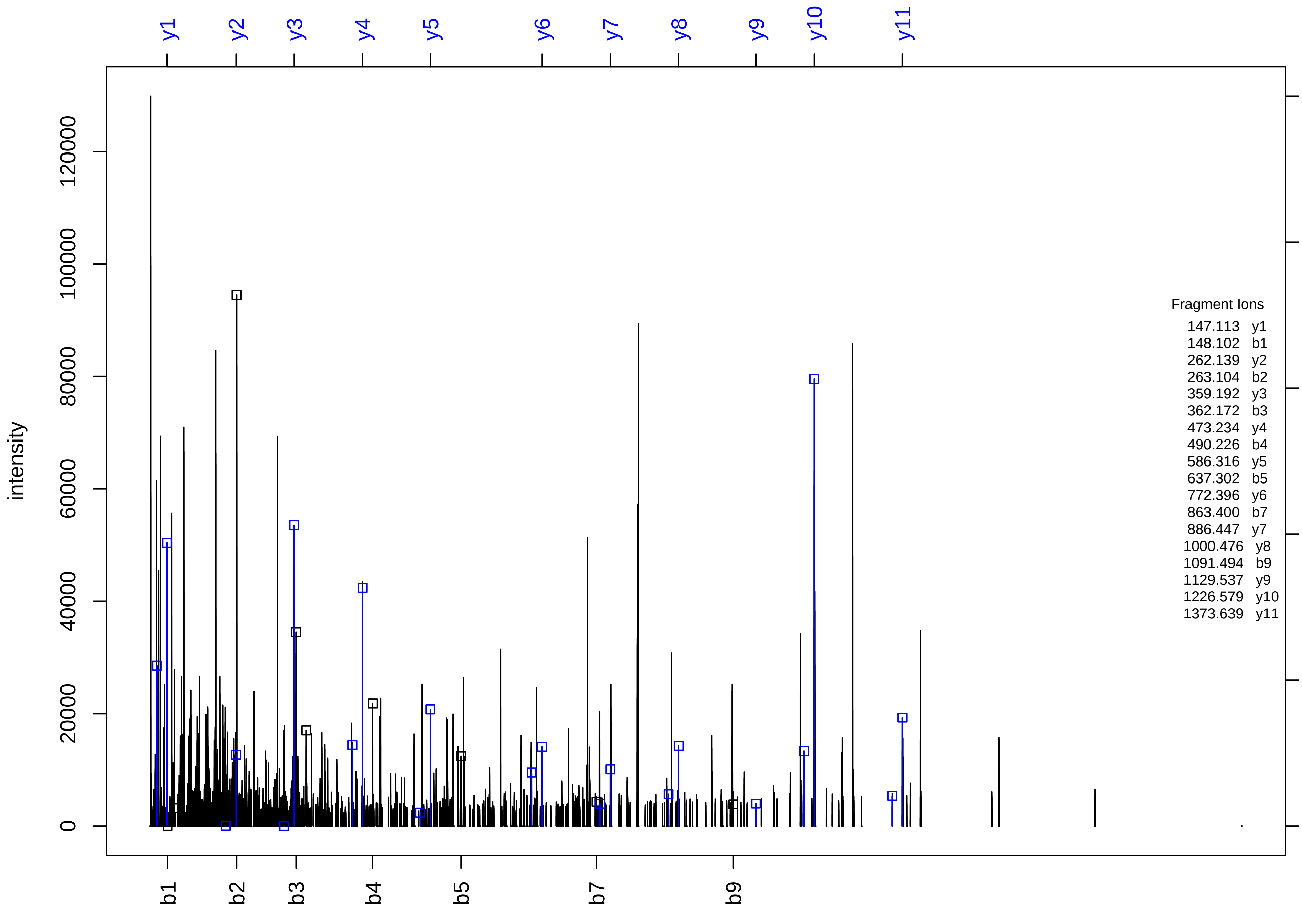

FDVQFPENNWINPDK / File: sydney_ip_23.raw; SpectrumID: ; scans: 15808

KVVCVDQ^Q^TPTIPN^R

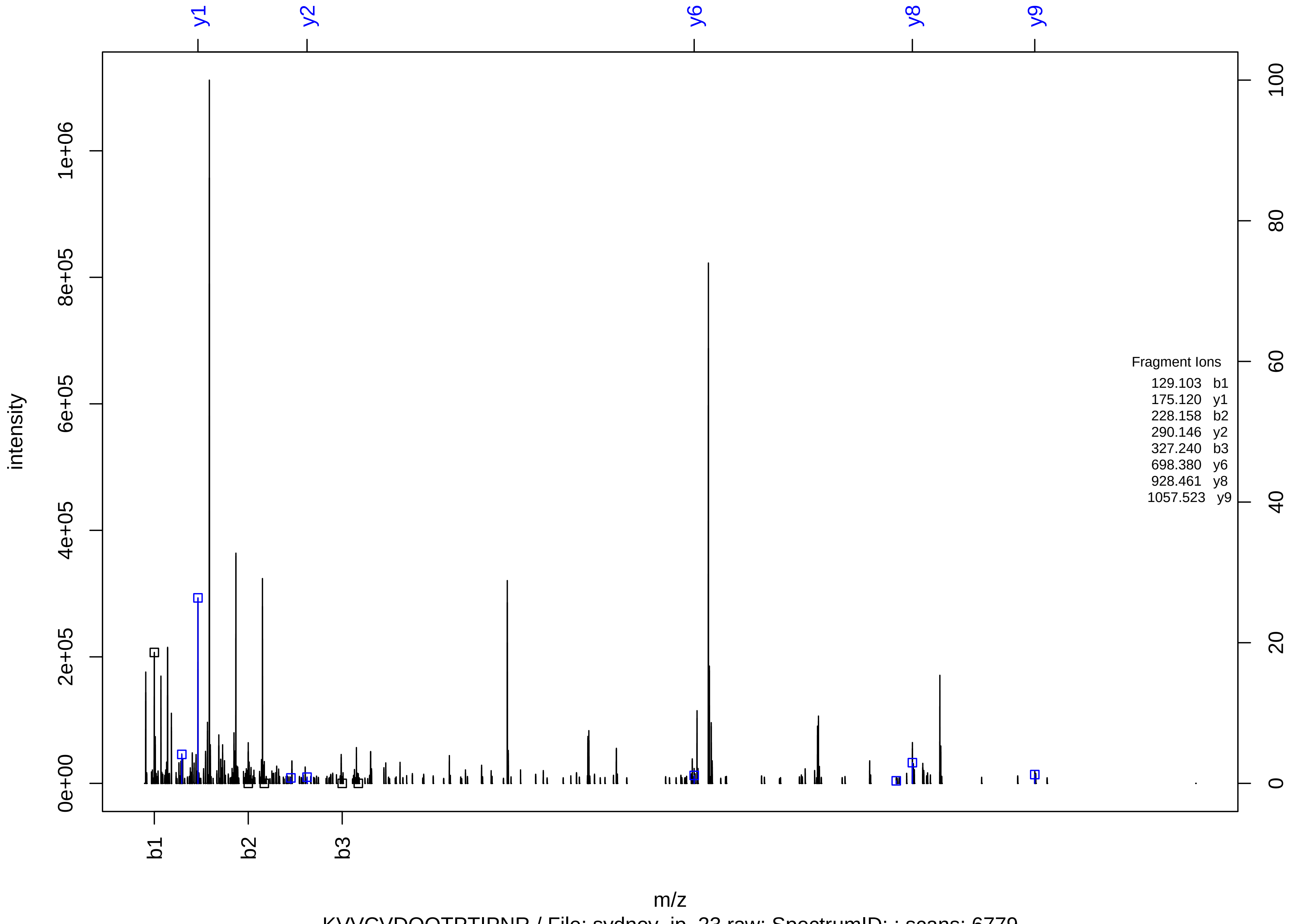

KVVCVDQQTPTIPNR / File: sydney_ip_23.raw; SpectrumID: ; scans: 6779

VLTEHVADAELEPR

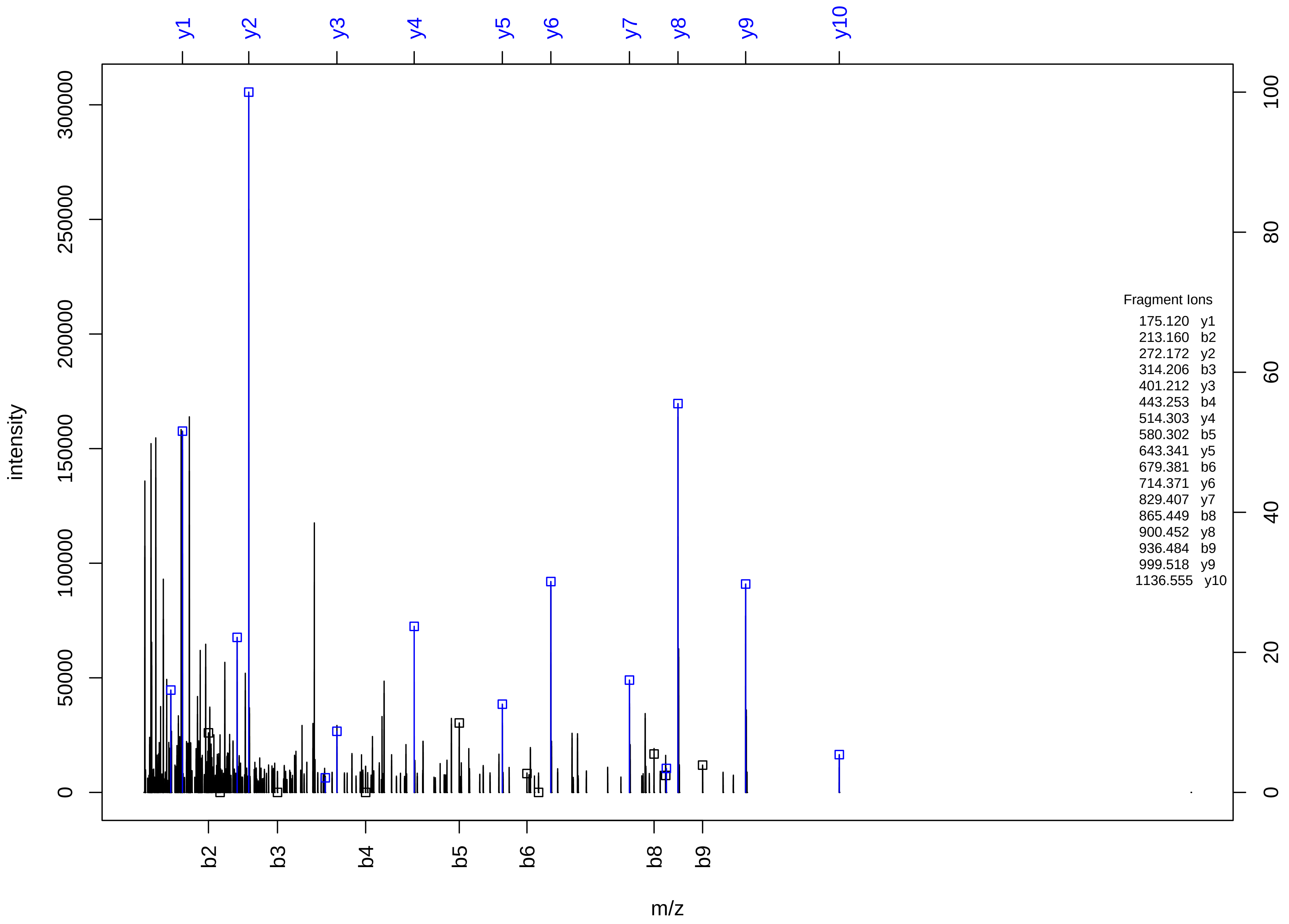

VLTEHVADAELEPR / File: sydney_ip_23.raw; SpectrumID: ; scans: 6266

ELLAFLSEPEILAK

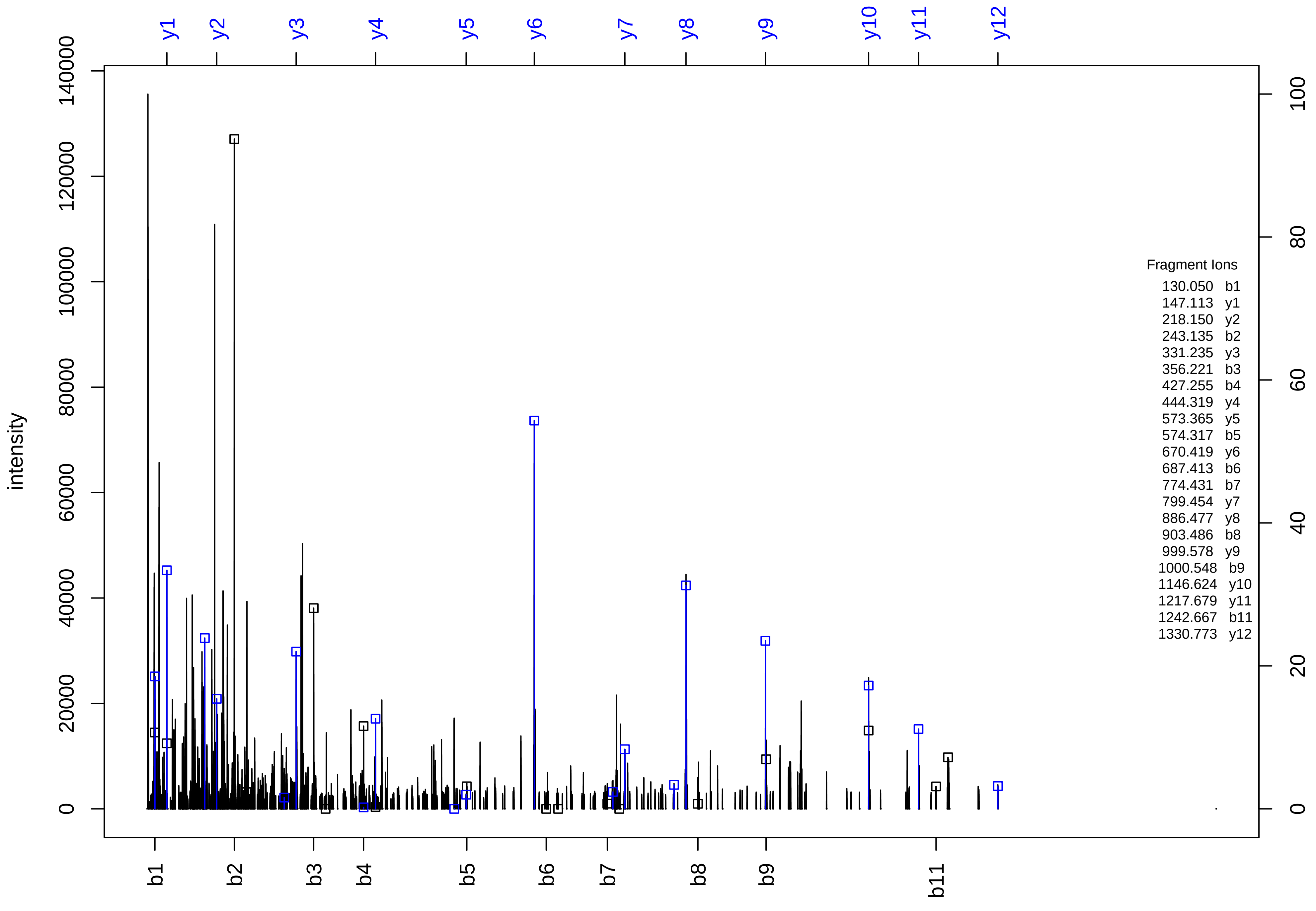

ELLAFLSEPEILAK / File: sydney_ip_23.raw; SpectrumID: ; scans: 19938

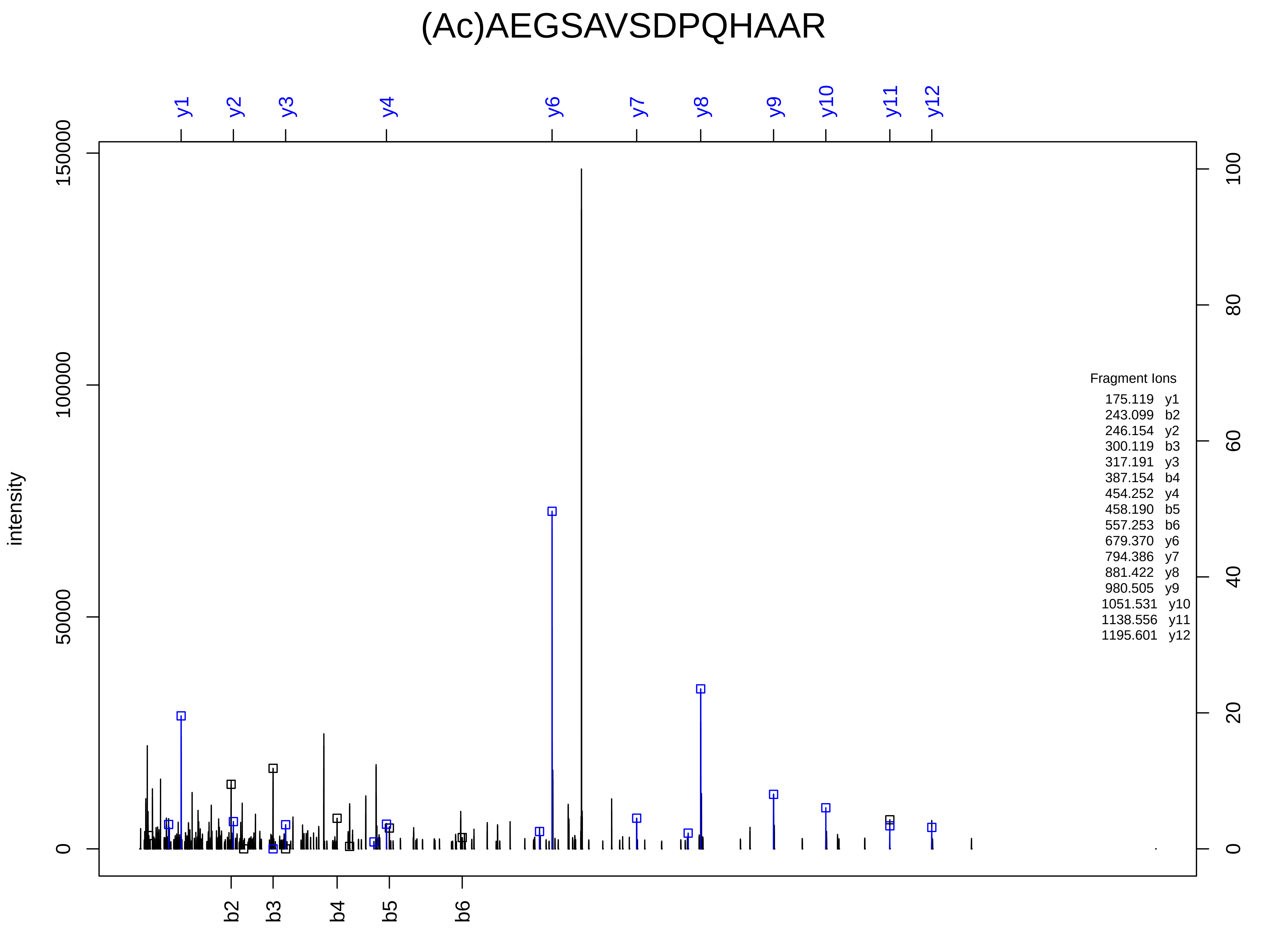

AEGSAVSDPQHAAR / File: sydney_ip_23.raw; SpectrumID: ; scans: 2383

GLSSLLYGSIPK

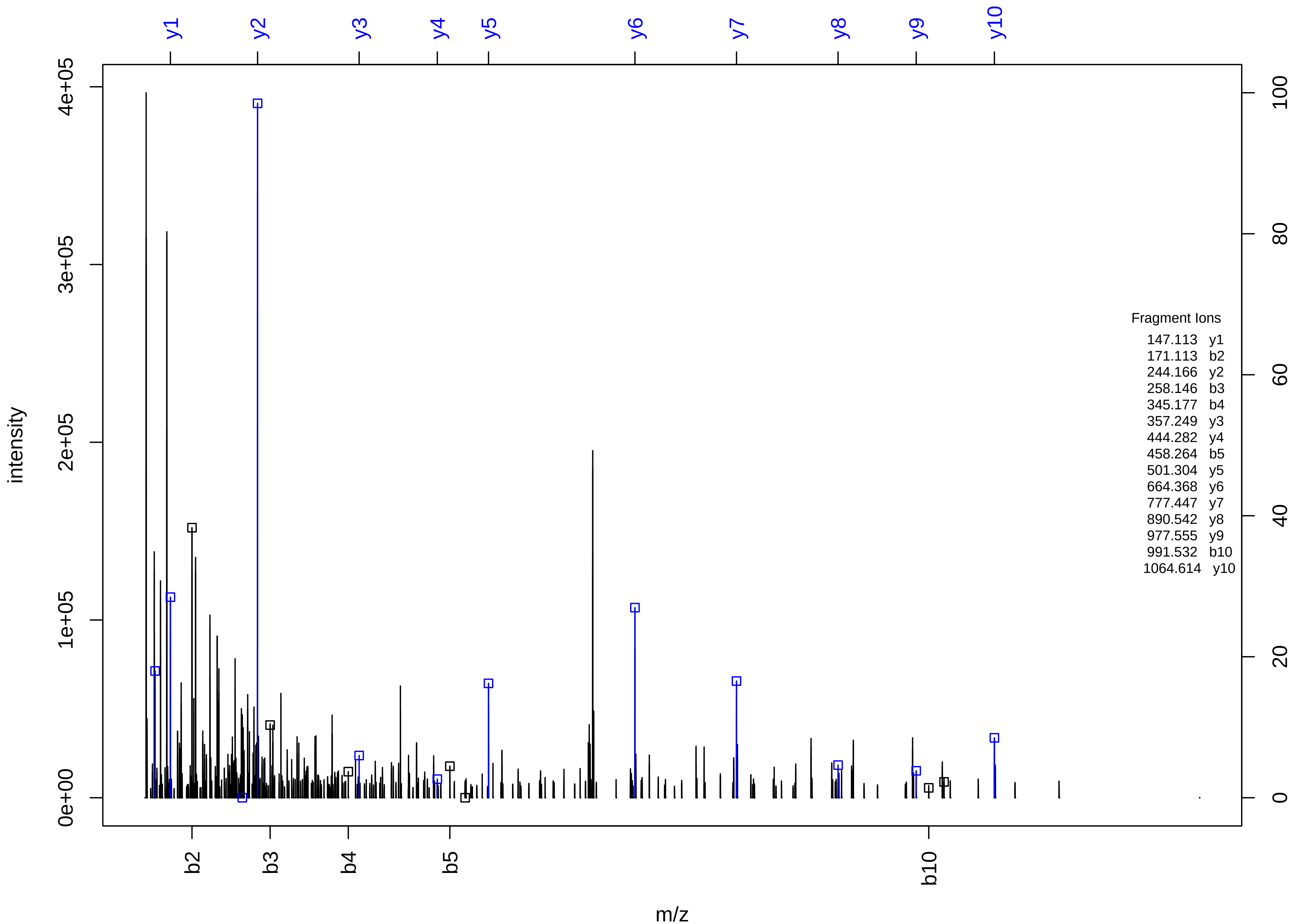

GLSSLLYGSIPK / File: sydney_ip_23.raw; SpectrumID: ; scans: 14520

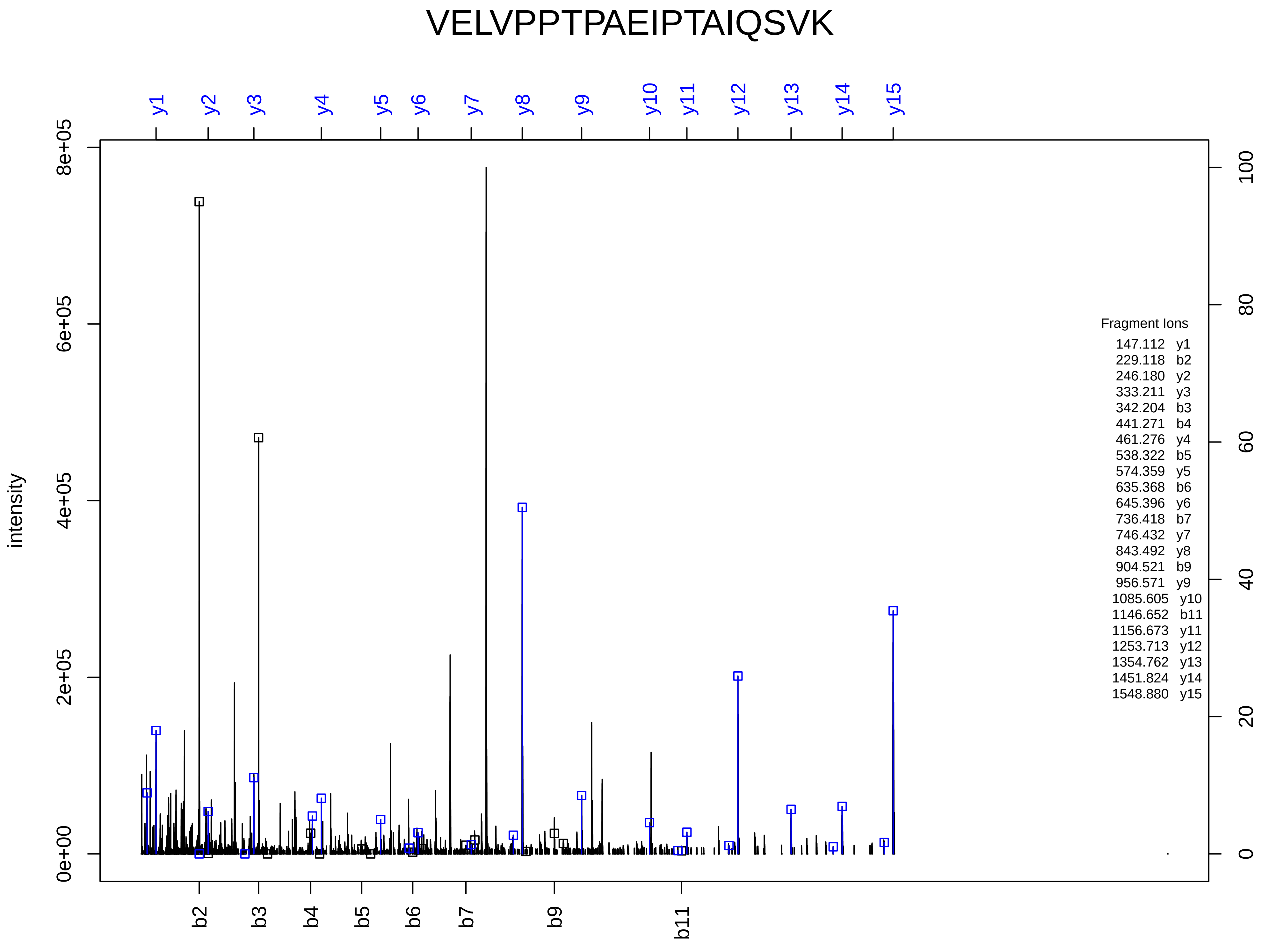

VELVPPTPAEIPTAIQSVK / File: sydney_ip_23.raw; SpectrumID: ; scans: 16663

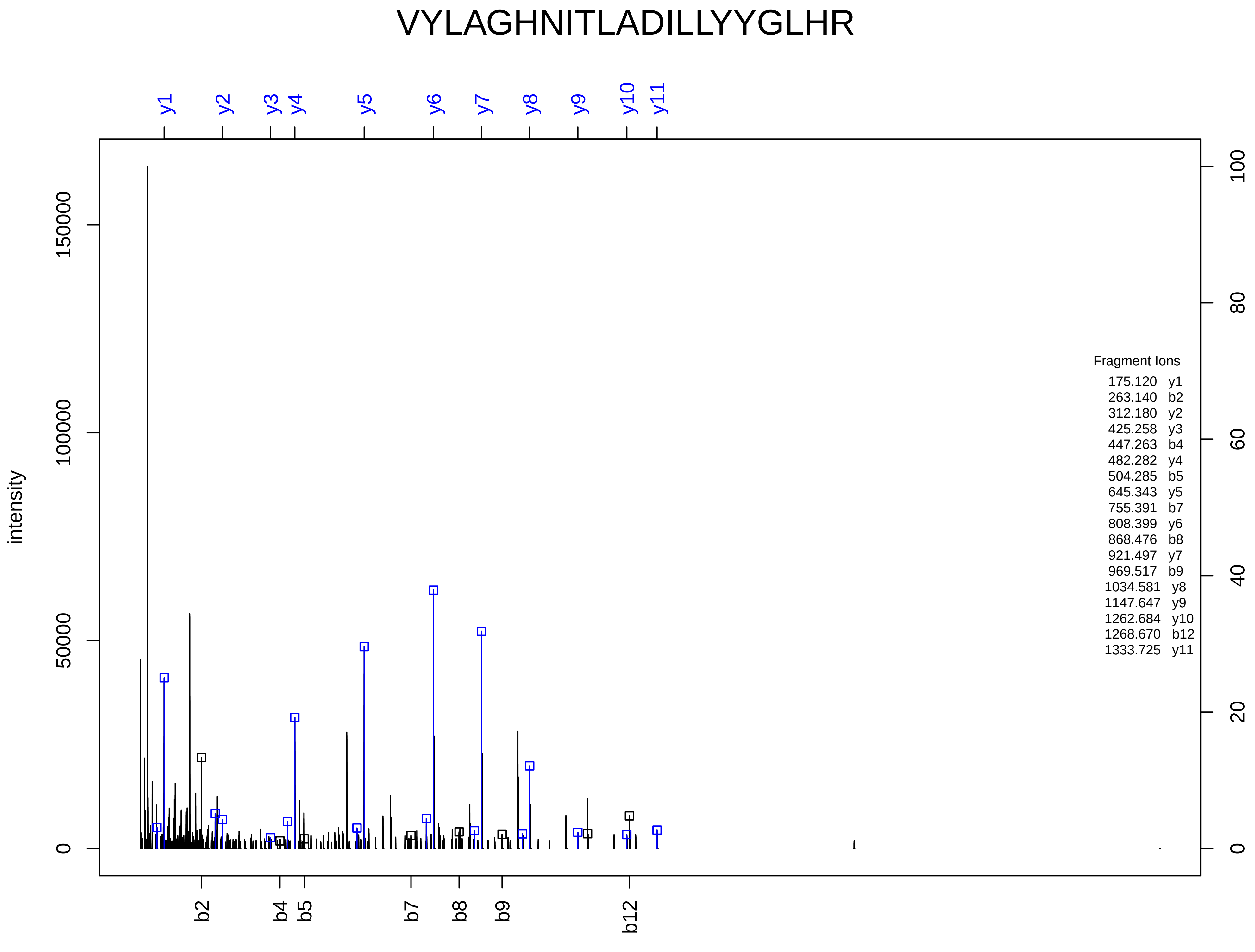

VYLAGHNITLADILLYYGLHR / File: sydney_ip_23.raw; SpectrumID: ; scans: 20125

IGGEEGGRPQEADDPAAGSQAR

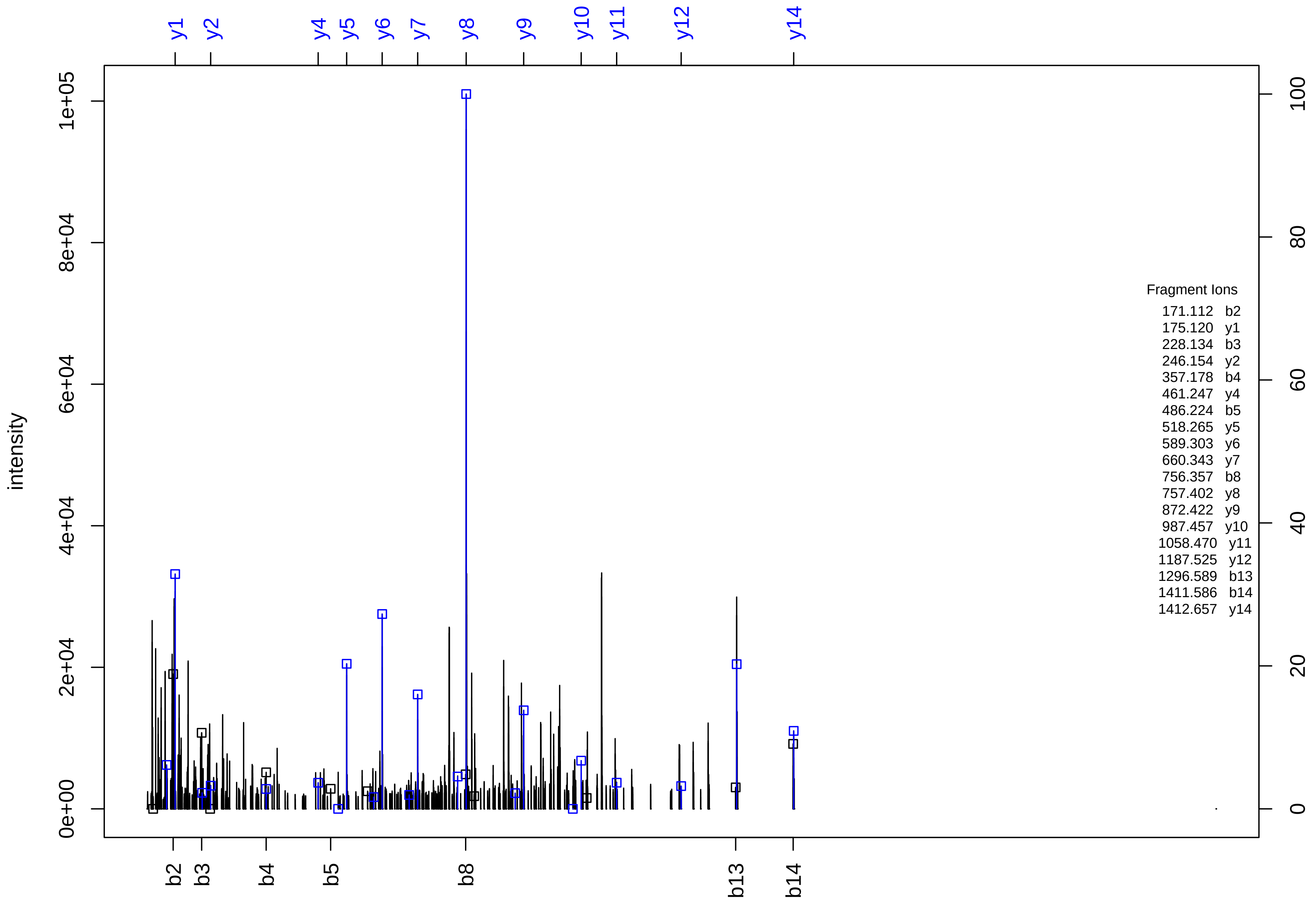

IGGEEGGRPQEADDPAAGSQAR / File: sydney_ip_23.raw; SpectrumID: ; scans: 2173

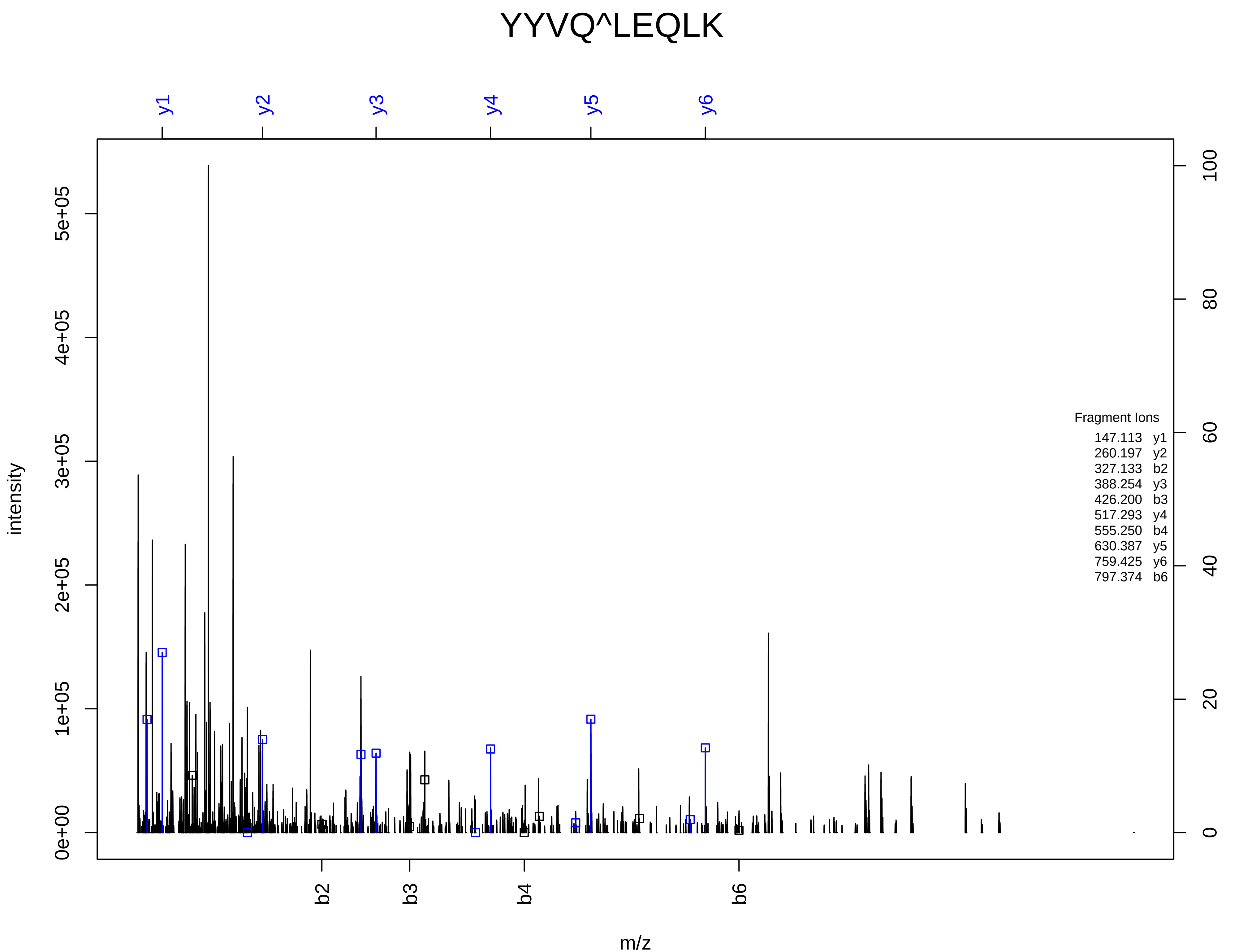
YYVQ^LEQLK
y1
y2
y3
y4
y5
y6
intensity
0e+00
1e+05
2e+05
3e+05
4e+05
5e+05
0
20
40
60
80
100
Fragment Ions
147.113 y1
260.197 y2
327.133 b2
388.254 y3
426.200 b3
517.293 y4
555.250 b4
630.387 y5
759.425 y6
797.374 b6
b2
b3
b4
b6
m/z
YYVQLEQLK / File: sydney_ip_23.raw; SpectrumID: ; scans: 14876

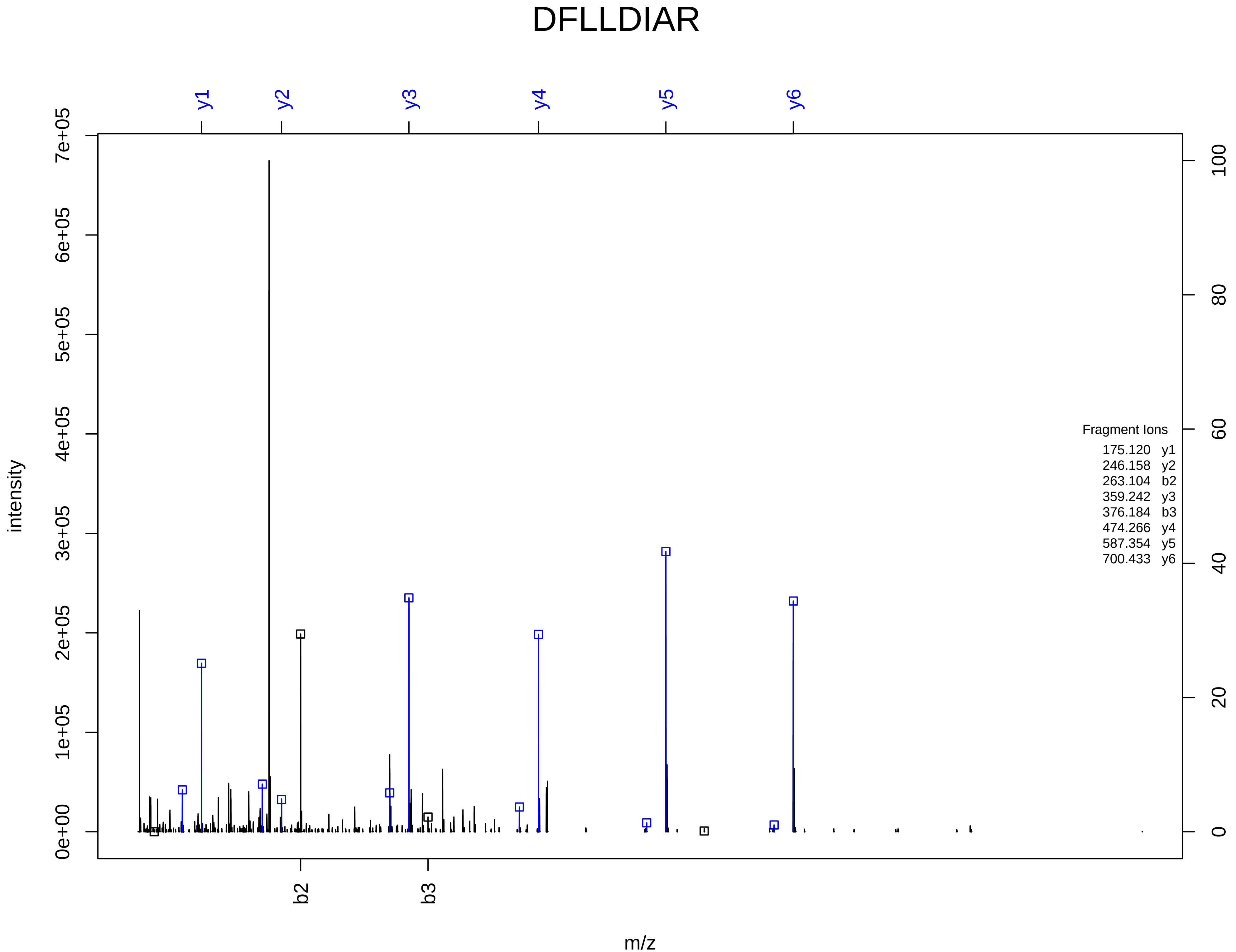
DFLLDIAR
y1
y2
y3
y4
y5
y6
b2
b3
intensity
m/z
Fragment Ions
175.120 y1
246.158 y2
263.104 b2
359.242 y3
376.184 b3
474.266 y4
587.354 y5
700.433 y6
0e+00
1e+05
2e+05
3e+05
4e+05
5e+05
6e+05
7e+05
0
20
40
60
80
100
DFLLDIAR / File: sydney_ip_24.raw; SpectrumID: ; scans: 15645

SLLDILTDPSK

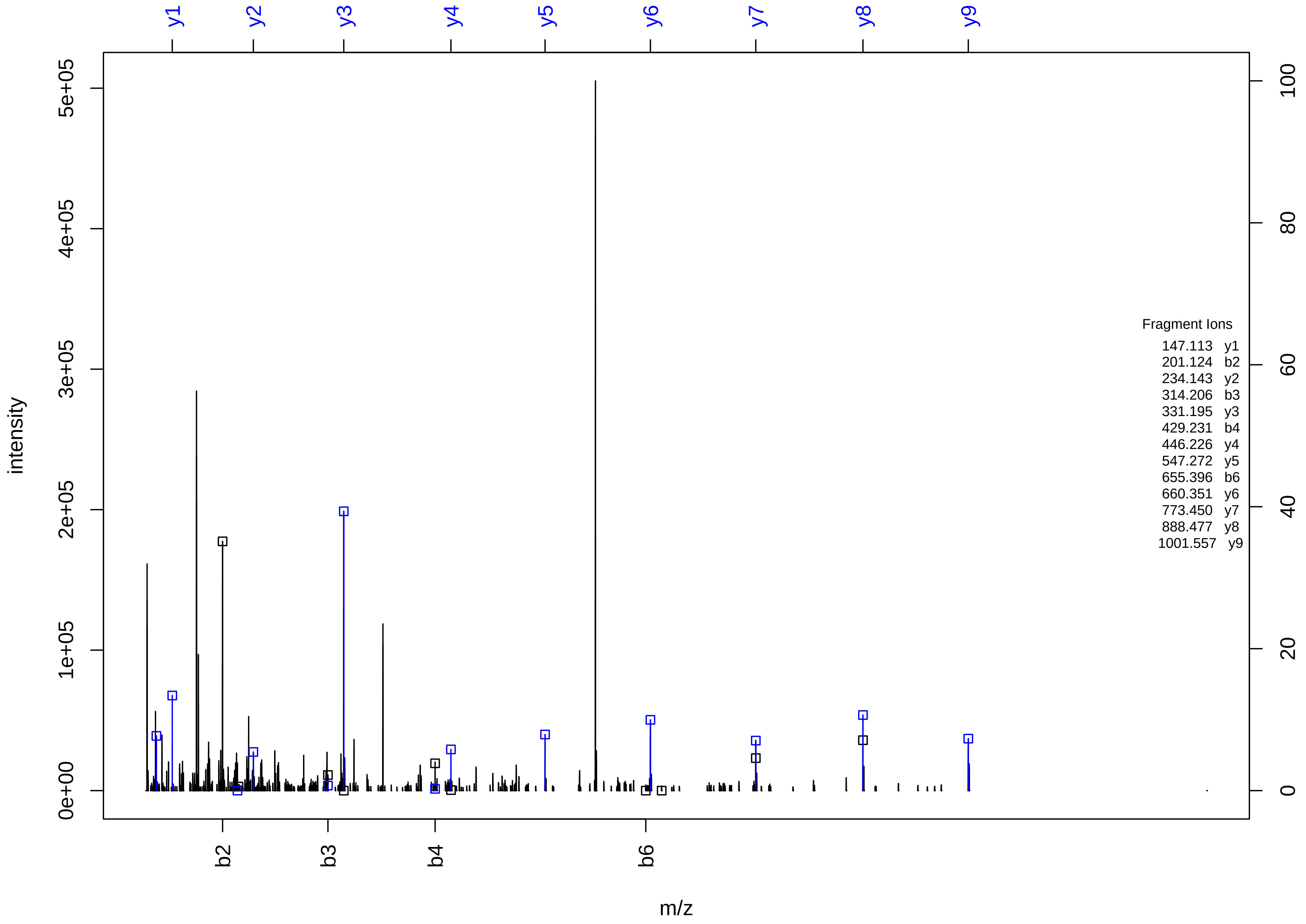

SLLDILTDPSK / File: sydney_ip_24.raw; SpectrumID: ; scans: 17338

LHLSVFDFDR

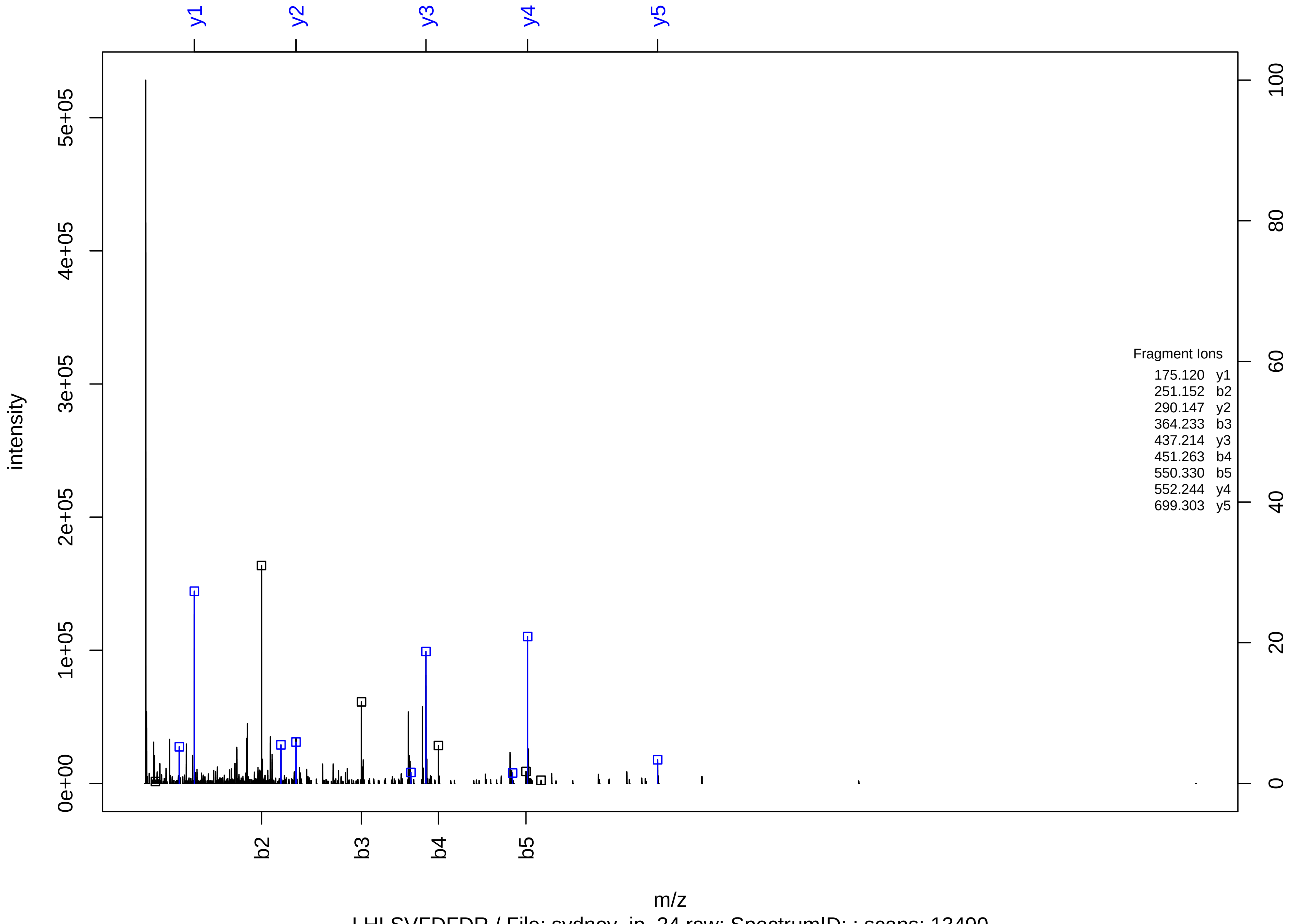

LHLSVFDFDR / File: sydney_ip_24.raw; SpectrumID: ; scans: 13490

ISQ^EIGN^LM*K

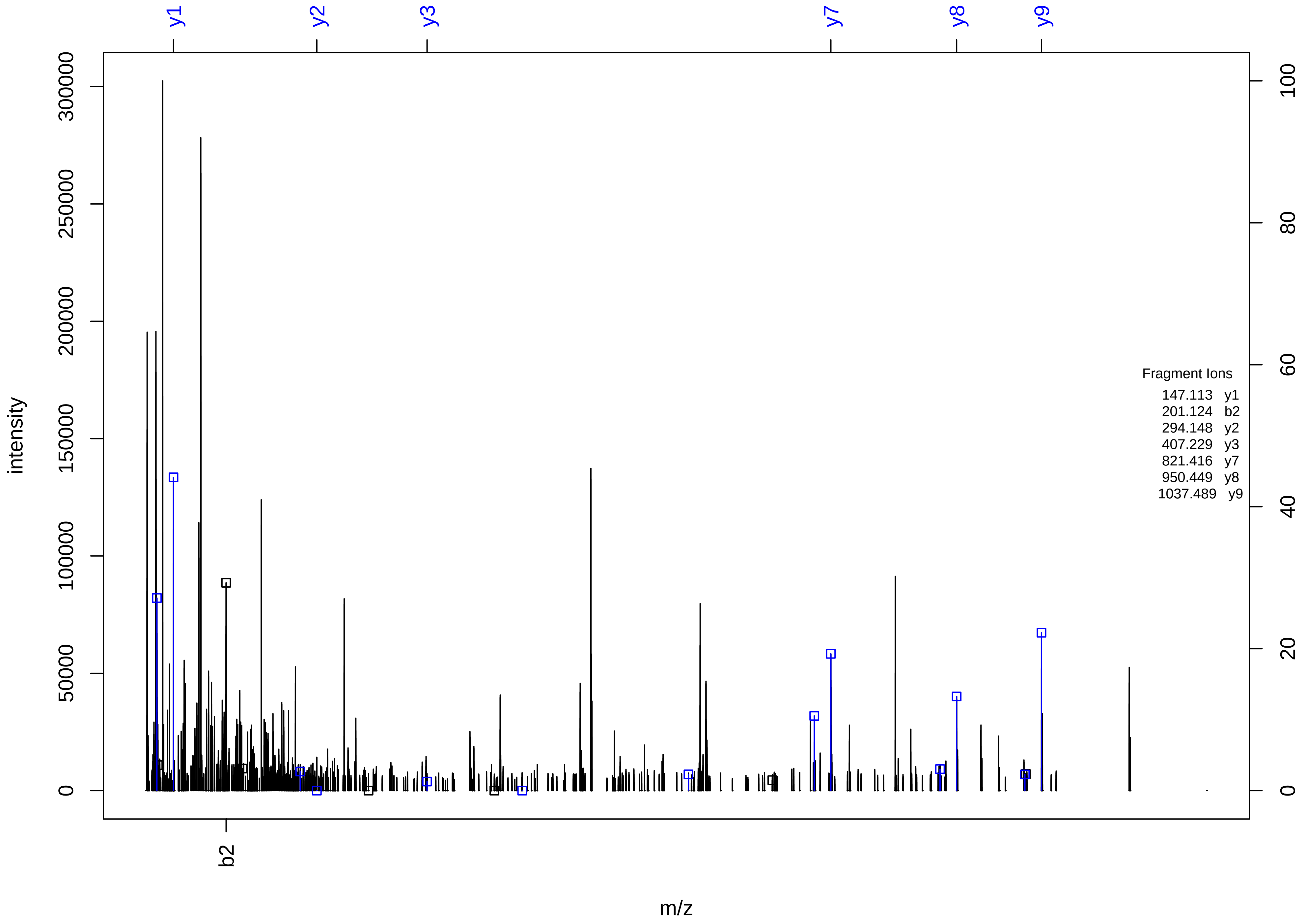

ISQEIGNLMK / File: sydney_ip_24.raw; SpectrumID: ; scans: 7822

EGDVLTPEQAR

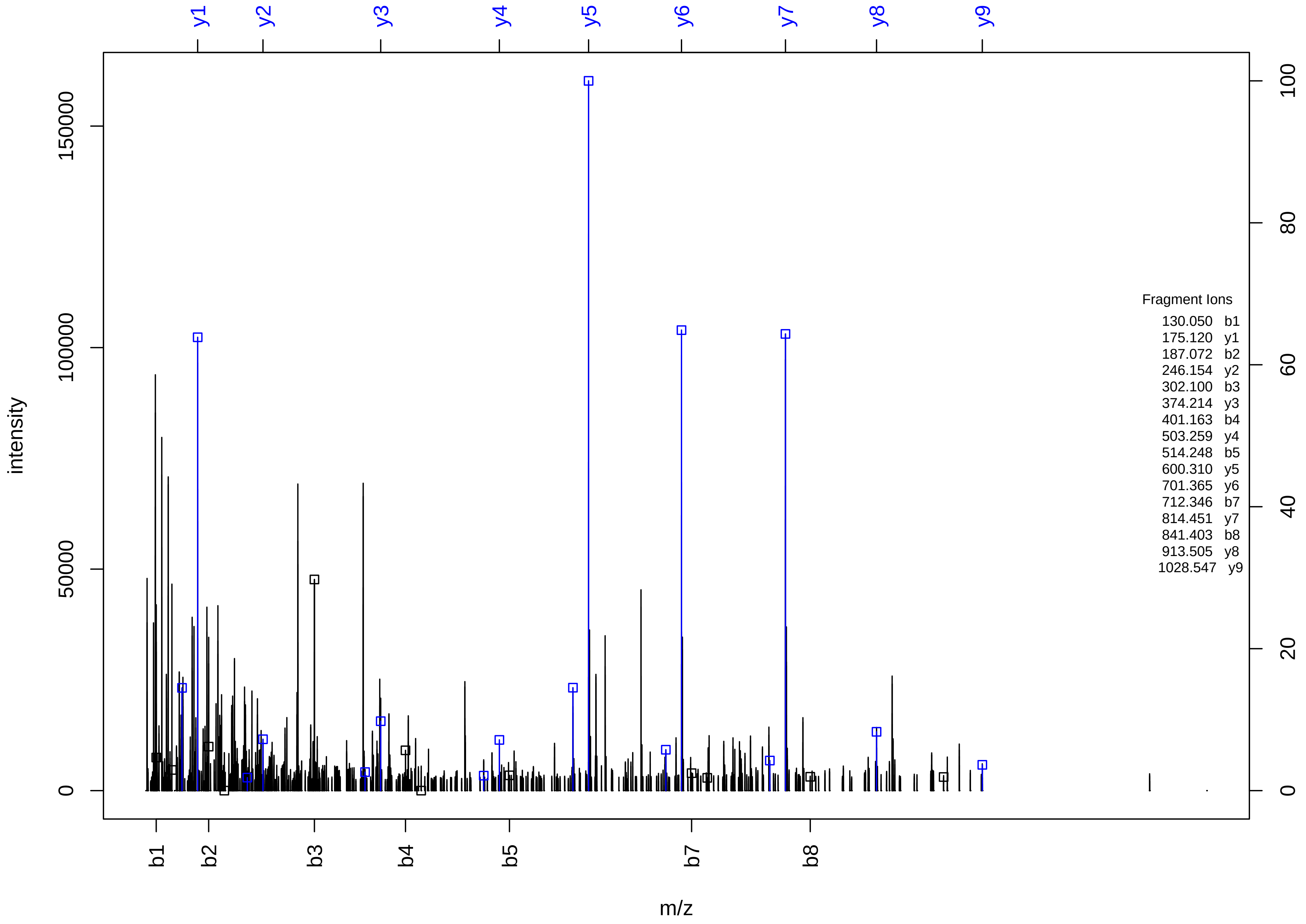

EGDVLTPEQAR / File: sydney_ip_24.raw; SpectrumID: ; scans: 3802

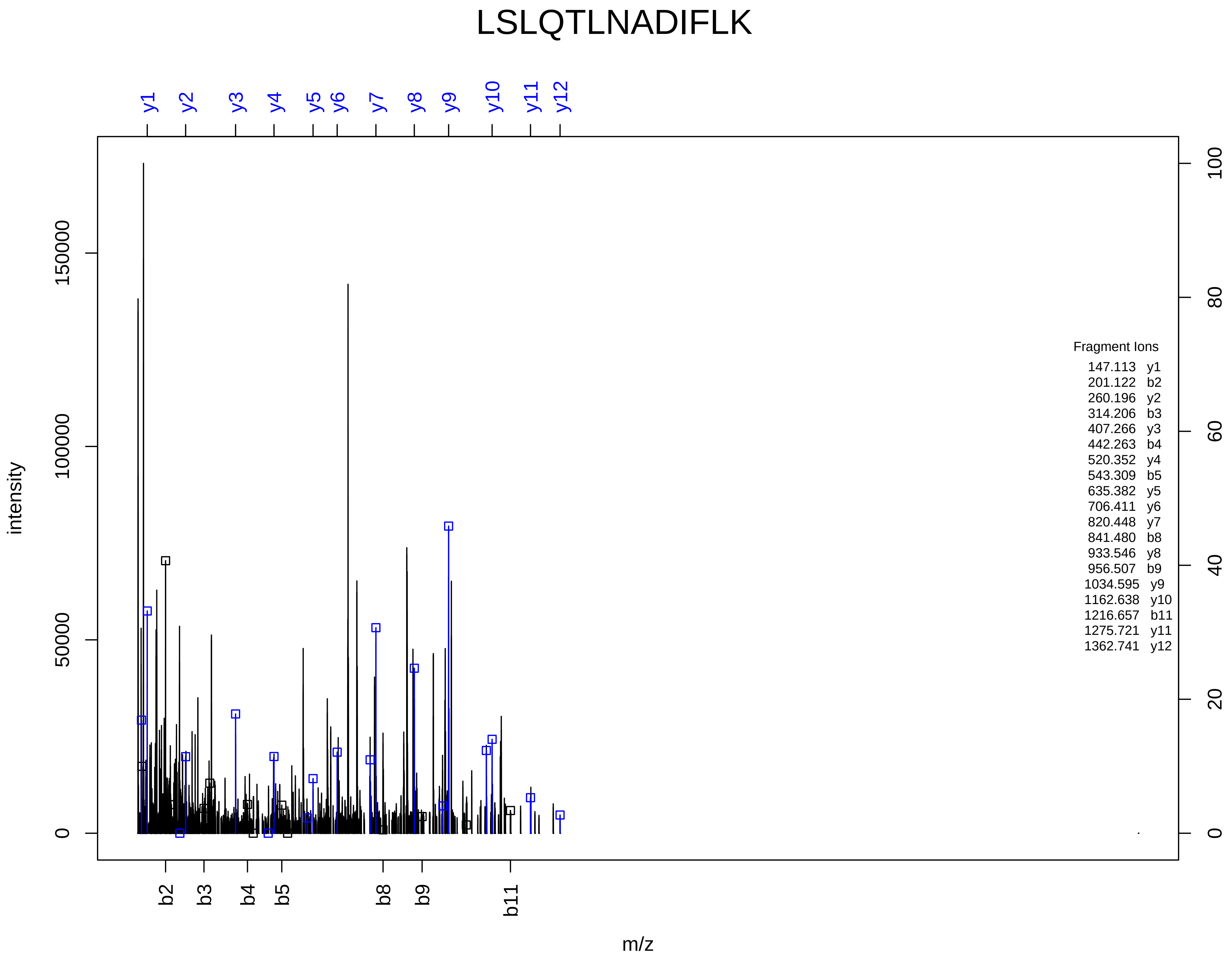

LSLQTLNADIFLK / File: sydney_ip_24.raw; SpectrumID: ; scans: 18201

FLIPNLAFIDK

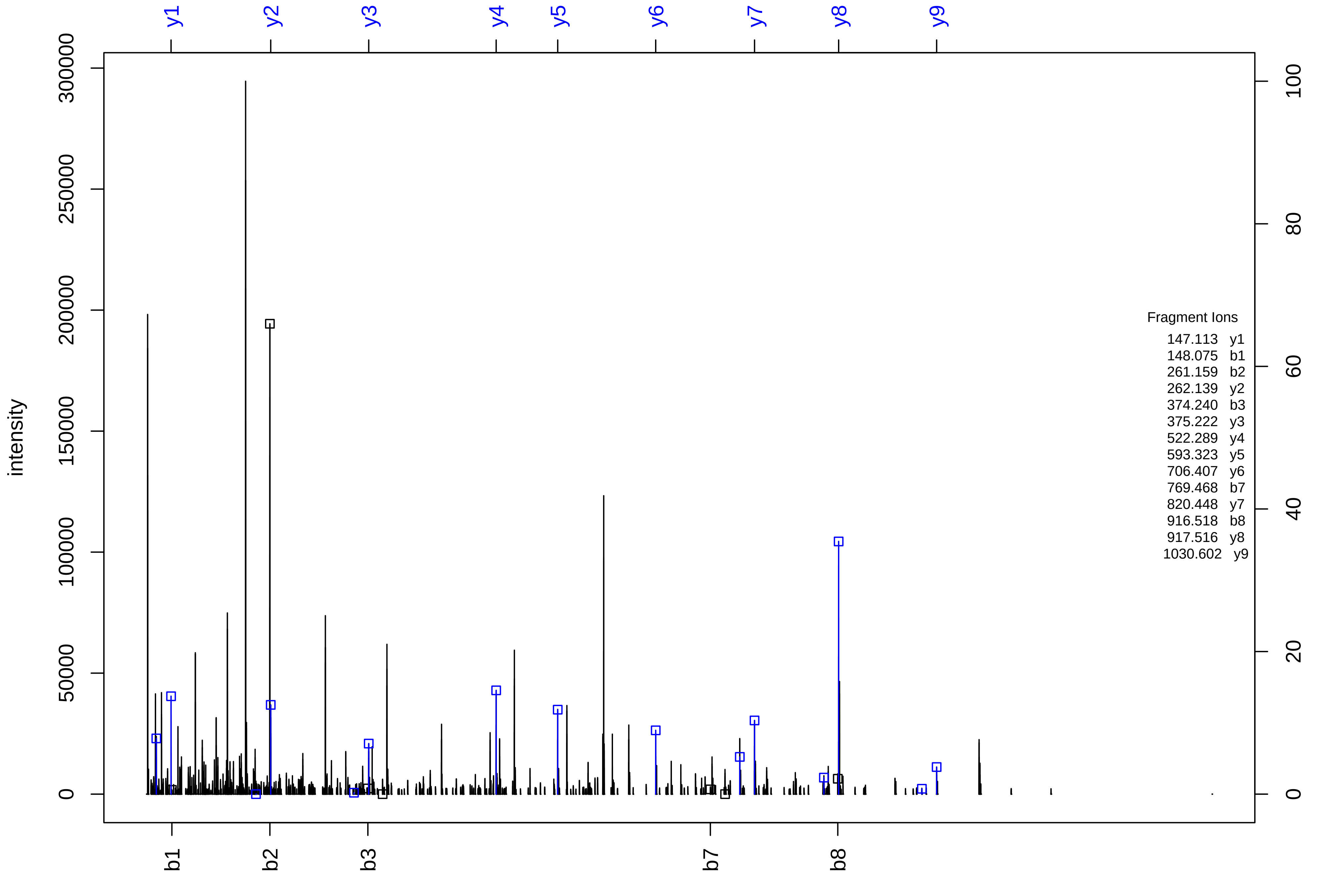

FLIPNLAFIDK / File: sydney_ip_24.raw; SpectrumID: ; scans: 19602

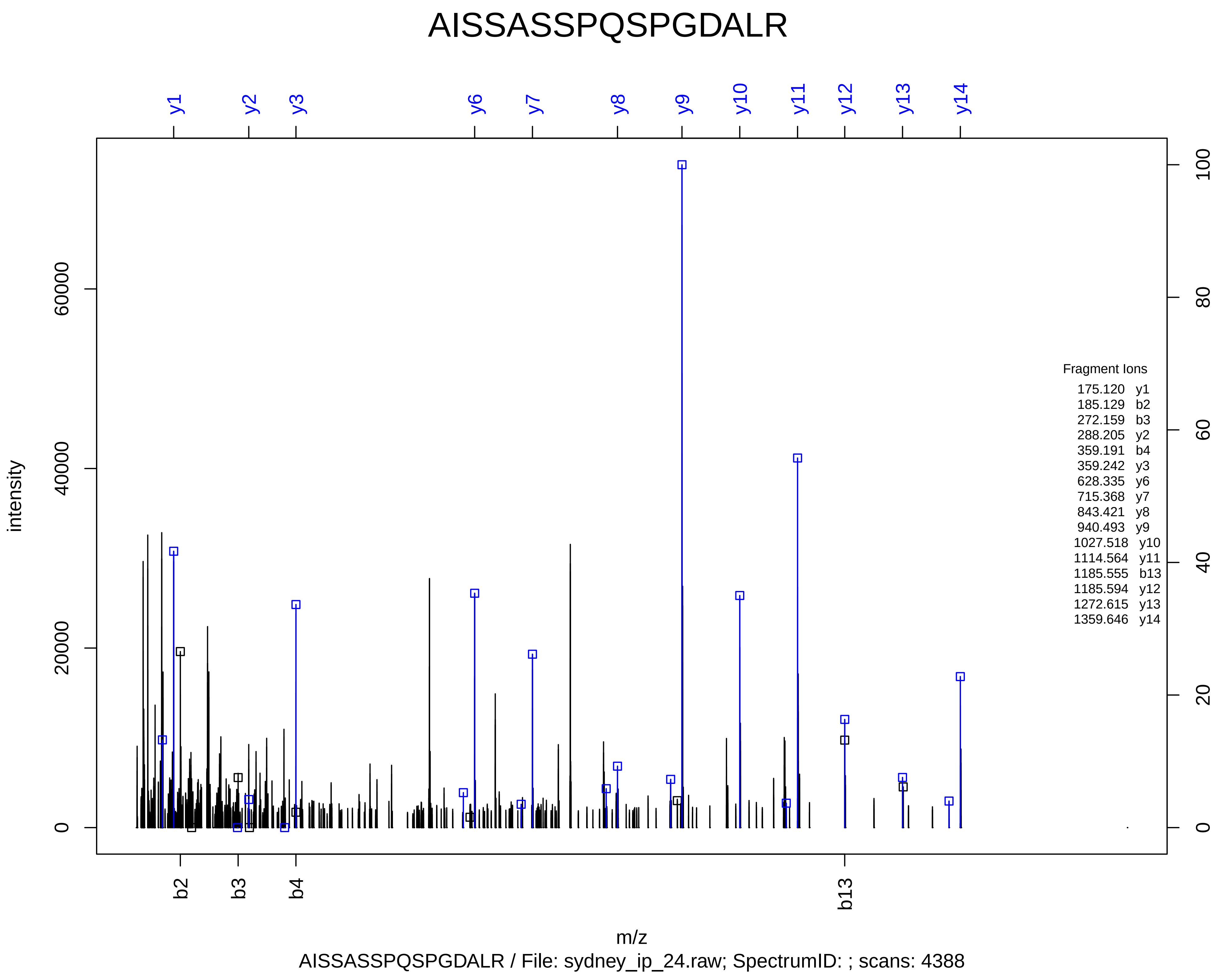
AISSASSPQSPGDALR
y1
y2
y3
y6
y7
y8
y9
y10
y11
y12
y13
y14
intensity
0
20000
40000
60000
0
20
40
60
80
100
Fragment Ions
175.120 y1
185.129 b2
272.159 b3
288.205 y2
359.191 b4
359.242 y3
628.335 y6
715.368 y7
843.421 y8
940.493 y9
1027.518 y10
1114.564 y11
1185.555 b13
1185.594 y12
1272.615 y13
1359.646 y14
b2
b3
b4
b13
m/z
AISSASSPQSPGDALR / File: sydney_ip_24.raw; SpectrumID: ; scans: 4388

AFGDVFSVIGVR

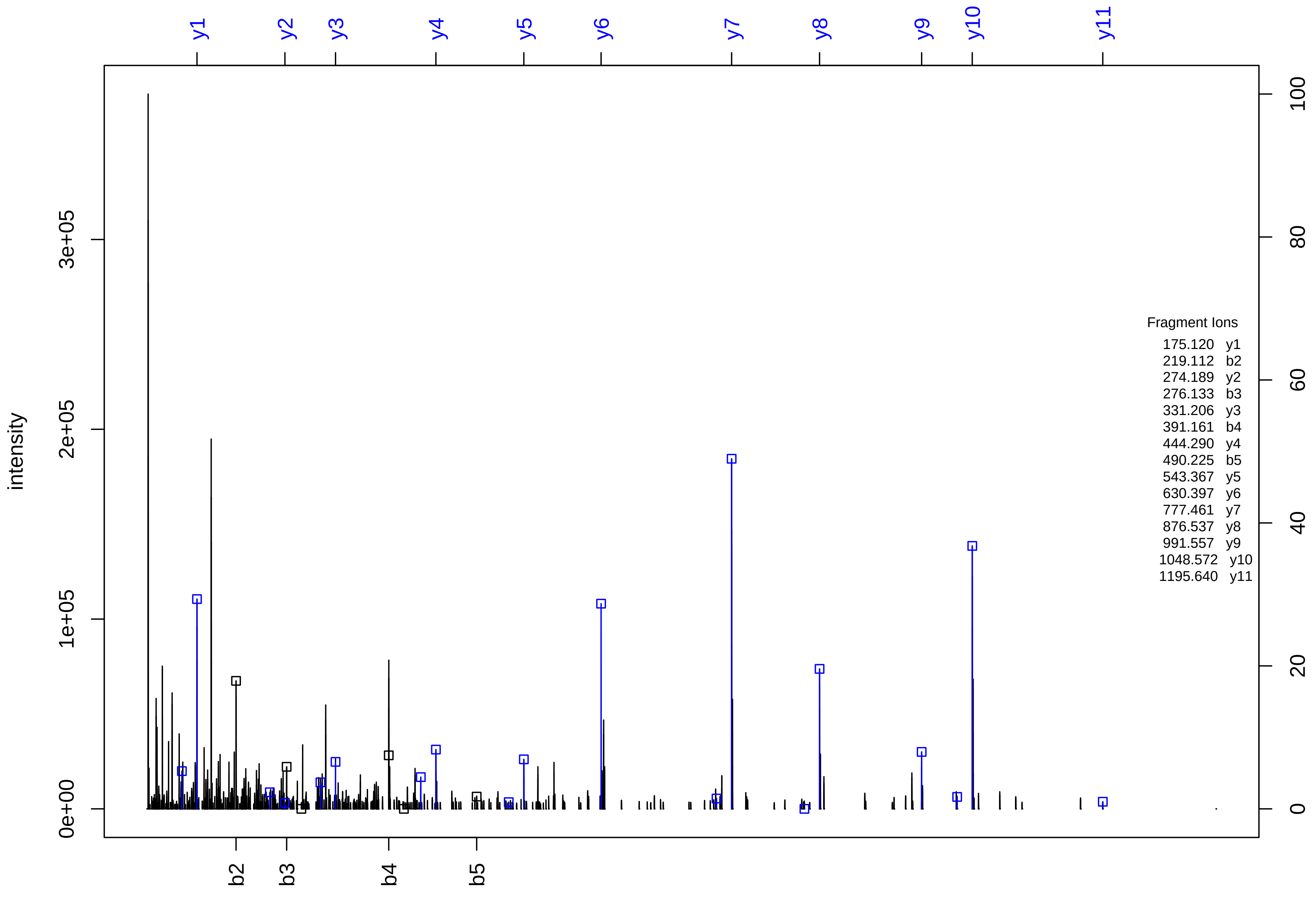

AFGDVFSVIGVR / File: sydney_ip_24.raw; SpectrumID: ; scans: 18044

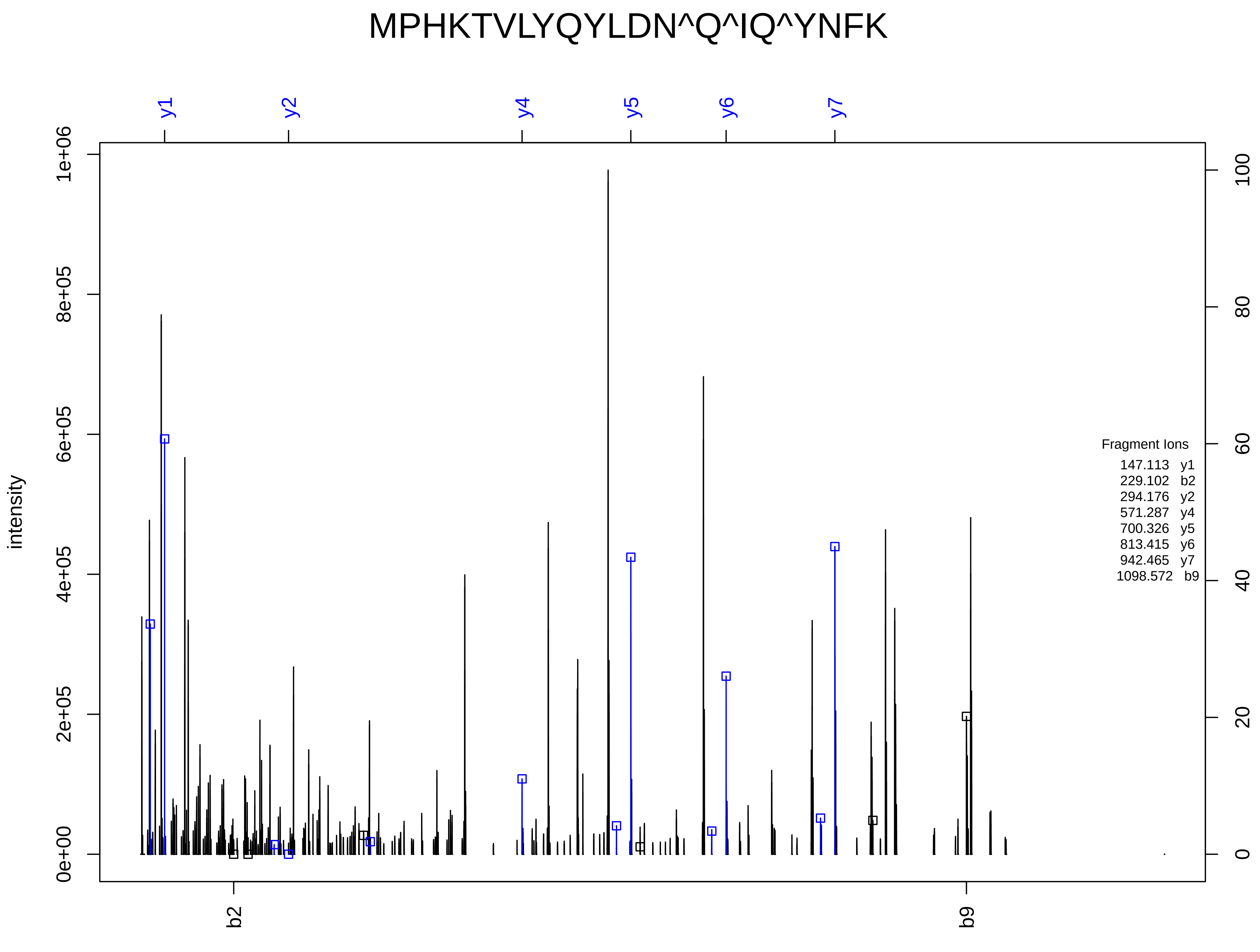

MPHKTVLYQYLDN^Q^IQ^YNFK
y1
y2
y4
y5
y6
y7
intensity
0e+00
2e+05
4e+05
6e+05
8e+05
1e+06
0
20
40
60
80
100
b2
b9
m/z
Fragment Ions
147.113 y1
229.102 b2
294.176 y2
571.287 y4
700.326 y5
813.415 y6
942.465 y7
1098.572 b9
MPHKTVLYQYLDNQIQYNFK / File: sydney_ip_24.raw; SpectrumID: ; scans: 9098

IQ^ELEGQ^LEK

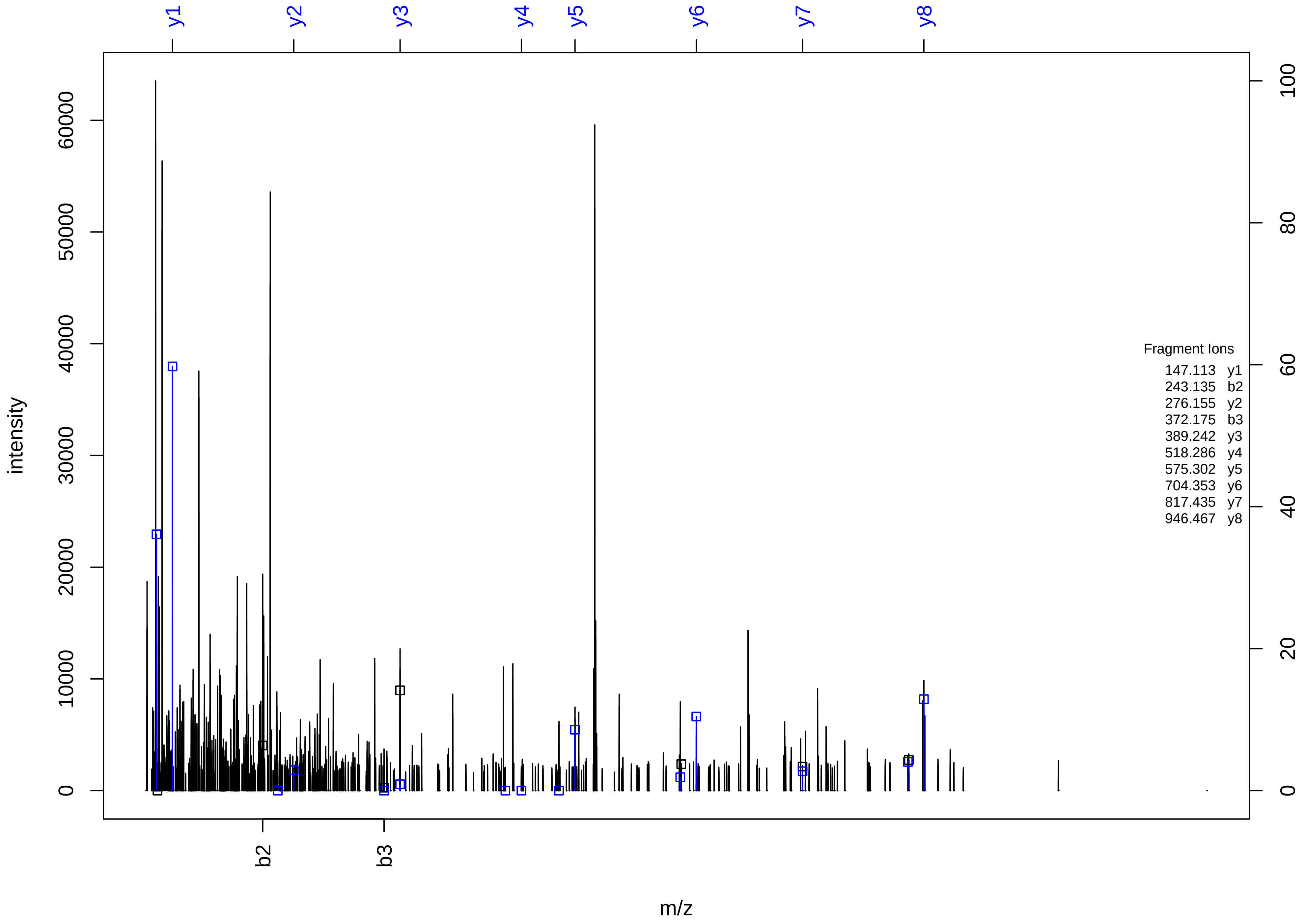

IQELEGQLEK / File: sydney_ip_24.raw; SpectrumID: ; scans: 2726

LLDM*AAQ^IAEGMAFIEEQ^NYIHR

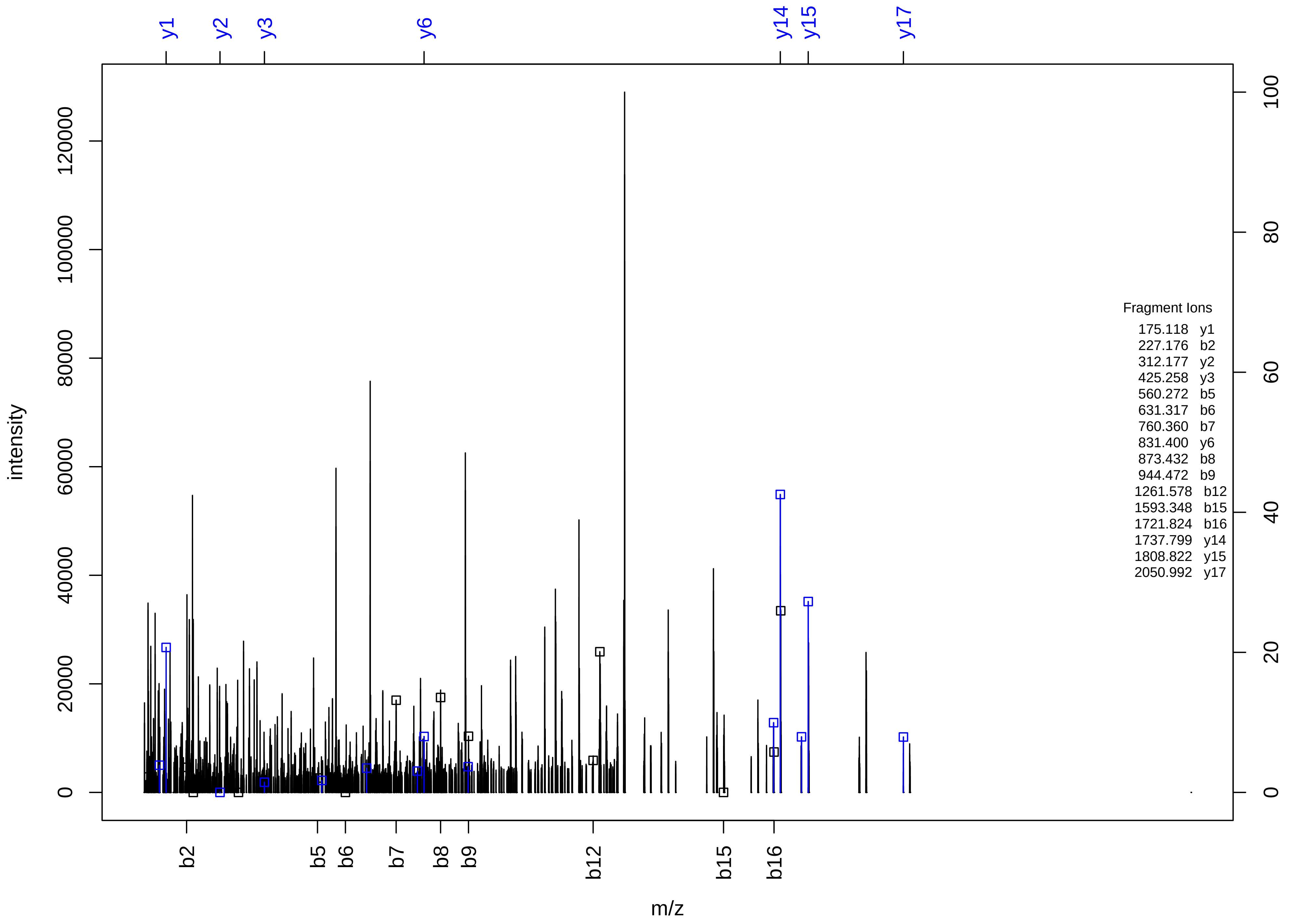

LLDMAAQIAEGMAFIEEQNYIHR / File: sydney_ip_24.raw; SpectrumID: ; scans: 13886

VLGYVLQ^SGLN^QK

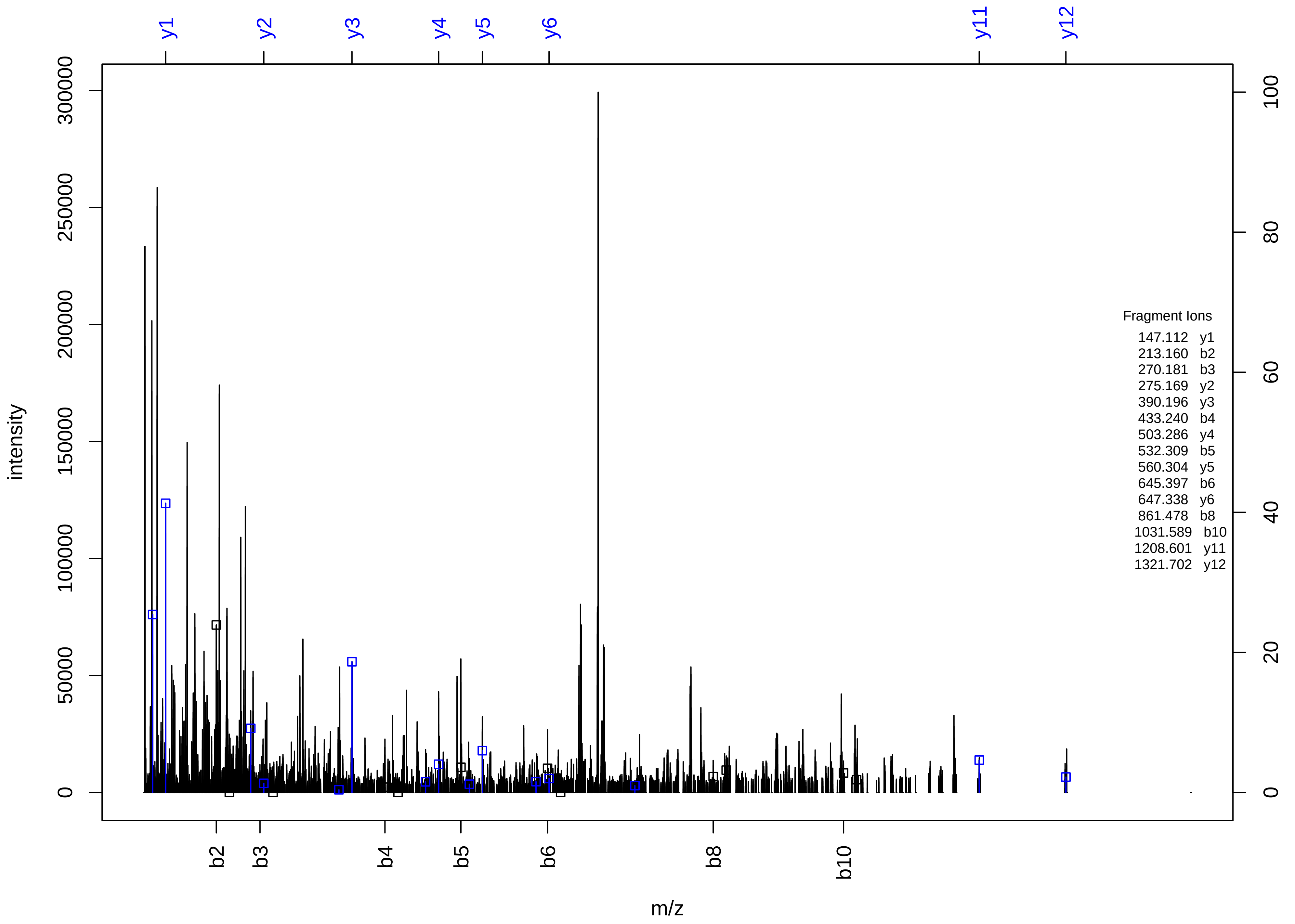

VLGYVLQSGLNQK / File: sydney_ip_24.raw; SpectrumID: ; scans: 8653

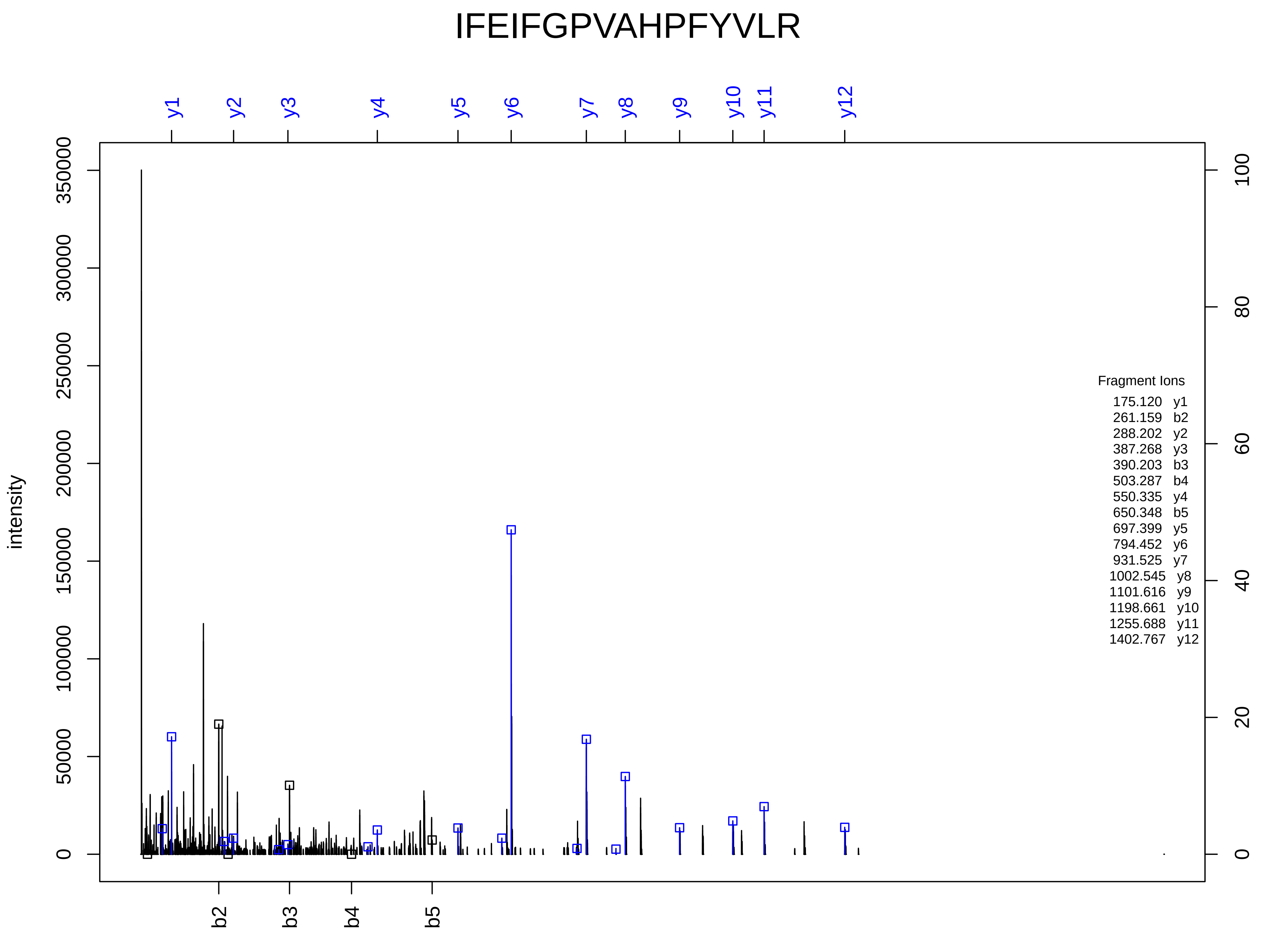

IFEIFGPVAHPFYVLR / File: sydney_ip_24.raw; SpectrumID: ; scans: 19589

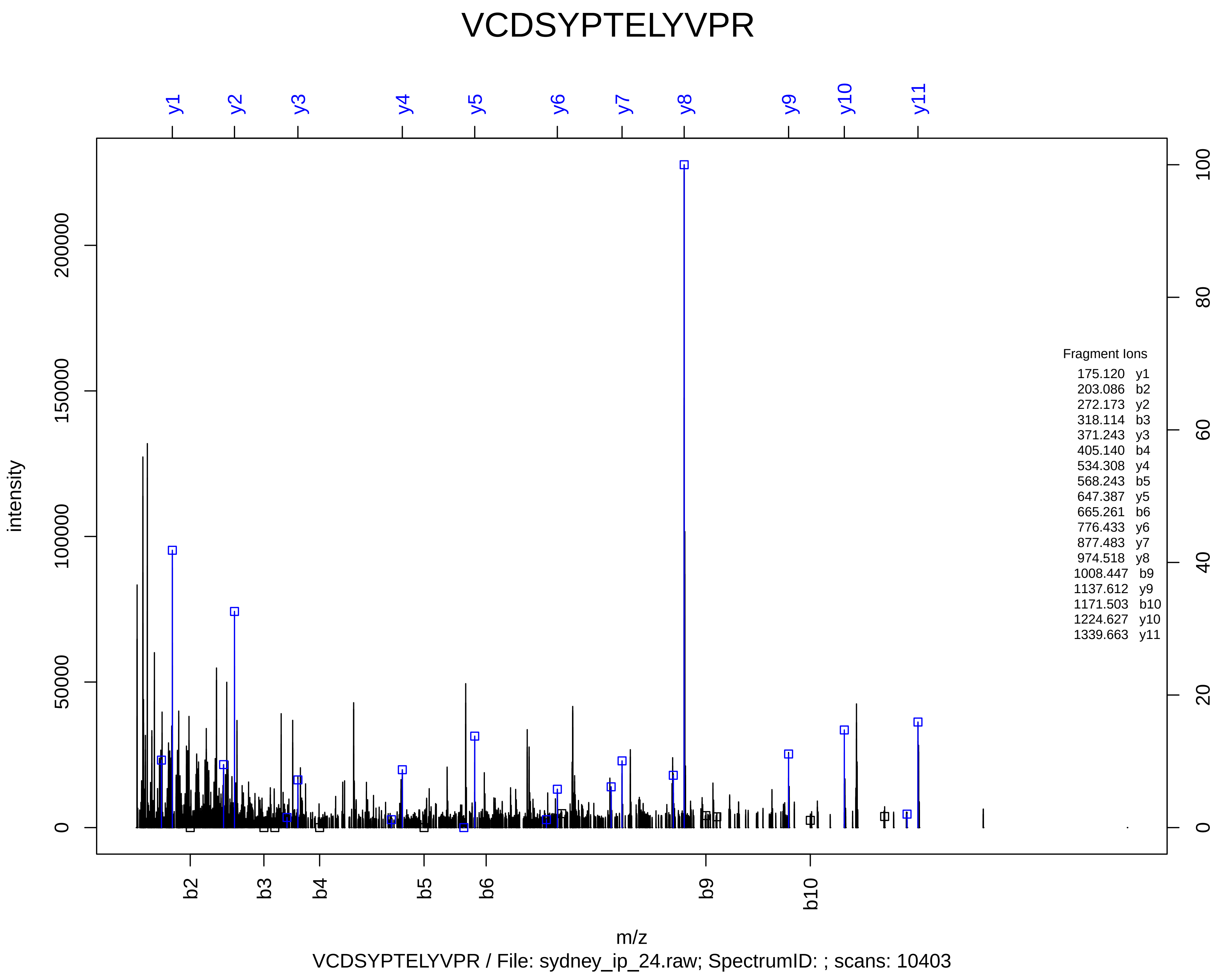
VCDSYPTELYVPR
y1
y2
y3
y4
y5
y6
y7
y8
y9
y10
y11
intensity
0
50000
100000
150000
200000
0
20
40
60
80
100
Fragment Ions
175.120 y1
203.086 b2
272.173 y2
318.114 b3
371.243 y3
405.140 b4
534.308 y4
568.243 b5
647.387 y5
665.261 b6
776.433 y6
877.483 y7
974.518 y8
1008.447 b9
1137.612 y9
1171.503 b10
1224.627 y10
1339.663 y11
b2
b3
b4
b5
b6
b9
b10
m/z
VCDSYPTELYVPR / File: sydney_ip_24.raw; SpectrumID: ; scans: 10403

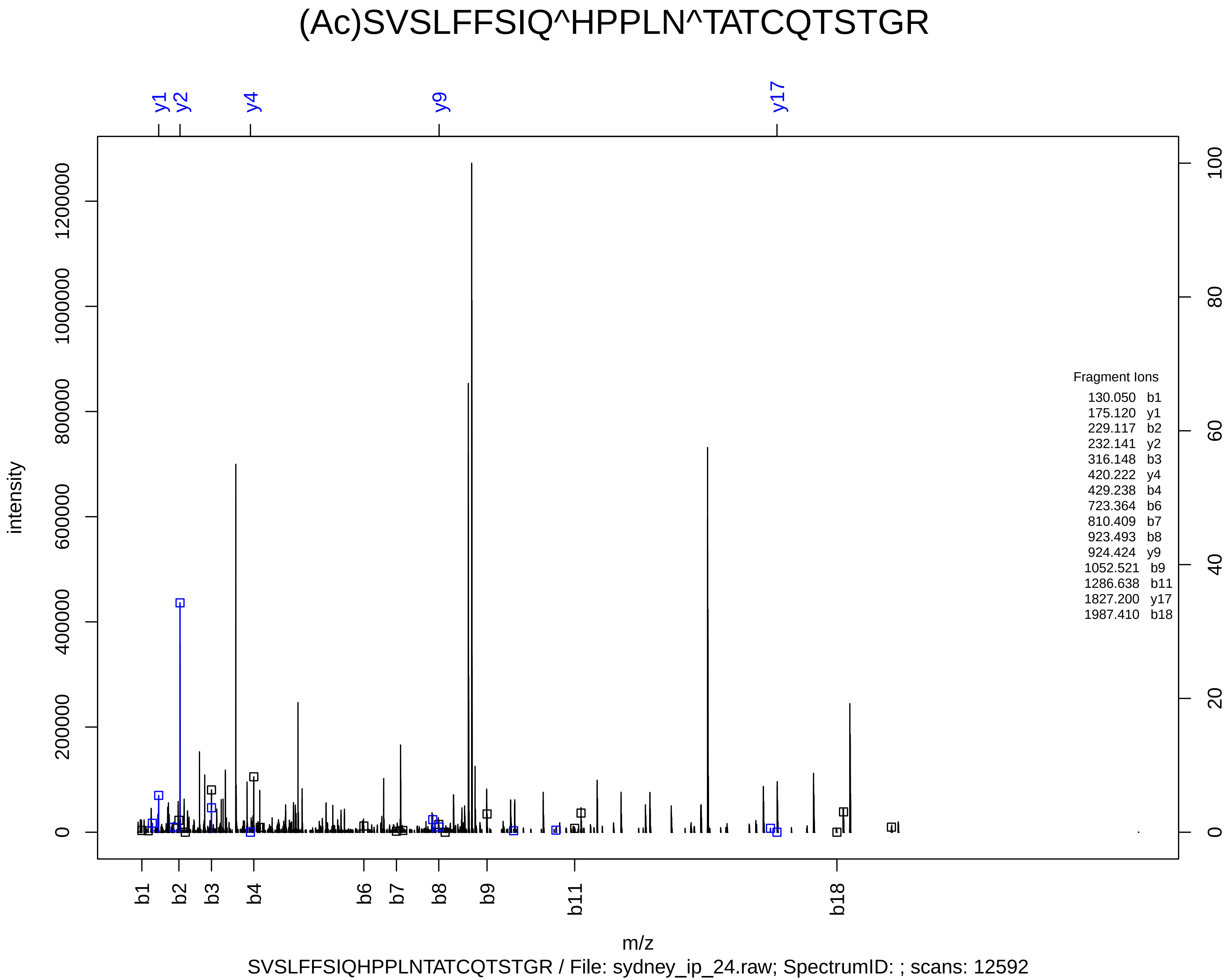
(Ac)SVSLFFSIQ^HPPLN^TATCQTSTGR
y1 y2 y4 y9 y17
intensity
0 200000 400000 600000 800000 1000000 1200000
0 20 40 60 80 100
b1 b2 b3 b4 b6 b7 b8 b9 b11 b18
m/z
Fragment Ions
130.050 b1
175.120 y1
229.117 b2
232.141 y2
316.148 b3
420.222 y4
429.238 b4
723.364 b6
810.409 b7
923.493 b8
924.424 y9
1052.521 b9
1286.638 b11
1827.200 y17
1987.410 b18
SVSLFFSIQHPPLNTATCQTSTGR / File: sydney_ip_24.raw; SpectrumID: ; scans: 12592

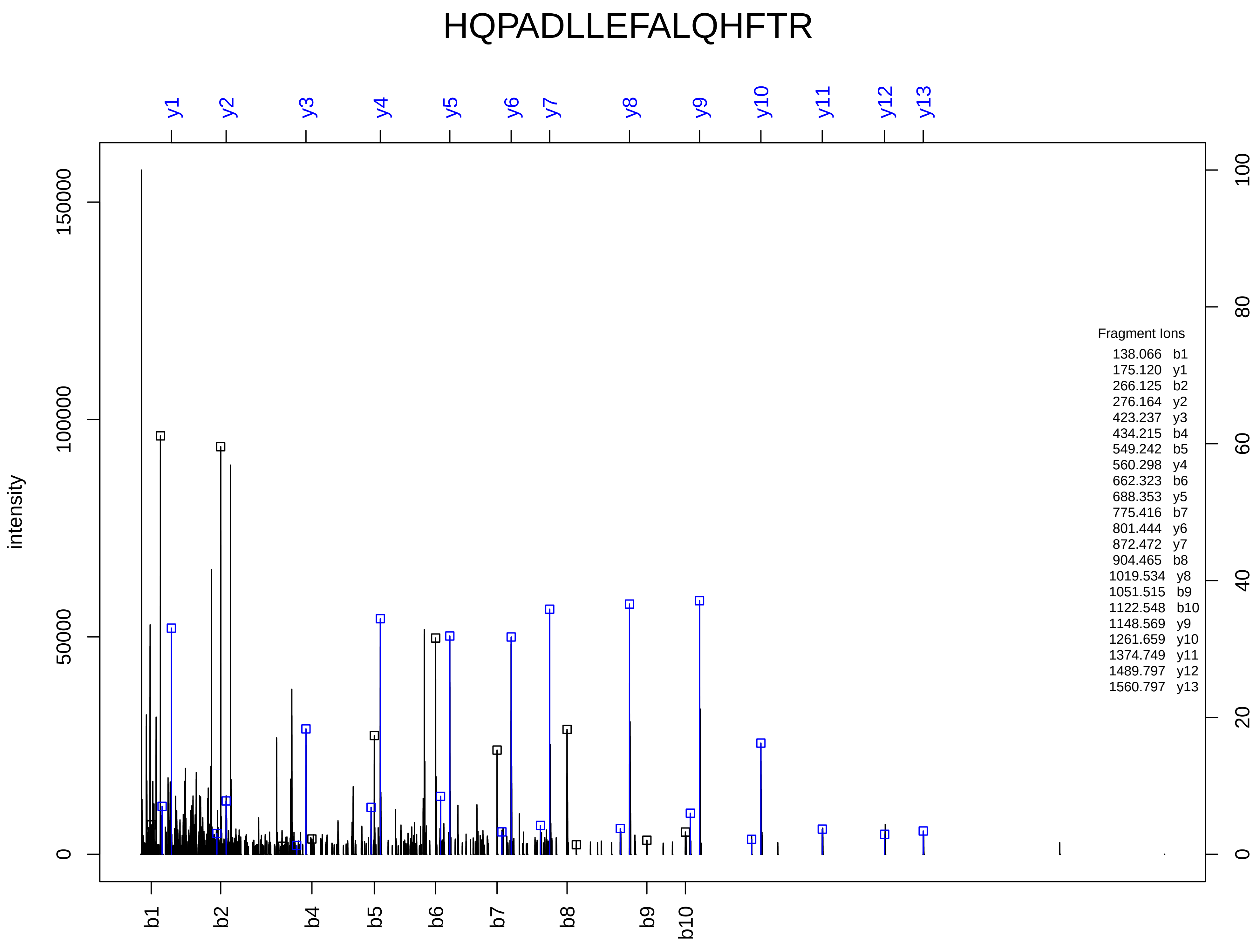

HQPADLLEFALQHFTR / File: sydney_ip_24.raw; SpectrumID: ; scans: 19051

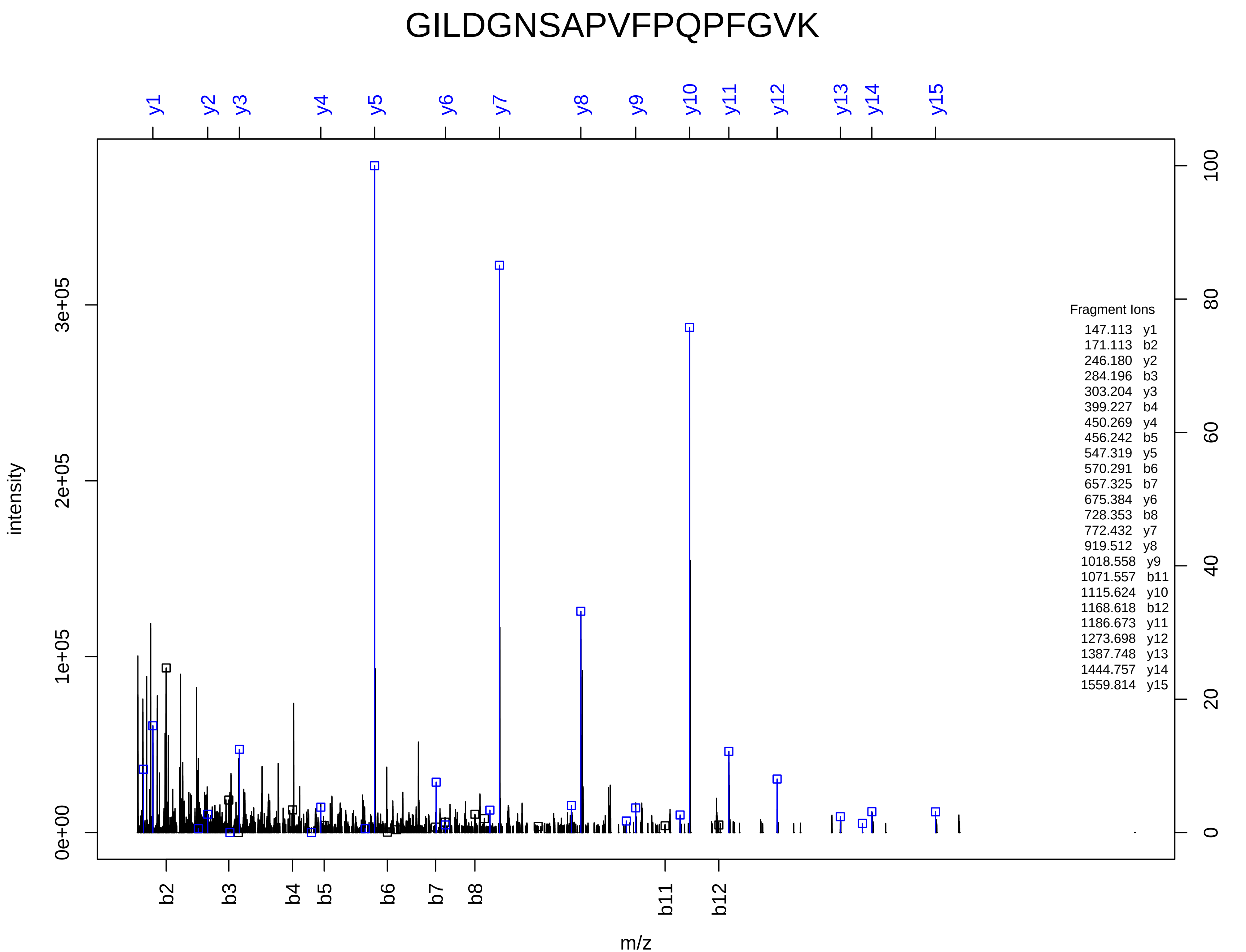
GILDGNSAPVFPQPFGVK
y1 y2 y3 y4 y5 y6 y7 y8 y9 y10 y11 y12 y13 y14 y15
b2 b3 b4 b5 b6 b7 b8 b11 b12
intensity
m/z
Fragment Ions
147.113 y1
171.113 b2
246.180 y2
284.196 b3
303.204 y3
399.227 b4
450.269 y4
456.242 b5
547.319 y5
570.291 b6
657.325 b7
675.384 y6
728.353 b8
772.432 y7
919.512 y8
1018.558 y9
1071.557 b11
1115.624 y10
1168.618 b12
1186.673 y11
1273.698 y12
1387.748 y13
1444.757 y14
1559.814 y15
GILDGNSAPVFPQPFGVK / File: sydney_ip_24.raw; SpectrumID: ; scans: 17183

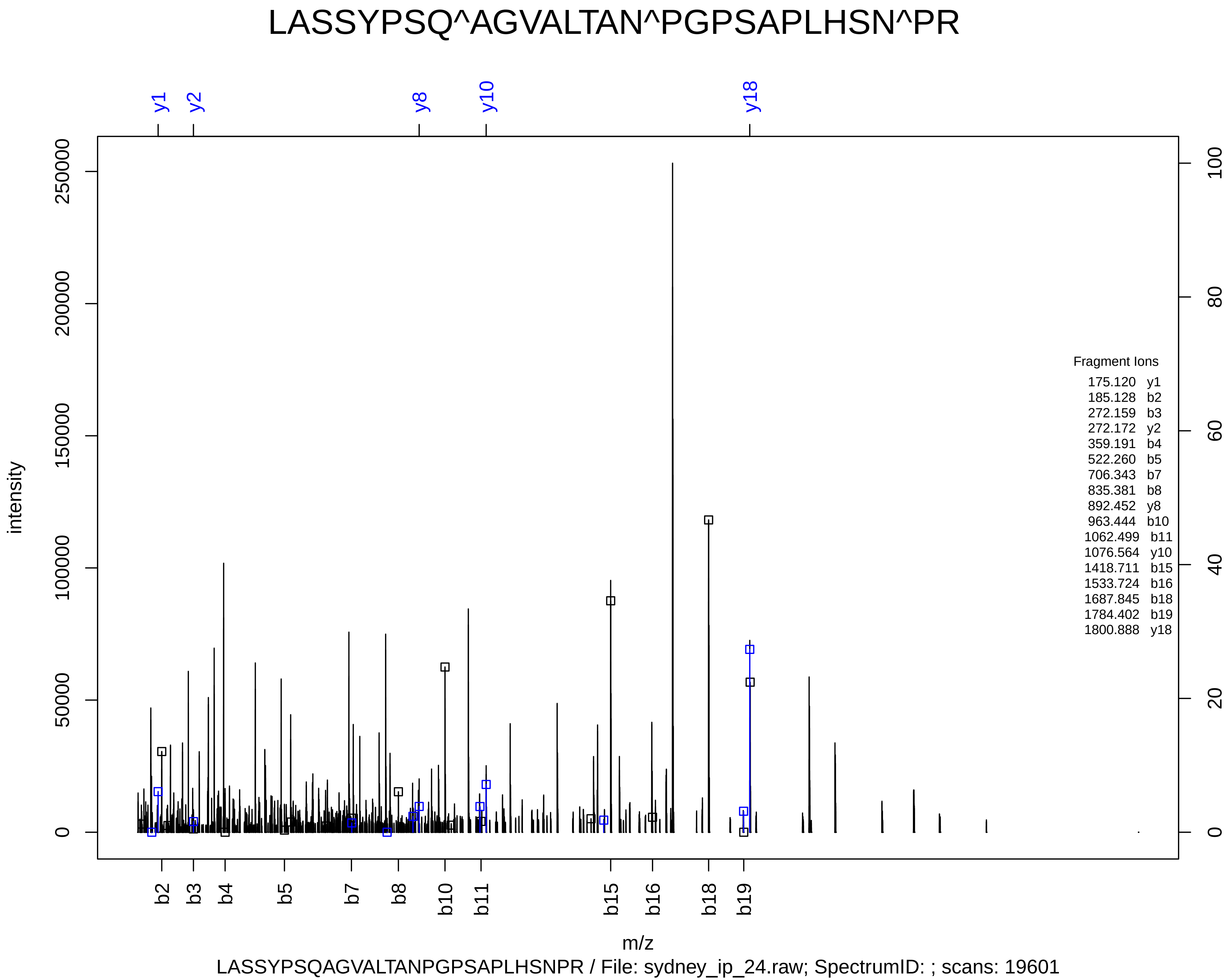

LASSYPSQ^AGVALTAN^PGPSAPLHSN^PR
y1
y2
y8
y10
y18
intensity
250000
200000
150000
100000
50000
0
100
80
60
40
20
0
Fragment Ions
175.120 y1
185.128 b2
272.159 b3
272.172 y2
359.191 b4
522.260 b5
706.343 b7
835.381 b8
892.452 y8
963.444 b10
1062.499 b11
1076.564 y10
1418.711 b15
1533.724 b16
1687.845 b18
1784.402 b19
1800.888 y18
b2
b3
b4
b5
b7
b8
b10
b11
b15
b16
b18
b19
m/z
LASSYPSQAGVALTANPGPSAPLHSNPR / File: sydney_ip_24.raw; SpectrumID: ; scans: 19601

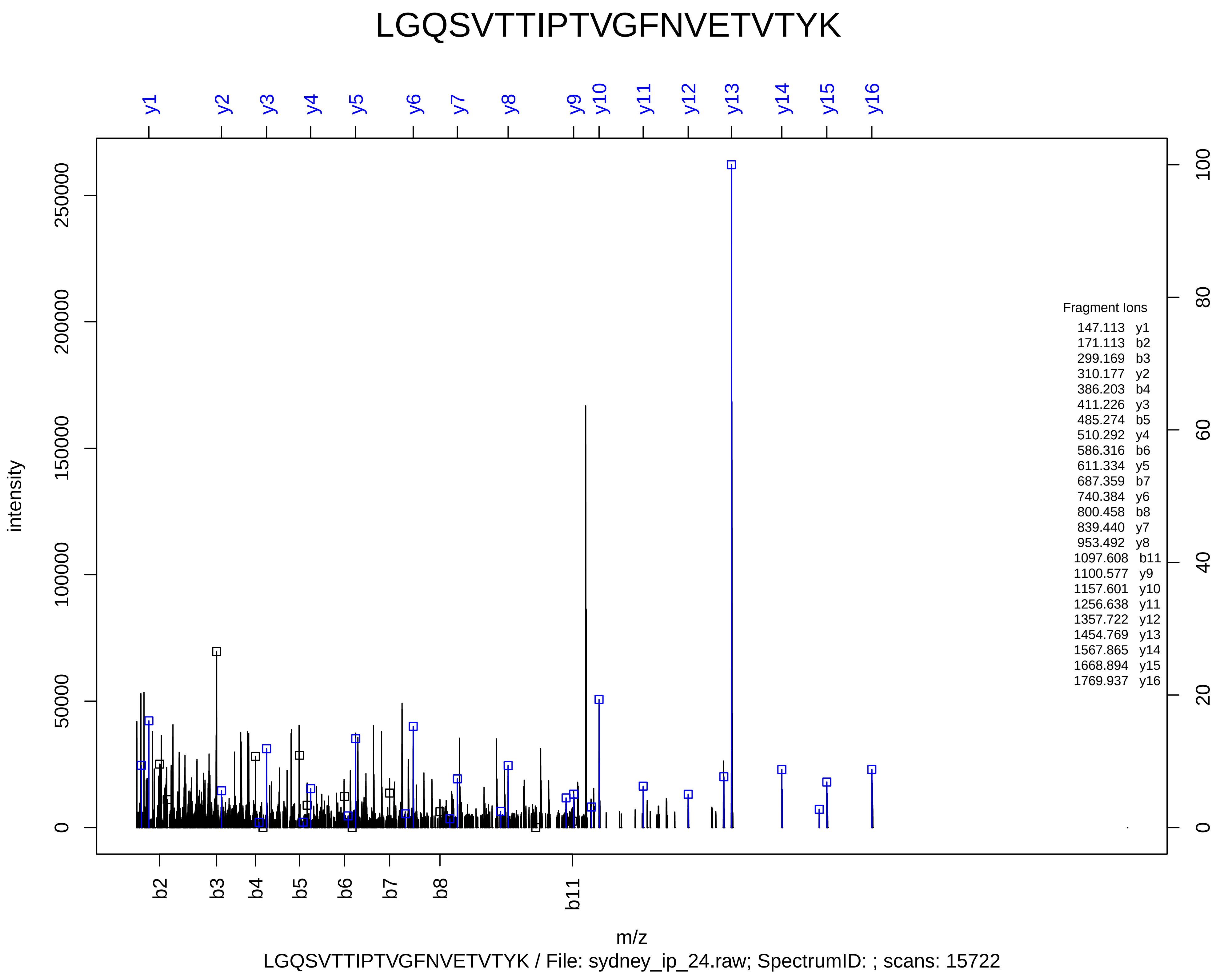
LGQSVTTIPTVGFNVETVTYK
y1 y2 y3 y4 y5 y6 y7 y8 y9 y10 y11 y12 y13 y14 y15 y16
b2 b3 b4 b5 b6 b7 b8 b11
intensity
m/z
Fragment Ions
147.113 y1
171.113 b2
299.169 b3
310.177 y2
386.203 b4
411.226 y3
485.274 b5
510.292 y4
586.316 b6
611.334 y5
687.359 b7
740.384 y6
800.458 b8
839.440 y7
953.492 y8
1097.608 b11
1100.577 y9
1157.601 y10
1256.638 y11
1357.722 y12
1454.769 y13
1567.865 y14
1668.894 y15
1769.937 y16
LGQSVTTIPTVGFNVETVTYK / File: sydney_ip_24.raw; SpectrumID: ; scans: 15722

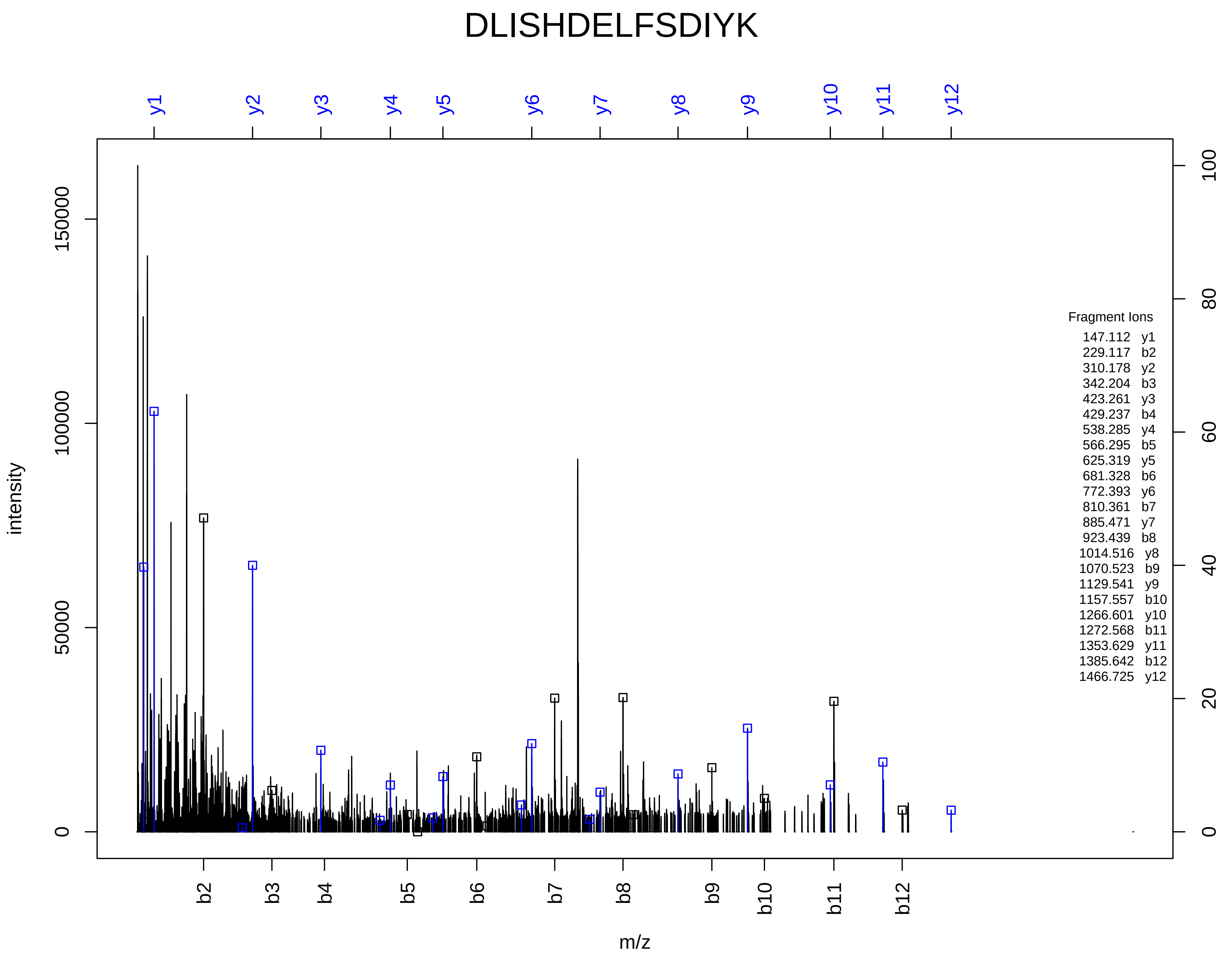

DLISHDELFSDIYK / File: sydney_ip_24.raw; SpectrumID: ; scans: 14457

M*AAN^VGSM*SQ^YWK

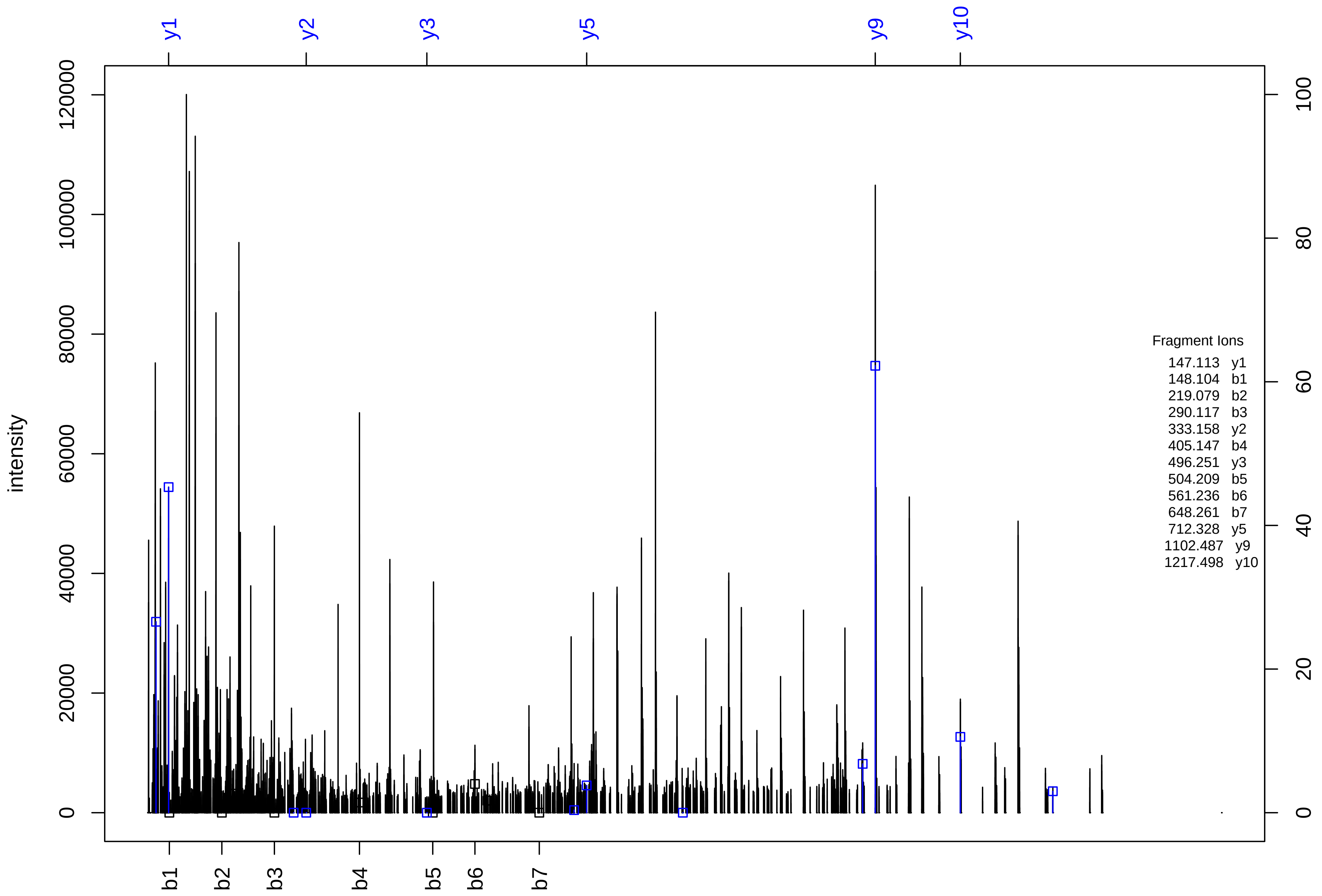

MAANVGSMSQYWK / File: sydney_ip_24.raw; SpectrumID: ; scans: 5508

GTGQSDDSDIWDDTALIK

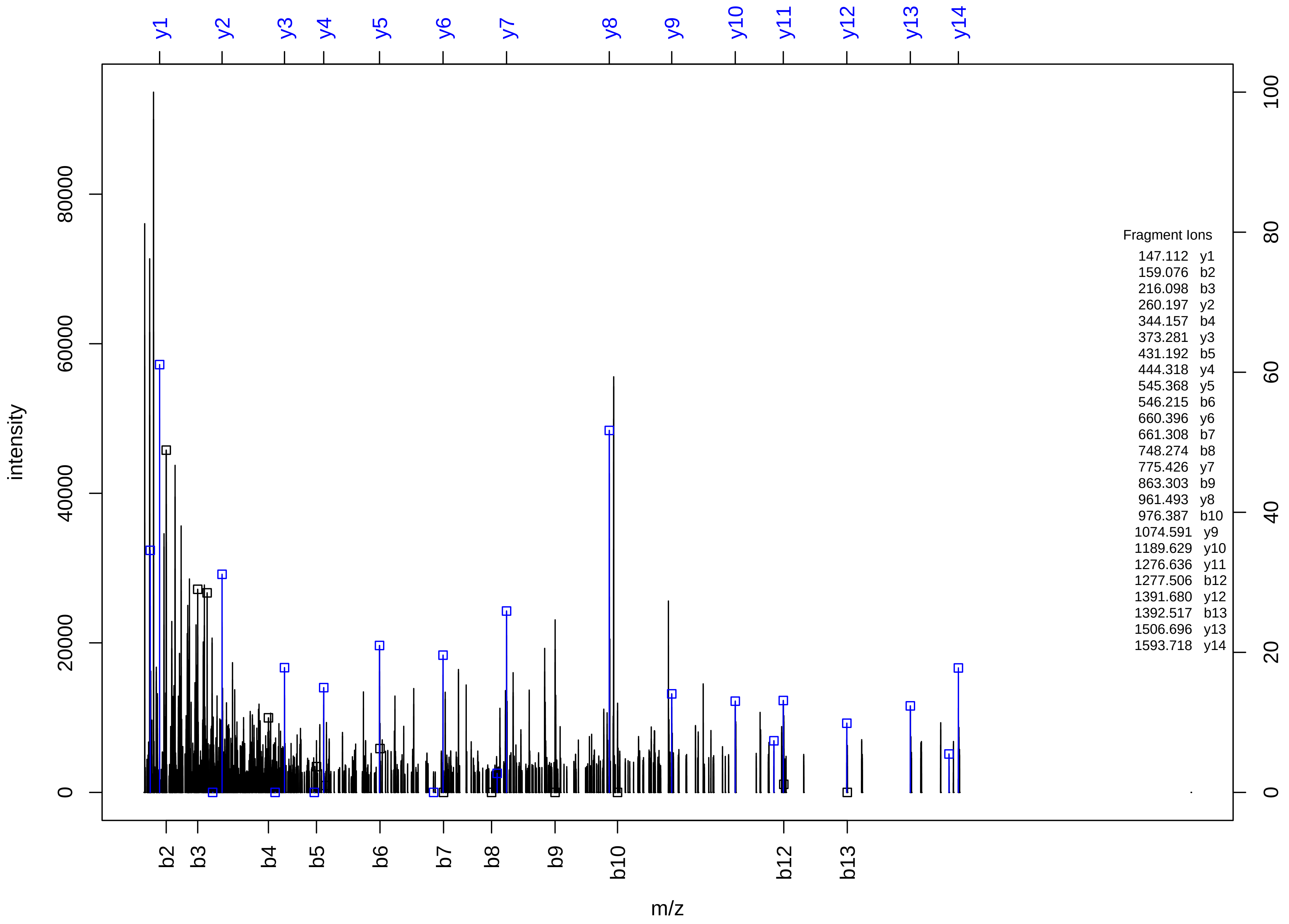

GTGQSDDSDIWDDTALIK / File: sydney_ip_24.raw; SpectrumID: ; scans: 13848

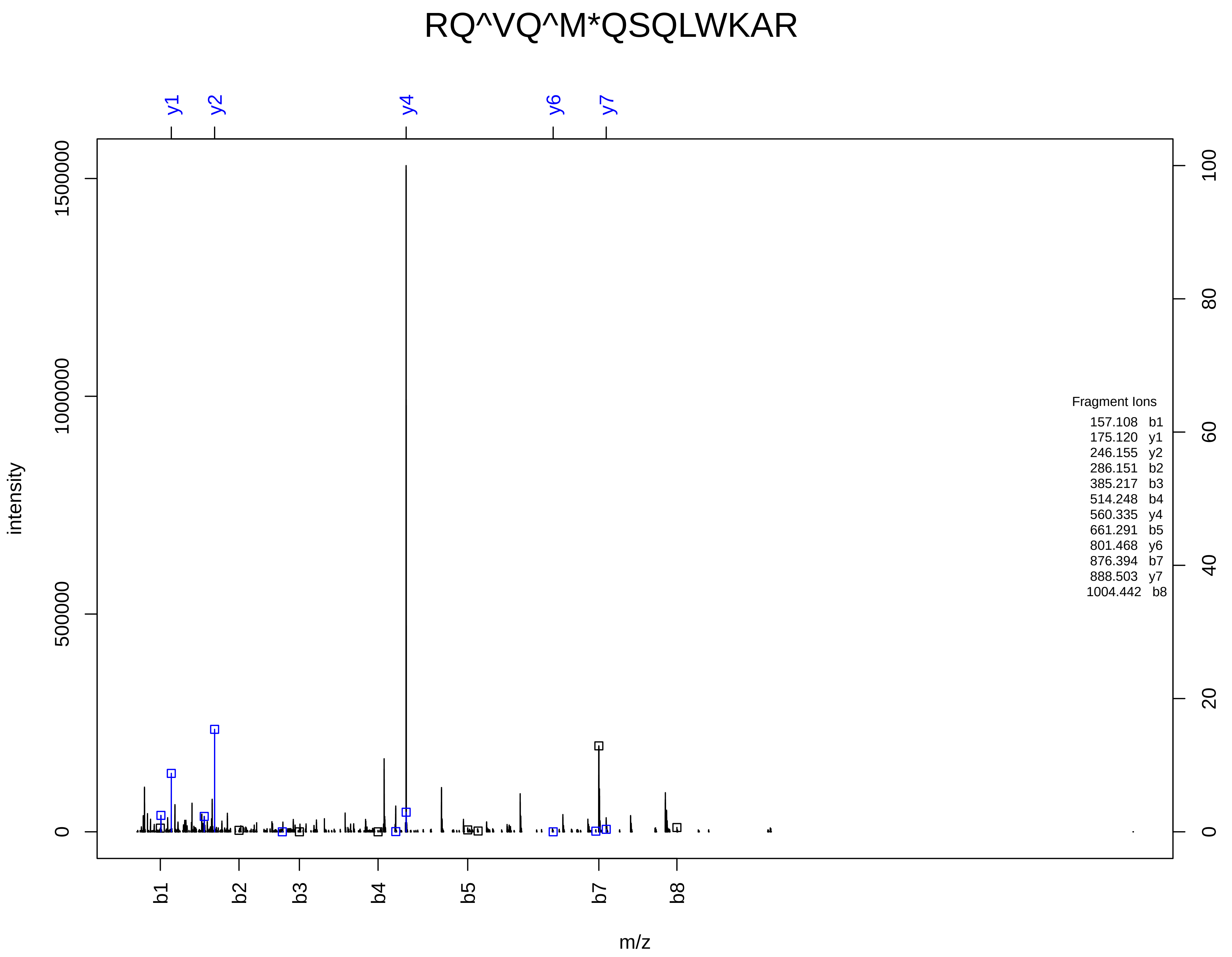
RQ^VQ^M*QSQLWKAR
y1
y2
y4
y6
y7
intensity
0
500000
1000000
1500000
0
20
40
60
80
100
Fragment Ions
157.108 b1
175.120 y1
246.155 y2
286.151 b2
385.217 b3
514.248 b4
560.335 y4
661.291 b5
801.468 y6
876.394 b7
888.503 y7
1004.442 b8
b1
b2
b3
b4
b5
b7
b8
m/z
RQVQMQSQLWKAR / File: sydney_ip_24.raw; SpectrumID: ; scans: 1512

VLLGETYTGTSVAR

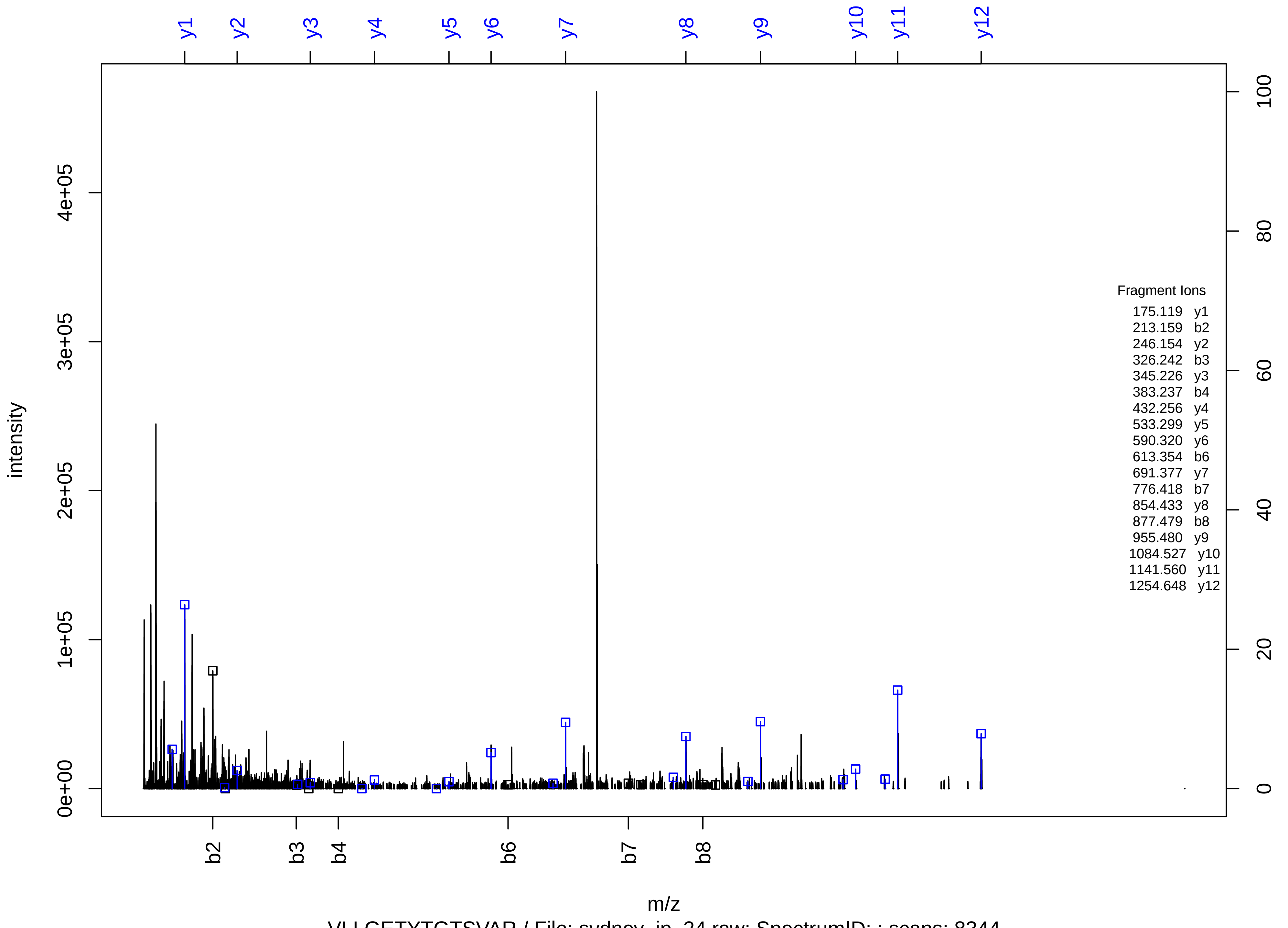

VLLGETYTGTSVAR / File: sydney_ip_24.raw; SpectrumID: ; scans: 8344

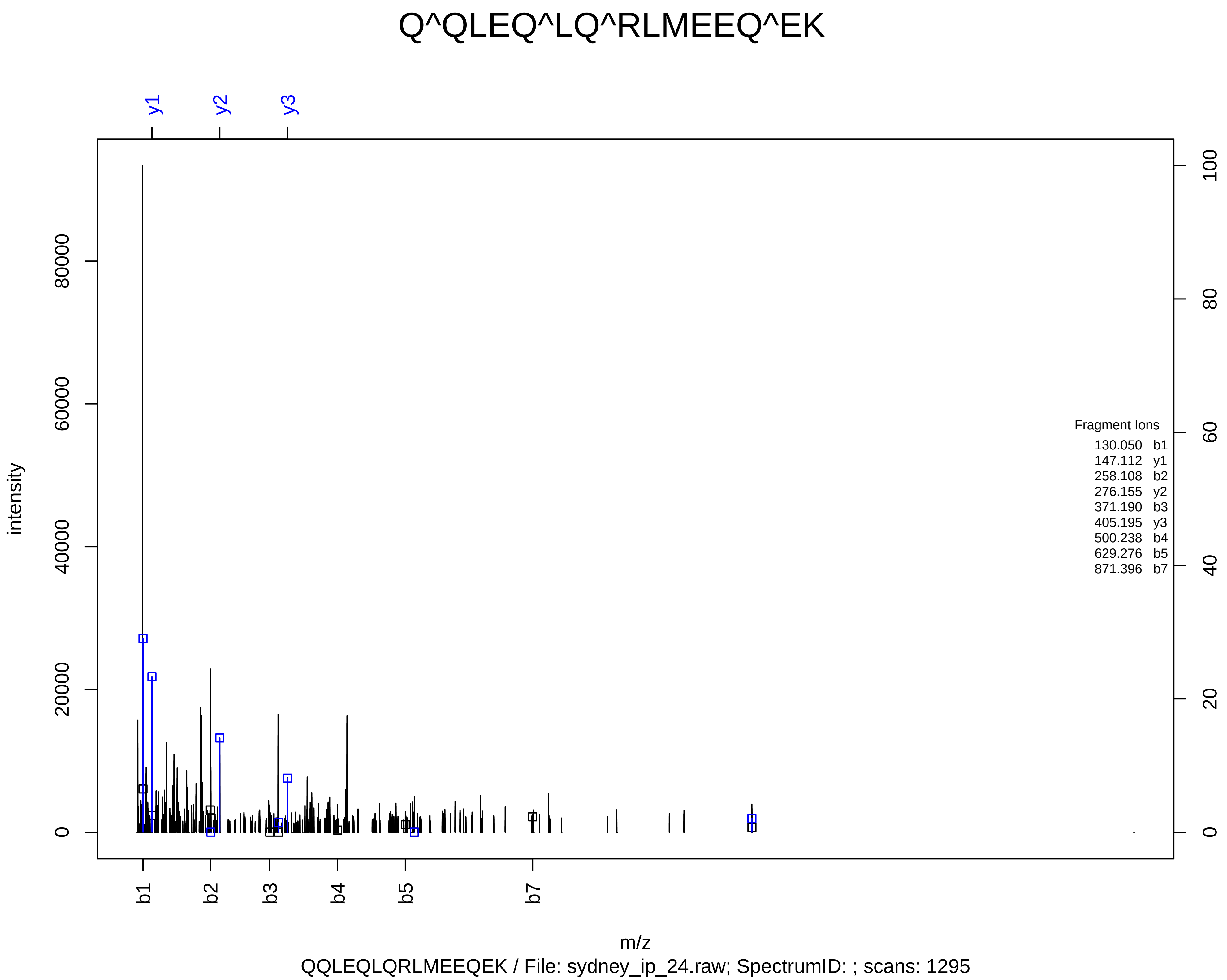

Q^QLEQ^LQ^RLMEEQ^EK
y1
y2
y3
intensity
0
20000
40000
60000
80000
0
20
40
60
80
100
Fragment Ions
130.050 b1
147.112 y1
258.108 b2
276.155 y2
371.190 b3
405.195 y3
500.238 b4
629.276 b5
871.396 b7
b1
b2
b3
b4
b5
b7
m/z
QQLEQLQRLMEEQEK / File: sydney_ip_24.raw; SpectrumID: ; scans: 1295

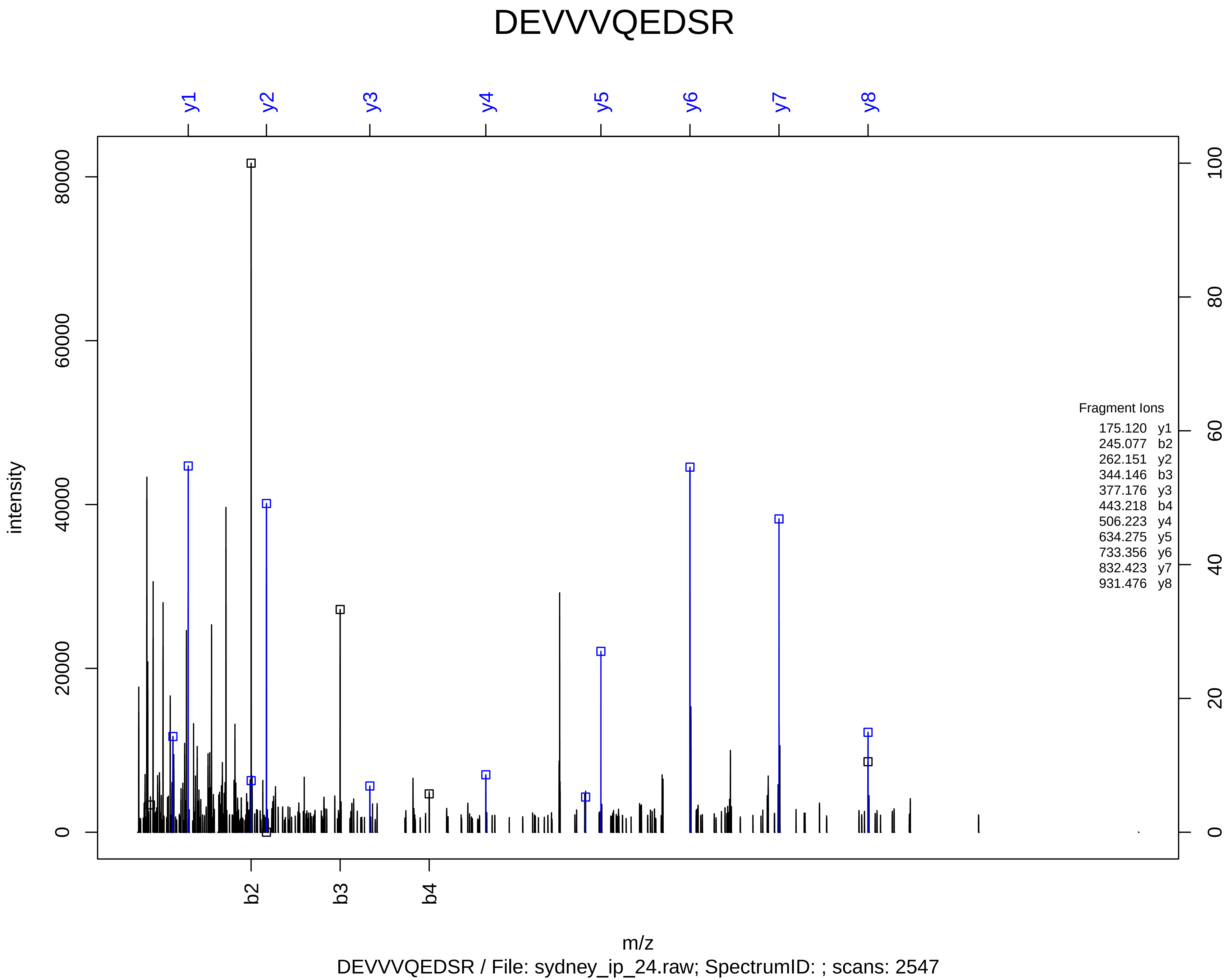
DEVVVQEDSR
y1
y2
y3
y4
y5
y6
y7
y8
intensity
0
20000
40000
60000
80000
0
20
40
60
80
100
b2
b3
b4
m/z
Fragment Ions
175.120 y1
245.077 b2
262.151 y2
344.146 b3
377.176 y3
443.218 b4
506.223 y4
634.275 y5
733.356 y6
832.423 y7
931.476 y8
DEVVVQEDSR / File: sydney_ip_24.raw; SpectrumID: ; scans: 2547

VQ^EQQ^KQLQDQ^DQ^K

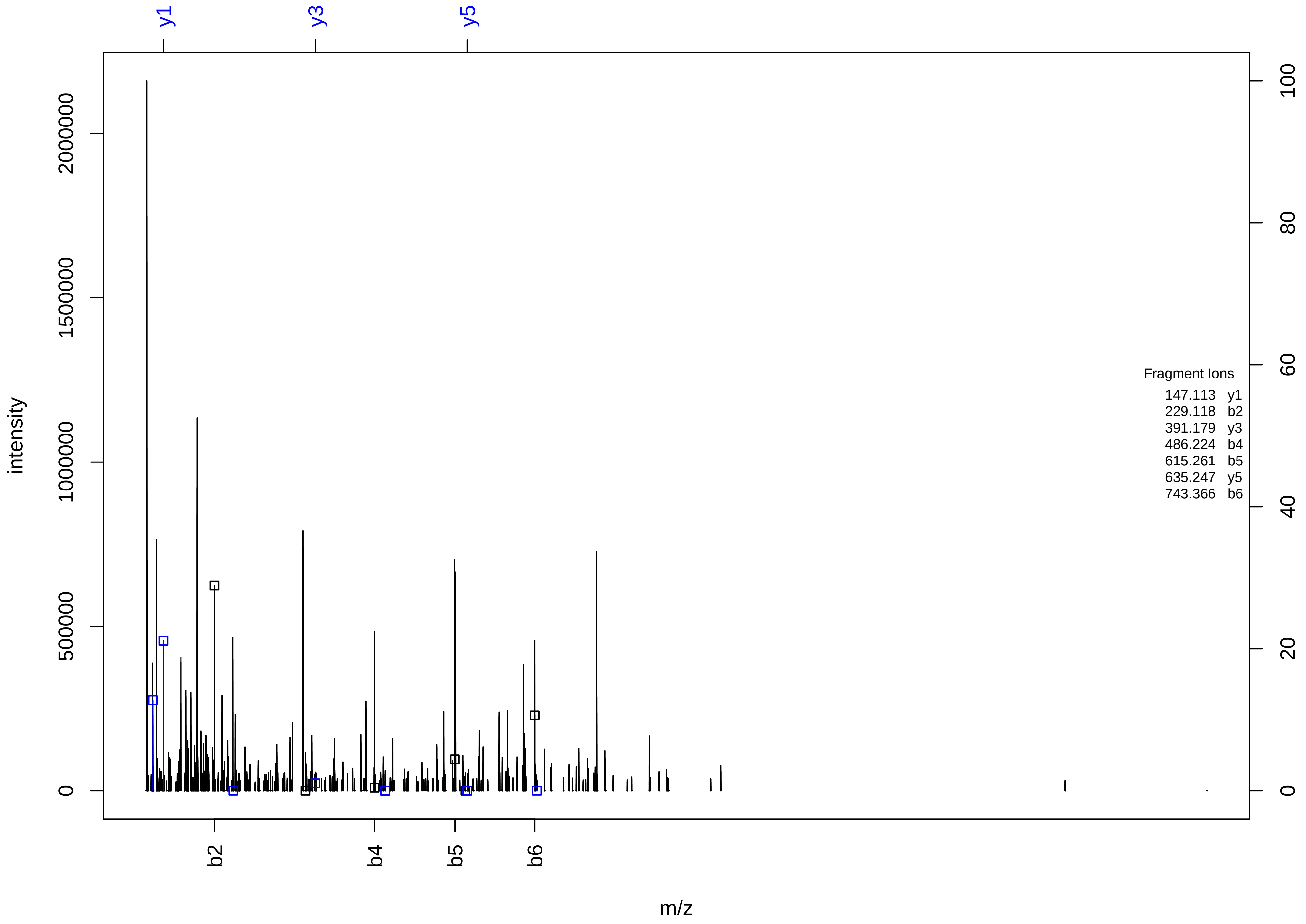

VQEQQKQLQDQDQK / File: sydney_ip_24.raw; SpectrumID: ; scans: 4197

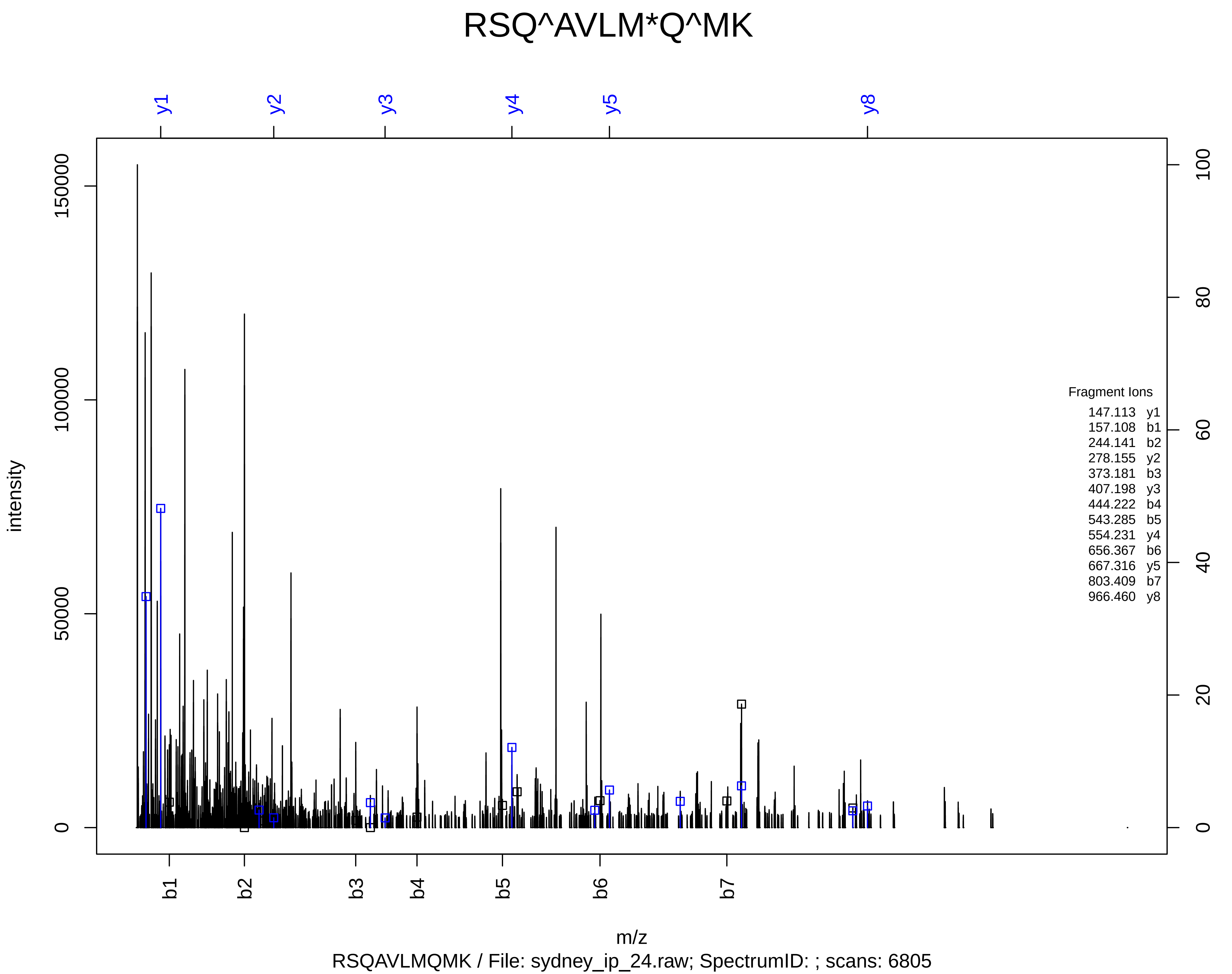
RSQ^AVLM*Q^MK
y1
y2
y3
y4
y5
y8
b1
b2
b3
b4
b5
b6
b7
intensity
m/z
150000
100000
50000
0
100
80
60
40
20
0
Fragment Ions
147.113 y1
157.108 b1
244.141 b2
278.155 y2
373.181 b3
407.198 y3
444.222 b4
543.285 b5
554.231 y4
656.367 b6
667.316 y5
803.409 b7
966.460 y8
RSQAVLMQMK / File: sydney_ip_24.raw; SpectrumID: ; scans: 6805

(Ac)TTNVQ^MSIIR

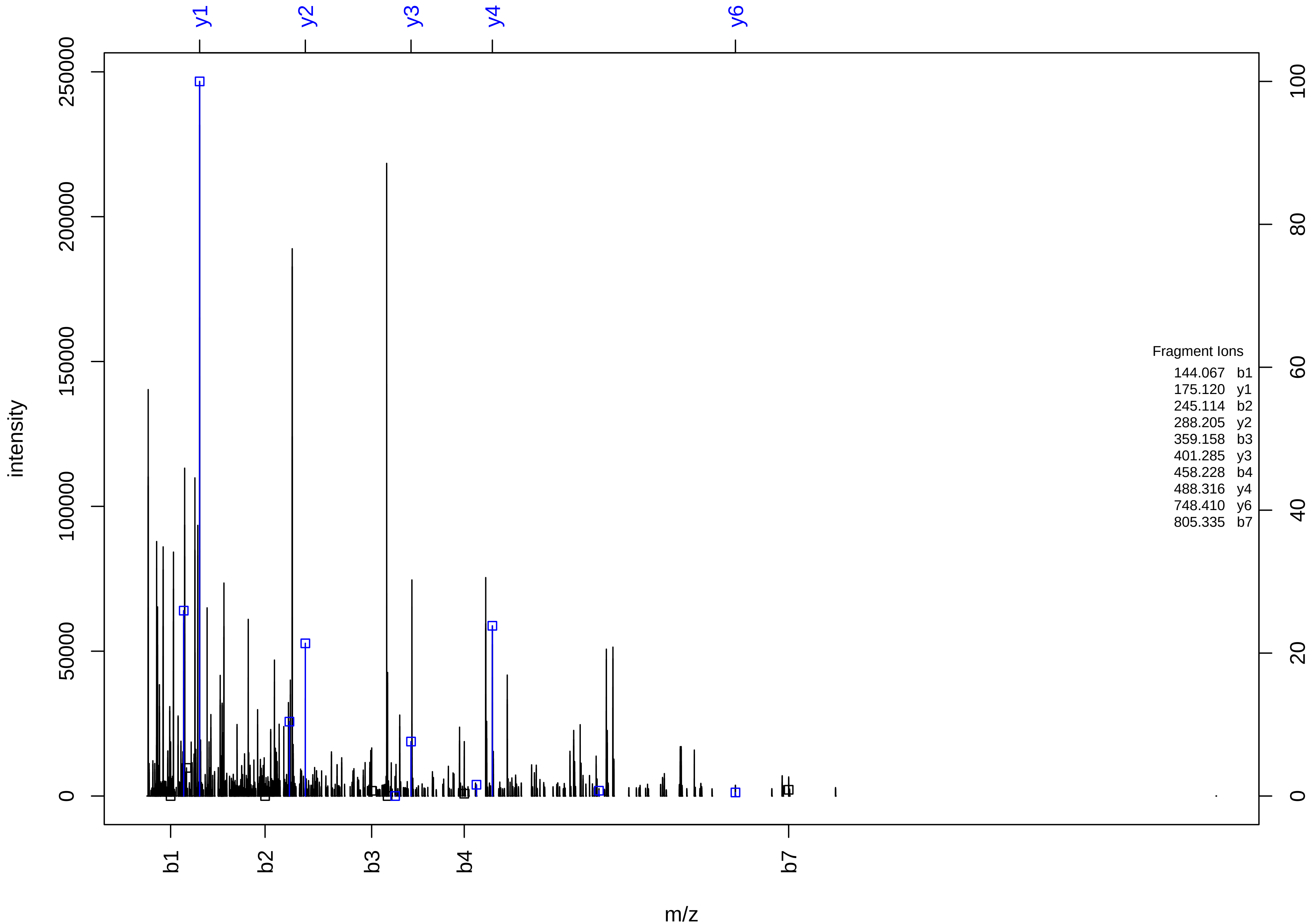

TTNVQMSIIR / File: sydney_ip_24.raw; SpectrumID: ; scans: 5239

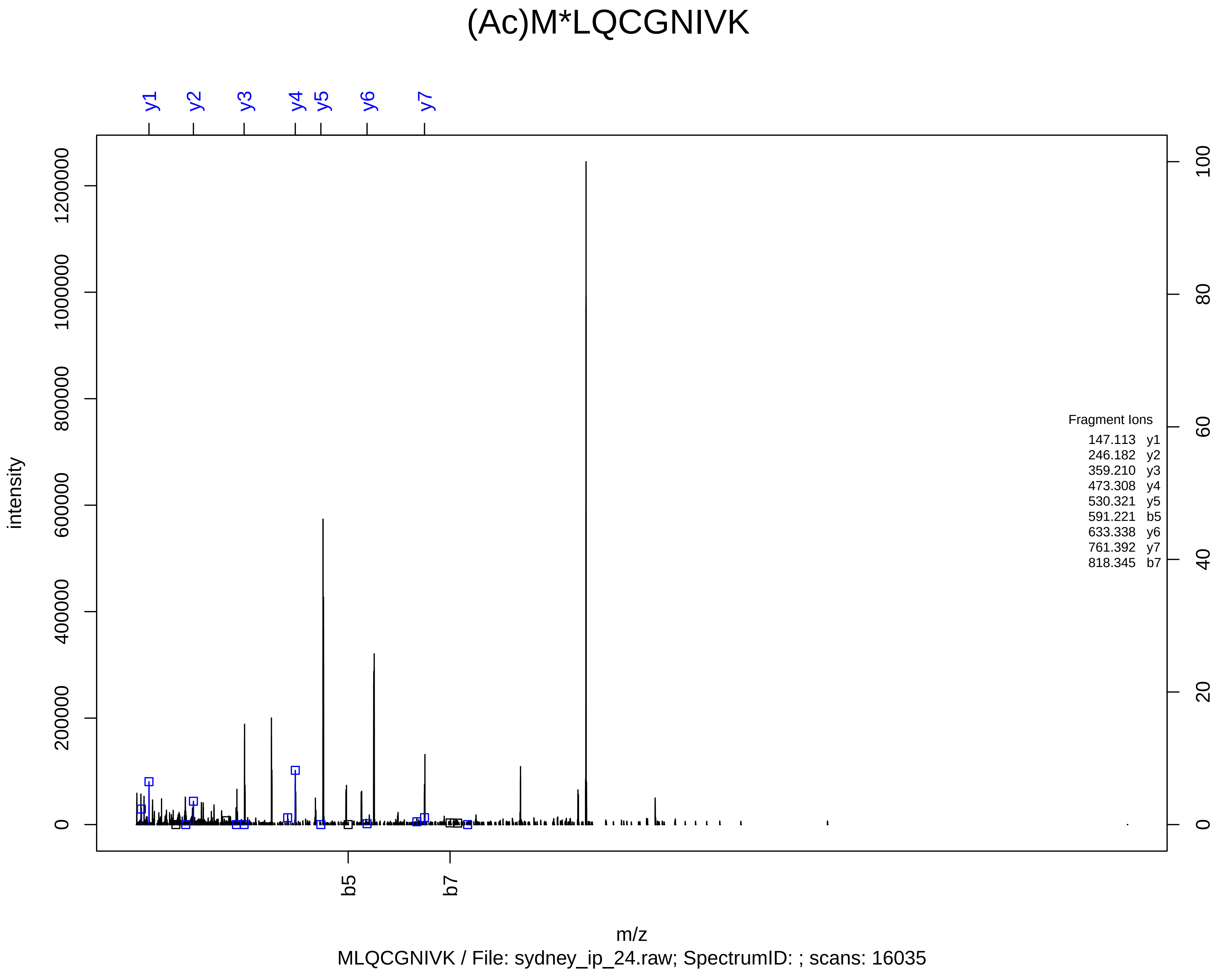
(Ac)M*LQCGNIVK
y1
y2
y3
y4
y5
y6
y7
intensity
0
200000
400000
600000
800000
1000000
1200000
0
20
40
60
80
100
Fragment Ions
147.113 y1
246.182 y2
359.210 y3
473.308 y4
530.321 y5
591.221 b5
633.338 y6
761.392 y7
818.345 b7
b5
b7
m/z
MLQCGNIVK / File: sydney_ip_24.raw; SpectrumID: ; scans: 16035

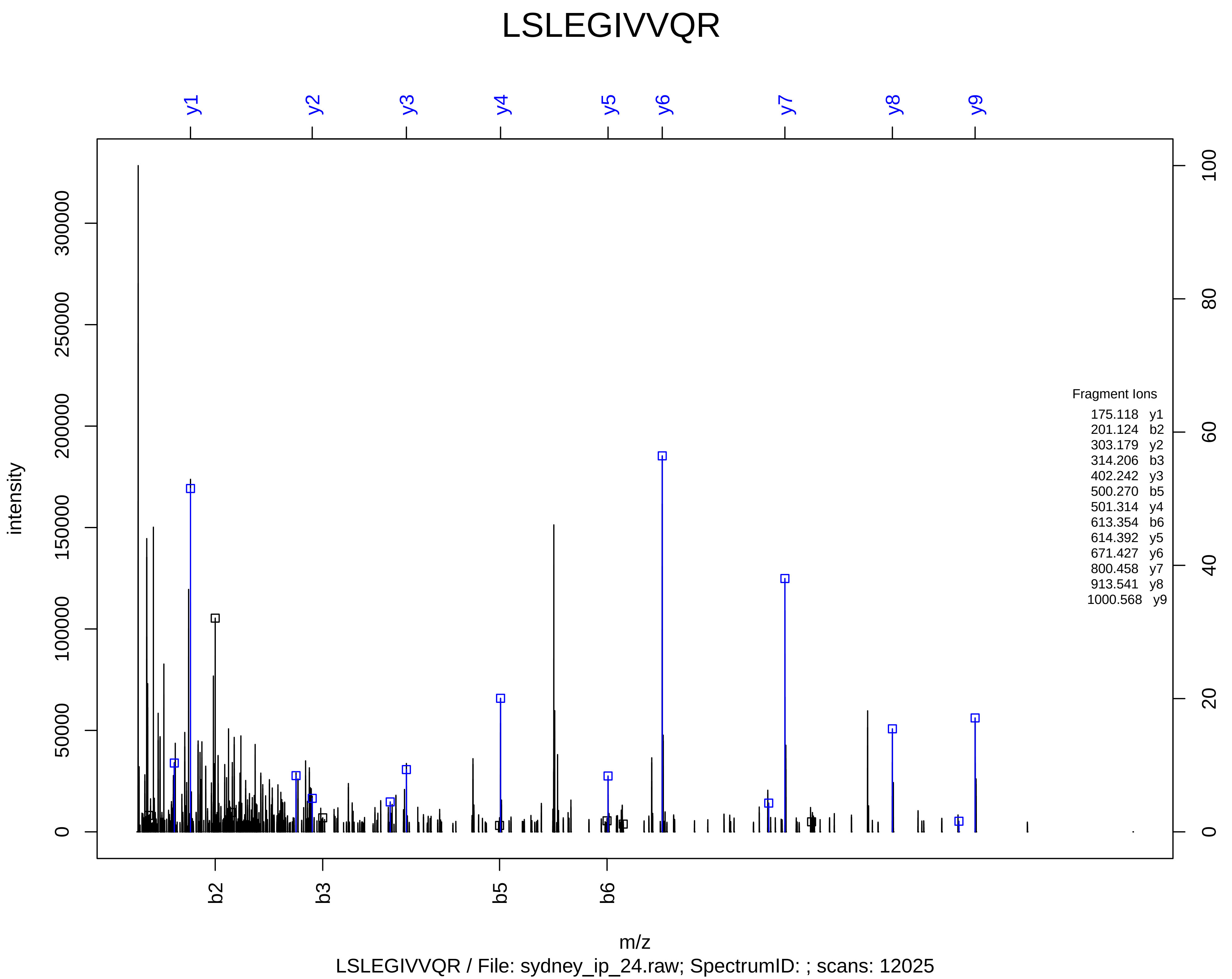
LSLEGIVVQR
y1
y2
y3
y4
y5
y6
y7
y8
y9
b2
b3
b5
b6
intensity
m/z
Fragment Ions
175.118 y1
201.124 b2
303.179 y2
314.206 b3
402.242 y3
500.270 b5
501.314 y4
613.354 b6
614.392 y5
671.427 y6
800.458 y7
913.541 y8
1000.568 y9
0
50000
100000
150000
200000
250000
300000
0
20
40
60
80
100
LSLEGIVVQR / File: sydney_ip_24.raw; SpectrumID: ; scans: 12025

EQASHLGGAVFSGAGNIAAATGLVK

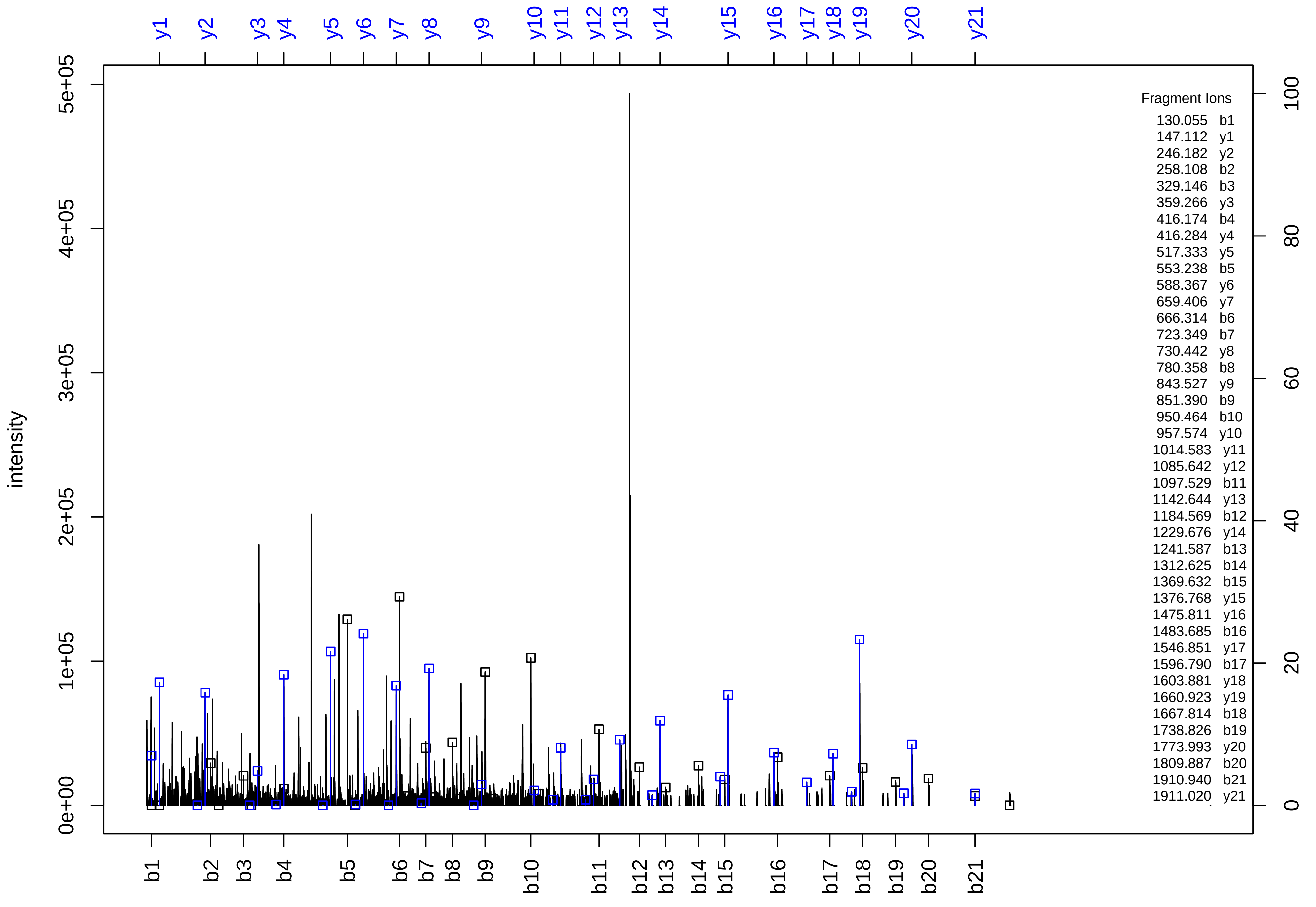

EQASHLGGAVFSGAGNIAAATGLVK / File: sydney_ip_24.raw; SpectrumID: ; scans: 14057

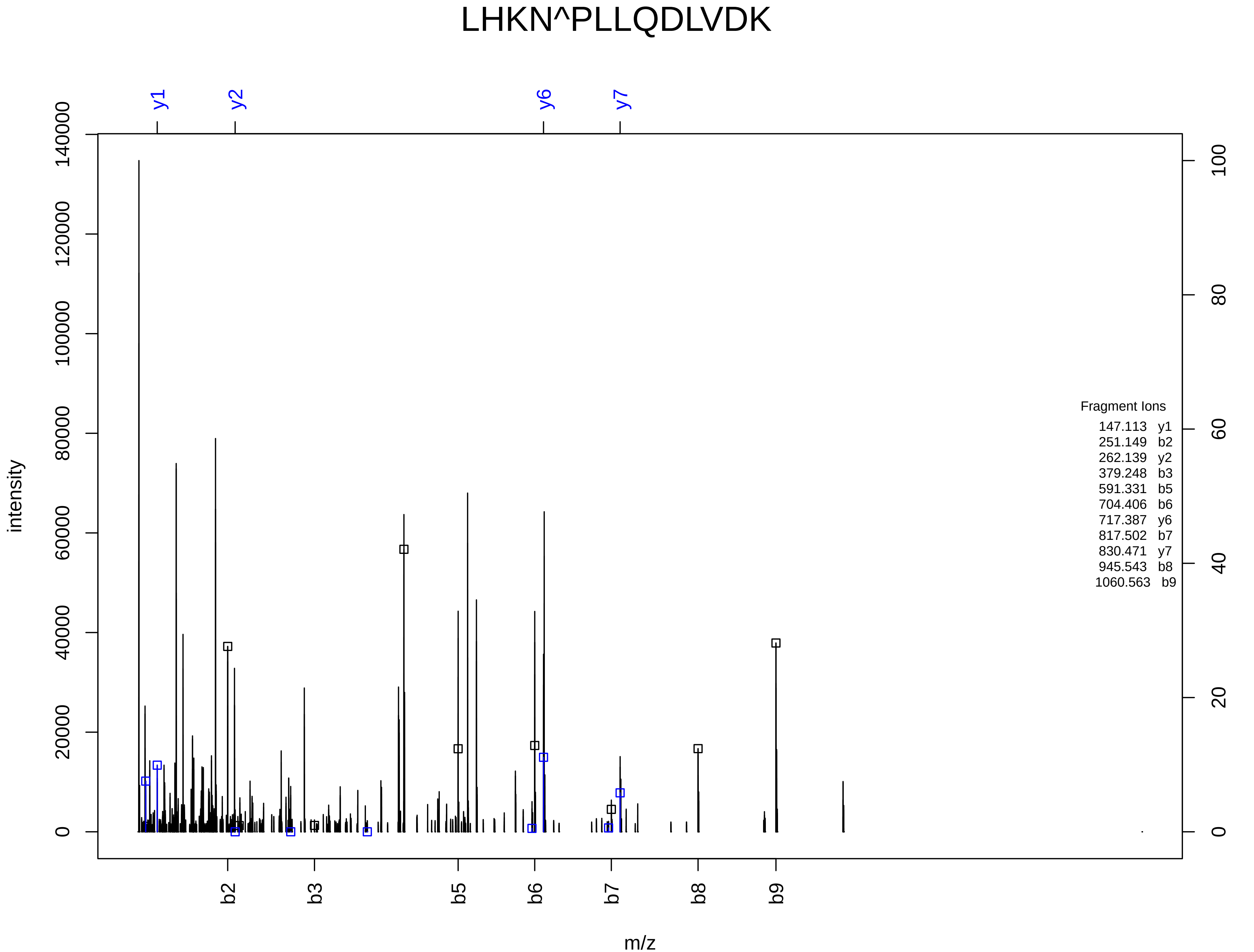

LHKN^PLLQDLVDK
intensity
m/z
y1
y2
y6
y7
b2
b3
b5
b6
b7
b8
b9
Fragment Ions
147.113 y1
251.149 b2
262.139 y2
379.248 b3
591.331 b5
704.406 b6
717.387 y6
817.502 b7
830.471 y7
945.543 b8
1060.563 b9
LHKNPLLQDLVDK / File: sydney_ip_24.raw; SpectrumID: ; scans: 19519

LAGATLLIFANK

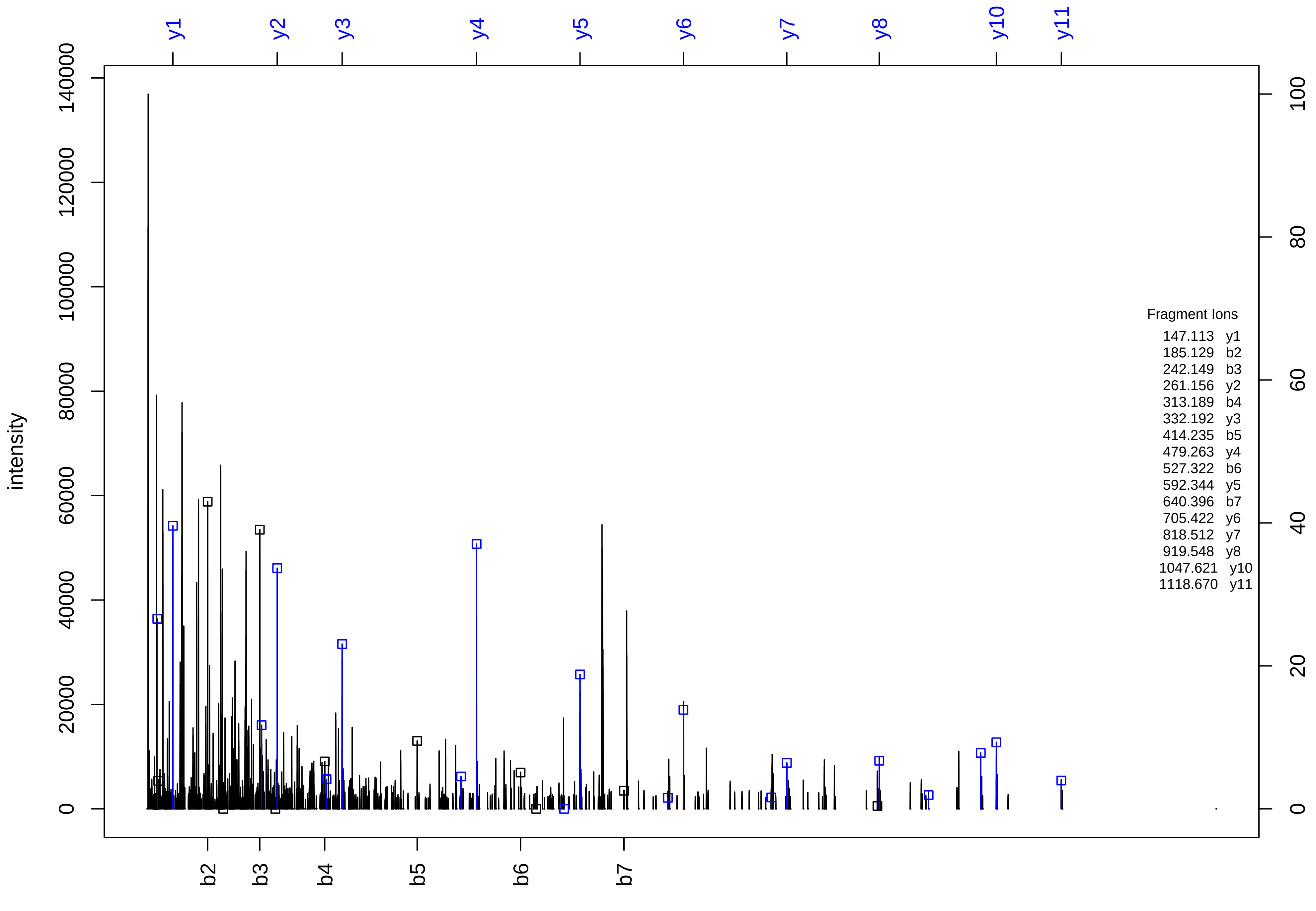

LAGATLLIFANK / File: sydney_ip_24.raw; SpectrumID: ; scans: 15631

EKM*Q^M*ELSHK

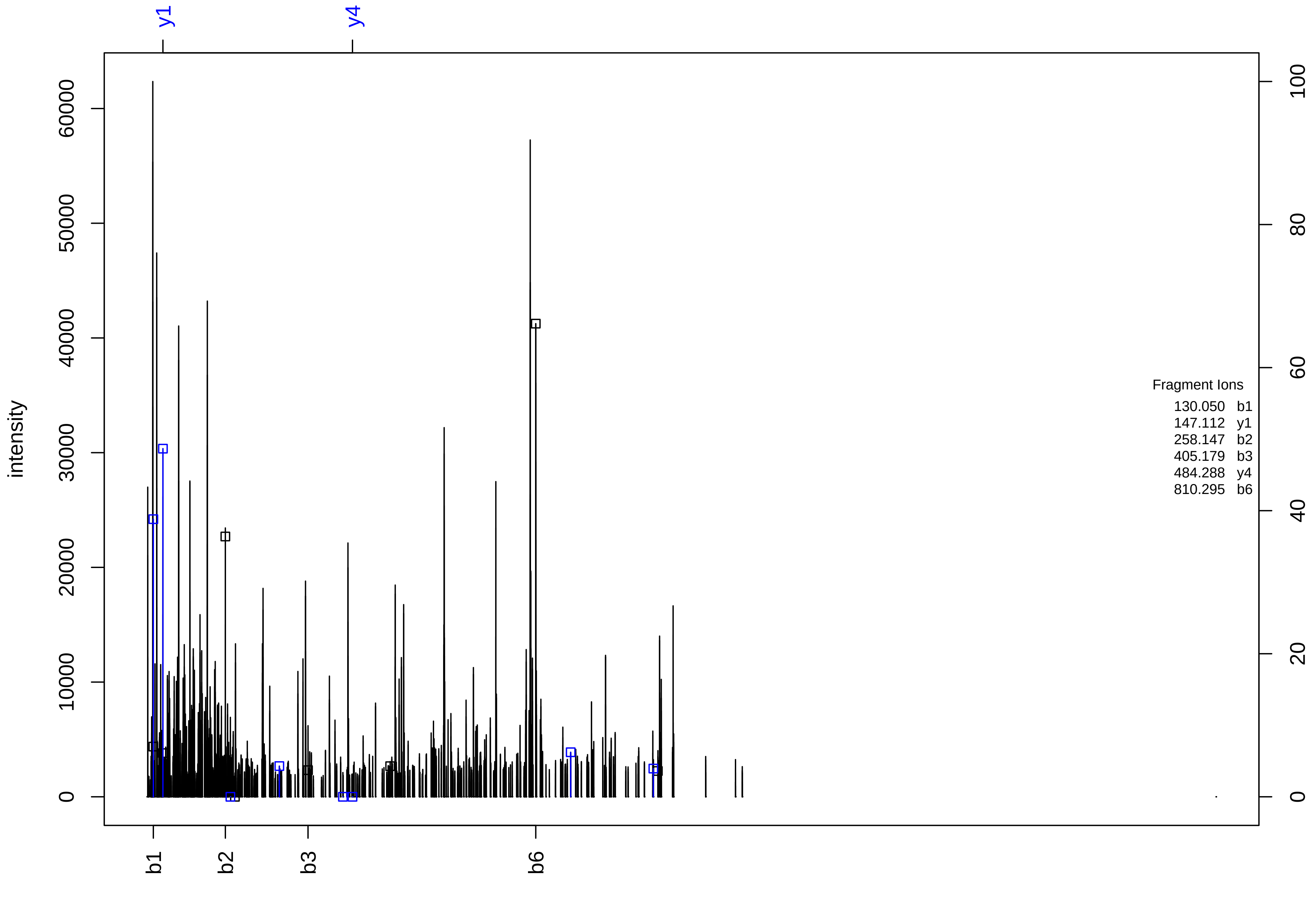

EKMQMELSHK / File: sydney_ip_24.raw; SpectrumID: ; scans: 2675

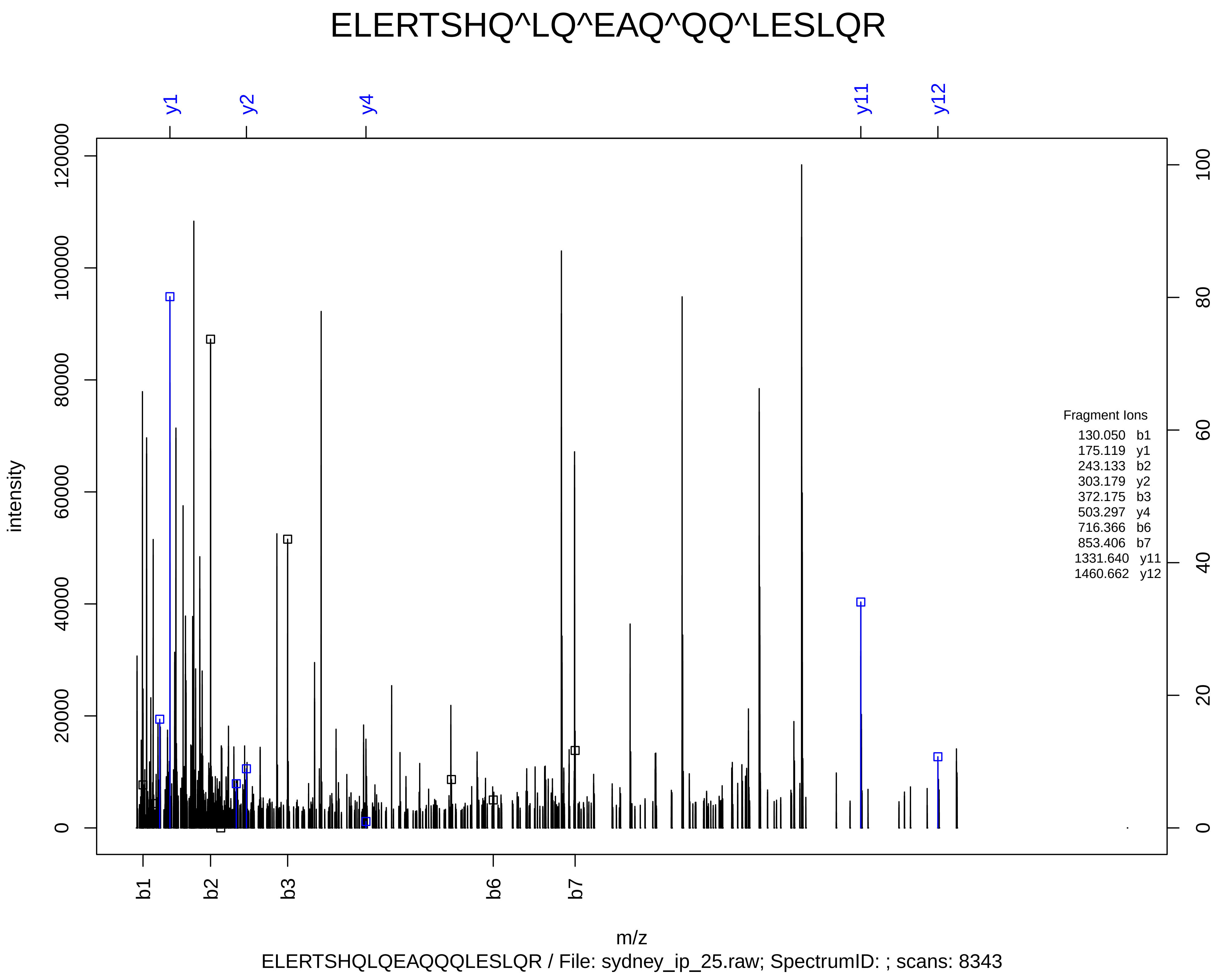
ELERTSHQ^LQ^EAQ^QQ^LESLQR
intensity
m/z
y1
y2
y4
y11
y12
b1
b2
b3
b6
b7
Fragment Ions
130.050 b1
175.119 y1
243.133 b2
303.179 y2
372.175 b3
503.297 y4
716.366 b6
853.406 b7
1331.640 y11
1460.662 y12
ELERTSHQLQEAQQQLESLQR / File: sydney_ip_25.raw; SpectrumID: ; scans: 8343

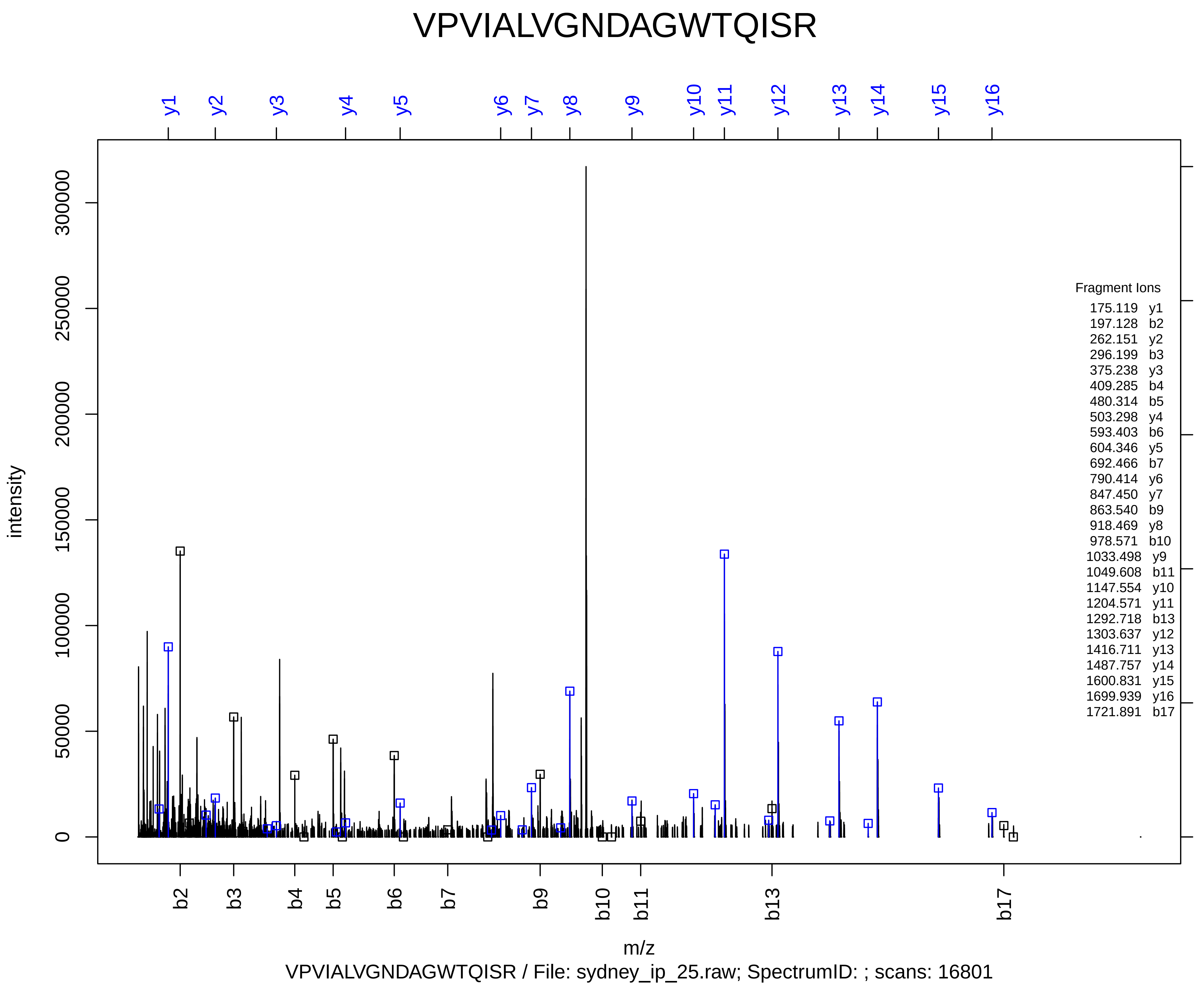
VPVIALVGNDAGWTQISR
intensity
m/z
y1 y2 y3 y4 y5 y6 y7 y8 y9 y10 y11 y12 y13 y14 y15 y16
b2 b3 b4 b5 b6 b7 b9 b10 b11 b13 b17
Fragment Ions
175.119 y1
197.128 b2
262.151 y2
296.199 b3
375.238 y3
409.285 b4
480.314 b5
503.298 y4
593.403 b6
604.346 y5
692.466 b7
790.414 y6
847.450 y7
863.540 b9
918.469 y8
978.571 b10
1033.498 y9
1049.608 b11
1147.554 y10
1204.571 y11
1292.718 b13
1303.637 y12
1416.711 y13
1487.757 y14
1600.831 y15
1699.939 y16
1721.891 b17
VPVIALVGNDAGWTQISR / File: sydney_ip_25.raw; SpectrumID: ; scans: 16801

GQQDPGSEASESHGLEAR

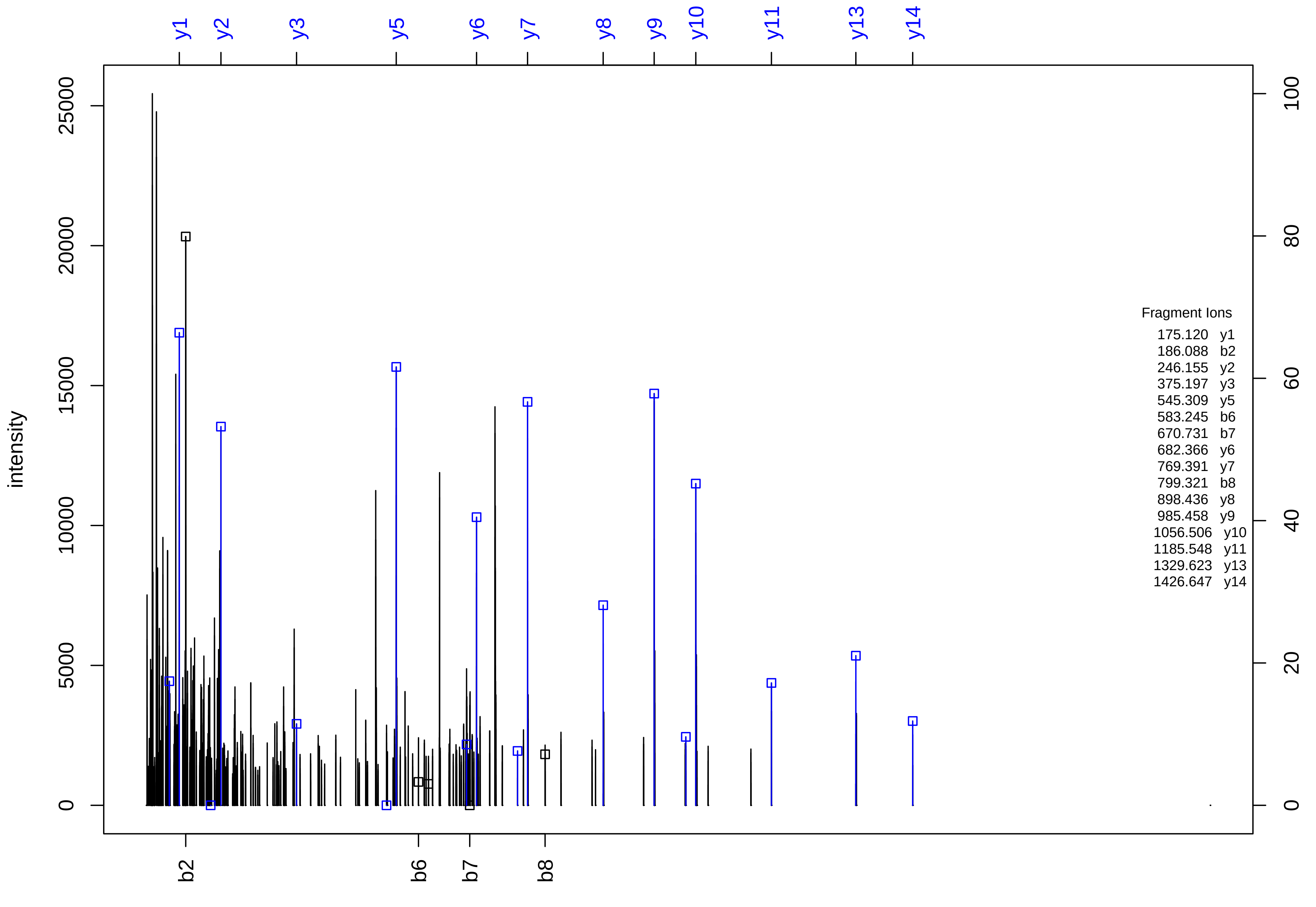

GQQDPGSEASESHGLEAR / File: sydney_ip_25.raw; SpectrumID: ; scans: 1970

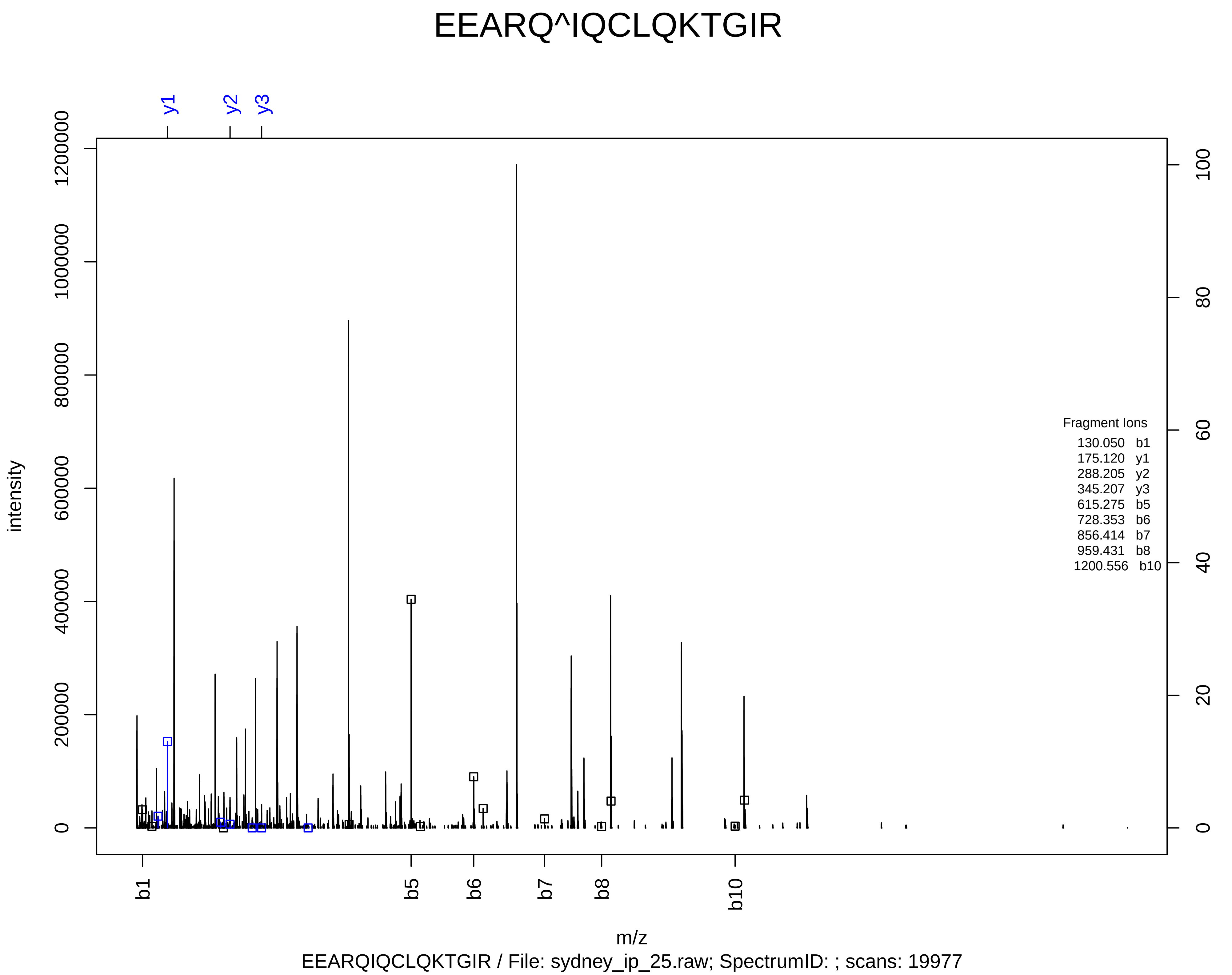
EEARQ^IQCLQKTGIR
y1
y2
y3
intensity
1200000
1000000
800000
600000
400000
200000
0
100
80
60
40
20
0
Fragment Ions
130.050 b1
175.120 y1
288.205 y2
345.207 y3
615.275 b5
728.353 b6
856.414 b7
959.431 b8
1200.556 b10
b1
b5
b6
b7
b8
b10
m/z
EEARQIQCLQKTGIR / File: sydney_ip_25.raw; SpectrumID: ; scans: 19977

LTHYIEEAN^VGR

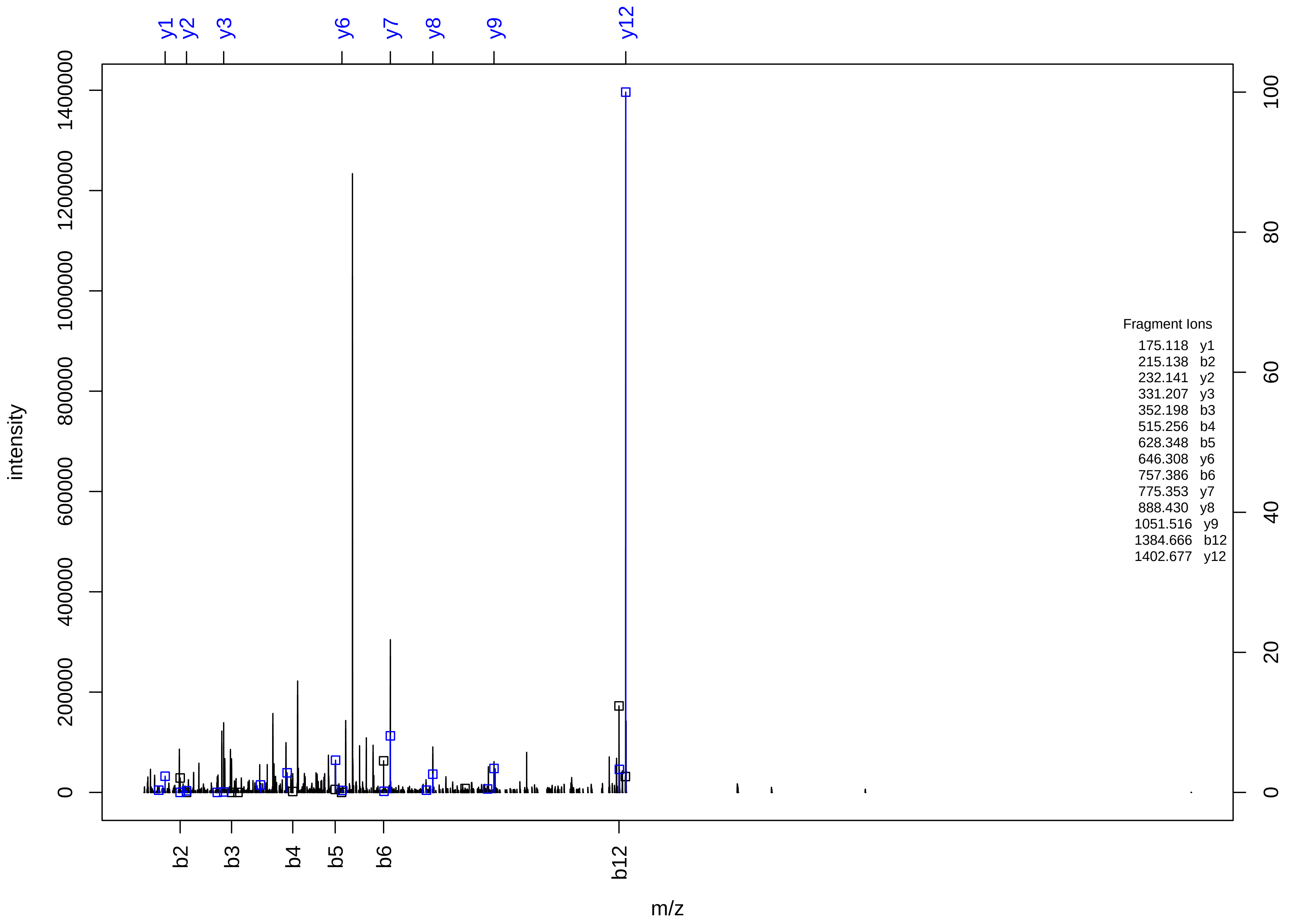

LTHYIEEANVGR / File: sydney_ip_25.raw; SpectrumID: ; scans: 12959

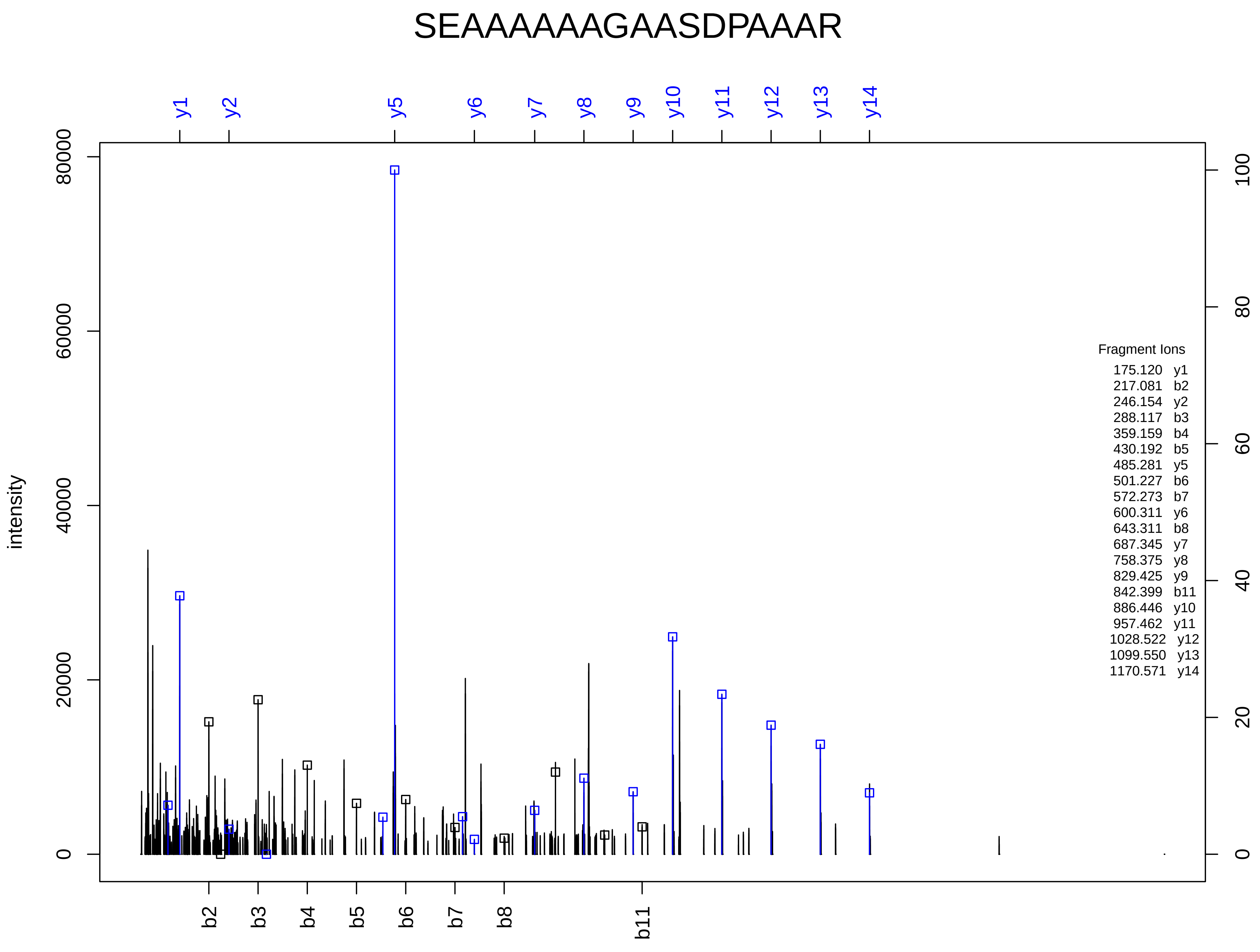

SEAAAAAAGAASDPAAAR / File: sydney_ip_25.raw; SpectrumID: ; scans: 2997

LLEELEEGQK

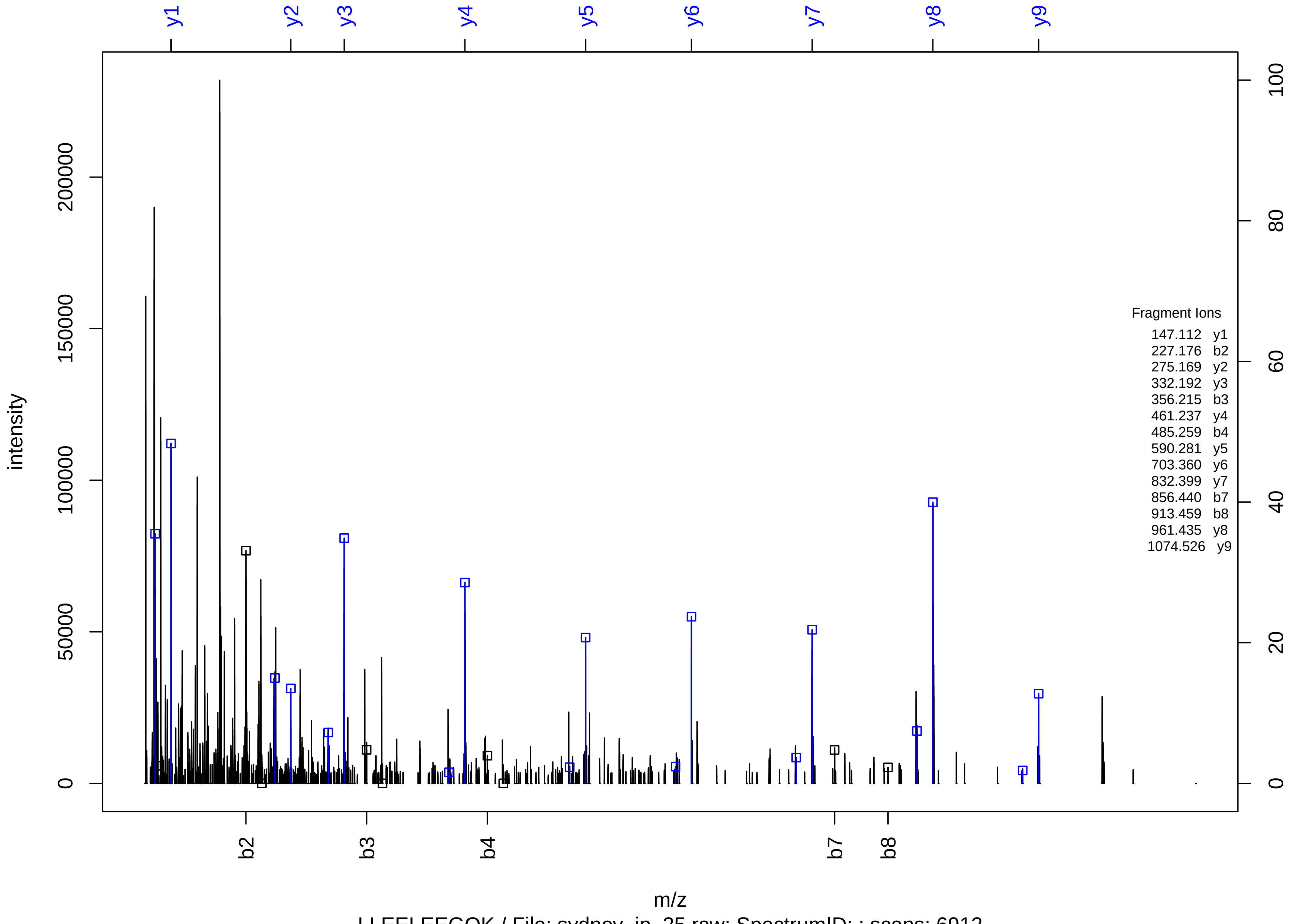

LLEELEEGQK / File: sydney_ip_25.raw; SpectrumID: ; scans: 6912

APPNATLEHFYQTSGK

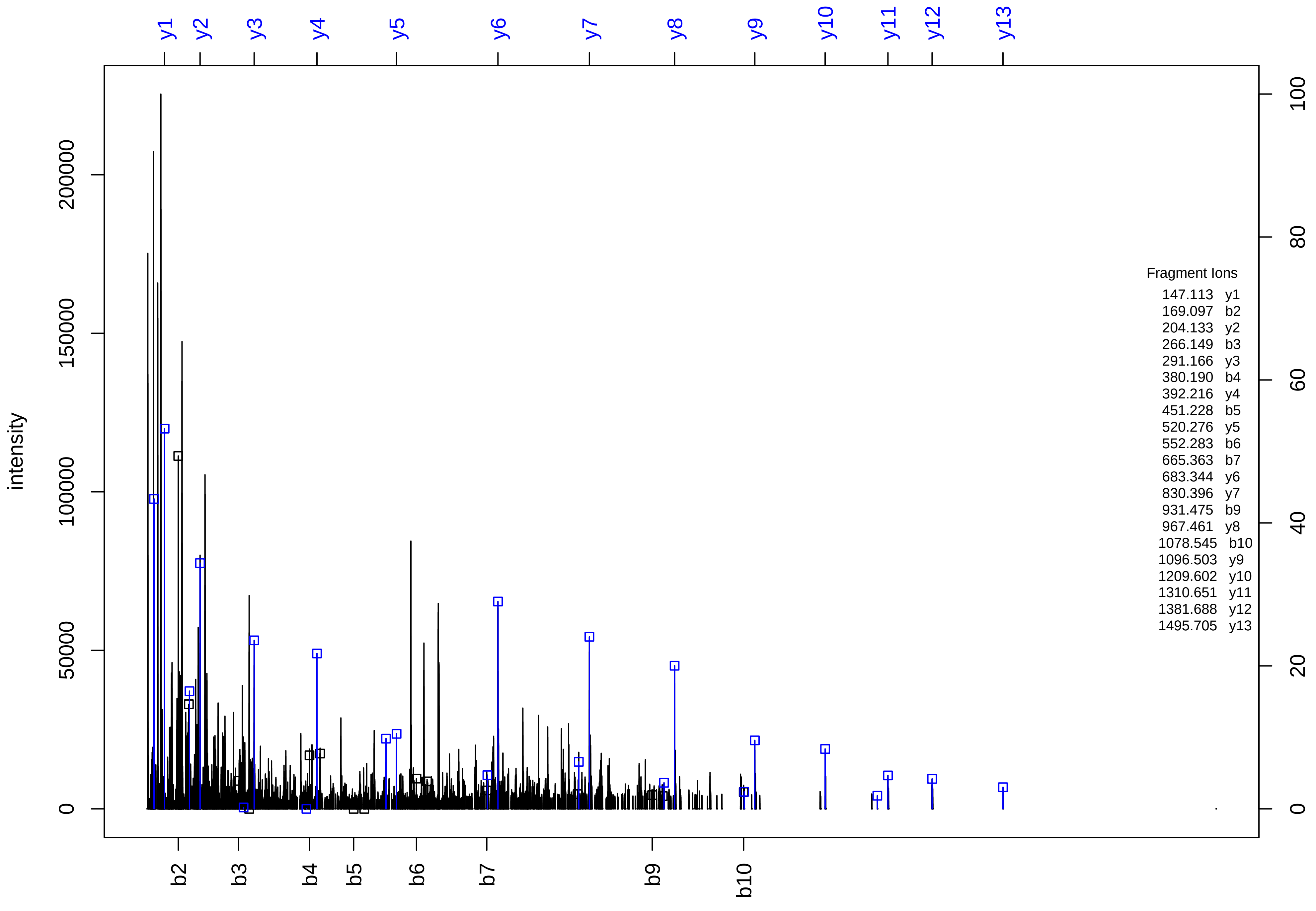

APPNATLEHFYQTSGK / File: sydney_ip_25.raw; SpectrumID: ; scans: 6739

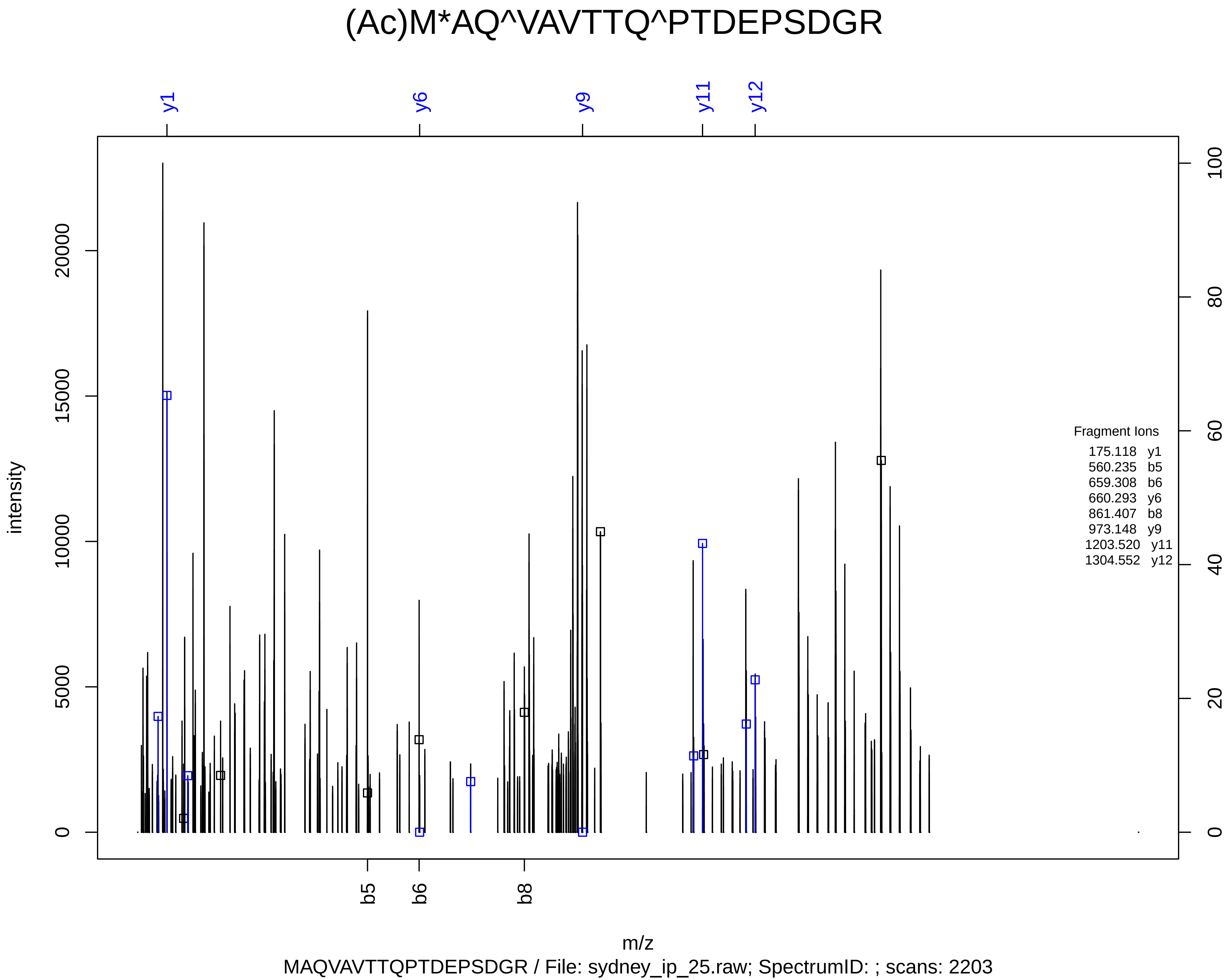
(Ac)M*AQ^VAVTTQ^PTDEPSDGR
y1
y6
y9
y11
y12
intensity
0
5000
10000
15000
20000
0
20
40
60
80
100
Fragment Ions
175.118 y1
560.235 b5
659.308 b6
660.293 y6
861.407 b8
973.148 y9
1203.520 y11
1304.552 y12
b5
b6
b8
m/z
MAQVAVTTQPTDEPSDGR / File: sydney_ip_25.raw; SpectrumID: ; scans: 2203

RAIQ^LSM*Q^GSSR

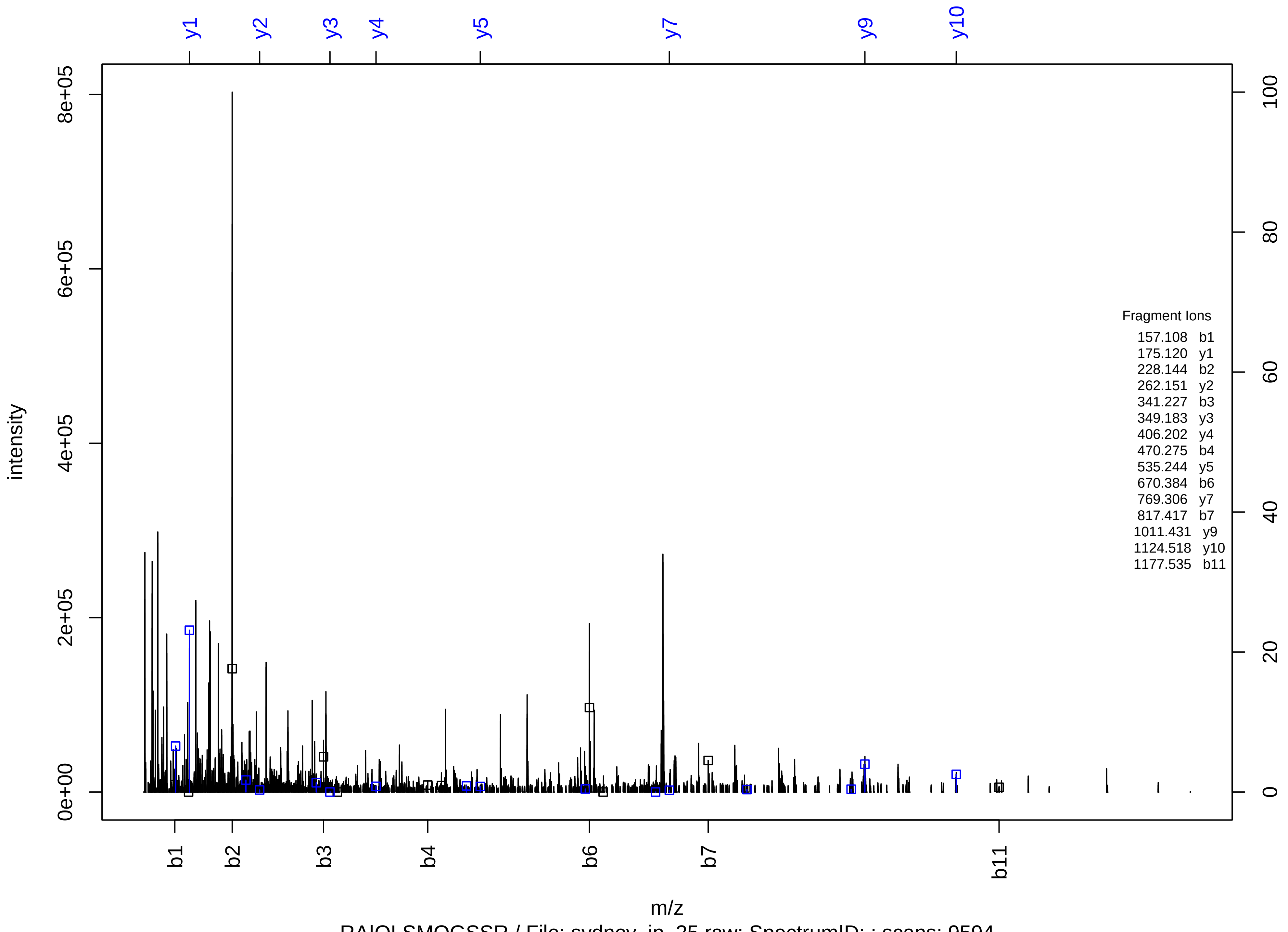

RAIQLSMQGSSR / File: sydney_ip_25.raw; SpectrumID: ; scans: 9594

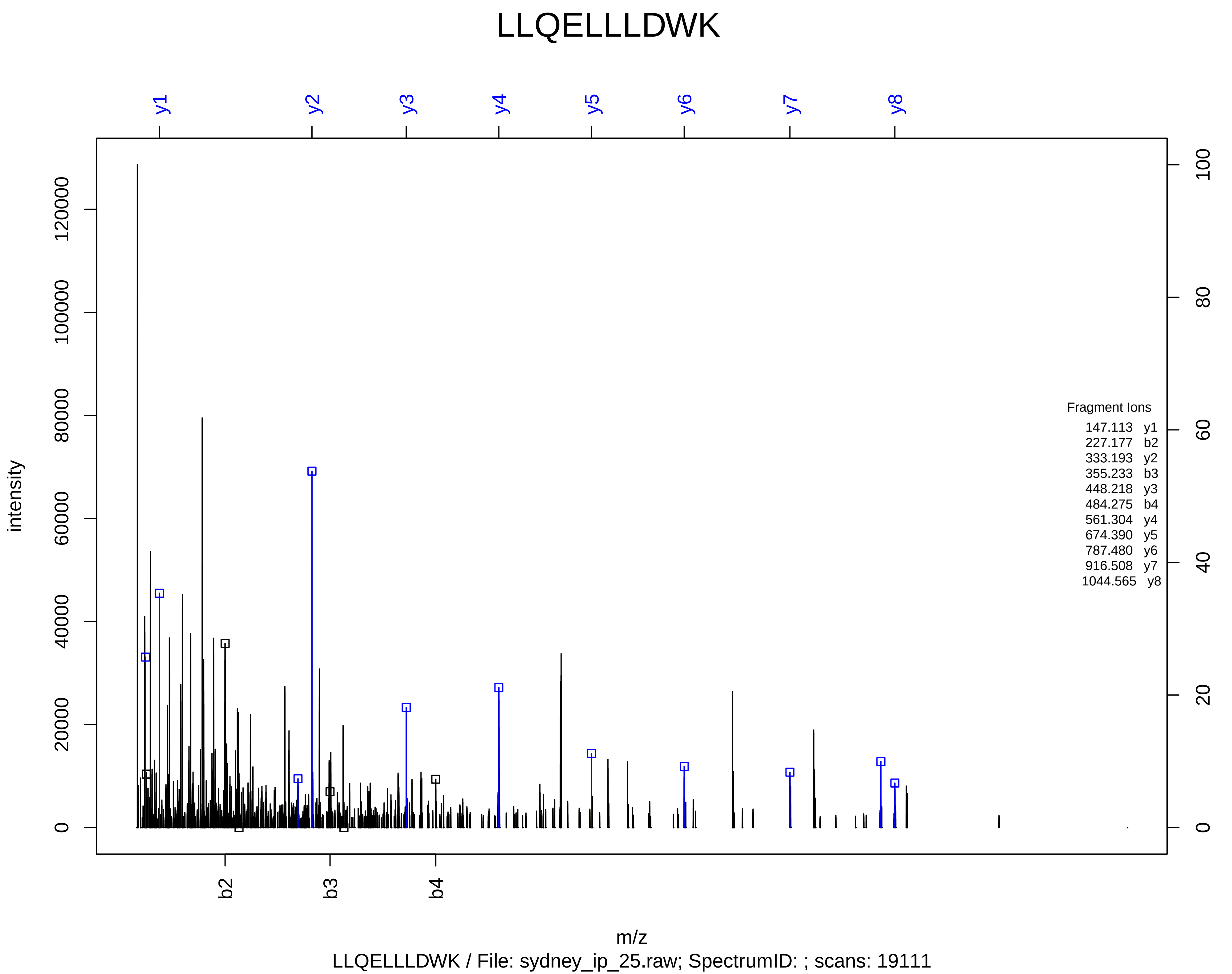
LLQELLLDWK
y1
y2
y3
y4
y5
y6
y7
y8
intensity
0
20000
40000
60000
80000
100000
120000
0
20
40
60
80
100
b2
b3
b4
m/z
Fragment Ions
147.113 y1
227.177 b2
333.193 y2
355.233 b3
448.218 y3
484.275 b4
561.304 y4
674.390 y5
787.480 y6
916.508 y7
1044.565 y8
LLQELLLDWK / File: sydney_ip_25.raw; SpectrumID: ; scans: 19111

VLLITPGFLR

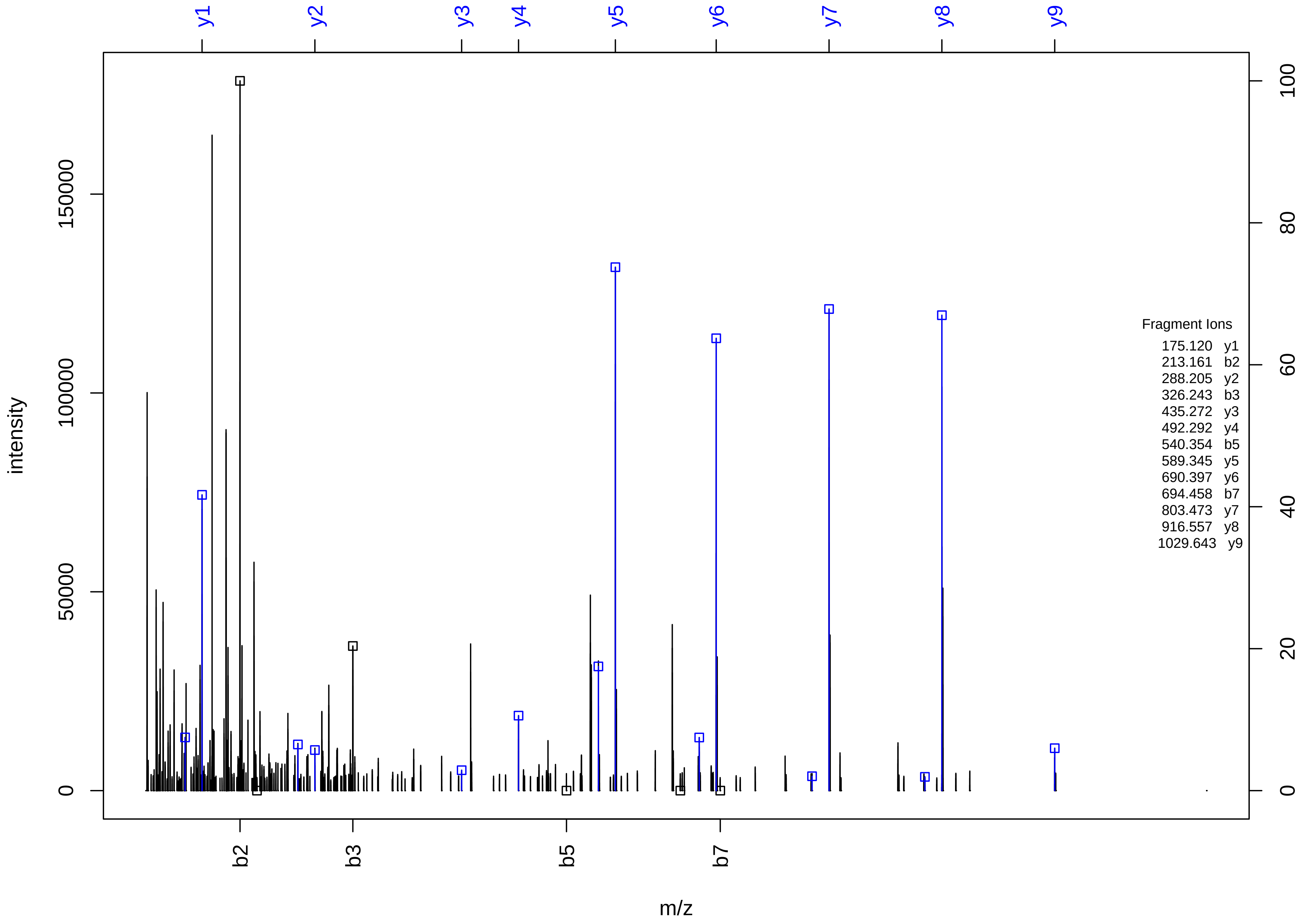

VLLITPGFLR / File: sydney_ip_25.raw; SpectrumID: ; scans: 16999

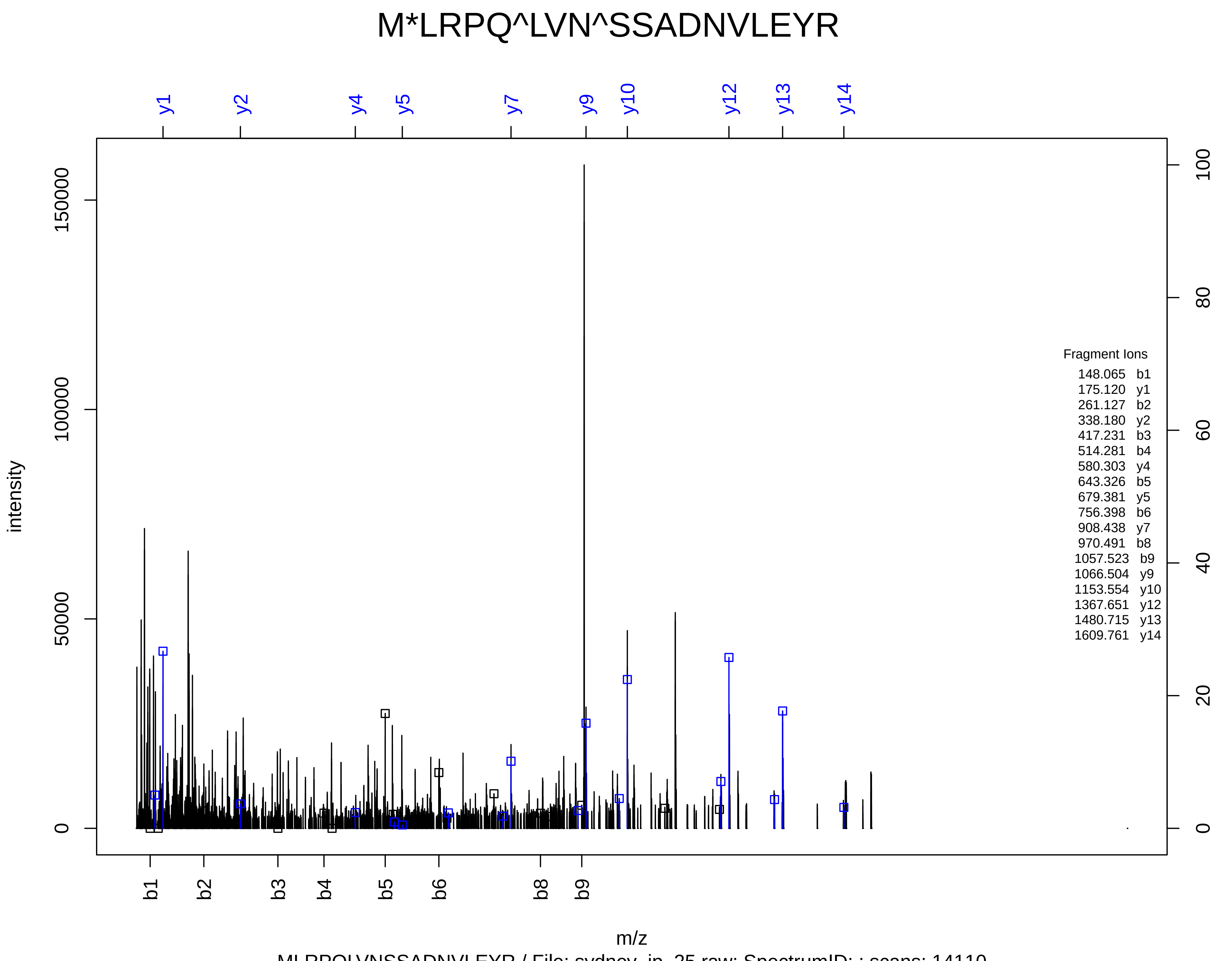

M*LRPQ^LVN^SSADNVLEYR
y1
y2
y4
y5
y7
y9
y10
y12
y13
y14
intensity
150000
100000
50000
0
100
80
60
40
20
0
Fragment Ions
148.065 b1
175.120 y1
261.127 b2
338.180 y2
417.231 b3
514.281 b4
580.303 y4
643.326 b5
679.381 y5
756.398 b6
908.438 y7
970.491 b8
1057.523 b9
1066.504 y9
1153.554 y10
1367.651 y12
1480.715 y13
1609.761 y14
b1
b2
b3
b4
b5
b6
b8
b9
m/z
MLRPQLVNSSADNVLEYR / File: sydney_ip_25.raw; SpectrumID: ; scans: 14110

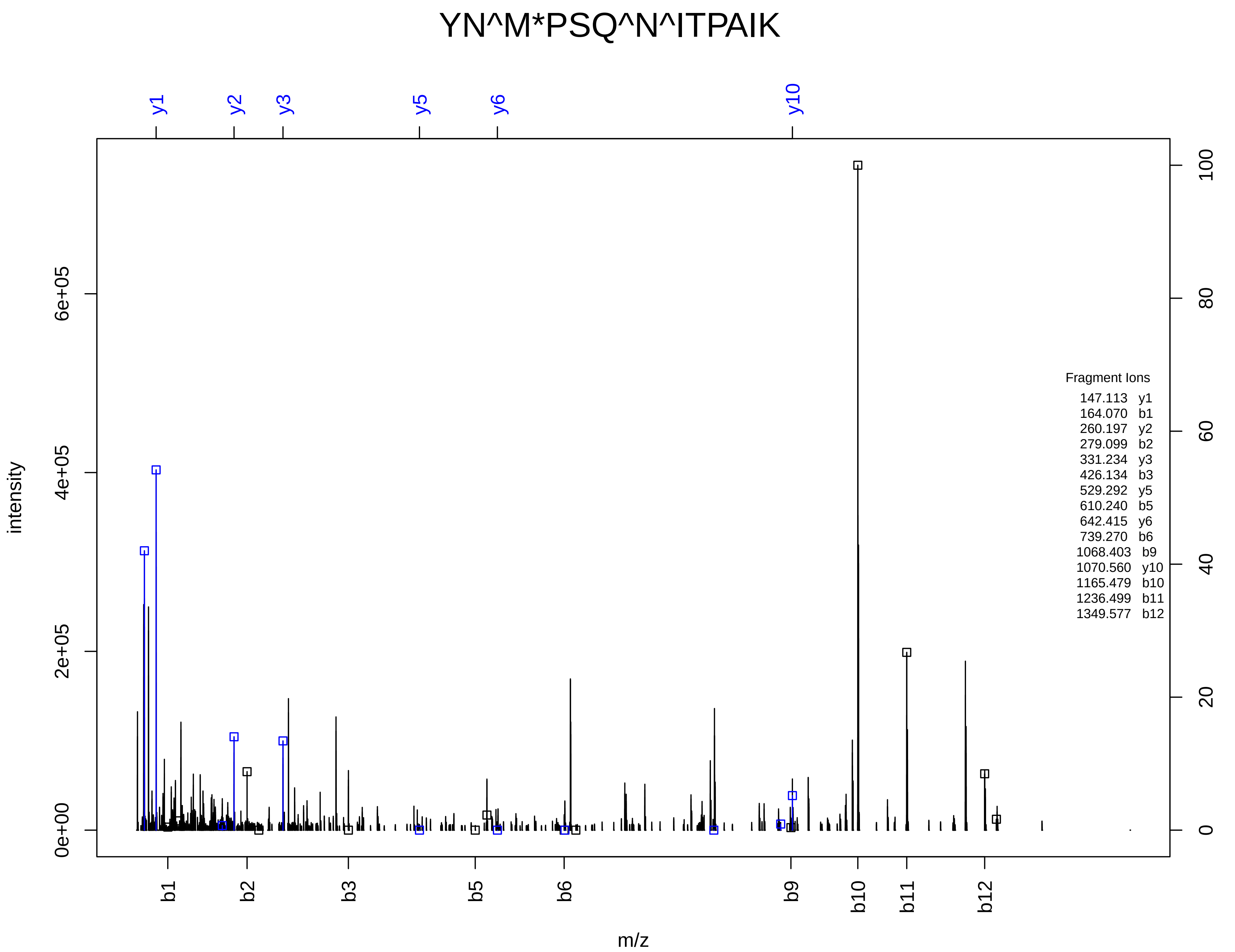

YNMPSQNITPAIK / File: sydney_ip_25.raw; SpectrumID: ; scans: 15044

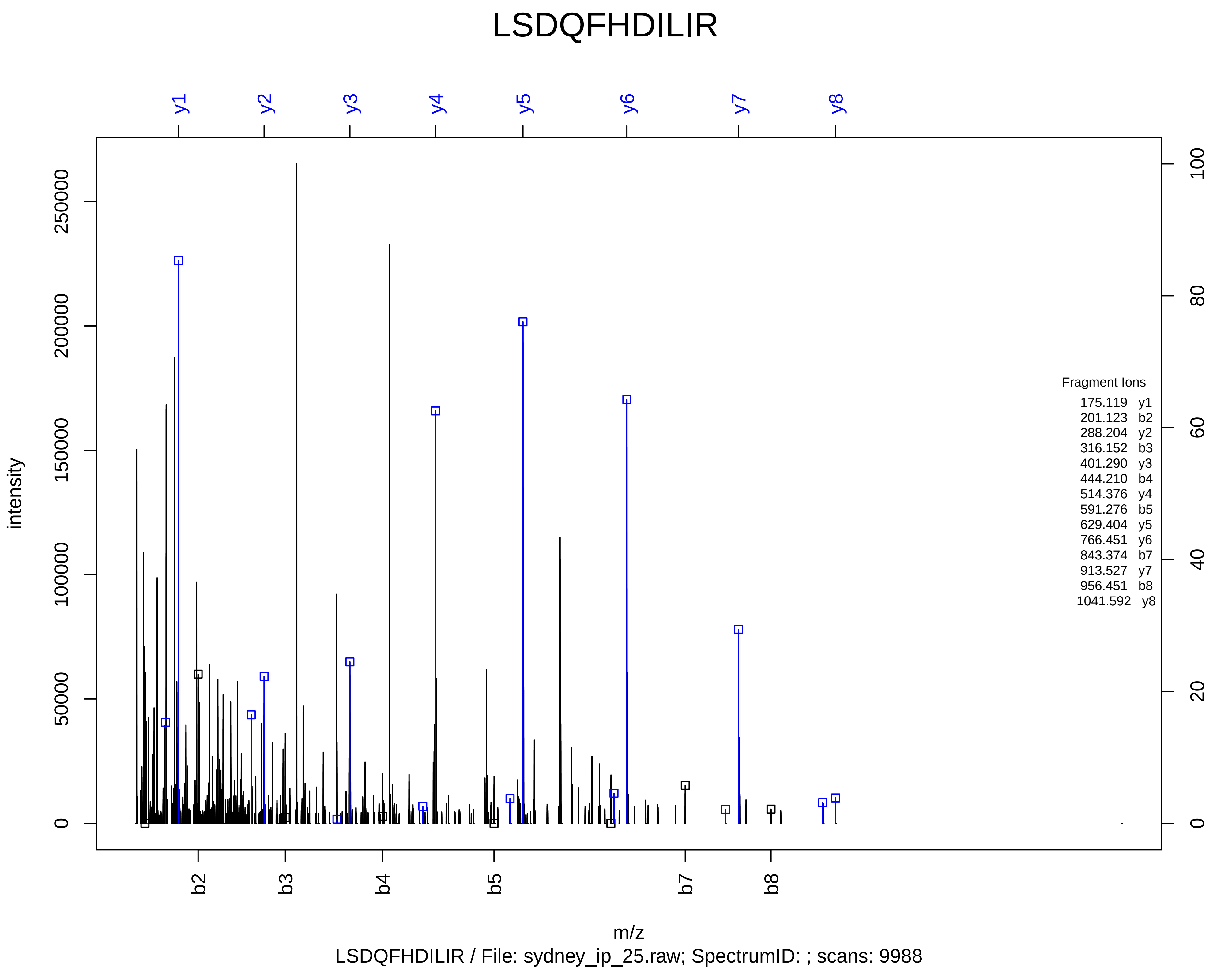

LSDQFHDILIR
y1
y2
y3
y4
y5
y6
y7
y8
intensity
0
50000
100000
150000
200000
250000
0
20
40
60
80
100
b2
b3
b4
b5
b7
b8
m/z
Fragment Ions
175.119 y1
201.123 b2
288.204 y2
316.152 b3
401.290 y3
444.210 b4
514.376 y4
591.276 b5
629.404 y5
766.451 y6
843.374 b7
913.527 y7
956.451 b8
1041.592 y8
LSDQFHDILIR / File: sydney_ip_25.raw; SpectrumID: ; scans: 9988

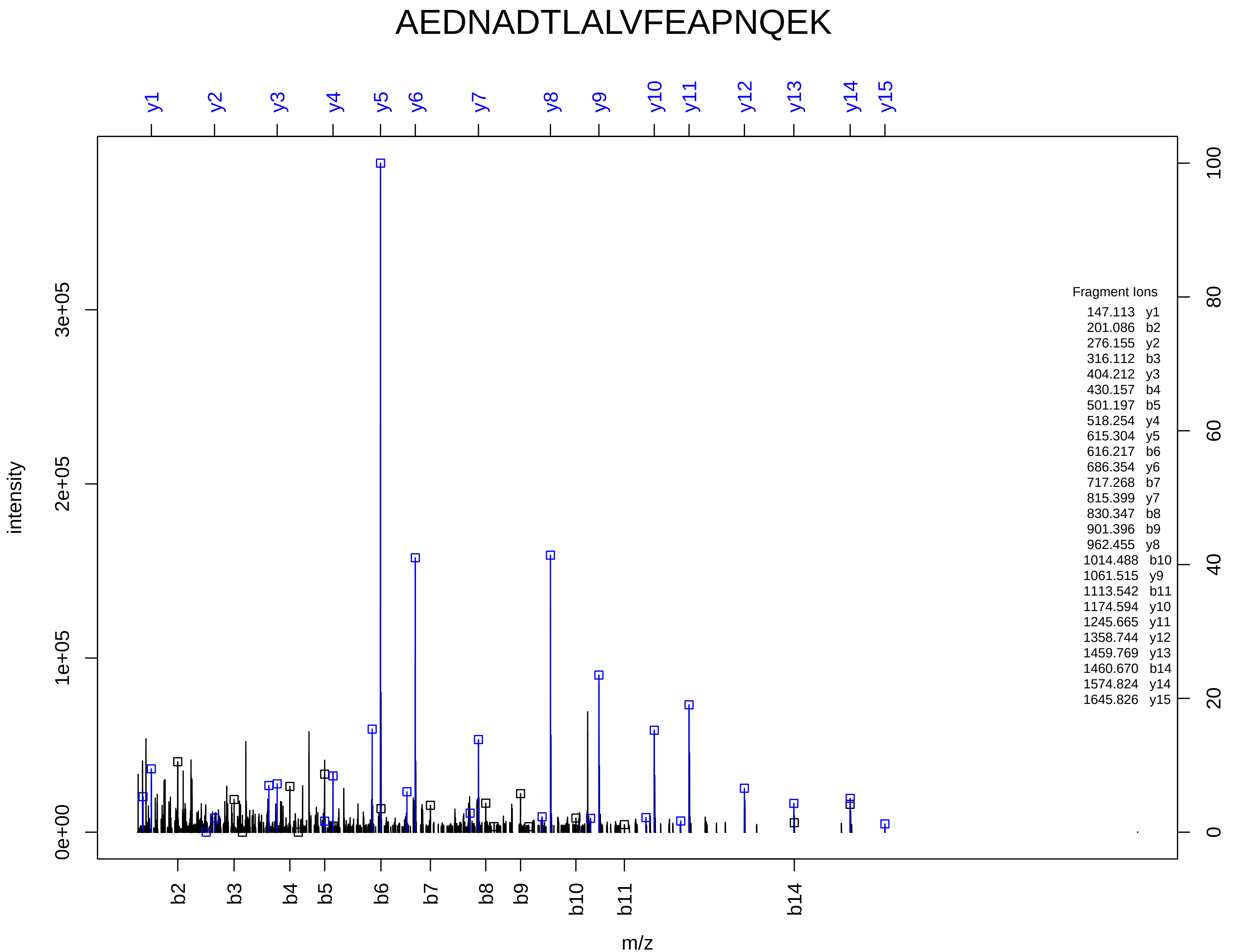

AEDNADTLALVFEAPNQEK / File: sydney_ip_25.raw; SpectrumID: ; scans: 14926

ILIQFFDTAR

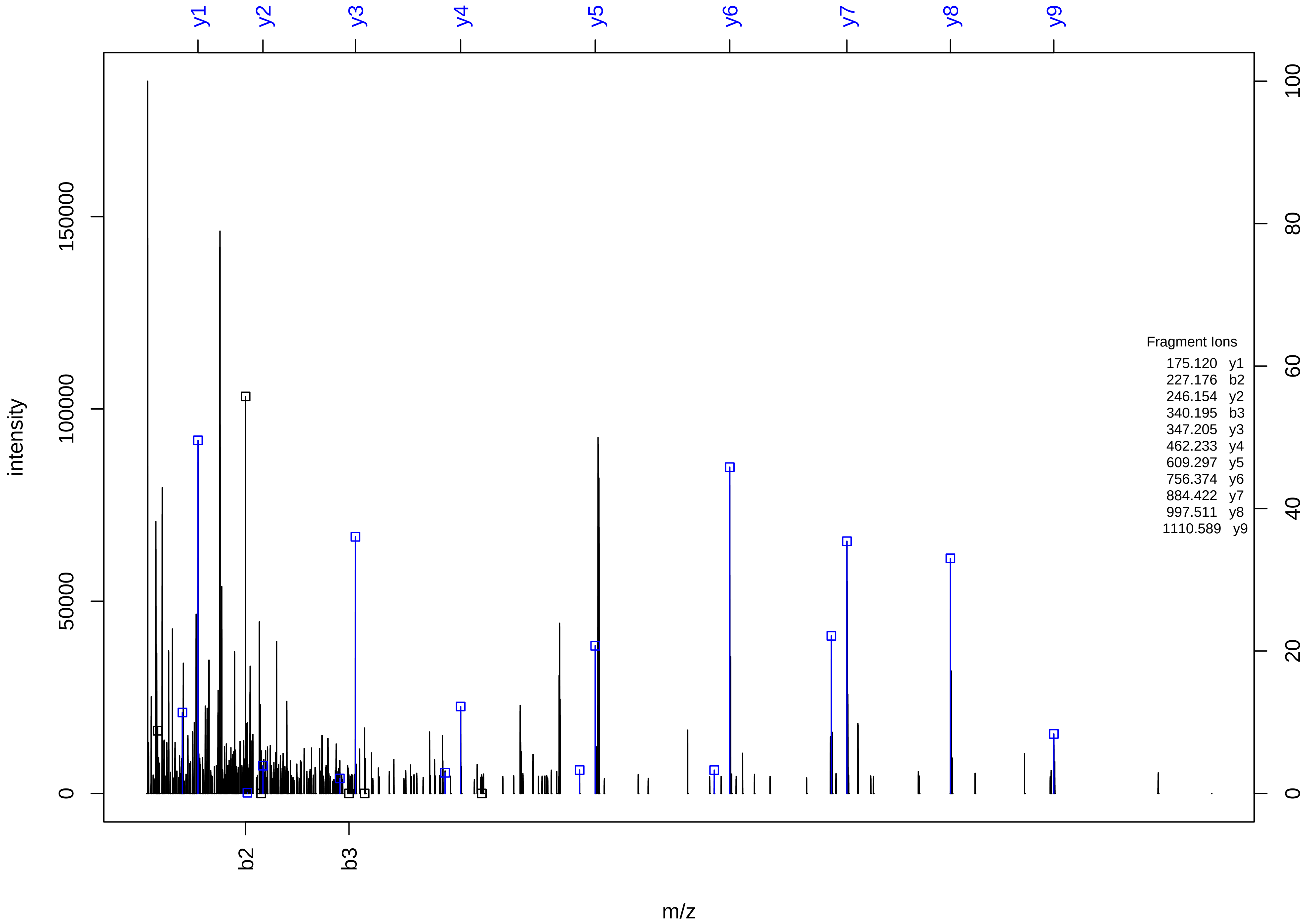

ILIQFFDTAR / File: sydney_ip_25.raw; SpectrumID: ; scans: 15890

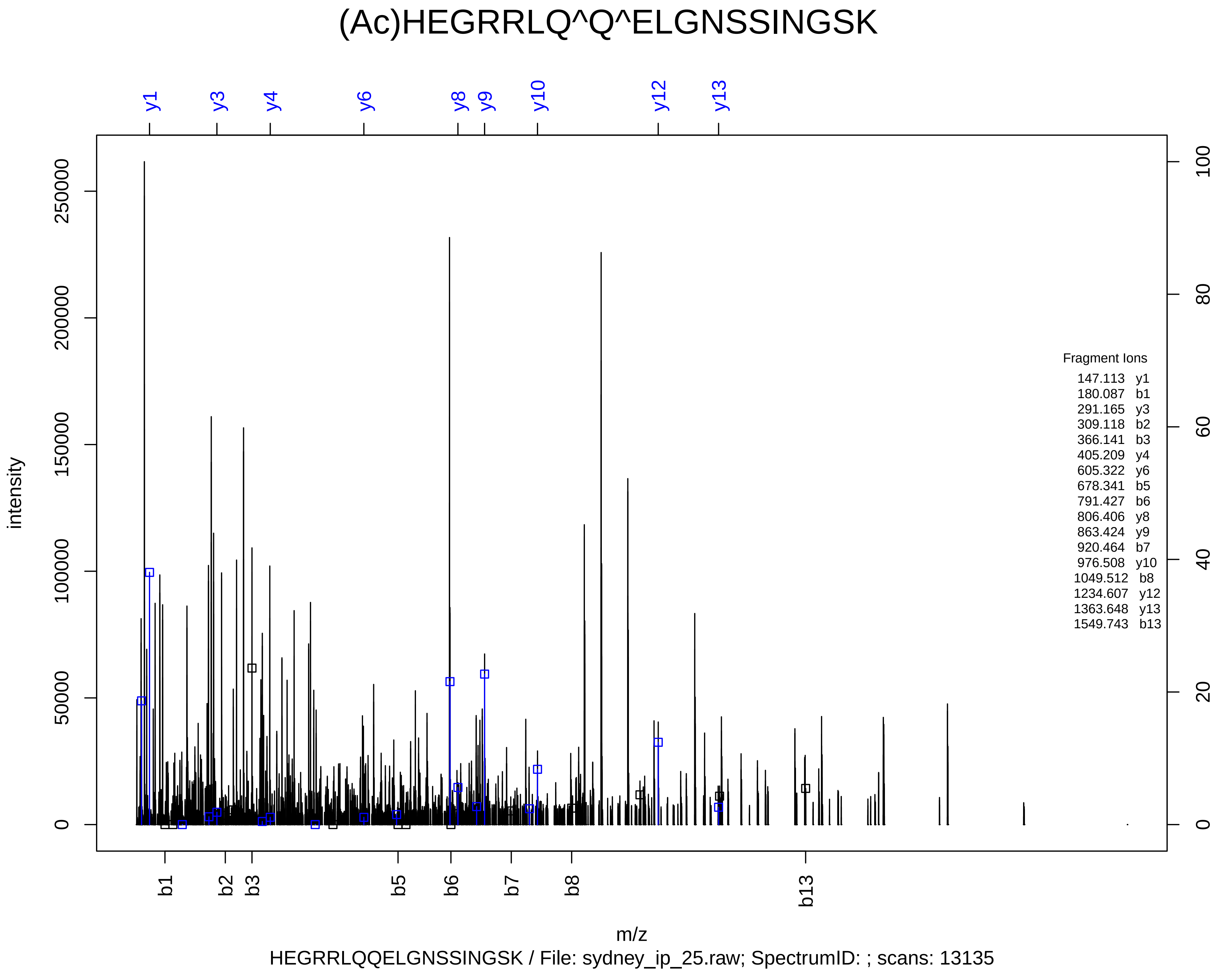
(Ac)HEGRRLQ^Q^ELGNSSINGSK
y1
y3
y4
y6
y8
y9
y10
y12
y13
intensity
0
50000
100000
150000
200000
250000
0
20
40
60
80
100
Fragment Ions
147.113 y1
180.087 b1
291.165 y3
309.118 b2
366.141 b3
405.209 y4
605.322 y6
678.341 b5
791.427 b6
806.406 y8
863.424 y9
920.464 b7
976.508 y10
1049.512 b8
1234.607 y12
1363.648 y13
1549.743 b13
b1
b2
b3
b5
b6
b7
b8
b13
m/z
HEGRRLQQELGNSSINGSK / File: sydney_ip_25.raw; SpectrumID: ; scans: 13135

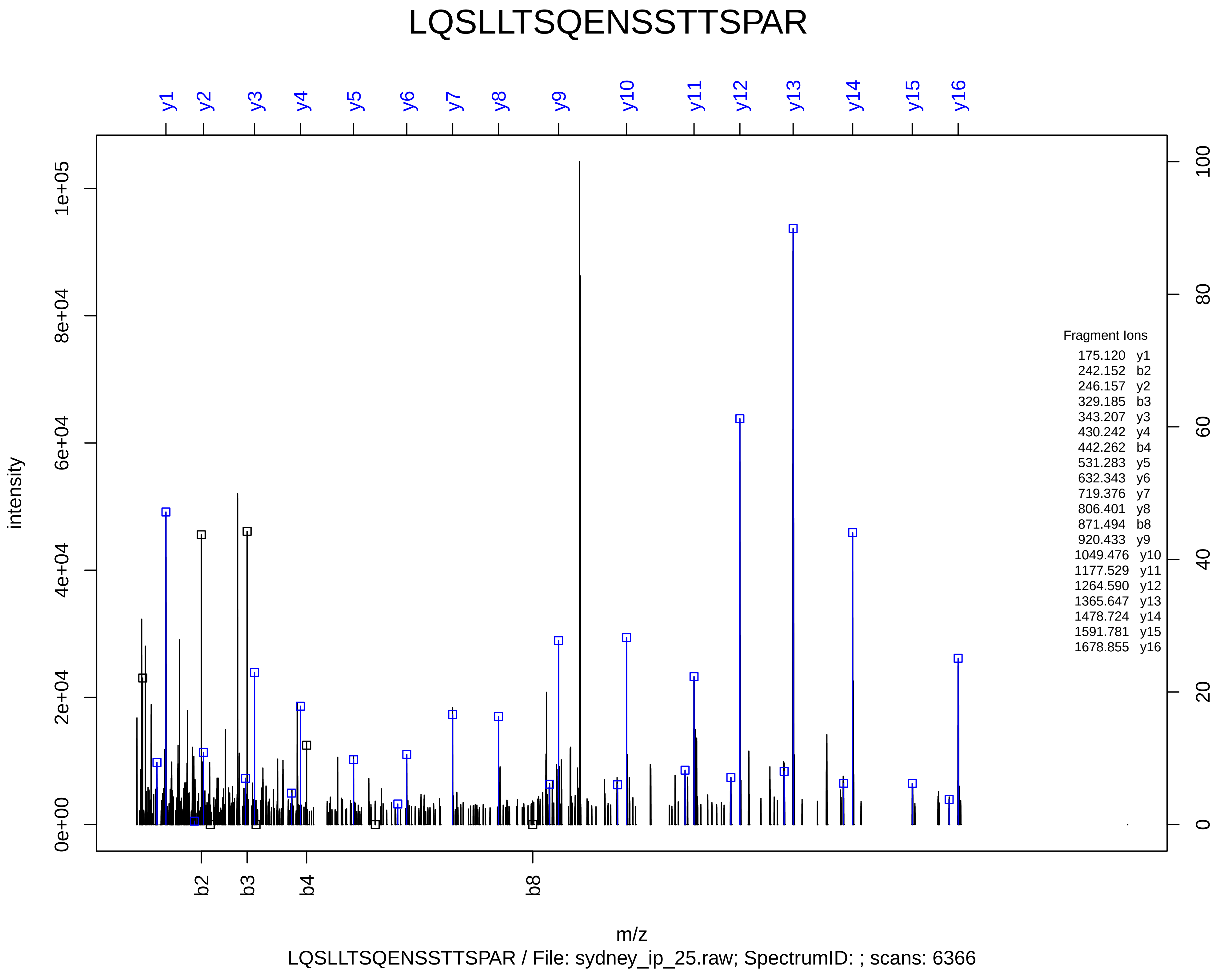

LQSLLTSQENSSTTSPAR
intensity
m/z
y1 y2 y3 y4 y5 y6 y7 y8 y9 y10 y11 y12 y13 y14 y15 y16
b2 b3 b4 b8
0e+00 2e+04 4e+04 6e+04 8e+04 1e+05
0 20 40 60 80 100
Fragment Ions
175.120 y1
242.152 b2
246.157 y2
329.185 b3
343.207 y3
430.242 y4
442.262 b4
531.283 y5
632.343 y6
719.376 y7
806.401 y8
871.494 b8
920.433 y9
1049.476 y10
1177.529 y11
1264.590 y12
1365.647 y13
1478.724 y14
1591.781 y15
1678.855 y16
LQSLLTSQENSSTTSPAR / File: sydney_ip_25.raw; SpectrumID: ; scans: 6366

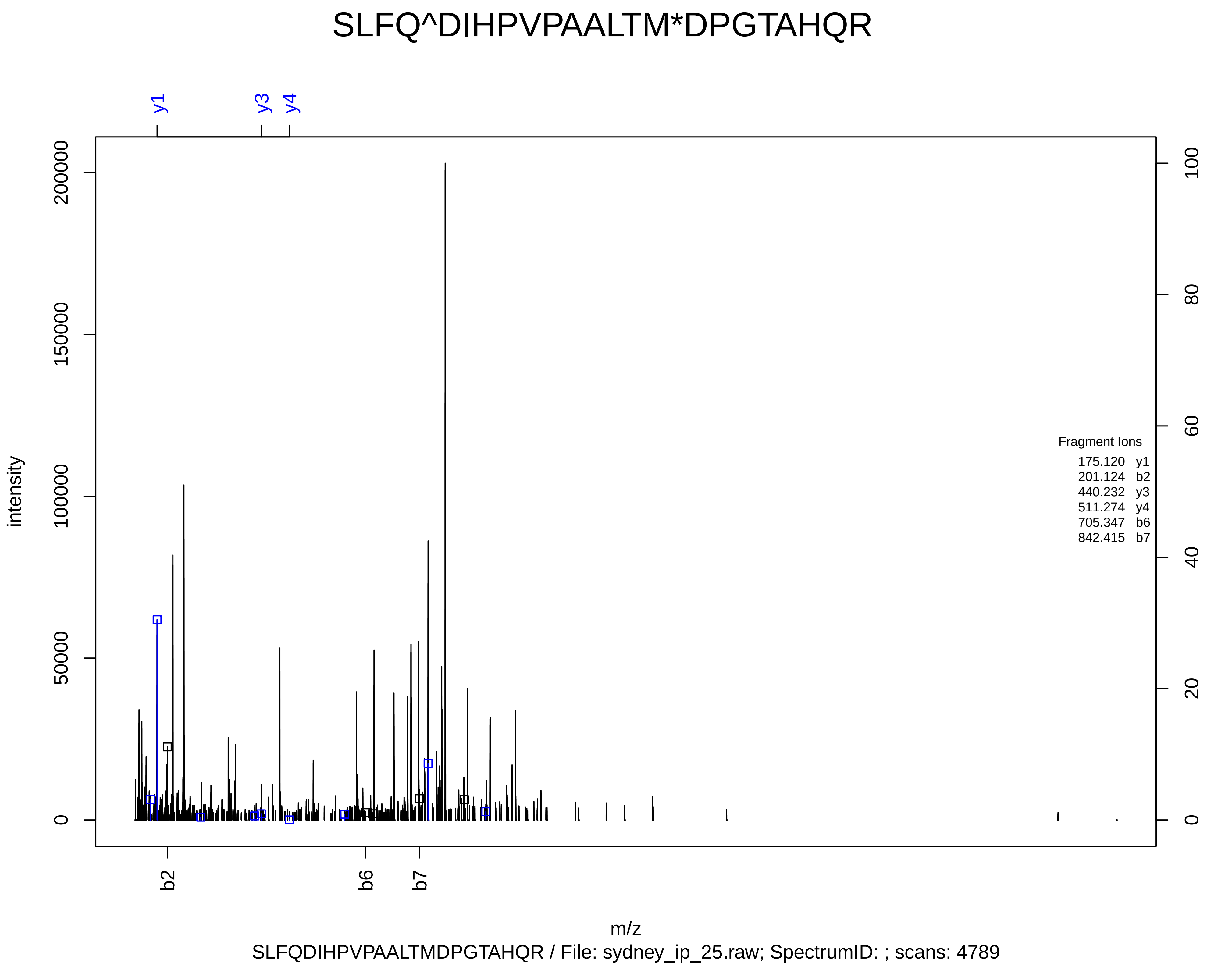
SLFQ^DIHPVPAALTM*DPGTAHQR
y1
y3
y4
intensity
200000
150000
100000
50000
0
100
80
60
40
20
0
Fragment Ions
175.120 y1
201.124 b2
440.232 y3
511.274 y4
705.347 b6
842.415 b7
b2
b6
b7
m/z
SLFQDIHPVPAALTMDPGTAHQR / File: sydney_ip_25.raw; SpectrumID: ; scans: 4789

KEKNQ^IN^N^N^K

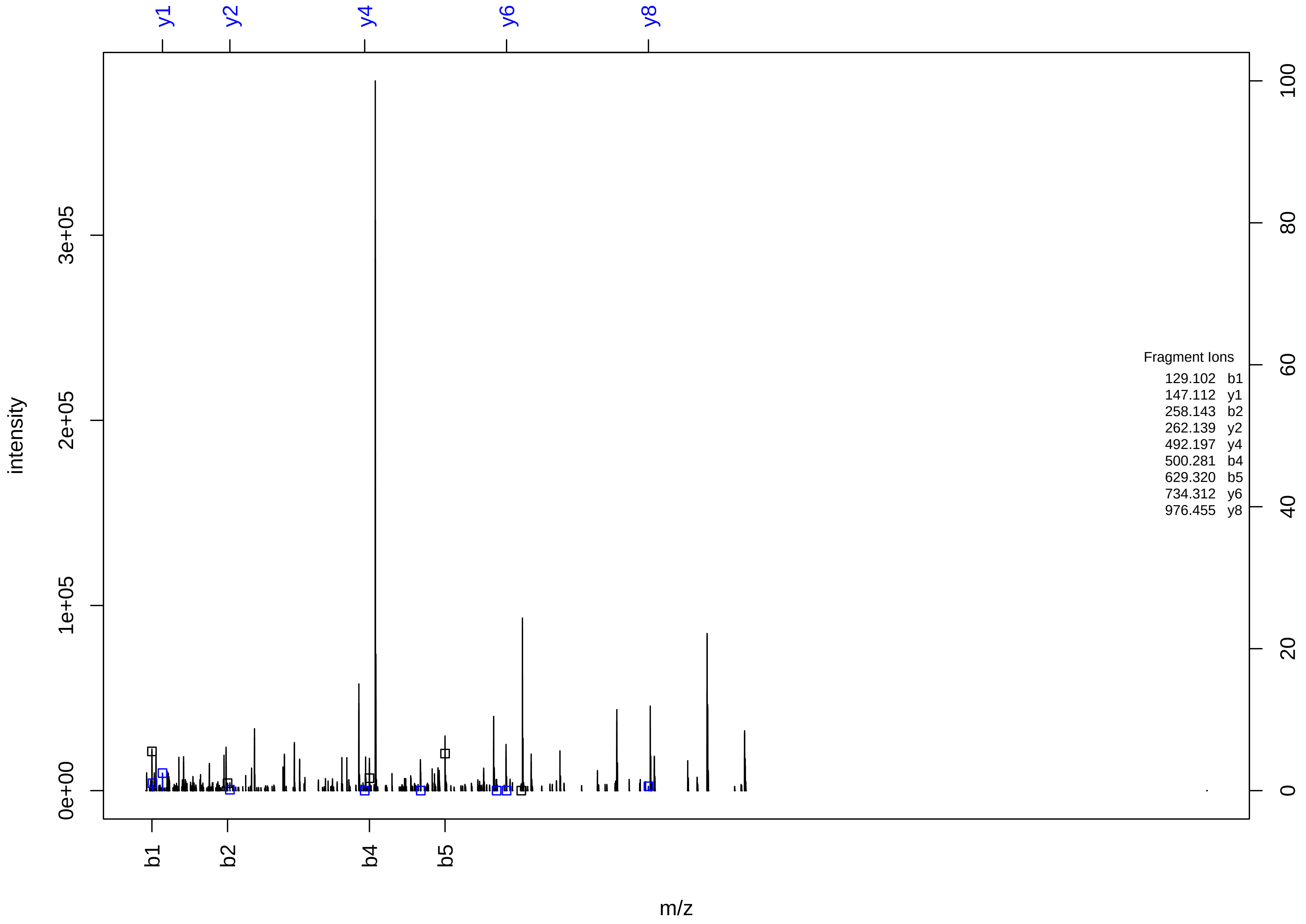

KEKNQINNNK / File: sydney_ip_25.raw; SpectrumID: ; scans: 1770

VFEDHENVVEVLSDWTR

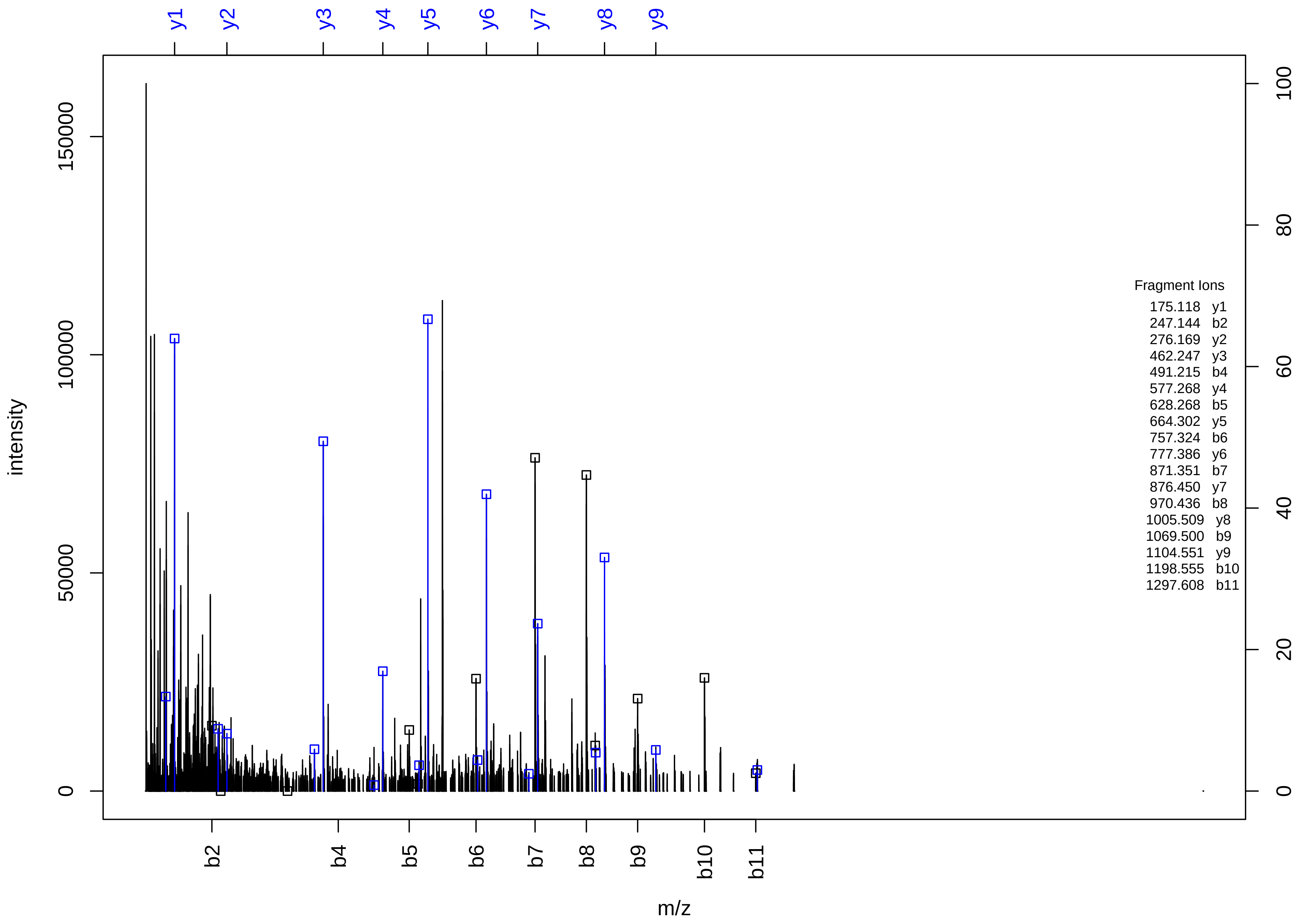

VFEDHENVVEVLSDWTR / File: sydney_ip_25.raw; SpectrumID: ; scans: 16737

VYELPFIVVLDHR

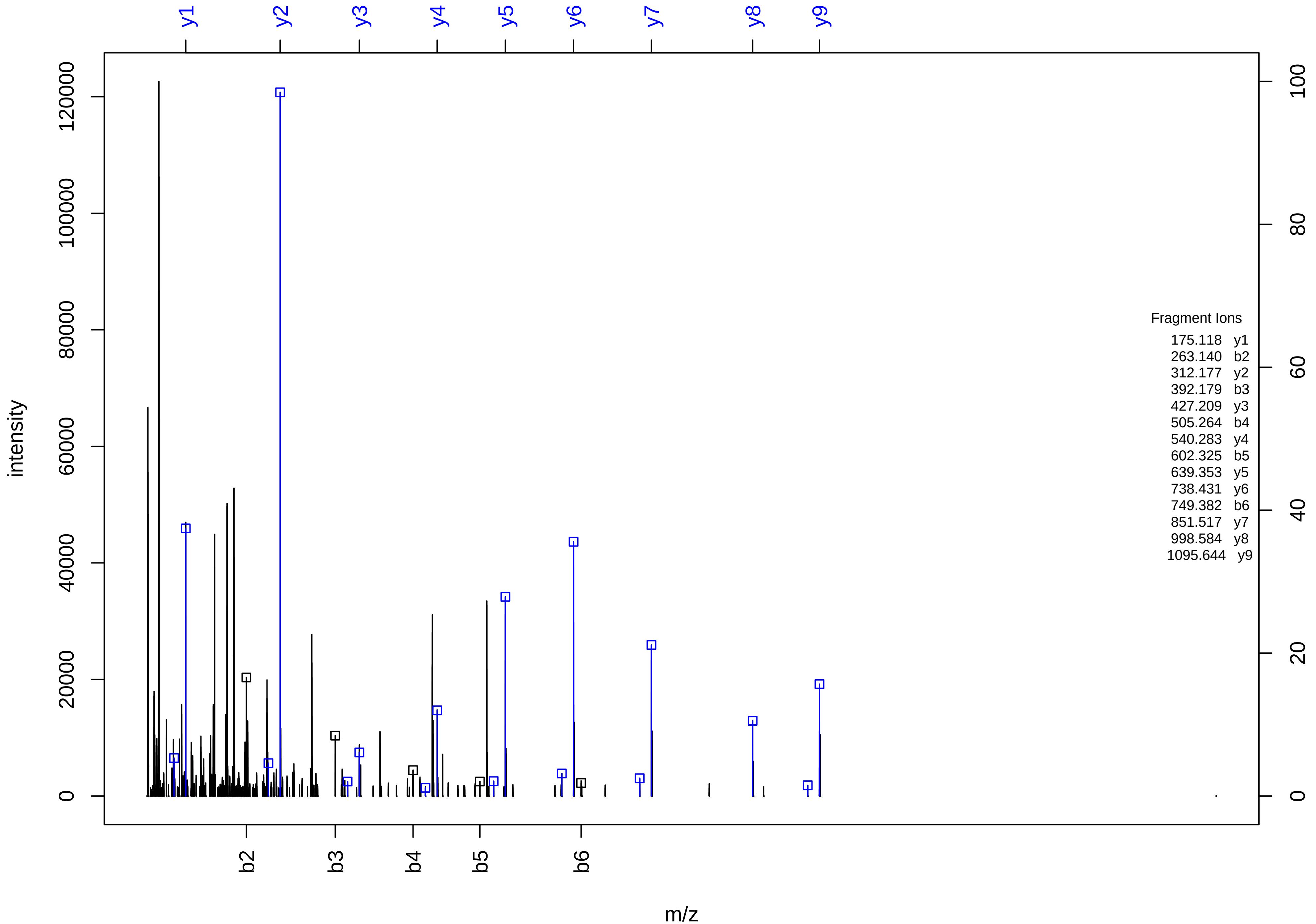

VYELPFIVVLDHR / File: sydney_ip_25.raw; SpectrumID: ; scans: 18918

IGIIGGTGLDDPEILEGR

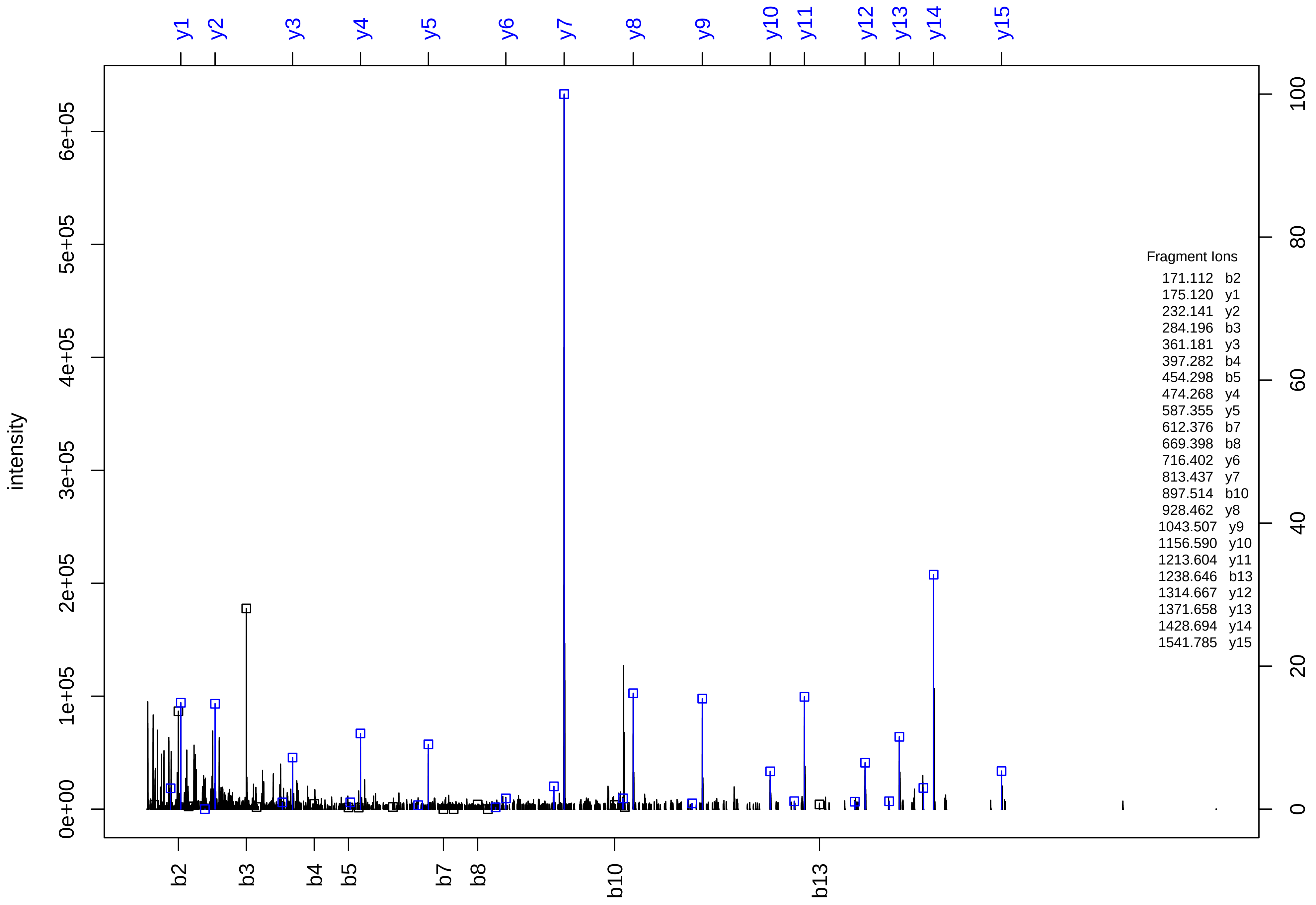

IGIIGGTGLDDPEILEGR / File: sydney_ip_25.raw; SpectrumID: ; scans: 16243

IEGTPLETVQK

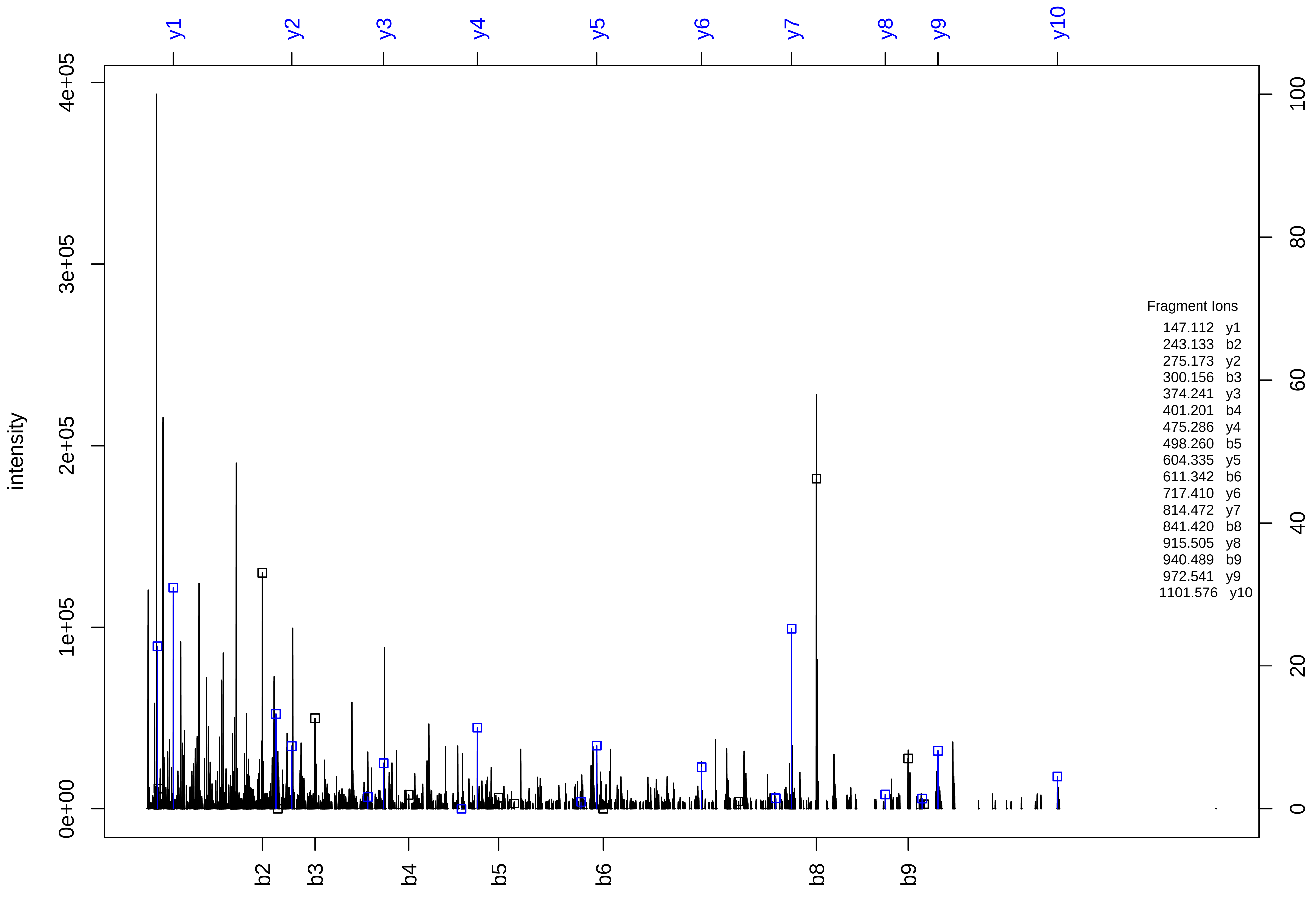

IEGTPLETVQK / File: sydney_ip_25.raw; SpectrumID: ; scans: 5252

M*KALN^IDRCQ^SLIR

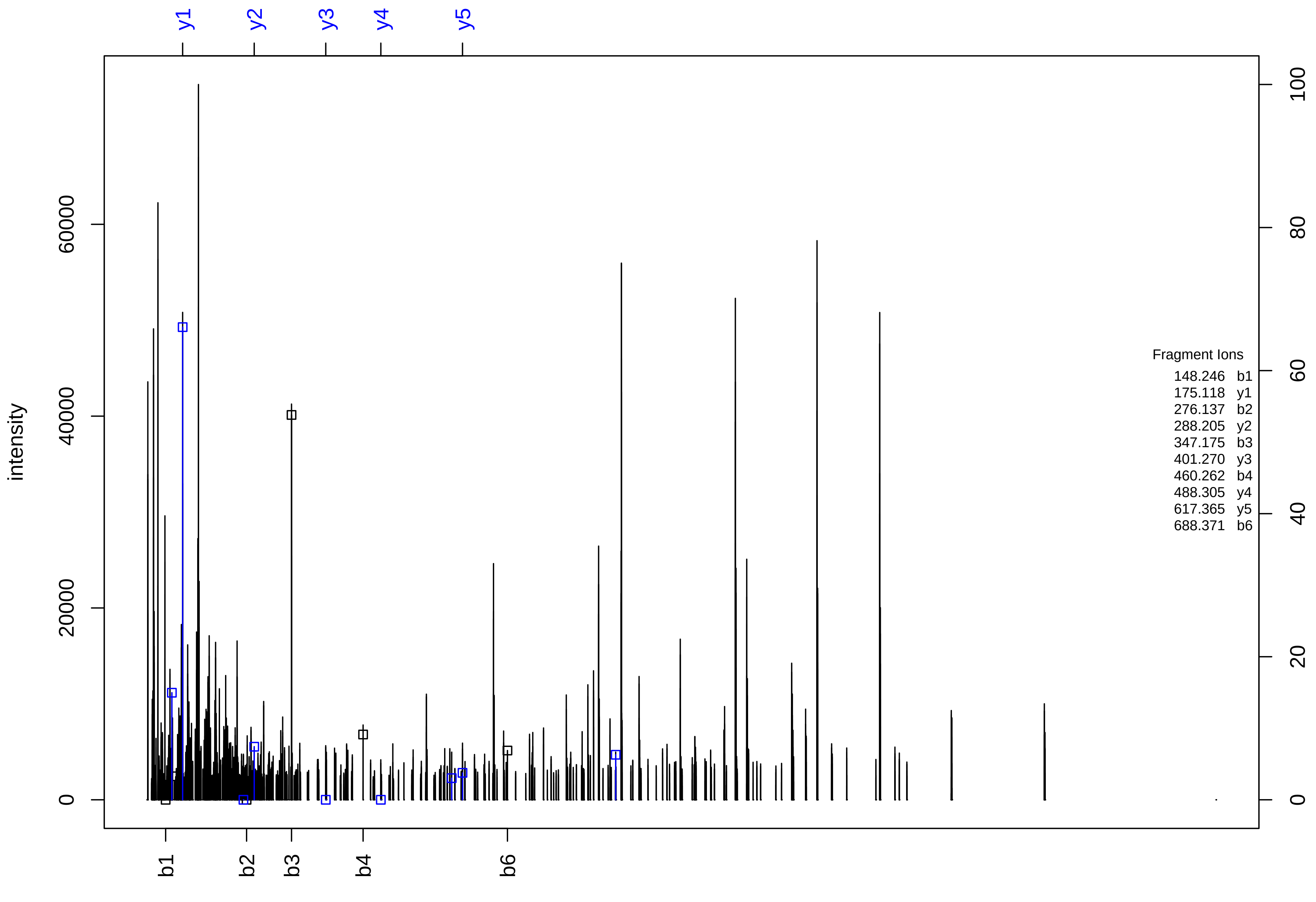

MKALNIDRCQSLIR / File: sydney_ip_25.raw; SpectrumID: ; scans: 6520

ELVFLQFR

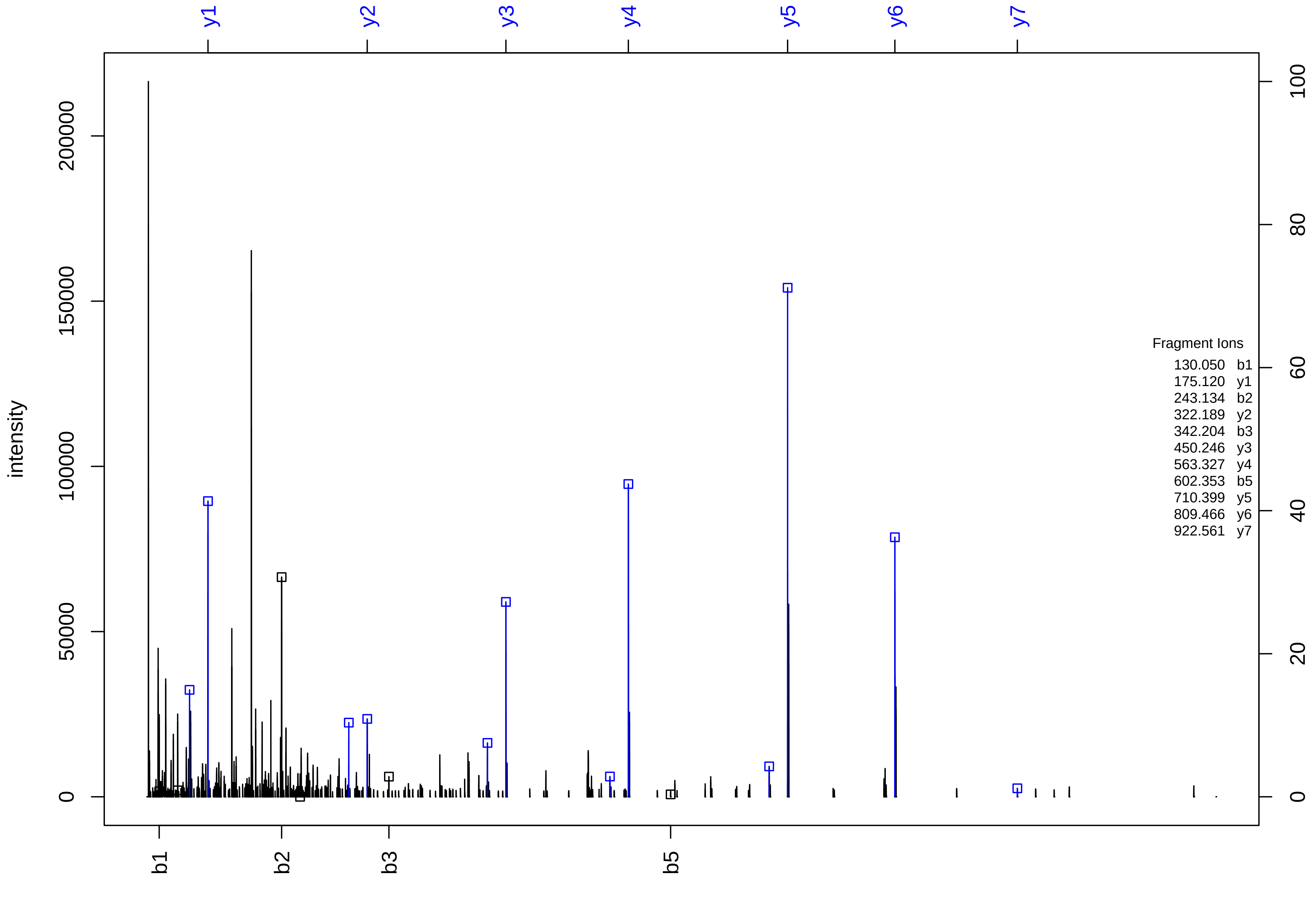

ELVFLQFR / File: sydney_ip_25.raw; SpectrumID: ; scans: 15932

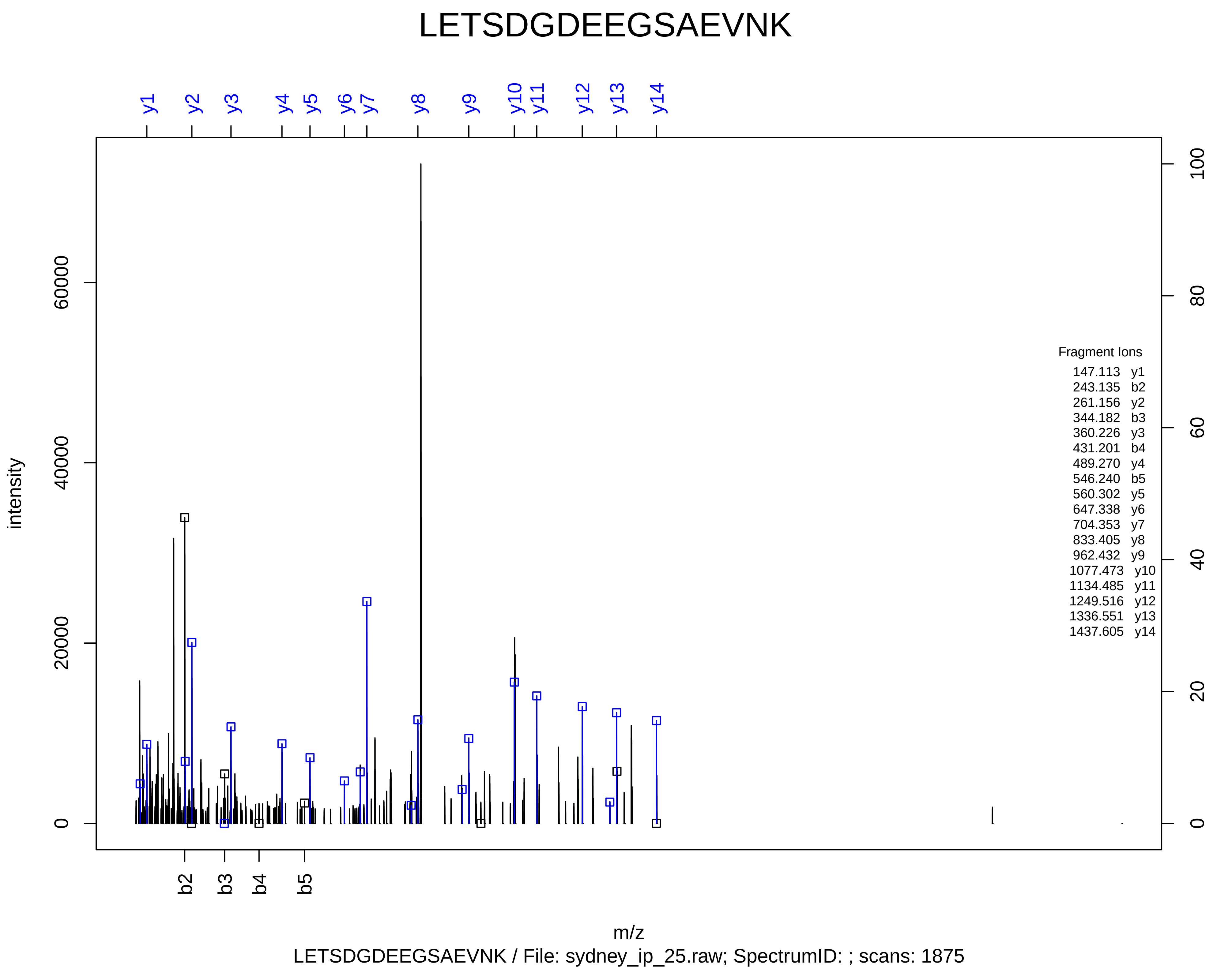

LETSDGDEEGSAEVNK
y1
y2
y3
y4
y5
y6
y7
y8
y9
y10
y11
y12
y13
y14
intensity
0
20000
40000
60000
0
20
40
60
80
100
b2
b3
b4
b5
m/z
Fragment Ions
147.113 y1
243.135 b2
261.156 y2
344.182 b3
360.226 y3
431.201 b4
489.270 y4
546.240 b5
560.302 y5
647.338 y6
704.353 y7
833.405 y8
962.432 y9
1077.473 y10
1134.485 y11
1249.516 y12
1336.551 y13
1437.605 y14
LETSDGDEEGSAEVNK / File: sydney_ip_25.raw; SpectrumID: ; scans: 1875

N^KREQ^LEQ^IQ^K

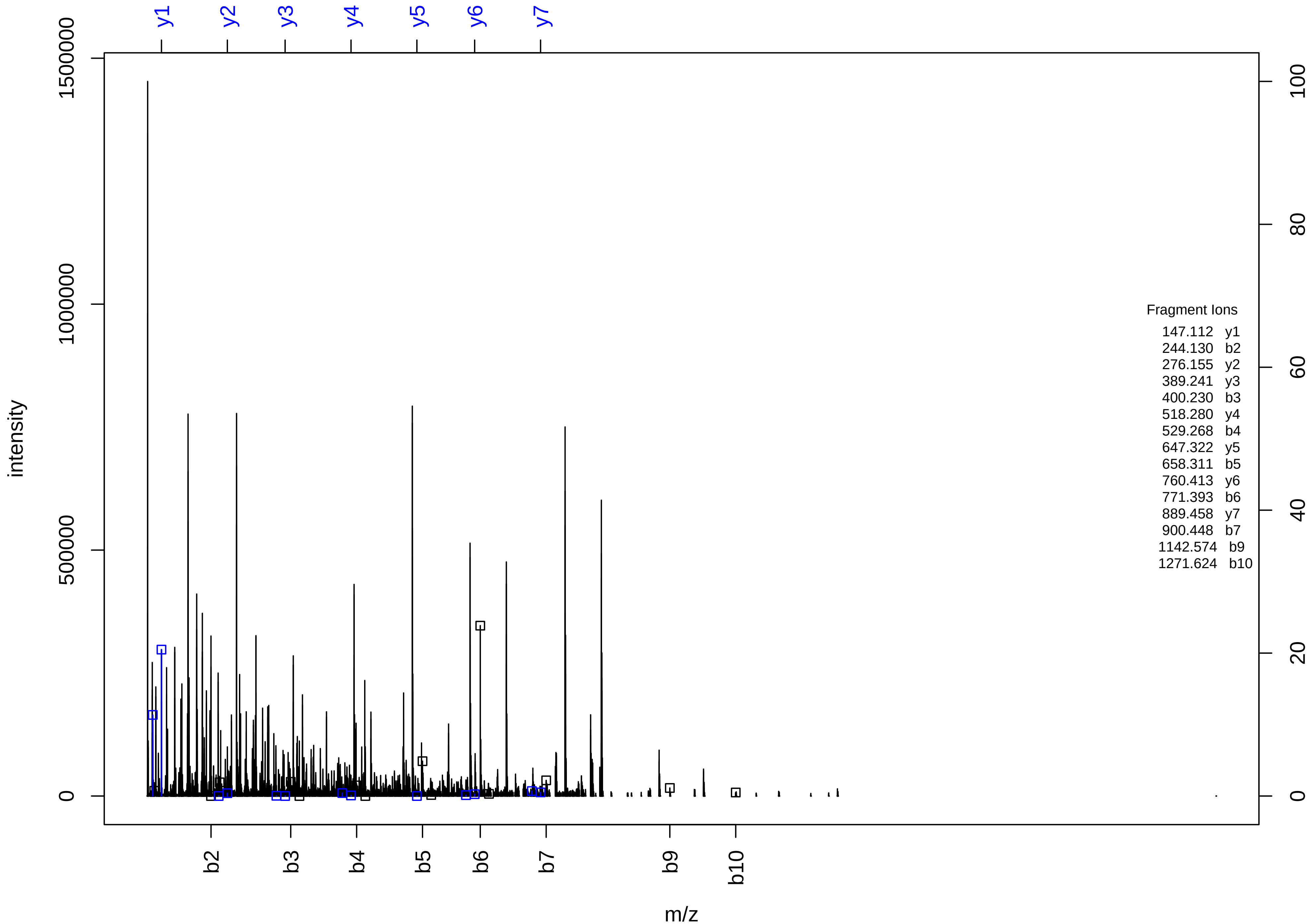

NKREQLEQIQK / File: sydney_ip_26.raw; SpectrumID: ; scans: 19483

LLPDFLLER

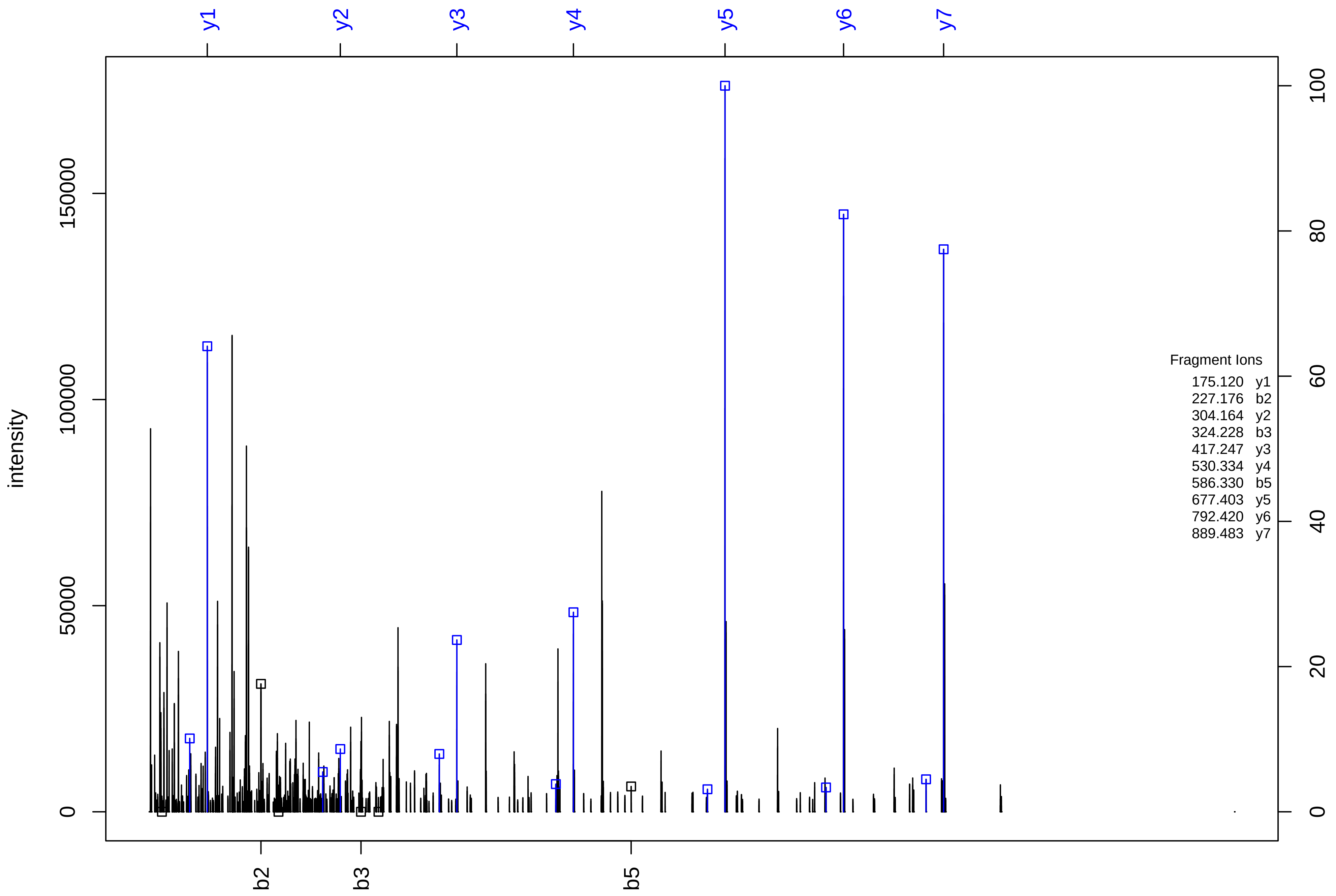

LLPDFLLER / File: sydney_ip_26.raw; SpectrumID: ; scans: 16082

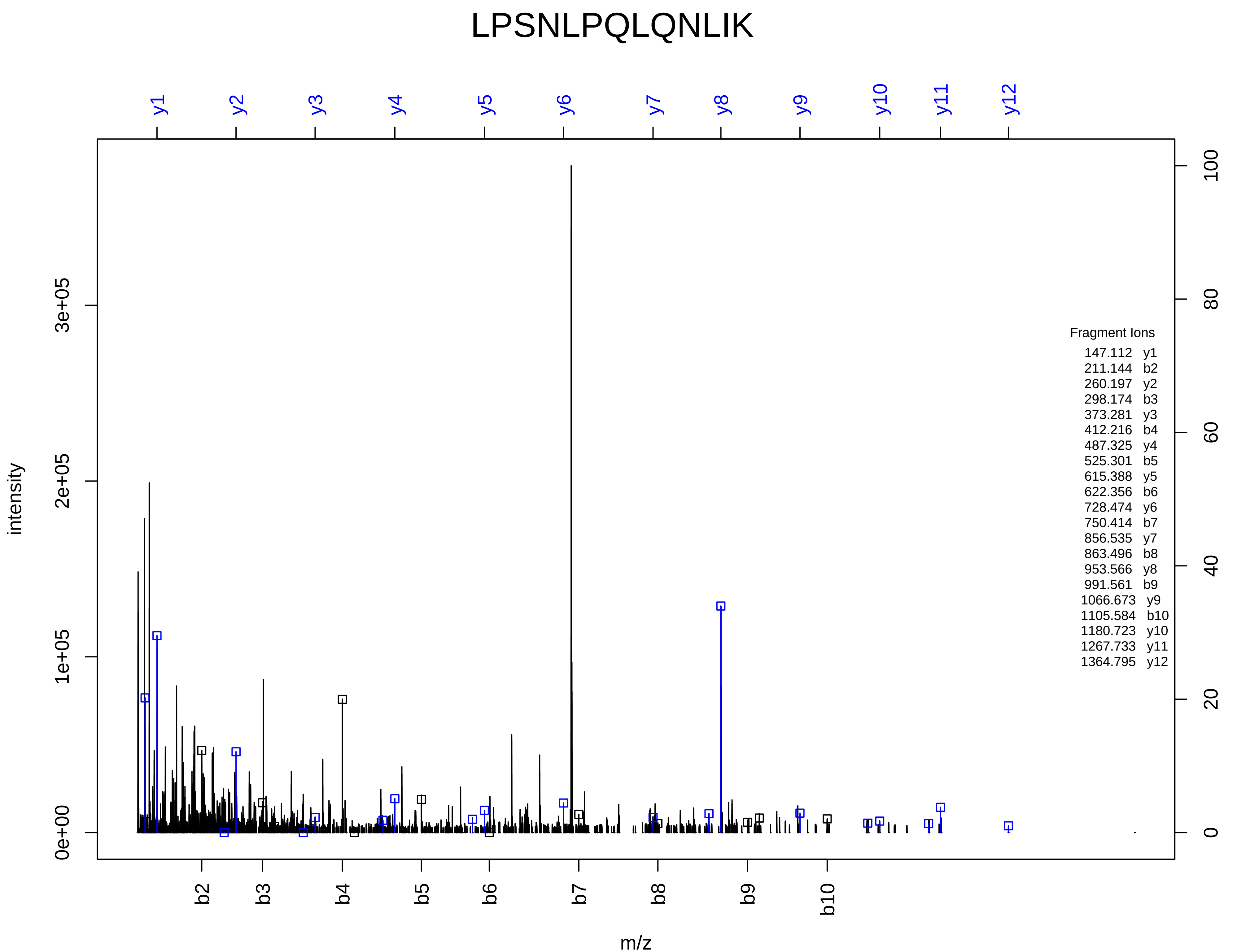
LPSNLPQLQNLIK
y1 y2 y3 y4 y5 y6 y7 y8 y9 y10 y11 y12
b2 b3 b4 b5 b6 b7 b8 b9 b10
intensity
m/z
Fragment Ions
147.112 y1
211.144 b2
260.197 y2
298.174 b3
373.281 y3
412.216 b4
487.325 y4
525.301 b5
615.388 y5
622.356 b6
728.474 y6
750.414 b7
856.535 y7
863.496 b8
953.566 y8
991.561 b9
1066.673 y9
1105.584 b10
1180.723 y10
1267.733 y11
1364.795 y12
LPSNLPQLQNLIK / File: sydney_ip_26.raw; SpectrumID: ; scans: 15197

GTEDFIVESLDASFR

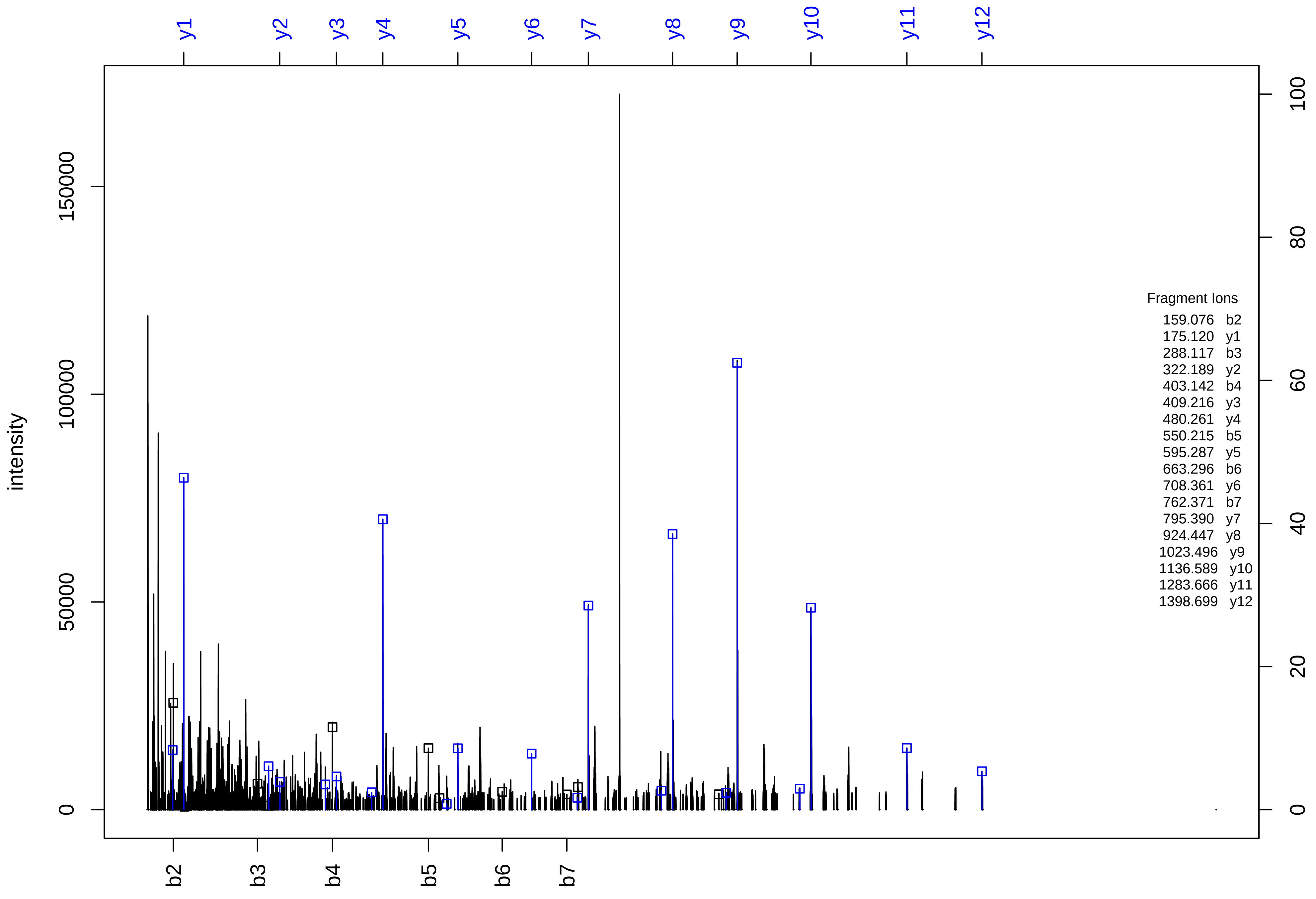

GTEDFIVESLDASFR / File: sydney_ip_26.raw; SpectrumID: ; scans: 18161

LFLSGLELVQQGAEAR

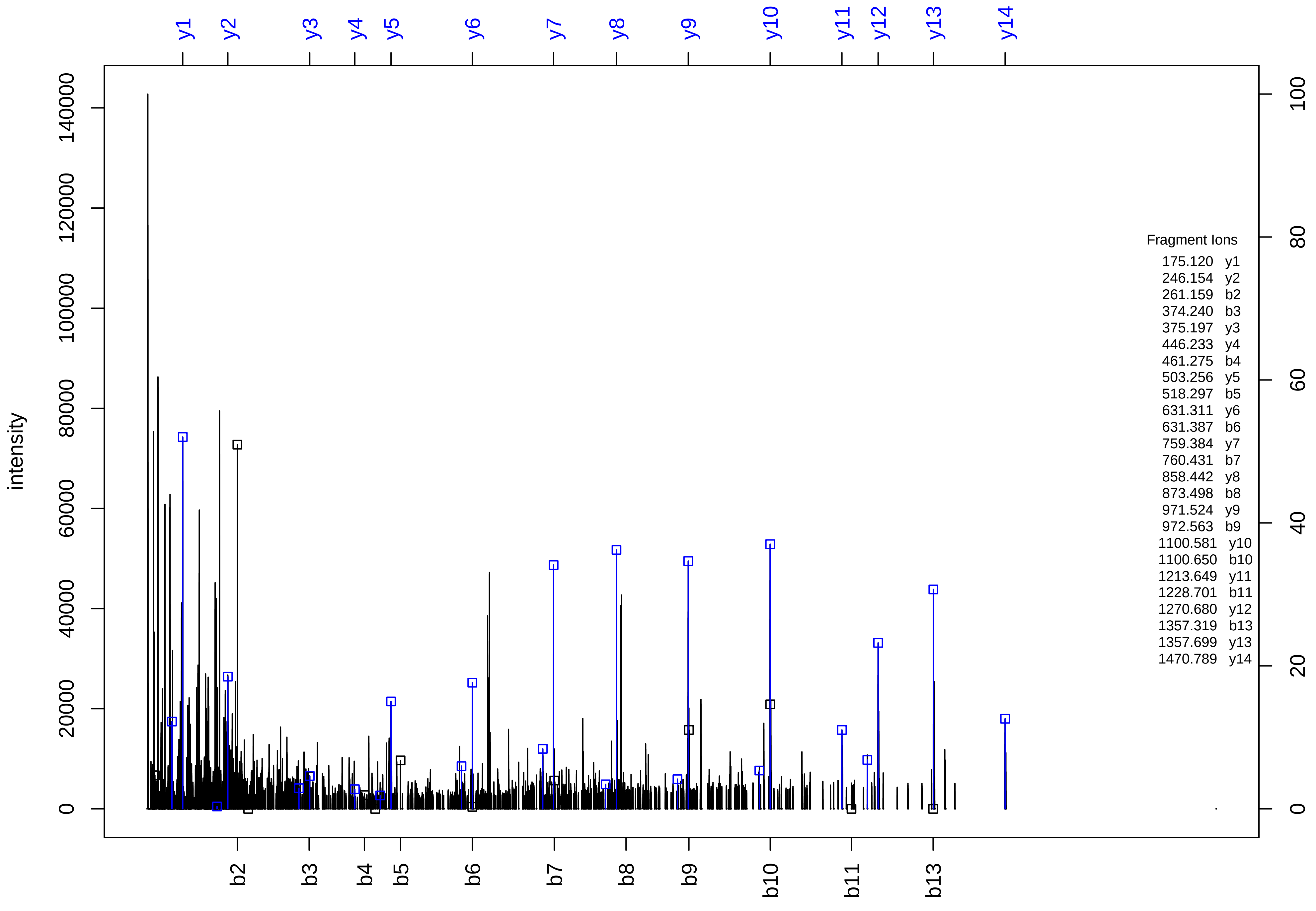

LFLSGLELVQQGAEAR / File: sydney_ip_26.raw; SpectrumID: ; scans: 16337

(Ac)QRTKGQ^DN^K

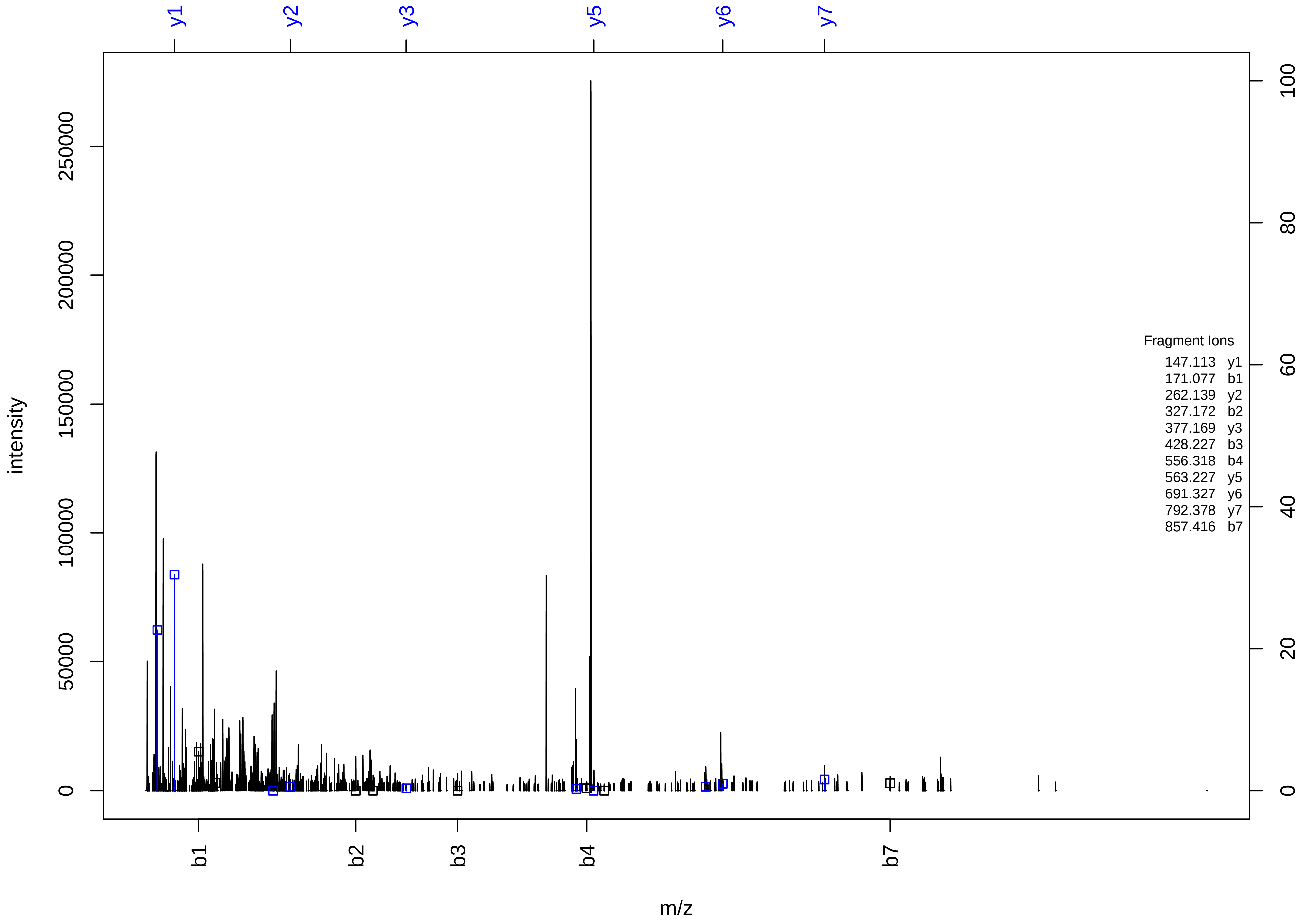

QRTKGQDNK / File: sydney_ip_26.raw; SpectrumID: ; scans: 2616

LQ^LLM*RM*M*AR

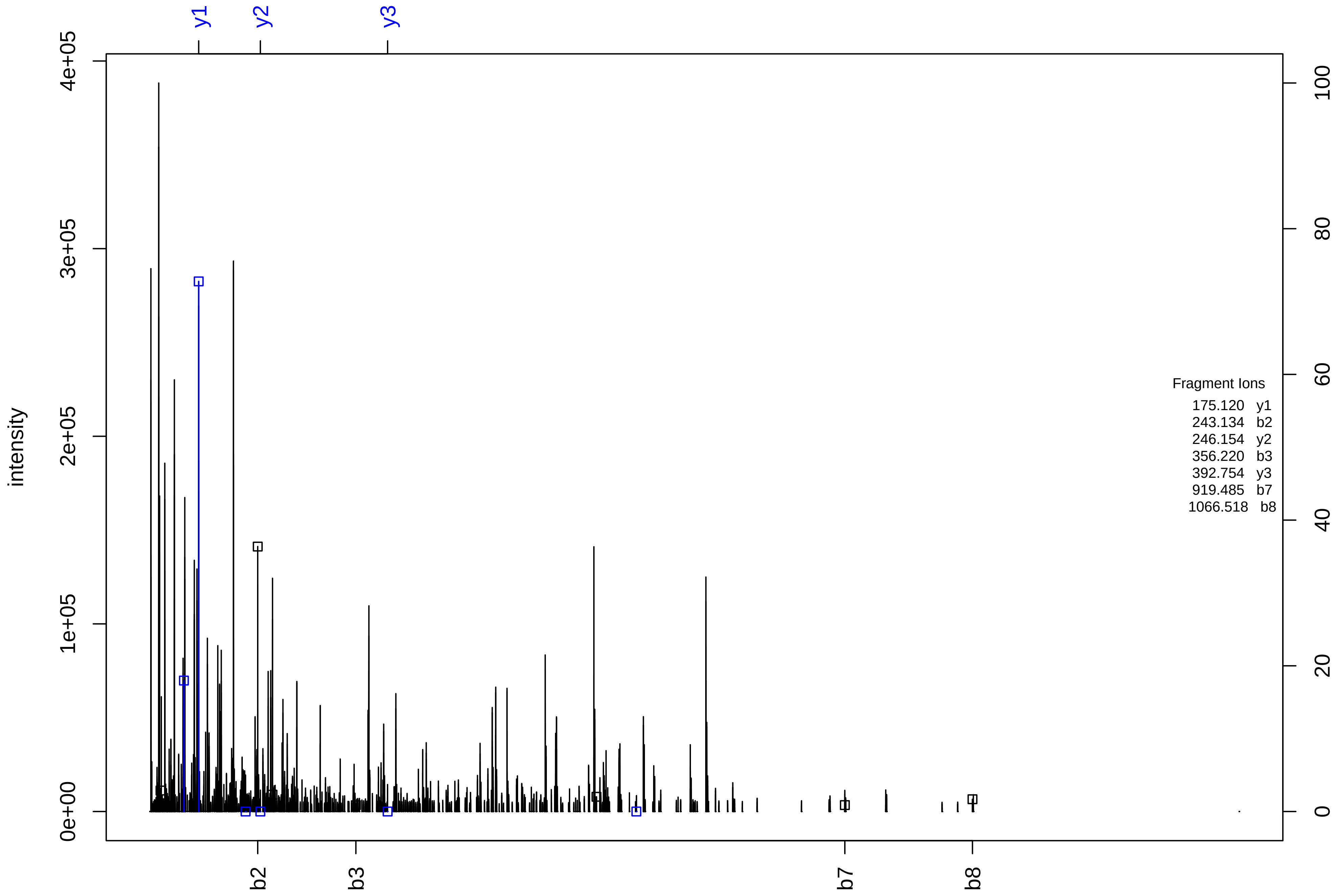

LQLLMRMMAR / File: sydney_ip_26.raw; SpectrumID: ; scans: 6104

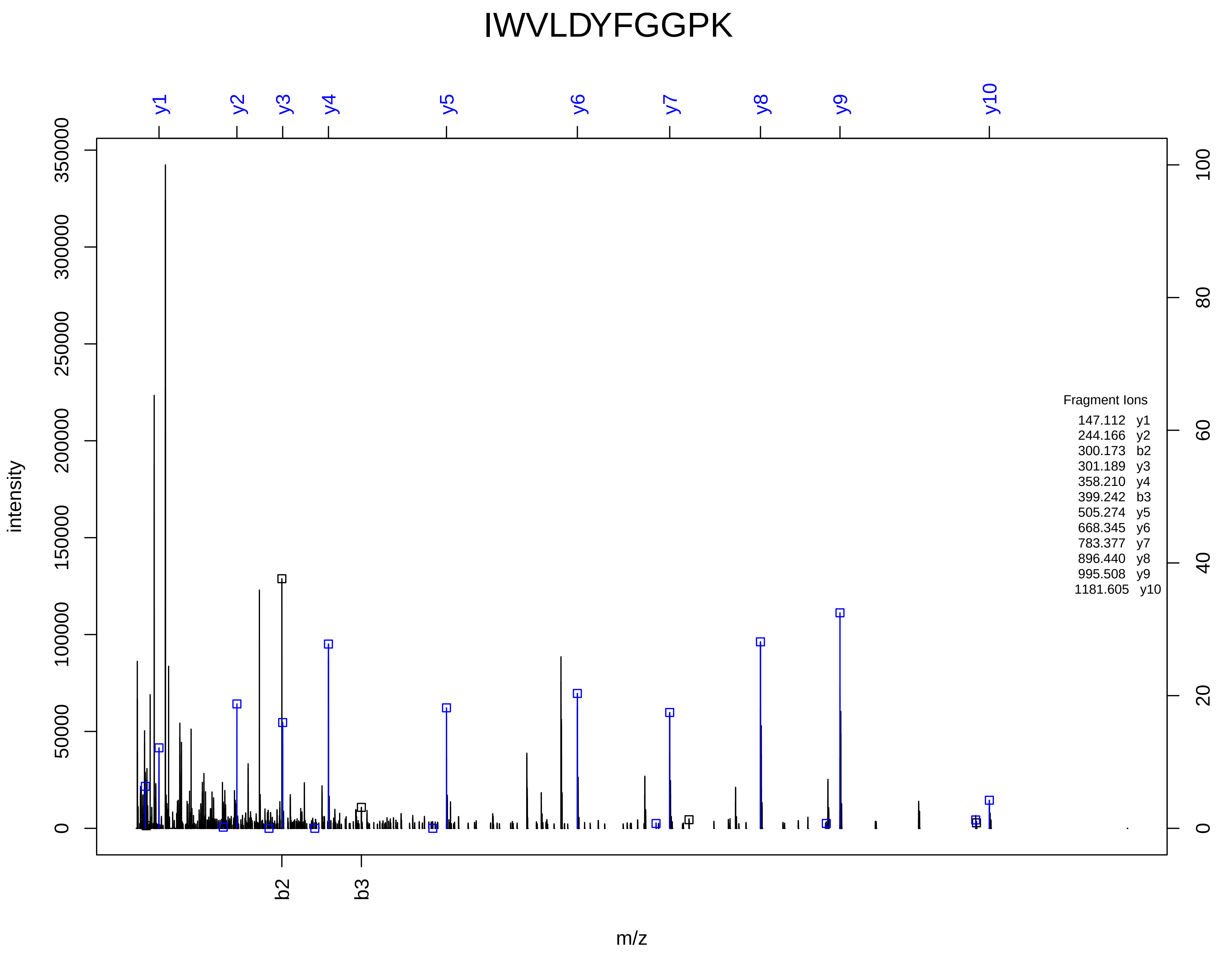
IWVLDYFGGPK
y1
y2
y3
y4
y5
y6
y7
y8
y9
y10
b2
b3
intensity
m/z
Fragment Ions
147.112 y1
244.166 y2
300.173 b2
301.189 y3
358.210 y4
399.242 b3
505.274 y5
668.345 y6
783.377 y7
896.440 y8
995.508 y9
1181.605 y10
IWVLDYFGGPK / File: sydney_ip_26.raw; SpectrumID: ; scans: 18097

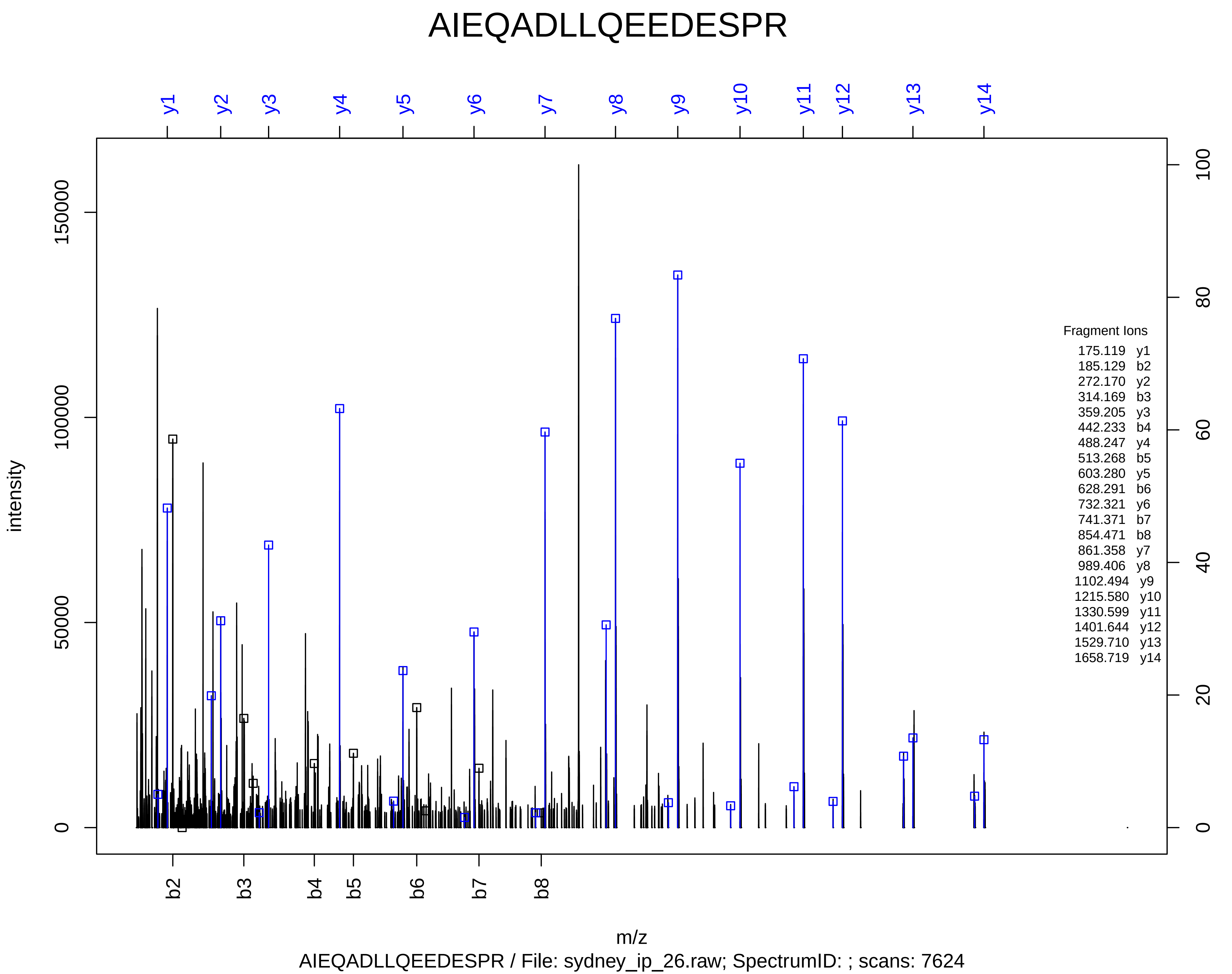
AIEQADLLQEEDESPR
y1
y2
y3
y4
y5
y6
y7
y8
y9
y10
y11
y12
y13
y14
intensity
0
50000
100000
150000
0
20
40
60
80
100
b2
b3
b4
b5
b6
b7
b8
m/z
Fragment Ions
175.119 y1
185.129 b2
272.170 y2
314.169 b3
359.205 y3
442.233 b4
488.247 y4
513.268 b5
603.280 y5
628.291 b6
732.321 y6
741.371 b7
854.471 b8
861.358 y7
989.406 y8
1102.494 y9
1215.580 y10
1330.599 y11
1401.644 y12
1529.710 y13
1658.719 y14
AIEQADLLQEEDESPR / File: sydney_ip_26.raw; SpectrumID: ; scans: 7624

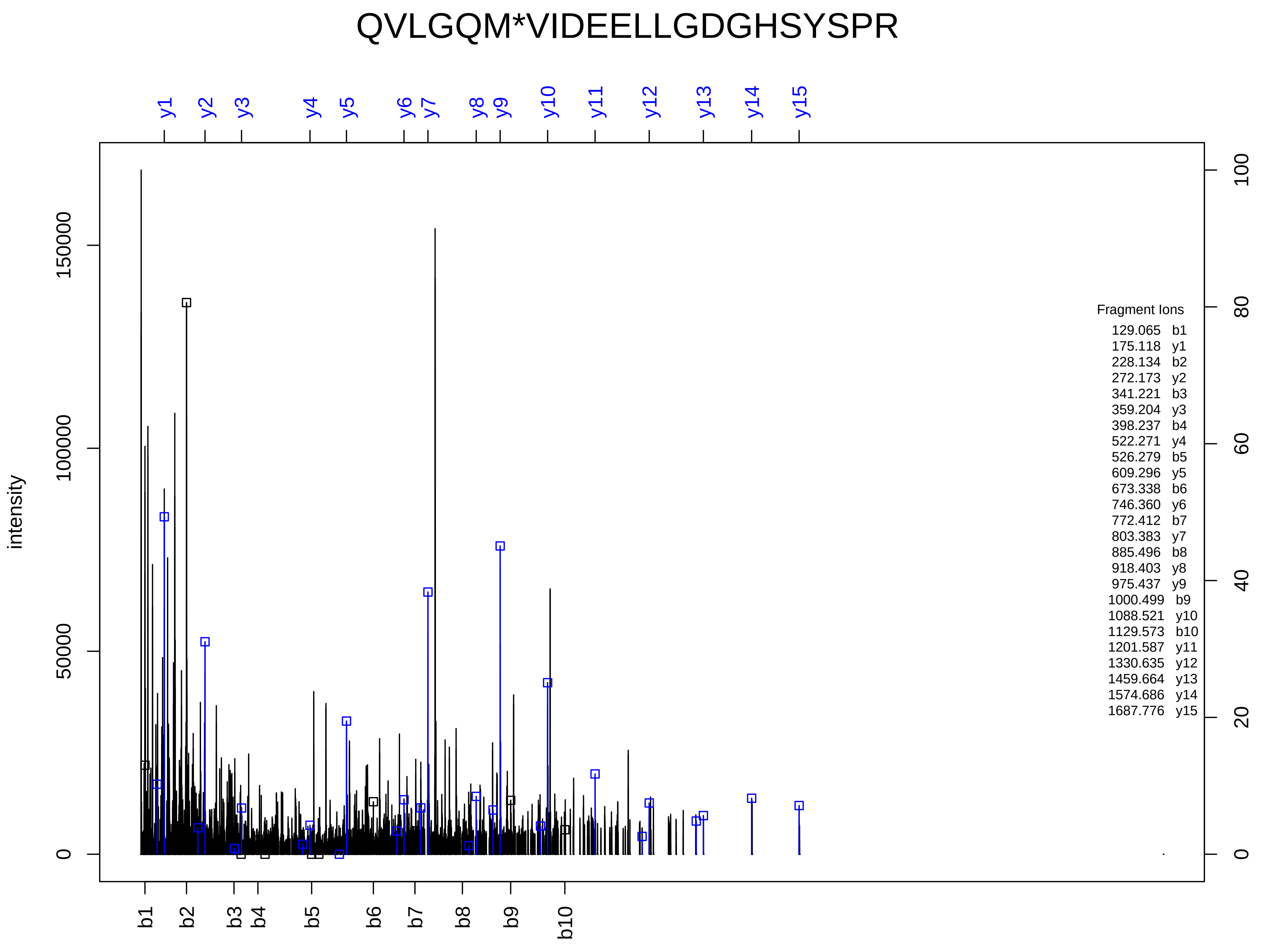

QVLGQMVIDEELLGDGHSYSPR / File: sydney_ip_26.raw; SpectrumID: ; scans: 12515

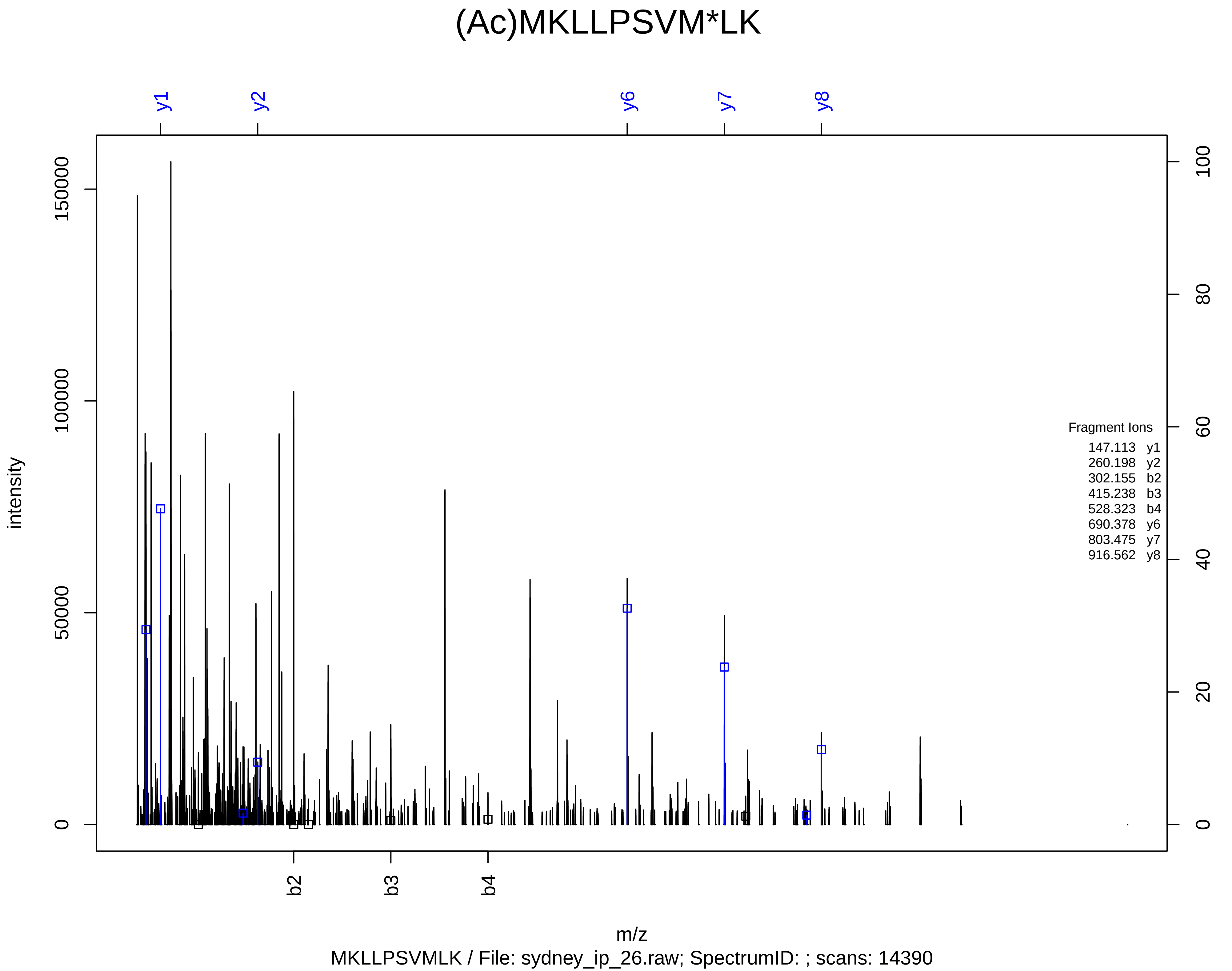
(Ac)MKLLPSVM*LK
y1
y2
y6
y7
y8
b2
b3
b4
intensity
0
50000
100000
150000
0
20
40
60
80
100
Fragment Ions
147.113 y1
260.198 y2
302.155 b2
415.238 b3
528.323 b4
690.378 y6
803.475 y7
916.562 y8
m/z
MKLLPSVMLK / File: sydney_ip_26.raw; SpectrumID: ; scans: 14390

(Ac)MLKTVQ^TDLR

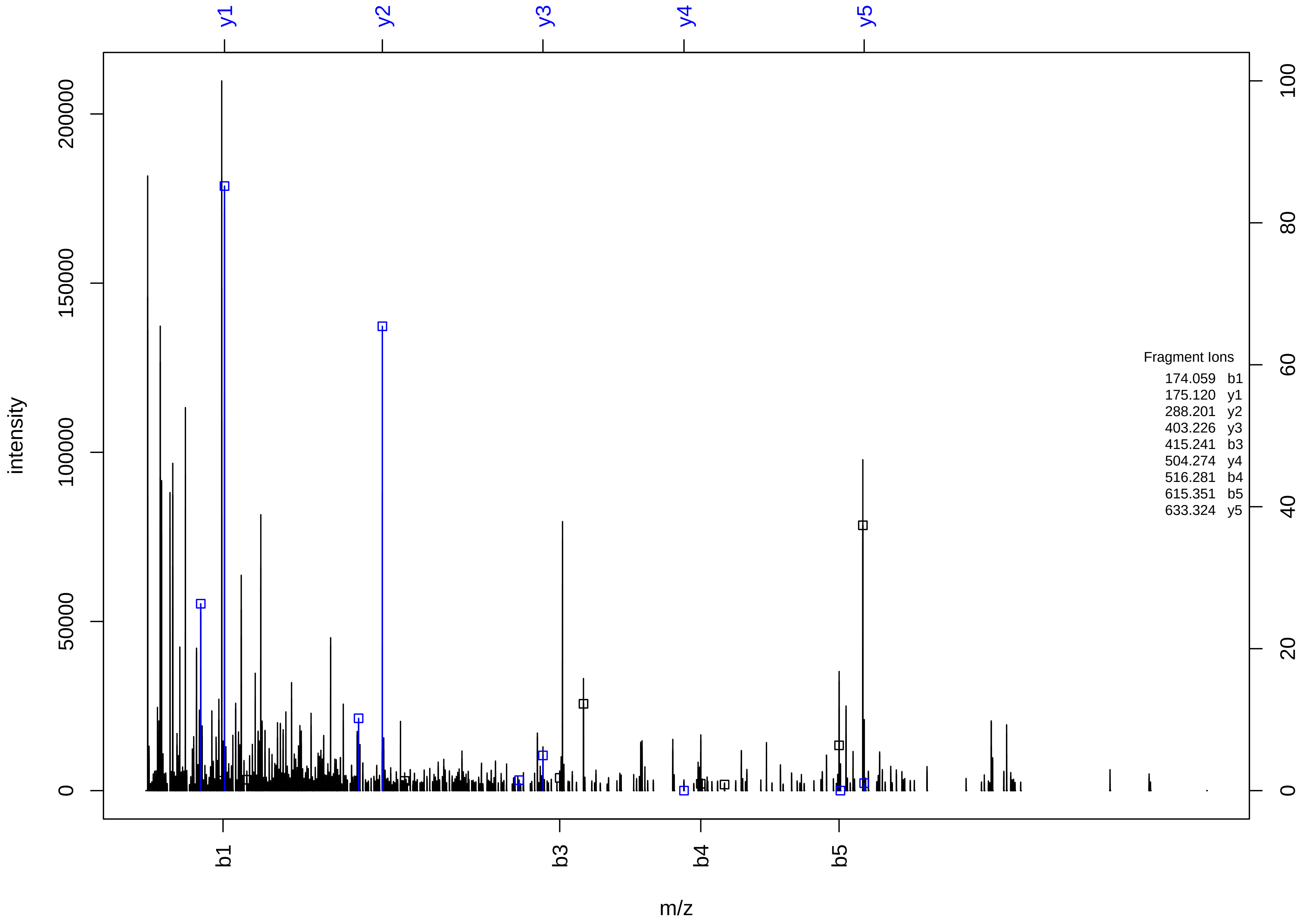

MLKTVQTDLR / File: sydney_ip_26.raw; SpectrumID: ; scans: 5134

(Ac)M*VTEQQ^EEVR

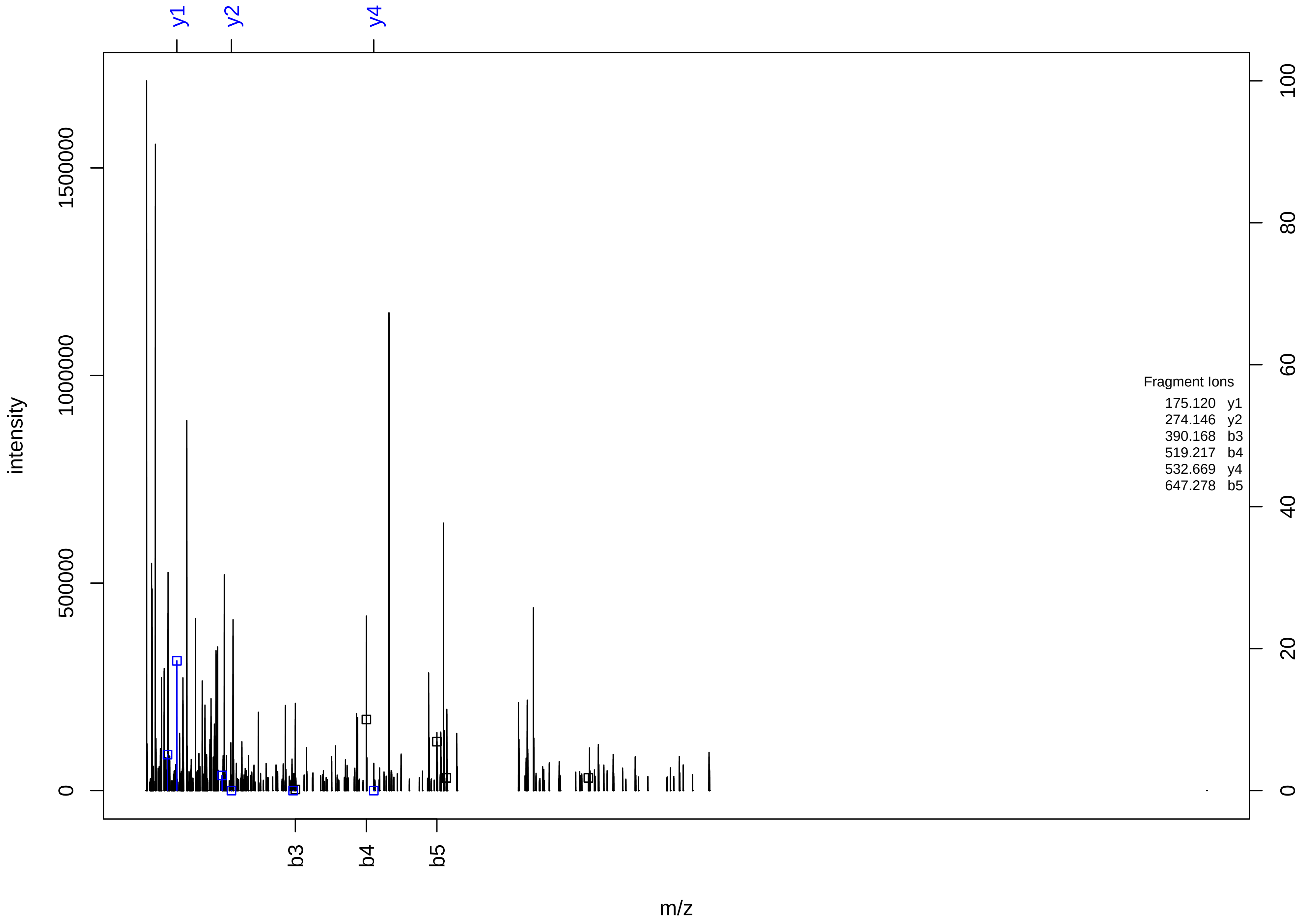

MVTEQQEEVR / File: sydney_ip_26.raw; SpectrumID: ; scans: 8928

ILPIM*FPSLYR

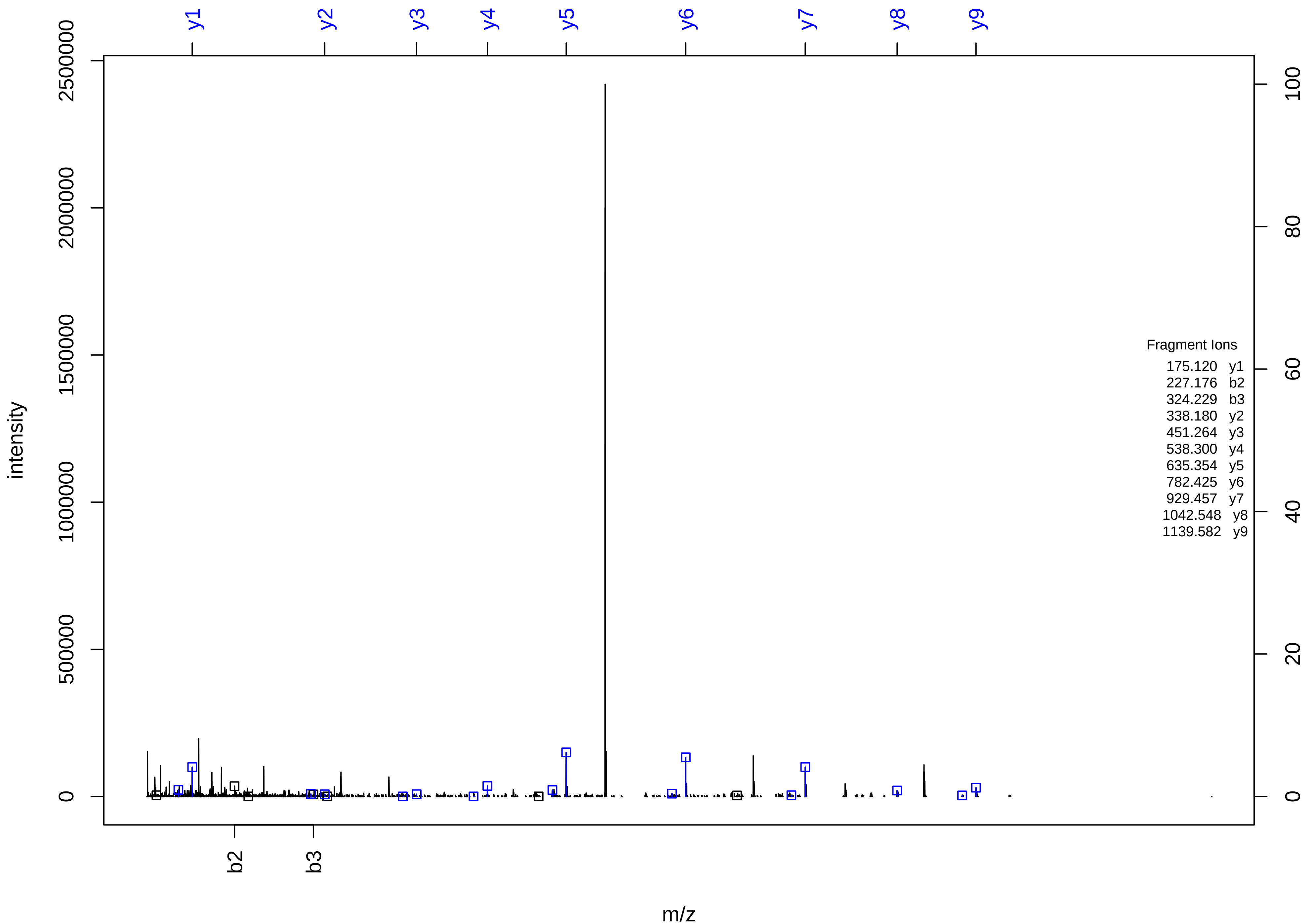

ILPIMFPSLYR / File: sydney_ip_26.raw; SpectrumID: ; scans: 17444

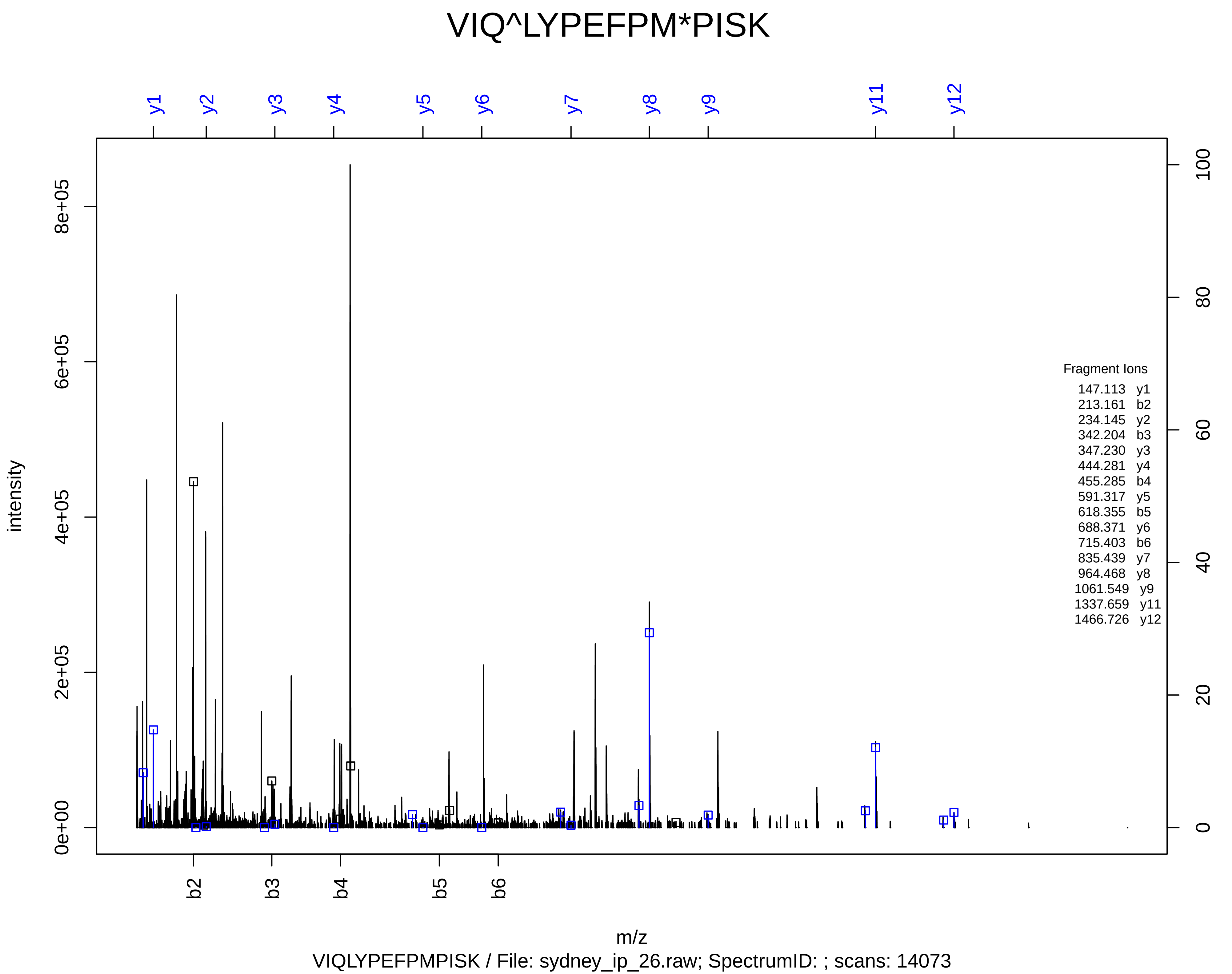
VIQ^LYPEFPM*PISK
y1
y2
y3
y4
y5
y6
y7
y8
y9
y11
y12
intensity
0e+00
2e+05
4e+05
6e+05
8e+05
0
20
40
60
80
100
Fragment Ions
147.113 y1
213.161 b2
234.145 y2
342.204 b3
347.230 y3
444.281 y4
455.285 b4
591.317 y5
618.355 b5
688.371 y6
715.403 b6
835.439 y7
964.468 y8
1061.549 y9
1337.659 y11
1466.726 y12
b2
b3
b4
b5
b6
m/z
VIQLYPEFPMPISK / File: sydney_ip_26.raw; SpectrumID: ; scans: 14073

LLPQFPLHLDK

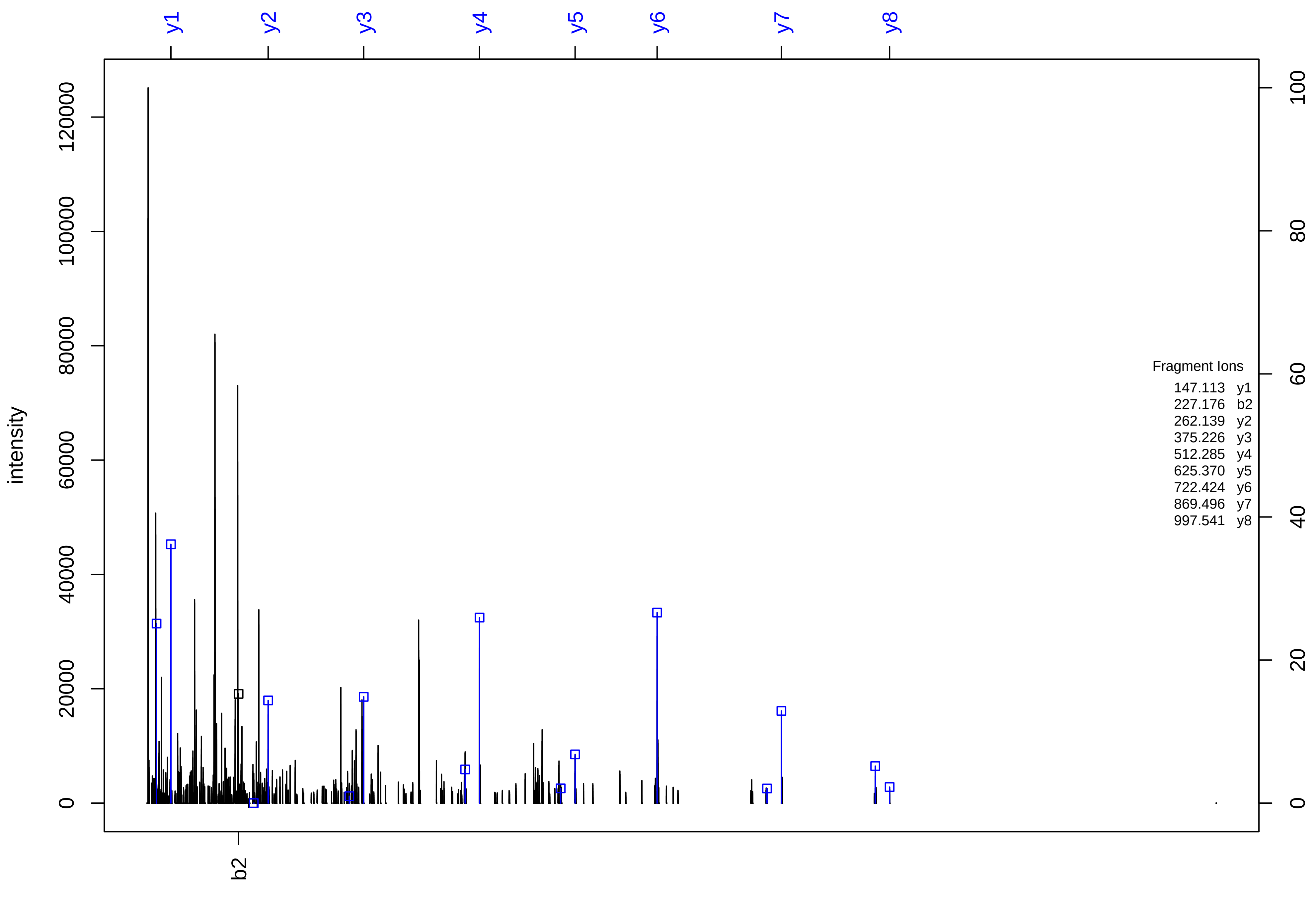

LLPQFPLHLDK / File: sydney_ip_26.raw; SpectrumID: ; scans: 12901

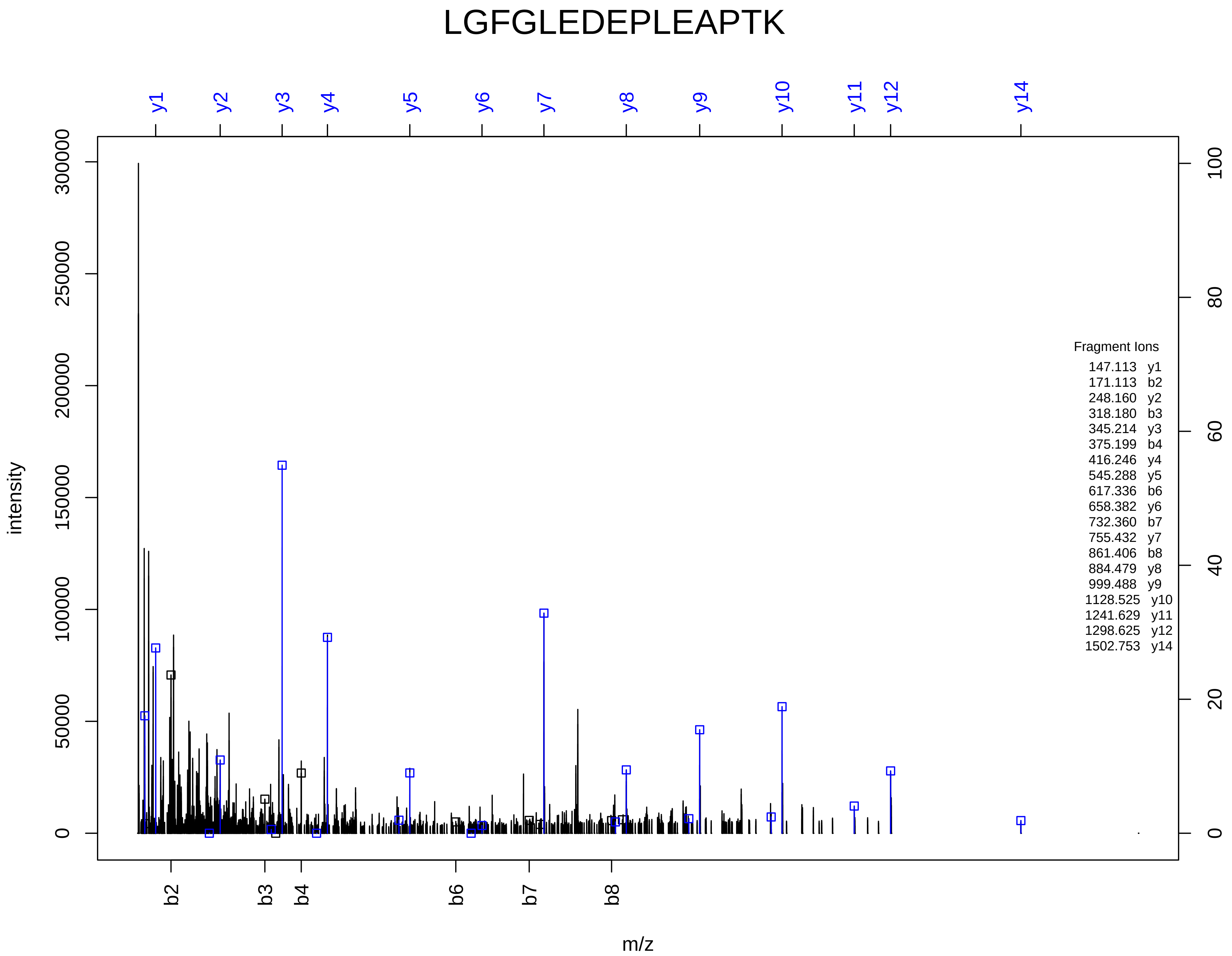

LGFGLEDEPLEAPTK / File: sydney_ip_26.raw; SpectrumID: ; scans: 12968

GVN^GKNM*Q^N^RK

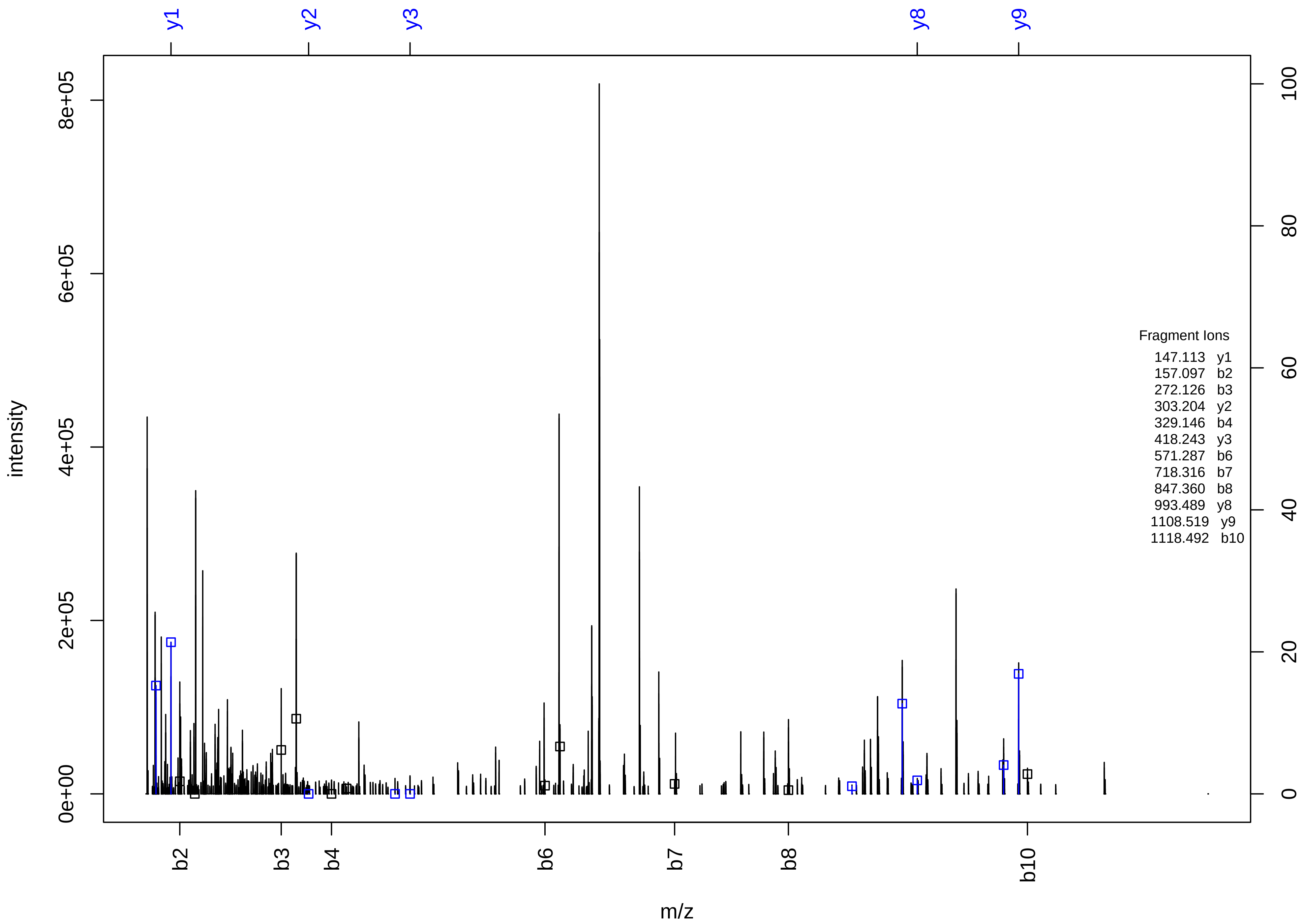

GVNGKNMQNRK / File: sydney_ip_26.raw; SpectrumID: ; scans: 10428

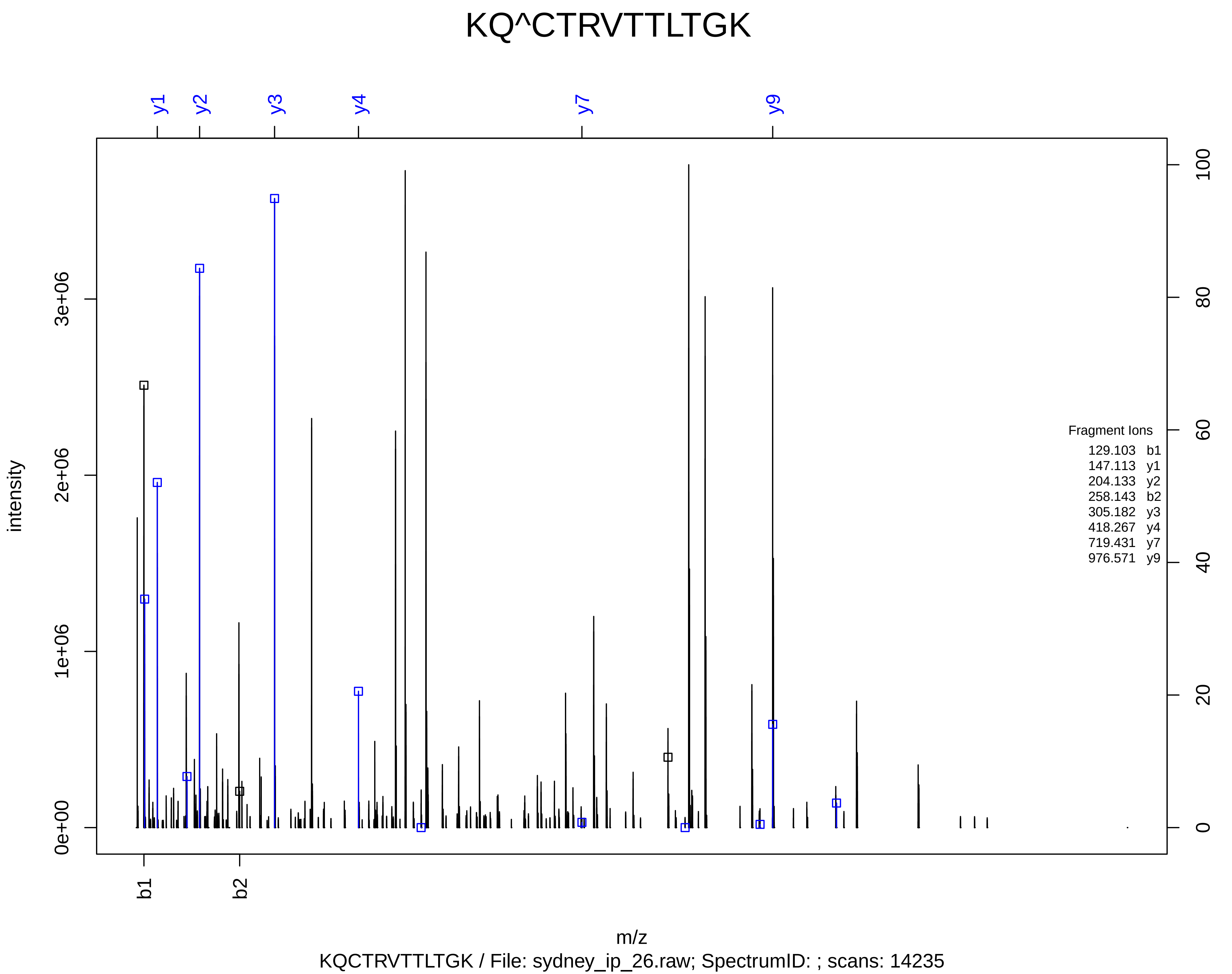
KQ^CTRVTTLTGK
y1
y2
y3
y4
y7
y9
intensity
0e+00
1e+06
2e+06
3e+06
0
20
40
60
80
100
b1
b2
m/z
Fragment Ions
129.103 b1
147.113 y1
204.133 y2
258.143 b2
305.182 y3
418.267 y4
719.431 y7
976.571 y9
KQCTRVTTLTGK / File: sydney_ip_26.raw; SpectrumID: ; scans: 14235

GNPSGIQPDLLISLTAPK

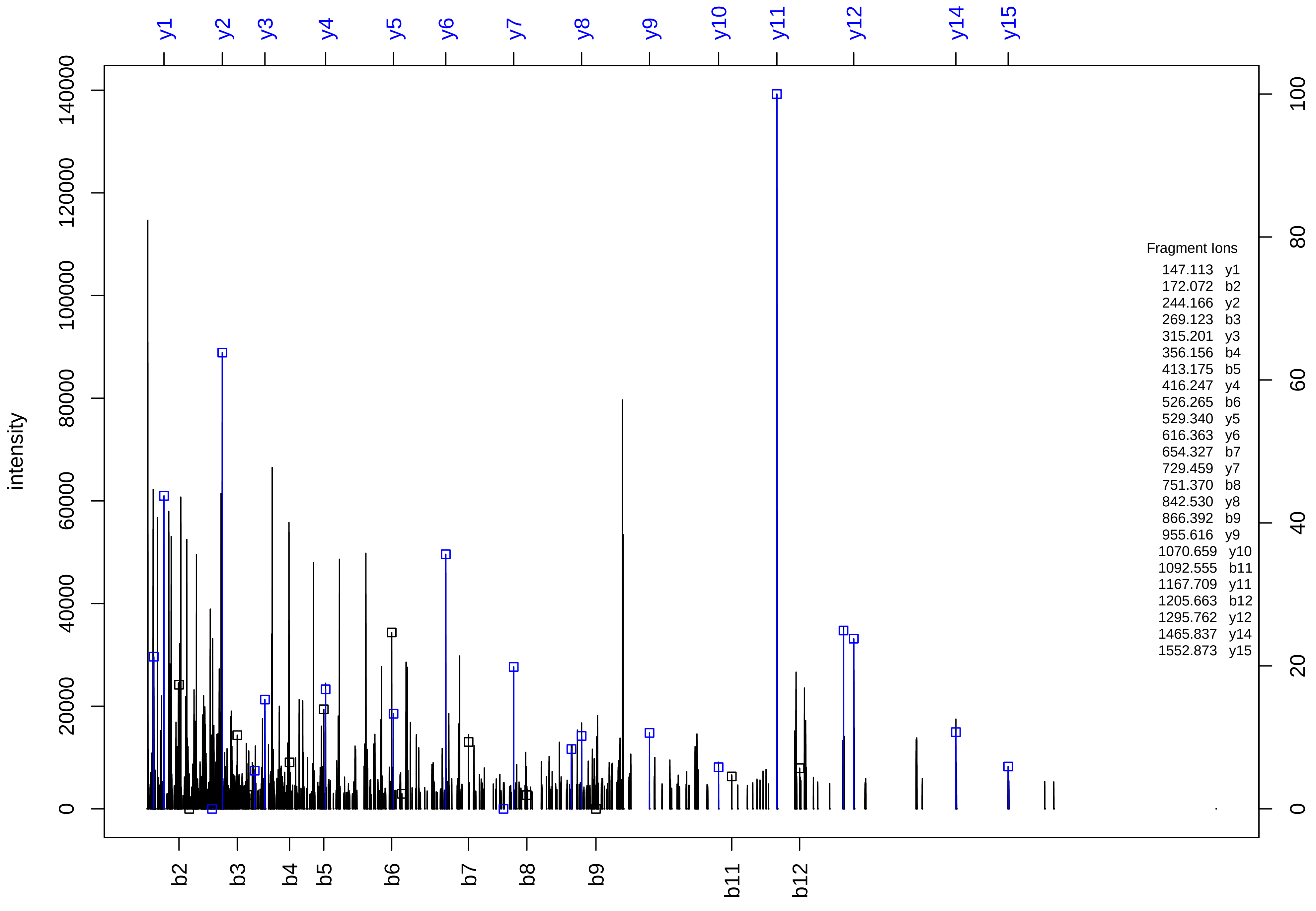

GNPSGIQPDLLISLTAPK / File: sydney_ip_26.raw; SpectrumID: ; scans: 16940

(Ac)M*SEEKPQ^Q^SAEEPDRGEPK

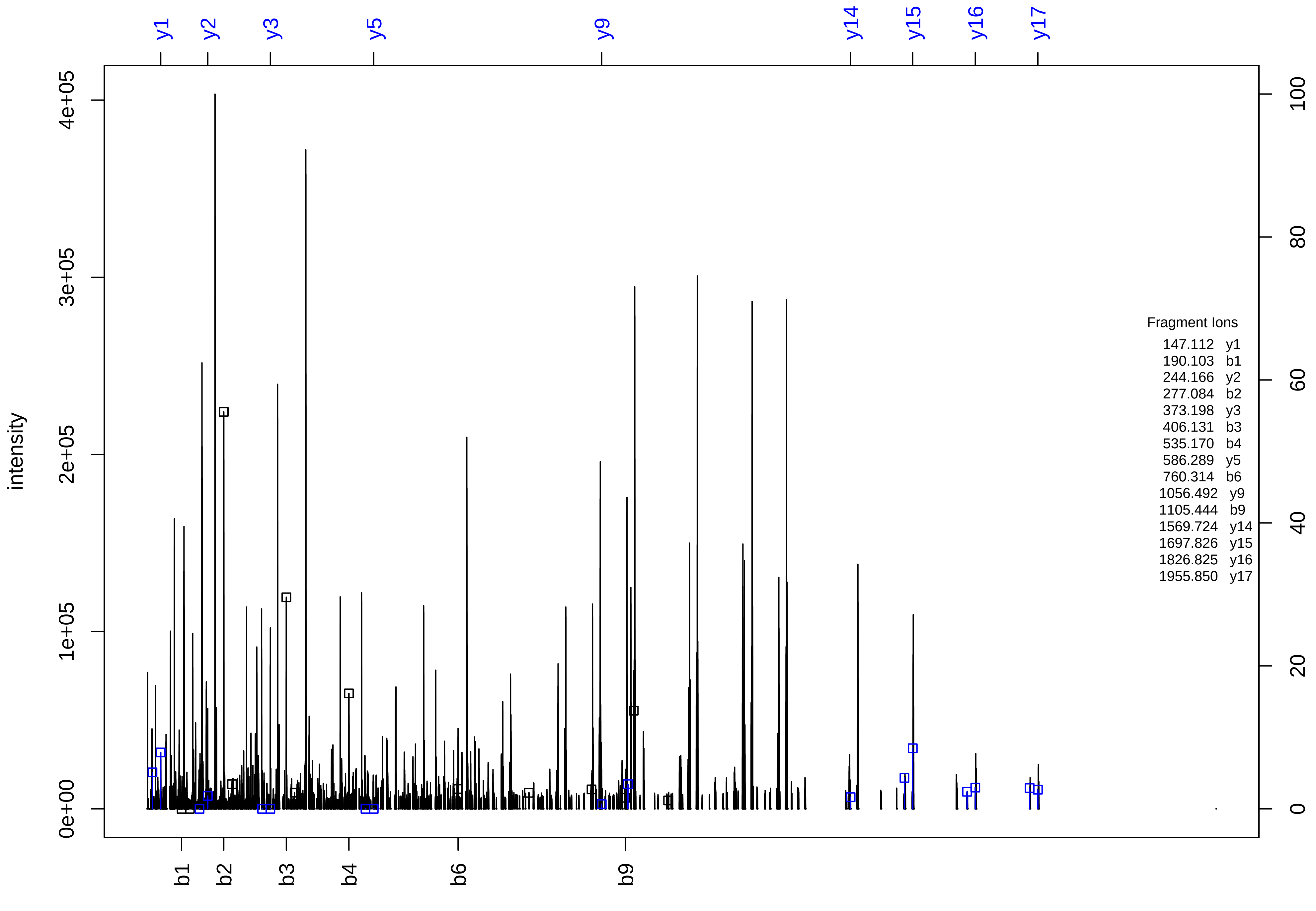

MSEEKPQQSAEEPDRGEPK / File: sydney_ip_26.raw; SpectrumID: ; scans: 11592

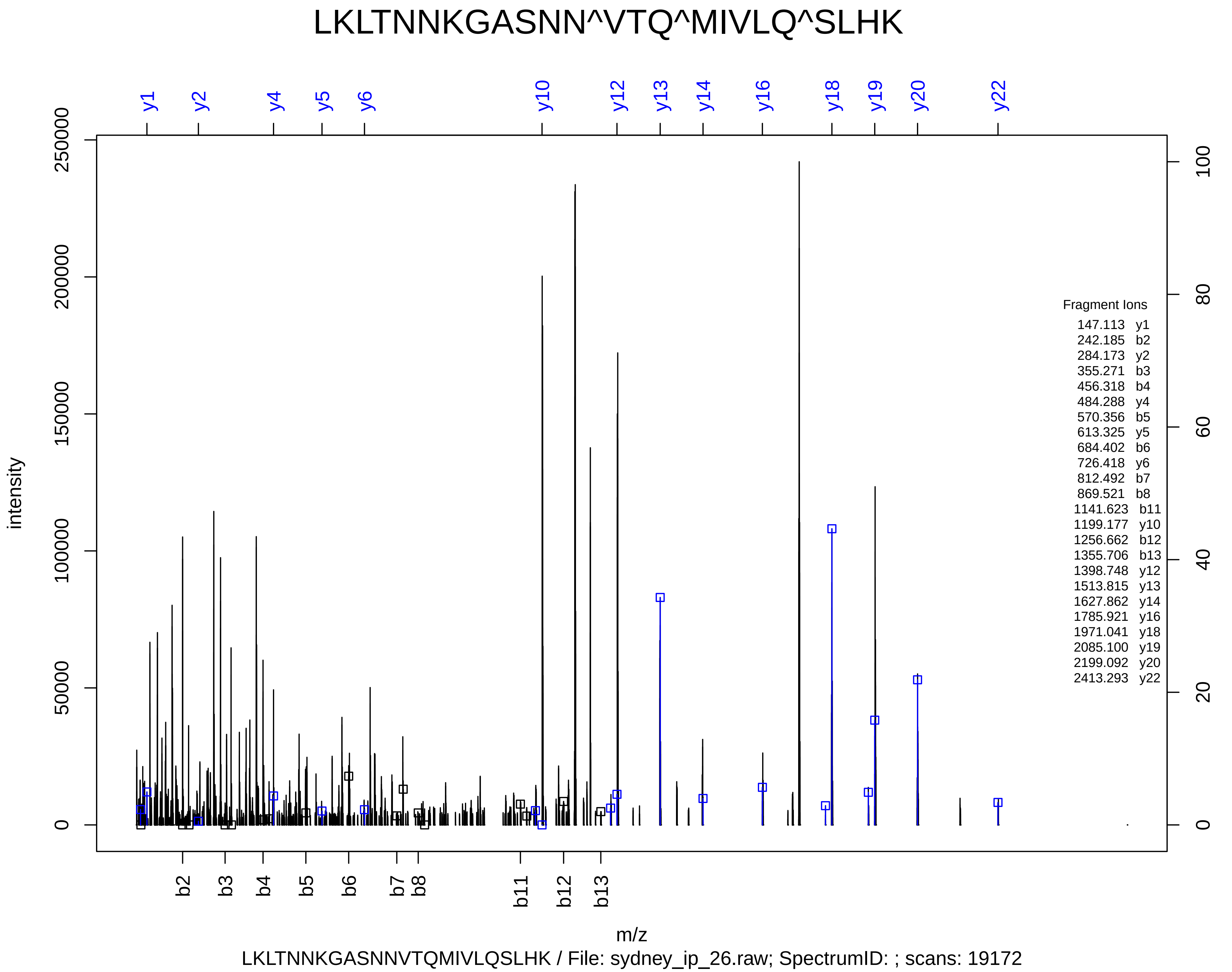

LKLTNNKGASNN^VTQ^MIVLQ^SLHK
y1
y2
y4
y5
y6
y10
y12
y13
y14
y16
y18
y19
y20
y22
intensity
250000
200000
150000
100000
50000
0
100
80
60
40
20
0
Fragment Ions
147.113 y1
242.185 b2
284.173 y2
355.271 b3
456.318 b4
484.288 y4
570.356 b5
613.325 y5
684.402 b6
726.418 y6
812.492 b7
869.521 b8
1141.623 b11
1199.177 y10
1256.662 b12
1355.706 b13
1398.748 y12
1513.815 y13
1627.862 y14
1785.921 y16
1971.041 y18
2085.100 y19
2199.092 y20
2413.293 y22
b2
b3
b4
b5
b6
b7
b8
b11
b12
b13
m/z
LKLTNNKGASNNVTQMIVLQSLHK / File: sydney_ip_26.raw; SpectrumID: ; scans: 19172

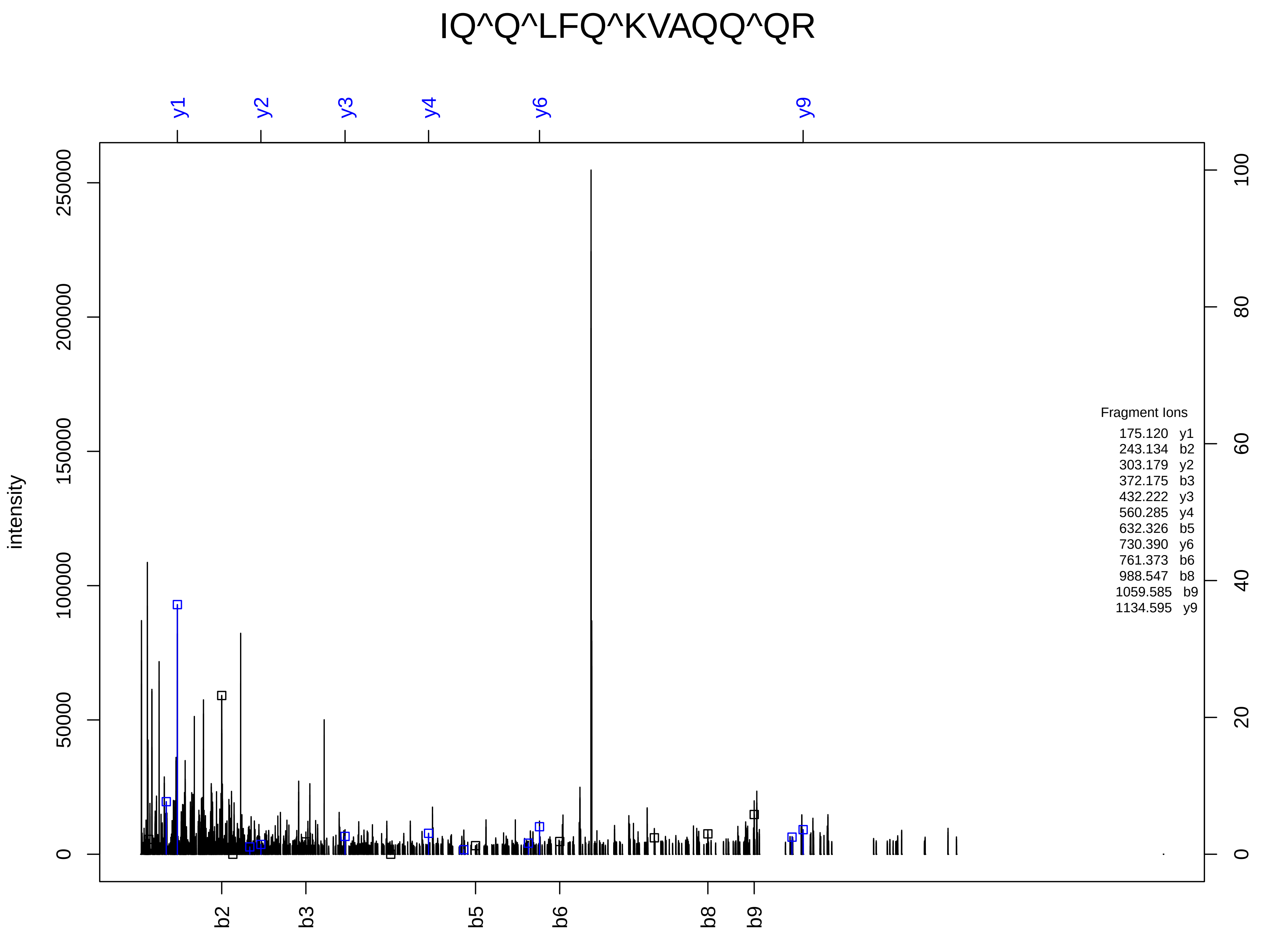

IQQLFQKVAQQQR / File: sydney_ip_27.raw; SpectrumID: ; scans: 9213

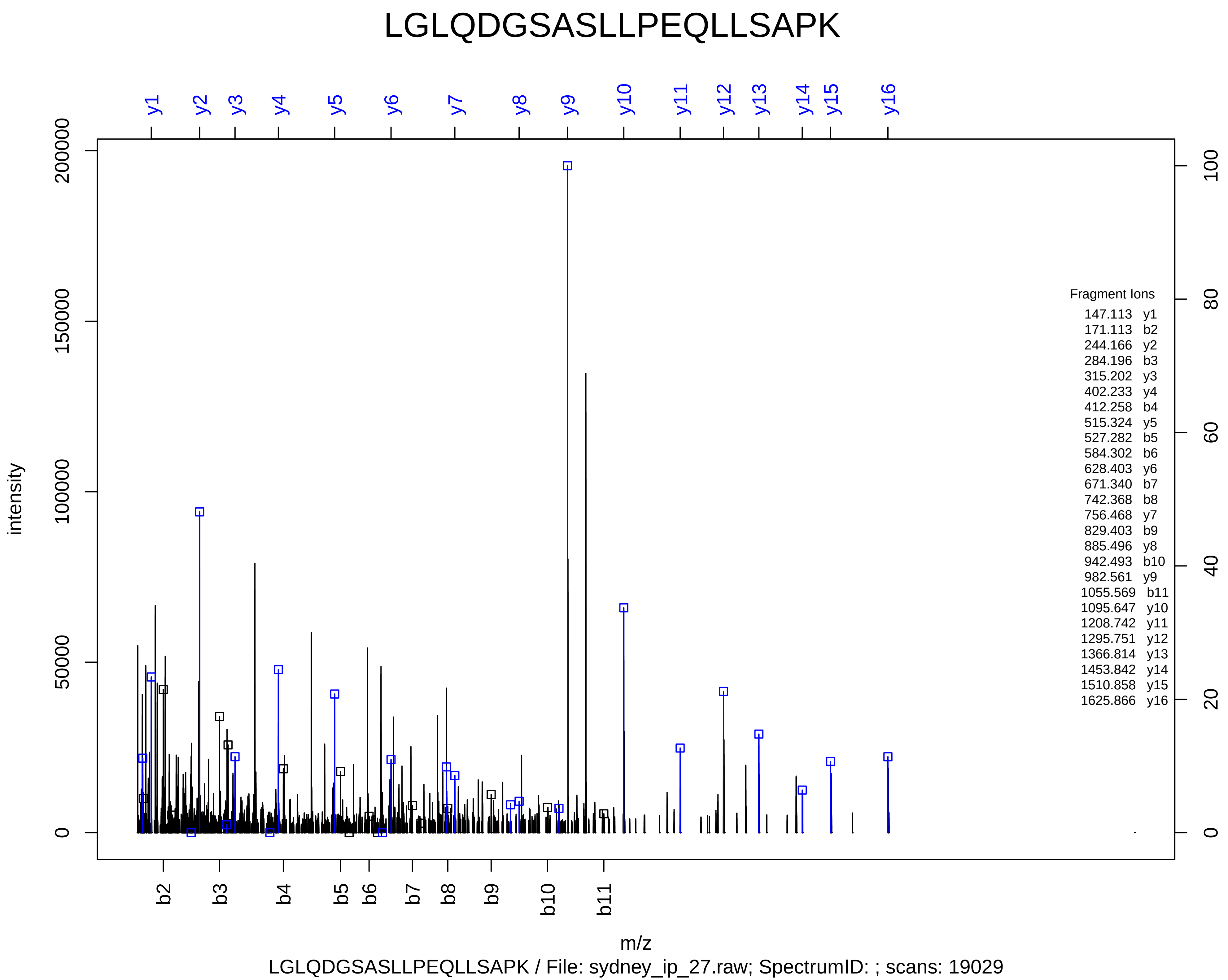
LGLQDGSASLLPEQLLSAPK
y1 y2 y3 y4 y5 y6 y7 y8 y9 y10 y11 y12 y13 y14 y15 y16
b2 b3 b4 b5 b6 b7 b8 b9 b10 b11
intensity
m/z
0
50000
100000
150000
200000
0
20
40
60
80
100
Fragment Ions
147.113 y1
171.113 b2
244.166 y2
284.196 b3
315.202 y3
402.233 y4
412.258 b4
515.324 y5
527.282 b5
584.302 b6
628.403 y6
671.340 b7
742.368 b8
756.468 y7
829.403 b9
885.496 y8
942.493 b10
982.561 y9
1055.569 b11
1095.647 y10
1208.742 y11
1295.751 y12
1366.814 y13
1453.842 y14
1510.858 y15
1625.866 y16
LGLQDGSASLLPEQLLSAPK / File: sydney_ip_27.raw; SpectrumID: ; scans: 19029

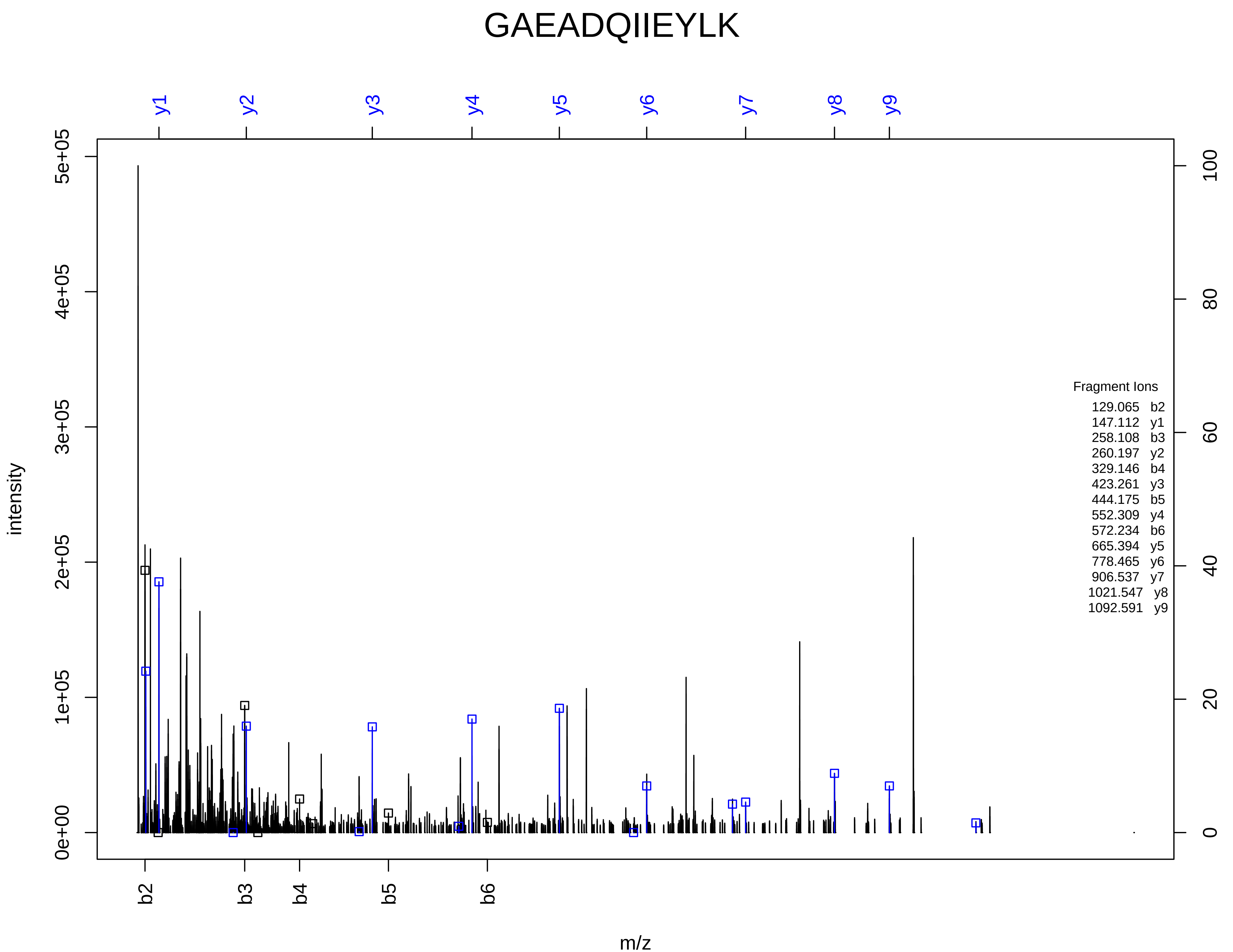

GAEADQIIEYLK
y1
y2
y3
y4
y5
y6
y7
y8
y9
b2
b3
b4
b5
b6
intensity
m/z
0e+00
1e+05
2e+05
3e+05
4e+05
5e+05
0
20
40
60
80
100
Fragment Ions
129.065 b2
147.112 y1
258.108 b3
260.197 y2
329.146 b4
423.261 y3
444.175 b5
552.309 y4
572.234 b6
665.394 y5
778.465 y6
906.537 y7
1021.547 y8
1092.591 y9
GAEADQIIEYLK / File: sydney_ip_27.raw; SpectrumID: ; scans: 14057

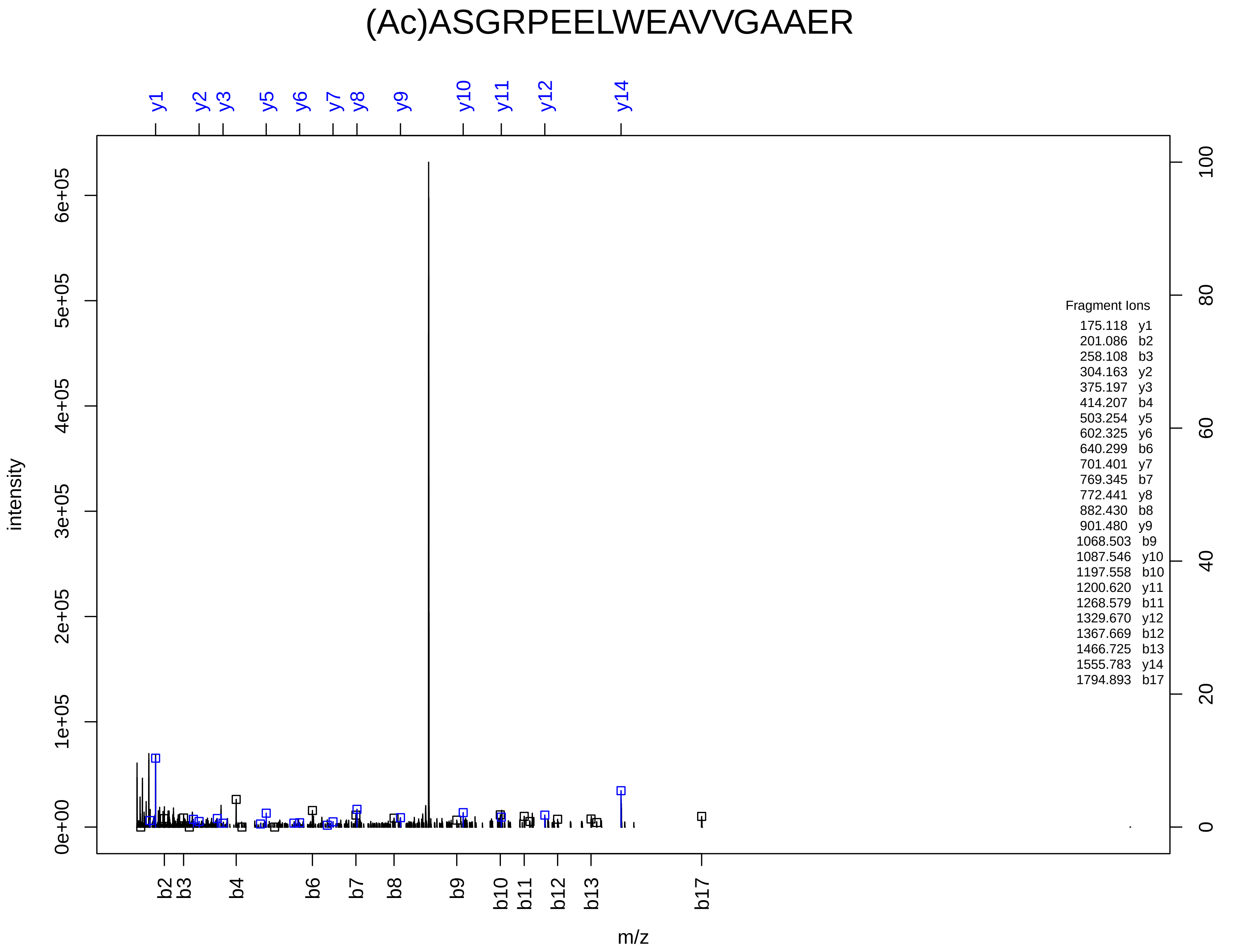

(Ac)ASGRPEELWEAVVGAAER
y1 y2 y3 y5 y6 y7 y8 y9 y10 y11 y12 y14
intensity
0e+00 1e+05 2e+05 3e+05 4e+05 5e+05 6e+05
0 20 40 60 80 100
Fragment Ions
175.118 y1
201.086 b2
258.108 b3
304.163 y2
375.197 y3
414.207 b4
503.254 y5
602.325 y6
640.299 b6
701.401 y7
769.345 b7
772.441 y8
882.430 b8
901.480 y9
1068.503 b9
1087.546 y10
1197.558 b10
1200.620 y11
1268.579 b11
1329.670 y12
1367.669 b12
1466.725 b13
1555.783 y14
1794.893 b17
b2 b3 b4 b6 b7 b8 b9 b10 b11 b12 b13 b17
m/z
ASGRPEELWEAVVGAAER / File: sydney_ip_27.raw; SpectrumID: ; scans: 19335

SLLSLLQR

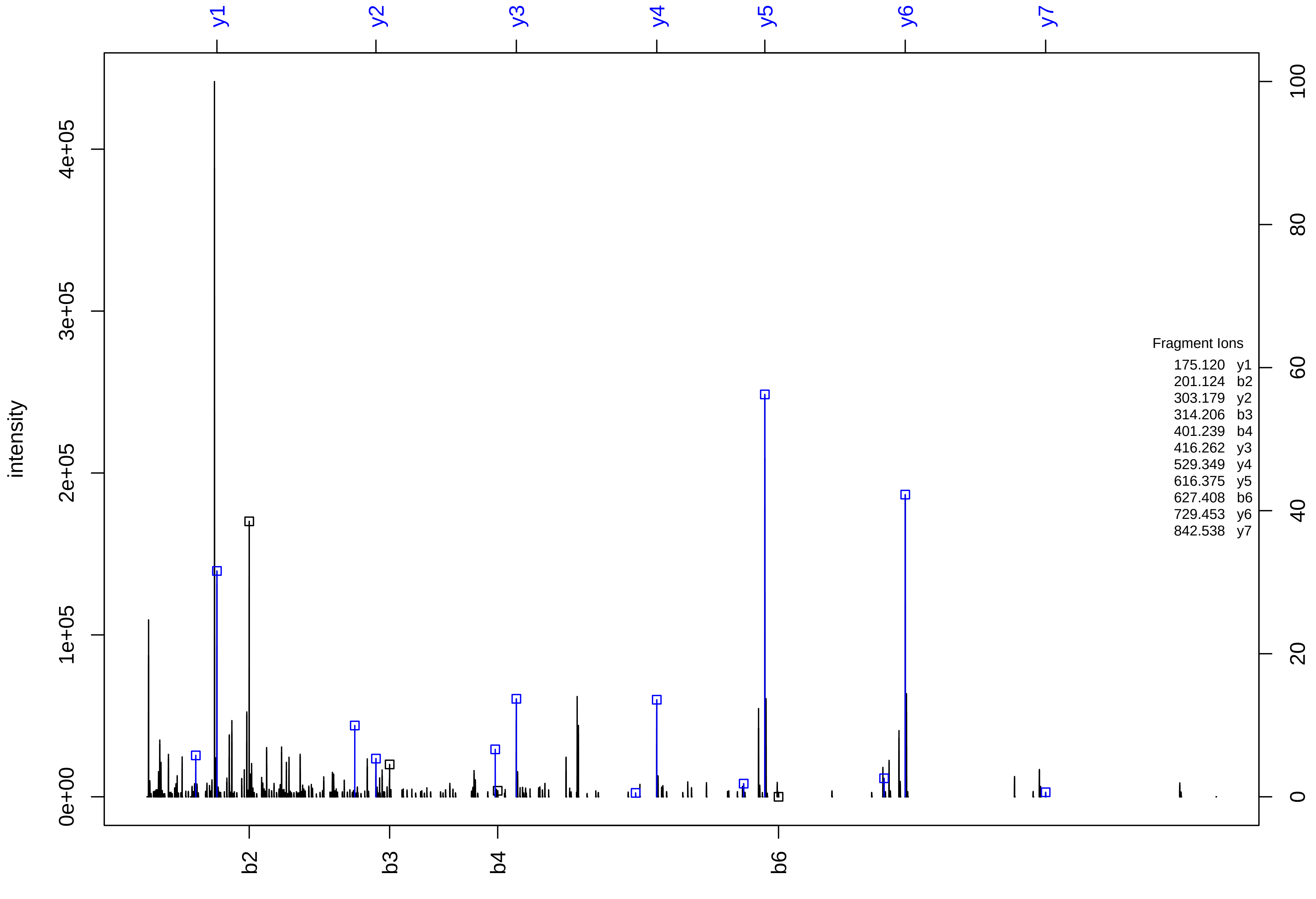

SLLSLLQR / File: sydney_ip_27.raw; SpectrumID: ; scans: 14510

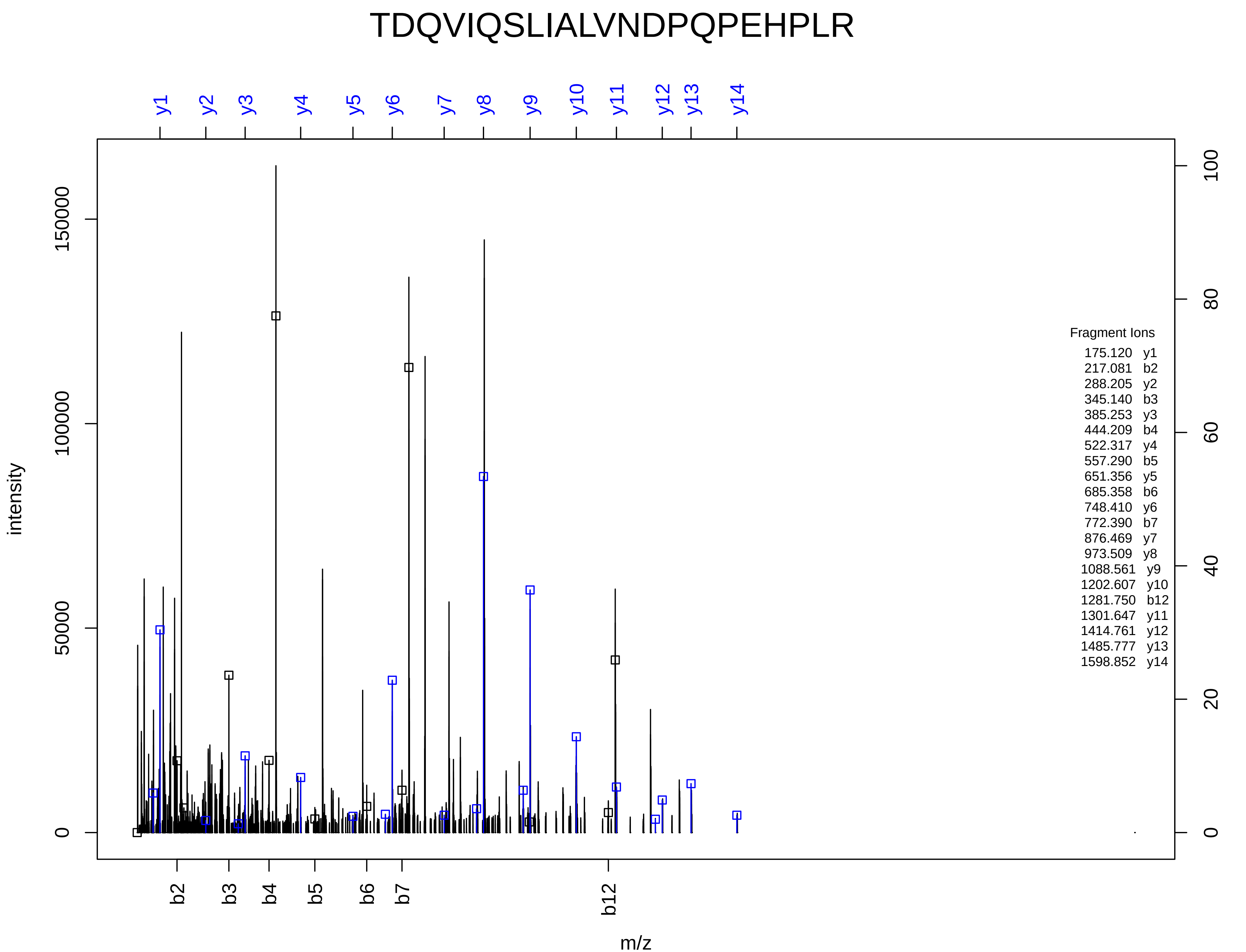

TDQVIQSLIALVNDPQPEHPLR / File: sydney_ip_27.raw; SpectrumID: ; scans: 20087

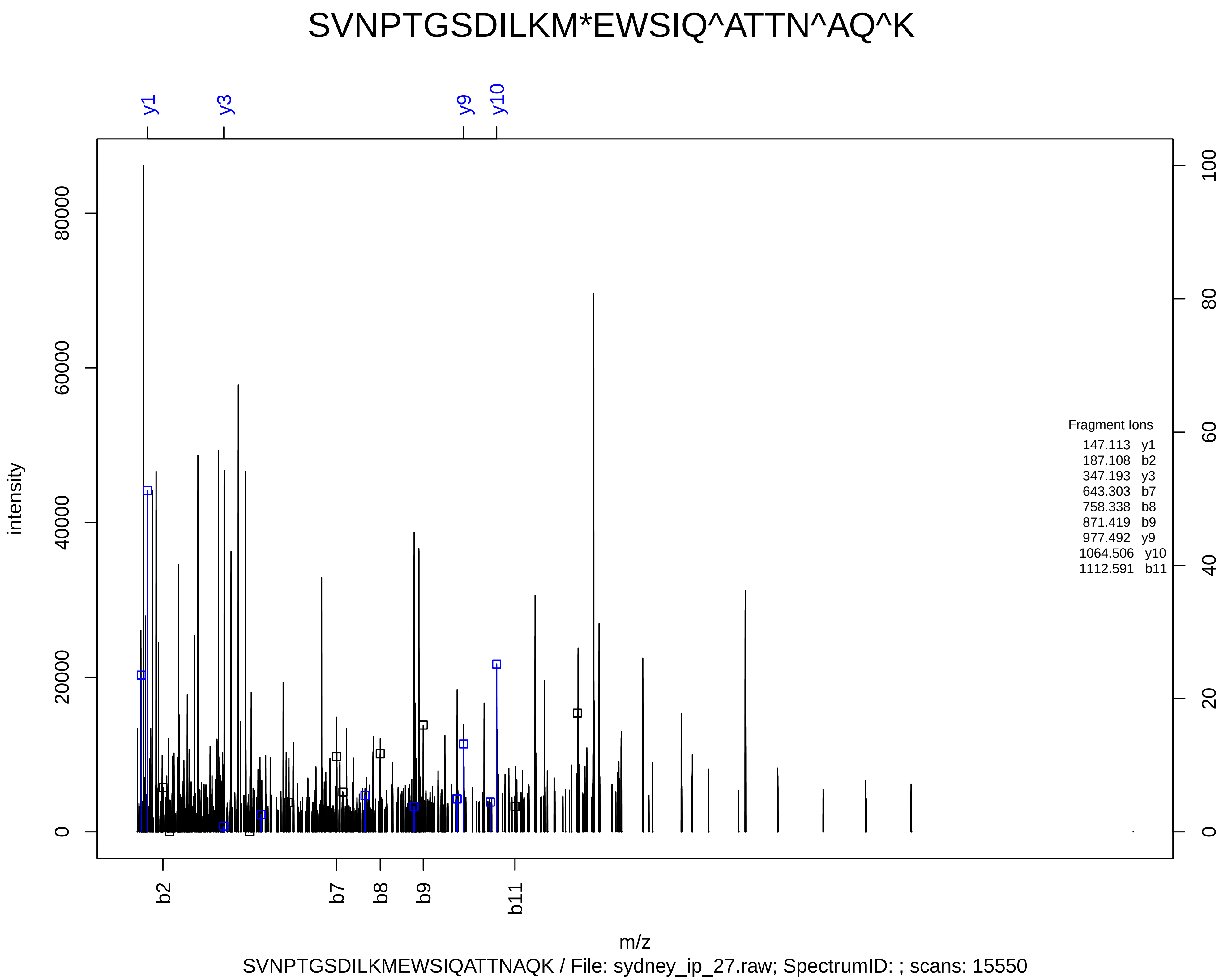
SVNPTGSDILKM*EWSIQ^ATTN^AQ^K
y1
y3
y9
y10
intensity
m/z
b2
b7
b8
b9
b11
Fragment Ions
147.113 y1
187.108 b2
347.193 y3
643.303 b7
758.338 b8
871.419 b9
977.492 y9
1064.506 y10
1112.591 b11
SVNPTGSDILKMEWSIQATTNAQK / File: sydney_ip_27.raw; SpectrumID: ; scans: 15550

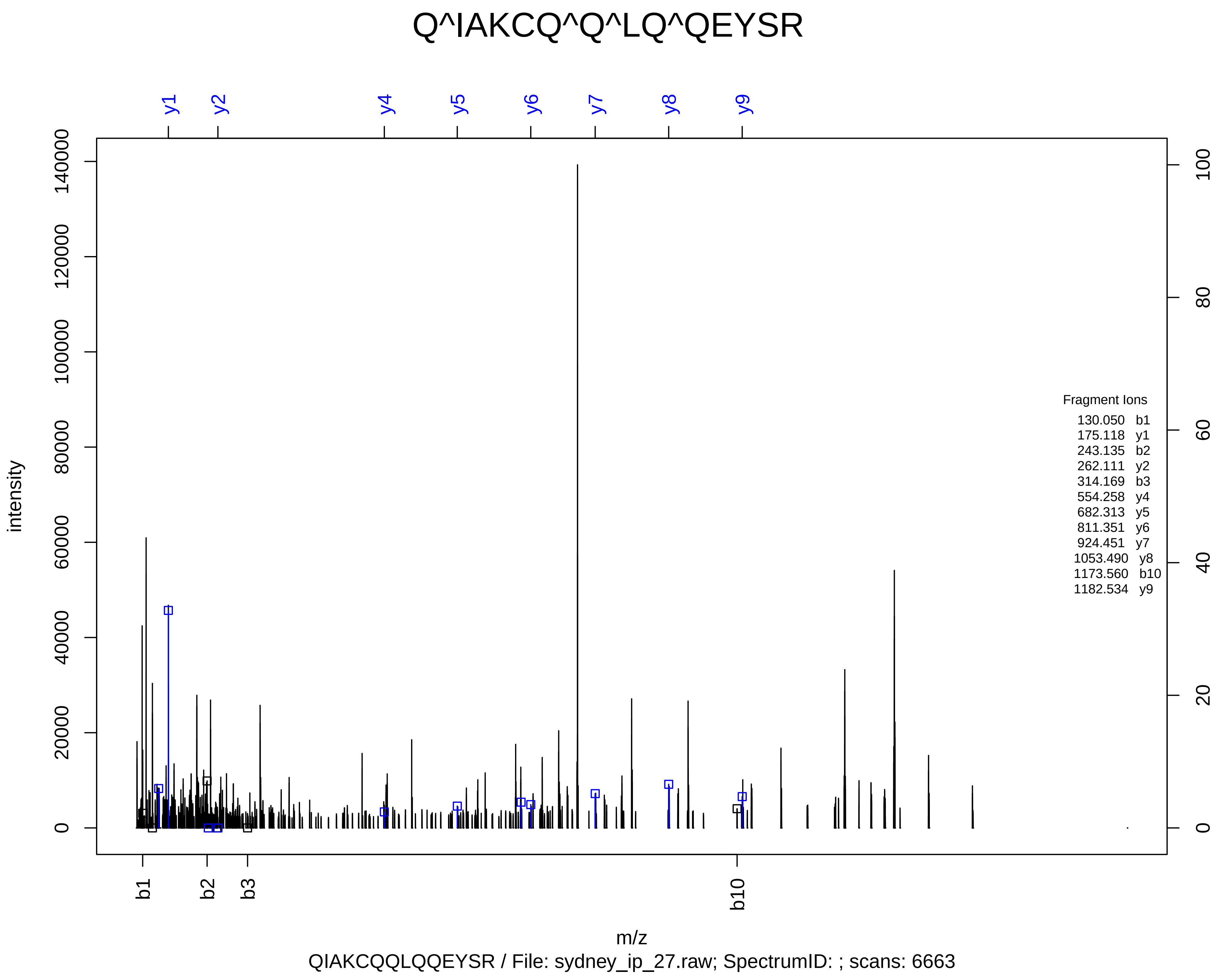
Q^IAKCQ^Q^LQ^QEYSR
y1 y2 y4 y5 y6 y7 y8 y9
intensity
0 20000 40000 60000 80000 100000 120000 140000
0 20 40 60 80 100
b1 b2 b3 b10
Fragment Ions
130.050 b1
175.118 y1
243.135 b2
262.111 y2
314.169 b3
554.258 y4
682.313 y5
811.351 y6
924.451 y7
1053.490 y8
1173.560 b10
1182.534 y9
m/z
QIAKCQQLQQEYSR / File: sydney_ip_27.raw; SpectrumID: ; scans: 6663

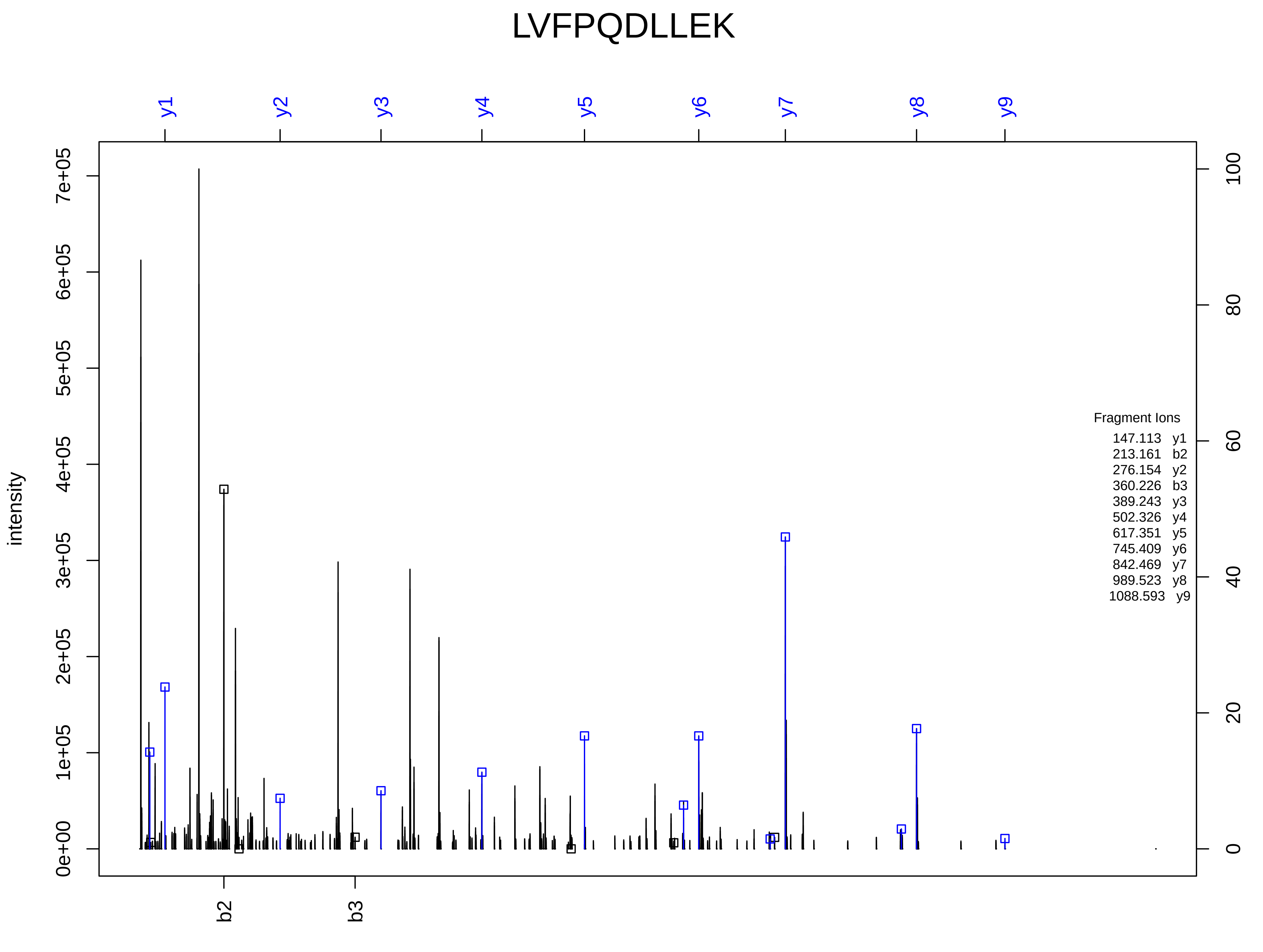

LVFPQDLLEK / File: sydney_ip_27.raw; SpectrumID: ; scans: 15742

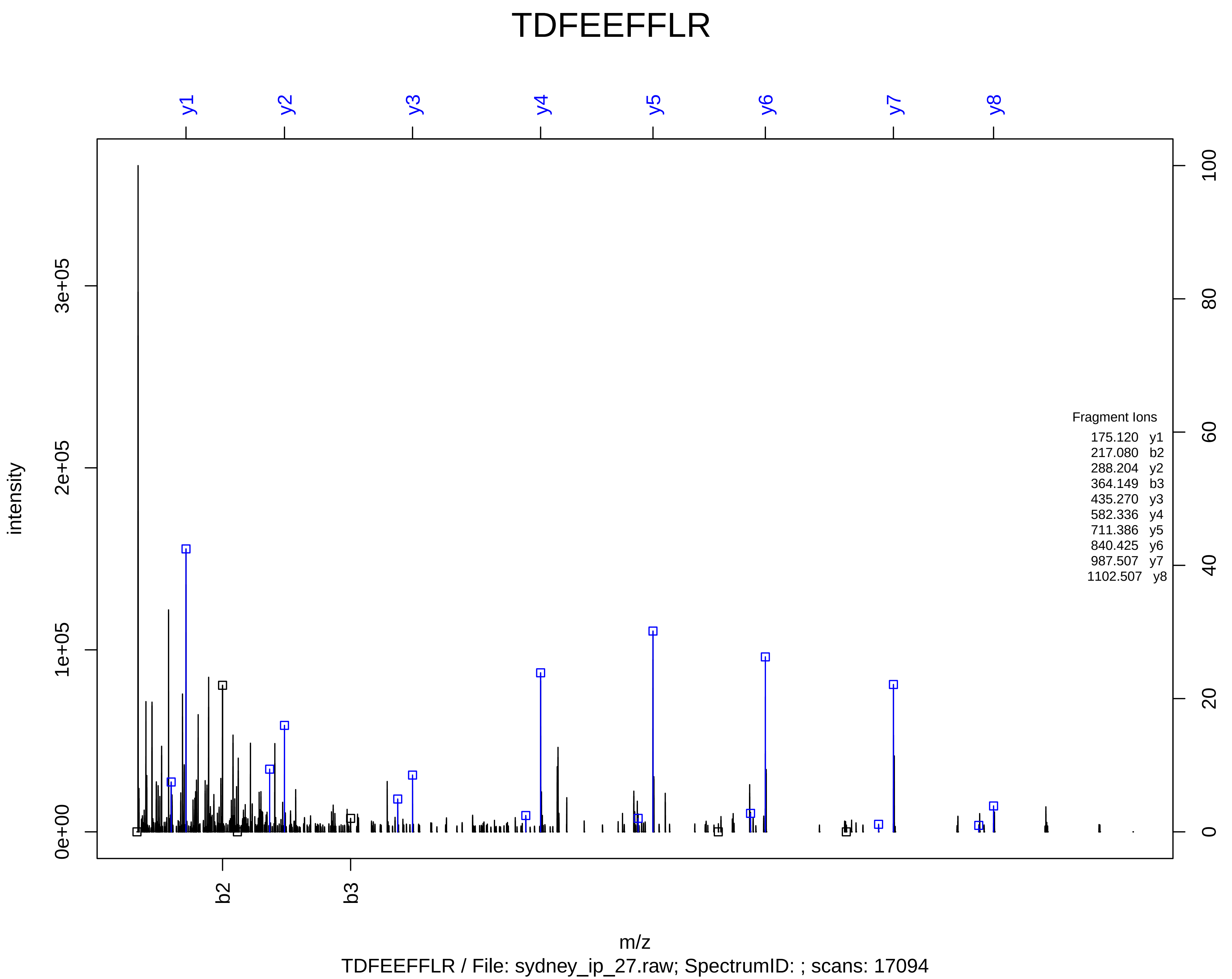
TDFEEFFLR
y1
y2
y3
y4
y5
y6
y7
y8
b2
b3
intensity
0e+00
1e+05
2e+05
3e+05
0
20
40
60
80
100
Fragment Ions
175.120 y1
217.080 b2
288.204 y2
364.149 b3
435.270 y3
582.336 y4
711.386 y5
840.425 y6
987.507 y7
1102.507 y8
m/z
TDFEEFFLR / File: sydney_ip_27.raw; SpectrumID: ; scans: 17094

QHGEQLGGGGSGGGGYNTSK

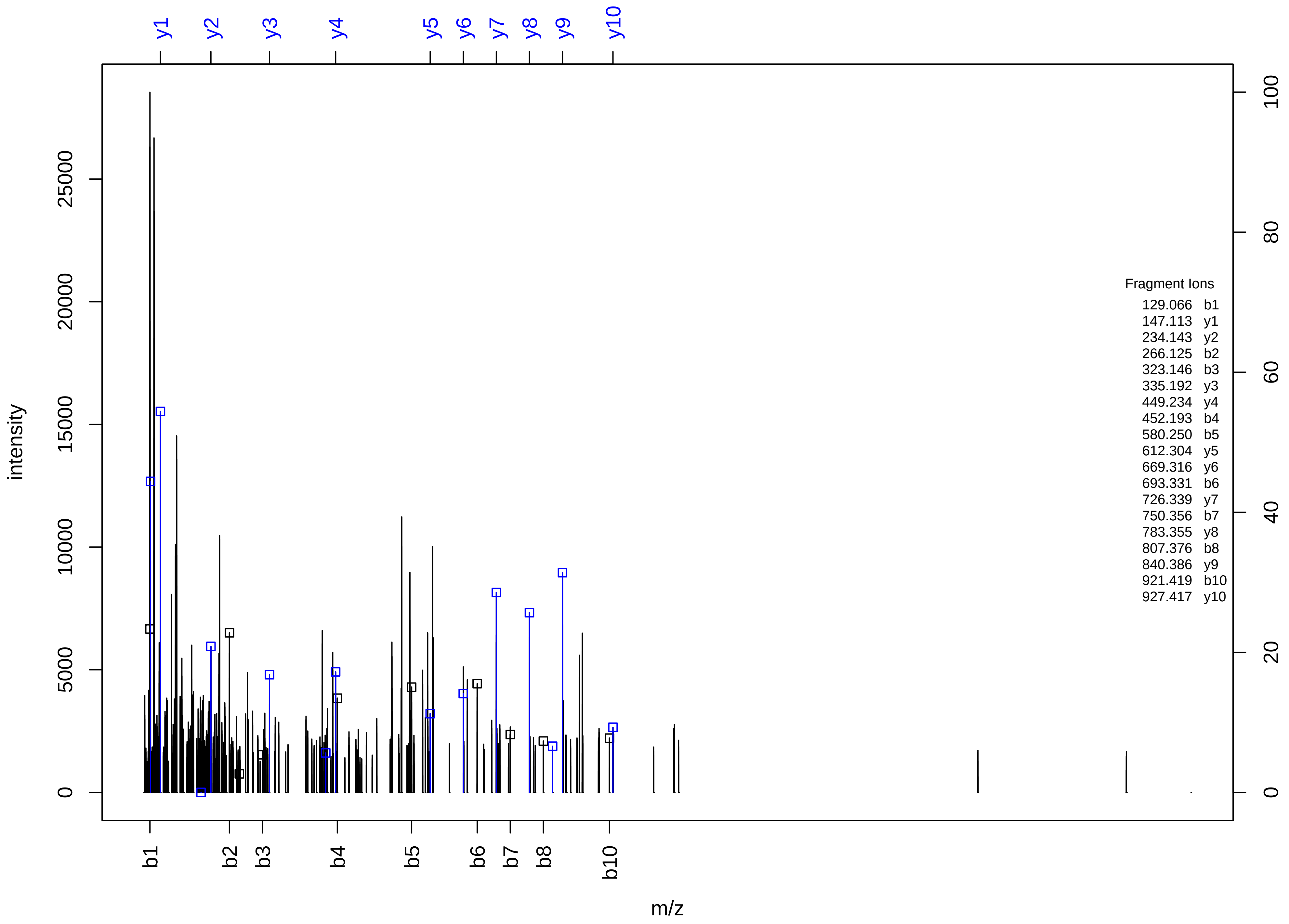

QHGEQLGGGGSGGGGYNTSK / File: sydney_ip_27.raw; SpectrumID: ; scans: 1412

(Ac)M*VEELGMEEN^QDK

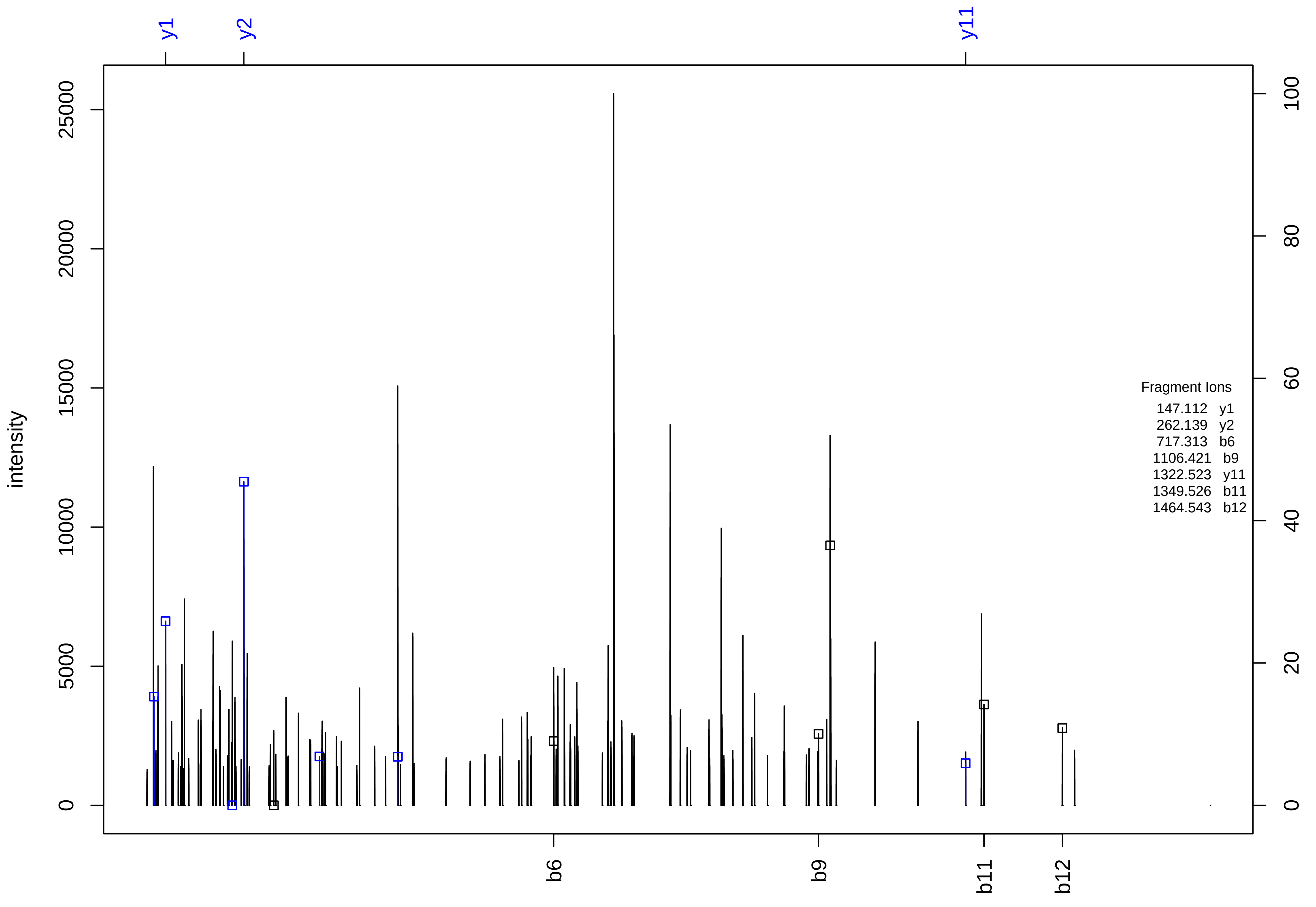

MVEELGMEENQDK / File: sydney_ip_27.raw; SpectrumID: ; scans: 1465

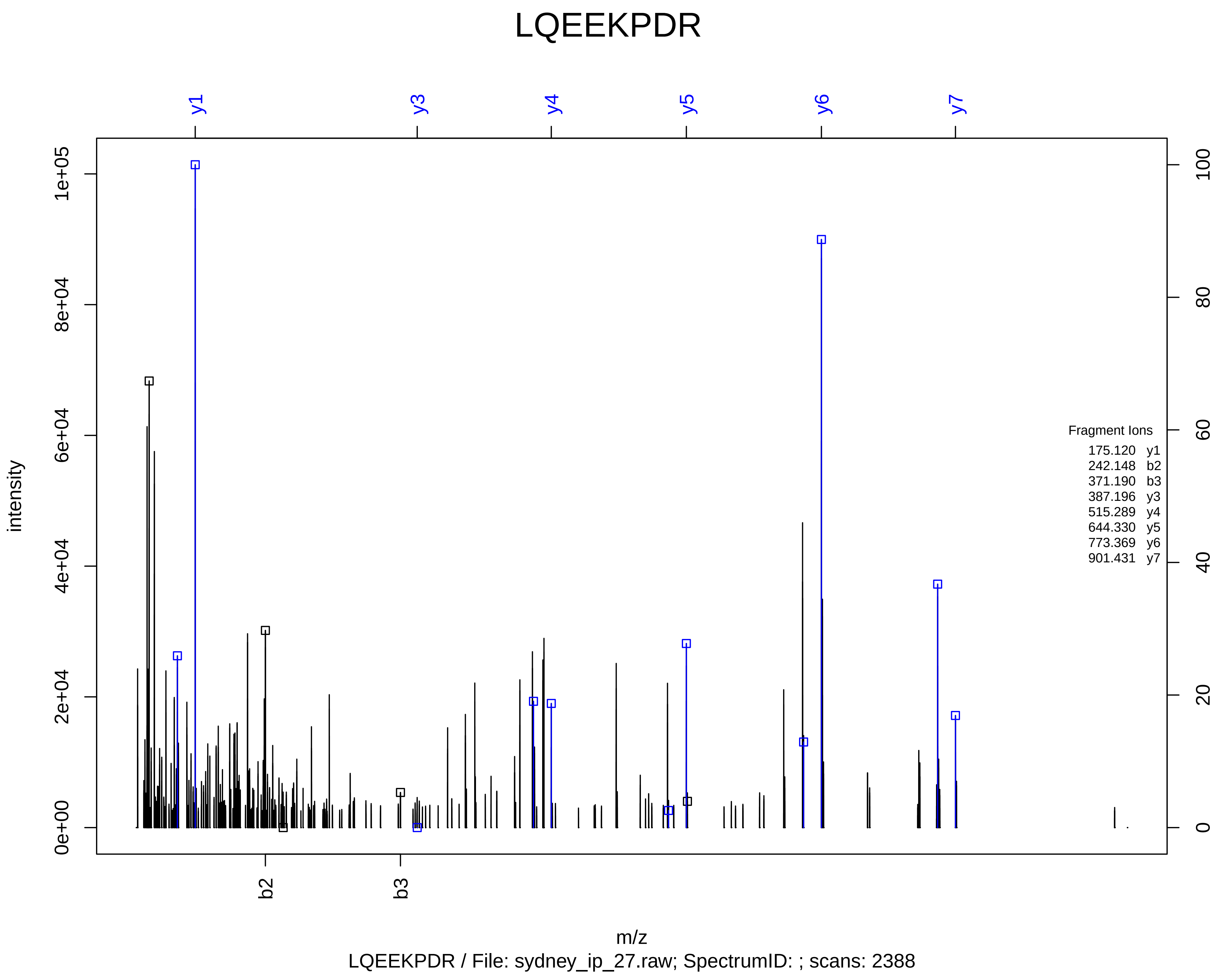
LQEEKPDR
y1
y3
y4
y5
y6
y7
b2
b3
intensity
0e+00
2e+04
4e+04
6e+04
8e+04
1e+05
0
20
40
60
80
100
Fragment Ions
175.120 y1
242.148 b2
371.190 b3
387.196 y3
515.289 y4
644.330 y5
773.369 y6
901.431 y7
m/z
LQEEKPDR / File: sydney_ip_27.raw; SpectrumID: ; scans: 2388

GEGLDGTFPAVIDYTPYLK

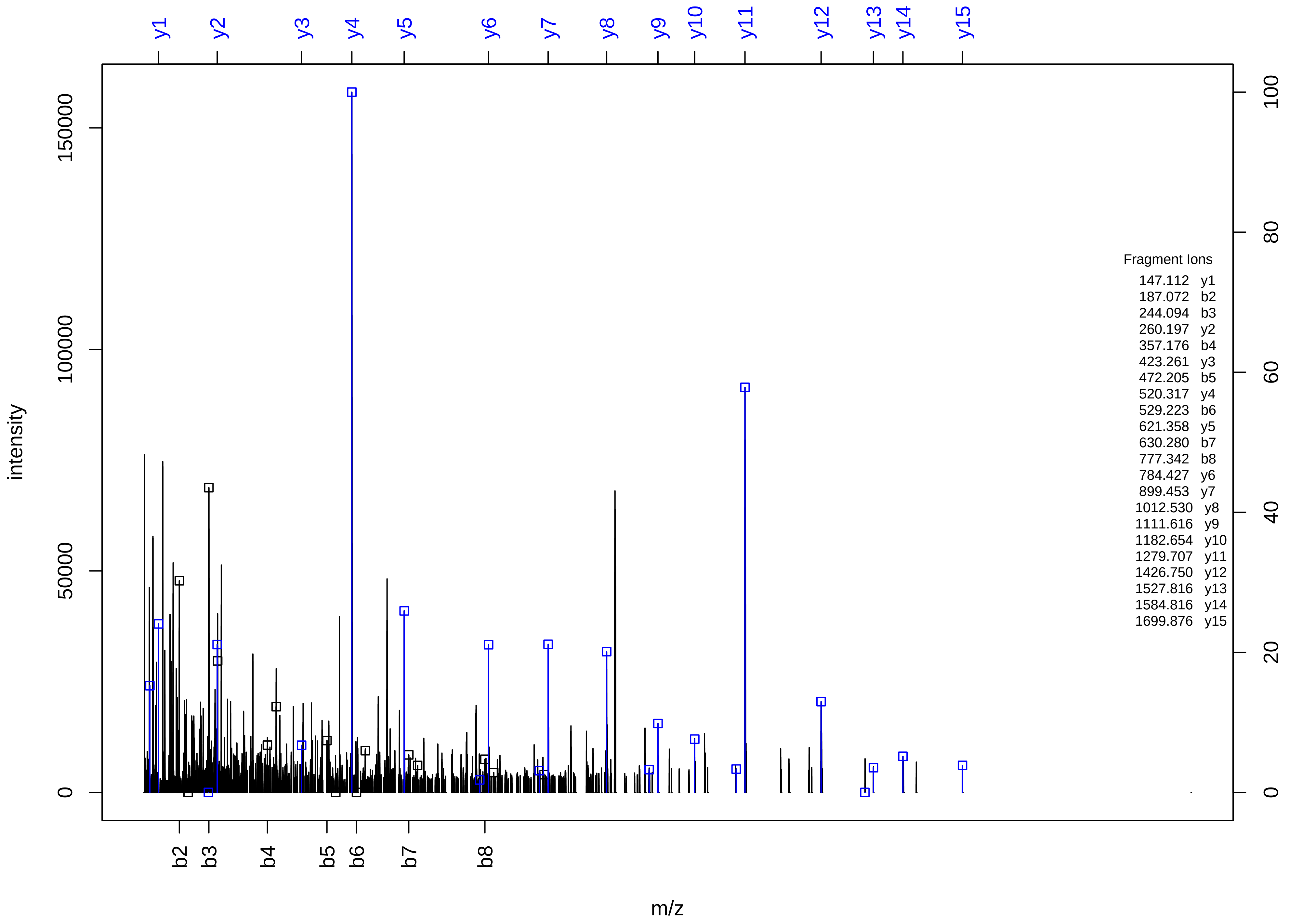

GEGLDGTFPAVIDYTPYLK / File: sydney_ip_27.raw; SpectrumID: ; scans: 19434

VVVLGLLPR

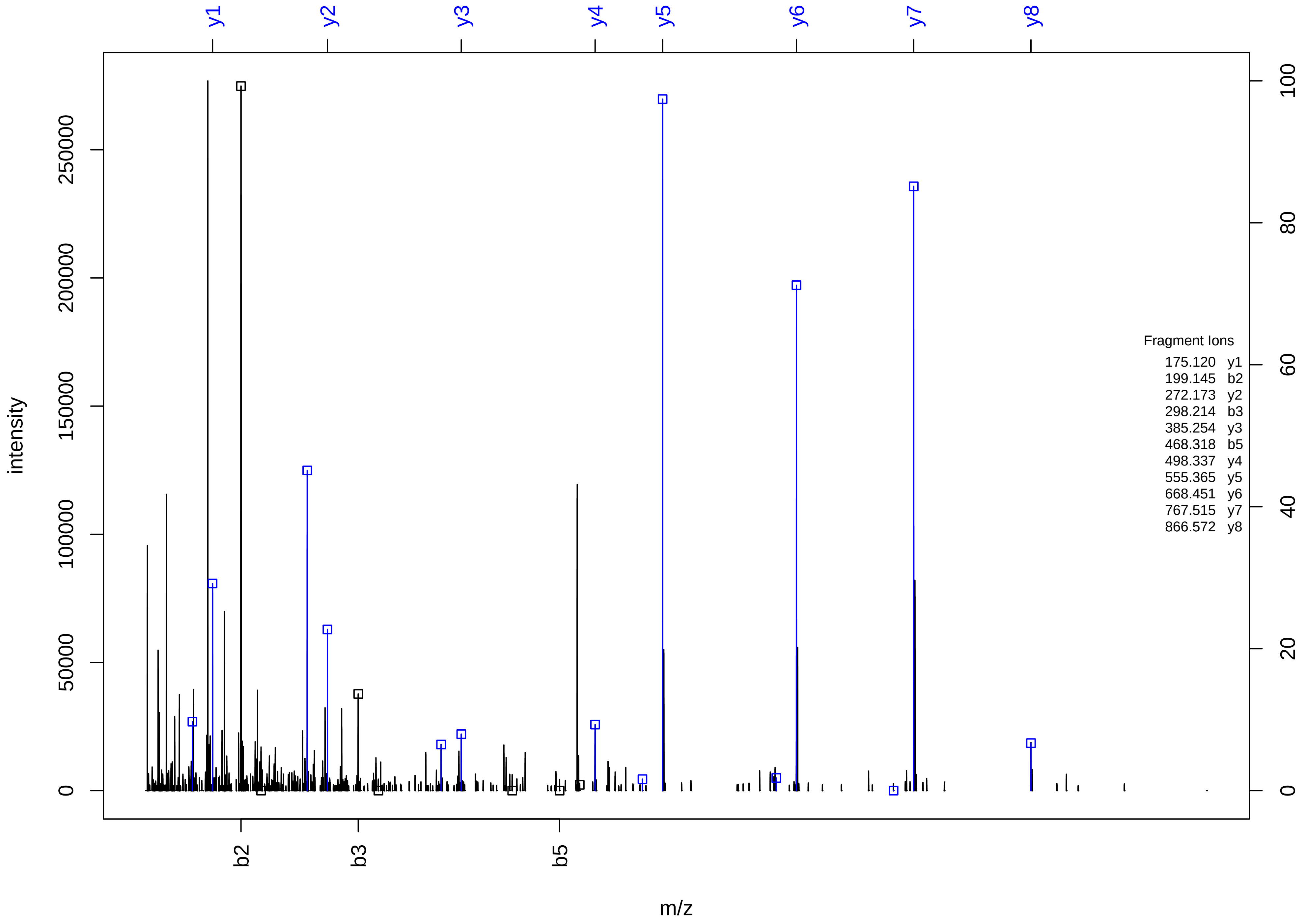

VVVLGLLPR / File: sydney_ip_27.raw; SpectrumID: ; scans: 15105

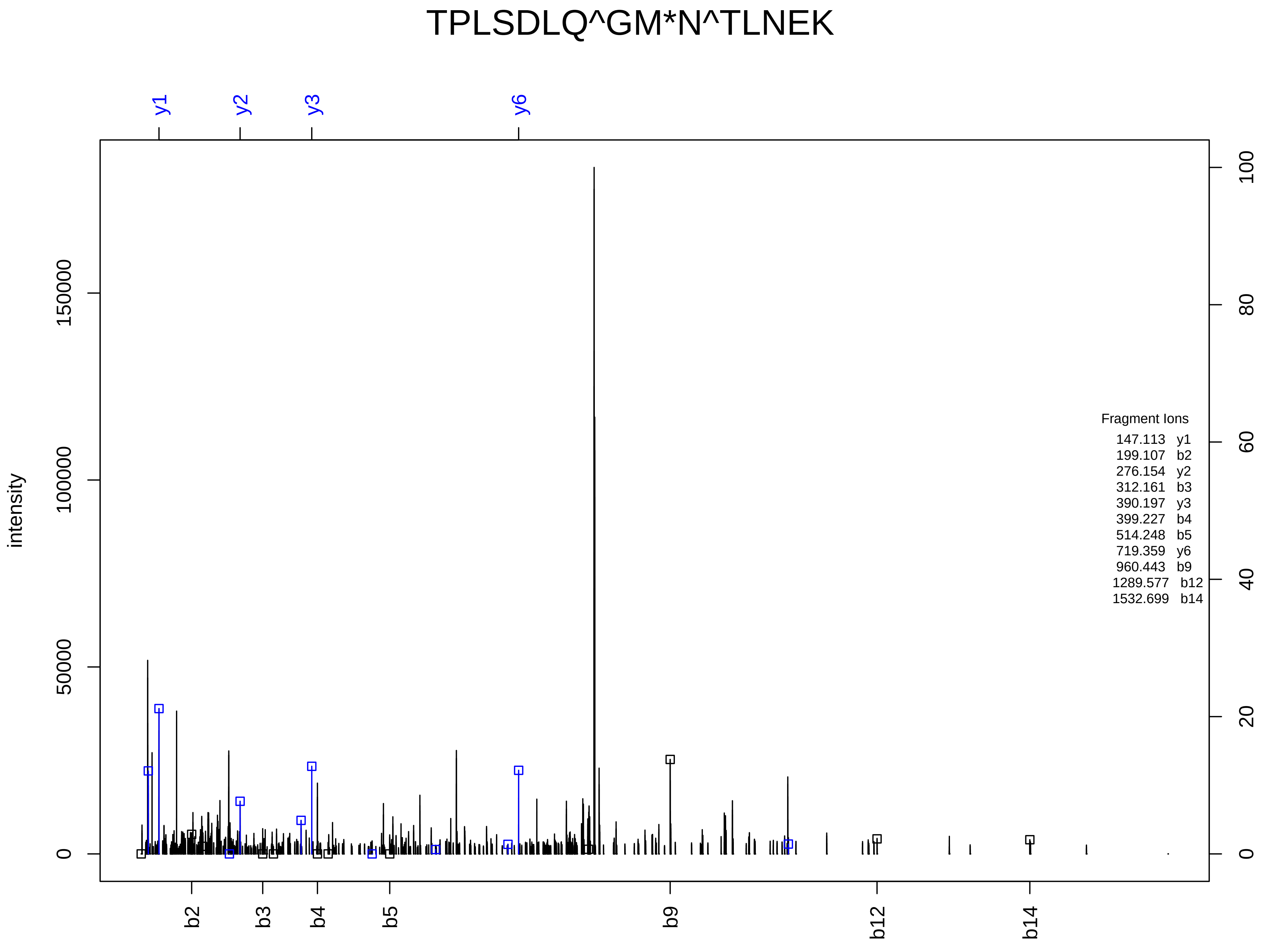

TPLSDLQGMNTLNEK / File: sydney_ip_27.raw; SpectrumID: ; scans: 3394

QVTIWFQ^N^RR

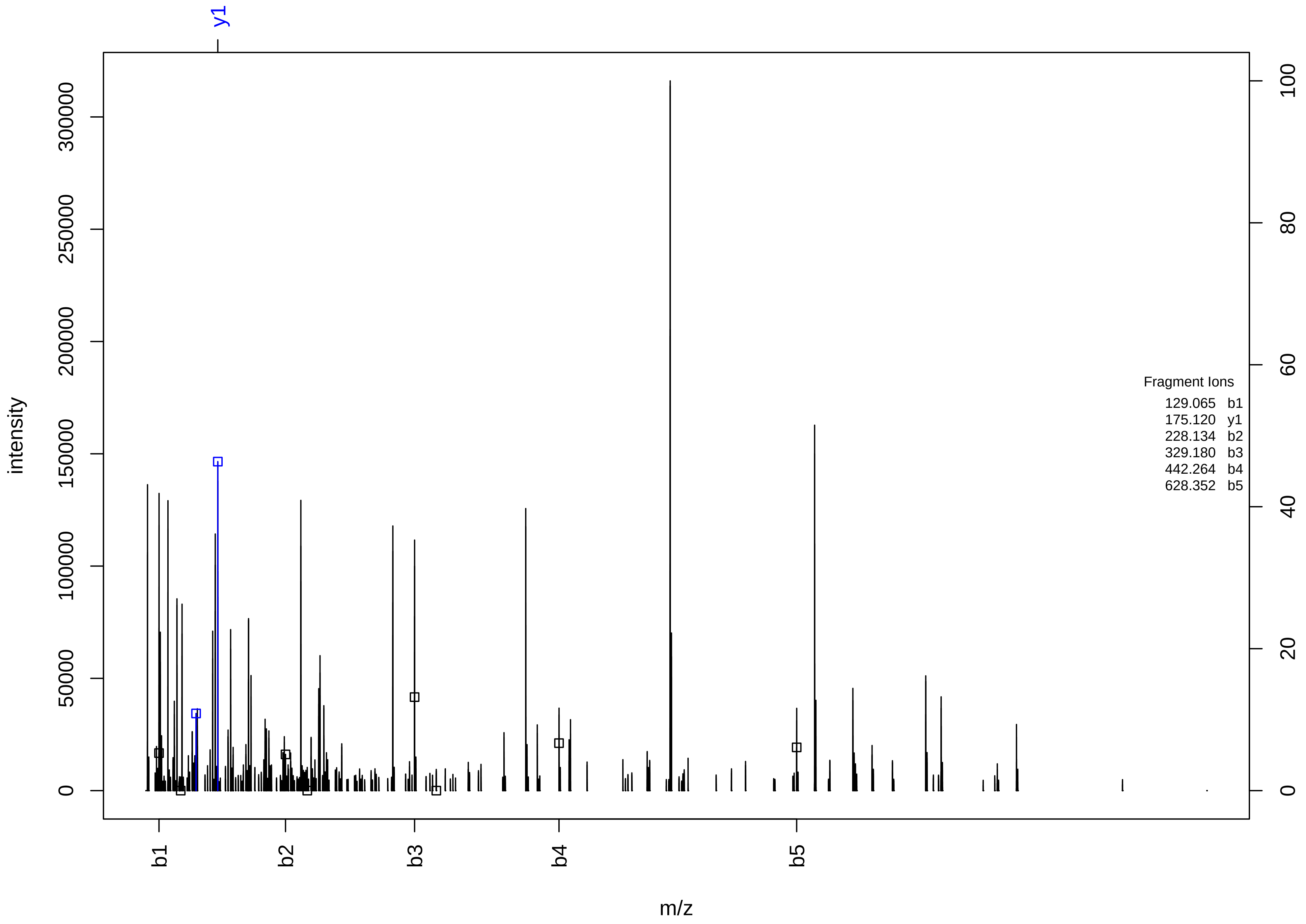

QVTIWFQNRR / File: sydney_ip_27.raw; SpectrumID: ; scans: 5715

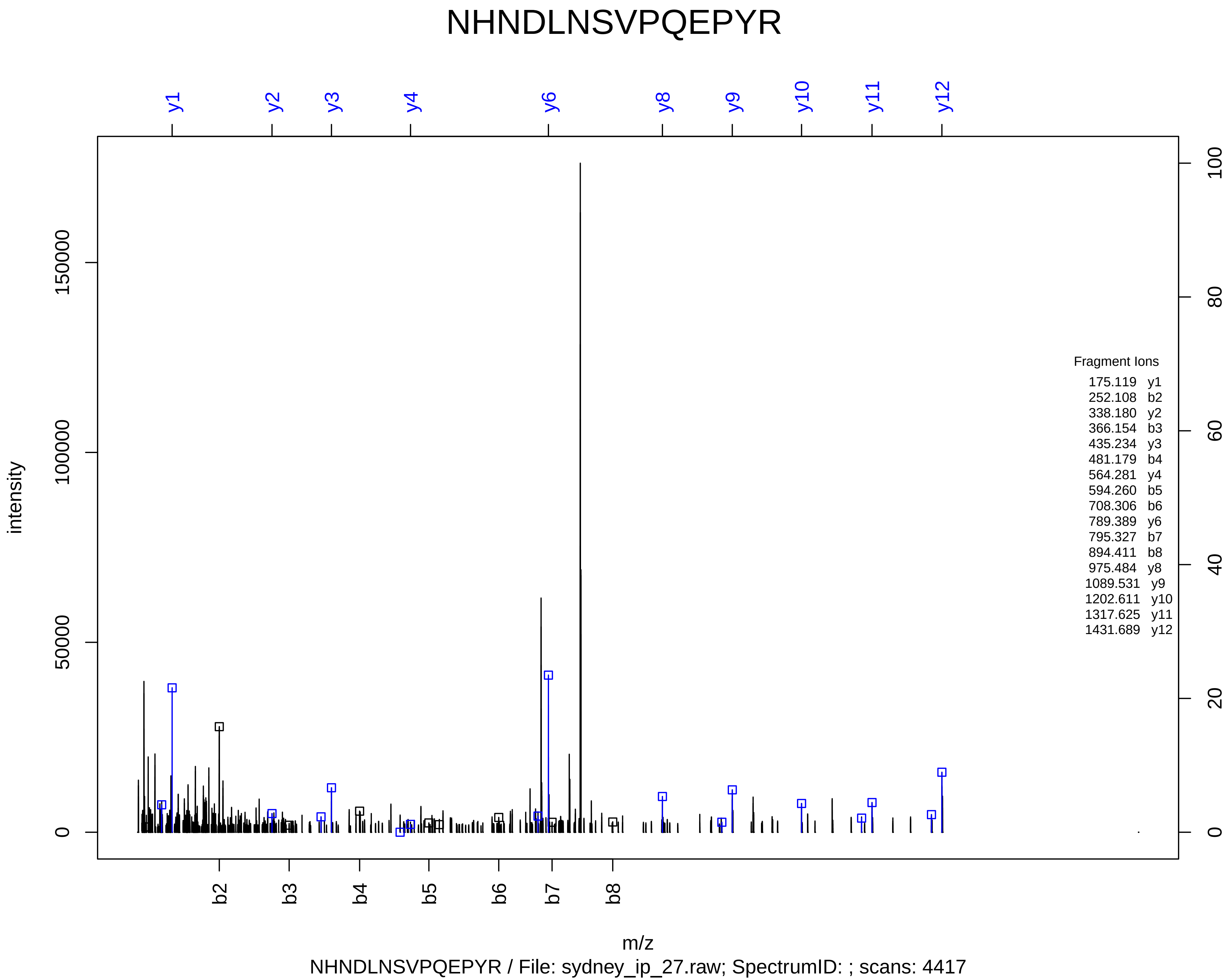
NHNDLNSVPQEPYR
y1
y2
y3
y4
y6
y8
y9
y10
y11
y12
intensity
0
50000
100000
150000
0
20
40
60
80
100
b2
b3
b4
b5
b6
b7
b8
m/z
Fragment Ions
175.119 y1
252.108 b2
338.180 y2
366.154 b3
435.234 y3
481.179 b4
564.281 y4
594.260 b5
708.306 b6
789.389 y6
795.327 b7
894.411 b8
975.484 y8
1089.531 y9
1202.611 y10
1317.625 y11
1431.689 y12
NHNDLNSVPQEPYR / File: sydney_ip_27.raw; SpectrumID: ; scans: 4417

LLLDTFEYQGLVK

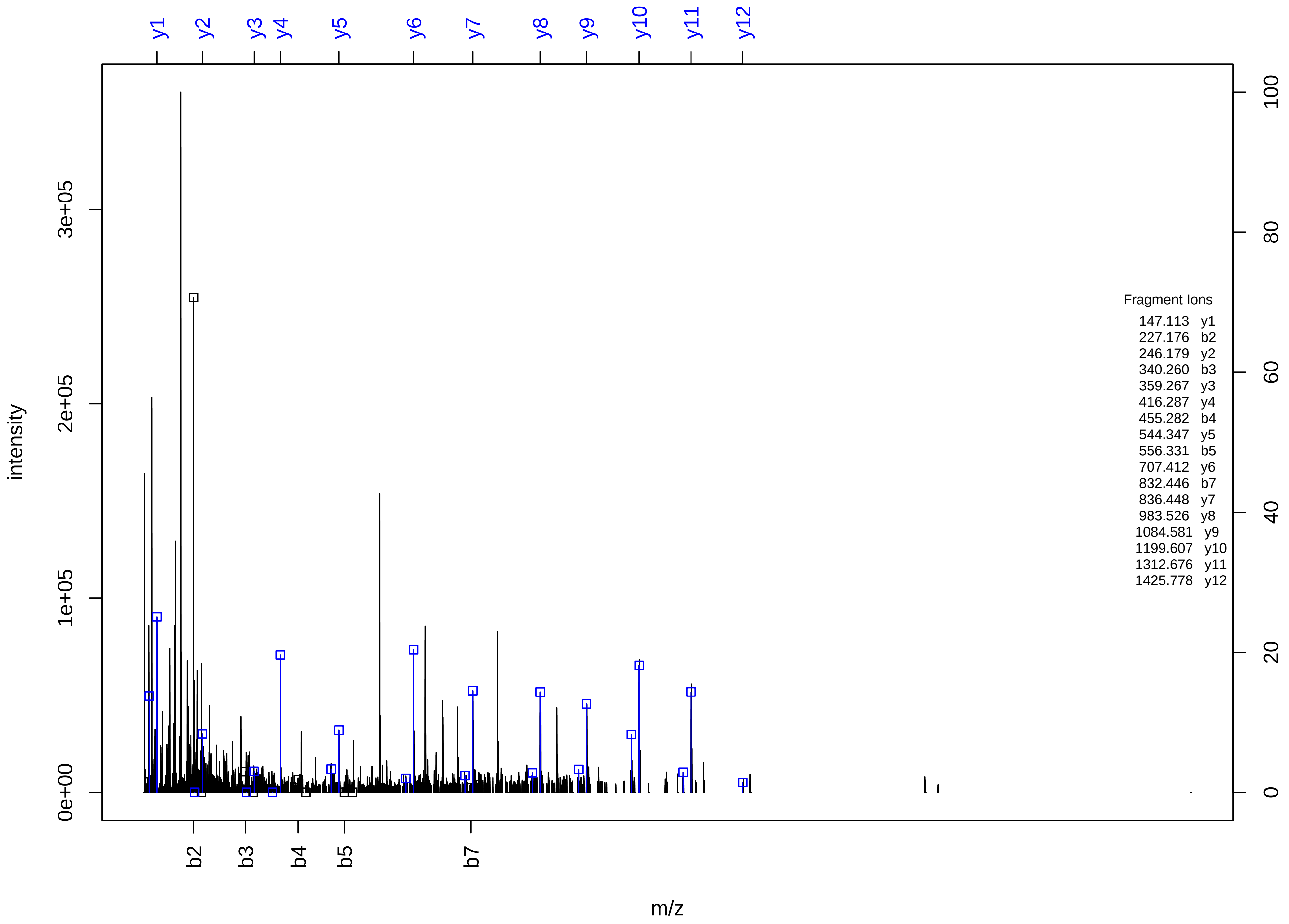

LLLDTFEYQGLVK / File: sydney_ip_27.raw; SpectrumID: ; scans: 17558

NTNDLM*LSFIVEDKPK

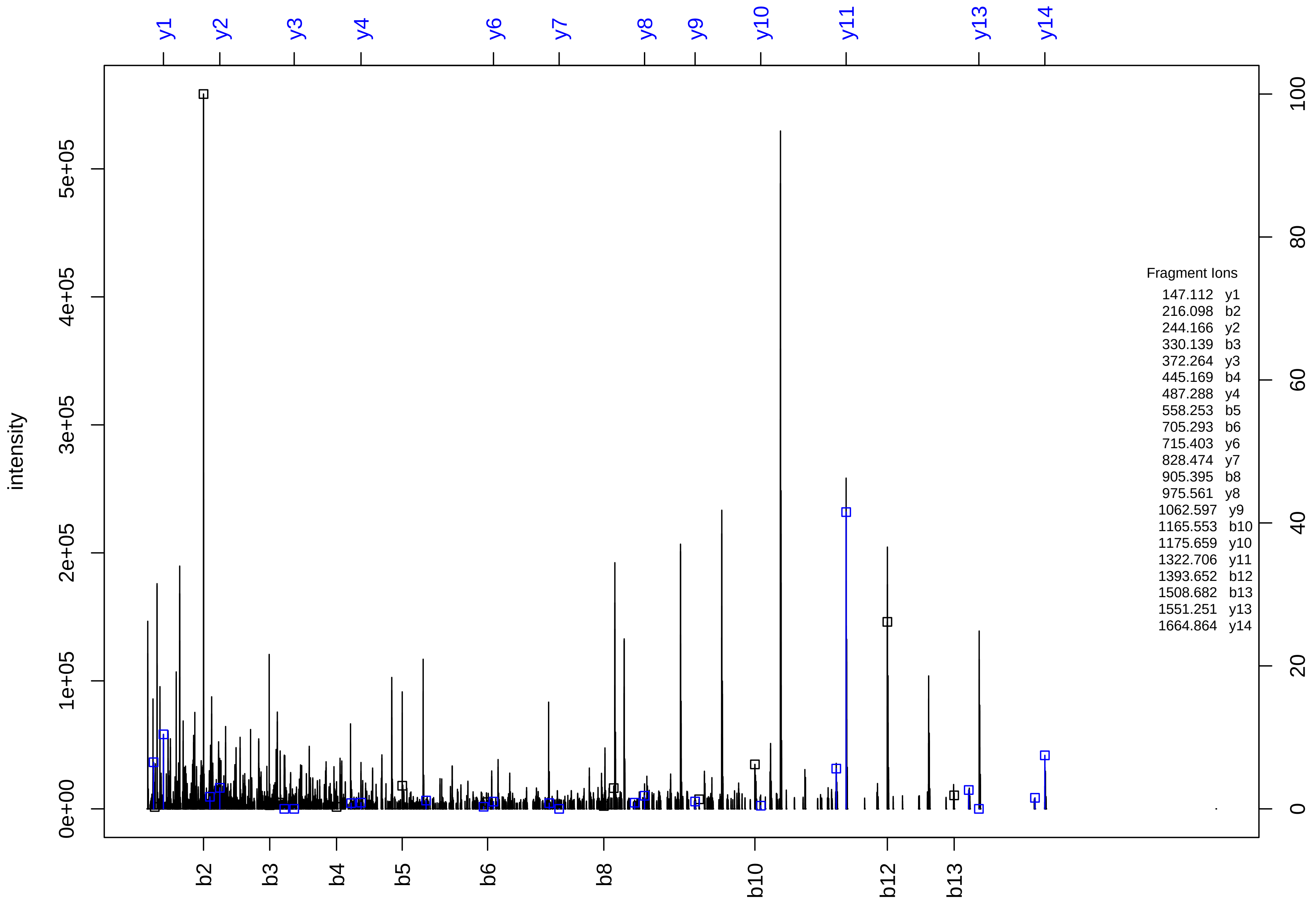

NTNDLMLSFIVEDKPK / File: sydney_ip_27.raw; SpectrumID: ; scans: 14824

SPATSAGPEQLTGLPR

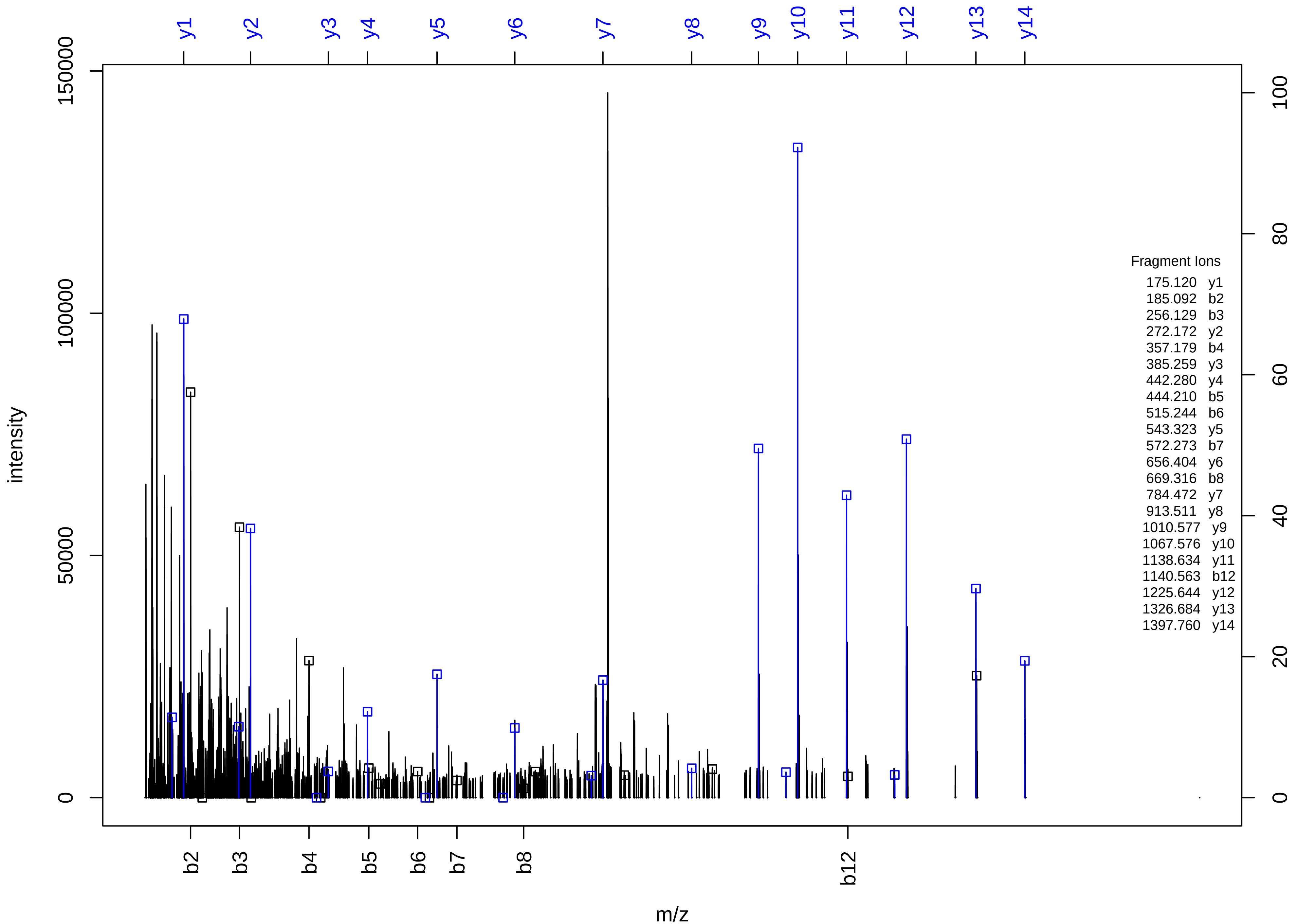

SPATSAGPEQLTGLPR / File: sydney_ip_27.raw; SpectrumID: ; scans: 8665

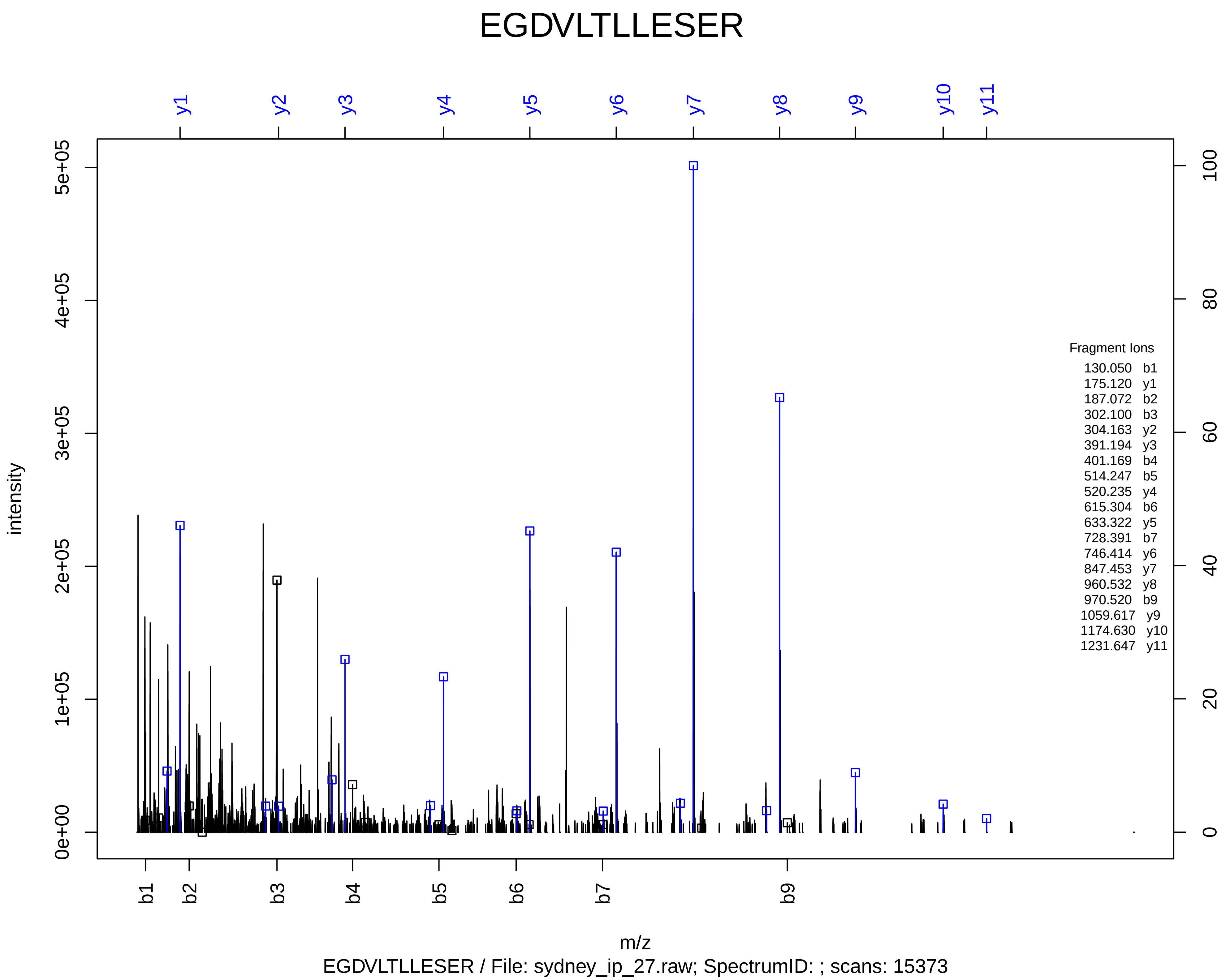

EGDVLTLLESER
y1
y2
y3
y4
y5
y6
y7
y8
y9
y10
y11
intensity
0e+00
1e+05
2e+05
3e+05
4e+05
5e+05
0
20
40
60
80
100
b1
b2
b3
b4
b5
b6
b7
b9
m/z
Fragment Ions
130.050 b1
175.120 y1
187.072 b2
302.100 b3
304.163 y2
391.194 y3
401.169 b4
514.247 b5
520.235 y4
615.304 b6
633.322 y5
728.391 b7
746.414 y6
847.453 y7
960.532 y8
970.520 b9
1059.617 y9
1174.630 y10
1231.647 y11
EGDVLTLLESER / File: sydney_ip_27.raw; SpectrumID: ; scans: 15373

SLTYLSILR

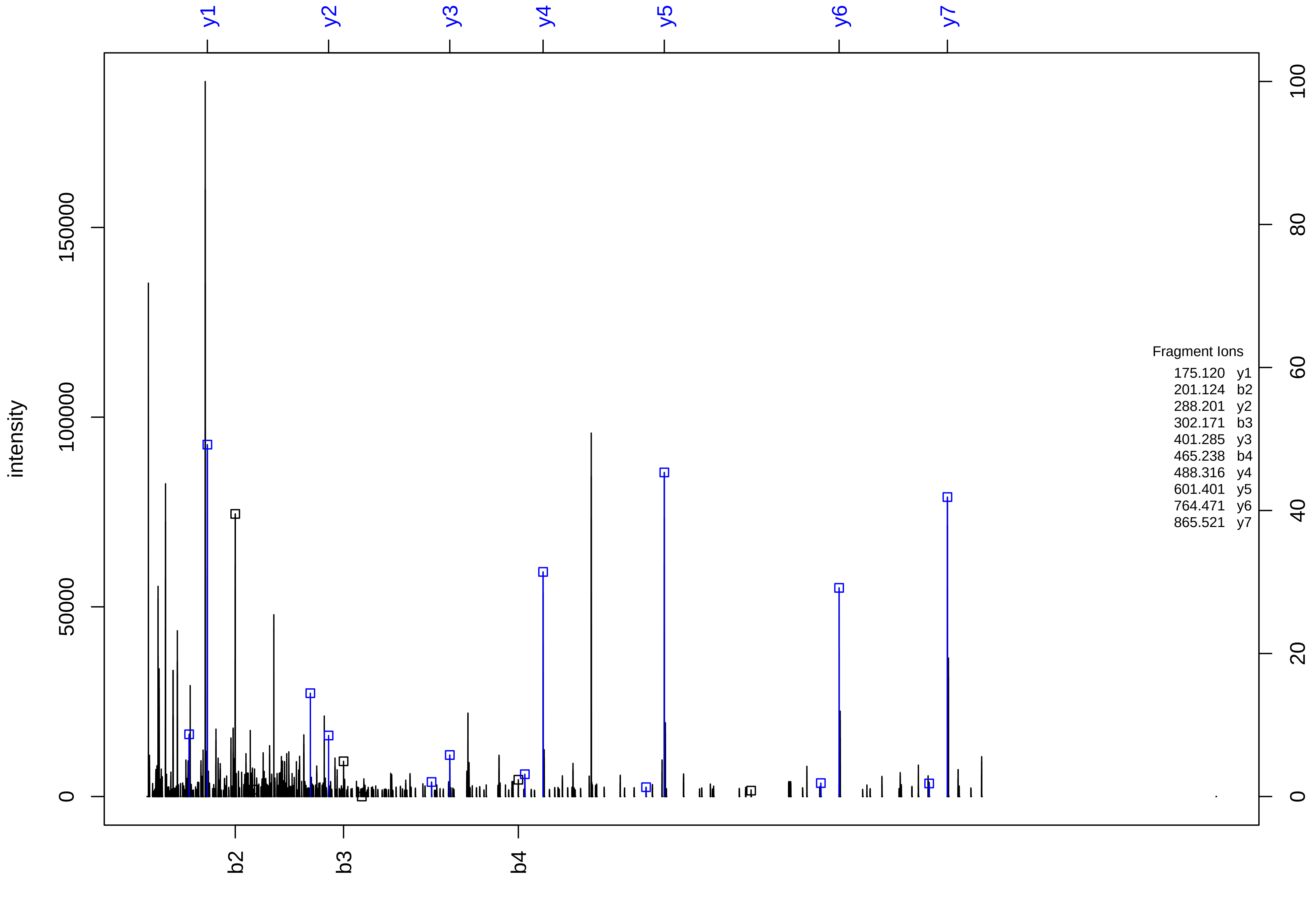

SLTYLSILR / File: sydney_ip_27.raw; SpectrumID: ; scans: 14938

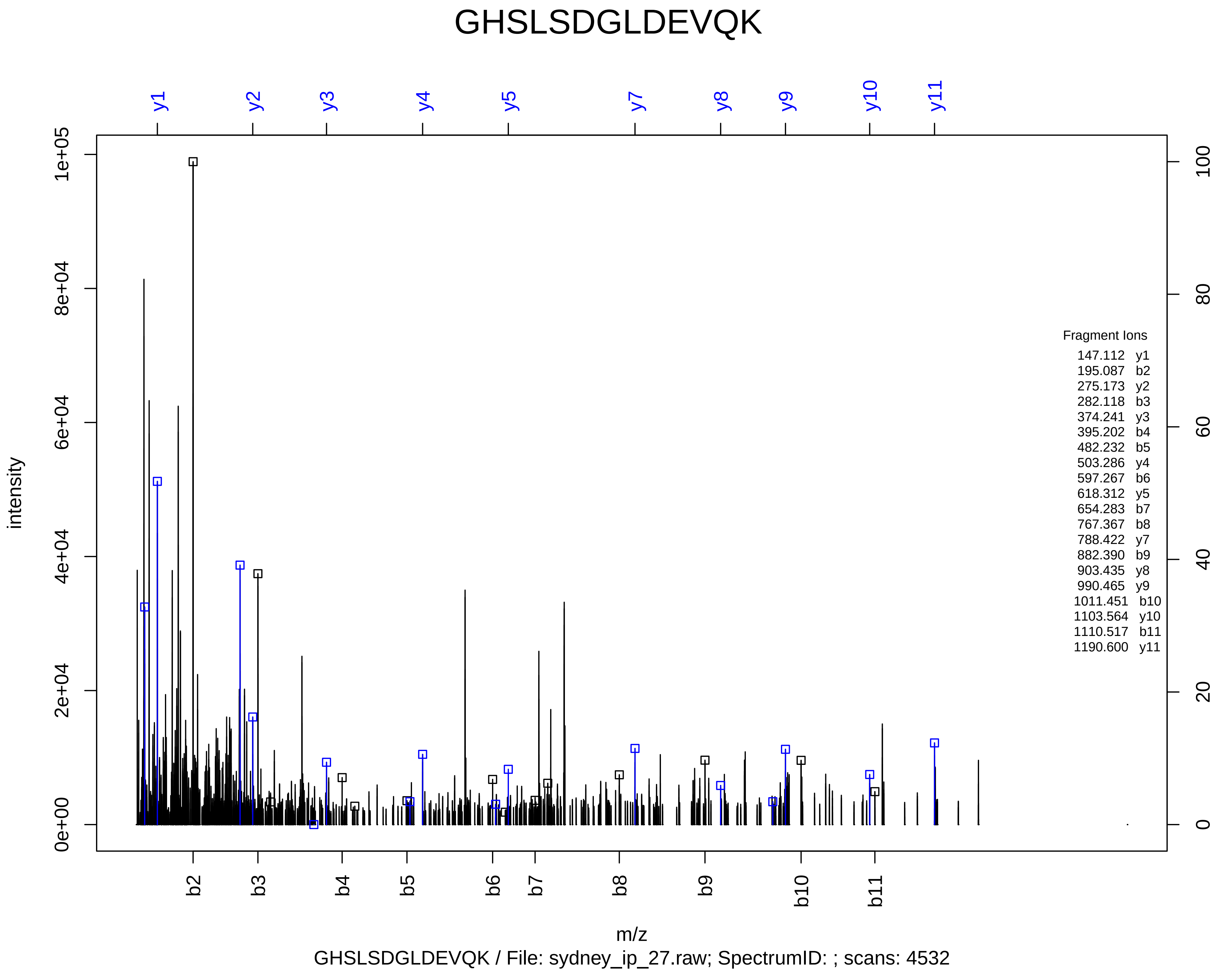
GHSLSDGLDEVQK
y1
y2
y3
y4
y5
y7
y8
y9
y10
y11
intensity
0e+00
2e+04
4e+04
6e+04
8e+04
1e+05
0
20
40
60
80
100
b2
b3
b4
b5
b6
b7
b8
b9
b10
b11
m/z
Fragment Ions
147.112 y1
195.087 b2
275.173 y2
282.118 b3
374.241 y3
395.202 b4
482.232 b5
503.286 y4
597.267 b6
618.312 y5
654.283 b7
767.367 b8
788.422 y7
882.390 b9
903.435 y8
990.465 y9
1011.451 b10
1103.564 y10
1110.517 b11
1190.600 y11
GHSLSDGLDEVQK / File: sydney_ip_27.raw; SpectrumID: ; scans: 4532

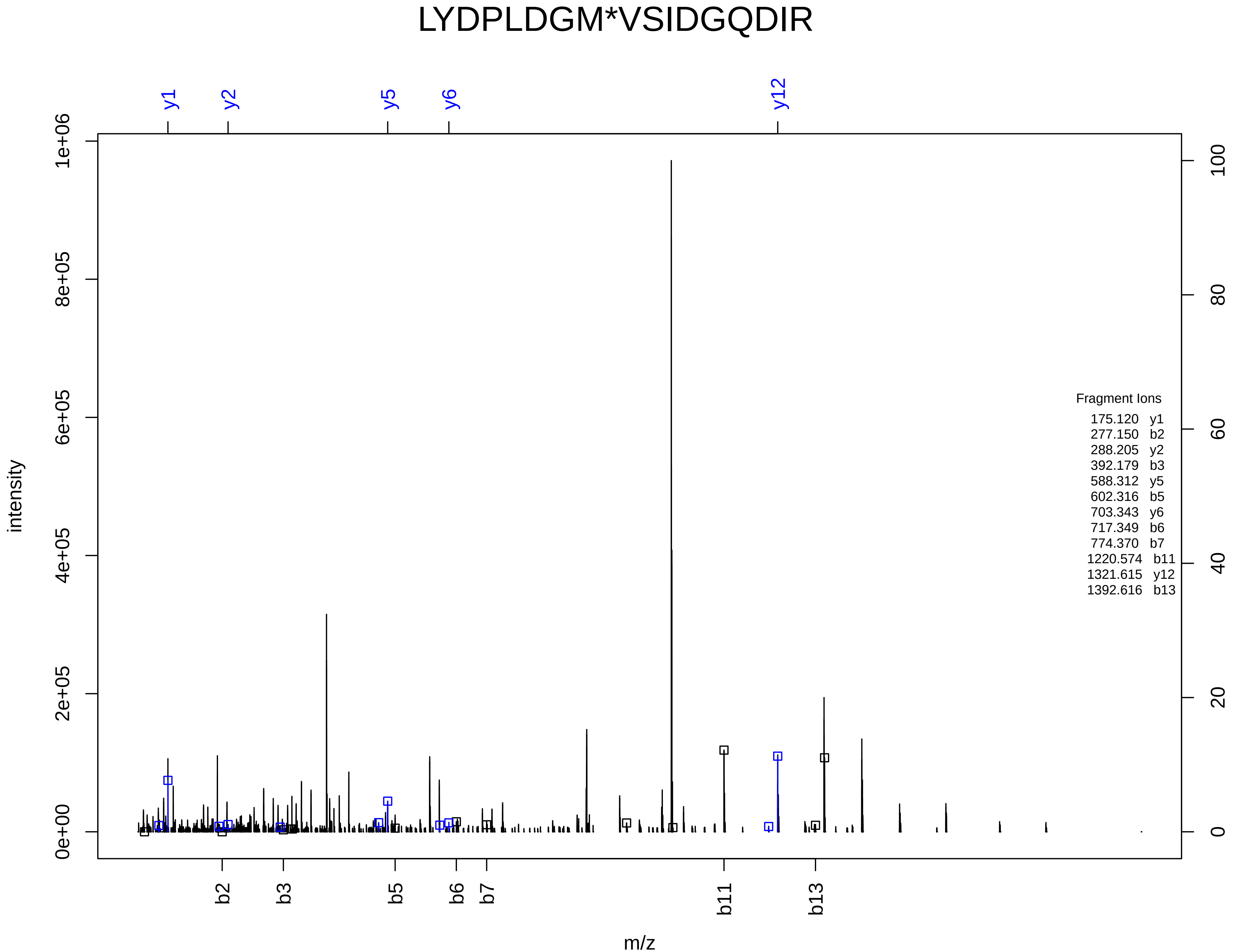

LYDPLDGMVSIDGQDIR / File: sydney_ip_27.raw; SpectrumID: ; scans: 6824

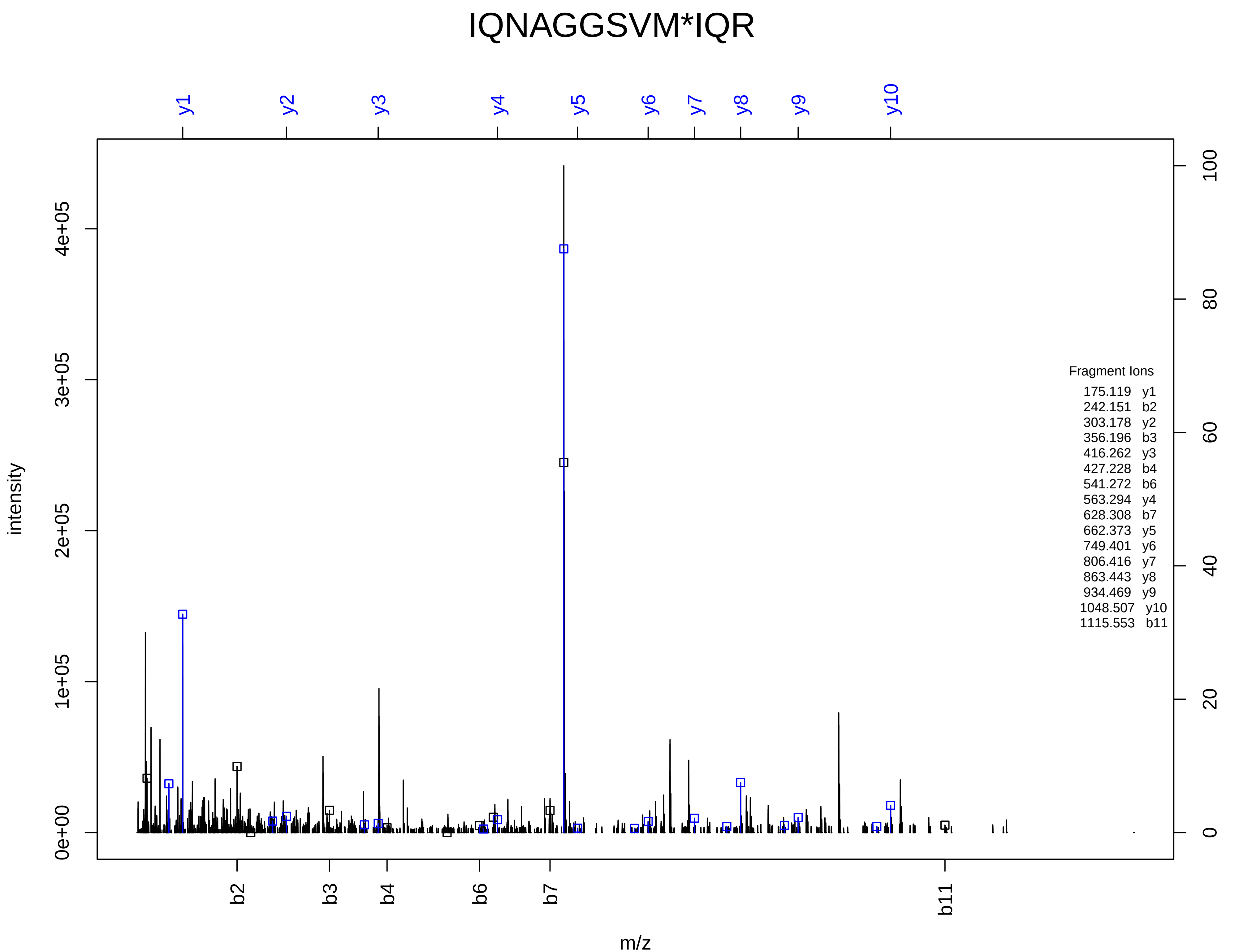
IQNAGGSVM*IQR
y1
y2
y3
y4
y5
y6
y7
y8
y9
y10
intensity
0e+00
1e+05
2e+05
3e+05
4e+05
0
20
40
60
80
100
Fragment Ions
175.119 y1
242.151 b2
303.178 y2
356.196 b3
416.262 y3
427.228 b4
541.272 b6
563.294 y4
628.308 b7
662.373 y5
749.401 y6
806.416 y7
863.443 y8
934.469 y9
1048.507 y10
1115.553 b11
b2
b3
b4
b6
b7
b11
m/z
IQNAGGSVMIQR / File: sydney_ip_27.raw; SpectrumID: ; scans: 3223

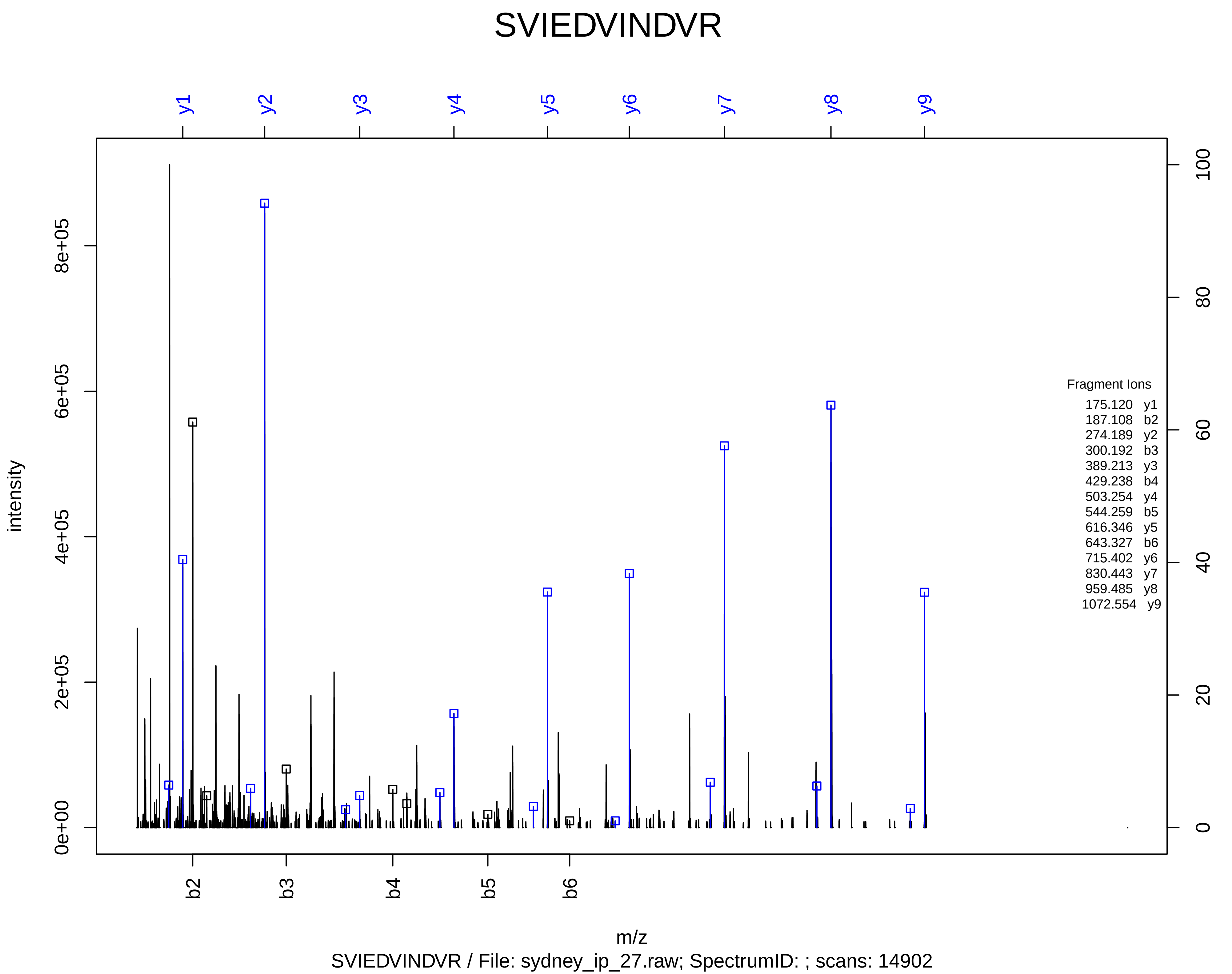
SVIEDVINDVR
y1
y2
y3
y4
y5
y6
y7
y8
y9
intensity
0e+00
2e+05
4e+05
6e+05
8e+05
0
20
40
60
80
100
b2
b3
b4
b5
b6
m/z
Fragment Ions
175.120 y1
187.108 b2
274.189 y2
300.192 b3
389.213 y3
429.238 b4
503.254 y4
544.259 b5
616.346 y5
643.327 b6
715.402 y6
830.443 y7
959.485 y8
1072.554 y9
SVIEDVINDVR / File: sydney_ip_27.raw; SpectrumID: ; scans: 14902

SCEVLFNPFDDITPR

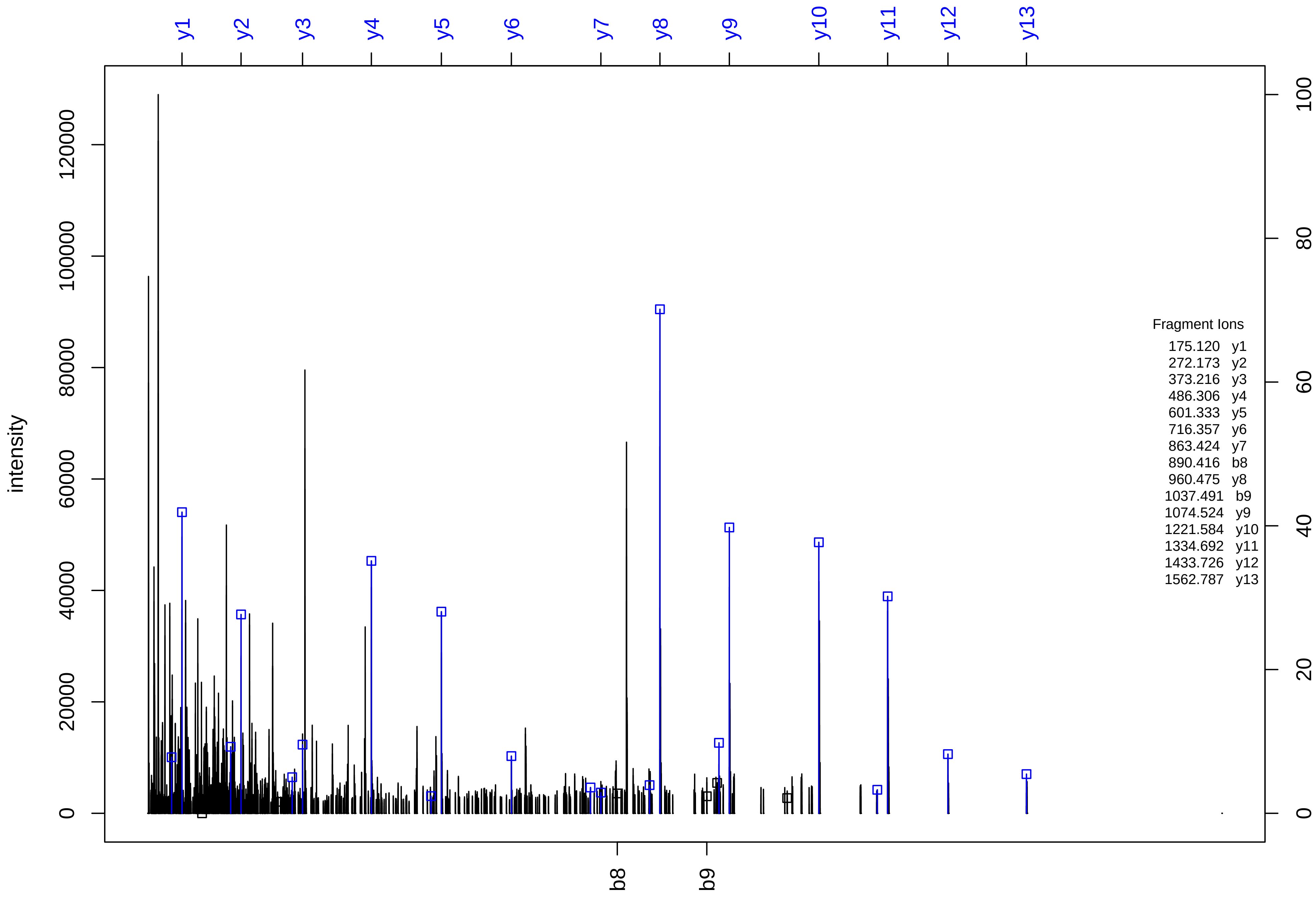

SCEVLFNPFDDITPR / File: sydney_ip_27.raw; SpectrumID: ; scans: 18659

YIHLENLLAR

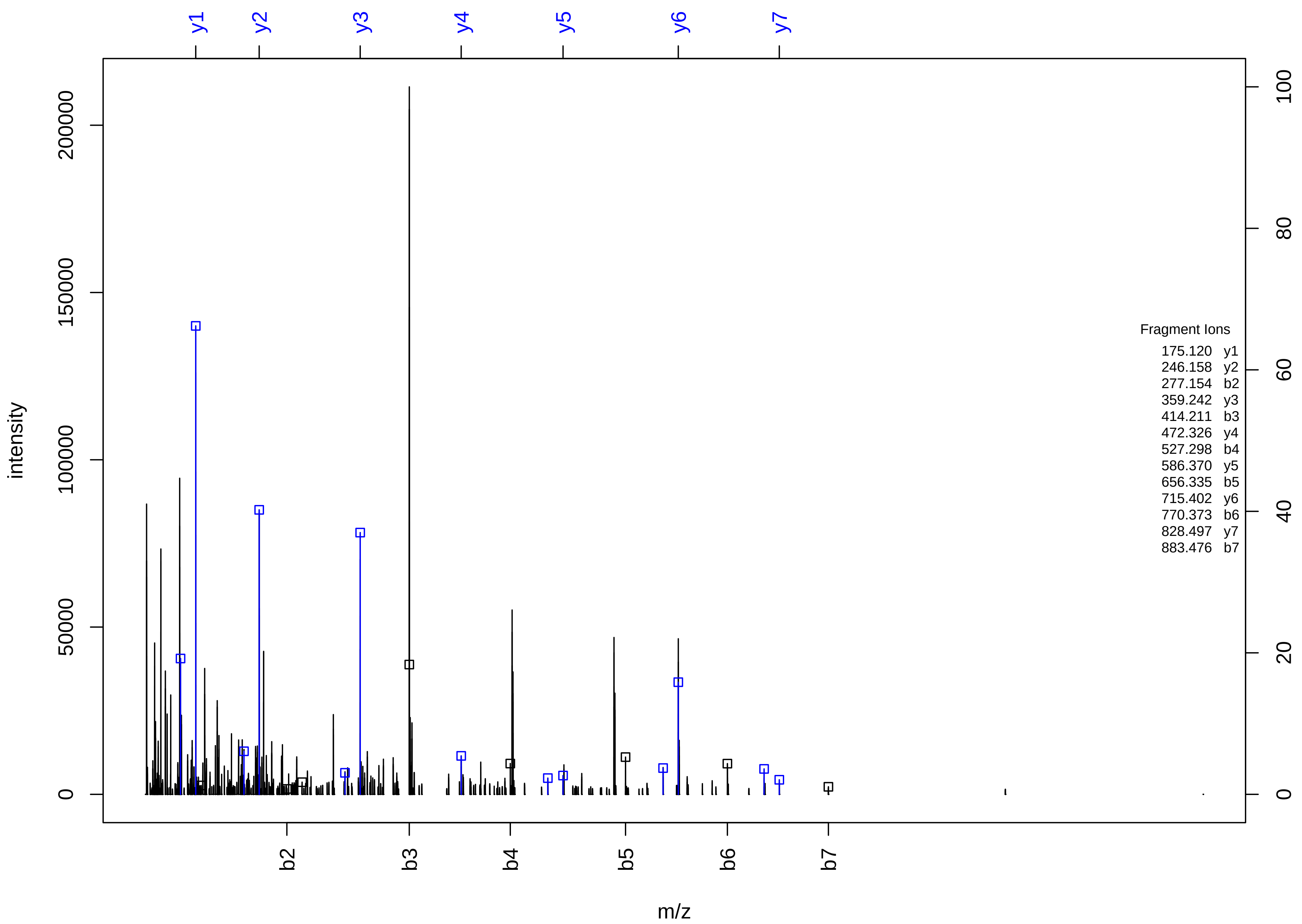

YIHLENLLAR / File: sydney_ip_27.raw; SpectrumID: ; scans: 11976

SGHDPERYISDSPILQ^Q^FQHN^IHLAR

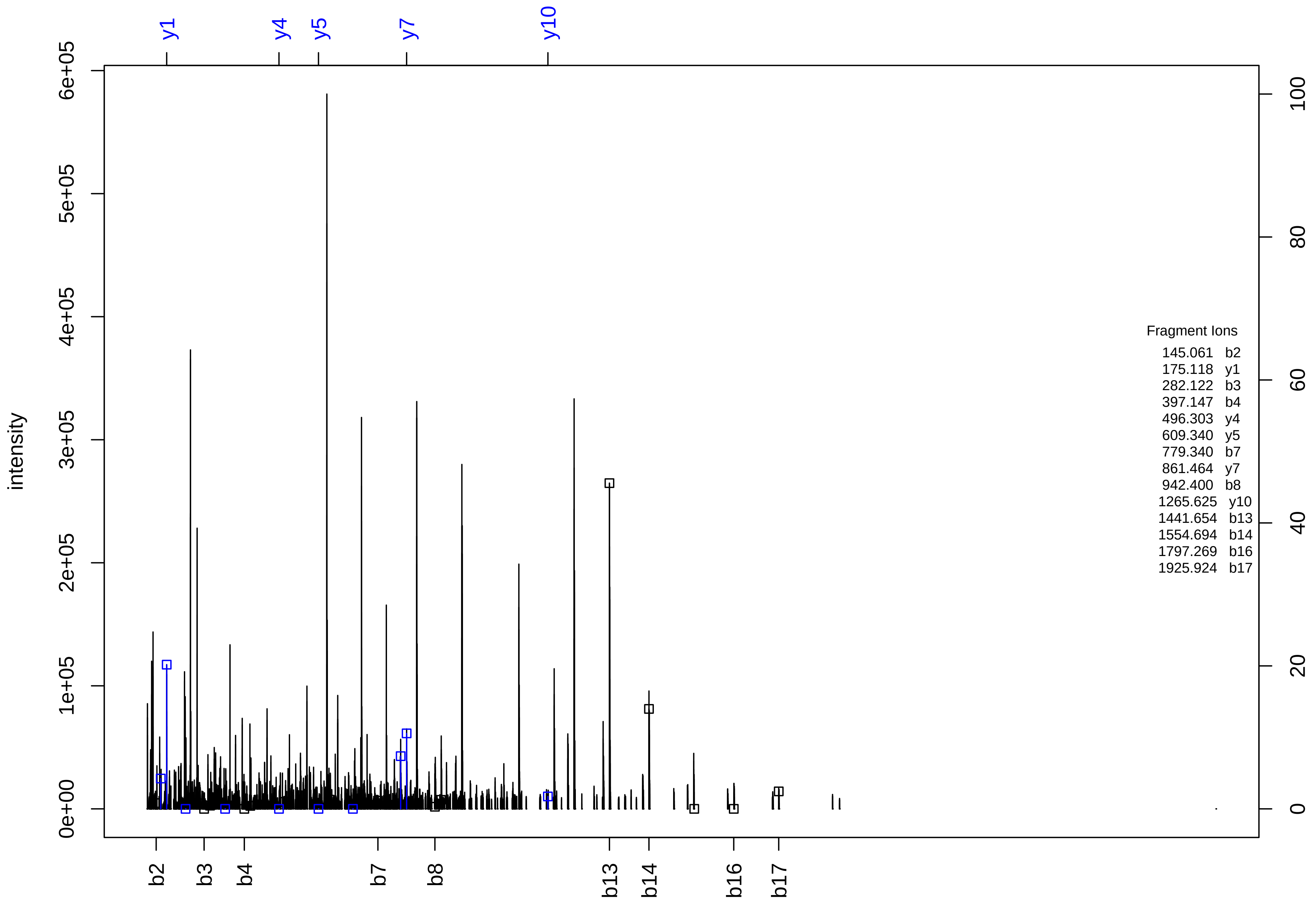

SGHDPERYISDSPILQQFQHNIHLAR / File: sydney_ip_27.raw; SpectrumID: ; scans: 16753

WFKNDAPVVQ^EPRR

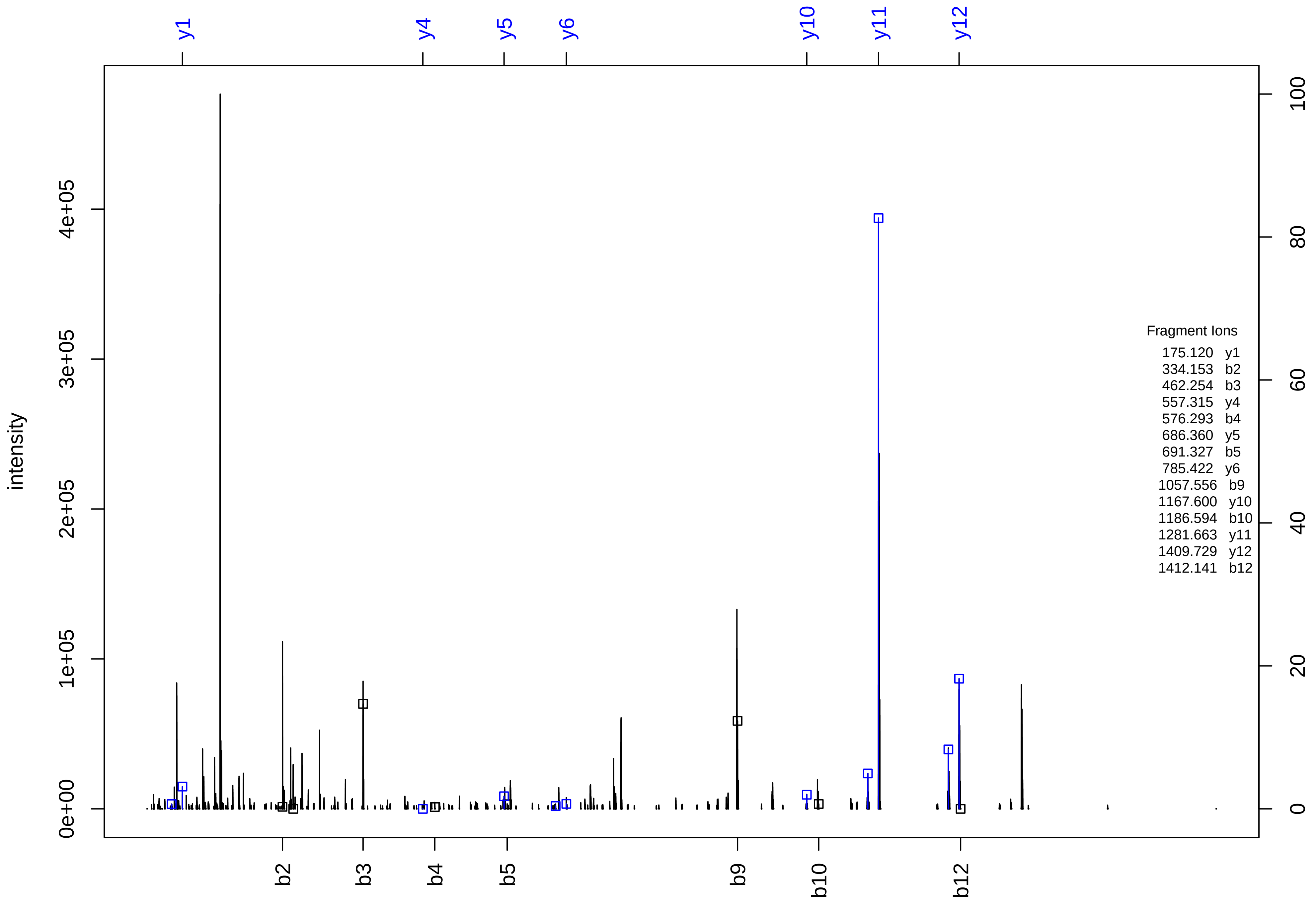

WFKNDAPVVQEPRR / File: sydney_ip_27.raw; SpectrumID: ; scans: 2105

EPEKQ^Q^RVQQ^LESELCK

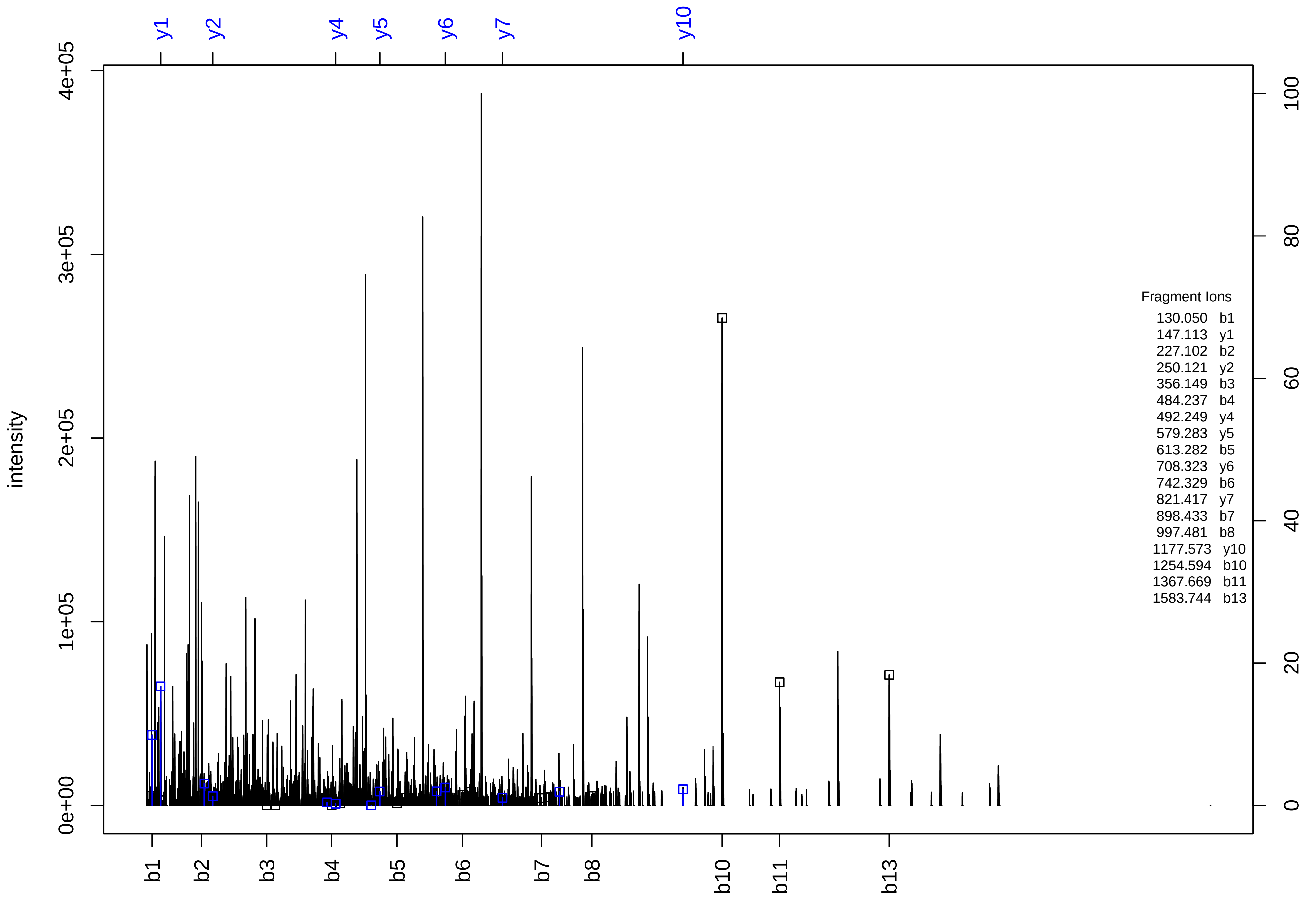

EPEKQQRVQQLESELCK / File: sydney_ip_27.raw; SpectrumID: ; scans: 20110

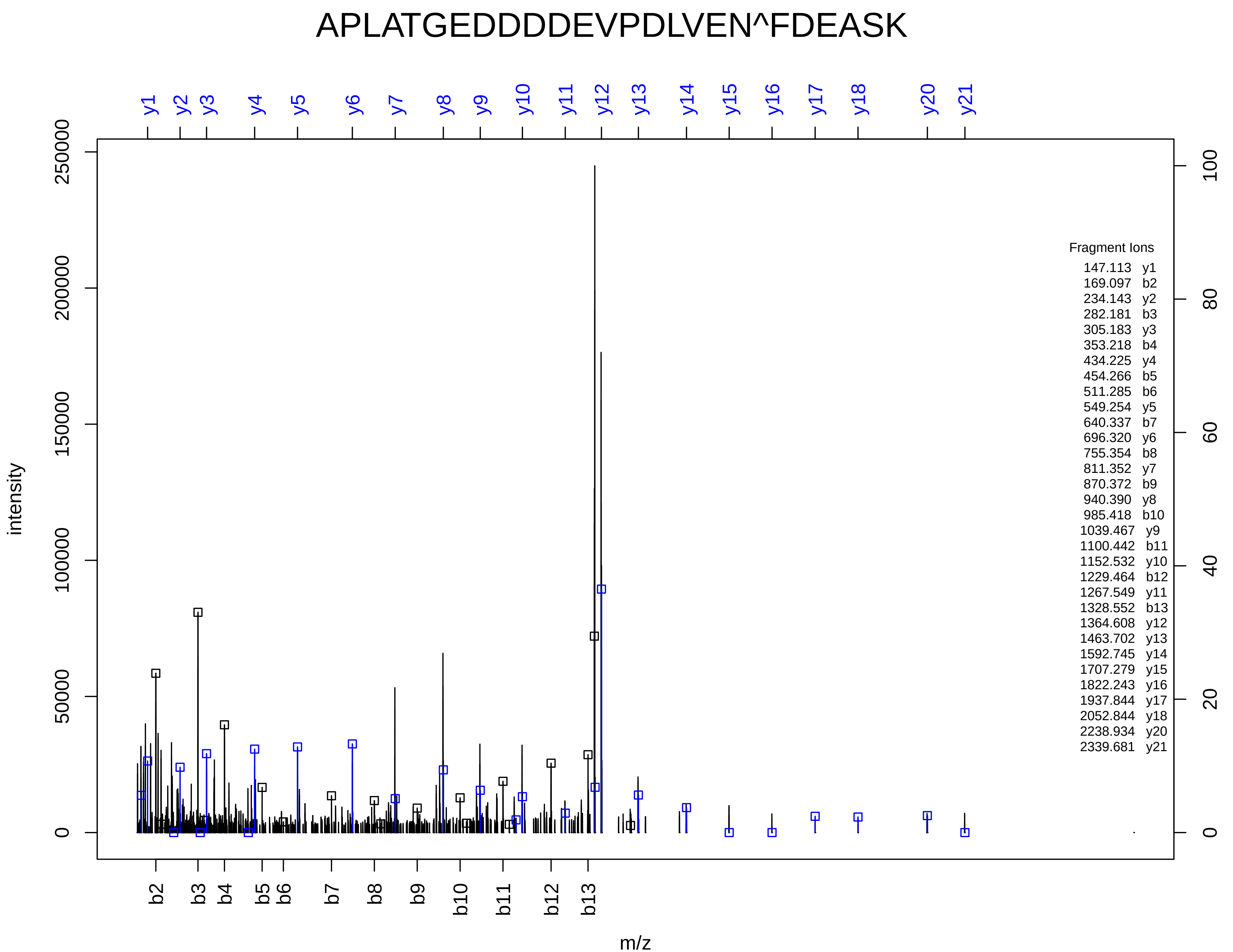
APLATGEDDDDEVPDLVEN^FDEASK
y1 y2 y3 y4 y5 y6 y7 y8 y9 y10 y11 y12 y13 y14 y15 y16 y17 y18 y20 y21
intensity
0
50000
100000
150000
200000
250000
0
20
40
60
80
100
b2 b3 b4 b5 b6 b7 b8 b9 b10 b11 b12 b13
m/z
Fragment Ions
147.113 y1
169.097 b2
234.143 y2
282.181 b3
305.183 y3
353.218 b4
434.225 y4
454.266 b5
511.285 b6
549.254 y5
640.337 b7
696.320 y6
755.354 b8
811.352 y7
870.372 b9
940.390 y8
985.418 b10
1039.467 y9
1100.442 b11
1152.532 y10
1229.464 b12
1267.549 y11
1328.552 b13
1364.608 y12
1463.702 y13
1592.745 y14
1707.279 y15
1822.243 y16
1937.844 y17
2052.844 y18
2238.934 y20
2339.681 y21
APLATGEDDDDEVPDLVENFDEASK / File: sydney_ip_27.raw; SpectrumID: ; scans: 16164

AAPWTSFLPPPPPM*PGSGLGPGK

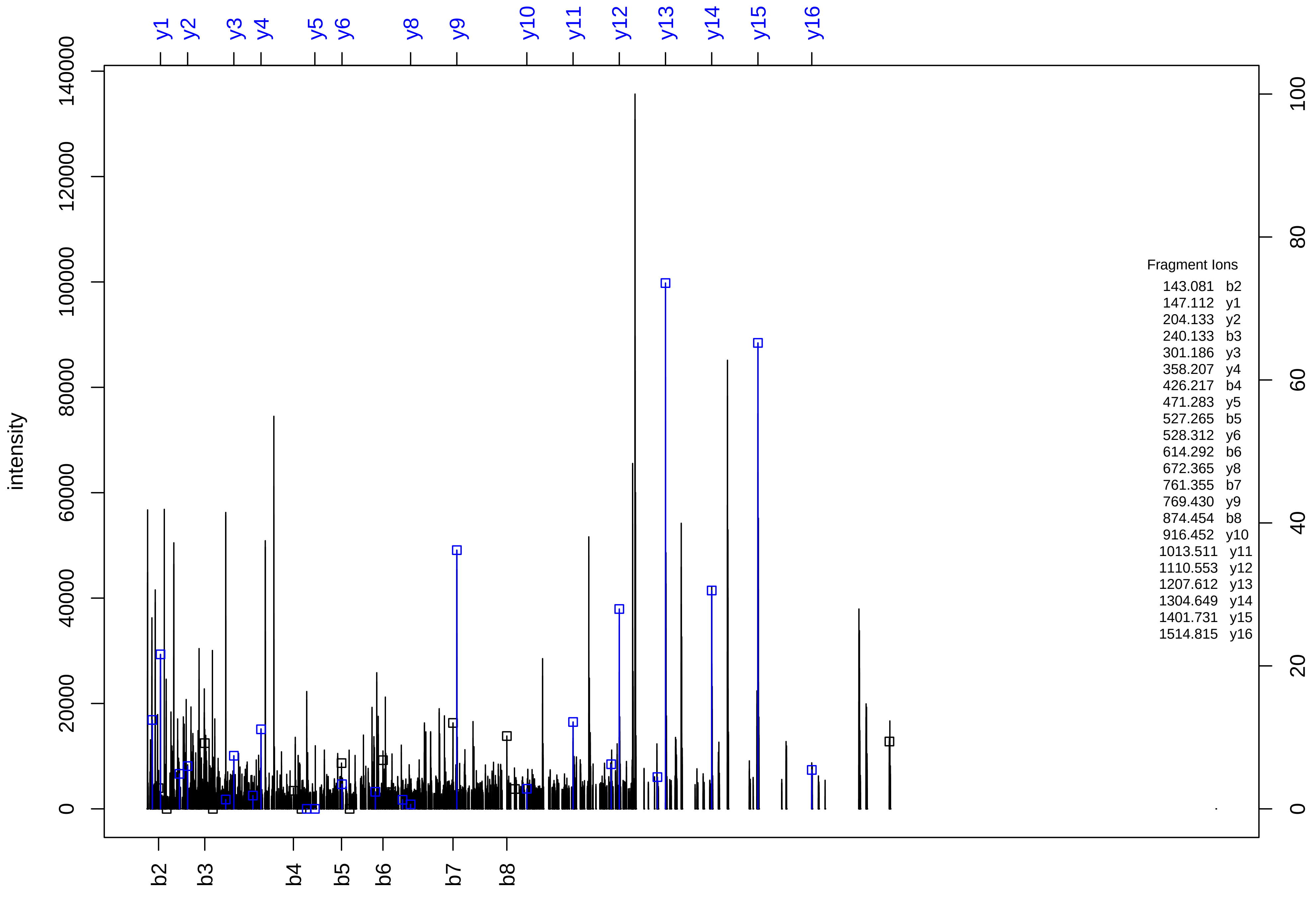

AAPWTSFLPPPPPMPGSGLGPGK / File: sydney_ip_27.raw; SpectrumID: ; scans: 17981

Q^VFPSADDVSIRR

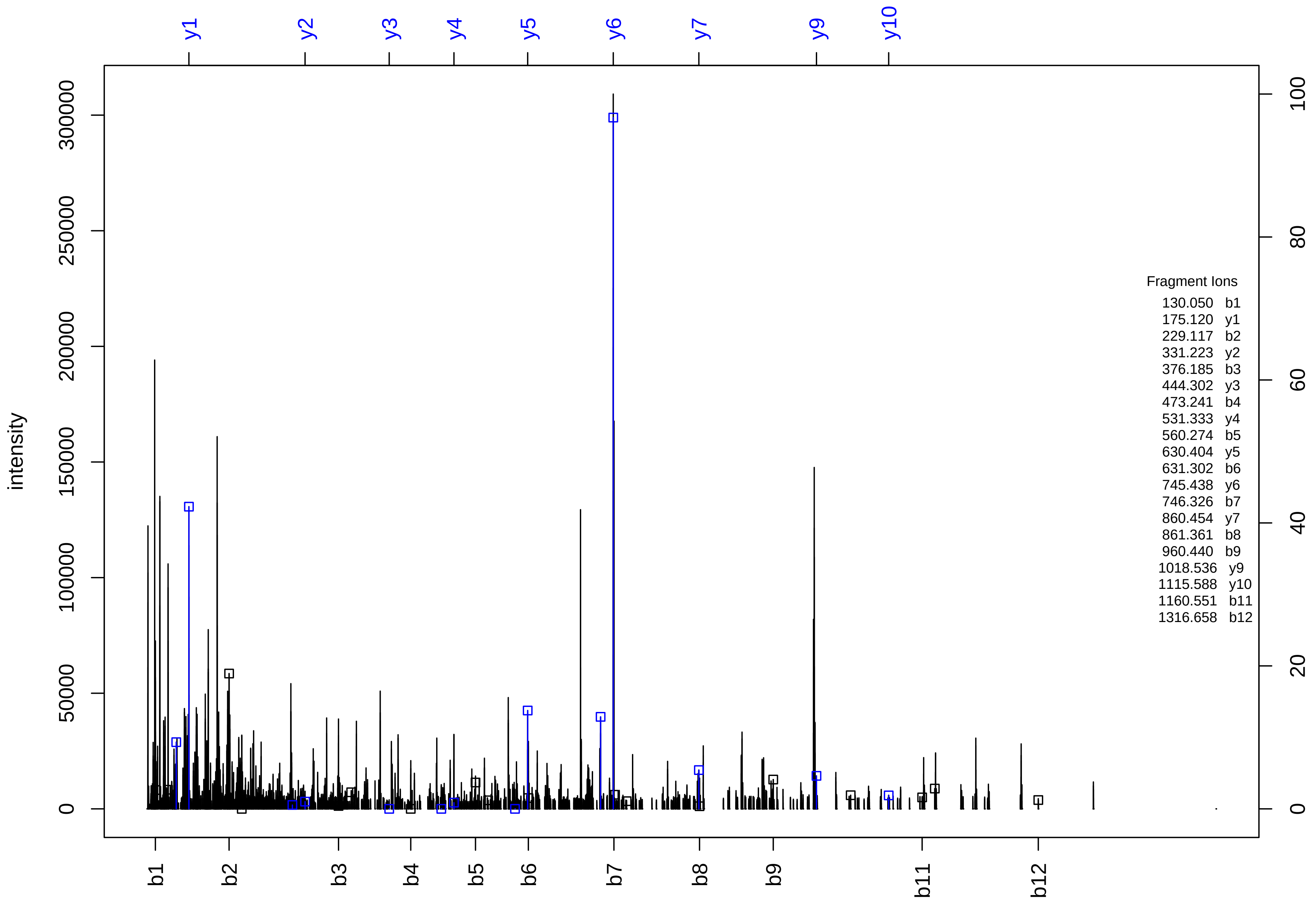

QVFPSADDVSIRR / File: sydney_ip_27.raw; SpectrumID: ; scans: 9429

Q^LQ^EM*KPTVSLK

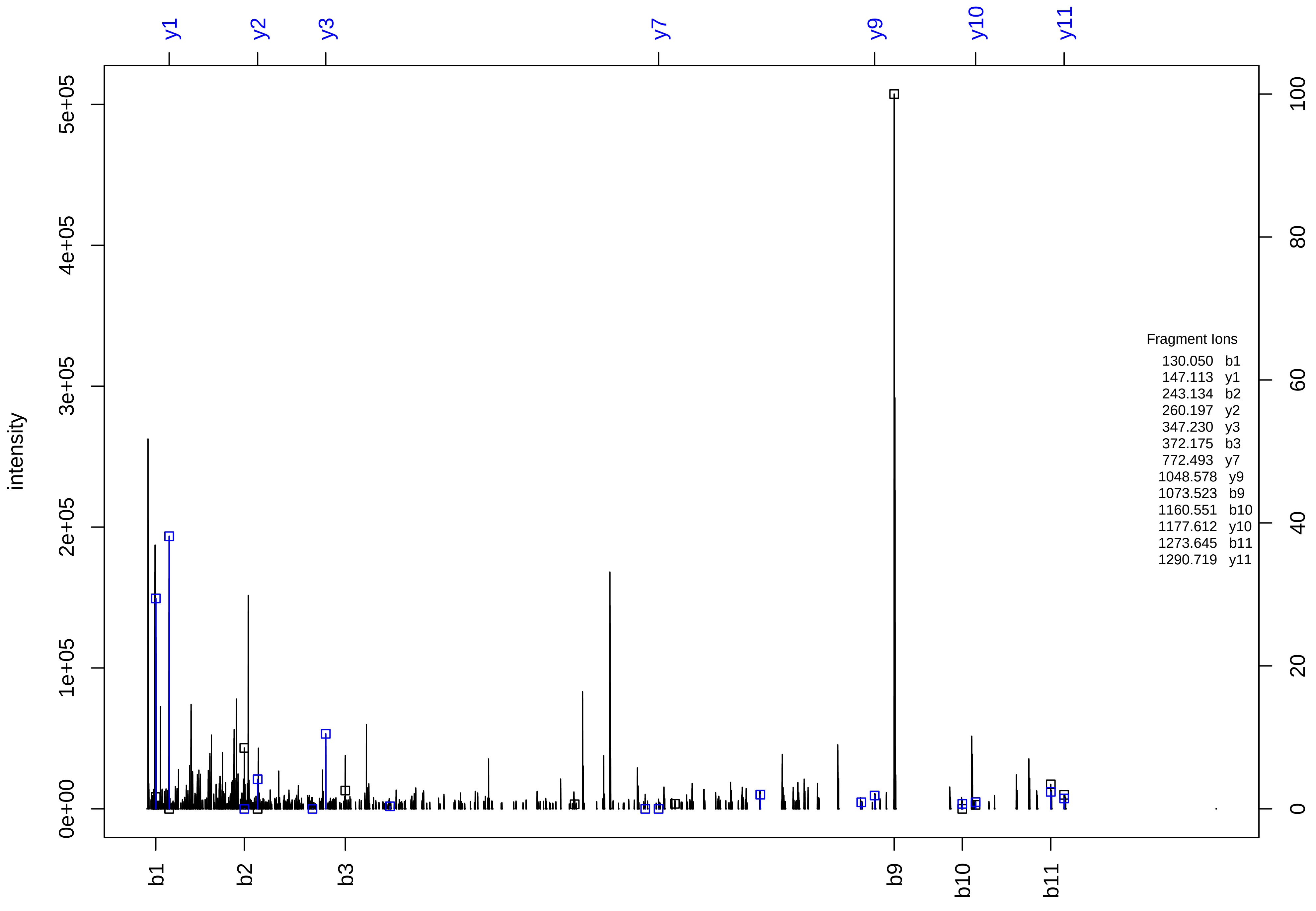

QLQEMKPTVSLK / File: sydney_ip_27.raw; SpectrumID: ; scans: 19149

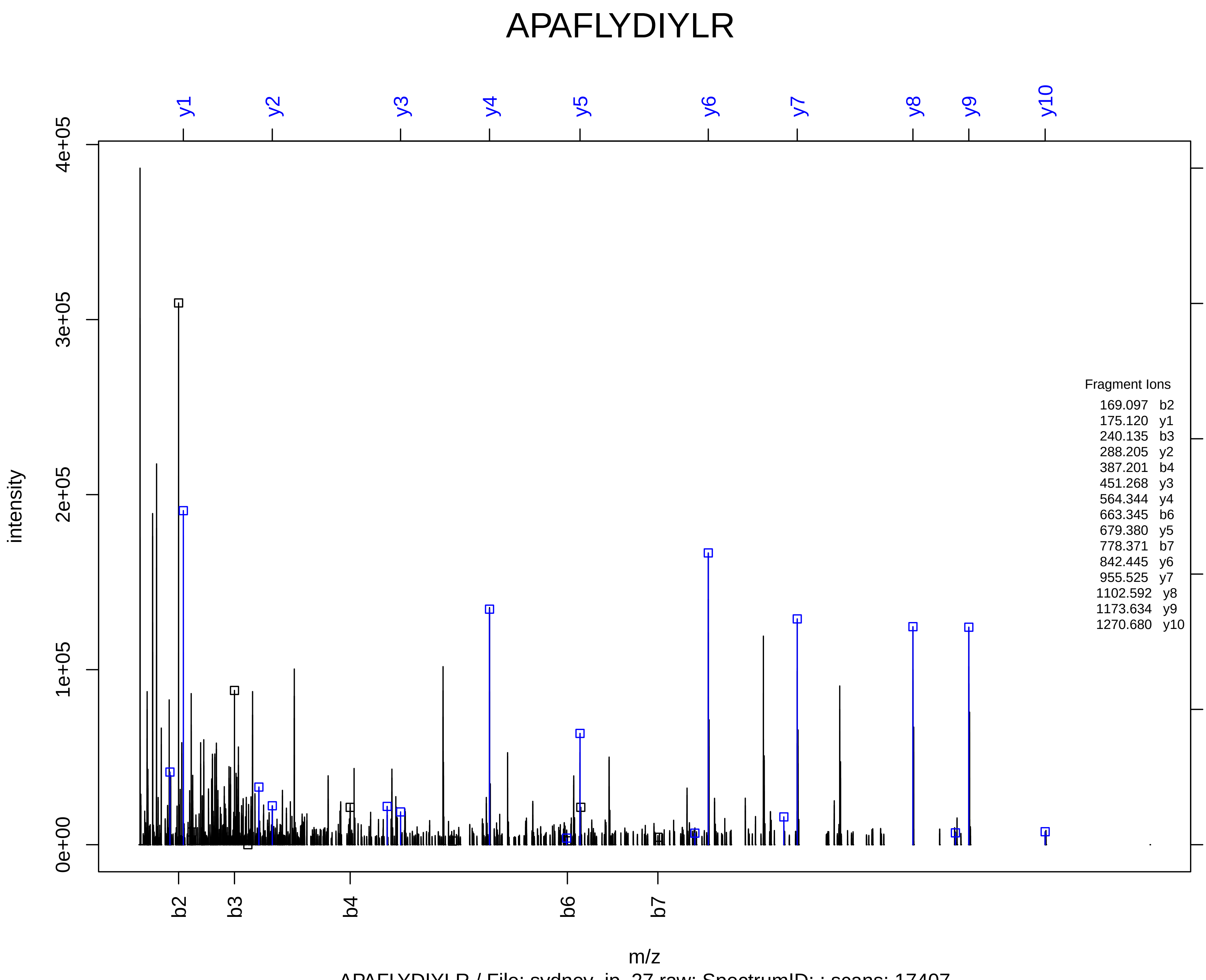
APAFLYDIYLR
y1
y2
y3
y4
y5
y6
y7
y8
y9
y10
intensity
4e+05
3e+05
2e+05
1e+05
0e+00
100
80
60
40
20
0
Fragment Ions
169.097 b2
175.120 y1
240.135 b3
288.205 y2
387.201 b4
451.268 y3
564.344 y4
663.345 b6
679.380 y5
778.371 b7
842.445 y6
955.525 y7
1102.592 y8
1173.634 y9
1270.680 y10
b2
b3
b4
b6
b7
m/z
APAFLYDIYLR / File: sydney_ip_27.raw; SpectrumID: ; scans: 17407

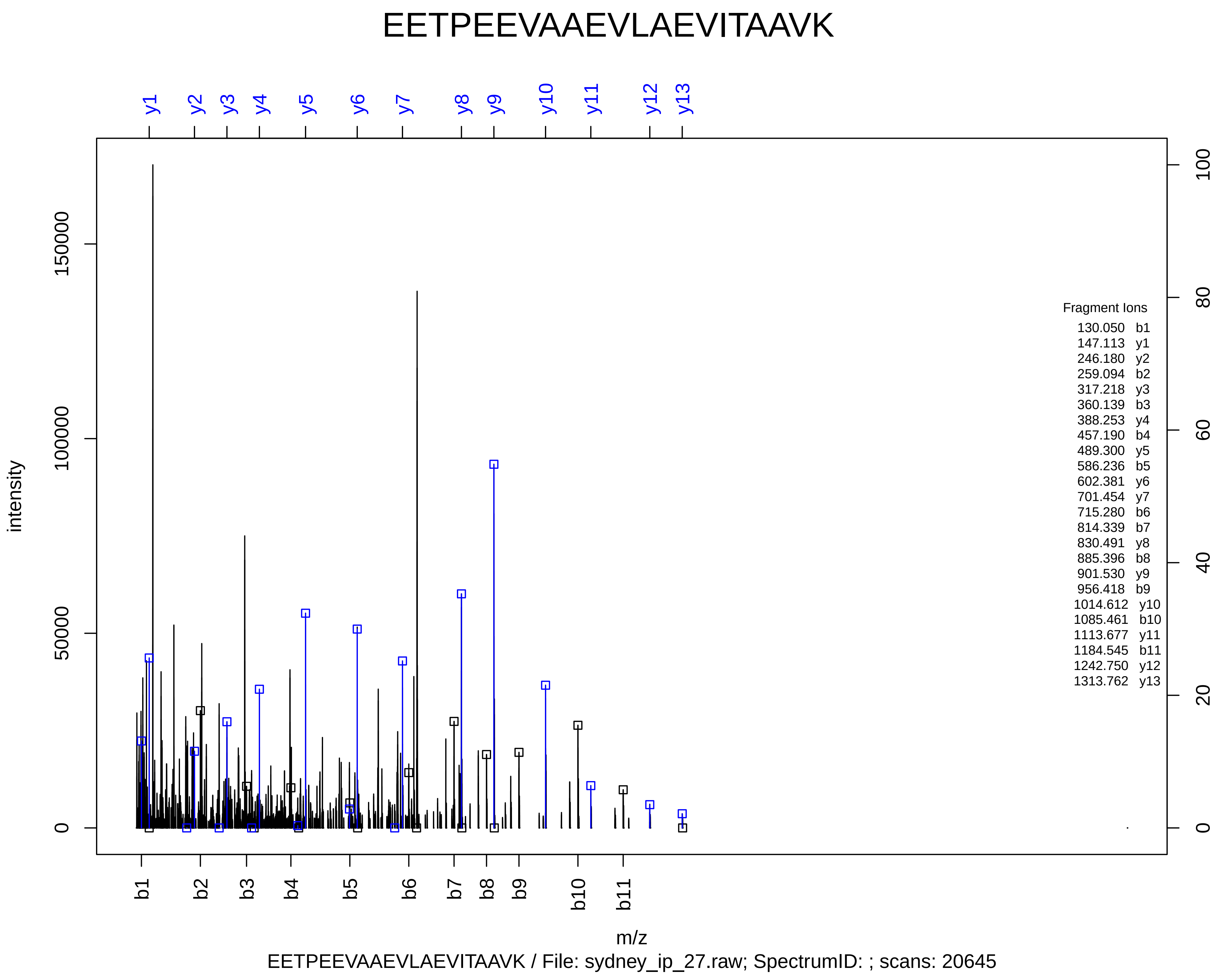

EETPEEVAAEVLAEVITAAVK
y1 y2 y3 y4 y5 y6 y7 y8 y9 y10 y11 y12 y13
b1 b2 b3 b4 b5 b6 b7 b8 b9 b10 b11
intensity
m/z
0
50000
100000
150000
0
20
40
60
80
100
Fragment Ions
130.050 b1
147.113 y1
246.180 y2
259.094 b2
317.218 y3
360.139 b3
388.253 y4
457.190 b4
489.300 y5
586.236 b5
602.381 y6
701.454 y7
715.280 b6
814.339 b7
830.491 y8
885.396 b8
901.530 y9
956.418 b9
1014.612 y10
1085.461 b10
1113.677 y11
1184.545 b11
1242.750 y12
1313.762 y13
EETPEEVAAEVLAEVITAAVK / File: sydney_ip_27.raw; SpectrumID: ; scans: 20645

APDIILLDEATSALDTSNER

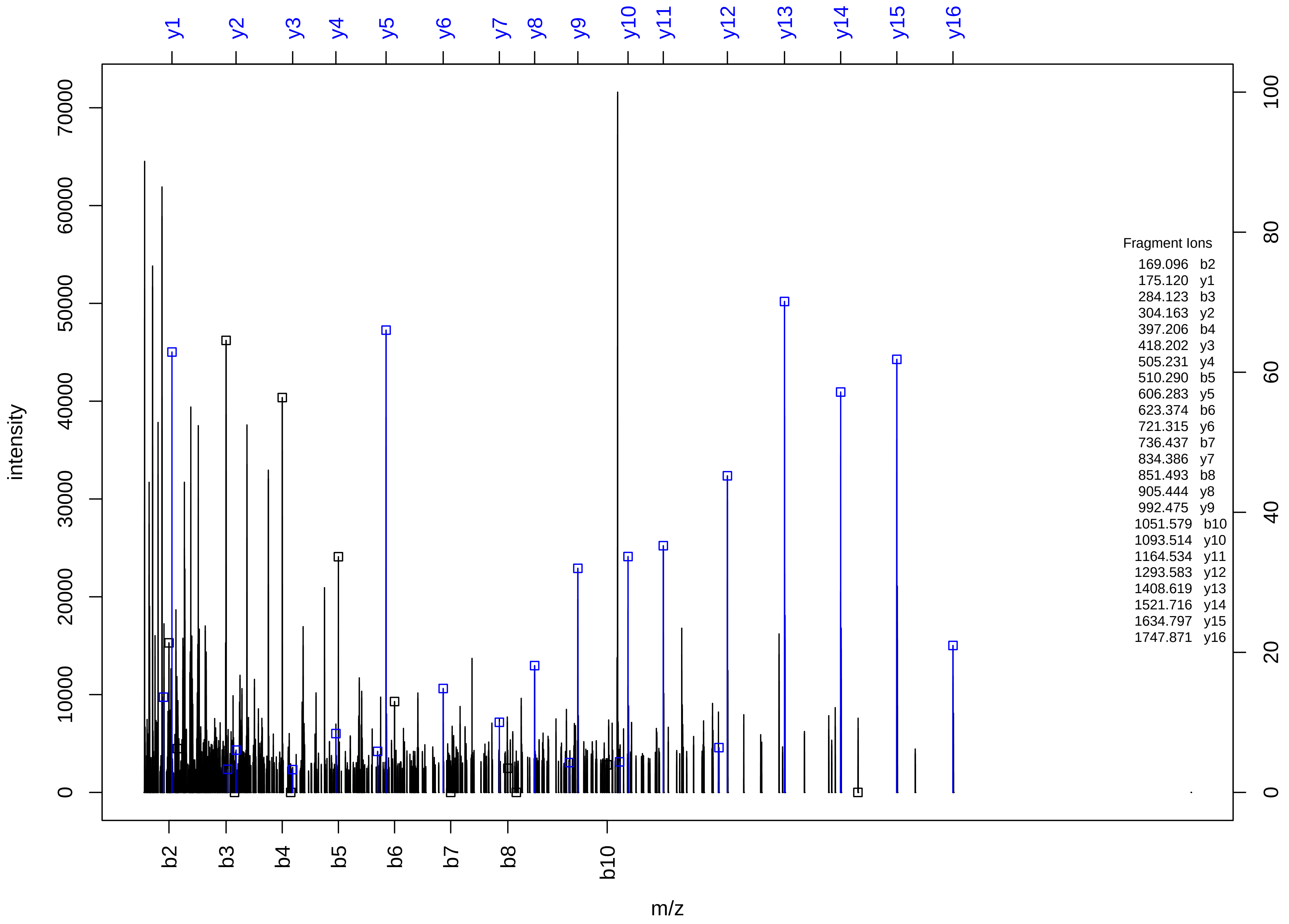

APDIILLDEATSALDTSNER / File: sydney_ip_27.raw; SpectrumID: ; scans: 19283

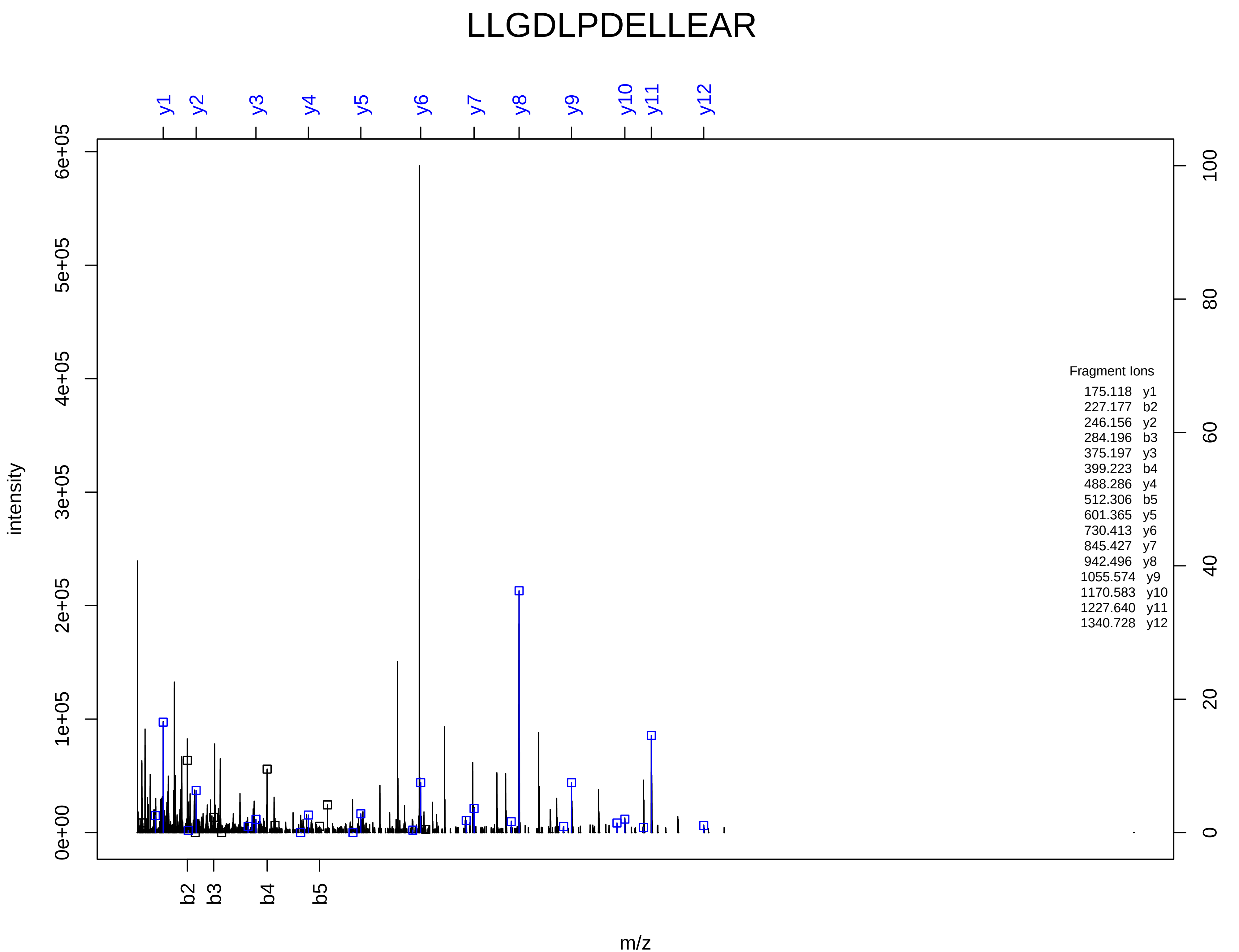

LLGDLPDELLEAR
y1 y2 y3 y4 y5 y6 y7 y8 y9 y10 y11 y12
b2 b3 b4 b5
intensity
0e+00 1e+05 2e+05 3e+05 4e+05 5e+05 6e+05
0 20 40 60 80 100
m/z
Fragment Ions
175.118 y1
227.177 b2
246.156 y2
284.196 b3
375.197 y3
399.223 b4
488.286 y4
512.306 b5
601.365 y5
730.413 y6
845.427 y7
942.496 y8
1055.574 y9
1170.583 y10
1227.640 y11
1340.728 y12
LLGDLPDELLEAR / File: sydney_ip_27.raw; SpectrumID: ; scans: 17979

SVISVIHLITAAR

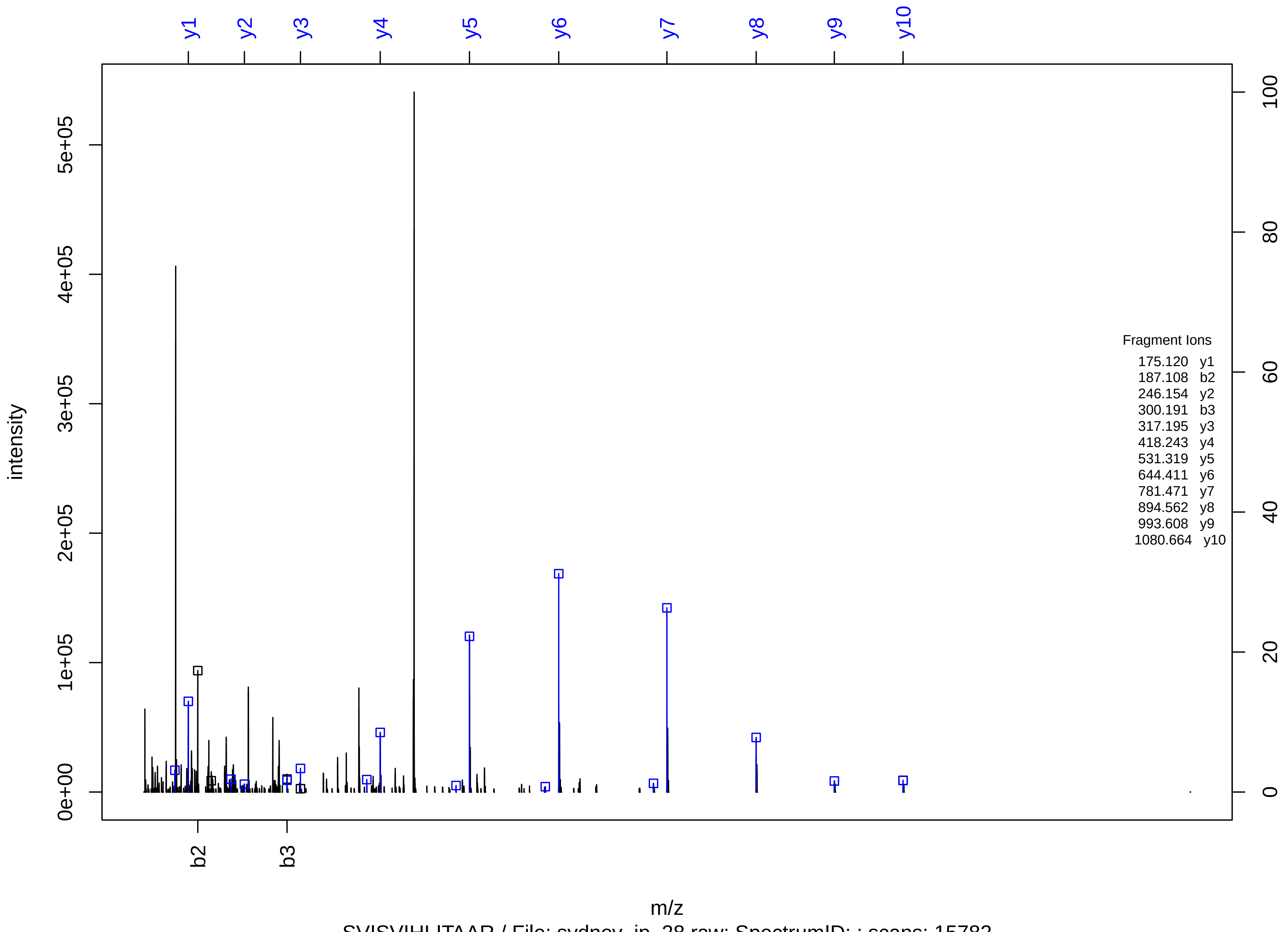

SVISVIHLITAAR / File: sydney_ip_28.raw; SpectrumID: ; scans: 15782

TAPAAAETTNESETVPK

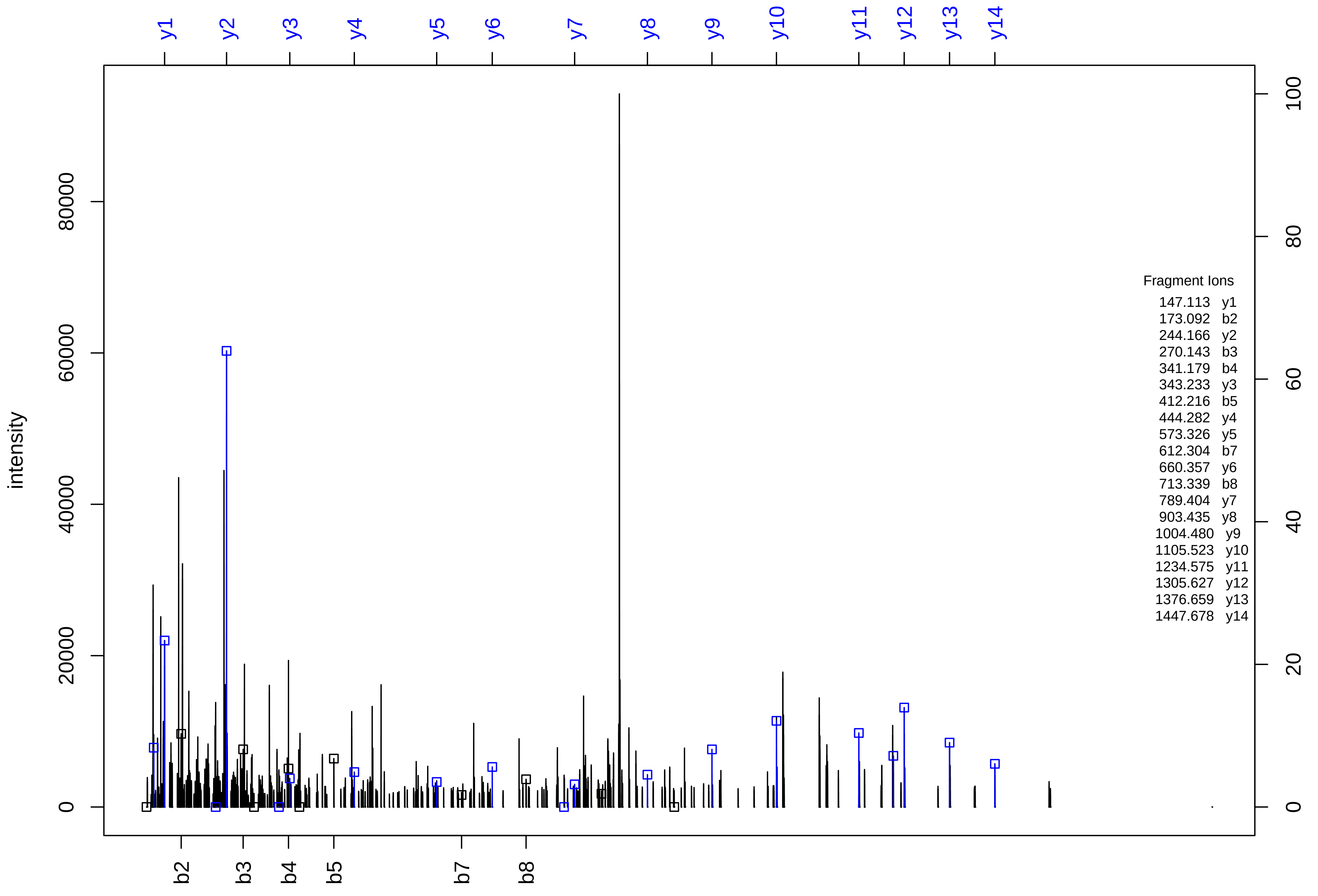

TAPAAAETTNESETVPK / File: sydney_ip_28.raw; SpectrumID: ; scans: 3378

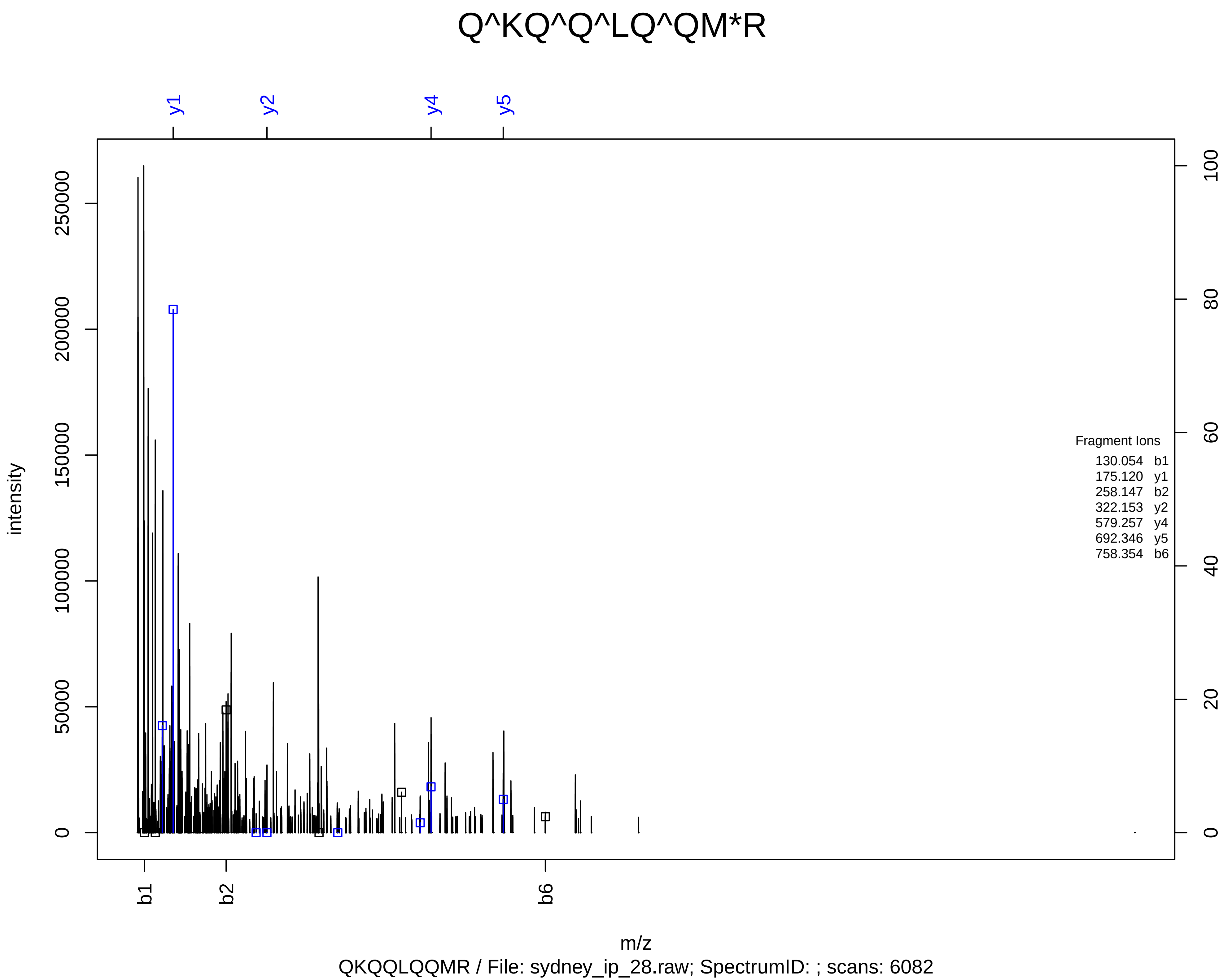
Q^KQ^Q^LQ^QM*R
y1
y2
y4
y5
intensity
250000
200000
150000
100000
50000
0
100
80
60
40
20
0
Fragment Ions
130.054 b1
175.120 y1
258.147 b2
322.153 y2
579.257 y4
692.346 y5
758.354 b6
b1
b2
b6
m/z
QKQQLQQMR / File: sydney_ip_28.raw; SpectrumID: ; scans: 6082

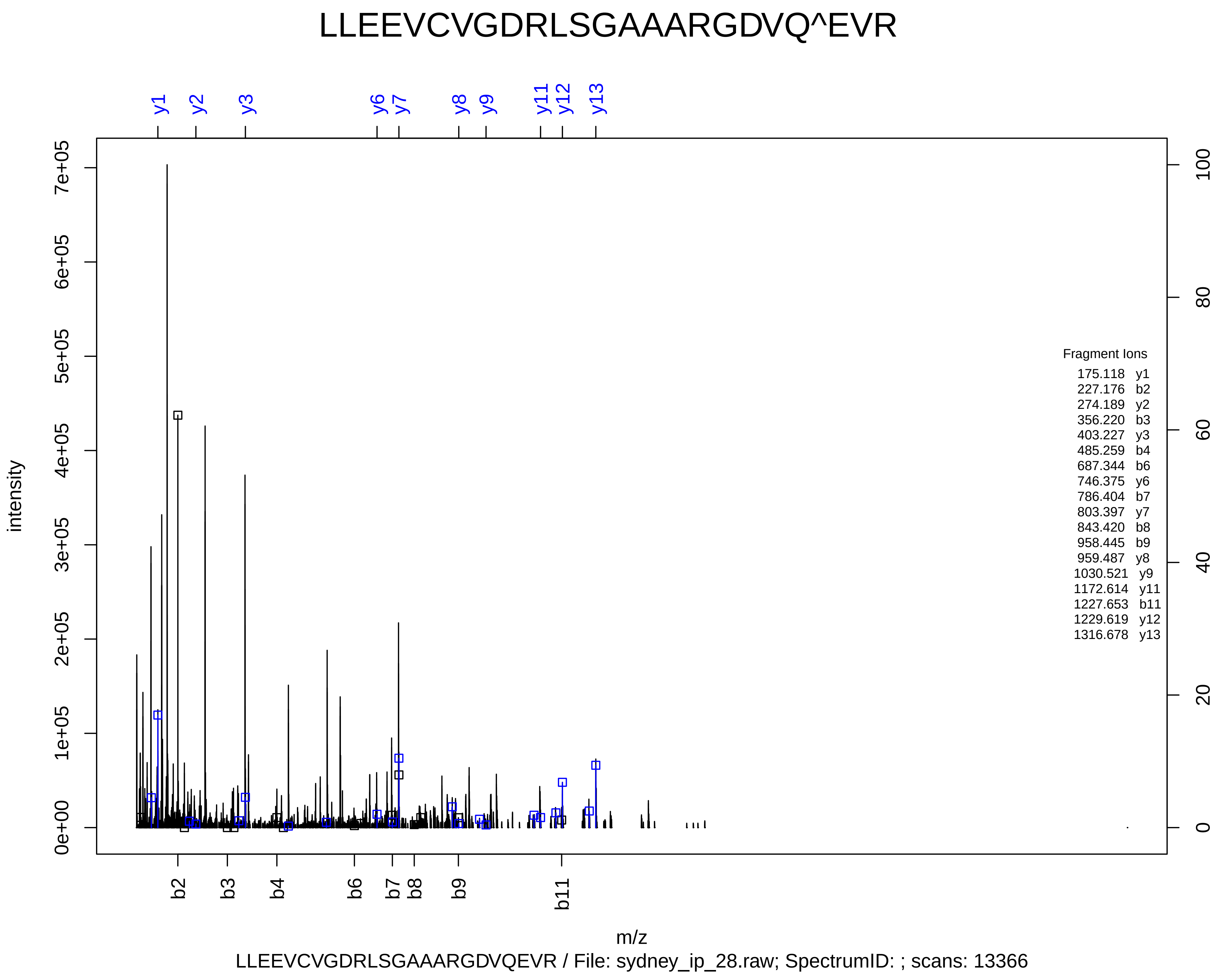
LLEEVCVGDRLSGAAARGDVQ^EVR
y1
y2
y3
y6
y7
y8
y9
y11
y12
y13
intensity
0e+00
1e+05
2e+05
3e+05
4e+05
5e+05
6e+05
7e+05
0
20
40
60
80
100
b2
b3
b4
b6
b7
b8
b9
b11
m/z
Fragment Ions
175.118 y1
227.176 b2
274.189 y2
356.220 b3
403.227 y3
485.259 b4
687.344 b6
746.375 y6
786.404 b7
803.397 y7
843.420 b8
958.445 b9
959.487 y8
1030.521 y9
1172.614 y11
1227.653 b11
1229.619 y12
1316.678 y13
LLEEVCVGDRLSGAAARGDVQEVR / File: sydney_ip_28.raw; SpectrumID: ; scans: 13366

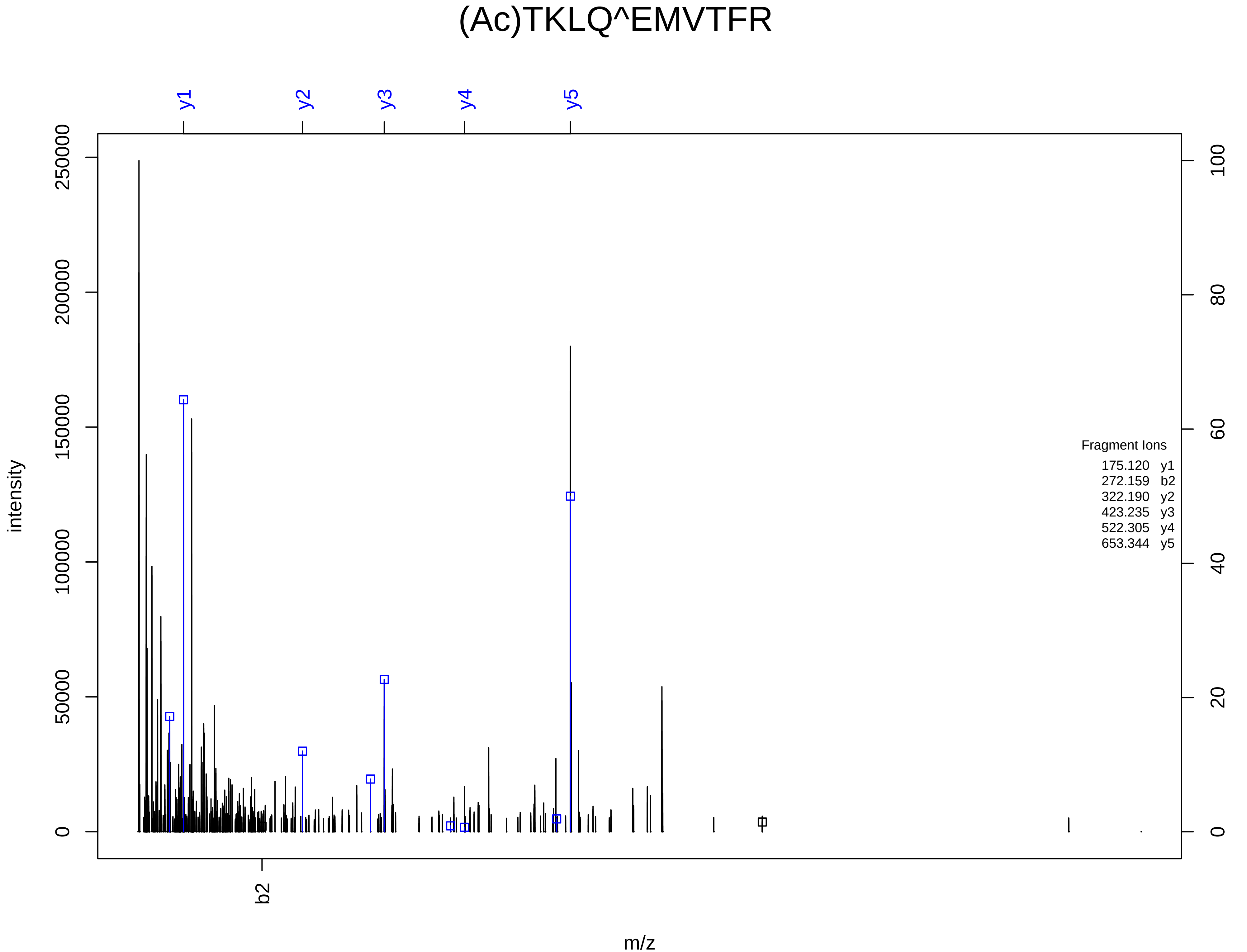

TKLQEMVTFR / File: sydney_ip_28.raw; SpectrumID: ; scans: 5647

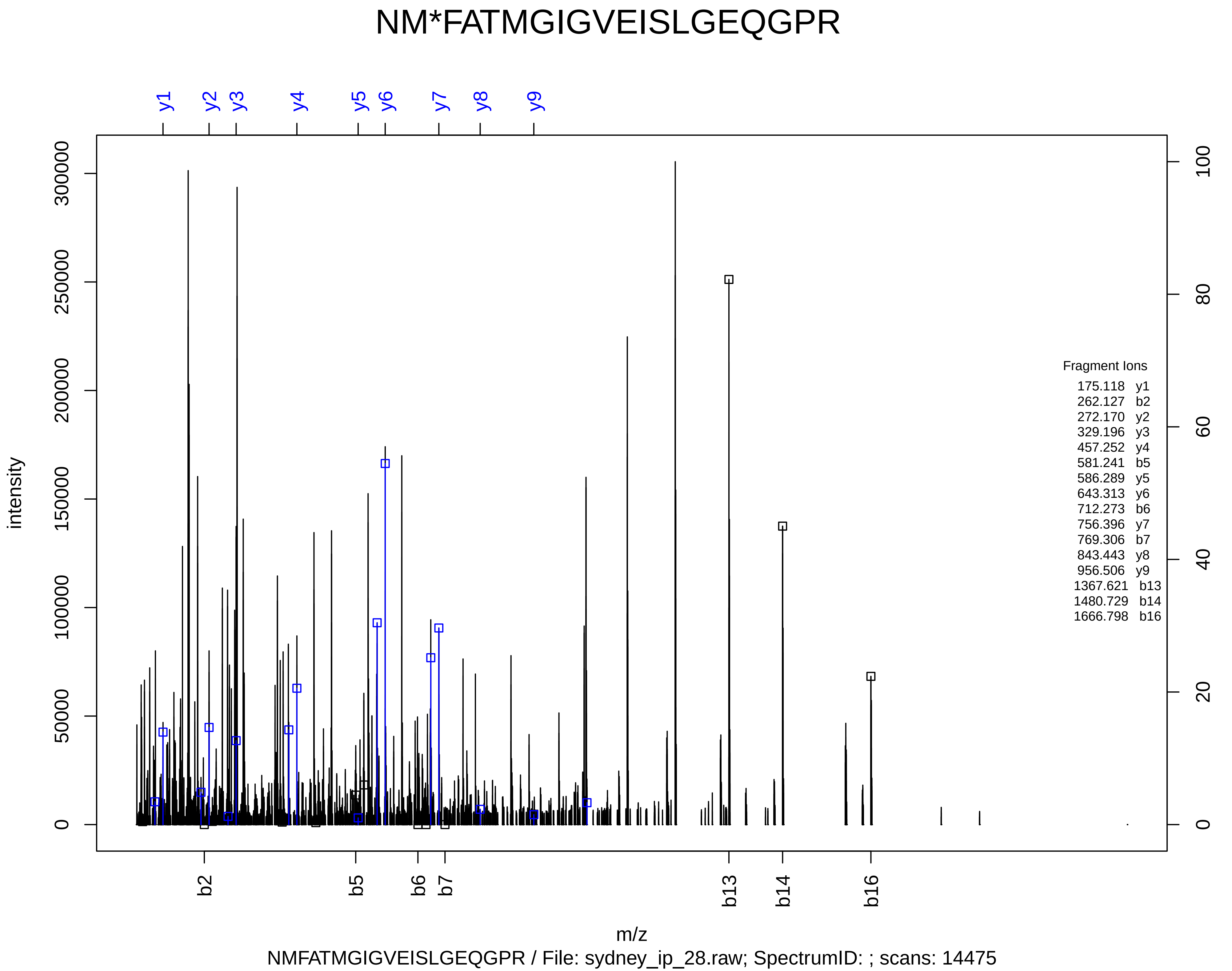
NM*FATMGIGVEISLGEQGPR
y1
y2
y3
y4
y5
y6
y7
y8
y9
intensity
300000
250000
200000
150000
100000
50000
0
100
80
60
40
20
0
Fragment Ions
175.118 y1
262.127 b2
272.170 y2
329.196 y3
457.252 y4
581.241 b5
586.289 y5
643.313 y6
712.273 b6
756.396 y7
769.306 b7
843.443 y8
956.506 y9
1367.621 b13
1480.729 b14
1666.798 b16
b2
b5
b6
b7
b13
b14
b16
m/z
NMFATMGIGVEISLGEQGPR / File: sydney_ip_28.raw; SpectrumID: ; scans: 14475

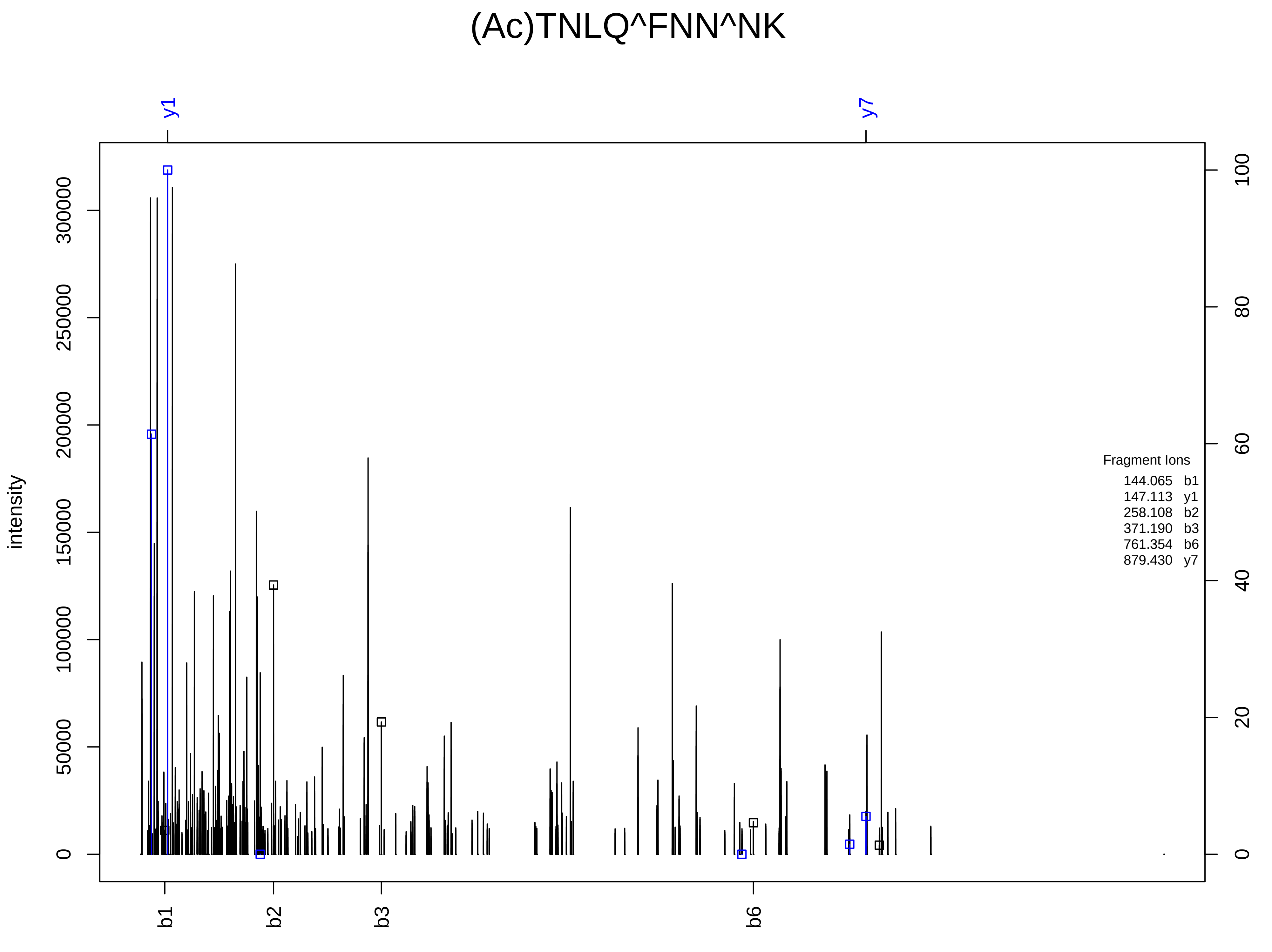

TNLQFNNNK / File: sydney_ip_28.raw; SpectrumID: ; scans: 6130

LQ^ISMCQ^RK

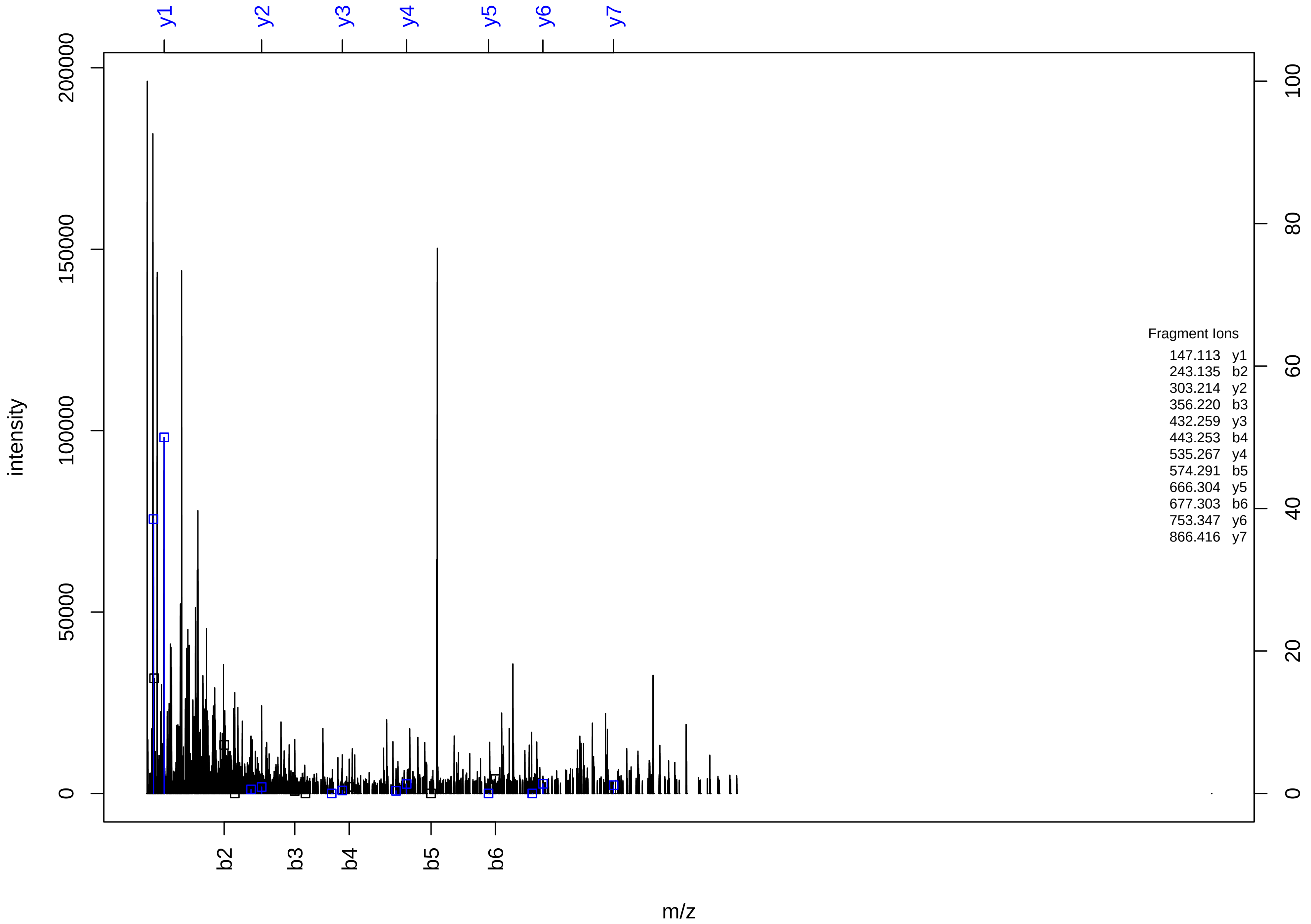

LQISMCQRK / File: sydney_ip_28.raw; SpectrumID: ; scans: 8141

Q^N^M*KKLLEQ^QYSN^K

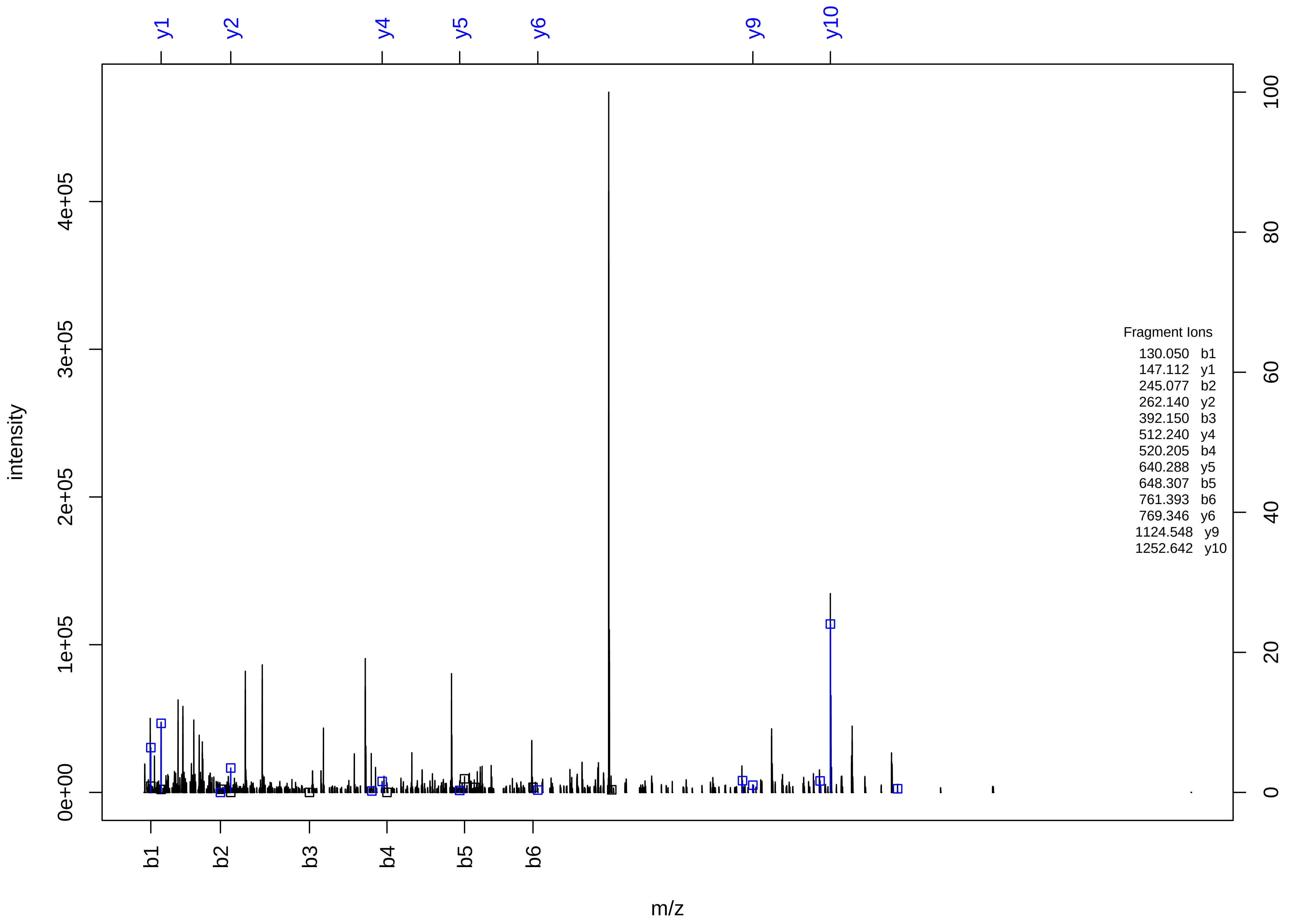

QNMKKLLEQQYSNK / File: sydney_ip_28.raw; SpectrumID: ; scans: 5982

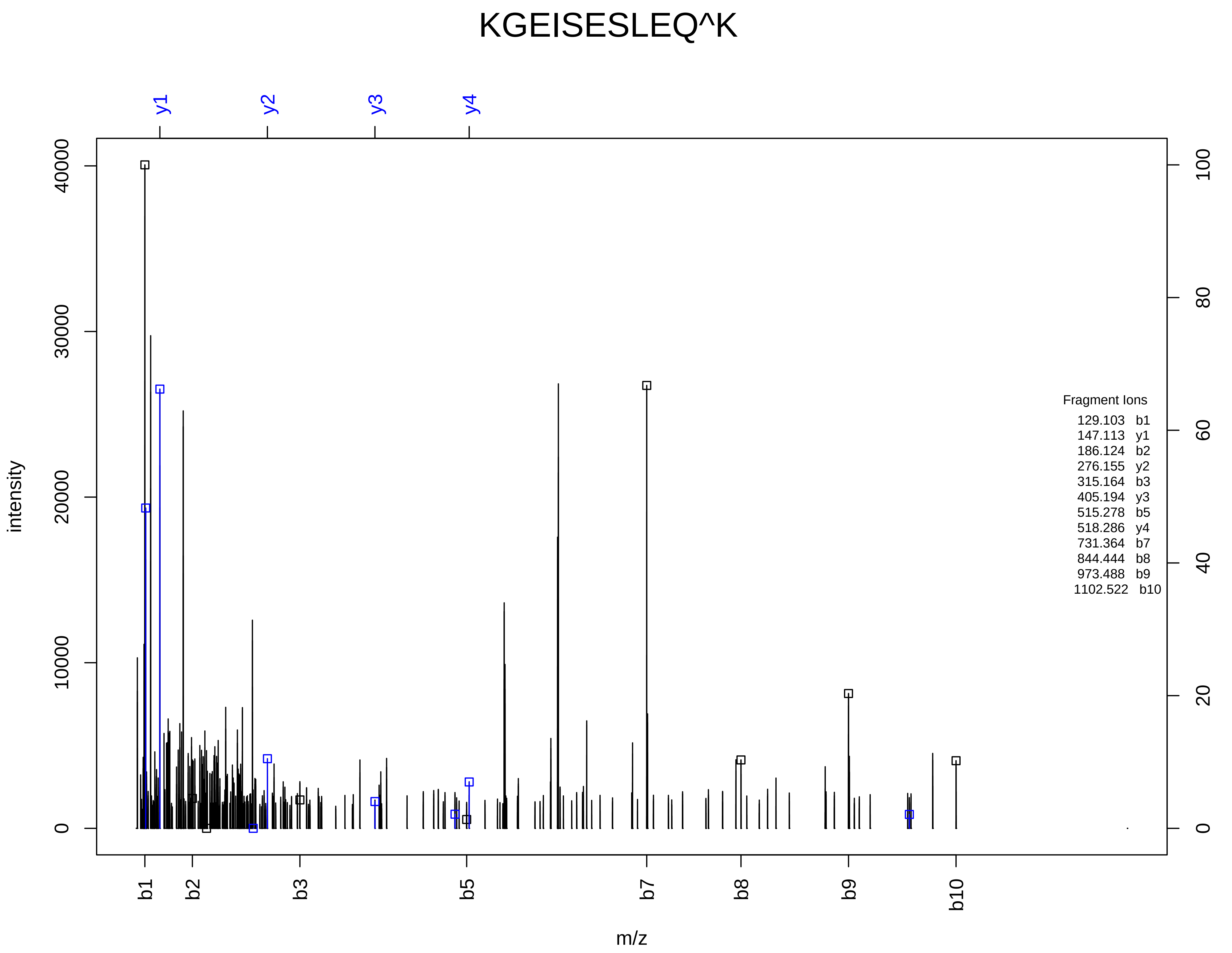
KGEISESLEQ^K
y1
y2
y3
y4
intensity
40000
30000
20000
10000
0
100
80
60
40
20
0
Fragment Ions
129.103 b1
147.113 y1
186.124 b2
276.155 y2
315.164 b3
405.194 y3
515.278 b5
518.286 y4
731.364 b7
844.444 b8
973.488 b9
1102.522 b10
b1
b2
b3
b5
b7
b8
b9
b10
m/z
KGEISESLEQK / File: sydney_ip_28.raw; SpectrumID: ; scans: 2070

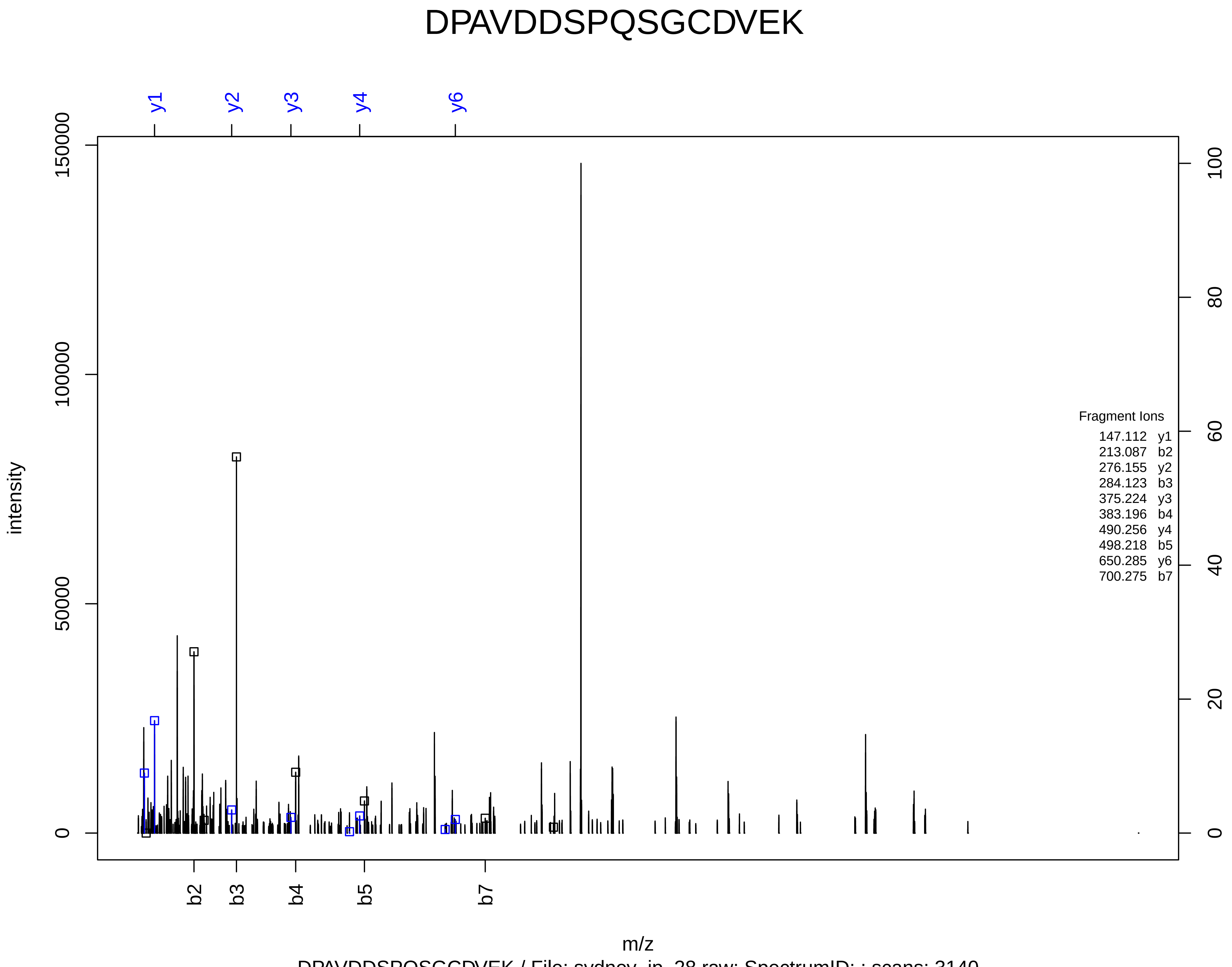

DPAVDDSPQSGCDVEK
y1
y2
y3
y4
y6
b2
b3
b4
b5
b7
intensity
m/z
0
50000
100000
150000
0
20
40
60
80
100
Fragment Ions
147.112 y1
213.087 b2
276.155 y2
284.123 b3
375.224 y3
383.196 b4
490.256 y4
498.218 b5
650.285 y6
700.275 b7
DPAVDDSPQSGCDVEK / File: sydney_ip_28.raw; SpectrumID: ; scans: 3140

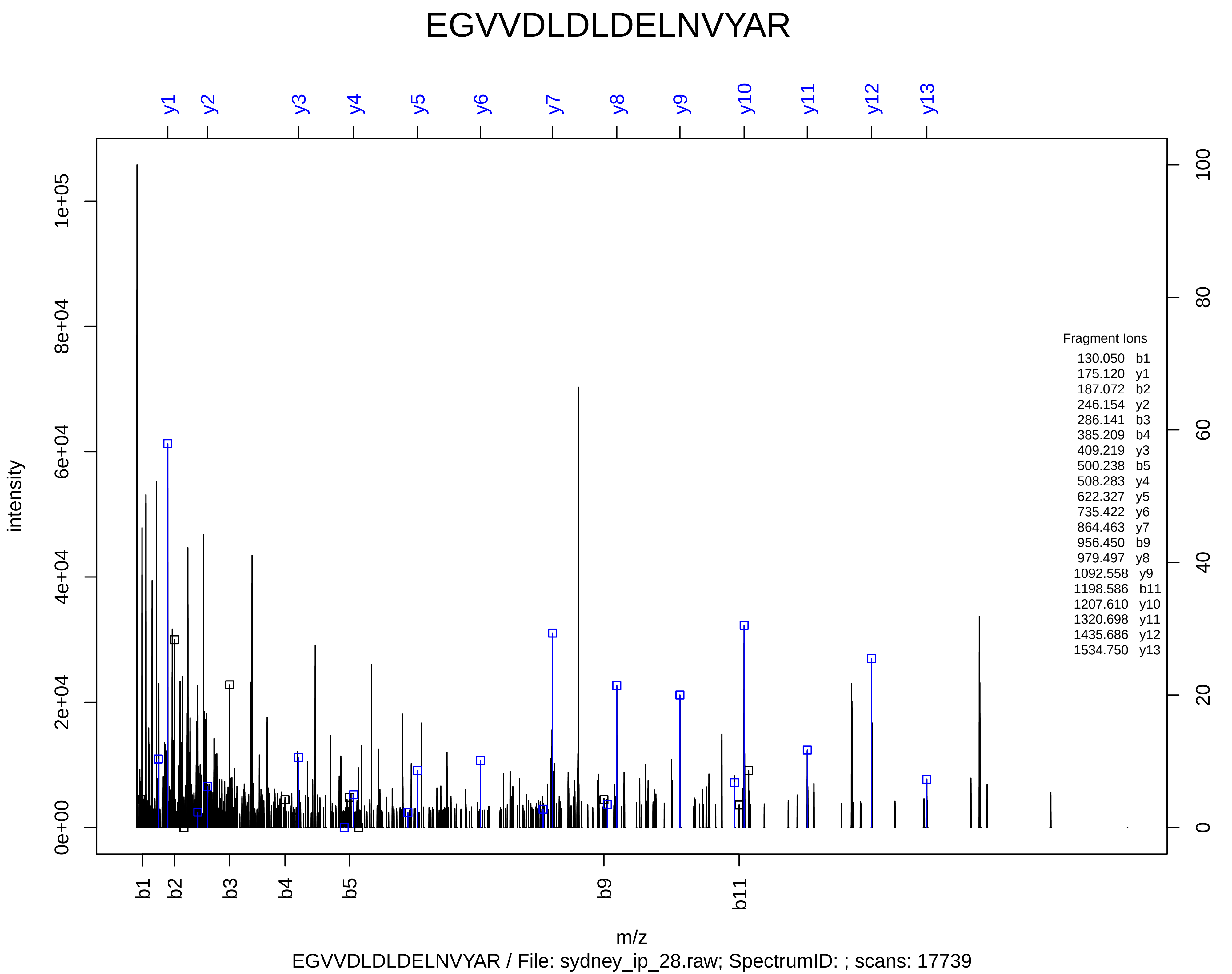
EGVVDLDLDELNVYAR
y1 y2 y3 y4 y5 y6 y7 y8 y9 y10 y11 y12 y13
b1 b2 b3 b4 b5 b9 b11
intensity
0e+00 2e+04 4e+04 6e+04 8e+04 1e+05
0 20 40 60 80 100
m/z
Fragment Ions
130.050 b1
175.120 y1
187.072 b2
246.154 y2
286.141 b3
385.209 b4
409.219 y3
500.238 b5
508.283 y4
622.327 y5
735.422 y6
864.463 y7
956.450 b9
979.497 y8
1092.558 y9
1198.586 b11
1207.610 y10
1320.698 y11
1435.686 y12
1534.750 y13
EGVVDLDLDELNVYAR / File: sydney_ip_28.raw; SpectrumID: ; scans: 17739

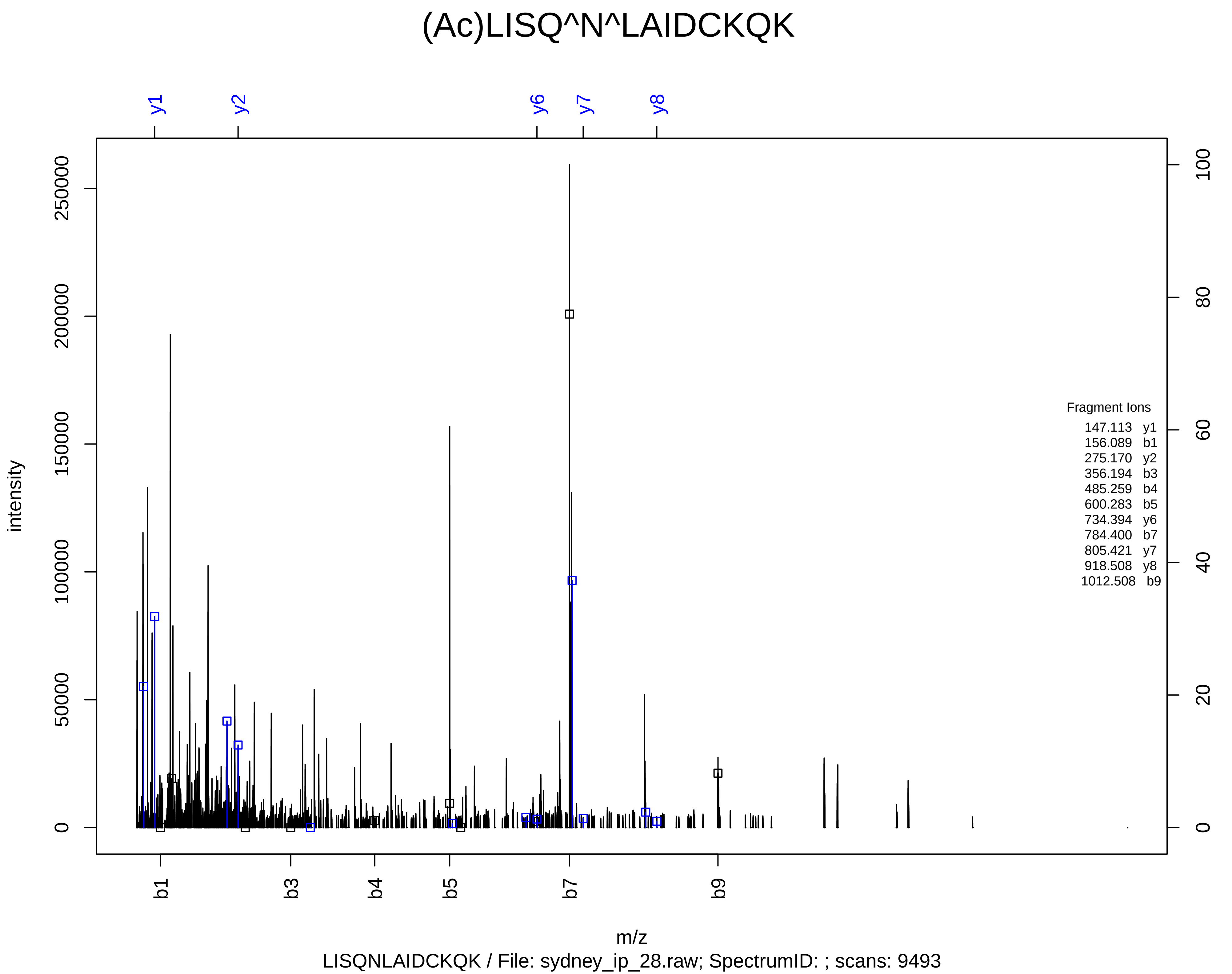

(Ac)LISQ^N^LAIDCKQK
y1
y2
y6
y7
y8
intensity
0
50000
100000
150000
200000
250000
0
20
40
60
80
100
b1
b3
b4
b5
b7
b9
m/z
Fragment Ions
147.113 y1
156.089 b1
275.170 y2
356.194 b3
485.259 b4
600.283 b5
734.394 y6
784.400 b7
805.421 y7
918.508 y8
1012.508 b9
LISQNLAIDCKQK / File: sydney_ip_28.raw; SpectrumID: ; scans: 9493

Q^LVQ^AWLQAK

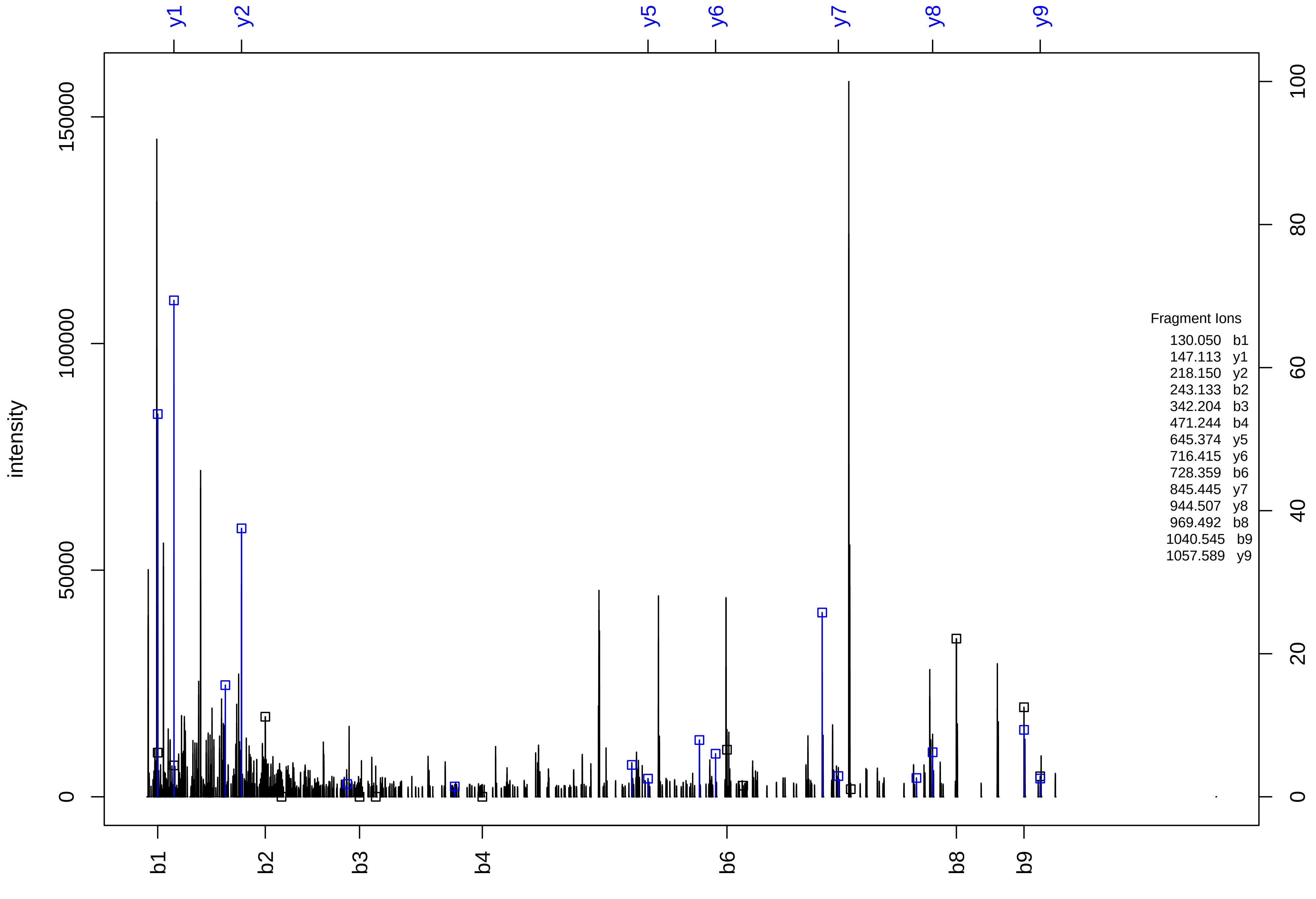

QLVQAWLQAK / File: sydney_ip_28.raw; SpectrumID: ; scans: 3496

NIGFQ^DSKN^LQ^R

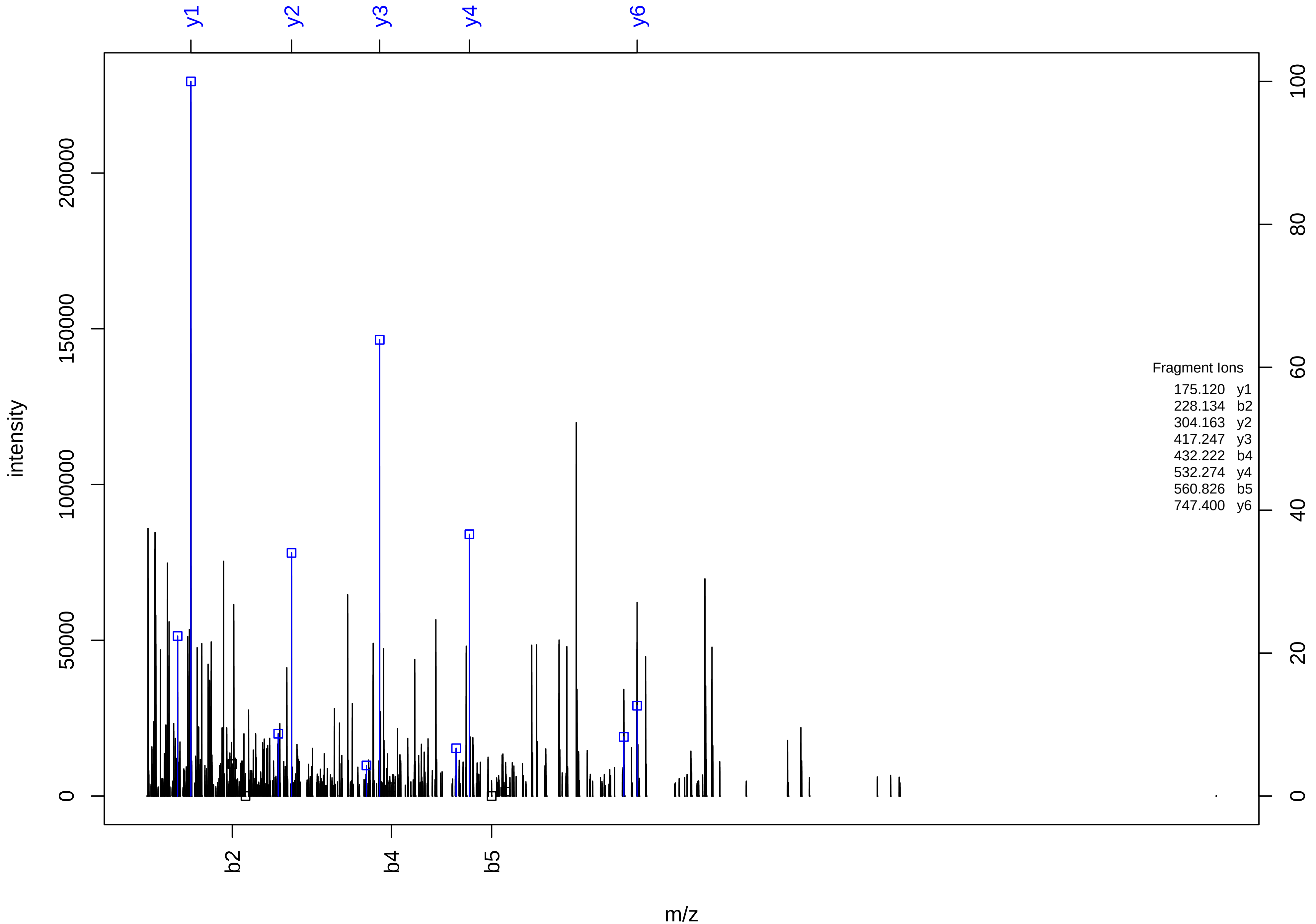

NIGFQDSKNLQR / File: sydney_ip_28.raw; SpectrumID: ; scans: 2627

YVALTSLLQLVQSDHSAVQR

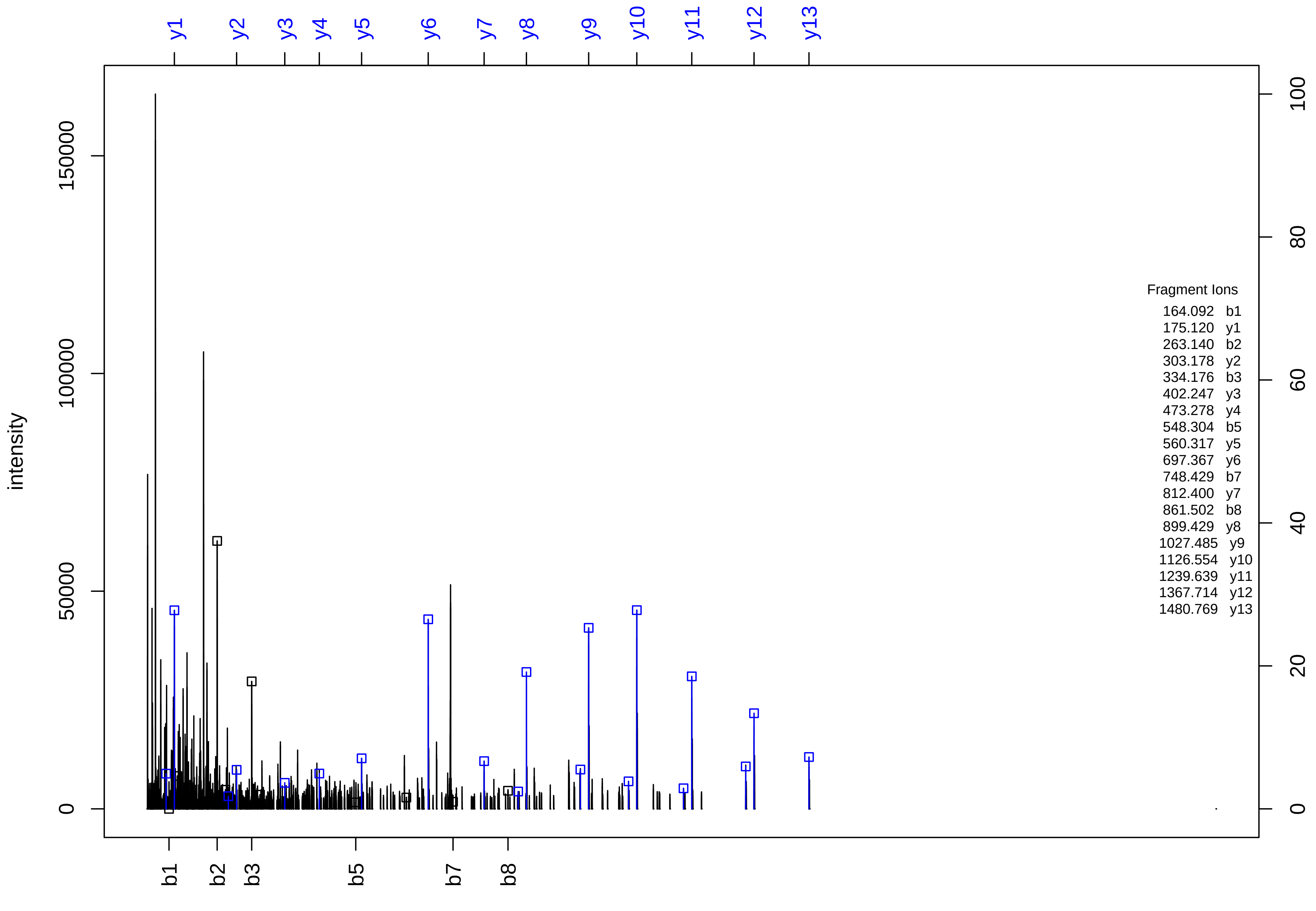

YVALTSLLQLVQSDHSAVQR / File: sydney_ip_28.raw; SpectrumID: ; scans: 19321

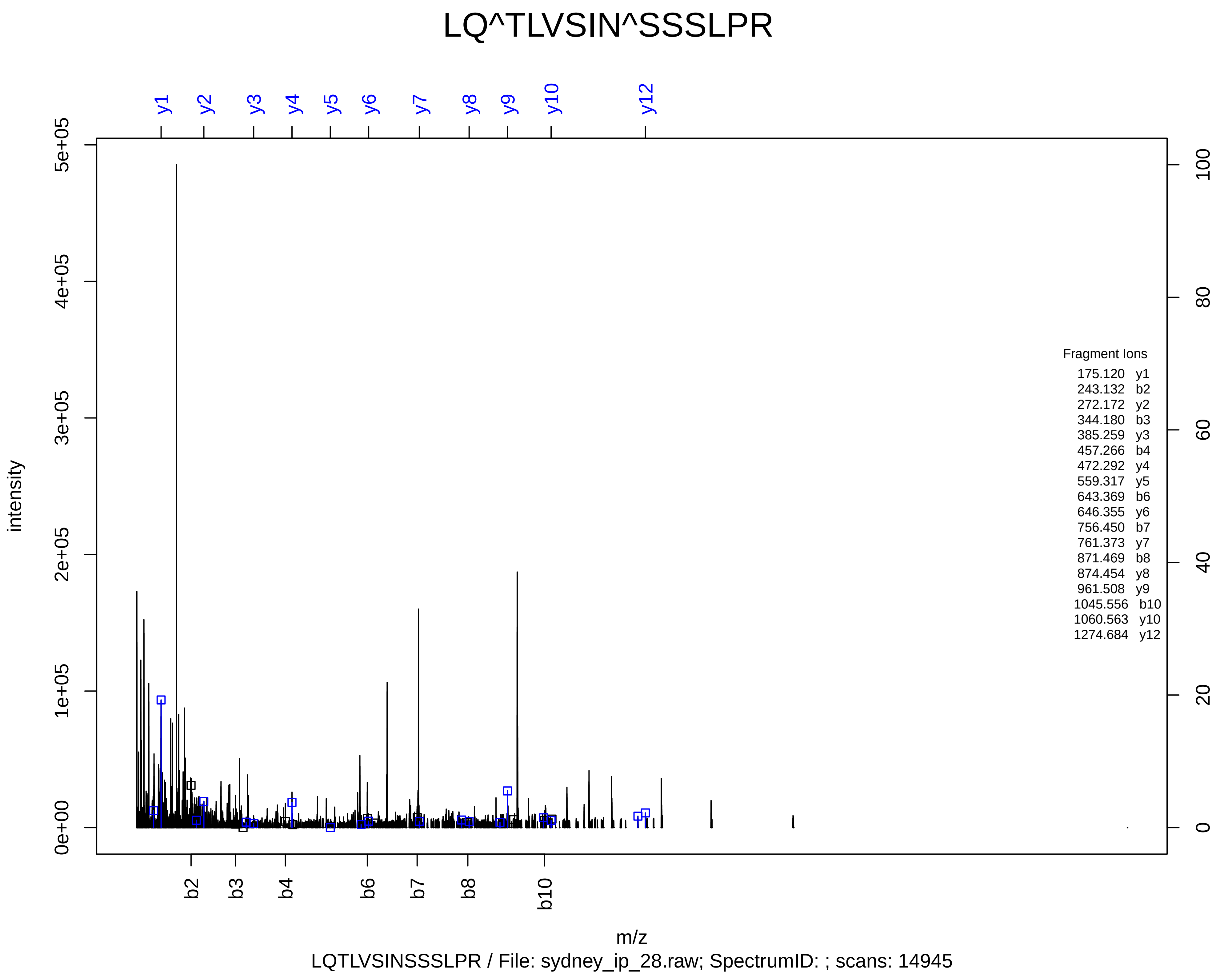
LQ^TLVSIN^SSSLPR
y1
y2
y3
y4
y5
y6
y7
y8
y9
y10
y12
intensity
0e+00
1e+05
2e+05
3e+05
4e+05
5e+05
0
20
40
60
80
100
b2
b3
b4
b6
b7
b8
b10
m/z
Fragment Ions
175.120 y1
243.132 b2
272.172 y2
344.180 b3
385.259 y3
457.266 b4
472.292 y4
559.317 y5
643.369 b6
646.355 y6
756.450 b7
761.373 y7
871.469 b8
874.454 y8
961.508 y9
1045.556 b10
1060.563 y10
1274.684 y12
LQTLVSINSSSLPR / File: sydney_ip_28.raw; SpectrumID: ; scans: 14945

RADQLADESLESTR

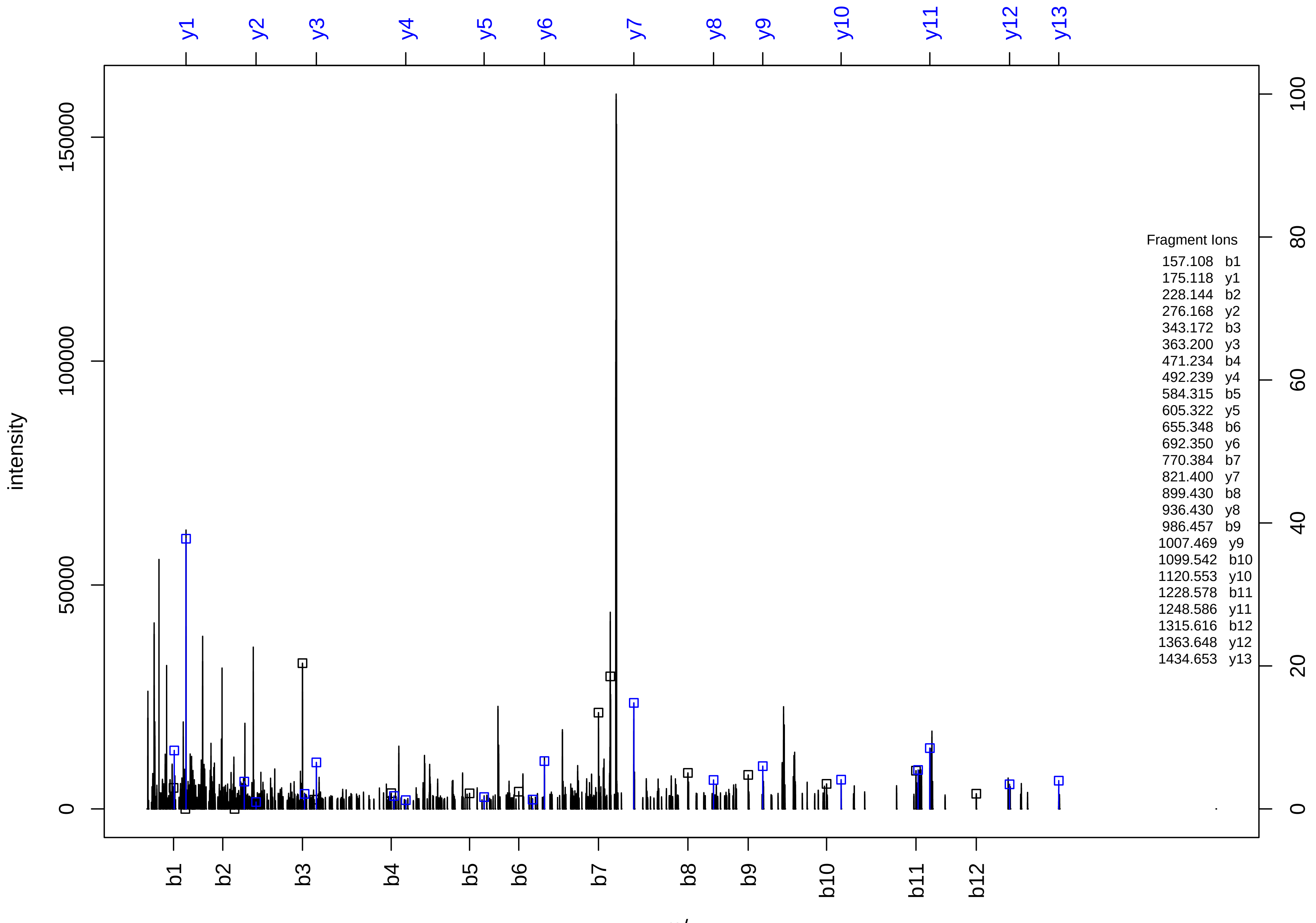

RADQLADESLESTR / File: sydney_ip_28.raw; SpectrumID: ; scans: 4227

GVEEEEEDGEM*RE

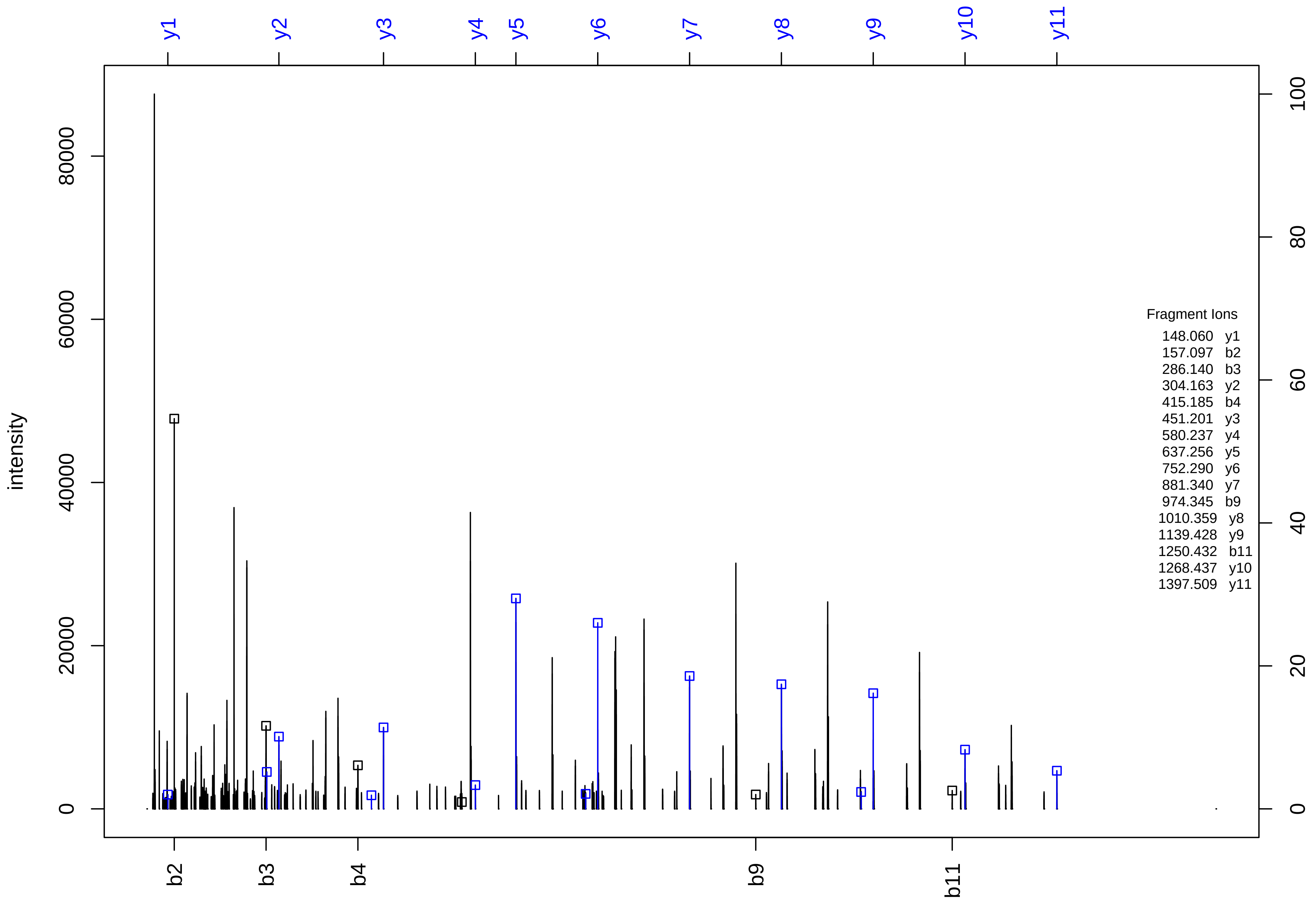

GVEEEEEDGEMRE / File: sydney_ip_28.raw; SpectrumID: ; scans: 1857

M*VDLSGSQLR

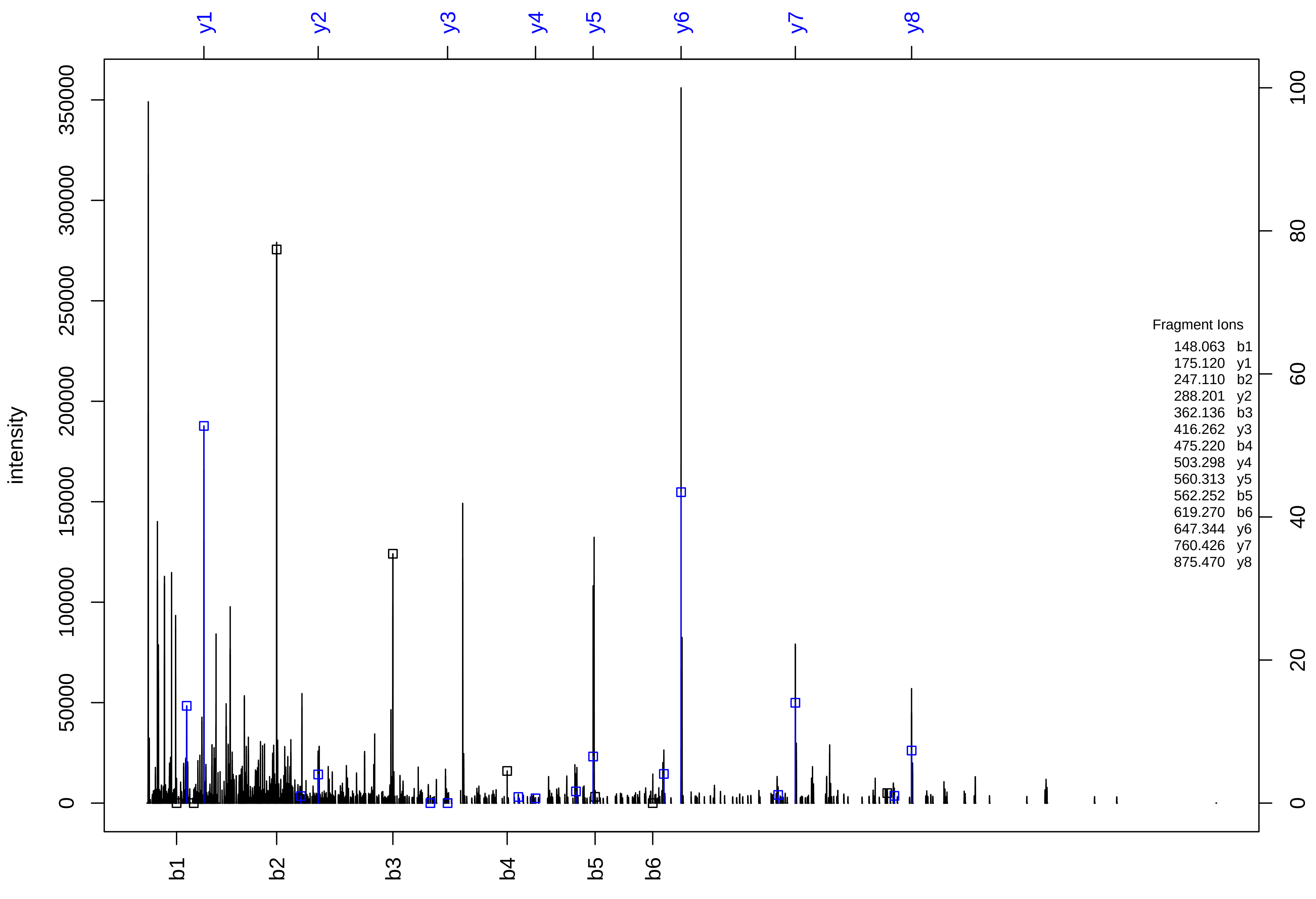

MVDLSGSQLR / File: sydney_ip_28.raw; SpectrumID: ; scans: 12613

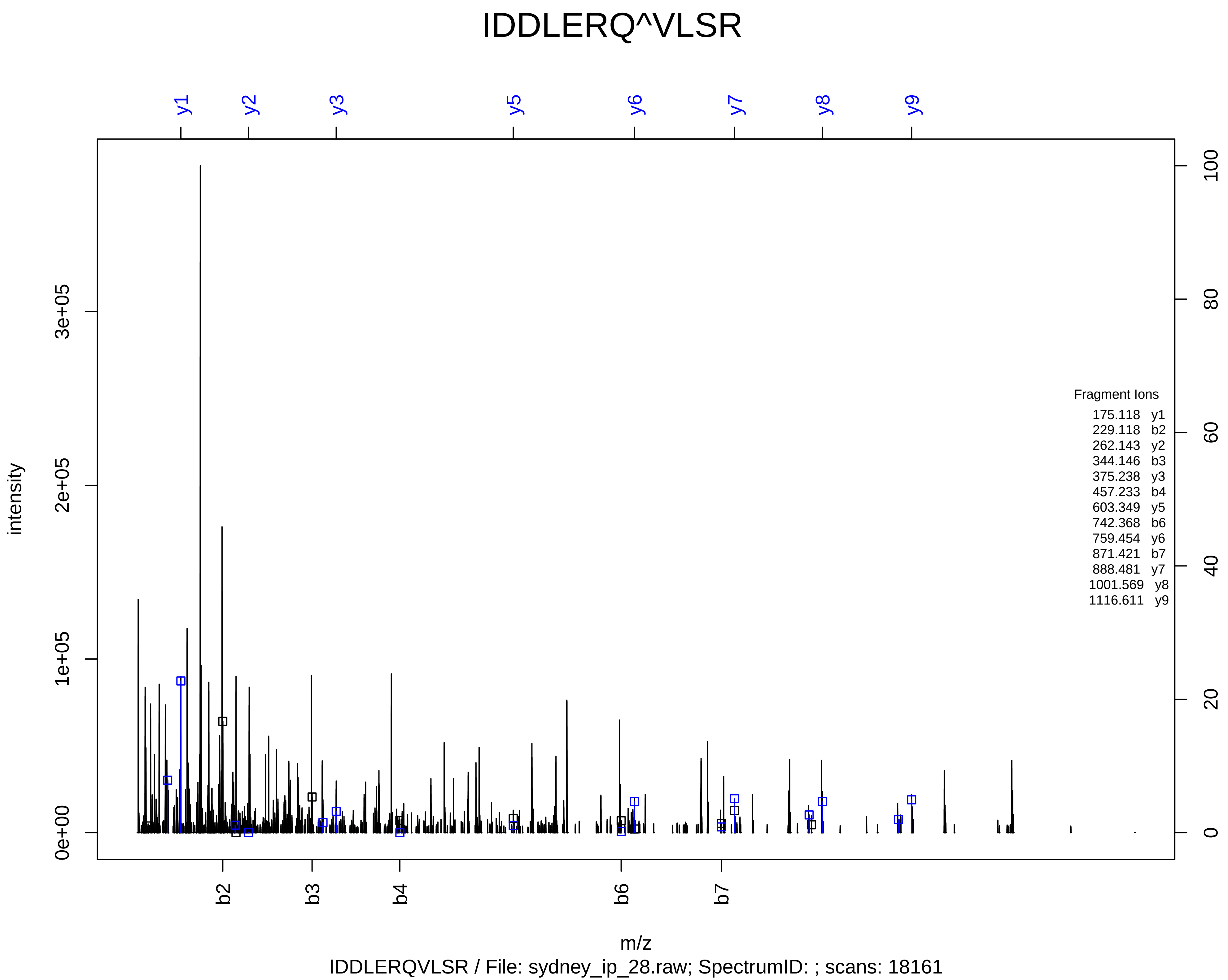
IDDLERQ^VLSR
y1
y2
y3
y5
y6
y7
y8
y9
b2
b3
b4
b6
b7
intensity
0e+00
1e+05
2e+05
3e+05
0
20
40
60
80
100
m/z
Fragment Ions
175.118 y1
229.118 b2
262.143 y2
344.146 b3
375.238 y3
457.233 b4
603.349 y5
742.368 b6
759.454 y6
871.421 b7
888.481 y7
1001.569 y8
1116.611 y9
IDDLERQVLSR / File: sydney_ip_28.raw; SpectrumID: ; scans: 18161

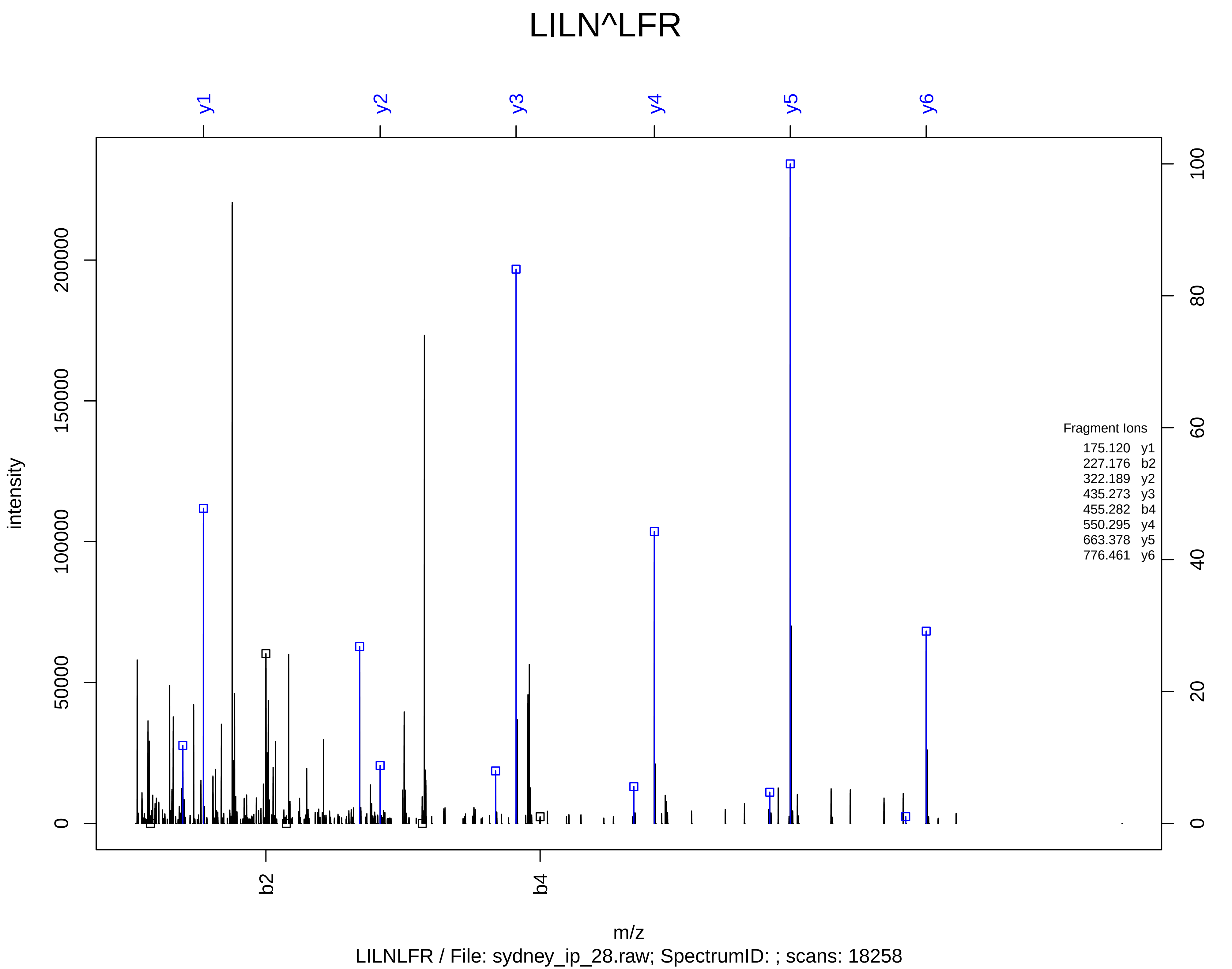

LILN^LFR
y1
y2
y3
y4
y5
y6
intensity
0
50000
100000
150000
200000
0
20
40
60
80
100
Fragment Ions
175.120 y1
227.176 b2
322.189 y2
435.273 y3
455.282 b4
550.295 y4
663.378 y5
776.461 y6
b2
b4
m/z
LILNLFR / File: sydney_ip_28.raw; SpectrumID: ; scans: 18258

ITVPLSVISQ^PM*KGK

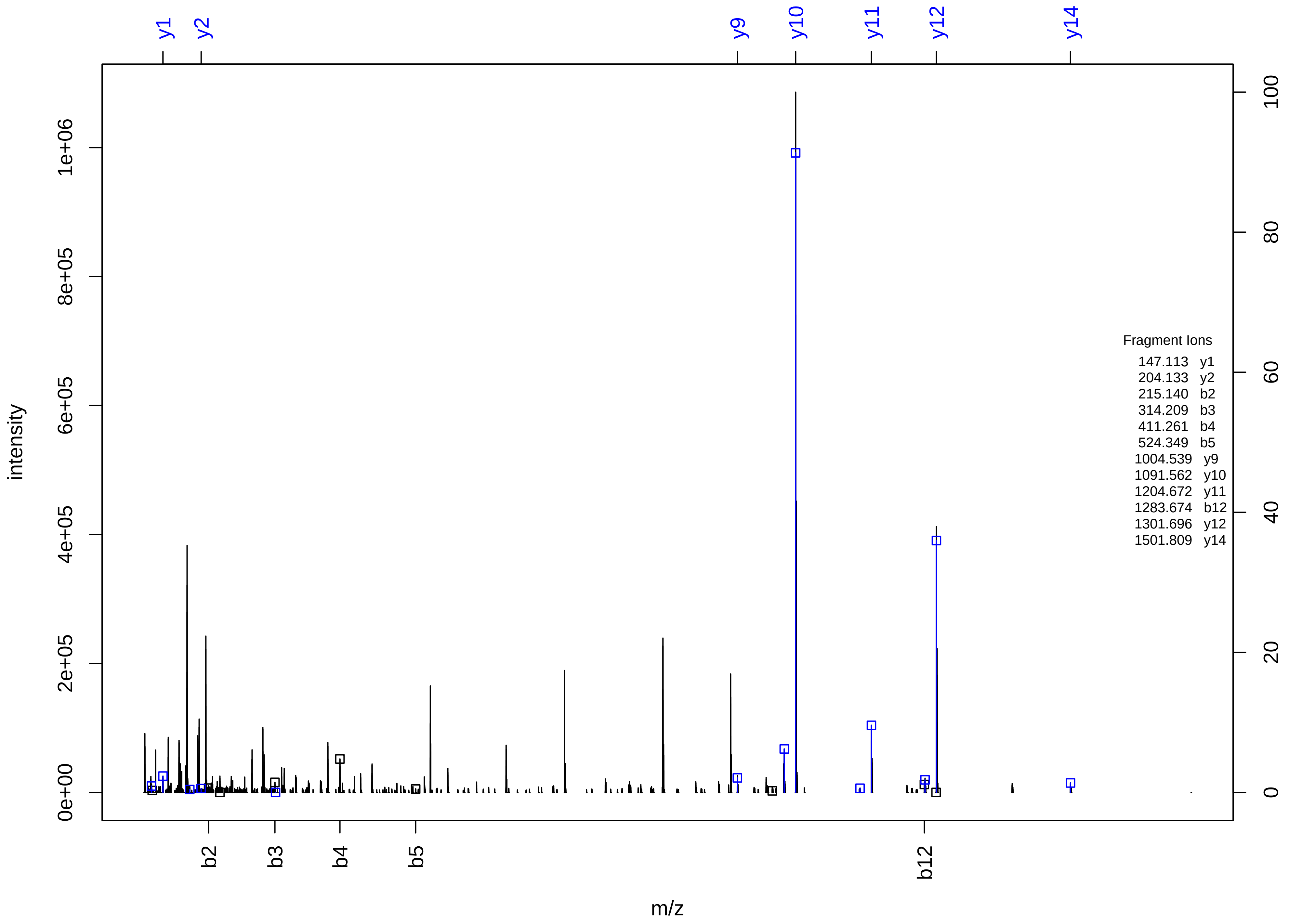

ITVPLSVISQPMKGK / File: sydney_ip_28.raw; SpectrumID: ; scans: 19727

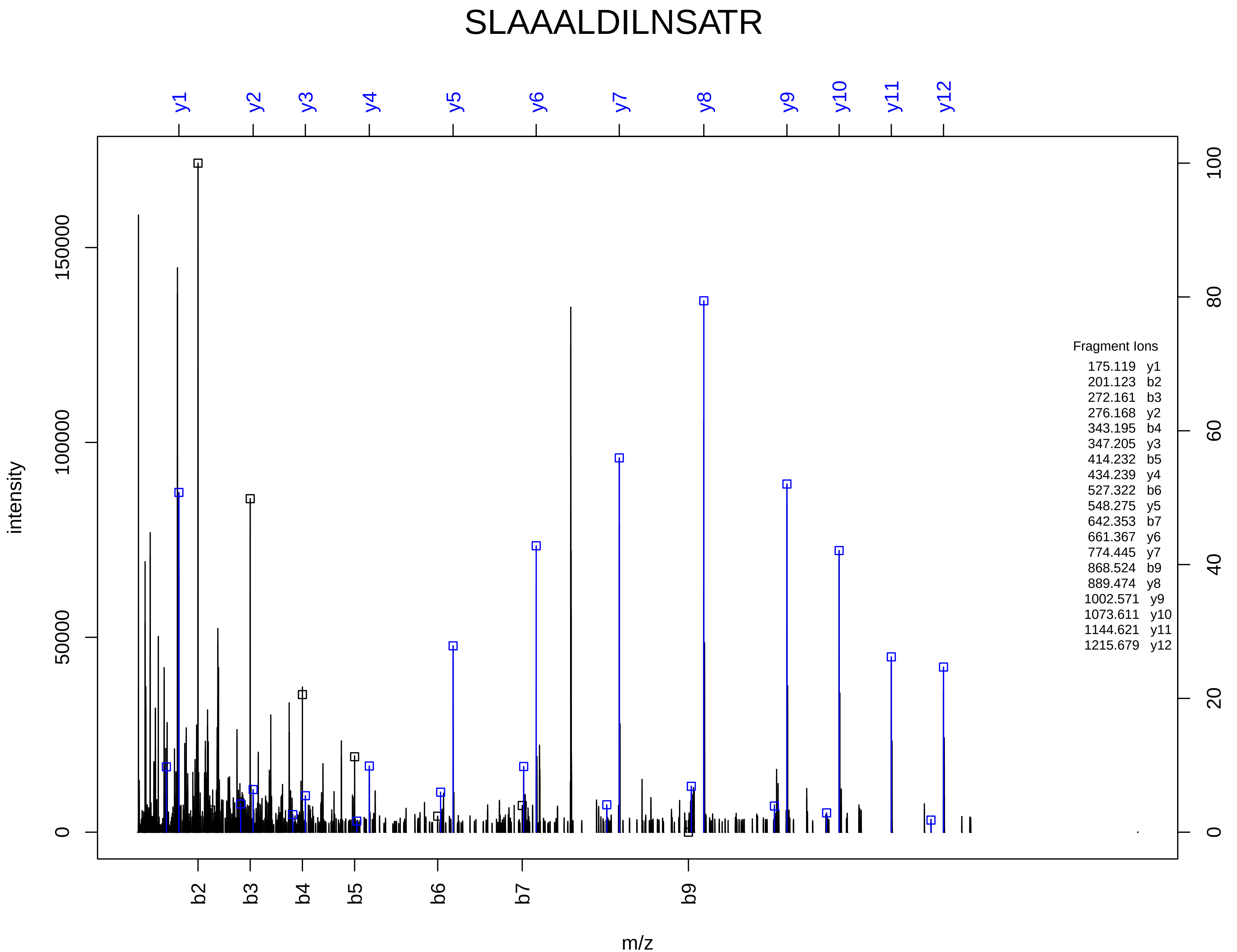

SLAAALDILNSATR / File: sydney_ip_28.raw; SpectrumID: ; scans: 17317

VTGAPVPAVSEPQDGDDFQSR

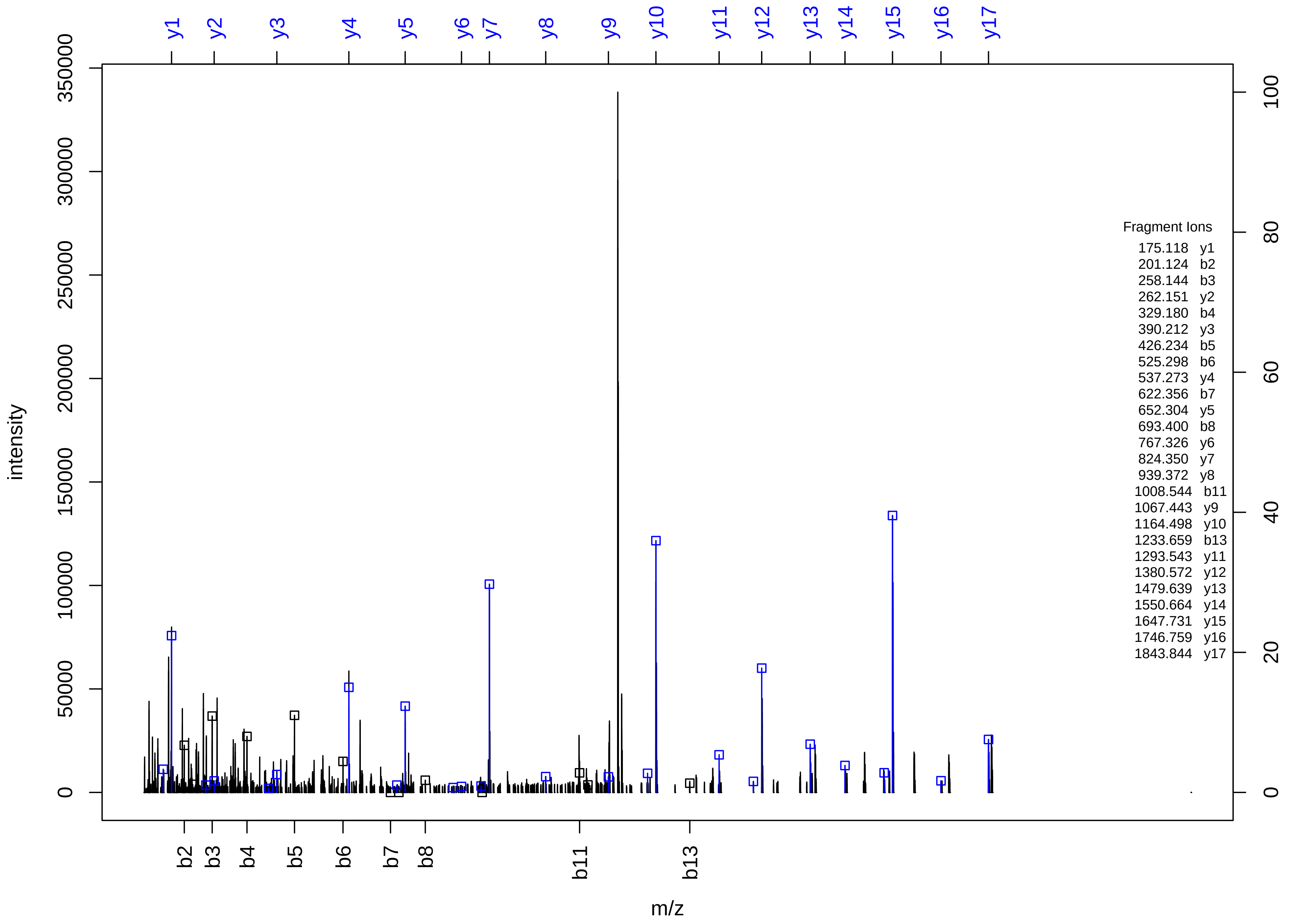

VTGAPVPAVSEPQDGDDFQSR / File: sydney_ip_28.raw; SpectrumID: ; scans: 8741

FIDDVVSAVLR

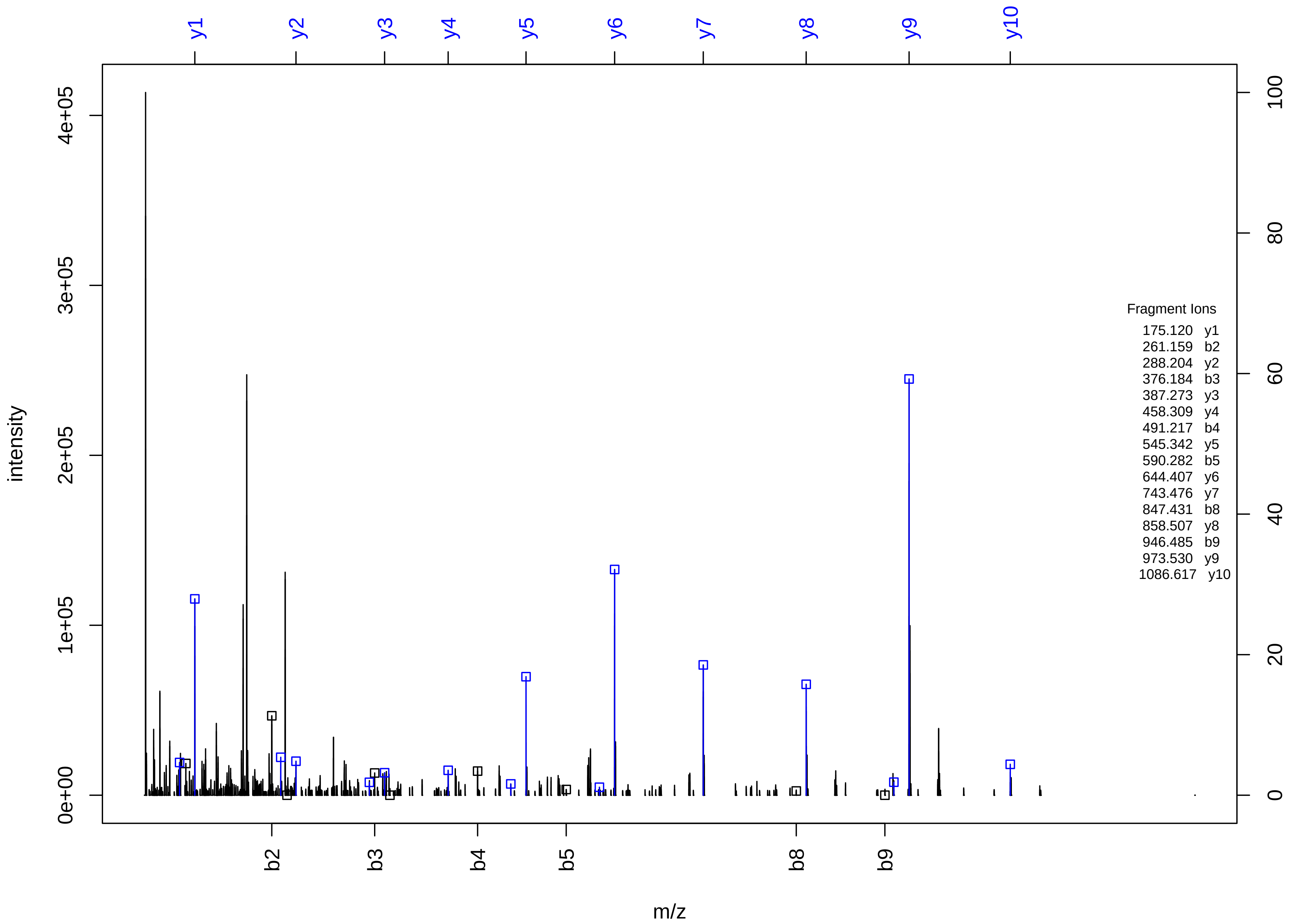

FIDDVVSAVLR / File: sydney_ip_28.raw; SpectrumID: ; scans: 17502

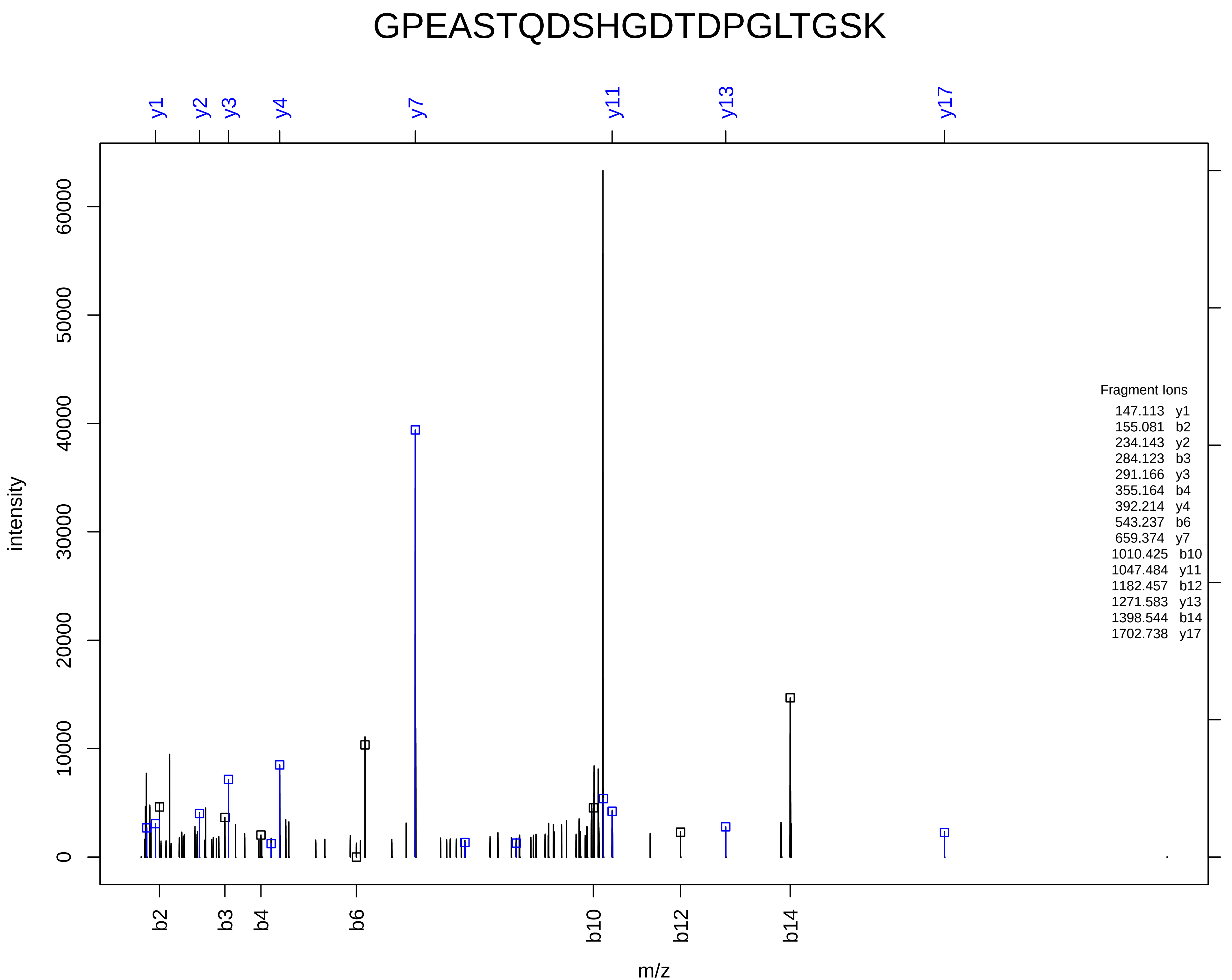
GPEASTQDSHGDTDPGLTGSK
y1
y2
y3
y4
y7
y11
y13
y17
intensity
0
10000
20000
30000
40000
50000
60000
0
20
40
60
80
100
b2
b3
b4
b6
b10
b12
b14
m/z
Fragment Ions
147.113 y1
155.081 b2
234.143 y2
284.123 b3
291.166 y3
355.164 b4
392.214 y4
543.237 b6
659.374 y7
1010.425 b10
1047.484 y11
1182.457 b12
1271.583 y13
1398.544 b14
1702.738 y17
GPEASTQDSHGDTDPGLTGSK / File: sydney_ip_28.raw; SpectrumID: ; scans: 2232

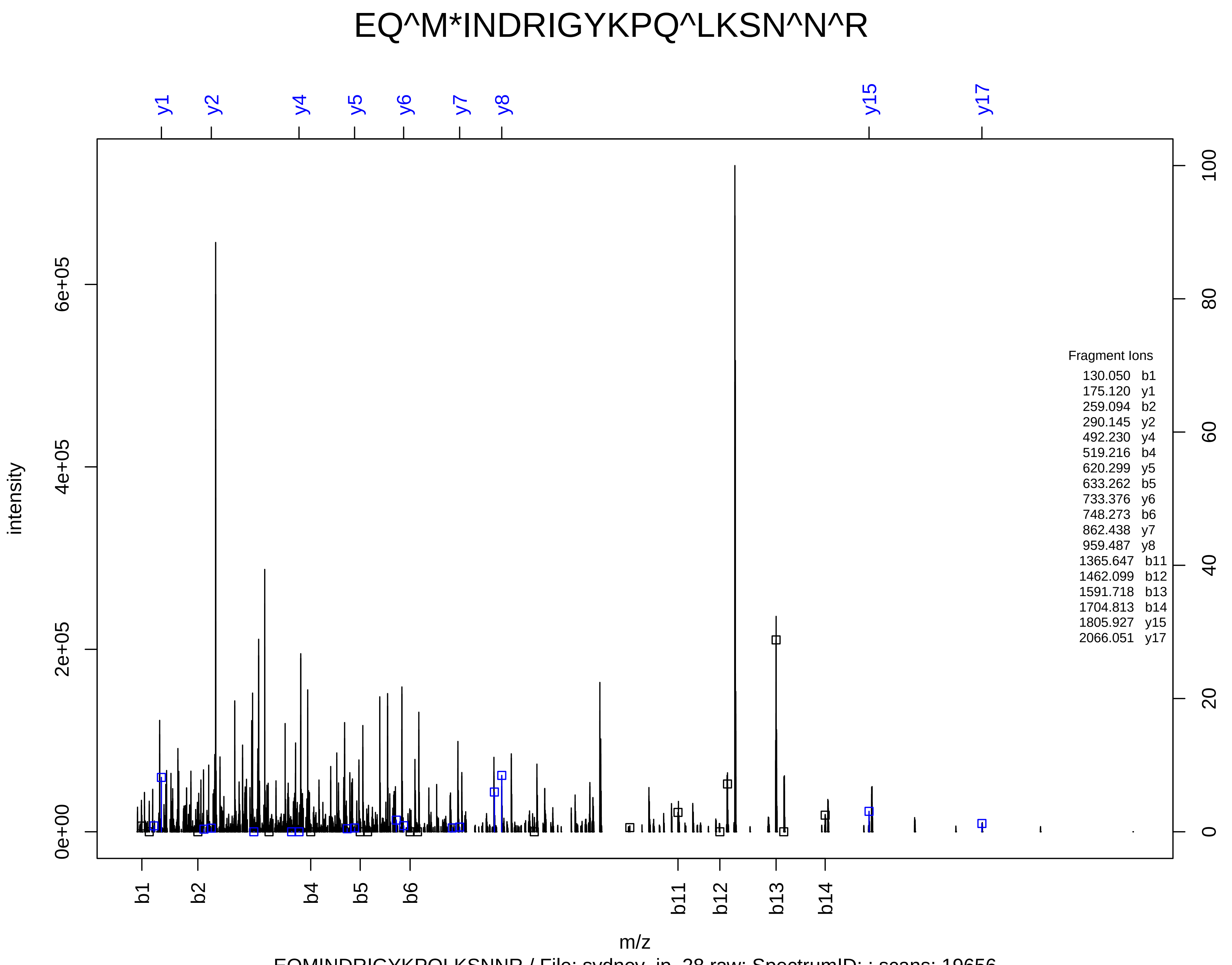

EQ^M*INDRIGYKPQ^LKSN^N^R
y1
y2
y4
y5
y6
y7
y8
y15
y17
intensity
0e+00
2e+05
4e+05
6e+05
0
20
40
60
80
100
Fragment Ions
130.050 b1
175.120 y1
259.094 b2
290.145 y2
492.230 y4
519.216 b4
620.299 y5
633.262 b5
733.376 y6
748.273 b6
862.438 y7
959.487 y8
1365.647 b11
1462.099 b12
1591.718 b13
1704.813 b14
1805.927 y15
2066.051 y17
b1
b2
b4
b5
b6
b11
b12
b13
b14
m/z
EQMINDRIGYKPQLKSNNR / File: sydney_ip_28.raw; SpectrumID: ; scans: 19656

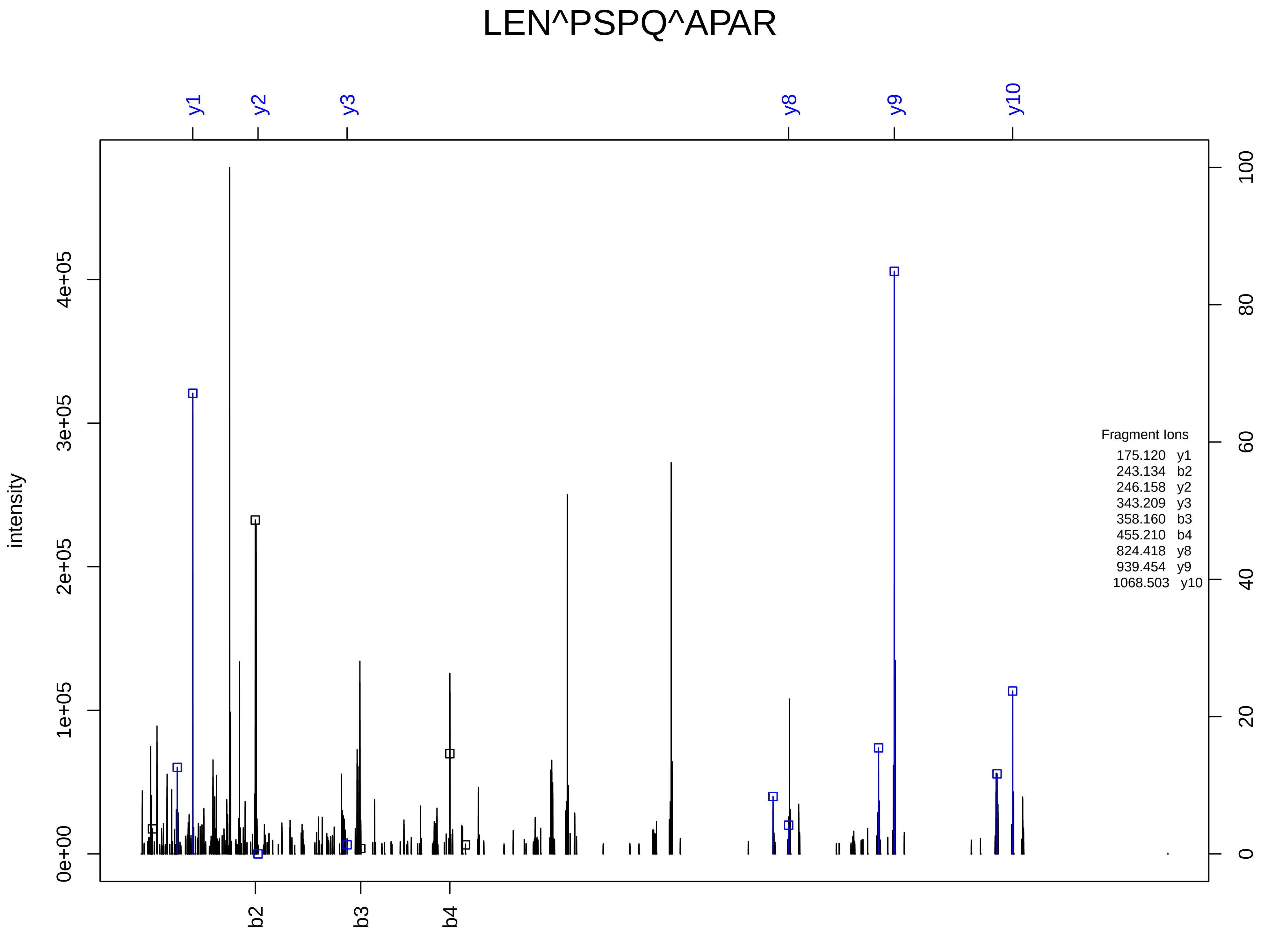

LENPSPQAPAR / File: sydney_ip_28.raw; SpectrumID: ; scans: 4367

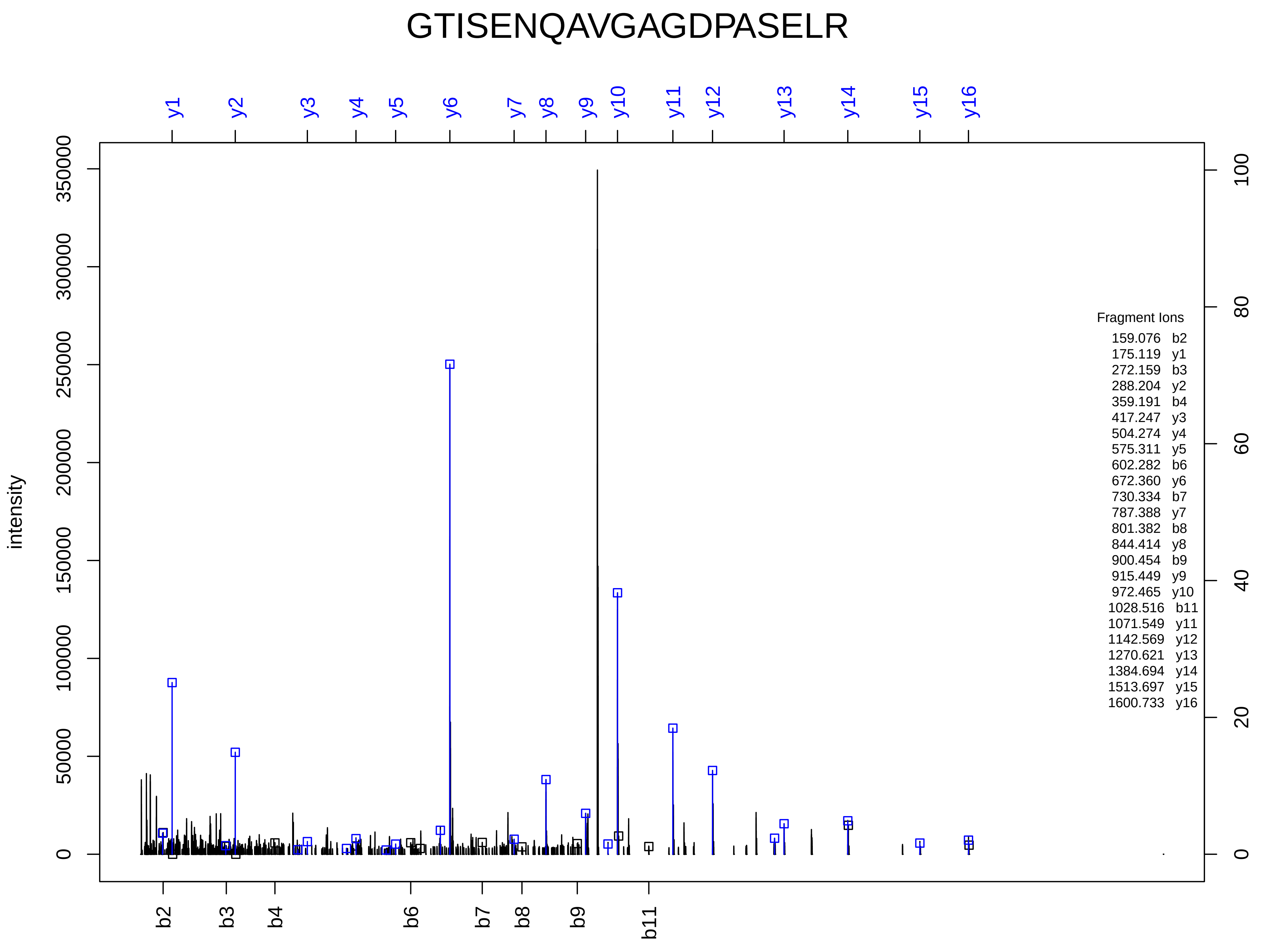

GTISENQAVGAGDPASELR
y1
y2
y3
y4
y5
y6
y7
y8
y9
y10
y11
y12
y13
y14
y15
y16
intensity
Fragment Ions
159.076 b2
175.119 y1
272.159 b3
288.204 y2
359.191 b4
417.247 y3
504.274 y4
575.311 y5
602.282 b6
672.360 y6
730.334 b7
787.388 y7
801.382 b8
844.414 y8
900.454 b9
915.449 y9
972.465 y10
1028.516 b11
1071.549 y11
1142.569 y12
1270.621 y13
1384.694 y14
1513.697 y15
1600.733 y16
b2
b3
b4
b6
b7
b8
b9
b11
m/z
GTISENQAVGAGDPASELR / File: sydney_ip_28.raw; SpectrumID: ; scans: 7496

CQRPRTLMRQ^QSLQQPLSQN^Q^R

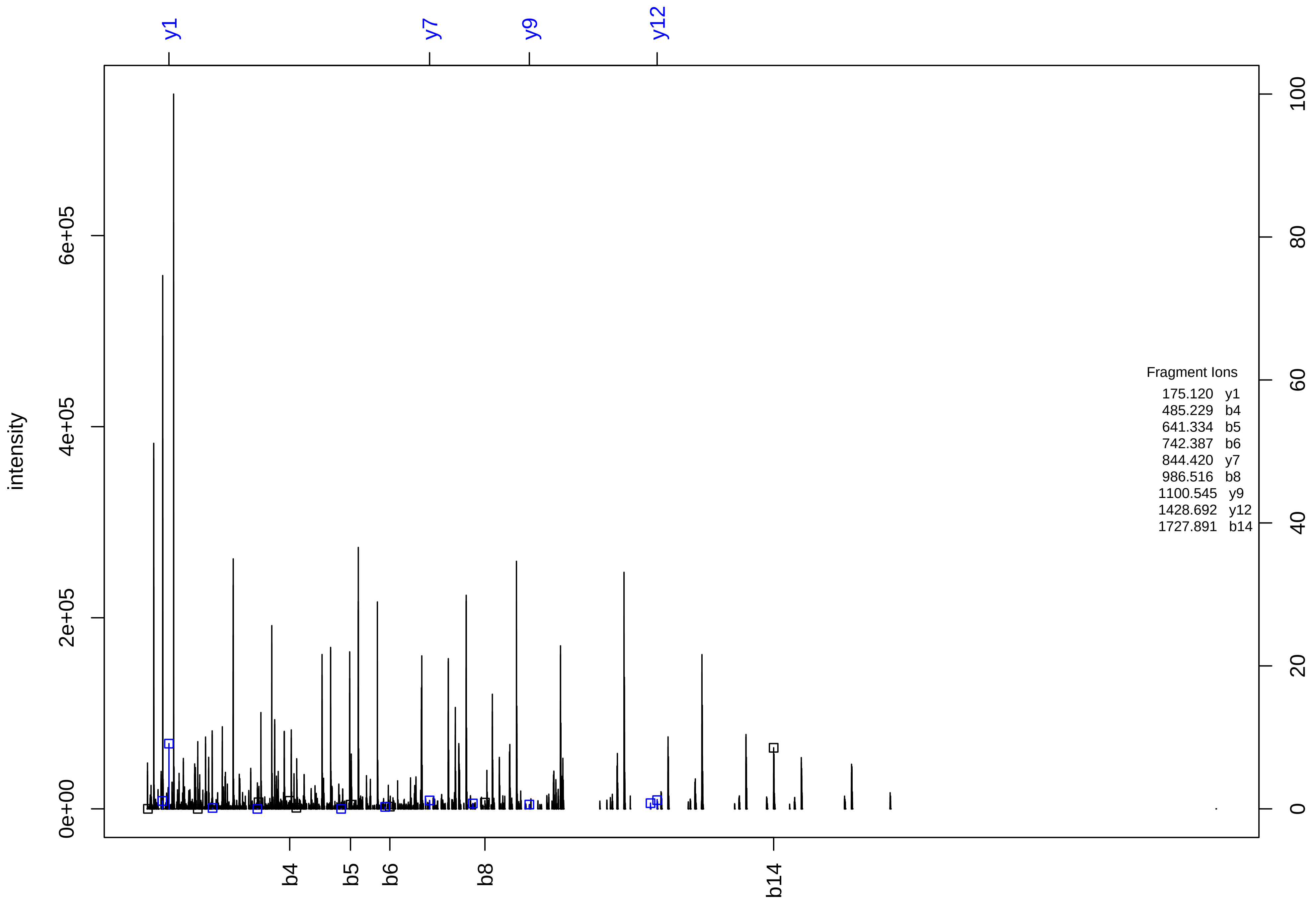

CQRPRTLMRQQSLQQPLSQNQR / File: sydney_ip_28.raw; SpectrumID: ; scans: 19834

SLLQ^Q^LEN^VTKERQ^M*K

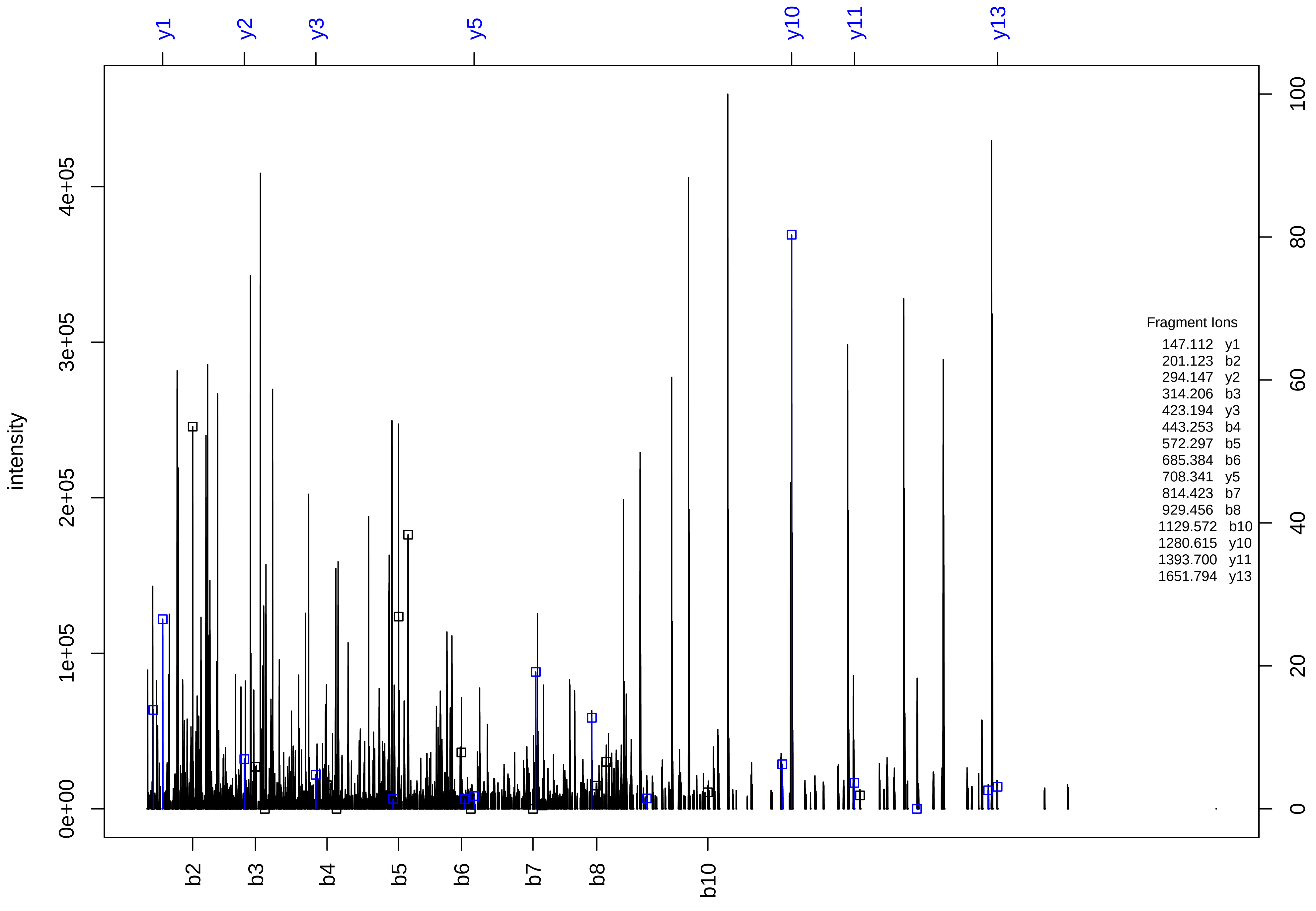

SLLQQLENVTKERQMK / File: sydney_ip_29.raw; SpectrumID: ; scans: 13219

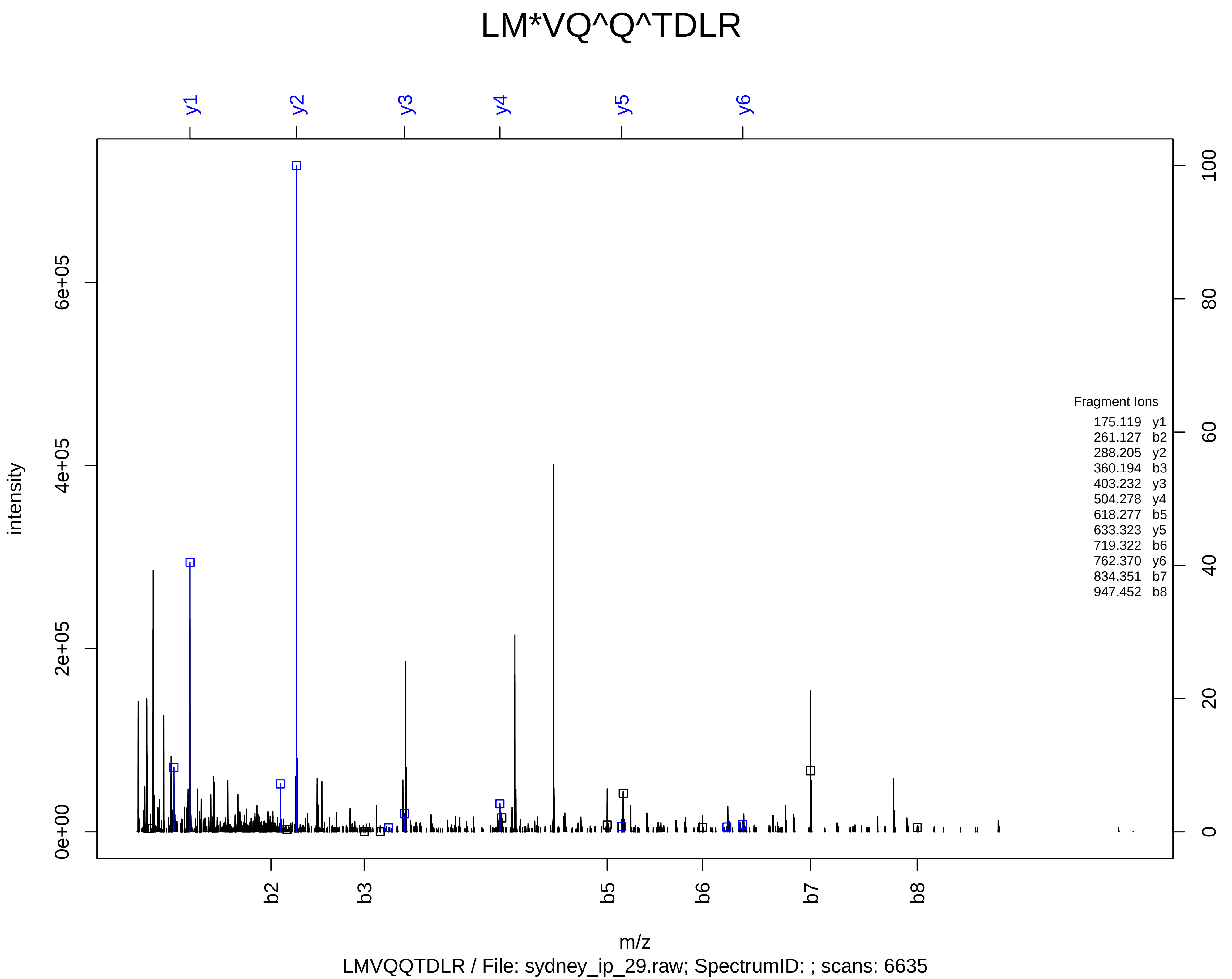
LM*VQ^Q^TDLR
y1
y2
y3
y4
y5
y6
intensity
0e+00
2e+05
4e+05
6e+05
0
20
40
60
80
100
Fragment Ions
175.119 y1
261.127 b2
288.205 y2
360.194 b3
403.232 y3
504.278 y4
618.277 b5
633.323 y5
719.322 b6
762.370 y6
834.351 b7
947.452 b8
b2
b3
b5
b6
b7
b8
m/z
LMVQQTDLR / File: sydney_ip_29.raw; SpectrumID: ; scans: 6635

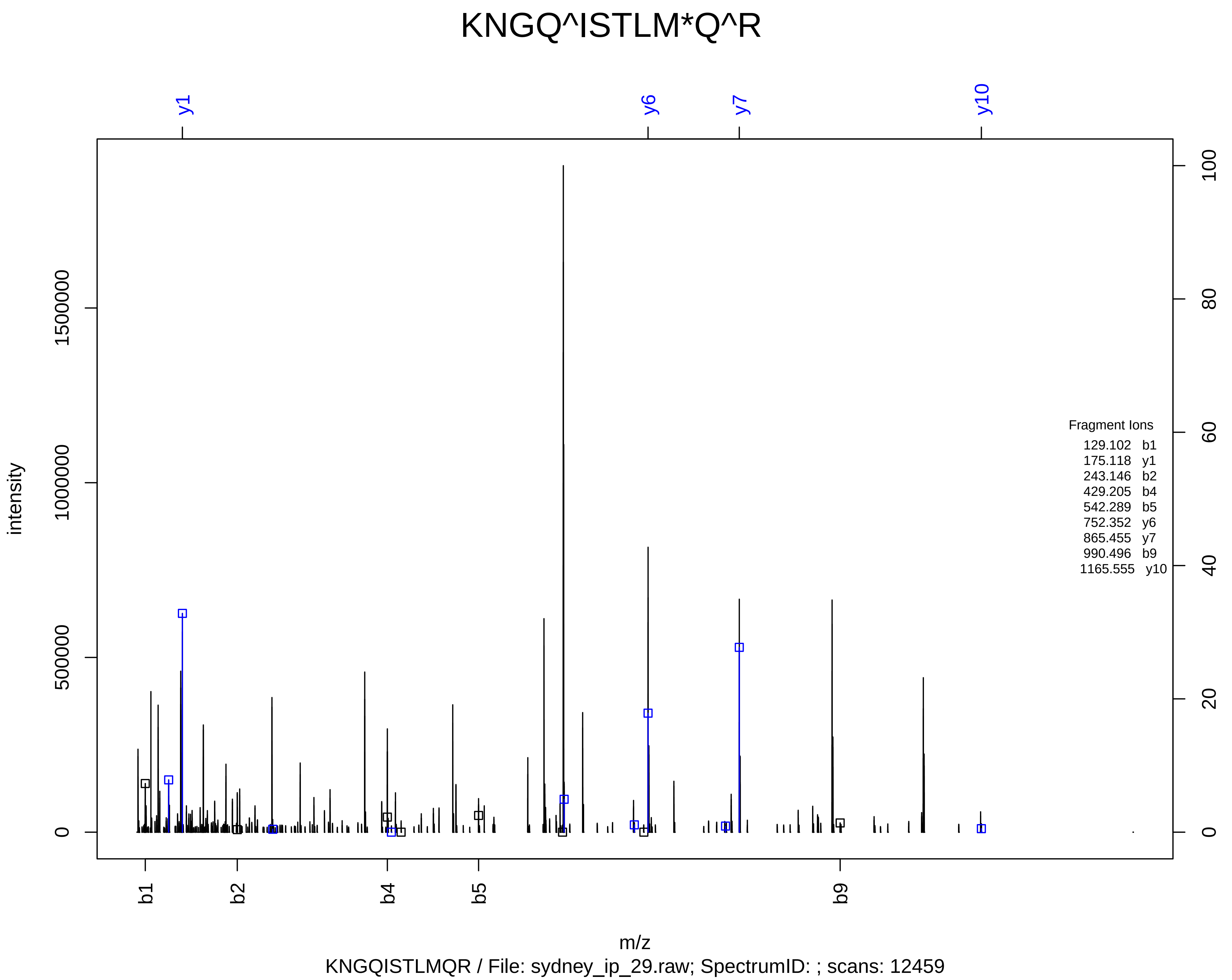
KNGQ^ISTLM*Q^R
y1
y6
y7
y10
intensity
0
500000
1000000
1500000
0
20
40
60
80
100
Fragment Ions
129.102 b1
175.118 y1
243.146 b2
429.205 b4
542.289 b5
752.352 y6
865.455 y7
990.496 b9
1165.555 y10
b1
b2
b4
b5
b9
m/z
KNGQISTLMQR / File: sydney_ip_29.raw; SpectrumID: ; scans: 12459

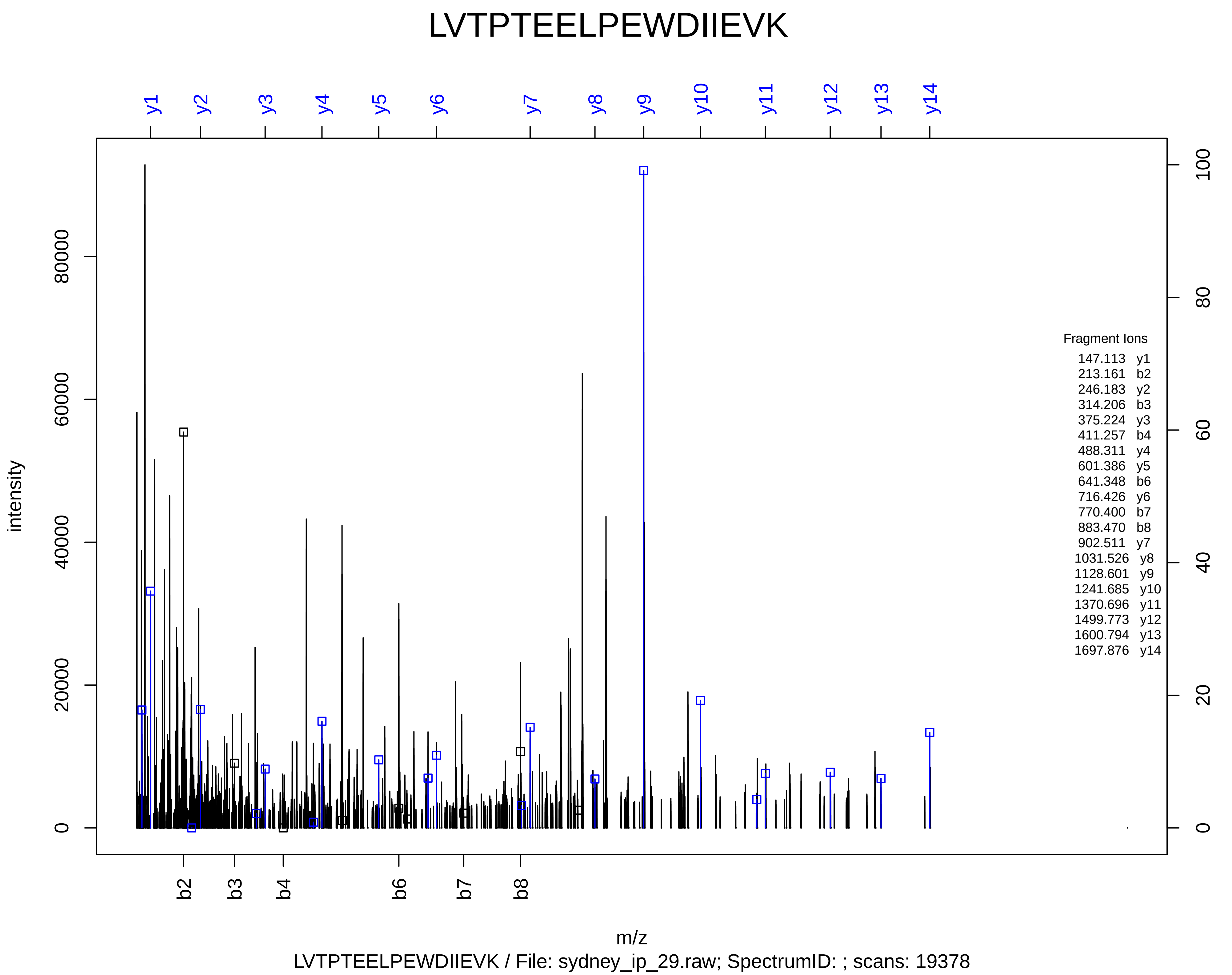
LVTPTEELPEWDIIEVK
intensity
m/z
Fragment Ions
147.113 y1
213.161 b2
246.183 y2
314.206 b3
375.224 y3
411.257 b4
488.311 y4
601.386 y5
641.348 b6
716.426 y6
770.400 b7
883.470 b8
902.511 y7
1031.526 y8
1128.601 y9
1241.685 y10
1370.696 y11
1499.773 y12
1600.794 y13
1697.876 y14
LVTPTEELPEWDIIEVK / File: sydney_ip_29.raw; SpectrumID: ; scans: 19378

VGDAIPSVEVFEGEPGK

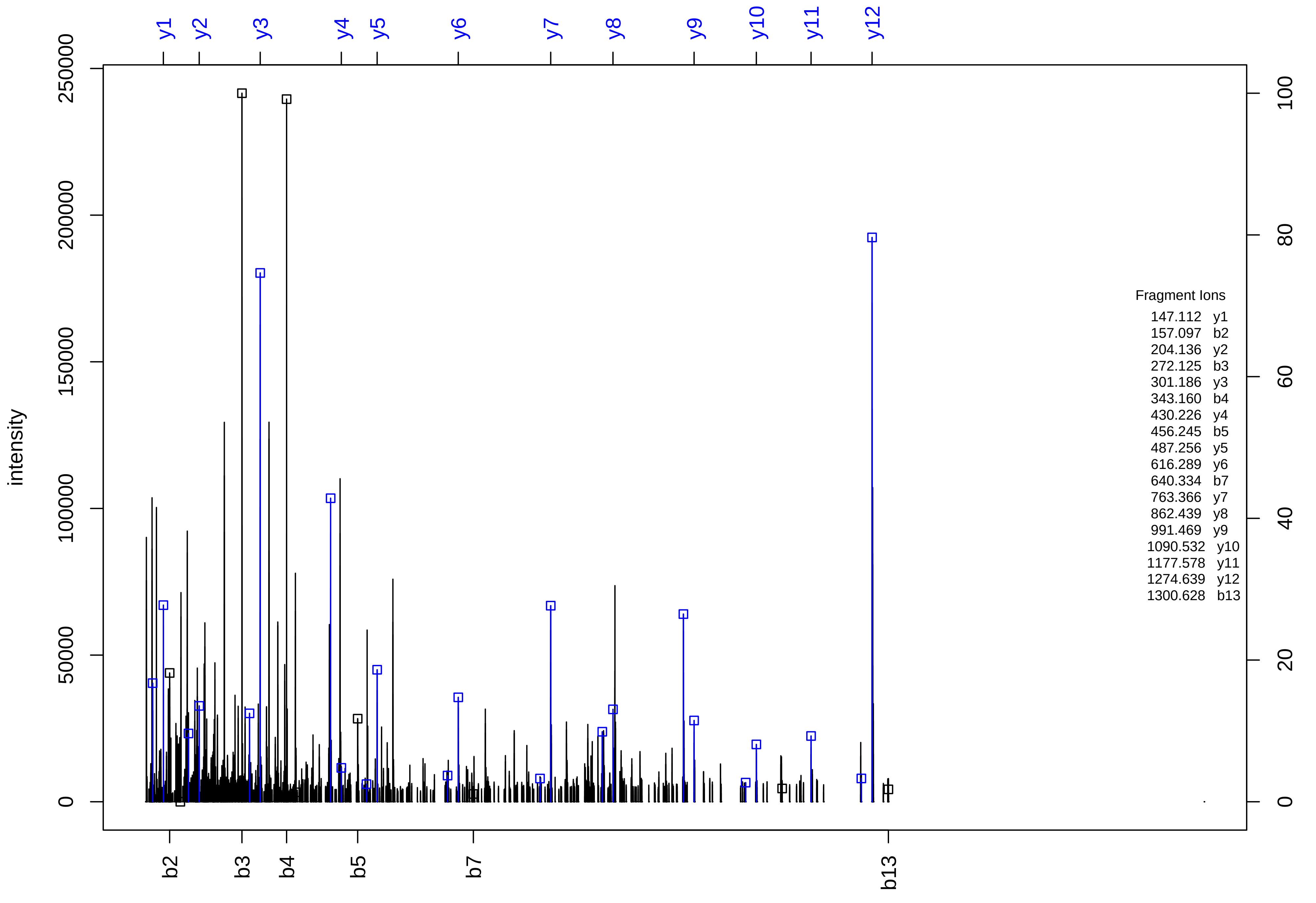

VGDAIPSVEVFEGEPGK / File: sydney_ip_29.raw; SpectrumID: ; scans: 13600

(Ac)MQ^GLPDPPCQR

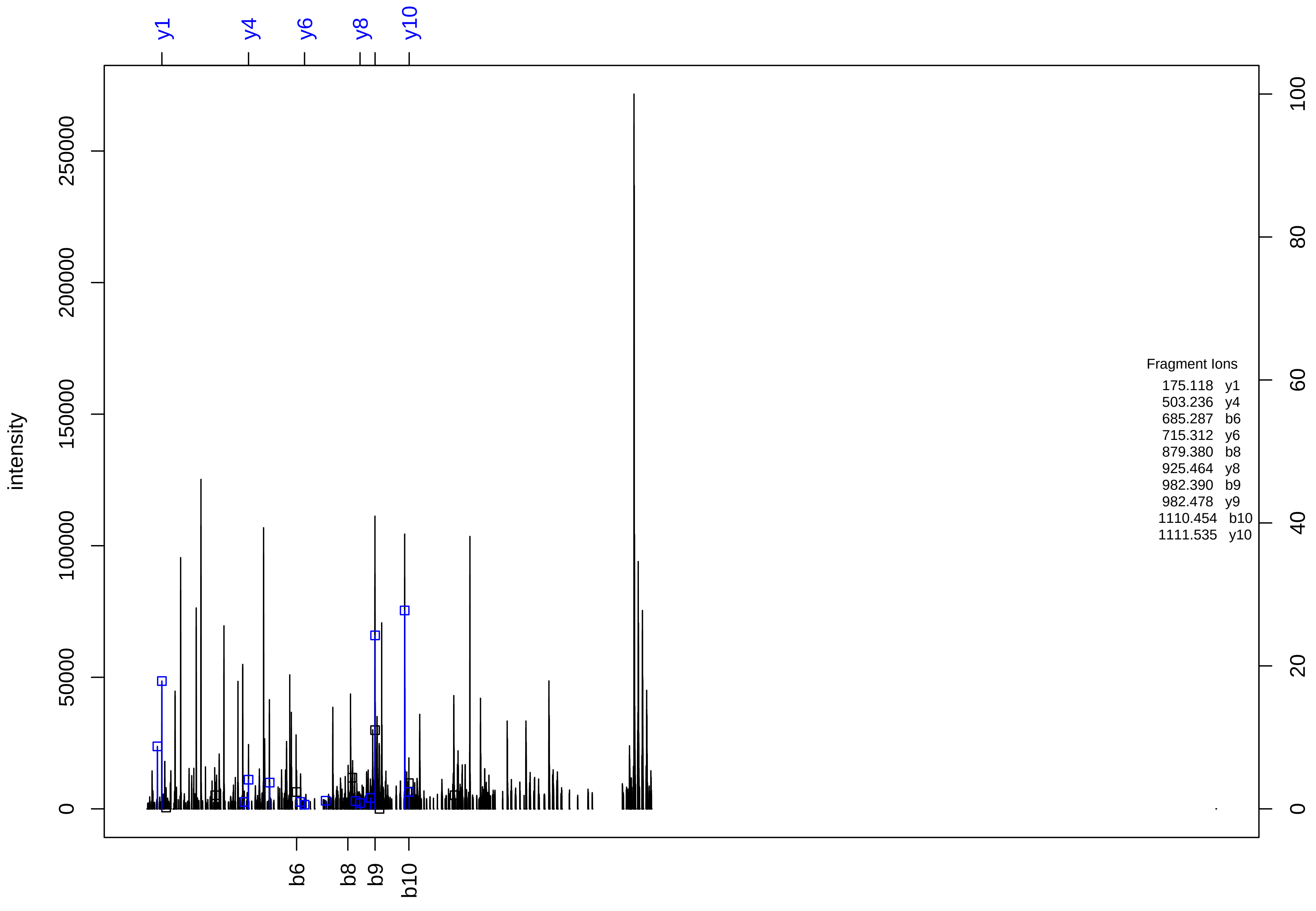

MQGLPDPPCQR / File: sydney_ip_29.raw; SpectrumID: ; scans: 4543

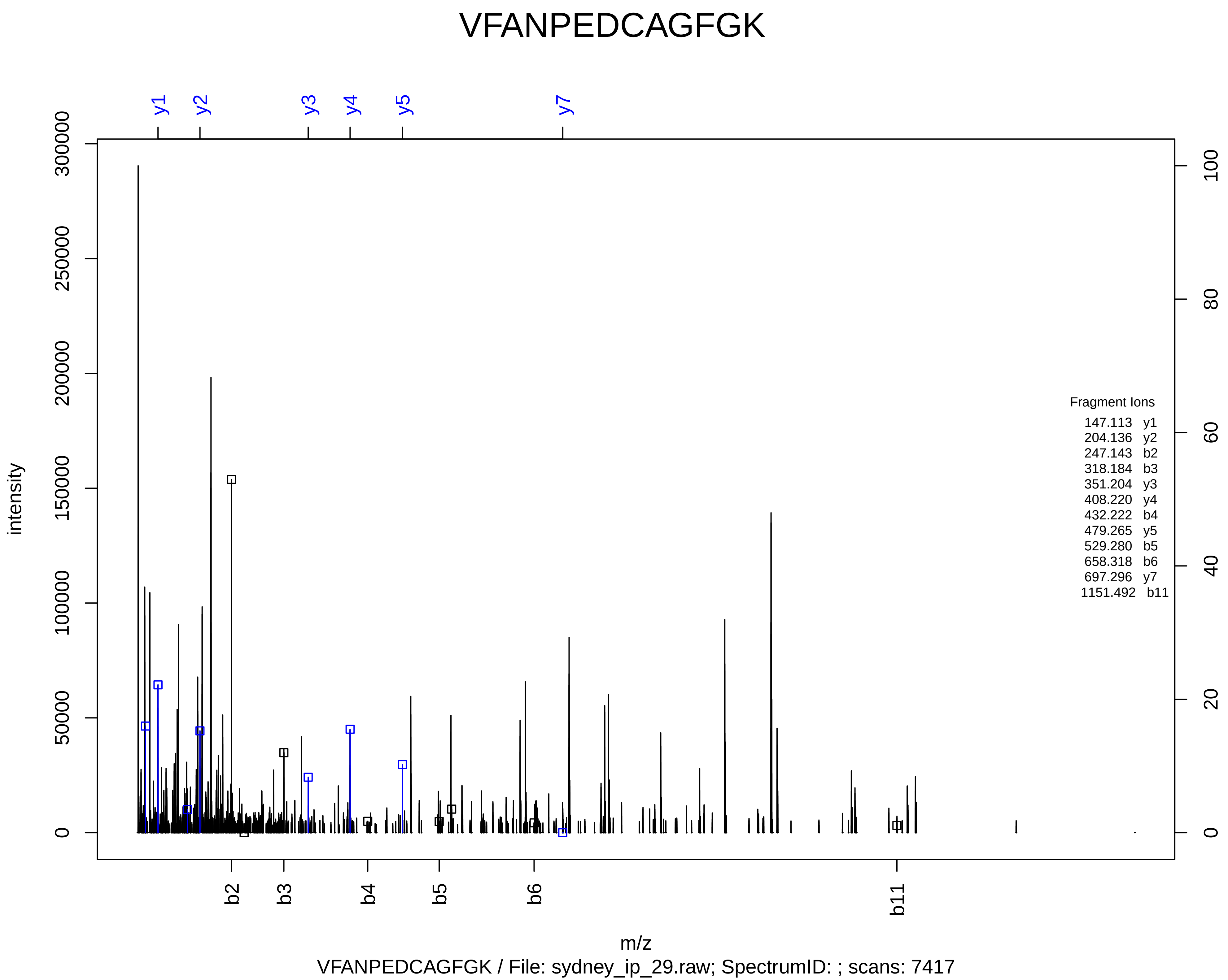

VFANPEDCAGFGK
y1
y2
y3
y4
y5
y7
intensity
0
50000
100000
150000
200000
250000
300000
0
20
40
60
80
100
b2
b3
b4
b5
b6
b11
m/z
Fragment Ions
147.113 y1
204.136 y2
247.143 b2
318.184 b3
351.204 y3
408.220 y4
432.222 b4
479.265 y5
529.280 b5
658.318 b6
697.296 y7
1151.492 b11
VFANPEDCAGFGK / File: sydney_ip_29.raw; SpectrumID: ; scans: 7417

DLIGIDNLVTPGR

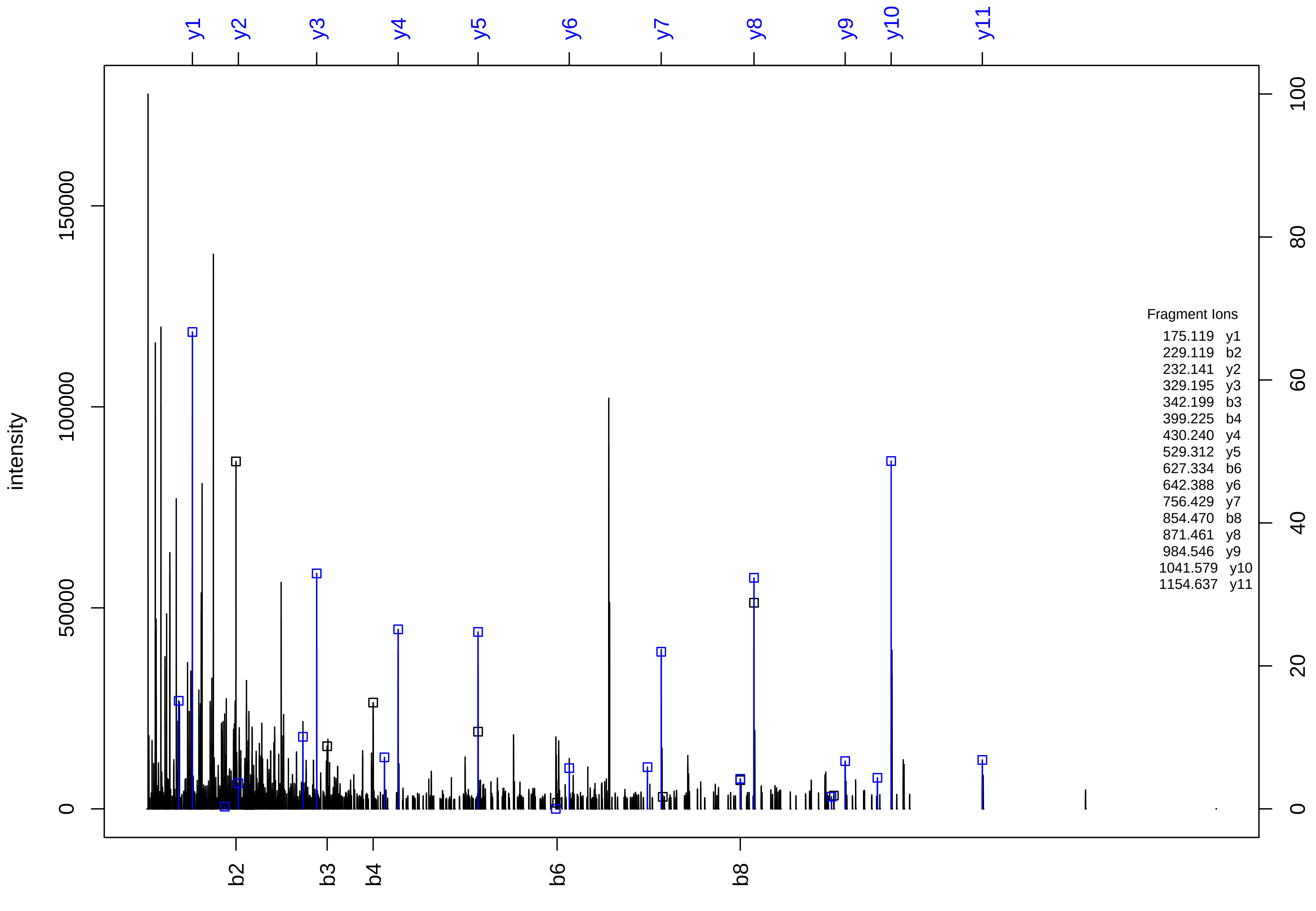

DLIGIDNLVTPGR / File: sydney_ip_29.raw; SpectrumID: ; scans: 16924

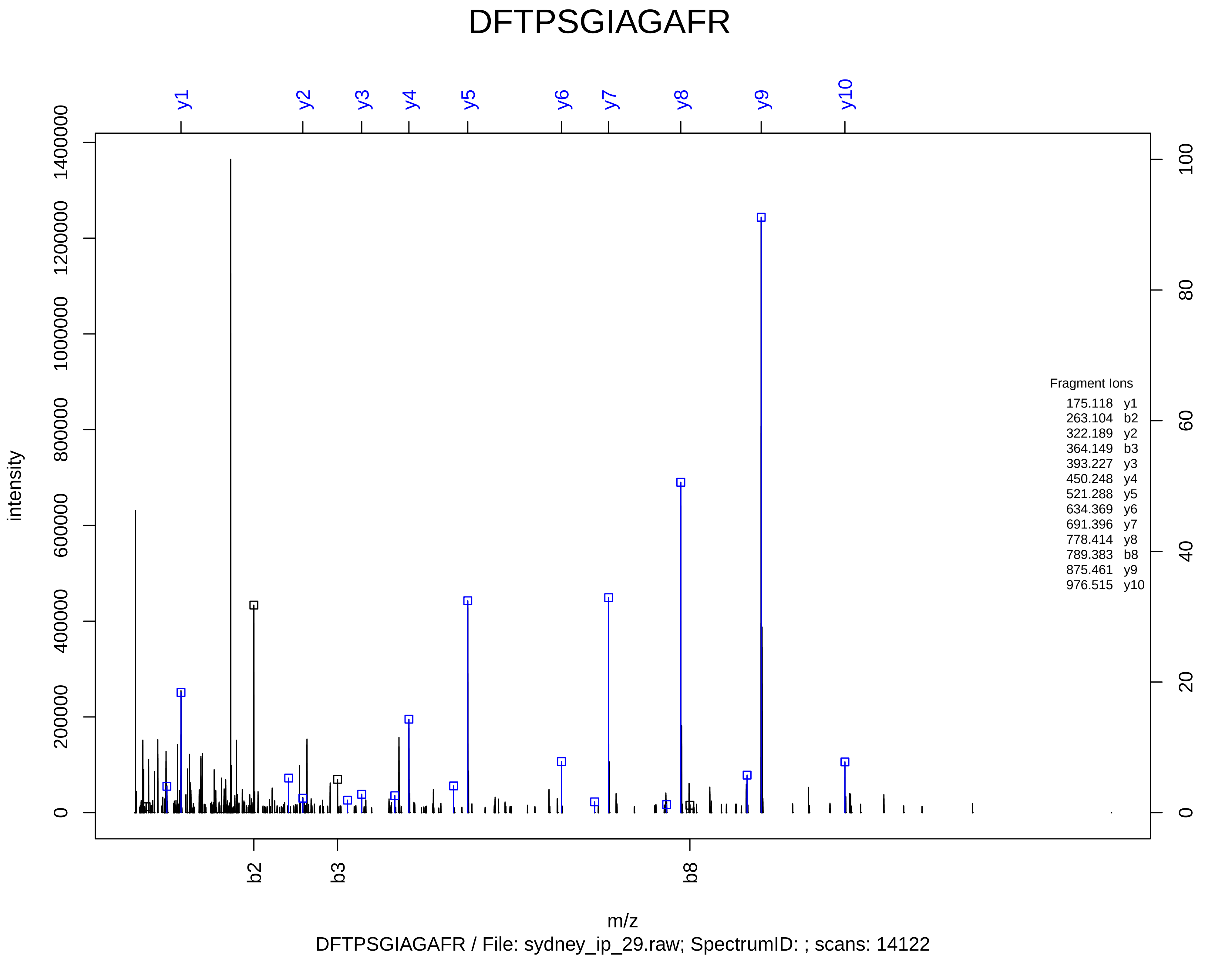
DFTPSGIAGAFR
intensity
m/z
y1
y2
y3
y4
y5
y6
y7
y8
y9
y10
b2
b3
b8
0
200000
400000
600000
800000
1000000
1200000
1400000
0
20
40
60
80
100
Fragment Ions
175.118 y1
263.104 b2
322.189 y2
364.149 b3
393.227 y3
450.248 y4
521.288 y5
634.369 y6
691.396 y7
778.414 y8
789.383 b8
875.461 y9
976.515 y10
DFTPSGIAGAFR / File: sydney_ip_29.raw; SpectrumID: ; scans: 14122

(Ac)M*Q^VSIACTEHNLK

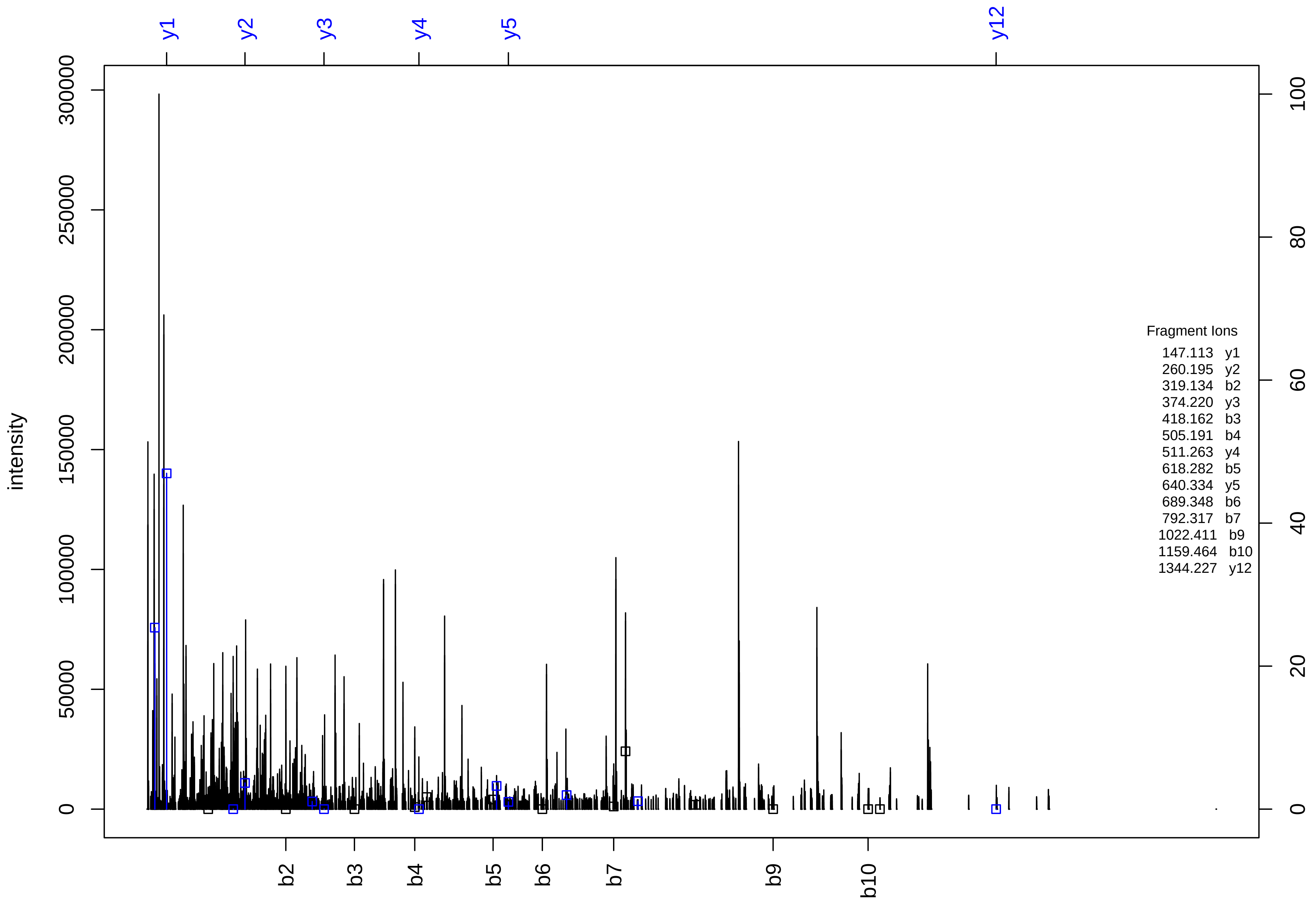

MQVSIACTEHNLK / File: sydney_ip_29.raw; SpectrumID: ; scans: 16714

EAQQLFLEPEVLDPESVELK

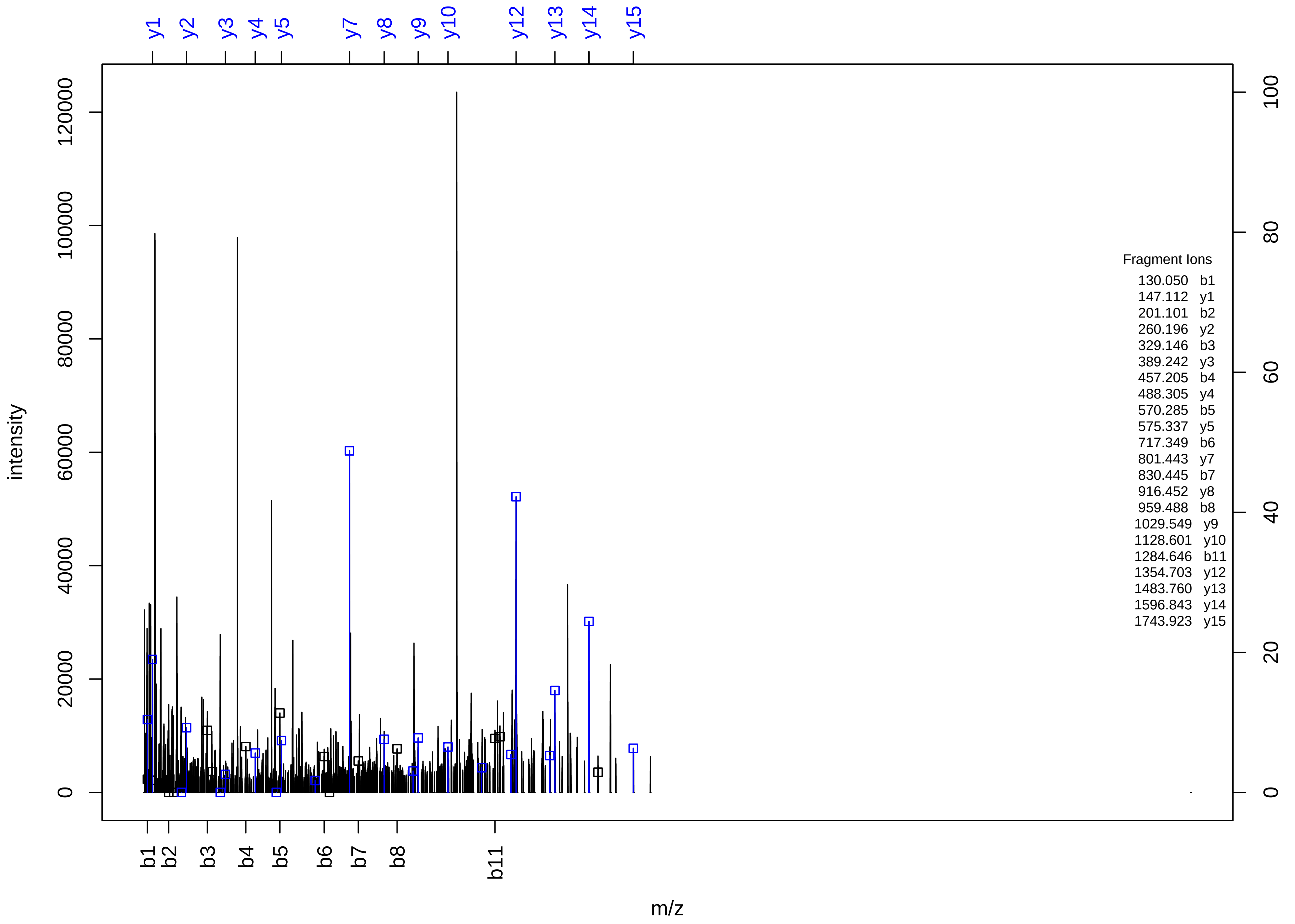

EAQQLFLEPEVLDPESVELK / File: sydney_ip_29.raw; SpectrumID: ; scans: 19421

IPRLLPRSQ^R

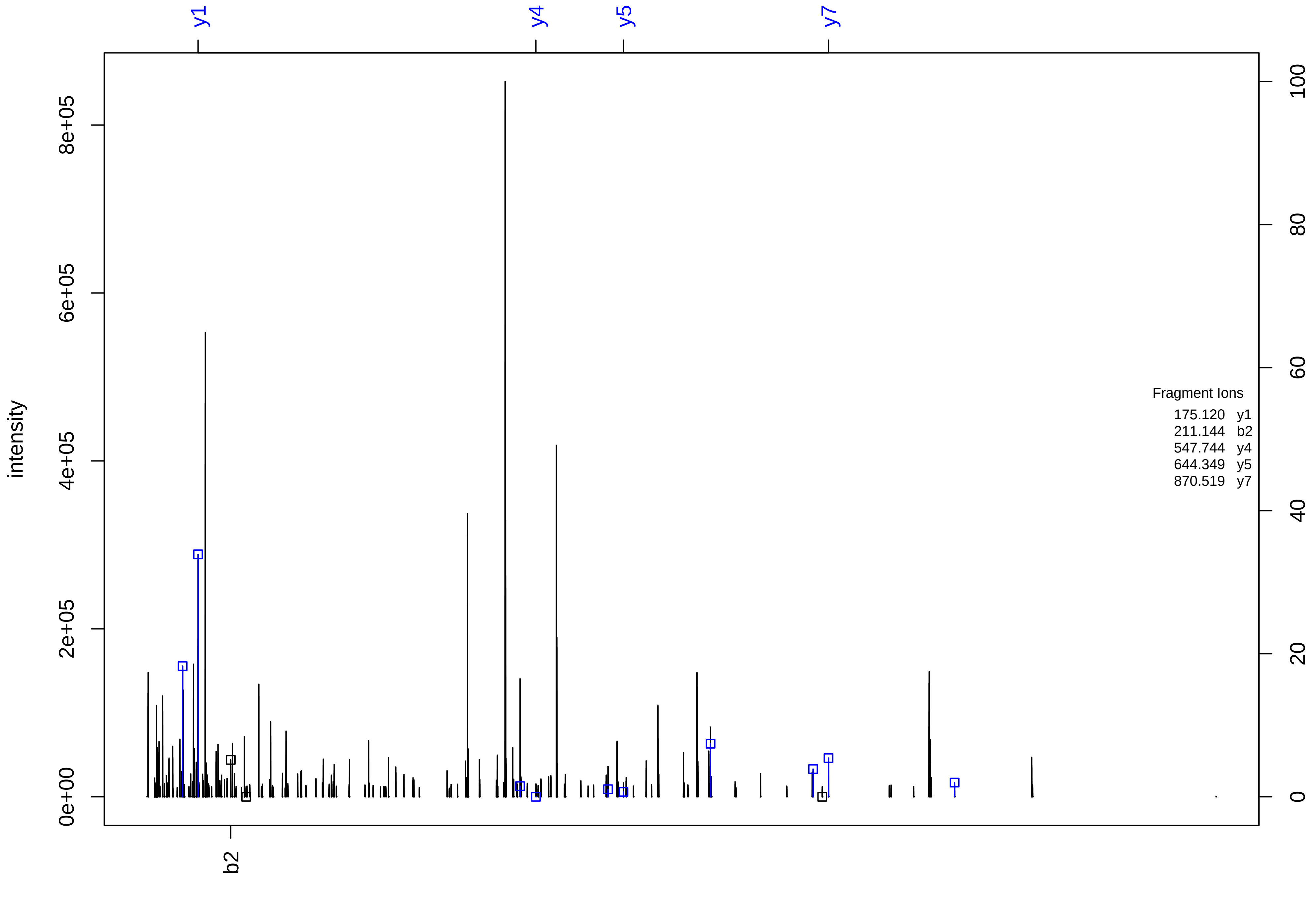

IPRLLPRSQR / File: sydney_ip_29.raw; SpectrumID: ; scans: 5640

VGISLLSSPHP

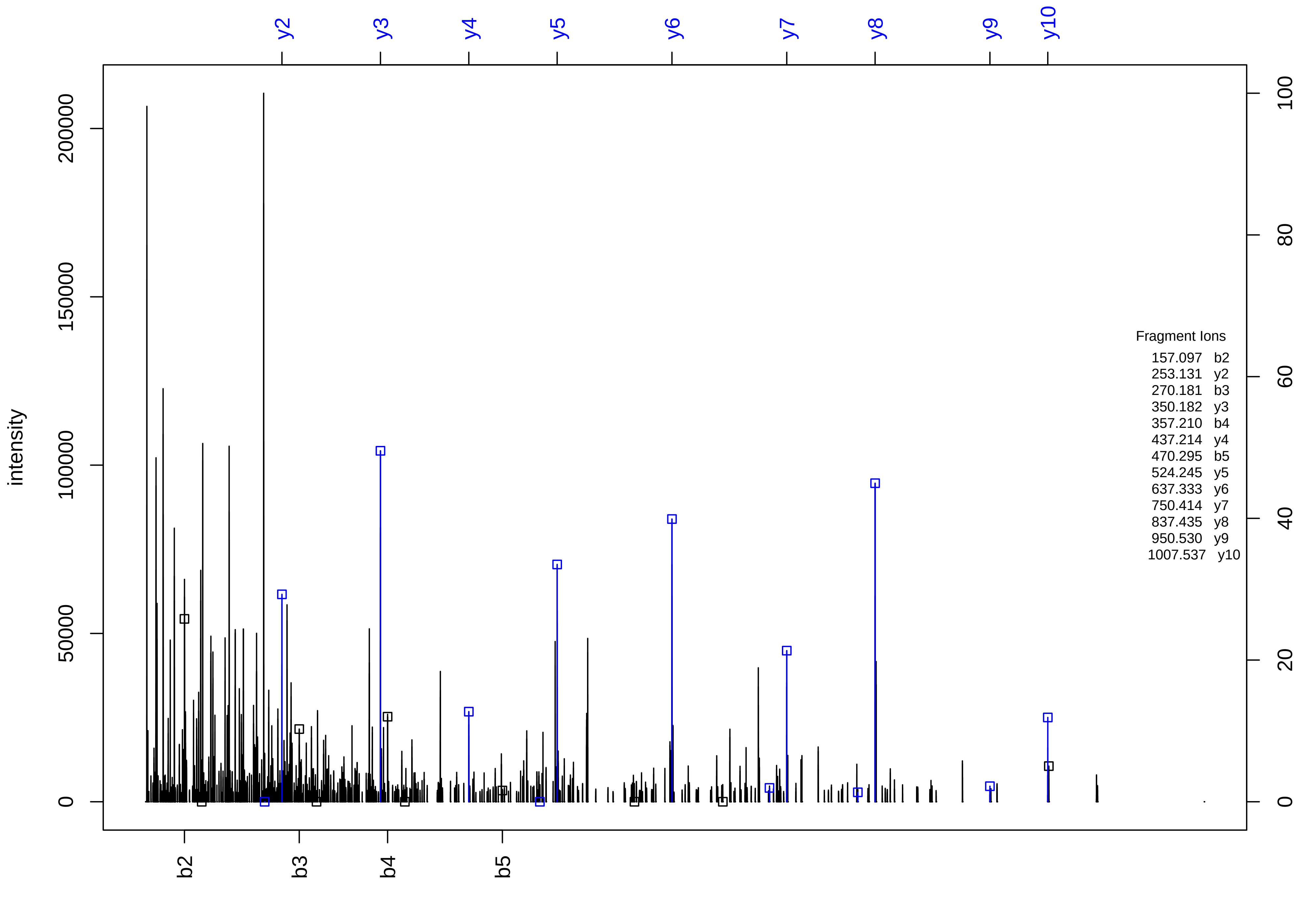

VGISLLSSPHP / File: sydney_ip_29.raw; SpectrumID: ; scans: 12236

Q^AAM*EKGN^R

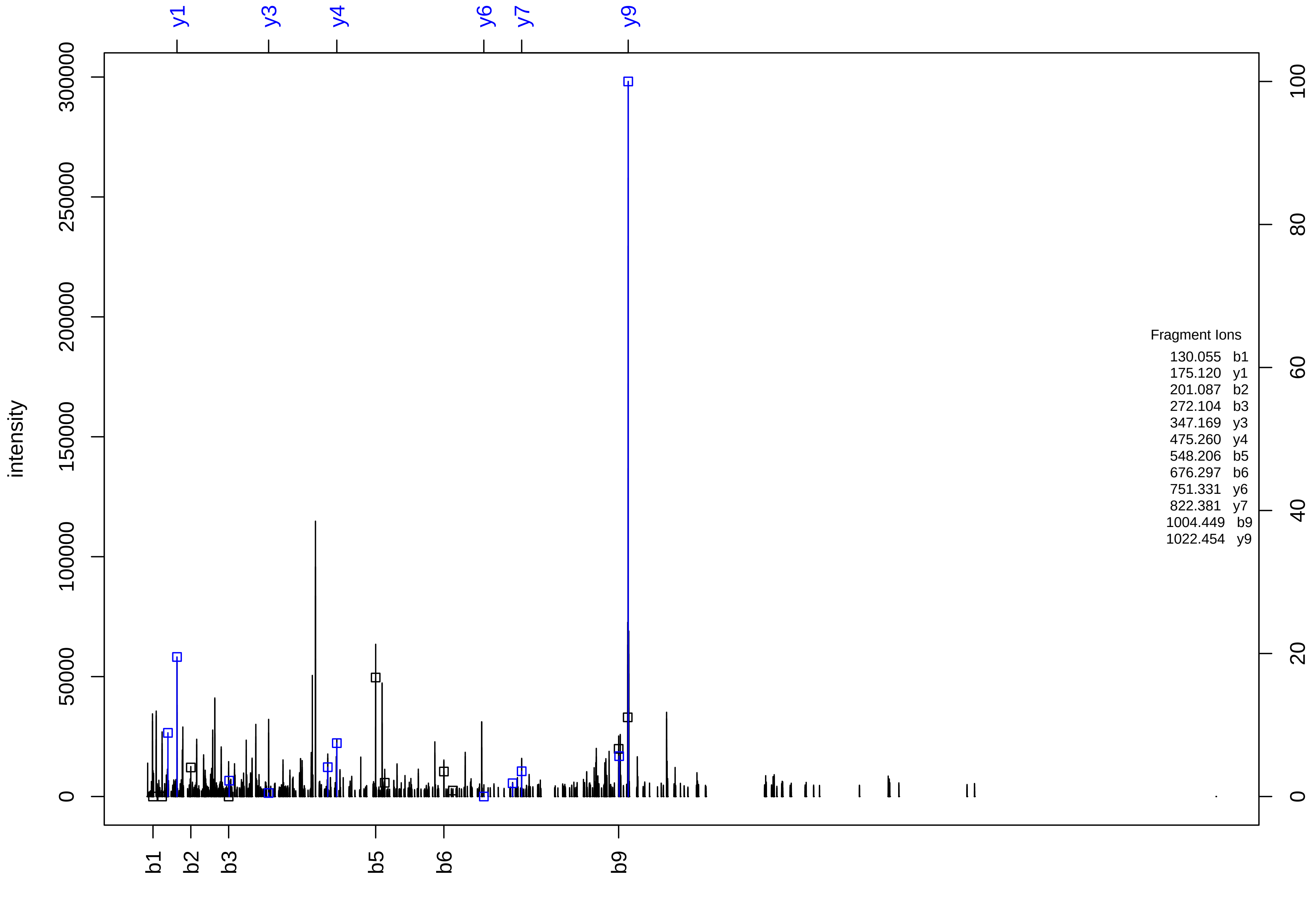

QAAMEKGNR / File: sydney_ip_29.raw; SpectrumID: ; scans: 6756

VIPTLALLR

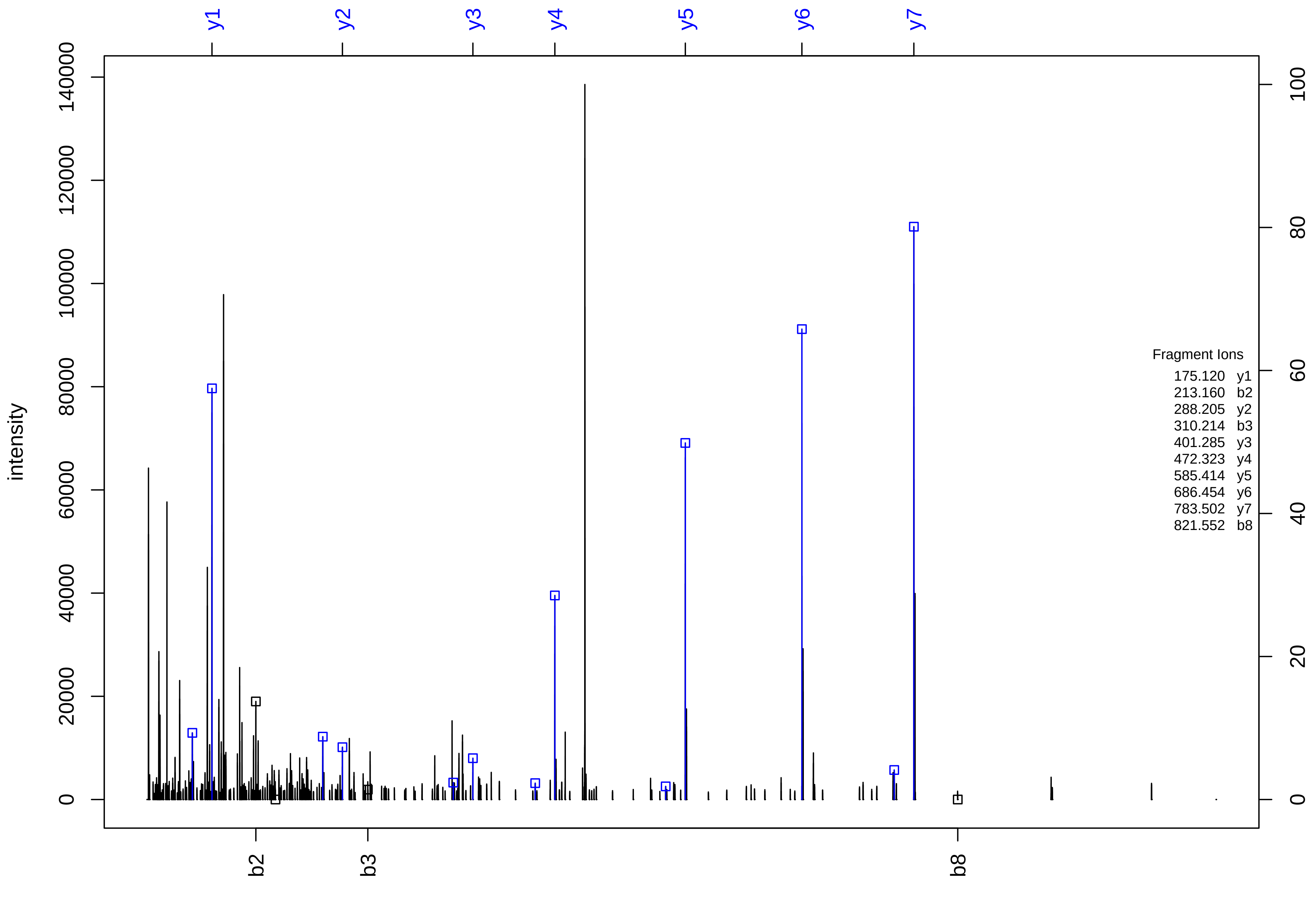

VIPTLALLR / File: sydney_ip_30.raw; SpectrumID: ; scans: 15456

N^RN^YQ^Q^M*SGYIIK

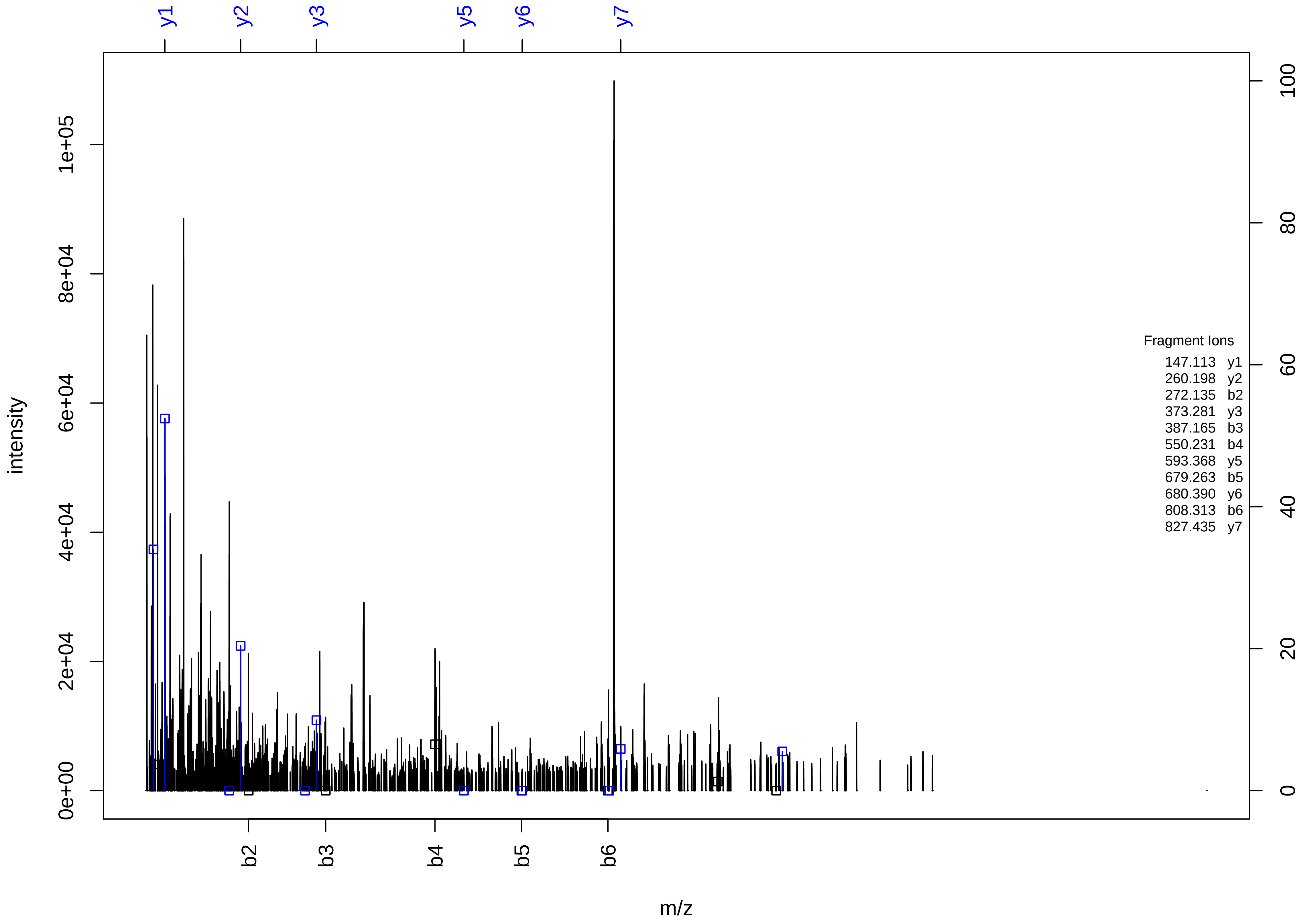

NRNYQQMSGYIIK / File: sydney_ip_30.raw; SpectrumID: ; scans: 8970

M*AGEVTQ^ALGGAQ^K

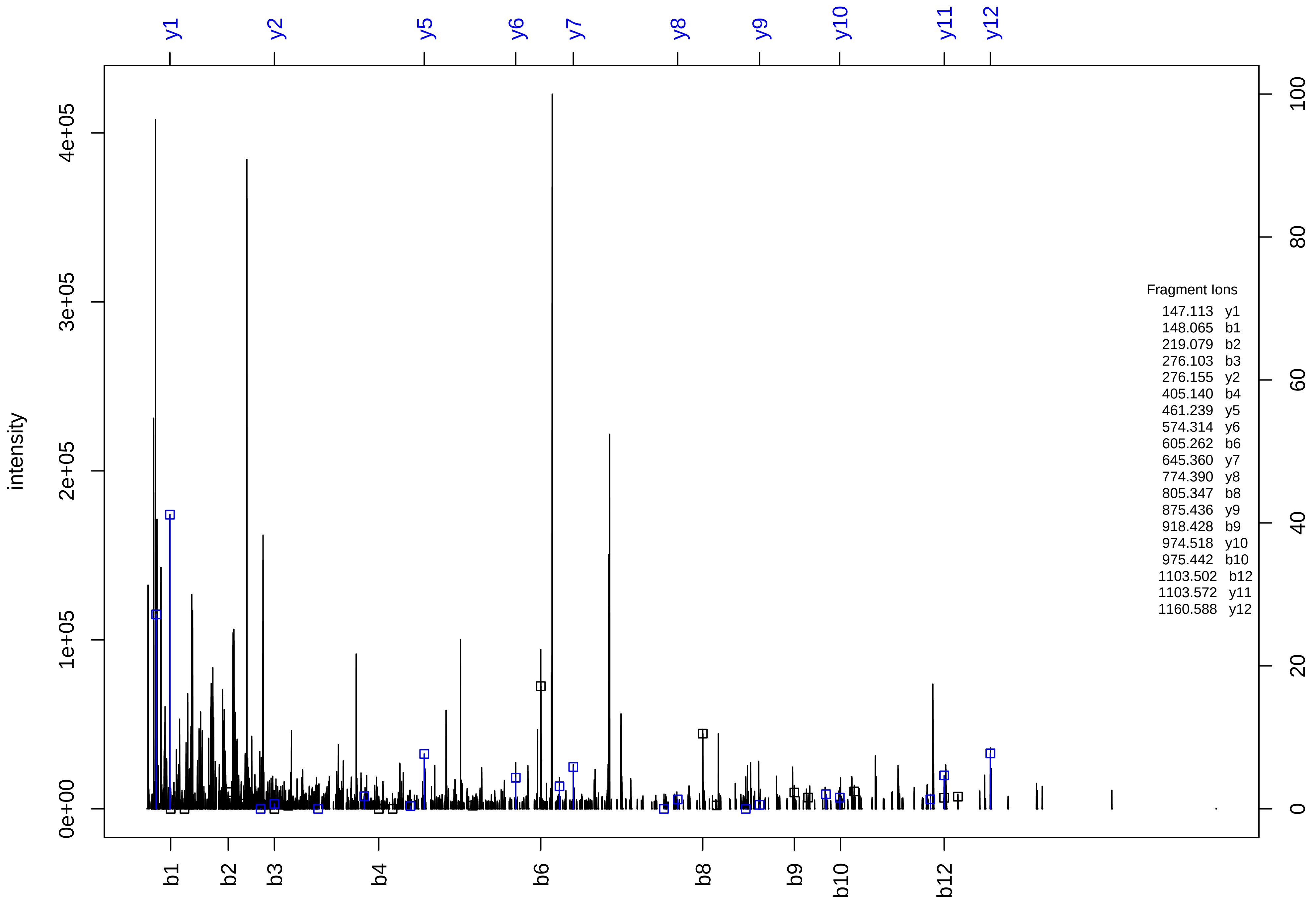

MAGEVTQALGGAQK / File: sydney_ip_30.raw; SpectrumID: ; scans: 8301

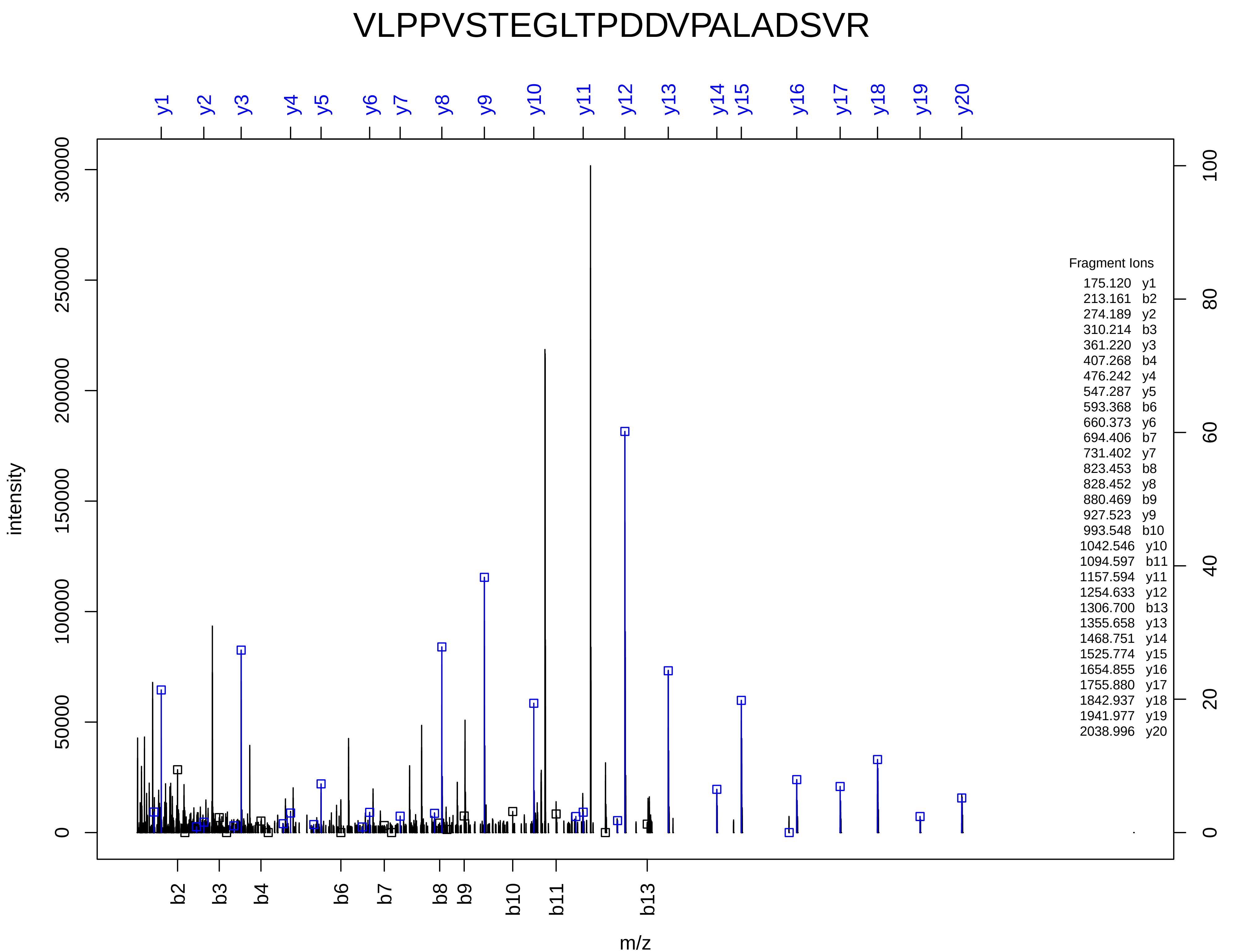

VLPPVSTEGLTPDDVPALADSVR
intensity
m/z
y1 y2 y3 y4 y5 y6 y7 y8 y9 y10 y11 y12 y13 y14 y15 y16 y17 y18 y19 y20
b2 b3 b4 b6 b7 b8 b9 b10 b11 b13
0 50000 100000 150000 200000 250000 300000
0 20 40 60 80 100
Fragment Ions
175.120 y1
213.161 b2
274.189 y2
310.214 b3
361.220 y3
407.268 b4
476.242 y4
547.287 y5
593.368 b6
660.373 y6
694.406 b7
731.402 y7
823.453 b8
828.452 y8
880.469 b9
927.523 y9
993.548 b10
1042.546 y10
1094.597 b11
1157.594 y11
1254.633 y12
1306.700 b13
1355.658 y13
1468.751 y14
1525.774 y15
1654.855 y16
1755.880 y17
1842.937 y18
1941.977 y19
2038.996 y20
VLPPVSTEGLTPDDVPALADSVR / File: sydney_ip_30.raw; SpectrumID: ; scans: 16773

SEQQPQEEEEPERR

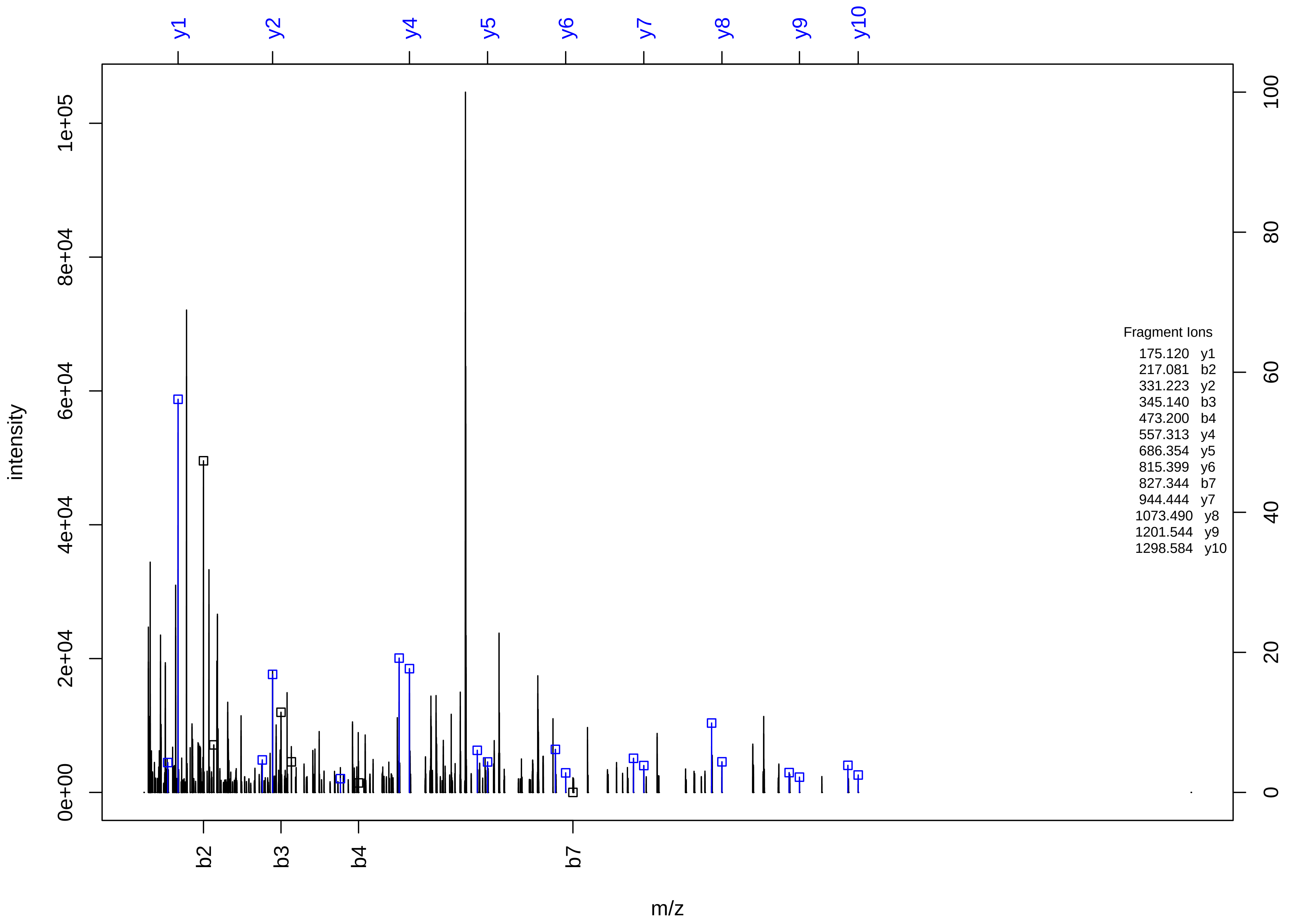

SEQQPQEEEEPERR / File: sydney_ip_30.raw; SpectrumID: ; scans: 1023

FETPLDLAALYGR

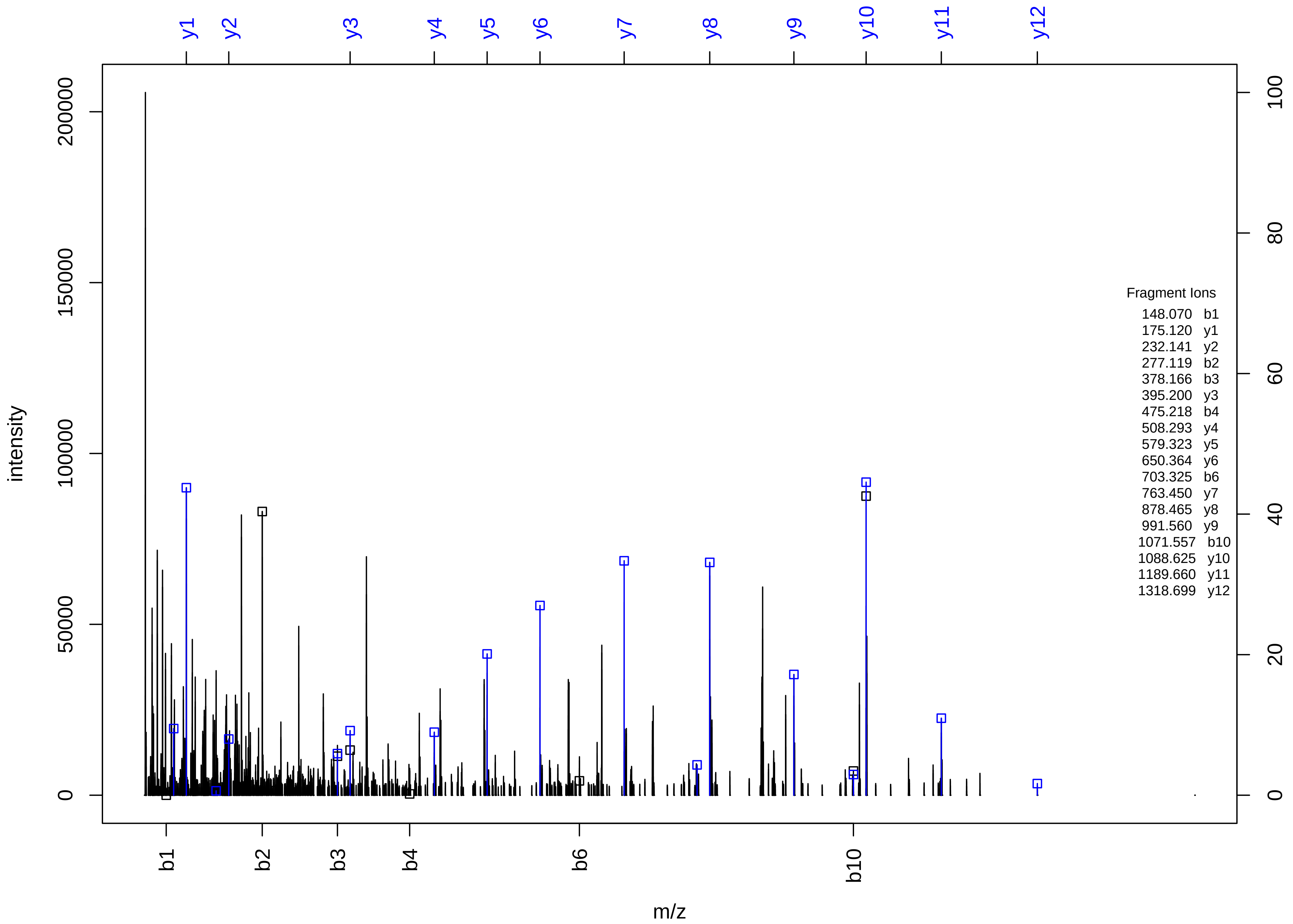

FETPLDLAALYGR / File: sydney_ip_30.raw; SpectrumID: ; scans: 18481

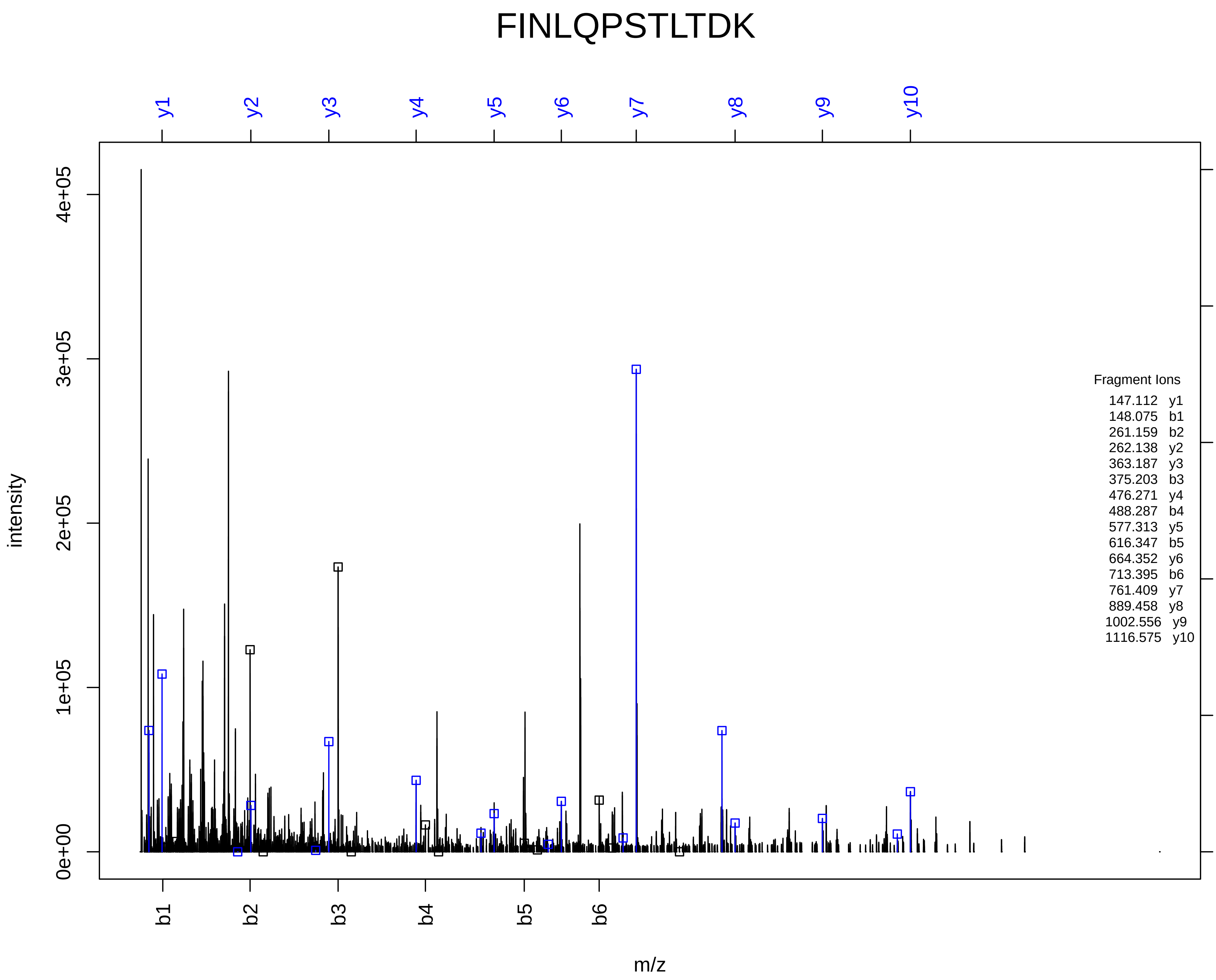

FINLQPSTLTDK / File: sydney_ip_30.raw; SpectrumID: ; scans: 11277

EELAPQEPQWSLPSK

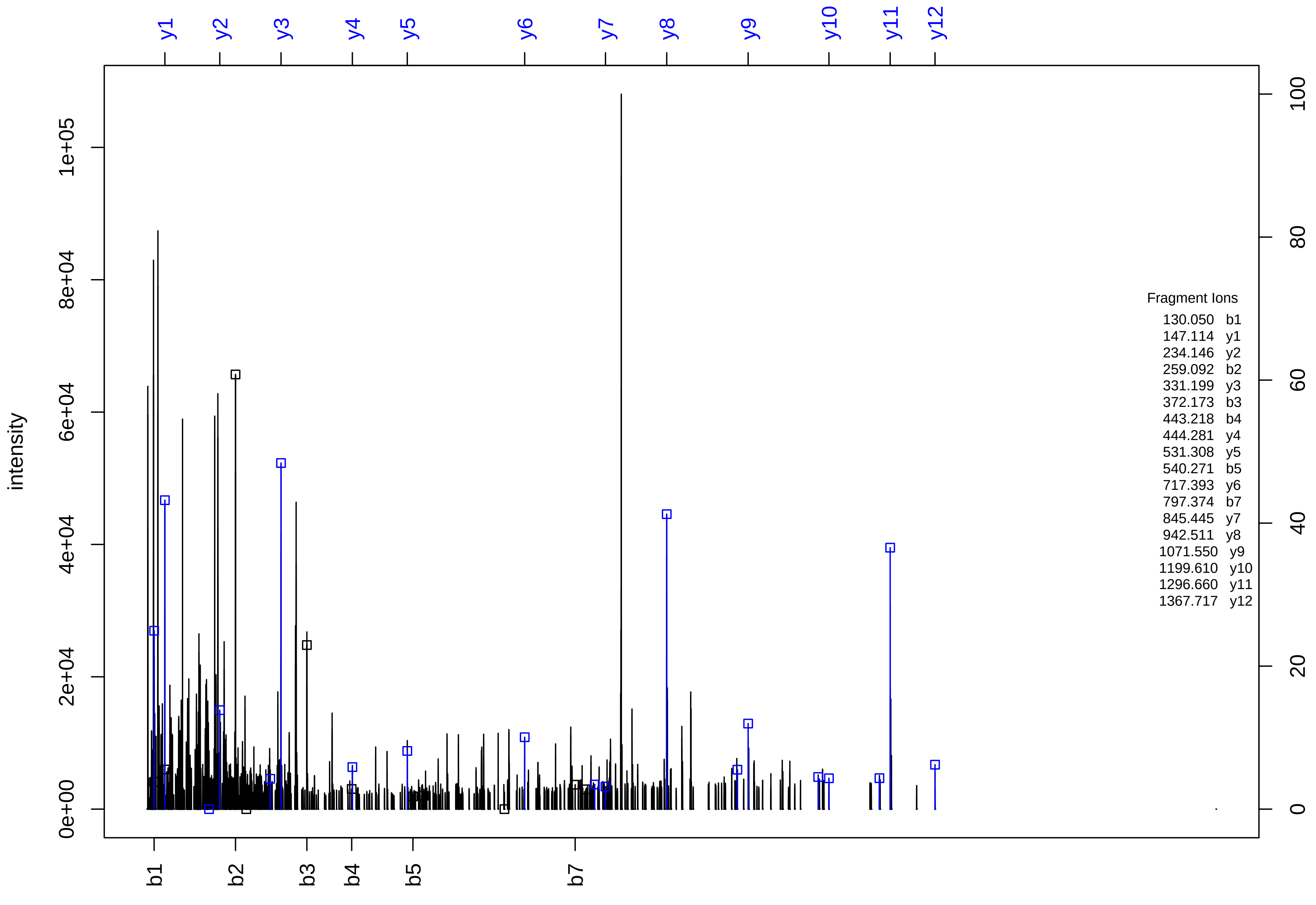

EELAPQEPQWSLPSK / File: sydney_ip_30.raw; SpectrumID: ; scans: 11348

EQ^Q^LLQDKQIEEEKQ^Q^TER

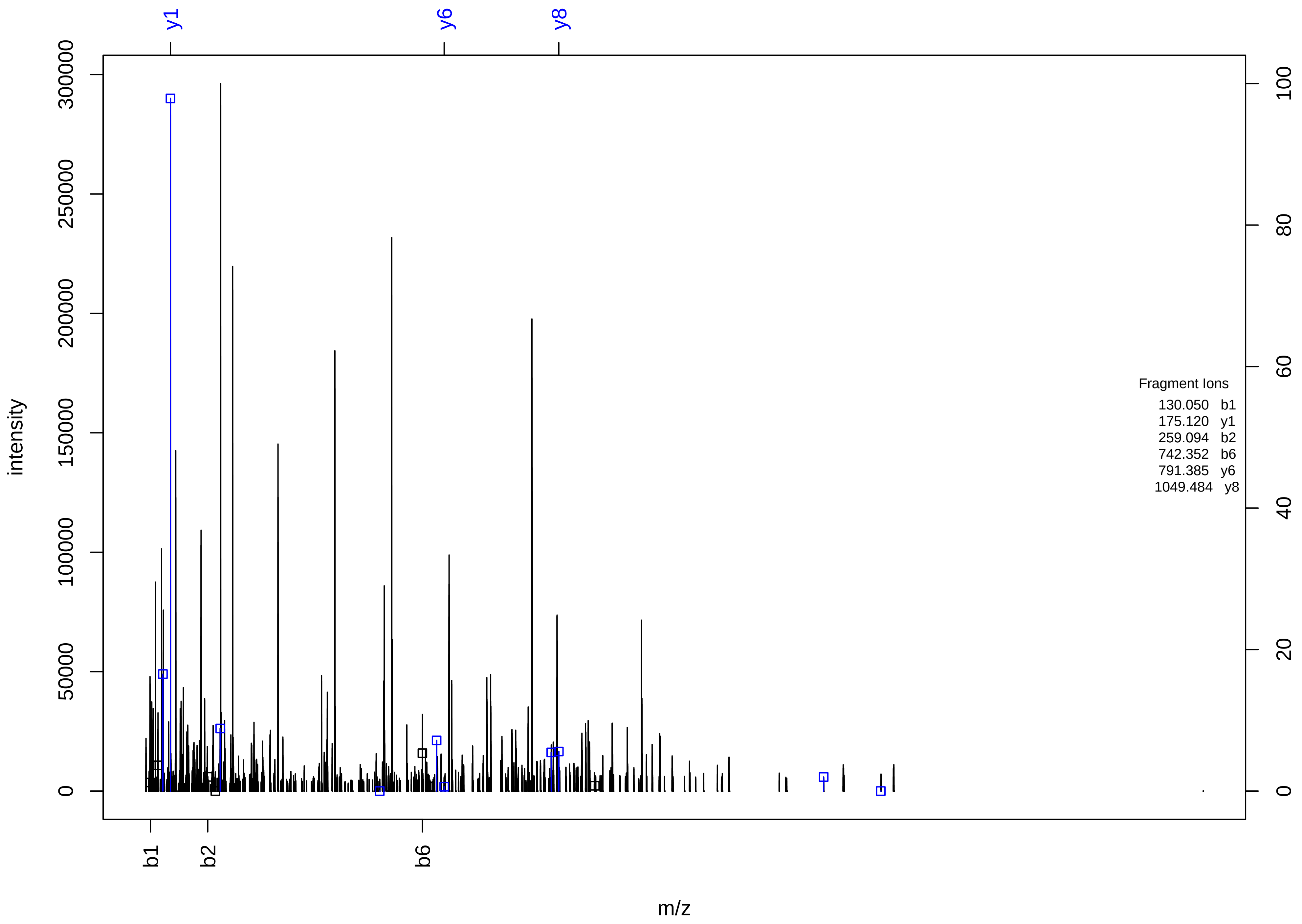

EQQLLQDKQIEEEKQQTER / File: sydney_ip_30.raw; SpectrumID: ; scans: 5614

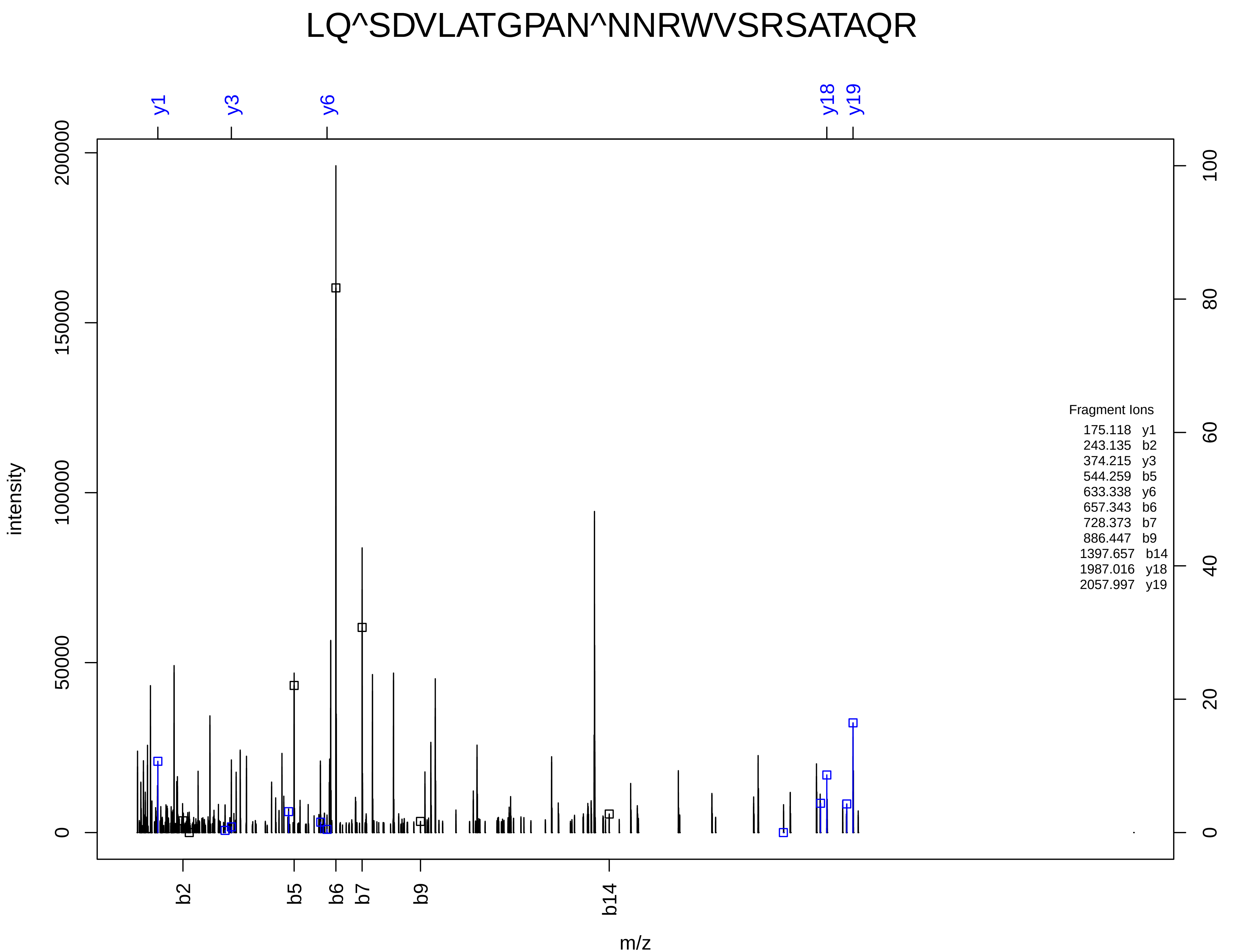

LQSDVLATGPANNNRWVSRSATAQR / File: sydney_ip_30.raw; SpectrumID: ; scans: 18570

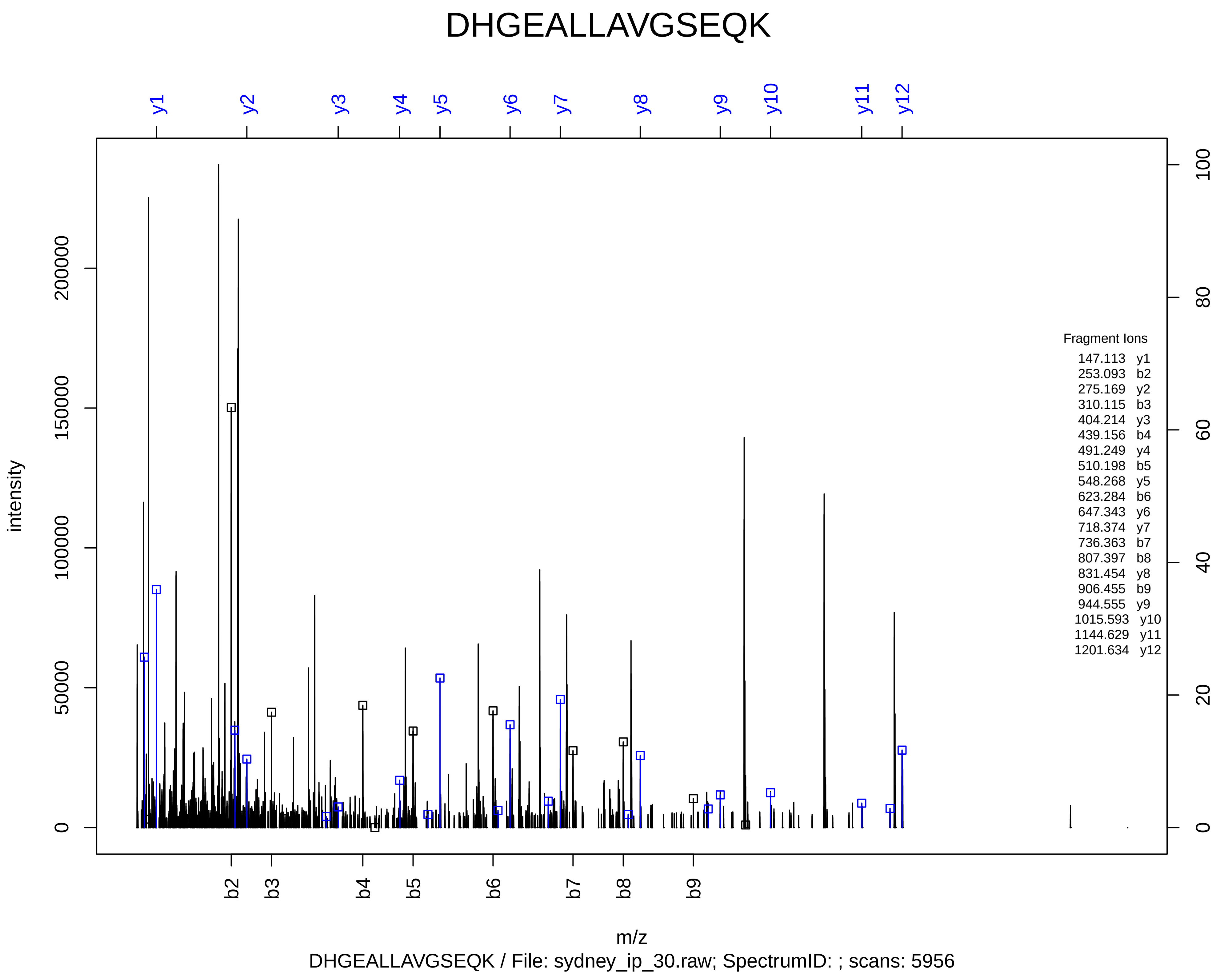
DHGEALLAVGSEQK
y1
y2
y3
y4
y5
y6
y7
y8
y9
y10
y11
y12
b2
b3
b4
b5
b6
b7
b8
b9
intensity
m/z
Fragment Ions
147.113 y1
253.093 b2
275.169 y2
310.115 b3
404.214 y3
439.156 b4
491.249 y4
510.198 b5
548.268 y5
623.284 b6
647.343 y6
718.374 y7
736.363 b7
807.397 b8
831.454 y8
906.455 b9
944.555 y9
1015.593 y10
1144.629 y11
1201.634 y12
DHGEALLAVGSEQK / File: sydney_ip_30.raw; SpectrumID: ; scans: 5956

M*VGTVIM*MKM*N^EDGLTPEQ^R

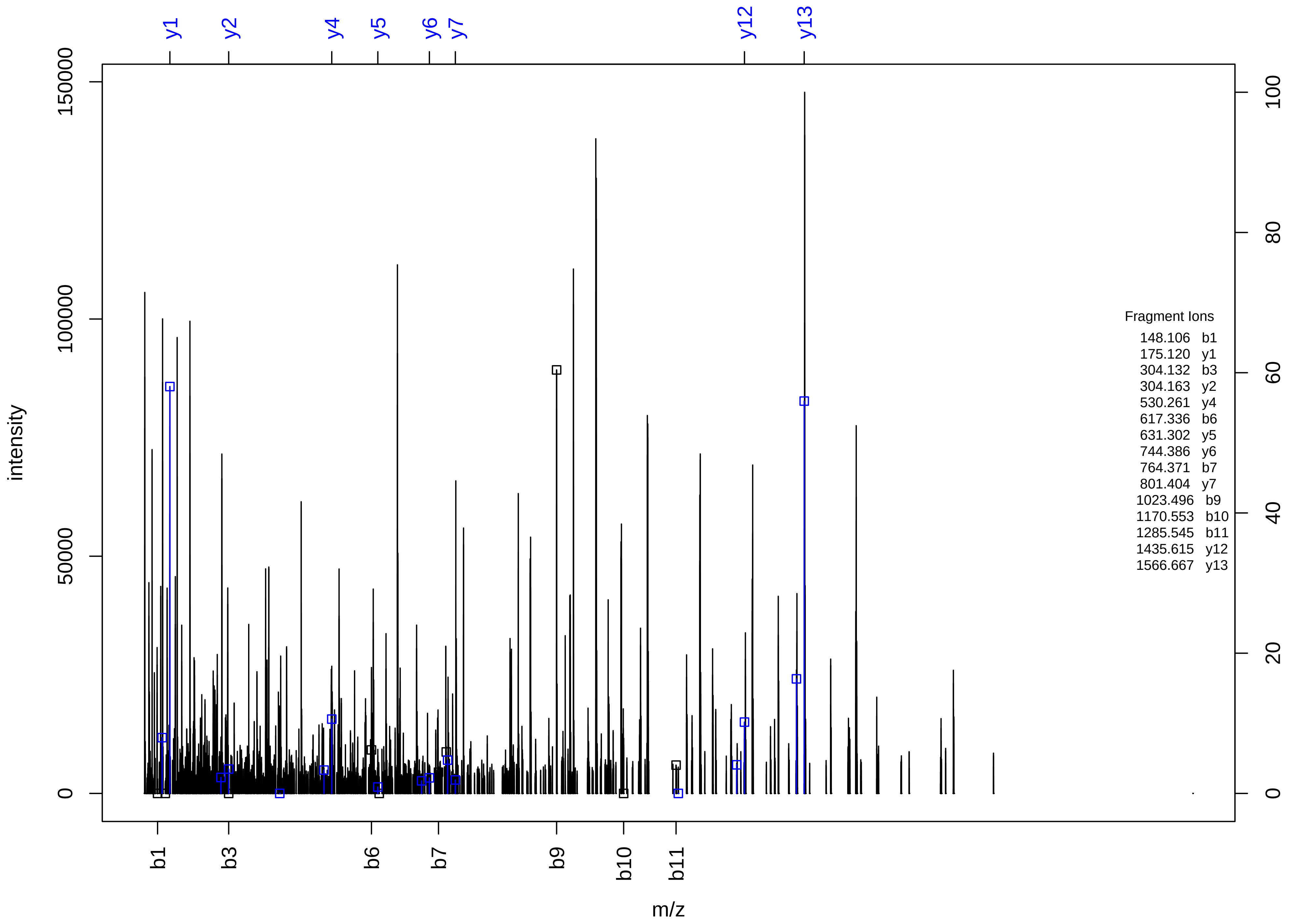

MVGTVIMMKMNEDGLTPEQR / File: sydney_ip_30.raw; SpectrumID: ; scans: 15027

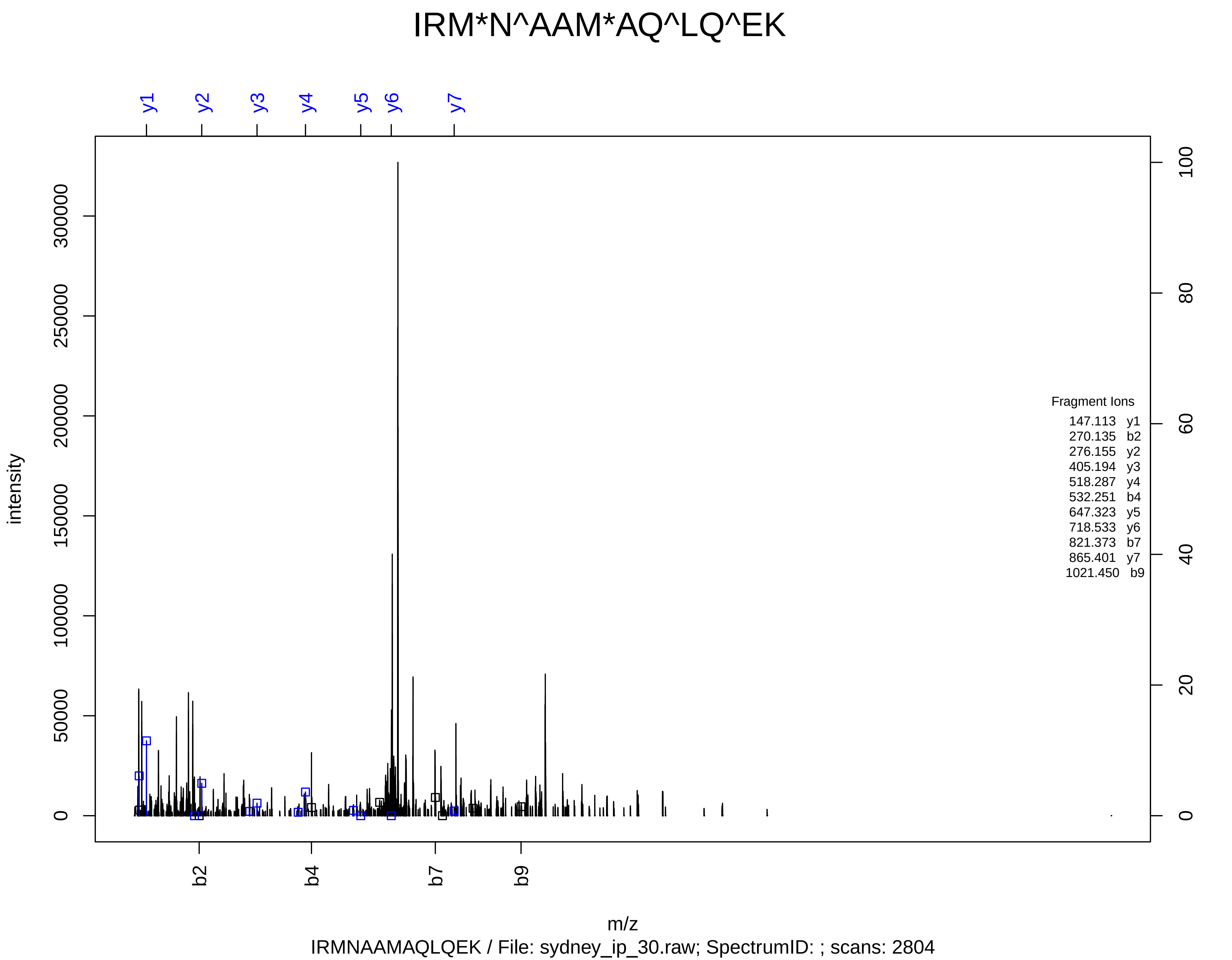
IRM*N^AAM*AQ^LQ^EK
y1
y2
y3
y4
y5
y6
y7
intensity
0
50000
100000
150000
200000
250000
300000
0
20
40
60
80
100
b2
b4
b7
b9
m/z
Fragment Ions
147.113 y1
270.135 b2
276.155 y2
405.194 y3
518.287 y4
532.251 b4
647.323 y5
718.533 y6
821.373 b7
865.401 y7
1021.450 b9
IRMNAAMAQLQEK / File: sydney_ip_30.raw; SpectrumID: ; scans: 2804

VAGQDGSVVQFK

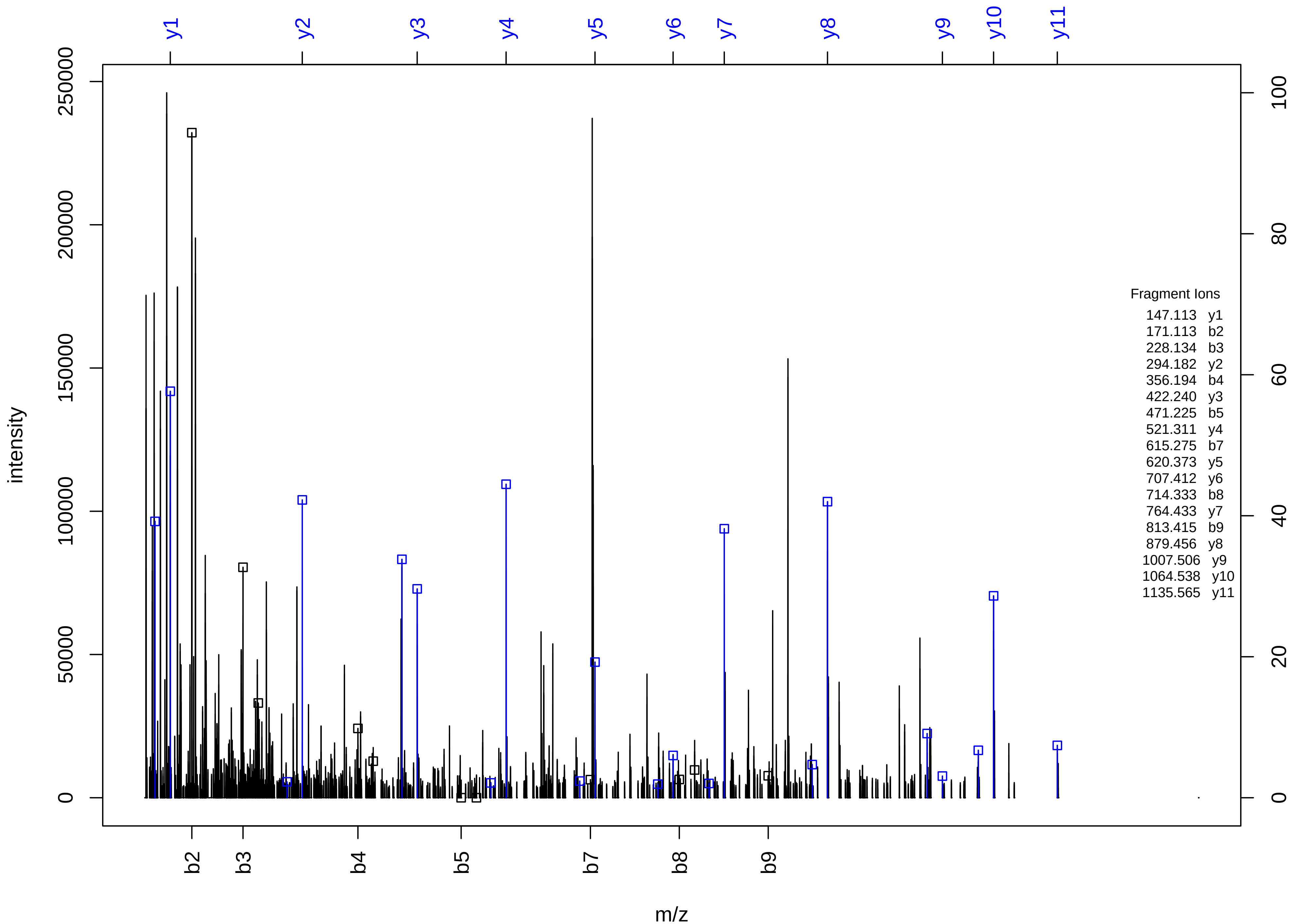

VAGQDGSVVQFK / File: sydney_ip_30.raw; SpectrumID: ; scans: 5716

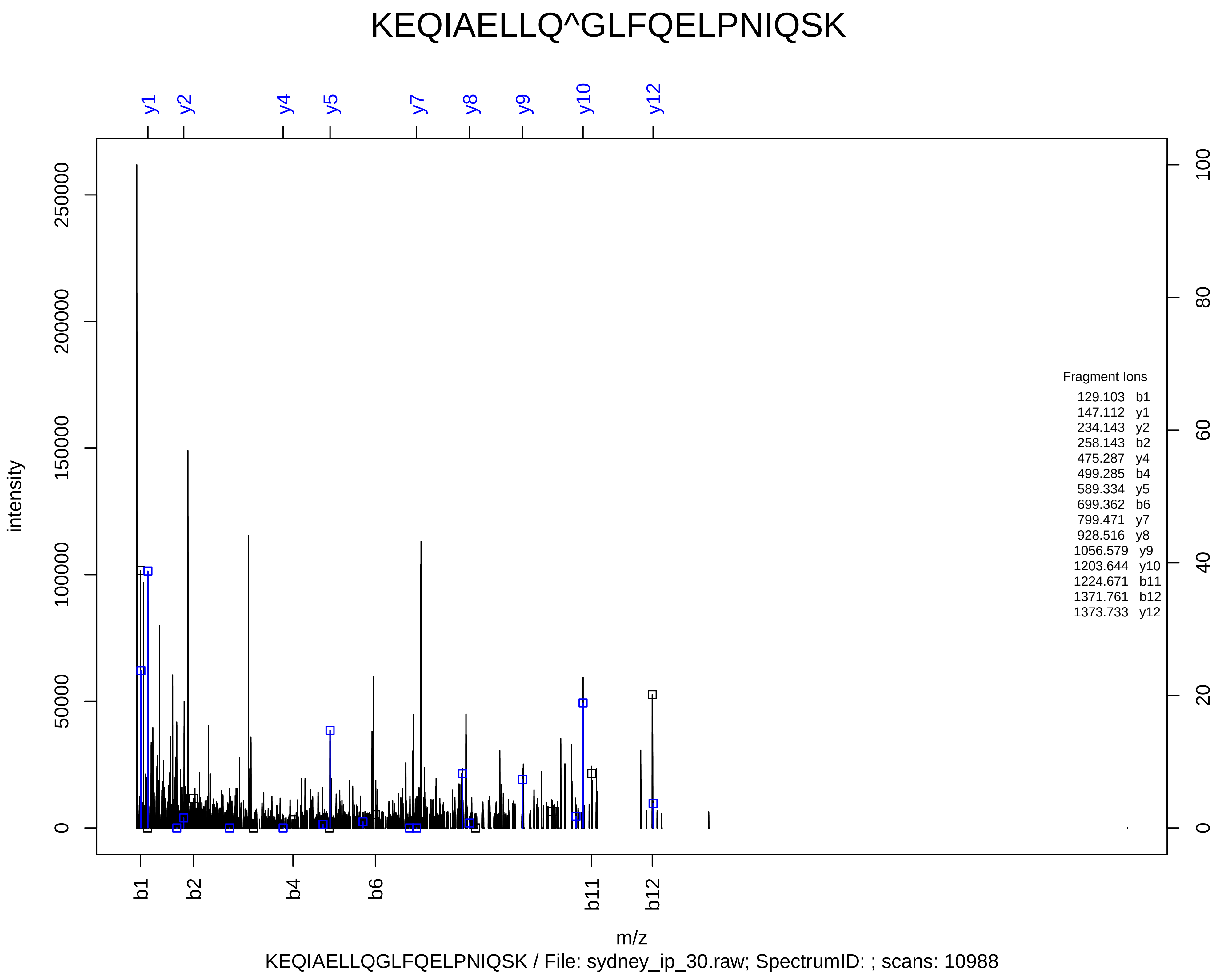
KEQIAELLQ^GLFQELPNIQSK
y1
y2
y4
y5
y7
y8
y9
y10
y12
intensity
0
50000
100000
150000
200000
250000
0
20
40
60
80
100
b1
b2
b4
b6
b11
b12
m/z
Fragment Ions
129.103 b1
147.112 y1
234.143 y2
258.143 b2
475.287 y4
499.285 b4
589.334 y5
699.362 b6
799.471 y7
928.516 y8
1056.579 y9
1203.644 y10
1224.671 b11
1371.761 b12
1373.733 y12
KEQIAELLQGLFQELPNIQSK / File: sydney_ip_30.raw; SpectrumID: ; scans: 10988

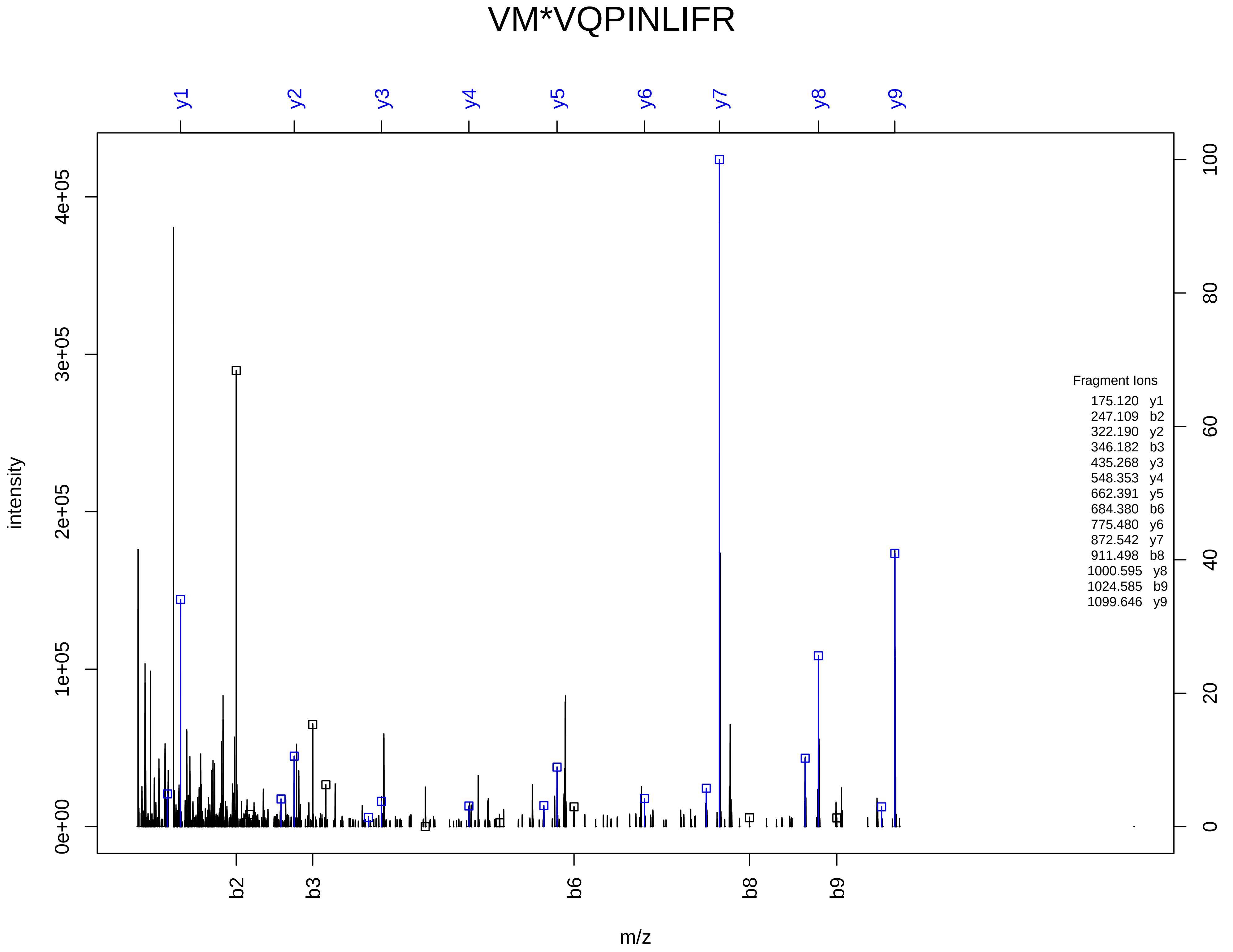

VM*VQPINLIFR
y1
y2
y3
y4
y5
y6
y7
y8
y9
intensity
0e+00
1e+05
2e+05
3e+05
4e+05
0
20
40
60
80
100
b2
b3
b6
b8
b9
m/z
Fragment Ions
175.120 y1
247.109 b2
322.190 y2
346.182 b3
435.268 y3
548.353 y4
662.391 y5
684.380 b6
775.480 y6
872.542 y7
911.498 b8
1000.595 y8
1024.585 b9
1099.646 y9
VMVQPINLIFR / File: sydney_ip_30.raw; SpectrumID: ; scans: 16549

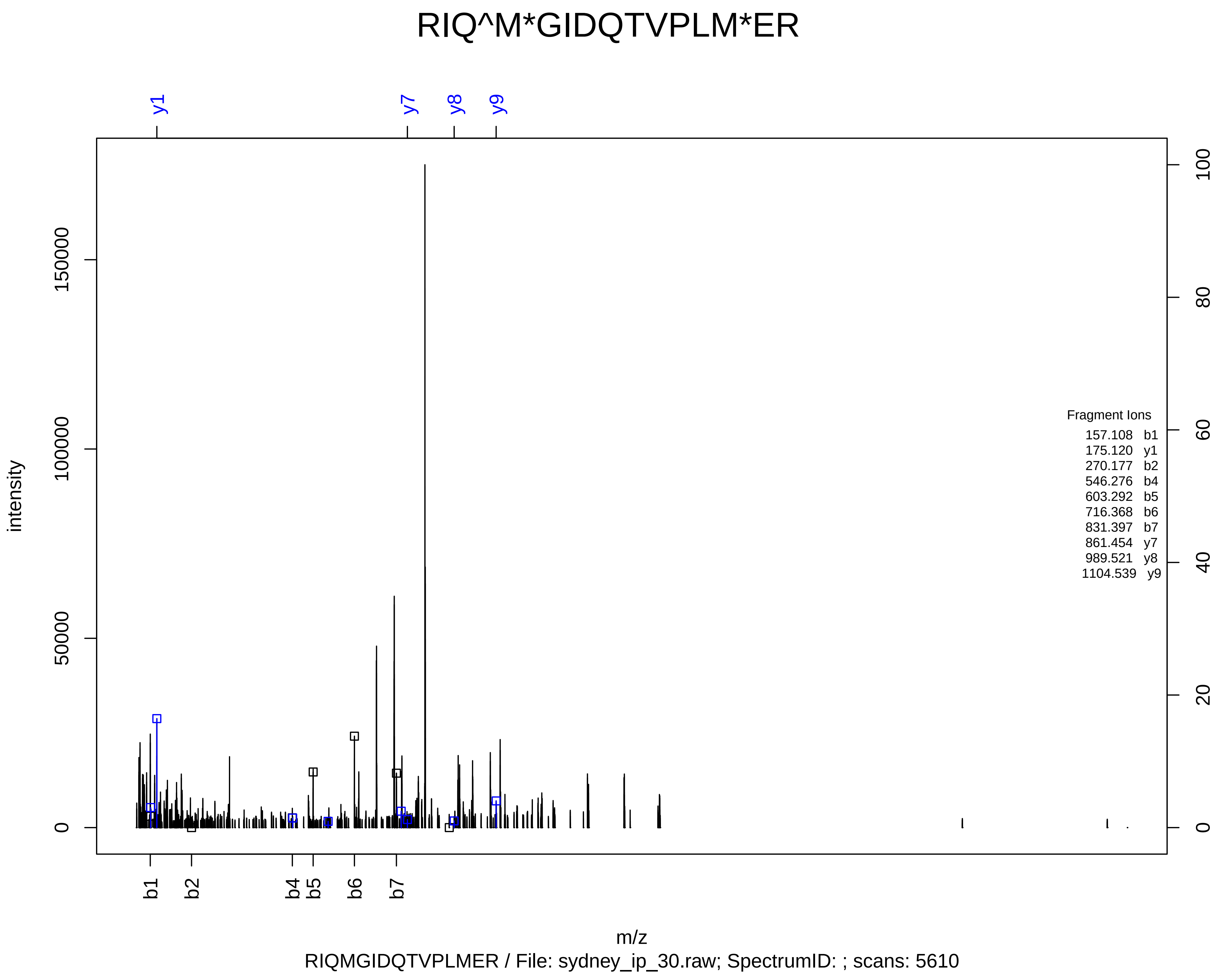
RIQ^M*GIDQTVPLM*ER
y1
y7
y8
y9
intensity
0
50000
100000
150000
0
20
40
60
80
100
b1
b2
b4
b5
b6
b7
m/z
Fragment Ions
157.108 b1
175.120 y1
270.177 b2
546.276 b4
603.292 b5
716.368 b6
831.397 b7
861.454 y7
989.521 y8
1104.539 y9
RIQMGIDQTVPLMER / File: sydney_ip_30.raw; SpectrumID: ; scans: 5610

IQ^LM*LN^Q^RK

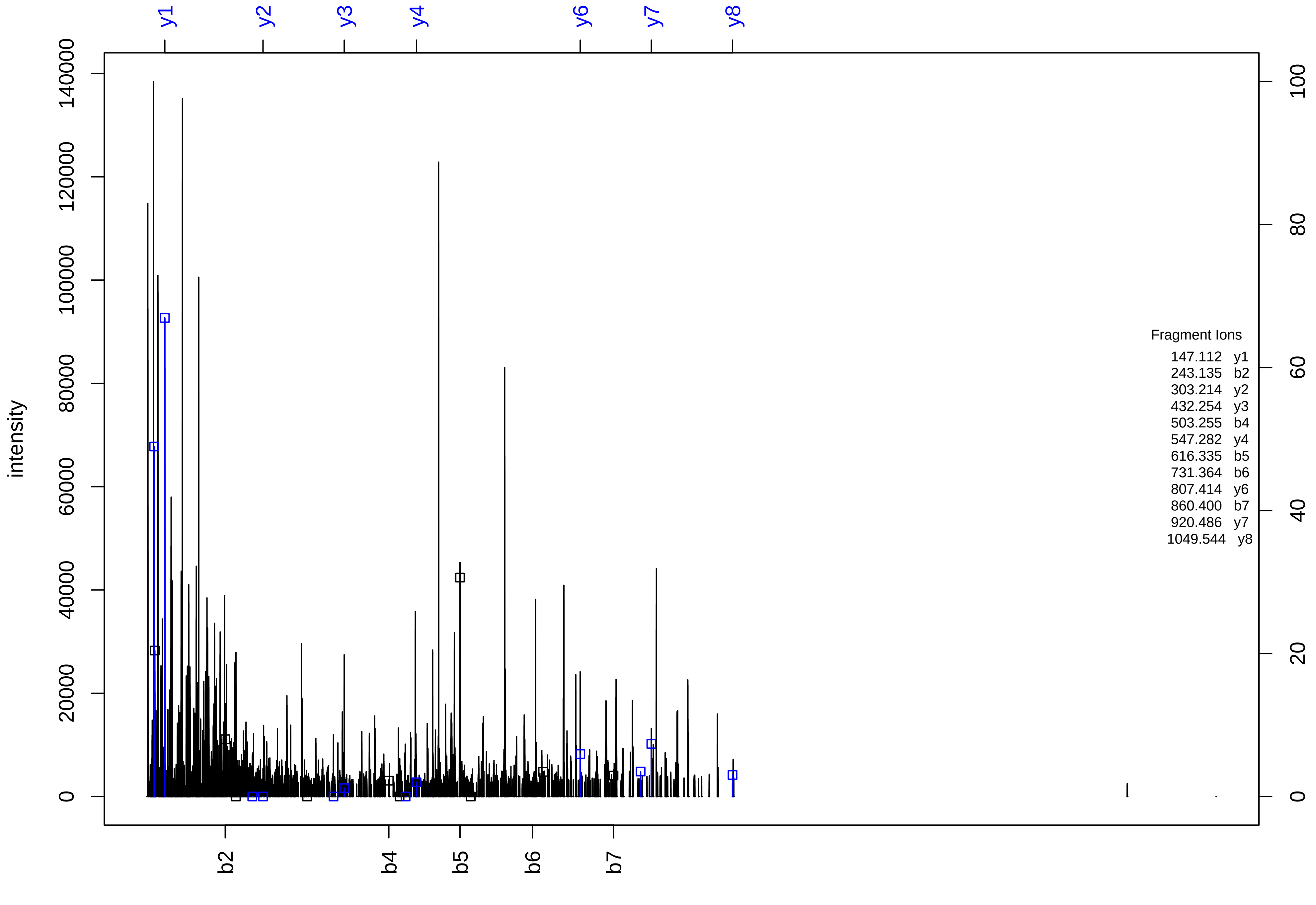

IQLMLNQRK / File: sydney_ip_30.raw; SpectrumID: ; scans: 8032

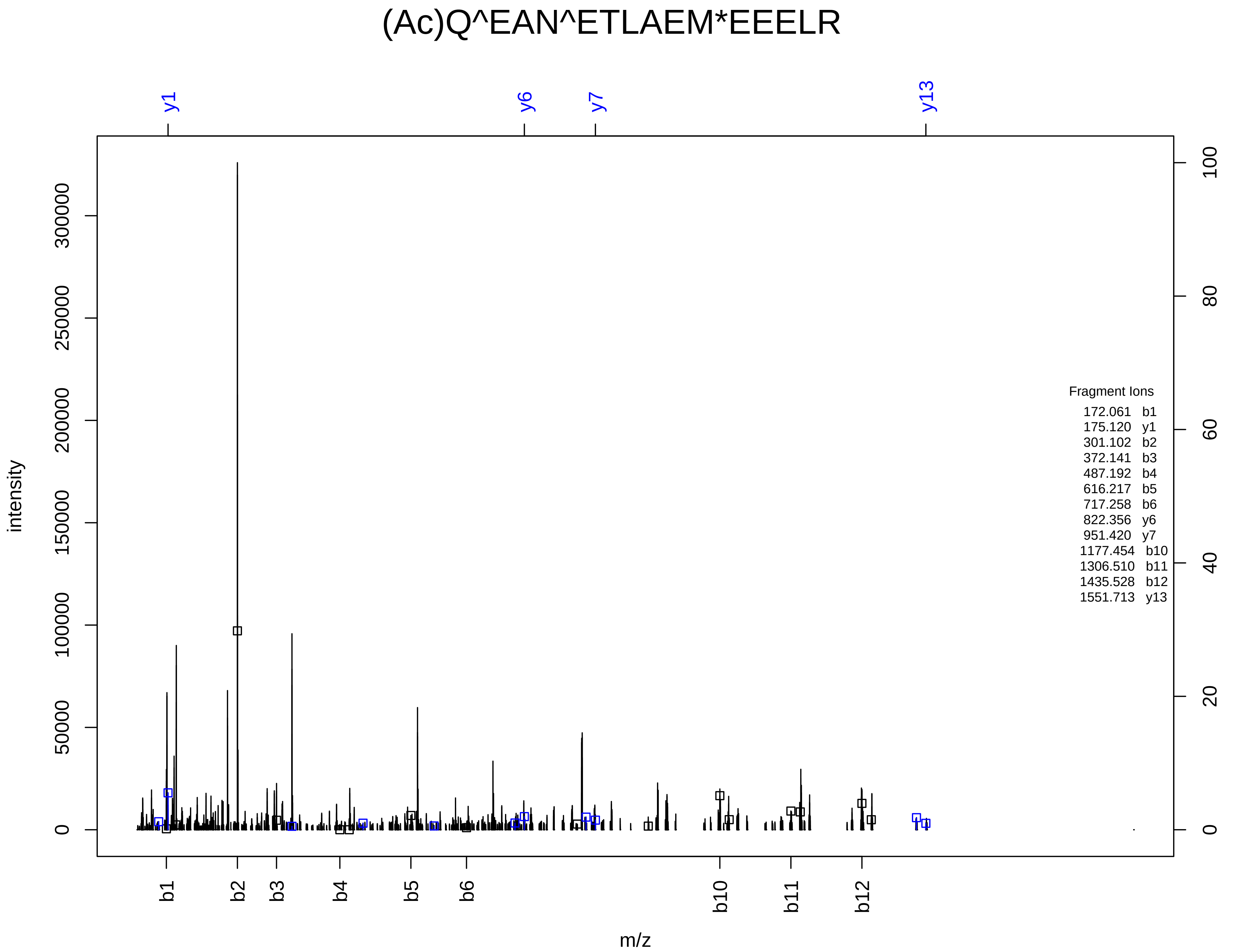

(Ac)Q^EAN^ETLAEM*EEELR
y1
y6
y7
y13
intensity
0
50000
100000
150000
200000
250000
300000
0
20
40
60
80
100
Fragment Ions
172.061 b1
175.120 y1
301.102 b2
372.141 b3
487.192 b4
616.217 b5
717.258 b6
822.356 y6
951.420 y7
1177.454 b10
1306.510 b11
1435.528 b12
1551.713 y13
b1
b2
b3
b4
b5
b6
b10
b11
b12
m/z
QEANETLAEMEEELR / File: sydney_ip_30.raw; SpectrumID: ; scans: 3814

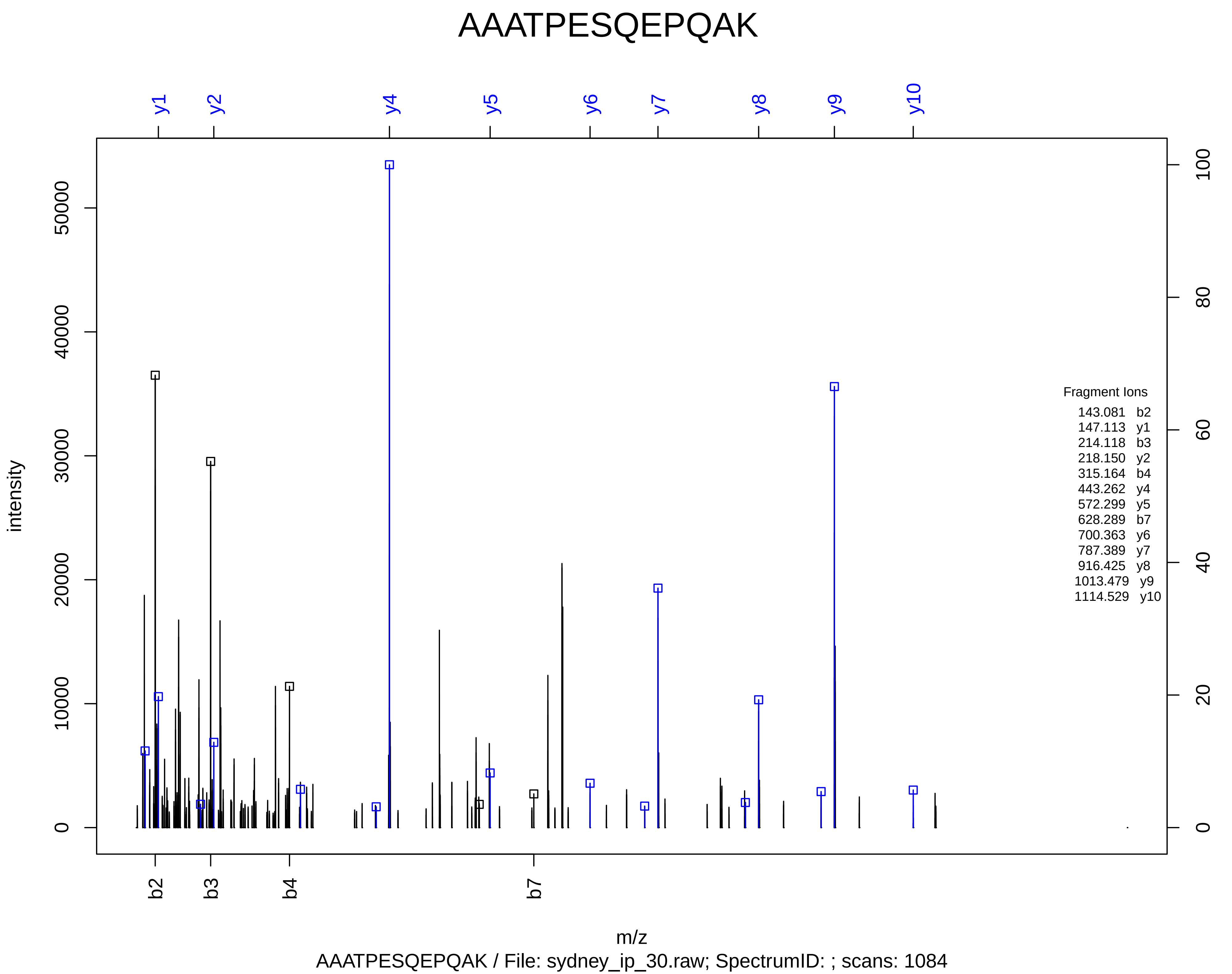
AAATPESQEPQAK
y1
y2
y4
y5
y6
y7
y8
y9
y10
intensity
0
10000
20000
30000
40000
50000
0
20
40
60
80
100
b2
b3
b4
b7
Fragment Ions
143.081 b2
147.113 y1
214.118 b3
218.150 y2
315.164 b4
443.262 y4
572.299 y5
628.289 b7
700.363 y6
787.389 y7
916.425 y8
1013.479 y9
1114.529 y10
m/z
AAATPESQEPQAK / File: sydney_ip_30.raw; SpectrumID: ; scans: 1084

DNYVPEVSALDQEIIEVDPDTK

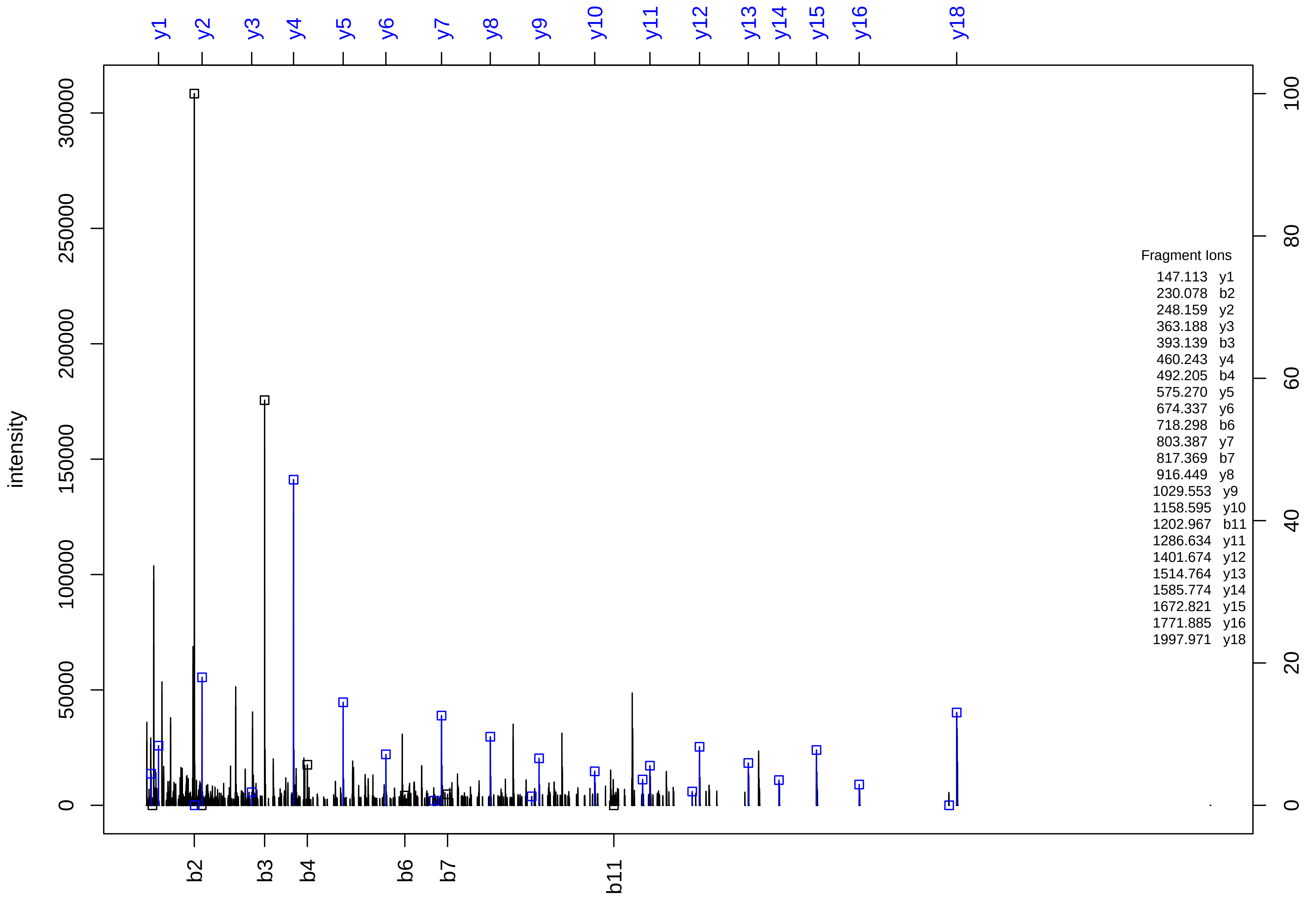

DNYVPEVSALDQEIIEVDPDTK / File: sydney_ip_30.raw; SpectrumID: ; scans: 18403

Q^LAAQFMN^GSLSEEDKR

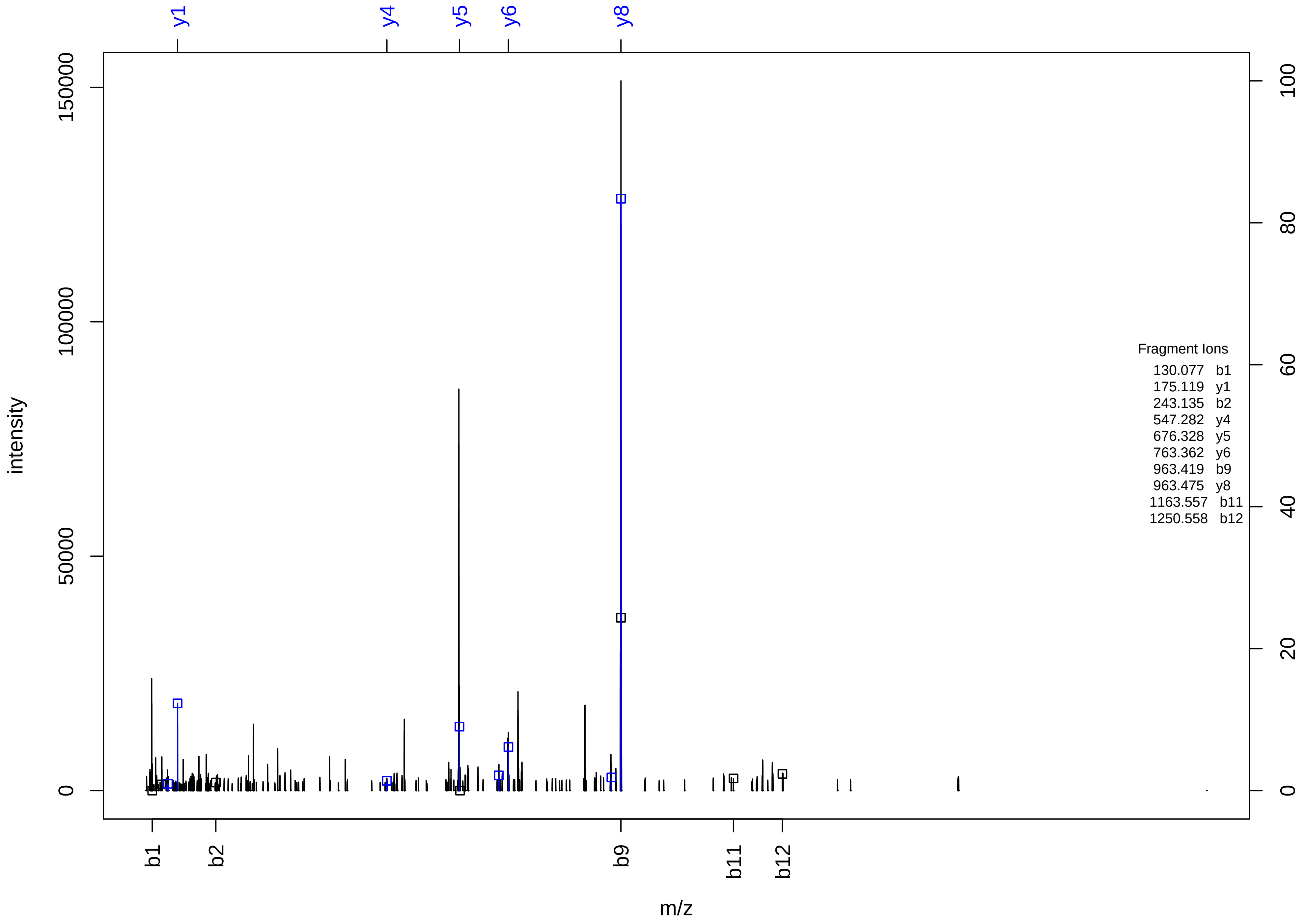

QLAAQFMNGSLSEEDKR / File: sydney_ip_30.raw; SpectrumID: ; scans: 4033

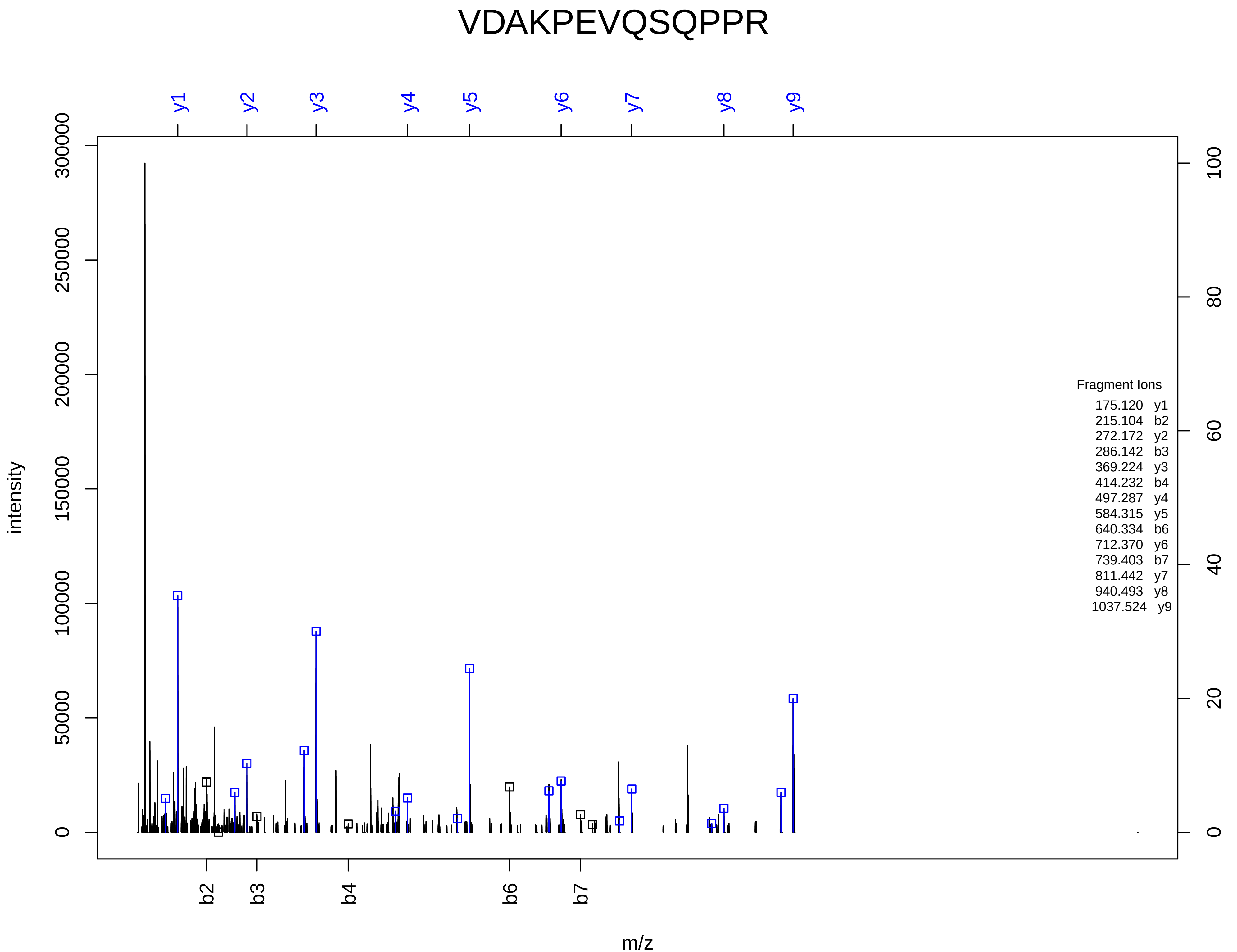

VDAKPEVQSQPPR / File: sydney_ip_30.raw; SpectrumID: ; scans: 1964

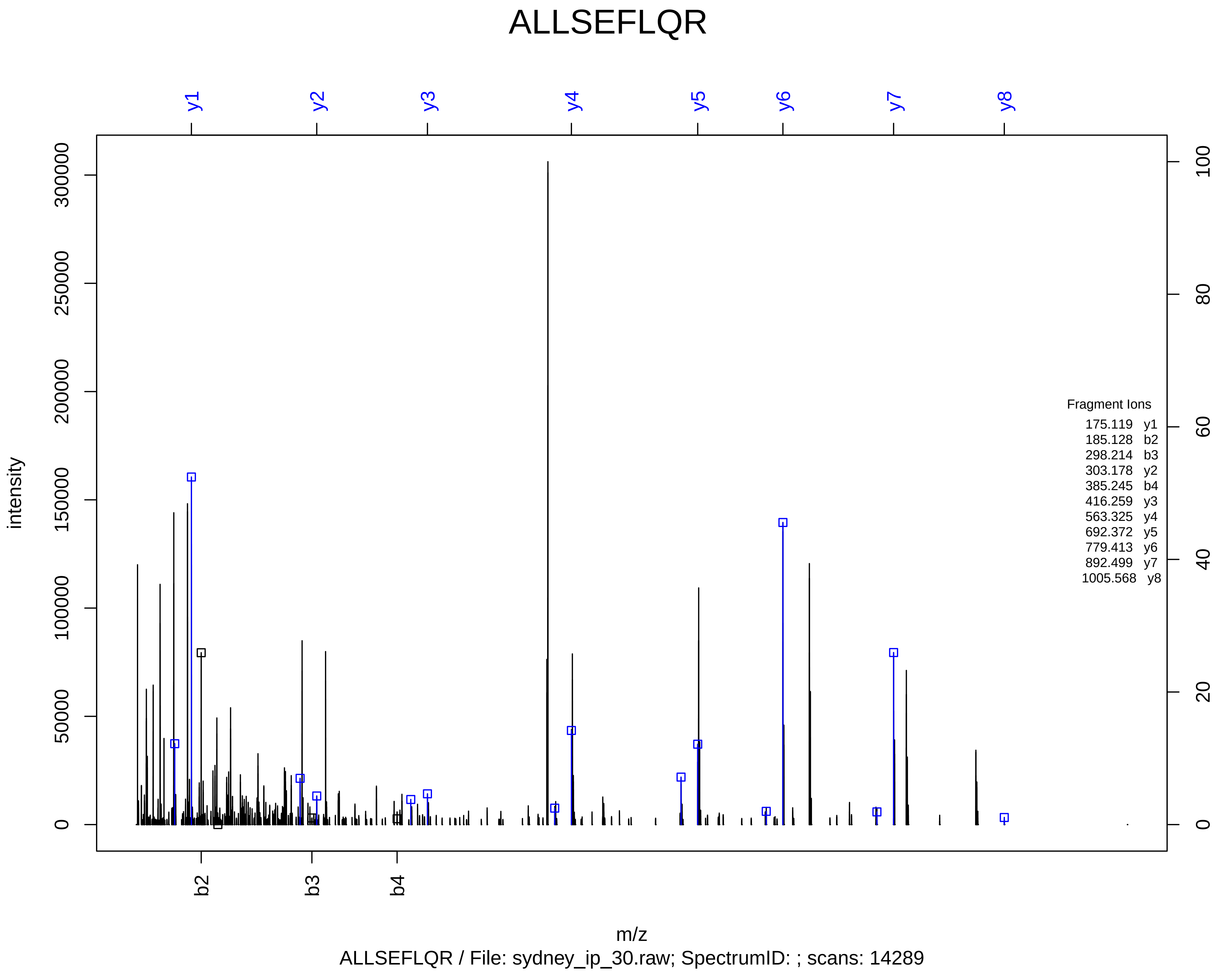
ALLSEFLQR
y1
y2
y3
y4
y5
y6
y7
y8
b2
b3
b4
intensity
0
50000
100000
150000
200000
250000
300000
0
20
40
60
80
100
m/z
Fragment Ions
175.119 y1
185.128 b2
298.214 b3
303.178 y2
385.245 b4
416.259 y3
563.325 y4
692.372 y5
779.413 y6
892.499 y7
1005.568 y8
ALLSEFLQR / File: sydney_ip_30.raw; SpectrumID: ; scans: 14289

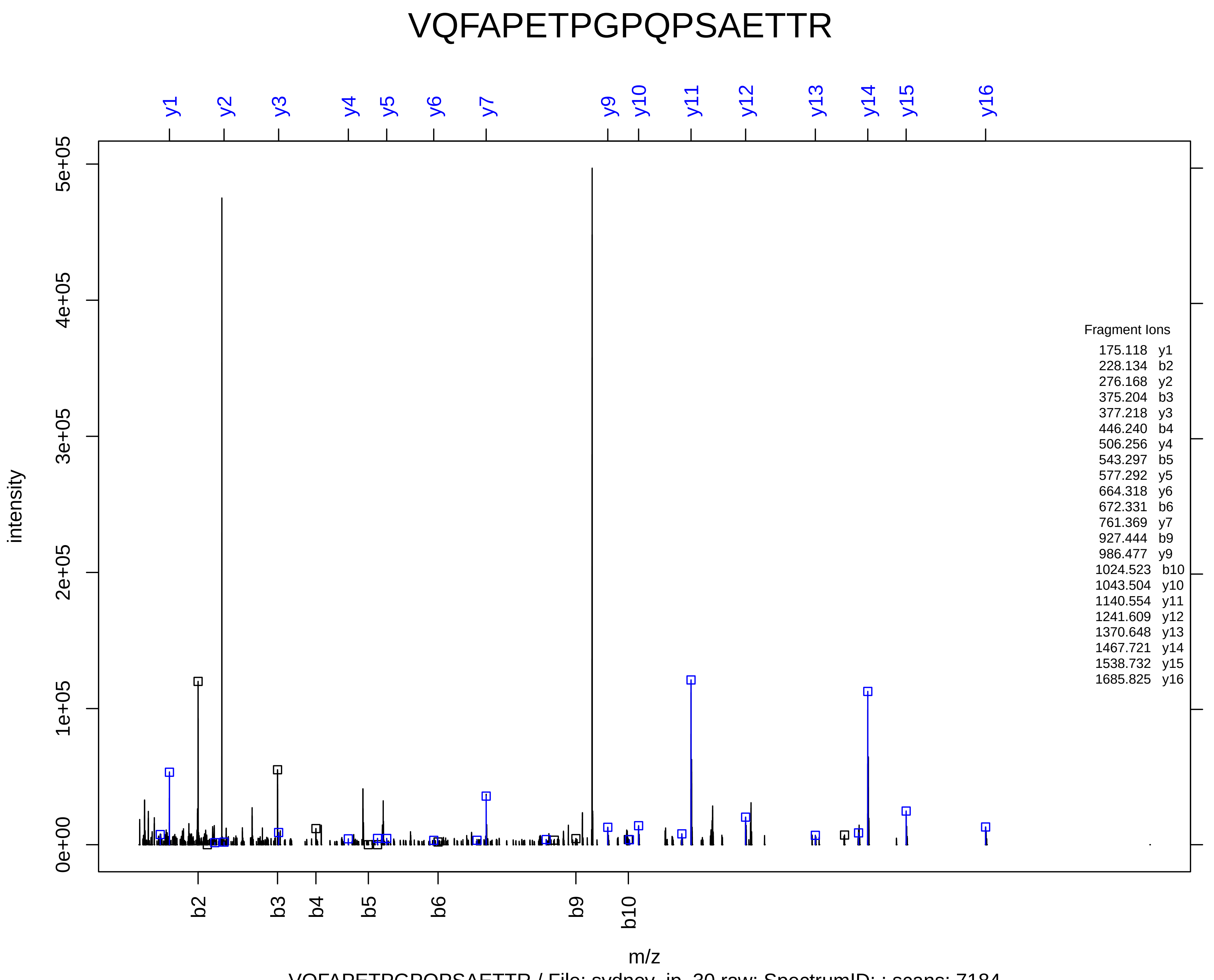

VQFAPETPGPQPSAETTR
y1 y2 y3 y4 y5 y6 y7 y9 y10 y11 y12 y13 y14 y15 y16
b2 b3 b4 b5 b6 b9 b10
intensity
m/z
Fragment Ions
175.118 y1
228.134 b2
276.168 y2
375.204 b3
377.218 y3
446.240 b4
506.256 y4
543.297 b5
577.292 y5
664.318 y6
672.331 b6
761.369 y7
927.444 b9
986.477 y9
1024.523 b10
1043.504 y10
1140.554 y11
1241.609 y12
1370.648 y13
1467.721 y14
1538.732 y15
1685.825 y16
VQFAPETPGPQPSAETTR / File: sydney_ip_30.raw; SpectrumID: ; scans: 7184

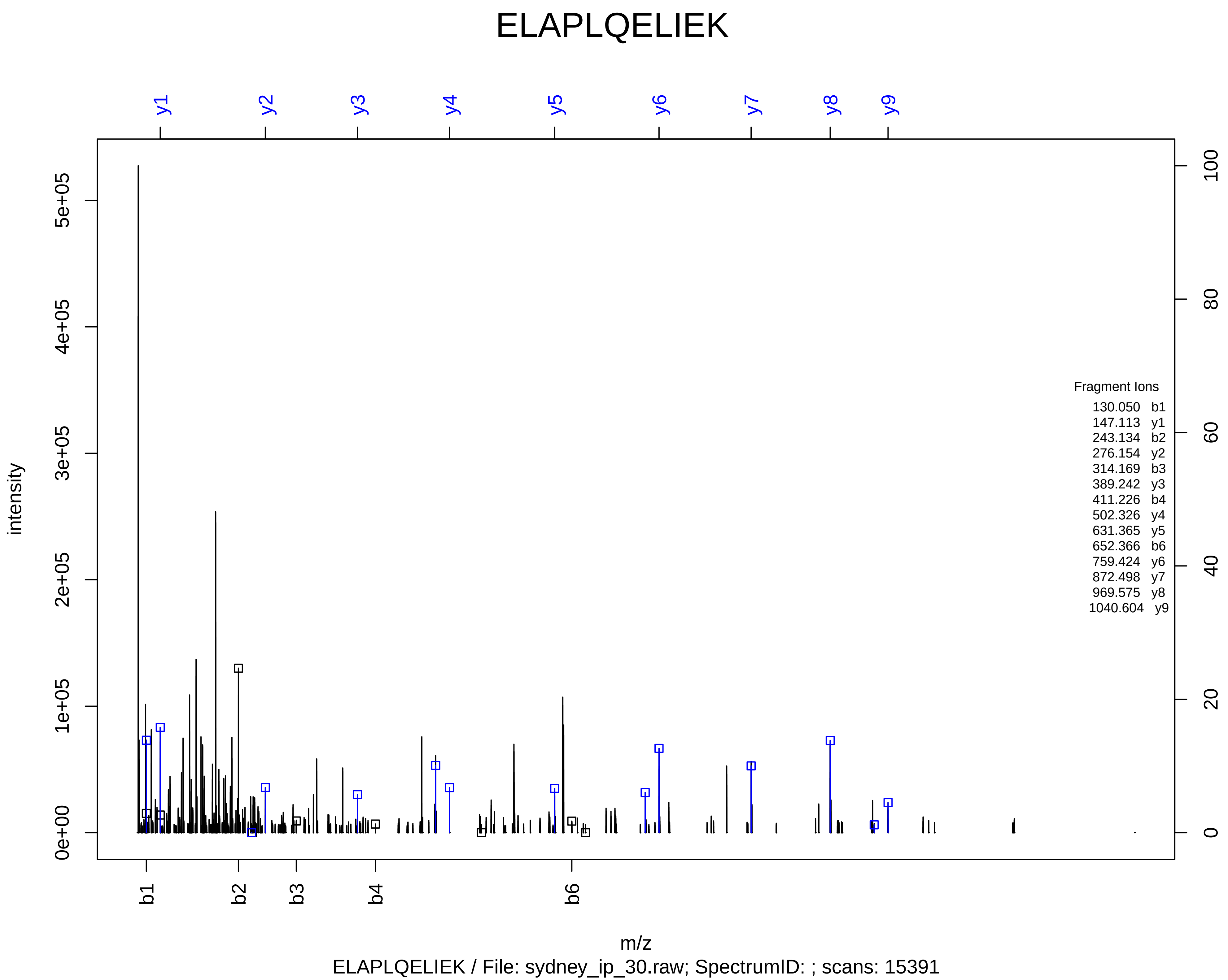
ELAPLQELIEK
y1
y2
y3
y4
y5
y6
y7
y8
y9
intensity
0e+00
1e+05
2e+05
3e+05
4e+05
5e+05
0
20
40
60
80
100
b1
b2
b3
b4
b6
m/z
Fragment Ions
130.050 b1
147.113 y1
243.134 b2
276.154 y2
314.169 b3
389.242 y3
411.226 b4
502.326 y4
631.365 y5
652.366 b6
759.424 y6
872.498 y7
969.575 y8
1040.604 y9
ELAPLQELIEK / File: sydney_ip_30.raw; SpectrumID: ; scans: 15391

SLLNEIESIYDER

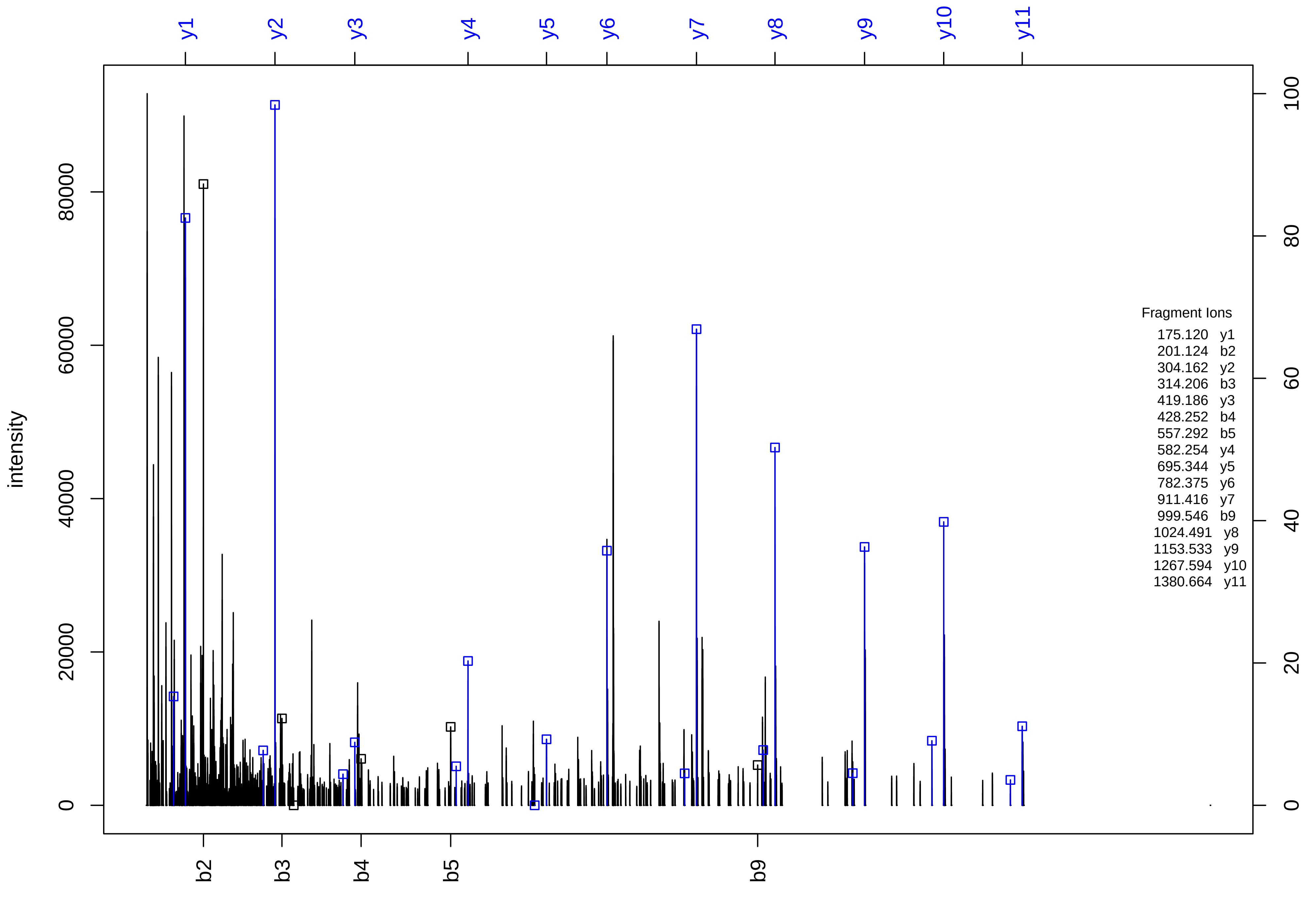

SLLNEIESIYDER / File: sydney_ip_30.raw; SpectrumID: ; scans: 18543

GPLPSGFSAVNDPQDIK

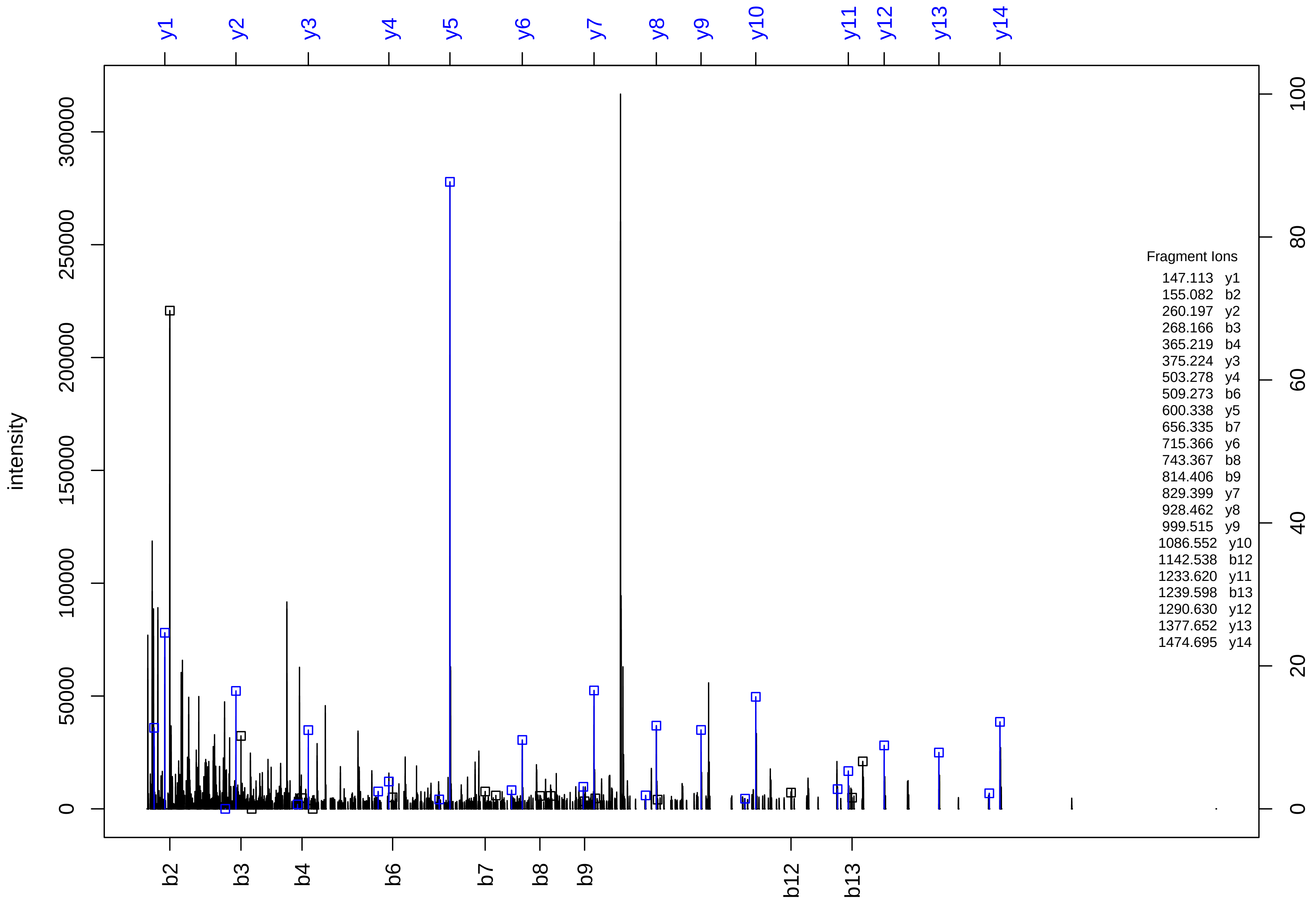

GPLPSGFSAVNDPQDIK / File: sydney_ip_30.raw; SpectrumID: ; scans: 11161

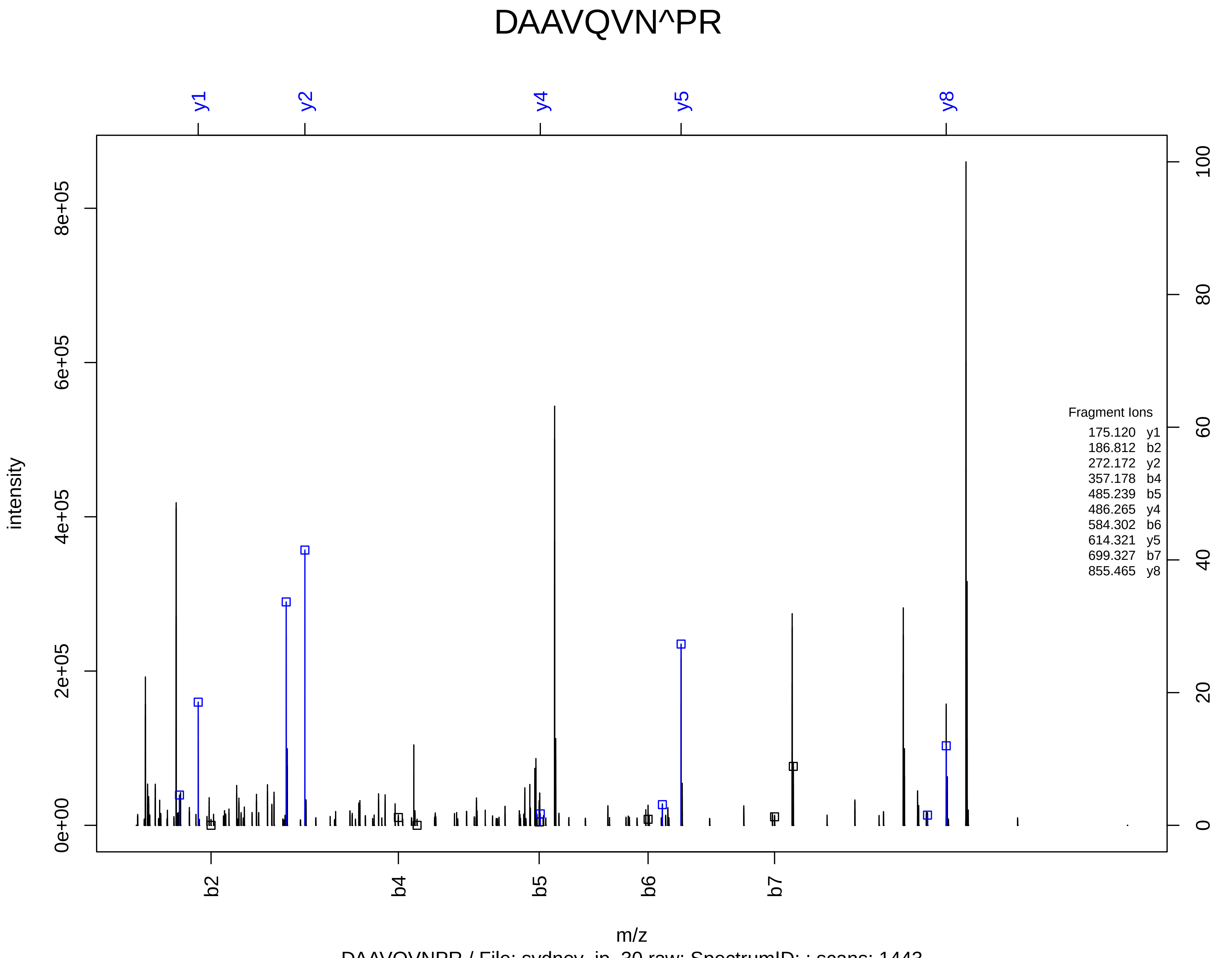
DAAVQVN^PR
y1
y2
y4
y5
y8
intensity
0e+00
2e+05
4e+05
6e+05
8e+05
0
20
40
60
80
100
b2
b4
b5
b6
b7
m/z
Fragment Ions
175.120 y1
186.812 b2
272.172 y2
357.178 b4
485.239 b5
486.265 y4
584.302 b6
614.321 y5
699.327 b7
855.465 y8
DAAVQVNPR / File: sydney_ip_30.raw; SpectrumID: ; scans: 1443

(Ac)MDRSLTTLMM*LVM*TATAR

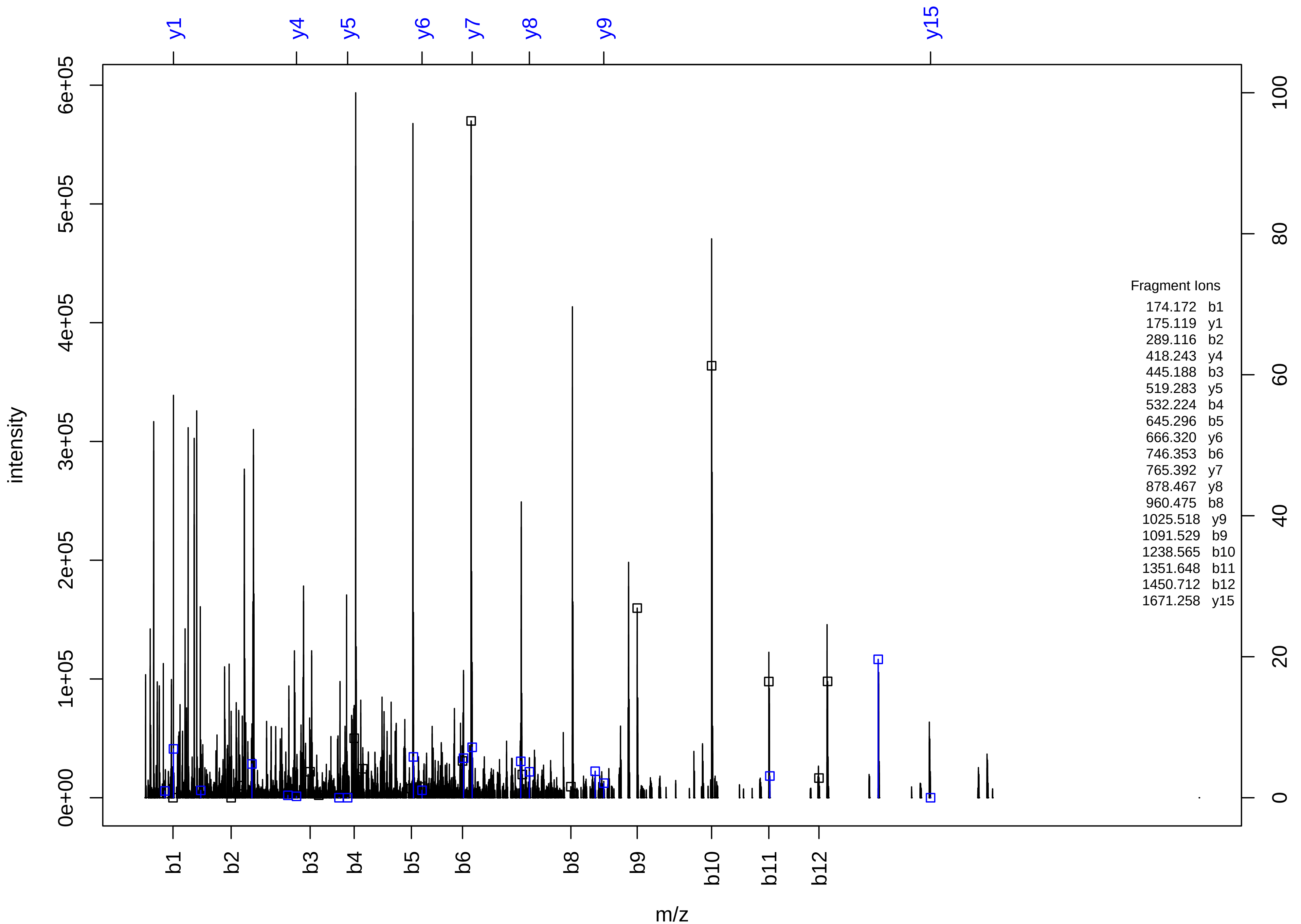

MDRSLTTLMMLVMTATAR / File: sydney_ip_30.raw; SpectrumID: ; scans: 19480

KLHETSCSSEQ^NQKTR

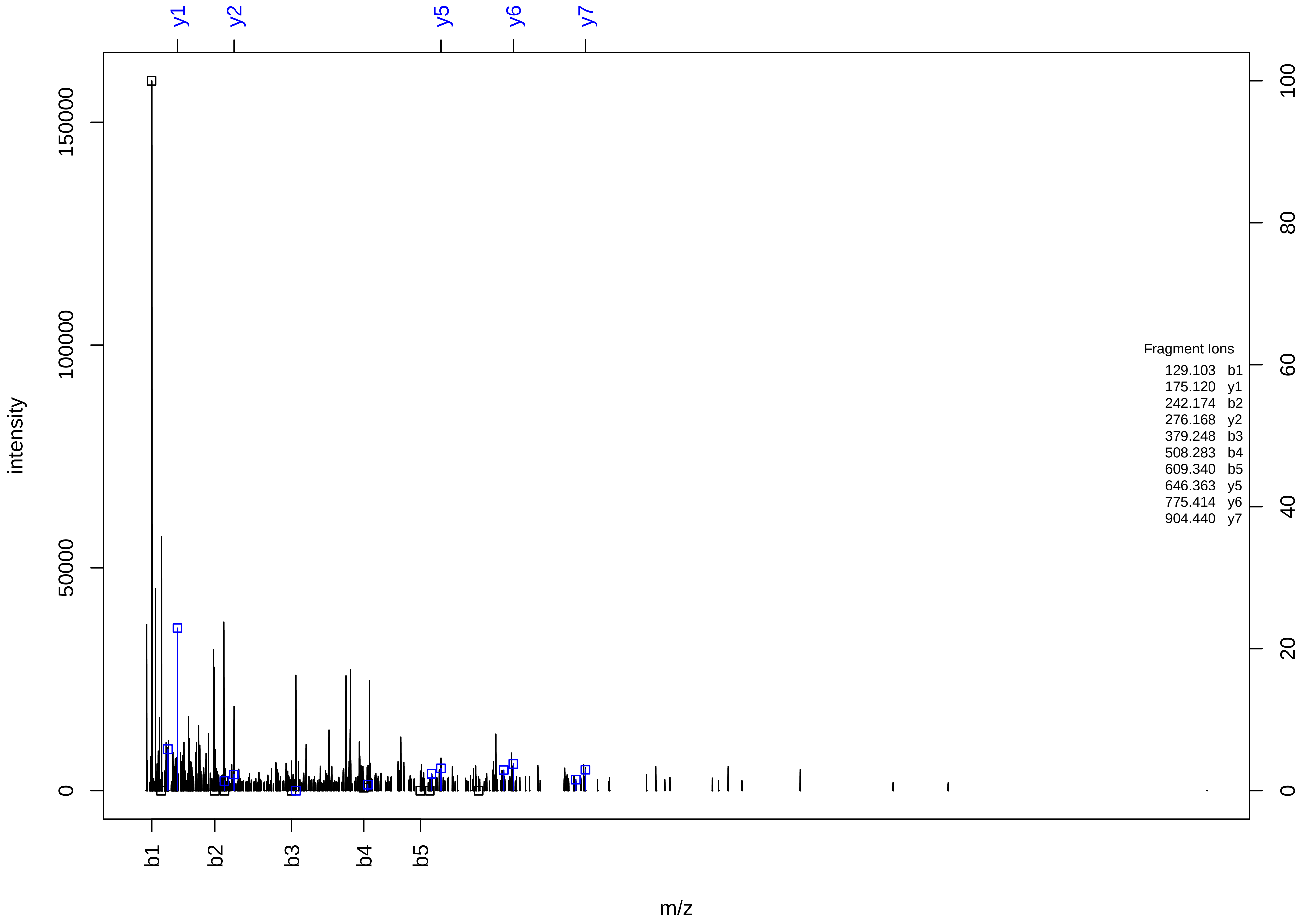

KLHETSCSSEQNQKTR / File: sydney_ip_30.raw; SpectrumID: ; scans: 1548

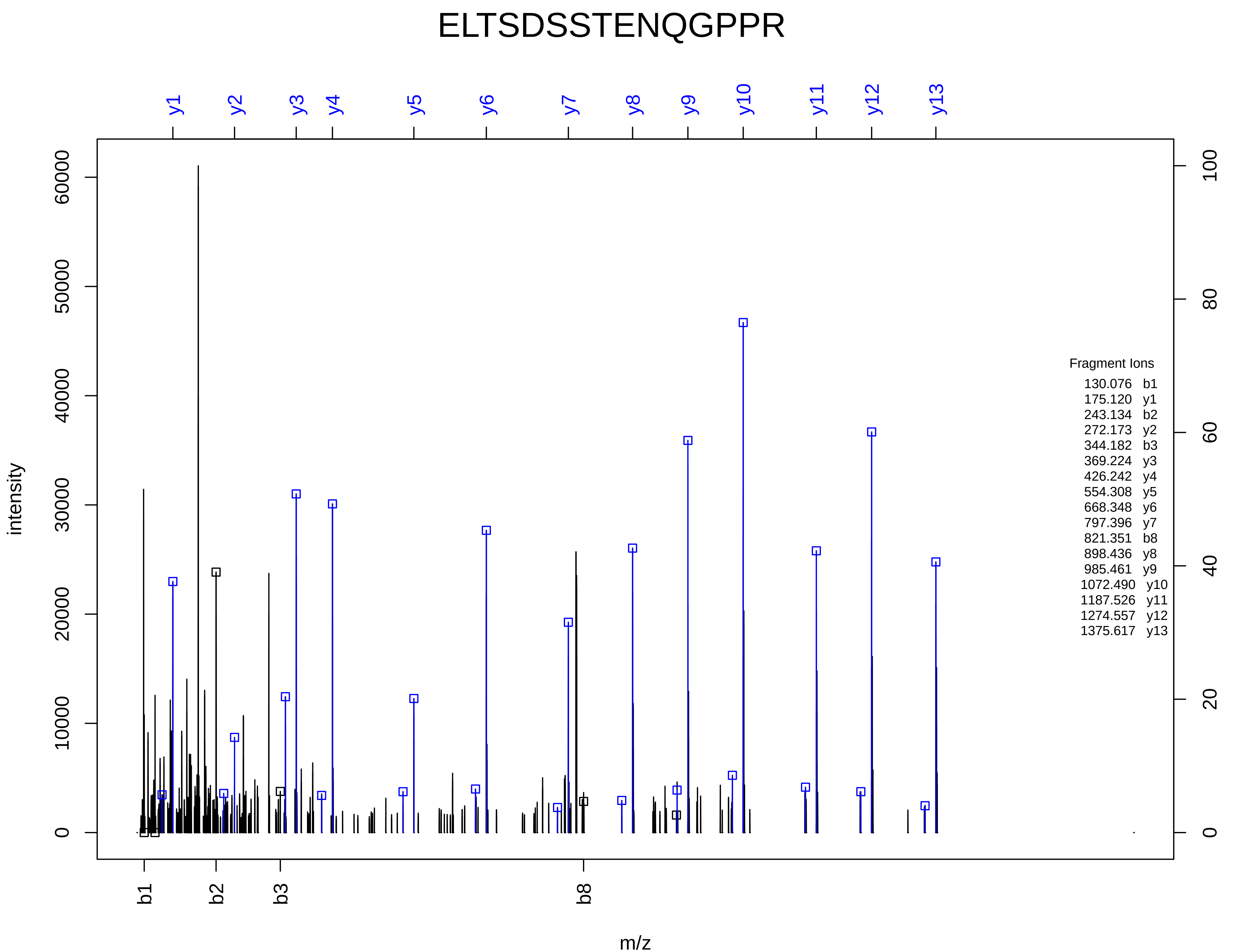

ELTSDSSTENQGPPR
y1 y2 y3 y4 y5 y6 y7 y8 y9 y10 y11 y12 y13
intensity
0 10000 20000 30000 40000 50000 60000
0 20 40 60 80 100
b1 b2 b3 b8
m/z
Fragment Ions
130.076 b1
175.120 y1
243.134 b2
272.173 y2
344.182 b3
369.224 y3
426.242 y4
554.308 y5
668.348 y6
797.396 y7
821.351 b8
898.436 y8
985.461 y9
1072.490 y10
1187.526 y11
1274.557 y12
1375.617 y13
ELTSDSSTENQGPPR / File: sydney_ip_30.raw; SpectrumID: ; scans: 2267

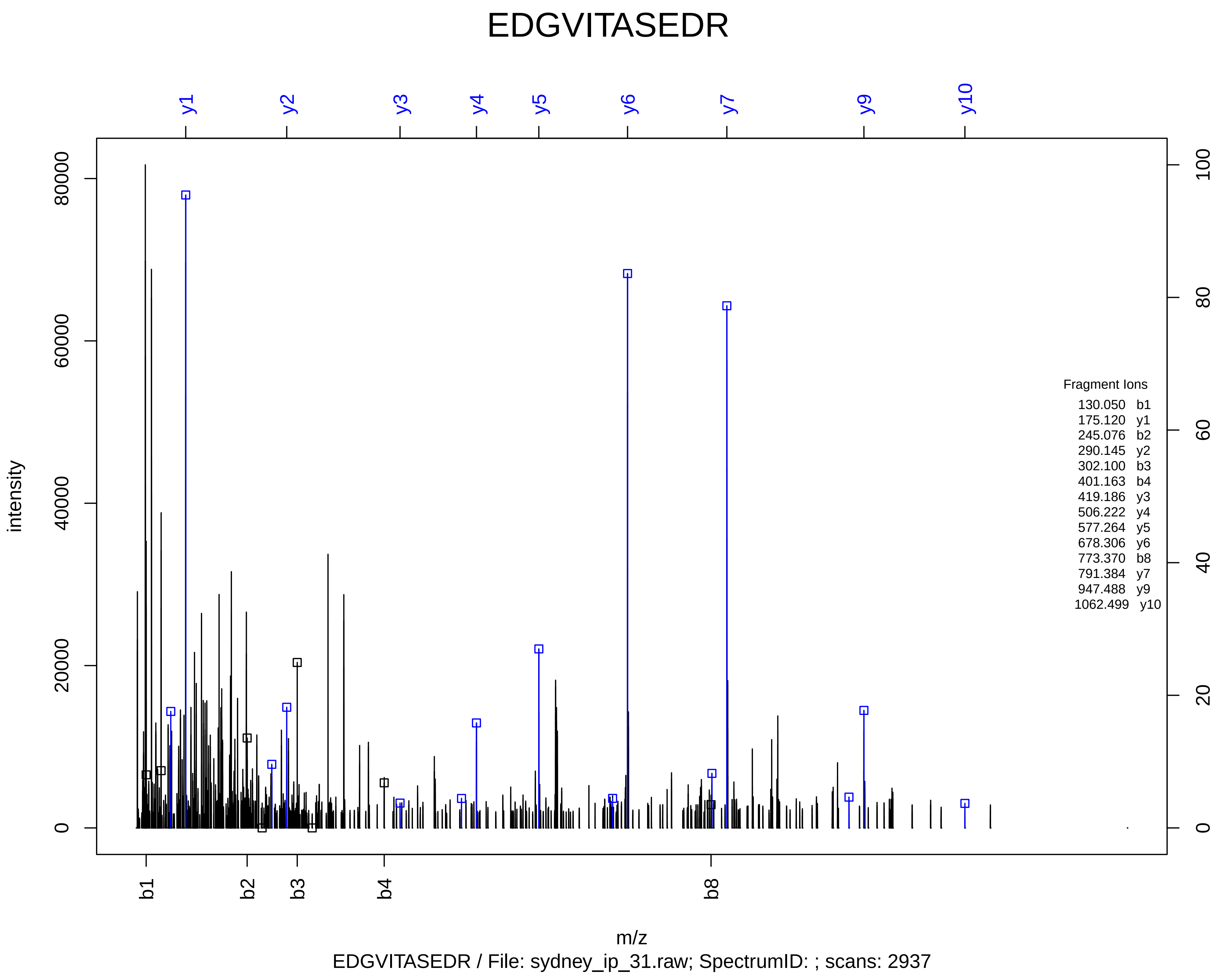

EDGVITASEDR
y1
y2
y3
y4
y5
y6
y7
y9
y10
intensity
0
20000
40000
60000
80000
0
20
40
60
80
100
Fragment Ions
130.050 b1
175.120 y1
245.076 b2
290.145 y2
302.100 b3
401.163 b4
419.186 y3
506.222 y4
577.264 y5
678.306 y6
773.370 b8
791.384 y7
947.488 y9
1062.499 y10
b1
b2
b3
b4
b8
m/z
EDGVITASEDR / File: sydney_ip_31.raw; SpectrumID: ; scans: 2937

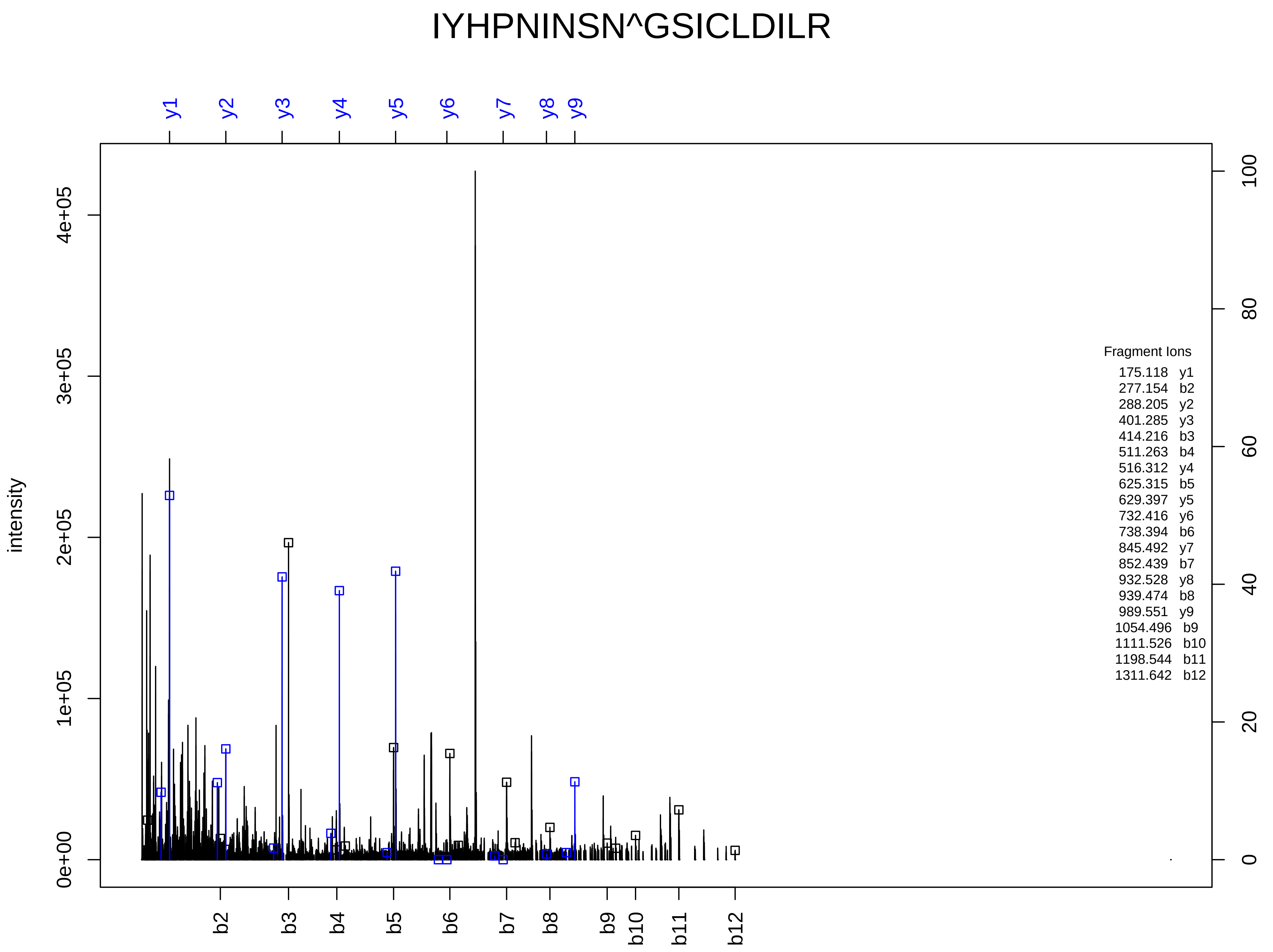

IYHPNINSNGSICLDILR / File: sydney_ip_31.raw; SpectrumID: ; scans: 13887

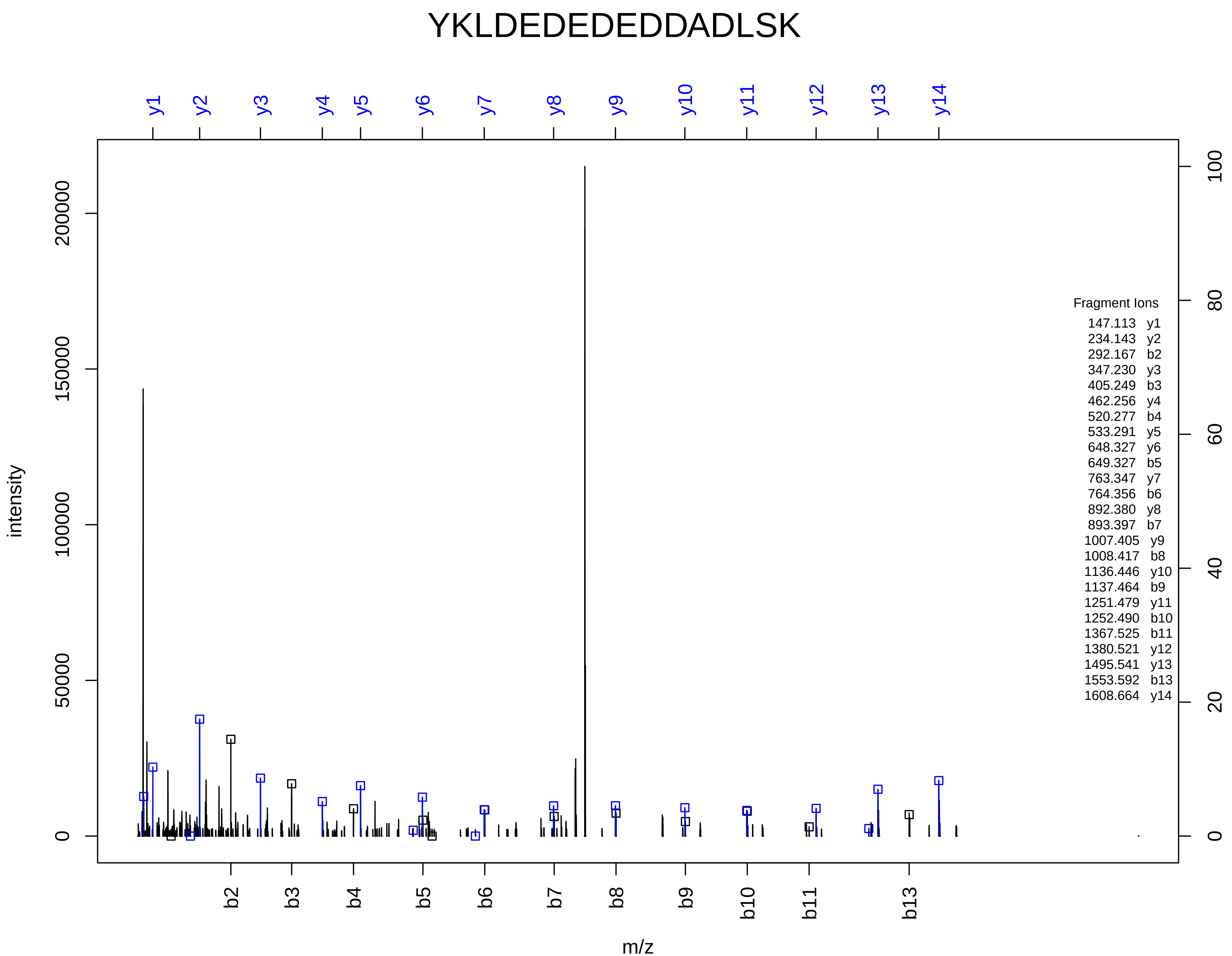
YKLDEDEDEDDADLSK
y1
y2
y3
y4
y5
y6
y7
y8
y9
y10
y11
y12
y13
y14
intensity
0
50000
100000
150000
200000
0
20
40
60
80
100
b2
b3
b4
b5
b6
b7
b8
b9
b10
b11
b13
m/z
Fragment Ions
147.113 y1
234.143 y2
292.167 b2
347.230 y3
405.249 b3
462.256 y4
520.277 b4
533.291 y5
648.327 y6
649.327 b5
763.347 y7
764.356 b6
892.380 y8
893.397 b7
1007.405 y9
1008.417 b8
1136.446 y10
1137.464 b9
1251.479 y11
1252.490 b10
1367.525 b11
1380.521 y12
1495.541 y13
1553.592 b13
1608.664 y14
YKLDEDEDEDDADLSK / File: sydney_ip_31.raw; SpectrumID: ; scans: 4562

ILAAQAQLSAQGGAQPSVEAPAPPRPTATQLTR

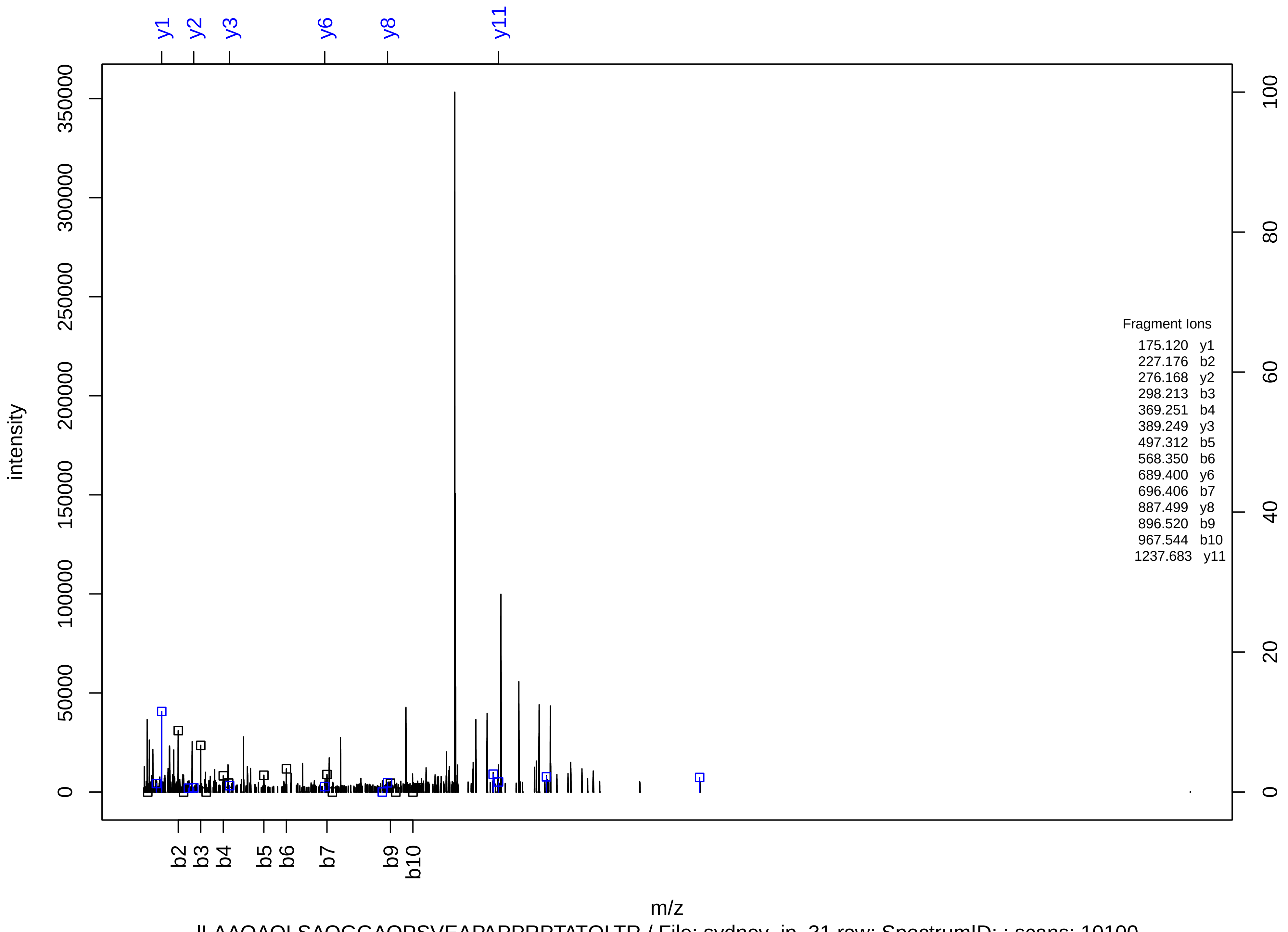

ILAAQAQLSAQGGAQPSVEAPAPPRPTATQLTR / File: sydney_ip_31.raw; SpectrumID: ; scans: 10100

TTITTTTSSSTTVGSAR

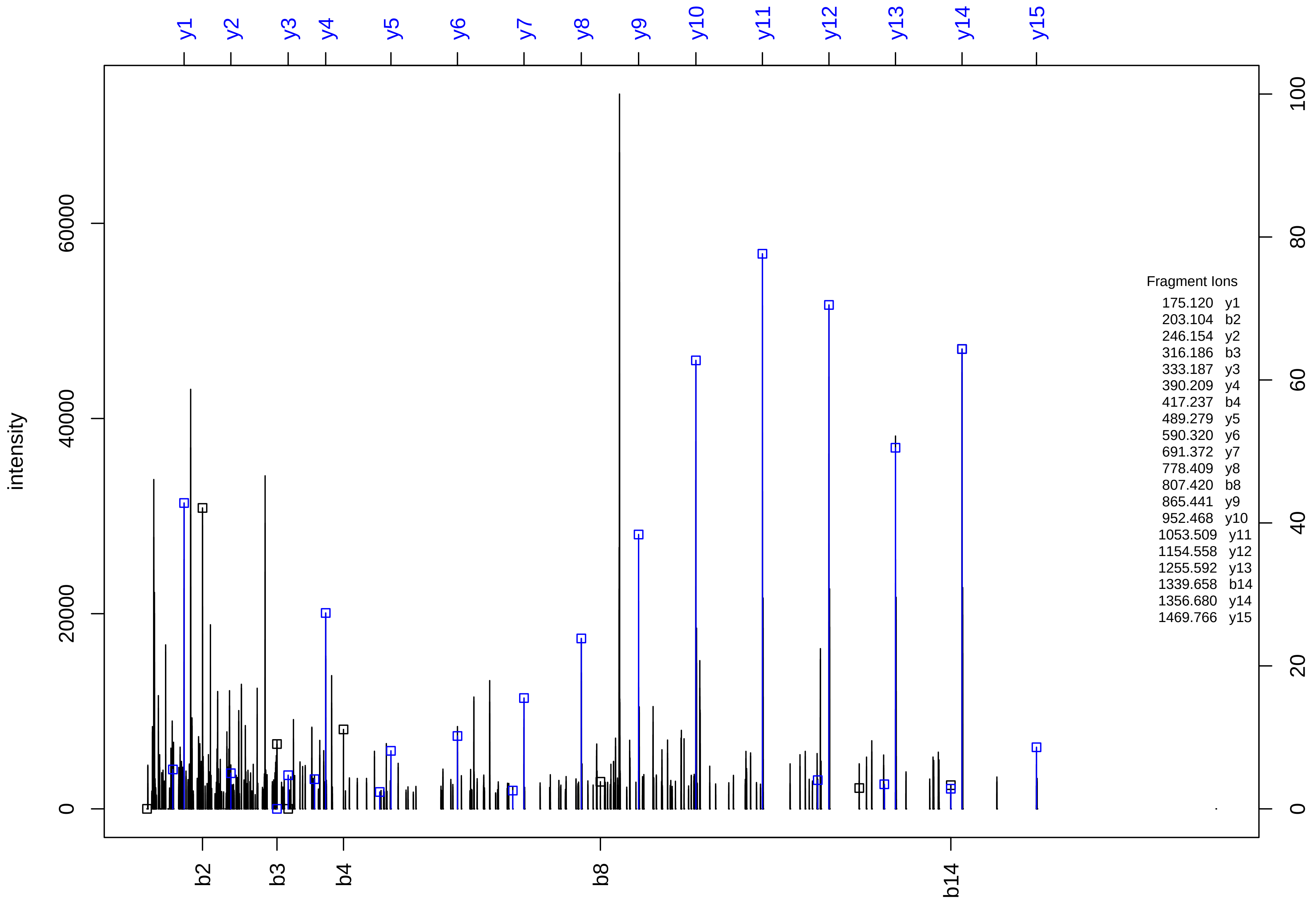

TTITTTTSSSTTVGSAR / File: sydney_ip_31.raw; SpectrumID: ; scans: 3130

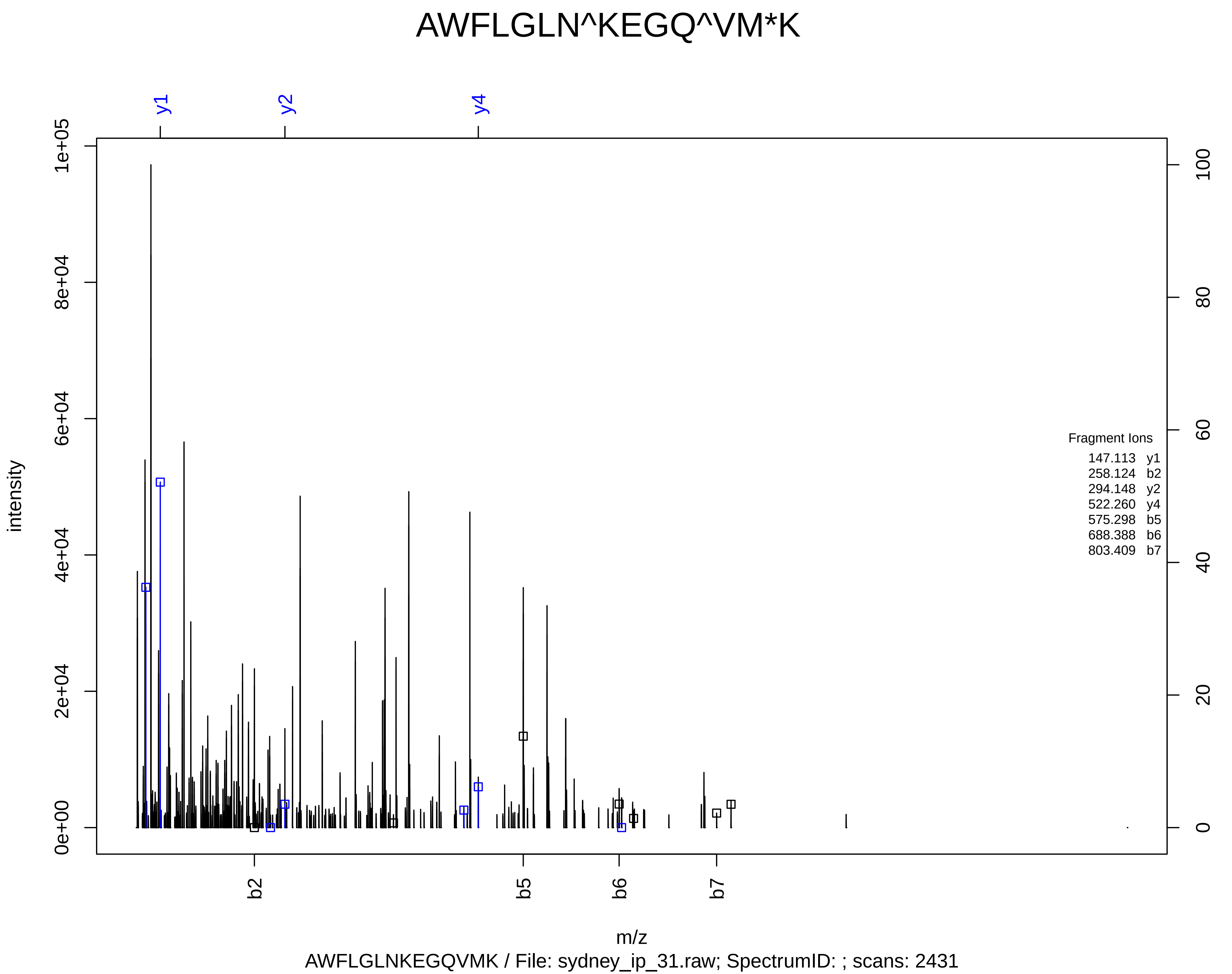
AWFLGLN^KEGQ^VM*K
y1
y2
y4
intensity
1e+05
8e+04
6e+04
4e+04
2e+04
0e+00
100
80
60
40
20
0
Fragment Ions
147.113 y1
258.124 b2
294.148 y2
522.260 y4
575.298 b5
688.388 b6
803.409 b7
b2
b5
b6
b7
m/z
AWFLGLNKEGQVMK / File: sydney_ip_31.raw; SpectrumID: ; scans: 2431

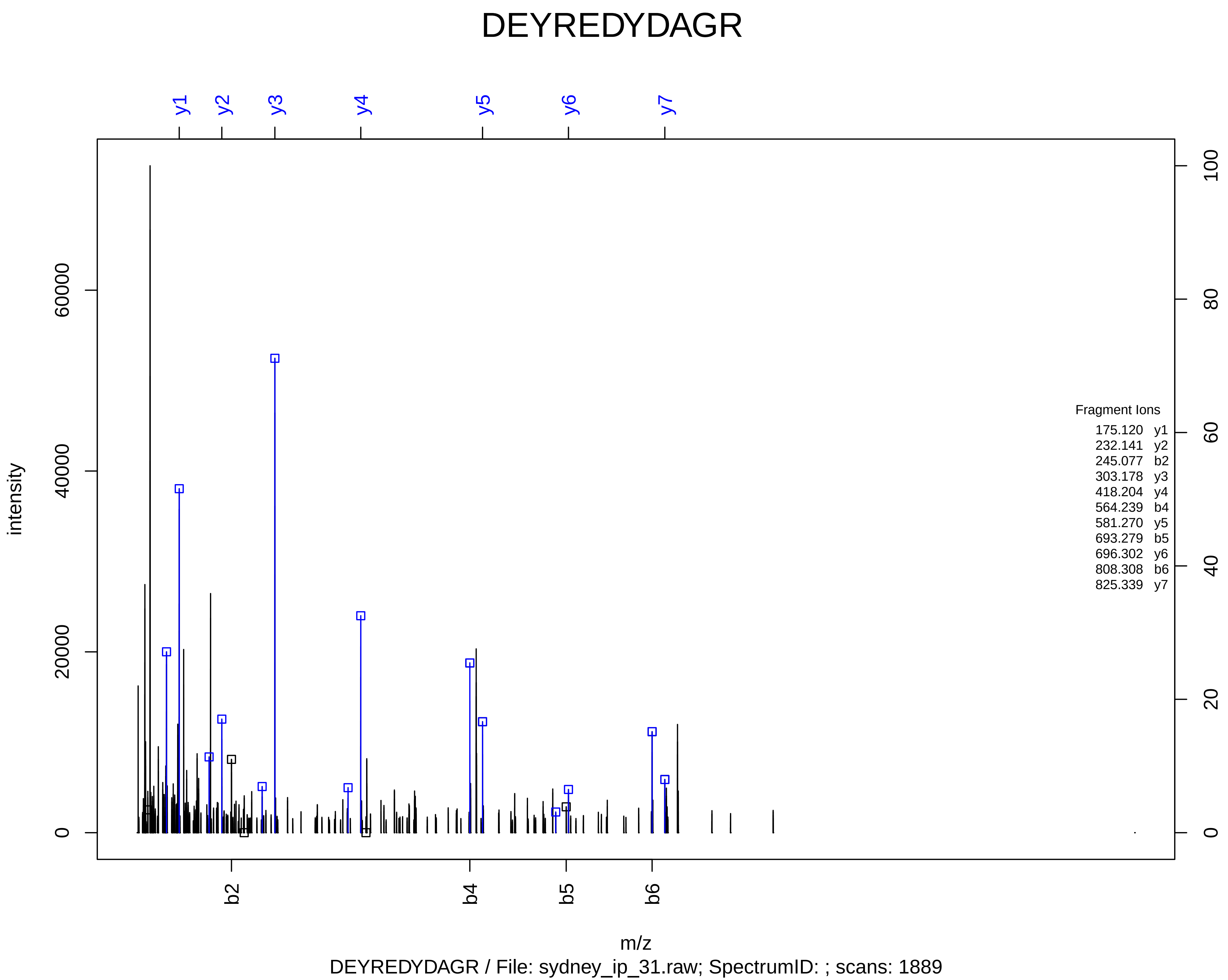
DEYREDYDAGR
y1
y2
y3
y4
y5
y6
y7
b2
b4
b5
b6
intensity
m/z
Fragment Ions
175.120 y1
232.141 y2
245.077 b2
303.178 y3
418.204 y4
564.239 b4
581.270 y5
693.279 b5
696.302 y6
808.308 b6
825.339 y7
DEYREDYDAGR / File: sydney_ip_31.raw; SpectrumID: ; scans: 1889

VLIQANQEDNFGTATPR

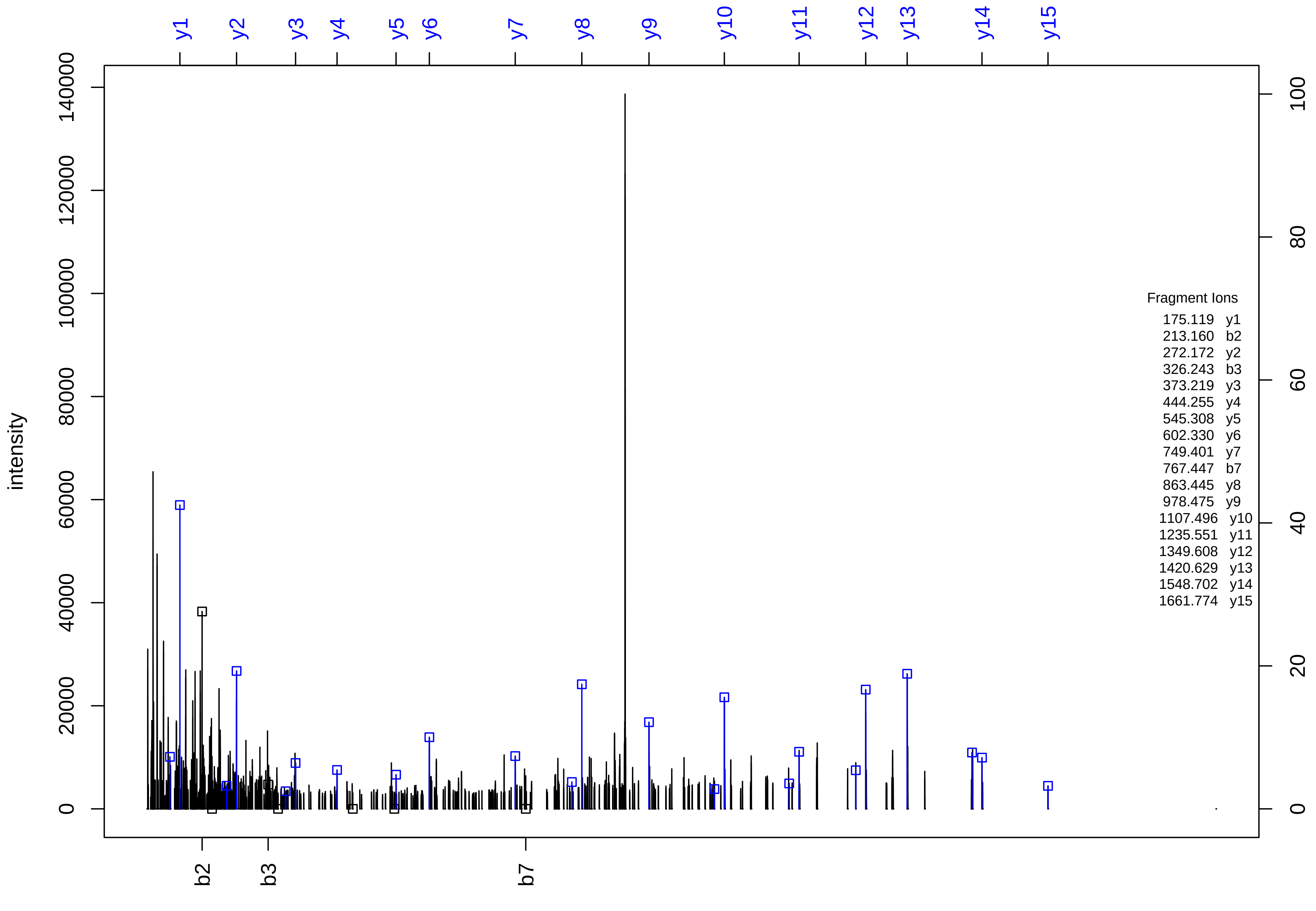

VLIQANQEDNFGTATPR / File: sydney_ip_31.raw; SpectrumID: ; scans: 8485

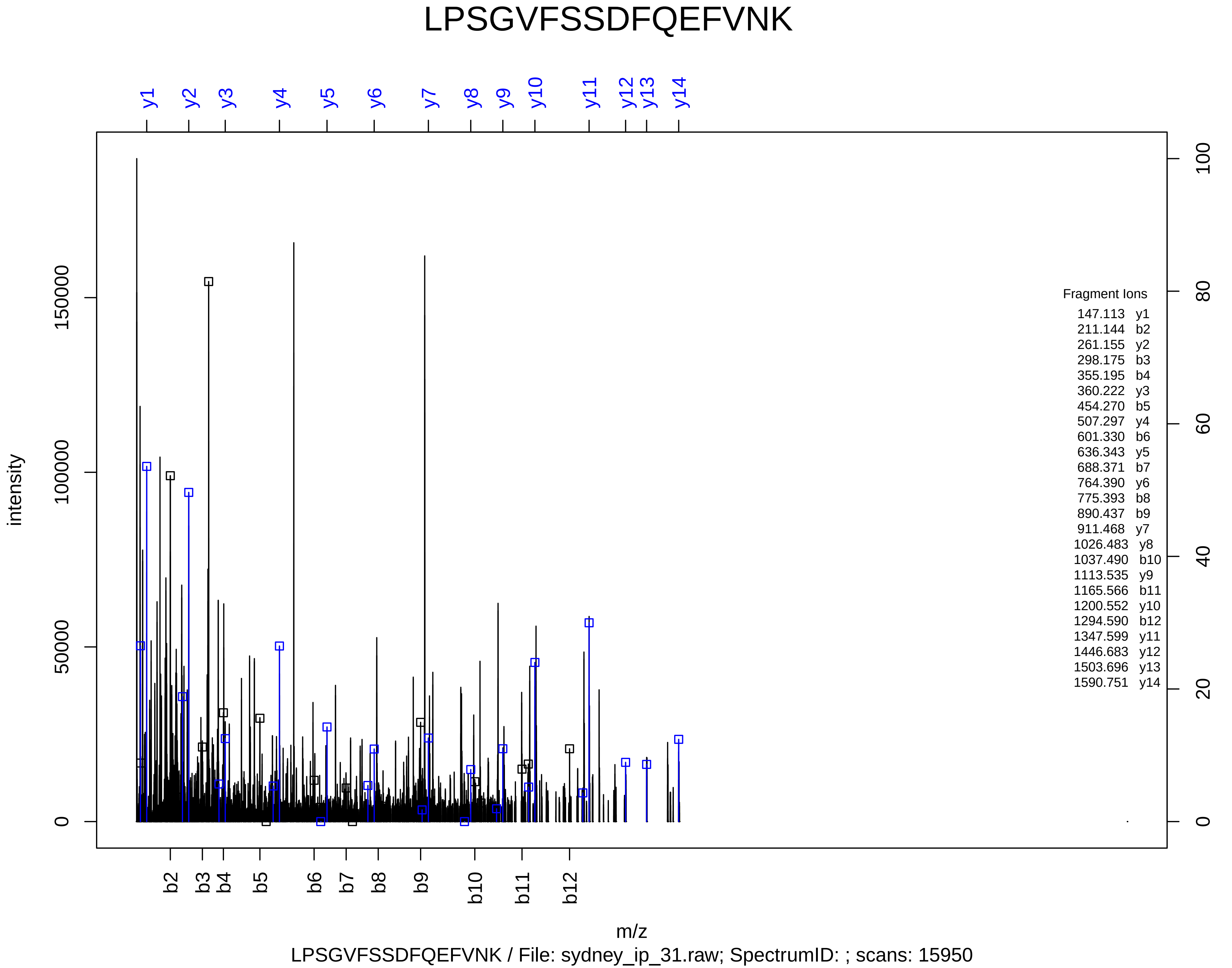
LPSGVFSSDFQEFVNK
y1 y2 y3 y4 y5 y6 y7 y8 y9 y10 y11 y12 y13 y14
b2 b3 b4 b5 b6 b7 b8 b9 b10 b11 b12
intensity
m/z
Fragment Ions
147.113 y1
211.144 b2
261.155 y2
298.175 b3
355.195 b4
360.222 y3
454.270 b5
507.297 y4
601.330 b6
636.343 y5
688.371 b7
764.390 y6
775.393 b8
890.437 b9
911.468 y7
1026.483 y8
1037.490 b10
1113.535 y9
1165.566 b11
1200.552 y10
1294.590 b12
1347.599 y11
1446.683 y12
1503.696 y13
1590.751 y14
LPSGVFSSDFQEFVNK / File: sydney_ip_31.raw; SpectrumID: ; scans: 15950

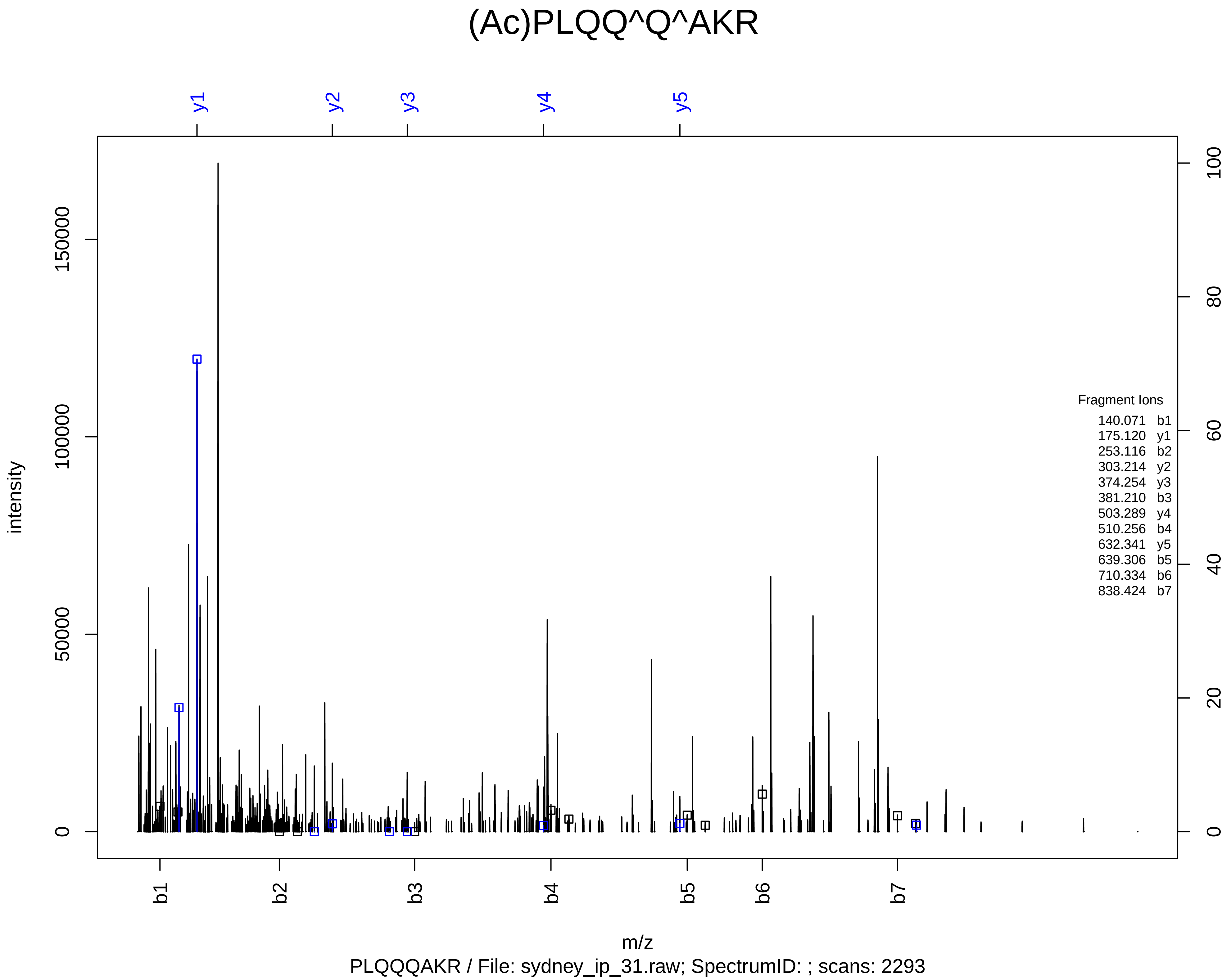
(Ac)PLQQ^Q^AKR
y1
y2
y3
y4
y5
intensity
0
50000
100000
150000
0
20
40
60
80
100
b1
b2
b3
b4
b5
b6
b7
m/z
Fragment Ions
140.071 b1
175.120 y1
253.116 b2
303.214 y2
374.254 y3
381.210 b3
503.289 y4
510.256 b4
632.341 y5
639.306 b5
710.334 b6
838.424 b7
PLQQQAKR / File: sydney_ip_31.raw; SpectrumID: ; scans: 2293

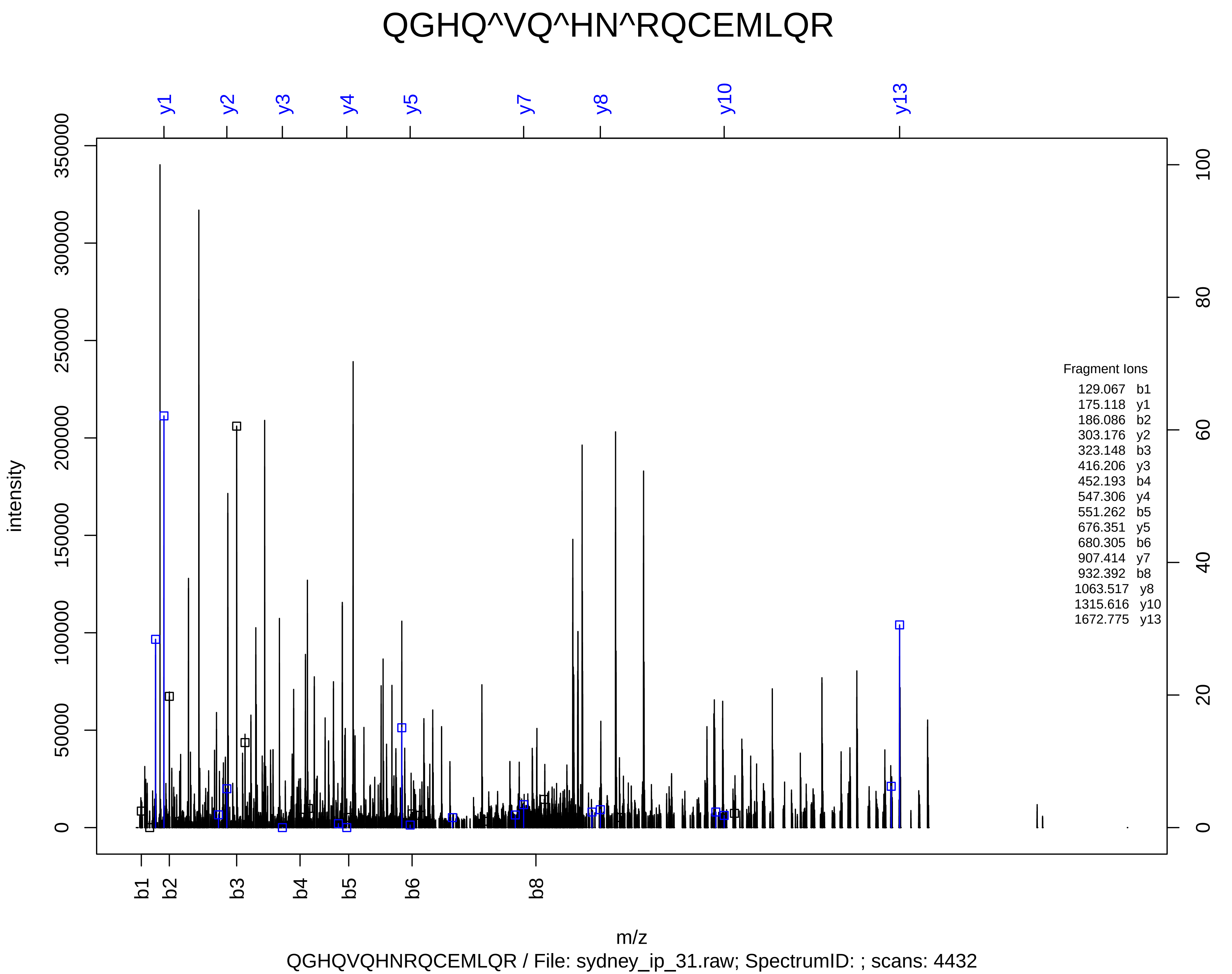
QGHQ^VQ^HN^RQCEMLQR
y1
y2
y3
y4
y5
y7
y8
y10
y13
intensity
350000
300000
250000
200000
150000
100000
50000
0
100
80
60
40
20
0
Fragment Ions
129.067 b1
175.118 y1
186.086 b2
303.176 y2
323.148 b3
416.206 y3
452.193 b4
547.306 y4
551.262 b5
676.351 y5
680.305 b6
907.414 y7
932.392 b8
1063.517 y8
1315.616 y10
1672.775 y13
b1
b2
b3
b4
b5
b6
b8
m/z
QGHQVQHNRQCEMLQR / File: sydney_ip_31.raw; SpectrumID: ; scans: 4432

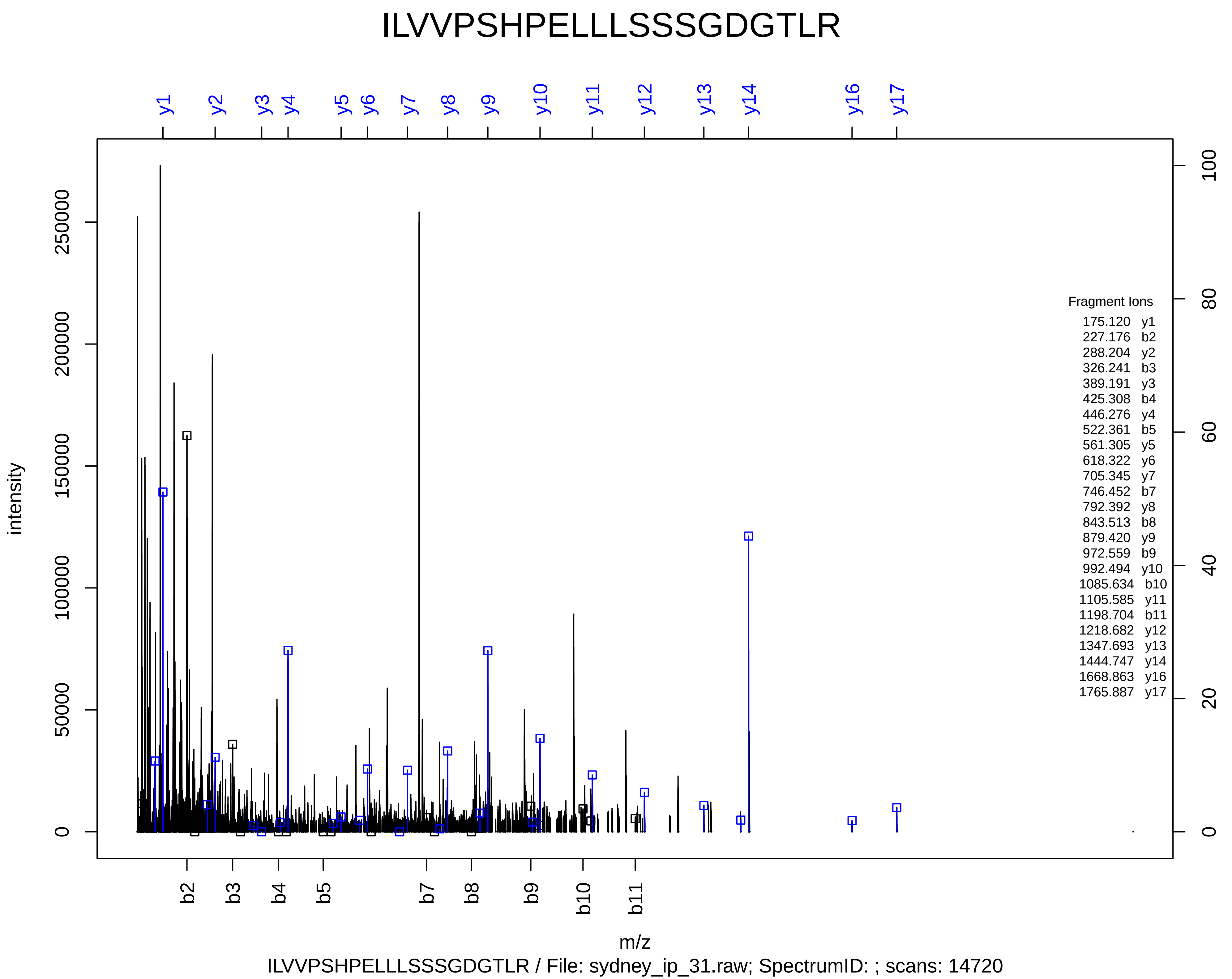

ILVVPSHPELLLSSSGDGTLR
y1
y2
y3
y4
y5
y6
y7
y8
y9
y10
y11
y12
y13
y14
y16
y17
b2
b3
b4
b5
b7
b8
b9
b10
b11
intensity
m/z
Fragment Ions
175.120 y1
227.176 b2
288.204 y2
326.241 b3
389.191 y3
425.308 b4
446.276 y4
522.361 b5
561.305 y5
618.322 y6
705.345 y7
746.452 b7
792.392 y8
843.513 b8
879.420 y9
972.559 b9
992.494 y10
1085.634 b10
1105.585 y11
1198.704 b11
1218.682 y12
1347.693 y13
1444.747 y14
1668.863 y16
1765.887 y17
ILVVPSHPELLLSSSGDGTLR / File: sydney_ip_31.raw; SpectrumID: ; scans: 14720

GLIAAICAGPTALLAHEVGFGCK

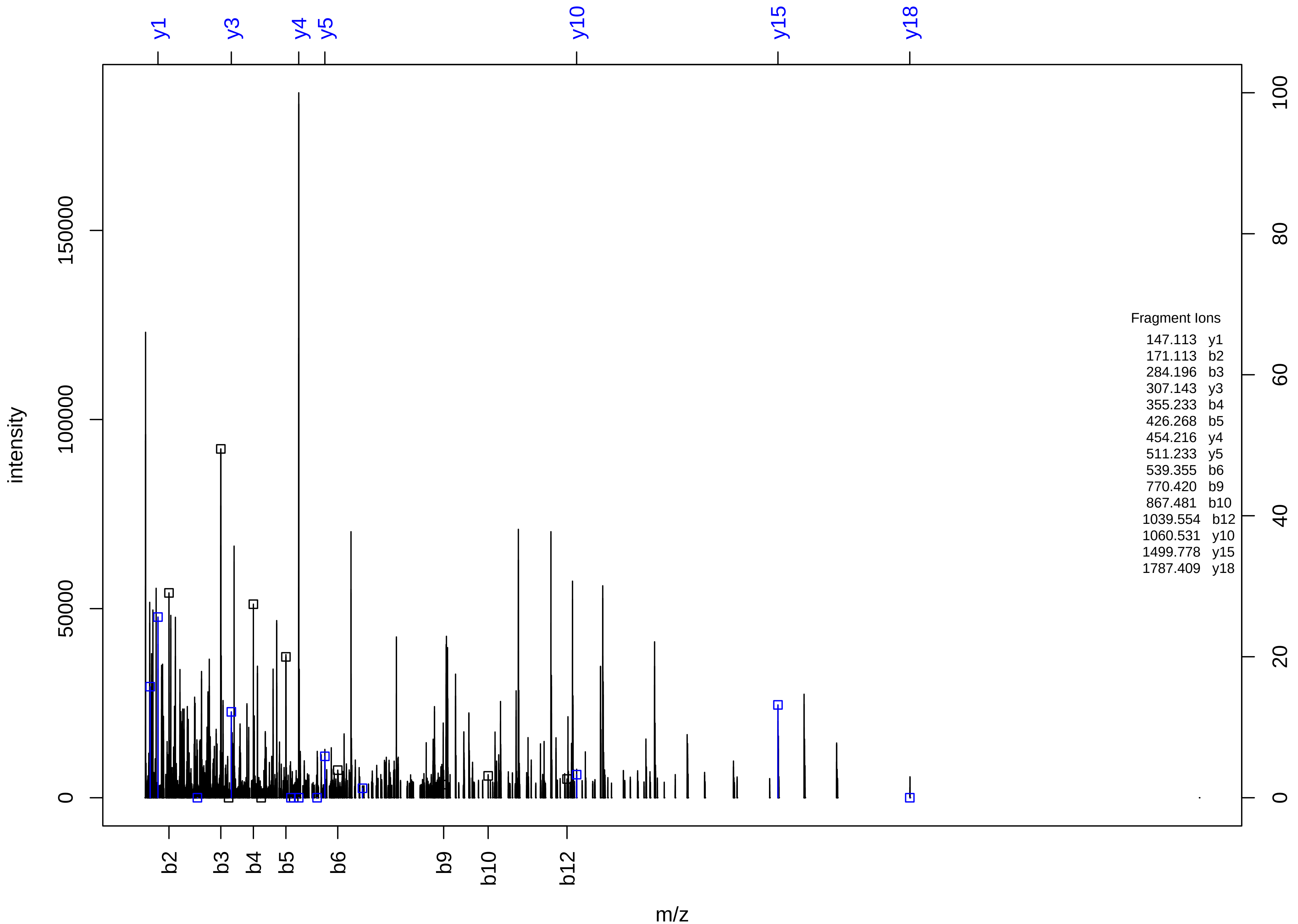

GLIAAICAGPTALLAHEVGFGCK / File: sydney_ip_31.raw; SpectrumID: ; scans: 18968

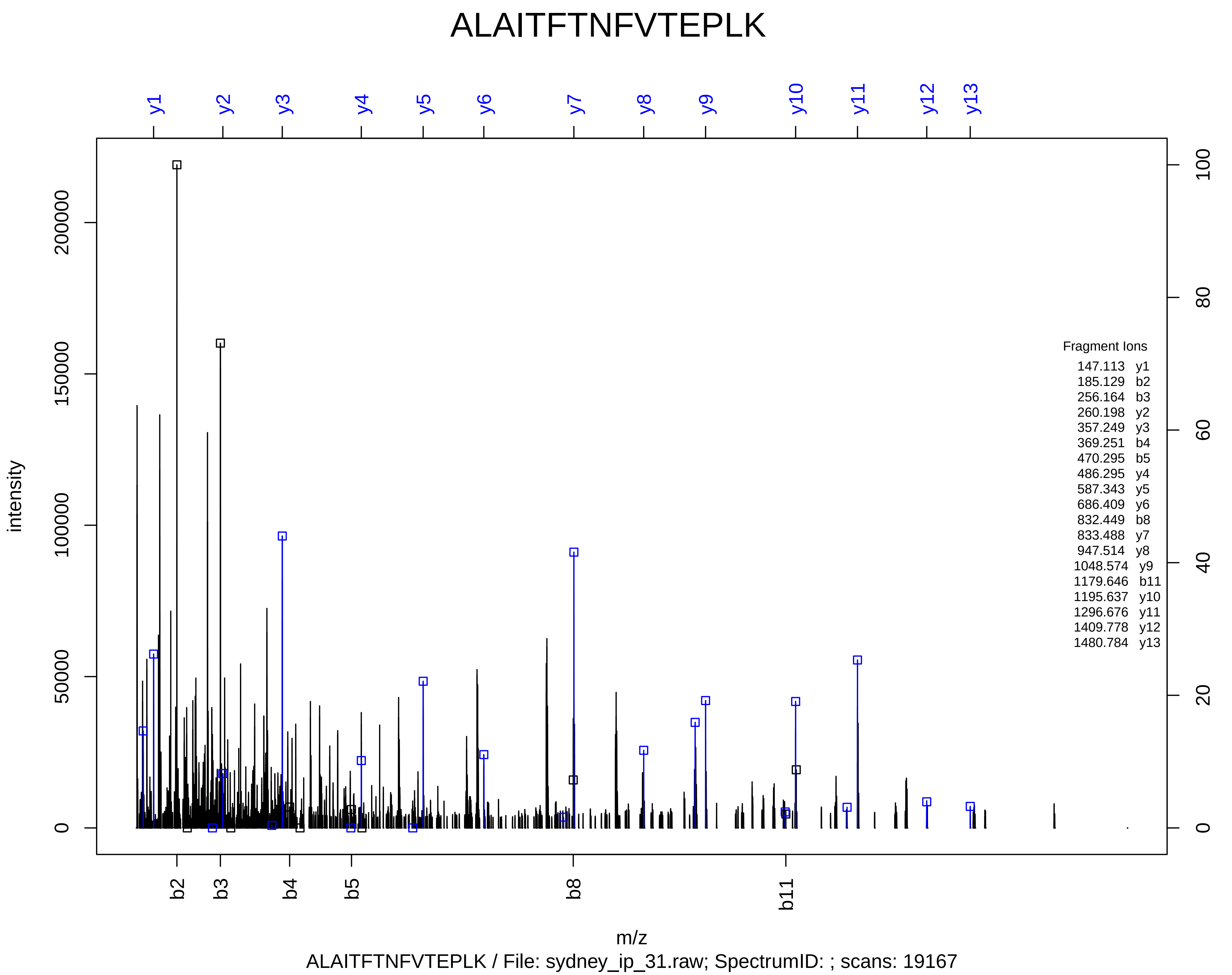
ALAITFTNFVTEPLK
y1
y2
y3
y4
y5
y6
y7
y8
y9
y10
y11
y12
y13
intensity
0
50000
100000
150000
200000
0
20
40
60
80
100
b2
b3
b4
b5
b8
b11
m/z
Fragment Ions
147.113 y1
185.129 b2
256.164 b3
260.198 y2
357.249 y3
369.251 b4
470.295 b5
486.295 y4
587.343 y5
686.409 y6
832.449 b8
833.488 y7
947.514 y8
1048.574 y9
1179.646 b11
1195.637 y10
1296.676 y11
1409.778 y12
1480.784 y13
ALAITFTNFVTEPLK / File: sydney_ip_31.raw; SpectrumID: ; scans: 19167

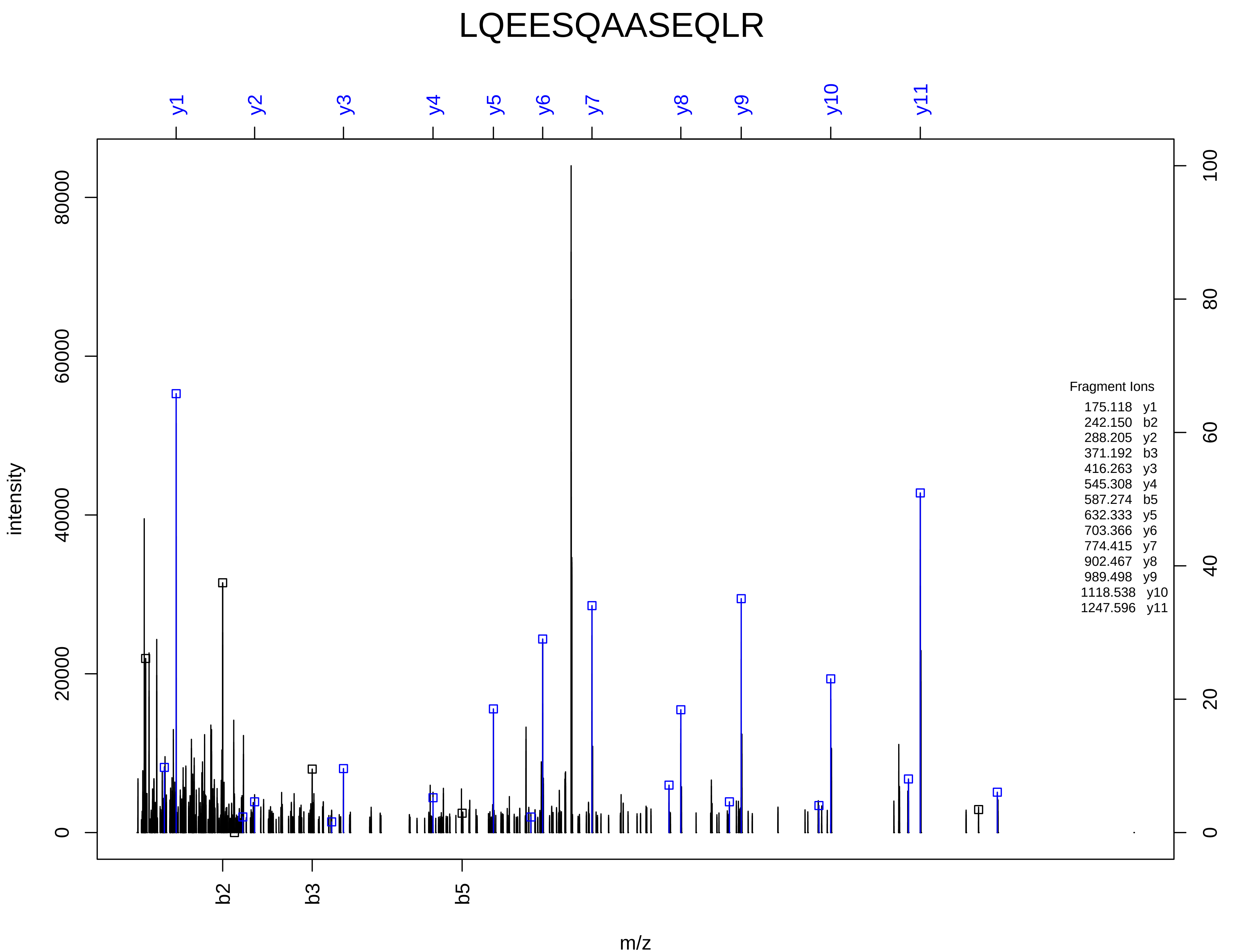

LQEESQAASEQLR / File: sydney_ip_31.raw; SpectrumID: ; scans: 3227

(Ac)NSKRN^SASQQ^N^K

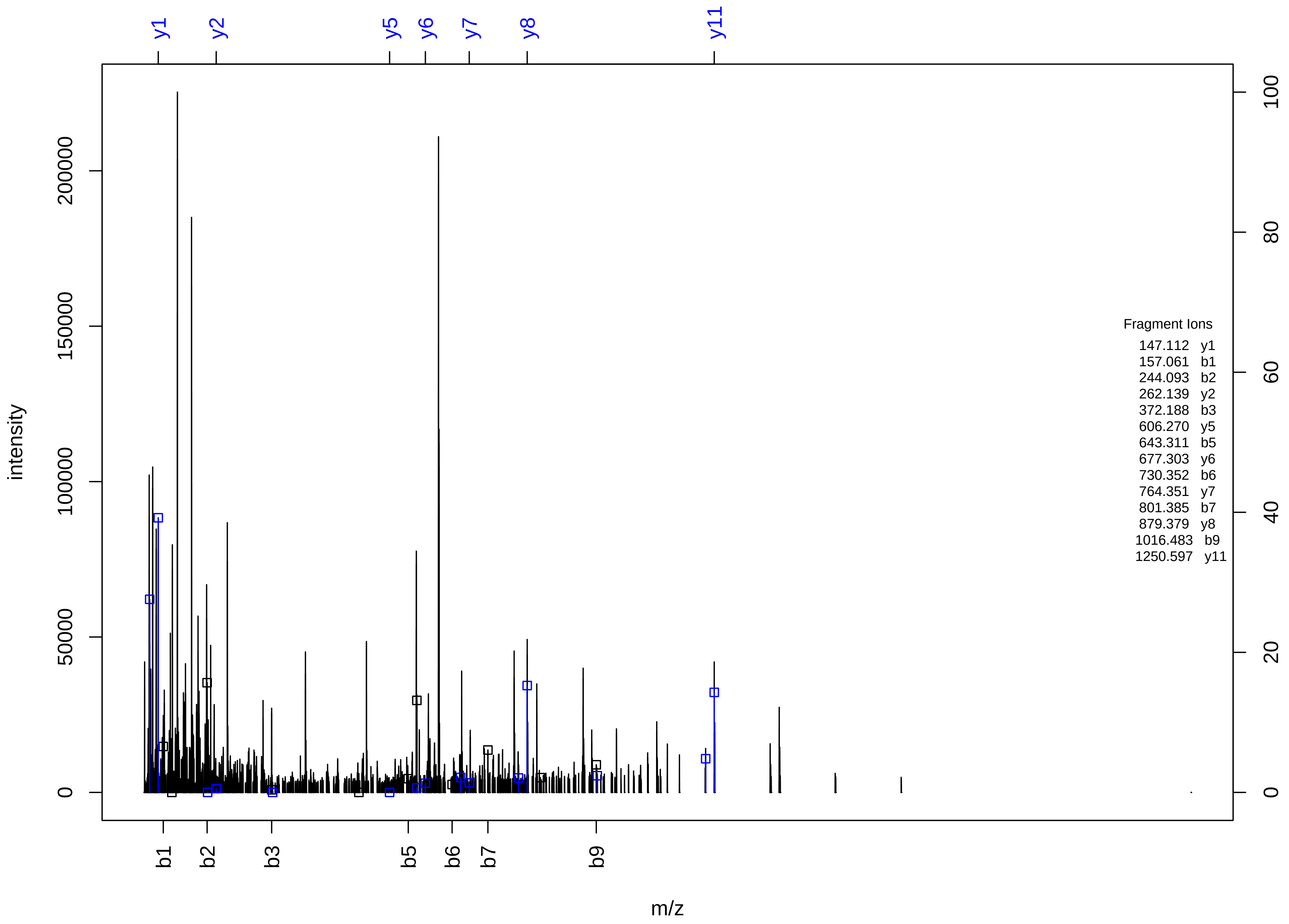

NSKRNSASQQNK / File: sydney_ip_31.raw; SpectrumID: ; scans: 5492

EYVAAQLGQLPAQLLK

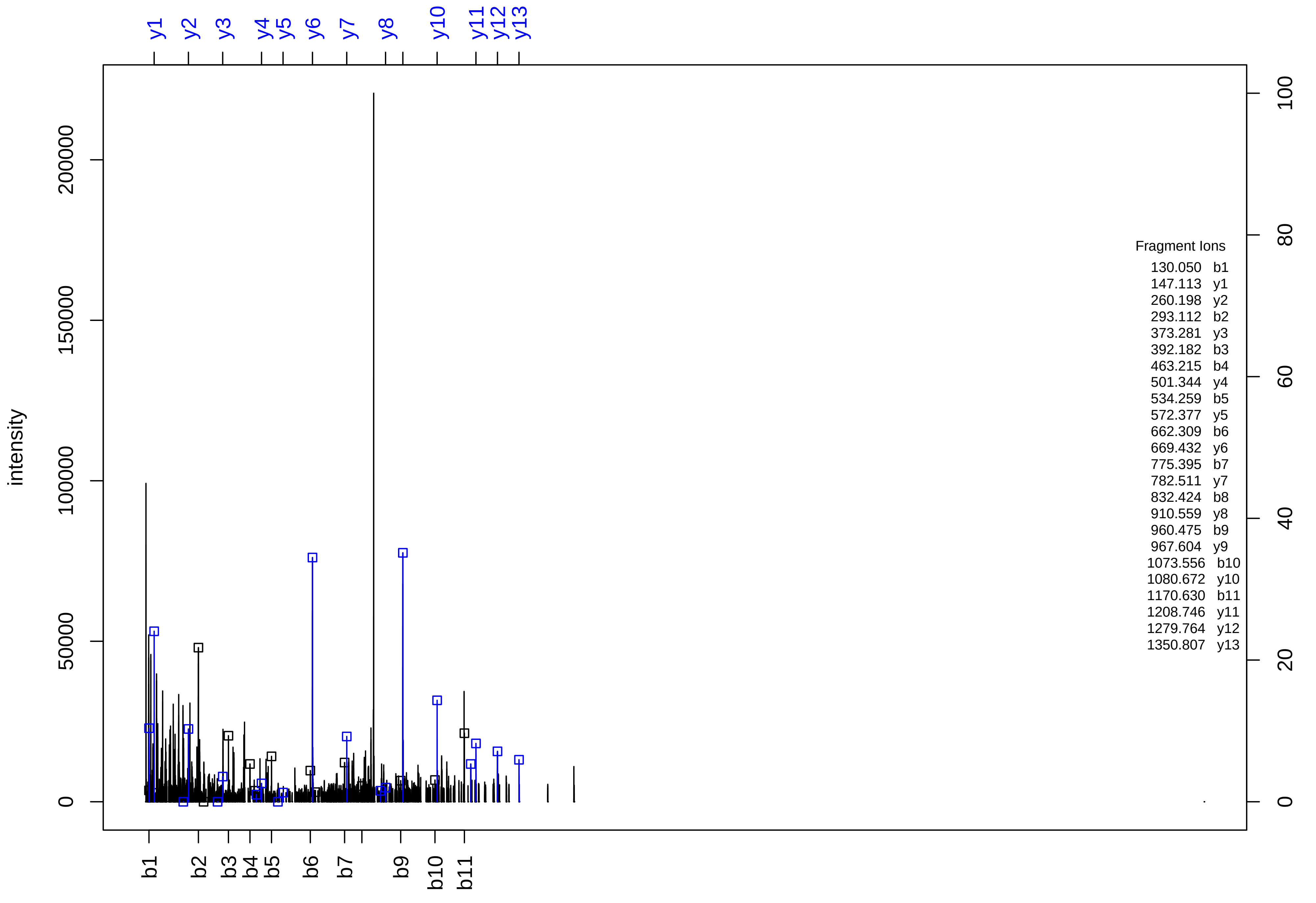

EYVAAQLGQLPAQLLK / File: sydney_ip_31.raw; SpectrumID: ; scans: 17297

ILLNPVQVFDVTK

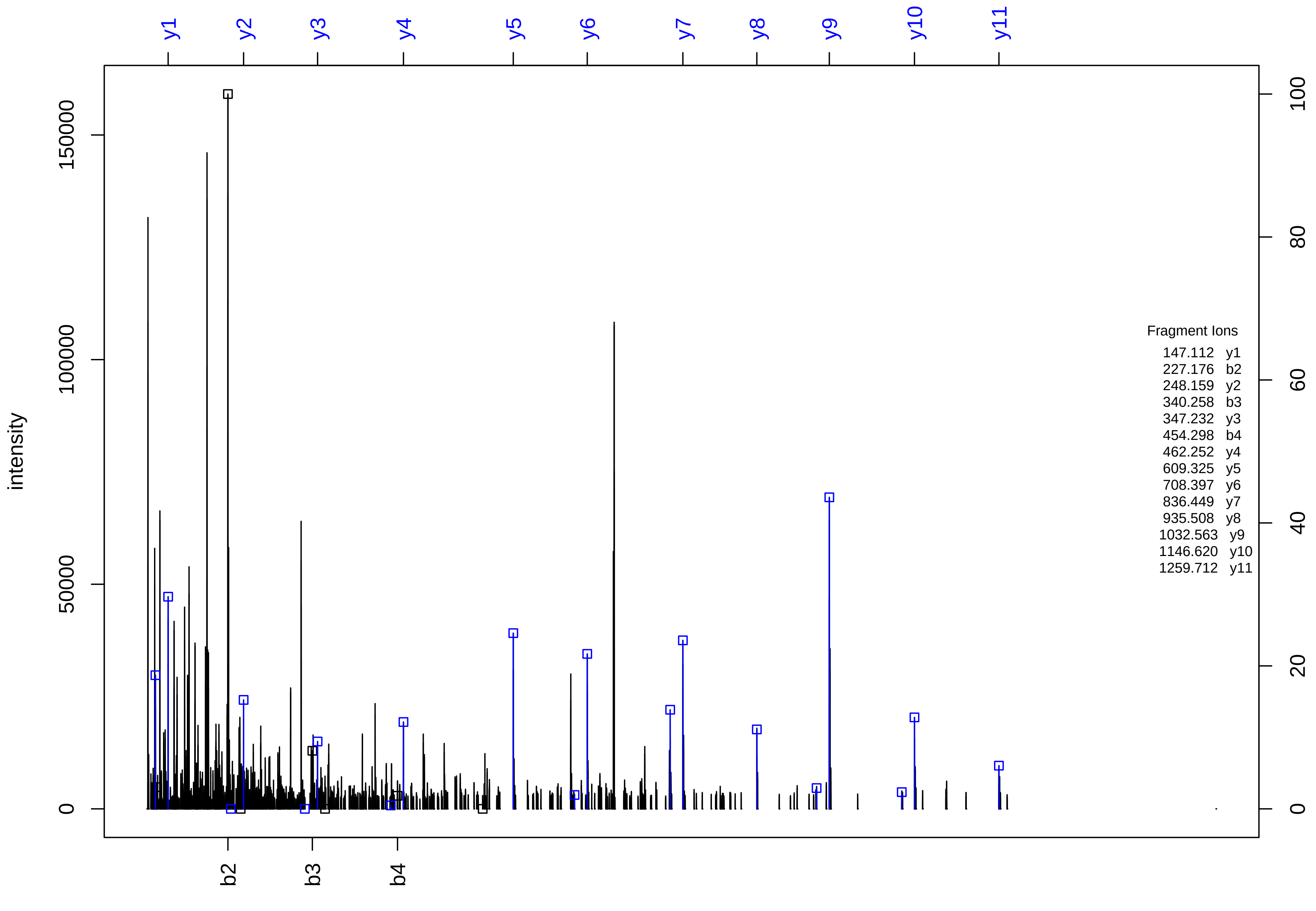

ILLNPVQVFDVTK / File: sydney_ip_31.raw; SpectrumID: ; scans: 18779

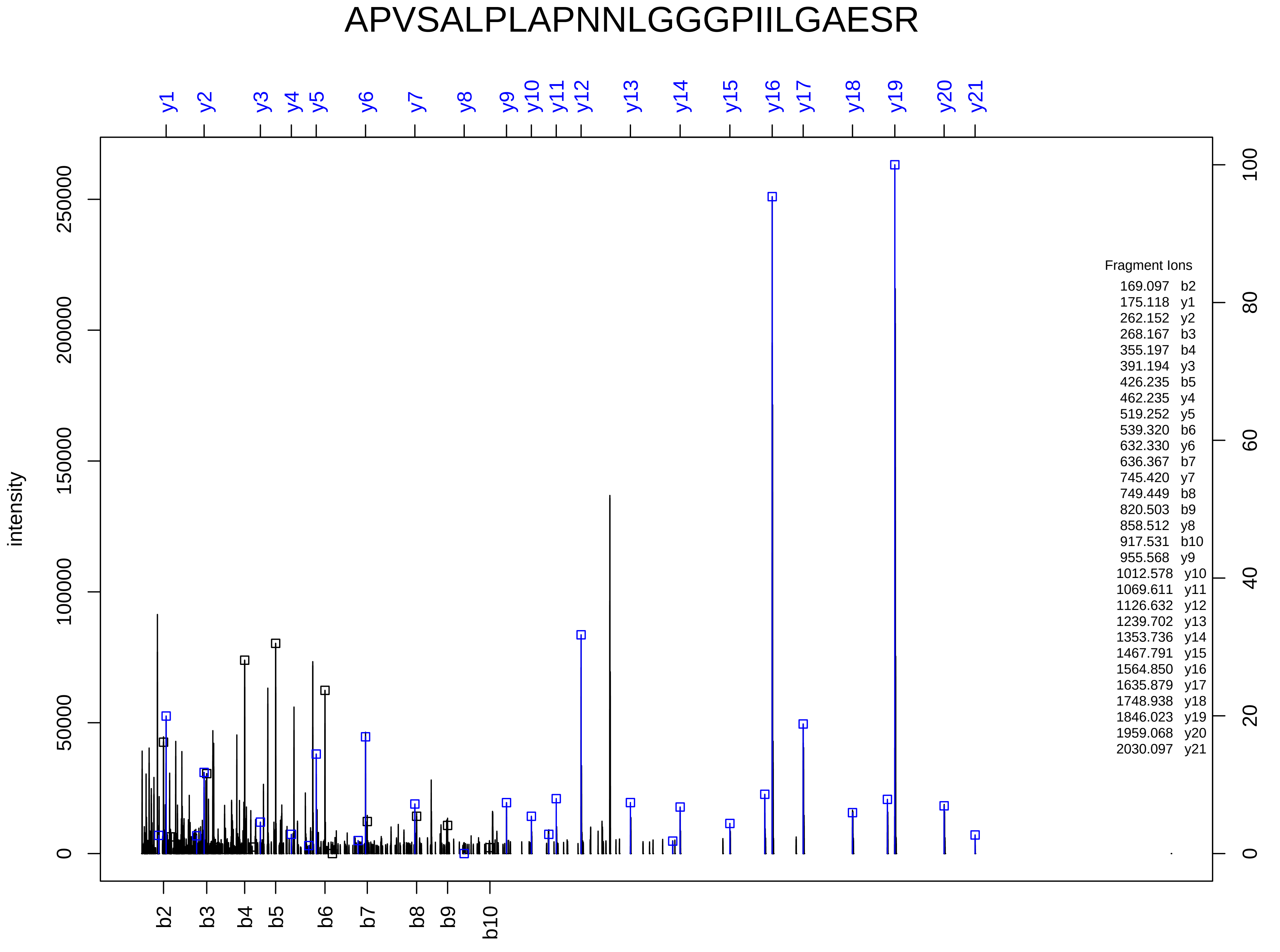

APVSALPLAPNNLGGGPIILGAESR / File: sydney_ip_31.raw; SpectrumID: ; scans: 18434

VLYDFVTDDTISPYSR

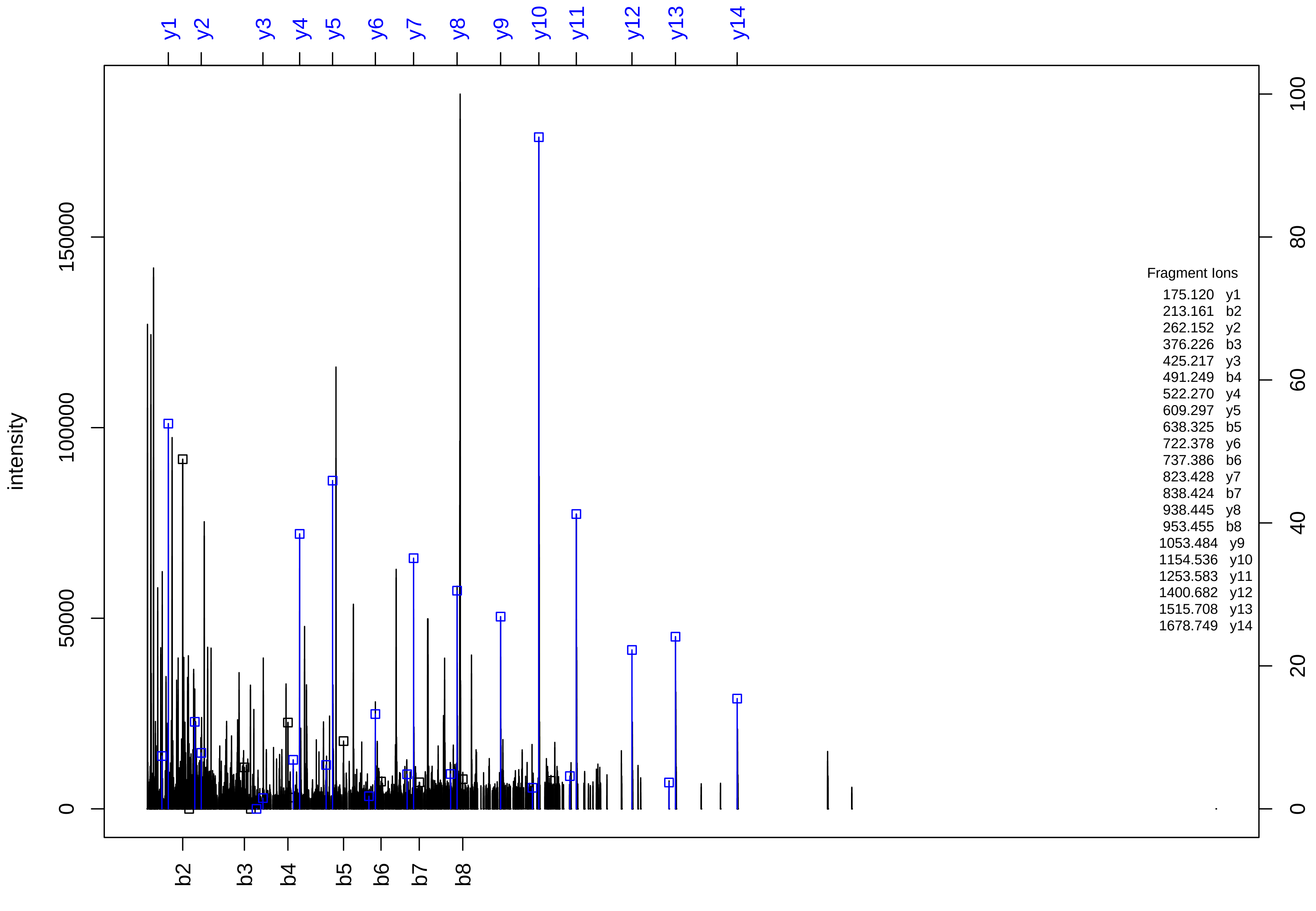

VLYDFVTDDTISPYSR / File: sydney_ip_31.raw; SpectrumID: ; scans: 14823

GDQ^FM*FRR

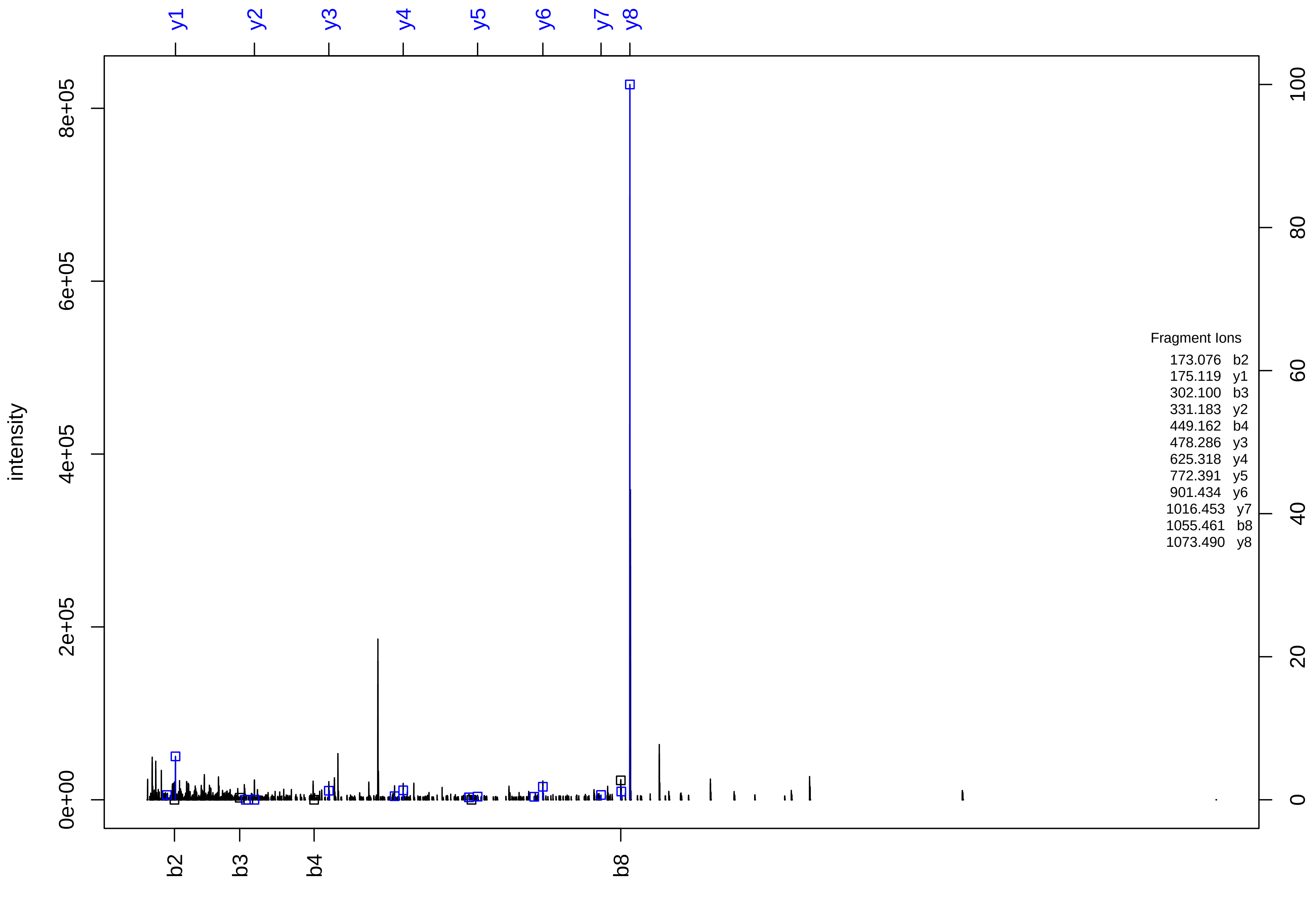

GDQFMFRR / File: sydney_ip_31.raw; SpectrumID: ; scans: 10813

VAQ^M*Q^GQ^SKIVQ^ER

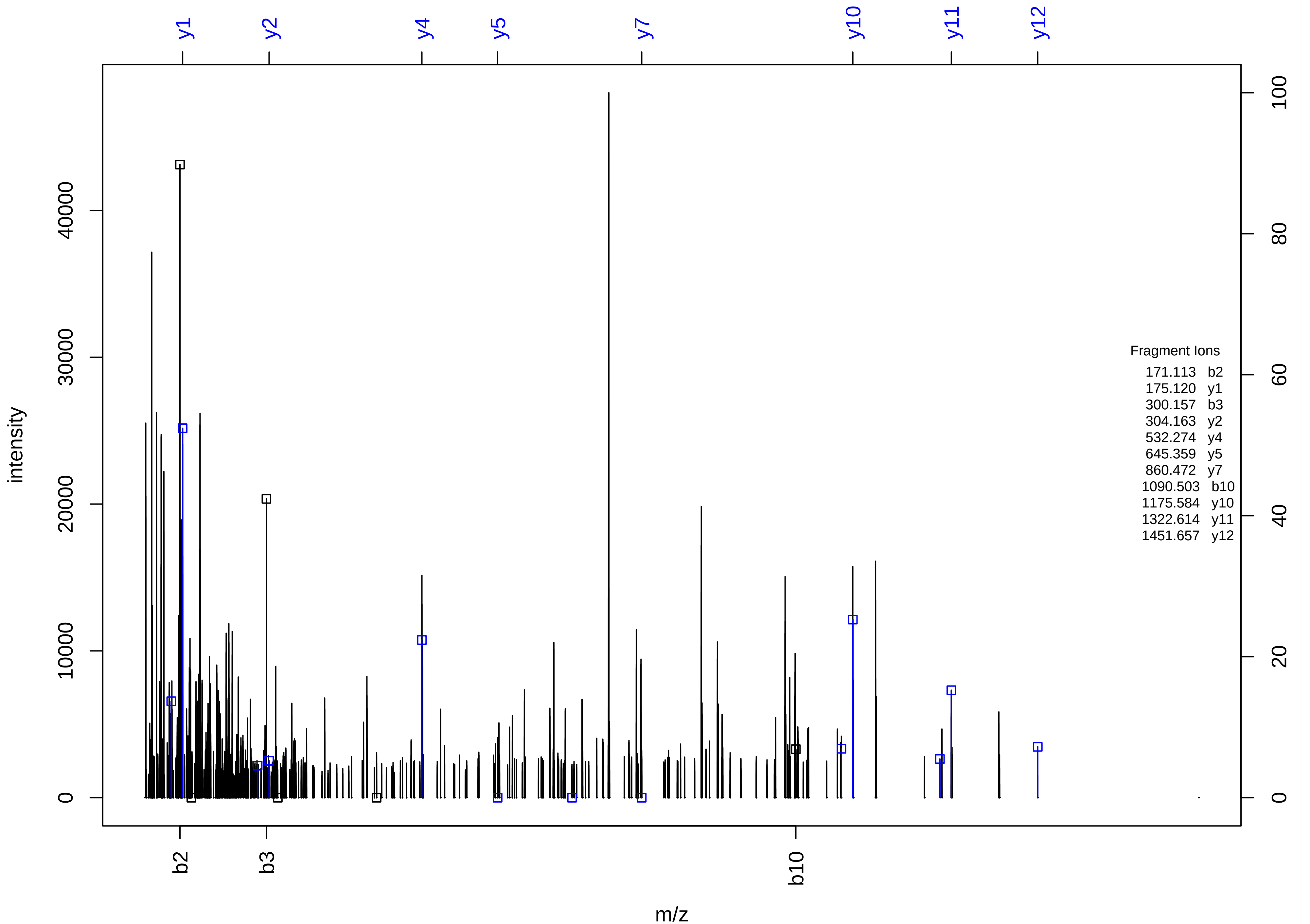

VAQMQGQSKIVQER / File: sydney_ip_31.raw; SpectrumID: ; scans: 5006

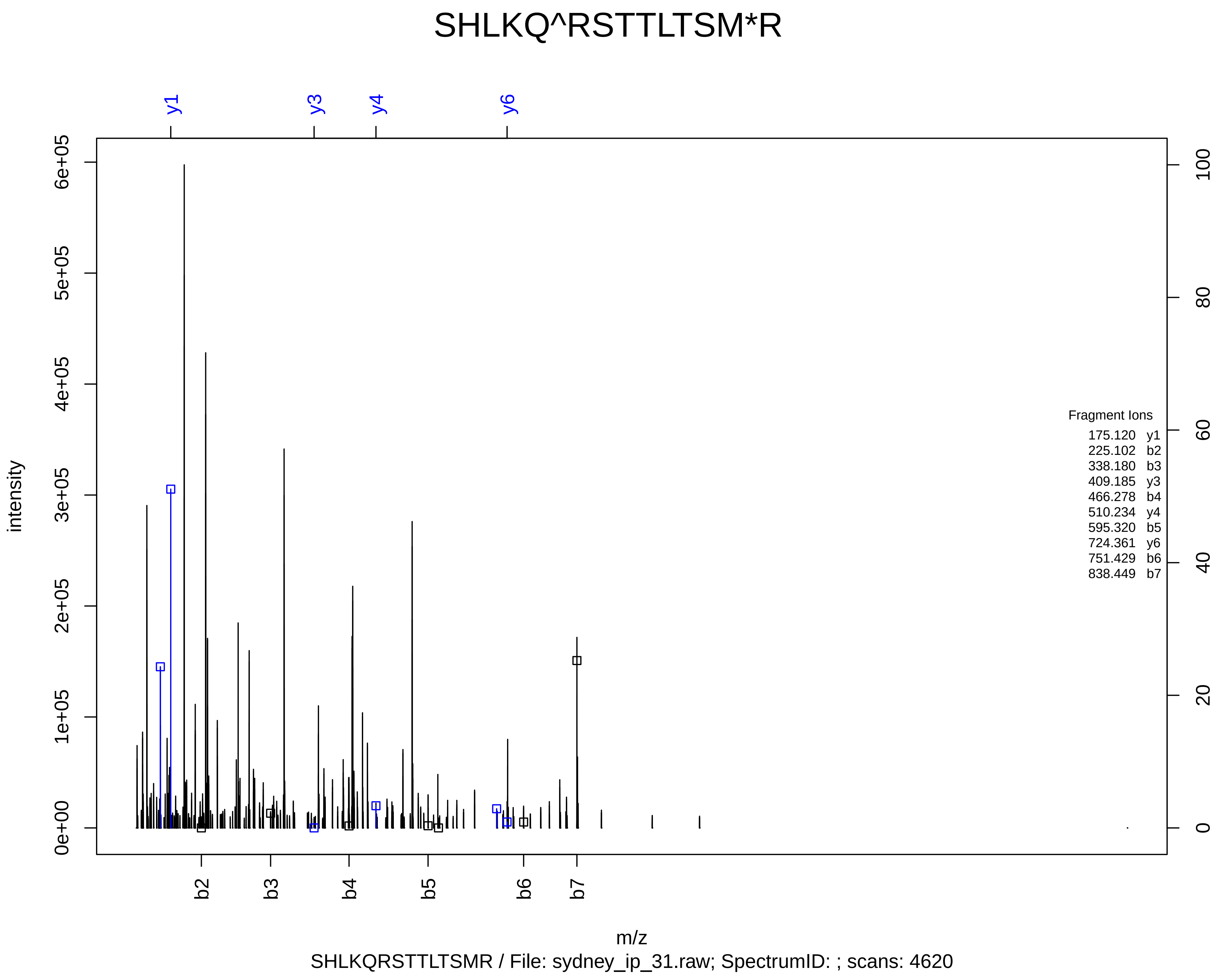
SHLKQ^RSTTLTSM*R
y1
y3
y4
y6
intensity
6e+05
5e+05
4e+05
3e+05
2e+05
1e+05
0e+00
100
80
60
40
20
0
Fragment Ions
175.120 y1
225.102 b2
338.180 b3
409.185 y3
466.278 b4
510.234 y4
595.320 b5
724.361 y6
751.429 b6
838.449 b7
b2
b3
b4
b5
b6
b7
m/z
SHLKQRSTTLTSMR / File: sydney_ip_31.raw; SpectrumID: ; scans: 4620

CLPSQLFLGQEK

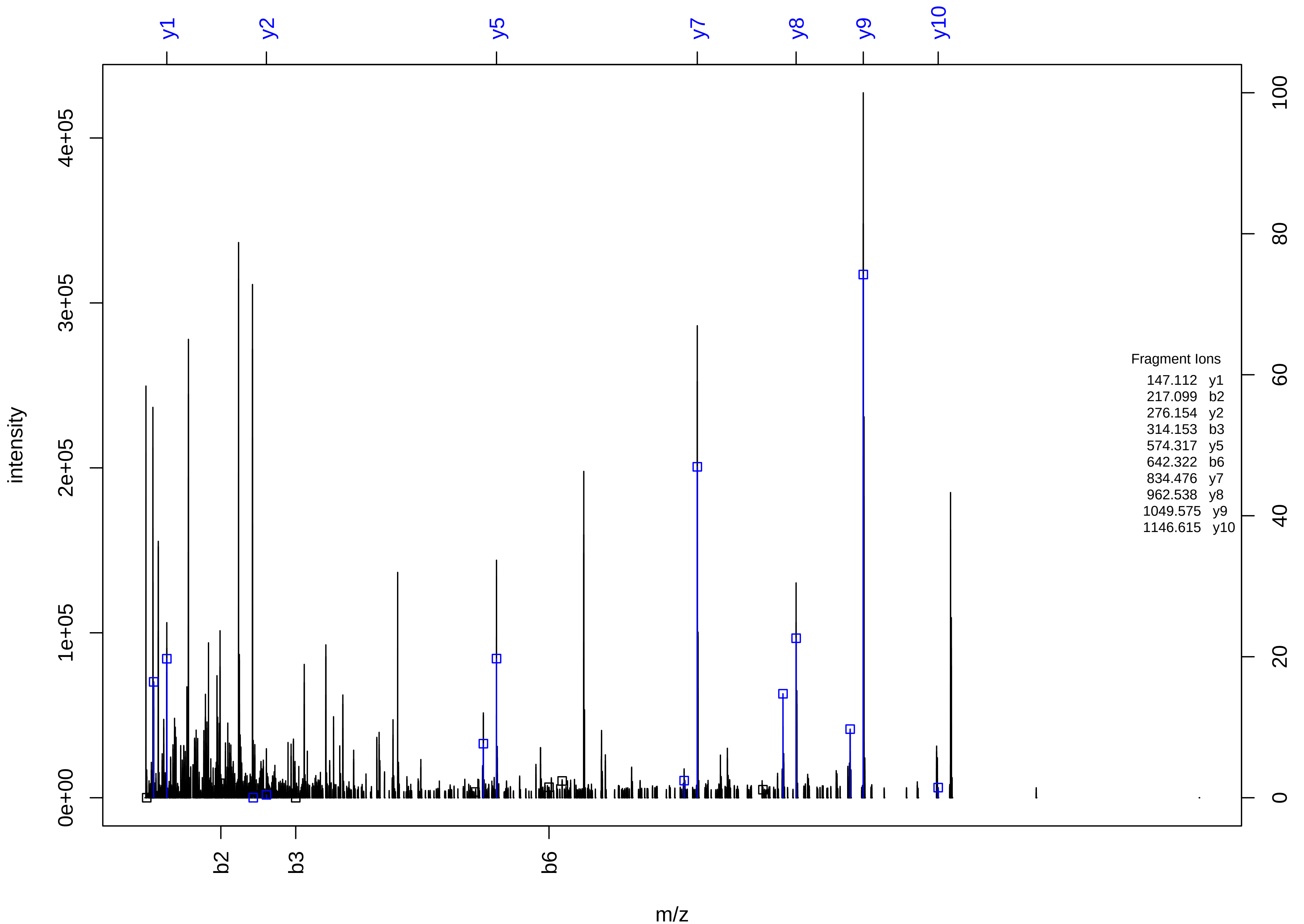

CLPSQLFLGQEK / File: sydney_ip_31.raw; SpectrumID: ; scans: 12168

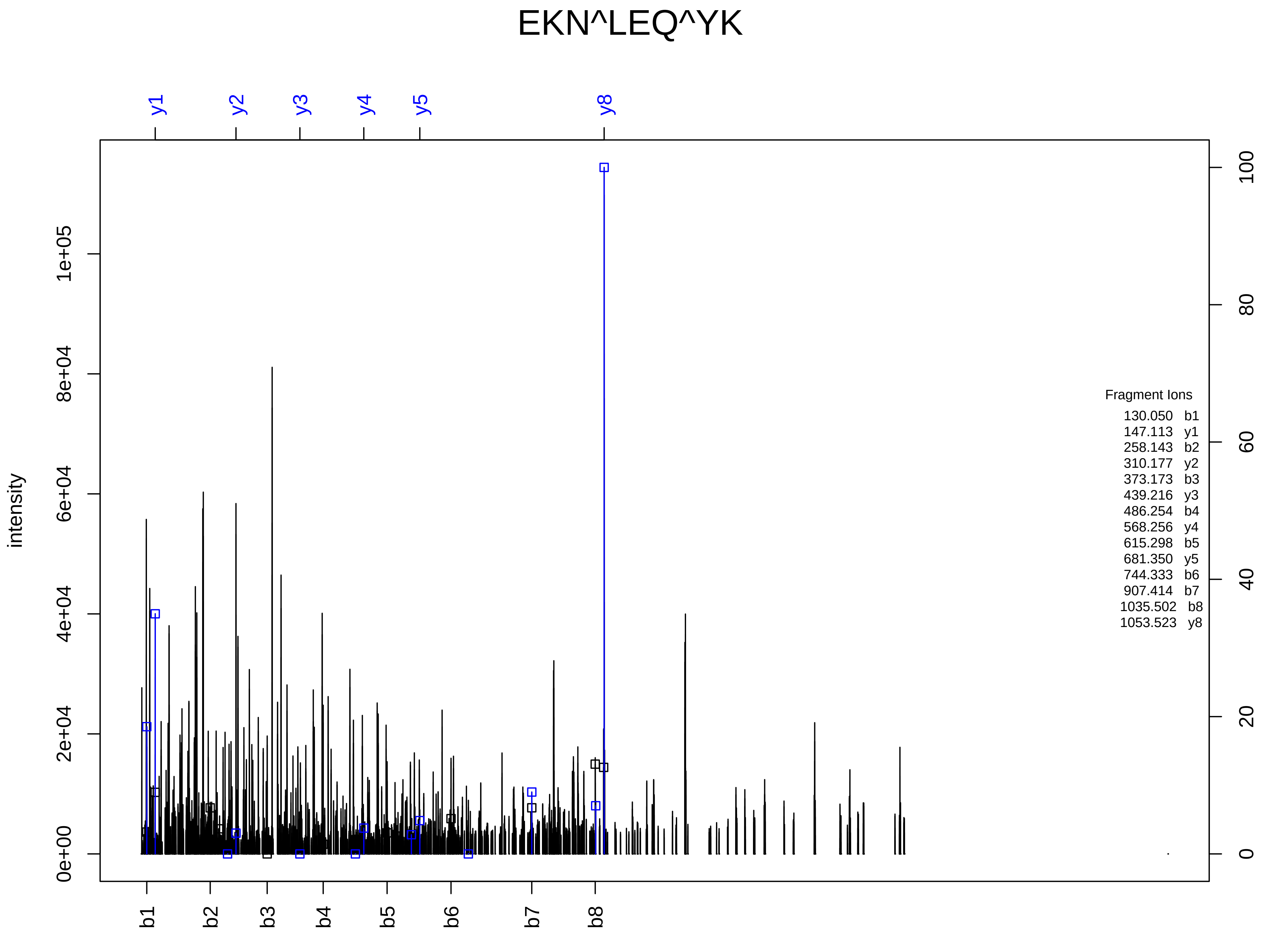

EKNLEQYK / File: sydney_ip_31.raw; SpectrumID: ; scans: 10957

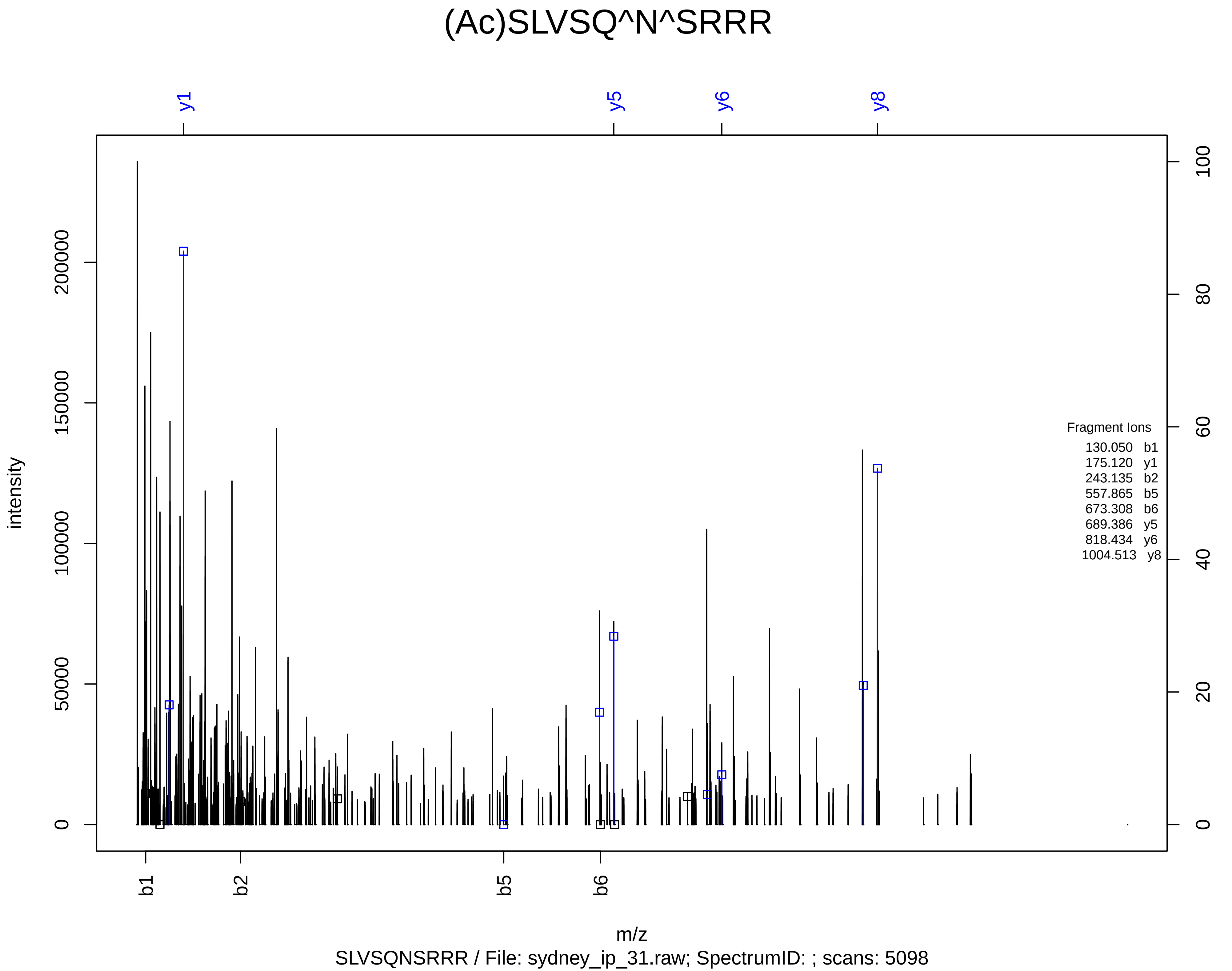
(Ac)SLVSQ^N^SRRR
y1
y5
y6
y8
intensity
0
50000
100000
150000
200000
0
20
40
60
80
100
b1
b2
b5
b6
m/z
Fragment Ions
130.050 b1
175.120 y1
243.135 b2
557.865 b5
673.308 b6
689.386 y5
818.434 y6
1004.513 y8
SLVSQNSRRR / File: sydney_ip_31.raw; SpectrumID: ; scans: 5098

LDLFSALGDAK

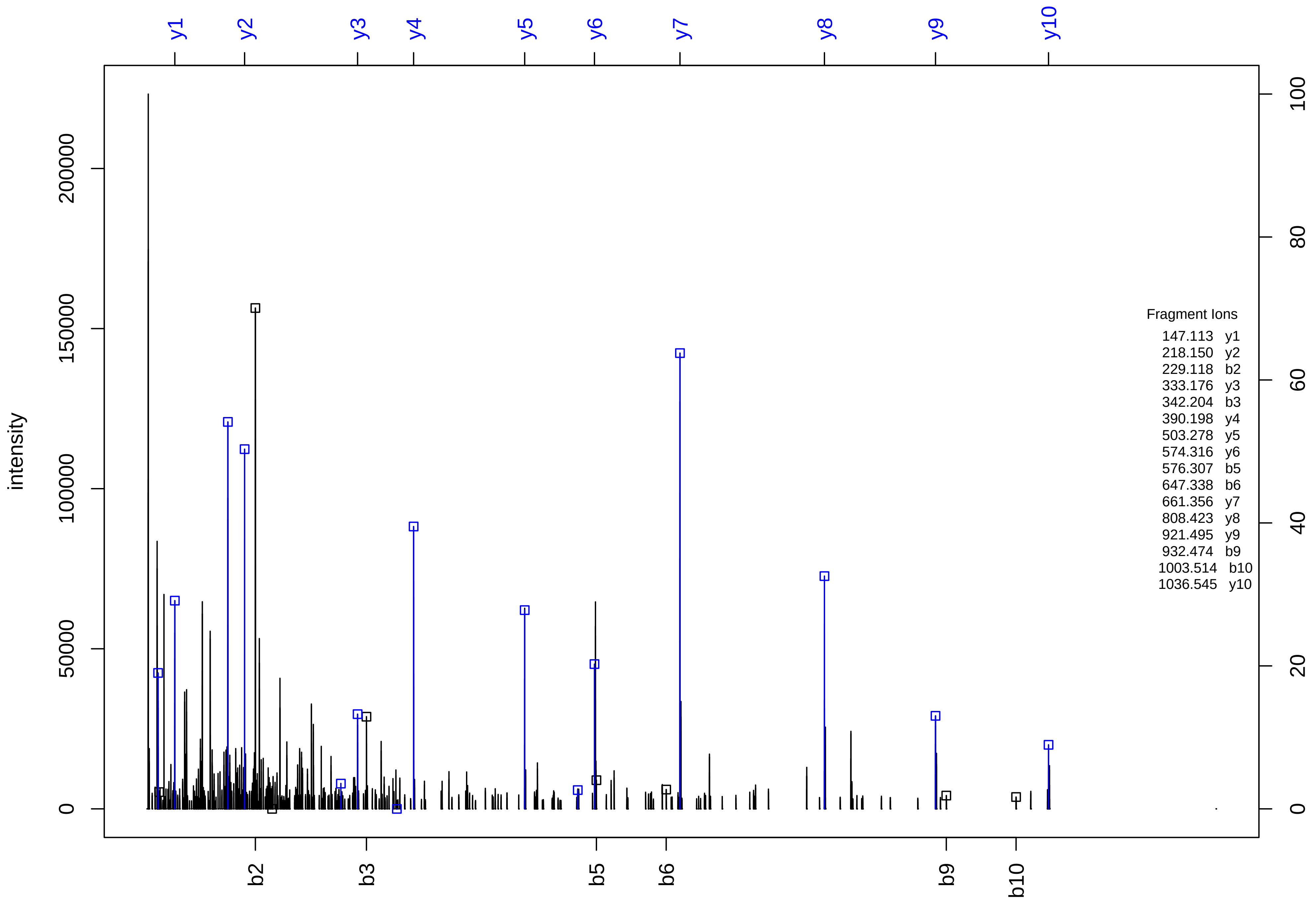

LDLFSALGDAK / File: sydney_ip_31.raw; SpectrumID: ; scans: 16305

VPFLVLECPNLK

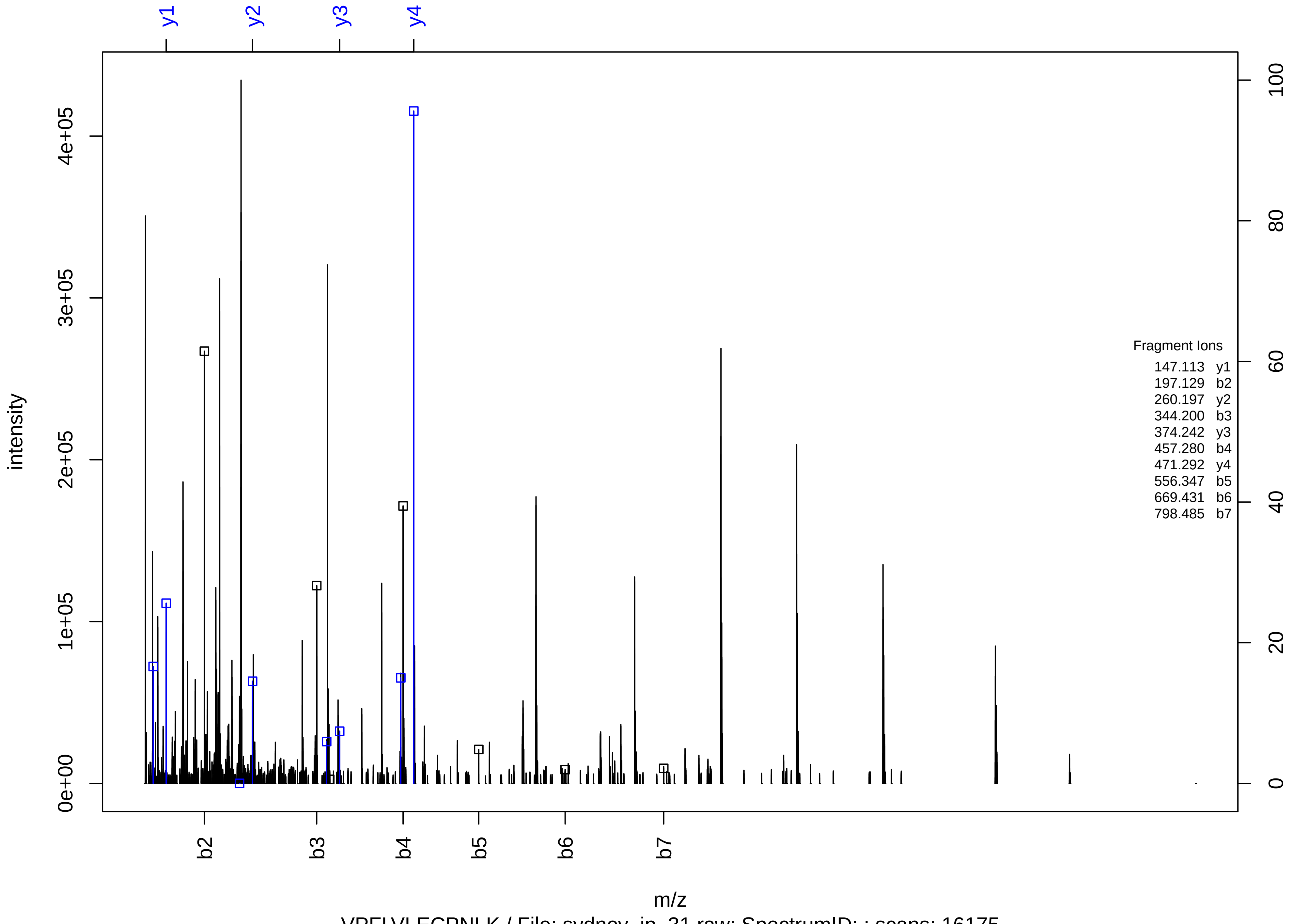

VPFLVLECPNLK / File: sydney_ip_31.raw; SpectrumID: ; scans: 16175

VQPPDADPVEIENR

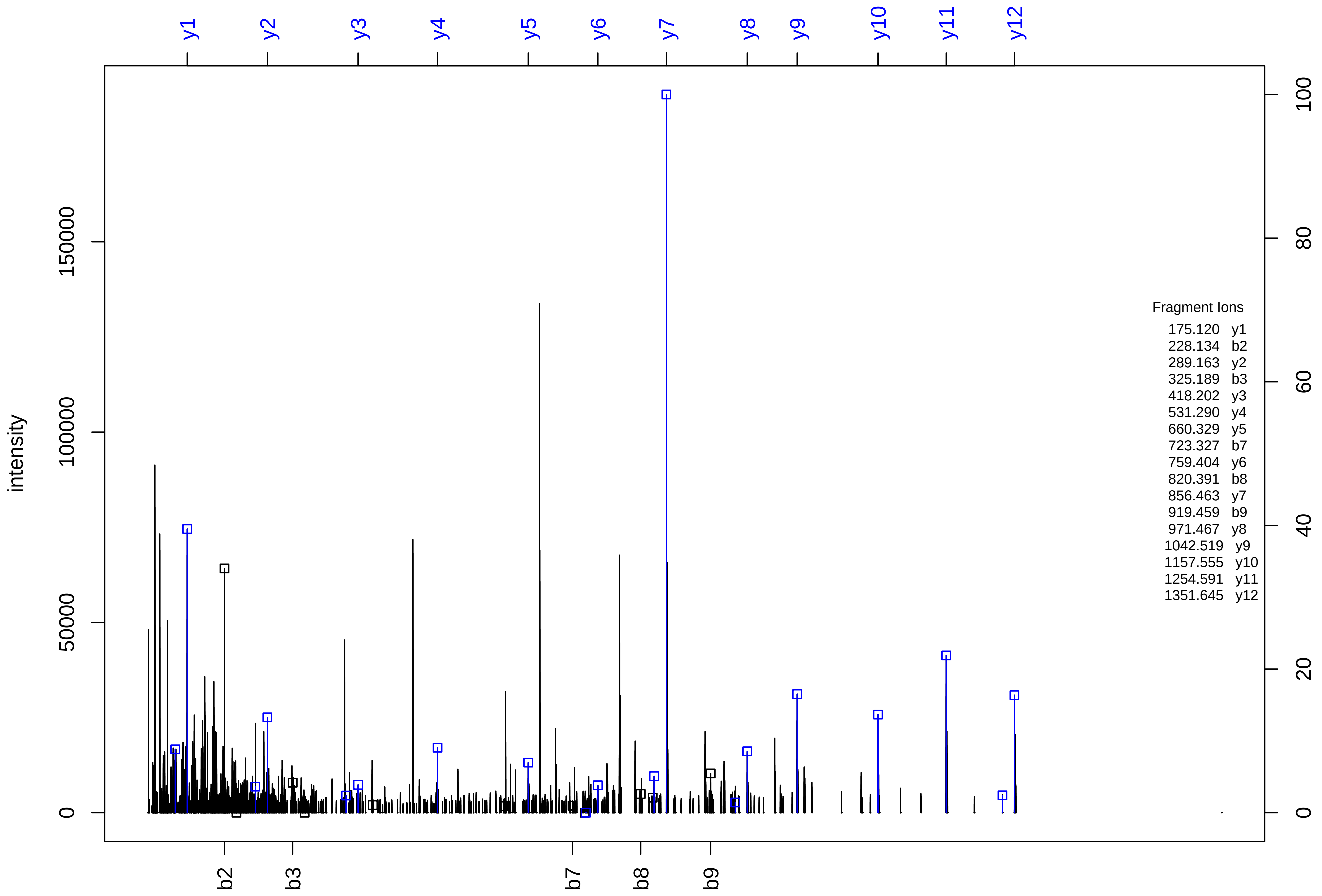

VQPPDADPVEIENR / File: sydney_ip_31.raw; SpectrumID: ; scans: 7233

IIIPQDVGLTILK

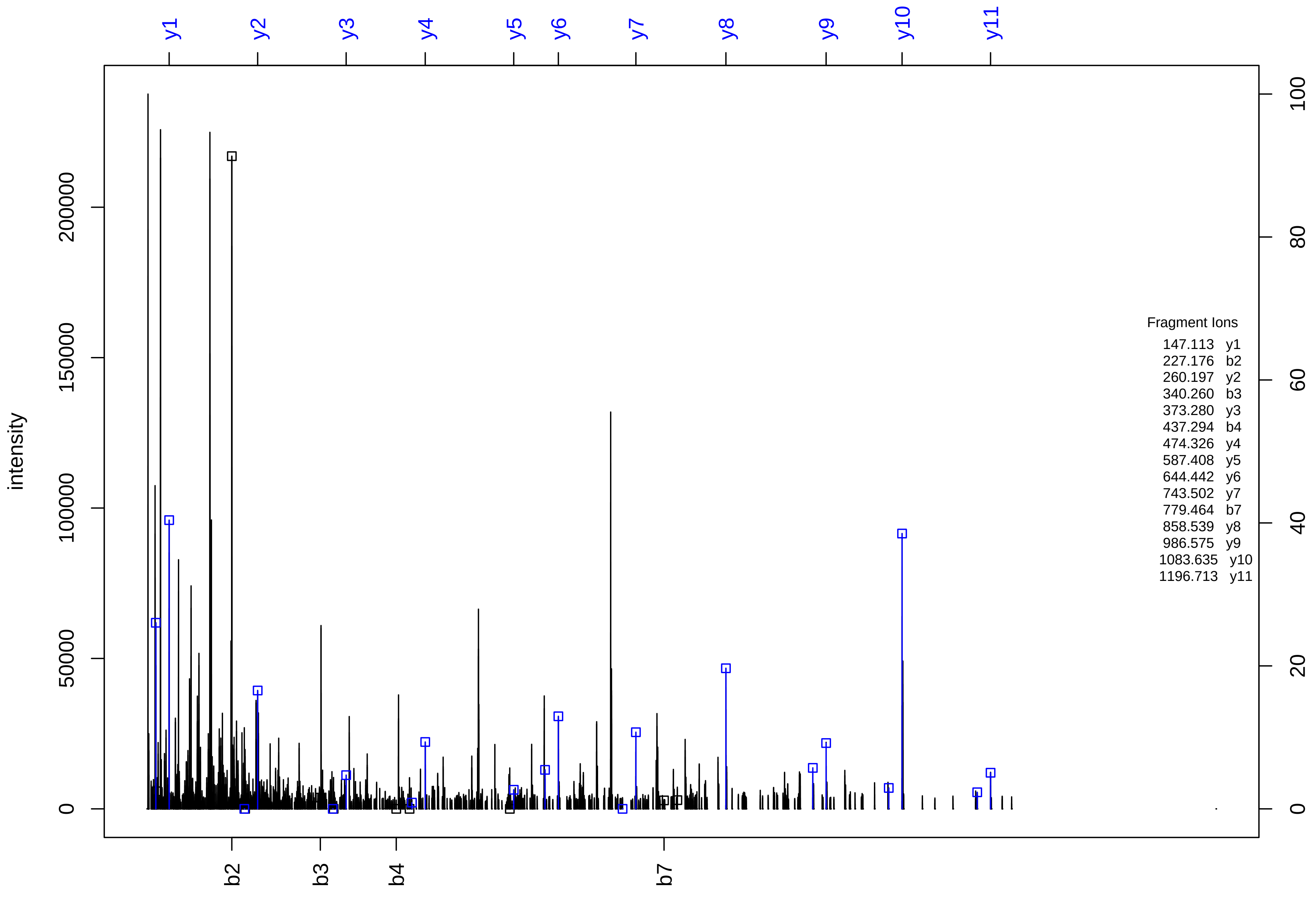

IIIPQDVGLTILK / File: sydney_ip_31.raw; SpectrumID: ; scans: 17981

DESGGGGGPEVQSHQEAASGELR

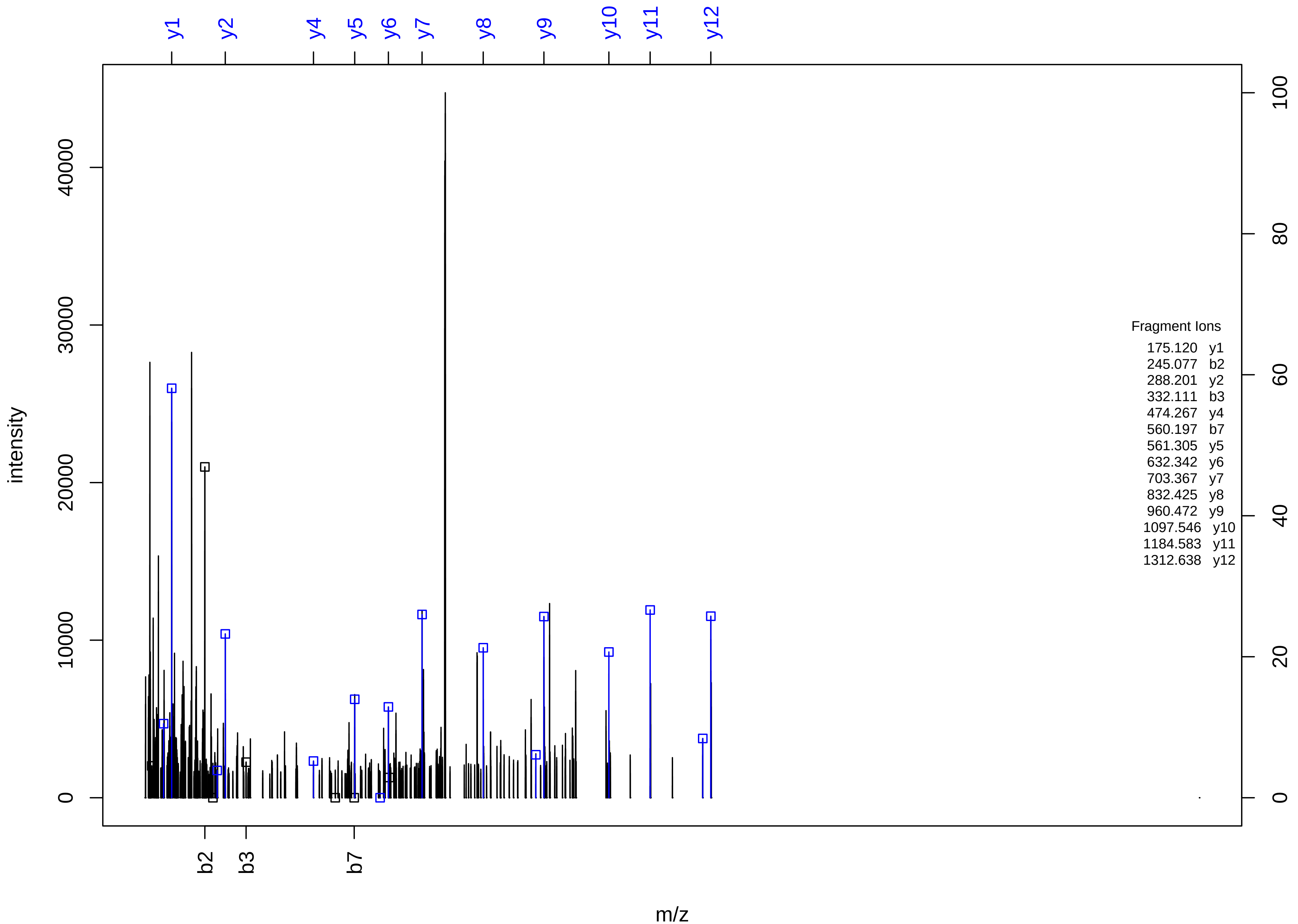

DESGGGGGPEVQSHQEAASGELR / File: sydney_ip_31.raw; SpectrumID: ; scans: 3175

TYAEILEPFHPVR

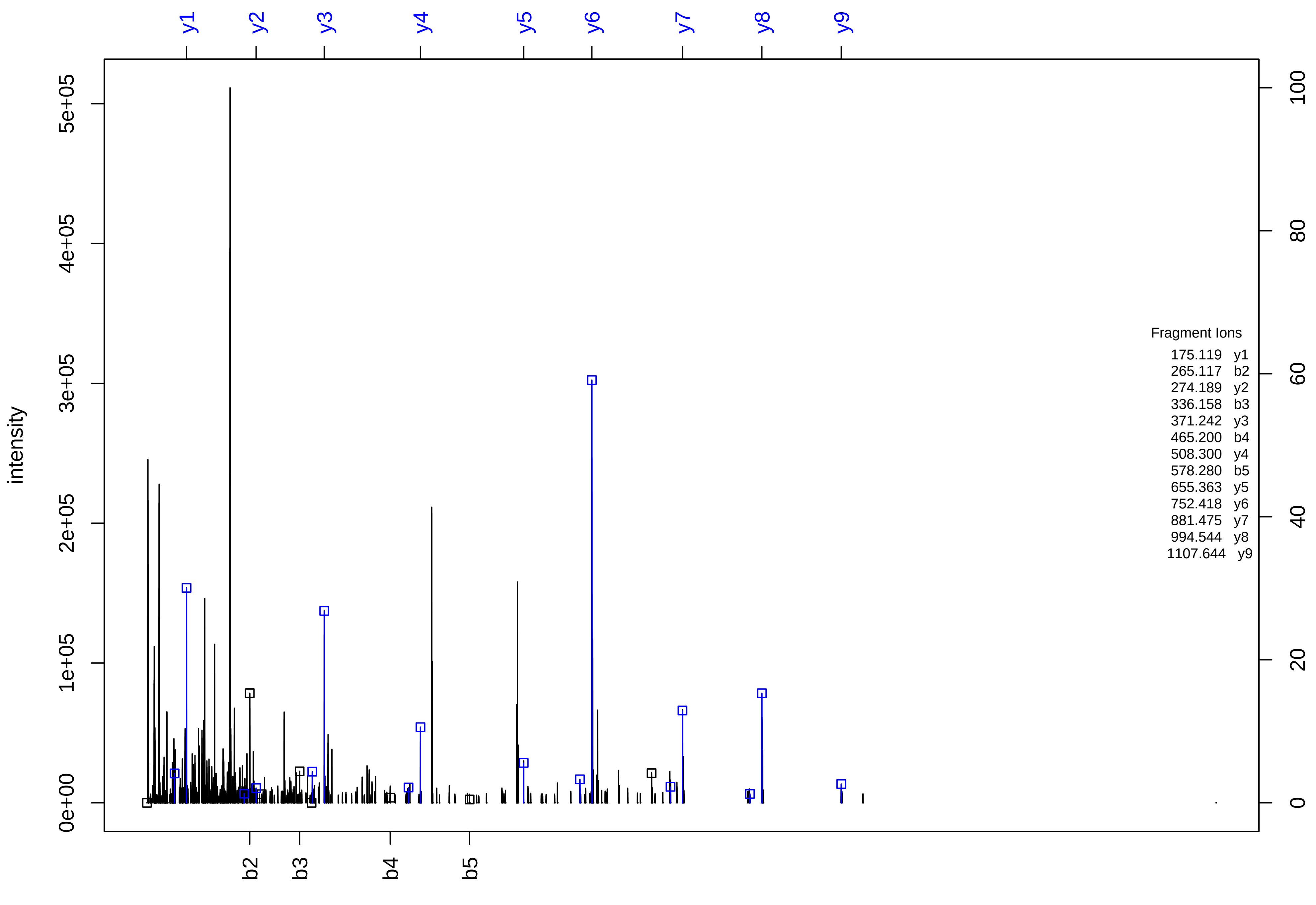

TYAEILEPFHPVR / File: sydney_ip_31.raw; SpectrumID: ; scans: 12241

LNEIVGNEDTVSR

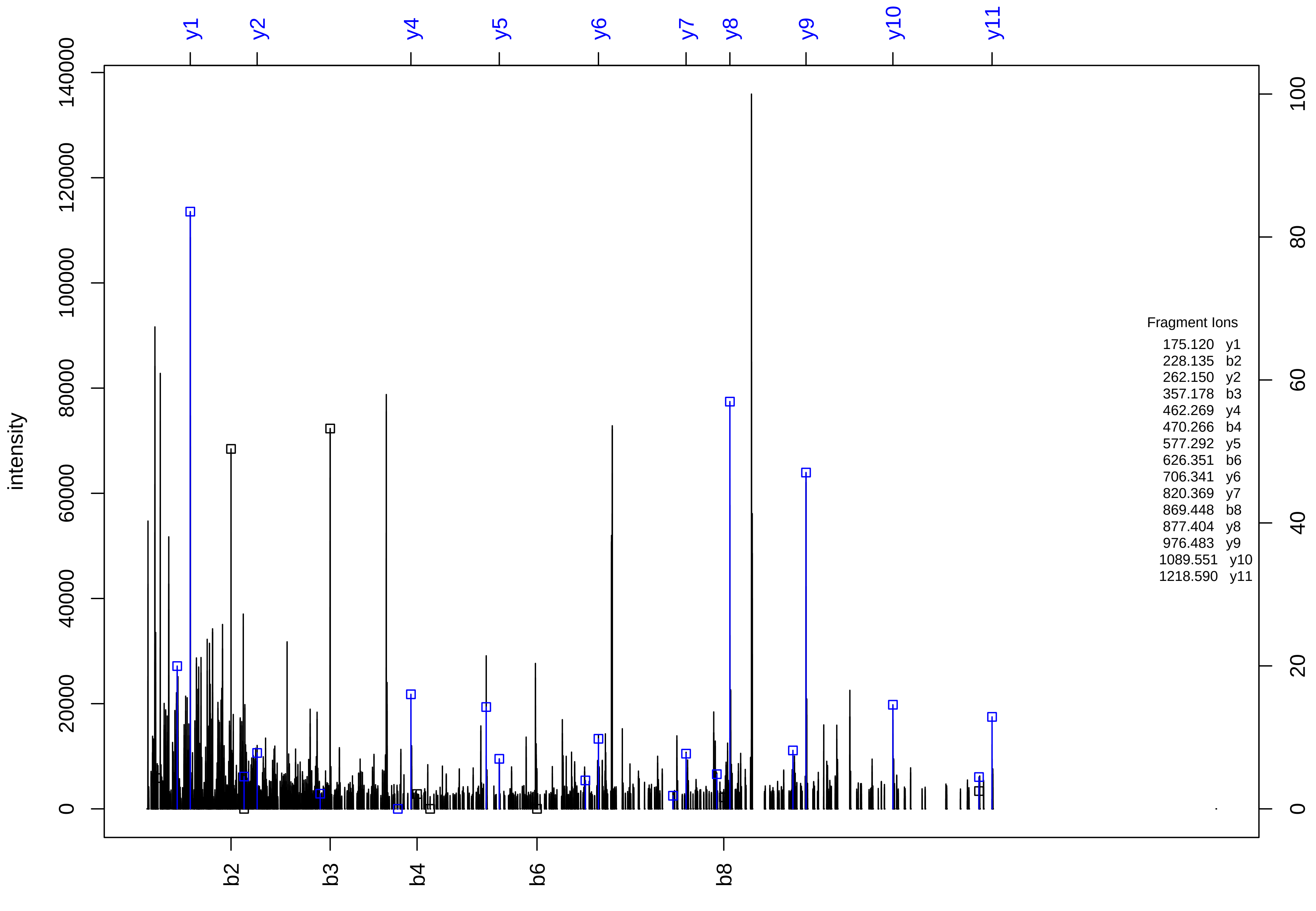

LNEIVGNEDTVSR / File: sydney_ip_31.raw; SpectrumID: ; scans: 5332

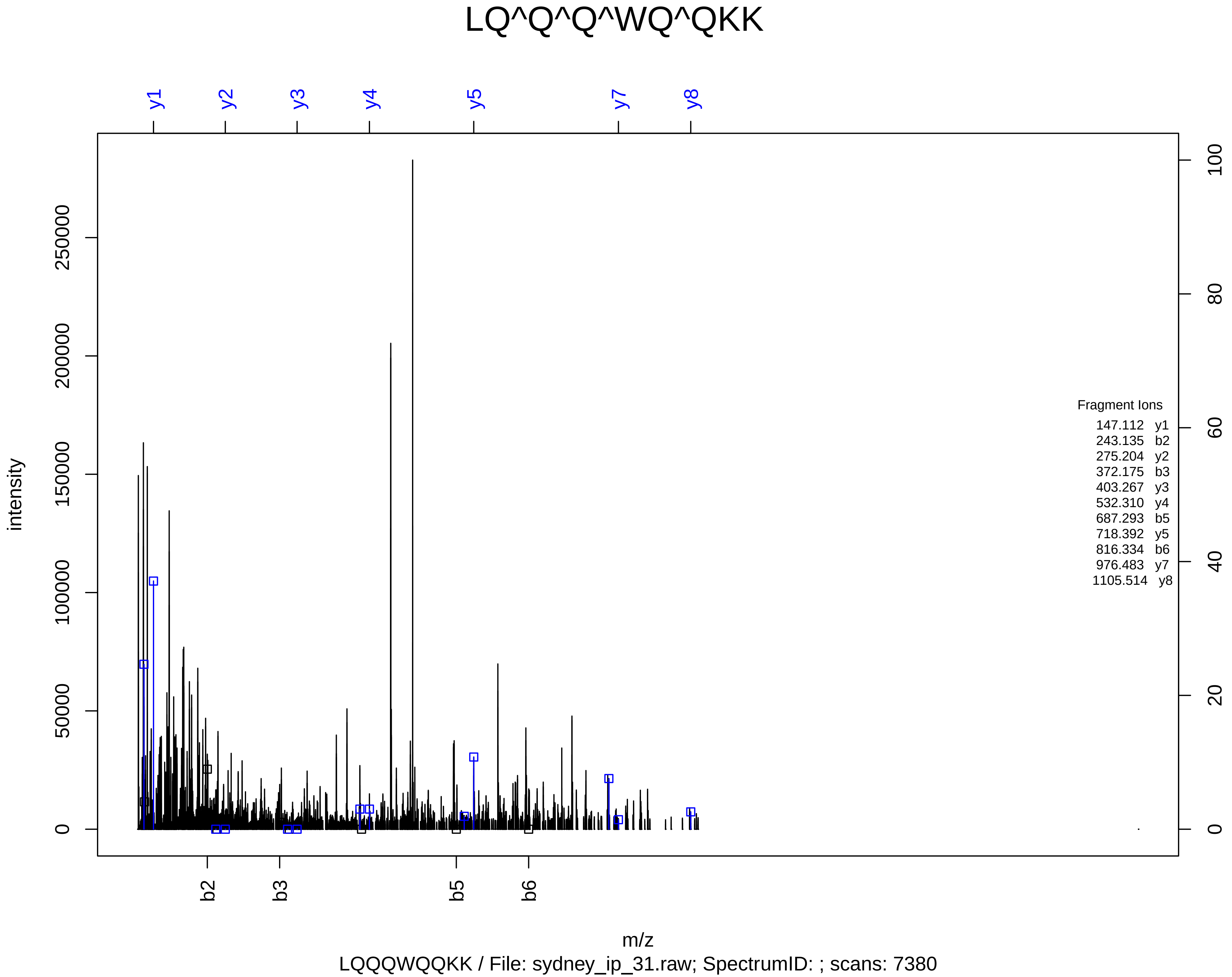
LQ^Q^Q^WQ^QKK
y1
y2
y3
y4
y5
y7
y8
intensity
0
50000
100000
150000
200000
250000
0
20
40
60
80
100
Fragment Ions
147.112 y1
243.135 b2
275.204 y2
372.175 b3
403.267 y3
532.310 y4
687.293 b5
718.392 y5
816.334 b6
976.483 y7
1105.514 y8
b2
b3
b5
b6
m/z
LQQQWQQKK / File: sydney_ip_31.raw; SpectrumID: ; scans: 7380

(Ac)AAPSLLNWR

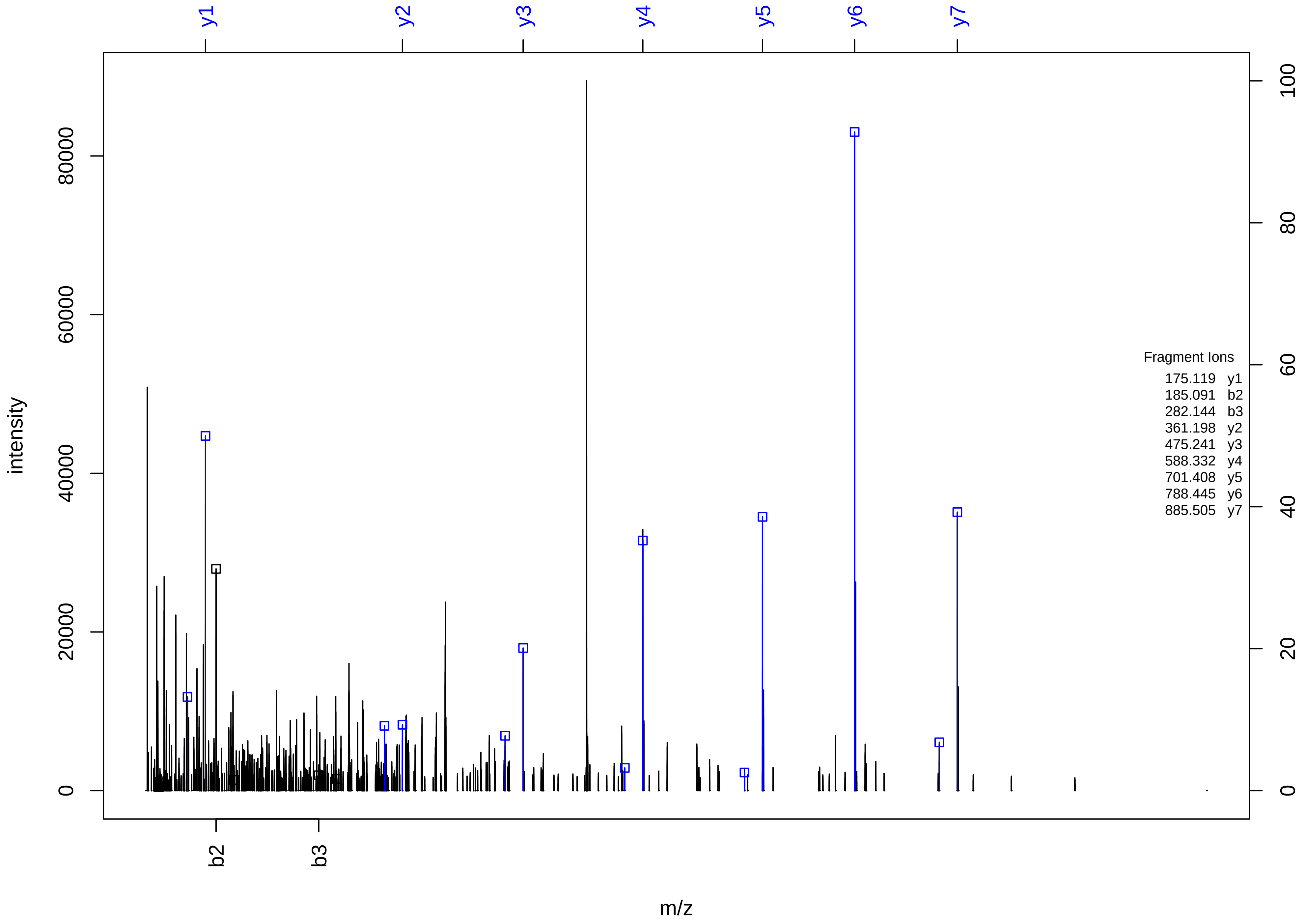

AAPSLLNWR / File: sydney_ip_31.raw; SpectrumID: ; scans: 17291

EGPSSIVQAPPVLEVVPPLGR

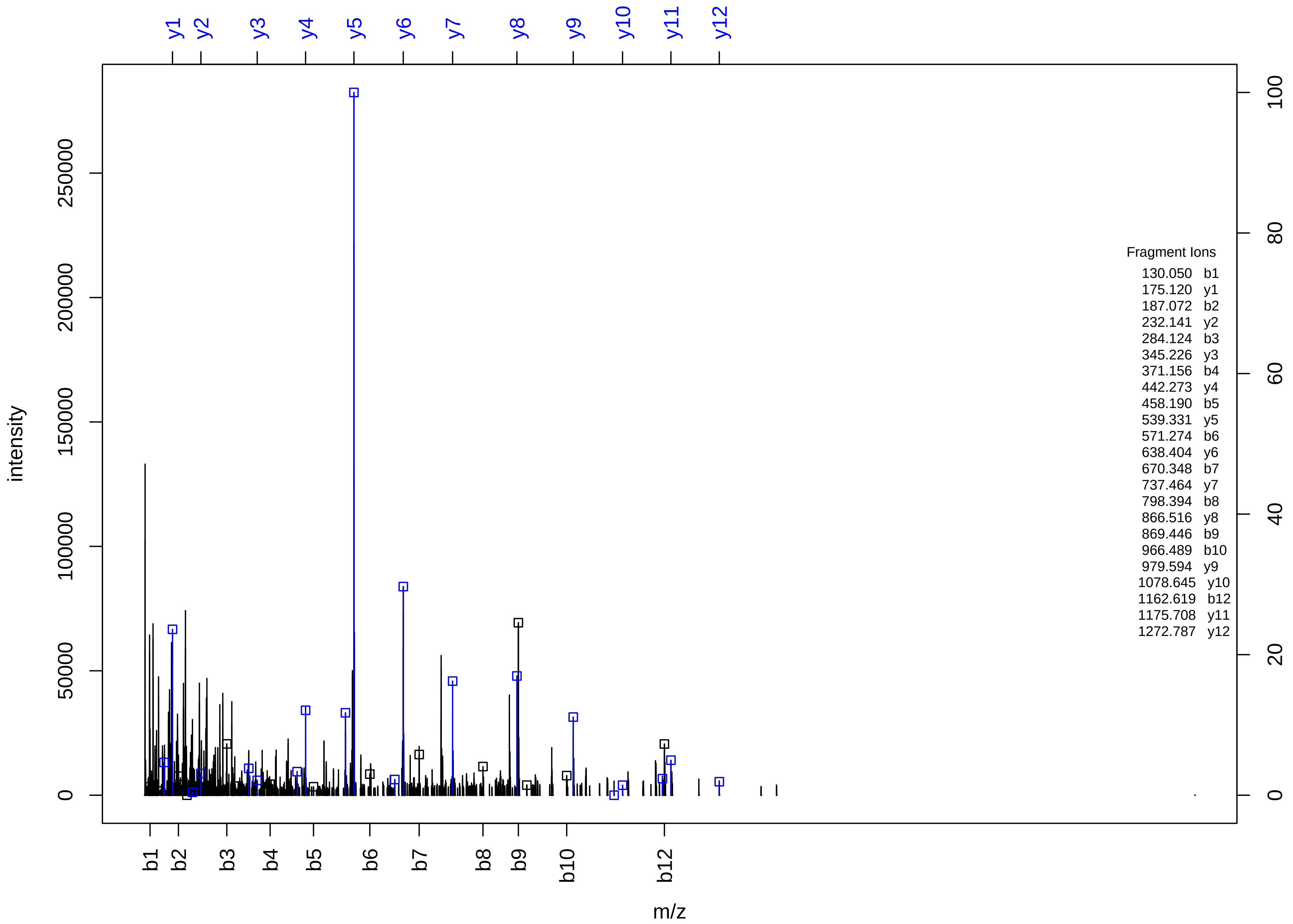

EGPSSIVQAPPVLEVVPPLGR / File: sydney_ip_31.raw; SpectrumID: ; scans: 19593

AM*LSGPGQFAENETNEVNFR

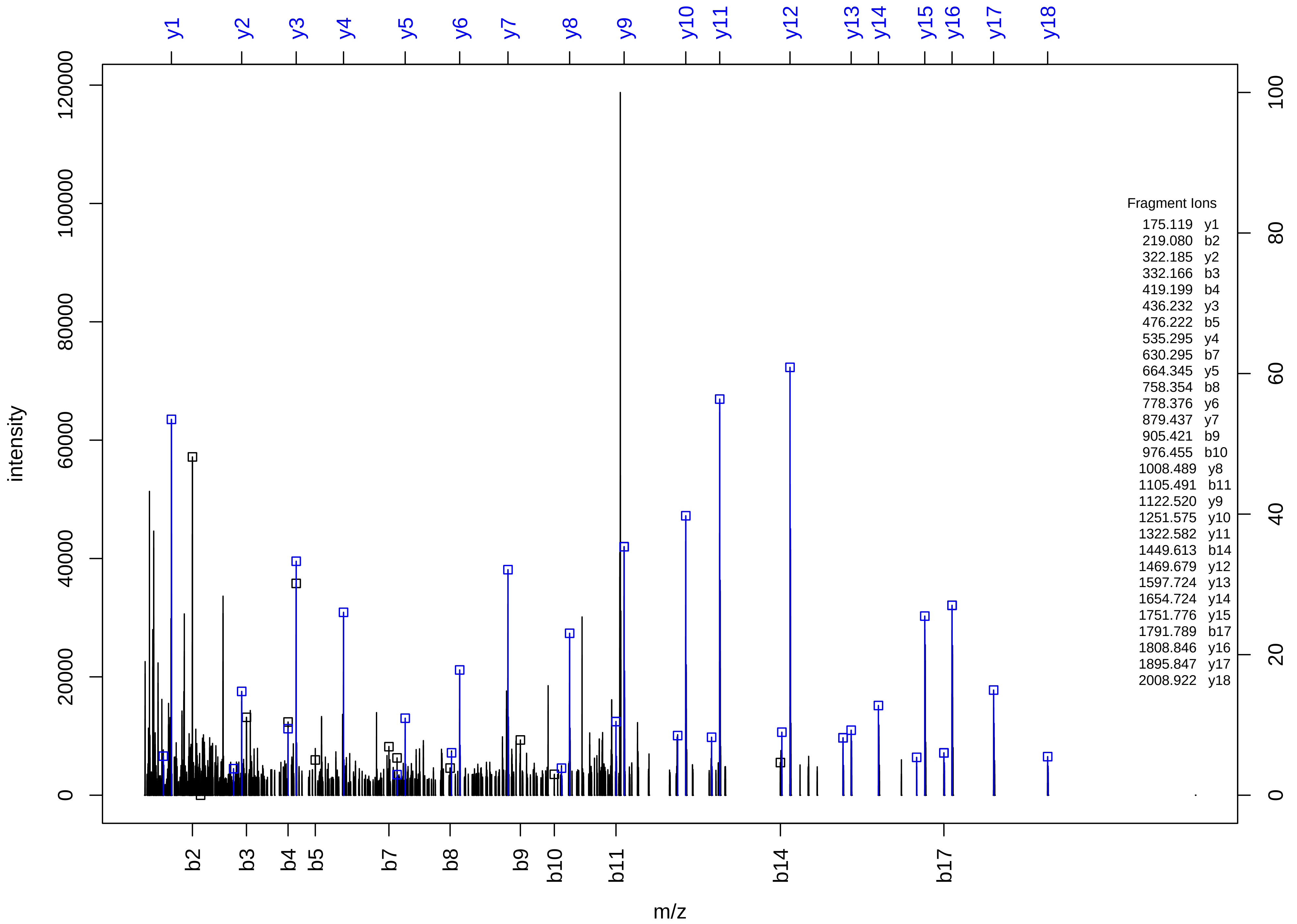

AMLSGPGQFAENETNEVNFR / File: sydney_ip_32.raw; SpectrumID: ; scans: 10231

YIDLEPDTPLSPEELK

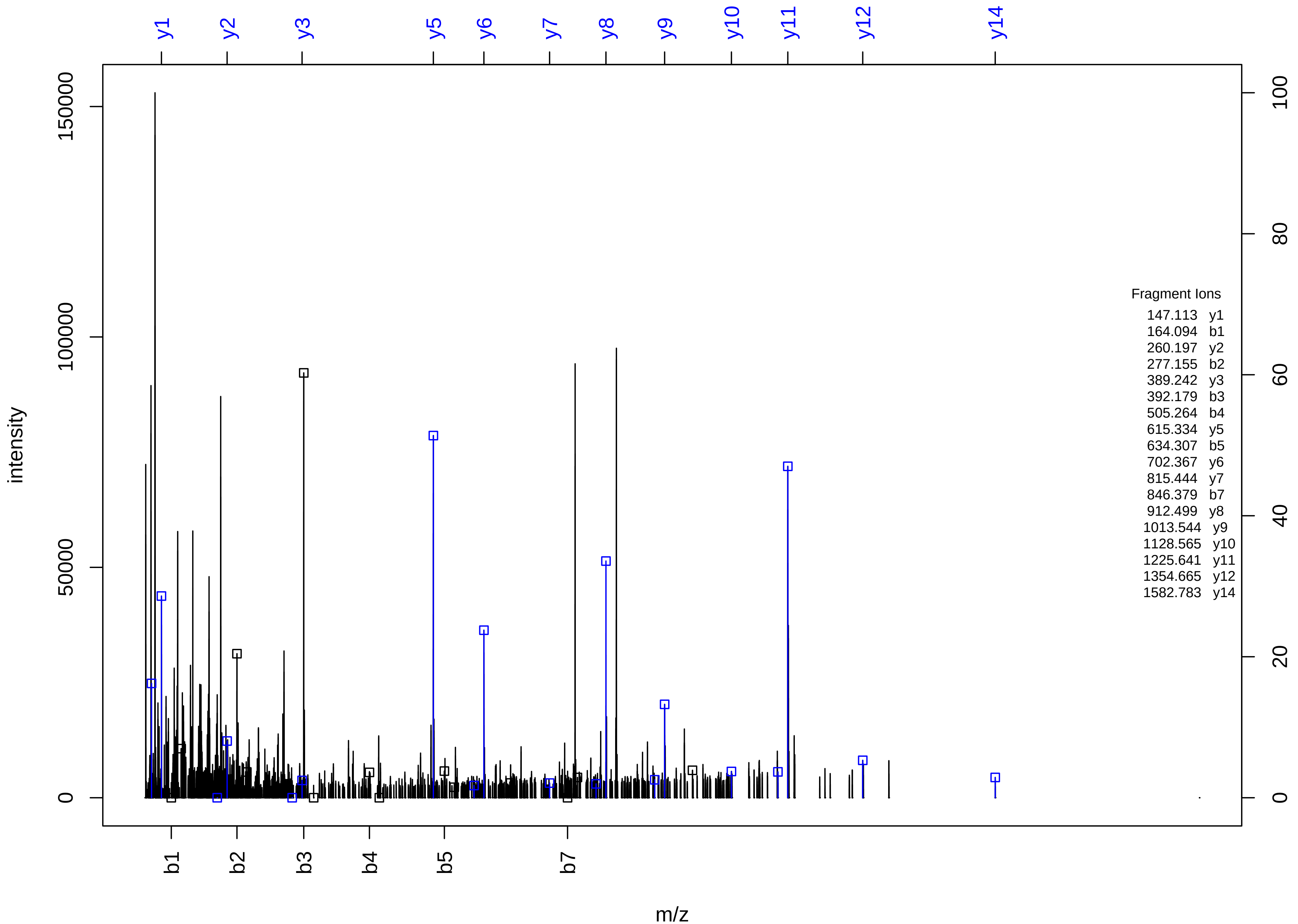

YIDLEPDTPLSPEELK / File: sydney_ip_32.raw; SpectrumID: ; scans: 13985

EQSSFPSEPTPNPELR

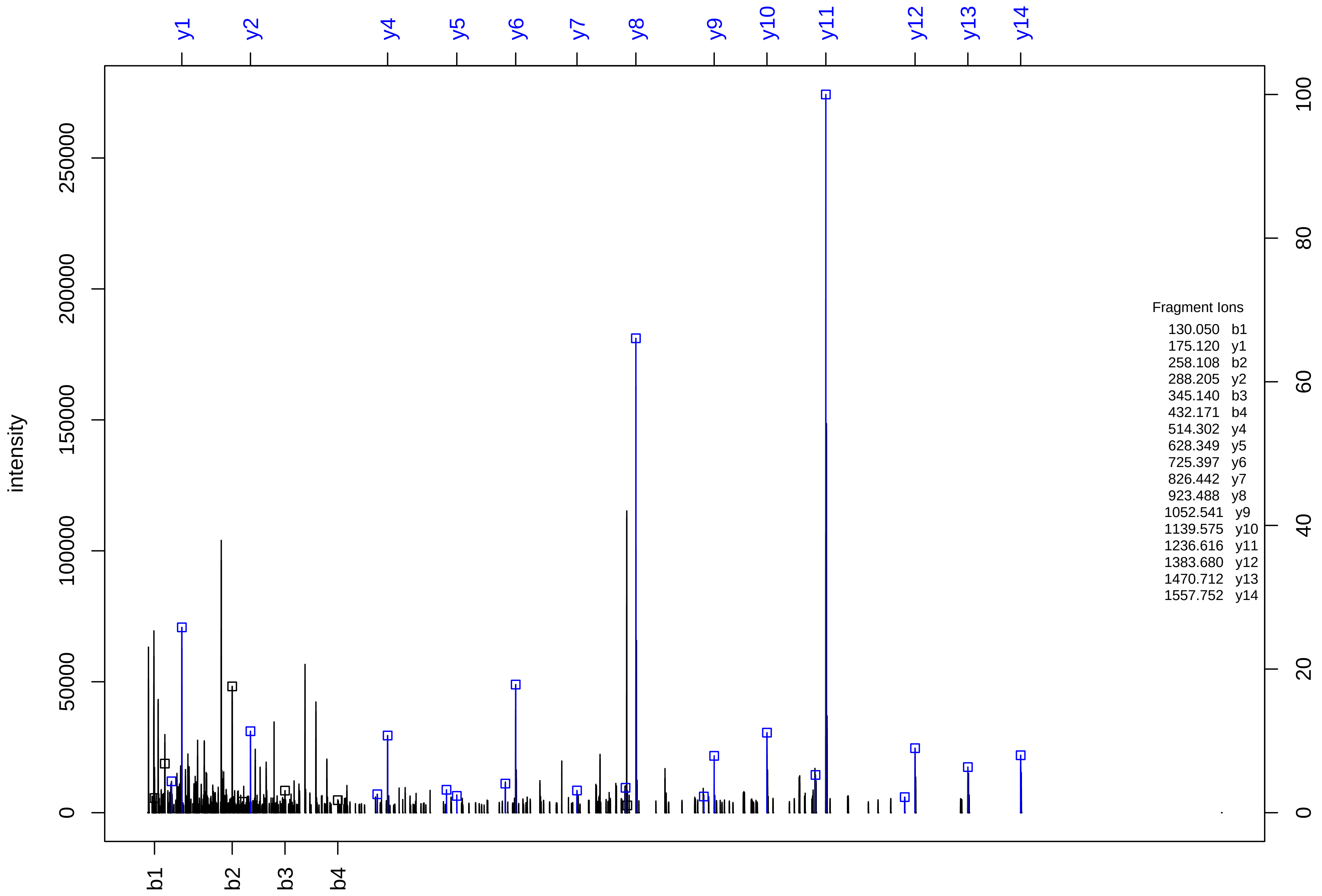

EQSSFPSEPTPNPELR / File: sydney_ip_32.raw; SpectrumID: ; scans: 7264

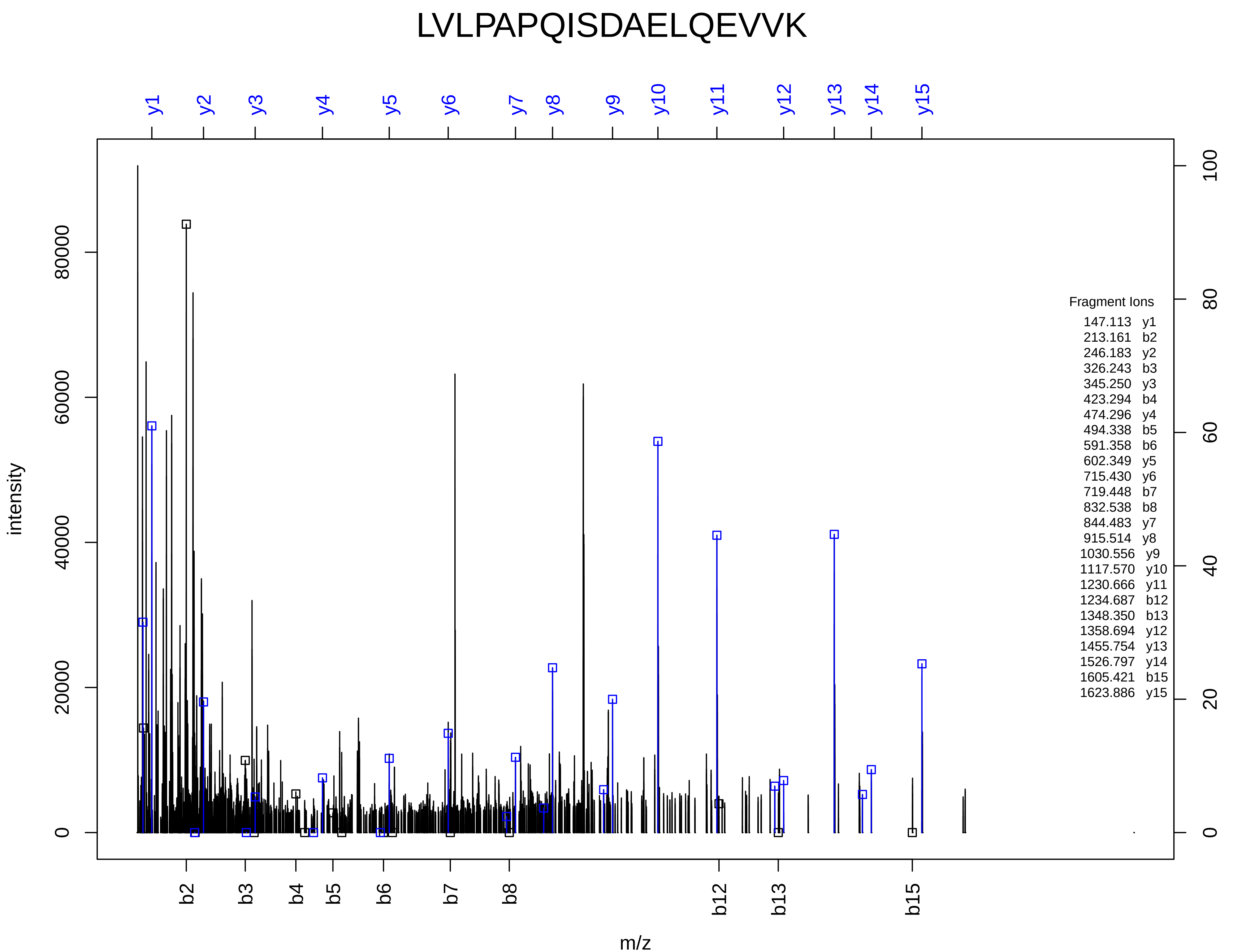

LVLPAPQISDAELQEVVK / File: sydney_ip_32.raw; SpectrumID: ; scans: 16597

DILLLPLR

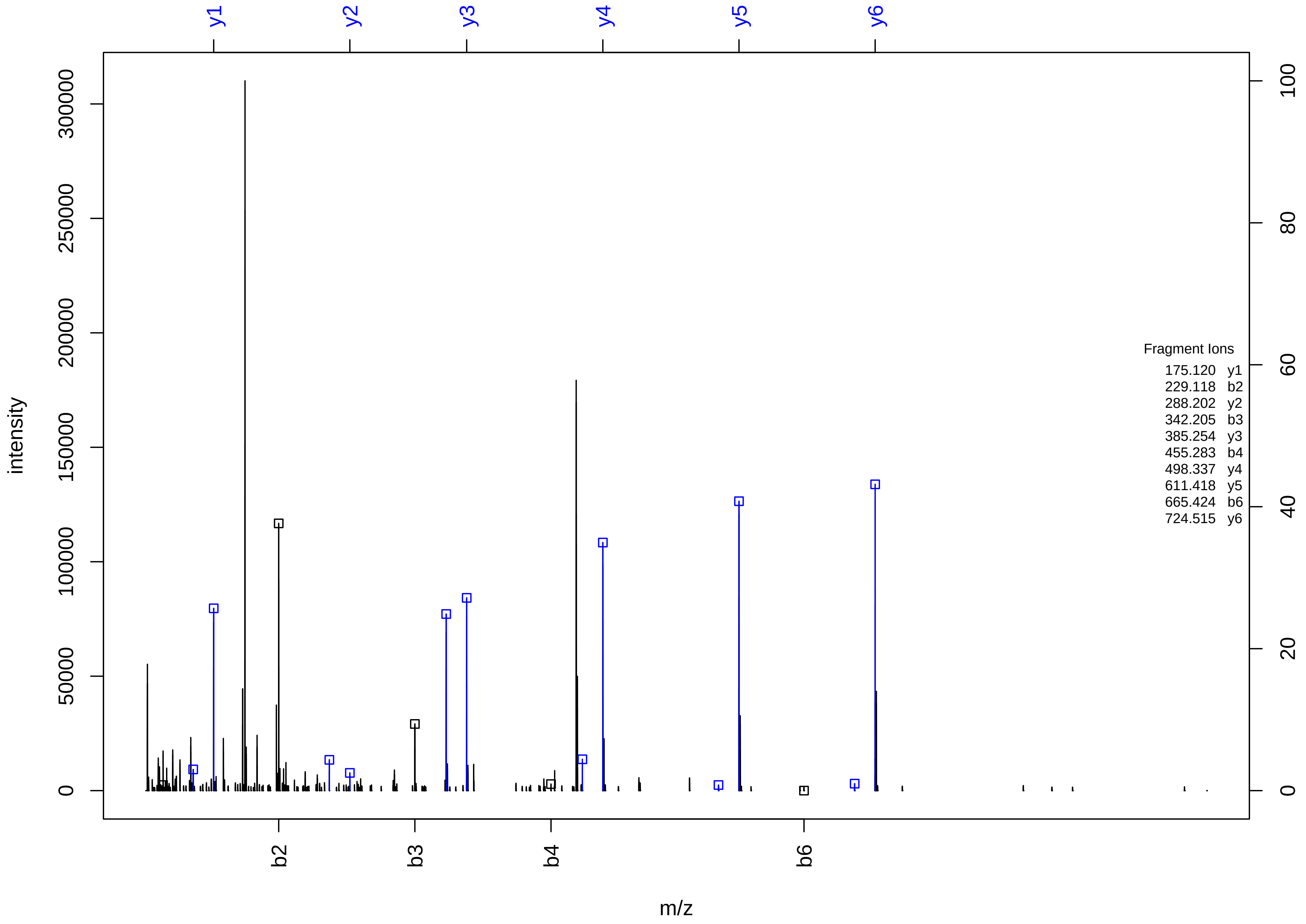

DILLLPLR / File: sydney_ip_32.raw; SpectrumID: ; scans: 17641

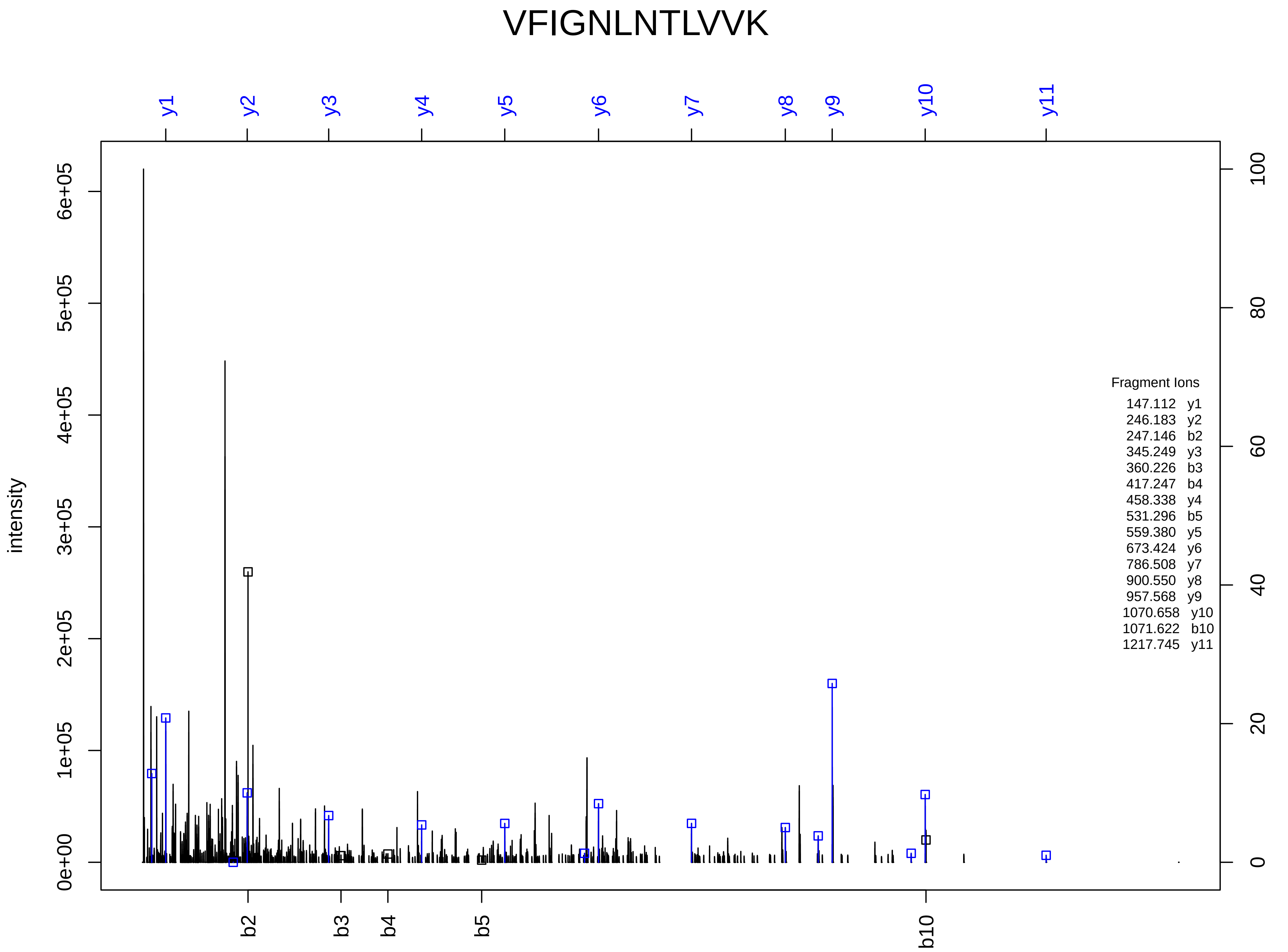

VFIGNLNTLVVK / File: sydney_ip_32.raw; SpectrumID: ; scans: 14167

M*KEAVTRSEM*LAVVN^K

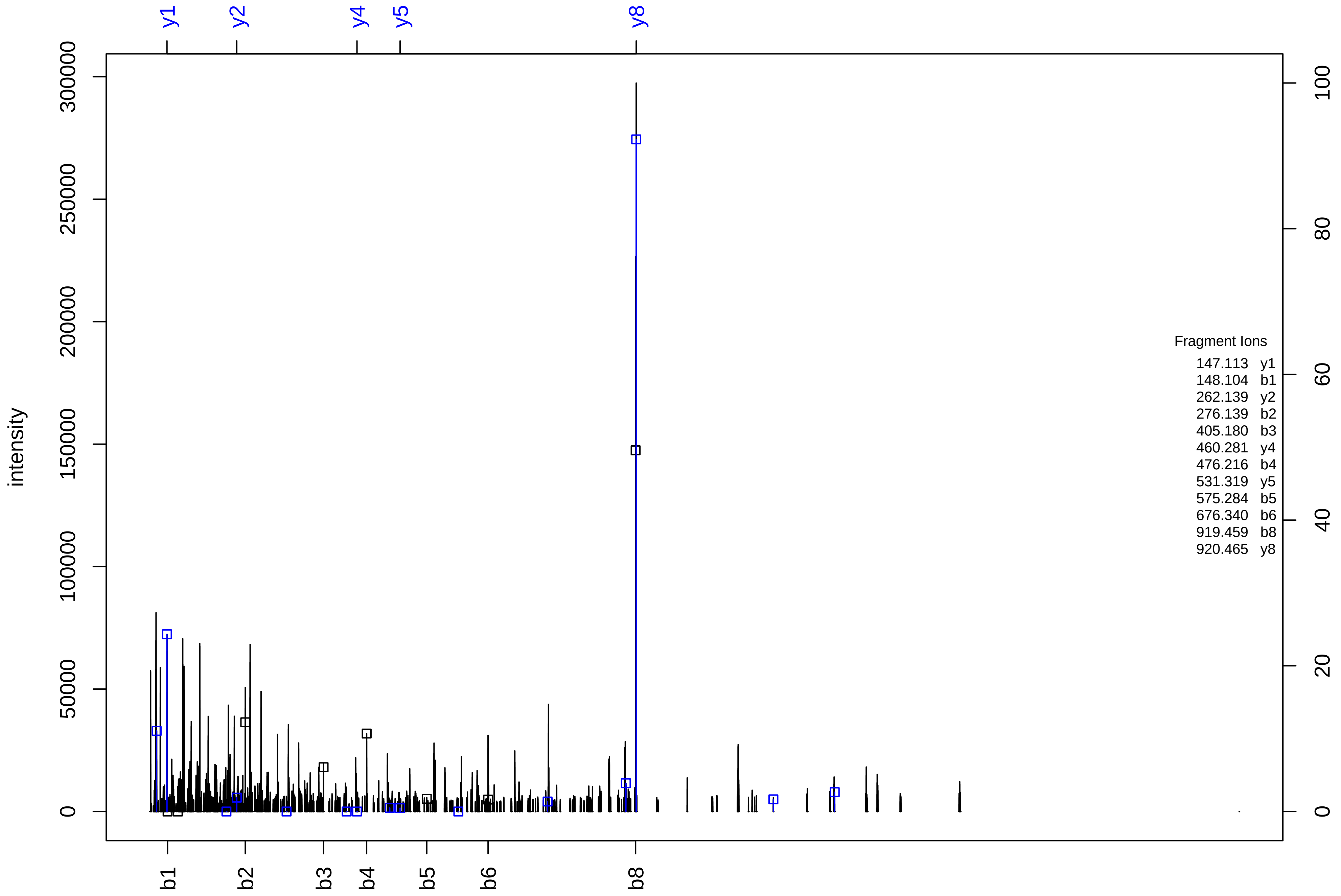

MKEAVTRSEMLAVVNK / File: sydney_ip_32.raw; SpectrumID: ; scans: 8324

M*DQ^SPEEM*Q^GAVR

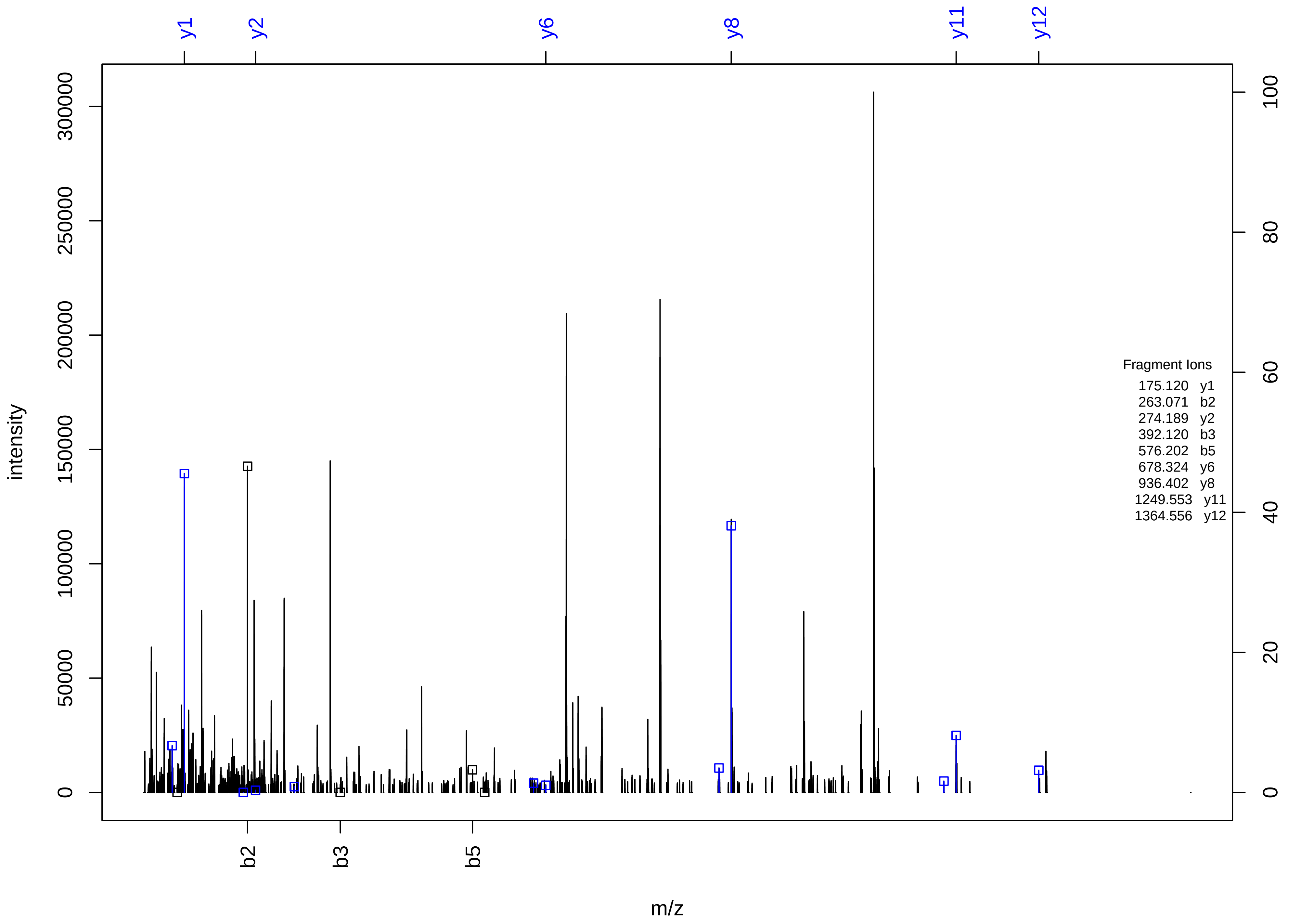

MDQSPEEMQGAVR / File: sydney_ip_32.raw; SpectrumID: ; scans: 3762

VETPVLPPVLVPR

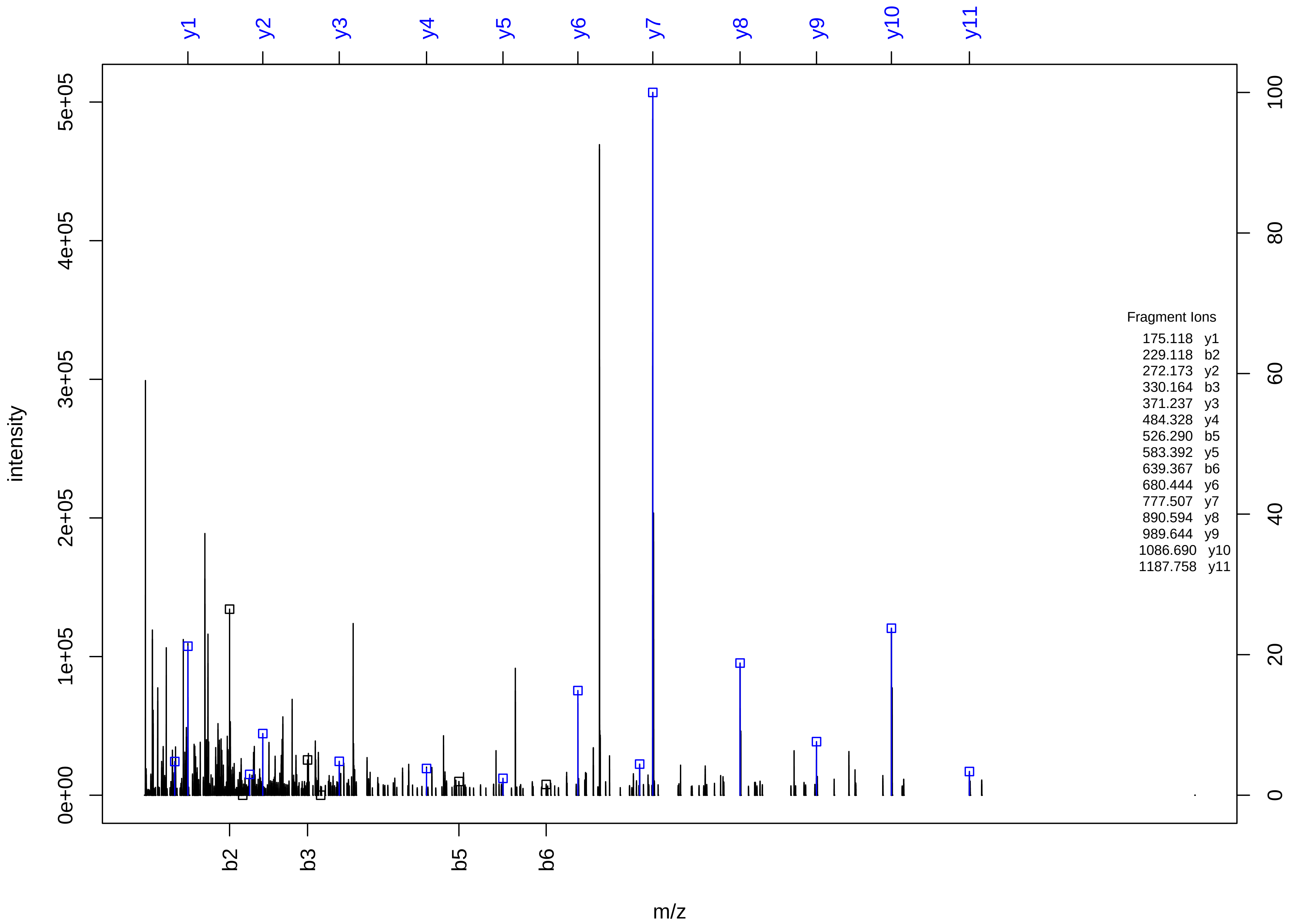

VETPVLPPVLVPR / File: sydney_ip_32.raw; SpectrumID: ; scans: 15835

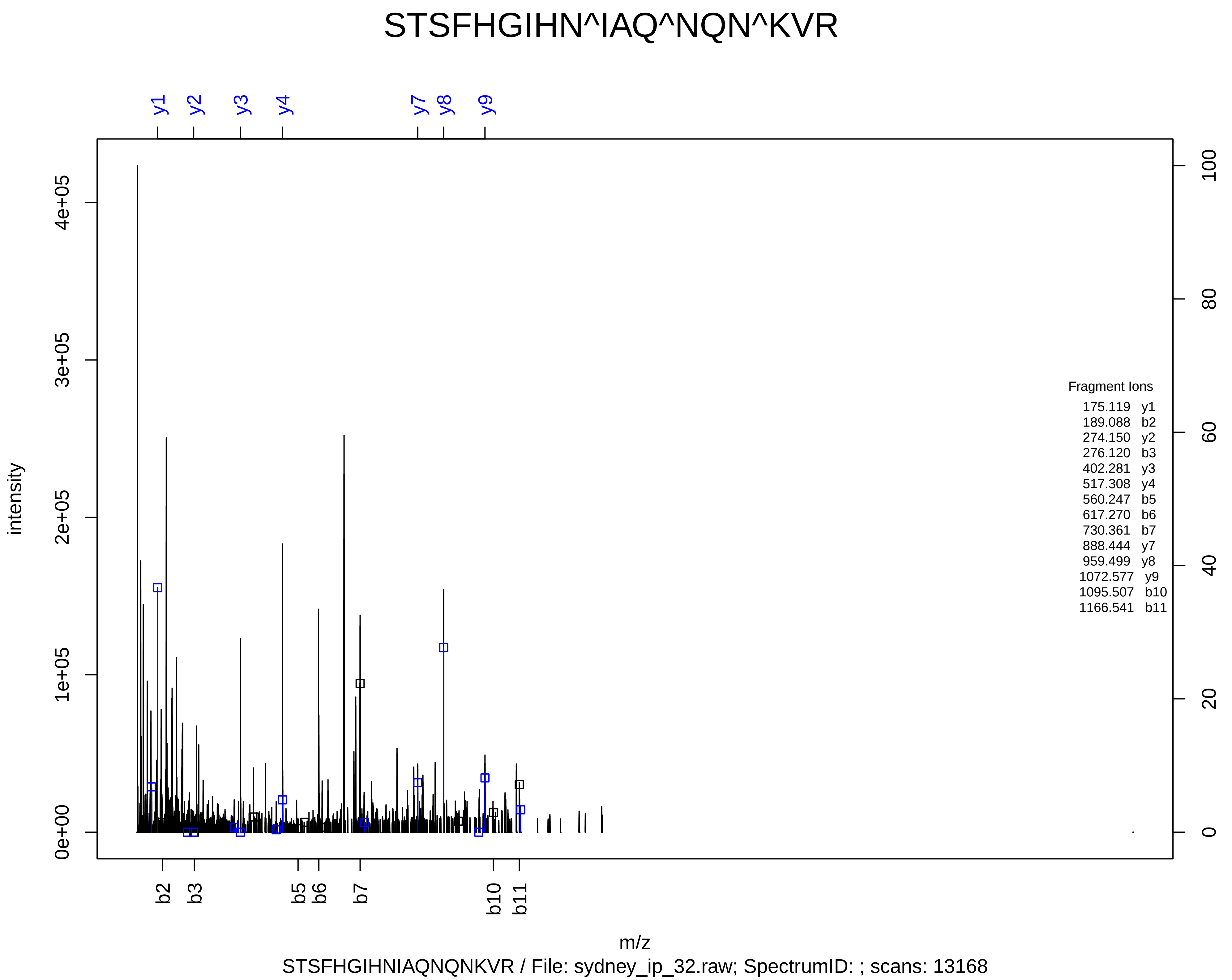
STSFHGIHN^IAQ^NQN^KVR
y1
y2
y3
y4
y7
y8
y9
intensity
0e+00
1e+05
2e+05
3e+05
4e+05
0
20
40
60
80
100
b2
b3
b5
b6
b7
b10
b11
Fragment Ions
175.119 y1
189.088 b2
274.150 y2
276.120 b3
402.281 y3
517.308 y4
560.247 b5
617.270 b6
730.361 b7
888.444 y7
959.499 y8
1072.577 y9
1095.507 b10
1166.541 b11
m/z
STSFHGIHNIAQNQNKVR / File: sydney_ip_32.raw; SpectrumID: ; scans: 13168

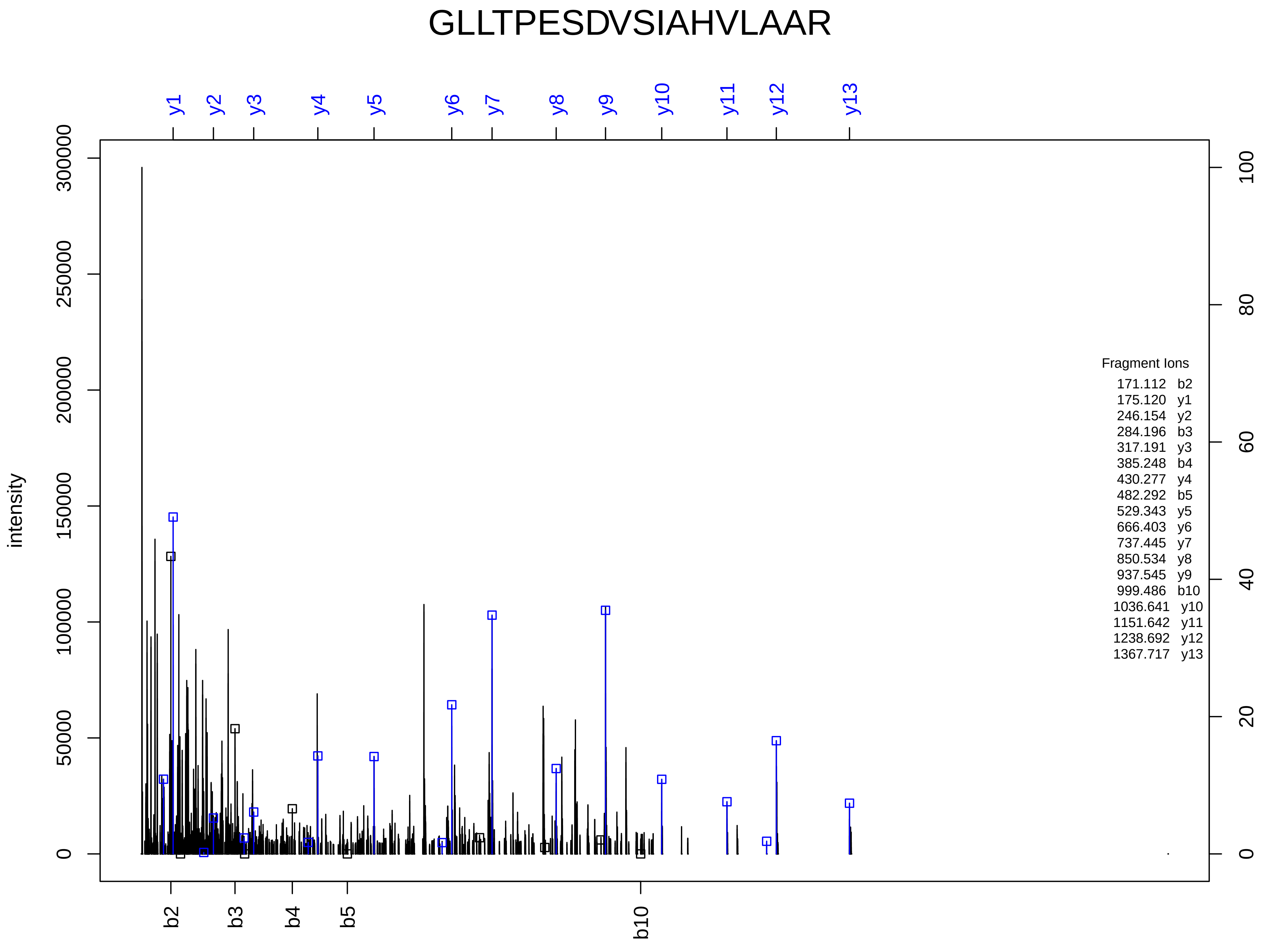

GLLTPESDVSIAHVLAAR / File: sydney_ip_32.raw; SpectrumID: ; scans: 14140

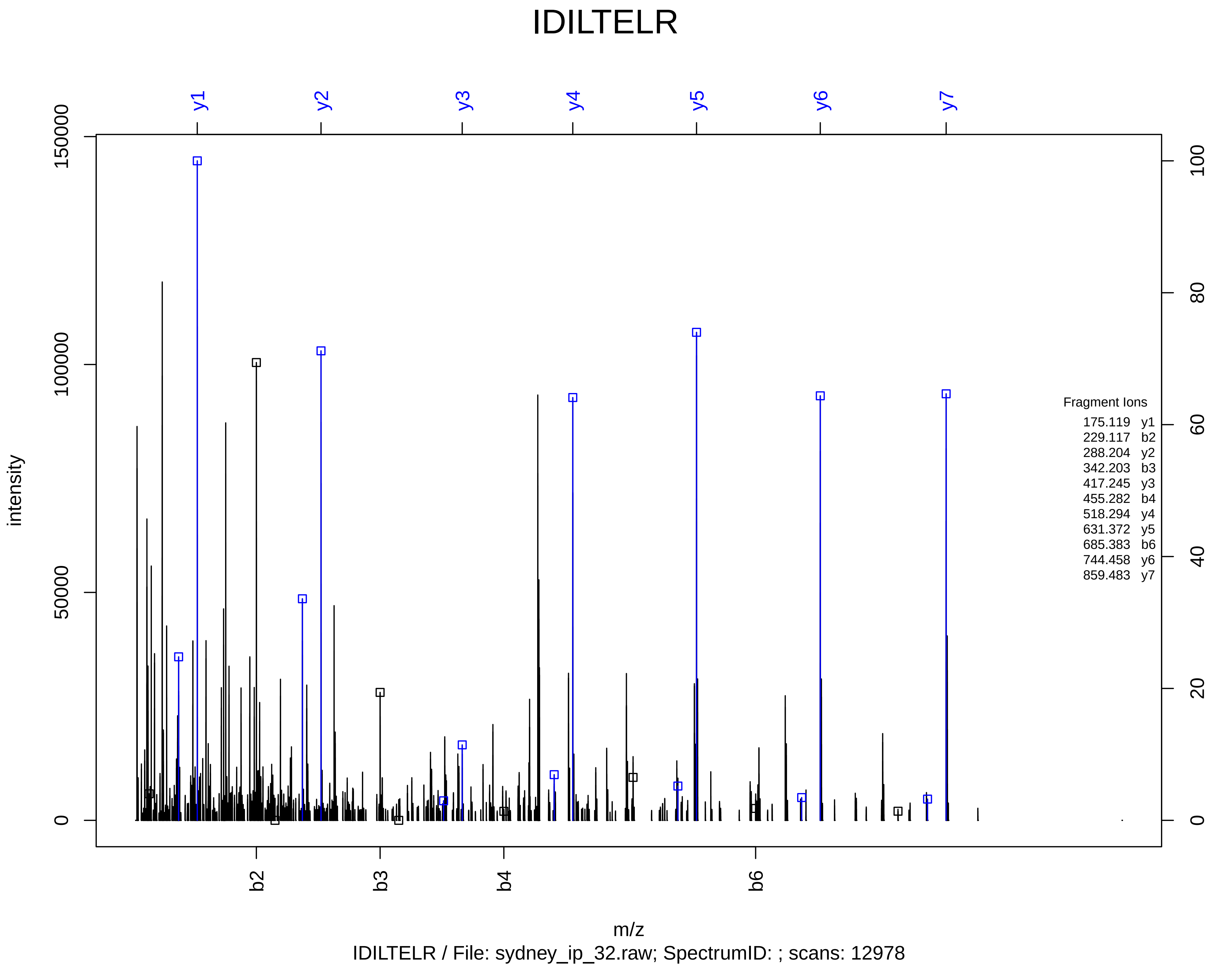

IDILTELR
y1
y2
y3
y4
y5
y6
y7
intensity
150000
100000
50000
0
100
80
60
40
20
0
b2
b3
b4
b6
m/z
Fragment Ions
175.119 y1
229.117 b2
288.204 y2
342.203 b3
417.245 y3
455.282 b4
518.294 y4
631.372 y5
685.383 b6
744.458 y6
859.483 y7
IDILTELR / File: sydney_ip_32.raw; SpectrumID: ; scans: 12978

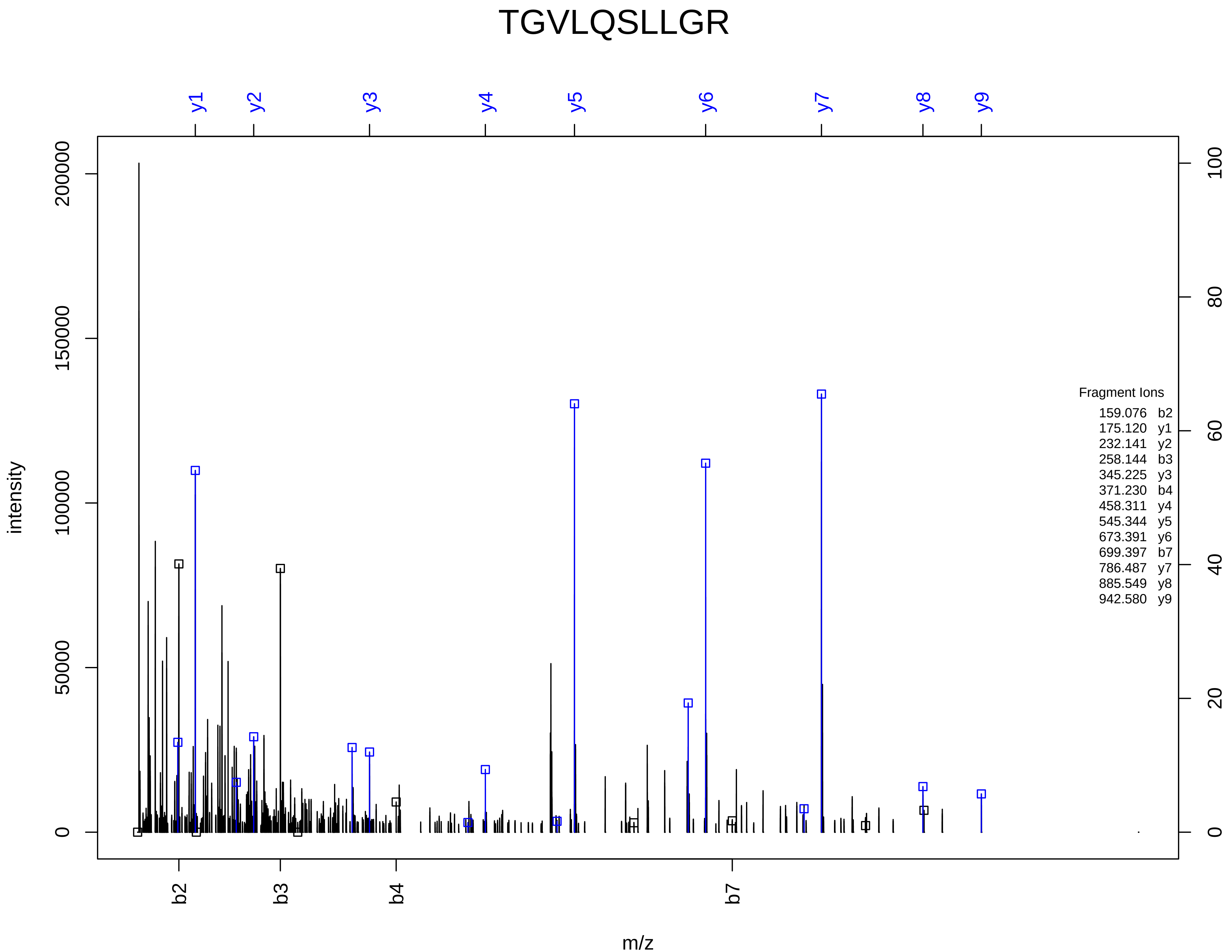

TGVLQSLLGR / File: sydney_ip_32.raw; SpectrumID: ; scans: 13304

SLIAQ^N^IQ^EATSGCFTTAQ^KR

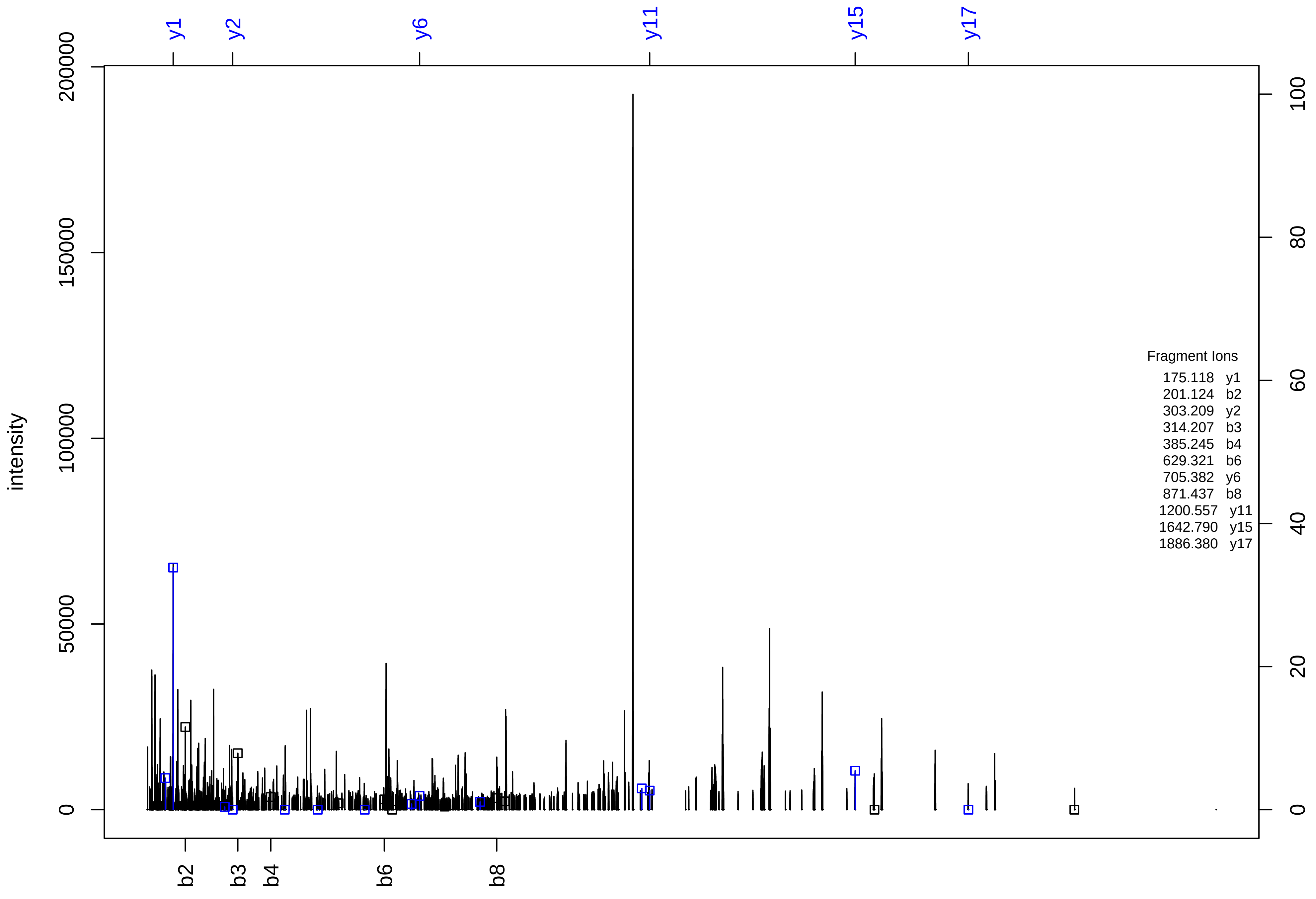

SLIAQNIQEATSGCFTTAQKR / File: sydney_ip_32.raw; SpectrumID: ; scans: 10523

TPEIVTVNSIGQLK

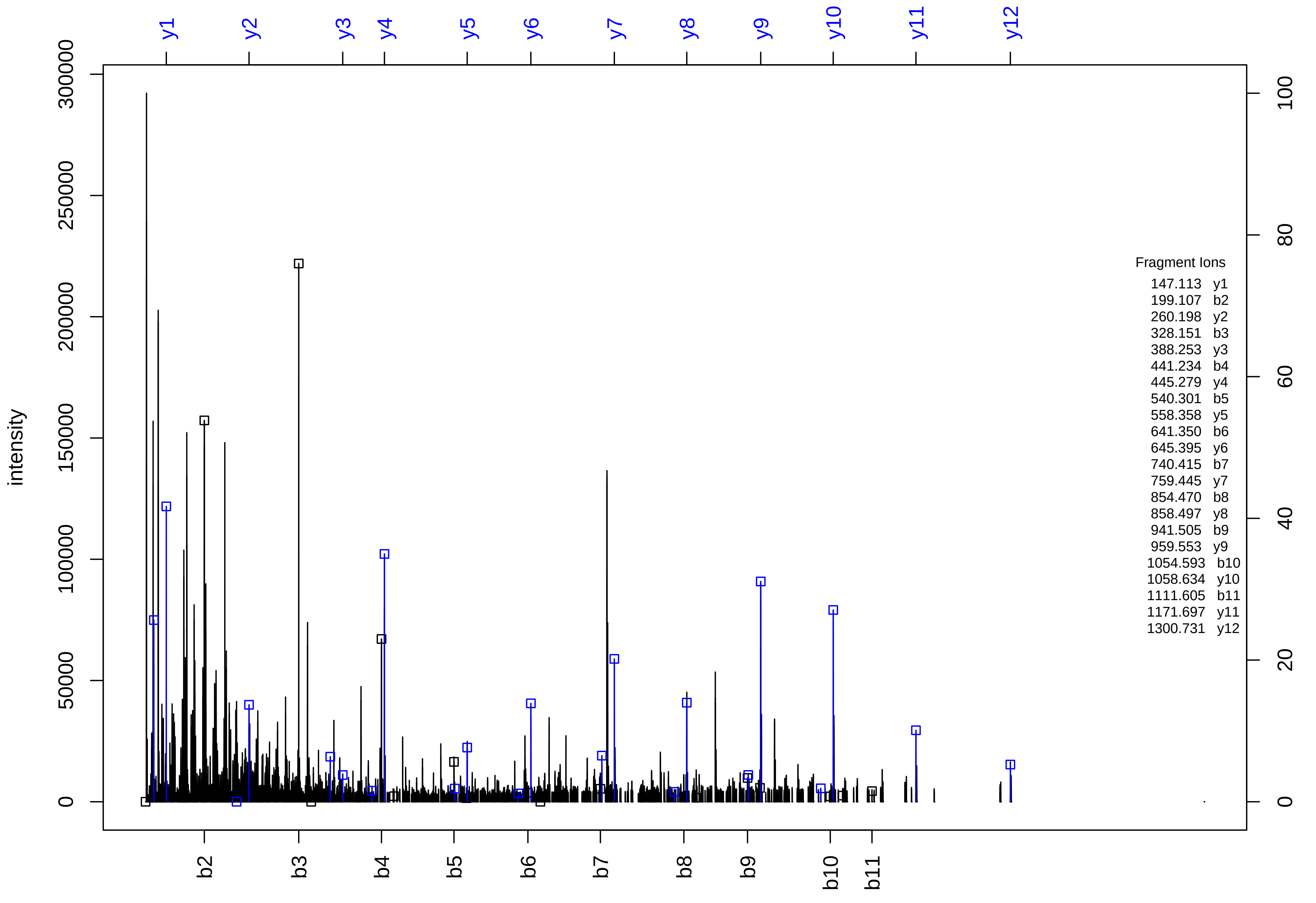

TPEIVTVNSIGQLK / File: sydney_ip_32.raw; SpectrumID: ; scans: 11333

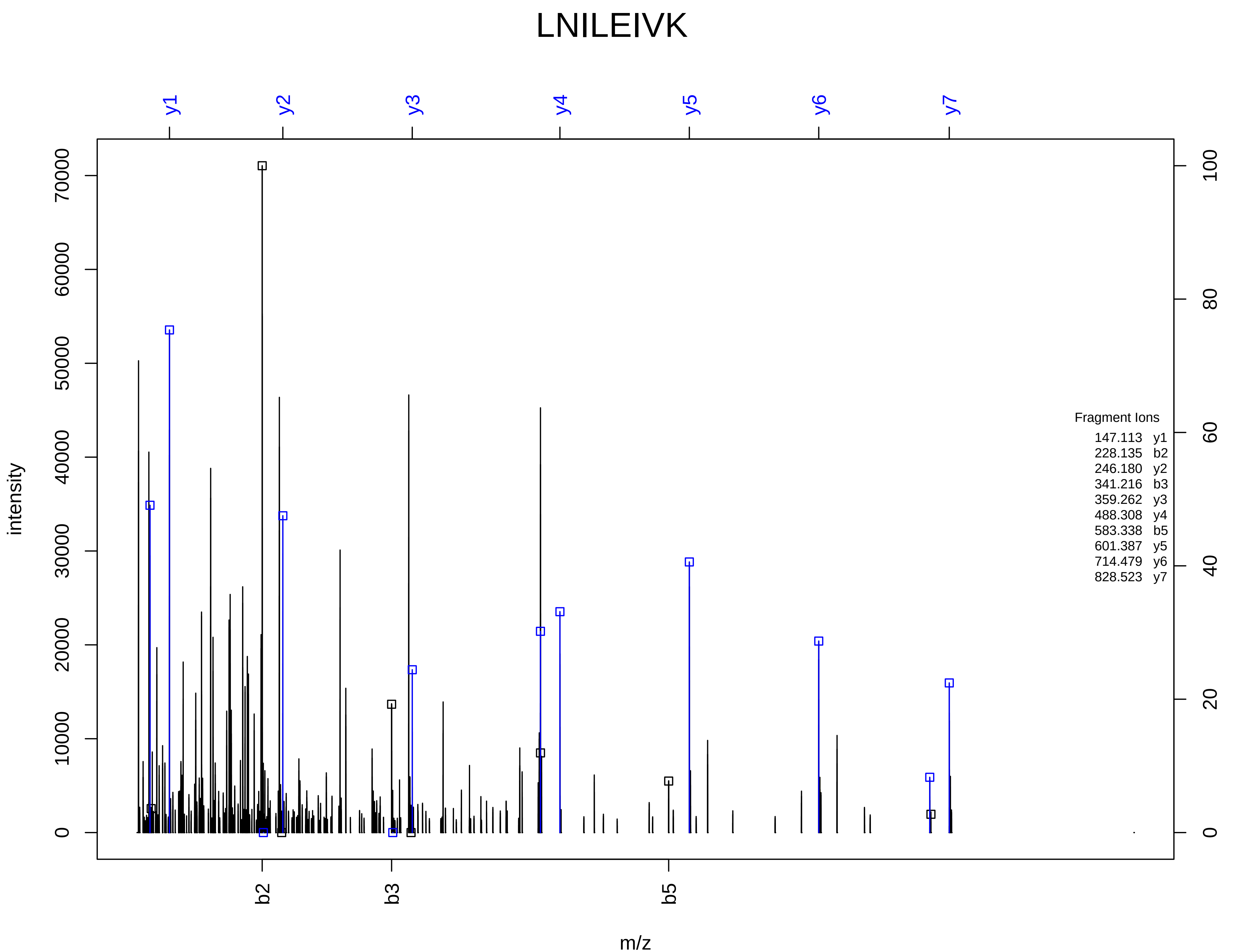
LNILEIVK
y1
y2
y3
y4
y5
y6
y7
intensity
70000
60000
50000
40000
30000
20000
10000
0
100
80
60
40
20
0
Fragment Ions
147.113 y1
228.135 b2
246.180 y2
341.216 b3
359.262 y3
488.308 y4
583.338 b5
601.387 y5
714.479 y6
828.523 y7
b2
b3
b5
m/z
LNILEIVK / File: sydney_ip_32.raw; SpectrumID: ; scans: 14584

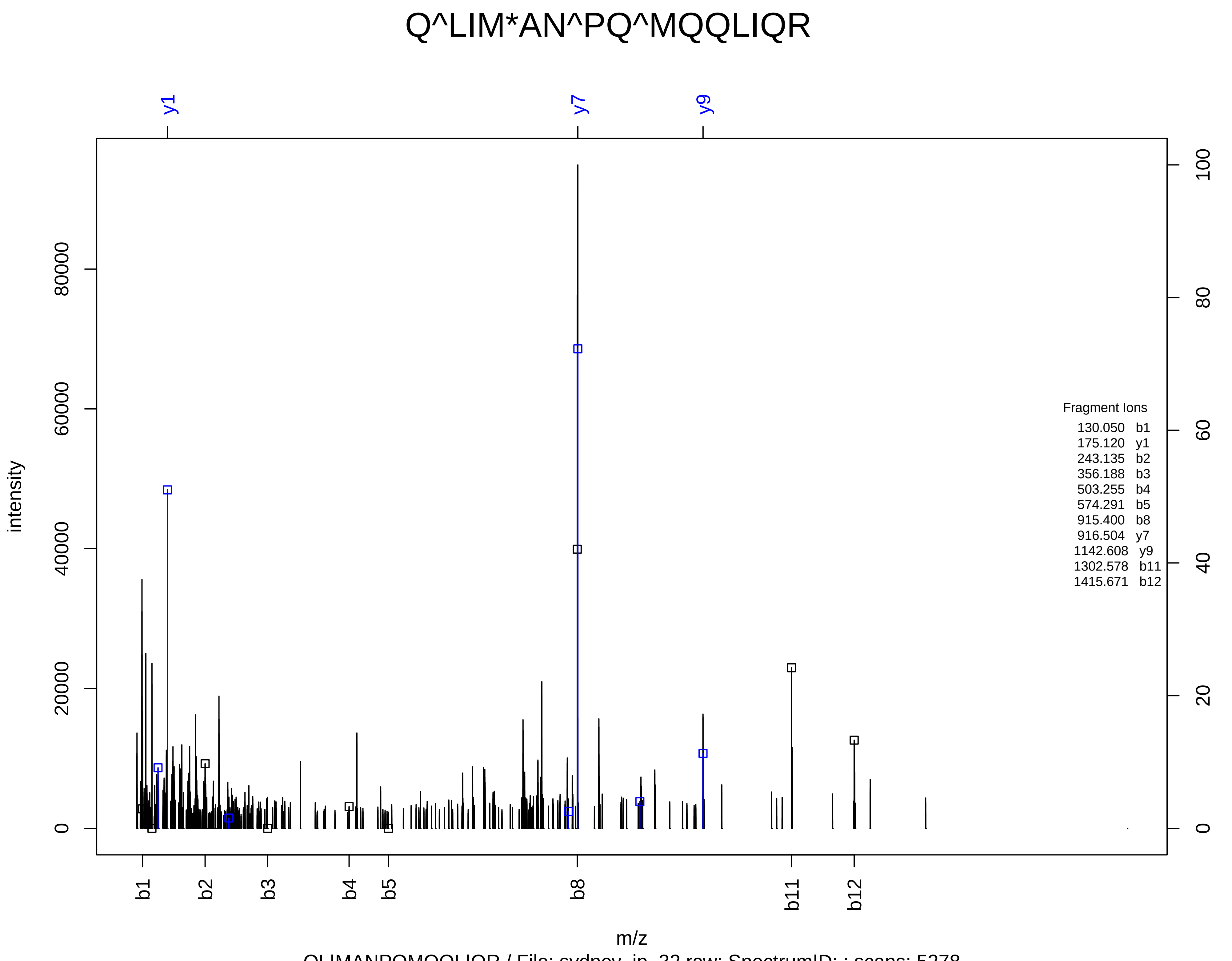
Q^LIM*AN^PQ^MQQLIQR
y1
y7
y9
intensity
80000
60000
40000
20000
0
100
80
60
40
20
0
Fragment Ions
130.050 b1
175.120 y1
243.135 b2
356.188 b3
503.255 b4
574.291 b5
915.400 b8
916.504 y7
1142.608 y9
1302.578 b11
1415.671 b12
b1
b2
b3
b4
b5
b8
b11
b12
m/z
QLIMANPQMQQLIQR / File: sydney_ip_32.raw; SpectrumID: ; scans: 5278

SSFPTQ^AEVER

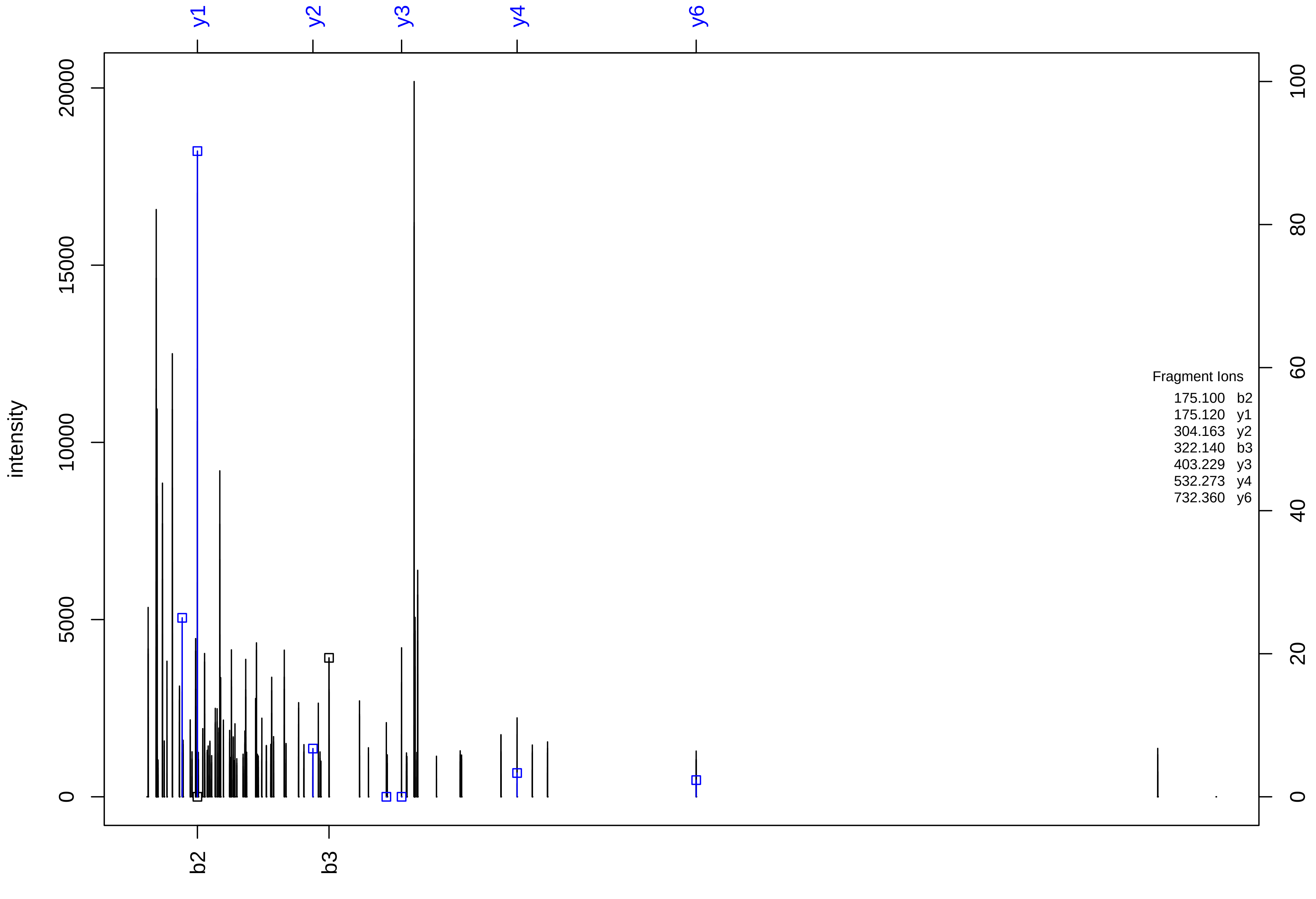

SSFPTQAEVER / File: sydney_ip_32.raw; SpectrumID: ; scans: 1498

TLVAVERPLDDIIAQLPPPIK

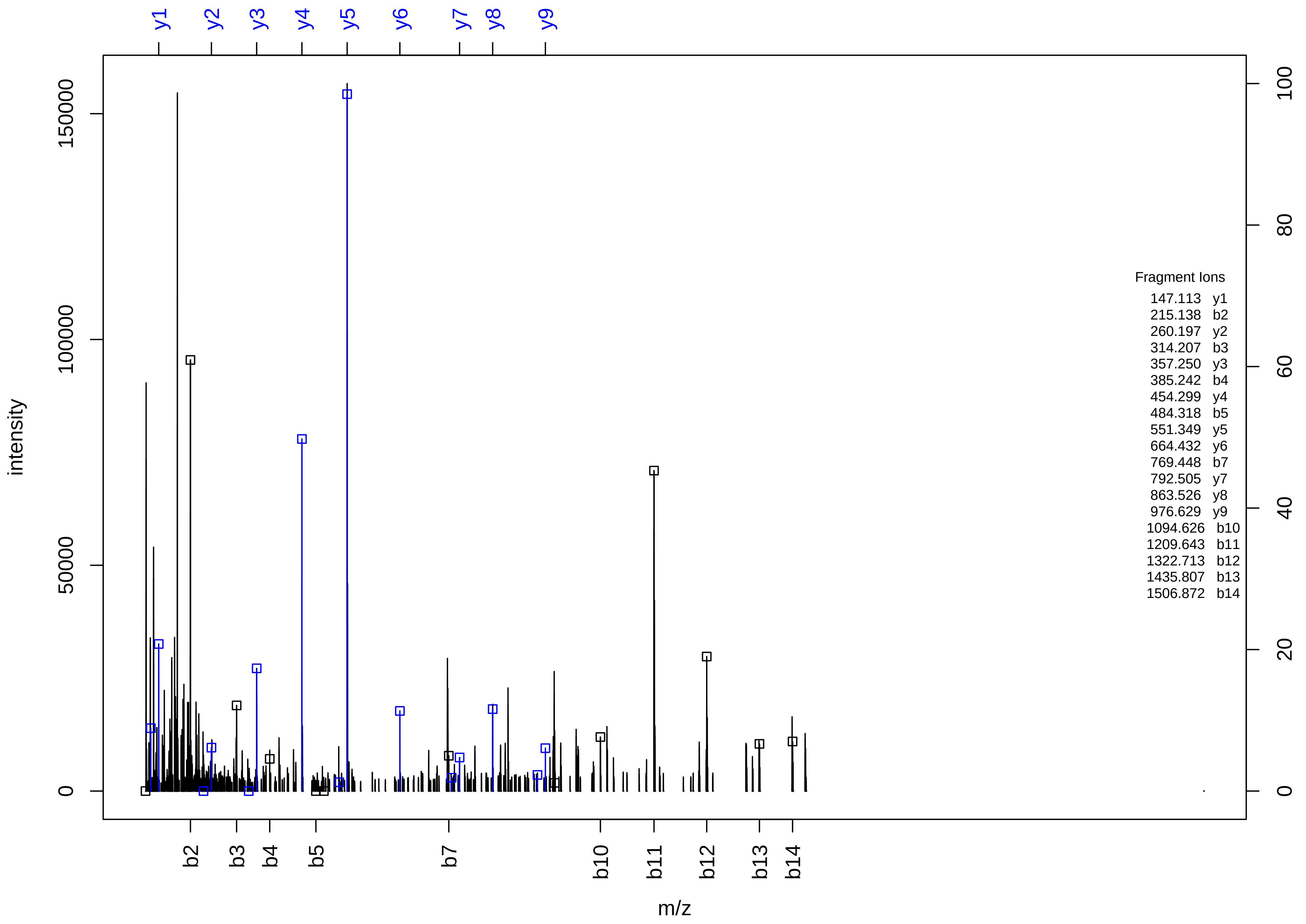

TLVAVERPLDDIIAQLPPPIK / File: sydney_ip_32.raw; SpectrumID: ; scans: 19152

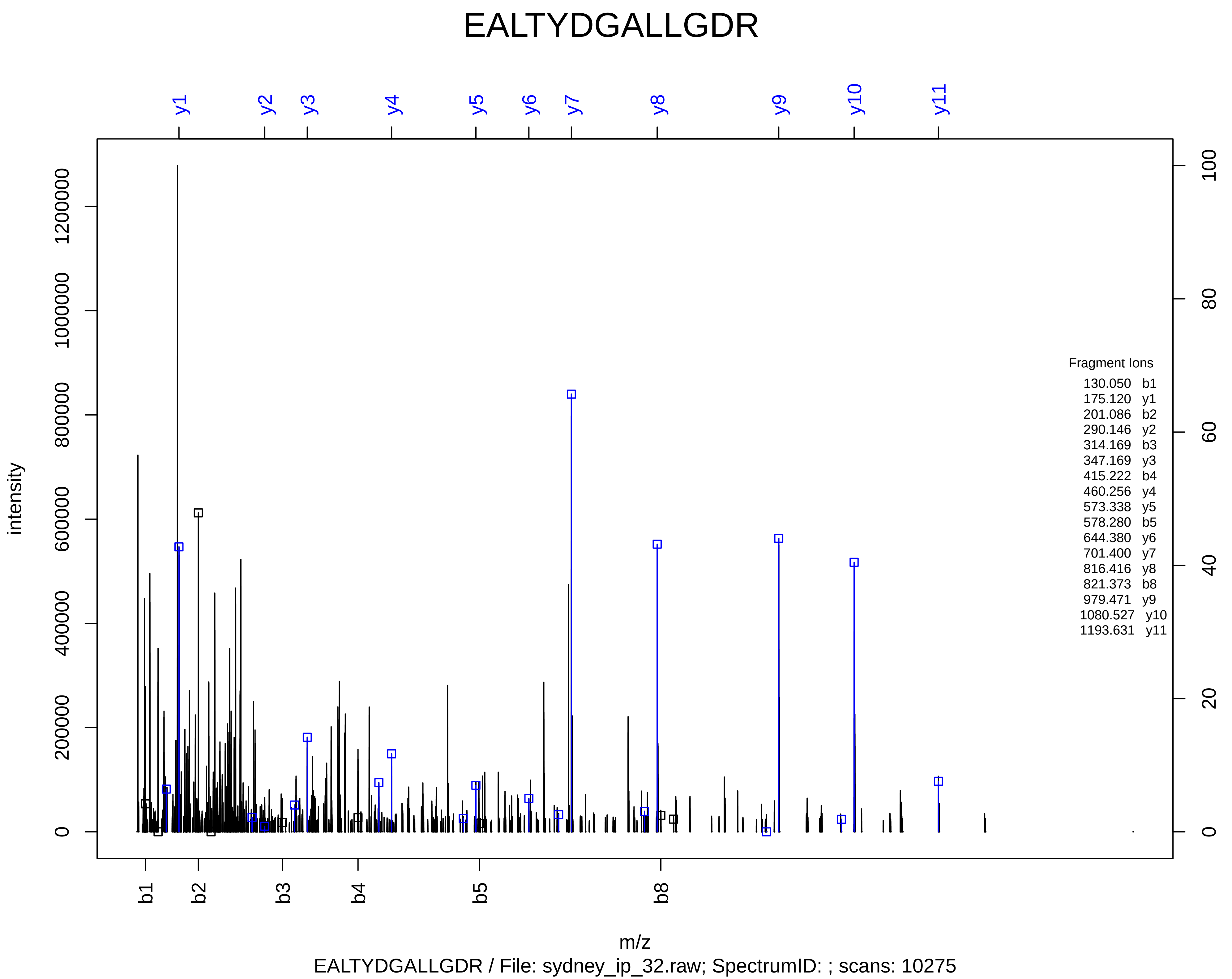
EALTYDGALLGDR
y1
y2
y3
y4
y5
y6
y7
y8
y9
y10
y11
intensity
0
200000
400000
600000
800000
1000000
1200000
0
20
40
60
80
100
Fragment Ions
130.050 b1
175.120 y1
201.086 b2
290.146 y2
314.169 b3
347.169 y3
415.222 b4
460.256 y4
573.338 y5
578.280 b5
644.380 y6
701.400 y7
816.416 y8
821.373 b8
979.471 y9
1080.527 y10
1193.631 y11
b1
b2
b3
b4
b5
b8
m/z
EALTYDGALLGDR / File: sydney_ip_32.raw; SpectrumID: ; scans: 10275

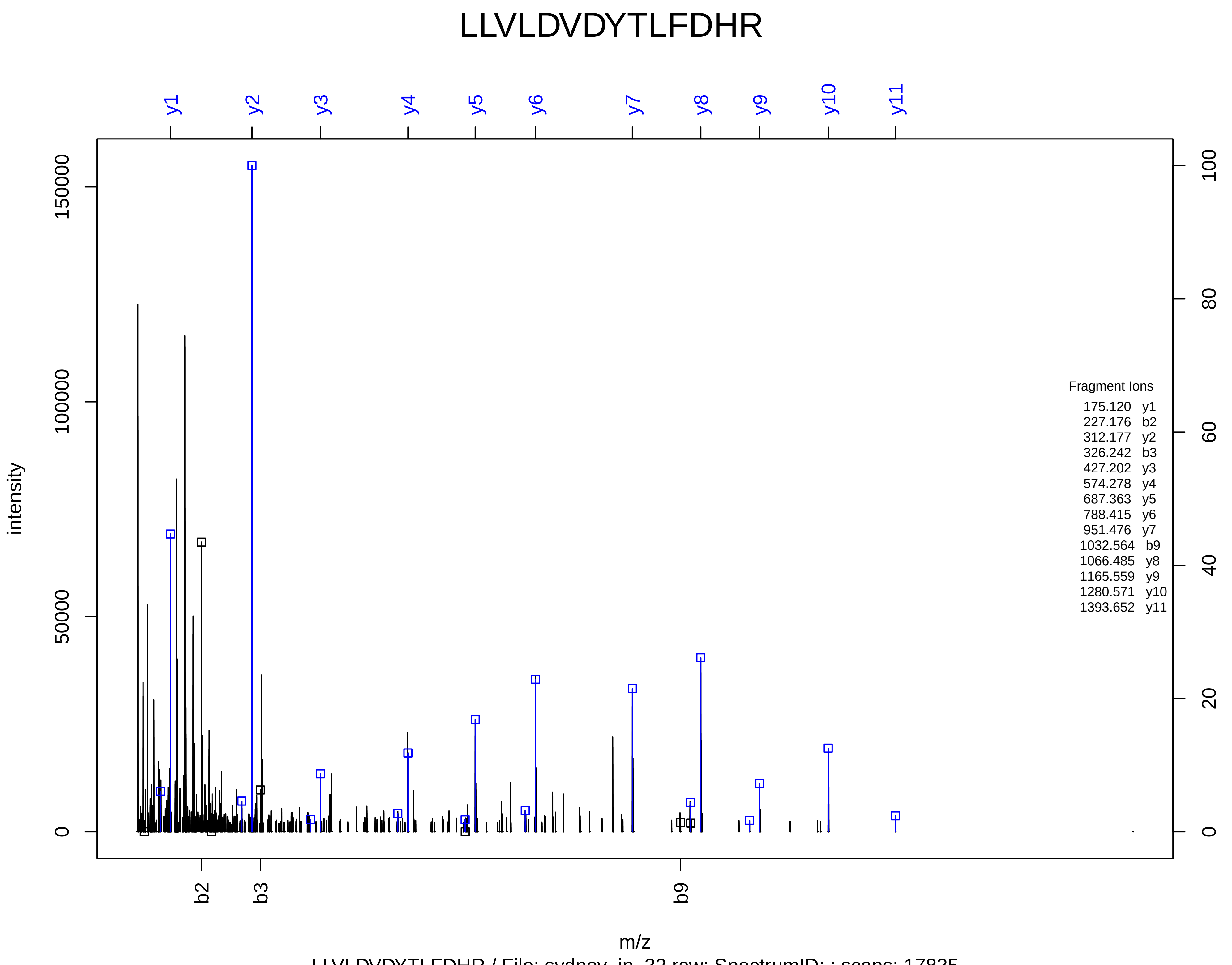

LLVLDVDYTLFDHR
y1
y2
y3
y4
y5
y6
y7
y8
y9
y10
y11
intensity
0
50000
100000
150000
0
20
40
60
80
100
b2
b3
b9
Fragment Ions
175.120 y1
227.176 b2
312.177 y2
326.242 b3
427.202 y3
574.278 y4
687.363 y5
788.415 y6
951.476 y7
1032.564 b9
1066.485 y8
1165.559 y9
1280.571 y10
1393.652 y11
m/z
LLVLDVDYTLFDHR / File: sydney_ip_32.raw; SpectrumID: ; scans: 17835

(Ac)MDVNLAPLR

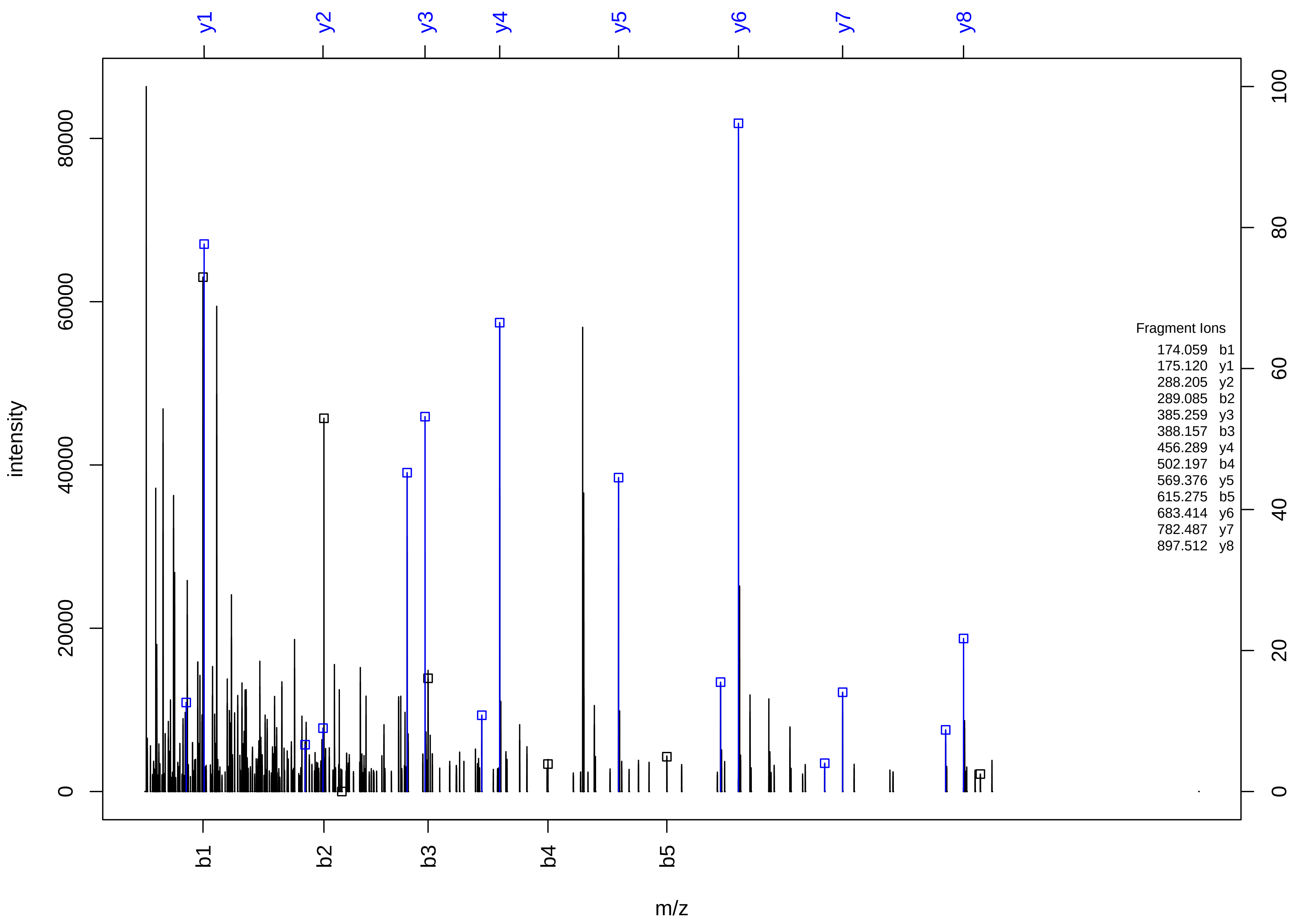

MDVNLAPLR / File: sydney_ip_32.raw; SpectrumID: ; scans: 15814

ILVTILR

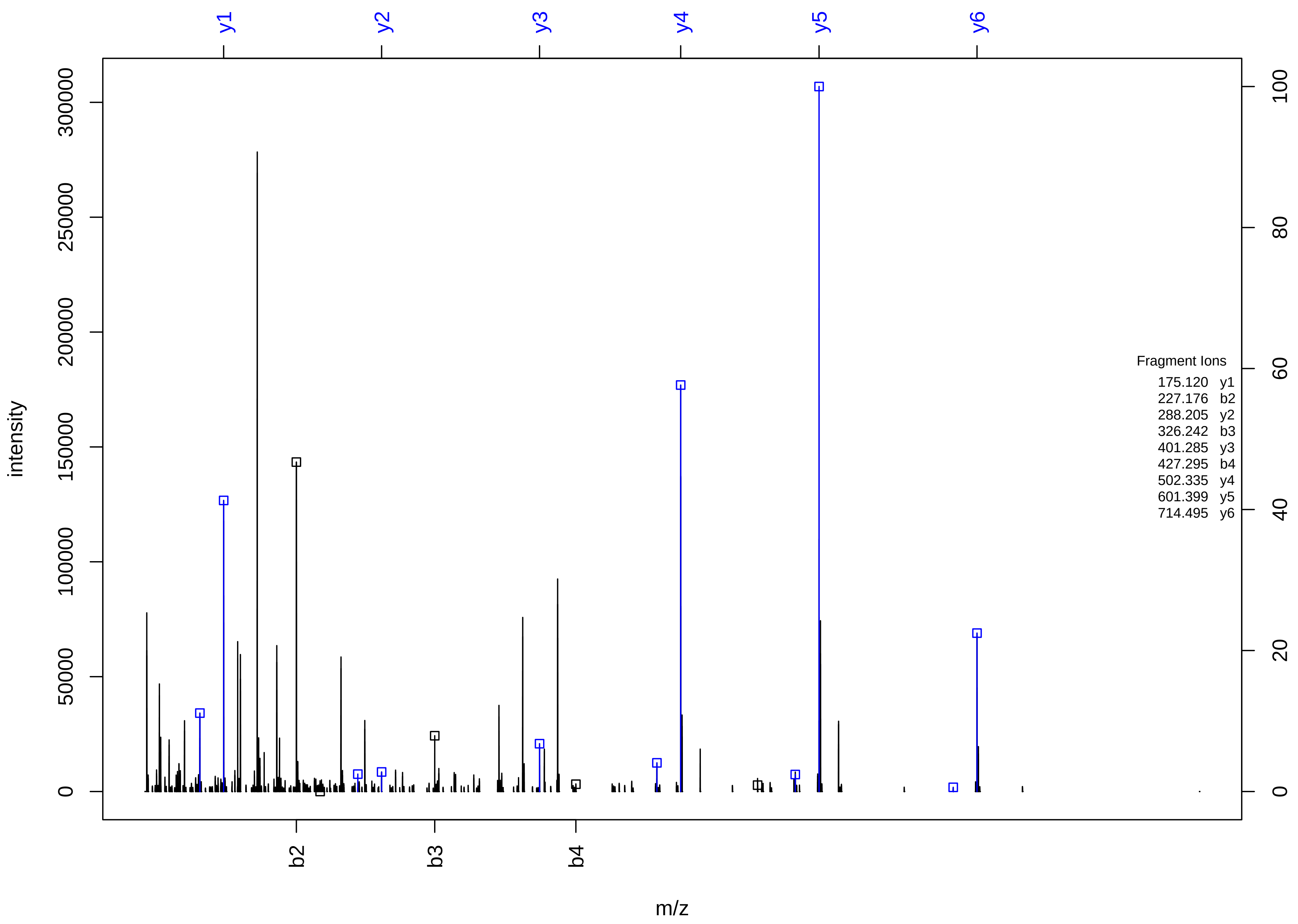

ILVTILR / File: sydney_ip_32.raw; SpectrumID: ; scans: 11660

TILFIDEIHR

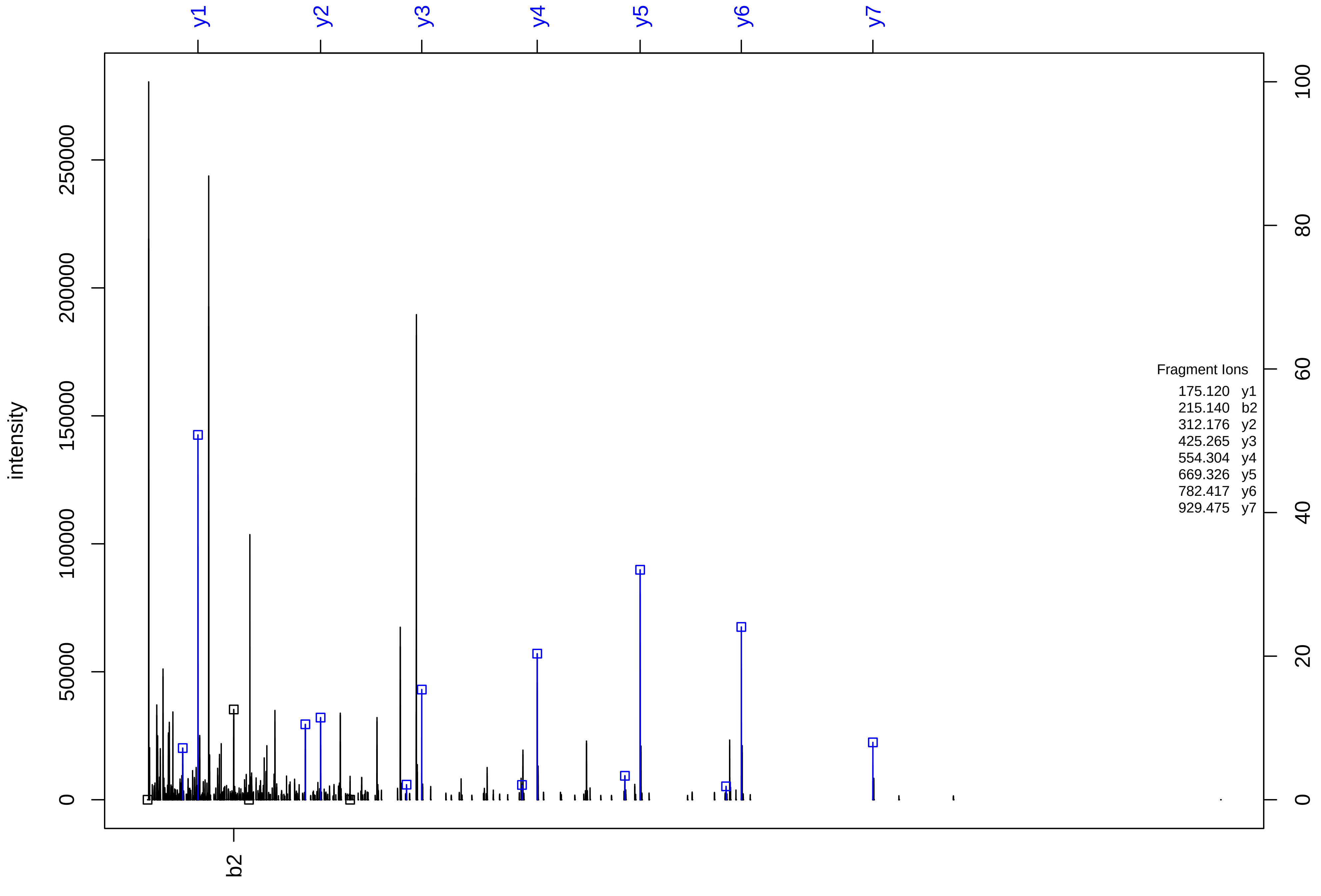

TILFIDEIHR / File: sydney_ip_32.raw; SpectrumID: ; scans: 11741

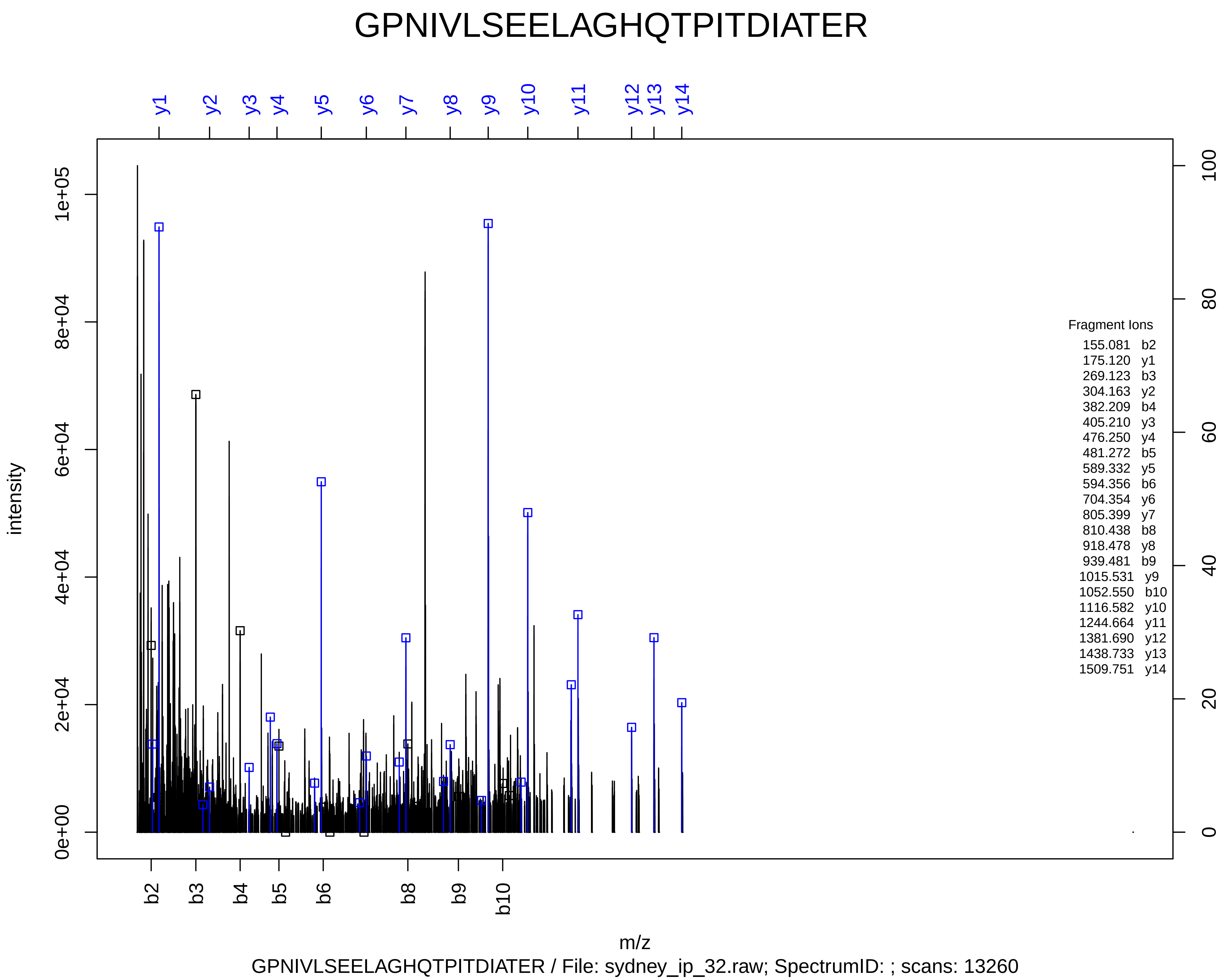
GPNIVLSEELAGHQTPITDIATER
y1 y2 y3 y4 y5 y6 y7 y8 y9 y10 y11 y12 y13 y14
b2 b3 b4 b5 b6 b8 b9 b10
intensity
0e+00 2e+04 4e+04 6e+04 8e+04 1e+05
0 20 40 60 80 100
m/z
Fragment Ions
155.081 b2
175.120 y1
269.123 b3
304.163 y2
382.209 b4
405.210 y3
476.250 y4
481.272 b5
589.332 y5
594.356 b6
704.354 y6
805.399 y7
810.438 b8
918.478 y8
939.481 b9
1015.531 y9
1052.550 b10
1116.582 y10
1244.664 y11
1381.690 y12
1438.733 y13
1509.751 y14
GPNIVLSEELAGHQTPITDIATER / File: sydney_ip_32.raw; SpectrumID: ; scans: 13260
