# Supplementary material for: Cellular Proteomic Profiling Using Proximity Labeling by TurboID-NES in Microglial and Neuronal Cell Lines
Source: Mol Cell Proteomics. 2023 Apr 14;22(6):100546. doi: 10.1016/j.mcpro.2023.100546 (PMC10205547; doi:10.1016/j.mcpro.2023.100546)
Supplement: Supplementary Figures [file mmc6.pdf]

Supplemental:

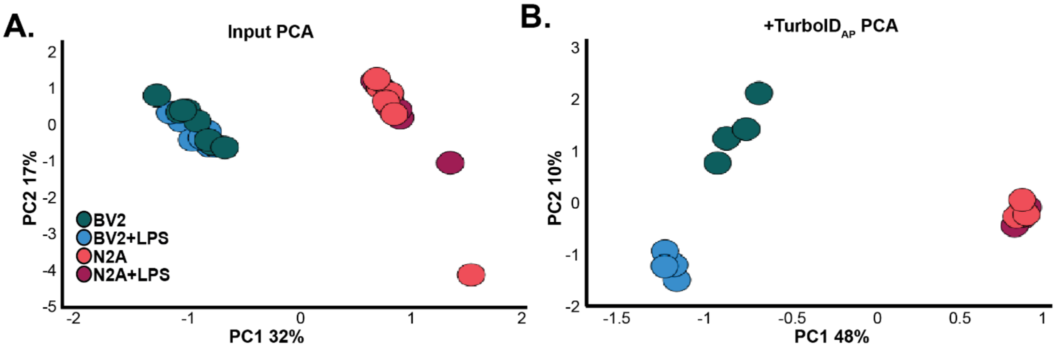

SF1.Dimension reduction of whole-cell and transduced AP samples. A. Principal component analysis (PCA) of whole-cell samples. Principal component 1 (PC1) accounts for ~32% of variance across which samples distinguish by cell type. **B.**PCA on TurboID labeled and biotin enriched samples indicates that PC1 accounts for ~48% of the variance and along this axis samples cluster by cell-type. PC2 accounts for 10% of the variance, along this axis, BV2 samples and not N2A samples cluster according to LPS treatment.

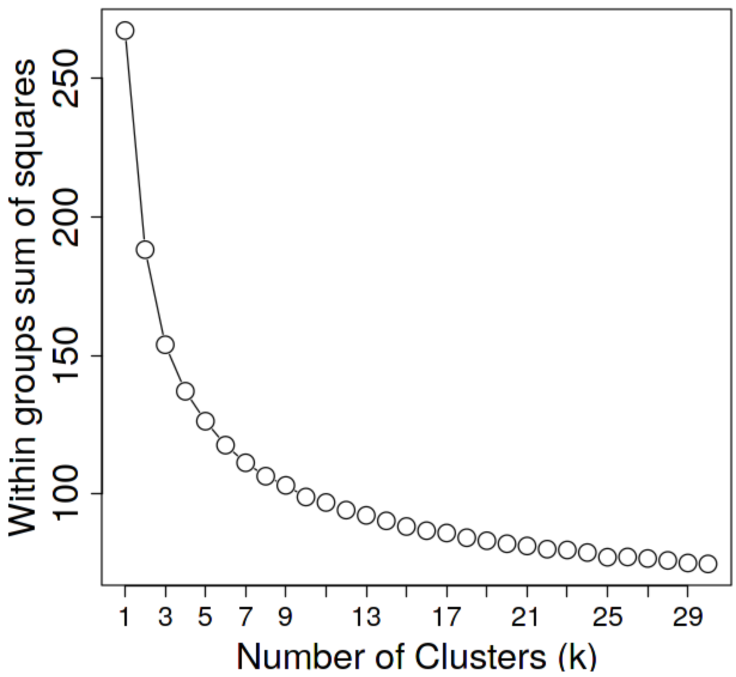

SF2. Optimization of cluster number using the Elbow-Curve Method. Mathematical determination of the optimal number of clusters for the clustering analysis performed in Figure 3.

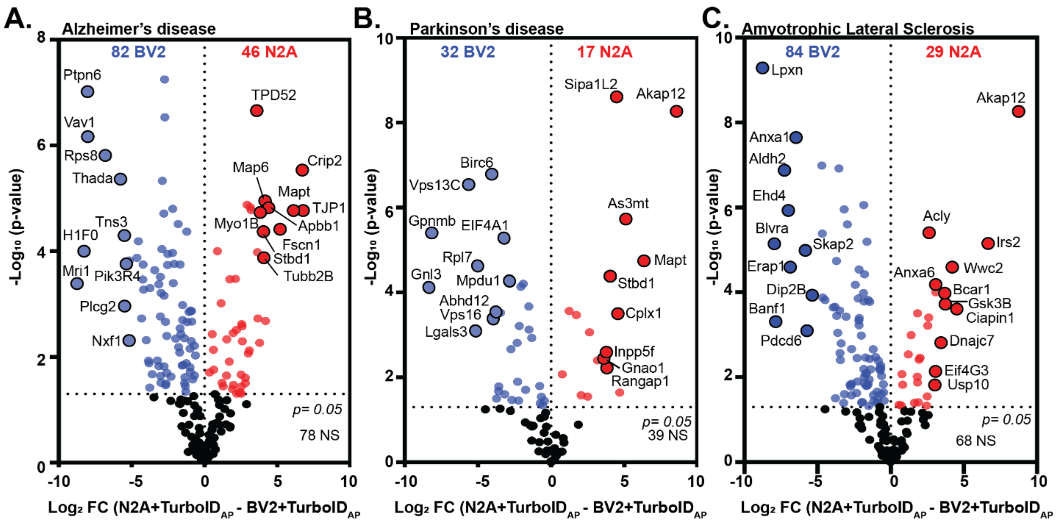

SF3. TurboID labels cellularly distinct proteins with relevance to neurodegenerative disease. Volcano plot representations of cellularly-distinct proteins labeled by TurboID (derived from significant DEP dataset in Fig 4A), and respective disease relevance. **A.** Mapping proteins onto the Alzheimer's disease (AD) AD MAGMA dataset, there are 82 AD-associated proteins enriched in biotin-labeled BV2 proteins and 46 AD-related proteins enriched in biotin-labeled N2A proteins. **B.** Mapping biotin-labeled and cellularly distinct proteins onto the Parkinson's disease (PD) MAGMA dataset, there are 32 PD-relevant proteins in the BV2 biotin-labeled proteome and 17 PD-relevant proteins in the N2A biotin-labeled proteome. **C.** Mapping proteins onto the Amyotrophic lateral Sclerosis (ALS) and Frontotemporal dementia (FTD) MAGMA dataset, there are 84 ALS/FTD-relevant BV2 proteins and 29 proteins in the N2A biotin-labeled proteins

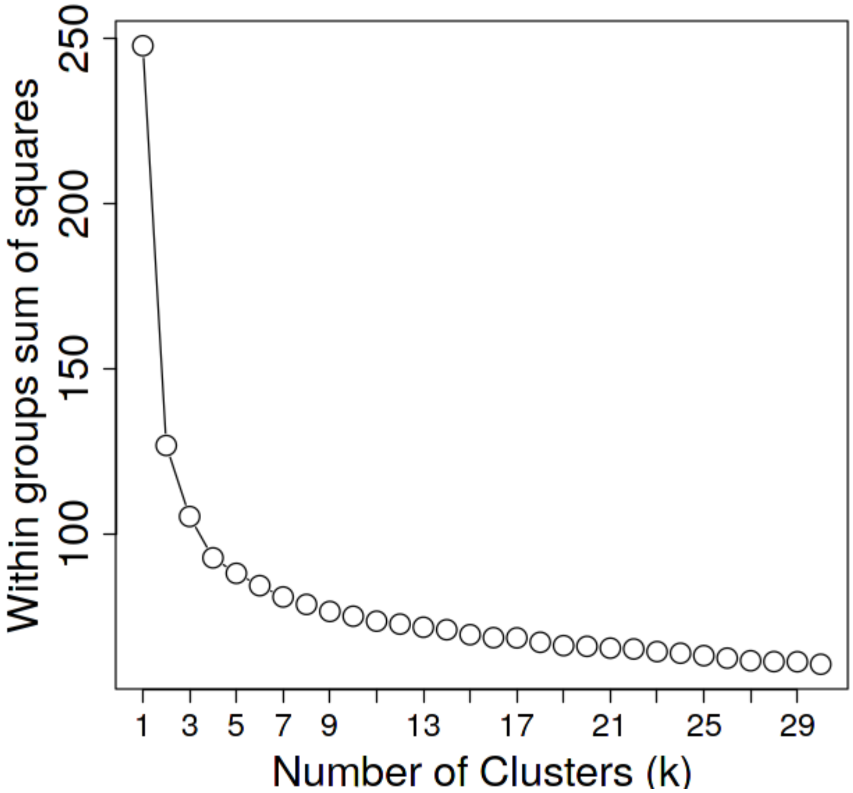

.

SF4. Optimization of cluster number using the Elbow-Curve Method. Mathematical determination of the optimal number of clusters for the clustering analysis performed in Figure 6

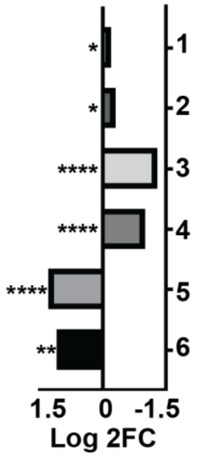

SF5. Log 2 FC of LPS driven changes between AP samples across all clusters. Bar-graph representation of the average Log2FC between BV2+TurboID+LPS AP and BV2+TurboID AP

samples at the cluster-level. Clusters 1 and 2 show a minimal impact of LPS on the transduced AP proteomes (***Cluster 1:*** Log2FC = -0.2, p = 0.015 **&** ***Cluster 2:*** Log2FC = -0.3, p = 0.015). Clusters 3 and 4 show a robust LPS-mediated decrease in proteins in BV2 AP (***Cluster 3:*** Log2FC = -1.3, p = 1.49 E^{-5} **&** ***Cluster 4:*** Log2FC = -1.0, p = 9.55 E^{-5}). Clusters 5 and 6 identify an LPS-mediated increase in protein abundance within BV2 AP samples (***Cluster 5:*** Log2FC = 1.3, p = 1.37 E^{-5} **&** ***Cluster 6:*** Log2FC = 1.1, p = 0.005).

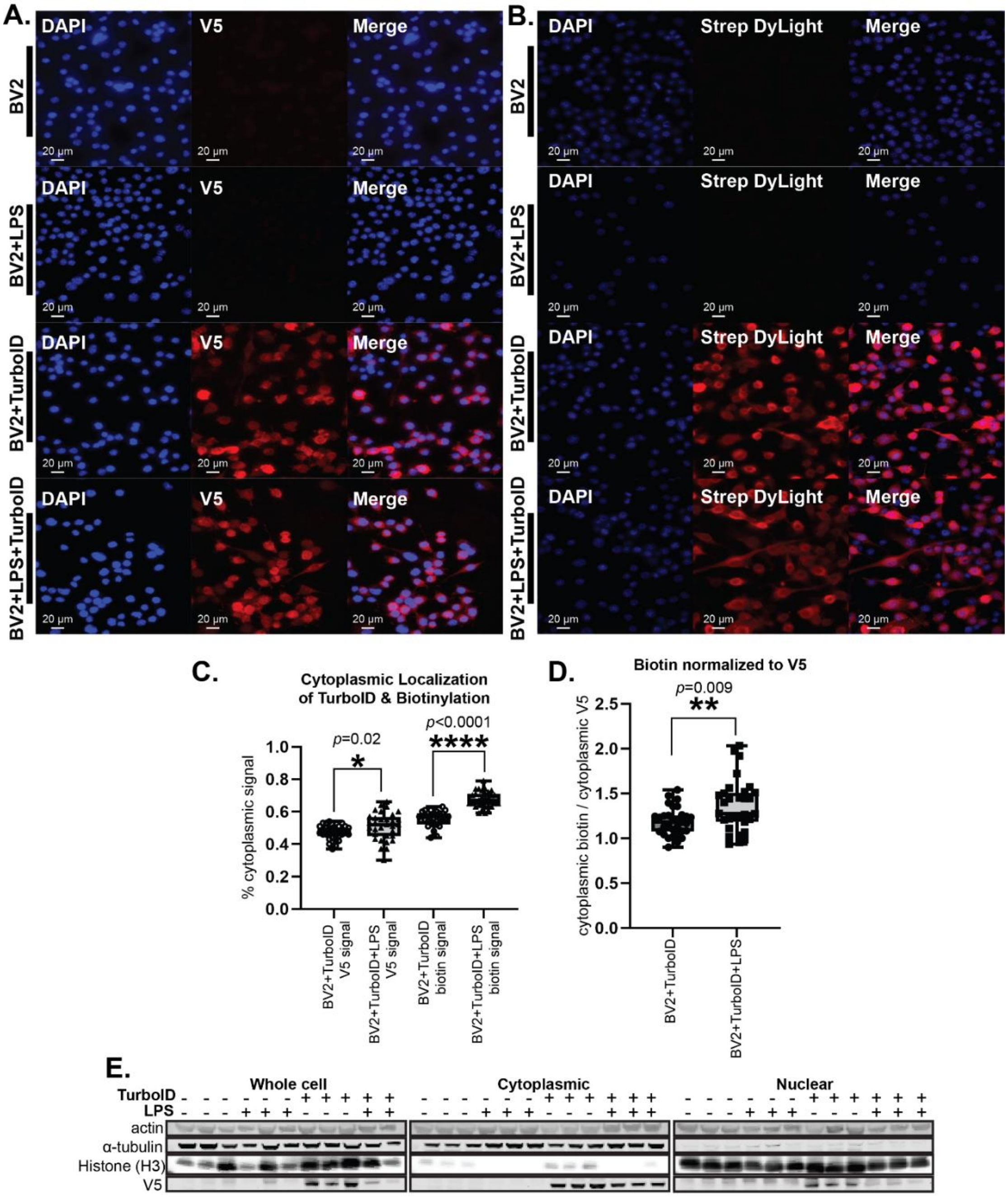

SF6 LPS challenge slightly increases cytosolic direction of TurboID and biotinylation of proteins. A. Immunofluorescence (IF) visualizing TurboID (V5, red) and nuclei (DAPI, blue) localization in transduced and untransduced BV2 cells with and without LPS challenge. Localization of V5-TurboID remains cytosolic under LPS challenge. **B.** IF visualizing biotinylation

of proteins (StrepDylight, red) and nuclei (DAPI, blue) in contexts of LPS challenge in transduced and untransduced BV2 cells. Biotinylation of proteins remains cytosolic under LPS challenge. **C.** Colocalization analysis of the area of cytoplasmic V5 signal (*left two* box-*plots*) and cytoplasmic biotinylation signal (*right two box-plots*) indicates a significant increase in cytosolic V5 and biotinylated proteins with LPS challenge. Significant p values were determined using the two-tailed Mann-Whitney test. **D.** Biotinylation signals normalized to V5 signals within the cytoplasm. After normalizing the biotinylation intensity values to V5 intensity values, there is a significant increase in cytosolic biotinylation of proteins with LPS. Significant p values were determined using the two-tailed Mann-Whitney test. **E.** Western blot (WB) verification of subcellular-fractionation experiments of transduced and untransduced BV2 cells with and without LPS challenge (n=3 /group). Using β-actin as a loading control, α-tubulin as a cytoplasmic marker, histone H3 as a nuclear marker, and V5 as a marker for TurboID, we can confirm purification of sub-cellular fractions with the decrease in α-tubulin signal in the nuclear fraction as compared with the whole cell and cytoplasmic fractions and a decrease in histone H3 in the cytoplasmic fraction as compared with the whole cell and cytoplasmic fractions. With actin signal intensity remaining constant as a loading control, there is no apparent difference in cytoplasmic V5 intensity with LPS challenge.
